# Matrisome Profiling During Intervertebral Disc Development And Ageing

**DOI:** 10.1038/s41598-017-11960-0

**Published:** 2017-09-14

**Authors:** Joana Caldeira, Cátia Santa, Hugo Osório, Maria Molinos, Bruno Manadas, Raquel Gonçalves, Mário Barbosa

**Affiliations:** 1 i3S - Instituto de Investigação e Inovação em Saúde, Universidade do Porto, Porto, Portugal; 2 INEB - Instituto de Engenharia Biomédica, Universidade do Porto, Rua Alfredo Allen, 208, 4200-180 Porto, Portugal; 3 IPATIMUP - Institute of Molecular Pathology and Immunology, University of Porto, Rua Júlio Amaral de Carvalho, 45, 4200-135 Porto, Portugal; 4 III – Institute for Interdisciplinary Research, University of Coimbra, Casa Costa Alemão – Pólo II, Rua Dom Francisco de Lemos, 3030-789 Coimbra, Portugal; 5 CNC – Center for Neuroscience and Cell Biology, University of Coimbra, 3004-504 Coimbra, Portugal; 6 Department of Pathology and Oncology, Faculty of Medicine, University of Porto, 4200-319 Porto, Portugal; 7 ICBAS - Instituto de Ciências Biomédicas de Abel Salazar, Universidade do Porto, Rua de Jorge Viterbo Ferreira n. 228, 4050-313 Porto, Portugal

## Abstract

Intervertebral disc (IVD) degeneration is often the cause of low back pain. Degeneration occurs with age and is accompanied by extracellular matrix (ECM) depletion, culminating in nucleus pulpous (NP) extrusion and IVD destruction. The changes that occur in the disc with age have been under investigation. However, a thorough study of ECM profiling is needed, to better understand IVD development and age-associated degeneration. As so, iTRAQ LC-MS/MS analysis of foetus, young and old bovine NPs, was performed to define the NP matrisome. The enrichment of Collagen XII and XIV in foetus, Fibronectin and Prolargin in elder NPs and Collagen XI in young ones was independently validated. This study provides the first matrisome database of healthy discs during development and ageing, which is key to determine the pathways and processes that maintain disc homeostasis. The factors identified may help to explain age-associated IVD degeneration or constitute putative effectors for disc regeneration.

## Introduction

The intervertebral disc (IVD) is a complex structure capable of resisting spinal compression while allowing motion of intervertebral segments^1, 2^. Besides water, it is mainly composed by extracellular matrix (ECM) molecules. These include collagens, proteoglycans (PGs) and other matrix proteins that contribute to the structural and mechanical function of the disc^3, 4^. Matrix degrading enzymes are also present to regulate matrix breakdown, maintaining disc homeostasis^5^.

A young healthy disc consists of a highly plastic and hydrated region – the nucleus pulposus (NP) – and a network of collagen fibres oriented in sheets around the nucleus – the annulus fibrosus (AF), which provides tensile strength and confines the NP, limiting bulging^6^.

During disc degeneration and ageing, significant changes are observed in the IVD at both cell and tissue level. From birth, notochordal cells gradually disappear from the NP^7^. Loss of cell density is accompanied by a shift towards a chondrocyte-like cell population^3^, less effective in NP-specific matrix synthesis^8^. Ultimately this results in NP fibrous transformation, from a translucent gel to a more solid and cartilaginous tissue^1^ making it difficult to distinguish between NP and AF^6^. Alterations in the composition and mechanical properties of the surrounding environment will in turn influence NP cell function and behaviour, in terms of differentiation, metabolism, proliferation and survival^8^.

Along with cellular changes, NP matrix remodeling is also an early step in the ageing process. Apart from overall matrix breakdown caused by MMPs (matrix metalloproteinases) and ADAMTS (a desintegrin and metalloprotease with thrombospondin motifs) overexpression^7^, PG and collagen synthesis patterns^9, 10^, as well as fibre crosslinking^1^ are also altered. This inhibits matrix turnover and, together with the already limited repair response, leads to dehydration and progressive ECM disorganization. Furthermore, it promotes mechanical failure, annular tears and many of the characteristic features of disc degeneration^6^. Over time, type II collagen is replaced by type I collagen in the NP^7^ and aggrecan content decreases^3^. Along with structural changes, soluble factors, and cytokines may also be released^11^, further affecting cell activity and tissue homoeostasis^7^. The availability of oxygen, nutrients and growth factors^12^, and the acidity of the environment, as well as the removal of metabolites, are also influenced by ECM calcification and impermeabilization^7^. With increasing age, this imbalance of the normal homeostatic mechanism impairs normal disc function, particularly in the NP^13^, ultimately resulting in reduced disc height, hernia formation and spinal pain, as nerve roots become compressed^2^.

Low back pain (LBP) causes disability and life quality deterioration, constituting a tremendous social and economic burden^14^. In more than 40% of the cases, it is triggered by IVD degeneration, which mimics disc ageing but occurs at an accelerated rate^1, 7^. Conventional therapies for LBP predominantly involve treatments based on pain modulators and invasive surgeries, like spine fusion or arthroplasty. However, spine surgeries have a high risk of complications associated^15, 16^, and recurrent interventions are many times needed (15–30% of cases)^17^, increasing the personal and financial costs even further^18^. Of note, the underlying pathophysiology is not being addressed, nor is the restoration of IVD’s function or the slowing down of disease progression. To date, promising strategies for disc regeneration, based on the maintenance and/or increase of matrix synthesis, are being explored *in vivo*: protein injection^19, 20^, gene transfer^21, 22^ and cell implantation^23, 24^. Although rapid advances are being made in understanding and regulating the degenerative process, many challenges remain^2^.

Understanding IVD pathophysiology (particularly in terms of IVD matrix constituents and their alterations in development and disease) is key to unveil molecular cues that might be used to slow, halt or reverse the age-associated degenerative cascade^2, 4^.

In this report, we have investigated matrisome changes observed with development and ageing in healthy bovine NPs, with the purpose of validating candidate molecules that might constitute novel therapeutic alternatives to treat IVD degeneration.

## Methods

### Sample preparation and iTRAQ analysis

Bovine caudal IVDs from foetus (around 7 months of gestation), young (12 months) and old animals (16 to 18 years old) were obtained from the local abattoir and dissected within 3–4 hours after slaughter. The NPs from 7–8 discs from Cd1 to Cd7 or Cd8 were collected as described by Molinos *et al*.^25^ and stored at −80 °C in a batch of 500 to 800 mg. For protein extraction, 1100 µL of guanidine extraction buffer were used. Further details on the iTRAQ analysis, including technicalities of protein extraction, precipitation and quantification, reduction, alkylation and trypsin digestion, as well as iTRAQ labelling, sample distribution, fractionation and LC-MS/MS analysis, database searching and protein identification, bioinformatics analysis and candidate selection criteria, can be found as Supplementary Data 1 and Supplementary Table 1. For cell extracts, cells were first isolated by Collagenase Type XI (2 mg/mL) treatment and posteriorly filtered to remove ECM contaminants, as previously reported^25^. Proteins were then extracted using the same guanidine hydrochloride based protocol that was used for whole tissue extracts (Supplementary Data 1). The mass spectrometry proteomics data have been deposited to the ProteomeXchange Consortium via the PRIDE partner repository with the dataset identifiers PXD005616 and PXD004922^26^.

### Macroscopic characterization and scanning electron microscopy (SEM)

For qualitative macroscopic evaluation of the different age groups, IVDs were excised and photographed under an Olympus SZX16 stereomicroscope coupled with a DP71 camera (Olympus, Tokyo, Japan) at 10X magnification.

For SEM analysis, samples were fixed using 2.5% (v/v) glutaraldehyde (Agar Scientific) in 0.1 M sodium cacodylate solution (Sigma) and then stored in sodium cacodylate buffer 0.1 M at 4 °C until further use. IVDs were then dehydrated in serial diluted ethanol solutions of 50, 60, 70, 80, 90, and 99% v/v, being incubated for 10 min in each dilution. Following critical point drying, samples were sputtered-coated with a Au/Pd thin film, using the SPI Module Sputter Coater equipment. Samples were examined at CEMUP (Materials Centre of the University of Porto), using a High resolution Scanning Electron Microscope with X-Ray Microanalysis - JEOL JSM 6301 F/ Oxford INCA Energy 350 - at 300X and 5000X magnification. The following parameters (mean fibril diameter, mean pore area, number of pores and number of intersections) were obtained using “DiameterJ”, a plugin for ImageJ/FIJI software version 1.46r (NIH) for topographic comparison of samples under study^27^.

### Western Blotting

Following denaturation for 10 min at 65 °C, protein samples were separated by sodium dodecyl sulphate (SDS) 9% polyacrylamide gel electrophoresis (PAGE), and electroblotted onto a Hybond enhanced chemiluminescence (ECL) membrane (Amersham Biosciences). Antibodies for Collagen Type XII, Collagen Type XIV, Collagen Type XI alpha 2, Fibronectin and Prolargin were used. Further details on blocking solutions, primary and secondary antibodies used, as well as their respective host species, working dilutions and commercial suppliers can be found in Supplementary Table 2.

After ECL detection (Amersham Biosciences), bands were quantified using Quantity One 4.6.8 Software (Bio-Rad) and values were normalized to the total protein loading (density value of each complete lane, obtained after staining of the membrane following immunodetection with Page Blue Protein Staining Solution (ThermoFisher Scientific), using a protocol adapted from Welinder and Ekblad^28^. All samples were run in the same SDS-PAGE gel, and background signal was measured in several different areas of the membrane. Average background signal was then subtracted to the band intensity signal to minimize background variation and interference.

### Statistical Analysis

Statistical analysis was performed using non-parametric Mann-Whitney test in GraphPad Prism software 5.0, to compare two groups of non-related samples (e.g. foetus *versus* young or young *versus* old NPs). The parametric distribution of the data was evaluated by D’Agostino and Pearson normality test. Results (from at least three independent biological samples) are expressed as median ± Interquartile Range (IQR) in box and whiskers plots or as mean ± Standard Error of the Mean (SEM). Values from p* ≤ *0.05 were considered statistically significant.

## Results

### Structural characterization of different aged IVDs by SEM

**Figure 1:**
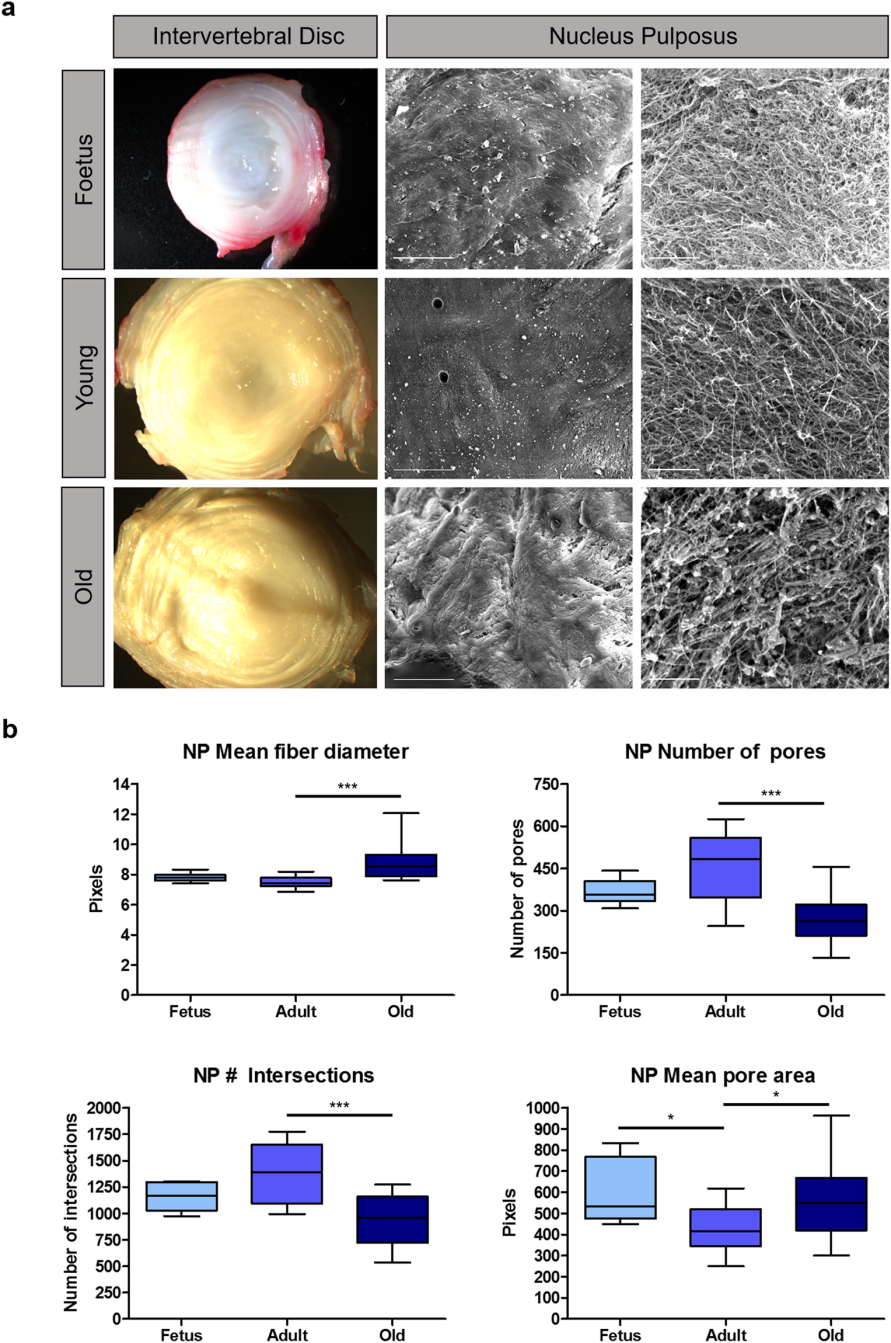
Gross view and topographical characterization of different aged IVDs. (a) - Macroscopic pictures from IVD transversal sections (left panels) and microscopy images from scanning electron microscopy (SEM) (central and right panels, with scale bars representing 200 µm and 10 µm, respectively) of foetal, young and old IVDs are presented. On the right panels are magnifications of the NP, representing the organization of ECM fibres. NP parameters such as mean fibril diameter, mean pore area, number of pores and number of intersections were also plotted regarding each age group (**b**). Box-plot lines represent median and interquartile ranges of the different parameters evaluated. (*) stands for p ≤ 0.05 (**) for p ≤ 0.01 and (***) for p ≤ 0.001, using the non-parametric Mann-Whitney test.

At the macroscopic level, several differences could be identified (Fig. 1, left panel). Foetal intervertebral discs (IVDs) consisted of a well-distinguished annulus fibrosus (AF) formed of concentric lamellae delimiting a highly hydrated gel-like nucleus pulposus (NP). During ageing and degeneration, the boundary between AF and NP became less obvious. In addition, discs started looking increasingly dry and fibrous.

Scanning electron microscopy (SEM) has also been optimized, for a more detailed comparison of NP topography from the three different age groups. NP SEM images clearly showed a fine network of randomly oriented fibres (Fig. 1, central and right panels).

Further quantitative characterization of the SEM images obtained for different aged NP samples revealed age-associated changes in matrix architecture. Using DiameterJ, a plugin of ImageJ/Fiji software, we evaluated the following parameters: mean fibre diameter, number of pores, number of intersections and mean pore area (Fig. 1b). Mean fibre diameter of elder (8.826 pixels) NPs was statistically higher than that of the adults (7.482 pxels), whereas no differences were observed between foetal (7.803 pixels) and young NPs. Elder bovine NPs also presented significantly fewer (275.2 pores) but bigger (566.5 pixels^2^) pores in comparison to young animals (459 pores and 432.6 pixels^2^, respectively) and also less fibre intersections (927 *vs* 1391 intersections). Foetal NP mean pore area (602.6 pixels^2^) was also significantly bigger than that of young IVDs.

### Optimization of the proteomics workflow

**Figure 2:**
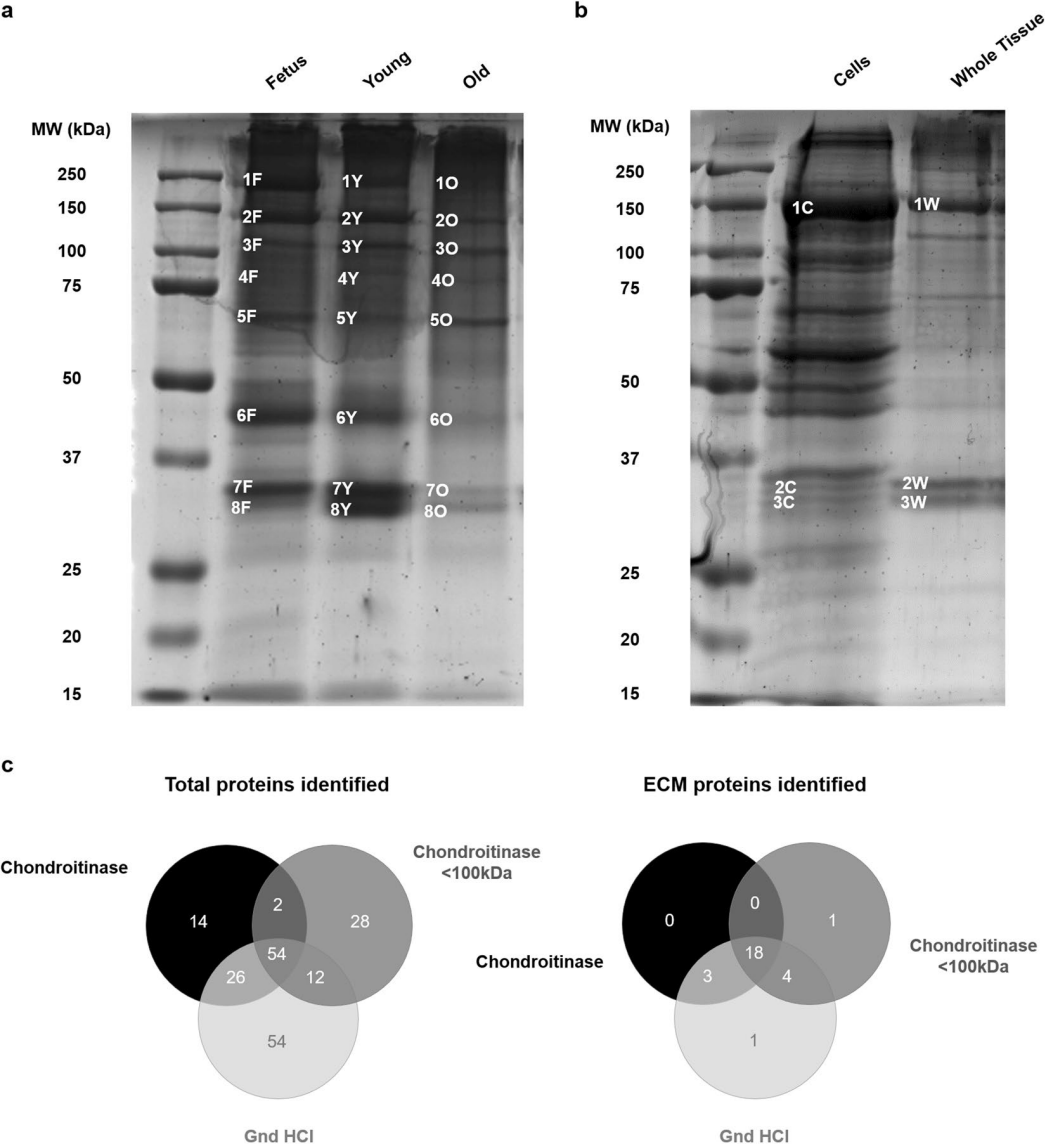
Sample complexity, age-associated SDS-PAGE band profile and extraction buffer evaluation. (**a**) - By comparing the band profiles observed in one dimension (1D) SDS-PAGE gels, for the three distinct age groups, there were already clear differences in the intensity of some of the bands identified by MS + MS/MS. Information on band identification from 1 F to 8 O can be found in Supplementary Table 3. (**b**) - With the same extraction buffer, we observed more bands within the cellular rather than the tissue extract. However, identification by MS + MS/MS (Supplementary Table 4) showed that the bands corresponding to ECM proteins were mainly present in the tissue extract (1 C–COL6A1/COL6A2; 1 W–COL6A1/COL2A1; 2 C–ANXA1/ANXA2/GAPDH; 2 W–COL2A1/CHAD/OGN; 3 C–ANXA8/LDHA; 3 W–CHAD). (**c**) - We compared the number of proteins identified by LC-MS/MS when using distinct buffers recommended from the literature: guanidine hydrochloride buffer (Gnd HCl), the same buffer with a prior chondroitinase 6 h treatment (chondroitinase) and this last condition followed by a centrifugal filtration, using a molecular weight cut-off of 100 kDa (chondroitinase <100 kDa). The extraction buffer with which more proteins in total were identified, and more ECM proteins, in particular, was Gnd HCl.

**Figure 3:**
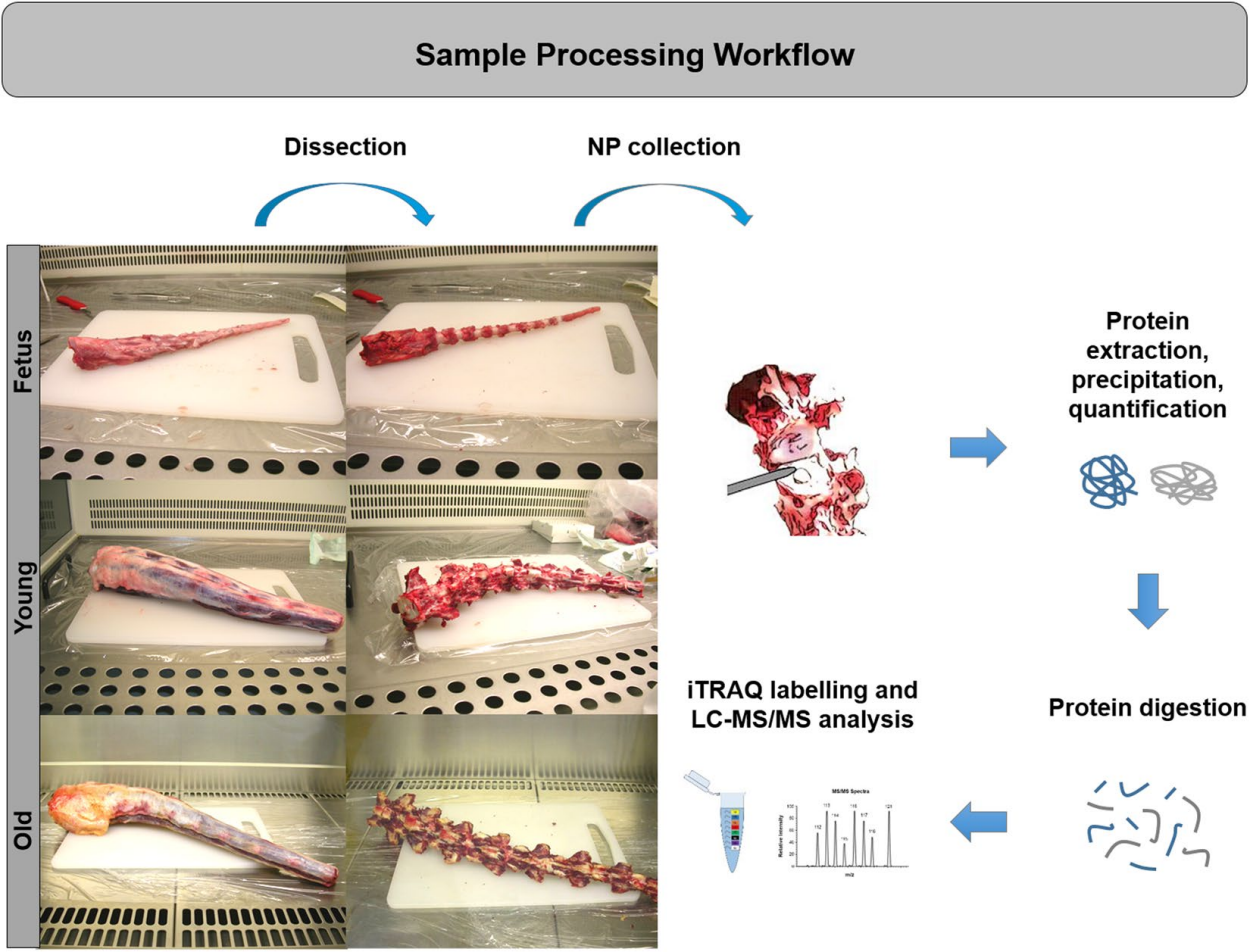
Proteomic sample processing workflow. Bovine caudal IVDs from foetus, young and old animals were dissected within a few hours after slaughter. The NPs were collected and protein was further extracted, precipitated and quantified. After digestion, peptides were marked with isobaric tags for relative and absolute quantitation (iTRAQ) prior to liquid chromatography – tandem mass spectrometry (LC-MS/MS).

**Figure 4:**
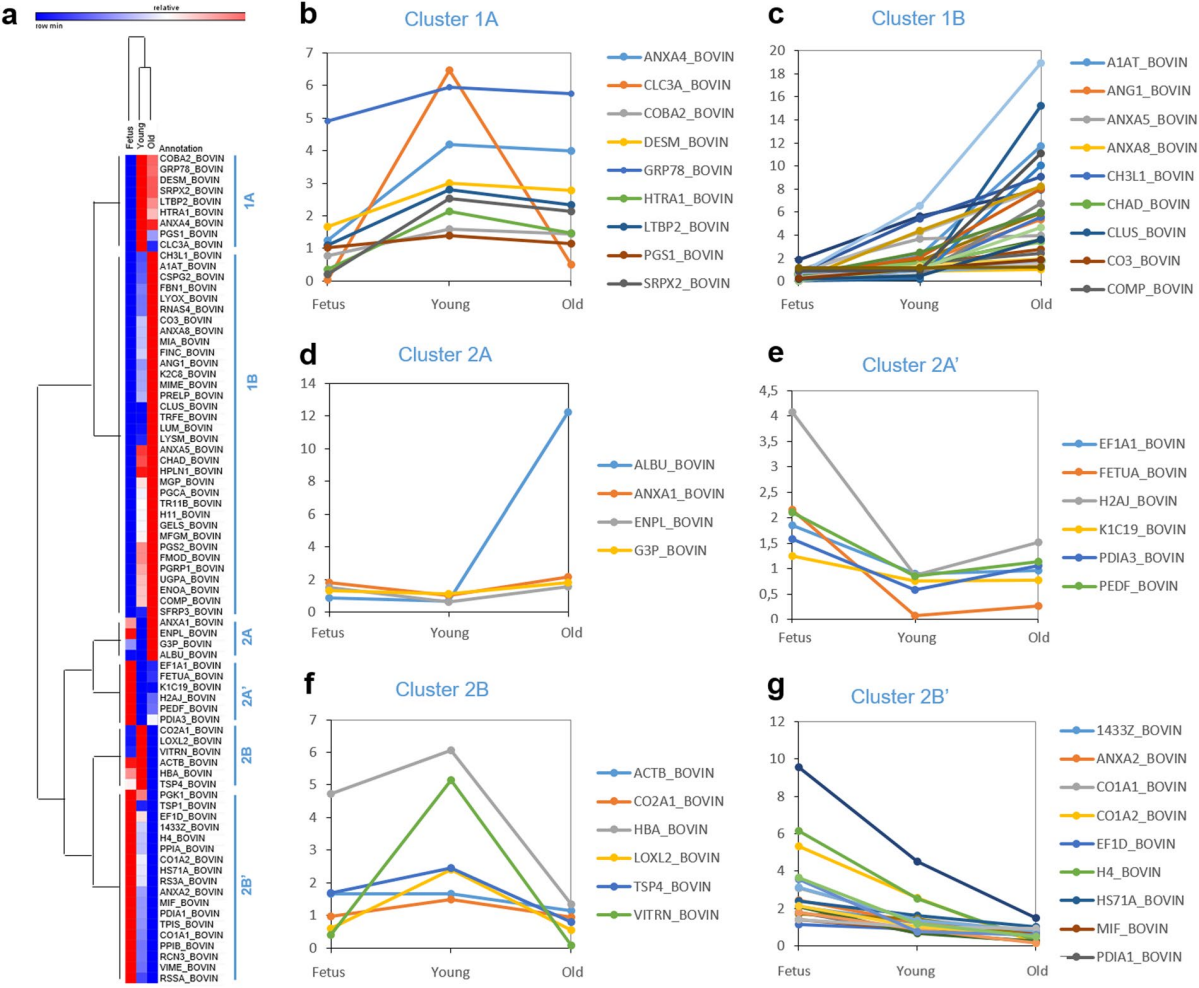
iTRAQ data analysis. (**a**) - Heatmap with the respective dendrogram representing sample-based hierarchical clusters. Average expression levels were represented by colour scale from blue (low) to red (high). In terms of protein expression, there were six main clusters. (**b–g**) - Graphical representation of iTRAQ relative protein expression profiles for the molecules within each of the 6 main clusters: cluster 1 A (**b**), cluster 1B (**c**), cluster 2A (**d**), cluster 2A’ (**e**), cluster 2B (**f**) and cluster 2B’ (**g**).

**Figure 5:**
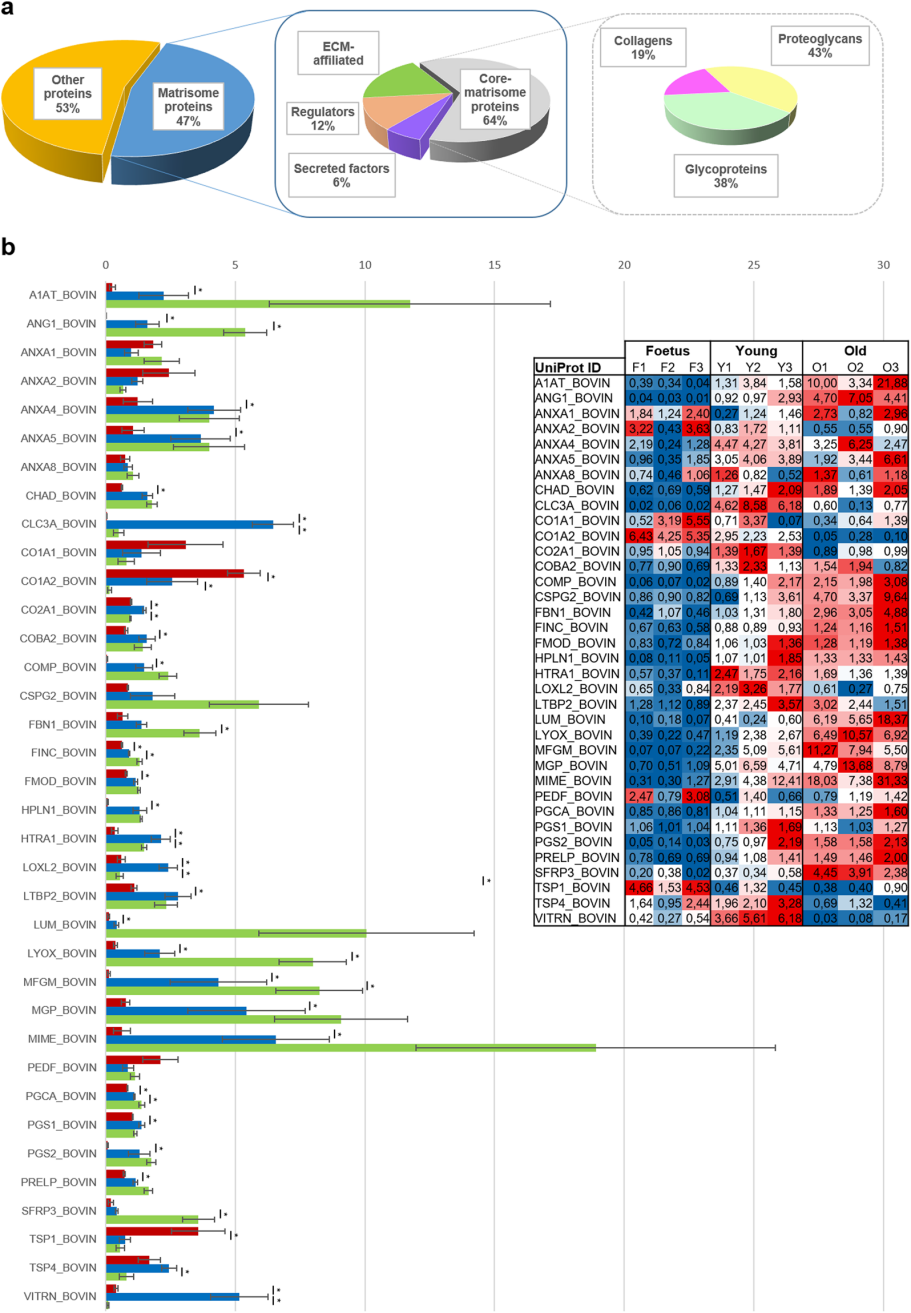
Characterization of the NP matrisome. (**a**) - The pie charts exhibit percentages of identified proteins distributed by matrisome categories. (**b**) - Comparison of the different aged NP matrisome signatures. iTRAQ relative protein expression levels (x axis) are displayed for each of the molecules identified (y axis). Foetal samples are in red, young in blue and old in green. iTRAQ protein quantification scores for individual samples can be found on the embedded table (colour scale from blue – low expression – to red – high expression). In the case of Young animals, the values represent an average of the technical replicates. The non-parametric Mann-Whitney test was used to compare two groups of non-related samples. Standard error of the mean (SEM) is represented as the error bar. (*) stands for p ≤ 0.05, using the non-parametric Mann-Whitney test.

**Figure 6:**
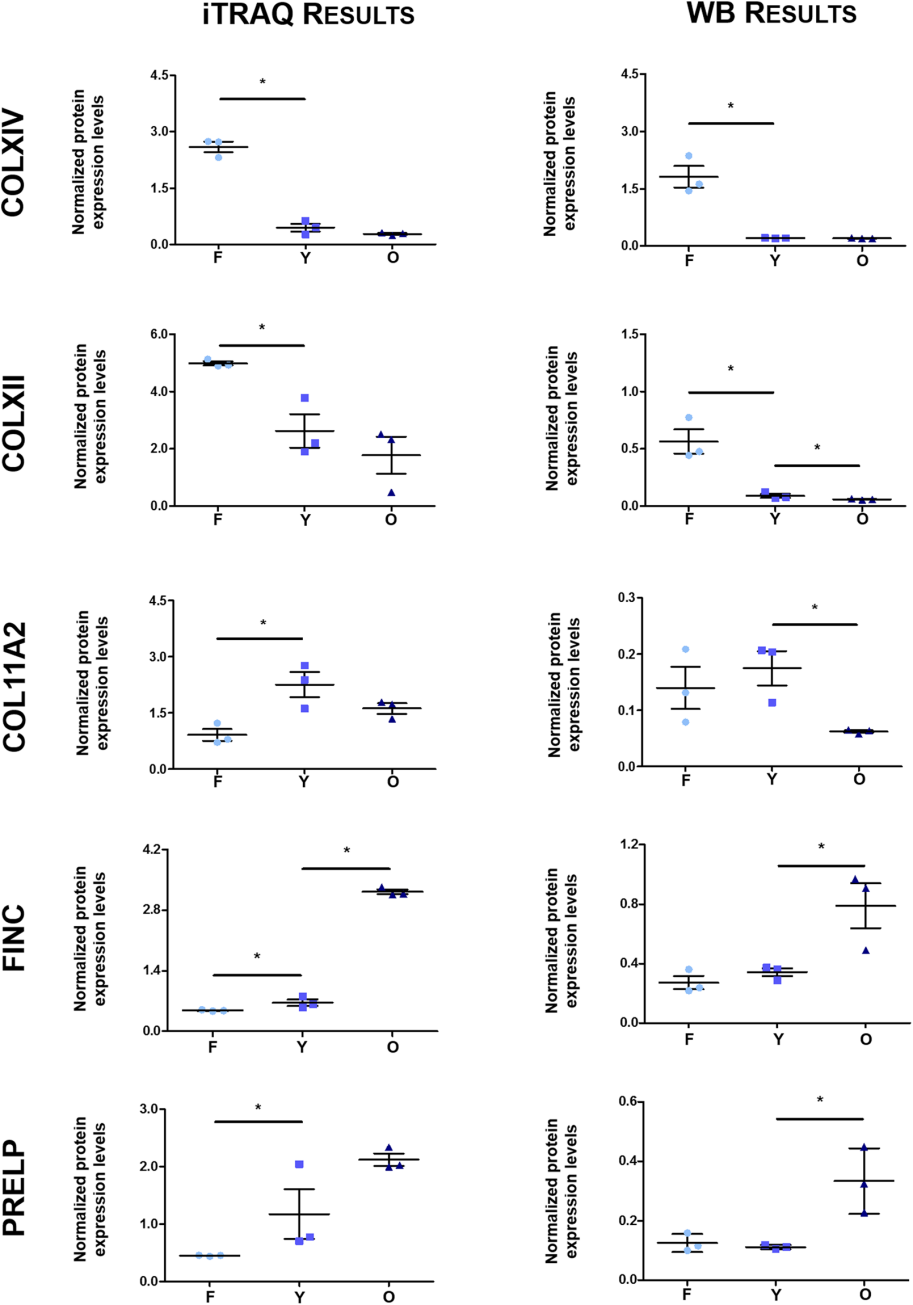
Validation of the candidates’ protein expression levels. On the left are the graphics representing normalized protein expression levels from the iTRAQ LC-MS/MS analysis and on the right, of Western Blots (WBs), from Foetus, Young and Old animals. Collagen Type XII (COLXII) and XIV (COLXIV) expression levels are higher in NPs from foetus, Collagen Type 11 (COL11A2) is mostly expressed in Young animals, whereas Fibronectin (FINC) and Prolargin (PRELP) are typical from elder NPs. The non-parametric Mann-Whitney test was used to compare two groups of non-related samples (Foetus *vs* Young and Young *vs* Old). Graphs correspond to the average of protein expression levels obtained by band quantification and subsequent normalization to the total protein loading (Supplementary Figure 3). Standard error of the mean (SEM) is represented as the error bar. (*) stands for p ≤ 0.05, using the non-parametric Mann-Whitney test.

**Figure 7:**
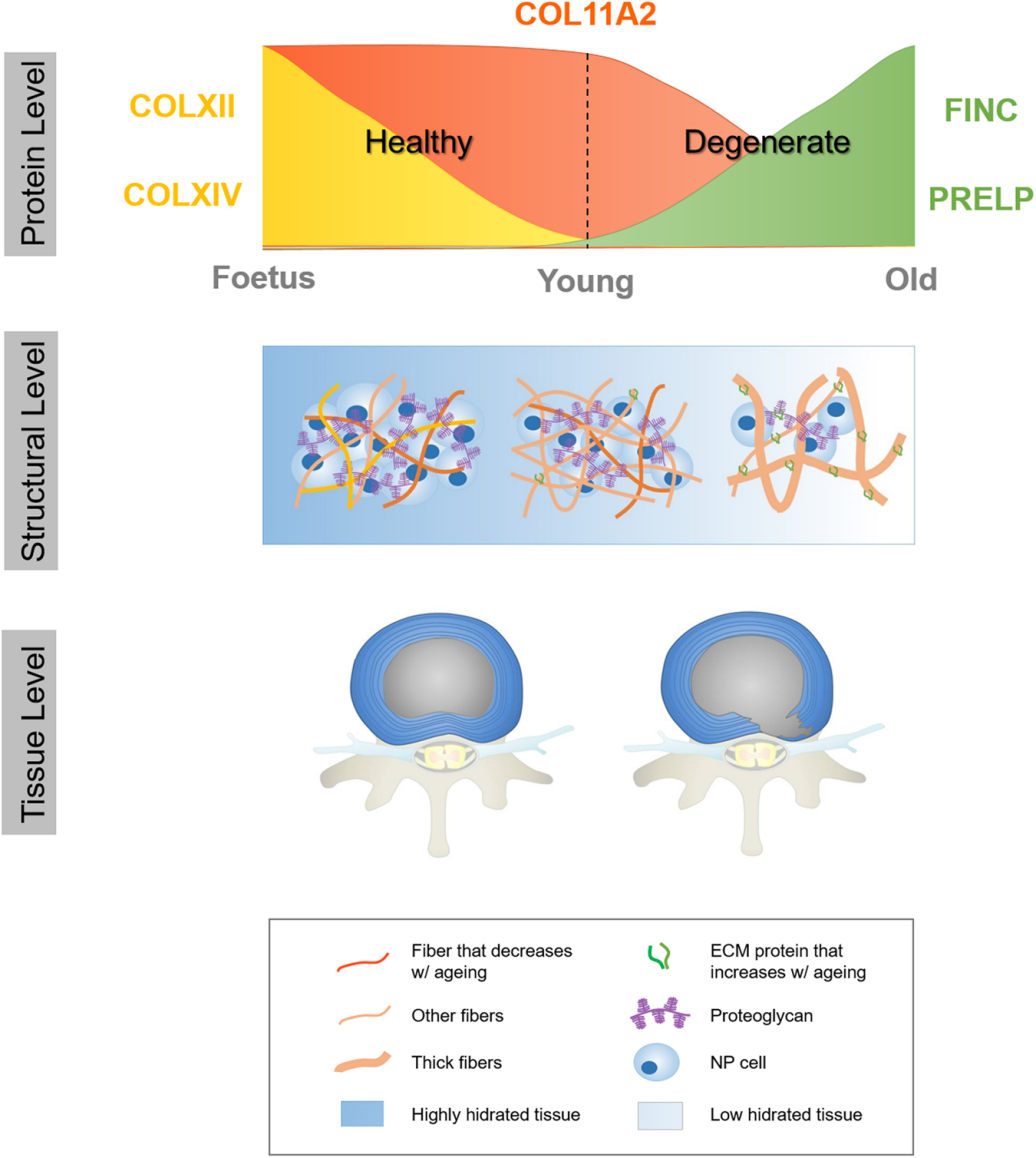
Working model for the changes that occur in the IVD microenvironment with development and ageing. From the foetal stages to adulthood, collagen type XII and XIV expression is lost and only collagen type XI is maintained. With increasing age, there is an enrichment in fibronectin and prolargin. Proteomic alterations are accompanied by matrix remodeling (fibrillogenesis and fibril organization are both affected), concomitant with water loss and a cell population decline. This ultimately causes age-associated degeneration, hernia formation and low back pain.

Age-associated protein expression profiles were identified in the first place by the distinct one dimension (1D) SDS-PAGE band signatures obtained. By comparing foetal, young and old NPs, we found that band intensities differed depending on disc age (Fig. 2a). Foetus and young samples presented an enrichment of high molecular weight proteins, between 150 and 250 kDa, where some of the molecules identified were tenascin, collagen type VI and biglycan (Fig. 2a–1F and Y and Supplementary Data 4). This trend was also visible among proteins around 50 kDa that corresponded to actin, link protein (HAPLN1), biglycan and decorin among others (Fig. 2a–6F,Y and O, Supplementary Table 3 and Supplementary Data 4). Noteworthy, we observed that young NPs had increased protein expression levels at around 30 kDa (Fig. 2a–7F,Y,O and 8F,Y,O). These bands corresponded to collagen type II and chondroadherin (Supplementary Table 3 and Supplementary Data 4). Moreover, above 100 kDa a band presented a trend to increase in Young and Old animals when compared to Foetus (Fig. 2a and 3F,Y,O). Fibromodulin, biglycan, aggrecan and cartilage oligomeric matrix protein (COMP) were identified within these bands (Supplementary Table 3 and Supplementary Data 4). In addition, prolargin (PRELP) was only identified in old samples (Fig. 2a–5O, Supplementary Table 3 and Supplementary Data 4) and not in the same molecular weight bands of Foetus or Young animals (Fig. 2a–5F and Y, Supplementary Table 3 and Supplementary Data 4). Nevertheless, the differences detected were only semi-quantitative and the fact that after MS identification of the molecules present in each of the gel bands, protein mixtures were found (Supplementary Table 3 and Supplementary Data 4), made it hard to dissect which were the ones accounting for the observed differences.

For a comprehensive age comparative analysis of the NP matrisome, we used a gel-free MS based high throughput proteomic approach. The first optimization step involved the decision on whether we could use the whole tissue extract or whether decellularization was needed. Comparing the protein band profiles of the whole tissue and of cell extracts obtained using the same buffer, more bands were observed within the cellular rather than the tissue extract. Nevertheless, MS/MS identification showed that the bands corresponding to ECM proteins (namely collagen type II, chondroadherin and mimecan) were mainly present in the tissue extract (Fig. 2b–1w, 2w and 3w, respectively, Supplementary Table 4 and Supplementary Data 5). In fact, most of the disc is made up of water, while ECM and cells represent a very small percentage in terms of tissue volume^12^. For this reason, we did not pursue further the laborious task of improving the decellularization step, given that it brought no added value.

We tested 3 different buffers taken from the literature^29^. For each of them, we evaluated the total number of bovine proteins identified, as well as the number of ECM-associated molecules obtained. For that we took advantage of the Functional Annotation Clustering Tool from DAVID (https://david.ncifcrf.gov/). Qualitative results from Liquid Chromatography coupled to tandem Mass Spectrometry (LC-MS/MS) enabled us to select Guanidine Hydrochloride as the best buffer for our analysis, given that it enabled the identification of more bovine total proteins (146) and, particularly, more ECM molecules (26) than the chondroitinase-containing buffers (Fig. 2c and Supplementary Data 6).

The optimized workflow that was used for sample processing is summarized in Fig. 3. Briefly, NPs were excised from foetal, young and old bovine tails and stored at −80 °C until further use. Samples were then snap frozen in liquid nitrogen and pulverized prior to the 24 hour protein extraction with the 4 M Guanidine Hydrochloride buffer. Protein was then precipitated, quantified, and digested. Following 8-plex isobaric tag for relative and absolute quantitation (iTRAQ) labelling, samples were pooled and fractionated by LC. Finally, the peptide mixture was analysed by LC-MS/MS and protein identification was performed using Protein Pilot. Of note, the labelled samples of each of the two batches were combined into one sample mixture (Batch 1: Y1, Y2, Y3, F1, F2, F3, O Pool, Total Pool; Batch 2: Y1, Y2, Y3, O1, O2, O3, F Pool, Total Pool – Supplementary Data 1 and Supplementary Table 1).

### Identification of NP age related proteomic signatures by iTRAQ analysis

PCA-DA plots, obtained with MarkerView, show significant clustering and differentiation among foetus (green), young (blue) and old (red) animals (Supplementary Figure 2a). Despite biological variability within the distinct age groups, and the fact that iTRAQ data was obtained from two independent runs, the three major groups of samples (Foetus, Young and Old) were well separated from each other, suggesting that NPs from different age sets presented distinct proteomes.

Averaged values of relative protein expression data from iTRAQ based LC-MS/MS (8-plex) assays were subjected to hierarchical clustering with Morpheus software. The heatmap and respective dendrogram generated are represented in Fig. 4a. This supervised analysis was performed by applying Spearman rank standardization. Foetuses were grouped together. Although they were part of the same cluster (showing that they both have similar protein expression profiles), Young and Old animals were also grouped according to their age status. Clustering data analysis demonstrated the ability to conduct global proteomics profiling on NP disc tissues, revealing that significantly different arrays of proteins were expressed depending on the age of the individuals, despite the fact that samples were collected from the same avascular organs in the bovine body.

To explore the biological processes affected by the 77 (out of 161) common proteins identified in all the samples, and thus related to NP function, we performed Gene Ontology (GO) and Pathway term enrichment using the Functional Annotation Clustering Tool from DAVID Database. This allowed us to determine GO and Pathway terms that occurred more frequently than expected by chance. Proteins were then clustered according to functional similarity (Supplementary Tables 5 and 6).

The most significant cluster of proteins, in comparison to *Bos taurus* proteome, included GO terms implicated in extracellular matrix and GAG binding, which was in accordance to what we expected. Interestingly, we also detected a statistically significant group of interactors involved in collagen fibril organization, cartilage and blood vessel development, and also proteins implicated in lipid binding and vesicles. Other statistically significant biological terms that appeared were glycolysis, regulation of phagocytosis, response to wounding, inflammatory response and calcium ion binding, among others. Redoing the analysis concerning pathway term enrichment, we obtained “ECM-receptor interaction”, “Focal adhesion” and “integrin signalling”.

To integrate known and predicted protein-protein interactions and better understand the relationships between the 77 distinct proteins commonly identified in all 3 age groups, we used STRING (Supplementary Figure 2b).

The interactome obtained highlighted six different clusters marked in same colour circles, broadly representing: cytoskeleton (light green), fibril/collagen organization and skeletal/cartilage development (beige), GAG binding/crosslinking (dark green), glycolysis (red) and vesicle-associated proteins (magenta). Large protein interaction networks illustrated the high degree of connectivity and the presence of promiscuous hub proteins.

Finally, to further highlight correlations between the proteins within each of the 6 main heatmap clusters (cluster 1A, cluster 1B, cluster 2A, cluster 2A’, cluster 2B and cluster 2B’ – Fig. 4), we performed gene ontology enrichment analysis based on the proteins identified in the different clusters. Among the proteins within cluster 1B (which increase with development and ageing), we found a significant enrichment of Gene Ontology terms related to extracellular matrix, glycosaminoglycan, polyssacharide, carbohydrate, hyaluronic acid and ion binding, as well as cell adhesion and membrane bound organelles. Among the proteins from cluster 2A, there was an enrichment of the term ion binding, whereas among the cluster 2B’ (proteins which decrease with development and ageing) there was an enrichment of terms such as melanosomes, cytoplasmic vesicles, collagen fibril organization, blood vessel development, endoplasmic reticulum and also ion binding. No other significantly enriched GO terms were found for other groups of samples. A detailed list of the enriched GO terms in each cluster can be found in the Supplementary Data 7.

### Definition of NP matrisome changes during development and ageing

Given that GO analysis revealed enrichment of several ECM-related categories, we examined the overlap of the NP proteomic signature with the matrisome, a comprehensive list of genes coding for ECM molecules and regulators, which is significantly more comprehensive for data mining and for posing questions relevant to matrix biology than GO terms^30^.

Of note, 47% of the genes composing the NP signature encode for matrisomal proteins (Fig. 5a), and ECM-associated molecules. Core matrisomal proteins (64%) are over-represented, particularly in terms of proteoglycans (44%) and glycoproteins (39%). We further defined the NP matrisome as the 36 matrisomal proteins identified in the 3 age groups (relative protein quantification of such molecules is summarized in Fig. 5b).

### Candidate validation by Western Blot

The rationale behind the identification of potential age-related matrix components deregulated during NP development and choice of candidates for further investigation was: the analysis of iTRAQ data (e.g. differential protein expression ratio cut-off >1.3 and <0.77 and ProteinPilot protein identification confidence), literature mining concerning promising associations with IVD ageing and degeneration or lack of published data suggesting novel potential candidate biomolecules. Given that most proteins overexpressed in Foetus had not been identified with the highest confidence scores, we decided to further explore additional results obtained by searching the original MS/MS data against the proteomes from all organisms available on Swiss-Prot (results available upon request), instead of only using the *Bos taurus* complete proteome set.

Following these criteria, 5 proteins were selected for further validation: Collagen type XII, Collagen type XIV, Collagen type XI, Prolargin and Fibronectin. To confirm if these NP matrisome candidates were expressed in an age-dependent manner, Western blot analysis was performed (Fig. 6, right panel and Supplementary Figure 3). Collagen type XII and Collagen type XIV were significantly more expressed (6-fold and 9-fold, respectively) in NPs from Foetus rather than from Young animals (0.56 to 0.09 in Collagen type XII and 1.81 to 0.21 in Collagen type XIV). No other relevant protein expression differences were found for Collagen type XIV, when comparing Young and Old samples. Nevertheless, there was also a slight but significant 1.5-fold decrease of Collagen type XII from Young to Old age groups (0.09 to 0.06). Collagen type XI was shown to be expressed at significantly higher levels in Young NPs (0.17) in comparison to Old ones (0.06) and this enrichment at younger stages was also verified by immunofluorescence (Supplementary Figure 4). Fibronectin and Prolargin results reflected a significant (2-fold and 3-fold, respectively) overexpression (from 0.34 to 0.79 in Fibronectin and from 0.11 to 0.33 in Prolargin) only in older tissues. The results of this analysis were consistent with the trends obtained by exploring iTRAQ data (Fig. 6, left panel) and thus highlight the implication of Collagen type XII, Collagen type XIV, Collagen type XI, Prolargin and Fibronectin in age mediated IVD degeneration.

## Discussion

Disc degeneration and ageing are intimately associated. While disc ageing is a natural and gradually occurring process, disc degeneration involves more rapid and severe changes. In this study, we aimed to characterize NP proteomic changes that occur with development and ageing and that are closely related to disc degeneration, with a special focus on the ECM. For that, we have used iTRAQ labelling coupled to LC-based tandem mass spectrometry. This technique infers the relative abundance of individual proteins from peptide MS signal intensities and has emerged as an effective tool for quantitative proteomic profiling of complex tissue extracts, like cartilage^31^. SEM analysis and Western blotting also helped us to highlight and validate structural and molecular differences between the different age groups. The significance of our results is summarized as follows: several well-known effector proteins and a number of novel putative players were identified, 5 of which were independently validated.

Recently, proteomic-based studies have risen sharply and have started to be used to characterize normal and/or degenerated disc cells^32, 33^, secreted factors^34, 35^ and tissue composition^36^, showing promising results. To date, however, limited studies have used proteomic strategies to study IVD matrix composition. One of the few existing studies focuses on the comparison of matrix proteomic signatures in different tissues^37^, while another is centred on the identification of cartilage matrix patterns of zonal distribution^38^. To the best of our knowledge, Yee *et al*. were the only ones that dissected ECM changes of human discs in age and degeneration^39^. Nevertheless, they used scoliotic samples as controls, which have been shown to present a gene expression profile that differs from healthy tissues, and signs of calcification that might reflect a premature degenerative process^40, 41^. In addition, the age range of non-degenerated samples under study was very limited and not representative of elder individuals.

Unlike existing approaches, we used healthy samples from different age groups, and included, for the first time in this type of analysis, foetal tissue and NPs from very old animals. At the macroscopic level, we demonstrated that tissues, particularly the NP, tend to gradually become less translucent and AF lamellae appears increasingly disorganized, as has been previously reported for degeneration^1^. These observations were supported by SEM analysis through which we thoroughly characterized NP collagen fibres. Mean fibril diameter was reduced in younger samples, indicating that fibrillogenesis was somehow affected at later developmental stages. Fibril organization in elder individuals was also disrupted. Matrix was sparser, with less intersections and reduced numbers of pores, which were bigger in size. Foetal pore area was also larger, possibly due to imbibed water^42^. This may explain the gel-like appearance of foetal NPs and their greater capacity to absorb load and tension.

iTRAQ technology enabled the identification of 161 proteins in total, 77 of which were detected in all samples. From the bioinformatics analysis of the common hits, a few observations were predictable. The highest ranking functional cluster of genes was for those involved in extracellular matrix, which was consistent with the guanidine extraction method selected^43^. Additional cytosolic components equally important for disc homeostasis and function might not have been identified, but these were not the focus of this study. A significant enrichment of other proteins and protein classes like GAG, polysaccharide and carbohydrate binding, collagen fibril organization, blood vessel, skeletal system and cartilage development, as well as glycolysis, was also found and reflected a complex network of interactions, involving more than pure ECM biosynthesis. With regard to response to wounding, its link to disc de- and regeneration has been well established^4^. Interestingly, melanosome regulation and vesicle-mediated transport were also enriched and seem to have a role in embryonic elongation and spine morphogenesis^44^.

With respect to pathway term enrichment, ECM-receptor interaction, focal adhesion, integrin and TGF- β signalling, were expected, given that most of these pathways are connected. In most of the tissues, integrin cell surface receptors mediate cell-matrix interactions, which are key to control adhesion, survival and differentiation, among others, in response to environmental cues, like mechanical stimuli^45, 46^. Moreover, focal adhesion kinase (FAK) has been shown to be activated in response to strain in non-degenerate disc cells, in an integrin dependent manner^47^. TGF-β can also be activated by integrin signalling, which, in turn, is affected by TGF-β, whose bioavailability is controlled by ECM binding^48^ Most of these NP-associated functions might be deregulated not only in development but also during ageing and degeneration.

By assessing the overlap of the NP proteomic signature with a list of nearly 300 ECM and ECM-associated molecules generated by Naba and co-workers^30^, we defined the NP matrisome and how it is changed during development and ageing. Meta-analysis of such proteomic data was used to identify potential age-related components deregulated during NP development and select candidates for further investigation. Collagen Type XI, XII, XIV Fibronectin and Prolargin expression profiles were confirmed by Western Blot analysis. Collagen Type XI overexpression in young animals was also validated by immunofluorescence. Moreover, PRELP increase with age was additionally verified by SDS-PAGE band identification. Other proteins like Actin, Collagen Type II, Fibromodulin, Aggrecan and COMP, among others, presented the same trend in iTRAQ analysis as in gel band profiling.

Fibronectin (FN) mediates a wide variety of cellular interactions with the matrix and plays important roles in cell adhesion, migration, growth, differentiation and survival, particularly through integrin interactions^4, 46^. It interacts with a broad range of collagens (type I, II, III, IV, V and X)^49^ and, in line with our results, FN has been shown to be upregulated in numerous models of disc ageing and degeneration^35, 39, 50^. Usually, 30 to 40%^51^ of the protein content is in the form of fragments from enzymatic cleavage^4, 52, 53^, which in turn promote further degeneration^54^. While excessive FN deposition has been linked to fibrosis^55^, polymorphisms have also been associated with disc degeneration^56^, given that it mediates collagen deposition and thus preserves matrix structural integrity^57^. FN may also be involved in the clearance of tissue denatured collagen in age and degeneration, or of circulating fibrin after trauma or in inflammation^58, 59^.

Prolargin (PRELP) is an ECM structural component that anchors the basement membrane to the underlying connective tissue^4^. Apart from binding GAG chains, it also binds type I and type II collagen^60^. PRELP overexpression in mice disrupts collagen fibres (which decrease in content and size), with no influence in fibril diameter^61^. In accordance, we have shown that the matrix of aged samples presented a lower density of fibres, resulting in a reduced number of fibre intersections, as well as in fewer but larger pores. Interestingly, PRELP is absent in neonatal articular cartilage ECM, in contrast to its abundance later in life^62^. This age-related accumulation agrees with our bovine data. In turn, human scoliotic NPs, also present PRELP increase with age^39^, whereas in dogs it has been associated with degeneration^35^.

Collagen Type XI is a fibril-forming collagen required for embryonic development and its abundance is inversely correlated with fibre diameter^4, 63^. This explains our observations (no differences between Foetuses and Young samples, where it is expressed, but an increase in fibre diameter in elder individuals, where it is absent). Apart from having a role in fibrillogenesis (controlling lateral growth of collagen type II fibrils) and in mineralization, Collagen XI also binds PGs, particularly at the cell surface, being important to maintain tissue integrity and cohesion, particularly during matrix remodeling^4, 64^. In line with our results, Collagen XI polymorphisms have been associated with disc degeneration^56^. COL11A1 expression levels tend to decrease with the severity of degeneration, at least in part due to MMP-mediated degradation^6, 65^. Recent data from Yee *et al*. also seem to indicate that collagen type XI declines in the human scoliotic NPs with ageing, in agreement with our bovine model^39^.

Collagen Type XII is a typical collagen-organizer molecule that binds to collagen I containing fibrils, as well as to other matrix proteins, like COMP, modulating fibril organization and mechanical properties^4, 66^. It has been suggested to take part in the development of stromal architecture and tissue cohesion^67^, particularly by promoting matrix bridges formation essential for network communication^68^. In addition, Collagen XII seems to have a pro-regenerative role, at least in other tissues^69, 70^.

Collagen Type XIV is a fibril-associated collagen, transiently expressed in several epithelia, including those undergoing rapid remodeling. At later developmental stages, it only persists in the BM, where it co-localizes with Collagen XII^71^. Collagen XIV is thought to control collagen I fibrillogenesis during embryonic development (as has been supported by our results, it inversely correlates with fibril diameter and premature growth), as well as differentiation^4, 66, 67^. Interestingly, in the chick embryo it is expressed in a gradient around the spinal cord^72^. Like Collagen XII, it is key for the hydration and thickness (and therefore transparency) of tissues^73^, supporting our macroscopic observations of a gel-like appearance in foetal NPs. In other settings, type XIV collagen also appears to play a role in regeneration^74, 75^.

Overall, this study provides the first matrisome database of healthy discs during development and ageing, which is key to determine the pathways and processes required to maintain disc homeostasis. The integrated analysis of the proteomic datasets enabled us to discover novel components and characterize the developmental system in greater detail. The data herein presented may establish a solid foundation for better understanding the complex microenvironment of the IVD. Furthermore, they provide a starting point from which potential biomarkers and pathways that are altered during the dynamic disc degeneration process may be recapitulated or resumed, opening new possibilities for the development of novel therapeutic solutions for the disease. For instance, using these cues to modulate the ECM, recreating a microenvironment similar to early developmental stages, may enable the expansion and differentiation of autologous NP cells *in vivo*. In fact, others have shown that cell-free tissue engineering strategies are sufficient to promote disc regeneration^76^. Alternatively, the same approach could be combined with cell-based therapies currently under study^23, 24^.

Evidence about the use of bovine coccygeal discs as a model of ageing is still scarce. To date, existing studies have failed to use very old individuals. Nevertheless, a higher incidence of degenerative disc changes has been registered in elder specimens, indicating their clinical relevance^77–79^. In fact, ageing similarities between the two species were found, particularly in terms of extracellular matrix alterations^77^.

Moreover, although interspecies differences may naturally occur, bovine coccygeal discs are becoming increasingly accepted tissues for large animal organ culture, majorly because of their large size, low cost and availability. Given the similar aspect ratios, transport distances, and cell content, especially concerning the absence of notochordal cells during adulthood, between bovine and human lumbar discs, and contrarily to what happens in the vast majority of small animal models, they have been proposed as a suitable model to study several aspects of lumbar discs pathobiology^12, 79–81^. In particular, comparable types and distribution, synthesis and deposition of extracellular matrix molecules have been found in both human lumbar and bovine coccygeal discs^82–86^.

The work herein presented paves the way for future studies using human samples to validate the observed differences and investigating these constituents, thus further elucidating about their functional roles. Importantly, and since it is known that extractability of NP proteins reduces with age due to increased crosslinking and resistance to degradation, particularly concerning the collagen fibrillar network, chemical digestion proposed by Chan and co-workers should also be addressed to analyze the insoluble fractions obtained^39^.

In addition, reanalysis of this dataset focusing on the numerous cellular proteins identified will be of major importance, given that there are many other proteins equally important for disc homeostasis and function, which are still to be unveiled.

As a model (Fig. 7), we propose that remodeling of NP tissue architecture, which affects IVD mechanical properties and biological function of the tissue, reflects changes that occur in terms of matrix biochemical composition during development and ageing. Integrating information from the protein to the tissue level, taking into account the cell-matrix crosstalk, will provide helpful cues for disc regeneration^26^.

## Electronic supplementary material

Supplementary Data 1

Supplementary Data 2

Supplementary Data 3

Supplementary Data 4

Supplementary Data 5

Supplementary Data 6

Supplementary Data 7
