# Supplementary material for: Matrisome Profiling During Intervertebral Disc Development And Ageing
Source: Sci Rep. 2017 Sep 14;7:11629. doi: 10.1038/s41598-017-11960-0 (PMC5599645; doi:10.1038/s41598-017-11960-0)
Supplement: Supplementary file 1 — Supplementary Data 1 [file 41598_2017_11960_MOESM1_ESM.pdf]

Title: **Matrisome profiling during Intervertebral Disc development and ageing**

Joana Caldeira[1,2,3*], Cátia Santa[4,5], Hugo Osório[1,3,6], Maria Molinos[1,2,7], Bruno Manadas[5], Raquel Gonçalves[1,2], Mário Barbosa[1,2,7]

[1] i3S - Instituto de Investigação e Inovação em Saúde, Universidade do Porto

[2] INEB - Instituto de Engenharia Biomédica, Universidade do Porto, Rua Alfredo Allen, 208, 4200-180 Porto, Portugal

[3] IPATIMUP - Institute of Molecular Pathology and Immunology, University of Porto, Rua Júlio Amaral de Carvalho, 45, 4200-135 Porto, Portugal

[4] III – Institute for Interdisciplinary Research, University of Coimbra, Casa Costa Alemão – Pólo II, Rua Dom Francisco de Lemos, 3030-789 Coimbra, Portugal

[5] CNC – Center for Neuroscience and Cell Biology, University of Coimbra, 3004-504 Coimbra, Portugal

[6] Department of Pathology and Oncology, Faculty of Medicine, University of Porto, 4200-319 Porto, Portugal.

[7] ICBAS - Instituto de Ciências Biomédicas de Abel Salazar, Universidade do Porto, Rua de Jorge Viterbo Ferreira n. 228, 4050-313 Porto, Portugal

*Corresponding author: Joana Caldeira, Instituto de Investigação e Inovação em Saúde (i3S), Rua Alfredo Allen, 208, 4200-135 Porto, Portugal E-mail: joana.caldeira@ineb.up.pt

Telephone: +351 220 408 800

SUPPLEMENTARY DATA 1

Protein extraction, precipitation and quantification

Protein was further extracted for 24 h at 4 ºC in 1100 µL of buffer containing guanidine hydrochloride 4M, Ethylenediamine tetraacetic acid (EDTA) 10mM (Sigma), sodium acetate 50 mM (Merck), dithiothreitol (DTT) 10mg/mL (Sigma), phenylmethylsulfonyl fluoride (PMSF) 1mM (Sigma) and cOmplete, Mini, EDTA-free Protease Inhibitor Cocktail inhibitor 20µL/mL (Sigma). After centrifugation, supernatants were precipitated in 9 volumes of ethanol for 2h at -20 ºC. Samples were kept at -80 ºC until the iTRAQ assay was performed. Otherwise, samples were centrifuged and the pellets ressolubilized in a 7M urea (Sigma), 2M thiourea (Sigma) and 2% 3-[(3-cholamidopropyl)dimethylammonio]-1-propanesulfonate (CHAPS) (Sigma) buffer for Western blot analysis. Protein quantification was performed using 2D Quant Kit (GE Healthcare), following manufacturer's instructions.

SDS-PAGE protein band identification by PMF+MS/MS

The selected protein bands from (Figure 2) were excised from the Coomassie Blue-stained gels, with a spotpicker (OneTouch 2-DE gel spotpicker, Gel Company) and then processed for Matrix Assisted Laser Desorption/Ionization (MALDI-TOF/TOF) mass spectrometry analysis following an already published procedure.[1] Briefly, gel protein bands were sequentially washed with ultrapure water, 50% acetonitrile in 50 ammonium bicarbonate 50 mM, and dehydrated with 100% acetonitrile. Afterwards, protein spots were reduced with 25 mM dithiothreitol, 0.1 mL, at 56ºC for 20 min and consecutively alkylated with 55 mM iodoacetamide, 0.1mL, for 20 min at room temperature in the dark, followed by the above described washing/dehydration procedures. Next, protein in gel enzymatic digestion was performed by the addition of 10 ng of trypsin in the presence of 0.01% surfactant (Promega) for 3h at 37ºC. The resulting peptides were extracted from the gel bands with 2.5% TFA for 15 min at 1400 rpm (Thermomixer, Eppendorf), dried under vacuum, (SpeedVac, Thermo Scientific) and resuspended in 0.1% TFA.

Protein identification was performed by MALDI TOF/TOF mass spectrometry (4700 Proteomics Analyzer and 4800 Plus Analyzer, SCIEX). Protein digests were purified by reversed-phase C18

chromatography (ZipTips, Millipore) following the manufacturer instructions and eluted in the MALDI sample plate. The elution solution composition was the MALDI matrix alpha-Cyano-4-hydroxycinnamic acid (CHCA) at 8 mg/mL in 50% ACN, 0.1% TFA, 6 mM ammonium phosphate. Peptide mass spectra were acquired in reflector positive mode in the mass range of *m/z* 700 to 4000 and internally calibrated with trypsin autolysis peaks. Some of the highest intensity MS peptide peaks were selected for MS/MS analysis in each spot. Proteins were identified using the combined information of PMF (Peptide Mass FingerPrint) and MS/MS peptide sequencing with the Mascot protein search software (v2.5.1, Matrix Science, Mascot) using the UniProt protein sequence database for the taxonomic selection *Bos taurus* (2016_06 release, Reference Proteome, 24214 sequences). The protein search settings were: cysteine carbamidomethylation (constant modification), methionine oxidation (variable modification), up to two missed trypsin cleavages, and maximum error tolerance of 25 ppm. Protein scores greater than 56 were considered significant ($p<0.05$) by the Mascot software.

Protein reduction, alkylation and trypsin digestion

Precipitated proteins were centrifuged at 20000 g for 20 min and resuspended in 0.5 M of Triethylammonium bicarbonate (TEAB) buffer (Sigma) pH 8.5, and vortexed. To better dissolve the pellet, samples were sonicated (Vibra Cell 750 watt, Sonics) for 2 min in a cuphorn at 20% to 40% amplitude, 1 second on and 1 second off cycles until all protein was solubilized. Protein content was quantified using 2D Quant Kit (GE Healthcare), according to manufacturer´s instructions.

The volume corresponding to 100 µg of protein from each of the 8 samples was concentrated in a rotary evaporator (Concentrator Plus, Eppendorf) at 60°C and resuspended in TEAB 0.5M to a final volume of 45 µL. A pool of all samples was performed in order to reach a total of 200 µg and two other pools (one of foetus and other of old samples), were constituted with 100 µg of protein in each. Pooled samples were also concentrated and adjusted to 45 µL with TEAB 0.5 M and 8 µL of the reducing agent tris(2-carboxyethyl)phosphine (TCEP) 50 mM (Sigma) were added. Samples were then vortexed, spinned and sonicated (Vibra Cell 750 watt, Sonics) in a cuphorn for 1 min at 20% amplitude to facilitate protein denaturation. To block cysteines, 4 µL of 200 mM

methyl methanethiosulfonate (MMTS) (Sigma) were added for 10 min at room temperature. Following addition of TEAB 0.5M to a final volume of 95 µL, the samples were vortexed. Protein digestion was performed by adding to each sample 5 µL of trypsin (Roche) at 0.5 µg/µL diluted in TEAB 0.5 M, using a 1:40 (w:w) enzyme:protein ratio. Next, samples were incubated for 16h at 37 °C. After digestion, 2 µL of formic acid (FA) 100% were added to each sample. Next, samples were dried for 1 h at 60°C by rotary evaporation under vacuum. Samples were then solubilized in 75 µL of 70% isopropanol and 30% TEAB 1M and sonicated in a cuphorn (Vibra Cell 750 watt, Sonics) for 10 min at 20 % amplitude, with pulses of 1 sec on and 1 second off.

iTRAQ labelling, sample fractionation and LC-MS/MS analysis

The digested peptides were then labelled with the iTRAQ (8-plex) tags according to manufacturer´s instructions (Sciex). Briefly, the peptide mixture was added to each label and incubated for 2 h at room temperature, then the reaction was quenched by incubation with 100 µl of water for 30 min. Two iTRAQ 8-plex batches were used where the pool of all samples was repeated in both to serve as normalization channel (distribution of samples in the iTRAQ channels detailed in Supplementary Table 1). The labelled samples of each batch were combined into one sample mixture of around 800 µg (100 µg from each of the 8 samples).

Only about 650 µg of the latter mixture were solubilized in 2% ACN in 72 mM TEAB and analysed by 2D LC-MS/MS, with a high pH reverse phase chromatography as first dimension and then analysed by LC-MS as described below.[2] The first dimension chromatography was performed in Ultimate™3000 LC (LC Packings, Dionex) with two online Aeris 3.6 µm XB-C18 columns (15 cm x 2.10 mm) (Phenomenex), using 72 mM TEAB pH 8.5 as mobile phase A and 72 mM TEAB in ACN pH 8.5 as mobile phase B (10 minutes with 2% mobile phase B followed by a linear gradient until 45% mobile phase B during 60 minutes, then followed by column wash and reequilibration). Throughout chromatographic separation, 75 fractions were collected and then joined into 18 samples (Supplementary Figure 1), evaporated and prepared for LC-MS/MS as described above.[3] Such prefractionation enables the depletion of highly dominant or interfering components, avoiding under-representation of less frequent ones.[4]

For the LC-MS analysis the peptides were resolved by liquid chromatography (nanoLC Ultra 2D, Eksigent) on a ChromXP™ C18AR reverse phase column (300 µm ID × 15 cm length, 3 µm particles, 120 Å pore size, Eksigent) at 5 µL/min. Peptides were eluted into the mass spectrometer with an ACN gradient in 0.1% FA (2% to 30% ACN, in a linear gradient for 40 min, followed by a column wash and equilibration step), using an electrospray ionization source (DuoSpray™ Source, AB Sciex). The mass spectrometer (Triple TOF™ 5600 System; AB Sciex) was programmed for scanning full spectra (350-1250 m/z) for 250 ms, followed by up to 30 MS/MS scans (100–1500 m/z for 100 ms each). Candidate ions with a charge state between +2 and +5 and a minimum threshold of 70 counts per second were isolated for fragmentation and two MS/MS spectra were collected before adding those ions to the exclusion list for 15 seconds (mass spectrometer operated by Analyst TF 1.6, AB Sciex). Specific iTRAQ rolling collision energy was used.

Database searching and protein identification

Following LC-MS analysis, the peptide and protein identification and protein relative quantification was performed with ProteinPilot™ software (v4.5, AB Sciex). The search parameters used were the following: Swiss-Prot database (release 2012_06), against *Bos taurus* or all species, using MMTS alkylated cysteines and iTRAQ labelled peptides as fixed modifications. An independent False Discovery Rate (FDR) analysis using the target-decoy approach provided with ProteinPilot software was used to assess the quality of the identifications and positive identifications were considered when identified proteins reached a 5% local FDR with at least one peptide that reached a 5% local FDR. Peptides are used for quantitation by the program algorithm if at least one Ratio is shown. Some of the ratios for a particular peptide may be blank and in this case they are not used, but instead they are excluded from the analysis. Some variable modifications are identified because the standard modification set of Protein Pilot establishes default probabilities for the occurrence of modifications, such as oxidation on several residues (for example: methionine or proline). Raw data from the search against *Bos taurus* and all species is available as Supplementary Data 2 and 3, respectively.

Bioinformatic Analysis

MarkerView software version 1.2.1 (AB Sciex) was used for differential comparison and visualization of data sets through principal component analysis and discriminant analysis (PCA-DA).

The Gene-E software version 3.0.206 (http://www.broadinstitute.org/cancer/software/GENE-E/) was used for data analysis and visualization. Hierarchical clustering was performed using Spearman correlation, a non-parametric measure of statistical dependence between two variables.

To explore the types of functions and pathways affected by the proteins and corresponding genes identified, we performed Gene Ontology (GO) and Pathway term enrichment, using the Functional Annotation Clustering Tool from Database for Annotation, Visualization and Integrated Discovery (DAVID) Bioinformatics Resources version 6.7 (https://david.ncifcrf.gov/).[5] This allowed us to determine whether particular GO terms and pathways occurred more frequently than expected by chance, in a given set of genes. Statistical enrichment of GO terms was calculated using the default background set provided by DAVID. Genes were then clustered according to functional similarity. The software uses the Fisher Exact test to measure functional enrichment in annotation categories from numerous public databases. The over-representation p-value is calculated based on, EASE Score, a modified Fisher Exact Test.

Identified proteins were further analysed using the Markov Cluster (MCL) clustering function of STRING (Search Tool for the Retrieval of Interacting Genes/Proteins database) (version 9.1) (http://string-db.org/), which uses both known and predicted protein interactions to quantitatively integrate interaction data from physical (direct) and functional (indirect) associations into a protein-protein interaction network.[6]

Candidate selection criteria

The rationale behind the identification of potential age-related matrix components deregulated during Nucleus Pulposus (NP) development and choice of candidates for further investigation

was: the analysis of iTRAQ data (e.g. differential protein expression ratio cut-off >1.3 and <0.77 and ProteinPilot protein identification confidence), literature mining concerning promising associations with IVD ageing and degeneration or lack of published data suggesting novel potential candidate biomolecules, and the fact that the proteins were classified as being part of the core matrisome envisaging interesting candidates to create a novel scaffold biomaterial. Given that most proteins overexpressed in Foetus had not been identified with the highest confidence scores, we explored additional results obtained by searching the original MS/MS data against the proteomes from all organisms available on Swiss-Prot (results available upon request), instead of only using the *Bos taurus* complete proteome set.

Immunofluorescence

Immunofluorescen of NPs was performed as previously reported by Antunes *et al.*[7] Briefly, antigen retrieval was performed in paraffin-embedded IVD slices through incubation with a 20 µg/mL proteinase K (Sigma-Aldrich) solution for 15 min at 37°C. A blocking step was introduced to reduce non-specific background staining with 1 h incubation in PBS containing 5% donkey serum (Santa Cruz Biotechnology) and 0.1% Triton X-100 (Sigma). Sections were then incubated overnight at 4°C with goat anti-COL11A2 (1:50) sc-131134 from Santa Cruz Biotechnology, which had been previously used in the literature[8] to evaluate chondrogenic differentiation of mesenchymal stem cells and nasal chondrocytes within 3D pellet cultures. Anti-goat Alexa Fluor 488 (Invitrogen) was used for fluorescent secondary antibody detection and samples were mounted in Fluoroshield mounting medium with DAPI (Sigma). Representative images were taken in an inverted fluorescent microscope (Axiovert 200M, Zeiss) using the 20x objective.

REFERENCES

1 Gomes, C. *et al.* Glycoproteomic analysis of serum from patients with gastric precancerous lesions. *Journal of proteome research* **12**, 1454-1466, doi:10.1021/pr301112x (2013).

2 Manadas, B., English, J. A., Wynne, K. J., Cotter, D. R. & Dunn, M. J. Comparative analysis of OFFGel, strong cation exchange with pH gradient, and RP at high pH for first-dimensional separation of peptides from a membrane-enriched protein fraction. *Proteomics* **9**, 5194-5198, doi:10.1002/pmic.200900349 (2009).

3 Silva, C., Santa, C., Anjo, S. I. & Manadas, B. A reference library of peripheral blood mononuclear cells for SWATH-MS analysis. *Proteomics Clin Appl* **10**, 760-764, doi:10.1002/prca.201600070 (2016).

4 Wilson, R., Whitelock, J. M. & Bateman, J. F. Proteomics makes progress in cartilage and arthritis research. *Matrix Biol* **28**, 121-128, doi:10.1016/j.matbio.2009.01.004 (2009).

5 Huang da, W., Sherman, B. T. & Lempicki, R. A. Systematic and integrative analysis of large gene lists using DAVID bioinformatics resources. *Nature protocols* **4**, 44-57, doi:10.1038/nprot.2008.211 (2009).

6 von Mering, C. *et al.* STRING: known and predicted protein-protein associations, integrated and transferred across organisms. *Nucleic acids research* **33**, D433-437, doi:10.1093/nar/gki005 (2005).

7 Antunes, J. C. *et al.* Poly(gamma-glutamic acid) and poly(gamma-glutamic acid)-based nanocomplexes enhance type II collagen production in intervertebral disc. *Journal of materials science. Materials in medicine* **28**, 6, doi:10.1007/s10856-016-5787-1 (2017).

8 Antunes, J. C. *et al.* Poly(gamma-Glutamic Acid) as an Exogenous Promoter of Chondrogenic Differentiation of Human Mesenchymal Stem/Stromal Cells. *Tissue engineering. Part A* **21**, 1869-1885, doi:10.1089/ten.TEA.2014.0386 (2015).

SUPPLEMENTARY FIGURES AND TABLES

a b

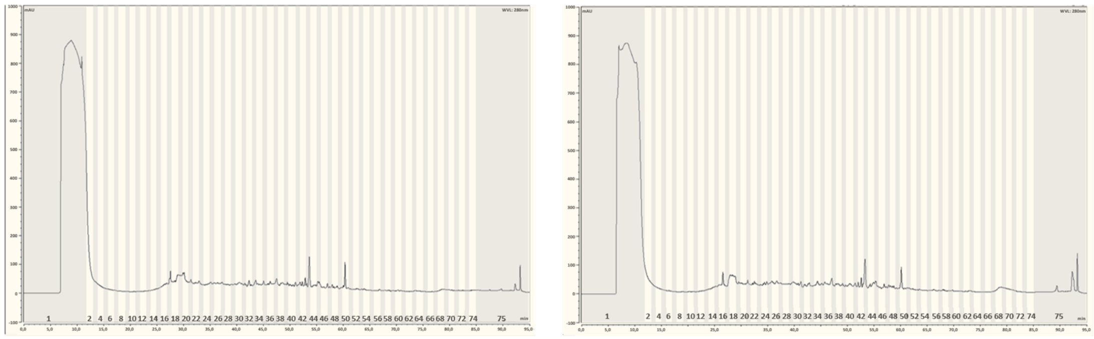

c

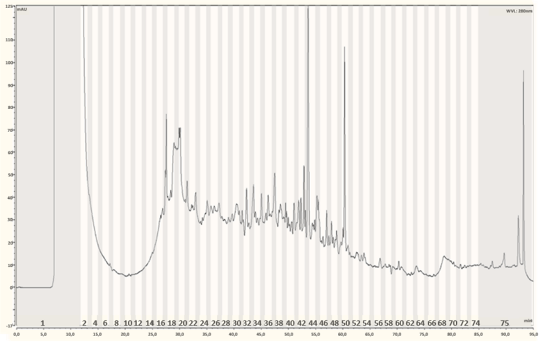

d

Fraction LC-MS	Joined collected fractions (1st dimension)																
**1**	3	4	5	6	7	8	9	10	11	12	13	14	15	16	17	18	19
**2**	20	35	50														
**3**	21	36	51														
**4**	22	37	52														
**5**	23	39	53														
**6**	24	38	54														
**7**	25	40	55														
**8**	26	41	56														
**9**	27	42	57														
**10**	28	43	58														
**11**	29	44	59														
**12**	30	45	60														
**13**	31	46	61														
**14**	32	47	62														
**15**	33	48	63														
**16**	34	49	64														
**17**	65	66	67	68	69	70	71	72	73	74							
**18**	75																

Supplementary Figure 1 - Details of the first LC dimension and of fraction combination for LC-MS. First dimension chromatograms of the iTRAQ labelled sample mixtures detected with UV lamp at 280 nm, where 75 fractions were collected, as indicated over the x axis. Fractions were afterwards pooled together as indicated on (d). (a) - Sample mixture from the 1st and 2nd batch (b); (c) - Zoom in of the gradient area of the chromatogram from the 1st batch.

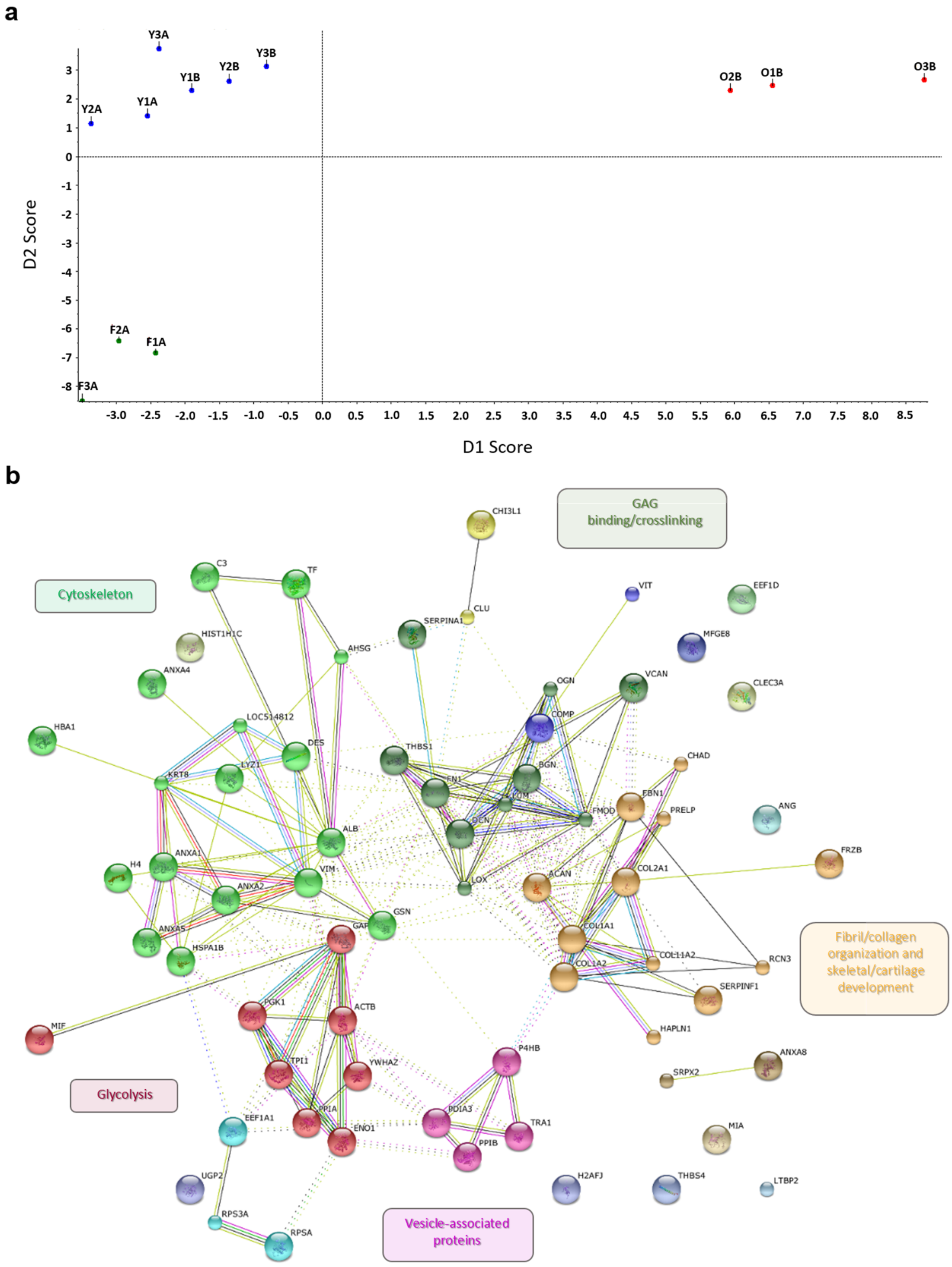

Supplementary Figure 2 - Complementary iTRAQ analysis. (a) - Comparison of the proteomic profiles of the different samples by Principal Component Analysis and Discriminant Analysis (PCA-DA). Sample age groups were colour-coded in accordance with the embedded legend Foetus (F) in green, Young (Y) in blue and Old (O) in red. (b) - Graphical view of the interactome deduced from STRING. Six clusters were found among the proteins identified by iTRAQ analysis

(clustered proteins are marked in same colour circles). Each protein is represented by a node. Lines between nodes indicate a connection such as experimental interaction (pink), co-expression (black), interaction databases (blue), literature (green), and homology (purple).

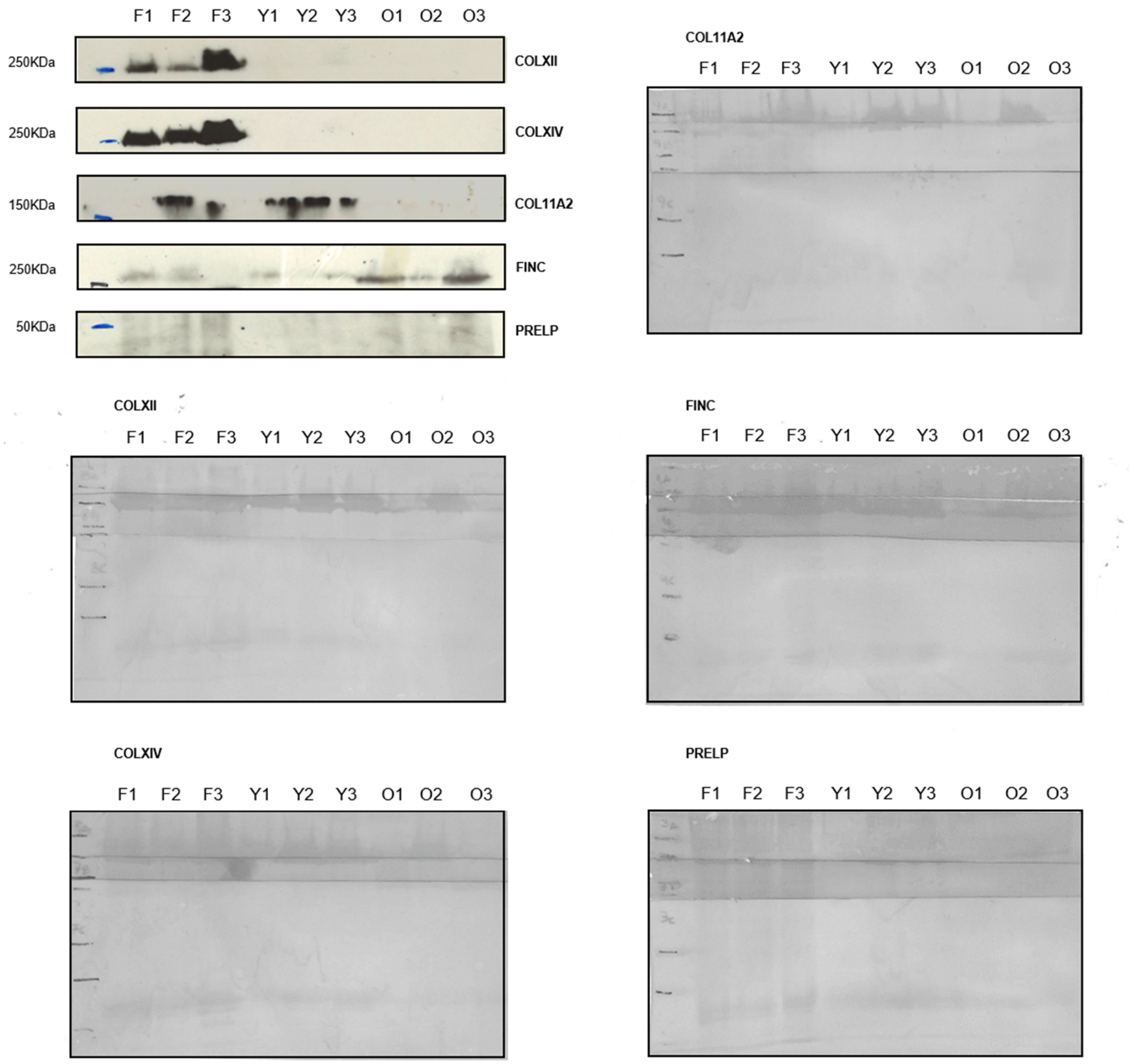

Supplementary Figure 3 - Western blot analysis of extracellular matrix proteins on NP extracts from different age groups. Collagen Type XII (COLXII) and XIV (COLXIV) are almost exclusively expressed in Foetus (F1-F3). Collagen Type XI is mostly present in Young (Y1-Y3) NPs and Old (O1-O3) animals show an overexpression of Fibronectin (FINC) and Prolargin (PRELP), as verified after band quantification and normalization of the data by to the total protein loading. Respective Page Blue stained membranes are shown. Relative protein expression levels are presented in Figure 6.

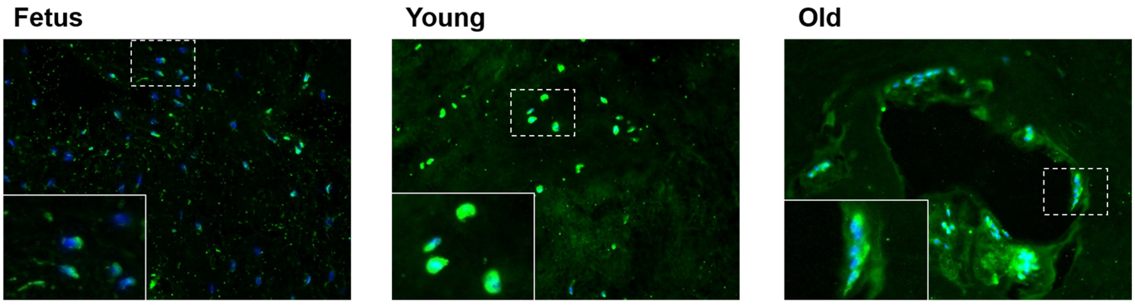

Supplementary Figure 4 – Paraffin-sections of NPs from Foetus, Young and Old animals, stained for Collagen Type XI. Representative immunofluorescent images demonstrate that Collagen Type XI is highly expressed in the pericellular matrix of Young NPs but not so much in that of Foetus or Old animals. Interestingly, in foetus, traces of fibrillar matrix deposition are also observed. Images on the bottom left hand corner represent magnifications of the squares delimited by a dashed line.

1st kit		2nd kit	
**Label**	**Sample**	**Label**	**Sample**
**113**	Young 1	**113**	Young 1
**114**	Young 2	**114**	Young 2
**115**	Young 3	**115**	Young 3
**116**	Foetus 1	**116**	Old 1
**117**	Foetus 2	**117**	Old 2
**118**	Foetus 3	**118**	Old 3
**119**	**P Old**	**119**	**P Foetus**
**121**	**Pool**	**121**	**Pool**

Supplementary Table 1 - Sample distribution in the iTRAQ 8-plex batches.

Primary Antibody	Primary Ab Supplier	Host species	Blocking	Primary Ab dilution	Secondary Ab Supplier	Secondary Ab Dilution
Collagen Type XII	gift from Dr. Nick Morris	rabbit	5% milk in PBS with 5% Tween	1:9	Amersham Biosciences	1:10000
Collagen Type XIV	gift from Dr. Nick Morris	rabbit	5% milk in PBS with 5% Tween	1:9	Amersham Biosciences	1:10000
Collagen Type XI alpha 2	Santa Cruz Biotechnology	goat	5% donkey serum in PBS without Tween	1:500	Santa Cruz Biotechnology	1:5000
Fibronectin	Santa Cruz Biotechnology	rabbit	5% milk in PBS with 5% Tween	1:50	Amersham Biosciences	1:20000
Prolargin	Santa Cruz Biotechnology	goat	5% donkey serum in PBS without Tween	1:1000	Santa Cruz Biotechnology	1:3000

Supplementary Table 2 - List of antibodies used for Western Blot validation of the selected iTRAQ candidates.

Gel Band[a]	UniProt Protein name	UniProt Entry	Entry name[b]	Gene name	Protein Score[c]	Peptide count	MW[d]	pI[d]
1F	Uncharacterized protein	F1MRZ5	F1MRZ5_BOVIN	TNC	384	75	196677	5,03
	Uncharacterized protein	F1MRZ6	F1MRZ6_BOVIN	TNC	352	78	250106	5,01
	Uncharacterized protein	E1BB91	E1BB91_BOVIN	COL6A3	110	83	343908	6,11
2F	Uncharacterized protein	E1BI98	E1BI98_BOVIN	COL6A1	246	42	109744	5,24
	Uncharacterized protein	F1MKG2	F1MKG2_BOVIN	COL6A2	111	36	110451	6,26
	Uncharacterized protein	G3MXU3	G3MXU3_BOVIN	COL6A2	103	13	25162	5,75
3F	Fibromodulin	E1BI02	E1BI02_BOVIN	FMOD	309	19	43291	5,57
	Fibromodulin	P13605	FMOD_BOVIN	FMOD	309	19	43410	5,57
	Biglycan	P21809	PGS1_BOVIN	BGN	168	25	41963	6,83
4F	Fibromodulin	E1BI02	E1BI02_BOVIN	FMOD	421	22	43291	5,57
	Fibromodulin	P13605	FMOD_BOVIN	FMOD	421	22	43410	5,57
5F	Fibromodulin	E1BI02	E1BI02_BOVIN	FMOD	404	25	43291	5,57
	Fibromodulin	P13605	FMOD_BOVIN	FMOD	403	25	43410	5,57
	Transforming growth factor-beta-induced protein ig-h3	P55906	BGH3_BOVIN	TGFBI	74	29	74988	6,82
	Transforming growth factor-beta-induced protein ig-h3	F1MBS3	F1MBS3_BOVIN	TGFBI	71	28	72834	6,70
6F	Actin, cytoplasmic 2	P63258	ACTG_BOVIN	ACTG1	201	21	42108	5,31
	Actin, cytoplasmic 1	P60712	ACTB_BOVIN	ACTB	196	20	42052	5,29
	Actin, cytoplasmic 1	F1MRD0	F1MRD0_BOVIN	ACTB	186	19	42167	5,16
	Actin, alpha cardiac muscle 1	Q3ZC07	ACTC_BOVIN	ACTC1	174	14	42334	5,23
	Actin, alpha skeletal muscle	P68138	ACTS_BOVIN	ACTA1	173	14	42366	5,23
	Actin, aortic smooth muscle	P62739	ACTA_BOVIN	ACTA2	171	13	42381	5,24
	Actin, aortic smooth muscle	G8JKX4	G8JKX4_BOVIN	ACTA2	169	13	45859	5,71
	Actin, gamma-enteric smooth muscle	Q5E9B5	ACTH_BOVIN	ACTG2	168	12	42249	5,31
	Actin, gamma-enteric smooth muscle	F1MKC4	F1MKC4_BOVIN	ACTG2	164	10	42355	5,74
	Hyaluronan and proteoglycan link protein 1	P55252	HPLN1_BOVIN	HAPLN1	67	21	40889	7,93
7F	Collagen alpha-1(II) chain	F1MSR8	F1MSR8_BOVIN	COL2A1	171	31	134858	8,66
	Collagen alpha-1(II) chain	P02459	CO2A1_BOVIN	COL2A1	170	32	142825	6,82
8F	Chondroadherin	F1MYE4	F1MYE4_BOVIN	CHAD	203	23	41319	9,49
	Collagen alpha-1(II)	F1MSR8	F1MSR8_BOVIN	COL2A1	164	34	134858	8,66
	Collagen alpha-1(II)	P02459	CO2A1_BOVIN	COL2A1	162	34	142825	6,82
	Chondroadherin	Q27972	CHAD_BOVIN	CHAD	110	20	41373	9,49
1Y	Uncharacterized protein	F1MRZ5	F1MRZ5_BOVIN	TNC	126	69	196677	5,03
	Uncharacterized protein	F1MRZ6	F1MRZ6_BOVIN	TNC	108	73	250106	5,01
	Biglycan	P21809	PGS1_BOVIN	BGN	103	27	41963	6,83
2Y	Aggrecan core protein	P13608	PGCA_BOVIN	ACAN	55	42	248150	4.16
	Aggrecan core protein	F1N368	F1N368_BOVIN	ACAN	55	45	248513	4.16
	Aggrecan core protein	F1N367	F1N367_BOVIN	ACAN	55	45	244177	4.18
3Y	Fibromodulin	E1BI02	E1BI02_BOVIN	FMOD	84	17	43291	5,57
	Fibromodulin	P13605	FMOD_BOVIN	FMOD	84	17	43410	5,57
	Aggrecan core protein	F1N367	F1N367_BOVIN	ACAN	78	32	244177	4,18
	Aggrecan core protein	F1N368	F1N368_BOVIN	ACAN	78	32	248513	4,16
	Aggrecan core protein	P13608	PGCA_BOVIN	ACAN	69	30	248150	4,16
	Biglycan	P21809	PGS1_BOVIN	BGN	65	20	41963	6,83
4Y	Fibromodulin	E1BI02	E1BI02_BOVIN	FMOD	334	27	43291	5,57
	Fibromodulin	P13605	FMOD_BOVIN	FMOD	334	27	43410	5,57
5Y	Fibromodulin	E1BI02	E1BI02_BOVIN	FMOD	275	24	43291	5,57
	Fibromodulin	P13605	FMOD_BOVIN	FMOD	275	24	43410	5,57
6Y	Hyaluronan and proteoglycan link protein 1	P55252	HPLN1_BOVIN	HAPLN1	149	30	40889	7,93
	Biglycan	P21809	PGS1_BOVIN	BGN	139	21	41963	6,83
	HAPLN1 protein	A8E4P9	A8E4P9_BOVIN	HAPLN1	72	13	20587	6,7
7Y	Chondroadherin	F1MYE4	F1MYE4_BOVIN	CHAD	200	23	41319	9,49
	Collagen alpha-1(II) chain	F1MSR8	F1MSR8_BOVIN	COL2A1	147	33	134858	8,66
	Collagen alpha-1(II) chain	P02459	CO2A1_BOVIN	COL2A1	145	33	142825	6,82
	Chondroadherin	Q27972	CHAD_BOVIN	CHAD	113	21	41373	9,49
8Y	Chondroadherin	F1MYE4	F1MYE4_BOVIN	CHAD	302	32	41319	9,49
	Chondroadherin	Q27972	CHAD_BOVIN	CHAD	220	30	41373	9,49
1O	Aggrecan core protein	P13608	PGCA_BOVIN	ACAN	88	49	248150	4,16
	Aggrecan core protein	F1N367	F1N367_BOVIN	ACAN	87	50	244177	4,18
	Aggrecan core protein	F1N368	F1N368_BOVIN	ACAN	86	50	248513	4,16
2O	Aggrecan core protein	P13608	PGCA_BOVIN	ACAN	89	36	248150	4,18
	Aggrecan core protein	F1N368	F1N368_BOVIN	ACAN	89	38	248513	4,16
	Aggrecan core protein	F1N367	F1N367_BOVIN	ACAN	89	38	244177	4,16
3O	Cartilage oligomeric matrix protein	P35445	COMP_BOVIN	COMP	81	14	84876	4,37
	Cartilage oligomeric matrix protein	F1MTZ9	F1MTZ9_BOVIN	COMP	79	12	84415	4,6
4O	Aggrecan core protein	P13608	PGCA_BOVIN	ACAN	174	27	248150	4,18
	Aggrecan core protein	F1N368	F1N368_BOVIN	ACAN	174	27	248513	4,16
	Aggrecan core protein	F1N367	F1N367_BOVIN	ACAN	174	27	244177	4,16
	Cartilage oligomeric matrix protein	P35445	COMP_BOVIN	COMP	119	27	84876	4,37
	Cartilage oligomeric matrix protein	F1MTZ9	F1MTZ9_BOVIN	COMP	118	23	84415	4,6
5O	Aggrecan core protein	P13608	PGCA_BOVIN	ACAN	218	28	248150	4,16
	Aggrecan core protein	F1N368	F1N368_BOVIN	ACAN	218	30	248513	4,16
	Aggrecan core protein	F1N367	F1N367_BOVIN	ACAN	218	30	244177	4,18
	Serum albumin	A0A140T897	A0A140T897_BOVIN	ALB	116	58	71274	5,82
	Serum albumin	P02769	ALBU_BOVIN	ALB	108	56	71244	5,82
	Prolargin	F1MX63	F1MX63_BOVIN	PRELP	57	16	44102	9,59
	Prolargin	Q9GKN8	PRELP_BOVIN	PRELP	57	16	44054	9,59
6O	Hyaluronan and proteoglycan link protein 1	P55252	HPLN1_BOVIN	HAPLN1	135	28	40889	7,93
	Decorin	P21793	PGS2_BOVIN	DCN	81	20	40196	8,72
	Biglycan	P21809	PGS1_BOVIN	BGN	68	11	41963	6,83
7O	Chondroadherin	F1MYE4	F1MYE4_BOVIN	CHAD	153	21	40319	9,49
	Collagen alpha-1(II) chain	P02459	CO2A1_BOVIN	COL2A1	138	48	134858	6,82
	Collagen alpha-1(II) chain	F1MSR8	F1MSR8_BOVIN	COL2A1	136	48	142825	6,82
	Chondroadherin	Q27972	CHAD_BOVIN	CHAD	86	19	41373	9,49
8O	Chondroadherin	F1MYE4	F1MYE4_BOVIN	CHAD	193	28	41318	9,49
	Chondroadherin	Q27972	CHAD_BOVIN	CHAD	96	26	41373	9,49

[a] Gel bands are named as indicated on the electrophoresis gel shown in Figure 2a.
[b] UniProtKB entry name.
[c] Protein score is -10*Log(P), where P is the probability that the observed match is a random event. Protein scores greater than 56 were considered significant (p<0.05) by the Protein Search algorithm (Mascot Matrix Science, UK),
[d] Protein theoretical molecular weight (MW) and isoelectric point (pI).

Supplementary Table 3 - Protein gel bands of the different age groups, identified by PMF+MS/MS using MALDI-TOF/TOF mass spectrometry.

Gel Band[a]	UniProt Protein name	Uniprot Entry	Entry name[b]	Gene name	Protein score[c]	Peptide count	MW [d]	pI [d]
1c	Uncharacterized protein	E1BI98	E1BI98_BOVIN	COL6A1	294	48	109744	5.18
	Uncharacterized protein	F1MKG2	F1MKG2_BOVIN	COL6A2	179	50	110451	5.89
	Collagen, type VI, alpha 2	Q1JQB0	Q1JQB0_BOVIN	COL6A2	122	42	98215	5.54
	Uncharacterized protein	G3MXU3	G3MXU3_BOVIN	COL6A2	68	12	25162	5.75
2c	Annexin A1	P46193	ANXA1_BOVIN	ANXA1	123	27	39212	6.41
	Annexin	F1N650	F1N650_BOVIN	ANXA1	123	27	39240	6.37
	Annexin A2	P04272	ANXA2_BOVIN	ANXA2	109	37	38873	6.90
	Glyceraldehyde-3-phosphate dehydrogenase	P10096	G3P_BOVIN	GAPDH	107	28	36073	8.52
3c	Annexin A8	Q95L54	ANXA8_BOVIN	ANXA8	184	34	36992	5.30
	L-lactate dehydrogenase A chain	P19858	LDHA_BOVIN	LDHA	98	29	36916	8.17
1w	Uncharacterized protein	E1BI98	E1BI98_BOVIN	COL6A1	106	41	109744	5.18
	Collagen alpha-1(II) chain	P02459	CO2A1_BOVIN	COL2A1	101	36	142825	8.38
	Collagen alpha-1(II) chain	F1MSR8	F1MSR8_BOVIN	COL2A1	101	36	134858	8.49
2w	Collagen alpha-1(II) chain	F1MSR8	F1MSR8_BOVIN	COL2A1	91	40	134858	8.49
	Collagen alpha-1(II) chain	P02459	CO2A1_BOVIN	COL2A1	91	41	142825	8.38
	Chondroadherin	F1MYE4	F1MYE4_BOVIN	CHAD	63	20	41319	9.43
	Mimecan	P19879	MIME_BOVIN	OGN	62	15	34530	5.21
	Mimecan	A5D9E8	A5D9E8_BOVIN	OGN	62	15	34517	5.21
	Mimecan	G3N088	G3N088_BOVIN	OGN	61	16	40915	6.76
	Chondroadherin	Q27972	CHAD_BOVIN	CHAD	58	19	41373	9.43
3w	Chondroadherin	F1MYE4	F1MYE4_BOVIN	CHAD	269	29	41319	9.43
	Chondroadherin	Q27972	CHAD_BOVIN	CHAD	183	27	41373	9.43

Detailed information about the mass spectra, identified proteins, and the peptides sequenced by MS/MS can be found in the supplementary material section.

[a] Gel bands are named as indicated on the electrophoresis gel shown in Figure 2b.
[b] UniProtKB entry name.
[c] Protein score is -10*Log(P), where P is the probability that the observed match is a random event. Protein scores greater than 56 were considered significant (p<0.05) by the Protein Search algorithm (Mascot, Matrix Science, UK).
[d] Protein theoretical molecular weight (MW) and isoelectric point (pI).

Supplementary Table 4 - Protein gel bands of the different extracts, identified by PMF+MS/MS using MALDI-TOF/TOF mass spectrometry.

GO Term	p-value	No. of genes	%
**Enrichment Score: 18,64357601541482**			
extracellular region	1,95E-20	39	51
extracellular region part	2,75E-20	30	39
proteinaceous extracellular matrix	9,15E-19	21	28
extracellular matrix	5,43E-18	21	28
**Enrichment Score: 9,01063624973187**			
glycosaminoglycan binding	1,59E-10	10	13
polysaccharide binding	9,66E-10	10	13
pattern binding	9,66E-10	10	13
carbohydrate binding	6,13E-09	12	16
**Enrichment Score: 4,452983838716395**			
extracellular matrix part	5,60E-10	10	13
collagen fibril organization	1,53E-08	6	8
extracellular matrix organization	5,28E-08	8	11
collagen	2,89E-07	6	8
extracellular structure organization	3,01E-07	8	11
extracellular matrix structural constituent	1,29E-05	5	7
blood vessel development	8,18E-04	6	8
skeletal system development	8,82E-04	6	8
vasculature development	9,16E-04	6	8
cartilage development	1,21E-03	4	5
skeletal system morphogenesis	3,89E-02	3	4
**Enrichment Score: 3,9685725330578823**			
calcium-dependent phospholipid binding	1,12E-06	5	7
phospholipid binding	2,83E-04	6	8
lipid binding	3,91E-03	7	9
**Enrichment Score: 3,4140560968157283**			
melanosome	3,64E-07	8	11
pigment granule	3,64E-07	8	11
endoplasmic reticulum lumen	8,46E-06	6	8
cytoplasmic membrane-bounded vesicle	1,20E-05	11	14
membrane-bounded vesicle	1,47E-05	11	14
cytoplasmic vesicle	6,47E-05	11	14
vesicle	8,31E-05	11	14
endoplasmic reticulum part	5,16E-03	6	8
**Enrichment Score: 2,65452169597921**			
aminoglycan metabolic process	4,28E-04	4	5
polysaccharide metabolic process	4,13E-03	4	5
glycosaminoglycan metabolic process	6,15E-03	3	4
**Enrichment Score: 2,233930191630517**			
carbohydrate catabolic process	4,96E-05	6	8
glycolysis	1,68E-03	4	5
glucose catabolic process	2,91E-03	4	5
hexose catabolic process	3,10E-03	4	5
monosaccharide catabolic process	3,29E-03	4	5
cellular carbohydrate catabolic process	4,84E-03	4	5
alcohol catabolic process	5,62E-03	4	5
glucose metabolic process	1,97E-02	4	5
hexose metabolic process	3,28E-02	4	5
monosaccharide metabolic process	4,23E-02	4	5
**Enrichment Score: 2,171813913175872**			
positive regulation of phagocytosis	1,05E-03	3	4
regulation of phagocytosis	1,35E-03	3	4
positive regulation of endocytosis	2,87E-03	3	4
regulation of endocytosis	1,05E-02	3	4
positive regulation of transport	1,10E-02	4	5
regulation of vesicle-mediated transport	2,46E-02	3	4
**Enrichment Score: 2,0277122158782714**			
response to wounding	7,40E-04	7	9
defense response	2,57E-03	7	9
inflammatory response	3,98E-03	5	7
regulation of response to external stimulus	8,73E-03	4	5
negative regulation of multicellular organismal process	1,18E-02	4	5
acute inflammatory response	2,95E-02	3	4
**Enrichment Score: 2,0259003501224484**			
calcium ion binding	3,27E-06	16	21
**Enrichment Score: 1,9175467397321206**			
skeletal system development	8,82E-04	6	8
ossification	4,48E-02	3	4
bone development	4,48E-02	3	4
**Enrichment Score: 1,6014159192745054**			
sarcolemma	2,11E-06	6	8
Z disc	1,59E-02	3	4
I band	1,87E-02	3	4
intermediate filament	4,03E-02	4	5
intermediate filament cytoskeleton	4,03E-02	4	5
sarcomere	4,48E-02	3	4
**Enrichment Score: 1,5971951870040029**			
enzyme inhibitor activity	6,17E-03	6	8
endopeptidase inhibitor activity	4,56E-02	4	5
**Enrichment Score: 1,274977605087997**			
actin cytoskeleton organization	1,55E-02	4	5
actin filament-based process	1,65E-02	4	5
**Enrichment Score: 0,8294386941389481**			
cellular macromolecular complex assembly	3,07E-02	5	7
macromolecular complex assembly	3,48E-02	6	8
cellular macromolecular complex subunit organization	3,78E-02	5	7
macromolecular complex subunit organization	4,28E-02	6	8

Supplementary Table 5 - Enriched Gene Ontology clusters (following DAVID analysis), based on the identified proteins common to the three age groups.

Pathway Term	p-value	No. of genes	%
Enrichment Score: 4,541530286435589			
ECM-receptor interaction	8,64E-09	9	12
Focal adhesion	7,52E-07	10	13
Integrin signaling pathway	8,96E-03	5	7
TGF-beta signaling pathway	1,17E-02	4	5

Supplementary Table 6 - Enriched Pathway Term cluster (following DAVID analysis), based on the identified proteins common to the three age groups.

SUPPLEMENTARY LEGENDS

Supplementary Data 1 - Text file containing detailed methodologies concerning iTRAQ sample preparation (protein extraction, precipitation, quantification, reduction, alkylation, trypsin digestion, labelling and fractionation) and LC-MS/MS analysis (database searching, protein identification, bioinformatic tools and parameters used, as well as candidate selection criteria).

Supplementary Data 2 - Excel book containing tables with information about the proteins and peptides identified with 95% confidence and quantified by iTRAQ, using the reviewed Swiss-Prot *Bos taurus* search database.

Supplementary Data 3 - Excel book containing tables with information about the proteins and peptides identified with 95% confidence and quantified by iTRAQ, using the reviewed all species Swiss-Prot database.

Supplementary Data 4 - Mascot protein gel band identification results and MALDI-TOF/TOF mass spectra for the different age groups.

Supplementary Data 5 - Mascot protein gel band identification results and MALDI-TOF/TOF mass spectra for the different extracts.

Supplementary Data 6 - Excel book containing tables with information about the proteins and peptides identified with 95% confidence in the preliminary test that compared the use of different protein extraction conditions. Detailed information about LC-MS/MS acquisition and database searches are given in the first page.

Supplementary Data 7 - Excel book containing Gene Ontology term enrichment data obtained using DAVID Functional Annotation Clustering Tool for the 3 major clusters of the NP proteome.
