# Supplementary material for: Matrisome Profiling During Intervertebral Disc Development And Ageing
Source: Sci Rep. 2017 Sep 14;7:11629. doi: 10.1038/s41598-017-11960-0 (PMC5599645; doi:10.1038/s41598-017-11960-0)
Supplement: Supplementary file 5 — Supplementary Data 5 [file 41598_2017_11960_MOESM5_ESM.pdf]

MATRIX SCIENCE Mascot Search Results

```
User          : 1c_(G1@11273_12Mar2012)
Email         : proteomics@ipatimup.pt
Search title  : Project: Proteomica, Spot Set: Proteomica\12Jan2012, Label: G1, Spot Id: 221888, Peak List Id: 435349, MS Job Run Id: 303
MS data file  : C:\Documents and Settings\Administrator\Desktop\xu\xu\xu\Proteomica\12Jan2012\ppw_G1_146796935503.txt
Database      : OrganismSpecie Bos_taurus_Reference_Proteome_2016_06 (24214 sequences; 12839866 residues)
Timestamp     : 8 Jul 2016 at 10:08:25 GMT
Warning       : A Peptide summary report will usually give a much clearer picture of MS/MS search results.
Top Score     : 294 for E1BI98, Uncharacterized protein OS=Bos taurus GN=COL6A1 PE=1 SV=1
```

Mascot Score Histogram

Protein score is -10*Log(P), where P is the probability that the observed match is a random event.
Protein scores greater than 56 are significant (p<0.05).
Protein scores are derived from ions scores as a non-probabilistic basis for ranking protein hits.

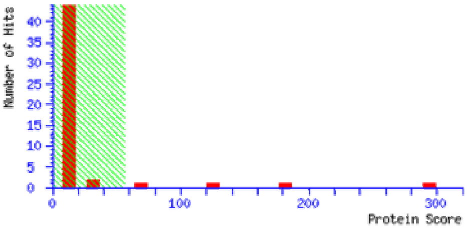

Protein Summary Report

Format As: Protein Summary (deprecated) | Help

Significance threshold p< 0.05 | Max. number of hits 50

Preferred taxonomy: All entries

Re-Search All | Search Unmatched

Index

	Accession	Mass	Score	Description
1.	E1BI98	109744	294	Uncharacterized protein OS=Bos taurus GN=COL6A1 PE=1 SV=1
2.	F1MKG2	110451	179	Uncharacterized protein OS=Bos taurus GN=COL6A2 PE=1 SV=2
3.	Q1JQB0	98215	122	Collagen, type VI, alpha 2 OS=Bos taurus GN=COL6A2 PE=1 SV=1
4.	G3MXU3	25162	68	Uncharacterized protein OS=Bos taurus GN=COL6A2 PE=4 SV=1
5.	G3MYC4	19371	24	Zinc finger protein 143 OS=Bos taurus GN=ZNF143 PE=4 SV=1
6.	F1MHI2	73699	23	Uncharacterized protein OS=Bos taurus GN=ARHGAP24 PE=4 SV=2
7.	E1BJM6	69143	19	Uncharacterized protein OS=Bos taurus GN=ZBTB7C PE=4 SV=2
8.	K7DXW4	10241	18	Uncharacterized protein OS=Bos taurus PE=3 SV=1
9.	Q2NKR1	10554	18	Mitochondrial import inner membrane translocase subunit Tim10 OS=Bos taurus GN=TIMM10 PE=3 SV=1
10.	F1MIS2	9764	18	Uncharacterized protein OS=Bos taurus PE=3 SV=2
11.	E1BC56	10970	18	Uncharacterized protein OS=Bos taurus GN=LOC785479 PE=4 SV=1
12.	F1MJH0	128067	17	Uncharacterized protein OS=Bos taurus GN=SPINK5 PE=4 SV=2
13.	F1MYI2	58389	17	Uncharacterized protein OS=Bos taurus GN=HNRNPM PE=1 SV=2
14.	P02698	8709	17	Guanine nucleotide-binding protein G(T) subunit gamma-T1 OS=Bos taurus GN=GNGT1 PE=1 SV=2
15.	Q3MHX9	23497	16	Nudix (Nucleoside diphosphate linked moiety X)-type motif 16-like 1 OS=Bos taurus GN=NUDT16L1 PE=2 SV=1
16.	F1MSW3	36190	16	Uncharacterized protein OS=Bos taurus GN=MAGIX PE=4 SV=2
17.	Q32PB8	9220	15	40S ribosomal protein S21 OS=Bos taurus GN=RPS21 PE=3 SV=1
18.	F1N7K9	123826	15	Uncharacterized protein OS=Bos taurus GN=TBC1D8 PE=4 SV=2
19.	Q05717	31377	15	Insulin-like growth factor-binding protein 5 OS=Bos taurus GN=IGFBP5 PE=2 SV=2
20.	F1MWQ0	17662	15	Complexin-3 OS=Bos taurus GN=CPLX3 PE=4 SV=1
21.	Q3ZBJ9	13284	15	Protein BEX5 OS=Bos taurus GN=BEX5 PE=3 SV=1
22.	Q08DP2	22300	15	Loss of heterozygosity 12 chromosomal region 1 protein homolog OS=Bos taurus GN=LOH12CR1 PE=2 SV=1
23.	Q3ZBC0	7228	15	DNA-directed RNA polymerases I, II, and III subunit RPABC4 OS=Bos taurus GN=POLR2K PE=1 SV=1
24.	P00129	13468	15	Cytochrome b-c1 complex subunit 7 OS=Bos taurus GN=UQCRB PE=1 SV=3
25.	A4FV50	40277	15	MGC142792 protein OS=Bos taurus GN=MGC142792 PE=2 SV=1
26.	E1BKM8	51997	14	Uncharacterized protein OS=Bos taurus GN=CYP24A1 PE=4 SV=2
27.	E1BJL9	65973	14	Uncharacterized protein OS=Bos taurus GN=CCDC173 PE=4 SV=2
28.	Q0VCY3	40674	14	Surfeit locus protein 6 OS=Bos taurus GN=SURF6 PE=2 SV=1
29.	G3N3F0	23102	14	Uncharacterized protein OS=Bos taurus PE=4 SV=1
30.	Q3T2L0	42579	14	Caspase-15 OS=Bos taurus GN=LOC515736 PE=2 SV=1
31.	E1BDX9	40138	14	Uncharacterized protein OS=Bos taurus GN=FAM50A PE=4 SV=1
32.	P84081	20790	14	ADP-ribosylation factor 2 OS=Bos taurus GN=ARF2 PE=2 SV=1
33.	F1MNB0	17016	14	Cystatin OS=Bos taurus GN=CST8 PE=3 SV=2
34.	Q3SZ59	12718	14	60S ribosomal protein L36a OS=Bos taurus GN=RPL36A PE=3 SV=3
35.	G3MYU4	73163	14	Centrosomal protein of 290 kDa OS=Bos taurus GN=CEP290 PE=4 SV=1
36.	P84088	15499	14	Complexin-2 OS=Bos taurus GN=CPLX2 PE=1 SV=1
37.	Q2TBI9	19064	14	M-phase phosphoprotein 6 OS=Bos taurus GN=MPHOSPH6 PE=2 SV=1
38.	P80209	42920	14	Cathepsin D OS=Bos taurus GN=CTSD PE=1 SV=2
39.	F1MMR6	45132	14	Cathepsin D OS=Bos taurus GN=CTSD PE=1 SV=1
40.	E1BNB8	12329	13	Uncharacterized protein OS=Bos taurus PE=4 SV=2
41.	E1B9N9	39688	13	Uncharacterized protein OS=Bos taurus GN=WISP3 PE=4 SV=1
42.	Q5E9I6	20645	13	ADP-ribosylation factor 3 OS=Bos taurus GN=ARF3 PE=2 SV=3
43.	F1MIV1	57360	13	Uncharacterized protein OS=Bos taurus GN=ZBTB34 PE=4 SV=2
44.	F1MK70	59648	13	Uncharacterized protein OS=Bos taurus GN=SPATS2 PE=4 SV=2
45.	F1MY44	77716	13	Uncharacterized protein OS=Bos taurus GN=HNRNPM PE=1 SV=2
46.	E1BHM6	206984	13	Uncharacterized protein OS=Bos taurus GN=SCN11A PE=4 SV=2
47.	Q58DW6	23636	13	Ras-related protein Rab-25 OS=Bos taurus GN=RAB25 PE=2 SV=1
48.	Q08D88	51575	13	Nuclear factor interleukin-3-regulated protein OS=Bos taurus GN=NFIL3 PE=2 SV=1

49. O02691	27294	13	3-hydroxyacyl-CoA dehydrogenase type-2 OS=Bos taurus GN=HSD17B10 PE=1 SV=3
50. F1MEV2	44049	13	Uncharacterized protein OS=Bos taurus GN=KIAA0930 PE=4 SV=2

Results List

1. E1BI98 **Mass:** 109744 **Score:** 294 **Expect:** 9.6e-026 **Matches:** 48

Uncharacterized protein OS=Bos taurus GN=COL6A1 PE=1 SV=1

Observed	Mr(expt)	Mr(calc)	ppm	Start	End	Miss	Ions	Peptide
801.4262	800.4189	800.4140	6.03	957 -	963	0	---	K.AVQEAQR.A
887.4245	886.4172	886.4145	3.10	864 -	871	0	---	R.TDPGQDVR.V
957.5774	956.5701	956.5655	4.85	723 -	731	0	---	R.IALVITDGR.S
978.5100	977.5027	977.5043	-1.58	976 -	983	0	46	R.QVNEPHVR.V
978.5146	977.5073	977.5043	3.11	976 -	983	0	---	R.QVNEPHVR.V
1006.5014	1005.4941	1005.4879	6.14	69 -	76	0	---	R.FIDNLNDR.Y
1021.5699	1020.5626	1020.5716	-8.80	714 -	722	0	---	R.LLPPTPNNR.I
1032.5238	1031.5165	1031.5070	9.27	106 -	114	1	---	R.MPSGRDELK.S
1046.6018	1045.5945	1045.5920	2.40	1005 -	1013	0	---	R.VPSYQALLR.G
1056.5515	1055.5442	1055.5400	3.99	1014 -	1022	0	---	R.GVFYQTVSR.K
1103.6257	1102.6184	1102.6386	-18.32	52 -	61	0	---	R.LKPYGALVDK.V
1134.3574	1133.3501	1133.3432	6.08	603 -	610	0	---	K.MCSCCECK.C
1162.6174	1161.6101	1161.5891	18.1	68 -	76	1	---	K.RFIDNLNDR.Y
1184.6412	1183.6339	1183.6350	-0.88	1014 -	1023	1	---	R.GVFYQTVSRK.V
1216.7081	1215.7008	1215.6976	2.68	964 -	975	0	---	R.AGVEIFAVVVGR.Q
1243.6161	1242.6088	1242.5993	7.70	925 -	935	1	---	R.FYREASSNAAK.K
1255.5972	1254.5899	1254.5881	1.49	990 -	1000	0	---	K.AAEYDVVFGER.H
1255.6000	1254.5927	1254.5881	3.72	990 -	1000	0	81	K.AAEYDVVFGER.H
1264.7130	1263.7057	1263.7047	0.78	712 -	722	1	---	R.SRLLPPTPNNR.I
1289.6875	1288.6802	1288.6775	2.09	200 -	210	0	---	R.LSIIATDHTYR.R
1414.8191	1413.8118	1413.8191	-5.15	641 -	652	2	---	K.VIDRLSKDELVK.F
1445.7839	1444.7766	1444.7786	-1.40	200 -	211	1	---	R.LSIIATDHTYRR.N
1475.7633	1474.7560	1474.7562	-0.12	102 -	114	2	---	R.GLTRMPSGRDELK.S + Oxidation (M)
1488.7194	1487.7121	1487.7157	-2.40	69 -	79	1	---	R.FIDNLNDRYYR.C
1516.6807	1515.6734	1515.6954	-14.49	476 -	490	1	---	K.GYRGDEGPPGTEGPK.G
1526.7343	1525.7270	1525.7300	-1.96	790 -	802	0	---	K.ENYAELLDDGFLK.N
1540.7114	1539.7041	1539.7066	-1.62	257 -	271	1	---	R.GPPGPRGDPGYEGER.G
1580.8400	1579.8327	1579.8358	-1.98	186 -	199	0	81	K.VFSVAITPDHLEPR.L
1580.8423	1579.8350	1579.8358	-0.52	186 -	199	0	---	K.VFSVAITPDHLEPR.L
1644.7915	1643.7842	1643.8168	-19.82	68 -	79	2	---	K.RFIDNLNDRYYR.C
1718.8790	1717.8717	1717.8669	2.83	106 -	121	2	---	R.MPSGRDELKSSVDAVK.Y
1722.9375	1721.9302	1721.9312	-0.55	136 -	151	1	---	K.GLEELLVGGSHLKENK.Y
1774.9180	1773.9107	1773.9122	-0.83	872 -	887	0	---	R.VAVVQYSGTGQQRPER.A
1774.9200	1773.9127	1773.9122	0.29	872 -	887	0	68	R.VAVVQYSGTGQQRPER.A
1932.0050	1930.9977	1931.0000	-1.17	938 -	956	0	---	R.LLLFSDGNSQGATPAAIEK.A
1997.8312	1996.8239	1996.8190	2.48	241 -	256	0	---	K.NNVEQVCCSFECQPAR.G
2015.9408	2014.9335	2014.9571	-11.70	694 -	711	0	---	K.LQWMGGGTFTGEALQYTR.S
2031.9606	2030.9533	2030.9520	0.64	694 -	711	0	---	K.LQWMGGGTFTGEALQYTR.S + Oxidation (M)
2088.1108	2087.1035	2087.1011	1.17	937 -	956	1	---	K.RLLLFSDGNSQGATPAAIEK.A
2144.0718	2143.0645	2143.0521	5.81	693 -	711	1	---	K.KLQWMGGGTFTGEALQYTR.S
2160.0554	2159.0481	2159.0470	0.53	693 -	711	1	---	K.KLQWMGGGTFTGEALQYTR.S + Oxidation (M)
2198.1831	2197.1758	2197.1665	4.26	737 -	757	0	---	R.DTTPLSVLCGPDIQVVSVGIK.D
2199.1499	2198.1426	2198.1120	13.9	83 -	101	0	---	R.NLVWNAGALHYSDEVEIIR.G
2559.1470	2558.1397	2558.1213	7.20	241 -	262	1	---	K.NNVEQVCCSFECQPARGPPGPR.G
2754.2197	2753.2124	2753.2715	-21.47	479 -	508	1	---	R.GDEGPPGTEGPKGAPGPAGPPGDPGLMGER.G
3159.4985	3158.4912	3158.5230	-10.07	152 -	180	1	---	K.YLVVVTDGHPLEGYKEPCGGLEDAVNEAK.H
3488.6545	3487.6472	3487.6440	0.93	653 -	682	1	---	K.FEPGQSHAGVVQYSHNQMQEHVDLRDPNIR.N
3504.6423	3503.6350	3503.6389	-1.11	653 -	682	1	---	K.FEPGQSHAGVVQYSHNQMQEHVDLRDPNIR.N + Oxidation (M)

No match to: 716.4763, 723.4098, 724.2417, 725.2533, 726.3622, 728.3046, 729.4749, 734.4570, 736.3904, 737.4152, 738.3791, 744.8651, 745.3838, 747.4550, 768.3673, 788.4571, 805.4606, 813.4481, 814.4558, 820.4053, 823.4778, 829.4589, 830.4522, 839.3830, 842.5110, 857.4382, 860.4207, 865.4518, 870.4165, 872.5054, 880.4424, 882.4292, 887.9669, 888.4561, 891.4898, 894.4144, 894.8973, 897.5006, 898.4523, 912.5132, 913.5293, 925.4620, 927.4981, 931.4992, 934.5098, 960.5294, 961.4949, 963.4734, 965.4692, 970.5181, 972.4899, 975.5167, 976.5063, 977.5212, 983.6283, 992.4958, 993.5046, 995.4181, 998.5911, 1000.5959, 1002.5007, 1011.5474, 1012.5447, 1014.5485, 1020.5577, 1024.5516, 1027.5438, 1028.5397, 1030.5978, 1036.5260, 1037.5405, 1039.5641, 1040.5908, 1042.5388, 1043.5178, 1045.5568, 1057.6100, 1057.6107, 1061.6168, 1074.6073, 1074.6100, 1076.6123, 1102.6007, 1105.6136, 1107.1058, 1113.5486, 1120.5498, 1122.5322, 1123.5986, 1128.5724, 1129.5924, 1137.5463, 1145.6348, 1149.6057, 1153.6460, 1156.4957, 1161.6295, 1164.1815, 1170.5497, 1172.6156, 1177.6410, 1180.6428, 1188.5603, 1193.6041, 1206.5107, 1209.5889, 1211.6099, 1212.6160, 1215.6743, 1219.6886, 1227.6234, 1229.7098, 1234.6414, 1240.5974, 1242.6003, 1253.6216, 1260.6227, 1265.7064, 1269.6498, 1270.6575, 1273.6556, 1277.6305, 1286.6099, 1293.6401, 1303.6455, 1304.6677, 1307.6788, 1309.6780, 1318.6750, 1319.6589, 1328.6600, 1328.6620, 1331.7888, 1333.7644, 1336.0798, 1336.6155, 1350.6853, 1351.7202, 1356.5811, 1359.1302, 1359.6246, 1365.6489, 1374.7588, 1383.7037, 1390.6974, 1393.7992, 1400.6683, 1401.6989, 1429.3142, 1436.7889, 1444.7122, 1450.7223, 1456.7717, 1462.6630, 1467.7329, 1470.7394, 1471.2693, 1471.7250, 1472.2531, 1474.5243, 1483.7141, 1488.5333, 1493.7366, 1502.5601, 1513.7441, 1514.6849, 1517.6437, 1518.7053, 1523.7611, 1528.6973, 1530.6893, 1536.8230, 1551.7063, 1553.7252, 1554.7474, 1557.6992, 1558.7140, 1563.6658, 1564.3619, 1565.7223, 1566.7162, 1572.7062, 1586.8090, 1597.7913, 1599.8337, 1601.8030, 1614.8046, 1616.7968, 1628.7136, 1630.8103, 1635.7697, 1642.7682, 1643.7688, 1646.8154, 1651.7681, 1657.7932, 1663.8466, 1664.8557, 1666.7545, 1695.8208, 1707.8933, 1721.9192, 1728.8976, 1730.9116, 1732.2391, 1734.1844, 1739.7894, 1743.8966, 1755.2666, 1755.7703, 1757.4702, 1757.9089, 1758.4524, 1758.8987, 1761.9023, 1765.7578, 1768.8339, 1769.8287, 1770.3949, 1772.8273, 1773.8792, 1786.8268, 1787.8239, 1809.0499, 1810.0303, 1829.8895, 1830.8380, 1833.3470, 1833.8501, 1851.8885, 1852.0399, 1862.9515, 1881.7703, 1895.9634, 1906.8195, 1908.8512, 1920.7972, 1936.9479, 1939.8563, 1940.8103, 1943.8293, 1954.8351, 1956.8574, 1958.8956, 1965.7998, 1967.9565, 1969.9998, 1979.8927, 1981.3531, 1982.8179, 1995.8232, 2003.8595, 2008.9971, 2011.8464, 2019.8779, 2025.8735, 2047.9397, 2062.9578, 2067.0020, 2076.8943, 2081.9827, 2082.9966, 2087.0103, 2089.1162, 2096.0430, 2097.0200, 2108.9771, 2110.0061, 2139.0991, 2140.0918, 2143.1006, 2149.8516, 2174.0378, 2178.0471, 2179.0574, 2188.0547, 2203.0386, 2211.1052, 2223.1582, 2225.1470, 2227.1042, 2228.0725, 2238.1086, 2239.1538, 2243.1243, 2244.0684, 2254.0896, 2255.1482, 2260.0779, 2270.2183, 2272.1736, 2284.1841, 2287.1108, 2307.0549, 2309.0659, 2310.0798, 2310.5271, 2311.0454, 2319.0945, 2337.6050, 2341.5510, 2353.0518, 2354.0767, 2355.0564, 2371.0581, 2383.9592, 2388.0437, 2392.1462, 2404.0471, 2412.0872, 2419.5879, 2420.0864, 2420.5930, 2423.2439, 2426.5667, 2427.1211, 2438.1724, 2455.1553, 2458.1648, 2459.1794, 2478.1587, 2516.1133, 2533.1262, 2542.1650, 2546.1746, 2549.1316, 2550.1252, 2561.1729, 2562.1765, 2567.1514, 2572.1523, 2574.2678, 2576.1628, 2583.1218, 2587.2722, 2604.1682, 2612.1438, 2653.7173, 2655.1897, 2671.1787, 2687.1833, 2701.2820, 2706.2766, 2712.2104, 2714.2156, 2716.1953, 2717.2725, 2725.2354, 2729.1675, 2739.2351, 2743.2642, 2745.2900, 2755.2358, 2759.2573, 2769.2219, 2771.2437, 2776.2644, 2785.2388, 2786.2617, 2787.2400, 2793.2439, 2801.2378, 2803.2302, 2809.2549, 2815.2153, 2817.2322, 2819.2236, 2830.2788, 2833.2280, 2836.2397, 2862.2927, 2873.2961, 2889.2024, 2894.3218, 2927.2520, 2931.3428, 2955.3005, 2974.4124, 2975.4084, 2990.3921, 3006.3206, 3011.2810, 3020.3557, 3021.2412, 3035.3281, 3053.3447, 3062.2456, 3086.4507, 3091.2622, 3103.3140, 3110.7285, 3113.8591, 3130.3557, 3140.4265, 3144.3657, 3173.5386, 3174.5486, 3185.4236, 3218.3301, 3259.3625, 3312.3267, 3331.4678, 3347.4761, 3363.4788, 3390.4675, 3431.4570, 3447.4666, 3463.4895, 3485.6985,

3489.6521, 3493.0195, 3494.5571, 3509.5613, 3525.6025, 3538.4521, 3553.6230, 3579.5403, 3648.1956, 3655.4729, 3664.7500, 3695.6177, 3712.5720, 3738.5837, 3816.7043, 3937.8308, 4010.8696

2. F1MKG2 **Mass:** 110451 **Score:** 179 **Expect:** 3e-014 **Matches:** 50

Uncharacterized protein OS=Bos taurus GN=COL6A2 PE=1 SV=2

Observed	Mr(expt)	Mr(calc)	ppm	Start	End	Miss	Ions	Peptide
729.4749	728.4676	728.4657	2.68	870	875	1	---	R.RLTLAR.K
829.4589	828.4516	828.4453	7.61	204	210	0	---	K.LNEQGLR.D
830.4522	829.4449	829.4406	5.25	188	194	1	---	R.AREEGIR.L
860.4207	859.4134	859.4035	11.5	747	753	0	---	R.DDDLNLR.A
865.4518	864.4445	864.4341	12.0	863	869	0	---	R.FVEEVSR.R
972.4899	971.4826	971.4825	0.13	852	859	0	---	R.LGEQNFHK.V
998.5911	997.5838	997.5960	-12.28	195	203	0	---	R.LFAVPPNLK.L
1024.5516	1023.5443	1023.5349	9.21	129	137	0	---	K.SLQSISSFR.R
1043.5178	1042.5105	1042.5043	5.95	876	884	1	---	R.KDDDPLNAR.V
1057.6100	1056.6027	1056.6080	-5.01	885	894	0	56	R.VALLQFGGPR.E
1057.6107	1056.6034	1056.6080	-4.35	885	894	0	---	R.VALLQFGGPR.E
1074.6073	1073.6000	1073.5982	1.72	644	652	0	---	K.NFVINVVNR.L
1074.6100	1073.6027	1073.5982	4.24	644	652	0	50	K.NFVINVVNR.L
1076.6123	1075.6050	1075.6026	2.24	733	742	0	---	R.VFAVVITDGR.H
1122.5322	1121.5249	1121.5101	13.2	315	325	1	---	K.GEKGEFGADGR.K
1145.6348	1144.6275	1144.6241	3.03	996	1006	0	---	K.ISLGDPAAVFR.E
1156.4957	1155.4884	1155.4655	19.9	248	256	0	---	K.HEAYGECYK.V
1177.6410	1176.6337	1176.6363	-2.22	862	870	2	---	R.RFVEEVSRR.L
1180.6428	1179.6355	1179.6360	-0.41	129	138	1	---	K.SLQSISSFRR.G
1234.6414	1233.6341	1233.6102	19.4	548	559	2	---	R.GDFGSKGEPGRK.G
1318.6750	1317.6677	1317.6902	-17.05	420	433	2	---	K.GGPGPRGPKGEPGR.R
1328.6600	1327.6527	1327.6520	0.52	211	221	0	49	R.DIANTPHELYR.N
1328.6620	1327.6547	1327.6520	2.02	211	221	0	---	R.DIANTPHELYR.N
1333.7644	1332.7571	1332.7514	4.29	731	742	1	---	K.TRVFAVVITDGR.H
1350.6853	1349.6780	1349.7013	-17.26	257	269	0	---	K.VSCLEIPGPPGPK.G
1365.6489	1364.6416	1364.6433	-1.21	743	753	1	---	R.HDPRDDDLNLR.A
1383.7037	1382.6964	1382.6752	15.4	793	804	0	---	R.NMTLFSDLVAEK.F + Oxidation (M)
1474.5243	1473.5170	1473.5105	4.42	608	618	1	---	R.ETCGCCDCEKR.C
1514.6849	1513.6776	1513.6693	5.47	245	256	1	---	K.VMKHEAYGECYK.V
1530.6893	1529.6820	1529.6787	2.19	1009	1021	0	---	K.DYDSLAQPGFFDR.F
1743.8966	1742.8893	1742.8992	-5.64	701	716	0	---	K.NLEWIAGGTWTPSALK.F
1769.8287	1768.8214	1768.8526	-17.63	273	290	2	---	R.GQKGAKGNMGEPGEPGQK.G
1774.9180	1773.9107	1773.8983	7.02	381	398	2	---	K.GDPGRPGRRGPPGENGAK.G
1774.9200	1773.9127	1773.8983	8.15	381	398	2	---	K.GDPGRPGRRGPPGENGAK.G
1787.8239	1786.8166	1786.8162	0.22	1007	1021	1	---	R.EKDYDSLAQPGFFDR.F
1809.0499	1808.0426	1808.0308	6.53	195	210	1	---	R.LFAVPPNLKLNEQGLR.D
1810.0303	1809.0230	1809.0261	-1.68	188	203	2	---	R.AREEGIRLFAVPPNLK.L
1932.0050	1930.9977	1930.9973	0.20	474	493	2	---	K.GDRGLPGPRGPQGTVGEPGK.Q
2019.8779	2018.8706	2018.8930	-11.08	360	380	1	---	R.GPDGYVGEAGSPGERGDQGSK.G
2139.0991	2138.0918	2138.0868	2.34	204	221	1	---	K.LNEQGLRDIANTPHELYR.N
2174.0378	2173.0305	2173.0222	3.84	775	792	0	---	K.HESENLYSIACDKPQQVR.N
2179.0574	2178.0501	2178.0382	5.47	104	123	0	---	R.YGGLHFSDLVEVFSPPGSDR.A
2270.2183	2269.2110	2269.2166	-2.45	974	995	1	---	R.KQNVVPTVVAVGSDVDTDVLSK.I
2284.1841	2283.1768	2283.1682	3.77	160	182	0	---	K.GVVNFAVVITDGHVTGSPCGGIK.L
2354.0767	2353.0694	2353.0604	3.82	222	241	0	---	R.NNYATMRPDSTEIDQDTINR.I
2392.1462	2391.1389	2391.1343	1.94	668	688	0	---	R.VGVVQYSHEGTFEAIQLDDER.I
2423.2439	2422.2366	2422.2353	0.53	733	753	2	---	R.VFAVVITDGRHDPRDDDLNLR.A
2755.2358	2754.2285	2754.2266	0.71	581	607	0	---	R.GAPGPEGEPGPPGDPGLTECDVMTYVR.E
2771.2437	2770.2364	2770.2215	5.39	581	607	0	---	R.GAPGPEGEPGPPGDPGLTECDVMTYVR.E + Oxidation (M)
2862.2927	2861.2854	2861.3550	-24.32	245	269	2	---	K.VMKHEAYGECYKVSCLEIPGPPGPK.G + Oxidation (M)

No match to: 716.4763, 723.4098, 724.2417, 725.2533, 726.3622, 728.3046, 734.4570, 736.3904, 737.4152, 738.3791, 744.8651, 745.3838, 747.4550, 768.3673, 788.4571, 801.4262, 805.4606, 813.4481, 814.4558, 820.4053, 823.4778, 839.3830, 842.5110, 857.4382, 870.4165, 872.5054, 880.4424, 882.4292, 887.4245, 887.9669, 888.4561, 891.4898, 894.4144, 894.8973, 897.5006, 898.4523, 912.5132, 913.5293, 925.4620, 927.4981, 931.4992, 934.5098, 957.5774, 960.5294, 961.4949, 963.4734, 965.4692, 970.5181, 975.5167, 976.5063, 977.5212, 978.5100, 978.5146, 983.6283, 992.4958, 993.5046, 995.4181, 1000.5959, 1002.5007, 1006.5014, 1011.5474, 1012.5447, 1014.5485, 1020.5577, 1021.5699, 1027.5438, 1028.5397, 1030.5978, 1032.5238, 1036.5260, 1037.5405, 1039.5641, 1040.5908, 1042.5388, 1045.5568, 1046.6018, 1056.5515, 1061.6168, 1102.6007, 1103.6257, 1105.6136, 1107.1058, 1113.5486, 1120.5498, 1123.5986, 1128.5724, 1129.5924, 1134.3574, 1137.5463, 1149.6057, 1153.6460, 1161.6295, 1162.6174, 1164.1815, 1170.5497, 1172.6156, 1184.6412, 1188.5603, 1193.6041, 1206.5107, 1209.5889, 1211.6099, 1212.6160, 1215.6743, 1216.7081, 1219.6886, 1227.6234, 1229.7098, 1240.5974, 1242.6003, 1243.6161, 1253.6216, 1255.5972, 1255.6000, 1260.6227, 1264.7130, 1265.7064, 1269.6498, 1270.6575, 1273.6556, 1277.6305, 1286.6099, 1289.6875, 1293.6401, 1303.6455, 1304.6677, 1307.6788, 1309.6780, 1319.6589, 1331.7888, 1336.0798, 1336.6155, 1351.7202, 1356.5811, 1359.1302, 1359.6246, 1374.7588, 1390.6974, 1393.7992, 1400.6683, 1401.6989, 1414.8191, 1429.3142, 1436.7889, 1444.7122, 1445.7839, 1450.7223, 1456.7717, 1462.6630, 1467.7329, 1470.7394, 1471.2693, 1471.7250, 1472.2531, 1475.7633, 1483.7141, 1488.5333, 1488.7194, 1493.7366, 1502.5601, 1513.7441, 1516.6807, 1517.6437, 1518.7053, 1523.7611, 1526.7343, 1528.6973, 1536.8230, 1540.7114, 1551.7063, 1553.7252, 1554.7474, 1557.6992, 1558.7140, 1563.6658, 1564.3619, 1565.7223, 1566.7162, 1572.7062, 1580.8400, 1580.8423, 1586.8090, 1597.7913, 1599.8337, 1601.8030, 1614.8046, 1616.7968, 1628.7136, 1630.8103, 1635.7697, 1642.7682, 1643.7688, 1644.7915, 1646.8154, 1651.7681, 1657.7932, 1663.8466, 1664.8557, 1666.7545, 1695.8208, 1707.8933, 1718.8790, 1721.9192, 1722.9375, 1728.8976, 1730.9116, 1732.2391, 1734.1844, 1739.7894, 1755.2666, 1755.7703, 1757.4702, 1757.9089, 1758.4524, 1758.8987, 1761.9023, 1765.7578, 1768.8339, 1770.3949, 1772.8273, 1773.8792, 1786.8268, 1829.8895, 1830.8380, 1833.3470, 1833.8501, 1851.8885, 1852.0399, 1862.9515, 1881.7703, 1895.9634, 1906.8195, 1908.8512, 1920.7972, 1936.9479, 1939.8563, 1940.8103, 1943.8293, 1954.8351, 1956.8574, 1958.8956, 1965.7998, 1967.9565, 1969.9998, 1979.8927, 1981.3531, 1982.8179, 1995.8232, 1997.8312, 2003.8595, 2008.9971, 2011.8464, 2015.9408, 2025.8735, 2031.9606, 2047.9397, 2062.9578, 2067.0020, 2076.8943, 2081.9827, 2082.9966, 2087.0103, 2088.1108, 2089.1162, 2096.0430, 2097.0200, 2108.9771, 2110.0061, 2140.0918, 2143.1006, 2144.0718, 2149.8516, 2160.0554, 2178.0471, 2188.0547, 2191.0457, 2198.1831, 2199.1499, 2203.0386, 2211.1052, 2223.1582, 2225.1470, 2227.1042, 2228.0725, 2238.1086, 2239.1538, 2243.1243, 2244.0684, 2254.0896, 2255.1482, 2260.0779, 2272.1736, 2287.1108, 2307.0549, 2309.0659, 2310.0798, 2310.5271, 2311.0454, 2319.0945, 2337.6050, 2341.5510, 2353.0518, 2355.0564, 2371.0581, 2383.9592, 2388.0437, 2404.0471, 2412.0872, 2419.5879, 2420.0864, 2420.5930, 2426.5667, 2427.1211, 2438.1724, 2455.1553, 2458.1648, 2459.1794, 2478.1587, 2516.1133, 2533.1262, 2542.1650, 2546.1746, 2549.1316, 2550.1252, 2559.1470, 2561.1729, 2562.1765, 2567.1514, 2572.1523, 2574.2678, 2576.1628, 2583.1218, 2587.2722, 2604.1682, 2612.1438, 2653.7173, 2655.1897, 2671.1787, 2687.1833, 2701.2820, 2706.2766, 2712.2104, 2714.2156, 2716.1953, 2717.2725, 2725.2354, 2729.1675, 2739.2351, 2743.2642, 2745.2900, 2754.2197, 2759.2573, 2769.2219, 2776.2644, 2785.2388, 2786.2617, 2787.2400, 2793.2439, 2801.2378, 2803.2302, 2809.2549, 2815.2153, 2817.2322, 2819.2236, 2830.2788, 2833.2280, 2836.2397, 2873.2961, 2889.2024, 2894.3218, 2927.2520, 2931.3428, 2955.3005, 2974.4124, 2975.4084, 2990.3921, 3006.3206, 3011.2810, 3020.3557, 3021.2412, 3035.3281, 3053.3447, 3062.2456, 3086.4507, 3091.2622, 3103.3140, 3110.7285, 3113.8591, 3130.3557, 3140.4265, 3144.3657, 3159.4985, 3173.5386, 3174.5486, 3185.4236, 3218.3301, 3259.3625, 3312.3267, 3331.4678, 3347.4761, 3363.4788, 3390.4675, 3431.4570, 3447.4666, 3463.4895, 3485.6985, 3488.6545, 3489.6521, 3493.0195, 3494.5571,

3504.6423, 3509.5613, 3525.6025, 3538.4521, 3553.6230, 3579.5403, 3648.1956, 3655.4729, 3664.7500, 3695.6177, 3712.5720, 3738.5837, 3816.7043, 3937.8308, 4010.8696

3. Q1JQB0 **Mass:** 98215 **Score:** 122 **Expect:** 1.5e-008 **Matches:** 42

Collagen, type VI, alpha 2 OS=Bos taurus GN=COL6A2 PE=1 SV=1

Observed	Mr(expt)	Mr(calc)	ppm	Start	End	Miss	Ions	Peptide
829.4589	828.4516	828.4453	7.61	195	201	0	---	K.LNEQGLR.D
830.4522	829.4449	829.4406	5.25	179	185	1	---	R.AREEGIR.L
860.4207	859.4134	859.4035	11.5	738	744	0	---	R.DDDLNLR.A
993.5046	992.4973	992.5039	-6.67	880	888	1	---	R.AKFATGNER.Q
998.5911	997.5838	997.5960	-12.28	186	194	0	---	R.LFAVPPNLK.L
1024.5516	1023.5443	1023.5349	9.21	120	128	0	---	K.SLQSISSFR.R
1074.6073	1073.6000	1073.5982	1.72	635	643	0	---	K.NFVINVVNR.L
1074.6100	1073.6027	1073.5982	4.24	635	643	0	50	K.NFVINVVNR.L
1076.6123	1075.6050	1075.6026	2.24	724	733	0	---	R.VFAVVITDGR.H
1122.5322	1121.5249	1121.5101	13.2	306	316	1	---	K.GEKGEFGADGR.K
1156.4957	1155.4884	1155.4655	19.9	239	247	0	---	K.HEAYGECYK.V
1180.6428	1179.6355	1179.6360	-0.41	120	129	1	---	K.SLQSISSFRR.G
1234.6414	1233.6341	1233.6102	19.4	539	550	2	---	R.GDFGSKGEPGRK.G
1318.6750	1317.6677	1317.6902	-17.05	411	424	2	---	K.GGPGPRGPKGEPGR.R
1328.6600	1327.6527	1327.6520	0.52	202	212	0	49	R.DIANTPHELYR.N
1328.6620	1327.6547	1327.6520	2.02	202	212	0	---	R.DIANTPHELYR.N
1333.7644	1332.7571	1332.7514	4.29	722	733	1	---	K.TRVFAVVITDGR.H
1350.6853	1349.6780	1349.7013	-17.26	248	260	0	---	K.VSCLEIPGPPGPK.G
1365.6489	1364.6416	1364.6433	-1.21	734	744	1	---	R.HDPRDDDLNLR.A
1383.7037	1382.6964	1382.6752	15.4	784	795	0	---	R.NMTLFSDLVAEK.F + Oxidation (M)
1474.5243	1473.5170	1473.5105	4.42	599	609	1	---	R.ETCGCCDCEKR.C
1514.6849	1513.6776	1513.6693	5.47	236	247	1	---	K.VMKHEAYGECYK.V
1530.6893	1529.6820	1529.6643	11.6	236	247	1	---	K.VMKHEAYGECYK.V + Oxidation (M)
1558.7140	1557.7067	1557.7174	-6.84	889	900	0	---	R.QDWMDLFIDTFK.L
1743.8966	1742.8893	1742.8992	-5.64	692	707	0	---	K.NLEWIAGGTWTPSALK.F
1769.8287	1768.8214	1768.8526	-17.63	264	281	2	---	R.GQKGAKGNMGEPGEPGQK.G
1774.9180	1773.9107	1773.8983	7.02	372	389	2	---	K.GDPGRPGRRGPPGENGAK.G
1774.9200	1773.9127	1773.8983	8.15	372	389	2	---	K.GDPGRPGRRGPPGENGAK.G
1809.0499	1808.0426	1808.0308	6.53	186	201	1	---	R.LFAVPPNLKLNEQGLR.D
1810.0303	1809.0230	1809.0261	-1.68	179	194	2	---	R.AREEGIRLFAVPPNLK.L
1932.0050	1930.9977	1930.9973	0.20	465	484	2	---	K.GDRGLPGPRGPQGTVGEPGK.Q
2019.8779	2018.8706	2018.8930	-11.08	351	371	1	---	R.GPDGYVGEAGSPGERGDQGSK.G
2139.0991	2138.0918	2138.0868	2.34	195	212	1	---	K.LNEQGLRDIANTPHELYR.N
2174.0378	2173.0305	2173.0222	3.84	766	783	0	---	K.HESENLYSIACDKPQQVR.N
2179.0574	2178.0501	2178.0382	5.47	95	114	0	---	R.YGGLHFSDLVEVFSPPGSDR.A
2284.1841	2283.1768	2283.1682	3.77	151	173	0	---	K.GVVNFAVVITDGHVTGSPCGGIK.L
2354.0767	2353.0694	2353.0604	3.82	213	232	0	---	R.NNYATMRPDSTEIDQDTINR.I
2392.1462	2391.1389	2391.1343	1.94	659	679	0	---	R.VGVVQYSHEGTFEAIQLDDER.I
2423.2439	2422.2366	2422.2353	0.53	724	744	2	---	R.VFAVVITDGRHDPRDDDLNLR.A
2755.2358	2754.2285	2754.2266	0.71	572	598	0	---	R.GAPGPEGEPGPPGDPGLTECDVMTYVR.E
2771.2437	2770.2364	2770.2215	5.39	572	598	0	---	R.GAPGPEGEPGPPGDPGLTECDVMTYVR.E + Oxidation (M)
2862.2927	2861.2854	2861.3550	-24.32	236	260	2	---	K.VMKHEAYGECYKVSCLEIPGPPGPK.G + Oxidation (M)

No match to: 716.4763, 723.4098, 724.2417, 725.2533, 726.3622, 728.3046, 729.4749, 734.4570, 736.3904, 737.4152, 738.3791, 744.8651, 745.3838, 747.4550, 768.3673, 788.4571, 801.4262, 805.4606, 813.4481, 814.4558, 820.4053, 823.4778, 839.3830, 842.5110, 857.4382, 865.4518, 870.4165, 872.5054, 880.4424, 882.4292, 887.4245, 887.9669, 888.4561, 891.4898, 894.4144, 894.8973, 897.5006, 898.4523, 912.5132, 913.5293, 925.4620, 927.4981, 931.4992, 934.5098, 957.5774, 960.5294, 961.4949, 963.4734, 965.4692, 970.5181, 972.4899, 975.5167, 976.5063, 977.5212, 978.5100, 978.5146, 983.6283, 992.4958, 995.4181, 1000.5959, 1002.5007, 1006.5014, 1011.5474, 1012.5447, 1014.5485, 1020.5577, 1021.5699, 1027.5438, 1028.5397, 1030.5978, 1032.5238, 1036.5260, 1037.5405, 1039.5641, 1040.5908, 1042.5388, 1043.5178, 1045.5568, 1046.6018, 1056.5515, 1057.6100, 1057.6107, 1061.6168, 1102.6007, 1103.6257, 1105.6136, 1107.1058, 1113.5486, 1120.5498, 1123.5986, 1128.5724, 1129.5924, 1134.3574, 1137.5463, 1145.6348, 1149.6057, 1153.6460, 1161.6295, 1162.6174, 1164.1815, 1170.5497, 1172.6156, 1177.6410, 1184.6412, 1188.5603, 1193.6041, 1206.5107, 1209.5889, 1211.6099, 1212.6160, 1215.6743, 1216.7081, 1219.6886, 1227.6234, 1229.7098, 1240.5974, 1242.6003, 1243.6161, 1253.6216, 1255.5972, 1255.6000, 1260.6227, 1264.7130, 1265.7064, 1269.6498, 1270.6575, 1273.6556, 1277.6305, 1286.6099, 1289.6875, 1293.6401, 1303.6455, 1304.6677, 1307.6788, 1309.6780, 1319.6589, 1331.7888, 1336.0798, 1336.6155, 1351.7202, 1356.5811, 1359.1302, 1359.6246, 1374.7588, 1390.6974, 1393.7992, 1400.6683, 1401.6989, 1414.8191, 1429.3142, 1436.7889, 1444.7122, 1445.7839, 1450.7223, 1456.7717, 1462.6630, 1467.7329, 1470.7394, 1471.2693, 1471.7250, 1472.2531, 1475.7633, 1483.7141, 1488.5333, 1488.7194, 1493.7366, 1502.5601, 1513.7441, 1516.6807, 1517.6437, 1518.7053, 1523.7611, 1526.7343, 1528.6973, 1536.8230, 1540.7114, 1551.7063, 1553.7252, 1554.7474, 1557.6992, 1563.6658, 1564.3619, 1565.7223, 1566.7162, 1572.7062, 1580.8400, 1580.8423, 1586.8090, 1597.7913, 1599.8337, 1601.8030, 1614.8046, 1616.7968, 1628.7136, 1630.8103, 1635.7697, 1642.7682, 1643.7688, 1644.7915, 1646.8154, 1651.7681, 1657.7932, 1663.8466, 1664.8557, 1666.7545, 1695.8208, 1707.8933, 1718.8790, 1721.9192, 1722.9375, 1728.8976, 1730.9116, 1732.2391, 1734.1844, 1739.7894, 1755.2666, 1755.7703, 1757.4702, 1757.9089, 1758.4524, 1758.8987, 1761.9023, 1765.7578, 1768.8339, 1770.3949, 1772.8273, 1773.8792, 1786.8268, 1787.8239, 1829.8895, 1830.8380, 1833.3470, 1833.8501, 1851.8885, 1852.0399, 1862.9515, 1881.7703, 1895.9634, 1906.8195, 1908.8512, 1920.7972, 1936.9479, 1939.8563, 1940.8103, 1943.8293, 1954.8351, 1956.8574, 1958.8956, 1965.7998, 1967.9565, 1969.9998, 1979.8927, 1981.3531, 1982.8179, 1995.8232, 1997.8312, 2003.8595, 2008.9971, 2011.8464, 2015.9408, 2025.8735, 2031.9606, 2047.9397, 2062.9578, 2067.0020, 2076.8943, 2081.9827, 2082.9966, 2087.0103, 2088.1108, 2089.1162, 2096.0430, 2097.0200, 2108.9771, 2110.0061, 2140.0918, 2143.1006, 2144.0718, 2149.8516, 2160.0554, 2178.0471, 2188.0547, 2191.0457, 2198.1831, 2199.1499, 2203.0386, 2211.1052, 2223.1582, 2225.1470, 2227.1042, 2228.0725, 2238.1086, 2239.1538, 2243.1243, 2244.0684, 2254.0896, 2255.1482, 2260.0779, 2270.2183, 2272.1736, 2287.1108, 2307.0549, 2309.0659, 2310.0798, 2310.5271, 2311.0454, 2319.0945, 2337.6050, 2341.5510, 2353.0518, 2355.0564, 2371.0581, 2383.9592, 2388.0437, 2404.0471, 2412.0872, 2419.5879, 2420.0864, 2420.5930, 2426.5667, 2427.1211, 2438.1724, 2455.1553, 2458.1648, 2459.1794, 2478.1587, 2516.1133, 2533.1262, 2542.1650, 2546.1746, 2549.1316, 2550.1252, 2559.1470, 2561.1729, 2562.1765, 2567.1514, 2572.1523, 2574.2678, 2576.1628, 2583.1218, 2587.2722, 2604.1682, 2612.1438, 2653.7173, 2655.1897, 2671.1787, 2687.1833, 2701.2820, 2706.2766, 2712.2104, 2714.2156, 2716.1953, 2717.2725, 2725.2354, 2729.1675, 2739.2351, 2743.2642, 2745.2900, 2754.2197, 2759.2573, 2769.2219, 2776.2644, 2785.2388, 2786.2617, 2787.2400, 2793.2439, 2801.2378, 2803.2302, 2809.2549, 2815.2153, 2817.2322, 2819.2236, 2830.2788, 2833.2280, 2836.2397, 2873.2961, 2889.2024, 2894.3218, 2927.2520, 2931.3428, 2955.3005, 2974.4124, 2975.4084, 2990.3921, 3006.3206, 3011.2810, 3020.3557, 3021.2412, 3035.3281, 3053.3447, 3062.2456, 3086.4507, 3091.2622, 3103.3140, 3110.7285, 3113.8591, 3130.3557, 3140.4265, 3144.3657, 3159.4985, 3173.5386, 3174.5486, 3185.4236, 3218.3301, 3259.3625, 3312.3267, 3331.4678, 3347.4761, 3363.4788, 3390.4675, 3431.4570, 3447.4666, 3463.4895, 3485.6985, 3488.6545, 3489.6521, 3493.0195, 3494.5571, 3504.6423, 3509.5613, 3525.6025, 3538.4521, 3553.6230, 3579.5403, 3648.1956, 3655.4729, 3664.7500, 3695.6177, 3712.5720, 3738.5837, 3816.7043, 3937.8308, 4010.8696

4. G3MXU3 **Mass:** 25162 **Score:** 68 **Expect:** 0.0034 **Matches:** 12

Uncharacterized protein OS=Bos taurus GN=COL6A2 PE=4 SV=1

Observed	Mr(expt)	Mr(calc)	ppm	Start	End	Miss	Ions	Peptide
729.4749	728.4676	728.4657	2.68	72	77	1	---	R.RLTLAR.K
865.4518	864.4445	864.4341	12.0	65	71	0	---	R.FVEEVSR.R
972.4899	971.4826	971.4825	0.13	54	61	0	---	R.LGEQNFHK.V

1043.5178	1042.5105	1042.5043	5.95	78	86	1	---	R.KDDDPLNAR.V
1057.6100	1056.6027	1056.6080	-5.01	87	96	0	56	R.VALLQFGGPR.E
1057.6107	1056.6034	1056.6080	-4.35	87	96	0	---	R.VALLQFGGPR.E
1145.6348	1144.6275	1144.6241	3.03	198	208	0	---	K.ISLGDPAAVFR.E
1149.6057	1148.5984	1148.5900	7.36	21	30	0	---	K.QDFPLMIAAK.E + Oxidation (M)
1177.6410	1176.6337	1176.6363	-2.22	64	72	2	---	R.RFVEEVSRR.L
1530.6893	1529.6820	1529.6787	2.19	211	223	0	---	K.DYDSLAQPGFFDR.F
1787.8239	1786.8166	1786.8162	0.22	209	223	1	---	R.EKDYDSLAQPGFFDR.F
2270.2183	2269.2110	2269.2166	-2.45	176	197	1	---	R.KQNVVPTVVAVGSDVDTDVLSK.I

No match to: 716.4763, 723.4098, 724.2417, 725.2533, 726.3622, 728.3046, 734.4570, 736.3904, 737.4152, 738.3791, 744.8651, 745.3838, 747.4550, 768.3673, 788.4571, 801.4262, 805.4606, 813.4481, 814.4558, 820.4053, 823.4778, 829.4589, 830.4522, 839.3830, 842.5110, 857.4382, 860.4207, 870.4165, 872.5054, 880.4424, 882.4292, 887.4245, 887.9669, 888.4561, 891.4898, 894.4144, 894.8973, 897.5006, 898.4523, 912.5132, 913.5293, 925.4620, 927.4981, 931.4992, 934.5098, 957.5774, 960.5294, 961.4949, 963.4734, 965.4692, 970.5181, 975.5167, 976.5063, 977.5212, 978.5100, 978.5146, 983.6283, 992.4958, 993.5046, 995.4181, 998.5911, 1000.5959, 1002.5007, 1006.5014, 1011.5474, 1012.5447, 1014.5485, 1020.5577, 1021.5699, 1024.5516, 1027.5438, 1028.5397, 1030.5978, 1032.5238, 1036.5260, 1037.5405, 1039.5641, 1040.5908, 1042.5388, 1045.5568, 1046.6018, 1056.5515, 1061.6168, 1074.6073, 1074.6100, 1076.6123, 1102.6007, 1103.6257, 1105.6136, 1107.1058, 1113.5486, 1120.5498, 1122.5322, 1123.5986, 1128.5724, 1129.5924, 1134.3574, 1137.5463, 1153.6460, 1156.4957, 1161.6295, 1162.6174, 1164.1815, 1170.5497, 1172.6156, 1180.6428, 1184.6412, 1188.5603, 1193.6041, 1206.5107, 1209.5889, 1211.6099, 1212.6160, 1215.6743, 1216.7081, 1219.6886, 1227.6234, 1229.7098, 1234.6414, 1240.5974, 1242.6003, 1243.6161, 1253.6216, 1255.5972, 1255.6000, 1260.6227, 1264.7130, 1265.7064, 1269.6498, 1270.6575, 1273.6556, 1277.6305, 1286.6099, 1289.6875, 1293.6401, 1303.6455, 1304.6677, 1307.6788, 1309.6780, 1318.6750, 1319.6589, 1328.6600, 1328.6620, 1331.7888, 1333.7644, 1336.0798, 1336.6155, 1350.6853, 1351.7202, 1356.5811, 1359.1302, 1359.6246, 1365.6489, 1374.7588, 1383.7037, 1390.6974, 1393.7992, 1400.6683, 1401.6989, 1414.8191, 1429.3142, 1436.7889, 1444.7122, 1445.7839, 1450.7223, 1456.7717, 1462.6630, 1467.7329, 1470.7394, 1471.2693, 1471.7250, 1472.2531, 1474.5243, 1475.7633, 1483.7141, 1488.5333, 1488.7194, 1493.7366, 1502.5601, 1513.7441, 1514.6849, 1516.6807, 1517.6437, 1518.7053, 1523.7611, 1526.7343, 1528.6973, 1536.8230, 1540.7114, 1551.7063, 1553.7252, 1554.7474, 1557.6992, 1558.7140, 1563.6658, 1564.3619, 1565.7223, 1566.7162, 1572.7062, 1580.8400, 1580.8423, 1586.8090, 1597.7913, 1599.8337, 1601.8030, 1614.8046, 1616.7968, 1628.7136, 1630.8103, 1635.7697, 1642.7682, 1643.7688, 1644.7915, 1646.8154, 1651.7681, 1657.7932, 1663.8466, 1664.8557, 1666.7545, 1695.8208, 1707.8933, 1718.8790, 1721.9192, 1722.9375, 1728.8976, 1730.9116, 1732.2391, 1734.1844, 1739.7894, 1743.8966, 1755.2666, 1755.7703, 1757.4702, 1757.9089, 1758.4524, 1758.8987, 1761.9023, 1765.7578, 1768.8339, 1769.8287, 1770.3949, 1772.8273, 1773.8792, 1774.9180, 1774.9200, 1786.8268, 1809.0499, 1810.0303, 1829.8895, 1830.8380, 1833.3470, 1833.8501, 1851.8885, 1852.0399, 1862.9515, 1881.7703, 1895.9634, 1906.8195, 1908.8512, 1920.7972, 1932.0050, 1936.9479, 1939.8563, 1940.8103, 1943.8293, 1954.8351, 1956.8574, 1958.8956, 1965.7998, 1967.9565, 1969.9998, 1979.8927, 1981.3531, 1982.8179, 1995.8232, 1997.8312, 2003.8595, 2008.9971, 2011.8464, 2015.9408, 2019.8779, 2025.8735, 2031.9606, 2047.9397, 2062.9578, 2067.0020, 2076.8943, 2081.9827, 2082.9966, 2087.0103, 2088.1108, 2089.1162, 2096.0430, 2097.0200, 2108.9771, 2110.0061, 2139.0991, 2140.0918, 2143.1006, 2144.0718, 2149.8516, 2160.0554, 2174.0378, 2178.0471, 2179.0574, 2188.0547, 2191.0457, 2198.1831, 2199.1499, 2203.0386, 2211.1052, 2223.1582, 2225.1470, 2227.1042, 2228.0725, 2238.1086, 2239.1538, 2243.1243, 2244.0684, 2254.0896, 2255.1482, 2260.0779, 2272.1736, 2284.1841, 2287.1108, 2307.0549, 2309.0659, 2310.0798, 2310.5271, 2311.0454, 2319.0945, 2337.6050, 2341.5510, 2353.0518, 2354.0767, 2355.0564, 2371.0581, 2383.9592, 2388.0437, 2392.1462, 2404.0471, 2412.0872, 2419.5879, 2420.0864, 2420.5930, 2423.2439, 2426.5667, 2427.1211, 2438.1724, 2455.1553, 2458.1648, 2459.1794, 2478.1587, 2516.1133, 2533.1262, 2542.1650, 2546.1746, 2549.1316, 2550.1252, 2559.1470, 2561.1729, 2562.1765, 2567.1514, 2572.1523, 2574.2678, 2576.1628, 2583.1218, 2587.2722, 2604.1682, 2612.1438, 2653.7173, 2655.1897, 2671.1787, 2687.1833, 2701.2820, 2706.2766, 2712.2104, 2714.2156, 2716.1953, 2717.2725, 2725.2354, 2729.1675, 2739.2351, 2743.2642, 2745.2900, 2754.2197, 2755.2358, 2759.2573, 2769.2219, 2771.2437, 2776.2644, 2785.2388, 2786.2617, 2787.2400, 2793.2439, 2801.2378, 2803.2302, 2809.2549, 2815.2153, 2817.2322, 2819.2236, 2830.2788, 2833.2280, 2836.2397, 2862.2927, 2873.2961, 2889.2024, 2894.3218, 2927.2520, 2931.3428, 2955.3005, 2974.4124, 2975.4084, 2990.3921, 3006.3206, 3011.2810, 3020.3557, 3021.2412, 3035.3281, 3053.3447, 3062.2456, 3086.4507, 3091.2622, 3103.3140, 3110.7285, 3113.8591, 3130.3557, 3140.4265, 3144.3657, 3159.4985, 3173.5386, 3174.5486, 3185.4236, 3218.3301, 3259.3625, 3312.3267, 3331.4678, 3347.4761, 3363.4788, 3390.4675, 3431.4570, 3447.4666, 3463.4895, 3485.6985, 3488.6545, 3489.6521, 3493.0195, 3494.5571, 3504.6423, 3509.5613, 3525.6025, 3538.4521, 3553.6230, 3579.5403, 3648.1956, 3655.4729, 3664.7500, 3695.6177, 3712.5720, 3738.5837, 3816.7043, 3937.8308, 4010.8696

5. G3MYC4 **Mass:** 19371 **Score:** 24 **Expect:** 86 **Matches:** 11

Zinc finger protein 143 OS=Bos taurus GN=ZNF143 PE=4 SV=1

Observed	Mr(expt)	Mr(calc)	ppm	Start	End	Miss	Ions	Peptide
925.4620	924.4548	924.4665	-12.69	122	129	0	---	R.SFTTSNIR.K
927.4981	926.4908	926.4862	4.98	62	70	0	---	K.AFATGYGLK.S
972.4899	971.4826	971.4712	11.7	152	160	0	---	R.AFASATNYK.N
1137.5463	1136.5390	1136.5574	-16.18	14	23	1	---	K.SQQSGEKAFR.C
1162.6174	1161.6101	1161.5990	9.62	10	20	1	---	R.VTAKSQQSGEK.A
1393.7992	1392.7919	1392.8201	-20.25	1	13	1	---	-.IVLQGHATRVTAK.S
1536.8230	1535.8157	1535.8056	6.60	10	23	2	---	R.VTAKSQQSGEKAFR.C
2355.0564	2354.0491	2354.1048	-23.67	21	40	2	---	K.AFRCGYDGCGKLYTTAHHLK.V
2546.1746	2545.1673	2545.1703	-1.17	131	151	1	---	K.VHIRTHTGERPYYCTEPGCGR.A
2562.1765	2561.1692	2561.1499	7.53	71	91	2	---	K.SHVRTHTGEKPYRCSEDNCTK.S
3347.4761	3346.4688	3346.5473	-23.44	45	74	2	---	R.SHTGDRPYQCEHAGCGKAFATGYGLKSHVR.T

No match to: 716.4763, 723.4098, 724.2417, 725.2533, 726.3622, 728.3046, 729.4749, 734.4570, 736.3904, 737.4152, 738.3791, 744.8651, 745.3838, 747.4550, 768.3673, 788.4571, 801.4262, 805.4606, 813.4481, 814.4558, 820.4053, 823.4778, 829.4589, 830.4522, 839.3830, 842.5110, 857.4382, 860.4207, 865.4518, 870.4165, 872.5054, 880.4424, 882.4292, 887.4245, 887.9669, 888.4561, 891.4898, 894.4144, 894.8973, 897.5006, 898.4523, 912.5132, 913.5293, 931.4992, 934.5098, 957.5774, 960.5294, 961.4949, 963.4734, 965.4692, 970.5181, 975.5167, 976.5063, 977.5212, 978.5100, 978.5146, 983.6283, 992.4958, 993.5046, 995.4181, 998.5911, 1000.5959, 1002.5007, 1006.5014, 1011.5474, 1012.5447, 1014.5485, 1020.5577, 1021.5699, 1024.5516, 1027.5438, 1028.5397, 1030.5978, 1032.5238, 1036.5260, 1037.5405, 1039.5641, 1040.5908, 1042.5388, 1043.5178, 1045.5568, 1046.6018, 1056.5515, 1057.6100, 1057.6107, 1061.6168, 1074.6073, 1074.6100, 1076.6123, 1102.6007, 1103.6257, 1105.6136, 1107.1058, 1113.5486, 1120.5498, 1122.5322, 1123.5986, 1128.5724, 1129.5924, 1134.3574, 1145.6348, 1149.6057, 1153.6460, 1156.4957, 1161.6295, 1164.1815, 1170.5497, 1172.6156, 1177.6410, 1180.6428, 1184.6412, 1188.5603, 1193.6041, 1206.5107, 1209.5889, 1211.6099, 1212.6160, 1215.6743, 1216.7081, 1219.6886, 1227.6234, 1229.7098, 1234.6414, 1240.5974, 1242.6003, 1243.6161, 1253.6216, 1255.5972, 1255.6000, 1260.6227, 1264.7130, 1265.7064, 1269.6498, 1270.6575, 1273.6556, 1277.6305, 1286.6099, 1289.6875, 1293.6401, 1303.6455, 1304.6677, 1307.6788, 1309.6780, 1318.6750, 1319.6589, 1328.6600, 1328.6620, 1331.7888, 1333.7644, 1336.0798, 1336.6155, 1350.6853, 1351.7202, 1356.5811, 1359.1302, 1359.6246, 1365.6489, 1374.7588, 1383.7037, 1390.6974, 1400.6683, 1401.6989, 1414.8191, 1429.3142, 1436.7889, 1444.7122, 1445.7839, 1450.7223, 1456.7717, 1462.6630, 1467.7329, 1470.7394, 1471.2693, 1471.7250, 1472.2531, 1474.5243, 1475.7633, 1483.7141, 1488.5333, 1488.7194, 1493.7366, 1502.5601, 1513.7441, 1514.6849, 1516.6807, 1517.6437, 1518.7053, 1523.7611, 1526.7343, 1528.6973, 1530.6893, 1540.7114, 1551.7063, 1553.7252, 1554.7474, 1557.6992, 1558.7140, 1563.6658, 1564.3619, 1565.7223, 1566.7162, 1572.7062, 1580.8400, 1580.8423, 1586.8090, 1597.7913, 1599.8337, 1601.8030, 1614.8046, 1616.7968, 1628.7136, 1630.8103, 1635.7697, 1642.7682, 1643.7688, 1644.7915, 1646.8154, 1651.7681, 1657.7932, 1663.8466, 1664.8557, 1666.7545, 1695.8208, 1707.8933, 1718.8790, 1721.9192, 1722.9375, 1728.8976, 1730.9116, 1732.2391, 1734.1844, 1739.7894, 1743.8966, 1755.2666, 1755.7703, 1757.4702, 1757.9089, 1758.4524, 1758.8987, 1761.9023, 1765.7578, 1768.8339, 1769.8287, 1770.3949, 1772.8273, 1773.8792, 1774.9180, 1774.9200, 1786.8268, 1787.8239, 1809.0499, 1810.0303, 1829.8895, 1830.8380, 1833.3470, 1833.8501, 1851.8885, 1852.0399, 1862.9515, 1881.7703, 1895.9634, 1906.8195, 1908.8512, 1920.7972, 1932.0050, 1936.9479, 1939.8563, 1940.8103, 1943.8293, 1954.8351, 1956.8574, 1958.8956, 1965.7998, 1967.9565, 1969.9998, 1979.8927, 1981.3531, 1982.8179, 1995.8232, 1997.8312, 2003.8595, 2008.9971, 2011.8464, 2015.9408, 2019.8779, 2025.8735, 2031.9606, 2047.9397, 2062.9578, 2067.0020, 2076.8943, 2081.9827, 2082.9966, 2087.0103, 2088.1108, 2089.1162, 2096.0430, 2097.0200, 2108.9771, 2110.0061, 2139.0991, 2140.0918, 2143.1006, 2144.0718, 2149.8516, 2160.0554, 2174.0378, 2178.0471, 2179.0574, 2188.0547, 2191.0457, 2198.1831, 2199.1499, 2203.0386, 2211.1052, 2223.1582, 2225.1470, 2227.1042, 2228.0725, 2238.1086, 2239.1538, 2243.1243, 2244.0684, 2254.0896, 2255.1482, 2260.0779, 2270.2183, 2272.1736, 2284.1841, 2287.1108, 2307.0549, 2309.0659, 2310.0798, 2310.5271, 2311.0454, 2319.0945, 2337.6050, 2341.5510, 2353.0518, 2354.0767, 2371.0581, 2383.9592, 2388.0437, 2392.1462, 2404.0471, 2412.0872, 2419.5879, 2420.0864, 2420.5930, 2423.2439, 2426.5667, 2427.1211, 2438.1724, 2455.1553, 2458.1648, 2459.1794, 2478.1587, 2516.1133, 2533.1262, 2542.1650, 2549.1316, 2550.1252, 2559.1470, 2561.1729, 2567.1514, 2572.1523, 2574.2678, 2576.1628, 2583.1218, 2587.2722, 2604.1682, 2612.1438, 2653.7173, 2655.1897, 2671.1787, 2687.1833, 2701.2820, 2706.2766, 2712.2104, 2714.2156, 2716.1953, 2717.2725, 2725.2354, 2729.1675, 2739.2351, 2743.2642, 2745.2900, 2754.2197, 2755.2358, 2759.2573, 2769.2219, 2771.2437, 2776.2644, 2785.2388, 2786.2617, 2787.2400, 2793.2439, 2801.2378, 2803.2302,

2809.2549, 2815.2153, 2817.2322, 2819.2236, 2830.2788, 2833.2280, 2836.2397, 2862.2927, 2873.2961, 2889.2024, 2894.3218, 2927.2520, 2931.3428, 2955.3005, 2974.4124, 2975.4084, 2990.3921, 3006.3206, 3011.2810, 3020.3557, 3021.2412, 3035.3281, 3053.3447, 3062.2456, 3086.4507, 3091.2622, 3103.3140, 3110.7285, 3113.8591, 3130.3557, 3140.4265, 3144.3657, 3159.4985, 3173.5386, 3174.5486, 3185.4236, 3218.3301, 3259.3625, 3312.3267, 3331.4678, 3363.4788, 3390.4675, 3431.4570, 3447.4666, 3463.4895, 3485.6985, 3488.6545, 3489.6521, 3493.0195, 3494.5571, 3504.6423, 3509.5613, 3525.6025, 3538.4521, 3553.6230, 3579.5403, 3648.1956, 3655.4729, 3664.7500, 3695.6177, 3712.5720, 3738.5837, 3816.7043, 3937.8308, 4010.8696

6. F1MHI2 **Mass:** 73699 **Score:** 23 **Expect:** 1.3e+002 **Matches:** 25

Uncharacterized protein OS=Bos taurus GN=ARHGAP24 PE=4 SV=2

Observed	Mr(expt)	Mr(calc)	ppm	Start	End	Miss	Ions	Peptide
912.5132	911.5060	911.4977	9.04	230	236	1	---	K.HDRLFPK.D
975.5167	974.5094	974.5079	1.55	607	614	2	---	K.MRNAERAK.E
1037.5405	1036.5332	1036.5553	-21.27	566	573	1	---	K.IEYESRIK.S
1153.6460	1152.6387	1152.6576	-16.41	598	606	2	---	R.KKFTMIEIK.M + Oxidation (M)
1184.6412	1183.6339	1183.6093	20.8	600	608	1	---	K.FTMIEIKMR.N + Oxidation (M)
1243.6161	1242.6088	1242.6317	-18.37	355	366	0	---	K.AQTTPNGSLQAR.R
1260.6227	1259.6154	1259.6105	3.88	609	619	2	---	R.NAERAKEDAEK.R
1286.6099	1285.6026	1285.6197	-13.31	378	389	0	---	K.MGTHSVQNGTVR.M
1523.7611	1522.7538	1522.7602	-4.20	406	418	0	---	R.SMSWLPNGYVTLR.D
1852.0399	1851.0326	1851.0076	13.5	160	174	1	---	K.SLPVVNYNLLKYICR.F
2062.9578	2061.9505	2061.9354	7.35	627	643	0	---	K.EMEQFFSTFGELTVEPR.R + Oxidation (M)
2203.0386	2202.0313	2202.0415	-4.64	627	644	1	---	K.EMEQFFSTFGELTVEPRR.T
2243.1243	2242.1170	2242.0760	18.3	259	278	1	---	K.ALMGQLQNKENNNTNDSPVR.R
2583.1218	2582.1145	2582.1417	-10.53	2	23	0	---	M.TANHESYLLMASTQNDMEDWVK.S
2714.2156	2713.2083	2713.1822	9.63	1	23	0	---	-.MTANHESYLLMASTQNDMEDWVK.S
2776.2644	2775.2571	2775.2884	-11.27	621	643	1	---	R.NDMLQKEMEQFFSTFGELTVEPR.R
2786.2617	2785.2544	2785.2586	-1.51	268	290	2	---	K.ENNNTNDSPVRRCSWDKPESPQR.S
2819.2236	2818.2163	2818.2650	-17.28	280	305	1	---	R.CSWDKPESPQRSSMDNGSPTALPGSK.T
2955.3005	2954.2932	2954.3538	-20.51	2	26	1	---	M.TANHESYLLMASTQNDMEDWVKSIR.R + Oxidation (M)
2975.4084	2974.4011	2974.3661	11.8	279	305	2	---	R.RCSWDKPESPQRSSMDNGSPTALPGSK.T
3086.4507	3085.4434	3085.3943	15.9	1	26	1	---	-.MTANHESYLLMASTQNDMEDWVKSIR.R + Oxidation (M)
3174.5486	3173.5413	3173.5332	2.55	572	597	2	---	R.IKSLEQRNLTLETEMMSLHDELDQER.K + Oxidation (M)
3390.4675	3389.4602	3389.5365	-22.49	280	310	2	---	R.CSWDKPESPQRSSMDNGSPTALPGSKTNSPR.N + Oxidation (M)
3664.7500	3663.7427	3663.6608	22.4	422	453	2	---	K.QKEQAGESGQHNRLSTYDNVHQQFSAMSLDDK.Q + Oxidation (M)
4010.8696	4009.8623	4009.8923	-7.48	529	565	2	---	R.ATSSSDNSETFVSNNTSNHSALHSLVSSLKQEMTKQK.I + Oxidation (M)

No match to: 716.4763, 723.4098, 724.2417, 725.2533, 726.3622, 728.3046, 729.4749, 734.4570, 736.3904, 737.4152, 738.3791, 744.8651, 745.3838, 747.4550, 768.3673, 788.4571, 801.4262, 805.4606, 813.4481, 814.4558, 820.4053, 823.4778, 829.4589, 830.4522, 839.3830, 842.5110, 857.4382, 860.4207, 865.4518, 870.4165, 872.5054, 880.4424, 882.4292, 887.4245, 887.9669, 888.4561, 891.4898, 894.4144, 894.8973, 897.5006, 898.4523, 913.5293, 925.4620, 927.4981, 931.4992, 934.5098, 957.5774, 960.5294, 961.4949, 963.4734, 965.4692, 970.5181, 972.4899, 976.5063, 977.5212, 978.5100, 978.5146, 983.6283, 992.4958, 993.5046, 995.4181, 998.5911, 1000.5959, 1002.5007, 1006.5014, 1011.5474, 1012.5447, 1014.5485, 1020.5577, 1021.5699, 1024.5516, 1027.5438, 1028.5397, 1030.5978, 1032.5238, 1036.5260, 1039.5641, 1040.5908, 1042.5388, 1043.5178, 1045.5568, 1046.6018, 1056.5515, 1057.6100, 1057.6107, 1061.6168, 1074.6073, 1074.6100, 1076.6123, 1102.6007, 1103.6257, 1105.6136, 1107.1058, 1113.5486, 1120.5498, 1122.5322, 1123.5986, 1128.5724, 1129.5924, 1134.3574, 1137.5463, 1145.6348, 1149.6057, 1156.4957, 1161.6295, 1162.6174, 1164.1815, 1170.5497, 1172.6156, 1177.6410, 1180.6428, 1188.5603, 1193.6041, 1206.5107, 1209.5889, 1211.6099, 1212.6160, 1215.6743, 1216.7081, 1219.6886, 1227.6234, 1229.7098, 1234.6414, 1240.5974, 1242.6003, 1253.6216, 1255.5972, 1255.6000, 1264.7130, 1265.7064, 1269.6498, 1270.6575, 1273.6556, 1277.6305, 1289.6875, 1293.6401, 1303.6455, 1304.6677, 1307.6788, 1309.6780, 1318.6750, 1319.6589, 1328.6600, 1328.6620, 1331.7888, 1333.7644, 1336.0798, 1336.6155, 1350.6853, 1351.7202, 1356.5811, 1359.1302, 1359.6246, 1365.6489, 1374.7588, 1383.7037, 1390.6974, 1393.7992, 1400.6683, 1401.6989, 1414.8191, 1429.3142, 1436.7889, 1444.7122, 1445.7839, 1450.7223, 1456.7717, 1462.6630, 1467.7329, 1470.7394, 1471.2693, 1471.7250, 1472.2531, 1474.5243, 1475.7633, 1483.7141, 1488.5333, 1488.7194, 1493.7366, 1502.5601, 1513.7441, 1514.6849, 1516.6807, 1517.6437, 1518.7053, 1526.7343, 1528.6973, 1530.6893, 1536.8230, 1540.7114, 1551.7063, 1553.7252, 1554.7474, 1557.6992, 1558.7140, 1563.6658, 1564.3619, 1565.7223, 1566.7162, 1572.7062, 1580.8400, 1580.8423, 1586.8090, 1597.7913, 1599.8337, 1601.8030, 1614.8046, 1616.7968, 1628.7136, 1630.8103, 1635.7697, 1642.7682, 1643.7688, 1644.7915, 1646.8154, 1651.7681, 1657.7932, 1663.8466, 1664.8557, 1666.7545, 1695.8208, 1707.8933, 1718.8790, 1721.9192, 1722.9375, 1728.8976, 1730.9116, 1732.2391, 1734.1844, 1739.7894, 1743.8966, 1755.2666, 1755.7703, 1757.4702, 1757.9089, 1758.4524, 1758.8987, 1761.9023, 1765.7578, 1768.8339, 1769.8287, 1770.3949, 1772.8273, 1773.8792, 1774.9180, 1774.9200, 1786.8268, 1787.8239, 1809.0499, 1810.0303, 1829.8895, 1830.8380, 1833.3470, 1833.8501, 1851.8885, 1862.9515, 1881.7703, 1895.9634, 1906.8195, 1908.8512, 1920.7972, 1932.0050, 1936.9479, 1939.8563, 1940.8103, 1943.8293, 1954.8351, 1956.8574, 1958.8956, 1965.7998, 1967.9565, 1969.9998, 1979.8927, 1981.3531, 1982.8179, 1995.8232, 1997.8312, 2003.8595, 2008.9971, 2011.8464, 2015.9408, 2019.8779, 2025.8735, 2031.9606, 2047.9397, 2067.0020, 2076.8943, 2081.9827, 2082.9966, 2087.0103, 2088.1108, 2089.1162, 2096.0430, 2097.0200, 2108.9771, 2110.0061, 2139.0991, 2140.0918, 2143.1006, 2144.0718, 2149.8516, 2160.0554, 2174.0378, 2178.0471, 2179.0574, 2188.0547, 2191.0457, 2198.1831, 2199.1499, 2211.1052, 2223.1582, 2225.1470, 2227.1042, 2228.0725, 2238.1086, 2239.1538, 2244.0684, 2254.0896, 2255.1482, 2260.0779, 2270.2183, 2272.1736, 2284.1841, 2287.1108, 2307.0549, 2309.0659, 2310.0798, 2310.5271, 2311.0454, 2319.0945, 2337.6050, 2341.5510, 2353.0518, 2354.0767, 2355.0564, 2371.0581, 2383.9592, 2388.0437, 2392.1462, 2404.0471, 2412.0872, 2419.5879, 2420.0864, 2420.5930, 2423.2439, 2426.5667, 2427.1211, 2438.1724, 2455.1553, 2458.1648, 2459.1794, 2478.1587, 2516.1133, 2533.1262, 2542.1650, 2546.1746, 2549.1316, 2550.1252, 2559.1470, 2561.1729, 2562.1765, 2567.1514, 2572.1523, 2574.2678, 2576.1628, 2587.2722, 2604.1682, 2612.1438, 2653.7173, 2655.1897, 2671.1787, 2687.1833, 2701.2820, 2706.2766, 2712.2104, 2716.1953, 2717.2725, 2725.2354, 2729.1675, 2739.2351, 2743.2642, 2745.2900, 2754.2197, 2755.2358, 2759.2573, 2769.2219, 2771.2437, 2785.2388, 2787.2400, 2793.2439, 2801.2378, 2803.2302, 2809.2549, 2815.2153, 2817.2322, 2830.2788, 2833.2280, 2836.2397, 2862.2927, 2873.2961, 2889.2024, 2894.3218, 2927.2520, 2931.3428, 2974.4124, 2990.3921, 3006.3206, 3011.2810, 3020.3557, 3021.2412, 3035.3281, 3053.3447, 3062.2456, 3091.2622, 3103.3140, 3110.7285, 3113.8591, 3130.3557, 3140.4265, 3144.3657, 3159.4985, 3173.5386, 3185.4236, 3218.3301, 3259.3625, 3312.3267, 3331.4678, 3347.4761, 3363.4788, 3431.4570, 3447.4666, 3463.4895, 3485.6985, 3488.6545, 3489.6521, 3493.0195, 3494.5571, 3504.6423, 3509.5613, 3525.6025, 3538.4521, 3553.6230, 3579.5403, 3648.1956, 3655.4729, 3695.6177, 3712.5720, 3738.5837, 3816.7043, 3937.8308

7. E1BJM6 **Mass:** 69143 **Score:** 19 **Expect:** 3.3e+002 **Matches:** 8

Uncharacterized protein OS=Bos taurus GN=ZBTB7C PE=4 SV=2

Observed	Mr(expt)	Mr(calc)	ppm	Start	End	Miss	Ions	Peptide
1057.6100	1056.6027	1056.6114	-8.19	365	374	1	19	K.VIMGAGKLPR.H + Oxidation (M)
1057.6107	1056.6034	1056.6114	-7.52	365	374	1	---	K.VIMGAGKLPR.H + Oxidation (M)
1184.6412	1183.6339	1183.6495	-13.19	397	405	2	---	R.QDKLKIHMR.K + Oxidation (M)
1255.6000	1254.5927	1254.6218	-23.15	449	458	1	---	K.SFTRSDHLHR.H
1273.6556	1272.6483	1272.6536	-4.17	50	60	1	---	R.SVLAACSKYFK.K
1289.6875	1288.6802	1288.6888	-6.62	551	561	1	---	K.LFGRAQLEAER.N
1513.7441	1512.7368	1512.7395	-1.76	543	554	1	---	R.QFEETQMKLFGR.A
2786.2617	2785.2544	2785.2734	-6.83	378	399	2	---	R.THTGEKPYMCNICEVRFTRQDK.L + Oxidation (M)

No match to: 716.4763, 723.4098, 724.2417, 725.2533, 726.3622, 728.3046, 729.4749, 734.4570, 736.3904, 737.4152, 738.3791, 744.8651, 745.3838, 747.4550, 768.3673, 788.4571, 801.4262, 805.4606, 813.4481, 814.4558, 820.4053, 823.4778, 829.4589, 830.4522, 839.3830, 842.5110, 857.4382, 860.4207, 865.4518, 870.4165, 872.5054, 880.4424, 882.4292, 887.4245, 887.9669, 888.4561, 891.4898, 894.4144, 894.8973, 897.5006, 898.4523, 912.5132, 913.5293, 925.4620, 927.4981, 931.4992, 934.5098, 957.5774, 960.5294, 961.4949, 963.4734, 965.4692, 970.5181, 972.4899, 975.5167, 976.5063, 977.5212, 978.5100, 978.5146, 983.6283, 992.4958, 993.5046, 995.4181, 998.5911, 1000.5959, 1002.5007, 1006.5014, 1011.5474, 1012.5447, 1014.5485, 1020.5577, 1021.5699, 1024.5516, 1027.5438, 1028.5397, 1030.5978, 1032.5238, 1036.5260, 1037.5405, 1039.5641, 1040.5908, 1042.5388, 1043.5178, 1045.5568, 1046.6018, 1056.5515, 1061.6168, 1074.6073, 1074.6100, 1076.6123, 1102.6007, 1103.6257, 1105.6136, 1107.1058, 1113.5486, 1120.5498, 1122.5322, 1123.5986, 1128.5724,

1129.5924, 1134.3574, 1137.5463, 1145.6348, 1149.6057, 1153.6460, 1156.4957, 1161.6295, 1162.6174, 1164.1815, 1170.5497, 1172.6156,
1177.6410, 1180.6428, 1188.5603, 1193.6041, 1206.5107, 1209.5889, 1211.6099, 1212.6160, 1215.6743, 1216.7081, 1219.6886, 1227.6234,
1229.7098, 1234.6414, 1240.5974, 1242.6003, 1243.6161, 1253.6216, 1255.5972, 1260.6227, 1264.7130, 1265.7064, 1269.6498, 1270.6575,
1277.6305, 1286.6099, 1293.6401, 1303.6455, 1304.6677, 1307.6788, 1309.6780, 1318.6750, 1319.6589, 1328.6600, 1328.6620, 1331.7888,
1333.7644, 1336.0798, 1336.6155, 1350.6853, 1351.7202, 1356.5811, 1359.1302, 1359.6246, 1365.6489, 1374.7588, 1383.7037, 1390.6974,
1393.7992, 1400.6683, 1401.6989, 1414.8191, 1429.3142, 1436.7889, 1444.7122, 1445.7839, 1450.7223, 1456.7717, 1462.6630, 1467.7329,
1470.7394, 1471.2693, 1471.7250, 1472.2531, 1474.5243, 1475.7633, 1483.7141, 1488.5333, 1488.7194, 1493.7366, 1502.5601, 1514.6849,
1516.6807, 1517.6437, 1518.7053, 1523.7611, 1526.7343, 1528.6973, 1530.6893, 1536.8230, 1540.7114, 1551.7063, 1553.7252, 1554.7474,
1557.6992, 1558.7140, 1563.6658, 1564.3619, 1565.7223, 1566.7162, 1572.7062, 1580.8400, 1580.8423, 1586.8090, 1597.7913, 1599.8337,
1601.8030, 1614.8046, 1616.7968, 1628.7136, 1630.8103, 1635.7697, 1642.7682, 1643.7688, 1644.7915, 1646.8154, 1651.7681, 1657.7932,
1663.8466, 1664.8557, 1666.7545, 1695.8208, 1707.8933, 1718.8790, 1721.9192, 1722.9375, 1728.8976, 1730.9116, 1732.2391, 1734.1844,
1739.7894, 1743.8966, 1755.2666, 1755.7703, 1757.4702, 1757.9089, 1758.4524, 1758.8987, 1761.9023, 1765.7578, 1768.8339, 1769.8287,
1770.3949, 1772.8273, 1773.8792, 1774.9180, 1774.9200, 1786.8268, 1787.8239, 1809.0499, 1810.0303, 1829.8895, 1830.8380, 1833.3470,
1833.8501, 1851.8885, 1852.0399, 1862.9515, 1881.7703, 1895.9634, 1906.8195, 1908.8512, 1920.7972, 1932.0050, 1936.9479, 1939.8563,
1940.8103, 1943.8293, 1954.8351, 1956.8574, 1958.8956, 1965.7998, 1967.9565, 1969.9998, 1979.8927, 1981.3531, 1982.8179, 1995.8232,
1997.8312, 2003.8595, 2008.9971, 2011.8464, 2015.9408, 2019.8779, 2025.8735, 2031.9606, 2047.9397, 2062.9578, 2067.0020, 2076.8943,
2081.9827, 2082.9966, 2087.0103, 2088.1108, 2089.1162, 2096.0430, 2097.0200, 2108.9771, 2110.0061, 2139.0991, 2140.0918, 2143.1006,
2144.0718, 2149.8516, 2160.0554, 2174.0378, 2178.0471, 2179.0574, 2188.0547, 2191.0457, 2198.1831, 2199.1499, 2203.0386, 2211.1052,
2223.1582, 2225.1470, 2227.1042, 2228.0725, 2238.1086, 2239.1538, 2243.1243, 2244.0684, 2254.0896, 2255.1482, 2260.0779, 2270.2183,
2272.1736, 2284.1841, 2287.1108, 2307.0549, 2309.0659, 2310.0798, 2310.5271, 2311.0454, 2319.0945, 2337.6050, 2341.5510, 2353.0518,
2354.0767, 2355.0564, 2371.0581, 2383.9592, 2388.0437, 2392.1462, 2404.0471, 2412.0872, 2419.5879, 2420.0864, 2420.5930, 2423.2439,
2426.5667, 2427.1211, 2438.1724, 2455.1553, 2458.1648, 2459.1794, 2478.1587, 2516.1133, 2533.1262, 2542.1650, 2546.1746, 2549.1316,
2550.1252, 2559.1470, 2561.1729, 2562.1765, 2567.1514, 2572.1523, 2574.2678, 2576.1628, 2583.1218, 2587.2722, 2604.1682, 2612.1438,
2653.7173, 2655.1897, 2671.1787, 2687.1833, 2701.2820, 2706.2766, 2712.2104, 2714.2156, 2716.1953, 2717.2725, 2725.2354, 2729.1675,
2739.2351, 2743.2642, 2745.2900, 2754.2197, 2755.2358, 2759.2573, 2769.2219, 2771.2437, 2776.2644, 2785.2388, 2787.2400, 2793.2439,
2801.2378, 2803.2302, 2809.2549, 2815.2153, 2817.2322, 2819.2236, 2830.2788, 2833.2280, 2836.2397, 2862.2927, 2873.2961, 2889.2024,
2894.3218, 2927.2520, 2931.3428, 2955.3005, 2974.4124, 2975.4084, 2990.3921, 3006.3206, 3011.2810, 3020.3557, 3021.2412, 3035.3281,
3053.3447, 3062.2456, 3086.4507, 3091.2622, 3103.3140, 3110.7285, 3113.8591, 3130.3557, 3140.4265, 3144.3657, 3159.4985, 3173.5386,
3174.5486, 3185.4236, 3218.3301, 3259.3625, 3312.3267, 3331.4678, 3347.4761, 3363.4788, 3390.4675, 3431.4570, 3447.4666, 3463.4895,
3485.6985, 3488.6545, 3489.6521, 3493.0195, 3494.5571, 3504.6423, 3509.5613, 3525.6025, 3538.4521, 3553.6230, 3579.5403, 3648.1956,
3655.4729, 3664.7500, 3695.6177, 3712.5720, 3738.5837, 3816.7043, 3937.8308, 4010.8696

8. K7DXW4 **Mass:** 10241 **Score:** 18 **Expect:** 3.4e+002 **Matches:** 6

Uncharacterized protein OS=Bos taurus PE=3 SV=1

Observed	Mr(expt)	Mr(calc)	ppm	Start	End	Miss	Ions	Peptide
1128.5724	1127.5651	1127.5611	3.56	2 -	10	1	---	M.GKITFYEDR.G
1488.7194	1487.7121	1487.7157	-2.42	4 -	15	1	---	K.ITFYEDRGFQGR.R
1644.7915	1643.7842	1643.8168	-19.84	4 -	16	2	---	K.ITFYEDRGFQGRR.Y
2776.2644	2775.2571	2775.2242	11.9	16 -	37	2	---	R.RYECSSDHSNLQPYFSRCNSIR.V
2819.2236	2818.2163	2818.2744	-20.61	38 -	59	0	---	R.VDSGCWMLYERPNYQGHQYFLR.R
2975.4084	2974.4011	2974.3755	8.61	38 -	60	1	---	R.VDSGCWMLYERPNYQGHQYFLRR.G

No match to: 716.4763, 723.4098, 724.2417, 725.2533, 726.3622, 728.3046, 729.4749, 734.4570, 736.3904, 737.4152, 738.3791,
744.8651, 745.3838, 747.4550, 768.3673, 788.4571, 801.4262, 805.4606, 813.4481, 814.4558, 820.4053, 823.4778, 829.4589, 830.4522,
839.3830, 842.5110, 857.4382, 860.4207, 865.4518, 870.4165, 872.5054, 880.4424, 882.4292, 887.4245, 887.9669, 888.4561, 891.4898,
894.4144, 894.8973, 897.5006, 898.4523, 912.5132, 913.5293, 925.4620, 927.4981, 931.4992, 934.5098, 957.5774, 960.5294, 961.4949,
963.4734, 965.4692, 970.5181, 972.4899, 975.5167, 976.5063, 977.5212, 978.5100, 978.5146, 983.6283, 992.4958, 993.5046, 995.4181,
998.5911, 1000.5959, 1002.5007, 1006.5014, 1011.5474, 1012.5447, 1014.5485, 1020.5577, 1021.5699, 1024.5516, 1027.5438, 1028.5397,
1030.5978, 1032.5238, 1036.5260, 1037.5405, 1039.5641, 1040.5908, 1042.5388, 1043.5178, 1045.5568, 1046.6018, 1056.5515, 1057.6100,
1057.6107, 1061.6168, 1074.6073, 1074.6100, 1076.6123, 1102.6007, 1103.6257, 1105.6136, 1107.1058, 1113.5486, 1120.5498, 1122.5322,
1123.5986, 1129.5924, 1134.3574, 1137.5463, 1145.6348, 1149.6057, 1153.6460, 1156.4957, 1161.6295, 1162.6174, 1164.1815, 1170.5497,
1172.6156, 1177.6410, 1180.6428, 1184.6412, 1188.5603, 1193.6041, 1206.5107, 1209.5889, 1211.6099, 1212.6160, 1215.6743, 1216.7081,
1219.6886, 1227.6234, 1229.7098, 1234.6414, 1240.5974, 1242.6003, 1243.6161, 1253.6216, 1255.5972, 1255.6000, 1260.6227, 1264.7130,
1265.7064, 1269.6498, 1270.6575, 1273.6556, 1277.6305, 1286.6099, 1289.6875, 1293.6401, 1303.6455, 1304.6677, 1307.6788, 1309.6780,
1318.6750, 1319.6589, 1328.6600, 1328.6620, 1331.7888, 1333.7644, 1336.0798, 1336.6155, 1350.6853, 1351.7202, 1356.5811, 1359.1302,
1359.6246, 1365.6489, 1374.7588, 1383.7037, 1390.6974, 1393.7992, 1400.6683, 1401.6989, 1414.8191, 1429.3142, 1436.7889, 1444.7122,
1445.7839, 1450.7223, 1456.7717, 1462.6630, 1467.7329, 1470.7394, 1471.2693, 1471.7250, 1472.2531, 1474.5243, 1475.7633, 1483.7141,
1488.5333, 1493.7366, 1502.5601, 1513.7441, 1514.6849, 1516.6807, 1517.6437, 1518.7053, 1523.7611, 1526.7343, 1528.6973, 1530.6893,
1536.8230, 1540.7114, 1551.7063, 1553.7252, 1554.7474, 1557.6992, 1558.7140, 1563.6658, 1564.3619, 1565.7223, 1566.7162, 1572.7062,
1580.8400, 1580.8423, 1586.8090, 1597.7913, 1599.8337, 1601.8030, 1614.8046, 1616.7968, 1628.7136, 1630.8103, 1635.7697, 1642.7682,
1643.7688, 1646.8154, 1651.7681, 1657.7932, 1663.8466, 1664.8557, 1666.7545, 1695.8208, 1707.8933, 1718.8790, 1721.9192, 1722.9375,
1728.8976, 1730.9116, 1732.2391, 1734.1844, 1739.7894, 1743.8966, 1755.2666, 1755.7703, 1757.4702, 1757.9089, 1758.4524, 1758.8987,
1761.9023, 1765.7578, 1768.8339, 1769.8287, 1770.3949, 1772.8273, 1773.8792, 1774.9180, 1774.9200, 1786.8268, 1787.8239, 1809.0499,
1810.0303, 1829.8895, 1830.8380, 1833.3470, 1833.8501, 1851.8885, 1852.0399, 1862.9515, 1881.7703, 1895.9634, 1906.8195, 1908.8512,
1920.7972, 1932.0050, 1936.9479, 1939.8563, 1940.8103, 1943.8293, 1954.8351, 1956.8574, 1958.8956, 1965.7998, 1967.9565, 1969.9998,
1979.8927, 1981.3531, 1982.8179, 1995.8232, 1997.8312, 2003.8595, 2008.9971, 2011.8464, 2015.9408, 2019.8779, 2025.8735, 2031.9606,
2047.9397, 2062.9578, 2067.0020, 2076.8943, 2081.9827, 2082.9966, 2087.0103, 2088.1108, 2089.1162, 2096.0430, 2097.0200, 2108.9771,
2110.0061, 2139.0991, 2140.0918, 2143.1006, 2144.0718, 2149.8516, 2160.0554, 2174.0378, 2178.0471, 2179.0574, 2188.0547, 2191.0457,
2198.1831, 2199.1499, 2203.0386, 2211.1052, 2223.1582, 2225.1470, 2227.1042, 2228.0725, 2238.1086, 2239.1538, 2243.1243, 2244.0684,
2254.0896, 2255.1482, 2260.0779, 2270.2183, 2272.1736, 2284.1841, 2287.1108, 2307.0549, 2309.0659, 2310.0798, 2310.5271, 2311.0454,
2319.0945, 2337.6050, 2341.5510, 2353.0518, 2354.0767, 2355.0564, 2371.0581, 2383.9592, 2388.0437, 2392.1462, 2404.0471, 2412.0872,
2419.5879, 2420.0864, 2420.5930, 2423.2439, 2426.5667, 2427.1211, 2438.1724, 2455.1553, 2458.1648, 2459.1794, 2478.1587, 2516.1133,
2533.1262, 2542.1650, 2546.1746, 2549.1316, 2550.1252, 2559.1470, 2561.1729, 2562.1765, 2567.1514, 2572.1523, 2574.2678, 2576.1628,
2583.1218, 2587.2722, 2604.1682, 2612.1438, 2653.7173, 2655.1897, 2671.1787, 2687.1833, 2701.2820, 2706.2766, 2712.2104, 2714.2156,
2716.1953, 2717.2725, 2725.2354, 2729.1675, 2739.2351, 2743.2642, 2745.2900, 2754.2197, 2755.2358, 2759.2573, 2769.2219, 2771.2437,
2785.2388, 2786.2617, 2787.2400, 2793.2439, 2801.2378, 2803.2302, 2809.2549, 2815.2153, 2817.2322, 2830.2788, 2833.2280, 2836.2397,
2862.2927, 2873.2961, 2889.2024, 2894.3218, 2927.2520, 2931.3428, 2955.3005, 2974.4124, 2990.3921, 3006.3206, 3011.2810, 3020.3557,
3021.2412, 3035.3281, 3053.3447, 3062.2456, 3086.4507, 3091.2622, 3103.3140, 3110.7285, 3113.8591, 3130.3557, 3140.4265, 3144.3657,
3159.4985, 3173.5386, 3174.5486, 3185.4236, 3218.3301, 3259.3625, 3312.3267, 3331.4678, 3347.4761, 3363.4788, 3390.4675, 3431.4570,
3447.4666, 3463.4895, 3485.6985, 3488.6545, 3489.6521, 3493.0195, 3494.5571, 3504.6423, 3509.5613, 3525.6025, 3538.4521, 3553.6230,
3579.5403, 3648.1956, 3655.4729, 3664.7500, 3695.6177, 3712.5720, 3738.5837, 3816.7043, 3937.8308, 4010.8696

9. Q2NKR1 **Mass:** 10554 **Score:** 18 **Expect:** 3.6e+002 **Matches:** 6

Mitochondrial import inner membrane translocase subunit Tim10 OS=Bos taurus GN=TIMM10 PE=3 SV=1

Observed	Mr(expt)	Mr(calc)	ppm	Start	End	Miss	Ions	Peptide
1028.5397	1027.5324	1027.5273	4.97	32 -	39	1	---	R.KCVPPHYK.E
1277.6305	1276.6232	1276.6234	-0.12	58 -	67	1	---	K.YLDIHERMGK.K + Oxidation (M)
1566.7162	1565.7089	1565.7317	-14.52	69 -	81	0	---	K.LTELSMQDEELMK.R
2067.0020	2065.9947	2065.9772	8.47	40 -	57	2	---	K.EAELSKGESVCLDRCVSK.Y
2873.2961	2872.2888	2872.2863	0.87	1 -	24	1	---	-.MDPLRAQQLAAELEVEMMADMYNR.M + 3 Oxidation (M)
3553.6230	3552.6157	3552.6040	3.31	2 -	31	2	---	M.DPLRAQQLAAELEVEMMADMYNRMTSACHR.K + Oxidation (M)

No match to: 716.4763, 723.4098, 724.2417, 725.2533, 726.3622, 728.3046, 729.4749, 734.4570, 736.3904, 737.4152, 738.3791,
744.8651, 745.3838, 747.4550, 768.3673, 788.4571, 801.4262, 805.4606, 813.4481, 814.4558, 820.4053, 823.4778, 829.4589, 830.4522,
839.3830, 842.5110, 857.4382, 860.4207, 865.4518, 870.4165, 872.5054, 880.4424, 882.4292, 887.4245, 887.9669, 888.4561, 891.4898,
894.4144, 894.8973, 897.5006, 898.4523, 912.5132, 913.5293, 925.4620, 927.4981, 931.4992, 934.5098, 957.5774, 960.5294, 961.4949,
963.4734, 965.4692, 970.5181, 972.4899, 975.5167, 976.5063, 977.5212, 978.5100, 978.5146, 983.6283, 992.4958, 993.5046, 995.4181,
998.5911, 1000.5959, 1002.5007, 1006.5014, 1011.5474, 1012.5447, 1014.5485, 1020.5577, 1021.5699, 1024.5516, 1027.5438, 1030.5978,
1032.5238, 1036.5260, 1037.5405, 1039.5641, 1040.5908, 1042.5388, 1043.5178, 1045.5568, 1046.6018, 1056.5515, 1057.6100, 1057.6107,

1061.6168, 1074.6073, 1074.6100, 1076.6123, 1102.6007, 1103.6257, 1105.6136, 1107.1058, 1113.5486, 1120.5498, 1122.5322, 1123.5986, 1128.5724, 1129.5924, 1134.3574, 1137.5463, 1145.6348, 1149.6057, 1153.6460, 1156.4957, 1161.6295, 1162.6174, 1164.1815, 1170.5497, 1172.6156, 1177.6410, 1180.6428, 1184.6412, 1188.5603, 1193.6041, 1206.5107, 1209.5889, 1211.6099, 1212.6160, 1215.6743, 1216.7081, 1219.6886, 1227.6234, 1229.7098, 1234.6414, 1240.5974, 1242.6003, 1243.6161, 1253.6216, 1255.5972, 1255.6000, 1260.6227, 1264.7130, 1265.7064, 1269.6498, 1270.6575, 1273.6556, 1286.6099, 1289.6875, 1293.6401, 1303.6455, 1304.6677, 1307.6788, 1309.6780, 1318.6750, 1319.6589, 1328.6600, 1328.6620, 1331.7888, 1333.7644, 1336.0798, 1336.6155, 1350.6853, 1351.7202, 1356.5811, 1359.1302, 1359.6246, 1365.6489, 1374.7588, 1383.7037, 1390.6974, 1393.7992, 1400.6683, 1401.6989, 1414.8191, 1429.3142, 1436.7889, 1444.7122, 1445.7839, 1450.7223, 1456.7717, 1462.6630, 1467.7329, 1470.7394, 1471.2693, 1471.7250, 1472.2531, 1474.5243, 1475.7633, 1483.7141, 1488.5333, 1488.7194, 1493.7366, 1502.5601, 1513.7441, 1514.6849, 1516.6807, 1517.6437, 1518.7053, 1523.7611, 1526.7343, 1528.6973, 1530.6893, 1536.8230, 1540.7114, 1551.7063, 1553.7252, 1554.7474, 1557.6992, 1558.7140, 1563.6658, 1564.3619, 1565.7223, 1572.7062, 1580.8400, 1580.8423, 1586.8090, 1597.7913, 1599.8337, 1601.8030, 1614.8046, 1616.7968, 1628.7136, 1630.8103, 1635.7697, 1642.7682, 1643.7688, 1644.7915, 1646.8154, 1651.7681, 1657.7932, 1663.8466, 1664.8557, 1666.7545, 1695.8208, 1707.8933, 1718.8790, 1721.9192, 1722.9375, 1728.8976, 1730.9116, 1732.2391, 1734.1844, 1739.7894, 1743.8966, 1755.2666, 1755.7703, 1757.4702, 1757.9089, 1758.4524, 1758.8987, 1761.9023, 1765.7578, 1768.8339, 1769.8287, 1770.3949, 1772.8273, 1773.8792, 1774.9180, 1774.9200, 1786.8268, 1787.8239, 1809.0499, 1810.0303, 1829.8895, 1830.8380, 1833.3470, 1833.8501, 1851.8885, 1852.0399, 1862.9515, 1881.7703, 1895.9634, 1906.8195, 1908.8512, 1920.7972, 1932.0050, 1936.9479, 1939.8563, 1940.8103, 1943.8293, 1954.8351, 1956.8574, 1958.8956, 1965.7998, 1967.9565, 1969.9998, 1979.8927, 1981.3531, 1982.8179, 1995.8232, 1997.8312, 2003.8595, 2008.9971, 2011.8464, 2015.9408, 2019.8779, 2025.8735, 2031.9606, 2047.9397, 2062.9578, 2076.8943, 2081.9827, 2082.9966, 2087.0103, 2088.1108, 2089.1162, 2096.0430, 2097.0200, 2108.9771, 2110.0061, 2139.0991, 2140.0918, 2143.1006, 2144.0718, 2149.8516, 2160.0554, 2174.0378, 2178.0471, 2179.0574, 2188.0547, 2191.0457, 2198.1831, 2199.1499, 2203.0386, 2211.1052, 2223.1582, 2225.1470, 2227.1042, 2228.0725, 2238.1086, 2239.1538, 2243.1243, 2244.0684, 2254.0896, 2255.1482, 2260.0779, 2270.2183, 2272.1736, 2284.1841, 2287.1108, 2307.0549, 2309.0659, 2310.0798, 2310.5271, 2311.0454, 2319.0945, 2337.6050, 2341.5510, 2353.0518, 2354.0767, 2355.0564, 2371.0581, 2383.9592, 2388.0437, 2392.1462, 2404.0471, 2412.0872, 2419.5879, 2420.0864, 2420.5930, 2423.2439, 2426.5667, 2427.1211, 2438.1724, 2455.1553, 2458.1648, 2459.1794, 2478.1587, 2516.1133, 2533.1262, 2542.1650, 2546.1746, 2549.1316, 2550.1252, 2559.1470, 2561.1729, 2562.1765, 2567.1514, 2572.1523, 2574.2678, 2576.1628, 2583.1218, 2587.2722, 2604.1682, 2612.1438, 2653.7173, 2655.1897, 2671.1787, 2687.1833, 2701.2820, 2706.2766, 2712.2104, 2714.2156, 2716.1953, 2717.2725, 2725.2354, 2729.1675, 2739.2351, 2743.2642, 2745.2900, 2754.2197, 2755.2358, 2759.2573, 2769.2219, 2771.2437, 2776.2644, 2785.2388, 2786.2617, 2787.2400, 2793.2439, 2801.2378, 2803.2302, 2809.2549, 2815.2153, 2817.2322, 2819.2236, 2830.2788, 2833.2280, 2836.2397, 2862.2927, 2889.2024, 2894.3218, 2927.2520, 2931.3428, 2955.3005, 2974.4124, 2975.4084, 2990.3921, 3006.3206, 3011.2810, 3020.3557, 3021.2412, 3035.3281, 3053.3447, 3062.2456, 3086.4507, 3091.2622, 3103.3140, 3110.7285, 3113.8591, 3130.3557, 3140.4265, 3144.3657, 3159.4985, 3173.5386, 3174.5486, 3185.4236, 3218.3301, 3259.3625, 3312.3267, 3331.4678, 3347.4761, 3363.4788, 3390.4675, 3431.4570, 3447.4666, 3463.4895, 3485.6985, 3488.6545, 3489.6521, 3493.0195, 3494.5571, 3504.6423, 3509.5613, 3525.6025, 3538.4521, 3579.5403, 3648.1956, 3655.4729, 3664.7500, 3695.6177, 3712.5720, 3738.5837, 3816.7043, 3937.8308, 4010.8696

10. F1MIS2 **Mass:** 9764 **Score:** 18 **Expect:** 3.8e+002 **Matches:** 6

Uncharacterized protein OS=Bos taurus PE=3 SV=2

Observed	Mr(expt)	Mr(calc)	ppm	Start		End	Miss	Ions	Peptide
801.4262	800.4189	800.4028	20.0	78	-	85	0	---	R.VQQSSGPV.-
993.5046	992.4973	992.4862	11.2	20	-	27	1	---	R.MTSAYHRK.C
2096.0430	2095.0357	2095.0051	14.6	1	-	19	2	---	-.TQGLAGRRAGVEMADMYNR.M
2307.0549	2306.0476	2305.9990	21.1	8	-	26	2	---	R.RAGVEMADMYNRMTSAYHR.K + 3 Oxidation (M)
2572.1523	2571.1450	2571.1444	0.26	41	-	62	1	---	K.GESVCLDPYASKYLDIHECMGK.K
2716.1953	2715.1880	2715.2342	-17.02	41	-	63	2	---	K.GESVCLDPYASKYLDIHECMGKK.L + Oxidation (M)

No match to: 716.4763, 723.4098, 724.2417, 725.2533, 726.3622, 728.3046, 729.4749, 734.4570, 736.3904, 737.4152, 738.3791, 744.8651, 745.3838, 747.4550, 768.3673, 788.4571, 805.4606, 813.4481, 814.4558, 820.4053, 823.4778, 829.4589, 830.4522, 839.3830, 842.5110, 857.4382, 860.4207, 865.4518, 870.4165, 872.5054, 880.4424, 882.4292, 887.4245, 887.9669, 888.4561, 891.4898, 894.4144, 894.8973, 897.5006, 898.4523, 912.5132, 913.5293, 925.4620, 927.4981, 931.4992, 934.5098, 957.5774, 960.5294, 961.4949, 963.4734, 965.4692, 970.5181, 972.4899, 975.5167, 976.5063, 977.5212, 978.5100, 978.5146, 983.6283, 992.4958, 995.4181, 998.5911, 1000.5959, 1002.5007, 1006.5014, 1011.5474, 1012.5447, 1014.5485, 1020.5577, 1021.5699, 1024.5516, 1027.5438, 1028.5337, 1030.5978, 1032.5238, 1036.5260, 1037.5405, 1039.5641, 1040.5908, 1042.5388, 1043.5178, 1045.5568, 1046.6018, 1056.5515, 1057.6100, 1057.6107, 1061.6168, 1074.6073, 1074.6100, 1076.6123, 1102.6007, 1103.6257, 1105.6136, 1107.1058, 1113.5486, 1120.5498, 1122.5322, 1123.5986, 1128.5724, 1129.5924, 1134.3574, 1137.5463, 1145.6348, 1149.6057, 1153.6460, 1156.4957, 1161.6295, 1162.6174, 1164.1815, 1170.5497, 1172.6156, 1177.6410, 1180.6428, 1184.6412, 1188.5603, 1193.6041, 1206.5107, 1209.5889, 1211.6099, 1212.6160, 1215.6743, 1216.7081, 1219.6886, 1227.6234, 1229.7098, 1234.6414, 1240.5974, 1242.6003, 1243.6161, 1253.6216, 1255.5972, 1255.6000, 1260.6227, 1264.7130, 1265.7064, 1269.6498, 1270.6575, 1273.6556, 1277.6305, 1286.6099, 1289.6875, 1293.6401, 1303.6455, 1304.6677, 1307.6788, 1309.6780, 1318.6750, 1319.6589, 1328.6600, 1328.6620, 1331.7888, 1333.7644, 1336.0798, 1336.6155, 1350.6853, 1351.7202, 1356.5811, 1359.1302, 1359.6246, 1365.6489, 1374.7588, 1383.7037, 1390.6974, 1393.7992, 1400.6683, 1401.6989, 1414.8191, 1429.3142, 1436.7889, 1444.7122, 1445.7839, 1450.7223, 1456.7717, 1462.6630, 1467.7329, 1470.7394, 1471.2693, 1471.7250, 1472.2531, 1474.5243, 1475.7633, 1483.7141, 1488.5333, 1488.7194, 1493.7366, 1502.5601, 1513.7441, 1514.6849, 1516.6807, 1517.6437, 1518.7053, 1523.7611, 1526.7343, 1528.6973, 1530.6893, 1536.8230, 1540.7114, 1551.7063, 1553.7252, 1554.7474, 1557.6992, 1558.7140, 1563.6658, 1564.3619, 1565.7223, 1566.7162, 1572.7062, 1580.8400, 1580.8423, 1586.8090, 1597.7913, 1599.8337, 1601.8030, 1614.8046, 1616.7968, 1628.7136, 1630.8103, 1635.7697, 1642.7682, 1643.7688, 1644.7915, 1646.8154, 1651.7681, 1657.7932, 1663.8466, 1664.8557, 1666.7545, 1695.8208, 1707.8933, 1718.8790, 1721.9192, 1722.9375, 1728.8976, 1730.9116, 1732.2391, 1734.1844, 1739.7894, 1743.8966, 1755.2666, 1755.7703, 1757.4702, 1757.9089, 1758.4524, 1758.8987, 1761.9023, 1765.7578, 1768.8339, 1769.8287, 1770.3949, 1772.8273, 1773.8792, 1774.9180, 1774.9200, 1786.8268, 1787.8239, 1809.0499, 1810.0303, 1829.8895, 1830.8380, 1833.3470, 1833.8501, 1851.8885, 1852.0399, 1862.9515, 1881.7703, 1895.9634, 1906.8195, 1908.8512, 1920.7972, 1932.0050, 1936.9479, 1939.8563, 1940.8103, 1943.8293, 1954.8351, 1956.8574, 1958.8956, 1965.7998, 1967.9565, 1969.9998, 1979.8927, 1981.3531, 1982.8179, 1995.8232, 1997.8312, 2003.8595, 2008.9971, 2011.8464, 2015.9408, 2019.8779, 2025.8735, 2031.9606, 2047.9397, 2062.9578, 2067.0020, 2076.8943, 2081.9827, 2082.9966, 2087.0103, 2088.1108, 2089.1162, 2097.0200, 2108.9771, 2110.0061, 2139.0991, 2140.0918, 2143.1006, 2144.0718, 2149.8516, 2160.0554, 2174.0378, 2178.0471, 2179.0574, 2188.0547, 2191.0457, 2198.1831, 2199.1499, 2203.0386, 2211.1052, 2223.1582, 2225.1470, 2227.1042, 2228.0725, 2238.1086, 2239.1538, 2243.1243, 2244.0684, 2254.0896, 2255.1482, 2260.0779, 2270.2183, 2272.1736, 2284.1841, 2287.1108, 2309.0659, 2310.0798, 2310.5271, 2311.0454, 2319.0945, 2337.6050, 2341.5510, 2353.0518, 2354.0767, 2355.0564, 2371.0581, 2383.9592, 2388.0437, 2392.1462, 2404.0471, 2412.0872, 2419.5879, 2420.0864, 2420.5930, 2423.2439, 2426.5667, 2427.1211, 2438.1724, 2455.1553, 2458.1648, 2459.1794, 2478.1587, 2516.1133, 2533.1262, 2542.1650, 2546.1746, 2549.1316, 2550.1252, 2559.1470, 2561.1729, 2562.1765, 2567.1514, 2574.2678, 2576.1628, 2583.1218, 2587.2722, 2604.1682, 2612.1438, 2653.7173, 2655.1897, 2671.1787, 2687.1833, 2701.2820, 2706.2766, 2712.2104, 2714.2156, 2717.2725, 2725.2354, 2729.1675, 2739.2351, 2743.2642, 2745.2900, 2754.2197, 2755.2358, 2759.2573, 2769.2219, 2771.2437, 2776.2644, 2785.2388, 2786.2617, 2787.2400, 2793.2439, 2801.2378, 2803.2302, 2809.2549, 2815.2153, 2817.2322, 2819.2236, 2830.2788, 2833.2280, 2836.2397, 2862.2927, 2873.2961, 2889.2024, 2894.3218, 2927.2520, 2931.3428, 2955.3005, 2974.4124, 2975.4084, 2990.3921, 3006.3206, 3011.2810, 3020.3557, 3021.2412, 3035.3281, 3053.3447, 3062.2456, 3086.4507, 3091.2622, 3103.3140, 3110.7285, 3113.8591, 3130.3557, 3140.4265, 3144.3657, 3159.4985, 3173.5386, 3174.5486, 3185.4236, 3218.3301, 3259.3625, 3312.3267, 3331.4678, 3347.4761, 3363.4788, 3390.4675, 3431.4570, 3447.4666, 3463.4895, 3485.6985, 3488.6545, 3489.6521, 3493.0195, 3494.5571, 3504.6423, 3509.5613, 3525.6025, 3538.4521, 3553.6230, 3579.5403, 3648.1956, 3655.4729, 3664.7500, 3695.6177, 3712.5720, 3738.5837, 3816.7043, 3937.8308, 4010.8696

11. E1BC56 **Mass:** 10970 **Score:** 18 **Expect:** 4.2e+002 **Matches:** 7

Uncharacterized protein OS=Bos taurus GN=LOC785479 PE=4 SV=1

Observed	Mr(expt)	Mr(calc)	ppm	Start		End	Miss	Ions	Peptide
814.4558	813.4485	813.4457	3.53	15	-	20	2	---	K.EEPKRR.S
993.5046	992.4973	992.5138	-16.64	5	-	14	1	---	R.KVSSTEGSAK.E
1020.5577	1019.5504	1019.5611	-10.48	46	-	54	2	---	K.SSDKKVQTK.G
1149.6057	1148.5984	1148.6149	-14.37	4	-	14	2	---	K.RKVSSTEGSAK.E
1162.6174	1161.6101	1161.6063	3.27	33	-	43	1	---	K.VEIEAKMAAGK.V + Oxidation (M)
1852.0399	1851.0326	1851.0577	-13.57	21	-	38	2	---	R.SGRLSAKPAPAKVEIEAK.M
2931.3428	2930.3355	2930.2900	15.5	73	-	99	2	---	K.EDLPAENGETKNEESPASDEAENKEAK.S

No match to: 716.4763, 723.4098, 724.2417, 725.2533, 726.3622, 728.3046, 729.4749, 734.4570, 736.3904, 737.4152, 738.3791, 744.8651, 745.3838, 747.4550, 768.3673, 788.4571, 801.4262, 805.4606, 813.4481, 820.4053, 823.4778, 829.4589, 830.4522, 839.3830, 842.5110, 857.4382, 860.4207, 865.4518, 870.4165, 872.5054, 880.4424, 882.4292, 887.4245, 887.9669, 888.4561, 891.4898, 894.4144, 894.8973, 897.5006, 898.4523, 912.5132, 913.5293, 925.4620, 927.4981, 931.4992, 934.5098, 957.5774, 960.5294, 961.4949, 963.4734,

965.4692, 970.5181, 972.4899, 975.5167, 976.5063, 977.5212, 978.5100, 978.5146, 983.6283, 992.4958, 995.4181, 998.5911, 1000.5959, 1002.5007, 1006.5014, 1011.5474, 1012.5447, 1014.5485, 1021.5699, 1024.5516, 1027.5438, 1028.5397, 1030.5978, 1032.5238, 1036.5260, 1037.5405, 1039.5641, 1040.5908, 1042.5388, 1043.5178, 1045.5568, 1046.6018, 1056.5515, 1057.6100, 1057.6107, 1061.6168, 1074.6073, 1074.6100, 1076.6123, 1102.6007, 1103.6257, 1105.6136, 1107.1058, 1113.5486, 1120.5498, 1122.5322, 1123.5986, 1128.5724, 1129.5924, 1134.3574, 1137.5463, 1145.6348, 1153.6460, 1156.4957, 1161.6295, 1164.1815, 1170.5497, 1172.6156, 1177.6410, 1180.6428, 1184.6412, 1188.5603, 1193.6041, 1206.5107, 1209.5889, 1211.6099, 1212.6160, 1215.6743, 1216.7081, 1219.6886, 1227.6234, 1229.7098, 1234.6414, 1240.5974, 1242.6003, 1243.6161, 1253.6216, 1255.5972, 1255.6000, 1260.6227, 1264.7130, 1265.7064, 1269.6498, 1270.6575, 1273.6556, 1277.6305, 1286.6099, 1289.6875, 1293.6401, 1303.6455, 1304.6677, 1307.6788, 1309.6780, 1318.6750, 1319.6589, 1328.6600, 1328.6620, 1331.7888, 1333.7644, 1336.0798, 1336.6155, 1350.6853, 1351.7202, 1356.5811, 1359.1302, 1359.6246, 1365.6489, 1374.7588, 1383.7037, 1390.6974, 1393.7992, 1400.6683, 1401.6989, 1414.8191, 1429.3142, 1436.7889, 1444.7122, 1445.7839, 1450.7223, 1456.7717, 1462.6630, 1467.7329, 1470.7394, 1471.2693, 1471.7250, 1472.2531, 1474.5243, 1475.7633, 1483.7141, 1488.5333, 1488.7194, 1493.7366, 1502.5601, 1513.7441, 1514.6849, 1516.6807, 1517.6437, 1518.7053, 1523.7611, 1526.7343, 1528.6973, 1530.6893, 1536.8230, 1540.7114, 1551.7063, 1553.7252, 1554.7474, 1557.6992, 1558.7140, 1563.6658, 1564.3619, 1565.7223, 1566.7162, 1572.7062, 1580.8400, 1580.8423, 1586.8090, 1597.7913, 1599.8337, 1601.8030, 1614.8046, 1616.7968, 1628.7136, 1630.8103, 1635.7697, 1642.7682, 1643.7688, 1644.7915, 1646.8154, 1651.7681, 1657.7932, 1663.8466, 1664.8557, 1666.7545, 1695.8208, 1707.8933, 1718.8790, 1721.9192, 1722.9375, 1728.8976, 1730.9116, 1732.2391, 1734.1844, 1739.7894, 1743.8966, 1755.2666, 1755.7703, 1757.4702, 1757.9089, 1758.4524, 1758.8987, 1761.9023, 1765.7578, 1768.8339, 1769.8287, 1770.3949, 1772.8273, 1773.8792, 1774.9180, 1774.9200, 1786.8268, 1787.8239, 1809.0499, 1810.0303, 1829.8895, 1830.8380, 1833.3470, 1833.8501, 1851.8885, 1862.9515, 1881.7703, 1895.9634, 1906.8195, 1908.8512, 1920.7972, 1932.0050, 1936.9479, 1939.8563, 1940.8103, 1943.8293, 1954.8351, 1956.8574, 1958.8956, 1965.7998, 1967.9565, 1969.9998, 1979.8927, 1981.3531, 1982.8179, 1995.8232, 1997.8312, 2003.8595, 2008.9971, 2011.8464, 2015.9408, 2019.8779, 2025.8735, 2031.9606, 2047.9397, 2062.9578, 2067.0020, 2076.8943, 2081.9827, 2082.9966, 2087.0103, 2088.1108, 2089.1162, 2096.0430, 2097.0200, 2108.9771, 2110.0061, 2139.0991, 2140.0918, 2143.1006, 2144.0718, 2149.8516, 2160.0554, 2174.0378, 2178.0471, 2179.0574, 2188.0547, 2191.0457, 2198.1831, 2199.1499, 2203.0386, 2211.1052, 2223.1582, 2225.1470, 2227.1042, 2228.0725, 2238.1086, 2239.1538, 2243.1243, 2244.0684, 2254.0896, 2255.1482, 2260.0779, 2270.2183, 2272.1736, 2284.1841, 2287.1108, 2307.0549, 2309.0659, 2310.0798, 2310.5271, 2311.0454, 2319.0945, 2337.6050, 2341.5510, 2353.0518, 2354.0767, 2355.0564, 2371.0581, 2383.9592, 2388.0437, 2392.1462, 2404.0471, 2412.0872, 2419.5879, 2420.0864, 2420.5930, 2423.2439, 2426.5667, 2427.1211, 2438.1724, 2455.1553, 2458.1648, 2459.1794, 2478.1587, 2516.1133, 2533.1262, 2542.1650, 2546.1746, 2549.1316, 2550.1252, 2559.1470, 2561.1729, 2562.1765, 2567.1514, 2572.1523, 2574.2678, 2576.1628, 2583.1218, 2587.2722, 2604.1682, 2612.1438, 2653.7173, 2655.1897, 2671.1787, 2687.1833, 2701.2820, 2706.2766, 2712.2104, 2714.2156, 2716.1953, 2717.2725, 2725.2354, 2729.1675, 2739.2351, 2743.2642, 2745.2900, 2754.2197, 2755.2358, 2759.2573, 2769.2219, 2771.2437, 2776.2644, 2785.2388, 2786.2617, 2787.2400, 2793.2439, 2801.2378, 2803.2302, 2809.2549, 2815.2153, 2817.2322, 2819.2236, 2830.2788, 2833.2280, 2836.2397, 2862.2927, 2873.2961, 2889.2024, 2894.3218, 2927.2520, 2955.3005, 2974.4124, 2975.4084, 2990.3921, 3006.3206, 3011.2810, 3020.3557, 3021.2412, 3035.3281, 3053.3447, 3062.2456, 3086.4507, 3091.2622, 3103.3140, 3110.7285, 3113.8591, 3130.3557, 3140.4265, 3144.3657, 3159.4985, 3173.5386, 3174.5486, 3185.4236, 3218.3301, 3259.3625, 3312.3267, 3331.4678, 3347.4761, 3363.4788, 3390.4675, 3431.4570, 3447.4666, 3463.4895, 3485.6985, 3488.6545, 3489.6521, 3493.0195, 3494.5571, 3504.6423, 3509.5613, 3525.6025, 3538.4521, 3553.6230, 3579.5403, 3648.1956, 3655.4729, 3664.7500, 3695.6177, 3712.5720, 3738.5837, 3816.7043, 3937.8308, 4010.8696

12. F1MJH0 **Mass:** 128067 **Score:** 17 **Expect:** 4.4e+002 **Matches:** 41

Uncharacterized protein OS=Bos taurus GN=SPINK5 PE=4 SV=2

Observed	Mr(expt)	Mr(calc)	ppm	Start	End	Miss	Ions	Peptide
716.4763	715.4690	715.4704	-2.04	491 -	496	2	---	R.SVKVKR.E
788.4571	787.4498	787.4552	-6.78	219 -	225	2	---	R.KANEKAK.R
805.4606	804.4534	804.4527	0.76	35 -	41	1	---	R.VLMKNGK.L + Oxidation (M)
830.4522	829.4449	829.4294	18.8	906 -	913	0	---	R.AKPSNDAK.D
872.5054	871.4982	871.4875	12.2	989 -	995	1	---	K.EALDRIR.L
934.5098	933.5026	933.4953	7.72	858 -	865	1	---	R.DGKLICTK.E
960.5294	959.5221	959.5400	-18.63	560 -	567	2	---	K.AETEKVKR.E
961.4949	960.4877	960.4811	6.83	381 -	388	0	---	K.NGQLLCTR.E
975.5167	974.5094	974.4893	20.7	353 -	360	2	---	K.EGESRNKR.Q
977.5212	976.5139	976.5263	-12.67	68 -	75	1	---	K.MILEKEAK.S + Oxidation (M)
1137.5463	1136.5390	1136.5462	-6.29	204 -	212	1	---	K.QQFSEEKNK.A
1170.5497	1169.5424	1169.5200	19.1	591 -	601	0	---	R.ENDPIEGPDGK.I
1188.5603	1187.5530	1187.5418	9.42	730 -	740	1	---	R.ESDPVRDADGK.S
1206.5107	1205.5034	1205.5104	-5.75	808 -	817	1	---	K.THGNKCAMCK.E
1211.6099	1210.6026	1210.5798	18.8	720 -	729	2	---	K.MKDGKLSCTR.E + Oxidation (M)
1242.6003	1241.5930	1241.5719	17.0	671 -	680	1	---	K.CAMCKAVFQK.E
1319.6589	1318.6516	1318.6299	16.4	725 -	735	1	---	K.LSCTRESDPVR.D
1450.7223	1449.7150	1449.7432	-19.43	854 -	865	2	---	R.NMVRDGKLICTK.E + Oxidation (M)
1475.7633	1474.7560	1474.7827	-18.09	307 -	318	2	---	R.QFVRNGKLPCTR.E
1558.7140	1557.7067	1557.7140	-4.68	951 -	962	1	---	R.QHVRNDELMCTR.E
1635.7697	1634.7624	1634.7367	15.7	977 -	988	2	---	K.NKCYMCRSIFEK.E
1643.7688	1642.7615	1642.7551	3.94	63 -	75	2	---	K.CAMCKMILEKEAK.S + 2 Oxidation (M)
1651.7681	1650.7608	1650.7316	17.7	977 -	988	2	---	K.NKCYMCRSIFEK.E + Oxidation (M)
1768.8339	1767.8266	1767.8461	-11.03	227 -	240	2	---	R.DTEKLCIEYQDRAK.N
1773.8792	1772.8719	1772.8587	7.43	906 -	920	2	---	R.AKPSNDAKDQCREVR.N
2081.9827	2080.9754	2080.9960	-9.89	311 -	329	2	---	R.NGKLPCTRENDPVQGPDGK.M
2108.9771	2107.9698	2107.9424	13.0	454 -	472	2	---	R.GPDGKMHGNKCAMCASLFK.L
2143.1006	2142.0933	2142.0462	22.0	185 -	203	2	---	R.GPDGRIHGNKCALCAEIFK.Q
2178.0471	2177.0398	2177.0059	15.6	361 -	377	2	---	R.QTETTTSFEELCREYRK.S
2188.0547	2187.0474	2187.0637	-7.44	436 -	453	2	---	R.NQVRNGMLICTRENDPVR.G + Oxidation (M)
2243.1243	2242.1170	2242.0987	8.18	231 -	248	2	---	K.LCIEYQDRAKNGVLFCTR.E
2353.0518	2352.0445	2352.0847	-17.06	531 -	549	2	---	K.MHVNKCAMCASLFRLEEEK.K
2412.0872	2411.0799	2411.0741	2.40	459 -	478	2	---	K.MHGNKCAMCASLFKLEEEEK.K
2533.1262	2532.1189	2532.0905	11.2	260 -	280	1	---	K.MHGNLCSMCLAFYQAEAEEKK.K + Oxidation (M)
2549.1316	2548.1243	2548.0854	15.3	260 -	280	1	---	K.MHGNLCSMCLAFYQAEAEEKK.K + 2 Oxidation (M)
2567.1514	2566.1441	2566.1614	-6.72	125 -	146	2	---	R.THGNKCAMCAELFLKEAEENAK.R + Oxidation (M)
2587.2722	2586.2649	2586.2609	1.56	643 -	665	2	---	R.SLLQNGNLFCTRENDPVRGPDGK.T
2745.2900	2744.2827	2744.2646	6.60	632 -	654	2	---	R.EAEKDACSEFRSLLQNGNLFCTR.E
2754.2197	2753.2124	2753.2068	2.04	941 -	962	2	---	R.ISADECSNFRQHVRNDELMCTR.E + Oxidation (M)
2862.2927	2861.2854	2861.2643	7.39	568 -	590	2	---	R.EAAQELCSEYRNYMRNGQLPCTR.E + Oxidation (M)
3553.6230	3552.6157	3552.5530	17.7	249 -	279	2	---	R.ENDPVRGPDGKMHGNLCSMCLAFYQAEAEEK.K

No match to: 723.4098, 724.2417, 725.2533, 726.3622, 728.3046, 729.4749, 734.4570, 736.3904, 737.4152, 738.3791, 744.8651, 745.3838, 747.4550, 768.3673, 801.4262, 813.4481, 814.4558, 820.4053, 823.4778, 829.4589, 839.3830, 842.5110, 857.4382, 860.4207, 865.4518, 870.4165, 880.4424, 882.4292, 887.4245, 887.9669, 888.4561, 891.4898, 894.4144, 894.8973, 897.5006, 898.4523, 912.5132, 913.5293, 925.4620, 927.4981, 931.4992, 957.5774, 963.4734, 965.4692, 970.5181, 972.4899, 976.5063, 978.5100, 978.5146, 983.6283, 992.4958, 993.5046, 995.4181, 998.5911, 1000.5959, 1002.5007, 1006.5014, 1011.5474, 1012.5447, 1014.5485, 1020.5577, 1021.5699, 1024.5516, 1027.5438, 1028.5397, 1030.5978, 1032.5238, 1036.5260, 1037.5405, 1039.5641, 1040.5908, 1042.5388, 1043.5178, 1045.5568, 1046.6018, 1056.5515, 1057.6100, 1057.6107, 1061.6168, 1074.6073, 1074.6100, 1076.6123, 1102.6007, 1103.6257, 1105.6136, 1107.1058, 1113.5486, 1120.5498, 1122.5322, 1123.5986, 1128.5724, 1129.5924, 1134.3574, 1145.6348, 1149.6057, 1153.6460, 1156.4957, 1161.6295, 1162.6174, 1164.1815, 1172.6156, 1177.6410, 1180.6428, 1184.6412, 1193.6041, 1209.5889, 1212.6160, 1215.6743, 1216.7081, 1219.6886, 1227.6234, 1229.7098, 1234.6414, 1240.5974, 1243.6161, 1253.6216, 1255.5972, 1255.6000, 1260.6227, 1264.7130, 1265.7064, 1269.6498, 1270.6575, 1273.6556, 1277.6305, 1286.6099, 1289.6875, 1293.6401, 1303.6455, 1304.6677, 1307.6788, 1309.6780, 1318.6750, 1328.6600, 1328.6620, 1331.7888, 1333.7644, 1336.0798, 1336.6155, 1350.6853, 1351.7202, 1356.5811, 1359.1302, 1359.6246, 1365.6489, 1374.7588,

1383.7037, 1390.6974, 1393.7992, 1400.6683, 1401.6989, 1414.8191, 1429.3142, 1436.7889, 1444.7122, 1445.7839, 1456.7717, 1462.6630, 1467.7329, 1470.7394, 1471.2693, 1471.7250, 1472.2531, 1474.5243, 1483.7141, 1488.5333, 1488.7194, 1493.7366, 1502.5601, 1513.7441, 1514.6849, 1516.6807, 1517.6437, 1518.7053, 1523.7611, 1526.7343, 1528.6973, 1530.6893, 1536.8230, 1540.7114, 1551.7063, 1553.7252, 1554.7474, 1557.6992, 1563.6658, 1564.3619, 1565.7223, 1566.7162, 1572.7062, 1580.8400, 1580.8423, 1586.8090, 1597.7913, 1599.8337, 1601.8030, 1614.8046, 1616.7968, 1628.7136, 1630.8103, 1642.7682, 1644.7915, 1646.8154, 1657.7932, 1663.8466, 1664.8557, 1666.7545, 1695.8208, 1707.8933, 1718.8790, 1721.9192, 1722.9375, 1728.8976, 1730.9116, 1732.2391, 1734.1844, 1739.7894, 1743.8966, 1755.2666, 1755.7703, 1757.4702, 1757.9089, 1758.4524, 1758.8987, 1761.9023, 1765.7578, 1769.8287, 1770.3949, 1772.8273, 1774.9180, 1774.9200, 1786.8268, 1787.8239, 1809.0499, 1810.0303, 1829.8895, 1830.8380, 1833.3470, 1833.8501, 1851.8885, 1852.0399, 1862.9515, 1881.7703, 1895.9634, 1906.8195, 1908.8512, 1920.7972, 1932.0050, 1936.9479, 1939.8563, 1940.8103, 1943.8293, 1954.8351, 1956.8574, 1958.8956, 1965.7998, 1967.9565, 1969.9998, 1979.8927, 1981.3531, 1982.8179, 1995.8232, 1997.8312, 2003.8595, 2008.9971, 2011.8464, 2015.9408, 2019.8779, 2025.8735, 2031.9606, 2047.9397, 2062.9578, 2067.0020, 2076.8943, 2082.9966, 2087.0103, 2088.1108, 2089.1162, 2096.0430, 2097.0200, 2110.0061, 2139.0991, 2140.0918, 2144.0718, 2149.8516, 2160.0554, 2174.0378, 2179.0574, 2191.0457, 2198.1831, 2199.1499, 2203.0386, 2211.1052, 2223.1582, 2225.1470, 2227.1042, 2228.0725, 2238.1086, 2239.1538, 2244.0684, 2254.0896, 2255.1482, 2260.0779, 2270.2183, 2272.1736, 2284.1841, 2287.1108, 2307.0549, 2309.0659, 2310.0798, 2310.5271, 2311.0454, 2319.0945, 2337.6050, 2341.5510, 2354.0767, 2355.0564, 2371.0581, 2383.9592, 2388.0437, 2392.1462, 2404.0471, 2419.5879, 2420.0864, 2420.5930, 2423.2439, 2426.5667, 2427.1211, 2438.1724, 2455.1553, 2458.1648, 2459.1794, 2478.1587, 2516.1133, 2542.1650, 2546.1746, 2550.1252, 2559.1470, 2561.1729, 2562.1765, 2572.1523, 2574.2678, 2576.1628, 2583.1218, 2604.1682, 2612.1438, 2653.7173, 2655.1897, 2671.1787, 2687.1833, 2701.2820, 2706.2766, 2712.2104, 2714.2156, 2716.1953, 2717.2725, 2725.2354, 2729.1675, 2739.2351, 2743.2642, 2755.2358, 2759.2573, 2769.2219, 2771.2437, 2776.2644, 2785.2388, 2786.2617, 2787.2400, 2793.2439, 2801.2378, 2803.2302, 2809.2549, 2815.2153, 2817.2322, 2819.2236, 2830.2788, 2833.2280, 2836.2397, 2873.2961, 2889.2024, 2894.3218, 2927.2520, 2931.3428, 2955.3005, 2974.4124, 2975.4084, 2990.3921, 3006.3206, 3011.2810, 3020.3557, 3021.2412, 3035.3281, 3053.3447, 3062.2456, 3086.4507, 3091.2622, 3103.3140, 3110.7285, 3113.8591, 3130.3557, 3140.4265, 3144.3657, 3159.4985, 3173.5386, 3174.5486, 3185.4236, 3218.3301, 3259.3625, 3312.3267, 3331.4678, 3347.4761, 3363.4788, 3390.4675, 3431.4570, 3447.4666, 3463.4895, 3485.6985, 3488.6545, 3489.6521, 3493.0195, 3494.5571, 3504.6423, 3509.5613, 3525.6025, 3538.4521, 3579.5403, 3648.1956, 3655.4729, 3664.7500, 3695.6177, 3712.5720, 3738.5837, 3816.7043, 3937.8308, 4010.8696

13. F1MYI2 **Mass:** 58389 **Score:** 17 **Expect:** 4.5e+002 **Matches:** 20

Uncharacterized protein OS=Bos taurus GN=HNRNPM PE=1 SV=2

Observed	Mr(expt)	Mr(calc)	ppm	Start	End	Miss	Ions	Peptide
745.3838	744.3765	744.3589	23.7	223 -	229	0	---	R.MGPGIDR.I
872.5054	871.4982	871.5127	-16.67	3 -	10	1	---	K.KAAEVLNK.H
975.5167	974.5094	974.4855	24.6	115 -	122	1	---	K.MDERALPK.G + Oxidation (M)
1000.5959	999.5886	999.6076	-19.02	2 -	10	2	---	M.KKAAEVLNK.H
1021.5699	1020.5626	1020.5539	8.58	470 -	477	1	---	R.KACQIFVR.N
1188.5603	1187.5530	1187.5791	-21.95	351 -	362	0	---	R.MVPAGMGAGLER.M
1269.6498	1268.6425	1268.6473	-3.78	209 -	222	0	---	R.INGGGGGSVPGIER.M
1528.6973	1527.6900	1527.6844	3.70	256 -	268	1	---	R.MGLVMDRMGSVER.M + 3 Oxidation (M)
1565.7223	1564.7150	1564.7344	-12.39	492 -	504	0	---	K.FNECGHVLYADIK.M
1566.7162	1565.7089	1565.7290	-12.82	291 -	304	1	---	R.MGQTMERIGSGVER.M + Oxidation (M)
1663.8466	1662.8393	1662.8036	21.5	512 -	526	1	---	K.GCGVVKFESPEVAER.A
1768.8339	1767.8266	1767.8430	-9.25	530 -	544	2	---	R.MMNGMKLSGREIDVR.I + 2 Oxidation (M)
2081.9827	2080.9754	2080.9816	-2.95	385 -	402	2	---	R.MGLERMGANSLERMGLER.M + 2 Oxidation (M)
2178.0471	2177.0398	2177.0432	-1.53	160 -	182	0	---	K.GIGMGNLGPAGMGMEGIGFGINK.M
2211.1052	2210.0979	2210.0428	24.9	256 -	275	2	---	R.MGLVMDRMGSVERMGSGIER.M
2354.0767	2353.0694	2353.0936	-10.29	390 -	410	2	---	R.MGANSLERMGLERMGANSLER.M + 2 Oxidation (M)
2392.1462	2391.1389	2391.0981	17.1	269 -	290	1	---	R.MGSGIERMGPLGLDHMASSIER.M + 3 Oxidation (M)
2706.2766	2705.2693	2705.2506	6.92	238 -	262	2	---	R.MGAGLGHGMDRVGSEIERMGLVMDR.M + 2 Oxidation (M)
2717.2725	2716.2652	2716.3103	-16.60	337 -	362	2	---	R.MGPAIERMGLGMERMVPAGMGAGLER.M
3553.6230	3552.6157	3552.6404	-6.94	411 -	446	2	---	R.MGPAMGPALGAGIERMGLAMGGGGGASFDRAIEMER.G + 2 Oxidation (M)

No match to: 716.4763, 723.4098, 724.2417, 725.2533, 726.3622, 728.3046, 729.4749, 734.4570, 736.3904, 737.4152, 738.3791, 744.8651, 747.4550, 768.3673, 788.4571, 801.4262, 805.4606, 813.4481, 814.4558, 820.4053, 823.4778, 829.4589, 830.4522, 839.3830, 842.5110, 857.4382, 860.4207, 865.4518, 870.4165, 880.4424, 882.4292, 887.4245, 887.9669, 888.4561, 891.4898, 894.4144, 894.8973, 897.5006, 898.4523, 912.5132, 913.5293, 925.4620, 927.4981, 931.4992, 934.5098, 957.5774, 960.5294, 961.4949, 963.4734, 965.4692, 970.5181, 972.4899, 976.5063, 977.5212, 978.5100, 978.5146, 983.6283, 992.4958, 993.5046, 995.4181, 998.5911, 1002.5007, 1006.5014, 1011.5474, 1012.5447, 1014.5485, 1020.5577, 1024.5516, 1027.5438, 1028.5397, 1030.5978, 1032.5238, 1036.5260, 1037.5405, 1039.5641, 1040.5908, 1042.5388, 1043.5178, 1045.5568, 1046.6018, 1056.5515, 1057.6100, 1057.6107, 1061.6168, 1074.6073, 1074.6100, 1076.6123, 1102.6007, 1103.6257, 1105.6136, 1107.1058, 1113.5486, 1120.5498, 1122.5322, 1123.5986, 1128.5724, 1129.5924, 1134.3574, 1137.5463, 1145.6348, 1149.6057, 1153.6460, 1156.4957, 1161.6295, 1162.6174, 1164.1815, 1170.5497, 1172.6156, 1177.6410, 1180.6428, 1184.6412, 1193.6041, 1206.5107, 1209.5889, 1211.6099, 1212.6160, 1215.6743, 1216.7081, 1219.6886, 1227.6234, 1229.7098, 1234.6414, 1240.5974, 1242.6003, 1243.6161, 1253.6216, 1255.5972, 1255.6000, 1260.6227, 1264.7130, 1265.7064, 1270.6575, 1273.6556, 1277.6305, 1286.6099, 1289.6875, 1293.6401, 1303.6455, 1304.6677, 1307.6788, 1309.6780, 1318.6750, 1319.6589, 1328.6600, 1328.6620, 1331.7888, 1333.7644, 1336.0798, 1336.6155, 1350.6853, 1351.7202, 1356.5811, 1359.1302, 1359.6246, 1365.6489, 1374.7588, 1383.7037, 1390.6974, 1393.7992, 1400.6683, 1401.6989, 1414.8191, 1429.3142, 1436.7889, 1444.7122, 1445.7839, 1450.7223, 1456.7717, 1462.6630, 1467.7329, 1470.7394, 1471.2693, 1471.7250, 1472.2531, 1474.5243, 1475.7633, 1483.7141, 1488.5333, 1488.7194, 1493.7366, 1502.5601, 1513.7441, 1514.6849, 1516.6807, 1517.6437, 1518.7053, 1523.7611, 1526.7343, 1530.6893, 1536.8230, 1540.7114, 1551.7063, 1553.7252, 1554.7474, 1557.6992, 1558.7140, 1563.6658, 1564.3619, 1572.7062, 1580.8400, 1580.8423, 1586.8090, 1597.7913, 1599.8337, 1601.8030, 1614.8046, 1616.7968, 1628.7136, 1630.8103, 1635.7697, 1642.7682, 1643.7688, 1644.7915, 1646.8154, 1651.7681, 1657.7932, 1664.8557, 1666.7545, 1695.8208, 1707.8933, 1718.8790, 1721.9192, 1722.9375, 1728.8976, 1730.9116, 1732.2391, 1734.1844, 1739.7894, 1743.8966, 1755.2666, 1755.7703, 1757.4702, 1757.9089, 1758.4524, 1758.8987, 1761.9023, 1765.7578, 1769.8287, 1770.3949, 1772.8273, 1773.8792, 1774.9180, 1774.9200, 1786.8268, 1787.8239, 1809.0499, 1810.0303, 1829.8895, 1830.8380, 1833.3470, 1833.8501, 1851.8885, 1852.0399, 1862.9515, 1881.7703, 1895.9634, 1906.8195, 1908.8512, 1920.7972, 1932.0050, 1936.9479, 1939.8563, 1940.8103, 1943.8293, 1954.8351, 1956.8574, 1958.8956, 1965.7998, 1967.9565, 1969.9998, 1979.8927, 1981.3531, 1982.8179, 1995.8232, 1997.8312, 2003.8595, 2008.9971, 2011.8464, 2015.9408, 2019.8779, 2025.8735, 2031.9606, 2047.9397, 2062.9578, 2067.0020, 2076.8943, 2082.9966, 2087.0103, 2088.1108, 2089.1162, 2096.0430, 2097.0200, 2108.9771, 2110.0061, 2139.0991, 2140.0918, 2143.1006, 2144.0718, 2149.8516, 2160.0554, 2174.0378, 2179.0574, 2188.0547, 2191.0457, 2198.1831, 2199.1499, 2203.0386, 2223.1582, 2225.1470, 2227.1042, 2228.0725, 2238.1086, 2239.1538, 2243.1243, 2244.0684, 2254.0896, 2255.1482, 2260.0779, 2270.2183, 2272.1736, 2284.1841, 2287.1108, 2307.0549, 2309.0659, 2310.0798, 2310.5271, 2311.0454, 2319.0945, 2337.6050, 2341.5510, 2353.0518, 2355.0564, 2371.0581, 2383.9592, 2388.0437, 2404.0471, 2412.0872, 2419.5879, 2420.0864, 2420.5930, 2423.2439, 2426.5667, 2427.1211, 2438.1724, 2455.1553, 2458.1648, 2459.1794, 2478.1587, 2516.1133, 2533.1262, 2542.1650, 2546.1746, 2549.1316, 2550.1252, 2559.1470, 2561.1729, 2562.1765, 2567.1514, 2572.1523, 2574.2678, 2576.1628, 2583.1218, 2587.2722, 2604.1682, 2612.1438, 2653.7173, 2655.1897, 2671.1787, 2687.1833, 2701.2820, 2712.2104, 2714.2156, 2716.1953, 2725.2354, 2729.1675, 2739.2351, 2743.2642, 2745.2900, 2754.2197, 2755.2358, 2759.2573, 2769.2219, 2771.2437, 2776.2644, 2785.2388, 2786.2617, 2787.2400, 2793.2439, 2801.2378, 2803.2302, 2809.2549, 2815.2153, 2817.2322, 2819.2236, 2830.2788, 2833.2280, 2836.2397, 2862.2927, 2873.2961, 2889.2024, 2894.3218, 2927.2520, 2931.3428, 2955.3005, 2974.4124, 2975.4084, 2990.3921, 3006.3206, 3011.2810, 3020.3557, 3021.2412, 3035.3281, 3053.3447, 3062.2456, 3086.4507, 3091.2622, 3103.3140, 3110.7285, 3113.8591, 3130.3557, 3140.4265, 3144.3657, 3159.4985, 3173.5386, 3174.5486, 3185.4236, 3218.3301, 3259.3625, 3312.3267, 3331.4678, 3347.4761, 3363.4788, 3390.4675, 3431.4570, 3447.4666, 3463.4895, 3485.6985, 3488.6545, 3489.6521, 3493.0195, 3494.5571, 3504.6423, 3509.5613, 3525.6025, 3538.4521, 3579.5403, 3648.1956, 3655.4729, 3664.7500, 3695.6177, 3712.5720, 3738.5837, 3816.7043, 3937.8308, 4010.8696

14. P02698 **Mass:** 8709 **Score:** 17 **Expect:** 5.3e+002 **Matches:** 6

Guanine nucleotide-binding protein G(T) subunit gamma-T1 OS=Bos taurus GN=GNGT1 PE=1 SV=2

Observed	Mr(expt)	Mr(calc)	ppm	Start	End	Miss	Ions	Peptide
1006.5014	1005.4941	1005.5165	-22.22	17 -	24	1	---	K.MEVDQLKK.E + Oxidation (M)
1103.6257	1102.6184	1102.6056	11.6	15 -	23	1	---	K.LKMEVDQLK.K
1270.6575	1269.6502	1269.6816	-24.72	2 -	12	0	---	M.PVINIEDLTEK.D
1401.6989	1400.6916	1400.7221	-21.75	1 -	12	0	---	-.MPVINIEDLTEK.D

1635.7697	1634.7624	1634.7788	-9.99	42 -	55	1	---	R.DYVEERSGEDPLVK.G
1969.9998	1968.9925	1969.0156	-11.74	48 -	65	2	---	R.SGEDPLVKGIPEDKNPFK.E

No match to: 716.4763, 723.4098, 724.2417, 725.2533, 726.3622, 728.3046, 729.4749, 734.4570, 736.3904, 737.4152, 738.3791, 744.8651, 745.3838, 747.4550, 768.3673, 788.4571, 801.4262, 805.4606, 813.4481, 814.4558, 820.4053, 823.4778, 829.4589, 830.4522, 839.3830, 842.5110, 857.4382, 860.4207, 865.4518, 870.4165, 872.5054, 880.4424, 882.4292, 887.4245, 887.9669, 888.4561, 891.4898, 894.4144, 894.8973, 897.5006, 898.4523, 912.5132, 913.5293, 925.4620, 927.4981, 931.4992, 934.5098, 957.5774, 960.5294, 961.4949, 963.4734, 965.4692, 970.5181, 972.4899, 975.5167, 976.5063, 977.5212, 978.5100, 978.5146, 983.6283, 992.4958, 993.5046, 995.4181, 998.5911, 1000.5959, 1002.5007, 1011.5474, 1012.5447, 1014.5485, 1020.5577, 1021.5699, 1024.5516, 1027.5438, 1028.5397, 1030.5978, 1032.5238, 1036.5260, 1037.5405, 1039.5641, 1040.5908, 1042.5388, 1043.5178, 1045.5568, 1046.6018, 1056.5515, 1057.6100, 1057.6107, 1061.6168, 1074.6073, 1074.6100, 1076.6123, 1102.6007, 1105.6136, 1107.1058, 1113.5486, 1120.5498, 1122.5322, 1123.5986, 1128.5724, 1129.5924, 1134.3574, 1137.5463, 1145.6348, 1149.6057, 1153.6460, 1156.4957, 1161.6295, 1162.6174, 1164.1815, 1170.5497, 1172.6156, 1177.6410, 1180.6428, 1184.6412, 1188.5603, 1193.6041, 1206.5107, 1209.5889, 1211.6099, 1212.6160, 1215.6743, 1216.7081, 1219.6886, 1227.6234, 1229.7098, 1234.6414, 1240.5974, 1242.6003, 1243.6161, 1253.6216, 1255.5972, 1255.6000, 1260.6227, 1264.7130, 1265.7064, 1269.6498, 1273.6556, 1277.6305, 1286.6099, 1289.6875, 1293.6401, 1303.6455, 1304.6677, 1307.6788, 1309.6780, 1318.6750, 1319.6589, 1328.6600, 1328.6620, 1331.7888, 1333.7644, 1336.0798, 1336.6155, 1350.6853, 1351.7202, 1356.5811, 1359.1302, 1359.6246, 1365.6489, 1374.7588, 1383.7037, 1390.6974, 1393.7992, 1400.6683, 1414.8191, 1429.3142, 1436.7889, 1444.7122, 1445.7839, 1450.7223, 1456.7717, 1462.6630, 1467.7329, 1470.7394, 1471.2693, 1471.7250, 1472.2531, 1474.5243, 1475.7633, 1483.7141, 1488.5333, 1488.7194, 1493.7366, 1502.5601, 1513.7441, 1514.6849, 1516.6807, 1517.6437, 1518.7053, 1523.7611, 1526.7343, 1528.6973, 1530.6893, 1536.8230, 1540.7114, 1551.7063, 1553.7252, 1554.7474, 1557.6992, 1558.7140, 1563.6658, 1564.3619, 1565.7223, 1566.7162, 1572.7062, 1580.8400, 1580.8423, 1586.8090, 1597.7913, 1599.8337, 1601.8030, 1614.8046, 1616.7968, 1628.7136, 1630.8103, 1642.7682, 1643.7688, 1644.7915, 1646.8154, 1651.7681, 1657.7932, 1663.8466, 1664.8557, 1666.7545, 1695.8208, 1707.8933, 1718.8790, 1721.9192, 1722.9375, 1728.8976, 1730.9116, 1732.2391, 1734.1844, 1739.7894, 1743.8966, 1755.2666, 1755.7703, 1757.4702, 1757.9089, 1758.4524, 1758.8987, 1761.9023, 1765.7578, 1768.8339, 1769.8287, 1770.3949, 1772.8273, 1773.8792, 1774.9180, 1774.9200, 1786.8268, 1787.8239, 1809.0499, 1810.0303, 1829.8895, 1830.8380, 1833.3470, 1833.8501, 1851.8885, 1852.0399, 1862.9515, 1881.7703, 1895.9634, 1906.8195, 1908.8512, 1920.7972, 1932.0050, 1936.9479, 1939.8563, 1940.8103, 1943.8293, 1954.8351, 1956.8574, 1958.8956, 1965.7998, 1967.9565, 1979.8927, 1981.3531, 1982.8179, 1995.8232, 1997.8312, 2003.8595, 2008.9971, 2011.8464, 2015.9408, 2019.8779, 2025.8735, 2031.9606, 2047.9397, 2062.9578, 2067.0020, 2076.8943, 2081.9827, 2082.9966, 2087.0103, 2088.1108, 2089.1162, 2096.0430, 2097.0200, 2108.9771, 2110.0061, 2139.0991, 2140.0918, 2143.1006, 2144.0718, 2149.8516, 2160.0554, 2174.0378, 2178.0471, 2179.0574, 2188.0547, 2191.0457, 2198.1831, 2199.1499, 2203.0386, 2211.1052, 2223.1582, 2225.1470, 2227.1042, 2228.0725, 2238.1086, 2239.1538, 2243.1243, 2244.0684, 2254.0896, 2255.1482, 2260.0779, 2270.2183, 2272.1736, 2284.1841, 2287.1108, 2307.0549, 2309.0659, 2310.0798, 2310.5271, 2311.0454, 2319.0945, 2337.6050, 2341.5510, 2353.0518, 2354.0767, 2355.0564, 2371.0581, 2383.9592, 2388.0437, 2392.1462, 2404.0471, 2412.0872, 2419.5879, 2420.0864, 2420.5930, 2423.2439, 2426.5667, 2427.1211, 2438.1724, 2455.1553, 2458.1648, 2459.1794, 2478.1587, 2516.1133, 2533.1262, 2542.1650, 2546.1746, 2549.1316, 2550.1252, 2559.1470, 2561.1729, 2562.1765, 2567.1514, 2572.1523, 2574.2678, 2576.1628, 2583.1218, 2587.2722, 2604.1682, 2612.1438, 2653.7173, 2655.1897, 2671.1787, 2687.1833, 2701.2820, 2706.2766, 2712.2104, 2714.2156, 2716.1953, 2717.2725, 2725.2354, 2729.1675, 2739.2351, 2743.2642, 2745.2900, 2754.2197, 2755.2358, 2759.2573, 2769.2219, 2771.2437, 2776.2644, 2785.2388, 2786.2617, 2787.2400, 2793.2439, 2801.2378, 2803.2302, 2809.2549, 2815.2153, 2817.2322, 2819.2236, 2830.2788, 2833.2280, 2836.2397, 2862.2927, 2873.2961, 2889.2024, 2894.3218, 2927.2520, 2931.3428, 2955.3005, 2974.4124, 2975.4084, 2990.3921, 3006.3206, 3011.2810, 3020.3557, 3021.2412, 3035.3281, 3053.3447, 3062.2456, 3086.4507, 3091.2622, 3103.3140, 3110.7285, 3113.8591, 3130.3557, 3140.4265, 3144.3657, 3159.4985, 3173.5386, 3174.5486, 3185.4236, 3218.3301, 3259.3625, 3312.3267, 3331.4678, 3347.4761, 3363.4788, 3390.4675, 3431.4570, 3447.4666, 3463.4895, 3485.6985, 3488.6545, 3489.6521, 3493.0195, 3494.5571, 3504.6423, 3509.5613, 3525.6025, 3538.4521, 3553.6230, 3579.5403, 3648.1956, 3655.4729, 3664.7500, 3695.6177, 3712.5720, 3738.5837, 3816.7043, 3937.8308, 4010.8696

15. Q3MHX9 **Mass:** 23497 **Score:** 16 **Expect:** 6.8e+002 **Matches:** 7

Nudix (Nucleoside diphosphate linked moiety X)-type motif 16-like 1 OS=Bos taurus GN=NUDT16L1 PE=2 SV=1

Observed	Mr(expt)	Mr(calc)	ppm	Start	End	Miss	Ions	Peptide
977.5212	976.5139	976.5124	1.57	12 -	19	1	---	R.ISRVEAMR.L + Oxidation (M)
1032.5238	1031.5165	1031.5321	-15.10	1 -	10	0	---	-.MSAAAVPELK.R + Oxidation (M)
1043.5178	1042.5105	1042.4940	15.9	48 -	55	0	---	R.FSVLMQMR.F + 2 Oxidation (M)
1057.6100	1056.6027	1056.6114	-8.20	81 -	90	0	10	R.VLGLGLGCLR.L
1057.6107	1056.6034	1056.6114	-7.54	81 -	90	0	---	R.VLGLGLGCLR.L
1122.5322	1121.5249	1121.5097	13.6	179 -	187	0	---	K.VLNMMPEEK.L + 2 Oxidation (M)
2191.0457	2190.0384	2190.0911	-24.07	179 -	198	1	---	K.VLNMMPEEKLAEALAAATEK.Q + 2 Oxidation (M)

No match to: 716.4763, 723.4098, 724.2417, 725.2533, 726.3622, 728.3046, 729.4749, 734.4570, 736.3904, 737.4152, 738.3791, 744.8651, 745.3838, 747.4550, 768.3673, 788.4571, 801.4262, 805.4606, 813.4481, 814.4558, 820.4053, 823.4778, 829.4589, 830.4522, 839.3830, 842.5110, 857.4382, 860.4207, 865.4518, 870.4165, 872.5054, 880.4424, 882.4292, 887.4245, 887.9669, 888.4561, 891.4898, 894.4144, 894.8973, 897.5006, 898.4523, 912.5132, 913.5293, 925.4620, 927.4981, 931.4992, 934.5098, 957.5774, 960.5294, 961.4949, 963.4734, 965.4692, 970.5181, 972.4899, 975.5167, 976.5063, 978.5100, 978.5146, 983.6283, 992.4958, 993.5046, 995.4181, 998.5911, 1000.5959, 1002.5007, 1006.5014, 1011.5474, 1012.5447, 1014.5485, 1020.5577, 1021.5699, 1024.5516, 1027.5438, 1028.5397, 1030.5978, 1036.5260, 1037.5405, 1039.5641, 1040.5908, 1042.5388, 1045.5568, 1046.6018, 1056.5515, 1061.6168, 1074.6073, 1074.6100, 1076.6123, 1102.6007, 1103.6257, 1105.6136, 1107.1058, 1113.5486, 1120.5498, 1123.5986, 1128.5724, 1129.5924, 1134.3574, 1137.5463, 1145.6348, 1149.6057, 1153.6460, 1156.4957, 1161.6295, 1162.6174, 1164.1815, 1170.5497, 1172.6156, 1177.6410, 1180.6428, 1184.6412, 1188.5603, 1193.6041, 1206.5107, 1209.5889, 1211.6099, 1212.6160, 1215.6743, 1216.7081, 1219.6886, 1227.6234, 1229.7098, 1234.6414, 1240.5974, 1242.6003, 1243.6161, 1253.6216, 1255.5972, 1255.6000, 1260.6227, 1264.7130, 1265.7064, 1269.6498, 1270.6575, 1273.6556, 1277.6305, 1286.6099, 1289.6875, 1293.6401, 1303.6455, 1304.6677, 1307.6788, 1309.6780, 1318.6750, 1319.6589, 1328.6600, 1328.6620, 1331.7888, 1333.7644, 1336.0798, 1336.6155, 1350.6853, 1351.7202, 1356.5811, 1359.1302, 1359.6246, 1365.6489, 1374.7588, 1383.7037, 1390.6974, 1393.7992, 1400.6683, 1401.6989, 1414.8191, 1429.3142, 1436.7889, 1444.7122, 1445.7839, 1450.7223, 1456.7717, 1462.6630, 1467.7329, 1470.7394, 1471.2693, 1471.7250, 1472.2531, 1474.5243, 1475.7633, 1483.7141, 1488.5333, 1488.7194, 1493.7366, 1502.5601, 1513.7441, 1514.6849, 1516.6807, 1517.6437, 1518.7053, 1523.7611, 1526.7343, 1528.6973, 1530.6893, 1536.8230, 1540.7114, 1551.7063, 1553.7252, 1554.7474, 1557.6992, 1558.7140, 1563.6658, 1564.3619, 1565.7223, 1566.7162, 1572.7062, 1580.8400, 1580.8423, 1586.8090, 1597.7913, 1599.8337, 1601.8030, 1614.8046, 1616.7968, 1628.7136, 1630.8103, 1635.7697, 1642.7682, 1643.7688, 1644.7915, 1646.8154, 1651.7681, 1657.7932, 1663.8466, 1664.8557, 1666.7545, 1695.8208, 1707.8933, 1718.8790, 1721.9192, 1722.9375, 1728.8976, 1730.9116, 1732.2391, 1734.1844, 1739.7894, 1743.8966, 1755.2666, 1755.7703, 1757.4702, 1757.9089, 1758.4524, 1758.8987, 1761.9023, 1765.7578, 1768.8339, 1769.8287, 1770.3949, 1772.8273, 1773.8792, 1774.9180, 1774.9200, 1786.8268, 1787.8239, 1809.0499, 1810.0303, 1829.8895, 1830.8380, 1833.3470, 1833.8501, 1851.8885, 1852.0399, 1862.9515, 1881.7703, 1895.9634, 1906.8195, 1908.8512, 1920.7972, 1932.0050, 1936.9479, 1939.8563, 1940.8103, 1943.8293, 1954.8351, 1956.8574, 1958.8956, 1965.7998, 1967.9565, 1969.9998, 1979.8927, 1981.3531, 1982.8179, 1995.8232, 1997.8312, 2003.8595, 2008.9971, 2011.8464, 2015.9408, 2019.8779, 2025.8735, 2031.9606, 2047.9397, 2062.9578, 2067.0020, 2076.8943, 2081.9827, 2082.9966, 2087.0103, 2088.1108, 2089.1162, 2096.0430, 2097.0200, 2108.9771, 2110.0061, 2139.0991, 2140.0918, 2143.1006, 2144.0718, 2149.8516, 2160.0554, 2174.0378, 2178.0471, 2179.0574, 2188.0547, 2198.1831, 2199.1499, 2203.0386, 2211.1052, 2223.1582, 2225.1470, 2227.1042, 2228.0725, 2238.1086, 2239.1538, 2243.1243, 2244.0684, 2254.0896, 2255.1482, 2260.0779, 2270.2183, 2272.1736, 2284.1841, 2287.1108, 2307.0549, 2309.0659, 2310.0798, 2310.5271, 2311.0454, 2319.0945, 2337.6050, 2341.5510, 2353.0518, 2354.0767, 2355.0564, 2371.0581, 2383.9592, 2388.0437, 2392.1462, 2404.0471, 2412.0872, 2419.5879, 2420.0864, 2420.5930, 2423.2439, 2426.5667, 2427.1211, 2438.1724, 2455.1553, 2458.1648, 2459.1794, 2478.1587, 2516.1133, 2533.1262, 2542.1650, 2546.1746, 2549.1316, 2550.1252, 2559.1470, 2561.1729, 2562.1765, 2567.1514, 2572.1523, 2574.2678, 2576.1628, 2583.1218, 2587.2722, 2604.1682, 2612.1438, 2653.7173, 2655.1897, 2671.1787, 2687.1833, 2701.2820, 2706.2766, 2712.2104, 2714.2156, 2716.1953, 2717.2725, 2725.2354, 2729.1675, 2739.2351, 2743.2642, 2745.2900, 2754.2197, 2755.2358, 2759.2573, 2769.2219, 2771.2437, 2776.2644, 2785.2388, 2786.2617, 2787.2400, 2793.2439, 2801.2378, 2803.2302, 2809.2549, 2815.2153, 2817.2322, 2819.2236, 2830.2788, 2833.2280, 2836.2397, 2862.2927, 2873.2961, 2889.2024, 2894.3218, 2927.2520, 2931.3428, 2955.3005, 2974.4124, 2975.4084, 2990.3921, 3006.3206, 3011.2810, 3020.3557, 3021.2412, 3035.3281, 3053.3447, 3062.2456, 3086.4507, 3091.2622, 3103.3140, 3110.7285, 3113.8591, 3130.3557, 3140.4265, 3144.3657, 3159.4985, 3173.5386, 3174.5486, 3185.4236, 3218.3301, 3259.3625, 3312.3267, 3331.4678, 3347.4761, 3363.4788, 3390.4675, 3431.4570, 3447.4666, 3463.4895, 3485.6985, 3488.6545, 3489.6521, 3493.0195, 3494.5571, 3504.6423, 3509.5613, 3525.6025, 3538.4521, 3553.6230, 3579.5403, 3648.1956, 3655.4729, 3664.7500, 3695.6177, 3712.5720, 3738.5837, 3816.7043, 3937.8308, 4010.8696

16. F1MSW3 **Mass:** 36190 **Score:** 16 **Expect:** 6.8e+002 **Matches:** 11

Uncharacterized protein OS=Bos taurus GN=MAGIX PE=4 SV=2

Observed	Mr(expt)	Mr(calc)	ppm	Start	End	Miss	Ions	Peptide
965.4692	964.4619	964.4760	-14.61	96 -	103	1	---	R.RSETMGIR.T + Oxidation (M)
1184.6412	1183.6339	1183.6058	23.8	4 -	15	1	---	R.RPGGGTDPRGSK.G

1243.6161	1242.6088	1242.5993	7.65	134 - 146	0	---	R.SSAGFGFTLSGGR.D
1255.5972	1254.5899	1254.5589	24.7	16 - 29	0	---	K.GGGGGPQPEDASAR.Q
1328.6600	1327.6527	1327.6415	8.45	1 - 12	1	7	-.MERRPGGGTDPR.G
1328.6620	1327.6547	1327.6415	9.95	1 - 12	1	---	-.MERRPGGGTDPR.G
1470.7394	1469.7321	1469.7184	9.33	237 - 249	1	---	R.IPDPGEPEMTKTR.S
1616.7968	1615.7895	1615.7849	2.87	1 - 15	2	---	-.MERRPGGGTDPRGSK.G + Oxidation (M)
2143.1006	2142.0933	2142.0566	17.1	13 - 34	2	---	R.GSKGGGGGPQPEDASARQFLAR.L
2238.1086	2237.1013	2237.0522	22.0	228 - 247	1	---	K.EDVLPSPDRIPDPGEPEMTK.T + Oxidation (M)
3173.5386	3172.5313	3172.6048	-23.15	168 - 196	1	---	R.CGRLQAGDLVLHINGESTQGLTHVEVVDR.I

No match to: 716.4763, 723.4098, 724.2417, 725.2533, 726.3622, 728.3046, 729.4749, 734.4570, 736.3904, 737.4152, 738.3791, 744.8651, 745.3838, 747.4550, 768.3673, 788.4571, 801.4262, 805.4606, 813.4481, 814.4558, 820.4053, 823.4778, 829.4589, 830.4522, 839.3830, 842.5110, 857.4382, 860.4207, 865.4518, 870.4165, 872.5054, 880.4424, 882.4292, 887.4245, 887.9669, 888.4561, 891.4898, 894.4144, 894.8973, 897.5006, 898.4523, 912.5132, 913.5293, 925.4620, 927.4981, 931.4992, 934.5098, 957.5774, 960.5294, 961.4949, 963.4734, 970.5181, 972.4899, 975.5167, 976.5063, 977.5212, 978.5100, 978.5146, 983.6283, 992.4958, 993.5046, 995.4181, 998.5911, 1000.5959, 1002.5007, 1006.5014, 1011.5474, 1012.5447, 1014.5485, 1020.5577, 1021.5699, 1024.5516, 1027.5438, 1028.5397, 1030.5978, 1032.5238, 1036.5260, 1037.5405, 1039.5641, 1040.5908, 1042.5388, 1043.5178, 1045.5568, 1046.6018, 1056.5515, 1057.6100, 1057.6107, 1061.6168, 1074.6073, 1074.6100, 1076.6123, 1102.6007, 1103.6257, 1105.6136, 1107.1058, 1113.5486, 1120.5498, 1122.5322, 1123.5986, 1128.5724, 1129.5924, 1134.3574, 1137.5463, 1145.6348, 1149.6057, 1153.6460, 1156.4957, 1161.6295, 1162.6174, 1164.1815, 1170.5497, 1172.6156, 1177.6410, 1180.6428, 1188.5603, 1193.6041, 1206.5107, 1209.5889, 1211.6099, 1212.6160, 1215.6743, 1216.7081, 1219.6886, 1227.6234, 1229.7098, 1234.6414, 1240.5974, 1242.6003, 1253.6216, 1255.6000, 1260.6227, 1264.7130, 1265.7064, 1269.6498, 1270.6575, 1273.6556, 1277.6305, 1286.6099, 1289.6875, 1293.6401, 1303.6455, 1304.6677, 1307.6788, 1309.6780, 1318.6750, 1319.6589, 1331.7888, 1333.7644, 1336.0798, 1336.6155, 1350.6853, 1351.7202, 1356.5811, 1359.1302, 1359.6246, 1365.6489, 1374.7588, 1383.7037, 1390.6974, 1393.7992, 1400.6683, 1401.6989, 1414.8191, 1429.3142, 1436.7889, 1444.7122, 1445.7839, 1450.7223, 1456.7717, 1462.6630, 1467.7329, 1471.2693, 1471.7250, 1472.2531, 1474.5243, 1475.7633, 1483.7141, 1488.5333, 1488.7194, 1493.7366, 1502.5601, 1513.7441, 1514.6849, 1516.6807, 1517.6437, 1518.7053, 1523.7611, 1526.7343, 1528.6973, 1530.6893, 1536.8230, 1540.7114, 1551.7063, 1553.7252, 1554.7474, 1557.6992, 1558.7140, 1563.6658, 1564.3619, 1565.7223, 1566.7162, 1572.7062, 1580.8400, 1580.8423, 1586.8090, 1597.7913, 1599.8337, 1601.8030, 1614.8046, 1628.7136, 1630.8103, 1635.7697, 1642.7682, 1643.7688, 1644.7915, 1646.8154, 1651.7681, 1657.7932, 1663.8466, 1664.8557, 1666.7545, 1695.8208, 1707.8933, 1718.8790, 1721.9192, 1722.9375, 1728.8976, 1730.9116, 1732.2391, 1734.1844, 1739.7894, 1743.8966, 1755.2666, 1755.7703, 1757.4702, 1757.9089, 1758.4524, 1758.8987, 1761.9023, 1765.7578, 1768.8339, 1769.8287, 1770.3949, 1772.8273, 1773.8792, 1774.9180, 1774.9200, 1786.8268, 1787.8239, 1809.0499, 1810.0303, 1829.8895, 1830.8380, 1833.3470, 1833.8501, 1851.8885, 1852.0399, 1862.9515, 1881.7703, 1895.9634, 1906.8195, 1908.8512, 1920.7972, 1932.0050, 1936.9479, 1939.8563, 1940.8103, 1943.8293, 1954.8351, 1956.8574, 1958.8956, 1965.7998, 1967.9565, 1969.9998, 1979.8927, 1981.3531, 1982.8179, 1995.8232, 1997.8312, 2003.8595, 2008.9971, 2011.8464, 2015.9408, 2019.8779, 2025.8735, 2031.9606, 2047.9397, 2062.9578, 2067.0020, 2076.8943, 2081.9827, 2082.9966, 2087.0103, 2088.1108, 2089.1162, 2096.0430, 2097.0200, 2108.9771, 2110.0061, 2139.0991, 2140.0918, 2144.0718, 2149.8516, 2160.0554, 2174.0378, 2178.0471, 2179.0574, 2188.0547, 2191.0457, 2198.1831, 2199.1499, 2203.0386, 2211.1052, 2223.1582, 2225.1470, 2227.1042, 2228.0725, 2239.1538, 2243.1243, 2244.0684, 2254.0896, 2255.1482, 2260.0779, 2270.2183, 2272.1736, 2284.1841, 2287.1108, 2307.0549, 2309.0659, 2310.0798, 2310.5271, 2311.0454, 2319.0945, 2337.6050, 2341.5510, 2353.0518, 2354.0767, 2355.0564, 2371.0581, 2383.9592, 2388.0437, 2392.1462, 2404.0471, 2412.0872, 2419.5879, 2420.0864, 2420.5930, 2423.2439, 2426.5667, 2427.1211, 2438.1724, 2455.1553, 2458.1648, 2459.1794, 2478.1587, 2516.1133, 2533.1262, 2542.1650, 2546.1746, 2549.1316, 2550.1252, 2559.1470, 2561.1729, 2562.1765, 2567.1514, 2572.1523, 2574.2678, 2576.1628, 2583.1218, 2587.2722, 2604.1682, 2612.1438, 2653.7173, 2655.1897, 2671.1787, 2687.1833, 2701.2820, 2706.2766, 2712.2104, 2714.2156, 2716.1953, 2717.2725, 2725.2354, 2729.1675, 2739.2351, 2743.2642, 2745.2900, 2754.2197, 2755.2358, 2759.2573, 2769.2219, 2771.2437, 2776.2644, 2785.2388, 2786.2617, 2787.2400, 2793.2439, 2801.2378, 2803.2302, 2809.2549, 2815.2153, 2817.2322, 2819.2236, 2830.2788, 2833.2280, 2836.2397, 2862.2927, 2873.2961, 2889.2024, 2894.3218, 2927.2520, 2931.3428, 2955.3005, 2974.4124, 2975.4084, 2990.3921, 3006.3206, 3011.2810, 3020.3557, 3021.2412, 3035.3281, 3053.3447, 3062.2456, 3086.4507, 3091.2622, 3103.3140, 3110.7285, 3113.8591, 3130.3557, 3140.4265, 3144.3657, 3159.4985, 3174.5486, 3185.4236, 3218.3301, 3259.3625, 3312.3267, 3331.4678, 3347.4761, 3363.4788, 3390.4675, 3431.4570, 3447.4666, 3463.4895, 3485.6985, 3488.6545, 3489.6521, 3493.0195, 3494.5571, 3504.6423, 3509.5613, 3525.6025, 3538.4521, 3553.6230, 3579.5403, 3648.1956, 3655.4729, 3664.7500, 3695.6177, 3712.5720, 3738.5837, 3816.7043, 3937.8308, 4010.8696

17. Q32PB8 **Mass:** 9220 **Score:** 15 **Expect:** 7.1e+002 **Matches:** 6

40S ribosomal protein S21 OS=Bos taurus GN=RPS21 PE=3 SV=1

Observed	Mr(expt)	Mr(calc)	ppm	Start - End	Miss	Ions	Peptide
1122.5322	1121.5249	1121.5023	20.2	62 - 71	0	---	R.MGESDDSILR.L
1180.6428	1179.6355	1179.6182	14.7	52 - 61	1	---	K.TYAICGAIRR.M
1450.7223	1449.7150	1449.7133	1.18	62 - 74	1	---	R.MGESDDSILRLAK.A + Oxidation (M)
1572.7062	1571.6989	1571.7250	-16.57	28 - 41	0	---	K.DHASIQMNVAEVDK.V + Oxidation (M)
1769.8287	1768.8214	1768.8090	7.00	1 - 15	0	---	-.MQNDAGEFVDLYVPR.K + Oxidation (M)
1969.9998	1968.9925	1968.9687	12.1	28 - 45	1	---	K.DHASIQMNVAEVDKVTGR.F

No match to: 716.4763, 723.4098, 724.2417, 725.2533, 726.3622, 728.3046, 729.4749, 734.4570, 736.3904, 737.4152, 738.3791, 744.8651, 745.3838, 747.4550, 768.3673, 788.4571, 801.4262, 805.4606, 813.4481, 814.4558, 820.4053, 823.4778, 829.4589, 830.4522, 839.3830, 842.5110, 857.4382, 860.4207, 865.4518, 870.4165, 872.5054, 880.4424, 882.4292, 887.4245, 887.9669, 888.4561, 891.4898, 894.4144, 894.8973, 897.5006, 898.4523, 912.5132, 913.5293, 925.4620, 927.4981, 931.4992, 934.5098, 957.5774, 960.5294, 961.4949, 963.4734, 965.4692, 970.5181, 972.4899, 975.5167, 976.5063, 977.5212, 978.5100, 978.5146, 983.6283, 992.4958, 993.5046, 995.4181, 998.5911, 1000.5959, 1002.5007, 1006.5014, 1011.5474, 1012.5447, 1014.5485, 1020.5577, 1021.5699, 1024.5516, 1027.5438, 1028.5397, 1030.5978, 1032.5238, 1036.5260, 1037.5405, 1039.5641, 1040.5908, 1042.5388, 1043.5178, 1045.5568, 1046.6018, 1056.5515, 1057.6100, 1057.6107, 1061.6168, 1074.6073, 1074.6100, 1076.6123, 1102.6007, 1103.6257, 1105.6136, 1107.1058, 1113.5486, 1120.5498, 1123.5986, 1128.5724, 1129.5924, 1134.3574, 1137.5463, 1145.6348, 1149.6057, 1153.6460, 1156.4957, 1161.6295, 1162.6174, 1164.1815, 1170.5497, 1172.6156, 1177.6410, 1184.6412, 1188.5603, 1193.6041, 1206.5107, 1209.5889, 1211.6099, 1212.6160, 1215.6743, 1216.7081, 1219.6886, 1227.6234, 1229.7098, 1234.6414, 1240.5974, 1242.6003, 1243.6161, 1253.6216, 1255.5972, 1255.6000, 1260.6227, 1264.7130, 1265.7064, 1269.6498, 1270.6575, 1273.6556, 1277.6305, 1286.6099, 1289.6875, 1293.6401, 1303.6455, 1304.6677, 1307.6788, 1309.6780, 1318.6750, 1319.6589, 1328.6600, 1328.6620, 1331.7888, 1333.7644, 1336.0798, 1336.6155, 1350.6853, 1351.7202, 1356.5811, 1359.1302, 1359.6246, 1365.6489, 1374.7588, 1383.7037, 1390.6974, 1393.7992, 1400.6683, 1401.6989, 1414.8191, 1429.3142, 1436.7889, 1444.7122, 1445.7839, 1456.7717, 1462.6630, 1467.7329, 1470.7394, 1471.2693, 1471.7250, 1472.2531, 1474.5243, 1475.7633, 1483.7141, 1488.5333, 1488.7194, 1493.7366, 1502.5601, 1513.7441, 1514.6849, 1516.6807, 1517.6437, 1518.7053, 1523.7611, 1526.7343, 1528.6973, 1530.6893, 1536.8230, 1540.7114, 1551.7063, 1553.7252, 1554.7474, 1557.6992, 1558.7140, 1563.6658, 1564.3619, 1565.7223, 1566.7162, 1580.8400, 1580.8423, 1586.8090, 1597.7913, 1599.8337, 1601.8030, 1614.8046, 1616.7968, 1628.7136, 1630.8103, 1635.7697, 1642.7682, 1643.7688, 1644.7915, 1646.8154, 1651.7681, 1657.7932, 1663.8466, 1664.8557, 1666.7545, 1695.8208, 1707.8933, 1718.8790, 1721.9192, 1722.9375, 1728.8976, 1730.9116, 1732.2391, 1734.1844, 1739.7894, 1743.8966, 1755.2666, 1755.7703, 1757.4702, 1757.9089, 1758.4524, 1758.8987, 1761.9023, 1765.7578, 1768.8339, 1770.3949, 1772.8273, 1773.8792, 1774.9180, 1774.9200, 1786.8268, 1787.8239, 1809.0499, 1810.0303, 1829.8895, 1830.8380, 1833.3470, 1833.8501, 1851.8885, 1852.0399, 1862.9515, 1881.7703, 1895.9634, 1906.8195, 1908.8512, 1920.7972, 1932.0050, 1936.9479, 1939.8563, 1940.8103, 1943.8293, 1954.8351, 1956.8574, 1958.8956, 1965.7998, 1967.9565, 1979.8927, 1981.3531, 1982.8179, 1995.8232, 1997.8312, 2003.8595, 2008.9971, 2011.8464, 2015.9408, 2019.8779, 2025.8735, 2031.9606, 2047.9397, 2062.9578, 2067.0020, 2076.8943, 2081.9827, 2082.9966, 2087.0103, 2088.1108, 2089.1162, 2096.0430, 2097.0200, 2108.9771, 2110.0061, 2139.0991, 2140.0918, 2143.1006, 2144.0718, 2149.8516, 2160.0554, 2174.0378, 2178.0471, 2179.0574, 2188.0547, 2191.0457, 2198.1831, 2199.1499, 2203.0386, 2211.1052, 2223.1582, 2225.1470, 2227.1042, 2228.0725, 2238.1086, 2239.1538, 2243.1243, 2244.0684, 2254.0896, 2255.1482, 2260.0779, 2270.2183, 2272.1736, 2284.1841, 2287.1108, 2307.0549, 2309.0659, 2310.0798, 2310.5271, 2311.0454, 2319.0945, 2337.6050, 2341.5510, 2353.0518, 2354.0767, 2355.0564, 2371.0581, 2383.9592, 2388.0437, 2392.1462, 2404.0471, 2412.0872, 2419.5879, 2420.0864, 2420.5930, 2423.2439, 2426.5667, 2427.1211, 2438.1724, 2455.1553, 2458.1648, 2459.1794, 2478.1587, 2516.1133, 2533.1262, 2542.1650, 2546.1746, 2549.1316, 2550.1252, 2559.1470, 2561.1729, 2562.1765, 2567.1514, 2572.1523, 2574.2678, 2576.1628, 2583.1218, 2587.2722, 2604.1682, 2612.1438, 2653.7173, 2655.1897, 2671.1787, 2687.1833, 2701.2820, 2706.2766, 2712.2104, 2714.2156, 2716.1953, 2717.2725, 2725.2354, 2729.1675, 2739.2351, 2743.2642, 2745.2900, 2754.2197, 2755.2358, 2759.2573, 2769.2219, 2771.2437, 2776.2644, 2785.2388, 2786.2617, 2787.2400, 2793.2439, 2801.2378, 2803.2302, 2809.2549, 2815.2153, 2817.2322, 2819.2236, 2830.2788, 2833.2280, 2836.2397, 2862.2927, 2873.2961, 2889.2024, 2894.3218, 2927.2520, 2931.3428, 2955.3005, 2974.4124, 2975.4084, 2990.3921, 3006.3206, 3011.2810, 3020.3557, 3021.2412, 3035.3281, 3053.3447, 3062.2456, 3086.4507, 3091.2622, 3103.3140, 3110.7285, 3113.8591, 3130.3557, 3140.4265, 3144.3657, 3159.4985, 3173.5386, 3174.5486, 3185.4236, 3218.3301, 3259.3625, 3312.3267, 3331.4678, 3347.4761, 3363.4788, 3390.4675, 3431.4570, 3447.4666, 3463.4895, 3485.6985, 3488.6545, 3489.6521, 3493.0195, 3494.5571, 3504.6423, 3509.5613, 3525.6025, 3538.4521, 3553.6230, 3579.5403, 3648.1956, 3655.4729, 3664.7500, 3695.6177, 3712.5720, 3738.5837, 3816.7043, 3937.8308, 4010.8696

18. F1N7K9 **Mass:** 123826 **Score:** 15 **Expect:** 7.1e+002 **Matches:** 18

Uncharacterized protein OS=Bos taurus GN=TBC1D8 PE=4 SV=2

Observed	Mr(expt)	Mr(calc)	ppm	Start	End	Miss	Ions	Peptide
829.4589	828.4516	828.4341	21.2	1060 -	1066	0	---	R.QSQPELK.L
887.4245	886.4172	886.4119	5.96	370 -	376	0	---	R.MHPEAFR.Q
897.5006	896.4933	896.4875	6.50	709 -	714	2	---	R.CRHRIR.V
1219.6886	1218.6813	1218.6642	14.1	894 -	903	2	---	K.QMIKDLAKEK.D + Oxidation (M)
1304.6677	1303.6604	1303.6806	-15.46	164 -	174	0	---	K.IMEQLADVTLR.R + Oxidation (M)
1331.7888	1330.7815	1330.7642	13.0	891 -	901	2	---	K.QLKQMIKDLAK.E + Oxidation (M)
1456.7717	1455.7644	1455.7326	21.8	407 -	417	2	---	R.TVCMFRTEKIR.K + Oxidation (M)
1518.7053	1517.6980	1517.7011	-2.03	769 -	780	0	---	R.HDPSRPYAEQYR.I
1616.7968	1615.7895	1615.7916	-1.26	812 -	824	0	---	R.LLDENMDHLIEFK.A
1664.8557	1663.8484	1663.8893	-24.58	129 -	142	1	---	R.LERTSNVFLTDTIR.I
1774.9180	1773.9107	1773.9513	-22.85	554 -	569	0	---	R.VIGAQVDQSVFEELIK.E
1774.9200	1773.9127	1773.9513	-21.72	554 -	569	0	15	R.VIGAQVDQSVFEELIK.E
1829.8895	1828.8822	1828.8560	14.3	467 -	480	1	---	K.CCLVTEEIERDLHR.S
1833.8501	1832.8428	1832.8410	0.98	696 -	710	1	---	K.FGDQSVAQIEHMRCR.H
2174.0378	2173.0305	2172.9971	15.4	370 -	389	1	---	R.MHPEAFRQSGSQSPDSGLSR.E
2516.1133	2515.1060	2515.1010	1.98	72 -	90	1	---	R.FNFPEAEKLITYYSCCCWK.G
2729.1675	2728.1602	2728.2236	-23.24	72 -	92	2	---	R.FNFPEAEKLITYYSCCCWKGR.V
4010.8696	4009.8623	4009.9619	-24.83	924 -	958	0	---	K.TLYSMFHEDPEENDLYQAIATVTTLLLQIGEVGQR.G + Oxidation (M)

No match to: 716.4763, 723.4098, 724.2417, 725.2533, 726.3622, 728.3046, 729.4749, 734.4570, 736.3904, 737.4152, 738.3791, 744.8651, 745.3838, 747.4550, 768.3673, 788.4571, 801.4262, 805.4606, 813.4481, 814.4558, 820.4053, 823.4778, 830.4522, 839.3830, 842.5110, 857.4382, 860.4207, 865.4518, 870.4165, 872.5054, 880.4424, 882.4292, 887.9669, 888.4561, 891.4898, 894.4144, 894.8973, 898.4523, 912.5132, 913.5293, 925.4620, 927.4981, 931.4992, 934.5098, 957.5774, 960.5294, 961.4949, 963.4734, 965.4692, 970.5181, 972.4899, 975.5167, 976.5063, 977.5212, 978.5100, 978.5146, 983.6283, 992.4958, 993.5046, 995.4181, 998.5911, 1000.5959, 1002.5007, 1006.5014, 1011.5474, 1012.5447, 1014.5485, 1020.5577, 1021.5699, 1024.5516, 1027.5438, 1028.5397, 1030.5978, 1032.5238, 1036.5260, 1037.5405, 1039.5641, 1040.5908, 1042.5388, 1043.5178, 1045.5568, 1046.6018, 1056.5515, 1057.6100, 1057.6107, 1061.6168, 1074.6073, 1074.6100, 1076.6123, 1102.6007, 1103.6257, 1105.6136, 1107.1058, 1113.5486, 1120.5498, 1122.5322, 1123.5986, 1128.5724, 1129.5924, 1134.3574, 1137.5463, 1145.6348, 1149.6057, 1153.6460, 1156.4957, 1161.6295, 1162.6174, 1164.1815, 1170.5497, 1172.6156, 1177.6410, 1180.6428, 1184.6412, 1188.5603, 1193.6041, 1206.5107, 1209.5889, 1211.6099, 1212.6160, 1215.6743, 1216.7081, 1227.6234, 1229.7098, 1234.6414, 1240.5974, 1242.6003, 1243.6161, 1253.6216, 1255.5972, 1255.6000, 1260.6227, 1264.7130, 1265.7064, 1269.6498, 1270.6575, 1273.6556, 1277.6305, 1286.6099, 1289.6875, 1293.6401, 1303.6455, 1307.6788, 1309.6780, 1318.6750, 1319.6589, 1328.6600, 1328.6620, 1333.7644, 1336.0798, 1336.6155, 1350.6853, 1351.7202, 1356.5811, 1359.1302, 1359.6246, 1365.6489, 1374.7588, 1383.7037, 1390.6974, 1393.7992, 1400.6683, 1401.6989, 1414.8191, 1429.3142, 1436.7889, 1444.7122, 1445.7839, 1450.7223, 1462.6630, 1467.7329, 1470.7394, 1471.2693, 1471.7250, 1472.2531, 1474.5243, 1475.7633, 1483.7141, 1488.5333, 1488.7194, 1493.7366, 1502.5601, 1513.7441, 1514.6849, 1516.6807, 1517.6437, 1523.7611, 1526.7343, 1528.6973, 1530.6893, 1536.8230, 1540.7114, 1551.7063, 1553.7252, 1554.7474, 1557.6992, 1558.7140, 1563.6658, 1564.3619, 1565.7223, 1566.7162, 1572.7062, 1580.8400, 1580.8423, 1586.8090, 1597.7913, 1599.8337, 1601.8030, 1614.8046, 1628.7136, 1630.8103, 1635.7697, 1642.7862, 1643.7688, 1644.7915, 1646.8154, 1651.7681, 1657.7932, 1663.8466, 1666.7545, 1695.8208, 1707.8933, 1718.8790, 1721.9192, 1722.9375, 1728.8976, 1730.9116, 1732.2391, 1734.1844, 1739.7894, 1743.8966, 1755.2666, 1755.7703, 1757.4702, 1757.9089, 1758.4524, 1758.8987, 1761.9023, 1765.7578, 1768.8339, 1769.8287, 1770.3949, 1772.8273, 1773.8792, 1786.8268, 1787.8239, 1809.0499, 1810.0303, 1830.8380, 1833.3470, 1851.8885, 1852.0399, 1862.9515, 1881.7703, 1895.9634, 1906.8195, 1908.8512, 1920.7972, 1932.0050, 1936.9479, 1939.8563, 1940.8103, 1943.8293, 1954.8351, 1956.8574, 1958.8956, 1965.7998, 1967.9565, 1969.9998, 1979.8927, 1981.3531, 1982.8179, 1995.8232, 1997.8312, 2003.8595, 2008.9971, 2011.8464, 2015.9408, 2019.8779, 2025.8735, 2031.9606, 2047.9397, 2062.9578, 2067.0020, 2076.8943, 2081.9827, 2082.9966, 2087.0103, 2088.1108, 2089.1162, 2096.0430, 2097.0200, 2108.9771, 2110.0061, 2139.0991, 2140.0918, 2143.1006, 2144.0718, 2149.8516, 2160.0554, 2178.0471, 2179.0574, 2188.0547, 2191.0457, 2198.1831, 2199.1499, 2203.0386, 2211.1052, 2223.1582, 2225.1470, 2227.1042, 2228.0725, 2238.1086, 2239.1538, 2243.1243, 2244.0684, 2254.0896, 2255.1482, 2260.0779, 2270.2183, 2272.1736, 2284.1841, 2287.1108, 2307.0549, 2309.0659, 2310.0798, 2310.5271, 2311.0454, 2319.0945, 2337.6050, 2341.5510, 2353.0518, 2354.0767, 2355.0564, 2371.0581, 2383.9592, 2388.0437, 2392.1462, 2404.0471, 2412.0872, 2419.5879, 2420.0864, 2420.5930, 2423.2439, 2426.5667, 2427.1211, 2438.1724, 2455.1553, 2458.1648, 2459.1794, 2478.1587, 2533.1262, 2542.1650, 2546.1746, 2549.1316, 2550.1252, 2559.1470, 2561.1729, 2562.1765, 2567.1514, 2572.1523, 2574.2678, 2576.1628, 2583.1218, 2587.2722, 2604.1682, 2612.1438, 2653.7173, 2655.1897, 2671.1787, 2687.1833, 2701.2820, 2706.2766, 2712.2104, 2714.2156, 2716.1953, 2717.2725, 2725.2354, 2739.2351, 2743.2642, 2745.2900, 2754.2197, 2755.2358, 2759.2573, 2769.2219, 2771.2437, 2776.2644, 2785.2388, 2786.2617, 2787.2400, 2793.2439, 2801.2378, 2803.2302, 2809.2549, 2815.2153, 2817.2322, 2819.2236, 2830.2788, 2833.2280, 2836.2397, 2862.2927, 2873.2961, 2889.2024, 2894.3218, 2927.2520, 2931.3428, 2955.3005, 2974.4124, 2975.4084, 2990.3921, 3006.3206, 3011.2810, 3020.3557, 3021.2412, 3035.3281, 3053.3447, 3062.2456, 3086.4507, 3091.2622, 3103.3140, 3110.7285, 3113.8591, 3130.3557, 3140.4265, 3144.3657, 3159.4985, 3173.5386, 3174.5486, 3185.4236, 3218.3301, 3259.3625, 3312.3267, 3331.4678, 3347.4761, 3363.4788, 3390.4675, 3431.4570, 3447.4666, 3463.4895, 3485.6985, 3488.6545, 3489.6521, 3493.0195, 3494.5571, 3504.6423, 3509.5613, 3525.6025, 3538.4521, 3553.6230, 3579.5403, 3648.1956, 3655.4729, 3664.7500, 3695.6177, 3712.5720, 3738.5837, 3816.7043, 3937.8308

19. Q05717 **Mass:** 31377 **Score:** 15 **Expect:** 7.3e+002 **Matches:** 7

Insulin-like growth factor-binding protein 5 OS=Bos taurus GN=IGFBP5 PE=2 SV=2

Observed	Mr(expt)	Mr(calc)	ppm	Start	End	Miss	Ions	Peptide
1255.5972	1254.5899	1254.6105	-16.43	164 -	175	0	---	K.FVGGAENTAHPR.V
1255.6000	1254.5927	1254.6105	-14.20	164 -	175	0	15	K.FVGGAENTAHPR.V
1516.6807	1515.6734	1515.6673	4.06	238 -	249	1	---	R.GICWCVDKYGMK.L
1718.8790	1717.8717	1717.8756	-2.25	208 -	221	2	---	R.MVPRAVYLPNCDRK.G
1758.8987	1757.8914	1757.8514	22.8	33 -	48	0	---	K.ALSMCPPSPLGCELVK.E
2096.0430	2095.0357	2095.0666	-14.75	194 -	211	2	---	R.HMEASLQELKASPRMVPR.A + Oxidation (M)
2139.0991	2138.0918	2138.0691	10.6	164 -	183	1	---	K.FVGGAENTAHPRVISAPEMR.Q

No match to: 716.4763, 723.4098, 724.2417, 725.2533, 726.3622, 728.3046, 729.4749, 734.4570, 736.3904, 737.4152, 738.3791, 744.8651, 745.3838, 747.4550, 768.3673, 788.4571, 801.4262, 805.4606, 813.4481, 814.4558, 820.4053, 823.4778, 829.4589, 830.4522, 839.3830, 842.5110, 857.4382, 860.4207, 865.4518, 870.4165, 872.5054, 880.4424, 882.4292, 887.4245, 887.9669, 888.4561, 891.4898, 894.4144, 894.8973, 897.5006, 898.4523, 912.5132, 913.5293, 925.4620, 927.4981, 931.4992, 934.5098, 957.5774, 960.5294, 961.4949, 963.4734, 965.4692, 970.5181, 972.4899, 975.5167, 976.5063, 977.5212, 978.5100, 978.5146, 983.6283, 992.4958, 993.5046, 995.4181, 998.5911, 1000.5959, 1002.5007, 1006.5014, 1011.5474, 1012.5447, 1014.5485, 1020.5577, 1021.5699, 1024.5516, 1027.5438, 1028.5397, 1030.5978, 1032.5238, 1036.5260, 1037.5405, 1039.5641, 1040.5908, 1042.5388, 1043.5178, 1045.5568, 1046.6018, 1056.5515, 1057.6100, 1057.6107, 1061.6168, 1074.6073, 1074.6100, 1076.6123, 1102.6007, 1103.6257, 1105.6136, 1107.1058, 1113.5486, 1120.5498, 1122.5322, 1123.5986, 1128.5724, 1129.5924, 1134.3574, 1137.5463, 1145.6348, 1149.6057, 1153.6460, 1156.4957, 1161.6295, 1162.6174, 1164.1815, 1170.5497, 1172.6156, 1177.6410, 1180.6428, 1184.6412, 1188.5603, 1193.6041, 1206.5107, 1209.5889, 1211.6099, 1212.6160, 1215.6743, 1216.7081, 1219.6886, 1227.6234, 1229.7098, 1234.6414, 1240.5974, 1242.6003, 1243.6161, 1253.6216, 1260.6227, 1264.7130, 1265.7064, 1269.6498, 1270.6575, 1273.6556, 1277.6305, 1286.6099, 1289.6875, 1293.6401, 1303.6455, 1304.6677, 1307.6788, 1309.6780, 1318.6750, 1319.6589, 1328.6600, 1328.6620, 1331.7888, 1333.7644, 1336.0798, 1336.6155, 1350.6853, 1351.7202, 1356.5811, 1359.1302, 1359.6246, 1365.6489, 1374.7588, 1383.7037, 1390.6974, 1393.7992, 1400.6683, 1401.6989, 1414.8191, 1429.3142, 1436.7889, 1444.7122, 1445.7839, 1450.7223, 1456.7717, 1462.6630, 1467.7329, 1470.7394, 1471.2693, 1471.7250, 1472.2531, 1474.5243, 1475.7633, 1483.7141, 1488.5333, 1488.7194, 1493.7366, 1502.5601, 1513.7441, 1514.6849, 1517.6437, 1518.7053, 1523.7611, 1526.7343, 1528.6973, 1530.6893, 1536.8230, 1540.7114, 1551.7063, 1553.7252, 1554.7474, 1557.6992, 1558.7140, 1563.6658, 1564.3619, 1565.7223, 1566.7162, 1572.7062, 1580.8400, 1580.8423, 1586.8090, 1597.7913, 1599.8337, 1601.8030, 1614.8046, 1616.7968, 1628.7136, 1630.8103, 1635.7697, 1642.7862, 1643.7688, 1644.7915, 1646.8154, 1651.7681, 1657.7932, 1663.8466, 1664.8557, 1666.7545, 1695.8208, 1707.8933, 1721.9192, 1722.9375, 1728.8976, 1730.9116, 1732.2391, 1734.1844, 1739.7894, 1743.8966, 1755.2666, 1755.7703, 1757.4702, 1757.9089, 1758.4524, 1761.9023, 1765.7578, 1768.8339, 1769.8287, 1770.3949, 1772.8273, 1773.8792, 1774.9180, 1774.9200, 1786.8268, 1787.8239, 1809.0499, 1810.0303, 1829.8895, 1830.8380, 1833.3470, 1833.8501, 1851.8885, 1852.0399, 1862.9515, 1881.7703, 1895.9634, 1906.8195, 1908.8512, 1920.7972, 1932.0050, 1936.9479, 1939.8563, 1940.8103, 1943.8293, 1954.8351, 1956.8574, 1958.8956, 1965.7998, 1967.9565, 1969.9998, 1979.8927, 1981.3531,

1982.8179, 1995.8232, 1997.8312, 2003.8595, 2008.9971, 2011.8464, 2015.9408, 2019.8779, 2025.8735, 2031.9606, 2047.9397, 2062.9578, 2067.0020, 2076.8943, 2081.9827, 2082.9966, 2087.0103, 2088.1108, 2089.1162, 2097.0200, 2108.9771, 2110.0061, 2140.0918, 2143.1006, 2144.0718, 2149.8516, 2160.0554, 2174.0378, 2178.0471, 2179.0574, 2188.0547, 2191.0457, 2198.1831, 2199.1499, 2203.0386, 2211.1052, 2223.1582, 2225.1470, 2227.1042, 2228.0725, 2238.1086, 2239.1538, 2243.1243, 2244.0684, 2254.0896, 2255.1482, 2260.0779, 2270.2183, 2272.1736, 2284.1841, 2287.1108, 2307.0549, 2309.0659, 2310.0798, 2310.5271, 2311.0454, 2319.0945, 2337.6050, 2341.5510, 2353.0518, 2354.0767, 2355.0564, 2371.0581, 2383.9592, 2388.0437, 2392.1462, 2404.0471, 2412.0872, 2419.5879, 2420.0864, 2420.5930, 2423.2439, 2426.5667, 2427.1211, 2438.1724, 2455.1553, 2458.1648, 2459.1794, 2478.1587, 2516.1133, 2533.1262, 2542.1650, 2546.1746, 2549.1316, 2550.1252, 2559.1470, 2561.1729, 2562.1765, 2567.1514, 2572.1523, 2574.2678, 2576.1628, 2583.1218, 2587.2722, 2604.1682, 2612.1438, 2653.7173, 2655.1897, 2671.1787, 2687.1833, 2701.2820, 2706.2766, 2712.2104, 2714.2156, 2716.1953, 2717.2725, 2725.2354, 2729.1675, 2739.2351, 2743.2642, 2745.2900, 2754.2197, 2755.2358, 2759.2573, 2769.2219, 2771.2437, 2776.2644, 2785.2388, 2786.2617, 2787.2400, 2793.2439, 2801.2378, 2803.2302, 2809.2549, 2815.2153, 2817.2322, 2819.2236, 2830.2788, 2833.2280, 2836.2397, 2862.2927, 2873.2961, 2889.2024, 2894.3218, 2927.2520, 2931.3428, 2955.3005, 2974.4124, 2975.4084, 2990.3921, 3006.3206, 3011.2810, 3020.3557, 3021.2412, 3035.3281, 3053.3447, 3062.2456, 3086.4507, 3091.2622, 3103.3140, 3110.7285, 3113.8591, 3130.3557, 3140.4265, 3144.3657, 3159.4985, 3173.5386, 3174.5486, 3185.4236, 3218.3301, 3259.3625, 3312.3267, 3331.4678, 3347.4761, 3363.4788, 3390.4675, 3431.4570, 3447.4666, 3463.4895, 3485.6985, 3488.6545, 3489.6521, 3493.0195, 3494.5571, 3504.6423, 3509.5613, 3525.6025, 3538.4521, 3553.6230, 3579.5403, 3648.1956, 3655.4729, 3664.7500, 3695.6177, 3712.5720, 3738.5837, 3816.7043, 3937.8308, 4010.8696

20. F1MWQ0 **Mass:** 17662 **Score:** 15 **Expect:** 7.7e+002 **Matches:** 10

Complexin-3 OS=Bos taurus GN=CPLX3 PE=4 SV=1

Observed	Mr(expt)	Mr(calc)	ppm	Start	End	Miss	Ions	Peptide
726.3622	725.3550	725.3604	-7.53	1 -	6	0	---	-.MAFMVK.T
993.5046	992.4973	992.5039	-6.68	59 -	66	1	---	R.DAQFTQRK.A
1475.7633	1474.7560	1474.7627	-4.53	141 -	154	1	---	K.AQSTLGDLKQSAEK.C
1718.8790	1717.8717	1717.8846	-7.50	139 -	154	2	---	K.DKAQSTLGDLKQSAEK.C
1979.8927	1978.8854	1978.8677	8.98	104 -	119	1	---	R.ELAKMIEEDTEEEEER.A
1995.8232	1994.8159	1994.8626	-23.39	104 -	119	1	---	R.ELAKMIEEDTEEEEER.A + Oxidation (M)
2260.0779	2259.0706	2259.0365	15.1	42 -	58	2	---	R.EEYEEYQKQLVEEKMER.D
2546.1746	2545.1673	2545.1966	-11.49	85 -	107	1	---	K.NETDESQIQMAGGDVELPRELAK.M + Oxidation (M)
2701.2820	2700.2747	2700.3285	-19.92	1 -	26	2	---	-.MAFMVKTMVGGQLKNLTGSLGGGEDK.G + 2 Oxidation (M)
2717.2725	2716.2652	2716.3234	-21.43	1 -	26	2	---	-.MAFMVKTMVGGQLKNLTGSLGGGEDK.G + 3 Oxidation (M)

No match to: 716.4763, 723.4098, 724.2417, 725.2533, 728.3046, 729.4749, 734.4570, 736.3904, 737.4152, 738.3791, 744.8651, 745.3838, 747.4550, 768.3673, 788.4571, 801.4262, 805.4606, 813.4481, 814.4558, 820.4053, 823.4778, 829.4589, 830.4522, 839.3830, 842.5110, 857.4382, 860.4207, 865.4518, 870.4165, 872.5054, 880.4424, 882.4292, 887.4245, 887.9669, 888.4561, 891.4898, 894.4144, 894.8973, 897.5006, 898.4523, 912.5132, 913.5293, 925.4620, 927.4981, 931.4992, 934.5098, 957.5774, 960.5294, 961.4949, 963.4734, 965.4692, 970.5181, 972.4899, 975.5167, 976.5063, 977.5212, 978.5100, 978.5146, 983.6283, 992.4958, 995.4181, 998.5911, 1000.5959, 1002.5007, 1006.5014, 1011.5474, 1012.5447, 1014.5485, 1020.5577, 1021.5699, 1024.5516, 1027.5438, 1028.5397, 1030.5978, 1032.5238, 1036.5260, 1037.5405, 1039.5641, 1040.5908, 1042.5388, 1043.5178, 1045.5568, 1046.6018, 1056.5515, 1057.6100, 1057.6107, 1061.6168, 1074.6073, 1074.6100, 1076.6123, 1102.6007, 1103.6257, 1105.6136, 1107.1058, 1113.5486, 1120.5498, 1122.5322, 1123.5986, 1128.5724, 1129.5924, 1134.3574, 1137.5463, 1145.6348, 1149.6057, 1153.6460, 1156.4957, 1161.6295, 1162.6174, 1164.1815, 1170.5497, 1172.6156, 1177.6410, 1180.6428, 1184.6412, 1188.5603, 1193.6041, 1206.5107, 1209.5889, 1211.6099, 1212.6160, 1215.6743, 1216.7081, 1219.6886, 1227.6234, 1229.7098, 1234.6414, 1240.5974, 1242.6003, 1243.6161, 1253.6216, 1255.5972, 1255.6000, 1260.6227, 1264.7130, 1265.7064, 1269.6498, 1270.6575, 1273.6556, 1277.6305, 1286.6099, 1289.6875, 1293.6401, 1303.6455, 1304.6677, 1307.6788, 1309.6780, 1318.6750, 1319.6589, 1328.6600, 1328.6620, 1331.7888, 1333.7644, 1336.0798, 1336.6155, 1350.6853, 1351.7202, 1356.5811, 1359.1302, 1359.6246, 1365.6489, 1374.7588, 1383.7037, 1390.6974, 1393.7992, 1400.6683, 1401.6989, 1414.8191, 1429.3142, 1436.7889, 1444.7122, 1445.7839, 1450.7223, 1456.7717, 1462.6630, 1467.7329, 1470.7394, 1471.2693, 1471.7250, 1472.2531, 1474.5243, 1483.7141, 1488.5333, 1488.7194, 1493.7366, 1502.5601, 1513.7441, 1514.6849, 1516.6807, 1517.6437, 1518.7053, 1523.7611, 1526.7343, 1528.6973, 1530.6893, 1536.8230, 1540.7114, 1551.7063, 1553.7252, 1554.7474, 1557.6992, 1558.7140, 1563.6658, 1564.3619, 1565.7223, 1566.7162, 1572.7062, 1580.8400, 1580.8423, 1586.8090, 1597.7913, 1599.8337, 1601.8030, 1614.8046, 1616.7968, 1628.7136, 1630.8103, 1635.7697, 1642.7682, 1643.7688, 1644.7915, 1646.8154, 1651.7681, 1657.7932, 1663.8466, 1664.8557, 1666.7545, 1695.8208, 1707.8933, 1721.9192, 1722.9375, 1728.8976, 1730.9116, 1732.2391, 1734.1844, 1739.7894, 1743.8966, 1755.2666, 1755.7703, 1757.4702, 1757.9089, 1758.4524, 1758.8987, 1761.9023, 1765.7578, 1768.8339, 1769.8287, 1770.3949, 1772.8273, 1773.8792, 1774.9180, 1774.9200, 1786.8268, 1787.8239, 1809.0499, 1810.0303, 1829.8895, 1830.8380, 1833.3470, 1833.8501, 1851.8885, 1852.0399, 1862.9515, 1881.7703, 1895.9634, 1906.8195, 1908.8512, 1920.7972, 1932.0050, 1936.9479, 1939.8563, 1940.8103, 1943.8293, 1954.8351, 1956.8574, 1958.8956, 1965.7998, 1967.9565, 1969.9998, 1981.3531, 1982.8179, 1997.8312, 2003.8595, 2008.9971, 2011.8464, 2015.9408, 2019.8779, 2025.8735, 2031.9606, 2047.9397, 2062.9578, 2067.0020, 2076.8943, 2081.9827, 2082.9966, 2087.0103, 2088.1108, 2089.1162, 2096.0430, 2097.0200, 2108.9771, 2110.0061, 2139.0991, 2140.0918, 2143.1006, 2144.0718, 2149.8516, 2160.0554, 2174.0378, 2178.0471, 2179.0574, 2188.0547, 2191.0457, 2198.1831, 2199.1499, 2203.0386, 2211.1052, 2223.1582, 2225.1470, 2227.1042, 2228.0725, 2238.1086, 2239.1538, 2243.1243, 2244.0684, 2254.0896, 2255.1482, 2270.2183, 2272.1736, 2284.1841, 2287.1108, 2307.0549, 2309.0659, 2310.0798, 2310.5271, 2311.0454, 2319.0945, 2337.6050, 2341.5510, 2353.0518, 2354.0767, 2355.0564, 2371.0581, 2383.9592, 2388.0437, 2392.1462, 2404.0471, 2412.0872, 2419.5879, 2420.0864, 2420.5930, 2423.2439, 2426.5667, 2427.1211, 2438.1724, 2455.1553, 2458.1648, 2459.1794, 2478.1587, 2516.1133, 2533.1262, 2542.1650, 2549.1316, 2550.1252, 2559.1470, 2561.1729, 2562.1765, 2567.1514, 2572.1523, 2574.2678, 2576.1628, 2583.1218, 2587.2722, 2604.1682, 2612.1438, 2653.7173, 2655.1897, 2671.1787, 2687.1833, 2706.2766, 2712.2104, 2714.2156, 2716.1953, 2725.2354, 2729.1675, 2739.2351, 2743.2642, 2745.2900, 2754.2197, 2755.2358, 2759.2573, 2769.2219, 2771.2437, 2776.2644, 2785.2388, 2786.2617, 2787.2400, 2793.2439, 2801.2378, 2803.2302, 2809.2549, 2815.2153, 2817.2322, 2819.2236, 2830.2788, 2833.2280, 2836.2397, 2862.2927, 2873.2961, 2889.2024, 2894.3218, 2927.2520, 2931.3428, 2955.3005, 2974.4124, 2975.4084, 2990.3921, 3006.3206, 3011.2810, 3020.3557, 3021.2412, 3035.3281, 3053.3447, 3062.2456, 3086.4507, 3091.2622, 3103.3140, 3110.7285, 3113.8591, 3130.3557, 3140.4265, 3144.3657, 3159.4985, 3173.5386, 3174.5486, 3185.4236, 3218.3301, 3259.3625, 3312.3267, 3331.4678, 3347.4761, 3363.4788, 3390.4675, 3431.4570, 3447.4666, 3463.4895, 3485.6985, 3488.6545, 3489.6521, 3493.0195, 3494.5571, 3504.6423, 3509.5613, 3525.6025, 3538.4521, 3553.6230, 3579.5403, 3648.1956, 3655.4729, 3664.7500, 3695.6177, 3712.5720, 3738.5837, 3816.7043, 3937.8308, 4010.8696

21. Q3ZBJ9 **Mass:** 13284 **Score:** 15 **Expect:** 7.8e+002 **Matches:** 6

Protein BEX5 OS=Bos taurus GN=BEX5 PE=3 SV=1

Observed	Mr(expt)	Mr(calc)	ppm	Start	End	Miss	Ions	Peptide
1032.5238	1031.5165	1031.5069	9.29	1 -	8	2	---	-.MEKDPKER.R
1177.6410	1176.6337	1176.6615	-23.58	85 -	93	1	---	R.ELQLRYSLR.I
1240.5974	1239.5901	1239.5740	13.0	72 -	80	1	---	R.FMEEMRELR.R
2561.1729	2560.1656	2560.1057	23.4	49 -	71	0	---	R.EDMPNGLVNNIDIIDGDADDMER.F
2612.1438	2611.1365	2611.1279	3.32	10 -	33	0	---	R.EEEQAPVQNEEACPMGGGEGPKPR.E + Oxidation (M)
3053.3447	3052.3374	3052.3726	-11.54	7 -	33	2	---	K.ERREEEQAPVQNEEACPMGGGEGPKPR.E + Oxidation (M)

No match to: 716.4763, 723.4098, 724.2417, 725.2533, 726.3622, 728.3046, 729.4749, 734.4570, 736.3904, 737.4152, 738.3791, 744.8651, 745.3838, 747.4550, 768.3673, 788.4571, 801.4262, 805.4606, 813.4481, 814.4558, 820.4053, 823.4778, 829.4589, 830.4522, 839.3830, 842.5110, 857.4382, 860.4207, 865.4518, 870.4165, 872.5054, 880.4424, 882.4292, 887.4245, 887.9669, 888.4561, 891.4898, 894.4144, 894.8973, 897.5006, 898.4523, 912.5132, 913.5293, 925.4620, 927.4981, 931.4992, 934.5098, 957.5774, 960.5294, 961.4949, 963.4734, 965.4692, 970.5181, 972.4899, 975.5167, 976.5063, 977.5212, 978.5100, 978.5146, 983.6283, 992.4958, 993.5046, 995.4181, 998.5911, 1000.5959, 1002.5007, 1006.5014, 1011.5474, 1012.5447, 1014.5485, 1020.5577, 1021.5699, 1024.5516, 1027.5438, 1028.5397, 1030.5978, 1036.5260, 1037.5405, 1039.5641, 1040.5908, 1042.5388, 1043.5178, 1045.5568, 1046.6018, 1056.5515, 1057.6100, 1057.6107, 1061.6168, 1074.6073, 1074.6100, 1076.6123, 1102.6007, 1103.6257, 1105.6136, 1107.1058, 1113.5486, 1120.5498, 1122.5322, 1123.5986, 1128.5724, 1129.5924, 1134.3574, 1137.5463, 1145.6348, 1149.6057, 1153.6460, 1156.4957, 1161.6295, 1162.6174, 1164.1815, 1170.5497, 1172.6156, 1180.6428, 1184.6412, 1188.5603, 1193.6041, 1206.5107, 1209.5889, 1211.6099, 1212.6160, 1215.6743, 1216.7081, 1219.6886, 1227.6234, 1229.7098, 1234.6414, 1242.6003, 1243.6161, 1253.6216, 1255.5972, 1255.6000, 1260.6227, 1264.7130, 1265.7064, 1269.6498, 1270.6575, 1273.6556, 1277.6305, 1286.6099, 1289.6875, 1293.6401, 1303.6455, 1304.6677, 1307.6788, 1309.6780, 1318.6750, 1319.6589, 1328.6600, 1328.6620, 1331.7888, 1333.7644, 1336.0798, 1336.6155, 1350.6853, 1351.7202, 1356.5811, 1359.1302, 1359.6246, 1365.6489, 1374.7588, 1383.7037, 1390.6974, 1393.7992, 1400.6683, 1401.6989, 1414.8191, 1429.3142, 1436.7889, 1444.7122, 1445.7839, 1450.7223, 1456.7717, 1462.6630, 1467.7329, 1470.7394, 1471.2693, 1471.7250, 1472.2531, 1474.5243, 1475.7633, 1483.7141, 1488.5333, 1488.7194, 1493.7366, 1502.5601, 1513.7441, 1514.6849, 1516.6807, 1517.6437, 1518.7053, 1523.7611, 1526.7343, 1528.6973, 1530.6893, 1536.8230, 1540.7114, 1551.7063, 1553.7252, 1554.7474, 1557.6992, 1558.7140, 1563.6658, 1564.3619, 1565.7223, 1566.7162, 1572.7062, 1580.8400,

1580.8423, 1586.8090, 1597.7913, 1599.8337, 1601.8030, 1614.8046, 1616.7968, 1628.7136, 1630.8103, 1635.7697, 1642.7682, 1643.7688, 1644.7915, 1646.8154, 1651.7681, 1657.7932, 1663.8466, 1664.8557, 1666.7545, 1695.8208, 1707.8933, 1718.8790, 1721.9192, 1722.9375, 1728.8976, 1730.9116, 1732.2391, 1734.1844, 1739.7894, 1743.8966, 1755.2666, 1755.7703, 1757.4702, 1757.9089, 1758.4524, 1758.8987, 1761.9023, 1765.7578, 1768.8339, 1769.8287, 1770.3949, 1772.8273, 1773.8792, 1774.9180, 1774.9200, 1786.8268, 1787.8239, 1809.0499, 1810.0303, 1829.8895, 1830.8380, 1833.3470, 1833.8501, 1851.8885, 1852.0399, 1862.9515, 1881.7703, 1895.9634, 1906.8195, 1908.8512, 1920.7972, 1932.0050, 1936.9479, 1939.8563, 1940.8103, 1943.8293, 1954.8351, 1956.8574, 1958.8956, 1965.7998, 1967.9565, 1969.9998, 1979.8927, 1981.3531, 1982.8179, 1995.8232, 1997.8312, 2003.8595, 2008.9971, 2011.8464, 2015.9408, 2019.8779, 2025.8735, 2031.9606, 2047.9397, 2062.9578, 2067.0020, 2076.8943, 2081.9827, 2082.9966, 2087.0103, 2088.1108, 2089.1162, 2096.0430, 2097.0200, 2108.9771, 2110.0061, 2139.0991, 2140.0918, 2143.1006, 2144.0718, 2149.8516, 2160.0554, 2174.0378, 2178.0471, 2179.0574, 2188.0547, 2191.0457, 2198.1831, 2199.1499, 2203.0386, 2211.1052, 2223.1582, 2225.1470, 2227.1042, 2228.0725, 2238.1086, 2239.1538, 2243.1243, 2244.0684, 2254.0896, 2255.1482, 2260.0779, 2270.2183, 2272.1736, 2284.1841, 2287.1108, 2307.0549, 2309.0659, 2310.0798, 2310.5271, 2311.0454, 2319.0945, 2337.6050, 2341.5510, 2353.0518, 2354.0767, 2355.0564, 2371.0581, 2383.9592, 2388.0437, 2392.1462, 2404.0471, 2412.0872, 2419.5879, 2420.0864, 2420.5930, 2423.2439, 2426.5667, 2427.1211, 2438.1724, 2455.1553, 2458.1648, 2459.1794, 2478.1587, 2516.1133, 2533.1262, 2542.1650, 2546.1746, 2549.1316, 2550.1252, 2559.1470, 2562.1765, 2567.1514, 2572.1523, 2574.2678, 2576.1628, 2583.1218, 2587.2722, 2604.1682, 2653.7173, 2655.1897, 2671.1787, 2687.1833, 2701.2820, 2706.2766, 2712.2104, 2714.2156, 2716.1953, 2717.2725, 2725.2354, 2729.1675, 2739.2351, 2743.2642, 2745.2900, 2754.2197, 2755.2358, 2759.2573, 2769.2219, 2771.2437, 2776.2644, 2785.2388, 2786.2617, 2787.2400, 2793.2439, 2801.2378, 2803.2302, 2809.2549, 2815.2153, 2817.2322, 2819.2236, 2830.2788, 2833.2280, 2836.2397, 2862.2927, 2873.2961, 2889.2024, 2894.3218, 2927.2520, 2931.3428, 2955.3005, 2974.4124, 2975.4084, 2990.3921, 3006.3206, 3011.2810, 3020.3557, 3021.2412, 3035.3281, 3062.2456, 3086.4507, 3091.2622, 3103.3140, 3110.7285, 3113.8591, 3130.3557, 3140.4265, 3144.3657, 3159.4985, 3173.5386, 3174.5486, 3185.4236, 3218.3301, 3259.3625, 3312.3267, 3331.4678, 3347.4761, 3363.4788, 3390.4675, 3431.4570, 3447.4666, 3463.4895, 3485.6985, 3488.6545, 3489.6521, 3493.0195, 3494.5571, 3504.6423, 3509.5613, 3525.6025, 3538.4521, 3553.6230, 3579.5403, 3648.1956, 3655.4729, 3664.7500, 3695.6177, 3712.5720, 3738.5837, 3816.7043, 3937.8308, 4010.8696

22. Q08DP2 **Mass:** 22300 **Score:** 15 **Expect:** 8e+002 **Matches:** 6

Loss of heterozygosity 12 chromosomal region 1 protein homolog OS=Bos taurus GN=LOH12CR1 PE=2 SV=1

Observed	Mr(expt)	Mr(calc)	ppm	Start	End	Miss	Ions	Peptide
1273.6556	1272.6483	1272.6674	-14.97	68 -	80	0	---	K.GLLSGQTSPTNAK.L
1774.9180	1773.9107	1773.9043	3.61	29 -	45	1	---	R.AKMDDIVVVAQGSQASR.N
1774.9200	1773.9127	1773.9043	4.73	29 -	45	1	8	R.AKMDDIVVVAQGSQASR.N
2203.0386	2202.0313	2202.0701	-17.59	118 -	135	1	---	R.IKEMDLSVETLFSFMQER.Q
2255.1482	2254.1409	2254.1779	-16.41	139 -	157	2	---	R.YAKYAEQIQKVNEMSAILR.R
2459.1794	2458.1721	2458.2236	-20.94	118 -	137	2	---	R.IKEMDLSVETLFSFMQERQK.R

No match to: 716.4763, 723.4098, 724.2417, 725.2533, 726.3622, 728.3046, 729.4749, 734.4570, 736.3904, 737.4152, 738.3791, 744.8651, 745.3838, 747.4550, 768.3673, 788.4571, 801.4262, 805.4606, 813.4481, 814.4558, 820.4053, 823.4778, 829.4589, 830.4522, 839.3830, 842.5110, 857.4382, 860.4207, 865.4518, 870.4165, 872.5054, 880.4424, 882.4292, 887.4245, 887.9669, 888.4561, 891.4898, 894.4144, 894.8973, 897.5006, 898.4523, 912.5132, 913.5293, 925.4620, 927.4981, 931.4992, 934.5098, 957.5774, 960.5294, 961.4949, 963.4734, 965.4692, 970.5181, 972.4899, 975.5167, 976.5063, 977.5212, 978.5100, 978.5146, 983.6283, 992.4958, 993.5046, 995.4181, 998.5911, 1000.5959, 1002.5007, 1006.5014, 1011.5474, 1012.5447, 1014.5485, 1020.5577, 1021.5699, 1024.5516, 1027.5438, 1028.5397, 1030.5978, 1032.5238, 1036.5260, 1037.5405, 1039.5641, 1040.5908, 1042.5388, 1043.5178, 1045.5568, 1046.6018, 1056.5515, 1057.6100, 1057.6107, 1061.6168, 1074.6073, 1074.6100, 1076.6123, 1102.6007, 1103.6257, 1105.6136, 1107.1058, 1113.5486, 1120.5498, 1122.5322, 1123.5986, 1128.5724, 1129.5924, 1134.3574, 1137.5463, 1145.6348, 1149.6057, 1153.6460, 1156.4957, 1161.6295, 1162.6174, 1164.1815, 1170.5497, 1172.6156, 1177.6410, 1180.6428, 1184.6412, 1188.5603, 1193.6041, 1206.5107, 1209.5889, 1211.6099, 1212.6160, 1215.6743, 1216.7081, 1219.6886, 1227.6234, 1229.7098, 1234.6414, 1240.5974, 1242.6003, 1243.6161, 1253.6216, 1255.5972, 1255.6000, 1260.6227, 1264.7130, 1265.7064, 1269.6498, 1270.6575, 1277.6305, 1286.6099, 1289.6875, 1293.6401, 1303.6455, 1304.6677, 1307.6788, 1309.6780, 1318.6750, 1319.6589, 1328.6600, 1328.6620, 1331.7888, 1333.7644, 1336.0798, 1336.6155, 1350.6853, 1351.7202, 1356.5811, 1359.1302, 1359.6246, 1365.6489, 1374.7588, 1383.7037, 1390.6974, 1393.7992, 1400.6683, 1401.6989, 1414.8191, 1429.3142, 1436.7889, 1444.7122, 1445.7839, 1450.7223, 1456.7717, 1462.6630, 1467.7329, 1470.7394, 1471.2693, 1471.7250, 1472.2531, 1474.5243, 1475.7633, 1483.7141, 1488.5333, 1488.7194, 1493.7366, 1502.5601, 1513.7441, 1514.6849, 1516.6807, 1517.6437, 1518.7053, 1523.7611, 1526.7343, 1528.6973, 1530.6893, 1536.8230, 1540.7114, 1551.7063, 1553.7252, 1554.7474, 1557.6992, 1558.7140, 1563.6658, 1564.3619, 1565.7223, 1566.7162, 1572.7062, 1580.8400, 1580.8423, 1586.8090, 1597.7913, 1599.8337, 1601.8030, 1614.8046, 1616.7968, 1628.7136, 1630.8103, 1635.7697, 1642.7682, 1643.7688, 1644.7915, 1646.8154, 1651.7681, 1657.7932, 1663.8466, 1664.8557, 1666.7545, 1695.8208, 1707.8933, 1718.8790, 1721.9192, 1722.9375, 1728.8976, 1730.9116, 1732.2391, 1734.1844, 1739.7894, 1743.8966, 1755.2666, 1755.7703, 1757.4702, 1757.9089, 1758.4524, 1758.8987, 1761.9023, 1765.7578, 1768.8339, 1769.8287, 1770.3949, 1772.8273, 1773.8792, 1786.8268, 1787.8239, 1809.0499, 1810.0303, 1829.8895, 1830.8380, 1833.3470, 1833.8501, 1851.8885, 1852.0399, 1862.9515, 1881.7703, 1895.9634, 1906.8195, 1908.8512, 1920.7972, 1932.0050, 1936.9479, 1939.8563, 1940.8103, 1943.8293, 1954.8351, 1956.8574, 1958.8956, 1965.7998, 1967.9565, 1969.9998, 1979.8927, 1981.3531, 1982.8179, 1995.8232, 1997.8312, 2003.8595, 2008.9971, 2011.8464, 2015.9408, 2019.8779, 2025.8735, 2031.9606, 2047.9397, 2062.9578, 2067.0020, 2076.8943, 2081.9827, 2082.9966, 2087.0103, 2088.1108, 2089.1162, 2096.0430, 2097.0200, 2108.9771, 2110.0061, 2139.0991, 2140.0918, 2143.1006, 2144.0718, 2149.8516, 2160.0554, 2174.0378, 2178.0471, 2179.0574, 2188.0547, 2191.0457, 2198.1831, 2199.1499, 2211.1052, 2223.1582, 2225.1470, 2227.1042, 2228.0725, 2238.1086, 2239.1538, 2243.1243, 2244.0684, 2254.0896, 2260.0779, 2270.2183, 2272.1736, 2284.1841, 2287.1108, 2307.0549, 2309.0659, 2310.0798, 2310.5271, 2311.0454, 2319.0945, 2337.6050, 2341.5510, 2353.0518, 2354.0767, 2355.0564, 2371.0581, 2383.9592, 2388.0437, 2392.1462, 2404.0471, 2412.0872, 2419.5879, 2420.0864, 2420.5930, 2423.2439, 2426.5667, 2427.1211, 2438.1724, 2455.1553, 2458.1648, 2478.1587, 2516.1133, 2533.1262, 2542.1650, 2546.1746, 2549.1316, 2550.1252, 2559.1470, 2561.1729, 2562.1765, 2567.1514, 2572.1523, 2574.2678, 2576.1628, 2583.1218, 2587.2722, 2604.1682, 2612.1438, 2653.7173, 2655.1897, 2671.1787, 2687.1833, 2701.2820, 2706.2766, 2712.2104, 2714.2156, 2716.1953, 2717.2725, 2725.2354, 2729.1675, 2739.2351, 2743.2642, 2745.2900, 2754.2197, 2755.2358, 2759.2573, 2769.2219, 2771.2437, 2776.2644, 2785.2388, 2786.2617, 2787.2400, 2793.2439, 2801.2378, 2803.2302, 2809.2549, 2815.2153, 2817.2322, 2819.2236, 2830.2788, 2833.2280, 2836.2397, 2862.2927, 2873.2961, 2889.2024, 2894.3218, 2927.2520, 2931.3428, 2955.3005, 2974.4124, 2975.4084, 2990.3921, 3006.3206, 3011.2810, 3020.3557, 3021.2412, 3035.3281, 3053.3447, 3062.2456, 3086.4507, 3091.2622, 3103.3140, 3110.7285, 3113.8591, 3130.3557, 3140.4265, 3144.3657, 3159.4985, 3173.5386, 3174.5486, 3185.4236, 3218.3301, 3259.3625, 3312.3267, 3331.4678, 3347.4761, 3363.4788, 3390.4675, 3431.4570, 3447.4666, 3463.4895, 3485.6985, 3488.6545, 3489.6521, 3493.0195, 3494.5571, 3504.6423, 3509.5613, 3525.6025, 3538.4521, 3553.6230, 3579.5403, 3648.1956, 3655.4729, 3664.7500, 3695.6177, 3712.5720, 3738.5837, 3816.7043, 3937.8308, 4010.8696

23. Q3ZBC0 **Mass:** 7228 **Score:** 15 **Expect:** 8e+002 **Matches:** 5

DNA-directed RNA polymerases I, II, and III subunit RPABC4 OS=Bos taurus GN=POLR2K PE=1 SV=1

Observed	Mr(expt)	Mr(calc)	ppm	Start	End	Miss	Ions	Peptide
1286.6099	1285.6026	1285.6336	-24.12	1 -	11	1	---	-.MDTQKDVQPPK.Q
1551.7063	1550.6990	1550.6792	12.8	36 -	46	2	---	R.CRECGYRIMYK.K + Oxidation (M)
2974.4124	2973.4051	2973.3895	5.26	12 -	35	2	---	K.QQPMIYICGECHTENEIKSRDPIR.C
2990.3921	2989.3848	2989.3844	0.14	12 -	35	2	---	K.QQPMIYICGECHTENEIKSRDPIR.C + Oxidation (M)
3173.5386	3172.5313	3172.4740	18.1	6 -	31	2	---	K.DVQPPKQQPMIYICGECHTENEIKSR.D + Oxidation (M)

No match to: 716.4763, 723.4098, 724.2417, 725.2533, 726.3622, 728.3046, 729.4749, 734.4570, 736.3904, 737.4152, 738.3791, 744.8651, 745.3838, 747.4550, 768.3673, 788.4571, 801.4262, 805.4606, 813.4481, 814.4558, 820.4053, 823.4778, 829.4589, 830.4522, 839.3830, 842.5110, 857.4382, 860.4207, 865.4518, 870.4165, 872.5054, 880.4424, 882.4292, 887.4245, 887.9669, 888.4561, 891.4898, 894.4144, 894.8973, 897.5006, 898.4523, 912.5132, 913.5293, 925.4620, 927.4981, 931.4992, 934.5098, 957.5774, 960.5294, 961.4949, 963.4734, 965.4692, 970.5181, 972.4899, 975.5167, 976.5063, 977.5212, 978.5100, 978.5146, 983.6283, 992.4958, 993.5046, 995.4181, 998.5911, 1000.5959, 1002.5007, 1006.5014, 1011.5474, 1012.5447, 1014.5485, 1020.5577, 1021.5699, 1024.5516, 1027.5438, 1028.5397, 1030.5978, 1032.5238, 1036.5260, 1037.5405, 1039.5641, 1040.5908, 1042.5388, 1043.5178, 1045.5568, 1046.6018, 1056.5515, 1057.6100, 1057.6107, 1061.6168, 1074.6073, 1074.6100, 1076.6123, 1102.6007, 1103.6257, 1105.6136, 1107.1058, 1113.5486, 1120.5498, 1122.5322, 1123.5986, 1128.5724, 1129.5924, 1134.3574, 1137.5463, 1145.6348, 1149.6057, 1153.6460, 1156.4957, 1161.6295, 1162.6174, 1164.1815, 1170.5497, 1172.6156, 1177.6410, 1180.6428, 1184.6412, 1188.5603, 1193.6041, 1206.5107, 1209.5889, 1211.6099, 1212.6160, 1215.6743, 1216.7081, 1219.6886, 1227.6234, 1229.7098, 1234.6414, 1240.5974, 1242.6003, 1243.6161, 1253.6216, 1255.5972, 1255.6000, 1260.6227, 1264.7130, 1265.7064, 1269.6498, 1270.6575, 1273.6556, 1277.6305, 1289.6875, 1293.6401, 1303.6455, 1304.6677, 1307.6788, 1309.6780, 1318.6750, 1319.6589, 1328.6600, 1328.6620, 1331.7888, 1333.7644, 1336.0798, 1336.6155, 1350.6853, 1351.7202, 1356.5811, 1359.1302, 1359.6246, 1365.6489, 1374.7588, 1383.7037, 1390.6974, 1393.7992, 1400.6683, 1401.6989, 1414.8191, 1429.3142, 1436.7889, 1444.7122, 1445.7839, 1450.7223, 1456.7717, 1462.6630, 1467.7329, 1470.7394, 1471.2693, 1471.7250, 1472.2531, 1474.5243, 1475.7633, 1483.7141, 1488.5333, 1488.7194, 1493.7366, 1502.5601, 1513.7441, 1514.6849, 1516.6807, 1517.6437, 1518.7053, 1523.7611, 1526.7343, 1528.6973, 1530.6893, 1536.8230, 1540.7114, 1553.7252, 1554.7474, 1557.6992, 1558.7140, 1563.6658, 1564.3619, 1565.7223, 1566.7162, 1572.7062,

1580.8400, 1580.8423, 1586.8090, 1597.7913, 1599.8337, 1601.8030, 1614.8046, 1616.7968, 1628.7136, 1630.8103, 1635.7697, 1642.7682, 1643.7688, 1644.7915, 1646.8154, 1651.7681, 1657.7932, 1663.8466, 1664.8557, 1666.7545, 1695.8208, 1707.8933, 1718.8790, 1721.9192, 1722.9375, 1728.8976, 1730.9116, 1732.2391, 1734.1844, 1739.7894, 1743.8966, 1755.2666, 1755.7703, 1757.4702, 1757.9089, 1758.4524, 1758.8987, 1761.9023, 1765.7578, 1768.8339, 1769.8287, 1770.3949, 1772.8273, 1773.8792, 1774.9180, 1774.9200, 1786.8268, 1787.8239, 1809.0499, 1810.0303, 1829.8895, 1830.8380, 1833.3470, 1833.8501, 1851.8885, 1852.0399, 1862.9515, 1881.7703, 1895.9634, 1906.8195, 1908.8512, 1920.7972, 1932.0050, 1936.9479, 1939.8563, 1940.8103, 1943.8293, 1954.8351, 1956.8574, 1958.8956, 1965.7998, 1967.9565, 1969.9998, 1979.8927, 1981.3531, 1982.8179, 1995.8232, 1997.8312, 2003.8595, 2008.9971, 2011.8464, 2015.9408, 2019.8779, 2025.8735, 2031.9606, 2047.9397, 2062.9578, 2067.0020, 2076.8943, 2081.9827, 2082.9966, 2087.0103, 2088.1108, 2089.1162, 2096.0430, 2097.0200, 2108.9771, 2110.0061, 2139.0991, 2140.0918, 2143.1006, 2144.0718, 2149.8516, 2160.0554, 2174.0378, 2178.0471, 2179.0574, 2188.0547, 2191.0457, 2198.1831, 2199.1499, 2203.0386, 2211.1052, 2223.1582, 2225.1470, 2227.1042, 2228.0725, 2238.1086, 2239.1538, 2243.1243, 2244.0684, 2254.0896, 2255.1482, 2260.0779, 2270.2183, 2272.1736, 2284.1841, 2287.1108, 2307.0549, 2309.0659, 2310.0798, 2310.5271, 2311.0454, 2319.0945, 2337.6050, 2341.5510, 2353.0518, 2354.0767, 2355.0564, 2371.0581, 2383.9592, 2388.0437, 2392.1462, 2404.0471, 2412.0872, 2419.5879, 2420.0864, 2420.5930, 2423.2439, 2426.5667, 2427.1211, 2438.1724, 2455.1553, 2458.1648, 2459.1794, 2478.1587, 2516.1133, 2533.1262, 2542.1650, 2546.1746, 2549.1316, 2550.1252, 2559.1470, 2561.1729, 2562.1765, 2567.1514, 2572.1523, 2574.2678, 2576.1628, 2583.1218, 2587.2722, 2604.1682, 2612.1438, 2653.7173, 2655.1897, 2671.1787, 2687.1833, 2701.2820, 2706.2766, 2712.2104, 2714.2156, 2716.1953, 2717.2725, 2725.2354, 2729.1675, 2739.2351, 2743.2642, 2745.2900, 2754.2197, 2755.2358, 2759.2573, 2769.2219, 2771.2437, 2776.2644, 2785.2388, 2786.2617, 2787.2400, 2793.2439, 2801.2378, 2803.2302, 2809.2549, 2815.2153, 2817.2322, 2819.2236, 2830.2788, 2833.2280, 2836.2397, 2862.2927, 2873.2961, 2889.2024, 2894.3218, 2927.2520, 2931.3428, 2955.3005, 2975.4084, 3006.3206, 3011.2810, 3020.3557, 3021.2412, 3035.3281, 3053.3447, 3062.2456, 3086.4507, 3091.2622, 3103.3140, 3110.7285, 3113.8591, 3130.3557, 3140.4265, 3144.3657, 3159.4985, 3174.5486, 3185.4236, 3218.3301, 3259.3625, 3312.3267, 3331.4678, 3347.4761, 3363.4788, 3390.4675, 3431.4570, 3447.4666, 3463.4895, 3485.6985, 3488.6545, 3489.6521, 3493.0195, 3494.5571, 3504.6423, 3509.5613, 3525.6025, 3538.4521, 3553.6230, 3579.5403, 3648.1956, 3655.4729, 3664.7500, 3695.6177, 3712.5720, 3738.5837, 3816.7043, 3937.8308, 4010.8696

24. P00129 **Mass:** 13468 **Score:** 15 **Expect:** 8.2e+002 **Matches:** 7

Cytochrome b-c1 complex subunit 7 OS=Bos taurus GN=UQCRB PE=1 SV=3

Observed	Mr(expt)	Mr(calc)	ppm	Start	End	Miss	Ions	Peptide
977.5212	976.5139	976.5124	1.57	65 -	72	1	---	K.RALDLSMR.Q + Oxidation (M)
1012.5447	1011.5374	1011.5277	9.65	89 -	96	0	---	K.SYLEPYLK.E
1289.6875	1288.6802	1288.6524	21.6	50 -	59	1	---	R.RLPENLYNDR.V
1758.8987	1757.8914	1757.9172	-14.67	46 -	59	2	---	K.EAIRRLPENLYNDR.V
1769.8287	1768.8214	1768.8228	-0.75	35 -	49	1	---	R.DDTIHENDDVKEAIR.R
1932.0050	1930.9977	1930.9876	5.26	19 -	34	2	---	R.KWYYNAAGFNKLGLMR.D
2088.1108	2087.1035	2087.1058	-1.09	1 -	19	2	---	-.MAGRPAVSASSRWLEGIRK.W + Oxidation (M)

No match to: 716.4763, 723.4098, 724.2417, 725.2533, 726.3622, 728.3046, 729.4749, 734.4570, 736.3904, 737.4152, 738.3791, 744.8651, 745.3838, 747.4550, 768.3673, 788.4571, 801.4262, 805.4606, 813.4481, 814.4558, 820.4053, 823.4778, 829.4589, 830.4522, 839.3830, 842.5110, 857.4382, 860.4207, 865.4518, 870.4165, 872.5054, 880.4424, 882.4292, 887.4245, 887.9669, 888.4561, 891.4898, 894.4144, 894.8973, 897.5006, 898.4523, 912.5132, 913.5293, 925.4620, 927.4981, 931.4992, 934.5098, 957.5774, 960.5294, 961.4949, 963.4734, 965.4692, 970.5181, 972.4899, 975.5167, 976.5063, 978.5100, 978.5146, 983.6283, 992.4958, 993.5046, 995.4181, 998.5911, 1000.5959, 1002.5007, 1006.5014, 1011.5474, 1014.5485, 1020.5577, 1021.5699, 1024.5516, 1027.5438, 1028.5397, 1030.5978, 1032.5238, 1036.5260, 1037.5405, 1039.5641, 1040.5908, 1042.5388, 1043.5178, 1045.5568, 1046.6018, 1056.5515, 1057.6100, 1057.6107, 1061.6168, 1074.6073, 1074.6100, 1076.6123, 1102.6007, 1103.6257, 1105.6136, 1107.1058, 1113.5486, 1120.5498, 1122.5322, 1123.5986, 1128.5724, 1129.5924, 1134.3574, 1137.5463, 1145.6348, 1149.6057, 1153.6460, 1156.4957, 1161.6295, 1162.6174, 1164.1815, 1170.5497, 1172.6156, 1177.6410, 1180.6428, 1184.6412, 1188.5603, 1193.6041, 1206.5107, 1209.5889, 1211.6099, 1212.6160, 1215.6743, 1216.7081, 1219.6886, 1227.6234, 1229.7098, 1234.6414, 1240.5974, 1242.6003, 1243.6161, 1253.6216, 1255.5972, 1255.6000, 1260.6227, 1264.7130, 1265.7064, 1269.6498, 1270.6575, 1273.6556, 1277.6305, 1286.6099, 1293.6401, 1303.6455, 1304.6677, 1307.6788, 1309.6780, 1318.6750, 1319.6589, 1328.6600, 1328.6620, 1331.7888, 1333.7644, 1336.0798, 1336.6155, 1350.6853, 1351.7202, 1356.5811, 1359.1302, 1359.6246, 1365.6489, 1374.7588, 1383.7037, 1390.6974, 1393.7992, 1400.6683, 1401.6989, 1414.8191, 1429.3142, 1436.7889, 1444.7122, 1445.7839, 1450.7223, 1456.7717, 1462.6630, 1467.7329, 1470.7394, 1471.2693, 1471.7250, 1472.2531, 1474.5243, 1475.7633, 1483.7141, 1488.5333, 1488.7194, 1493.7366, 1502.5601, 1513.7441, 1514.6849, 1516.6807, 1517.6437, 1518.7053, 1523.7611, 1526.7343, 1528.6973, 1530.6893, 1536.8230, 1540.7114, 1551.7063, 1553.7252, 1554.7474, 1557.6992, 1558.7140, 1563.6658, 1564.3619, 1565.7223, 1566.7162, 1572.7062, 1580.8400, 1580.8423, 1586.8090, 1597.7913, 1599.8337, 1601.8030, 1614.8046, 1616.7968, 1628.7136, 1630.8103, 1635.7697, 1642.7682, 1643.7688, 1644.7915, 1646.8154, 1651.7681, 1657.7932, 1663.8466, 1664.8557, 1666.7545, 1695.8208, 1707.8933, 1718.8790, 1721.9192, 1722.9375, 1728.8976, 1730.9116, 1732.2391, 1734.1844, 1739.7894, 1743.8966, 1755.2666, 1755.7703, 1757.4702, 1757.9089, 1758.4524, 1761.9023, 1765.7578, 1768.8339, 1770.3949, 1772.8273, 1773.8792, 1774.9180, 1774.9200, 1786.8268, 1787.8239, 1809.0499, 1810.0303, 1829.8895, 1830.8380, 1833.3470, 1833.8501, 1851.8885, 1852.0399, 1862.9515, 1881.7703, 1895.9634, 1906.8195, 1908.8512, 1920.7972, 1936.9479, 1939.8563, 1940.8103, 1943.8293, 1954.8351, 1956.8574, 1958.8956, 1965.7998, 1967.9565, 1969.9998, 1979.8927, 1981.3531, 1982.8179, 1995.8232, 1997.8312, 2003.8595, 2008.9971, 2011.8464, 2015.9408, 2019.8779, 2025.8735, 2031.9606, 2047.9397, 2062.9578, 2067.0020, 2076.8943, 2081.9827, 2082.9966, 2087.0103, 2089.1162, 2096.0430, 2097.0200, 2108.9771, 2110.0061, 2139.0991, 2140.0918, 2143.1006, 2144.0718, 2149.8516, 2160.0554, 2174.0378, 2178.0471, 2179.0574, 2188.0547, 2191.0457, 2198.1831, 2199.1499, 2203.0386, 2211.1052, 2223.1582, 2225.1470, 2227.1042, 2228.0725, 2238.1086, 2239.1538, 2243.1243, 2244.0684, 2254.0896, 2255.1482, 2260.0779, 2270.2183, 2272.1736, 2284.1841, 2287.1108, 2307.0549, 2309.0659, 2310.0798, 2310.5271, 2311.0454, 2319.0945, 2337.6050, 2341.5510, 2353.0518, 2354.0767, 2355.0564, 2371.0581, 2383.9592, 2388.0437, 2392.1462, 2404.0471, 2412.0872, 2419.5879, 2420.0864, 2420.5930, 2423.2439, 2426.5667, 2427.1211, 2438.1724, 2455.1553, 2458.1648, 2459.1794, 2478.1587, 2516.1133, 2533.1262, 2542.1650, 2546.1746, 2549.1316, 2550.1252, 2559.1470, 2561.1729, 2562.1765, 2567.1514, 2572.1523, 2574.2678, 2576.1628, 2583.1218, 2587.2722, 2604.1682, 2612.1438, 2653.7173, 2655.1897, 2671.1787, 2687.1833, 2701.2820, 2706.2766, 2712.2104, 2714.2156, 2716.1953, 2717.2725, 2725.2354, 2729.1675, 2739.2351, 2743.2642, 2745.2900, 2754.2197, 2755.2358, 2759.2573, 2769.2219, 2771.2437, 2776.2644, 2785.2388, 2786.2617, 2787.2400, 2793.2439, 2801.2378, 2803.2302, 2809.2549, 2815.2153, 2817.2322, 2819.2236, 2830.2788, 2833.2280, 2836.2397, 2862.2927, 2873.2961, 2889.2024, 2894.3218, 2927.2520, 2931.3428, 2955.3005, 2974.4124, 2975.4084, 2990.3921, 3006.3206, 3011.2810, 3020.3557, 3021.2412, 3035.3281, 3053.3447, 3062.2456, 3086.4507, 3091.2622, 3103.3140, 3110.7285, 3113.8591, 3130.3557, 3140.4265, 3144.3657, 3159.4985, 3173.5386, 3174.5486, 3185.4236, 3218.3301, 3259.3625, 3312.3267, 3331.4678, 3347.4761, 3363.4788, 3390.4675, 3431.4570, 3447.4666, 3463.4895, 3485.6985, 3488.6545, 3489.6521, 3493.0195, 3494.5571, 3504.6423, 3509.5613, 3525.6025, 3538.4521, 3553.6230, 3579.5403, 3648.1956, 3655.4729, 3664.7500, 3695.6177, 3712.5720, 3738.5837, 3816.7043, 3937.8308, 4010.8696

25. A4FV50 **Mass:** 40277 **Score:** 15 **Expect:** 8.4e+002 **Matches:** 14

MGC142792 protein OS=Bos taurus GN=MGC142792 PE=2 SV=1

Observed	Mr(expt)	Mr(calc)	ppm	Start	End	Miss	Ions	Peptide
716.4763	715.4690	715.4592	13.7	297 -	302	1	---	K.LTLKNK.F
1145.6348	1144.6275	1144.6088	16.4	179 -	188	1	---	K.ELTADARELK.G
1657.7932	1656.7859	1656.8004	-8.73	301 -	313	1	---	K.NKFVEFDMKPVCK.K + Oxidation (M)
2140.0918	2139.0845	2139.0506	15.8	216 -	233	1	---	R.VVNAMGKQWHVEHFVCAK.C
2355.0564	2354.0491	2354.0413	3.33	2 -	24	0	---	M.LGVASGMTNSNMANALANASCER.C + Oxidation (M)
2371.0581	2370.0508	2370.0362	6.17	2 -	24	0	---	M.LGVASGMTNSNMANALANASCER.C + 2 Oxidation (M)
2542.1650	2541.1577	2541.1749	-6.76	282 -	302	2	---	K.AWCVHCFACSTCSAKLTLKNK.F
2655.1897	2654.1824	2654.1781	1.62	2 -	26	1	---	M.LGVASGMTNSNMANALANASCERCR.G
2671.1787	2670.1714	2670.1730	-0.60	2 -	26	1	---	M.LGVASGMTNSNMANALANASCERCR.G + Oxidation (M)
2687.1833	2686.1760	2686.1680	3.00	2 -	26	1	---	M.LGVASGMTNSNMANALANASCERCR.G + 2 Oxidation (M)
2786.2617	2785.2544	2785.2186	12.9	1 -	26	1	---	-.MLGVASGMTNSNMANALANASCERCR.G
2889.2024	2888.1951	2888.2581	-21.82	247 -	269	1	---	R.KGLAYCETHYNQLFGDVCFHCNR.V
3091.2622	3090.2549	3090.2704	-5.00	70 -	93	0	---	K.YCEHDFQMLFAPCCHQCGEFIIGR.V + Oxidation (M)
3431.4570	3430.4497	3430.5178	-19.85	70 -	96	1	---	K.YCEHDFQMLFAPCCHQCGEFIIGRVIK.A + Oxidation (M)

No match to: 723.4098, 724.2417, 725.2533, 726.3622, 728.3046, 729.4749, 734.4570, 736.3904, 737.4152, 738.3791, 744.8651, 745.3838, 747.4550, 768.3673, 788.4571, 801.4262, 805.4606, 813.4481, 814.4558, 820.4053, 823.4778, 829.4589, 830.4522, 839.3830, 842.5110, 857.4382, 860.4207, 865.4518, 870.4165, 872.5054, 880.4424, 882.4292, 887.4245, 887.9669, 888.4561, 891.4898, 894.4144, 894.8973, 897.5006, 898.4523, 912.5132, 913.5293, 925.4620, 927.4981, 931.4992, 934.5098, 957.5774, 960.5294, 961.4949, 963.4734, 965.4692, 970.5181, 972.4899, 975.5167, 976.5063, 977.5212, 978.5100, 978.5146, 983.6283, 992.4958, 993.5046, 995.4181, 998.5911,

1000.5959, 1002.5007, 1006.5014, 1011.5474, 1012.5447, 1014.5485, 1020.5577, 1021.5699, 1024.5516, 1027.5438, 1028.5397, 1030.5978,
1032.5238, 1036.5260, 1037.5405, 1039.5641, 1040.5908, 1042.5388, 1043.5178, 1045.5568, 1046.6018, 1056.5515, 1057.6100, 1057.6107,
1061.6168, 1074.6073, 1074.6100, 1076.6123, 1102.6007, 1103.6257, 1105.6136, 1107.1058, 1113.5486, 1120.5498, 1122.5322, 1123.5986,
1128.5724, 1129.5924, 1134.3574, 1137.5463, 1149.6057, 1153.6460, 1156.4957, 1161.6295, 1162.6174, 1164.1815, 1170.5497, 1172.6156,
1177.6410, 1180.6428, 1184.6412, 1188.5603, 1193.6041, 1206.5107, 1209.5889, 1211.6099, 1212.6160, 1215.6743, 1216.7081, 1219.6886,
1227.6234, 1229.7098, 1234.6414, 1240.5974, 1242.6003, 1243.6161, 1253.6216, 1255.5972, 1255.6000, 1260.6227, 1264.7130, 1265.7064,
1269.6498, 1270.6575, 1273.6556, 1277.6305, 1286.6099, 1289.6875, 1293.6401, 1303.6455, 1304.6677, 1307.6788, 1309.6780, 1318.6750,
1319.6589, 1328.6600, 1328.6620, 1331.7888, 1333.7644, 1336.0798, 1336.6155, 1350.6853, 1351.7202, 1356.5811, 1359.1302, 1359.6246,
1365.6489, 1374.7588, 1383.7037, 1390.6974, 1393.7992, 1400.6683, 1401.6989, 1414.8191, 1429.3142, 1436.7889, 1444.7122, 1445.7839,
1450.7223, 1456.7717, 1462.6630, 1467.7329, 1470.7394, 1471.2693, 1471.7250, 1472.2531, 1474.5243, 1475.7633, 1483.7141, 1488.5333,
1488.7194, 1493.7366, 1502.5601, 1513.7441, 1514.6849, 1516.6807, 1517.6437, 1518.7053, 1523.7611, 1526.7343, 1528.6973, 1530.6893,
1536.8230, 1540.7114, 1551.7063, 1553.7252, 1554.7474, 1557.6992, 1558.7140, 1563.6658, 1564.3619, 1565.7223, 1566.7162, 1572.7062,
1580.8400, 1580.8423, 1586.8090, 1597.7913, 1599.8337, 1601.8030, 1614.8046, 1616.7968, 1628.7136, 1630.8103, 1635.7697, 1642.7682,
1643.7688, 1644.7915, 1646.8154, 1651.7681, 1663.8466, 1664.8557, 1666.7545, 1695.8208, 1707.8933, 1718.8790, 1721.9192, 1722.9375,
1728.8976, 1730.9116, 1732.2391, 1734.1844, 1739.7894, 1743.8966, 1755.2666, 1755.7703, 1757.4702, 1757.9089, 1758.4524, 1758.8987,
1761.9023, 1765.7578, 1768.8339, 1769.8287, 1770.3949, 1772.8273, 1773.8792, 1774.9180, 1774.9200, 1786.8268, 1787.8239, 1809.0499,
1810.0303, 1829.8895, 1830.8380, 1833.3470, 1833.8501, 1851.8885, 1852.0399, 1862.9515, 1881.7703, 1895.9634, 1906.8195, 1908.8512,
1920.7972, 1932.0050, 1936.9479, 1939.8563, 1940.8103, 1943.8293, 1954.8351, 1956.8574, 1958.8956, 1965.7998, 1967.9565, 1969.9998,
1979.8927, 1981.3531, 1982.8179, 1995.8232, 1997.8312, 2003.8595, 2008.9971, 2011.8464, 2015.9408, 2019.8779, 2025.8735, 2031.9606,
2047.9397, 2062.9578, 2067.0020, 2076.8943, 2081.9827, 2082.9966, 2087.0103, 2088.1108, 2089.1162, 2096.0430, 2097.0200, 2108.9771,
2110.0061, 2139.0991, 2143.1006, 2144.0718, 2149.8516, 2160.0554, 2174.0378, 2178.0471, 2179.0574, 2188.0547, 2191.0457, 2198.1831,
2199.1499, 2203.0386, 2211.1052, 2223.1582, 2225.1470, 2227.1042, 2228.0725, 2238.1086, 2239.1538, 2243.1243, 2244.0684, 2254.0896,
2255.1482, 2260.0779, 2270.2183, 2272.1736, 2284.1841, 2287.1108, 2307.0549, 2309.0659, 2310.0798, 2310.5271, 2311.0454, 2319.0945,
2337.6050, 2341.5510, 2353.0518, 2354.0767, 2383.9592, 2388.0437, 2392.1462, 2404.0471, 2412.0872, 2419.5879, 2420.0864, 2420.5930,
2423.2439, 2426.5667, 2427.1211, 2438.1724, 2455.1553, 2458.1648, 2459.1794, 2478.1587, 2516.1133, 2533.1262, 2546.1746, 2549.1316,
2550.1252, 2559.1470, 2561.1729, 2562.1765, 2567.1514, 2572.1523, 2574.2678, 2576.1628, 2583.1218, 2587.2722, 2604.1682, 2612.1438,
2653.7173, 2701.2820, 2706.2766, 2712.2104, 2714.2156, 2716.1953, 2717.2725, 2725.2354, 2729.1675, 2739.2351, 2743.2642, 2745.2900,
2754.2197, 2755.2358, 2759.2573, 2769.2219, 2771.2437, 2776.2644, 2785.2388, 2787.2400, 2793.2439, 2801.2378, 2803.2302, 2809.2549,
2815.2153, 2817.2322, 2819.2236, 2830.2788, 2833.2280, 2836.2397, 2862.2927, 2873.2961, 2894.3218, 2927.2520, 2931.3428, 2955.3005,
2974.4124, 2975.4084, 2990.3921, 3006.3206, 3011.2810, 3020.3557, 3021.2412, 3035.3281, 3053.3447, 3062.2456, 3086.4507, 3103.3140,
3110.7285, 3113.8591, 3130.3557, 3140.4265, 3144.3657, 3159.4985, 3173.5386, 3174.5486, 3185.4236, 3218.3301, 3259.3625, 3312.3267,
3331.4678, 3347.4761, 3363.4788, 3390.4675, 3447.4666, 3463.4895, 3485.6985, 3488.6545, 3489.6521, 3493.0195, 3494.5571, 3504.6423,
3509.5613, 3525.6025, 3538.4521, 3553.6230, 3579.5403, 3648.1956, 3655.4729, 3664.7500, 3695.6177, 3712.5720, 3738.5837, 3816.7043,
3937.8308, 4010.8696

26. E1BKM8 **Mass:** 51997 **Score:** 14 **Expect:** 8.8e+002 **Matches:** 19

Uncharacterized protein OS=Bos taurus GN=CYP24A1 PE=4 SV=2

Observed	Mr(expt)	Mr(calc)	ppm	Start	End	Miss	Ions	Peptide
913.5293	912.5220	912.5433	-23.27	129 -	135	0	---	R.LEIKPWK.A
925.4620	924.4548	924.4413	14.5	121 -	128	0	---	R.TESAHPQR.L
992.4958	991.4885	991.5120	-23.73	1 -	9	1	---	-.MSSPISKSR.S
1120.5498	1119.5425	1119.5342	7.40	284 -	292	1	---	K.SCVDNRLEK.Y
1145.6348	1144.6275	1144.6241	3.02	387 -	396	0	---	R.LNPTVPFTTR.T
1212.6160	1211.6087	1211.6155	-5.58	252 -	261	0	---	K.MMVTPVELHR.S
1216.7081	1215.7008	1215.7083	-6.13	165 -	174	1	---	K.KLMKPVEIMK.L
1253.6216	1252.6143	1252.6308	-13.13	373 -	382	1	---	R.NMPYLKACLK.E + Oxidation (M)
1269.6498	1268.6425	1268.6149	21.7	82 -	91	0	---	K.QHDTLVEYHK.K
1450.7223	1449.7150	1449.7463	-21.59	198 -	209	1	---	R.GRIEDLYTELNK.W
1664.8557	1663.8484	1663.8352	7.95	383 -	396	1	---	K.ESMRLNPTVPFTTR.T + Oxidation (M)
1758.8987	1757.8914	1757.8638	15.7	267 -	280	0	---	R.VWQAHTQAWDTIFR.S
1772.8273	1771.8200	1771.8419	-12.34	373 -	386	2	---	R.NMPYLKACLKESMR.L + 2 Oxidation (M)
1810.0303	1809.0230	1808.9892	18.7	160 -	174	2	---	R.SAFQKKLMKPVEIMK.L + 2 Oxidation (M)
1829.8895	1828.8822	1828.9182	-19.65	210 -	223	1	---	K.WSFESICLVLYEKR.F
2174.0378	2173.0305	2172.9862	20.4	244 -	261	1	---	K.TMMSMFGKMMVTPVELHR.S + 3 Oxidation (M)
2311.0454	2310.0381	2310.0620	-10.34	179 -	197	1	---	K.INEVLADFMGRIDELCDER.G + Oxidation (M)
2612.1438	2611.1365	2611.1471	-4.07	293 -	313	0	---	K.YSEQPSMDFLCDIYHHNQLSK.K
2745.2900	2744.2827	2744.2940	-4.12	244 -	266	2	---	K.TMMSMFGKMMVTPVELHRSLNTR.V + 3 Oxidation (M)

No match to: 716.4763, 723.4098, 724.2417, 725.2533, 726.3622, 728.3046, 729.4749, 734.4570, 736.3904, 737.4152, 738.3791,
744.8651, 745.3838, 747.4550, 768.3673, 788.4571, 801.4262, 805.4606, 813.4481, 814.4558, 820.4053, 823.4778, 829.4589, 830.4522,
839.3830, 842.5110, 857.4382, 860.4207, 865.4518, 870.4165, 872.5054, 880.4424, 882.4292, 887.4245, 887.9669, 888.4561, 891.4898,
894.4144, 894.8973, 897.5006, 898.4523, 912.5132, 927.4981, 931.4992, 934.5098, 957.5774, 960.5294, 961.4949, 963.4734, 965.4692,
970.5181, 972.4899, 975.5167, 976.5063, 977.5212, 978.5100, 978.5146, 983.6283, 993.5046, 995.4181, 998.5911, 1000.5959, 1002.5007,
1006.5014, 1011.5474, 1012.5447, 1014.5485, 1020.5577, 1021.5699, 1024.5516, 1027.5438, 1028.5397, 1030.5978, 1032.5238, 1036.5260,
1037.5405, 1039.5641, 1040.5908, 1042.5388, 1043.5178, 1045.5568, 1046.6018, 1056.5515, 1057.6100, 1057.6107, 1061.6168, 1074.6073,
1074.6100, 1076.6123, 1102.6007, 1103.6257, 1105.6136, 1107.1058, 1113.5486, 1122.5322, 1123.5986, 1128.5724, 1129.5924, 1134.3574,
1137.5463, 1149.6057, 1153.6460, 1156.4957, 1161.6295, 1162.6174, 1164.1815, 1170.5497, 1172.6156, 1177.6410, 1180.6428, 1184.6412,
1188.5603, 1193.6041, 1206.5107, 1209.5889, 1211.6099, 1215.6743, 1219.6886, 1227.6234, 1229.7098, 1234.6414, 1240.5974, 1242.6003,
1243.6161, 1255.5972, 1255.6000, 1260.6227, 1264.7130, 1265.7064, 1270.6575, 1273.6556, 1277.6305, 1286.6099, 1289.6875, 1293.6401,
1303.6455, 1304.6677, 1307.6788, 1309.6780, 1318.6750, 1319.6589, 1328.6600, 1328.6620, 1331.7888, 1333.7644, 1336.0798, 1336.6155,
1350.6853, 1351.7202, 1356.5811, 1359.1302, 1359.6246, 1365.6489, 1374.7588, 1383.7037, 1390.6974, 1393.7992, 1400.6683, 1401.6989,
1414.8191, 1429.3142, 1436.7889, 1444.7122, 1445.7839, 1456.7717, 1462.6630, 1467.7329, 1470.7394, 1471.2693, 1471.7250, 1472.2531,
1474.5243, 1475.7633, 1483.7141, 1488.5333, 1488.7194, 1493.7366, 1502.5601, 1513.7441, 1514.6849, 1516.6807, 1517.6437, 1518.7053,
1523.7611, 1526.7343, 1528.6973, 1530.6893, 1536.8230, 1540.7114, 1551.7063, 1553.7252, 1554.7474, 1557.6992, 1558.7140, 1563.6658,
1564.3619, 1565.7223, 1566.7162, 1572.7062, 1580.8400, 1580.8423, 1586.8090, 1597.7913, 1599.8337, 1601.8030, 1614.8046, 1616.7968,
1628.7136, 1630.8103, 1635.7697, 1642.7682, 1643.7688, 1644.7915, 1646.8154, 1651.7681, 1657.7932, 1663.8466, 1666.7545, 1695.8208,
1707.8933, 1718.8790, 1721.9192, 1722.9375, 1728.8976, 1730.9116, 1732.2391, 1734.1844, 1739.7894, 1743.8966, 1755.2666, 1755.7703,
1757.4702, 1757.9089, 1758.4524, 1761.9023, 1765.7578, 1768.8339, 1769.8287, 1770.3949, 1773.8792, 1774.9180, 1774.9200, 1786.8268,
1787.8239, 1809.0499, 1830.8380, 1833.3470, 1833.8501, 1851.8885, 1852.0399, 1862.9515, 1881.7703, 1895.9634, 1906.8195, 1908.8512,
1920.7972, 1932.0050, 1936.9479, 1939.8563, 1940.8103, 1943.8293, 1954.8351, 1956.8574, 1958.8956, 1965.7998, 1967.9565, 1969.9998,
1979.8927, 1981.3531, 1982.8179, 1995.8232, 1997.8312, 2003.8595, 2008.9971, 2011.8464, 2015.9408, 2019.8779, 2025.8735, 2031.9606,
2047.9397, 2062.9578, 2067.0020, 2076.8943, 2081.9827, 2082.9966, 2087.0103, 2088.1108, 2089.1162, 2096.0430, 2097.0200, 2108.9771,
2110.0061, 2139.0991, 2140.0918, 2143.1006, 2144.0718, 2149.8516, 2160.0554, 2178.0471, 2179.0574, 2188.0547, 2191.0457, 2198.1831,
2199.1499, 2203.0386, 2211.1052, 2223.1582, 2225.1470, 2227.1042, 2228.0725, 2238.1086, 2239.1538, 2243.1243, 2244.0684, 2254.0896,
2255.1482, 2260.0779, 2270.2183, 2272.1736, 2284.1841, 2287.1108, 2307.0549, 2309.0659, 2310.0798, 2310.5271, 2319.0945, 2337.6050,
2341.5510, 2353.0518, 2354.0767, 2355.0564, 2371.0581, 2383.9592, 2388.0437, 2392.1462, 2404.0471, 2412.0872, 2419.5879, 2420.0864,
2420.5930, 2423.2439, 2426.5667, 2427.1211, 2438.1724, 2455.1553, 2458.1648, 2459.1794, 2478.1587, 2516.1133, 2533.1262, 2542.1650,
2546.1746, 2549.1316, 2550.1252, 2559.1470, 2561.1729, 2562.1765, 2567.1514, 2572.1523, 2574.2678, 2576.1628, 2583.1218, 2587.2722,
2604.1682, 2653.7173, 2655.1897, 2671.1787, 2687.1833, 2701.2820, 2706.2766, 2712.2104, 2714.2156, 2716.1953, 2717.2725, 2725.2354,
2729.1675, 2739.2351, 2743.2642, 2754.2197, 2755.2358, 2759.2573, 2769.2219, 2771.2437, 2776.2644, 2785.2388, 2786.2617, 2787.2400,
2793.2439, 2801.2378, 2803.2302, 2809.2549, 2815.2153, 2817.2322, 2819.2236, 2830.2788, 2833.2280, 2836.2397, 2862.2927, 2873.2961,
2889.2024, 2894.3218, 2927.2520, 2931.3428, 2955.3005, 2974.4124, 2975.4084, 2990.3921, 3006.3206, 3011.2810, 3020.3557, 3021.2412,
3035.3281, 3053.3447, 3062.2456, 3086.4507, 3091.2622, 3103.3140, 3110.7285, 3113.8591, 3130.3557, 3140.4265, 3144.3657, 3159.4985,
3173.5386, 3174.5486, 3185.4236, 3218.3301, 3259.3625, 3312.3267, 3331.4678, 3347.4761, 3363.4788, 3390.4675, 3431.4570, 3447.4666,
3463.4895, 3485.6985, 3488.6545, 3489.6521, 3493.0195, 3494.5571, 3504.6423, 3509.5613, 3525.6025, 3538.4521, 3553.6230, 3579.5403,
3648.1956, 3655.4729, 3664.7500, 3695.6177, 3712.5720, 3738.5837, 3816.7043, 3937.8308, 4010.8696

27. E1BJL9 **Mass:** 65973 **Score:** 14 **Expect:** 9.2e+002 **Matches:** 23

Uncharacterized protein OS=Bos taurus GN=CCDC173 PE=4 SV=2

Observed	Mr(expt)	Mr(calc)	ppm	Start	End	Miss	Ions	Peptide
716.4763	715.4690	715.4592	13.7	105	110	2	---	K.LEAKKK.R
805.4606	804.4534	804.4705	-21.30	177	183	2	---	K.SKIKSDK.K
813.4481	812.4408	812.4327	10.1	80	85	1	---	K.KEMHLR.S
934.5098	933.5026	933.4953	7.74	307	314	1	---	R.LTQMGKEK.E
977.5212	976.5139	976.5124	1.58	321	327	2	---	R.LMEERRK.R + Oxidation (M)
978.5100	977.5027	977.4818	21.4	169	176	0	---	R.DAQIEFQK.S
1028.5397	1027.5324	1027.5087	23.1	539	546	1	---	K.YNNFQKSK.G
1032.5238	1031.5165	1031.4995	16.5	263	270	1	---	K.EEINESRR.L
1036.5260	1035.5187	1035.5349	-15.59	196	203	2	---	K.AFKEEREK.A
1162.6174	1161.6101	1161.5818	24.3	271	279	0	---	R.LFFEHLNDK.H
1193.6041	1192.5968	1192.6088	-10.03	169	178	1	---	R.DAQIEFQKSK.I
1318.6750	1317.6677	1317.6830	-11.56	270	279	1	---	R.RLFFEHLNDK.H
1365.6489	1364.6416	1364.6671	-18.64	477	488	1	---	R.EVIESESESTKK.Y
1400.6683	1399.6610	1399.6514	6.90	315	325	1	---	K.EAETHRLMEER.R
1436.7889	1435.7816	1435.7606	14.7	1	13	1	---	-.AAGTLEMLVRFGR.R + Oxidation (M)
1475.7633	1474.7560	1474.7449	7.53	382	393	2	---	R.ALVMKNKEEEER.Q
1657.7932	1656.7859	1656.7889	-1.80	313	325	2	---	K.EKEAETHRLMEER.R
1663.8466	1662.8393	1662.8802	-24.57	496	512	1	---	K.AVQEGPGGGRGPVLVDR.G
1810.0303	1809.0230	1809.0049	9.99	270	283	2	---	R.RLFFEHLNDKHIIK.A
2096.0430	2095.0357	2095.0255	4.88	239	255	2	---	K.DAEEIKRQNSLYEIEMK.K
2140.0918	2139.0845	2139.0847	-0.10	459	476	2	---	R.LTEALVAEKEKEFQDYAR.E
2254.0896	2253.0823	2253.0947	-5.47	451	469	2	---	K.QAESEYCRLTEALVAEKEK.E
2576.1628	2575.1555	2575.2186	-24.47	20	41	1	---	K.ESAEMKNCEEDPVLYPPLLPTK.V + Oxidation (M)

No match to: 723.4098, 724.2417, 725.2533, 726.3622, 728.3046, 729.4749, 734.4570, 736.3904, 737.4152, 738.3791, 744.8651, 745.3838, 747.4550, 768.3673, 788.4571, 801.4262, 814.4558, 820.4053, 823.4778, 829.4589, 830.4522, 839.3830, 842.5110, 857.4382, 860.4207, 865.4518, 870.4165, 872.5054, 880.4424, 882.4292, 887.4245, 887.9669, 888.4561, 891.4898, 894.4144, 894.8973, 897.5006, 898.4523, 912.5132, 913.5293, 925.4620, 927.4981, 931.4992, 957.5774, 960.5294, 961.4949, 963.4734, 965.4692, 970.5181, 972.4899, 975.5167, 976.5063, 978.5146, 983.6283, 992.4958, 993.5046, 995.4181, 998.5911, 1000.5959, 1002.5007, 1006.5014, 1011.5474, 1012.5447, 1014.5485, 1020.5577, 1021.5699, 1024.5516, 1027.5438, 1030.5978, 1037.5405, 1039.5641, 1040.5908, 1042.5388, 1043.5178, 1045.5568, 1046.6018, 1056.5515, 1057.6100, 1057.6107, 1061.6168, 1074.6073, 1074.6100, 1076.6123, 1102.6007, 1103.6257, 1105.6136, 1107.1058, 1113.5486, 1120.5498, 1122.5322, 1123.5986, 1128.5724, 1129.5924, 1134.3574, 1137.5463, 1145.6348, 1149.6057, 1153.6460, 1156.4957, 1161.6295, 1164.1815, 1170.5497, 1172.6156, 1177.6410, 1180.6428, 1184.6412, 1188.5603, 1206.5107, 1209.5889, 1211.6099, 1212.6160, 1215.6743, 1216.7081, 1219.6886, 1227.6234, 1229.7098, 1234.6414, 1240.5974, 1242.6003, 1243.6161, 1253.6216, 1255.5972, 1255.6000, 1260.6227, 1264.7130, 1265.7064, 1269.6498, 1270.6575, 1273.6556, 1277.6305, 1286.6099, 1289.6875, 1293.6401, 1303.6455, 1304.6677, 1307.6788, 1309.6780, 1319.6589, 1328.6600, 1328.6620, 1331.7888, 1333.7644, 1336.0798, 1336.6155, 1350.6853, 1351.7202, 1356.5811, 1359.1302, 1359.6246, 1374.7588, 1383.7037, 1390.6974, 1393.7992, 1401.6989, 1414.8191, 1429.3142, 1444.7122, 1445.7839, 1450.7223, 1456.7717, 1462.6630, 1467.7329, 1470.7394, 1471.2693, 1471.7250, 1472.2531, 1474.5243, 1483.7141, 1488.5333, 1488.7194, 1493.7366, 1502.5601, 1513.7441, 1514.6849, 1516.6807, 1517.6437, 1518.7053, 1523.7611, 1526.7343, 1528.6973, 1530.6893, 1536.8230, 1540.7114, 1551.7063, 1553.7252, 1554.7474, 1557.6992, 1558.7140, 1563.6658, 1564.3619, 1565.7223, 1566.7162, 1572.7062, 1580.8400, 1580.8423, 1586.8090, 1597.7913, 1599.8337, 1601.8030, 1614.8046, 1616.7968, 1628.7136, 1630.8103, 1635.7697, 1642.7682, 1643.7688, 1644.7915, 1646.8154, 1651.7681, 1664.8557, 1666.7545, 1695.8208, 1707.8933, 1718.8790, 1721.9192, 1722.9375, 1728.8976, 1730.9116, 1732.2391, 1734.1844, 1739.7894, 1743.8966, 1755.2666, 1755.7703, 1757.4702, 1757.9089, 1758.4524, 1758.8987, 1761.9023, 1765.7578, 1768.8339, 1769.8287, 1770.3949, 1772.8273, 1773.8792, 1774.9180, 1774.9200, 1786.8268, 1787.8239, 1809.0499, 1829.8895, 1830.8380, 1833.3470, 1833.8501, 1851.8885, 1852.0399, 1862.9515, 1881.7703, 1895.9634, 1906.8195, 1908.8512, 1920.7972, 1932.0050, 1936.9479, 1939.8563, 1940.8103, 1943.8293, 1954.8351, 1956.8574, 1958.8956, 1965.7998, 1967.9565, 1969.9998, 1979.8927, 1981.3531, 1982.8179, 1995.8232, 1997.8312, 2003.8595, 2008.9971, 2011.8464, 2015.9408, 2019.8779, 2025.8735, 2031.9606, 2047.9397, 2062.9578, 2067.0020, 2076.8943, 2081.9827, 2082.9966, 2087.0103, 2088.1108, 2089.1162, 2097.0200, 2108.9771, 2110.0061, 2139.0991, 2143.1006, 2144.0718, 2149.8516, 2160.0554, 2174.0378, 2178.0471, 2179.0574, 2188.0547, 2191.0457, 2198.1831, 2199.1499, 2203.0386, 2211.1052, 2223.1582, 2225.1470, 2227.1042, 2228.0725, 2238.1086, 2239.1538, 2243.1243, 2244.0684, 2255.1482, 2260.0779, 2270.2183, 2272.1736, 2284.1841, 2287.1108, 2307.0549, 2309.0659, 2310.0798, 2310.5271, 2311.0454, 2319.0945, 2337.6050, 2341.5510, 2353.0518, 2354.0767, 2355.0564, 2371.0581, 2383.9592, 2388.0437, 2392.1462, 2404.0471, 2412.0872, 2419.5879, 2420.0864, 2420.5930, 2423.2439, 2426.5667, 2427.1211, 2438.1724, 2455.1553, 2458.1648, 2459.1794, 2478.1587, 2516.1133, 2533.1262, 2542.1650, 2546.1746, 2549.1316, 2550.1252, 2559.1470, 2561.1729, 2562.1765, 2567.1514, 2572.1523, 2574.2678, 2583.1218, 2587.2722, 2604.1682, 2612.1438, 2653.7173, 2655.1897, 2671.1787, 2687.1833, 2701.2820, 2706.2766, 2712.2104, 2714.2156, 2716.1953, 2717.2725, 2725.2354, 2729.1675, 2739.2351, 2743.2642, 2745.2900, 2754.2197, 2755.2358, 2759.2573, 2769.2219, 2771.2437, 2776.2644, 2785.2388, 2786.2617, 2787.2400, 2793.2439, 2801.2378, 2803.2302, 2809.2549, 2815.2153, 2817.2322, 2819.2236, 2830.2788, 2833.2280, 2836.2397, 2862.2927, 2873.2961, 2889.2024, 2894.3218, 2927.2520, 2931.3428, 2955.3005, 2974.4124, 2975.4084, 2990.3921, 3006.3206, 3011.2810, 3020.3557, 3021.2412, 3035.3281, 3053.3447, 3062.2456, 3086.4507, 3091.2622, 3103.3140, 3110.7285, 3113.8591, 3130.3557, 3140.4265, 3144.3657, 3159.4985, 3173.5386, 3174.5486, 3185.4236, 3218.3301, 3259.3625, 3312.3267, 3331.4678, 3347.4761, 3363.4788, 3390.4675, 3431.4570, 3447.4666, 3463.4895, 3485.6985, 3488.6545, 3489.6521, 3493.0195, 3494.5571, 3504.6423, 3509.5613, 3525.6025, 3538.4521, 3553.6230, 3579.5403, 3648.1956, 3655.4729, 3664.7500, 3695.6177, 3712.5720, 3738.5837, 3816.7043, 3937.8308, 4010.8696

28. Q0VCY3 **Mass:** 40674 **Score:** 14 **Expect:** 9.6e+002 **Matches:** 17

Surfeit locus protein 6 OS=Bos taurus GN=SURF6 PE=2 SV=1

Observed	Mr(expt)	Mr(calc)	ppm	Start	End	Miss	Ions	Peptide
814.4558	813.4485	813.4681	-24.10	292	297	2	---	R.RAQRQR.A
842.5110	841.5038	841.4882	18.5	235	241	1	---	R.LQARQAR.L
957.5774	956.5701	956.5767	-6.86	59	66	2	---	R.ERKTAKPK.A
978.5100	977.5027	977.5182	-15.80	8	16	0	---	K.DAYLQGLAK.K
978.5146	977.5073	977.5182	-11.11	8	16	0	---	K.DAYLQGLAK.K
1123.5986	1122.5913	1122.6145	-20.66	116	124	1	---	R.LHEKIQEAR.G
1172.6156	1171.6083	1171.6210	-10.82	293	301	2	---	R.AQRQRAWEK.R
1229.7098	1228.7025	1228.6775	20.4	62	73	1	---	K.TAKPKAQASAEK.S
1270.6575	1269.6502	1269.6612	-8.64	303	313	1	---	R.TAHVVGKMQQR.Q + Oxidation (M)
1400.6683	1399.6610	1399.6878	-19.11	18	29	1	---	K.ICSQPSAEPQKR.K
1444.7122	1443.7049	1443.7358	-21.36	182	194	0	---	R.EEAQAQPGLLFNK.V
1462.6630	1461.6557	1461.6882	-22.19	249	261	1	---	R.DAGQAQELEAKMR.W + Oxidation (M)
1599.8337	1598.8264	1598.7900	22.8	195	208	1	---	K.VEVTEEEPANKAQR.R
1695.8208	1694.8135	1694.8475	-20.05	166	181	0	---	K.ALEGAEATEPDLQVPR.E
1718.8790	1717.8717	1717.8482	13.7	78	93	1	---	R.KPEVAKEEEGATSSTR.V
1722.9375	1721.9302	1721.9386	-4.84	1	16	1	---	-.MTSLLAKDAYLQGLAK.K
2223.1582	2222.1509	2222.1542	-1.48	161	181	2	---	K.EKAAKALEGAEATEPDLQVPR.E

No match to: 716.4763, 723.4098, 724.2417, 725.2533, 726.3622, 728.3046, 729.4749, 734.4570, 736.3904, 737.4152, 738.3791, 744.8651, 745.3838, 747.4550, 768.3673, 788.4571, 801.4262, 805.4606, 813.4481, 820.4053, 823.4778, 829.4589, 830.4522, 839.3830, 857.4382, 860.4207, 865.4518, 870.4165, 872.5054, 880.4424, 882.4292, 887.4245, 887.9669, 888.4561, 891.4898, 894.4144, 894.8973, 897.5006, 898.4523, 912.5132, 913.5293, 925.4620, 927.4981, 931.4992, 934.5098, 960.5294, 961.4949, 963.4734, 965.4692, 970.5181, 972.4899, 975.5167, 976.5063, 977.5212, 983.6283, 992.4958, 993.5046, 995.4181, 998.5911, 1000.5959, 1002.5007, 1006.5014, 1011.5474, 1012.5447, 1014.5485, 1020.5577, 1021.5699, 1024.5516, 1027.5438, 1028.5397, 1030.5978, 1032.5238, 1036.5260, 1037.5405, 1039.5641, 1040.5908, 1042.5388, 1043.5178, 1045.5568, 1046.6018, 1056.5515, 1057.6100, 1057.6107, 1061.6168, 1074.6073, 1074.6100,

1076.6123, 1102.6007, 1103.6257, 1105.6136, 1107.1058, 1113.5486, 1120.5498, 1122.5322, 1128.5724, 1129.5924, 1134.3574, 1137.5463, 1145.6348, 1149.6057, 1153.6460, 1156.4957, 1161.6295, 1162.6174, 1164.1815, 1170.5497, 1177.6410, 1180.6428, 1184.6412, 1188.5603, 1193.6041, 1206.5107, 1209.5889, 1211.6099, 1212.6160, 1215.6743, 1216.7081, 1219.6886, 1227.6234, 1234.6414, 1240.5974, 1242.6003, 1243.6161, 1253.6216, 1255.5972, 1255.6000, 1260.6227, 1264.7130, 1265.7064, 1269.6498, 1273.6556, 1277.6305, 1286.6099, 1289.6875, 1293.6401, 1303.6455, 1304.6677, 1307.6788, 1309.6780, 1318.6750, 1319.6589, 1328.6600, 1328.6620, 1331.7888, 1333.7644, 1336.0798, 1336.6155, 1350.6853, 1351.7202, 1356.5811, 1359.1302, 1359.6246, 1365.6489, 1374.7588, 1383.7037, 1390.6974, 1393.7992, 1401.6989, 1414.8191, 1429.3142, 1436.7889, 1445.7839, 1450.7223, 1456.7717, 1467.7329, 1470.7394, 1471.2693, 1471.7250, 1472.2531, 1474.5243, 1475.7633, 1483.7141, 1488.5333, 1488.7194, 1493.7366, 1502.5601, 1513.7441, 1514.6849, 1516.6807, 1517.6437, 1518.7053, 1523.7611, 1526.7343, 1528.6973, 1530.6893, 1536.8230, 1540.7114, 1551.7063, 1553.7252, 1554.7474, 1557.6992, 1558.7140, 1563.6658, 1564.3619, 1565.7223, 1566.7162, 1572.7062, 1580.8400, 1580.8423, 1586.8090, 1597.7913, 1601.8030, 1614.8046, 1616.7968, 1628.7136, 1630.8103, 1635.7697, 1642.7682, 1643.7688, 1644.7915, 1646.8154, 1651.7681, 1657.7932, 1663.8466, 1664.8557, 1666.7545, 1707.8933, 1721.9192, 1728.8976, 1730.9116, 1732.2391, 1734.1844, 1739.7894, 1743.8966, 1755.2666, 1755.7703, 1757.4702, 1757.9089, 1758.4524, 1758.8987, 1761.9023, 1765.7578, 1768.8339, 1769.8287, 1770.3949, 1772.8273, 1773.8792, 1774.9180, 1774.9200, 1786.8268, 1787.8239, 1809.0499, 1810.0303, 1829.8895, 1830.8380, 1833.3470, 1833.8501, 1851.8885, 1852.0399, 1862.9515, 1881.7703, 1895.9634, 1906.8195, 1908.8512, 1920.7972, 1932.0050, 1936.9479, 1939.8563, 1940.8103, 1943.8293, 1954.8351, 1956.8574, 1958.8956, 1965.7998, 1967.9565, 1969.9998, 1979.8927, 1981.3531, 1982.8179, 1995.8232, 1997.8312, 2003.8595, 2008.9971, 2011.8464, 2015.9408, 2019.8779, 2025.8735, 2031.9606, 2047.9397, 2062.9578, 2067.0020, 2076.8943, 2081.9827, 2082.9966, 2087.0103, 2088.1108, 2089.1162, 2096.0430, 2097.0200, 2108.9771, 2110.0061, 2139.0991, 2140.0918, 2143.1006, 2144.0718, 2149.8516, 2160.0554, 2174.0378, 2178.0471, 2179.0574, 2188.0547, 2191.0457, 2198.1831, 2199.1499, 2203.0386, 2211.1052, 2225.1470, 2227.1042, 2228.0725, 2238.1086, 2239.1538, 2243.1243, 2244.0684, 2254.0896, 2255.1482, 2260.0779, 2270.2183, 2272.1736, 2284.1841, 2287.1108, 2307.0549, 2309.0659, 2310.0798, 2310.5271, 2311.0454, 2319.0945, 2337.6050, 2341.5510, 2353.0518, 2354.0767, 2355.0564, 2371.0581, 2383.9592, 2388.0437, 2392.1462, 2404.0471, 2412.0872, 2419.5879, 2420.0864, 2420.5930, 2423.2439, 2426.5667, 2427.1211, 2438.1724, 2455.1553, 2458.1648, 2459.1794, 2478.1587, 2516.1133, 2533.1262, 2542.1650, 2546.1746, 2549.1316, 2550.1252, 2559.1470, 2561.1729, 2562.1765, 2567.1514, 2572.1523, 2574.2678, 2576.1628, 2583.1218, 2587.2722, 2604.1682, 2612.1438, 2653.7173, 2655.1897, 2671.1787, 2687.1833, 2701.2820, 2706.2766, 2712.2104, 2714.2156, 2716.1953, 2717.2725, 2725.2354, 2729.1675, 2739.2351, 2743.2642, 2745.2900, 2754.2197, 2755.2358, 2759.2573, 2769.2219, 2771.2437, 2776.2644, 2785.2388, 2786.2617, 2787.2400, 2793.2439, 2801.2378, 2803.2302, 2809.2549, 2815.2153, 2817.2322, 2819.2236, 2830.2788, 2833.2280, 2836.2397, 2862.2927, 2873.2961, 2889.2024, 2894.3218, 2927.2520, 2931.3428, 2955.3005, 2974.4124, 2975.4084, 2990.3921, 3006.3206, 3011.2810, 3020.3557, 3021.2412, 3035.3281, 3053.3447, 3062.2456, 3086.4507, 3091.2622, 3103.3140, 3110.7285, 3113.8591, 3130.3557, 3140.4265, 3144.3657, 3159.4985, 3173.5386, 3174.5486, 3185.4236, 3218.3301, 3259.3625, 3312.3267, 3331.4678, 3347.4761, 3363.4788, 3390.4675, 3431.4570, 3447.4666, 3463.4895, 3485.6985, 3488.6545, 3489.6521, 3493.0195, 3494.5571, 3504.6423, 3509.5613, 3525.6025, 3538.4521, 3553.6230, 3579.5403, 3648.1956, 3655.4729, 3664.7500, 3695.6177, 3712.5720, 3738.5837, 3816.7043, 3937.8308, 4010.8696

29. G3N3F0 **Mass:** 23102 **Score:** 14 **Expect:** 9.9e+002 **Matches:** 9

Uncharacterized protein OS=Bos taurus PE=4 SV=1

Observed	Mr(expt)	Mr(calc)	ppm	Start	End	Miss	Ions	Peptide
1234.6414	1233.6341	1233.6070	22.0	25 -	36	2	---	R.MAGAMPARGGKR.R + 2 Oxidation (M)
1666.7545	1665.7472	1665.7749	-16.62	98 -	112	2	---	R.MAVSHMKSMRGTGNK.S + 2 Oxidation (M)
2287.1108	2286.1035	2286.0694	14.9	69 -	88	0	---	K.MTQYITELSDMVPTCSALAR.K
2319.0945	2318.0872	2318.0592	12.1	69 -	88	0	---	K.MTQYITELSDMVPTCSALAR.K + 2 Oxidation (M)
2561.1729	2560.1656	2560.1971	-12.31	67 -	88	1	---	R.NKMTQYITELSDMVPTCSALAR.K + 2 Oxidation (M)
2701.2820	2700.2747	2700.3033	-10.59	66 -	88	2	---	R.RNKMTQYITELSDMVPTCSALAR.K + Oxidation (M)
2717.2725	2716.2652	2716.2982	-12.15	66 -	88	2	---	R.RNKMTQYITELSDMVPTCSALAR.K + 2 Oxidation (M)
2745.2900	2744.2827	2744.3439	-22.30	105 -	129	2	---	K.SMRGTGNKSTDGAYKPSFLTEQELK.H
3489.6521	3488.6448	3488.6884	-12.50	1 -	35	2	---	-.EMASDIPGSVTLPVAPMAATGQVRMAGAMPARGGK.R + 4 Oxidation (M)

No match to: 716.4763, 723.4098, 724.2417, 725.2533, 726.3622, 728.3046, 729.4749, 734.4570, 736.3904, 737.4152, 738.3791, 744.8651, 745.3838, 747.4550, 768.3673, 788.4571, 801.4262, 805.4606, 813.4481, 814.4558, 820.4053, 823.4778, 829.4589, 830.4522, 839.3830, 842.5110, 857.4382, 860.4207, 865.4518, 870.4165, 872.5054, 880.4424, 882.4292, 887.4245, 887.9669, 888.4561, 891.4898, 894.4144, 894.8973, 897.5006, 898.4523, 912.5132, 913.5293, 925.4620, 927.4981, 931.4992, 934.5098, 957.5774, 960.5294, 961.4949, 963.4734, 965.4692, 970.5181, 972.4899, 975.5167, 976.5063, 977.5212, 978.5100, 978.5146, 983.6283, 992.4958, 993.5046, 995.4181, 998.5911, 1000.5959, 1002.5007, 1006.5014, 1011.5474, 1012.5447, 1014.5485, 1020.5577, 1021.5699, 1024.5516, 1027.5438, 1028.5397, 1030.5978, 1032.5238, 1036.5260, 1037.5405, 1039.5641, 1040.5908, 1042.5388, 1043.5178, 1045.5568, 1046.6018, 1056.5515, 1057.6100, 1057.6107, 1061.6168, 1074.6073, 1074.6100, 1076.6123, 1102.6007, 1103.6257, 1105.6136, 1107.1058, 1113.5486, 1120.5498, 1122.5322, 1123.5986, 1128.5724, 1129.5924, 1134.3574, 1137.5463, 1145.6348, 1149.6057, 1153.6460, 1156.4957, 1161.6295, 1162.6174, 1164.1815, 1170.5497, 1172.6156, 1177.6410, 1180.6428, 1184.6412, 1188.5603, 1193.6041, 1206.5107, 1209.5889, 1211.6099, 1212.6160, 1215.6743, 1216.7081, 1219.6886, 1227.6234, 1229.7098, 1240.5974, 1242.6003, 1243.6161, 1253.6216, 1255.5972, 1255.6000, 1260.6227, 1264.7130, 1265.7064, 1269.6498, 1270.6575, 1273.6556, 1277.6305, 1286.6099, 1289.6875, 1293.6401, 1303.6455, 1304.6677, 1307.6788, 1309.6780, 1318.6750, 1319.6589, 1328.6600, 1328.6620, 1331.7888, 1333.7644, 1336.0798, 1336.6155, 1350.6853, 1351.7202, 1356.5811, 1359.1302, 1359.6246, 1365.6489, 1374.7588, 1383.7037, 1390.6974, 1393.7992, 1400.6683, 1401.6989, 1414.8191, 1429.3142, 1436.7889, 1444.7122, 1445.7839, 1450.7223, 1456.7717, 1462.6630, 1467.7329, 1470.7394, 1471.2693, 1471.7250, 1472.2531, 1474.5243, 1475.7633, 1483.7141, 1488.5333, 1488.7194, 1493.7366, 1502.5601, 1513.7441, 1514.6849, 1516.6807, 1517.6437, 1518.7053, 1523.7611, 1526.7343, 1528.6973, 1530.6893, 1536.8230, 1540.7114, 1551.7063, 1553.7252, 1554.7474, 1557.6992, 1558.7140, 1563.6658, 1564.3619, 1565.7223, 1566.7162, 1572.7062, 1580.8400, 1580.8423, 1586.8090, 1597.7913, 1599.8337, 1601.8030, 1614.8046, 1616.7968, 1628.7136, 1630.8103, 1635.7697, 1642.7682, 1643.7688, 1644.7915, 1646.8154, 1651.7681, 1657.7932, 1663.8466, 1664.8557, 1695.8208, 1707.8933, 1718.8790, 1721.9192, 1722.9375, 1728.8976, 1730.9116, 1732.2391, 1734.1844, 1739.7894, 1743.8966, 1755.2666, 1755.7703, 1757.4702, 1757.9089, 1758.4524, 1758.8987, 1761.9023, 1765.7578, 1768.8339, 1769.8287, 1770.3949, 1772.8273, 1773.8792, 1774.9180, 1774.9200, 1786.8268, 1787.8239, 1809.0499, 1810.0303, 1829.8895, 1830.8380, 1833.3470, 1833.8501, 1851.8885, 1852.0399, 1862.9515, 1881.7703, 1895.9634, 1906.8195, 1908.8512, 1920.7972, 1932.0050, 1936.9479, 1939.8563, 1940.8103, 1943.8293, 1954.8351, 1956.8574, 1958.8956, 1965.7998, 1967.9565, 1969.9998, 1979.8927, 1981.3531, 1982.8179, 1995.8232, 1997.8312, 2003.8595, 2008.9971, 2011.8464, 2015.9408, 2019.8779, 2025.8735, 2031.9606, 2047.9397, 2062.9578, 2067.0020, 2076.8943, 2081.9827, 2082.9966, 2087.0103, 2088.1108, 2089.1162, 2096.0430, 2097.0200, 2108.9771, 2110.0061, 2139.0991, 2140.0918, 2143.1006, 2144.0718, 2149.8516, 2160.0554, 2174.0378, 2178.0471, 2179.0574, 2188.0547, 2191.0457, 2198.1831, 2199.1499, 2203.0386, 2211.1052, 2223.1582, 2225.1470, 2227.1042, 2228.0725, 2238.1086, 2239.1538, 2243.1243, 2244.0684, 2254.0896, 2255.1482, 2260.0779, 2270.2183, 2272.1736, 2284.1841, 2307.0549, 2309.0659, 2310.0798, 2310.5271, 2311.0454, 2337.6050, 2341.5510, 2353.0518, 2354.0767, 2355.0564, 2371.0581, 2383.9592, 2388.0437, 2392.1462, 2404.0471, 2412.0872, 2419.5879, 2420.0864, 2420.5930, 2423.2439, 2426.5667, 2427.1211, 2438.1724, 2455.1553, 2458.1648, 2459.1794, 2478.1587, 2516.1133, 2533.1262, 2542.1650, 2546.1746, 2549.1316, 2550.1252, 2559.1470, 2562.1765, 2567.1514, 2572.1523, 2574.2678, 2576.1628, 2583.1218, 2587.2722, 2604.1682, 2612.1438, 2653.7173, 2655.1897, 2671.1787, 2687.1833, 2706.2766, 2712.2104, 2714.2156, 2716.1953, 2725.2354, 2729.1675, 2739.2351, 2743.2642, 2754.2197, 2755.2358, 2759.2573, 2769.2219, 2771.2437, 2776.2644, 2785.2388, 2786.2617, 2787.2400, 2793.2439, 2801.2378, 2803.2302, 2809.2549, 2815.2153, 2817.2322, 2819.2236, 2830.2788, 2833.2280, 2836.2397, 2862.2927, 2873.2961, 2889.2024, 2894.3218, 2927.2520, 2931.3428, 2955.3005, 2974.4124, 2975.4084, 2990.3921, 3006.3206, 3011.2810, 3020.3557, 3021.2412, 3035.3281, 3053.3447, 3062.2456, 3086.4507, 3091.2622, 3103.3140, 3110.7285, 3113.8591, 3130.3557, 3140.4265, 3144.3657, 3159.4985, 3173.5386, 3174.5486, 3185.4236, 3218.3301, 3259.3625, 3312.3267, 3331.4678, 3347.4761, 3363.4788, 3390.4675, 3431.4570, 3447.4666, 3463.4895, 3485.6985, 3488.6545, 3493.0195, 3494.5571, 3504.6423, 3509.5613, 3525.6025, 3538.4521, 3553.6230, 3579.5403, 3648.1956, 3655.4729, 3664.7500, 3695.6177, 3712.5720, 3738.5837, 3816.7043, 3937.8308, 4010.8696

30. Q3T2L0 **Mass:** 42579 **Score:** 14 **Expect:** 9.9e+002 **Matches:** 15

Caspase-15 OS=Bos taurus GN=LOC515736 PE=2 SV=1

Observed	Mr(expt)	Mr(calc)	ppm	Start	End	Miss	Ions	Peptide
860.4207	859.4134	859.4287	-17.73	195 -	201	0	---	R.DELNEIK.D
1012.5447	1011.5374	1011.5245	12.7	145 -	152	1	---	K.AFLMCVKK.N + Oxidation (M)
1020.5577	1019.5504	1019.5586	-8.03	251 -	258	0	---	K.IFIIQACR.G
1030.5978	1029.5905	1029.5719	18.0	47 -	54	2	---	R.SFRDHLKK.V
1234.6414	1233.6341	1233.6176	13.4	344 -	353	0	---	R.DFNLCNKPVK.V
1260.6227	1259.6154	1259.6357	-16.12	22 -	32	0	---	K.ISPTTEELQSR.L
1526.7343	1525.7270	1525.7348	-5.07	108 -	120	0	---	R.WQSAPTDCPVIPR.K
1599.8337	1598.8264	1598.8048	13.5	308 -	321	1	---	R.TGSVMIKEMTEVFK.Q
1967.9565	1966.9492	1966.9856	-18.50	305 -	321	2	---	R.DPRTGSVMIKEMTEVFK.Q

2087.0103	2086.0030	2085.9862	8.08	1 -	21	2	---	-.MEGSLGSGRRSGSDGQPAPPK.I + Oxidation (M)
2199.1499	2198.1426	2198.0976	20.5	155 -	172	2	---	R.LGAHQDVQLMKDWLKECK.F
2223.1582	2222.1509	2222.1021	22.0	322 -	339	1	---	K.QYGNKWHLADFFTIVNNR.V
2423.2439	2422.2366	2422.2600	-9.65	344 -	364	1	---	R.DFNLCNKPVKVSLVMESTLTK.F
2438.1724	2437.1651	2437.2197	-22.40	10 -	32	2	---	R.RSGSDGQPAPPKISPTTEELQSR.L
2572.1523	2571.1450	2571.1478	-1.07	202 -	223	1	---	K.DDIGCCLVTLMSHGEEGFIKMK.D + 2 Oxidation (M)

No match to: 716.4763, 723.4098, 724.2417, 725.2533, 726.3622, 728.3046, 729.4749, 734.4570, 736.3904, 737.4152, 738.3791, 744.8651, 745.3838, 747.4550, 768.3673, 788.4571, 801.4262, 805.4606, 813.4481, 814.4558, 820.4053, 823.4778, 829.4589, 830.4522, 839.3830, 842.5110, 857.4382, 865.4518, 870.4165, 872.5054, 880.4424, 882.4292, 887.4245, 887.9669, 888.4561, 891.4898, 894.4144, 894.8973, 897.5006, 898.4523, 912.5132, 913.5293, 925.4620, 927.4981, 931.4992, 934.5098, 957.5774, 960.5294, 961.4949, 963.4734, 965.4692, 970.5181, 972.4899, 975.5167, 976.5063, 977.5212, 978.5100, 978.5146, 983.6283, 992.4958, 993.5046, 995.4181, 998.5911, 1000.5959, 1002.5007, 1006.5014, 1011.5474, 1014.5485, 1021.5699, 1024.5516, 1027.5438, 1028.5397, 1032.5238, 1036.5260, 1037.5405, 1039.5641, 1040.5908, 1042.5388, 1043.5178, 1045.5568, 1046.6018, 1056.5515, 1057.6100, 1057.6107, 1061.6168, 1074.6073, 1074.6100, 1076.6123, 1102.6007, 1103.6257, 1105.6136, 1107.1058, 1113.5486, 1120.5498, 1122.5322, 1123.5986, 1128.5724, 1129.5924, 1134.3574, 1137.5463, 1145.6348, 1149.6057, 1153.6460, 1156.4957, 1161.6295, 1162.6174, 1164.1815, 1170.5497, 1172.6156, 1177.6410, 1180.6428, 1184.6412, 1188.5603, 1193.6041, 1206.5107, 1209.5889, 1211.6099, 1212.6160, 1215.6743, 1216.7081, 1219.6886, 1227.6234, 1229.7098, 1240.5974, 1242.6003, 1243.6161, 1253.6216, 1255.5972, 1255.6000, 1264.7130, 1265.7064, 1269.6498, 1270.6575, 1273.6556, 1277.6305, 1286.6099, 1289.6875, 1293.6401, 1303.6455, 1304.6677, 1307.6788, 1309.6780, 1318.6750, 1319.6589, 1328.6600, 1328.6620, 1331.7888, 1333.7644, 1336.0798, 1336.6155, 1350.6853, 1351.7202, 1356.5811, 1359.1302, 1359.6246, 1365.6489, 1374.7588, 1383.7037, 1390.6974, 1393.7992, 1400.6683, 1401.6989, 1414.8191, 1429.3142, 1436.7889, 1444.7122, 1445.7839, 1450.7223, 1456.7717, 1462.6630, 1467.7329, 1470.7394, 1471.2693, 1471.7250, 1472.2531, 1474.5243, 1475.7633, 1483.7141, 1488.5333, 1488.7194, 1493.7366, 1502.5601, 1513.7441, 1514.6849, 1516.6807, 1517.6437, 1518.7053, 1523.7611, 1528.6973, 1530.6893, 1536.8230, 1540.7114, 1551.7063, 1553.7252, 1554.7474, 1557.6992, 1558.7140, 1563.6658, 1564.3619, 1565.7223, 1566.7162, 1572.7062, 1580.8400, 1580.8423, 1586.8090, 1597.7913, 1601.8030, 1614.8046, 1616.7968, 1628.7136, 1630.8103, 1635.7697, 1642.7682, 1643.7688, 1644.7915, 1646.8154, 1651.7681, 1657.7932, 1663.8466, 1664.8557, 1666.7545, 1695.8208, 1707.8933, 1718.8790, 1721.9192, 1722.9375, 1728.8976, 1730.9116, 1732.2391, 1734.1844, 1739.7894, 1743.8966, 1755.2666, 1755.7703, 1757.4702, 1757.9089, 1758.4524, 1758.8987, 1761.9023, 1765.7578, 1768.8339, 1769.8287, 1770.3949, 1772.8273, 1773.8792, 1774.9180, 1774.9200, 1786.8268, 1787.8239, 1809.0499, 1810.0303, 1829.8895, 1830.8380, 1833.3470, 1833.8501, 1851.8885, 1852.0399, 1862.9515, 1881.7703, 1895.9634, 1906.8195, 1908.8512, 1920.7972, 1932.0050, 1936.9479, 1939.8563, 1940.8103, 1943.8293, 1954.8351, 1956.8574, 1958.8956, 1965.7998, 1969.9998, 1979.8927, 1981.3531, 1982.8179, 1995.8232, 1997.8312, 2003.8595, 2008.9971, 2011.8464, 2015.9408, 2019.8779, 2025.8735, 2031.9606, 2047.9397, 2062.9578, 2067.0020, 2076.8943, 2081.9827, 2082.9966, 2088.1108, 2089.1162, 2096.0430, 2097.0200, 2108.9771, 2110.0061, 2139.0991, 2140.0918, 2143.1006, 2144.0718, 2149.8516, 2160.0554, 2174.0378, 2178.0471, 2179.0574, 2188.0547, 2191.0457, 2198.1831, 2203.0386, 2211.1052, 2225.1470, 2227.1042, 2228.0725, 2238.1086, 2239.1538, 2243.1243, 2244.0684, 2254.0896, 2255.1482, 2260.0779, 2270.2183, 2272.1736, 2284.1841, 2287.1108, 2307.0549, 2309.0659, 2310.0798, 2310.5271, 2311.0454, 2319.0945, 2337.6050, 2341.5510, 2353.0518, 2354.0767, 2355.0564, 2371.0581, 2383.9592, 2388.0437, 2392.1462, 2404.0471, 2412.0872, 2419.5879, 2420.0864, 2420.5930, 2426.5667, 2427.1211, 2455.1553, 2458.1648, 2459.1794, 2478.1587, 2516.1133, 2533.1262, 2542.1650, 2546.1746, 2549.1316, 2550.1252, 2559.1470, 2561.1729, 2562.1765, 2567.1514, 2574.2678, 2576.1628, 2583.1218, 2587.2722, 2604.1682, 2612.1438, 2653.7173, 2655.1897, 2671.1787, 2687.1833, 2701.2820, 2706.2766, 2712.2104, 2714.2156, 2716.1953, 2717.2725, 2725.2354, 2729.1675, 2739.2351, 2743.2642, 2745.2900, 2754.2197, 2755.2358, 2759.2573, 2769.2219, 2771.2437, 2776.2644, 2785.2388, 2786.2617, 2787.2400, 2793.2439, 2801.2378, 2803.2302, 2809.2549, 2815.2153, 2817.2322, 2819.2236, 2830.2788, 2833.2280, 2836.2397, 2862.2927, 2873.2961, 2889.2024, 2894.3218, 2927.2520, 2931.3428, 2955.3005, 2974.4124, 2975.4084, 2990.3921, 3006.3206, 3011.2810, 3020.3557, 3021.2412, 3035.3281, 3053.3447, 3062.2456, 3086.4507, 3091.2622, 3103.3140, 3110.7285, 3113.8591, 3130.3557, 3140.4265, 3144.3657, 3159.4985, 3173.5386, 3174.5486, 3185.4236, 3218.3301, 3259.3625, 3312.3267, 3331.4678, 3347.4761, 3363.4788, 3390.4675, 3431.4570, 3447.4666, 3463.4895, 3485.6985, 3488.6545, 3489.6521, 3493.0195, 3494.5571, 3504.6423, 3509.5613, 3525.6025, 3538.4521, 3553.6230, 3579.5403, 3648.1956, 3655.4729, 3664.7500, 3695.6177, 3712.5720, 3738.5837, 3816.7043, 3937.8308, 4010.8696

31. E1BDX9 **Mass:** 40138 **Score:** 14 **Expect:** 9.9e+002 **Matches:** 16

Uncharacterized protein OS=Bos taurus GN=FAM50A PE=4 SV=1

Observed	Mr(expt)	Mr(calc)	ppm	Start	End	Miss	Ions	Peptide
734.4570	733.4497	733.4520	-3.16	214 -	219	2	---	R.TVKMKK.G
963.4734	962.4661	962.4742	-8.45	35 -	42	0	---	R.IAEENIMK.S + Oxidation (M)
975.5167	974.5094	974.5284	-19.46	290 -	298	0	---	R.LLSDATVEK.D
1000.5959	999.5886	999.5964	-7.78	96 -	103	1	---	K.ELQLKLEK.L
1014.5485	1013.5412	1013.5626	-21.11	14 -	21	2	---	R.AMHLMKKR.E
1102.6007	1101.5934	1101.6030	-8.65	92 -	100	1	---	K.EQSKELQLK.L
1229.7098	1228.7025	1228.6775	20.4	77 -	86	2	---	K.QEALVKEREK.Q
1586.8090	1585.8017	1585.8212	-12.28	179 -	190	2	---	R.LREELRQEWEAK.Q
1597.7913	1596.7840	1596.8116	-17.27	217 -	229	2	---	K.MKKGNTMQQFLQK.A + Oxidation (M)
1601.8030	1600.7957	1600.7804	9.55	173 -	184	2	---	R.EEEENRLREELR.Q
1646.8154	1645.8081	1645.7696	23.4	159 -	172	1	---	K.NPDVDTSFLPDRDR.E
2067.0020	2065.9947	2065.9859	4.26	1 -	19	2	---	-.MAQYKGAASEAGRAMHLMK.K + Oxidation (M)
2082.9966	2081.9893	2081.9808	4.08	1 -	19	2	---	-.MAQYKGAASEAGRAMHLMK.K + 2 Oxidation (M)
2211.1052	2210.0979	2210.1160	-8.20	254 -	271	0	---	K.EDLIIPHHHSFYDFIVTK.A
2254.0896	2253.0823	2253.0702	5.39	194 -	212	1	---	K.IKSEEIEITFSYWDGSGHR.R
2574.2678	2573.2605	2573.2762	-6.09	276 -	298	1	---	K.SGPLFNFDVHDDVRLLSDATVEK.D

No match to: 716.4763, 723.4098, 724.2417, 725.2533, 726.3622, 728.3046, 729.4749, 736.3904, 737.4152, 738.3791, 744.8651, 745.3838, 747.4550, 768.3673, 788.4571, 801.4262, 805.4606, 813.4481, 814.4558, 820.4053, 823.4778, 829.4589, 830.4522, 839.3830, 842.5110, 857.4382, 860.4207, 865.4518, 870.4165, 872.5054, 880.4424, 882.4292, 887.4245, 887.9669, 888.4561, 891.4898, 894.4144, 894.8973, 897.5006, 898.4523, 912.5132, 913.5293, 925.4620, 927.4981, 931.4992, 934.5098, 957.5774, 960.5294, 961.4949, 965.4692, 970.5181, 972.4899, 976.5063, 977.5212, 978.5100, 978.5146, 983.6283, 992.4958, 993.5046, 995.4181, 998.5911, 1002.5007, 1006.5014, 1011.5474, 1012.5447, 1020.5577, 1021.5699, 1024.5516, 1027.5438, 1028.5397, 1030.5978, 1032.5238, 1036.5260, 1037.5405, 1039.5641, 1040.5908, 1042.5388, 1043.5178, 1045.5568, 1046.6018, 1056.5515, 1057.6100, 1057.6107, 1061.6168, 1074.6073, 1074.6100, 1076.6123, 1103.6257, 1105.6136, 1107.1058, 1113.5486, 1120.5498, 1122.5322, 1123.5986, 1128.5724, 1129.5924, 1134.3574, 1137.5463, 1145.6348, 1149.6057, 1153.6460, 1156.4957, 1161.6295, 1162.6174, 1164.1815, 1170.5497, 1172.6156, 1177.6410, 1180.6428, 1184.6412, 1188.5603, 1193.6041, 1206.5107, 1209.5889, 1211.6099, 1212.6160, 1215.6743, 1216.7081, 1219.6886, 1227.6234, 1234.6414, 1240.5974, 1242.6003, 1243.6161, 1253.6216, 1255.5972, 1255.6000, 1260.6227, 1264.7130, 1265.7064, 1269.6498, 1270.6575, 1273.6556, 1277.6305, 1286.6099, 1289.6875, 1293.6401, 1303.6455, 1304.6677, 1307.6788, 1309.6780, 1318.6750, 1319.6589, 1328.6600, 1328.6620, 1331.7888, 1333.7644, 1336.0798, 1336.6155, 1350.6853, 1351.7202, 1356.5811, 1359.1302, 1359.6246, 1365.6489, 1374.7588, 1383.7037, 1390.6974, 1393.7992, 1400.6683, 1401.6989, 1414.8191, 1429.3142, 1436.7889, 1444.7122, 1445.7839, 1450.7223, 1456.7717, 1462.6630, 1467.7329, 1470.7394, 1471.2693, 1471.7250, 1472.2531, 1474.5243, 1475.7633, 1483.7141, 1488.5333, 1488.7194, 1493.7366, 1502.5601, 1513.7441, 1514.6849, 1516.6807, 1517.6437, 1518.7053, 1523.7611, 1526.7343, 1528.6973, 1530.6893, 1536.8230, 1540.7114, 1551.7063, 1553.7252, 1554.7474, 1557.6992, 1558.7140, 1563.6658, 1564.3619, 1565.7223, 1566.7162, 1572.7062, 1580.8400, 1580.8423, 1599.8337, 1614.8046, 1616.7968, 1628.7136, 1630.8103, 1635.7697, 1642.7682, 1643.7688, 1644.7915, 1651.7681, 1657.7932, 1663.8466, 1664.8557, 1666.7545, 1695.8208, 1707.8933, 1718.8790, 1721.9192, 1722.9375, 1728.8976, 1730.9116, 1732.2391, 1734.1844, 1739.7894, 1743.8966, 1755.2666, 1755.7703, 1757.4702, 1757.9089, 1758.4524, 1758.8987, 1761.9023, 1765.7578, 1768.8339, 1769.8287, 1770.3949, 1772.8273, 1773.8792, 1774.9180, 1774.9200, 1786.8268, 1787.8239, 1809.0499, 1810.0303, 1829.8895, 1830.8380, 1833.3470, 1833.8501, 1851.8885, 1852.0399, 1862.9515, 1881.7703, 1895.9634, 1906.8195, 1908.8512, 1920.7972, 1932.0050, 1936.9479, 1939.8563, 1940.8103, 1943.8293, 1954.8351, 1956.8574, 1958.8956, 1965.7998, 1967.9565, 1969.9998, 1979.8927, 1981.3531, 1982.8179, 1995.8232, 1997.8312, 2003.8595, 2008.9971, 2011.8464, 2015.9408, 2019.8779, 2025.8735, 2031.9606, 2047.9397, 2062.9578, 2076.8943, 2081.9827, 2087.0103, 2088.1108, 2089.1162, 2096.0430, 2097.0200, 2108.9771, 2110.0061, 2139.0991, 2140.0918, 2143.1006, 2144.0718, 2149.8516, 2160.0554, 2174.0378, 2178.0471, 2179.0574, 2188.0547, 2191.0457, 2198.1831, 2199.1499, 2203.0386, 2223.1582, 2225.1470, 2227.1042, 2228.0725, 2238.1086, 2239.1538, 2243.1243, 2244.0684, 2255.1482, 2260.0779, 2270.2183, 2272.1736, 2284.1841, 2287.1108, 2307.0549, 2309.0659, 2310.0798, 2310.5271, 2311.0454, 2319.0945, 2337.6050, 2341.5510, 2353.0518, 2354.0767, 2355.0564, 2371.0581, 2383.9592, 2388.0437, 2392.1462, 2404.0471, 2412.0872, 2419.5879, 2420.0864, 2420.5930, 2423.2439, 2426.5667, 2427.1211, 2438.1724, 2455.1553, 2458.1648, 2459.1794, 2478.1587, 2516.1133, 2533.1262, 2542.1650, 2546.1746, 2549.1316, 2550.1252, 2559.1470, 2561.1729, 2562.1765, 2567.1514, 2572.1523, 2576.1628, 2583.1218, 2587.2722, 2604.1682, 2612.1438, 2653.7173, 2655.1897, 2671.1787, 2687.1833, 2701.2820, 2706.2766, 2712.2104, 2714.2156, 2716.1953,

2717.2725, 2725.2354, 2729.1675, 2739.2351, 2743.2642, 2745.2900, 2754.2197, 2755.2358, 2759.2573, 2769.2219, 2771.2437, 2776.2644, 2785.2388, 2786.2617, 2787.2400, 2793.2439, 2801.2378, 2803.2302, 2809.2549, 2815.2153, 2817.2322, 2819.2236, 2830.2788, 2833.2280, 2836.2397, 2862.2927, 2873.2961, 2889.2024, 2894.3218, 2927.2520, 2931.3428, 2955.3005, 2974.4124, 2975.4084, 2990.3921, 3006.3206, 3011.2810, 3020.3557, 3021.2412, 3035.3281, 3053.3447, 3062.2456, 3086.4507, 3091.2622, 3103.3140, 3110.7285, 3113.8591, 3130.3557, 3140.4265, 3144.3657, 3159.4985, 3173.5386, 3174.5486, 3185.4236, 3218.3301, 3259.3625, 3312.3267, 3331.4678, 3347.4761, 3363.4788, 3390.4675, 3431.4570, 3447.4666, 3463.4895, 3485.6985, 3488.6545, 3489.6521, 3493.0195, 3494.5571, 3504.6423, 3509.5613, 3525.6025, 3538.4521, 3553.6230, 3579.5403, 3648.1956, 3655.4729, 3664.7500, 3695.6177, 3712.5720, 3738.5837, 3816.7043, 3937.8308, 4010.8696

32. P84081 **Mass:** 20790 **Score:** 14 **Expect:** 1e+003 **Matches:** 9
ADP-ribosylation factor 2 OS=Bos taurus GN=ARF2 PE=2 SV=1

Observed	Mr(expt)	Mr(calc)	ppm	Start	End	Miss	Ions	Peptide
992.4958	991.4885	991.4644	24.3	110 -	117	0	---	R.MLAEDELR.D + Oxidation (M)
1103.6257	1102.6184	1102.6056	11.6	20 -	30	0	---	R.ILMVGLDAAGK.T + Oxidation (M)
1212.6160	1211.6087	1211.6373	-23.55	1 -	10	1	---	-.MGNVFEKLFK.S
1565.7223	1564.7150	1564.7522	-23.76	60 -	73	0	---	K.NISFTVWDVGGQDK.I
1630.8103	1629.8030	1629.7668	22.2	128 -	142	0	---	K.QDLPNAMNAAEITDK.L
1663.8466	1662.8393	1662.8797	-24.26	16 -	30	2	---	K.KEMRILMVGLDAAGK.T + 2 Oxidation (M)
2174.0378	2173.0305	2173.0797	-22.61	100 -	117	2	---	R.VNEAREELTRMLAEDELR.D
2423.2439	2422.2366	2422.2274	3.80	128 -	149	1	---	K.QDLPNAMNAAEITDKLGLHSLR.Q + Oxidation (M)
2438.1724	2437.1651	2437.1775	-5.07	80 -	99	1	---	R.HYFQNTQGLIFVVDSNDRER.V

No match to: 716.4763, 723.4098, 724.2417, 725.2533, 726.3622, 728.3046, 729.4749, 734.4570, 736.3904, 737.4152, 738.3791, 744.8651, 745.3838, 747.4550, 768.3673, 788.4571, 801.4262, 805.4606, 813.4481, 814.4558, 820.4053, 823.4778, 829.4589, 830.4522, 839.3830, 842.5110, 857.4382, 860.4207, 865.4518, 870.4165, 872.5054, 880.4424, 882.4292, 887.4245, 887.9669, 888.4561, 891.4898, 894.4144, 894.8973, 897.5006, 898.4523, 912.5132, 913.5293, 925.4620, 927.4981, 931.4992, 934.5098, 957.5774, 960.5294, 961.4949, 963.4734, 965.4692, 970.5181, 972.4899, 975.5167, 976.5063, 977.5212, 978.5100, 978.5146, 983.6283, 993.5046, 995.4181, 998.5911, 1000.5959, 1002.5007, 1006.5014, 1011.5474, 1012.5447, 1014.5485, 1020.5577, 1021.5699, 1024.5516, 1027.5438, 1028.5397, 1030.5978, 1032.5238, 1036.5260, 1037.5405, 1039.5641, 1040.5908, 1042.5388, 1043.5178, 1045.5568, 1046.6018, 1056.5515, 1057.6100, 1057.6107, 1061.6168, 1074.6073, 1074.6100, 1076.6123, 1102.6007, 1105.6136, 1107.1058, 1113.5486, 1120.5498, 1122.5322, 1123.5986, 1128.5724, 1129.5924, 1134.3574, 1137.5463, 1145.6348, 1149.6057, 1153.6460, 1156.4957, 1161.6295, 1162.6174, 1164.1815, 1170.5497, 1172.6156, 1177.6410, 1180.6428, 1184.6412, 1188.5603, 1193.6041, 1206.5107, 1209.5889, 1211.6099, 1215.6743, 1216.7081, 1219.6886, 1227.6234, 1229.7098, 1234.6414, 1240.5974, 1242.6003, 1243.6161, 1253.6216, 1255.5972, 1255.6000, 1260.6227, 1264.7130, 1265.7064, 1269.6498, 1270.6575, 1273.6556, 1277.6305, 1286.6099, 1289.6875, 1293.6401, 1303.6455, 1304.6677, 1307.6788, 1309.6780, 1318.6750, 1319.6589, 1328.6600, 1328.6620, 1331.7888, 1333.7644, 1336.0798, 1336.6155, 1350.6853, 1351.7202, 1356.5811, 1359.1302, 1359.6246, 1365.6489, 1374.7588, 1383.7037, 1390.6974, 1393.7992, 1400.6683, 1401.6989, 1414.8191, 1429.3142, 1436.7889, 1444.7122, 1445.7839, 1450.7223, 1456.7717, 1462.6630, 1467.7329, 1470.7394, 1471.2693, 1471.7250, 1472.2531, 1474.5243, 1475.7633, 1483.7141, 1488.5333, 1488.7194, 1493.7366, 1502.5601, 1513.7441, 1514.6849, 1516.6807, 1517.6437, 1518.7053, 1523.7611, 1526.7343, 1528.6973, 1530.6893, 1536.8230, 1540.7114, 1551.7063, 1553.7252, 1554.7474, 1557.6992, 1558.7140, 1563.6658, 1564.3619, 1566.7162, 1572.7062, 1580.8400, 1580.8423, 1586.8090, 1597.7913, 1599.8337, 1601.8030, 1614.8046, 1616.7968, 1628.7136, 1635.7697, 1642.7682, 1643.7688, 1644.7915, 1646.8154, 1651.7681, 1657.7932, 1664.8557, 1666.7545, 1695.8208, 1707.8933, 1718.8790, 1721.9192, 1722.9375, 1728.8976, 1730.9116, 1732.2391, 1734.1844, 1739.7894, 1743.8966, 1755.2666, 1755.7703, 1757.4702, 1757.9089, 1758.4524, 1758.8987, 1761.9023, 1765.7578, 1768.8339, 1769.8287, 1770.3949, 1772.8273, 1773.8792, 1774.9180, 1774.9200, 1786.8268, 1787.8239, 1809.0499, 1810.0303, 1829.8895, 1830.8380, 1833.3470, 1833.8501, 1851.8885, 1852.0399, 1862.9515, 1881.7703, 1895.9634, 1906.8195, 1908.8512, 1920.7972, 1932.0050, 1936.9479, 1939.8563, 1940.8103, 1943.8293, 1954.8351, 1956.8574, 1958.8956, 1965.7998, 1967.9565, 1969.9998, 1979.8927, 1981.3531, 1982.8179, 1995.8232, 1997.8312, 2003.8595, 2008.9971, 2011.8464, 2015.9408, 2019.8779, 2025.8735, 2031.9606, 2047.9397, 2062.9578, 2067.0020, 2076.8943, 2081.9827, 2082.9966, 2087.0103, 2088.1108, 2089.1162, 2096.0430, 2097.0200, 2108.9771, 2110.0061, 2139.0991, 2140.0918, 2143.1006, 2144.0718, 2149.8516, 2160.0554, 2178.0471, 2179.0574, 2188.0547, 2191.0457, 2198.1831, 2199.1499, 2203.0386, 2211.1052, 2223.1582, 2225.1470, 2227.1042, 2228.0725, 2238.1086, 2239.1538, 2243.1243, 2244.0684, 2254.0896, 2255.1482, 2260.0779, 2270.2183, 2272.1736, 2284.1841, 2287.1108, 2307.0549, 2309.0659, 2310.0798, 2310.5271, 2311.0454, 2319.0945, 2337.6050, 2341.5510, 2353.0518, 2354.0767, 2355.0564, 2371.0581, 2383.9592, 2388.0437, 2392.1462, 2404.0471, 2412.0872, 2419.5879, 2420.0864, 2420.5930, 2426.5667, 2427.1211, 2455.1553, 2458.1648, 2459.1794, 2478.1587, 2516.1133, 2533.1262, 2542.1650, 2546.1746, 2549.1316, 2550.1252, 2559.1470, 2561.1729, 2562.1765, 2567.1514, 2572.1523, 2574.2678, 2576.1628, 2583.1218, 2587.2722, 2604.1682, 2612.1438, 2653.7173, 2655.1897, 2671.1787, 2687.1833, 2701.2820, 2706.2766, 2712.2104, 2714.2156, 2716.1953, 2717.2725, 2725.2354, 2729.1675, 2739.2351, 2743.2642, 2745.2900, 2754.2197, 2755.2358, 2759.2573, 2769.2219, 2771.2437, 2776.2644, 2785.2388, 2786.2617, 2787.2400, 2793.2439, 2801.2378, 2803.2302, 2809.2549, 2815.2153, 2817.2322, 2819.2236, 2830.2788, 2833.2280, 2836.2397, 2862.2927, 2873.2961, 2889.2024, 2894.3218, 2927.2520, 2931.3428, 2955.3005, 2974.4124, 2975.4084, 2990.3921, 3006.3206, 3011.2810, 3020.3557, 3021.2412, 3035.3281, 3053.3447, 3062.2456, 3086.4507, 3091.2622, 3103.3140, 3110.7285, 3113.8591, 3130.3557, 3140.4265, 3144.3657, 3159.4985, 3173.5386, 3174.5486, 3185.4236, 3218.3301, 3259.3625, 3312.3267, 3331.4678, 3347.4761, 3363.4788, 3390.4675, 3431.4570, 3447.4666, 3463.4895, 3485.6985, 3488.6545, 3489.6521, 3493.0195, 3494.5571, 3504.6423, 3509.5613, 3525.6025, 3538.4521, 3553.6230, 3579.5403, 3648.1956, 3655.4729, 3664.7500, 3695.6177, 3712.5720, 3738.5837, 3816.7043, 3937.8308, 4010.8696

33. F1MNB0 **Mass:** 17016 **Score:** 14 **Expect:** 1e+003 **Matches:** 7
Cystatin OS=Bos taurus GN=CST8 PE=3 SV=2

Observed	Mr(expt)	Mr(calc)	ppm	Start	End	Miss	Ions	Peptide
976.5063	975.4990	975.4808	18.7	77 -	84	0	---	R.VQMQVTDR.L
992.4958	991.4885	991.4757	12.9	77 -	84	0	---	R.VQMQVTDR.L + Oxidation (M)
1401.6989	1400.6916	1400.7082	-11.82	100 -	111	1	---	R.KLPNSNENCVVK.N
2561.1729	2560.1656	2560.1888	-9.05	120 -	140	1	---	R.QMCTFCVGALPWHGYFTVMKK.Q
2717.2725	2716.2652	2716.2899	-9.09	119 -	140	2	---	K.RQMCTFCVGALPWHGYFTVMKK.Q
2975.4084	2974.4011	2974.4115	-3.48	116 -	139	2	---	K.LEKRQMCTFCVGALPWHGYFTVMK.K + Oxidation (M)
3493.0195	3492.0122	3491.9868	7.28	2 -	33	2	---	M.TRLWGTYQLLLALLVALVALGGNTLVHKDTDK.V

No match to: 716.4763, 723.4098, 724.2417, 725.2533, 726.3622, 728.3046, 729.4749, 734.4570, 736.3904, 737.4152, 738.3791, 744.8651, 745.3838, 747.4550, 768.3673, 788.4571, 801.4262, 805.4606, 813.4481, 814.4558, 820.4053, 823.4778, 829.4589, 830.4522, 839.3830, 842.5110, 857.4382, 860.4207, 865.4518, 870.4165, 872.5054, 880.4424, 882.4292, 887.4245, 887.9669, 888.4561, 891.4898, 894.4144, 894.8973, 897.5006, 898.4523, 912.5132, 913.5293, 925.4620, 927.4981, 931.4992, 934.5098, 957.5774, 960.5294, 961.4949, 963.4734, 965.4692, 970.5181, 972.4899, 975.5167, 977.5212, 978.5100, 978.5146, 983.6283, 993.5046, 995.4181, 998.5911, 1000.5959, 1002.5007, 1006.5014, 1011.5474, 1012.5447, 1014.5485, 1020.5577, 1021.5699, 1024.5516, 1027.5438, 1028.5397, 1030.5978, 1032.5238, 1036.5260, 1037.5405, 1039.5641, 1040.5908, 1042.5388, 1043.5178, 1045.5568, 1046.6018, 1056.5515, 1057.6100, 1057.6107, 1061.6168, 1074.6073, 1074.6100, 1076.6123, 1102.6007, 1103.6257, 1105.6136, 1107.1058, 1113.5486, 1120.5498, 1122.5322, 1123.5986, 1128.5724, 1129.5924, 1134.3574, 1137.5463, 1145.6348, 1149.6057, 1153.6460, 1156.4957, 1161.6295, 1162.6174, 1164.1815, 1170.5497, 1172.6156, 1177.6410, 1180.6428, 1184.6412, 1188.5603, 1193.6041, 1206.5107, 1209.5889, 1211.6099, 1212.6160, 1215.6743, 1216.7081, 1219.6886, 1227.6234, 1229.7098, 1234.6414, 1240.5974, 1242.6003, 1243.6161, 1253.6216, 1255.5972, 1255.6000, 1260.6227, 1264.7130, 1265.7064, 1269.6498, 1270.6575, 1273.6556, 1277.6305, 1286.6099, 1289.6875, 1293.6401, 1303.6455, 1304.6677, 1307.6788, 1309.6780, 1318.6750, 1319.6589, 1328.6600, 1328.6620, 1331.7888, 1333.7644, 1336.0798, 1336.6155, 1350.6853, 1351.7202, 1356.5811, 1359.1302, 1359.6246, 1365.6489, 1374.7588, 1383.7037, 1390.6974, 1393.7992, 1400.6683, 1414.8191, 1429.3142, 1436.7889, 1444.7122, 1445.7839, 1450.7223, 1456.7717, 1462.6630, 1467.7329, 1470.7394, 1471.2693, 1471.7250, 1472.2531, 1474.5243, 1475.7633, 1483.7141, 1488.5333, 1488.7194, 1493.7366, 1502.5601, 1513.7441, 1514.6849, 1516.6807, 1517.6437, 1518.7053, 1523.7611, 1526.7343, 1528.6973, 1530.6893, 1536.8230, 1540.7114, 1551.7063, 1553.7252, 1554.7474, 1557.6992, 1558.7140, 1563.6658, 1564.3619, 1565.7223, 1566.7162, 1572.7062, 1580.8400, 1580.8423, 1586.8090, 1597.7913, 1599.8337, 1601.8030, 1614.8046, 1616.7968, 1628.7136, 1630.8103, 1635.7697, 1642.7682, 1643.7688, 1644.7915, 1646.8154, 1651.7681, 1657.7932, 1663.8466, 1664.8557, 1666.7545, 1695.8208, 1707.8933, 1718.8790, 1721.9192, 1722.9375, 1728.8976, 1730.9116, 1732.2391, 1734.1844, 1739.7894, 1743.8966, 1755.2666, 1755.7703, 1757.4702, 1757.9089, 1758.4524, 1758.8987, 1761.9023, 1765.7578, 1768.8339, 1769.8287, 1770.3949, 1772.8273, 1773.8792, 1774.9180, 1774.9200, 1786.8268, 1787.8239, 1809.0499, 1810.0303, 1829.8895, 1830.8380, 1833.3470, 1833.8501, 1851.8885, 1852.0399, 1862.9515, 1881.7703, 1895.9634, 1906.8195, 1908.8512, 1920.7972, 1932.0050, 1936.9479, 1939.8563, 1940.8103, 1943.8293, 1954.8351, 1956.8574, 1958.8956, 1965.7998, 1967.9565, 1969.9998, 1979.8927, 1981.3531, 1982.8179, 1995.8232, 1997.8312, 2003.8595, 2008.9971, 2011.8464, 2015.9408, 2019.8779, 2025.8735, 2031.9606, 2047.9397, 2062.9578, 2067.0020, 2076.8943, 2081.9827, 2082.9966, 2087.0103, 2088.1108, 2089.1162, 2096.0430, 2097.0200, 2108.9771, 2110.0061, 2139.0991, 2140.0918, 2143.1006, 2144.0718, 2149.8516, 2160.0554, 2174.0378, 2178.0471, 2179.0574, 2188.0547, 2191.0457,

2198.1831, 2199.1499, 2203.0386, 2211.1052, 2223.1582, 2225.1470, 2227.1042, 2228.0725, 2238.1086, 2239.1538, 2243.1243, 2244.0684, 2254.0896, 2255.1482, 2260.0779, 2270.2183, 2272.1736, 2284.1841, 2287.1108, 2307.0549, 2309.0659, 2310.0798, 2310.5271, 2311.0454, 2319.0945, 2337.6050, 2341.5510, 2353.0518, 2354.0767, 2355.0564, 2371.0581, 2383.9592, 2388.0437, 2392.1462, 2404.0471, 2412.0872, 2419.5879, 2420.0864, 2420.5930, 2423.2439, 2426.5667, 2427.1211, 2438.1724, 2455.1553, 2458.1648, 2459.1794, 2478.1587, 2516.1133, 2533.1262, 2542.1650, 2546.1746, 2549.1316, 2550.1252, 2559.1470, 2562.1765, 2567.1514, 2572.1523, 2574.2678, 2576.1628, 2583.1218, 2587.2722, 2604.1682, 2612.1438, 2653.7173, 2655.1897, 2671.1787, 2687.1833, 2701.2820, 2706.2766, 2712.2104, 2714.2156, 2716.1953, 2725.2354, 2729.1675, 2739.2351, 2743.2642, 2745.2900, 2754.2197, 2755.2358, 2759.2573, 2769.2219, 2771.2437, 2776.2644, 2785.2388, 2786.2617, 2787.2400, 2793.2439, 2801.2378, 2803.2302, 2809.2549, 2815.2153, 2817.2322, 2819.2236, 2830.2788, 2833.2280, 2836.2397, 2862.2927, 2873.2961, 2889.2024, 2894.3218, 2927.2520, 2931.3428, 2955.3005, 2974.4124, 2990.3921, 3006.3206, 3011.2810, 3020.3557, 3021.2412, 3035.3281, 3053.3447, 3062.2456, 3086.4507, 3091.2622, 3103.3140, 3110.7285, 3113.8591, 3130.3557, 3140.4265, 3144.3657, 3159.4985, 3173.5386, 3174.5486, 3185.4236, 3218.3301, 3259.3625, 3312.3267, 3331.4678, 3347.4761, 3363.4788, 3390.4675, 3431.4570, 3447.4666, 3463.4895, 3485.6985, 3488.6545, 3489.6521, 3494.5571, 3504.6423, 3509.5613, 3525.6025, 3538.4521, 3553.6230, 3579.5403, 3648.1956, 3655.4729, 3664.7500, 3695.6177, 3712.5720, 3738.5837, 3816.7043, 3937.8308, 4010.8696

34. Q3SZ59 **Mass:** 12718 **Score:** 14 **Expect:** 1e+003 **Matches:** 7
60S ribosomal protein L36a OS=Bos taurus GN=RPL36A PE=3 SV=3

Observed	Mr(expt)	Mr(calc)	ppm	Start	End	Miss	Ions	Peptide
737.4152	736.4079	736.3980	13.4	40 -	44	2	---	R.RYDRK.Q
747.4550	746.4477	746.4473	0.58	82 -	87	1	---	R.MLAIKR.C + Oxidation (M)
960.5294	959.5221	959.5222	-0.15	1 -	8	1	---	-.MVNVPKTR.R + Oxidation (M)
1037.5405	1036.5332	1036.5301	2.98	31 -	39	1	---	K.DSLYAQGKR.R
1265.7064	1264.6991	1264.6676	24.9	18 -	27	1	---	K.HQPHKVTQYK.K
1318.6750	1317.6677	1317.6499	13.5	88 -	98	2	---	R.CKHFELGGDKK.R
1393.7992	1392.7919	1392.7626	21.1	18 -	28	2	---	K.HQPHKVTQYKK.G

No match to: 716.4763, 723.4098, 724.2417, 725.2533, 726.3622, 728.3046, 729.4749, 734.4570, 736.3904, 738.3791, 744.8651, 745.3838, 768.3673, 788.4571, 801.4262, 805.4606, 813.4481, 814.4558, 820.4053, 823.4778, 829.4589, 830.4522, 839.3830, 842.5110, 857.4382, 860.4207, 865.4518, 870.4165, 872.5054, 880.4424, 882.4292, 887.4245, 887.9669, 888.4561, 891.4898, 894.4144, 894.8973, 897.5006, 898.4523, 912.5132, 913.5293, 925.4620, 927.4981, 931.4992, 934.5098, 957.5774, 961.4949, 963.4734, 965.4692, 970.5181, 972.4899, 975.5167, 976.5063, 977.5212, 978.5100, 978.5146, 983.6283, 992.4958, 993.5046, 995.4181, 998.5911, 1000.5959, 1002.5007, 1006.5014, 1011.5474, 1012.5447, 1014.5485, 1020.5577, 1021.5699, 1024.5516, 1027.5438, 1028.5397, 1030.5978, 1032.5238, 1036.5260, 1039.5641, 1040.5908, 1042.5388, 1043.5178, 1045.5568, 1046.6018, 1056.5515, 1057.6100, 1057.6107, 1061.6168, 1074.6073, 1074.6100, 1076.6123, 1102.6007, 1103.6257, 1105.6136, 1107.1058, 1113.5486, 1120.5498, 1122.5322, 1123.5986, 1128.5724, 1129.5924, 1134.3574, 1137.5463, 1145.6348, 1149.6057, 1153.6460, 1156.4957, 1161.6295, 1162.6174, 1164.1815, 1170.5497, 1172.6156, 1177.6410, 1180.6428, 1184.6412, 1188.5603, 1193.6041, 1206.5107, 1209.5889, 1211.6099, 1212.6160, 1215.6743, 1216.7081, 1219.6886, 1227.6234, 1229.7098, 1234.6414, 1240.5974, 1242.6003, 1243.6161, 1253.6216, 1255.5972, 1255.6000, 1260.6227, 1264.7130, 1269.6498, 1270.6575, 1273.6556, 1277.6305, 1286.6099, 1289.6875, 1293.6401, 1303.6455, 1304.6677, 1307.6788, 1309.6780, 1319.6589, 1328.6600, 1331.7888, 1333.7644, 1336.0798, 1336.6155, 1350.6853, 1351.7202, 1356.5811, 1359.1302, 1359.6246, 1365.6489, 1374.7588, 1383.7037, 1390.6974, 1400.6683, 1401.6989, 1414.8191, 1429.3142, 1436.7889, 1444.7122, 1445.7839, 1450.7223, 1456.7717, 1462.6630, 1467.7329, 1470.7394, 1471.2693, 1471.7250, 1472.2531, 1474.5243, 1475.7633, 1483.7141, 1488.5333, 1488.7194, 1493.7366, 1502.5601, 1513.7441, 1514.6849, 1516.6807, 1517.6437, 1518.7053, 1523.7611, 1526.7343, 1528.6973, 1530.6893, 1536.8230, 1540.7114, 1551.7063, 1553.7252, 1554.7474, 1557.6992, 1558.7140, 1563.6658, 1564.3619, 1565.7223, 1566.7162, 1572.7062, 1580.8400, 1580.8423, 1586.8090, 1597.7913, 1599.8337, 1601.8030, 1614.8046, 1616.7968, 1628.7136, 1630.8103, 1635.7697, 1642.7682, 1643.7688, 1644.7915, 1646.8154, 1651.7681, 1657.7932, 1663.8466, 1664.8557, 1666.7545, 1695.8208, 1707.8933, 1718.8790, 1721.9192, 1722.9375, 1728.8976, 1730.9116, 1732.2391, 1734.1844, 1739.7894, 1743.8966, 1755.2666, 1755.7703, 1757.4702, 1757.9089, 1758.4524, 1758.8987, 1761.9023, 1765.7578, 1768.8339, 1769.8287, 1770.3949, 1772.8273, 1773.8792, 1774.9180, 1774.9200, 1786.8268, 1787.8239, 1809.0499, 1810.0303, 1829.8895, 1830.8380, 1833.3470, 1833.8501, 1851.8885, 1852.0399, 1862.9515, 1881.7703, 1895.9634, 1906.8195, 1908.8512, 1920.7972, 1932.0050, 1936.9479, 1939.8563, 1940.8103, 1943.8293, 1954.8351, 1956.8574, 1958.8956, 1965.7998, 1967.9565, 1969.9998, 1979.8927, 1981.3531, 1982.8179, 1995.8232, 1997.8312, 2003.8595, 2008.9971, 2011.8464, 2015.9408, 2019.8779, 2025.8735, 2031.9606, 2047.9397, 2062.9578, 2067.0020, 2076.8943, 2081.9827, 2082.9966, 2087.0103, 2088.1108, 2089.1162, 2096.0430, 2097.0200, 2108.9771, 2110.0061, 2139.0991, 2140.0918, 2143.1006, 2144.0718, 2149.8516, 2160.0554, 2174.0378, 2178.0471, 2179.0574, 2188.0547, 2191.0457, 2198.1831, 2199.1499, 2203.0386, 2211.1052, 2223.1582, 2225.1470, 2227.1042, 2228.0725, 2238.1086, 2239.1538, 2243.1243, 2244.0684, 2254.0896, 2255.1482, 2260.0779, 2270.2183, 2272.1736, 2284.1841, 2287.1108, 2307.0549, 2309.0659, 2310.0798, 2310.5271, 2311.0454, 2319.0945, 2337.6050, 2341.5510, 2353.0518, 2354.0767, 2355.0564, 2371.0581, 2383.9592, 2388.0437, 2392.1462, 2404.0471, 2412.0872, 2419.5879, 2420.0864, 2420.5930, 2423.2439, 2426.5667, 2427.1211, 2438.1724, 2455.1553, 2458.1648, 2459.1794, 2478.1587, 2516.1133, 2533.1262, 2542.1650, 2546.1746, 2549.1316, 2550.1252, 2559.1470, 2561.1729, 2562.1765, 2567.1514, 2572.1523, 2574.2678, 2576.1628, 2583.1218, 2587.2722, 2604.1682, 2612.1438, 2653.7173, 2655.1897, 2671.1787, 2687.1833, 2701.2820, 2706.2766, 2712.2104, 2714.2156, 2716.1953, 2717.2725, 2725.2354, 2729.1675, 2739.2351, 2743.2642, 2745.2900, 2754.2197, 2755.2358, 2759.2573, 2769.2219, 2771.2437, 2776.2644, 2785.2388, 2786.2617, 2787.2400, 2793.2439, 2801.2378, 2803.2302, 2809.2549, 2815.2153, 2817.2322, 2819.2236, 2830.2788, 2833.2280, 2836.2397, 2862.2927, 2873.2961, 2889.2024, 2894.3218, 2927.2520, 2931.3428, 2955.3005, 2974.4124, 2975.4084, 2990.3921, 3006.3206, 3011.2810, 3020.3557, 3021.2412, 3035.3281, 3053.3447, 3062.2456, 3086.4507, 3091.2622, 3103.3140, 3110.7285, 3113.8591, 3130.3557, 3140.4265, 3144.3657, 3159.4985, 3173.5386, 3174.5486, 3185.4236, 3218.3301, 3259.3625, 3312.3267, 3331.4678, 3347.4761, 3363.4788, 3390.4675, 3431.4570, 3447.4666, 3463.4895, 3485.6985, 3488.6545, 3489.6521, 3493.0195, 3494.5571, 3504.6423, 3509.5613, 3525.6025, 3538.4521, 3553.6230, 3579.5403, 3648.1956, 3655.4729, 3664.7500, 3695.6177, 3712.5720, 3738.5837, 3816.7043, 3937.8308, 4010.8696

35. G3MYU4 **Mass:** 73163 **Score:** 14 **Expect:** 1e+003 **Matches:** 28
Centrosomal protein of 290 kDa OS=Bos taurus GN=CEP290 PE=4 SV=1

Observed	Mr(expt)	Mr(calc)	ppm	Start	End	Miss	Ions	Peptide
830.4522	829.4449	829.4545	-11.55	35 -	41	0	---	K.VEVNELK.S
1074.6073	1073.6000	1073.5968	2.99	432 -	440	1	---	K.DKELVETLK.R
1074.6100	1073.6027	1073.5968	5.50	432 -	440	1	---	K.DKELVETLK.R
1102.6007	1101.5934	1101.6030	-8.66	470 -	478	1	---	R.DREIEVLTK.E
1303.6455	1302.6382	1302.6415	-2.52	420 -	431	1	---	R.TAELAEADAREK.D
1304.6677	1303.6604	1303.6554	3.86	225 -	234	1	---	K.IELQNQEMRK.N + Oxidation (M)
1331.7888	1330.7815	1330.7496	24.0	303 -	313	1	---	K.VEEWKLILSSK.D
1444.7122	1443.7049	1443.6850	13.8	583 -	594	2	---	R.MGERKSDFMSLK.N + Oxidation (M)
1493.7366	1492.7293	1492.7456	-10.93	390 -	402	1	---	K.GASTLTQRTHYMK.I
1516.6807	1515.6734	1515.6875	-9.28	289 -	302	0	---	K.NEEDDPIMAAVNAK.V
1540.7114	1539.7041	1539.7277	-15.33	191 -	203	2	---	R.RGEDSDYRSQLSK.K
1657.7932	1656.7859	1656.7525	20.1	94 -	108	0	---	K.LENELEMAQHSAGGR.D + Oxidation (M)
1730.9116	1729.9043	1729.9097	-3.12	535 -	548	2	---	K.EIESLEEERLDLKK.K
1761.9023	1760.8950	1760.8866	4.80	61 -	75	1	---	K.MKAQEVELALEEVEK.A + Oxidation (M)
1769.8287	1768.8214	1768.8376	-9.12	501 -	515	0	---	R.MGLEPTTMIDLTEFR.N + Oxidation (M)
1932.0050	1930.9977	1931.0000	-1.15	483 -	498	1	---	K.LELKINDFLDENEALR.E
2015.9408	2014.9335	2014.9564	-11.36	91 -	108	1	---	K.VMKLENELEMAQHSAGGR.D + Oxidation (M)
2031.9606	2030.9533	2030.9513	0.98	91 -	108	1	---	K.VMKLENELEMAQHSAGGR.D + 2 Oxidation (M)
2087.0103	2086.0030	2085.9926	4.99	144 -	161	1	---	K.VNEQLALRNEEAENENSK.L
2097.0200	2096.0127	2096.0282	-7.38	501 -	518	1	---	R.MGLEPTTMIDLTEFRNTK.S
2188.0547	2187.0474	2187.0154	14.7	289 -	307	1	---	K.NEEDDPIMAAVNAKVEEWK.L
2458.1648	2457.1575	2457.1767	-7.80	354 -	373	2	---	R.DSQIKMLTEQVEQYTKEMEK.N
2459.1794	2458.1721	2458.2260	-21.93	61 -	82	2	---	K.MKAQEVELALEEVEKAGEEQAK.F
2561.1729	2560.1656	2560.1798	-5.55	255 -	275	2	---	K.AVVHQTDSMMDQLKKENDHYR.L + Oxidation (M)
2771.2437	2770.2364	2770.2547	-6.58	246 -	268	2	---	K.MTDEYNRMKAVVHQTDSMMDQLK.K
2787.2400	2786.2327	2786.2496	-6.05	246 -	268	2	---	K.MTDEYNRMKAVVHQTDSMMDQLK.K + Oxidation (M)

2803.2302	2802.2229	2802.2445	-7.70	246 -	268	2	---	K.MTDEYNRMKAVVHQTDSMMDQLK.K + 2 Oxidation (M)
2819.2236	2818.2163	2818.2394	-8.19	246 -	268	2	---	K.MTDEYNRMKAVVHQTDSMMDQLK.K + 3 Oxidation (M)

No match to: 716.4763, 723.4098, 724.2417, 725.2533, 726.3622, 728.3046, 729.4749, 734.4570, 736.3904, 737.4152, 738.3791, 744.8651, 745.3838, 747.4550, 768.3673, 788.4571, 801.4262, 805.4606, 813.4481, 814.4558, 820.4053, 823.4778, 829.4589, 839.3830, 842.5110, 857.4382, 860.4207, 865.4518, 870.4165, 872.5054, 880.4424, 882.4292, 887.4245, 887.9669, 888.4561, 891.4898, 894.4144, 894.8973, 897.5006, 898.4523, 912.5132, 913.5293, 925.4620, 927.4981, 931.4992, 934.5098, 957.5774, 960.5294, 961.4949, 963.4734, 965.4692, 970.5181, 972.4899, 975.5167, 976.5063, 977.5212, 978.5100, 978.5146, 983.6283, 992.4958, 993.5046, 995.4181, 998.5911, 1000.5959, 1002.5007, 1006.5014, 1011.5474, 1012.5447, 1014.5485, 1020.5577, 1021.5699, 1024.5516, 1027.5438, 1028.5397, 1030.5978, 1032.5238, 1036.5260, 1037.5405, 1039.5641, 1040.5908, 1042.5388, 1043.5178, 1045.5568, 1046.6018, 1056.5515, 1057.6100, 1057.6107, 1061.6168, 1076.6123, 1103.6257, 1105.6136, 1107.1058, 1113.5486, 1120.5498, 1122.5322, 1123.5986, 1128.5724, 1129.5924, 1134.3574, 1137.5463, 1145.6348, 1149.6057, 1153.6460, 1156.4957, 1161.6295, 1162.6174, 1164.1815, 1170.5497, 1172.6156, 1177.6410, 1180.6428, 1184.6412, 1188.5603, 1193.6041, 1206.5107, 1209.5889, 1211.6099, 1212.6160, 1215.6743, 1216.7081, 1219.6886, 1227.6234, 1229.7098, 1234.6414, 1240.5974, 1242.6003, 1243.6161, 1253.6216, 1255.5972, 1255.6000, 1260.6227, 1264.7130, 1265.7064, 1269.6498, 1270.6575, 1273.6556, 1277.6305, 1286.6099, 1289.6875, 1293.6401, 1307.6788, 1309.6780, 1318.6750, 1319.6589, 1328.6600, 1328.6620, 1333.7644, 1336.0798, 1336.6155, 1350.6853, 1351.7202, 1356.5811, 1359.1302, 1359.6246, 1365.6489, 1374.7588, 1383.7037, 1390.6974, 1393.7992, 1400.6683, 1401.6989, 1414.8191, 1429.3142, 1436.7889, 1445.7839, 1450.7223, 1456.7717, 1462.6630, 1467.7329, 1470.7394, 1471.2693, 1471.7250, 1472.2531, 1474.5243, 1475.7633, 1483.7141, 1488.5333, 1488.7194, 1502.5601, 1513.7441, 1514.6849, 1517.6437, 1518.7053, 1523.7611, 1526.7343, 1528.6973, 1530.6893, 1536.8230, 1551.7063, 1553.7252, 1554.7474, 1557.6992, 1558.7140, 1563.6658, 1564.3619, 1565.7223, 1566.7162, 1572.7062, 1580.8400, 1580.8423, 1586.8090, 1597.7913, 1599.8337, 1601.8030, 1614.8046, 1616.7968, 1628.7136, 1630.8103, 1635.7697, 1642.7682, 1643.7688, 1644.7915, 1646.8154, 1651.7681, 1663.8466, 1664.8557, 1666.7545, 1695.8208, 1707.8933, 1718.8790, 1721.9192, 1722.9375, 1728.8976, 1732.2391, 1734.1844, 1739.7894, 1743.8966, 1755.2666, 1755.7703, 1757.4702, 1757.9089, 1758.4524, 1758.8987, 1765.7578, 1768.8339, 1770.3949, 1772.8273, 1773.8792, 1774.9180, 1774.9200, 1786.8268, 1787.8239, 1809.0499, 1810.0303, 1829.8895, 1830.8380, 1833.3470, 1833.8501, 1851.8885, 1852.0399, 1862.9515, 1881.7703, 1895.9634, 1906.8195, 1908.8512, 1920.7972, 1936.9479, 1939.8563, 1940.8103, 1943.8293, 1954.8351, 1956.8574, 1958.8956, 1965.7998, 1967.9565, 1969.9998, 1979.8927, 1981.3531, 1982.8179, 1995.8232, 1997.8312, 2003.8595, 2008.9971, 2011.8464, 2019.8779, 2025.8735, 2047.9397, 2062.9578, 2067.0020, 2076.8943, 2081.9827, 2082.9966, 2088.1108, 2089.1162, 2096.0430, 2108.9771, 2110.0061, 2139.0991, 2140.0918, 2143.1006, 2144.0718, 2149.8516, 2160.0554, 2174.0378, 2178.0471, 2179.0574, 2191.0457, 2198.1831, 2199.1499, 2203.0386, 2211.1052, 2223.1582, 2225.1470, 2227.1042, 2228.0725, 2238.1086, 2239.1538, 2243.1243, 2244.0684, 2254.0896, 2255.1482, 2260.0779, 2270.2183, 2272.1736, 2284.1841, 2287.1108, 2307.0549, 2309.0659, 2310.0798, 2310.5271, 2311.0454, 2319.0945, 2337.6050, 2341.5510, 2353.0518, 2354.0767, 2355.0564, 2371.0581, 2383.9592, 2388.0437, 2392.1462, 2404.0471, 2412.0872, 2419.5879, 2420.0864, 2420.5930, 2423.2439, 2426.5667, 2427.1211, 2438.1724, 2455.1553, 2478.1587, 2516.1133, 2533.1262, 2542.1650, 2546.1746, 2549.1316, 2550.1252, 2559.1470, 2562.1765, 2567.1514, 2572.1523, 2574.2678, 2576.1628, 2583.1218, 2587.2722, 2604.1682, 2612.1438, 2653.7173, 2655.1897, 2671.1787, 2687.1833, 2701.2820, 2706.2766, 2712.2104, 2714.2156, 2716.1953, 2717.2725, 2725.2354, 2729.1675, 2739.2351, 2743.2642, 2745.2900, 2754.2197, 2755.2358, 2759.2573, 2769.2219, 2776.2644, 2785.2388, 2786.2617, 2793.2439, 2801.2378, 2809.2549, 2815.2153, 2817.2322, 2830.2788, 2833.2280, 2836.2397, 2862.2927, 2873.2961, 2889.2024, 2894.3218, 2927.2520, 2931.3428, 2955.3005, 2974.4124, 2975.4084, 2990.3921, 3006.3206, 3011.2810, 3020.3557, 3021.2412, 3035.3281, 3053.3447, 3062.2456, 3086.4507, 3091.2622, 3103.3140, 3110.7285, 3113.8591, 3130.3557, 3140.4265, 3144.3657, 3159.4985, 3173.5386, 3174.5486, 3185.4236, 3218.3301, 3259.3625, 3312.3267, 3331.4678, 3347.4761, 3363.4788, 3390.4675, 3431.4570, 3447.4666, 3463.4895, 3485.6985, 3488.6545, 3489.6521, 3493.0195, 3494.5571, 3504.6423, 3509.5613, 3525.6025, 3538.4521, 3553.6230, 3579.5403, 3648.1956, 3655.4729, 3664.7500, 3695.6177, 3712.5720, 3738.5837, 3816.7043, 3937.8308, 4010.8696

36. P84088 **Mass:** 15499 **Score:** 14 **Expect:** 1.1e+003 **Matches:** 7

Complexin-2 OS=Bos taurus GN=CPLX2 PE=1 SV=1

Observed	Mr(expt)	Mr(calc)	ppm	Start	End	Miss	Ions	Peptide
723.4098	722.4025	722.3963	8.64	68 -	73	1	---	R.DKYGLK.K
801.4262	800.4189	800.4028	20.1	27 -	33	1	---	K.DPDAQKK.E
1436.7889	1435.7816	1435.7533	19.7	123 -	134	1	---	K.YLPGPLQDMFKK.-
1958.8956	1957.8883	1957.9089	-10.50	34 -	48	2	---	K.EEERQEALRQQEEER.K
2081.9827	2080.9754	2080.9769	-0.70	7 -	26	2	---	K.QALGGATKDMGKMLGGEEEK.D + 2 Oxidation (M)
2243.1243	2242.1170	2242.0899	12.1	79 -	98	1	---	K.EAEEKAALEQPCEGSLTRPK.K
2533.1262	2532.1189	2532.1425	-9.30	100 -	122	0	---	K.AIPAGCGDEEEEEEESILDTVLK.Y

No match to: 716.4763, 724.2417, 725.2533, 726.3622, 728.3046, 729.4749, 734.4570, 736.3904, 737.4152, 738.3791, 744.8651, 745.3838, 747.4550, 768.3673, 788.4571, 805.4606, 813.4481, 814.4558, 820.4053, 823.4778, 829.4589, 830.4522, 839.3830, 842.5110, 857.4382, 860.4207, 865.4518, 870.4165, 872.5054, 880.4424, 882.4292, 887.4245, 887.9669, 888.4561, 891.4898, 894.4144, 894.8973, 897.5006, 898.4523, 912.5132, 913.5293, 925.4620, 927.4981, 931.4992, 934.5098, 957.5774, 960.5294, 961.4949, 963.4734, 965.4692, 970.5181, 972.4899, 975.5167, 976.5063, 977.5212, 978.5100, 978.5146, 983.6283, 992.4958, 993.5046, 995.4181, 998.5911, 1000.5959, 1002.5007, 1006.5014, 1011.5474, 1012.5447, 1014.5485, 1020.5577, 1021.5699, 1024.5516, 1027.5438, 1028.5397, 1030.5978, 1032.5238, 1036.5260, 1037.5405, 1039.5641, 1040.5908, 1042.5388, 1043.5178, 1045.5568, 1046.6018, 1056.5515, 1057.6100, 1057.6107, 1061.6168, 1074.6073, 1074.6100, 1076.6123, 1102.6007, 1103.6257, 1105.6136, 1107.1058, 1113.5486, 1120.5498, 1122.5322, 1123.5986, 1128.5724, 1129.5924, 1134.3574, 1137.5463, 1145.6348, 1149.6057, 1153.6460, 1156.4957, 1161.6295, 1162.6174, 1164.1815, 1170.5497, 1172.6156, 1177.6410, 1180.6428, 1184.6412, 1188.5603, 1193.6041, 1206.5107, 1209.5889, 1211.6099, 1212.6160, 1215.6743, 1216.7081, 1219.6886, 1227.6234, 1229.7098, 1234.6414, 1240.5974, 1242.6003, 1243.6161, 1253.6216, 1255.5972, 1255.6000, 1260.6227, 1264.7130, 1265.7064, 1269.6498, 1270.6575, 1273.6556, 1277.6305, 1286.6099, 1289.6875, 1293.6401, 1303.6455, 1304.6677, 1307.6788, 1309.6780, 1318.6750, 1319.6589, 1328.6600, 1328.6620, 1331.7888, 1333.7644, 1336.0798, 1336.6155, 1350.6853, 1351.7202, 1356.5811, 1359.1302, 1359.6246, 1365.6489, 1374.7588, 1383.7037, 1390.6974, 1393.7992, 1400.6683, 1401.6989, 1414.8191, 1429.3142, 1444.7122, 1445.7839, 1450.7223, 1456.7717, 1462.6630, 1467.7329, 1470.7394, 1471.2693, 1471.7250, 1472.2531, 1474.5243, 1475.7633, 1483.7141, 1488.5333, 1488.7194, 1493.7366, 1502.5601, 1513.7441, 1514.6849, 1516.6807, 1517.6437, 1518.7053, 1523.7611, 1526.7343, 1528.6973, 1530.6893, 1536.8230, 1540.7114, 1551.7063, 1553.7252, 1554.7474, 1557.6992, 1558.7140, 1563.6658, 1564.3619, 1565.7223, 1566.7162, 1572.7062, 1580.8400, 1580.8423, 1586.8090, 1597.7913, 1599.8337, 1601.8030, 1614.8046, 1616.7968, 1628.7136, 1630.8103, 1635.7697, 1642.7682, 1643.7688, 1644.7915, 1646.8154, 1651.7681, 1657.7932, 1663.8466, 1664.8557, 1666.7545, 1695.8208, 1707.8933, 1718.8790, 1721.9192, 1722.9375, 1728.8976, 1730.9116, 1732.2391, 1734.1844, 1739.7894, 1743.8966, 1755.2666, 1755.7703, 1757.4702, 1757.9089, 1758.4524, 1758.8987, 1761.9023, 1765.7578, 1768.8339, 1769.8287, 1770.3949, 1772.8273, 1773.8792, 1774.9180, 1774.9200, 1786.8268, 1787.8239, 1809.0499, 1810.0303, 1829.8895, 1830.8380, 1833.3470, 1833.8501, 1851.8885, 1852.0399, 1862.9515, 1881.7703, 1895.9634, 1906.8195, 1908.8512, 1920.7972, 1932.0050, 1936.9479, 1939.8563, 1940.8103, 1943.8293, 1954.8351, 1956.8574, 1965.7998, 1967.9565, 1969.9998, 1979.8927, 1981.3531, 1982.8179, 1995.8232, 1997.8312, 2003.8595, 2008.9971, 2011.8464, 2015.9408, 2019.8779, 2025.8735, 2031.9606, 2047.9397, 2062.9578, 2067.0020, 2076.8943, 2082.9966, 2087.0103, 2088.1108, 2089.1162, 2096.0430, 2097.0200, 2108.9771, 2110.0061, 2139.0991, 2140.0918, 2143.1006, 2144.0718, 2149.8516, 2160.0554, 2174.0378, 2178.0471, 2179.0574, 2188.0547, 2191.0457, 2198.1831, 2199.1499, 2203.0386, 2211.1052, 2223.1582, 2225.1470, 2227.1042, 2228.0725, 2238.1086, 2239.1538, 2244.0684, 2254.0896, 2255.1482, 2260.0779, 2270.2183, 2272.1736, 2284.1841, 2287.1108, 2307.0549, 2309.0659, 2310.0798, 2310.5271, 2311.0454, 2319.0945, 2337.6050, 2341.5510, 2353.0518, 2354.0767, 2355.0564, 2371.0581, 2383.9592, 2388.0437, 2392.1462, 2404.0471, 2412.0872, 2419.5879, 2420.0864, 2420.5930, 2423.2439, 2426.5667, 2427.1211, 2438.1724, 2455.1553, 2458.1648, 2459.1794, 2478.1587, 2516.1133, 2542.1650, 2546.1746, 2549.1316, 2550.1252, 2559.1470, 2561.1729, 2562.1765, 2567.1514, 2572.1523, 2574.2678, 2576.1628, 2583.1218, 2587.2722, 2604.1682, 2612.1438, 2653.7173, 2655.1897, 2671.1787, 2687.1833, 2701.2820, 2706.2766, 2712.2104, 2714.2156, 2716.1953, 2717.2725, 2725.2354, 2729.1675, 2739.2351, 2743.2642, 2745.2900, 2754.2197, 2755.2358, 2759.2573, 2769.2219, 2771.2437, 2776.2644, 2785.2388, 2786.2617, 2787.2400, 2793.2439, 2801.2378, 2803.2302, 2809.2549, 2815.2153, 2817.2322, 2819.2236, 2830.2788, 2833.2280, 2836.2397, 2862.2927, 2873.2961, 2889.2024, 2894.3218, 2927.2520, 2931.3428, 2955.3005, 2974.4124, 2975.4084, 2990.3921, 3006.3206, 3011.2810, 3020.3557, 3021.2412, 3035.3281, 3053.3447, 3062.2456, 3086.4507, 3091.2622, 3103.3140, 3110.7285, 3113.8591, 3130.3557, 3140.4265, 3144.3657, 3159.4985, 3173.5386, 3174.5486, 3185.4236, 3218.3301, 3259.3625, 3312.3267, 3331.4678, 3347.4761, 3363.4788, 3390.4675, 3431.4570, 3447.4666, 3463.4895, 3485.6985, 3488.6545, 3489.6521, 3493.0195, 3494.5571, 3504.6423, 3509.5613, 3525.6025, 3538.4521, 3553.6230, 3579.5403, 3648.1956, 3655.4729, 3664.7500, 3695.6177, 3712.5720, 3738.5837, 3816.7043, 3937.8308, 4010.8696

37. Q2TBI9 **Mass:** 19064 **Score:** 14 **Expect:** 1.1e+003 **Matches:** 9

M-phase phosphoprotein 6 OS=Bos taurus GN=MPHOSPH6 PE=2 SV=1

Observed	Mr(expt)	Mr(calc)	ppm	Start	End	Miss	Ions	Peptide
934.5098	933.5026	933.5065	-4.28	1 -	8	2	---	-.MAAERKTK.L
978.5100	977.5027	977.5038	-1.08	87 -	94	0	---	K.LMLQMNTK.N
978.5146	977.5073	977.5038	3.61	87 -	94	0	---	K.LMLQMNTK.N

1032.5238	1031.5165	1031.5135	2.97	30 -	37	1	---	K.QLEEEEKK.I
2270.2183	2269.2110	2269.1994	5.14	37 -	54	2	---	K.KIISEEHWYLDLPELKEK.E
2550.1252	2549.1179	2549.1439	-10.17	95 -	116	1	---	K.NKAEEVEEQTVELDVSDEEMAR.R
2706.2766	2705.2693	2705.2450	9.00	95 -	117	2	---	K.NKAEEVEEQTVELDVSDEEMARR.Y
3509.5613	3508.5540	3508.6371	-23.67	87 -	116	2	---	K.LMLQMNTKNKAEEVEEQTVELDVSDEEMAR.R
3525.6025	3524.5952	3524.6320	-10.44	87 -	116	2	---	K.LMLQMNTKNKAEEVEEQTVELDVSDEEMAR.R + Oxidation (M)

No match to: 716.4763, 723.4098, 724.2417, 725.2533, 726.3622, 728.3046, 729.4749, 734.4570, 736.3904, 737.4152, 738.3791, 744.8651, 745.3838, 747.4550, 768.3673, 788.4571, 801.4262, 805.4606, 813.4481, 814.4558, 820.4053, 823.4778, 829.4589, 830.4522, 839.3830, 842.5110, 857.4382, 860.4207, 865.4518, 870.4165, 872.5054, 880.4424, 882.4292, 887.4245, 887.9669, 888.4561, 891.4898, 894.4144, 894.8973, 897.5006, 898.4523, 912.5132, 913.5293, 925.4620, 927.4981, 931.4992, 957.5774, 960.5294, 961.4949, 963.4734, 965.4692, 970.5181, 972.4899, 975.5167, 976.5063, 977.5212, 983.6283, 992.4958, 993.5046, 995.4181, 998.5911, 1000.5959, 1002.5007, 1006.5014, 1011.5474, 1012.5447, 1014.5485, 1020.5577, 1021.5699, 1024.5516, 1027.5438, 1028.5397, 1030.5978, 1036.5260, 1037.5405, 1039.5641, 1040.5908, 1042.5388, 1043.5178, 1045.5568, 1046.6018, 1056.5515, 1057.6100, 1057.6107, 1061.6168, 1074.6073, 1074.6100, 1076.6123, 1102.6007, 1103.6257, 1105.6136, 1107.1058, 1113.5486, 1120.5498, 1122.5322, 1123.5986, 1128.5724, 1129.5924, 1134.3574, 1137.5463, 1145.6348, 1149.6057, 1153.6460, 1156.4957, 1161.6295, 1162.6174, 1164.1815, 1170.5497, 1172.6156, 1177.6410, 1180.6428, 1184.6412, 1188.5603, 1193.6041, 1206.5107, 1209.5889, 1211.6099, 1212.6160, 1215.6743, 1216.7081, 1219.6886, 1227.6234, 1229.7098, 1234.6414, 1240.5974, 1242.6003, 1243.6161, 1253.6216, 1255.5972, 1255.6000, 1260.6227, 1264.7130, 1265.7064, 1269.6498, 1270.6575, 1273.6556, 1277.6305, 1286.6099, 1289.6875, 1293.6401, 1303.6455, 1304.6677, 1307.6788, 1309.6780, 1318.6750, 1319.6589, 1328.6600, 1328.6620, 1331.7888, 1333.7644, 1336.0798, 1336.6155, 1350.6853, 1351.7202, 1356.5811, 1359.1302, 1359.6246, 1365.6489, 1374.7588, 1383.7037, 1390.6974, 1393.7992, 1400.6683, 1401.6989, 1414.8191, 1429.3142, 1436.7889, 1444.7122, 1445.7839, 1450.7223, 1456.7717, 1462.6630, 1467.7329, 1470.7394, 1471.2693, 1471.7250, 1472.2531, 1474.5243, 1475.7633, 1483.7141, 1488.5333, 1488.7194, 1493.7366, 1502.5601, 1513.7441, 1514.6849, 1516.6807, 1517.6437, 1518.7053, 1523.7611, 1526.7343, 1528.6973, 1530.6893, 1536.8230, 1540.7114, 1551.7063, 1553.7252, 1554.7474, 1557.6992, 1558.7140, 1563.6658, 1564.3619, 1565.7223, 1566.7162, 1572.7062, 1580.8400, 1580.8423, 1586.8090, 1597.7913, 1599.8337, 1601.8030, 1614.8046, 1616.7968, 1628.7136, 1630.8103, 1635.7697, 1642.7682, 1643.7688, 1644.7915, 1646.8154, 1651.7681, 1657.7932, 1663.8466, 1664.8557, 1666.7545, 1695.8208, 1707.8933, 1718.8790, 1721.9192, 1722.9375, 1728.8976, 1730.9116, 1732.2391, 1734.1844, 1739.7894, 1743.8966, 1755.2666, 1755.7703, 1757.4702, 1757.9089, 1758.4524, 1758.8987, 1761.9023, 1765.7578, 1768.8339, 1769.8287, 1770.3949, 1772.8273, 1773.8792, 1774.9180, 1774.9200, 1786.8268, 1787.8239, 1809.0499, 1810.0303, 1829.8895, 1830.8380, 1833.3470, 1833.8501, 1851.8885, 1852.0399, 1862.9515, 1881.7703, 1895.9634, 1906.8195, 1908.8512, 1920.7972, 1932.0050, 1936.9479, 1939.8563, 1940.8103, 1943.8293, 1954.8351, 1956.8574, 1958.8956, 1965.7998, 1967.9565, 1969.9998, 1979.8927, 1981.3531, 1982.8179, 1995.8232, 1997.8312, 2003.8595, 2008.9971, 2011.8464, 2015.9408, 2019.8779, 2025.8735, 2031.9606, 2047.9397, 2062.9578, 2067.0020, 2076.8943, 2081.9827, 2082.9966, 2087.0103, 2088.1108, 2089.1162, 2096.0430, 2097.0200, 2108.9771, 2110.0061, 2139.0991, 2140.0918, 2143.1006, 2144.0718, 2149.8516, 2160.0554, 2174.0378, 2178.0471, 2179.0574, 2188.0547, 2191.0457, 2198.1831, 2199.1499, 2203.0386, 2211.1052, 2223.1582, 2225.1470, 2227.1042, 2228.0725, 2238.1086, 2239.1538, 2243.1243, 2244.0684, 2254.0896, 2255.1482, 2260.0779, 2272.1736, 2284.1841, 2287.1108, 2307.0549, 2309.0659, 2310.0798, 2310.5271, 2311.0454, 2319.0945, 2337.6050, 2341.5510, 2353.0518, 2354.0767, 2355.0564, 2371.0581, 2383.9592, 2388.0437, 2392.1462, 2404.0471, 2412.0872, 2419.5879, 2420.0864, 2420.5930, 2423.2439, 2426.5667, 2427.1211, 2438.1724, 2455.1553, 2458.1648, 2459.1794, 2478.1587, 2516.1133, 2533.1262, 2542.1650, 2546.1746, 2549.1316, 2559.1470, 2561.1729, 2562.1765, 2567.1514, 2572.1523, 2574.2678, 2576.1628, 2583.1218, 2587.2722, 2604.1682, 2612.1438, 2653.7173, 2655.1897, 2671.1787, 2687.1833, 2701.2820, 2712.2104, 2714.2156, 2716.1953, 2717.2725, 2725.2354, 2729.1675, 2739.2351, 2743.2642, 2745.2900, 2754.2197, 2755.2358, 2759.2573, 2769.2219, 2771.2437, 2776.2644, 2785.2388, 2786.2617, 2787.2400, 2793.2439, 2801.2378, 2803.2302, 2809.2549, 2815.2153, 2817.2322, 2819.2236, 2830.2788, 2833.2280, 2836.2397, 2862.2927, 2873.2961, 2889.2024, 2894.3218, 2927.2520, 2931.3428, 2955.3005, 2974.4124, 2975.4084, 2990.3921, 3006.3206, 3011.2810, 3020.3557, 3021.2412, 3035.3281, 3053.3447, 3062.2456, 3086.4507, 3091.2622, 3103.3140, 3110.7285, 3113.8591, 3130.3557, 3140.4265, 3144.3657, 3159.4985, 3173.5386, 3174.5486, 3185.4236, 3218.3301, 3259.3625, 3312.3267, 3331.4678, 3347.4761, 3363.4788, 3390.4675, 3431.4570, 3447.4666, 3463.4895, 3485.6985, 3488.6545, 3489.6521, 3493.0195, 3494.5571, 3504.6423, 3538.4521, 3553.6230, 3579.5403, 3648.1956, 3655.4729, 3664.7500, 3695.6177, 3712.5720, 3738.5837, 3816.7043, 3937.8308, 4010.8696

38. P80209 **Mass:** 42920 **Score:** 14 **Expect:** 1.1e+003 **Matches:** 6

Cathepsin D OS=Bos taurus GN=CTSD PE=1 SV=2

Observed	Mr(expt)	Mr(calc)	ppm	Start	End	Miss	Ions	Peptide
963.4734	962.4661	962.4821	-16.64	35 -	43	0	---	K.YATGEPAVR.Q
1255.5972	1254.5899	1254.6067	-13.36	173 -	183	0	---	K.FDGILGMAYPR.I + Oxidation (M)
1255.6000	1254.5927	1254.6067	-11.13	173 -	183	0	13	K.FDGILGMAYPR.I + Oxidation (M)
1445.7839	1444.7766	1444.7674	6.38	30 -	43	1	---	K.GPISKYATGEPAVR.Q
1958.8956	1957.8883	1957.9204	-16.36	217 -	234	1	---	K.AQPGGELMLGGTDSKYYR.G + Oxidation (M)
2244.0684	2243.0611	2243.1083	-21.03	371 -	389	2	---	R.YYTVFDRDQNRVGLAEAAR.L

No match to: 716.4763, 723.4098, 724.2417, 725.2533, 726.3622, 728.3046, 729.4749, 734.4570, 736.3904, 737.4152, 738.3791, 744.8651, 745.3838, 747.4550, 768.3673, 788.4571, 801.4262, 805.4606, 813.4481, 814.4558, 820.4053, 823.4778, 829.4589, 830.4522, 839.3830, 842.5110, 857.4382, 860.4207, 865.4518, 870.4165, 872.5054, 880.4424, 882.4292, 887.4245, 887.9669, 888.4561, 891.4898, 894.4144, 894.8973, 897.5006, 898.4523, 912.5132, 913.5293, 925.4620, 927.4981, 931.4992, 934.5098, 957.5774, 960.5294, 961.4949, 965.4692, 970.5181, 972.4899, 975.5167, 976.5063, 977.5212, 978.5100, 978.5146, 983.6283, 992.4958, 993.5046, 995.4181, 998.5911, 1000.5959, 1002.5007, 1006.5014, 1011.5474, 1012.5447, 1014.5485, 1020.5577, 1021.5699, 1024.5516, 1027.5438, 1028.5397, 1030.5978, 1032.5238, 1036.5260, 1037.5405, 1039.5641, 1040.5908, 1042.5388, 1043.5178, 1045.5568, 1046.6018, 1056.5515, 1057.6100, 1057.6107, 1061.6168, 1074.6073, 1074.6100, 1076.6123, 1102.6007, 1103.6257, 1105.6136, 1107.1058, 1113.5486, 1120.5498, 1122.5322, 1123.5986, 1128.5724, 1129.5924, 1134.3574, 1137.5463, 1145.6348, 1149.6057, 1153.6460, 1156.4957, 1161.6295, 1162.6174, 1164.1815, 1170.5497, 1172.6156, 1177.6410, 1180.6428, 1184.6412, 1188.5603, 1193.6041, 1206.5107, 1209.5889, 1211.6099, 1212.6160, 1215.6743, 1216.7081, 1219.6886, 1227.6234, 1229.7098, 1234.6414, 1240.5974, 1242.6003, 1243.6161, 1253.6216, 1260.6227, 1264.7130, 1265.7064, 1269.6498, 1270.6575, 1273.6556, 1277.6305, 1286.6099, 1289.6875, 1293.6401, 1303.6455, 1304.6677, 1307.6788, 1309.6780, 1318.6750, 1319.6589, 1328.6600, 1328.6620, 1331.7888, 1333.7644, 1336.0798, 1336.6155, 1350.6853, 1351.7202, 1356.5811, 1359.1302, 1359.6246, 1365.6489, 1374.7588, 1383.7037, 1390.6974, 1393.7992, 1400.6683, 1401.6989, 1414.8191, 1429.3142, 1436.7889, 1444.7122, 1450.7223, 1456.7717, 1462.6630, 1467.7329, 1470.7394, 1471.2693, 1471.7250, 1472.2531, 1474.5243, 1475.7633, 1483.7141, 1488.5333, 1488.7194, 1493.7366, 1502.5601, 1513.7441, 1514.6849, 1516.6807, 1517.6437, 1518.7053, 1523.7611, 1526.7343, 1528.6973, 1530.6893, 1536.8230, 1540.7114, 1551.7063, 1553.7252, 1554.7474, 1557.6992, 1558.7140, 1563.6658, 1564.3619, 1565.7223, 1566.7162, 1572.7062, 1580.8400, 1580.8423, 1586.8090, 1597.7913, 1599.8337, 1601.8030, 1614.8046, 1616.7968, 1628.7136, 1630.8103, 1635.7697, 1642.7682, 1643.7688, 1644.7915, 1646.8154, 1651.7681, 1657.7932, 1663.8466, 1664.8557, 1666.7545, 1695.8208, 1707.8933, 1718.8790, 1721.9192, 1722.9375, 1728.8976, 1730.9116, 1732.2391, 1734.1844, 1739.7894, 1743.8966, 1755.2666, 1755.7703, 1757.4702, 1757.9089, 1758.4524, 1758.8987, 1761.9023, 1765.7578, 1768.8339, 1769.8287, 1770.3949, 1772.8273, 1773.8792, 1774.9180, 1774.9200, 1786.8268, 1787.8239, 1809.0499, 1810.0303, 1829.8895, 1830.8380, 1833.3470, 1833.8501, 1851.8885, 1852.0399, 1862.9515, 1881.7703, 1895.9634, 1906.8195, 1908.8512, 1920.7972, 1932.0050, 1936.9479, 1939.8563, 1940.8103, 1943.8293, 1954.8351, 1956.8574, 1965.7998, 1967.9565, 1969.9998, 1979.8927, 1981.3531, 1982.8179, 1995.8232, 1997.8312, 2003.8595, 2008.9971, 2011.8464, 2015.9408, 2019.8779, 2025.8735, 2031.9606, 2047.9397, 2062.9578, 2067.0020, 2076.8943, 2081.9827, 2082.9966, 2087.0103, 2088.1108, 2089.1162, 2096.0430, 2097.0200, 2108.9771, 2110.0061, 2139.0991, 2140.0918, 2143.1006, 2144.0718, 2149.8516, 2160.0554, 2174.0378, 2178.0471, 2179.0574, 2188.0547, 2191.0457, 2198.1831, 2199.1499, 2203.0386, 2211.1052, 2223.1582, 2225.1470, 2227.1042, 2228.0725, 2238.1086, 2239.1538, 2243.1243, 2254.0896, 2255.1482, 2260.0779, 2270.2183, 2272.1736, 2284.1841, 2287.1108, 2307.0549, 2309.0659, 2310.0798, 2310.5271, 2311.0454, 2319.0945, 2337.6050, 2341.5510, 2353.0518, 2354.0767, 2355.0564, 2371.0581, 2383.9592, 2388.0437, 2392.1462, 2404.0471, 2412.0872, 2419.5879, 2420.0864, 2420.5930, 2423.2439, 2426.5667, 2427.1211, 2438.1724, 2455.1553, 2458.1648, 2459.1794, 2478.1587, 2516.1133, 2533.1262, 2542.1650, 2546.1746, 2549.1316, 2550.1252, 2559.1470, 2561.1729, 2562.1765, 2567.1514, 2572.1523, 2574.2678, 2576.1628, 2583.1218, 2587.2722, 2604.1682, 2612.1438, 2653.7173, 2655.1897, 2671.1787, 2687.1833, 2701.2820, 2706.2766, 2712.2104, 2714.2156, 2716.1953, 2717.2725, 2725.2354, 2729.1675, 2739.2351, 2743.2642, 2745.2900, 2754.2197, 2755.2358, 2759.2573, 2769.2219, 2771.2437, 2776.2644, 2785.2388, 2786.2617, 2787.2400, 2793.2439, 2801.2378, 2803.2302, 2809.2549, 2815.2153, 2817.2322, 2819.2236, 2830.2788, 2833.2280, 2836.2397, 2862.2927, 2873.2961, 2889.2024, 2894.3218, 2927.2520, 2931.3428, 2955.3005, 2974.4124, 2975.4084, 2990.3921, 3006.3206, 3011.2810, 3020.3557, 3021.2412, 3035.3281, 3053.3447, 3062.2456, 3086.4507, 3091.2622, 3103.3140, 3110.7285, 3113.8591, 3130.3557, 3140.4265, 3144.3657, 3159.4985, 3173.5386, 3174.5486, 3185.4236, 3218.3301, 3259.3625, 3312.3267, 3331.4678, 3347.4761, 3363.4788, 3390.4675, 3431.4570, 3447.4666, 3463.4895, 3485.6985, 3488.6545, 3489.6521, 3493.0195, 3494.5571, 3504.6423, 3509.5613, 3525.6025, 3538.4521, 3553.6230, 3579.5403, 3648.1956, 3655.4729, 3664.7500, 3695.6177, 3712.5720, 3738.5837, 3816.7043, 3937.8308, 4010.8696

39. F1MMR6 **Mass:** 45132 **Score:** 14 **Expect:** 1.1e+003 **Matches:** 10

Cathepsin D OS=Bos taurus GN=CTSD PE=1 SV=1

Observed	Mr(expt)	Mr(calc)	ppm	Start	End	Miss	Ions	Peptide
963.4734	962.4661	962.4821	-16.64	57 -	65	0	---	K.YATGEPAVR.Q
1057.6100	1056.6027	1056.6179	-14.38	332 -	341	0	---	K.VSSLPQVTVK.L
1057.6107	1056.6034	1056.6179	-13.72	332 -	341	0	---	K.VSSLPQVTVK.L
1255.5972	1254.5899	1254.6067	-13.36	195 -	205	0	---	K.FDGILGMAYPR.I + Oxidation (M)
1255.6000	1254.5927	1254.6067	-11.13	195 -	205	0	13	K.FDGILGMAYPR.I + Oxidation (M)
1445.7839	1444.7766	1444.7674	6.38	52 -	65	1	---	K.GPISKYATGEPAVR.Q
1958.8956	1957.8883	1957.9204	-16.36	239 -	256	1	---	K.AQPGGELMLGGTDSKYYR.G + Oxidation (M)
2096.0430	2095.0357	2095.0806	-21.40	37 -	56	1	---	R.TMSEAMGPVEHLIAKGPISK.Y
2244.0684	2243.0611	2243.1083	-21.03	393 -	411	2	---	R.YYTVFDRDQNRVGLAEAAR.L
2284.1841	2283.1768	2283.1715	2.33	36 -	56	2	---	R.RTMSEAMGPVEHLIAKGPISK.Y + 2 Oxidation (M)

No match to: 716.4763, 723.4098, 724.2417, 725.2533, 726.3622, 728.3046, 729.4749, 734.4570, 736.3904, 737.4152, 738.3791, 744.8651, 745.3838, 747.4550, 768.3673, 788.4571, 801.4262, 805.4606, 813.4481, 814.4558, 820.4053, 823.4778, 829.4589, 830.4522, 839.3830, 842.5110, 857.4382, 860.4207, 865.4518, 870.4165, 872.5054, 880.4424, 882.4292, 887.4245, 887.9669, 888.4561, 891.4898, 894.4144, 894.8973, 897.5006, 898.4523, 912.5132, 913.5293, 925.4620, 927.4981, 931.4992, 934.5098, 957.5774, 960.5294, 961.4949, 965.4692, 970.5181, 972.4899, 975.5167, 976.5063, 977.5212, 978.5100, 978.5146, 983.6283, 992.4958, 993.5046, 995.4181, 998.5911, 1000.5959, 1002.5007, 1006.5014, 1011.5474, 1012.5447, 1014.5485, 1020.5577, 1021.5699, 1024.5516, 1027.5438, 1028.5397, 1030.5978, 1032.5238, 1036.5260, 1037.5405, 1039.5641, 1040.5908, 1042.5388, 1043.5178, 1045.5568, 1046.6018, 1056.5515, 1061.6168, 1074.6073, 1074.6100, 1076.6123, 1102.6007, 1103.6257, 1105.6136, 1107.1058, 1113.5486, 1120.5498, 1122.5322, 1123.5986, 1128.5724, 1129.5924, 1134.3574, 1137.5463, 1145.6348, 1149.6057, 1153.6460, 1156.4957, 1161.6295, 1162.6174, 1164.1815, 1170.5497, 1172.6156, 1177.6410, 1180.6428, 1184.6412, 1188.5603, 1193.6041, 1206.5107, 1209.5889, 1211.6099, 1212.6160, 1215.6743, 1216.7081, 1219.6886, 1227.6234, 1229.7098, 1234.6414, 1240.5974, 1242.6003, 1243.6161, 1253.6216, 1260.6227, 1264.7130, 1265.7064, 1269.6498, 1270.6575, 1273.6556, 1277.6305, 1286.6099, 1289.6875, 1293.6401, 1303.6455, 1304.6677, 1307.6788, 1309.6780, 1318.6750, 1319.6589, 1328.6600, 1328.6620, 1331.7888, 1333.7644, 1336.0798, 1336.6155, 1350.6853, 1351.7202, 1356.5811, 1359.1302, 1359.6246, 1365.6489, 1374.7588, 1383.7037, 1390.6974, 1393.7992, 1400.6683, 1401.6989, 1414.8191, 1429.3142, 1436.7889, 1444.7122, 1450.7223, 1456.7717, 1462.6630, 1467.7329, 1470.7394, 1471.2693, 1471.7250, 1472.2531, 1474.5243, 1475.7633, 1483.7141, 1488.5333, 1488.7194, 1493.7366, 1502.5601, 1513.7441, 1514.6849, 1516.6807, 1517.6437, 1518.7053, 1523.7611, 1526.7343, 1528.6973, 1530.6893, 1536.8230, 1540.7114, 1551.7063, 1553.7252, 1554.7474, 1557.6992, 1558.7140, 1563.6658, 1564.3619, 1565.7223, 1566.7162, 1572.7062, 1580.8400, 1580.8423, 1586.8090, 1597.7913, 1599.8337, 1601.8030, 1614.8046, 1616.7968, 1628.7136, 1630.8103, 1635.7697, 1642.7682, 1643.7688, 1644.7915, 1646.8154, 1651.7681, 1657.7932, 1663.8466, 1664.8557, 1666.7545, 1695.8208, 1707.8933, 1718.8790, 1721.9192, 1722.9375, 1728.8976, 1730.9116, 1732.2391, 1734.1844, 1739.7894, 1743.8966, 1755.2666, 1755.7703, 1757.4702, 1757.9089, 1758.4524, 1758.8987, 1761.9023, 1765.7578, 1768.8339, 1769.8287, 1770.3949, 1772.8273, 1773.8792, 1774.9180, 1774.9200, 1786.8268, 1787.8239, 1809.0499, 1810.0303, 1829.8895, 1830.8380, 1833.3470, 1833.8501, 1851.8885, 1852.0399, 1862.9515, 1881.7703, 1895.9634, 1906.8195, 1908.8512, 1920.7972, 1932.0050, 1936.9479, 1939.8563, 1940.8103, 1943.8293, 1954.8351, 1956.8574, 1965.7998, 1967.9565, 1969.9998, 1979.8927, 1981.3531, 1982.8179, 1995.8232, 1997.8312, 2003.8595, 2008.9971, 2011.8464, 2015.9408, 2019.8779, 2025.8735, 2031.9606, 2047.9397, 2062.9578, 2067.0020, 2076.8943, 2081.9827, 2082.9966, 2087.0103, 2088.1108, 2089.1162, 2097.0200, 2108.9771, 2110.0061, 2139.0991, 2140.0918, 2143.1006, 2144.0718, 2149.8516, 2160.0554, 2174.0378, 2178.0471, 2179.0574, 2188.0547, 2191.0457, 2198.1831, 2199.1499, 2203.0386, 2211.1052, 2223.1582, 2225.1470, 2227.1042, 2228.0725, 2238.1086, 2239.1538, 2243.1243, 2254.0896, 2255.1482, 2260.0779, 2270.2183, 2272.1736, 2287.1108, 2307.0549, 2309.0659, 2310.0798, 2310.5271, 2311.0454, 2319.0945, 2337.6050, 2341.5510, 2353.0518, 2354.0767, 2355.0564, 2371.0581, 2383.9592, 2388.0437, 2392.1462, 2404.0471, 2412.0872, 2419.5879, 2420.0864, 2420.5930, 2423.2439, 2426.5667, 2427.1211, 2438.1724, 2455.1553, 2458.1648, 2459.1794, 2478.1587, 2516.1133, 2533.1262, 2542.1650, 2546.1746, 2549.1316, 2550.1252, 2559.1470, 2561.1729, 2562.1765, 2567.1514, 2572.1523, 2574.2678, 2576.1628, 2583.1218, 2587.2722, 2604.1682, 2612.1438, 2653.7173, 2655.1897, 2671.1787, 2687.1833, 2701.2820, 2706.2766, 2712.2104, 2714.2156, 2716.1953, 2717.2725, 2725.2354, 2729.1675, 2739.2351, 2743.2642, 2745.2900, 2754.2197, 2755.2358, 2759.2573, 2769.2219, 2771.2437, 2776.2644, 2785.2388, 2786.2617, 2787.2400, 2793.2439, 2801.2378, 2803.2302, 2809.2549, 2815.2153, 2817.2322, 2819.2236, 2830.2788, 2833.2280, 2836.2397, 2862.2927, 2873.2961, 2889.2024, 2894.3218, 2927.2520, 2931.3428, 2955.3005, 2974.4124, 2975.4084, 2990.3921, 3006.3206, 3011.2810, 3020.3557, 3021.2412, 3035.3281, 3053.3447, 3062.2456, 3086.4507, 3091.2622, 3103.3140, 3110.7285, 3113.8591, 3130.3557, 3140.4265, 3144.3657, 3159.4985, 3173.5386, 3174.5486, 3185.4236, 3218.3301, 3259.3625, 3312.3267, 3331.4678, 3347.4761, 3363.4788, 3390.4675, 3431.4570, 3447.4666, 3463.4895, 3485.6985, 3488.6545, 3489.6521, 3493.0195, 3494.5571, 3504.6423, 3509.5613, 3525.6025, 3538.4521, 3553.6230, 3579.5403, 3648.1956, 3655.4729, 3664.7500, 3695.6177, 3712.5720, 3738.5837, 3816.7043, 3937.8308, 4010.8696

40. E1BNB8 **Mass:** 12329 **Score:** 13 **Expect:** 1.1e+003 **Matches:** 5

Uncharacterized protein OS=Bos taurus PE=4 SV=2

Observed	Mr(expt)	Mr(calc)	ppm	Start	End	Miss	Ions	Peptide
1557.6992	1556.6919	1556.6929	-0.66	100 -	113	0	---	R.VDPADSSLYFCGAR.-
2088.1108	2087.1035	2087.0629	19.5	41 -	57	1	---	R.ALDFQATAVFWYRQFPK.R
2561.1729	2560.1656	2560.2115	-17.93	59 -	82	1	---	R.GLVLMATSNVGSDATYEQGYNKDK.I
2717.2725	2716.2652	2716.3126	-17.45	58 -	82	2	---	K.RGLVLMATSNVGSDATYEQGYNKDK.I
2974.4124	2973.4051	2973.4654	-20.28	54 -	80	2	---	R.QFPKRGLVLMATSNVGSDATYEQGYNK.D

No match to: 716.4763, 723.4098, 724.2417, 725.2533, 726.3622, 728.3046, 729.4749, 734.4570, 736.3904, 737.4152, 738.3791, 744.8651, 745.3838, 747.4550, 768.3673, 788.4571, 801.4262, 805.4606, 813.4481, 814.4558, 820.4053, 823.4778, 829.4589, 830.4522, 839.3830, 842.5110, 857.4382, 860.4207, 865.4518, 870.4165, 872.5054, 880.4424, 882.4292, 887.4245, 887.9669, 888.4561, 891.4898, 894.4144, 894.8973, 897.5006, 898.4523, 912.5132, 913.5293, 925.4620, 927.4981, 931.4992, 934.5098, 957.5774, 960.5294, 961.4949, 963.4734, 965.4692, 970.5181, 972.4899, 975.5167, 976.5063, 977.5212, 978.5100, 978.5146, 983.6283, 992.4958, 993.5046, 995.4181, 998.5911, 1000.5959, 1002.5007, 1006.5014, 1011.5474, 1012.5447, 1014.5485, 1020.5577, 1021.5699, 1024.5516, 1027.5438, 1028.5397, 1030.5978, 1032.5238, 1036.5260, 1037.5405, 1039.5641, 1040.5908, 1042.5388, 1043.5178, 1045.5568, 1046.6018, 1056.5515, 1057.6100, 1057.6107, 1061.6168, 1074.6073, 1074.6100, 1076.6123, 1102.6007, 1103.6257, 1105.6136, 1107.1058, 1113.5486, 1120.5498, 1122.5322, 1123.5986, 1128.5724, 1129.5924, 1134.3574, 1137.5463, 1145.6348, 1149.6057, 1153.6460, 1156.4957, 1161.6295, 1162.6174, 1164.1815, 1170.5497, 1172.6156, 1177.6410, 1180.6428, 1184.6412, 1188.5603, 1193.6041, 1206.5107, 1209.5889, 1211.6099, 1212.6160, 1215.6743, 1216.7081, 1219.6886, 1227.6234, 1229.7098, 1234.6414, 1240.5974, 1242.6003, 1243.6161, 1253.6216, 1255.5972, 1255.6000, 1260.6227, 1264.7130, 1265.7064, 1269.6498, 1270.6575, 1273.6556, 1277.6305, 1286.6099, 1289.6875, 1293.6401, 1303.6455, 1304.6677, 1307.6788, 1309.6780, 1318.6750, 1319.6589, 1328.6600, 1328.6620, 1331.7888, 1333.7644, 1336.0798, 1336.6155, 1350.6853, 1351.7202, 1356.5811, 1359.1302, 1359.6246, 1365.6489, 1374.7588, 1383.7037, 1390.6974, 1393.7992, 1400.6683, 1401.6989, 1414.8191, 1429.3142, 1436.7889, 1444.7122, 1445.7839, 1450.7223, 1456.7717, 1462.6630, 1467.7329, 1470.7394, 1471.2693, 1471.7250, 1472.2531, 1474.5243, 1475.7633, 1483.7141, 1488.5333, 1488.7194, 1493.7366, 1502.5601, 1513.7441, 1514.6849, 1516.6807, 1517.6437, 1518.7053, 1523.7611, 1526.7343, 1528.6973, 1530.6893, 1536.8230, 1540.7114, 1551.7063, 1553.7252, 1554.7474, 1558.7140, 1563.6658, 1564.3619, 1565.7223, 1566.7162, 1572.7062, 1580.8400, 1580.8423, 1586.8090, 1597.7913, 1599.8337, 1601.8030, 1614.8046, 1616.7968, 1628.7136, 1630.8103, 1635.7697, 1642.7682, 1643.7688, 1644.7915, 1646.8154, 1651.7681, 1657.7932, 1663.8466, 1664.8557, 1666.7545, 1695.8208, 1707.8933, 1718.8790, 1721.9192, 1722.9375, 1728.8976, 1730.9116, 1732.2391, 1734.1844, 1739.7894, 1743.8966, 1755.2666, 1755.7703, 1757.4702, 1757.9089, 1758.4524, 1758.8987, 1761.9023, 1765.7578, 1768.8339, 1769.8287, 1770.3949, 1772.8273, 1773.8792, 1774.9180, 1774.9200, 1786.8268, 1787.8239, 1809.0499, 1810.0303, 1829.8895, 1830.8380, 1833.3470, 1833.8501, 1851.8885, 1852.0399, 1862.9515, 1881.7703, 1895.9634, 1906.8195, 1908.8512, 1920.7972, 1932.0050, 1936.9479, 1939.8563, 1940.8103, 1943.8293, 1954.8351, 1956.8574, 1958.8956, 1965.7998, 1967.9565, 1969.9998, 1979.8927, 1981.3531, 1982.8179, 1995.8232, 1997.8312, 2003.8595, 2008.9971, 2011.8464, 2015.9408, 2019.8779, 2025.8735, 2031.9606, 2047.9397, 2062.9578, 2067.0020, 2076.8943, 2081.9827, 2082.9966, 2087.0103, 2089.1162, 2096.0430, 2097.0200, 2108.9771, 2110.0061, 2139.0991, 2140.0918, 2143.1006, 2144.0718, 2149.8516, 2160.0554, 2174.0378, 2178.0471, 2179.0574, 2188.0547, 2191.0457, 2198.1831, 2199.1499, 2203.0386, 2211.1052, 2223.1582, 2225.1470, 2227.1042, 2228.0725, 2238.1086, 2239.1538, 2243.1243, 2244.0684, 2254.0896, 2255.1482, 2260.0779, 2270.2183, 2272.1736, 2284.1841, 2287.1108, 2307.0549, 2309.0659, 2310.0798, 2310.5271, 2311.0454, 2319.0945, 2337.6050, 2341.5510, 2353.0518, 2354.0767, 2355.0564, 2371.0581, 2383.9592, 2388.0437, 2392.1462, 2404.0471, 2412.0872, 2419.5879, 2420.0864, 2420.5930, 2423.2439, 2426.5667, 2427.1211, 2438.1724, 2455.1553, 2458.1648, 2459.1794, 2478.1587, 2516.1133, 2533.1262, 2542.1650, 2546.1746, 2549.1316, 2550.1252, 2559.1470, 2562.1765, 2567.1514, 2572.1523, 2574.2678, 2576.1628, 2583.1218, 2587.2722, 2604.1682, 2612.1438, 2653.7173, 2655.1897, 2671.1787, 2687.1833, 2701.2820, 2706.2766, 2712.2104, 2714.2156, 2716.1953, 2725.2354, 2729.1675, 2739.2351, 2743.2642, 2745.2900, 2754.2197, 2755.2358, 2759.2573, 2769.2219, 2771.2437, 2776.2644, 2785.2388, 2786.2617, 2787.2400, 2793.2439, 2801.2378, 2803.2302, 2809.2549, 2815.2153, 2817.2322, 2819.2236, 2830.2788, 2833.2280, 2836.2397, 2862.2927, 2873.2961, 2889.2024, 2894.3218, 2927.2520, 2931.3428, 2955.3005, 2975.4084, 2990.3921, 3006.3206, 3011.2810, 3020.3557, 3021.2412, 3035.3281, 3053.3447, 3062.2456, 3086.4507, 3091.2622, 3103.3140, 3110.7285, 3113.8591, 3130.3557, 3140.4265, 3144.3657, 3159.4985, 3173.5386, 3174.5486, 3185.4236, 3218.3301, 3259.3625, 3312.3267, 3331.4678, 3347.4761, 3363.4788, 3390.4675,

3431.4570, 3447.4666, 3463.4895, 3485.6985, 3488.6545, 3489.6521, 3493.0195, 3494.5571, 3504.6423, 3509.5613, 3525.6025, 3538.4521, 3553.6230, 3579.5403, 3648.1956, 3655.4729, 3664.7500, 3695.6177, 3712.5720, 3738.5837, 3816.7043, 3937.8308, 4010.8696

41. E1B9N9 **Mass:** 39688 **Score:** 13 **Expect:** 1.1e+003 **Matches:** 12
Uncharacterized protein OS=Bos taurus GN=WISP3 PE=4 SV=1

Observed	Mr(expt)	Mr(calc)	ppm	Start	End	Miss	Ions	Peptide
995.4181	994.4108	994.4324	-21.74	208 -	216	0	---	R.TCGMGISNR.V
1103.6257	1102.6184	1102.6281	-8.76	193 -	201	2	---	K.RKCLVQATK.W
1390.6974	1389.6901	1389.7075	-12.50	251 -	262	1	---	K.GKTCQPTFQPVK.A
1643.7688	1642.7615	1642.7985	-22.48	165 -	179	1	---	K.CGLGSSQKQLSTSYK.T
1718.8790	1717.8717	1717.8821	-6.06	251 -	265	2	---	K.GKTCQPTFQPVKAEK.F
1772.8273	1771.8200	1771.7910	16.4	298 -	312	0	---	K.MITVQFDCPNEGSFK.W
2087.0103	2086.0030	2085.9652	18.1	298 -	314	1	---	K.MITVQFDCPNEGSFKWK.M
2203.0386	2202.0313	2202.0587	-12.41	160 -	179	2	---	K.TDQSKCGLGSSQKQLSTSYK.T
2701.2820	2700.2747	2700.2135	22.7	266 -	288	0	---	K.FVFSGCASTQSYRPTFCGICLDK.R
2889.2024	2888.1951	2888.2554	-20.89	19 -	42	2	---	K.GSPAEGAEGHHRKQFCHWPCQCPR.R
3185.4236	3184.4163	3184.4893	-22.90	263 -	289	2	---	K.AEKFVFSGCASTQSYRPTFCGICLDKR.C
3553.6230	3552.6157	3552.6121	1.02	298 -	325	2	---	K.MITVQFDCPNEGSFKWKMLWITSCVCQR.N + 2 Oxidation (M)

No match to: 716.4763, 723.4098, 724.2417, 725.2533, 726.3622, 728.3046, 729.4749, 734.4570, 736.3904, 737.4152, 738.3791, 744.8651, 745.3838, 747.4550, 768.3673, 788.4571, 801.4262, 805.4606, 813.4481, 814.4558, 820.4053, 823.4778, 829.4589, 830.4522, 839.3830, 842.5110, 857.4382, 860.4207, 865.4518, 870.4165, 872.5054, 880.4424, 882.4292, 887.4245, 887.9669, 888.4561, 891.4898, 894.4144, 894.8973, 897.5006, 898.4523, 912.5132, 913.5293, 925.4620, 927.4981, 931.4992, 934.5098, 957.5774, 960.5294, 961.4949, 963.4734, 965.4692, 970.5181, 972.4899, 975.5167, 976.5063, 977.5212, 978.5100, 978.5146, 983.6283, 992.4958, 993.5046, 998.5911, 1000.5959, 1002.5007, 1006.5014, 1011.5474, 1012.5447, 1014.5485, 1020.5577, 1021.5699, 1024.5516, 1027.5438, 1028.5397, 1030.5978, 1032.5238, 1036.5260, 1037.5405, 1039.5641, 1040.5908, 1042.5388, 1043.5178, 1045.5568, 1046.6018, 1056.5515, 1057.6100, 1057.6107, 1061.6168, 1074.6073, 1074.6100, 1076.6123, 1102.6007, 1105.6136, 1107.1058, 1113.5486, 1120.5498, 1122.5322, 1123.5986, 1128.5724, 1129.5924, 1134.3574, 1137.5463, 1145.6348, 1149.6057, 1153.6460, 1156.4957, 1161.6295, 1162.6174, 1164.1815, 1170.5497, 1172.6156, 1177.6410, 1180.6428, 1184.6412, 1188.5603, 1193.6041, 1206.5107, 1209.5889, 1211.6099, 1212.6160, 1215.6743, 1216.7081, 1219.6886, 1227.6234, 1229.7098, 1234.6414, 1240.5974, 1242.6003, 1243.6161, 1253.6216, 1255.5972, 1255.6000, 1260.6227, 1264.7130, 1265.7064, 1269.6498, 1270.6575, 1273.6556, 1277.6305, 1286.6099, 1289.6875, 1293.6401, 1303.6455, 1304.6677, 1307.6788, 1309.6780, 1318.6750, 1319.6589, 1328.6600, 1328.6620, 1331.7888, 1333.7644, 1336.0798, 1336.6155, 1350.6853, 1351.7202, 1356.5811, 1359.1302, 1359.6246, 1365.6489, 1374.7588, 1383.7037, 1393.7992, 1400.6683, 1401.6989, 1414.8191, 1429.3142, 1436.7889, 1444.7122, 1445.7839, 1450.7223, 1456.7717, 1462.6630, 1467.7329, 1470.7394, 1471.2693, 1471.7250, 1472.2531, 1474.5243, 1475.7633, 1483.7141, 1488.5333, 1488.7194, 1493.7366, 1502.5601, 1513.7441, 1514.6849, 1516.6807, 1517.6437, 1518.7053, 1523.7611, 1526.7343, 1528.6973, 1530.6893, 1536.8230, 1540.7114, 1551.7063, 1553.7252, 1554.7474, 1557.6992, 1558.7140, 1563.6658, 1564.3619, 1565.7223, 1566.7162, 1572.7062, 1580.8400, 1580.8423, 1586.8090, 1597.7913, 1599.8337, 1601.8030, 1614.8046, 1616.7968, 1628.7136, 1630.8103, 1635.7697, 1642.7682, 1644.7915, 1646.8154, 1651.7681, 1657.7932, 1663.8466, 1664.8557, 1666.7545, 1695.8208, 1707.8933, 1721.9192, 1722.9375, 1728.8976, 1730.9116, 1732.2391, 1734.1844, 1739.7894, 1743.8966, 1755.2666, 1755.7703, 1757.4702, 1757.9089, 1758.4524, 1758.8987, 1761.9023, 1765.7578, 1768.8339, 1769.8287, 1770.3949, 1773.8792, 1774.9180, 1774.9200, 1786.8268, 1787.8239, 1809.0499, 1810.0303, 1829.8895, 1830.8380, 1833.3470, 1833.8501, 1851.8885, 1852.0399, 1862.9515, 1881.7703, 1895.9634, 1906.8195, 1908.8512, 1920.7972, 1932.0050, 1936.9479, 1939.8563, 1940.8103, 1943.8293, 1954.8351, 1956.8574, 1958.8956, 1965.7998, 1967.9565, 1969.9998, 1979.8927, 1981.3531, 1982.8179, 1995.8232, 1997.8312, 2003.8595, 2008.9971, 2011.8464, 2015.9408, 2019.8779, 2025.8735, 2031.9606, 2047.9397, 2062.9578, 2067.0020, 2076.8943, 2081.9827, 2082.9966, 2088.1108, 2089.1162, 2096.0430, 2097.0200, 2108.9771, 2110.0061, 2139.0991, 2140.0918, 2143.1006, 2144.0718, 2149.8516, 2160.0554, 2174.0378, 2178.0471, 2179.0574, 2188.0547, 2191.0457, 2198.1831, 2199.1499, 2211.1052, 2223.1582, 2225.1470, 2227.1042, 2228.0725, 2238.1086, 2239.1538, 2243.1243, 2244.0684, 2254.0896, 2255.1482, 2260.0779, 2270.2183, 2272.1736, 2284.1841, 2287.1108, 2307.0549, 2309.0659, 2310.0798, 2310.5271, 2311.0454, 2319.0945, 2337.6050, 2341.5510, 2353.0518, 2354.0767, 2355.0564, 2371.0581, 2383.9592, 2388.0437, 2392.1462, 2404.0471, 2412.0872, 2419.5879, 2420.0864, 2420.5930, 2423.2439, 2426.5667, 2427.1211, 2438.1724, 2455.1553, 2458.1648, 2459.1794, 2478.1587, 2516.1133, 2533.1262, 2542.1650, 2546.1746, 2549.1316, 2550.1252, 2559.1470, 2561.1729, 2562.1765, 2567.1514, 2572.1523, 2574.2678, 2576.1628, 2583.1218, 2587.2722, 2604.1682, 2612.1438, 2653.7173, 2655.1897, 2671.1787, 2687.1833, 2706.2766, 2712.2104, 2714.2156, 2716.1953, 2717.2725, 2725.2354, 2729.1675, 2739.2351, 2743.2642, 2745.2900, 2754.2197, 2755.2358, 2759.2573, 2769.2219, 2771.2437, 2776.2644, 2785.2388, 2786.2617, 2787.2400, 2793.2439, 2801.2378, 2803.2302, 2809.2549, 2815.2153, 2817.2322, 2819.2236, 2830.2788, 2833.2280, 2836.2397, 2862.2927, 2873.2961, 2894.3218, 2927.2520, 2931.3428, 2955.3005, 2974.4124, 2975.4084, 2990.3921, 3006.3206, 3011.2810, 3020.3557, 3021.2412, 3035.3281, 3053.3447, 3062.2456, 3086.4507, 3091.2622, 3103.3140, 3110.7285, 3113.8591, 3130.3557, 3140.4265, 3144.3657, 3159.4985, 3173.5386, 3174.5486, 3218.3301, 3259.3625, 3312.3267, 3331.4678, 3347.4761, 3363.4788, 3390.4675, 3431.4570, 3447.4666, 3463.4895, 3485.6985, 3488.6545, 3489.6521, 3493.0195, 3494.5571, 3504.6423, 3509.5613, 3525.6025, 3538.4521, 3579.5403, 3648.1956, 3655.4729, 3664.7500, 3695.6177, 3712.5720, 3738.5837, 3816.7043, 3937.8308, 4010.8696

42. Q5E9I6 **Mass:** 20645 **Score:** 13 **Expect:** 1.1e+003 **Matches:** 9
ADP-ribosylation factor 3 OS=Bos taurus GN=ARF3 PE=2 SV=3

Observed	Mr(expt)	Mr(calc)	ppm	Start	End	Miss	Ions	Peptide
992.4958	991.4885	991.4644	24.3	110 -	117	0	---	R.MLAEDELR.D + Oxidation (M)
1061.6168	1060.6095	1060.6063	3.08	11 -	19	2	---	K.SLIGKKEMR.I
1103.6257	1102.6184	1102.6056	11.6	20 -	30	0	---	R.ILMVGLDAAGK.T + Oxidation (M)
1565.7223	1564.7150	1564.7522	-23.76	60 -	73	0	---	K.NISFTVWDVGGQDK.I
1630.8103	1629.8030	1629.7668	22.2	128 -	142	0	---	K.QDLPNAMNAAEITDK.L
1663.8466	1662.8393	1662.8797	-24.26	16 -	30	2	---	K.KEMRILMVGLDAAGK.T + 2 Oxidation (M)
1666.7545	1665.7472	1665.7702	-13.77	105 -	117	1	---	R.EELMRMLAEDELR.D + 2 Oxidation (M)
2423.2439	2422.2366	2422.2274	3.80	128 -	149	1	---	K.QDLPNAMNAAEITDKLGLHSLR.H + Oxidation (M)
2438.1724	2437.1651	2437.1775	-5.07	80 -	99	1	---	R.HYFQNTQGLIFVVDSNDRER.V

No match to: 716.4763, 723.4098, 724.2417, 725.2533, 726.3622, 728.3046, 729.4749, 734.4570, 736.3904, 737.4152, 738.3791, 744.8651, 745.3838, 747.4550, 768.3673, 788.4571, 801.4262, 805.4606, 813.4481, 814.4558, 820.4053, 823.4778, 829.4589, 830.4522, 839.3830, 842.5110, 857.4382, 860.4207, 865.4518, 870.4165, 872.5054, 880.4424, 882.4292, 887.4245, 887.9669, 888.4561, 891.4898, 894.4144, 894.8973, 897.5006, 898.4523, 912.5132, 913.5293, 925.4620, 927.4981, 931.4992, 934.5098, 957.5774, 960.5294, 961.4949, 963.4734, 965.4692, 970.5181, 972.4899, 975.5167, 976.5063, 977.5212, 978.5100, 978.5146, 983.6283, 993.5046, 995.4181, 998.5911, 1000.5959, 1002.5007, 1006.5014, 1011.5474, 1012.5447, 1014.5485, 1020.5577, 1021.5699, 1024.5516, 1027.5438, 1028.5397, 1030.5978, 1032.5238, 1036.5260, 1037.5405, 1039.5641, 1040.5908, 1042.5388, 1043.5178, 1045.5568, 1046.6018, 1056.5515, 1057.6100, 1057.6107, 1074.6073, 1074.6100, 1076.6123, 1102.6007, 1105.6136, 1107.1058, 1113.5486, 1120.5498, 1122.5322, 1123.5986, 1128.5724, 1129.5924, 1134.3574, 1137.5463, 1145.6348, 1149.6057, 1153.6460, 1156.4957, 1161.6295, 1162.6174, 1164.1815, 1170.5497, 1172.6156, 1177.6410, 1180.6428, 1184.6412, 1188.5603, 1193.6041, 1206.5107, 1209.5889, 1211.6099, 1212.6160, 1215.6743, 1216.7081, 1219.6886, 1227.6234, 1229.7098, 1234.6414, 1240.5974, 1242.6003, 1243.6161, 1253.6216, 1255.5972, 1255.6000, 1260.6227, 1264.7130, 1265.7064, 1269.6498, 1270.6575, 1273.6556, 1277.6305, 1286.6099, 1289.6875, 1293.6401, 1303.6455, 1304.6677, 1307.6788, 1309.6780, 1318.6750, 1319.6589, 1328.6600, 1328.6620, 1331.7888, 1333.7644, 1336.0798, 1336.6155, 1350.6853, 1351.7202, 1356.5811, 1359.1302, 1359.6246, 1365.6489, 1374.7588, 1383.7037, 1390.6974, 1393.7992, 1400.6683, 1401.6989, 1414.8191, 1429.3142, 1436.7889, 1444.7122, 1445.7839, 1450.7223, 1456.7717, 1462.6630, 1467.7329, 1470.7394, 1471.2693, 1471.7250, 1472.2531, 1474.5243, 1475.7633, 1483.7141, 1488.5333, 1488.7194, 1493.7366, 1502.5601, 1513.7441, 1514.6849, 1516.6807, 1517.6437, 1518.7053, 1523.7611, 1526.7343, 1528.6973, 1530.6893, 1536.8230, 1540.7114, 1551.7063, 1553.7252, 1554.7474, 1557.6992, 1558.7140, 1563.6658, 1564.3619, 1566.7162, 1572.7062, 1580.8400, 1580.8423, 1586.8090, 1597.7913, 1599.8337, 1601.8030, 1614.8046, 1616.7968, 1628.7136, 1635.7697, 1642.7682, 1643.7688, 1644.7915, 1646.8154, 1651.7681, 1657.7932, 1664.8557, 1695.8208, 1707.8933, 1718.8790, 1721.9192, 1722.9375, 1728.8976, 1730.9116, 1732.2391, 1734.1844, 1739.7894, 1743.8966, 1755.2666, 1755.7703, 1757.4702, 1757.9089, 1758.4524, 1758.8987, 1761.9023, 1765.7578, 1768.8339, 1769.8287, 1770.3949, 1772.8273, 1773.8792, 1774.9180, 1774.9200, 1786.8268, 1787.8239, 1809.0499, 1810.0303, 1829.8895, 1830.8380, 1833.3470, 1833.8501, 1851.8885, 1852.0399, 1862.9515, 1881.7703, 1895.9634, 1906.8195, 1908.8512, 1920.7972, 1932.0050, 1936.9479, 1939.8563, 1940.8103, 1943.8293, 1954.8351, 1956.8574, 1958.8956, 1965.7998, 1967.9565, 1969.9998, 1979.8927, 1981.3531, 1982.8179, 1995.8232, 1997.8312, 2003.8595, 2008.9971, 2011.8464, 2015.9408, 2019.8779, 2025.8735, 2031.9606, 2047.9397, 2062.9578, 2067.0020, 2076.8943, 2081.9827, 2082.9966, 2087.0103, 2088.1108, 2089.1162, 2096.0430, 2097.0200, 2108.9771, 2110.0061, 2139.0991, 2140.0918, 2143.1006,

2144.0718, 2149.8516, 2160.0554, 2174.0378, 2178.0471, 2179.0574, 2188.0547, 2191.0457, 2198.1831, 2199.1499, 2203.0386, 2211.1052, 2223.1582, 2225.1470, 2227.1042, 2228.0725, 2238.1086, 2239.1538, 2243.1243, 2244.0684, 2254.0896, 2255.1482, 2260.0779, 2270.2183, 2272.1736, 2284.1841, 2287.1108, 2307.0549, 2309.0659, 2310.0798, 2310.5271, 2311.0454, 2319.0945, 2337.6050, 2341.5510, 2353.0518, 2354.0767, 2355.0564, 2371.0581, 2383.9592, 2388.0437, 2392.1462, 2404.0471, 2412.0872, 2419.5879, 2420.0864, 2420.5930, 2426.5667, 2427.1211, 2455.1553, 2458.1648, 2459.1794, 2478.1587, 2516.1133, 2533.1262, 2542.1650, 2546.1746, 2549.1316, 2550.1252, 2559.1470, 2561.1729, 2562.1765, 2567.1514, 2572.1523, 2574.2678, 2576.1628, 2583.1218, 2587.2722, 2604.1682, 2612.1438, 2653.7173, 2655.1897, 2671.1787, 2687.1833, 2701.2820, 2706.2766, 2712.2104, 2714.2156, 2716.1953, 2717.2725, 2725.2354, 2729.1675, 2739.2351, 2743.2642, 2745.2900, 2754.2197, 2755.2358, 2759.2573, 2769.2219, 2771.2437, 2776.2644, 2785.2388, 2786.2617, 2787.2400, 2793.2439, 2801.2378, 2803.2302, 2809.2549, 2815.2153, 2817.2322, 2819.2236, 2830.2788, 2833.2280, 2836.2397, 2862.2927, 2873.2961, 2889.2024, 2894.3218, 2927.2520, 2931.3428, 2955.3005, 2974.4124, 2975.4084, 2990.3921, 3006.3206, 3011.2810, 3020.3557, 3021.2412, 3035.3281, 3053.3447, 3062.2456, 3086.4507, 3091.2622, 3103.3140, 3110.7285, 3113.8591, 3130.3557, 3140.4265, 3144.3657, 3159.4985, 3173.5386, 3174.5486, 3185.4236, 3218.3301, 3259.3625, 3312.3267, 3331.4678, 3347.4761, 3363.4788, 3390.4675, 3431.4570, 3447.4666, 3463.4895, 3485.6985, 3488.6545, 3489.6521, 3493.0195, 3494.5571, 3504.6423, 3509.5613, 3525.6025, 3538.4521, 3553.6230, 3579.5403, 3648.1956, 3655.4729, 3664.7500, 3695.6177, 3712.5720, 3738.5837, 3816.7043, 3937.8308, 4010.8696

43. F1MIV1 **Mass:** 57360 **Score:** 13 **Expect:** 1.1e+003 **Matches:** 16

Uncharacterized protein OS=Bos taurus GN=ZBTB34 PE=4 SV=2

Observed	Mr(expt)	Mr(calc)	ppm	Start	End	Miss	Ions	Peptide
978.5100	977.5027	977.5043	-1.58	460 -	468	0	---	K.NHPGVAEVR.S
978.5146	977.5073	977.5043	3.11	460 -	468	0	---	K.NHPGVAEVR.S
1149.6057	1148.5984	1148.5859	10.9	190 -	199	1	---	R.METTPSKALR.S + Oxidation (M)
1170.5497	1169.5424	1169.5434	-0.82	320 -	329	1	---	R.SMLSCFRGGR.A
1318.6750	1317.6677	1317.6574	7.86	403 -	413	0	---	R.LHMGITPFVCK.F + Oxidation (M)
1400.6683	1399.6610	1399.6440	12.2	200 -	211	1	---	R.SSRLQEEGHSDR.G
1630.8103	1629.8030	1629.8007	1.42	382 -	394	1	---	R.LICIYCGKSFNQK.G
1758.8987	1757.8914	1757.8528	22.0	400 -	413	1	---	R.HMRLHMGITPFVCK.F + 2 Oxidation (M)
1761.9023	1760.8950	1760.8978	-1.60	67 -	83	0	---	R.DHSALSTMSGLSISVIK.N + Oxidation (M)
2255.1482	2254.1409	2254.1286	5.49	395 -	413	2	---	K.GSLDRHMRLHMGITPFVCK.F
2272.1736	2271.1663	2271.1279	16.9	106 -	125	0	---	K.DVVSFLTAASFLQMQCVIDK.C
2287.1108	2286.1035	2286.1184	-6.50	395 -	413	2	---	K.GSLDRHMRLHMGITPFVCK.F + 2 Oxidation (M)
2559.1470	2558.1397	2558.1442	-1.75	477 -	499	1	---	R.TDVYAEQKLENDASASEMALDSR.M + Oxidation (M)
2836.2397	2835.2324	2835.2175	5.28	485 -	510	1	---	K.LENDASASEMALDSRMEIHTVSDAPD.- + 2 Oxidation (M)
3738.5837	3737.5764	3737.6672	-24.29	477 -	510	2	---	R.TDVYAEQKLENDASASEMALDSRMEIHTVSDAPD.-
4010.8696	4009.8623	4009.9554	-23.20	1 -	34	2	---	-.RLRFMSVEMDSSSFIQFDVPEYSSTVLSQLNELR.L

No match to: 716.4763, 723.4098, 724.2417, 725.2533, 726.3622, 728.3046, 729.4749, 734.4570, 736.3904, 737.4152, 738.3791, 744.8651, 745.3838, 747.4550, 768.3673, 788.4571, 801.4262, 805.4606, 813.4481, 814.4558, 820.4053, 823.4778, 829.4589, 830.4522, 839.3830, 842.5110, 857.4382, 860.4207, 865.4518, 870.4165, 872.5054, 880.4424, 882.4292, 887.4245, 887.9669, 888.4561, 891.4898, 894.4144, 894.8973, 897.5006, 898.4523, 912.5132, 913.5293, 925.4620, 927.4981, 931.4992, 934.5098, 957.5774, 960.5294, 961.4949, 963.4734, 965.4692, 970.5181, 972.4899, 975.5167, 976.5063, 977.5212, 983.6283, 992.4958, 993.5046, 995.4181, 998.5911, 1000.5959, 1002.5007, 1006.5014, 1011.5474, 1012.5447, 1014.5485, 1020.5577, 1021.5699, 1024.5516, 1027.5438, 1028.5327, 1030.5978, 1032.5238, 1036.5260, 1037.5405, 1039.5641, 1040.5908, 1042.5388, 1043.5178, 1045.5568, 1046.6018, 1056.5515, 1057.6100, 1057.6107, 1061.6168, 1074.6073, 1074.6100, 1076.6123, 1102.6007, 1103.6257, 1105.6136, 1107.1058, 1113.5486, 1120.5498, 1122.5322, 1123.5986, 1128.5724, 1129.5924, 1134.3574, 1137.5463, 1145.6348, 1153.6460, 1156.4957, 1161.6295, 1162.6174, 1164.1815, 1172.6156, 1177.6410, 1180.6428, 1184.6412, 1188.5603, 1193.6041, 1206.5107, 1209.5889, 1211.6099, 1212.6160, 1215.6743, 1216.7081, 1219.6886, 1227.6234, 1229.7098, 1234.6414, 1240.5974, 1242.6003, 1243.6161, 1253.6216, 1255.5972, 1255.6000, 1260.6227, 1264.7130, 1265.7064, 1269.6498, 1270.6575, 1273.6556, 1277.6305, 1286.6099, 1289.6875, 1293.6401, 1303.6455, 1304.6677, 1307.6788, 1309.6780, 1319.6589, 1328.6600, 1328.6620, 1331.7888, 1333.7644, 1336.0798, 1336.6155, 1350.6853, 1351.7202, 1356.5811, 1359.1302, 1359.6246, 1365.6489, 1374.7588, 1383.7037, 1390.6974, 1393.7992, 1401.6989, 1414.8191, 1429.3142, 1436.7889, 1444.7122, 1445.7839, 1450.7223, 1456.7717, 1462.6630, 1467.7329, 1470.7394, 1471.2693, 1471.7250, 1472.2531, 1474.5243, 1475.7633, 1483.7141, 1488.5333, 1488.7194, 1493.7366, 1502.5601, 1513.7441, 1514.6849, 1516.6807, 1517.6437, 1518.7053, 1523.7611, 1526.7343, 1528.6973, 1530.6893, 1536.8230, 1540.7114, 1551.7063, 1553.7252, 1554.7474, 1557.6992, 1558.7140, 1563.6658, 1564.3619, 1565.7223, 1566.7162, 1572.7062, 1580.8400, 1580.8423, 1586.8090, 1597.7913, 1599.8337, 1601.8030, 1614.8046, 1616.7968, 1628.7136, 1635.7697, 1642.7682, 1643.7688, 1644.7915, 1646.8154, 1651.7681, 1657.7932, 1663.8466, 1664.8557, 1666.7545, 1695.8208, 1707.8933, 1718.8790, 1721.9192, 1722.9375, 1728.8976, 1730.9116, 1732.2391, 1734.1844, 1739.7894, 1743.8966, 1755.2666, 1755.7703, 1757.4702, 1757.9089, 1758.4524, 1765.7578, 1768.8339, 1769.8287, 1770.3949, 1772.8273, 1773.8792, 1774.9180, 1774.9200, 1786.8268, 1787.8239, 1809.0499, 1810.0303, 1829.8895, 1830.8380, 1833.3470, 1833.8501, 1851.8885, 1852.0399, 1862.9515, 1881.7703, 1895.9634, 1906.8195, 1908.8512, 1920.7972, 1932.0050, 1936.9479, 1939.8563, 1940.8103, 1943.8293, 1954.8351, 1956.8574, 1958.8956, 1965.7998, 1967.9565, 1969.9998, 1979.8927, 1981.3531, 1982.8179, 1995.8232, 1997.8312, 2003.8595, 2008.9971, 2011.8464, 2015.9408, 2019.8779, 2025.8735, 2031.9606, 2047.9397, 2062.9578, 2067.0020, 2076.8943, 2081.9827, 2082.9966, 2087.0103, 2088.1108, 2089.1162, 2096.0430, 2097.0200, 2108.9771, 2110.0061, 2139.0991, 2140.0918, 2143.1006, 2144.0718, 2149.8516, 2160.0554, 2174.0378, 2178.0471, 2179.0574, 2188.0547, 2191.0457, 2198.1831, 2199.1499, 2203.0386, 2211.1052, 2223.1582, 2225.1470, 2227.1042, 2228.0725, 2238.1086, 2239.1538, 2243.1243, 2244.0684, 2254.0896, 2260.0779, 2270.2183, 2284.1841, 2307.0549, 2309.0659, 2310.0798, 2310.5271, 2311.0454, 2319.0945, 2337.6050, 2341.5510, 2353.0518, 2354.0767, 2355.0564, 2371.0581, 2383.9592, 2388.0437, 2392.1462, 2404.0471, 2412.0872, 2419.5879, 2420.0864, 2420.5930, 2423.2439, 2426.5667, 2427.1211, 2438.1724, 2455.1553, 2458.1648, 2459.1794, 2478.1587, 2516.1133, 2533.1262, 2542.1650, 2546.1746, 2549.1316, 2550.1252, 2561.1729, 2562.1765, 2567.1514, 2572.1523, 2574.2678, 2576.1628, 2583.1218, 2587.2722, 2604.1682, 2612.1438, 2653.7173, 2655.1897, 2671.1787, 2687.1833, 2701.2820, 2706.2766, 2712.2104, 2714.2156, 2716.1953, 2717.2725, 2725.2354, 2729.1675, 2739.2351, 2743.2642, 2745.2900, 2754.2197, 2755.2358, 2759.2573, 2769.2219, 2771.2437, 2776.2644, 2785.2388, 2786.2617, 2787.2400, 2793.2439, 2801.2378, 2803.2302, 2809.2549, 2815.2153, 2817.2322, 2819.2236, 2830.2788, 2833.2280, 2862.2927, 2873.2961, 2889.2024, 2894.3218, 2927.2520, 2931.3428, 2955.3005, 2974.4124, 2975.4084, 2990.3921, 3006.3206, 3011.2810, 3020.3557, 3021.2412, 3035.3281, 3053.3447, 3062.2456, 3086.4507, 3091.2622, 3103.3140, 3110.7285, 3113.8591, 3130.3557, 3140.4265, 3144.3657, 3159.4985, 3173.5386, 3174.5486, 3185.4236, 3218.3301, 3259.3625, 3312.3267, 3331.4678, 3347.4761, 3363.4788, 3390.4675, 3431.4570, 3447.4666, 3463.4895, 3485.6985, 3488.6545, 3489.6521, 3493.0195, 3494.5571, 3504.6423, 3509.5613, 3525.6025, 3538.4521, 3553.6230, 3579.5403, 3648.1956, 3655.4729, 3664.7500, 3695.6177, 3712.5720, 3816.7043, 3937.8308

44. F1MK70 **Mass:** 59648 **Score:** 13 **Expect:** 1.1e+003 **Matches:** 21

Uncharacterized protein OS=Bos taurus GN=SPATS2 PE=4 SV=2

Observed	Mr(expt)	Mr(calc)	ppm	Start	End	Miss	Ions	Peptide
820.4053	819.3981	819.4127	-17.84	80 -	86	0	---	K.EWTVTGK.K
912.5132	911.5060	911.4937	13.5	443 -	450	1	---	R.EALPGNRR.G
1074.6073	1073.6000	1073.6080	-7.47	235 -	244	1	---	K.LGSNIEKSVK.D
1074.6100	1073.6027	1073.6080	-4.96	235 -	244	1	---	K.LGSNIEKSVK.D
1076.6123	1075.6050	1075.6026	2.27	80 -	88	2	---	K.EWTVTGKKK.N
1193.6041	1192.5968	1192.5836	11.1	345 -	354	1	---	K.YDEDLGRVAR.F
1240.5974	1239.5901	1239.6169	-21.62	355 -	364	1	---	R.FTCDVETLKK.N
1309.6780	1308.6707	1308.6417	22.2	262 -	272	2	---	K.EEMDASIKKMK.Q
1318.6750	1317.6677	1317.6823	-11.07	245 -	255	1	---	K.DLQRCTVSLAR.Y
1328.6600	1327.6527	1327.6521	0.49	365 -	376	0	---	K.NIDSFGQVSHPK.N
1328.6620	1327.6547	1327.6521	2.00	365 -	376	0	---	K.NIDSFGQVSHPK.N
1456.7717	1455.7644	1455.7470	12.0	364 -	376	1	---	K.KNIDSFGQVSHPK.N
1628.7136	1627.7063	1627.7148	-5.21	175 -	189	0	---	R.TGSMLENGVSDFESR.S
1969.9998	1968.9925	1968.9905	1.03	4 -	20	2	---	R.KQNQKDSSGFIFDLQSK.T
2087.0103	2086.0030	2085.9940	4.31	451 -	470	1	---	R.GGQGYRPQGPKSSDPTNQGR.H
2243.1243	2242.1170	2242.0951	9.76	450 -	470	2	---	R.RGGQGYRPQGPKSSDPTNQGR.H
2287.1108	2286.1035	2286.0950	3.71	345 -	363	2	---	K.YDEDLGRVARFTCDVETLK.K

2455.1553	2454.1480	2454.1346	5.46	504	528	0	---	R.GQAHSAGTNGTGASMEPGPPKPSFR.K + Oxidation (M)
2706.2766	2705.2693	2705.3007	-11.59	9	33	1	---	K.DSSGFIFDLQSKTVLAQGGAFENMK.E + Oxidation (M)
2714.2156	2713.2083	2713.2117	-1.23	479	503	1	---	R.NSSWYSSGSRYQSAPPQAPGNTSER.G
2975.4084	2974.4011	2974.3908	3.47	271	295	2	---	K.MKQAFAELQSCLMDREVALLAEMDK.V + 3 Oxidation (M)

No match to: 716.4763, 723.4098, 724.2417, 725.2533, 726.3622, 728.3046, 729.4749, 734.4570, 736.3904, 737.4152, 738.3791, 744.8651, 745.3838, 747.4550, 768.3673, 788.4571, 801.4262, 805.4606, 813.4481, 814.4558, 823.4778, 829.4589, 830.4522, 839.3830, 842.5110, 857.4382, 860.4207, 865.4518, 870.4165, 872.5054, 880.4424, 882.4292, 887.4245, 887.9669, 888.4561, 891.4898, 894.4144, 894.8973, 897.5006, 898.4523, 913.5293, 925.4620, 927.4981, 931.4992, 934.5098, 957.5774, 960.5294, 961.4949, 963.4734, 965.4692, 970.5181, 972.4899, 975.5167, 976.5063, 977.5212, 978.5100, 978.5146, 983.6283, 992.4958, 993.5046, 995.4181, 998.5911, 1000.5959, 1002.5007, 1006.5014, 1011.5474, 1012.5447, 1014.5485, 1020.5577, 1021.5699, 1024.5516, 1027.5438, 1028.5397, 1030.5978, 1032.5238, 1036.5260, 1037.5405, 1039.5641, 1040.5908, 1042.5388, 1043.5178, 1045.5568, 1046.6018, 1056.5515, 1057.6100, 1057.6107, 1061.6168, 1102.6007, 1103.6257, 1105.6136, 1107.1058, 1113.5486, 1120.5498, 1122.5322, 1123.5986, 1128.5724, 1129.5924, 1134.3574, 1137.5463, 1145.6348, 1149.6057, 1153.6460, 1156.4957, 1161.6295, 1162.6174, 1164.1815, 1170.5497, 1172.6156, 1177.6410, 1180.6428, 1184.6412, 1188.5603, 1206.5107, 1209.5889, 1211.6099, 1212.6160, 1215.6743, 1216.7081, 1219.6886, 1227.6234, 1229.7098, 1234.6414, 1242.6003, 1243.6161, 1253.6216, 1255.5972, 1255.6000, 1260.6227, 1264.7130, 1265.7064, 1269.6498, 1270.6575, 1273.6556, 1277.6305, 1286.6099, 1289.6875, 1293.6401, 1303.6455, 1304.6677, 1307.6788, 1319.6589, 1331.7888, 1333.7644, 1336.0798, 1336.6155, 1350.6853, 1351.7202, 1356.5811, 1359.1302, 1359.6246, 1365.6489, 1374.7588, 1383.7037, 1390.6974, 1393.7992, 1400.6683, 1401.6989, 1414.8191, 1429.3142, 1436.7889, 1444.7122, 1445.7839, 1450.7223, 1462.6630, 1467.7329, 1470.7394, 1471.2693, 1471.7250, 1472.2531, 1474.5243, 1475.7633, 1483.7141, 1488.5333, 1488.7194, 1493.7366, 1502.5601, 1513.7441, 1514.6849, 1516.6807, 1517.6437, 1518.7053, 1523.7611, 1526.7343, 1528.6973, 1530.6893, 1536.8230, 1540.7114, 1551.7063, 1553.7252, 1554.7474, 1557.6992, 1558.7140, 1563.6658, 1564.3619, 1565.7223, 1566.7162, 1572.7062, 1580.8400, 1580.8423, 1586.8090, 1597.7913, 1599.8337, 1601.8030, 1614.8046, 1616.7968, 1630.8103, 1635.7697, 1642.7682, 1643.7688, 1644.7915, 1646.8154, 1651.7681, 1657.7932, 1663.8466, 1664.8557, 1666.7545, 1695.8208, 1707.8933, 1718.8790, 1721.9192, 1722.9375, 1728.8976, 1730.9116, 1732.2391, 1734.1844, 1739.7894, 1743.8966, 1755.2666, 1755.7703, 1757.4702, 1757.9089, 1758.4524, 1758.8987, 1761.9023, 1765.7578, 1768.8339, 1769.8287, 1770.3949, 1772.8273, 1773.8792, 1774.9180, 1774.9200, 1786.8268, 1787.8239, 1809.0499, 1810.0303, 1829.8895, 1830.8380, 1833.3470, 1833.8501, 1851.8885, 1852.0399, 1862.9515, 1881.7703, 1895.9634, 1906.8195, 1908.8512, 1920.7972, 1932.0050, 1936.9479, 1939.8563, 1940.8103, 1943.8293, 1954.8351, 1956.8574, 1958.8956, 1965.7998, 1967.9565, 1979.8927, 1981.3531, 1982.8179, 1995.8232, 1997.8312, 2003.8595, 2008.9971, 2011.8464, 2015.9408, 2019.8779, 2025.8735, 2031.9606, 2047.9397, 2062.9578, 2067.0020, 2076.8943, 2081.9827, 2082.9966, 2088.1108, 2089.1162, 2096.0430, 2097.0200, 2108.9771, 2110.0061, 2139.0991, 2140.0918, 2143.1006, 2144.0718, 2149.8516, 2160.0554, 2174.0378, 2178.0471, 2179.0574, 2188.0547, 2191.0457, 2198.1831, 2199.1499, 2203.0386, 2211.1052, 2223.1582, 2225.1470, 2227.1042, 2228.0725, 2238.1086, 2239.1538, 2244.0684, 2254.0896, 2255.1482, 2260.0779, 2270.2183, 2272.1736, 2284.1841, 2307.0549, 2309.0659, 2310.0798, 2310.5271, 2311.0454, 2319.0945, 2337.6050, 2341.5510, 2353.0518, 2354.0767, 2355.0564, 2371.0581, 2383.9592, 2388.0437, 2392.1462, 2404.0471, 2412.0872, 2419.5879, 2420.0864, 2420.5930, 2423.2439, 2426.5667, 2427.1211, 2438.1724, 2458.1648, 2459.1794, 2478.1587, 2516.1133, 2533.1262, 2542.1650, 2546.1746, 2549.1316, 2550.1252, 2559.1470, 2561.1729, 2562.1765, 2567.1514, 2572.1523, 2574.2678, 2576.1628, 2583.1218, 2587.2722, 2604.1682, 2612.1438, 2653.7173, 2655.1897, 2671.1787, 2687.1833, 2701.2820, 2712.2104, 2716.1953, 2717.2725, 2725.2354, 2729.1675, 2739.2351, 2743.2642, 2745.2900, 2754.2197, 2755.2358, 2759.2573, 2769.2219, 2771.2437, 2776.2644, 2785.2388, 2786.2617, 2787.2400, 2793.2439, 2801.2378, 2803.2302, 2809.2549, 2815.2153, 2817.2322, 2819.2236, 2830.2788, 2833.2280, 2836.2397, 2862.2927, 2873.2961, 2889.2024, 2894.3218, 2927.2520, 2931.3428, 2955.3005, 2974.4124, 2990.3921, 3006.3206, 3011.2810, 3020.3557, 3021.2412, 3035.3281, 3053.3447, 3062.2456, 3086.4507, 3091.2622, 3103.3140, 3110.7285, 3113.8591, 3130.3557, 3140.4265, 3144.3657, 3159.4985, 3173.5386, 3174.5486, 3185.4236, 3218.3301, 3259.3625, 3312.3267, 3331.4678, 3347.4761, 3363.4788, 3390.4675, 3431.4570, 3447.4666, 3463.4895, 3485.6985, 3488.6545, 3489.6521, 3493.0195, 3494.5571, 3504.6423, 3509.5613, 3525.6025, 3538.4521, 3553.6230, 3579.5403, 3648.1956, 3655.4729, 3664.7500, 3695.6177, 3712.5720, 3738.5837, 3816.7043, 3937.8308, 4010.8696

45. F1MY44 **Mass:** 77716 **Score:** 13 **Expect:** 1.2e+003 **Matches:** 22

Uncharacterized protein OS=Bos taurus GN=HNRNPM PE=1 SV=2

Observed	Mr(expt)	Mr(calc)	ppm	Start	End	Miss	Ions	Peptide
745.3838	744.3765	744.3589	23.7	404	410	0	---	R.MGPGIDR.I
872.5054	871.4982	871.5127	-16.67	127	134	1	---	K.KAAEVLNK.H
975.5167	974.5094	974.4855	24.6	278	285	1	---	K.MDERALPK.G + Oxidation (M)
1021.5699	1020.5626	1020.5539	8.58	651	658	1	---	R.KACQIFVR.N
1188.5603	1187.5530	1187.5791	-21.95	532	543	0	---	R.MVPAGMGAGLER.M
1264.7130	1263.7057	1263.6863	15.4	73	83	0	---	R.AFITNIPFDVK.W
1450.7223	1449.7150	1449.7001	10.3	57	69	1	---	R.GGNRFEPYANPTK.R
1528.6973	1527.6900	1527.6844	3.70	437	449	1	---	R.MGLVMDRMGSVER.M + 3 Oxidation (M)
1565.7223	1564.7150	1564.7344	-12.39	673	685	0	---	K.FNECGHVLYADIK.M
1566.7162	1565.7089	1565.7290	-12.82	472	485	1	---	R.MGQTMERIGSGVER.M + Oxidation (M)
1663.8466	1662.8393	1662.8036	21.5	693	707	1	---	K.GCGVVKFESPEVAER.A
1768.8339	1767.8266	1767.8430	-9.25	711	725	2	---	R.MMNGMKLSGREIDVR.I + 2 Oxidation (M)
1772.8273	1771.8200	1771.8307	-6.01	113	127	2	---	R.GCAVVEFKMEESMKK.A
1895.9634	1894.9561	1894.9861	-15.82	383	403	1	---	R.GEIIAKQGGGGGGGSVPGIER.M
2081.9827	2080.9754	2080.9816	-2.95	566	583	2	---	R.MGLERMGANSLERMGLER.M + 2 Oxidation (M)
2178.0471	2177.0398	2177.0432	-1.53	323	345	0	---	K.GIGMGNLGPAGMGMEGIGFGINK.M
2211.1052	2210.0979	2210.0428	24.9	437	456	2	---	R.MGLVMDRMGSVERMGSGIER.M
2354.0767	2353.0694	2353.0936	-10.29	571	591	2	---	R.MGANSLERMGLERMGANSLER.M + 2 Oxidation (M)
2392.1462	2391.1389	2391.0981	17.1	450	471	1	---	R.MGSGIERMGPLGLDHMASSIER.M + 3 Oxidation (M)
2706.2766	2705.2693	2705.2506	6.92	419	443	2	---	R.MGAGLGHGMDRVGSEIERMGLVMDR.M + 2 Oxidation (M)
2717.2725	2716.2652	2716.3103	-16.60	518	543	2	---	R.MGPAIERMGLGMERMVPAGMGAGLER.M
3553.6230	3552.6157	3552.6404	-6.94	592	627	2	---	R.MGPAMGPALGAGIERMGLAMGGGGGASFDRAIEMER.G + 2 Oxidation (M)

No match to: 716.4763, 723.4098, 724.2417, 725.2533, 726.3622, 728.3046, 729.4749, 734.4570, 736.3904, 737.4152, 738.3791, 744.8651, 747.4550, 768.3673, 788.4571, 801.4262, 805.4606, 813.4481, 814.4558, 820.4053, 823.4778, 829.4589, 830.4522, 839.3830, 842.5110, 857.4382, 860.4207, 865.4518, 870.4165, 880.4424, 882.4292, 887.4245, 887.9669, 888.4561, 891.4898, 894.4144, 894.8973, 897.5006, 898.4523, 912.5132, 913.5293, 925.4620, 927.4981, 931.4992, 934.5098, 957.5774, 960.5294, 961.4949, 963.4734, 965.4692, 970.5181, 972.4899, 976.5063, 977.5212, 978.5100, 978.5146, 983.6283, 992.4958, 993.5046, 995.4181, 998.5911, 1000.5959, 1002.5007, 1006.5014, 1011.5474, 1012.5447, 1014.5485, 1020.5577, 1024.5516, 1027.5438, 1028.5397, 1030.5978, 1032.5238, 1036.5260, 1037.5405, 1039.5641, 1040.5908, 1042.5388, 1043.5178, 1045.5568, 1046.6018, 1056.5515, 1057.6100, 1057.6107, 1061.6168, 1074.6073, 1074.6100, 1076.6123, 1102.6007, 1103.6257, 1105.6136, 1107.1058, 1113.5486, 1120.5498, 1122.5322, 1123.5986, 1128.5724, 1129.5924, 1134.3574, 1137.5463, 1145.6348, 1149.6057, 1153.6460, 1156.4957, 1161.6295, 1162.6174, 1164.1815, 1170.5497, 1172.6156, 1177.6410, 1180.6428, 1184.6412, 1193.6041, 1206.5107, 1209.5889, 1211.6099, 1212.6160, 1215.6743, 1216.7081, 1219.6886, 1227.6234, 1229.7098, 1234.6414, 1240.5974, 1242.6003, 1243.6161, 1253.6216, 1255.5972, 1255.6000, 1260.6227, 1265.7064, 1269.6498, 1270.6575, 1273.6556, 1277.6305, 1286.6099, 1289.6875, 1293.6401, 1303.6455, 1304.6677, 1307.6788, 1309.6780, 1318.6750, 1319.6589, 1328.6600, 1328.6620, 1331.7888, 1333.7644, 1336.0798, 1336.6155, 1350.6853, 1351.7202, 1356.5811, 1359.1302, 1359.6246, 1365.6489, 1374.7588, 1383.7037, 1390.6974, 1393.7992, 1400.6683, 1401.6989, 1414.8191, 1429.3142, 1436.7889, 1444.7122, 1445.7839, 1456.7717, 1462.6630, 1467.7329, 1470.7394, 1471.2693, 1471.7250, 1472.2531, 1474.5243, 1475.7633, 1483.7141, 1488.5333, 1488.7194, 1493.7366, 1502.5601, 1513.7441, 1514.6849, 1516.6807, 1517.6437, 1518.7053, 1523.7611, 1526.7343, 1530.6893, 1536.8230, 1540.7114, 1551.7063, 1553.7252, 1554.7474, 1557.6992, 1558.7140, 1563.6658, 1564.3619, 1572.7062, 1580.8400, 1580.8423, 1586.8090, 1597.7913, 1599.8337, 1601.8030, 1614.8046, 1616.7968, 1628.7136, 1630.8103, 1635.7697, 1642.7682, 1643.7688, 1644.7915, 1646.8154, 1651.7681, 1657.7932, 1664.8557, 1666.7545, 1695.8208, 1707.8933, 1718.8790, 1721.9192, 1722.9375, 1728.8976, 1730.9116, 1732.2391, 1734.1844, 1739.7894, 1743.8966, 1755.2666, 1755.7703, 1757.4702, 1757.9089, 1758.4524, 1758.8987, 1761.9023, 1765.7578, 1769.8287, 1770.3949, 1773.8792, 1774.9180, 1774.9200, 1786.8268, 1787.8239, 1809.0499, 1810.0303, 1829.8895, 1830.8380, 1833.3470, 1833.8501, 1851.8885, 1852.0399, 1862.9515, 1881.7703, 1906.8195, 1908.8512, 1920.7972, 1932.0050, 1936.9479, 1939.8563, 1940.8103, 1943.8293, 1954.8351, 1956.8574, 1958.8956, 1965.7998, 1967.9565, 1969.9998, 1979.8927, 1981.3531, 1982.8179, 1995.8232, 1997.8312, 2003.8595, 2008.9971, 2011.8464, 2015.9408, 2019.8779, 2025.8735, 2031.9606, 2047.9397, 2062.9578, 2067.0020, 2076.8943, 2082.9966, 2087.0103, 2088.1108, 2089.1162, 2096.0430, 2097.0200, 2108.9771, 2110.0061, 2139.0991, 2140.0918, 2143.1006, 2144.0718, 2149.8516, 2160.0554, 2174.0378, 2179.0574, 2188.0547, 2191.0457, 2198.1831, 2199.1499, 2203.0386, 2223.1582, 2225.1470, 2227.1042, 2228.0725, 2238.1086, 2239.1538, 2243.1243, 2244.0684, 2254.0896, 2255.1482, 2260.0779, 2270.2183, 2272.1736, 2284.1841, 2287.1108, 2307.0549, 2309.0659, 2310.0798, 2310.5271, 2311.0454, 2319.0945, 2337.6050,

2341.5510, 2353.0518, 2355.0564, 2371.0581, 2383.9592, 2388.0437, 2404.0471, 2412.0872, 2419.5879, 2420.0864, 2420.5930, 2423.2439, 2426.5667, 2427.1211, 2438.1724, 2455.1553, 2458.1648, 2459.1794, 2478.1587, 2516.1133, 2533.1262, 2542.1650, 2546.1746, 2549.1316, 2550.1252, 2559.1470, 2561.1729, 2562.1765, 2567.1514, 2572.1523, 2574.2678, 2576.1628, 2583.1218, 2587.2722, 2604.1682, 2612.1438, 2653.7173, 2655.1897, 2671.1787, 2687.1833, 2701.2820, 2712.2104, 2714.2156, 2716.1953, 2725.2354, 2729.1675, 2739.2351, 2743.2642, 2745.2900, 2754.2197, 2755.2358, 2759.2573, 2769.2219, 2771.2437, 2776.2644, 2785.2388, 2786.2617, 2787.2400, 2793.2439, 2801.2378, 2803.2302, 2809.2549, 2815.2153, 2817.2322, 2819.2236, 2830.2788, 2833.2280, 2836.2397, 2862.2927, 2873.2961, 2889.2024, 2894.3218, 2927.2520, 2931.3428, 2955.3005, 2974.4124, 2975.4084, 2990.3921, 3006.3206, 3011.2810, 3020.3557, 3021.2412, 3035.3281, 3053.3447, 3062.2456, 3086.4507, 3091.2622, 3103.3140, 3110.7285, 3113.8591, 3130.3557, 3140.4265, 3144.3657, 3159.4985, 3173.5386, 3174.5486, 3185.4236, 3218.3301, 3259.3625, 3312.3267, 3331.4678, 3347.4761, 3363.4788, 3390.4675, 3431.4570, 3447.4666, 3463.4895, 3485.6985, 3488.6545, 3489.6521, 3493.0195, 3494.5571, 3504.6423, 3509.5613, 3525.6025, 3538.4521, 3579.5403, 3648.1956, 3655.4729, 3664.7500, 3695.6177, 3712.5720, 3738.5837, 3816.7043, 3937.8308, 4010.8696

46. E1BHM6 **Mass:** 206984 **Score:** 13 **Expect:** 1.2e+003 **Matches:** 19

Uncharacterized protein OS=Bos taurus GN=SCN11A PE=4 SV=2

Observed	Mr(expt)	Mr(calc)	ppm	Start		End	Miss	Ions	Peptide
957.5774	956.5701	956.5728	-2.85	1002	-	1009	1	---	R.VLEMIPKK.E
1045.5568	1044.5495	1044.5426	6.64	1329	-	1336	2	---	K.KYYNAMKK.L
1057.6100	1056.6027	1056.6291	-24.98	829	-	837	1	13	K.TKIQIALDR.F
1057.6107	1056.6034	1056.6291	-24.32	829	-	837	1	---	K.TKIQIALDR.F
1137.5463	1136.5390	1136.5172	19.2	357	-	365	0	---	R.LMTQDSWEK.L
1145.6348	1144.6275	1144.6492	-18.94	686	-	695	0	---	K.SWPTLSTLIK.I
1172.6156	1171.6083	1171.6085	-0.12	867	-	877	1	---	K.EVTEDPGGKIK.D
1318.6750	1317.6677	1317.6751	-5.57	1330	-	1340	2	---	K.YYNAMKKLGSK.K + Oxidation (M)
1493.7366	1492.7293	1492.7409	-7.78	1316	-	1328	0	---	K.ISGQDVFLTEEQK.K
1526.7343	1525.7270	1525.7558	-18.89	45	-	57	0	---	K.AAECEPRPQLDLK.V
2008.9971	2007.9898	2007.9917	-0.93	556	-	570	1	---	R.YLVWDCSPWWLDIKK.V
2097.0200	2096.0127	2096.0203	-3.63	749	-	764	1	---	R.RWHMGDFYHSFLVVFR.I
2284.1841	2283.1768	2283.2144	-16.47	867	-	887	2	---	K.EVTEDPGGKIKDIIPLVTGMR.K + Oxidation (M)
2287.1108	2286.1035	2286.0979	2.45	838	-	854	1	---	R.FCRAFCFVIHTLEHFFR.K
2392.1462	2391.1389	2391.1780	-16.35	1316	-	1335	2	---	K.ISGQDVFLTEEQKKYYNAMK.K
2412.0872	2411.0799	2411.1097	-12.35	980	-	1001	1	---	K.KSDAASVLSECSTIGPQDPFMR.V + Oxidation (M)
2759.2573	2758.2500	2758.2003	18.0	1256	-	1279	0	---	K.GWMDIMYAAVDSTEEGQQPQFEAR.S
3110.7285	3109.7212	3109.6860	11.3	1394	-	1419	1	---	K.HLLESLNMAFVVIFAIECFIKIFALR.Q + Oxidation (M)
3218.3301	3217.3228	3217.2490	22.9	307	-	331	2	---	K.EESTEFKMCGMWMGNRACPEQYECR.D + 2 Oxidation (M)

No match to: 716.4763, 723.4098, 724.2417, 725.2533, 726.3622, 728.3046, 729.4749, 734.4570, 736.3904, 737.4152, 738.3791, 744.8651, 745.3838, 747.4550, 768.3673, 788.4571, 801.4262, 805.4606, 813.4481, 814.4558, 820.4053, 823.4778, 829.4589, 830.4522, 839.3830, 842.5110, 857.4382, 860.4207, 865.4518, 870.4165, 872.5054, 880.4424, 882.4292, 887.4245, 887.9669, 888.4561, 891.4898, 894.4144, 894.8973, 897.5006, 898.4523, 912.5132, 913.5293, 925.4620, 927.4981, 931.4992, 934.5098, 960.5294, 961.4949, 963.4734, 965.4692, 970.5181, 972.4899, 975.5167, 976.5063, 977.5212, 978.5100, 978.5146, 983.6283, 992.4958, 993.5046, 995.4181, 998.5911, 1000.5959, 1002.5007, 1006.5014, 1011.5474, 1012.5447, 1014.5485, 1020.5577, 1021.5699, 1024.5516, 1027.5438, 1028.5397, 1030.5978, 1032.5238, 1036.5260, 1037.5405, 1039.5641, 1040.5908, 1042.5388, 1043.5178, 1046.6018, 1056.5515, 1061.6168, 1074.6073, 1074.6100, 1076.6123, 1102.6007, 1103.6257, 1105.6136, 1107.1058, 1113.5486, 1120.5498, 1122.5322, 1123.5986, 1128.5724, 1129.5924, 1134.3574, 1149.6057, 1153.6460, 1156.4957, 1161.6295, 1162.6174, 1164.1815, 1170.5497, 1177.6410, 1180.6428, 1184.6412, 1188.5603, 1193.6041, 1206.5107, 1209.5889, 1211.6099, 1212.6160, 1215.6743, 1216.7081, 1219.6886, 1227.6234, 1229.7098, 1234.6414, 1240.5974, 1242.6003, 1243.6161, 1253.6216, 1255.5972, 1255.6000, 1260.6227, 1264.7130, 1265.7064, 1269.6498, 1270.6575, 1273.6556, 1277.6305, 1286.6099, 1289.6875, 1293.6401, 1303.6455, 1304.6677, 1307.6788, 1309.6780, 1319.6589, 1328.6600, 1328.6620, 1331.7888, 1333.7644, 1336.0798, 1336.6155, 1350.6853, 1351.7202, 1356.5811, 1359.1302, 1359.6246, 1365.6489, 1374.7588, 1383.7037, 1390.6974, 1393.7992, 1400.6683, 1401.6989, 1414.8191, 1429.3142, 1436.7889, 1444.7122, 1445.7839, 1450.7223, 1456.7717, 1462.6630, 1467.7329, 1470.7394, 1471.2693, 1471.7250, 1472.2531, 1474.5243, 1475.7633, 1483.7141, 1488.5333, 1488.7194, 1502.5601, 1513.7441, 1514.6849, 1516.6807, 1517.6437, 1518.7053, 1523.7611, 1528.6973, 1530.6893, 1536.8230, 1540.7114, 1551.7063, 1553.7252, 1554.7474, 1557.6992, 1558.7140, 1563.6658, 1564.3619, 1565.7223, 1566.7162, 1572.7062, 1580.8400, 1580.8423, 1586.8090, 1597.7913, 1599.8337, 1601.8030, 1614.8046, 1616.7968, 1628.7136, 1630.8103, 1635.7697, 1642.7682, 1643.7688, 1644.7915, 1646.8154, 1651.7681, 1657.7932, 1663.8466, 1664.8557, 1666.7545, 1695.8208, 1707.8933, 1718.8790, 1721.9192, 1722.9375, 1728.8976, 1730.9116, 1732.2391, 1734.1844, 1739.7894, 1743.8966, 1755.2666, 1755.7703, 1757.4702, 1757.9089, 1758.4524, 1758.8987, 1761.9023, 1765.7578, 1768.8339, 1769.8287, 1770.3949, 1772.8273, 1773.8792, 1774.9180, 1774.9200, 1786.8268, 1787.8239, 1809.0499, 1810.0303, 1829.8895, 1830.8380, 1833.3470, 1833.8501, 1851.8885, 1852.0399, 1862.9515, 1881.7703, 1895.9634, 1906.8195, 1908.8512, 1920.7972, 1932.0050, 1936.9479, 1939.8563, 1940.8103, 1943.8293, 1954.8351, 1956.8574, 1958.8956, 1965.7998, 1967.9565, 1969.9998, 1979.8927, 1981.3531, 1982.8179, 1995.8232, 1997.8312, 2003.8595, 2011.8464, 2015.9408, 2019.8779, 2025.8735, 2031.9606, 2047.9397, 2062.9578, 2067.0020, 2076.8943, 2081.9827, 2082.9966, 2087.0103, 2088.1108, 2089.1162, 2096.0430, 2108.9771, 2110.0061, 2139.0991, 2140.0918, 2143.1006, 2144.0718, 2149.8516, 2160.0554, 2174.0378, 2178.0471, 2179.0574, 2188.0547, 2191.0457, 2198.1831, 2199.1499, 2203.0386, 2211.1052, 2223.1582, 2225.1470, 2227.1042, 2228.0725, 2238.1086, 2239.1538, 2243.1243, 2244.0684, 2254.0896, 2255.1482, 2260.0779, 2270.2183, 2272.1736, 2307.0549, 2309.0659, 2310.0798, 2310.5271, 2311.0454, 2319.0945, 2337.6050, 2341.5510, 2353.0518, 2354.0767, 2355.0564, 2371.0581, 2383.9592, 2388.0437, 2404.0471, 2419.5879, 2420.0864, 2420.5930, 2423.2439, 2426.5667, 2427.1211, 2438.1724, 2455.1553, 2458.1648, 2459.1794, 2478.1587, 2516.1133, 2533.1262, 2542.1650, 2546.1746, 2549.1316, 2550.1252, 2559.1470, 2561.1729, 2562.1765, 2567.1514, 2572.1523, 2574.2678, 2576.1628, 2583.1218, 2587.2722, 2604.1682, 2612.1438, 2653.7173, 2655.1897, 2671.1787, 2687.1833, 2701.2820, 2706.2766, 2712.2104, 2714.2156, 2716.1953, 2717.2725, 2725.2354, 2729.1675, 2739.2351, 2743.2642, 2745.2900, 2754.2197, 2755.2358, 2769.2219, 2771.2437, 2776.2644, 2785.2388, 2786.2617, 2787.2400, 2793.2439, 2801.2378, 2803.2302, 2809.2549, 2815.2153, 2817.2322, 2819.2236, 2830.2788, 2833.2280, 2836.2397, 2862.2927, 2873.2961, 2889.2024, 2894.3218, 2927.2520, 2931.3428, 2955.3005, 2974.4124, 2975.4084, 2990.3921, 3006.3206, 3011.2810, 3020.3557, 3021.2412, 3035.3281, 3053.3447, 3062.2456, 3086.4507, 3091.2622, 3103.3140, 3113.8591, 3130.3557, 3140.4265, 3144.3657, 3159.4985, 3173.5386, 3174.5486, 3185.4236, 3259.3625, 3312.3267, 3331.4678, 3347.4761, 3363.4788, 3390.4675, 3431.4570, 3447.4666, 3463.4895, 3485.6985, 3488.6545, 3489.6521, 3493.0195, 3494.5571, 3504.6423, 3509.5613, 3525.6025, 3538.4521, 3553.6230, 3579.5403, 3648.1956, 3655.4729, 3664.7500, 3695.6177, 3712.5720, 3738.5837, 3816.7043, 3937.8308, 4010.8696

47. Q58DW6 **Mass:** 23636 **Score:** 13 **Expect:** 1.2e+003 **Matches:** 4

Ras-related protein Rab-25 OS=Bos taurus GN=RAB25 PE=2 SV=1

Observed	Mr(expt)	Mr(calc)	ppm	Start		End	Miss	Ions	Peptide
1020.5577	1019.5504	1019.5685	-17.74	53	-	62	0	---	R.TVMLGTAAIK.A + Oxidation (M)
1057.6100	1056.6027	1056.6179	-14.38	15	-	25	0	13	K.VVLIGESGVGK.T
1057.6107	1056.6034	1056.6179	-13.72	15	-	25	0	---	K.VVLIGESGVGK.T
2082.9966	2081.9893	2081.9767	6.09	35	-	52	1	---	R.NEFSHDSRTTIGVEFSTR.T

No match to: 716.4763, 723.4098, 724.2417, 725.2533, 726.3622, 728.3046, 729.4749, 734.4570, 736.3904, 737.4152, 738.3791, 744.8651, 745.3838, 747.4550, 768.3673, 788.4571, 801.4262, 805.4606, 813.4481, 814.4558, 820.4053, 823.4778, 829.4589, 830.4522, 839.3830, 842.5110, 857.4382, 860.4207, 865.4518, 870.4165, 872.5054, 880.4424, 882.4292, 887.4245, 887.9669, 888.4561, 891.4898, 894.4144, 894.8973, 897.5006, 898.4523, 912.5132, 913.5293, 925.4620, 927.4981, 931.4992, 934.5098, 957.5774, 960.5294, 961.4949, 963.4734, 965.4692, 970.5181, 972.4899, 975.5167, 976.5063, 977.5212, 978.5100, 978.5146, 983.6283, 992.4958, 993.5046, 995.4181, 998.5911, 1000.5959, 1002.5007, 1006.5014, 1011.5474, 1012.5447, 1014.5485, 1021.5699, 1024.5516, 1027.5438, 1028.5397, 1030.5978, 1032.5238, 1036.5260, 1037.5405, 1039.5641, 1040.5908, 1042.5388, 1043.5178, 1045.5568, 1046.6018, 1056.5515, 1061.6168, 1074.6073, 1074.6100, 1076.6123, 1102.6007, 1103.6257, 1105.6136, 1107.1058, 1113.5486, 1120.5498, 1122.5322, 1123.5986, 1128.5724, 1129.5924, 1134.3574, 1137.5463, 1145.6348, 1149.6057, 1153.6460, 1156.4957, 1161.6295, 1162.6174, 1164.1815, 1170.5497, 1172.6156, 1177.6410, 1180.6428, 1184.6412, 1188.5603, 1193.6041, 1206.5107, 1209.5889, 1211.6099, 1212.6160, 1215.6743, 1216.7081, 1219.6886, 1227.6234, 1229.7098, 1234.6414, 1240.5974, 1242.6003, 1243.6161, 1253.6216, 1255.5972, 1255.6000, 1260.6227, 1264.7130, 1265.7064, 1269.6498, 1270.6575, 1273.6556, 1277.6305, 1286.6099, 1289.6875, 1293.6401, 1303.6455, 1304.6677, 1307.6788, 1309.6780, 1318.6750, 1319.6589, 1328.6600, 1328.6620, 1331.7888, 1333.7644, 1336.0798, 1336.6155, 1350.6853, 1351.7202, 1356.5811, 1359.1302, 1359.6246, 1365.6489, 1374.7588, 1383.7037, 1390.6974, 1393.7992, 1400.6683, 1401.6989, 1414.8191, 1429.3142, 1436.7889, 1444.7122, 1445.7839, 1450.7223, 1456.7717, 1462.6630, 1467.7329, 1470.7394, 1471.2693, 1471.7250, 1472.2531, 1474.5243, 1475.7633, 1483.7141, 1488.5333, 1488.7194,

1493.7366, 1502.5601, 1513.7441, 1514.6849, 1516.6807, 1517.6437, 1518.7053, 1523.7611, 1526.7343, 1528.6973, 1530.6893, 1536.8230, 1540.7114, 1551.7063, 1553.7252, 1554.7474, 1557.6992, 1558.7140, 1563.6658, 1564.3619, 1565.7223, 1566.7162, 1572.7062, 1580.8400, 1580.8423, 1586.8090, 1597.7913, 1599.8337, 1601.8030, 1614.8046, 1616.7968, 1628.7136, 1630.8103, 1635.7697, 1642.7682, 1643.7688, 1644.7915, 1646.8154, 1651.7681, 1657.7932, 1663.8466, 1664.8557, 1666.7545, 1695.8208, 1707.8933, 1718.8790, 1721.9192, 1722.9375, 1728.8976, 1730.9116, 1732.2391, 1734.1844, 1739.7894, 1743.8966, 1755.2666, 1755.7703, 1757.4702, 1757.9089, 1758.4524, 1758.8987, 1761.9023, 1765.7578, 1768.8339, 1769.8287, 1770.3949, 1772.8273, 1773.8792, 1774.9180, 1774.9200, 1786.8268, 1787.8239, 1809.0499, 1810.0303, 1829.8895, 1830.8380, 1833.3470, 1833.8501, 1851.8885, 1852.0399, 1862.9515, 1881.7703, 1895.9634, 1906.8195, 1908.8512, 1920.7972, 1932.0050, 1936.9479, 1939.8563, 1940.8103, 1943.8293, 1954.8351, 1956.8574, 1958.8956, 1965.7998, 1967.9565, 1969.9998, 1979.8927, 1981.3531, 1982.8179, 1995.8232, 1997.8312, 2003.8595, 2008.9971, 2011.8464, 2015.9408, 2019.8779, 2025.8735, 2031.9606, 2047.9397, 2062.9578, 2067.0020, 2076.8943, 2081.9827, 2087.0103, 2088.1108, 2089.1162, 2096.0430, 2097.0200, 2108.9771, 2110.0061, 2139.0991, 2140.0918, 2143.1006, 2144.0718, 2149.8516, 2160.0554, 2174.0378, 2178.0471, 2179.0574, 2188.0547, 2191.0457, 2198.1831, 2199.1499, 2203.0386, 2211.1052, 2223.1582, 2225.1470, 2227.1042, 2228.0725, 2238.1086, 2239.1538, 2243.1243, 2244.0684, 2254.0896, 2255.1482, 2260.0779, 2270.2183, 2272.1736, 2284.1841, 2287.1108, 2307.0549, 2309.0659, 2310.0798, 2310.5271, 2311.0454, 2319.0945, 2337.6050, 2341.5510, 2353.0518, 2354.0767, 2355.0564, 2371.0581, 2383.9592, 2388.0437, 2392.1462, 2404.0471, 2412.0872, 2419.5879, 2420.0864, 2420.5930, 2423.2439, 2426.5667, 2427.1211, 2438.1724, 2455.1553, 2458.1648, 2459.1794, 2478.1587, 2516.1133, 2533.1262, 2542.1650, 2546.1746, 2549.1316, 2550.1252, 2559.1470, 2561.1729, 2562.1765, 2567.1514, 2572.1523, 2574.2678, 2576.1628, 2583.1218, 2587.2722, 2604.1682, 2612.1438, 2653.7173, 2655.1897, 2671.1787, 2687.1833, 2701.2820, 2706.2766, 2712.2104, 2714.2156, 2716.1953, 2717.2725, 2725.2354, 2729.1675, 2739.2351, 2743.2642, 2745.2900, 2754.2197, 2755.2358, 2759.2573, 2769.2219, 2771.2437, 2776.2644, 2785.2388, 2786.2617, 2787.2400, 2793.2439, 2801.2378, 2803.2302, 2809.2549, 2815.2153, 2817.2322, 2819.2236, 2830.2788, 2833.2280, 2836.2397, 2862.2927, 2873.2961, 2889.2024, 2894.3218, 2927.2520, 2931.3428, 2955.3005, 2974.4124, 2975.4084, 2990.3921, 3006.3206, 3011.2810, 3020.3557, 3021.2412, 3035.3281, 3053.3447, 3062.2456, 3086.4507, 3091.2622, 3103.3140, 3110.7285, 3113.8591, 3130.3557, 3140.4265, 3144.3657, 3159.4985, 3173.5386, 3174.5486, 3185.4236, 3218.3301, 3259.3625, 3312.3267, 3331.4678, 3347.4761, 3363.4788, 3390.4675, 3431.4570, 3447.4666, 3463.4895, 3485.6985, 3488.6545, 3489.6521, 3493.0195, 3494.5571, 3504.6423, 3509.5613, 3525.6025, 3538.4521, 3553.6230, 3579.5403, 3648.1956, 3655.4729, 3664.7500, 3695.6177, 3712.5720, 3738.5837, 3816.7043, 3937.8308, 4010.8696

48. Q08D88 **Mass:** 51575 **Score:** 13 **Expect:** 1.2e+003 **Matches:** 13
Nuclear factor interleukin-3-regulated protein OS=Bos taurus GN=NFIL3 PE=2 SV=1

Observed	Mr(expt)	Mr(calc)	ppm	Start		End	Miss	Ions	Peptide
805.4606	804.4534	804.4527	0.78	331	-	337	1	---	K.AKAMQIK.V + Oxidation (M)
978.5100	977.5027	977.4825	20.7	55	-	62	2	6	K.NKSSACRR.K
1045.5568	1044.5495	1044.5424	6.82	83	-	91	2	---	K.NNEAAKRSR.E
1057.6100	1056.6027	1056.5815	20.1	96	-	104	0	---	R.LNDLVLENK.L
1057.6107	1056.6034	1056.5815	20.8	96	-	104	0	---	R.LNDLVLENK.L
1161.6295	1160.6222	1160.6189	2.83	64	-	72	2	---	K.REFIPDEKK.D
1242.6003	1241.5930	1241.5863	5.45	72	-	80	2	---	K.KDAMYWEKR.R + Oxidation (M)
1475.7633	1474.7560	1474.7263	20.1	11	-	24	1	---	K.KEQASLDAGTNVDK.M
1851.8885	1850.8812	1850.8687	6.79	141	-	156	0	---	K.LSNSTAVYFQDYQTSK.S
1967.9565	1966.9492	1966.9822	-16.77	215	-	230	1	---	K.FQVIKQEPMELESYAR.E
2110.0061	2108.9988	2108.9837	7.17	333	-	350	1	---	K.AMQIKVEAFDHEFDGTQK.L + Oxidation (M)
2309.0659	2308.0586	2308.1158	-24.76	331	-	350	2	---	K.AKAMQIKVEAFDHEFDGTQK.L + Oxidation (M)
3488.6545	3487.6472	3487.7147	-19.35	126	-	156	1	---	K.FGLISSTAYAQEIQKLSNSTAVYFQDYQTSK.S

No match to: 716.4763, 723.4098, 724.2417, 725.2533, 726.3622, 728.3046, 729.4749, 734.4570, 736.3904, 737.4152, 738.3791, 744.8651, 745.3838, 747.4550, 768.3673, 788.4571, 801.4262, 813.4481, 814.4558, 820.4053, 823.4778, 829.4589, 830.4522, 839.3830, 842.5110, 857.4382, 860.4207, 865.4518, 870.4165, 872.5054, 880.4424, 882.4292, 887.4245, 887.9669, 888.4561, 891.4898, 894.4144, 894.8973, 897.5006, 898.4523, 912.5132, 913.5293, 925.4620, 927.4981, 931.4992, 934.5098, 957.5774, 960.5294, 961.4949, 963.4734, 965.4692, 970.5181, 972.4899, 975.5167, 976.5063, 977.5212, 978.5146, 983.6283, 992.4958, 993.5046, 995.4181, 998.5911, 1000.5959, 1002.5007, 1006.5014, 1011.5474, 1012.5447, 1014.5485, 1020.5577, 1021.5699, 1024.5516, 1027.5438, 1028.5397, 1030.5978, 1032.5238, 1036.5260, 1037.5405, 1039.5641, 1040.5908, 1042.5388, 1043.5178, 1046.6018, 1056.5515, 1061.6168, 1074.6073, 1074.6100, 1076.6123, 1102.6007, 1103.6257, 1105.6136, 1107.1058, 1113.5486, 1120.5498, 1122.5322, 1123.5986, 1128.5724, 1129.5924, 1134.3574, 1137.5463, 1145.6348, 1149.6057, 1153.6460, 1156.4957, 1162.6174, 1164.1815, 1170.5497, 1172.6156, 1177.6410, 1180.6428, 1184.6412, 1188.5603, 1193.6041, 1206.5107, 1209.5889, 1211.6099, 1212.6160, 1215.6743, 1216.7081, 1219.6886, 1227.6234, 1229.7098, 1234.6414, 1240.5974, 1243.6161, 1253.6216, 1255.5972, 1255.6000, 1260.6227, 1264.7130, 1265.7064, 1269.6498, 1270.6575, 1273.6556, 1277.6305, 1286.6099, 1289.6875, 1293.6401, 1303.6455, 1304.6677, 1307.6788, 1309.6780, 1318.6750, 1319.6589, 1328.6600, 1328.6620, 1331.7888, 1333.7644, 1336.0798, 1336.6155, 1350.6853, 1351.7202, 1356.5811, 1359.1302, 1359.6246, 1365.6489, 1374.7588, 1383.7037, 1390.6974, 1393.7992, 1400.6683, 1401.6989, 1414.8191, 1429.3142, 1436.7889, 1444.7122, 1445.7839, 1450.7223, 1456.7717, 1462.6630, 1467.7329, 1470.7394, 1471.2693, 1471.7250, 1472.2531, 1474.5243, 1483.7141, 1488.5333, 1488.7194, 1493.7366, 1502.5601, 1513.7441, 1514.6849, 1516.6807, 1517.6437, 1518.7053, 1523.7611, 1526.7343, 1528.6973, 1530.6893, 1536.8230, 1540.7114, 1551.7063, 1553.7252, 1554.7474, 1557.6992, 1558.7140, 1563.6658, 1564.3619, 1565.7223, 1566.7162, 1572.7062, 1580.8400, 1580.8423, 1586.8090, 1597.7913, 1599.8337, 1601.8030, 1614.8046, 1616.7968, 1628.7136, 1630.8103, 1635.7697, 1642.7682, 1643.7688, 1644.7915, 1646.8154, 1651.7681, 1657.7932, 1663.8466, 1664.8557, 1666.7545, 1695.8208, 1707.8933, 1718.8790, 1721.9192, 1722.9375, 1728.8976, 1730.9116, 1732.2391, 1734.1844, 1739.7894, 1743.8966, 1755.2666, 1755.7703, 1757.4702, 1757.9089, 1758.4524, 1758.8987, 1761.9023, 1765.7578, 1768.8339, 1769.8287, 1770.3949, 1772.8273, 1773.8792, 1774.9180, 1774.9200, 1786.8268, 1787.8239, 1809.0499, 1810.0303, 1829.8895, 1830.8380, 1833.3470, 1833.8501, 1852.0399, 1862.9515, 1881.7703, 1895.9634, 1906.8195, 1908.8512, 1920.7972, 1932.0050, 1936.9479, 1939.8563, 1940.8103, 1943.8293, 1954.8351, 1956.8574, 1958.8956, 1965.7998, 1969.9998, 1979.8927, 1981.3531, 1982.8179, 1995.8232, 1997.8312, 2003.8595, 2008.9971, 2011.8464, 2015.9408, 2019.8779, 2025.8735, 2031.9606, 2047.9397, 2062.9578, 2067.0020, 2076.8943, 2081.9827, 2082.9966, 2087.0103, 2088.1108, 2089.1162, 2096.0430, 2097.0200, 2108.9771, 2139.0991, 2140.0918, 2143.1006, 2144.0718, 2149.8516, 2160.0554, 2174.0378, 2178.0471, 2179.0574, 2188.0547, 2191.0457, 2198.1831, 2199.1499, 2203.0386, 2211.1052, 2223.1582, 2225.1470, 2227.1042, 2228.0725, 2238.1086, 2239.1538, 2243.1243, 2244.0684, 2254.0896, 2255.1482, 2260.0779, 2270.2183, 2272.1736, 2284.1841, 2287.1108, 2307.0549, 2310.0798, 2310.5271, 2311.0454, 2319.0945, 2337.6050, 2341.5510, 2353.0518, 2354.0767, 2355.0564, 2371.0581, 2383.9592, 2388.0437, 2392.1462, 2404.0471, 2412.0872, 2419.5879, 2420.0864, 2420.5930, 2423.2439, 2426.5667, 2427.1211, 2438.1724, 2455.1553, 2458.1648, 2459.1794, 2478.1587, 2516.1133, 2533.1262, 2542.1650, 2546.1746, 2549.1316, 2550.1252, 2559.1470, 2561.1729, 2562.1765, 2567.1514, 2572.1523, 2574.2678, 2576.1628, 2583.1218, 2587.2722, 2604.1682, 2612.1438, 2653.7173, 2655.1897, 2671.1787, 2687.1833, 2701.2820, 2706.2766, 2712.2104, 2714.2156, 2716.1953, 2717.2725, 2725.2354, 2729.1675, 2739.2351, 2743.2642, 2745.2900, 2754.2197, 2755.2358, 2759.2573, 2769.2219, 2771.2437, 2776.2644, 2785.2388, 2786.2617, 2787.2400, 2793.2439, 2801.2378, 2803.2302, 2809.2549, 2815.2153, 2817.2322, 2819.2236, 2830.2788, 2833.2280, 2836.2397, 2862.2927, 2873.2961, 2889.2024, 2894.3218, 2927.2520, 2931.3428, 2955.3005, 2974.4124, 2975.4084, 2990.3921, 3006.3206, 3011.2810, 3020.3557, 3021.2412, 3035.3281, 3053.3447, 3062.2456, 3086.4507, 3091.2622, 3103.3140, 3110.7285, 3113.8591, 3130.3557, 3140.4265, 3144.3657, 3159.4985, 3173.5386, 3174.5486, 3185.4236, 3218.3301, 3259.3625, 3312.3267, 3331.4678, 3347.4761, 3363.4788, 3390.4675, 3431.4570, 3447.4666, 3463.4895, 3485.6985, 3489.6521, 3493.0195, 3494.5571, 3504.6423, 3509.5613, 3525.6025, 3538.4521, 3553.6230, 3579.5403, 3648.1956, 3655.4729, 3664.7500, 3695.6177, 3712.5720, 3738.5837, 3816.7043, 3937.8308, 4010.8696

49. O02691 **Mass:** 27294 **Score:** 13 **Expect:** 1.2e+003 **Matches:** 4
3-hydroxyacyl-CoA dehydrogenase type-2 OS=Bos taurus GN=HSD17B10 PE=1 SV=3

Observed	Mr(expt)	Mr(calc)	ppm	Start		End	Miss	Ions	Peptide
888.4561	887.4488	887.4535	-5.22	185	-	192	0	---	R.DLAPMGIR.V + Oxidation (M)
993.5046	992.4973	992.4895	7.83	1	-	9	1	---	-.MAAACRSVK.G
1057.6100	1056.6027	1056.5927	9.45	70	-	79	0	13	K.DVQAALTLAR.E
1057.6107	1056.6034	1056.5927	10.1	70	-	79	0	---	K.DVQAALTLAR.E

No match to: 716.4763, 723.4098, 724.2417, 725.2533, 726.3622, 728.3046, 729.4749, 734.4570, 736.3904, 737.4152, 738.3791, 744.8651, 745.3838, 747.4550, 768.3673, 788.4571, 801.4262, 805.4606, 813.4481, 814.4558, 820.4053, 823.4778, 829.4589, 830.4522, 839.3830, 842.5110, 857.4382, 860.4207, 865.4518, 870.4165, 872.5054, 880.4424, 882.4292, 887.4245, 887.9669, 891.4898, 894.4144, 894.8973, 897.5006, 898.4523, 912.5132, 913.5293, 925.4620, 927.4981, 931.4992, 934.5098, 957.5774, 960.5294, 961.4949, 963.4734, 965.4692, 970.5181, 972.4899, 975.5167, 976.5063, 977.5212, 978.5100, 978.5146, 983.6283, 992.4958, 995.4181, 998.5911, 1000.5959, 1002.5007, 1006.5014, 1011.5474, 1012.5447, 1014.5485, 1020.5577, 1021.5699, 1024.5516, 1027.5438, 1028.5397, 1030.5978, 1032.5238, 1036.5260, 1037.5405, 1039.5641, 1040.5908, 1042.5388, 1043.5178, 1045.5568, 1046.6018, 1056.5515, 1061.6168, 1074.6073, 1074.6100, 1076.6123, 1102.6007, 1103.6257, 1105.6136, 1107.1058, 1113.5486, 1120.5498, 1122.5322, 1123.5986, 1128.5724, 1129.5924, 1134.3574,

1137.5463, 1145.6348, 1149.6057, 1153.6460, 1156.4957, 1161.6295, 1162.6174, 1164.1815, 1170.5497, 1172.6156, 1177.6410, 1180.6428,
1184.6412, 1188.5603, 1193.6041, 1206.5107, 1209.5889, 1211.6099, 1212.6160, 1215.6743, 1216.7081, 1219.6886, 1227.6234, 1229.7098,
1234.6414, 1240.5974, 1242.6003, 1243.6161, 1253.6216, 1255.5972, 1255.6000, 1260.6227, 1264.7130, 1265.7064, 1269.6498, 1270.6575,
1273.6556, 1277.6305, 1286.6099, 1289.6875, 1293.6401, 1303.6455, 1304.6677, 1307.6788, 1309.6780, 1318.6750, 1319.6589, 1328.6600,
1328.6620, 1331.7888, 1333.7644, 1336.0798, 1336.6155, 1350.6853, 1351.7202, 1356.5811, 1359.1302, 1359.6246, 1365.6489, 1374.7588,
1383.7037, 1390.6974, 1393.7992, 1400.6683, 1401.6989, 1414.8191, 1429.3142, 1436.7889, 1444.7122, 1445.7839, 1450.7223, 1456.7717,
1462.6630, 1467.7329, 1470.7394, 1471.2693, 1471.7250, 1472.2531, 1474.5243, 1475.7633, 1483.7141, 1488.5333, 1488.7194, 1493.7366,
1502.5601, 1513.7441, 1514.6849, 1516.6807, 1517.6437, 1518.7053, 1523.7611, 1526.7343, 1528.6973, 1530.6893, 1536.8230, 1540.7114,
1551.7063, 1553.7252, 1554.7474, 1557.6992, 1558.7140, 1563.6658, 1564.3619, 1565.7223, 1566.7162, 1572.7062, 1580.8400, 1580.8423,
1586.8090, 1597.7913, 1599.8337, 1601.8030, 1614.8046, 1616.7968, 1628.7136, 1630.8103, 1635.7697, 1642.7682, 1643.7688, 1644.7915,
1646.8154, 1651.7681, 1657.7932, 1663.8466, 1664.8557, 1666.7545, 1695.8208, 1707.8933, 1718.8790, 1721.9192, 1722.9375, 1728.8976,
1730.9116, 1732.2391, 1734.1844, 1739.7894, 1743.8966, 1755.2666, 1755.7703, 1757.4702, 1757.9089, 1758.4524, 1758.8987, 1761.9023,
1765.7578, 1768.8339, 1769.8287, 1770.3949, 1772.8273, 1773.8792, 1774.9180, 1774.9200, 1786.8268, 1787.8239, 1809.0499, 1810.0303,
1829.8895, 1830.8380, 1833.3470, 1833.8501, 1851.8885, 1852.0399, 1862.9515, 1881.7703, 1895.9634, 1906.8195, 1908.8512, 1920.7972,
1932.0050, 1936.9479, 1939.8563, 1940.8103, 1943.8293, 1954.8351, 1956.8574, 1958.8956, 1965.7998, 1967.9565, 1969.9998, 1979.8927,
1981.3531, 1982.8179, 1995.8232, 1997.8312, 2003.8595, 2008.9971, 2011.8464, 2015.9408, 2019.8779, 2025.8735, 2031.9606, 2047.9397,
2062.9578, 2067.0020, 2076.8943, 2081.9827, 2082.9966, 2087.0103, 2088.1108, 2089.1162, 2096.0430, 2097.0200, 2108.9771, 2110.0061,
2139.0991, 2140.0918, 2143.1006, 2144.0718, 2149.8516, 2160.0554, 2174.0378, 2178.0471, 2179.0574, 2188.0547, 2191.0457, 2198.1831,
2199.1499, 2203.0386, 2211.1052, 2223.1582, 2225.1470, 2227.1042, 2228.0725, 2238.1086, 2239.1538, 2243.1243, 2244.0684, 2254.0896,
2255.1482, 2260.0779, 2270.2183, 2272.1736, 2284.1841, 2287.1108, 2307.0549, 2309.0659, 2310.0798, 2310.5271, 2311.0454, 2319.0945,
2337.6050, 2341.5510, 2353.0518, 2354.0767, 2355.0564, 2371.0581, 2383.9592, 2388.0437, 2392.1462, 2404.0471, 2412.0872, 2419.5879,
2420.0864, 2420.5930, 2423.2439, 2426.5667, 2427.1211, 2438.1724, 2455.1553, 2458.1648, 2459.1794, 2478.1587, 2516.1133, 2533.1262,
2542.1650, 2546.1746, 2549.1316, 2550.1252, 2559.1470, 2561.1729, 2562.1765, 2567.1514, 2572.1523, 2574.2678, 2576.1628, 2583.1218,
2587.2722, 2604.1682, 2612.1438, 2653.7173, 2655.1897, 2671.1787, 2687.1833, 2701.2820, 2706.2766, 2712.2104, 2714.2156, 2716.1953,
2717.2725, 2725.2354, 2729.1675, 2739.2351, 2743.2642, 2745.2900, 2754.2197, 2755.2358, 2759.2573, 2769.2219, 2771.2437, 2776.2644,
2785.2388, 2786.2617, 2787.2400, 2793.2439, 2801.2378, 2803.2302, 2809.2549, 2815.2153, 2817.2322, 2819.2236, 2830.2788, 2833.2280,
2836.2397, 2862.2927, 2873.2961, 2889.2024, 2894.3218, 2927.2520, 2931.3428, 2955.3005, 2974.4124, 2975.4084, 2990.3921, 3006.3206,
3011.2810, 3020.3557, 3021.2412, 3035.3281, 3053.3447, 3062.2456, 3086.4507, 3091.2622, 3103.3140, 3110.7285, 3113.8591, 3130.3557,
3140.4265, 3144.3657, 3159.4985, 3173.5386, 3174.5486, 3185.4236, 3218.3301, 3259.3625, 3312.3267, 3331.4678, 3347.4761, 3363.4788,
3390.4675, 3431.4570, 3447.4666, 3463.4895, 3485.6985, 3488.6545, 3489.6521, 3493.0195, 3494.5571, 3504.6423, 3509.5613, 3525.6025,
3538.4521, 3553.6230, 3579.5403, 3648.1956, 3655.4729, 3664.7500, 3695.6177, 3712.5720, 3738.5837, 3816.7043, 3937.8308, 4010.8696

50. F1MEV2 **Mass:** 44049 **Score:** 13 **Expect:** 1.2e+003 **Matches:** 18

Uncharacterized protein OS=Bos taurus GN=KIAA0930 PE=4 SV=2

Observed	Mr(expt)	Mr(calc)	ppm	Start	End	Miss	Ions	Peptide
891.4898	890.4825	890.4644	20.4	296	302	1	---	R.NRITEMK.K
1076.6123	1075.6050	1075.5808	22.5	202	211	1	---	R.VSVAARMAQK.M + Oxidation (M)
1234.6414	1233.6341	1233.6466	-10.09	118	128	1	---	K.RSQQVFASPSK.H
1318.6750	1317.6677	1317.6565	8.52	267	278	0	---	R.VTSFSTPPTPER.N
1336.6155	1335.6082	1335.5951	9.81	219	228	1	---	K.YSNMEFVRMK.G + 2 Oxidation (M)
1374.7588	1373.7515	1373.7276	17.4	107	118	2	---	R.ADGGDIHIHRKR.S
1470.7394	1469.7321	1469.7449	-8.70	28	38	2	---	R.QDDMLFYVRRK.L
1553.7252	1552.7179	1552.6906	17.6	303	315	1	---	K.KSHSANDSEEFFR.E
1707.8933	1706.8860	1706.8886	-1.51	197	211	2	---	K.VYDNRVSVAARMAQK.M
1773.8792	1772.8719	1772.8516	11.5	119	134	1	---	R.SQQVFASPSKHPMDSK.G
1787.8239	1786.8166	1786.8494	-18.37	219	233	2	---	K.YSNMEFVRMKGPQGK.G + Oxidation (M)
1936.9479	1935.9406	1935.9699	-15.12	202	218	2	---	R.VSVAARMAQKMSFGFYK.Y + Oxidation (M)
2015.9408	2014.9335	2014.9362	-1.31	9	23	0	---	R.IVFWTWMFSTYFMEK.W
2031.9606	2030.9533	2030.9311	11.0	9	23	0	---	R.IVFWTWMFSTYFMEK.W + Oxidation (M)
2047.9397	2046.9324	2046.9260	3.14	9	23	0	---	R.IVFWTWMFSTYFMEK.W + 2 Oxidation (M)
2412.0872	2411.0799	2411.0748	2.11	208	226	2	---	R.MAQKMSFGFYKYSNMEFVR.M + 3 Oxidation (M)
2542.1650	2541.1577	2541.2014	-17.18	9	27	1	---	R.IVFWTWMFSTYFMEKWAPR.Q + Oxidation (M)
2576.1628	2575.1555	2575.1730	-6.79	95	116	1	---	K.LDYMVTCAVCTRADGGDIHIHR.K + Oxidation (M)

No match to: 716.4763, 723.4098, 724.2417, 725.2533, 726.3622, 728.3046, 729.4749, 734.4570, 736.3904, 737.4152, 738.3791,
744.8651, 745.3838, 747.4550, 768.3673, 788.4571, 801.4262, 805.4606, 813.4481, 814.4558, 820.4053, 823.4778, 829.4589, 830.4522,
839.3830, 842.5110, 857.4382, 860.4207, 865.4518, 870.4165, 872.5054, 880.4424, 882.4292, 887.4245, 887.9669, 888.4561, 894.4144,
894.8973, 897.5006, 898.4523, 912.5132, 913.5293, 925.4620, 927.4981, 931.4992, 934.5098, 957.5774, 960.5294, 961.4949, 963.4734,
965.4692, 970.5181, 972.4899, 975.5167, 976.5063, 977.5212, 978.5100, 978.5146, 983.6283, 992.4958, 993.5046, 995.4181, 998.5911,
1000.5959, 1002.5007, 1006.5014, 1011.5474, 1012.5447, 1014.5485, 1020.5577, 1021.5699, 1024.5516, 1027.5438, 1028.5397, 1030.5978,
1032.5238, 1036.5260, 1037.5405, 1039.5641, 1040.5908, 1042.5388, 1043.5178, 1045.5568, 1046.6018, 1056.5515, 1057.6100, 1057.6107,
1061.6168, 1074.6073, 1074.6100, 1102.6007, 1103.6257, 1105.6136, 1107.1058, 1113.5486, 1120.5498, 1122.5322, 1123.5986, 1128.5724,
1129.5924, 1134.3574, 1137.5463, 1145.6348, 1149.6057, 1153.6460, 1156.4957, 1161.6295, 1162.6174, 1164.1815, 1170.5497, 1172.6156,
1177.6410, 1180.6428, 1184.6412, 1188.5603, 1193.6041, 1206.5107, 1209.5889, 1211.6099, 1212.6160, 1215.6743, 1216.7081, 1219.6886,
1227.6234, 1229.7098, 1240.5974, 1242.6003, 1243.6161, 1253.6216, 1255.5972, 1255.6000, 1260.6227, 1264.7130, 1265.7064, 1269.6498,
1270.6575, 1273.6556, 1277.6305, 1286.6099, 1289.6875, 1293.6401, 1303.6455, 1304.6677, 1307.6788, 1309.6780, 1319.6589, 1328.6600,
1328.6620, 1331.7888, 1333.7644, 1336.0798, 1350.6853, 1351.7202, 1356.5811, 1359.1302, 1359.6246, 1365.6489, 1383.7037, 1390.6974,
1393.7992, 1400.6683, 1401.6989, 1414.8191, 1429.3142, 1436.7889, 1444.7122, 1445.7839, 1450.7223, 1456.7717, 1462.6630, 1467.7329,
1471.2693, 1471.7250, 1472.2531, 1474.5243, 1475.7633, 1483.7141, 1488.5333, 1488.7194, 1493.7366, 1502.5601, 1513.7441, 1514.6849,
1516.6807, 1517.6437, 1518.7053, 1523.7611, 1526.7343, 1528.6973, 1530.6893, 1536.8230, 1540.7114, 1551.7063, 1554.7474, 1557.6992,
1558.7140, 1563.6658, 1564.3619, 1565.7223, 1566.7162, 1572.7062, 1580.8400, 1580.8423, 1586.8090, 1597.7913, 1599.8337, 1601.8030,
1614.8046, 1616.7968, 1628.7136, 1630.8103, 1635.7697, 1642.7682, 1643.7688, 1644.7915, 1646.8154, 1651.7681, 1657.7932, 1663.8466,
1664.8557, 1666.7545, 1695.8208, 1718.8790, 1721.9192, 1722.9375, 1728.8976, 1730.9116, 1732.2391, 1734.1844, 1739.7894, 1743.8966,
1755.2666, 1755.7703, 1757.4702, 1757.9089, 1758.4524, 1758.8987, 1761.9023, 1765.7578, 1768.8339, 1769.8287, 1770.3949, 1772.8273,
1774.9180, 1774.9200, 1786.8268, 1809.0499, 1810.0303, 1829.8895, 1830.8380, 1833.3470, 1833.8501, 1851.8885, 1852.0399, 1862.9515,
1881.7703, 1895.9634, 1906.8195, 1908.8512, 1920.7972, 1932.0050, 1939.8563, 1940.8103, 1943.8293, 1954.8351, 1956.8574, 1958.8956,
1965.7998, 1967.9565, 1969.9998, 1979.8927, 1981.3531, 1982.8179, 1995.8232, 1997.8312, 2003.8595, 2008.9971, 2011.8464, 2019.8779,
2025.8735, 2062.9578, 2067.0020, 2076.8943, 2081.9827, 2082.9966, 2087.0103, 2088.1108, 2089.1162, 2096.0430, 2097.0200, 2108.9771,
2110.0061, 2139.0991, 2140.0918, 2143.1006, 2144.0718, 2149.8516, 2160.0554, 2174.0378, 2178.0471, 2179.0574, 2188.0547, 2191.0457,
2198.1831, 2199.1499, 2203.0386, 2211.1052, 2223.1582, 2225.1470, 2227.1042, 2228.0725, 2238.1086, 2239.1538, 2243.1243, 2244.0684,
2254.0896, 2255.1482, 2260.0779, 2270.2183, 2272.1736, 2284.1841, 2287.1108, 2307.0549, 2309.0659, 2310.0798, 2310.5271, 2311.0454,
2319.0945, 2337.6050, 2341.5510, 2353.0518, 2354.0767, 2355.0564, 2371.0581, 2383.9592, 2388.0437, 2392.1462, 2404.0471, 2419.5879,
2420.0864, 2420.5930, 2423.2439, 2426.5667, 2427.1211, 2438.1724, 2455.1553, 2458.1648, 2459.1794, 2478.1587, 2516.1133, 2533.1262,
2546.1746, 2549.1316, 2550.1252, 2559.1470, 2561.1729, 2562.1765, 2567.1514, 2572.1523, 2574.2678, 2583.1218, 2587.2722, 2604.1682,
2612.1438, 2653.7173, 2655.1897, 2671.1787, 2687.1833, 2701.2820, 2706.2766, 2712.2104, 2714.2156, 2716.1953, 2717.2725, 2725.2354,
2729.1675, 2739.2351, 2743.2642, 2745.2900, 2754.2197, 2755.2358, 2759.2573, 2769.2219, 2771.2437, 2776.2644, 2785.2388, 2786.2617,
2787.2400, 2793.2439, 2801.2378, 2803.2302, 2809.2549, 2815.2153, 2817.2322, 2819.2236, 2830.2788, 2833.2280, 2836.2397, 2862.2927,
2873.2961, 2889.2024, 2894.3218, 2927.2520, 2931.3428, 2955.3005, 2974.4124, 2975.4084, 2990.3921, 3006.3206, 3011.2810, 3020.3557,
3021.2412, 3035.3281, 3053.3447, 3062.2456, 3086.4507, 3091.2622, 3103.3140, 3110.7285, 3113.8591, 3130.3557, 3140.4265, 3144.3657,
3159.4985, 3173.5386, 3174.5486, 3185.4236, 3218.3301, 3259.3625, 3312.3267, 3331.4678, 3347.4761, 3363.4788, 3390.4675, 3431.4570,
3447.4666, 3463.4895, 3485.6985, 3488.6545, 3489.6521, 3493.0195, 3494.5571, 3504.6423, 3509.5613, 3525.6025, 3538.4521, 3553.6230,
3579.5403, 3648.1956, 3655.4729, 3664.7500, 3695.6177, 3712.5720, 3738.5837, 3816.7043, 3937.8308, 4010.8696

Search Parameters

```
Type of search         : MS/MS Ion Search
Enzyme                 : Trypsin
Fixed modifications    : Carbamidomethyl (C)
Variable modifications : Oxidation (M)
Mass values            : Monoisotopic
Protein Mass           : Unrestricted
Peptide Mass Tolerance : ± 25 ppm
Fragment Mass Tolerance: ± 0.5 Da
Max Missed Cleavages   : 2
Instrument type        : MALDI-TOF-TOF
   Query1 (716.4763,1+): <no title>
   Query2 (723.4098,1+): <no title>
   Query3 (724.2417,1+): <no title>
   Query4 (725.2533,1+): <no title>
   Query5 (726.3622,1+): <no title>
   Query6 (728.3046,1+): <no title>
   Query7 (729.4749,1+): <no title>
   Query8 (734.4570,1+): <no title>
   Query9 (736.3904,1+): <no title>
  Query10 (737.4152,1+): <no title>
  Query11 (738.3791,1+): <no title>
  Query12 (744.8651,1+): <no title>
  Query13 (745.3838,1+): <no title>
  Query14 (747.4550,1+): <no title>
  Query15 (768.3673,1+): <no title>
  Query16 (788.4571,1+): <no title>
  Query17 (801.4262,1+): <no title>
  Query18 (805.4606,1+): <no title>
  Query19 (813.4481,1+): <no title>
  Query20 (814.4558,1+): <no title>
  Query21 (820.4053,1+): <no title>
  Query22 (823.4778,1+): <no title>
  Query23 (829.4589,1+): <no title>
  Query24 (830.4522,1+): <no title>
  Query25 (839.3830,1+): <no title>
  Query26 (842.5110,1+): <no title>
  Query27 (857.4382,1+): <no title>
  Query28 (860.4207,1+): <no title>
  Query29 (865.4518,1+): <no title>
  Query30 (870.4165,1+): <no title>
  Query31 (872.5054,1+): <no title>
  Query32 (880.4424,1+): <no title>
  Query33 (882.4292,1+): <no title>
  Query34 (887.4245,1+): <no title>
  Query35 (887.9669,1+): <no title>
  Query36 (888.4561,1+): <no title>
  Query37 (891.4898,1+): <no title>
  Query38 (894.4144,1+): <no title>
  Query39 (894.8973,1+): <no title>
  Query40 (897.5006,1+): <no title>
  Query41 (898.4523,1+): <no title>
  Query42 (912.5132,1+): <no title>
  Query43 (913.5293,1+): <no title>
  Query44 (925.4620,1+): <no title>
  Query45 (927.4981,1+): <no title>
  Query46 (931.4992,1+): <no title>
  Query47 (934.5098,1+): <no title>
  Query48 (957.5774,1+): <no title>
  Query49 (960.5294,1+): <no title>
  Query50 (961.4949,1+): <no title>
  Query51 (963.4734,1+): <no title>
  Query52 (965.4692,1+): <no title>
  Query53 (970.5181,1+): <no title>
  Query54 (972.4899,1+): <no title>
  Query55 (975.5167,1+): <no title>
  Query56 (976.5063,1+): <no title>
  Query57 (977.5212,1+): <no title>
  Query58 (978.5100,1+): Label: G1, Spot_Id: 221888, Peak_List_Id: 435515, MSMS Job_Run_Id: 30377, Comment:
  Query59 (978.5146,1+): <no title>
  Query60 (983.6283,1+): <no title>
  Query61 (992.4958,1+): <no title>
  Query62 (993.5046,1+): <no title>
  Query63 (995.4181,1+): <no title>
  Query64 (998.5911,1+): <no title>
 Query65 (1000.5959,1+): <no title>
 Query66 (1002.5007,1+): <no title>
 Query67 (1006.5014,1+): <no title>
 Query68 (1011.5474,1+): <no title>
 Query69 (1012.5447,1+): <no title>
 Query70 (1014.5485,1+): <no title>
 Query71 (1020.5577,1+): <no title>
 Query72 (1021.5699,1+): <no title>
 Query73 (1024.5516,1+): <no title>
 Query74 (1027.5438,1+): <no title>
 Query75 (1028.5397,1+): <no title>
 Query76 (1030.5978,1+): <no title>
 Query77 (1032.5238,1+): <no title>
 Query78 (1036.5260,1+): <no title>
 Query79 (1037.5405,1+): <no title>
 Query80 (1039.5641,1+): <no title>
 Query81 (1040.5908,1+): <no title>
 Query82 (1042.5388,1+): <no title>
 Query83 (1043.5178,1+): <no title>
 Query84 (1045.5568,1+): <no title>
 Query85 (1046.6018,1+): <no title>
 Query86 (1056.5515,1+): <no title>
 Query87 (1057.6100,1+): Label: G1, Spot_Id: 221888, Peak_List_Id: 435412, MSMS Job_Run_Id: 30377, Comment:
 Query88 (1057.6107,1+): <no title>
 Query89 (1061.6168,1+): <no title>
 Query90 (1074.6073,1+): <no title>
```

```
 Query91 (1074.6100,1+): Label: G1, Spot_Id: 221888, Peak_List_Id: 435413, MSMS Job_Run_Id: 30377, Comment:
 Query92 (1076.6123,1+): <no title>
 Query93 (1102.6007,1+): <no title>
 Query94 (1103.6257,1+): <no title>
 Query95 (1105.6136,1+): <no title>
 Query96 (1107.1058,1+): <no title>
 Query97 (1113.5486,1+): <no title>
 Query98 (1120.5498,1+): <no title>
 Query99 (1122.5322,1+): <no title>
Query100 (1123.5986,1+): <no title>
Query101 (1128.5724,1+): <no title>
Query102 (1129.5924,1+): <no title>
Query103 (1134.3574,1+): <no title>
Query104 (1137.5463,1+): <no title>
Query105 (1145.6348,1+): <no title>
Query106 (1149.6057,1+): <no title>
Query107 (1153.6460,1+): <no title>
Query108 (1156.4957,1+): <no title>
Query109 (1161.6295,1+): <no title>
Query110 (1162.6174,1+): <no title>
Query111 (1164.1815,1+): <no title>
Query112 (1170.5497,1+): <no title>
Query113 (1172.6156,1+): <no title>
Query114 (1177.6410,1+): <no title>
Query115 (1180.6428,1+): <no title>
Query116 (1184.6412,1+): <no title>
Query117 (1188.5603,1+): <no title>
Query118 (1193.6041,1+): <no title>
Query119 (1206.5107,1+): <no title>
Query120 (1209.5889,1+): <no title>
Query121 (1211.6099,1+): <no title>
Query122 (1212.6160,1+): <no title>
Query123 (1215.6743,1+): <no title>
Query124 (1216.7081,1+): <no title>
Query125 (1219.6886,1+): <no title>
Query126 (1227.6234,1+): <no title>
Query127 (1229.7098,1+): <no title>
Query128 (1234.6414,1+): <no title>
Query129 (1240.5974,1+): <no title>
Query130 (1242.6003,1+): <no title>
Query131 (1243.6161,1+): <no title>
Query132 (1253.6216,1+): <no title>
Query133 (1255.5972,1+): <no title>
Query134 (1255.6000,1+): Label: G1, Spot_Id: 221888, Peak_List_Id: 435409, MSMS Job_Run_Id: 30377, Comment:
Query135 (1260.6227,1+): <no title>
Query136 (1264.7130,1+): <no title>
Query137 (1265.7064,1+): <no title>
Query138 (1269.6498,1+): <no title>
Query139 (1270.6575,1+): <no title>
Query140 (1273.6556,1+): <no title>
Query141 (1277.6305,1+): <no title>
Query142 (1286.6099,1+): <no title>
Query143 (1289.6875,1+): <no title>
Query144 (1293.6401,1+): <no title>
Query145 (1303.6455,1+): <no title>
Query146 (1304.6677,1+): <no title>
Query147 (1307.6788,1+): <no title>
Query148 (1309.6780,1+): <no title>
Query149 (1318.6750,1+): <no title>
Query150 (1319.6589,1+): <no title>
Query151 (1328.6600,1+): Label: G1, Spot_Id: 221888, Peak_List_Id: 435411, MSMS Job_Run_Id: 30377, Comment:
Query152 (1328.6620,1+): <no title>
Query153 (1331.7888,1+): <no title>
Query154 (1333.7644,1+): <no title>
Query155 (1336.0798,1+): <no title>
Query156 (1336.6155,1+): <no title>
Query157 (1350.6853,1+): <no title>
Query158 (1351.7202,1+): <no title>
Query159 (1356.5811,1+): <no title>
Query160 (1359.1302,1+): <no title>
Query161 (1359.6246,1+): <no title>
Query162 (1365.6489,1+): <no title>
Query163 (1374.7588,1+): <no title>
Query164 (1383.7037,1+): <no title>
Query165 (1390.6974,1+): <no title>
Query166 (1393.7992,1+): <no title>
Query167 (1400.6683,1+): <no title>
Query168 (1401.6989,1+): <no title>
Query169 (1414.8191,1+): <no title>
Query170 (1429.3142,1+): <no title>
Query171 (1436.7889,1+): <no title>
Query172 (1444.7122,1+): <no title>
Query173 (1445.7839,1+): <no title>
Query174 (1450.7223,1+): <no title>
Query175 (1456.7717,1+): <no title>
Query176 (1462.6630,1+): <no title>
Query177 (1467.7329,1+): <no title>
Query178 (1470.7394,1+): <no title>
Query179 (1471.2693,1+): <no title>
Query180 (1471.7250,1+): <no title>
Query181 (1472.2531,1+): <no title>
Query182 (1474.5243,1+): <no title>
Query183 (1475.7633,1+): <no title>
Query184 (1483.7141,1+): <no title>
Query185 (1488.5333,1+): <no title>
Query186 (1488.7194,1+): <no title>
Query187 (1493.7366,1+): <no title>
Query188 (1502.5601,1+): <no title>
Query189 (1513.7441,1+): <no title>
Query190 (1514.6849,1+): <no title>
```

```
Query191 (1516.6807,1+): <no title>
Query192 (1517.6437,1+): <no title>
Query193 (1518.7053,1+): <no title>
Query194 (1523.7611,1+): <no title>
Query195 (1526.7343,1+): <no title>
Query196 (1528.6973,1+): <no title>
Query197 (1530.6893,1+): <no title>
Query198 (1536.8230,1+): <no title>
Query199 (1540.7114,1+): <no title>
Query200 (1551.7063,1+): <no title>
Query201 (1553.7252,1+): <no title>
Query202 (1554.7474,1+): <no title>
Query203 (1557.6992,1+): <no title>
Query204 (1558.7140,1+): <no title>
Query205 (1563.6658,1+): <no title>
Query206 (1564.3619,1+): <no title>
Query207 (1565.7223,1+): <no title>
Query208 (1566.7162,1+): <no title>
Query209 (1572.7062,1+): <no title>
Query210 (1580.8400,1+): Label: G1, Spot_Id: 221888, Peak_List_Id: 435414, MSMS Job_Run_Id: 30377, Comment:
Query211 (1580.8423,1+): <no title>
Query212 (1586.8090,1+): <no title>
Query213 (1597.7913,1+): <no title>
Query214 (1599.8337,1+): <no title>
Query215 (1601.8030,1+): <no title>
Query216 (1614.8046,1+): <no title>
Query217 (1616.7968,1+): <no title>
Query218 (1628.7136,1+): <no title>
Query219 (1630.8103,1+): <no title>
Query220 (1635.7697,1+): <no title>
Query221 (1642.7682,1+): <no title>
Query222 (1643.7688,1+): <no title>
Query223 (1644.7915,1+): <no title>
Query224 (1646.8154,1+): <no title>
Query225 (1651.7681,1+): <no title>
Query226 (1657.7932,1+): <no title>
Query227 (1663.8466,1+): <no title>
Query228 (1664.8557,1+): <no title>
Query229 (1666.7545,1+): <no title>
Query230 (1695.8208,1+): <no title>
Query231 (1707.8933,1+): <no title>
Query232 (1718.8790,1+): <no title>
Query233 (1721.9192,1+): <no title>
Query234 (1722.9375,1+): <no title>
Query235 (1728.8976,1+): <no title>
Query236 (1730.9116,1+): <no title>
Query237 (1732.2391,1+): <no title>
Query238 (1734.1844,1+): <no title>
Query239 (1739.7894,1+): <no title>
Query240 (1743.8966,1+): <no title>
Query241 (1755.2666,1+): <no title>
Query242 (1755.7703,1+): <no title>
Query243 (1757.4702,1+): <no title>
Query244 (1757.9089,1+): <no title>
Query245 (1758.4524,1+): <no title>
Query246 (1758.8987,1+): <no title>
Query247 (1761.9023,1+): <no title>
Query248 (1765.7578,1+): <no title>
Query249 (1768.8339,1+): <no title>
Query250 (1769.8287,1+): <no title>
Query251 (1770.3949,1+): <no title>
Query252 (1772.8273,1+): <no title>
Query253 (1773.8792,1+): <no title>
Query254 (1774.9180,1+): <no title>
Query255 (1774.9200,1+): Label: G1, Spot_Id: 221888, Peak_List_Id: 435410, MSMS Job_Run_Id: 30377, Comment:
Query256 (1786.8268,1+): <no title>
Query257 (1787.8239,1+): <no title>
Query258 (1809.0499,1+): <no title>
Query259 (1810.0303,1+): <no title>
Query260 (1829.8895,1+): <no title>
Query261 (1830.8380,1+): <no title>
Query262 (1833.3470,1+): <no title>
Query263 (1833.8501,1+): <no title>
Query264 (1851.8885,1+): <no title>
Query265 (1852.0399,1+): <no title>
Query266 (1862.9515,1+): <no title>
Query267 (1881.7703,1+): <no title>
Query268 (1895.9634,1+): <no title>
Query269 (1906.8195,1+): <no title>
Query270 (1908.8512,1+): <no title>
Query271 (1920.7972,1+): <no title>
Query272 (1932.0050,1+): <no title>
Query273 (1936.9479,1+): <no title>
Query274 (1939.8563,1+): <no title>
Query275 (1940.8103,1+): <no title>
Query276 (1943.8293,1+): <no title>
Query277 (1954.8351,1+): <no title>
Query278 (1956.8574,1+): <no title>
Query279 (1958.8956,1+): <no title>
Query280 (1965.7998,1+): <no title>
Query281 (1967.9565,1+): <no title>
Query282 (1969.9998,1+): <no title>
Query283 (1979.8927,1+): <no title>
Query284 (1981.3531,1+): <no title>
Query285 (1982.8179,1+): <no title>
Query286 (1995.8232,1+): <no title>
Query287 (1997.8312,1+): <no title>
Query288 (2003.8595,1+): <no title>
Query289 (2008.9971,1+): <no title>
Query290 (2011.8464,1+): <no title>
```

```
Query291 (2015.9408,1+): <no title>
Query292 (2019.8779,1+): <no title>
Query293 (2025.8735,1+): <no title>
Query294 (2031.9606,1+): <no title>
Query295 (2047.9397,1+): <no title>
Query296 (2062.9578,1+): <no title>
Query297 (2067.0020,1+): <no title>
Query298 (2076.8943,1+): <no title>
Query299 (2081.9827,1+): <no title>
Query300 (2082.9966,1+): <no title>
Query301 (2087.0103,1+): <no title>
Query302 (2088.1108,1+): <no title>
Query303 (2089.1162,1+): <no title>
Query304 (2096.0430,1+): <no title>
Query305 (2097.0200,1+): <no title>
Query306 (2108.9771,1+): <no title>
Query307 (2110.0061,1+): <no title>
Query308 (2139.0991,1+): <no title>
Query309 (2140.0918,1+): <no title>
Query310 (2143.1006,1+): <no title>
Query311 (2144.0718,1+): <no title>
Query312 (2149.8516,1+): <no title>
Query313 (2160.0554,1+): <no title>
Query314 (2174.0378,1+): <no title>
Query315 (2178.0471,1+): <no title>
Query316 (2179.0574,1+): <no title>
Query317 (2188.0547,1+): <no title>
Query318 (2191.0457,1+): <no title>
Query319 (2198.1831,1+): <no title>
Query320 (2199.1499,1+): <no title>
Query321 (2203.0386,1+): <no title>
Query322 (2211.1052,1+): <no title>
Query323 (2223.1582,1+): <no title>
Query324 (2225.1470,1+): <no title>
Query325 (2227.1042,1+): <no title>
Query326 (2228.0725,1+): <no title>
Query327 (2238.1086,1+): <no title>
Query328 (2239.1538,1+): <no title>
Query329 (2243.1243,1+): <no title>
Query330 (2244.0684,1+): <no title>
Query331 (2254.0896,1+): <no title>
Query332 (2255.1482,1+): <no title>
Query333 (2260.0779,1+): <no title>
Query334 (2270.2183,1+): <no title>
Query335 (2272.1736,1+): <no title>
Query336 (2284.1841,1+): <no title>
Query337 (2287.1108,1+): <no title>
Query338 (2307.0549,1+): <no title>
Query339 (2309.0659,1+): <no title>
Query340 (2310.0798,1+): <no title>
Query341 (2310.5271,1+): <no title>
Query342 (2311.0454,1+): <no title>
Query343 (2319.0945,1+): <no title>
Query344 (2337.6050,1+): <no title>
Query345 (2341.5510,1+): <no title>
Query346 (2353.0518,1+): <no title>
Query347 (2354.0767,1+): <no title>
Query348 (2355.0564,1+): <no title>
Query349 (2371.0581,1+): <no title>
Query350 (2383.9592,1+): <no title>
Query351 (2388.0437,1+): <no title>
Query352 (2392.1462,1+): <no title>
Query353 (2404.0471,1+): <no title>
Query354 (2412.0872,1+): <no title>
Query355 (2419.5879,1+): <no title>
Query356 (2420.0864,1+): <no title>
Query357 (2420.5930,1+): <no title>
Query358 (2423.2439,1+): <no title>
Query359 (2426.5667,1+): <no title>
Query360 (2427.1211,1+): <no title>
Query361 (2438.1724,1+): <no title>
Query362 (2455.1553,1+): <no title>
Query363 (2458.1648,1+): <no title>
Query364 (2459.1794,1+): <no title>
Query365 (2478.1587,1+): <no title>
Query366 (2516.1133,1+): <no title>
Query367 (2533.1262,1+): <no title>
Query368 (2542.1650,1+): <no title>
Query369 (2546.1746,1+): <no title>
Query370 (2549.1316,1+): <no title>
Query371 (2550.1252,1+): <no title>
Query372 (2559.1470,1+): <no title>
Query373 (2561.1729,1+): <no title>
Query374 (2562.1765,1+): <no title>
Query375 (2567.1514,1+): <no title>
Query376 (2572.1523,1+): <no title>
Query377 (2574.2678,1+): <no title>
Query378 (2576.1628,1+): <no title>
Query379 (2583.1218,1+): <no title>
Query380 (2587.2722,1+): <no title>
Query381 (2604.1682,1+): <no title>
Query382 (2612.1438,1+): <no title>
Query383 (2653.7173,1+): <no title>
Query384 (2655.1897,1+): <no title>
Query385 (2671.1787,1+): <no title>
Query386 (2687.1833,1+): <no title>
Query387 (2701.2820,1+): <no title>
Query388 (2706.2766,1+): <no title>
Query389 (2712.2104,1+): <no title>
Query390 (2714.2156,1+): <no title>
```

```
Query391 (2716.1953,1+): <no title>
Query392 (2717.2725,1+): <no title>
Query393 (2725.2354,1+): <no title>
Query394 (2729.1675,1+): <no title>
Query395 (2739.2351,1+): <no title>
Query396 (2743.2642,1+): <no title>
Query397 (2745.2900,1+): <no title>
Query398 (2754.2197,1+): <no title>
Query399 (2755.2358,1+): <no title>
Query400 (2759.2573,1+): <no title>
Query401 (2769.2219,1+): <no title>
Query402 (2771.2437,1+): <no title>
Query403 (2776.2644,1+): <no title>
Query404 (2785.2388,1+): <no title>
Query405 (2786.2617,1+): <no title>
Query406 (2787.2400,1+): <no title>
Query407 (2793.2439,1+): <no title>
Query408 (2801.2378,1+): <no title>
Query409 (2803.2302,1+): <no title>
Query410 (2809.2549,1+): <no title>
Query411 (2815.2153,1+): <no title>
Query412 (2817.2322,1+): <no title>
Query413 (2819.2236,1+): <no title>
Query414 (2830.2788,1+): <no title>
Query415 (2833.2280,1+): <no title>
Query416 (2836.2397,1+): <no title>
Query417 (2862.2927,1+): <no title>
Query418 (2873.2961,1+): <no title>
Query419 (2889.2024,1+): <no title>
Query420 (2894.3218,1+): <no title>
Query421 (2927.2520,1+): <no title>
Query422 (2931.3428,1+): <no title>
Query423 (2955.3005,1+): <no title>
Query424 (2974.4124,1+): <no title>
Query425 (2975.4084,1+): <no title>
Query426 (2990.3921,1+): <no title>
Query427 (3006.3206,1+): <no title>
Query428 (3011.2810,1+): <no title>
Query429 (3020.3557,1+): <no title>
Query430 (3021.2412,1+): <no title>
Query431 (3035.3281,1+): <no title>
Query432 (3053.3447,1+): <no title>
Query433 (3062.2456,1+): <no title>
Query434 (3086.4507,1+): <no title>
Query435 (3091.2622,1+): <no title>
Query436 (3103.3140,1+): <no title>
Query437 (3110.7285,1+): <no title>
Query438 (3113.8591,1+): <no title>
Query439 (3130.3557,1+): <no title>
Query440 (3140.4265,1+): <no title>
Query441 (3144.3657,1+): <no title>
Query442 (3159.4985,1+): <no title>
Query443 (3173.5386,1+): <no title>
Query444 (3174.5486,1+): <no title>
Query445 (3185.4236,1+): <no title>
Query446 (3218.3301,1+): <no title>
Query447 (3259.3625,1+): <no title>
Query448 (3312.3267,1+): <no title>
Query449 (3331.4678,1+): <no title>
Query450 (3347.4761,1+): <no title>
Query451 (3363.4788,1+): <no title>
Query452 (3390.4675,1+): <no title>
Query453 (3431.4570,1+): <no title>
Query454 (3447.4666,1+): <no title>
Query455 (3463.4895,1+): <no title>
Query456 (3485.6985,1+): <no title>
Query457 (3488.6545,1+): <no title>
Query458 (3489.6521,1+): <no title>
Query459 (3493.0195,1+): <no title>
Query460 (3494.5571,1+): <no title>
Query461 (3504.6423,1+): <no title>
Query462 (3509.5613,1+): <no title>
Query463 (3525.6025,1+): <no title>
Query464 (3538.4521,1+): <no title>
Query465 (3553.6230,1+): <no title>
Query466 (3579.5403,1+): <no title>
Query467 (3648.1956,1+): <no title>
Query468 (3655.4729,1+): <no title>
Query469 (3664.7500,1+): <no title>
Query470 (3695.6177,1+): <no title>
Query471 (3712.5720,1+): <no title>
Query472 (3738.5837,1+): <no title>
Query473 (3816.7043,1+): <no title>
Query474 (3937.8308,1+): <no title>
Query475 (4010.8696,1+): <no title>
```

Mascot: http://www.matrixscience.com/

MATRIX SCIENCE Mascot Search Results

```
User          : 2c_(G5@11273_12Mar2012)
Email         : proteomics@ipatimup.pt
Search title  : Project: Proteomica, Spot Set: Proteomica\12Jan2012, Label: G5, Spot Id: 221892, Peak List Id: 435350, MS Job Run Id: 303
MS data file  : C:\Documents and Settings\Administrator\Desktop\xu\xu\xu\Proteomica\12Jan2012\ppw_G5_146796935705.txt
Database      : OrganismSpecie Bos_taurus_Reference_Proteome_2016_06 (24214 sequences; 12839866 residues)
Timestamp     : 8 Jul 2016 at 10:10:07 GMT
Warning       : A Peptide summary report will usually give a much clearer picture of MS/MS search results.
Top Score     : 123 for P46193, Annexin A1 OS=Bos taurus GN=ANXA1 PE=1 SV=2
```

Mascot Score Histogram

Protein score is -10*Log(P), where P is the probability that the observed match is a random event.
Protein scores greater than 56 are significant (p<0.05).
Protein scores are derived from ions scores as a non-probabilistic basis for ranking protein hits.

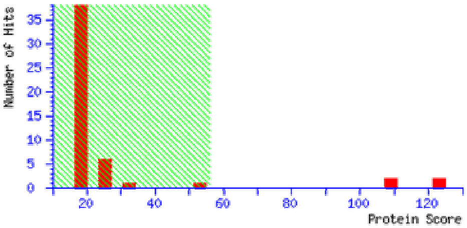

Protein Summary Report

Format As | Protein Summary (deprecated) | Help

Significance threshold p< 0.05 | Max. number of hits 50

Preferred taxonomy | All entries

Re-Search All | Search Unmatched

Index

	Accession	Mass	Score	Description
1.	P46193	39212	123	Annexin A1 OS=Bos taurus GN=ANXA1 PE=1 SV=2
2.	F1N650	39240	123	Annexin OS=Bos taurus GN=ANXA1 PE=1 SV=1
3.	P04272	38873	109	Annexin A2 OS=Bos taurus GN=ANXA2 PE=1 SV=2
4.	P10096	36073	107	Glyceraldehyde-3-phosphate dehydrogenase OS=Bos taurus GN=GAPDH PE=1 SV=4
5.	Q2HJ60	36041	50	Heterogeneous nuclear ribonucleoproteins A2/B1 OS=Bos taurus GN=HNRNPA2B1 PE=2 SV=1
6.	E1BB48	65458	34	Polypeptide N-acetylgalactosaminyltransferase OS=Bos taurus GN=GALNT2 PE=3 SV=2
7.	P02687	18312	26	Myelin basic protein OS=Bos taurus GN=MBP PE=1 SV=1
8.	F1N0N9	36314	26	Uncharacterized protein OS=Bos taurus PE=4 SV=1
9.	F6QBP1	18443	26	Myelin basic protein OS=Bos taurus GN=MBP PE=4 SV=1
10.	G3MZA4	83962	25	Uncharacterized protein OS=Bos taurus GN=CXorf23 PE=4 SV=1
11.	Q3T087	20468	24	60S ribosomal protein L11 OS=Bos taurus GN=RPL11 PE=2 SV=3
12.	F1MI98	52161	23	Uncharacterized protein OS=Bos taurus GN=KRT36 PE=3 SV=2
13.	Q58DL0	26401	22	Chromosome X open reading frame 23 OS=Bos taurus GN=CXorf23 PE=2 SV=1
14.	Q3SZ43	16409	22	Ubiquitin-conjugating enzyme E2 variant 2 OS=Bos taurus GN=UBE2V2 PE=2 SV=3
15.	E1BE64	104496	22	Uncharacterized protein OS=Bos taurus GN=TSGA10 PE=4 SV=2
16.	Q2KIJ9	18752	21	Transcription elongation factor A protein-like 1 OS=Bos taurus GN=TCEAL1 PE=2 SV=1
17.	G3MYE2	34779	21	Uncharacterized protein OS=Bos taurus PE=3 SV=1
18.	E1BJY0	22866	20	Guanylyl cyclase-activating protein 2 OS=Bos taurus GN=GUCA1B PE=4 SV=2
19.	F1MTD8	31878	20	Tyrosine-protein kinase OS=Bos taurus GN=HCK PE=3 SV=1
20.	Q2YDN4	58176	20	Coiled-coil domain-containing protein 105 OS=Bos taurus GN=CCDC105 PE=2 SV=1
21.	F1MNJ2	65953	20	Signal recognition particle receptor subunit alpha OS=Bos taurus GN=SRPRA PE=4 SV=1
22.	Q3MHN7	69222	20	Endonuclease 8-like 3 OS=Bos taurus GN=NEIL3 PE=2 SV=2
23.	G3MZK6	94689	20	Uncharacterized protein OS=Bos taurus GN=MAP7D3 PE=4 SV=1
24.	P02548	62665	19	Neurofilament light polypeptide OS=Bos taurus GN=NEFL PE=1 SV=3
25.	E1BF55	69481	19	Uncharacterized protein OS=Bos taurus GN=MAP7D2 PE=4 SV=2
26.	E1BGS4	63649	19	Uncharacterized protein OS=Bos taurus GN=TXLNA PE=4 SV=1
27.	Q32LI7	30313	19	Putative uncharacterized protein MGC133880 OS=Bos taurus GN=MGC133880 PE=2 SV=1
28.	Q8MKH7	29800	19	Troponin T fast skeletal muscle type OS=Bos taurus GN=Tnnt3 PE=2 SV=1
29.	G5E6L5	47201	19	Centrosomal protein of 290 kDa OS=Bos taurus GN=CEP290 PE=4 SV=1
30.	F1MJU1	14824	19	Histone H2B OS=Bos taurus PE=3 SV=2
31.	G3MYI2	118628	19	Uncharacterized protein OS=Bos taurus PE=4 SV=1
32.	E1BP00	59905	19	PC4 and SFRS1-interacting protein OS=Bos taurus GN=PSIP1 PE=4 SV=2
33.	A4FV84	27623	19	mRNA turnover protein 4 homolog OS=Bos taurus GN=MRTO4 PE=2 SV=1
34.	G8JL04	5563	19	Uncharacterized protein OS=Bos taurus PE=4 SV=1
35.	Q02366	15044	19	NADH dehydrogenase [ubiquinone] 1 alpha subcomplex subunit 6 OS=Bos taurus GN=NDUFA6 PE=1 SV=2
36.	G3MZ91	14728	19	Histone H2B OS=Bos taurus PE=3 SV=1
37.	F1MEY1	167004	19	Uncharacterized protein OS=Bos taurus GN=ATAD2B PE=4 SV=2
38.	F1N469	96792	19	Uncharacterized protein OS=Bos taurus GN=MAP7D3 PE=4 SV=2
39.	F1MIS2	9764	19	Uncharacterized protein OS=Bos taurus PE=3 SV=2
40.	F1MHH2	64063	19	Outer dense fiber protein 2-like OS=Bos taurus GN=ODF2L PE=4 SV=2
41.	E1BPQ8	60653	19	Uncharacterized protein OS=Bos taurus GN=CDC73 PE=4 SV=1
42.	Q2TA00	49021	18	Coiled-coil domain-containing protein 83 OS=Bos taurus GN=CCDC83 PE=2 SV=1
43.	E1B992	72756	18	Uncharacterized protein OS=Bos taurus PE=4 SV=2
44.	F1MB20	119646	18	Oral-facial-digital syndrome 1 isoform 1 OS=Bos taurus GN=OFD1 PE=2 SV=2
45.	E1BI42	127633	18	Uncharacterized protein OS=Bos taurus GN=CCDC158 PE=4 SV=2
46.	G3MWK4	30649	18	Uncharacterized protein OS=Bos taurus GN=SRSF12 PE=4 SV=1
47.	E1BLN1	117436	18	Uncharacterized protein OS=Bos taurus GN=SYCP1 PE=4 SV=1
48.	G3N2Q8	20708	18	Uncharacterized protein OS=Bos taurus GN=ISG20 PE=4 SV=1

49. Q32L31 23052 18 High mobility group protein B3 OS=Bos taurus GN=HMGB3 PE=2 SV=2
50. I7FVA7 23052 18 HMGB3 transcript variant 1/2 OS=Bos taurus GN=HMGB3 PE=4 SV=1

Results List

1. P46193 **Mass:** 39212 **Score:** 123 **Expect:** 1.2e-008 **Matches:** 27

Annexin A1 OS=Bos taurus GN=ANXA1 PE=1 SV=2

Observed	Mr(expt)	Mr(calc)	ppm	Start	End	Miss	Ions	Peptide
767.4740	766.4667	766.4813	-19.07	293	298	1	---	R.HKTLIR.I
772.4178	771.4105	771.4028	10.1	229	234	0	---	R.SYPHLR.R
788.4225	787.4152	787.4300	-18.81	145	150	1	---	R.TNREIR.E
822.4321	821.4249	821.4283	-4.18	82	88	0	---	K.AAYLQEK.G
908.4577	907.4504	907.4399	11.6	205	212	0	---	R.ALYEAGER.R
928.5031	927.4958	927.5039	-8.72	229	235	1	---	R.SYPHLRR.V
940.4773	939.4700	939.4735	-3.75	2	9	0	---	M.AMVSEFLK.Q + Oxidation (M)
1016.5522	1015.5449	1015.5484	-3.47	338	346	1	---	K.ILVALCGRD.-
1064.5358	1063.5285	1063.5410	-11.75	205	213	1	---	R.ALYEAGERR.K
1071.5361	1070.5288	1070.5140	13.8	1	9	0	---	-.MAMVSEFLK.Q + Oxidation (M)
1092.6000	1091.5927	1091.6087	-14.64	155	162	2	21	R.VYREELKR.D
1092.6002	1091.5929	1091.6087	-14.46	155	162	2	---	R.VYREELKR.D
1276.5977	1275.5904	1275.6095	-14.96	114	124	0	---	K.TPAQFDAEELR.A
1276.6000	1275.5927	1275.6095	-13.16	114	124	0	76	K.TPAQFDAEELR.A
1400.6588	1399.6515	1399.6554	-2.77	240	250	2	---	K.YSKYSKHDMNK.V
1416.6047	1415.5974	1415.6214	-16.90	270	281	0	---	K.CATSQPMFFAEK.L
1677.8074	1676.8001	1676.8192	-11.36	114	128	1	---	K.TPAQFDAEELRAAMK.G
1703.8214	1702.8141	1702.8373	-13.63	129	144	0	---	K.GLGTDEDTLNEILASR.T
1719.8075	1718.8002	1718.7595	23.7	189	204	0	---	R.SEELAVNDDLADSDAR.A
1723.8743	1722.8670	1722.8862	-11.13	318	332	0	---	K.LYGISLCQAILDETK.G
1826.8450	1825.8377	1825.8522	-7.95	10	23	0	---	K.QAWFIENEEQEYIK.T
2047.9097	2046.9024	2046.9090	-3.21	186	204	1	---	K.GDRSEELAVNDDLADSDAR.A
2155.0667	2154.0594	2154.0633	-1.80	10	26	1	---	K.QAWFIENEEQEYIKTVK.G
2166.0911	2165.0838	2165.1184	-15.97	295	312	2	---	K.TLIRIMVSRSEIDMNDIK.A + 2 Oxidation (M)
2414.1833	2413.1760	2413.1550	8.71	30	53	0	---	K.GGPGSAVSPYPTFNPSSDVEALHK.A
2686.3572	2685.3499	2685.3035	17.3	27	53	1	---	K.GSKGGPGSAVSPYPTFNPSSDVEALHK.A
2788.3362	2787.3289	2787.3645	-12.78	258	281	2	---	K.GDIEKCLTVIVKCATSQPMFFAEK.L + Oxidation (M)

No match to: 724.2539, 725.2472, 728.2482, 730.2519, 732.2390, 733.2623, 739.3115, 742.4642, 743.4729, 745.4483, 747.4419, 752.3972, 754.3827, 756.3716, 758.3382, 760.3329, 764.3364, 766.3494, 768.3865, 770.3671, 779.4436, 784.3415, 787.2366, 790.4196, 795.4260, 799.2464, 802.4372, 805.4478, 808.4353, 811.4178, 814.4170, 819.4304, 821.4247, 829.4457, 837.4396, 840.4623, 842.5109, 854.4525, 856.5045, 858.5442, 860.4092, 863.4233, 863.9185, 869.4846, 870.5100, 872.4994, 880.4412, 904.4613, 906.4929, 913.5685, 919.5024, 925.5089, 927.4999, 942.4820, 950.4946, 957.5654, 960.4840, 966.4929, 969.4786, 972.5046, 974.5551, 977.5352, 978.5240, 981.4474, 989.2700, 991.5198, 993.5112, 1001.5782, 1007.5089, 1009.5097, 1013.4454, 1015.5150, 1018.5189, 1021.5313, 1024.5028, 1025.5192, 1029.5330, 1032.5870, 1033.5739, 1035.5203, 1045.5564, 1046.5759, 1051.5074, 1056.5415, 1057.6005, 1073.5377, 1079.5398, 1083.4637, 1086.4774, 1094.5596, 1096.5302, 1106.5431, 1110.5461, 1111.5398, 1111.5400, 1115.5790, 1118.5620, 1120.5724, 1123.5819, 1128.5537, 1132.5682, 1134.5841, 1135.5942, 1137.5565, 1143.5844, 1145.6155, 1149.5597, 1153.5929, 1158.6288, 1163.5771, 1173.6008, 1175.5867, 1177.6178, 1178.6053, 1179.5962, 1181.6432, 1192.5721, 1199.6315, 1203.5826, 1211.5984, 1215.6246, 1216.6786, 1221.5699, 1222.5789, 1227.6271, 1228.6411, 1229.6412, 1231.6102, 1233.6571, 1237.6270, 1238.5917, 1244.6069, 1248.5958, 1250.6042, 1255.5922, 1258.6344, 1268.6414, 1271.6490, 1275.6388, 1285.5864, 1294.6315, 1299.6810, 1300.6566, 1309.6340, 1320.6268, 1328.6292, 1333.7025, 1336.6038, 1338.6589, 1341.6488, 1343.6613, 1345.6910, 1349.6838, 1350.6704, 1352.6350, 1355.6870, 1357.7130, 1358.6704, 1369.7067, 1371.7366, 1374.6705, 1377.6100, 1377.6146, 1384.7109, 1386.7134, 1390.6848, 1393.7169, 1399.6119, 1406.7207, 1410.6736, 1411.6647, 1418.6714, 1421.6632, 1425.7395, 1426.6846, 1427.7076, 1430.7102, 1432.6718, 1434.6552, 1438.6991, 1439.7164, 1443.7589, 1444.7375, 1445.7306, 1454.6836, 1461.7990, 1472.7026, 1475.7224, 1485.6906, 1489.7172, 1490.7058, 1493.7157, 1510.7383, 1515.7393, 1523.6987, 1524.7383, 1527.7585, 1529.7466, 1530.6902, 1531.6953, 1537.7804, 1538.7736, 1539.7621, 1541.7805, 1542.8290, 1549.6758, 1553.7277, 1555.7928, 1556.7874, 1560.7551, 1563.7314, 1568.7372, 1570.8015, 1572.8151, 1576.7402, 1577.7395, 1578.7518, 1581.8373, 1591.7852, 1600.8068, 1603.8092, 1604.8136, 1606.8130, 1608.7599, 1613.7793, 1615.8575, 1615.8600, 1624.7632, 1628.7653, 1633.7913, 1634.7969, 1635.7941, 1639.8157, 1643.8085, 1646.8591, 1647.8268, 1651.8206, 1653.8284, 1681.8087, 1688.8287, 1690.8313, 1694.7357, 1697.8672, 1706.8364, 1707.8435, 1708.3533, 1709.3307, 1710.8229, 1715.8053, 1721.8296, 1724.8828, 1725.8090, 1730.8199, 1748.7931, 1757.8975, 1763.7841, 1768.8075, 1771.9686, 1774.9059, 1777.8727, 1779.8081, 1786.8824, 1787.8207, 1790.8423, 1793.9556, 1796.8109, 1797.8339, 1800.8578, 1815.9008, 1819.8838, 1821.8926, 1822.9185, 1829.8655, 1833.8975, 1836.9054, 1843.8765, 1844.8785, 1851.9031, 1859.8494, 1862.9265, 1863.9319, 1866.8857, 1868.8972, 1879.9445, 1880.9215, 1887.9023, 1888.8057, 1890.8025, 1896.8954, 1901.0060, 1906.8872, 1908.8663, 1913.8895, 1916.9475, 1924.9314, 1925.9336, 1928.0060, 1931.9956, 1932.9833, 1968.8563, 1984.9392, 2008.0485, 2008.9869, 2009.9747, 2012.9506, 2015.9661, 2020.9644, 2030.0151, 2030.4648, 2046.9839, 2047.5186, 2051.9133, 2059.0122, 2064.9746, 2070.9209, 2077.0454, 2082.9409, 2085.9888, 2090.9844, 2097.9778, 2103.1526, 2108.9897, 2118.8926, 2140.0432, 2142.0310, 2142.9895, 2152.0510, 2159.0752, 2160.0703, 2161.0813, 2167.0676, 2174.0425, 2178.0034, 2181.0369, 2184.0767, 2189.9133, 2194.9761, 2199.0986, 2203.0464, 2205.9102, 2208.9729, 2211.1077, 2213.1116, 2214.1145, 2218.1018, 2221.1035, 2223.2205, 2225.1592, 2229.1064, 2233.1023, 2245.0991, 2269.1128, 2273.0393, 2277.0576, 2279.1094, 2280.1204, 2283.1279, 2285.1169, 2292.1465, 2296.1479, 2312.0664, 2323.1892, 2326.0947, 2334.1531, 2335.1282, 2337.1343, 2369.2256, 2372.2229, 2383.9978, 2385.2129, 2392.1528, 2400.1094, 2401.1360, 2409.1914, 2411.1907, 2429.1475, 2436.1699, 2437.1758, 2440.2007, 2447.2368, 2477.1301, 2495.0542, 2498.0898, 2510.2229, 2522.1980, 2524.1689, 2527.2310, 2535.2598, 2541.2075, 2543.1907, 2544.1514, 2553.1934, 2557.2300, 2560.1125, 2569.2915, 2572.0935, 2612.1936, 2963.6167, 3056.4641, 3058.5842, 3180.5432, 3313.5032, 3330.6311, 3347.6077, 3511.9668, 3761.1558

2. F1N650 **Mass:** 39240 **Score:** 123 **Expect:** 1.2e-008 **Matches:** 27

Annexin OS=Bos taurus GN=ANXA1 PE=1 SV=1

Observed	Mr(expt)	Mr(calc)	ppm	Start	End	Miss	Ions	Peptide
767.4740	766.4667	766.4813	-19.07	293	298	1	---	R.HKTLIR.I
772.4178	771.4105	771.4028	10.1	229	234	0	---	R.SYPHLR.R
788.4225	787.4152	787.4300	-18.81	145	150	1	---	R.TNREIR.E
822.4321	821.4249	821.4283	-4.18	82	88	0	---	K.AAYLQEK.G
908.4577	907.4504	907.4399	11.6	205	212	0	---	R.ALYEAGER.R
928.5031	927.4958	927.5039	-8.72	229	235	1	---	R.SYPHLRR.V
940.4773	939.4700	939.4735	-3.75	2	9	0	---	M.AMVSEFLK.Q + Oxidation (M)
1016.5522	1015.5449	1015.5484	-3.47	338	346	1	---	K.ILVALCGRD.-
1064.5358	1063.5285	1063.5410	-11.75	205	213	1	---	R.ALYEAGERR.K
1071.5361	1070.5288	1070.5140	13.8	1	9	0	---	-.MAMVSEFLK.Q + Oxidation (M)
1092.6000	1091.5927	1091.6087	-14.64	155	162	2	21	R.VYREELKR.D
1092.6002	1091.5929	1091.6087	-14.46	155	162	2	---	R.VYREELKR.D
1276.5977	1275.5904	1275.6095	-14.96	114	124	0	---	K.TPAQFDAEELR.A
1276.6000	1275.5927	1275.6095	-13.16	114	124	0	76	K.TPAQFDAEELR.A
1400.6588	1399.6515	1399.6554	-2.77	240	250	2	---	K.YSKYSKHDMNK.V
1416.6047	1415.5974	1415.6214	-16.90	270	281	0	---	K.CATSQPMFFAEK.L
1677.8074	1676.8001	1676.8192	-11.36	114	128	1	---	K.TPAQFDAEELRAAMK.G

1703.8214	1702.8141	1702.8373	-13.63	129	144	0	---	K.GLGTDEDTLNEILASR.T
1719.8075	1718.8002	1718.7595	23.7	189	204	0	---	R.SEELAVNDDLADSDAR.A
1723.8743	1722.8670	1722.8862	-11.13	318	332	0	---	K.LYGISLCQAILDETK.G
1826.8450	1825.8377	1825.8522	-7.95	10	23	0	---	K.QAWFIENEEQEYIK.T
2047.9097	2046.9024	2046.9090	-3.21	186	204	1	---	K.GDRSEELAVNDDLADSDAR.A
2155.0667	2154.0594	2154.0633	-1.80	10	26	1	---	K.QAWFIENEEQEYIKTVK.G
2166.0911	2165.0838	2165.1184	-15.97	295	312	2	---	K.TLIRIMVSRSEIDMNDIK.A + 2 Oxidation (M)
2414.1833	2413.1760	2413.1550	8.71	30	53	0	---	K.GGPGSAVSPYPTFNPSSDVEALHK.A
2686.3572	2685.3499	2685.3035	17.3	27	53	1	---	K.GSKGGPGSAVSPYPTFNPSSDVEALHK.A
2788.3362	2787.3289	2787.3645	-12.78	258	281	2	---	K.GDIEKCLTVIVKCATSQPMFFAEK.L + Oxidation (M)

No match to: 724.2539, 725.2472, 728.2482, 730.2519, 732.2390, 733.2623, 739.3115, 742.4642, 743.4729, 745.4483, 747.4419, 752.3972, 754.3827, 756.3716, 758.3382, 760.3329, 764.3364, 766.3494, 768.3865, 770.3671, 779.4436, 784.3415, 787.2366, 790.4196, 795.4260, 799.2464, 802.4372, 805.4478, 808.4353, 811.4178, 814.4170, 819.4304, 821.4247, 829.4457, 837.4396, 840.4623, 842.5109, 854.4525, 856.5045, 858.5442, 860.4092, 863.4233, 863.9185, 869.4846, 870.5100, 872.4994, 880.4412, 904.4613, 906.4929, 913.5685, 919.5024, 925.5089, 927.4999, 942.4820, 950.4946, 957.5654, 960.4840, 966.4929, 969.4786, 972.5046, 974.5551, 977.5352, 978.5240, 981.4474, 989.2700, 991.5198, 993.5112, 1001.5782, 1007.5089, 1009.5097, 1013.4454, 1015.5150, 1018.5189, 1021.5313, 1024.5028, 1025.5192, 1029.5330, 1032.5870, 1033.5739, 1035.5203, 1045.5564, 1046.5759, 1051.5074, 1056.5415, 1057.6005, 1073.5377, 1079.5398, 1083.4637, 1086.4774, 1094.5596, 1096.5302, 1106.5431, 1110.5461, 1111.5398, 1111.5400, 1115.5790, 1118.5620, 1120.5724, 1123.5819, 1128.5537, 1132.5682, 1134.5841, 1135.5942, 1137.5565, 1143.5844, 1145.6155, 1149.5597, 1153.5929, 1158.6288, 1163.5771, 1173.6008, 1175.5867, 1177.6178, 1178.6053, 1179.5962, 1181.6432, 1192.5721, 1199.6315, 1203.5826, 1211.5984, 1215.6246, 1216.6786, 1221.5699, 1222.5789, 1227.6271, 1228.6411, 1229.6412, 1231.6102, 1233.6571, 1237.6270, 1238.5917, 1244.6069, 1248.5958, 1250.6042, 1255.5922, 1258.6344, 1268.6414, 1271.6490, 1275.6388, 1285.5864, 1294.6315, 1299.6810, 1300.6566, 1309.6340, 1320.6268, 1328.6292, 1333.7025, 1336.6038, 1338.6589, 1341.6488, 1343.6613, 1345.6910, 1349.6838, 1350.6704, 1352.6350, 1355.6870, 1357.7130, 1358.6704, 1369.7067, 1371.7366, 1374.6705, 1377.6100, 1377.6146, 1384.7109, 1386.7134, 1390.6848, 1393.7169, 1399.6119, 1406.7207, 1410.6736, 1411.6647, 1418.6714, 1421.6632, 1425.7395, 1426.6846, 1427.7076, 1430.7102, 1432.6718, 1434.6552, 1438.6991, 1439.7164, 1443.7589, 1444.7375, 1445.7306, 1454.6836, 1461.7990, 1472.7026, 1475.7224, 1485.6906, 1489.7172, 1490.7058, 1493.7157, 1510.7383, 1515.7393, 1523.6987, 1524.7383, 1527.7585, 1529.7466, 1530.6902, 1531.6953, 1537.7804, 1538.7736, 1539.7621, 1541.7805, 1542.8290, 1549.6758, 1553.7277, 1555.7928, 1556.7874, 1560.7551, 1563.7314, 1568.7372, 1570.8015, 1572.8151, 1576.7402, 1577.7395, 1578.7518, 1581.8373, 1591.7852, 1600.8068, 1603.8092, 1604.8136, 1606.8130, 1608.7599, 1613.7793, 1615.8575, 1615.8600, 1624.7632, 1628.7653, 1633.7913, 1634.7969, 1635.7941, 1639.8157, 1643.8085, 1646.8591, 1647.8268, 1651.8206, 1653.8284, 1681.8087, 1688.8287, 1690.8313, 1694.7357, 1697.8672, 1706.8364, 1707.8435, 1708.3533, 1709.3307, 1710.8229, 1715.8053, 1721.8296, 1724.8828, 1725.8090, 1730.8199, 1748.7931, 1757.8975, 1763.7841, 1768.8075, 1771.9686, 1774.9059, 1777.8727, 1779.8081, 1786.8824, 1787.8207, 1790.8423, 1793.9556, 1796.8109, 1797.8339, 1800.8578, 1815.9008, 1819.8838, 1821.8926, 1822.9185, 1829.8655, 1833.8975, 1836.9054, 1843.8765, 1844.8785, 1851.9031, 1859.8494, 1862.9265, 1863.9319, 1866.8857, 1868.8972, 1879.9445, 1880.9215, 1887.9023, 1888.8057, 1890.8025, 1896.8954, 1901.0060, 1906.8872, 1908.8663, 1913.8895, 1916.9475, 1924.9314, 1925.9336, 1928.0060, 1931.9956, 1932.9833, 1968.8563, 1984.9392, 2008.0485, 2008.9869, 2009.9747, 2012.9506, 2015.9661, 2020.9644, 2030.0151, 2030.4648, 2046.9839, 2047.5186, 2051.9133, 2059.0122, 2064.9746, 2070.9209, 2077.0454, 2082.9409, 2085.9888, 2090.9844, 2097.9778, 2103.1526, 2108.9897, 2118.8926, 2140.0432, 2142.0310, 2142.9895, 2152.0510, 2159.0752, 2160.0703, 2161.0813, 2167.0676, 2174.0425, 2178.0034, 2181.0369, 2184.0767, 2189.9133, 2194.9761, 2199.0986, 2203.0464, 2205.9102, 2208.9729, 2211.1077, 2213.1116, 2214.1145, 2218.1018, 2221.1035, 2223.2205, 2225.1592, 2229.1064, 2233.1023, 2245.0991, 2269.1128, 2273.0393, 2277.0576, 2279.1094, 2280.1204, 2283.1279, 2285.1169, 2292.1465, 2296.1479, 2312.0664, 2323.1892, 2326.0947, 2334.1531, 2335.1282, 2337.1343, 2369.2256, 2372.2229, 2383.9978, 2385.2129, 2392.1528, 2400.1094, 2401.1360, 2409.1914, 2411.1907, 2429.1475, 2436.1699, 2437.1758, 2440.2007, 2447.2368, 2477.1301, 2495.0542, 2498.0898, 2510.2229, 2522.1980, 2524.1689, 2527.2310, 2535.2598, 2541.2075, 2543.1907, 2544.1514, 2553.1934, 2557.2300, 2560.1125, 2569.2915, 2572.0935, 2612.1936, 2963.6167, 3056.4641, 3058.5842, 3180.5432, 3313.5032, 3330.6311, 3347.6077, 3511.9668, 3761.1558

3. P04272 **Mass:** 38873 **Score:** 109 **Expect:** 3e-007 **Matches:** 37

Annexin A2 OS=Bos taurus GN=ANXA2 PE=1 SV=2

Observed	Mr(expt)	Mr(calc)	ppm	Start	End	Miss	Ions	Peptide
743.4729	742.4656	742.4701	-6.04	285	290	1	---	R.DKVLIR.I
745.4483	744.4410	744.4568	-21.13	170	176	0	---	K.LMVALAK.G
756.3716	755.3643	755.3524	15.8	274	279	0	---	R.LYDSMK.G
779.4436	778.4363	778.4337	3.35	303	308	1	---	K.IRSEFK.K
821.4247	820.4174	820.4000	21.2	296	302	0	---	R.SEVDMLK.I
880.4412	879.4339	879.4338	0.17	197	204	0	---	R.DLYDAGVK.R
960.4840	959.4767	959.4786	-2.01	146	152	1	---	R.VYKEMYK.T
1035.5203	1034.5130	1034.5219	-8.56	213	220	0	---	K.WISIMTER.S
1051.5074	1050.5001	1050.5168	-15.87	213	220	0	---	K.WISIMTER.S + Oxidation (M)
1086.4774	1085.4701	1085.4778	-7.04	29	37	0	---	K.AYTNFDAER.D
1111.5398	1110.5325	1110.5458	-11.95	69	77	0	---	R.QDIAFAYQR.R
1111.5400	1110.5327	1110.5458	-11.77	69	77	0	63	R.QDIAFAYQR.R
1222.5789	1221.5716	1221.5877	-13.17	105	115	0	---	K.TPAQYDASELK.A
1244.6069	1243.5996	1243.6156	-12.88	136	145	0	---	R.TNQELQEINR.V
1271.6490	1270.6417	1270.6340	6.11	274	284	2	---	R.LYDSMKGKGTR.D + Oxidation (M)
1421.6632	1420.6559	1420.6874	-22.17	314	324	0	---	K.SLYYYIQQDTK.G
1432.6718	1431.6645	1431.6591	3.75	234	245	0	---	K.SYSPYDMLESIK.K
1439.7164	1438.7091	1438.7160	-4.76	291	302	1	---	R.IMVSRSEVDMLK.I + 2 Oxidation (M)
1542.8290	1541.8217	1541.8413	-12.71	50	63	0	---	K.GVDEVTIVNILTNR.S
1560.7551	1559.7478	1559.7541	-4.03	234	246	1	---	K.SYSPYDMLESIKK.E
1576.7402	1575.7329	1575.7490	-10.22	234	246	1	---	K.SYSPYDMLESIKK.E + Oxidation (M)
1581.8373	1580.8300	1580.8232	4.32	296	308	2	---	R.SEVDMLKIRSEFK.K
1639.8157	1638.8084	1638.7923	9.85	105	119	1	---	K.TPAQYDASELKASMK.G
1725.8090	1724.8017	1724.8230	-12.34	64	77	1	---	R.SNEQRQDIAFAYQR.R
1771.9686	1770.9613	1770.9840	-12.78	48	63	1	---	K.TKGVDEVTIVNILTNR.S
1777.8727	1776.8654	1776.8564	5.10	120	135	0	---	K.GLGTDEDSLIEIICSR.T
1797.8339	1796.8266	1796.8429	-9.03	153	168	1	---	K.TDLEKDIVSDTSGDFR.K
1844.8785	1843.8712	1843.8952	-13.00	11	28	0	---	K.LSLEGDHSTPPSAYGSVK.A
1851.9031	1850.8958	1850.9124	-8.95	232	246	2	---	R.YKSYSPYDMLESIKK.E
1908.8663	1907.8590	1907.8748	-8.29	180	196	0	---	R.AEDGSVIDYELIDQDAR.D
1916.9475	1915.9402	1915.9601	-10.36	234	249	2	---	K.SYSPYDMLESIKKEVK.G
1925.9336	1924.9263	1924.9378	-5.96	153	169	2	---	K.TDLEKDIVSDTSGDFRK.L
1932.9833	1931.9760	1931.9550	10.9	234	249	2	---	K.SYSPYDMLESIKKEVK.G + Oxidation (M)
2012.9506	2011.9433	2011.9527	-4.66	314	329	1	---	K.SLYYYIQQDTKGDYQK.A
2064.9746	2063.9673	2063.9760	-4.18	179	196	1	---	R.RAEDGSVIDYELIDQDAR.D
2155.0667	2154.0594	2154.0593	0.07	29	47	1	---	K.AYTNFDAERDALNIETAIK.T
2211.1077	2210.1004	2210.0559	20.2	116	135	1	---	K.ASMKGLGTDEDSLIEIICSR.T + Oxidation (M)

No match to: 724.2539, 725.2472, 728.2482, 730.2519, 732.2390, 733.2623, 739.3115, 742.4642, 747.4419, 752.3972, 754.3827, 758.3382, 760.3329, 764.3364, 766.3494, 767.4740, 768.3865, 770.3671, 772.4178, 784.3415, 787.2366, 788.4225, 790.4196, 795.4260, 799.2464, 802.4372, 805.4478, 808.4353, 811.4178, 814.4170, 819.4304, 822.4321, 829.4457, 837.4396, 840.4623, 842.5109, 854.4525, 856.5045, 858.5442, 860.4092, 863.4233, 863.9185, 869.4846, 870.5100, 872.4994, 904.4613, 906.4929, 908.4577, 913.5685, 919.5024, 925.5089, 927.4999, 928.5031, 940.4773, 942.4820, 950.4946, 957.5654, 966.4929, 969.4786, 972.5046, 974.5551, 977.5352, 978.5240, 981.4474, 989.2700, 991.5198, 993.5112, 1001.5782, 1007.5089, 1009.5097, 1013.4454, 1015.5150, 1016.5522, 1018.5189, 1021.5313,

1024.5028, 1025.5192, 1029.5330, 1032.5870, 1033.5739, 1045.5564, 1046.5759, 1056.5415, 1057.6005, 1064.5358, 1071.5361, 1073.5377, 1079.5398, 1083.4637, 1092.6000, 1092.6002, 1094.5596, 1096.5302, 1106.5431, 1110.5461, 1115.5790, 1118.5620, 1120.5724, 1123.5819, 1128.5537, 1132.5682, 1134.5841, 1135.5942, 1137.5565, 1143.5844, 1145.6155, 1149.5597, 1153.5929, 1158.6288, 1163.5771, 1173.6008, 1175.5867, 1177.6178, 1178.6053, 1179.5962, 1181.6432, 1192.5721, 1199.6315, 1203.5826, 1211.5984, 1215.6246, 1216.6786, 1221.5699, 1227.6271, 1228.6411, 1229.6412, 1231.6102, 1233.6571, 1237.6270, 1238.5917, 1248.5958, 1250.6042, 1255.5922, 1258.6344, 1268.6414, 1275.6388, 1276.5977, 1276.6000, 1285.5864, 1294.6315, 1299.6810, 1300.6566, 1309.6340, 1320.6268, 1328.6292, 1333.7025, 1336.6038, 1338.6589, 1341.6488, 1343.6613, 1345.6910, 1349.6838, 1350.6704, 1352.6350, 1355.6870, 1357.7130, 1358.6704, 1369.7067, 1371.7366, 1374.6705, 1377.6100, 1377.6146, 1384.7109, 1386.7134, 1390.6848, 1393.7169, 1399.6119, 1400.6588, 1406.7207, 1410.6736, 1411.6647, 1416.6047, 1418.6714, 1425.7395, 1426.6846, 1427.7076, 1430.7102, 1434.6552, 1438.6991, 1443.7589, 1444.7375, 1445.7306, 1454.6836, 1461.7990, 1472.7026, 1475.7224, 1485.6906, 1489.7172, 1490.7058, 1493.7157, 1510.7383, 1515.7393, 1523.6987, 1524.7383, 1527.7585, 1529.7466, 1530.6902, 1531.6953, 1537.7804, 1538.7736, 1539.7621, 1541.7805, 1549.6758, 1553.7277, 1555.7928, 1556.7874, 1563.7314, 1568.7372, 1570.8015, 1572.8151, 1577.7395, 1578.7518, 1591.7852, 1600.8068, 1603.8092, 1604.8136, 1606.8130, 1608.7599, 1613.7793, 1615.8575, 1615.8600, 1624.7632, 1628.7653, 1633.7913, 1634.7969, 1635.7941, 1643.8085, 1646.8591, 1647.8268, 1651.8206, 1653.8284, 1677.8074, 1681.8087, 1688.8287, 1690.8313, 1694.7357, 1697.8672, 1703.8214, 1706.8364, 1707.8435, 1708.3533, 1709.3307, 1710.8229, 1715.8053, 1719.8075, 1721.8296, 1723.8743, 1724.8828, 1730.8199, 1748.7931, 1757.8975, 1763.7841, 1768.8075, 1774.9059, 1779.8081, 1786.8824, 1787.8207, 1790.8423, 1793.9556, 1796.8109, 1800.8578, 1815.9008, 1819.8838, 1821.8926, 1822.9185, 1826.8450, 1829.8655, 1833.8975, 1836.9054, 1843.8765, 1859.8494, 1862.9265, 1863.9319, 1866.8857, 1868.8972, 1879.9445, 1880.9215, 1887.9023, 1888.8057, 1890.8025, 1896.8954, 1901.0060, 1906.8872, 1913.8895, 1924.9314, 1928.0060, 1931.9956, 1968.8563, 1984.9392, 2008.0485, 2008.9869, 2009.9747, 2015.9661, 2020.9644, 2030.0151, 2030.4648, 2046.9839, 2047.5186, 2047.9097, 2051.9133, 2059.0122, 2070.9209, 2077.0454, 2082.9409, 2085.9888, 2090.9844, 2097.9778, 2103.1526, 2108.9897, 2118.8926, 2140.0432, 2142.0310, 2142.9895, 2152.0510, 2159.0752, 2160.0703, 2161.0813, 2166.0911, 2167.0676, 2174.0425, 2178.0034, 2181.0369, 2184.0767, 2189.9133, 2194.9761, 2199.0986, 2203.0464, 2205.9102, 2208.9729, 2213.1116, 2214.1145, 2218.1018, 2221.1035, 2223.2205, 2225.1592, 2229.1064, 2233.1023, 2245.0991, 2269.1128, 2273.0393, 2277.0576, 2279.1094, 2280.1204, 2283.1279, 2285.1169, 2292.1465, 2296.1479, 2312.0664, 2323.1892, 2326.0947, 2334.1531, 2335.1282, 2337.1343, 2369.2256, 2372.2229, 2383.9978, 2385.2129, 2392.1528, 2400.1094, 2401.1360, 2409.1914, 2411.1907, 2414.1833, 2429.1475, 2436.1699, 2437.1758, 2440.2007, 2447.2368, 2477.1301, 2495.0542, 2498.0898, 2510.2229, 2522.1980, 2524.1689, 2527.2310, 2535.2598, 2541.2075, 2543.1907, 2544.1514, 2553.1934, 2557.2300, 2560.1125, 2569.2915, 2572.0935, 2612.1936, 2686.3572, 2788.3362, 2963.6167, 3056.4641, 3058.5842, 3180.5432, 3313.5032, 3330.6311, 3347.6077, 3511.9668, 3761.1558

4. P10096 **Mass:** 36073 **Score:** 107 **Expect:** 4.8e-007 **Matches:** 28

Glyceraldehyde-3-phosphate dehydrogenase OS=Bos taurus GN=GAPDH PE=1 SV=4

Observed	Mr(expt)	Mr(calc)	ppm	Start	End	Miss	Ions	Peptide
752.3972	751.3899	751.3864	4.60	138 -	143	0	---	K.YNNTLK.I
795.4260	794.4187	794.4174	1.63	253 -	258	1	---	K.YDEIKK.V
802.4372	801.4299	801.4246	6.68	193 -	198	1	---	K.LWRDGR.G
805.4478	804.4405	804.4243	20.2	4 -	11	0	---	K.VGVNGFGR.I
811.4178	810.4105	810.4058	5.81	226 -	232	0	---	K.LTGMAFR.V + Oxidation (M)
829.4457	828.4385	828.4341	5.25	262 -	269	0	---	K.QASEGPLK.G
977.5352	976.5279	976.5342	-6.43	71 -	78	0	---	K.AITIFQER.D
1032.5870	1031.5797	1031.5876	-7.66	2 -	11	1	---	M.VKVGVNGFGR.I
1215.6246	1214.6173	1214.6408	-19.31	185 -	195	1	---	K.TVDGPSGKLWR.D
1229.6412	1228.6339	1228.6308	2.54	322 -	332	0	---	R.VVDLMVHMASK.E
1358.6704	1357.6631	1357.6734	-7.56	322 -	333	1	---	R.VVDLMVHMASKE.-
1374.6705	1373.6632	1373.6683	-3.70	322 -	333	1	---	R.VVDLMVHMASKE.- + Oxidation (M)
1390.6848	1389.6775	1389.6632	10.3	322 -	333	1	---	R.VVDLMVHMASKE.- + 2 Oxidation (M)
1461.7990	1460.7917	1460.8238	-21.97	247 -	258	2	---	R.LEKPAKYDEIKK.V
1556.7874	1555.7801	1555.8029	-14.61	233 -	246	0	---	R.VPTPNVSVVDLTCR.L
1615.8575	1614.8502	1614.8729	-14.06	71 -	84	1	---	K.AITIFQERDPANIK.W
1615.8600	1614.8527	1614.8729	-12.51	71 -	84	1	60	K.AITIFQERDPANIK.W
1697.8672	1696.8599	1696.8856	-15.14	196 -	213	1	---	R.DGRGAAQNIIPASTGAAK.A
1763.7841	1762.7768	1762.7951	-10.35	308 -	321	0	---	K.LISWYDNEFGYSNR.V
1819.8838	1818.8765	1818.8968	-11.14	144 -	160	0	---	K.IVSNASCTTNCLAPLAK.V
2213.1116	2212.1043	2212.1020	1.03	117 -	137	0	---	R.VIISAPSADAPMFVMGVNHEK.Y
2223.2205	2222.2132	2222.2093	1.77	233 -	252	1	---	R.VPTPNVSVVDLTCRLEKPAK.Y
2229.1064	2228.0991	2228.0970	0.97	117 -	137	0	---	R.VIISAPSADAPMFVMGVNHEK.Y + Oxidation (M)
2245.0991	2244.0918	2244.0919	-0.02	117 -	137	0	---	R.VIISAPSADAPMFVMGVNHEK.Y + 2 Oxidation (M)
2277.0576	2276.0503	2276.0307	8.61	85 -	105	0	---	K.WGDAGAEYVVESTGVFTTMEK.A
2369.2256	2368.2183	2368.2031	6.41	116 -	137	1	---	K.RVIISAPSADAPMFVMGVNHEK.Y
2385.2129	2384.2056	2384.1981	3.17	116 -	137	1	---	K.RVIISAPSADAPMFVMGVNHEK.Y + Oxidation (M)
3347.6077	3346.6004	3346.5679	9.72	26 -	53	0	---	K.VDIVAINDPFIDLHYMVYMFQYDSTHGK.F + Oxidation (M)

No match to: 724.2539, 725.2472, 728.2482, 730.2519, 732.2390, 733.2623, 739.3115, 742.4642, 743.4729, 745.4483, 747.4419, 754.3827, 756.3716, 758.3382, 760.3329, 764.3364, 766.3494, 767.4740, 768.3865, 770.3671, 772.4178, 779.4436, 784.3415, 787.2366, 788.4225, 790.4196, 799.2464, 808.4353, 814.4170, 819.4304, 821.4247, 822.4321, 837.4396, 840.4623, 842.5109, 854.4525, 856.5045, 858.5442, 860.4092, 863.4233, 863.9185, 869.4846, 870.5100, 872.4994, 880.4412, 904.4613, 906.4929, 908.4577, 913.5685, 919.5024, 925.5089, 927.4999, 928.5031, 940.4773, 942.4820, 950.4946, 957.5654, 960.4840, 966.4929, 969.4786, 972.5046, 974.5551, 978.5240, 981.4474, 989.2700, 991.5198, 993.5112, 1001.5782, 1007.5089, 1009.5097, 1013.4454, 1015.5150, 1016.5522, 1018.5189, 1021.5313, 1024.5028, 1025.5192, 1029.5330, 1033.5739, 1035.5203, 1045.5564, 1046.5759, 1051.5074, 1056.5415, 1057.6005, 1064.5358, 1071.5361, 1073.5377, 1079.5398, 1083.4637, 1086.4774, 1092.6000, 1092.6002, 1094.5596, 1096.5302, 1106.5431, 1110.5461, 1111.5398, 1111.5400, 1115.5790, 1118.5620, 1120.5724, 1123.5819, 1128.5537, 1132.5682, 1134.5841, 1135.5942, 1137.5565, 1143.5844, 1145.6155, 1149.5597, 1153.5929, 1158.6288, 1163.5771, 1173.6008, 1175.5867, 1177.6178, 1178.6053, 1179.5962, 1181.6432, 1192.5721, 1199.6315, 1203.5826, 1211.5984, 1216.6786, 1221.5699, 1222.5789, 1227.6271, 1228.6411, 1231.6102, 1233.6571, 1237.6270, 1238.5917, 1244.6069, 1248.5958, 1250.6042, 1255.5922, 1258.6344, 1268.6414, 1271.6490, 1275.6388, 1276.5977, 1276.6000, 1285.5864, 1294.6315, 1299.6810, 1300.6566, 1309.6340, 1320.6268, 1328.6292, 1333.7025, 1336.6038, 1338.6589, 1341.6488, 1343.6613, 1345.6910, 1349.6838, 1350.6704, 1352.6350, 1355.6870, 1357.7130, 1369.7067, 1371.7366, 1377.6100, 1377.6146, 1384.7109, 1386.7134, 1393.7169, 1399.6119, 1400.6588, 1406.7207, 1410.6736, 1411.6647, 1416.6047, 1418.6714, 1421.6632, 1425.7395, 1426.6846, 1427.7076, 1430.7102, 1432.6718, 1434.6552, 1438.6991, 1439.7164, 1443.7589, 1444.7375, 1445.7306, 1454.6836, 1472.7026, 1475.7224, 1485.6906, 1489.7172, 1490.7058, 1493.7157, 1510.7383, 1515.7393, 1523.6987, 1524.7383, 1527.7585, 1529.7466, 1530.6902, 1531.6953, 1537.7804, 1538.7736, 1539.7621, 1541.7805, 1542.8290, 1549.6758, 1553.7277, 1555.7928, 1560.7551, 1563.7314, 1568.7372, 1570.8015, 1572.8151, 1576.7402, 1577.7395, 1578.7518, 1581.8373, 1591.7852, 1600.8068, 1603.8092, 1604.8136, 1606.8130, 1608.7599, 1613.7793, 1624.7632, 1628.7653, 1633.7913, 1634.7969, 1635.7941, 1639.8157, 1643.8085, 1646.8591, 1647.8268, 1651.8206, 1653.8284, 1677.8074, 1681.8087, 1688.8287, 1690.8313, 1694.7357, 1703.8214, 1706.8364, 1707.8435, 1708.3533, 1709.3307, 1710.8229, 1715.8053, 1719.8075, 1721.8296, 1723.8743, 1724.8828, 1725.8090, 1730.8199, 1748.7931, 1757.8975, 1768.8075, 1771.9686, 1774.9059, 1777.8727, 1779.8081, 1786.8824, 1787.8207, 1790.8423, 1793.9556, 1796.8109, 1797.8339, 1800.8578, 1815.9008, 1821.8926, 1822.9185, 1826.8450, 1829.8655, 1833.8975, 1836.9054, 1843.8765, 1844.8785, 1851.9031, 1859.8494, 1862.9265, 1863.9319, 1866.8857, 1868.8972, 1879.9445, 1880.9215, 1887.9023, 1888.8057, 1890.8025, 1896.8954, 1901.0060, 1906.8872, 1908.8663, 1913.8895, 1916.9475, 1924.9314, 1925.9336, 1928.0060, 1931.9956, 1932.9833, 1968.8563, 1984.9392, 2008.0485, 2008.9869, 2009.9747, 2012.9506, 2015.9661, 2020.9644, 2030.0151, 2030.4648, 2046.9839, 2047.5186, 2047.9097, 2051.9133, 2059.0122, 2064.9746, 2070.9209, 2077.0454, 2082.9409, 2085.9888, 2090.9844, 2097.9778, 2103.1526, 2108.9897, 2118.8926, 2140.0432, 2142.0310, 2142.9895, 2152.0510, 2155.0667, 2159.0752, 2160.0703, 2161.0813, 2166.0911, 2167.0676, 2174.0425, 2178.0034, 2181.0369, 2184.0767, 2189.9133, 2194.9761, 2199.0986, 2203.0464, 2205.9102, 2208.9729, 2211.1077, 2214.1145, 2218.1018, 2221.1035, 2225.1592, 2233.1023, 2269.1128, 2273.0393, 2279.1094, 2280.1204, 2283.1279, 2285.1169, 2292.1465, 2296.1479, 2312.0664, 2323.1892, 2326.0947, 2334.1531, 2335.1282, 2337.1343, 2372.2229, 2383.9978, 2392.1528, 2400.1094, 2401.1360, 2409.1914, 2411.1907, 2414.1833, 2429.1475, 2436.1699, 2437.1758, 2440.2007, 2447.2368, 2477.1301, 2495.0542, 2498.0898, 2510.2229, 2522.1980, 2524.1689, 2527.2310, 2535.2598, 2541.2075, 2543.1907, 2544.1514, 2553.1934, 2557.2300, 2560.1125, 2569.2915, 2572.0935, 2612.1936, 2686.3572, 2788.3362, 2963.6167, 3056.4641, 3058.5842, 3180.5432, 3313.5032, 3330.6311, 3511.9668, 3761.1558

5. Q2HJ60 **Mass:** 36041 **Score:** 50 **Expect:** 0.24 **Matches:** 13

Heterogeneous nuclear ribonucleoproteins A2/B1 OS=Bos taurus GN=HNRNPA2B1 PE=2 SV=1

Observed	Mr(expt)	Mr(calc)	ppm	Start	End	Miss	Ions	Peptide
1013.4454	1012.4381	1012.4363	1.82	192 -	201	0	---	R.GGNFGFGDSR.G
1221.5699	1220.5626	1220.5455	14.0	179 -	188	0	---	R.QEMQEVQSSR.S
1377.6100	1376.6027	1376.6222	-14.14	202 -	216	0	38	R.GGGGNFGPGPGSNFR.G
1377.6146	1376.6073	1376.6222	-10.80	202 -	216	0	---	R.GGGGNFGPGPGSNFR.G
1410.6736	1409.6663	1409.6800	-9.70	162 -	173	0	---	K.YHTINGHNAEVR.K
1538.7736	1537.7663	1537.7749	-5.61	162 -	174	1	---	K.YHTINGHNAEVRK.A
1647.8268	1646.8195	1646.8232	-2.26	35 -	48	2	---	K.LTDCVVMRDPASKR.S
1851.9031	1850.8958	1850.8588	20.0	141 -	156	1	---	K.RGFGFVTFDDHDPVDK.I
1879.9445	1878.9372	1878.9588	-11.48	102 -	117	1	---	K.LFVGGIKEDTEEHHLR.D
2008.0485	2007.0412	2007.0538	-6.24	101 -	117	2	---	K.KLFVGGIKEDTEEHHLR.D
2189.9133	2188.9060	2188.8981	3.61	314 -	338	0	---	R.NMGGPYGGGNYGPGGSGGSGGYGGR.S
2205.9102	2204.9029	2204.8930	4.48	314 -	338	0	---	R.NMGGPYGGGNYGPGGSGGSGGYGGR.S + Oxidation (M)
2495.0542	2494.0469	2494.0323	5.85	227 -	254	0	---	R.GFGDGYNGYGGGPGGGNFGGSPGYGGGR.G

No match to: 724.2539, 725.2472, 728.2482, 730.2519, 732.2390, 733.2623, 739.3115, 742.4642, 743.4729, 745.4483, 747.4419, 752.3972, 754.3827, 756.3716, 758.3382, 760.3329, 764.3364, 766.3494, 767.4740, 768.3865, 770.3671, 772.4178, 779.4436, 784.3415, 787.2366, 788.4225, 790.4196, 795.4260, 799.2464, 802.4372, 805.4478, 808.4353, 811.4178, 814.4170, 819.4304, 821.4247, 822.4321, 829.4457, 837.4396, 840.4623, 842.5109, 854.4525, 856.5045, 858.5442, 860.4092, 863.4233, 863.9185, 869.4846, 870.5100, 872.4994, 880.4412, 904.4613, 906.4929, 908.4577, 913.5685, 919.5024, 925.5089, 927.4999, 928.5031, 940.4773, 942.4820, 950.4946, 957.5654, 960.4840, 966.4929, 969.4786, 972.5046, 974.5551, 977.5352, 978.5240, 981.4474, 989.2700, 991.5198, 993.5112, 1001.5782, 1007.5089, 1009.5097, 1015.5150, 1016.5522, 1018.5189, 1021.5313, 1024.5028, 1025.5192, 1029.5330, 1032.5870, 1033.5739, 1035.5203, 1045.5564, 1046.5759, 1051.5074, 1056.5415, 1057.6005, 1064.5358, 1071.5361, 1073.5377, 1079.5398, 1083.4637, 1086.4774, 1092.6000, 1092.6002, 1094.5596, 1096.5302, 1106.5431, 1110.5461, 1111.5398, 1111.5400, 1115.5790, 1118.5620, 1120.5724, 1123.5819, 1128.5537, 1132.5682, 1134.5841, 1135.5942, 1137.5565, 1143.5844, 1145.6155, 1149.5597, 1153.5929, 1158.6288, 1163.5771, 1173.6008, 1175.5867, 1177.6178, 1178.6053, 1179.5962, 1181.6432, 1192.5721, 1199.6315, 1203.5826, 1211.5984, 1215.6246, 1216.6786, 1222.5789, 1227.6271, 1228.6411, 1229.6412, 1231.6102, 1233.6571, 1237.6270, 1238.5917, 1244.6069, 1248.5958, 1250.6042, 1255.5922, 1258.6344, 1268.6414, 1271.6490, 1275.6388, 1276.5977, 1276.6000, 1285.5864, 1294.6315, 1299.6810, 1300.6566, 1309.6340, 1320.6268, 1328.6292, 1333.7025, 1336.6038, 1338.6589, 1341.6488, 1343.6613, 1345.6910, 1349.6838, 1350.6704, 1352.6350, 1355.6870, 1357.7130, 1358.6704, 1369.7067, 1371.7366, 1374.6705, 1384.7109, 1386.7134, 1390.6848, 1393.7169, 1399.6119, 1400.6588, 1406.7207, 1411.6647, 1416.6047, 1418.6714, 1421.6632, 1425.7395, 1426.6846, 1427.7076, 1430.7102, 1432.6718, 1434.6552, 1438.6991, 1439.7164, 1443.7589, 1444.7375, 1445.7306, 1454.6836, 1461.7990, 1472.7026, 1475.7224, 1485.6906, 1489.7172, 1490.7058, 1493.7157, 1510.7383, 1515.7393, 1523.6987, 1524.7383, 1527.7585, 1529.7466, 1530.6902, 1531.6953, 1537.7804, 1539.7621, 1541.7805, 1542.8290, 1549.6758, 1553.7277, 1555.7928, 1556.7874, 1560.7551, 1563.7314, 1568.7372, 1570.8015, 1572.8151, 1576.7402, 1577.7395, 1578.7518, 1581.8373, 1591.7852, 1600.8068, 1603.8092, 1604.8136, 1606.8130, 1608.7599, 1613.7793, 1615.8575, 1615.8600, 1624.7632, 1628.7653, 1633.7913, 1634.7969, 1635.7941, 1639.8157, 1643.8085, 1646.8591, 1651.8206, 1653.8284, 1677.8074, 1681.8087, 1688.8287, 1690.8313, 1694.7357, 1697.8672, 1703.8214, 1706.8364, 1707.8435, 1708.3533, 1709.3307, 1710.8229, 1715.8053, 1719.8075, 1721.8296, 1723.8743, 1724.8828, 1725.8090, 1730.8199, 1748.7931, 1757.8975, 1763.7841, 1768.8075, 1771.9686, 1774.9059, 1777.8727, 1779.8081, 1786.8824, 1787.8207, 1790.8423, 1793.9556, 1796.8109, 1797.8339, 1800.8578, 1815.9008, 1819.8838, 1821.8926, 1822.9185, 1826.8450, 1829.8655, 1833.8975, 1836.9054, 1843.8765, 1844.8785, 1859.8494, 1862.9265, 1863.9319, 1866.8857, 1868.8972, 1880.9215, 1887.9023, 1888.8027, 1890.8025, 1896.8954, 1901.0060, 1906.8872, 1908.8663, 1913.8895, 1916.9475, 1924.9314, 1925.9336, 1928.0060, 1931.9956, 1932.9833, 1968.8563, 1984.9392, 2008.9869, 2009.9747, 2012.9506, 2015.9661, 2020.9644, 2030.0151, 2030.4648, 2046.9839, 2047.5186, 2047.9097, 2051.9133, 2059.0122, 2064.9746, 2070.9209, 2077.0454, 2082.9409, 2085.9888, 2090.9844, 2097.9778, 2103.1526, 2108.9897, 2118.8926, 2140.0432, 2142.0310, 2142.9895, 2152.0510, 2155.0667, 2159.0752, 2160.0703, 2161.0813, 2166.0911, 2167.0676, 2174.0425, 2178.0034, 2181.0369, 2184.0767, 2194.9761, 2199.0986, 2203.0464, 2208.9729, 2211.1077, 2213.1116, 2214.1145, 2218.1018, 2221.1035, 2223.2205, 2225.1592, 2229.1064, 2233.1023, 2245.0991, 2269.1128, 2273.0393, 2277.0576, 2279.1094, 2280.1204, 2283.1279, 2285.1169, 2292.1465, 2296.1479, 2312.0664, 2323.1892, 2326.0947, 2334.1531, 2335.1282, 2337.1343, 2369.2256, 2372.2229, 2383.9978, 2385.2129, 2392.1528, 2400.1094, 2401.1360, 2409.1914, 2411.1907, 2414.1833, 2429.1475, 2436.1699, 2437.1758, 2440.2007, 2447.2368, 2477.1301, 2498.0898, 2510.2229, 2522.1980, 2524.1689, 2527.2310, 2535.2598, 2541.2075, 2543.1907, 2544.1514, 2553.1934, 2557.2300, 2560.1125, 2569.2915, 2572.0935, 2612.1936, 2686.3572, 2788.3362, 2963.6167, 3056.4641, 3058.5842, 3180.5432, 3313.5032, 3330.6311, 3347.6077, 3511.9668, 3761.1558

6. E1BB48 **Mass:** 65458 **Score:** 34 **Expect:** 9 **Matches:** 28

Polypeptide N-acetylgalactosaminyltransferase OS=Bos taurus GN=GALNT2 PE=3 SV=2

Observed	Mr(expt)	Mr(calc)	ppm	Start	End	Miss	Ions	Peptide
842.5109	841.5036	841.4923	13.5	357 -	363	1	---	R.VGHVFRK.Q
906.4929	905.4856	905.4793	6.95	421 -	427	0	---	K.LNCKPFK.W
1229.6412	1228.6339	1228.6636	-24.16	207 -	218	2	---	R.SRVRGADAAQAK.V
1244.6069	1243.5996	1243.5721	22.2	43 -	52	0	---	K.EDWNDIDPIK.K
1258.6344	1257.6271	1257.6142	10.3	394 -	404	0	---	K.NFYYAAVPSAR.N
1345.6910	1344.6837	1344.6932	-7.05	112 -	122	2	---	K.LRMDRAVPDTR.H + Oxidation (M)
1358.6704	1357.6631	1357.6483	11.0	492 -	502	0	---	K.HMDLCLTVVDR.A
1374.6705	1373.6632	1373.6432	14.6	492 -	502	0	---	K.HMDLCLTVVDR.A + Oxidation (M)
1411.6647	1410.6574	1410.6887	-22.19	303 -	315	0	---	K.TPMIAGGLFVMDK.F + 2 Oxidation (M)
1490.7058	1489.6985	1489.7056	-4.72	510 -	521	2	---	K.LQGCRENDSRQK.W
1527.7585	1526.7512	1526.7623	-7.28	533 -	546	1	---	R.HVGSNLCLDSRAAK.T
1615.8575	1614.8502	1614.8478	1.52	520 -	532	2	---	R.QKWEQIEGNSKLR.H
1615.8600	1614.8527	1614.8478	3.07	520 -	532	2	4	R.QKWEQIEGNSKLR.H
1624.7632	1623.7559	1623.7576	-1.02	282 -	293	2	---	K.WDYMTPEQRRSR.Q
1653.8284	1652.8211	1652.7940	16.4	104 -	116	2	---	K.FNQVESDKLRMDR.A + Oxidation (M)
1688.8287	1687.8214	1687.8386	-10.16	489 -	502	1	---	K.SVKHMDLCLTVVDR.A + Oxidation (M)
1721.8296	1720.8223	1720.8322	-5.73	364 -	379	0	---	K.QHPYTFPGGSGTVFAR.N
1768.8075	1767.8002	1767.8362	-20.36	380 -	393	2	---	R.NTRRAAEVWMDEYK.N
1779.8081	1778.8008	1778.7716	16.4	219 -	232	0	---	K.VLTFLDSHCECNER.W
1901.0060	1899.9987	1899.9585	21.2	503 -	519	2	---	R.APGSLIKLQGCRENDSR.Q
1916.9475	1915.9402	1915.9462	-3.13	547 -	564	0	---	K.TGGLSVEVCGPALSQQWK.F
2082.9409	2081.9336	2081.9766	-20.65	94 -	111	2	---	R.SGQDPYARNKFNQVESDK.L
2184.0767	2183.0694	2183.0694	-0.01	76 -	93	2	---	K.VRWRDFNQEAYVGGTMVR.S
2208.9729	2207.9656	2207.9326	15.0	324 -	341	0	---	K.YDMMMDVWGGENLEISFR.V + Oxidation (M)
2221.1035	2220.0962	2220.1189	-10.19	363 -	382	2	---	R.KQHPYTFPGGSGTVFARNTR.R
2369.2256	2368.2183	2368.2038	6.15	421 -	438	1	---	K.LNCKPFKWYLENVYPELR.V
2409.1914	2408.1841	2408.1796	1.87	303 -	323	1	---	K.TPMIAGGLFVMDKFYFEELGK.Y + Oxidation (M)
2477.1301	2476.1228	2476.1077	6.09	80 -	101	1	---	R.DFNQEAYVGGTMVRSGQDPYAR.N + Oxidation (M)

No match to: 724.2539, 725.2472, 728.2482, 730.2519, 732.2390, 733.2623, 739.3115, 742.4642, 743.4729, 745.4483, 747.4419, 752.3972, 754.3827, 756.3716, 758.3382, 760.3329, 764.3364, 766.3494, 767.4740, 768.3865, 770.3671, 772.4178, 779.4436, 784.3415, 787.2366, 788.4225, 790.4196, 795.4260, 799.2464, 802.4372, 805.4478, 808.4353, 811.4178, 814.4170, 819.4304, 821.4247, 822.4321, 829.4457, 837.4396, 840.4623, 854.4525, 856.5045, 858.5442, 860.4092, 863.4233, 863.9185, 869.4846, 870.5100, 872.4994, 880.4412, 904.4613, 908.4577, 913.5685, 919.5024, 925.5089, 927.4999, 928.5031, 940.4773, 942.4820, 950.4946, 957.5654, 960.4840, 966.4929, 969.4786, 972.5046, 974.5551, 977.5352, 978.5240, 981.4474, 989.2700, 991.5198, 993.5112, 1001.5782, 1007.5089, 1009.5097, 1013.4454, 1015.5150, 1016.5522, 1018.5189, 1021.5313, 1024.5028, 1025.5192, 1029.5330, 1032.5870, 1033.5739, 1035.5203, 1045.5564, 1046.5759, 1051.5074, 1056.5415, 1057.6005, 1064.5358, 1071.5361, 1073.5377, 1079.5398, 1083.4637, 1086.4774, 1092.6000, 1092.6002, 1094.5596, 1096.5302, 1106.5431, 1110.5461, 1111.5398, 1111.5400, 1115.5790, 1118.5620, 1120.5724, 1123.5819, 1128.5537, 1132.5682,

1134.5841, 1135.5942, 1137.5565, 1143.5844, 1145.6155, 1149.5597, 1153.5929, 1158.6288, 1163.5771, 1173.6008, 1175.5867, 1177.6178, 1178.6053, 1179.5962, 1181.6432, 1192.5721, 1199.6315, 1203.5826, 1211.5984, 1215.6246, 1216.6786, 1221.5699, 1222.5789, 1227.6271, 1228.6411, 1231.6102, 1233.6571, 1237.6270, 1238.5917, 1248.5958, 1250.6042, 1255.5922, 1268.6414, 1271.6490, 1275.6388, 1276.5977, 1276.6000, 1285.5864, 1294.6315, 1299.6810, 1300.6566, 1309.6340, 1320.6268, 1328.6292, 1333.7025, 1336.6038, 1338.6589, 1341.6488, 1343.6613, 1349.6838, 1350.6704, 1352.6350, 1355.6870, 1357.7130, 1369.7067, 1371.7366, 1377.6100, 1377.6146, 1384.7109, 1386.7134, 1390.6848, 1393.7169, 1399.6119, 1400.6588, 1406.7207, 1410.6736, 1416.6047, 1418.6714, 1421.6632, 1425.7395, 1426.6846, 1427.7076, 1430.7102, 1432.6718, 1434.6552, 1438.6991, 1439.7164, 1443.7589, 1444.7375, 1445.7306, 1454.6836, 1461.7990, 1472.7026, 1475.7224, 1485.6906, 1489.7172, 1493.7157, 1510.7383, 1515.7393, 1523.6987, 1524.7383, 1529.7466, 1530.6902, 1531.6953, 1537.7804, 1538.7736, 1539.7621, 1541.7805, 1542.8290, 1549.6758, 1553.7277, 1555.7928, 1556.7874, 1560.7551, 1563.7314, 1568.7372, 1570.8015, 1572.8151, 1576.7402, 1577.7395, 1578.7518, 1581.8373, 1591.7852, 1600.8068, 1603.8092, 1604.8136, 1606.8130, 1608.7599, 1613.7793, 1628.7653, 1633.7913, 1634.7969, 1635.7941, 1639.8157, 1643.8085, 1646.8591, 1647.8268, 1651.8206, 1677.8074, 1681.8087, 1690.8313, 1694.7357, 1697.8672, 1703.8214, 1706.8364, 1707.8435, 1708.3533, 1709.3307, 1710.8229, 1715.8053, 1719.8075, 1723.8743, 1724.8828, 1725.8090, 1730.8199, 1748.7931, 1757.8975, 1763.7841, 1771.9686, 1774.9059, 1777.8727, 1786.8824, 1787.8207, 1790.8423, 1793.9556, 1796.8109, 1797.8339, 1800.8578, 1815.9008, 1819.8838, 1821.8926, 1822.9185, 1826.8450, 1829.8655, 1833.8975, 1836.9054, 1843.8765, 1844.8785, 1851.9031, 1859.8494, 1862.9265, 1863.9319, 1866.8857, 1868.8972, 1879.9445, 1880.9215, 1887.9023, 1888.8057, 1890.8025, 1896.8954, 1906.8872, 1908.8663, 1913.8895, 1924.9314, 1925.9336, 1928.0060, 1931.9956, 1932.9833, 1968.8563, 1984.9392, 2008.0485, 2008.9869, 2009.9747, 2012.9506, 2015.9661, 2020.9644, 2030.0151, 2030.4648, 2046.9839, 2047.5186, 2047.9097, 2051.9133, 2059.0122, 2064.9746, 2070.9209, 2077.0454, 2085.9888, 2090.9844, 2097.9778, 2103.1526, 2108.9897, 2118.8926, 2140.0432, 2142.0310, 2142.9895, 2152.0510, 2155.0667, 2159.0752, 2160.0703, 2161.0813, 2166.0911, 2167.0676, 2174.0425, 2178.0034, 2181.0369, 2189.9133, 2194.9761, 2199.0986, 2203.0464, 2205.9102, 2211.1077, 2213.1116, 2214.1145, 2218.1018, 2223.2205, 2225.1592, 2229.1064, 2233.1023, 2245.0991, 2269.1128, 2273.0393, 2277.0576, 2279.1094, 2280.1204, 2283.1279, 2285.1169, 2292.1465, 2296.1479, 2312.0664, 2323.1892, 2326.0947, 2334.1531, 2335.1282, 2337.1343, 2372.2229, 2383.9978, 2385.2129, 2392.1528, 2400.1094, 2401.1360, 2411.1907, 2414.1833, 2429.1475, 2436.1699, 2437.1758, 2440.2007, 2447.2368, 2495.0542, 2498.0898, 2510.2229, 2522.1980, 2524.1689, 2527.2310, 2535.2598, 2541.2075, 2543.1907, 2544.1514, 2553.1934, 2557.2300, 2560.1125, 2569.2915, 2572.0935, 2612.1936, 2686.3572, 2788.3362, 2963.6167, 3056.4641, 3058.5842, 3180.5432, 3313.5032, 3330.6311, 3347.6077, 3511.9668, 3761.1558

7. P02687 **Mass:** 18312 **Score:** 26 **Expect:** 61 **Matches:** 11

Myelin basic protein OS=Bos taurus GN=MBP PE=1 SV=1

Observed	Mr(expt)	Mr(calc)	ppm	Start	End	Miss	Ions	Peptide
811.4178	810.4105	810.4236	-16.11	97 -	104	0	---	R.TPPPSQGK.G
1237.6270	1236.6197	1236.6364	-13.45	42 -	52	2	---	R.FFGSDRGAPKR.G
1537.7804	1536.7731	1536.7354	24.5	10 -	23	1	---	R.SKYLASASTMDHAR.H
1553.7277	1552.7204	1552.7304	-6.40	10 -	23	1	---	R.SKYLASASTMDHAR.H + Oxidation (M)
1800.8578	1799.8505	1799.8380	6.97	113 -	129	0	---	R.FSWGAEGQKPGFGYGGR.A
2030.0151	2029.0078	2028.9952	6.23	12 -	29	1	---	K.YLASASTMDHARHGFLPR.H
2245.0991	2244.0918	2244.1222	-13.53	10 -	29	2	---	R.SKYLASASTMDHARHGFLPR.H
2323.1892	2322.1819	2322.1552	11.5	12 -	31	2	---	K.YLASASTMDHARHGFLPRHR.D
2414.1833	2413.1760	2413.1927	-6.92	107 -	129	1	---	R.GLSLSRFSWGAEGQKPGFGYGGR.A
2686.3572	2685.3499	2685.3888	-14.48	74 -	96	1	---	K.AQGHRPQDENPVVHFFKNIVTPR.T
2788.3362	2787.3289	2787.3154	4.87	113 -	138	2	---	R.FSWGAEGQKPGFGYGGRASDYKSAHK.G

No match to: 724.2539, 725.2472, 728.2482, 730.2519, 732.2390, 733.2623, 739.3115, 742.4642, 743.4729, 745.4483, 747.4419, 752.3972, 754.3827, 756.3716, 758.3382, 760.3329, 764.3364, 766.3494, 767.4740, 768.3865, 770.3671, 772.4178, 779.4436, 784.3415, 787.2366, 788.4225, 790.4196, 795.4260, 799.2464, 802.4372, 805.4478, 808.4353, 814.4170, 819.4304, 821.4247, 822.4321, 829.4457, 837.4396, 840.4623, 842.5109, 854.4525, 856.5045, 858.5442, 860.4092, 863.4233, 863.9185, 869.4846, 870.5100, 872.4994, 880.4412, 904.4613, 906.4929, 908.4577, 913.5685, 919.5024, 925.5089, 927.4999, 928.5031, 940.4773, 942.4820, 950.4946, 957.5654, 960.4840, 966.4929, 969.4786, 972.5046, 974.5551, 977.5352, 978.5240, 981.4474, 989.2700, 991.5198, 993.5112, 1001.5782, 1007.5089, 1009.5097, 1013.4454, 1015.5150, 1016.5522, 1018.5189, 1021.5313, 1024.5028, 1025.5192, 1029.5330, 1032.5870, 1033.5739, 1035.5203, 1045.5564, 1046.5759, 1051.5074, 1056.5415, 1057.6005, 1064.5358, 1071.5361, 1073.5377, 1079.5398, 1083.4637, 1086.4774, 1092.6000, 1092.6002, 1094.5596, 1096.5302, 1106.5431, 1110.5461, 1111.5398, 1111.5400, 1115.5790, 1118.5620, 1120.5724, 1123.5819, 1128.5537, 1132.5682, 1134.5841, 1135.5942, 1137.5565, 1143.5844, 1145.6155, 1149.5597, 1153.5929, 1158.6288, 1163.5771, 1173.6008, 1175.5867, 1177.6178, 1178.6053, 1179.5962, 1181.6432, 1192.5721, 1199.6315, 1203.5826, 1211.5984, 1215.6246, 1216.6786, 1221.5699, 1222.5789, 1227.6271, 1228.6411, 1229.6412, 1231.6102, 1233.6571, 1238.5917, 1244.6069, 1248.5958, 1250.6042, 1255.5922, 1258.6344, 1268.6414, 1271.6490, 1275.6388, 1276.5977, 1276.6000, 1285.5864, 1294.6315, 1299.6810, 1300.6566, 1309.6340, 1320.6268, 1328.6292, 1333.7025, 1336.6038, 1338.6589, 1341.6488, 1343.6613, 1345.6910, 1349.6838, 1350.6704, 1352.6350, 1355.6870, 1357.7130, 1358.6704, 1369.7067, 1371.7366, 1374.6705, 1377.6100, 1377.6146, 1384.7109, 1386.7134, 1390.6848, 1393.7169, 1399.6119, 1400.6588, 1406.7207, 1410.6736, 1411.6647, 1416.6047, 1418.6714, 1421.6632, 1425.7395, 1426.6846, 1427.7076, 1430.7102, 1432.6718, 1434.6552, 1438.6991, 1439.7164, 1443.7589, 1444.7375, 1445.7306, 1454.6836, 1461.7990, 1472.7026, 1475.7224, 1485.6906, 1489.7172, 1490.7058, 1493.7157, 1510.7383, 1515.7393, 1523.6987, 1524.7383, 1527.7585, 1529.7466, 1530.6902, 1531.6953, 1538.7736, 1539.7621, 1541.7805, 1542.8290, 1549.6758, 1555.7928, 1556.7874, 1560.7551, 1563.7314, 1568.7372, 1570.8015, 1572.8151, 1576.7402, 1577.7395, 1578.7518, 1581.8373, 1591.7852, 1600.8068, 1603.8092, 1604.8136, 1606.8130, 1608.7599, 1613.7793, 1615.8575, 1615.8600, 1624.7632, 1628.7653, 1633.7913, 1634.7969, 1635.7941, 1639.8157, 1643.8085, 1646.8591, 1647.8268, 1651.8206, 1653.8284, 1677.8074, 1681.8087, 1688.8287, 1690.8313, 1694.7357, 1697.8672, 1703.8214, 1706.8364, 1707.8435, 1708.3533, 1709.3307, 1710.8229, 1715.8053, 1719.8075, 1721.8296, 1723.8743, 1724.8828, 1725.8090, 1730.8199, 1748.7931, 1757.8975, 1763.7841, 1768.8075, 1771.9686, 1774.9059, 1777.8727, 1779.8081, 1786.8824, 1787.8207, 1790.8423, 1793.9556, 1796.8109, 1797.8339, 1815.9008, 1819.8838, 1821.8926, 1822.9185, 1826.8450, 1829.8655, 1833.8975, 1836.9054, 1843.8765, 1844.8785, 1851.9031, 1859.8494, 1862.9265, 1863.9319, 1866.8857, 1868.8972, 1879.9445, 1880.9215, 1887.9023, 1888.8057, 1890.8025, 1896.8954, 1901.0060, 1906.8872, 1908.8663, 1913.8895, 1916.9475, 1924.9314, 1925.9336, 1928.0060, 1931.9956, 1932.9833, 1968.8563, 1984.9392, 2008.0485, 2008.9869, 2009.9747, 2012.9506, 2015.9661, 2020.9644, 2030.4648, 2046.9839, 2047.5186, 2047.9097, 2051.9133, 2059.0122, 2064.9746, 2070.9209, 2077.0454, 2082.9409, 2085.9888, 2090.9844, 2097.9778, 2103.1526, 2108.9897, 2118.8926, 2140.0432, 2142.0310, 2142.9895, 2152.0510, 2155.0667, 2159.0752, 2160.0703, 2161.0813, 2166.0911, 2167.0676, 2174.0425, 2178.0034, 2181.0369, 2184.0767, 2189.9133, 2194.9761, 2199.0986, 2203.0464, 2205.9102, 2208.9729, 2211.1077, 2213.1116, 2214.1145, 2218.1018, 2221.1035, 2223.2205, 2225.1592, 2229.1064, 2233.1023, 2269.1128, 2273.0393, 2277.0576, 2279.1094, 2280.1204, 2283.1279, 2285.1169, 2292.1465, 2296.1479, 2312.0664, 2326.0947, 2334.1531, 2335.1282, 2337.1343, 2369.2256, 2372.2229, 2383.9978, 2385.2129, 2392.1528, 2400.1094, 2401.1360, 2409.1914, 2411.1907, 2429.1475, 2436.1699, 2437.1758, 2440.2007, 2447.2368, 2477.1301, 2495.0542, 2498.0898, 2510.2229, 2522.1980, 2524.1689, 2527.2310, 2535.2598, 2541.2075, 2543.1907, 2544.1514, 2553.1934, 2557.2300, 2560.1125, 2569.2915, 2572.0935, 2612.1936, 2963.6167, 3056.4641, 3058.5842, 3180.5432, 3313.5032, 3330.6311, 3347.6077, 3511.9668, 3761.1558

8. F1N0N9 **Mass:** 36314 **Score:** 26 **Expect:** 62 **Matches:** 12

Uncharacterized protein OS=Bos taurus PE=4 SV=1

Observed	Mr(expt)	Mr(calc)	ppm	Start	End	Miss	Ions	Peptide
752.3972	751.3899	751.3752	19.5	144 -	149	0	---	K.YDLDVK.S
919.5024	918.4952	918.5175	-24.27	172 -	179	0	---	K.NYPVVSIK.D
1328.6292	1327.6219	1327.5932	21.6	157 -	167	0	---	R.YTPEQTSDPYK.S
1349.6838	1348.6765	1348.7099	-24.74	312 -	322	1	---	K.SFSEQRNIQIK.-
1604.8136	1603.8063	1603.7664	24.9	71 -	84	0	---	R.EAMHIGAEVYNNLK.N + Oxidation (M)
1615.8575	1614.8502	1614.8803	-18.64	20 -	33	0	---	R.GVLYLHIADMLNLK.V + Oxidation (M)
1615.8600	1614.8527	1614.8803	-17.09	20 -	33	0	16	R.GVLYLHIADMLNLK.V + Oxidation (M)
1646.8591	1645.8518	1645.8788	-16.37	94 -	109	1	---	K.DATNVGVKGNFLLGNK.E
1771.9686	1770.9613	1770.9814	-11.35	19 -	33	1	---	R.RGVLYLHIADMLNLK.V + Oxidation (M)
1800.8578	1799.8505	1799.8149	19.8	250 -	265	0	---	R.WGVMVSYNSGETEGIR.I + Oxidation (M)
2012.9506	2011.9433	2011.9487	-2.66	150 -	167	1	---	K.SPSGLSRYTPEQTSDPYK.S
2181.0369	2180.0296	2180.0075	10.1	180 -	196	1	---	K.DLFNPNDWENWQKFTAR.T

No match to: 724.2539, 725.2472, 728.2482, 730.2519, 732.2390, 733.2623, 739.3115, 742.4642, 743.4729, 745.4483, 747.4419, 754.3827, 756.3716, 758.3382, 760.3329, 764.3364, 766.3494, 767.4740, 768.3865, 770.3671, 772.4178, 779.4436, 784.3415, 787.2366, 788.4225, 790.4196, 795.4260, 799.2464, 802.4372, 805.4478, 808.4353, 811.4178, 814.4170, 819.4304, 821.4247, 822.4321, 829.4457, 837.4396, 840.4623, 842.5109, 854.4525, 856.5045, 858.5442, 860.4092, 863.4233, 863.9185, 869.4846, 870.5100, 872.4994, 880.4412,

904.4613, 906.4929, 908.4577, 913.5685, 925.5089, 927.4999, 928.5031, 940.4773, 942.4820, 950.4946, 957.5654, 960.4840, 966.4929, 969.4786, 972.5046, 974.5551, 977.5352, 978.5240, 981.4474, 989.2700, 991.5198, 993.5112, 1001.5782, 1007.5089, 1009.5097, 1013.4454, 1015.5150, 1016.5522, 1018.5189, 1021.5313, 1024.5028, 1025.5192, 1029.5330, 1032.5870, 1033.5739, 1035.5203, 1045.5564, 1046.5759, 1051.5074, 1056.5415, 1057.6005, 1064.5358, 1071.5361, 1073.5377, 1079.5398, 1083.4637, 1086.4774, 1092.6000, 1092.6002, 1094.5596, 1096.5302, 1106.5431, 1110.5461, 1111.5398, 1111.5400, 1115.5790, 1118.5620, 1120.5724, 1123.5819, 1128.5537, 1132.5682, 1134.5841, 1135.5942, 1137.5565, 1143.5844, 1145.6155, 1149.5597, 1153.5929, 1158.6288, 1163.5771, 1173.6008, 1175.5867, 1177.6178, 1178.6053, 1179.5962, 1181.6432, 1192.5721, 1199.6315, 1203.5826, 1211.5984, 1215.6246, 1216.6786, 1221.5699, 1222.5789, 1227.6271, 1228.6411, 1229.6412, 1231.6102, 1233.6571, 1237.6270, 1238.5917, 1244.6069, 1248.5958, 1250.6042, 1255.5922, 1258.6344, 1268.6414, 1271.6490, 1275.6388, 1276.5977, 1276.6000, 1285.5864, 1294.6315, 1299.6810, 1300.6566, 1309.6340, 1320.6268, 1333.7025, 1336.6038, 1338.6589, 1341.6488, 1343.6613, 1345.6910, 1350.6704, 1352.6350, 1355.6870, 1357.7130, 1358.6704, 1369.7067, 1371.7366, 1374.6705, 1377.6100, 1377.6146, 1384.7109, 1386.7134, 1390.6848, 1393.7169, 1399.6119, 1400.6588, 1406.7207, 1410.6736, 1411.6647, 1416.6047, 1418.6714, 1421.6632, 1425.7395, 1426.6846, 1427.7076, 1430.7102, 1432.6718, 1434.6552, 1438.6991, 1439.7164, 1443.7589, 1444.7375, 1445.7306, 1454.6836, 1461.7990, 1472.7026, 1475.7224, 1485.6906, 1489.7172, 1490.7058, 1493.7157, 1510.7383, 1515.7393, 1523.6987, 1524.7383, 1527.7585, 1529.7466, 1530.6902, 1531.6953, 1537.7804, 1538.7736, 1539.7621, 1541.7805, 1542.8290, 1549.6758, 1553.7277, 1555.7928, 1556.7874, 1560.7551, 1563.7314, 1568.7372, 1570.8015, 1572.8151, 1576.7402, 1577.7395, 1578.7518, 1581.8373, 1591.7852, 1600.8068, 1603.8092, 1606.8130, 1608.7599, 1613.7793, 1624.7632, 1628.7653, 1633.7913, 1634.7969, 1635.7941, 1639.8157, 1643.8085, 1647.8268, 1651.8206, 1653.8284, 1677.8074, 1681.8087, 1688.8287, 1690.8313, 1694.7357, 1697.8672, 1703.8214, 1706.8364, 1707.8435, 1708.3533, 1709.3307, 1710.8229, 1715.8053, 1719.8075, 1721.8296, 1723.8743, 1724.8828, 1725.8090, 1730.8199, 1748.7931, 1757.8975, 1763.7841, 1768.8075, 1774.9059, 1777.8727, 1779.8081, 1786.8824, 1787.8207, 1790.8423, 1793.9556, 1796.8109, 1797.8339, 1815.9008, 1819.8838, 1821.8926, 1822.9185, 1826.8450, 1829.8655, 1833.8975, 1836.9054, 1843.8765, 1844.8785, 1851.9031, 1859.8494, 1862.9265, 1863.9319, 1866.8857, 1868.8972, 1879.9445, 1880.9215, 1887.9023, 1888.8057, 1890.8025, 1896.8954, 1901.0060, 1906.8872, 1908.8663, 1913.8895, 1916.9475, 1924.9314, 1925.9336, 1928.0060, 1931.9956, 1932.9833, 1968.8563, 1984.9392, 2008.0485, 2008.9869, 2009.9747, 2015.9661, 2020.9644, 2030.0151, 2030.4648, 2046.9839, 2047.5186, 2047.9097, 2051.9133, 2059.0122, 2064.9746, 2070.9209, 2077.0454, 2082.9409, 2085.9888, 2090.9844, 2097.9778, 2103.1526, 2108.9897, 2118.8926, 2140.0432, 2142.0310, 2142.9895, 2152.0510, 2155.0667, 2159.0752, 2160.0703, 2161.0813, 2166.0911, 2167.0676, 2174.0425, 2178.0034, 2184.0767, 2189.9133, 2194.9761, 2199.0986, 2203.0464, 2205.9102, 2208.9729, 2211.1077, 2213.1116, 2214.1145, 2218.1018, 2221.1035, 2223.2205, 2225.1592, 2229.1064, 2233.1023, 2245.0991, 2269.1128, 2273.0393, 2277.0576, 2279.1094, 2280.1204, 2283.1279, 2285.1169, 2292.1465, 2296.1479, 2312.0664, 2323.1892, 2326.0947, 2334.1531, 2335.1282, 2337.1343, 2369.2256, 2372.2229, 2383.9978, 2385.2129, 2392.1528, 2400.1094, 2401.1360, 2409.1914, 2411.1907, 2414.1833, 2429.1475, 2436.1699, 2437.1758, 2440.2007, 2447.2368, 2477.1301, 2495.0542, 2498.0898, 2510.2229, 2522.1980, 2524.1689, 2527.2310, 2535.2598, 2541.2075, 2543.1907, 2544.1514, 2553.1934, 2557.2300, 2560.1125, 2569.2915, 2572.0935, 2612.1936, 2686.3572, 2788.3362, 2963.6167, 3056.4641, 3058.5842, 3180.5432, 3313.5032, 3330.6311, 3347.6077, 3511.9668, 3761.1558

9. F6QBP1 **Mass:** 18443 **Score:** 26 **Expect:** 62 **Matches:** 11

Myelin basic protein OS=Bos taurus GN=MBP PE=4 SV=1

Observed	Mr(expt)	Mr(calc)	ppm	Start	End	Miss	Ions	Peptide
811.4178	810.4105	810.4236	-16.11	98	- 105	0	---	R.TPPPSQGK.G
1237.6270	1236.6197	1236.6364	-13.45	43	- 53	2	---	R.FFGSDRGAPKR.G
1537.7804	1536.7731	1536.7354	24.5	11	- 24	1	---	R.SKYLASASTMDHAR.H
1553.7277	1552.7204	1552.7304	-6.40	11	- 24	1	---	R.SKYLASASTMDHAR.H + Oxidation (M)
1800.8578	1799.8505	1799.8380	6.97	114	- 130	0	---	R.FSWGAEGQKPGFGYGGR.A
2030.0151	2029.0078	2028.9952	6.23	13	- 30	1	---	K.YLASASTMDHARHGFLPR.H
2245.0991	2244.0918	2244.1222	-13.53	11	- 30	2	---	R.SKYLASASTMDHARHGFLPR.H
2323.1892	2322.1819	2322.1552	11.5	13	- 32	2	---	K.YLASASTMDHARHGFLPRHR.D
2414.1833	2413.1760	2413.1927	-6.92	108	- 130	1	---	R.GLSLSRFSWGAEGQKPGFGYGGR.A
2686.3572	2685.3499	2685.3888	-14.48	75	- 97	1	---	K.AQHGRPQDENPVVHFFKNIVTPR.T
2788.3362	2787.3289	2787.3154	4.87	114	- 139	2	---	R.FSWGAEGQKPGFGYGGRASDYKSAHK.G

No match to: 724.2539, 725.2472, 728.2482, 730.2519, 732.2390, 733.2623, 739.3115, 742.4642, 743.4729, 745.4483, 747.4419, 752.3972, 754.3827, 756.3716, 758.3382, 760.3329, 764.3364, 766.3494, 767.4740, 768.3865, 770.3671, 772.4178, 779.4436, 784.3415, 787.2366, 788.4225, 790.4196, 795.4260, 799.2464, 802.4372, 805.4478, 808.4353, 814.4170, 819.4304, 821.4247, 822.4321, 829.4457, 837.4396, 840.4623, 842.5109, 854.4525, 856.5045, 858.5442, 860.4092, 863.4233, 863.9185, 869.4846, 870.5100, 872.4994, 880.4412, 904.4613, 906.4929, 908.4577, 913.5685, 919.5024, 925.5089, 927.4999, 928.5031, 940.4773, 942.4820, 950.4946, 957.5654, 960.4840, 966.4929, 969.4786, 972.5046, 974.5551, 977.5352, 978.5240, 981.4474, 989.2700, 991.5198, 993.5112, 1001.5782, 1007.5089, 1009.5097, 1013.4454, 1015.5150, 1016.5522, 1018.5189, 1021.5313, 1024.5028, 1025.5192, 1029.5330, 1032.5870, 1033.5739, 1035.5203, 1045.5564, 1046.5759, 1051.5074, 1056.5415, 1057.6005, 1064.5358, 1071.5361, 1073.5377, 1079.5398, 1083.4637, 1086.4774, 1092.6000, 1092.6002, 1094.5596, 1096.5302, 1106.5431, 1110.5461, 1111.5398, 1111.5400, 1115.5790, 1118.5620, 1120.5724, 1123.5819, 1128.5537, 1132.5682, 1134.5841, 1135.5942, 1137.5565, 1143.5844, 1145.6155, 1149.5597, 1153.5929, 1158.6288, 1163.5771, 1173.6008, 1175.5867, 1177.6178, 1178.6053, 1179.5962, 1181.6432, 1192.5721, 1199.6315, 1203.5826, 1211.5984, 1215.6246, 1216.6786, 1221.5699, 1222.5789, 1227.6271, 1228.6411, 1229.6412, 1231.6102, 1233.6571, 1238.5917, 1244.6069, 1248.5958, 1250.6042, 1255.5922, 1258.6344, 1268.6414, 1271.6490, 1275.6388, 1276.5977, 1276.6000, 1285.5864, 1294.6315, 1299.6810, 1300.6566, 1309.6340, 1320.6268, 1328.6292, 1333.7025, 1336.6038, 1338.6589, 1341.6488, 1343.6613, 1345.6910, 1349.6838, 1350.6704, 1352.6350, 1355.6870, 1357.7130, 1358.6704, 1369.7067, 1371.7366, 1374.6705, 1377.6100, 1377.6146, 1384.7109, 1386.7134, 1390.6848, 1393.7169, 1399.6119, 1400.6588, 1406.7207, 1410.6736, 1411.6647, 1416.6047, 1418.6714, 1421.6632, 1425.7395, 1426.6846, 1427.7076, 1430.7102, 1432.6718, 1434.6552, 1438.6991, 1439.7164, 1443.7589, 1444.7375, 1445.7306, 1454.6836, 1461.7990, 1472.7026, 1475.7224, 1485.6906, 1489.7172, 1490.7058, 1493.7157, 1510.7383, 1515.7393, 1523.6987, 1524.7383, 1527.7585, 1529.7466, 1530.6902, 1531.6953, 1538.7736, 1539.7621, 1541.7805, 1542.8290, 1549.6758, 1555.7928, 1556.7874, 1560.7551, 1563.7314, 1568.7372, 1570.8015, 1572.8151, 1576.7402, 1577.7395, 1578.7518, 1581.8373, 1591.7852, 1600.8068, 1603.8092, 1604.8136, 1606.8130, 1608.7599, 1613.7793, 1615.8575, 1615.8600, 1624.7632, 1628.7653, 1633.7913, 1634.7969, 1635.7941, 1639.8157, 1643.8085, 1646.8591, 1647.8268, 1651.8206, 1653.8284, 1677.8074, 1681.8087, 1688.8287, 1690.8313, 1694.7357, 1697.8672, 1703.8214, 1706.8364, 1707.8435, 1708.3533, 1709.3307, 1710.8229, 1715.8053, 1719.8075, 1721.8296, 1723.8743, 1724.8828, 1725.8090, 1730.8199, 1748.7931, 1757.8975, 1763.7841, 1768.8075, 1771.9686, 1774.9059, 1777.8727, 1779.8081, 1786.8824, 1787.8207, 1790.8423, 1793.9556, 1796.8109, 1797.8339, 1815.9008, 1819.8838, 1821.8926, 1822.9185, 1826.8450, 1829.8655, 1833.8975, 1836.9054, 1843.8765, 1844.8785, 1851.9031, 1859.8494, 1862.9265, 1863.9319, 1866.8857, 1868.8972, 1879.9445, 1880.9215, 1887.9023, 1888.8057, 1890.8025, 1896.8954, 1901.0060, 1906.8872, 1908.8663, 1913.8895, 1916.9475, 1924.9314, 1925.9336, 1928.0060, 1931.9956, 1932.9833, 1968.8563, 1984.9392, 2008.0485, 2008.9869, 2009.9747, 2012.9506, 2015.9661, 2020.9644, 2030.4648, 2046.9839, 2047.5186, 2047.9097, 2051.9133, 2059.0122, 2064.9746, 2070.9209, 2077.0454, 2082.9409, 2085.9888, 2090.9844, 2097.9778, 2103.1526, 2108.9897, 2118.8926, 2140.0432, 2142.0310, 2142.9895, 2152.0510, 2155.0667, 2159.0752, 2160.0703, 2161.0813, 2166.0911, 2167.0676, 2174.0425, 2178.0034, 2181.0369, 2184.0767, 2189.9133, 2194.9761, 2199.0986, 2203.0464, 2205.9102, 2208.9729, 2211.1077, 2213.1116, 2214.1145, 2218.1018, 2221.1035, 2223.2205, 2225.1592, 2229.1064, 2233.1023, 2269.1128, 2273.0393, 2277.0576, 2279.1094, 2280.1204, 2283.1279, 2285.1169, 2292.1465, 2296.1479, 2312.0664, 2326.0947, 2334.1531, 2335.1282, 2337.1343, 2369.2256, 2372.2229, 2383.9978, 2385.2129, 2392.1528, 2400.1094, 2401.1360, 2409.1914, 2411.1907, 2429.1475, 2436.1699, 2437.1758, 2440.2007, 2447.2368, 2477.1301, 2495.0542, 2498.0898, 2510.2229, 2522.1980, 2524.1689, 2527.2310, 2535.2598, 2541.2075, 2543.1907, 2544.1514, 2553.1934, 2557.2300, 2560.1125, 2569.2915, 2572.0935, 2612.1936, 2963.6167, 3056.4641, 3058.5842, 3180.5432, 3313.5032, 3330.6311, 3347.6077, 3511.9668, 3761.1558

10. G3MZA4 **Mass:** 83962 **Score:** 25 **Expect:** 75 **Matches:** 34

Uncharacterized protein OS=Bos taurus GN=CXorf23 PE=4 SV=1

Observed	Mr(expt)	Mr(calc)	ppm	Start	End	Miss	Ions	Peptide
766.3494	765.3422	765.3439	-2.32	364	- 369	1	---	R.SCKDTR.K
908.4577	907.4504	907.4624	-13.20	220	- 226	0	---	K.RPHDVER.Y
928.5031	927.4958	927.5025	-7.29	546	- 553	0	---	K.IIDPSDLR.H
1015.5150	1014.5077	1014.5094	-1.68	413	- 421	0	---	K.VDVQQGQNK.S
1024.5028	1023.4955	1023.4985	-2.91	255	- 262	0	---	R.NLETQTYR.Y
1064.5358	1063.5285	1063.5298	-1.19	303	- 310	2	---	K.YPKEEDRK.L
1079.5398	1078.5325	1078.5407	-7.58	176	- 183	1	---	R.YQRIQDEK.Y
1106.5431	1105.5358	1105.5192	15.0	242	- 250	0	---	R.HFSAPYQEK.K
1250.6042	1249.5969	1249.6224	-20.37	522	- 532	0	---	R.IDMSLAELQSK.Q + Oxidation (M)
1258.6344	1257.6271	1257.6426	-12.28	413	- 423	1	---	K.VDVQQGQNKSR.V

1349.6838	1348.6765	1348.6524	17.9	240 - 250	1	---	K.SRHFSAPYQEK.K
1377.6100	1376.6027	1376.6149	-8.87	30 - 39	1	---	R.FEHYGYDYRK.D
1377.6146	1376.6073	1376.6149	-5.52	30 - 39	1	---	R.FEHYGYDYRK.D
1406.7207	1405.7134	1405.7235	-7.15	521 - 532	1	---	R.RIDMSLAELQSK.Q + Oxidation (M)
1553.7277	1552.7204	1552.7521	-20.42	263 - 276	1	---	R.YAPREFPETSSAAK.V
1556.7874	1555.7801	1555.7916	-7.37	533 - 545	1	---	K.QTMVYDSKQTLVK.I + Oxidation (M)
1570.8015	1569.7942	1569.7787	9.89	191 - 203	2	---	R.GSEDFEKRISFQK.R
1576.7402	1575.7329	1575.7001	20.8	116 - 127	1	---	R.HSEGMFYREHQR.S
1613.7793	1612.7720	1612.7338	23.7	612 - 624	0	---	K.FIQKPCSSDTMQR.K + Oxidation (M)
1633.7913	1632.7840	1632.8008	-10.29	242 - 254	2	---	R.HFSAPYQEKKDQR.N
1725.8090	1724.8017	1724.8338	-18.60	612 - 625	1	---	K.FIQKPCSSDTMQRK.D
1786.8824	1785.8751	1785.8832	-4.51	40 - 53	2	---	K.DPKRPITWRMDDEK.Y
1821.8926	1820.8853	1820.9017	-8.99	148 - 164	2	---	R.SEKGGTPPVRSTTDSFR.F
1896.8954	1895.8881	1895.9213	-17.50	109 - 123	2	---	R.RAQYVPRHSEGMFYR.E
2009.9747	2008.9674	2008.9926	-12.52	504 - 521	2	---	K.AADANETKLSSDPEIHRR.I
2178.0034	2176.9961	2176.9841	5.52	348 - 366	2	---	K.DCAALTHSDKSDVDLRSCK.D
2203.0464	2202.0391	2202.0548	-7.12	522 - 540	1	---	R.IDMSLAELQSKQTMVYDSK.Q + Oxidation (M)
2279.1094	2278.1021	2278.1277	-11.21	43 - 60	2	---	K.RPITWRMDDEKYGQSKPR.I + Oxidation (M)
2312.0664	2311.0591	2311.0869	-12.03	592 - 610	1	---	K.DTHVDEFQKPTSFEKSNFR.K
2337.1343	2336.1270	2336.1543	-11.67	424 - 444	2	---	R.VASSYSTERQMSHDLVAVGRK.S + Oxidation (M)
2429.1475	2428.1402	2428.1996	-24.45	626 - 645	1	---	K.DVITHRPFVEENNQNTRGFR.R
2436.1699	2435.1626	2435.1975	-14.34	422 - 443	2	---	K.SRVASSYSTERQMSHDLVAVGR.K
2440.2007	2439.1934	2439.1819	4.73	592 - 611	2	---	K.DTHVDEFQKPTSFEKSNFRK.F
2527.2310	2526.2237	2526.2139	3.88	590 - 610	2	---	R.SKDTHVDEFQKPTSFEKSNFR.K

No match to: 724.2539, 725.2472, 728.2482, 730.2519, 732.2390, 733.2623, 739.3115, 742.4642, 743.4729, 745.4483, 747.4419, 752.3972, 754.3827, 756.3716, 758.3382, 760.3329, 764.3364, 767.4740, 768.3865, 770.3671, 772.4178, 779.4436, 784.3415, 787.2366, 788.4225, 790.4196, 795.4260, 799.2464, 802.4372, 805.4478, 808.4353, 811.4178, 814.4170, 819.4304, 821.4247, 822.4321, 829.4457, 837.4396, 840.4623, 842.5109, 854.4525, 856.5045, 858.5442, 860.4092, 863.4233, 863.9185, 869.4846, 870.5100, 872.4994, 880.4412, 904.4613, 906.4929, 913.5685, 919.5024, 925.5089, 927.4999, 940.4773, 942.4820, 950.4946, 957.5654, 960.4840, 966.4929, 969.4786, 972.5046, 974.5551, 977.5352, 978.5240, 981.4474, 989.2700, 991.5198, 993.5112, 1001.5782, 1007.5089, 1009.5097, 1013.4454, 1016.5522, 1018.5189, 1021.5313, 1025.5192, 1029.5330, 1032.5870, 1033.5739, 1035.5203, 1045.5564, 1046.5759, 1051.5074, 1056.5415, 1057.6005, 1071.5361, 1073.5377, 1083.4637, 1086.4774, 1092.6000, 1092.6002, 1094.5596, 1096.5302, 1110.5461, 1111.5398, 1111.5400, 1115.5790, 1118.5620, 1120.5724, 1123.5819, 1128.5537, 1132.5682, 1134.5841, 1135.5942, 1137.5565, 1143.5844, 1145.6155, 1149.5597, 1153.5929, 1158.6288, 1163.5771, 1173.6008, 1175.5867, 1177.6178, 1178.6053, 1179.5962, 1181.6432, 1192.5721, 1199.6315, 1203.5826, 1211.5984, 1215.6246, 1216.6786, 1221.5699, 1222.5789, 1227.6271, 1228.6411, 1229.6412, 1231.6102, 1233.6571, 1237.6270, 1238.5917, 1244.6069, 1248.5958, 1255.5922, 1268.6414, 1271.6490, 1275.6388, 1276.5977, 1276.6000, 1285.5864, 1294.6315, 1299.6810, 1300.6566, 1309.6340, 1320.6268, 1328.6292, 1333.7025, 1336.6038, 1338.6589, 1341.6488, 1343.6613, 1345.6910, 1350.6704, 1352.6350, 1355.6870, 1357.7130, 1358.6704, 1369.7067, 1371.7366, 1374.6705, 1384.7109, 1386.7134, 1390.6848, 1393.7169, 1399.6119, 1400.6588, 1410.6736, 1411.6647, 1416.6047, 1418.6714, 1421.6632, 1425.7395, 1426.6846, 1427.7076, 1430.7102, 1432.6718, 1434.6552, 1438.6991, 1439.7164, 1443.7589, 1444.7375, 1445.7306, 1454.6836, 1461.7990, 1472.7026, 1475.7224, 1485.6906, 1489.7172, 1490.7058, 1493.7157, 1510.7383, 1515.7393, 1523.6987, 1524.7383, 1527.7585, 1529.7466, 1530.6902, 1531.6953, 1537.7804, 1538.7736, 1539.7621, 1541.7805, 1542.8290, 1549.6758, 1555.7928, 1560.7551, 1563.7314, 1568.7372, 1572.8151, 1577.7395, 1578.7518, 1581.8373, 1591.7852, 1600.8068, 1603.8092, 1604.8136, 1606.8130, 1608.7599, 1615.8575, 1615.8600, 1624.7632, 1628.7653, 1634.7969, 1635.7941, 1639.8157, 1643.8085, 1646.8591, 1647.8268, 1651.8206, 1653.8284, 1677.8074, 1681.8087, 1688.8287, 1690.8313, 1694.7357, 1697.8672, 1703.8214, 1706.8364, 1707.8435, 1708.3533, 1709.3307, 1710.8229, 1715.8053, 1719.8075, 1721.8296, 1723.8743, 1724.8828, 1730.8199, 1748.7931, 1757.8975, 1763.7841, 1768.8075, 1771.9686, 1774.9059, 1777.8727, 1779.8081, 1787.8207, 1790.8423, 1793.9556, 1796.8109, 1797.8339, 1800.8578, 1815.9008, 1819.8838, 1822.9185, 1826.8450, 1829.8655, 1833.8975, 1836.9054, 1843.8765, 1844.8785, 1851.9031, 1859.8494, 1862.9265, 1863.9319, 1866.8857, 1868.8972, 1879.9445, 1880.9215, 1887.9023, 1888.8057, 1890.8025, 1901.0060, 1906.8872, 1908.8663, 1913.8895, 1916.9475, 1924.9314, 1925.9336, 1928.0060, 1931.9956, 1932.9833, 1968.8563, 1984.9392, 2008.0485, 2008.9869, 2012.9506, 2015.9661, 2020.9644, 2030.0151, 2030.4648, 2046.9839, 2047.5186, 2047.9097, 2051.9133, 2059.0122, 2064.9746, 2070.9209, 2077.0454, 2082.9409, 2085.9888, 2090.9844, 2097.9778, 2103.1526, 2108.9897, 2118.8926, 2140.0432, 2142.0310, 2142.9895, 2152.0510, 2155.0667, 2159.0752, 2160.0703, 2161.0813, 2166.0911, 2167.0676, 2174.0425, 2181.0369, 2184.0767, 2189.9133, 2194.9761, 2199.0986, 2205.9102, 2208.9729, 2211.1077, 2213.1116, 2214.1145, 2218.1018, 2221.1035, 2223.2205, 2225.1592, 2229.1064, 2233.1023, 2245.0991, 2269.1128, 2273.0393, 2277.0576, 2280.1204, 2283.1279, 2285.1169, 2292.1465, 2296.1479, 2323.1892, 2326.0947, 2334.1531, 2335.1282, 2369.2256, 2372.2229, 2383.9978, 2385.2129, 2392.1528, 2400.1094, 2401.1360, 2409.1914, 2411.1907, 2414.1833, 2437.1758, 2447.2368, 2477.1301, 2495.0542, 2498.0898, 2510.2229, 2522.1980, 2524.1689, 2535.2598, 2541.2075, 2543.1907, 2544.1514, 2553.1934, 2557.2300, 2560.1125, 2569.2915, 2572.0935, 2612.1936, 2686.3572, 2788.3362, 2963.6167, 3056.4641, 3058.5842, 3180.5432, 3313.5032, 3330.6311, 3347.6077, 3511.9668, 3761.1558

11. Q3T087 **Mass:** 20468 **Score:** 24 **Expect:** 99 **Matches:** 7

60S ribosomal protein L11 OS=Bos taurus GN=RPL11 PE=2 SV=3

Observed	Mr(expt)	Mr(calc)	ppm	Start - End	Miss	Ions	Peptide
837.4396	836.4323	836.4392	-8.22	91 - 96	1	---	R.EYELRK.N
1092.6000	1091.5927	1091.6087	-14.64	89 - 96	2	16	K.VREYELRK.N
1092.6002	1091.5929	1091.6087	-14.46	89 - 96	2	---	K.VREYELRK.N
1352.6350	1351.6277	1351.6343	-4.85	160 - 169	1	---	K.EEAMRWFQQK.Y
1549.6758	1548.6685	1548.6660	1.60	1 - 13	1	---	-.MAQDQGEKENPMR.E + Oxidation (M)
1800.8578	1799.8505	1799.8584	-4.37	2 - 16	2	---	M.AQDQGEKENPMRELR.I
1862.9265	1861.9192	1861.9138	2.89	20 - 35	1	---	K.LCLNICVGESGDRLTR.A

No match to: 724.2539, 725.2472, 728.2482, 730.2519, 732.2390, 733.2623, 739.3115, 742.4642, 743.4729, 745.4483, 747.4419, 752.3972, 754.3827, 756.3716, 758.3382, 760.3329, 764.3364, 766.3494, 767.4740, 768.3865, 770.3671, 772.4178, 779.4436, 784.3415, 787.2366, 788.4225, 790.4196, 795.4260, 799.2464, 802.4372, 805.4478, 808.4353, 811.4178, 814.4170, 819.4304, 821.4247, 822.4321, 829.4457, 840.4623, 842.5109, 854.4525, 856.5045, 858.5442, 860.4092, 863.4233, 863.9185, 869.4846, 870.5100, 872.4994, 880.4412, 904.4613, 906.4929, 908.4577, 913.5685, 919.5024, 925.5089, 927.4999, 928.5031, 940.4773, 942.4820, 950.4946, 957.5654, 960.4840, 966.4929, 969.4786, 972.5046, 974.5551, 977.5352, 978.5240, 981.4474, 989.2700, 991.5198, 993.5112, 1001.5782, 1007.5089, 1009.5097, 1013.4454, 1015.5150, 1016.5522, 1018.5189, 1021.5313, 1024.5028, 1025.5192, 1029.5330, 1032.5870, 1033.5739, 1035.5203, 1045.5564, 1046.5759, 1051.5074, 1056.5415, 1057.6005, 1064.5358, 1071.5361, 1073.5377, 1079.5398, 1083.4637, 1086.4774, 1094.5596, 1096.5302, 1106.5431, 1110.5461, 1111.5398, 1111.5400, 1115.5790, 1118.5620, 1120.5724, 1123.5819, 1128.5537, 1132.5682, 1134.5841, 1135.5942, 1137.5565, 1143.5844, 1145.6155, 1149.5597, 1153.5929, 1158.6288, 1163.5771, 1173.6008, 1175.5867, 1177.6178, 1178.6053, 1179.5962, 1181.6432, 1192.5721, 1199.6315, 1203.5826, 1211.5984, 1215.6246, 1216.6786, 1221.5699, 1222.5789, 1227.6271, 1228.6411, 1229.6412, 1231.6102, 1233.6571, 1237.6270, 1238.5917, 1244.6069, 1248.5958, 1250.6042, 1255.5922, 1258.6344, 1268.6414, 1271.6490, 1275.6388, 1276.5977, 1276.6000, 1285.5864, 1294.6315, 1299.6810, 1300.6566, 1309.6340, 1320.6268, 1328.6292, 1333.7025, 1336.6038, 1338.6589, 1341.6488, 1343.6613, 1345.6910, 1349.6838, 1350.6704, 1355.6870, 1357.7130, 1358.6704, 1369.7067, 1371.7366, 1374.6705, 1377.6100, 1377.6146, 1384.7109, 1386.7134, 1390.6848, 1393.7169, 1399.6119, 1400.6588, 1406.7207, 1410.6736, 1411.6647, 1416.6047, 1418.6714, 1421.6632, 1425.7395, 1426.6846, 1427.7076, 1430.7102, 1432.6718, 1434.6552, 1438.6991, 1439.7164, 1443.7589, 1444.7375, 1445.7306, 1454.6836, 1461.7990, 1472.7026, 1475.7224, 1485.6906, 1489.7172, 1490.7058, 1493.7157, 1510.7383, 1515.7393, 1523.6987, 1524.7383, 1527.7585, 1529.7466, 1530.6902, 1531.6953, 1537.7804, 1538.7736, 1539.7621, 1541.7805, 1542.8290, 1553.7277, 1555.7928, 1556.7874, 1560.7551, 1563.7314, 1568.7372, 1570.8015, 1572.8151, 1576.7402, 1577.7395, 1578.7518, 1581.8373, 1591.7852, 1600.8068, 1603.8092, 1604.8136, 1606.8130, 1608.7599, 1613.7793, 1615.8575, 1615.8600, 1624.7632, 1628.7653, 1633.7913, 1634.7969, 1635.7941, 1639.8157, 1643.8085, 1646.8591, 1647.8268, 1651.8206, 1653.8284, 1677.8074, 1681.8087, 1688.8287, 1690.8313, 1694.7357, 1697.8672, 1703.8214, 1706.8364, 1707.8435, 1708.3533, 1709.3307, 1710.8229, 1715.8053, 1719.8075, 1721.8296, 1723.8743, 1724.8828, 1725.8090, 1730.8199, 1748.7931, 1757.8975, 1763.7841, 1768.8075, 1771.9686, 1774.9059, 1777.8727, 1779.8081, 1786.8824, 1787.8207, 1790.8423, 1793.9556, 1796.8109, 1797.8339, 1815.9008, 1819.8838, 1821.8926, 1822.9185, 1826.8450, 1829.8655, 1833.8975, 1836.9054, 1843.8765, 1844.8785, 1851.9031, 1859.8494, 1863.9319, 1866.8857, 1868.8972, 1879.9445, 1880.9215, 1887.9023, 1888.8057, 1890.8025, 1896.8954, 1901.0060, 1906.8872, 1908.8663, 1913.8895, 1916.9475, 1924.9314, 1925.9336, 1928.0060, 1931.9956, 1932.9833, 1968.8563, 1984.9392,

2008.0485, 2008.9869, 2009.9747, 2012.9506, 2015.9661, 2020.9644, 2030.0151, 2030.4648, 2046.9839, 2047.5186, 2047.9097, 2051.9133, 2059.0122, 2064.9746, 2070.9209, 2077.0454, 2082.9409, 2085.9888, 2090.9844, 2097.9778, 2103.1526, 2108.9897, 2118.8926, 2140.0432, 2142.0310, 2142.9895, 2152.0510, 2155.0667, 2159.0752, 2160.0703, 2161.0813, 2166.0911, 2167.0676, 2174.0425, 2178.0034, 2181.0369, 2184.0767, 2189.9133, 2194.9761, 2199.0986, 2203.0464, 2205.9102, 2208.9729, 2211.1077, 2213.1116, 2214.1145, 2218.1018, 2221.1035, 2223.2205, 2225.1592, 2229.1064, 2233.1023, 2245.0991, 2269.1128, 2273.0393, 2277.0576, 2279.1094, 2280.1204, 2283.1279, 2285.1169, 2292.1465, 2296.1479, 2312.0664, 2323.1892, 2326.0947, 2334.1531, 2335.1282, 2337.1343, 2369.2256, 2372.2229, 2383.9978, 2385.2129, 2392.1528, 2400.1094, 2401.1360, 2409.1914, 2411.1907, 2414.1833, 2429.1475, 2436.1699, 2437.1758, 2440.2007, 2447.2368, 2477.1301, 2495.0542, 2498.0898, 2510.2229, 2522.1980, 2524.1689, 2527.2310, 2535.2598, 2541.2075, 2543.1907, 2544.1514, 2553.1934, 2557.2300, 2560.1125, 2569.2915, 2572.0935, 2612.1936, 2686.3572, 2788.3362, 2963.6167, 3056.4641, 3058.5842, 3180.5432, 3313.5032, 3330.6311, 3347.6077, 3511.9668, 3761.1558

12. F1MI98 **Mass:** 52161 **Score:** 23 **Expect:** 1.2e+002 **Matches:** 20
Uncharacterized protein OS=Bos taurus GN=KRT36 PE=3 SV=2

Observed	Mr(expt)	Mr(calc)	ppm	Start	End	Miss	Ions	Peptide
904.4613	903.4540	903.4735	-21.59	219	225	0	---	K.EELICLK.K
1032.5870	1031.5797	1031.5685	10.9	219	226	1	---	K.EELICLKK.N
1215.6246	1214.6173	1214.6255	-6.73	187	197	0	---	R.QLVEADTNGLR.Q
1227.6271	1226.6198	1226.5965	19.0	177	186	1	---	R.TKYETELGMR.Q
1237.6270	1236.6197	1236.6462	-21.42	383	392	1	---	R.LESEIATYRR.L
1643.8085	1642.8012	1642.7773	14.5	2	16	0	---	M.ASQFCSPIFSSGSIR.G
1703.8214	1702.8141	1702.7992	8.78	17	32	1	---	R.GHCGATSGISRVSCVR.S
1706.8364	1705.8291	1705.8392	-5.92	401	415	0	---	K.LPAHPCATECKPAVR.V
1790.8423	1789.8350	1789.8127	12.4	1	16	0	---	-.MASQFCSPIFSSGSIR.G + Oxidation (M)
1868.8972	1867.8899	1867.9210	-16.64	312	327	0	---	R.NVNALEIELQAQHSMR.N + Oxidation (M)
2008.9869	2007.9796	2008.0272	-23.69	311	327	1	---	R.RNVNALEIELQAQHSMR.N
2015.9661	2014.9588	2014.9782	-9.61	170	186	2	---	K.LAADDFRTKYETELGMR.Q
2097.9778	2096.9705	2097.0021	-15.07	226	242	2	---	K.KNHEEEVNTLRCQLGDR.L
2211.1077	2210.1004	2210.0637	16.6	179	197	1	---	K.YETELGMRQLVEADTNGLR.Q + Oxidation (M)
2213.1116	2212.1043	2212.1310	-12.06	92	109	2	---	K.ETMQFLNDRLASYLEKVR.Q
2229.1064	2228.0991	2228.1259	-12.02	92	109	2	---	K.ETMQFLNDRLASYLEKVR.Q + Oxidation (M)
2292.1465	2291.1392	2291.1752	-15.71	208	226	2	---	K.ADLEMQVESLKEELICLKK.N + Oxidation (M)
2323.1892	2322.1819	2322.1638	7.81	237	257	1	---	R.CQLGDRLNVEVDAAPPVDLNK.I
2440.2007	2439.1934	2439.2064	-5.30	177	197	2	---	R.TKYETELGMRQLVEADTNGLR.Q + Oxidation (M)
2535.2598	2534.2525	2534.2798	-10.78	341	363	0	---	R.YSSQLAQMQGLIGNVEAQLAEIR.C + Oxidation (M)

No match to: 724.2539, 725.2472, 728.2482, 730.2519, 732.2390, 733.2623, 739.3115, 742.4642, 743.4729, 745.4483, 747.4419, 752.3972, 754.3827, 756.3716, 758.3382, 760.3329, 764.3364, 766.3494, 767.4740, 768.3865, 770.3671, 772.4178, 779.4436, 784.3415, 787.2366, 788.4225, 790.4196, 795.4260, 799.2464, 802.4372, 805.4478, 808.4353, 811.4178, 814.4170, 819.4304, 821.4247, 822.4321, 829.4457, 837.4396, 840.4623, 842.5109, 854.4525, 856.5045, 858.5442, 860.4092, 863.4233, 863.9185, 869.4846, 870.5100, 872.4994, 880.4412, 906.4929, 908.4577, 913.5685, 919.5024, 925.5089, 927.4999, 928.5031, 940.4773, 942.4820, 950.4946, 957.5654, 960.4840, 966.4929, 969.4786, 972.5046, 974.5551, 977.5352, 978.5240, 981.4474, 989.2700, 991.5198, 993.5112, 1001.5782, 1007.5089, 1009.5097, 1013.4454, 1015.5150, 1016.5522, 1018.5189, 1021.5313, 1024.5028, 1025.5192, 1029.5330, 1033.5739, 1035.5203, 1045.5564, 1046.5759, 1051.5074, 1056.5415, 1057.6005, 1064.5358, 1071.5361, 1073.5377, 1079.5398, 1083.4637, 1086.4774, 1092.6000, 1092.6002, 1094.5596, 1096.5302, 1106.5431, 1110.5461, 1111.5398, 1111.5400, 1115.5790, 1118.5620, 1120.5724, 1123.5819, 1128.5537, 1132.5682, 1134.5841, 1135.5942, 1137.5565, 1143.5844, 1145.6155, 1149.5597, 1153.5929, 1158.6288, 1163.5771, 1173.6008, 1175.5867, 1177.6178, 1178.6053, 1179.5962, 1181.6432, 1192.5721, 1199.6315, 1203.5826, 1211.5984, 1216.6786, 1221.5699, 1222.5789, 1228.6411, 1229.6412, 1231.6102, 1233.6571, 1238.5917, 1244.6069, 1248.5958, 1250.6042, 1255.5922, 1258.6344, 1268.6414, 1271.6490, 1275.6388, 1276.5977, 1276.6000, 1285.5864, 1294.6315, 1299.6810, 1300.6566, 1309.6340, 1320.6268, 1328.6292, 1333.7025, 1336.6038, 1338.6589, 1341.6488, 1343.6613, 1345.6910, 1349.6838, 1350.6704, 1352.6350, 1355.6870, 1357.7130, 1358.6704, 1369.7067, 1371.7366, 1374.6705, 1377.6100, 1377.6146, 1384.7109, 1386.7134, 1390.6848, 1393.7169, 1399.6119, 1400.6588, 1406.7207, 1410.6736, 1411.6647, 1416.6047, 1418.6714, 1421.6632, 1425.7395, 1426.6846, 1427.7076, 1430.7102, 1432.6718, 1434.6552, 1438.6991, 1439.7164, 1443.7589, 1444.7375, 1445.7306, 1454.6836, 1461.7990, 1472.7026, 1475.7224, 1485.6906, 1489.7172, 1490.7058, 1493.7157, 1510.7383, 1515.7393, 1523.6987, 1524.7383, 1527.7585, 1529.7466, 1530.6902, 1531.6953, 1537.7804, 1538.7736, 1539.7621, 1541.7805, 1542.8290, 1549.6758, 1553.7277, 1555.7928, 1556.7874, 1560.7551, 1563.7314, 1568.7372, 1570.8015, 1572.8151, 1576.7402, 1577.7395, 1578.7518, 1581.8373, 1591.7852, 1600.8068, 1603.8092, 1604.8136, 1606.8130, 1608.7599, 1613.7793, 1615.8575, 1615.8600, 1624.7632, 1628.7653, 1633.7913, 1634.7969, 1635.7941, 1639.8157, 1646.8591, 1647.8268, 1651.8206, 1653.8284, 1677.8074, 1681.8087, 1688.8287, 1690.8313, 1694.7357, 1697.8672, 1707.8435, 1708.3533, 1709.3307, 1710.8229, 1715.8053, 1719.8075, 1721.8296, 1723.8743, 1724.8828, 1725.8090, 1730.8199, 1748.7931, 1757.8975, 1763.7841, 1768.8075, 1771.9686, 1774.9059, 1777.8727, 1779.8081, 1786.8824, 1787.8207, 1793.9556, 1796.8109, 1797.8339, 1800.8578, 1815.9008, 1819.8838, 1821.8926, 1822.9185, 1826.8450, 1829.8655, 1833.8975, 1836.9054, 1843.8765, 1844.8785, 1851.9031, 1859.8494, 1862.9265, 1863.9319, 1866.8857, 1879.9445, 1880.9215, 1887.9023, 1888.8027, 1890.8025, 1896.8954, 1901.0060, 1906.8872, 1908.8663, 1913.8895, 1916.9475, 1924.9314, 1925.9336, 1928.0060, 1931.9956, 1932.9833, 1968.8563, 1984.9392, 2008.0485, 2009.9747, 2012.9506, 2020.9644, 2030.0151, 2030.4648, 2046.9839, 2047.5186, 2047.9097, 2051.9133, 2059.0122, 2064.9746, 2070.9209, 2077.0454, 2082.9409, 2085.9888, 2090.9844, 2103.1526, 2108.9897, 2118.8926, 2140.0432, 2142.0310, 2142.9895, 2152.0510, 2155.0667, 2159.0752, 2160.0703, 2161.0813, 2166.0911, 2167.0676, 2174.0425, 2178.0034, 2181.0369, 2184.0767, 2189.9133, 2194.9761, 2199.0986, 2203.0464, 2205.9102, 2208.9729, 2214.1145, 2218.1018, 2221.1035, 2223.2205, 2225.1592, 2233.1023, 2245.0991, 2269.1128, 2273.0393, 2277.0576, 2279.1094, 2280.1204, 2283.1279, 2285.1169, 2296.1479, 2312.0664, 2326.0947, 2334.1531, 2335.1282, 2337.1343, 2369.2256, 2372.2229, 2383.9978, 2385.2129, 2392.1528, 2400.1094, 2401.1360, 2409.1914, 2411.1907, 2414.1833, 2429.1475, 2436.1699, 2437.1758, 2447.2368, 2477.1301, 2495.0542, 2498.0898, 2510.2229, 2522.1980, 2524.1689, 2527.2310, 2541.2075, 2543.1907, 2544.1514, 2553.1934, 2557.2300, 2560.1125, 2569.2915, 2572.0935, 2612.1936, 2686.3572, 2788.3362, 2963.6167, 3056.4641, 3058.5842, 3180.5432, 3313.5032, 3330.6311, 3347.6077, 3511.9668, 3761.1558

13. Q58DL0 **Mass:** 26401 **Score:** 22 **Expect:** 1.6e+002 **Matches:** 15
Chromosome X open reading frame 23 OS=Bos taurus GN=CXorf23 PE=2 SV=1

Observed	Mr(expt)	Mr(calc)	ppm	Start	End	Miss	Ions	Peptide
928.5031	927.4958	927.5025	-7.29	113	120	0	---	K.IIDPSDLR.H
1035.5203	1034.5130	1034.4920	20.3	217	225	0	---	K.EYTNIDPAI.-
1228.6411	1227.6338	1227.6394	-4.53	1	11	1	---	-.MSHDLVAVGRK.S + Oxidation (M)
1250.6042	1249.5969	1249.6224	-20.37	89	99	0	---	R.IDMSLAELQSK.Q + Oxidation (M)
1406.7207	1405.7134	1405.7235	-7.15	88	99	1	---	R.RIDMSLAELQSK.Q + Oxidation (M)
1556.7874	1555.7801	1555.7916	-7.37	100	112	1	---	K.QTMVYDSKQTLVK.I + Oxidation (M)
1572.8151	1571.8078	1571.8090	-0.72	1	14	2	---	-.MSHDLVAVGRKSEK.F + Oxidation (M)
1613.7793	1612.7720	1612.7338	23.7	179	191	0	---	K.FIQKPCSSDTMQR.K + Oxidation (M)
1725.8090	1724.8017	1724.8338	-18.60	179	192	1	---	K.FIQKPCSSDTMQRK.D
2009.9747	2008.9674	2008.9926	-12.52	71	88	2	---	K.AADANETKLSSDPEIHRR.I
2203.0464	2202.0391	2202.0548	-7.12	89	107	1	---	R.IDMSLAELQSKQTMVYDSK.Q + Oxidation (M)
2312.0664	2311.0591	2311.0869	-12.03	159	177	1	---	K.DTHVDEFQKPTSFEKSNFR.K
2429.1475	2428.1402	2428.1996	-24.45	193	212	1	---	K.DVITHRPFVEENNQNTRGFR.R
2440.2007	2439.1934	2439.1819	4.73	159	178	2	---	K.DTHVDEFQKPTSFEKSNFRK.F
2527.2310	2526.2237	2526.2139	3.88	157	177	2	---	R.SKDTHVDEFQKPTSFEKSNFR.K

No match to: 724.2539, 725.2472, 728.2482, 730.2519, 732.2390, 733.2623, 739.3115, 742.4642, 743.4729, 745.4483, 747.4419, 752.3972, 754.3827, 756.3716, 758.3382, 760.3329, 764.3364, 766.3494, 767.4740, 768.3865, 770.3671, 772.4178, 779.4436, 784.3415, 787.2366, 788.4225, 790.4196, 795.4260, 799.2464, 802.4372, 805.4478, 808.4353, 811.4178, 814.4170, 819.4304, 821.4247, 822.4321, 829.4457, 837.4396, 840.4623, 842.5109, 854.4525, 856.5045, 858.5442, 860.4092, 863.4233, 863.9185, 869.4846, 870.5100, 872.4994, 880.4412, 904.4613, 906.4929, 908.4577, 913.5685, 919.5024, 925.5089, 927.4999, 940.4773, 942.4820, 950.4946, 957.5654, 960.4840,

966.4929, 969.4786, 972.5046, 974.5551, 977.5352, 978.5240, 981.4474, 989.2700, 991.5198, 993.5112, 1001.5782, 1007.5089, 1009.5097, 1013.4454, 1015.5150, 1016.5522, 1018.5189, 1021.5313, 1024.5028, 1025.5192, 1029.5330, 1032.5870, 1033.5739, 1045.5564, 1046.5759, 1051.5074, 1056.5415, 1057.6005, 1064.5358, 1071.5361, 1073.5377, 1079.5398, 1083.4637, 1086.4774, 1092.6000, 1092.6002, 1094.5596, 1096.5302, 1106.5431, 1110.5461, 1111.5398, 1111.5400, 1115.5790, 1118.5620, 1120.5724, 1123.5819, 1128.5537, 1132.5682, 1134.5841, 1135.5942, 1137.5565, 1143.5844, 1145.6155, 1149.5597, 1153.5929, 1158.6288, 1163.5771, 1173.6008, 1175.5867, 1177.6178, 1178.6053, 1179.5962, 1181.6432, 1192.5721, 1199.6315, 1203.5826, 1211.5984, 1215.6246, 1216.6786, 1221.5699, 1222.5789, 1227.6271, 1229.6412, 1231.6102, 1233.6571, 1237.6270, 1238.5917, 1244.6069, 1248.5958, 1255.5922, 1258.6344, 1268.6414, 1271.6490, 1275.6388, 1276.5977, 1276.6000, 1285.5864, 1294.6315, 1299.6810, 1300.6566, 1309.6340, 1320.6268, 1328.6292, 1333.7025, 1336.6038, 1338.6589, 1341.6488, 1343.6613, 1345.6910, 1349.6838, 1350.6704, 1352.6350, 1355.6870, 1357.7130, 1358.6704, 1369.7067, 1371.7366, 1374.6705, 1377.6100, 1377.6146, 1384.7109, 1386.7134, 1390.6848, 1393.7169, 1399.6119, 1400.6588, 1410.6736, 1411.6647, 1416.6047, 1418.6714, 1421.6632, 1425.7395, 1426.6846, 1427.7076, 1430.7102, 1432.6718, 1434.6552, 1438.6991, 1439.7164, 1443.7589, 1444.7375, 1445.7306, 1454.6836, 1461.7990, 1472.7026, 1475.7224, 1485.6906, 1489.7172, 1490.7058, 1493.7157, 1510.7383, 1515.7393, 1523.6987, 1524.7383, 1527.7585, 1529.7466, 1530.6902, 1531.6953, 1537.7804, 1538.7736, 1539.7621, 1541.7805, 1542.8290, 1549.6758, 1553.7277, 1555.7928, 1560.7551, 1563.7314, 1568.7372, 1570.8015, 1576.7402, 1577.7395, 1578.7518, 1581.8373, 1591.7852, 1600.8068, 1603.8092, 1604.8136, 1606.8130, 1608.7599, 1615.8575, 1615.8600, 1624.7632, 1628.7653, 1633.7913, 1634.7969, 1635.7941, 1639.8157, 1643.8085, 1646.8591, 1647.8268, 1651.8206, 1653.8284, 1677.8074, 1681.8087, 1688.8287, 1690.8313, 1694.7357, 1697.8672, 1703.8214, 1706.8364, 1707.8435, 1708.3533, 1709.3307, 1710.8229, 1715.8053, 1719.8075, 1721.8296, 1723.8743, 1724.8828, 1730.8199, 1748.7931, 1757.8975, 1763.7841, 1768.8075, 1771.9686, 1774.9059, 1777.8727, 1779.8081, 1786.8824, 1787.8207, 1790.8423, 1793.9556, 1796.8109, 1797.8339, 1800.8578, 1815.9008, 1819.8838, 1821.8926, 1822.9185, 1826.8450, 1829.8655, 1833.8975, 1836.9054, 1843.8765, 1844.8785, 1851.9031, 1859.8494, 1862.9265, 1863.9319, 1866.8857, 1868.8972, 1879.9445, 1880.9215, 1887.9023, 1888.8057, 1890.8025, 1896.8954, 1901.0060, 1906.8872, 1908.8663, 1913.8895, 1916.9475, 1924.9314, 1925.9336, 1928.0060, 1931.9956, 1932.9833, 1968.8563, 1984.9392, 2008.0485, 2008.9869, 2012.9506, 2015.9661, 2020.9644, 2030.0151, 2030.4648, 2046.9839, 2047.5186, 2047.9097, 2051.9133, 2059.0122, 2064.9746, 2070.9209, 2077.0454, 2082.9409, 2085.9888, 2090.9844, 2097.9778, 2103.1526, 2108.9897, 2118.8926, 2140.0432, 2142.0310, 2142.9895, 2152.0510, 2155.0667, 2159.0752, 2160.0703, 2161.0813, 2166.0911, 2167.0676, 2174.0425, 2178.0034, 2181.0369, 2184.0767, 2189.9133, 2194.9761, 2199.0986, 2205.9102, 2208.9729, 2211.1077, 2213.1116, 2214.1145, 2218.1018, 2221.1035, 2223.2205, 2225.1592, 2229.1064, 2233.1023, 2245.0991, 2269.1128, 2273.0393, 2277.0576, 2279.1094, 2280.1204, 2283.1279, 2285.1169, 2292.1465, 2296.1479, 2323.1892, 2326.0947, 2334.1531, 2335.1282, 2337.1343, 2369.2256, 2372.2229, 2383.9978, 2385.2129, 2392.1528, 2400.1094, 2401.1360, 2409.1914, 2411.1907, 2414.1833, 2436.1699, 2437.1758, 2447.2368, 2477.1301, 2495.0542, 2498.0898, 2510.2229, 2522.1980, 2524.1689, 2535.2598, 2541.2075, 2543.1907, 2544.1514, 2553.1934, 2557.2300, 2560.1125, 2569.2915, 2572.0935, 2612.1936, 2686.3572, 2788.3362, 2963.6167, 3056.4641, 3058.5842, 3180.5432, 3313.5032, 3330.6311, 3347.6077, 3511.9668, 3761.1558

14. Q3SZ43 **Mass:** 16409 **Score:** 22 **Expect:** 1.7e+002 **Matches:** 13

Ubiquitin-conjugating enzyme E2 variant 2 OS=Bos taurus GN=UBE2V2 PE=2 SV=3

Observed	Mr(expt)	Mr(calc)	ppm	Start	End	Miss	Ions	Peptide
808.4353	807.4280	807.4160	14.8	1 -	8	0	---	-.MAVSTGVK.V + Oxidation (M)
856.5045	855.4972	855.5178	-24.02	117 -	123	0	---	K.VVLQELR.R
1015.5150	1014.5077	1014.5134	-5.62	73 -	81	0	---	K.YPEAPPSVR.F
1025.5192	1024.5119	1024.4978	13.8	109 -	116	0	---	K.WQNSYSIK.V
1111.5398	1110.5325	1110.5235	8.11	125 -	133	1	---	R.LMMSKENMK.L
1111.5400	1110.5327	1110.5235	8.29	125 -	133	1	---	R.LMMSKENMK.L
1143.5844	1142.5771	1142.5907	-11.84	46 -	55	0	---	R.WTGMIIGPPR.T + Oxidation (M)
1604.8136	1603.8063	1603.8205	-8.86	12 -	24	1	---	R.NFRLLEELEEGQK.G
1859.8494	1858.8421	1858.8519	-5.27	130 -	145	1	---	K.ENMKLPQPPEGQTYNN.-
2161.0813	2160.0740	2160.1038	-13.77	67 -	85	2	---	K.VECGPKYPEAPPSVRFVTK.I
2184.0767	2183.0694	2183.0463	10.6	82 -	101	1	---	R.FVTKINMNGINNSSGMVDAR.S + Oxidation (M)
2498.0898	2497.0825	2497.1287	-18.49	125 -	145	2	---	R.LMMSKENMKLPQPPEGQTYNN.- + 3 Oxidation (M)
3180.5432	3179.5359	3179.5492	-4.17	73 -	101	2	---	K.YPEAPPSVRFVTKINMNGINNSSGMVDAR.S + Oxidation (M)

No match to: 724.2539, 725.2472, 728.2482, 730.2519, 732.2390, 733.2623, 739.3115, 742.4642, 743.4729, 745.4483, 747.4419, 752.3972, 754.3827, 756.3716, 758.3382, 760.3329, 764.3364, 766.3494, 767.4740, 768.3865, 770.3671, 772.4178, 779.4436, 784.3415, 787.2366, 788.4225, 790.4196, 795.4260, 799.2464, 802.4372, 805.4478, 811.4178, 814.4170, 819.4304, 821.4247, 822.4321, 829.4457, 837.4396, 840.4623, 842.5109, 854.4525, 858.5442, 860.4092, 863.4233, 863.9185, 869.4846, 870.5100, 872.4994, 880.4412, 904.4613, 906.4929, 908.4577, 913.5685, 919.5024, 925.5089, 927.4999, 928.5031, 940.4773, 942.4820, 950.4946, 957.5654, 960.4840, 966.4929, 969.4786, 972.5046, 974.5551, 977.5352, 978.5240, 981.4474, 989.2700, 991.5198, 993.5112, 1001.5782, 1007.5089, 1009.5097, 1013.4454, 1016.5522, 1018.5189, 1021.5313, 1024.5028, 1029.5330, 1032.5870, 1033.5739, 1035.5203, 1045.5564, 1046.5759, 1051.5074, 1056.5415, 1057.6005, 1064.5358, 1071.5361, 1073.5377, 1079.5398, 1083.4637, 1086.4774, 1092.6000, 1092.6002, 1094.5596, 1096.5302, 1106.5431, 1110.5461, 1115.5790, 1118.5620, 1120.5724, 1123.5819, 1128.5537, 1132.5682, 1134.5841, 1135.5942, 1137.5565, 1145.6155, 1149.5597, 1153.5929, 1158.6288, 1163.5771, 1173.6008, 1175.5867, 1177.6178, 1178.6053, 1179.5962, 1181.6432, 1192.5721, 1199.6315, 1203.5826, 1211.5984, 1215.6246, 1216.6786, 1221.5699, 1222.5789, 1227.6271, 1228.6411, 1229.6412, 1231.6102, 1233.6571, 1237.6270, 1238.5917, 1244.6069, 1248.5958, 1250.6042, 1255.5922, 1258.6344, 1268.6414, 1271.6490, 1275.6388, 1276.5977, 1276.6000, 1285.5864, 1294.6315, 1299.6810, 1300.6566, 1309.6340, 1320.6268, 1328.6292, 1333.7025, 1336.6038, 1338.6589, 1341.6488, 1343.6613, 1345.6910, 1349.6838, 1350.6704, 1352.6350, 1355.6870, 1357.7130, 1358.6704, 1369.7067, 1371.7366, 1374.6705, 1377.6100, 1377.6146, 1384.7109, 1386.7134, 1390.6848, 1393.7169, 1399.6119, 1400.6588, 1406.7207, 1410.6736, 1411.6647, 1416.6047, 1418.6714, 1421.6632, 1425.7395, 1426.6846, 1427.7076, 1430.7102, 1432.6718, 1434.6552, 1438.6991, 1439.7164, 1443.7589, 1444.7375, 1445.7306, 1454.6836, 1461.7990, 1472.7026, 1475.7224, 1485.6906, 1489.7172, 1490.7058, 1493.7157, 1510.7383, 1515.7393, 1523.6987, 1524.7383, 1527.7585, 1529.7466, 1530.6902, 1531.6953, 1537.7804, 1538.7736, 1539.7621, 1541.7805, 1542.8290, 1549.6758, 1553.7277, 1555.7928, 1556.7874, 1560.7551, 1563.7314, 1568.7372, 1570.8015, 1572.8151, 1576.7402, 1577.7395, 1578.7518, 1581.8373, 1591.7852, 1600.8068, 1603.8092, 1606.8130, 1608.7599, 1613.7793, 1615.8575, 1615.8600, 1624.7632, 1628.7653, 1633.7913, 1634.7969, 1635.7941, 1639.8157, 1643.8085, 1646.8591, 1647.8268, 1651.8206, 1653.8284, 1677.8074, 1681.8087, 1688.8287, 1690.8313, 1694.7357, 1697.8672, 1703.8214, 1706.8364, 1707.8435, 1708.3533, 1709.3307, 1710.8229, 1715.8053, 1719.8075, 1721.8296, 1723.8743, 1724.8828, 1725.8090, 1730.8199, 1748.7931, 1757.8975, 1763.7841, 1768.8075, 1771.9686, 1774.9059, 1777.8727, 1779.8081, 1786.8824, 1787.8207, 1790.8423, 1793.9556, 1796.8109, 1797.8339, 1800.8578, 1815.9008, 1819.8838, 1821.8926, 1822.9185, 1826.8450, 1829.8655, 1833.8975, 1836.9054, 1843.8765, 1844.8785, 1851.9031, 1862.9265, 1863.9319, 1866.8857, 1868.8972, 1879.9445, 1880.9215, 1887.9023, 1888.8057, 1890.8025, 1896.8954, 1901.0060, 1906.8872, 1908.8663, 1913.8895, 1916.9475, 1924.9314, 1925.9336, 1928.0060, 1931.9956, 1932.9833, 1968.8563, 1984.9392, 2008.0485, 2008.9869, 2009.9747, 2012.9506, 2015.9661, 2020.9644, 2030.0151, 2030.4648, 2046.9839, 2047.5186, 2047.9097, 2051.9133, 2059.0122, 2064.9746, 2070.9209, 2077.0454, 2082.9409, 2085.9888, 2090.9844, 2097.9778, 2103.1526, 2108.9897, 2118.8926, 2140.0432, 2142.0310, 2142.9895, 2152.0510, 2155.0667, 2159.0752, 2160.0703, 2166.0911, 2167.0676, 2174.0425, 2178.0034, 2181.0369, 2189.9133, 2194.9761, 2199.0986, 2203.0464, 2205.9102, 2208.9729, 2211.1077, 2213.1116, 2214.1145, 2218.1018, 2221.1035, 2223.2205, 2225.1592, 2229.1064, 2233.1023, 2245.0991, 2269.1128, 2273.0393, 2277.0576, 2279.1094, 2280.1204, 2283.1279, 2285.1169, 2292.1465, 2296.1479, 2312.0664, 2323.1892, 2326.0947, 2334.1531, 2335.1282, 2337.1343, 2369.2256, 2372.2229, 2383.9978, 2385.2129, 2392.1528, 2400.1094, 2401.1360, 2409.1914, 2411.1907, 2414.1833, 2429.1475, 2436.1699, 2437.1758, 2440.2007, 2447.2368, 2477.1301, 2495.0542, 2510.2229, 2522.1980, 2524.1689, 2527.2310, 2535.2598, 2541.2075, 2543.1907, 2544.1514, 2553.1934, 2557.2300, 2560.1125, 2569.2915, 2572.0935, 2612.1936, 2686.3572, 2788.3362, 2963.6167, 3056.4641, 3058.5842, 3313.5032, 3330.6311, 3347.6077, 3511.9668, 3761.1558

15. E1BE64 **Mass:** 104496 **Score:** 22 **Expect:** 1.7e+002 **Matches:** 32

Uncharacterized protein OS=Bos taurus GN=TSGA10 PE=4 SV=2

Observed	Mr(expt)	Mr(calc)	ppm	Start	End	Miss	Ions	Peptide
978.5240	977.5168	977.5328	-16.35	371 -	378	2	---	K.EMKSLARK.A + Oxidation (M)
1009.5097	1008.5024	1008.5127	-10.23	398 -	405	0	---	R.LLYENTEK.D
1045.5564	1044.5491	1044.5563	-6.90	559 -	567	1	---	K.QENQALSKK.L
1092.6000	1091.5927	1091.5724	18.7	299 -	307	1	6	R.DVAFTDLRR.M
1092.6002	1091.5929	1091.5724	18.8	299 -	307	1	---	R.DVAFTDLRR.M
1135.5942	1134.5869	1134.5782	7.73	826 -	834	1	---	R.QLGTERFER.E
1179.5962	1178.5889	1178.5675	18.2	227 -	235	1	---	R.EELKCMLEK.Y
1393.7169	1392.7096	1392.7249	-10.96	171 -	182	1	---	R.LDSFVKTLEADR.D
1443.7589	1442.7516	1442.7616	-6.93	47 -	58	1	---	K.IDQLAEQLEKEK.N

1445.7306	1444.7233	1444.7310	-5.33	7 -	18	1	---	K.QTNHGLEEYVKK.L
1537.7804	1536.7731	1536.7494	15.5	683 -	694	1	---	K.MEEELQKVQFEK.V
1553.7277	1552.7204	1552.7443	-15.36	683 -	694	1	---	K.MEEELQKVQFEK.V + Oxidation (M)
1556.7874	1555.7801	1555.8107	-19.62	236 -	248	1	---	K.YERHLAEIQGNVK.V
1624.7632	1623.7559	1623.7675	-7.11	877 -	889	1	---	R.GLDRSLEENLCYR.D
1643.8085	1642.8012	1642.7694	19.4	227 -	238	2	---	R.EELKCMLEKYER.H + Oxidation (M)
1706.8364	1705.8291	1705.8556	-15.52	474 -	489	1	---	K.SENIASLGESLAMKEK.T
1707.8435	1706.8362	1706.8363	-0.03	59 -	73	1	---	K.NFVVDTADKELEEAK.K
1710.8229	1709.8156	1709.8519	-21.21	299 -	312	2	---	R.DVAFTDLRRMTTER.D
1721.8296	1720.8223	1720.8414	-11.06	741 -	754	1	---	R.SEIELLRSQMTNER.I + Oxidation (M)
1777.8727	1776.8654	1776.8788	-7.55	212 -	226	2	---	R.GTNCDVELLKTTRDR.E
1793.9556	1792.9483	1792.9795	-17.40	239 -	254	1	---	R.HLAEIQGNVKVLTSER.D
1796.8109	1795.8036	1795.8080	-2.45	507 -	521	0	---	R.QSTEALIMCEQDISR.M + Oxidation (M)
1815.9008	1814.8935	1814.8767	9.27	790 -	804	1	---	K.EHLCLAENKMAIQSR.D + Oxidation (M)
1896.8954	1895.8881	1895.8465	21.9	598 -	612	2	---	K.SEESENRQMMDQLRK.A + Oxidation (M)
2059.0122	2058.0049	2058.0276	-11.01	522 -	538	2	---	R.MRRQLDETNDELAQIAR.E
2152.0510	2151.0437	2151.0663	-10.51	488 -	506	2	---	K.EKTISGMKNIIAEMEQASR.Q + Oxidation (M)
2218.1018	2217.0945	2217.1034	-4.00	231 -	248	2	---	K.CMLEKYERHLAEIQGNVK.V
2221.1035	2220.0962	2220.0626	15.1	594 -	611	2	---	K.NILKSEESENRQMMDQLR.K
2223.2205	2222.2132	2222.1695	19.7	255 -	272	2	---	R.DKTFLLYEQAQEEIARLR.R
2245.0991	2244.0918	2244.0510	18.2	352 -	370	1	---	R.MEQMSNMTLMKETITTVEK.E
2277.0576	2276.0503	2276.0408	4.18	352 -	370	1	---	R.MEQMSNMTLMKETITTVEK.E + 2 Oxidation (M)
2369.2256	2368.2183	2368.1771	17.4	539 -	558	2	---	R.ERDILAHENDNLQEQFAKAK.Q

No match to: 724.2539, 725.2472, 728.2482, 730.2519, 732.2390, 733.2623, 739.3115, 742.4642, 743.4729, 745.4483, 747.4419, 752.3972, 754.3827, 756.3716, 758.3382, 760.3329, 764.3364, 766.3494, 767.4740, 768.3865, 770.3671, 772.4178, 779.4436, 784.3415, 787.2366, 788.4225, 790.4196, 795.4260, 799.2464, 802.4372, 805.4478, 808.4353, 811.4178, 814.4170, 819.4304, 821.4247, 822.4321, 829.4457, 837.4396, 840.4623, 842.5109, 854.4525, 856.5045, 858.5442, 860.4092, 863.4233, 863.9185, 869.4846, 870.5100, 872.4994, 880.4412, 904.4613, 906.4929, 908.4577, 913.5685, 919.5024, 925.5089, 927.4999, 928.5031, 940.4773, 942.4820, 950.4946, 957.5654, 960.4840, 966.4929, 969.4786, 972.5046, 974.5551, 977.5352, 981.4474, 989.2700, 991.5198, 993.5112, 1001.5782, 1007.5089, 1013.4454, 1015.5150, 1016.5522, 1018.5189, 1021.5313, 1024.5028, 1025.5192, 1029.5330, 1032.5870, 1033.5739, 1035.5203, 1046.5759, 1051.5074, 1056.5415, 1057.6005, 1064.5358, 1071.5361, 1073.5377, 1079.5398, 1083.4637, 1086.4774, 1094.5596, 1096.5302, 1106.5431, 1110.5461, 1111.5398, 1111.5400, 1115.5790, 1118.5620, 1120.5724, 1123.5819, 1128.5537, 1132.5682, 1134.5841, 1137.5565, 1143.5844, 1145.6155, 1149.5597, 1153.5929, 1158.6288, 1163.5771, 1173.6008, 1175.5867, 1177.6178, 1178.6053, 1181.6432, 1192.5721, 1199.6315, 1203.5826, 1211.5984, 1215.6246, 1216.6786, 1221.5699, 1222.5789, 1227.6271, 1228.6411, 1229.6412, 1231.6102, 1233.6571, 1237.6270, 1238.5917, 1244.6069, 1248.5958, 1250.6042, 1255.5922, 1258.6344, 1268.6414, 1271.6490, 1275.6388, 1276.5977, 1276.6000, 1285.5864, 1294.6315, 1299.6810, 1300.6566, 1309.6340, 1320.6268, 1328.6292, 1333.7025, 1336.6038, 1338.6589, 1341.6488, 1343.6613, 1345.6910, 1349.6838, 1350.6704, 1352.6350, 1355.6870, 1357.7130, 1358.6704, 1369.7067, 1371.7366, 1374.6705, 1377.6100, 1377.6146, 1384.7109, 1386.7134, 1390.6848, 1399.6119, 1400.6588, 1406.7207, 1410.6736, 1411.6647, 1416.6047, 1418.6714, 1421.6632, 1425.7395, 1426.6846, 1427.7076, 1430.7102, 1432.6718, 1434.6552, 1438.6991, 1439.7164, 1444.7375, 1454.6836, 1461.7990, 1472.7026, 1475.7224, 1485.6906, 1489.7172, 1490.7058, 1493.7157, 1510.7383, 1515.7393, 1523.6987, 1524.7383, 1527.7585, 1529.7466, 1530.6902, 1531.6953, 1538.7736, 1539.7621, 1541.7805, 1542.8290, 1549.6758, 1555.7928, 1560.7551, 1563.7314, 1568.7372, 1570.8015, 1572.8151, 1576.7402, 1577.7395, 1578.7518, 1581.8373, 1591.7852, 1600.8068, 1603.8092, 1604.8136, 1606.8130, 1608.7599, 1613.7793, 1615.8575, 1615.8600, 1628.7653, 1633.7913, 1634.7969, 1635.7941, 1639.8157, 1646.8591, 1647.8268, 1651.8206, 1653.8284, 1677.8074, 1681.8087, 1688.8287, 1690.8313, 1694.7357, 1697.8672, 1703.8214, 1708.3533, 1709.3307, 1715.8053, 1719.8075, 1723.8743, 1724.8828, 1725.8090, 1730.8199, 1748.7931, 1757.8975, 1763.7841, 1768.8075, 1771.9686, 1774.9059, 1779.8081, 1786.8824, 1787.8207, 1790.8423, 1797.8339, 1800.8578, 1819.8838, 1821.8926, 1822.9185, 1826.8450, 1829.8655, 1833.8975, 1836.9054, 1843.8765, 1844.8785, 1851.9031, 1859.8494, 1862.9265, 1863.9319, 1866.8857, 1868.8972, 1879.9445, 1880.9215, 1887.9023, 1888.8057, 1890.8025, 1901.0060, 1906.8872, 1908.8663, 1913.8895, 1916.9475, 1924.9314, 1925.9336, 1928.0060, 1931.9956, 1932.9833, 1968.8563, 1984.9392, 2008.0485, 2008.9869, 2009.9747, 2012.9506, 2015.9661, 2020.9644, 2030.0151, 2030.4648, 2046.9839, 2047.5186, 2047.9097, 2051.9133, 2064.9746, 2070.9209, 2077.0454, 2082.9409, 2085.9888, 2090.9844, 2097.9778, 2103.1526, 2108.9897, 2118.8926, 2140.0432, 2142.0310, 2142.9895, 2155.0667, 2159.0752, 2160.0703, 2161.0813, 2166.0911, 2167.0676, 2174.0425, 2178.0034, 2181.0369, 2184.0767, 2189.9133, 2194.9761, 2199.0986, 2203.0464, 2205.9102, 2208.9729, 2211.1077, 2213.1116, 2214.1145, 2225.1592, 2229.1064, 2233.1023, 2269.1128, 2273.0393, 2279.1094, 2280.1204, 2283.1279, 2285.1169, 2292.1465, 2296.1479, 2312.0664, 2323.1892, 2326.0947, 2334.1531, 2335.1282, 2337.1343, 2372.2229, 2383.9978, 2385.2129, 2392.1528, 2400.1094, 2401.1360, 2409.1914, 2411.1907, 2414.1833, 2429.1475, 2436.1699, 2437.1758, 2440.2007, 2447.2368, 2477.1301, 2495.0542, 2498.0898, 2510.2229, 2522.1980, 2524.1689, 2527.2310, 2535.2598, 2541.2075, 2543.1907, 2544.1514, 2553.1934, 2557.2300, 2560.1125, 2569.2915, 2572.0935, 2612.1936, 2686.3572, 2788.3362, 2963.6167, 3056.4641, 3058.5842, 3180.5432, 3313.5032, 3330.6311, 3347.6077, 3511.9668, 3761.1558

16. Q2KIJ9 **Mass:** 18752 **Score:** 21 **Expect:** 1.8e+002 **Matches:** 11

Transcription elongation factor A protein-like 1 OS=Bos taurus GN=TCEAL1 PE=2 SV=1

Observed	Mr(expt)	Mr(calc)	ppm	Start	End	Miss	Ions	Peptide
766.3494	765.3422	765.3513	-11.95	1 -	6	1	---	-.MEKACK.E
790.4196	789.4123	789.4246	-15.48	109 -	114	0	---	R.SRPQFR.G
960.4840	959.4767	959.4721	4.76	144 -	150	0	---	K.LMVMHWK.A + Oxidation (M)
1094.5596	1093.5523	1093.5404	10.9	97 -	105	1	---	K.LEEGSFKER.L
1203.5826	1202.5753	1202.6052	-24.88	144 -	152	1	---	K.LMVMHWKAR.R + 2 Oxidation (M)
1425.7395	1424.7322	1424.7385	-4.42	109 -	120	1	---	R.SRPQFRGDIHGR.N
1445.7306	1444.7233	1444.7431	-13.70	142 -	152	2	---	R.NKLMVMHWKAR.R + 2 Oxidation (M)
1624.7632	1623.7559	1623.7562	-0.20	4 -	17	1	---	K.ACKEPEEQPQSPPK.A
2012.9506	2011.9433	2011.9343	4.50	1 -	17	2	---	-.MEKACKEPEEQPQSPPK.A
2326.0947	2325.0874	2325.0650	9.65	121 -	139	2	---	R.NLSNEEMIKVAEEMEEMKR.V + Oxidation (M)
2437.1758	2436.1685	2436.2042	-14.65	76 -	96	2	---	R.KDLFEARPPMEQPPCGVGKHK.L + Oxidation (M)

No match to: 724.2539, 725.2472, 728.2482, 730.2519, 732.2390, 733.2623, 739.3115, 742.4642, 743.4729, 745.4483, 747.4419, 752.3972, 754.3827, 756.3716, 758.3382, 760.3329, 764.3364, 767.4740, 768.3865, 770.3671, 772.4178, 779.4436, 784.3415, 787.2366, 788.4225, 795.4260, 799.2464, 802.4372, 805.4478, 808.4353, 811.4178, 814.4170, 819.4304, 821.4247, 822.4321, 829.4457, 837.4396, 840.4623, 842.5109, 854.4525, 856.5045, 858.5442, 860.4092, 863.4233, 863.9185, 869.4846, 870.5100, 872.4994, 880.4412, 904.4613, 906.4929, 908.4577, 913.5685, 919.5024, 925.5089, 927.4999, 928.5031, 940.4773, 942.4820, 950.4946, 957.5654, 966.4929, 969.4786, 972.5046, 974.5551, 977.5352, 978.5240, 981.4474, 989.2700, 991.5198, 993.5112, 1001.5782, 1007.5089, 1009.5097, 1013.4454, 1015.5150, 1016.5522, 1018.5189, 1021.5313, 1024.5028, 1025.5192, 1029.5330, 1032.5870, 1033.5739, 1035.5203, 1045.5564, 1046.5759, 1051.5074, 1056.5415, 1057.6005, 1064.5358, 1071.5361, 1073.5377, 1079.5398, 1083.4637, 1086.4774, 1092.6000, 1092.6002, 1096.5302, 1106.5431, 1110.5461, 1111.5398, 1111.5400, 1115.5790, 1118.5620, 1120.5724, 1123.5819, 1128.5537, 1132.5682, 1134.5841, 1135.5942, 1137.5565, 1143.5844, 1145.6155, 1149.5597, 1153.5929, 1158.6288, 1163.5771, 1173.6008, 1175.5867, 1177.6178, 1178.6053, 1179.5962, 1181.6432, 1192.5721, 1199.6315, 1211.5984, 1215.6246, 1216.6786, 1221.5699, 1222.5789, 1227.6271, 1228.6411, 1229.6412, 1231.6102, 1233.6571, 1237.6270, 1238.5917, 1244.6069, 1248.5958, 1250.6042, 1255.5922, 1258.6344, 1268.6414, 1271.6490, 1275.6388, 1276.5977, 1276.6000, 1285.5864, 1294.6315, 1299.6810, 1300.6566, 1309.6340, 1320.6268, 1328.6292, 1333.7025, 1336.6038, 1338.6589, 1341.6488, 1343.6613, 1345.6910, 1349.6838, 1350.6704, 1352.6350, 1355.6870, 1357.7130, 1358.6704, 1369.7067, 1371.7366, 1374.6705, 1377.6100, 1377.6146, 1384.7109, 1386.7134, 1390.6848, 1393.7169, 1399.6119, 1400.6588, 1406.7207, 1410.6736, 1411.6647, 1416.6047, 1418.6714, 1421.6632, 1426.6846, 1427.7076, 1430.7102, 1432.6718, 1434.6552, 1438.6991, 1439.7164, 1443.7589, 1444.7375, 1454.6836, 1461.7990, 1472.7026, 1475.7224, 1485.6906, 1489.7172, 1490.7058, 1493.7157, 1510.7383, 1515.7393, 1523.6987, 1524.7383, 1527.7585, 1529.7466, 1530.6902, 1531.6953, 1537.7804, 1538.7736, 1539.7621, 1541.7805, 1542.8290, 1549.6758, 1553.7277, 1555.7928, 1556.7874, 1560.7551, 1563.7314, 1568.7372, 1570.8015, 1572.8151, 1576.7402, 1577.7395, 1578.7518, 1581.8373, 1591.7852, 1600.8068, 1603.8092, 1604.8136, 1606.8130, 1608.7599, 1613.7793, 1615.8575, 1615.8600, 1628.7653, 1633.7913, 1634.7969, 1635.7941, 1639.8157, 1643.8085, 1646.8591, 1647.8268, 1651.8206, 1653.8284, 1677.8074, 1681.8087, 1688.8287, 1690.8313, 1694.7357, 1697.8672, 1703.8214, 1706.8364, 1707.8435,

1708.3533, 1709.3307, 1710.8229, 1715.8053, 1719.8075, 1721.8296, 1723.8743, 1724.8828, 1725.8090, 1730.8199, 1748.7931, 1757.8975, 1763.7841, 1768.8075, 1771.9686, 1774.9059, 1777.8727, 1779.8081, 1786.8824, 1787.8207, 1790.8423, 1793.9556, 1796.8109, 1797.8339, 1800.8578, 1815.9008, 1819.8838, 1821.8926, 1822.9185, 1826.8450, 1829.8655, 1833.8975, 1836.9054, 1843.8765, 1844.8785, 1851.9031, 1859.8494, 1862.9265, 1863.9319, 1866.8857, 1868.8972, 1879.9445, 1880.9215, 1887.9023, 1888.8057, 1890.8025, 1896.8954, 1901.0060, 1906.8872, 1908.8663, 1913.8895, 1916.9475, 1924.9314, 1925.9336, 1928.0060, 1931.9956, 1932.9833, 1968.8563, 1984.9392, 2008.0485, 2008.9869, 2009.9747, 2015.9661, 2020.9644, 2030.0151, 2030.4648, 2046.9839, 2047.5186, 2047.9097, 2051.9133, 2059.0122, 2064.9746, 2070.9209, 2077.0454, 2082.9409, 2085.9888, 2090.9844, 2097.9778, 2103.1526, 2108.9897, 2118.8926, 2140.0432, 2142.0310, 2142.9895, 2152.0510, 2155.0667, 2159.0752, 2160.0703, 2161.0813, 2166.0911, 2167.0676, 2174.0425, 2178.0034, 2181.0369, 2184.0767, 2189.9133, 2194.9761, 2199.0986, 2203.0464, 2205.9102, 2208.9729, 2211.1077, 2213.1116, 2214.1145, 2218.1018, 2221.1035, 2223.2205, 2225.1592, 2229.1064, 2233.1023, 2245.0991, 2269.1128, 2273.0393, 2277.0576, 2279.1094, 2280.1204, 2283.1279, 2285.1169, 2292.1465, 2296.1479, 2312.0664, 2323.1892, 2334.1531, 2335.1282, 2337.1343, 2369.2256, 2372.2229, 2383.9978, 2385.2129, 2392.1528, 2400.1094, 2401.1360, 2409.1914, 2411.1907, 2414.1833, 2429.1475, 2436.1699, 2440.2007, 2447.2368, 2477.1301, 2495.0542, 2498.0898, 2510.2229, 2522.1980, 2524.1689, 2527.2310, 2535.2598, 2541.2075, 2543.1907, 2544.1514, 2553.1934, 2557.2300, 2560.1125, 2569.2915, 2572.0935, 2612.1936, 2686.3572, 2788.3362, 2963.6167, 3056.4641, 3058.5842, 3180.5432, 3313.5032, 3330.6311, 3347.6077, 3511.9668, 3761.1558

17. G3MYE2 **Mass:** 34779 **Score:** 21 **Expect:** 2e+002 **Matches:** 15

Uncharacterized protein OS=Bos taurus PE=3 SV=1

Observed	Mr(expt)	Mr(calc)	ppm	Start	End	Miss	Ions	Peptide
840.4623	839.4550	839.4654	-12.34	20	- 25	1	---	R.YQVKFR.R
856.5045	855.4972	855.4827	17.0	267	- 272	1	---	K.RWNRPK.M
957.5654	956.5581	956.5655	-7.70	40	- 47	1	---	R.LVIQDKNK.Y
1341.6488	1340.6415	1340.6693	-20.72	53	- 62	1	---	K.YRMIICVTNR.D + Oxidation (M)
1358.6704	1357.6631	1357.6626	0.39	28	- 38	2	---	R.QEGKTDYYARK.R
1374.6705	1373.6632	1373.6350	20.5	214	- 224	1	---	R.YLTEDEDAYKK.Q
1434.6552	1433.6479	1433.6544	-4.50	202	- 213	0	---	K.HIMGQNVADYMR.Y
1475.7224	1474.7151	1474.7198	-3.17	273	- 285	1	---	K.MSLGQENDRVAQK.K
1578.7518	1577.7445	1577.7442	0.18	201	- 213	1	---	R.KHIMGQNVADYMR.Y + Oxidation (M)
1603.8092	1602.8019	1602.8148	-8.01	273	- 286	2	---	K.MSLGQENDRVAQKK.A
1730.8199	1729.8126	1729.8318	-11.09	268	- 281	1	---	R.WNRPKMSLGQENDR.V
1916.9475	1915.9402	1915.9172	12.0	73	- 89	0	---	R.IEGDMIVCAAYAHELPK.Y
2218.1018	2217.0945	2217.0670	12.4	232	- 250	2	---	K.NNVTPDMEEMYKKAHAAIR.E
2269.1128	2268.1055	2268.0950	4.65	214	- 231	2	---	R.YLTEDEDAYKKQFSQYIK.N
3058.5842	3057.5769	3057.6256	-15.91	90	- 116	2	---	K.YGVKVGLTNYAVAYCTGLLLACRLLNR.F

No match to: 724.2539, 725.2472, 728.2482, 730.2519, 732.2390, 733.2623, 739.3115, 742.4642, 743.4729, 745.4483, 747.4419, 752.3972, 754.3827, 756.3716, 758.3382, 760.3329, 764.3364, 766.3494, 767.4740, 768.3865, 770.3671, 772.4178, 779.4436, 784.3415, 787.2366, 788.4225, 790.4196, 795.4260, 799.2464, 802.4372, 805.4478, 808.4353, 811.4178, 814.4170, 819.4304, 821.4247, 822.4321, 829.4457, 837.4396, 842.5109, 854.4525, 858.5442, 860.4092, 863.4233, 863.9185, 869.4846, 870.5100, 872.4994, 880.4412, 904.4613, 906.4929, 908.4577, 913.5685, 919.5024, 925.5089, 927.4999, 928.5031, 940.4773, 942.4820, 950.4946, 960.4840, 966.4929, 969.4786, 972.5046, 974.5551, 977.5352, 978.5240, 981.4474, 989.2700, 991.5198, 993.5112, 1001.5782, 1007.5089, 1009.5097, 1013.4454, 1015.5150, 1016.5522, 1018.5189, 1021.5313, 1024.5028, 1025.5192, 1029.5330, 1032.5870, 1033.5739, 1035.5203, 1045.5564, 1046.5759, 1051.5074, 1056.5415, 1057.6005, 1064.5358, 1071.5361, 1073.5377, 1079.5398, 1083.4637, 1086.4774, 1092.6000, 1092.6002, 1094.5596, 1096.5302, 1106.5431, 1110.5461, 1111.5398, 1111.5400, 1115.5790, 1118.5620, 1120.5724, 1123.5819, 1128.5537, 1132.5682, 1134.5841, 1135.5942, 1137.5565, 1143.5844, 1145.6155, 1149.5597, 1153.5929, 1158.6288, 1163.5771, 1173.6008, 1175.5867, 1177.6178, 1178.6053, 1179.5962, 1181.6432, 1192.5721, 1199.6315, 1203.5826, 1211.5984, 1215.6246, 1216.6786, 1221.5699, 1222.5789, 1227.6271, 1228.6411, 1229.6412, 1231.6102, 1233.6571, 1237.6270, 1238.5917, 1244.6069, 1248.5958, 1250.6042, 1255.5922, 1258.6344, 1268.6414, 1271.6490, 1275.6388, 1276.5977, 1276.6000, 1285.5864, 1294.6315, 1299.6810, 1300.6566, 1309.6340, 1320.6268, 1328.6292, 1333.7025, 1336.6038, 1338.6589, 1343.6613, 1345.6910, 1349.6838, 1350.6704, 1352.6350, 1355.6870, 1357.7130, 1369.7067, 1371.7366, 1377.6100, 1377.6146, 1384.7109, 1386.7134, 1390.6848, 1393.7169, 1399.6119, 1400.6588, 1406.7207, 1410.6736, 1411.6647, 1416.6047, 1418.6714, 1421.6632, 1425.7395, 1426.6846, 1427.7076, 1430.7102, 1432.6718, 1438.6991, 1439.7164, 1443.7589, 1444.7375, 1445.7306, 1454.6836, 1461.7990, 1472.7026, 1485.6906, 1489.7172, 1490.7058, 1493.7157, 1510.7383, 1515.7393, 1523.6987, 1524.7383, 1527.7585, 1529.7466, 1530.6902, 1531.6953, 1537.7804, 1538.7736, 1539.7621, 1541.7805, 1542.8290, 1549.6758, 1553.7277, 1555.7928, 1556.7874, 1560.7551, 1563.7314, 1568.7372, 1570.8015, 1572.8151, 1576.7402, 1577.7395, 1581.8373, 1591.7852, 1600.8068, 1604.8136, 1606.8130, 1608.7599, 1613.7793, 1615.8575, 1615.8600, 1624.7632, 1628.7653, 1633.7913, 1634.7969, 1635.7941, 1639.8157, 1643.8085, 1646.8591, 1647.8268, 1651.8206, 1653.8284, 1677.8074, 1681.8087, 1688.8287, 1690.8313, 1694.7357, 1697.8672, 1703.8214, 1706.8364, 1707.8435, 1708.3533, 1709.3307, 1710.8229, 1715.8053, 1719.8075, 1721.8296, 1723.8743, 1724.8828, 1725.8090, 1748.7931, 1757.8975, 1763.7841, 1768.8075, 1771.9686, 1774.9059, 1777.8727, 1779.8081, 1786.8824, 1787.8207, 1790.8423, 1793.9556, 1796.8109, 1797.8339, 1800.8578, 1815.9008, 1819.8838, 1821.8926, 1822.9185, 1826.8450, 1829.8655, 1833.8975, 1836.9054, 1843.8765, 1844.8785, 1851.9031, 1859.8494, 1862.9265, 1863.9319, 1866.8857, 1868.8972, 1879.9445, 1880.9215, 1887.9023, 1888.8057, 1890.8025, 1896.8954, 1901.0060, 1906.8872, 1908.8663, 1913.8895, 1924.9314, 1925.9336, 1928.0060, 1931.9956, 1932.9833, 1968.8563, 1984.9392, 2008.0485, 2008.9869, 2009.9747, 2012.9506, 2015.9661, 2020.9644, 2030.0151, 2030.4648, 2046.9839, 2047.5186, 2047.9097, 2051.9133, 2059.0122, 2064.9746, 2070.9209, 2077.0454, 2082.9409, 2085.9888, 2090.9844, 2097.9778, 2103.1526, 2108.9897, 2118.8926, 2140.0432, 2142.0310, 2142.9895, 2152.0510, 2155.0667, 2159.0752, 2160.0703, 2161.0813, 2166.0911, 2167.0676, 2174.0425, 2178.0034, 2181.0369, 2184.0767, 2189.9133, 2194.9761, 2199.0986, 2203.0464, 2205.9102, 2208.9729, 2211.1077, 2213.1116, 2214.1145, 2221.1035, 2223.2205, 2225.1592, 2229.1064, 2233.1023, 2245.0991, 2273.0393, 2277.0576, 2279.1094, 2280.1204, 2283.1279, 2285.1169, 2292.1465, 2296.1479, 2312.0664, 2323.1892, 2326.0947, 2334.1531, 2335.1282, 2337.1343, 2369.2256, 2372.2229, 2383.9978, 2385.2129, 2392.1528, 2400.1094, 2401.1360, 2409.1914, 2411.1907, 2414.1833, 2429.1475, 2436.1699, 2437.1758, 2440.2007, 2447.2368, 2477.1301, 2495.0542, 2498.0898, 2510.2229, 2522.1980, 2524.1689, 2527.2310, 2535.2598, 2541.2075, 2543.1907, 2544.1514, 2553.1934, 2557.2300, 2560.1125, 2569.2915, 2572.0935, 2612.1936, 2686.3572, 2788.3362, 2963.6167, 3056.4641, 3180.5432, 3313.5032, 3330.6311, 3347.6077, 3511.9668, 3761.1558

18. E1BJY0 **Mass:** 22866 **Score:** 20 **Expect:** 2.5e+002 **Matches:** 10

Guanylyl cyclase-activating protein 2 OS=Bos taurus GN=GUCA1B PE=4 SV=2

Observed	Mr(expt)	Mr(calc)	ppm	Start	End	Miss	Ions	Peptide
822.4321	821.4249	821.4106	17.4	168	- 173	1	---	R.DKWVMK.M + Oxidation (M)
978.5240	977.5168	977.5117	5.23	167	- 173	2	---	R.RDKWVMK.M + Oxidation (M)
1371.7366	1370.7293	1370.6983	22.7	99	- 108	2	---	K.WTFKIYDKDR.N
1524.7383	1523.7310	1523.7151	10.5	103	- 114	2	---	K.IYDKDRNGCIDR.Q
1901.0060	1899.9987	1899.9724	13.9	109	- 124	1	---	R.NGCIDRQELLDIVEVK.R
2218.1018	2217.0945	2217.1146	-9.07	174	- 191	2	---	K.MLQMDLNPSSWISQQRRK.S
2337.1343	2336.1270	2336.0783	20.8	48	- 66	1	---	R.FFKVPDNEEATQYVEAMFR.A + Oxidation (M)
2477.1301	2476.1228	2476.1845	-24.91	47	- 66	2	---	K.RFFKVPDNEEATQYVEAMFR.A
2510.2229	2509.2156	2509.1916	9.58	170	- 189	1	---	K.WVMKMLQMDLNPSSWISQQR.R + 2 Oxidation (M)
2535.2598	2534.2525	2534.2653	-5.03	144	- 166	0	---	R.IFLLVDENGDGQLSLNEFVEGAR.R

No match to: 724.2539, 725.2472, 728.2482, 730.2519, 732.2390, 733.2623, 739.3115, 742.4642, 743.4729, 745.4483, 747.4419, 752.3972, 754.3827, 756.3716, 758.3382, 760.3329, 764.3364, 766.3494, 767.4740, 768.3865, 770.3671, 772.4178, 779.4436, 784.3415, 787.2366, 788.4225, 790.4196, 795.4260, 799.2464, 802.4372, 805.4478, 808.4353, 811.4178, 814.4170, 819.4304, 821.4247, 829.4457, 837.4396, 840.4623, 842.5109, 854.4525, 856.5045, 858.5442, 860.4092, 863.4233, 863.9185, 869.4846, 870.5100, 872.4994, 880.4412, 904.4613, 906.4929, 908.4577, 913.5685, 919.5024, 925.5089, 927.4999, 928.5031, 940.4773, 942.4820, 950.4946, 957.5654, 960.4840, 966.4929, 969.4786, 972.5046, 974.5551, 977.5352, 981.4474, 989.2700, 991.5198, 993.5112, 1001.5782, 1007.5089, 1009.5097, 1013.4454, 1015.5150, 1016.5522, 1018.5189, 1021.5313, 1024.5028, 1025.5192, 1029.5330, 1032.5870, 1033.5739, 1035.5203, 1045.5564, 1046.5759, 1051.5074, 1056.5415, 1057.6005, 1064.5358, 1071.5361, 1073.5377, 1079.5398, 1083.4637, 1086.4774, 1092.6000, 1092.6002, 1094.5596, 1096.5302, 1106.5431, 1110.5461, 1111.5398, 1111.5400, 1115.5790, 1118.5620, 1120.5724, 1123.5819, 1128.5537, 1132.5682, 1134.5841, 1135.5942, 1137.5565, 1143.5844, 1145.6155, 1149.5597, 1153.5929, 1158.6288, 1163.5771, 1173.6008, 1175.5867, 1177.6178, 1178.6053, 1179.5962, 1181.6432, 1192.5721, 1199.6315, 1203.5826, 1211.5984, 1215.6246, 1216.6786, 1221.5699, 1222.5789, 1227.6271, 1228.6411, 1229.6412, 1231.6102, 1233.6571, 1237.6270, 1238.5917, 1244.6069, 1248.5958, 1250.6042, 1255.5922, 1258.6344, 1268.6414,

1271.6490, 1275.6388, 1276.5977, 1276.6000, 1285.5864, 1294.6315, 1299.6810, 1300.6566, 1309.6340, 1320.6268, 1328.6292, 1333.7025, 1336.6038, 1338.6589, 1341.6488, 1343.6613, 1345.6910, 1349.6838, 1350.6704, 1352.6350, 1355.6870, 1357.7130, 1358.6704, 1369.7067, 1374.6705, 1377.6100, 1377.6146, 1384.7109, 1386.7134, 1390.6848, 1393.7169, 1399.6119, 1400.6588, 1406.7207, 1410.6736, 1411.6647, 1416.6047, 1418.6714, 1421.6632, 1425.7395, 1426.6846, 1427.7076, 1430.7102, 1432.6718, 1434.6552, 1438.6991, 1439.7164, 1443.7589, 1444.7375, 1445.7306, 1454.6836, 1461.7990, 1472.7026, 1475.7224, 1485.6906, 1489.7172, 1490.7058, 1493.7157, 1510.7383, 1515.7393, 1523.6987, 1527.7585, 1529.7466, 1530.6902, 1531.6953, 1537.7804, 1538.7736, 1539.7621, 1541.7805, 1542.8290, 1549.6758, 1553.7277, 1555.7928, 1556.7874, 1560.7551, 1563.7314, 1568.7372, 1570.8015, 1572.8151, 1576.7402, 1577.7395, 1578.7518, 1581.8373, 1591.7852, 1600.8068, 1603.8092, 1604.8136, 1606.8130, 1608.7599, 1613.7793, 1615.8575, 1615.8600, 1624.7632, 1628.7653, 1633.7913, 1634.7969, 1635.7941, 1639.8157, 1643.8085, 1646.8591, 1647.8268, 1651.8206, 1653.8284, 1677.8074, 1681.8087, 1688.8287, 1690.8313, 1694.7357, 1697.8672, 1703.8214, 1706.8364, 1707.8435, 1708.3533, 1709.3307, 1710.8229, 1715.8053, 1719.8075, 1721.8296, 1723.8743, 1724.8828, 1725.8090, 1730.8199, 1748.7931, 1757.8975, 1763.7841, 1768.8075, 1771.9686, 1774.9059, 1777.8727, 1779.8081, 1786.8824, 1787.8207, 1790.8423, 1793.9556, 1796.8109, 1797.8339, 1800.8578, 1815.9008, 1819.8838, 1821.8926, 1822.9185, 1826.8450, 1829.8655, 1833.8975, 1836.9054, 1843.8765, 1844.8785, 1851.9031, 1859.8494, 1862.9265, 1863.9319, 1866.8857, 1868.8972, 1879.9445, 1880.9215, 1887.9023, 1888.8057, 1890.8025, 1896.8954, 1906.8872, 1908.8663, 1913.8895, 1916.9475, 1924.9314, 1925.9336, 1928.0060, 1931.9956, 1932.9833, 1968.8563, 1984.9392, 2008.0485, 2008.9869, 2009.9747, 2012.9506, 2015.9661, 2020.9644, 2030.0151, 2030.4648, 2046.9839, 2047.5186, 2047.9097, 2051.9133, 2059.0122, 2064.9746, 2070.9209, 2077.0454, 2082.9409, 2085.9888, 2090.9844, 2097.9778, 2103.1526, 2108.9897, 2118.8926, 2140.0432, 2142.0310, 2142.9895, 2152.0510, 2155.0667, 2159.0752, 2160.0703, 2161.0813, 2166.0911, 2167.0676, 2174.0425, 2178.0034, 2181.0369, 2184.0767, 2189.9133, 2194.9761, 2199.0986, 2203.0464, 2205.9102, 2208.9729, 2211.1077, 2213.1116, 2214.1145, 2221.1035, 2223.2205, 2225.1592, 2229.1064, 2233.1023, 2245.0991, 2269.1128, 2273.0393, 2277.0576, 2279.1094, 2280.1204, 2283.1279, 2285.1169, 2292.1465, 2296.1479, 2312.0664, 2323.1892, 2326.0947, 2334.1531, 2335.1282, 2369.2256, 2372.2229, 2383.9978, 2385.2129, 2392.1528, 2400.1094, 2401.1360, 2409.1914, 2411.1907, 2414.1833, 2429.1475, 2436.1699, 2437.1758, 2440.2007, 2447.2368, 2495.0542, 2498.0898, 2522.1980, 2524.1689, 2527.2310, 2541.2075, 2543.1907, 2544.1514, 2553.1934, 2557.2300, 2560.1125, 2569.2915, 2572.0935, 2612.1936, 2686.3572, 2788.3362, 2963.6167, 3056.4641, 3058.5842, 3180.5432, 3313.5032, 3330.6311, 3347.6077, 3511.9668, 3761.1558

19. F1MTD8 **Mass:** 31878 **Score:** 20 **Expect:** 2.6e+002 **Matches:** 14
Tyrosine-protein kinase OS=Bos taurus GN=HCK PE=3 SV=1

Observed	Mr(expt)	Mr(calc)	ppm	Start	End	Miss	Ions	Peptide
880.4412	879.4339	879.4371	-3.63	157 -	163	1	---	R.ESLKMEK.K + Oxidation (M)
906.4929	905.4856	905.4640	23.8	1 -	7	0	---	-.MEQTLLR.S + Oxidation (M)
1268.6414	1267.6341	1267.6197	11.4	22 -	32	0	---	K.EGYIPSNYVAR.V
1529.7466	1528.7393	1528.7369	1.58	69 -	82	1	---	R.DSETTKGSYSLSVR.D
1560.7551	1559.7478	1559.7845	-23.48	2 -	13	1	---	M.EQTLLRSGEWWR.A
1615.8600	1614.8527	1614.8916	-24.06	54 -	68	0	---	R.QLLAPGNVLGSFMIR.D
1707.8435	1706.8362	1706.8199	9.59	1 -	13	1	---	-.MEQTLLRSGEWWR.A + Oxidation (M)
1779.8081	1778.8008	1778.8336	-18.43	75 -	89	1	---	K.GSYSLSVRDYDPQHR.D
1787.8207	1786.8134	1786.8349	-11.99	165 -	180	0	---	K.LGAGQFGEVWMATYNK.H + Oxidation (M)
1931.9956	1930.9883	1930.9645	12.3	134 -	149	0	---	K.LTVPCMSSKPQKPWEK.D + Oxidation (M)
2103.1526	2102.1453	2102.0942	24.3	50 -	68	1	---	K.DAERQLLAPGNVLGSFMIR.D + Oxidation (M)
2155.0667	2154.0594	2154.0523	3.32	188 -	207	0	---	K.TMKPGSMSVEAFLAEANLMK.T
2203.0464	2202.0391	2202.0370	0.95	188 -	207	0	---	K.TMKPGSMSVEAFLAEANLMK.T + 3 Oxidation (M)
2535.2598	2534.2525	2534.3104	-22.83	165 -	187	2	---	K.LGAGQFGEVWMATYNKHTKVAVK.T

No match to: 724.2539, 725.2472, 728.2482, 730.2519, 732.2390, 733.2623, 739.3115, 742.4642, 743.4729, 745.4483, 747.4419, 752.3972, 754.3827, 756.3716, 758.3382, 760.3329, 764.3364, 766.3494, 767.4740, 768.3865, 770.3671, 772.4178, 779.4436, 784.3415, 787.2366, 788.4225, 790.4196, 795.4260, 799.2464, 802.4372, 805.4478, 808.4353, 811.4178, 814.4170, 819.4304, 821.4247, 822.4321, 829.4457, 837.4396, 840.4623, 842.5109, 854.4525, 856.5045, 858.5442, 860.4092, 863.4233, 863.9185, 869.4846, 870.5100, 872.4994, 904.4613, 908.4577, 913.5685, 919.5024, 925.5089, 927.4999, 928.5031, 940.4773, 942.4820, 950.4946, 957.5654, 960.4840, 966.4929, 969.4786, 972.5046, 974.5551, 977.5352, 978.5240, 981.4474, 989.2700, 991.5198, 993.5112, 1001.5782, 1007.5089, 1009.5097, 1013.4454, 1015.5150, 1016.5522, 1018.5189, 1021.5313, 1024.5028, 1025.5192, 1029.5330, 1032.5870, 1033.5739, 1035.5203, 1045.5564, 1046.5759, 1051.5074, 1056.5415, 1057.6005, 1064.5358, 1071.5361, 1073.5377, 1079.5398, 1083.4637, 1086.4774, 1092.6000, 1092.6002, 1094.5596, 1096.5302, 1106.5431, 1110.5461, 1111.5398, 1111.5400, 1115.5790, 1118.5620, 1120.5724, 1123.5819, 1128.5537, 1132.5682, 1134.5841, 1135.5942, 1137.5565, 1143.5844, 1145.6155, 1149.5597, 1158.6288, 1163.5771, 1173.6008, 1175.5867, 1177.6178, 1178.6053, 1179.5962, 1181.6432, 1192.5721, 1199.6315, 1203.5826, 1211.5984, 1215.6246, 1216.6786, 1221.5699, 1222.5789, 1227.6271, 1228.6411, 1229.6412, 1231.6102, 1233.6571, 1237.6270, 1238.5917, 1244.6069, 1248.5958, 1250.6042, 1255.5922, 1258.6344, 1271.6490, 1275.6388, 1276.5977, 1276.6000, 1285.5864, 1294.6315, 1299.6810, 1300.6566, 1309.6340, 1320.6268, 1328.6292, 1333.7025, 1336.6038, 1338.6589, 1341.6488, 1343.6613, 1345.6910, 1349.6838, 1350.6704, 1352.6350, 1355.6870, 1357.7130, 1358.6704, 1369.7067, 1371.7366, 1374.6705, 1377.6100, 1377.6146, 1384.7109, 1386.7134, 1390.6848, 1393.7169, 1399.6119, 1400.6588, 1406.7207, 1410.6736, 1411.6647, 1416.6047, 1418.6714, 1421.6632, 1425.7395, 1426.6846, 1427.7076, 1430.7102, 1432.6718, 1434.6552, 1438.6991, 1439.7164, 1443.7589, 1444.7375, 1445.7306, 1454.6836, 1461.7990, 1472.7026, 1475.7224, 1485.6906, 1489.7172, 1490.7058, 1493.7157, 1510.7383, 1515.7393, 1523.6987, 1524.7383, 1527.7585, 1530.6902, 1531.6953, 1537.7804, 1538.7736, 1539.7621, 1541.7805, 1542.8290, 1549.6758, 1553.7277, 1555.7928, 1556.7874, 1563.7314, 1568.7372, 1570.8015, 1572.8151, 1576.7402, 1577.7395, 1578.7518, 1581.8373, 1591.7852, 1600.8068, 1603.8092, 1604.8136, 1606.8130, 1608.7599, 1613.7793, 1615.8575, 1624.7632, 1628.7653, 1633.7913, 1634.7969, 1635.7941, 1639.8157, 1643.8085, 1646.8591, 1647.8268, 1651.8206, 1653.8284, 1677.8074, 1681.8087, 1688.8287, 1690.8313, 1694.7357, 1697.8672, 1703.8214, 1706.8364, 1708.3533, 1709.3307, 1710.8229, 1715.8053, 1719.8075, 1721.8296, 1723.8743, 1724.8828, 1725.8090, 1730.8199, 1748.7931, 1757.8975, 1763.7841, 1768.8075, 1771.9686, 1774.9059, 1777.8727, 1786.8824, 1790.8423, 1793.9556, 1796.8109, 1797.8339, 1800.8578, 1815.9008, 1819.8838, 1821.8926, 1822.9185, 1826.8450, 1829.8655, 1833.8975, 1836.9054, 1843.8765, 1844.8785, 1851.9031, 1859.8494, 1862.9265, 1863.9319, 1866.8857, 1868.8972, 1879.9445, 1880.9215, 1887.9023, 1888.8057, 1890.8025, 1896.8954, 1901.0060, 1906.8872, 1908.8663, 1913.8895, 1916.9475, 1924.9314, 1925.9336, 1928.0060, 1932.9833, 1968.8563, 1984.9392, 2008.0485, 2008.9869, 2009.9747, 2012.9506, 2015.9661, 2020.9644, 2030.0151, 2030.4648, 2046.9839, 2047.5186, 2047.9097, 2051.9133, 2059.0122, 2064.9746, 2070.9209, 2077.0454, 2082.9409, 2085.9888, 2090.9844, 2097.9778, 2108.9897, 2118.8926, 2140.0432, 2142.0310, 2142.9895, 2152.0510, 2159.0752, 2160.0703, 2161.0813, 2166.0911, 2167.0676, 2174.0425, 2178.0034, 2181.0369, 2184.0767, 2189.9133, 2194.9761, 2199.0986, 2205.9102, 2208.9729, 2211.1077, 2213.1116, 2214.1145, 2218.1018, 2221.1035, 2223.2205, 2225.1592, 2229.1064, 2233.1023, 2245.0991, 2269.1128, 2273.0393, 2277.0576, 2279.1094, 2280.1204, 2283.1279, 2285.1169, 2292.1465, 2296.1479, 2312.0664, 2323.1892, 2326.0947, 2334.1531, 2335.1282, 2337.1343, 2369.2256, 2372.2229, 2383.9978, 2385.2129, 2392.1528, 2400.1094, 2401.1360, 2409.1914, 2411.1907, 2414.1833, 2429.1475, 2436.1699, 2437.1758, 2440.2007, 2447.2368, 2477.1301, 2495.0542, 2498.0898, 2510.2229, 2522.1980, 2524.1689, 2527.2310, 2541.2075, 2543.1907, 2544.1514, 2553.1934, 2557.2300, 2560.1125, 2569.2915, 2572.0935, 2612.1936, 2686.3572, 2788.3362, 2963.6167, 3056.4641, 3058.5842, 3180.5432, 3313.5032, 3330.6311, 3347.6077, 3511.9668, 3761.1558

20. Q2YDN4 **Mass:** 58176 **Score:** 20 **Expect:** 2.6e+002 **Matches:** 22
Coiled-coil domain-containing protein 105 OS=Bos taurus GN=CCDC105 PE=2 SV=1

Observed	Mr(expt)	Mr(calc)	ppm	Start	End	Miss	Ions	Peptide
772.4178	771.4105	771.4239	-17.34	255 -	261	0	---	K.VLEQAGR.H
822.4321	821.4249	821.4297	-5.85	16 -	22	0	---	R.VGHPAWR.E
880.4412	879.4339	879.4371	-3.63	204 -	210	1	---	K.EELKSMK.R + Oxidation (M)
972.5046	971.4974	971.4971	0.29	359 -	366	1	---	R.GTIHRCTK.F
981.4474	980.4401	980.4307	9.64	212 -	219	1	---	R.KMEADMEK.S
1013.4454	1012.4381	1012.4205	17.4	212 -	219	1	---	R.KMEADMEK.S + 2 Oxidation (M)
1064.5358	1063.5285	1063.5518	-21.89	350 -	358	0	---	R.LNMTLGLMR.G + Oxidation (M)
1106.5431	1105.5358	1105.5437	-7.15	432 -	440	0	---	K.NLDELLSMR.K + Oxidation (M)
1137.5565	1136.5492	1136.5318	15.4	211 -	219	2	---	K.RKMEADMEK.S
1163.5771	1162.5698	1162.5951	-21.71	332 -	341	1	---	R.VCASLAQKMR.E
1178.6053	1177.5980	1177.6204	-18.97	404 -	414	0	---	R.HVGTQLPEAAR.L
1179.5962	1178.5889	1178.5900	-0.90	332 -	341	1	---	R.VCASLAQKMR.E + Oxidation (M)
1258.6344	1257.6271	1257.6466	-15.48	452 -	462	0	---	K.IGHNVDYSVVR.L
1510.7383	1509.7310	1509.7054	17.0	213 -	225	1	---	K.MEADMEKSEALLK.T + Oxidation (M)

1639.8157	1638.8084	1638.8208	-7.56	303	316	1	---	R.LLMESKDTLLDMAK.N + 2 Oxidation (M)
1887.9023	1886.8950	1886.8718	12.3	317	331	1	---	K.NEEDIREQQQQISDR.V
1925.9336	1924.9263	1924.9274	-0.55	195	210	2	---	K.ADSMLTWEKEELKSMK.R
2155.0667	2154.0594	2154.0462	6.12	359	375	2	---	R.GTIHRCTKFNQEMYITR.G
2199.0986	2198.0913	2198.0381	24.2	213	231	2	---	K.MEADMEKSEALLKTLASCR.D + Oxidation (M)
2292.1465	2291.1392	2291.1110	12.3	323	341	2	---	R.EQQQQISDRVCASLAQKMR.E + Oxidation (M)
2411.1907	2410.1834	2410.1237	24.8	469	487	1	---	R.HPQVCYEQAQRLVNDWDPR.T
2686.3572	2685.3499	2685.3870	-13.80	75	99	2	---	R.VEMLKGGSTMEKPPPGEGVTLWKSK.M

No match to: 724.2539, 725.2472, 728.2482, 730.2519, 732.2390, 733.2623, 739.3115, 742.4642, 743.4729, 745.4483, 747.4419, 752.3972, 754.3827, 756.3716, 758.3382, 760.3329, 764.3364, 766.3494, 767.4740, 768.3865, 770.3671, 779.4436, 784.3415, 787.2366, 788.4225, 790.4196, 795.4260, 799.2464, 802.4372, 805.4478, 808.4353, 811.4178, 814.4170, 819.4304, 821.4247, 829.4457, 837.4396, 840.4623, 842.5109, 854.4525, 856.5045, 858.5442, 860.4092, 863.4233, 863.9185, 869.4846, 870.5100, 872.4994, 904.4613, 906.4929, 908.4577, 913.5685, 919.5024, 925.5089, 927.4999, 928.5031, 940.4773, 942.4820, 950.4946, 957.5654, 960.4840, 966.4929, 969.4786, 974.5551, 977.5352, 978.5240, 989.2700, 991.5198, 993.5112, 1001.5782, 1007.5089, 1009.5097, 1015.5150, 1016.5522, 1018.5189, 1021.5313, 1024.5028, 1025.5192, 1029.5330, 1032.5870, 1033.5739, 1035.5203, 1045.5564, 1046.5759, 1051.5074, 1056.5415, 1057.6005, 1071.5361, 1073.5377, 1079.5398, 1083.4637, 1086.4774, 1092.6000, 1092.6002, 1094.5596, 1096.5302, 1110.5461, 1111.5398, 1111.5400, 1115.5790, 1118.5620, 1120.5724, 1123.5819, 1128.5537, 1132.5682, 1134.5841, 1135.5942, 1143.5844, 1145.6155, 1149.5597, 1153.5929, 1158.6288, 1173.6008, 1175.5867, 1177.6178, 1181.6432, 1192.5721, 1199.6315, 1203.5826, 1211.5984, 1215.6246, 1216.6786, 1221.5699, 1222.5789, 1227.6271, 1228.6411, 1229.6412, 1231.6102, 1233.6571, 1237.6270, 1238.5917, 1244.6069, 1248.5958, 1250.6042, 1255.5922, 1268.6414, 1271.6490, 1275.6388, 1276.5977, 1276.6000, 1285.5864, 1294.6315, 1299.6810, 1300.6566, 1309.6340, 1320.6268, 1328.6292, 1333.7025, 1336.6038, 1338.6589, 1341.6488, 1343.6613, 1345.6910, 1349.6838, 1350.6704, 1352.6350, 1355.6870, 1357.7130, 1358.6704, 1369.7067, 1371.7366, 1374.6705, 1377.6100, 1377.6146, 1384.7109, 1386.7134, 1390.6848, 1393.7169, 1399.6119, 1400.6588, 1406.7207, 1410.6736, 1411.6647, 1416.6047, 1418.6714, 1421.6632, 1425.7395, 1426.6846, 1427.7076, 1430.7102, 1432.6718, 1434.6552, 1438.6991, 1439.7164, 1443.7589, 1444.7375, 1445.7306, 1454.6836, 1461.7990, 1472.7026, 1475.7224, 1485.6906, 1489.7172, 1490.7058, 1493.7157, 1515.7393, 1523.6987, 1524.7383, 1527.7585, 1529.7466, 1530.6902, 1531.6953, 1537.7804, 1538.7736, 1539.7621, 1541.7805, 1542.8290, 1549.6758, 1553.7277, 1555.7928, 1556.7874, 1560.7551, 1563.7314, 1568.7372, 1570.8015, 1572.8151, 1576.7402, 1577.7395, 1578.7518, 1581.8373, 1591.7852, 1600.8068, 1603.8092, 1604.8136, 1606.8130, 1608.7599, 1613.7793, 1615.8575, 1615.8600, 1624.7632, 1628.7653, 1633.7913, 1634.7969, 1635.7941, 1643.8085, 1646.8591, 1647.8268, 1651.8206, 1653.8284, 1677.8074, 1681.8087, 1688.8287, 1690.8313, 1694.7357, 1697.8672, 1703.8214, 1706.8364, 1707.8435, 1708.3533, 1709.3307, 1710.8229, 1715.8053, 1719.8075, 1721.8296, 1723.8743, 1724.8828, 1725.8090, 1730.8199, 1748.7931, 1757.8975, 1763.7841, 1768.8075, 1771.9686, 1774.9059, 1777.8727, 1779.8081, 1786.8824, 1787.8207, 1790.8423, 1793.9556, 1796.8109, 1797.8339, 1800.8578, 1815.9008, 1819.8838, 1821.8926, 1822.9185, 1826.8450, 1829.8655, 1833.8975, 1836.9054, 1843.8765, 1844.8785, 1851.9031, 1859.8494, 1862.9265, 1863.9319, 1866.8857, 1868.8972, 1879.9445, 1880.9215, 1888.8057, 1890.8025, 1896.8954, 1901.0060, 1906.8872, 1908.8663, 1913.8895, 1916.9475, 1924.9314, 1928.0060, 1931.9956, 1932.9833, 1968.8563, 1984.9392, 2008.0485, 2008.9869, 2009.9747, 2012.9506, 2015.9661, 2020.9644, 2030.0151, 2030.4648, 2046.9839, 2047.5186, 2047.9097, 2051.9133, 2059.0122, 2064.9746, 2070.9209, 2077.0454, 2082.9409, 2085.9888, 2090.9844, 2097.9778, 2103.1526, 2108.9897, 2118.8926, 2140.0432, 2142.0310, 2142.9895, 2152.0510, 2159.0752, 2160.0703, 2161.0813, 2166.0911, 2167.0676, 2174.0425, 2178.0034, 2181.0369, 2184.0767, 2189.9133, 2194.9761, 2203.0464, 2205.9102, 2208.9729, 2211.1077, 2213.1116, 2214.1145, 2218.1018, 2221.1035, 2223.2205, 2225.1592, 2229.1064, 2233.1023, 2245.0991, 2269.1128, 2273.0393, 2277.0576, 2279.1094, 2280.1204, 2283.1279, 2285.1169, 2296.1479, 2312.0664, 2323.1892, 2326.0947, 2334.1531, 2335.1282, 2337.1343, 2369.2256, 2372.2229, 2383.9978, 2385.2129, 2392.1528, 2400.1094, 2401.1360, 2409.1914, 2414.1833, 2429.1475, 2436.1699, 2437.1758, 2440.2007, 2447.2368, 2477.1301, 2495.0542, 2498.0898, 2510.2229, 2522.1980, 2524.1689, 2527.2310, 2535.2598, 2541.2075, 2543.1907, 2544.1514, 2553.1934, 2557.2300, 2560.1125, 2569.2915, 2572.0935, 2612.1936, 2788.3362, 2963.6167, 3056.4641, 3058.5842, 3180.5432, 3313.5032, 3330.6311, 3347.6077, 3511.9668, 3761.1558

21. F1MNJ2 **Mass:** 65953 **Score:** 20 **Expect:** 2.7e+002 **Matches:** 24

Signal recognition particle receptor subunit alpha OS=Bos taurus GN=SRPRA PE=4 SV=1

**Observed**	**Mr(expt)**	**Mr(calc)**	**ppm**	**Start**	**End**	**Miss**	**Ions**	**Peptide**
745.4483	744.4410	744.4242	22.6	166	171	2	---	K.NNKKNK.G
805.4478	804.4405	804.4276	16.1	403	408	1	---	R.RVDMLR.D + Oxidation (M)
814.4170	813.4097	813.3915	22.3	222	228	1	---	K.HGRGMEK.S
819.4304	818.4231	818.4134	11.9	232	239	1	---	K.SSKSDAPK.E
1111.5398	1110.5325	1110.5566	-21.66	316	326	0	---	K.GTLGGMFGMLK.G
1111.5400	1110.5327	1110.5566	-21.48	316	326	0	---	K.GTLGGMFGMLK.G
1132.5682	1131.5609	1131.5455	13.7	222	231	2	---	K.HGRGMEKSSK.S + Oxidation (M)
1143.5844	1142.5771	1142.5832	-5.35	216	224	1	---	R.EEFIQKHGR.G
1216.6786	1215.6713	1215.6758	-3.64	147	156	1	---	K.KPVRSMIETR.G
1299.6810	1298.6737	1298.6843	-8.18	215	224	2	---	K.REEFIQKHGR.G
1352.6350	1351.6277	1351.6112	12.3	337	347	1	---	R.EDMESVLDKMR.D
1357.7130	1356.7057	1356.6959	7.21	375	387	0	---	K.VMGTFSTVTSTVK.Q
1406.7207	1405.7134	1405.7023	7.88	498	511	0	---	K.DAAGIAMEAIAFAR.N
1445.7306	1444.7233	1444.7522	-19.95	172	186	2	---	K.GAKKEGSDGPLATSK.A
1527.7585	1526.7512	1526.7286	14.8	132	144	2	---	R.APTTMKKFEDSEK.A + Oxidation (M)
1538.7736	1537.7663	1537.7294	24.0	333	345	1	---	K.SLTREDMESVLDK.M + Oxidation (M)
1604.8136	1603.8063	1603.7776	17.9	216	228	2	---	R.EEFIQKHGRGMEK.S + Oxidation (M)
1634.7969	1633.7896	1633.8028	-8.08	403	415	2	---	R.RVDMLRDIMDAQR.H + Oxidation (M)
1677.8074	1676.8001	1676.8403	-23.95	123	137	2	---	R.EAEESSKIRAPTTMK.K
1730.8199	1729.8126	1729.8318	-11.10	567	581	1	---	K.FNRALADHSMAQTPR.L + Oxidation (M)
1771.9686	1770.9613	1770.9774	-9.08	147	161	2	---	K.KPVRSMIETRGEKPK.E + Oxidation (M)
2030.0151	2029.0078	2028.9972	5.23	337	353	2	---	R.EDMESVLDKMRDHLIAK.N
2229.1064	2228.0991	2228.1470	-21.51	354	374	1	---	K.NVAADIAVQLCESVANKLEGK.V
3347.6077	3346.6004	3346.6582	-17.26	92	119	2	---	R.DKYRTEIQQQSALSLLNGTFDFQNDFLR.L

No match to: 724.2539, 725.2472, 728.2482, 730.2519, 732.2390, 733.2623, 739.3115, 742.4642, 743.4729, 747.4419, 752.3972, 754.3827, 756.3716, 758.3382, 760.3329, 764.3364, 766.3494, 767.4740, 768.3865, 770.3671, 772.4178, 779.4436, 784.3415, 787.2366, 788.4225, 790.4196, 795.4260, 799.2464, 802.4372, 808.4353, 811.4178, 821.4247, 822.4321, 829.4457, 837.4396, 840.4623, 842.5109, 854.4525, 856.5045, 858.5442, 860.4092, 863.4233, 863.9185, 869.4846, 870.5100, 872.4994, 880.4412, 904.4613, 906.4929, 908.4577, 913.5685, 919.5024, 925.5089, 927.4999, 928.5031, 940.4773, 942.4820, 950.4946, 957.5654, 960.4840, 966.4929, 969.4786, 972.5046, 974.5551, 977.5352, 978.5240, 981.4474, 989.2700, 991.5198, 993.5112, 1001.5782, 1007.5089, 1009.5097, 1013.4454, 1015.5150, 1016.5522, 1018.5189, 1021.5313, 1024.5028, 1025.5192, 1029.5330, 1032.5870, 1033.5739, 1035.5203, 1045.5564, 1046.5759, 1051.5074, 1056.5415, 1057.6005, 1064.5358, 1071.5361, 1073.5377, 1079.5398, 1083.4637, 1086.4774, 1092.6000, 1092.6002, 1094.5596, 1096.5302, 1106.5431, 1110.5461, 1115.5790, 1118.5620, 1120.5724, 1123.5819, 1128.5537, 1134.5841, 1135.5942, 1137.5565, 1145.6155, 1149.5597, 1153.5929, 1158.6288, 1163.5771, 1173.6008, 1175.5867, 1177.6178, 1178.6053, 1179.5962, 1181.6432, 1192.5721, 1199.6315, 1203.5826, 1211.5984, 1215.6246, 1221.5699, 1222.5789, 1227.6271, 1228.6411, 1229.6412, 1231.6102, 1233.6571, 1237.6270, 1238.5917, 1244.6069, 1248.5958, 1250.6042, 1255.5922, 1258.6344, 1268.6414, 1271.6490, 1275.6388, 1276.5977, 1276.6000, 1285.5864, 1294.6315, 1300.6566, 1309.6340, 1320.6268, 1328.6292, 1333.7025, 1336.6038, 1338.6589, 1341.6488, 1343.6613, 1345.6910, 1349.6838, 1350.6704, 1355.6870, 1358.6704, 1369.7067, 1371.7366, 1374.6705, 1377.6100, 1377.6146, 1384.7109, 1386.7134, 1390.6848, 1393.7169, 1399.6119, 1400.6588, 1410.6736, 1411.6647, 1416.6047, 1418.6714, 1421.6632, 1425.7395, 1426.6846, 1427.7076, 1430.7102, 1432.6718, 1434.6552, 1438.6991, 1439.7164, 1443.7589, 1444.7375, 1454.6836, 1461.7990, 1472.7026, 1475.7224, 1485.6906, 1489.7172, 1490.7058, 1493.7157, 1510.7383, 1515.7393, 1523.6987, 1524.7383, 1529.7466, 1530.6902, 1531.6953, 1537.7804, 1539.7621, 1541.7805, 1542.8290, 1549.6758, 1553.7277, 1555.7928, 1556.7874, 1560.7551, 1563.7314, 1568.7372, 1570.8015, 1572.8151, 1576.7402, 1577.7395, 1578.7518, 1581.8373, 1591.7852, 1600.8068, 1603.8092, 1606.8130, 1608.7599, 1613.7793, 1615.8575, 1615.8600, 1624.7632, 1628.7653, 1633.7913, 1635.7941, 1639.8157, 1643.8085, 1646.8591, 1647.8268, 1651.8206, 1653.8284, 1681.8087, 1688.8287, 1690.8313, 1694.7357, 1697.8672, 1703.8214, 1706.8364, 1707.8435, 1708.3533, 1709.3307, 1710.8229, 1715.8053, 1719.8075, 1721.8296, 1723.8743, 1724.8828, 1725.8090, 1748.7931, 1757.8975, 1763.7841, 1768.8075, 1774.9059, 1777.8727, 1779.8081, 1786.8824, 1787.8207, 1790.8423, 1793.9556, 1796.8109, 1797.8339, 1800.8578, 1815.9008, 1819.8838, 1821.8926, 1822.9185, 1826.8450, 1829.8655, 1833.8975, 1836.9054, 1843.8765, 1844.8785, 1851.9031, 1859.8494, 1862.9265, 1863.9319, 1866.8857, 1868.8972, 1879.9445, 1880.9215, 1887.9023, 1888.8057, 1890.8025, 1896.8954, 1901.0060, 1906.8872,

1908.8663, 1913.8895, 1916.9475, 1924.9314, 1925.9336, 1928.0060, 1931.9956, 1932.9833, 1968.8563, 1984.9392, 2008.0485, 2008.9869, 2009.9747, 2012.9506, 2015.9661, 2020.9644, 2030.4648, 2046.9839, 2047.5186, 2047.9097, 2051.9133, 2059.0122, 2064.9746, 2070.9209, 2077.0454, 2082.9409, 2085.9888, 2090.9844, 2097.9778, 2103.1526, 2108.9897, 2118.8926, 2140.0432, 2142.0310, 2142.9895, 2152.0510, 2155.0667, 2159.0752, 2160.0703, 2161.0813, 2166.0911, 2167.0676, 2174.0425, 2178.0034, 2181.0369, 2184.0767, 2189.9133, 2194.9761, 2199.0986, 2203.0464, 2205.9102, 2208.9729, 2211.1077, 2213.1116, 2214.1145, 2218.1018, 2221.1035, 2223.2205, 2225.1592, 2233.1023, 2245.0991, 2269.1128, 2273.0393, 2277.0576, 2279.1094, 2280.1204, 2283.1279, 2285.1169, 2292.1465, 2296.1479, 2312.0664, 2323.1892, 2326.0947, 2334.1531, 2335.1282, 2337.1343, 2369.2256, 2372.2229, 2383.9978, 2385.2129, 2392.1528, 2400.1094, 2401.1360, 2409.1914, 2411.1907, 2414.1833, 2429.1475, 2436.1699, 2437.1758, 2440.2007, 2447.2368, 2477.1301, 2495.0542, 2498.0898, 2510.2229, 2522.1980, 2524.1689, 2527.2310, 2535.2598, 2541.2075, 2543.1907, 2544.1514, 2553.1934, 2557.2300, 2560.1125, 2569.2915, 2572.0935, 2612.1936, 2686.3572, 2788.3362, 2963.6167, 3056.4641, 3058.5842, 3180.5432, 3313.5032, 3330.6311, 3511.9668, 3761.1558

22. Q3MHN7 **Mass:** 69222 **Score:** 20 **Expect:** 2.7e+002 **Matches:** 23

Endonuclease 8-like 3 OS=Bos taurus GN=NEIL3 PE=2 SV=2

Observed	Mr(expt)	Mr(calc)	ppm	Start	End	Miss	Ions	Peptide
790.4196	789.4123	789.4232	-13.76	167 -	173	1	---	R.AESEVKK.Q
966.4929	965.4856	965.4977	-12.53	511 -	518	0	---	K.HGRPCALR.V
1046.5759	1045.5686	1045.5767	-7.76	167 -	175	2	---	R.AESEVKKQK.G
1338.6589	1337.6516	1337.6802	-21.36	576 -	586	1	---	K.NFFVCPLGKEK.Q
1341.6488	1340.6415	1340.6554	-10.34	508 -	518	1	---	R.CSKHGRPCALR.V
1386.7134	1385.7061	1385.6761	21.6	367 -	378	2	---	K.YPCNSFGKSKAK.V
1393.7169	1392.7096	1392.7071	1.79	448 -	459	0	---	K.VNISPVVCSQYK.L
1489.7172	1488.7099	1488.7395	-19.85	354 -	366	0	---	R.NEGNPIVFNNLMK.Y
1529.7466	1528.7393	1528.7668	-17.95	2 -	15	1	---	M.VEGPGCTLNGEKIR.A
1537.7804	1536.7731	1536.7541	12.4	237 -	249	2	---	R.CCKVGSALSKHYK.V
1578.7518	1577.7445	1577.7838	-24.90	69 -	83	0	---	R.LFNGHGYSGVETLGK.E
1600.8068	1599.7995	1599.7603	24.5	128 -	140	0	---	K.DLICFFDSSVEIR.N
1603.8092	1602.8019	1602.7858	10.1	149 -	161	1	---	R.IRVMEELDVCSPR.F
1721.8296	1720.8223	1720.8423	-11.58	174 -	187	2	---	K.QKGRMLCDVLMDQK.V
1757.8975	1756.8902	1756.9043	-7.99	293 -	307	1	---	R.MLPVRNTTVNWPSSR.E
1821.8926	1820.8853	1820.8509	18.9	338 -	353	0	---	K.TCDACLTSRPADSVLR.N
1862.9265	1861.9192	1861.9006	10.0	523 -	538	2	---	K.SGENKGRHFYACPLAR.E
1968.8563	1967.8490	1967.8369	6.16	253 -	267	1	---	K.RPNCGQCCCKITVCR.L
2059.0122	2058.0049	2058.0429	-18.44	293 -	309	2	---	R.MLPVRNTTVNWPSSRER.H + Oxidation (M)
2070.9209	2069.9136	2069.8870	12.9	268 -	283	1	---	R.LGENNRMTYFCPHCQK.E + Oxidation (M)
2312.0664	2311.0591	2311.0824	-10.08	319 -	337	1	---	K.SEEQWTCEVCTLINKLSSK.T
2409.1914	2408.1841	2408.2336	-20.54	385 -	406	1	---	K.TAFGTTTLVLTDFSNKHSALER.E
3056.4641	3055.4568	3055.4401	5.47	274 -	297	2	---	R.MTYFCPHCQKENPQHVDIRMLPVR.N

No match to: 724.2539, 725.2472, 728.2482, 730.2519, 732.2390, 733.2623, 739.3115, 742.4642, 743.4729, 745.4483, 747.4419, 752.3972, 754.3827, 756.3716, 758.3382, 760.3329, 764.3364, 766.3494, 767.4740, 768.3865, 770.3671, 772.4178, 779.4436, 784.3415, 787.2366, 788.4225, 795.4260, 799.2464, 802.4372, 805.4478, 808.4353, 811.4178, 814.4170, 819.4304, 821.4247, 822.4321, 829.4457, 837.4396, 840.4623, 842.5109, 854.4525, 856.5045, 858.5442, 860.4092, 863.4233, 863.9185, 869.4846, 870.5100, 872.4994, 880.4412, 904.4613, 906.4929, 908.4577, 913.5685, 919.5024, 925.5089, 927.4999, 928.5031, 940.4773, 942.4820, 950.4946, 957.5654, 960.4840, 969.4786, 972.5046, 974.5551, 977.5352, 978.5240, 981.4474, 989.2700, 991.5198, 993.5112, 1001.5782, 1007.5089, 1009.5097, 1013.4454, 1015.5150, 1016.5522, 1018.5189, 1021.5313, 1024.5028, 1025.5192, 1029.5330, 1032.5870, 1033.5739, 1035.5203, 1045.5564, 1051.5074, 1056.5415, 1057.6005, 1064.5358, 1071.5361, 1073.5377, 1079.5398, 1083.4637, 1086.4774, 1092.6000, 1092.6002, 1094.5596, 1096.5302, 1106.5431, 1110.5461, 1111.5398, 1111.5400, 1115.5790, 1118.5620, 1120.5724, 1123.5819, 1128.5537, 1132.5682, 1134.5841, 1135.5942, 1137.5565, 1143.5844, 1145.6155, 1149.5597, 1153.5929, 1158.6288, 1163.5771, 1173.6008, 1175.5867, 1177.6178, 1178.6053, 1179.5962, 1181.6432, 1192.5721, 1199.6315, 1203.5826, 1211.5984, 1215.6246, 1216.6786, 1221.5699, 1222.5789, 1227.6271, 1228.6411, 1229.6412, 1231.6102, 1233.6571, 1237.6270, 1238.5917, 1244.6069, 1248.5958, 1250.6042, 1255.5922, 1258.6344, 1268.6414, 1271.6490, 1275.6388, 1276.5977, 1276.6000, 1285.5864, 1294.6315, 1299.6810, 1300.6566, 1309.6340, 1320.6268, 1328.6292, 1333.7025, 1336.6038, 1343.6613, 1345.6910, 1349.6838, 1350.6704, 1352.6350, 1355.6870, 1357.7130, 1358.6704, 1369.7067, 1371.7366, 1374.6705, 1377.6100, 1377.6146, 1384.7109, 1390.6848, 1399.6119, 1400.6588, 1406.7207, 1410.6736, 1411.6647, 1416.6047, 1418.6714, 1421.6632, 1425.7395, 1426.6846, 1427.7076, 1430.7102, 1432.6718, 1434.6552, 1438.6991, 1439.7164, 1443.7589, 1444.7375, 1445.7306, 1454.6836, 1461.7990, 1472.7026, 1475.7224, 1485.6906, 1490.7058, 1493.7157, 1510.7383, 1515.7393, 1523.6987, 1524.7383, 1527.7585, 1530.6902, 1531.6953, 1538.7736, 1539.7621, 1541.7805, 1542.8290, 1549.6758, 1553.7277, 1555.7928, 1556.7874, 1560.7551, 1563.7314, 1568.7372, 1570.8015, 1572.8151, 1576.7402, 1577.7395, 1581.8373, 1591.7852, 1604.8136, 1606.8130, 1608.7599, 1613.7793, 1615.8575, 1615.8600, 1624.7632, 1628.7653, 1633.7913, 1634.7969, 1635.7941, 1639.8157, 1643.8085, 1646.8591, 1647.8268, 1651.8206, 1653.8284, 1677.8074, 1681.8087, 1688.8287, 1690.8313, 1694.7357, 1697.8672, 1703.8214, 1706.8364, 1707.8435, 1708.3533, 1709.3307, 1710.8229, 1715.8053, 1719.8075, 1723.8743, 1724.8828, 1725.8090, 1730.8199, 1748.7931, 1763.7841, 1768.8075, 1771.9686, 1774.9059, 1777.8727, 1779.8081, 1786.8824, 1787.8207, 1790.8423, 1793.9556, 1796.8109, 1797.8339, 1800.8578, 1815.9008, 1819.8838, 1822.9185, 1826.8450, 1829.8655, 1833.8975, 1836.9054, 1843.8765, 1844.8785, 1851.9031, 1859.8494, 1863.9319, 1866.8857, 1868.8972, 1879.9445, 1880.9215, 1887.9023, 1888.8057, 1890.8025, 1896.8954, 1901.0060, 1906.8872, 1908.8663, 1913.8895, 1916.9475, 1924.9314, 1925.9336, 1928.0060, 1931.9956, 1932.9833, 1984.9392, 2008.0485, 2008.9869, 2009.9747, 2012.9506, 2015.9661, 2020.9644, 2030.0151, 2030.4648, 2046.9839, 2047.5186, 2047.9097, 2051.9133, 2064.9746, 2077.0454, 2082.9409, 2085.9888, 2090.9844, 2097.9778, 2103.1526, 2108.9897, 2118.8926, 2140.0432, 2142.0310, 2142.9895, 2152.0510, 2155.0667, 2159.0752, 2160.0703, 2161.0813, 2166.0911, 2167.0676, 2174.0425, 2178.0034, 2181.0369, 2184.0767, 2189.9133, 2194.9761, 2199.0986, 2203.0464, 2205.9102, 2208.9729, 2211.1077, 2213.1116, 2214.1145, 2218.1018, 2221.1035, 2223.2205, 2225.1592, 2229.1064, 2233.1023, 2245.0991, 2269.1128, 2273.0393, 2277.0576, 2279.1094, 2280.1204, 2283.1279, 2285.1169, 2292.1465, 2296.1479, 2323.1892, 2326.0947, 2334.1531, 2335.1282, 2337.1343, 2369.2256, 2372.2229, 2383.9978, 2385.2129, 2392.1528, 2400.1094, 2401.1360, 2411.1907, 2414.1833, 2429.1475, 2436.1699, 2437.1758, 2440.2007, 2447.2368, 2477.1301, 2495.0542, 2498.0898, 2510.2229, 2522.1980, 2524.1689, 2527.2310, 2535.2598, 2541.2075, 2543.1907, 2544.1514, 2553.1934, 2557.2300, 2560.1125, 2569.2915, 2572.0935, 2612.1936, 2686.3572, 2788.3362, 2963.6167, 3058.5842, 3180.5432, 3313.5032, 3330.6311, 3347.6077, 3511.9668, 3761.1558

23. G3MZK6 **Mass:** 94689 **Score:** 20 **Expect:** 2.7e+002 **Matches:** 34

Uncharacterized protein OS=Bos taurus GN=MAP7D3 PE=4 SV=1

Observed	Mr(expt)	Mr(calc)	ppm	Start	End	Miss	Ions	Peptide
802.4372	801.4299	801.4344	-5.64	547 -	552	1	---	K.EERLQK.E
819.4304	818.4231	818.4358	-15.58	162 -	168	1	---	R.SSSLNRR.C
870.5100	869.5027	869.5235	-24.00	120 -	126	1	---	K.FKAVLHR.T
960.4840	959.4767	959.4784	-1.82	96 -	102	2	---	K.EKDEQRR.I
1001.5782	1000.5709	1000.5665	4.42	539 -	546	2	---	R.LAREQKEK.E
1016.5522	1015.5449	1015.5410	3.83	154 -	161	2	---	K.TETPEKRR.S
1046.5759	1045.5686	1045.5628	5.54	739 -	747	1	---	R.SSTKRPTNR.A
1071.5361	1070.5288	1070.5356	-6.36	514 -	524	1	---	K.ESGNKGTPGPK.N
1145.6155	1144.6082	1144.6088	-0.47	66 -	74	1	---	K.ETQLLEKER.K
1221.5699	1220.5626	1220.5455	14.0	109 -	117	1	---	K.RNEMLEEER.E + Oxidation (M)
1222.5789	1221.5716	1221.5772	-4.54	754 -	764	0	---	K.AETSSTMRPSR.S
1231.6102	1230.6029	1230.6316	-23.32	98 -	107	2	---	K.DEQRRISAEK.K
1238.5917	1237.5844	1237.5721	9.97	754 -	764	0	---	K.AETSSTMRPSR.S + Oxidation (M)
1276.5977	1275.5904	1275.5691	16.7	592 -	601	1	---	K.RESQEQEDQK.V
1276.6000	1275.5927	1275.5691	18.5	592 -	601	1	---	K.RESQEQEDQK.V
1299.6810	1298.6737	1298.6466	20.9	519 -	531	1	---	K.GTPGPKNAEEATK.I
1343.6613	1342.6540	1342.6703	-12.16	713 -	723	1	---	K.AFLNTDMKTFR.Q

1386.7134	1385.7061	1385.7238	-12.73	422	432	1	---	K.RIPPSQIPCYR.W
1426.6846	1425.6773	1425.7108	-23.48	553	563	2	---	K.EMEQSFIRKMK.D
1443.7589	1442.7516	1442.7841	-22.53	726	738	2	---	K.GVKDPLNQTKSTR.S
1524.7383	1523.7310	1523.7362	-3.39	751	764	1	---	K.TGKAETSSTMRPSR.S + Oxidation (M)
1527.7585	1526.7512	1526.7650	-9.02	76	87	2	---	K.SKIVYEKQMEEK.Q + Oxidation (M)
1530.6902	1529.6829	1529.7209	-24.83	172	184	0	---	R.LHSSTETEQIEEK.R
1542.8290	1541.8217	1541.8347	-8.45	627	638	1	---	R.IMLQNLQERLER.K
1604.8136	1603.8063	1603.7817	15.3	823	837	1	---	K.SVPAPSGKALSCFHF.-
1615.8575	1614.8502	1614.8188	19.5	713	725	2	---	K.AFLNTDMKTFRQK.G + Oxidation (M)
1615.8600	1614.8527	1614.8188	21.0	713	725	2	---	K.AFLNTDMKTFRQK.G + Oxidation (M)
1715.8053	1714.7980	1714.8382	-23.44	483	498	1	---	R.TVFAQNALGTMGMKSK.A + 2 Oxidation (M)
1774.9059	1773.8986	1773.8857	7.30	497	513	1	---	K.SKAVSNTINNSEAPSQK.E
1797.8339	1796.8266	1796.8594	-18.25	139	153	1	---	K.RWSWAGYTTVNSQNK.T
1819.8838	1818.8765	1818.9087	-17.68	821	837	2	---	R.SKSVPAPSGKALSCFHF.-
1822.9185	1821.9112	1821.9043	3.81	547	560	2	---	K.EERLQKEMEQSFIR.K
2009.9747	2008.9674	2008.9749	-3.71	803	820	1	---	R.GSVTSCLSHQPPIDNKNR.S
2225.1592	2224.1519	2224.1019	22.5	803	822	2	---	R.GSVTSCLSHQPPIDNKNRSK.S

No match to: 724.2539, 725.2472, 728.2482, 730.2519, 732.2390, 733.2623, 739.3115, 742.4642, 743.4729, 745.4483, 747.4419, 752.3972, 754.3827, 756.3716, 758.3382, 760.3329, 764.3364, 766.3494, 767.4740, 768.3865, 770.3671, 772.4178, 779.4436, 784.3415, 787.2366, 788.4225, 790.4196, 795.4260, 799.2464, 805.4478, 808.4353, 811.4178, 814.4170, 821.4247, 822.4321, 829.4457, 837.4396, 840.4623, 842.5109, 854.4525, 856.5045, 858.5442, 860.4092, 863.4233, 863.9185, 869.4846, 872.4994, 880.4412, 904.4613, 906.4929, 908.4577, 913.5685, 919.5024, 925.5089, 927.4999, 928.5031, 940.4773, 942.4820, 950.4946, 957.5654, 966.4929, 969.4786, 972.5046, 974.5551, 977.5352, 978.5240, 981.4474, 989.2700, 991.5198, 993.5112, 1007.5089, 1009.5097, 1013.4454, 1015.5150, 1018.5189, 1021.5313, 1024.5028, 1025.5192, 1029.5330, 1032.5870, 1033.5739, 1035.5203, 1045.5564, 1051.5074, 1056.5415, 1057.6005, 1064.5358, 1073.5377, 1079.5398, 1083.4637, 1086.4774, 1092.6000, 1092.6002, 1094.5596, 1096.5302, 1106.5431, 1110.5461, 1111.5398, 1111.5400, 1115.5790, 1118.5620, 1120.5724, 1123.5819, 1128.5537, 1132.5682, 1134.5841, 1135.5942, 1137.5565, 1143.5844, 1149.5597, 1153.5929, 1158.6288, 1163.5771, 1173.6008, 1175.5867, 1177.6178, 1178.6053, 1179.5962, 1181.6432, 1192.5721, 1199.6315, 1203.5826, 1211.5984, 1215.6246, 1216.6786, 1227.6271, 1228.6411, 1229.6412, 1233.6571, 1237.6270, 1244.6069, 1248.5958, 1250.6042, 1255.5922, 1258.6344, 1268.6414, 1271.6490, 1275.6388, 1285.5864, 1294.6315, 1300.6566, 1309.6340, 1320.6268, 1328.6292, 1333.7025, 1336.6038, 1338.6589, 1341.6488, 1345.6910, 1349.6838, 1350.6704, 1352.6350, 1355.6870, 1357.7130, 1358.6704, 1369.7067, 1371.7366, 1374.6705, 1377.6100, 1377.6146, 1384.7109, 1390.6848, 1393.7169, 1399.6119, 1400.6588, 1406.7207, 1410.6736, 1411.6647, 1416.6047, 1418.6714, 1421.6632, 1425.7395, 1427.7076, 1430.7102, 1432.6718, 1434.6552, 1438.6991, 1439.7164, 1444.7375, 1445.7306, 1454.6836, 1461.7990, 1472.7026, 1475.7224, 1485.6906, 1489.7172, 1490.7058, 1493.7157, 1510.7383, 1515.7393, 1523.6987, 1529.7466, 1531.6953, 1537.7804, 1538.7736, 1539.7621, 1541.7805, 1549.6758, 1553.7277, 1555.7928, 1556.7874, 1560.7551, 1563.7314, 1568.7372, 1570.8015, 1572.8151, 1576.7402, 1577.7395, 1578.7518, 1581.8373, 1591.7852, 1600.8068, 1603.8092, 1606.8130, 1608.7599, 1613.7793, 1624.7632, 1628.7653, 1633.7913, 1634.7969, 1635.7941, 1639.8157, 1643.8085, 1646.8591, 1647.8268, 1651.8206, 1653.8284, 1677.8074, 1681.8087, 1688.8287, 1690.8313, 1694.7357, 1697.8672, 1703.8214, 1706.8364, 1707.8435, 1708.3533, 1709.3307, 1710.8229, 1719.8075, 1721.8296, 1723.8743, 1724.8828, 1725.8090, 1730.8199, 1748.7931, 1757.8975, 1763.7841, 1768.8075, 1771.9686, 1777.8727, 1779.8081, 1786.8824, 1787.8207, 1790.8423, 1793.9556, 1796.8109, 1800.8578, 1815.9008, 1821.8926, 1826.8450, 1829.8655, 1833.8975, 1836.9054, 1843.8765, 1844.8785, 1851.9031, 1859.8494, 1862.9265, 1863.9319, 1866.8857, 1868.8972, 1879.9445, 1880.9215, 1887.9023, 1888.8057, 1890.8025, 1896.8954, 1901.0060, 1906.8872, 1908.8663, 1913.8895, 1916.9475, 1924.9314, 1925.9336, 1928.0060, 1931.9956, 1932.9833, 1968.8563, 1984.9392, 2008.0485, 2008.9869, 2012.9506, 2015.9661, 2020.9644, 2030.0151, 2030.4648, 2046.9839, 2047.5186, 2047.9097, 2051.9133, 2059.0122, 2064.9746, 2070.9209, 2077.0454, 2082.9409, 2085.9888, 2090.9844, 2097.9778, 2103.1526, 2108.9897, 2118.8926, 2140.0432, 2142.0310, 2142.9895, 2152.0510, 2155.0667, 2159.0752, 2160.0703, 2161.0813, 2166.0911, 2167.0676, 2174.0425, 2178.0034, 2181.0369, 2184.0767, 2189.9133, 2194.9761, 2199.0986, 2203.0464, 2205.9102, 2208.9729, 2211.1077, 2213.1116, 2214.1145, 2218.1018, 2221.1035, 2223.2205, 2229.1064, 2233.1023, 2245.0991, 2269.1128, 2273.0393, 2277.0576, 2279.1094, 2280.1204, 2283.1279, 2285.1169, 2292.1465, 2296.1479, 2312.0664, 2323.1892, 2326.0947, 2334.1531, 2335.1282, 2337.1343, 2369.2256, 2372.2229, 2383.9978, 2385.2129, 2392.1528, 2400.1094, 2401.1360, 2409.1914, 2411.1907, 2414.1833, 2429.1475, 2436.1699, 2437.1758, 2440.2007, 2447.2368, 2477.1301, 2495.0542, 2498.0898, 2510.2229, 2522.1980, 2524.1689, 2527.2310, 2535.2598, 2541.2075, 2543.1907, 2544.1514, 2553.1934, 2557.2300, 2560.1125, 2569.2915, 2572.0935, 2612.1936, 2686.3572, 2788.3362, 2963.6167, 3056.4641, 3058.5842, 3180.5432, 3313.5032, 3330.6311, 3347.6077, 3511.9668, 3761.1558

24. P02548 **Mass:** 62665 **Score:** 19 **Expect:** 2.8e+002 **Matches:** 18

Neurofilament light polypeptide OS=Bos taurus GN=NEFL PE=1 SV=3

Observed	Mr(expt)	Mr(calc)	ppm	Start	End	Miss	Ions	Peptide
1015.5150	1014.5077	1014.4995	8.09	129	136	1	---	K.HSEPSRFR.A
1021.5313	1020.5240	1020.5240	0.05	137	144	0	---	R.ALYEQEIR.D
1024.5028	1023.4955	1023.4873	8.08	178	185	0	---	R.YEEEVLSR.E
1271.6490	1270.6417	1270.6530	-8.91	127	136	2	---	R.QKHSEPSRFR.A
1276.5977	1275.5904	1275.5877	2.13	186	196	1	---	R.EDAEGRLMEAR.K
1276.6000	1275.5927	1275.5877	3.93	186	196	1	8	R.EDAEGRLMEAR.K
1390.6848	1389.6775	1389.6736	2.86	542	554	2	---	K.DEGAGEEQATKKK.D
1393.7169	1392.7096	1392.7435	-24.30	381	392	1	---	K.MALDIEIAAYRK.L
1443.7589	1442.7516	1442.7365	10.5	198	211	1	---	K.GADEAALARAELEK.R
1445.7306	1444.7233	1444.7157	5.25	145	157	1	---	R.DLRLAAEDATNEK.Q
1542.8290	1541.8217	1541.8314	-6.29	18	30	1	---	R.YVETPRVHISSVR.S
1606.8130	1605.8057	1605.8110	-3.31	173	185	1	---	R.NLQARYEEEVLSR.E
1843.8765	1842.8692	1842.9071	-20.56	148	164	1	---	R.LAAEDATNEKQALQGER.E
1863.9319	1862.9246	1862.8866	20.4	317	332	1	---	K.TLEIEACRGMNEALEK.Q
2020.9644	2019.9571	2019.9326	12.1	2	17	2	---	M.SSFSYEPYYSTSYKRR.Y
2323.1892	2322.1819	2322.1492	14.1	401	422	0	---	R.LSFTSVGSLTTGYTQSSQVFGR.S
2409.1914	2408.1841	2408.2084	-10.08	88	107	2	---	R.TQEKAQLQDLNDRFASFIER.V
2495.0542	2494.0469	2494.0354	4.63	520	541	2	---	K.EEEGGEGEEAEETKEAEEEEKK.D

No match to: 724.2539, 725.2472, 728.2482, 730.2519, 732.2390, 733.2623, 739.3115, 742.4642, 743.4729, 745.4483, 747.4419, 752.3972, 754.3827, 756.3716, 758.3382, 760.3329, 764.3364, 766.3494, 767.4740, 768.3865, 770.3671, 772.4178, 779.4436, 784.3415, 787.2366, 788.4225, 790.4196, 795.4260, 799.2464, 802.4372, 805.4478, 808.4353, 811.4178, 814.4170, 819.4304, 821.4247, 822.4321, 829.4457, 837.4396, 840.4623, 842.5109, 854.4525, 856.5045, 858.5442, 860.4092, 863.4233, 863.9185, 869.4846, 870.5100, 872.4994, 880.4412, 904.4613, 906.4929, 908.4577, 913.5685, 919.5024, 925.5089, 927.4999, 928.5031, 940.4773, 942.4820, 950.4946, 957.5654, 960.4840, 966.4929, 969.4786, 972.5046, 974.5551, 977.5352, 978.5240, 981.4474, 989.2700, 991.5198, 993.5112, 1001.5782, 1007.5089, 1009.5097, 1013.4454, 1016.5522, 1018.5189, 1025.5192, 1029.5330, 1032.5870, 1033.5739, 1035.5203, 1045.5564, 1046.5759, 1051.5074, 1056.5415, 1057.6005, 1064.5358, 1071.5361, 1073.5377, 1079.5398, 1083.4637, 1086.4774, 1092.6000, 1092.6002, 1094.5596, 1096.5302, 1106.5431, 1110.5461, 1111.5398, 1111.5400, 1115.5790, 1118.5620, 1120.5724, 1123.5819, 1128.5537, 1132.5682, 1134.5841, 1135.5942, 1137.5565, 1143.5844, 1145.6155, 1149.5597, 1153.5929, 1158.6288, 1163.5771, 1173.6008, 1175.5867, 1177.6178, 1178.6053, 1179.5962, 1181.6432, 1192.5721, 1199.6315, 1203.5826, 1211.5984, 1215.6246, 1216.6786, 1221.5699, 1222.5789, 1227.6271, 1228.6411, 1229.6412, 1231.6102, 1233.6571, 1237.6270, 1238.5917, 1244.6069, 1248.5958, 1250.6042, 1255.5922, 1258.6344, 1268.6414, 1275.6388, 1285.5864, 1294.6315, 1299.6810, 1300.6566, 1309.6340, 1320.6268, 1328.6292, 1333.7025, 1336.6038, 1338.6589, 1341.6488, 1343.6613, 1345.6910, 1349.6838, 1350.6704, 1352.6350, 1355.6870, 1357.7130, 1358.6704, 1369.7067, 1371.7366, 1374.6705, 1377.6100, 1377.6146, 1384.7109, 1386.7134, 1399.6119, 1400.6588, 1406.7207, 1410.6736, 1411.6647, 1416.6047, 1418.6714, 1421.6632, 1425.7395, 1426.6846, 1427.7076, 1430.7102, 1432.6718, 1434.6552, 1438.6991, 1439.7164, 1444.7375, 1454.6836, 1461.7990, 1472.7026, 1475.7224, 1485.6906, 1489.7172, 1490.7058, 1493.7157, 1510.7383, 1515.7393, 1523.6987, 1524.7383, 1527.7585, 1529.7466, 1530.6902, 1531.6953, 1537.7804, 1538.7736, 1539.7621, 1541.7805, 1549.6758, 1553.7277, 1555.7928, 1556.7874, 1560.7551, 1563.7314, 1568.7372, 1570.8015, 1572.8151, 1576.7402, 1577.7395, 1578.7518, 1581.8373, 1591.7852, 1600.8068, 1603.8092, 1604.8136, 1608.7599, 1613.7793, 1615.8575, 1615.8600, 1624.7632, 1628.7653, 1633.7913, 1634.7969, 1635.7941, 1639.8157, 1643.8085, 1646.8591, 1647.8268, 1651.8206, 1653.8284, 1677.8074, 1681.8087, 1688.8287, 1690.8313, 1694.7357, 1697.8672, 1703.8214, 1706.8364, 1707.8435, 1708.3533, 1709.3307, 1710.8229, 1715.8053, 1719.8075,

1721.8296, 1723.8743, 1724.8828, 1725.8090, 1730.8199, 1748.7931, 1757.8975, 1763.7841, 1768.8075, 1771.9686, 1774.9059, 1777.8727, 1779.8081, 1786.8824, 1787.8207, 1790.8423, 1793.9556, 1796.8109, 1797.8339, 1800.8578, 1815.9008, 1819.8838, 1821.8926, 1822.9185, 1826.8450, 1829.8655, 1833.8975, 1836.9054, 1844.8785, 1851.9031, 1859.8494, 1862.9265, 1866.8857, 1868.8972, 1879.9445, 1880.9215, 1887.9023, 1888.8057, 1890.8025, 1896.8954, 1901.0060, 1906.8872, 1908.8663, 1913.8895, 1916.9475, 1924.9314, 1925.9336, 1928.0060, 1931.9956, 1932.9833, 1968.8563, 1984.9392, 2008.0485, 2008.9869, 2009.9747, 2012.9506, 2015.9661, 2030.0151, 2030.4648, 2046.9839, 2047.5186, 2047.9097, 2051.9133, 2059.0122, 2064.9746, 2070.9209, 2077.0454, 2082.9409, 2085.9888, 2090.9844, 2097.9778, 2103.1526, 2108.9897, 2118.8926, 2140.0432, 2142.0310, 2142.9895, 2152.0510, 2155.0667, 2159.0752, 2160.0703, 2161.0813, 2166.0911, 2167.0676, 2174.0425, 2178.0034, 2181.0369, 2184.0767, 2189.9133, 2194.9761, 2199.0986, 2203.0464, 2205.9102, 2208.9729, 2211.1077, 2213.1116, 2214.1145, 2218.1018, 2221.1035, 2223.2205, 2225.1592, 2229.1064, 2233.1023, 2245.0991, 2269.1128, 2273.0393, 2277.0576, 2279.1094, 2280.1204, 2283.1279, 2285.1169, 2292.1465, 2296.1479, 2312.0664, 2326.0947, 2334.1531, 2335.1282, 2337.1343, 2369.2256, 2372.2229, 2383.9978, 2385.2129, 2392.1528, 2400.1094, 2401.1360, 2411.1907, 2414.1833, 2429.1475, 2436.1699, 2437.1758, 2440.2007, 2447.2368, 2477.1301, 2498.0898, 2510.2229, 2522.1980, 2524.1689, 2527.2310, 2535.2598, 2541.2075, 2543.1907, 2544.1514, 2553.1934, 2557.2300, 2560.1125, 2569.2915, 2572.0935, 2612.1936, 2686.3572, 2788.3362, 2963.6167, 3056.4641, 3058.5842, 3180.5432, 3313.5032, 3330.6311, 3347.6077, 3511.9668, 3761.1558

25. E1BF55 **Mass:** 69481 **Score:** 19 **Expect:** 2.8e+002 **Matches:** 24

Uncharacterized protein OS=Bos taurus GN=MAP7D2 PE=4 SV=2

Observed	Mr(expt)	Mr(calc)	ppm	Start	End	Miss	Ions	Peptide
745.4483	744.4410	744.4494	-11.19	302 -	307	1	---	K.ILSEKR.R
766.3494	765.3422	765.3513	-11.95	4 -	9	0	---	R.LEAMMR.R + Oxidation (M)
790.4196	789.4123	789.4021	13.0	195 -	201	0	---	K.AYPQSPK.N
802.4372	801.4299	801.4344	-5.64	368 -	374	1	---	K.VAEEAKR.K
919.5024	918.4952	918.4770	19.8	229 -	236	2	---	K.ADKEKSNK.E
1032.5870	1031.5797	1031.5797	0.01	439 -	446	2	---	K.KRIDEIMK.R
1045.5564	1044.5491	1044.5312	17.2	121 -	129	1	---	K.SSPTRNVER.K
1073.5377	1072.5304	1072.5335	-2.88	87 -	95	1	---	K.QTEPPMSKR.L
1163.5771	1162.5698	1162.5587	9.61	1 -	9	1	---	-.QERLEAMMR.R
1177.6178	1176.6105	1176.6139	-2.84	191 -	201	1	---	K.ATSKAYPQSPK.N
1179.5962	1178.5889	1178.6143	-21.51	183 -	194	1	---	R.SSSPVTSKATSK.A
1432.6718	1431.6645	1431.6889	-17.00	23 -	38	1	---	K.KCSSGGASLAAGPGGR.D
1434.6552	1433.6479	1433.6642	-11.38	424 -	434	0	---	R.EQIMLQMEQER.L
1454.6836	1453.6763	1453.7049	-19.63	96 -	109	0	---	R.LSSSTAAISYSPDR.A
1510.7383	1509.7310	1509.7535	-14.91	117 -	129	2	---	K.SSYKSSPTRNVER.K
1560.7551	1559.7478	1559.7838	-23.07	22 -	38	2	---	K.KKCSSGGASLAAGPGGR.D
1570.8015	1569.7942	1569.7998	-3.56	237 -	251	1	---	K.EKEGTLPQQAASSPK.E
1647.8268	1646.8195	1646.8046	9.09	406 -	420	2	---	K.EAAEAKAQEAAKQMR.L + Oxidation (M)
1748.7931	1747.7858	1747.8224	-20.95	283 -	301	0	---	R.DAASGKPTAGTTDAGEATK.I
1771.9686	1770.9613	1770.9556	3.22	582 -	597	0	---	R.ALQDLLDFTGPPIFPK.R
1863.9319	1862.9246	1862.9342	-5.14	421 -	434	2	---	R.LKREQIMLQMEQER.L + 2 Oxidation (M)
1901.0060	1899.9987	1900.0452	-24.44	163 -	181	1	---	R.ATASVPSVGLMSPLRSISK.R
2020.9644	2019.9571	2019.9829	-12.78	423 -	437	2	---	K.REQIMLQMEQERLER.K + 2 Oxidation (M)
2218.1018	2217.0945	2217.0985	-1.81	278 -	301	2	---	K.AKGGRDAASGKPTAGTTDAGEATK.I

No match to: 724.2539, 725.2472, 728.2482, 730.2519, 732.2390, 733.2623, 739.3115, 742.4642, 743.4729, 747.4419, 752.3972, 754.3827, 756.3716, 758.3382, 760.3329, 764.3364, 767.4740, 768.3865, 770.3671, 772.4178, 779.4436, 784.3415, 787.2366, 788.4225, 795.4260, 799.2464, 805.4478, 808.4353, 811.4178, 814.4170, 819.4304, 821.4247, 822.4321, 829.4457, 837.4396, 840.4623, 842.5109, 854.4525, 856.5045, 858.5442, 860.4092, 863.4233, 863.9185, 869.4846, 870.5100, 872.4994, 880.4412, 904.4613, 906.4929, 908.4577, 913.5685, 925.5089, 927.4999, 928.5031, 940.4773, 942.4820, 950.4946, 957.5654, 960.4840, 966.4929, 969.4786, 972.5046, 974.5551, 977.5352, 978.5240, 981.4474, 989.2700, 991.5198, 993.5112, 1001.5782, 1007.5089, 1009.5097, 1013.4454, 1015.5150, 1016.5522, 1018.5189, 1021.5313, 1024.5028, 1025.5192, 1029.5330, 1033.5739, 1035.5203, 1046.5759, 1051.5074, 1056.5415, 1057.6005, 1064.5358, 1071.5361, 1079.5398, 1083.4637, 1086.4774, 1092.6000, 1092.6002, 1094.5596, 1096.5302, 1106.5431, 1110.5461, 1111.5398, 1111.5400, 1115.5790, 1118.5620, 1120.5724, 1123.5819, 1128.5537, 1132.5682, 1134.5841, 1135.5942, 1137.5565, 1143.5844, 1145.6155, 1149.5597, 1153.5929, 1158.6288, 1173.6008, 1175.5867, 1178.6053, 1181.6432, 1192.5721, 1199.6315, 1203.5826, 1211.5984, 1215.6246, 1216.6786, 1221.5699, 1222.5789, 1227.6271, 1228.6411, 1229.6412, 1231.6102, 1233.6571, 1237.6270, 1238.5917, 1244.6069, 1248.5958, 1250.6042, 1255.5922, 1258.6344, 1268.6414, 1271.6490, 1275.6388, 1276.5977, 1276.6000, 1285.5864, 1294.6315, 1299.6810, 1300.6566, 1309.6340, 1320.6268, 1328.6292, 1333.7025, 1336.6038, 1338.6589, 1341.6488, 1343.6613, 1345.6910, 1349.6838, 1350.6704, 1352.6350, 1355.6870, 1357.7130, 1358.6704, 1369.7067, 1371.7366, 1374.6705, 1377.6100, 1377.6146, 1384.7109, 1386.7134, 1390.6848, 1393.7169, 1399.6119, 1400.6588, 1406.7207, 1410.6736, 1411.6647, 1416.6047, 1418.6714, 1421.6632, 1425.7395, 1426.6846, 1427.7076, 1430.7102, 1438.6991, 1439.7164, 1443.7589, 1444.7375, 1445.7306, 1461.7990, 1472.7026, 1475.7224, 1485.6906, 1489.7172, 1490.7058, 1493.7157, 1515.7393, 1523.6987, 1524.7383, 1527.7585, 1529.7466, 1530.6902, 1531.6953, 1537.7804, 1538.7736, 1539.7621, 1541.7805, 1542.8290, 1549.6758, 1553.7277, 1555.7928, 1556.7874, 1563.7314, 1568.7372, 1572.8151, 1576.7402, 1577.7395, 1578.7518, 1581.8373, 1591.7852, 1600.8068, 1603.8092, 1604.8136, 1606.8130, 1608.7599, 1613.7793, 1615.8575, 1615.8600, 1624.7632, 1628.7653, 1633.7913, 1634.7969, 1635.7941, 1639.8157, 1643.8085, 1646.8591, 1651.8206, 1653.8284, 1677.8074, 1681.8087, 1688.8287, 1690.8313, 1694.7357, 1697.8672, 1703.8214, 1706.8364, 1707.8435, 1708.3533, 1709.3307, 1710.8229, 1715.8053, 1719.8075, 1721.8296, 1723.8743, 1724.8828, 1725.8090, 1730.8199, 1757.8975, 1763.7841, 1768.8075, 1774.9059, 1777.8727, 1779.8081, 1786.8824, 1787.8207, 1790.8423, 1793.9556, 1796.8109, 1797.8339, 1800.8578, 1815.9008, 1819.8838, 1821.8926, 1822.9185, 1826.8450, 1829.8655, 1833.8975, 1836.9054, 1843.8765, 1844.8785, 1851.9031, 1859.8494, 1862.9265, 1866.8857, 1868.8972, 1879.9445, 1880.9215, 1887.9023, 1888.8057, 1890.8025, 1896.8954, 1906.8872, 1908.8663, 1913.8895, 1916.9475, 1924.9314, 1925.9336, 1928.0060, 1931.9956, 1932.9833, 1968.8563, 1984.9392, 2008.0485, 2008.9869, 2009.9747, 2012.9506, 2015.9661, 2030.0151, 2030.4648, 2046.9839, 2047.5186, 2047.9097, 2051.9133, 2059.0122, 2064.9746, 2070.9209, 2077.0454, 2082.9409, 2085.9888, 2090.9844, 2097.9778, 2103.1526, 2108.9897, 2118.8926, 2140.0432, 2142.0310, 2142.9895, 2152.0510, 2155.0667, 2159.0752, 2160.0703, 2161.0813, 2166.0911, 2167.0676, 2174.0425, 2178.0034, 2181.0369, 2184.0767, 2189.9133, 2194.9761, 2199.0986, 2203.0464, 2205.9102, 2208.9729, 2211.1077, 2213.1116, 2214.1145, 2221.1035, 2223.2205, 2225.1592, 2229.1064, 2233.1023, 2245.0991, 2269.1128, 2273.0393, 2277.0576, 2279.1094, 2280.1204, 2283.1279, 2285.1169, 2292.1465, 2296.1479, 2312.0664, 2323.1892, 2326.0947, 2334.1531, 2335.1282, 2337.1343, 2369.2256, 2372.2229, 2383.9978, 2385.2129, 2392.1528, 2400.1094, 2401.1360, 2409.1914, 2411.1907, 2414.1833, 2429.1475, 2436.1699, 2437.1758, 2440.2007, 2447.2368, 2477.1301, 2495.0542, 2498.0898, 2510.2229, 2522.1980, 2524.1689, 2527.2310, 2535.2598, 2541.2075, 2543.1907, 2544.1514, 2553.1934, 2557.2300, 2560.1125, 2569.2915, 2572.0935, 2612.1936, 2686.3572, 2788.3362, 2963.6167, 3056.4641, 3058.5842, 3180.5432, 3313.5032, 3330.6311, 3347.6077, 3511.9668, 3761.1558

26. E1BGS4 **Mass:** 63649 **Score:** 19 **Expect:** 2.8e+002 **Matches:** 23

Uncharacterized protein OS=Bos taurus GN=TXLNA PE=4 SV=1

Observed	Mr(expt)	Mr(calc)	ppm	Start	End	Miss	Ions	Peptide
770.3671	769.3599	769.3606	-0.98	324 -	329	0	---	R.EEHIDK.V
863.4233	862.4161	862.4331	-19.71	209 -	215	1	---	R.NSQKQMK.L
913.5685	912.5613	912.5505	11.8	162 -	168	2	---	R.RPQEKKK.A
969.4786	968.4713	968.4563	15.5	142 -	150	0	---	K.GEPGPDEIR.T
1024.5028	1023.4955	1023.4729	22.1	417 -	424	1	---	K.QEMEKMTK.K
1045.5564	1044.5491	1044.5564	-6.92	258 -	266	1	---	R.SLKEEGVQR.A
1115.5790	1114.5717	1114.5731	-1.20	3 -	12	2	---	K.NQDKKNGPSK.Q
1132.5682	1131.5609	1131.5818	-18.49	474 -	482	1	---	K.LCRALQSER.N
1158.6288	1157.6215	1157.6292	-6.61	458 -	467	1	---	R.DKELEGLQVK.I
1328.6292	1327.6219	1327.6117	7.73	151 -	161	1	---	R.TSDEVVDRDHR.R
1426.6846	1425.6773	1425.7099	-22.88	138 -	150	1	---	K.EISKGEPGPDEIR.T
1443.7589	1442.7516	1442.7589	-5.07	477 -	488	2	---	R.ALQSERNDLNKR.V

1444.7375	1443.7302	1443.7430	-8.83	58	72	1	---	K.AEGAQAKSAQSGALR.D
1646.8591	1645.8518	1645.8709	-11.56	446	459	2	---	K.ALLEMAEEKTLRDK.E
1690.8313	1689.8240	1689.7920	19.0	408	421	1	---	K.SSEVFTTFKQEMEK.M
1706.8364	1705.8291	1705.7869	24.8	408	421	1	---	K.SSEVFTTFKQEMEK.M + Oxidation (M)
1790.8423	1789.8350	1789.8053	16.6	432	445	2	---	K.ETTMYRSRWESSNK.A + Oxidation (M)
2077.0454	2076.0381	2076.0660	-13.43	175	192	0	---	K.EITLLMQTLNTLSTPEEK.L + Oxidation (M)
2082.9409	2081.9336	2081.9649	-15.04	408	424	2	---	K.SSEVFTTFKQEMEKMTK.K + 2 Oxidation (M)
2140.0432	2139.0359	2139.0490	-6.13	345	361	2	---	K.LQQAQEMLKEAEERHQR.E + Oxidation (M)
2174.0425	2173.0352	2173.0287	2.99	118	137	1	---	R.TYASRNGEPEPETPVVNGEK.E
2269.1128	2268.1055	2268.0950	4.63	398	416	1	---	K.FEEFQNTLSKSSEVFTTFK.Q
2279.1094	2278.1021	2278.0574	19.6	142	161	2	---	K.GEPGPDEIRTSDEVVDRDHR.R

No match to: 724.2539, 725.2472, 728.2482, 730.2519, 732.2390, 733.2623, 739.3115, 742.4642, 743.4729, 745.4483, 747.4419, 752.3972, 754.3827, 756.3716, 758.3382, 760.3329, 764.3364, 766.3494, 767.4740, 768.3865, 772.4178, 779.4436, 784.3415, 787.2366, 788.4225, 790.4196, 795.4260, 799.2464, 802.4372, 805.4478, 808.4353, 811.4178, 814.4170, 819.4304, 821.4247, 822.4321, 829.4457, 837.4396, 840.4623, 842.5109, 854.4525, 856.5045, 858.5442, 860.4092, 863.9185, 869.4846, 870.5100, 872.4994, 880.4412, 904.4613, 906.4929, 908.4577, 919.5024, 925.5089, 927.4999, 928.5031, 940.4773, 942.4820, 950.4946, 957.5654, 960.4840, 966.4929, 972.5046, 974.5551, 977.5352, 978.5240, 981.4474, 989.2700, 991.5198, 993.5112, 1001.5782, 1007.5089, 1009.5097, 1013.4454, 1015.5150, 1016.5522, 1018.5189, 1021.5313, 1025.5192, 1029.5330, 1032.5870, 1033.5739, 1035.5203, 1046.5759, 1051.5074, 1056.5415, 1057.6005, 1064.5358, 1071.5361, 1073.5377, 1079.5398, 1083.4637, 1086.4774, 1092.6000, 1092.6002, 1094.5596, 1096.5302, 1106.5431, 1110.5461, 1111.5398, 1111.5400, 1118.5620, 1120.5724, 1123.5819, 1128.5537, 1134.5841, 1135.5942, 1137.5565, 1143.5844, 1145.6155, 1149.5597, 1153.5929, 1163.5771, 1173.6008, 1175.5867, 1177.6178, 1178.6053, 1179.5962, 1181.6432, 1192.5721, 1199.6315, 1203.5826, 1211.5984, 1215.6246, 1216.6786, 1221.5699, 1222.5789, 1227.6271, 1228.6411, 1229.6412, 1231.6102, 1233.6571, 1237.6270, 1238.5917, 1244.6069, 1248.5958, 1250.6042, 1255.5922, 1258.6344, 1268.6414, 1271.6490, 1275.6388, 1276.5977, 1276.6000, 1285.5864, 1294.6315, 1299.6810, 1300.6566, 1309.6340, 1320.6268, 1333.7025, 1336.6038, 1338.6589, 1341.6488, 1343.6613, 1345.6910, 1349.6838, 1350.6704, 1352.6350, 1355.6870, 1357.7130, 1358.6704, 1369.7067, 1371.7366, 1374.6705, 1377.6100, 1377.6146, 1384.7109, 1386.7134, 1390.6848, 1393.7169, 1399.6119, 1400.6588, 1406.7207, 1410.6736, 1411.6647, 1416.6047, 1418.6714, 1421.6632, 1425.7395, 1427.7076, 1430.7102, 1432.6718, 1434.6552, 1438.6991, 1439.7164, 1445.7306, 1454.6836, 1461.7990, 1472.7026, 1475.7224, 1485.6906, 1489.7172, 1490.7058, 1493.7157, 1510.7383, 1515.7393, 1523.6987, 1524.7383, 1527.7585, 1529.7466, 1530.6902, 1531.6953, 1537.7804, 1538.7736, 1539.7621, 1541.7805, 1542.8290, 1549.6758, 1553.7277, 1555.7928, 1556.7874, 1560.7551, 1563.7314, 1568.7372, 1570.8015, 1572.8151, 1576.7402, 1577.7395, 1578.7518, 1581.8373, 1591.7852, 1600.8068, 1603.8092, 1604.8136, 1606.8130, 1608.7599, 1613.7793, 1615.8575, 1615.8600, 1624.7632, 1628.7653, 1633.7913, 1634.7969, 1635.7941, 1639.8157, 1643.8085, 1647.8268, 1651.8206, 1653.8284, 1677.8074, 1681.8087, 1688.8287, 1694.7357, 1697.8672, 1703.8214, 1707.8435, 1708.3533, 1709.3307, 1710.8229, 1715.8053, 1719.8075, 1721.8296, 1723.8743, 1724.8828, 1725.8090, 1730.8199, 1748.7931, 1757.8975, 1763.7841, 1768.8075, 1771.9686, 1774.9059, 1777.8727, 1779.8081, 1786.8824, 1787.8207, 1793.9556, 1796.8109, 1797.8339, 1800.8578, 1815.9008, 1819.8838, 1821.8926, 1822.9185, 1826.8450, 1829.8655, 1833.8975, 1836.9054, 1843.8765, 1844.8785, 1851.9031, 1859.8494, 1862.9265, 1863.9319, 1866.8857, 1868.8972, 1879.9445, 1880.9215, 1887.9023, 1888.8057, 1890.8025, 1896.8954, 1901.0060, 1906.8872, 1908.8663, 1913.8895, 1916.9475, 1924.9314, 1925.9336, 1928.0060, 1931.9956, 1932.9833, 1968.8563, 1984.9392, 2008.0485, 2008.9869, 2009.9747, 2012.9506, 2015.9661, 2020.9644, 2030.0151, 2030.4648, 2046.9839, 2047.5186, 2047.9097, 2051.9133, 2059.0122, 2064.9746, 2070.9209, 2085.9888, 2090.9844, 2097.9778, 2103.1526, 2108.9897, 2118.8926, 2142.0310, 2142.9895, 2152.0510, 2155.0667, 2159.0752, 2160.0703, 2161.0813, 2166.0911, 2167.0676, 2178.0034, 2181.0369, 2184.0767, 2189.9133, 2194.9761, 2199.0986, 2203.0464, 2205.9102, 2208.9729, 2211.1077, 2213.1116, 2214.1145, 2218.1018, 2221.1035, 2223.2205, 2225.1592, 2229.1064, 2233.1023, 2245.0991, 2273.0393, 2277.0576, 2280.1204, 2283.1279, 2285.1169, 2292.1465, 2296.1479, 2312.0664, 2323.1892, 2326.0947, 2334.1531, 2335.1282, 2337.1343, 2369.2256, 2372.2229, 2383.9978, 2385.2129, 2392.1528, 2400.1094, 2401.1360, 2409.1914, 2411.1907, 2414.1833, 2429.1475, 2436.1699, 2437.1758, 2440.2007, 2447.2368, 2477.1301, 2495.0542, 2498.0898, 2510.2229, 2522.1980, 2524.1689, 2527.2310, 2535.2598, 2541.2075, 2543.1907, 2544.1514, 2553.1934, 2557.2300, 2560.1125, 2569.2915, 2572.0935, 2612.1936, 2686.3572, 2788.3362, 2963.6167, 3056.4641, 3058.5842, 3180.5432, 3313.5032, 3330.6311, 3347.6077, 3511.9668, 3761.1558

27. Q32LI7 **Mass:** 30313 **Score:** 19 **Expect:** 2.8e+002 **Matches:** 13

Putative uncharacterized protein MGC133880 OS=Bos taurus GN=MGC133880 PE=2 SV=1

Observed	Mr(expt)	Mr(calc)	ppm	Start	End	Miss	Ions	Peptide
805.4478	804.4405	804.4453	-5.96	266	272	2	---	K.SKNKNSK.-
1018.5189	1017.5116	1017.4978	13.6	89	97	1	---	R.DSDPESLKK.G
1021.5313	1020.5240	1020.5465	-21.99	97	105	1	---	K.KGSFRPSSR.E
1118.5620	1117.5547	1117.5476	6.38	57	67	1	---	R.GDSPRSSSLGR.A
1255.5922	1254.5849	1254.6105	-20.41	240	249	1	---	K.TDDLGRWHQK.N
1393.7169	1392.7096	1392.7222	-9.02	97	108	2	---	K.KGSFRPSSRESR.A
1489.7172	1488.7099	1488.7209	-7.34	84	96	1	---	R.GSLWRDSDPESLK.K
1635.7941	1634.7868	1634.7756	6.88	250	263	1	---	K.NAMNMNLQKALDEK.E + Oxidation (M)
1646.8591	1645.8518	1645.8675	-9.53	68	83	0	---	R.ASLEEIPPPPPSAVSR.G
1777.8727	1776.8654	1776.8642	0.66	145	160	0	---	K.FGSNVVDQLVSEEQAR.K
2199.0986	2198.0913	2198.0724	8.60	246	263	2	---	R.WHQKNAMNMNLQKALDEK.E
2214.1145	2213.1072	2213.1413	-15.42	2	21	1	---	M.ELGHRPGTTTLTRAHPNNNK.E
2277.0576	2276.0503	2276.0894	-17.15	123	144	1	---	K.EDPNAGAQGQSGSSIPNNIRHK.F

No match to: 724.2539, 725.2472, 728.2482, 730.2519, 732.2390, 733.2623, 739.3115, 742.4642, 743.4729, 745.4483, 747.4419, 752.3972, 754.3827, 756.3716, 758.3382, 760.3329, 764.3364, 766.3494, 767.4740, 768.3865, 770.3671, 772.4178, 779.4436, 784.3415, 787.2366, 788.4225, 790.4196, 795.4260, 799.2464, 802.4372, 808.4353, 811.4178, 814.4170, 819.4304, 821.4247, 822.4321, 829.4457, 837.4396, 840.4623, 842.5109, 854.4525, 856.5045, 858.5442, 860.4092, 863.4233, 863.9185, 869.4846, 870.5100, 872.4994, 880.4412, 904.4613, 906.4929, 908.4577, 913.5685, 919.5024, 925.5089, 927.4999, 928.5031, 940.4773, 942.4820, 950.4946, 957.5654, 960.4840, 966.4929, 969.4786, 972.5046, 974.5551, 977.5352, 978.5240, 981.4474, 989.2700, 991.5198, 993.5112, 1001.5782, 1007.5089, 1009.5097, 1013.4454, 1015.5150, 1016.5522, 1024.5028, 1025.5192, 1029.5330, 1032.5870, 1033.5739, 1035.5203, 1045.5564, 1046.5759, 1051.5074, 1056.5415, 1057.6005, 1064.5358, 1071.5361, 1073.5377, 1079.5398, 1083.4637, 1086.4774, 1092.6000, 1092.6002, 1094.5596, 1096.5302, 1106.5431, 1110.5461, 1111.5398, 1111.5400, 1115.5790, 1120.5724, 1123.5819, 1128.5537, 1132.5682, 1134.5841, 1135.5942, 1137.5565, 1143.5844, 1145.6155, 1149.5597, 1153.5929, 1158.6288, 1163.5771, 1173.6008, 1175.5867, 1177.6178, 1178.6053, 1179.5962, 1181.6432, 1192.5721, 1199.6315, 1203.5826, 1211.5984, 1215.6246, 1216.6786, 1221.5699, 1222.5789, 1227.6271, 1228.6411, 1229.6412, 1231.6102, 1233.6571, 1237.6270, 1238.5917, 1244.6069, 1248.5958, 1250.6042, 1258.6344, 1268.6414, 1271.6490, 1275.6388, 1276.5977, 1276.6000, 1285.5864, 1294.6315, 1299.6810, 1300.6566, 1309.6340, 1320.6268, 1328.6292, 1333.7025, 1336.6038, 1338.6589, 1341.6488, 1343.6613, 1345.6910, 1349.6838, 1350.6704, 1352.6350, 1355.6870, 1357.7130, 1358.6704, 1369.7067, 1371.7366, 1374.6705, 1377.6100, 1377.6146, 1384.7109, 1386.7134, 1390.6848, 1399.6119, 1400.6588, 1406.7207, 1410.6736, 1411.6647, 1416.6047, 1418.6714, 1421.6632, 1425.7395, 1426.6846, 1427.7076, 1430.7102, 1432.6718, 1434.6552, 1438.6991, 1439.7164, 1443.7589, 1444.7375, 1445.7306, 1454.6836, 1461.7990, 1472.7026, 1475.7224, 1485.6906, 1490.7058, 1493.7157, 1510.7383, 1515.7393, 1523.6987, 1524.7383, 1527.7585, 1529.7466, 1530.6902, 1531.6953, 1537.7804, 1538.7736, 1539.7621, 1541.7805, 1542.8290, 1549.6758, 1553.7277, 1555.7928, 1556.7874, 1560.7551, 1563.7314, 1568.7372, 1570.8015, 1572.8151, 1576.7402, 1577.7395, 1578.7518, 1581.8373, 1591.7852, 1600.8068, 1603.8092, 1604.8136, 1606.8130, 1608.7599, 1613.7793, 1615.8575, 1615.8600, 1624.7632, 1628.7653, 1633.7913, 1634.7969, 1639.8157, 1643.8085, 1647.8268, 1651.8206, 1653.8284, 1677.8074, 1681.8087, 1688.8287, 1690.8313, 1694.7357, 1697.8672, 1703.8214, 1706.8364, 1707.8435, 1708.3533, 1709.3307, 1710.8229, 1715.8053, 1719.8075, 1721.8296, 1723.8743, 1724.8828, 1725.8090, 1730.8199, 1748.7931, 1757.8975, 1763.7841, 1768.8075, 1771.9686, 1774.9059, 1779.8081, 1786.8824, 1787.8207, 1790.8423, 1793.9556, 1796.8109, 1797.8339, 1800.8578, 1815.9008, 1819.8838, 1821.8926, 1822.9185, 1826.8450, 1829.8655, 1833.8975, 1836.9054, 1843.8765, 1844.8785, 1851.9031, 1859.8494, 1862.9265, 1863.9319, 1866.8857, 1868.8972, 1879.9445, 1880.9215, 1887.9023, 1888.8057, 1890.8025, 1896.8954, 1901.0060, 1906.8872, 1908.8663, 1913.8895, 1916.9475, 1924.9314, 1925.9336, 1928.0060, 1931.9956, 1932.9833, 1968.8563, 1984.9392, 2008.0485, 2008.9869, 2009.9747, 2012.9506, 2015.9661, 2020.9644, 2030.0151, 2030.4648, 2046.9839, 2047.5186, 2047.9097, 2051.9133, 2059.0122, 2064.9746, 2070.9209, 2077.0454, 2082.9409, 2085.9888, 2090.9844, 2097.9778, 2103.1526, 2108.9897, 2118.8926, 2140.0432, 2142.0310, 2142.9895, 2152.0510, 2155.0667, 2159.0752, 2160.0703, 2161.0813, 2166.0911, 2167.0676, 2174.0425, 2178.0034, 2181.0369, 2184.0767, 2189.9133, 2194.9761, 2203.0464, 2205.9102, 2208.9729, 2211.1077, 2213.1116, 2218.1018, 2221.1035, 2223.2205, 2225.1592, 2229.1064, 2233.1023, 2245.0991, 2269.1128, 2273.0393, 2279.1094, 2280.1204, 2283.1279, 2285.1169, 2292.1465, 2296.1479, 2312.0664, 2323.1892, 2326.0947, 2334.1531, 2335.1282, 2337.1343, 2369.2256, 2372.2229, 2383.9978, 2385.2129, 2392.1528, 2400.1094, 2401.1360, 2409.1914, 2411.1907, 2414.1833, 2429.1475, 2436.1699, 2437.1758, 2440.2007, 2447.2368, 2477.1301, 2495.0542, 2498.0898, 2510.2229, 2522.1980, 2524.1689, 2527.2310,

2535.2598, 2541.2075, 2543.1907, 2544.1514, 2553.1934, 2557.2300, 2560.1125, 2569.2915, 2572.0935, 2612.1936, 2686.3572, 2788.3362, 2963.6167, 3056.4641, 3058.5842, 3180.5432, 3313.5032, 3330.6311, 3347.6077, 3511.9668, 3761.1558

28. Q8MKH7 **Mass:** 29800 **Score:** 19 **Expect:** 2.9e+002 **Matches:** 14

Troponin T fast skeletal muscle type OS=Bos taurus GN=Tnnt3 PE=2 SV=1

Observed	Mr(expt)	Mr(calc)	ppm	Start	End	Miss	Ions	Peptide
802.4372	801.4299	801.4457	-19.67	153	159	2	---	K.ADQKRGK.K
863.4233	862.4161	862.4331	-19.71	161	167	1	---	K.QTAREMK.K
991.5198	990.5125	990.5280	-15.66	161	168	2	---	K.QTAREMKK.K
1007.5089	1006.5016	1006.5229	-21.17	161	168	2	---	K.QTAREMKK.K + Oxidation (M)
1143.5844	1142.5771	1142.5904	-11.64	92	100	2	---	R.RAERAEQQR.I
1578.7518	1577.7445	1577.7395	3.17	138	152	0	---	K.ALSSMGANYSSYLAK.A + Oxidation (M)
1653.8284	1652.8211	1652.7933	16.8	194	206	0	---	K.ELWDTLYQLETDK.F
1690.8313	1689.8240	1689.8396	-9.19	137	152	1	---	K.KALSSMGANYSSYLAK.A
1706.8364	1705.8291	1705.8345	-3.14	137	152	1	---	K.KALSSMGANYSSYLAK.A + Oxidation (M)
1887.9023	1886.8950	1886.9196	-13.03	59	74	0	---	K.DLMELQALIDSHFEAR.K
2020.9644	2019.9571	2019.9571	0.01	138	156	1	---	K.ALSSMGANYSSYLAKADQK.R + Oxidation (M)
2160.0703	2159.0630	2159.1044	-19.18	59	76	2	---	K.DLMELQALIDSHFEARKK.E + Oxidation (M)
2161.0813	2160.0740	2160.0633	4.97	138	157	2	---	K.ALSSMGANYSSYLAKADQKR.G
2414.1833	2413.1760	2413.2172	-17.05	55	74	2	---	K.RQNKDLMELQALIDSHFEAR.K

No match to: 724.2539, 725.2472, 728.2482, 730.2519, 732.2390, 733.2623, 739.3115, 742.4642, 743.4729, 745.4483, 747.4419, 752.3972, 754.3827, 756.3716, 758.3382, 760.3329, 764.3364, 766.3494, 767.4740, 768.3865, 770.3671, 772.4178, 779.4436, 784.3415, 787.2366, 788.4225, 790.4196, 795.4260, 799.2464, 805.4478, 808.4353, 811.4178, 814.4170, 819.4304, 821.4247, 822.4321, 829.4457, 837.4396, 840.4623, 842.5109, 854.4525, 856.5045, 858.5442, 860.4092, 863.9185, 869.4846, 870.5100, 872.4994, 880.4412, 904.4613, 906.4929, 908.4577, 913.5685, 919.5024, 925.5089, 927.4999, 928.5031, 940.4773, 942.4820, 950.4946, 957.5654, 960.4840, 966.4929, 969.4786, 972.5046, 974.5551, 977.5352, 978.5240, 981.4474, 989.2700, 993.5112, 1001.5782, 1009.5097, 1013.4454, 1015.5150, 1016.5522, 1018.5189, 1021.5313, 1024.5028, 1025.5192, 1029.5330, 1032.5870, 1033.5739, 1035.5203, 1045.5564, 1046.5759, 1051.5074, 1056.5415, 1057.6005, 1064.5358, 1071.5361, 1073.5377, 1079.5398, 1083.4637, 1086.4774, 1092.6000, 1092.6002, 1094.5596, 1096.5302, 1106.5431, 1110.5461, 1111.5398, 1111.5400, 1115.5790, 1118.5620, 1120.5724, 1123.5819, 1128.5537, 1132.5682, 1134.5841, 1135.5942, 1137.5565, 1145.6155, 1149.5597, 1153.5929, 1158.6288, 1163.5771, 1173.6008, 1175.5867, 1177.6178, 1178.6053, 1179.5962, 1181.6432, 1192.5721, 1199.6315, 1203.5826, 1211.5984, 1215.6246, 1216.6786, 1221.5699, 1222.5789, 1227.6271, 1228.6411, 1229.6412, 1231.6102, 1233.6571, 1237.6270, 1238.5917, 1244.6069, 1248.5958, 1250.6042, 1255.5922, 1258.6344, 1268.6414, 1271.6490, 1275.6388, 1276.5977, 1276.6000, 1285.5864, 1294.6315, 1299.6810, 1300.6566, 1309.6340, 1320.6268, 1328.6292, 1333.7025, 1336.6038, 1338.6589, 1341.6488, 1343.6613, 1345.6910, 1349.6838, 1350.6704, 1352.6350, 1355.6870, 1357.7130, 1358.6704, 1369.7067, 1371.7366, 1374.6705, 1377.6100, 1377.6146, 1384.7109, 1386.7134, 1390.6848, 1393.7169, 1399.6119, 1400.6588, 1406.7207, 1410.6736, 1411.6647, 1416.6047, 1418.6714, 1421.6632, 1425.7395, 1426.6846, 1427.7076, 1430.7102, 1432.6718, 1434.6552, 1438.6991, 1439.7164, 1443.7589, 1444.7375, 1445.7306, 1454.6836, 1461.7990, 1472.7026, 1475.7224, 1485.6906, 1489.7172, 1490.7058, 1493.7157, 1510.7383, 1515.7393, 1523.6987, 1524.7383, 1527.7585, 1529.7466, 1530.6902, 1531.6953, 1537.7804, 1538.7736, 1539.7621, 1541.7805, 1542.8290, 1549.6758, 1553.7277, 1555.7928, 1556.7874, 1560.7551, 1563.7314, 1568.7372, 1570.8015, 1572.8151, 1576.7402, 1577.7395, 1581.8373, 1591.7852, 1600.8068, 1603.8092, 1604.8136, 1606.8130, 1608.7599, 1613.7793, 1615.8575, 1615.8600, 1624.7632, 1628.7653, 1633.7913, 1634.7969, 1635.7941, 1639.8157, 1643.8085, 1646.8591, 1647.8268, 1651.8206, 1677.8074, 1681.8087, 1688.8287, 1694.7357, 1697.8672, 1703.8214, 1707.8435, 1708.3533, 1709.3307, 1710.8229, 1715.8053, 1719.8075, 1721.8296, 1723.8743, 1724.8828, 1725.8090, 1730.8199, 1748.7931, 1757.8975, 1763.7841, 1768.8075, 1771.9686, 1774.9059, 1777.8727, 1779.8081, 1786.8824, 1787.8207, 1790.8423, 1793.9556, 1796.8109, 1797.8339, 1800.8578, 1815.9008, 1819.8838, 1821.8926, 1822.9185, 1826.8450, 1829.8655, 1833.8975, 1836.9054, 1843.8765, 1844.8785, 1851.9031, 1859.8494, 1862.9265, 1863.9319, 1866.8857, 1868.8972, 1879.9445, 1880.9215, 1888.8057, 1890.8025, 1896.8954, 1901.0060, 1906.8872, 1908.8663, 1913.8895, 1916.9475, 1924.9314, 1925.9336, 1928.0060, 1931.9956, 1932.9833, 1968.8563, 1984.9392, 2008.0485, 2008.9869, 2009.9747, 2012.9506, 2015.9661, 2030.0151, 2030.4648, 2046.9839, 2047.5186, 2047.9097, 2051.9133, 2059.0122, 2064.9746, 2070.9209, 2077.0454, 2082.9409, 2085.9888, 2090.9844, 2097.9778, 2103.1526, 2108.9897, 2118.8926, 2140.0432, 2142.0310, 2142.9895, 2152.0510, 2155.0667, 2159.0752, 2166.0911, 2167.0676, 2174.0425, 2178.0034, 2181.0369, 2184.0767, 2189.9133, 2194.9761, 2199.0986, 2203.0464, 2205.9102, 2208.9729, 2211.1077, 2213.1116, 2214.1145, 2218.1018, 2221.1035, 2223.2205, 2225.1592, 2229.1064, 2233.1023, 2245.0991, 2269.1128, 2273.0393, 2277.0576, 2279.1094, 2280.1204, 2283.1279, 2285.1169, 2292.1465, 2296.1479, 2312.0664, 2323.1892, 2326.0947, 2334.1531, 2335.1282, 2337.1343, 2369.2256, 2372.2229, 2383.9978, 2385.2129, 2392.1528, 2400.1094, 2401.1360, 2409.1914, 2411.1907, 2429.1475, 2436.1699, 2437.1758, 2440.2007, 2447.2368, 2477.1301, 2495.0542, 2498.0898, 2510.2229, 2522.1980, 2524.1689, 2527.2310, 2535.2598, 2541.2075, 2543.1907, 2544.1514, 2553.1934, 2557.2300, 2560.1125, 2569.2915, 2572.0935, 2612.1936, 2686.3572, 2788.3362, 2963.6167, 3056.4641, 3058.5842, 3180.5432, 3313.5032, 3330.6311, 3347.6077, 3511.9668, 3761.1558

29. G5E6L5 **Mass:** 47201 **Score:** 19 **Expect:** 3e+002 **Matches:** 19

Centrosomal protein of 290 kDa OS=Bos taurus GN=CEP290 PE=4 SV=1

Observed	Mr(expt)	Mr(calc)	ppm	Start	End	Miss	Ions	Peptide
940.4773	939.4700	939.4662	4.11	13	20	0	---	K.VDPDELPR.Q
1007.5089	1006.5016	1006.5196	-17.84	388	396	1	---	R.NKGNSVFNK.Y
1015.5150	1014.5077	1014.4957	11.9	1	8	0	---	-.MPPNINWK.E + Oxidation (M)
1092.6000	1091.5927	1091.5863	5.92	374	382	0	---	K.NTFIIEDLK.N
1092.6002	1091.5929	1091.5863	6.11	374	382	0	---	K.NTFIIEDLK.N
1115.5790	1114.5717	1114.5982	-23.76	331	340	1	---	K.LKNAQLDADK.S
1432.6718	1431.6645	1431.6589	3.90	152	163	1	---	R.NEEAENENSKLR.R
1489.7172	1488.7099	1488.7355	-17.15	341	353	0	---	K.SNVMALQQGIQER.D + Oxidation (M)
1490.7058	1489.6985	1489.6983	0.13	42	53	0	---	K.SESQENMIHLFR.I
1572.8151	1571.8078	1571.8406	-20.86	21	34	0	---	R.QEELADNLLISLSK.V
1647.8268	1646.8195	1646.8488	-17.80	383	396	2	---	K.NELQRNKGNSVFNK.Y
1715.8053	1714.7980	1714.8195	-12.55	287	302	1	---	K.AKNEEDDPIMAAVNAK.V
1721.8296	1720.8223	1720.8632	-23.73	171	184	1	---	R.QDIIDYQKQIDSQK.E
1730.8199	1729.8126	1729.8491	-21.10	255	269	1	---	K.AVVHQTDSMMDQLKK.E
1790.8423	1789.8350	1789.8516	-9.25	123	136	2	---	K.QLEQKDRELEDMEK.E
2015.9661	2014.9588	2014.9564	1.20	91	108	1	---	K.VMKLENELEMAQHSAGGR.D + Oxidation (M)
2229.1064	2228.0991	2228.1041	-2.24	89	108	2	---	K.TKVMKLENELEMAQHSAGGR.D
2245.0991	2244.0918	2244.0990	-3.22	89	108	2	---	K.TKVMKLENELEMAQHSAGGR.D + Oxidation (M)
2292.1465	2291.1392	2291.1402	-0.42	42	60	1	---	K.SESQENMIHLFRITQSLMK.M

No match to: 724.2539, 725.2472, 728.2482, 730.2519, 732.2390, 733.2623, 739.3115, 742.4642, 743.4729, 745.4483, 747.4419, 752.3972, 754.3827, 756.3716, 758.3382, 760.3329, 764.3364, 766.3494, 767.4740, 768.3865, 770.3671, 772.4178, 779.4436, 784.3415, 787.2366, 788.4225, 790.4196, 795.4260, 799.2464, 802.4372, 805.4478, 808.4353, 811.4178, 814.4170, 819.4304, 821.4247, 822.4321, 829.4457, 837.4396, 840.4623, 842.5109, 854.4525, 856.5045, 858.5442, 860.4092, 863.4233, 863.9185, 869.4846, 870.5100, 872.4994, 880.4412, 904.4613, 906.4929, 908.4577, 913.5685, 919.5024, 925.5089, 927.4999, 928.5031, 942.4820, 950.4946, 957.5654, 960.4840, 966.4929, 969.4786, 972.5046, 974.5551, 977.5352, 978.5240, 981.4474, 989.2700, 991.5198, 993.5112, 1001.5782, 1009.5097, 1013.4454, 1016.5522, 1018.5189, 1021.5313, 1024.5028, 1025.5192, 1029.5330, 1032.5870, 1033.5739, 1035.5203, 1045.5564, 1046.5759, 1051.5074, 1056.5415, 1057.6005, 1064.5358, 1071.5361, 1073.5377, 1079.5398, 1083.4637, 1086.4774, 1094.5596, 1096.5302, 1106.5431, 1110.5461, 1111.5398, 1111.5400, 1118.5620, 1120.5724, 1123.5819, 1128.5537, 1132.5682, 1134.5841, 1135.5942, 1137.5565, 1143.5844, 1145.6155, 1149.5597, 1153.5929, 1158.6288, 1163.5771, 1173.6008, 1175.5867, 1177.6178, 1178.6053, 1179.5962, 1181.6432, 1192.5721, 1199.6315, 1203.5826, 1211.5984, 1215.6246, 1216.6786, 1221.5699, 1222.5789, 1227.6271, 1228.6411, 1229.6412, 1231.6102, 1233.6571, 1237.6270, 1238.5917, 1244.6069, 1248.5958, 1250.6042, 1255.5922, 1258.6344, 1268.6414, 1271.6490, 1275.6388, 1276.5977, 1276.6000, 1285.5864, 1294.6315, 1299.6810, 1300.6566, 1309.6340, 1320.6268, 1328.6292, 1333.7025, 1336.6038, 1338.6589, 1341.6488, 1343.6613, 1345.6910, 1349.6838, 1350.6704, 1352.6350, 1355.6870, 1357.7130, 1358.6704, 1369.7067, 1371.7366, 1374.6705, 1377.6100, 1377.6146, 1384.7109, 1386.7134, 1390.6848, 1393.7169, 1399.6119, 1400.6588, 1406.7207, 1410.6736, 1411.6647, 1416.6047, 1418.6714, 1421.6632,

1425.7395, 1426.6846, 1427.7076, 1430.7102, 1434.6552, 1438.6991, 1439.7164, 1443.7589, 1444.7375, 1445.7306, 1454.6836, 1461.7990, 1472.7026, 1475.7224, 1485.6906, 1493.7157, 1510.7383, 1515.7393, 1523.6987, 1524.7383, 1527.7585, 1529.7466, 1530.6902, 1531.6953, 1537.7804, 1538.7736, 1539.7621, 1541.7805, 1542.8290, 1549.6758, 1553.7277, 1555.7928, 1556.7874, 1560.7551, 1563.7314, 1568.7372, 1570.8015, 1576.7402, 1577.7395, 1578.7518, 1581.8373, 1591.7852, 1600.8068, 1603.8092, 1604.8136, 1606.8130, 1608.7599, 1613.7793, 1615.8575, 1615.8600, 1624.7632, 1628.7653, 1633.7913, 1634.7969, 1635.7941, 1639.8157, 1643.8085, 1646.8591, 1651.8206, 1653.8284, 1677.8074, 1681.8087, 1688.8287, 1690.8313, 1694.7357, 1697.8672, 1703.8214, 1706.8364, 1707.8435, 1708.3533, 1709.3307, 1710.8229, 1719.8075, 1723.8743, 1724.8828, 1725.8090, 1748.7931, 1757.8975, 1763.7841, 1768.8075, 1771.9686, 1774.9059, 1777.8727, 1779.8081, 1786.8824, 1787.8207, 1793.9556, 1796.8109, 1797.8339, 1800.8578, 1815.9008, 1819.8838, 1821.8926, 1822.9185, 1826.8450, 1829.8655, 1833.8975, 1836.9054, 1843.8765, 1844.8785, 1851.9031, 1859.8494, 1862.9265, 1863.9319, 1866.8857, 1868.8972, 1879.9445, 1880.9215, 1887.9023, 1888.8057, 1890.8025, 1896.8954, 1901.0060, 1906.8872, 1908.8663, 1913.8895, 1916.9475, 1924.9314, 1925.9336, 1928.0060, 1931.9956, 1932.9833, 1968.8563, 1984.9392, 2008.0485, 2008.9869, 2009.9747, 2012.9506, 2020.9644, 2030.0151, 2030.4648, 2046.9839, 2047.5186, 2047.9097, 2051.9133, 2059.0122, 2064.9746, 2070.9209, 2077.0454, 2082.9409, 2085.9888, 2090.9844, 2097.9778, 2103.1526, 2108.9897, 2118.8926, 2140.0432, 2142.0310, 2142.9895, 2152.0510, 2155.0667, 2159.0752, 2160.0703, 2161.0813, 2166.0911, 2167.0676, 2174.0425, 2178.0034, 2181.0369, 2184.0767, 2189.9133, 2194.9761, 2199.0986, 2203.0464, 2205.9102, 2208.9729, 2211.1077, 2213.1116, 2214.1145, 2218.1018, 2221.1035, 2223.2205, 2225.1592, 2233.1023, 2269.1128, 2273.0393, 2277.0576, 2279.1094, 2280.1204, 2283.1279, 2285.1169, 2296.1479, 2312.0664, 2323.1892, 2326.0947, 2334.1531, 2335.1282, 2337.1343, 2369.2256, 2372.2229, 2383.9978, 2385.2129, 2392.1528, 2400.1094, 2401.1360, 2409.1914, 2411.1907, 2414.1833, 2429.1475, 2436.1699, 2437.1758, 2440.2007, 2447.2368, 2477.1301, 2495.0542, 2498.0898, 2510.2229, 2522.1980, 2524.1689, 2527.2310, 2535.2598, 2541.2075, 2543.1907, 2544.1514, 2553.1934, 2557.2300, 2560.1125, 2569.2915, 2572.0935, 2612.1936, 2686.3572, 2788.3362, 2963.6167, 3056.4641, 3058.5842, 3180.5432, 3313.5032, 3330.6311, 3347.6077, 3511.9668, 3761.1558

30. F1MJU1 **Mass:** 14824 **Score:** 19 **Expect:** 3e+002 **Matches:** 9

Histone H2B OS=Bos taurus PE=3 SV=2

Observed	Mr(expt)	Mr(calc)	ppm	Start		End	Miss	Ions	Peptide
745.4483	744.4410	744.4494	-11.21	18	-	24	1	---	K.AVTKAQK.D
1092.6000	1091.5927	1091.5975	-4.35	2	-	12	1	---	M.PEPAKSAPAPK.K
1092.6002	1091.5929	1091.5975	-4.17	2	-	12	1	---	M.PEPAKSAPAPK.K
1137.5565	1136.5492	1136.5390	9.02	35	-	43	0	---	K.ESYSVYVYK.V
1199.6315	1198.6242	1198.6306	-5.31	108	-	119	1	---	K.HAVSEGTKAATK.Y
1604.8136	1603.8063	1603.8430	-22.88	79	-	91	2	---	R.LEHYNKRSTITSR.E
1628.7653	1627.7580	1627.7334	15.1	58	-	71	0	---	K.AMGIMNSVNDIFER.I + 2 Oxidation (M)
1793.9556	1792.9483	1792.9068	23.2	108	-	124	2	---	K.HAVSEGTKAATKYTSSR.F
2788.3362	2787.3289	2787.3684	-14.15	47	-	71	1	---	K.QVHPDTGILSKAMGIMNSVNDIFER.I + Oxidation (M)

No match to: 724.2539, 725.2472, 728.2482, 730.2519, 732.2390, 733.2623, 739.3115, 742.4642, 743.4729, 747.4419, 752.3972, 754.3827, 756.3716, 758.3382, 760.3329, 764.3364, 766.3494, 767.4740, 768.3865, 770.3671, 772.4178, 779.4436, 784.3415, 787.2366, 788.4225, 790.4196, 795.4260, 799.2464, 802.4372, 805.4478, 808.4353, 811.4178, 814.4170, 819.4304, 821.4247, 822.4321, 829.4457, 837.4396, 840.4623, 842.5109, 854.4525, 856.5045, 858.5442, 860.4092, 863.4233, 863.9185, 869.4846, 870.5100, 872.4994, 880.4412, 904.4613, 906.4929, 908.4577, 913.5685, 919.5024, 925.5089, 927.4999, 928.5031, 940.4773, 942.4820, 950.4946, 957.5654, 960.4840, 966.4929, 969.4786, 972.5046, 974.5551, 977.5352, 978.5240, 981.4474, 989.2700, 991.5198, 993.5112, 1001.5782, 1007.5089, 1009.5097, 1013.4454, 1015.5150, 1016.5522, 1018.5189, 1021.5313, 1024.5028, 1025.5192, 1029.5330, 1032.5870, 1033.5739, 1035.5203, 1045.5564, 1046.5759, 1051.5074, 1056.5415, 1057.6005, 1064.5358, 1071.5361, 1073.5377, 1079.5398, 1083.4637, 1086.4774, 1094.5596, 1096.5302, 1106.5431, 1110.5461, 1111.5398, 1111.5400, 1115.5790, 1118.5620, 1120.5724, 1123.5819, 1128.5537, 1132.5682, 1134.5841, 1135.5942, 1143.5844, 1145.6155, 1149.5597, 1153.5929, 1158.6288, 1163.5771, 1173.6008, 1175.5867, 1177.6178, 1178.6053, 1179.5962, 1181.6432, 1192.5721, 1203.5826, 1211.5984, 1215.6246, 1216.6786, 1221.5699, 1222.5789, 1227.6271, 1228.6411, 1229.6412, 1231.6102, 1233.6571, 1237.6270, 1238.5917, 1244.6069, 1248.5958, 1250.6042, 1255.5922, 1258.6344, 1268.6414, 1271.6490, 1275.6388, 1276.5977, 1276.6000, 1285.5864, 1294.6315, 1299.6810, 1300.6566, 1309.6340, 1320.6268, 1328.6292, 1333.7025, 1336.6038, 1338.6589, 1341.6488, 1343.6613, 1345.6910, 1349.6838, 1350.6704, 1352.6350, 1355.6870, 1357.7130, 1358.6704, 1369.7067, 1371.7366, 1374.6705, 1377.6100, 1377.6146, 1384.7109, 1386.7134, 1390.6848, 1393.7169, 1399.6119, 1400.6588, 1406.7207, 1410.6736, 1411.6647, 1416.6047, 1418.6714, 1421.6632, 1425.7395, 1426.6846, 1427.7076, 1430.7102, 1432.6718, 1434.6552, 1438.6991, 1439.7164, 1443.7589, 1444.7375, 1445.7306, 1454.6836, 1461.7990, 1472.7026, 1475.7224, 1485.6906, 1489.7172, 1490.7058, 1493.7157, 1510.7383, 1515.7393, 1523.6987, 1524.7383, 1527.7585, 1529.7466, 1530.6902, 1531.6953, 1537.7804, 1538.7736, 1539.7621, 1541.7805, 1542.8290, 1549.6758, 1553.7277, 1555.7928, 1556.7874, 1560.7551, 1563.7314, 1568.7372, 1570.8015, 1572.8151, 1576.7402, 1577.7395, 1578.7518, 1581.8373, 1591.7852, 1600.8068, 1603.8092, 1606.8130, 1608.7599, 1613.7793, 1615.8575, 1615.8600, 1624.7632, 1633.7913, 1634.7969, 1635.7941, 1639.8157, 1643.8085, 1646.8591, 1647.8268, 1651.8206, 1653.8284, 1677.8074, 1681.8087, 1688.8287, 1690.8313, 1694.7357, 1697.8672, 1703.8214, 1706.8364, 1707.8435, 1708.3533, 1709.3307, 1710.8229, 1715.8053, 1719.8075, 1721.8296, 1723.8743, 1724.8828, 1725.8090, 1730.8199, 1748.7931, 1757.8975, 1763.7841, 1768.8075, 1771.9686, 1774.9059, 1777.8727, 1779.8081, 1786.8824, 1787.8207, 1790.8423, 1796.8109, 1797.8339, 1800.8578, 1815.9008, 1819.8838, 1821.8926, 1822.9185, 1826.8450, 1829.8655, 1833.8975, 1836.9054, 1843.8765, 1844.8785, 1851.9031, 1859.8494, 1862.9265, 1863.9319, 1866.8857, 1868.8972, 1879.9445, 1880.9215, 1887.9023, 1888.8057, 1890.8025, 1896.8954, 1901.0060, 1906.8872, 1908.8663, 1913.8895, 1916.9475, 1924.9314, 1925.9336, 1928.0060, 1931.9956, 1932.9833, 1968.8563, 1984.9392, 2008.0485, 2008.9869, 2009.9747, 2012.9506, 2015.9661, 2020.9644, 2030.0151, 2030.4648, 2046.9839, 2047.5186, 2047.9097, 2051.9133, 2059.0122, 2064.9746, 2070.9209, 2077.0454, 2082.9409, 2085.9888, 2090.9844, 2097.9778, 2103.1526, 2108.9897, 2118.8926, 2140.0432, 2142.0310, 2142.9895, 2152.0510, 2155.0667, 2159.0752, 2160.0703, 2161.0813, 2166.0911, 2167.0676, 2174.0425, 2178.0034, 2181.0369, 2184.0767, 2189.9133, 2194.9761, 2199.0986, 2203.0464, 2205.9102, 2208.9729, 2211.1077, 2213.1116, 2214.1145, 2218.1018, 2221.1035, 2223.2205, 2225.1592, 2229.1064, 2233.1023, 2245.0991, 2269.1128, 2273.0393, 2277.0576, 2279.1094, 2280.1204, 2283.1279, 2285.1169, 2292.1465, 2296.1479, 2312.0664, 2323.1892, 2326.0947, 2334.1531, 2335.1282, 2337.1343, 2369.2256, 2372.2229, 2383.9978, 2385.2129, 2392.1528, 2400.1094, 2401.1360, 2409.1914, 2411.1907, 2414.1833, 2429.1475, 2436.1699, 2437.1758, 2440.2007, 2447.2368, 2477.1301, 2495.0542, 2498.0898, 2510.2229, 2522.1980, 2524.1689, 2527.2310, 2535.2598, 2541.2075, 2543.1907, 2544.1514, 2553.1934, 2557.2300, 2560.1125, 2569.2915, 2572.0935, 2612.1936, 2686.3572, 2963.6167, 3056.4641, 3058.5842, 3180.5432, 3313.5032, 3330.6311, 3347.6077, 3511.9668, 3761.1558

31. G3MYI2 **Mass:** 118628 **Score:** 19 **Expect:** 3e+002 **Matches:** 34

Uncharacterized protein OS=Bos taurus PE=4 SV=1

Observed	Mr(expt)	Mr(calc)	ppm	Start		End	Miss	Ions	Peptide
747.4419	746.4346	746.4174	23.1	231	-	236	1	---	K.IKTEEK.S
802.4372	801.4299	801.4344	-5.64	299	-	305	1	---	R.GREAELK.Q
805.4478	804.4405	804.4341	7.98	258	-	264	0	---	K.SETLISR.E
819.4304	818.4231	818.4068	19.8	201	-	206	1	---	K.LNECKR.K
942.4820	941.4747	941.4930	-19.43	681	-	689	0	---	K.NITAAAPER.C
957.5654	956.5581	956.5403	18.6	293	-	300	1	---	K.DIDLLRGR.E
1175.5867	1174.5794	1174.5578	18.4	894	-	902	1	---	R.QEEEQRQTK.R
1250.6042	1249.5969	1249.5860	8.76	407	-	416	0	---	K.ELELEMESVR.A + Oxidation (M)
1299.6810	1298.6737	1298.6983	-18.90	21	-	30	2	---	R.KKLYQTFTDR.G
1355.6870	1354.6797	1354.6802	-0.37	433	-	443	1	---	K.VKEMDNYSLLK.E + Oxidation (M)
1400.6588	1399.6515	1399.6805	-20.73	214	-	224	1	---	R.AEMNQELKYFK.D
1426.6846	1425.6773	1425.6809	-2.54	435	-	446	1	---	K.EMDNYSLLKEGK.L
1485.6906	1484.6833	1484.6639	13.1	949	-	960	1	---	K.NFEMSVQEMKDK.S
1523.6987	1522.6914	1522.6722	12.6	744	-	756	0	---	R.SHDSEMYLEGLSR.S
1539.7621	1538.7548	1538.7246	19.6	712	-	726	0	---	K.SVVSEVPSSMASTSR.K + Oxidation (M)
1570.8015	1569.7942	1569.8038	-6.12	353	-	364	1	---	K.YQLELKEDYITR.T
1578.7518	1577.7445	1577.7144	19.1	910	-	921	1	---	R.EWGELEMLDRER.R + Oxidation (M)
1651.8206	1650.8133	1650.8247	-6.88	712	-	727	1	---	K.SVVSEVPSSMASTSRK.S
1653.8284	1652.8211	1652.8443	-14.03	433	-	446	2	---	K.VKEMDNYSLLKEGK.L
1721.8296	1720.8223	1720.8454	-13.40	961	-	975	1	---	K.SISVSGENHLEKYMK.I
1730.8199	1729.8126	1729.8080	2.67	332	-	345	1	---	R.EMTIKSLEETYDQK.L + Oxidation (M)

1815.9008	1814.8935	1814.8911	1.34	880	893	2	---	K.EEDKLWEQQAKQQR.Q
1822.9185	1821.9112	1821.8679	23.8	239	252	2	---	K.CEKELAEFRNELER.A
1887.9023	1886.8950	1886.9342	-20.77	417	432	1	---	R.AHCLALTKQNQMLSEK.V + Oxidation (M)
1984.9392	1983.9319	1983.9208	5.63	973	988	1	---	K.YMKILQQNQDQDTTDK.C + Oxidation (M)
2008.0485	2007.0412	2007.0385	1.38	248	264	2	---	R.NELERAYQAKSETLISR.E
2108.9897	2107.9824	2108.0320	-23.53	757	776	2	---	R.SPVAAPCPDRKSQPSPAESK.C
2152.0510	2151.0437	2151.0954	-24.01	712	732	2	---	K.SVVSEVPSSMASTSRKSAVSR.W
2184.0767	2183.0694	2183.1044	-16.04	208	224	2	---	K.IEQQLRAEMNQELKYFK.D + Oxidation (M)
2334.1531	2333.1458	2333.1070	16.7	904	921	2	---	R.QETLQREWGELEMLDRER.R + Oxidation (M)
2372.2229	2371.2156	2371.2239	-3.49	405	424	2	---	R.VKELELEMESVRAHCLALTK.Q + Oxidation (M)
2409.1914	2408.1841	2408.2084	-10.07	491	511	2	---	K.AENAIASEHKEFETHKQALQK.Q
2498.0898	2497.0825	2497.1406	-23.26	940	960	2	---	K.QFFSFGNGKNFEMSVQEMKDK.S
2544.1514	2543.1441	2543.1697	-10.06	155	176	1	---	K.ESRNMETQTSSTFPIEDSLVEK.L + Oxidation (M)

No match to: 724.2539, 725.2472, 728.2482, 730.2519, 732.2390, 733.2623, 739.3115, 742.4642, 743.4729, 745.4483, 752.3972, 754.3827, 756.3716, 758.3382, 760.3329, 764.3364, 766.3494, 767.4740, 768.3865, 770.3671, 772.4178, 779.4436, 784.3415, 787.2366, 788.4225, 790.4196, 795.4260, 799.2464, 808.4353, 811.4178, 814.4170, 821.4247, 822.4321, 829.4457, 837.4396, 840.4623, 842.5109, 854.4525, 856.5045, 858.5442, 860.4092, 863.4233, 863.9185, 869.4846, 870.5100, 872.4994, 880.4412, 904.4613, 906.4929, 908.4577, 913.5685, 919.5024, 925.5089, 927.4999, 928.5031, 940.4773, 950.4946, 960.4840, 966.4929, 969.4786, 972.5046, 974.5551, 977.5352, 978.5240, 981.4474, 989.2700, 991.5198, 993.5112, 1001.5782, 1007.5089, 1009.5097, 1013.4454, 1015.5150, 1016.5522, 1018.5189, 1021.5313, 1024.5028, 1025.5192, 1029.5330, 1032.5870, 1033.5739, 1035.5203, 1045.5564, 1046.5759, 1051.5074, 1056.5415, 1057.6005, 1064.5358, 1071.5361, 1073.5377, 1079.5398, 1083.4637, 1086.4774, 1092.6000, 1092.6002, 1094.5596, 1096.5302, 1106.5431, 1110.5461, 1111.5398, 1111.5400, 1115.5790, 1118.5620, 1120.5724, 1123.5819, 1128.5537, 1132.5682, 1134.5841, 1135.5942, 1137.5565, 1143.5844, 1145.6155, 1149.5597, 1153.5929, 1158.6288, 1163.5771, 1173.6008, 1177.6178, 1178.6053, 1179.5962, 1181.6432, 1192.5721, 1199.6315, 1203.5826, 1211.5984, 1215.6246, 1216.6786, 1221.5699, 1222.5789, 1227.6271, 1228.6411, 1229.6412, 1231.6102, 1233.6571, 1237.6270, 1238.5917, 1244.6069, 1248.5958, 1255.5922, 1258.6344, 1268.6414, 1271.6490, 1275.6388, 1276.5977, 1276.6000, 1285.5864, 1294.6315, 1300.6566, 1309.6340, 1320.6268, 1328.6292, 1333.7025, 1336.6038, 1338.6589, 1341.6488, 1343.6613, 1345.6910, 1349.6838, 1350.6704, 1352.6350, 1357.7130, 1358.6704, 1369.7067, 1371.7366, 1374.6705, 1377.6100, 1377.6146, 1384.7109, 1386.7134, 1390.6848, 1393.7169, 1399.6119, 1406.7207, 1410.6736, 1411.6647, 1416.6047, 1418.6714, 1421.6632, 1425.7395, 1427.7076, 1430.7102, 1432.6718, 1434.6552, 1438.6991, 1439.7164, 1443.7589, 1444.7375, 1445.7306, 1454.6836, 1461.7990, 1472.7026, 1475.7224, 1489.7172, 1490.7058, 1493.7157, 1510.7383, 1515.7393, 1524.7383, 1527.7585, 1529.7466, 1530.6902, 1531.6953, 1537.7804, 1538.7736, 1541.7805, 1542.8290, 1549.6758, 1553.7277, 1555.7928, 1556.7874, 1560.7551, 1563.7314, 1568.7372, 1572.8151, 1576.7402, 1577.7395, 1581.8373, 1591.7852, 1600.8068, 1603.8092, 1604.8136, 1606.8130, 1608.7599, 1613.7793, 1615.8575, 1615.8600, 1624.7632, 1628.7653, 1633.7913, 1634.7969, 1635.7941, 1639.8157, 1643.8085, 1646.8591, 1647.8268, 1677.8074, 1681.8087, 1688.8287, 1690.8313, 1694.7357, 1697.8672, 1703.8214, 1706.8364, 1707.8435, 1708.3533, 1709.3307, 1710.8229, 1715.8053, 1719.8075, 1723.8743, 1724.8828, 1725.8090, 1748.7931, 1757.8975, 1763.7841, 1768.8075, 1771.9686, 1774.9059, 1777.8727, 1779.8081, 1786.8824, 1787.8207, 1790.8423, 1793.9556, 1796.8109, 1797.8339, 1800.8578, 1819.8838, 1821.8926, 1826.8450, 1829.8655, 1833.8975, 1836.9054, 1843.8765, 1844.8785, 1851.9031, 1859.8494, 1862.9265, 1863.9319, 1866.8857, 1868.8972, 1879.9445, 1880.9215, 1888.8057, 1890.8025, 1896.8954, 1901.0060, 1906.8872, 1908.8663, 1913.8895, 1916.9475, 1924.9314, 1925.9336, 1928.0060, 1931.9956, 1932.9833, 1968.8563, 2008.9869, 2009.9747, 2012.9506, 2015.9661, 2020.9644, 2030.0151, 2030.4648, 2046.9839, 2047.5186, 2047.9097, 2051.9133, 2059.0122, 2064.9746, 2070.9209, 2077.0454, 2082.9409, 2085.9888, 2090.9844, 2097.9778, 2103.1526, 2118.8926, 2140.0432, 2142.0310, 2142.9895, 2155.0667, 2159.0752, 2160.0703, 2161.0813, 2166.0911, 2167.0676, 2174.0425, 2178.0034, 2181.0369, 2189.9133, 2194.9761, 2199.0986, 2203.0464, 2205.9102, 2208.9729, 2211.1077, 2213.1116, 2214.1145, 2218.1018, 2221.1035, 2223.2205, 2225.1592, 2229.1064, 2233.1023, 2245.0991, 2269.1128, 2273.0393, 2277.0576, 2279.1094, 2280.1204, 2283.1279, 2285.1169, 2292.1465, 2296.1479, 2312.0664, 2323.1892, 2326.0947, 2335.1282, 2337.1343, 2369.2256, 2383.9978, 2385.2129, 2392.1528, 2400.1094, 2401.1360, 2411.1907, 2414.1833, 2429.1475, 2436.1699, 2437.1758, 2440.2007, 2447.2368, 2477.1301, 2495.0542, 2510.2229, 2522.1980, 2524.1689, 2527.2310, 2535.2598, 2541.2075, 2543.1907, 2553.1934, 2557.2300, 2560.1125, 2569.2915, 2572.0935, 2612.1936, 2686.3572, 2788.3362, 2963.6167, 3056.4641, 3058.5842, 3180.5432, 3313.5032, 3330.6311, 3347.6077, 3511.9668, 3761.1558

32. E1BP00 **Mass:** 59905 **Score:** 19 **Expect:** 3e+002 **Matches:** 22

PC4 and SFRS1-interacting protein OS=Bos taurus GN=PSIP1 PE=4 SV=2

Observed	Mr(expt)	Mr(calc)	ppm	Start	End	Miss	Ions	Peptide
819.4304	818.4231	818.4385	-18.86	518	524	0	---	R.ETEISLK.D
863.4233	862.4161	862.4032	14.9	128	135	0	---	K.ASNEDVTK.A
913.5685	912.5613	912.5392	24.1	463	470	2	---	K.GPNKKLEK.E
928.5031	927.4958	927.5138	-19.39	181	189	0	---	R.GRPAATEVK.I
981.4474	980.4401	980.4345	5.70	344	351	1	---	K.RETSMDSR.L
1029.5330	1028.5257	1028.5264	-0.66	293	300	1	---	R.NFQTAHRR.N
1057.6005	1056.5932	1056.6040	-10.17	220	228	2	---	R.NVRLDKQGK.N
1143.5844	1142.5771	1142.5693	6.82	290	299	1	---	K.GGRNFQTAHR.R
1222.5789	1221.5716	1221.5772	-4.54	345	354	1	---	R.ETSMDSRLQR.I
1238.5917	1237.5844	1237.5721	9.97	345	354	1	---	R.ETSMDSRLQR.I + Oxidation (M)
1258.6344	1257.6271	1257.6077	15.5	15	24	1	---	K.MKGYPHWPAR.V + Oxidation (M)
1268.6414	1267.6341	1267.6269	5.72	305	315	2	---	K.GQHEKEAADRK.R
1271.6490	1270.6417	1270.6643	-17.76	289	299	2	---	R.KGGRNFQTAHR.R
1299.6810	1298.6737	1298.6704	2.53	290	300	2	---	K.GGRNFQTAHRR.N
1406.7207	1405.7134	1405.7089	3.21	229	241	0	---	K.NFVGITVESSPEK.L
1421.6632	1420.6559	1420.6318	17.0	101	113	0	---	K.QSNASSDVEVEEK.E
1539.7621	1538.7548	1538.7437	7.24	443	455	1	---	K.SLAEQRQHEEANK.T
1563.7314	1562.7241	1562.7035	13.2	202	214	0	---	K.QPCPSESDIFLDR.N
1786.8824	1785.8751	1785.9005	-14.19	408	422	1	---	K.VSQVIMEKSTMLYNK.F + Oxidation (M)
1916.9475	1915.9402	1915.9540	-7.20	74	89	2	---	K.RKGFNEGLWEIDNNPK.V
2292.1465	2291.1392	2291.1137	11.1	373	392	0	---	R.CIEALDELASLQVTMQQAQK.H + Oxidation (M)
3330.6311	3329.6238	3329.6669	-12.94	373	401	1	---	R.CIEALDELASLQVTMQQAQKHTEMITTLK.K

No match to: 724.2539, 725.2472, 728.2482, 730.2519, 732.2390, 733.2623, 739.3115, 742.4642, 743.4729, 745.4483, 747.4419, 752.3972, 754.3827, 756.3716, 758.3382, 760.3329, 764.3364, 766.3494, 767.4740, 768.3865, 770.3671, 772.4178, 779.4436, 784.3415, 787.2366, 788.4225, 790.4196, 795.4260, 799.2464, 802.4372, 805.4478, 808.4353, 811.4178, 814.4170, 821.4247, 822.4321, 829.4457, 837.4396, 840.4623, 842.5109, 854.4525, 856.5045, 858.5442, 860.4092, 863.9185, 869.4846, 870.5100, 872.4994, 880.4412, 904.4613, 906.4929, 908.4577, 919.5024, 925.5089, 927.4999, 940.4773, 942.4820, 950.4946, 957.5654, 960.4840, 966.4929, 969.4786, 972.5046, 974.5551, 977.5352, 978.5240, 989.2700, 991.5198, 993.5112, 1001.5782, 1007.5089, 1009.5097, 1013.4454, 1015.5150, 1016.5522, 1018.5189, 1021.5313, 1024.5028, 1025.5192, 1032.5870, 1033.5739, 1035.5203, 1045.5564, 1046.5759, 1051.5074, 1056.5415, 1064.5358, 1071.5361, 1073.5377, 1079.5398, 1083.4637, 1086.4774, 1092.6000, 1092.6002, 1094.5596, 1096.5302, 1106.5431, 1110.5461, 1111.5398, 1111.5400, 1115.5790, 1118.5620, 1120.5724, 1123.5819, 1128.5537, 1132.5682, 1134.5841, 1135.5942, 1137.5565, 1145.6155, 1149.5597, 1153.5929, 1158.6288, 1163.5771, 1173.6008, 1175.5867, 1177.6178, 1178.6053, 1179.5962, 1181.6432, 1192.5721, 1199.6315, 1203.5826, 1211.5984, 1215.6246, 1216.6786, 1221.5699, 1227.6271, 1228.6411, 1229.6412, 1231.6102, 1233.6571, 1237.6270, 1244.6069, 1248.5958, 1250.6042, 1255.5922, 1275.6388, 1276.5977, 1276.6000, 1285.5864, 1294.6315, 1300.6566, 1309.6340, 1320.6268, 1328.6292, 1333.7025, 1336.6038, 1338.6589, 1341.6488, 1343.6613, 1345.6910, 1349.6838, 1350.6704, 1352.6350, 1355.6870, 1357.7130, 1358.6704, 1369.7067, 1371.7366, 1374.6705, 1377.6100, 1377.6146, 1384.7109, 1386.7134, 1390.6848, 1393.7169, 1399.6119, 1400.6588, 1410.6736, 1411.6647, 1416.6047, 1418.6714, 1425.7395, 1426.6846, 1427.7076, 1430.7102, 1432.6718, 1434.6552, 1438.6991, 1439.7164, 1443.7589, 1444.7375, 1445.7306, 1454.6836, 1461.7990, 1472.7026, 1475.7224, 1485.6906, 1489.7172, 1490.7058, 1493.7157, 1510.7383, 1515.7393, 1523.6987, 1524.7383, 1527.7585, 1529.7466, 1530.6902, 1531.6953, 1537.7804, 1538.7736, 1541.7805, 1542.8290, 1549.6758, 1553.7277, 1555.7928, 1556.7874, 1560.7551, 1568.7372, 1570.8015, 1572.8151, 1576.7402, 1577.7395, 1578.7518, 1581.8373, 1591.7852, 1600.8068, 1603.8092, 1604.8136, 1606.8130, 1608.7599, 1613.7793, 1615.8575, 1615.8600, 1624.7632, 1628.7653, 1633.7913, 1634.7969, 1635.7941, 1639.8157, 1643.8085, 1646.8591, 1647.8268, 1651.8206, 1653.8284, 1677.8074, 1681.8087, 1688.8287, 1690.8313, 1694.7357, 1697.8672, 1703.8214, 1706.8364, 1707.8435, 1708.3533, 1709.3307, 1710.8229, 1715.8053, 1719.8075, 1721.8296, 1723.8743, 1724.8828, 1725.8090, 1730.8199,

1748.7931, 1757.8975, 1763.7841, 1768.8075, 1771.9686, 1774.9059, 1777.8727, 1779.8081, 1787.8207, 1790.8423, 1793.9556, 1796.8109, 1797.8339, 1800.8578, 1815.9008, 1819.8838, 1821.8926, 1822.9185, 1826.8450, 1829.8655, 1833.8975, 1836.9054, 1843.8765, 1844.8785, 1851.9031, 1859.8494, 1862.9265, 1863.9319, 1866.8857, 1868.8972, 1879.9445, 1880.9215, 1887.9023, 1888.8057, 1890.8025, 1896.8954, 1901.0060, 1906.8872, 1908.8663, 1913.8895, 1924.9314, 1925.9336, 1928.0060, 1931.9956, 1932.9833, 1968.8563, 1984.9392, 2008.0485, 2008.9869, 2009.9747, 2012.9506, 2015.9661, 2020.9644, 2030.0151, 2030.4648, 2046.9839, 2047.5186, 2047.9097, 2051.9133, 2059.0122, 2064.9746, 2070.9209, 2077.0454, 2082.9409, 2085.9888, 2090.9844, 2097.9778, 2103.1526, 2108.9897, 2118.8926, 2140.0432, 2142.0310, 2142.9895, 2152.0510, 2155.0667, 2159.0752, 2160.0703, 2161.0813, 2166.0911, 2167.0676, 2174.0425, 2178.0034, 2181.0369, 2184.0767, 2189.9133, 2194.9761, 2199.0986, 2203.0464, 2205.9102, 2208.9729, 2211.1077, 2213.1116, 2214.1145, 2218.1018, 2221.1035, 2223.2205, 2225.1592, 2229.1064, 2233.1023, 2245.0991, 2269.1128, 2273.0393, 2277.0576, 2279.1094, 2280.1204, 2283.1279, 2285.1169, 2296.1479, 2312.0664, 2323.1892, 2326.0947, 2334.1531, 2335.1282, 2337.1343, 2369.2256, 2372.2229, 2383.9978, 2385.2129, 2392.1528, 2400.1094, 2401.1360, 2409.1914, 2411.1907, 2414.1833, 2429.1475, 2436.1699, 2437.1758, 2440.2007, 2447.2368, 2477.1301, 2495.0542, 2498.0898, 2510.2229, 2522.1980, 2524.1689, 2527.2310, 2535.2598, 2541.2075, 2543.1907, 2544.1514, 2553.1934, 2557.2300, 2560.1125, 2569.2915, 2572.0935, 2612.1936, 2686.3572, 2788.3362, 2963.6167, 3056.4641, 3058.5842, 3180.5432, 3313.5032, 3347.6077, 3511.9668, 3761.1558

33. A4FV84 **Mass:** 27623 **Score:** 19 **Expect:** 3e+002 **Matches:** 14

mRNA turnover protein 4 homolog OS=Bos taurus GN=MRTO4 PE=2 SV=1

Observed	Mr(expt)	Mr(calc)	ppm	Start	End	Miss	Ions	Peptide
908.4577	907.4504	907.4619	-12.70	72 -	79	0	---	K.VMMVALGR.S + 2 Oxidation (M)
940.4773	939.4700	939.4774	-7.85	87 -	94	0	---	K.DNLHQVSK.K
1009.5097	1008.5024	1008.4964	6.02	62 -	69	1	---	K.HSRMFFGK.N
1025.5192	1024.5119	1024.4913	20.2	62 -	69	1	---	K.HSRMFFGK.N + Oxidation (M)
1134.5841	1133.5768	1133.6049	-24.77	70 -	79	1	---	K.NKVMMVALGR.S + Oxidation (M)
1250.6042	1249.5969	1249.5689	22.4	192 -	201	0	---	K.LFGYEMAEFK.V + Oxidation (M)
1411.6647	1410.6574	1410.6667	-6.58	108 -	118	1	---	R.TKEEVDEWFTK.Y
1418.6714	1417.6641	1417.6296	24.4	119 -	130	1	---	K.YTEMDYARAGNK.A
1434.6552	1433.6479	1433.6245	16.3	119 -	130	1	---	K.YTEMDYARAGNK.A + Oxidation (M)
1524.7383	1523.7310	1523.7456	-9.55	58 -	69	2	---	R.NAWKHSRMFFGK.N + Oxidation (M)
1556.7874	1555.7801	1555.7453	22.4	202 -	214	1	---	K.VSIKYMWDAQSGR.F + Oxidation (M)
1924.9314	1923.9241	1923.9546	-15.85	70 -	86	2	---	K.NKVMMVALGRSPSDEYK.D
2142.9895	2141.9822	2142.0278	-21.28	33 -	49	1	---	K.CVDTYKYLFIFSVANMR.N + Oxidation (M)
2788.3362	2787.3289	2787.3036	9.07	192 -	214	2	---	K.LFGYEMAEFKVSIKYMWDAQSGR.F + 2 Oxidation (M)

No match to: 724.2539, 725.2472, 728.2482, 730.2519, 732.2390, 733.2623, 739.3115, 742.4642, 743.4729, 745.4483, 747.4419, 752.3972, 754.3827, 756.3716, 758.3382, 760.3329, 764.3364, 766.3494, 767.4740, 768.3865, 770.3671, 772.4178, 779.4436, 784.3415, 787.2366, 788.4225, 790.4196, 795.4260, 799.2464, 802.4372, 805.4478, 808.4353, 811.4178, 814.4170, 819.4304, 821.4247, 822.4321, 829.4457, 837.4396, 840.4623, 842.5109, 854.4525, 856.5045, 858.5442, 860.4092, 863.4233, 863.9185, 869.4846, 870.5100, 872.4994, 880.4412, 904.4613, 906.4929, 913.5685, 919.5024, 925.5089, 927.4999, 928.5031, 942.4820, 950.4946, 957.5654, 960.4840, 966.4929, 969.4786, 972.5046, 974.5551, 977.5352, 978.5240, 981.4474, 989.2700, 991.5198, 993.5112, 1001.5782, 1007.5089, 1013.4454, 1015.5150, 1016.5522, 1018.5189, 1021.5313, 1024.5028, 1029.5330, 1032.5870, 1033.5739, 1035.5203, 1045.5564, 1046.5759, 1051.5074, 1056.5415, 1057.6005, 1064.5358, 1071.5361, 1073.5377, 1079.5398, 1083.4637, 1086.4774, 1092.6000, 1092.6002, 1094.5596, 1096.5302, 1106.5431, 1110.5461, 1111.5398, 1111.5400, 1115.5790, 1118.5620, 1120.5724, 1123.5819, 1128.5537, 1132.5682, 1135.5942, 1137.5565, 1143.5844, 1145.6155, 1149.5597, 1153.5929, 1158.6288, 1163.5771, 1173.6008, 1175.5867, 1177.6178, 1178.6053, 1179.5962, 1181.6432, 1192.5721, 1199.6315, 1203.5826, 1211.5984, 1215.6246, 1216.6786, 1221.5699, 1222.5789, 1227.6271, 1228.6411, 1229.6412, 1231.6102, 1233.6571, 1237.6270, 1238.5917, 1244.6069, 1248.5958, 1255.5922, 1258.6344, 1268.6414, 1271.6490, 1275.6388, 1276.5977, 1276.6000, 1285.5864, 1294.6315, 1299.6810, 1300.6566, 1309.6340, 1320.6268, 1328.6292, 1333.7025, 1336.6038, 1338.6589, 1341.6488, 1343.6613, 1345.6910, 1349.6838, 1350.6704, 1352.6350, 1355.6870, 1357.7130, 1358.6704, 1369.7067, 1371.7366, 1374.6705, 1377.6100, 1377.6146, 1384.7109, 1386.7134, 1390.6848, 1393.7169, 1399.6119, 1400.6588, 1406.7207, 1410.6736, 1416.6047, 1421.6632, 1425.7395, 1426.6846, 1427.7076, 1430.7102, 1432.6718, 1438.6991, 1439.7164, 1443.7589, 1444.7375, 1445.7306, 1454.6836, 1461.7990, 1472.7026, 1475.7224, 1485.6906, 1489.7172, 1490.7058, 1493.7157, 1510.7383, 1515.7393, 1523.6987, 1527.7585, 1529.7466, 1530.6902, 1531.6953, 1537.7804, 1538.7736, 1539.7621, 1541.7805, 1542.8290, 1549.6758, 1553.7277, 1555.7928, 1560.7551, 1563.7314, 1568.7372, 1570.8015, 1572.8151, 1576.7402, 1577.7395, 1578.7518, 1581.8373, 1591.7852, 1600.8068, 1603.8092, 1604.8136, 1606.8130, 1608.7599, 1613.7793, 1615.8575, 1615.8600, 1624.7632, 1628.7653, 1633.7913, 1634.7969, 1635.7941, 1639.8157, 1643.8085, 1646.8591, 1647.8268, 1651.8206, 1653.8284, 1677.8074, 1681.8087, 1688.8287, 1690.8313, 1694.7357, 1697.8672, 1703.8214, 1706.8364, 1707.8435, 1708.3533, 1709.3307, 1710.8229, 1715.8053, 1719.8075, 1721.8296, 1723.8743, 1724.8828, 1725.8090, 1730.8199, 1748.7931, 1757.8975, 1763.7841, 1768.8075, 1771.9686, 1774.9059, 1777.8727, 1779.8081, 1786.8824, 1787.8207, 1790.8423, 1793.9556, 1796.8109, 1797.8339, 1800.8578, 1815.9008, 1819.8838, 1821.8926, 1822.9185, 1826.8450, 1829.8655, 1833.8975, 1836.9054, 1843.8765, 1844.8785, 1851.9031, 1859.8494, 1862.9265, 1863.9319, 1866.8857, 1868.8972, 1879.9445, 1880.9215, 1887.9023, 1888.8057, 1890.8025, 1896.8954, 1901.0060, 1906.8872, 1908.8663, 1913.8895, 1916.9475, 1925.9336, 1928.0060, 1931.9956, 1932.9833, 1968.8563, 1984.9392, 2008.0485, 2008.9869, 2009.9747, 2012.9506, 2015.9661, 2020.9644, 2030.0151, 2030.4648, 2046.9839, 2047.5186, 2047.9097, 2051.9133, 2059.0122, 2064.9746, 2070.9209, 2077.0454, 2082.9409, 2085.9888, 2090.9844, 2097.9778, 2103.1526, 2108.9897, 2118.8926, 2140.0432, 2142.0310, 2152.0510, 2155.0667, 2159.0752, 2160.0703, 2161.0813, 2166.0911, 2167.0676, 2174.0425, 2178.0034, 2181.0369, 2184.0767, 2189.9133, 2194.9761, 2199.0986, 2203.0464, 2205.9102, 2208.9729, 2211.1077, 2213.1116, 2214.1145, 2218.1018, 2221.1035, 2223.2205, 2225.1592, 2229.1064, 2233.1023, 2245.0991, 2269.1128, 2273.0393, 2277.0576, 2279.1094, 2280.1204, 2283.1279, 2285.1169, 2292.1465, 2296.1479, 2312.0664, 2323.1892, 2326.0947, 2334.1531, 2335.1282, 2337.1343, 2369.2256, 2372.2229, 2383.9978, 2385.2129, 2392.1528, 2400.1094, 2401.1360, 2409.1914, 2411.1907, 2414.1833, 2429.1475, 2436.1699, 2437.1758, 2440.2007, 2447.2368, 2477.1301, 2495.0542, 2498.0898, 2510.2229, 2522.1980, 2524.1689, 2527.2310, 2535.2598, 2541.2075, 2543.1907, 2544.1514, 2553.1934, 2557.2300, 2560.1125, 2569.2915, 2572.0935, 2612.1936, 2686.3572, 2963.6167, 3056.4641, 3058.5842, 3180.5432, 3313.5032, 3330.6311, 3347.6077, 3511.9668, 3761.1558

34. G8JL04 **Mass:** 5563 **Score:** 19 **Expect:** 3.1e+002 **Matches:** 5

Uncharacterized protein OS=Bos taurus PE=4 SV=1

Observed	Mr(expt)	Mr(calc)	ppm	Start	End	Miss	Ions	Peptide
1371.7366	1370.7293	1370.7041	18.4	27 -	38	1	---	K.TETQEKNPLPSK.E
1906.8872	1905.8799	1905.8812	-0.65	1 -	18	0	---	-.SAPSATMSDKPNMAEIEK.F
1984.9392	1983.9319	1983.9749	-21.65	33 -	50	2	---	K.NPLPSKETIEQEKQAGES.-
2229.1064	2228.0991	2228.1172	-8.10	27 -	45	2	---	K.TETQEKNPLPSKETIEQEK.Q
2544.1514	2543.1441	2543.1883	-17.37	1 -	23	2	---	-.SAPSATMSDKPNMAEIEKFDKSK.L + 2 Oxidation (M)

No match to: 724.2539, 725.2472, 728.2482, 730.2519, 732.2390, 733.2623, 739.3115, 742.4642, 743.4729, 745.4483, 747.4419, 752.3972, 754.3827, 756.3716, 758.3382, 760.3329, 764.3364, 766.3494, 767.4740, 768.3865, 770.3671, 772.4178, 779.4436, 784.3415, 787.2366, 788.4225, 790.4196, 795.4260, 799.2464, 802.4372, 805.4478, 808.4353, 811.4178, 814.4170, 819.4304, 821.4247, 822.4321, 829.4457, 837.4396, 840.4623, 842.5109, 854.4525, 856.5045, 858.5442, 860.4092, 863.4233, 863.9185, 869.4846, 870.5100, 872.4994, 880.4412, 904.4613, 906.4929, 908.4577, 913.5685, 919.5024, 925.5089, 927.4999, 928.5031, 940.4773, 942.4820, 950.4946, 957.5654, 960.4840, 966.4929, 969.4786, 972.5046, 974.5551, 977.5352, 978.5240, 981.4474, 989.2700, 991.5198, 993.5112, 1001.5782, 1007.5089, 1009.5097, 1013.4454, 1015.5150, 1016.5522, 1018.5189, 1021.5313, 1024.5028, 1025.5192, 1029.5330, 1032.5870, 1033.5739, 1035.5203, 1045.5564, 1046.5759, 1051.5074, 1056.5415, 1057.6005, 1064.5358, 1071.5361, 1073.5377, 1079.5398, 1083.4637, 1086.4774, 1092.6000, 1092.6002, 1094.5596, 1096.5302, 1106.5431, 1110.5461, 1111.5398, 1111.5400, 1115.5790, 1118.5620, 1120.5724, 1123.5819, 1128.5537, 1132.5682, 1134.5841, 1135.5942, 1137.5565, 1143.5844, 1145.6155, 1149.5597, 1153.5929, 1158.6288, 1163.5771, 1173.6008, 1175.5867, 1177.6178, 1178.6053, 1179.5962, 1181.6432, 1192.5721, 1199.6315, 1203.5826, 1211.5984, 1215.6246, 1216.6786, 1221.5699, 1222.5789, 1227.6271, 1228.6411, 1229.6412, 1231.6102, 1233.6571, 1237.6270, 1238.5917, 1244.6069, 1248.5958, 1250.6042, 1255.5922, 1258.6344, 1268.6414, 1271.6490, 1275.6388, 1276.5977, 1276.6000, 1285.5864, 1294.6315, 1299.6810, 1300.6566, 1309.6340, 1320.6268, 1328.6292, 1333.7025, 1336.6038, 1338.6589, 1341.6488, 1343.6613, 1345.6910, 1349.6838, 1350.6704, 1352.6350, 1355.6870, 1357.7130, 1358.6704, 1369.7067, 1374.6705, 1377.6100, 1377.6146, 1384.7109, 1386.7134, 1390.6848, 1393.7169, 1399.6119, 1400.6588, 1406.7207, 1410.6736, 1411.6647, 1416.6047, 1418.6714, 1421.6632, 1425.7395, 1426.6846, 1427.7076, 1430.7102, 1432.6718, 1434.6552, 1438.6991, 1439.7164, 1443.7589, 1444.7375, 1445.7306, 1454.6836, 1461.7990, 1472.7026, 1475.7224, 1485.6906, 1489.7172, 1490.7058, 1493.7157, 1510.7383, 1515.7393, 1523.6987, 1524.7383, 1527.7585, 1529.7466, 1530.6902, 1531.6953, 1537.7804, 1538.7736, 1539.7621, 1541.7805, 1542.8290, 1549.6758, 1553.7277, 1555.7928, 1556.7874, 1560.7551, 1563.7314, 1568.7372, 1570.8015, 1572.8151, 1576.7402, 1577.7395, 1578.7518, 1581.8373, 1591.7852, 1600.8068, 1603.8092, 1604.8136, 1606.8130, 1608.7599, 1613.7793, 1615.8575, 1615.8600, 1624.7632, 1628.7653,

1633.7913, 1634.7969, 1635.7941, 1639.8157, 1643.8085, 1646.8591, 1647.8268, 1651.8206, 1653.8284, 1677.8074, 1681.8087, 1688.8287, 1690.8313, 1694.7357, 1697.8672, 1703.8214, 1706.8364, 1707.8435, 1708.3533, 1709.3307, 1710.8229, 1715.8053, 1719.8075, 1721.8296, 1723.8743, 1724.8828, 1725.8090, 1730.8199, 1748.7931, 1757.8975, 1763.7841, 1768.8075, 1771.9686, 1774.9059, 1777.8727, 1779.8081, 1786.8824, 1787.8207, 1790.8423, 1793.9556, 1796.8109, 1797.8339, 1800.8578, 1815.9008, 1819.8838, 1821.8926, 1822.9185, 1826.8450, 1829.8655, 1833.8975, 1836.9054, 1843.8765, 1844.8785, 1851.9031, 1859.8494, 1862.9265, 1863.9319, 1866.8857, 1868.8972, 1879.9445, 1880.9215, 1887.9023, 1888.8057, 1890.8025, 1896.8954, 1901.0060, 1908.8663, 1913.8895, 1916.9475, 1924.9314, 1925.9336, 1928.0060, 1931.9956, 1932.9833, 1968.8563, 2008.0485, 2008.9869, 2009.9747, 2012.9506, 2015.9661, 2020.9644, 2030.0151, 2030.4648, 2046.9839, 2047.5186, 2047.9097, 2051.9133, 2059.0122, 2064.9746, 2070.9209, 2077.0454, 2082.9409, 2085.9888, 2090.9844, 2097.9778, 2103.1526, 2108.9897, 2118.8926, 2140.0432, 2142.0310, 2142.9895, 2152.0510, 2155.0667, 2159.0752, 2160.0703, 2161.0813, 2166.0911, 2167.0676, 2174.0425, 2178.0034, 2181.0369, 2184.0767, 2189.9133, 2194.9761, 2199.0986, 2203.0464, 2205.9102, 2208.9729, 2211.1077, 2213.1116, 2214.1145, 2218.1018, 2221.1035, 2223.2205, 2225.1592, 2233.1023, 2245.0991, 2269.1128, 2273.0393, 2277.0576, 2279.1094, 2280.1204, 2283.1279, 2285.1169, 2292.1465, 2296.1479, 2312.0664, 2323.1892, 2326.0947, 2334.1531, 2335.1282, 2337.1343, 2369.2256, 2372.2229, 2383.9978, 2385.2129, 2392.1528, 2400.1094, 2401.1360, 2409.1914, 2411.1907, 2414.1833, 2429.1475, 2436.1699, 2437.1758, 2440.2007, 2447.2368, 2477.1301, 2495.0542, 2498.0898, 2510.2229, 2522.1980, 2524.1689, 2527.2310, 2535.2598, 2541.2075, 2543.1907, 2553.1934, 2557.2300, 2560.1125, 2569.2915, 2572.0935, 2612.1936, 2686.3572, 2788.3362, 2963.6167, 3056.4641, 3058.5842, 3180.5432, 3313.5032, 3330.6311, 3347.6077, 3511.9668, 3761.1558

35. Q02366 **Mass:** 15044 **Score:** 19 **Expect:** 3.1e+002 **Matches:** 8

NADH dehydrogenase [ubiquinone] 1 alpha subcomplex subunit 6 OS=Bos taurus GN=NDUFA6 PE=1 SV=2

Observed	Mr(expt)	Mr(calc)	ppm	Start	End	Miss	Ions	Peptide
863.4233	862.4161	862.3967	22.5	24 -	30	1	---	R.DMNEAKR.R
927.4999	926.4926	926.4868	6.21	99 -	105	1	---	K.QRTHVMR.F
1035.5203	1034.5130	1034.4927	19.7	24 -	31	2	---	R.DMNEAKRR.V + Oxidation (M)
1177.6178	1176.6105	1176.6060	3.86	86 -	95	1	---	K.GKMELEETIK.V
1358.6704	1357.6631	1357.6779	-10.86	106 -	116	0	---	R.FFHETEAPRPK.D
1606.8130	1605.8057	1605.8436	-23.58	86 -	98	2	---	K.GKMELEETIKVWK.Q + Oxidation (M)
2283.1279	2282.1206	2282.1491	-12.47	99 -	116	2	---	K.QRTHVMRFFHETEAPRPK.D + Oxidation (M)
2337.1343	2336.1270	2336.1794	-22.43	8 -	29	1	---	R.QAAVAASTSVKPIFSRDMNEAK.R + Oxidation (M)

No match to: 724.2539, 725.2472, 728.2482, 730.2519, 732.2390, 733.2623, 739.3115, 742.4642, 743.4729, 745.4483, 747.4419, 752.3972, 754.3827, 756.3716, 758.3382, 760.3329, 764.3364, 766.3494, 767.4740, 768.3865, 770.3671, 772.4178, 779.4436, 784.3415, 787.2366, 788.4225, 790.4196, 795.4260, 799.2464, 802.4372, 805.4478, 808.4353, 811.4178, 814.4170, 819.4304, 821.4247, 822.4321, 829.4457, 837.4396, 840.4623, 842.5109, 854.4525, 856.5045, 858.5442, 860.4092, 863.9185, 869.4846, 870.5100, 872.4994, 880.4412, 904.4613, 906.4929, 908.4577, 913.5685, 919.5024, 925.5089, 928.5031, 940.4773, 942.4820, 950.4946, 957.5654, 960.4840, 966.4929, 969.4786, 972.5046, 974.5551, 977.5352, 978.5240, 981.4474, 989.2700, 991.5198, 993.5112, 1001.5782, 1007.5089, 1009.5097, 1013.4454, 1015.5150, 1016.5522, 1018.5189, 1021.5313, 1024.5028, 1025.5192, 1029.5330, 1032.5870, 1033.5739, 1045.5564, 1046.5759, 1051.5074, 1056.5415, 1057.6005, 1064.5358, 1071.5361, 1073.5377, 1079.5398, 1083.4637, 1086.4774, 1092.6000, 1092.6002, 1094.5596, 1096.5302, 1106.5431, 1110.5461, 1111.5398, 1111.5400, 1115.5790, 1118.5620, 1120.5724, 1123.5819, 1128.5537, 1132.5682, 1134.5841, 1135.5942, 1137.5565, 1143.5844, 1145.6155, 1149.5597, 1153.5929, 1158.6288, 1163.5771, 1173.6008, 1175.5867, 1178.6053, 1179.5962, 1181.6432, 1192.5721, 1199.6315, 1203.5826, 1211.5984, 1215.6246, 1216.6786, 1221.5699, 1222.5789, 1227.6271, 1228.6411, 1229.6412, 1231.6102, 1233.6571, 1237.6270, 1238.5917, 1244.6069, 1248.5958, 1250.6042, 1255.5922, 1258.6344, 1268.6414, 1271.6490, 1275.6388, 1276.5977, 1276.6000, 1285.5864, 1294.6315, 1299.6810, 1300.6566, 1309.6340, 1320.6268, 1328.6292, 1333.7025, 1336.6038, 1338.6589, 1341.6488, 1343.6613, 1345.6910, 1349.6838, 1350.6704, 1352.6350, 1355.6870, 1357.7130, 1369.7067, 1371.7366, 1374.6705, 1377.6100, 1377.6146, 1384.7109, 1386.7134, 1390.6848, 1393.7169, 1399.6119, 1400.6588, 1406.7207, 1410.6736, 1411.6647, 1416.6047, 1418.6714, 1421.6632, 1425.7395, 1426.6846, 1427.7076, 1430.7102, 1432.6718, 1434.6552, 1438.6991, 1439.7164, 1443.7589, 1444.7375, 1445.7306, 1454.6836, 1461.7990, 1472.7026, 1475.7224, 1485.6906, 1489.7172, 1490.7058, 1493.7157, 1510.7383, 1515.7393, 1523.6987, 1524.7383, 1527.7585, 1529.7466, 1530.6902, 1531.6953, 1537.7804, 1538.7736, 1539.7621, 1541.7805, 1542.8290, 1549.6758, 1553.7277, 1555.7928, 1556.7874, 1560.7551, 1563.7314, 1568.7372, 1570.8015, 1572.8151, 1576.7402, 1577.7395, 1578.7518, 1581.8373, 1591.7852, 1600.8068, 1603.8092, 1604.8136, 1608.7599, 1613.7793, 1615.8575, 1615.8600, 1624.7632, 1628.7653, 1633.7913, 1634.7969, 1635.7941, 1639.8157, 1643.8085, 1646.8591, 1647.8268, 1651.8206, 1653.8284, 1677.8074, 1681.8087, 1688.8287, 1690.8313, 1694.7357, 1697.8672, 1703.8214, 1706.8364, 1707.8435, 1708.3533, 1709.3307, 1710.8229, 1715.8053, 1719.8075, 1721.8296, 1723.8743, 1724.8828, 1725.8090, 1730.8199, 1748.7931, 1757.8975, 1763.7841, 1768.8075, 1771.9686, 1774.9059, 1777.8727, 1779.8081, 1786.8824, 1787.8207, 1790.8423, 1793.9556, 1796.8109, 1797.8339, 1800.8578, 1815.9008, 1819.8838, 1821.8926, 1822.9185, 1826.8450, 1829.8655, 1833.8975, 1836.9054, 1843.8765, 1844.8785, 1851.9031, 1859.8494, 1862.9265, 1863.9319, 1866.8857, 1868.8972, 1879.9445, 1880.9215, 1887.9023, 1888.8057, 1890.8025, 1896.8954, 1901.0060, 1906.8872, 1908.8663, 1913.8895, 1916.9475, 1924.9314, 1925.9336, 1928.0060, 1931.9956, 1932.9833, 1968.8563, 1984.9392, 2008.0485, 2008.9869, 2009.9747, 2012.9506, 2015.9661, 2020.9644, 2030.0151, 2030.4648, 2046.9839, 2047.9097, 2051.9133, 2059.0122, 2064.9746, 2070.9209, 2077.0454, 2082.9409, 2085.9888, 2090.9844, 2097.9778, 2103.1526, 2108.9897, 2118.8926, 2140.0432, 2142.0310, 2142.9895, 2152.0510, 2155.0667, 2159.0752, 2160.0703, 2161.0813, 2166.0911, 2167.0676, 2174.0425, 2178.0034, 2181.0369, 2184.0767, 2189.9133, 2194.9761, 2199.0986, 2203.0464, 2205.9102, 2208.9729, 2211.1077, 2213.1116, 2214.1145, 2218.1018, 2221.1035, 2223.2205, 2225.1592, 2229.1064, 2233.1023, 2245.0991, 2269.1128, 2273.0393, 2277.0576, 2279.1094, 2280.1204, 2285.1169, 2292.1465, 2296.1479, 2312.0664, 2323.1892, 2326.0947, 2334.1531, 2335.1282, 2369.2256, 2372.2229, 2383.9978, 2385.2129, 2392.1528, 2400.1094, 2401.1360, 2409.1914, 2411.1907, 2414.1833, 2429.1475, 2436.1699, 2437.1758, 2440.2007, 2447.2368, 2477.1301, 2495.0542, 2498.0898, 2510.2229, 2522.1980, 2524.1689, 2527.2310, 2535.2598, 2541.2075, 2543.1907, 2544.1514, 2553.1934, 2557.2300, 2560.1125, 2569.2915, 2572.0935, 2612.1936, 2686.3572, 2788.3362, 2963.6167, 3056.4641, 3058.5842, 3180.5432, 3313.5032, 3330.6311, 3347.6077, 3511.9668, 3761.1558

36. G3MZ91 **Mass:** 14728 **Score:** 19 **Expect:** 3.1e+002 **Matches:** 9

Histone H2B OS=Bos taurus PE=3 SV=1

Observed	Mr(expt)	Mr(calc)	ppm	Start	End	Miss	Ions	Peptide
745.4483	744.4410	744.4494	-11.21	18 -	24	1	---	K.AVTKAQK.D
1092.6000	1091.5927	1091.5975	-4.35	2 -	12	1	---	M.PEPAKSAPAPK.K
1092.6002	1091.5929	1091.5975	-4.17	2 -	12	1	---	M.PEPAKSAPAPK.K
1137.5565	1136.5492	1136.5390	9.02	35 -	43	0	---	K.ESYSVYVYK.V
1199.6315	1198.6242	1198.6306	-5.31	108 -	119	1	---	K.HAVSEGTKAATK.Y
1604.8136	1603.8063	1603.8430	-22.88	79 -	91	2	---	R.LEHYNKRSTITSR.E
1628.7653	1627.7580	1627.7334	15.1	58 -	71	0	---	K.AMGIMNSVNDIFER.I + 2 Oxidation (M)
1793.9556	1792.9483	1792.9068	23.2	108 -	124	2	---	K.HAVSEGTKAATKYTSSR.I
2788.3362	2787.3289	2787.3684	-14.15	47 -	71	1	---	K.QVHPDTGILSKAMGIMNSVNDIFER.I + Oxidation (M)

No match to: 724.2539, 725.2472, 728.2482, 730.2519, 732.2390, 733.2623, 739.3115, 742.4642, 743.4729, 747.4419, 752.3972, 754.3827, 756.3716, 758.3382, 760.3329, 764.3364, 766.3494, 767.4740, 768.3865, 770.3671, 772.4178, 779.4436, 784.3415, 787.2366, 788.4225, 790.4196, 795.4260, 799.2464, 802.4372, 805.4478, 808.4353, 811.4178, 814.4170, 819.4304, 821.4247, 822.4321, 829.4457, 837.4396, 840.4623, 842.5109, 854.4525, 856.5045, 858.5442, 860.4092, 863.4233, 863.9185, 869.4846, 870.5100, 872.4994, 880.4412, 904.4613, 906.4929, 908.4577, 913.5685, 919.5024, 925.5089, 927.4999, 928.5031, 940.4773, 942.4820, 950.4946, 957.5654, 960.4840, 966.4929, 969.4786, 972.5046, 974.5551, 977.5352, 978.5240, 981.4474, 989.2700, 991.5198, 993.5112, 1001.5782, 1007.5089, 1009.5097, 1013.4454, 1015.5150, 1016.5522, 1018.5189, 1021.5313, 1024.5028, 1025.5192, 1029.5330, 1032.5870, 1033.5739, 1035.5203, 1045.5564, 1046.5759, 1051.5074, 1056.5415, 1057.6005, 1064.5358, 1071.5361, 1073.5377, 1079.5398, 1083.4637, 1086.4774, 1094.5596, 1096.5302, 1106.5431, 1110.5461, 1111.5398, 1111.5400, 1115.5790, 1118.5620, 1120.5724, 1123.5819, 1128.5537, 1132.5682, 1134.5841, 1135.5942, 1143.5844, 1145.6155, 1149.5597, 1153.5929, 1158.6288, 1163.5771, 1173.6008, 1175.5867, 1177.6178, 1178.6053, 1179.5962, 1181.6432, 1192.5721, 1203.5826, 1211.5984, 1215.6246, 1216.6786, 1221.5699, 1222.5789, 1227.6271, 1228.6411, 1229.6412, 1231.6102, 1233.6571, 1237.6270, 1238.5917, 1244.6069, 1248.5958, 1250.6042, 1255.5922, 1258.6344, 1268.6414, 1271.6490, 1275.6388, 1276.5977, 1276.6000, 1285.5864, 1294.6315, 1299.6810, 1300.6566, 1309.6340, 1320.6268, 1328.6292, 1333.7025, 1336.6038, 1338.6589, 1341.6488, 1343.6613, 1345.6910, 1349.6838, 1350.6704, 1352.6350, 1355.6870, 1357.7130, 1358.6704, 1369.7067, 1371.7366, 1374.6705, 1377.6100, 1377.6146, 1384.7109, 1386.7134, 1390.6848, 1393.7169, 1399.6119, 1400.6588, 1406.7207, 1410.6736, 1411.6647, 1416.6047, 1418.6714, 1421.6632, 1425.7395, 1426.6846, 1427.7076, 1430.7102, 1432.6718, 1434.6552, 1438.6991, 1439.7164, 1443.7589, 1444.7375, 1445.7306, 1454.6836, 1461.7990, 1472.7026, 1475.7224, 1485.6906, 1489.7172, 1490.7058, 1493.7157, 1510.7383, 1515.7393, 1523.6987, 1524.7383, 1527.7585, 1529.7466, 1530.6902, 1531.6953, 1537.7804, 1538.7736, 1539.7621, 1541.7805, 1542.8290, 1549.6758, 1553.7277, 1555.7928, 1556.7874, 1560.7551, 1563.7314, 1568.7372, 1570.8015, 1572.8151, 1576.7402, 1577.7395, 1578.7518, 1581.8373, 1591.7852, 1600.8068,

1603.8092, 1606.8130, 1608.7599, 1613.7793, 1615.8575, 1615.8600, 1624.7632, 1633.7913, 1634.7969, 1635.7941, 1639.8157, 1643.8085, 1646.8591, 1647.8268, 1651.8206, 1653.8284, 1677.8074, 1681.8087, 1688.8287, 1690.8313, 1694.7357, 1697.8672, 1703.8214, 1706.8364, 1707.8435, 1708.3533, 1709.3307, 1710.8229, 1715.8053, 1719.8075, 1721.8296, 1723.8743, 1724.8828, 1725.8090, 1730.8199, 1748.7931, 1757.8975, 1763.7841, 1768.8075, 1771.9686, 1774.9059, 1777.8727, 1779.8081, 1786.8824, 1787.8207, 1790.8423, 1796.8109, 1797.8339, 1800.8578, 1815.9008, 1819.8838, 1821.8926, 1822.9185, 1826.8450, 1829.8655, 1833.8975, 1836.9054, 1843.8765, 1844.8785, 1851.9031, 1859.8494, 1862.9265, 1863.9319, 1866.8857, 1868.8972, 1879.9445, 1880.9215, 1887.9023, 1888.8057, 1890.8025, 1896.8954, 1901.0060, 1906.8872, 1908.8663, 1913.8895, 1916.9475, 1924.9314, 1925.9336, 1928.0060, 1931.9956, 1932.9833, 1968.8563, 1984.9392, 2008.0485, 2008.9869, 2009.9747, 2012.9506, 2015.9661, 2020.9644, 2030.0151, 2030.4648, 2046.9839, 2047.5186, 2047.9097, 2051.9133, 2059.0122, 2064.9746, 2070.9209, 2077.0454, 2082.9409, 2085.9888, 2090.9844, 2097.9778, 2103.1526, 2108.9897, 2118.8926, 2140.0432, 2142.0310, 2142.9895, 2152.0510, 2155.0667, 2159.0752, 2160.0703, 2161.0813, 2166.0911, 2167.0676, 2174.0425, 2178.0034, 2181.0369, 2184.0767, 2189.9133, 2194.9761, 2199.0986, 2203.0464, 2205.9102, 2208.9729, 2211.1077, 2213.1116, 2214.1145, 2218.1018, 2221.1035, 2223.2205, 2225.1592, 2229.1064, 2233.1023, 2245.0991, 2269.1128, 2273.0393, 2277.0576, 2279.1094, 2280.1204, 2283.1279, 2285.1169, 2292.1465, 2296.1479, 2312.0664, 2323.1892, 2326.0947, 2334.1531, 2335.1282, 2337.1343, 2369.2256, 2372.2229, 2383.9978, 2385.2129, 2392.1528, 2400.1094, 2401.1360, 2409.1914, 2411.1907, 2414.1833, 2429.1475, 2436.1699, 2437.1758, 2440.2007, 2447.2368, 2477.1301, 2495.0542, 2498.0898, 2510.2229, 2522.1980, 2524.1689, 2527.2310, 2535.2598, 2541.2075, 2543.1907, 2544.1514, 2553.1934, 2557.2300, 2560.1125, 2569.2915, 2572.0935, 2612.1936, 2686.3572, 2963.6167, 3056.4641, 3058.5842, 3180.5432, 3313.5032, 3330.6311, 3347.6077, 3511.9668, 3761.1558

37. F1MEY1 **Mass:** 167004 **Score:** 19 **Expect:** 3.2e+002 **Matches:** 33

Uncharacterized protein OS=Bos taurus GN=ATAD2B PE=4 SV=2

Observed	Mr(expt)	Mr(calc)	ppm	Start		End	Miss	Ions	Peptide
808.4353	807.4280	807.4174	13.2	1135	-	1140	1	---	K.CAFRVR.R
906.4929	905.4856	905.4865	-0.99	355	-	362	2	---	K.SKSMARAR.N
1015.5150	1014.5077	1014.5247	-16.69	1113	-	1120	1	---	R.VEEAFRHK.Q
1173.6008	1172.5935	1172.5906	2.46	363	-	371	1	---	R.NRCLPMNLR.A
1175.5867	1174.5794	1174.6016	-18.89	92	-	101	1	---	R.TLKQPDSVCK.D
1233.6571	1232.6498	1232.6322	14.3	1006	-	1016	0	---	K.EPMDLSTVITK.I
1237.6270	1236.6197	1236.6098	7.99	1109	-	1118	1	---	K.TETRVEEAFR.H
1258.6344	1257.6271	1257.6313	-3.32	1419	-	1429	0	---	K.SNNLAVDQLER.L
1276.5977	1275.5904	1275.5844	4.76	113	-	123	0	---	R.EEWNISSGQAR.L
1276.6000	1275.5927	1275.5844	6.56	113	-	123	0	19	R.EEWNISSGQAR.L
1299.6810	1298.6737	1298.6843	-8.18	1113	-	1122	2	---	R.VEEAFRHKQR.N
1300.6566	1299.6493	1299.6684	-14.65	313	-	322	1	---	K.RENTLFDIHR.S
1320.6268	1319.6195	1319.6139	4.23	458	-	469	1	---	R.ALANECSQGDKK.V
1349.6838	1348.6765	1348.6888	-9.07	633	-	643	1	---	R.RYPQIYASSHK.L
1445.7306	1444.7233	1444.7270	-2.55	58	-	73	1	---	K.AGGSGSAGVALDEARK.A
1555.7928	1554.7855	1554.7903	-3.05	314	-	326	1	---	R.ENTLFDIHRSPAR.R
1560.7551	1559.7478	1559.7501	-1.47	386	-	400	0	---	K.VGASLADVDPMNIDK.S + Oxidation (M)
1707.8435	1706.8362	1706.8700	-19.79	1091	-	1107	0	---	R.GLSVTAEQINPHGTGAR.K
1721.8296	1720.8223	1720.8203	1.20	476	-	490	2	---	R.KGADCLSKWVGESER.Q
1771.9686	1770.9613	1770.9186	24.1	384	-	400	1	---	R.VKVGASLADVDPMNIDK.S
1779.8081	1778.8008	1778.7927	4.55	152	-	167	1	---	K.GDGELSCINGDMEVRK.S
1793.9556	1792.9483	1792.9294	10.5	441	-	457	1	---	R.GCLFYGPPGTGKTLVAR.A
1819.8838	1818.8765	1818.8609	8.61	108	-	123	1	---	R.TTGQREEWNISSGQAR.L
1863.9319	1862.9246	1862.9711	-24.95	1090	-	1107	1	---	K.RGLSVTAEQINPHGTGAR.K
1931.9956	1930.9883	1930.9530	18.3	955	-	969	2	---	K.IRMEDQEENTLRELR.L
1932.9833	1931.9760	1931.9629	6.81	593	-	609	1	---	R.DWNPKLSDAFLGELAEK.C
2166.0911	2165.0838	2165.1076	-11.00	74	-	94	2	---	K.AEADGSLSDSHVSPPAKRTLK.Q
2199.0986	2198.0913	2198.1412	-22.68	363	-	381	2	---	R.NRCLPMNLRAEDLASGILR.E
2214.1145	2213.1072	2213.1408	-15.19	365	-	383	2	---	R.CLPMNLRAEDLASGILRER.V
2323.1892	2322.1819	2322.2040	-9.51	1091	-	1112	2	---	R.GLSVTAEQINPHGTGARKTETR.V
2335.1282	2334.1209	2334.1121	3.78	948	-	966	2	---	R.QLSESEKIRMEDQEENTLR.E
2392.1528	2391.1455	2391.0900	23.2	1031	-	1051	1	---	K.DIDLICSNALEYNPDKDPGDK.I
3056.4641	3055.4568	3055.4419	4.88	593	-	619	2	---	R.DWNPKLSDAFLGELAEKCVGYCGADIK.A

No match to: 724.2539, 725.2472, 728.2482, 730.2519, 732.2390, 733.2623, 739.3115, 742.4642, 743.4729, 745.4483, 747.4419, 752.3972, 754.3827, 756.3716, 758.3382, 760.3329, 764.3364, 766.3494, 767.4740, 768.3865, 770.3671, 772.4178, 779.4436, 784.3415, 787.2366, 788.4225, 790.4196, 795.4260, 799.2464, 802.4372, 805.4478, 811.4178, 814.4170, 819.4304, 821.4247, 822.4321, 829.4457, 837.4396, 840.4623, 842.5109, 854.4525, 856.5045, 858.5442, 860.4092, 863.4233, 863.9185, 869.4846, 870.5100, 872.4994, 880.4412, 904.4613, 908.4577, 913.5685, 919.5024, 925.5089, 927.4999, 928.5031, 940.4773, 942.4820, 950.4946, 957.5654, 960.4840, 966.4929, 969.4786, 972.5046, 974.5551, 977.5352, 978.5240, 981.4474, 989.2700, 991.5198, 993.5112, 1001.5782, 1007.5089, 1009.5097, 1013.4454, 1016.5522, 1018.5189, 1021.5313, 1024.5028, 1025.5192, 1029.5330, 1032.5870, 1033.5739, 1035.5203, 1045.5564, 1046.5759, 1051.5074, 1056.5415, 1057.6005, 1064.5358, 1071.5361, 1073.5377, 1079.5398, 1083.4637, 1086.4774, 1092.6000, 1092.6002, 1094.5596, 1096.5302, 1106.5431, 1110.5461, 1111.5398, 1111.5400, 1115.5790, 1118.5620, 1120.5724, 1123.5819, 1128.5537, 1132.5682, 1134.5841, 1135.5942, 1137.5565, 1143.5844, 1145.6155, 1149.5597, 1153.5929, 1158.6288, 1163.5771, 1177.6178, 1178.6053, 1179.5962, 1181.6432, 1192.5721, 1199.6315, 1203.5826, 1211.5984, 1215.6246, 1216.6786, 1221.5699, 1222.5789, 1227.6271, 1228.6411, 1229.6412, 1231.6102, 1238.5917, 1244.6069, 1248.5958, 1250.6042, 1255.5922, 1268.6414, 1271.6490, 1275.6388, 1285.5864, 1294.6315, 1309.6340, 1328.6292, 1333.7025, 1336.6038, 1338.6589, 1341.6488, 1343.6613, 1345.6910, 1350.6704, 1352.6350, 1355.6870, 1357.7130, 1358.6704, 1369.7067, 1371.7366, 1374.6705, 1377.6100, 1377.6146, 1384.7109, 1386.7134, 1390.6848, 1393.7169, 1399.6119, 1400.6588, 1406.7207, 1410.6736, 1411.6647, 1416.6047, 1418.6714, 1421.6632, 1425.7395, 1426.6846, 1427.7076, 1430.7102, 1432.6718, 1434.6552, 1438.6991, 1439.7164, 1443.7589, 1444.7375, 1454.6836, 1461.7990, 1472.7026, 1475.7224, 1485.6906, 1489.7172, 1490.7058, 1493.7157, 1510.7383, 1515.7393, 1523.6987, 1524.7383, 1527.7585, 1529.7466, 1530.6902, 1531.6953, 1537.7804, 1538.7736, 1539.7621, 1541.7805, 1542.8290, 1549.6758, 1553.7277, 1556.7874, 1563.7314, 1568.7372, 1570.8015, 1572.8151, 1576.7402, 1577.7395, 1578.7518, 1581.8373, 1591.7852, 1600.8068, 1603.8092, 1604.8136, 1606.8130, 1608.7599, 1613.7793, 1615.8575, 1615.8600, 1624.7632, 1628.7653, 1633.7913, 1634.7969, 1635.7941, 1639.8157, 1643.8085, 1646.8591, 1647.8268, 1651.8206, 1653.8284, 1677.8074, 1681.8087, 1688.8287, 1690.8313, 1694.7357, 1697.8672, 1703.8214, 1706.8364, 1708.3533, 1709.3307, 1710.8229, 1715.8053, 1719.8075, 1723.8743, 1724.8828, 1725.8090, 1730.8199, 1748.7931, 1757.8975, 1763.7841, 1768.8075, 1774.9059, 1777.8727, 1786.8824, 1787.8207, 1790.8423, 1796.8109, 1797.8339, 1800.8578, 1815.9008, 1821.8926, 1822.9185, 1826.8450, 1829.8655, 1833.8975, 1836.9054, 1843.8765, 1844.8785, 1851.9031, 1859.8494, 1862.9265, 1866.8857, 1868.8972, 1879.9445, 1880.9215, 1887.9023, 1888.8057, 1890.8025, 1896.8954, 1901.0060, 1906.8872, 1908.8663, 1913.8895, 1916.9475, 1924.9314, 1925.9336, 1928.0060, 1968.8563, 1984.9392, 2008.0485, 2008.9869, 2009.9747, 2012.9506, 2015.9661, 2020.9644, 2030.0151, 2030.4648, 2046.9839, 2047.5186, 2047.9097, 2051.9133, 2059.0122, 2064.9746, 2070.9209, 2077.0454, 2082.9409, 2085.9888, 2090.9844, 2097.9778, 2103.1526, 2108.9897, 2118.8926, 2140.0432, 2142.0310, 2142.9895, 2152.0510, 2155.0667, 2159.0752, 2160.0703, 2161.0813, 2167.0676, 2174.0425, 2178.0034, 2181.0369, 2184.0767, 2189.9133, 2194.9761, 2203.0464, 2205.9102, 2208.9729, 2211.1077, 2213.1116, 2218.1018, 2221.1035, 2223.2205, 2225.1592, 2229.1064, 2233.1023, 2245.0991, 2269.1128, 2273.0393, 2277.0576, 2279.1094, 2280.1204, 2283.1279, 2285.1169, 2292.1465, 2296.1479, 2312.0664, 2326.0947, 2334.1531, 2337.1343, 2369.2256, 2372.2229, 2383.9978, 2385.2129, 2400.1094, 2401.1360, 2409.1914, 2411.1907, 2414.1833, 2429.1475, 2436.1699, 2437.1758, 2440.2007, 2447.2368, 2477.1301, 2495.0542, 2498.0898, 2510.2229, 2522.1980, 2524.1689, 2527.2310, 2535.2598, 2541.2075, 2543.1907, 2544.1514, 2553.1934, 2557.2300, 2560.1125, 2569.2915, 2572.0935, 2612.1936, 2686.3572, 2788.3362, 2963.6167, 3058.5842, 3180.5432, 3313.5032, 3330.6311, 3347.6077, 3511.9668, 3761.1558

38. F1N469 **Mass:** 96792 **Score:** 19 **Expect:** 3.2e+002 **Matches:** 34

Uncharacterized protein OS=Bos taurus GN=MAP7D3 PE=4 SV=2

Observed	Mr(expt)	Mr(calc)	ppm	Start		End	Miss	Ions	Peptide
802.4372	801.4299	801.4344	-5.64	599	-	604	1	---	K.EERLQK.E

819.4304	818.4231	818.4358	-15.58	195 - 201	1	---	R.SSSLNRR.C
870.5100	869.5027	869.5235	-24.00	120 - 126	1	---	K.FKAVLHR.T
960.4840	959.4767	959.4784	-1.82	96 - 102	2	---	K.EKDEQRR.I
1001.5782	1000.5709	1000.5665	4.42	591 - 598	2	---	R.LAREQKEK.E
1046.5759	1045.5686	1045.5628	5.54	809 - 817	1	---	R.SSTKRPTNR.A
1145.6155	1144.6082	1144.6088	-0.47	66 - 74	1	---	K.ETQLLEKER.K
1221.5699	1220.5626	1220.5455	14.0	109 - 117	1	---	K.RNEMLEEER.E + Oxidation (M)
1222.5789	1221.5716	1221.5772	-4.54	824 - 834	0	---	K.AETSSTMRPSR.S
1231.6102	1230.6029	1230.6204	-14.22	154 - 164	1	---	K.TVNKHSTSTEK.L
1238.5917	1237.5844	1237.5721	9.97	824 - 834	0	---	K.AETSSTMRPSR.S + Oxidation (M)
1276.5977	1275.5904	1275.5691	16.7	644 - 653	1	---	K.RESQEQEDQK.V
1276.6000	1275.5927	1275.5691	18.5	644 - 653	1	---	K.RESQEQEDQK.V
1299.6810	1298.6737	1298.6466	20.9	571 - 583	1	---	K.GTPGPKNAEEATK.I
1343.6613	1342.6540	1342.6703	-12.16	783 - 793	1	---	K.AFLNTDMKTFR.Q
1350.6704	1349.6631	1349.6609	1.64	175 - 187	0	---	R.QMSSTGLQNSVAK.K
1384.7109	1383.7036	1383.6956	5.82	328 - 339	0	---	K.VSVETFLEVSMK.E + Oxidation (M)
1386.7134	1385.7061	1385.7238	-12.73	468 - 478	1	---	K.RIPPSQIPCYR.W
1426.6846	1425.6773	1425.7108	-23.48	605 - 615	2	---	K.EMEQSFIRKMK.D
1443.7589	1442.7516	1442.7841	-22.53	796 - 808	2	---	K.GVKDPLNQTKSTR.S
1524.7383	1523.7310	1523.7362	-3.39	821 - 834	1	---	K.TGKAETSSTMRPSR.S + Oxidation (M)
1527.7585	1526.7512	1526.7650	-9.02	76 - 87	2	---	K.SKIVYEKQMEEK.Q + Oxidation (M)
1530.6902	1529.6829	1529.7209	-24.83	205 - 217	0	---	R.LHSSTETEQIEEK.R
1542.8290	1541.8217	1541.8347	-8.45	679 - 690	1	---	R.IMLQNLQERLER.K
1615.8575	1614.8502	1614.8188	19.5	783 - 795	2	---	K.AFLNTDMKTFRQK.G + Oxidation (M)
1615.8600	1614.8527	1614.8188	21.0	783 - 795	2	---	K.AFLNTDMKTFRQK.G + Oxidation (M)
1715.8053	1714.7980	1714.8382	-23.44	530 - 545	1	---	R.TVFAQNALGTMGMKSK.A + 2 Oxidation (M)
1719.8075	1718.8002	1718.7604	23.2	561 - 576	1	---	K.DMICEESGNKGTPGPK.N
1774.9059	1773.8986	1773.8857	7.30	544 - 560	1	---	K.SKAVSNTINNSEAPSQK.D
1797.8339	1796.8266	1796.8594	-18.25	139 - 153	1	---	K.RWSWAGYTTVNSQNK.T
1822.9185	1821.9112	1821.9043	3.81	599 - 612	2	---	K.EERLQKEMEQSFIR.K
2159.0752	2158.0679	2158.0212	21.7	449 - 467	2	---	K.DSPQKSEMDEQPSNPITKK.R
2203.0464	2202.0391	2202.0739	-15.78	356 - 375	1	---	K.EHPAVNVEALAEASMKEHPK.V + Oxidation (M)
2211.1077	2210.1004	2210.1457	-20.48	479 - 497	2	---	R.WSSSPTRRWRPPSPISASR.Q

No match to: 724.2539, 725.2472, 728.2482, 730.2519, 732.2390, 733.2623, 739.3115, 742.4642, 743.4729, 745.4483, 747.4419, 752.3972, 754.3827, 756.3716, 758.3382, 760.3329, 764.3364, 766.3494, 767.4740, 768.3865, 770.3671, 772.4178, 779.4436, 784.3415, 787.2366, 788.4225, 790.4196, 795.4260, 799.2464, 805.4478, 808.4353, 811.4178, 814.4170, 821.4247, 822.4321, 829.4457, 837.4396, 840.4623, 842.5109, 854.4525, 856.5045, 858.5442, 860.4092, 863.4233, 863.9185, 869.4846, 872.4994, 880.4412, 904.4613, 906.4929, 908.4577, 913.5685, 919.5024, 925.5089, 927.4999, 928.5031, 940.4773, 942.4820, 950.4946, 957.5654, 966.4929, 969.4786, 972.5046, 974.5551, 977.5352, 978.5240, 981.4474, 989.2700, 991.5198, 993.5112, 1007.5089, 1009.5097, 1013.4454, 1015.5150, 1016.5522, 1018.5189, 1021.5313, 1024.5028, 1025.5192, 1029.5330, 1032.5870, 1033.5739, 1035.5203, 1045.5564, 1051.5074, 1056.5415, 1057.6005, 1064.5358, 1071.5361, 1073.5377, 1079.5398, 1083.4637, 1086.4774, 1092.6000, 1092.6002, 1094.5596, 1096.5302, 1106.5431, 1110.5461, 1111.5398, 1111.5400, 1115.5790, 1118.5620, 1120.5724, 1123.5819, 1128.5537, 1132.5682, 1134.5841, 1135.5942, 1137.5565, 1143.5844, 1149.5597, 1153.5929, 1158.6288, 1163.5771, 1173.6008, 1175.5867, 1177.6178, 1178.6053, 1179.5962, 1181.6432, 1192.5721, 1199.6315, 1203.5826, 1211.5984, 1215.6246, 1216.6786, 1227.6271, 1228.6411, 1229.6412, 1233.6571, 1237.6270, 1244.6069, 1248.5958, 1250.6042, 1255.5922, 1258.6344, 1268.6414, 1271.6490, 1275.6388, 1285.5864, 1294.6315, 1300.6566, 1309.6340, 1320.6268, 1328.6292, 1333.7025, 1336.6038, 1338.6589, 1341.6488, 1345.6910, 1349.6838, 1352.6350, 1355.6870, 1357.7130, 1358.6704, 1369.7067, 1371.7366, 1374.6705, 1377.6100, 1377.6146, 1390.6848, 1393.7169, 1399.6119, 1400.6588, 1406.7207, 1410.6736, 1411.6647, 1416.6047, 1418.6714, 1421.6632, 1425.7395, 1427.7076, 1430.7102, 1432.6718, 1434.6552, 1438.6991, 1439.7164, 1444.7375, 1445.7306, 1454.6836, 1461.7990, 1472.7026, 1475.7224, 1485.6906, 1489.7172, 1490.7058, 1493.7157, 1510.7383, 1515.7393, 1523.6987, 1529.7466, 1531.6953, 1537.7804, 1538.7736, 1539.7621, 1541.7805, 1549.6758, 1553.7277, 1555.7928, 1556.7874, 1560.7551, 1563.7314, 1568.7372, 1570.8015, 1572.8151, 1576.7402, 1577.7395, 1578.7518, 1581.8373, 1591.7852, 1600.8068, 1603.8092, 1604.8136, 1606.8130, 1608.7599, 1613.7793, 1624.7632, 1628.7653, 1633.7913, 1634.7969, 1635.7941, 1639.8157, 1643.8085, 1646.8591, 1647.8268, 1651.8206, 1653.8284, 1677.8074, 1681.8087, 1688.8287, 1690.8313, 1694.7357, 1697.8672, 1703.8214, 1706.8364, 1707.8435, 1708.3533, 1709.3307, 1710.8229, 1721.8296, 1723.8743, 1724.8828, 1725.8090, 1730.8199, 1748.7931, 1757.8975, 1763.7841, 1768.8075, 1771.9686, 1777.8727, 1779.8081, 1786.8824, 1787.8207, 1790.8423, 1793.9556, 1796.8109, 1800.8578, 1815.9008, 1819.8838, 1821.8926, 1826.8450, 1829.8655, 1833.8975, 1836.9054, 1843.8765, 1844.8785, 1851.9031, 1859.8494, 1862.9265, 1863.9319, 1866.8857, 1868.8972, 1879.9445, 1880.9215, 1887.9023, 1888.8057, 1890.8025, 1896.8954, 1901.0060, 1906.8872, 1908.8663, 1913.8895, 1916.9475, 1924.9314, 1925.9336, 1928.0060, 1931.9956, 1932.9833, 1968.8563, 1984.9392, 2008.0485, 2008.9869, 2009.9747, 2012.9506, 2015.9661, 2020.9644, 2030.0151, 2030.4648, 2046.9839, 2047.5186, 2047.9097, 2051.9133, 2059.0122, 2064.9746, 2070.9209, 2077.0454, 2082.9409, 2085.9888, 2090.9844, 2097.9778, 2103.1526, 2108.9897, 2118.8926, 2140.0432, 2142.0310, 2142.9895, 2152.0510, 2155.0667, 2160.0703, 2161.0813, 2166.0911, 2167.0676, 2174.0425, 2178.0034, 2181.0369, 2184.0767, 2189.9133, 2194.9761, 2199.0986, 2205.9102, 2208.9729, 2213.1116, 2214.1145, 2218.1018, 2221.1035, 2223.2205, 2225.1592, 2229.1064, 2233.1023, 2245.0991, 2269.1128, 2273.0393, 2277.0576, 2279.1094, 2280.1204, 2283.1279, 2285.1169, 2292.1465, 2296.1479, 2312.0664, 2323.1892, 2326.0947, 2334.1531, 2335.1282, 2337.1343, 2369.2256, 2372.2229, 2383.9978, 2385.2129, 2392.1528, 2400.1094, 2401.1360, 2409.1914, 2411.1907, 2414.1833, 2429.1475, 2436.1699, 2437.1758, 2440.2007, 2447.2368, 2477.1301, 2495.0542, 2498.0898, 2510.2229, 2522.1980, 2524.1689, 2527.2310, 2535.2598, 2541.2075, 2543.1907, 2544.1514, 2553.1934, 2557.2300, 2560.1125, 2569.2915, 2572.0935, 2612.1936, 2686.3572, 2788.3362, 2963.6167, 3056.4641, 3058.5842, 3180.5432, 3313.5032, 3330.6311, 3347.6077, 3511.9668, 3761.1558

39. F1MIS2 **Mass:** 9764 **Score:** 19 **Expect:** 3.3e+002 **Matches:** 7

Uncharacterized protein OS=Bos taurus PE=3 SV=2

Observed	Mr(expt)	Mr(calc)	ppm	Start	End	Miss	Ions	Peptide
993.5112	992.5039	992.4862	17.9	20	27	1	---	R.MTSAYHRK.C
1009.5097	1008.5024	1008.4811	21.2	20	27	1	---	R.MTSAYHRK.C + Oxidation (M)
1563.7314	1562.7241	1562.7221	1.30	28	40	1	---	K.CMPAHYKEAELSK.G
1707.8435	1706.8362	1706.8120	14.2	27	40	2	---	R.KCMPAHYKEAELSK.G + Oxidation (M)
1896.8954	1895.8881	1895.8593	15.2	20	34	2	---	R.MTSAYHRKCMPAHYK.E + Oxidation (M)
2118.8926	2117.8853	2117.9081	-10.75	9	26	1	---	R.AGVEMADMYNRMTSAYHR.K + Oxidation (M)
2572.0935	2571.0862	2571.1444	-22.61	41	62	1	---	K.GESVCLDPYASKYLDIHECMGK.K

No match to: 724.2539, 725.2472, 728.2482, 730.2519, 732.2390, 733.2623, 739.3115, 742.4642, 743.4729, 745.4483, 747.4419, 752.3972, 754.3827, 756.3716, 758.3382, 760.3329, 764.3364, 766.3494, 767.4740, 768.3865, 770.3671, 772.4178, 779.4436, 784.3415, 787.2366, 788.4225, 790.4196, 795.4260, 799.2464, 802.4372, 805.4478, 808.4353, 811.4178, 814.4170, 819.4304, 821.4247, 822.4321, 829.4457, 837.4396, 840.4623, 842.5109, 854.4525, 856.5045, 858.5442, 860.4092, 863.4233, 863.9185, 869.4846, 870.5100, 872.4994, 880.4412, 904.4613, 906.4929, 908.4577, 913.5685, 919.5024, 925.5089, 927.4999, 928.5031, 940.4773, 942.4820, 950.4946, 957.5654, 960.4840, 966.4929, 969.4786, 972.5046, 974.5551, 977.5352, 978.5240, 981.4474, 989.2700, 991.5198, 1001.5782, 1007.5089, 1013.4454, 1015.5150, 1016.5522, 1018.5189, 1021.5313, 1024.5028, 1025.5192, 1029.5330, 1032.5870, 1033.5739, 1035.5203, 1045.5564, 1046.5759, 1051.5074, 1056.5415, 1057.6005, 1064.5358, 1071.5361, 1073.5377, 1079.5398, 1083.4637, 1086.4774, 1092.6000, 1092.6002, 1094.5596, 1096.5302, 1106.5431, 1110.5461, 1111.5398, 1111.5400, 1115.5790, 1118.5620, 1120.5724, 1123.5819, 1128.5537, 1132.5682, 1134.5841, 1135.5942, 1137.5565, 1143.5844, 1145.6155, 1149.5597, 1153.5929, 1158.6288, 1163.5771, 1173.6008, 1175.5867, 1177.6178, 1178.6053, 1179.5962, 1181.6432, 1192.5721, 1199.6315, 1203.5826, 1211.5984, 1215.6246, 1216.6786, 1221.5699, 1222.5789, 1227.6271, 1228.6411, 1229.6412, 1231.6102, 1233.6571, 1237.6270, 1238.5917, 1244.6069, 1248.5958, 1250.6042, 1255.5922, 1258.6344, 1268.6414, 1271.6490, 1275.6388, 1276.5977, 1276.6000, 1285.5864, 1294.6315, 1299.6810, 1300.6566, 1309.6340, 1320.6268, 1328.6292, 1333.7025, 1336.6038, 1338.6589, 1341.6488, 1343.6613, 1345.6910, 1349.6838, 1350.6704, 1352.6350, 1355.6870, 1357.7130, 1358.6704, 1369.7067, 1371.7366, 1374.6705, 1377.6100, 1377.6146, 1384.7109, 1386.7134, 1390.6848, 1393.7169, 1399.6119, 1400.6588, 1406.7207, 1410.6736,

1411.6647, 1416.6047, 1418.6714, 1421.6632, 1425.7395, 1426.6846, 1427.7076, 1430.7102, 1432.6718, 1434.6552, 1438.6991, 1439.7164, 1443.7589, 1444.7375, 1445.7306, 1454.6836, 1461.7990, 1472.7026, 1475.7224, 1485.6906, 1489.7172, 1490.7058, 1493.7157, 1510.7383, 1515.7393, 1523.6987, 1524.7383, 1527.7585, 1529.7466, 1530.6902, 1531.6953, 1537.7804, 1538.7736, 1539.7621, 1541.7805, 1542.8290, 1549.6758, 1553.7277, 1555.7928, 1556.7874, 1560.7551, 1568.7372, 1570.8015, 1572.8151, 1576.7402, 1577.7395, 1578.7518, 1581.8373, 1591.7852, 1600.8068, 1603.8092, 1604.8136, 1606.8130, 1608.7599, 1613.7793, 1615.8575, 1615.8600, 1624.7632, 1628.7653, 1633.7913, 1634.7969, 1635.7941, 1639.8157, 1643.8085, 1646.8591, 1647.8268, 1651.8206, 1653.8284, 1677.8074, 1681.8087, 1688.8287, 1690.8313, 1694.7357, 1697.8672, 1703.8214, 1706.8364, 1708.3533, 1709.3307, 1710.8229, 1715.8053, 1719.8075, 1721.8296, 1723.8743, 1724.8828, 1725.8090, 1730.8199, 1748.7931, 1757.8975, 1763.7841, 1768.8075, 1771.9686, 1774.9059, 1777.8727, 1779.8081, 1786.8824, 1787.8207, 1790.8423, 1793.9556, 1796.8109, 1797.8339, 1800.8578, 1815.9008, 1819.8838, 1821.8926, 1822.9185, 1826.8450, 1829.8655, 1833.8975, 1836.9054, 1843.8765, 1844.8785, 1851.9031, 1859.8494, 1862.9265, 1863.9319, 1866.8857, 1868.8972, 1879.9445, 1880.9215, 1887.9023, 1888.8057, 1890.8025, 1901.0060, 1906.8872, 1908.8663, 1913.8895, 1916.9475, 1924.9314, 1925.9336, 1928.0060, 1931.9956, 1932.9833, 1968.8563, 1984.9392, 2008.0485, 2008.9869, 2009.9747, 2012.9506, 2015.9661, 2020.9644, 2030.0151, 2030.4648, 2046.9839, 2047.5186, 2047.9097, 2051.9133, 2059.0122, 2064.9746, 2070.9209, 2077.0454, 2082.9409, 2085.9888, 2090.9844, 2097.9778, 2103.1526, 2108.9897, 2140.0432, 2142.0310, 2142.9895, 2152.0510, 2155.0667, 2159.0752, 2160.0703, 2161.0813, 2166.0911, 2167.0676, 2174.0425, 2178.0034, 2181.0369, 2184.0767, 2189.9133, 2194.9761, 2199.0986, 2203.0464, 2205.9102, 2208.9729, 2211.1077, 2213.1116, 2214.1145, 2218.1018, 2221.1035, 2223.2205, 2225.1592, 2229.1064, 2233.1023, 2245.0991, 2269.1128, 2273.0393, 2277.0576, 2279.1094, 2280.1204, 2283.1279, 2285.1169, 2292.1465, 2296.1479, 2312.0664, 2323.1892, 2326.0947, 2334.1531, 2335.1282, 2337.1343, 2369.2256, 2372.2229, 2383.9978, 2385.2129, 2392.1528, 2400.1094, 2401.1360, 2409.1914, 2411.1907, 2414.1833, 2429.1475, 2436.1699, 2437.1758, 2440.2007, 2447.2368, 2477.1301, 2495.0542, 2498.0898, 2510.2229, 2522.1980, 2524.1689, 2527.2310, 2535.2598, 2541.2075, 2543.1907, 2544.1514, 2553.1934, 2557.2300, 2560.1125, 2569.2915, 2612.1936, 2686.3572, 2788.3362, 2963.6167, 3056.4641, 3058.5842, 3180.5432, 3313.5032, 3330.6311, 3347.6077, 3511.9668, 3761.1558

40. F1MHH2 **Mass:** 64063 **Score:** 19 **Expect:** 3.3e+002 **Matches:** 22

Outer dense fiber protein 2-like OS=Bos taurus GN=ODF2L PE=4 SV=2

Observed	Mr(expt)	Mr(calc)	ppm	Start	End	Miss	Ions	Peptide
767.4740	766.4667	766.4701	-4.40	160	165	1	---	K.LKEHIK.S
811.4178	810.4105	810.3945	19.7	183	189	0	---	K.YEAVAMK.E
904.4613	903.4540	903.4661	-13.39	350	357	0	---	K.AELSSELR.K
925.5089	924.5016	924.5069	-5.74	534	540	0	---	K.NYLQFLK.T
960.4840	959.4767	959.4923	-16.31	154	161	1	---	K.EAENEKLK.E
1032.5870	1031.5797	1031.5611	18.1	350	358	1	---	K.AELSSELRK.D
1115.5790	1114.5717	1114.5618	8.89	453	462	0	---	R.QLEAALEEGR.Q
1181.6432	1180.6359	1180.6274	7.24	180	189	2	---	K.KNKYEAVAMK.E
1294.6315	1293.6242	1293.6234	0.62	465	475	1	---	K.VSEEIEKMSSR.E
1352.6350	1351.6277	1351.6255	1.61	73	83	0	---	K.DTIEEISFENR.G
1371.7366	1370.7293	1370.7153	10.2	453	464	1	---	R.QLEAALEEGRQK.V
1377.6100	1376.6027	1376.6354	-23.73	410	420	1	---	K.QKLEQMDAENR.E + Oxidation (M)
1377.6146	1376.6073	1376.6354	-20.39	410	420	1	---	K.QKLEQMDAENR.E + Oxidation (M)
1721.8296	1720.8223	1720.7872	20.4	494	507	1	---	K.NEEQNQLVCKMNSK.A
1730.8199	1729.8126	1729.8127	-0.04	276	289	1	---	K.MEEHGKISCEEILR.K
1906.8872	1905.8799	1905.8891	-4.80	210	225	0	---	R.IEHFTGDMENLTSQIR.E + Oxidation (M)
2012.9506	2011.9433	2011.9810	-18.73	517	533	1	---	K.EIQHSLEKSENQNESIK.N
2046.9839	2045.9766	2045.9905	-6.79	291	307	0	---	K.LHSIEYENETLNLENTK.L
2077.0454	2076.0381	2076.0422	-1.96	508	524	1	---	K.AQHQEVCLKEIQHSLEK.S
2334.1531	2333.1458	2333.1975	-22.13	444	464	2	---	K.SAEYTALARQLEAALEEGRQK.V
2522.1980	2521.1907	2521.2084	-7.02	226	247	2	---	R.EQEAKLSETVSASNAWKSHYEK.I
2553.1934	2552.1861	2552.2475	-24.05	504	524	2	---	K.MNSKAQHQEVCLKEIQHSLEK.S + Oxidation (M)

No match to: 724.2539, 725.2472, 728.2482, 730.2519, 732.2390, 733.2623, 739.3115, 742.4642, 743.4729, 745.4483, 747.4419, 752.3972, 754.3827, 756.3716, 758.3382, 760.3329, 764.3364, 766.3494, 768.3865, 770.3671, 772.4178, 779.4436, 784.3415, 787.2366, 788.4225, 790.4196, 795.4260, 799.2464, 802.4372, 805.4478, 808.4353, 814.4170, 819.4304, 821.4247, 822.4321, 829.4457, 837.4396, 840.4623, 842.5109, 854.4525, 856.5045, 858.5442, 860.4092, 863.4233, 863.9185, 869.4846, 870.5100, 872.4994, 880.4412, 906.4929, 908.4577, 913.5685, 919.5024, 927.4999, 928.5031, 940.4773, 942.4820, 950.4946, 957.5654, 966.4929, 969.4786, 972.5046, 974.5551, 977.5352, 978.5240, 981.4474, 989.2700, 991.5198, 993.5112, 1001.5782, 1007.5089, 1009.5097, 1013.4454, 1015.5150, 1016.5522, 1018.5189, 1021.5313, 1024.5028, 1025.5192, 1029.5330, 1033.5739, 1035.5203, 1045.5564, 1046.5759, 1051.5074, 1056.5415, 1057.6005, 1064.5358, 1071.5361, 1073.5377, 1079.5398, 1083.4637, 1086.4774, 1092.6000, 1092.6002, 1094.5596, 1096.5302, 1106.5431, 1110.5461, 1111.5398, 1111.5400, 1118.5620, 1120.5724, 1123.5819, 1128.5537, 1132.5682, 1134.5841, 1135.5942, 1137.5565, 1143.5844, 1145.6155, 1149.5597, 1153.5929, 1158.6288, 1163.5771, 1173.6008, 1175.5867, 1177.6178, 1178.6053, 1179.5962, 1192.5721, 1199.6315, 1203.5826, 1211.5984, 1215.6246, 1216.6786, 1221.5699, 1222.5789, 1227.6271, 1228.6411, 1229.6412, 1231.6102, 1233.6571, 1237.6270, 1238.5917, 1244.6069, 1248.5958, 1250.6042, 1255.5922, 1258.6344, 1268.6414, 1271.6490, 1275.6388, 1276.5977, 1276.6000, 1285.5864, 1299.6810, 1300.6566, 1309.6340, 1320.6268, 1328.6292, 1333.7025, 1336.6038, 1338.6589, 1341.6488, 1343.6613, 1345.6910, 1349.5956, 1350.6704, 1355.6870, 1357.7130, 1358.6704, 1369.7067, 1374.6705, 1384.7109, 1386.7134, 1390.6848, 1393.7169, 1399.6119, 1400.6588, 1406.7207, 1410.6736, 1411.6647, 1416.6047, 1418.6714, 1421.6632, 1425.7395, 1426.6846, 1427.7076, 1430.7102, 1432.6718, 1434.6552, 1438.6991, 1439.7164, 1443.7589, 1444.7375, 1445.7306, 1454.6836, 1461.7990, 1472.7026, 1475.7224, 1485.6906, 1489.7172, 1490.7058, 1493.7157, 1510.7383, 1515.7393, 1523.6987, 1524.7383, 1527.7585, 1529.7466, 1530.6902, 1531.6953, 1537.7804, 1538.7736, 1539.7621, 1541.7805, 1542.8290, 1549.6758, 1553.7277, 1555.7928, 1556.7874, 1560.7551, 1563.7314, 1568.7372, 1570.8015, 1572.8151, 1576.7402, 1577.7395, 1578.7518, 1581.8373, 1591.7852, 1600.8068, 1603.8092, 1604.8136, 1606.8130, 1608.7599, 1613.7793, 1615.8575, 1615.8600, 1624.7632, 1628.7653, 1633.7913, 1634.7969, 1635.7941, 1639.8157, 1643.8085, 1646.8591, 1647.8268, 1651.8206, 1653.8284, 1677.8074, 1681.8087, 1688.8287, 1690.8313, 1694.7357, 1697.8672, 1703.8214, 1706.8364, 1707.8435, 1708.3533, 1709.3307, 1710.8229, 1715.8053, 1719.8075, 1723.8743, 1724.8828, 1725.8090, 1748.7931, 1757.8975, 1763.7841, 1768.8075, 1771.9686, 1774.9059, 1777.8727, 1779.8081, 1786.8824, 1787.8207, 1790.8423, 1793.9556, 1796.8109, 1797.8339, 1800.8578, 1815.9008, 1819.8838, 1821.8926, 1822.9185, 1826.8450, 1829.8655, 1833.8975, 1836.9054, 1843.8765, 1844.8785, 1851.9031, 1859.8494, 1862.9265, 1863.9319, 1866.8857, 1868.8972, 1879.9445, 1880.9215, 1887.9023, 1888.8057, 1890.8025, 1896.8954, 1901.0060, 1908.8663, 1913.8895, 1916.9475, 1924.9314, 1925.9336, 1928.0060, 1931.9956, 1932.9833, 1968.8563, 1984.9392, 2008.0485, 2008.9869, 2009.9747, 2015.9661, 2020.9644, 2030.0151, 2030.4648, 2047.5186, 2047.9097, 2051.9133, 2059.0122, 2064.9746, 2070.9209, 2082.9409, 2085.9888, 2090.9844, 2097.9778, 2103.1526, 2108.9897, 2118.8926, 2140.0432, 2142.0310, 2142.9895, 2152.0510, 2155.0667, 2159.0752, 2160.0703, 2161.0813, 2166.0911, 2167.0676, 2174.0425, 2178.0034, 2181.0369, 2184.0767, 2189.9133, 2194.9761, 2199.0986, 2203.0464, 2205.9102, 2208.9729, 2211.1077, 2213.1116, 2214.1145, 2218.1018, 2221.1035, 2223.2205, 2225.1592, 2229.1064, 2233.1023, 2245.0991, 2269.1128, 2273.0393, 2277.0576, 2279.1094, 2280.1204, 2283.1279, 2285.1169, 2292.1465, 2296.1479, 2312.0664, 2323.1892, 2326.0947, 2335.1282, 2337.1343, 2369.2256, 2372.2229, 2383.9978, 2385.2129, 2392.1528, 2400.1094, 2401.1360, 2409.1914, 2411.1907, 2414.1833, 2429.1475, 2436.1699, 2437.1758, 2440.2007, 2447.2368, 2477.1301, 2495.0542, 2498.0898, 2510.2229, 2524.1689, 2527.2310, 2535.2598, 2541.2075, 2543.1907, 2544.1514, 2557.2300, 2560.1125, 2569.2915, 2572.0935, 2612.1936, 2686.3572, 2788.3362, 2963.6167, 3056.4641, 3058.5842, 3180.5432, 3313.5032, 3330.6311, 3347.6077, 3511.9668, 3761.1558

41. E1BPQ8 **Mass:** 60653 **Score:** 19 **Expect:** 3.3e+002 **Matches:** 22

Uncharacterized protein OS=Bos taurus GN=CDC73 PE=4 SV=1

Observed	Mr(expt)	Mr(calc)	ppm	Start	End	Miss	Ions	Peptide
805.4478	804.4405	804.4316	11.1	521	526	1	---	R.YMVKHK.S
821.4247	820.4174	820.4265	-11.12	521	526	1	---	R.YMVKHK.S + Oxidation (M)
872.4994	871.4921	871.5127	-23.63	2	9	0	---	M.ADVLSVLR.Q
904.4613	903.4540	903.4709	-18.63	393	399	2	---	K.KKQGCQR.E
1024.5028	1023.4955	1023.4873	8.06	300	308	1	---	K.GKEETEGFK.I
1029.5330	1028.5257	1028.5403	-14.17	284	292	0	---	K.QPIPAAYNR.Y
1096.5302	1095.5229	1095.5118	10.2	172	181	0	---	R.SLSEAMSVEK.I + Oxidation (M)
1158.6288	1157.6215	1157.6153	5.42	400	408	1	---	R.ENETLIQRR.K

1237.6270	1236.6197	1236.5986	17.0	212	222	0	---	R.SFVDAEVDVTR.D
1299.6810	1298.6737	1298.6507	17.8	298	308	2	---	R.FKGKEETEGFK.I
1430.7102	1429.7029	1429.6983	3.21	137	147	1	---	K.KPRIEDEECVR.L
1475.7224	1474.7151	1474.7416	-17.95	480	491	1	---	K.YDEVRLDPNVQK.W
1510.7383	1509.7310	1509.7140	11.3	22	34	0	---	K.GDEVIFGEFSWPK.N
1646.8591	1645.8518	1645.8563	-2.72	379	392	1	---	K.DLLQDLKFVPSDEK.K
1690.8313	1689.8240	1689.7992	14.7	140	152	2	---	R.IEDEECVRLDKER.L
1786.8824	1785.8751	1785.9043	-16.34	137	150	2	---	K.KPRIEDEECVRLDK.E
1793.9556	1792.9483	1792.9505	-1.23	1	15	1	---	-.MADVLSVLRQYNIQK.K + Oxidation (M)
1819.8838	1818.8765	1818.8934	-9.30	410	426	0	---	K.DQMQPGGTAISVTVPYR.V
1851.9031	1850.8958	1850.9203	-13.23	22	37	1	---	K.GDEVIFGEFSWPKNVK.T
1932.9833	1931.9760	1932.0164	-20.88	195	211	2	---	R.STIKTDLDDDITALKQR.S
2009.9747	2008.9674	2009.0065	-19.46	92	110	1	---	R.KDLLGYLNGEASTSASIDR.S
2326.0947	2325.0874	2325.1093	-9.41	309	330	1	---	K.IDTMGTYHGMTLKSVTEGASAR.K

No match to: 724.2539, 725.2472, 728.2482, 730.2519, 732.2390, 733.2623, 739.3115, 742.4642, 743.4729, 745.4483, 747.4419, 752.3972, 754.3827, 756.3716, 758.3382, 760.3329, 764.3364, 766.3494, 767.4740, 768.3865, 770.3671, 772.4178, 779.4436, 784.3415, 787.2366, 788.4225, 790.4196, 795.4260, 799.2464, 802.4372, 808.4353, 811.4178, 814.4170, 819.4304, 822.4321, 829.4457, 837.4396, 840.4623, 842.5109, 854.4525, 856.5045, 858.5442, 860.4092, 863.4233, 863.9185, 869.4846, 870.5100, 880.4412, 906.4929, 908.4577, 913.5685, 919.5024, 925.5089, 927.4999, 928.5031, 940.4773, 942.4820, 950.4946, 957.5654, 960.4840, 966.4929, 969.4786, 972.5046, 974.5551, 977.5352, 978.5240, 981.4474, 989.2700, 991.5198, 993.5112, 1001.5782, 1007.5089, 1009.5097, 1013.4454, 1015.5150, 1016.5522, 1018.5189, 1021.5313, 1025.5192, 1032.5870, 1033.5739, 1035.5203, 1045.5564, 1046.5759, 1051.5074, 1056.5415, 1057.6005, 1064.5358, 1071.5361, 1073.5377, 1079.5398, 1083.4637, 1086.4774, 1092.6000, 1092.6002, 1094.5596, 1106.5431, 1110.5461, 1111.5398, 1111.5400, 1115.5790, 1118.5620, 1120.5724, 1123.5819, 1128.5537, 1132.5682, 1134.5841, 1135.5942, 1137.5565, 1143.5844, 1145.6155, 1149.5597, 1153.5929, 1163.5771, 1173.6008, 1175.5867, 1177.6178, 1178.6053, 1179.5962, 1181.6432, 1192.5721, 1199.6315, 1203.5826, 1211.5984, 1215.6246, 1216.6786, 1221.5699, 1222.5789, 1227.6271, 1228.6411, 1229.6412, 1231.6102, 1233.6571, 1238.5917, 1244.6069, 1248.5958, 1250.6042, 1255.5922, 1258.6344, 1268.6414, 1271.6490, 1275.6388, 1276.5977, 1276.6000, 1285.5864, 1294.6315, 1300.6566, 1309.6340, 1320.6268, 1328.6292, 1333.7025, 1336.6038, 1338.6589, 1341.6488, 1343.6613, 1345.6910, 1349.6838, 1350.6704, 1352.6350, 1355.6870, 1357.7130, 1358.6704, 1369.7067, 1371.7366, 1374.6705, 1377.6100, 1377.6146, 1384.7109, 1386.7134, 1390.6848, 1393.7169, 1399.6119, 1400.6588, 1406.7207, 1410.6736, 1411.6647, 1416.6047, 1418.6714, 1421.6632, 1425.7395, 1426.6846, 1427.7076, 1432.6718, 1434.6552, 1438.6991, 1439.7164, 1443.7589, 1444.7375, 1445.7306, 1454.6836, 1461.7990, 1472.7026, 1485.6906, 1489.7172, 1490.7058, 1493.7157, 1515.7393, 1523.6987, 1524.7383, 1527.7585, 1529.7466, 1530.6902, 1531.6953, 1537.7804, 1538.7736, 1539.7621, 1541.7805, 1542.8290, 1549.6758, 1553.7277, 1555.7928, 1556.7874, 1560.7551, 1563.7314, 1568.7372, 1570.8015, 1572.8151, 1576.7402, 1577.7395, 1578.7518, 1581.8373, 1591.7852, 1600.8068, 1603.8092, 1604.8136, 1606.8130, 1608.7599, 1613.7793, 1615.8575, 1615.8600, 1624.7632, 1628.7653, 1633.7913, 1634.7969, 1635.7941, 1639.8157, 1643.8085, 1647.8268, 1651.8206, 1653.8284, 1677.8074, 1681.8087, 1688.8287, 1694.7357, 1697.8672, 1703.8214, 1706.8364, 1707.8435, 1708.3533, 1709.3307, 1710.8229, 1715.8053, 1719.8075, 1721.8296, 1723.8743, 1724.8828, 1725.8090, 1730.8199, 1748.7931, 1757.8975, 1763.7841, 1768.8075, 1771.9686, 1774.9059, 1777.8727, 1779.8081, 1787.8207, 1790.8423, 1796.8109, 1797.8339, 1800.8578, 1815.9008, 1821.8926, 1822.9185, 1826.8450, 1829.8655, 1833.8975, 1836.9054, 1843.8765, 1844.8785, 1859.8494, 1862.9265, 1863.9319, 1866.8857, 1868.8972, 1879.9445, 1880.9215, 1887.9023, 1888.8057, 1890.8025, 1896.8954, 1901.0060, 1906.8872, 1908.8663, 1913.8895, 1916.9475, 1924.9314, 1925.9336, 1928.0060, 1931.9956, 1968.8563, 1984.9392, 2008.0485, 2008.9869, 2012.9506, 2015.9661, 2020.9644, 2030.0151, 2030.4648, 2046.9839, 2047.5186, 2047.9097, 2051.9133, 2059.0122, 2064.9746, 2070.9209, 2077.0454, 2082.9409, 2085.9888, 2090.9844, 2097.9778, 2103.1526, 2108.9897, 2118.8926, 2140.0432, 2142.0310, 2142.9895, 2152.0510, 2155.0667, 2159.0752, 2160.0703, 2161.0813, 2166.0911, 2167.0676, 2174.0425, 2178.0034, 2181.0369, 2184.0767, 2189.9133, 2194.9761, 2199.0986, 2203.0464, 2205.9102, 2208.9729, 2211.1077, 2213.1116, 2214.1145, 2218.1018, 2221.1035, 2223.2205, 2225.1592, 2229.1064, 2233.1023, 2245.0991, 2269.1128, 2273.0393, 2277.0576, 2279.1094, 2280.1204, 2283.1279, 2285.1169, 2292.1465, 2296.1479, 2312.0664, 2323.1892, 2334.1531, 2335.1282, 2337.1343, 2369.2256, 2372.2229, 2383.9978, 2385.2129, 2392.1528, 2400.1094, 2401.1360, 2409.1914, 2411.1907, 2414.1833, 2429.1475, 2436.1699, 2437.1758, 2440.2007, 2447.2368, 2477.1301, 2495.0542, 2498.0898, 2510.2229, 2522.1980, 2524.1689, 2527.2310, 2535.2598, 2541.2075, 2543.1907, 2544.1514, 2553.1934, 2557.2300, 2560.1125, 2569.2915, 2572.0935, 2612.1936, 2686.3572, 2788.3362, 2963.6167, 3056.4641, 3058.5842, 3180.5432, 3313.5032, 3330.6311, 3347.6077, 3511.9668, 3761.1558

42. Q2TA00 **Mass:** 49021 **Score:** 18 **Expect:** 3.4e+002 **Matches:** 20

Coiled-coil domain-containing protein 83 OS=Bos taurus GN=CCDC83 PE=2 SV=1

Observed	Mr(expt)	Mr(calc)	ppm	Start	End	Miss	Ions	Peptide
756.3716	755.3643	755.3710	-8.85	408	413	1	---	K.MMKSFL.-
993.5112	992.5039	992.4960	7.91	1	9	1	---	-.METAAKTNK.R
1009.5097	1008.5024	1008.4910	11.4	1	9	1	---	-.METAAKTNK.R + Oxidation (M)
1025.5192	1024.5119	1024.5302	-17.79	10	18	1	---	K.RDIQDGPPK.E
1029.5330	1028.5257	1028.5113	14.0	380	387	0	---	K.MPIHFQEK.E
1096.5302	1095.5229	1095.5461	-21.18	66	73	0	---	K.DEQIWHIR.N
1173.6008	1172.5935	1172.6012	-6.54	379	387	1	---	K.KMPIHFQEK.E + Oxidation (M)
1222.5789	1221.5716	1221.5547	13.9	93	102	1	---	R.EEVENAMKEK.W + Oxidation (M)
1231.6102	1230.6029	1230.6105	-6.15	52	60	2	---	R.EKNQKYHER.N
1276.5977	1275.5904	1275.5877	2.12	108	117	1	---	R.DQEQNLKDMR.I
1276.6000	1275.5927	1275.5877	3.92	108	117	1	---	R.DQEQNLKDMR.I
1320.6268	1319.6195	1319.6503	-23.33	115	125	1	---	K.DMRIQISNAEK.L + Oxidation (M)
1863.9319	1862.9246	1862.9044	10.9	84	100	1	---	K.SEGSVVVTREEVENAMK.E
1879.9445	1878.9372	1878.8993	20.2	84	100	1	---	K.SEGSVVVTREEVENAMK.E + Oxidation (M)
2155.0667	2154.0594	2154.0262	15.4	164	181	2	---	K.ENAEKMSEQYKITLEDAR.K
2160.0703	2159.0630	2159.0640	-0.46	108	125	2	---	R.DQEQNLKDMRIQISNAEK.L
2199.0986	2198.0913	2198.0796	5.32	206	222	2	---	R.FIDKGSYREIWENDWLK.K
2277.0576	2276.0503	2276.0605	-4.49	393	410	2	---	K.FYEDVRSPESHITYKMMK.S + Oxidation (M)
2409.1914	2408.1841	2408.1835	0.28	380	398	2	---	K.MPIHFQEKETPVKFYEDVR.S + Oxidation (M)
2440.2007	2439.1934	2439.1342	24.3	131	150	2	---	K.LSEKEYWEEYKNVGSEQHGK.L

No match to: 724.2539, 725.2472, 728.2482, 730.2519, 732.2390, 733.2623, 739.3115, 742.4642, 743.4729, 745.4483, 747.4419, 752.3972, 754.3827, 758.3382, 760.3329, 764.3364, 766.3494, 767.4740, 768.3865, 770.3671, 772.4178, 779.4436, 784.3415, 787.2366, 788.4225, 790.4196, 795.4260, 799.2464, 802.4372, 805.4478, 808.4353, 811.4178, 814.4170, 819.4304, 821.4247, 822.4321, 829.4457, 837.4396, 840.4623, 842.5109, 854.4525, 856.5045, 858.5442, 860.4092, 863.4233, 863.9185, 869.4846, 870.5100, 872.4994, 880.4412, 904.4613, 906.4929, 908.4577, 913.5685, 919.5024, 925.5089, 927.4999, 928.5031, 940.4773, 942.4820, 950.4946, 957.5654, 960.4840, 966.4929, 969.4786, 972.5046, 974.5551, 977.5352, 978.5240, 981.4474, 989.2700, 991.5198, 1001.5782, 1007.5089, 1013.4454, 1015.5150, 1016.5522, 1018.5189, 1021.5313, 1024.5028, 1032.5870, 1033.5739, 1035.5203, 1045.5564, 1046.5759, 1051.5074, 1056.5415, 1057.6005, 1064.5358, 1071.5361, 1073.5377, 1079.5398, 1083.4637, 1086.4774, 1092.6000, 1092.6002, 1094.5596, 1106.5431, 1110.5461, 1111.5398, 1111.5400, 1115.5790, 1118.5620, 1120.5724, 1123.5819, 1128.5537, 1132.5682, 1134.5841, 1135.5942, 1137.5565, 1143.5844, 1145.6155, 1149.5597, 1153.5929, 1158.6288, 1163.5771, 1175.5867, 1177.6178, 1178.6053, 1179.5962, 1181.6432, 1192.5721, 1199.6315, 1203.5826, 1211.5984, 1215.6246, 1216.6786, 1221.5699, 1227.6271, 1228.6411, 1229.6412, 1233.6571, 1237.6270, 1238.5917, 1244.6069, 1248.5958, 1250.6042, 1255.5922, 1258.6344, 1268.6414, 1271.6490, 1275.6388, 1285.5864, 1294.6315, 1299.6810, 1300.6566, 1309.6340, 1328.6292, 1333.7025, 1336.6038, 1338.6589, 1341.6488, 1343.6613, 1345.6910, 1349.6838, 1350.6704, 1352.6350, 1355.6870, 1357.7130, 1358.6704, 1369.7067, 1371.7366, 1374.6705, 1377.6100, 1377.6146, 1384.7109, 1386.7134, 1390.6848, 1393.7169, 1399.6119, 1400.6588, 1406.7207, 1410.6736, 1411.6647, 1416.6047, 1418.6714, 1421.6632, 1425.7395, 1426.6846, 1427.7076, 1430.7102, 1432.6718, 1434.6552, 1438.6991, 1439.7164, 1443.7589, 1444.7375, 1445.7306, 1454.6836, 1461.7990, 1472.7026, 1475.7224, 1485.6906, 1489.7172, 1490.7058, 1493.7157, 1510.7383, 1515.7393, 1523.6987, 1524.7383, 1527.7585, 1529.7466, 1530.6902, 1531.6953, 1537.7804, 1538.7736, 1539.7621, 1541.7805, 1542.8290, 1549.6758, 1553.7277, 1555.7928, 1556.7874, 1560.7551, 1563.7314, 1568.7372, 1570.8015, 1572.8151, 1576.7402, 1577.7395, 1578.7518, 1581.8373, 1591.7852, 1600.8068, 1603.8092, 1604.8136, 1606.8130, 1608.7599, 1613.7793, 1615.8575, 1615.8600, 1624.7632, 1628.7653, 1633.7913, 1634.7969, 1635.7941, 1639.8157, 1643.8085, 1646.8591, 1647.8268, 1651.8206, 1653.8284, 1677.8074, 1681.8087, 1688.8287, 1690.8313, 1694.7357, 1697.8672, 1703.8214, 1706.8364, 1707.8435, 1708.3533, 1709.3307, 1710.8229, 1715.8053,

1719.8075, 1721.8296, 1723.8743, 1724.8828, 1725.8090, 1730.8199, 1748.7931, 1757.8975, 1763.7841, 1768.8075, 1771.9686, 1774.9059, 1777.8727, 1779.8081, 1786.8824, 1787.8207, 1790.8423, 1793.9556, 1796.8109, 1797.8339, 1800.8578, 1815.9008, 1819.8838, 1821.8926, 1822.9185, 1826.8450, 1829.8655, 1833.8975, 1836.9054, 1843.8765, 1844.8785, 1851.9031, 1859.8494, 1862.9265, 1866.8857, 1868.8972, 1880.9215, 1887.9023, 1888.8057, 1890.8025, 1896.8954, 1901.0060, 1906.8872, 1908.8663, 1913.8895, 1916.9475, 1924.9314, 1925.9336, 1928.0060, 1931.9956, 1932.9833, 1968.8563, 1984.9392, 2008.0485, 2008.9869, 2009.9747, 2012.9506, 2015.9661, 2020.9644, 2030.0151, 2030.4648, 2046.9839, 2047.5186, 2047.9097, 2051.9133, 2059.0122, 2064.9746, 2070.9209, 2077.0454, 2082.9409, 2085.9888, 2090.9844, 2097.9778, 2103.1526, 2108.9897, 2118.8926, 2140.0432, 2142.0310, 2142.9895, 2152.0510, 2159.0752, 2161.0813, 2166.0911, 2167.0676, 2174.0425, 2178.0034, 2181.0369, 2184.0767, 2189.9133, 2194.9761, 2203.0464, 2205.9102, 2208.9729, 2211.1077, 2213.1116, 2214.1145, 2218.1018, 2221.1035, 2223.2205, 2225.1592, 2229.1064, 2233.1023, 2245.0991, 2269.1128, 2273.0393, 2279.1094, 2280.1204, 2283.1279, 2285.1169, 2292.1465, 2296.1479, 2312.0664, 2323.1892, 2326.0947, 2334.1531, 2335.1282, 2337.1343, 2369.2256, 2372.2229, 2383.9978, 2385.2129, 2392.1528, 2400.1094, 2401.1360, 2411.1907, 2414.1833, 2429.1475, 2436.1699, 2437.1758, 2447.2368, 2477.1301, 2495.0542, 2498.0898, 2510.2229, 2522.1980, 2524.1689, 2527.2310, 2535.2598, 2541.2075, 2543.1907, 2544.1514, 2553.1934, 2557.2300, 2560.1125, 2569.2915, 2572.0935, 2612.1936, 2686.3572, 2788.3362, 2963.6167, 3056.4641, 3058.5842, 3180.5432, 3313.5032, 3330.6311, 3347.6077, 3511.9668, 3761.1558

43. E1B992 **Mass:** 72756 **Score:** 18 **Expect:** 3.4e+002 **Matches:** 21

Uncharacterized protein OS=Bos taurus PE=4 SV=2

Observed	Mr(expt)	Mr(calc)	ppm	Start	End	Miss	Ions	Peptide
1025.5192	1024.5119	1024.4937	17.7	67	74	1	---	K.SYDRLSER.K
1153.5929	1152.5856	1152.5887	-2.67	67	75	2	---	K.SYDRLSERK.D
1299.6810	1298.6737	1298.6441	22.8	424	433	1	---	R.KPYQCKQYGK.T
1393.7169	1392.7096	1392.7105	-0.64	487	498	0	---	K.CSQVLSSVCLLK.N
1400.6588	1399.6515	1399.6554	-2.80	333	344	0	---	K.HMVTHSGDLPYK.C + Oxidation (M)
1485.6906	1484.6833	1484.7194	-24.30	131	141	2	---	R.EHQKCHEKVYK.C
1524.7383	1523.7310	1523.7225	5.62	404	415	1	---	K.CEKTFISPNCLR.M
1568.7372	1567.7299	1567.7235	4.08	96	109	1	---	K.TAGFKPRECSACGK.F
1606.8130	1605.8057	1605.8086	-1.77	474	486	2	---	K.RFHIGETPYACKK.C
1613.7793	1612.7720	1612.7490	14.3	528	541	0	---	K.HMIIHTGDGPFTCK.E
1688.8287	1687.8214	1687.7988	13.4	259	271	1	---	R.EIIHTREQPYDCK.Q
1703.8214	1702.8141	1702.7845	17.4	157	170	2	---	R.THKRSHTGEAYECK.Q
1793.9556	1792.9483	1792.9189	16.4	284	298	2	---	R.LHRGKTPHVCITCSK.E
1800.8578	1799.8505	1799.8447	3.23	333	347	1	---	K.HMVTHSGDLPYKCQK.C
2159.0752	2158.0679	2158.0769	-4.15	434	451	1	---	K.TFKTPKPVGYMEEFTEVR.K
2199.0986	2198.0913	2198.1266	-16.04	255	271	2	---	R.IQIREIIHTREQPYDCK.Q
2277.0576	2276.0503	2276.0790	-12.61	289	307	1	---	K.TPHVCITCSKELTTAHHER.I
2436.1699	2435.1626	2435.1652	-1.04	299	318	1	---	K.ELTTAHHERIHTGEKPYECK.E
2524.1689	2523.1616	2523.1965	-13.81	180	200	1	---	K.NTHTGVKPYEYKECGNALTWR.S
3180.5432	3179.5359	3179.5571	-6.65	180	206	2	---	K.NTHTGVKPYEYKECGNALTWRSNVVTR.T
3347.6077	3346.6004	3346.6292	-8.60	2	29	2	---	M.DSLVFEDVAMNFTEQWGLLDSSQRKLYR.D

No match to: 724.2539, 725.2472, 728.2482, 730.2519, 732.2390, 733.2623, 739.3115, 742.4642, 743.4729, 745.4483, 747.4419, 752.3972, 754.3827, 756.3716, 758.3382, 760.3329, 764.3364, 766.3494, 767.4740, 768.3865, 770.3671, 772.4178, 779.4436, 784.3415, 787.2366, 788.4225, 790.4196, 795.4260, 799.2464, 802.4372, 805.4478, 808.4353, 811.4178, 814.4170, 819.4304, 821.4247, 822.4321, 829.4457, 837.4396, 840.4623, 842.5109, 854.4525, 856.5045, 858.5442, 860.4092, 863.4233, 863.9185, 869.4846, 870.5100, 872.4994, 880.4412, 904.4613, 906.4929, 908.4577, 913.5685, 919.5024, 925.5089, 927.4999, 928.5031, 940.4773, 942.4820, 950.4946, 957.5654, 960.4840, 966.4929, 969.4786, 972.5046, 974.5551, 977.5352, 978.5240, 981.4474, 989.2700, 991.5198, 993.5112, 1001.5782, 1007.5089, 1009.5097, 1013.4454, 1015.5150, 1016.5522, 1018.5189, 1021.5313, 1024.5028, 1029.5330, 1032.5870, 1033.5739, 1035.5203, 1045.5564, 1046.5759, 1051.5074, 1056.5415, 1057.6005, 1064.5358, 1071.5361, 1073.5377, 1079.5398, 1083.4637, 1086.4774, 1092.6000, 1092.6002, 1094.5596, 1096.5302, 1106.5431, 1110.5461, 1111.5398, 1111.5400, 1115.5790, 1118.5620, 1120.5724, 1123.5819, 1128.5537, 1132.5682, 1134.5841, 1135.5942, 1137.5565, 1143.5844, 1145.6155, 1149.5597, 1158.6288, 1163.5771, 1173.6008, 1175.5867, 1177.6178, 1178.6053, 1179.5962, 1181.6432, 1192.5721, 1199.6315, 1203.5826, 1211.5984, 1215.6246, 1216.6786, 1221.5699, 1222.5789, 1227.6271, 1228.6411, 1229.6412, 1231.6102, 1233.6571, 1237.6270, 1238.5917, 1244.6069, 1248.5958, 1250.6042, 1255.5922, 1258.6344, 1268.6414, 1271.6490, 1275.6388, 1276.5977, 1276.6000, 1285.5864, 1294.6315, 1300.6566, 1309.6340, 1320.6268, 1328.6292, 1333.7025, 1336.6038, 1338.6589, 1341.6488, 1343.6613, 1345.6910, 1349.6838, 1350.6704, 1352.6350, 1355.6870, 1357.7130, 1358.6704, 1369.7067, 1371.7366, 1374.6705, 1377.6100, 1377.6146, 1384.7109, 1386.7134, 1390.6848, 1399.6119, 1406.7207, 1410.6736, 1411.6647, 1416.6047, 1418.6714, 1421.6632, 1425.7395, 1426.6846, 1427.7076, 1430.7102, 1432.6718, 1434.6552, 1438.6991, 1439.7164, 1443.7589, 1444.7375, 1445.7306, 1454.6836, 1461.7990, 1472.7026, 1475.7224, 1489.7172, 1490.7058, 1493.7157, 1510.7383, 1515.7393, 1523.6987, 1527.7585, 1529.7466, 1530.6902, 1531.6953, 1537.7804, 1538.7736, 1539.7621, 1541.7805, 1542.8290, 1549.6758, 1553.7277, 1555.7928, 1556.7874, 1560.7551, 1563.7314, 1570.8015, 1572.8151, 1576.7402, 1577.7395, 1578.7518, 1581.8373, 1591.7852, 1600.8068, 1603.8092, 1604.8136, 1608.7599, 1615.8575, 1615.8600, 1624.7632, 1628.7653, 1633.7913, 1634.7969, 1635.7941, 1639.8157, 1643.8085, 1646.8591, 1647.8268, 1651.8206, 1653.8284, 1677.8074, 1681.8087, 1690.8313, 1694.7357, 1697.8672, 1706.8364, 1707.8435, 1708.3533, 1709.3307, 1710.8229, 1715.8053, 1719.8075, 1721.8296, 1723.8743, 1724.8828, 1725.8090, 1730.8199, 1748.7931, 1757.8975, 1763.7841, 1768.8075, 1771.9686, 1774.9059, 1777.8727, 1779.8081, 1786.8824, 1787.8207, 1790.8423, 1796.8109, 1797.8339, 1815.9008, 1819.8838, 1821.8926, 1822.9185, 1826.8450, 1829.8655, 1833.8975, 1836.9054, 1843.8765, 1844.8785, 1851.9031, 1859.8494, 1862.9265, 1863.9319, 1866.8857, 1868.8972, 1879.9445, 1880.9215, 1887.9023, 1888.8057, 1890.8025, 1896.8954, 1901.0060, 1906.8872, 1908.8663, 1913.8895, 1916.9475, 1924.9314, 1925.9336, 1928.0060, 1931.9956, 1932.9833, 1968.8563, 1984.9392, 2008.0485, 2008.9869, 2009.9747, 2012.9506, 2015.9661, 2020.9644, 2030.0151, 2030.4648, 2046.9839, 2047.5186, 2047.9097, 2051.9133, 2059.0122, 2064.9746, 2070.9209, 2077.0454, 2082.9409, 2085.9888, 2090.9844, 2097.9778, 2103.1526, 2108.9897, 2118.8926, 2140.0432, 2142.0310, 2142.9895, 2152.0510, 2155.0667, 2160.0703, 2161.0813, 2166.0911, 2167.0676, 2174.0425, 2178.0034, 2181.0369, 2184.0767, 2189.9133, 2194.9761, 2203.0464, 2205.9102, 2208.9729, 2211.1077, 2213.1116, 2214.1145, 2218.1018, 2221.1035, 2223.2205, 2225.1592, 2229.1064, 2233.1023, 2245.0991, 2269.1128, 2273.0393, 2279.1094, 2280.1204, 2283.1279, 2285.1169, 2292.1465, 2296.1479, 2312.0664, 2323.1892, 2326.0947, 2334.1531, 2335.1282, 2337.1343, 2369.2256, 2372.2229, 2383.9978, 2385.2129, 2392.1528, 2400.1094, 2401.1360, 2409.1914, 2411.1907, 2414.1833, 2429.1475, 2437.1758, 2440.2007, 2447.2368, 2477.1301, 2495.0542, 2498.0898, 2510.2229, 2522.1980, 2527.2310, 2535.2598, 2541.2075, 2543.1907, 2544.1514, 2553.1934, 2557.2300, 2560.1125, 2569.2915, 2572.0935, 2612.1936, 2686.3572, 2788.3362, 2963.6167, 3056.4641, 3058.5842, 3313.5032, 3330.6311, 3511.9668, 3761.1558

44. F1MB20 **Mass:** 119646 **Score:** 18 **Expect:** 3.4e+002 **Matches:** 39

Oral-facial-digital syndrome 1 isoform 1 OS=Bos taurus GN=OFD1 PE=2 SV=2

Observed	Mr(expt)	Mr(calc)	ppm	Start	End	Miss	Ions	Peptide
745.4483	744.4410	744.4382	3.85	55	61	0	---	R.GVLDTLK.T
802.4372	801.4299	801.4344	-5.64	323	329	1	---	R.GREAELK.Q
822.4321	821.4249	821.4395	-17.85	225	230	1	---	K.LNEYKR.E
950.4946	949.4873	949.4902	-3.10	238	245	1	---	R.AEMSQKLK.Y + Oxidation (M)
957.5654	956.5581	956.5403	18.6	317	324	1	---	K.DIDLLRGR.E
960.4840	959.4767	959.4825	-6.04	925	931	0	---	K.WQETLQR.E
977.5352	976.5279	976.5263	1.63	249	256	1	---	K.DTEIMKIK.M
993.5112	992.5039	992.5212	-17.45	249	256	1	---	K.DTEIMKIK.M + Oxidation (M)
1016.5522	1015.5449	1015.5662	-20.96	53	61	1	---	K.DRGVLDTLK.T
1021.5313	1020.5240	1020.5022	21.4	346	354	1	---	K.NKSMTDGLR.R
1029.5330	1028.5257	1028.5475	-21.19	750	758	2	---	R.SHKSTASRR.L
1057.6005	1056.5932	1056.5676	24.3	230	237	1	---	K.REVEQQLR.A
1079.5398	1078.5325	1078.5519	-18.00	2	12	0	---	M.AHVPSPAASSR.R
1145.6155	1144.6082	1144.5836	21.5	390	398	1	---	R.TNRLIEDER.K
1158.6288	1157.6215	1157.6040	15.1	935	943	1	---	R.QLEKLDQER.R

1173.6008	1172.5935	1172.5646	24.6	913 -	921	2	---	R.ERRQGDEQR.Q
1177.6178	1176.6105	1176.6033	6.13	346 -	355	2	---	K.NKSMTDGLRR.R
1199.6315	1198.6242	1198.6306	-5.33	722 -	732	0	---	R.VTSQQPPVSTR.G
1229.6412	1228.6339	1228.6234	8.58	448 -	457	1	---	K.QNHMLSEKVK.E + Oxidation (M)
1231.6102	1230.6029	1230.6065	-2.90	915 -	924	2	---	R.RQGDEQRQSK.W
1271.6490	1270.6417	1270.6420	-0.23	25 -	34	0	---	R.FLSWSIMFPK.S + Oxidation (M)
1275.6388	1274.6315	1274.6466	-11.86	35 -	45	1	---	K.SDVLSQDDLRK.K
1377.6100	1376.6027	1376.6129	-7.40	662 -	672	0	---	R.ELEQEAECLEK.A
1377.6146	1376.6073	1376.6129	-4.06	662 -	672	0	---	R.ELEQEAECLEK.A
1439.7164	1438.7091	1438.6940	10.5	769 -	781	0	---	R.SLDSETYLEGLGR.S
1445.7306	1444.7233	1444.7521	-19.93	896 -	907	2	---	K.SLGEQKEEEKLR.E
1510.7383	1509.7310	1509.7418	-7.15	249 -	260	2	---	K.DTEIMKIKMEEK.R + Oxidation (M)
1570.8015	1569.7942	1569.8038	-6.12	378 -	389	1	---	K.YQLELKEDYITR.T
1591.7852	1590.7779	1590.7671	6.78	231 -	243	1	---	R.EVEQQLRAEMSQK.L + Oxidation (M)
1703.8214	1702.8141	1702.7719	24.8	957 -	970	2	---	K.EYESSVQEMKDKSK.S + Oxidation (M)
1723.8743	1722.8670	1722.8676	-0.31	357 -	370	1	---	R.ELTIKNIEETYDQK.L
1859.8494	1858.8421	1858.8328	5.01	952 -	966	1	---	K.LEMEKEYESSVQEMK.D
1863.9319	1862.9246	1862.8846	21.5	673 -	687	1	---	K.AFQNYHQRVSQCPTK.S
1879.9445	1878.9372	1878.9687	-16.73	356 -	370	2	---	R.RELTIKNIEETYDQK.L
1887.9023	1886.8950	1886.9342	-20.77	440 -	455	1	---	R.AQCLALTKQNHMLSEK.V + Oxidation (M)
2280.1204	2279.1131	2279.1289	-6.94	448 -	466	2	---	K.QNHMLSEKVKEMSDYSLLK.E
2296.1479	2295.1406	2295.1239	7.31	448 -	466	2	---	K.QNHMLSEKVKEMSDYSLLK.E + Oxidation (M)
2411.1907	2410.1834	2410.1877	-1.76	573 -	593	0	---	K.QTQTALENEVYPNPSHSLLNR.S
2527.2310	2526.2237	2526.1874	14.4	636 -	659	1	---	R.YPNASAEGSSPDSDLEFVASTKVR.V

No match to: 724.2539, 725.2472, 728.2482, 730.2519, 732.2390, 733.2623, 739.3115, 742.4642, 743.4729, 747.4419, 752.3972, 754.3827, 756.3716, 758.3382, 760.3329, 764.3364, 766.3494, 767.4740, 768.3865, 770.3671, 772.4178, 779.4436, 784.3415, 787.2366, 788.4225, 790.4196, 795.4260, 799.2464, 805.4478, 808.4353, 811.4178, 814.4170, 819.4304, 821.4247, 829.4457, 837.4396, 840.4623, 842.5109, 854.4525, 856.5045, 858.5442, 860.4092, 863.4233, 863.9185, 869.4846, 870.5100, 872.4994, 880.4412, 904.4613, 906.4929, 908.4577, 913.5685, 919.5024, 925.5089, 927.4999, 928.5031, 940.4773, 942.4820, 966.4929, 969.4786, 972.5046, 974.5551, 978.5240, 981.4474, 989.2700, 991.5198, 1001.5782, 1007.5089, 1009.5097, 1013.4454, 1015.5150, 1018.5189, 1024.5028, 1025.5192, 1032.5870, 1033.5739, 1035.5203, 1045.5564, 1046.5759, 1051.5074, 1056.5415, 1064.5358, 1071.5361, 1073.5377, 1083.4637, 1086.4774, 1092.6000, 1092.6002, 1094.5596, 1096.5302, 1106.5431, 1110.5461, 1111.5398, 1111.5400, 1115.5790, 1118.5620, 1120.5724, 1123.5819, 1128.5537, 1132.5682, 1134.5841, 1135.5942, 1137.5565, 1143.5844, 1149.5597, 1153.5929, 1163.5771, 1175.5867, 1178.6053, 1179.5962, 1181.6432, 1192.5721, 1203.5826, 1211.5984, 1215.6246, 1216.6786, 1221.5699, 1222.5789, 1227.6271, 1228.6411, 1233.6571, 1237.6270, 1238.5917, 1244.6069, 1248.5958, 1250.6042, 1255.5922, 1258.6344, 1268.6414, 1276.5977, 1276.6000, 1285.5864, 1294.6315, 1299.6810, 1300.6566, 1309.6340, 1320.6268, 1328.6292, 1333.7025, 1336.6038, 1338.6589, 1341.6488, 1343.6613, 1345.6910, 1349.6838, 1350.6704, 1352.6350, 1355.6870, 1357.7130, 1358.6704, 1369.7067, 1371.7366, 1374.6705, 1384.7109, 1386.7134, 1390.6848, 1393.7169, 1399.6119, 1400.6588, 1406.7207, 1410.6736, 1411.6647, 1416.6047, 1418.6714, 1421.6632, 1425.7395, 1426.6846, 1427.7076, 1430.7102, 1432.6718, 1434.6552, 1438.6991, 1443.7589, 1444.7375, 1454.6836, 1461.7990, 1472.7026, 1475.7224, 1485.6906, 1489.7172, 1490.7058, 1493.7157, 1515.7393, 1523.6987, 1524.7383, 1527.7585, 1529.7466, 1530.6902, 1531.6953, 1537.7804, 1538.7736, 1539.7621, 1541.7805, 1542.8290, 1549.6758, 1553.7277, 1555.7928, 1556.7874, 1560.7551, 1563.7314, 1568.7372, 1572.8151, 1576.7402, 1577.7395, 1578.7518, 1581.8373, 1600.8068, 1603.8092, 1604.8136, 1606.8130, 1608.7599, 1613.7793, 1615.8575, 1615.8600, 1624.7632, 1628.7653, 1633.7913, 1634.7969, 1635.7941, 1639.8157, 1643.8085, 1646.8591, 1647.8268, 1651.8206, 1653.8284, 1677.8074, 1681.8087, 1688.8287, 1690.8313, 1694.7357, 1697.8672, 1706.8364, 1707.8435, 1708.3533, 1709.3307, 1710.8229, 1715.8053, 1719.8075, 1721.8296, 1724.8828, 1725.8090, 1730.8199, 1748.7931, 1757.8975, 1763.7841, 1768.8075, 1771.9686, 1774.9059, 1777.8727, 1779.8081, 1786.8824, 1787.8207, 1790.8423, 1793.9556, 1796.8109, 1797.8339, 1800.8578, 1815.9008, 1819.8838, 1821.8926, 1822.9185, 1826.8450, 1829.8655, 1833.8975, 1836.9054, 1843.8765, 1844.8785, 1851.9031, 1862.9265, 1866.8857, 1868.8972, 1880.9215, 1888.8057, 1890.8025, 1896.8954, 1901.0060, 1906.8872, 1908.8663, 1913.8895, 1916.9475, 1924.9314, 1925.9336, 1928.0060, 1931.9956, 1932.9833, 1968.8563, 1984.9392, 2008.0485, 2008.9869, 2009.9747, 2012.9506, 2015.9661, 2020.9644, 2030.0151, 2030.4648, 2046.9839, 2047.5186, 2047.9097, 2051.9133, 2059.0122, 2064.9746, 2070.9209, 2077.0454, 2082.9409, 2085.9888, 2090.9844, 2097.9778, 2103.1526, 2108.9897, 2118.8926, 2140.0432, 2142.0310, 2142.9895, 2152.0510, 2155.0667, 2159.0752, 2160.0703, 2161.0813, 2166.0911, 2167.0676, 2174.0425, 2178.0034, 2181.0369, 2184.0767, 2189.9133, 2194.9761, 2199.0986, 2203.0464, 2205.9102, 2208.9729, 2211.1077, 2213.1116, 2214.1145, 2218.1018, 2221.1035, 2223.2205, 2225.1592, 2229.1064, 2233.1023, 2245.0991, 2269.1128, 2273.0393, 2277.0576, 2279.1094, 2283.1279, 2285.1169, 2292.1465, 2312.0664, 2323.1892, 2326.0947, 2334.1531, 2335.1282, 2337.1343, 2369.2256, 2372.2229, 2383.9978, 2385.2129, 2392.1528, 2400.1094, 2401.1360, 2409.1914, 2414.1833, 2429.1475, 2436.1699, 2437.1758, 2440.2007, 2447.2368, 2477.1301, 2495.0542, 2498.0898, 2510.2229, 2522.1980, 2524.1689, 2535.2598, 2541.2075, 2543.1907, 2544.1514, 2553.1934, 2557.2300, 2560.1125, 2569.2915, 2572.0935, 2612.1936, 2686.3572, 2788.3362, 2963.6167, 3056.4641, 3058.5842, 3180.5432, 3313.5032, 3330.6311, 3347.6077, 3511.9668, 3761.1558

45. E1BI42 **Mass:** 127633 **Score:** 18 **Expect:** 3.4e+002 **Matches:** 37

Uncharacterized protein OS=Bos taurus GN=CCDC158 PE=4 SV=2

Observed	Mr(expt)	Mr(calc)	ppm	Start	End	Miss	Ions	Peptide
742.4642	741.4570	741.4749	-24.14	563 -	568	0	---	K.VIEILR.Q
788.4225	787.4152	787.4188	-4.55	650 -	655	1	---	K.DIKQER.D
821.4247	820.4174	820.4113	7.50	217 -	224	0	---	R.NMASAISK.V
1032.5870	1031.5797	1031.6049	-24.36	431 -	439	1	---	R.LEALLKAMK.S + Oxidation (M)
1096.5302	1095.5229	1095.5230	-0.06	488 -	496	1	---	K.KMTLESSER.T + Oxidation (M)
1215.6246	1214.6173	1214.6255	-6.71	593 -	602	1	---	K.AQLEKEINDR.R
1231.6102	1230.6029	1230.6244	-17.48	79 -	88	0	---	R.VLEEYSHQVK.D
1275.6388	1274.6315	1274.6255	4.74	57 -	66	1	---	K.YEVELDSHRK.V
1341.6488	1340.6415	1340.6684	-20.08	407 -	418	0	---	R.DTGNSITIDHLR.R
1371.7366	1370.7293	1370.7266	2.00	593 -	603	2	---	K.AQLEKEINDRR.L
1410.6736	1409.6663	1409.6708	-3.16	687 -	698	1	---	K.SEEMETTTNKLK.M
1411.6647	1410.6574	1410.6296	19.7	685 -	696	1	---	R.NKSEEMETTTNK.L
1426.6846	1425.6773	1425.6697	5.35	339 -	349	1	---	R.MYEDKIEELEK.Q
1443.7589	1442.7516	1442.7729	-14.73	350 -	362	0	---	K.QLVLANSELTEAR.T
1445.7306	1444.7233	1444.7521	-19.93	509 -	521	1	---	K.ERAIEATNAEITK.L
1461.7990	1460.7917	1460.7955	-2.62	728 -	740	2	---	K.VAMGMQKQITAKR.G
1524.7383	1523.7310	1523.7290	1.36	750 -	762	0	---	K.IQFLEEAMTNANK.E + Oxidation (M)
1531.6953	1530.6880	1530.6798	5.39	964 -	977	1	---	R.ESTEGSKSSETFSR.E
1560.7551	1559.7478	1559.7474	0.27	419 -	430	2	---	R.RELDDRNMEVQR.L
1568.7372	1567.7299	1567.7082	13.8	1087 -	1099	1	---	K.NQAMSSMIRNQEK.R + 2 Oxidation (M)
1576.7402	1575.7329	1575.7423	-5.97	419 -	430	2	---	R.RELDDRNMEVQR.L + Oxidation (M)
1604.8136	1603.8063	1603.8165	-6.36	703 -	716	1	---	K.SAQSELEQTRNTLK.S
1646.8591	1645.8518	1645.8597	-4.75	1073 -	1086	0	---	R.LESLQTLVEDLQMK.N
1721.8296	1720.8223	1720.8162	3.54	938 -	951	2	---	K.DDRVRDCITESSLR.L
1730.8199	1729.8126	1729.8231	-6.05	925 -	940	2	---	K.SEEDGRAPSLGVKDDR.V
1822.9185	1821.9112	1821.9108	0.23	667 -	681	1	---	R.NELNSLTEDYEVLKR.N
1844.8785	1843.8712	1843.8370	18.6	682 -	696	2	---	R.NFRNKSEEMETTTNK.L + Oxidation (M)
1880.9215	1879.9142	1879.9500	-19.02	89 -	103	2	---	K.DLQRRLNESNELHEK.Q
2077.0454	2076.0381	2076.0343	1.83	110 -	126	1	---	R.QSVIDLQTKLQEMQMER.D

2108.9897	2107.9824	2108.0242	-19.80	110 - 126	1	---	R.QSVIDLQTKLQEMQMER.D + 2 Oxidation (M)
2203.0464	2202.0391	2202.0148	11.0	958 - 977	2	---	R.SNNSLRESTEGSKSSETFSR.E
2369.2256	2368.2183	2368.1791	16.5	488 - 508	2	---	K.KMTLESSERTVSDLTASLQEK.E + Oxidation (M)
2436.1699	2435.1626	2435.1429	8.09	717 - 739	2	---	K.SMEGSDGHAMKVAMGMQKQITAK.R
2510.2229	2509.2156	2509.2330	-6.91	489 - 510	2	---	K.MTLESSERTVSDLTASLQEKER.A
2535.2598	2534.2525	2534.2183	13.5	816 - 836	1	---	K.ASLQFAECQDIIQRQEQESVR.L
2686.3572	2685.3499	2685.2963	20.0	569 - 592	1	---	R.QQIENMTQLVGQHGRTAGAMQVEK.A + 2 Oxidation (M)
3180.5432	3179.5359	3179.5624	-8.33	1073 - 1099	2	---	R.LESLQTLVEDLQMKNQAMSSMIRNQEK.R + Oxidation (M)

No match to: 724.2539, 725.2472, 728.2482, 730.2519, 732.2390, 733.2623, 739.3115, 743.4729, 745.4483, 747.4419, 752.3972, 754.3827, 756.3716, 758.3382, 760.3329, 764.3364, 766.3494, 767.4740, 768.3865, 770.3671, 772.4178, 779.4436, 784.3415, 787.2366, 790.4196, 795.4260, 799.2464, 802.4372, 805.4478, 808.4353, 811.4178, 814.4170, 819.4304, 822.4321, 829.4457, 837.4396, 840.4623, 842.5109, 854.4525, 856.5045, 858.5442, 860.4092, 863.4233, 863.9185, 869.4846, 870.5100, 872.4994, 880.4412, 904.4613, 906.4929, 908.4577, 913.5685, 919.5024, 925.5089, 927.4999, 928.5031, 940.4773, 942.4820, 950.4946, 957.5654, 960.4840, 966.4929, 969.4786, 972.5046, 974.5551, 977.5352, 978.5240, 981.4474, 989.2700, 991.5198, 993.5112, 1001.5782, 1007.5089, 1009.5097, 1013.4454, 1015.5150, 1016.5522, 1018.5189, 1021.5313, 1024.5028, 1025.5192, 1029.5330, 1033.5739, 1035.5203, 1045.5564, 1046.5759, 1051.5074, 1056.5415, 1057.6005, 1064.5358, 1071.5361, 1073.5377, 1079.5398, 1083.4637, 1086.4774, 1092.6000, 1092.6002, 1094.5596, 1106.5431, 1110.5461, 1111.5398, 1111.5400, 1115.5790, 1118.5620, 1120.5724, 1123.5819, 1128.5537, 1132.5682, 1134.5841, 1135.5942, 1137.5565, 1143.5844, 1145.6155, 1149.5597, 1153.5929, 1158.6288, 1163.5771, 1173.6008, 1175.5867, 1177.6178, 1178.6053, 1179.5962, 1181.6432, 1192.5721, 1199.6315, 1203.5826, 1211.5984, 1216.6786, 1221.5699, 1222.5789, 1227.6271, 1228.6411, 1229.6412, 1233.6571, 1237.6270, 1238.5917, 1244.6069, 1248.5958, 1250.6042, 1255.5922, 1258.6344, 1268.6414, 1271.6490, 1276.5977, 1276.6000, 1285.5864, 1294.6315, 1299.6810, 1300.6566, 1309.6340, 1320.6268, 1328.6292, 1333.7025, 1336.6038, 1338.6589, 1343.6613, 1345.6910, 1349.6838, 1350.6704, 1352.6350, 1355.6870, 1357.7130, 1358.6704, 1369.7067, 1374.6705, 1377.6100, 1377.6146, 1384.7109, 1386.7134, 1390.6848, 1393.7169, 1399.6119, 1400.6588, 1406.7207, 1416.6047, 1418.6714, 1421.6632, 1425.7395, 1427.7076, 1430.7102, 1432.6718, 1434.6552, 1438.6991, 1439.7164, 1444.7375, 1454.6836, 1472.7026, 1475.7224, 1485.6906, 1489.7172, 1490.7058, 1493.7157, 1510.7383, 1515.7393, 1523.6987, 1527.7585, 1529.7466, 1530.6902, 1537.7804, 1538.7736, 1539.7621, 1541.7805, 1542.8290, 1549.6758, 1553.7277, 1555.7928, 1556.7874, 1563.7314, 1570.8015, 1572.8151, 1577.7395, 1578.7518, 1581.8373, 1591.7852, 1600.8068, 1603.8092, 1606.8130, 1608.7599, 1613.7793, 1615.8575, 1615.8600, 1624.7632, 1628.7653, 1633.7913, 1634.7969, 1635.7941, 1639.8157, 1643.8085, 1647.8268, 1651.8206, 1653.8284, 1677.8074, 1681.8087, 1688.8287, 1690.8313, 1694.7357, 1697.8672, 1703.8214, 1706.8364, 1707.8435, 1708.3533, 1709.3307, 1710.8229, 1715.8053, 1719.8075, 1723.8743, 1724.8828, 1725.8090, 1748.7931, 1757.8975, 1763.7841, 1768.8075, 1771.9686, 1774.9059, 1777.8727, 1779.8081, 1786.8824, 1787.8207, 1790.8423, 1793.9556, 1796.8109, 1797.8339, 1800.8578, 1815.9008, 1819.8838, 1821.8926, 1826.8450, 1829.8655, 1833.8975, 1836.9054, 1843.8765, 1851.9031, 1859.8494, 1862.9265, 1863.9319, 1866.8857, 1868.8972, 1879.9445, 1887.9023, 1888.8057, 1890.8025, 1896.8954, 1901.0060, 1906.8872, 1908.8663, 1913.8895, 1916.9475, 1924.9314, 1925.9336, 1928.0060, 1931.9956, 1932.9833, 1968.8563, 1984.9392, 2008.0485, 2008.9869, 2009.9747, 2012.9506, 2015.9661, 2020.9644, 2030.0151, 2030.4648, 2046.9839, 2047.5186, 2047.9097, 2051.9133, 2059.0122, 2064.9746, 2070.9209, 2082.9409, 2085.9888, 2090.9844, 2097.9778, 2103.1526, 2118.8926, 2140.0432, 2142.0310, 2142.9895, 2152.0510, 2155.0667, 2159.0752, 2160.0703, 2161.0813, 2166.0911, 2167.0676, 2174.0425, 2178.0034, 2181.0369, 2184.0767, 2189.9133, 2194.9761, 2199.0986, 2205.9102, 2208.9729, 2211.1077, 2213.1116, 2214.1145, 2218.1018, 2221.1035, 2223.2205, 2225.1592, 2229.1064, 2233.1023, 2245.0991, 2269.1128, 2273.0393, 2277.0576, 2279.1094, 2280.1204, 2283.1279, 2285.1169, 2292.1465, 2296.1479, 2312.0664, 2323.1892, 2326.0947, 2334.1531, 2335.1282, 2337.1343, 2372.2229, 2383.9978, 2385.2129, 2392.1528, 2400.1094, 2401.1360, 2409.1914, 2411.1907, 2414.1833, 2429.1475, 2437.1758, 2440.2007, 2447.2368, 2477.1301, 2495.0542, 2498.0898, 2522.1980, 2524.1689, 2527.2310, 2541.2075, 2543.1907, 2544.1514, 2553.1934, 2557.2300, 2560.1125, 2569.2915, 2572.0935, 2612.1936, 2788.3362, 2963.6167, 3056.4641, 3058.5842, 3313.5032, 3330.6311, 3347.6077, 3511.9668, 3761.1558

46. G3MWK4 **Mass:** 30649 **Score:** 18 **Expect:** 3.4e+002 **Matches:** 14

Uncharacterized protein OS=Bos taurus GN=SRSF12 PE=4 SV=1

Observed	Mr(expt)	Mr(calc)	ppm	Start End	Miss	Ions	Peptide
840.4623	839.4550	839.4474	9.05	139 - 144	2	---	K.ESRHRR.F
1135.5942	1134.5869	1134.5642	20.0	248 - 256	2	---	R.SHSRSRSYR.H
1177.6178	1176.6105	1176.5921	15.7	92 - 101	2	---	K.TPGQMKSKER.H + Oxidation (M)
1179.5962	1178.5889	1178.5905	-1.30	120 - 129	2	---	R.TRSRSSSWGR.N
1192.5721	1191.5648	1191.5857	-17.51	122 - 131	2	---	R.SRSSSWGRNR.R
1222.5789	1221.5716	1221.5963	-20.17	212 - 222	1	---	R.SHGRHSDSIAR.S
1233.6571	1232.6498	1232.6361	11.2	191 - 202	1	---	K.SIGKSQSSSPQK.Q
1248.5958	1247.5885	1247.5578	24.6	102 - 111	1	---	R.HPCSPSDHRR.S
1377.6100	1376.6027	1376.6004	1.71	100 - 110	1	---	K.ERHPCSPSDHR.R
1377.6146	1376.6073	1376.6004	5.05	100 - 110	1	---	K.ERHPCSPSDHR.R
1393.7169	1392.7096	1392.6871	16.1	243 - 253	2	---	K.HSHFRSHSRSR.S
1537.7804	1536.7731	1536.7380	22.9	195 - 209	1	---	K.SQSSSPQKQTSSGTK.S
1724.8828	1723.8755	1723.8740	0.86	227 - 242	2	---	K.SPKAYTNSETKAQTAK.H
2077.0454	2076.0381	2076.0137	11.8	230 - 247	2	---	K.AYTNSETKAQTAKHSHFR.S

No match to: 724.2539, 725.2472, 728.2482, 730.2519, 732.2390, 733.2623, 739.3115, 742.4642, 743.4729, 745.4483, 747.4419, 752.3972, 754.3827, 756.3716, 758.3382, 760.3329, 764.3364, 766.3494, 767.4740, 768.3865, 770.3671, 772.4178, 779.4436, 784.3415, 787.2366, 788.4225, 790.4196, 795.4260, 799.2464, 802.4372, 805.4478, 808.4353, 811.4178, 814.4170, 819.4304, 821.4247, 822.4321, 829.4457, 837.4396, 842.5109, 854.4525, 856.5045, 858.5442, 860.4092, 863.4233, 863.9185, 869.4846, 870.5100, 872.4994, 880.4412, 904.4613, 906.4929, 908.4577, 913.5685, 919.5024, 925.5089, 927.4999, 928.5031, 940.4773, 942.4820, 950.4946, 957.5654, 960.4840, 966.4929, 969.4786, 972.5046, 974.5551, 977.5352, 978.5240, 981.4474, 989.2700, 991.5198, 993.5112, 1001.5782, 1007.5089, 1009.5097, 1013.4454, 1015.5150, 1016.5522, 1018.5189, 1021.5313, 1024.5028, 1025.5192, 1029.5330, 1032.5870, 1033.5739, 1035.5203, 1045.5564, 1046.5759, 1051.5074, 1056.5415, 1057.6005, 1064.5358, 1071.5361, 1073.5377, 1079.5398, 1083.4637, 1086.4774, 1092.6000, 1092.6002, 1094.5596, 1096.5302, 1106.5431, 1110.5461, 1111.5398, 1111.5400, 1115.5790, 1118.5620, 1120.5724, 1123.5819, 1128.5537, 1132.5682, 1134.5841, 1137.5565, 1143.5844, 1145.6155, 1149.5597, 1153.5929, 1158.6288, 1163.5771, 1173.6008, 1175.5867, 1178.6053, 1181.6432, 1199.6315, 1203.5826, 1211.5984, 1215.6246, 1216.6786, 1221.5699, 1227.6271, 1228.6411, 1229.6412, 1231.6102, 1237.6270, 1238.5917, 1244.6069, 1250.6042, 1255.5922, 1258.6344, 1268.6414, 1271.6490, 1275.6388, 1276.5977, 1276.6000, 1285.5864, 1294.6315, 1299.6810, 1300.6566, 1309.6340, 1320.6268, 1328.6292, 1333.7025, 1336.6038, 1338.6589, 1341.6488, 1343.6613, 1345.6910, 1349.6838, 1350.6704, 1352.6350, 1355.6870, 1357.7130, 1358.6704, 1369.7067, 1371.7366, 1374.6705, 1384.7109, 1386.7134, 1390.6848, 1399.6119, 1400.6588, 1406.7207, 1410.6736, 1411.6647, 1416.6047, 1418.6714, 1421.6632, 1425.7395, 1426.6846, 1427.7076, 1430.7102, 1432.6718, 1434.6552, 1438.6991, 1439.7164, 1443.7589, 1444.7375, 1445.7306, 1454.6836, 1461.7990, 1472.7026, 1475.7224, 1485.6906, 1489.7172, 1490.7058, 1493.7157, 1510.7383, 1515.7393, 1523.6987, 1524.7383, 1527.7585, 1529.7466, 1530.6902, 1531.6953, 1538.7736, 1539.7621, 1541.7805, 1542.8290, 1549.6758, 1553.7277, 1555.7928, 1556.7874, 1560.7551, 1563.7314, 1568.7372, 1570.8015, 1572.8151, 1576.7402, 1577.7395, 1578.7518, 1581.8373, 1591.7852, 1600.8068, 1603.8092, 1604.8136, 1606.8130, 1608.7599, 1613.7793, 1615.8575, 1615.8600, 1624.7632, 1628.7653, 1633.7913, 1634.7969, 1635.7941, 1639.8157, 1643.8085, 1646.8591, 1647.8268, 1651.8206, 1653.8284, 1677.8074, 1681.8087, 1688.8287, 1690.8313, 1694.7357, 1697.8672, 1703.8214, 1706.8364, 1707.8435, 1708.3533, 1709.3307, 1710.8229, 1715.8053, 1719.8075, 1721.8296, 1723.8743, 1725.8090, 1730.8199, 1748.7931, 1757.8975, 1763.7841, 1768.8075, 1771.9686, 1774.9059, 1777.8727, 1779.8081, 1786.8824, 1787.8207, 1790.8423, 1793.9556, 1796.8109, 1797.8339, 1800.8578, 1815.9008, 1819.8838, 1821.8926, 1822.9185, 1826.8450, 1829.8655, 1833.8975, 1836.9054, 1843.8765, 1844.8785, 1851.9031, 1859.8494, 1862.9265, 1863.9319, 1866.8857, 1868.8972, 1879.9445, 1880.9215, 1887.9023, 1888.8057, 1890.8025, 1896.8954, 1901.0060, 1906.8872, 1908.8663, 1913.8895, 1916.9475, 1924.9314, 1925.9336, 1928.0060, 1931.9956, 1932.9833, 1968.8563, 1984.9392, 2008.0485, 2008.9869, 2009.9747, 2012.9506, 2015.9661, 2020.9644, 2030.0151, 2030.4648, 2046.9839, 2047.5186, 2047.9097, 2051.9133, 2059.0122, 2064.9746, 2070.9209, 2082.9409, 2085.9888, 2090.9844, 2097.9778, 2103.1526, 2108.9897, 2118.8926, 2140.0432, 2142.0310, 2142.9895, 2152.0510, 2155.0667, 2159.0752, 2160.0703, 2161.0813, 2166.0911, 2167.0676, 2174.0425, 2178.0034, 2181.0369, 2184.0767, 2189.9133, 2194.9761, 2199.0986, 2203.0464, 2205.9102, 2208.9729, 2211.1077, 2213.1116, 2214.1145, 2218.1018, 2221.1035, 2223.2205, 2225.1592, 2229.1064, 2233.1023, 2245.0991, 2269.1128, 2273.0393, 2277.0576, 2279.1094, 2280.1204, 2283.1279, 2285.1169, 2292.1465, 2296.1479, 2312.0664, 2323.1892, 2326.0947, 2334.1531, 2335.1282, 2337.1343, 2369.2256, 2372.2229, 2383.9978, 2385.2129, 2392.1528, 2400.1094, 2401.1360, 2409.1914, 2411.1907, 2414.1833, 2429.1475, 2436.1699, 2437.1758, 2440.2007, 2447.2368, 2477.1301, 2495.0542, 2498.0898, 2510.2229, 2522.1980, 2524.1689, 2527.2310, 2535.2598, 2541.2075, 2543.1907, 2544.1514, 2553.1934, 2557.2300, 2560.1125, 2569.2915, 2572.0935, 2612.1936, 2686.3572, 2788.3362, 2963.6167, 3056.4641, 3058.5842, 3180.5432, 3313.5032, 3330.6311, 3347.6077, 3511.9668, 3761.1558

47. E1BLN1 **Mass:** 117436 **Score:** 18 **Expect:** 3.5e+002 **Matches:** 36

Uncharacterized protein OS=Bos taurus GN=SYCP1 PE=4 SV=1

Observed	Mr(expt)	Mr(calc)	ppm	Start	End	Miss	Ions	Peptide
745.4483	744.4410	744.4381	3.89	447	452	1	---	K.LLDEKK.Q
747.4419	746.4346	746.4174	23.1	505	510	1	---	K.TELEKK.N
904.4613	903.4540	903.4549	-0.95	795	801	1	---	R.EEKEELK.R
977.5352	976.5279	976.5375	-9.88	383	390	1	---	K.TTICNLKK.L
1001.5782	1000.5709	1000.5917	-20.72	445	452	2	---	K.QKLLDEKK.Q
1051.5074	1050.5001	1050.4764	22.6	189	197	1	---	K.ETCARSAEK.T
1115.5790	1114.5717	1114.5982	-23.74	295	303	1	---	R.NKINQLEEK.T
1134.5841	1133.5768	1133.5824	-4.93	730	739	0	---	K.IAEMVALMEK.H
1158.6288	1157.6215	1157.6152	5.43	132	140	2	---	K.ENKLQENRK.I
1173.6008	1172.5935	1172.5859	6.47	372	382	0	---	K.AAHSSMVTELK.T
1203.5826	1202.5753	1202.5891	-11.45	545	554	1	---	K.KQQEDINNSK.R
1216.6786	1215.6713	1215.6910	-16.18	978	987	2	---	R.WAVIAKMNRK.Q
1231.6102	1230.6029	1230.5952	6.24	546	555	1	---	K.QQEDINNSKR.Q
1294.6315	1293.6242	1293.6486	-18.82	417	427	1	---	K.SSELEEMTKLK.N
1345.6910	1344.6837	1344.6885	-3.52	576	586	1	---	R.NELESVREELK.Q
1350.6704	1349.6631	1349.6497	9.95	954	966	0	---	K.TPSSLTTPGSMQK.S + Oxidation (M)
1371.7366	1370.7293	1370.7445	-11.08	106	116	2	---	R.LYSKLYKEAEK.I
1393.7169	1392.7096	1392.7282	-13.37	803	814	2	---	R.EMKENTVTLKGK.K + Oxidation (M)
1400.6588	1399.6515	1399.6579	-4.56	58	70	0	---	K.NGENIDSDPALQK.V
1406.7207	1405.7134	1405.6983	10.7	513	524	1	---	R.LKNTELTASCNR.L
1421.6632	1420.6559	1420.6881	-22.67	938	949	0	---	K.DNNPPISHLCVR.T
1427.7076	1426.7003	1426.6940	4.45	318	329	0	---	K.QDHLTSELEDIK.M
1445.7306	1444.7233	1444.7157	5.26	306	317	1	---	K.LQNENLKESNEK.Q
1538.7736	1537.7663	1537.7485	11.6	847	860	2	---	R.SFPSANQSRSSKDK.R
1577.7395	1576.7322	1576.7654	-21.04	531	544	0	---	K.ELAQETSDMALELK.K
1581.8373	1580.8300	1580.8059	15.3	740	751	2	---	K.HKHQYDKIVEER.D
1703.8214	1702.8141	1702.8204	-3.71	726	739	1	---	R.CQHKIAEMVALMEK.H + Oxidation (M)
1719.8075	1718.8002	1718.8154	-8.80	726	739	1	---	R.CQHKIAEMVALMEK.H + 2 Oxidation (M)
1721.8296	1720.8223	1720.8553	-19.15	531	545	1	---	K.ELAQETSDMALELKK.Q + Oxidation (M)
1725.8090	1724.8017	1724.8443	-24.69	238	250	2	---	R.LEMHFKLKEDYEK.I + Oxidation (M)
1822.9185	1821.9112	1821.8978	7.37	935	949	1	---	K.LCKDNNPPISHLCVR.T
1984.9392	1983.9319	1983.9207	5.64	206	221	1	---	R.EETRQVYMDLNSNIEK.M + Oxidation (M)
2059.0122	2058.0049	2058.0051	-0.11	318	334	1	---	K.QDHLTSELEDIKMSLQR.S + Oxidation (M)
2214.1145	2213.1072	2213.1287	-9.71	163	181	2	---	K.LEEGIQENKDLIKENNATR.H
2477.1301	2476.1228	2476.1686	-18.47	760	780	2	---	K.SKEQEHSSMKASLETELCNLR.N
2522.1980	2521.1907	2521.2516	-24.14	330	351	1	---	K.MSLQRSVNTQMALEEDLQIATK.N + Oxidation (M)

No match to: 724.2539, 725.2472, 728.2482, 730.2519, 732.2390, 733.2623, 739.3115, 742.4642, 743.4729, 752.3972, 754.3827, 756.3716, 758.3382, 760.3329, 764.3364, 766.3494, 767.4740, 768.3865, 770.3671, 772.4178, 779.4436, 784.3415, 787.2366, 788.4225, 790.4196, 795.4260, 799.2464, 802.4372, 805.4478, 808.4353, 811.4178, 814.4170, 819.4304, 821.4247, 822.4321, 829.4457, 837.4396, 840.4623, 842.5109, 854.4525, 856.5045, 858.5442, 860.4092, 863.4233, 863.9185, 869.4846, 870.5100, 872.4994, 880.4412, 906.4929, 908.4577, 913.5685, 919.5024, 925.5089, 927.4999, 928.5031, 940.4773, 942.4820, 950.4946, 957.5654, 960.4840, 966.4929, 969.4786, 972.5046, 974.5551, 978.5240, 981.4474, 989.2700, 991.5198, 993.5112, 1007.5089, 1009.5097, 1013.4454, 1015.5150, 1016.5522, 1018.5189, 1021.5313, 1024.5028, 1025.5192, 1029.5330, 1032.5870, 1033.5739, 1035.5203, 1045.5564, 1046.5759, 1056.5415, 1057.6005, 1064.5358, 1071.5361, 1073.5377, 1079.5398, 1083.4637, 1086.4774, 1092.6000, 1092.6002, 1094.5596, 1096.5302, 1106.5431, 1110.5461, 1111.5398, 1111.5400, 1118.5620, 1120.5724, 1123.5819, 1128.5537, 1132.5682, 1135.5942, 1137.5565, 1143.5844, 1145.6155, 1149.5597, 1153.5929, 1163.5771, 1175.5867, 1177.6178, 1178.6053, 1179.5962, 1181.6432, 1192.5721, 1199.6315, 1211.5984, 1215.6246, 1221.5699, 1222.5789, 1227.6271, 1228.6411, 1229.6412, 1233.6571, 1237.6270, 1238.5917, 1244.6069, 1248.5958, 1250.6042, 1255.5922, 1258.6344, 1268.6414, 1271.6490, 1275.6388, 1276.5977, 1276.6000, 1285.5864, 1299.6810, 1300.6566, 1309.6340, 1320.6268, 1328.6292, 1333.7025, 1336.6038, 1338.6589, 1341.6488, 1343.6613, 1349.6838, 1352.6350, 1355.6870, 1357.7130, 1358.6704, 1369.7067, 1374.6705, 1377.6100, 1377.6146, 1384.7109, 1386.7134, 1390.6848, 1399.6119, 1410.6736, 1411.6647, 1416.6047, 1418.6714, 1425.7395, 1426.6846, 1430.7102, 1432.6718, 1434.6552, 1438.6991, 1439.7164, 1443.7589, 1444.7375, 1454.6836, 1461.7990, 1472.7026, 1475.7224, 1485.6906, 1489.7172, 1490.7058, 1493.7157, 1510.7383, 1515.7393, 1523.6987, 1524.7383, 1527.7585, 1529.7466, 1530.6902, 1531.6953, 1537.7804, 1539.7621, 1541.7805, 1542.8290, 1549.6758, 1553.7277, 1555.7928, 1556.7874, 1560.7551, 1563.7314, 1568.7372, 1570.8015, 1572.8151, 1576.7402, 1578.7518, 1591.7852, 1600.8068, 1603.8092, 1604.8136, 1606.8130, 1608.7599, 1613.7793, 1615.8575, 1615.8600, 1624.7632, 1628.7653, 1633.7913, 1634.7969, 1635.7941, 1639.8157, 1643.8085, 1646.8591, 1647.8268, 1651.8206, 1653.8284, 1677.8074, 1681.8087, 1688.8287, 1690.8313, 1694.7357, 1697.8672, 1706.8364, 1707.8435, 1708.3533, 1709.3307, 1710.8229, 1715.8053, 1723.8743, 1724.8828, 1730.8199, 1748.7931, 1757.8975, 1763.7841, 1768.8075, 1771.9686, 1774.9059, 1777.8727, 1779.8081, 1786.8824, 1787.8207, 1790.8423, 1793.9556, 1796.8109, 1797.8339, 1800.8578, 1815.9008, 1819.8838, 1821.8926, 1826.8450, 1829.8655, 1833.8975, 1836.9054, 1843.8765, 1844.8785, 1851.9031, 1859.8494, 1862.9265, 1863.9319, 1866.8857, 1868.8972, 1879.9445, 1880.9215, 1887.9023, 1888.8057, 1890.8025, 1896.8954, 1901.0060, 1906.8872, 1908.8663, 1913.8895, 1916.9475, 1924.9314, 1925.9336, 1928.0060, 1931.9956, 1932.9833, 1968.8563, 2008.0485, 2008.9869, 2009.9747, 2012.9506, 2015.9661, 2020.9644, 2030.0151, 2030.4648, 2046.9839, 2047.5186, 2047.9097, 2051.9133, 2064.9746, 2070.9209, 2077.0454, 2082.9409, 2085.9888, 2090.9844, 2097.9778, 2103.1526, 2108.9897, 2118.8926, 2140.0432, 2142.0310, 2142.9895, 2152.0510, 2155.0667, 2159.0752, 2160.0703, 2161.0813, 2166.0911, 2167.0676, 2174.0425, 2178.0034, 2181.0369, 2184.0767, 2189.9133, 2194.9761, 2199.0986, 2203.0464, 2205.9102, 2208.9729, 2211.1077, 2213.1116, 2218.1018, 2221.1035, 2223.2205, 2225.1592, 2229.1064, 2233.1023, 2245.0991, 2269.1128, 2273.0393, 2277.0576, 2279.1094, 2280.1204, 2283.1279, 2285.1169, 2292.1465, 2296.1479, 2312.0664, 2323.1892, 2326.0947, 2334.1531, 2335.1282, 2337.1343, 2369.2256, 2372.2229, 2383.9978, 2385.2129, 2392.1528, 2400.1094, 2401.1360, 2409.1914, 2411.1907, 2414.1833, 2429.1475, 2436.1699, 2437.1758, 2440.2007, 2447.2368, 2495.0542, 2498.0898, 2510.2229, 2524.1689, 2527.2310, 2535.2598, 2541.2075, 2543.1907, 2544.1514, 2553.1934, 2557.2300, 2560.1125, 2569.2915, 2572.0935, 2612.1936, 2686.3572, 2788.3362, 2963.6167, 3056.4641, 3058.5842, 3180.5432, 3313.5032, 3330.6311, 3347.6077, 3511.9668, 3761.1558

48. G3N2Q8 **Mass:** 20708 **Score:** 18 **Expect:** 3.5e+002 **Matches:** 9

Uncharacterized protein OS=Bos taurus GN=ISG20 PE=4 SV=1

Observed	Mr(expt)	Mr(calc)	ppm	Start	End	Miss	Ions	Peptide
1421.6632	1420.6559	1420.6769	-14.76	170	181	1	---	R.EQRGNCFLSITP.-
1639.8157	1638.8084	1638.7962	7.48	142	156	0	---	R.IQDSVLGHNSVEDAR.A
1690.8313	1689.8240	1689.8621	-22.51	168	181	2	---	R.IREQRGNCFLSITP.-
1890.8025	1889.7952	1889.8213	-13.82	100	115	1	---	K.ENMSDYAIYDTAADRR.L
2046.9839	2045.9766	2045.9364	19.7	97	114	1	---	K.ALKENMSDYAIYDTAADR.R
2181.0369	2180.0296	2180.0177	5.48	2	21	1	---	M.ARSADVVAMDCEMVGLGPFR.E
2203.0464	2202.0391	2202.0375	0.75	97	115	2	---	K.ALKENMSDYAIYDTAADRR.L
2269.1128	2268.1055	2268.0845	9.28	22	41	1	---	R.ESGLARCSLVDYHGSVLYDK.F
2312.0664	2311.0591	2311.0582	0.42	1	21	1	---	-.MARSADVVAMDCEMVGLGPFR.E

No match to: 724.2539, 725.2472, 728.2482, 730.2519, 732.2390, 733.2623, 739.3115, 742.4642, 743.4729, 745.4483, 747.4419, 752.3972, 754.3827, 756.3716, 758.3382, 760.3329, 764.3364, 766.3494, 767.4740, 768.3865, 770.3671, 772.4178, 779.4436, 784.3415, 787.2366, 788.4225, 790.4196, 795.4260, 799.2464, 802.4372, 805.4478, 808.4353, 811.4178, 814.4170, 819.4304, 821.4247, 822.4321, 829.4457, 837.4396, 840.4623, 842.5109, 854.4525, 856.5045, 858.5442, 860.4092, 863.4233, 863.9185, 869.4846, 870.5100, 872.4994, 880.4412, 904.4613, 906.4929, 908.4577, 913.5685, 919.5024, 925.5089, 927.4999, 928.5031, 940.4773, 942.4820, 950.4946, 957.5654, 960.4840, 966.4929, 969.4786, 972.5046, 974.5551, 977.5352, 978.5240, 981.4474, 989.2700, 991.5198, 993.5112, 1001.5782, 1007.5089,

1009.5097, 1013.4454, 1015.5150, 1016.5522, 1018.5189, 1021.5313, 1024.5028, 1025.5192, 1029.5330, 1032.5870, 1033.5739, 1035.5203, 1045.5564, 1046.5759, 1051.5074, 1056.5415, 1057.6005, 1064.5358, 1071.5361, 1073.5377, 1079.5398, 1083.4637, 1086.4774, 1092.6000, 1092.6002, 1094.5596, 1096.5302, 1106.5431, 1110.5461, 1111.5398, 1111.5400, 1115.5790, 1118.5620, 1120.5724, 1123.5819, 1128.5537, 1132.5682, 1134.5841, 1135.5942, 1137.5565, 1143.5844, 1145.6155, 1149.5597, 1153.5929, 1158.6288, 1163.5771, 1173.6008, 1175.5867, 1177.6178, 1178.6053, 1179.5962, 1181.6432, 1192.5721, 1199.6315, 1203.5826, 1211.5984, 1215.6246, 1216.6786, 1221.5699, 1222.5789, 1227.6271, 1228.6411, 1229.6412, 1231.6102, 1233.6571, 1237.6270, 1238.5917, 1244.6069, 1248.5958, 1250.6042, 1255.5922, 1258.6344, 1268.6414, 1271.6490, 1275.6388, 1276.5977, 1276.6000, 1285.5864, 1294.6315, 1299.6810, 1300.6566, 1309.6340, 1320.6268, 1328.6292, 1333.7025, 1336.6038, 1338.6589, 1341.6488, 1343.6613, 1345.6910, 1349.6838, 1350.6704, 1352.6350, 1355.6870, 1357.7130, 1358.6704, 1369.7067, 1371.7366, 1374.6705, 1377.6100, 1377.6146, 1384.7109, 1386.7134, 1390.6848, 1393.7169, 1399.6119, 1400.6588, 1406.7207, 1410.6736, 1411.6647, 1416.6047, 1418.6714, 1425.7395, 1426.6846, 1427.7076, 1430.7102, 1432.6718, 1434.6552, 1438.6991, 1439.7164, 1443.7589, 1444.7375, 1445.7306, 1454.6836, 1461.7990, 1472.7026, 1475.7224, 1485.6906, 1489.7172, 1490.7058, 1493.7157, 1510.7383, 1515.7393, 1523.6987, 1524.7383, 1527.7585, 1529.7466, 1530.6902, 1531.6953, 1537.7804, 1538.7736, 1539.7621, 1541.7805, 1542.8290, 1549.6758, 1553.7277, 1555.7928, 1556.7874, 1560.7551, 1563.7314, 1568.7372, 1570.8015, 1572.8151, 1576.7402, 1577.7395, 1578.7518, 1581.8373, 1591.7852, 1600.8068, 1603.8092, 1604.8136, 1606.8130, 1608.7599, 1613.7793, 1615.8575, 1615.8600, 1624.7632, 1628.7653, 1633.7913, 1634.7969, 1635.7941, 1643.8085, 1646.8591, 1647.8268, 1651.8206, 1653.8284, 1677.8074, 1681.8087, 1688.8287, 1694.7357, 1697.8672, 1703.8214, 1706.8364, 1707.8435, 1708.3533, 1709.3307, 1710.8229, 1715.8053, 1719.8075, 1721.8296, 1723.8743, 1724.8828, 1725.8090, 1730.8199, 1748.7931, 1757.8975, 1763.7841, 1768.8075, 1771.9686, 1774.9059, 1777.8727, 1779.8081, 1786.8824, 1787.8207, 1790.8423, 1793.9556, 1796.8109, 1797.8339, 1800.8578, 1815.9008, 1819.8838, 1821.8926, 1822.9185, 1826.8450, 1829.8655, 1833.8975, 1836.9054, 1843.8765, 1844.8785, 1851.9031, 1859.8494, 1862.9265, 1863.9319, 1866.8857, 1868.8972, 1879.9445, 1880.9215, 1887.9023, 1888.8057, 1896.8954, 1901.0060, 1906.8872, 1908.8663, 1913.8895, 1916.9475, 1924.9314, 1925.9336, 1928.0060, 1931.9956, 1932.9833, 1968.8563, 1984.9392, 2008.0485, 2008.9869, 2009.9747, 2012.9506, 2015.9661, 2020.9644, 2030.0151, 2030.4648, 2047.5186, 2047.9097, 2051.9133, 2059.0122, 2064.9746, 2070.9209, 2077.0454, 2082.9409, 2085.9888, 2090.9844, 2097.9778, 2103.1526, 2108.9897, 2118.8926, 2140.0432, 2142.0310, 2142.9895, 2152.0510, 2155.0667, 2159.0752, 2160.0703, 2161.0813, 2166.0911, 2167.0676, 2174.0425, 2178.0034, 2184.0767, 2189.9133, 2194.9761, 2199.0986, 2205.9102, 2208.9729, 2211.1077, 2213.1116, 2214.1145, 2218.1018, 2221.1035, 2223.2205, 2225.1592, 2229.1064, 2233.1023, 2245.0991, 2273.0393, 2277.0576, 2279.1094, 2280.1204, 2283.1279, 2285.1169, 2292.1465, 2296.1479, 2323.1892, 2326.0947, 2334.1531, 2335.1282, 2337.1343, 2369.2256, 2372.2229, 2383.9978, 2385.2129, 2392.1528, 2400.1094, 2401.1360, 2409.1914, 2411.1907, 2414.1833, 2429.1475, 2436.1699, 2437.1758, 2440.2007, 2447.2368, 2477.1301, 2495.0542, 2498.0898, 2510.2229, 2522.1980, 2524.1689, 2527.2310, 2535.2598, 2541.2075, 2543.1907, 2544.1514, 2553.1934, 2557.2300, 2560.1125, 2569.2915, 2572.0935, 2612.1936, 2686.3572, 2788.3362, 2963.6167, 3056.4641, 3058.5842, 3180.5432, 3313.5032, 3330.6311, 3347.6077, 3511.9668, 3761.1558

49. Q32L31 **Mass:** 23052 **Score:** 18 **Expect:** 3.5e+002 **Matches:** 12

High mobility group protein B3 OS=Bos taurus GN=HMGB3 PE=2 SV=2

Observed	Mr(expt)	Mr(calc)	ppm	Start	End	Miss	Ions	Peptide
837.4396	836.4323	836.4214	13.0	49	55	1	---	R.WKTMSGK.E
993.5112	992.5039	992.4862	17.9	44	50	2	---	K.KCSERWK.T
1032.5870	1031.5797	1031.5763	3.28	140	148	1	---	K.QPYINKAAK.L
1094.5596	1093.5523	1093.5590	-6.10	49	57	2	---	R.WKTMSGKEK.S
1096.5302	1095.5229	1095.5495	-24.24	69	76	2	---	K.VRYDREMK.D
1110.5461	1109.5388	1109.5539	-13.60	49	57	2	---	R.WKTMSGKEK.S + Oxidation (M)
1228.6411	1227.6338	1227.6459	-9.84	113	125	0	---	K.SANPGISIGDVAK.K
1285.5864	1284.5791	1284.6020	-17.79	58	68	2	---	K.SKFDEMAKADK.V + Oxidation (M)
1309.6340	1308.6267	1308.6496	-17.48	60	70	2	---	K.FDEMAKADKVR.Y
1345.6910	1344.6837	1344.6561	20.5	153	163	2	---	K.YEKDVADYKSK.G
1472.7026	1471.6953	1471.6765	12.8	71	82	2	---	R.YDREMKDYGPAK.G
1681.8087	1680.8014	1680.7752	15.6	11	24	1	---	K.GKMSAYAFFVQTCR.E + Oxidation (M)

No match to: 724.2539, 725.2472, 728.2482, 730.2519, 732.2390, 733.2623, 739.3115, 742.4642, 743.4729, 745.4483, 747.4419, 752.3972, 754.3827, 756.3716, 758.3382, 760.3329, 764.3364, 766.3494, 767.4740, 768.3865, 770.3671, 772.4178, 779.4436, 784.3415, 787.2366, 788.4225, 790.4196, 795.4260, 799.2464, 802.4372, 805.4478, 808.4353, 811.4178, 814.4170, 819.4304, 821.4247, 822.4321, 829.4457, 840.4623, 842.5109, 854.4525, 856.5045, 858.5442, 860.4092, 863.4233, 863.9185, 869.4846, 870.5100, 872.4994, 880.4412, 904.4613, 906.4929, 908.4577, 913.5685, 919.5024, 925.5089, 927.4999, 928.5031, 940.4773, 942.4820, 950.4946, 957.5654, 960.4840, 966.4929, 969.4786, 972.5046, 974.5551, 977.5352, 978.5240, 981.4474, 989.2700, 991.5198, 1001.5782, 1007.5089, 1009.5097, 1013.4454, 1015.5150, 1016.5522, 1018.5189, 1021.5313, 1024.5028, 1025.5192, 1029.5330, 1033.5739, 1035.5203, 1045.5564, 1046.5759, 1051.5074, 1056.5415, 1057.6005, 1064.5358, 1071.5361, 1073.5377, 1079.5398, 1083.4637, 1086.4774, 1092.6000, 1092.6002, 1106.5431, 1111.5398, 1111.5400, 1115.5790, 1118.5620, 1120.5724, 1123.5819, 1128.5537, 1132.5682, 1134.5841, 1135.5942, 1137.5565, 1143.5844, 1145.6155, 1149.5597, 1153.5929, 1158.6288, 1163.5771, 1173.6008, 1175.5867, 1177.6178, 1178.6053, 1179.5962, 1181.6432, 1192.5721, 1199.6315, 1203.5826, 1211.5984, 1215.6246, 1216.6786, 1221.5699, 1222.5789, 1227.6271, 1229.6412, 1231.6102, 1237.6270, 1238.5917, 1244.6069, 1248.5958, 1250.6042, 1255.5922, 1258.6344, 1268.6414, 1271.6490, 1275.6388, 1276.5977, 1276.6000, 1294.6315, 1299.6810, 1300.6566, 1320.6268, 1328.6292, 1333.7025, 1336.6038, 1338.6589, 1341.6488, 1343.6613, 1349.6838, 1350.6704, 1352.6350, 1355.6870, 1357.7130, 1358.6704, 1369.7067, 1371.7366, 1374.6705, 1377.6100, 1377.6146, 1384.7109, 1386.7134, 1390.6848, 1393.7169, 1399.6119, 1400.6588, 1406.7207, 1410.6736, 1411.6647, 1416.6047, 1418.6714, 1421.6632, 1425.7395, 1426.6846, 1427.7076, 1430.7102, 1432.6718, 1434.6552, 1438.6991, 1439.7164, 1443.7589, 1444.7375, 1445.7306, 1454.6836, 1461.7990, 1475.7224, 1485.6906, 1489.7172, 1490.7058, 1493.7157, 1510.7383, 1515.7393, 1523.6987, 1524.7383, 1527.7585, 1529.7466, 1530.6902, 1531.6953, 1537.7804, 1538.7736, 1539.7621, 1541.7805, 1542.8290, 1549.6758, 1553.7277, 1555.7928, 1556.7874, 1560.7551, 1563.7314, 1568.7372, 1570.8015, 1572.8151, 1576.7402, 1577.7395, 1578.7518, 1581.8373, 1591.7852, 1600.8068, 1603.8092, 1604.8136, 1606.8130, 1608.7599, 1613.7793, 1615.8575, 1615.8600, 1624.7632, 1628.7653, 1633.7913, 1634.7969, 1635.7941, 1639.8157, 1643.8085, 1646.8591, 1647.8268, 1651.8206, 1653.8284, 1677.8074, 1688.8287, 1690.8313, 1694.7357, 1697.8672, 1703.8214, 1706.8364, 1707.8435, 1708.3533, 1709.3307, 1710.8229, 1715.8053, 1719.8075, 1721.8296, 1723.8743, 1724.8828, 1725.8090, 1730.8199, 1748.7931, 1757.8975, 1763.7841, 1768.8075, 1771.9686, 1774.9059, 1777.8727, 1779.8081, 1786.8824, 1787.8207, 1790.8423, 1793.9556, 1796.8109, 1797.8339, 1800.8578, 1815.9008, 1819.8838, 1821.8926, 1822.9185, 1826.8450, 1829.8655, 1833.8975, 1836.9054, 1843.8765, 1844.8785, 1851.9031, 1859.8494, 1862.9265, 1863.9319, 1866.8857, 1868.8972, 1879.9445, 1880.9215, 1887.9023, 1888.8057, 1890.8025, 1896.8954, 1901.0060, 1906.8872, 1908.8663, 1913.8895, 1916.9475, 1924.9314, 1925.9336, 1928.0060, 1931.9956, 1932.9833, 1968.8563, 1984.9392, 2008.0485, 2008.9869, 2009.9747, 2012.9506, 2015.9661, 2020.9644, 2030.0151, 2030.4648, 2046.9839, 2047.5186, 2047.9097, 2051.9133, 2059.0122, 2064.9746, 2070.9209, 2077.0454, 2082.9409, 2085.9888, 2090.9844, 2097.9778, 2103.1526, 2108.9897, 2118.8926, 2140.0432, 2142.0310, 2142.9895, 2152.0510, 2155.0667, 2159.0752, 2160.0703, 2161.0813, 2166.0911, 2167.0676, 2174.0425, 2178.0034, 2181.0369, 2184.0767, 2189.9133, 2194.9761, 2199.0986, 2203.0464, 2205.9102, 2208.9729, 2211.1077, 2213.1116, 2214.1145, 2218.1018, 2221.1035, 2223.2205, 2225.1592, 2229.1064, 2233.1023, 2245.0991, 2269.1128, 2273.0393, 2277.0576, 2279.1094, 2280.1204, 2283.1279, 2285.1169, 2292.1465, 2296.1479, 2312.0664, 2323.1892, 2326.0947, 2334.1531, 2335.1282, 2337.1343, 2369.2256, 2372.2229, 2383.9978, 2385.2129, 2392.1528, 2400.1094, 2401.1360, 2409.1914, 2411.1907, 2414.1833, 2429.1475, 2436.1699, 2437.1758, 2440.2007, 2447.2368, 2477.1301, 2495.0542, 2498.0898, 2510.2229, 2522.1980, 2524.1689, 2527.2310, 2535.2598, 2541.2075, 2543.1907, 2544.1514, 2553.1934, 2557.2300, 2560.1125, 2569.2915, 2572.0935, 2612.1936, 2686.3572, 2788.3362, 2963.6167, 3056.4641, 3058.5842, 3180.5432, 3313.5032, 3330.6311, 3347.6077, 3511.9668, 3761.1558

50. I7FVA7 **Mass:** 23052 **Score:** 18 **Expect:** 3.5e+002 **Matches:** 12

HMGB3 transcript variant 1/2 OS=Bos taurus GN=HMGB3 PE=4 SV=1

Observed	Mr(expt)	Mr(calc)	ppm	Start	End	Miss	Ions	Peptide
837.4396	836.4323	836.4214	13.0	49	55	1	---	R.WKTMSGK.E
993.5112	992.5039	992.4862	17.9	44	50	2	---	K.KCSERWK.T
1032.5870	1031.5797	1031.5763	3.28	140	148	1	---	K.QPYINKAAK.L
1094.5596	1093.5523	1093.5590	-6.10	49	57	2	---	R.WKTMSGKEK.S
1096.5302	1095.5229	1095.5495	-24.24	69	76	2	---	K.VRYDREMK.D
1110.5461	1109.5388	1109.5539	-13.60	49	57	2	---	R.WKTMSGKEK.S + Oxidation (M)
1228.6411	1227.6338	1227.6459	-9.84	113	125	0	---	K.SANPGISIGDVAK.K
1285.5864	1284.5791	1284.6020	-17.79	58	68	2	---	K.SKFDEMAKADK.V + Oxidation (M)
1309.6340	1308.6267	1308.6496	-17.48	60	70	2	---	K.FDEMAKADKVR.Y
1345.6910	1344.6837	1344.6561	20.5	153	163	2	---	K.YEKDVADYKSK.G
1472.7026	1471.6953	1471.6765	12.8	71	82	2	---	R.YDREMKDYGPAK.G

1681.8087 1680.8014 1680.7752 15.6 11 - 24 1 --- K.GKMSAYAFFVQTCR.E + Oxidation (M)

No match to: 724.2539, 725.2472, 728.2482, 730.2519, 732.2390, 733.2623, 739.3115, 742.4642, 743.4729, 745.4483, 747.4419,
752.3972, 754.3827, 756.3716, 758.3382, 760.3329, 764.3364, 766.3494, 767.4740, 768.3865, 770.3671, 772.4178, 779.4436, 784.3415,
787.2366, 788.4225, 790.4196, 795.4260, 799.2464, 802.4372, 805.4478, 808.4353, 811.4178, 814.4170, 819.4304, 821.4247, 822.4321,
829.4457, 840.4623, 842.5109, 854.4525, 856.5045, 858.5442, 860.4092, 863.4233, 863.9185, 869.4846, 870.5100, 872.4994, 880.4412,
904.4613, 906.4929, 908.4577, 913.5685, 919.5024, 925.5089, 927.4999, 928.5031, 940.4773, 942.4820, 950.4946, 957.5654, 960.4840,
966.4929, 969.4786, 972.5046, 974.5551, 977.5352, 978.5240, 981.4474, 989.2700, 991.5198, 1001.5782, 1007.5089, 1009.5097,
1013.4454, 1015.5150, 1016.5522, 1018.5189, 1021.5313, 1024.5028, 1025.5192, 1029.5330, 1033.5739, 1035.5203, 1045.5564, 1046.5759,
1051.5074, 1056.5415, 1057.6005, 1064.5358, 1071.5361, 1073.5377, 1079.5398, 1083.4637, 1086.4774, 1092.6000, 1092.6002, 1106.5431,
1111.5398, 1111.5400, 1115.5790, 1118.5620, 1120.5724, 1123.5819, 1128.5537, 1132.5682, 1134.5841, 1135.5942, 1137.5565, 1143.5844,
1145.6155, 1149.5597, 1153.5929, 1158.6288, 1163.5771, 1173.6008, 1175.5867, 1177.6178, 1178.6053, 1179.5962, 1181.6432, 1192.5721,
1199.6315, 1203.5826, 1211.5984, 1215.6246, 1216.6786, 1221.5699, 1222.5789, 1227.6271, 1229.6412, 1231.6102, 1233.6571, 1237.6270,
1238.5917, 1244.6069, 1248.5958, 1250.6042, 1255.5922, 1258.6344, 1268.6414, 1271.6490, 1275.6388, 1276.5977, 1276.6000, 1294.6315,
1299.6810, 1300.6566, 1320.6268, 1328.6292, 1333.7025, 1336.6038, 1338.6589, 1341.6488, 1343.6613, 1349.6838, 1350.6704, 1352.6350,
1355.6870, 1357.7130, 1358.6704, 1369.7067, 1371.7366, 1374.6705, 1377.6100, 1377.6146, 1384.7109, 1386.7134, 1390.6848, 1393.7169,
1399.6119, 1400.6588, 1406.7207, 1410.6736, 1411.6647, 1416.6047, 1418.6714, 1421.6632, 1425.7395, 1426.6846, 1427.7076, 1430.7102,
1432.6718, 1434.6552, 1438.6991, 1439.7164, 1443.7589, 1444.7375, 1445.7306, 1454.6836, 1461.7990, 1475.7224, 1485.6906, 1489.7172,
1490.7058, 1493.7157, 1510.7383, 1515.7393, 1523.6987, 1524.7383, 1527.7585, 1529.7466, 1530.6902, 1531.6953, 1537.7804, 1538.7736,
1539.7621, 1541.7805, 1542.8290, 1549.6758, 1553.7277, 1555.7928, 1556.7874, 1560.7551, 1563.7314, 1568.7372, 1570.8015, 1572.8151,
1576.7402, 1577.7395, 1578.7518, 1581.8373, 1591.7852, 1600.8068, 1603.8092, 1604.8136, 1606.8130, 1608.7599, 1613.7793, 1615.8575,
1615.8600, 1624.7632, 1628.7653, 1633.7913, 1634.7969, 1635.7941, 1639.8157, 1643.8085, 1646.8591, 1647.8268, 1651.8206, 1653.8284,
1677.8074, 1688.8287, 1690.8313, 1694.7357, 1697.8672, 1703.8214, 1706.8364, 1707.8435, 1708.3533, 1709.3307, 1710.8229, 1715.8053,
1719.8075, 1721.8296, 1723.8743, 1724.8828, 1725.8090, 1730.8199, 1748.7931, 1757.8975, 1763.7841, 1768.8075, 1771.9686, 1774.9059,
1777.8727, 1779.8081, 1786.8824, 1787.8207, 1790.8423, 1793.9556, 1796.8109, 1797.8339, 1800.8578, 1815.9008, 1819.8838, 1821.8926,
1822.9185, 1826.8450, 1829.8655, 1833.8975, 1836.9054, 1843.8765, 1844.8785, 1851.9031, 1859.8494, 1862.9265, 1863.9319, 1866.8857,
1868.8972, 1879.9445, 1880.9215, 1887.9023, 1888.8057, 1890.8025, 1896.8954, 1901.0060, 1906.8872, 1908.8663, 1913.8895, 1916.9475,
1924.9314, 1925.9336, 1928.0060, 1931.9956, 1932.9833, 1968.8563, 1984.9392, 2008.0485, 2008.9869, 2009.9747, 2012.9506, 2015.9661,
2020.9644, 2030.0151, 2030.4648, 2046.9839, 2047.5186, 2047.9097, 2051.9133, 2059.0122, 2064.9746, 2070.9209, 2077.0454, 2082.9409,
2085.9888, 2090.9844, 2097.9778, 2103.1526, 2108.9897, 2118.8926, 2140.0432, 2142.0310, 2142.9895, 2152.0510, 2155.0667, 2159.0752,
2160.0703, 2161.0813, 2166.0911, 2167.0676, 2174.0425, 2178.0034, 2181.0369, 2184.0767, 2189.9133, 2194.9761, 2199.0986, 2203.0464,
2205.9102, 2208.9729, 2211.1077, 2213.1116, 2214.1145, 2218.1018, 2221.1035, 2223.2205, 2225.1592, 2229.1064, 2233.1023, 2245.0991,
2269.1128, 2273.0393, 2277.0576, 2279.1094, 2280.1204, 2283.1279, 2285.1169, 2292.1465, 2296.1479, 2312.0664, 2323.1892, 2326.0947,
2334.1531, 2335.1282, 2337.1343, 2369.2256, 2372.2229, 2383.9978, 2385.2129, 2392.1528, 2400.1094, 2401.1360, 2409.1914, 2411.1907,
2414.1833, 2429.1475, 2436.1699, 2437.1758, 2440.2007, 2447.2368, 2477.1301, 2495.0542, 2498.0898, 2510.2229, 2522.1980, 2524.1689,
2527.2310, 2535.2598, 2541.2075, 2543.1907, 2544.1514, 2553.1934, 2557.2300, 2560.1125, 2569.2915, 2572.0935, 2612.1936, 2686.3572,
2788.3362, 2963.6167, 3056.4641, 3058.5842, 3180.5432, 3313.5032, 3330.6311, 3347.6077, 3511.9668, 3761.1558

Search Parameters

Type of search : MS/MS Ion Search
Enzyme : Trypsin
Fixed modifications : Carbamidomethyl (C)
Variable modifications : Oxidation (M)
Mass values : Monoisotopic
Protein Mass : Unrestricted
Peptide Mass Tolerance : ± 25 ppm
Fragment Mass Tolerance: ± 0.5 Da
Max Missed Cleavages : 2
Instrument type : MALDI-TOF-TOF
Query1 (724.2539,1+): <no title>
Query2 (725.2472,1+): <no title>
Query3 (728.2482,1+): <no title>
Query4 (730.2519,1+): <no title>
Query5 (732.2390,1+): <no title>
Query6 (733.2623,1+): <no title>
Query7 (739.3115,1+): <no title>
Query8 (742.4642,1+): <no title>
Query9 (743.4729,1+): <no title>
Query10 (745.4483,1+): <no title>
Query11 (747.4419,1+): <no title>
Query12 (752.3972,1+): <no title>
Query13 (754.3827,1+): <no title>
Query14 (756.3716,1+): <no title>
Query15 (758.3382,1+): <no title>
Query16 (760.3329,1+): <no title>
Query17 (764.3364,1+): <no title>
Query18 (766.3494,1+): <no title>
Query19 (767.4740,1+): <no title>
Query20 (768.3865,1+): <no title>
Query21 (770.3671,1+): <no title>
Query22 (772.4178,1+): <no title>
Query23 (779.4436,1+): <no title>
Query24 (784.3415,1+): <no title>
Query25 (787.2366,1+): <no title>
Query26 (788.4225,1+): <no title>
Query27 (790.4196,1+): <no title>
Query28 (795.4260,1+): <no title>
Query29 (799.2464,1+): <no title>
Query30 (802.4372,1+): <no title>
Query31 (805.4478,1+): <no title>
Query32 (808.4353,1+): <no title>
Query33 (811.4178,1+): <no title>
Query34 (814.4170,1+): <no title>
Query35 (819.4304,1+): <no title>
Query36 (821.4247,1+): <no title>
Query37 (822.4321,1+): <no title>
Query38 (829.4457,1+): <no title>
Query39 (837.4396,1+): <no title>
Query40 (840.4623,1+): <no title>
Query41 (842.5109,1+): <no title>
Query42 (854.4525,1+): <no title>
Query43 (856.5045,1+): <no title>
Query44 (858.5442,1+): <no title>
Query45 (860.4092,1+): <no title>
Query46 (863.4233,1+): <no title>
Query47 (863.9185,1+): <no title>
Query48 (869.4846,1+): <no title>
Query49 (870.5100,1+): <no title>

Query50 (872.4994,1+): <no title>
Query51 (880.4412,1+): <no title>
Query52 (904.4613,1+): <no title>
Query53 (906.4929,1+): <no title>
Query54 (908.4577,1+): <no title>
Query55 (913.5685,1+): <no title>
Query56 (919.5024,1+): <no title>
Query57 (925.5089,1+): <no title>
Query58 (927.4999,1+): <no title>
Query59 (928.5031,1+): <no title>
Query60 (940.4773,1+): <no title>
Query61 (942.4820,1+): <no title>
Query62 (950.4946,1+): <no title>
Query63 (957.5654,1+): <no title>
Query64 (960.4840,1+): <no title>
Query65 (966.4929,1+): <no title>
Query66 (969.4786,1+): <no title>
Query67 (972.5046,1+): <no title>
Query68 (974.5551,1+): <no title>
Query69 (977.5352,1+): <no title>
Query70 (978.5240,1+): <no title>
Query71 (981.4474,1+): <no title>
Query72 (989.2700,1+): <no title>
Query73 (991.5198,1+): <no title>
Query74 (993.5112,1+): <no title>
Query75 (1001.5782,1+): <no title>
Query76 (1007.5089,1+): <no title>
Query77 (1009.5097,1+): <no title>
Query78 (1013.4454,1+): <no title>
Query79 (1015.5150,1+): <no title>
Query80 (1016.5522,1+): <no title>
Query81 (1018.5189,1+): <no title>
Query82 (1021.5313,1+): <no title>
Query83 (1024.5028,1+): <no title>
Query84 (1025.5192,1+): <no title>
Query85 (1029.5330,1+): <no title>
Query86 (1032.5870,1+): <no title>
Query87 (1033.5739,1+): <no title>
Query88 (1035.5203,1+): <no title>
Query89 (1045.5564,1+): <no title>
Query90 (1046.5759,1+): <no title>
Query91 (1051.5074,1+): <no title>
Query92 (1056.5415,1+): <no title>
Query93 (1057.6005,1+): <no title>
Query94 (1064.5358,1+): <no title>
Query95 (1071.5361,1+): <no title>
Query96 (1073.5377,1+): <no title>
Query97 (1079.5398,1+): <no title>
Query98 (1083.4637,1+): <no title>
Query99 (1086.4774,1+): <no title>
Query100 (1092.6000,1+): Label: G5, Spot_Id: 221892, Peak_List_Id: 435422, MSMS Job_Run_Id: 30377, Comment:
Query101 (1092.6002,1+): <no title>
Query102 (1094.5596,1+): <no title>
Query103 (1096.5302,1+): <no title>
Query104 (1106.5431,1+): <no title>
Query105 (1110.5461,1+): <no title>
Query106 (1111.5398,1+): <no title>
Query107 (1111.5400,1+): Label: G5, Spot_Id: 221892, Peak_List_Id: 435420, MSMS Job_Run_Id: 30377, Comment:
Query108 (1115.5790,1+): <no title>
Query109 (1118.5620,1+): <no title>
Query110 (1120.5724,1+): <no title>
Query111 (1123.5819,1+): <no title>
Query112 (1128.5537,1+): <no title>
Query113 (1132.5682,1+): <no title>
Query114 (1134.5841,1+): <no title>
Query115 (1135.5942,1+): <no title>
Query116 (1137.5565,1+): <no title>
Query117 (1143.5844,1+): <no title>
Query118 (1145.6155,1+): <no title>
Query119 (1149.5597,1+): <no title>
Query120 (1153.5929,1+): <no title>
Query121 (1158.6288,1+): <no title>
Query122 (1163.5771,1+): <no title>
Query123 (1173.6008,1+): <no title>
Query124 (1175.5867,1+): <no title>
Query125 (1177.6178,1+): <no title>
Query126 (1178.6053,1+): <no title>
Query127 (1179.5962,1+): <no title>
Query128 (1181.6432,1+): <no title>
Query129 (1192.5721,1+): <no title>
Query130 (1199.6315,1+): <no title>
Query131 (1203.5826,1+): <no title>
Query132 (1211.5984,1+): <no title>
Query133 (1215.6246,1+): <no title>
Query134 (1216.6786,1+): <no title>
Query135 (1221.5699,1+): <no title>
Query136 (1222.5789,1+): <no title>
Query137 (1227.6271,1+): <no title>
Query138 (1228.6411,1+): <no title>
Query139 (1229.6412,1+): <no title>
Query140 (1231.6102,1+): <no title>
Query141 (1233.6571,1+): <no title>
Query142 (1237.6270,1+): <no title>
Query143 (1238.5917,1+): <no title>
Query144 (1244.6069,1+): <no title>
Query145 (1248.5958,1+): <no title>
Query146 (1250.6042,1+): <no title>
Query147 (1255.5922,1+): <no title>
Query148 (1258.6344,1+): <no title>
Query149 (1268.6414,1+): <no title>

```
Query150 (1271.6490,1+): <no title>
Query151 (1275.6388,1+): <no title>
Query152 (1276.5977,1+): <no title>
Query153 (1276.6000,1+): Label: G5, Spot_Id: 221892, Peak_List_Id: 435423, MSMS Job_Run_Id: 30377, Comment:
Query154 (1285.5864,1+): <no title>
Query155 (1294.6315,1+): <no title>
Query156 (1299.6810,1+): <no title>
Query157 (1300.6566,1+): <no title>
Query158 (1309.6340,1+): <no title>
Query159 (1320.6268,1+): <no title>
Query160 (1328.6292,1+): <no title>
Query161 (1333.7025,1+): <no title>
Query162 (1336.6038,1+): <no title>
Query163 (1338.6589,1+): <no title>
Query164 (1341.6488,1+): <no title>
Query165 (1343.6613,1+): <no title>
Query166 (1345.6910,1+): <no title>
Query167 (1349.6838,1+): <no title>
Query168 (1350.6704,1+): <no title>
Query169 (1352.6350,1+): <no title>
Query170 (1355.6870,1+): <no title>
Query171 (1357.7130,1+): <no title>
Query172 (1358.6704,1+): <no title>
Query173 (1369.7067,1+): <no title>
Query174 (1371.7366,1+): <no title>
Query175 (1374.6705,1+): <no title>
Query176 (1377.6100,1+): Label: G5, Spot_Id: 221892, Peak_List_Id: 435518, MSMS Job_Run_Id: 30377, Comment:
Query177 (1377.6146,1+): <no title>
Query178 (1384.7109,1+): <no title>
Query179 (1386.7134,1+): <no title>
Query180 (1390.6848,1+): <no title>
Query181 (1393.7169,1+): <no title>
Query182 (1399.6119,1+): <no title>
Query183 (1400.6588,1+): <no title>
Query184 (1406.7207,1+): <no title>
Query185 (1410.6736,1+): <no title>
Query186 (1411.6647,1+): <no title>
Query187 (1416.6047,1+): <no title>
Query188 (1418.6714,1+): <no title>
Query189 (1421.6632,1+): <no title>
Query190 (1425.7395,1+): <no title>
Query191 (1426.6846,1+): <no title>
Query192 (1427.7076,1+): <no title>
Query193 (1430.7102,1+): <no title>
Query194 (1432.6718,1+): <no title>
Query195 (1434.6552,1+): <no title>
Query196 (1438.6991,1+): <no title>
Query197 (1439.7164,1+): <no title>
Query198 (1443.7589,1+): <no title>
Query199 (1444.7375,1+): <no title>
Query200 (1445.7306,1+): <no title>
Query201 (1454.6836,1+): <no title>
Query202 (1461.7990,1+): <no title>
Query203 (1472.7026,1+): <no title>
Query204 (1475.7224,1+): <no title>
Query205 (1485.6906,1+): <no title>
Query206 (1489.7172,1+): <no title>
Query207 (1490.7058,1+): <no title>
Query208 (1493.7157,1+): <no title>
Query209 (1510.7383,1+): <no title>
Query210 (1515.7393,1+): <no title>
Query211 (1523.6987,1+): <no title>
Query212 (1524.7383,1+): <no title>
Query213 (1527.7585,1+): <no title>
Query214 (1529.7466,1+): <no title>
Query215 (1530.6902,1+): <no title>
Query216 (1531.6953,1+): <no title>
Query217 (1537.7804,1+): <no title>
Query218 (1538.7736,1+): <no title>
Query219 (1539.7621,1+): <no title>
Query220 (1541.7805,1+): <no title>
Query221 (1542.8290,1+): <no title>
Query222 (1549.6758,1+): <no title>
Query223 (1553.7277,1+): <no title>
Query224 (1555.7928,1+): <no title>
Query225 (1556.7874,1+): <no title>
Query226 (1560.7551,1+): <no title>
Query227 (1563.7314,1+): <no title>
Query228 (1568.7372,1+): <no title>
Query229 (1570.8015,1+): <no title>
Query230 (1572.8151,1+): <no title>
Query231 (1576.7402,1+): <no title>
Query232 (1577.7395,1+): <no title>
Query233 (1578.7518,1+): <no title>
Query234 (1581.8373,1+): <no title>
Query235 (1591.7852,1+): <no title>
Query236 (1600.8068,1+): <no title>
Query237 (1603.8092,1+): <no title>
Query238 (1604.8136,1+): <no title>
Query239 (1606.8130,1+): <no title>
Query240 (1608.7599,1+): <no title>
Query241 (1613.7793,1+): <no title>
Query242 (1615.8575,1+): <no title>
Query243 (1615.8600,1+): Label: G5, Spot_Id: 221892, Peak_List_Id: 435421, MSMS Job_Run_Id: 30377, Comment:
Query244 (1624.7632,1+): <no title>
Query245 (1628.7653,1+): <no title>
Query246 (1633.7913,1+): <no title>
Query247 (1634.7969,1+): <no title>
Query248 (1635.7941,1+): <no title>
Query249 (1639.8157,1+): <no title>
```

```
Query250 (1643.8085,1+): <no title>
Query251 (1646.8591,1+): <no title>
Query252 (1647.8268,1+): <no title>
Query253 (1651.8206,1+): <no title>
Query254 (1653.8284,1+): <no title>
Query255 (1677.8074,1+): <no title>
Query256 (1681.8087,1+): <no title>
Query257 (1688.8287,1+): <no title>
Query258 (1690.8313,1+): <no title>
Query259 (1694.7357,1+): <no title>
Query260 (1697.8672,1+): <no title>
Query261 (1703.8214,1+): <no title>
Query262 (1706.8364,1+): <no title>
Query263 (1707.8435,1+): <no title>
Query264 (1708.3533,1+): <no title>
Query265 (1709.3307,1+): <no title>
Query266 (1710.8229,1+): <no title>
Query267 (1715.8053,1+): <no title>
Query268 (1719.8075,1+): <no title>
Query269 (1721.8296,1+): <no title>
Query270 (1723.8743,1+): <no title>
Query271 (1724.8828,1+): <no title>
Query272 (1725.8090,1+): <no title>
Query273 (1730.8199,1+): <no title>
Query274 (1748.7931,1+): <no title>
Query275 (1757.8975,1+): <no title>
Query276 (1763.7841,1+): <no title>
Query277 (1768.8075,1+): <no title>
Query278 (1771.9686,1+): <no title>
Query279 (1774.9059,1+): <no title>
Query280 (1777.8727,1+): <no title>
Query281 (1779.8081,1+): <no title>
Query282 (1786.8824,1+): <no title>
Query283 (1787.8207,1+): <no title>
Query284 (1790.8423,1+): <no title>
Query285 (1793.9556,1+): <no title>
Query286 (1796.8109,1+): <no title>
Query287 (1797.8339,1+): <no title>
Query288 (1800.8578,1+): <no title>
Query289 (1815.9008,1+): <no title>
Query290 (1819.8838,1+): <no title>
Query291 (1821.8926,1+): <no title>
Query292 (1822.9185,1+): <no title>
Query293 (1826.8450,1+): <no title>
Query294 (1829.8655,1+): <no title>
Query295 (1833.8975,1+): <no title>
Query296 (1836.9054,1+): <no title>
Query297 (1843.8765,1+): <no title>
Query298 (1844.8785,1+): <no title>
Query299 (1851.9031,1+): <no title>
Query300 (1859.8494,1+): <no title>
Query301 (1862.9265,1+): <no title>
Query302 (1863.9319,1+): <no title>
Query303 (1866.8857,1+): <no title>
Query304 (1868.8972,1+): <no title>
Query305 (1879.9445,1+): <no title>
Query306 (1880.9215,1+): <no title>
Query307 (1887.9023,1+): <no title>
Query308 (1888.8057,1+): <no title>
Query309 (1890.8025,1+): <no title>
Query310 (1896.8954,1+): <no title>
Query311 (1901.0060,1+): <no title>
Query312 (1906.8872,1+): <no title>
Query313 (1908.8663,1+): <no title>
Query314 (1913.8895,1+): <no title>
Query315 (1916.9475,1+): <no title>
Query316 (1924.9314,1+): <no title>
Query317 (1925.9336,1+): <no title>
Query318 (1928.0060,1+): <no title>
Query319 (1931.9956,1+): <no title>
Query320 (1932.9833,1+): <no title>
Query321 (1968.8563,1+): <no title>
Query322 (1984.9392,1+): <no title>
Query323 (2008.0485,1+): <no title>
Query324 (2008.9869,1+): <no title>
Query325 (2009.9747,1+): <no title>
Query326 (2012.9506,1+): <no title>
Query327 (2015.9661,1+): <no title>
Query328 (2020.9644,1+): <no title>
Query329 (2030.0151,1+): <no title>
Query330 (2030.4648,1+): <no title>
Query331 (2046.9839,1+): <no title>
Query332 (2047.5186,1+): <no title>
Query333 (2047.9097,1+): <no title>
Query334 (2051.9133,1+): <no title>
Query335 (2059.0122,1+): <no title>
Query336 (2064.9746,1+): <no title>
Query337 (2070.9209,1+): <no title>
Query338 (2077.0454,1+): <no title>
Query339 (2082.9409,1+): <no title>
Query340 (2085.9888,1+): <no title>
Query341 (2090.9844,1+): <no title>
Query342 (2097.9778,1+): <no title>
Query343 (2103.1526,1+): <no title>
Query344 (2108.9897,1+): <no title>
Query345 (2118.8926,1+): <no title>
Query346 (2140.0432,1+): <no title>
Query347 (2142.0310,1+): <no title>
Query348 (2142.9895,1+): <no title>
Query349 (2152.0510,1+): <no title>
```

```
Query350 (2155.0667,1+): <no title>
Query351 (2159.0752,1+): <no title>
Query352 (2160.0703,1+): <no title>
Query353 (2161.0813,1+): <no title>
Query354 (2166.0911,1+): <no title>
Query355 (2167.0676,1+): <no title>
Query356 (2174.0425,1+): <no title>
Query357 (2178.0034,1+): <no title>
Query358 (2181.0369,1+): <no title>
Query359 (2184.0767,1+): <no title>
Query360 (2189.9133,1+): <no title>
Query361 (2194.9761,1+): <no title>
Query362 (2199.0986,1+): <no title>
Query363 (2203.0464,1+): <no title>
Query364 (2205.9102,1+): <no title>
Query365 (2208.9729,1+): <no title>
Query366 (2211.1077,1+): <no title>
Query367 (2213.1116,1+): <no title>
Query368 (2214.1145,1+): <no title>
Query369 (2218.1018,1+): <no title>
Query370 (2221.1035,1+): <no title>
Query371 (2223.2205,1+): <no title>
Query372 (2225.1592,1+): <no title>
Query373 (2229.1064,1+): <no title>
Query374 (2233.1023,1+): <no title>
Query375 (2245.0991,1+): <no title>
Query376 (2269.1128,1+): <no title>
Query377 (2273.0393,1+): <no title>
Query378 (2277.0576,1+): <no title>
Query379 (2279.1094,1+): <no title>
Query380 (2280.1204,1+): <no title>
Query381 (2283.1279,1+): <no title>
Query382 (2285.1169,1+): <no title>
Query383 (2292.1465,1+): <no title>
Query384 (2296.1479,1+): <no title>
Query385 (2312.0664,1+): <no title>
Query386 (2323.1892,1+): <no title>
Query387 (2326.0947,1+): <no title>
Query388 (2334.1531,1+): <no title>
Query389 (2335.1282,1+): <no title>
Query390 (2337.1343,1+): <no title>
Query391 (2369.2256,1+): <no title>
Query392 (2372.2229,1+): <no title>
Query393 (2383.9978,1+): <no title>
Query394 (2385.2129,1+): <no title>
Query395 (2392.1528,1+): <no title>
Query396 (2400.1094,1+): <no title>
Query397 (2401.1360,1+): <no title>
Query398 (2409.1914,1+): <no title>
Query399 (2411.1907,1+): <no title>
Query400 (2414.1833,1+): <no title>
Query401 (2429.1475,1+): <no title>
Query402 (2436.1699,1+): <no title>
Query403 (2437.1758,1+): <no title>
Query404 (2440.2007,1+): <no title>
Query405 (2447.2368,1+): <no title>
Query406 (2477.1301,1+): <no title>
Query407 (2495.0542,1+): <no title>
Query408 (2498.0898,1+): <no title>
Query409 (2510.2229,1+): <no title>
Query410 (2522.1980,1+): <no title>
Query411 (2524.1689,1+): <no title>
Query412 (2527.2310,1+): <no title>
Query413 (2535.2598,1+): <no title>
Query414 (2541.2075,1+): <no title>
Query415 (2543.1907,1+): <no title>
Query416 (2544.1514,1+): <no title>
Query417 (2553.1934,1+): <no title>
Query418 (2557.2300,1+): <no title>
Query419 (2560.1125,1+): <no title>
Query420 (2569.2915,1+): <no title>
Query421 (2572.0935,1+): <no title>
Query422 (2612.1936,1+): <no title>
Query423 (2686.3572,1+): <no title>
Query424 (2788.3362,1+): <no title>
Query425 (2963.6167,1+): <no title>
Query426 (3056.4641,1+): <no title>
Query427 (3058.5842,1+): <no title>
Query428 (3180.5432,1+): <no title>
Query429 (3313.5032,1+): <no title>
Query430 (3330.6311,1+): <no title>
Query431 (3347.6077,1+): <no title>
Query432 (3511.9668,1+): <no title>
Query433 (3761.1558,1+): <no title>
```

Mascot: http://www.matrixscience.com/

MATRIX SCIENCE Mascot Search Results

```
User          : 3c_(G3@11273_12Mar2012)
Email         : proteomics@ipatimup.pt
Search title  : Project: Proteomica, Spot Set: Proteomica\12Jan2012, Label: G3, Spot Id: 221890, Peak List Id: 435351, MS Job Run Id: 303
MS data file  : C:\Documents and Settings\Administrator\Desktop\xu\xu\xu\Proteomica\12Jan2012\ppw_G3_146796935604.txt
Database      : OrganismSpecie Bos_taurus_Reference_Proteome_2016_06 (24214 sequences; 12839866 residues)
Timestamp     : 8 Jul 2016 at 10:11:59 GMT
Warning       : A Peptide summary report will usually give a much clearer picture of MS/MS search results.
Top Score     : 184 for Q95L54, Annexin A8 OS=Bos taurus GN=ANXA8 PE=2 SV=1
```

Mascot Score Histogram

Protein score is -10*Log(P), where P is the probability that the observed match is a random event.
Protein scores greater than 56 are significant (p<0.05).
Protein scores are derived from ions scores as a non-probabilistic basis for ranking protein hits.

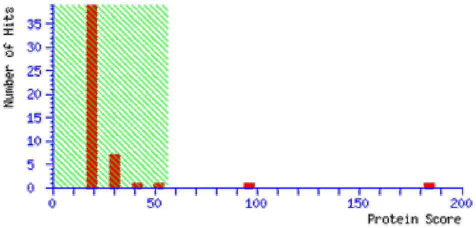

Protein Summary Report

Format As | Protein Summary (deprecated) | Help

Significance threshold p< 0.05 | Max. number of hits 50

Preferred taxonomy | All entries

Re-Search All | Search Unmatched

Index

	Accession	Mass	Score	Description
1.	Q95L54	36992	184	Annexin A8 OS=Bos taurus GN=ANXA8 PE=2 SV=1
2.	P19858	36916	98	L-lactate dehydrogenase A chain OS=Bos taurus GN=LDHA PE=2 SV=2
3.	P04272	38873	52	Annexin A2 OS=Bos taurus GN=ANXA2 PE=1 SV=2
4.	F1MK19	26274	42	L-lactate dehydrogenase OS=Bos taurus GN=LDHC PE=3 SV=1
5.	E1BNS9	36334	34	L-lactate dehydrogenase OS=Bos taurus GN=LDHC PE=3 SV=2
6.	E1BFB5	68315	33	Uncharacterized protein OS=Bos taurus GN=KIAA0391 PE=4 SV=2
7.	P09867	34289	31	Heterogeneous nuclear ribonucleoprotein A1 OS=Bos taurus GN=HNRNPA1 PE=1 SV=2
8.	Q32LI7	30313	29	Putative uncharacterized protein MGC133880 OS=Bos taurus GN=MGC133880 PE=2 SV=1
9.	F6QVC9	36109	26	Annexin OS=Bos taurus GN=ANXA5 PE=1 SV=1
10.	E1BPX8	222520	26	Uncharacterized protein OS=Bos taurus GN=MYH7B PE=3 SV=2
11.	Q29RH6	55738	26	Chromosome 6 open reading frame 182 ortholog OS=Bos taurus GN=LOC519522 PE=2 SV=1
12.	Q2HJF2	26289	24	F-box and leucine-rich protein 22 OS=Bos taurus GN=FBXL22 PE=2 SV=1
13.	G5E523	28080	24	Uncharacterized protein OS=Bos taurus GN=STAU2 PE=4 SV=1
14.	F1N024	7899	24	Uncharacterized protein OS=Bos taurus GN=FAM19A4 PE=4 SV=1
15.	F1MS41	14310	24	Beta-synuclein OS=Bos taurus GN=SNCB PE=3 SV=2
16.	P81287	36124	24	Annexin A5 OS=Bos taurus GN=ANXA5 PE=1 SV=3
17.	E1BM24	57270	23	CCDC64B protein-like OS=Bos taurus GN=CCDC64B PE=4 SV=1
18.	F1MHM8	57999	22	Uncharacterized protein OS=Bos taurus GN=TIGD4 PE=4 SV=2
19.	P08057	24153	22	Troponin I, cardiac muscle OS=Bos taurus GN=TNNI3 PE=1 SV=2
20.	G3X720	22128	22	Uncharacterized protein OS=Bos taurus GN=C19orf81 PE=4 SV=1
21.	Q58DU0	26366	21	Multiple myeloma tumor-associated protein 2 homolog OS=Bos taurus GN=MMTAG2 PE=2 SV=1
22.	Q28024	8166	21	Guanine nucleotide-binding protein G(I)/G(S)/G(O) subunit gamma-12 OS=Bos taurus GN=GNG12 PE=1 SV=2
23.	E1BD03	66968	21	Uncharacterized protein OS=Bos taurus GN=PPM1D PE=3 SV=2
24.	F1N6S6	28266	21	Placental prolactin-related protein 3 OS=Bos taurus GN=PRP3 PE=3 SV=2
25.	F6QYE2	72629	20	Uncharacterized protein OS=Bos taurus GN=CFAP100 PE=4 SV=1
26.	G1K1U4	51632	20	DNA polymerase delta subunit 3 OS=Bos taurus GN=POLD3 PE=4 SV=2
27.	G3MX17	200058	20	Uncharacterized protein OS=Bos taurus PE=3 SV=1
28.	G3N2D2	41913	20	Testis-specific serine/threonine-protein kinase 1 OS=Bos taurus GN=TSSK1B PE=3 SV=1
29.	Q32PB1	14905	20	Profilin-3 OS=Bos taurus GN=PFN3 PE=2 SV=1
30.	G3X7E1	19619	20	Histone deacetylase complex subunit SAP18 OS=Bos taurus GN=SAP18 PE=4 SV=1
31.	Q58DQ3	32727	20	60S ribosomal protein L6 OS=Bos taurus GN=RPL6 PE=2 SV=3
32.	P80311	23786	20	Peptidyl-prolyl cis-trans isomerase B OS=Bos taurus GN=PPIB PE=1 SV=4
33.	G1K1R3	40420	20	Tropomodulin-1 OS=Bos taurus GN=TMOD1 PE=4 SV=2
34.	E1B871	21420	20	Uncharacterized protein OS=Bos taurus PE=3 SV=2
35.	A0JNC0	40548	20	Tropomodulin-1 OS=Bos taurus GN=TMOD1 PE=2 SV=1
36.	F1MZ00	14021	19	Uncharacterized protein OS=Bos taurus GN=SNRPD3 PE=4 SV=2
37.	A3KMZ9	18849	19	SNRNP27 protein OS=Bos taurus GN=SNRNP27 PE=2 SV=1
38.	P42899	11695	19	60S acidic ribosomal protein P2 OS=Bos taurus GN=RPLP2 PE=3 SV=1
39.	Q0VCY7	27842	19	Serine/arginine-rich splicing factor 1 OS=Bos taurus GN=SRSF1 PE=2 SV=1
40.	Q0P5K9	54202	19	Chromosome 3 open reading frame 19 ortholog OS=Bos taurus GN=C22H3ORF19 PE=2 SV=1
41.	F1MB56	24689	19	Uncharacterized protein OS=Bos taurus GN=TEX35 PE=4 SV=1
42.	P61257	17248	19	60S ribosomal protein L26 OS=Bos taurus GN=RPL26 PE=2 SV=1
43.	Q861S4	18652	19	Similar to ribosomal protein L21 (Fragment) OS=Bos taurus GN=RPL21 PE=2 SV=1
44.	Q00361	8315	19	ATP synthase subunit e, mitochondrial OS=Bos taurus GN=ATP5I PE=1 SV=2
45.	F1N1S3	20635	19	Uncharacterized protein OS=Bos taurus GN=LOC100294792 PE=4 SV=2
46.	A5PK00	44048	19	Vacuolar protein-sorting-associated protein 36 OS=Bos taurus GN=VPS36 PE=2 SV=1
47.	A0JNH6	63280	19	Coiled-coil domain-containing protein 102A OS=Bos taurus GN=CCDC102A PE=2 SV=1
48.	G3MYC1	17221	19	Uncharacterized protein OS=Bos taurus PE=3 SV=1

49. F1MYD0	90289	19	Uncharacterized protein OS=Bos taurus GN=CUL1 PE=3 SV=2
50. F1MHT3	217593	19	Uncharacterized protein OS=Bos taurus GN=MYO5A PE=4 SV=2

Results List

1. Q95L54 **Mass:** 36992 **Score:** 184 **Expect:** 9.6e-015 **Matches:** 34

Annexin A8 OS=Bos taurus GN=ANXA8 PE=2 SV=1

Observed	Mr(expt)	Mr(calc)	ppm	Start	End	Miss	Ions	Peptide
731.4017	730.3945	730.3834	15.1	51 -	56	1	---	K.RSNAQR.Q
772.4093	771.4020	771.3989	4.04	262 -	267	0	---	R.LYFAMK.G
815.4332	814.4260	814.4119	17.2	209 -	215	0	---	R.SATHLMR.V
869.4231	868.4158	868.4000	18.2	101 -	107	0	---	K.ELYDAMK.G
943.4289	942.4216	942.4334	-12.55	216 -	222	0	---	R.VFEEYEK.I
1023.5663	1022.5590	1022.5583	0.72	201 -	208	0	---	K.FITILCTR.S
1023.5700	1022.5627	1022.5583	4.34	201 -	208	0	37	K.FITILCTR.S
1044.5751	1043.5678	1043.5862	-17.65	284 -	292	0	---	R.SEIDLNLIK.N
1073.5902	1072.5829	1072.5877	-4.43	268 -	278	0	---	K.GAGTLDGTLIR.N
1106.5391	1105.5318	1105.5305	1.22	253 -	261	0	---	R.NLHGYFAER.L
1143.6318	1142.6245	1142.6156	7.82	52 -	61	1	---	R.SNAQRQQIAK.S
1158.6685	1157.6612	1157.6591	1.87	158 -	167	0	---	R.ILVCLLQGSR.D
1199.7006	1198.6933	1198.6921	1.01	113 -	123	0	---	K.EGVIIEILASR.T
1231.6387	1230.6314	1230.6245	5.67	6 -	16	0	---	K.AWVEQEGVSVK.G
1232.6447	1231.6374	1231.6594	-17.86	124 -	133	1	---	R.TKNQLQEIMK.A
1360.6638	1359.6565	1359.6380	13.6	97 -	107	1	---	R.YEAKELYDAMK.G
1365.7379	1364.7306	1364.7526	-16.10	86 -	96	0	---	R.LIIALMYPPYR.Y + Oxidation (M)
1369.7043	1368.6970	1368.6925	3.31	216 -	226	1	---	R.VFEEYEKIANK.S
1505.8177	1504.8104	1504.8110	-0.38	52 -	64	2	---	R.SNAQRQQIAKSFK.A
1561.8401	1560.8328	1560.8511	-11.72	284 -	296	1	---	R.SEIDLNLIKNQFK.K
1571.8400	1570.8327	1570.8566	-15.22	36 -	50	0	---	K.GIGTNEQAIIDVLTK.R
1689.9465	1688.9392	1688.9461	-4.06	284 -	297	2	---	R.SEIDLNLIKNQFKK.M
1730.8931	1729.8858	1729.8556	17.5	234 -	249	0	---	K.SETHGSLEEAMLTVVK.C
1774.7610	1773.7537	1773.7581	-2.44	134 -	148	0	---	K.AYEEDYGSNLEEDIK.A
1787.8188	1786.8115	1786.8162	-2.63	17 -	32	0	---	K.GSPHFNPDPDAETLYK.A
1787.8200	1786.8127	1786.8162	-1.96	17 -	32	0	85	K.GSPHFNPDPDAETLYK.A
1840.9827	1839.9754	1839.9957	-10.99	86 -	100	1	---	R.LIIALMYPPYRYEAK.E
1856.9908	1855.9835	1855.9906	-3.80	86 -	100	1	---	R.LIIALMYPPYRYEAK.E + Oxidation (M)
1933.9950	1932.9877	1932.9556	16.6	1 -	16	1	---	-.MAWWKAWVEQEGVSVK.G
1949.9595	1948.9522	1948.9505	0.86	1 -	16	1	---	-.MAWWKAWVEQEGVSVK.G + Oxidation (M)
1973.9845	1972.9772	1972.9420	17.8	193 -	208	1	---	K.ICGTDEMKFITILCTR.S + Oxidation (M)
2277.0823	2276.0750	2276.0983	-10.21	250 -	267	2	---	K.CTRNLHGYFAERLYFAMK.G
2284.1990	2283.1917	2283.2295	-16.53	36 -	56	2	---	K.GIGTNEQAIIDVLTKRSNAQR.Q
2767.2666	2766.2593	2766.2144	16.3	134 -	157	1	---	K.AYEEDYGSNLEEDIKADTSGYLER.I

No match to: 721.4365, 725.4001, 726.4063, 728.3681, 730.3848, 732.3573, 734.4151, 735.4143, 741.4101, 742.4515, 744.4171, 745.4167, 746.4169, 747.4125, 750.4153, 752.4127, 755.4100, 756.4199, 757.4313, 761.4177, 767.4266, 768.3700, 768.3737, 770.3966, 775.4307, 779.4308, 784.3688, 786.4000, 787.4221, 788.4210, 792.4376, 795.4350, 798.4351, 800.4296, 812.4388, 822.4482, 825.4540, 831.4382, 832.4557, 835.4719, 837.4463, 842.5060, 844.4760, 854.4837, 856.4911, 860.4691, 862.4852, 885.4465, 905.4955, 907.4991, 908.4929, 913.5700, 913.5747, 916.5284, 918.5050, 919.5053, 922.5070, 928.5117, 934.5172, 940.4840, 942.4854, 943.5453, 946.5259, 950.4959, 951.5076, 953.5164, 954.5388, 959.5266, 966.5410, 969.5256, 972.5305, 974.5395, 976.5310, 978.5314, 979.5442, 991.5409, 993.5307, 1001.5854, 1003.5496, 1011.5262, 1014.5215, 1021.5579, 1031.5903, 1035.5381, 1036.5447, 1037.5573, 1039.5327, 1041.5658, 1045.5729, 1046.5897, 1051.5448, 1056.5658, 1057.6078, 1067.5753, 1086.5509, 1092.5961, 1100.6071, 1111.5615, 1113.5729, 1118.5818, 1120.6045, 1122.5912, 1123.5848, 1125.5823, 1132.6047, 1134.5714, 1139.5746, 1144.6497, 1145.6407, 1147.6393, 1150.6108, 1155.6215, 1157.6199, 1162.6182, 1163.6152, 1168.6215, 1173.6418, 1175.6089, 1178.6356, 1179.6157, 1181.6819, 1188.6544, 1197.6704, 1208.6415, 1216.6714, 1227.6595, 1234.6672, 1235.6445, 1244.6453, 1247.6211, 1248.6174, 1250.6554, 1255.6096, 1256.6531, 1261.6592, 1262.6453, 1266.6777, 1273.6260, 1275.6362, 1276.6343, 1277.6531, 1301.7079, 1303.7076, 1307.7042, 1308.6376, 1310.6146, 1312.6418, 1315.7098, 1320.6659, 1322.6886, 1323.6771, 1324.6964, 1328.7089, 1331.7202, 1335.7318, 1338.6581, 1339.6637, 1349.7253, 1352.7205, 1356.7230, 1363.7153, 1367.7319, 1377.6947, 1379.7268, 1383.7137, 1385.7534, 1388.7404, 1390.7284, 1393.7428, 1407.7563, 1417.7010, 1421.7061, 1422.7019, 1425.7727, 1427.7742, 1429.7365, 1431.7412, 1433.7228, 1434.7446, 1437.7584, 1461.7778, 1467.7521, 1472.7633, 1475.7632, 1477.7545, 1484.7889, 1488.7537, 1491.7965, 1493.7579, 1500.7737, 1513.8079, 1515.8213, 1517.7854, 1519.7544, 1522.7811, 1523.7953, 1529.8099, 1530.7523, 1531.7472, 1537.7974, 1539.8005, 1542.8422, 1545.8091, 1547.8051, 1549.7339, 1551.7778, 1553.8065, 1555.8101, 1556.8109, 1557.8010, 1563.8109, 1565.8199, 1579.7939, 1580.8077, 1583.8444, 1587.7620, 1592.8066, 1594.8335, 1604.8323, 1605.8586, 1608.8497, 1615.8754, 1624.8062, 1628.7900, 1628.7921, 1633.7985, 1640.9384, 1642.8099, 1646.8297, 1649.8447, 1657.8101, 1667.8622, 1677.7988, 1680.8746, 1694.7288, 1698.9225, 1699.8862, 1704.8773, 1706.8701, 1707.8302, 1710.9279, 1716.8671, 1719.9058, 1723.9011, 1725.8300, 1725.8346, 1727.8986, 1737.9006, 1751.8656, 1754.8580, 1765.8463, 1766.8040, 1767.8138, 1769.8741, 1770.3567, 1772.9315, 1774.9131, 1777.8770, 1780.8777, 1783.9246, 1791.8137, 1793.9050, 1795.9353, 1798.9340, 1800.9336, 1804.9142, 1805.9069, 1809.8469, 1815.9044, 1821.9144, 1828.9227, 1837.9396, 1843.9688, 1844.9012, 1846.9153, 1852.9364, 1861.8881, 1867.9218, 1874.9695, 1880.9265, 1884.9417, 1891.9622, 1894.9313, 1896.9078, 1896.9100, 1900.9546, 1905.9313, 1906.9501, 1908.9043, 1911.9130, 1912.9154, 1916.9923, 1918.9587, 1921.9825, 1923.9718, 1925.9573, 1928.0469, 1932.0060, 1937.9630, 1939.9740, 1944.0040, 1946.9679, 1954.0236, 1955.9738, 1958.9556, 1960.0034, 1968.8600, 1976.9861, 1978.9910, 1981.9854, 1983.9884, 1987.9784, 1995.9408, 1998.9894, 2000.9926, 2008.0460, 2012.9590, 2015.0104, 2017.9747, 2021.0170, 2026.0275, 2027.0256, 2031.0166, 2044.0123, 2047.4957, 2047.9515, 2052.0496, 2060.0520, 2062.0073, 2064.9880, 2066.9873, 2094.0376, 2097.0454, 2102.0667, 2103.1641, 2111.0305, 2127.0930, 2139.0630, 2144.0493, 2148.1023, 2155.0950, 2156.0796, 2160.0632, 2176.0664, 2190.9895, 2195.0774, 2199.0723, 2202.1624, 2203.1221, 2208.0862, 2211.1042, 2216.1062, 2225.1274, 2226.1272, 2229.0972, 2233.0984, 2239.0225, 2242.0435, 2268.1489, 2270.1287, 2271.1294, 2292.1426, 2309.0994, 2334.1719, 2342.9915, 2350.1746, 2383.9607, 2398.2400, 2400.0862, 2413.0549, 2414.1897, 2417.0959, 2497.2800, 2501.2637, 2511.1460, 2513.1421, 2528.1750, 2537.2012, 2541.1948, 2542.1836, 2556.2058, 2562.1487, 2612.2666, 2706.2109, 2717.0869, 2888.2532, 3221.1733, 3224.2917, 3312.3306, 3916.9434

2. P19858 **Mass:** 36916 **Score:** 98 **Expect:** 4.1e-006 **Matches:** 29

L-lactate dehydrogenase A chain OS=Bos taurus GN=LDHA PE=2 SV=2

Observed	Mr(expt)	Mr(calc)	ppm	Start	End	Miss	Ions	Peptide
734.4151	733.4078	733.4123	-6.05	113 -	118	0	---	R.NVNIFK.F
742.4515	741.4442	741.4497	-7.42	107 -	112	0	---	R.LNLVQR.N
768.3700	767.3627	767.3636	-1.12	172 -	177	0	25	R.YLMGER.L
768.3737	767.3664	767.3636	3.65	172 -	177	0	---	R.YLMGER.L
784.3688	783.3615	783.3585	3.82	172 -	177	0	---	R.YLMGER.L + Oxidation (M)
913.5700	912.5627	912.5756	-14.15	91 -	99	0	22	R.LVIITAGAR.Q
913.5747	912.5674	912.5756	-9.06	91 -	99	0	---	R.LVIITAGAR.Q
918.5050	917.4977	917.5083	-11.51	150 -	157	1	---	K.ISGFPKNR.V
1041.5658	1040.5585	1040.5688	-9.92	270 -	278	0	---	R.VHPISTMIK.G + Oxidation (M)
1118.5818	1117.5745	1117.5768	-2.01	319 -	328	0	---	K.SADTLWGIQK.E
1181.6819	1180.6746	1180.6750	-0.35	269 -	278	1	---	R.RVHPISTMIK.G

1197.6704	1196.6631	1196.6700	-5.71	269	278	1	---	R.RVHPISTMIK.G + Oxidation (M)
1248.6174	1247.6101	1247.5928	13.9	158	169	0	---	R.VIGSGCNLDSAR.F
1250.6554	1249.6481	1249.6554	-5.83	233	243	0	---	K.QVVDSAYEVIK.L
1523.7953	1522.7880	1522.7740	9.24	77	90	1	---	K.IVSGKDYNVTANSR.L
1551.7778	1550.7705	1550.7624	5.26	158	171	1	---	R.VIGSGCNLDSARFR.Y
1556.8109	1555.8036	1555.8066	-1.94	100	112	1	---	R.QQEGESRLNLVQR.N
1557.8010	1556.7937	1556.7868	4.44	306	318	1	---	K.VTLTHEEEACLKK.S
1633.7985	1632.7912	1632.7752	9.80	60	73	0	---	K.GEMMDLQHGSLFLR.T
1677.7988	1676.7915	1676.7815	5.99	43	57	0	---	K.DLADEVALVDVMEDK.L + Oxidation (M)
1727.8986	1726.8913	1726.9326	-23.88	91	106	1	---	R.LVIITAGARQQEGESR.L
1821.9144	1820.9071	1820.9064	0.40	156	171	2	---	K.NRVIGSGCNLDSARFR.Y
1874.9695	1873.9622	1873.9542	4.26	58	73	1	---	K.LKGEMMDLQHGSLFLR.T
1880.9265	1879.9192	1879.9064	6.81	213	228	1	---	K.NLHPELGTDADKEQWK.A
1906.9501	1905.9428	1905.9441	-0.66	58	73	1	---	K.LKGEMMDLQHGSLFLR.T + 2 Oxidation (M)
1918.9587	1917.9514	1917.9605	-4.73	43	59	1	---	K.DLADEVALVDVMEDKLK.G + Oxidation (M)
1960.0034	1958.9961	1958.9706	13.0	60	76	1	---	K.GEMMDLQHGSLFLRTPK.I
2148.1023	2147.0950	2147.0906	2.08	150	169	2	---	K.ISGFPKNRVIGSGCNLDSAR.F
2233.0984	2232.0911	2232.1395	-21.66	58	76	2	---	K.LKGEMMDLQHGSLFLRTPK.I + 2 Oxidation (M)

No match to: 721.4365, 725.4001, 726.4063, 728.3681, 730.3848, 731.4017, 732.3573, 735.4143, 741.4101, 744.4171, 745.4167, 746.4169, 747.4125, 750.4153, 752.4127, 755.4100, 756.4199, 757.4313, 761.4177, 767.4266, 770.3966, 772.4093, 775.4307, 779.4308, 786.4000, 787.4221, 788.4210, 792.4376, 795.4350, 798.4351, 800.4296, 812.4388, 815.4332, 822.4482, 825.4540, 831.4382, 832.4557, 835.4719, 837.4463, 842.5060, 844.4760, 854.4837, 856.4911, 860.4691, 862.4852, 869.4231, 885.4465, 905.4955, 907.4991, 908.4929, 916.5284, 919.5053, 922.5070, 928.5117, 934.5172, 940.4840, 942.4854, 943.4289, 943.5453, 946.5259, 950.4959, 951.5076, 953.5164, 954.5388, 959.5266, 966.5410, 969.5256, 972.5305, 974.5395, 976.5310, 978.5314, 979.5442, 991.5409, 993.5307, 1001.5854, 1003.5496, 1011.5262, 1014.5215, 1021.5579, 1023.5663, 1023.5700, 1031.5903, 1035.5381, 1036.5447, 1037.5573, 1039.5327, 1044.5751, 1045.5729, 1046.5897, 1051.5448, 1056.5658, 1057.6078, 1067.5753, 1073.5902, 1086.5509, 1092.5961, 1100.6071, 1106.5391, 1111.5615, 1113.5729, 1120.6045, 1122.5912, 1123.5848, 1125.5823, 1132.6047, 1134.5714, 1139.5746, 1143.6318, 1144.6497, 1145.6407, 1147.6393, 1150.6108, 1155.6215, 1157.6199, 1158.6685, 1162.6182, 1163.6152, 1168.6215, 1173.6418, 1175.6089, 1178.6356, 1179.6157, 1188.6544, 1199.7006, 1208.6415, 1216.6714, 1227.6595, 1231.6387, 1232.6447, 1234.6672, 1235.6445, 1244.6453, 1247.6211, 1255.6096, 1256.6531, 1261.6592, 1262.6453, 1266.6777, 1273.6260, 1275.6362, 1276.6343, 1277.6531, 1301.7079, 1303.7076, 1307.7042, 1308.6376, 1310.6146, 1312.6418, 1315.7098, 1320.6659, 1322.6886, 1323.6771, 1324.6964, 1328.7089, 1331.7202, 1335.7318, 1338.6581, 1339.6637, 1349.7253, 1352.7205, 1356.7230, 1360.6638, 1363.7153, 1365.7379, 1367.7319, 1369.7043, 1377.6947, 1379.7268, 1383.7137, 1385.7534, 1388.7404, 1390.7284, 1393.7428, 1407.7563, 1417.7010, 1421.7061, 1422.7019, 1425.7727, 1427.7742, 1429.7365, 1431.7412, 1433.7228, 1434.7446, 1437.7584, 1461.7778, 1467.7521, 1472.7633, 1475.7632, 1477.7545, 1484.7889, 1488.7537, 1491.7965, 1493.7579, 1500.7737, 1505.8177, 1513.8079, 1515.8213, 1517.7854, 1519.7544, 1522.7811, 1529.8099, 1530.7523, 1531.7472, 1537.7974, 1539.8005, 1542.8422, 1545.8091, 1547.8051, 1549.7339, 1553.8065, 1555.8101, 1561.8401, 1563.8109, 1565.8199, 1571.8400, 1579.7939, 1580.8077, 1583.8444, 1587.7620, 1592.8066, 1594.8335, 1604.8323, 1605.8586, 1608.8497, 1615.8754, 1624.8062, 1628.7900, 1628.7921, 1640.9384, 1642.8099, 1646.8297, 1649.8447, 1657.8101, 1667.8622, 1680.8746, 1689.9465, 1694.7288, 1698.9225, 1699.8862, 1704.8773, 1706.8701, 1707.8302, 1710.9279, 1716.8671, 1719.9058, 1723.9011, 1725.8300, 1725.8346, 1730.8931, 1737.9006, 1751.8656, 1754.8580, 1765.8463, 1766.8040, 1767.8138, 1769.8741, 1770.3567, 1772.9315, 1774.7610, 1774.9131, 1777.8770, 1780.8777, 1783.9246, 1787.8188, 1787.8200, 1791.8137, 1793.9050, 1795.9353, 1798.9340, 1800.9336, 1804.9142, 1805.9069, 1809.8469, 1815.9044, 1828.9227, 1837.9396, 1840.9827, 1843.9688, 1844.9012, 1846.9153, 1852.9364, 1856.9908, 1861.8881, 1867.9218, 1884.9417, 1891.9622, 1894.9313, 1896.9078, 1896.9100, 1900.9546, 1905.9313, 1908.9043, 1911.9130, 1912.9154, 1916.9923, 1921.9825, 1923.9718, 1925.9573, 1928.0469, 1932.0060, 1933.9950, 1937.9630, 1939.9740, 1944.0040, 1946.9679, 1949.9595, 1954.0236, 1955.9738, 1958.9556, 1968.8600, 1973.9845, 1976.9861, 1978.9910, 1981.9854, 1983.9884, 1987.9784, 1995.9408, 1998.9894, 2000.9926, 2008.0460, 2012.9590, 2015.0104, 2017.9747, 2021.0170, 2026.0275, 2027.0256, 2031.0166, 2044.0123, 2047.4957, 2047.9515, 2052.0496, 2060.0520, 2062.0073, 2064.9880, 2066.9873, 2094.0376, 2097.0454, 2102.0667, 2103.1641, 2111.0305, 2127.0930, 2139.0630, 2144.0493, 2155.0950, 2156.0796, 2160.0632, 2176.0664, 2190.9895, 2195.0774, 2199.0723, 2202.1624, 2203.1221, 2208.0862, 2211.1042, 2216.1062, 2225.1274, 2226.1272, 2229.0972, 2239.0225, 2242.0435, 2268.1489, 2270.1287, 2271.1294, 2277.0823, 2284.1990, 2292.1426, 2309.0994, 2334.1719, 2342.9915, 2350.1746, 2383.9607, 2398.2400, 2400.0862, 2413.0549, 2414.1897, 2417.0959, 2497.2800, 2501.2637, 2511.1460, 2513.1421, 2528.1750, 2537.2012, 2541.1948, 2542.1836, 2556.2058, 2562.1487, 2612.2666, 2706.2109, 2717.0869, 2767.2666, 2888.2532, 3221.1733, 3224.2917, 3312.3306, 3916.9434

3. P04272 **Mass:** 38873 **Score:** 52 **Expect:** 0.17 **Matches:** 29

Annexin A2 OS=Bos taurus GN=ANXA2 PE=1 SV=2

Observed	Mr(expt)	Mr(calc)	ppm	Start	End	Miss	Ions	Peptide
744.4171	743.4098	743.4178	-10.74	206	212	1	---	R.KGTDVPK.W
761.4177	760.4104	760.4191	-11.44	280	286	2	---	K.GKGTRDK.V
779.4308	778.4235	778.4337	-13.11	303	308	1	---	K.IRSEFK.K
1035.5381	1034.5308	1034.5219	8.64	213	220	0	---	K.WISIMTER.S
1036.5447	1035.5374	1035.5349	2.44	197	205	1	---	R.DLYDAGVKR.K
1051.5448	1050.5375	1050.5168	19.7	213	220	0	---	K.WISIMTER.S + Oxidation (M)
1086.5509	1085.5436	1085.5539	-9.49	2	10	0	---	M.STVHEILCK.L
1111.5615	1110.5542	1110.5458	7.59	69	77	0	---	R.QDIAFAYQR.R
1244.6453	1243.6380	1243.6156	18.0	136	145	0	---	R.TNQELQEINR.V
1339.6637	1338.6564	1338.6416	11.1	158	169	1	---	K.DIVSDTSGDFRK.L
1407.7563	1406.7490	1406.7261	16.3	291	302	1	---	R.IMVSRSEVDMLK.I
1421.7061	1420.6988	1420.6874	8.02	314	324	0	---	K.SLYYYIQQDTK.G
1542.8422	1541.8349	1541.8413	-4.15	50	63	0	---	K.GVDEVTIVNILTNR.S
1725.8300	1724.8227	1724.8230	-0.17	64	77	1	21	R.SNEQRQDIAFAYQR.R
1725.8346	1724.8273	1724.8230	2.50	64	77	1	---	R.SNEQRQDIAFAYQR.R
1769.8741	1768.8668	1768.8672	-0.20	311	324	1	---	K.YGKSLYYYIQQDTK.G
1777.8770	1776.8697	1776.8564	7.52	120	135	0	---	K.GLGTDEDSLIEIICSR.T
1844.9012	1843.8939	1843.8952	-0.69	11	28	0	---	K.LSLEGDHSTPPSAYGSVK.A
1867.9218	1866.9145	1866.9073	3.87	232	246	2	---	R.YKSYSPYDMLESIKK.E + Oxidation (M)
1908.9043	1907.8970	1907.8748	11.6	180	196	0	---	R.AEDGSVIDYELIDQDAR.D
1916.9923	1915.9850	1915.9601	13.0	234	249	2	---	K.SYSPYDMLESIKKEVK.G
1925.9573	1924.9500	1924.9378	6.35	153	169	2	---	K.TDLEKDIVSDTSGDFRK.L
2012.9590	2011.9517	2011.9527	-0.48	314	329	1	---	K.SLYYYIQQDTKGDYQK.A
2064.9880	2063.9807	2063.9760	2.31	179	196	1	---	R.RAEDGSVIDYELIDQDAR.D
2155.0950	2154.0877	2154.0593	13.2	29	47	1	---	K.AYTNFDAERDALNIETAIK.T
2195.0774	2194.0701	2194.0609	4.18	116	135	1	---	K.ASMKGLGTDEDSLIEIICSR.T
2211.1042	2210.0969	2210.0559	18.6	116	135	1	---	K.ASMKGLGTDEDSLIEIICSR.T + Oxidation (M)
2271.1294	2270.1221	2270.0929	12.9	228	245	2	---	K.VFERYKSYSPYDMLESIK.K + Oxidation (M)
2501.2637	2500.2564	2500.2567	-0.09	207	227	2	---	K.GTDVPKWISIMTERSVCHLQK.V + Oxidation (M)

No match to: 721.4365, 725.4001, 726.4063, 728.3681, 730.3848, 731.4017, 732.3573, 734.4151, 735.4143, 741.4101, 742.4515, 745.4167, 746.4169, 747.4125, 750.4153, 752.4127, 755.4100, 756.4199, 757.4313, 767.4266, 768.3700, 768.3737, 770.3966, 772.4093, 775.4307, 784.3688, 786.4000, 787.4221, 788.4210, 792.4376, 795.4350, 798.4351, 800.4296, 812.4388, 815.4332, 822.4482, 825.4540, 831.4382, 832.4557, 835.4719, 837.4463, 842.5060, 844.4760, 854.4837, 856.4911, 860.4691, 862.4852, 869.4231, 885.4465, 905.4955, 907.4991, 908.4929, 913.5700, 913.5747, 916.5284, 918.5050, 919.5053, 922.5070, 928.5117, 934.5172, 940.4840, 942.4854, 943.4289, 943.5453, 946.5259, 950.4959, 951.5076, 953.5164, 954.5388, 959.5266, 966.5410, 969.5256, 972.5305, 974.5395, 976.5310, 978.5314, 979.5442, 991.5409, 993.5307, 1001.5854, 1003.5496, 1011.5262, 1014.5215, 1021.5579, 1023.5663, 1023.5700, 1031.5903, 1037.5573,

1039.5327, 1041.5658, 1044.5751, 1045.5729, 1046.5897, 1056.5658, 1057.6078, 1067.5753, 1073.5902, 1092.5961, 1100.6071, 1106.5391, 1113.5729, 1118.5818, 1120.6045, 1122.5912, 1123.5848, 1125.5823, 1132.6047, 1134.5714, 1139.5746, 1143.6318, 1144.6497, 1145.6407, 1147.6393, 1150.6108, 1155.6215, 1157.6199, 1158.6685, 1162.6182, 1163.6152, 1168.6215, 1173.6418, 1175.6089, 1178.6356, 1179.6157, 1181.6819, 1188.6544, 1197.6704, 1199.7006, 1208.6415, 1216.6714, 1227.6595, 1231.6387, 1232.6447, 1234.6672, 1235.6445, 1247.6211, 1248.6174, 1250.6554, 1255.6096, 1256.6531, 1261.6592, 1262.6453, 1266.6777, 1273.6260, 1275.6362, 1276.6343, 1277.6531, 1301.7079, 1303.7076, 1307.7042, 1308.6376, 1310.6146, 1312.6418, 1315.7098, 1320.6659, 1322.6886, 1323.6771, 1324.6964, 1328.7089, 1331.7202, 1335.7318, 1338.6581, 1349.7253, 1352.7205, 1356.7230, 1360.6638, 1363.7153, 1365.7379, 1367.7319, 1369.7043, 1377.6947, 1379.7268, 1383.7137, 1385.7534, 1388.7404, 1390.7284, 1393.7428, 1417.7010, 1422.7019, 1425.7727, 1427.7742, 1429.7365, 1431.7412, 1433.7228, 1434.7446, 1437.7584, 1461.7778, 1467.7521, 1472.7633, 1475.7632, 1477.7545, 1484.7889, 1488.7537, 1491.7965, 1493.7579, 1500.7737, 1505.8177, 1513.8079, 1515.8213, 1517.7854, 1519.7544, 1522.7811, 1523.7953, 1529.8099, 1530.7523, 1531.7472, 1537.7974, 1539.8005, 1545.8091, 1547.8051, 1549.7339, 1551.7778, 1553.8065, 1555.8101, 1556.8109, 1557.8010, 1561.8401, 1563.8109, 1565.8199, 1571.8400, 1579.7939, 1580.8077, 1583.8444, 1587.7620, 1592.8066, 1594.8335, 1604.8323, 1605.8586, 1608.8497, 1615.8754, 1624.8062, 1628.7900, 1628.7921, 1633.7985, 1640.9384, 1642.8099, 1646.8297, 1649.8447, 1657.8101, 1667.8622, 1677.7988, 1680.8746, 1689.9465, 1694.7288, 1698.9225, 1699.8862, 1704.8773, 1706.8701, 1707.8302, 1710.9279, 1716.8671, 1719.9058, 1723.9011, 1727.8986, 1730.8931, 1737.9006, 1751.8656, 1754.8580, 1765.8463, 1766.8040, 1767.8138, 1770.3567, 1772.9315, 1774.7610, 1774.9131, 1780.8777, 1783.9246, 1787.8188, 1787.8200, 1791.8137, 1793.9050, 1795.9353, 1798.9340, 1800.9336, 1804.9142, 1805.9069, 1809.8469, 1815.9044, 1821.9144, 1828.9227, 1837.9396, 1840.9827, 1843.9688, 1846.9153, 1852.9364, 1856.9908, 1861.8881, 1874.9695, 1880.9265, 1884.9417, 1891.9622, 1894.9313, 1896.9078, 1896.9100, 1900.9546, 1905.9313, 1906.9501, 1911.9130, 1912.9154, 1918.9587, 1921.9825, 1923.9718, 1928.0469, 1932.0060, 1933.9950, 1937.9630, 1939.9740, 1944.0040, 1946.9679, 1949.9595, 1954.0236, 1955.9738, 1958.9556, 1960.0034, 1968.8600, 1973.9845, 1976.9861, 1978.9910, 1981.9854, 1983.9884, 1987.9784, 1995.9408, 1998.9894, 2000.9926, 2008.0460, 2015.0104, 2017.9747, 2021.0170, 2026.0275, 2027.0256, 2031.0166, 2044.0123, 2047.4957, 2047.9515, 2052.0496, 2060.0520, 2062.0073, 2066.9873, 2094.0376, 2097.0454, 2102.0667, 2103.1641, 2111.0305, 2127.0930, 2139.0630, 2144.0493, 2148.1023, 2156.0796, 2160.0632, 2176.0664, 2190.9895, 2199.0723, 2202.1624, 2203.1221, 2208.0862, 2216.1062, 2225.1274, 2226.1272, 2229.0972, 2233.0984, 2239.0225, 2242.0435, 2268.1489, 2270.1287, 2277.0823, 2284.1990, 2292.1426, 2309.0994, 2334.1719, 2342.9915, 2350.1746, 2383.9607, 2398.2400, 2400.0862, 2413.0549, 2414.1897, 2417.0959, 2497.2800, 2511.1460, 2513.1421, 2528.1750, 2537.2012, 2541.1948, 2542.1836, 2556.2058, 2562.1487, 2612.2666, 2706.2109, 2717.0869, 2767.2666, 2888.2532, 3221.1733, 3224.2917, 3312.3306, 3916.9434

4. F1MK19 **Mass:** 26274 **Score:** 42 **Expect:** 1.7 **Matches:** 13

L-lactate dehydrogenase OS=Bos taurus GN=LDHC PE=3 SV=1

Observed	Mr(expt)	Mr(calc)	ppm	Start	End	Miss	Ions	Peptide
721.4365	720.4292	720.4170	17.0	172 -	177	0	---	R.YLIGQK.L
742.4515	741.4442	741.4497	-7.42	107 -	112	0	---	R.LNLVQR.N
913.5700	912.5627	912.5756	-14.15	91 -	99	0	22	K.LVIITAGAR.Q
913.5747	912.5674	912.5756	-9.06	91 -	99	0	---	K.LVIITAGAR.Q
1248.6174	1247.6101	1247.5928	13.9	158 -	169	0	---	R.VIGSGCNLDSAR.F
1519.7544	1518.7471	1518.7725	-16.71	229 -	241	2	---	K.NIHKEVVGRAHME.-
1551.7778	1550.7705	1550.7624	5.26	158 -	171	1	---	R.VIGSGCNLDSARFR.Y
1556.8109	1555.8036	1555.8066	-1.94	100 -	112	1	---	R.QQEGESRLNLVQR.N
1615.8754	1614.8681	1614.8352	20.4	43 -	57	0	---	K.DLADELALVDVVTDK.L
1727.8986	1726.8913	1726.9326	-23.88	91 -	106	1	---	K.LVIITAGARQQEGESR.L
1777.8770	1776.8697	1776.8530	9.41	213 -	228	2	---	K.SLDPKLGSDSDKDSWK.N
1856.9908	1855.9835	1856.0143	-16.56	43 -	59	1	---	K.DLADELALVDVVTDKLK.G
2044.0123	2043.0050	2043.0531	-23.54	150 -	169	1	---	K.LSGLPATRVIGSGCNLDSAR.F

No match to: 725.4001, 726.4063, 728.3681, 730.3848, 731.4017, 732.3573, 734.4151, 735.4143, 741.4101, 744.4171, 745.4167, 746.4169, 747.4125, 750.4153, 752.4127, 755.4100, 756.4199, 757.4313, 761.4177, 767.4266, 768.3700, 768.3737, 770.3966, 772.4093, 775.4307, 779.4308, 784.3688, 786.4000, 787.4221, 788.4210, 792.4376, 795.4350, 798.4351, 800.4296, 812.4388, 815.4332, 822.4482, 825.4540, 831.4382, 832.4557, 835.4719, 837.4463, 842.5060, 844.4760, 854.4837, 856.4911, 860.4691, 862.4852, 869.4231, 885.4465, 905.4955, 907.4991, 908.4929, 916.5284, 918.5050, 919.5053, 922.5070, 928.5117, 934.5172, 940.4840, 942.4854, 943.4289, 943.5453, 946.5259, 950.4959, 951.5076, 953.5164, 954.5388, 959.5266, 966.5410, 969.5256, 972.5305, 974.5395, 976.5310, 978.5314, 979.5442, 991.5409, 993.5307, 1001.5854, 1003.5496, 1011.5262, 1014.5215, 1021.5579, 1023.5663, 1023.5700, 1031.5903, 1035.5381, 1036.5447, 1037.5573, 1039.5327, 1041.5658, 1044.5751, 1045.5729, 1046.5897, 1051.5448, 1056.5658, 1057.6078, 1067.5753, 1073.5902, 1086.5509, 1092.5961, 1100.6071, 1106.5391, 1111.5615, 1113.5729, 1118.5818, 1120.6045, 1122.5912, 1123.5848, 1125.5823, 1132.6047, 1134.5714, 1139.5746, 1143.6318, 1144.6497, 1145.6407, 1147.6393, 1150.6108, 1155.6215, 1157.6199, 1158.6685, 1162.6182, 1163.6152, 1168.6215, 1173.6418, 1175.6089, 1178.6356, 1179.6157, 1181.6819, 1188.6544, 1197.6704, 1199.7006, 1208.6415, 1216.6714, 1227.6595, 1231.6387, 1232.6447, 1234.6672, 1235.6445, 1244.6453, 1247.6211, 1250.6554, 1255.6096, 1256.6531, 1261.6592, 1262.6453, 1266.6777, 1273.6260, 1275.6362, 1276.6343, 1277.6531, 1301.7079, 1303.7076, 1307.7042, 1308.6376, 1310.6146, 1312.6418, 1315.7098, 1320.6659, 1322.6886, 1323.6771, 1324.6964, 1328.7089, 1331.7202, 1335.7318, 1338.6581, 1339.6637, 1349.7253, 1352.7205, 1356.7230, 1360.6638, 1363.7153, 1365.7379, 1367.7319, 1369.7043, 1377.6947, 1379.7268, 1383.7137, 1385.7534, 1388.7404, 1390.7284, 1393.7428, 1407.7563, 1417.7010, 1421.7061, 1422.7019, 1425.7727, 1427.7742, 1429.7365, 1431.7412, 1433.7228, 1434.7446, 1437.7584, 1461.7778, 1467.7521, 1472.7633, 1475.7632, 1477.7545, 1484.7889, 1488.7537, 1491.7965, 1493.7579, 1500.7737, 1505.8177, 1513.8079, 1515.8213, 1517.7854, 1522.7811, 1523.7953, 1529.8099, 1530.7523, 1531.7472, 1537.7974, 1539.8005, 1542.8422, 1545.8091, 1547.8051, 1549.7339, 1553.8065, 1555.8101, 1557.8010, 1561.8401, 1563.8109, 1565.8199, 1571.8400, 1579.7939, 1580.8077, 1583.8444, 1587.7620, 1592.8066, 1594.8335, 1604.8323, 1605.8586, 1608.8497, 1624.8062, 1628.7900, 1628.7921, 1633.7985, 1640.9384, 1642.8099, 1646.8297, 1649.8447, 1657.8101, 1667.8622, 1677.7988, 1680.8746, 1689.9465, 1694.7288, 1698.9225, 1699.8862, 1704.8773, 1706.8701, 1707.8302, 1710.9279, 1716.8671, 1719.9058, 1723.9011, 1725.8300, 1725.8346, 1730.8931, 1737.9006, 1751.8656, 1754.8580, 1765.8463, 1766.8040, 1767.8138, 1769.8741, 1770.3567, 1772.9315, 1774.7610, 1774.9131, 1780.8777, 1783.9246, 1787.8188, 1787.8200, 1791.8137, 1793.9050, 1795.9353, 1798.9340, 1800.9336, 1804.9142, 1805.9069, 1809.8469, 1815.9044, 1821.9144, 1828.9227, 1837.9396, 1840.9827, 1843.9688, 1844.9012, 1846.9153, 1852.9364, 1861.8881, 1867.9218, 1874.9695, 1880.9265, 1884.9417, 1891.9622, 1894.9313, 1896.9078, 1896.9100, 1900.9546, 1905.9313, 1906.9501, 1908.9043, 1911.9130, 1912.9154, 1916.9923, 1918.9587, 1921.9825, 1923.9718, 1925.9573, 1928.0469, 1932.0060, 1933.9950, 1937.9630, 1939.9740, 1944.0040, 1946.9679, 1949.9595, 1954.0236, 1955.9738, 1958.9556, 1960.0034, 1968.8600, 1973.9845, 1976.9861, 1978.9910, 1981.9854, 1983.9884, 1987.9784, 1995.9408, 1998.9894, 2000.9926, 2008.0460, 2012.9590, 2015.0104, 2017.9747, 2021.0170, 2026.0275, 2027.0256, 2031.0166, 2047.4957, 2047.9515, 2052.0496, 2060.0520, 2062.0073, 2064.9880, 2066.9873, 2094.0376, 2097.0454, 2102.0667, 2103.1641, 2111.0305, 2127.0930, 2139.0630, 2144.0493, 2148.1023, 2155.0950, 2156.0796, 2160.0632, 2176.0664, 2190.9895, 2195.0774, 2199.0723, 2202.1624, 2203.1221, 2208.0862, 2211.1042, 2216.1062, 2225.1274, 2226.1272, 2229.0972, 2233.0984, 2239.0225, 2242.0435, 2268.1489, 2270.1287, 2271.1294, 2277.0823, 2284.1990, 2292.1426, 2309.0994, 2334.1719, 2342.9915, 2350.1746, 2383.9607, 2398.2400, 2400.0862, 2413.0549, 2414.1897, 2417.0959, 2497.2800, 2501.2637, 2511.1460, 2513.1421, 2528.1750, 2537.2012, 2541.1948, 2542.1836, 2556.2058, 2562.1487, 2612.2666, 2706.2109, 2717.0869, 2767.2666, 2888.2532, 3221.1733, 3224.2917, 3312.3306, 3916.9434

5. E1BNS9 **Mass:** 36334 **Score:** 34 **Expect:** 9.6 **Matches:** 13

L-lactate dehydrogenase OS=Bos taurus GN=LDHC PE=3 SV=2

Observed	Mr(expt)	Mr(calc)	ppm	Start	End	Miss	Ions	Peptide
721.4365	720.4292	720.4170	17.0	172 -	177	0	---	R.YLIGQK.L
742.4515	741.4442	741.4497	-7.42	107 -	112	0	---	R.LNLVQR.N
913.5700	912.5627	912.5756	-14.15	91 -	99	0	22	K.LVIITAGAR.Q
913.5747	912.5674	912.5756	-9.06	91 -	99	0	---	K.LVIITAGAR.Q
1248.6174	1247.6101	1247.5928	13.9	158 -	169	0	---	R.VIGSGCNLDSAR.F
1261.6592	1260.6519	1260.6826	-24.35	318 -	328	1	---	K.KSASTLWNVQK.D
1551.7778	1550.7705	1550.7624	5.26	158 -	171	1	---	R.VIGSGCNLDSARFR.Y
1556.8109	1555.8036	1555.8066	-1.94	100 -	112	1	---	R.QQEGESRLNLVQR.N
1615.8754	1614.8681	1614.8352	20.4	43 -	57	0	---	K.DLADELALVDVVTDK.L
1727.8986	1726.8913	1726.9326	-23.88	91 -	106	1	---	K.LVIITAGARQQEGESR.L
1777.8770	1776.8697	1776.8530	9.41	213 -	228	2	---	K.SLDPKLGSDSDKDSWK.N
1856.9908	1855.9835	1856.0143	-16.56	43 -	59	1	---	K.DLADELALVDVVTDKLK.G
2044.0123	2043.0050	2043.0531	-23.54	150 -	169	1	---	K.LSGLPATRVIGSGCNLDSAR.F

No match to: 725.4001, 726.4063, 728.3681, 730.3848, 731.4017, 732.3573, 734.4151, 735.4143, 741.4101, 744.4171, 745.4167, 746.4169, 747.4125, 750.4153, 752.4127, 755.4100, 756.4199, 757.4313, 761.4177, 767.4266, 768.3700, 768.3737, 770.3966, 772.4093, 775.4307, 779.4308, 784.3688, 786.4000, 787.4221, 788.4210, 792.4376, 795.4350, 798.4351, 800.4296, 812.4388, 815.4332, 822.4482, 825.4540, 831.4382, 832.4557, 835.4719, 837.4463, 842.5060, 844.4760, 854.4837, 856.4911, 860.4691, 862.4852, 869.4231, 885.4465, 905.4955, 907.4991, 908.4929, 916.5284, 918.5050, 919.5053, 922.5070, 928.5117, 934.5172, 940.4840, 942.4854, 943.4289, 943.5453, 946.5259, 950.4959, 951.5076, 953.5164, 954.5388, 959.5266, 966.5410, 969.5256, 972.5305, 974.5395, 976.5310, 978.5314, 979.5442, 991.5409, 993.5307, 1001.5854, 1003.5496, 1011.5262, 1014.5215, 1021.5579, 1023.5663, 1023.5700, 1031.5903, 1035.5381, 1036.5447, 1037.5573, 1039.5327, 1041.5658, 1044.5751, 1045.5729, 1046.5897, 1051.5448, 1056.5658, 1057.6078, 1067.5753, 1073.5902, 1086.5509, 1092.5961, 1100.6071, 1106.5391, 1111.5615, 1113.5729, 1118.5818, 1120.6045, 1122.5912, 1123.5848, 1125.5823, 1132.6047, 1134.5714, 1139.5746, 1143.6318, 1144.6497, 1145.6407, 1147.6393, 1150.6108, 1155.6215, 1157.6199, 1158.6685, 1162.6182, 1163.6152, 1168.6215, 1173.6418, 1175.6089, 1178.6356, 1179.6157, 1181.6819, 1188.6544, 1197.6704, 1199.7006, 1208.6415, 1216.6714, 1227.6595, 1231.6387, 1232.6447, 1234.6672, 1235.6445, 1244.6453, 1247.6211, 1250.6554, 1255.6096, 1256.6531, 1262.6453, 1266.6777, 1273.6260, 1275.6362, 1276.6343, 1277.6531, 1301.7079, 1303.7076, 1307.7042, 1308.6376, 1310.6146, 1312.6418, 1315.7098, 1320.6659, 1322.6886, 1323.6771, 1324.6964, 1328.7089, 1331.7202, 1335.7318, 1338.6581, 1339.6637, 1349.7253, 1352.7205, 1356.7230, 1360.6638, 1363.7153, 1365.7379, 1367.7319, 1369.7043, 1377.6947, 1379.7268, 1383.7137, 1385.7534, 1388.7404, 1390.7284, 1393.7428, 1407.7563, 1417.7010, 1421.7061, 1422.7019, 1425.7727, 1427.7742, 1429.7365, 1431.7412, 1433.7228, 1434.7446, 1437.7584, 1461.7778, 1467.7521, 1472.7633, 1475.7632, 1477.7545, 1484.7889, 1488.7537, 1491.7965, 1493.7579, 1500.7737, 1505.8177, 1513.8079, 1515.8213, 1517.7854, 1519.7544, 1522.7811, 1523.7953, 1529.8099, 1530.7523, 1531.7472, 1537.7974, 1539.8005, 1542.8422, 1545.8091, 1547.8051, 1549.7339, 1553.8065, 1555.8101, 1557.8010, 1561.8401, 1563.8109, 1565.8199, 1571.8400, 1579.7939, 1580.8077, 1583.8444, 1587.7620, 1592.8066, 1594.8335, 1604.8323, 1605.8586, 1608.8497, 1624.8062, 1628.7900, 1628.7921, 1633.7985, 1640.9384, 1642.8099, 1646.8297, 1649.8447, 1657.8101, 1667.8622, 1677.7988, 1680.8746, 1689.9465, 1694.7288, 1698.9225, 1699.8862, 1704.8773, 1706.8701, 1707.8302, 1710.9279, 1716.8671, 1719.9058, 1723.9011, 1725.8300, 1725.8346, 1730.8931, 1737.9006, 1751.8656, 1754.8580, 1765.8463, 1766.8040, 1767.8138, 1769.8741, 1770.3567, 1772.9315, 1774.7610, 1774.9131, 1780.8777, 1783.9246, 1787.8188, 1787.8200, 1791.8137, 1793.9050, 1795.9353, 1798.9340, 1800.9336, 1804.9142, 1805.9069, 1809.8469, 1815.9044, 1821.9144, 1828.9227, 1837.9396, 1840.9827, 1843.9688, 1844.9012, 1846.9153, 1852.9364, 1861.8881, 1867.9218, 1874.9695, 1880.9265, 1884.9417, 1891.9622, 1894.9313, 1896.9078, 1896.9100, 1900.9546, 1905.9313, 1906.9501, 1908.9043, 1911.9130, 1912.9154, 1916.9923, 1918.9587, 1921.9825, 1923.9718, 1925.9573, 1928.0469, 1932.0060, 1933.9950, 1937.9630, 1939.9740, 1944.0040, 1946.9679, 1949.9595, 1954.0236, 1955.9738, 1958.9556, 1960.0034, 1968.8600, 1973.9845, 1976.9861, 1978.9910, 1981.9854, 1983.9884, 1987.9784, 1995.9408, 1998.9894, 2000.9926, 2008.0460, 2012.9590, 2015.0104, 2017.9747, 2021.0170, 2026.0275, 2027.0256, 2031.0166, 2047.4957, 2047.9515, 2052.0496, 2060.0520, 2062.0073, 2064.9880, 2066.9873, 2094.0376, 2097.0454, 2102.0667, 2103.1641, 2111.0305, 2127.0930, 2139.0630, 2144.0493, 2148.1023, 2155.0950, 2156.0796, 2160.0632, 2176.0664, 2190.9895, 2195.0774, 2199.0723, 2202.1624, 2203.1221, 2208.0862, 2211.1042, 2216.1062, 2225.1274, 2226.1272, 2229.0972, 2233.0984, 2239.0225, 2242.0435, 2268.1489, 2270.1287, 2271.1294, 2277.0823, 2284.1990, 2292.1426, 2309.0994, 2334.1719, 2342.9915, 2350.1746, 2383.9607, 2398.2400, 2400.0862, 2413.0549, 2414.1897, 2417.0959, 2497.2800, 2501.2637, 2511.1460, 2513.1421, 2528.1750, 2537.2012, 2541.1948, 2542.1836, 2556.2058, 2562.1487, 2612.2666, 2706.2109, 2717.0869, 2767.2666, 2888.2532, 3221.1733, 3224.2917, 3312.3306, 3916.9434

6. E1BFB5 **Mass:** 68315 **Score:** 33 **Expect:** 13 **Matches:** 21

Uncharacterized protein OS=Bos taurus GN=KIAA0391 PE=4 SV=2

Observed	Mr(expt)	Mr(calc)	ppm	Start	End	Miss	Ions	Peptide
837.4463	836.4390	836.4392	-0.23	220	226	0	---	R.GLIYTDR.W
972.5305	971.5232	971.5400	-17.22	387	394	1	---	K.TTPQELKR.F
1023.5663	1022.5590	1022.5583	0.73	496	503	1	---	K.FITKDLMR.D
1023.5700	1022.5627	1022.5583	4.35	496	503	1	20	K.FITKDLMR.D
1113.5729	1112.5656	1112.5913	-23.09	577	584	2	---	K.WLCLHRKT.-
1139.5746	1138.5673	1138.5441	20.4	42	50	1	---	R.NQEKCFSVK.T
1162.6182	1161.6109	1161.5924	15.9	96	105	2	---	K.KRSQMNPSSK.D
1179.6157	1178.6084	1178.6196	-9.51	220	228	1	---	R.GLIYTDRWR.E
1425.7727	1424.7654	1424.7446	14.6	208	219	1	---	K.RLESGAYTLLMR.G + Oxidation (M)
1472.7633	1471.7560	1471.7466	6.38	447	457	2	---	R.KHMLTQNSRWR.R + Oxidation (M)
1484.7889	1483.7816	1483.7579	16.0	448	458	2	---	K.HMLTQNSRWRR.V
1500.7737	1499.7664	1499.7528	9.10	448	458	2	---	K.HMLTQNSRWRR.V + Oxidation (M)
1539.8005	1538.7932	1538.7623	20.1	504	516	2	---	R.DHKACLPDAKTQR.L
1594.8335	1593.8262	1593.8014	15.6	1	13	1	---	-.MTFYFTGIRSFPK.L
1605.8586	1604.8513	1604.8385	8.02	139	151	0	---	K.ANFENWIILQMVK.C
1783.9246	1782.9173	1782.9305	-7.38	401	416	0	---	K.YHPPFDVVIDGLNVAK.M
1795.9353	1794.9280	1794.9185	5.28	191	205	1	---	K.QTSEIIDVYEIMKAR.Y
1861.8881	1860.8808	1860.9264	-24.51	80	97	2	---	K.QVSADSSHMFAAGAAKKR.S
1928.0469	1927.0396	1926.9986	21.3	152	169	1	---	K.CHSSVDVAKSVLAWVAAK.N
2000.9926	1999.9853	1999.9455	19.9	42	58	2	---	R.NQEKCFSVKTMSAQNTK.A
2501.2637	2500.2564	2500.3188	-24.96	170	190	1	---	K.NSGIVGYDLLVKYLYLCVFHK.Q

No match to: 721.4365, 725.4001, 726.4063, 728.3681, 730.3848, 731.4017, 732.3573, 734.4151, 735.4143, 741.4101, 742.4515, 744.4171, 745.4167, 746.4169, 747.4125, 750.4153, 752.4127, 755.4100, 756.4199, 757.4313, 761.4177, 767.4266, 768.3700, 768.3737, 770.3966, 772.4093, 775.4307, 779.4308, 784.3688, 786.4000, 787.4221, 788.4210, 792.4376, 795.4350, 798.4351, 800.4296, 812.4388, 815.4332, 822.4482, 825.4540, 831.4382, 832.4557, 835.4719, 842.5060, 844.4760, 854.4837, 856.4911, 860.4691, 862.4852, 869.4231, 885.4465, 905.4955, 907.4991, 908.4929, 913.5700, 913.5747, 916.5284, 918.5050, 919.5053, 922.5070, 928.5117, 934.5172, 940.4840, 942.4854, 943.4289, 943.5453, 946.5259, 950.4959, 951.5076, 953.5164, 954.5388, 959.5266, 966.5410, 969.5256, 974.5395, 976.5310, 978.5314, 979.5442, 991.5409, 993.5307, 1001.5854, 1003.5496, 1011.5262, 1014.5215, 1021.5579, 1031.5903, 1035.5381, 1036.5447, 1037.5573, 1039.5327, 1041.5658, 1044.5751, 1045.5729, 1046.5897, 1051.5448, 1056.5658, 1057.6078, 1067.5753, 1073.5902, 1086.5509, 1092.5961, 1100.6071, 1106.5391, 1111.5615, 1118.5818, 1120.6045, 1122.5912, 1123.5848, 1125.5823, 1132.6047, 1134.5714, 1143.6318, 1144.6497, 1145.6407, 1147.6393, 1150.6108, 1155.6215, 1157.6199, 1158.6685, 1163.6152, 1168.6215, 1173.6418, 1175.6089, 1178.6356, 1181.6819, 1188.6544, 1197.6704, 1199.7006, 1208.6415, 1216.6714, 1227.6595, 1231.6387, 1232.6447, 1234.6672, 1235.6445, 1244.6453, 1247.6211, 1248.6174, 1250.6554, 1255.6096, 1256.6531, 1261.6592, 1262.6453, 1266.6777, 1273.6260, 1275.6362, 1276.6343, 1277.6531, 1301.7079, 1303.7076, 1307.7042, 1308.6376, 1310.6146, 1312.6418, 1315.7098, 1320.6659, 1322.6886, 1323.6771, 1324.6964, 1328.7089, 1331.7202, 1335.7318, 1338.6581, 1339.6637, 1349.7253, 1352.7205, 1356.7230, 1360.6638, 1363.7153, 1365.7379, 1367.7319, 1369.7043, 1377.6947, 1379.7268, 1383.7137, 1385.7534, 1388.7404, 1390.7284, 1393.7428, 1407.7563, 1417.7010, 1421.7061, 1422.7019, 1427.7742, 1429.7365, 1431.7412, 1433.7228, 1434.7446, 1437.7584, 1461.7778, 1467.7521, 1475.7632, 1477.7545, 1488.7537, 1491.7965, 1493.7579, 1505.8177, 1513.8079, 1515.8213, 1517.7854, 1519.7544, 1522.7811, 1523.7953, 1529.8099, 1530.7523, 1531.7472, 1537.7974, 1542.8422, 1545.8091, 1547.8051, 1549.7339, 1551.7778, 1553.8065, 1555.8101, 1556.8109, 1557.8010, 1561.8401, 1563.8109, 1565.8199, 1571.8400, 1579.7939, 1580.8077, 1583.8444, 1587.7620, 1592.8066, 1604.8323, 1608.8497, 1615.8754, 1624.8062, 1628.7900, 1628.7921, 1633.7985, 1640.9384, 1642.8099, 1646.8297, 1649.8447, 1657.8101, 1667.8622, 1677.7988, 1680.8746, 1689.9465, 1694.7288, 1698.9225, 1699.8862, 1704.8773, 1706.8701, 1707.8302, 1710.9279, 1716.8671, 1719.9058, 1723.9011, 1725.8300, 1725.8346, 1727.8986, 1730.8931, 1737.9006, 1751.8656, 1754.8580, 1765.8463, 1766.8040, 1767.8138, 1769.8741, 1770.3567, 1772.9315, 1774.7610, 1774.9131, 1777.8770, 1780.8777, 1787.8188, 1787.8200, 1791.8137, 1793.9050, 1798.9340, 1800.9336, 1804.9142, 1805.9069, 1809.8469, 1815.9044, 1821.9144, 1828.9227, 1837.9396, 1840.9827, 1843.9688, 1844.9012, 1846.9153, 1852.9364, 1856.9908, 1867.9218, 1874.9695, 1880.9265, 1884.9417, 1891.9622, 1894.9313, 1896.9078, 1896.9100, 1900.9546, 1905.9313, 1906.9501, 1908.9043, 1911.9130, 1912.9154, 1916.9923, 1918.9587, 1921.9825, 1923.9718, 1925.9573, 1932.0060, 1933.9950, 1937.9630, 1939.9740, 1944.0040, 1946.9679, 1949.9595, 1954.0236, 1955.9738, 1958.9556, 1960.0034, 1968.8600, 1973.9845, 1976.9861, 1978.9910, 1981.9854, 1983.9884, 1987.9784, 1995.9408, 1998.9894, 2008.0460, 2012.9590, 2015.0104, 2017.9747, 2021.0170, 2026.0275, 2027.0256, 2031.0166, 2044.0123, 2047.4957, 2047.9515, 2052.0496, 2060.0520, 2062.0073, 2064.9880, 2066.9873, 2094.0376, 2097.0454, 2102.0667, 2103.1641, 2111.0305, 2127.0930, 2139.0630, 2144.0493, 2148.1023, 2155.0950, 2156.0796, 2160.0632, 2176.0664, 2190.9895, 2195.0774, 2199.0723, 2202.1624, 2203.1221, 2208.0862, 2211.1042, 2216.1062, 2225.1274, 2226.1272, 2229.0972, 2233.0984, 2239.0225, 2242.0435, 2268.1489, 2270.1287, 2271.1294, 2277.0823, 2284.1990, 2292.1426, 2309.0994, 2334.1719, 2342.9915, 2350.1746, 2383.9607, 2398.2400, 2400.0862, 2413.0549, 2414.1897, 2417.0959, 2497.2800, 2511.1460, 2513.1421, 2528.1750, 2537.2012, 2541.1948, 2542.1836, 2556.2058, 2562.1487, 2612.2666, 2706.2109, 2717.0869, 2767.2666, 2888.2532, 3221.1733, 3224.2917, 3312.3306, 3916.9434

7. P09867 **Mass:** 34289 **Score:** 31 **Expect:** 20 **Matches:** 18

Heterogeneous nuclear ribonucleoprotein A1 OS=Bos taurus GN=HNRNPA1 PE=1 SV=2

Observed	Mr(expt)	Mr(calc)	ppm	Start	End	Miss	Ions	Peptide
730.3848	729.3775	729.3769	0.76	48	53	1	---	R.DPNTKR.S
1312.6418	1311.6345	1311.6069	21.1	219	232	1	---	R.GGNFSGRGGFGGSR.G
1427.7742	1426.7669	1426.7416	17.8	4	15	2	---	K.SESPKEPEQLRK.L
1437.7584	1436.7511	1436.7372	9.70	93	105	0	---	R.EDSQRPGAHLTVK.K
1547.8051	1546.7978	1546.8025	-3.01	131	144	1	---	K.IEVIEIMTDRGSGK.K
1563.8109	1562.8036	1562.7974	3.99	131	144	1	---	K.IEVIEIMTDRGSGK.K + Oxidation (M)
1565.8199	1564.8126	1564.8321	-12.47	93	106	1	---	R.EDSQRPGAHLTVKK.I
1580.8077	1579.8004	1579.7624	24.1	180	194	1	---	K.ALSKQEMASASSSQR.G
1628.7900	1627.7827	1627.7743	5.17	285	300	0	13	R.SSGPYGGGGQYFAKPR.N
1628.7921	1627.7848	1627.7743	6.46	285	300	0	---	R.SSGPYGGGGQYFAKPR.N
1694.7288	1693.7215	1693.6928	16.9	301	318	0	---	R.NQGGYGGSSSSSSYGSGR.R
1793.9050	1792.8977	1792.8849	7.13	180	196	2	---	K.ALSKQEMASASSSQRGR.S
1809.8469	1808.8396	1808.8799	-22.25	180	196	2	---	K.ALSKQEMASASSSQRGR.S + Oxidation (M)
1983.9884	1982.9811	1982.9487	16.4	145	161	2	---	K.KRGFAFVTFDDHDSVDK.I
2008.0460	2007.0387	2007.0538	-7.49	106	122	2	---	K.KIFVGGIKEDTEEHHLR.D
2012.9590	2011.9517	2012.0010	-24.49	167	183	2	---	K.YHTVNGHNCEVRKALSK.Q
2537.2012	2536.1939	2536.1475	18.3	32	52	1	---	R.SHFEQWGTLTDCVVMRDPNTK.R + Oxidation (M)
2706.2109	2705.2036	2705.1828	7.68	197	225	2	---	R.SGSGNFGGGRGGGFGGNDNFGRGGNFSGR.G

No match to: 721.4365, 725.4001, 726.4063, 728.3681, 731.4017, 732.3573, 734.4151, 735.4143, 741.4101, 742.4515, 744.4171, 745.4167, 746.4169, 747.4125, 750.4153, 752.4127, 755.4100, 756.4199, 757.4313, 761.4177, 767.4266, 768.3700, 768.3737, 770.3966, 772.4093, 775.4307, 779.4308, 784.3688, 786.4000, 787.4221, 788.4210, 792.4376, 795.4350, 798.4351, 800.4296, 812.4388, 815.4332, 822.4482, 825.4540, 831.4382, 832.4557, 835.4719, 837.4463, 842.5060, 844.4760, 854.4837, 856.4911, 860.4691, 862.4852, 869.4231, 885.4465, 905.4955, 907.4991, 908.4929, 913.5700, 913.5747, 916.5284, 918.5050, 919.5053, 922.5070, 928.5117, 934.5172, 940.4840, 942.4854, 943.4289, 943.5453, 946.5259, 950.4959, 951.5076, 953.5164, 954.5388, 959.5266, 966.5410, 969.5256, 972.5305, 974.5395, 976.5310, 978.5314, 979.5442, 991.5409, 993.5307, 1001.5854, 1003.5496, 1011.5262, 1014.5215, 1021.5579, 1023.5663, 1023.5700, 1031.5903, 1035.5381, 1036.5447, 1037.5573, 1039.5327, 1041.5658, 1044.5751, 1045.5729, 1046.5897, 1051.5448, 1056.5658, 1057.6078, 1067.5753, 1073.5902, 1086.5509, 1092.5961, 1100.6071, 1106.5391, 1111.5615, 1113.5729, 1118.5818, 1120.6045, 1122.5912, 1123.5848, 1125.5823, 1132.6047, 1134.5714, 1139.5746, 1143.6318, 1144.6497, 1145.6407, 1147.6393, 1150.6108, 1155.6215, 1157.6199, 1158.6685, 1162.6182, 1163.6152, 1168.6215, 1173.6418, 1175.6089, 1178.6356, 1179.6157, 1181.6819, 1188.6544, 1197.6704, 1199.7006, 1208.6415, 1216.6714, 1227.6595, 1231.6387, 1232.6447, 1234.6672, 1235.6445, 1244.6453, 1247.6211, 1248.6174, 1250.6554, 1255.6096, 1256.6531, 1261.6592, 1262.6453, 1266.6777, 1273.6260, 1275.6362, 1276.6343, 1277.6531, 1301.7079, 1303.7076, 1307.7042, 1308.6376, 1310.6146, 1315.7098, 1320.6659, 1322.6886, 1323.6771, 1324.6964, 1328.7089, 1331.7202, 1335.7318, 1338.6581, 1339.6637, 1349.7253, 1352.7205, 1356.7230, 1360.6638, 1363.7153, 1365.7379, 1367.7319, 1369.7043, 1377.6947, 1379.7268, 1383.7137, 1385.7534, 1388.7404, 1390.7284, 1393.7428, 1407.7563, 1417.7010, 1421.7061, 1422.7019, 1425.7727, 1429.7365, 1431.7412, 1433.7228, 1434.7446, 1461.7778, 1467.7521, 1472.7633, 1475.7632, 1477.7545, 1484.7889, 1488.7537, 1491.7965, 1493.7579, 1500.7737, 1505.8177, 1513.8079, 1515.8213, 1517.7854, 1519.7544, 1522.7811, 1523.7953, 1529.8099, 1530.7523, 1531.7472, 1537.7974, 1539.8005, 1542.8422, 1545.8091, 1549.7339, 1551.7778, 1553.8065, 1555.8101, 1556.8109, 1557.8010, 1561.8401, 1571.8400, 1579.7939, 1583.8444, 1587.7620, 1592.8066, 1594.8335, 1604.8323, 1605.8586, 1608.8497, 1615.8754, 1624.8062, 1633.7985, 1640.9384, 1642.8099, 1646.8297, 1649.8447, 1657.8101, 1667.8622, 1677.7988, 1680.8746, 1689.9465, 1698.9225, 1699.8862, 1704.8773, 1706.8701, 1707.8302, 1710.9279, 1716.8671, 1719.9058, 1723.9011, 1725.8300, 1725.8346, 1727.8986, 1730.8931, 1737.9006, 1751.8656, 1754.8580, 1765.8463, 1766.8040, 1767.8138, 1769.8741, 1770.3567, 1772.9315, 1774.7610, 1774.9131, 1777.8770, 1780.8777, 1783.9246, 1787.8188, 1787.8200, 1791.8137, 1795.9353, 1798.9340, 1800.9336, 1804.9142, 1805.9069, 1815.9044, 1821.9144, 1828.9227, 1837.9396, 1840.9827, 1843.9688, 1844.9012, 1846.9153, 1852.9364, 1856.9908, 1861.8881, 1867.9218, 1874.9695, 1880.9265, 1884.9417, 1891.9622, 1894.9313, 1896.9078, 1896.9100, 1900.9546, 1905.9313, 1906.9501, 1908.9043, 1911.9130, 1912.9154, 1916.9923, 1918.9587, 1921.9825, 1923.9718, 1925.9573, 1928.0469, 1932.0060, 1933.9950, 1937.9630, 1939.9740, 1944.0040, 1946.9679, 1949.9595, 1954.0236, 1955.9738, 1958.9556, 1960.0034, 1968.8600, 1973.9845, 1976.9861, 1978.9910, 1981.9854, 1987.9784, 1995.9408, 1998.9894, 2000.9926, 2015.0104, 2017.9747, 2021.0170, 2026.0275, 2027.0256, 2031.0166, 2044.0123, 2047.4957, 2047.9515, 2052.0496, 2060.0520, 2062.0073, 2064.9880, 2066.9873, 2094.0376, 2097.0454, 2102.0667, 2103.1641, 2111.0305, 2127.0930, 2139.0630, 2144.0493, 2148.1023, 2155.0950, 2156.0796, 2160.0632, 2176.0664, 2190.9895, 2195.0774, 2199.0723, 2202.1624, 2203.1221, 2208.0862, 2211.1042, 2216.1062, 2225.1274, 2226.1272, 2229.0972, 2233.0984, 2239.0225, 2242.0435, 2268.1489, 2270.1287, 2271.1294, 2277.0823, 2284.1990, 2292.1426, 2309.0994, 2334.1719, 2342.9915, 2350.1746, 2383.9607, 2398.2400, 2400.0862, 2413.0549, 2414.1897, 2417.0959, 2497.2800, 2501.2637, 2511.1460, 2513.1421, 2528.1750, 2541.1948, 2542.1836, 2556.2058, 2562.1487, 2612.2666, 2717.0869, 2767.2666, 2888.2532, 3221.1733, 3224.2917, 3312.3306, 3916.9434

8. Q32LI7 **Mass:** 30313 **Score:** 29 **Expect:** 32 **Matches:** 17

Putative uncharacterized protein MGC133880 OS=Bos taurus GN=MGC133880 PE=2 SV=1

Observed	Mr(expt)	Mr(calc)	ppm	Start	End	Miss	Ions	Peptide
832.4557	831.4485	831.4338	17.7	259	265	1	---	K.ALDEKEK.S
1021.5579	1020.5506	1020.5465	4.08	97	105	1	---	K.KGSFRPSSR.E
1118.5818	1117.5745	1117.5476	24.1	57	67	1	---	R.GDSPRSSSLGR.A
1143.6318	1142.6245	1142.6295	-4.36	161	171	1	---	R.KAIGEALEGQK.R
1255.6096	1254.6023	1254.6105	-6.54	240	249	1	---	K.TDDLGRWHQK.N
1383.7137	1382.7064	1382.7055	0.68	239	249	2	---	R.KTDDLGRWHQK.N
1393.7428	1392.7355	1392.7222	9.58	97	108	2	---	K.KGSFRPSSRESR.A
1519.7544	1518.7471	1518.7804	-21.90	228	239	2	---	K.SVRDLEHWHGRK.T
1642.8099	1641.8026	1641.7868	9.66	246	258	1	---	R.WHQKNAMNMNLQK.A
1777.8770	1776.8697	1776.8642	3.08	145	160	0	---	K.FGSNVVDQLVSEEQAR.K
1884.9417	1883.9344	1883.9272	3.86	106	122	2	---	R.ESRASLREGAQPCQAPK.E
1905.9313	1904.9240	1904.9592	-18.46	145	161	1	---	K.FGSNVVDQLVSEEQARK.A
1908.9043	1907.8970	1907.9080	-5.77	250	265	2	---	K.NAMNMNLQKALDEKEK.S + 2 Oxidation (M)
1954.0236	1953.0163	1953.0241	-3.97	214	230	2	---	K.SLMKASYTPEVIEKSVR.D + Oxidation (M)
2199.0723	2198.0650	2198.0724	-3.36	246	263	2	---	R.WHQKNAMNMNLQKALDEK.E
2239.0225	2238.0152	2238.0301	-6.66	22	41	1	---	K.EGQQDTDPWRAAHSPLDTSK.L
2277.0823	2276.0750	2276.0894	-6.30	123	144	1	---	K.EDPNAGAQGQSGSSIPNNIRHK.F

No match to: 721.4365, 725.4001, 726.4063, 728.3681, 730.3848, 731.4017, 732.3573, 734.4151, 735.4143, 741.4101, 742.4515, 744.4171, 745.4167, 746.4169, 747.4125, 750.4153, 752.4127, 755.4100, 756.4199, 757.4313, 761.4177, 767.4266, 768.3700, 768.3737, 770.3966, 772.4093, 775.4307, 779.4308, 784.3688, 786.4000, 787.4221, 788.4210, 792.4376, 795.4350, 798.4351, 800.4296, 812.4388, 815.4332, 822.4482, 825.4540, 831.4382, 835.4719, 837.4463, 842.5060, 844.4760, 854.4837, 856.4911, 860.4691, 862.4852, 869.4231, 885.4465, 905.4955, 907.4991, 908.4929, 913.5700, 913.5747, 916.5284, 918.5050, 919.5053, 922.5070, 928.5117, 934.5172, 940.4840, 942.4854, 943.4289, 943.5453, 946.5259, 950.4959, 951.5076, 953.5164, 954.5388, 959.5266, 966.5410, 969.5256, 972.5305, 974.5395, 976.5310, 978.5314, 979.5442, 991.5409, 993.5307, 1001.5854, 1003.5496, 1011.5262, 1014.5215, 1023.5663, 1023.5700, 1031.5903, 1035.5381, 1036.5447, 1037.5573, 1039.5327, 1041.5658, 1044.5751, 1045.5729, 1046.5897, 1051.5448, 1056.5658, 1057.6078, 1067.5753, 1073.5902, 1086.5509, 1092.5961, 1100.6071, 1106.5391, 1111.5615, 1113.5729, 1120.6045, 1122.5912, 1123.5848, 1125.5823, 1132.6047, 1134.5714, 1139.5746, 1144.6497, 1145.6407, 1147.6393, 1150.6108, 1155.6215, 1157.6199, 1158.6685, 1162.6182, 1163.6152, 1168.6215, 1173.6418, 1175.6089, 1178.6356, 1179.6157, 1181.6819, 1188.6544, 1197.6704, 1199.7006, 1208.6415, 1216.6714, 1227.6595, 1231.6387, 1232.6447, 1234.6672, 1235.6445, 1244.6453, 1247.6211, 1248.6174, 1250.6554, 1256.6531, 1261.6592, 1262.6453, 1266.6777, 1273.6260, 1275.6362, 1276.6343, 1277.6531, 1301.7079, 1303.7076, 1307.7042, 1308.6376, 1310.6146, 1312.6418, 1315.7098, 1320.6659, 1322.6886, 1323.6771, 1324.6964, 1328.7089, 1331.7202, 1335.7318, 1338.6581, 1339.6637, 1349.7253, 1352.7205, 1356.7230, 1360.6638, 1363.7153, 1365.7379, 1367.7319, 1369.7043, 1377.6947, 1379.7268, 1385.7534, 1388.7404, 1390.7284, 1407.7563, 1417.7010, 1421.7061, 1422.7019, 1425.7727, 1427.7742, 1429.7365, 1431.7412, 1433.7228, 1434.7446, 1437.7584, 1461.7778, 1467.7521, 1472.7633, 1475.7632, 1477.7545, 1484.7889, 1488.7537, 1491.7965, 1493.7579, 1500.7737, 1505.8177, 1513.8079, 1515.8213, 1517.7854, 1522.7811, 1523.7953, 1529.8099, 1530.7523, 1531.7472, 1537.7974, 1539.8005, 1542.8422, 1545.8091, 1547.8051, 1549.7339, 1551.7778, 1553.8065, 1555.8101, 1556.8109,

1557.8010, 1561.8401, 1563.8109, 1565.8199, 1571.8400, 1579.7939, 1580.8077, 1583.8444, 1587.7620, 1592.8066, 1594.8335, 1604.8323, 1605.8586, 1608.8497, 1615.8754, 1624.8062, 1628.7900, 1628.7921, 1633.7985, 1640.9384, 1646.8297, 1649.8447, 1657.8101, 1667.8622, 1677.7988, 1680.8746, 1689.9465, 1694.7288, 1698.9225, 1699.8862, 1704.8773, 1706.8701, 1707.8302, 1710.9279, 1716.8671, 1719.9058, 1723.9011, 1725.8300, 1725.8346, 1727.8986, 1730.8931, 1737.9006, 1751.8656, 1754.8580, 1765.8463, 1766.8040, 1767.8138, 1769.8741, 1770.3567, 1772.9315, 1774.7610, 1774.9131, 1780.8777, 1783.9246, 1787.8188, 1787.8200, 1791.8137, 1793.9050, 1795.9353, 1798.9340, 1800.9336, 1804.9142, 1805.9069, 1809.8469, 1815.9044, 1821.9144, 1828.9227, 1837.9396, 1840.9827, 1843.9688, 1844.9012, 1846.9153, 1852.9364, 1856.9908, 1861.8881, 1867.9218, 1874.9695, 1880.9265, 1891.9622, 1894.9313, 1896.9078, 1896.9100, 1900.9546, 1906.9501, 1911.9130, 1912.9154, 1916.9923, 1918.9587, 1921.9825, 1923.9718, 1925.9573, 1928.0469, 1932.0060, 1933.9950, 1937.9630, 1939.9740, 1944.0040, 1946.9679, 1949.9595, 1955.9738, 1958.9556, 1960.0034, 1968.8600, 1973.9845, 1976.9861, 1978.9910, 1981.9854, 1983.9884, 1987.9784, 1995.9408, 1998.9894, 2000.9926, 2008.0460, 2012.9590, 2015.0104, 2017.9747, 2021.0170, 2026.0275, 2027.0256, 2031.0166, 2044.0123, 2047.4957, 2047.9515, 2052.0496, 2060.0520, 2062.0073, 2064.9880, 2066.9873, 2094.0376, 2097.0454, 2102.0667, 2103.1641, 2111.0305, 2127.0930, 2139.0630, 2144.0493, 2148.1023, 2155.0950, 2156.0796, 2160.0632, 2176.0664, 2190.9895, 2195.0774, 2202.1624, 2203.1221, 2208.0862, 2211.1042, 2216.1062, 2225.1274, 2226.1272, 2229.0972, 2233.0984, 2242.0435, 2268.1489, 2270.1287, 2271.1294, 2284.1990, 2292.1426, 2309.0994, 2334.1719, 2342.9915, 2350.1746, 2383.9607, 2398.2400, 2400.0862, 2413.0549, 2414.1897, 2417.0959, 2497.2800, 2501.2637, 2511.1460, 2513.1421, 2528.1750, 2537.2012, 2541.1948, 2542.1836, 2556.2058, 2562.1487, 2612.2666, 2706.2109, 2717.0869, 2767.2666, 2888.2532, 3221.1733, 3224.2917, 3312.3306, 3916.9434

9. F6QVC9 **Mass:** 36109 **Score:** 26 **Expect:** 58 **Matches:** 18

Annexin OS=Bos taurus GN=ANXA5 PE=1 SV=1

Observed	Mr(expt)	Mr(calc)	ppm	Start	End	Miss	Ions	Peptide
831.4382	830.4309	830.4385	-9.20	64 -	70	0	---	R.DLLDDLK.S
854.4837	853.4764	853.4882	-13.82	202 -	208	1	---	R.SVSHLRR.V
905.4955	904.4883	904.5018	-14.97	51 -	58	0	---	R.QEIAVAFK.T
954.5388	953.5315	953.5335	-2.01	194 -	201	0	---	K.FITIFGTR.S
1001.5854	1000.5781	1000.5917	-13.54	109 -	117	0	---	K.VLTEIIASR.T
1014.5215	1013.5142	1013.5069	7.20	90 -	97	0	---	R.LYDAYELK.H
1122.5912	1121.5839	1121.5717	10.9	277 -	285	0	---	R.SEIDLYNIR.K
1250.6554	1249.6481	1249.6666	-14.79	277 -	286	1	---	R.SEIDLYNIRK.E
1276.6343	1275.6270	1275.6169	7.93	291 -	301	0	---	K.NFGTSLYSMIK.G + Oxidation (M)
1310.6146	1309.6073	1309.5939	10.2	7 -	18	0	---	R.GTVADFPGFDER.A
1388.7404	1387.7331	1387.7169	11.7	290 -	301	1	---	R.KNFGTSLYSMIK.G
1461.7778	1460.7705	1460.7735	-2.07	46 -	58	1	---	R.SNAQRQEIAVAFK.T
1704.8773	1703.8700	1703.8941	-14.15	30 -	45	0	---	K.GLGTDEESILTLLTSR.S
1710.9279	1709.9206	1709.9247	-2.36	152 -	166	1	---	R.MLVVLLQANRDPDAR.I
2066.9873	2065.9800	2065.9705	4.61	7 -	25	1	---	R.GTVADFPGFDERADAETLR.K
2195.0774	2194.0701	2194.0655	2.13	7 -	26	2	---	R.GTVADFPGFDERADAETLRK.A
2203.1221	2202.1148	2202.0804	15.6	167 -	186	0	---	R.IDEAQVEQDAQALFQAGELK.W
2292.1426	2291.1353	2291.1144	9.14	209 -	227	1	---	R.VFDKYMTISGFQIEETIDR.E

No match to: 721.4365, 725.4001, 726.4063, 728.3681, 730.3848, 731.4017, 732.3573, 734.4151, 735.4143, 741.4101, 742.4515, 744.4171, 745.4167, 746.4169, 747.4125, 750.4153, 752.4127, 755.4100, 756.4199, 757.4313, 761.4177, 767.4266, 768.3700, 768.3737, 770.3966, 772.4093, 775.4307, 779.4308, 784.3688, 786.4000, 787.4221, 788.4210, 792.4376, 795.4350, 798.4351, 800.4296, 812.4388, 815.4332, 822.4482, 825.4540, 832.4557, 835.4719, 837.4463, 842.5060, 844.4760, 856.4911, 860.4691, 862.4852, 869.4231, 885.4465, 907.4991, 908.4929, 913.5700, 913.5747, 916.5284, 918.5050, 919.5053, 922.5070, 928.5117, 934.5172, 940.4840, 942.4854, 943.4289, 943.5453, 946.5259, 950.4959, 951.5076, 953.5164, 959.5266, 966.5410, 969.5256, 972.5305, 974.5395, 976.5310, 978.5314, 979.5442, 991.5409, 993.5307, 1003.5496, 1011.5262, 1021.5579, 1023.5663, 1023.5700, 1031.5903, 1035.5381, 1036.5447, 1037.5573, 1039.5327, 1041.5658, 1044.5751, 1045.5729, 1046.5897, 1051.5448, 1056.5658, 1057.6078, 1067.5753, 1073.5902, 1086.5509, 1092.5961, 1100.6071, 1106.5391, 1111.5615, 1113.5729, 1118.5818, 1120.6045, 1123.5848, 1125.5823, 1132.6047, 1134.5714, 1139.5746, 1143.6318, 1144.6497, 1145.6407, 1147.6393, 1150.6108, 1155.6215, 1157.6199, 1158.6685, 1162.6182, 1163.6152, 1168.6215, 1173.6418, 1175.6089, 1178.6356, 1179.6157, 1181.6819, 1188.6544, 1197.6704, 1199.7006, 1208.6415, 1216.6714, 1227.6595, 1231.6387, 1232.6447, 1234.6672, 1235.6445, 1244.6453, 1247.6211, 1248.6174, 1255.6096, 1256.6531, 1261.6592, 1262.6453, 1266.6777, 1273.6260, 1275.6362, 1277.6531, 1301.7079, 1303.7076, 1307.7042, 1308.6376, 1312.6418, 1315.7098, 1320.6659, 1322.6886, 1323.6771, 1324.6964, 1328.7089, 1331.7202, 1335.7318, 1338.6581, 1339.6637, 1349.7253, 1352.7205, 1356.7230, 1360.6638, 1363.7153, 1365.7379, 1367.7319, 1369.7043, 1377.6947, 1379.7268, 1383.7137, 1385.7534, 1390.7284, 1393.7428, 1407.7563, 1417.7010, 1421.7061, 1422.7019, 1425.7727, 1427.7742, 1429.7365, 1431.7412, 1433.7228, 1434.7446, 1437.7584, 1467.7521, 1472.7633, 1475.7632, 1477.7545, 1484.7889, 1488.7537, 1491.7965, 1493.7579, 1500.7737, 1505.8177, 1513.8079, 1515.8213, 1517.7854, 1519.7544, 1522.7811, 1523.7953, 1529.8099, 1530.7523, 1531.7472, 1537.7974, 1539.8005, 1542.8422, 1545.8091, 1547.8051, 1549.7339, 1551.7778, 1553.8065, 1555.8101, 1556.8109, 1557.8010, 1561.8401, 1563.8109, 1565.8199, 1571.8400, 1579.7939, 1580.8077, 1583.8444, 1587.7620, 1592.8066, 1594.8335, 1604.8323, 1605.8586, 1608.8497, 1615.8754, 1624.8062, 1628.7900, 1628.7921, 1633.7985, 1640.9384, 1642.8099, 1646.8297, 1649.8447, 1657.8101, 1667.8622, 1677.7988, 1680.8746, 1689.9465, 1694.7288, 1698.9225, 1699.8862, 1706.8701, 1707.8302, 1716.8671, 1719.9058, 1723.9011, 1725.8300, 1725.8346, 1727.8986, 1730.8931, 1737.9006, 1751.8656, 1754.8580, 1765.8463, 1766.8040, 1767.8138, 1769.8741, 1770.3567, 1772.9315, 1774.7610, 1774.9131, 1777.8770, 1780.8777, 1783.9246, 1787.8188, 1787.8200, 1791.8137, 1793.9050, 1795.9353, 1798.9340, 1800.9336, 1804.9142, 1805.9069, 1809.8469, 1815.9044, 1821.9144, 1828.9227, 1837.9396, 1840.9827, 1843.9688, 1844.9012, 1846.9153, 1852.9364, 1856.9908, 1861.8881, 1867.9218, 1874.9695, 1880.9265, 1884.9417, 1891.9622, 1894.9313, 1896.9078, 1896.9100, 1900.9546, 1905.9313, 1906.9501, 1908.9043, 1911.9130, 1912.9154, 1916.9923, 1918.9587, 1921.9825, 1923.9718, 1925.9573, 1928.0469, 1932.0060, 1933.9950, 1937.9630, 1939.9740, 1944.0040, 1946.9679, 1949.9595, 1954.0236, 1955.9738, 1958.9556, 1960.0034, 1968.8600, 1973.9845, 1976.9861, 1978.9910, 1981.9854, 1983.9884, 1987.9784, 1995.9408, 1998.9894, 2000.9926, 2008.0460, 2012.9590, 2015.0104, 2017.9747, 2021.0170, 2026.0275, 2027.0256, 2031.0166, 2044.0123, 2047.4957, 2047.9515, 2052.0496, 2060.0520, 2062.0073, 2064.9880, 2094.0376, 2097.0454, 2102.0667, 2103.1641, 2111.0305, 2127.0930, 2139.0630, 2144.0493, 2148.1023, 2155.0950, 2156.0796, 2160.0632, 2176.0664, 2190.9895, 2199.0723, 2202.1624, 2208.0862, 2211.1042, 2216.1062, 2225.1274, 2226.1272, 2229.0972, 2233.0984, 2239.0225, 2242.0435, 2268.1489, 2270.1287, 2271.1294, 2277.0823, 2284.1990, 2309.0994, 2334.1719, 2342.9915, 2350.1746, 2383.9607, 2398.2400, 2400.0862, 2413.0549, 2414.1897, 2417.0959, 2497.2800, 2501.2637, 2511.1460, 2513.1421, 2528.1750, 2537.2012, 2541.1948, 2542.1836, 2556.2058, 2562.1487, 2612.2666, 2706.2109, 2717.0869, 2767.2666, 2888.2532, 3221.1733, 3224.2917, 3312.3306, 3916.9434

10. E1BPX8 **Mass:** 222520 **Score:** 26 **Expect:** 64 **Matches:** 54

Uncharacterized protein OS=Bos taurus GN=MYH7B PE=3 SV=2

Observed	Mr(expt)	Mr(calc)	ppm	Start	End	Miss	Ions	Peptide
725.4001	724.3928	724.4092	-22.65	1850 -	1854	2	---	R.KHERR.V
747.4125	746.4052	746.4109	-7.60	666 -	671	0	---	K.LMTNLR.A
761.4177	760.4104	760.4079	3.37	1228 -	1233	1	---	K.EKSELR.M
831.4382	830.4309	830.4498	-22.71	869 -	877	0	---	R.GALATAEAK.R
832.4557	831.4485	831.4338	17.7	1530 -	1536	0	---	K.SVQELEK.A
860.4691	859.4618	859.4763	-16.82	927 -	933	1	---	K.VKELSER.L
913.5700	912.5627	912.5756	-14.15	792 -	799	0	10	K.VLTLLQAR.S
913.5747	912.5674	912.5756	-9.06	792 -	799	0	---	K.VLTLLQAR.S
918.5050	917.4977	917.4818	17.4	1443 -	1452	0	---	R.ATSAAAALDK.K
922.5070	921.4997	921.4920	8.37	1484 -	1491	0	---	R.SLGTELFR.L
943.5453	942.5381	942.5498	-12.45	1819 -	1826	1	---	K.QVQKLEAK.V
966.5410	965.5337	965.5519	-18.77	1453 -	1459	2	---	K.KQRHLER.A
974.5395	973.5323	973.5556	-23.99	1792 -	1799	1	---	K.KTLEQTVR.E
1031.5903	1030.5830	1030.5658	16.7	1530 -	1538	1	---	K.SVQELEKAK.K
1041.5658	1040.5585	1040.5403	17.5	434 -	441	1	---	K.ATYDRLFR.W
1046.5897	1045.5824	1045.5767	5.44	1443 -	1453	1	---	R.ATSAAAALDKK.Q
1057.6078	1056.6005	1056.6179	-16.43	1569 -	1577	0	---	R.IQLELSQVK.A

1100.6071	1099.5998	1099.5734	24.0	1677	1685	1	---	R.EQAQALERR.A
1157.6199	1156.6126	1156.6312	-16.08	1140	1149	2	---	R.VEKQRAEAAR.E
1158.6685	1157.6612	1157.6404	18.0	1578	1587	2	---	K.AEVDRKLAEK.D
1275.6362	1274.6289	1274.6466	-13.86	972	982	2	---	K.AEKEKQATENK.V
1301.7079	1300.7006	1300.7252	-18.86	275	285	1	---	K.SRVIFQLPGER.G
1315.7098	1314.7025	1314.7118	-7.07	356	367	0	---	K.IVGALLHFGNMK.F + Oxidation (M)
1320.6659	1319.6586	1319.6755	-12.78	1872	1882	1	---	R.MQDLVDKLQSK.V + Oxidation (M)
1437.7584	1436.7511	1436.7398	7.86	262	274	0	---	K.LASADIDSYLLEK.S
1529.8099	1528.8026	1528.7733	19.2	1827	1839	1	---	K.VRELEAELDAEQK.K
1537.7974	1536.7901	1536.7527	24.3	1766	1779	1	---	K.AITDAAMMAEELKK.E + Oxidation (M)
1557.8010	1556.7937	1556.8158	-14.17	1708	1720	1	---	R.RLAEQELLEATER.L
1633.7985	1632.7912	1632.7889	1.41	1584	1596	2	---	K.LAEKDEECTNLRR.N
1649.8447	1648.8374	1648.8528	-9.30	1765	1779	2	---	K.KAITDAAMMAEELKK.E
1680.8746	1679.8673	1679.8730	-3.36	262	276	1	---	K.LASADIDSYLLEKSR.V
1699.8862	1698.8789	1698.8988	-11.69	666	679	1	---	K.LMTNLRATQPHFVR.C + Oxidation (M)
1716.8671	1715.8598	1715.8802	-11.87	1601	1616	1	---	R.AVESLQASLDAETRAR.N
1774.9131	1773.9058	1773.9407	-19.65	1868	1882	2	---	K.NLARMQDLVDKLQSK.V + Oxidation (M)
1791.8137	1790.8064	1790.8509	-24.83	732	747	0	---	R.ILNPSAIPDDTFMDSR.K
1800.9336	1799.9263	1799.9489	-12.55	1706	1720	2	---	R.SRRLAEQELLEATER.L
1805.9069	1804.8996	1804.9067	-3.92	1857	1871	2	---	K.ELAYQAEEDRKNLAR.M
1840.9827	1839.9754	1840.0166	-22.39	1643	1659	1	---	R.QATEAQAATRLLQAQLK.E
1894.9313	1893.9240	1893.8955	15.0	1383	1398	1	---	K.YEADAIQRTEELEEAK.K
1911.9130	1910.9057	1910.9156	-5.17	1626	1642	0	---	K.MEGDLNDLELQLGHATR.Q
1981.9854	1980.9781	1980.9736	2.28	351	367	1	---	K.CACYKIVGALLHFGNMK.F
2031.0166	2030.0093	2030.0168	-3.66	1063	1081	1	---	K.LEGDLKLTQESVADAAQDK.Q
2044.0123	2043.0050	2042.9756	14.4	1118	1134	1	---	K.ELQARAEELEEELEAER.A
2052.0496	2051.0423	2051.0534	-5.41	1829	1846	2	---	R.ELEAELDAEQKKHAEALK.G
2060.0520	2059.0447	2059.0922	-23.07	1492	1508	2	---	R.LRHSHEEALEALETLRR.E
2064.9880	2063.9807	2064.0157	-16.95	174	193	1	---	R.ENQSMLITGESGAGKTVNTK.R
2111.0305	2110.0232	2110.0153	3.75	730	747	1	---	R.YRILNPSAIPDDTFMDSR.K
2148.1023	2147.0950	2147.0528	19.7	1230	1248	1	---	K.SELRMEVDDLSANVETLAR.G
2160.0632	2159.0559	2159.0481	3.64	1546	1565	0	---	K.SELQAALEEAEGALELEETK.T
2270.1287	2269.1214	2269.0822	17.3	893	912	0	---	K.NDLALQLQAEQENLADAEER.C
2292.1426	2291.1353	2291.1678	-14.19	985	1004	2	---	K.NLTEEMAALDESVVRLTKEK.K + Oxidation (M)
2334.1719	2333.1646	2333.1532	4.88	1234	1255	2	---	R.MEVDDLSANVETLARGKASAEK.L
2350.1746	2349.1673	2349.1482	8.16	1234	1255	2	---	R.MEVDDLSANVETLARGKASAEK.L + Oxidation (M)
2706.2109	2705.2036	2705.2099	-2.33	1346	1367	1	---	R.HDCDLLREQHEEEAEAQAELQR.L

No match to: 721.4365, 726.4063, 728.3681, 730.3848, 731.4017, 732.3573, 734.4151, 735.4143, 741.4101, 742.4515, 744.4171, 745.4167, 746.4169, 750.4153, 752.4127, 755.4100, 756.4199, 757.4313, 767.4266, 768.3700, 768.3737, 770.3966, 772.4093, 775.4307, 779.4308, 784.3688, 786.4000, 787.4221, 788.4210, 792.4376, 795.4350, 798.4351, 800.4296, 812.4388, 815.4332, 822.4482, 825.4540, 835.4719, 837.4463, 842.5060, 844.4760, 854.4837, 856.4911, 862.4852, 869.4231, 885.4465, 905.4955, 907.4991, 908.4929, 916.5284, 919.5053, 928.5117, 934.5172, 940.4840, 942.4854, 943.4289, 946.5259, 950.4959, 951.5076, 953.5164, 954.5388, 959.5266, 969.5256, 972.5305, 976.5310, 978.5314, 979.5442, 991.5409, 993.5307, 1001.5854, 1003.5496, 1011.5262, 1014.5215, 1021.5579, 1023.5663, 1023.5700, 1035.5381, 1036.5447, 1037.5573, 1039.5327, 1044.5751, 1045.5729, 1051.5448, 1056.5658, 1067.5753, 1073.5902, 1086.5509, 1092.5961, 1106.5391, 1111.5615, 1113.5729, 1118.5818, 1120.6045, 1122.5912, 1123.5848, 1125.5823, 1132.6047, 1134.5714, 1139.5746, 1143.6318, 1144.6497, 1145.6407, 1147.6393, 1150.6108, 1155.6215, 1162.6182, 1163.6152, 1168.6215, 1173.6418, 1175.6089, 1178.6356, 1179.6157, 1181.6819, 1188.6544, 1197.6704, 1199.7006, 1208.6415, 1216.6714, 1227.6595, 1231.6387, 1232.6447, 1234.6672, 1235.6445, 1244.6453, 1247.6211, 1248.6174, 1250.6554, 1255.6096, 1256.6531, 1261.6592, 1262.6453, 1266.6777, 1273.6260, 1276.6343, 1277.6531, 1303.7076, 1307.7042, 1308.6376, 1310.6146, 1312.6418, 1322.6886, 1323.6771, 1324.6964, 1328.7089, 1331.7202, 1335.7318, 1338.6581, 1339.6637, 1349.7253, 1352.7205, 1356.7230, 1360.6638, 1363.7153, 1365.7379, 1367.7319, 1369.7043, 1377.6947, 1379.7268, 1383.7137, 1385.7534, 1388.7404, 1390.7284, 1393.7428, 1407.7563, 1417.7010, 1421.7061, 1422.7019, 1425.7727, 1427.7742, 1429.7365, 1431.7412, 1433.7228, 1434.7446, 1461.7778, 1467.7521, 1472.7633, 1475.7632, 1477.7545, 1484.7889, 1488.7537, 1491.7965, 1493.7579, 1500.7737, 1505.8177, 1513.8079, 1515.8213, 1517.7854, 1519.7544, 1522.7811, 1523.7953, 1530.7523, 1531.7472, 1539.8005, 1542.8422, 1545.8091, 1547.8051, 1549.7339, 1551.7778, 1553.8065, 1555.8101, 1556.8109, 1561.8401, 1563.8109, 1565.8199, 1571.8400, 1579.7939, 1580.8077, 1583.8444, 1587.7620, 1592.8066, 1594.8335, 1604.8323, 1605.8586, 1608.8497, 1615.8754, 1624.8062, 1628.7900, 1628.7921, 1640.9384, 1642.8099, 1646.8297, 1657.8101, 1667.8622, 1677.7988, 1689.9465, 1694.7288, 1698.9225, 1704.8773, 1706.8701, 1707.8302, 1710.9279, 1719.9058, 1723.9011, 1725.8300, 1725.8346, 1727.8986, 1730.8931, 1737.9006, 1751.8656, 1754.8580, 1765.8463, 1766.8040, 1767.8138, 1769.8741, 1770.3567, 1772.9315, 1774.7610, 1777.8770, 1780.8777, 1783.9246, 1787.8188, 1787.8200, 1793.9050, 1795.9353, 1798.9340, 1804.9142, 1809.8469, 1815.9044, 1821.9144, 1828.9227, 1837.9396, 1843.9688, 1844.9012, 1846.9153, 1852.9364, 1856.9908, 1861.8881, 1867.9218, 1874.9695, 1880.9265, 1884.9417, 1891.9622, 1896.9078, 1896.9100, 1900.9546, 1905.9313, 1906.9501, 1908.9043, 1912.9154, 1916.9923, 1918.9587, 1921.9825, 1923.9718, 1925.9573, 1928.0469, 1932.0060, 1933.9950, 1937.9630, 1939.9740, 1944.0040, 1946.9679, 1949.9595, 1954.0236, 1955.9738, 1958.9556, 1960.0034, 1968.8600, 1973.9845, 1976.9861, 1978.9910, 1983.9884, 1987.9784, 1995.9408, 1998.9894, 2000.9926, 2008.0460, 2012.9590, 2015.0104, 2017.9747, 2021.0170, 2026.0275, 2027.0256, 2047.4957, 2047.9515, 2062.0073, 2066.9873, 2094.0376, 2097.0454, 2102.0667, 2103.1641, 2127.0930, 2139.0630, 2144.0493, 2155.0950, 2156.0796, 2176.0664, 2190.9895, 2195.0774, 2199.0723, 2202.1624, 2203.1221, 2208.0862, 2211.1042, 2216.1062, 2225.1274, 2226.1272, 2229.0972, 2233.0984, 2239.0225, 2242.0435, 2268.1489, 2271.1294, 2277.0823, 2284.1990, 2309.0994, 2342.9915, 2383.9607, 2398.2400, 2400.0862, 2413.0549, 2414.1897, 2417.0959, 2497.2800, 2501.2637, 2511.1460, 2513.1421, 2528.1750, 2537.2012, 2541.1948, 2542.1836, 2556.2058, 2562.1487, 2612.2666, 2717.0869, 2767.2666, 2888.2532, 3221.1733, 3224.2917, 3312.3306, 3916.9434

11. Q29RH6 **Mass:** 55738 **Score:** 26 **Expect:** 68 **Matches:** 24

Chromosome 6 open reading frame 182 ortholog OS=Bos taurus GN=LOC519522 PE=2 SV=1

Observed	Mr(expt)	Mr(calc)	ppm	Start	End	Miss	Ions	Peptide
775.4307	774.4235	774.4309	-9.67	127	132	0	---	R.CILLEK.Q
837.4463	836.4390	836.4392	-0.20	87	93	1	---	R.EAAQYKK.A
860.4691	859.4618	859.4586	3.82	140	146	0	---	R.MVLNVER.E
885.4465	884.4393	884.4538	-16.46	460	466	0	---	R.VHNLQMK.L + Oxidation (M)
946.5259	945.5186	945.5355	-17.91	435	442	2	---	K.NIKNSSRK.C
1113.5729	1112.5656	1112.5760	-9.37	257	265	0	---	R.RPPQQICSK.F
1162.6182	1161.6109	1161.6063	3.96	388	397	1	---	K.MEIKGEQISK.L
1178.6356	1177.6283	1177.6053	19.6	266	275	0	---	K.FETLPIMAEK.S
1235.6445	1234.6372	1234.6405	-2.64	186	196	1	---	K.LTTTQKTAEDK.I
1255.6096	1254.6023	1254.6278	-20.32	182	191	1	---	K.ECFKLTTTQK.T
1338.6581	1337.6508	1337.6245	19.7	115	126	0	---	K.DISMQLSSAQSR.C + Oxidation (M)
1493.7579	1492.7506	1492.7456	3.36	161	172	2	---	R.EKEQDHMKLHAK.L
1529.8099	1528.8026	1528.8184	-10.32	230	242	1	---	R.ILMSSVTNPRHFK.E
1537.7974	1536.7901	1536.8221	-20.82	127	138	1	---	R.CILLEKQLEYTK.R
1545.8091	1544.8018	1544.8133	-7.44	230	242	1	---	R.ILMSSVTNPRHFK.E + Oxidation (M)
1628.7900	1627.7827	1627.7810	1.04	312	324	1	---	R.CLYKPSRTTSQCK.A
1628.7921	1627.7848	1627.7810	2.33	312	324	1	---	R.CLYKPSRTTSQCK.A
1704.8773	1703.8700	1703.8590	6.45	199	211	2	---	K.HLEEKLKEEEHQR.K

1706.8701	1705.8628	1705.8781	-8.94	112 - 126	2	---	K.QKKDISMQLSSAQSR.C
1769.8741	1768.8668	1768.8890	-12.53	392 - 406	1	---	K.GEQISKLMQHQESVR.K
1933.9950	1932.9877	1933.0269	-20.25	213 - 229	1	---	K.LFQDKASQLQTGLEISR.I
2271.1294	2270.1221	2270.1511	-12.75	388 - 406	2	---	K.MEIKGEQISKLMQHQESVR.K
2497.2800	2496.2727	2496.2213	20.6	149 - 168	2	---	K.NMILEQQAQLQREKEQDHMK.L
2528.1750	2527.1677	2527.1496	7.15	28 - 49	0	---	R.SDESSQTHYSVNLELTSSQMLR.S + Oxidation (M)

No match to: 721.4365, 725.4001, 726.4063, 728.3681, 730.3848, 731.4017, 732.3573, 734.4151, 735.4143, 741.4101, 742.4515, 744.4171, 745.4167, 746.4169, 747.4125, 750.4153, 752.4127, 755.4100, 756.4199, 757.4313, 761.4177, 767.4266, 768.3700, 768.3737, 770.3966, 772.4093, 779.4308, 784.3688, 786.4000, 787.4221, 788.4210, 792.4376, 795.4350, 798.4351, 800.4296, 812.4388, 815.4332, 822.4482, 825.4540, 831.4382, 832.4557, 835.4719, 842.5060, 844.4760, 854.4837, 856.4911, 862.4852, 869.4231, 905.4955, 907.4991, 908.4929, 913.5700, 913.5747, 916.5284, 918.5050, 919.5053, 922.5070, 928.5117, 934.5172, 940.4840, 942.4854, 943.4289, 943.5453, 950.4959, 951.5076, 953.5164, 954.5388, 959.5266, 966.5410, 969.5256, 972.5305, 974.5395, 976.5310, 978.5314, 979.5442, 991.5409, 993.5307, 1001.5854, 1003.5496, 1011.5262, 1014.5215, 1021.5579, 1023.5663, 1023.5700, 1031.5903, 1035.5381, 1036.5447, 1037.5573, 1039.5327, 1041.5658, 1044.5751, 1045.5729, 1046.5897, 1051.5448, 1056.5658, 1057.6078, 1067.5753, 1073.5902, 1086.5509, 1092.5961, 1100.6071, 1106.5391, 1111.5615, 1118.5818, 1120.6045, 1122.5912, 1123.5848, 1125.5823, 1132.6047, 1134.5714, 1139.5746, 1143.6318, 1144.6497, 1145.6407, 1147.6393, 1150.6108, 1155.6215, 1157.6199, 1158.6685, 1163.6152, 1168.6215, 1173.6418, 1175.6089, 1179.6157, 1181.6819, 1188.6544, 1197.6704, 1199.7006, 1208.6415, 1216.6714, 1227.6595, 1231.6387, 1232.6447, 1234.6672, 1244.6453, 1247.6211, 1248.6174, 1250.6554, 1256.6531, 1261.6592, 1262.6453, 1266.6777, 1273.6260, 1275.6362, 1276.6343, 1277.6531, 1301.7079, 1303.7076, 1307.7042, 1308.6376, 1310.6146, 1312.6418, 1315.7098, 1320.6659, 1322.6886, 1323.6771, 1324.6964, 1328.7089, 1331.7202, 1335.7318, 1339.6637, 1349.7253, 1352.7205, 1356.7230, 1360.6638, 1363.7153, 1365.7379, 1367.7319, 1369.7043, 1377.6947, 1379.7268, 1383.7137, 1385.7534, 1388.7404, 1390.7284, 1393.7428, 1407.7563, 1417.7010, 1421.7061, 1422.7019, 1425.7727, 1427.7742, 1429.7365, 1431.7412, 1433.7228, 1434.7446, 1437.7584, 1461.7778, 1467.7521, 1472.7633, 1475.7632, 1477.7545, 1484.7889, 1488.7537, 1491.7965, 1500.7737, 1505.8177, 1513.8079, 1515.8213, 1517.7854, 1519.7544, 1522.7811, 1523.7953, 1530.7523, 1531.7472, 1539.8005, 1542.8422, 1547.8051, 1549.7339, 1551.7778, 1553.8065, 1555.8101, 1556.8109, 1557.8010, 1561.8401, 1563.8109, 1565.8199, 1571.8400, 1579.7939, 1580.8077, 1583.8444, 1587.7620, 1592.8066, 1594.8335, 1604.8323, 1605.8586, 1608.8497, 1615.8754, 1624.8062, 1633.7985, 1640.9384, 1642.8099, 1646.8297, 1649.8447, 1657.8101, 1667.8622, 1677.7988, 1680.8746, 1689.9465, 1694.7288, 1698.9225, 1699.8862, 1707.8302, 1710.9279, 1716.8671, 1719.9058, 1723.9011, 1725.8300, 1725.8346, 1727.8986, 1730.8931, 1737.9006, 1751.8656, 1754.8580, 1765.8463, 1766.8040, 1767.8138, 1770.3567, 1772.9315, 1774.7610, 1774.9131, 1777.8770, 1780.8777, 1783.9246, 1787.8188, 1787.8200, 1791.8137, 1793.9050, 1795.9353, 1798.9340, 1800.9336, 1804.9142, 1805.9069, 1809.8469, 1815.9044, 1821.9144, 1828.9227, 1837.9396, 1840.9827, 1843.9688, 1844.9012, 1846.9153, 1852.9364, 1856.9908, 1861.8881, 1867.9218, 1874.9695, 1880.9265, 1884.9417, 1891.9622, 1894.9313, 1896.9078, 1896.9100, 1900.9546, 1905.9313, 1906.9501, 1908.9043, 1911.9130, 1912.9154, 1916.9923, 1918.9587, 1921.9825, 1923.9718, 1925.9573, 1928.0469, 1932.0060, 1937.9630, 1939.9740, 1944.0040, 1946.9679, 1949.9595, 1954.0236, 1955.9738, 1958.9556, 1960.0034, 1968.8600, 1973.9845, 1976.9861, 1978.9910, 1981.9854, 1983.9884, 1987.9784, 1995.9408, 1998.9894, 2000.9926, 2008.0460, 2012.9590, 2015.0104, 2017.9747, 2021.0170, 2026.0275, 2027.0256, 2031.0166, 2044.0123, 2047.4957, 2047.9515, 2052.0496, 2060.0520, 2062.0073, 2064.9880, 2066.9873, 2094.0376, 2097.0454, 2102.0667, 2103.1641, 2111.0305, 2127.0930, 2139.0630, 2144.0493, 2148.1023, 2155.0950, 2156.0796, 2160.0632, 2176.0664, 2190.9895, 2195.0774, 2199.0723, 2202.1624, 2203.1221, 2208.0862, 2211.1042, 2216.1062, 2225.1274, 2226.1272, 2229.0972, 2233.0984, 2239.0225, 2242.0435, 2268.1489, 2270.1287, 2277.0823, 2284.1990, 2292.1426, 2309.0994, 2334.1719, 2342.9915, 2350.1746, 2383.9607, 2398.2400, 2400.0862, 2413.0549, 2414.1897, 2417.0959, 2501.2637, 2511.1460, 2513.1421, 2537.2012, 2541.1948, 2542.1836, 2556.2058, 2562.1487, 2612.2666, 2706.2109, 2717.0869, 2767.2666, 2888.2532, 3221.1733, 3224.2917, 3312.3306, 3916.9434

12. Q2HJF2 **Mass:** 26289 **Score:** 24 **Expect:** 88 **Matches:** 13

F-box and leucine-rich protein 22 OS=Bos taurus GN=FBXL22 PE=2 SV=1

Observed	Mr(expt)	Mr(calc)	ppm	Start	End	Miss	Ions	Peptide
943.5453	942.5381	942.5359	2.32	178 -	186	1	---	R.NVSAAGLRR.L
946.5259	945.5186	945.5144	4.41	212 -	221	0	---	R.GPHAPGAALR.K
959.5266	958.5193	958.5308	-11.95	21 -	28	2	---	K.DSRKNLAR.T
1057.6078	1056.6005	1056.5862	13.5	189 -	197	1	---	R.AACPRLTLR.A
1145.6407	1144.6334	1144.6240	8.20	57 -	66	0	---	K.DNFLLSPALR.S
1352.7205	1351.7132	1351.7142	-0.76	142 -	152	1	---	R.LRALHLENCAR.V
1551.7778	1550.7705	1550.7511	12.5	198 -	211	0	---	R.AEHSAAMIPDQLPR.G + Oxidation (M)
1553.8065	1552.7992	1552.7892	6.45	144 -	156	1	---	R.ALHLENCARVTNR.T
1844.9012	1843.8939	1843.8887	2.84	98 -	112	0	---	R.HESLVNDFLLQVCDR.C
1925.9573	1924.9500	1924.9294	10.7	138 -	152	2	---	R.CCPRLRALHLENCAR.V
1932.0060	1930.9987	1930.9935	2.72	77 -	92	1	---	R.VQVCSIEDWLKSALQR.N
2008.0460	2007.0387	2007.0248	6.96	9 -	24	2	---	R.ECLLHLFSFLDKDSRK.N
2284.1990	2283.1917	2283.2018	-4.43	167 -	186	2	---	R.ALQTLHVDFCRNVSAAGLRR.L

No match to: 721.4365, 725.4001, 726.4063, 728.3681, 730.3848, 731.4017, 732.3573, 734.4151, 735.4143, 741.4101, 742.4515, 744.4171, 745.4167, 746.4169, 747.4125, 750.4153, 752.4127, 755.4100, 756.4199, 757.4313, 761.4177, 767.4266, 768.3700, 768.3737, 770.3966, 772.4093, 775.4307, 779.4308, 784.3688, 786.4000, 787.4221, 788.4210, 792.4376, 795.4350, 798.4351, 800.4296, 812.4388, 815.4332, 822.4482, 825.4540, 831.4382, 832.4557, 835.4719, 837.4463, 842.5060, 844.4760, 854.4837, 856.4911, 860.4691, 862.4852, 869.4231, 885.4465, 905.4955, 907.4991, 908.4929, 913.5700, 913.5747, 916.5284, 918.5050, 919.5053, 922.5070, 928.5117, 934.5172, 940.4840, 942.4854, 943.4289, 950.4959, 951.5076, 953.5164, 954.5388, 966.5410, 969.5256, 972.5305, 974.5395, 976.5310, 978.5314, 979.5442, 991.5409, 993.5307, 1001.5854, 1003.5496, 1011.5262, 1014.5215, 1021.5579, 1023.5663, 1023.5700, 1031.5903, 1035.5381, 1036.5447, 1037.5573, 1039.5327, 1041.5658, 1044.5751, 1045.5729, 1046.5897, 1051.5448, 1056.5658, 1067.5753, 1073.5902, 1086.5509, 1092.5961, 1100.6071, 1106.5391, 1111.5615, 1113.5729, 1118.5818, 1120.6045, 1122.5912, 1123.5848, 1125.5823, 1132.6047, 1134.5714, 1139.5746, 1143.6318, 1144.6497, 1147.6393, 1150.6108, 1155.6215, 1157.6199, 1158.6685, 1162.6182, 1163.6152, 1168.6215, 1173.6418, 1175.6089, 1178.6356, 1179.6157, 1181.6819, 1188.6544, 1197.6704, 1199.7006, 1208.6415, 1216.6714, 1227.6595, 1231.6387, 1232.6447, 1234.6672, 1235.6445, 1244.6453, 1247.6211, 1248.6174, 1250.6554, 1255.6096, 1256.6531, 1261.6592, 1262.6453, 1266.6777, 1273.6260, 1275.6362, 1276.6343, 1277.6531, 1301.7079, 1303.7076, 1307.7042, 1308.6376, 1310.6146, 1312.6418, 1315.7098, 1320.6659, 1322.6886, 1323.6771, 1324.6964, 1328.7089, 1331.7202, 1335.7318, 1338.6581, 1339.6637, 1349.7253, 1356.7230, 1360.6638, 1363.7153, 1365.7379, 1367.7319, 1369.7043, 1377.6947, 1379.7268, 1383.7137, 1385.7534, 1388.7404, 1390.7284, 1393.7428, 1407.7563, 1417.7010, 1421.7061, 1422.7019, 1425.7727, 1427.7742, 1429.7365, 1431.7412, 1433.7228, 1434.7446, 1437.7584, 1461.7778, 1467.7521, 1472.7633, 1475.7632, 1477.7545, 1484.7889, 1488.7537, 1491.7965, 1493.7579, 1500.7737, 1505.8177, 1513.8079, 1515.8213, 1517.7854, 1519.7544, 1522.7811, 1523.7953, 1529.8099, 1530.7523, 1531.7472, 1537.7974, 1539.8005, 1542.8422, 1545.8091, 1547.8051, 1549.7339, 1555.8101, 1556.8109, 1557.8010, 1561.8401, 1563.8109, 1565.8199, 1571.8400, 1579.7939, 1580.8077, 1583.8444, 1587.7620, 1592.8066, 1594.8335, 1604.8323, 1605.8586, 1608.8497, 1615.8754, 1624.8062, 1628.7900, 1628.7921, 1633.7985, 1640.9384, 1642.8099, 1646.8297, 1649.8447, 1657.8101, 1667.8622, 1677.7988, 1680.8746, 1689.9465, 1694.7288, 1698.9225, 1699.8862, 1704.8773, 1706.8701, 1707.8302, 1710.9279, 1716.8671, 1719.9058, 1723.9011, 1725.8300, 1725.8346, 1727.8986, 1730.8931, 1737.9006, 1751.8656, 1754.8580, 1765.8463, 1766.8040, 1767.8138, 1769.8741, 1770.3567, 1772.9315, 1774.7610, 1774.9131, 1777.8770, 1780.8777, 1783.9246, 1787.8188, 1787.8200, 1791.8137, 1793.9050, 1795.9353, 1798.9340, 1800.9336, 1804.9142, 1805.9069, 1809.8469, 1815.9044, 1821.9144, 1828.9227, 1837.9396, 1840.9827, 1843.9688, 1846.9153, 1852.9364, 1856.9908, 1861.8881, 1867.9218, 1874.9695, 1880.9265, 1884.9417, 1891.9622, 1894.9313, 1896.9078, 1896.9100, 1900.9546, 1905.9313, 1906.9501, 1908.9043, 1911.9130, 1912.9154, 1916.9923, 1918.9587, 1921.9825, 1923.9718, 1928.0469, 1933.9950, 1937.9630, 1939.9740, 1944.0040, 1946.9679, 1949.9595, 1954.0236, 1955.9738, 1958.9556, 1960.0034, 1968.8600, 1973.9845, 1976.9861, 1978.9910, 1981.9854, 1983.9884, 1987.9784, 1995.9408, 1998.9894, 2000.9926, 2012.9590, 2015.0104, 2017.9747, 2021.0170, 2026.0275, 2027.0256, 2031.0166, 2044.0123, 2047.4957, 2047.9515, 2052.0496, 2060.0520, 2062.0073, 2064.9880, 2066.9873, 2094.0376, 2097.0454, 2102.0667, 2103.1641, 2111.0305, 2127.0930, 2139.0630, 2144.0493, 2148.1023, 2155.0950, 2156.0796, 2160.0632, 2176.0664, 2190.9895, 2195.0774, 2199.0723, 2202.1624, 2203.1221, 2208.0862, 2211.1042, 2216.1062, 2225.1274, 2226.1272, 2229.0972, 2233.0984, 2239.0225, 2242.0435, 2268.1489, 2270.1287, 2271.1294, 2277.0823, 2292.1426, 2309.0994, 2334.1719, 2342.9915, 2350.1746, 2383.9607, 2398.2400, 2400.0862, 2413.0549, 2414.1897, 2417.0959, 2497.2800, 2501.2637, 2511.1460, 2513.1421, 2528.1750, 2537.2012, 2541.1948, 2542.1836, 2556.2058, 2562.1487, 2612.2666, 2706.2109, 2717.0869, 2767.2666, 2888.2532, 3221.1733, 3224.2917, 3312.3306, 3916.9434

13. G5E523 **Mass:** 28080 **Score:** 24 **Expect:** 94 **Matches:** 22

Uncharacterized protein OS=Bos taurus GN=STAU2 PE=4 SV=1

Observed	Mr(expt)	Mr(calc)	ppm	Start	End	Miss	Ions	Peptide
788.4210	787.4137	787.4235	-12.48	197 -	202	2	---	R.GMPRRR.E + Oxidation (M)

1036.5447	1035.5374	1035.5535	-15.55	202 -	209	1	---	R.REFVMQVK.V
1056.5658	1055.5585	1055.5335	23.7	1 -	8	1	---	-.RYHCPVPK.I
1113.5729	1112.5656	1112.5509	13.3	29 -	38	1	---	R.QAARHNAAMK.A + Oxidation (M)
1163.6152	1162.6079	1162.6056	1.98	84 -	93	0	---	R.NMPVSFEVIK.E
1179.6157	1178.6084	1178.6005	6.69	84 -	93	0	---	R.NMPVSFEVIK.E + Oxidation (M)
1208.6415	1207.6342	1207.6496	-12.69	201 -	209	2	---	R.RREFVMQVK.V + Oxidation (M)
1335.7318	1334.7245	1334.7016	17.1	83 -	93	1	---	K.RNMPVSFEVIK.E + Oxidation (M)
1437.7584	1436.7511	1436.7333	12.4	228 -	240	0	---	K.NAAEAMLLQLGYK.A + Oxidation (M)
1472.7633	1471.7560	1471.7242	21.6	94 -	106	1	---	K.ESGPPHMKSFVTR.V
1477.7545	1476.7472	1476.7460	0.84	185 -	196	1	---	K.EKEPDYVLLSER.G
1488.7537	1487.7464	1487.7191	18.4	94 -	106	1	---	K.ESGPPHMKSFVTR.V + Oxidation (M)
1565.8199	1564.8126	1564.8283	-9.99	227 -	240	1	---	K.KNAAEAMLLQLGYK.A + Oxidation (M)
1605.8586	1604.8513	1604.8409	6.47	184 -	196	2	---	K.KEKEPDYVLLSER.G
1624.8062	1623.7989	1623.7740	15.3	107 -	122	1	---	R.VSVGEFSAEGEGNSKK.L
1677.7988	1676.7915	1676.8192	-16.49	187 -	200	1	---	K.EPDYVLLSERGMPR.R + Oxidation (M)
1918.9587	1917.9514	1917.9618	-5.41	185 -	200	2	---	K.EKEPDYVLLSERGMPR.R
1933.9950	1932.9877	1932.9727	7.76	158 -	175	1	---	K.TIVKAGPEYGQGMNPISR.L + Oxidation (M)
2027.0256	2026.0183	2026.0016	8.26	84 -	101	1	---	R.NMPVSFEVIKESGPPHMK.S
2062.0073	2061.0000	2061.0208	-10.06	9 -	26	0	---	K.IFYVQLTVGNNEFFGEGK.T
2199.0723	2198.0650	2198.0976	-14.82	83 -	101	2	---	K.RNMPVSFEVIKESGPPHMK.S + Oxidation (M)
2501.2637	2500.2564	2500.2856	-11.67	162 -	184	2	---	K.AGPEYGQGMNPISRLAQIQQAKK.E + Oxidation (M)

No match to: 721.4365, 725.4001, 726.4063, 728.3681, 730.3848, 731.4017, 732.3573, 734.4151, 735.4143, 741.4101, 742.4515, 744.4171, 745.4167, 746.4169, 747.4125, 750.4153, 752.4127, 755.4100, 756.4199, 757.4313, 761.4177, 767.4266, 768.3700, 768.3737, 770.3966, 772.4093, 775.4307, 779.4308, 784.3688, 786.4000, 787.4221, 792.4376, 795.4350, 798.4351, 800.4296, 812.4388, 815.4332, 822.4482, 825.4540, 831.4382, 832.4557, 835.4719, 837.4463, 842.5060, 844.4760, 854.4837, 856.4911, 860.4691, 862.4852, 869.4231, 885.4465, 905.4955, 907.4991, 908.4929, 913.5700, 913.5747, 916.5284, 918.5050, 919.5053, 922.5070, 928.5117, 934.5172, 940.4840, 942.4854, 943.4289, 943.5453, 946.5259, 950.4959, 951.5076, 953.5164, 954.5388, 959.5266, 966.5410, 969.5256, 972.5305, 974.5395, 976.5310, 978.5314, 979.5442, 991.5409, 993.5307, 1001.5854, 1003.5496, 1011.5262, 1014.5215, 1021.5579, 1023.5663, 1023.5700, 1031.5903, 1035.5381, 1037.5573, 1039.5327, 1041.5658, 1044.5751, 1045.5729, 1046.5897, 1051.5448, 1057.6078, 1067.5753, 1073.5902, 1086.5509, 1092.5961, 1100.6071, 1106.5391, 1111.5615, 1118.5818, 1120.6045, 1122.5912, 1123.5848, 1125.5823, 1132.6047, 1134.5714, 1139.5746, 1143.6318, 1144.6497, 1145.6407, 1147.6393, 1150.6108, 1155.6215, 1157.6199, 1158.6685, 1162.6182, 1168.6215, 1173.6418, 1175.6089, 1178.6356, 1181.6819, 1188.6544, 1197.6704, 1199.7006, 1216.6714, 1227.6595, 1231.6387, 1232.6447, 1234.6672, 1235.6445, 1244.6453, 1247.6211, 1248.6174, 1250.6554, 1255.6096, 1256.6531, 1261.6592, 1262.6453, 1266.6777, 1273.6260, 1275.6362, 1276.6343, 1277.6531, 1301.7079, 1303.7076, 1307.7042, 1308.6376, 1310.6146, 1312.6418, 1315.7098, 1320.6659, 1322.6886, 1323.6771, 1324.6964, 1328.7089, 1331.7202, 1338.6581, 1339.6637, 1349.7253, 1352.7205, 1356.7230, 1360.6638, 1363.7153, 1365.7379, 1367.7319, 1369.7043, 1377.6947, 1379.7268, 1383.7137, 1385.7534, 1388.7404, 1390.7284, 1393.7428, 1407.7563, 1417.7010, 1421.7061, 1422.7019, 1425.7727, 1427.7742, 1429.7365, 1431.7412, 1433.7228, 1434.7446, 1461.7778, 1467.7521, 1475.7632, 1484.7889, 1491.7965, 1493.7579, 1500.7737, 1505.8177, 1513.8079, 1515.8213, 1517.7854, 1519.7544, 1522.7811, 1523.7953, 1529.8099, 1530.7523, 1531.7472, 1537.7974, 1539.8005, 1542.8422, 1545.8091, 1547.8051, 1549.7339, 1551.7778, 1553.8065, 1555.8101, 1556.8109, 1557.8010, 1561.8401, 1563.8109, 1571.8400, 1579.7939, 1580.8077, 1583.8444, 1587.7620, 1592.8066, 1594.8335, 1604.8323, 1608.8497, 1615.8754, 1628.7900, 1628.7921, 1633.7985, 1640.9384, 1642.8099, 1646.8297, 1649.8447, 1657.8101, 1667.8622, 1680.8746, 1689.9465, 1694.7288, 1698.9225, 1699.8862, 1704.8773, 1706.8701, 1707.8302, 1710.9279, 1716.8671, 1719.9058, 1723.9011, 1725.8300, 1725.8346, 1727.8986, 1730.8931, 1737.9006, 1751.8656, 1754.8580, 1765.8463, 1766.8040, 1767.8138, 1769.8741, 1770.3567, 1772.9315, 1774.7610, 1774.9131, 1777.8770, 1780.8777, 1783.9246, 1787.8188, 1787.8200, 1791.8137, 1793.9050, 1795.9353, 1798.9340, 1800.9336, 1804.9142, 1805.9069, 1809.8469, 1815.9044, 1821.9144, 1828.9227, 1837.9396, 1840.9827, 1843.9688, 1844.9012, 1846.9153, 1852.9364, 1856.9908, 1861.8881, 1867.9218, 1874.9695, 1880.9265, 1884.9417, 1891.9622, 1894.9313, 1896.9078, 1896.9100, 1900.9546, 1905.9313, 1906.9501, 1908.9043, 1911.9130, 1912.9154, 1916.9923, 1921.9825, 1923.9718, 1925.9573, 1928.0469, 1932.0060, 1937.9630, 1939.9740, 1944.0040, 1946.9679, 1949.9595, 1954.0236, 1955.9738, 1958.9556, 1960.0034, 1968.8600, 1973.9845, 1976.9861, 1978.9910, 1981.9854, 1983.9884, 1987.9784, 1995.9408, 1998.9894, 2000.9926, 2008.0460, 2012.9590, 2015.0104, 2017.9747, 2021.0170, 2026.0275, 2031.0166, 2044.0123, 2047.4957, 2047.9515, 2052.0496, 2060.0520, 2064.9880, 2066.9873, 2094.0376, 2097.0454, 2102.0667, 2103.1641, 2111.0305, 2127.0930, 2139.0630, 2144.0493, 2148.1023, 2155.0950, 2156.0796, 2160.0632, 2176.0664, 2190.9895, 2195.0774, 2202.1624, 2203.1221, 2208.0862, 2211.1042, 2216.1062, 2225.1274, 2226.1272, 2229.0972, 2233.0984, 2239.0225, 2242.0435, 2268.1489, 2270.1287, 2271.1294, 2277.0823, 2284.1990, 2292.1426, 2309.0994, 2334.1719, 2342.9915, 2350.1746, 2383.9607, 2398.2400, 2400.0862, 2413.0549, 2414.1897, 2417.0959, 2497.2800, 2511.1460, 2513.1421, 2528.1750, 2537.2012, 2541.1948, 2542.1836, 2556.2058, 2562.1487, 2612.2666, 2706.2109, 2717.0869, 2767.2666, 2888.2532, 3221.1733, 3224.2917, 3312.3306, 3916.9434

14. F1N024 **Mass:** 7899 **Score:** 24 **Expect:** 1e+002 **Matches:** 6

Uncharacterized protein OS=Bos taurus GN=FAM19A4 PE=4 SV=1

Observed	Mr(expt)	Mr(calc)	ppm	Start	End	Miss	Ions	Peptide
885.4465	884.4393	884.4464	-8.09	2 -	9	0	---	M.SASSQHLR.G
1014.5215	1013.5142	1013.5155	-1.28	10 -	18	0	---	R.GHTGHHQIK.Q
1255.6096	1254.6023	1254.6139	-9.24	19 -	29	0	---	K.QGTCEVVAVHR.C
1880.9265	1879.9192	1879.9514	-17.10	2 -	18	1	---	M.SASSQHLRGHTGHHQIK.Q
2511.1460	2510.1387	2510.2006	-24.65	36 -	57	2	---	R.IEERSQTVKCSCFPGQVAGTTR.A
2767.2666	2766.2593	2766.2313	10.1	45 -	69	1	---	K.CSCFPGQVAGTTRAQPSCVEAAWNL.-

No match to: 721.4365, 725.4001, 726.4063, 728.3681, 730.3848, 731.4017, 732.3573, 734.4151, 735.4143, 741.4101, 742.4515, 744.4171, 745.4167, 746.4169, 747.4125, 750.4153, 752.4127, 755.4100, 756.4199, 757.4313, 761.4177, 767.4266, 768.3700, 768.3737, 770.3966, 772.4093, 775.4307, 779.4308, 784.3688, 786.4000, 787.4221, 788.4210, 792.4376, 795.4350, 798.4351, 800.4296, 812.4388, 815.4332, 822.4482, 825.4540, 831.4382, 832.4557, 835.4719, 837.4463, 842.5060, 844.4760, 854.4837, 856.4911, 860.4691, 862.4852, 869.4231, 905.4955, 907.4991, 908.4929, 913.5700, 913.5747, 916.5284, 918.5050, 919.5053, 922.5070, 928.5117, 934.5172, 940.4840, 942.4854, 943.4289, 943.5453, 946.5259, 950.4959, 951.5076, 953.5164, 954.5388, 959.5266, 966.5410, 969.5256, 972.5305, 974.5395, 976.5310, 978.5314, 979.5442, 991.5409, 993.5307, 1001.5854, 1003.5496, 1011.5262, 1021.5579, 1023.5663, 1023.5700, 1031.5903, 1035.5381, 1036.5447, 1037.5573, 1039.5327, 1041.5658, 1044.5751, 1045.5729, 1046.5897, 1051.5448, 1056.5658, 1057.6078, 1067.5753, 1073.5902, 1086.5509, 1092.5961, 1100.6071, 1106.5391, 1111.5615, 1113.5729, 1118.5818, 1120.6045, 1122.5912, 1123.5848, 1125.5823, 1132.6047, 1134.5714, 1139.5746, 1143.6318, 1144.6497, 1145.6407, 1147.6393, 1150.6108, 1155.6215, 1157.6199, 1158.6685, 1162.6182, 1163.6152, 1168.6215, 1173.6418, 1175.6089, 1178.6356, 1179.6157, 1181.6819, 1188.6544, 1197.6704, 1199.7006, 1208.6415, 1216.6714, 1227.6595, 1231.6387, 1232.6447, 1234.6672, 1235.6445, 1244.6453, 1247.6211, 1248.6174, 1250.6554, 1256.6531, 1261.6592, 1262.6453, 1266.6777, 1273.6260, 1275.6362, 1276.6343, 1277.6531, 1301.7079, 1303.7076, 1307.7042, 1308.6376, 1310.6146, 1312.6418, 1315.7098, 1320.6659, 1322.6886, 1323.6771, 1324.6964, 1328.7089, 1331.7202, 1335.7318, 1338.6581, 1339.6637, 1349.7253, 1352.7205, 1356.7230, 1360.6638, 1363.7153, 1365.7379, 1367.7319, 1369.7043, 1377.6947, 1379.7268, 1383.7137, 1385.7534, 1388.7404, 1390.7284, 1393.7428, 1407.7563, 1417.7010, 1421.7061, 1422.7019, 1425.7727, 1427.7742, 1429.7365, 1431.7412, 1433.7228, 1434.7446, 1437.7584, 1461.7778, 1467.7521, 1472.7633, 1475.7632, 1477.7545, 1484.7889, 1488.7537, 1491.7965, 1493.7579, 1500.7737, 1505.8177, 1513.8079, 1515.8213, 1517.7854, 1519.7544, 1522.7811, 1523.7953, 1529.8099, 1530.7523, 1531.7472, 1537.7974, 1539.8005, 1542.8422, 1545.8091, 1547.8051, 1549.7339, 1551.7778, 1553.8065, 1555.8101, 1556.8109, 1557.8010, 1561.8401, 1563.8109, 1565.8199, 1571.8400, 1579.7939, 1580.8077, 1583.8444, 1587.7620, 1592.8066, 1594.8335, 1604.8323, 1605.8586, 1608.8497, 1615.8754, 1624.8062, 1628.7900, 1628.7921, 1633.7985, 1640.9384, 1642.8099, 1646.8297, 1649.8447, 1657.8101, 1667.8622, 1677.7988, 1680.8746, 1689.9465, 1694.7288, 1698.9225, 1699.8862, 1704.8773, 1706.8701, 1707.8302, 1710.9279, 1716.8671, 1719.9058, 1723.9011, 1725.8300, 1725.8346, 1727.8986, 1730.8931, 1737.9006, 1751.8656, 1754.8580, 1765.8463, 1766.8040, 1767.8138, 1769.8741, 1770.3567, 1772.9315, 1774.7610, 1774.9131, 1777.8770, 1780.8777, 1783.9246, 1787.8188, 1787.8200, 1791.8137, 1793.9050, 1795.9353, 1798.9340, 1800.9336, 1804.9142, 1805.9069, 1809.8469, 1815.9044, 1821.9144, 1828.9227, 1837.9396, 1840.9827, 1843.9688, 1844.9012, 1846.9153, 1852.9364, 1856.9908, 1861.8881, 1867.9218, 1874.9695, 1884.9417, 1891.9622, 1894.9313, 1896.9078, 1896.9100, 1900.9546, 1905.9313, 1906.9501, 1908.9043, 1911.9130, 1912.9154, 1916.9923, 1918.9587, 1921.9825, 1923.9718, 1925.9573, 1928.0469, 1932.0060, 1933.9950, 1937.9630, 1939.9740, 1944.0040, 1946.9679, 1949.9595, 1954.0236, 1955.9738, 1958.9556, 1960.0034, 1968.8600, 1973.9845, 1976.9861, 1978.9910, 1981.9854, 1983.9884, 1987.9784, 1995.9408, 1998.9894, 2000.9926, 2008.0460, 2012.9590, 2015.0104, 2017.9747, 2021.0170, 2026.0275, 2027.0256, 2031.0166, 2044.0123, 2047.4957, 2047.9515, 2052.0496, 2060.0520, 2062.0073, 2064.9880, 2066.9873, 2094.0376, 2097.0454, 2102.0667, 2103.1641, 2111.0305, 2127.0930,

2139.0630, 2144.0493, 2148.1023, 2155.0950, 2156.0796, 2160.0632, 2176.0664, 2190.9895, 2195.0774, 2199.0723, 2202.1624, 2203.1221, 2208.0862, 2211.1042, 2216.1062, 2225.1274, 2226.1272, 2229.0972, 2233.0984, 2239.0225, 2242.0435, 2268.1489, 2270.1287, 2271.1294, 2277.0823, 2284.1990, 2292.1426, 2309.0994, 2334.1719, 2342.9915, 2350.1746, 2383.9607, 2398.2400, 2400.0862, 2413.0549, 2414.1897, 2417.0959, 2497.2800, 2501.2637, 2513.1421, 2528.1750, 2537.2012, 2541.1948, 2542.1836, 2556.2058, 2562.1487, 2612.2666, 2706.2109, 2717.0869, 2888.2532, 3221.1733, 3224.2917, 3312.3306, 3916.9434

15. F1MS41 **Mass:** 14310 **Score:** 24 **Expect:** 1e+002 **Matches:** 9

Beta-synuclein OS=Bos taurus GN=SNCB PE=3 SV=2

Observed	Mr(expt)	Mr(calc)	ppm	Start	End	Miss	Ions	Peptide
784.3688	783.3615	783.3659	-5.63	1 -	6	0	---	-.MEVFMK.G
951.5076	950.5004	950.5073	-7.30	35 -	43	0	---	K.EGVLYVGSK.T
1208.6415	1207.6342	1207.6561	-18.10	35 -	45	1	---	K.EGVLYVGSKTR.E
1256.6531	1255.6458	1255.6304	12.2	2 -	12	1	---	M.EVFMKGLSMAK.E + Oxidation (M)
1390.7284	1389.7211	1389.7463	-18.14	22 -	34	2	---	K.TKQGVTEAAEKTK.E
1529.8099	1528.8026	1528.8209	-11.97	44 -	58	1	---	K.TREGVVQGVASVAEK.T
1689.9465	1688.9392	1688.9131	15.5	7 -	23	2	---	K.GLSMAKEGVVAAAEKTK.Q
2127.0930	2126.0857	2126.0751	4.98	2 -	21	2	---	M.EVFMKGLSMAKEGVVAAAEK.T + 2 Oxidation (M)
2226.1272	2225.1199	2225.1258	-2.63	1 -	21	2	---	-.MEVFMKGLSMAKEGVVAAAEK.T

No match to: 721.4365, 725.4001, 726.4063, 728.3681, 730.3848, 731.4017, 732.3573, 734.4151, 735.4143, 741.4101, 742.4515, 744.4171, 745.4167, 746.4169, 747.4125, 750.4153, 752.4127, 755.4100, 756.4199, 757.4313, 761.4177, 767.4266, 768.3700, 768.3737, 770.3966, 772.4093, 775.4307, 779.4308, 786.4000, 787.4221, 788.4210, 792.4376, 795.4350, 798.4351, 800.4296, 812.4388, 815.4332, 822.4482, 825.4540, 831.4382, 832.4557, 835.4719, 837.4463, 842.5060, 844.4760, 854.4837, 856.4911, 860.4691, 862.4852, 869.4231, 885.4465, 905.4955, 907.4991, 908.4929, 913.5700, 913.5747, 916.5284, 918.5050, 919.5053, 922.5070, 928.5117, 934.5172, 940.4840, 942.4854, 943.4289, 943.5453, 946.5259, 950.4959, 953.5164, 954.5388, 959.5266, 966.5410, 969.5256, 972.5305, 974.5395, 976.5310, 978.5314, 979.5442, 991.5409, 993.5307, 1001.5854, 1003.5496, 1011.5262, 1014.5215, 1021.5579, 1023.5663, 1023.5700, 1031.5903, 1035.5381, 1036.5447, 1037.5573, 1039.5327, 1041.5658, 1044.5751, 1045.5729, 1046.5897, 1051.5448, 1056.5658, 1057.6078, 1067.5753, 1073.5902, 1086.5509, 1092.5961, 1100.6071, 1106.5391, 1111.5615, 1113.5729, 1118.5818, 1120.6045, 1122.5912, 1123.5848, 1125.5823, 1132.6047, 1134.5714, 1139.5746, 1143.6318, 1144.6497, 1145.6407, 1147.6393, 1150.6108, 1155.6215, 1157.6199, 1158.6685, 1162.6182, 1163.6152, 1168.6215, 1173.6418, 1175.6089, 1178.6356, 1179.6157, 1181.6819, 1188.6544, 1197.6704, 1199.7006, 1216.6714, 1227.6595, 1231.6387, 1232.6447, 1234.6672, 1235.6445, 1244.6453, 1247.6211, 1248.6174, 1250.6554, 1255.6096, 1261.6592, 1262.6453, 1266.6777, 1273.6260, 1275.6362, 1276.6343, 1277.6531, 1301.7079, 1303.7076, 1307.7042, 1308.6376, 1310.6146, 1312.6418, 1315.7098, 1320.6659, 1322.6886, 1323.6771, 1324.6964, 1328.7089, 1331.7202, 1335.7318, 1338.6581, 1339.6637, 1349.7253, 1352.7205, 1356.7230, 1360.6638, 1363.7153, 1365.7379, 1367.7319, 1369.7043, 1377.6947, 1379.7268, 1383.7137, 1385.7534, 1388.7404, 1393.7428, 1407.7563, 1417.7010, 1421.7061, 1422.7019, 1425.7727, 1427.7742, 1429.7365, 1431.7412, 1433.7228, 1434.7446, 1437.7584, 1461.7778, 1467.7521, 1472.7633, 1475.7632, 1477.7545, 1484.7889, 1488.7537, 1491.7965, 1493.7579, 1500.7737, 1505.8177, 1513.8079, 1515.8213, 1517.7854, 1519.7544, 1522.7811, 1523.7953, 1530.7523, 1531.7472, 1537.7974, 1539.8005, 1542.8422, 1545.8091, 1547.8051, 1549.7339, 1551.7778, 1553.8065, 1555.8101, 1556.8109, 1557.8010, 1561.8401, 1563.8109, 1565.8199, 1571.8400, 1579.7939, 1580.8077, 1583.8444, 1587.7620, 1592.8066, 1594.8335, 1604.8323, 1605.8586, 1608.8497, 1615.8754, 1624.8062, 1628.7900, 1628.7921, 1633.7985, 1640.9384, 1642.8099, 1646.8297, 1649.8447, 1657.8101, 1667.8622, 1677.7988, 1680.8746, 1694.7288, 1698.9225, 1699.8862, 1704.8773, 1706.8701, 1707.8302, 1710.9279, 1716.8671, 1719.9058, 1723.9011, 1725.8300, 1725.8346, 1727.8986, 1730.8931, 1737.9006, 1751.8656, 1754.8580, 1765.8463, 1766.8040, 1767.8138, 1769.8741, 1770.3567, 1772.9315, 1774.7610, 1774.9131, 1777.8770, 1780.8777, 1783.9246, 1787.8188, 1787.8200, 1791.8137, 1793.9050, 1795.9353, 1798.9340, 1800.9336, 1804.9142, 1805.9069, 1809.8469, 1815.9044, 1821.9144, 1828.9227, 1837.9396, 1840.9827, 1843.9688, 1844.9012, 1846.9153, 1852.9364, 1856.9908, 1861.8881, 1867.9218, 1874.9695, 1880.9265, 1884.9417, 1891.9622, 1894.9313, 1896.9078, 1896.9100, 1900.9546, 1905.9313, 1906.9501, 1908.9043, 1911.9130, 1912.9154, 1916.9923, 1918.9587, 1921.9825, 1923.9718, 1925.9573, 1928.0469, 1932.0060, 1933.9950, 1937.9630, 1939.9740, 1944.0040, 1946.9679, 1949.9595, 1954.0236, 1955.9738, 1958.9556, 1960.0034, 1968.8600, 1973.9845, 1976.9861, 1978.9910, 1981.9854, 1983.9884, 1987.9784, 1995.9408, 1998.9894, 2000.9926, 2008.0460, 2012.9590, 2015.0104, 2017.9747, 2021.0170, 2026.0275, 2027.0256, 2031.0166, 2044.0123, 2047.4957, 2047.9515, 2052.0496, 2060.0520, 2062.0073, 2064.9880, 2066.9873, 2094.0376, 2097.0454, 2102.0667, 2103.1641, 2111.0305, 2139.0630, 2144.0493, 2148.1023, 2155.0950, 2156.0796, 2160.0632, 2176.0664, 2190.9895, 2195.0774, 2199.0723, 2202.1624, 2203.1221, 2208.0862, 2211.1042, 2216.1062, 2225.1274, 2229.0972, 2233.0984, 2239.0225, 2242.0435, 2268.1489, 2270.1287, 2271.1294, 2277.0823, 2284.1990, 2292.1426, 2309.0994, 2334.1719, 2342.9915, 2350.1746, 2383.9607, 2398.2400, 2400.0862, 2413.0549, 2414.1897, 2417.0959, 2497.2800, 2501.2637, 2511.1460, 2513.1421, 2528.1750, 2537.2012, 2541.1948, 2542.1836, 2556.2058, 2562.1487, 2612.2666, 2706.2109, 2717.0869, 2767.2666, 2888.2532, 3221.1733, 3224.2917, 3312.3306, 3916.9434

16. P81287 **Mass:** 36124 **Score:** 24 **Expect:** 1e+002 **Matches:** 17

Annexin A5 OS=Bos taurus GN=ANXA5 PE=1 SV=3

Observed	Mr(expt)	Mr(calc)	ppm	Start	End	Miss	Ions	Peptide
831.4382	830.4309	830.4385	-9.20	64 -	70	0	---	R.DLLDDLK.S
854.4837	853.4764	853.4882	-13.82	202 -	208	1	---	R.SVSHLRR.V
905.4955	904.4883	904.5018	-14.97	51 -	58	0	---	R.QEIAVAFK.T
954.5388	953.5315	953.5335	-2.01	194 -	201	0	---	K.FITIFGTR.S
1001.5854	1000.5781	1000.5917	-13.54	109 -	117	0	---	K.VLTEIIASR.T
1014.5215	1013.5142	1013.5069	7.20	90 -	97	0	---	R.LYDAYELK.H
1122.5912	1121.5839	1121.5717	10.9	277 -	285	0	---	R.SEIDLYNIR.K
1250.6554	1249.6481	1249.6666	-14.79	277 -	286	1	---	R.SEIDLYNIRK.E
1276.6343	1275.6270	1275.6169	7.93	291 -	301	0	---	K.NFGTSLYSMIK.G + Oxidation (M)
1310.6146	1309.6073	1309.5939	10.2	7 -	18	0	---	R.GTVADFPGFDER.A
1388.7404	1387.7331	1387.7169	11.7	290 -	301	1	---	R.KNFGTSLYSMIK.G
1461.7778	1460.7705	1460.7735	-2.07	46 -	58	1	---	R.SNAQRQEIAVAFK.T
1710.9279	1709.9206	1709.9247	-2.36	152 -	166	1	---	R.MLVVLLQANRDPDAR.I
2066.9873	2065.9800	2065.9705	4.61	7 -	25	1	---	R.GTVADFPGFDERADAETLR.K
2195.0774	2194.0701	2194.0655	2.13	7 -	26	2	---	R.GTVADFPGFDERADAETLRK.A
2203.1221	2202.1148	2202.0804	15.6	167 -	186	0	---	R.IDEAQVEQDAQALFQAGELK.W
2292.1426	2291.1353	2291.1144	9.14	209 -	227	1	---	R.VFDKYMTISGFQIEETIDR.E

No match to: 721.4365, 725.4001, 726.4063, 728.3681, 730.3848, 731.4017, 732.3573, 734.4151, 735.4143, 741.4101, 742.4515, 744.4171, 745.4167, 746.4169, 747.4125, 750.4153, 752.4127, 755.4100, 756.4199, 757.4313, 761.4177, 767.4266, 768.3700, 768.3737, 770.3966, 772.4093, 775.4307, 779.4308, 784.3688, 786.4000, 787.4221, 788.4210, 792.4376, 795.4350, 798.4351, 800.4296, 812.4388, 815.4332, 822.4482, 825.4540, 832.4557, 835.4719, 837.4463, 842.5060, 844.4760, 856.4911, 860.4691, 862.4852, 869.4231, 885.4465, 907.4991, 908.4929, 913.5700, 913.5747, 916.5284, 918.5050, 919.5053, 922.5070, 928.5117, 934.5172, 940.4840, 942.4854, 943.4289, 943.5453, 946.5259, 950.4959, 951.5076, 953.5164, 959.5266, 966.5410, 969.5256, 972.5305, 974.5395, 976.5310, 978.5314, 979.5442, 991.5409, 993.5307, 1003.5496, 1011.5262, 1021.5579, 1023.5663, 1023.5700, 1031.5903, 1035.5381, 1036.5447, 1037.5573, 1039.5327, 1041.5658, 1044.5751, 1045.5729, 1046.5897, 1051.5448, 1056.5658, 1057.6078, 1067.5753, 1073.5902, 1086.5509, 1092.5961, 1100.6071, 1106.5391, 1111.5615, 1113.5729, 1118.5818, 1120.6045, 1123.5848, 1125.5823, 1132.6047, 1134.5714, 1139.5746, 1143.6318, 1144.6497, 1145.6407, 1147.6393, 1150.6108, 1155.6215, 1157.6199, 1158.6685, 1162.6182, 1163.6152, 1168.6215, 1173.6418, 1175.6089, 1178.6356, 1179.6157, 1181.6819, 1188.6544, 1197.6704, 1199.7006, 1208.6415, 1216.6714, 1227.6595, 1231.6387, 1232.6447, 1234.6672, 1235.6445, 1244.6453, 1247.6211, 1248.6174, 1255.6096, 1256.6531, 1261.6592, 1262.6453, 1266.6777, 1273.6260, 1275.6362, 1277.6531, 1301.7079, 1303.7076, 1307.7042, 1308.6376, 1312.6418, 1315.7098, 1320.6659, 1322.6886, 1323.6771, 1324.6964, 1328.7089, 1331.7202, 1335.7318, 1338.6581, 1339.6637, 1349.7253, 1352.7205, 1356.7230, 1360.6638, 1363.7153, 1365.7379, 1367.7319, 1369.7043, 1377.6947, 1379.7268, 1383.7137, 1385.7534, 1390.7284, 1393.7428, 1407.7563, 1417.7010, 1421.7061, 1422.7019, 1425.7727, 1427.7742, 1429.7365, 1431.7412, 1433.7228, 1434.7446, 1437.7584, 1467.7521, 1472.7633, 1475.7632, 1477.7545, 1484.7889, 1488.7537, 1491.7965, 1493.7579, 1500.7737, 1505.8177, 1513.8079, 1515.8213, 1517.7854, 1519.7544, 1522.7811, 1523.7953, 1529.8099, 1530.7523, 1531.7472, 1537.7974, 1539.8005, 1542.8422, 1545.8091, 1547.8051, 1549.7339, 1551.7778, 1553.8065, 1555.8101, 1556.8109, 1557.8010, 1561.8401, 1563.8109, 1565.8199, 1571.8400, 1579.7939, 1580.8077, 1583.8444, 1587.7620, 1592.8066, 1594.8335, 1604.8323, 1605.8586, 1608.8497, 1615.8754, 1624.8062, 1628.7900, 1628.7921, 1633.7985, 1640.9384, 1642.8099, 1646.8297, 1649.8447, 1657.8101, 1667.8622, 1677.7988, 1680.8746, 1689.9465,

1694.7288, 1698.9225, 1699.8862, 1704.8773, 1706.8701, 1707.8302, 1716.8671, 1719.9058, 1723.9011, 1725.8300, 1725.8346, 1727.8986, 1730.8931, 1737.9006, 1751.8656, 1754.8580, 1765.8463, 1766.8040, 1767.8138, 1769.8741, 1770.3567, 1772.9315, 1774.7610, 1774.9131, 1777.8770, 1780.8777, 1783.9246, 1787.8188, 1787.8200, 1791.8137, 1793.9050, 1795.9353, 1798.9340, 1800.9336, 1804.9142, 1805.9069, 1809.8469, 1815.9044, 1821.9144, 1828.9227, 1837.9396, 1840.9827, 1843.9688, 1844.9012, 1846.9153, 1852.9364, 1856.9908, 1861.8881, 1867.9218, 1874.9695, 1880.9265, 1884.9417, 1891.9622, 1894.9313, 1896.9078, 1896.9100, 1900.9546, 1905.9313, 1906.9501, 1908.9043, 1911.9130, 1912.9154, 1916.9923, 1918.9587, 1921.9825, 1923.9718, 1925.9573, 1928.0469, 1932.0060, 1933.9950, 1937.9630, 1939.9740, 1944.0040, 1946.9679, 1949.9595, 1954.0236, 1955.9738, 1958.9556, 1960.0034, 1968.8600, 1973.9845, 1976.9861, 1978.9910, 1981.9854, 1983.9884, 1987.9784, 1995.9408, 1998.9894, 2000.9926, 2008.0460, 2012.9590, 2015.0104, 2017.9747, 2021.0170, 2026.0275, 2027.0256, 2031.0166, 2044.0123, 2047.4957, 2047.9515, 2052.0496, 2060.0520, 2062.0073, 2064.9880, 2094.0376, 2097.0454, 2102.0667, 2103.1641, 2111.0305, 2127.0930, 2139.0630, 2144.0493, 2148.1023, 2155.0950, 2156.0796, 2160.0632, 2176.0664, 2190.9895, 2199.0723, 2202.1624, 2208.0862, 2211.1042, 2216.1062, 2225.1274, 2226.1272, 2229.0972, 2233.0984, 2239.0225, 2242.0435, 2268.1489, 2270.1287, 2271.1294, 2277.0823, 2284.1990, 2309.0994, 2334.1719, 2342.9915, 2350.1746, 2383.9607, 2398.2400, 2400.0862, 2413.0549, 2414.1897, 2417.0959, 2497.2800, 2501.2637, 2511.1460, 2513.1421, 2528.1750, 2537.2012, 2541.1948, 2542.1836, 2556.2058, 2562.1487, 2612.2666, 2706.2109, 2717.0869, 2767.2666, 2888.2532, 3221.1733, 3224.2917, 3312.3306, 3916.9434

17. E1BM24 **Mass:** 57270 **Score:** 23 **Expect:** 1.3e+002 **Matches:** 23
CCDC64B protein-like OS=Bos taurus GN=CCDC64B PE=4 SV=1

Observed	Mr(expt)	Mr(calc)	ppm	Start	End	Miss	Ions	Peptide
972.5305	971.5232	971.5036	20.2	214 -	221	0	---	R.QDLEAQIR.G
1045.5729	1044.5656	1044.5563	8.89	225 -	233	2	---	R.EELEKGRGK.L
1158.6685	1157.6612	1157.6656	-3.74	414 -	423	0	---	K.VLELSLELSR.V
1328.7089	1327.7016	1327.7208	-14.42	383 -	392	2	---	R.EELQRQKELR.V
1338.6581	1337.6508	1337.6575	-4.99	336 -	346	1	---	R.SPEEERLEPPR.K
1365.7379	1364.7306	1364.7272	2.47	249 -	259	2	---	R.REHGLELERAR.S
1484.7889	1483.7816	1483.7743	4.95	124 -	136	1	---	R.DVEALRAQLGEQR.S
1553.8065	1552.7992	1552.8038	-2.97	490 -	504	0	---	R.LSPGPAGGFFSTLFR.R
1804.9142	1803.9069	1803.8897	9.54	199 -	213	1	---	R.LESLQGENQMLQSRR.Q + Oxidation (M)
1846.9153	1845.9080	1845.9506	-23.04	408 -	423	1	---	R.DEAMNKVLELSLELSR.V
1874.9695	1873.9622	1873.9390	12.4	367 -	382	0	---	R.LQVEMAMQQAELQSLR.E
1905.9313	1904.9240	1904.9374	-7.02	197 -	212	1	---	R.TRLESLQGENQMLQSR.R + Oxidation (M)
1906.9501	1905.9428	1905.9288	7.36	367 -	382	0	---	R.LQVEMAMQQAELQSLR.E + 2 Oxidation (M)
1908.9043	1907.8970	1907.9085	-6.02	90 -	104	1	---	R.AEREEQLQQENHELR.R
1973.9845	1972.9772	1972.9311	23.4	130 -	146	2	---	R.AQLGEQRSEQQDSGRER.A
1983.9884	1982.9811	1983.0286	-23.92	106 -	123	2	---	R.GLAARGAEWEARAVELER.D
2015.0104	2014.0031	2013.9967	3.20	390 -	407	1	---	K.ELRVQDPEEALSSALSDR.D
2047.9515	2046.9442	2046.9818	-18.35	316 -	335	0	---	R.SGGALTTLSPETQEASSQQR.S
2062.0073	2061.0000	2061.0385	-18.67	197 -	213	2	---	R.TRLESLQGENQMLQSRR.Q + Oxidation (M)
2064.9880	2063.9807	2064.0096	-14.00	90 -	105	2	---	R.AEREEQLQQENHELRR.G
2199.0723	2198.0650	2198.1079	-19.52	111 -	129	2	---	R.GAEWEARAVELERDVEALR.A
2271.1294	2270.1221	2270.1502	-12.37	388 -	407	2	---	R.QKELRVQDPEEALSSALSDR.D
2284.1990	2283.1917	2283.2083	-7.28	469 -	489	1	---	R.QQELSAATSAPRPAPTRFSLR.L

No match to: 721.4365, 725.4001, 726.4063, 728.3681, 730.3848, 731.4017, 732.3573, 734.4151, 735.4143, 741.4101, 742.4515, 744.4171, 745.4167, 746.4169, 747.4125, 750.4153, 752.4127, 755.4100, 756.4199, 757.4313, 761.4177, 767.4266, 768.3700, 768.3737, 770.3966, 772.4093, 775.4307, 779.4308, 784.3688, 786.4000, 787.4221, 788.4210, 792.4376, 795.4350, 798.4351, 800.4296, 812.4388, 815.4332, 822.4482, 825.4540, 831.4382, 832.4557, 835.4719, 837.4463, 842.5060, 844.4760, 854.4837, 856.4911, 860.4691, 862.4852, 869.4231, 885.4465, 905.4955, 907.4991, 908.4929, 913.5700, 913.5747, 916.5284, 918.5050, 919.5053, 922.5070, 928.5117, 934.5172, 940.4840, 942.4854, 943.4289, 943.5453, 946.5259, 950.4959, 951.5076, 953.5164, 954.5388, 959.5266, 966.5410, 969.5256, 974.5395, 976.5310, 978.5314, 979.5442, 991.5409, 993.5307, 1001.5854, 1003.5496, 1011.5262, 1014.5215, 1021.5579, 1023.5663, 1023.5700, 1031.5903, 1035.5381, 1036.5447, 1037.5573, 1039.5327, 1041.5658, 1044.5751, 1046.5897, 1051.5448, 1056.5658, 1057.6078, 1067.5753, 1073.5902, 1086.5509, 1092.5961, 1100.6071, 1106.5391, 1111.5615, 1113.5729, 1118.5818, 1120.6045, 1122.5912, 1123.5848, 1125.5823, 1132.6047, 1134.5714, 1139.5746, 1143.6318, 1144.6497, 1145.6407, 1147.6393, 1150.6108, 1155.6215, 1157.6199, 1162.6182, 1163.6152, 1168.6215, 1173.6418, 1175.6089, 1178.6356, 1179.6157, 1181.6819, 1188.6544, 1197.6704, 1199.7006, 1208.6415, 1216.6714, 1227.6595, 1231.6387, 1232.6447, 1234.6672, 1235.6445, 1244.6453, 1247.6211, 1248.6174, 1250.6554, 1255.6096, 1256.6531, 1261.6592, 1262.6453, 1266.6777, 1273.6260, 1275.6362, 1276.6343, 1277.6531, 1301.7079, 1303.7076, 1307.7042, 1308.6376, 1310.6146, 1312.6418, 1315.7098, 1320.6659, 1322.6886, 1323.6771, 1324.6964, 1331.7202, 1335.7318, 1339.6637, 1349.7253, 1352.7205, 1356.7230, 1360.6638, 1363.7153, 1367.7319, 1369.7043, 1377.6947, 1379.7268, 1383.7137, 1385.7534, 1388.7404, 1390.7284, 1393.7428, 1407.7563, 1417.7010, 1421.7061, 1422.7019, 1425.7727, 1427.7742, 1429.7365, 1431.7412, 1433.7228, 1434.7446, 1437.7584, 1461.7778, 1467.7521, 1472.7633, 1475.7632, 1477.7545, 1488.7537, 1491.7965, 1493.7579, 1500.7737, 1505.8177, 1513.8079, 1515.8213, 1517.7854, 1519.7544, 1522.7811, 1523.7953, 1529.8099, 1530.7523, 1531.7472, 1537.7974, 1539.8005, 1542.8422, 1545.8091, 1547.8051, 1549.7339, 1551.7778, 1555.8101, 1556.8109, 1557.8010, 1561.8401, 1563.8109, 1565.8199, 1571.8400, 1579.7939, 1580.8077, 1583.8444, 1587.7620, 1592.8066, 1594.8335, 1604.8323, 1605.8586, 1608.8497, 1615.8754, 1624.8062, 1628.7900, 1628.7921, 1633.7985, 1640.9384, 1642.8099, 1646.8297, 1649.8447, 1657.8101, 1667.8622, 1677.7988, 1680.8746, 1689.9465, 1694.7288, 1698.9225, 1699.8862, 1704.8773, 1706.8701, 1707.8302, 1710.9279, 1716.8671, 1719.9058, 1723.9011, 1725.8300, 1725.8346, 1727.8986, 1730.8931, 1737.9006, 1751.8656, 1754.8580, 1765.8463, 1766.8040, 1767.8138, 1769.8741, 1770.3567, 1772.9315, 1774.7610, 1774.9131, 1777.8770, 1780.8777, 1783.9246, 1787.8188, 1787.8200, 1791.8137, 1793.9050, 1795.9353, 1798.9340, 1800.9336, 1805.9069, 1809.8469, 1815.9044, 1821.9144, 1828.9227, 1837.9396, 1840.9827, 1843.9688, 1844.9012, 1852.9364, 1856.9908, 1861.8881, 1867.9218, 1880.9265, 1884.9417, 1891.9622, 1894.9313, 1896.9078, 1896.9100, 1900.9546, 1911.9130, 1912.9154, 1916.9923, 1918.9587, 1921.9825, 1923.9718, 1925.9573, 1928.0469, 1932.0060, 1933.9950, 1937.9630, 1939.9740, 1944.0040, 1946.9679, 1949.9595, 1954.0236, 1955.9738, 1958.9556, 1960.0034, 1968.8600, 1976.9861, 1978.9910, 1981.9854, 1987.9784, 1995.9408, 1998.9894, 2000.9926, 2008.0460, 2012.9590, 2017.9747, 2021.0170, 2026.0275, 2027.0256, 2031.0166, 2044.0123, 2047.4957, 2052.0496, 2060.0520, 2066.9873, 2094.0376, 2097.0454, 2102.0667, 2103.1641, 2111.0305, 2127.0930, 2139.0630, 2144.0493, 2148.1023, 2155.0950, 2156.0796, 2160.0632, 2176.0664, 2190.9895, 2195.0774, 2202.1624, 2203.1221, 2208.0862, 2211.1042, 2216.1062, 2225.1274, 2226.1272, 2229.0972, 2233.0984, 2239.0225, 2242.0435, 2268.1489, 2270.1287, 2277.0823, 2292.1426, 2309.0994, 2334.1719, 2342.9915, 2350.1746, 2383.9607, 2398.2400, 2400.0862, 2413.0549, 2414.1897, 2417.0959, 2497.2800, 2501.2637, 2511.1460, 2513.1421, 2528.1750, 2537.2012, 2541.1948, 2542.1836, 2556.2058, 2562.1487, 2612.2666, 2706.2109, 2717.0869, 2767.2666, 2888.2532, 3221.1733, 3224.2917, 3312.3306, 3916.9434

18. F1MHM8 **Mass:** 57999 **Score:** 22 **Expect:** 1.6e+002 **Matches:** 18
Uncharacterized protein OS=Bos taurus GN=TIGD4 PE=4 SV=2

Observed	Mr(expt)	Mr(calc)	ppm	Start	End	Miss	Ions	Peptide
953.5164	952.5091	952.4866	23.7	338 -	346	0	---	K.FLSSVEGSK.E
1023.5663	1022.5590	1022.5430	15.7	50 -	58	1	---	K.KNSLSSIMK.N + Oxidation (M)
1023.5700	1022.5627	1022.5430	19.3	50 -	58	1	9	K.KNSLSSIMK.N + Oxidation (M)
1188.6544	1187.6471	1187.6397	6.22	473 -	483	0	---	K.NEAITALDTLK.T
1772.9315	1771.9242	1771.9390	-8.33	1 -	17	1	---	-.MAEAPVDASTLPVTVKK.K + Oxidation (M)
1793.9050	1792.8977	1792.9359	-21.31	61 -	75	2	---	K.DKVLEAFESLRFDPK.R
1828.9227	1827.9154	1827.9189	-1.89	79 -	93	1	---	R.LRTAFYTDLEEALMR.W
1844.9012	1843.8939	1843.9138	-10.78	79 -	93	1	---	R.LRTAFYTDLEEALMR.W + Oxidation (M)
1916.9923	1915.9850	1915.9502	18.2	241 -	256	1	---	R.CFKGIQSLPVYYEANK.M
2000.9926	1999.9853	2000.0149	-14.79	78 -	93	2	---	K.RLRTAFYTDLEEALMR.W + Oxidation (M)
2017.9747	2016.9674	2016.9649	1.26	191 -	208	1	---	R.MLPTNTFAFKGETCSIGK.L + Oxidation (M)
2064.9880	2063.9807	2063.9775	1.58	81 -	96	1	---	R.TAFYTDLEEALMRWYR.I
2094.0376	2093.0303	2092.9901	19.2	121 -	137	2	---	K.LGHNDFKCSNGWLDRFK.S

2176.0664	2175.0591	2175.1108	-23.76	301 -	319	1	---	K.SIELAFFPSCLSSKFIAMK.Q
2203.1221	2202.1148	2202.1190	-1.90	94 -	111	1	---	R.WYRIAQCLNVPVNGPMLR.L + Oxidation (M)
2284.1990	2283.1917	2283.1470	19.6	238 -	256	2	---	R.NPRCFKGIQSLPVYYEANK.M
2334.1719	2333.1646	2333.1626	0.86	79 -	96	2	---	R.LRTAFYTDLEEALMRWYR.I
2350.1746	2349.1673	2349.1575	4.16	79 -	96	2	---	R.LRTAFYTDLEEALMRWYR.I + Oxidation (M)

No match to: 721.4365, 725.4001, 726.4063, 728.3681, 730.3848, 731.4017, 732.3573, 734.4151, 735.4143, 741.4101, 742.4515, 744.4171, 745.4167, 746.4169, 747.4125, 750.4153, 752.4127, 755.4100, 756.4199, 757.4313, 761.4177, 767.4266, 768.3700, 768.3737, 770.3966, 772.4093, 775.4307, 779.4308, 784.3688, 786.4000, 787.4221, 788.4210, 792.4376, 795.4350, 798.4351, 800.4296, 812.4388, 815.4332, 822.4482, 825.4540, 831.4382, 832.4557, 835.4719, 837.4463, 842.5060, 844.4760, 854.4837, 856.4911, 860.4691, 862.4852, 869.4231, 885.4465, 905.4955, 907.4991, 908.4929, 913.5700, 913.5747, 916.5284, 918.5050, 919.5053, 922.5070, 928.5117, 934.5172, 940.4840, 942.4854, 943.4289, 943.5453, 946.5259, 950.4959, 951.5076, 954.5388, 959.5266, 966.5410, 969.5256, 972.5305, 974.5395, 976.5310, 978.5314, 979.5442, 991.5409, 993.5307, 1001.5854, 1003.5496, 1011.5262, 1014.5215, 1021.5579, 1031.5903, 1035.5381, 1036.5447, 1037.5573, 1039.5327, 1041.5658, 1044.5751, 1045.5729, 1046.5897, 1051.5448, 1056.5658, 1057.6078, 1067.5753, 1073.5902, 1086.5509, 1092.5961, 1100.6071, 1106.5391, 1111.5615, 1113.5729, 1118.5818, 1120.6045, 1122.5912, 1123.5848, 1125.5823, 1132.6047, 1134.5714, 1139.5746, 1143.6318, 1144.6497, 1145.6407, 1147.6393, 1150.6108, 1155.6215, 1157.6199, 1158.6685, 1162.6182, 1163.6152, 1168.6215, 1173.6418, 1175.6089, 1178.6356, 1179.6157, 1181.6819, 1197.6704, 1199.7006, 1208.6415, 1216.6714, 1227.6595, 1231.6387, 1232.6447, 1234.6672, 1235.6445, 1244.6453, 1247.6211, 1248.6174, 1250.6554, 1255.6096, 1256.6531, 1261.6592, 1262.6453, 1266.6777, 1273.6260, 1275.6362, 1276.6343, 1277.6531, 1301.7079, 1303.7076, 1307.7042, 1308.6376, 1310.6146, 1312.6418, 1315.7098, 1320.6659, 1322.6886, 1323.6771, 1324.6964, 1328.7089, 1331.7202, 1335.7318, 1338.6581, 1339.6637, 1349.7253, 1352.7205, 1356.7230, 1360.6638, 1363.7153, 1365.7379, 1367.7319, 1369.7043, 1377.6947, 1379.7268, 1383.7137, 1385.7534, 1388.7404, 1390.7284, 1393.7428, 1407.7563, 1417.7010, 1421.7061, 1422.7019, 1425.7727, 1427.7742, 1429.7365, 1431.7412, 1433.7228, 1434.7446, 1437.7584, 1461.7778, 1467.7521, 1472.7633, 1475.7632, 1477.7545, 1484.7889, 1488.7537, 1491.7965, 1493.7579, 1500.7737, 1505.8177, 1513.8079, 1515.8213, 1517.7854, 1519.7544, 1522.7811, 1523.7953, 1529.8099, 1530.7523, 1531.7472, 1537.7974, 1539.8005, 1542.8422, 1545.8091, 1547.8051, 1549.7339, 1551.7778, 1553.8065, 1555.8101, 1556.8109, 1557.8010, 1561.8401, 1563.8109, 1565.8199, 1571.8400, 1579.7939, 1580.8077, 1583.8444, 1587.7620, 1592.8066, 1594.8335, 1604.8323, 1605.8586, 1608.8497, 1615.8754, 1624.8062, 1628.7900, 1628.7921, 1633.7985, 1640.9384, 1642.8099, 1646.8297, 1649.8447, 1657.8101, 1667.8622, 1677.7988, 1680.8746, 1689.9465, 1694.7288, 1698.9225, 1699.8862, 1704.8773, 1706.8701, 1707.8302, 1710.9279, 1716.8671, 1719.9058, 1723.9011, 1725.8300, 1725.8346, 1727.8986, 1730.8931, 1737.9006, 1751.8656, 1754.8580, 1765.8463, 1766.8040, 1767.8138, 1769.8741, 1770.3567, 1774.7610, 1774.9131, 1777.8770, 1780.8777, 1783.9246, 1787.8188, 1787.8200, 1791.8137, 1795.9353, 1798.9340, 1800.9336, 1804.9142, 1805.9069, 1809.8469, 1815.9044, 1821.9144, 1837.9396, 1840.9827, 1843.9688, 1846.9153, 1852.9364, 1856.9908, 1861.8881, 1867.9218, 1874.9695, 1880.9265, 1884.9417, 1891.9622, 1894.9313, 1896.9078, 1896.9100, 1900.9546, 1905.9313, 1906.9501, 1908.9043, 1911.9130, 1912.9154, 1918.9587, 1921.9825, 1923.9718, 1925.9573, 1928.0469, 1932.0060, 1933.9950, 1937.9630, 1939.9740, 1944.0040, 1946.9679, 1949.9595, 1954.0236, 1955.9738, 1958.9556, 1960.0034, 1968.8600, 1973.9845, 1976.9861, 1978.9910, 1981.9854, 1983.9884, 1987.9784, 1995.9408, 1998.9894, 2008.0460, 2012.9590, 2015.0104, 2021.0170, 2026.0275, 2027.0256, 2031.0166, 2044.0123, 2047.4957, 2047.9515, 2052.0496, 2060.0520, 2062.0073, 2066.9873, 2097.0454, 2102.0667, 2103.1641, 2111.0305, 2127.0930, 2139.0630, 2144.0493, 2148.1023, 2155.0950, 2156.0796, 2160.0632, 2190.9895, 2195.0774, 2199.0723, 2202.1624, 2208.0862, 2211.1042, 2216.1062, 2225.1274, 2226.1272, 2229.0972, 2233.0984, 2239.0225, 2242.0435, 2268.1489, 2270.1287, 2271.1294, 2277.0823, 2292.1426, 2309.0994, 2342.9915, 2383.9607, 2398.2400, 2400.0862, 2413.0549, 2414.1897, 2417.0959, 2497.2800, 2501.2637, 2511.1460, 2513.1421, 2528.1750, 2537.2012, 2541.1948, 2542.1836, 2556.2058, 2562.1487, 2612.2666, 2706.2109, 2717.0869, 2767.2666, 2888.2532, 3221.1733, 3224.2917, 3312.3306, 3916.9434

19. P08057 **Mass:** 24153 **Score:** 22 **Expect:** 1.7e+002 **Matches:** 13

Troponin I, cardiac muscle OS=Bos taurus GN=TNNI3 PE=1 SV=2

Observed	Mr(expt)	Mr(calc)	ppm	Start	End	Miss	Ions	Peptide
746.4169	745.4096	745.3970	16.9	167 -	172	0	---	K.ETLDLR.A
966.5410	965.5337	965.5406	-7.15	101 -	108	1	---	R.QLHARVDK.V
1434.7446	1433.7373	1433.7296	5.37	196 -	208	2	---	K.NIDALSGMEGRKK.K + Oxidation (M)
1565.8199	1564.8126	1564.7846	17.9	5 -	21	0	---	R.SGGSTAGDTVPAPPPVR.R
1594.8335	1593.8262	1593.8223	2.46	101 -	113	2	---	R.QLHARVDKVDEER.Y
1680.8746	1679.8673	1679.8366	18.3	109 -	122	2	---	K.VDEERYDVEAKVTK.N
1793.9050	1792.8977	1792.8856	6.77	24 -	39	2	---	R.SSANYRAYATEPHAKK.K
1821.9144	1820.9071	1820.8917	8.45	23 -	38	2	---	R.RSSANYRAYATEPHAK.K
1874.9695	1873.9622	1873.9978	-18.99	148 -	164	2	---	R.RVRISADAMMQALLGAR.A + Oxidation (M)
1933.9950	1932.9877	1933.0237	-18.61	149 -	166	2	---	R.VRISADAMMQALLGARAK.E + 2 Oxidation (M)
2195.0774	2194.0701	2194.0913	-9.66	1 -	22	2	---	-.MADRSGGSTAGDTVPAPPPVRR.R
2203.1221	2202.1148	2202.1314	-7.51	53 -	70	2	---	K.TLMLQIAKQELEREAEER.R + Oxidation (M)
2211.1042	2210.0969	2210.0862	4.84	1 -	22	2	---	-.MADRSGGSTAGDTVPAPPPVRR.R + Oxidation (M)

No match to: 721.4365, 725.4001, 726.4063, 728.3681, 730.3848, 731.4017, 732.3573, 734.4151, 735.4143, 741.4101, 742.4515, 744.4171, 745.4167, 747.4125, 750.4153, 752.4127, 755.4100, 756.4199, 757.4313, 761.4177, 767.4266, 768.3700, 768.3737, 770.3966, 772.4093, 775.4307, 779.4308, 784.3688, 786.4000, 787.4221, 788.4210, 792.4376, 795.4350, 798.4351, 800.4296, 812.4388, 815.4332, 822.4482, 825.4540, 831.4382, 832.4557, 835.4719, 837.4463, 842.5060, 844.4760, 854.4837, 856.4911, 860.4691, 862.4852, 869.4231, 885.4465, 905.4955, 907.4991, 908.4929, 913.5700, 913.5747, 916.5284, 918.5050, 919.5053, 922.5070, 928.5117, 934.5172, 940.4840, 942.4854, 943.4289, 943.5453, 946.5259, 950.4959, 951.5076, 953.5164, 954.5388, 959.5266, 969.5256, 972.5305, 974.5395, 976.5310, 978.5314, 979.5442, 991.5409, 993.5307, 1001.5854, 1003.5496, 1011.5262, 1014.5215, 1021.5579, 1023.5663, 1023.5700, 1031.5903, 1035.5381, 1036.5447, 1037.5573, 1039.5327, 1041.5658, 1044.5751, 1045.5729, 1046.5897, 1051.5448, 1056.5658, 1057.6078, 1067.5753, 1073.5902, 1086.5509, 1092.5961, 1100.6071, 1106.5391, 1111.5615, 1113.5729, 1118.5818, 1120.6045, 1122.5912, 1125.5823, 1132.6047, 1134.5714, 1139.5746, 1143.6318, 1144.6497, 1145.6407, 1147.6393, 1150.6108, 1155.6215, 1157.6199, 1158.6685, 1162.6182, 1163.6152, 1168.6215, 1173.6418, 1175.6089, 1178.6356, 1179.6157, 1181.6819, 1188.6544, 1197.6704, 1199.7006, 1208.6415, 1216.6714, 1227.6595, 1231.6387, 1232.6447, 1234.6672, 1235.6445, 1244.6453, 1247.6211, 1248.6174, 1250.6554, 1255.6096, 1256.6531, 1261.6592, 1262.6453, 1266.6777, 1273.6260, 1275.6362, 1276.6343, 1277.6531, 1301.7079, 1303.7076, 1307.7042, 1308.6376, 1310.6146, 1312.6418, 1315.7098, 1320.6659, 1322.6886, 1323.6771, 1324.6964, 1328.7089, 1331.7202, 1335.7318, 1338.6581, 1339.6637, 1349.7253, 1352.7205, 1356.7230, 1360.6638, 1363.7153, 1365.7379, 1367.7319, 1369.7043, 1377.6947, 1379.7268, 1383.7137, 1385.7534, 1388.7404, 1390.7284, 1393.7428, 1407.7563, 1417.7010, 1421.7061, 1422.7019, 1425.7727, 1427.7742, 1429.7365, 1431.7412, 1433.7228, 1437.7584, 1461.7778, 1467.7521, 1472.7633, 1475.7632, 1477.7545, 1484.7889, 1488.7537, 1491.7965, 1493.7579, 1500.7737, 1505.8177, 1513.8079, 1515.8213, 1517.7854, 1519.7544, 1522.7811, 1523.7953, 1529.8099, 1530.7523, 1531.7472, 1537.7974, 1539.8005, 1542.8422, 1545.8091, 1547.8051, 1549.7339, 1551.7778, 1553.8065, 1555.8101, 1556.8109, 1557.8010, 1561.8401, 1563.8109, 1571.8400, 1579.7939, 1580.8077, 1583.8444, 1587.7620, 1592.8066, 1604.8323, 1605.8586, 1608.8497, 1615.8754, 1624.8062, 1628.7900, 1628.7921, 1633.7985, 1640.9384, 1642.8099, 1646.8297, 1649.8447, 1657.8101, 1667.8622, 1677.7988, 1689.9465, 1694.7288, 1698.9225, 1699.8862, 1704.8773, 1706.8701, 1707.8302, 1710.9279, 1716.8671, 1719.9058, 1723.9011, 1725.8300, 1725.8346, 1727.8986, 1730.8931, 1737.9006, 1751.8656, 1754.8580, 1765.8463, 1766.8040, 1767.8138, 1769.8741, 1770.3567, 1772.9315, 1774.7610, 1774.9131, 1777.8770, 1780.8777, 1783.9246, 1787.8188, 1787.8200, 1791.8137, 1795.9353, 1798.9340, 1800.9336, 1804.9142, 1805.9069, 1809.8469, 1815.9044, 1828.9227, 1837.9396, 1840.9827, 1843.9688, 1844.9012, 1846.9153, 1852.9364, 1856.9908, 1861.8881, 1867.9218, 1880.9265, 1884.9417, 1891.9622, 1894.9313, 1896.9078, 1896.9100, 1900.9546, 1905.9313, 1906.9501, 1908.9043, 1911.9130, 1912.9154, 1916.9923, 1918.9587, 1921.9825, 1923.9718, 1925.9573, 1928.0469, 1932.0060, 1937.9630, 1939.9740, 1944.0040, 1946.9679, 1949.9595, 1954.0236, 1955.9738, 1958.9556, 1960.0034, 1968.8600, 1973.9845, 1976.9861, 1978.9910, 1981.9854, 1983.9884, 1987.9784, 1995.9408, 1998.9894, 2000.9926, 2008.0460, 2012.9590, 2015.0104, 2017.9747, 2021.0170, 2026.0275, 2027.0256, 2031.0166, 2044.0123, 2047.4957, 2047.9515, 2052.0496, 2060.0520, 2062.0073, 2064.9880, 2066.9873, 2094.0376, 2097.0454, 2102.0667, 2103.1641, 2111.0305, 2127.0930, 2139.0630, 2144.0493, 2148.1023, 2155.0950, 2156.0796, 2160.0632, 2176.0664, 2190.9895, 2199.0723, 2202.1624, 2208.0862, 2216.1062, 2225.1274, 2226.1272, 2229.0972, 2233.0984, 2239.0225, 2242.0435, 2268.1489, 2270.1287, 2271.1294, 2277.0823, 2284.1990, 2292.1426, 2309.0994, 2334.1719, 2342.9915, 2350.1746, 2383.9607, 2398.2400, 2400.0862, 2413.0549, 2414.1897, 2417.0959, 2497.2800, 2501.2637, 2511.1460, 2513.1421, 2528.1750, 2537.2012, 2541.1948, 2542.1836, 2556.2058, 2562.1487, 2612.2666, 2706.2109, 2717.0869, 2767.2666, 2888.2532, 3221.1733, 3224.2917, 3312.3306, 3916.9434

20. G3X720 **Mass:** 22128 **Score:** 22 **Expect:** 1.7e+002 **Matches:** 12

Uncharacterized protein OS=Bos taurus GN=C19orf81 PE=4 SV=1

Observed	Mr(expt)	Mr(calc)	ppm	Start	End	Miss	Ions	Peptide
787.4221	786.4148	786.4058	11.5	64 -	69	0	---	R.ELPCIR.K

1519.7544	1518.7471	1518.7096	24.7	30 -	42	1	---	R.ETPEEMQARSLGR.S + Oxidation (M)
1624.8062	1623.7989	1623.7610	23.4	119 -	132	1	---	R.FENMNVICGTAGRR.D
1680.8746	1679.8673	1679.8454	13.1	133 -	145	1	---	R.DRWLIMVTDFQTR.S
1852.9364	1851.9291	1851.9414	-6.62	132 -	145	2	---	R.RDRWLIMVTDFQTR.S + Oxidation (M)
1911.9130	1910.9057	1910.8839	11.4	119 -	134	2	---	R.FENMNVICGTAGRRDR.W + Oxidation (M)
1923.9718	1922.9645	1922.9785	-7.27	133 -	147	2	---	R.DRWLIMVTDFQTRSR.L
1939.9740	1938.9667	1938.9734	-3.45	133 -	147	2	---	R.DRWLIMVTDFQTRSR.L + Oxidation (M)
2000.9926	1999.9853	1999.9633	11.0	21 -	38	1	---	R.DAGALLVDRETPEEMQAR.S
2027.0256	2026.0183	2025.9724	22.7	114 -	131	1	---	R.VSSIRFENMNVICGTAGR.R + Oxidation (M)
2203.1221	2202.1148	2202.1466	-14.45	53 -	70	2	---	R.QVIAEYEALDRELPCIRK.F
2414.1897	2413.1824	2413.2019	-8.09	21 -	42	2	---	R.DAGALLVDRETPEEMQARSLGR.S

No match to: 721.4365, 725.4001, 726.4063, 728.3681, 730.3848, 731.4017, 732.3573, 734.4151, 735.4143, 741.4101, 742.4515, 744.4171, 745.4167, 746.4169, 747.4125, 750.4153, 752.4127, 755.4100, 756.4199, 757.4313, 761.4177, 767.4266, 768.3700, 768.3737, 770.3966, 772.4093, 775.4307, 779.4308, 784.3688, 786.4000, 788.4210, 792.4376, 795.4350, 798.4351, 800.4296, 812.4388, 815.4332, 822.4482, 825.4540, 831.4382, 832.4557, 835.4719, 837.4463, 842.5060, 844.4760, 854.4837, 856.4911, 860.4691, 862.4852, 869.4231, 885.4465, 905.4955, 907.4991, 908.4929, 913.5700, 913.5747, 916.5284, 918.5050, 919.5053, 922.5070, 928.5117, 934.5172, 940.4840, 942.4854, 943.4289, 943.5453, 946.5259, 950.4959, 951.5076, 953.5164, 954.5388, 959.5266, 966.5410, 969.5256, 972.5305, 974.5395, 976.5310, 978.5314, 979.5442, 991.5409, 993.5307, 1001.5854, 1003.5496, 1011.5262, 1014.5215, 1021.5579, 1023.5663, 1023.5700, 1031.5903, 1035.5381, 1036.5447, 1037.5573, 1039.5327, 1041.5658, 1044.5751, 1045.5729, 1046.5897, 1051.5448, 1056.5658, 1057.6078, 1067.5753, 1073.5902, 1086.5509, 1092.5961, 1100.6071, 1106.5391, 1111.5615, 1113.5729, 1118.5818, 1120.6045, 1122.5912, 1123.5848, 1125.5823, 1132.6047, 1134.5714, 1139.5746, 1143.6318, 1144.6497, 1145.6407, 1147.6393, 1150.6108, 1155.6215, 1157.6199, 1158.6685, 1162.6182, 1163.6152, 1168.6215, 1173.6418, 1175.6089, 1178.6356, 1179.6157, 1181.6819, 1188.6544, 1197.6704, 1199.7006, 1208.6415, 1216.6714, 1227.6595, 1231.6387, 1232.6447, 1234.6672, 1235.6445, 1244.6453, 1247.6211, 1248.6174, 1250.6554, 1255.6096, 1256.6531, 1261.6592, 1262.6453, 1266.6777, 1273.6260, 1275.6362, 1276.6343, 1277.6531, 1301.7079, 1303.7076, 1307.7042, 1308.6376, 1310.6146, 1312.6418, 1315.7098, 1320.6659, 1322.6886, 1323.6771, 1324.6964, 1328.7089, 1331.7202, 1335.7318, 1338.6581, 1339.6637, 1349.7253, 1352.7205, 1356.7230, 1360.6638, 1363.7153, 1365.7379, 1367.7319, 1369.7043, 1377.6947, 1379.7268, 1383.7137, 1385.7534, 1388.7404, 1390.7284, 1393.7428, 1407.7563, 1417.7010, 1421.7061, 1422.7019, 1425.7727, 1427.7742, 1429.7365, 1431.7412, 1433.7228, 1434.7446, 1437.7584, 1461.7778, 1467.7521, 1472.7633, 1475.7632, 1477.7545, 1484.7889, 1488.7537, 1491.7965, 1493.7579, 1500.7737, 1505.8177, 1513.8079, 1515.8213, 1517.7854, 1522.7811, 1523.7953, 1529.8099, 1530.7523, 1531.7472, 1537.7974, 1539.8005, 1542.8422, 1545.8091, 1547.8051, 1549.7339, 1551.7778, 1553.8065, 1555.8101, 1556.8109, 1557.8010, 1561.8401, 1563.8109, 1565.8199, 1571.8400, 1579.7939, 1580.8077, 1583.8444, 1587.7620, 1592.8066, 1594.8335, 1604.8323, 1605.8586, 1608.8497, 1615.8754, 1628.7900, 1628.7921, 1633.7985, 1640.9384, 1642.8099, 1646.8297, 1649.8447, 1657.8101, 1667.8622, 1677.7988, 1689.9465, 1694.7288, 1698.9225, 1699.8862, 1704.8773, 1706.8701, 1707.8302, 1710.9279, 1716.8671, 1719.9058, 1723.9011, 1725.8300, 1725.8346, 1727.8986, 1730.8931, 1737.9006, 1751.8656, 1754.8580, 1765.8463, 1766.8040, 1767.8138, 1769.8741, 1770.3567, 1772.9315, 1774.7610, 1774.9131, 1777.8770, 1780.8777, 1783.9246, 1787.8188, 1787.8200, 1791.8137, 1793.9050, 1795.9353, 1798.9340, 1800.9336, 1804.9142, 1805.9069, 1809.8469, 1815.9044, 1821.9144, 1828.9227, 1837.9396, 1840.9827, 1843.9688, 1844.9012, 1846.9153, 1856.9908, 1861.8881, 1867.9218, 1874.9695, 1880.9265, 1884.9417, 1891.9622, 1894.9313, 1896.9078, 1896.9100, 1900.9546, 1905.9313, 1906.9501, 1908.9043, 1912.9154, 1916.9923, 1918.9587, 1921.9825, 1925.9573, 1928.0469, 1932.0060, 1933.9950, 1937.9630, 1944.0040, 1946.9679, 1949.9595, 1954.0236, 1955.9738, 1958.9556, 1960.0034, 1968.8600, 1973.9845, 1976.9861, 1978.9910, 1981.9854, 1983.9884, 1987.9784, 1995.9408, 1998.9894, 2008.0460, 2012.9590, 2015.0104, 2017.9747, 2021.0170, 2026.0275, 2031.0166, 2044.0123, 2047.4957, 2047.9515, 2052.0496, 2060.0520, 2062.0073, 2064.9880, 2066.9873, 2094.0376, 2097.0454, 2102.0667, 2103.1641, 2111.0305, 2127.0930, 2139.0630, 2144.0493, 2148.1023, 2155.0950, 2156.0796, 2160.0632, 2176.0664, 2190.9895, 2195.0774, 2199.0723, 2202.1624, 2208.0862, 2211.1042, 2216.1062, 2225.1274, 2226.1272, 2229.0972, 2233.0984, 2239.0225, 2242.0435, 2268.1489, 2270.1287, 2271.1294, 2277.0823, 2284.1990, 2292.1426, 2309.0994, 2334.1719, 2342.9915, 2350.1746, 2383.9607, 2398.2400, 2400.0862, 2413.0549, 2417.0959, 2497.2800, 2501.2637, 2511.1460, 2513.1421, 2528.1750, 2537.2012, 2541.1948, 2542.1836, 2556.2058, 2562.1487, 2612.2666, 2706.2109, 2717.0869, 2767.2666, 2888.2532, 3221.1733, 3224.2917, 3312.3306, 3916.9434

21. Q58DU0 **Mass:** 26366 **Score:** 21 **Expect:** 1.8e+002 **Matches:** 12

Multiple myeloma tumor-associated protein 2 homolog OS=Bos taurus GN=MMTAG2 PE=2 SV=1

Observed	Mr(expt)	Mr(calc)	ppm	Start	End	Miss	Ions	Peptide
761.4177	760.4104	760.4079	3.37	168 -	173	1	---	K.LRSEEK.V
922.5070	921.4997	921.4781	23.5	2 -	10	1	---	M.FGSSRGGVR.G
1199.7006	1198.6933	1198.7034	-8.37	86 -	96	2	---	K.NVKKQPTGLSK.E
1322.6886	1321.6813	1321.6891	-5.88	45 -	55	2	---	K.GRDLTWYAKGR.A
1522.7811	1521.7738	1521.7497	15.9	72 -	85	0	---	R.QAEQEALMAALGYK.N
1551.7778	1550.7705	1550.7994	-18.61	42 -	53	2	---	R.WQKGRDLTWYAK.G
1565.8199	1564.8126	1564.8474	-22.22	138 -	151	1	---	K.EAAKLGLSVFTHHR.V
1804.9142	1803.9069	1803.9050	1.08	26 -	41	1	---	K.QRENYLGNSLMAPVGR.W
1978.9910	1977.9837	1977.9731	5.39	28 -	44	1	---	R.ENYLGNSLMAPVGRWQK.G + Oxidation (M)
2008.0460	2007.0387	2007.0459	-3.55	72 -	89	2	---	R.QAEQEALMAALGYKNVKK.Q + Oxidation (M)
2176.0664	2175.0591	2175.1007	-19.12	28 -	46	2	---	R.ENYLGNSLMAPVGRWQKGR.D
2417.0959	2416.0886	2416.0999	-4.65	208 -	229	1	---	R.LDSSLAPAPIQHDSDSSTCCKK.R

No match to: 721.4365, 725.4001, 726.4063, 728.3681, 730.3848, 731.4017, 732.3573, 734.4151, 735.4143, 741.4101, 742.4515, 744.4171, 745.4167, 746.4169, 747.4125, 750.4153, 752.4127, 755.4100, 756.4199, 757.4313, 767.4266, 768.3700, 768.3737, 770.3966, 772.4093, 775.4307, 779.4308, 784.3688, 786.4000, 787.4221, 788.4210, 792.4376, 795.4350, 798.4351, 800.4296, 812.4388, 815.4332, 822.4482, 825.4540, 831.4382, 832.4557, 835.4719, 837.4463, 842.5060, 844.4760, 854.4837, 856.4911, 860.4691, 862.4852, 869.4231, 885.4465, 905.4955, 907.4991, 908.4929, 913.5700, 913.5747, 916.5284, 918.5050, 919.5053, 928.5117, 934.5172, 940.4840, 942.4854, 943.4289, 943.5453, 946.5259, 950.4959, 951.5076, 953.5164, 954.5388, 959.5266, 966.5410, 969.5256, 972.5305, 974.5395, 976.5310, 978.5314, 979.5442, 991.5409, 993.5307, 1001.5854, 1003.5496, 1011.5262, 1014.5215, 1021.5579, 1023.5663, 1023.5700, 1031.5903, 1035.5381, 1036.5447, 1037.5573, 1039.5327, 1041.5658, 1044.5751, 1045.5729, 1046.5897, 1051.5448, 1056.5658, 1057.6078, 1067.5753, 1073.5902, 1086.5509, 1092.5961, 1100.6071, 1106.5391, 1111.5615, 1113.5729, 1118.5818, 1120.6045, 1122.5912, 1123.5848, 1125.5823, 1132.6047, 1134.5714, 1139.5746, 1143.6318, 1144.6497, 1145.6407, 1147.6393, 1150.6108, 1155.6215, 1157.6199, 1158.6685, 1162.6182, 1163.6152, 1168.6215, 1173.6418, 1175.6089, 1178.6356, 1179.6157, 1181.6819, 1188.6544, 1197.6704, 1208.6415, 1216.6714, 1227.6595, 1231.6387, 1232.6447, 1234.6672, 1235.6445, 1244.6453, 1247.6211, 1248.6174, 1250.6554, 1255.6096, 1256.6531, 1261.6592, 1262.6453, 1266.6777, 1273.6260, 1275.6362, 1276.6343, 1277.6531, 1301.7079, 1303.7076, 1307.7042, 1308.6376, 1310.6146, 1312.6418, 1315.7098, 1320.6659, 1323.6771, 1324.6964, 1328.7089, 1331.7202, 1335.7318, 1338.6581, 1339.6637, 1349.7253, 1352.7205, 1356.7230, 1360.6638, 1363.7153, 1365.7379, 1367.7319, 1369.7043, 1377.6947, 1379.7268, 1383.7137, 1385.7534, 1388.7404, 1390.7284, 1393.7428, 1407.7563, 1417.7010, 1421.7061, 1422.7019, 1425.7727, 1427.7742, 1429.7365, 1431.7412, 1433.7228, 1434.7446, 1437.7584, 1461.7778, 1467.7521, 1472.7633, 1475.7632, 1477.7545, 1484.7889, 1488.7537, 1491.7965, 1493.7579, 1500.7737, 1505.8177, 1513.8079, 1515.8213, 1517.7854, 1519.7544, 1523.7953, 1529.8099, 1530.7523, 1531.7472, 1537.7974, 1539.8005, 1542.8422, 1545.8091, 1547.8051, 1549.7339, 1553.8065, 1555.8101, 1556.8109, 1557.8010, 1561.8401, 1563.8109, 1571.8400, 1579.7939, 1580.8077, 1583.8444, 1587.7620, 1592.8066, 1594.8335, 1604.8323, 1605.8586, 1608.8497, 1615.8754, 1624.8062, 1628.7900, 1628.7921, 1633.7985, 1640.9384, 1642.8099, 1646.8297, 1649.8447, 1657.8101, 1667.8622, 1677.7988, 1680.8746, 1689.9465, 1694.7288, 1698.9225, 1699.8862, 1704.8773, 1706.8701, 1707.8302, 1710.9279, 1716.8671, 1719.9058, 1723.9011, 1725.8300, 1725.8346, 1727.8986, 1730.8931, 1737.9006, 1751.8656, 1754.8580, 1765.8463, 1766.8040, 1767.8138, 1769.8741, 1770.3567, 1772.9315, 1774.7610, 1774.9131, 1777.8770, 1780.8777, 1783.9246, 1787.8188, 1787.8200, 1791.8137, 1793.9050, 1795.9353, 1798.9340, 1800.9336, 1805.9069, 1809.8469, 1815.9044, 1821.9144, 1828.9227, 1837.9396, 1840.9827, 1843.9688, 1844.9012, 1846.9153, 1852.9364, 1856.9908, 1861.8881, 1867.9218, 1874.9695, 1880.9265, 1884.9417, 1891.9622, 1894.9313, 1896.9078, 1896.9100, 1900.9546, 1905.9313, 1906.9501, 1908.9043, 1911.9130, 1912.9154, 1916.9923, 1918.9587, 1921.9825, 1923.9718, 1925.9573, 1928.0469, 1932.0060, 1933.9950, 1937.9630, 1939.9740, 1944.0040, 1946.9679, 1949.9595, 1954.0236, 1955.9738, 1958.9556, 1960.0034, 1968.8600, 1973.9845, 1976.9861, 1981.9854, 1983.9884, 1987.9784, 1995.9408, 1998.9894, 2000.9926, 2012.9590, 2015.0104, 2017.9747, 2021.0170, 2026.0275, 2027.0256, 2031.0166, 2044.0123, 2047.4957, 2047.9515, 2052.0496, 2060.0520, 2062.0073, 2064.9880, 2066.9873, 2094.0376, 2097.0454, 2102.0667, 2103.1641, 2111.0305, 2127.0930, 2139.0630, 2144.0493, 2148.1023, 2155.0950, 2156.0796, 2160.0632, 2190.9895, 2195.0774, 2199.0723, 2202.1624, 2203.1221, 2208.0862, 2211.1042, 2216.1062, 2225.1274, 2226.1272, 2229.0972, 2233.0984, 2239.0225, 2242.0435, 2268.1489, 2270.1287, 2271.1294, 2277.0823, 2284.1990, 2292.1426, 2309.0994, 2334.1719, 2342.9915, 2350.1746, 2383.9607, 2398.2400, 2400.0862, 2413.0549, 2414.1897, 2497.2800, 2501.2637, 2511.1460, 2513.1421, 2528.1750, 2537.2012, 2541.1948, 2542.1836, 2556.2058, 2562.1487, 2612.2666, 2706.2109, 2717.0869, 2767.2666, 2888.2532, 3221.1733, 3224.2917, 3312.3306, 3916.9434

22. Q28024 **Mass:** 8166 **Score:** 21 **Expect:** 1.8e+002 **Matches:** 10
Guanine nucleotide-binding protein G(I)/G(S)/G(O) subunit gamma-12 OS=Bos taurus GN=GNG12 PE=1 SV=2

Observed	Mr(expt)	Mr(calc)	ppm	Start	End	Miss	Ions	Peptide
735.4143	734.4070	734.3997	9.96	67 -	72	1	---	K.KTCTIL.-
978.5314	977.5241	977.5216	2.59	65 -	72	2	---	K.DKKTCTIL.-
1092.5961	1091.5888	1091.5645	22.3	23 -	31	1	---	R.MEASIERIK.V + Oxidation (M)
1579.7939	1578.7866	1578.7784	5.23	1 -	15	1	---	-.MSSKTASTNNIAQAR.R
1604.8323	1603.8250	1603.8390	-8.71	2 -	16	2	---	M.SSKTASTNNIAQARR.T
1751.8656	1750.8583	1750.8744	-9.18	1 -	16	2	---	-.MSSKTASTNNIAQARR.T + Oxidation (M)
1772.9315	1771.9242	1771.8815	24.1	49 -	64	0	---	R.NDPLLMGIPTSENPFK.D
2160.0632	2159.0559	2159.0932	-17.28	49 -	67	2	---	R.NDPLLMGIPTSENPFKDKK.T + Oxidation (M)
2195.0774	2194.0701	2194.0510	8.70	30 -	48	2	---	R.IKVSKASADLMSYCEEHAR.N
2211.1042	2210.0969	2210.0459	23.1	30 -	48	2	---	R.IKVSKASADLMSYCEEHAR.N + Oxidation (M)

No match to: 721.4365, 725.4001, 726.4063, 728.3681, 730.3848, 731.4017, 732.3573, 734.4151, 741.4101, 742.4515, 744.4171, 745.4167, 746.4169, 747.4125, 750.4153, 752.4127, 755.4100, 756.4199, 757.4313, 761.4177, 767.4266, 768.3700, 768.3737, 770.3966, 772.4093, 775.4307, 779.4308, 784.3688, 786.4000, 787.4221, 788.4210, 792.4376, 795.4350, 798.4351, 800.4296, 812.4388, 815.4332, 822.4482, 825.4540, 831.4382, 832.4557, 835.4719, 837.4463, 842.5060, 844.4760, 854.4837, 856.4911, 860.4691, 862.4852, 869.4231, 885.4465, 905.4955, 907.4991, 908.4929, 913.5700, 913.5747, 916.5284, 918.5050, 919.5053, 922.5070, 928.5117, 934.5172, 940.4840, 942.4854, 943.4289, 943.5453, 946.5259, 950.4959, 951.5076, 953.5164, 954.5388, 959.5266, 966.5410, 969.5256, 972.5305, 974.5395, 976.5310, 979.5442, 991.5409, 993.5307, 1001.5854, 1003.5496, 1011.5262, 1014.5215, 1021.5579, 1023.5663, 1023.5700, 1031.5903, 1035.5381, 1036.5447, 1037.5573, 1039.5327, 1041.5658, 1044.5751, 1045.5729, 1046.5897, 1051.5448, 1056.5658, 1057.6078, 1067.5753, 1073.5902, 1086.5509, 1100.6071, 1106.5391, 1111.5615, 1113.5729, 1118.5818, 1120.6045, 1122.5912, 1123.5848, 1125.5823, 1132.6047, 1134.5714, 1139.5746, 1143.6318, 1144.6497, 1145.6407, 1147.6393, 1150.6108, 1155.6215, 1157.6199, 1158.6685, 1162.6182, 1163.6152, 1168.6215, 1173.6418, 1175.6089, 1178.6356, 1179.6157, 1181.6819, 1188.6544, 1197.6704, 1199.7006, 1208.6415, 1216.6714, 1227.6595, 1231.6387, 1232.6447, 1234.6672, 1235.6445, 1244.6453, 1247.6211, 1248.6174, 1250.6554, 1255.6096, 1256.6531, 1261.6592, 1262.6453, 1266.6777, 1273.6260, 1275.6362, 1276.6343, 1277.6531, 1301.7079, 1303.7076, 1307.7042, 1308.6376, 1310.6146, 1312.6418, 1315.7098, 1320.6659, 1322.6886, 1323.6771, 1324.6964, 1328.7089, 1331.7202, 1335.7318, 1338.6581, 1339.6637, 1349.7253, 1352.7205, 1356.7230, 1360.6638, 1363.7153, 1365.7379, 1367.7319, 1369.7043, 1377.6947, 1379.7268, 1383.7137, 1385.7534, 1388.7404, 1390.7284, 1393.7428, 1407.7563, 1417.7010, 1421.7061, 1422.7019, 1425.7727, 1427.7742, 1429.7365, 1431.7412, 1433.7228, 1434.7446, 1437.7584, 1461.7778, 1467.7521, 1472.7633, 1475.7632, 1477.7545, 1484.7889, 1488.7537, 1491.7965, 1493.7579, 1500.7737, 1505.8177, 1513.8079, 1515.8213, 1517.7854, 1519.7544, 1522.7811, 1523.7953, 1529.8099, 1530.7523, 1531.7472, 1537.7974, 1539.8005, 1542.8422, 1545.8091, 1547.8051, 1549.7339, 1551.7778, 1553.8065, 1555.8101, 1556.8109, 1557.8010, 1561.8401, 1563.8109, 1565.8199, 1571.8400, 1580.8077, 1583.8444, 1587.7620, 1592.8066, 1594.8335, 1605.8586, 1608.8497, 1615.8754, 1624.8062, 1628.7900, 1628.7921, 1633.7985, 1640.9384, 1642.8099, 1646.8297, 1649.8447, 1657.8101, 1667.8622, 1677.7988, 1680.8746, 1689.9465, 1694.7288, 1698.9225, 1699.8862, 1704.8773, 1706.8701, 1707.8302, 1710.9279, 1716.8671, 1719.9058, 1723.9011, 1725.8300, 1725.8346, 1727.8986, 1730.8931, 1737.9006, 1754.8580, 1765.8463, 1766.8040, 1767.8138, 1769.8741, 1770.3567, 1774.7610, 1774.9131, 1777.8770, 1780.8777, 1783.9246, 1787.8188, 1787.8200, 1791.8137, 1793.9050, 1795.9353, 1798.9340, 1800.9336, 1804.9142, 1805.9069, 1809.8469, 1815.9044, 1821.9144, 1828.9227, 1837.9396, 1840.9827, 1843.9688, 1844.9012, 1846.9153, 1852.9364, 1856.9908, 1861.8881, 1867.9218, 1874.9695, 1880.9265, 1884.9417, 1891.9622, 1894.9313, 1896.9078, 1896.9100, 1900.9546, 1905.9313, 1906.9501, 1908.9043, 1911.9130, 1912.9154, 1916.9923, 1918.9587, 1921.9825, 1923.9718, 1925.9573, 1928.0469, 1932.0060, 1933.9950, 1937.9630, 1939.9740, 1944.0040, 1946.9679, 1949.9595, 1954.0236, 1955.9738, 1958.9556, 1960.0034, 1968.8600, 1973.9845, 1976.9861, 1978.9910, 1981.9854, 1983.9884, 1987.9784, 1995.9408, 1998.9894, 2000.9926, 2008.0460, 2012.9590, 2015.0104, 2017.9747, 2021.0170, 2026.0275, 2027.0256, 2031.0166, 2044.0123, 2047.4957, 2047.9515, 2052.0496, 2060.0520, 2062.0073, 2064.9880, 2066.9873, 2094.0376, 2097.0454, 2102.0667, 2103.1641, 2111.0305, 2127.0930, 2139.0630, 2144.0493, 2148.1023, 2155.0950, 2156.0796, 2176.0664, 2190.9895, 2199.0723, 2202.1624, 2203.1221, 2208.0862, 2216.1062, 2225.1274, 2226.1272, 2229.0972, 2233.0984, 2239.0225, 2242.0435, 2268.1489, 2270.1287, 2271.1294, 2277.0823, 2284.1990, 2292.1426, 2309.0994, 2334.1719, 2342.9915, 2350.1746, 2383.9607, 2398.2400, 2400.0862, 2413.0549, 2414.1897, 2417.0959, 2497.2800, 2501.2637, 2511.1460, 2513.1421, 2528.1750, 2537.2012, 2541.1948, 2542.1836, 2556.2058, 2562.1487, 2612.2666, 2706.2109, 2717.0869, 2767.2666, 2888.2532, 3221.1733, 3224.2917, 3312.3306, 3916.9434

23. E1BD03 **Mass:** 66968 **Score:** 21 **Expect:** 1.8e+002 **Matches:** 26
Uncharacterized protein OS=Bos taurus GN=PPM1D PE=3 SV=2

Observed	Mr(expt)	Mr(calc)	ppm	Start	End	Miss	Ions	Peptide
1044.5751	1043.5678	1043.5876	-18.97	239 -	247	1	---	K.SGVNRVVWK.R
1045.5729	1044.5656	1044.5763	-10.23	358 -	364	2	---	R.WRQRMLR.A
1073.5902	1072.5829	1072.5877	-4.42	459 -	469	0	---	R.GNVQAAVISSK.D
1157.6199	1156.6126	1156.6393	-23.06	118 -	126	1	---	R.EHLWGFIKK.Q
1188.6544	1187.6471	1187.6332	11.7	512 -	522	1	---	K.NVKISSPGQMK.A
1335.7318	1334.7245	1334.6942	22.7	126 -	137	2	---	K.KQRGFTSSEPAK.V
1433.7228	1432.7155	1432.7231	-5.32	537 -	549	1	---	R.TLEESNSGPLMKK.H
1461.7778	1460.7705	1460.7421	19.5	145 -	156	1	---	K.GFLACHLAMWKK.L
1477.7545	1476.7472	1476.7370	6.93	145 -	156	1	---	K.GFLACHLAMWKK.L + Oxidation (M)
1605.8586	1604.8513	1604.8319	12.1	144 -	156	2	---	R.KGFLACHLAMWKK.L + Oxidation (M)
1615.8754	1614.8681	1614.8325	22.0	558 -	573	1	---	R.SSGAQPASLPTTSQRK.N
1633.7985	1632.7912	1632.8253	-20.90	228 -	243	1	---	R.IEGLGGSVMNKSGVNR.V + Oxidation (M)
1840.9827	1839.9754	1839.9479	14.9	2 -	19	1	---	M.AGLYSLGVSVFSDQGGRK.Y
1900.9546	1899.9473	1899.9836	-19.10	512 -	528	2	---	K.NVKISSPGQMKAQEVER.T
1916.9923	1915.9850	1915.9785	3.39	512 -	528	2	---	K.NVKISSPGQMKAQEVER.T + Oxidation (M)
1918.9587	1917.9514	1917.9690	-9.18	226 -	243	2	---	R.ERIEGLGGSVMNKSGVNR.V + Oxidation (M)
1932.0060	1930.9987	1930.9982	0.27	590 -	605	2	---	K.IGNPLLHQHRKTVCVC.-
1987.9784	1986.9711	1986.9833	-6.14	1 -	19	1	---	-.MAGLYSLGVSVFSDQGGRK.Y + Oxidation (M)
2015.0104	2014.0031	2014.0192	-7.96	553 -	572	1	---	R.NGLSRSSGAQPASLPTTSQR.K
2066.9873	2065.9800	2065.9534	12.9	74 -	93	2	---	R.EARAAPPDAGASPAPGRCCR.R
2102.0667	2101.0594	2101.0777	-8.68	54 -	76	2	---	R.SPPPPAGGEVAGRGAAGAAREAR.A
2148.1023	2147.0950	2147.0954	-0.20	138 -	155	2	---	K.VCAAIRKGFLACHLAMWK.K + Oxidation (M)
2202.1624	2201.1551	2201.1278	12.4	145 -	162	2	---	K.GFLACHLAMWKKLAEWPK.T + Oxidation (M)
2233.0984	2232.0911	2232.1208	-13.31	529 -	548	2	---	R.TPPANFKRTLEESNSGPLMK.K + Oxidation (M)
2398.2400	2397.2327	2397.2363	-1.47	182 -	204	1	---	R.GLKMYVAHVGDSGVVLGIQDDPK.D
2414.1897	2413.1824	2413.2312	-20.20	182 -	204	1	---	R.GLKMYVAHVGDSGVVLGIQDDPK.D + Oxidation (M)

No match to: 721.4365, 725.4001, 726.4063, 728.3681, 730.3848, 731.4017, 732.3573, 734.4151, 735.4143, 741.4101, 742.4515, 744.4171, 745.4167, 746.4169, 747.4125, 750.4153, 752.4127, 755.4100, 756.4199, 757.4313, 761.4177, 767.4266, 768.3700, 768.3737, 770.3966, 772.4093, 775.4307, 779.4308, 784.3688, 786.4000, 787.4221, 788.4210, 792.4376, 795.4350, 798.4351, 800.4296, 812.4388, 815.4332, 822.4482, 825.4540, 831.4382, 832.4557, 835.4719, 837.4463, 842.5060, 844.4760, 854.4837, 856.4911, 860.4691, 862.4852, 869.4231, 885.4465, 905.4955, 907.4991, 908.4929, 913.5700, 913.5747, 916.5284, 918.5050, 919.5053, 922.5070, 928.5117, 934.5172, 940.4840, 942.4854, 943.4289, 943.5453, 946.5259, 950.4959, 951.5076, 953.5164, 954.5388, 959.5266, 966.5410, 969.5256, 972.5305, 974.5395, 976.5310, 978.5314, 979.5442, 991.5409, 993.5307, 1001.5854, 1003.5496, 1011.5262, 1014.5215, 1021.5579, 1023.5663, 1023.5700, 1031.5903, 1035.5381, 1036.5447, 1037.5573, 1039.5327, 1041.5658, 1046.5897, 1051.5448, 1056.5658, 1057.6078, 1067.5753, 1086.5509, 1092.5961, 1100.6071, 1106.5391, 1111.5615, 1113.5729, 1118.5818, 1120.6045, 1122.5912, 1123.5848, 1125.5823, 1132.6047, 1134.5714, 1139.5746, 1143.6318, 1144.6497, 1145.6407, 1147.6393, 1150.6108, 1155.6215, 1158.6685, 1162.6182, 1163.6152, 1168.6215, 1173.6418, 1175.6089, 1178.6356, 1179.6157, 1181.6819, 1197.6704, 1199.7006, 1208.6415, 1216.6714, 1227.6595, 1231.6387, 1232.6447, 1234.6672, 1235.6445, 1244.6453, 1247.6211, 1248.6174, 1250.6554, 1255.6096, 1256.6531, 1261.6592, 1262.6453, 1266.6777, 1273.6260, 1275.6362, 1276.6343, 1277.6531, 1301.7079, 1303.7076, 1307.7042, 1308.6376, 1310.6146, 1312.6418, 1315.7098, 1320.6659, 1322.6886,

1323.6771, 1324.6964, 1328.7089, 1331.7202, 1338.6581, 1339.6637, 1349.7253, 1352.7205, 1356.7230, 1360.6638, 1363.7153, 1365.7379, 1367.7319, 1369.7043, 1377.6947, 1379.7268, 1383.7137, 1385.7534, 1388.7404, 1390.7284, 1393.7428, 1407.7563, 1417.7010, 1421.7061, 1422.7019, 1425.7727, 1427.7742, 1429.7365, 1431.7412, 1434.7446, 1437.7584, 1467.7521, 1472.7633, 1475.7632, 1484.7889, 1488.7537, 1491.7965, 1493.7579, 1500.7737, 1505.8177, 1513.8079, 1515.8213, 1517.7854, 1519.7544, 1522.7811, 1523.7953, 1529.8099, 1530.7523, 1531.7472, 1537.7974, 1539.8005, 1542.8422, 1545.8091, 1547.8051, 1549.7339, 1551.7778, 1553.8065, 1555.8101, 1556.8109, 1557.8010, 1561.8401, 1563.8109, 1565.8199, 1571.8400, 1579.7939, 1580.8077, 1583.8444, 1587.7620, 1592.8066, 1594.8335, 1604.8323, 1608.8497, 1624.8062, 1628.7900, 1628.7921, 1640.9384, 1642.8099, 1646.8297, 1649.8447, 1657.8101, 1667.8622, 1677.7988, 1680.8746, 1689.9465, 1694.7288, 1698.9225, 1699.8862, 1704.8773, 1706.8701, 1707.8302, 1710.9279, 1716.8671, 1719.9058, 1723.9011, 1725.8300, 1725.8346, 1727.8986, 1730.8931, 1737.9006, 1751.8656, 1754.8580, 1765.8463, 1766.8040, 1767.8138, 1769.8741, 1770.3567, 1772.9315, 1774.7610, 1774.9131, 1777.8770, 1780.8777, 1783.9246, 1787.8188, 1787.8200, 1791.8137, 1793.9050, 1795.9353, 1798.9340, 1800.9336, 1804.9142, 1805.9069, 1809.8469, 1815.9044, 1821.9144, 1828.9227, 1837.9396, 1843.9688, 1844.9012, 1846.9153, 1852.9364, 1856.9908, 1861.8881, 1867.9218, 1874.9695, 1880.9265, 1884.9417, 1891.9622, 1894.9313, 1896.9078, 1896.9100, 1905.9313, 1906.9501, 1908.9043, 1911.9130, 1912.9154, 1921.9825, 1923.9718, 1925.9573, 1928.0469, 1933.9950, 1937.9630, 1939.9740, 1944.0040, 1946.9679, 1949.9595, 1954.0236, 1955.9738, 1958.9556, 1960.0034, 1968.8600, 1973.9845, 1976.9861, 1978.9910, 1981.9854, 1983.9884, 1995.9408, 1998.9894, 2000.9926, 2008.0460, 2012.9590, 2017.9747, 2021.0170, 2026.0275, 2027.0256, 2031.0166, 2044.0123, 2047.4957, 2047.9515, 2052.0496, 2060.0520, 2062.0073, 2064.9880, 2094.0376, 2097.0454, 2103.1641, 2111.0305, 2127.0930, 2139.0630, 2144.0493, 2155.0950, 2156.0796, 2160.0632, 2176.0664, 2190.9895, 2195.0774, 2199.0723, 2203.1221, 2208.0862, 2211.1042, 2216.1062, 2225.1274, 2226.1272, 2229.0972, 2239.0225, 2242.0435, 2268.1489, 2270.1287, 2271.1294, 2277.0823, 2284.1990, 2292.1426, 2309.0994, 2334.1719, 2342.9915, 2350.1746, 2383.9607, 2400.0862, 2413.0549, 2417.0959, 2497.2800, 2501.2637, 2511.1460, 2513.1421, 2528.1750, 2537.2012, 2541.1948, 2542.1836, 2556.2058, 2562.1487, 2612.2666, 2706.2109, 2717.0869, 2767.2666, 2888.2532, 3221.1733, 3224.2917, 3312.3306, 3916.9434

24. F1N6S6 **Mass:** 28266 **Score:** 21 **Expect:** 2.1e+002 **Matches:** 13

Placental prolactin-related protein 3 OS=Bos taurus GN=PRP3 PE=3 SV=2

Observed	Mr(expt)	Mr(calc)	ppm	Start	End	Miss	Ions	Peptide
745.4167	744.4094	744.3919	23.6	2 -	8	0	---	M.APAPSFR.G
1349.7253	1348.7180	1348.7503	-23.95	172 -	182	0	---	R.QFSQVIYPVIR.T
1360.6638	1359.6565	1359.6427	10.1	212 -	221	1	---	K.LFMCLYRDSR.K
1488.7537	1487.7464	1487.7377	5.86	212 -	222	2	---	K.LFMCLYRDSRK.L
1547.8051	1546.7978	1546.7773	13.3	109 -	121	2	---	R.EKALRMNNEDLSK.W
1563.8109	1562.8036	1562.7722	20.1	109 -	121	2	---	R.EKALRMNNEDLSK.W + Oxidation (M)
1751.8656	1750.8583	1750.8323	14.8	206 -	218	1	---	R.HSAFYKLFMCLYR.D + Oxidation (M)
1867.9218	1866.9145	1866.9244	-5.31	140 -	156	1	---	K.DLQSMKEVSDTILSSAK.E + Oxidation (M)
1921.9825	1920.9752	1920.9727	1.32	157 -	171	2	---	K.ENMRKIEELQAFIER.Q + Oxidation (M)
1923.9718	1922.9645	1922.9374	14.1	189 -	205	0	---	R.IYWSGLASLVSNDEDVR.H
1983.9884	1982.9811	1982.9864	-2.64	2 -	18	1	---	M.APAPSFRGHQWTYNPVR.G
2094.0376	2093.0303	2092.9975	15.7	206 -	221	2	---	R.HSAFYKLFMCLYRDSR.K
2414.1897	2413.1824	2413.1465	14.9	140 -	160	2	---	K.DLQSMKEVSDTILSSAKENMR.K + 2 Oxidation (M)

No match to: 721.4365, 725.4001, 726.4063, 728.3681, 730.3848, 731.4017, 732.3573, 734.4151, 735.4143, 741.4101, 742.4515, 744.4171, 746.4169, 747.4125, 750.4153, 752.4127, 755.4100, 756.4199, 757.4313, 761.4177, 767.4266, 768.3700, 768.3737, 770.3966, 772.4093, 775.4307, 779.4308, 784.3688, 786.4000, 787.4221, 788.4210, 792.4376, 795.4350, 798.4351, 800.4296, 812.4388, 815.4332, 822.4482, 825.4540, 831.4382, 832.4557, 835.4719, 837.4463, 842.5060, 844.4760, 854.4837, 856.4911, 860.4691, 862.4852, 869.4231, 885.4465, 905.4955, 907.4991, 908.4929, 913.5700, 913.5747, 916.5284, 918.5050, 919.5053, 922.5070, 928.5117, 934.5172, 940.4840, 942.4854, 943.4289, 943.5453, 946.5259, 950.4959, 951.5076, 953.5164, 954.5388, 959.5266, 966.5410, 969.5256, 972.5305, 974.5395, 976.5310, 978.5314, 979.5442, 991.5409, 993.5307, 1001.5854, 1003.5496, 1011.5262, 1014.5215, 1021.5579, 1023.5663, 1023.5700, 1031.5903, 1035.5381, 1036.5447, 1037.5573, 1039.5327, 1041.5658, 1044.5751, 1045.5729, 1046.5897, 1051.5448, 1056.5658, 1057.6078, 1067.5753, 1073.5902, 1086.5509, 1092.5961, 1100.6071, 1106.5391, 1111.5615, 1113.5729, 1118.5818, 1120.6045, 1122.5912, 1123.5848, 1125.5823, 1132.6047, 1134.5714, 1139.5746, 1143.6318, 1144.6497, 1145.6407, 1147.6393, 1150.6108, 1155.6215, 1157.6199, 1158.6685, 1162.6182, 1163.6152, 1168.6215, 1173.6418, 1175.6089, 1178.6356, 1179.6157, 1181.6819, 1188.6544, 1197.6704, 1199.7006, 1208.6415, 1216.6714, 1227.6595, 1231.6387, 1232.6447, 1234.6672, 1235.6445, 1244.6453, 1247.6211, 1248.6174, 1250.6554, 1255.6096, 1256.6531, 1261.6592, 1262.6453, 1266.6777, 1273.6260, 1275.6362, 1276.6343, 1277.6531, 1301.7079, 1303.7076, 1307.7042, 1308.6376, 1310.6146, 1312.6418, 1315.7098, 1320.6659, 1322.6886, 1323.6771, 1324.6964, 1328.7089, 1331.7202, 1335.7318, 1338.6581, 1339.6637, 1352.7205, 1356.7230, 1363.7153, 1365.7379, 1367.7319, 1369.7043, 1377.6947, 1379.7268, 1383.7137, 1385.7534, 1388.7404, 1390.7284, 1393.7428, 1407.7563, 1417.7010, 1421.7061, 1422.7019, 1425.7727, 1427.7742, 1429.7365, 1431.7412, 1433.7228, 1434.7446, 1437.7584, 1461.7778, 1467.7521, 1472.7633, 1475.7632, 1477.7545, 1484.7889, 1491.7965, 1493.7579, 1500.7737, 1505.8177, 1513.8079, 1515.8213, 1517.7854, 1519.7544, 1522.7811, 1523.7953, 1529.8099, 1530.7523, 1531.7472, 1537.7974, 1539.8005, 1542.8422, 1545.8091, 1549.7339, 1551.7778, 1553.8065, 1555.8101, 1556.8109, 1557.8010, 1561.8401, 1565.8199, 1571.8400, 1579.7939, 1580.8077, 1583.8444, 1587.7620, 1592.8066, 1594.8335, 1604.8323, 1605.8586, 1608.8497, 1615.8754, 1624.8062, 1628.7900, 1628.7921, 1633.7985, 1640.9384, 1642.8099, 1646.8297, 1649.8447, 1657.8101, 1667.8622, 1677.7988, 1680.8746, 1689.9465, 1694.7288, 1698.9225, 1699.8862, 1704.8773, 1706.8701, 1707.8302, 1710.9279, 1716.8671, 1719.9058, 1723.9011, 1725.8300, 1725.8346, 1727.8986, 1730.8931, 1737.9006, 1754.8580, 1765.8463, 1766.8040, 1767.8138, 1769.8741, 1770.3567, 1772.9315, 1774.7610, 1774.9131, 1777.8770, 1780.8777, 1783.9246, 1787.8188, 1787.8200, 1791.8137, 1793.9050, 1795.9353, 1798.9340, 1800.9336, 1804.9142, 1805.9069, 1809.8469, 1815.9044, 1821.9144, 1828.9227, 1837.9396, 1840.9827, 1843.9688, 1844.9012, 1846.9153, 1852.9364, 1856.9908, 1861.8881, 1874.9695, 1880.9265, 1884.9417, 1891.9622, 1894.9313, 1896.9078, 1896.9100, 1900.9546, 1905.9313, 1906.9501, 1908.9043, 1911.9130, 1912.9154, 1916.9923, 1918.9587, 1925.9573, 1928.0469, 1932.0060, 1933.9950, 1937.9630, 1939.9740, 1944.0040, 1946.9679, 1949.9595, 1954.0236, 1955.9738, 1958.9556, 1960.0034, 1968.8600, 1973.9845, 1976.9861, 1978.9910, 1981.9854, 1987.9784, 1995.9408, 1998.9894, 2000.9926, 2008.0460, 2012.9590, 2015.0104, 2017.9747, 2021.0170, 2026.0275, 2027.0256, 2031.0166, 2044.0123, 2047.4957, 2047.9515, 2052.0496, 2060.0520, 2062.0073, 2064.9880, 2066.9873, 2097.0454, 2102.0667, 2103.1641, 2111.0305, 2127.0930, 2139.0630, 2144.0493, 2148.1023, 2155.0950, 2156.0796, 2160.0632, 2176.0664, 2190.9895, 2195.0774, 2199.0723, 2202.1624, 2203.1221, 2208.0862, 2211.1042, 2216.1062, 2225.1274, 2226.1272, 2229.0972, 2233.0984, 2239.0225, 2242.0435, 2268.1489, 2270.1287, 2271.1294, 2277.0823, 2284.1990, 2292.1426, 2309.0994, 2334.1719, 2342.9915, 2350.1746, 2383.9607, 2398.2400, 2400.0862, 2413.0549, 2417.0959, 2497.2800, 2501.2637, 2511.1460, 2513.1421, 2528.1750, 2537.2012, 2541.1948, 2542.1836, 2556.2058, 2562.1487, 2612.2666, 2706.2109, 2717.0869, 2767.2666, 2888.2532, 3221.1733, 3224.2917, 3312.3306, 3916.9434

25. F6QYE2 **Mass:** 72629 **Score:** 20 **Expect:** 2.5e+002 **Matches:** 25

Uncharacterized protein OS=Bos taurus GN=CFAP100 PE=4 SV=1

Observed	Mr(expt)	Mr(calc)	ppm	Start	End	Miss	Ions	Peptide
788.4210	787.4137	787.4300	-20.75	167 -	172	1	---	K.RSEIQR.L
916.5284	915.5211	915.5185	2.87	588 -	594	2	---	R.GRRLVCR.S
943.5453	942.5381	942.5610	-24.34	554 -	560	2	---	R.LRLREEK.A
950.4959	949.4886	949.4902	-1.73	219 -	226	2	---	K.ESKAKMEK.I
976.5310	975.5237	975.5059	18.3	489 -	496	0	---	K.LLESLNCK.V
1031.5903	1030.5830	1030.5845	-1.39	224 -	231	1	---	K.MEKILEIR.E
1173.6418	1172.6345	1172.6400	-4.71	541 -	550	2	---	R.IEQAEKAKEK.E
1175.6089	1174.6016	1174.6194	-15.09	292 -	302	0	---	K.ESLLLSSVGDR.A
1199.7006	1198.6933	1198.6782	12.6	575 -	585	2	---	R.AQARAQAEVKK.K
1248.6174	1247.6101	1247.5890	17.0	1 -	11	0	---	-.MSENLPPMVSK.S + Oxidation (M)
1488.7537	1487.7464	1487.7467	-0.18	462 -	474	1	---	K.EEETAAELELKAR.V
1500.7737	1499.7664	1499.7516	9.87	155 -	166	0	---	R.QMFLIQYALDMK.R
1513.8079	1512.8006	1512.8372	-24.18	73 -	84	2	---	K.SLLDREQKQQLR.V
1531.7472	1530.7399	1530.7096	19.8	604 -	615	2	---	K.EQSKHELMDKDR.E + Oxidation (M)
1539.8005	1538.7932	1538.8165	-15.11	292 -	305	1	---	K.ESLLLSSVGDRAHR.L
1565.8199	1564.8126	1564.7879	15.8	208 -	221	2	---	R.SSVQALRMADKESK.A + Oxidation (M)
1587.7620	1586.7547	1586.7787	-15.12	173 -	186	0	---	R.LESLAAQEEAELER.A
1594.8335	1593.8262	1593.8185	4.88	85 -	98	2	---	R.VHEKMTYSSKVSAK.H

1719.9058	1718.8985	1718.8562	24.6	124 - 137	1	---	R.SFHEQVAWKLAMTR.E + Oxidation (M)
1777.8770	1776.8697	1776.9080	-21.54	2 - 17	1	---	M.SENLPPMVSKSVTFNK.H
1793.9050	1792.8977	1792.9029	-2.90	2 - 17	1	---	M.SENLPPMVSKSVTFNK.H + Oxidation (M)
1955.9738	1954.9665	1954.9432	12.0	303 - 318	1	---	R.AHRLWEVGQLSEQSCR.L
1976.9861	1975.9788	1975.9938	-7.57	124 - 139	2	---	R.SFHEQVAWKLAMTREK.K + Oxidation (M)
2270.1287	2269.1214	2269.1711	-21.88	155 - 172	2	---	R.QMFLIQYALDMKRSEIQR.L
2513.1421	2512.1348	2512.1789	-17.56	99 - 118	2	---	K.HTSLRRELQLEDETEDQEER.A

No match to: 721.4365, 725.4001, 726.4063, 728.3681, 730.3848, 731.4017, 732.3573, 734.4151, 735.4143, 741.4101, 742.4515, 744.4171, 745.4167, 746.4169, 747.4125, 750.4153, 752.4127, 755.4100, 756.4199, 757.4313, 761.4177, 767.4266, 768.3700, 768.3737, 770.3966, 772.4093, 775.4307, 779.4308, 784.3688, 786.4000, 787.4221, 792.4376, 795.4350, 798.4351, 800.4296, 812.4388, 815.4332, 822.4482, 825.4540, 831.4382, 832.4557, 835.4719, 837.4463, 842.5060, 844.4760, 854.4837, 856.4911, 860.4691, 862.4852, 869.4231, 885.4465, 905.4955, 907.4991, 908.4929, 913.5700, 913.5747, 918.5050, 919.5053, 922.5070, 928.5117, 934.5172, 940.4840, 942.4854, 943.4289, 946.5259, 951.5076, 953.5164, 954.5388, 959.5266, 966.5410, 969.5256, 972.5305, 974.5395, 978.5314, 979.5442, 991.5409, 993.5307, 1001.5854, 1003.5496, 1011.5262, 1014.5215, 1021.5579, 1023.5663, 1023.5700, 1035.5381, 1036.5447, 1037.5573, 1039.5327, 1041.5658, 1044.5751, 1045.5729, 1046.5897, 1051.5448, 1056.5658, 1057.6078, 1067.5753, 1073.5902, 1086.5509, 1092.5961, 1100.6071, 1106.5391, 1111.5615, 1113.5729, 1118.5818, 1120.6045, 1122.5912, 1123.5848, 1125.5823, 1132.6047, 1134.5714, 1139.5746, 1143.6318, 1144.6497, 1145.6407, 1147.6393, 1150.6108, 1155.6215, 1157.6199, 1158.6685, 1162.6182, 1163.6152, 1168.6215, 1178.6356, 1179.6157, 1181.6819, 1188.6544, 1197.6704, 1208.6415, 1216.6714, 1227.6595, 1231.6387, 1232.6447, 1234.6672, 1235.6445, 1244.6453, 1247.6211, 1250.6554, 1255.6096, 1256.6531, 1261.6592, 1262.6453, 1266.6777, 1273.6260, 1275.6362, 1276.6343, 1277.6531, 1301.7079, 1303.7076, 1307.7042, 1308.6376, 1310.6146, 1312.6418, 1315.7098, 1320.6659, 1322.6886, 1323.6771, 1324.6964, 1328.7089, 1331.7202, 1335.7318, 1338.6581, 1339.6637, 1349.7253, 1352.7205, 1356.7230, 1360.6638, 1363.7153, 1365.7379, 1367.7319, 1369.7043, 1377.6947, 1379.7268, 1383.7137, 1385.7534, 1388.7404, 1390.7284, 1393.7428, 1407.7563, 1417.7010, 1421.7061, 1422.7019, 1425.7727, 1427.7742, 1429.7365, 1431.7412, 1433.7228, 1434.7446, 1437.7584, 1461.7778, 1467.7521, 1472.7633, 1475.7632, 1477.7545, 1484.7889, 1491.7965, 1493.7579, 1505.8177, 1515.8213, 1517.7854, 1519.7544, 1522.7811, 1523.7953, 1529.8099, 1530.7523, 1537.7974, 1542.8422, 1545.8091, 1547.8051, 1549.7339, 1551.7778, 1553.8065, 1555.8101, 1556.8109, 1557.8010, 1561.8401, 1563.8109, 1571.8400, 1579.7939, 1580.8077, 1583.8444, 1592.8066, 1604.8323, 1605.8586, 1608.8497, 1615.8754, 1624.8062, 1628.7900, 1628.7921, 1633.7985, 1640.9384, 1642.8099, 1646.8297, 1649.8447, 1657.8101, 1667.8622, 1677.7988, 1680.8746, 1689.9465, 1694.7288, 1698.9225, 1699.8862, 1704.8773, 1706.8701, 1707.8302, 1710.9279, 1716.8671, 1723.9011, 1725.8300, 1725.8346, 1727.8986, 1730.8931, 1737.9006, 1751.8656, 1754.8580, 1765.8463, 1766.8040, 1767.8138, 1769.8741, 1770.3567, 1772.9315, 1774.7610, 1774.9131, 1780.8777, 1783.9246, 1787.8188, 1787.8200, 1791.8137, 1795.9353, 1798.9340, 1800.9336, 1804.9142, 1805.9069, 1809.8469, 1815.9044, 1821.9144, 1828.9227, 1837.9396, 1840.9827, 1843.9688, 1844.9012, 1846.9153, 1852.9364, 1856.9908, 1861.8881, 1867.9218, 1874.9695, 1880.9265, 1884.9417, 1891.9622, 1894.9313, 1896.9078, 1896.9100, 1900.9546, 1905.9313, 1906.9501, 1908.9043, 1911.9130, 1912.9154, 1916.9923, 1918.9587, 1921.9825, 1923.9718, 1925.9573, 1928.0469, 1932.0060, 1933.9950, 1937.9630, 1939.9740, 1944.0040, 1946.9679, 1949.9595, 1954.0236, 1958.9556, 1960.0034, 1968.8600, 1973.9845, 1978.9910, 1981.9854, 1983.9884, 1987.9784, 1995.9408, 1998.9894, 2000.9926, 2008.0460, 2012.9590, 2015.0104, 2017.9747, 2021.0170, 2026.0275, 2027.0256, 2031.0166, 2044.0123, 2047.4957, 2047.9515, 2052.0496, 2060.0520, 2062.0073, 2064.9880, 2066.9873, 2094.0376, 2097.0454, 2102.0667, 2103.1641, 2111.0305, 2127.0930, 2139.0630, 2144.0493, 2148.1023, 2155.0950, 2156.0796, 2160.0632, 2176.0664, 2190.9895, 2195.0774, 2199.0723, 2202.1624, 2203.1221, 2208.0862, 2211.1042, 2216.1062, 2225.1274, 2226.1272, 2229.0972, 2233.0984, 2239.0225, 2242.0435, 2268.1489, 2271.1294, 2277.0823, 2284.1990, 2292.1426, 2309.0994, 2334.1719, 2342.9915, 2350.1746, 2383.9607, 2398.2400, 2400.0862, 2413.0549, 2414.1897, 2417.0959, 2497.2800, 2501.2637, 2511.1460, 2528.1750, 2537.2012, 2541.1948, 2542.1836, 2556.2058, 2562.1487, 2612.2666, 2706.2109, 2717.0869, 2767.2666, 2888.2532, 3221.1733, 3224.2917, 3312.3306, 3916.9434

26. G1K1U4 **Mass:** 51632 **Score:** 20 **Expect:** 2.5e+002 **Matches:** 21

DNA polymerase delta subunit 3 OS=Bos taurus GN=POLD3 PE=4 SV=2

Observed	Mr(expt)	Mr(calc)	ppm	Start - End	Miss	Ions	Peptide
775.4307	774.4235	774.4422	-24.15	241 - 247	1	---	K.AAMNKLK.V
919.5053	918.4980	918.4957	2.53	292 - 298	2	---	R.MQQKEKK.R
993.5307	992.5234	992.5179	5.61	363 - 371	0	---	K.TEPEPPPVK.G
1001.5854	1000.5781	1000.5665	11.6	283 - 291	2	---	K.SSKKAEPVR.M
1045.5729	1044.5656	1044.5750	-8.96	440 - 450	2	---	K.GPKKGTAAMGK.A
1086.5509	1085.5436	1085.5651	-19.82	287 - 295	1	---	K.AEPVRMQQK.E
1120.6045	1119.5972	1119.5818	13.7	443 - 453	2	---	K.KGTAAMGKANR.Q + Oxidation (M)
1134.5714	1133.5641	1133.5387	22.5	216 - 226	0	---	K.EVMNASSVGPK.A + Oxidation (M)
1360.6638	1359.6565	1359.6493	5.34	39 - 48	1	---	K.QMLYDYVERK.R + Oxidation (M)
1433.7228	1432.7155	1432.7157	-0.15	203 - 215	2	---	K.AQDANKETKTEAK.E
1500.7737	1499.7664	1499.7554	7.32	39 - 49	2	---	K.QMLYDYVERKR.K
1515.8213	1514.8140	1514.8457	-20.89	92 - 105	0	---	K.LAVTASVHVYSIQK.A
1547.8051	1546.7978	1546.7661	20.5	212 - 226	1	---	K.TEAKEVMNASSVGPK.A
1615.8754	1614.8681	1614.8518	10.1	25 - 38	0	---	K.WLSYTLGVHVNQAK.Q
1783.9246	1782.9173	1782.9597	-23.74	417 - 432	2	---	K.MKTSSVHRPPAMAVKK.E + Oxidation (M)
1900.9546	1899.9473	1899.9724	-13.19	212 - 230	2	---	K.TEAKEVMNASSVGPKAPGK.G
1905.9313	1904.9240	1904.9513	-14.32	209 - 226	2	---	K.ETKTEAKEVMNASSVGPK.A
1916.9923	1915.9850	1915.9673	9.25	212 - 230	2	---	K.TEAKEVMNASSVGPKAPGK.G + Oxidation (M)
1921.9825	1920.9752	1920.9462	15.1	209 - 226	2	---	K.ETKTEAKEVMNASSVGPK.A + Oxidation (M)
1925.9573	1924.9500	1924.9499	0.09	190 - 208	2	---	K.GIMGMFASKAASKAQDANK.E
2102.0667	2101.0594	2101.0071	24.9	384 - 401	2	---	R.VLKSKTFTDEEGCMVTEK.V

No match to: 721.4365, 725.4001, 726.4063, 728.3681, 730.3848, 731.4017, 732.3573, 734.4151, 735.4143, 741.4101, 742.4515, 744.4171, 745.4167, 746.4169, 747.4125, 750.4153, 752.4127, 755.4100, 756.4199, 757.4313, 761.4177, 767.4266, 768.3700, 768.3737, 770.3966, 772.4093, 779.4308, 784.3688, 786.4000, 787.4221, 788.4210, 792.4376, 795.4350, 798.4351, 800.4296, 812.4388, 815.4332, 822.4482, 825.4540, 831.4382, 832.4557, 835.4719, 837.4463, 842.5060, 844.4760, 854.4837, 856.4911, 860.4691, 862.4852, 869.4231, 885.4465, 905.4955, 907.4991, 908.4929, 913.5700, 913.5747, 916.5284, 918.5050, 922.5070, 928.5117, 934.5172, 940.4840, 942.4854, 943.4289, 943.5453, 946.5259, 950.4959, 951.5076, 953.5164, 954.5388, 959.5266, 966.5410, 969.5256, 972.5305, 974.5395, 976.5310, 978.5314, 979.5442, 991.5409, 1003.5496, 1011.5262, 1014.5215, 1021.5579, 1023.5663, 1023.5700, 1031.5903, 1035.5381, 1036.5447, 1037.5573, 1039.5327, 1041.5658, 1044.5751, 1046.5897, 1051.5448, 1056.5658, 1057.6078, 1067.5753, 1073.5902, 1092.5961, 1100.6071, 1106.5391, 1111.5615, 1113.5729, 1118.5818, 1122.5912, 1123.5848, 1125.5823, 1132.6047, 1139.5746, 1143.6318, 1144.6497, 1145.6407, 1147.6393, 1150.6108, 1155.6215, 1157.6199, 1158.6685, 1162.6182, 1163.6152, 1168.6215, 1173.6418, 1175.6089, 1178.6356, 1179.6157, 1181.6819, 1188.6544, 1197.6704, 1199.7006, 1208.6415, 1216.6714, 1227.6595, 1231.6387, 1232.6447, 1234.6672, 1235.6445, 1244.6453, 1247.6211, 1248.6174, 1250.6554, 1255.6096, 1256.6531, 1261.6592, 1262.6453, 1266.6777, 1273.6260, 1275.6362, 1276.6343, 1277.6531, 1301.7079, 1303.7076, 1307.7042, 1308.6376, 1310.6146, 1312.6418, 1315.7098, 1320.6659, 1322.6886, 1323.6771, 1324.6964, 1328.7089, 1331.7202, 1335.7318, 1338.6581, 1339.6637, 1349.7253, 1352.7205, 1356.7230, 1363.7153, 1365.7379, 1367.7319, 1369.7043, 1377.6947, 1379.7268, 1383.7137, 1385.7534, 1388.7404, 1390.7284, 1393.7428, 1407.7563, 1417.7010, 1421.7061, 1422.7019, 1425.7727, 1427.7742, 1429.7365, 1431.7412, 1434.7446, 1437.7584, 1461.7778, 1467.7521, 1472.7633, 1475.7632, 1477.7545, 1484.7889, 1488.7537, 1491.7965, 1493.7579, 1505.8177, 1513.8079, 1517.7854, 1519.7544, 1522.7811, 1523.7953, 1529.8099, 1530.7523, 1531.7472, 1537.7974, 1539.8005, 1542.8422, 1545.8091, 1549.7339, 1551.7778, 1553.8065, 1555.8101, 1556.8109, 1557.8010, 1561.8401, 1563.8109, 1565.8199, 1571.8400, 1579.7939, 1580.8077, 1583.8444, 1587.7620, 1592.8066, 1594.8335, 1604.8323, 1605.8586, 1608.8497, 1624.8062, 1628.7900, 1628.7921, 1633.7985, 1640.9384, 1642.8099, 1646.8297, 1649.8447, 1657.8101, 1667.8622, 1677.7988, 1680.8746, 1689.9465, 1694.7288, 1698.9225, 1699.8862, 1704.8773, 1706.8701, 1707.8302, 1710.9279, 1716.8671, 1719.9058, 1723.9011, 1725.8300, 1725.8346, 1727.8986, 1730.8931, 1737.9006, 1751.8656, 1754.8580, 1765.8463, 1766.8040, 1767.8138, 1769.8741, 1770.3567, 1772.9315, 1774.7610, 1774.9131, 1777.8770, 1780.8777, 1787.8188, 1787.8200, 1791.8137, 1793.9050, 1795.9353, 1798.9340, 1800.9336, 1804.9142, 1805.9069, 1809.8469, 1815.9044, 1821.9144, 1828.9227, 1837.9396, 1840.9827, 1843.9688, 1844.9012, 1846.9153, 1852.9364, 1856.9908, 1861.8881, 1867.9218, 1874.9695, 1880.9265, 1884.9417, 1891.9622, 1894.9313, 1896.9078, 1896.9100, 1906.9501, 1908.9043, 1911.9130, 1912.9154, 1918.9587, 1923.9718, 1928.0469, 1932.0060, 1933.9950, 1937.9630, 1939.9740, 1944.0040, 1946.9679, 1949.9595, 1954.0236, 1955.9738, 1958.9556, 1960.0034, 1968.8600, 1973.9845, 1976.9861, 1978.9910, 1981.9854, 1983.9884, 1987.9784, 1995.9408, 1998.9894, 2000.9926, 2008.0460, 2012.9590, 2015.0104, 2017.9747, 2021.0170, 2026.0275, 2027.0256, 2031.0166, 2044.0123, 2047.4957, 2047.9515, 2052.0496, 2060.0520, 2062.0073, 2064.9880, 2066.9873, 2094.0376, 2097.0454, 2103.1641, 2111.0305, 2127.0930, 2139.0630, 2144.0493, 2148.1023, 2155.0950, 2156.0796,

2160.0632, 2176.0664, 2190.9895, 2195.0774, 2199.0723, 2202.1624, 2203.1221, 2208.0862, 2211.1042, 2216.1062, 2225.1274, 2226.1272, 2229.0972, 2233.0984, 2239.0225, 2242.0435, 2268.1489, 2270.1287, 2271.1294, 2277.0823, 2284.1990, 2292.1426, 2309.0994, 2334.1719, 2342.9915, 2350.1746, 2383.9607, 2398.2400, 2400.0862, 2413.0549, 2414.1897, 2417.0959, 2497.2800, 2501.2637, 2511.1460, 2513.1421, 2528.1750, 2537.2012, 2541.1948, 2542.1836, 2556.2058, 2562.1487, 2612.2666, 2706.2109, 2717.0869, 2767.2666, 2888.2532, 3221.1733, 3224.2917, 3312.3306, 3916.9434

27. G3MX17 **Mass:** 200058 **Score:** 20 **Expect:** 2.5e+002 **Matches:** 53

Uncharacterized protein OS=Bos taurus PE=3 SV=1

Observed	Mr(expt)	Mr(calc)	ppm	Start	End	Miss	Ions	Peptide
745.4167	744.4094	744.4017	10.3	1218	- 1223	0	---	K.INELEK.K
767.4266	766.4194	766.4337	-18.73	1598	- 1603	1	---	K.KTYSLR.S
812.4388	811.4316	811.4188	15.7	738	- 744	1	---	K.TKTYSGR.K
832.4557	831.4485	831.4490	-0.66	1359	- 1364	1	---	K.KWLEEK.M
854.4837	853.4764	853.4658	12.5	1590	- 1597	0	---	K.VSIHPSSK.K
916.5284	915.5211	915.5138	8.02	140	- 147	1	---	K.DLLKGQSR.L
951.5076	950.5004	950.4929	7.85	1365	- 1372	0	---	K.MMLITQAK.E + Oxidation (M)
1001.5854	1000.5781	1000.5916	-13.51	1218	- 1225	2	---	K.INELEKKK.N
1011.5262	1010.5189	1010.4954	23.3	1643	- 1651	0	---	K.IIETMSSSK.F + Oxidation (M)
1118.5818	1117.5745	1117.5655	8.07	1302	- 1310	0	---	R.LATELEEWK.E
1123.5848	1122.5775	1122.5920	-12.93	1311	- 1319	2	---	K.EKYKDLEAK.H
1125.5823	1124.5750	1124.5938	-16.69	1696	- 1704	1	---	K.ESDHQILKR.R
1139.5746	1138.5673	1138.5903	-20.22	1642	- 1651	1	---	K.KIIETMSSSK.F + Oxidation (M)
1147.6393	1146.6320	1146.6397	-6.68	677	- 686	1	---	K.FNQVKAELAK.T
1175.6089	1174.6016	1174.5982	2.91	1151	- 1159	1	---	R.NWQETEKLK.E
1199.7006	1198.6933	1198.7173	-19.96	577	- 586	1	---	K.LLDLIEDLKK.L
1216.6714	1215.6641	1215.6645	-0.31	897	- 906	1	---	K.LMQAKIDELR.R
1232.6447	1231.6374	1231.6594	-17.86	897	- 906	1	---	K.LMQAKIDELR.R + Oxidation (M)
1367.7319	1366.7246	1366.7568	-23.57	1587	- 1598	2	---	K.EQKVSIHPSSKK.T
1377.6947	1376.6874	1376.6936	-4.46	201	- 211	1	---	R.EYLRLSPDQEK.E
1388.7404	1387.7331	1387.7605	-19.75	897	- 907	2	---	K.LMQAKIDELRR.L + Oxidation (M)
1434.7446	1433.7373	1433.7514	-9.84	176	- 187	0	---	R.TLNVLFDSLQER.L
1467.7521	1466.7448	1466.7365	5.67	762	- 775	0	---	K.GLTHASPAITEDQK.K
1488.7537	1487.7464	1487.7198	17.9	437	- 448	0	---	K.LTHYFQSFFNGK.G
1519.7544	1518.7471	1518.7824	-23.21	865	- 876	2	---	K.SINQEQKERIMK.L + Oxidation (M)
1624.8062	1623.7989	1623.7740	15.4	747	- 760	0	---	R.LNENELQQDEPPAK.K
1642.8099	1641.8026	1641.8223	-11.98	1404	- 1417	1	---	K.GQLAEKDGNLQQWR.E
1699.8862	1698.8789	1698.9100	-18.28	192	- 204	2	---	K.MNLKPHRSREYLR.L
1707.8302	1706.8229	1706.8475	-14.38	1174	- 1187	2	---	K.ADLQRKEEDYAELK.E
1719.9058	1718.8985	1718.8951	1.98	1652	- 1666	1	---	K.FSNVEVSKENVSRPK.R
1723.9011	1722.8938	1722.9264	-18.92	761	- 776	2	---	K.KGLTHASPAITEDQKK.S
1751.8656	1750.8583	1750.8624	-2.35	1180	- 1194	2	---	K.EEDYAELKEKLADAK.K
1774.9131	1773.9058	1773.8931	7.20	1395	- 1409	1	---	K.QQNEMEILKGQLAEK.D + Oxidation (M)
1777.8770	1776.8697	1776.9039	-19.26	872	- 886	2	---	K.ERIMKLSEEIETAGR.N + Oxidation (M)
1780.8777	1779.8704	1779.8751	-2.62	746	- 760	1	---	K.RLNENELQQDEPPAK.K
1793.9050	1792.8977	1792.8703	15.3	996	- 1009	2	---	K.DYTNENNRLKIEER.E
1815.9044	1814.8971	1814.8655	17.4	1390	- 1403	1	---	R.EHCLKQQNEMEILK.G + Oxidation (M)
1821.9144	1820.9071	1820.9057	0.79	315	- 329	1	---	K.DLQWIQVSDAKEAYR.L
1896.9078	1895.9005	1895.8723	14.9	1551	- 1565	2	---	K.SHEMEEDFVKFENKK.N
1896.9100	1895.9027	1895.8723	16.0	1550	- 1564	2	---	R.KSHEMEEDFVKFENK.K
1905.9313	1904.9240	1904.9302	-3.24	102	- 118	2	---	R.LSEKSSGQMAQKFSFSK.V + Oxidation (M)
1912.9154	1911.9081	1911.8672	21.4	1551	- 1565	2	---	K.SHEMEEDFVKFENKK.N + Oxidation (M)
1916.9923	1915.9850	1916.0156	-15.95	1625	- 1641	1	---	K.FGDFLQHSPTILQSKAK.K
1921.9825	1920.9752	1920.9761	-0.43	1365	- 1380	2	---	K.MMLITQAKEAENLRNK.E + 2 Oxidation (M)
1944.0040	1942.9967	1943.0211	-12.54	1020	- 1035	2	---	K.EKDSLIQQLKEEVQEK.T
1954.0236	1953.0163	1953.0176	-0.66	1528	- 1545	2	---	R.MAVKHPGCTSPVTVKTPK.A + Oxidation (M)
1987.9784	1986.9711	1986.9541	8.57	1098	- 1114	1	---	R.NLNECQANLKDSIQNAR.D
2017.9747	2016.9674	2017.0058	-19.00	434	- 450	2	---	R.ESKLTHYFQSFFNGKGK.I
2021.0170	2020.0097	2020.0112	-0.75	201	- 217	2	---	R.EYLRLSPDQEKEEVASK.S
2062.0073	2061.0000	2060.9506	24.0	1072	- 1088	2	---	K.KDECSHSAKLEQEIMEK.E
2097.0454	2096.0381	2096.0134	11.8	1342	- 1358	2	---	K.LQDELQESEQKHEAERK.K
2216.1062	2215.0989	2215.1444	-20.53	877	- 896	1	---	K.LSEEIETAGRNITNNVSQIK.L
3916.9434	3915.9361	3915.9312	1.25	40	- 73	2	---	R.EFSLIASSTEANSSESKDYLQVCLRVRPFTQPEK.E

No match to: 721.4365, 725.4001, 726.4063, 728.3681, 730.3848, 731.4017, 732.3573, 734.4151, 735.4143, 741.4101, 742.4515, 744.4171, 746.4169, 747.4125, 750.4153, 752.4127, 755.4100, 756.4199, 757.4313, 761.4177, 768.3700, 768.3737, 770.3966, 772.4093, 775.4307, 779.4308, 784.3688, 786.4000, 787.4221, 788.4210, 792.4376, 795.4350, 798.4351, 800.4296, 815.4332, 822.4482, 825.4540, 831.4382, 835.4719, 837.4463, 842.5060, 844.4760, 856.4911, 860.4691, 862.4852, 869.4231, 885.4465, 905.4955, 907.4991, 908.4929, 913.5700, 913.5747, 918.5050, 919.5053, 922.5070, 928.5117, 934.5172, 940.4840, 942.4854, 943.4289, 943.5453, 946.5259, 950.4959, 953.5164, 954.5388, 959.5266, 966.5410, 969.5256, 972.5305, 974.5395, 976.5310, 978.5314, 979.5442, 991.5409, 993.5307, 1003.5496, 1014.5215, 1021.5579, 1023.5663, 1023.5700, 1031.5903, 1035.5381, 1036.5447, 1037.5573, 1039.5327, 1041.5658, 1044.5751, 1045.5729, 1046.5897, 1051.5448, 1056.5658, 1057.6078, 1067.5753, 1073.5902, 1086.5509, 1092.5961, 1100.6071, 1106.5391, 1111.5615, 1113.5729, 1120.6045, 1122.5912, 1132.6047, 1134.5714, 1143.6318, 1144.6497, 1145.6407, 1150.6108, 1155.6215, 1157.6199, 1158.6685, 1162.6182, 1163.6152, 1168.6215, 1173.6418, 1178.6356, 1179.6157, 1181.6819, 1188.6544, 1197.6704, 1208.6415, 1227.6595, 1231.6387, 1234.6672, 1235.6445, 1244.6453, 1247.6211, 1248.6174, 1250.6554, 1255.6096, 1256.6531, 1261.6592, 1262.6453, 1266.6777, 1273.6260, 1275.6362, 1276.6343, 1277.6531, 1301.7079, 1303.7076, 1307.7042, 1308.6376, 1310.6146, 1312.6418, 1315.7098, 1320.6659, 1322.6886, 1323.6771, 1324.6964, 1328.7089, 1331.7202, 1335.7318, 1338.6581, 1339.6637, 1349.7253, 1352.7205, 1356.7230, 1360.6638, 1363.7153, 1365.7379, 1369.7043, 1379.7268, 1383.7137, 1385.7534, 1390.7284, 1393.7428, 1407.7563, 1417.7010, 1421.7061, 1422.7019, 1425.7727, 1427.7742, 1429.7365, 1431.7412, 1433.7228, 1437.7584, 1461.7778, 1472.7633, 1475.7632, 1477.7545, 1484.7889, 1491.7965, 1493.7579, 1500.7737, 1505.8177, 1513.8079, 1515.8213, 1517.7854, 1522.7811, 1523.7953, 1529.8099, 1530.7523, 1531.7472, 1537.7974, 1539.8005, 1542.8422, 1545.8091, 1547.8051, 1549.7339, 1551.7778, 1553.8065, 1555.8101, 1556.8109, 1557.8010, 1561.8401, 1563.8109, 1565.8199, 1571.8400, 1579.7939, 1580.8077, 1583.8444, 1587.7620, 1592.8066, 1594.8335, 1604.8323, 1605.8586, 1608.8497, 1615.8754, 1628.7900, 1628.7921, 1633.7985, 1640.9384, 1646.8297, 1649.8447, 1657.8101, 1667.8622, 1677.7988, 1680.8746, 1689.9465, 1694.7288, 1698.9225, 1704.8773, 1706.8701, 1710.9279, 1716.8671, 1725.8300, 1725.8346, 1727.8986, 1730.8931, 1737.9006, 1754.8580, 1765.8463, 1766.8040, 1767.8138, 1769.8741, 1770.3567, 1772.9315, 1774.7610, 1783.9246, 1787.8188, 1787.8200, 1791.8137, 1795.9353, 1798.9340, 1800.9336, 1804.9142, 1805.9069, 1809.8469, 1828.9227, 1837.9396, 1840.9827, 1843.9688, 1844.9012, 1846.9153, 1852.9364, 1856.9908, 1861.8881, 1867.9218, 1874.9695, 1880.9265, 1884.9417, 1891.9622, 1894.9313, 1900.9546, 1906.9501, 1908.9043, 1911.9130, 1918.9587, 1923.9718, 1925.9573, 1928.0469, 1932.0060, 1933.9950, 1937.9630, 1939.9740, 1946.9679, 1949.9595, 1955.9738, 1958.9556, 1960.0034, 1968.8600, 1973.9845, 1976.9861, 1978.9910, 1981.9854, 1983.9884, 1995.9408, 1998.9894, 2000.9926, 2008.0460, 2012.9590, 2015.0104, 2026.0275, 2027.0256, 2031.0166, 2044.0123, 2047.4957, 2047.9515, 2052.0496, 2060.0520, 2064.9880, 2066.9873, 2094.0376, 2102.0667, 2103.1641, 2111.0305, 2127.0930, 2139.0630, 2144.0493, 2148.1023, 2155.0950, 2156.0796, 2160.0632, 2176.0664, 2190.9895, 2195.0774, 2199.0723, 2202.1624, 2203.1221, 2208.0862, 2211.1042, 2225.1274, 2226.1272, 2229.0972, 2233.0984, 2239.0225, 2242.0435, 2268.1489, 2270.1287, 2271.1294,

2277.0823, 2284.1990, 2292.1426, 2309.0994, 2334.1719, 2342.9915, 2350.1746, 2383.9607, 2398.2400, 2400.0862, 2413.0549, 2414.1897, 2417.0959, 2497.2800, 2501.2637, 2511.1460, 2513.1421, 2528.1750, 2537.2012, 2541.1948, 2542.1836, 2556.2058, 2562.1487, 2612.2666, 2706.2109, 2717.0869, 2767.2666, 2888.2532, 3221.1733, 3224.2917, 3312.3306

28. G3N2D2 **Mass:** 41913 **Score:** 20 **Expect:** 2.5e+002 **Matches:** 17

Testis-specific serine/threonine-protein kinase 1 OS=Bos taurus GN=TSSK1B PE=3 SV=1

Observed	Mr(expt)	Mr(calc)	ppm	Start	End	Miss	Ions	Peptide
731.4017	730.3945	730.3861	11.4	2 -	8	0	---	M.DDAAVLK.R
953.5164	952.5091	952.4978	11.9	28 -	35	1	---	K.SAYSERLK.F
1056.5658	1055.5585	1055.5400	17.6	152 -	160	1	---	K.LSDFGFSKR.C
1073.5902	1072.5829	1072.5989	-14.87	276 -	286	1	---	K.ARGLSSAAVNK.E
1134.5714	1133.5641	1133.5360	24.8	160 -	168	2	---	K.RCLRDDSGR.L
1158.6685	1157.6612	1157.6557	4.80	116 -	125	1	---	K.KFHQLSSAIK.Y
1517.7854	1516.7781	1516.7562	14.5	147 -	159	1	---	K.DFNIKLSDFGFSK.R
1561.8401	1560.8328	1560.8664	-21.51	47 -	59	2	---	K.KAPTDFLEKFLPR.E
1719.9058	1718.8985	1718.8774	12.3	238 -	251	2	---	R.SKHLTGECKDLIYR.M
1730.8931	1729.8858	1729.8774	4.86	71 -	85	1	---	R.SIIKTYEIFETSDGK.V
1815.9044	1814.8971	1814.9236	-14.61	11 -	27	1	---	R.GYIMGINLGEGSYAKVK.S + Oxidation (M)
1874.9695	1873.9622	1873.9509	6.05	261 -	275	0	---	R.LHIDEILSHCWVQPK.A
1884.9417	1883.9344	1883.9676	-17.59	9 -	25	2	---	K.RRGYIMGINLGEGSYAK.V
1900.9546	1899.9473	1899.9625	-7.98	9 -	25	2	---	K.RRGYIMGINLGEGSYAK.V + Oxidation (M)
2027.0256	2026.0183	2026.0007	8.70	293 -	311	2	---	R.AAEPPWTPEPSSDKKSATK.L
2031.0166	2030.0093	2030.0520	-21.02	260 -	275	1	---	R.RLHIDEILSHCWVQPK.A
2102.0667	2101.0594	2101.0891	-14.12	261 -	277	1	---	R.LHIDEILSHCWVQPKAR.G

No match to: 721.4365, 725.4001, 726.4063, 728.3681, 730.3848, 732.3573, 734.4151, 735.4143, 741.4101, 742.4515, 744.4171, 745.4167, 746.4169, 747.4125, 750.4153, 752.4127, 755.4100, 756.4199, 757.4313, 761.4177, 767.4266, 768.3700, 768.3737, 770.3966, 772.4093, 775.4307, 779.4308, 784.3688, 786.4000, 787.4221, 788.4210, 792.4376, 795.4350, 798.4351, 800.4296, 812.4388, 815.4332, 822.4482, 825.4540, 831.4382, 832.4557, 835.4719, 837.4463, 842.5060, 844.4760, 854.4837, 856.4911, 860.4691, 862.4852, 869.4231, 885.4465, 905.4955, 907.4991, 908.4929, 913.5700, 913.5747, 916.5284, 918.5050, 919.5053, 922.5070, 928.5117, 934.5172, 940.4840, 942.4854, 943.4289, 943.5453, 946.5259, 950.4959, 951.5076, 954.5388, 959.5266, 966.5410, 969.5256, 972.5305, 974.5395, 976.5310, 978.5314, 979.5442, 991.5409, 993.5307, 1001.5854, 1003.5496, 1011.5262, 1014.5215, 1021.5579, 1023.5663, 1023.5700, 1031.5903, 1035.5381, 1036.5447, 1037.5573, 1039.5327, 1041.5658, 1044.5751, 1045.5729, 1046.5897, 1051.5448, 1057.6078, 1067.5753, 1086.5509, 1092.5961, 1100.6071, 1106.5391, 1111.5615, 1113.5729, 1118.5818, 1120.6045, 1122.5912, 1123.5848, 1125.5823, 1132.6047, 1139.5746, 1143.6318, 1144.6497, 1145.6407, 1147.6393, 1150.6108, 1155.6215, 1157.6199, 1162.6182, 1163.6152, 1168.6215, 1173.6418, 1175.6089, 1178.6356, 1179.6157, 1181.6819, 1188.6544, 1197.6704, 1199.7006, 1208.6415, 1216.6714, 1227.6595, 1231.6387, 1232.6447, 1234.6672, 1235.6445, 1244.6453, 1247.6211, 1248.6174, 1250.6554, 1255.6096, 1256.6531, 1261.6592, 1262.6453, 1266.6777, 1273.6260, 1275.6362, 1276.6343, 1277.6531, 1301.7079, 1303.7076, 1307.7042, 1308.6376, 1310.6146, 1312.6418, 1315.7098, 1320.6659, 1322.6886, 1323.6771, 1324.6964, 1328.7089, 1331.7202, 1335.7318, 1338.6581, 1339.6637, 1349.7253, 1352.7205, 1356.7230, 1360.6638, 1363.7153, 1365.7379, 1367.7319, 1369.7043, 1377.6947, 1379.7268, 1383.7137, 1385.7534, 1388.7404, 1390.7284, 1393.7428, 1407.7563, 1417.7010, 1421.7061, 1422.7019, 1425.7727, 1427.7742, 1429.7365, 1431.7412, 1433.7228, 1434.7446, 1437.7584, 1461.7778, 1467.7521, 1472.7633, 1475.7632, 1477.7545, 1484.7889, 1488.7537, 1491.7965, 1493.7579, 1500.7737, 1505.8177, 1513.8079, 1515.8213, 1519.7544, 1522.7811, 1523.7953, 1529.8099, 1530.7523, 1531.7472, 1537.7974, 1539.8005, 1542.8422, 1545.8091, 1547.8051, 1549.7339, 1551.7778, 1553.8065, 1555.8101, 1556.8109, 1557.8010, 1563.8109, 1565.8199, 1571.8400, 1579.7939, 1580.8077, 1583.8444, 1587.7620, 1592.8066, 1594.8335, 1604.8323, 1605.8586, 1608.8497, 1615.8754, 1624.8062, 1628.7900, 1628.7921, 1633.7985, 1640.9384, 1642.8099, 1646.8297, 1649.8447, 1657.8101, 1667.8622, 1677.7988, 1680.8746, 1689.9465, 1694.7288, 1698.9225, 1699.8862, 1704.8773, 1706.8701, 1707.8302, 1710.9279, 1716.8671, 1723.9011, 1725.8300, 1725.8346, 1727.8986, 1737.9006, 1751.8656, 1754.8580, 1765.8463, 1766.8040, 1767.8138, 1769.8741, 1770.3567, 1772.9315, 1774.7610, 1774.9131, 1777.8770, 1780.8777, 1783.9246, 1787.8188, 1787.8200, 1791.8137, 1793.9050, 1795.9353, 1798.9340, 1800.9336, 1804.9142, 1805.9069, 1809.8469, 1821.9144, 1828.9227, 1837.9396, 1840.9827, 1843.9688, 1844.9012, 1846.9153, 1852.9364, 1856.9908, 1861.8881, 1867.9218, 1880.9265, 1891.9622, 1894.9313, 1896.9078, 1896.9100, 1905.9313, 1906.9501, 1908.9043, 1911.9130, 1912.9154, 1916.9923, 1918.9587, 1921.9825, 1923.9718, 1925.9573, 1928.0469, 1932.0060, 1933.9950, 1937.9630, 1939.9740, 1944.0040, 1946.9679, 1949.9595, 1954.0236, 1955.9738, 1958.9556, 1960.0034, 1968.8600, 1973.9845, 1976.9861, 1978.9910, 1981.9854, 1983.9884, 1987.9784, 1995.9408, 1998.9894, 2000.9926, 2008.0460, 2012.9590, 2015.0104, 2017.9747, 2021.0170, 2026.0275, 2044.0123, 2047.4957, 2047.9515, 2052.0496, 2060.0520, 2062.0073, 2064.9880, 2066.9873, 2094.0376, 2097.0454, 2103.1641, 2111.0305, 2127.0930, 2139.0630, 2144.0493, 2148.1023, 2155.0950, 2156.0796, 2160.0632, 2176.0664, 2190.9895, 2195.0774, 2199.0723, 2202.1624, 2203.1221, 2208.0862, 2211.1042, 2216.1062, 2225.1274, 2226.1272, 2229.0972, 2233.0984, 2239.0225, 2242.0435, 2268.1489, 2270.1287, 2271.1294, 2277.0823, 2284.1990, 2292.1426, 2309.0994, 2334.1719, 2342.9915, 2350.1746, 2383.9607, 2398.2400, 2400.0862, 2413.0549, 2414.1897, 2417.0959, 2497.2800, 2501.2637, 2511.1460, 2513.1421, 2528.1750, 2537.2012, 2541.1948, 2542.1836, 2556.2058, 2562.1487, 2612.2666, 2706.2109, 2717.0869, 2767.2666, 2888.2532, 3221.1733, 3224.2917, 3312.3306, 3916.9434

29. Q32PB1 **Mass:** 14905 **Score:** 20 **Expect:** 2.5e+002 **Matches:** 8

Profilin-3 OS=Bos taurus GN=PFN3 PE=2 SV=1

Observed	Mr(expt)	Mr(calc)	ppm	Start	End	Miss	Ions	Peptide
746.4169	745.4096	745.4082	1.80	90 -	96	1	---	R.TKGLDGR.A
1379.7268	1378.7195	1378.7027	12.2	57 -	69	0	---	R.STFLQAGLCVAGR.R
1579.7939	1578.7866	1578.7937	-4.45	124 -	137	1	---	K.TMHELIHGLRSQGT.-
1737.9006	1736.8933	1736.9104	-9.85	90 -	105	2	---	R.TKGLDGRAICVGHTPR.A
1809.8469	1808.8396	1808.8765	-20.41	14 -	29	1	---	R.DQRIDDVAIVGHSDNR.C
1894.9313	1893.9240	1893.9519	-14.74	1 -	16	2	---	-.MGDWKGYISAVLRDQR.I
2208.0862	2207.0789	2207.1295	-22.89	76 -	96	2	---	R.DHLLAEGDGVLDARTKGLDGR.A
2398.2400	2397.2327	2397.1893	18.1	71 -	91	2	---	R.CCVIRDHLLAEGDGVLDARTK.G

No match to: 721.4365, 725.4001, 726.4063, 728.3681, 730.3848, 731.4017, 732.3573, 734.4151, 735.4143, 741.4101, 742.4515, 744.4171, 745.4167, 747.4125, 750.4153, 752.4127, 755.4100, 756.4199, 757.4313, 761.4177, 767.4266, 768.3700, 768.3737, 770.3966, 772.4093, 775.4307, 779.4308, 784.3688, 786.4000, 787.4221, 788.4210, 792.4376, 795.4350, 798.4351, 800.4296, 812.4388, 815.4332, 822.4482, 825.4540, 831.4382, 832.4557, 835.4719, 837.4463, 842.5060, 844.4760, 854.4837, 856.4911, 860.4691, 862.4852, 869.4231, 885.4465, 905.4955, 907.4991, 908.4929, 913.5700, 913.5747, 916.5284, 918.5050, 919.5053, 922.5070, 928.5117, 934.5172, 940.4840, 942.4854, 943.4289, 943.5453, 946.5259, 950.4959, 951.5076, 953.5164, 954.5388, 959.5266, 966.5410, 969.5256, 972.5305, 974.5395, 976.5310, 978.5314, 979.5442, 991.5409, 993.5307, 1001.5854, 1003.5496, 1011.5262, 1014.5215, 1021.5579, 1023.5663, 1023.5700, 1031.5903, 1035.5381, 1036.5447, 1037.5573, 1039.5327, 1041.5658, 1044.5751, 1045.5729, 1046.5897, 1051.5448, 1056.5658, 1057.6078, 1067.5753, 1073.5902, 1086.5509, 1092.5961, 1100.6071, 1106.5391, 1111.5615, 1113.5729, 1118.5818, 1120.6045, 1122.5912, 1123.5848, 1125.5823, 1132.6047, 1134.5714, 1139.5746, 1143.6318, 1144.6497, 1145.6407, 1147.6393, 1150.6108, 1155.6215, 1157.6199, 1158.6685, 1162.6182, 1163.6152, 1168.6215, 1173.6418, 1175.6089, 1178.6356, 1179.6157, 1181.6819, 1188.6544, 1197.6704, 1199.7006, 1208.6415, 1216.6714, 1227.6595, 1231.6387, 1232.6447, 1234.6672, 1235.6445, 1244.6453, 1247.6211, 1248.6174, 1250.6554, 1255.6096, 1256.6531, 1261.6592, 1262.6453, 1266.6777, 1273.6260, 1275.6362, 1276.6343, 1277.6531, 1301.7079, 1303.7076, 1307.7042, 1308.6376, 1310.6146, 1312.6418, 1315.7098, 1320.6659, 1322.6886, 1323.6771, 1324.6964, 1328.7089, 1331.7202, 1335.7318, 1338.6581, 1339.6637, 1349.7253, 1352.7205, 1356.7230, 1360.6638, 1363.7153, 1365.7379, 1367.7319, 1369.7043, 1377.6947, 1383.7137, 1385.7534, 1388.7404, 1390.7284, 1393.7428, 1407.7563, 1417.7010, 1421.7061, 1422.7019, 1425.7727, 1427.7742, 1429.7365, 1431.7412, 1433.7228, 1434.7446, 1437.7584, 1461.7778, 1467.7521, 1472.7633, 1475.7632, 1477.7545, 1484.7889, 1488.7537, 1491.7965, 1493.7579, 1500.7737, 1505.8177, 1513.8079, 1515.8213, 1517.7854, 1519.7544, 1522.7811, 1523.7953, 1529.8099, 1530.7523, 1531.7472, 1537.7974, 1539.8005, 1542.8422, 1545.8091, 1547.8051, 1549.7339, 1551.7778, 1553.8065, 1555.8101, 1556.8109, 1557.8010, 1561.8401, 1563.8109, 1565.8199, 1571.8400, 1580.8077, 1583.8444, 1587.7620, 1592.8066, 1594.8335, 1604.8323, 1605.8586, 1608.8497, 1615.8754, 1624.8062, 1628.7900, 1628.7921, 1633.7985, 1640.9384, 1642.8099, 1646.8297, 1649.8447, 1657.8101, 1667.8622, 1677.7988, 1680.8746, 1689.9465, 1694.7288, 1698.9225, 1699.8862, 1704.8773, 1706.8701, 1707.8302, 1710.9279, 1716.8671, 1719.9058, 1723.9011, 1725.8300, 1725.8346, 1727.8986, 1730.8931, 1751.8656, 1754.8580, 1765.8463, 1766.8040, 1767.8138, 1769.8741, 1770.3567, 1772.9315, 1774.7610, 1774.9131, 1777.8770, 1780.8777, 1783.9246, 1787.8188, 1787.8200, 1791.8137, 1793.9050, 1795.9353, 1798.9340, 1800.9336, 1804.9142, 1805.9069, 1815.9044, 1821.9144, 1828.9227,

1837.9396, 1840.9827, 1843.9688, 1844.9012, 1846.9153, 1852.9364, 1856.9908, 1861.8881, 1867.9218, 1874.9695, 1880.9265, 1884.9417, 1891.9622, 1896.9078, 1896.9100, 1900.9546, 1905.9313, 1906.9501, 1908.9043, 1911.9130, 1912.9154, 1916.9923, 1918.9587, 1921.9825, 1923.9718, 1925.9573, 1928.0469, 1932.0060, 1933.9950, 1937.9630, 1939.9740, 1944.0040, 1946.9679, 1949.9595, 1954.0236, 1955.9738, 1958.9556, 1960.0034, 1968.8600, 1973.9845, 1976.9861, 1978.9910, 1981.9854, 1983.9884, 1987.9784, 1995.9408, 1998.9894, 2000.9926, 2008.0460, 2012.9590, 2015.0104, 2017.9747, 2021.0170, 2026.0275, 2027.0256, 2031.0166, 2044.0123, 2047.4957, 2047.9515, 2052.0496, 2060.0520, 2062.0073, 2064.9880, 2066.9873, 2094.0376, 2097.0454, 2102.0667, 2103.1641, 2111.0305, 2127.0930, 2139.0630, 2144.0493, 2148.1023, 2155.0950, 2156.0796, 2160.0632, 2176.0664, 2190.9895, 2195.0774, 2199.0723, 2202.1624, 2203.1221, 2211.1042, 2216.1062, 2225.1274, 2226.1272, 2229.0972, 2233.0984, 2239.0225, 2242.0435, 2268.1489, 2270.1287, 2271.1294, 2277.0823, 2284.1990, 2292.1426, 2309.0994, 2334.1719, 2342.9915, 2350.1746, 2383.9607, 2400.0862, 2413.0549, 2414.1897, 2417.0959, 2497.2800, 2501.2637, 2511.1460, 2513.1421, 2528.1750, 2537.2012, 2541.1948, 2542.1836, 2556.2058, 2562.1487, 2612.2666, 2706.2109, 2717.0869, 2767.2666, 2888.2532, 3221.1733, 3224.2917, 3312.3306, 3916.9434

30. G3X7E1 **Mass:** 19619 **Score:** 20 **Expect:** 2.5e+002 **Matches:** 11

Histone deacetylase complex subunit SAP18 OS=Bos taurus GN=SAP18 PE=4 SV=1

Observed	Mr(expt)	Mr(calc)	ppm	Start	End	Miss	Ions	Peptide
974.5395	973.5323	973.5444	-12.44	26 -	33	1	---	R.VTQEEIKK.E
976.5310	975.5237	975.5284	-4.73	18 -	25	2	---	R.RKMAVESR.V
991.5409	990.5336	990.5134	20.4	94 -	101	1	---	K.EVYPEARK.K
1175.6089	1174.6016	1174.5764	21.4	1 -	12	0	---	-.MLAAGVGGQSER.L
1232.6447	1231.6374	1231.6132	19.7	162 -	172	1	---	R.APPTSGRMRPY.-
1248.6174	1247.6101	1247.6081	1.64	162 -	172	1	---	R.APPTSGRMRPY.- + Oxidation (M)
1310.6146	1309.6073	1309.5820	19.3	134 -	145	0	---	K.GTDDSMTLQSQK.F
1338.6581	1337.6508	1337.6701	-14.43	51 -	61	1	---	R.VFTTNNGRHHR.M
1519.7544	1518.7471	1518.7712	-15.83	20 -	32	1	---	K.MAVESRVTQEEIK.K
1737.9006	1736.8933	1736.9250	-18.24	102 -	116	1	---	K.KGTHFNFAIVFTDLK.R
1793.9050	1792.8977	1792.9366	-21.69	117 -	132	2	---	K.RPGYRVKEIGSTMSGR.K

No match to: 721.4365, 725.4001, 726.4063, 728.3681, 730.3848, 731.4017, 732.3573, 734.4151, 735.4143, 741.4101, 742.4515, 744.4171, 745.4167, 746.4169, 747.4125, 750.4153, 752.4127, 755.4100, 756.4199, 757.4313, 761.4177, 767.4266, 768.3700, 768.3737, 770.3966, 772.4093, 775.4307, 779.4308, 784.3688, 786.4000, 787.4221, 788.4210, 792.4376, 795.4350, 798.4351, 800.4296, 812.4388, 815.4332, 822.4482, 825.4540, 831.4382, 832.4557, 835.4719, 837.4463, 842.5060, 844.4760, 854.4837, 856.4911, 860.4691, 862.4852, 869.4231, 885.4465, 905.4955, 907.4991, 908.4929, 913.5700, 913.5747, 916.5284, 918.5050, 919.5053, 922.5070, 928.5117, 934.5172, 940.4840, 942.4854, 943.4289, 943.5453, 946.5259, 950.4959, 951.5076, 953.5164, 954.5388, 959.5266, 966.5410, 969.5256, 972.5305, 978.5314, 979.5442, 993.5307, 1001.5854, 1003.5496, 1011.5262, 1014.5215, 1021.5579, 1023.5663, 1023.5700, 1031.5903, 1035.5381, 1036.5447, 1037.5573, 1039.5327, 1041.5658, 1044.5751, 1045.5729, 1046.5897, 1051.5448, 1056.5658, 1057.6078, 1067.5753, 1073.5902, 1086.5509, 1092.5961, 1100.6071, 1106.5391, 1111.5615, 1113.5729, 1118.5818, 1120.6045, 1122.5912, 1123.5848, 1125.5823, 1132.6047, 1134.5714, 1139.5746, 1143.6318, 1144.6497, 1145.6407, 1147.6393, 1150.6108, 1155.6215, 1157.6199, 1158.6685, 1162.6182, 1163.6152, 1168.6215, 1173.6418, 1178.6356, 1179.6157, 1181.6819, 1188.6544, 1197.6704, 1199.7006, 1208.6415, 1216.6714, 1227.6595, 1231.6387, 1234.6672, 1235.6445, 1244.6453, 1247.6211, 1250.6554, 1255.6096, 1256.6531, 1261.6592, 1262.6453, 1266.6777, 1273.6260, 1275.6362, 1276.6343, 1277.6531, 1301.7079, 1303.7076, 1307.7042, 1308.6376, 1312.6418, 1315.7098, 1320.6659, 1322.6886, 1323.6771, 1324.6964, 1328.7089, 1331.7202, 1335.7318, 1339.6637, 1349.7253, 1352.7205, 1356.7230, 1360.6638, 1363.7153, 1365.7379, 1367.7319, 1369.7043, 1377.6947, 1379.7268, 1383.7137, 1385.7534, 1388.7404, 1390.7284, 1393.7428, 1407.7563, 1417.7010, 1421.7061, 1422.7019, 1425.7727, 1427.7742, 1429.7365, 1431.7412, 1433.7228, 1434.7446, 1437.7584, 1461.7778, 1467.7521, 1472.7633, 1475.7632, 1477.7545, 1484.7889, 1488.7537, 1491.7965, 1493.7579, 1500.7737, 1505.8177, 1513.8079, 1515.8213, 1517.7854, 1522.7811, 1523.7953, 1529.8099, 1530.7523, 1531.7472, 1537.7974, 1539.8005, 1542.8422, 1545.8091, 1547.8051, 1549.7339, 1551.7778, 1553.8065, 1555.8101, 1556.8109, 1557.8010, 1561.8401, 1563.8109, 1565.8199, 1571.8400, 1579.7939, 1580.8077, 1583.8444, 1587.7620, 1592.8066, 1594.8335, 1604.8323, 1605.8586, 1608.8497, 1615.8754, 1624.8062, 1628.7900, 1628.7921, 1633.7985, 1640.9384, 1642.8099, 1646.8297, 1649.8447, 1657.8101, 1667.8622, 1677.7988, 1680.8746, 1689.9465, 1694.7288, 1698.9225, 1699.8862, 1704.8773, 1706.8701, 1707.8302, 1710.9279, 1716.8671, 1719.9058, 1723.9011, 1725.8300, 1725.8346, 1727.8986, 1730.8931, 1751.8656, 1754.8580, 1765.8463, 1766.8040, 1767.8138, 1769.8741, 1770.3567, 1772.9315, 1774.7610, 1774.9131, 1777.8770, 1780.8777, 1783.9246, 1787.8188, 1787.8200, 1791.8137, 1795.9353, 1798.9340, 1800.9336, 1804.9142, 1805.9069, 1809.8469, 1815.9044, 1821.9144, 1828.9227, 1837.9396, 1840.9827, 1843.9688, 1844.9012, 1846.9153, 1852.9364, 1856.9908, 1861.8881, 1867.9218, 1874.9695, 1880.9265, 1884.9417, 1891.9622, 1894.9313, 1896.9078, 1896.9100, 1900.9546, 1905.9313, 1906.9501, 1908.9043, 1911.9130, 1912.9154, 1916.9923, 1918.9587, 1921.9825, 1923.9718, 1925.9573, 1928.0469, 1932.0060, 1933.9950, 1937.9630, 1939.9740, 1944.0040, 1946.9679, 1949.9595, 1954.0236, 1955.9738, 1958.9556, 1960.0034, 1968.8600, 1973.9845, 1976.9861, 1978.9910, 1981.9854, 1983.9884, 1987.9784, 1995.9408, 1998.9894, 2000.9926, 2008.0460, 2012.9590, 2015.0104, 2017.9747, 2021.0170, 2026.0275, 2027.0256, 2031.0166, 2044.0123, 2047.4957, 2047.9515, 2052.0496, 2060.0520, 2062.0073, 2064.9880, 2066.9873, 2094.0376, 2097.0454, 2102.0667, 2103.1641, 2111.0305, 2127.0930, 2139.0630, 2144.0493, 2148.1023, 2155.0950, 2156.0796, 2160.0632, 2176.0664, 2190.9895, 2195.0774, 2199.0723, 2202.1624, 2203.1221, 2208.0862, 2211.1042, 2216.1062, 2225.1274, 2226.1272, 2229.0972, 2233.0984, 2239.0225, 2242.0435, 2268.1489, 2270.1287, 2271.1294, 2277.0823, 2284.1990, 2292.1426, 2309.0994, 2334.1719, 2342.9915, 2350.1746, 2383.9607, 2398.2400, 2400.0862, 2413.0549, 2414.1897, 2417.0959, 2497.2800, 2501.2637, 2511.1460, 2513.1421, 2528.1750, 2537.2012, 2541.1948, 2542.1836, 2556.2058, 2562.1487, 2612.2666, 2706.2109, 2717.0869, 2767.2666, 2888.2532, 3221.1733, 3224.2917, 3312.3306, 3916.9434

31. Q58DQ3 **Mass:** 32727 **Score:** 20 **Expect:** 2.5e+002 **Matches:** 14

60S ribosomal protein L6 OS=Bos taurus GN=RPL6 PE=2 SV=3

Observed	Mr(expt)	Mr(calc)	ppm	Start	End	Miss	Ions	Peptide
788.4210	787.4137	787.4076	7.77	6 -	12	0	---	K.AEKPDTK.E
969.5256	968.5183	968.4974	21.6	38 -	45	1	---	K.KGKPHCSR.N
1045.5729	1044.5656	1044.5451	19.6	6 -	14	1	---	K.AEKPDTKEK.K
1057.6078	1056.6005	1056.6040	-3.26	251 -	259	1	---	K.AVDSQILRR.I
1144.6497	1143.6424	1143.6400	2.09	262 -	271	0	---	K.AVPQLQGYLR.S
1248.6174	1247.6101	1247.6080	1.66	56 -	65	2	---	R.YSRSAMYSRK.A
1320.6659	1319.6586	1319.6391	14.8	1 -	12	1	---	-.MAGEKAEKPDTK.E + Oxidation (M)
1323.6771	1322.6698	1322.6830	-9.95	237 -	246	2	---	R.EKYEITEQRK.V
1363.7153	1362.7080	1362.6820	19.1	207 -	217	0	---	K.IPEHLTDTYFK.K
1491.7965	1490.7892	1490.7769	8.26	207 -	218	1	---	K.IPEHLTDTYFKK.K
1519.7544	1518.7471	1518.7361	7.25	52 -	64	2	---	R.GIGRYSRSAMYSR.K + Oxidation (M)
1667.8622	1666.8549	1666.8137	24.7	111 -	123	2	---	K.MPRYYPTEDVPRK.L + Oxidation (M)
1998.9894	1997.9821	1997.9919	-4.88	223 -	238	2	---	R.KPRHQEGEIFDTEREK.Y
2537.2012	2536.1939	2536.1830	4.32	226 -	245	2	---	R.HQEGEIFDTEREKYEITEQR.K

No match to: 721.4365, 725.4001, 726.4063, 728.3681, 730.3848, 731.4017, 732.3573, 734.4151, 735.4143, 741.4101, 742.4515, 744.4171, 745.4167, 746.4169, 747.4125, 750.4153, 752.4127, 755.4100, 756.4199, 757.4313, 761.4177, 767.4266, 768.3700, 768.3737, 770.3966, 772.4093, 775.4307, 779.4308, 784.3688, 786.4000, 787.4221, 792.4376, 795.4350, 798.4351, 800.4296, 812.4388, 815.4332, 822.4482, 825.4540, 831.4382, 832.4557, 835.4719, 837.4463, 842.5060, 844.4760, 854.4837, 856.4911, 860.4691, 862.4852, 869.4231, 885.4465, 905.4955, 907.4991, 908.4929, 913.5700, 913.5747, 916.5284, 918.5050, 919.5053, 922.5070, 928.5117, 934.5172, 940.4840, 942.4854, 943.4289, 943.5453, 946.5259, 950.4959, 951.5076, 953.5164, 954.5388, 959.5266, 966.5410, 972.5305, 974.5395, 976.5310, 978.5314, 979.5442, 991.5409, 993.5307, 1001.5854, 1003.5496, 1011.5262, 1014.5215, 1021.5579, 1023.5663, 1023.5700, 1031.5903, 1035.5381, 1036.5447, 1037.5573, 1039.5327, 1041.5658, 1044.5751, 1046.5897, 1051.5448, 1056.5658, 1067.5753, 1073.5902, 1086.5509, 1092.5961, 1100.6071, 1106.5391, 1111.5615, 1113.5729, 1118.5818, 1120.6045, 1122.5912, 1123.5848, 1125.5823, 1132.6047, 1134.5714, 1139.5746, 1143.6318, 1145.6407, 1147.6393, 1150.6108, 1155.6215, 1157.6199, 1158.6685, 1162.6182, 1163.6152, 1168.6215, 1173.6418, 1175.6089, 1178.6356, 1179.6157, 1181.6819, 1188.6544, 1197.6704, 1199.7006, 1208.6415, 1216.6714, 1227.6595, 1231.6387, 1232.6447, 1234.6672, 1235.6445, 1244.6453, 1247.6211, 1250.6554, 1255.6096, 1256.6531, 1261.6592, 1262.6453, 1266.6777, 1273.6260, 1275.6362, 1276.6343, 1277.6531, 1301.7079, 1303.7076, 1307.7042, 1308.6376, 1310.6146, 1312.6418, 1315.7098, 1322.6886, 1324.6964, 1328.7089, 1331.7202, 1335.7318, 1338.6581, 1339.6637, 1349.7253, 1352.7205, 1356.7230, 1360.6638, 1365.7379, 1367.7319, 1369.7043, 1377.6947, 1379.7268, 1383.7137, 1385.7534, 1388.7404, 1390.7284, 1393.7428, 1407.7563, 1417.7010, 1421.7061, 1422.7019, 1425.7727, 1427.7742,

1429.7365, 1431.7412, 1433.7228, 1434.7446, 1437.7584, 1461.7778, 1467.7521, 1472.7633, 1475.7632, 1477.7545, 1484.7889, 1488.7537, 1493.7579, 1500.7737, 1505.8177, 1513.8079, 1515.8213, 1517.7854, 1522.7811, 1523.7953, 1529.8099, 1530.7523, 1531.7472, 1537.7974, 1539.8005, 1542.8422, 1545.8091, 1547.8051, 1549.7339, 1551.7778, 1553.8065, 1555.8101, 1556.8109, 1557.8010, 1561.8401, 1563.8109, 1565.8199, 1571.8400, 1579.7939, 1580.8077, 1583.8444, 1587.7620, 1592.8066, 1594.8335, 1604.8323, 1605.8586, 1608.8497, 1615.8754, 1624.8062, 1628.7900, 1628.7921, 1633.7985, 1640.9384, 1642.8099, 1646.8297, 1649.8447, 1657.8101, 1677.7988, 1680.8746, 1689.9465, 1694.7288, 1698.9225, 1699.8862, 1704.8773, 1706.8701, 1707.8302, 1710.9279, 1716.8671, 1719.9058, 1723.9011, 1725.8300, 1725.8346, 1727.8986, 1730.8931, 1737.9006, 1751.8656, 1754.8580, 1765.8463, 1766.8040, 1767.8138, 1769.8741, 1770.3567, 1772.9315, 1774.7610, 1774.9131, 1777.8770, 1780.8777, 1783.9246, 1787.8188, 1787.8200, 1791.8137, 1793.9050, 1795.9353, 1798.9340, 1800.9336, 1804.9142, 1805.9069, 1809.8469, 1815.9044, 1821.9144, 1828.9227, 1837.9396, 1840.9827, 1843.9688, 1844.9012, 1846.9153, 1852.9364, 1856.9908, 1861.8881, 1867.9218, 1874.9695, 1880.9265, 1884.9417, 1891.9622, 1894.9313, 1896.9078, 1896.9100, 1900.9546, 1905.9313, 1906.9501, 1908.9043, 1911.9130, 1912.9154, 1916.9923, 1918.9587, 1921.9825, 1923.9718, 1925.9573, 1928.0469, 1932.0060, 1933.9950, 1937.9630, 1939.9740, 1944.0040, 1946.9679, 1949.9595, 1954.0236, 1955.9738, 1958.9556, 1960.0034, 1968.8600, 1973.9845, 1976.9861, 1978.9910, 1981.9854, 1983.9884, 1987.9784, 1995.9408, 2000.9926, 2008.0460, 2012.9590, 2015.0104, 2017.9747, 2021.0170, 2026.0275, 2027.0256, 2031.0166, 2044.0123, 2047.4957, 2047.9515, 2052.0496, 2060.0520, 2062.0073, 2064.9880, 2066.9873, 2094.0376, 2097.0454, 2102.0667, 2103.1641, 2111.0305, 2127.0930, 2139.0630, 2144.0493, 2148.1023, 2155.0950, 2156.0796, 2160.0632, 2176.0664, 2190.9895, 2195.0774, 2199.0723, 2202.1624, 2203.1221, 2208.0862, 2211.1042, 2216.1062, 2225.1274, 2226.1272, 2229.0972, 2233.0984, 2239.0225, 2242.0435, 2268.1489, 2270.1287, 2271.1294, 2277.0823, 2284.1990, 2292.1426, 2309.0994, 2334.1719, 2342.9915, 2350.1746, 2383.9607, 2398.2400, 2400.0862, 2413.0549, 2414.1897, 2417.0959, 2497.2800, 2501.2637, 2511.1460, 2513.1421, 2528.1750, 2541.1948, 2542.1836, 2556.2058, 2562.1487, 2612.2666, 2706.2109, 2717.0869, 2767.2666, 2888.2532, 3221.1733, 3224.2917, 3312.3306, 3916.9434

32. P80311 **Mass:** 23786 **Score:** 20 **Expect:** 2.6e+002 **Matches:** 11

Peptidyl-prolyl cis-trans isomerase B OS=Bos taurus GN=PPIB PE=1 SV=4

Observed	Mr(expt)	Mr(calc)	ppm	Start	End	Miss	Ions	Peptide
812.4388	811.4316	811.4228	10.8	46 -	51	0	---	K.VYFDLR.I
1120.6045	1119.5972	1119.5884	7.89	181 -	190	2	---	R.KVESTKTDGR.D
1145.6407	1144.6334	1144.6274	5.24	172 -	181	1	---	K.VLEGMDVVRK.V
1162.6182	1161.6109	1161.6288	-15.37	2 -	10	2	---	M.LRLSERNMK.I + Oxidation (M)
1277.6531	1276.6458	1276.6743	-22.34	1 -	10	2	---	-.MLRLSERNMK.I
1642.8099	1641.8026	1641.8185	-9.67	96 -	109	1	---	R.VIKDFMIQGGDFTR.G + Oxidation (M)
1667.8622	1666.8549	1666.8315	14.1	46 -	59	1	---	K.VYFDLRIGDEDIGR.V
1689.9465	1688.9392	1688.9131	15.5	172 -	186	2	---	K.VLEGMDVVRKVESTK.T
1916.9923	1915.9850	1915.9680	8.89	72 -	89	1	---	K.TVDNFVALATGEKGFGYK.D
2199.0723	2198.0650	2198.0790	-6.38	96 -	116	2	---	R.VIKDFMIQGGDFTRGDGTGGK.S
2229.0972	2228.0899	2228.1114	-9.63	146 -	165	1	---	K.DTNGSQFFITTVKTAWLDGK.H

No match to: 721.4365, 725.4001, 726.4063, 728.3681, 730.3848, 731.4017, 732.3573, 734.4151, 735.4143, 741.4101, 742.4515, 744.4171, 745.4167, 746.4169, 747.4125, 750.4153, 752.4127, 755.4100, 756.4199, 757.4313, 761.4177, 767.4266, 768.3700, 768.3737, 770.3966, 772.4093, 775.4307, 779.4308, 784.3688, 786.4000, 787.4221, 788.4210, 792.4376, 795.4350, 798.4351, 800.4296, 815.4332, 822.4482, 825.4540, 831.4382, 832.4557, 835.4719, 837.4463, 842.5060, 844.4760, 854.4837, 856.4911, 860.4691, 862.4852, 869.4231, 885.4465, 905.4955, 907.4991, 908.4929, 913.5700, 913.5747, 916.5284, 918.5050, 919.5053, 922.5070, 928.5117, 934.5172, 940.4840, 942.4854, 943.4289, 943.5453, 946.5259, 950.4959, 951.5076, 953.5164, 954.5388, 959.5266, 966.5410, 969.5256, 972.5305, 974.5395, 976.5310, 978.5314, 979.5442, 991.5409, 993.5307, 1001.5854, 1003.5496, 1011.5262, 1014.5215, 1021.5579, 1023.5663, 1023.5700, 1031.5903, 1035.5381, 1036.5447, 1037.5573, 1039.5327, 1041.5658, 1044.5751, 1045.5729, 1046.5897, 1051.5448, 1056.5658, 1057.6078, 1067.5753, 1073.5902, 1086.5509, 1092.5961, 1100.6071, 1106.5391, 1111.5615, 1113.5729, 1118.5818, 1122.5912, 1123.5848, 1125.5823, 1132.6047, 1134.5714, 1139.5746, 1143.6318, 1144.6497, 1147.6393, 1150.6108, 1155.6215, 1157.6199, 1158.6685, 1163.6152, 1168.6215, 1173.6418, 1175.6089, 1178.6356, 1179.6157, 1181.6819, 1188.6544, 1197.6704, 1199.7006, 1208.6415, 1216.6714, 1227.6595, 1231.6387, 1232.6447, 1234.6672, 1235.6445, 1244.6453, 1247.6211, 1248.6174, 1250.6554, 1255.6096, 1256.6531, 1261.6592, 1262.6453, 1266.6777, 1273.6260, 1275.6362, 1276.6343, 1301.7079, 1303.7076, 1307.7042, 1308.6376, 1310.6146, 1312.6418, 1315.7098, 1320.6659, 1322.6886, 1323.6771, 1324.6964, 1328.7089, 1331.7202, 1335.7318, 1338.6581, 1339.6637, 1349.7253, 1352.7205, 1356.7230, 1360.6638, 1363.7153, 1365.7379, 1367.7319, 1369.7043, 1377.6947, 1379.7268, 1383.7137, 1385.7534, 1388.7404, 1390.7284, 1393.7428, 1407.7563, 1417.7010, 1421.7061, 1422.7019, 1425.7727, 1427.7742, 1429.7365, 1431.7412, 1433.7228, 1434.7446, 1437.7584, 1461.7778, 1467.7521, 1472.7633, 1475.7632, 1477.7545, 1484.7889, 1488.7537, 1491.7965, 1493.7579, 1500.7737, 1505.8177, 1513.8079, 1515.8213, 1517.7854, 1519.7544, 1522.7811, 1523.7953, 1529.8099, 1530.7523, 1531.7472, 1537.7974, 1539.8005, 1542.8422, 1545.8091, 1547.8051, 1549.7339, 1551.7778, 1553.8065, 1555.8101, 1556.8109, 1557.8010, 1561.8401, 1563.8109, 1565.8199, 1571.8400, 1579.7939, 1580.8077, 1583.8444, 1587.7620, 1592.8066, 1594.8335, 1604.8323, 1605.8586, 1608.8497, 1615.8754, 1624.8062, 1628.7900, 1628.7921, 1633.7985, 1640.9384, 1646.8297, 1649.8447, 1657.8101, 1677.7988, 1680.8746, 1694.7288, 1698.9225, 1699.8862, 1704.8773, 1706.8701, 1707.8302, 1710.9279, 1716.8671, 1719.9058, 1723.9011, 1725.8300, 1725.8346, 1727.8986, 1730.8931, 1737.9006, 1751.8656, 1754.8580, 1765.8463, 1766.8040, 1767.8138, 1769.8741, 1770.3567, 1772.9315, 1774.7610, 1774.9131, 1777.8770, 1780.8777, 1783.9246, 1787.8188, 1787.8200, 1791.8137, 1793.9050, 1795.9353, 1798.9340, 1800.9336, 1804.9142, 1805.9069, 1809.8469, 1815.9044, 1821.9144, 1828.9227, 1837.9396, 1840.9827, 1843.9688, 1844.9012, 1846.9153, 1852.9364, 1856.9908, 1861.8881, 1867.9218, 1874.9695, 1880.9265, 1884.9417, 1891.9622, 1894.9313, 1896.9078, 1896.9100, 1900.9546, 1905.9313, 1906.9501, 1908.9043, 1911.9130, 1912.9154, 1918.9587, 1921.9825, 1923.9718, 1925.9573, 1928.0469, 1932.0060, 1933.9950, 1937.9630, 1939.9740, 1944.0040, 1946.9679, 1949.9595, 1954.0236, 1955.9738, 1958.9556, 1960.0034, 1968.8600, 1973.9845, 1976.9861, 1978.9910, 1981.9854, 1983.9884, 1987.9784, 1995.9408, 1998.9894, 2000.9926, 2008.0460, 2012.9590, 2015.0104, 2017.9747, 2021.0170, 2026.0275, 2027.0256, 2031.0166, 2044.0123, 2047.4957, 2047.9515, 2052.0496, 2060.0520, 2062.0073, 2064.9880, 2066.9873, 2094.0376, 2097.0454, 2102.0667, 2103.1641, 2111.0305, 2127.0930, 2139.0630, 2144.0493, 2148.1023, 2155.0950, 2156.0796, 2160.0632, 2176.0664, 2190.9895, 2195.0774, 2202.1624, 2203.1221, 2208.0862, 2211.1042, 2216.1062, 2225.1274, 2226.1272, 2233.0984, 2239.0225, 2242.0435, 2268.1489, 2270.1287, 2271.1294, 2277.0823, 2284.1990, 2292.1426, 2309.0994, 2334.1719, 2342.9915, 2350.1746, 2383.9607, 2398.2400, 2400.0862, 2413.0549, 2414.1897, 2417.0959, 2497.2800, 2501.2637, 2511.1460, 2513.1421, 2528.1750, 2537.2012, 2541.1948, 2542.1836, 2556.2058, 2562.1487, 2612.2666, 2706.2109, 2717.0869, 2767.2666, 2888.2532, 3221.1733, 3224.2917, 3312.3306, 3916.9434

33. G1K1R3 **Mass:** 40420 **Score:** 20 **Expect:** 2.7e+002 **Matches:** 17

Tropomodulin-1 OS=Bos taurus GN=TMOD1 PE=4 SV=2

Observed	Mr(expt)	Mr(calc)	ppm	Start	End	Miss	Ions	Peptide
728.3681	727.3608	727.3435	23.8	1 -	5	1	---	-.MSYRR.E + Oxidation (M)
745.4167	744.4094	744.3919	23.6	57 -	63	0	---	K.APTGPFR.R
750.4153	749.4081	749.4072	1.20	74 -	79	1	---	K.QAKEFK.D
779.4308	778.4235	778.4337	-13.14	229 -	235	0	---	K.FSIVGTR.S
837.4463	836.4390	836.4392	-0.20	6 -	11	1	---	R.ELEKYR.D
993.5307	992.5234	992.5403	-16.99	5 -	11	2	---	R.RELEKYR.D
1021.5579	1020.5506	1020.5352	15.1	74 -	81	2	---	K.QAKEFKDR.E
1125.5823	1124.5750	1124.5713	3.29	65 -	73	0	---	R.EELLDHLEK.Q
1323.6771	1322.6698	1322.6461	17.9	298 -	308	0	---	K.VEMEIVSMLEK.N + Oxidation (M)
1339.6637	1338.6564	1338.6411	11.5	298 -	308	0	---	K.VEMEIVSMLEK.N + 2 Oxidation (M)
1421.7061	1420.6988	1420.6834	10.8	80 -	91	1	---	K.DREDLVPYTGEK.R
1491.7965	1490.7892	1490.7729	11.0	82 -	94	2	---	R.EDLVPYTGEKRGK.V
1608.8497	1607.8424	1607.8631	-12.84	64 -	76	2	---	R.REELLDHLEKQAK.E
1765.8463	1764.8390	1764.8359	1.77	327 -	341	2	---	R.ASNAMMNNNDLVRKR.R + 2 Oxidation (M)
1846.9153	1845.9080	1845.9301	-11.97	325 -	340	2	---	R.LRASNAMMNNNDLVRK.R
1856.9908	1855.9835	1855.9679	8.40	65 -	79	2	---	R.EELLDHLEKQAKEFK.D
2008.0460	2007.0387	2007.0537	-7.48	57 -	73	2	---	K.APTGPFRREELLDHLEK.Q

No match to: 721.4365, 725.4001, 726.4063, 730.3848, 731.4017, 732.3573, 734.4151, 735.4143, 741.4101, 742.4515, 744.4171, 746.4169, 747.4125, 752.4127, 755.4100, 756.4199, 757.4313, 761.4177, 767.4266, 768.3700, 768.3737, 770.3966, 772.4093, 775.4307, 784.3688, 786.4000, 787.4221, 788.4210, 792.4376, 795.4350, 798.4351, 800.4296, 812.4388, 815.4332, 822.4482, 825.4540, 831.4382, 832.4557, 835.4719, 842.5060, 844.4760, 854.4837, 856.4911, 860.4691, 862.4852, 869.4231, 885.4465, 905.4955, 907.4991, 908.4929,

913.5700, 913.5747, 916.5284, 918.5050, 919.5053, 922.5070, 928.5117, 934.5172, 940.4840, 942.4854, 943.4289, 943.5453, 946.5259, 950.4959, 951.5076, 953.5164, 954.5388, 959.5266, 966.5410, 969.5256, 972.5305, 974.5395, 976.5310, 978.5314, 979.5442, 991.5409, 1001.5854, 1003.5496, 1011.5262, 1014.5215, 1023.5663, 1023.5700, 1031.5903, 1035.5381, 1036.5447, 1037.5573, 1039.5327, 1041.5658, 1044.5751, 1045.5729, 1046.5897, 1051.5448, 1056.5658, 1057.6078, 1067.5753, 1073.5902, 1086.5509, 1092.5961, 1100.6071, 1106.5391, 1111.5615, 1113.5729, 1118.5818, 1120.6045, 1122.5912, 1123.5848, 1132.6047, 1134.5714, 1139.5746, 1143.6318, 1144.6497, 1145.6407, 1147.6393, 1150.6108, 1155.6215, 1157.6199, 1158.6685, 1162.6182, 1163.6152, 1168.6215, 1173.6418, 1175.6089, 1178.6356, 1179.6157, 1181.6819, 1188.6544, 1197.6704, 1199.7006, 1208.6415, 1216.6714, 1227.6595, 1231.6387, 1232.6447, 1234.6672, 1235.6445, 1244.6453, 1247.6211, 1248.6174, 1250.6554, 1255.6096, 1256.6531, 1261.6592, 1262.6453, 1266.6777, 1273.6260, 1275.6362, 1276.6343, 1277.6531, 1301.7079, 1303.7076, 1307.7042, 1308.6376, 1310.6146, 1312.6418, 1315.7098, 1320.6659, 1322.6886, 1324.6964, 1328.7089, 1331.7202, 1335.7318, 1338.6581, 1349.7253, 1352.7205, 1356.7230, 1360.6638, 1363.7153, 1365.7379, 1367.7319, 1369.7043, 1377.6947, 1379.7268, 1383.7137, 1385.7534, 1388.7404, 1390.7284, 1393.7428, 1407.7563, 1417.7010, 1422.7019, 1425.7727, 1427.7742, 1429.7365, 1431.7412, 1433.7228, 1434.7446, 1437.7584, 1461.7778, 1467.7521, 1472.7633, 1475.7632, 1477.7545, 1484.7889, 1488.7537, 1493.7579, 1500.7737, 1505.8177, 1513.8079, 1515.8213, 1517.7854, 1519.7544, 1522.7811, 1523.7953, 1529.8099, 1530.7523, 1531.7472, 1537.7974, 1539.8005, 1542.8422, 1545.8091, 1547.8051, 1549.7339, 1551.7778, 1553.8065, 1555.8101, 1556.8109, 1557.8010, 1561.8401, 1563.8109, 1565.8199, 1571.8400, 1579.7939, 1580.8077, 1583.8444, 1587.7620, 1592.8066, 1594.8335, 1604.8323, 1605.8586, 1615.8754, 1624.8062, 1628.7900, 1628.7921, 1633.7985, 1640.9384, 1642.8099, 1646.8297, 1649.8447, 1657.8101, 1667.8622, 1677.7988, 1680.8746, 1689.9465, 1694.7288, 1698.9225, 1699.8862, 1704.8773, 1706.8701, 1707.8302, 1710.9279, 1716.8671, 1719.9058, 1723.9011, 1725.8300, 1725.8346, 1727.8986, 1730.8931, 1737.9006, 1751.8656, 1754.8580, 1766.8040, 1767.8138, 1769.8741, 1770.3567, 1772.9315, 1774.7610, 1774.9131, 1777.8770, 1780.8777, 1783.9246, 1787.8188, 1787.8200, 1791.8137, 1793.9050, 1795.9353, 1798.9340, 1800.9336, 1804.9142, 1805.9069, 1809.8469, 1815.9044, 1821.9144, 1828.9227, 1837.9396, 1840.9827, 1843.9688, 1844.9012, 1852.9364, 1861.8881, 1867.9218, 1874.9695, 1880.9265, 1884.9417, 1891.9622, 1894.9313, 1896.9078, 1896.9100, 1900.9546, 1905.9313, 1906.9501, 1908.9043, 1911.9130, 1912.9154, 1916.9923, 1918.9587, 1921.9825, 1923.9718, 1925.9573, 1928.0469, 1932.0060, 1933.9950, 1937.9630, 1939.9740, 1944.0040, 1946.9679, 1949.9595, 1954.0236, 1955.9738, 1958.9556, 1960.0034, 1968.8600, 1973.9845, 1976.9861, 1978.9910, 1981.9854, 1983.9884, 1987.9784, 1995.9408, 1998.9894, 2000.9926, 2012.9590, 2015.0104, 2017.9747, 2021.0170, 2026.0275, 2027.0256, 2031.0166, 2044.0123, 2047.4957, 2047.9515, 2052.0496, 2060.0520, 2062.0073, 2064.9880, 2066.9873, 2094.0376, 2097.0454, 2102.0667, 2103.1641, 2111.0305, 2127.0930, 2139.0630, 2144.0493, 2148.1023, 2155.0950, 2156.0796, 2160.0632, 2176.0664, 2190.9895, 2195.0774, 2199.0723, 2202.1624, 2203.1221, 2208.0862, 2211.1042, 2216.1062, 2225.1274, 2226.1272, 2229.0972, 2233.0984, 2239.0225, 2242.0435, 2268.1489, 2270.1287, 2271.1294, 2277.0823, 2284.1990, 2292.1426, 2309.0994, 2334.1719, 2342.9915, 2350.1746, 2383.9607, 2398.2400, 2400.0862, 2413.0549, 2414.1897, 2417.0959, 2497.2800, 2501.2637, 2511.1460, 2513.1421, 2528.1750, 2537.2012, 2541.1948, 2542.1836, 2556.2058, 2562.1487, 2612.2666, 2706.2109, 2717.0869, 2767.2666, 2888.2532, 3221.1733, 3224.2917, 3312.3306, 3916.9434

34. E1B871 **Mass:** 21420 **Score:** 20 **Expect:** 2.7e+002 **Matches:** 11

Uncharacterized protein OS=Bos taurus PE=3 SV=2

Observed	Mr(expt)	Mr(calc)	ppm	Start	End	Miss	Ions	Peptide
741.4101	740.4028	740.4115	-11.78	38 -	43	1	---	K.GMHIRK.A
757.4313	756.4241	756.4065	23.3	38 -	43	1	---	K.GMHIRK.A + Oxidation (M)
832.4557	831.4485	831.4450	4.15	177 -	184	1	---	K.KQTLTAGN.-
835.4719	834.4646	834.4599	5.58	87 -	93	0	---	K.SAEFLLR.M
860.4691	859.4618	859.4413	23.9	129 -	135	1	---	R.TYRAHGR.I
1057.6078	1056.6005	1056.5862	13.5	38 -	46	2	---	K.GMHIRKATK.Y + Oxidation (M)
1522.7811	1521.7738	1521.7861	-8.06	141 -	153	0	---	R.SSPCHIEIILPEK.E
1549.7339	1548.7266	1548.7606	-21.94	1 -	13	1	---	-.MVRYSLDPENPTK.S
1793.9050	1792.8977	1792.8778	11.1	2 -	16	2	---	M.VRYSLDPENPTKSCK.S
2103.1641	2102.1568	2102.1484	4.01	106 -	124	1	---	K.GLDVDSLVIEHIQVNKAPR.M
2413.0549	2412.0476	2412.0998	-21.63	56 -	76	2	---	K.QCVPFCRYNGGVGGCAQAKQR.G

No match to: 721.4365, 725.4001, 726.4063, 728.3681, 730.3848, 731.4017, 732.3573, 734.4151, 735.4143, 742.4515, 744.4171, 745.4167, 746.4169, 747.4125, 750.4153, 752.4127, 755.4100, 756.4199, 761.4177, 767.4266, 768.3700, 768.3737, 770.3966, 772.4093, 775.4307, 779.4308, 784.3688, 786.4000, 787.4221, 788.4210, 792.4376, 795.4350, 798.4351, 800.4296, 812.4388, 815.4332, 822.4482, 825.4540, 831.4382, 837.4463, 842.5060, 844.4760, 854.4837, 856.4911, 862.4852, 869.4231, 885.4465, 905.4955, 907.4991, 908.4929, 913.5700, 913.5747, 916.5284, 918.5050, 919.5053, 922.5070, 928.5117, 934.5172, 940.4840, 942.4854, 943.4289, 943.5453, 946.5259, 950.4959, 951.5076, 953.5164, 954.5388, 959.5266, 966.5410, 969.5256, 972.5305, 974.5395, 976.5310, 978.5314, 979.5442, 991.5409, 993.5307, 1001.5854, 1003.5496, 1011.5262, 1014.5215, 1021.5579, 1023.5663, 1023.5700, 1031.5903, 1035.5381, 1036.5447, 1037.5573, 1039.5327, 1041.5658, 1044.5751, 1045.5729, 1046.5897, 1051.5448, 1056.5658, 1067.5753, 1073.5902, 1086.5509, 1092.5961, 1100.6071, 1106.5391, 1111.5615, 1113.5729, 1118.5818, 1120.6045, 1122.5912, 1123.5848, 1125.5823, 1132.6047, 1134.5714, 1139.5746, 1143.6318, 1144.6497, 1145.6407, 1147.6393, 1150.6108, 1155.6215, 1157.6199, 1158.6685, 1162.6182, 1163.6152, 1168.6215, 1173.6418, 1175.6089, 1178.6356, 1179.6157, 1181.6819, 1188.6544, 1197.6704, 1199.7006, 1208.6415, 1216.6714, 1227.6595, 1231.6387, 1232.6447, 1234.6672, 1235.6445, 1244.6453, 1247.6211, 1248.6174, 1250.6554, 1255.6096, 1256.6531, 1261.6592, 1262.6453, 1266.6777, 1273.6260, 1275.6362, 1276.6343, 1277.6531, 1301.7079, 1303.7076, 1307.7042, 1308.6376, 1310.6146, 1312.6418, 1315.7098, 1320.6659, 1322.6886, 1323.6771, 1324.6964, 1328.7089, 1331.7202, 1335.7318, 1338.6581, 1339.6637, 1349.7253, 1352.7205, 1356.7230, 1360.6638, 1363.7153, 1365.7379, 1367.7319, 1369.7043, 1377.6947, 1379.7268, 1383.7137, 1385.7534, 1388.7404, 1390.7284, 1393.7428, 1407.7563, 1417.7010, 1421.7061, 1422.7019, 1425.7727, 1427.7742, 1429.7365, 1431.7412, 1433.7228, 1434.7446, 1437.7584, 1461.7778, 1467.7521, 1472.7633, 1475.7632, 1477.7545, 1484.7889, 1488.7537, 1491.7965, 1493.7579, 1500.7737, 1505.8177, 1513.8079, 1515.8213, 1517.7854, 1519.7544, 1523.7953, 1529.8099, 1530.7523, 1531.7472, 1537.7974, 1539.8005, 1542.8422, 1545.8091, 1547.8051, 1551.7778, 1553.8065, 1555.8101, 1556.8109, 1557.8010, 1561.8401, 1563.8109, 1565.8199, 1571.8400, 1579.7939, 1580.8077, 1583.8444, 1587.7620, 1592.8066, 1594.8335, 1604.8323, 1605.8586, 1608.8497, 1615.8754, 1624.8062, 1628.7900, 1628.7921, 1633.7985, 1640.9384, 1642.8099, 1646.8297, 1649.8447, 1657.8101, 1667.8622, 1677.7988, 1680.8746, 1689.9465, 1694.7288, 1698.9225, 1699.8862, 1704.8773, 1706.8701, 1707.8302, 1710.9279, 1716.8671, 1719.9058, 1723.9011, 1725.8300, 1725.8346, 1727.8986, 1730.8931, 1737.9006, 1751.8656, 1754.8580, 1765.8463, 1766.8040, 1767.8138, 1769.8741, 1770.3567, 1772.9315, 1774.7610, 1774.9131, 1777.8770, 1780.8777, 1783.9246, 1787.8188, 1787.8200, 1791.8137, 1795.9353, 1798.9340, 1800.9336, 1804.9142, 1805.9069, 1809.8469, 1815.9044, 1821.9144, 1828.9227, 1837.9396, 1840.9827, 1843.9688, 1844.9012, 1846.9153, 1852.9364, 1856.9908, 1861.8881, 1867.9218, 1874.9695, 1880.9265, 1884.9417, 1891.9622, 1894.9313, 1896.9078, 1896.9100, 1900.9546, 1905.9313, 1906.9501, 1908.9043, 1911.9130, 1912.9154, 1916.9923, 1918.9587, 1921.9825, 1923.9718, 1925.9923, 1928.0469, 1932.0060, 1933.9950, 1937.9630, 1939.9740, 1944.0040, 1946.9679, 1949.9595, 1954.0236, 1955.9738, 1958.9556, 1960.0034, 1968.8600, 1973.9845, 1976.9861, 1978.9910, 1981.9854, 1983.9884, 1987.9784, 1995.9408, 1998.9894, 2000.9926, 2008.0460, 2012.9590, 2015.0104, 2017.9747, 2021.0170, 2026.0275, 2027.0256, 2031.0166, 2044.0123, 2047.4957, 2047.9515, 2052.0496, 2060.0520, 2062.0073, 2064.9880, 2066.9873, 2094.0376, 2097.0454, 2102.0667, 2111.0305, 2127.0930, 2139.0630, 2144.0493, 2148.1023, 2155.0950, 2156.0796, 2160.0632, 2176.0664, 2190.9895, 2195.0774, 2199.0723, 2202.1624, 2203.1221, 2208.0862, 2211.1042, 2216.1062, 2225.1274, 2226.1272, 2229.0972, 2233.0984, 2239.0225, 2242.0435, 2268.1489, 2270.1287, 2271.1294, 2277.0823, 2284.1990, 2292.1426, 2309.0994, 2334.1719, 2342.9915, 2350.1746, 2383.9607, 2398.2400, 2400.0862, 2414.1897, 2417.0959, 2497.2800, 2501.2637, 2511.1460, 2513.1421, 2528.1750, 2537.2012, 2541.1948, 2542.1836, 2556.2058, 2562.1487, 2612.2666, 2706.2109, 2717.0869, 2767.2666, 2888.2532, 3221.1733, 3224.2917, 3312.3306, 3916.9434

35. A0JNC0 **Mass:** 40548 **Score:** 20 **Expect:** 2.7e+002 **Matches:** 17

Tropomodulin-1 OS=Bos taurus GN=TMOD1 PE=2 SV=1

Observed	Mr(expt)	Mr(calc)	ppm	Start	End	Miss	Ions	Peptide
728.3681	727.3608	727.3435	23.8	1 -	5	1	---	-.MSYRR.E + Oxidation (M)
745.4167	744.4094	744.3919	23.6	57 -	63	0	---	K.APTGPFR.R
750.4153	749.4081	749.4072	1.20	74 -	79	1	---	K.QAKEFK.D
779.4308	778.4235	778.4337	-13.14	229 -	235	0	---	K.FSIVGTR.S
837.4463	836.4390	836.4392	-0.20	6 -	11	1	---	R.ELEKYR.D
993.5307	992.5234	992.5403	-16.99	5 -	11	2	---	R.RELEKYR.D
1021.5579	1020.5506	1020.5352	15.1	74 -	81	2	---	K.QAKEFKDR.E
1125.5823	1124.5750	1124.5713	3.29	65 -	73	0	---	R.EELLDHLEK.Q
1323.6771	1322.6698	1322.6461	17.9	298 -	308	0	---	K.VEMEIVSMLEK.N + Oxidation (M)
1339.6637	1338.6564	1338.6411	11.5	298 -	308	0	---	K.VEMEIVSMLEK.N + 2 Oxidation (M)
1421.7061	1420.6988	1420.6834	10.8	80 -	91	1	---	K.DREDLVPYTGEK.R

1491.7965	1490.7892	1490.7729	11.0	82 -	94	2	---	R.EDLVPYTGEKRGK.V
1608.8497	1607.8424	1607.8631	-12.84	64 -	76	2	---	R.REELLDHLEKQAK.E
1765.8463	1764.8390	1764.8359	1.77	328 -	342	2	---	R.ASNAMMNNNDLVRKR.R + 2 Oxidation (M)
1846.9153	1845.9080	1845.9301	-11.97	326 -	341	2	---	R.LRASNAMMNNNDLVRK.R
1856.9908	1855.9835	1855.9679	8.40	65 -	79	2	---	R.EELLDHLEKQAKEFK.D
2008.0460	2007.0387	2007.0537	-7.48	57 -	73	2	---	K.APTGPFRREELLDHLEK.Q

No match to: 721.4365, 725.4001, 726.4063, 730.3848, 731.4017, 732.3573, 734.4151, 735.4143, 741.4101, 742.4515, 744.4171, 746.4169, 747.4125, 752.4127, 755.4100, 756.4199, 757.4313, 761.4177, 767.4266, 768.3700, 768.3737, 770.3966, 772.4093, 775.4307, 784.3688, 786.4000, 787.4221, 788.4210, 792.4376, 795.4350, 798.4351, 800.4296, 812.4388, 815.4332, 822.4482, 825.4540, 831.4382, 832.4557, 835.4719, 842.5060, 844.4760, 854.4837, 856.4911, 860.4691, 862.4852, 869.4231, 885.4465, 905.4955, 907.4991, 908.4929, 913.5700, 913.5747, 916.5284, 918.5050, 919.5053, 922.5070, 928.5117, 934.5172, 940.4840, 942.4854, 943.4289, 943.5453, 946.5259, 950.4959, 951.5076, 953.5164, 954.5388, 959.5266, 966.5410, 969.5256, 972.5305, 974.5395, 976.5310, 978.5314, 979.5442, 991.5409, 1001.5854, 1003.5496, 1011.5262, 1014.5215, 1023.5663, 1023.5700, 1031.5903, 1035.5381, 1036.5447, 1037.5573, 1039.5327, 1041.5658, 1044.5751, 1045.5729, 1046.5897, 1051.5448, 1056.5658, 1057.6078, 1067.5753, 1073.5902, 1086.5509, 1092.5961, 1100.6071, 1106.5391, 1111.5615, 1113.5729, 1118.5818, 1120.6045, 1122.5912, 1123.5848, 1132.6047, 1134.5714, 1139.5746, 1143.6318, 1144.6497, 1145.6407, 1147.6393, 1150.6108, 1155.6215, 1157.6199, 1158.6685, 1162.6182, 1163.6152, 1168.6215, 1173.6418, 1175.6089, 1178.6356, 1179.6157, 1181.6819, 1188.6544, 1197.6704, 1199.7006, 1208.6415, 1216.6714, 1227.6595, 1231.6387, 1232.6447, 1234.6672, 1235.6445, 1244.6453, 1247.6211, 1248.6174, 1250.6554, 1255.6096, 1256.6531, 1261.6592, 1262.6453, 1266.6777, 1273.6260, 1275.6362, 1276.6343, 1277.6531, 1301.7079, 1303.7076, 1307.7042, 1308.6376, 1310.6146, 1312.6418, 1315.7098, 1320.6659, 1322.6886, 1324.6964, 1328.7089, 1331.7202, 1335.7318, 1338.6581, 1349.7253, 1352.7205, 1356.7230, 1360.6638, 1363.7153, 1365.7379, 1367.7319, 1369.7043, 1377.6947, 1379.7268, 1383.7137, 1385.7534, 1388.7404, 1390.7284, 1393.7428, 1407.7563, 1417.7010, 1422.7019, 1425.7727, 1427.7742, 1429.7365, 1431.7412, 1433.7228, 1434.7446, 1437.7584, 1461.7778, 1467.7521, 1472.7633, 1475.7632, 1477.7545, 1484.7889, 1488.7537, 1493.7579, 1500.7737, 1505.8177, 1513.8079, 1515.8213, 1517.7854, 1519.7544, 1522.7811, 1523.7953, 1529.8099, 1530.7523, 1531.7472, 1537.7974, 1539.8005, 1542.8422, 1545.8091, 1547.8051, 1549.7339, 1551.7778, 1553.8065, 1555.8101, 1556.8109, 1557.8010, 1561.8401, 1563.8109, 1565.8199, 1571.8400, 1579.7939, 1580.8077, 1583.8444, 1587.7620, 1592.8066, 1594.8335, 1604.8323, 1605.8586, 1615.8754, 1624.8062, 1628.7900, 1628.7921, 1633.7985, 1640.9384, 1642.8099, 1646.8297, 1649.8447, 1657.8101, 1667.8622, 1677.7988, 1680.8746, 1689.9465, 1694.7288, 1698.9225, 1699.8862, 1704.8773, 1706.8701, 1707.8302, 1710.9279, 1716.8671, 1719.9058, 1723.9011, 1725.8300, 1725.8346, 1727.8986, 1730.8931, 1737.9006, 1751.8656, 1754.8580, 1766.8040, 1767.8138, 1769.8741, 1770.3567, 1772.9315, 1774.7610, 1774.9131, 1777.8770, 1780.8777, 1783.9246, 1787.8188, 1787.8200, 1791.8137, 1793.9050, 1795.9353, 1798.9340, 1800.9336, 1804.9142, 1805.9069, 1809.8469, 1815.9044, 1821.9144, 1828.9227, 1837.9396, 1840.9827, 1843.9688, 1844.9012, 1852.9364, 1861.8881, 1867.9218, 1874.9695, 1880.9265, 1884.9417, 1891.9622, 1894.9313, 1896.9078, 1896.9100, 1900.9546, 1905.9313, 1906.9501, 1908.9043, 1911.9130, 1912.9154, 1916.9923, 1918.9587, 1921.9825, 1923.9718, 1925.9573, 1928.0469, 1932.0060, 1933.9950, 1937.9630, 1939.9740, 1944.0040, 1946.9679, 1949.9595, 1954.0236, 1955.9738, 1958.9556, 1960.0034, 1968.8600, 1973.9845, 1976.9861, 1978.9910, 1981.9854, 1983.9884, 1987.9784, 1995.9408, 1998.9894, 2000.9926, 2012.9590, 2015.0104, 2017.9747, 2021.0170, 2026.0275, 2027.0256, 2031.0166, 2044.0123, 2047.4957, 2047.9515, 2052.0496, 2060.0520, 2062.0073, 2064.9880, 2066.9873, 2094.0376, 2097.0454, 2102.0667, 2103.1641, 2111.0305, 2127.0930, 2139.0630, 2144.0493, 2148.1023, 2155.0950, 2156.0796, 2160.0632, 2176.0664, 2190.9895, 2195.0774, 2199.0723, 2202.1624, 2203.1221, 2208.0862, 2211.1042, 2216.1062, 2225.1274, 2226.1272, 2229.0972, 2233.0984, 2239.0225, 2242.0435, 2268.1489, 2270.1287, 2271.1294, 2277.0823, 2284.1990, 2292.1426, 2309.0994, 2334.1719, 2342.9915, 2350.1746, 2383.9607, 2398.2400, 2400.0862, 2413.0549, 2414.1897, 2417.0959, 2497.2800, 2501.2637, 2511.1460, 2513.1421, 2528.1750, 2537.2012, 2541.1948, 2542.1836, 2556.2058, 2562.1487, 2612.2666, 2706.2109, 2717.0869, 2767.2666, 2888.2532, 3221.1733, 3224.2917, 3312.3306, 3916.9434

36. F1MZ00 **Mass:** 14021 **Score:** 19 **Expect:** 2.8e+002 **Matches:** 10

Uncharacterized protein OS=Bos taurus GN=SNRPD3 PE=4 SV=2

Observed	Mr(expt)	Mr(calc)	ppm	Start	End	Miss	Ions	Peptide
844.4760	843.4687	843.4888	-23.79	1 -	8	0	---	-.MSIGVPIK.V
862.4852	861.4779	861.4821	-4.85	119 -	125	1	---	R.GNIFQKR.R
1035.5381	1034.5308	1034.5252	5.40	79 -	87	1	---	K.NAPMLKSMK.N + Oxidation (M)
1051.5448	1050.5375	1050.5202	16.5	79 -	87	1	---	K.NAPMLKSMK.N + 2 Oxidation (M)
1123.5848	1122.5775	1122.5604	15.2	115 -	124	1	---	R.GMGRGNIFQK.R + Oxidation (M)
1261.6592	1260.6519	1260.6682	-12.91	79 -	89	2	---	K.NAPMLKSMKNK.N
1277.6531	1276.6458	1276.6631	-13.54	79 -	89	2	---	K.NAPMLKSMKNK.N + Oxidation (M)
1320.6659	1319.6586	1319.6881	-22.31	113 -	124	2	---	R.GRGMGRGNIFQK.R
1427.7742	1426.7669	1426.8004	-23.48	90 -	104	2	---	K.NQGSGAGRGKAAILK.A
2414.1897	2413.1824	2413.1332	20.4	9 -	29	0	---	K.VLHEAEGHIVTCETNTGEVYR.G

No match to: 721.4365, 725.4001, 726.4063, 728.3681, 730.3848, 731.4017, 732.3573, 734.4151, 735.4143, 741.4101, 742.4515, 744.4171, 745.4167, 746.4169, 747.4125, 750.4153, 752.4127, 755.4100, 756.4199, 757.4313, 761.4177, 767.4266, 768.3700, 768.3737, 770.3966, 772.4093, 775.4307, 779.4308, 784.3688, 786.4000, 787.4221, 788.4210, 792.4376, 795.4350, 798.4351, 800.4296, 812.4388, 815.4332, 822.4482, 825.4540, 831.4382, 832.4557, 835.4719, 837.4463, 842.5060, 854.4837, 856.4911, 860.4691, 869.4231, 885.4465, 905.4955, 907.4991, 908.4929, 913.5700, 913.5747, 916.5284, 918.5050, 919.5053, 922.5070, 928.5117, 934.5172, 940.4840, 942.4854, 943.4289, 943.5453, 946.5259, 950.4959, 951.5076, 953.5164, 954.5388, 959.5266, 966.5410, 969.5256, 972.5305, 974.5395, 976.5310, 978.5314, 979.5442, 991.5409, 993.5307, 1001.5854, 1003.5496, 1011.5262, 1014.5215, 1021.5579, 1023.5663, 1023.5700, 1031.5903, 1036.5447, 1037.5573, 1039.5327, 1041.5658, 1044.5751, 1045.5729, 1046.5897, 1056.5658, 1057.6078, 1067.5753, 1073.5902, 1086.5509, 1092.5961, 1100.6071, 1106.5391, 1111.5615, 1113.5729, 1118.5818, 1120.6045, 1122.5912, 1125.5823, 1132.6047, 1134.5714, 1139.5746, 1143.6318, 1144.6497, 1145.6407, 1147.6393, 1150.6108, 1155.6215, 1157.6199, 1158.6685, 1162.6182, 1163.6152, 1168.6215, 1173.6418, 1175.6089, 1178.6356, 1179.6157, 1181.6819, 1188.6544, 1197.6704, 1199.7006, 1208.6415, 1216.6714, 1227.6595, 1231.6387, 1232.6447, 1234.6672, 1235.6445, 1244.6453, 1247.6211, 1248.6174, 1250.6554, 1255.6096, 1256.6531, 1262.6453, 1266.6777, 1273.6260, 1275.6362, 1276.6343, 1301.7079, 1303.7076, 1307.7042, 1308.6376, 1310.6146, 1312.6418, 1315.7098, 1322.6886, 1323.6771, 1324.6964, 1328.7089, 1331.7202, 1335.7318, 1338.6581, 1339.6637, 1349.7253, 1352.7205, 1356.7230, 1360.6638, 1363.7153, 1365.7379, 1367.7319, 1369.7043, 1377.6947, 1379.7268, 1383.7137, 1385.7534, 1388.7404, 1390.7284, 1393.7428, 1407.7563, 1417.7010, 1421.7061, 1422.7019, 1425.7727, 1429.7365, 1431.7412, 1433.7228, 1434.7446, 1437.7584, 1461.7778, 1467.7521, 1472.7633, 1475.7632, 1477.7545, 1484.7889, 1488.7537, 1491.7965, 1493.7579, 1500.7737, 1505.8177, 1513.8079, 1515.8213, 1517.7854, 1519.7544, 1522.7811, 1523.7953, 1529.8099, 1530.7523, 1531.7472, 1537.7974, 1539.8005, 1542.8422, 1545.8091, 1547.8051, 1549.7339, 1551.7778, 1553.8065, 1555.8101, 1556.8109, 1557.8010, 1561.8401, 1563.8109, 1565.8199, 1571.8400, 1579.7939, 1580.8077, 1583.8444, 1587.7620, 1592.8066, 1594.8335, 1604.8323, 1605.8586, 1608.8497, 1615.8754, 1624.8062, 1628.7900, 1628.7921, 1633.7985, 1640.9384, 1642.8099, 1646.8297, 1649.8447, 1657.8101, 1667.8622, 1677.7988, 1680.8746, 1689.9465, 1694.7288, 1698.9225, 1699.8862, 1704.8773, 1706.8701, 1707.8302, 1710.9279, 1716.8671, 1719.9058, 1723.9011, 1725.8300, 1725.8346, 1727.8986, 1730.8931, 1737.9006, 1751.8656, 1754.8580, 1765.8463, 1766.8040, 1767.8138, 1769.8741, 1770.3567, 1772.9315, 1774.7610, 1774.9131, 1777.8770, 1780.8777, 1783.9246, 1787.8188, 1787.8200, 1791.8137, 1793.9050, 1795.9353, 1798.9340, 1800.9336, 1804.9142, 1805.9069, 1809.8469, 1815.9044, 1821.9144, 1828.9227, 1837.9396, 1840.9827, 1843.9688, 1844.9012, 1846.9153, 1852.9364, 1856.9908, 1861.8881, 1867.9218, 1874.9695, 1880.9265, 1884.9417, 1891.9622, 1894.9313, 1896.9078, 1896.9100, 1900.9546, 1905.9313, 1906.9501, 1908.9043, 1911.9130, 1912.9154, 1916.9923, 1918.9587, 1921.9825, 1923.9718, 1925.9573, 1928.0469, 1932.0060, 1933.9950, 1937.9630, 1939.9740, 1944.0040, 1946.9679, 1949.9595, 1954.0236, 1955.9738, 1958.9556, 1960.0034, 1968.8600, 1973.9845, 1976.9861, 1978.9910, 1981.9854, 1983.9884, 1987.9784, 1995.9408, 1998.9894, 2000.9926, 2008.0460, 2012.9590, 2015.0104, 2017.9747, 2021.0170, 2026.0275, 2027.0256, 2031.0166, 2044.0123, 2047.4957, 2047.9515, 2052.0496, 2060.0520, 2062.0073, 2064.9880, 2066.9873, 2094.0376, 2097.0454, 2102.0667, 2103.1641, 2111.0305, 2127.0930, 2139.0630, 2144.0493, 2148.1023, 2155.0950, 2156.0796, 2160.0632, 2176.0664, 2190.9895, 2195.0774, 2199.0723, 2202.1624, 2203.1221, 2208.0862, 2211.1042, 2216.1062, 2225.1274, 2226.1272, 2229.0972, 2233.0984, 2239.0225, 2242.0435, 2268.1489, 2270.1287, 2271.1294, 2277.0823, 2284.1990, 2292.1426, 2309.0994, 2334.1719, 2342.9915, 2350.1746, 2383.9607, 2398.2400, 2400.0862, 2413.0549, 2417.0959, 2497.2800, 2501.2637, 2511.1460, 2513.1421, 2528.1750, 2537.2012, 2541.1948, 2542.1836, 2556.2058, 2562.1487, 2612.2666, 2706.2109, 2717.0869, 2767.2666, 2888.2532, 3221.1733, 3224.2917, 3312.3306, 3916.9434

37. A3KMZ9 **Mass:** 18849 **Score:** 19 **Expect:** 2.8e+002 **Matches:** 9

SNRNP27 protein OS=Bos taurus GN=SNRNP27 PE=2 SV=1

Observed	Mr(expt)	Mr(calc)	ppm	Start	End	Miss	Ions	Peptide
731.4017	730.3945	730.3834	15.1	35 -	39	2	---	R.ERDRR.R
745.4167	744.4094	744.3990	13.9	22 -	26	2	---	R.ERERR.R

800.4296	799.4223	799.4413	-23.72	8 - 13	2	---	R.SPRRER.R
950.4959	949.4886	949.5080	-20.45	77 - 84	2	---	K.ETKETKSK.E
978.5314	977.5241	977.5002	24.4	16 - 23	2	---	R.SRSTSRER.E
1111.5615	1110.5542	1110.5530	1.09	57 - 66	1	---	R.HRSTSPSPSR.L
1352.7205	1351.7132	1351.7320	-13.91	57 - 68	2	---	R.HRSTSPSPSRLK.E
2027.0256	2026.0183	2026.0207	-1.16	139 - 155	2	---	R.QYMNRKGGFNRPLDFIA.-
2541.1948	2540.1875	2540.1484	15.4	97 - 118	2	---	K.TEEEIEMMKLMGFASFDSTKGK.K + 2 Oxidation (M)

No match to: 721.4365, 725.4001, 726.4063, 728.3681, 730.3848, 732.3573, 734.4151, 735.4143, 741.4101, 742.4515, 744.4171, 746.4169, 747.4125, 750.4153, 752.4127, 755.4100, 756.4199, 757.4313, 761.4177, 767.4266, 768.3700, 768.3737, 770.3966, 772.4093, 775.4307, 779.4308, 784.3688, 786.4000, 787.4221, 788.4210, 792.4376, 795.4350, 798.4351, 812.4388, 815.4332, 822.4482, 825.4540, 831.4382, 832.4557, 835.4719, 837.4463, 842.5060, 844.4760, 854.4837, 856.4911, 860.4691, 862.4852, 869.4231, 885.4465, 905.4955, 907.4991, 908.4929, 913.5700, 913.5747, 916.5284, 918.5050, 919.5053, 922.5070, 928.5117, 934.5172, 940.4840, 942.4854, 943.4289, 943.5453, 946.5259, 951.5076, 953.5164, 954.5388, 959.5266, 966.5410, 969.5256, 972.5305, 974.5395, 976.5310, 979.5442, 991.5409, 993.5307, 1001.5854, 1003.5496, 1011.5262, 1014.5215, 1021.5579, 1023.5663, 1023.5700, 1031.5903, 1035.5381, 1036.5447, 1037.5573, 1039.5327, 1041.5658, 1044.5751, 1045.5729, 1046.5897, 1051.5448, 1056.5658, 1057.6078, 1067.5753, 1073.5902, 1086.5509, 1092.5961, 1100.6071, 1106.5391, 1113.5729, 1118.5818, 1120.6045, 1122.5912, 1123.5848, 1125.5823, 1132.6047, 1134.5714, 1139.5746, 1143.6318, 1144.6497, 1145.6407, 1147.6393, 1150.6108, 1155.6215, 1157.6199, 1158.6685, 1162.6182, 1163.6152, 1168.6215, 1173.6418, 1175.6089, 1178.6356, 1179.6157, 1181.6819, 1188.6544, 1197.6704, 1199.7006, 1208.6415, 1216.6714, 1227.6595, 1231.6387, 1232.6447, 1234.6672, 1235.6445, 1244.6453, 1247.6211, 1248.6174, 1250.6554, 1255.6096, 1256.6531, 1261.6592, 1262.6453, 1266.6777, 1273.6260, 1275.6362, 1276.6343, 1277.6531, 1301.7079, 1303.7076, 1307.7042, 1308.6376, 1310.6146, 1312.6418, 1315.7098, 1320.6659, 1322.6886, 1323.6771, 1324.6964, 1328.7089, 1331.7202, 1335.7318, 1338.6581, 1339.6637, 1349.7253, 1356.7230, 1360.6638, 1363.7153, 1365.7379, 1367.7319, 1369.7043, 1377.6947, 1379.7268, 1383.7137, 1385.7534, 1388.7404, 1390.7284, 1393.7428, 1407.7563, 1417.7010, 1421.7061, 1422.7019, 1425.7727, 1427.7742, 1429.7365, 1431.7412, 1433.7228, 1434.7446, 1437.7584, 1461.7778, 1467.7521, 1472.7633, 1475.7632, 1477.7545, 1484.7889, 1488.7537, 1491.7965, 1493.7579, 1500.7737, 1505.8177, 1513.8079, 1515.8213, 1517.7854, 1519.7544, 1522.7811, 1523.7953, 1529.8099, 1530.7523, 1531.7472, 1537.7974, 1539.8005, 1542.8422, 1545.8091, 1547.8051, 1549.7339, 1551.7778, 1553.8065, 1555.8101, 1556.8109, 1557.8010, 1561.8401, 1563.8109, 1565.8199, 1571.8400, 1579.7939, 1580.8077, 1583.8444, 1587.7620, 1592.8066, 1594.8335, 1604.8323, 1605.8586, 1608.8497, 1615.8754, 1624.8062, 1628.7900, 1628.7921, 1633.7985, 1640.9384, 1642.8099, 1646.8297, 1649.8447, 1657.8101, 1667.8622, 1677.7988, 1680.8746, 1689.9465, 1694.7288, 1698.9225, 1699.8862, 1704.8773, 1706.8701, 1707.8302, 1710.9279, 1716.8671, 1719.9058, 1723.9011, 1725.8300, 1725.8346, 1727.8986, 1730.8931, 1737.9006, 1751.8656, 1754.8580, 1765.8463, 1766.8040, 1767.8138, 1769.8741, 1770.3567, 1772.9315, 1774.7610, 1774.9131, 1777.8770, 1780.8777, 1783.9246, 1787.8188, 1787.8200, 1791.8137, 1793.9050, 1795.9353, 1798.9340, 1800.9336, 1804.9142, 1805.9069, 1809.8469, 1815.9044, 1821.9144, 1828.9227, 1837.9396, 1840.9827, 1843.9688, 1844.9012, 1846.9153, 1852.9364, 1856.9908, 1861.8881, 1867.9218, 1874.9695, 1880.9265, 1884.9417, 1891.9622, 1894.9313, 1896.9078, 1896.9100, 1900.9546, 1905.9313, 1906.9501, 1908.9043, 1911.9130, 1912.9154, 1916.9923, 1918.9587, 1921.9825, 1923.9718, 1925.9573, 1928.0469, 1932.0060, 1933.9950, 1937.9630, 1939.9740, 1944.0040, 1946.9679, 1949.9595, 1954.0236, 1955.9738, 1958.9556, 1960.0034, 1968.8600, 1973.9845, 1976.9861, 1978.9910, 1981.9854, 1983.9884, 1987.9784, 1995.9408, 1998.9894, 2000.9926, 2008.0460, 2012.9590, 2015.0104, 2017.9747, 2021.0170, 2026.0275, 2031.0166, 2044.0123, 2047.4957, 2047.9515, 2052.0496, 2060.0520, 2062.0073, 2064.9880, 2066.9873, 2094.0376, 2097.0454, 2102.0667, 2103.1641, 2111.0305, 2127.0930, 2139.0630, 2144.0493, 2148.1023, 2155.0950, 2156.0796, 2160.0632, 2176.0664, 2190.9895, 2195.0774, 2199.0723, 2202.1624, 2203.1221, 2208.0862, 2211.1042, 2216.1062, 2225.1274, 2226.1272, 2229.0972, 2233.0984, 2239.0225, 2242.0435, 2268.1489, 2270.1287, 2271.1294, 2277.0823, 2284.1990, 2292.1426, 2309.0994, 2334.1719, 2342.9915, 2350.1746, 2383.9607, 2398.2400, 2400.0862, 2413.0549, 2414.1897, 2417.0959, 2497.2800, 2501.2637, 2511.1460, 2513.1421, 2528.1750, 2537.2012, 2542.1836, 2556.2058, 2562.1487, 2612.2666, 2706.2109, 2717.0869, 2767.2666, 2888.2532, 3221.1733, 3224.2917, 3312.3306, 3916.9434

38. P42899 **Mass:** 11695 **Score:** 19 **Expect:** 2.8e+002 **Matches:** 7

60S acidic ribosomal protein P2 OS=Bos taurus GN=RPLP2 PE=3 SV=1

Observed	Mr(expt)	Mr(calc)	ppm	Start - End	Miss	Ions	Peptide
1256.6531	1255.6458	1255.6772	-24.99	50 - 61	0	---	K.NIEDVIAQGIGK.L
1417.7010	1416.6937	1416.6732	14.5	26 - 38	0	---	K.ILDSVGIEADDDR.L
1545.8091	1544.8018	1544.7682	21.8	25 - 38	1	---	K.KILDSVGIEADDDR.L
1772.9315	1771.9242	1771.8952	16.4	26 - 41	1	---	K.ILDSVGIEADDDRLNK.V
1900.9546	1899.9473	1899.9901	-22.54	25 - 41	2	---	K.KILDSVGIEADDDRLNK.V
2156.0796	2155.0723	2155.1095	-17.26	1 - 21	1	---	-.MRYVASYLLAALGGNSSPSAK.D
2225.1274	2224.1201	2224.1739	-24.18	3 - 24	1	---	R.YVASYLLAALGGNSSPSAKDIK.K

No match to: 721.4365, 725.4001, 726.4063, 728.3681, 730.3848, 731.4017, 732.3573, 734.4151, 735.4143, 741.4101, 742.4515, 744.4171, 745.4167, 746.4169, 747.4125, 750.4153, 752.4127, 755.4100, 756.4199, 757.4313, 761.4177, 767.4266, 768.3700, 768.3737, 770.3966, 772.4093, 775.4307, 779.4308, 784.3688, 786.4000, 787.4221, 788.4210, 792.4376, 795.4350, 798.4351, 800.4296, 812.4388, 815.4332, 822.4482, 825.4540, 831.4382, 832.4557, 835.4719, 837.4463, 842.5060, 844.4760, 854.4837, 856.4911, 860.4691, 862.4852, 869.4231, 885.4465, 905.4955, 907.4991, 908.4929, 913.5700, 913.5747, 916.5284, 918.5050, 919.5053, 922.5070, 928.5117, 934.5172, 940.4840, 942.4854, 943.4289, 943.5453, 946.5259, 950.4959, 951.5076, 953.5164, 954.5388, 959.5266, 966.5410, 969.5256, 972.5305, 974.5395, 976.5310, 978.5314, 979.5442, 991.5409, 993.5307, 1001.5854, 1003.5496, 1011.5262, 1014.5215, 1021.5579, 1023.5663, 1023.5700, 1031.5903, 1035.5381, 1036.5447, 1037.5573, 1039.5327, 1041.5658, 1044.5751, 1045.5729, 1046.5897, 1051.5448, 1056.5658, 1057.6078, 1067.5753, 1073.5902, 1086.5509, 1092.5961, 1100.6071, 1106.5391, 1111.5615, 1113.5729, 1118.5818, 1120.6045, 1122.5912, 1123.5848, 1125.5823, 1132.6047, 1134.5714, 1139.5746, 1143.6318, 1144.6497, 1145.6407, 1147.6393, 1150.6108, 1155.6215, 1157.6199, 1158.6685, 1162.6182, 1163.6152, 1168.6215, 1173.6418, 1175.6089, 1178.6356, 1179.6157, 1181.6819, 1188.6544, 1197.6704, 1199.7006, 1208.6415, 1216.6714, 1227.6595, 1231.6387, 1232.6447, 1234.6672, 1235.6445, 1244.6453, 1247.6211, 1248.6174, 1250.6554, 1255.6096, 1261.6592, 1262.6453, 1266.6777, 1273.6260, 1275.6362, 1276.6343, 1277.6531, 1301.7079, 1303.7076, 1307.7042, 1308.6376, 1310.6146, 1312.6418, 1315.7098, 1320.6659, 1322.6886, 1323.6771, 1324.6964, 1328.7089, 1331.7202, 1335.7318, 1338.6581, 1339.6637, 1349.7253, 1352.7205, 1356.7230, 1360.6638, 1363.7153, 1365.7379, 1367.7319, 1369.7043, 1377.6947, 1379.7268, 1383.7137, 1385.7534, 1388.7404, 1390.7284, 1393.7428, 1407.7563, 1421.7061, 1422.7019, 1425.7727, 1427.7742, 1429.7365, 1431.7412, 1433.7228, 1434.7446, 1437.7584, 1461.7778, 1467.7521, 1472.7633, 1475.7632, 1477.7545, 1484.7889, 1488.7537, 1491.7965, 1493.7579, 1500.7737, 1505.8177, 1513.8079, 1515.8213, 1517.7854, 1519.7544, 1522.7811, 1523.7953, 1529.8099, 1530.7523, 1531.7472, 1537.7974, 1539.8005, 1542.8422, 1547.8051, 1549.7339, 1551.7778, 1553.8065, 1555.8101, 1556.8109, 1557.8010, 1561.8401, 1563.8109, 1565.8199, 1571.8400, 1579.7939, 1580.8077, 1583.8444, 1587.7620, 1592.8066, 1594.8335, 1604.8323, 1605.8586, 1608.8497, 1615.8754, 1624.8062, 1628.7900, 1628.7921, 1633.7985, 1640.9384, 1642.8099, 1646.8297, 1649.8447, 1657.8101, 1667.8622, 1677.7988, 1680.8746, 1689.9465, 1694.7288, 1698.9225, 1699.8862, 1704.8773, 1706.8701, 1707.8302, 1710.9279, 1716.8671, 1719.9058, 1723.9011, 1725.8300, 1725.8346, 1727.8986, 1730.8931, 1737.9006, 1751.8656, 1754.8580, 1765.8463, 1766.8040, 1767.8138, 1769.8741, 1770.3567, 1774.7610, 1774.9131, 1777.8770, 1780.8777, 1783.9246, 1787.8188, 1787.8200, 1791.8137, 1793.9050, 1795.9353, 1798.9340, 1800.9336, 1804.9142, 1805.9069, 1809.8469, 1815.9044, 1821.9144, 1828.9227, 1837.9396, 1840.9827, 1843.9688, 1844.9012, 1846.9153, 1852.9364, 1856.9908, 1861.8881, 1867.9218, 1874.9695, 1880.9265, 1884.9417, 1891.9622, 1894.9313, 1896.9078, 1896.9100, 1905.9313, 1906.9501, 1908.9043, 1911.9130, 1912.9154, 1916.9923, 1918.9587, 1921.9825, 1923.9718, 1925.9573, 1928.0469, 1932.0060, 1933.9950, 1937.9630, 1939.9740, 1944.0040, 1946.9679, 1949.9595, 1954.0236, 1955.9738, 1958.9556, 1960.0034, 1968.8600, 1973.9845, 1976.9861, 1978.9910, 1981.9854, 1983.9884, 1987.9784, 1995.9408, 1998.9894, 2000.9926, 2008.0460, 2012.9590, 2015.0104, 2017.9747, 2021.0170, 2026.0275, 2027.0256, 2031.0166, 2044.0123, 2047.4957, 2047.9515, 2052.0496, 2060.0520, 2062.0073, 2064.9880, 2066.9873, 2094.0376, 2097.0454, 2102.0667, 2103.1641, 2111.0305, 2127.0930, 2139.0630, 2144.0493, 2148.1023, 2155.0950, 2160.0632, 2176.0664, 2190.9895, 2195.0774, 2199.0723, 2202.1624, 2203.1221, 2208.0862, 2211.1042, 2216.1062, 2226.1272, 2229.0972, 2233.0984, 2239.0225, 2242.0435, 2268.1489, 2270.1287, 2271.1294, 2277.0823, 2284.1990, 2292.1426, 2309.0994, 2334.1719, 2342.9915, 2350.1746, 2383.9607, 2398.2400, 2400.0862, 2413.0549, 2414.1897, 2417.0959, 2497.2800, 2501.2637, 2511.1460, 2513.1421, 2528.1750, 2537.2012, 2541.1948, 2542.1836, 2556.2058, 2562.1487, 2612.2666, 2706.2109, 2717.0869, 2767.2666, 2888.2532, 3221.1733, 3224.2917, 3312.3306, 3916.9434

39. Q0VCY7 **Mass:** 27842 **Score:** 19 **Expect:** 2.8e+002 **Matches:** 12

Serine/arginine-rich splicing factor 1 OS=Bos taurus GN=SRSF1 PE=2 SV=1

Observed	Mr(expt)	Mr(calc)	ppm	Start - End	Miss	Ions	Peptide
832.4557	831.4485	831.4351	16.1	112 - 118	1	---	R.YGPPSRR.S
916.5284	915.5211	915.5290	-8.65	84 - 90	1	---	R.LRVEFPR.S
1021.5579	1020.5506	1020.5716	-20.56	174 - 181	2	---	R.KLDNTKFR.S
1045.5729	1044.5656	1044.5577	7.59	110 - 118	2	---	R.GRYGPPSRR.S

1162.6182	1161.6109	1161.6003	9.16	231	240	2	---	R.SRGSPRYSPR.H
1163.6152	1162.6079	1162.6346	-22.94	39	48	1	---	K.YGAIRDIDLK.N
1216.6714	1215.6641	1215.6836	-16.04	84	93	2	---	R.LRVEFPRSGR.G
1256.6531	1255.6458	1255.6230	18.1	165	174	2	---	R.KEDMTYAVRK.L + Oxidation (M)
1433.7228	1432.7155	1432.7171	-1.11	194	206	2	---	K.VDGPRSPSYGRSR.S
2155.0950	2154.0877	2154.1069	-8.92	119	138	1	---	R.SENRVVVSGLPPSGSWQDLK.D
2208.0862	2207.0789	2207.1157	-16.68	123	142	1	---	R.VVVSGLPPSGSWQDLKDHMR.E
2541.1948	2540.1875	2540.1608	10.5	52	74	1	---	R.GGPPFAFVEFEDPRDAEDAVYGR.D

No match to: 721.4365, 725.4001, 726.4063, 728.3681, 730.3848, 731.4017, 732.3573, 734.4151, 735.4143, 741.4101, 742.4515, 744.4171, 745.4167, 746.4169, 747.4125, 750.4153, 752.4127, 755.4100, 756.4199, 757.4313, 761.4177, 767.4266, 768.3700, 768.3737, 770.3966, 772.4093, 775.4307, 779.4308, 784.3688, 786.4000, 787.4221, 788.4210, 792.4376, 795.4350, 798.4351, 800.4296, 812.4388, 815.4332, 822.4482, 825.4540, 831.4382, 835.4719, 837.4463, 842.5060, 844.4760, 854.4837, 856.4911, 860.4691, 862.4852, 869.4231, 885.4465, 905.4955, 907.4991, 908.4929, 913.5700, 913.5747, 918.5050, 919.5053, 922.5070, 928.5117, 934.5172, 940.4840, 942.4854, 943.4289, 943.5453, 946.5259, 950.4959, 951.5076, 953.5164, 954.5388, 959.5266, 966.5410, 969.5256, 972.5305, 974.5395, 976.5310, 978.5314, 979.5442, 991.5409, 993.5307, 1001.5854, 1003.5496, 1011.5262, 1014.5215, 1023.5663, 1023.5700, 1031.5903, 1035.5381, 1036.5447, 1037.5573, 1039.5327, 1041.5658, 1044.5751, 1046.5897, 1051.5448, 1056.5658, 1057.6078, 1067.5753, 1073.5902, 1086.5509, 1092.5961, 1100.6071, 1106.5391, 1111.5615, 1113.5729, 1118.5818, 1120.6045, 1122.5912, 1123.5848, 1125.5823, 1132.6047, 1134.5714, 1139.5746, 1143.6318, 1144.6497, 1145.6407, 1147.6393, 1150.6108, 1155.6215, 1157.6199, 1158.6685, 1168.6215, 1173.6418, 1175.6089, 1178.6356, 1179.6157, 1181.6819, 1188.6544, 1197.6704, 1199.7006, 1208.6415, 1227.6595, 1231.6387, 1232.6447, 1234.6672, 1235.6445, 1244.6453, 1247.6211, 1248.6174, 1250.6554, 1255.6096, 1261.6592, 1262.6453, 1266.6777, 1273.6260, 1275.6362, 1276.6343, 1277.6531, 1301.7079, 1303.7076, 1307.7042, 1308.6376, 1310.6146, 1312.6418, 1315.7098, 1320.6659, 1322.6886, 1323.6771, 1324.6964, 1328.7089, 1331.7202, 1335.7318, 1338.6581, 1339.6637, 1349.7253, 1352.7205, 1356.7230, 1360.6638, 1363.7153, 1365.7379, 1367.7319, 1369.7043, 1377.6947, 1379.7268, 1383.7137, 1385.7534, 1388.7404, 1390.7284, 1393.7428, 1407.7563, 1417.7010, 1421.7061, 1422.7019, 1425.7727, 1427.7742, 1429.7365, 1431.7412, 1434.7446, 1437.7584, 1461.7778, 1467.7521, 1472.7633, 1475.7632, 1477.7545, 1484.7889, 1488.7537, 1491.7965, 1493.7579, 1500.7737, 1505.8177, 1513.8079, 1515.8213, 1517.7854, 1519.7544, 1522.7811, 1523.7953, 1529.8099, 1530.7523, 1531.7472, 1537.7974, 1539.8005, 1542.8422, 1545.8091, 1547.8051, 1549.7339, 1551.7778, 1553.8065, 1555.8101, 1556.8109, 1557.8010, 1561.8401, 1563.8109, 1565.8199, 1571.8400, 1579.7939, 1580.8077, 1583.8444, 1587.7620, 1592.8066, 1594.8335, 1604.8323, 1605.8586, 1608.8497, 1615.8754, 1624.8062, 1628.7900, 1628.7921, 1633.7985, 1640.9384, 1642.8099, 1646.8297, 1649.8447, 1657.8101, 1667.8622, 1677.7988, 1680.8746, 1689.9465, 1694.7288, 1698.9225, 1699.8862, 1704.8773, 1706.8701, 1707.8302, 1710.9279, 1716.8671, 1719.9058, 1723.9011, 1725.8300, 1725.8346, 1727.8986, 1730.8931, 1737.9006, 1751.8656, 1754.8580, 1765.8463, 1766.8040, 1767.8138, 1769.8741, 1770.3567, 1772.9315, 1774.7610, 1774.9131, 1777.8770, 1780.8777, 1783.9246, 1787.8188, 1787.8200, 1791.8137, 1793.9050, 1795.9353, 1798.9340, 1800.9336, 1804.9142, 1805.9069, 1809.8469, 1815.9044, 1821.9144, 1828.9227, 1837.9396, 1840.9827, 1843.9688, 1844.9012, 1846.9153, 1852.9364, 1856.9908, 1861.8881, 1867.9218, 1874.9695, 1880.9265, 1884.9417, 1891.9622, 1894.9313, 1896.9078, 1896.9100, 1900.9546, 1905.9313, 1906.9501, 1908.9043, 1911.9130, 1912.9154, 1916.9923, 1918.9587, 1921.9825, 1923.9718, 1925.9573, 1928.0469, 1932.0060, 1933.9950, 1937.9630, 1939.9740, 1944.0040, 1946.9679, 1949.9595, 1954.0236, 1955.9738, 1958.9556, 1960.0034, 1968.8600, 1973.9845, 1976.9861, 1978.9910, 1981.9854, 1983.9884, 1987.9784, 1995.9408, 1998.9894, 2000.9926, 2008.0460, 2012.9590, 2015.0104, 2017.9747, 2021.0170, 2026.0275, 2027.0256, 2031.0166, 2044.0123, 2047.4957, 2047.9515, 2052.0496, 2060.0520, 2062.0073, 2064.9880, 2066.9873, 2094.0376, 2097.0454, 2102.0667, 2103.1641, 2111.0305, 2127.0930, 2139.0630, 2144.0493, 2148.1023, 2156.0796, 2160.0632, 2176.0664, 2190.9895, 2195.0774, 2199.0723, 2202.1624, 2203.1221, 2211.1042, 2216.1062, 2225.1274, 2226.1272, 2229.0972, 2233.0984, 2239.0225, 2242.0435, 2268.1489, 2270.1287, 2271.1294, 2277.0823, 2284.1990, 2292.1426, 2309.0994, 2334.1719, 2342.9915, 2350.1746, 2383.9607, 2398.2400, 2400.0862, 2413.0549, 2414.1897, 2417.0959, 2497.2800, 2501.2637, 2511.1460, 2513.1421, 2528.1750, 2537.2012, 2542.1836, 2556.2058, 2562.1487, 2612.2666, 2706.2109, 2717.0869, 2767.2666, 2888.2532, 3221.1733, 3224.2917, 3312.3306, 3916.9434

40. Q0P5K9 **Mass:** 54202 **Score:** 19 **Expect:** 2.8e+002 **Matches:** 20

Chromosome 3 open reading frame 19 ortholog OS=Bos taurus GN=C22H3ORF19 PE=2 SV=1

Observed	Mr(expt)	Mr(calc)	ppm	Start	End	Miss	Ions	Peptide
775.4307	774.4235	774.4123	14.4	84	89	1	---	R.EKLEEK.A
916.5284	915.5211	915.5137	8.05	282	288	2	---	R.ENIKEKR.K
974.5395	973.5323	973.5444	-12.41	84	91	2	---	R.EKLEEKAK.L
1046.5897	1045.5824	1045.5702	11.7	258	265	2	---	K.ELRNKQMK.T
1092.5961	1091.5888	1091.5645	22.3	203	211	1	---	K.TLLSEDMRK.E
1106.5391	1105.5318	1105.5364	-4.10	272	280	1	---	R.EQTTDQRTK.R
1262.6453	1261.6380	1261.6375	0.44	272	281	2	---	R.EQTTDQRTKR.E
1339.6637	1338.6564	1338.6350	16.0	124	135	1	---	K.EMEAIGASRGYR.K
1407.7563	1406.7490	1406.7374	8.30	261	271	2	---	R.NKQMKTLEMLR.E + Oxidation (M)
1467.7521	1466.7448	1466.7299	10.1	124	136	2	---	K.EMEAIGASRGYRK.T
1477.7545	1476.7472	1476.7150	21.8	367	378	2	---	R.GKDFSFGYWSKR.Q
1545.8091	1544.8018	1544.7682	21.8	65	77	2	---	R.AEKDAEQKIEEQK.T
1706.8701	1705.8628	1705.8491	8.06	118	132	2	---	K.MIDKQKEMEAIGASR.G
1719.9058	1718.8985	1718.8601	22.4	353	366	1	---	K.DTRPGVPHVREWDR.G
1884.9417	1883.9344	1883.9775	-22.84	178	193	2	---	R.KDLPDLLEMDKNLQGR.L
1900.9546	1899.9473	1899.9724	-13.19	178	193	2	---	R.KDLPDLLEMDKNLQGR.L + Oxidation (M)
1949.9595	1948.9522	1948.9928	-20.81	194	210	1	---	R.LFVSPANEKTLLSEDMR.K
1955.9738	1954.9665	1955.0000	-17.13	318	336	0	---	R.DGDVIGPLPPEPEVTPAPR.T
1958.9556	1957.9483	1957.9210	13.9	387	402	1	---	R.DPEFAPPSYYFVDQKR.T
2094.0376	2093.0303	2093.0827	-25.00	194	211	2	---	R.LFVSPANEKTLLSEDMRK.E + Oxidation (M)

No match to: 721.4365, 725.4001, 726.4063, 728.3681, 730.3848, 731.4017, 732.3573, 734.4151, 735.4143, 741.4101, 742.4515, 744.4171, 745.4167, 746.4169, 747.4125, 750.4153, 752.4127, 755.4100, 756.4199, 757.4313, 761.4177, 767.4266, 768.3700, 768.3737, 770.3966, 772.4093, 779.4308, 784.3688, 786.4000, 787.4221, 788.4210, 792.4376, 795.4350, 798.4351, 800.4296, 812.4388, 815.4332, 822.4482, 825.4540, 831.4382, 832.4557, 835.4719, 837.4463, 842.5060, 844.4760, 854.4837, 856.4911, 860.4691, 862.4852, 869.4231, 885.4465, 905.4955, 907.4991, 908.4929, 913.5700, 913.5747, 918.5050, 919.5053, 922.5070, 928.5117, 934.5172, 940.4840, 942.4854, 943.4289, 943.5453, 946.5259, 950.4959, 951.5076, 953.5164, 954.5388, 959.5266, 966.5410, 969.5256, 972.5305, 976.5310, 978.5314, 979.5442, 991.5409, 993.5307, 1001.5854, 1003.5496, 1011.5262, 1014.5215, 1021.5579, 1023.5663, 1023.5700, 1031.5903, 1035.5381, 1036.5447, 1037.5573, 1039.5327, 1041.5658, 1044.5751, 1045.5729, 1051.5448, 1056.5658, 1057.6078, 1067.5753, 1073.5902, 1086.5509, 1100.6071, 1111.5615, 1113.5729, 1118.5818, 1120.6045, 1122.5912, 1123.5848, 1125.5823, 1132.6047, 1134.5714, 1139.5746, 1143.6318, 1144.6497, 1145.6407, 1147.6393, 1150.6108, 1155.6215, 1157.6199, 1158.6685, 1162.6182, 1163.6152, 1168.6215, 1173.6418, 1175.6089, 1178.6356, 1179.6157, 1181.6819, 1188.6544, 1197.6704, 1199.7006, 1208.6415, 1216.6714, 1227.6595, 1231.6387, 1232.6447, 1234.6672, 1235.6445, 1244.6453, 1247.6211, 1248.6174, 1250.6554, 1255.6096, 1256.6531, 1261.6592, 1266.6777, 1273.6260, 1275.6362, 1276.6343, 1277.6531, 1301.7079, 1303.7076, 1307.7042, 1308.6376, 1310.6146, 1312.6418, 1315.7098, 1320.6659, 1322.6886, 1323.6771, 1324.6964, 1328.7089, 1331.7202, 1335.7318, 1338.6581, 1349.7253, 1352.7205, 1356.7230, 1360.6638, 1363.7153, 1365.7379, 1367.7319, 1369.7043, 1377.6947, 1379.7268, 1383.7137, 1385.7534, 1388.7404, 1390.7284, 1393.7428, 1417.7010, 1421.7061, 1422.7019, 1425.7727, 1427.7742, 1429.7365, 1431.7412, 1433.7228, 1434.7446, 1437.7584, 1461.7778, 1472.7633, 1475.7632, 1484.7889, 1488.7537, 1491.7965, 1493.7579, 1500.7737, 1505.8177, 1513.8079, 1515.8213, 1517.7854, 1519.7544, 1522.7811, 1523.7953, 1529.8099, 1530.7523, 1531.7472, 1537.7974, 1539.8005, 1542.8422, 1547.8051, 1549.7339, 1551.7778, 1553.8065, 1555.8101, 1556.8109, 1557.8010, 1561.8401, 1563.8109, 1565.8199, 1571.8400, 1579.7939, 1580.8077, 1583.8444, 1587.7620, 1592.8066, 1594.8335, 1604.8323, 1605.8586, 1608.8497, 1615.8754, 1624.8062, 1628.7900, 1628.7921, 1633.7985, 1640.9384, 1642.8099, 1646.8297, 1649.8447, 1657.8101, 1667.8622, 1677.7988, 1680.8746, 1689.9465, 1694.7288, 1698.9225, 1699.8862, 1704.8773, 1707.8302, 1710.9279, 1716.8671, 1723.9011, 1725.8300, 1725.8346, 1727.8986, 1730.8931, 1737.9006, 1751.8656, 1754.8580, 1765.8463, 1766.8040, 1767.8138, 1769.8741, 1770.3567, 1772.9315, 1774.7610, 1774.9131, 1777.8770, 1780.8777, 1783.9246, 1787.8188, 1787.8200, 1791.8137, 1793.9050, 1795.9353, 1798.9340, 1800.9336, 1804.9142, 1805.9069, 1809.8469, 1815.9044, 1821.9144, 1828.9227, 1837.9396, 1840.9827, 1843.9688, 1844.9012, 1846.9153, 1852.9364, 1856.9908, 1861.8881, 1867.9218, 1874.9695, 1880.9265, 1891.9622, 1894.9313, 1896.9078, 1896.9100, 1905.9313, 1906.9501, 1908.9043, 1911.9130, 1912.9154, 1916.9923, 1918.9587, 1921.9825, 1923.9718, 1925.9573, 1928.0469, 1932.0060, 1933.9950, 1937.9630, 1939.9740, 1944.0040, 1946.9679, 1954.0236, 1960.0034, 1968.8600, 1973.9845, 1976.9861, 1978.9910, 1981.9854, 1983.9884, 1987.9784, 1995.9408, 1998.9894, 2000.9926, 2008.0460, 2012.9590, 2015.0104, 2017.9747, 2021.0170, 2026.0275, 2027.0256, 2031.0166, 2044.0123, 2047.4957, 2047.9515, 2052.0496, 2060.0520,

2062.0073, 2064.9880, 2066.9873, 2097.0454, 2102.0667, 2103.1641, 2111.0305, 2127.0930, 2139.0630, 2144.0493, 2148.1023, 2155.0950, 2156.0796, 2160.0632, 2176.0664, 2190.9895, 2195.0774, 2199.0723, 2202.1624, 2203.1221, 2208.0862, 2211.1042, 2216.1062, 2225.1274, 2226.1272, 2229.0972, 2233.0984, 2239.0225, 2242.0435, 2268.1489, 2270.1287, 2271.1294, 2277.0823, 2284.1990, 2292.1426, 2309.0994, 2334.1719, 2342.9915, 2350.1746, 2383.9607, 2398.2400, 2400.0862, 2413.0549, 2414.1897, 2417.0959, 2497.2800, 2501.2637, 2511.1460, 2513.1421, 2528.1750, 2537.2012, 2541.1948, 2542.1836, 2556.2058, 2562.1487, 2612.2666, 2706.2109, 2717.0869, 2767.2666, 2888.2532, 3221.1733, 3224.2917, 3312.3306, 3916.9434

41. F1MB56 **Mass:** 24689 **Score:** 19 **Expect:** 2.9e+002 **Matches:** 11

Uncharacterized protein OS=Bos taurus GN=TEX35 PE=4 SV=1

Observed	Mr(expt)	Mr(calc)	ppm	Start	End	Miss	Ions	Peptide
775.4307	774.4235	774.4123	14.4	61 -	66	1	---	R.EELKEK.M
976.5310	975.5237	975.5059	18.3	72 -	79	1	---	K.QIKDVMDK.D
1045.5729	1044.5656	1044.5750	-8.94	1 -	9	2	---	-.MSAKRVEPK.K
1073.5902	1072.5829	1072.5950	-11.30	102 -	110	0	---	K.MDVLINIQK.N
1118.5818	1117.5745	1117.5914	-15.06	119 -	127	1	---	R.GLKMQQELR.L + Oxidation (M)
1421.7061	1420.6988	1420.7119	-9.19	61 -	71	2	---	R.EELKEKMEEIK.Q + Oxidation (M)
1657.8101	1656.8028	1656.8069	-2.46	80 -	92	1	---	K.DFDKLQEFVEIMK.E + Oxidation (M)
1707.8302	1706.8229	1706.8219	0.61	97 -	110	1	---	K.DMDEKMDVLINIQK.N + Oxidation (M)
1918.9587	1917.9514	1917.9414	5.25	194 -	211	0	---	K.LPYHAWASFSPLASGPAF.-
2021.0170	2020.0097	2019.9969	6.36	97 -	113	2	---	K.DMDEKMDVLINIQKNSK.F
2208.0862	2207.0789	2207.0636	6.96	93 -	110	2	---	K.EMQKDMDEKMDVLINIQK.N

No match to: 721.4365, 725.4001, 726.4063, 728.3681, 730.3848, 731.4017, 732.3573, 734.4151, 735.4143, 741.4101, 742.4515, 744.4171, 745.4167, 746.4169, 747.4125, 750.4153, 752.4127, 755.4100, 756.4199, 757.4313, 761.4177, 767.4266, 768.3700, 768.3737, 770.3966, 772.4093, 779.4308, 784.3688, 786.4000, 787.4221, 788.4210, 792.4376, 795.4350, 798.4351, 800.4296, 812.4388, 815.4332, 822.4482, 825.4540, 831.4382, 832.4557, 835.4719, 837.4463, 842.5060, 844.4760, 854.4837, 856.4911, 860.4691, 862.4852, 869.4231, 885.4465, 905.4955, 907.4991, 908.4929, 913.5700, 913.5747, 916.5284, 918.5050, 919.5053, 922.5070, 928.5117, 934.5172, 940.4840, 942.4854, 943.4289, 943.5453, 946.5259, 950.4959, 951.5076, 953.5164, 954.5388, 959.5266, 966.5410, 969.5256, 972.5305, 974.5395, 978.5314, 979.5442, 991.5409, 993.5307, 1001.5854, 1003.5496, 1011.5262, 1014.5215, 1021.5579, 1023.5663, 1023.5700, 1031.5903, 1035.5381, 1036.5447, 1037.5573, 1039.5327, 1041.5658, 1044.5751, 1046.5897, 1051.5448, 1056.5658, 1057.6078, 1067.5753, 1086.5509, 1092.5961, 1100.6071, 1106.5391, 1111.5615, 1113.5729, 1120.6045, 1122.5912, 1123.5848, 1125.5823, 1132.6047, 1134.5714, 1139.5746, 1143.6318, 1144.6497, 1145.6407, 1147.6393, 1150.6108, 1155.6215, 1157.6199, 1158.6685, 1162.6182, 1163.6152, 1168.6215, 1173.6418, 1175.6089, 1178.6356, 1179.6157, 1181.6819, 1188.6544, 1197.6704, 1199.7006, 1208.6415, 1216.6714, 1227.6595, 1231.6387, 1232.6447, 1234.6672, 1235.6445, 1244.6453, 1247.6211, 1248.6174, 1250.6554, 1255.6096, 1256.6531, 1261.6592, 1262.6453, 1266.6777, 1273.6260, 1275.6362, 1276.6343, 1277.6531, 1301.7079, 1303.7076, 1307.7042, 1308.6376, 1310.6146, 1312.6418, 1315.7098, 1320.6659, 1322.6886, 1323.6771, 1324.6964, 1328.7089, 1331.7202, 1335.7318, 1338.6581, 1339.6637, 1349.7253, 1352.7205, 1356.7230, 1360.6638, 1363.7153, 1365.7379, 1367.7319, 1369.7043, 1377.6947, 1379.7268, 1383.7137, 1385.7534, 1388.7404, 1390.7284, 1393.7428, 1407.7563, 1417.7010, 1422.7019, 1425.7727, 1427.7742, 1429.7365, 1431.7412, 1433.7228, 1434.7446, 1437.7584, 1461.7778, 1467.7521, 1472.7633, 1475.7632, 1477.7545, 1484.7889, 1488.7537, 1491.7965, 1493.7579, 1500.7737, 1505.8177, 1513.8079, 1515.8213, 1517.7854, 1519.7544, 1522.7811, 1523.7953, 1529.8099, 1530.7523, 1531.7472, 1537.7974, 1539.8005, 1542.8422, 1545.8091, 1547.8051, 1549.7339, 1551.7778, 1553.8065, 1555.8101, 1556.8109, 1557.8010, 1561.8401, 1563.8109, 1565.8199, 1571.8400, 1579.7939, 1580.8077, 1583.8444, 1587.7620, 1592.8066, 1594.8335, 1604.8323, 1605.8586, 1608.8497, 1615.8754, 1624.8062, 1628.7900, 1628.7921, 1633.7985, 1640.9384, 1642.8099, 1646.8297, 1649.8447, 1667.8622, 1677.7988, 1680.8746, 1689.9465, 1694.7288, 1698.9225, 1699.8862, 1704.8773, 1706.8701, 1710.9279, 1716.8671, 1719.9058, 1723.9011, 1725.8300, 1725.8346, 1727.8986, 1730.8931, 1737.9006, 1751.8656, 1754.8580, 1765.8463, 1766.8040, 1767.8138, 1769.8741, 1770.3567, 1772.9315, 1774.7610, 1774.9131, 1777.8770, 1780.8777, 1783.9246, 1787.8188, 1787.8200, 1791.8137, 1793.9050, 1795.9353, 1798.9340, 1800.9336, 1804.9142, 1805.9069, 1809.8469, 1815.9044, 1821.9144, 1828.9227, 1837.9396, 1840.9827, 1843.9688, 1844.9012, 1846.9153, 1852.9364, 1856.9908, 1861.8881, 1867.9218, 1874.9695, 1880.9265, 1884.9417, 1891.9622, 1894.9313, 1896.9078, 1896.9100, 1900.9546, 1905.9313, 1906.9501, 1908.9043, 1911.9130, 1912.9154, 1916.9923, 1921.9825, 1923.9718, 1925.9573, 1928.0469, 1932.0060, 1933.9950, 1937.9630, 1939.9740, 1944.0040, 1946.9679, 1949.9595, 1954.0236, 1955.9738, 1958.9556, 1960.0034, 1968.8600, 1973.9845, 1976.9861, 1978.9910, 1981.9854, 1983.9884, 1987.9784, 1995.9408, 1998.9894, 2000.9926, 2008.0460, 2012.9590, 2015.0104, 2017.9747, 2026.0275, 2027.0256, 2031.0166, 2044.0123, 2047.4957, 2047.9515, 2052.0496, 2060.0520, 2062.0073, 2064.9880, 2066.9873, 2094.0376, 2097.0454, 2102.0667, 2103.1641, 2111.0305, 2127.0930, 2139.0630, 2144.0493, 2148.1023, 2155.0950, 2156.0796, 2160.0632, 2176.0664, 2190.9895, 2195.0774, 2199.0723, 2202.1624, 2203.1221, 2211.1042, 2216.1062, 2225.1274, 2226.1272, 2229.0972, 2233.0984, 2239.0225, 2242.0435, 2268.1489, 2270.1287, 2271.1294, 2277.0823, 2284.1990, 2292.1426, 2309.0994, 2334.1719, 2342.9915, 2350.1746, 2383.9607, 2398.2400, 2400.0862, 2413.0549, 2414.1897, 2417.0959, 2497.2800, 2501.2637, 2511.1460, 2513.1421, 2528.1750, 2537.2012, 2541.1948, 2542.1836, 2556.2058, 2562.1487, 2612.2666, 2706.2109, 2717.0869, 2767.2666, 2888.2532, 3221.1733, 3224.2917, 3312.3306, 3916.9434

42. P61257 **Mass:** 17248 **Score:** 19 **Expect:** 2.9e+002 **Matches:** 9

60S ribosomal protein L26 OS=Bos taurus GN=RPL26 PE=2 SV=1

Observed	Mr(expt)	Mr(calc)	ppm	Start	End	Miss	Ions	Peptide
747.4125	746.4052	746.4109	-7.60	46 -	51	1	---	R.SMPIRK.D + Oxidation (M)
752.4127	751.4054	751.4228	-23.11	131 -	136	2	---	K.EKGKYK.E
862.4852	861.4779	861.4630	17.3	29 -	36	0	---	K.IMSSPLSK.E
1039.5327	1038.5254	1038.5233	2.05	135 -	142	1	---	K.YKEETIEK.M
1234.6672	1233.6599	1233.6479	9.74	18 -	27	1	---	R.HFNAPSHIRR.K
1390.7284	1389.7211	1389.7490	-20.06	17 -	27	2	---	K.RHFNAPSHIRR.K
1407.7563	1406.7490	1406.7452	2.70	40 -	50	2	---	R.QKYNVRSMPIR.K + Oxidation (M)
1425.7727	1424.7654	1424.7412	17.0	2 -	13	2	---	M.KFNPFVTSDRSK.N
1556.8109	1555.8036	1555.7817	14.1	1 -	13	2	---	-.MKFNPFVTSDRSK.N

No match to: 721.4365, 725.4001, 726.4063, 728.3681, 730.3848, 731.4017, 732.3573, 734.4151, 735.4143, 741.4101, 742.4515, 744.4171, 745.4167, 746.4169, 750.4153, 755.4100, 756.4199, 757.4313, 761.4177, 767.4266, 768.3700, 768.3737, 770.3966, 772.4093, 775.4307, 779.4308, 784.3688, 786.4000, 787.4221, 788.4210, 792.4376, 795.4350, 798.4351, 800.4296, 812.4388, 815.4332, 822.4482, 825.4540, 831.4382, 832.4557, 835.4719, 837.4463, 842.5060, 844.4760, 854.4837, 856.4911, 860.4691, 869.4231, 885.4465, 905.4955, 907.4991, 908.4929, 913.5700, 913.5747, 916.5284, 918.5050, 919.5053, 922.5070, 928.5117, 934.5172, 940.4840, 942.4854, 943.4289, 943.5453, 946.5259, 950.4959, 951.5076, 953.5164, 954.5388, 959.5266, 966.5410, 969.5256, 972.5305, 974.5395, 976.5310, 978.5314, 979.5442, 991.5409, 993.5307, 1001.5854, 1003.5496, 1011.5262, 1014.5215, 1021.5579, 1023.5663, 1023.5700, 1031.5903, 1035.5381, 1036.5447, 1037.5573, 1041.5658, 1044.5751, 1045.5729, 1046.5897, 1051.5448, 1056.5658, 1057.6078, 1067.5753, 1073.5902, 1086.5509, 1092.5961, 1100.6071, 1106.5391, 1111.5615, 1113.5729, 1118.5818, 1120.6045, 1122.5912, 1123.5848, 1125.5823, 1132.6047, 1134.5714, 1139.5746, 1143.6318, 1144.6497, 1145.6407, 1147.6393, 1150.6108, 1155.6215, 1157.6199, 1158.6685, 1162.6182, 1163.6152, 1168.6215, 1173.6418, 1175.6089, 1178.6356, 1179.6157, 1181.6819, 1188.6544, 1197.6704, 1199.7006, 1208.6415, 1216.6714, 1227.6595, 1231.6387, 1232.6447, 1235.6445, 1244.6453, 1247.6211, 1248.6174, 1250.6554, 1255.6096, 1256.6531, 1261.6592, 1262.6453, 1266.6777, 1273.6260, 1275.6362, 1276.6343, 1277.6531, 1301.7079, 1303.7076, 1307.7042, 1308.6376, 1310.6146, 1312.6418, 1315.7098, 1320.6659, 1322.6886, 1323.6771, 1324.6964, 1328.7089, 1331.7202, 1335.7318, 1338.6581, 1339.6637, 1349.7253, 1352.7205, 1356.7230, 1360.6638, 1363.7153, 1365.7379, 1367.7319, 1369.7043, 1377.6947, 1379.7268, 1383.7137, 1385.7534, 1388.7404, 1393.7428, 1417.7010, 1421.7061, 1422.7019, 1427.7742, 1429.7365, 1431.7412, 1433.7228, 1434.7446, 1437.7584, 1461.7778, 1467.7521, 1472.7633, 1475.7632, 1477.7545, 1484.7889, 1488.7537, 1491.7965, 1493.7579, 1500.7737, 1505.8177, 1513.8079, 1515.8213, 1517.7854, 1519.7544, 1522.7811, 1523.7953, 1529.8099, 1530.7523, 1531.7472, 1537.7974, 1539.8005, 1542.8422, 1545.8091, 1547.8051, 1549.7339, 1551.7778, 1553.8065, 1555.8101, 1557.8010, 1561.8401, 1563.8109, 1565.8199, 1571.8400, 1579.7939, 1580.8077, 1583.8444, 1587.7620, 1592.8066, 1594.8335, 1604.8323, 1605.8586, 1608.8497, 1615.8754, 1624.8062, 1628.7900, 1628.7921, 1633.7985, 1640.9384, 1642.8099, 1646.8297, 1649.8447, 1657.8101, 1667.8622, 1677.7988, 1680.8746, 1689.9465, 1694.7288, 1698.9225, 1699.8862, 1704.8773, 1706.8701, 1707.8302, 1710.9279, 1716.8671, 1719.9058, 1723.9011, 1725.8300, 1725.8346, 1727.8986, 1730.8931, 1737.9006, 1751.8656, 1754.8580, 1765.8463, 1766.8040, 1767.8138, 1769.8741, 1770.3567, 1772.9315, 1774.7610, 1774.9131, 1777.8770, 1780.8777, 1783.9246, 1787.8188, 1787.8200, 1791.8137, 1793.9050, 1795.9353, 1798.9340, 1800.9336, 1804.9142, 1805.9069, 1809.8469, 1815.9044, 1821.9144, 1828.9227, 1837.9396, 1840.9827, 1843.9688, 1844.9012, 1846.9153, 1852.9364, 1856.9908, 1861.8881, 1867.9218, 1874.9695, 1880.9265, 1884.9417, 1891.9622, 1894.9313, 1896.9078, 1896.9100, 1900.9546, 1905.9313, 1906.9501, 1908.9043, 1911.9130, 1912.9154, 1916.9923, 1918.9587, 1921.9825, 1923.9718, 1925.9573, 1928.0469,

1932.0060, 1933.9950, 1937.9630, 1939.9740, 1944.0040, 1946.9679, 1949.9595, 1954.0236, 1955.9738, 1958.9556, 1960.0034, 1968.8600, 1973.9845, 1976.9861, 1978.9910, 1981.9854, 1983.9884, 1987.9784, 1995.9408, 1998.9894, 2000.9926, 2008.0460, 2012.9590, 2015.0104, 2017.9747, 2021.0170, 2026.0275, 2027.0256, 2031.0166, 2044.0123, 2047.4957, 2047.9515, 2052.0496, 2060.0520, 2062.0073, 2064.9880, 2066.9873, 2094.0376, 2097.0454, 2102.0667, 2103.1641, 2111.0305, 2127.0930, 2139.0630, 2144.0493, 2148.1023, 2155.0950, 2156.0796, 2160.0632, 2176.0664, 2190.9895, 2195.0774, 2199.0723, 2202.1624, 2203.1221, 2208.0862, 2211.1042, 2216.1062, 2225.1274, 2226.1272, 2229.0972, 2233.0984, 2239.0225, 2242.0435, 2268.1489, 2270.1287, 2271.1294, 2277.0823, 2284.1990, 2292.1426, 2309.0994, 2334.1719, 2342.9915, 2350.1746, 2383.9607, 2398.2400, 2400.0862, 2413.0549, 2414.1897, 2417.0959, 2497.2800, 2501.2637, 2511.1460, 2513.1421, 2528.1750, 2537.2012, 2541.1948, 2542.1836, 2556.2058, 2562.1487, 2612.2666, 2706.2109, 2717.0869, 2767.2666, 2888.2532, 3221.1733, 3224.2917, 3312.3306, 3916.9434

43. Q861S4 **Mass:** 18652 **Score:** 19 **Expect:** 2.9e+002 **Matches:** 12
Similar to ribosomal protein L21 (Fragment) OS=Bos taurus GN=RPL21 PE=2 SV=1

Observed	Mr(expt)	Mr(calc)	ppm	Start	End	Miss	Ions	Peptide
951.5076	950.5004	950.4967	3.81	1 -	8	2	---	-.MTNTKGKR.R + Oxidation (M)
1231.6387	1230.6314	1230.6332	-1.41	13 -	21	1	---	R.YMFSRPFRK.H
1247.6211	1246.6138	1246.6281	-11.44	13 -	21	1	---	R.YMFSRPFRK.H + Oxidation (M)
1417.7010	1416.6937	1416.7085	-10.41	10 -	20	1	---	R.GTRYMFSRPFR.K
1433.7228	1432.7155	1432.7034	8.47	10 -	20	1	---	R.GTRYMFSRPFR.K + Oxidation (M)
1545.8091	1544.8018	1544.8034	-1.03	10 -	21	2	---	R.GTRYMFSRPFRK.H
1561.8401	1560.8328	1560.7983	22.1	10 -	21	2	---	R.GTRYMFSRPFRK.H + Oxidation (M)
1604.8323	1603.8250	1603.8604	-22.03	36 -	50	2	---	R.KGDIVDIKGMGTVQK.G + Oxidation (M)
1677.7988	1676.7915	1676.8007	-5.48	147 -	160	0	---	K.EPELLEPIPYEFMA.-
1804.9142	1803.9069	1803.9492	-23.45	131 -	146	2	---	R.QPAPPREAHFVRTNGK.E
2027.0256	2026.0183	2026.0340	-7.73	37 -	55	2	---	K.GDIVDIKGMGTVQKGMPHK.C + Oxidation (M)
2094.0376	2093.0303	2093.0027	13.2	143 -	160	1	---	R.TNGKEPELLEPIPYEFMA.- + Oxidation (M)

No match to: 721.4365, 725.4001, 726.4063, 728.3681, 730.3848, 731.4017, 732.3573, 734.4151, 735.4143, 741.4101, 742.4515, 744.4171, 745.4167, 746.4169, 747.4125, 750.4153, 752.4127, 755.4100, 756.4199, 757.4313, 761.4177, 767.4266, 768.3700, 768.3737, 770.3966, 772.4093, 775.4307, 779.4308, 784.3688, 786.4000, 787.4221, 788.4210, 792.4376, 795.4350, 798.4351, 800.4296, 812.4388, 815.4332, 822.4482, 825.4540, 831.4382, 832.4557, 835.4719, 837.4463, 842.5060, 844.4760, 854.4837, 856.4911, 860.4691, 862.4852, 869.4231, 885.4465, 905.4955, 907.4991, 908.4929, 913.5700, 913.5747, 916.5284, 918.5050, 919.5053, 922.5070, 928.5117, 934.5172, 940.4840, 942.4854, 943.4289, 943.5453, 946.5259, 950.4959, 953.5164, 954.5388, 959.5266, 966.5410, 969.5256, 972.5305, 974.5395, 976.5310, 978.5314, 979.5442, 991.5409, 993.5307, 1001.5854, 1003.5496, 1011.5262, 1014.5215, 1021.5579, 1023.5663, 1023.5700, 1031.5903, 1035.5381, 1036.5447, 1037.5573, 1039.5327, 1041.5658, 1044.5751, 1045.5729, 1046.5897, 1051.5448, 1056.5658, 1057.6078, 1067.5753, 1073.5902, 1086.5509, 1092.5961, 1100.6071, 1106.5391, 1111.5615, 1113.5729, 1118.5818, 1120.6045, 1122.5912, 1123.5848, 1125.5823, 1132.6047, 1134.5714, 1139.5746, 1143.6318, 1144.6497, 1145.6407, 1147.6393, 1150.6108, 1155.6215, 1157.6199, 1158.6685, 1162.6182, 1163.6152, 1168.6215, 1173.6418, 1175.6089, 1178.6356, 1179.6157, 1181.6819, 1188.6544, 1197.6704, 1199.7006, 1208.6415, 1216.6714, 1227.6595, 1232.6447, 1234.6672, 1235.6445, 1244.6453, 1248.6174, 1250.6554, 1255.6096, 1256.6531, 1261.6592, 1262.6453, 1266.6777, 1273.6260, 1275.6362, 1276.6343, 1277.6531, 1301.7079, 1303.7076, 1307.7042, 1308.6376, 1310.6146, 1312.6418, 1315.7098, 1320.6659, 1322.6886, 1323.6771, 1324.6964, 1328.7089, 1331.7202, 1335.7318, 1338.6581, 1339.6637, 1349.7253, 1352.7205, 1356.7230, 1360.6638, 1363.7153, 1365.7379, 1367.7319, 1369.7043, 1377.6947, 1379.7268, 1383.7137, 1385.7534, 1388.7404, 1390.7284, 1393.7428, 1407.7563, 1421.7061, 1422.7019, 1425.7727, 1427.7742, 1429.7365, 1431.7412, 1434.7446, 1437.7584, 1461.7778, 1467.7521, 1472.7633, 1475.7632, 1477.7545, 1484.7889, 1488.7537, 1491.7965, 1493.7579, 1500.7737, 1505.8177, 1513.8079, 1515.8213, 1517.7854, 1519.7544, 1522.7811, 1523.7953, 1529.8099, 1530.7523, 1531.7472, 1537.7974, 1539.8005, 1542.8422, 1547.8051, 1549.7339, 1551.7778, 1553.8065, 1555.8101, 1556.8109, 1557.8010, 1563.8109, 1565.8199, 1571.8400, 1579.7939, 1580.8077, 1583.8444, 1587.7620, 1592.8066, 1594.8335, 1605.8586, 1608.8497, 1615.8754, 1624.8062, 1628.7900, 1628.7921, 1633.7985, 1640.9384, 1642.8099, 1646.8297, 1649.8447, 1657.8101, 1667.8622, 1680.8746, 1689.9465, 1694.7288, 1698.9225, 1699.8862, 1704.8773, 1706.8701, 1707.8302, 1710.9279, 1716.8671, 1719.9058, 1723.9011, 1725.8300, 1725.8346, 1727.8986, 1730.8931, 1737.9006, 1751.8656, 1754.8580, 1765.8463, 1766.8040, 1767.8138, 1769.8741, 1770.3567, 1772.9315, 1774.7610, 1774.9131, 1777.8770, 1780.8777, 1783.9246, 1787.8188, 1787.8200, 1791.8137, 1793.9050, 1795.9353, 1798.9340, 1800.9336, 1805.9069, 1809.8469, 1815.9044, 1821.9144, 1828.9227, 1837.9396, 1840.9827, 1843.9688, 1844.9012, 1846.9153, 1852.9364, 1856.9908, 1861.8881, 1867.9218, 1874.9695, 1880.9265, 1884.9417, 1891.9622, 1894.9313, 1896.9078, 1896.9100, 1900.9546, 1905.9313, 1906.9501, 1908.9043, 1911.9130, 1912.9154, 1916.9923, 1918.9587, 1921.9825, 1923.9718, 1925.9573, 1928.0469, 1932.0060, 1933.9950, 1937.9630, 1939.9740, 1944.0040, 1946.9679, 1949.9595, 1954.0236, 1955.9738, 1958.9556, 1960.0034, 1968.8600, 1973.9845, 1976.9861, 1978.9910, 1981.9854, 1983.9884, 1987.9784, 1995.9408, 1998.9894, 2000.9926, 2008.0460, 2012.9590, 2015.0104, 2017.9747, 2021.0170, 2026.0275, 2031.0166, 2044.0123, 2047.4957, 2047.9515, 2052.0496, 2060.0520, 2062.0073, 2064.9880, 2066.9873, 2097.0454, 2102.0667, 2103.1641, 2111.0305, 2127.0930, 2139.0630, 2144.0493, 2148.1023, 2155.0950, 2156.0796, 2160.0632, 2176.0664, 2190.9895, 2195.0774, 2199.0723, 2202.1624, 2203.1221, 2208.0862, 2211.1042, 2216.1062, 2225.1274, 2226.1272, 2229.0972, 2233.0984, 2239.0225, 2242.0435, 2268.1489, 2270.1287, 2271.1294, 2277.0823, 2284.1990, 2292.1426, 2309.0994, 2334.1719, 2342.9915, 2350.1746, 2383.9607, 2398.2400, 2400.0862, 2413.0549, 2414.1897, 2417.0959, 2497.2800, 2501.2637, 2511.1460, 2513.1421, 2528.1750, 2537.2012, 2541.1948, 2542.1836, 2556.2058, 2562.1487, 2612.2666, 2706.2109, 2717.0869, 2767.2666, 2888.2532, 3221.1733, 3224.2917, 3312.3306, 3916.9434

44. Q00361 **Mass:** 8315 **Score:** 19 **Expect:** 2.9e+002 **Matches:** 7
ATP synthase subunit e, mitochondrial OS=Bos taurus GN=ATP5I PE=1 SV=2

Observed	Mr(expt)	Mr(calc)	ppm	Start	End	Miss	Ions	Peptide
831.4382	830.4309	830.4358	-5.95	51 -	56	2	---	K.RDEQKR.I
916.5284	915.5211	915.5389	-19.41	43 -	50	2	---	R.LAAEEKKK.R
953.5164	952.5091	952.5130	-4.13	30 -	36	0	---	R.YNYLKPR.A
1073.5902	1072.5829	1072.5625	19.1	52 -	59	2	---	R.DEQKRIER.E
1547.8051	1546.7978	1546.7966	0.80	16 -	29	1	---	R.YSALFLGMAYGAKR.Y
1563.8109	1562.8036	1562.7915	7.76	16 -	29	1	---	R.YSALFLGMAYGAKR.Y + Oxidation (M)
1723.9011	1722.8938	1722.8801	7.97	30 -	42	2	---	R.YNYLKPRAEEERR.L

No match to: 721.4365, 725.4001, 726.4063, 728.3681, 730.3848, 731.4017, 732.3573, 734.4151, 735.4143, 741.4101, 742.4515, 744.4171, 745.4167, 746.4169, 747.4125, 750.4153, 752.4127, 755.4100, 756.4199, 757.4313, 761.4177, 767.4266, 768.3700, 768.3737, 770.3966, 772.4093, 775.4307, 779.4308, 784.3688, 786.4000, 787.4221, 788.4210, 792.4376, 795.4350, 798.4351, 800.4296, 812.4388, 815.4332, 822.4482, 825.4540, 832.4557, 835.4719, 837.4463, 842.5060, 844.4760, 854.4837, 856.4911, 860.4691, 862.4852, 869.4231, 885.4465, 905.4955, 907.4991, 908.4929, 913.5700, 913.5747, 918.5050, 919.5053, 922.5070, 928.5117, 934.5172, 940.4840, 942.4854, 943.4289, 943.5453, 946.5259, 950.4959, 951.5076, 954.5388, 959.5266, 966.5410, 969.5256, 972.5305, 974.5395, 976.5310, 978.5314, 979.5442, 991.5409, 993.5307, 1001.5854, 1003.5496, 1011.5262, 1014.5215, 1021.5579, 1023.5663, 1023.5700, 1031.5903, 1035.5381, 1036.5447, 1037.5573, 1039.5327, 1041.5658, 1044.5751, 1045.5729, 1046.5897, 1051.5448, 1056.5658, 1057.6078, 1067.5753, 1086.5509, 1092.5961, 1100.6071, 1106.5391, 1111.5615, 1113.5729, 1118.5818, 1120.6045, 1122.5912, 1123.5848, 1125.5823, 1132.6047, 1134.5714, 1139.5746, 1143.6318, 1144.6497, 1145.6407, 1147.6393, 1150.6108, 1155.6215, 1157.6199, 1158.6685, 1162.6182, 1163.6152, 1168.6215, 1173.6418, 1175.6089, 1178.6356, 1179.6157, 1181.6819, 1188.6544, 1197.6704, 1199.7006, 1208.6415, 1216.6714, 1227.6595, 1231.6387, 1232.6447, 1234.6672, 1235.6445, 1244.6453, 1247.6211, 1248.6174, 1250.6554, 1255.6096, 1256.6531, 1261.6592, 1262.6453, 1266.6777, 1273.6260, 1275.6362, 1276.6343, 1277.6531, 1301.7079, 1303.7076, 1307.7042, 1308.6376, 1310.6146, 1312.6418, 1315.7098, 1320.6659, 1322.6886, 1323.6771, 1324.6964, 1328.7089, 1331.7202, 1335.7318, 1338.6581, 1339.6637, 1349.7253, 1352.7205, 1356.7230, 1360.6638, 1363.7153, 1365.7379, 1367.7319, 1369.7043, 1377.6947, 1379.7268, 1383.7137, 1385.7534, 1388.7404, 1390.7284, 1393.7428, 1407.7563, 1417.7010, 1421.7061, 1422.7019, 1425.7727, 1427.7742, 1429.7365, 1431.7412, 1433.7228, 1434.7446, 1437.7584, 1461.7778, 1467.7521, 1472.7633, 1475.7632, 1477.7545, 1484.7889, 1488.7537, 1491.7965, 1493.7579, 1500.7737, 1505.8177, 1513.8079, 1515.8213, 1517.7854, 1519.7544, 1522.7811, 1523.7953, 1529.8099, 1530.7523, 1531.7472, 1537.7974, 1539.8005, 1542.8422, 1545.8091, 1549.7339, 1551.7778, 1553.8065, 1555.8101, 1556.8109, 1557.8010, 1561.8401, 1565.8199, 1571.8400, 1579.7939, 1580.8077, 1583.8444, 1587.7620, 1592.8066, 1594.8335, 1604.8323, 1605.8586, 1608.8497, 1615.8754, 1624.8062, 1628.7900, 1628.7921, 1633.7985, 1640.9384, 1642.8099, 1646.8297, 1649.8447, 1657.8101, 1667.8622, 1677.7988, 1680.8746, 1689.9465, 1694.7288, 1698.9225, 1699.8862, 1704.8773, 1706.8701, 1707.8302, 1710.9279, 1716.8671, 1719.9058, 1725.8300, 1725.8346, 1727.8986, 1730.8931, 1737.9006, 1751.8656, 1754.8580, 1765.8463, 1766.8040, 1767.8138, 1769.8741, 1770.3567, 1772.9315, 1774.7610, 1774.9131, 1777.8770, 1780.8777, 1783.9246, 1787.8188, 1787.8200, 1791.8137, 1793.9050, 1795.9353, 1798.9340, 1800.9336, 1804.9142, 1805.9069, 1809.8469, 1815.9044, 1821.9144, 1828.9227, 1837.9396, 1840.9827, 1843.9688, 1844.9012, 1846.9153, 1852.9364, 1856.9908, 1861.8881, 1867.9218, 1874.9695, 1880.9265, 1884.9417, 1891.9622, 1894.9313,

1896.9078, 1896.9100, 1900.9546, 1905.9313, 1906.9501, 1908.9043, 1911.9130, 1912.9154, 1916.9923, 1918.9587, 1921.9825, 1923.9718, 1925.9573, 1928.0469, 1932.0060, 1933.9950, 1937.9630, 1939.9740, 1944.0040, 1946.9679, 1949.9595, 1954.0236, 1955.9738, 1958.9556, 1960.0034, 1968.8600, 1973.9845, 1976.9861, 1978.9910, 1981.9854, 1983.9884, 1987.9784, 1995.9408, 1998.9894, 2000.9926, 2008.0460, 2012.9590, 2015.0104, 2017.9747, 2021.0170, 2026.0275, 2027.0256, 2031.0166, 2044.0123, 2047.4957, 2047.9515, 2052.0496, 2060.0520, 2062.0073, 2064.9880, 2066.9873, 2094.0376, 2097.0454, 2102.0667, 2103.1641, 2111.0305, 2127.0930, 2139.0630, 2144.0493, 2148.1023, 2155.0950, 2156.0796, 2160.0632, 2176.0664, 2190.9895, 2195.0774, 2199.0723, 2202.1624, 2203.1221, 2208.0862, 2211.1042, 2216.1062, 2225.1274, 2226.1272, 2229.0972, 2233.0984, 2239.0225, 2242.0435, 2268.1489, 2270.1287, 2271.1294, 2277.0823, 2284.1990, 2292.1426, 2309.0994, 2334.1719, 2342.9915, 2350.1746, 2383.9607, 2398.2400, 2400.0862, 2413.0549, 2414.1897, 2417.0959, 2497.2800, 2501.2637, 2511.1460, 2513.1421, 2528.1750, 2537.2012, 2541.1948, 2542.1836, 2556.2058, 2562.1487, 2612.2666, 2706.2109, 2717.0869, 2767.2666, 2888.2532, 3221.1733, 3224.2917, 3312.3306, 3916.9434

45. F1N1S3 **Mass:** 20635 **Score:** 19 **Expect:** 2.9e+002 **Matches:** 10

Uncharacterized protein OS=Bos taurus GN=LOC100294792 PE=4 SV=2

Observed	Mr(expt)	Mr(calc)	ppm	Start	End	Miss	Ions	Peptide
770.3966	769.3893	769.4010	-15.18	45 -	50	0	---	K.AEYFLK.W
772.4093	771.4020	771.3875	18.8	107 -	113	0	---	R.DAADKPR.G
922.5070	921.4997	921.4953	4.71	6 -	13	1	---	K.TTLQKMGK.K + Oxidation (M)
928.5117	927.5044	927.4886	17.1	106 -	113	1	---	K.RDAADKPR.G
1056.5658	1055.5585	1055.5836	-23.71	105 -	113	2	---	K.KRDAADKPR.G
1197.6704	1196.6631	1196.6626	0.48	107 -	117	1	---	R.DAADKPRGLVR.G
1322.6886	1321.6813	1321.7024	-15.92	2 -	13	2	---	M.ASNKTTLQKMGK.K + Oxidation (M)
1437.7584	1436.7511	1436.7479	2.23	1 -	13	2	---	-.MASNKTTLQKMGK.K
1646.8297	1645.8224	1645.8087	8.37	21 -	34	0	---	K.IVEEAEPEEFVVEK.V
2350.1746	2349.1673	2349.1708	-1.50	118 -	139	1	---	R.GLDPERIIGATDGSGELMFLMK.W

No match to: 721.4365, 725.4001, 726.4063, 728.3681, 730.3848, 731.4017, 732.3573, 734.4151, 735.4143, 741.4101, 742.4515, 744.4171, 745.4167, 746.4169, 747.4125, 750.4153, 752.4127, 755.4100, 756.4199, 757.4313, 761.4177, 767.4266, 768.3700, 768.3737, 775.4307, 779.4308, 784.3688, 786.4000, 787.4221, 788.4210, 792.4376, 795.4350, 798.4351, 800.4296, 812.4388, 815.4332, 822.4482, 825.4540, 831.4382, 832.4557, 835.4719, 837.4463, 842.5060, 844.4760, 854.4837, 856.4911, 860.4691, 862.4852, 869.4231, 885.4465, 905.4955, 907.4991, 908.4929, 913.5700, 913.5747, 916.5284, 918.5050, 919.5053, 934.5172, 940.4840, 942.4854, 943.4289, 943.5453, 946.5259, 950.4959, 951.5076, 953.5164, 954.5388, 959.5266, 966.5410, 969.5256, 972.5305, 974.5395, 976.5310, 978.5314, 979.5442, 991.5409, 993.5307, 1001.5854, 1003.5496, 1011.5262, 1014.5215, 1021.5579, 1023.5663, 1023.5700, 1031.5903, 1035.5381, 1036.5447, 1037.5573, 1039.5327, 1041.5658, 1044.5751, 1045.5729, 1046.5897, 1051.5448, 1057.6078, 1067.5753, 1073.5902, 1086.5509, 1092.5961, 1100.6071, 1106.5391, 1111.5615, 1113.5729, 1118.5818, 1120.6045, 1122.5912, 1123.5848, 1125.5823, 1132.6047, 1134.5714, 1139.5746, 1143.6318, 1144.6497, 1145.6407, 1147.6393, 1150.6108, 1155.6215, 1157.6199, 1158.6685, 1162.6182, 1163.6152, 1168.6215, 1173.6418, 1175.6089, 1178.6356, 1179.6157, 1181.6819, 1188.6544, 1199.7006, 1208.6415, 1216.6714, 1227.6595, 1231.6387, 1232.6447, 1234.6672, 1235.6445, 1244.6453, 1247.6211, 1248.6174, 1250.6554, 1255.6096, 1256.6531, 1261.6592, 1262.6453, 1266.6777, 1273.6260, 1275.6362, 1276.6343, 1277.6531, 1301.7079, 1303.7076, 1307.7042, 1308.6376, 1310.6146, 1312.6418, 1315.7098, 1320.6659, 1323.6771, 1324.6964, 1328.7089, 1331.7202, 1335.7318, 1338.6581, 1339.6637, 1349.7253, 1352.7205, 1356.7230, 1360.6638, 1363.7153, 1365.7379, 1367.7319, 1369.7043, 1377.6947, 1379.7268, 1383.7137, 1385.7534, 1388.7404, 1390.7284, 1393.7428, 1407.7563, 1417.7010, 1421.7061, 1422.7019, 1425.7727, 1427.7742, 1429.7365, 1431.7412, 1433.7228, 1434.7446, 1461.7778, 1467.7521, 1472.7633, 1475.7632, 1477.7545, 1484.7889, 1488.7537, 1491.7965, 1493.7579, 1500.7737, 1505.8177, 1513.8079, 1515.8213, 1517.7854, 1519.7544, 1522.7811, 1523.7953, 1529.8099, 1530.7523, 1531.7472, 1537.7974, 1539.8005, 1542.8422, 1545.8091, 1547.8051, 1549.7339, 1551.7778, 1553.8065, 1555.8101, 1556.8109, 1557.8010, 1561.8401, 1563.8109, 1565.8199, 1571.8400, 1579.7939, 1580.8077, 1583.8444, 1587.7620, 1592.8066, 1594.8335, 1604.8323, 1605.8586, 1608.8497, 1615.8754, 1624.8062, 1628.7900, 1628.7921, 1633.7985, 1640.9384, 1642.8099, 1649.8447, 1657.8101, 1667.8622, 1677.7988, 1680.8746, 1689.9465, 1694.7288, 1698.9225, 1699.8862, 1704.8773, 1706.8701, 1707.8302, 1710.9279, 1716.8671, 1719.9058, 1723.9011, 1725.8300, 1725.8346, 1727.8986, 1730.8931, 1737.9006, 1751.8656, 1754.8580, 1765.8463, 1766.8040, 1767.8138, 1769.8741, 1770.3567, 1772.9315, 1774.7610, 1774.9131, 1777.8770, 1780.8777, 1783.9246, 1787.8188, 1787.8200, 1791.8137, 1793.9050, 1795.9353, 1798.9340, 1800.9336, 1804.9142, 1805.9069, 1809.8469, 1815.9044, 1821.9144, 1828.9227, 1837.9396, 1840.9827, 1843.9688, 1844.9012, 1846.9153, 1852.9364, 1856.9908, 1861.8881, 1867.9218, 1874.9695, 1880.9265, 1884.9417, 1891.9622, 1894.9313, 1896.9078, 1896.9100, 1900.9546, 1905.9313, 1906.9501, 1908.9043, 1911.9130, 1912.9154, 1916.9923, 1918.9587, 1921.9825, 1923.9718, 1925.9573, 1928.0469, 1932.0060, 1933.9950, 1937.9630, 1939.9740, 1944.0040, 1946.9679, 1949.9595, 1954.0236, 1955.9738, 1958.9556, 1960.0034, 1968.8600, 1973.9845, 1976.9861, 1978.9910, 1981.9854, 1983.9884, 1987.9784, 1995.9408, 1998.9894, 2000.9926, 2008.0460, 2012.9590, 2015.0104, 2017.9747, 2021.0170, 2026.0275, 2027.0256, 2031.0166, 2044.0123, 2047.4957, 2047.9515, 2052.0496, 2060.0520, 2062.0073, 2064.9880, 2066.9873, 2094.0376, 2097.0454, 2102.0667, 2103.1641, 2111.0305, 2127.0930, 2139.0630, 2144.0493, 2148.1023, 2155.0950, 2156.0796, 2160.0632, 2176.0664, 2190.9895, 2195.0774, 2199.0723, 2202.1624, 2203.1221, 2208.0862, 2211.1042, 2216.1062, 2225.1274, 2226.1272, 2229.0972, 2233.0984, 2239.0225, 2242.0435, 2268.1489, 2270.1287, 2271.1294, 2277.0823, 2284.1990, 2292.1426, 2309.0994, 2334.1719, 2342.9915, 2383.9607, 2398.2400, 2400.0862, 2413.0549, 2414.1897, 2417.0959, 2497.2800, 2501.2637, 2511.1460, 2513.1421, 2528.1750, 2537.2012, 2541.1948, 2542.1836, 2556.2058, 2562.1487, 2612.2666, 2706.2109, 2717.0869, 2767.2666, 2888.2532, 3221.1733, 3224.2917, 3312.3306, 3916.9434

46. A5PK00 **Mass:** 44048 **Score:** 19 **Expect:** 2.9e+002 **Matches:** 18

Vacuolar protein-sorting-associated protein 36 OS=Bos taurus GN=VPS36 PE=2 SV=1

Observed	Mr(expt)	Mr(calc)	ppm	Start	End	Miss	Ions	Peptide
1199.7006	1198.6933	1198.7107	-14.52	293 -	302	1	---	K.MLEALKLPLR.L + Oxidation (M)
1356.7230	1355.7157	1355.7198	-2.97	35 -	46	0	---	K.FDPGTLLLSTHR.L
1363.7153	1362.7080	1362.7363	-20.75	345 -	356	1	---	K.LMGMSVLLAKER.L + Oxidation (M)
1379.7268	1378.7195	1378.7312	-8.48	345 -	356	1	---	K.LMGMSVLLAKER.L + 2 Oxidation (M)
1505.8177	1504.8104	1504.8357	-16.77	184 -	196	2	---	K.LMVKAKEMVELSK.S
1519.7544	1518.7471	1518.7162	20.4	204 -	216	1	---	K.DKQGDITEDETIR.F
1537.7974	1536.7901	1536.7970	-4.47	219 -	232	0	---	K.SYLLSMGIANPVTR.E + Oxidation (M)
1551.7778	1550.7705	1550.7576	8.30	206 -	218	1	---	K.QGDITEDETIRFK.S
1563.8109	1562.8036	1562.8337	-19.27	188 -	201	2	---	K.AKEMVELSKSIANK.I + Oxidation (M)
1605.8586	1604.8513	1604.8807	-18.30	190 -	203	2	---	K.EMVELSKSIANKIK.D + Oxidation (M)
1704.8773	1703.8700	1703.8553	8.66	305 -	319	0	---	R.VFDSGVMVIELQSHK.E + Oxidation (M)
1723.9011	1722.8938	1722.8862	4.43	173 -	187	1	---	K.NISEAFEDLSKLMVK.A
1725.8300	1724.8227	1724.8104	7.12	169 -	183	1	---	K.ETDKNISEAFEDLSK.L
1725.8346	1724.8273	1724.8104	9.79	169 -	183	1	---	K.ETDKNISEAFEDLSK.L
1918.9587	1917.9514	1917.9288	11.8	276 -	292	1	---	R.ARGMELLSPEDLVNACK.M + Oxidation (M)
2144.0493	2143.0420	2143.0905	-22.60	335 -	354	1	---	K.GSLTSEEFAKLMGMSVLLAK.E + 2 Oxidation (M)
2292.1426	2291.1353	2291.1229	5.41	131 -	150	1	---	R.WENMPVPQSLQTNRGPQPGR.I
2309.0994	2308.0921	2308.1018	-4.20	112 -	129	2	---	K.EHGQIEFYRRLSEEMTQR.R

No match to: 721.4365, 725.4001, 726.4063, 728.3681, 730.3848, 731.4017, 732.3573, 734.4151, 735.4143, 741.4101, 742.4515, 744.4171, 745.4167, 746.4169, 747.4125, 750.4153, 752.4127, 755.4100, 756.4199, 757.4313, 761.4177, 767.4266, 768.3700, 768.3737, 770.3966, 772.4093, 775.4307, 779.4308, 784.3688, 786.4000, 787.4221, 788.4210, 792.4376, 795.4350, 798.4351, 800.4296, 812.4388, 815.4332, 822.4482, 825.4540, 831.4382, 832.4557, 835.4719, 837.4463, 842.5060, 844.4760, 854.4837, 856.4911, 860.4691, 862.4852, 869.4231, 885.4465, 905.4955, 907.4991, 908.4929, 913.5700, 913.5747, 916.5284, 918.5050, 919.5053, 922.5070, 928.5117, 934.5172, 940.4840, 942.4854, 943.4289, 943.5453, 946.5259, 950.4959, 951.5076, 953.5164, 954.5388, 959.5266, 966.5410, 969.5256, 972.5305, 974.5395, 976.5310, 978.5314, 979.5442, 991.5409, 993.5307, 1001.5854, 1003.5496, 1011.5262, 1014.5215, 1021.5579, 1023.5663, 1023.5700, 1031.5903, 1035.5381, 1036.5447, 1037.5573, 1039.5327, 1041.5658, 1044.5751, 1045.5729, 1046.5897, 1051.5448, 1056.5658, 1057.6078, 1067.5753, 1073.5902, 1086.5509, 1092.5961, 1100.6071, 1106.5391, 1111.5615, 1113.5729, 1118.5818, 1120.6045, 1122.5912, 1123.5848, 1125.5823, 1132.6047, 1134.5714, 1139.5746, 1143.6318, 1144.6497, 1145.6407, 1147.6393, 1150.6108, 1155.6215, 1157.6199, 1158.6685, 1162.6182, 1163.6152, 1168.6215, 1173.6418, 1175.6089, 1178.6356, 1179.6157, 1181.6819, 1188.6544, 1197.6704, 1208.6415, 1216.6714, 1227.6595, 1231.6387, 1232.6447, 1234.6672, 1235.6445, 1244.6453, 1247.6211, 1248.6174, 1250.6554, 1255.6096, 1256.6531, 1261.6592, 1262.6453, 1266.6777, 1273.6260, 1275.6362, 1276.6343, 1277.6531, 1301.7079, 1303.7076, 1307.7042, 1308.6376, 1310.6146,

1312.6418, 1315.7098, 1320.6659, 1322.6886, 1323.6771, 1324.6964, 1328.7089, 1331.7202, 1335.7318, 1338.6581, 1339.6637, 1349.7253, 1352.7205, 1360.6638, 1365.7379, 1367.7319, 1369.7043, 1377.6947, 1383.7137, 1385.7534, 1388.7404, 1390.7284, 1393.7428, 1407.7563, 1417.7010, 1421.7061, 1422.7019, 1425.7727, 1427.7742, 1429.7365, 1431.7412, 1433.7228, 1434.7446, 1437.7584, 1461.7778, 1467.7521, 1472.7633, 1475.7632, 1477.7545, 1484.7889, 1488.7537, 1491.7965, 1493.7579, 1500.7737, 1513.8079, 1515.8213, 1517.7854, 1522.7811, 1523.7953, 1529.8099, 1530.7523, 1531.7472, 1539.8005, 1542.8422, 1545.8091, 1547.8051, 1549.7339, 1553.8065, 1555.8101, 1556.8109, 1557.8010, 1561.8401, 1565.8199, 1571.8400, 1579.7939, 1580.8077, 1583.8444, 1587.7620, 1592.8066, 1594.8335, 1604.8323, 1608.8497, 1615.8754, 1624.8062, 1628.7900, 1628.7921, 1633.7985, 1640.9384, 1642.8099, 1646.8297, 1649.8447, 1657.8101, 1667.8622, 1677.7988, 1680.8746, 1689.9465, 1694.7288, 1698.9225, 1699.8862, 1706.8701, 1707.8302, 1710.9279, 1716.8671, 1719.9058, 1727.8986, 1730.8931, 1737.9006, 1751.8656, 1754.8580, 1765.8463, 1766.8040, 1767.8138, 1769.8741, 1770.3567, 1772.9315, 1774.7610, 1774.9131, 1777.8770, 1780.8777, 1783.9246, 1787.8188, 1787.8200, 1791.8137, 1793.9050, 1795.9353, 1798.9340, 1800.9336, 1804.9142, 1805.9069, 1809.8469, 1815.9044, 1821.9144, 1828.9227, 1837.9396, 1840.9827, 1843.9688, 1844.9012, 1846.9153, 1852.9364, 1856.9908, 1861.8881, 1867.9218, 1874.9695, 1880.9265, 1884.9417, 1891.9622, 1894.9313, 1896.9078, 1896.9100, 1900.9546, 1905.9313, 1906.9501, 1908.9043, 1911.9130, 1912.9154, 1916.9923, 1921.9825, 1923.9718, 1925.9573, 1928.0469, 1932.0060, 1933.9950, 1937.9630, 1939.9740, 1944.0040, 1946.9679, 1949.9595, 1954.0236, 1955.9738, 1958.9556, 1960.0034, 1968.8600, 1973.9845, 1976.9861, 1978.9910, 1981.9854, 1983.9884, 1987.9784, 1995.9408, 1998.9894, 2000.9926, 2008.0460, 2012.9590, 2015.0104, 2017.9747, 2021.0170, 2026.0275, 2027.0256, 2031.0166, 2044.0123, 2047.4957, 2047.9515, 2052.0496, 2060.0520, 2062.0073, 2064.9880, 2066.9873, 2094.0376, 2097.0454, 2102.0667, 2103.1641, 2111.0305, 2127.0930, 2139.0630, 2148.1023, 2155.0950, 2156.0796, 2160.0632, 2176.0664, 2190.9895, 2195.0774, 2199.0723, 2202.1624, 2203.1221, 2208.0862, 2211.1042, 2216.1062, 2225.1274, 2226.1272, 2229.0972, 2233.0984, 2239.0225, 2242.0435, 2268.1489, 2270.1287, 2271.1294, 2277.0823, 2284.1990, 2334.1719, 2342.9915, 2350.1746, 2383.9607, 2398.2400, 2400.0862, 2413.0549, 2414.1897, 2417.0959, 2497.2800, 2501.2637, 2511.1460, 2513.1421, 2528.1750, 2537.2012, 2541.1948, 2542.1836, 2556.2058, 2562.1487, 2612.2666, 2706.2109, 2717.0869, 2767.2666, 2888.2532, 3221.1733, 3224.2917, 3312.3306, 3916.9434

47. A0JNH6 **Mass:** 63280 **Score:** 19 **Expect:** 3e+002 **Matches:** 24

Coiled-coil domain-containing protein 102A OS=Bos taurus GN=CCDC102A PE=2 SV=1

Observed	Mr(expt)	Mr(calc)	ppm	Start	End	Miss	Ions	Peptide
747.4125	746.4052	746.4109	-7.57	344	349	1	---	R.KMAELR.G
768.3700	767.3627	767.3701	-9.61	299	304	0	---	K.YEELSK.T
768.3737	767.3664	767.3701	-4.85	299	304	0	---	K.YEELSK.T
787.4221	786.4148	786.4097	6.60	162	169	1	---	R.GARGGVDR.T
953.5164	952.5091	952.4978	11.9	181	188	0	---	R.EQEPVPVR.D
972.5305	971.5232	971.5287	-5.65	9	17	0	---	R.LAESPQLSK.G
1031.5903	1030.5830	1030.5672	15.4	108	115	2	---	K.WSKVRAER.N
1057.6078	1056.6005	1056.5788	20.6	135	143	2	---	K.ELAGARRER.Q
1308.6376	1307.6303	1307.6438	-10.29	85	95	2	---	R.ARAAQMEKTMR.W + Oxidation (M)
1323.6771	1322.6698	1322.6434	19.9	339	349	2	---	R.SSMDRKMAELR.G
1339.6637	1338.6564	1338.6384	13.5	339	349	2	---	R.SSMDRKMAELR.G + Oxidation (M)
1352.7205	1351.7132	1351.7208	-5.59	450	460	2	---	R.RVEQHEAEVKK.L
1427.7742	1426.7669	1426.7528	9.89	385	397	1	---	R.AQVGDLEEALARR.R
1433.7228	1432.7155	1432.7344	-13.15	276	287	2	---	R.EDKMALSRNIEK.L
1542.8422	1541.8349	1541.8161	12.2	73	84	2	---	R.EELRLRELEEAR.A
1583.8444	1582.8371	1582.8539	-10.61	385	398	2	---	R.AQVGDLEEALARRR.R
1642.8099	1641.8026	1641.8283	-15.65	299	311	2	---	K.YEELSKTKQEMLK.Q + Oxidation (M)
1821.9144	1820.9071	1820.9203	-7.23	1	17	1	---	-.MSHGPSPRLAESPQLSK.G
1837.9396	1836.9323	1836.9152	9.32	1	17	1	---	-.MSHGPSPRLAESPQLSK.G + Oxidation (M)
1995.9408	1994.9335	1994.9406	-3.55	162	180	2	---	R.GARGGVDRTPDGPETEPER.E
2066.9873	2065.9800	2065.9664	6.59	471	488	1	---	K.ELAQAEDELDEAHNQARK.L
2148.1023	2147.0950	2147.1044	-4.38	279	296	2	---	K.MALSRNIEKLEGELSQWK.I + Oxidation (M)
2195.0774	2194.0701	2194.0688	0.60	400	419	1	---	R.QTASALDCDLRASQAALFEK.N
2400.0862	2399.0789	2399.0659	5.43	318	338	1	---	K.EAHQDELGRMSEDLEDELGAR.S

No match to: 721.4365, 725.4001, 726.4063, 728.3681, 730.3848, 731.4017, 732.3573, 734.4151, 735.4143, 741.4101, 742.4515, 744.4171, 745.4167, 746.4169, 750.4153, 752.4127, 755.4100, 756.4199, 757.4313, 761.4177, 767.4266, 770.3966, 772.4093, 775.4307, 779.4308, 784.3688, 786.4000, 788.4210, 792.4376, 795.4350, 798.4351, 800.4296, 812.4388, 815.4332, 822.4482, 825.4540, 831.4382, 832.4557, 835.4719, 837.4463, 842.5060, 844.4760, 854.4837, 856.4911, 860.4691, 862.4852, 869.4231, 885.4465, 905.4955, 907.4991, 908.4929, 913.5700, 913.5747, 916.5284, 918.5050, 919.5053, 922.5070, 928.5117, 934.5172, 940.4840, 942.4854, 943.4289, 943.5453, 946.5259, 950.4959, 951.5076, 954.5388, 959.5266, 966.5410, 969.5256, 974.5395, 976.5310, 978.5314, 979.5442, 991.5409, 993.5307, 1001.5854, 1003.5496, 1011.5262, 1014.5215, 1021.5579, 1023.5663, 1023.5700, 1035.5381, 1036.5447, 1037.5573, 1039.5327, 1041.5658, 1044.5751, 1045.5729, 1046.5897, 1051.5448, 1056.5658, 1067.5753, 1073.5902, 1086.5509, 1092.5961, 1100.6071, 1106.5391, 1111.5615, 1113.5729, 1118.5818, 1120.6045, 1122.5912, 1123.5848, 1125.5823, 1132.6047, 1134.5714, 1139.5746, 1143.6318, 1144.6497, 1145.6407, 1147.6393, 1150.6108, 1155.6215, 1157.6199, 1158.6685, 1162.6182, 1163.6152, 1168.6215, 1173.6418, 1175.6089, 1178.6356, 1179.6157, 1181.6819, 1188.6544, 1197.6704, 1199.7006, 1208.6415, 1216.6714, 1227.6595, 1231.6387, 1232.6447, 1234.6672, 1235.6445, 1244.6453, 1247.6211, 1248.6174, 1250.6554, 1255.6096, 1256.6531, 1261.6592, 1262.6453, 1266.6777, 1273.6260, 1275.6362, 1276.6343, 1277.6531, 1301.7079, 1303.7076, 1307.7042, 1310.6146, 1312.6418, 1315.7098, 1320.6659, 1322.6886, 1324.6964, 1328.7089, 1331.7202, 1335.7318, 1338.6581, 1349.7253, 1356.7230, 1360.6638, 1363.7153, 1365.7379, 1367.7319, 1369.7043, 1377.6947, 1379.7268, 1383.7137, 1385.7534, 1388.7404, 1390.7284, 1393.7428, 1407.7563, 1417.7010, 1421.7061, 1422.7019, 1425.7727, 1429.7365, 1431.7412, 1434.7446, 1437.7584, 1461.7778, 1467.7521, 1472.7633, 1475.7632, 1477.7545, 1484.7889, 1488.7537, 1491.7965, 1493.7579, 1500.7737, 1505.8177, 1513.8079, 1515.8213, 1517.7854, 1519.7544, 1522.7811, 1523.7953, 1529.8099, 1530.7523, 1531.7472, 1537.7974, 1539.8005, 1545.8091, 1547.8051, 1549.7339, 1551.7778, 1553.8065, 1555.8101, 1556.8109, 1557.8010, 1561.8401, 1563.8109, 1565.8199, 1571.8400, 1579.7939, 1580.8077, 1587.7620, 1592.8066, 1594.8335, 1604.8323, 1605.8586, 1608.8497, 1615.8754, 1624.8062, 1628.7900, 1628.7921, 1633.7985, 1640.9384, 1646.8297, 1649.8447, 1657.8101, 1667.8622, 1677.7988, 1680.8746, 1689.9465, 1694.7288, 1698.9225, 1699.8862, 1704.8773, 1706.8701, 1707.8302, 1710.9279, 1716.8671, 1719.9058, 1723.9011, 1725.8300, 1725.8346, 1727.8986, 1730.8931, 1737.9006, 1751.8656, 1754.8580, 1765.8463, 1766.8040, 1767.8138, 1769.8741, 1770.3567, 1772.9315, 1774.7610, 1774.9131, 1777.8770, 1780.8777, 1783.9246, 1787.8188, 1787.8200, 1791.8137, 1793.9050, 1795.9353, 1798.9340, 1800.9336, 1804.9142, 1805.9069, 1809.8469, 1815.9044, 1828.9227, 1840.9827, 1843.9688, 1844.9012, 1846.9153, 1852.9364, 1856.9908, 1861.8881, 1867.9218, 1874.9695, 1880.9265, 1884.9417, 1891.9622, 1894.9313, 1896.9078, 1896.9100, 1900.9546, 1905.9313, 1906.9501, 1908.9043, 1911.9130, 1912.9154, 1916.9923, 1918.9587, 1921.9825, 1923.9718, 1925.9573, 1928.0469, 1932.0060, 1933.9950, 1937.9630, 1939.9740, 1944.0040, 1946.9679, 1949.9595, 1954.0236, 1955.9738, 1958.9556, 1960.0034, 1968.8600, 1973.9845, 1976.9861, 1978.9910, 1981.9854, 1983.9884, 1987.9784, 1998.9894, 2000.9926, 2008.0460, 2012.9590, 2015.0104, 2017.9747, 2021.0170, 2026.0275, 2027.0256, 2031.0166, 2044.0123, 2047.4957, 2047.9515, 2052.0496, 2060.0520, 2062.0073, 2064.9880, 2094.0376, 2097.0454, 2102.0667, 2103.1641, 2111.0305, 2127.0930, 2139.0630, 2144.0493, 2155.0950, 2156.0796, 2160.0632, 2176.0664, 2190.9895, 2199.0723, 2202.1624, 2203.1221, 2208.0862, 2211.1042, 2216.1062, 2225.1274, 2226.1272, 2229.0972, 2233.0984, 2239.0225, 2242.0435, 2268.1489, 2270.1287, 2271.1294, 2277.0823, 2284.1990, 2292.1426, 2309.0994, 2334.1719, 2342.9915, 2350.1746, 2383.9607, 2398.2400, 2413.0549, 2414.1897, 2417.0959, 2497.2800, 2501.2637, 2511.1460, 2513.1421, 2528.1750, 2537.2012, 2541.1948, 2542.1836, 2556.2058, 2562.1487, 2612.2666, 2706.2109, 2717.0869, 2767.2666, 2888.2532, 3221.1733, 3224.2917, 3312.3306, 3916.9434

48. G3MYC1 **Mass:** 17221 **Score:** 19 **Expect:** 3e+002 **Matches:** 9

Uncharacterized protein OS=Bos taurus PE=3 SV=1

Observed	Mr(expt)	Mr(calc)	ppm	Start	End	Miss	Ions	Peptide
747.4125	746.4052	746.4109	-7.60	46	51	1	---	R.SMPIRK.D + Oxidation (M)
752.4127	751.4054	751.4228	-23.11	131	136	2	---	K.EKGKYK.E
862.4852	861.4779	861.4630	17.3	29	36	0	---	K.IMSSPLSK.E
1039.5327	1038.5254	1038.5233	2.05	135	142	1	---	K.YKEETIEK.M
1057.6078	1056.6005	1056.6080	-7.06	77	84	1	---	K.KHVIYIER.V
1234.6672	1233.6599	1233.6479	9.74	18	27	1	---	R.HFNAPSHIRR.K
1390.7284	1389.7211	1389.7490	-20.06	17	27	2	---	K.RHFNAPSHIRR.K

1407.7563	1406.7490	1406.7452	2.70	40 - 50	2	---	R.QKYNVRSMPIR.K + Oxidation (M)
1555.8101	1554.8028	1554.7977	3.32	1 - 13	2	---	-.MKFNPFVTSNRSK.N

No match to: 721.4365, 725.4001, 726.4063, 728.3681, 730.3848, 731.4017, 732.3573, 734.4151, 735.4143, 741.4101, 742.4515, 744.4171, 745.4167, 746.4169, 750.4153, 755.4100, 756.4199, 757.4313, 761.4177, 767.4266, 768.3700, 768.3737, 770.3966, 772.4093, 775.4307, 779.4308, 784.3688, 786.4000, 787.4221, 788.4210, 792.4376, 795.4350, 798.4351, 800.4296, 812.4388, 815.4332, 822.4482, 825.4540, 831.4382, 832.4557, 835.4719, 837.4463, 842.5060, 844.4760, 854.4837, 856.4911, 860.4691, 869.4231, 885.4465, 905.4955, 907.4991, 908.4929, 913.5700, 913.5747, 916.5284, 918.5050, 919.5053, 922.5070, 928.5117, 934.5172, 940.4840, 942.4854, 943.4289, 943.5453, 946.5259, 950.4959, 951.5076, 953.5164, 954.5388, 959.5266, 966.5410, 969.5256, 972.5305, 974.5395, 976.5310, 978.5314, 979.5442, 991.5409, 993.5307, 1001.5854, 1003.5496, 1011.5262, 1014.5215, 1021.5579, 1023.5663, 1023.5700, 1031.5903, 1035.5381, 1036.5447, 1037.5573, 1041.5658, 1044.5751, 1045.5729, 1046.5897, 1051.5448, 1056.5658, 1067.5753, 1073.5902, 1086.5509, 1092.5961, 1100.6071, 1106.5391, 1111.5615, 1113.5729, 1118.5818, 1120.6045, 1122.5912, 1123.5848, 1125.5823, 1132.6047, 1134.5714, 1139.5746, 1143.6318, 1144.6497, 1145.6407, 1147.6393, 1150.6108, 1155.6215, 1157.6199, 1158.6685, 1162.6182, 1163.6152, 1168.6215, 1173.6418, 1175.6089, 1178.6356, 1179.6157, 1181.6819, 1188.6544, 1197.6704, 1199.7006, 1208.6415, 1216.6714, 1227.6595, 1231.6387, 1232.6447, 1235.6445, 1244.6453, 1247.6211, 1248.6174, 1250.6554, 1255.6096, 1256.6531, 1261.6592, 1262.6453, 1266.6777, 1273.6260, 1275.6362, 1276.6343, 1277.6531, 1301.7079, 1303.7076, 1307.7042, 1308.6376, 1310.6146, 1312.6418, 1315.7098, 1320.6659, 1322.6886, 1323.6771, 1324.6964, 1328.7089, 1331.7202, 1335.7318, 1338.6581, 1339.6637, 1349.7253, 1352.7205, 1356.7230, 1360.6638, 1363.7153, 1365.7379, 1367.7319, 1369.7043, 1377.6947, 1379.7268, 1383.7137, 1385.7534, 1388.7404, 1393.7428, 1417.7010, 1421.7061, 1422.7019, 1425.7727, 1427.7742, 1429.7365, 1431.7412, 1433.7228, 1434.7446, 1437.7584, 1461.7778, 1467.7521, 1472.7633, 1475.7632, 1477.7545, 1484.7889, 1488.7537, 1491.7965, 1493.7579, 1500.7737, 1505.8177, 1513.8079, 1515.8213, 1517.7854, 1519.7544, 1522.7811, 1523.7953, 1529.8099, 1530.7523, 1531.7472, 1537.7974, 1539.8005, 1542.8422, 1545.8091, 1547.8051, 1549.7339, 1551.7778, 1553.8065, 1556.8109, 1557.8010, 1561.8401, 1563.8109, 1565.8199, 1571.8400, 1579.7939, 1580.8077, 1583.8444, 1587.7620, 1592.8066, 1594.8335, 1604.8323, 1605.8586, 1608.8497, 1615.8754, 1624.8062, 1628.7900, 1628.7921, 1633.7985, 1640.9384, 1642.8099, 1646.8297, 1649.8447, 1657.8101, 1667.8622, 1677.7988, 1680.8746, 1689.9465, 1694.7288, 1698.9225, 1699.8862, 1704.8773, 1706.8701, 1707.8302, 1710.9279, 1716.8671, 1719.9058, 1723.9011, 1725.8300, 1725.8346, 1727.8986, 1730.8931, 1737.9006, 1751.8656, 1754.8580, 1765.8463, 1766.8040, 1767.8138, 1769.8741, 1770.3567, 1772.9315, 1774.7610, 1774.9131, 1777.8770, 1780.8777, 1783.9246, 1787.8188, 1787.8200, 1791.8137, 1793.9050, 1795.9353, 1798.9340, 1800.9336, 1804.9142, 1805.9069, 1809.8469, 1815.9044, 1821.9144, 1828.9227, 1837.9396, 1840.9827, 1843.9688, 1844.9012, 1846.9153, 1852.9364, 1856.9908, 1861.8881, 1867.9218, 1874.9695, 1880.9265, 1884.9417, 1891.9622, 1894.9313, 1896.9078, 1896.9100, 1900.9546, 1905.9313, 1906.9501, 1908.9043, 1911.9130, 1912.9154, 1916.9923, 1918.9587, 1921.9825, 1923.9718, 1925.9573, 1928.0469, 1932.0060, 1933.9950, 1937.9630, 1939.9740, 1944.0040, 1946.9679, 1949.9595, 1954.0236, 1955.9738, 1958.9556, 1960.0034, 1968.8600, 1973.9845, 1976.9861, 1978.9910, 1981.9854, 1983.9884, 1987.9784, 1995.9408, 1998.9894, 2000.9926, 2008.0460, 2012.9590, 2015.0104, 2017.9747, 2021.0170, 2026.0275, 2027.0256, 2031.0166, 2044.0123, 2047.4957, 2047.9515, 2052.0496, 2060.0520, 2062.0073, 2064.9880, 2066.9873, 2094.0376, 2097.0454, 2102.0667, 2103.1641, 2111.0305, 2127.0930, 2139.0630, 2144.0493, 2148.1023, 2155.0950, 2156.0796, 2160.0632, 2176.0664, 2190.9895, 2195.0774, 2199.0723, 2202.1624, 2203.1221, 2208.0862, 2211.1042, 2216.1062, 2225.1274, 2226.1272, 2229.0972, 2233.0984, 2239.0225, 2242.0435, 2268.1489, 2270.1287, 2271.1294, 2277.0823, 2284.1990, 2292.1426, 2309.0994, 2334.1719, 2342.9915, 2350.1746, 2383.9607, 2398.2400, 2400.0862, 2413.0549, 2414.1897, 2417.0959, 2497.2800, 2501.2637, 2511.1460, 2513.1421, 2528.1750, 2537.2012, 2541.1948, 2542.1836, 2556.2058, 2562.1487, 2612.2666, 2706.2109, 2717.0869, 2767.2666, 2888.2532, 3221.1733, 3224.2917, 3312.3306, 3916.9434

49. F1MYD0 **Mass:** 90289 **Score:** 19 **Expect:** 3e+002 **Matches:** 28

Uncharacterized protein OS=Bos taurus GN=CUL1 PE=3 SV=2

Observed	Mr(expt)	Mr(calc)	ppm	Start - End	Miss	Ions	Peptide
756.4199	755.4126	755.4218	-12.14	671 - 676	0	---	K.LYLGYK.N
787.4221	786.4148	786.4235	-11.06	268 - 273	0	---	R.LLEEQR.R
943.5453	942.5381	942.5246	14.3	268 - 274	1	---	R.LLEEQRR.V
972.5305	971.5232	971.5287	-5.67	329 - 337	0	---	R.IQDGLGELK.K
1003.5496	1002.5423	1002.5419	0.38	752 - 759	0	---	K.CIDILIEK.E
1035.5381	1034.5308	1034.5509	-19.39	26 - 34	0	---	R.AGIQQVYTR.Q
1100.6071	1099.5998	1099.6237	-21.72	329 - 338	1	---	R.IQDGLGELKK.L
1143.6318	1142.6245	1142.6270	-2.17	465 - 473	2	---	K.FYAKMLAKR.L + Oxidation (M)
1162.6182	1161.6109	1161.5965	12.5	169 - 177	0	---	R.DCLFRPLNK.Q
1199.7006	1198.6933	1198.6856	6.45	680 - 689	1	---	K.LRVNINVPMK.T + Oxidation (M)
1324.6964	1323.6891	1323.6643	18.7	2 - 13	1	---	M.SSNRSQNPHGLK.Q
1328.7089	1327.7016	1327.6806	15.9	425 - 435	2	---	R.YCDSLLKKSSK.N
1437.7584	1436.7511	1436.7446	4.55	504 - 515	1	---	K.LQRMFQDIGVSK.D + Oxidation (M)
1580.8077	1579.8004	1579.8140	-8.62	26 - 39	1	---	R.AGIQQVYTRQSMAK.S
1587.7620	1586.7547	1586.7511	2.28	494 - 506	1	---	K.QACGFEYTSKLQR.M
1604.8323	1603.8250	1603.8432	-11.34	460 - 472	2	---	K.DVFQKFYAKMLAK.R + Oxidation (M)
1640.9384	1639.9311	1639.9621	-18.87	178 - 191	2	---	K.QVTNAVLKLIEKER.N
1793.9050	1792.8977	1792.9036	-3.26	455 - 468	2	---	K.YIEDKDVFQKFYAK.M
1828.9227	1827.9154	1827.9301	-8.05	492 - 506	2	---	K.LKQACGFEYTSKLQR.M
1884.9417	1883.9344	1883.8973	19.7	694 - 708	2	---	K.QEQETTHKNIEEDRK.L
1944.0040	1942.9967	1942.9861	5.48	274 - 289	1	---	R.RVQVYLHESTQDELAR.K
2008.0460	2007.0387	2007.0789	-20.03	72 - 90	2	---	K.SKKGQTPGGAQFVGLELYK.R
2027.0256	2026.0183	2026.0193	-0.51	507 - 523	2	---	R.MFQDIGVSKDLNEQFKK.H
2144.0493	2143.0420	2143.0579	-7.40	474 - 493	1	---	R.LVHQNSASDDAEASMISKLK.Q
2160.0632	2159.0559	2159.0528	1.44	474 - 493	1	---	R.LVHQNSASDDAEASMISKLK.Q + Oxidation (M)
2398.2400	2397.2327	2397.1958	15.4	682 - 701	2	---	R.VNINVPMKTEQKQEQETTHK.N + Oxidation (M)
2511.1460	2510.1387	2510.1788	-15.95	243 - 262	1	---	R.FYTRESTEFLQQNPVTEYMK.K
2767.2666	2766.2593	2766.2119	17.1	106 - 127	1	---	K.DGEDLMDESVLKFYTQQWEDYR.F

No match to: 721.4365, 725.4001, 726.4063, 728.3681, 730.3848, 731.4017, 732.3573, 734.4151, 735.4143, 741.4101, 742.4515, 744.4171, 745.4167, 746.4169, 747.4125, 750.4153, 752.4127, 755.4100, 757.4313, 761.4177, 767.4266, 768.3700, 768.3737, 770.3966, 772.4093, 775.4307, 779.4308, 784.3688, 786.4000, 788.4210, 792.4376, 795.4350, 798.4351, 800.4296, 812.4388, 815.4332, 822.4482, 825.4540, 831.4382, 832.4557, 835.4719, 837.4463, 842.5060, 844.4760, 854.4837, 856.4911, 860.4691, 862.4852, 869.4231, 885.4465, 905.4955, 907.4991, 908.4929, 913.5700, 913.5747, 916.5284, 918.5050, 919.5053, 922.5070, 928.5117, 934.5172, 940.4840, 942.4854, 943.4289, 946.5259, 950.4959, 951.5076, 953.5164, 954.5388, 959.5266, 966.5410, 969.5256, 974.5395, 976.5310, 978.5314, 979.5442, 991.5409, 993.5307, 1001.5854, 1011.5262, 1014.5215, 1021.5579, 1023.5663, 1023.5700, 1031.5903, 1036.5447, 1037.5573, 1039.5327, 1041.5658, 1044.5751, 1045.5729, 1046.5897, 1051.5448, 1056.5658, 1057.6078, 1067.5753, 1073.5902, 1086.5509, 1092.5961, 1106.5391, 1111.5615, 1113.5729, 1118.5818, 1120.6045, 1122.5912, 1123.5848, 1125.5823, 1132.6047, 1134.5714, 1139.5746, 1144.6497, 1145.6407, 1147.6393, 1150.6108, 1155.6215, 1157.6199, 1158.6685, 1163.6152, 1168.6215, 1173.6418, 1175.6089, 1178.6356, 1179.6157, 1181.6819, 1188.6544, 1197.6704, 1208.6415, 1216.6714, 1227.6595, 1231.6387, 1232.6447, 1234.6672, 1235.6445, 1244.6453, 1247.6211, 1248.6174, 1250.6554, 1255.6096, 1256.6531, 1261.6592, 1262.6453, 1266.6777, 1273.6260, 1275.6362, 1276.6343, 1277.6531, 1301.7079, 1303.7076, 1307.7042, 1308.6376, 1310.6146, 1312.6418, 1315.7098, 1320.6659, 1322.6886, 1323.6771, 1331.7202, 1335.7318, 1338.6581, 1339.6637, 1349.7253, 1352.7205, 1356.7230, 1360.6638, 1363.7153, 1365.7379, 1367.7319, 1369.7043, 1377.6947, 1379.7268, 1383.7137, 1385.7534, 1388.7404, 1390.7284, 1393.7428, 1407.7563, 1417.7010, 1421.7061, 1422.7019, 1425.7727, 1427.7742, 1429.7365, 1431.7412, 1433.7228, 1434.7446, 1461.7778, 1467.7521, 1472.7633, 1475.7632, 1477.7545, 1484.7889, 1488.7537, 1491.7965, 1493.7579, 1500.7737, 1505.8177, 1513.8079, 1515.8213, 1517.7854, 1519.7544, 1522.7811, 1523.7953, 1529.8099, 1530.7523, 1531.7472, 1537.7974, 1539.8005, 1542.8422, 1545.8091, 1547.8051, 1549.7339, 1551.7778, 1553.8065, 1555.8101, 1556.8109, 1557.8010, 1561.8401, 1563.8109, 1565.8199, 1571.8400, 1579.7939, 1583.8444, 1592.8066, 1594.8335, 1605.8586, 1608.8497, 1615.8754, 1624.8062, 1628.7900, 1628.7921, 1633.7985, 1642.8099, 1646.8297, 1649.8447, 1657.8101, 1667.8622, 1677.7988, 1680.8746, 1689.9465, 1694.7288, 1698.9225, 1699.8862, 1704.8773, 1706.8701, 1707.8302, 1710.9279, 1716.8671, 1719.9058, 1723.9011, 1725.8300, 1725.8346, 1727.8986, 1730.8931, 1737.9006, 1751.8656, 1754.8580, 1765.8463, 1766.8040, 1767.8138, 1769.8741, 1770.3567, 1772.9315, 1774.7610, 1774.9131, 1777.8770, 1780.8777, 1783.9246, 1787.8188, 1787.8200, 1791.8137, 1795.9353, 1798.9340, 1800.9336, 1804.9142, 1805.9069, 1809.8469, 1815.9044, 1821.9144, 1837.9396, 1840.9827, 1843.9688, 1844.9012, 1846.9153, 1852.9364, 1856.9908, 1861.8881, 1867.9218, 1874.9695, 1880.9265, 1891.9622, 1894.9313, 1896.9078, 1896.9100, 1900.9546, 1905.9313, 1906.9501, 1908.9043, 1911.9130, 1912.9154, 1916.9923, 1918.9587, 1921.9825, 1923.9718, 1925.9573,

1928.0469, 1932.0060, 1933.9950, 1937.9630, 1939.9740, 1946.9679, 1949.9595, 1954.0236, 1955.9738, 1958.9556, 1960.0034, 1968.8600, 1973.9845, 1976.9861, 1978.9910, 1981.9854, 1983.9884, 1987.9784, 1995.9408, 1998.9894, 2000.9926, 2012.9590, 2015.0104, 2017.9747, 2021.0170, 2026.0275, 2031.0166, 2044.0123, 2047.4957, 2047.9515, 2052.0496, 2060.0520, 2062.0073, 2064.9880, 2066.9873, 2094.0376, 2097.0454, 2102.0667, 2103.1641, 2111.0305, 2127.0930, 2139.0630, 2148.1023, 2155.0950, 2156.0796, 2176.0664, 2190.9895, 2195.0774, 2199.0723, 2202.1624, 2203.1221, 2208.0862, 2211.1042, 2216.1062, 2225.1274, 2226.1272, 2229.0972, 2233.0984, 2239.0225, 2242.0435, 2268.1489, 2270.1287, 2271.1294, 2277.0823, 2284.1990, 2292.1426, 2309.0994, 2334.1719, 2342.9915, 2350.1746, 2383.9607, 2400.0862, 2413.0549, 2414.1897, 2417.0959, 2497.2800, 2501.2637, 2513.1421, 2528.1750, 2537.2012, 2541.1948, 2542.1836, 2556.2058, 2562.1487, 2612.2666, 2706.2109, 2717.0869, 2888.2532, 3221.1733, 3224.2917, 3312.3306, 3916.9434

50. F1MHT3 **Mass:** 217593 **Score:** 19 **Expect:** 3e+002 **Matches:** 55

Uncharacterized protein OS=Bos taurus GN=MYO5A PE=4 SV=2

Observed	Mr(expt)	Mr(calc)	ppm	Start	End	Miss	Ions	Peptide
726.4063	725.3990	725.3894	13.2	836	840	1	---	R.YKIMR.T + Oxidation (M)
732.3573	731.3500	731.3450	6.91	938	943	0	---	K.VDEQNK.D
746.4169	745.4096	745.4082	1.80	1422	1427	1	---	K.QDKTVR.K
767.4266	766.4194	766.4086	14.1	1041	1046	0	---	K.EVLNHR.I
792.4376	791.4303	791.4258	5.75	900	905	2	---	R.RMMAKR.E
795.4350	794.4277	794.4221	7.07	829	834	1	---	R.MYVARR.R
825.4540	824.4467	824.4578	-13.49	710	715	1	---	R.YRVLMK.Q + Oxidation (M)
832.4557	831.4485	831.4450	4.15	718	724	1	---	K.DVLSDRK.Q
885.4465	884.4393	884.4392	0.05	741	747	1	---	K.DKYQFGK.T
905.4955	904.4883	904.4767	12.8	692	700	0	---	R.ISAAGFPSR.W
916.5284	915.5211	915.5324	-12.32	931	937	1	---	K.IMQLQRK.V
951.5076	950.5004	950.5232	-24.04	829	835	2	---	R.MYVARRR.Y
959.5266	958.5193	958.5196	-0.24	912	919	1	---	K.IEARSVER.Y
972.5305	971.5232	971.5301	-7.06	633	640	1	---	K.KTVGHQFR.N
978.5314	977.5241	977.5182	6.06	754	762	0	---	R.AGQVAYLEK.L
1056.5658	1055.5585	1055.5359	21.4	1192	1199	0	---	K.EEERPQIR.G
1106.5391	1105.5318	1105.5404	-7.73	249	257	0	---	R.VVFQAEEER.N
1113.5729	1112.5656	1112.5913	-23.07	809	816	2	---	R.CYAKFLRR.T
1134.5714	1133.5641	1133.5677	-3.12	598	609	0	---	K.AISPTSATSSGR.T
1162.6182	1161.6109	1161.6393	-24.46	1308	1318	0	---	K.GEIAQAYIGLK.E
1173.6418	1172.6345	1172.6401	-4.73	1323	1332	0	---	R.LLESQLQSQK.R
1178.6356	1177.6283	1177.6417	-11.34	1150	1159	0	---	K.VPLDMSLFLK.L + Oxidation (M)
1188.6544	1187.6471	1187.6557	-7.21	1807	1815	2	---	R.TIQMRLRDR.K
1208.6415	1207.6342	1207.6422	-6.56	622	632	1	---	K.GRPGQTAKEHK.K
1231.6387	1230.6314	1230.6455	-11.47	1031	1040	1	---	K.EENTLLKQEK.E
1247.6211	1246.6138	1246.5942	15.8	1010	1019	1	---	K.SIEEHADRYK.Q
1250.6554	1249.6481	1249.6778	-23.78	912	921	2	---	K.IEARSVERYK.K
1261.6592	1260.6519	1260.6384	10.8	1816	1826	1	---	R.KDSPQLLMDAK.H + Oxidation (M)
1320.6659	1319.6586	1319.6873	-21.77	1401	1410	1	---	K.YQSYRISLYK.R
1338.6581	1337.6508	1337.6775	-19.93	877	886	2	---	R.GWLARTCYRR.S
1417.7010	1416.6937	1416.7170	-16.44	503	514	0	---	K.LGILDLLDEECK.M
1421.7061	1420.6988	1420.7054	-4.62	1411	1421	1	---	K.RMIDLMEQLEK.Q + Oxidation (M)
1472.7633	1471.7560	1471.7823	-17.88	220	231	2	---	R.FGKYIEIGFDKR.Y
1513.8079	1512.8006	1512.7896	7.30	1068	1079	1	---	K.QLELDLNDERLR.Y
1531.7472	1530.7399	1530.7388	0.73	1474	1485	2	---	K.EKDFQGMLEYKK.E + Oxidation (M)
1561.8401	1560.8328	1560.8624	-18.92	754	767	2	---	R.AGQVAYLEKLRADK.L
1579.7939	1578.7866	1578.8253	-24.50	1018	1030	1	---	R.YKQETEQLVSNLK.E
1646.8297	1645.8224	1645.8158	4.00	968	981	1	---	R.SDLERLQLSEEEAK.I
1680.8746	1679.8673	1679.8916	-14.45	1304	1318	2	---	K.MKDKGEIAQAYIGLK.E + Oxidation (M)
1707.8302	1706.8229	1706.8397	-9.81	1289	1303	0	---	K.NTMTDSTILLEDVQK.M
1710.9279	1709.9206	1709.9069	8.04	923	936	1	---	K.LHIGMENKIMQLQR.K
1725.8300	1724.8227	1724.8589	-21.00	1099	1113	0	5	K.EEMTLMVNVPKPGHK.R + Oxidation (M)
1725.8346	1724.8273	1724.8589	-18.33	1099	1113	0	---	K.EEMTLMVNVPKPGHK.R + Oxidation (M)
1840.9827	1839.9754	1840.0029	-14.92	232	246	2	---	R.YRIIGANMRTYLLEK.S
1856.9908	1855.9835	1855.9978	-7.68	232	246	2	---	R.YRIIGANMRTYLLEK.S + Oxidation (M)
1944.0040	1942.9967	1942.9847	6.18	1062	1077	1	---	K.LVEETKQLELDLNDER.L
1976.9861	1975.9788	1976.0070	-14.28	1412	1427	2	---	R.MIDLMEQLEKQDKTVR.K
1998.9894	1997.9821	1997.9649	8.60	1289	1305	1	---	K.NTMTDSTILLEDVQKMK.D + 2 Oxidation (M)
2026.0275	2025.0202	2025.0638	-21.52	1406	1421	2	---	R.ISLYKRMIDLMEQLEK.Q + Oxidation (M)
2097.0454	2096.0381	2096.0902	-24.83	241	257	2	---	R.TYLLEKSRVVFQAEEER.N
2176.0664	2175.0591	2175.0807	-9.92	1192	1209	1	---	K.EEERPQIRGAELEYESLK.R
2226.1272	2225.1199	2225.1263	-2.85	641	659	0	---	R.NSLHLLMETLNATTPHYVR.C + Oxidation (M)
2233.0984	2232.0911	2232.0917	-0.24	149	169	2	---	K.QMARDERNQSIIVSGESGAGK.T
2242.0435	2241.0362	2241.0868	-22.58	1289	1307	2	---	K.NTMTDSTILLEDVQKMKDK.G + 2 Oxidation (M)
2501.2637	2500.2564	2500.3020	-18.23	1020	1040	2	---	K.QETEQLVSNLKEENTLLKQEK.E

No match to: 721.4365, 725.4001, 728.3681, 730.3848, 731.4017, 734.4151, 735.4143, 741.4101, 742.4515, 744.4171, 745.4167, 747.4125, 750.4153, 752.4127, 755.4100, 756.4199, 757.4313, 761.4177, 768.3700, 768.3737, 770.3966, 772.4093, 775.4307, 779.4308, 784.3688, 786.4000, 787.4221, 788.4210, 798.4351, 800.4296, 812.4388, 815.4332, 822.4482, 831.4382, 835.4719, 837.4463, 842.5060, 844.4760, 854.4837, 856.4911, 860.4691, 862.4852, 869.4231, 907.4991, 908.4929, 913.5700, 913.5747, 918.5050, 919.5053, 922.5070, 928.5117, 934.5172, 940.4840, 942.4854, 943.4289, 943.5453, 946.5259, 950.4959, 953.5164, 954.5388, 966.5410, 969.5256, 974.5395, 976.5310, 979.5442, 991.5409, 993.5307, 1001.5854, 1003.5496, 1011.5262, 1014.5215, 1021.5579, 1023.5663, 1023.5700, 1031.5903, 1035.5381, 1036.5447, 1037.5573, 1039.5327, 1041.5658, 1044.5751, 1045.5897, 1046.5897, 1051.5448, 1057.6078, 1067.5753, 1073.5902, 1086.5509, 1092.5961, 1100.6071, 1111.5615, 1118.5818, 1120.6045, 1122.5912, 1123.5848, 1125.5823, 1132.6047, 1139.5746, 1143.6318, 1144.6497, 1145.6407, 1147.6393, 1150.6108, 1155.6215, 1157.6199, 1158.6685, 1163.6152, 1168.6215, 1175.6089, 1179.6157, 1181.6819, 1197.6704, 1199.7006, 1216.6714, 1227.6595, 1232.6447, 1234.6672, 1235.6445, 1244.6453, 1248.6174, 1255.6096, 1256.6531, 1262.6453, 1266.6777, 1273.6260, 1275.6362, 1276.6343, 1277.6531, 1301.7079, 1303.7076, 1307.7042, 1308.6376, 1310.6146, 1312.6418, 1315.7098, 1322.6886, 1323.6771, 1324.6964, 1328.7089, 1331.7202, 1335.7318, 1339.6637, 1349.7253, 1352.7205, 1356.7230, 1360.6638, 1363.7153, 1365.7379, 1367.7319, 1369.7043, 1377.6947, 1379.7268, 1383.7137, 1385.7534, 1388.7404, 1390.7284, 1393.7428, 1407.7563, 1422.7019, 1425.7727, 1427.7742, 1429.7365, 1431.7412, 1433.7228, 1434.7446, 1437.7584, 1461.7778, 1467.7521, 1475.7632, 1477.7545, 1484.7889, 1488.7537, 1491.7965, 1493.7579, 1500.7737, 1505.8177, 1515.8213, 1517.7854, 1519.7544, 1522.7811, 1523.7953, 1529.8099, 1530.7523, 1537.7974, 1539.8005, 1542.8422, 1545.8091, 1547.8051, 1549.7339, 1551.7778, 1553.8065, 1555.8101, 1556.8109, 1557.8010, 1563.8109, 1565.8199, 1571.8400, 1580.8077, 1583.8444, 1587.7620, 1592.8066, 1594.8335, 1604.8323, 1605.8586, 1608.8497, 1615.8754, 1624.8062, 1628.7900, 1628.7921, 1633.7985, 1640.9384, 1642.8099, 1649.8447, 1657.8101, 1667.8622, 1677.7988, 1689.9465, 1694.7288, 1698.9225, 1699.8862, 1704.8773, 1706.8701, 1716.8671, 1719.9058, 1723.9011, 1727.8986, 1730.8931, 1737.9006, 1751.8656, 1754.8580, 1765.8463, 1766.8040, 1767.8138, 1769.8741, 1770.3567, 1772.9315, 1774.7610, 1774.9131, 1777.8770, 1780.8777, 1783.9246, 1787.8188, 1787.8200, 1791.8137, 1793.9050, 1795.9353, 1798.9340, 1800.9336, 1804.9142, 1805.9069, 1809.8469, 1815.9044, 1821.9144, 1828.9227, 1837.9396, 1843.9688, 1844.9012, 1846.9153, 1852.9364, 1861.8881, 1867.9218, 1874.9695, 1880.9265, 1884.9417, 1891.9622, 1894.9313, 1896.9078, 1896.9100, 1900.9546, 1905.9313, 1906.9501, 1908.9043, 1911.9130, 1912.9154, 1916.9923, 1918.9587, 1921.9825, 1923.9718, 1925.9573,

```
1928.0469, 1932.0060, 1933.9950, 1937.9630, 1939.9740, 1946.9679, 1949.9595, 1954.0236, 1955.9738, 1958.9556, 1960.0034, 1968.8600,
1973.9845, 1978.9910, 1981.9854, 1983.9884, 1987.9784, 1995.9408, 2000.9926, 2008.0460, 2012.9590, 2015.0104, 2017.9747, 2021.0170,
2027.0256, 2031.0166, 2044.0123, 2047.4957, 2047.9515, 2052.0496, 2060.0520, 2062.0073, 2064.9880, 2066.9873, 2094.0376, 2102.0667,
2103.1641, 2111.0305, 2127.0930, 2139.0630, 2144.0493, 2148.1023, 2155.0950, 2156.0796, 2160.0632, 2190.9895, 2195.0774, 2199.0723,
2202.1624, 2203.1221, 2208.0862, 2211.1042, 2216.1062, 2225.1274, 2229.0972, 2239.0225, 2268.1489, 2270.1287, 2271.1294, 2277.0823,
2284.1990, 2292.1426, 2309.0994, 2334.1719, 2342.9915, 2350.1746, 2383.9607, 2398.2400, 2400.0862, 2413.0549, 2414.1897, 2417.0959,
2497.2800, 2511.1460, 2513.1421, 2528.1750, 2537.2012, 2541.1948, 2542.1836, 2556.2058, 2562.1487, 2612.2666, 2706.2109, 2717.0869,
2767.2666, 2888.2532, 3221.1733, 3224.2917, 3312.3306, 3916.9434
```

Search Parameters

```
Type of search         : MS/MS Ion Search
Enzyme                 : Trypsin
Fixed modifications    : Carbamidomethyl (C)
Variable modifications : Oxidation (M)
Mass values            : Monoisotopic
Protein Mass           : Unrestricted
Peptide Mass Tolerance : ± 25 ppm
Fragment Mass Tolerance: ± 0.5 Da
Max Missed Cleavages   : 2
Instrument type        : MALDI-TOF-TOF
   Query1 (721.4365,1+): <no title>
   Query2 (725.4001,1+): <no title>
   Query3 (726.4063,1+): <no title>
   Query4 (728.3681,1+): <no title>
   Query5 (730.3848,1+): <no title>
   Query6 (731.4017,1+): <no title>
   Query7 (732.3573,1+): <no title>
   Query8 (734.4151,1+): <no title>
   Query9 (735.4143,1+): <no title>
  Query10 (741.4101,1+): <no title>
  Query11 (742.4515,1+): <no title>
  Query12 (744.4171,1+): <no title>
  Query13 (745.4167,1+): <no title>
  Query14 (746.4169,1+): <no title>
  Query15 (747.4125,1+): <no title>
  Query16 (750.4153,1+): <no title>
  Query17 (752.4127,1+): <no title>
  Query18 (755.4100,1+): <no title>
  Query19 (756.4199,1+): <no title>
  Query20 (757.4313,1+): <no title>
  Query21 (761.4177,1+): <no title>
  Query22 (767.4266,1+): <no title>
  Query23 (768.3700,1+): Label: G3, Spot_Id: 221890, Peak_List_Id: 435516, MSMS Job_Run_Id: 30377, Comment:
  Query24 (768.3737,1+): <no title>
  Query25 (770.3966,1+): <no title>
  Query26 (772.4093,1+): <no title>
  Query27 (775.4307,1+): <no title>
  Query28 (779.4308,1+): <no title>
  Query29 (784.3688,1+): <no title>
  Query30 (786.4000,1+): <no title>
  Query31 (787.4221,1+): <no title>
  Query32 (788.4210,1+): <no title>
  Query33 (792.4376,1+): <no title>
  Query34 (795.4350,1+): <no title>
  Query35 (798.4351,1+): <no title>
  Query36 (800.4296,1+): <no title>
  Query37 (812.4388,1+): <no title>
  Query38 (815.4332,1+): <no title>
  Query39 (822.4482,1+): <no title>
  Query40 (825.4540,1+): <no title>
  Query41 (831.4382,1+): <no title>
  Query42 (832.4557,1+): <no title>
  Query43 (835.4719,1+): <no title>
  Query44 (837.4463,1+): <no title>
  Query45 (842.5060,1+): <no title>
  Query46 (844.4760,1+): <no title>
  Query47 (854.4837,1+): <no title>
  Query48 (856.4911,1+): <no title>
  Query49 (860.4691,1+): <no title>
  Query50 (862.4852,1+): <no title>
  Query51 (869.4231,1+): <no title>
  Query52 (885.4465,1+): <no title>
  Query53 (905.4955,1+): <no title>
  Query54 (907.4991,1+): <no title>
  Query55 (908.4929,1+): <no title>
  Query56 (913.5700,1+): Label: G3, Spot_Id: 221890, Peak_List_Id: 435417, MSMS Job_Run_Id: 30377, Comment:
  Query57 (913.5747,1+): <no title>
  Query58 (916.5284,1+): <no title>
  Query59 (918.5050,1+): <no title>
  Query60 (919.5053,1+): <no title>
  Query61 (922.5070,1+): <no title>
  Query62 (928.5117,1+): <no title>
  Query63 (934.5172,1+): <no title>
  Query64 (940.4840,1+): <no title>
  Query65 (942.4854,1+): <no title>
  Query66 (943.4289,1+): <no title>
  Query67 (943.5453,1+): <no title>
  Query68 (946.5259,1+): <no title>
  Query69 (950.4959,1+): <no title>
  Query70 (951.5076,1+): <no title>
  Query71 (953.5164,1+): <no title>
  Query72 (954.5388,1+): <no title>
  Query73 (959.5266,1+): <no title>
  Query74 (966.5410,1+): <no title>
  Query75 (969.5256,1+): <no title>
  Query76 (972.5305,1+): <no title>
  Query77 (974.5395,1+): <no title>
```

Query78 (976.5310,1+): <no title>
Query79 (978.5314,1+): <no title>
Query80 (979.5442,1+): <no title>
Query81 (991.5409,1+): <no title>
Query82 (993.5307,1+): <no title>
Query83 (1001.5854,1+): <no title>
Query84 (1003.5496,1+): <no title>
Query85 (1011.5262,1+): <no title>
Query86 (1014.5215,1+): <no title>
Query87 (1021.5579,1+): <no title>
Query88 (1023.5663,1+): <no title>
Query89 (1023.5700,1+): Label: G3, Spot_Id: 221890, Peak_List_Id: 435416, MSMS Job_Run_Id: 30377, Comment:
Query90 (1031.5903,1+): <no title>
Query91 (1035.5381,1+): <no title>
Query92 (1036.5447,1+): <no title>
Query93 (1037.5573,1+): <no title>
Query94 (1039.5327,1+): <no title>
Query95 (1041.5658,1+): <no title>
Query96 (1044.5751,1+): <no title>
Query97 (1045.5729,1+): <no title>
Query98 (1046.5897,1+): <no title>
Query99 (1051.5448,1+): <no title>
Query100 (1056.5658,1+): <no title>
Query101 (1057.6078,1+): <no title>
Query102 (1067.5753,1+): <no title>
Query103 (1073.5902,1+): <no title>
Query104 (1086.5509,1+): <no title>
Query105 (1092.5961,1+): <no title>
Query106 (1100.6071,1+): <no title>
Query107 (1106.5391,1+): <no title>
Query108 (1111.5615,1+): <no title>
Query109 (1113.5729,1+): <no title>
Query110 (1118.5818,1+): <no title>
Query111 (1120.6045,1+): <no title>
Query112 (1122.5912,1+): <no title>
Query113 (1123.5848,1+): <no title>
Query114 (1125.5823,1+): <no title>
Query115 (1132.6047,1+): <no title>
Query116 (1134.5714,1+): <no title>
Query117 (1139.5746,1+): <no title>
Query118 (1143.6318,1+): <no title>
Query119 (1144.6497,1+): <no title>
Query120 (1145.6407,1+): <no title>
Query121 (1147.6393,1+): <no title>
Query122 (1150.6108,1+): <no title>
Query123 (1155.6215,1+): <no title>
Query124 (1157.6199,1+): <no title>
Query125 (1158.6685,1+): <no title>
Query126 (1162.6182,1+): <no title>
Query127 (1163.6152,1+): <no title>
Query128 (1168.6215,1+): <no title>
Query129 (1173.6418,1+): <no title>
Query130 (1175.6089,1+): <no title>
Query131 (1178.6356,1+): <no title>
Query132 (1179.6157,1+): <no title>
Query133 (1181.6819,1+): <no title>
Query134 (1188.6544,1+): <no title>
Query135 (1197.6704,1+): <no title>
Query136 (1199.7006,1+): <no title>
Query137 (1208.6415,1+): <no title>
Query138 (1216.6714,1+): <no title>
Query139 (1227.6595,1+): <no title>
Query140 (1231.6387,1+): <no title>
Query141 (1232.6447,1+): <no title>
Query142 (1234.6672,1+): <no title>
Query143 (1235.6445,1+): <no title>
Query144 (1244.6453,1+): <no title>
Query145 (1247.6211,1+): <no title>
Query146 (1248.6174,1+): <no title>
Query147 (1250.6554,1+): <no title>
Query148 (1255.6096,1+): <no title>
Query149 (1256.6531,1+): <no title>
Query150 (1261.6592,1+): <no title>
Query151 (1262.6453,1+): <no title>
Query152 (1266.6777,1+): <no title>
Query153 (1273.6260,1+): <no title>
Query154 (1275.6362,1+): <no title>
Query155 (1276.6343,1+): <no title>
Query156 (1277.6531,1+): <no title>
Query157 (1301.7079,1+): <no title>
Query158 (1303.7076,1+): <no title>
Query159 (1307.7042,1+): <no title>
Query160 (1308.6376,1+): <no title>
Query161 (1310.6146,1+): <no title>
Query162 (1312.6418,1+): <no title>
Query163 (1315.7098,1+): <no title>
Query164 (1320.6659,1+): <no title>
Query165 (1322.6886,1+): <no title>
Query166 (1323.6771,1+): <no title>
Query167 (1324.6964,1+): <no title>
Query168 (1328.7089,1+): <no title>
Query169 (1331.7202,1+): <no title>
Query170 (1335.7318,1+): <no title>
Query171 (1338.6581,1+): <no title>
Query172 (1339.6637,1+): <no title>
Query173 (1349.7253,1+): <no title>
Query174 (1352.7205,1+): <no title>
Query175 (1356.7230,1+): <no title>
Query176 (1360.6638,1+): <no title>
Query177 (1363.7153,1+): <no title>

Query178 (1365.7379,1+): <no title>
Query179 (1367.7319,1+): <no title>
Query180 (1369.7043,1+): <no title>
Query181 (1377.6947,1+): <no title>
Query182 (1379.7268,1+): <no title>
Query183 (1383.7137,1+): <no title>
Query184 (1385.7534,1+): <no title>
Query185 (1388.7404,1+): <no title>
Query186 (1390.7284,1+): <no title>
Query187 (1393.7428,1+): <no title>
Query188 (1407.7563,1+): <no title>
Query189 (1417.7010,1+): <no title>
Query190 (1421.7061,1+): <no title>
Query191 (1422.7019,1+): <no title>
Query192 (1425.7727,1+): <no title>
Query193 (1427.7742,1+): <no title>
Query194 (1429.7365,1+): <no title>
Query195 (1431.7412,1+): <no title>
Query196 (1433.7228,1+): <no title>
Query197 (1434.7446,1+): <no title>
Query198 (1437.7584,1+): <no title>
Query199 (1461.7778,1+): <no title>
Query200 (1467.7521,1+): <no title>
Query201 (1472.7633,1+): <no title>
Query202 (1475.7632,1+): <no title>
Query203 (1477.7545,1+): <no title>
Query204 (1484.7889,1+): <no title>
Query205 (1488.7537,1+): <no title>
Query206 (1491.7965,1+): <no title>
Query207 (1493.7579,1+): <no title>
Query208 (1500.7737,1+): <no title>
Query209 (1505.8177,1+): <no title>
Query210 (1513.8079,1+): <no title>
Query211 (1515.8213,1+): <no title>
Query212 (1517.7854,1+): <no title>
Query213 (1519.7544,1+): <no title>
Query214 (1522.7811,1+): <no title>
Query215 (1523.7953,1+): <no title>
Query216 (1529.8099,1+): <no title>
Query217 (1530.7523,1+): <no title>
Query218 (1531.7472,1+): <no title>
Query219 (1537.7974,1+): <no title>
Query220 (1539.8005,1+): <no title>
Query221 (1542.8422,1+): <no title>
Query222 (1545.8091,1+): <no title>
Query223 (1547.8051,1+): <no title>
Query224 (1549.7339,1+): <no title>
Query225 (1551.7778,1+): <no title>
Query226 (1553.8065,1+): <no title>
Query227 (1555.8101,1+): <no title>
Query228 (1556.8109,1+): <no title>
Query229 (1557.8010,1+): <no title>
Query230 (1561.8401,1+): <no title>
Query231 (1563.8109,1+): <no title>
Query232 (1565.8199,1+): <no title>
Query233 (1571.8400,1+): <no title>
Query234 (1579.7939,1+): <no title>
Query235 (1580.8077,1+): <no title>
Query236 (1583.8444,1+): <no title>
Query237 (1587.7620,1+): <no title>
Query238 (1592.8066,1+): <no title>
Query239 (1594.8335,1+): <no title>
Query240 (1604.8323,1+): <no title>
Query241 (1605.8586,1+): <no title>
Query242 (1608.8497,1+): <no title>
Query243 (1615.8754,1+): <no title>
Query244 (1624.8062,1+): <no title>
Query245 (1628.7900,1+): Label: G3, Spot_Id: 221890, Peak_List_Id: 435517, MSMS Job_Run_Id: 30377, Comment:
Query246 (1628.7921,1+): <no title>
Query247 (1633.7985,1+): <no title>
Query248 (1640.9384,1+): <no title>
Query249 (1642.8099,1+): <no title>
Query250 (1646.8297,1+): <no title>
Query251 (1649.8447,1+): <no title>
Query252 (1657.8101,1+): <no title>
Query253 (1667.8622,1+): <no title>
Query254 (1677.7988,1+): <no title>
Query255 (1680.8746,1+): <no title>
Query256 (1689.9465,1+): <no title>
Query257 (1694.7288,1+): <no title>
Query258 (1698.9225,1+): <no title>
Query259 (1699.8862,1+): <no title>
Query260 (1704.8773,1+): <no title>
Query261 (1706.8701,1+): <no title>
Query262 (1707.8302,1+): <no title>
Query263 (1710.9279,1+): <no title>
Query264 (1716.8671,1+): <no title>
Query265 (1719.9058,1+): <no title>
Query266 (1723.9011,1+): <no title>
Query267 (1725.8300,1+): Label: G3, Spot_Id: 221890, Peak_List_Id: 435419, MSMS Job_Run_Id: 30377, Comment:
Query268 (1725.8346,1+): <no title>
Query269 (1727.8986,1+): <no title>
Query270 (1730.8931,1+): <no title>
Query271 (1737.9006,1+): <no title>
Query272 (1751.8656,1+): <no title>
Query273 (1754.8580,1+): <no title>
Query274 (1765.8463,1+): <no title>
Query275 (1766.8040,1+): <no title>
Query276 (1767.8138,1+): <no title>
Query277 (1769.8741,1+): <no title>

```
Query278 (1770.3567,1+): <no title>
Query279 (1772.9315,1+): <no title>
Query280 (1774.7610,1+): <no title>
Query281 (1774.9131,1+): <no title>
Query282 (1777.8770,1+): <no title>
Query283 (1780.8777,1+): <no title>
Query284 (1783.9246,1+): <no title>
Query285 (1787.8188,1+): <no title>
Query286 (1787.8200,1+): Label: G3, Spot_Id: 221890, Peak_List_Id: 435415, MSMS Job_Run_Id: 30377, Comment:
Query287 (1791.8137,1+): <no title>
Query288 (1793.9050,1+): <no title>
Query289 (1795.9353,1+): <no title>
Query290 (1798.9340,1+): <no title>
Query291 (1800.9336,1+): <no title>
Query292 (1804.9142,1+): <no title>
Query293 (1805.9069,1+): <no title>
Query294 (1809.8469,1+): <no title>
Query295 (1815.9044,1+): <no title>
Query296 (1821.9144,1+): <no title>
Query297 (1828.9227,1+): <no title>
Query298 (1837.9396,1+): <no title>
Query299 (1840.9827,1+): <no title>
Query300 (1843.9688,1+): <no title>
Query301 (1844.9012,1+): <no title>
Query302 (1846.9153,1+): <no title>
Query303 (1852.9364,1+): <no title>
Query304 (1856.9908,1+): <no title>
Query305 (1861.8881,1+): <no title>
Query306 (1867.9218,1+): <no title>
Query307 (1874.9695,1+): <no title>
Query308 (1880.9265,1+): <no title>
Query309 (1884.9417,1+): <no title>
Query310 (1891.9622,1+): <no title>
Query311 (1894.9313,1+): <no title>
Query312 (1896.9078,1+): <no title>
Query313 (1896.9100,1+): Label: G3, Spot_Id: 221890, Peak_List_Id: 435418, MSMS Job_Run_Id: 30377, Comment:
Query314 (1900.9546,1+): <no title>
Query315 (1905.9313,1+): <no title>
Query316 (1906.9501,1+): <no title>
Query317 (1908.9043,1+): <no title>
Query318 (1911.9130,1+): <no title>
Query319 (1912.9154,1+): <no title>
Query320 (1916.9923,1+): <no title>
Query321 (1918.9587,1+): <no title>
Query322 (1921.9825,1+): <no title>
Query323 (1923.9718,1+): <no title>
Query324 (1925.9573,1+): <no title>
Query325 (1928.0469,1+): <no title>
Query326 (1932.0060,1+): <no title>
Query327 (1933.9950,1+): <no title>
Query328 (1937.9630,1+): <no title>
Query329 (1939.9740,1+): <no title>
Query330 (1944.0040,1+): <no title>
Query331 (1946.9679,1+): <no title>
Query332 (1949.9595,1+): <no title>
Query333 (1954.0236,1+): <no title>
Query334 (1955.9738,1+): <no title>
Query335 (1958.9556,1+): <no title>
Query336 (1960.0034,1+): <no title>
Query337 (1968.8600,1+): <no title>
Query338 (1973.9845,1+): <no title>
Query339 (1976.9861,1+): <no title>
Query340 (1978.9910,1+): <no title>
Query341 (1981.9854,1+): <no title>
Query342 (1983.9884,1+): <no title>
Query343 (1987.9784,1+): <no title>
Query344 (1995.9408,1+): <no title>
Query345 (1998.9894,1+): <no title>
Query346 (2000.9926,1+): <no title>
Query347 (2008.0460,1+): <no title>
Query348 (2012.9590,1+): <no title>
Query349 (2015.0104,1+): <no title>
Query350 (2017.9747,1+): <no title>
Query351 (2021.0170,1+): <no title>
Query352 (2026.0275,1+): <no title>
Query353 (2027.0256,1+): <no title>
Query354 (2031.0166,1+): <no title>
Query355 (2044.0123,1+): <no title>
Query356 (2047.4957,1+): <no title>
Query357 (2047.9515,1+): <no title>
Query358 (2052.0496,1+): <no title>
Query359 (2060.0520,1+): <no title>
Query360 (2062.0073,1+): <no title>
Query361 (2064.9880,1+): <no title>
Query362 (2066.9873,1+): <no title>
Query363 (2094.0376,1+): <no title>
Query364 (2097.0454,1+): <no title>
Query365 (2102.0667,1+): <no title>
Query366 (2103.1641,1+): <no title>
Query367 (2111.0305,1+): <no title>
Query368 (2127.0930,1+): <no title>
Query369 (2139.0630,1+): <no title>
Query370 (2144.0493,1+): <no title>
Query371 (2148.1023,1+): <no title>
Query372 (2155.0950,1+): <no title>
Query373 (2156.0796,1+): <no title>
Query374 (2160.0632,1+): <no title>
Query375 (2176.0664,1+): <no title>
Query376 (2190.9895,1+): <no title>
Query377 (2195.0774,1+): <no title>
```

```
Query378 (2199.0723,1+): <no title>
Query379 (2202.1624,1+): <no title>
Query380 (2203.1221,1+): <no title>
Query381 (2208.0862,1+): <no title>
Query382 (2211.1042,1+): <no title>
Query383 (2216.1062,1+): <no title>
Query384 (2225.1274,1+): <no title>
Query385 (2226.1272,1+): <no title>
Query386 (2229.0972,1+): <no title>
Query387 (2233.0984,1+): <no title>
Query388 (2239.0225,1+): <no title>
Query389 (2242.0435,1+): <no title>
Query390 (2268.1489,1+): <no title>
Query391 (2270.1287,1+): <no title>
Query392 (2271.1294,1+): <no title>
Query393 (2277.0823,1+): <no title>
Query394 (2284.1990,1+): <no title>
Query395 (2292.1426,1+): <no title>
Query396 (2309.0994,1+): <no title>
Query397 (2334.1719,1+): <no title>
Query398 (2342.9915,1+): <no title>
Query399 (2350.1746,1+): <no title>
Query400 (2383.9607,1+): <no title>
Query401 (2398.2400,1+): <no title>
Query402 (2400.0862,1+): <no title>
Query403 (2413.0549,1+): <no title>
Query404 (2414.1897,1+): <no title>
Query405 (2417.0959,1+): <no title>
Query406 (2497.2800,1+): <no title>
Query407 (2501.2637,1+): <no title>
Query408 (2511.1460,1+): <no title>
Query409 (2513.1421,1+): <no title>
Query410 (2528.1750,1+): <no title>
Query411 (2537.2012,1+): <no title>
Query412 (2541.1948,1+): <no title>
Query413 (2542.1836,1+): <no title>
Query414 (2556.2058,1+): <no title>
Query415 (2562.1487,1+): <no title>
Query416 (2612.2666,1+): <no title>
Query417 (2706.2109,1+): <no title>
Query418 (2717.0869,1+): <no title>
Query419 (2767.2666,1+): <no title>
Query420 (2888.2532,1+): <no title>
Query421 (3221.1733,1+): <no title>
Query422 (3224.2917,1+): <no title>
Query423 (3312.3306,1+): <no title>
Query424 (3916.9434,1+): <no title>
```

**Mascot:** http://www.matrixscience.com/

MATRIX SCIENCE Mascot Search Results

```
User          : 1w_(F23@11273_12Mar2012)
Email         : proteomics@ipatimup.pt
Search title  : Project: Proteomica, Spot Set: Proteomica\12Jan2012, Label: F23, Spot Id: 221886, Peak List Id: 435348, MS Job Run Id: 30
MS data file  : C:\Documents and Settings\Administrator\Desktop\xu\xu\xu\Proteomica\12Jan2012\ppw_F23_146796935502.txt
Database      : OrganismSpecie Bos_taurus_Reference_Proteome_2016_06 (24214 sequences; 12839866 residues)
Timestamp     : 8 Jul 2016 at 10:05:41 GMT
Warning       : A Peptide summary report will usually give a much clearer picture of MS/MS search results.
Top Score     : 106 for E1BI98, Uncharacterized protein OS=Bos taurus GN=COL6A1 PE=1 SV=1
```

Mascot Score Histogram

Protein score is -10*Log(P), where P is the probability that the observed match is a random event.
Protein scores greater than 56 are significant (p<0.05).
Protein scores are derived from ions scores as a non-probabilistic basis for ranking protein hits.

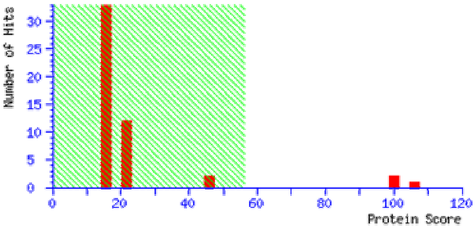

Protein Summary Report

Format As | Protein Summary (deprecated) | Help

Significance threshold p< 0.05 | Max. number of hits 50

Preferred taxonomy | All entries

Re-Search All | Search Unmatched

Index

	Accession	Mass	Score	Description
1.	E1BI98	109744	106	Uncharacterized protein OS=Bos taurus GN=COL6A1 PE=1 SV=1
2.	P02459	142825	101	Collagen alpha-1(II) chain OS=Bos taurus GN=COL2A1 PE=1 SV=4
3.	F1MSR8	134858	101	Collagen alpha-1(II) chain OS=Bos taurus GN=COL2A1 PE=4 SV=2
4.	F1MKG2	110451	45	Uncharacterized protein OS=Bos taurus GN=COL6A2 PE=1 SV=2
5.	G3MXU3	25162	45	Uncharacterized protein OS=Bos taurus GN=COL6A2 PE=4 SV=1
6.	Q32LI7	30313	24	Putative uncharacterized protein MGC133880 OS=Bos taurus GN=MGC133880 PE=2 SV=1
7.	P46171	4789	23	Beta-defensin 13 OS=Bos taurus GN=DEFB13 PE=1 SV=1
8.	Q0VC96	22800	21	Thymocyte nuclear protein 1 OS=Bos taurus GN=THYN1 PE=2 SV=1
9.	P62935	18086	20	Peptidyl-prolyl cis-trans isomerase A OS=Bos taurus GN=PPIA PE=1 SV=2
10.	Q29RM9	23940	20	Regulator of G-protein signaling 4 OS=Bos taurus GN=RGS4 PE=2 SV=1
11.	Q28024	8166	20	Guanine nucleotide-binding protein G(I)/G(S)/G(O) subunit gamma-12 OS=Bos taurus GN=GNG12 PE=1 SV=2
12.	P46170	4445	20	Beta-defensin 12 OS=Bos taurus GN=DEFB12 PE=1 SV=1
13.	Q29RZ4	49390	19	Zinc finger protein 2 OS=Bos taurus GN=ZNF2 PE=2 SV=1
14.	A7Z060	81637	19	RNF219 protein OS=Bos taurus GN=RNF219 PE=2 SV=1
15.	P48427	12756	19	Tubulin-specific chaperone A OS=Bos taurus GN=TBCA PE=1 SV=3
16.	Q3ZBD3	12035	19	Pterin-4-alpha-carbinolamine dehydratase OS=Bos taurus GN=PCBD1 PE=3 SV=2
17.	F1MK70	59648	19	Uncharacterized protein OS=Bos taurus GN=SPATS2 PE=4 SV=2
18.	Q5EAC6	45065	18	Hsp90 co-chaperone Cdc37 OS=Bos taurus GN=CDC37 PE=2 SV=1
19.	Q2HJG7	16553	18	Protein Churchill OS=Bos taurus GN=CHURC1 PE=2 SV=2
20.	Q0VCG0	10705	18	LYR motif-containing protein 4 OS=Bos taurus GN=LYRM4 PE=3 SV=1
21.	G3N0J4	6707	18	Uncharacterized protein OS=Bos taurus PE=4 SV=1
22.	F1N2B6	97976	18	Uncharacterized protein OS=Bos taurus GN=AR PE=3 SV=2
23.	A8QW39	16136	18	Protein FAM183A OS=Bos taurus GN=FAM183A PE=2 SV=1
24.	Q0VCW1	42846	18	Speckle-type POZ protein OS=Bos taurus GN=SPOP PE=2 SV=1
25.	Q95106	28555	17	Brain-derived neurotrophic factor OS=Bos taurus GN=BDNF PE=2 SV=2
26.	G5E620	21503	17	Uncharacterized protein OS=Bos taurus GN=LOC101904345 PE=4 SV=1
27.	Q0II95	50922	17	Inositol hexakisphosphate kinase 1 OS=Bos taurus GN=IP6K1 PE=2 SV=1
28.	F6QWT9	13281	17	Uncharacterized protein OS=Bos taurus GN=FAM229A PE=4 SV=1
29.	A4FV50	40277	17	MGC142792 protein OS=Bos taurus GN=MGC142792 PE=2 SV=1
30.	G3MXX8	36525	17	Uncharacterized protein OS=Bos taurus GN=SCRT1 PE=4 SV=1
31.	G5E5D2	21544	17	Uncharacterized protein OS=Bos taurus GN=TNNI2 PE=4 SV=1
32.	Q2YDE5	22966	17	Uncharacterized protein C14orf53 homolog OS=Bos taurus PE=2 SV=1
33.	F1N7K1	24749	17	Ropporin-1-like protein OS=Bos taurus GN=ROPN1L PE=4 SV=1
34.	F1N1Y9	49406	17	Zinc finger protein 2 OS=Bos taurus GN=ZNF2 PE=4 SV=2
35.	P79136	34176	17	F-actin-capping protein subunit beta OS=Bos taurus GN=CAPZB PE=1 SV=1
36.	Q0VC77	19543	17	CCDC49 protein OS=Bos taurus GN=CCDC49 PE=2 SV=1
37.	F1MHL1	40868	17	Hsp90 co-chaperone Cdc37 OS=Bos taurus GN=CDC37 PE=1 SV=2
38.	F1MQW7	14077	16	Uncharacterized protein OS=Bos taurus GN=C15H11orf97 PE=4 SV=2
39.	E1B9E7	17216	16	Uncharacterized protein OS=Bos taurus GN=LOC783279 PE=4 SV=2
40.	E1BA70	34295	16	Uncharacterized protein OS=Bos taurus PE=4 SV=1
41.	F1MR52	116761	16	Uncharacterized protein OS=Bos taurus PE=4 SV=2
42.	E1B705	50364	16	Uncharacterized protein OS=Bos taurus GN=RASSF9 PE=4 SV=2
43.	E1BDB7	30920	16	Uncharacterized protein OS=Bos taurus GN=CEBPE PE=4 SV=1
44.	Q0P584	23768	16	Uncharacterized protein OS=Bos taurus GN=ZMAT2 PE=2 SV=1
45.	F1MH60	51585	16	Uncharacterized protein OS=Bos taurus GN=SPRED1 PE=4 SV=2
46.	Q3ZCH0	73981	16	Stress-70 protein, mitochondrial OS=Bos taurus GN=HSPA9 PE=2 SV=1
47.	Q0P571	19114	16	Myosin regulatory light chain 2, skeletal muscle isoform OS=Bos taurus GN=MYLPF PE=2 SV=1
48.	G3N0L1	8718	16	Uncharacterized protein OS=Bos taurus GN=FGF12 PE=3 SV=1

49. **G3MYV8** 6868 16 Uncharacterized protein OS=Bos taurus PE=4 SV=1
50. **A2VDP3** 30659 16 RASD family, member 2 OS=Bos taurus GN=RASD2 PE=2 SV=1

Results List

1. E1BI98 **Mass:** 109744 **Score:** 106 **Expect:** 6.1e-007 **Matches:** 41

Uncharacterized protein OS=Bos taurus GN=COL6A1 PE=1 SV=1

Observed	Mr(expt)	Mr(calc)	ppm	Start	End	Miss	Ions	Peptide
801.4280	800.4207	800.4140	8.32	957 -	963	0	---	K.AVQEAQR.A
882.4470	881.4397	881.4468	-7.96	425 -	433	1	---	R.GGPGERGPR.G
887.4295	886.4222	886.4145	8.74	864 -	871	0	---	R.TDPGQDVR.V
905.4763	904.4690	904.4614	8.49	928 -	936	1	---	R.EASSNAAKK.R
949.5244	948.5171	948.5141	3.20	311 -	319	2	---	K.GSRGPKGYK.G
957.5792	956.5719	956.5655	6.76	723 -	731	0	---	R.IALVITDGR.S
978.5352	977.5279	977.5043	24.2	976 -	983	0	---	R.QVNEPHVR.V
1006.5120	1005.5047	1005.4879	16.7	69 -	76	0	---	R.FIDNLNDR.Y
1021.5762	1020.5689	1020.5716	-2.63	714 -	722	0	---	R.LLPPTPNNR.I
1046.6058	1045.5985	1045.5920	6.23	1005 -	1013	0	---	R.VPSYQALLR.G
1056.5575	1055.5502	1055.5400	9.68	1014 -	1022	0	---	R.GVFYQTVSR.K
1156.5652	1155.5579	1155.5594	-1.28	126 -	135	1	---	K.GTYTDCAIKK.G
1178.6371	1177.6298	1177.6428	-11.05	431 -	442	2	---	R.GPRGTPGARGPR.G
1184.6431	1183.6358	1183.6350	0.73	1014 -	1023	1	---	R.GVFYQTVSRK.V
1216.7178	1215.7105	1215.6976	10.7	964 -	975	0	---	R.AGVEIFAVVVGR.Q
1264.7139	1263.7066	1263.7047	1.49	712 -	722	1	---	R.SRLLPPTPNNR.I
1289.7029	1288.6956	1288.6775	14.0	200 -	210	0	---	R.LSIIATDHTYR.R
1445.7913	1444.7840	1444.7786	3.73	200 -	211	1	---	R.LSIIATDHTYRR.N
1475.7615	1474.7542	1474.7562	-1.34	102 -	114	2	---	R.GLTRMPSGRDELK.S + Oxidation (M)
1488.7366	1487.7293	1487.7157	9.16	69 -	79	1	---	R.FIDNLNDRYYR.C
1516.7281	1515.7208	1515.6954	16.8	476 -	490	1	---	K.GYRGDEGPPGTEGPK.G
1562.8008	1561.7935	1561.7849	5.53	858 -	871	1	---	R.FLTASRTDPGQDVR.V
1580.8580	1579.8507	1579.8358	9.42	186 -	199	0	---	K.VFSVAITPDHLEPR.L
1580.8600	1579.8527	1579.8358	10.7	186 -	199	0	46	K.VFSVAITPDHLEPR.L
1632.8120	1631.8047	1631.7726	19.7	491 -	508	0	---	K.GAPGPAGPPGDPGLMGER.G
1651.8369	1650.8296	1650.8076	13.4	122 -	135	2	---	K.YFGKGTYTDCAIKK.G
1718.9094	1717.9021	1717.8669	20.5	106 -	121	2	---	R.MPSGRDELKSSVDAVK.Y
1722.9003	1721.8930	1721.9312	-22.15	136 -	151	1	---	K.GLEELLVGGSHLKENK.Y
1724.9030	1723.8957	1723.9216	-15.04	678 -	692	2	---	R.DPNIRNAQDLKEAIK.K
1774.9300	1773.9227	1773.9122	5.93	872 -	887	0	46	R.VAVVQYSGTGQQRPER.A
1774.9319	1773.9246	1773.9122	7.00	872 -	887	0	---	R.VAVVQYSGTGQQRPER.A
1932.0068	1930.9995	1931.0000	-0.24	938 -	956	0	---	R.LLLFSDGNSQGATPAAIEK.A
1997.8479	1996.8406	1996.8190	10.8	241 -	256	0	---	K.NNVEQVCCSFECQPAR.G
2015.9694	2014.9621	2014.9571	2.49	694 -	711	0	---	K.LQWMGGGTFTGEALQYTR.S
2088.1033	2087.0960	2087.1011	-2.43	937 -	956	1	---	K.RLLLFSDGNSQGATPAAIEK.A
2144.0559	2143.0486	2143.0521	-1.60	693 -	711	1	---	K.KLQWMGGGTFTGEALQYTR.S
2199.1150	2198.1077	2198.1120	-1.94	83 -	101	0	---	R.NLVWNAGALHYSDEVEIIR.G
3070.3745	3069.3672	3069.4217	-17.76	509 -	540	1	---	R.GEDGPPGNGTEGFPGFPGYPGSRGPPGINGTK.G
3159.5210	3158.5137	3158.5230	-2.95	152 -	180	1	---	K.YLVVVTDGHPLEGYKEPCGGLEDAVNEAK.H
3380.5210	3379.5137	3379.5189	-1.53	371 -	406	1	---	K.GQKGEPGADGEPGRPGSTGPPGDEGEPGEPGPPGEK.G
3427.6099	3426.6026	3426.6189	-4.75	443 -	478	2	---	R.GDPGEAGPQGDQGREGPVGVPGDPGEAGPIGPKGYR.G

No match to: 716.3353, 718.2895, 724.2175, 726.3070, 728.2833, 729.4336, 730.2399, 732.2518, 734.4341, 743.3130, 745.3617, 747.4360, 753.3469, 756.3524, 758.3462, 759.3632, 768.3517, 779.4633, 781.3725, 789.3640, 794.3674, 796.3701, 799.2854, 805.4417, 807.4076, 810.3293, 812.3207, 818.3782, 820.3700, 828.4415, 829.4508, 830.4513, 834.3805, 842.5101, 849.3834, 852.4368, 857.4457, 860.4262, 868.4412, 870.4462, 873.4370, 877.4166, 880.4280, 883.4816, 889.4857, 891.4872, 894.4923, 898.4610, 900.5174, 907.4251, 912.5086, 914.4942, 920.4296, 922.4855, 928.4565, 929.4484, 933.4327, 936.4976, 938.5062, 944.4982, 946.4672, 950.5168, 961.5109, 965.5151, 967.4963, 972.5023, 974.4913, 976.5255, 981.5225, 983.6221, 986.5463, 988.4922, 993.5123, 995.4512, 998.5833, 1000.5594, 1007.5640, 1024.5542, 1031.5300, 1034.5703, 1037.5466, 1041.5637, 1043.5297, 1044.5375, 1045.5594, 1054.5287, 1057.6187, 1057.6200, 1068.5790, 1074.6162, 1076.6190, 1082.6056, 1105.5938, 1110.5851, 1113.5701, 1118.5687, 1120.6050, 1122.5892, 1123.6047, 1126.6241, 1128.5618, 1130.5756, 1135.5951, 1137.5647, 1143.5592, 1145.6373, 1148.6318, 1149.6230, 1150.6226, 1157.5676, 1159.5890, 1160.5966, 1169.5873, 1177.6421, 1180.6443, 1182.6539, 1185.6815, 1193.6090, 1194.6001, 1209.6238, 1213.7180, 1221.5953, 1227.6459, 1229.6874, 1234.6899, 1238.2251, 1239.6622, 1254.6993, 1255.6351, 1265.1680, 1265.7196, 1269.6532, 1276.6729, 1278.6892, 1281.6375, 1285.6674, 1293.6760, 1301.6362, 1303.6489, 1304.6685, 1306.6946, 1308.6797, 1314.7227, 1319.6770, 1320.6364, 1323.6815, 1326.6937, 1328.6799, 1333.7430, 1336.7301, 1337.6429, 1349.6729, 1351.6969, 1353.6891, 1356.6255, 1358.6919, 1359.7013, 1361.7147, 1364.7118, 1365.6619, 1366.6965, 1372.6228, 1375.6693, 1382.6771, 1383.7347, 1390.7089, 1393.8007, 1397.7449, 1403.7434, 1410.7849, 1416.7737, 1420.7114, 1423.7096, 1434.7847, 1436.7469, 1442.8257, 1456.7555, 1457.7440, 1467.7560, 1470.7466, 1479.8038, 1483.7579, 1484.7480, 1487.7660, 1493.7556, 1500.7393, 1500.7400, 1503.7648, 1504.7708, 1514.7063, 1519.7705, 1522.7722, 1524.8040, 1526.7924, 1528.7079, 1530.7058, 1536.7252, 1537.7859, 1540.8043, 1543.7938, 1550.7543, 1551.7465, 1553.7668, 1555.7836, 1556.8011, 1568.7620, 1572.7245, 1578.8058, 1584.8486, 1587.7675, 1608.8051, 1616.8165, 1621.7870, 1625.7886, 1628.7313, 1635.7964, 1637.7974, 1642.7417, 1647.8439, 1650.8429, 1655.8147, 1657.8483, 1658.8953, 1660.8379, 1662.8666, 1664.8490, 1666.7892, 1679.8091, 1680.8357, 1682.8409, 1696.8464, 1698.8180, 1701.8243, 1702.8386, 1707.8938, 1710.8904, 1714.7905, 1716.8219, 1717.9052, 1721.8976, 1728.8840, 1730.8910, 1734.1617, 1738.8267, 1739.8165, 1741.8934, 1743.9005, 1747.9127, 1758.4807, 1758.8918, 1761.8734, 1765.8154, 1772.0012, 1779.9012, 1781.8585, 1783.8062, 1787.8407, 1809.0583, 1810.0460, 1812.9126, 1829.8527, 1832.8584, 1833.8571, 1834.8650, 1836.8618, 1838.8677, 1845.8363, 1847.8600, 1848.8938, 1850.9128, 1851.8932, 1861.8531, 1862.9542, 1864.9258, 1880.9026, 1883.7704, 1887.8878, 1897.7863, 1902.9769, 1904.9276, 1908.8986, 1920.9131, 1923.9126, 1928.0443, 1936.9510, 1938.0171, 1947.9314, 1958.9393, 1959.9655, 1960.9813, 1961.9463, 1967.1549, 1969.9951, 1973.9971, 1975.9755, 1976.9708, 1990.4971, 1990.9373, 1991.4857, 2001.8607, 2003.8768, 2004.8850, 2007.9500, 2007.9532, 2011.9381, 2012.8951, 2017.9860, 2018.9740, 2020.1631, 2023.9482, 2026.9529, 2027.5033, 2029.9972, 2043.5356, 2044.9940, 2055.0205, 2057.0154, 2060.9878, 2066.9939, 2068.9661, 2082.9822, 2086.0254, 2097.0371, 2098.4941, 2102.9954, 2109.0095, 2112.9932, 2114.9707, 2122.9578, 2128.0444, 2130.9980, 2132.0200, 2136.9250, 2140.0063, 2143.0237, 2145.0781, 2148.0483, 2153.0049, 2153.9973, 2174.0508, 2175.0435, 2178.0483, 2188.0610, 2191.0464, 2192.0427, 2205.0615, 2211.1067, 2223.1533, 2225.1382, 2226.1472, 2239.1646, 2243.1448, 2246.9482, 2251.1108, 2255.1484, 2265.0837, 2271.1211, 2273.0735, 2277.0642, 2283.1606, 2285.1355, 2286.0986, 2289.0901, 2291.0173, 2309.0349, 2328.9888, 2343.0608, 2352.9583, 2355.0383, 2366.9595, 2371.0325, 2392.1594, 2400.0532, 2424.1577, 2434.2429, 2438.2283, 2449.2180, 2450.2451, 2451.2480, 2465.1592, 2466.2458, 2467.2458, 2478.1377, 2501.2207, 2523.2014, 2526.2585, 2540.2451, 2542.2578, 2545.2258, 2550.2373, 2561.6841, 2562.1826, 2562.6890, 2573.2495, 2576.1792, 2646.9758, 2659.2144, 2661.1409, 2662.0120, 2671.1924, 2681.2705, 2682.2888, 2685.3098, 2689.3059, 2695.1064, 2696.2695, 2699.2324, 2703.2529, 2705.2944, 2712.2937, 2717.2903, 2720.2839, 2721.2937, 2726.2935, 2727.2986, 2728.3079, 2739.2837, 2743.2729, 2744.2207, 2745.2256, 2746.2031, 2748.2295, 2755.2700, 2760.2590, 2763.1829, 2771.2400, 2778.1865, 2785.2969, 2786.2561, 2787.2524, 2790.2317, 2792.2166, 2800.3093, 2801.2932, 2803.2415, 2804.2429, 2807.2869, 2808.2227, 2812.2244, 2813.2373, 2817.2632, 2820.2441, 2824.2598, 2828.2454, 2833.2544, 2843.2900, 2844.3398, 2847.3630, 2851.3313, 2864.3188, 2890.3293, 2896.4324, 2908.3105, 2910.3325, 2911.3594, 2912.4265, 2925.3323, 2927.4434, 3023.3674, 3025.3865, 3026.3452, 3039.3838, 3042.3542, 3071.3933, 3086.3984, 3087.3970, 3093.4294, 3101.3831, 3103.4006, 3104.4041, 3110.4143, 3127.4749, 3141.4126, 3143.5066, 3144.5298, 3219.4854, 3236.5574, 3266.6021, 3280.5635, 3321.6216, 3344.4895, 3347.5515, 3348.5020, 3396.5195, 3412.5801, 3428.5874, 3444.5840, 3462.6301, 3478.6123, 3484.6497, 3489.7246, 3505.6919, 3510.5859, 3521.6392, 3552.6614, 3553.6028, 3554.5630, 3558.6460, 3573.6545, 3589.6523, 3608.6155, 3624.6685, 3630.5806, 3641.7090, 3651.7944, 3667.7266, 3683.6980, 3691.7681, 3707.7200, 3714.5493, 3715.6206, 3719.6914, 3720.7048, 3723.7041, 3735.7100, 3751.7539, 3830.8091, 3869.7393, 3876.6707, 3885.6946

2. P02459 **Mass:** 142825 **Score:** 101 **Expect:** 1.9e-006 **Matches:** 36

Collagen alpha-1(II) chain OS=Bos taurus GN=COL2A1 PE=1 SV=4

Observed	Mr(expt)	Mr(calc)	ppm	Start	End	Miss	Ions	Peptide
828.4415	827.4342	827.4250	11.2	335	344	0	---	R.TGPAGAAGAR.G
868.4412	867.4339	867.4199	16.1	384	392	0	---	R.GPEGAQGPR.G
949.5244	948.5171	948.5029	15.0	1164	1174	0	---	R.GPPGPVGPSGK.D
1041.5637	1040.5564	1040.5475	8.55	333	344	1	---	R.GRTGPAGAAGAR.G
1126.6241	1125.6168	1125.6183	-1.29	276	287	0	---	R.GFPGTPGLPGVK.G
1143.5592	1142.5519	1142.5720	-17.60	516	527	0	---	R.GFPGQDGLAGPK.G
1150.6226	1149.6153	1149.5931	19.4	1334	1342	2	---	K.KNWWSSKSK.D
1227.6459	1226.6386	1226.6156	18.8	288	299	1	---	K.GHRGYPGLDGAK.G
1293.6760	1292.6687	1292.6473	16.6	804	818	0	---	K.GEVGPPGPAGTAGAR.G
1349.6729	1348.6656	1348.6445	15.7	1230	1240	1	---	R.EKGPDPLQYMR.A + Oxidation (M)
1423.7096	1422.7023	1422.6964	4.17	926	940	2	---	K.DGPKGARGDSGPPGR.A
1503.7648	1502.7575	1502.7325	16.7	1116	1130	2	---	R.GDKGETGEAGERGLK.G
1526.7924	1525.7851	1525.7711	9.19	1445	1456	2	---	K.WGKTMIEYRSQK.T
1551.7465	1550.7392	1550.7260	8.55	1089	1106	0	---	R.GEAGAQGPMGPAGPAGAR.G
1553.7668	1552.7595	1552.7706	-7.13	1119	1133	2	---	K.GETGEAGERGLKGHR.G
1625.7886	1624.7813	1624.7805	0.50	765	781	2	---	K.GDRGDVGEKGPEGAPGK.D
1650.8429	1649.8356	1649.8710	-21.43	327	344	2	---	R.GLPGERGRTGPAGAAGAR.G
1664.8490	1663.8417	1663.8026	23.5	375	392	1	---	K.GEAGPTGARGPEGAQGPR.G
1682.8409	1681.8336	1681.8020	18.8	768	785	2	---	R.GDVGEKGPEGAPGKDGGR.G
1710.8904	1709.8831	1709.8485	20.2	516	533	1	---	R.GFPGQDGLAGPKGAPGER.G
1829.8527	1828.8454	1828.8205	13.6	191	209	0	---	K.AGGAQMGVMQGPMGPMGPR.G
1845.8363	1844.8290	1844.8154	7.38	191	209	0	---	K.AGGAQMGVMQGPMGPMGPR.G + Oxidation (M)
1861.8531	1860.8458	1860.8103	19.1	191	209	0	---	K.AGGAQMGVMQGPMGPMGPR.G + 2 Oxidation (M)
1973.9971	1972.9898	1972.9425	24.0	1232	1249	1	---	K.GPDPLQYMRADEAAGNLR.Q
2007.9500	2006.9427	2006.9341	4.31	1085	1106	1	101	K.QGDRGEAGAQGPMGPAGPAGAR.G
2007.9532	2006.9459	2006.9341	5.91	1085	1106	1	---	K.QGDRGEAGAQGPMGPAGPAGAR.G
2023.9482	2022.9409	2022.9290	5.90	1085	1106	1	---	K.QGDRGEAGAQGPMGPAGPAGAR.G + Oxidation (M)
2029.9972	2028.9899	2028.9726	8.55	120	140	2	---	K.GPPGPQGPAGEQGPRGDRGDK.G
2082.9822	2081.9749	2081.9549	9.63	1107	1127	2	---	R.GMPGPQGPRGDKGETGEAGER.G
2143.0237	2142.0164	2142.0276	-5.24	732	755	0	---	K.GAAGPAGPPGAQGPPGLQGMPGER.G + Oxidation (M)
2283.1606	2282.1533	2282.1114	18.4	597	620	2	---	R.GQPGVMGFPGPKGANGEPGKAGEK.G + Oxidation (M)
2400.0532	2399.0459	2399.0596	-5.70	1313	1333	0	---	K.VFCNMETGETCVYPNPASVPK.K
2844.3398	2843.3325	2843.3951	-22.01	1017	1048	1	---	R.GPPGPVGPPGLTGPAGEPGREGSPGADGPPGR.D
3023.3674	3022.3601	3022.3992	-12.93	210	242	0	---	R.GPPGPAGAPGPQGFQGNPGEPGEPGVSGPMGPR.G + Oxidation (M)
3321.6216	3320.6143	3320.6763	-18.67	1128	1163	2	---	R.GLKGHRGFTGLQGLPGPPGPSGDQGASGPAGPSGPR.G
3869.7393	3868.7320	3868.8056	-19.03	1346	1379	0	---	K.HIWFGETINGGFHFSYGDDNLAPNTANVQMTFLR.L

No match to: 716.3353, 718.2895, 724.2175, 726.3070, 728.2833, 729.4336, 730.2399, 732.2518, 734.4341, 743.3130, 745.3617, 747.4360, 753.3469, 756.3524, 758.3462, 759.3632, 768.3517, 779.4633, 781.3725, 789.3640, 794.3674, 796.3701, 799.2854, 801.4280, 805.4417, 807.4076, 810.3293, 812.3207, 818.3782, 820.3700, 829.4508, 830.4513, 834.3805, 842.5101, 849.3834, 852.4368, 857.4457, 860.4262, 870.4462, 873.4370, 877.4166, 880.4280, 882.4470, 883.4816, 887.4295, 889.4857, 891.4872, 894.4923, 898.4610, 900.5174, 905.4763, 907.4251, 912.5086, 914.4942, 920.4296, 922.4855, 928.4565, 929.4484, 933.4327, 936.4976, 938.5062, 944.4982, 946.4672, 950.5168, 957.5792, 961.5109, 965.5151, 967.4963, 972.5023, 974.4913, 976.5255, 978.5352, 981.5225, 983.6221, 986.5463, 988.4922, 993.5123, 995.4512, 998.5833, 1000.5594, 1006.5120, 1007.5640, 1021.5762, 1024.5542, 1031.5300, 1034.5703, 1037.5466, 1043.5297, 1044.5375, 1045.5594, 1046.6058, 1054.5287, 1056.5575, 1057.6187, 1057.6200, 1068.5790, 1074.6162, 1076.6190, 1082.6056, 1105.5938, 1110.5851, 1113.5701, 1118.5687, 1120.6050, 1122.5892, 1123.6047, 1128.5618, 1130.5756, 1135.5951, 1137.5647, 1145.6373, 1148.6318, 1149.6230, 1156.5652, 1157.5676, 1159.5890, 1160.5966, 1169.5873, 1177.6421, 1178.6371, 1180.6443, 1182.6539, 1184.6431, 1185.6815, 1193.6090, 1194.6001, 1209.6238, 1213.7180, 1216.7178, 1221.5953, 1229.6874, 1234.6899, 1238.2251, 1239.6622, 1254.6993, 1255.6351, 1264.7139, 1265.1680, 1265.7196, 1269.6532, 1276.6729, 1278.6892, 1281.6375, 1285.6674, 1289.7029, 1301.6362, 1303.6489, 1304.6685, 1306.6946, 1308.6797, 1314.7227, 1319.6770, 1320.6364, 1323.6815, 1326.6937, 1328.6799, 1333.7430, 1336.7301, 1337.6429, 1351.6969, 1353.6891, 1356.6255, 1358.6919, 1359.7013, 1361.7147, 1364.7118, 1365.6619, 1366.6965, 1372.6228, 1375.6693, 1382.6771, 1383.7347, 1390.7089, 1393.8007, 1397.7449, 1403.7434, 1410.7849, 1416.7737, 1420.7114, 1434.7847, 1436.7469, 1442.8257, 1445.7913, 1456.7555, 1457.7440, 1467.7560, 1470.7466, 1475.7615, 1479.8038, 1483.7579, 1484.7480, 1487.7660, 1488.7366, 1493.7556, 1500.7393, 1500.7400, 1504.7708, 1514.7063, 1516.7281, 1519.7705, 1522.7722, 1524.8040, 1528.7079, 1530.7058, 1536.7252, 1537.7859, 1540.8043, 1543.7938, 1550.7543, 1555.7836, 1556.8011, 1562.8008, 1568.7620, 1572.7245, 1578.8058, 1580.8580, 1580.8600, 1584.8486, 1587.7675, 1608.8051, 1616.8165, 1621.7870, 1628.7313, 1632.8120, 1635.7964, 1637.7974, 1642.7417, 1647.8439, 1651.8369, 1655.8147, 1657.8483, 1658.8953, 1660.8379, 1662.8666, 1666.7892, 1679.8091, 1680.8357, 1696.8464, 1698.8180, 1701.8243, 1702.8386, 1707.8938, 1714.7905, 1716.8219, 1717.9052, 1718.9094, 1721.8976, 1722.9003, 1724.9030, 1728.8840, 1730.8910, 1734.1617, 1738.8267, 1739.8165, 1741.8934, 1743.9005, 1747.9127, 1758.4807, 1758.8918, 1761.8734, 1765.8154, 1772.0012, 1774.9300, 1774.9319, 1779.9012, 1781.8585, 1783.8062, 1787.8407, 1809.0583, 1810.0460, 1812.9126, 1832.8584, 1833.8571, 1834.8650, 1836.8618, 1838.8677, 1847.8600, 1848.8938, 1850.9128, 1851.8932, 1862.9542, 1864.9258, 1880.9026, 1883.7704, 1887.8878, 1897.7863, 1902.9769, 1904.9276, 1908.8986, 1920.9131, 1923.9126, 1928.0443, 1932.0068, 1936.9510, 1938.0171, 1947.9314, 1958.9393, 1959.9655, 1960.9813, 1961.9463, 1967.1549, 1969.9951, 1975.9755, 1976.9708, 1990.4971, 1990.9373, 1991.4857, 1997.8479, 2001.8607, 2003.8768, 2004.8850, 2011.9381, 2012.8951, 2015.9694, 2017.9860, 2018.9740, 2020.1631, 2026.9529, 2027.5033, 2043.5356, 2044.9940, 2055.0205, 2057.0154, 2060.9878, 2066.9939, 2068.9661, 2086.0254, 2088.1033, 2097.0371, 2098.4941, 2102.9954, 2109.0095, 2112.9932, 2114.9707, 2122.9578, 2128.0444, 2130.9980, 2132.0200, 2136.9250, 2140.0063, 2144.0559, 2145.0781, 2148.0483, 2153.0049, 2153.9973, 2174.0508, 2175.0435, 2178.0483, 2188.0610, 2191.0464, 2192.0427, 2199.1150, 2205.0615, 2211.1067, 2223.1533, 2225.1382, 2226.1472, 2239.1646, 2243.1448, 2246.9482, 2251.1108, 2255.1484, 2265.0837, 2271.1211, 2273.0735, 2277.0642, 2285.1355, 2286.0986, 2289.0901, 2291.0173, 2309.0349, 2328.9888, 2343.0608, 2352.9583, 2355.0383, 2366.9595, 2371.0325, 2392.1594, 2424.1577, 2434.2429, 2438.2283, 2449.2180, 2450.2451, 2451.2480, 2465.1592, 2466.2458, 2467.2458, 2478.1377, 2501.2207, 2523.2014, 2526.2585, 2540.2451, 2542.2578, 2545.2258, 2550.2373, 2561.6841, 2562.1826, 2562.6890, 2573.2495, 2576.1792, 2646.9758, 2659.2144, 2661.1409, 2662.0120, 2671.1924, 2681.2705, 2682.2888, 2685.3098, 2689.3059, 2695.1064, 2696.2695, 2699.2324, 2703.2529, 2705.2944, 2712.2937, 2717.2903, 2720.2839, 2721.2937, 2726.2935, 2727.2986, 2728.3079, 2739.2837, 2743.2729, 2744.2207, 2745.2256, 2746.2031, 2748.2295, 2755.2700, 2760.2590, 2763.1829, 2771.2400, 2778.1865, 2785.2969, 2786.2561, 2787.2524, 2790.2317, 2792.2166, 2800.3093, 2801.2932, 2803.2415, 2804.2429, 2807.2869, 2808.2227, 2812.2244, 2813.2373, 2817.2632, 2820.2441, 2824.2598, 2828.2454, 2833.2544, 2843.2900, 2847.3630, 2851.3313, 2864.3188, 2890.3293, 2896.4324, 2908.3105, 2910.3325, 2911.3594, 2912.4265, 2925.3323, 2927.4434, 3025.3865, 3026.3452, 3039.3838, 3042.3542, 3070.3745, 3071.3933, 3086.3984, 3087.3970, 3093.4294, 3101.3831, 3103.4006, 3104.4041, 3110.4143, 3127.4749, 3141.4126, 3143.5066, 3144.5298, 3159.5210, 3219.4854, 3236.5574, 3266.6021, 3280.5635, 3344.4895, 3347.5515, 3348.5020, 3380.5210, 3396.5195, 3412.5801, 3427.6099, 3428.5874, 3444.5840, 3462.6301, 3478.6123, 3484.6497, 3489.7246, 3505.6919, 3510.5859, 3521.6392, 3552.6614, 3553.6028, 3554.5630, 3558.6460, 3573.6545, 3589.6523, 3608.6155, 3624.6685, 3630.5806, 3641.7090, 3651.7944, 3667.7266, 3683.6980, 3691.7681, 3707.7200, 3714.5493, 3715.6206, 3719.6914, 3720.7048, 3723.7041, 3735.7100, 3751.7539, 3830.8091, 3876.6707, 3885.6946

3. F1MSR8 **Mass:** 134858 **Score:** 101 **Expect:** 1.9e-006 **Matches:** 36

Collagen alpha-1(II) chain OS=Bos taurus GN=COL2A1 PE=4 SV=2

Observed	Mr(expt)	Mr(calc)	ppm	Start	End	Miss	Ions	Peptide
828.4415	827.4342	827.4250	11.2	266	275	0	---	R.TGPAGAAGAR.G
868.4412	867.4339	867.4199	16.1	315	323	0	---	R.GPEGAQGPR.G
949.5244	948.5171	948.5029	15.0	1095	1105	0	---	R.GPPGPVGPSGK.D
1041.5637	1040.5564	1040.5475	8.55	264	275	1	---	R.GRTGPAGAAGAR.G

Observed	Mr(expt)	Mr(calc)	ppm	Start	End	Miss	Ions	Peptide
1126.6241	1125.6168	1125.6183	-1.29	207 -	218	0	---	R.GFPGTPGLPGVK.G
1143.5592	1142.5519	1142.5720	-17.60	447 -	458	0	---	R.GFPGQDGLAGPK.G
1150.6226	1149.6153	1149.5931	19.4	1265 -	1273	2	---	K.KNWWSSKSK.D
1227.6459	1226.6386	1226.6156	18.8	219 -	230	1	---	K.GHRGYPGLDGAK.G
1293.6760	1292.6687	1292.6473	16.6	735 -	749	0	---	K.GEVGPPGPAGTAGAR.G
1349.6729	1348.6656	1348.6445	15.7	1161 -	1171	1	---	R.EKGPDPLQYMR.A + Oxidation (M)
1423.7096	1422.7023	1422.6964	4.17	857 -	871	2	---	K.DGPKGARGDSGPPGR.A
1503.7648	1502.7575	1502.7325	16.7	1047 -	1061	2	---	R.GDKGETGEAGERGLK.G
1526.7924	1525.7851	1525.7711	9.19	1376 -	1387	2	---	K.WGKTMIEYRSQK.T
1551.7465	1550.7392	1550.7260	8.55	1020 -	1037	0	---	R.GEAGAQGPMGPAGPAGAR.G
1553.7668	1552.7595	1552.7706	-7.13	1050 -	1064	2	---	K.GETGEAGERGLKGHR.G
1625.7886	1624.7813	1624.7805	0.50	696 -	712	2	---	K.GDRGDVGEKGPEGAPGK.D
1650.8429	1649.8356	1649.8710	-21.43	258 -	275	2	---	R.GLPGERGRTGPAGAAGAR.G
1664.8490	1663.8417	1663.8026	23.5	306 -	323	1	---	K.GEAGPTGARGPEGAQGPR.G
1682.8409	1681.8336	1681.8020	18.8	699 -	716	2	---	R.GDVGEKGPEGAPGKDGGR.G
1710.8904	1709.8831	1709.8485	20.2	447 -	464	1	---	R.GFPGQDGLAGPKGAPGER.G
1829.8527	1828.8454	1828.8205	13.6	122 -	140	0	---	K.AGGAQMGVMQGPMGPMGPR.G
1845.8363	1844.8290	1844.8154	7.38	122 -	140	0	---	K.AGGAQMGVMQGPMGPMGPR.G + Oxidation (M)
1861.8531	1860.8458	1860.8103	19.1	122 -	140	0	---	K.AGGAQMGVMQGPMGPMGPR.G + 2 Oxidation (M)
1973.9971	1972.9898	1972.9425	24.0	1163 -	1180	1	---	K.GPDPLQYMRADEAAGNLR.Q
2007.9500	2006.9427	2006.9341	4.31	1016 -	1037	1	101	K.QGDRGEAGAQGPMGPAGPAGAR.G
2007.9532	2006.9459	2006.9341	5.91	1016 -	1037	1	---	K.QGDRGEAGAQGPMGPAGPAGAR.G
2023.9482	2022.9409	2022.9290	5.90	1016 -	1037	1	---	K.QGDRGEAGAQGPMGPAGPAGAR.G + Oxidation (M)
2029.9972	2028.9899	2028.9726	8.55	51 -	71	2	---	K.GPPGPQGPAGEQGPRGDRGDK.G
2082.9822	2081.9749	2081.9549	9.63	1038 -	1058	2	---	R.GMPGPQGPRGDKGETGEAGER.G
2143.0237	2142.0164	2142.0276	-5.24	663 -	686	0	---	K.GAAGPAGPPGAQGPPGLQGMPGER.G + Oxidation (M)
2283.1606	2282.1533	2282.1114	18.4	528 -	551	2	---	R.GQPGVMGFPGPKGANGEPGKAGEK.G + Oxidation (M)
2400.0532	2399.0459	2399.0596	-5.70	1244 -	1264	0	---	K.VFCNMETGETCVYPNPASVPK.K
2844.3398	2843.3325	2843.3951	-22.01	948 -	979	1	---	R.GPPGPVGPPGLTGPAGEPGREGSPGADGPPGR.D
3023.3674	3022.3601	3022.3992	-12.93	141 -	173	0	---	R.GPPGPAGAPGPQGFQGNPGEPGEPGVSGPMGPR.G + Oxidation (M)
3321.6216	3320.6143	3320.6763	-18.67	1059 -	1094	2	---	R.GLKGHRGFTGLQGLPGPPGPSGDQGASGPAGPSGPR.G
3869.7393	3868.7320	3868.8056	-19.03	1277 -	1310	0	---	K.HIWFGETINGGFHFSYGDDNLAPNTANVQMTFLR.L

No match to: 716.3353, 718.2895, 724.2175, 726.3070, 728.2833, 729.4336, 730.2399, 732.2518, 734.4341, 743.3130, 745.3617, 747.4360, 753.3469, 756.3524, 758.3462, 759.3632, 768.3517, 779.4633, 781.3725, 789.3640, 794.3674, 796.3701, 799.2854, 801.4280, 805.4417, 807.4076, 810.3293, 812.3207, 818.3782, 820.3700, 829.4508, 830.4513, 834.3805, 842.5101, 849.3834, 852.4368, 857.4457, 860.4262, 870.4462, 873.4370, 877.4166, 880.4280, 882.4470, 883.4816, 887.4295, 889.4857, 891.4872, 894.4923, 898.4610, 900.5174, 905.4763, 907.4251, 912.5086, 914.4942, 920.4296, 922.4855, 928.4565, 929.4484, 933.4327, 936.4976, 938.5062, 944.4982, 946.4672, 950.5168, 957.5792, 961.5109, 965.5151, 967.4963, 972.5023, 974.4913, 976.5255, 978.5352, 981.5225, 983.6221, 986.5463, 988.4922, 993.5123, 995.4512, 998.5833, 1000.5594, 1006.5120, 1007.5640, 1021.5762, 1024.5542, 1031.5300, 1034.5703, 1037.5466, 1043.5297, 1044.5375, 1045.5594, 1046.6058, 1054.5287, 1056.5575, 1057.6187, 1057.6200, 1068.5790, 1074.6162, 1076.6190, 1082.6056, 1105.5938, 1110.5851, 1113.5701, 1118.5687, 1120.6050, 1122.5892, 1123.6047, 1128.5618, 1130.5756, 1135.5951, 1137.5647, 1145.6373, 1148.6318, 1149.6230, 1156.5652, 1157.5676, 1159.5890, 1160.5966, 1169.5873, 1177.6421, 1178.6371, 1180.6443, 1182.6539, 1184.6431, 1185.6815, 1193.6090, 1194.6001, 1209.6238, 1213.7180, 1216.7178, 1221.5953, 1229.6874, 1234.6899, 1238.2251, 1239.6622, 1254.6993, 1255.6351, 1264.7139, 1265.1680, 1265.7196, 1269.6532, 1276.6729, 1278.6892, 1281.6375, 1285.6674, 1289.7029, 1301.6362, 1303.6489, 1304.6685, 1306.6946, 1308.6797, 1314.7227, 1319.6770, 1320.6364, 1323.6815, 1326.6937, 1328.6799, 1333.7430, 1336.7301, 1337.6429, 1351.6969, 1353.6891, 1356.6255, 1358.6919, 1359.7013, 1361.7147, 1364.7118, 1365.6619, 1366.6965, 1372.6228, 1375.6693, 1382.6771, 1383.7347, 1390.7089, 1393.8007, 1397.7449, 1403.7434, 1410.7849, 1416.7737, 1420.7114, 1434.7847, 1436.7469, 1442.8257, 1445.7913, 1456.7555, 1457.7440, 1467.7560, 1470.7466, 1475.7615, 1479.8038, 1483.7579, 1484.7480, 1487.7660, 1488.7366, 1493.7556, 1500.7393, 1500.7400, 1504.7708, 1514.7063, 1516.7281, 1519.7705, 1522.7722, 1524.8040, 1528.7079, 1530.7058, 1536.7252, 1537.7859, 1540.8043, 1543.7938, 1550.7543, 1555.7836, 1556.8011, 1562.8008, 1568.7620, 1572.7245, 1578.8058, 1580.8580, 1580.8600, 1584.8486, 1587.7675, 1608.8051, 1616.8165, 1621.7870, 1628.7313, 1632.8120, 1635.7964, 1637.7974, 1642.7417, 1647.8439, 1651.8369, 1655.8147, 1657.8483, 1658.8953, 1660.8379, 1662.8666, 1666.7892, 1679.8091, 1680.8357, 1696.8464, 1698.8180, 1701.8243, 1702.8386, 1707.8938, 1714.7905, 1716.8219, 1717.9052, 1718.9094, 1721.8976, 1722.9003, 1724.9030, 1728.8840, 1730.8910, 1734.1617, 1738.8267, 1739.8165, 1741.8934, 1743.9005, 1747.9127, 1758.4807, 1758.8918, 1761.8734, 1765.8154, 1772.0012, 1774.9300, 1774.9319, 1779.9012, 1781.8585, 1783.8062, 1787.8407, 1809.0583, 1810.0460, 1812.9126, 1832.8584, 1833.8571, 1834.8650, 1836.8618, 1838.8677, 1847.8600, 1848.8938, 1850.9128, 1851.8932, 1862.9542, 1864.9258, 1880.9026, 1883.7704, 1887.8878, 1897.7863, 1902.9769, 1904.9276, 1908.8986, 1920.9131, 1923.9126, 1928.0443, 1932.0068, 1936.9510, 1938.0171, 1947.9314, 1958.9393, 1959.9655, 1960.9813, 1961.9463, 1967.1549, 1969.9951, 1975.9755, 1976.9708, 1990.4971, 1990.9373, 1991.4857, 1997.8479, 2001.8607, 2003.8768, 2004.8850, 2011.9381, 2012.8951, 2015.9694, 2017.9860, 2018.9740, 2020.1631, 2026.9529, 2027.5033, 2043.5356, 2044.9940, 2055.0205, 2057.0154, 2060.9878, 2066.9939, 2068.9661, 2086.0254, 2088.1033, 2097.0371, 2098.4941, 2102.9954, 2109.0095, 2112.9932, 2114.9707, 2122.9578, 2128.0444, 2130.9980, 2132.0200, 2136.9250, 2140.0063, 2144.0559, 2145.0781, 2148.0483, 2153.0049, 2153.9973, 2174.0508, 2175.0435, 2178.0483, 2188.0610, 2191.0464, 2192.0427, 2199.1150, 2205.0615, 2211.1067, 2223.1533, 2225.1382, 2226.1472, 2239.1646, 2243.1448, 2246.9482, 2251.1108, 2255.1484, 2265.0837, 2271.1211, 2273.0735, 2277.0642, 2285.1355, 2286.0986, 2289.0901, 2291.0173, 2309.0349, 2328.9888, 2343.0608, 2352.9583, 2355.0383, 2366.9595, 2371.0325, 2392.1594, 2424.1577, 2434.2429, 2438.2283, 2449.2180, 2450.2451, 2451.2480, 2465.1592, 2466.2458, 2467.2458, 2478.1377, 2501.2207, 2523.2014, 2526.2585, 2540.2451, 2542.2578, 2545.2258, 2550.2373, 2561.6841, 2562.1826, 2562.6890, 2573.2495, 2576.1792, 2646.9758, 2659.2144, 2661.1409, 2662.0120, 2671.1924, 2681.2705, 2682.2888, 2685.3098, 2689.3059, 2695.1064, 2696.2695, 2699.2324, 2703.2529, 2705.2944, 2712.2937, 2717.2903, 2720.2839, 2721.2937, 2726.2935, 2727.2986, 2728.3079, 2739.2837, 2743.2729, 2744.2207, 2745.2256, 2746.2031, 2748.2295, 2755.2700, 2760.2590, 2763.1829, 2771.2400, 2778.1865, 2785.2969, 2786.2561, 2787.2524, 2790.2317, 2792.2166, 2800.3093, 2801.2932, 2803.2415, 2804.2429, 2807.2869, 2808.2227, 2812.2244, 2813.2373, 2817.2632, 2820.2441, 2824.2598, 2828.2454, 2833.2544, 2843.2900, 2847.3630, 2851.3313, 2864.3188, 2890.3293, 2896.4324, 2908.3105, 2910.3325, 2911.3594, 2912.4265, 2925.3323, 2927.4434, 3025.3865, 3026.3452, 3039.3838, 3042.3542, 3070.3745, 3071.3933, 3086.3984, 3087.3970, 3093.4294, 3101.3831, 3103.4006, 3104.4041, 3110.4143, 3127.4749, 3141.4126, 3143.5066, 3144.5298, 3159.5210, 3219.4854, 3236.5574, 3266.6021, 3280.5635, 3344.4895, 3347.5515, 3348.5020, 3380.5210, 3396.5195, 3412.5801, 3427.6099, 3428.5874, 3444.5840, 3462.6301, 3478.6123, 3484.6497, 3489.7246, 3505.6919, 3510.5859, 3521.6392, 3552.6614, 3553.6028, 3554.5630, 3558.6460, 3573.6545, 3589.6523, 3608.6155, 3624.6685, 3630.5806, 3641.7090, 3651.7944, 3667.7266, 3683.6980, 3691.7681, 3707.7200, 3714.5493, 3715.6206, 3719.6914, 3720.7048, 3723.7041, 3735.7100, 3751.7539, 3830.8091, 3876.6707, 3885.6946

4. F1MKG2 **Mass:** 110451 **Score:** 45 **Expect:** 0.77 **Matches:** 39

Uncharacterized protein OS=Bos taurus GN=COL6A2 PE=1 SV=2

Observed	Mr(expt)	Mr(calc)	ppm	Start	End	Miss	Ions	Peptide
829.4508	828.4435	828.4453	-2.18	204 -	210	0	---	K.LNEQGLR.D
830.4513	829.4440	829.4406	4.14	188 -	194	1	---	R.AREEGIR.L
860.4262	859.4189	859.4035	17.8	747 -	753	0	---	R.DDDLNLR.A
967.4963	966.4891	966.5108	-22.44	381 -	389	1	---	K.GDPGRPGRR.G
972.5023	971.4950	971.4825	12.9	852 -	859	0	---	R.LGEQNFHK.V
998.5833	997.5760	997.5960	-20.05	195 -	203	0	---	R.LFAVPPNLK.L
1024.5542	1023.5469	1023.5349	11.8	129 -	137	0	---	K.SLQSISSFR.R
1043.5297	1042.5224	1042.5043	17.4	876 -	884	1	---	R.KDDDPLNAR.V
1057.6187	1056.6114	1056.6080	3.22	885 -	894	0	---	R.VALLQFGGPR.E
1057.6200	1056.6127	1056.6080	4.45	885 -	894	0	39	R.VALLQFGGPR.E
1074.6162	1073.6089	1073.5982	10.0	644 -	652	0	---	K.NFVINVVNR.L
1076.6190	1075.6117	1075.6026	8.47	733 -	742	0	---	R.VFAVVITDGR.H

1145.6373	1144.6300	1144.6241	5.22	996 -	1006	0	---	K.ISLGDPAAVFR.E
1177.6421	1176.6348	1176.6363	-1.29	862 -	870	2	---	R.RFVEEVSRR.L
1180.6443	1179.6370	1179.6360	0.87	129 -	138	1	---	K.SLQSISSFRR.G
1227.6459	1226.6386	1226.6520	-10.90	852 -	861	1	---	R.LGEQNFHKVR.R
1328.6799	1327.6726	1327.6520	15.5	211 -	221	0	---	R.DIANTPHELYR.N
1333.7430	1332.7357	1332.7514	-11.77	731 -	742	1	---	K.TRVFAVVITDGR.H
1365.6619	1364.6546	1364.6433	8.31	743 -	753	1	---	R.HDPRDDDLNLR.A
1383.7347	1382.7274	1382.7531	-18.57	852 -	862	2	---	R.LGEQNFHKVRR.F
1514.7063	1513.6990	1513.6693	19.6	245 -	256	1	---	K.VMKHEAYGECYK.V
1530.7058	1529.6985	1529.6787	13.0	1009 -	1021	0	---	K.DYDSLAQPGFFDR.F
1555.7836	1554.7763	1554.8114	-22.57	327 -	343	2	---	K.GAPGLAGKNGTDGQKGK.L
1655.8147	1654.8074	1654.8210	-8.18	344 -	359	2	---	K.LGRIGPPGCKGDTGDR.G
1743.9005	1742.8932	1742.8992	-3.40	701 -	716	0	---	K.NLEWIAGGTWTPSALK.F
1774.9300	1773.9227	1773.8983	13.8	381 -	398	2	---	K.GDPGRPGRRGPPGENGAK.G
1774.9319	1773.9246	1773.8983	14.9	381 -	398	2	---	K.GDPGRPGRRGPPGENGAK.G
1787.8407	1786.8334	1786.8162	9.63	1007 -	1021	1	---	R.EKDYDSLAQPGFFDR.F
1809.0583	1808.0510	1808.0308	11.2	195 -	210	1	---	R.LFAVPPNLKLNEQGLR.D
1810.0460	1809.0387	1809.0261	7.00	188 -	203	2	---	R.AREEGIRLFAVPPNLK.L
1932.0068	1930.9995	1930.9973	1.13	474 -	493	2	---	K.GDRGLPGPRGPQGTVGEPGK.Q
1947.9314	1946.9241	1946.9082	8.15	441 -	461	2	---	K.GGPGSDGPKGEKGDPGPEGPR.G
2174.0508	2173.0435	2173.0222	9.82	775 -	792	0	---	K.HESENLYSIACDKPQQVR.N
2277.0642	2276.0569	2276.0894	-14.28	483 -	506	2	---	R.GPQGTVGEPGKQGSRGDPGDAGPR.G
2392.1594	2391.1521	2391.1343	7.46	668 -	688	0	---	R.VGVVQYSHEGTFEAIQLDDER.I
2755.2700	2754.2627	2754.2266	13.1	581 -	607	0	---	R.GAPGPEGEPGPPGDPGLTECDVMTYVR.E
2771.2400	2770.2327	2770.2215	4.06	581 -	607	0	---	R.GAPGPEGEPGPPGDPGLTECDVMTYVR.E + Oxidation (M)
2864.3188	2863.3115	2863.3421	-10.70	248 -	272	2	---	K.HEAYGECYKVSCLEIPGPPGPKGYR.G
3023.3674	3022.3601	3022.4268	-22.07	662 -	688	1	---	K.SETGTRVGVVQYSHEGTFEAIQLDDER.I

No match to: 716.3353, 718.2895, 724.2175, 726.3070, 728.2833, 729.4336, 730.2399, 732.2518, 734.4341, 743.3130, 745.3617, 747.4360, 753.3469, 756.3524, 758.3462, 759.3632, 768.3517, 779.4633, 781.3725, 789.3640, 794.3674, 796.3701, 799.2854, 801.4280, 805.4417, 807.4076, 810.3293, 812.3207, 818.3782, 820.3700, 828.4415, 834.3805, 842.5101, 849.3834, 852.4368, 857.4457, 868.4412, 870.4462, 873.4370, 877.4166, 880.4280, 882.4470, 883.4816, 887.4295, 889.4857, 891.4872, 894.4923, 898.4610, 900.5174, 905.4763, 907.4251, 912.5086, 914.4942, 920.4296, 922.4855, 928.4565, 929.4484, 933.4327, 936.4976, 938.5062, 944.4982, 946.4672, 949.5244, 950.5168, 957.5792, 961.5109, 965.5151, 974.4913, 976.5255, 978.5352, 981.5225, 983.6221, 986.5463, 988.4922, 993.5123, 995.4512, 1000.5594, 1006.5120, 1007.5640, 1021.5762, 1031.5300, 1034.5703, 1037.5466, 1041.5637, 1044.5375, 1045.5594, 1046.6058, 1054.5287, 1056.5575, 1068.5790, 1082.6056, 1105.5938, 1110.5851, 1113.5701, 1118.5687, 1120.6050, 1122.5892, 1123.6047, 1126.6241, 1128.5618, 1130.5756, 1135.5951, 1137.5647, 1143.5592, 1148.6318, 1149.6230, 1150.6226, 1156.5652, 1157.5676, 1159.5890, 1160.5966, 1169.5873, 1178.6371, 1182.6539, 1184.6431, 1185.6815, 1193.6090, 1194.6001, 1209.6238, 1213.7180, 1216.7178, 1221.5953, 1229.6874, 1234.6899, 1238.2251, 1239.6622, 1254.6993, 1255.6351, 1264.7139, 1265.1680, 1265.7196, 1269.6532, 1276.6729, 1278.6892, 1281.6375, 1285.6674, 1289.7029, 1293.6760, 1301.6362, 1303.6489, 1304.6685, 1306.6946, 1308.6797, 1314.7227, 1319.6770, 1320.6364, 1323.6815, 1326.6937, 1336.7301, 1337.6429, 1349.6729, 1351.6969, 1353.6891, 1356.6255, 1358.6919, 1359.7013, 1361.7147, 1364.7118, 1366.6965, 1372.6228, 1375.6693, 1382.6771, 1390.7089, 1393.8007, 1397.7449, 1403.7434, 1410.7849, 1416.7737, 1420.7114, 1423.7096, 1434.7847, 1436.7469, 1442.8257, 1445.7913, 1456.7555, 1457.7440, 1467.7560, 1470.7466, 1475.7615, 1479.8038, 1483.7579, 1484.7480, 1487.7660, 1488.7366, 1493.7556, 1500.7393, 1500.7400, 1503.7648, 1504.7708, 1516.7281, 1519.7705, 1522.7722, 1524.8040, 1526.7924, 1528.7079, 1536.7252, 1537.7859, 1540.8043, 1543.7938, 1550.7543, 1551.7465, 1553.7668, 1556.8011, 1562.8008, 1568.7620, 1572.7245, 1578.8058, 1580.8580, 1580.8600, 1584.8486, 1587.7675, 1608.8051, 1616.8165, 1621.7870, 1625.7886, 1628.7313, 1632.8120, 1635.7964, 1637.7974, 1642.7417, 1647.8439, 1650.8429, 1651.8369, 1657.8483, 1658.8953, 1660.8379, 1662.8666, 1664.8490, 1666.7892, 1679.8091, 1680.8357, 1682.8409, 1696.8464, 1698.8180, 1701.8243, 1702.8386, 1707.8938, 1710.8904, 1714.7905, 1716.8219, 1717.9052, 1718.9094, 1721.8976, 1722.9003, 1724.9030, 1728.8840, 1730.8910, 1734.1617, 1738.8267, 1739.8165, 1741.8934, 1747.9127, 1758.4807, 1758.8918, 1761.8734, 1765.8154, 1772.0012, 1779.9012, 1781.8585, 1783.8062, 1812.9126, 1829.8527, 1832.8584, 1833.8571, 1834.8650, 1836.8618, 1838.8677, 1845.8363, 1847.8600, 1848.8938, 1850.9128, 1851.8932, 1861.8531, 1862.9542, 1864.9258, 1880.9026, 1883.7704, 1887.8878, 1897.7863, 1902.9769, 1904.9276, 1908.8986, 1920.9131, 1923.9126, 1928.0443, 1936.9510, 1938.0171, 1958.9393, 1959.9655, 1960.9813, 1961.9463, 1967.1549, 1969.9951, 1973.9971, 1975.9755, 1976.9708, 1990.4971, 1990.9373, 1991.4857, 1997.8479, 2001.8607, 2003.8768, 2004.8850, 2007.9500, 2007.9532, 2011.9381, 2012.8951, 2015.9694, 2017.9860, 2018.9740, 2020.1631, 2023.9482, 2026.9529, 2027.5033, 2029.9972, 2043.5356, 2044.9940, 2055.0205, 2057.0154, 2060.9878, 2066.9939, 2068.9661, 2082.9822, 2086.0254, 2088.1033, 2097.0371, 2098.4941, 2102.9954, 2109.0095, 2112.9932, 2114.9707, 2122.9578, 2128.0444, 2130.9980, 2132.0200, 2136.9250, 2140.0063, 2143.0237, 2144.0559, 2145.0781, 2148.0483, 2153.0049, 2153.9973, 2175.0435, 2178.0483, 2188.0610, 2191.0464, 2192.0427, 2199.1150, 2205.0615, 2211.1067, 2223.1533, 2225.1382, 2226.1472, 2239.1646, 2243.1448, 2246.9482, 2251.1108, 2255.1484, 2265.0837, 2271.1211, 2273.0735, 2283.1606, 2285.1355, 2286.0986, 2289.0901, 2291.0173, 2309.0349, 2328.9888, 2343.0608, 2352.9583, 2355.0383, 2366.9595, 2371.0325, 2400.0532, 2424.1577, 2434.2429, 2438.2283, 2449.2180, 2450.2451, 2451.2480, 2465.1592, 2466.2458, 2467.2458, 2478.1377, 2501.2207, 2523.2014, 2526.2585, 2540.2451, 2542.2578, 2545.2258, 2550.2373, 2561.6841, 2562.1826, 2562.6890, 2573.2495, 2576.1792, 2646.9758, 2659.2144, 2661.1409, 2662.0120, 2671.1924, 2681.2705, 2682.2888, 2685.3098, 2689.3059, 2695.1064, 2696.2695, 2699.2324, 2703.2529, 2705.2944, 2712.2937, 2717.2903, 2720.2839, 2721.2937, 2726.2935, 2727.2986, 2728.3079, 2739.2837, 2743.2729, 2744.2207, 2745.2256, 2746.2031, 2748.2295, 2760.2590, 2763.1829, 2778.1865, 2785.2969, 2786.2561, 2787.2524, 2790.2317, 2792.2166, 2800.3093, 2801.2932, 2803.2415, 2804.2429, 2807.2869, 2808.2227, 2812.2244, 2813.2373, 2817.2632, 2820.2441, 2824.2598, 2828.2454, 2833.2544, 2843.2900, 2844.3398, 2847.3630, 2851.3313, 2890.3293, 2896.4324, 2908.3105, 2910.3325, 2911.3594, 2912.4265, 2925.3323, 2927.4434, 3025.3865, 3026.3452, 3039.3838, 3042.3542, 3070.3745, 3071.3933, 3086.3984, 3087.3970, 3093.4294, 3101.3831, 3103.4006, 3104.4041, 3110.4143, 3127.4749, 3141.4126, 3143.5066, 3144.5298, 3159.5210, 3219.4854, 3236.5574, 3266.6021, 3280.5635, 3321.6216, 3344.4895, 3347.5515, 3348.5020, 3380.5210, 3396.5195, 3412.5801, 3427.6099, 3428.5874, 3444.5840, 3462.6301, 3478.6123, 3484.6497, 3489.7246, 3505.6919, 3510.5859, 3521.6392, 3552.6614, 3553.6028, 3554.5630, 3558.6460, 3573.6545, 3589.6523, 3608.6155, 3624.6685, 3630.5806, 3641.7090, 3651.7944, 3667.7266, 3683.6980, 3691.7681, 3707.7200, 3714.5493, 3715.6206, 3719.6914, 3720.7048, 3723.7041, 3735.7100, 3751.7539, 3830.8091, 3869.7393, 3876.6707, 3885.6946

5. G3MXU3 **Mass:** 25162 **Score:** 45 **Expect:** 0.78 **Matches:** 11

Uncharacterized protein OS=Bos taurus GN=COL6A2 PE=4 SV=1

Observed	Mr(expt)	Mr(calc)	ppm	Start	End	Miss	Ions	Peptide
972.5023	971.4950	971.4825	12.9	54 -	61	0	---	R.LGEQNFHK.V
1043.5297	1042.5224	1042.5043	17.4	78 -	86	1	---	R.KDDDPLNAR.V
1057.6187	1056.6114	1056.6080	3.22	87 -	96	0	---	R.VALLQFGGPR.E
1057.6200	1056.6127	1056.6080	4.45	87 -	96	0	39	R.VALLQFGGPR.E
1145.6373	1144.6300	1144.6241	5.22	198 -	208	0	---	K.ISLGDPAAVFR.E
1149.6230	1148.6157	1148.5900	22.4	21 -	30	0	---	K.QDFPLMIAAK.E + Oxidation (M)
1177.6421	1176.6348	1176.6363	-1.29	64 -	72	2	---	R.RFVEEVSRR.L
1227.6459	1226.6386	1226.6520	-10.90	54 -	63	1	---	R.LGEQNFHKVR.R
1383.7347	1382.7274	1382.7531	-18.57	54 -	64	2	---	R.LGEQNFHKVRR.F
1530.7058	1529.6985	1529.6787	13.0	211 -	223	0	---	K.DYDSLAQPGFFDR.F
1787.8407	1786.8334	1786.8162	9.63	209 -	223	1	---	R.EKDYDSLAQPGFFDR.F

No match to: 716.3353, 718.2895, 724.2175, 726.3070, 728.2833, 729.4336, 730.2399, 732.2518, 734.4341, 743.3130, 745.3617, 747.4360, 753.3469, 756.3524, 758.3462, 759.3632, 768.3517, 779.4633, 781.3725, 789.3640, 794.3674, 796.3701, 799.2854, 801.4280, 805.4417, 807.4076, 810.3293, 812.3207, 818.3782, 820.3700, 828.4415, 829.4508, 830.4513, 834.3805, 842.5101, 849.3834, 852.4368, 857.4457, 860.4262, 868.4412, 870.4462, 873.4370, 877.4166, 880.4280, 882.4470, 883.4816, 887.4295, 889.4857, 891.4872, 894.4923, 898.4610, 900.5174, 905.4763, 907.4251, 912.5086, 914.4942, 920.4296, 922.4855, 928.4565, 929.4484, 933.4327, 936.4976, 938.5062, 944.4982, 946.4672, 949.5244, 950.5168, 957.5792, 961.5109, 965.5151, 967.4963, 974.4913, 976.5255, 978.5352, 981.5225, 983.6221, 986.5463, 988.4922, 993.5123, 995.4512, 998.5833, 1000.5594, 1006.5120, 1007.5640, 1021.5762, 1024.5542, 1031.5300, 1034.5703,

1037.5466, 1041.5637, 1044.5375, 1045.5594, 1046.6058, 1054.5287, 1056.5575, 1068.5790, 1074.6162, 1076.6190, 1082.6056, 1105.5938, 1110.5851, 1113.5701, 1118.5687, 1120.6050, 1122.5892, 1123.6047, 1126.6241, 1128.5618, 1130.5756, 1135.5951, 1137.5647, 1143.5592, 1148.6318, 1150.6226, 1156.5652, 1157.5676, 1159.5890, 1160.5966, 1169.5873, 1178.6371, 1180.6443, 1182.6539, 1184.6431, 1185.6815, 1193.6090, 1194.6001, 1209.6238, 1213.7180, 1216.7178, 1221.5953, 1229.6874, 1234.6899, 1238.2251, 1239.6622, 1254.6993, 1255.6351, 1264.7139, 1265.1680, 1265.7196, 1269.6532, 1276.6729, 1278.6892, 1281.6375, 1285.6674, 1289.7029, 1293.6760, 1301.6362, 1303.6489, 1304.6685, 1306.6946, 1308.6797, 1314.7227, 1319.6770, 1320.6364, 1323.6815, 1326.6937, 1328.6799, 1333.7430, 1336.7301, 1337.6429, 1349.6729, 1351.6969, 1353.6891, 1356.6255, 1358.6919, 1359.7013, 1361.7147, 1364.7118, 1365.6619, 1366.6965, 1372.6228, 1375.6693, 1382.6771, 1390.7089, 1393.8007, 1397.7449, 1403.7434, 1410.7849, 1416.7737, 1420.7114, 1423.7096, 1434.7847, 1436.7469, 1442.8257, 1445.7913, 1456.7555, 1457.7440, 1467.7560, 1470.7466, 1475.7615, 1479.8038, 1483.7579, 1484.7480, 1487.7660, 1488.7366, 1493.7556, 1500.7393, 1500.7400, 1503.7648, 1504.7708, 1514.7063, 1516.7281, 1519.7705, 1522.7722, 1524.8040, 1526.7924, 1528.7079, 1536.7252, 1537.7859, 1540.8043, 1543.7938, 1550.7543, 1551.7465, 1553.7668, 1555.7836, 1556.8011, 1562.8008, 1568.7620, 1572.7245, 1578.8058, 1580.8580, 1580.8600, 1584.8486, 1587.7675, 1608.8051, 1616.8165, 1621.7870, 1625.7886, 1628.7313, 1632.8120, 1635.7964, 1637.7974, 1642.7417, 1647.8439, 1650.8429, 1651.8369, 1655.8147, 1657.8483, 1658.8953, 1660.8379, 1662.8666, 1664.8490, 1666.7892, 1679.8091, 1680.8357, 1682.8409, 1696.8464, 1698.8180, 1701.8243, 1702.8386, 1707.8938, 1710.8904, 1714.7905, 1716.8219, 1717.9052, 1718.9094, 1721.8976, 1722.9003, 1724.9030, 1728.8840, 1730.8910, 1734.1617, 1738.8267, 1739.8165, 1741.8934, 1743.9005, 1747.9127, 1758.4807, 1758.8918, 1761.8734, 1765.8154, 1772.0012, 1774.9300, 1774.9319, 1779.9012, 1781.8585, 1783.8062, 1809.0583, 1810.0460, 1812.9126, 1829.8527, 1832.8584, 1833.8571, 1834.8650, 1836.8618, 1838.8677, 1845.8363, 1847.8600, 1848.8938, 1850.9128, 1851.8932, 1861.8531, 1862.9542, 1864.9258, 1880.9026, 1883.7704, 1887.8878, 1897.7863, 1902.9769, 1904.9276, 1908.8986, 1920.9131, 1923.9126, 1928.0443, 1932.0068, 1936.9510, 1938.0171, 1947.9314, 1958.9393, 1959.9655, 1960.9813, 1961.9463, 1967.1549, 1969.9951, 1973.9971, 1975.9755, 1976.9708, 1990.4971, 1990.9373, 1991.4857, 1997.8479, 2001.8607, 2003.8768, 2004.8850, 2007.9500, 2007.9532, 2011.9381, 2012.8951, 2015.9694, 2017.9860, 2018.9740, 2020.1631, 2023.9482, 2026.9529, 2027.5033, 2029.9972, 2043.5356, 2044.9940, 2055.0205, 2057.0154, 2060.9878, 2066.9939, 2068.9661, 2082.9822, 2086.0254, 2088.1033, 2097.0371, 2098.4941, 2102.9954, 2109.0095, 2112.9932, 2114.9707, 2122.9578, 2128.0444, 2130.9980, 2132.0200, 2136.9250, 2140.0063, 2143.0237, 2144.0559, 2145.0781, 2148.0483, 2153.0049, 2153.9973, 2174.0508, 2175.0435, 2178.0483, 2188.0610, 2191.0464, 2192.0427, 2199.1150, 2205.0615, 2211.1067, 2223.1533, 2225.1382, 2226.1472, 2239.1646, 2243.1448, 2246.9482, 2251.1108, 2255.1484, 2265.0837, 2271.1211, 2273.0735, 2277.0642, 2283.1606, 2285.1355, 2286.0986, 2289.0901, 2291.0173, 2309.0349, 2328.9888, 2343.0608, 2352.9583, 2355.0383, 2366.9595, 2371.0325, 2392.1594, 2400.0532, 2424.1577, 2434.2429, 2438.2283, 2449.2180, 2450.2451, 2451.2480, 2465.1592, 2466.2458, 2467.2458, 2478.1377, 2501.2207, 2523.2014, 2526.2585, 2540.2451, 2542.2578, 2545.2258, 2550.2373, 2561.6841, 2562.1826, 2562.6890, 2573.2495, 2576.1792, 2646.9758, 2659.2144, 2661.1409, 2662.0120, 2671.1924, 2681.2705, 2682.2888, 2685.3098, 2689.3059, 2695.1064, 2696.2695, 2699.2324, 2703.2529, 2705.2944, 2712.2937, 2717.2903, 2720.2839, 2721.2937, 2726.2935, 2727.2986, 2728.3079, 2739.2837, 2743.2729, 2744.2207, 2745.2256, 2746.2031, 2748.2295, 2755.2700, 2760.2590, 2763.1829, 2771.2400, 2778.1865, 2785.2969, 2786.2561, 2787.2524, 2790.2317, 2792.2166, 2800.3093, 2801.2932, 2803.2415, 2804.2429, 2807.2869, 2808.2227, 2812.2244, 2813.2373, 2817.2632, 2820.2441, 2824.2598, 2828.2454, 2833.2544, 2843.2900, 2844.3398, 2847.3630, 2851.3313, 2864.3188, 2890.3293, 2896.4324, 2908.3105, 2910.3325, 2911.3594, 2912.4265, 2925.3323, 2927.4434, 3023.3674, 3025.3865, 3026.3452, 3039.3838, 3042.3542, 3070.3745, 3071.3933, 3086.3984, 3087.3970, 3093.4294, 3101.3831, 3103.4006, 3104.4041, 3110.4143, 3127.4749, 3141.4126, 3143.5066, 3144.5298, 3159.5210, 3219.4854, 3236.5574, 3266.6021, 3280.5635, 3321.6216, 3344.4895, 3347.5515, 3348.5020, 3380.5210, 3396.5195, 3412.5801, 3427.6099, 3428.5874, 3444.5840, 3462.6301, 3478.6123, 3484.6497, 3489.7246, 3505.6919, 3510.5859, 3521.6392, 3552.6614, 3553.6028, 3554.5630, 3558.6460, 3573.6545, 3589.6523, 3608.6155, 3624.6685, 3630.5806, 3641.7090, 3651.7944, 3667.7266, 3683.6980, 3691.7681, 3707.7200, 3714.5493, 3715.6206, 3719.6914, 3720.7048, 3723.7041, 3735.7100, 3751.7539, 3830.8091, 3869.7393, 3876.6707, 3885.6946

6. Q32LI7 **Mass:** 30313 **Score:** 24 **Expect:** 86 **Matches:** 18

Putative uncharacterized protein MGC133880 OS=Bos taurus GN=MGC133880 PE=2 SV=1

Observed	Mr(expt)	Mr(calc)	ppm	Start	End	Miss	Ions	Peptide
805.4417	804.4344	804.4453	-13.54	266 -	272	2	---	K.SKNKNSK.-
1021.5762	1020.5689	1020.5465	22.0	97 -	105	1	---	K.KGSFRPSSR.E
1118.5687	1117.5614	1117.5476	12.4	57 -	67	1	---	R.GDSPRSSSLGR.A
1255.6351	1254.6278	1254.6105	13.8	240 -	249	1	---	K.TDDLGRWHQK.N
1319.6770	1318.6697	1318.6517	13.7	202 -	213	0	---	R.SNLFQGAPQETK.S
1383.7347	1382.7274	1382.7055	15.9	239 -	249	2	---	R.KTDDLGRWHQK.N
1519.7705	1518.7632	1518.7804	-11.30	228 -	239	2	---	K.SVRDLEHWHGRK.T
1635.7964	1634.7891	1634.7756	8.29	250 -	263	1	---	K.NAMNMNLQKALDEK.E + Oxidation (M)
1834.8650	1833.8577	1833.8870	-15.99	231 -	245	2	---	R.DLEHWHGRKTDDLGR.W
1908.8986	1907.8913	1907.9080	-8.76	250 -	265	2	---	K.NAMNMNLQKALDEKEK.S + 2 Oxidation (M)
1938.0171	1937.0098	1937.0292	-9.98	214 -	230	2	---	K.SLMKASYTPEVIEKSVR.D
2011.9381	2010.9308	2010.9355	-2.32	123 -	142	0	---	K.EDPNAGAQGQSGSSIPNNIR.H
2199.1150	2198.1077	2198.0724	16.1	246 -	263	2	---	R.WHQKNAMNMNLQKALDEK.E
2277.0642	2276.0569	2276.0894	-14.25	123 -	144	1	---	K.EDPNAGAQGQSGSSIPNNIRHK.F
2896.4324	2895.4251	2895.4688	-15.07	202 -	227	2	---	R.SNLFQGAPQETKSLMKASYTPEVIEK.S
2912.4265	2911.4192	2911.4637	-15.27	202 -	227	2	---	R.SNLFQGAPQETKSLMKASYTPEVIEK.S + Oxidation (M)
3505.6919	3504.6846	3504.6764	2.36	109 -	142	2	---	R.ASLREGAQPCQAPKEDPNAGAQGQSGSSIPNNIR.H
3573.6545	3572.6472	3572.6927	-12.73	1 -	31	2	---	-.MELGHRPGTTTLTRAHPNNNKEGQQDTDPWR.A + Oxidation (M)

No match to: 716.3353, 718.2895, 724.2175, 726.3070, 728.2833, 729.4336, 730.2399, 732.2518, 734.4341, 743.3130, 745.3617, 747.4360, 753.3469, 756.3524, 758.3462, 759.3632, 768.3517, 779.4633, 781.3725, 789.3640, 794.3674, 796.3701, 799.2854, 801.4280, 807.4076, 810.3293, 812.3207, 818.3782, 820.3700, 828.4415, 829.4508, 830.4513, 834.3805, 842.5101, 849.3834, 852.4368, 857.4457, 860.4262, 868.4412, 870.4462, 873.4370, 877.4166, 880.4280, 882.4470, 883.4816, 887.4295, 889.4857, 891.4872, 894.4923, 898.4610, 900.5174, 905.4763, 907.4251, 912.5086, 914.4942, 920.4296, 922.4855, 928.4565, 929.4484, 933.4327, 936.4976, 938.5062, 944.4982, 946.4672, 949.5244, 950.5168, 957.5792, 961.5109, 965.5151, 967.4963, 972.5023, 974.4913, 976.5255, 978.5352, 981.5225, 983.6221, 986.5463, 988.4922, 993.5123, 995.4512, 998.5833, 1000.5594, 1006.5120, 1007.5640, 1024.5542, 1031.5300, 1034.5703, 1037.5466, 1041.5637, 1043.5297, 1044.5375, 1045.5594, 1046.6058, 1054.5287, 1056.5575, 1057.6187, 1057.6200, 1068.5790, 1074.6162, 1076.6190, 1082.6056, 1105.5938, 1110.5851, 1113.5701, 1120.6050, 1122.5892, 1123.6047, 1126.6241, 1128.5618, 1130.5756, 1135.5951, 1137.5647, 1143.5592, 1145.6373, 1148.6318, 1149.6230, 1150.6226, 1156.5652, 1157.5676, 1159.5890, 1160.5966, 1169.5873, 1177.6421, 1178.6371, 1180.6443, 1182.6539, 1184.6431, 1185.6815, 1193.6090, 1194.6001, 1209.6238, 1213.7180, 1216.7178, 1221.5953, 1227.6459, 1229.6874, 1234.6899, 1238.2251, 1239.6622, 1254.6993, 1264.7139, 1265.1680, 1265.7196, 1269.6532, 1276.6729, 1278.6892, 1281.6375, 1285.6674, 1289.7029, 1293.6760, 1301.6362, 1303.6489, 1304.6685, 1306.6946, 1308.6797, 1314.7227, 1320.6364, 1323.6815, 1326.6937, 1328.6799, 1333.7430, 1336.7301, 1337.6429, 1349.6729, 1351.6969, 1353.6891, 1356.6255, 1358.6919, 1359.7013, 1361.7147, 1364.7118, 1365.6619, 1366.6965, 1372.6228, 1375.6693, 1382.6771, 1390.7089, 1393.8007, 1397.7449, 1403.7434, 1410.7849, 1416.7737, 1420.7114, 1423.7096, 1434.7847, 1436.7469, 1442.8257, 1445.7913, 1456.7555, 1457.7440, 1467.7560, 1470.7466, 1475.7615, 1479.8038, 1483.7579, 1484.7480, 1487.7660, 1488.7366, 1493.7556, 1500.7393, 1500.7400, 1503.7648, 1504.7708, 1514.7063, 1516.7281, 1522.7722, 1524.8040, 1526.7924, 1528.7079, 1530.7058, 1536.7252, 1537.7859, 1540.8043, 1543.7938, 1550.7543, 1551.7465, 1553.7668, 1555.7836, 1556.8011, 1562.8008, 1568.7620, 1572.7245, 1578.8058, 1580.8580, 1580.8600, 1584.8486, 1587.7675, 1608.8051, 1616.8165, 1621.7870, 1625.7886, 1628.7313, 1632.8120, 1637.7974, 1642.7417, 1647.8439, 1650.8429, 1651.8369, 1655.8147, 1657.8483, 1658.8953, 1660.8379, 1662.8666, 1664.8490, 1666.7892, 1679.8091, 1680.8357, 1682.8409, 1696.8464, 1698.8180, 1701.8243, 1702.8386, 1707.8938, 1710.8904, 1714.7905, 1716.8219, 1717.9052, 1718.9094, 1721.8976, 1722.9003, 1724.9030, 1728.8840, 1730.8910, 1734.1617, 1738.8267, 1739.8165, 1741.8934, 1743.9005, 1747.9127, 1758.4807, 1758.8918, 1761.8734, 1765.8154, 1772.0012, 1774.9300, 1774.9319, 1779.9012, 1781.8585, 1783.8062, 1787.8407, 1809.0583, 1810.0460, 1812.9126, 1829.8527, 1832.8584, 1833.8571, 1836.8618, 1838.8677, 1845.8363, 1847.8600, 1848.8938, 1850.9128, 1851.8932, 1861.8531, 1862.9542, 1864.9258, 1880.9026, 1883.7704, 1887.8878, 1897.7863, 1902.9769, 1904.9276, 1920.9131, 1923.9126, 1928.0443, 1932.0068, 1936.9510, 1947.9314, 1958.9393, 1959.9655, 1960.9813, 1961.9463, 1967.1549, 1969.9951, 1973.9971, 1975.9755, 1976.9708, 1990.4971, 1990.9373, 1991.4857, 1997.8479, 2001.8607, 2003.8768, 2004.8850, 2007.9500, 2007.9532, 2012.8951, 2015.9694, 2017.9860, 2018.9740, 2020.1631, 2023.9482, 2026.9529, 2027.5033, 2029.9972, 2043.5356, 2044.9940, 2055.0205, 2057.0154, 2060.9878, 2066.9939, 2068.9661, 2082.9822, 2086.0254, 2088.1033, 2097.0371, 2098.4941, 2102.9954, 2109.0095, 2112.9932, 2114.9707, 2122.9578, 2128.0444, 2130.9980, 2132.0200, 2136.9250, 2140.0063, 2143.0237, 2144.0559, 2145.0781, 2148.0483, 2153.0049, 2153.9973, 2174.0508, 2175.0435, 2178.0483, 2188.0610, 2191.0464, 2192.0427, 2205.0615, 2211.1067, 2223.1533, 2225.1382, 2226.1472, 2239.1646, 2243.1448, 2246.9482, 2251.1108, 2255.1484, 2265.0837, 2271.1211, 2273.0735, 2283.1606, 2285.1355, 2286.0986, 2289.0901, 2291.0173, 2309.0349, 2328.9888, 2343.0608, 2352.9583, 2355.0383, 2366.9595, 2371.0325, 2392.1594, 2400.0532, 2424.1577, 2434.2429, 2438.2283, 2449.2180, 2450.2451, 2451.2480, 2465.1592, 2466.2458, 2467.2458, 2478.1377, 2501.2207, 2523.2014, 2526.2585, 2540.2451, 2542.2578, 2545.2258, 2550.2373, 2561.6841, 2562.1826, 2562.6890, 2573.2495, 2576.1792, 2646.9758, 2659.2144, 2661.1409, 2662.0120, 2671.1924, 2681.2705,

2682.2888, 2685.3098, 2689.3059, 2695.1064, 2696.2695, 2699.2324, 2703.2529, 2705.2944, 2712.2937, 2717.2903, 2720.2839, 2721.2937, 2726.2935, 2727.2986, 2728.3079, 2739.2837, 2743.2729, 2744.2207, 2745.2256, 2746.2031, 2748.2295, 2755.2700, 2760.2590, 2763.1829, 2771.2400, 2778.1865, 2785.2969, 2786.2561, 2787.2524, 2790.2317, 2792.2166, 2800.3093, 2801.2932, 2803.2415, 2804.2429, 2807.2869, 2808.2227, 2812.2244, 2813.2373, 2817.2632, 2820.2441, 2824.2598, 2828.2454, 2833.2544, 2843.2900, 2844.3398, 2847.3630, 2851.3313, 2864.3188, 2890.3293, 2908.3105, 2910.3325, 2911.3594, 2925.3323, 2927.4434, 3023.3674, 3025.3865, 3026.3452, 3039.3838, 3042.3542, 3070.3745, 3071.3933, 3086.3984, 3087.3970, 3093.4294, 3101.3831, 3103.4006, 3104.4041, 3110.4143, 3127.4749, 3141.4126, 3143.5066, 3144.5298, 3159.5210, 3219.4854, 3236.5574, 3266.6021, 3280.5635, 3321.6216, 3344.4895, 3347.5515, 3348.5020, 3380.5210, 3396.5195, 3412.5801, 3427.6099, 3428.5874, 3444.5840, 3462.6301, 3478.6123, 3484.6497, 3489.7246, 3510.5859, 3521.6392, 3552.6614, 3553.6028, 3554.5630, 3558.6460, 3589.6523, 3608.6155, 3624.6685, 3630.5806, 3641.7090, 3651.7944, 3667.7266, 3683.6980, 3691.7681, 3707.7200, 3714.5493, 3715.6206, 3719.6914, 3720.7048, 3723.7041, 3735.7100, 3751.7539, 3830.8091, 3869.7393, 3876.6707, 3885.6946

7. P46171 **Mass:** 4789 **Score:** 23 **Expect:** 1.3e+002 **Matches:** 6

Beta-defensin 13 OS=Bos taurus GN=DEFB13 PE=1 SV=1

Observed	Mr(expt)	Mr(calc)	ppm	Start	End	Miss	Ions	Peptide
759.3632	758.3559	758.3568	-1.14	21 -	26	0	---	R.CPVPMR.Q
1738.8267	1737.8194	1737.8226	-1.80	27 -	40	1	---	R.QIGTCFGRPVKCCR.S
1741.8934	1740.8861	1740.8586	15.8	12 -	26	1	---	R.NGGVCIPIRCPVPMR.Q + Oxidation (M)
2011.9381	2010.9308	2010.9339	-1.53	27 -	42	2	---	R.QIGTCFGRPVKCCRSW.-
2018.9740	2017.9667	2018.0013	-17.11	21 -	37	1	---	R.CPVPMRQIGTCFGRPVK.C + Oxidation (M)
2057.0154	2056.0081	2056.0306	-10.94	1 -	20	1	---	-.SGISGPLSCGRNGGVCIPIR.C

No match to: 716.3353, 718.2895, 724.2175, 726.3070, 728.2833, 729.4336, 730.2399, 732.2518, 734.4341, 743.3130, 745.3617, 747.4360, 753.3469, 756.3524, 758.3462, 768.3517, 779.4633, 781.3725, 789.3640, 794.3674, 796.3701, 799.2854, 801.4280, 805.4417, 807.4076, 810.3293, 812.3207, 818.3782, 820.3700, 828.4415, 829.4508, 830.4513, 834.3805, 842.5101, 849.3834, 852.4368, 857.4457, 860.4262, 868.4412, 870.4462, 873.4370, 877.4166, 880.4280, 882.4470, 883.4816, 887.4295, 889.4857, 891.4872, 894.4923, 898.4610, 900.5174, 905.4763, 907.4251, 912.5086, 914.4942, 920.4296, 922.4855, 928.4565, 929.4484, 933.4327, 936.4976, 938.5062, 944.4982, 946.4672, 949.5244, 950.5168, 957.5792, 961.5109, 965.5151, 967.4963, 972.5023, 974.4913, 976.5255, 978.5352, 981.5225, 983.6221, 986.5463, 988.4922, 993.5123, 995.4512, 998.5833, 1000.5594, 1006.5120, 1007.5640, 1021.5762, 1024.5542, 1031.5300, 1034.5703, 1037.5466, 1041.5637, 1043.5297, 1044.5375, 1045.5594, 1046.6058, 1054.5287, 1056.5575, 1057.6187, 1057.6200, 1068.5790, 1074.6162, 1076.6190, 1082.6056, 1105.5938, 1110.5851, 1113.5701, 1118.5687, 1120.6050, 1122.5892, 1123.6047, 1126.6241, 1128.5618, 1130.5756, 1135.5951, 1137.5647, 1143.5592, 1145.6373, 1148.6318, 1149.6230, 1150.6226, 1156.5652, 1157.5676, 1159.5890, 1160.5966, 1169.5873, 1177.6421, 1178.6371, 1180.6443, 1182.6539, 1184.6431, 1185.6815, 1193.6090, 1194.6001, 1209.6238, 1213.7180, 1216.7178, 1221.5953, 1227.6459, 1229.6874, 1234.6899, 1238.2251, 1239.6622, 1254.6993, 1255.6351, 1264.7139, 1265.1680, 1265.7196, 1269.6532, 1276.6729, 1278.6892, 1281.6375, 1285.6674, 1289.7029, 1293.6760, 1301.6362, 1303.6489, 1304.6685, 1306.6946, 1308.6797, 1314.7227, 1319.6770, 1320.6364, 1323.6815, 1326.6937, 1328.6799, 1333.7430, 1336.7301, 1337.6429, 1349.6729, 1351.6969, 1353.6891, 1356.6255, 1358.6919, 1359.7013, 1361.7147, 1364.7118, 1365.6619, 1366.6965, 1372.6228, 1375.6693, 1382.6771, 1383.7347, 1390.7089, 1393.8007, 1397.7449, 1403.7434, 1410.7849, 1416.7737, 1420.7114, 1423.7096, 1434.7847, 1436.7469, 1442.8257, 1445.7913, 1456.7555, 1457.7440, 1467.7560, 1470.7466, 1475.7615, 1479.8038, 1483.7579, 1484.7480, 1487.7660, 1488.7366, 1493.7556, 1500.7393, 1500.7400, 1503.7648, 1504.7708, 1514.7063, 1516.7281, 1519.7705, 1522.7722, 1524.8040, 1526.7924, 1528.7079, 1530.7058, 1536.7252, 1537.7859, 1540.8043, 1543.7938, 1550.7543, 1551.7465, 1553.7668, 1555.7836, 1556.8011, 1562.8008, 1568.7620, 1572.7245, 1578.8058, 1580.8580, 1580.8600, 1584.8486, 1587.7675, 1608.8051, 1616.8165, 1621.7870, 1625.7886, 1628.7313, 1632.8120, 1635.7964, 1637.7974, 1642.7417, 1647.8439, 1650.8429, 1651.8369, 1655.8147, 1657.8483, 1658.8953, 1660.8379, 1662.8666, 1664.8490, 1666.7892, 1679.8091, 1680.8357, 1682.8409, 1696.8464, 1698.8180, 1701.8243, 1702.8386, 1707.8938, 1710.8904, 1714.7905, 1716.8219, 1717.9052, 1718.9094, 1721.8976, 1722.9003, 1724.9030, 1728.8840, 1730.8910, 1734.1617, 1739.8165, 1743.9005, 1747.9127, 1758.4807, 1758.8918, 1761.8734, 1765.8154, 1772.0012, 1774.9300, 1774.9319, 1779.9012, 1781.8585, 1783.8062, 1787.8407, 1809.0583, 1810.0460, 1812.9126, 1829.8527, 1832.8584, 1833.8571, 1834.8650, 1836.8618, 1838.8677, 1845.8363, 1847.8600, 1848.8938, 1850.9128, 1851.8932, 1861.8531, 1862.9542, 1864.9258, 1880.9026, 1883.7704, 1887.8878, 1897.7863, 1902.9769, 1904.9276, 1908.8986, 1920.9131, 1923.9126, 1928.0443, 1932.0068, 1936.9510, 1938.0171, 1947.9314, 1958.9393, 1959.9655, 1960.9813, 1961.9463, 1967.1549, 1969.9951, 1973.9971, 1975.9755, 1976.9708, 1990.4971, 1990.9373, 1991.4857, 1997.8479, 2001.8607, 2003.8768, 2004.8850, 2007.9500, 2007.9532, 2012.8951, 2015.9694, 2017.9860, 2020.1631, 2023.9482, 2026.9529, 2027.5033, 2029.9972, 2043.5356, 2044.9940, 2055.0205, 2060.9878, 2066.9939, 2068.9661, 2082.9822, 2086.0254, 2088.1033, 2097.0371, 2098.4941, 2102.9954, 2109.0095, 2112.9932, 2114.9707, 2122.9578, 2128.0444, 2130.9980, 2132.0200, 2136.9250, 2140.0063, 2143.0237, 2144.0559, 2145.0781, 2148.0483, 2153.0049, 2153.9973, 2174.0508, 2175.0435, 2178.0483, 2188.0610, 2191.0464, 2192.0427, 2199.1150, 2205.0615, 2211.1067, 2223.1533, 2225.1382, 2226.1472, 2239.1646, 2243.1448, 2246.9482, 2251.1108, 2255.1484, 2265.0837, 2271.1211, 2273.0735, 2277.0642, 2283.1606, 2285.1355, 2286.0986, 2289.0901, 2291.0173, 2309.0349, 2328.9888, 2343.0608, 2352.9583, 2355.0383, 2366.9595, 2371.0325, 2392.1594, 2400.0532, 2424.1577, 2434.2429, 2438.2283, 2449.2180, 2450.2451, 2451.2480, 2465.1592, 2466.2458, 2467.2458, 2478.1377, 2501.2207, 2523.2014, 2526.2585, 2540.2451, 2542.2578, 2545.2258, 2550.2373, 2561.6841, 2562.1826, 2562.6890, 2573.2495, 2576.1792, 2646.9758, 2659.2144, 2661.1409, 2662.0120, 2671.1924, 2681.2705, 2682.2888, 2685.3098, 2689.3059, 2695.1064, 2696.2695, 2699.2324, 2703.2529, 2705.2944, 2712.2937, 2717.2903, 2720.2839, 2721.2937, 2726.2935, 2727.2986, 2728.3079, 2739.2837, 2743.2729, 2744.2207, 2745.2256, 2746.2031, 2748.2295, 2755.2700, 2760.2590, 2763.1829, 2771.2400, 2778.1865, 2785.2969, 2786.2561, 2787.2524, 2790.2317, 2792.2166, 2800.3093, 2801.2932, 2803.2415, 2804.2429, 2807.2869, 2808.2227, 2812.2244, 2813.2373, 2817.2632, 2820.2441, 2824.2598, 2828.2454, 2833.2544, 2843.2900, 2844.3398, 2847.3630, 2851.3313, 2864.3188, 2890.3293, 2896.4324, 2908.3105, 2910.3325, 2911.3594, 2912.4265, 2925.3323, 2927.4434, 3023.3674, 3025.3865, 3026.3452, 3039.3838, 3042.3542, 3070.3745, 3071.3933, 3086.3984, 3087.3970, 3093.4294, 3101.3831, 3103.4006, 3104.4041, 3110.4143, 3127.4749, 3141.4126, 3143.5066, 3144.5298, 3159.5210, 3219.4854, 3236.5574, 3266.6021, 3280.5635, 3321.6216, 3344.4895, 3347.5515, 3348.5020, 3380.5210, 3396.5195, 3412.5801, 3427.6099, 3428.5874, 3444.5840, 3462.6301, 3478.6123, 3484.6497, 3489.7246, 3505.6919, 3510.5859, 3521.6392, 3552.6614, 3553.6028, 3554.5630, 3558.6460, 3573.6545, 3589.6523, 3608.6155, 3624.6685, 3630.5806, 3641.7090, 3651.7944, 3667.7266, 3683.6980, 3691.7681, 3707.7200, 3714.5493, 3715.6206, 3719.6914, 3720.7048, 3723.7041, 3735.7100, 3751.7539, 3830.8091, 3869.7393, 3876.6707, 3885.6946

8. Q0VC96 **Mass:** 22800 **Score:** 21 **Expect:** 2.1e+002 **Matches:** 16

Thymocyte nuclear protein 1 OS=Bos taurus GN=THYN1 PE=2 SV=1

Observed	Mr(expt)	Mr(calc)	ppm	Start	End	Miss	Ions	Peptide
887.4295	886.4222	886.4331	-12.24	7 -	14	0	---	R.RPAGPDMK.G + Oxidation (M)
944.4982	943.4909	943.5087	-18.84	15 -	22	2	---	K.GPEEKRTK.T
1043.5297	1042.5224	1042.5342	-11.28	6 -	14	1	---	R.RRPAGPDMK.G + Oxidation (M)
1178.6371	1177.6298	1177.6026	23.1	156 -	164	1	---	K.WSMRLSVQR.L + Oxidation (M)
1254.6993	1253.6920	1253.6775	11.6	4 -	14	2	---	R.ARRRPAGPDMK.G
1303.6489	1302.6416	1302.6568	-11.65	73 -	83	0	---	K.FSIEDLQAQPR.Q
1555.7836	1554.7763	1554.7837	-4.76	93 -	104	2	---	R.NYQARNFLRAMR.L + Oxidation (M)
1761.8734	1760.8661	1760.8628	1.90	151 -	164	2	---	K.EDNPKWSMRLSVQR.L + Oxidation (M)
2015.9694	2014.9621	2014.9194	21.2	41 -	58	0	---	K.TPVSEDSGEALSSYWLMK.S + Oxidation (M)
2285.1355	2284.1282	2284.0920	15.9	84 -	101	2	---	R.QTTCWDGVRNYQARNFLR.A
2576.1792	2575.1719	2575.1848	-5.02	98 -	117	2	---	R.NFLRAMRLDEEAFFYHSNCR.E
2844.3398	2843.3325	2843.3402	-2.70	127 -	150	2	---	K.IVKEAYPDHTQFEKNNPYYDASSK.E
2910.3325	2909.3252	2909.3952	-24.06	102 -	126	2	---	R.AMRLDEEAFFYHSNCREPGIAGLVK.I
3039.3838	3038.3765	3038.4417	-21.45	73 -	97	2	---	K.FSIEDLQAQPRQTTCWDGVRNYQAR.N
3071.3933	3070.3860	3070.4441	-18.90	41 -	67	2	---	K.TPVSEDSGEALSSYWLMKSEPESRLEK.G + Oxidation (M)
3087.3970	3086.3897	3086.3529	11.9	130 -	155	2	---	K.EAYPDHTQFEKNNPYYDASSKEDNPK.W

No match to: 716.3353, 718.2895, 724.2175, 726.3070, 728.2833, 729.4336, 730.2399, 732.2518, 734.4341, 743.3130, 745.3617, 747.4360, 753.3469, 756.3524, 758.3462, 759.3632, 768.3517, 779.4633, 781.3725, 789.3640, 794.3674, 796.3701, 799.2854, 801.4280, 805.4417, 807.4076, 810.3293, 812.3207, 818.3782, 820.3700, 828.4415, 829.4508, 830.4513, 834.3805, 842.5101, 849.3834, 852.4368, 857.4457, 860.4262, 868.4412, 870.4462, 873.4370, 877.4166, 880.4280, 882.4470, 883.4816, 889.4857, 891.4872, 894.4923, 898.4610, 900.5174, 905.4763, 907.4251, 912.5086, 914.4942, 920.4296, 922.4855, 928.4565, 929.4484, 933.4327, 936.4976, 938.5062, 946.4672, 949.5244, 950.5168, 957.5792, 961.5109, 965.5151, 967.4963, 972.5023, 974.4913, 976.5255, 978.5352, 981.5225, 983.6221, 986.5463, 988.4922, 993.5123, 995.4512, 998.5833, 1000.5594, 1006.5120, 1007.5640, 1021.5762, 1024.5542, 1031.5300, 1034.5703, 1037.5466, 1041.5637, 1044.5375, 1045.5594, 1046.6058, 1054.5287, 1056.5575, 1057.6187, 1057.6200, 1068.5790, 1074.6162, 1076.6190, 1082.6056, 1105.5938, 1110.5851, 1113.5701, 1118.5687, 1120.6050, 1122.5892, 1123.6047, 1126.6241, 1128.5618, 1130.5756, 1135.5951, 1137.5647,

1143.5592, 1145.6373, 1148.6318, 1149.6230, 1150.6226, 1156.5652, 1157.5676, 1159.5890, 1160.5966, 1169.5873, 1177.6421, 1180.6443, 1182.6539, 1184.6431, 1185.6815, 1193.6090, 1194.6001, 1209.6238, 1213.7180, 1216.7178, 1221.5953, 1227.6459, 1229.6874, 1234.6899, 1238.2251, 1239.6622, 1255.6351, 1264.7139, 1265.1680, 1265.7196, 1269.6532, 1276.6729, 1278.6892, 1281.6375, 1285.6674, 1289.7029, 1293.6760, 1301.6362, 1304.6685, 1306.6946, 1308.6797, 1314.7227, 1319.6770, 1320.6364, 1323.6815, 1326.6937, 1328.6799, 1333.7430, 1336.7301, 1337.6429, 1349.6729, 1351.6969, 1353.6891, 1356.6255, 1358.6919, 1359.7013, 1361.7147, 1364.7118, 1365.6619, 1366.6965, 1372.6228, 1375.6693, 1382.6771, 1383.7347, 1390.7089, 1393.8007, 1397.7449, 1403.7434, 1410.7849, 1416.7737, 1420.7114, 1423.7096, 1434.7847, 1436.7469, 1442.8257, 1445.7913, 1456.7555, 1457.7440, 1467.7560, 1470.7466, 1475.7615, 1479.8038, 1483.7579, 1484.7480, 1487.7660, 1488.7366, 1493.7556, 1500.7393, 1500.7400, 1503.7648, 1504.7708, 1514.7063, 1516.7281, 1519.7705, 1522.7722, 1524.8040, 1526.7924, 1528.7079, 1530.7058, 1536.7252, 1537.7859, 1540.8043, 1543.7938, 1550.7543, 1551.7465, 1553.7668, 1556.8011, 1562.8008, 1568.7620, 1572.7245, 1578.8058, 1580.8580, 1580.8600, 1584.8486, 1587.7675, 1608.8051, 1616.8165, 1621.7870, 1625.7886, 1628.7313, 1632.8120, 1635.7964, 1637.7974, 1642.7417, 1647.8439, 1650.8429, 1651.8369, 1655.8147, 1657.8483, 1658.8953, 1660.8379, 1662.8666, 1664.8490, 1666.7892, 1679.8091, 1680.8357, 1682.8409, 1696.8464, 1698.8180, 1701.8243, 1702.8386, 1707.8938, 1710.8904, 1714.7905, 1716.8219, 1717.9052, 1718.9094, 1721.8976, 1722.9003, 1724.9030, 1728.8840, 1730.8910, 1734.1617, 1738.8267, 1739.8165, 1741.8934, 1743.9005, 1747.9127, 1758.4807, 1758.8918, 1765.8154, 1772.0012, 1774.9300, 1774.9319, 1779.9012, 1781.8585, 1783.8062, 1787.8407, 1809.0583, 1810.0460, 1812.9126, 1829.8527, 1832.8584, 1833.8571, 1834.8650, 1836.8618, 1838.8677, 1845.8363, 1847.8600, 1848.8938, 1850.9128, 1851.8932, 1861.8531, 1862.9542, 1864.9258, 1880.9026, 1883.7704, 1887.8878, 1897.7863, 1902.9769, 1904.9276, 1908.8986, 1920.9131, 1923.9126, 1928.0443, 1932.0068, 1936.9510, 1938.0171, 1947.9314, 1958.9393, 1959.9655, 1960.9813, 1961.9463, 1967.1549, 1969.9951, 1973.9971, 1975.9755, 1976.9708, 1990.4971, 1990.9373, 1991.4857, 1997.8479, 2001.8607, 2003.8768, 2004.8850, 2007.9500, 2007.9532, 2011.9381, 2012.8951, 2017.9860, 2018.9740, 2020.1631, 2023.9482, 2026.9529, 2027.5033, 2029.9972, 2043.5356, 2044.9940, 2055.0205, 2057.0154, 2060.9878, 2066.9939, 2068.9661, 2082.9822, 2086.0254, 2088.1033, 2097.0371, 2098.4941, 2102.9954, 2109.0095, 2112.9932, 2114.9707, 2122.9578, 2128.0444, 2130.9980, 2132.0200, 2136.9250, 2140.0063, 2143.0237, 2144.0559, 2145.0781, 2148.0483, 2153.0049, 2153.9973, 2174.0508, 2175.0435, 2178.0483, 2188.0610, 2191.0464, 2192.0427, 2199.1150, 2205.0615, 2211.1067, 2223.1533, 2225.1382, 2226.1472, 2239.1646, 2243.1448, 2246.9482, 2251.1108, 2255.1484, 2265.0837, 2271.1211, 2273.0735, 2277.0642, 2283.1606, 2286.0986, 2289.0901, 2291.0173, 2309.0349, 2328.9888, 2343.0608, 2352.9583, 2355.0383, 2366.9595, 2371.0325, 2392.1594, 2400.0532, 2424.1577, 2434.2429, 2438.2283, 2449.2180, 2450.2451, 2451.2480, 2465.1592, 2466.2458, 2467.2458, 2478.1377, 2501.2207, 2523.2014, 2526.2585, 2540.2451, 2542.2578, 2545.2258, 2550.2373, 2561.6841, 2562.1826, 2562.6890, 2573.2495, 2646.9758, 2659.2144, 2661.1409, 2662.0120, 2671.1924, 2681.2705, 2682.2888, 2685.3098, 2689.3059, 2695.1064, 2696.2695, 2699.2324, 2703.2529, 2705.2944, 2712.2937, 2717.2903, 2720.2839, 2721.2937, 2726.2935, 2727.2986, 2728.3079, 2739.2837, 2743.2729, 2744.2207, 2745.2256, 2746.2031, 2748.2295, 2755.2700, 2760.2590, 2763.1829, 2771.2400, 2778.1865, 2785.2969, 2786.2561, 2787.2524, 2790.2317, 2792.2166, 2800.3093, 2801.2932, 2803.2415, 2804.2429, 2807.2869, 2808.2227, 2812.2244, 2813.2373, 2817.2632, 2820.2441, 2824.2598, 2828.2454, 2833.2544, 2843.2900, 2847.3630, 2851.3313, 2864.3188, 2890.3293, 2896.4324, 2908.3105, 2911.3594, 2912.4265, 2925.3323, 2927.4434, 3023.3674, 3025.3865, 3026.3452, 3042.3542, 3070.3745, 3086.3984, 3093.4294, 3101.3831, 3103.4006, 3104.4041, 3110.4143, 3127.4749, 3141.4126, 3143.5066, 3144.5298, 3159.5210, 3219.4854, 3236.5574, 3266.6021, 3280.5635, 3321.6216, 3344.4895, 3347.5515, 3348.5020, 3380.5210, 3396.5195, 3412.5801, 3427.6099, 3428.5874, 3444.5840, 3462.6301, 3478.6123, 3484.6497, 3489.7246, 3505.6919, 3510.5859, 3521.6392, 3552.6614, 3553.6028, 3554.5630, 3558.6460, 3573.6545, 3589.6523, 3608.6155, 3624.6685, 3630.5806, 3641.7090, 3651.7944, 3667.7266, 3683.6980, 3691.7681, 3707.7200, 3714.5493, 3715.6206, 3719.6914, 3720.7048, 3723.7041, 3735.7100, 3751.7539, 3830.8091, 3869.7393, 3876.6707, 3885.6946

9. P62935 **Mass:** 18086 **Score:** 20 **Expect:** 2.4e+002 **Matches:** 10

Peptidyl-prolyl cis-trans isomerase A OS=Bos taurus GN=PPIA PE=1 SV=2

Observed	Mr(expt)	Mr(calc)	ppm	Start		End	Miss	Ions	Peptide
1118.5687	1117.5614	1117.5801	-16.75	155	-	164	1	---	K.KITIADCGQI.-
1423.7096	1422.7023	1422.7103	-5.59	32	-	44	1	---	K.TAENFRALSTGEK.G
1434.7847	1433.7774	1433.7548	15.8	152	-	164	2	---	K.TSKKITIADCGQI.-
1747.9127	1746.9054	1746.9264	-12.02	29	-	44	2	---	K.VPKTAENFRALSTGEK.G
1975.9755	1974.9682	1974.9799	-5.92	32	-	49	2	---	K.TAENFRALSTGEKGFGYK.G
2205.0615	2204.0542	2204.1082	-24.48	126	-	144	2	---	K.HVVFGKVKEGMNIVEAMER.F + 2 Oxidation (M)
2343.0608	2342.0535	2342.0507	1.19	50	-	69	1	---	K.GSCFHRIIPGFMCQGGDFTR.H
2807.2869	2806.2796	2806.3167	-13.21	92	-	118	0	---	K.HTGPGILSMANAGPNTNGSQFFICTAK.T + Oxidation (M)
2911.3594	2910.3521	2910.3153	12.7	45	-	69	2	---	K.GFGYKGSCFHRIIPGFMCQGGDFTR.H + Oxidation (M)
2927.4434	2926.4361	2926.3855	17.3	56	-	82	2	---	R.IIPGFMCQGGDFTRHNGTGGKSIYGEK.F

No match to: 716.3353, 718.2895, 724.2175, 726.3070, 728.2833, 729.4336, 730.2399, 732.2518, 734.4341, 743.3130, 745.3617, 747.4360, 753.3469, 756.3524, 758.3462, 759.3632, 768.3517, 779.4633, 781.3725, 789.3640, 794.3674, 796.3701, 799.2854, 801.4280, 805.4417, 807.4076, 810.3293, 812.3207, 818.3782, 820.3700, 828.4415, 829.4508, 830.4513, 834.3805, 842.5101, 849.3834, 852.4368, 857.4457, 860.4262, 868.4412, 870.4462, 873.4370, 877.4166, 880.4280, 882.4470, 883.4816, 887.4295, 889.4857, 891.4872, 894.4923, 898.4610, 900.5174, 905.4763, 907.4251, 912.5086, 914.4942, 920.4296, 922.4855, 928.4565, 929.4484, 933.4327, 936.4976, 938.5062, 944.4982, 946.4672, 949.5244, 950.5168, 957.5792, 961.5109, 965.5151, 967.4963, 972.5023, 974.4913, 976.5255, 978.5352, 981.5225, 983.6221, 986.5463, 988.4922, 993.5123, 995.4512, 998.5833, 1000.5594, 1006.5120, 1007.5640, 1021.5762, 1024.5542, 1031.5300, 1034.5703, 1037.5466, 1041.5637, 1043.5297, 1044.5375, 1045.5594, 1046.6058, 1054.5287, 1056.5575, 1057.6187, 1057.6200, 1068.5790, 1074.6162, 1076.6190, 1082.6056, 1105.5938, 1110.5851, 1113.5701, 1120.6050, 1122.5892, 1123.6047, 1126.6241, 1128.5618, 1130.5756, 1135.5951, 1137.5647, 1143.5592, 1145.6373, 1148.6318, 1149.6230, 1150.6226, 1156.5652, 1157.5676, 1159.5890, 1160.5966, 1169.5873, 1177.6421, 1178.6371, 1180.6443, 1182.6539, 1184.6431, 1185.6815, 1193.6090, 1194.6001, 1209.6238, 1213.7180, 1216.7178, 1221.5953, 1227.6459, 1229.6874, 1234.6899, 1238.2251, 1239.6622, 1254.6993, 1255.6351, 1264.7139, 1265.1680, 1265.7196, 1269.6532, 1276.6729, 1278.6892, 1281.6375, 1285.6674, 1289.7029, 1293.6760, 1301.6362, 1303.6489, 1304.6685, 1306.6946, 1308.6797, 1314.7227, 1319.6770, 1320.6364, 1323.6815, 1326.6937, 1328.6799, 1333.7430, 1336.7301, 1337.6429, 1349.6729, 1351.6969, 1353.6891, 1356.6255, 1358.6919, 1359.7013, 1361.7147, 1364.7118, 1365.6619, 1366.6965, 1372.6228, 1375.6693, 1382.6771, 1383.7347, 1390.7089, 1393.8007, 1397.7449, 1403.7434, 1410.7849, 1416.7737, 1420.7114, 1436.7469, 1442.8257, 1445.7913, 1456.7555, 1457.7440, 1467.7560, 1470.7466, 1475.7615, 1479.8038, 1483.7579, 1484.7480, 1487.7660, 1488.7366, 1493.7556, 1500.7393, 1500.7400, 1503.7648, 1504.7708, 1514.7063, 1516.7281, 1519.7705, 1522.7722, 1524.8040, 1526.7924, 1528.7079, 1530.7058, 1536.7252, 1537.7859, 1540.8043, 1543.7938, 1550.7543, 1551.7465, 1553.7668, 1555.7836, 1556.8011, 1562.8008, 1568.7620, 1572.7245, 1578.8058, 1580.8580, 1580.8600, 1584.8486, 1587.7675, 1608.8051, 1616.8165, 1621.7870, 1625.7886, 1628.7313, 1632.8120, 1635.7964, 1637.7974, 1642.7417, 1647.8439, 1650.8429, 1651.8369, 1655.8147, 1657.8483, 1658.8953, 1660.8379, 1662.8666, 1664.8490, 1666.7892, 1679.8091, 1680.8357, 1682.8409, 1696.8464, 1698.8180, 1701.8243, 1702.8386, 1707.8938, 1710.8904, 1714.7905, 1716.8219, 1717.9052, 1718.9094, 1721.8976, 1722.9003, 1724.9030, 1728.8840, 1730.8910, 1734.1617, 1738.8267, 1739.8165, 1741.8934, 1743.9005, 1758.4807, 1758.8918, 1761.8734, 1765.8154, 1772.0012, 1774.9300, 1774.9319, 1779.9012, 1781.8585, 1783.8062, 1787.8407, 1809.0583, 1810.0460, 1812.9126, 1829.8527, 1832.8584, 1833.8571, 1834.8650, 1836.8618, 1838.8677, 1845.8363, 1847.8600, 1848.8938, 1850.9128, 1851.8932, 1861.8531, 1862.9542, 1864.9258, 1880.9026, 1883.7704, 1887.8878, 1897.7863, 1902.9769, 1904.9276, 1908.8986, 1920.9131, 1923.9126, 1928.0443, 1932.0068, 1936.9510, 1938.0171, 1947.9314, 1958.9393, 1959.9655, 1960.9813, 1961.9463, 1967.1549, 1969.9951, 1973.9971, 1976.9708, 1990.4971, 1990.9373, 1991.4857, 1997.8479, 2001.8607, 2003.8768, 2004.8850, 2007.9500, 2007.9532, 2011.9381, 2012.8951, 2015.9694, 2017.9860, 2018.9740, 2020.1631, 2023.9482, 2026.9529, 2027.5033, 2029.9972, 2043.5356, 2044.9940, 2055.0205, 2057.0154, 2060.9878, 2066.9939, 2068.9661, 2082.9822, 2086.0254, 2088.1033, 2097.0371, 2098.4941, 2102.9954, 2109.0095, 2112.9932, 2114.9707, 2122.9578, 2128.0444, 2130.9980, 2132.0200, 2136.9250, 2140.0063, 2143.0237, 2144.0559, 2145.0781, 2148.0483, 2153.0049, 2153.9973, 2174.0508, 2175.0435, 2178.0483, 2188.0610, 2191.0464, 2192.0427, 2199.1150, 2211.1067, 2223.1533, 2225.1382, 2226.1472, 2239.1646, 2243.1448, 2246.9482, 2251.1108, 2255.1484, 2265.0837, 2271.1211, 2273.0735, 2277.0642, 2283.1606, 2285.1355, 2286.0986, 2289.0901, 2291.0173, 2309.0349, 2328.9888, 2352.9583, 2355.0383, 2366.9595, 2371.0325, 2392.1594, 2400.0532, 2424.1577, 2434.2429, 2438.2283, 2449.2180, 2450.2451, 2451.2480, 2465.1592, 2466.2458, 2467.2458, 2478.1377, 2501.2207, 2523.2014, 2526.2585, 2540.2451, 2542.2578, 2545.2258, 2550.2373, 2561.6841, 2562.1826, 2562.6890, 2573.2495, 2576.1792, 2646.9758, 2659.2144, 2661.1409, 2662.0120, 2671.1924, 2681.2705, 2682.2888, 2685.3098, 2689.3059, 2695.1064, 2696.2695, 2699.2324, 2703.2529, 2705.2944, 2712.2937, 2717.2903, 2720.2839, 2721.2937, 2726.2935, 2727.2986, 2728.3079, 2739.2837, 2743.2729, 2744.2207, 2745.2256, 2746.2031, 2748.2295, 2755.2700, 2760.2590, 2763.1829, 2771.2400, 2778.1865, 2785.2969, 2786.2561, 2787.2524, 2790.2317, 2792.2166, 2800.3093, 2801.2932, 2803.2415, 2804.2429, 2808.2227, 2812.2244, 2813.2373, 2817.2632, 2820.2441, 2824.2598, 2828.2454, 2833.2544, 2843.2900, 2844.3398, 2847.3630, 2851.3313, 2864.3188, 2890.3293, 2896.4324, 2908.3105, 2910.3325, 2912.4265, 2925.3323, 3023.3674, 3025.3865, 3026.3452, 3039.3838, 3042.3542, 3070.3745, 3071.3933, 3086.3984, 3087.3970, 3093.4294, 3101.3831, 3103.4006, 3104.4041, 3110.4143, 3127.4749, 3141.4126, 3143.5066, 3144.5298, 3159.5210, 3219.4854, 3236.5574, 3266.6021, 3280.5635, 3321.6216, 3344.4895, 3347.5515, 3348.5020, 3380.5210, 3396.5195, 3412.5801, 3427.6099, 3428.5874, 3444.5840, 3462.6301, 3478.6123, 3484.6497, 3489.7246, 3505.6919, 3510.5859, 3521.6392, 3552.6614, 3553.6028, 3554.5630, 3558.6460, 3573.6545, 3589.6523, 3608.6155, 3624.6685, 3630.5806, 3641.7090, 3651.7944, 3667.7266, 3683.6980, 3691.7681, 3707.7200, 3714.5493, 3715.6206, 3719.6914, 3720.7048, 3723.7041, 3735.7100, 3751.7539, 3830.8091, 3869.7393, 3876.6707, 3885.6946

10. Q29RM9 **Mass:** 23940 **Score:** 20 **Expect:** 2.4e+002 **Matches:** 15

Regulator of G-protein signaling 4 OS=Bos taurus GN=RGS4 PE=2 SV=1

Observed	Mr(expt)	Mr(calc)	ppm	Start	End	Miss	Ions	Peptide
894.4923	893.4850	893.4680	19.0	156 -	162	0	---	K.IFNLMEK.D
972.5023	971.4950	971.4971	-2.13	15 -	22	2	---	R.SAKDMKHR.L
988.4922	987.4849	987.4920	-7.15	15 -	22	2	---	R.SAKDMKHR.L + Oxidation (M)
1145.6373	1144.6300	1144.6387	-7.54	42 -	50	2	---	K.KDKVVICQR.V
1540.8043	1539.7970	1539.8297	-21.20	113 -	125	1	---	K.KIYNEFISVQATK.E
1543.7938	1542.7865	1542.7864	0.08	155 -	166	2	---	R.KIFNLMEKDSYR.R
1587.7675	1586.7602	1586.7875	-17.17	156 -	167	2	---	K.IFNLMEKDSYRR.F + Oxidation (M)
1710.8904	1709.8831	1709.9247	-24.30	45 -	58	2	---	K.VVICQRVSHEEVKK.W
1774.9300	1773.9227	1773.9229	-0.11	4 -	20	2	---	K.GLAGLPASCLRSAKDMK.H
1774.9319	1773.9246	1773.9229	0.96	4 -	20	2	---	K.GLAGLPASCLRSAKDMK.H
2174.0508	2173.0435	2173.0222	9.81	23 -	41	1	---	R.LGFLLQKSDSCEHNSSHSK.K
2175.0435	2174.0362	2174.0466	-4.77	59 -	77	0	---	K.WAESLENLISHECGLAAFK.A
2467.2458	2466.2385	2466.1822	22.8	21 -	41	2	---	K.HRLGFLLQKSDSCEHNSSHSK.K
2787.2524	2786.2451	2786.2421	1.08	78 -	99	1	---	K.AFLKSEYSEENIDFWISCEEYK.K
2844.3398	2843.3325	2843.3656	-11.62	140 -	162	2	---	R.NMLEPTITCFDEAQRKIFNLMEK.D + Oxidation (M)

No match to: 716.3353, 718.2895, 724.2175, 726.3070, 728.2833, 729.4336, 730.2399, 732.2518, 734.4341, 743.3130, 745.3617, 747.4360, 753.3469, 756.3524, 758.3462, 759.3632, 768.3517, 779.4633, 781.3725, 789.3640, 794.3674, 796.3701, 799.2854, 801.4280, 805.4417, 807.4076, 810.3293, 812.3207, 818.3782, 820.3700, 828.4415, 829.4508, 830.4513, 834.3805, 842.5101, 849.3834, 852.4368, 857.4457, 860.4262, 868.4412, 870.4462, 873.4370, 877.4166, 880.4280, 882.4470, 883.4816, 887.4295, 889.4857, 891.4872, 898.4610, 900.5174, 905.4763, 907.4251, 912.5086, 914.4942, 920.4296, 922.4855, 928.4565, 929.4484, 933.4327, 936.4976, 938.5062, 944.4982, 946.4672, 949.5244, 950.5168, 957.5792, 961.5109, 965.5151, 967.4963, 974.4913, 976.5255, 978.5352, 981.5225, 983.6221, 986.5463, 993.5123, 995.4512, 998.5833, 1000.5594, 1006.5120, 1007.5640, 1021.5762, 1024.5542, 1031.5300, 1034.5703, 1037.5466, 1041.5637, 1043.5297, 1044.5375, 1045.5594, 1046.6058, 1054.5287, 1056.5575, 1057.6187, 1057.6200, 1068.5790, 1074.6162, 1076.6190, 1082.6056, 1105.5938, 1110.5851, 1113.5701, 1118.5687, 1120.6050, 1122.5892, 1123.6047, 1126.6241, 1128.5618, 1130.5756, 1135.5951, 1137.5647, 1143.5592, 1148.6318, 1149.6230, 1150.6226, 1156.5652, 1157.5676, 1159.5890, 1160.5966, 1169.5873, 1177.6421, 1178.6371, 1180.6443, 1182.6539, 1184.6431, 1185.6815, 1193.6090, 1194.6001, 1209.6238, 1213.7180, 1216.7178, 1221.5953, 1227.6459, 1229.6874, 1234.6899, 1238.2251, 1239.6622, 1254.6993, 1255.6351, 1264.7139, 1265.1680, 1265.7196, 1269.6532, 1276.6729, 1278.6892, 1281.6375, 1285.6674, 1289.7029, 1293.6760, 1301.6362, 1303.6489, 1304.6685, 1306.6946, 1308.6797, 1314.7227, 1319.6770, 1320.6364, 1323.6815, 1326.6937, 1328.6799, 1333.7430, 1336.7301, 1337.6429, 1349.6729, 1351.6969, 1353.6891, 1356.6255, 1358.6919, 1359.7013, 1361.7147, 1364.7118, 1365.6619, 1366.6965, 1372.6228, 1375.6693, 1382.6771, 1383.7347, 1390.7089, 1393.8007, 1397.7449, 1403.7434, 1410.7849, 1416.7737, 1420.7114, 1423.7096, 1434.7847, 1436.7469, 1442.8257, 1445.7913, 1456.7555, 1457.7440, 1467.7560, 1470.7466, 1475.7615, 1479.8038, 1483.7579, 1484.7480, 1487.7660, 1488.7366, 1493.7556, 1500.7393, 1500.7400, 1503.7648, 1504.7708, 1514.7063, 1516.7281, 1519.7705, 1522.7722, 1524.8040, 1526.7924, 1528.7079, 1530.7058, 1536.7252, 1537.7859, 1550.7543, 1551.7465, 1553.7668, 1555.7836, 1556.8011, 1562.8008, 1568.7620, 1572.7245, 1578.8058, 1580.8580, 1580.8600, 1584.8486, 1608.8051, 1616.8165, 1621.7870, 1625.7886, 1628.7313, 1632.8120, 1635.7964, 1637.7974, 1642.7417, 1647.8439, 1650.8429, 1651.8369, 1655.8147, 1657.8483, 1658.8953, 1660.8379, 1662.8666, 1664.8490, 1666.7892, 1679.8091, 1680.8357, 1682.8409, 1696.8464, 1698.8180, 1701.8243, 1702.8386, 1707.8938, 1714.7905, 1716.8219, 1717.9052, 1718.9094, 1721.8976, 1722.9003, 1724.9030, 1728.8840, 1730.8910, 1734.1617, 1738.8267, 1739.8165, 1741.8934, 1743.9005, 1747.9127, 1758.4807, 1758.8918, 1761.8734, 1765.8154, 1772.0012, 1779.9012, 1781.8585, 1783.8062, 1787.8407, 1809.0583, 1810.0460, 1812.9126, 1829.8527, 1832.8584, 1833.8571, 1834.8650, 1836.8618, 1838.8677, 1845.8363, 1847.8600, 1848.8938, 1850.9128, 1851.8932, 1861.8531, 1862.9542, 1864.9258, 1880.9026, 1883.7704, 1887.8878, 1897.7863, 1902.9769, 1904.9276, 1908.8986, 1920.9131, 1923.9126, 1928.0443, 1932.0068, 1936.9510, 1938.0171, 1947.9314, 1958.9393, 1959.9655, 1960.9813, 1961.9463, 1967.1549, 1969.9951, 1973.9971, 1975.9755, 1976.9708, 1990.4971, 1990.9373, 1991.4857, 1997.8479, 2001.8607, 2003.8768, 2004.8850, 2007.9500, 2007.9532, 2011.9381, 2012.8951, 2015.9694, 2017.9860, 2018.9740, 2020.1631, 2023.9482, 2026.9529, 2027.5033, 2029.9972, 2043.5356, 2044.9940, 2055.0205, 2057.0154, 2060.9878, 2066.9939, 2068.9661, 2082.9822, 2086.0254, 2088.1033, 2097.0371, 2098.4941, 2102.9954, 2109.0095, 2112.9932, 2114.9707, 2122.9578, 2128.0444, 2130.9980, 2132.0200, 2136.9250, 2140.0063, 2143.0237, 2144.0559, 2145.0781, 2148.0483, 2153.0049, 2153.9973, 2178.0483, 2188.0610, 2191.0464, 2192.0427, 2199.1150, 2205.0615, 2211.1067, 2223.1533, 2225.1382, 2226.1472, 2239.1646, 2243.1448, 2246.9482, 2251.1108, 2255.1484, 2265.0837, 2271.1211, 2273.0735, 2277.0642, 2283.1606, 2285.1355, 2286.0986, 2289.0901, 2291.0173, 2309.0349, 2328.9888, 2343.0608, 2352.9583, 2355.0383, 2366.9595, 2371.0325, 2392.1594, 2400.0532, 2424.1577, 2434.2429, 2438.2283, 2449.2180, 2450.2451, 2451.2480, 2465.1592, 2466.2458, 2478.1377, 2501.2207, 2523.2014, 2526.2585, 2540.2451, 2542.2578, 2545.2258, 2550.2373, 2561.6841, 2562.1826, 2562.6890, 2573.2495, 2576.1792, 2646.9758, 2659.2144, 2661.1409, 2662.0120, 2671.1924, 2681.2705, 2682.2888, 2685.3098, 2689.3059, 2695.1064, 2696.2695, 2699.2324, 2703.2529, 2705.2944, 2712.2937, 2717.2903, 2720.2839, 2721.2937, 2726.2935, 2727.2986, 2728.3079, 2739.2837, 2743.2729, 2744.2207, 2745.2256, 2746.2031, 2748.2295, 2755.2700, 2760.2590, 2763.1829, 2771.2400, 2778.1865, 2785.2969, 2786.2561, 2790.2317, 2792.2166, 2800.3093, 2801.2932, 2803.2415, 2804.2429, 2807.2869, 2808.2227, 2812.2244, 2813.2373, 2817.2632, 2820.2441, 2824.2598, 2828.2454, 2833.2544, 2843.2900, 2847.3630, 2851.3313, 2864.3188, 2890.3293, 2896.4324, 2908.3105, 2910.3325, 2911.3594, 2912.4265, 2925.3323, 2927.4434, 3023.3674, 3025.3865, 3026.3452, 3039.3838, 3042.3542, 3070.3745, 3071.3933, 3086.3984, 3087.3970, 3093.4294, 3101.3831, 3103.4006, 3104.4041, 3110.4143, 3127.4749, 3141.4126, 3143.5066, 3144.5298, 3159.5210, 3219.4854, 3236.5574, 3266.6021, 3280.5635, 3321.6216, 3344.4895, 3347.5515, 3348.5020, 3380.5210, 3396.5195, 3412.5801, 3427.6099, 3428.5874, 3444.5840, 3462.6301, 3478.6123, 3484.6497, 3489.7246, 3505.6919, 3510.5859, 3521.6392, 3552.6614, 3553.6028, 3554.5630, 3558.6460, 3573.6545, 3589.6523, 3608.6155, 3624.6685, 3630.5806, 3641.7090, 3651.7944, 3667.7266, 3683.6980, 3691.7681, 3707.7200, 3714.5493, 3715.6206, 3719.6914, 3720.7048, 3723.7041, 3735.7100, 3751.7539, 3830.8091, 3869.7393, 3876.6707, 3885.6946

11. Q28024 **Mass:** 8166 **Score:** 20 **Expect:** 2.5e+002 **Matches:** 7

Guanine nucleotide-binding protein G(I)/G(S)/G(O) subunit gamma-12 OS=Bos taurus GN=GNG12 PE=1 SV=2

Observed	Mr(expt)	Mr(calc)	ppm	Start	End	Miss	Ions	Peptide
900.5174	899.5101	899.5301	-22.20	16 -	22	1	---	R.RTVQQLR.M
978.5352	977.5279	977.5216	6.53	65 -	72	2	---	K.DKKTCTIL.-
2015.9694	2014.9621	2015.0034	-20.46	49 -	66	1	---	R.NDPLLMGIPTSENPFKDK.K
2144.0559	2143.0486	2143.0983	-23.19	49 -	67	2	---	R.NDPLLMGIPTSENPFKDKK.T
2211.1067	2210.0994	2210.0459	24.2	30 -	48	2	---	R.IKVSKASADLMSYCEEHAR.N + Oxidation (M)
3707.7200	3706.7127	3706.7429	-8.14	32 -	64	2	---	K.VSKASADLMSYCEEHARNDPLLMGIPTSENPFK.D
3723.7041	3722.6968	3722.7378	-11.01	32 -	64	2	---	K.VSKASADLMSYCEEHARNDPLLMGIPTSENPFK.D + Oxidation (M)

No match to: 716.3353, 718.2895, 724.2175, 726.3070, 728.2833, 729.4336, 730.2399, 732.2518, 734.4341, 743.3130, 745.3617, 747.4360, 753.3469, 756.3524, 758.3462, 759.3632, 768.3517, 779.4633, 781.3725, 789.3640, 794.3674, 796.3701, 799.2854, 801.4280, 805.4417, 807.4076, 810.3293, 812.3207, 818.3782, 820.3700, 828.4415, 829.4508, 830.4513, 834.3805, 842.5101, 849.3834, 852.4368, 857.4457, 860.4262, 868.4412, 870.4462, 873.4370, 877.4166, 880.4280, 882.4470, 883.4816, 887.4295, 889.4857, 891.4872, 894.4923, 898.4610, 905.4763, 907.4251, 912.5086, 914.4942, 920.4296, 922.4855, 928.4565, 929.4484, 933.4327, 936.4976, 938.5062, 944.4982, 946.4672, 949.5244, 950.5168, 957.5792, 961.5109, 965.5151, 967.4963, 972.5023, 974.4913, 976.5255, 981.5225, 983.6221, 986.5463, 988.4922, 993.5123, 995.4512, 998.5833, 1000.5594, 1006.5120, 1007.5640, 1021.5762, 1024.5542, 1031.5300, 1034.5703, 1037.5466, 1041.5637, 1043.5297, 1044.5375, 1045.5594, 1046.6058, 1054.5287, 1056.5575, 1057.6187, 1057.6200, 1068.5790, 1074.6162, 1076.6190, 1082.6056, 1105.5938, 1110.5851, 1113.5701, 1118.5687, 1120.6050, 1122.5892, 1123.6047, 1126.6241, 1128.5618, 1130.5756, 1135.5951, 1137.5647, 1143.5592, 1145.6373, 1148.6318, 1149.6230, 1150.6226, 1156.5652, 1157.5676, 1159.5890, 1160.5966, 1169.5873, 1177.6421, 1178.6371, 1180.6443, 1182.6539, 1184.6431, 1185.6815, 1193.6090, 1194.6001, 1209.6238, 1213.7180, 1216.7178, 1221.5953, 1227.6459, 1229.6874, 1234.6899, 1238.2251, 1239.6622, 1254.6993, 1255.6351, 1264.7139, 1265.1680, 1265.7196, 1269.6532, 1276.6729, 1278.6892, 1281.6375, 1285.6674, 1289.7029, 1293.6760, 1301.6362, 1303.6489, 1304.6685, 1306.6946, 1308.6797, 1314.7227, 1319.6770, 1320.6364, 1323.6815, 1326.6937, 1328.6799, 1333.7430, 1336.7301, 1337.6429, 1349.6729, 1351.6969, 1353.6891, 1356.6255, 1358.6919, 1359.7013, 1361.7147, 1364.7118, 1365.6619, 1366.6965, 1372.6228, 1375.6693, 1382.6771, 1383.7347, 1390.7089, 1393.8007, 1397.7449, 1403.7434, 1410.7849, 1416.7737, 1420.7114, 1423.7096, 1434.7847, 1436.7469, 1442.8257, 1445.7913, 1456.7555, 1457.7440, 1467.7560, 1470.7466, 1475.7615, 1479.8038, 1483.7579, 1484.7480, 1487.7660, 1488.7366, 1493.7556, 1500.7393, 1500.7400, 1503.7648, 1504.7708, 1514.7063, 1516.7281, 1519.7705, 1522.7722, 1524.8040, 1526.7924, 1528.7079, 1530.7058, 1536.7252, 1537.7859, 1540.8043, 1543.7938, 1550.7543, 1551.7465, 1553.7668, 1555.7836, 1556.8011, 1562.8008, 1568.7620, 1572.7245, 1578.8058, 1580.8580, 1580.8600, 1584.8486, 1587.7675,

1608.8051, 1616.8165, 1621.7870, 1625.7886, 1628.7313, 1632.8120, 1635.7964, 1637.7974, 1642.7417, 1647.8439, 1650.8429, 1651.8369,
1655.8147, 1657.8483, 1658.8953, 1660.8379, 1662.8666, 1664.8490, 1666.7892, 1679.8091, 1680.8357, 1682.8409, 1696.8464, 1698.8180,
1701.8243, 1702.8386, 1707.8938, 1710.8904, 1714.7905, 1716.8219, 1717.9052, 1718.9094, 1721.8976, 1722.9003, 1724.9030, 1728.8840,
1730.8910, 1734.1617, 1738.8267, 1739.8165, 1741.8934, 1743.9005, 1747.9127, 1758.4807, 1758.8918, 1761.8734, 1765.8154, 1772.0012,
1774.9300, 1774.9319, 1779.9012, 1781.8585, 1783.8062, 1787.8407, 1809.0583, 1810.0460, 1812.9126, 1829.8527, 1832.8584, 1833.8571,
1834.8650, 1836.8618, 1838.8677, 1845.8363, 1847.8600, 1848.8938, 1850.9128, 1851.8932, 1861.8531, 1862.9542, 1864.9258, 1880.9026,
1883.7704, 1887.8878, 1897.7863, 1902.9769, 1904.9276, 1908.8986, 1920.9131, 1923.9126, 1928.0443, 1932.0068, 1936.9510, 1938.0171,
1947.9314, 1958.9393, 1959.9655, 1960.9813, 1961.9463, 1967.1549, 1969.9951, 1973.9971, 1975.9755, 1976.9708, 1990.4971, 1990.9373,
1991.4857, 1997.8479, 2001.8607, 2003.8768, 2004.8850, 2007.9500, 2007.9532, 2011.9381, 2012.8951, 2017.9860, 2018.9740, 2020.1631,
2023.9482, 2026.9529, 2027.5033, 2029.9972, 2043.5356, 2044.9940, 2055.0205, 2057.0154, 2060.9878, 2066.9939, 2068.9661, 2082.9822,
2086.0254, 2088.1033, 2097.0371, 2098.4941, 2102.9954, 2109.0095, 2112.9932, 2114.9707, 2122.9578, 2128.0444, 2130.9980, 2132.0200,
2136.9250, 2140.0063, 2143.0237, 2145.0781, 2148.0483, 2153.0049, 2153.9973, 2174.0508, 2175.0435, 2178.0483, 2188.0610, 2191.0464,
2192.0427, 2199.1150, 2205.0615, 2223.1533, 2225.1382, 2226.1472, 2239.1646, 2243.1448, 2246.9482, 2251.1108, 2255.1484, 2265.0837,
2271.1211, 2273.0735, 2277.0642, 2283.1606, 2285.1355, 2286.0986, 2289.0901, 2291.0173, 2309.0349, 2328.9888, 2343.0608, 2352.9583,
2355.0383, 2366.9595, 2371.0325, 2392.1594, 2400.0532, 2424.1577, 2434.2429, 2438.2283, 2449.2180, 2450.2451, 2451.2480, 2465.1592,
2466.2458, 2467.2458, 2478.1377, 2501.2207, 2523.2014, 2526.2585, 2540.2451, 2542.2578, 2545.2258, 2550.2373, 2561.6841, 2562.1826,
2562.6890, 2573.2495, 2576.1792, 2646.9758, 2659.2144, 2661.1409, 2662.0120, 2671.1924, 2681.2705, 2682.2888, 2685.3098, 2689.3059,
2695.1064, 2696.2695, 2699.2324, 2703.2529, 2705.2944, 2712.2937, 2717.2903, 2720.2839, 2721.2937, 2726.2935, 2727.2986, 2728.3079,
2739.2837, 2743.2729, 2744.2207, 2745.2256, 2746.2031, 2748.2295, 2755.2700, 2760.2590, 2763.1829, 2771.2400, 2778.1865, 2785.2969,
2786.2561, 2787.2524, 2790.2317, 2792.2166, 2800.3093, 2801.2932, 2803.2415, 2804.2429, 2807.2869, 2808.2227, 2812.2244, 2813.2373,
2817.2632, 2820.2441, 2824.2598, 2828.2454, 2833.2544, 2843.2900, 2844.3398, 2847.3630, 2851.3313, 2864.3188, 2890.3293, 2896.4324,
2908.3105, 2910.3325, 2911.3594, 2912.4265, 2925.3323, 2927.4434, 3023.3674, 3025.3865, 3026.3452, 3039.3838, 3042.3542, 3070.3745,
3071.3933, 3086.3984, 3087.3970, 3093.4294, 3101.3831, 3103.4006, 3104.4041, 3110.4143, 3127.4749, 3141.4126, 3143.5066, 3144.5298,
3159.5210, 3219.4854, 3236.5574, 3266.6021, 3280.5635, 3321.6216, 3344.4895, 3347.5515, 3348.5020, 3380.5210, 3396.5195, 3412.5801,
3427.6099, 3428.5874, 3444.5840, 3462.6301, 3478.6123, 3484.6497, 3489.7246, 3505.6919, 3510.5859, 3521.6392, 3552.6614, 3553.6028,
3554.5630, 3558.6460, 3573.6545, 3589.6523, 3608.6155, 3624.6685, 3630.5806, 3641.7090, 3651.7944, 3667.7266, 3683.6980, 3691.7681,
3714.5493, 3715.6206, 3719.6914, 3720.7048, 3735.7100, 3751.7539, 3830.8091, 3869.7393, 3876.6707, 3885.6946

12. P46170 **Mass:** 4445 **Score:** 20 **Expect:** 2.6e+002 **Matches:** 5

Beta-defensin 12 OS=Bos taurus GN=DEFB12 PE=1 SV=1

Observed	Mr(expt)	Mr(calc)	ppm	Start	End	Miss	Ions	Peptide
759.3632	758.3559	758.3568	-1.14	17 -	22	0	---	R.CPVPMR.Q
1738.8267	1737.8194	1737.8226	-1.80	23 -	36	1	---	R.QIGTCFGRPVKCCR.S
1741.8934	1740.8861	1740.8586	15.8	8 -	22	1	---	R.NGGVCIPIRCPVPMR.Q + Oxidation (M)
2011.9381	2010.9308	2010.9339	-1.53	23 -	38	2	---	R.QIGTCFGRPVKCCRSW.-
2018.9740	2017.9667	2018.0013	-17.11	17 -	33	1	---	R.CPVPMRQIGTCFGRPVK.C + Oxidation (M)

No match to: 716.3353, 718.2895, 724.2175, 726.3070, 728.2833, 729.4336, 730.2399, 732.2518, 734.4341, 743.3130, 745.3617,
747.4360, 753.3469, 756.3524, 758.3462, 768.3517, 779.4633, 781.3725, 789.3640, 794.3674, 796.3701, 799.2854, 801.4280, 805.4417,
807.4076, 810.3293, 812.3207, 818.3782, 820.3700, 828.4415, 829.4508, 830.4513, 834.3805, 842.5101, 849.3834, 852.4368, 857.4457,
860.4262, 868.4412, 870.4462, 873.4370, 877.4166, 880.4280, 882.4470, 883.4816, 887.4295, 889.4857, 891.4872, 894.4923, 898.4610,
900.5174, 905.4763, 907.4251, 912.5086, 914.4942, 920.4296, 922.4855, 928.4565, 929.4484, 933.4327, 936.4976, 938.5062, 944.4982,
946.4672, 949.5244, 950.5168, 957.5792, 961.5109, 965.5151, 967.4963, 972.5023, 974.4913, 976.5255, 978.5352, 981.5225, 983.6221,
986.5463, 988.4922, 993.5123, 995.4512, 998.5833, 1000.5594, 1006.5120, 1007.5640, 1021.5762, 1024.5542, 1031.5300, 1034.5703,
1037.5466, 1041.5637, 1043.5297, 1044.5375, 1045.5594, 1046.6058, 1054.5287, 1056.5575, 1057.6187, 1057.6200, 1068.5790, 1074.6162,
1076.6190, 1082.6056, 1105.5938, 1110.5851, 1113.5701, 1118.5687, 1120.6050, 1122.5892, 1123.6047, 1126.6241, 1128.5618, 1130.5756,
1135.5951, 1137.5647, 1143.5592, 1145.6373, 1148.6318, 1149.6230, 1150.6226, 1156.5652, 1157.5676, 1159.5890, 1160.5966, 1169.5873,
1177.6421, 1178.6371, 1180.6443, 1182.6539, 1184.6431, 1185.6815, 1193.6090, 1194.6001, 1209.6238, 1213.7180, 1216.7178, 1221.5953,
1227.6459, 1229.6874, 1234.6899, 1238.2251, 1239.6622, 1254.6993, 1255.6351, 1264.7139, 1265.1680, 1265.7196, 1269.6532, 1276.6729,
1278.6892, 1281.6375, 1285.6674, 1289.7029, 1293.6760, 1301.6362, 1303.6489, 1304.6685, 1306.6946, 1308.6797, 1314.7227, 1319.6770,
1320.6364, 1323.6815, 1326.6937, 1328.6799, 1333.7430, 1336.7301, 1337.6429, 1349.6729, 1351.6969, 1353.6891, 1356.6255, 1358.6919,
1359.7013, 1361.7147, 1364.7118, 1365.6619, 1366.6965, 1372.6228, 1375.6693, 1382.6771, 1383.7347, 1390.7089, 1393.8007, 1397.7449,
1403.7434, 1410.7849, 1416.7737, 1420.7114, 1423.7096, 1434.7847, 1436.7469, 1442.8257, 1445.7913, 1456.7555, 1457.7440, 1467.7560,
1470.7466, 1475.7615, 1479.8038, 1483.7579, 1484.7480, 1487.7660, 1488.7366, 1493.7556, 1500.7393, 1500.7400, 1503.7648, 1504.7708,
1514.7063, 1516.7281, 1519.7705, 1522.7722, 1524.8040, 1526.7924, 1528.7079, 1530.7058, 1536.7252, 1537.7859, 1540.8043, 1543.7938,
1550.7543, 1551.7465, 1553.7668, 1555.7836, 1556.8011, 1562.8008, 1568.7620, 1572.7245, 1578.8058, 1580.8580, 1580.8600, 1584.8486,
1587.7675, 1608.8051, 1616.8165, 1621.7870, 1625.7886, 1628.7313, 1632.8120, 1635.7964, 1637.7974, 1642.7417, 1647.8439, 1650.8429,
1651.8369, 1655.8147, 1657.8483, 1658.8953, 1660.8379, 1662.8666, 1664.8490, 1666.7892, 1679.8091, 1680.8357, 1682.8409, 1696.8464,
1698.8180, 1701.8243, 1702.8386, 1707.8938, 1710.8904, 1714.7905, 1716.8219, 1717.9052, 1718.9094, 1721.8976, 1722.9003, 1724.9030,
1728.8840, 1730.8910, 1734.1617, 1739.8165, 1743.9005, 1747.9127, 1758.4807, 1758.8918, 1761.8734, 1765.8154, 1772.0012, 1774.9300,
1774.9319, 1779.9012, 1781.8585, 1783.8062, 1787.8407, 1809.0583, 1810.0460, 1812.9126, 1829.8527, 1832.8584, 1833.8571, 1834.8650,
1836.8618, 1838.8677, 1845.8363, 1847.8600, 1848.8938, 1850.9128, 1851.8932, 1861.8531, 1862.9542, 1864.9258, 1880.9026, 1883.7704,
1887.8878, 1897.7863, 1902.9769, 1904.9276, 1908.8986, 1920.9131, 1923.9126, 1928.0443, 1932.0068, 1936.9510, 1938.0171, 1947.9314,
1958.9393, 1959.9655, 1960.9813, 1961.9463, 1967.1549, 1969.9951, 1973.9971, 1975.9755, 1976.9708, 1990.4971, 1990.9373, 1991.4857,
1997.8479, 2001.8607, 2003.8768, 2004.8850, 2007.9500, 2007.9532, 2012.8951, 2015.9694, 2017.9860, 2020.1631, 2023.9482, 2026.9529,
2027.5033, 2029.9972, 2043.5356, 2044.9940, 2055.0205, 2057.0154, 2060.9878, 2066.9939, 2068.9661, 2082.9822, 2086.0254, 2088.1033,
2097.0371, 2098.4941, 2102.9954, 2109.0095, 2112.9932, 2114.9707, 2122.9578, 2128.0444, 2130.9980, 2132.0200, 2136.9250, 2140.0063,
2143.0237, 2144.0559, 2145.0781, 2148.0483, 2153.0049, 2153.9973, 2174.0508, 2175.0435, 2178.0483, 2188.0610, 2191.0464, 2192.0427,
2199.1150, 2205.0615, 2211.1067, 2223.1533, 2225.1382, 2226.1472, 2239.1646, 2243.1448, 2246.9482, 2251.1108, 2255.1484, 2265.0837,
2271.1211, 2273.0735, 2277.0642, 2283.1606, 2285.1355, 2286.0986, 2289.0901, 2291.0173, 2309.0349, 2328.9888, 2343.0608, 2352.9583,
2355.0383, 2366.9595, 2371.0325, 2392.1594, 2400.0532, 2424.1577, 2434.2429, 2438.2283, 2449.2180, 2450.2451, 2451.2480, 2465.1592,
2466.2458, 2467.2458, 2478.1377, 2501.2207, 2523.2014, 2526.2585, 2540.2451, 2542.2578, 2545.2258, 2550.2373, 2561.6841, 2562.1826,
2562.6890, 2573.2495, 2576.1792, 2646.9758, 2659.2144, 2661.1409, 2662.0120, 2671.1924, 2681.2705, 2682.2888, 2685.3098, 2689.3059,
2695.1064, 2696.2695, 2699.2324, 2703.2529, 2705.2944, 2712.2937, 2717.2903, 2720.2839, 2721.2937, 2726.2935, 2727.2986, 2728.3079,
2739.2837, 2743.2729, 2744.2207, 2745.2256, 2746.2031, 2748.2295, 2755.2700, 2760.2590, 2763.1829, 2771.2400, 2778.1865, 2785.2969,
2786.2561, 2787.2524, 2790.2317, 2792.2166, 2800.3093, 2801.2932, 2803.2415, 2804.2429, 2807.2869, 2808.2227, 2812.2244, 2813.2373,
2817.2632, 2820.2441, 2824.2598, 2828.2454, 2833.2544, 2843.2900, 2844.3398, 2847.3630, 2851.3313, 2864.3188, 2890.3293, 2896.4324,
2908.3105, 2910.3325, 2911.3594, 2912.4265, 2925.3323, 2927.4434, 3023.3674, 3025.3865, 3026.3452, 3039.3838, 3042.3542, 3070.3745,
3071.3933, 3086.3984, 3087.3970, 3093.4294, 3101.3831, 3103.4006, 3104.4041, 3110.4143, 3127.4749, 3141.4126, 3143.5066, 3144.5298,
3159.5210, 3219.4854, 3236.5574, 3266.6021, 3280.5635, 3321.6216, 3344.4895, 3347.5515, 3348.5020, 3380.5210, 3396.5195, 3412.5801,
3427.6099, 3428.5874, 3444.5840, 3462.6301, 3478.6123, 3484.6497, 3489.7246, 3505.6919, 3510.5859, 3521.6392, 3552.6614, 3553.6028,
3554.5630, 3558.6460, 3573.6545, 3589.6523, 3608.6155, 3624.6685, 3630.5806, 3641.7090, 3651.7944, 3667.7266, 3683.6980, 3691.7681,
3707.7200, 3714.5493, 3715.6206, 3719.6914, 3720.7048, 3723.7041, 3735.7100, 3751.7539, 3830.8091, 3869.7393, 3876.6707, 3885.6946

13. Q29RZ4 **Mass:** 49390 **Score:** 19 **Expect:** 2.8e+002 **Matches:** 20

Zinc finger protein 2 OS=Bos taurus GN=ZNF2 PE=2 SV=1

Observed	Mr(expt)	Mr(calc)	ppm	Start	End	Miss	Ions	Peptide
950.5168	949.5096	949.4981	12.1	418 -	425	2	---	R.RYAKQGID.-
961.5109	960.5037	960.5253	-22.58	303 -	310	0	---	R.HQLIHTGR.K
1056.5575	1055.5502	1055.5764	-24.77	250 -	258	0	---	R.IHTGEKPFK.C
1110.5851	1109.5778	1109.6054	-24.83	410 -	418	1	---	K.SSVIQHQRR.Y
1113.5701	1112.5628	1112.5614	1.24	321 -	330	0	---	K.AFYGVSSLNR.H
1126.6241	1125.6168	1125.5938	20.5	382 -	389	2	---	R.CRLTRHQR.V
1130.5756	1129.5683	1129.5741	-5.08	331 -	340	1	---	R.HQKAHAGEPR.Y
1658.8953	1657.8880	1657.9012	-7.97	405 -	418	2	---	K.VFSSKSSVIQHQRR.Y
1920.9131	1919.9058	1919.8618	22.9	259 -	274	2	---	K.CSECGKAFFDRSSLTR.H
2225.1382	2224.1309	2224.1250	2.68	321 -	340	2	---	K.AFYGVSSLNRHQKAHAGEPR.Y

2239.1646	2238.1573	2238.1718	-6.47	30	47	2	---	R.LVPTQRDLYKEVMLENYK.S
2255.1484	2254.1411	2254.1667	-11.35	30	47	2	---	R.LVPTQRDLYKEVMLENYK.S + Oxidation (M)
2450.2451	2449.2378	2449.2383	-0.20	139	159	2	---	K.ESPTVEACKKPPSLEESLRHR.A
2526.2585	2525.2512	2525.2380	5.24	384	404	2	---	R.LTRHQRVHTGEKPFECSVCGK.V
2685.3098	2684.3025	2684.3690	-24.78	134	157	2	---	R.LGTEKESPTVEACKKPPSLEESLR.H
2745.2256	2744.2183	2744.2507	-11.80	270	292	2	---	R.SSLTRHQRIHTGESPYECNQCGK.A
2911.3594	2910.3521	2910.3752	-7.94	278	302	2	---	R.IHTGESPYECNQCGKAFSQKSILTR.H
3280.5635	3279.5562	3279.5619	-1.73	2	29	2	---	M.AAVSPTRCQDPVTFEDVAVVFTDEEWRR.L
3321.6216	3320.6143	3320.5931	6.39	303	330	2	---	R.HQLIHTGRKPYECNECGKAFYGVSSLNR.H
3427.6099	3426.6026	3426.5973	1.55	1	29	2	---	-.MAAVSPTRCQDPVTFEDVAVVFTDEEWRR.L + Oxidation (M)

No match to: 716.3353, 718.2895, 724.2175, 726.3070, 728.2833, 729.4336, 730.2399, 732.2518, 734.4341, 743.3130, 745.3617, 747.4360, 753.3469, 756.3524, 758.3462, 759.3632, 768.3517, 779.4633, 781.3725, 789.3640, 794.3674, 796.3701, 799.2854, 801.4280, 805.4417, 807.4076, 810.3293, 812.3207, 818.3782, 820.3700, 828.4415, 829.4508, 830.4513, 834.3805, 842.5101, 849.3834, 852.4368, 857.4457, 860.4262, 868.4412, 870.4462, 873.4370, 877.4166, 880.4280, 882.4470, 883.4816, 887.4295, 889.4857, 891.4872, 894.4923, 898.4610, 900.5174, 905.4763, 907.4251, 912.5086, 914.4942, 920.4296, 922.4855, 928.4565, 929.4484, 933.4327, 936.4976, 938.5062, 944.4982, 946.4672, 949.5244, 957.5792, 965.5151, 967.4963, 972.5023, 974.4913, 976.5255, 978.5352, 981.5225, 983.6221, 986.5463, 988.4922, 993.5123, 995.4512, 998.5833, 1000.5594, 1006.5120, 1007.5640, 1021.5762, 1024.5542, 1031.5300, 1034.5703, 1037.5466, 1041.5637, 1043.5297, 1044.5375, 1045.5594, 1046.6058, 1054.5287, 1057.6187, 1057.6200, 1068.5790, 1074.6162, 1076.6190, 1082.6056, 1105.5938, 1118.5687, 1120.6050, 1122.5892, 1123.6047, 1128.5618, 1135.5951, 1137.5647, 1143.5592, 1145.6373, 1148.6318, 1149.6230, 1150.6226, 1156.5652, 1157.5676, 1159.5890, 1160.5966, 1169.5873, 1177.6421, 1178.6371, 1180.6443, 1182.6539, 1184.6431, 1185.6815, 1193.6090, 1194.6001, 1209.6238, 1213.7180, 1216.7178, 1221.5953, 1227.6459, 1229.6874, 1234.6899, 1238.2251, 1239.6622, 1254.6993, 1255.6351, 1264.7139, 1265.1680, 1265.7196, 1269.6532, 1276.6729, 1278.6892, 1281.6375, 1285.6674, 1289.7029, 1293.6760, 1301.6362, 1303.6489, 1304.6685, 1306.6946, 1308.6797, 1314.7227, 1319.6770, 1320.6364, 1323.6815, 1326.6937, 1328.6799, 1333.7430, 1336.7301, 1337.6429, 1349.6729, 1351.6969, 1353.6891, 1356.6255, 1358.6919, 1359.7013, 1361.7147, 1364.7118, 1365.6619, 1366.6965, 1372.6228, 1375.6693, 1382.6771, 1383.7347, 1390.7089, 1393.8007, 1397.7449, 1403.7434, 1410.7849, 1416.7737, 1420.7114, 1423.7096, 1434.7847, 1436.7469, 1442.8257, 1445.7913, 1456.7555, 1457.7440, 1467.7560, 1470.7466, 1475.7615, 1479.8038, 1483.7579, 1484.7480, 1487.7660, 1488.7366, 1493.7556, 1500.7393, 1500.7400, 1503.7648, 1504.7708, 1514.7063, 1516.7281, 1519.7705, 1522.7722, 1524.8040, 1526.7924, 1528.7079, 1530.7058, 1536.7252, 1537.7859, 1540.8043, 1543.7938, 1550.7543, 1551.7465, 1553.7668, 1555.7836, 1556.8011, 1562.8008, 1568.7620, 1572.7245, 1578.8058, 1580.8580, 1580.8600, 1584.8486, 1587.7675, 1608.8051, 1616.8165, 1621.7870, 1625.7886, 1628.7313, 1632.8120, 1635.7964, 1637.7974, 1642.7417, 1647.8439, 1650.8429, 1651.8369, 1655.8147, 1657.8483, 1660.8379, 1662.8666, 1664.8490, 1666.7892, 1679.8091, 1680.8357, 1682.8409, 1696.8464, 1698.8180, 1701.8243, 1702.8386, 1707.8938, 1710.8904, 1714.7905, 1716.8219, 1717.9052, 1718.9094, 1721.8976, 1722.9003, 1724.9030, 1728.8840, 1730.8910, 1734.1617, 1738.8267, 1739.8165, 1741.8934, 1743.9005, 1747.9127, 1758.4807, 1758.8918, 1761.8734, 1765.8154, 1772.0012, 1774.9300, 1774.9319, 1779.9012, 1781.8585, 1783.8062, 1787.8407, 1809.0583, 1810.0460, 1812.9126, 1829.8527, 1832.8584, 1833.8571, 1834.8650, 1836.8618, 1838.8677, 1845.8363, 1847.8600, 1848.8938, 1850.9128, 1851.8932, 1861.8531, 1862.9542, 1864.9258, 1880.9026, 1883.7704, 1887.8878, 1897.7863, 1902.9769, 1904.9276, 1908.8986, 1923.9126, 1928.0443, 1932.0068, 1936.9510, 1938.0171, 1947.9314, 1958.9393, 1959.9655, 1960.9813, 1961.9463, 1967.1549, 1969.9951, 1973.9971, 1975.9755, 1976.9708, 1990.4971, 1990.9373, 1991.4857, 1997.8479, 2001.8607, 2003.8768, 2004.8850, 2007.9500, 2007.9532, 2011.9381, 2012.8951, 2015.9694, 2017.9860, 2018.9740, 2020.1631, 2023.9482, 2026.9529, 2027.5033, 2029.9972, 2043.5356, 2044.9940, 2055.0205, 2057.0154, 2060.9878, 2066.9939, 2068.9661, 2082.9822, 2086.0254, 2088.1033, 2097.0371, 2098.4941, 2102.9954, 2109.0095, 2112.9932, 2114.9707, 2122.9578, 2128.0444, 2130.9980, 2132.0200, 2136.9250, 2140.0063, 2143.0237, 2144.0559, 2145.0781, 2148.0483, 2153.0049, 2153.9973, 2174.0508, 2175.0435, 2178.0483, 2188.0610, 2191.0464, 2192.0427, 2199.1150, 2205.0615, 2211.1067, 2223.1533, 2226.1472, 2243.1448, 2246.9482, 2251.1108, 2265.0837, 2271.1211, 2273.0735, 2277.0642, 2283.1606, 2285.1355, 2286.0986, 2289.0901, 2291.0173, 2309.0349, 2328.9888, 2343.0608, 2352.9583, 2355.0383, 2366.9595, 2371.0325, 2392.1594, 2400.0532, 2424.1577, 2434.2429, 2438.2283, 2449.2180, 2451.2480, 2465.1592, 2466.2458, 2467.2458, 2478.1377, 2501.2207, 2523.2014, 2540.2451, 2542.2578, 2545.2258, 2550.2373, 2561.6841, 2562.1826, 2562.6890, 2573.2495, 2576.1792, 2646.9758, 2659.2144, 2661.1409, 2662.0120, 2671.1924, 2681.2705, 2682.2888, 2689.3059, 2695.1064, 2696.2695, 2699.2324, 2703.2529, 2705.2944, 2712.2937, 2717.2903, 2720.2839, 2721.2937, 2726.2935, 2727.2986, 2728.3079, 2739.2837, 2743.2729, 2744.2207, 2746.2031, 2748.2295, 2755.2700, 2760.2590, 2763.1829, 2771.2400, 2778.1865, 2785.2969, 2786.2561, 2787.2524, 2790.2317, 2792.2166, 2800.3093, 2801.2932, 2803.2415, 2804.2429, 2807.2869, 2808.2227, 2812.2244, 2813.2373, 2817.2632, 2820.2441, 2824.2598, 2828.2454, 2833.2544, 2843.2900, 2844.3398, 2847.3630, 2851.3313, 2864.3188, 2890.3293, 2896.4324, 2908.3105, 2910.3325, 2912.4265, 2925.3323, 2927.4434, 3023.3674, 3025.3865, 3026.3452, 3039.3838, 3042.3542, 3070.3745, 3071.3933, 3086.3984, 3087.3970, 3093.4294, 3101.3831, 3103.4006, 3104.4041, 3110.4143, 3127.4749, 3141.4126, 3143.5066, 3144.5298, 3159.5210, 3219.4854, 3236.5574, 3266.6021, 3344.4895, 3347.5515, 3348.5020, 3380.5210, 3396.5195, 3412.5801, 3428.5874, 3444.5840, 3462.6301, 3478.6123, 3484.6497, 3489.7246, 3505.6919, 3510.5859, 3521.6392, 3552.6614, 3553.6028, 3554.5630, 3558.6460, 3573.6545, 3589.6523, 3608.6155, 3624.6685, 3630.5806, 3641.7090, 3651.7944, 3667.7266, 3683.6980, 3691.7681, 3707.7200, 3714.5493, 3715.6206, 3719.6914, 3720.7048, 3723.7041, 3735.7100, 3751.7539, 3830.8091, 3869.7393, 3876.6707, 3885.6946

14. A7Z060 **Mass:** 81637 **Score:** 19 **Expect:** 2.8e+002 **Matches:** 31

RNF219 protein OS=Bos taurus GN=RNF219 PE=2 SV=1

Observed	Mr(expt)	Mr(calc)	ppm	Start	End	Miss	Ions	Peptide
807.4076	806.4003	806.3923	9.99	653	658	0	---	R.LFEDQR.F
944.4982	943.4909	943.5087	-18.84	202	209	1	---	R.LKAEVDNR.S
967.4963	966.4891	966.4658	24.1	166	173	0	---	R.TANEIYEK.V
1143.5592	1142.5519	1142.5680	-14.05	204	213	1	---	K.AEVDNRSPQK.F
1314.7227	1313.7154	1313.7303	-11.30	574	584	1	---	K.LEERTKPNLSK.G
1361.7147	1360.7074	1360.7133	-4.29	490	501	1	---	K.LSEKPGSCLSKR.L
1366.6965	1365.6892	1365.6823	5.08	502	512	1	---	R.LNSLRSFEMNR.T
1382.6771	1381.6698	1381.6772	-5.34	502	512	1	---	R.LNSLRSFEMNR.T + Oxidation (M)
1456.7555	1455.7482	1455.7793	-21.38	198	209	2	---	R.ENLRLKAEVDNR.S
1503.7648	1502.7575	1502.7590	-0.96	204	216	2	---	K.AEVDNRSPQKFGR.F
1522.7722	1521.7649	1521.7834	-12.13	501	512	2	---	K.RLNSLRSFEMNR.T
1851.8932	1850.8859	1850.8833	1.44	544	560	1	---	K.SPCNNGFKSLDLDGLSK.S
1880.9026	1879.8953	1879.8846	5.69	327	341	2	---	K.TEPNCSKNQDLYQKR.V
1958.9393	1957.9320	1957.9415	-4.84	67	84	0	---	K.EIIGGTSESEPMLSHTVR.K + Oxidation (M)
2003.8768	2002.8695	2002.8587	5.40	418	433	1	---	R.KLMFDEFCDSPNVCNK.D
2136.9250	2135.9177	2135.9603	-19.92	342	359	0	---	R.VETMLTVTDTSMDTYLER.E + 2 Oxidation (M)
2175.0435	2174.0362	2174.0565	-9.32	92	108	1	---	R.LELLHKEYEDEIDCLQK.E
2277.0642	2276.0569	2276.0665	-4.19	341	359	1	---	K.RVETMLTVTDTSMDTYLER.E + Oxidation (M)
2355.0383	2354.0310	2354.0808	-21.16	321	340	2	---	R.QEGTSKTEPNCSKNQDLYQK.R
2739.2837	2738.2764	2738.2363	14.6	412	433	2	---	K.QSNHLRKLMFDEFCDSPNVCNK.D
2745.2256	2744.2183	2744.2574	-14.26	666	689	0	---	K.MSSEMPGLHTHLQSPWSTSFVPEK.R + 2 Oxidation (M)
2755.2700	2754.2627	2754.2312	11.4	412	433	2	---	K.QSNHLRKLMFDEFCDSPNVCNK.D + Oxidation (M)
2808.2227	2807.2154	2807.1473	24.3	418	440	2	---	R.KLMFDEFCDSPNVCNKDSSEDDR.S
3127.4749	3126.4676	3126.5015	-10.84	666	692	2	---	K.MSSEMPGLHTHLQSPWSTSFVPEKRNK.N + Oxidation (M)
3143.5066	3142.4993	3142.4964	0.92	666	692	2	---	K.MSSEMPGLHTHLQSPWSTSFVPEKRNK.N + 2 Oxidation (M)
3521.6392	3520.6319	3520.5643	19.2	529	560	2	---	K.ISELDSMMSESDNSKSPCNNGFKSLDLDGLSK.S + Oxidation (M)
3651.7944	3650.7871	3650.7650	6.06	659	690	2	---	R.FGSSLFKMSSEMPGLHTHLQSPWSTSFVPEKR.N + Oxidation (M)
3667.7266	3666.7193	3666.7599	-11.07	659	690	2	---	R.FGSSLFKMSSEMPGLHTHLQSPWSTSFVPEKR.N + 2 Oxidation (M)
3720.7048	3719.6975	3719.7658	-18.36	373	405	1	---	K.DEELYDLPASCAPLSLSCLQLNTPDNRESSVVK.A
3869.7393	3868.7320	3868.7416	-2.48	341	372	2	---	K.RVETMLTVTDTSMDTYLEREWGNKPSDCMPYK.D + Oxidation (M)
3885.6946	3884.6873	3884.7365	-12.67	341	372	2	---	K.RVETMLTVTDTSMDTYLEREWGNKPSDCMPYK.D + 2 Oxidation (M)

No match to: 716.3353, 718.2895, 724.2175, 726.3070, 728.2833, 729.4336, 730.2399, 732.2518, 734.4341, 743.3130, 745.3617, 747.4360, 753.3469, 756.3524, 758.3462, 759.3632, 768.3517, 779.4633, 781.3725, 789.3640, 794.3674, 796.3701, 799.2854, 801.4280,

805.4417, 810.3293, 812.3207, 818.3782, 820.3700, 828.4415, 829.4508, 830.4513, 834.3805, 842.5101, 849.3834, 852.4368, 857.4457, 860.4262, 868.4412, 870.4462, 873.4370, 877.4166, 880.4280, 882.4470, 883.4816, 887.4295, 889.4857, 891.4872, 894.4923, 898.4610, 900.5174, 905.4763, 907.4251, 912.5086, 914.4942, 920.4296, 922.4855, 928.4565, 929.4484, 933.4327, 936.4976, 938.5062, 946.4672, 949.5244, 950.5168, 957.5792, 961.5109, 965.5151, 972.5023, 974.4913, 976.5255, 978.5352, 981.5225, 983.6221, 986.5463, 988.4922, 993.5123, 995.4512, 998.5833, 1000.5594, 1006.5120, 1007.5640, 1021.5762, 1024.5542, 1031.5300, 1034.5703, 1037.5466, 1041.5637, 1043.5297, 1044.5375, 1045.5594, 1046.6058, 1054.5287, 1056.5575, 1057.6187, 1057.6200, 1068.5790, 1074.6162, 1076.6190, 1082.6056, 1105.5938, 1110.5851, 1113.5701, 1118.5687, 1120.6050, 1122.5892, 1123.6047, 1126.6241, 1128.5618, 1130.5756, 1135.5951, 1137.5647, 1145.6373, 1148.6318, 1149.6230, 1150.6226, 1156.5652, 1157.5676, 1159.5890, 1160.5966, 1169.5873, 1177.6421, 1178.6371, 1180.6443, 1182.6539, 1184.6431, 1185.6815, 1193.6090, 1194.6001, 1209.6238, 1213.7180, 1216.7178, 1221.5953, 1227.6459, 1229.6874, 1234.6899, 1238.2251, 1239.6622, 1254.6993, 1255.6351, 1264.7139, 1265.1680, 1265.7196, 1269.6532, 1276.6729, 1278.6892, 1281.6375, 1285.6674, 1289.7029, 1293.6760, 1301.6362, 1303.6489, 1304.6685, 1306.6946, 1308.6797, 1319.6770, 1320.6364, 1323.6815, 1326.6937, 1328.6799, 1333.7430, 1336.7301, 1337.6429, 1349.6729, 1351.6969, 1353.6891, 1356.6255, 1358.6919, 1359.7013, 1364.7118, 1365.6619, 1372.6228, 1375.6693, 1383.7347, 1390.7089, 1393.8007, 1397.7449, 1403.7434, 1410.7849, 1416.7737, 1420.7114, 1423.7096, 1434.7847, 1436.7469, 1442.8257, 1445.7913, 1457.7440, 1467.7560, 1470.7466, 1475.7615, 1479.8038, 1483.7579, 1484.7480, 1487.7660, 1488.7366, 1493.7556, 1500.7393, 1500.7400, 1504.7708, 1514.7063, 1516.7281, 1519.7705, 1524.8040, 1526.7924, 1528.7079, 1530.7058, 1536.7252, 1537.7859, 1540.8043, 1543.7938, 1550.7543, 1551.7465, 1553.7668, 1555.7836, 1556.8011, 1562.8008, 1568.7620, 1572.7245, 1578.8058, 1580.8580, 1580.8600, 1584.8486, 1587.7675, 1608.8051, 1616.8165, 1621.7870, 1625.7886, 1628.7313, 1632.8120, 1635.7964, 1637.7974, 1642.7417, 1647.8439, 1650.8429, 1651.8369, 1655.8147, 1657.8483, 1658.8953, 1660.8379, 1662.8666, 1664.8490, 1666.7892, 1679.8091, 1680.8357, 1682.8409, 1696.8464, 1698.8180, 1701.8243, 1702.8386, 1707.8938, 1710.8904, 1714.7905, 1716.8219, 1717.9052, 1718.9094, 1721.8976, 1722.9003, 1724.9030, 1728.8840, 1730.8910, 1734.1617, 1738.8267, 1739.8165, 1741.8934, 1743.9005, 1747.9127, 1758.4807, 1758.8918, 1761.8734, 1765.8154, 1772.0012, 1774.9300, 1774.9319, 1779.9012, 1781.8585, 1783.8062, 1787.8407, 1809.0583, 1810.0460, 1812.9126, 1829.8527, 1832.8584, 1833.8571, 1834.8650, 1836.8618, 1838.8677, 1845.8363, 1847.8600, 1848.8938, 1850.9128, 1861.8531, 1862.9542, 1864.9258, 1883.7704, 1887.8878, 1897.7863, 1902.9769, 1904.9276, 1908.8986, 1920.9131, 1923.9126, 1928.0443, 1932.0068, 1936.9510, 1938.0171, 1947.9314, 1959.9655, 1960.9813, 1961.9463, 1967.1549, 1969.9951, 1973.9971, 1975.9755, 1976.9708, 1990.4971, 1990.9373, 1991.4857, 1997.8479, 2001.8607, 2004.8850, 2007.9500, 2007.9532, 2011.9381, 2012.8951, 2015.9694, 2017.9860, 2018.9740, 2020.1631, 2023.9482, 2026.9529, 2027.5033, 2029.9972, 2043.5356, 2044.9940, 2055.0205, 2057.0154, 2060.9878, 2066.9939, 2068.9661, 2082.9822, 2086.0254, 2088.1033, 2097.0371, 2098.4941, 2102.9954, 2109.0095, 2112.9932, 2114.9707, 2122.9578, 2128.0444, 2130.9980, 2132.0200, 2140.0063, 2143.0237, 2144.0559, 2145.0781, 2148.0483, 2153.0049, 2153.9973, 2174.0508, 2178.0483, 2188.0610, 2191.0464, 2192.0427, 2199.1150, 2205.0615, 2211.1067, 2223.1533, 2225.1382, 2226.1472, 2239.1646, 2243.1448, 2246.9482, 2251.1108, 2255.1484, 2265.0837, 2271.1211, 2273.0735, 2283.1606, 2285.1355, 2286.0986, 2289.0901, 2291.0173, 2309.0349, 2328.9888, 2343.0608, 2352.9583, 2366.9595, 2371.0325, 2392.1594, 2400.0532, 2424.1577, 2434.2429, 2438.2283, 2449.2180, 2450.2451, 2451.2480, 2465.1592, 2466.2458, 2467.2458, 2478.1377, 2501.2207, 2523.2014, 2526.2585, 2540.2451, 2542.2578, 2545.2258, 2550.2373, 2561.6841, 2562.1826, 2562.6890, 2573.2495, 2576.1792, 2646.9758, 2659.2144, 2661.1409, 2662.0120, 2671.1924, 2681.2705, 2682.2888, 2685.3098, 2689.3059, 2695.1064, 2696.2695, 2699.2324, 2703.2529, 2705.2944, 2712.2937, 2717.2903, 2720.2839, 2721.2937, 2726.2935, 2727.2986, 2728.3079, 2743.2729, 2744.2207, 2746.2031, 2748.2295, 2760.2590, 2763.1829, 2771.2400, 2778.1865, 2785.2969, 2786.2561, 2787.2524, 2790.2317, 2792.2166, 2800.3093, 2801.2932, 2803.2415, 2804.2429, 2807.2869, 2812.2244, 2813.2373, 2817.2632, 2820.2441, 2824.2598, 2828.2454, 2833.2544, 2843.2900, 2844.3398, 2847.3630, 2851.3313, 2864.3188, 2890.3293, 2896.4324, 2908.3105, 2910.3325, 2911.3594, 2912.4265, 2925.3323, 2927.4434, 3023.3674, 3025.3865, 3026.3452, 3039.3838, 3042.3542, 3070.3745, 3071.3933, 3086.3984, 3087.3970, 3093.4294, 3101.3831, 3103.4006, 3104.4041, 3110.4143, 3141.4126, 3144.5298, 3159.5210, 3219.4854, 3236.5574, 3266.6021, 3280.5635, 3321.6216, 3344.4895, 3347.5515, 3348.5020, 3380.5210, 3396.5195, 3412.5801, 3427.6099, 3428.5874, 3444.5840, 3462.6301, 3478.6123, 3484.6497, 3489.7246, 3505.6919, 3510.5859, 3552.6614, 3553.6028, 3554.5630, 3558.6460, 3573.6545, 3589.6523, 3608.6155, 3624.6685, 3630.5806, 3641.7090, 3683.6980, 3691.7681, 3707.7200, 3714.5493, 3715.6206, 3719.6914, 3723.7041, 3735.7100, 3751.7539, 3830.8091, 3876.6707

15. P48427 **Mass:** 12756 **Score:** 19 **Expect:** 2.9e+002 **Matches:** 9

Tubulin-specific chaperone A OS=Bos taurus GN=TBCA PE=1 SV=3

Observed	Mr(expt)	Mr(calc)	ppm	Start	End	Miss	Ions	Peptide
860.4262	859.4189	859.4334	-16.91	1 -	7	1	---	-.MADPRVR.Q + Oxidation (M)
1031.5300	1030.5227	1030.5294	-6.53	32 -	39	1	---	K.QQEEKIEK.M
1229.6874	1228.6801	1228.6710	7.42	1 -	10	2	---	-.MADPRVRQIK.I + Oxidation (M)
1306.6946	1305.6873	1305.6598	21.1	32 -	41	2	---	K.QQEEKIEKMK.A + Oxidation (M)
1359.7013	1358.6940	1358.7041	-7.42	29 -	39	2	---	K.EAKQQEEKIEK.M
1442.8257	1441.8184	1441.8140	3.06	96 -	108	2	---	K.EARLVLDSVKLEA.-
1738.8267	1737.8194	1737.8607	-23.73	37 -	51	2	---	K.IEKMKAEDGENYAIK.K
2265.0837	2264.0764	2264.0711	2.34	52 -	69	2	---	K.KQAEILQESRMMIPDCQR.R + 2 Oxidation (M)
2277.0642	2276.0569	2276.0824	-11.17	53 -	70	2	---	K.QAEILQESRMMIPDCQRR.L + Oxidation (M)

No match to: 716.3353, 718.2895, 724.2175, 726.3070, 728.2833, 729.4336, 730.2399, 732.2518, 734.4341, 743.3130, 745.3617, 747.4360, 753.3469, 756.3524, 758.3462, 759.3632, 768.3517, 779.4633, 781.3725, 789.3640, 794.3674, 796.3701, 799.2854, 801.4280, 805.4417, 807.4076, 810.3293, 812.3207, 818.3782, 820.3700, 828.4415, 829.4508, 830.4513, 834.3805, 842.5101, 849.3834, 852.4368, 857.4457, 868.4412, 870.4462, 873.4370, 877.4166, 880.4280, 882.4470, 883.4816, 887.4295, 889.4857, 891.4872, 894.4923, 898.4610, 900.5174, 905.4763, 907.4251, 912.5086, 914.4942, 920.4296, 922.4855, 928.4565, 929.4484, 933.4327, 936.4976, 938.5062, 944.4982, 946.4672, 949.5244, 950.5168, 957.5792, 961.5109, 965.5151, 967.4963, 972.5023, 974.4913, 976.5255, 978.5352, 981.5225, 983.6221, 986.5463, 988.4922, 993.5123, 995.4512, 998.5833, 1000.5594, 1006.5120, 1007.5640, 1021.5762, 1024.5542, 1034.5703, 1037.5466, 1041.5637, 1043.5297, 1044.5375, 1045.5594, 1046.6058, 1054.5287, 1056.5575, 1057.6187, 1057.6200, 1068.5790, 1074.6162, 1076.6190, 1082.6056, 1105.5938, 1110.5851, 1113.5701, 1118.5687, 1120.6050, 1122.5892, 1123.6047, 1126.6241, 1128.5618, 1130.5756, 1135.5951, 1137.5647, 1143.5592, 1145.6373, 1148.6318, 1149.6230, 1150.6226, 1156.5652, 1157.5676, 1159.5890, 1160.5966, 1169.5873, 1177.6421, 1178.6371, 1180.6443, 1182.6539, 1184.6431, 1185.6815, 1193.6090, 1194.6001, 1209.6238, 1213.7180, 1216.7178, 1221.5953, 1227.6459, 1234.6899, 1238.2251, 1239.6622, 1254.6993, 1255.6351, 1264.7139, 1265.1680, 1265.7196, 1269.6532, 1276.6729, 1278.6892, 1281.6375, 1285.6674, 1289.7029, 1293.6760, 1301.6362, 1303.6489, 1304.6685, 1308.6797, 1314.7227, 1319.6770, 1320.6364, 1323.6815, 1326.6937, 1328.6799, 1333.7430, 1336.7301, 1337.6429, 1349.6729, 1351.6969, 1353.6891, 1356.6255, 1358.6919, 1361.7147, 1364.7118, 1365.6619, 1366.6965, 1372.6228, 1375.6693, 1382.6771, 1383.7347, 1390.7089, 1393.8007, 1397.7449, 1403.7434, 1410.7849, 1416.7737, 1420.7114, 1423.7096, 1434.7847, 1436.7469, 1445.7913, 1456.7555, 1457.7440, 1467.7560, 1470.7466, 1475.7615, 1479.8038, 1483.7579, 1484.7480, 1487.7660, 1488.7366, 1493.7556, 1500.7393, 1500.7400, 1503.7648, 1504.7708, 1514.7063, 1516.7281, 1519.7705, 1522.7722, 1524.8040, 1526.7924, 1528.7079, 1530.7058, 1536.7252, 1537.7859, 1540.8043, 1543.7938, 1550.7543, 1551.7465, 1553.7668, 1555.7836, 1556.8011, 1562.8008, 1568.7620, 1572.7245, 1578.8058, 1580.8580, 1580.8600, 1584.8486, 1587.7675, 1608.8051, 1616.8165, 1621.7870, 1625.7886, 1628.7313, 1632.8120, 1635.7964, 1637.7974, 1642.7417, 1647.8439, 1650.8429, 1651.8369, 1655.8147, 1657.8483, 1658.8953, 1660.8379, 1662.8666, 1664.8490, 1666.7892, 1679.8091, 1680.8357, 1682.8409, 1696.8464, 1698.8180, 1701.8243, 1702.8386, 1707.8938, 1710.8904, 1714.7905, 1716.8219, 1717.9052, 1718.9094, 1721.8976, 1722.9003, 1724.9030, 1728.8840, 1730.8910, 1734.1617, 1739.8165, 1741.8934, 1743.9005, 1747.9127, 1758.4807, 1758.8918, 1761.8734, 1765.8154, 1772.0012, 1774.9300, 1774.9319, 1779.9012, 1781.8585, 1783.8062, 1787.8407, 1809.0583, 1810.0460, 1812.9126, 1829.8527, 1832.8584, 1833.8571, 1834.8650, 1836.8618, 1838.8677, 1845.8363, 1847.8600, 1848.8938, 1850.9128, 1851.8932, 1861.8531, 1862.9542, 1864.9258, 1880.9026, 1883.7704, 1887.8878, 1897.7863, 1902.9769, 1904.9276, 1908.8986, 1920.9131, 1923.9126, 1928.0443, 1932.0068, 1936.9510, 1938.0171, 1947.9314, 1958.9393, 1959.9655, 1960.9813, 1961.9463, 1967.1549, 1969.9951, 1973.9971, 1975.9755, 1976.9708, 1990.4971, 1990.9373, 1991.4857, 1997.8479, 2001.8607, 2003.8778, 2004.8850, 2007.9500, 2007.9532, 2011.9381, 2012.8951, 2015.9694, 2017.9860, 2018.9740, 2020.1631, 2023.9482, 2026.9529, 2027.5033, 2029.9972, 2043.5356, 2044.9940, 2055.0205, 2057.0154, 2060.9878, 2066.9939, 2068.9661, 2082.9822, 2086.0254, 2088.1033, 2097.0371, 2098.4941, 2102.9954, 2109.0095, 2112.9932, 2114.9707, 2122.9578, 2128.0444, 2130.9980, 2132.0200, 2136.9250, 2140.0063, 2143.0237, 2144.0559, 2145.0781, 2148.0483, 2153.0049, 2153.9973, 2174.0508, 2175.0435, 2178.0483, 2188.0610, 2191.0464, 2192.0427, 2199.1150, 2205.0615, 2211.1067, 2223.1533, 2225.1382, 2226.1472, 2239.1646, 2243.1448, 2246.9482, 2251.1108, 2255.1484, 2271.1211, 2273.0735, 2283.1606, 2285.1355, 2286.0986, 2289.0901, 2291.0173, 2309.0349, 2328.9888, 2343.0608, 2352.9583, 2355.0383, 2366.9595, 2371.0325, 2392.1594, 2400.0532, 2424.1577, 2434.2429, 2438.2283, 2449.2180, 2450.2451, 2451.2480, 2465.1592, 2466.2458, 2467.2458, 2478.1377, 2501.2207, 2523.2014, 2526.2585, 2540.2451, 2542.2578, 2545.2258, 2550.2373, 2561.6841, 2562.1826, 2562.6890, 2573.2495, 2576.1792, 2646.9758, 2659.2144, 2661.1409, 2662.0120, 2671.1924, 2681.2705, 2682.2888, 2685.3098, 2689.3059, 2695.1064, 2696.2695, 2699.2324, 2703.2529, 2705.2944, 2712.2937, 2717.2903, 2720.2839, 2721.2937, 2726.2935, 2727.2986, 2728.3079, 2739.2837, 2743.2729, 2744.2207, 2745.2256, 2746.2031, 2748.2295, 2755.2700, 2760.2590, 2763.1829, 2771.2400, 2778.1865, 2785.2969, 2786.2561, 2787.2524, 2790.2317, 2792.2166, 2800.3093, 2801.2932, 2803.2415, 2804.2429, 2807.2869, 2808.2227, 2812.2244, 2813.2373, 2817.2632, 2820.2441, 2824.2598, 2828.2454, 2833.2544, 2843.2900, 2844.3398, 2847.3630, 2851.3313, 2864.3188, 2890.3293, 2896.4324, 2908.3105, 2910.3325, 2911.3594, 2912.4265, 2925.3323, 2927.4434, 3023.3674, 3025.3865, 3026.3452, 3039.3838, 3042.3542, 3070.3745, 3071.3933, 3086.3984, 3087.3970, 3093.4294, 3101.3831, 3103.4006, 3104.4041, 3110.4143, 3127.4749, 3141.4126, 3143.5066, 3144.5298, 3159.5210, 3219.4854, 3236.5574, 3266.6021, 3280.5635, 3321.6216, 3344.4895, 3347.5515, 3348.5020, 3380.5210, 3396.5195, 3412.5801, 3427.6099, 3428.5874, 3444.5840, 3462.6301,

3478.6123, 3484.6497, 3489.7246, 3505.6919, 3510.5859, 3521.6392, 3552.6614, 3553.6028, 3554.5630, 3558.6460, 3573.6545, 3589.6523, 3608.6155, 3624.6685, 3630.5806, 3641.7090, 3651.7944, 3667.7266, 3683.6980, 3691.7681, 3707.7200, 3714.5493, 3715.6206, 3719.6914, 3720.7048, 3723.7041, 3735.7100, 3751.7539, 3830.8091, 3869.7393, 3876.6707, 3885.6946

16. Q3ZBD3 **Mass:** 12035 **Score:** 19 **Expect:** 2.9e+002 **Matches:** 8

Pterin-4-alpha-carbinolamine dehydratase OS=Bos taurus GN=PCBD1 PE=3 SV=2

Observed	Mr(expt)	Mr(calc)	ppm	Start	End	Miss	Ions	Peptide
1068.5790	1067.5717	1067.5472	23.0	5 -	13	1	---	K.AHRLSAEER.D
1130.5756	1129.5683	1129.5516	14.8	22 -	31	0	---	R.AVGWNELEGR.D
1584.8486	1583.8413	1583.8130	17.9	46 -	59	1	---	R.AFGFMTRVALQAEK.L + Oxidation (M)
1698.8180	1697.8107	1697.7950	9.24	60 -	72	0	---	K.LDHHPEWFNVYNK.V
1739.8165	1738.8092	1738.8448	-20.43	89 -	104	0	---	R.DVNLASFIEQVAVSMT.- + Oxidation (M)
1812.9126	1811.9053	1811.9107	-2.99	32 -	45	2	---	R.DAIFKQFHFKDFNR.A
2438.2283	2437.2210	2437.2179	1.30	53 -	72	1	---	R.VALQAEKLDHHPEWFNVYNK.V
3489.7246	3488.7173	3488.6684	14.0	60 -	88	1	---	K.LDHHPEWFNVYNKVHITLSTHECAGLSER.D

No match to: 716.3353, 718.2895, 724.2175, 726.3070, 728.2833, 729.4336, 730.2399, 732.2518, 734.4341, 743.3130, 745.3617, 747.4360, 753.3469, 756.3524, 758.3462, 759.3632, 768.3517, 779.4633, 781.3725, 789.3640, 794.3674, 796.3701, 799.2854, 801.4280, 805.4417, 807.4076, 810.3293, 812.3207, 818.3782, 820.3700, 828.4415, 829.4508, 830.4513, 834.3805, 842.5101, 849.3834, 852.4368, 857.4457, 860.4262, 868.4412, 870.4462, 873.4370, 877.4166, 880.4280, 882.4470, 883.4816, 887.4295, 889.4857, 891.4872, 894.4923, 898.4610, 900.5174, 905.4763, 907.4251, 912.5086, 914.4942, 920.4296, 922.4855, 928.4565, 929.4484, 933.4327, 936.4976, 938.5062, 944.4982, 946.4672, 949.5244, 950.5168, 957.5792, 961.5109, 965.5151, 967.4963, 972.5023, 974.4913, 976.5255, 978.5352, 981.5225, 983.6221, 986.5463, 988.4922, 993.5123, 995.4512, 998.5833, 1000.5594, 1006.5120, 1007.5640, 1021.5762, 1024.5542, 1031.5300, 1034.5703, 1037.5466, 1041.5637, 1043.5297, 1044.5375, 1045.5594, 1046.6058, 1054.5287, 1056.5575, 1057.6187, 1057.6200, 1074.6162, 1076.6190, 1082.6056, 1105.5938, 1110.5851, 1113.5701, 1118.5687, 1120.6050, 1122.5892, 1123.6047, 1126.6241, 1128.5618, 1135.5951, 1137.5647, 1143.5592, 1145.6373, 1148.6318, 1149.6230, 1150.6226, 1156.5652, 1157.5676, 1159.5890, 1160.5966, 1169.5873, 1177.6421, 1178.6371, 1180.6443, 1182.6539, 1184.6431, 1185.6815, 1193.6090, 1194.6001, 1209.6238, 1213.7180, 1216.7178, 1221.5953, 1227.6459, 1229.6874, 1234.6899, 1238.2251, 1239.6622, 1254.6993, 1255.6351, 1264.7139, 1265.1680, 1265.7196, 1269.6532, 1276.6729, 1278.6892, 1281.6375, 1285.6674, 1289.7029, 1293.6760, 1301.6362, 1303.6489, 1304.6685, 1306.6946, 1308.6797, 1314.7227, 1319.6770, 1320.6364, 1323.6815, 1326.6937, 1328.6799, 1333.7430, 1336.7301, 1337.6429, 1349.6729, 1351.6969, 1353.6891, 1356.6255, 1358.6919, 1359.7013, 1361.7147, 1364.7118, 1365.6619, 1366.6965, 1372.6228, 1375.6693, 1382.6771, 1383.7347, 1390.7089, 1393.8007, 1397.7449, 1403.7434, 1410.7849, 1416.7737, 1420.7114, 1423.7096, 1434.7847, 1436.7469, 1442.8257, 1445.7913, 1456.7555, 1457.7440, 1467.7560, 1470.7466, 1475.7615, 1479.8038, 1483.7579, 1484.7480, 1487.7660, 1488.7366, 1493.7556, 1500.7393, 1500.7400, 1503.7648, 1504.7708, 1514.7063, 1516.7281, 1519.7705, 1522.7722, 1524.8040, 1526.7924, 1528.7079, 1530.7058, 1536.7252, 1537.7859, 1540.8043, 1543.7938, 1550.7543, 1551.7465, 1553.7668, 1555.7836, 1556.8011, 1562.8008, 1568.7620, 1572.7245, 1578.8058, 1580.8580, 1580.8600, 1587.7675, 1608.8051, 1616.8165, 1621.7870, 1625.7886, 1628.7313, 1632.8120, 1635.7964, 1637.7974, 1642.7417, 1647.8439, 1650.8429, 1651.8369, 1655.8147, 1657.8483, 1658.8953, 1660.8379, 1662.8666, 1664.8490, 1666.7892, 1679.8091, 1680.8357, 1682.8409, 1696.8464, 1701.8243, 1702.8386, 1707.8938, 1710.8904, 1714.7905, 1716.8219, 1717.9052, 1718.9094, 1721.8976, 1722.9003, 1724.9030, 1728.8840, 1730.8910, 1734.1617, 1738.8267, 1741.8934, 1743.9005, 1747.9127, 1758.4807, 1758.8918, 1761.8734, 1765.8154, 1772.0012, 1774.9300, 1774.9319, 1779.9012, 1781.8585, 1783.8062, 1787.8407, 1809.0583, 1810.0460, 1829.8527, 1832.8584, 1833.8571, 1834.8650, 1836.8618, 1838.8677, 1845.8363, 1847.8600, 1848.8938, 1850.9128, 1851.8932, 1861.8531, 1862.9542, 1864.9258, 1880.9026, 1883.7704, 1887.8878, 1897.7863, 1902.9769, 1904.9276, 1908.8986, 1920.9131, 1923.9126, 1928.0443, 1932.0068, 1936.9510, 1938.0171, 1947.9314, 1958.9393, 1959.9655, 1960.9813, 1961.9463, 1967.1549, 1969.9951, 1973.9971, 1975.9755, 1976.9708, 1990.4971, 1990.9373, 1991.4857, 1997.8479, 2001.8607, 2003.8768, 2004.8850, 2007.9500, 2007.9532, 2011.9381, 2012.8951, 2015.9694, 2017.9860, 2018.9740, 2020.1631, 2023.9482, 2026.9529, 2027.5033, 2029.9972, 2043.5356, 2044.9940, 2055.0205, 2057.0154, 2060.9878, 2066.9939, 2068.9661, 2082.9822, 2086.0254, 2088.1033, 2097.0371, 2098.4941, 2102.9954, 2109.0095, 2112.9932, 2114.9707, 2122.9578, 2128.0444, 2130.9980, 2132.0200, 2136.9250, 2140.0063, 2143.0237, 2144.0559, 2145.0781, 2148.0483, 2153.0049, 2153.9973, 2174.0508, 2175.0435, 2178.0483, 2188.0610, 2191.0464, 2192.0427, 2199.1150, 2205.0615, 2211.1067, 2223.1533, 2225.1382, 2226.1472, 2239.1646, 2243.1448, 2246.9482, 2251.1108, 2255.1484, 2265.0837, 2271.1211, 2273.0735, 2277.0642, 2283.1606, 2285.1355, 2286.0986, 2289.0901, 2291.0173, 2309.0349, 2328.9888, 2343.0608, 2352.9583, 2355.0383, 2366.9595, 2371.0325, 2392.1594, 2400.0532, 2424.1577, 2434.2429, 2449.2180, 2450.2451, 2451.2480, 2465.1592, 2466.2458, 2467.2458, 2478.1377, 2501.2207, 2523.2014, 2526.2585, 2540.2451, 2542.2578, 2545.2258, 2550.2373, 2561.6841, 2562.1826, 2562.6890, 2573.2495, 2576.1792, 2646.9758, 2659.2144, 2661.1409, 2662.0120, 2671.1924, 2681.2705, 2682.2888, 2685.3098, 2689.3059, 2695.1064, 2696.2695, 2699.2324, 2703.2529, 2705.2944, 2712.2937, 2717.2903, 2720.2839, 2721.2937, 2726.2935, 2727.2986, 2728.3079, 2739.2837, 2743.2729, 2744.2207, 2745.2256, 2746.2031, 2748.2295, 2755.2700, 2760.2590, 2763.1829, 2771.2400, 2778.1865, 2785.2969, 2786.2561, 2787.2524, 2790.2317, 2792.2166, 2800.3093, 2801.2932, 2803.2415, 2804.2429, 2807.2869, 2808.2227, 2812.2244, 2813.2373, 2817.2632, 2820.2441, 2824.2598, 2828.2454, 2833.2544, 2843.2900, 2844.3398, 2847.3630, 2851.3313, 2864.3188, 2890.3293, 2896.4324, 2908.3105, 2910.3325, 2911.3594, 2912.4265, 2925.3323, 2927.4434, 3023.3674, 3025.3865, 3026.3452, 3039.3838, 3042.3542, 3070.3745, 3071.3933, 3086.3984, 3087.3970, 3093.4294, 3101.3831, 3103.4006, 3104.4041, 3110.4143, 3127.4749, 3141.4126, 3143.5066, 3144.5298, 3159.5210, 3219.4854, 3236.5574, 3266.6021, 3280.5635, 3321.6216, 3344.4895, 3347.5515, 3348.5020, 3380.5210, 3396.5195, 3412.5801, 3427.6099, 3428.5874, 3444.5840, 3462.6301, 3478.6123, 3484.6497, 3505.6919, 3510.5859, 3521.6392, 3552.6614, 3553.6028, 3554.5630, 3558.6460, 3573.6545, 3589.6523, 3608.6155, 3624.6685, 3630.5806, 3641.7090, 3651.7944, 3667.7266, 3683.6980, 3691.7681, 3707.7200, 3714.5493, 3715.6206, 3719.6914, 3720.7048, 3723.7041, 3735.7100, 3751.7539, 3830.8091, 3869.7393, 3876.6707, 3885.6946

17. F1MK70 **Mass:** 59648 **Score:** 19 **Expect:** 3e+002 **Matches:** 26

Uncharacterized protein OS=Bos taurus GN=SPATS2 PE=4 SV=2

Observed	Mr(expt)	Mr(calc)	ppm	Start	End	Miss	Ions	Peptide
807.4076	806.4003	806.3956	5.79	317 -	323	0	---	K.MTDVAVR.M + Oxidation (M)
912.5086	911.5013	911.4937	8.39	443 -	450	1	---	R.EALPGNRR.G
946.4672	945.4599	945.4404	20.7	538 -	546	0	---	R.TSQPDVVNS.-
1074.6162	1073.6089	1073.6080	0.82	235 -	244	1	---	K.LGSNIEKSVK.D
1076.6190	1075.6117	1075.6026	8.50	80 -	88	2	---	K.EWTVTGKKK.N
1118.5687	1117.5614	1117.5689	-6.67	286 -	295	0	---	R.EVALLAEMDK.V
1193.6090	1192.6017	1192.5836	15.2	345 -	354	1	---	K.YDEDLGRVAR.F
1328.6799	1327.6726	1327.6521	15.5	365 -	376	0	---	K.NIDSFGQVSHPK.N
1361.7147	1360.7074	1360.7272	-14.51	286 -	297	1	---	R.EVALLAEMDKVK.A + Oxidation (M)
1365.6619	1364.6546	1364.6758	-15.54	21 -	33	0	---	K.TVLAQGGAFENMK.E
1456.7555	1455.7482	1455.7470	0.82	364 -	376	1	---	K.KNIDSFGQVSHPK.N
1524.8040	1523.7967	1523.7593	24.6	437 -	449	1	---	R.SYQPHREALPGNR.R
1587.7675	1586.7602	1586.7285	20.0	420 -	436	0	---	K.NSAPGETPATTANSGGR.S
1628.7313	1627.7240	1627.7148	5.67	175 -	189	0	---	R.TGSMLENGVSDFESR.S
1660.8379	1659.8306	1659.8501	-11.75	324 -	337	1	---	R.MSEEQLVELRADIK.H
1680.8357	1679.8284	1679.8604	-19.03	437 -	450	2	---	R.SYQPHREALPGNRR.G
1682.8409	1681.8336	1681.8709	-22.15	256 -	269	2	---	R.YRVVVKEEMDASIK.K + Oxidation (M)
1969.9951	1968.9878	1968.9905	-1.36	4 -	20	2	---	R.KQNQKDSSGFIFDLQSK.T
2243.1448	2242.1375	2242.0951	18.9	450 -	470	2	---	R.RGGQGYRPQGPKSSDPTNQGR.H
2449.2180	2448.2107	2448.2352	-10.00	317 -	337	2	---	K.MTDVAVRMSEEQLVELRADIK.H + Oxidation (M)
2526.2585	2525.2512	2525.2836	-12.81	65 -	87	2	---	K.TVQAFMEGSASEVLKEWTVTGKK.K
2542.2578	2541.2505	2541.2785	-11.00	65 -	87	2	---	K.TVQAFMEGSASEVLKEWTVTGKK.K + Oxidation (M)
2550.2373	2549.2300	2549.2584	-11.15	355 -	376	2	---	R.FTCDVETLKKNIDSFGQVSHPK.N
2927.4434	2926.4361	2926.4238	4.21	273 -	297	2	---	K.QAFAELQSCLMDREVALLAEMDKVK.A + 2 Oxidation (M)
3093.4294	3092.4221	3092.4772	-17.81	420 -	449	2	---	K.NSAPGETPATTANSGGRSYQPHREALPGNR.R
3751.7539	3750.7466	3750.8233	-20.44	47 -	79	1	---	K.SNNEIILVLQHFDNCVDKTVQAFMEGSASEVLK.E + Oxidation (M)

No match to: 716.3353, 718.2895, 724.2175, 726.3070, 728.2833, 729.4336, 730.2399, 732.2518, 734.4341, 743.3130, 745.3617, 747.4360, 753.3469, 756.3524, 758.3462, 759.3632, 768.3517, 779.4633, 781.3725, 789.3640, 794.3674, 796.3701, 799.2854, 801.4280,

805.4417, 810.3293, 812.3207, 818.3782, 820.3700, 828.4415, 829.4508, 830.4513, 834.3805, 842.5101, 849.3834, 852.4368, 857.4457, 860.4262, 868.4412, 870.4462, 873.4370, 877.4166, 880.4280, 882.4470, 883.4816, 887.4295, 889.4857, 891.4872, 894.4923, 898.4610, 900.5174, 905.4763, 907.4251, 914.4942, 920.4296, 922.4855, 928.4565, 929.4484, 933.4327, 936.4976, 938.5062, 944.4982, 949.5244, 950.5168, 957.5792, 961.5109, 965.5151, 967.4963, 972.5023, 974.4913, 976.5255, 978.5352, 981.5225, 983.6221, 986.5463, 988.4922, 993.5123, 995.4512, 998.5833, 1000.5594, 1006.5120, 1007.5640, 1021.5762, 1024.5542, 1031.5300, 1034.5703, 1037.5466, 1041.5637, 1043.5297, 1044.5375, 1045.5594, 1046.6058, 1054.5287, 1056.5575, 1057.6187, 1057.6200, 1068.5790, 1082.6056, 1105.5938, 1110.5851, 1113.5701, 1120.6050, 1122.5892, 1123.6047, 1126.6241, 1128.5618, 1130.5756, 1135.5951, 1137.5647, 1143.5592, 1145.6373, 1148.6318, 1149.6230, 1150.6226, 1156.5652, 1157.5676, 1159.5890, 1160.5966, 1169.5873, 1177.6421, 1178.6371, 1180.6443, 1182.6539, 1184.6431, 1185.6815, 1194.6001, 1209.6238, 1213.7180, 1216.7178, 1221.5953, 1227.6459, 1229.6874, 1234.6899, 1238.2251, 1239.6622, 1254.6993, 1255.6351, 1264.7139, 1265.1680, 1265.7196, 1269.6532, 1276.6729, 1278.6892, 1281.6375, 1285.6674, 1289.7029, 1293.6760, 1301.6362, 1303.6489, 1304.6685, 1306.6946, 1308.6797, 1314.7227, 1319.6770, 1320.6364, 1323.6815, 1326.6937, 1333.7430, 1336.7301, 1337.6429, 1349.6729, 1351.6969, 1353.6891, 1356.6255, 1358.6919, 1359.7013, 1364.7118, 1366.6965, 1372.6228, 1375.6693, 1382.6771, 1383.7347, 1390.7089, 1393.8007, 1397.7449, 1403.7434, 1410.7849, 1416.7737, 1420.7114, 1423.7096, 1434.7847, 1436.7469, 1442.8257, 1445.7913, 1457.7440, 1467.7560, 1470.7466, 1475.7615, 1479.8038, 1483.7579, 1484.7480, 1487.7660, 1488.7366, 1493.7556, 1500.7393, 1500.7400, 1503.7648, 1504.7708, 1514.7063, 1516.7281, 1519.7705, 1522.7722, 1526.7924, 1528.7079, 1530.7058, 1536.7252, 1537.7859, 1540.8043, 1543.7938, 1550.7543, 1551.7465, 1553.7668, 1555.7836, 1556.8011, 1562.8008, 1568.7620, 1572.7245, 1578.8058, 1580.8580, 1580.8600, 1584.8486, 1608.8051, 1616.8165, 1621.7870, 1625.7886, 1632.8120, 1635.7964, 1637.7974, 1642.7417, 1647.8439, 1650.8429, 1651.8369, 1655.8147, 1657.8483, 1658.8953, 1662.8666, 1664.8490, 1666.7892, 1679.8091, 1696.8464, 1698.8180, 1701.8243, 1702.8386, 1707.8938, 1710.8904, 1714.7905, 1716.8219, 1717.9052, 1718.9094, 1721.8976, 1722.9003, 1724.9030, 1728.8840, 1730.8910, 1734.1617, 1738.8267, 1739.8165, 1741.8934, 1743.9005, 1747.9127, 1758.4807, 1758.8918, 1761.8734, 1765.8154, 1772.0012, 1774.9300, 1774.9319, 1779.9012, 1781.8585, 1783.8062, 1787.8407, 1809.0583, 1810.0460, 1812.9126, 1829.8527, 1832.8584, 1833.8571, 1834.8650, 1836.8618, 1838.8677, 1845.8363, 1847.8600, 1848.8938, 1850.9128, 1851.8932, 1861.8531, 1862.9542, 1864.9258, 1880.9026, 1883.7704, 1887.8878, 1897.7863, 1902.9769, 1904.9276, 1908.8986, 1920.9131, 1923.9126, 1928.0443, 1932.0068, 1936.9510, 1938.0171, 1947.9314, 1958.9393, 1959.9655, 1960.9813, 1961.9463, 1967.1549, 1973.9971, 1975.9755, 1976.9708, 1990.4971, 1990.9373, 1991.4857, 1997.8479, 2001.8607, 2003.8768, 2004.8850, 2007.9500, 2007.9532, 2011.9381, 2012.8951, 2015.9694, 2017.9860, 2018.9740, 2020.1631, 2023.9482, 2026.9529, 2027.5033, 2029.9972, 2043.5356, 2044.9940, 2055.0205, 2057.0154, 2060.9878, 2066.9939, 2068.9661, 2082.9822, 2086.0254, 2088.1033, 2097.0371, 2098.4941, 2102.9954, 2109.0095, 2112.9932, 2114.9707, 2122.9578, 2128.0444, 2130.9980, 2132.0200, 2136.9250, 2140.0063, 2143.0237, 2144.0559, 2145.0781, 2148.0483, 2153.0049, 2153.9973, 2174.0508, 2175.0435, 2178.0483, 2188.0610, 2191.0464, 2192.0427, 2199.1150, 2205.0615, 2211.1067, 2223.1533, 2225.1382, 2226.1472, 2239.1646, 2246.9482, 2251.1108, 2255.1484, 2265.0837, 2271.1211, 2273.0735, 2277.0642, 2283.1606, 2285.1355, 2286.0986, 2289.0901, 2291.0173, 2309.0349, 2328.9888, 2343.0608, 2352.9583, 2355.0383, 2366.9595, 2371.0325, 2392.1594, 2400.0532, 2424.1577, 2434.2429, 2438.2283, 2450.2451, 2451.2480, 2465.1592, 2466.2458, 2467.2458, 2478.1377, 2501.2207, 2523.2014, 2540.2451, 2545.2258, 2561.6841, 2562.1826, 2562.6890, 2573.2495, 2576.1792, 2646.9758, 2659.2144, 2661.1409, 2662.0120, 2671.1924, 2681.2705, 2682.2888, 2685.3098, 2689.3059, 2695.1064, 2696.2695, 2699.2324, 2703.2529, 2705.2944, 2712.2937, 2717.2903, 2720.2839, 2721.2937, 2726.2935, 2727.2986, 2728.3079, 2739.2837, 2743.2729, 2744.2207, 2745.2256, 2746.2031, 2748.2295, 2755.2700, 2760.2590, 2763.1829, 2771.2400, 2778.1865, 2785.2969, 2786.2561, 2787.2524, 2790.2317, 2792.2166, 2800.3093, 2801.2932, 2803.2415, 2804.2429, 2807.2869, 2808.2227, 2812.2244, 2813.2373, 2817.2632, 2820.2441, 2824.2598, 2828.2454, 2833.2544, 2843.2900, 2844.3398, 2847.3630, 2851.3313, 2864.3188, 2890.3293, 2896.4324, 2908.3105, 2910.3325, 2911.3594, 2912.4265, 2925.3323, 3023.3674, 3025.3865, 3026.3452, 3039.3838, 3042.3542, 3070.3745, 3071.3933, 3086.3984, 3087.3970, 3101.3831, 3103.4006, 3104.4041, 3110.4143, 3127.4749, 3141.4126, 3143.5066, 3144.5298, 3159.5210, 3219.4854, 3236.5574, 3266.6021, 3280.5635, 3321.6216, 3344.4895, 3347.5515, 3348.5020, 3380.5210, 3396.5195, 3412.5801, 3427.6099, 3428.5874, 3444.5840, 3462.6301, 3478.6123, 3484.6497, 3489.7246, 3505.6919, 3510.5859, 3521.6392, 3552.6614, 3553.6028, 3554.5630, 3558.6460, 3573.6545, 3589.6523, 3608.6155, 3624.6685, 3630.5806, 3641.7090, 3651.7944, 3667.7266, 3683.6980, 3691.7681, 3707.7200, 3714.5493, 3715.6206, 3719.6914, 3720.7048, 3723.7041, 3735.7100, 3830.8091, 3869.7393, 3876.6707, 3885.6946

18. Q5EAC6 **Mass:** 45065 **Score:** 18 **Expect:** 3.6e+002 **Matches:** 20

Hsp90 co-chaperone Cdc37 OS=Bos taurus GN=CDC37 PE=2 SV=1

Observed	Mr(expt)	Mr(calc)	ppm	Start	End	Miss	Ions	Peptide
805.4417	804.4344	804.4163	22.5	104 -	109	1	---	K.LEEMRK.K
949.5244	948.5171	948.5062	11.5	104 -	110	2	---	K.LEEMRKK.E + Oxidation (M)
1043.5297	1042.5224	1042.5335	-10.62	152 -	159	1	---	K.TFVEKYEK.Q
1056.5575	1055.5502	1055.5723	-20.94	86 -	94	0	---	R.LQAEAQQLR.K
1145.6373	1144.6300	1144.6175	10.9	160 -	168	1	---	K.QIKHFGMLR.R + Oxidation (M)
1194.6001	1193.5928	1193.5863	5.49	37 -	45	1	---	R.VERMEQFQK.E
1308.6797	1307.6724	1307.6874	-11.42	150 -	159	2	---	K.HKTFVEKYEK.Q
1358.6919	1357.6846	1357.7089	-17.86	68 -	80	1	---	K.LKELEVAEGEGGK.A
1375.6693	1374.6620	1374.6602	1.34	338 -	349	0	---	R.CIDSGLWVPNSK.S
1445.7913	1444.7840	1444.7649	13.2	234 -	244	2	---	R.ACFRQFFTKIK.T
1467.7560	1466.7487	1466.7187	20.4	37 -	47	2	---	R.VERMEQFQKEK.E + Oxidation (M)
1550.7543	1549.7470	1549.7446	1.55	111 -	123	1	---	K.EKSMPWNVDTLSK.D + Oxidation (M)
1662.8666	1661.8593	1661.8447	8.82	110 -	123	2	---	K.KEKSMPWNVDTLSK.D
1702.8386	1701.8313	1701.8369	-3.28	33 -	45	2	---	R.HQARVERMEQFQK.E + Oxidation (M)
1765.8154	1764.8081	1764.8100	-1.09	96 -	108	2	---	K.EERSWEQKLEEMR.K + Oxidation (M)
2068.9661	2067.9588	2067.9935	-16.78	111 -	128	2	---	K.EKSMPWNVDTLSKDGFSK.S
2192.0427	2191.0354	2191.0128	10.3	350 -	371	1	---	K.SSEAKDGEEAGTGDPLLEATSK.S
2659.2144	2658.2071	2658.1975	3.61	310 -	332	2	---	K.CFDAKDVQMLQDAISKMDPTDAK.Y + 2 Oxidation (M)
2721.2937	2720.2864	2720.2720	5.30	315 -	337	2	---	K.DVQMLQDAISKMDPTDAKYHMQR.C
3553.6028	3552.5955	3552.6792	-23.57	176 -	204	0	---	K.YLSDNVHLVCEETANYLVIWCIDLEVEEK.C

No match to: 716.3353, 718.2895, 724.2175, 726.3070, 728.2833, 729.4336, 730.2399, 732.2518, 734.4341, 743.3130, 745.3617, 747.4360, 753.3469, 756.3524, 758.3462, 759.3632, 768.3517, 779.4633, 781.3725, 789.3640, 794.3674, 796.3701, 799.2854, 801.4280, 807.4076, 810.3293, 812.3207, 818.3782, 820.3700, 828.4415, 829.4508, 830.4513, 834.3805, 842.5101, 849.3834, 852.4368, 857.4457, 860.4262, 868.4412, 870.4462, 873.4370, 877.4166, 880.4280, 882.4470, 883.4816, 887.4295, 889.4857, 891.4872, 894.4923, 898.4610, 900.5174, 905.4763, 907.4251, 912.5086, 914.4942, 920.4296, 922.4855, 928.4565, 929.4484, 933.4327, 936.4976, 938.5062, 944.4982, 946.4672, 950.5168, 957.5792, 961.5109, 965.5151, 967.4963, 972.5023, 974.4913, 976.5255, 978.5352, 981.5225, 983.6221, 986.5463, 988.4922, 993.5123, 995.4512, 998.5833, 1000.5594, 1006.5120, 1007.5640, 1021.5762, 1024.5542, 1031.5300, 1034.5703, 1037.5466, 1041.5637, 1044.5375, 1045.5594, 1046.6058, 1054.5287, 1057.6187, 1057.6200, 1068.5790, 1074.6162, 1076.6190, 1082.6056, 1105.5938, 1110.5851, 1113.5701, 1118.5687, 1120.6050, 1122.5892, 1123.6047, 1126.6241, 1128.5618, 1130.5756, 1135.5951, 1137.5647, 1143.5592, 1148.6318, 1149.6230, 1150.6226, 1156.5652, 1157.5676, 1159.5890, 1160.5966, 1169.5873, 1177.6421, 1178.6371, 1180.6443, 1182.6539, 1184.6431, 1185.6815, 1193.6090, 1209.6238, 1213.7180, 1216.7178, 1221.5953, 1227.6459, 1229.6874, 1234.6899, 1238.2251, 1239.6622, 1254.6993, 1255.6351, 1264.7139, 1265.1680, 1265.7196, 1269.6532, 1276.6729, 1278.6892, 1281.6375, 1285.6674, 1289.7029, 1293.6760, 1301.6362, 1303.6489, 1304.6685, 1306.6946, 1314.7227, 1319.6770, 1320.6364, 1323.6815, 1326.6937, 1328.6799, 1333.7430, 1336.7301, 1337.6429, 1349.6729, 1351.6969, 1353.6891, 1356.6255, 1359.7013, 1361.7147, 1364.7118, 1365.6619, 1366.6965, 1372.6228, 1382.6771, 1383.7347, 1390.7089, 1393.8007, 1397.7449, 1403.7434, 1410.7849, 1416.7737, 1420.7114, 1423.7096, 1434.7847, 1436.7469, 1442.8257, 1456.7555, 1457.7440, 1470.7466, 1475.7615, 1479.8038, 1483.7579, 1484.7480, 1487.7660, 1488.7366, 1493.7556, 1500.7393, 1500.7400, 1503.7648, 1504.7708, 1514.7063, 1516.7281, 1519.7705, 1522.7722, 1524.8040, 1526.7924, 1528.7079, 1530.7058, 1536.7252, 1537.7859, 1540.8043, 1543.7938, 1551.7465, 1553.7668, 1555.7836, 1556.8011, 1562.8008, 1568.7620, 1572.7245, 1578.8058, 1580.8580, 1580.8600, 1584.8486, 1587.7675, 1608.8051, 1616.8165, 1621.7870, 1625.7886, 1628.7313, 1632.8120, 1635.7964, 1637.7974, 1642.7417, 1647.8439, 1650.8429, 1651.8369, 1655.8147, 1657.8483, 1658.8953, 1660.8379, 1664.8490, 1666.7892, 1679.8091, 1680.8357, 1682.8409, 1696.8464, 1698.8180, 1701.8243, 1707.8938, 1710.8904, 1714.7905, 1716.8219, 1717.9052, 1718.9094, 1721.8976, 1722.9003, 1724.9030, 1728.8840, 1730.8910, 1734.1617, 1738.8267, 1739.8165, 1741.8934, 1743.9005, 1747.9127, 1758.4807, 1758.8918, 1761.8734, 1772.0012, 1774.9300, 1774.9319, 1779.9012, 1781.8585, 1783.8062, 1787.8407, 1809.0583, 1810.0460, 1812.9126, 1829.8527, 1832.8584, 1833.8571, 1834.8650, 1836.8618, 1838.8677, 1845.8363, 1847.8600, 1848.8938, 1850.9128, 1851.8932, 1861.8531, 1862.9542, 1864.9258, 1880.9026, 1883.7704, 1887.8878, 1897.7863, 1902.9769, 1904.9276, 1908.8986, 1920.9131, 1923.9126, 1928.0443, 1932.0068, 1936.9510, 1938.0171, 1947.9314, 1958.9393, 1959.9655, 1960.9813, 1961.9463, 1967.1549, 1969.9951, 1973.9971, 1975.9755, 1976.9708, 1990.4971, 1990.9373, 1991.4857, 1997.8479, 2001.8607, 2003.8768, 2004.8850, 2007.9500, 2007.9532, 2011.9381, 2012.8951, 2015.9694, 2017.9860, 2018.9740, 2020.1631, 2023.9482, 2026.9529, 2027.5033, 2029.9972, 2043.5356, 2044.9940, 2055.0205, 2057.0154, 2060.9878, 2066.9939, 2082.9822, 2086.0254, 2088.1033, 2097.0371, 2098.4941, 2102.9954, 2109.0095, 2112.9932, 2114.9707, 2122.9578, 2128.0444, 2130.9980, 2132.0200, 2136.9250,

2140.0063, 2143.0237, 2144.0559, 2145.0781, 2148.0483, 2153.0049, 2153.9973, 2174.0508, 2175.0435, 2178.0483, 2188.0610, 2191.0464, 2199.1150, 2205.0615, 2211.1067, 2223.1533, 2225.1382, 2226.1472, 2239.1646, 2243.1448, 2246.9482, 2251.1108, 2255.1484, 2265.0837, 2271.1211, 2273.0735, 2277.0642, 2283.1606, 2285.1355, 2286.0986, 2289.0901, 2291.0173, 2309.0349, 2328.9888, 2343.0608, 2352.9583, 2355.0383, 2366.9595, 2371.0325, 2392.1594, 2400.0532, 2424.1577, 2434.2429, 2438.2283, 2449.2180, 2450.2451, 2451.2480, 2465.1592, 2466.2458, 2467.2458, 2478.1377, 2501.2207, 2523.2014, 2526.2585, 2540.2451, 2542.2578, 2545.2258, 2550.2373, 2561.6841, 2562.1826, 2562.6890, 2573.2495, 2576.1792, 2646.9758, 2661.1409, 2662.0120, 2671.1924, 2681.2705, 2682.2888, 2685.3098, 2689.3059, 2695.1064, 2696.2695, 2699.2324, 2703.2529, 2705.2944, 2712.2937, 2717.2903, 2720.2839, 2726.2935, 2727.2986, 2728.3079, 2739.2837, 2743.2729, 2744.2207, 2745.2256, 2746.2031, 2748.2295, 2755.2700, 2760.2590, 2763.1829, 2771.2400, 2778.1865, 2785.2969, 2786.2561, 2787.2524, 2790.2317, 2792.2166, 2800.3093, 2801.2932, 2803.2415, 2804.2429, 2807.2869, 2808.2227, 2812.2244, 2813.2373, 2817.2632, 2820.2441, 2824.2598, 2828.2454, 2833.2544, 2843.2900, 2844.3398, 2847.3630, 2851.3313, 2864.3188, 2890.3293, 2896.4324, 2908.3105, 2910.3325, 2911.3594, 2912.4265, 2925.3323, 2927.4434, 3023.3674, 3025.3865, 3026.3452, 3039.3838, 3042.3542, 3070.3745, 3071.3933, 3086.3984, 3087.3970, 3093.4294, 3101.3831, 3103.4006, 3104.4041, 3110.4143, 3127.4749, 3141.4126, 3143.5066, 3144.5298, 3159.5210, 3219.4854, 3236.5574, 3266.6021, 3280.5635, 3321.6216, 3344.4895, 3347.5515, 3348.5020, 3380.5210, 3396.5195, 3412.5801, 3427.6099, 3428.5874, 3444.5840, 3462.6301, 3478.6123, 3484.6497, 3489.7246, 3505.6919, 3510.5859, 3521.6392, 3552.6614, 3554.5630, 3558.6460, 3573.6545, 3589.6523, 3608.6155, 3624.6685, 3630.5806, 3641.7090, 3651.7944, 3667.7266, 3683.6980, 3691.7681, 3707.7200, 3714.5493, 3715.6206, 3719.6914, 3720.7048, 3723.7041, 3735.7100, 3751.7539, 3830.8091, 3869.7393, 3876.6707, 3885.6946

19. Q2HJG7 **Mass:** 16553 **Score:** 18 **Expect:** 3.6e+002 **Matches:** 10

Protein Churchill OS=Bos taurus GN=CHURC1 PE=2 SV=2

Observed	Mr(expt)	Mr(calc)	ppm	Start	End	Miss	Ions	Peptide
829.4508	828.4435	828.4566	-15.74	3 -	9	0	---	R.RPSLNSR.E
981.5225	980.5152	980.5001	15.4	64 -	71	0	---	R.DFMLITNK.S
1006.5120	1005.5047	1005.4927	12.0	90 -	97	0	---	K.NCHHVIAR.H
1761.8734	1760.8661	1760.9064	-22.85	1 -	15	2	---	-.MRRPSLNSREVSSSR.K
2191.0464	2190.0391	2190.0878	-22.24	121 -	139	1	---	K.AEDTISILPDDPRQMTLLF.- + Oxidation (M)
2277.0642	2276.0569	2276.0613	-1.91	17 -	35	2	---	K.HRRIFADNGVAMCVDCVEK.E
2659.2144	2658.2071	2658.1989	3.11	19 -	40	2	---	R.RIFADNGVAMCVDCVEKEYPNR.G + Oxidation (M)
2824.2598	2823.2525	2823.2704	-6.35	75 -	97	1	---	K.EEDGEEIVTYDHLCKNCHHVIAR.H
3127.4749	3126.4676	3126.4889	-6.81	64 -	89	2	---	R.DFMLITNKSLKEEDGEEIVTYDHLCK.N
3143.5066	3142.4993	3142.4838	4.93	64 -	89	2	---	R.DFMLITNKSLKEEDGEEIVTYDHLCK.N + Oxidation (M)

No match to: 716.3353, 718.2895, 724.2175, 726.3070, 728.2833, 729.4336, 730.2399, 732.2518, 734.4341, 743.3130, 745.3617, 747.4360, 753.3469, 756.3524, 758.3462, 759.3632, 768.3517, 779.4633, 781.3725, 789.3640, 794.3674, 796.3701, 799.2854, 801.4280, 805.4417, 807.4076, 810.3293, 812.3207, 818.3782, 820.3700, 828.4415, 830.4513, 834.3805, 842.5101, 849.3834, 852.4368, 857.4457, 860.4262, 868.4412, 870.4462, 873.4370, 877.4166, 880.4280, 882.4470, 883.4816, 887.4295, 889.4857, 891.4872, 894.4923, 898.4610, 900.5174, 905.4763, 907.4251, 912.5086, 914.4942, 920.4296, 922.4855, 928.4565, 929.4484, 933.4327, 936.4976, 938.5062, 944.4982, 946.4672, 949.5244, 950.5168, 957.5792, 961.5109, 965.5151, 967.4963, 972.5023, 974.4913, 976.5255, 978.5352, 983.6221, 986.5463, 988.4922, 993.5123, 995.4512, 998.5833, 1000.5594, 1007.5640, 1021.5762, 1024.5542, 1031.5300, 1034.5703, 1037.5466, 1041.5637, 1043.5297, 1044.5375, 1045.5594, 1046.6058, 1054.5287, 1056.5575, 1057.6187, 1057.6200, 1068.5790, 1074.6162, 1076.6190, 1082.6056, 1105.5938, 1110.5851, 1113.5701, 1118.5687, 1120.6050, 1122.5892, 1123.6047, 1126.6241, 1128.5618, 1130.5756, 1135.5951, 1137.5647, 1143.5592, 1145.6373, 1148.6318, 1149.6230, 1150.6226, 1156.5652, 1157.5676, 1159.5890, 1160.5966, 1169.5873, 1177.6421, 1178.6371, 1180.6443, 1182.6539, 1184.6431, 1185.6815, 1193.6090, 1194.6001, 1209.6238, 1213.7180, 1216.7178, 1221.5953, 1227.6459, 1229.6874, 1234.6899, 1238.2251, 1239.6622, 1254.6993, 1255.6351, 1264.7139, 1265.1680, 1265.7196, 1269.6532, 1276.6729, 1278.6892, 1281.6375, 1285.6674, 1289.7029, 1293.6760, 1301.6362, 1303.6489, 1304.6685, 1306.6946, 1308.6797, 1314.7227, 1319.6770, 1320.6364, 1323.6815, 1326.6937, 1328.6799, 1333.7430, 1336.7301, 1337.6429, 1349.6729, 1351.6969, 1353.6891, 1356.6255, 1358.6919, 1359.7013, 1361.7147, 1364.7118, 1365.6619, 1366.6965, 1372.6228, 1375.6693, 1382.6771, 1383.7347, 1390.7089, 1393.8007, 1397.7449, 1403.7434, 1410.7849, 1416.7737, 1420.7114, 1423.7096, 1434.7847, 1436.7469, 1442.8257, 1445.7913, 1456.7555, 1457.7440, 1467.7560, 1470.7466, 1475.7615, 1479.8038, 1483.7579, 1484.7480, 1487.7660, 1488.7366, 1493.7556, 1500.7393, 1500.7400, 1503.7648, 1504.7708, 1514.7063, 1516.7281, 1519.7705, 1522.7722, 1524.8040, 1526.7924, 1528.7079, 1530.7058, 1536.7252, 1537.7859, 1540.8043, 1543.7938, 1550.7543, 1551.7465, 1553.7668, 1555.7836, 1556.8011, 1562.8008, 1568.7620, 1572.7245, 1578.8058, 1580.8580, 1580.8600, 1584.8486, 1587.7675, 1608.8051, 1616.8165, 1621.7870, 1625.7886, 1628.7313, 1632.8120, 1635.7964, 1637.7974, 1642.7417, 1647.8439, 1650.8429, 1651.8369, 1655.8147, 1657.8483, 1658.8953, 1660.8379, 1662.8666, 1664.8490, 1666.7892, 1679.8091, 1680.8357, 1682.8409, 1696.8464, 1698.8180, 1701.8243, 1702.8386, 1707.8938, 1710.8904, 1714.7905, 1716.8219, 1717.9052, 1718.9094, 1721.8976, 1722.9003, 1724.9030, 1728.8840, 1730.8910, 1734.1617, 1738.8267, 1739.8165, 1741.8934, 1743.9005, 1747.9127, 1758.4807, 1758.8918, 1765.8154, 1772.0012, 1774.9300, 1774.9319, 1779.9012, 1781.8585, 1783.8062, 1787.8407, 1809.0583, 1810.0460, 1812.9126, 1829.8527, 1832.8584, 1833.8571, 1834.8650, 1836.8618, 1838.8677, 1845.8363, 1847.8600, 1848.8938, 1850.9128, 1851.8932, 1861.8531, 1862.9542, 1864.9258, 1880.9026, 1883.7704, 1887.8878, 1897.7863, 1902.9769, 1904.9276, 1908.8986, 1920.9131, 1923.9126, 1928.0443, 1932.0068, 1936.9510, 1938.0171, 1947.9314, 1958.9393, 1959.9655, 1960.9813, 1961.9463, 1967.1549, 1969.9951, 1973.9971, 1975.9755, 1976.9708, 1990.4971, 1990.9373, 1991.4857, 1997.8479, 2001.8607, 2003.8768, 2004.8850, 2007.9500, 2007.9532, 2011.9381, 2012.8951, 2015.9694, 2017.9860, 2018.9740, 2020.1631, 2023.9482, 2026.9529, 2027.5033, 2029.9972, 2043.5356, 2044.9940, 2055.0205, 2057.0154, 2060.9878, 2066.9939, 2068.9661, 2082.9822, 2086.0254, 2088.1033, 2097.0371, 2098.4941, 2102.9954, 2109.0095, 2112.9932, 2114.9707, 2122.9578, 2128.0444, 2130.9980, 2132.0200, 2136.9250, 2140.0063, 2143.0237, 2144.0559, 2145.0781, 2148.0483, 2153.0049, 2153.9973, 2174.0508, 2175.0435, 2178.0483, 2188.0610, 2192.0427, 2199.1150, 2205.0615, 2211.1067, 2223.1533, 2225.1382, 2226.1472, 2239.1646, 2243.1448, 2246.9482, 2251.1108, 2255.1484, 2265.0837, 2271.1211, 2273.0735, 2283.1606, 2285.1355, 2286.0986, 2289.0901, 2291.0173, 2309.0349, 2328.9888, 2343.0608, 2352.9583, 2355.0383, 2366.9595, 2371.0325, 2392.1594, 2400.0532, 2424.1577, 2434.2429, 2438.2283, 2449.2180, 2450.2451, 2451.2480, 2465.1592, 2466.2458, 2467.2458, 2478.1377, 2501.2207, 2523.2014, 2526.2585, 2540.2451, 2542.2578, 2545.2258, 2550.2373, 2561.6841, 2562.1826, 2562.6890, 2573.2495, 2576.1792, 2646.9758, 2661.1409, 2662.0120, 2671.1924, 2681.2705, 2682.2888, 2685.3098, 2689.3059, 2695.1064, 2696.2695, 2699.2324, 2703.2529, 2705.2944, 2712.2937, 2717.2903, 2720.2839, 2721.2937, 2726.2935, 2727.2986, 2728.3079, 2739.2837, 2743.2729, 2744.2207, 2745.2256, 2746.2031, 2748.2295, 2755.2700, 2760.2590, 2763.1829, 2771.2400, 2778.1865, 2785.2969, 2786.2561, 2787.2524, 2790.2317, 2792.2166, 2800.3093, 2801.2932, 2803.2415, 2804.2429, 2807.2869, 2808.2227, 2812.2244, 2813.2373, 2817.2632, 2820.2441, 2828.2454, 2833.2544, 2843.2900, 2844.3398, 2847.3630, 2851.3313, 2864.3188, 2890.3293, 2896.4324, 2908.3105, 2910.3325, 2911.3594, 2912.4265, 2925.3323, 2927.4434, 3023.3674, 3025.3865, 3026.3452, 3039.3838, 3042.3542, 3070.3745, 3071.3933, 3086.3984, 3087.3970, 3093.4294, 3101.3831, 3103.4006, 3104.4041, 3110.4143, 3141.4126, 3144.5298, 3159.5210, 3219.4854, 3236.5574, 3266.6021, 3280.5635, 3321.6216, 3344.4895, 3347.5515, 3348.5020, 3380.5210, 3396.5195, 3412.5801, 3427.6099, 3428.5874, 3444.5840, 3462.6301, 3478.6123, 3484.6497, 3489.7246, 3505.6919, 3510.5859, 3521.6392, 3552.6614, 3553.6028, 3554.5630, 3558.6460, 3573.6545, 3589.6523, 3608.6155, 3624.6685, 3630.5806, 3641.7090, 3651.7944, 3667.7266, 3683.6980, 3691.7681, 3707.7200, 3714.5493, 3715.6206, 3719.6914, 3720.7048, 3723.7041, 3735.7100, 3751.7539, 3830.8091, 3869.7393, 3876.6707, 3885.6946

20. Q0VCG0 **Mass:** 10705 **Score:** 18 **Expect:** 3.7e+002 **Matches:** 10

LYR motif-containing protein 4 OS=Bos taurus GN=LYRM4 PE=3 SV=1

Observed	Mr(expt)	Mr(calc)	ppm	Start	End	Miss	Ions	Peptide
779.4633	778.4560	778.4449	14.3	30 -	35	1	---	R.TYAIRR.I
842.5101	841.5029	841.5134	-12.49	62 -	68	1	---	R.DLGIIRR.Q
1148.6318	1147.6245	1147.6098	12.9	36 -	44	2	---	R.IRDAFRENK.N
1390.7089	1389.7016	1389.6789	16.3	19 -	29	2	---	R.ESKRFGAYNYR.T
1562.8008	1561.7935	1561.7671	16.9	68 -	80	1	---	R.RQVHIGQMYSTDK.L
1578.8058	1577.7985	1577.7620	23.1	68 -	80	1	---	R.RQVHIGQMYSTDK.L + Oxidation (M)
1580.8580	1579.8507	1579.8140	23.2	1 -	14	1	---	-.MAASSRAQVLDLYR.A
1580.8600	1579.8527	1579.8140	24.5	1 -	14	1	---	-.MAASSRAQVLDLYR.A
1650.8429	1649.8356	1649.8426	-4.24	23 -	35	2	---	R.FGAYNYRTYAIRR.I
1938.0171	1937.0098	1937.0582	-24.95	42 -	58	2	---	R.ENKNVKDPVEIQALVNK.A

No match to: 716.3353, 718.2895, 724.2175, 726.3070, 728.2833, 729.4336, 730.2399, 732.2518, 734.4341, 743.3130, 745.3617, 747.4360, 753.3469, 756.3524, 758.3462, 759.3632, 768.3517, 781.3725, 789.3640, 794.3674, 796.3701, 799.2854, 801.4280, 805.4417, 807.4076, 810.3293, 812.3207, 818.3782, 820.3700, 828.4415, 829.4508, 830.4513, 834.3805, 849.3834, 852.4368, 857.4457, 860.4262, 868.4412, 870.4462, 873.4370, 877.4166, 880.4280, 882.4470, 883.4816, 887.4295, 889.4857, 891.4872, 894.4923, 898.4610, 900.5174, 905.4763, 907.4251, 912.5086, 914.4942, 920.4296, 922.4855, 928.4565, 929.4484, 933.4327, 936.4976, 938.5062, 944.4982, 946.4672,

949.5244, 950.5168, 957.5792, 961.5109, 965.5151, 967.4963, 972.5023, 974.4913, 976.5255, 978.5352, 981.5225, 983.6221, 986.5463, 988.4922, 993.5123, 995.4512, 998.5833, 1000.5594, 1006.5120, 1007.5640, 1021.5762, 1024.5542, 1031.5300, 1034.5703, 1037.5466, 1041.5637, 1043.5297, 1044.5375, 1045.5594, 1046.6058, 1054.5287, 1056.5575, 1057.6187, 1057.6200, 1068.5790, 1074.6162, 1076.6190, 1082.6056, 1105.5938, 1110.5851, 1113.5701, 1118.5687, 1120.6050, 1122.5892, 1123.6047, 1126.6241, 1128.5618, 1130.5756, 1135.5951, 1137.5647, 1143.5592, 1145.6373, 1149.6230, 1150.6226, 1156.5652, 1157.5676, 1159.5890, 1160.5966, 1169.5873, 1177.6421, 1178.6371, 1180.6443, 1182.6539, 1184.6431, 1185.6815, 1193.6090, 1194.6001, 1209.6238, 1213.7180, 1216.7178, 1221.5953, 1227.6459, 1229.6874, 1234.6899, 1238.2251, 1239.6622, 1254.6993, 1255.6351, 1264.7139, 1265.1680, 1265.7196, 1269.6532, 1276.6729, 1278.6892, 1281.6375, 1285.6674, 1289.7029, 1293.6760, 1301.6362, 1303.6489, 1304.6685, 1306.6946, 1308.6797, 1314.7227, 1319.6770, 1320.6364, 1323.6815, 1326.6937, 1328.6799, 1333.7430, 1336.7301, 1337.6429, 1349.6729, 1351.6969, 1353.6891, 1356.6255, 1358.6919, 1359.7013, 1361.7147, 1364.7118, 1365.6619, 1366.6965, 1372.6228, 1375.6693, 1382.6771, 1383.7347, 1393.8007, 1397.7449, 1403.7434, 1410.7849, 1416.7737, 1420.7114, 1423.7096, 1434.7847, 1436.7469, 1442.8257, 1445.7913, 1456.7555, 1457.7440, 1467.7560, 1470.7466, 1475.7615, 1479.8038, 1483.7579, 1484.7480, 1487.7660, 1488.7366, 1493.7556, 1500.7393, 1500.7400, 1503.7648, 1504.7708, 1514.7063, 1516.7281, 1519.7705, 1522.7722, 1524.8040, 1526.7924, 1528.7079, 1530.7058, 1536.7252, 1537.7859, 1540.8043, 1543.7938, 1550.7543, 1551.7465, 1553.7668, 1555.7836, 1556.8011, 1568.7620, 1572.7245, 1584.8486, 1587.7675, 1608.8051, 1616.8165, 1621.7870, 1625.7886, 1628.7313, 1632.8120, 1635.7964, 1637.7974, 1642.7417, 1647.8439, 1651.8369, 1655.8147, 1657.8483, 1658.8953, 1660.8379, 1662.8666, 1664.8490, 1666.7892, 1679.8091, 1680.8357, 1682.8409, 1696.8464, 1698.8180, 1701.8243, 1702.8386, 1707.8938, 1710.8904, 1714.7905, 1716.8219, 1717.9052, 1718.9094, 1721.8976, 1722.9003, 1724.9030, 1728.8840, 1730.8910, 1734.1617, 1738.8267, 1739.8165, 1741.8934, 1743.9005, 1747.9127, 1758.4807, 1758.8918, 1761.8734, 1765.8154, 1772.0012, 1774.9300, 1774.9319, 1779.9012, 1781.8585, 1783.8062, 1787.8407, 1809.0583, 1810.0460, 1812.9126, 1829.8527, 1832.8584, 1833.8571, 1834.8650, 1836.8618, 1838.8677, 1845.8363, 1847.8600, 1848.8938, 1850.9128, 1851.8932, 1861.8531, 1862.9542, 1864.9258, 1880.9026, 1883.7704, 1887.8878, 1897.7863, 1902.9769, 1904.9276, 1908.8986, 1920.9131, 1923.9126, 1928.0443, 1932.0068, 1936.9510, 1947.9314, 1958.9393, 1959.9655, 1960.9813, 1961.9463, 1967.1549, 1969.9951, 1973.9971, 1975.9755, 1976.9708, 1990.4971, 1990.9373, 1991.4857, 1997.8479, 2001.8607, 2003.8768, 2004.8850, 2007.9500, 2007.9532, 2011.9381, 2012.8951, 2015.9694, 2017.9860, 2018.9740, 2020.1631, 2023.9482, 2026.9529, 2027.5033, 2029.9972, 2043.5356, 2044.9940, 2055.0205, 2057.0154, 2060.9878, 2066.9939, 2068.9661, 2082.9822, 2086.0254, 2088.1033, 2097.0371, 2098.4941, 2102.9954, 2109.0095, 2112.9932, 2114.9707, 2122.9578, 2128.0444, 2130.9980, 2132.0200, 2136.9250, 2140.0063, 2143.0237, 2144.0559, 2145.0781, 2148.0483, 2153.0049, 2153.9973, 2174.0508, 2175.0435, 2178.0483, 2188.0610, 2191.0464, 2192.0427, 2199.1150, 2205.0615, 2211.1067, 2223.1533, 2225.1382, 2226.1472, 2239.1646, 2243.1448, 2246.9482, 2251.1108, 2255.1484, 2265.0837, 2271.1211, 2273.0735, 2277.0642, 2283.1606, 2285.1355, 2286.0986, 2289.0901, 2291.0173, 2309.0349, 2328.9888, 2343.0608, 2352.9583, 2355.0383, 2366.9595, 2371.0325, 2392.1594, 2400.0532, 2424.1577, 2434.2429, 2438.2283, 2449.2180, 2450.2451, 2451.2480, 2465.1592, 2466.2458, 2467.2458, 2478.1377, 2501.2207, 2523.2014, 2526.2585, 2540.2451, 2542.2578, 2545.2258, 2550.2373, 2561.6841, 2562.1826, 2562.6890, 2573.2495, 2576.1792, 2646.9758, 2659.2144, 2661.1409, 2662.0120, 2671.1924, 2681.2705, 2682.2888, 2685.3098, 2689.3059, 2695.1064, 2696.2695, 2699.2324, 2703.2529, 2705.2944, 2712.2937, 2717.2903, 2720.2839, 2721.2937, 2726.2935, 2727.2986, 2728.3079, 2739.2837, 2743.2729, 2744.2207, 2745.2256, 2746.2031, 2748.2295, 2755.2700, 2760.2590, 2763.1829, 2771.2400, 2778.1865, 2785.2969, 2786.2561, 2787.2524, 2790.2317, 2792.2166, 2800.3093, 2801.2932, 2803.2415, 2804.2429, 2807.2869, 2808.2227, 2812.2244, 2813.2373, 2817.2632, 2820.2441, 2824.2598, 2828.2454, 2833.2544, 2843.2900, 2844.3398, 2847.3630, 2851.3313, 2864.3188, 2890.3293, 2896.4324, 2908.3105, 2910.3325, 2911.3594, 2912.4265, 2925.3323, 2927.4434, 3023.3674, 3025.3865, 3026.3452, 3039.3838, 3042.3542, 3070.3745, 3071.3933, 3086.3984, 3087.3970, 3093.4294, 3101.3831, 3103.4006, 3104.4041, 3110.4143, 3127.4749, 3141.4126, 3143.5066, 3144.5298, 3159.5210, 3219.4854, 3236.5574, 3266.6021, 3280.5635, 3321.6216, 3344.4895, 3347.5515, 3348.5020, 3380.5210, 3396.5195, 3412.5801, 3427.6099, 3428.5874, 3444.5840, 3462.6301, 3478.6123, 3484.6497, 3489.7246, 3505.6919, 3510.5859, 3521.6392, 3552.6614, 3553.6028, 3554.5630, 3558.6460, 3573.6545, 3589.6523, 3608.6155, 3624.6685, 3630.5806, 3641.7090, 3651.7944, 3667.7266, 3683.6980, 3691.7681, 3707.7200, 3714.5493, 3715.6206, 3719.6914, 3720.7048, 3723.7041, 3735.7100, 3751.7539, 3830.8091, 3869.7393, 3876.6707, 3885.6946

21. G3N0J4 **Mass:** 6707 **Score:** 18 **Expect:** 4e+002 **Matches:** 6

Uncharacterized protein OS=Bos taurus PE=4 SV=1

Observed	Mr(expt)	Mr(calc)	ppm	Start	End	Miss	Ions	Peptide
1194.6001	1193.5928	1193.5750	14.9	17 -	26	0	---	R.LPEAMLEGYR.A + Oxidation (M)
1657.8483	1656.8410	1656.8431	-1.24	27 -	42	2	---	R.AESIPPTSSGRNEGKK.E
2277.0642	2276.0569	2276.1107	-23.61	17 -	37	1	---	R.LPEAMLEGYRAESIPPTSSGR.N + Oxidation (M)
2545.2258	2544.2185	2544.2754	-22.36	14 -	37	2	---	R.AGRLPEAMLEGYRAESIPPTSSGR.N
2689.3059	2688.2986	2688.3177	-7.09	17 -	41	2	---	R.LPEAMLEGYRAESIPPTSSGRNEGK.K
2705.2944	2704.2871	2704.3126	-9.42	17 -	41	2	---	R.LPEAMLEGYRAESIPPTSSGRNEGK.K + Oxidation (M)

No match to: 716.3353, 718.2895, 724.2175, 726.3070, 728.2833, 729.4336, 730.2399, 732.2518, 734.4341, 743.3130, 745.3617, 747.4360, 753.3469, 756.3524, 758.3462, 759.3632, 768.3517, 779.4633, 781.3725, 789.3640, 794.3674, 796.3701, 799.2854, 801.4280, 805.4417, 807.4076, 810.3293, 812.3207, 818.3782, 820.3700, 828.4415, 829.4508, 830.4513, 834.3805, 842.5101, 849.3834, 852.4368, 857.4457, 860.4262, 868.4412, 870.4462, 873.4370, 877.4166, 880.4280, 882.4470, 883.4816, 887.4295, 889.4857, 891.4872, 894.4923, 898.4610, 900.5174, 905.4763, 907.4251, 912.5086, 914.4942, 920.4296, 922.4855, 928.4565, 929.4484, 933.4327, 936.4976, 938.5062, 944.4982, 946.4672, 949.5244, 950.5168, 957.5792, 961.5109, 965.5151, 967.4963, 972.5023, 974.4913, 976.5255, 978.5352, 981.5225, 983.6221, 986.5463, 988.4922, 993.5123, 995.4512, 998.5833, 1000.5594, 1006.5120, 1007.5640, 1021.5762, 1024.5542, 1031.5300, 1034.5703, 1037.5466, 1041.5637, 1043.5297, 1044.5375, 1045.5594, 1046.6058, 1054.5287, 1056.5575, 1057.6187, 1057.6200, 1068.5790, 1074.6162, 1076.6190, 1082.6056, 1105.5938, 1110.5851, 1113.5701, 1118.5687, 1120.6050, 1122.5892, 1123.6047, 1126.6241, 1128.5618, 1130.5756, 1135.5951, 1137.5647, 1143.5592, 1145.6373, 1148.6318, 1149.6230, 1150.6226, 1156.5652, 1157.5676, 1159.5890, 1160.5966, 1169.5873, 1177.6421, 1178.6371, 1180.6443, 1182.6539, 1184.6431, 1185.6815, 1193.6090, 1209.6238, 1213.7180, 1216.7178, 1221.5953, 1227.6459, 1229.6874, 1234.6899, 1238.2251, 1239.6622, 1254.6993, 1255.6351, 1264.7139, 1265.1680, 1265.7196, 1269.6532, 1276.6729, 1278.6892, 1281.6375, 1285.6674, 1289.7029, 1293.6760, 1301.6362, 1303.6489, 1304.6685, 1306.6946, 1308.6797, 1314.7227, 1319.6770, 1320.6364, 1323.6815, 1326.6937, 1328.6799, 1333.7430, 1336.7301, 1337.6429, 1349.6729, 1351.6969, 1353.6891, 1356.6255, 1358.6919, 1359.7013, 1361.7147, 1364.7118, 1365.6619, 1366.6965, 1372.6228, 1375.6693, 1382.6771, 1383.7347, 1390.7089, 1393.8007, 1397.7449, 1403.7434, 1410.7849, 1416.7737, 1420.7114, 1423.7096, 1434.7847, 1436.7469, 1442.8257, 1445.7913, 1456.7555, 1457.7440, 1467.7560, 1470.7466, 1475.7615, 1479.8038, 1483.7579, 1484.7480, 1487.7660, 1488.7366, 1493.7556, 1500.7393, 1500.7400, 1503.7648, 1504.7708, 1514.7063, 1516.7281, 1519.7705, 1522.7722, 1524.8040, 1526.7924, 1528.7079, 1530.7058, 1536.7252, 1537.7859, 1540.8043, 1543.7938, 1550.7543, 1551.7465, 1553.7668, 1555.7836, 1556.8011, 1562.8008, 1568.7620, 1572.7245, 1578.8058, 1580.8580, 1580.8600, 1584.8486, 1587.7675, 1608.8051, 1616.8165, 1621.7870, 1625.7886, 1628.7313, 1632.8120, 1635.7964, 1637.7974, 1642.7417, 1647.8439, 1650.8429, 1651.8369, 1655.8147, 1658.8953, 1660.8379, 1662.8666, 1664.8490, 1666.7892, 1679.8091, 1680.8357, 1682.8409, 1696.8464, 1698.8180, 1701.8243, 1702.8386, 1707.8938, 1710.8904, 1714.7905, 1716.8219, 1717.9052, 1718.9094, 1721.8976, 1722.9003, 1724.9030, 1728.8840, 1730.8910, 1734.1617, 1738.8267, 1739.8165, 1741.8934, 1743.9005, 1747.9127, 1758.4807, 1758.8918, 1761.8734, 1765.8154, 1772.0012, 1774.9300, 1774.9319, 1779.9012, 1781.8585, 1783.8062, 1787.8407, 1809.0583, 1810.0460, 1812.9126, 1829.8527, 1832.8584, 1833.8571, 1834.8650, 1836.8618, 1838.8677, 1845.8363, 1847.8600, 1848.8938, 1850.9128, 1851.8932, 1861.8531, 1862.9542, 1864.9258, 1880.9026, 1883.7704, 1887.8878, 1897.7863, 1902.9769, 1904.9276, 1908.8986, 1920.9131, 1923.9126, 1928.0443, 1932.0068, 1936.9510, 1938.0171, 1947.9314, 1958.9393, 1959.9655, 1960.9813, 1961.9463, 1967.1549, 1969.9951, 1973.9971, 1975.9755, 1976.9708, 1990.4971, 1990.9373, 1991.4857, 1997.8479, 2001.8607, 2003.8768, 2004.8850, 2007.9500, 2007.9532, 2011.9381, 2012.8951, 2015.9694, 2017.9860, 2018.9740, 2020.1631, 2023.9482, 2026.9529, 2027.5033, 2029.9972, 2043.5356, 2044.9940, 2055.0205, 2057.0154, 2060.9878, 2066.9939, 2068.9661, 2082.9822, 2086.0254, 2088.1033, 2097.0371, 2098.4941, 2102.9954, 2109.0095, 2112.9932, 2114.9707, 2122.9578, 2128.0444, 2130.9980, 2132.0200, 2136.9250, 2140.0063, 2143.0237, 2144.0559, 2145.0781, 2148.0483, 2153.0049, 2153.9973, 2174.0508, 2175.0435, 2178.0483, 2188.0610, 2191.0464, 2192.0427, 2199.1150, 2205.0615, 2211.1067, 2223.1533, 2225.1382, 2226.1472, 2239.1646, 2243.1448, 2246.9482, 2251.1108, 2255.1484, 2265.0837, 2271.1211, 2273.0735, 2283.1606, 2285.1355, 2286.0986, 2289.0901, 2291.0173, 2309.0349, 2328.9888, 2343.0608, 2352.9583, 2355.0383, 2366.9595, 2371.0325, 2392.1594, 2400.0532, 2424.1577, 2434.2429, 2438.2283, 2449.2180, 2450.2451, 2451.2480, 2465.1592, 2466.2458, 2467.2458, 2478.1377, 2501.2207, 2523.2014, 2526.2585, 2540.2451, 2542.2578, 2550.2373, 2561.6841, 2562.1826, 2562.6890, 2573.2495, 2576.1792, 2646.9758, 2659.2144, 2661.1409, 2662.0120, 2671.1924, 2681.2705, 2682.2888, 2685.3098, 2695.1064, 2696.2695, 2699.2324, 2703.2529, 2712.2937, 2717.2903, 2720.2839, 2721.2937, 2726.2935, 2727.2986, 2728.3079, 2739.2837, 2743.2729, 2744.2207, 2745.2256, 2746.2031, 2748.2295, 2755.2700, 2760.2590, 2763.1829, 2771.2400, 2778.1865, 2785.2969, 2786.2561, 2787.2524, 2790.2317, 2792.2166, 2800.3093, 2801.2932, 2803.2415, 2804.2429, 2807.2869, 2808.2227, 2812.2244, 2813.2373, 2817.2632, 2820.2441, 2824.2598, 2828.2454, 2833.2544, 2843.2900, 2844.3398, 2847.3630, 2851.3313, 2864.3188, 2890.3293, 2896.4324, 2908.3105, 2910.3325, 2911.3594, 2912.4265, 2925.3323, 2927.4434, 3023.3674, 3025.3865, 3026.3452, 3039.3838, 3042.3542, 3070.3745, 3071.3933, 3086.3984, 3087.3970, 3093.4294, 3101.3831, 3103.4006, 3104.4041, 3110.4143, 3127.4749, 3141.4126, 3143.5066, 3144.5298, 3159.5210, 3219.4854, 3236.5574, 3266.6021, 3280.5635, 3321.6216, 3344.4895, 3347.5515, 3348.5020, 3380.5210, 3396.5195, 3412.5801, 3427.6099, 3428.5874, 3444.5840, 3462.6301, 3478.6123, 3484.6497, 3489.7246, 3505.6919, 3510.5859, 3521.6392, 3552.6614, 3553.6028, 3554.5630, 3558.6460, 3573.6545, 3589.6523, 3608.6155, 3624.6685, 3630.5806, 3641.7090, 3651.7944, 3667.7266, 3683.6980, 3691.7681, 3707.7200, 3714.5493, 3715.6206, 3719.6914, 3720.7048, 3723.7041, 3735.7100, 3751.7539, 3830.8091, 3869.7393, 3876.6707, 3885.6946

22. F1N2B6 **Mass:** 97976 **Score:** 18 **Expect:** 4.1e+002 **Matches:** 19
Uncharacterized protein OS=Bos taurus GN=AR PE=3 SV=2

Observed	Mr(expt)	Mr(calc)	ppm	Start	End	Miss	Ions	Peptide
988.4922	987.4849	987.4873	-2.41	274 -	283	0	---	K.GSLLDDGPSK.G
1356.6255	1355.6182	1355.6326	-10.58	590 -	601	1	---	K.CYEAGMTLGARK.L
1372.6228	1371.6155	1371.6275	-8.72	590 -	601	1	---	K.CYEAGMTLGARK.L + Oxidation (M)
1475.7615	1474.7542	1474.7278	17.9	797 -	807	1	---	K.FFDELRMNYIK.E
1484.7480	1483.7407	1483.7275	8.90	589 -	601	2	---	R.KCYEAGMTLGARK.L
1500.7393	1499.7320	1499.7224	6.39	589 -	601	2	---	R.KCYEAGMTLGARK.L + Oxidation (M)
1500.7400	1499.7327	1499.7224	6.86	589 -	601	2	18	R.KCYEAGMTLGARK.L + Oxidation (M)
1625.7886	1624.7813	1624.8177	-22.41	587 -	600	2	---	R.LRKCYEAGMTLGAR.K
2017.9860	2016.9787	2016.9310	23.7	205 -	223	1	---	K.DSYLGGSSTISDSAKELCK.A
2174.0508	2173.0435	2173.0489	-2.46	732 -	748	1	---	R.MLYFAPDLVFNEYRMHK.S
2449.2180	2448.2107	2448.1718	15.9	732 -	750	2	---	R.MLYFAPDLVFNEYRMHKSR.M + 2 Oxidation (M)
2699.2324	2698.2251	2698.2850	-22.17	724 -	745	1	---	R.SFTNVNSRMLYFAPDLVFNEYR.M + Oxidation (M)
2912.4265	2911.4192	2911.3853	11.7	247 -	273	0	---	R.GDCMYAPLLGPPAAVRPTPCAPLAECK.G
3478.6123	3477.6050	3477.6693	-18.49	54 -	82	2	---	R.LQQQQETSPRQQQQQQQQREDGSPQVQSR.G
3707.7200	3706.7127	3706.6706	11.4	83 -	116	0	---	R.GPTGYLALEEEQQPSQHHSAPEGHPESGCVPEPR.A
3719.6914	3718.6841	3718.7339	-13.39	751 -	779	2	---	R.MYSQCVRMRHLSQEFGWLQITPQEFLCMK.A + Oxidation (M)
3735.7100	3734.7027	3734.7288	-6.99	751 -	779	2	---	R.MYSQCVRMRHLSQEFGWLQITPQEFLCMK.A + 2 Oxidation (M)
3751.7539	3750.7466	3750.7238	6.10	751 -	779	2	---	R.MYSQCVRMRHLSQEFGWLQITPQEFLCMK.A + 3 Oxidation (M)
3869.7393	3868.7320	3868.6841	12.4	482 -	514	2	---	R.VPYPSPSCVKSEMGPWMENYSGPYGDMRLETTR.D + 3 Oxidation (M)

No match to: 716.3353, 718.2895, 724.2175, 726.3070, 728.2833, 729.4336, 730.2399, 732.2518, 734.4341, 743.3130, 745.3617, 747.4360, 753.3469, 756.3524, 758.3462, 759.3632, 768.3517, 779.4633, 781.3725, 789.3640, 794.3674, 796.3701, 799.2854, 801.4280, 805.4417, 807.4076, 810.3293, 812.3207, 818.3782, 820.3700, 828.4415, 829.4508, 830.4513, 834.3805, 842.5101, 849.3834, 852.4368, 857.4457, 860.4262, 868.4412, 870.4462, 873.4370, 877.4166, 880.4280, 882.4470, 883.4816, 887.4295, 889.4857, 891.4872, 894.4923, 898.4610, 900.5174, 905.4763, 907.4251, 912.5086, 914.4942, 920.4296, 922.4855, 928.4565, 929.4484, 933.4327, 936.4976, 938.5062, 944.4982, 946.4672, 949.5244, 950.5168, 957.5792, 961.5109, 965.5151, 967.4963, 972.5023, 974.4913, 976.5255, 978.5352, 981.5225, 983.6221, 986.5463, 993.5123, 995.4512, 998.5833, 1000.5594, 1006.5120, 1007.5640, 1021.5762, 1024.5542, 1031.5300, 1034.5703, 1037.5466, 1041.5637, 1043.5297, 1044.5375, 1045.5594, 1046.6058, 1054.5287, 1056.5575, 1057.6187, 1057.6200, 1068.5790, 1074.6162, 1076.6190, 1082.6056, 1105.5938, 1110.5851, 1113.5701, 1118.5687, 1120.6050, 1122.5892, 1123.6047, 1126.6241, 1128.5618, 1130.5756, 1135.5951, 1137.5647, 1143.5592, 1145.6373, 1148.6318, 1149.6230, 1150.6226, 1156.5652, 1157.5676, 1159.5890, 1160.5966, 1169.5873, 1177.6421, 1178.6371, 1180.6443, 1182.6539, 1184.6431, 1185.6815, 1193.6090, 1194.6001, 1209.6238, 1213.7180, 1216.7178, 1221.5953, 1227.6459, 1229.6874, 1234.6899, 1238.2251, 1239.6622, 1254.6993, 1255.6351, 1264.7139, 1265.1680, 1265.7196, 1269.6532, 1276.6729, 1278.6892, 1281.6375, 1285.6674, 1289.7029, 1293.6760, 1301.6362, 1303.6489, 1304.6685, 1306.6946, 1308.6797, 1314.7227, 1319.6770, 1320.6364, 1323.6815, 1326.6937, 1328.6799, 1333.7430, 1336.7301, 1337.6429, 1349.6729, 1351.6969, 1353.6891, 1358.6919, 1359.7013, 1361.7147, 1364.7118, 1365.6619, 1366.6965, 1375.6693, 1382.6771, 1383.7347, 1390.7089, 1393.8007, 1397.7449, 1403.7434, 1410.7849, 1416.7737, 1420.7114, 1423.7096, 1434.7847, 1436.7469, 1442.8257, 1445.7913, 1456.7555, 1457.7440, 1467.7560, 1470.7466, 1479.8038, 1483.7579, 1487.7660, 1488.7366, 1493.7556, 1503.7648, 1504.7708, 1514.7063, 1516.7281, 1519.7705, 1522.7722, 1524.8040, 1526.7924, 1528.7079, 1530.7058, 1536.7252, 1537.7859, 1540.8043, 1543.7938, 1550.7543, 1551.7465, 1553.7668, 1555.7836, 1556.8011, 1562.8008, 1568.7620, 1572.7245, 1578.8058, 1580.8580, 1580.8600, 1584.8486, 1587.7675, 1608.8051, 1616.8165, 1621.7870, 1628.7313, 1632.8120, 1635.7964, 1637.7974, 1642.7417, 1647.8439, 1650.8429, 1651.8369, 1655.8147, 1657.8483, 1658.8953, 1660.8379, 1662.8666, 1664.8490, 1666.7892, 1679.8091, 1680.8357, 1682.8409, 1696.8464, 1698.8180, 1701.8243, 1702.8386, 1707.8938, 1710.8904, 1714.7905, 1716.8219, 1717.9052, 1718.9094, 1721.8976, 1722.9003, 1724.9030, 1728.8840, 1730.8910, 1734.1617, 1738.8267, 1739.8165, 1741.8934, 1743.9005, 1747.9127, 1758.4807, 1758.8918, 1761.8734, 1765.8154, 1772.0012, 1774.9300, 1774.9319, 1779.9012, 1781.8585, 1783.8062, 1787.8407, 1809.0583, 1810.0460, 1812.9126, 1829.8527, 1832.8584, 1833.8571, 1834.8650, 1836.8618, 1838.8677, 1845.8363, 1847.8600, 1848.8938, 1850.9128, 1851.8932, 1861.8531, 1862.9542, 1864.9258, 1880.9026, 1883.7704, 1887.8878, 1897.7863, 1902.9769, 1904.9276, 1908.8986, 1920.9131, 1923.9126, 1928.0443, 1932.0068, 1936.9510, 1938.0171, 1947.9314, 1958.9393, 1959.9655, 1960.9813, 1961.9463, 1967.1549, 1969.9951, 1973.9971, 1975.9755, 1976.9708, 1990.4971, 1990.9373, 1991.4857, 1997.8479, 2001.8607, 2003.8768, 2004.8850, 2007.9500, 2007.9532, 2011.9381, 2012.8951, 2015.9694, 2018.9740, 2020.1631, 2023.9482, 2026.9529, 2027.5033, 2029.9972, 2043.5356, 2044.9940, 2055.0205, 2057.0154, 2060.9878, 2066.9939, 2068.9661, 2082.9822, 2086.0254, 2088.1033, 2097.0371, 2098.4941, 2102.9954, 2109.0095, 2112.9932, 2114.9707, 2122.9578, 2128.0444, 2130.9980, 2132.0200, 2136.9250, 2140.0063, 2143.0237, 2144.0559, 2145.0781, 2148.0483, 2153.0049, 2153.9973, 2175.0435, 2178.0483, 2188.0610, 2191.0464, 2192.0427, 2199.1150, 2205.0615, 2211.1067, 2223.1533, 2225.1382, 2226.1472, 2239.1646, 2243.1448, 2246.9482, 2251.1108, 2255.1484, 2265.0837, 2271.1211, 2273.0735, 2277.0642, 2283.1606, 2285.1355, 2286.0986, 2289.0901, 2291.0173, 2309.0349, 2328.9888, 2343.0608, 2352.9583, 2355.0383, 2366.9595, 2371.0325, 2392.1594, 2400.0532, 2424.1577, 2434.2429, 2438.2283, 2450.2451, 2451.2480, 2465.1592, 2466.2458, 2467.2458, 2478.1377, 2501.2207, 2523.2014, 2526.2585, 2540.2451, 2542.2578, 2545.2258, 2550.2373, 2561.6841, 2562.1826, 2562.6890, 2573.2495, 2576.1792, 2646.9758, 2659.2144, 2661.1409, 2662.0120, 2671.1924, 2681.2705, 2682.2888, 2685.3098, 2689.3059, 2695.1064, 2696.2695, 2703.2529, 2705.2944, 2712.2937, 2717.2903, 2720.2839, 2721.2937, 2726.2935, 2727.2986, 2728.3079, 2739.2837, 2743.2729, 2744.2207, 2745.2256, 2746.2031, 2748.2295, 2755.2700, 2760.2590, 2763.1829, 2771.2400, 2778.1865, 2785.2969, 2786.2561, 2787.2524, 2790.2317, 2792.2166, 2800.3093, 2801.2932, 2803.2415, 2804.2429, 2807.2869, 2808.2227, 2812.2244, 2813.2373, 2817.2632, 2820.2441, 2824.2598, 2828.2454, 2833.2544, 2843.2900, 2844.3398, 2847.3630, 2851.3313, 2864.3188, 2890.3293, 2896.4324, 2908.3105, 2910.3325, 2911.3594, 2925.3323, 2927.4434, 3023.3674, 3025.3865, 3026.3452, 3039.3838, 3042.3542, 3070.3745, 3071.3933, 3086.3984, 3087.3970, 3093.4294, 3101.3831, 3103.4006, 3104.4041, 3110.4143, 3127.4749, 3141.4126, 3143.5066, 3144.5298, 3159.5210, 3219.4854, 3236.5574, 3266.6021, 3280.5635, 3321.6216, 3344.4895, 3347.5515, 3348.5020, 3380.5210, 3396.5195, 3412.5801, 3427.6099, 3428.5874, 3444.5840, 3462.6301, 3484.6497, 3489.7246, 3505.6919, 3510.5859, 3521.6392, 3552.6614, 3553.6028, 3554.5630, 3558.6460, 3573.6545, 3589.6523, 3608.6155, 3624.6685, 3630.5806, 3641.7090, 3651.7944, 3667.7266, 3683.6980, 3691.7681, 3714.5493, 3715.6206, 3720.7048, 3723.7041, 3830.8091, 3876.6707, 3885.6946

23. A8QW39 **Mass:** 16136 **Score:** 18 **Expect:** 4.2e+002 **Matches:** 9
Protein FAM183A OS=Bos taurus GN=FAM183A PE=2 SV=1

Observed	Mr(expt)	Mr(calc)	ppm	Start	End	Miss	Ions	Peptide
928.4565	927.4493	927.4596	-11.16	1 -	8	1	---	-.MAGHQKEK.A
1076.6190	1075.6117	1075.5927	17.7	109 -	116	1	---	R.LNHFRVYK.D
1403.7434	1402.7361	1402.7357	0.29	33 -	43	0	---	K.LYTQYHVNPLR.K
1572.7245	1571.7172	1571.7514	-21.77	123 -	135	2	---	K.AKMWSLGEDDRHK.-
1810.0460	1809.0387	1808.9937	24.9	109 -	122	2	---	R.LNHFRVYKDITLYK.A
2057.0154	2056.0081	2055.9935	7.11	117 -	133	2	---	K.DITLYKAKMWSLGEDDR.H + Oxidation (M)
2573.2495	2572.2422	2572.2605	-7.09	45 -	66	1	---	K.VHTIARKPMSWHDNLEEPADAR.F
2717.2903	2716.2830	2716.3503	-24.78	44 -	66	2	---	R.KVHTIARKPMSWHDNLEEPADAR.F + Oxidation (M)
2801.2932	2800.2859	2800.3304	-15.88	81 -	104	1	---	K.KYPETQTEGQEIGWDSEPLVNPQR.D

No match to: 716.3353, 718.2895, 724.2175, 726.3070, 728.2833, 729.4336, 730.2399, 732.2518, 734.4341, 743.3130, 745.3617, 747.4360, 753.3469, 756.3524, 758.3462, 759.3632, 768.3517, 779.4633, 781.3725, 789.3640, 794.3674, 796.3701, 799.2854, 801.4280, 805.4417, 807.4076, 810.3293, 812.3207, 818.3782, 820.3700, 828.4415, 829.4508, 830.4513, 834.3805, 842.5101, 849.3834, 852.4368, 857.4457, 860.4262, 868.4412, 870.4462, 873.4370, 877.4166, 880.4280, 882.4470, 883.4816, 887.4295, 889.4857, 891.4872, 894.4923, 898.4610, 900.5174, 905.4763, 907.4251, 912.5086, 914.4942, 920.4296, 922.4855, 929.4484, 933.4327, 936.4976, 938.5062, 944.4982, 946.4672, 949.5244, 950.5168, 957.5792, 961.5109, 965.5151, 967.4963, 972.5023, 974.4913, 976.5255, 978.5352, 981.5225, 983.6221, 986.5463, 988.4922, 993.5123, 995.4512, 998.5833, 1000.5594, 1006.5120, 1007.5640, 1021.5762, 1024.5542, 1031.5300, 1034.5703, 1037.5466, 1041.5637, 1043.5297, 1044.5375, 1045.5594, 1046.6058, 1054.5287, 1056.5575, 1057.6187, 1057.6200, 1068.5790, 1074.6162, 1082.6056, 1105.5938, 1110.5851, 1113.5701, 1118.5687, 1120.6050, 1122.5892, 1123.6047, 1126.6241, 1128.5618, 1130.5756, 1135.5951, 1137.5647, 1143.5592, 1145.6373, 1148.6318, 1149.6230, 1150.6226, 1156.5652, 1157.5676, 1159.5890, 1160.5966, 1169.5873, 1177.6421, 1178.6371, 1180.6443, 1182.6539, 1184.6431, 1185.6815, 1193.6090, 1194.6001, 1209.6238, 1213.7180, 1216.7178, 1221.5953, 1227.6459, 1229.6874, 1234.6899, 1238.2251, 1239.6622, 1254.6993, 1255.6351, 1264.7139, 1265.1680, 1265.7196, 1269.6532, 1276.6729, 1278.6892, 1281.6375, 1285.6674, 1289.7029, 1293.6760, 1301.6362, 1303.6489, 1304.6685, 1306.6946, 1308.6797, 1314.7227, 1319.6770, 1320.6364, 1323.6815, 1326.6937, 1328.6799, 1333.7430, 1336.7301, 1337.6429, 1349.6729, 1351.6969, 1353.6891, 1356.6255, 1358.6919, 1359.7013,

1361.7147, 1364.7118, 1365.6619, 1366.6965, 1372.6228, 1375.6693, 1382.6771, 1383.7347, 1390.7089, 1393.8007, 1397.7449, 1410.7849, 1416.7737, 1420.7114, 1423.7096, 1434.7847, 1436.7469, 1442.8257, 1445.7913, 1456.7555, 1457.7440, 1467.7560, 1470.7466, 1475.7615, 1479.8038, 1483.7579, 1484.7480, 1487.7660, 1488.7366, 1493.7556, 1500.7393, 1500.7400, 1503.7648, 1504.7708, 1514.7063, 1516.7281, 1519.7705, 1522.7722, 1524.8040, 1526.7924, 1528.7079, 1530.7058, 1536.7252, 1537.7859, 1540.8043, 1543.7938, 1550.7543, 1551.7465, 1553.7668, 1555.7836, 1556.8011, 1562.8008, 1568.7620, 1578.8058, 1580.8580, 1580.8600, 1584.8486, 1587.7675, 1608.8051, 1616.8165, 1621.7870, 1625.7886, 1628.7313, 1632.8120, 1635.7964, 1637.7974, 1642.7417, 1647.8439, 1650.8429, 1651.8369, 1655.8147, 1657.8483, 1658.8953, 1660.8379, 1662.8666, 1664.8490, 1666.7892, 1679.8091, 1680.8357, 1682.8409, 1696.8464, 1698.8180, 1701.8243, 1702.8386, 1707.8938, 1710.8904, 1714.7905, 1716.8219, 1717.9052, 1718.9094, 1721.8976, 1722.9003, 1724.9030, 1728.8840, 1730.8910, 1734.1617, 1738.8267, 1739.8165, 1741.8934, 1743.9005, 1747.9127, 1758.4807, 1758.8918, 1761.8734, 1765.8154, 1772.0012, 1774.9300, 1774.9319, 1779.9012, 1781.8585, 1783.8062, 1787.8407, 1809.0583, 1812.9126, 1829.8527, 1832.8584, 1833.8571, 1834.8650, 1836.8618, 1838.8677, 1845.8363, 1847.8600, 1848.8938, 1850.9128, 1851.8932, 1861.8531, 1862.9542, 1864.9258, 1880.9026, 1883.7704, 1887.8878, 1897.7863, 1902.9769, 1904.9276, 1908.8986, 1920.9131, 1923.9126, 1928.0443, 1932.0068, 1936.9510, 1938.0171, 1947.9314, 1958.9393, 1959.9655, 1960.9813, 1961.9463, 1967.1549, 1969.9951, 1973.9971, 1975.9755, 1976.9708, 1990.4971, 1990.9373, 1991.4857, 1997.8479, 2001.8607, 2003.8768, 2004.8850, 2007.9500, 2007.9532, 2011.9381, 2012.8951, 2015.9694, 2017.9860, 2018.9740, 2020.1631, 2023.9482, 2026.9529, 2027.5033, 2029.9972, 2043.5356, 2044.9940, 2055.0205, 2060.9878, 2066.9939, 2068.9661, 2082.9822, 2086.0254, 2088.1033, 2097.0371, 2098.4941, 2102.9954, 2109.0095, 2112.9932, 2114.9707, 2122.9578, 2128.0444, 2130.9980, 2132.0200, 2136.9250, 2140.0063, 2143.0237, 2144.0559, 2145.0781, 2148.0483, 2153.0049, 2153.9973, 2174.0508, 2175.0435, 2178.0483, 2188.0610, 2191.0464, 2192.0427, 2199.1150, 2205.0615, 2211.1067, 2223.1533, 2225.1382, 2226.1472, 2239.1646, 2243.1448, 2246.9482, 2251.1108, 2255.1484, 2265.0837, 2271.1211, 2273.0735, 2277.0642, 2283.1606, 2285.1355, 2286.0986, 2289.0901, 2291.0173, 2309.0349, 2328.9888, 2343.0608, 2352.9583, 2355.0383, 2366.9595, 2371.0325, 2392.1594, 2400.0532, 2424.1577, 2434.2429, 2438.2283, 2449.2180, 2450.2451, 2451.2480, 2465.1592, 2466.2458, 2467.2458, 2478.1377, 2501.2207, 2523.2014, 2526.2585, 2540.2451, 2542.2578, 2545.2258, 2550.2373, 2561.6841, 2562.1826, 2562.6890, 2576.1792, 2646.9758, 2659.2144, 2661.1409, 2662.0120, 2671.1924, 2681.2705, 2682.2888, 2685.3098, 2689.3059, 2695.1064, 2696.2695, 2699.2324, 2703.2529, 2705.2944, 2712.2937, 2720.2839, 2721.2937, 2726.2935, 2727.2986, 2728.3079, 2739.2837, 2743.2729, 2744.2207, 2745.2256, 2746.2031, 2748.2295, 2755.2700, 2760.2590, 2763.1829, 2771.2400, 2778.1865, 2785.2969, 2786.2561, 2787.2524, 2790.2317, 2792.2166, 2800.3093, 2803.2415, 2804.2429, 2807.2869, 2808.2227, 2812.2244, 2813.2373, 2817.2632, 2820.2441, 2824.2598, 2828.2454, 2833.2544, 2843.2900, 2844.3398, 2847.3630, 2851.3313, 2864.3188, 2890.3293, 2896.4324, 2908.3105, 2910.3325, 2911.3594, 2912.4265, 2925.3323, 2927.4434, 3023.3674, 3025.3865, 3026.3452, 3039.3838, 3042.3542, 3070.3745, 3071.3933, 3086.3984, 3087.3970, 3093.4294, 3101.3831, 3103.4006, 3104.4041, 3110.4143, 3127.4749, 3141.4126, 3143.5066, 3144.5298, 3159.5210, 3219.4854, 3236.5574, 3266.6021, 3280.5635, 3321.6216, 3344.4895, 3347.5515, 3348.5020, 3380.5210, 3396.5195, 3412.5801, 3427.6099, 3428.5874, 3444.5840, 3462.6301, 3478.6123, 3484.6497, 3489.7246, 3505.6919, 3510.5859, 3521.6392, 3552.6614, 3553.6028, 3554.5630, 3558.6460, 3573.6545, 3589.6523, 3608.6155, 3624.6685, 3630.5806, 3641.7090, 3651.7944, 3667.7266, 3683.6980, 3691.7681, 3707.7200, 3714.5493, 3715.6206, 3719.6914, 3720.7048, 3723.7041, 3735.7100, 3751.7539, 3830.8091, 3869.7393, 3876.6707, 3885.6946

24. Q0VCW1 **Mass:** 42846 **Score:** 18 **Expect:** 4.2e+002 **Matches:** 17

Speckle-type POZ protein OS=Bos taurus GN=SPOP PE=2 SV=1

Observed	Mr(expt)	Mr(calc)	ppm	Start	End	Miss	Ions	Peptide
1336.7301	1335.7228	1335.7034	14.5	104 -	115	1	---	K.FSILNAKGEETK.A
1500.7393	1499.7320	1499.7555	-15.64	355 -	368	0	---	R.SLASAQCPFLGPPR.K
1500.7400	1499.7327	1499.7555	-15.17	355 -	368	0	---	R.SLASAQCPFLGPPR.K
1655.8147	1654.8074	1654.7733	20.6	111 -	124	2	---	K.GEETKAMESQRAYR.F
1781.8585	1780.8512	1780.8665	-8.58	269 -	284	1	---	K.MADDLLAAADKYALER.L + Oxidation (M)
1787.8407	1786.8334	1786.8771	-24.43	263 -	279	1	---	K.APNLDKMADDLLAAADK.Y + Oxidation (M)
2012.8951	2011.8878	2011.9019	-7.00	222 -	238	1	---	R.SPVFSAMFEHEMEESKK.N
2015.9694	2014.9621	2014.9153	23.2	46 -	64	1	---	R.EEMGEVIKSSTFSSGANDK.L
2055.0205	2054.0132	2054.0102	1.47	104 -	121	2	---	K.FSILNAKGEETKAMESQR.A + Oxidation (M)
2273.0735	2272.0662	2272.0893	-10.14	46 -	66	2	---	R.EEMGEVIKSSTFSSGANDKLK.W + Oxidation (M)
2804.2429	2803.2356	2803.2444	-3.14	32 -	53	1	---	K.FSYMWTINNFSFCREEMGEVIK.S + Oxidation (M)
2820.2441	2819.2368	2819.2393	-0.89	32 -	53	1	---	K.FSYMWTINNFSFCREEMGEVIK.S + 2 Oxidation (M)
3321.6216	3320.6143	3320.6910	-23.10	340 -	369	2	---	K.SMVVSHPHLVAEAYRSLASAQCPFLGPPRK.R + Oxidation (M)
3348.5020	3347.4947	3347.5764	-24.39	241 -	268	2	---	R.VEINDVEPEVFKEMMCFIYTGKAPNLDK.M + 2 Oxidation (M)
3558.6460	3557.6387	3557.7051	-18.66	287 -	318	0	---	K.VMCEDALCSNLSVENAAEILILADLHSADQLK.T + Oxidation (M)
3869.7393	3868.7320	3868.7171	3.85	32 -	64	2	---	K.FSYMWTINNFSFCREEMGEVIKSSTFSSGANDK.L
3885.6946	3884.6873	3884.7120	-6.36	32 -	64	2	---	K.FSYMWTINNFSFCREEMGEVIKSSTFSSGANDK.L + Oxidation (M)

No match to: 716.3353, 718.2895, 724.2175, 726.3070, 728.2833, 729.4336, 730.2399, 732.2518, 734.4341, 743.3130, 745.3617, 747.4360, 753.3469, 756.3524, 758.3462, 759.3632, 768.3517, 779.4633, 781.3725, 789.3640, 794.3674, 796.3701, 799.2854, 801.4280, 805.4417, 807.4076, 810.3293, 812.3207, 818.3782, 820.3700, 828.4415, 829.4508, 830.4513, 834.3805, 842.5101, 849.3834, 852.4368, 857.4457, 860.4262, 868.4412, 870.4462, 873.4370, 877.4166, 880.4280, 882.4470, 883.4816, 887.4295, 889.4857, 891.4872, 894.4923, 898.4610, 900.5174, 905.4763, 907.4251, 912.5086, 914.4942, 920.4296, 922.4855, 928.4565, 929.4484, 933.4327, 936.4976, 938.5062, 944.4982, 946.4672, 949.5244, 950.5168, 957.5792, 961.5109, 965.5151, 967.4963, 972.5023, 974.4913, 976.5255, 978.5352, 981.5225, 983.6221, 986.5463, 988.4922, 993.5123, 995.4512, 998.5833, 1000.5594, 1006.5120, 1007.5640, 1021.5762, 1024.5542, 1031.5300, 1034.5703, 1037.5466, 1041.5637, 1043.5297, 1044.5375, 1045.5594, 1046.6058, 1054.5287, 1056.5575, 1057.6187, 1057.6200, 1068.5790, 1074.6162, 1076.6190, 1082.6056, 1105.5938, 1110.5851, 1113.5701, 1118.5687, 1120.6050, 1122.5892, 1123.6047, 1126.6241, 1128.5618, 1130.5756, 1135.5951, 1137.5647, 1143.5592, 1145.6373, 1148.6318, 1149.6230, 1150.6226, 1156.5652, 1157.5676, 1159.5890, 1160.5966, 1169.5873, 1177.6421, 1178.6371, 1180.6443, 1182.6539, 1184.6431, 1185.6815, 1193.6090, 1194.6001, 1209.6238, 1213.7180, 1216.7178, 1221.5953, 1227.6459, 1229.6874, 1234.6899, 1238.2251, 1239.6622, 1254.6993, 1255.6351, 1264.7139, 1265.1680, 1265.7196, 1269.6532, 1276.6729, 1278.6892, 1281.6375, 1285.6674, 1289.7029, 1293.6760, 1301.6362, 1303.6489, 1304.6685, 1306.6946, 1308.6797, 1314.7227, 1319.6770, 1320.6364, 1323.6815, 1326.6937, 1328.6799, 1333.7430, 1337.6429, 1349.6729, 1351.6969, 1353.6891, 1356.6255, 1358.6919, 1359.7013, 1361.7147, 1364.7118, 1365.6619, 1366.6965, 1372.6228, 1375.6693, 1382.6771, 1383.7347, 1390.7089, 1393.8007, 1397.7449, 1403.7434, 1410.7849, 1416.7737, 1420.7114, 1423.7096, 1434.7847, 1436.7469, 1442.8257, 1445.7913, 1456.7555, 1457.7440, 1467.7560, 1470.7466, 1475.7615, 1479.8038, 1483.7579, 1484.7480, 1487.7660, 1488.7366, 1493.7556, 1503.7648, 1504.7708, 1514.7063, 1516.7281, 1519.7705, 1522.7722, 1524.8040, 1526.7924, 1528.7079, 1530.7058, 1536.7252, 1537.7859, 1540.8043, 1543.7938, 1550.7543, 1551.7465, 1553.7668, 1555.7836, 1556.8011, 1562.8008, 1568.7620, 1572.7245, 1578.8058, 1580.8580, 1580.8600, 1584.8486, 1587.7675, 1608.8051, 1616.8165, 1621.7870, 1625.7886, 1628.7313, 1632.8120, 1635.7964, 1637.7974, 1642.7417, 1647.8439, 1650.8429, 1651.8369, 1657.8483, 1658.8953, 1660.8379, 1662.8666, 1664.8490, 1666.7892, 1679.8091, 1680.8357, 1682.8409, 1696.8464, 1698.8180, 1701.8243, 1702.8386, 1707.8938, 1710.8904, 1714.7905, 1716.8219, 1717.9052, 1718.9094, 1721.8976, 1722.9003, 1724.9030, 1728.8840, 1730.8910, 1734.1617, 1738.8267, 1739.8165, 1741.8934, 1743.9005, 1747.9127, 1758.4807, 1758.8918, 1761.8734, 1765.8154, 1772.0012, 1774.9300, 1774.9319, 1779.9012, 1783.8062, 1809.0583, 1810.0460, 1812.9126, 1829.8527, 1832.8584, 1833.8571, 1834.8650, 1836.8618, 1838.8677, 1845.8363, 1847.8600, 1848.8938, 1850.9128, 1851.8932, 1861.8531, 1862.9542, 1864.9258, 1880.9026, 1883.7704, 1887.8878, 1897.7863, 1902.9769, 1904.9276, 1908.8986, 1920.9131, 1923.9126, 1928.0443, 1932.0068, 1936.9510, 1938.0171, 1947.9314, 1958.9393, 1959.9655, 1960.9813, 1961.9463, 1967.1549, 1969.9951, 1973.9971, 1975.9755, 1976.9708, 1990.4971, 1990.9373, 1991.4857, 1997.8479, 2001.8607, 2003.8768, 2004.8850, 2007.9500, 2007.9532, 2011.9381, 2017.9860, 2018.9740, 2020.1631, 2023.9482, 2026.9529, 2027.5033, 2029.9972, 2043.5356, 2044.9940, 2057.0154, 2060.9878, 2066.9939, 2068.9661, 2082.9822, 2086.0254, 2088.1033, 2097.0371, 2098.4941, 2102.9954, 2109.0095, 2112.9932, 2114.9707, 2122.9578, 2128.0444, 2130.9980, 2132.0200, 2136.9250, 2140.0063, 2143.0237, 2144.0559, 2145.0781, 2148.0483, 2153.0049, 2153.9973, 2174.0508, 2175.0435, 2178.0483, 2188.0610, 2191.0464, 2192.0427, 2199.1150, 2205.0615, 2211.1067, 2223.1533, 2225.1382, 2226.1472, 2239.1646, 2243.1448, 2246.9482, 2251.1108, 2255.1484, 2265.0837, 2271.1211, 2277.0642, 2283.1606, 2285.1355, 2286.0986, 2289.0901, 2291.0173, 2309.0349, 2328.9888, 2343.0608, 2352.9583, 2355.0383, 2366.9595, 2371.0325, 2392.1594, 2400.0532, 2424.1577, 2434.2429, 2438.2283, 2449.2180, 2450.2451, 2451.2480, 2465.1592, 2466.2458, 2467.2458, 2478.1377, 2501.2207, 2523.2014, 2526.2585, 2540.2451, 2542.2578, 2545.2258, 2550.2373, 2561.6841, 2562.1826, 2562.6890, 2573.2495, 2576.1792, 2646.9758, 2659.2144, 2661.1409, 2662.0120, 2671.1924, 2681.2705, 2682.2888, 2685.3098, 2689.3059, 2695.1064, 2696.2695, 2699.2324, 2703.2529, 2705.2944, 2712.2937, 2717.2903, 2720.2839, 2721.2937, 2726.2935, 2727.2986, 2728.3079, 2739.2837, 2743.2729, 2744.2207, 2745.2256, 2746.2031, 2748.2295, 2755.2700, 2760.2590, 2763.1829, 2771.2400, 2778.1865, 2785.2969, 2786.2561, 2787.2524, 2790.2317, 2792.2166, 2800.3093, 2801.2932, 2803.2415, 2807.2869, 2808.2227, 2812.2244, 2813.2373, 2817.2632, 2824.2598, 2828.2454, 2833.2544, 2843.2900, 2844.3398, 2847.3630, 2851.3313, 2864.3188, 2890.3293, 2896.4324, 2908.3105, 2910.3325, 2911.3594, 2912.4265, 2925.3323, 2927.4434, 3023.3674, 3025.3865, 3026.3452, 3039.3838, 3042.3542, 3070.3745, 3071.3933, 3086.3984, 3087.3970, 3093.4294, 3101.3831, 3103.4006, 3104.4041, 3110.4143, 3127.4749, 3141.4126, 3143.5066, 3144.5298, 3159.5210, 3219.4854, 3236.5574, 3266.6021, 3280.5635, 3344.4895, 3347.5515, 3380.5210, 3396.5195, 3412.5801, 3427.6099, 3428.5874, 3444.5840, 3462.6301, 3478.6123, 3484.6497, 3489.7246, 3505.6919, 3510.5859,

3521.6392, 3552.6614, 3553.6028, 3554.5630, 3573.6545, 3589.6523, 3608.6155, 3624.6685, 3630.5806, 3641.7090, 3651.7944, 3667.7266, 3683.6980, 3691.7681, 3707.7200, 3714.5493, 3715.6206, 3719.6914, 3720.7048, 3723.7041, 3735.7100, 3751.7539, 3830.8091, 3876.6707

25. Q95106 **Mass:** 28555 **Score:** 17 **Expect:** 4.5e+002 **Matches:** 15

Brain-derived neurotrophic factor OS=Bos taurus GN=BDNF PE=2 SV=2

Observed	Mr(expt)	Mr(calc)	ppm	Start	End	Miss	Ions	Peptide
781.3725	780.3652	780.3687	-4.51	220 -	226	0	---	R.ALTMDSK.K + Oxidation (M)
1118.5687	1117.5614	1117.5880	-23.78	28 -	38	0	---	R.AQGSLAYPGVR.T
1572.7245	1571.7172	1571.7184	-0.76	118 -	130	1	---	K.NYLDAANMSMRVR.R + 2 Oxidation (M)
1616.8165	1615.8092	1615.7876	13.4	213 -	226	1	---	R.TTQSYVRALTMDSK.K + Oxidation (M)
1651.8369	1650.8296	1650.8499	-12.25	157 -	172	1	---	K.KTAVDMSGGTVTVLEK.V + Oxidation (M)
1696.8464	1695.8391	1695.8297	5.57	118 -	131	2	---	K.NYLDAANMSMRVRR.H
1728.8840	1727.8767	1727.8876	-6.30	213 -	227	2	---	R.TTQSYVRALTMDSKK.R
1779.9012	1778.8939	1778.9131	-10.80	236 -	250	2	---	R.IDTSCVCTLTIKRGR.-
2011.9381	2010.9308	2010.9065	12.1	80 -	96	1	---	K.VRPSEENNKDADMYTSR.V
2055.0205	2054.0132	2054.0113	0.93	1 -	17	0	---	-.MTILFLTMVISYFGCMK.A
2102.9954	2101.9881	2101.9961	-3.78	1 -	17	0	---	-.MTILFLTMVISYFGCMK.A + 3 Oxidation (M)
2122.9578	2121.9505	2122.0001	-23.35	138 -	156	1	---	R.RGELSVCDSISEWVTAADK.K
2251.1108	2250.1035	2250.0950	3.77	138 -	157	2	---	R.RGELSVCDSISEWVTAADKK.T
2438.2283	2437.2210	2437.2282	-2.93	2 -	22	1	---	M.TILFLTMVISYFGCMKAAPMK.E + Oxidation (M)
2786.2561	2785.2488	2785.3089	-21.59	132 -	156	2	---	R.HSDPARRGELSVCDSISEWVTAADK.K

No match to: 716.3353, 718.2895, 724.2175, 726.3070, 728.2833, 729.4336, 730.2399, 732.2518, 734.4341, 743.3130, 745.3617, 747.4360, 753.3469, 756.3524, 758.3462, 759.3632, 768.3517, 779.4633, 789.3640, 794.3674, 796.3701, 799.2854, 801.4280, 805.4417, 807.4076, 810.3293, 812.3207, 818.3782, 820.3700, 828.4415, 829.4508, 830.4513, 834.3805, 842.5101, 849.3834, 852.4368, 857.4457, 860.4262, 868.4412, 870.4462, 873.4370, 877.4166, 880.4280, 882.4470, 883.4816, 887.4295, 889.4857, 891.4872, 894.4923, 898.4610, 900.5174, 905.4763, 907.4251, 912.5086, 914.4942, 920.4296, 922.4855, 928.4565, 929.4484, 933.4327, 936.4976, 938.5062, 944.4982, 946.4672, 949.5244, 950.5168, 957.5792, 961.5109, 965.5151, 967.4963, 972.5023, 974.4913, 976.5255, 978.5352, 981.5225, 983.6221, 986.5463, 988.4922, 993.5123, 995.4512, 998.5833, 1000.5594, 1006.5120, 1007.5640, 1021.5762, 1024.5542, 1031.5300, 1034.5703, 1037.5466, 1041.5637, 1043.5297, 1044.5375, 1045.5594, 1046.6058, 1054.5287, 1056.5575, 1057.6187, 1057.6200, 1068.5790, 1074.6162, 1076.6190, 1082.6056, 1105.5938, 1110.5851, 1113.5701, 1120.6050, 1122.5892, 1123.6047, 1126.6241, 1128.5618, 1130.5756, 1135.5951, 1137.5647, 1143.5592, 1145.6373, 1148.6318, 1149.6230, 1150.6226, 1156.5652, 1157.5676, 1159.5890, 1160.5966, 1169.5873, 1177.6421, 1178.6371, 1180.6443, 1182.6539, 1184.6431, 1185.6815, 1193.6090, 1194.6001, 1209.6238, 1213.7180, 1216.7178, 1221.5953, 1227.6459, 1229.6874, 1234.6899, 1238.2251, 1239.6622, 1254.6993, 1255.6351, 1264.7139, 1265.1680, 1265.7196, 1269.6532, 1276.6729, 1278.6892, 1281.6375, 1285.6674, 1289.7029, 1293.6760, 1301.6362, 1303.6489, 1304.6685, 1306.6946, 1308.6797, 1314.7227, 1319.6770, 1320.6364, 1323.6815, 1326.6937, 1328.6799, 1333.7430, 1336.7301, 1337.6429, 1349.6729, 1351.6969, 1353.6891, 1356.6255, 1358.6919, 1359.7013, 1361.7147, 1364.7118, 1365.6619, 1366.6965, 1372.6228, 1375.6693, 1382.6771, 1383.7347, 1390.7089, 1393.8007, 1397.7449, 1403.7434, 1410.7849, 1416.7737, 1420.7114, 1423.7096, 1434.7847, 1436.7469, 1442.8257, 1445.7913, 1456.7555, 1457.7440, 1467.7560, 1470.7466, 1475.7615, 1479.8038, 1483.7579, 1484.7480, 1487.7660, 1488.7366, 1493.7556, 1500.7393, 1500.7400, 1503.7648, 1504.7708, 1514.7063, 1516.7281, 1519.7705, 1522.7722, 1524.8040, 1526.7924, 1528.7079, 1530.7058, 1536.7252, 1537.7859, 1540.8043, 1543.7938, 1550.7543, 1551.7465, 1553.7668, 1555.7836, 1556.8011, 1562.8008, 1568.7620, 1578.8058, 1580.8580, 1580.8600, 1584.8486, 1587.7675, 1608.8051, 1621.7870, 1625.7886, 1628.7313, 1632.8120, 1635.7964, 1637.7974, 1642.7417, 1647.8439, 1650.8429, 1655.8147, 1657.8483, 1658.8953, 1660.8379, 1662.8666, 1664.8490, 1666.7892, 1679.8091, 1680.8357, 1682.8409, 1698.8180, 1701.8243, 1702.8386, 1707.8938, 1710.8904, 1714.7905, 1716.8219, 1717.9052, 1718.9094, 1721.8976, 1722.9003, 1724.9030, 1730.8910, 1734.1617, 1738.8267, 1739.8165, 1741.8934, 1743.9005, 1747.9127, 1758.4807, 1758.8918, 1761.8734, 1765.8154, 1772.0012, 1774.9300, 1774.9319, 1781.8585, 1783.8062, 1787.8407, 1809.0583, 1810.0460, 1812.9126, 1829.8527, 1832.8584, 1833.8571, 1834.8650, 1836.8618, 1838.8677, 1845.8363, 1847.8600, 1848.8938, 1850.9128, 1851.8932, 1861.8531, 1862.9542, 1864.9258, 1880.9026, 1883.7704, 1887.8878, 1897.7863, 1902.9769, 1904.9276, 1908.8986, 1920.9131, 1923.9126, 1928.0443, 1932.0068, 1936.9510, 1938.0171, 1947.9314, 1958.9393, 1959.9655, 1960.9813, 1961.9463, 1967.1549, 1969.9951, 1973.9971, 1975.9755, 1976.9708, 1990.4971, 1990.9373, 1991.4857, 1997.8479, 2001.8607, 2003.8768, 2004.8850, 2007.9500, 2007.9532, 2012.8951, 2015.9694, 2017.9860, 2018.9740, 2020.1631, 2023.9482, 2026.9529, 2027.5033, 2029.9972, 2043.5356, 2044.9940, 2057.0154, 2060.9878, 2066.9939, 2068.9661, 2082.9822, 2086.0254, 2088.1033, 2097.0371, 2098.4941, 2109.0095, 2112.9932, 2114.9707, 2128.0444, 2130.9980, 2132.0200, 2136.9250, 2140.0063, 2143.0237, 2144.0559, 2145.0781, 2148.0483, 2153.0049, 2153.9973, 2174.0508, 2175.0435, 2178.0483, 2188.0610, 2191.0464, 2192.0427, 2199.1150, 2205.0615, 2211.1067, 2223.1533, 2225.1382, 2226.1472, 2239.1646, 2243.1448, 2246.9482, 2255.1484, 2265.0837, 2271.1211, 2273.0735, 2277.0642, 2283.1606, 2285.1355, 2286.0986, 2289.0901, 2291.0173, 2309.0349, 2328.9888, 2343.0608, 2352.9583, 2355.0383, 2366.9595, 2371.0325, 2392.1594, 2400.0532, 2424.1577, 2434.2429, 2449.2180, 2450.2451, 2451.2480, 2465.1592, 2466.2458, 2467.2458, 2478.1377, 2501.2207, 2523.2014, 2526.2585, 2540.2451, 2542.2578, 2545.2258, 2550.2373, 2561.6841, 2562.1826, 2562.6890, 2573.2495, 2576.1792, 2646.9758, 2659.2144, 2661.1409, 2662.0120, 2671.1924, 2681.2705, 2682.2888, 2685.3098, 2689.3059, 2695.1064, 2696.2695, 2699.2324, 2703.2529, 2705.2944, 2712.2937, 2717.2903, 2720.2839, 2721.2937, 2726.2935, 2727.2986, 2728.3079, 2739.2837, 2743.2729, 2744.2207, 2745.2256, 2746.2031, 2748.2295, 2755.2700, 2760.2590, 2763.1829, 2771.2400, 2778.1865, 2785.2969, 2787.2524, 2790.2317, 2792.2166, 2800.3093, 2801.2932, 2803.2415, 2804.2429, 2807.2869, 2808.2227, 2812.2244, 2813.2373, 2817.2632, 2820.2441, 2824.2598, 2828.2454, 2833.2544, 2843.2900, 2844.3398, 2847.3630, 2851.3313, 2864.3188, 2890.3293, 2896.4324, 2908.3105, 2910.3325, 2911.3594, 2912.4265, 2925.3323, 2927.4434, 3023.3674, 3025.3865, 3026.3452, 3039.3838, 3042.3542, 3070.3745, 3071.3933, 3086.3984, 3087.3970, 3093.4294, 3101.3831, 3103.4006, 3104.4041, 3110.4143, 3127.4749, 3141.4126, 3143.5066, 3144.5298, 3159.5210, 3219.4854, 3236.5574, 3266.6021, 3280.5635, 3321.6216, 3344.4895, 3347.5515, 3348.5020, 3380.5210, 3396.5195, 3412.5801, 3427.6099, 3428.5874, 3444.5840, 3462.6301, 3478.6123, 3484.6497, 3489.7246, 3505.6919, 3510.5859, 3521.6392, 3552.6614, 3553.6028, 3554.5630, 3558.6460, 3573.6545, 3589.6523, 3608.6155, 3624.6685, 3630.5806, 3641.7090, 3651.7944, 3667.7266, 3683.6980, 3691.7681, 3707.7200, 3714.5493, 3715.6206, 3719.6914, 3720.7048, 3723.7041, 3735.7100, 3751.7539, 3830.8091, 3869.7393, 3876.6707, 3885.6946

26. G5E620 **Mass:** 21503 **Score:** 17 **Expect:** 4.6e+002 **Matches:** 11

Uncharacterized protein OS=Bos taurus GN=LOC101904345 PE=4 SV=1

Observed	Mr(expt)	Mr(calc)	ppm	Start	End	Miss	Ions	Peptide
820.3700	819.3627	819.3659	-3.88	160 -	165	0	---	R.MTMFYK.K
1356.6255	1355.6182	1355.6140	3.15	167 -	178	0	---	K.GQLQYDAMTSSR.M
1372.6228	1371.6155	1371.6089	4.85	167 -	178	0	---	K.GQLQYDAMTSSR.M + Oxidation (M)
1484.7480	1483.7407	1483.7089	21.4	166 -	178	1	---	K.KGQLQYDAMTSSR.M
1500.7393	1499.7320	1499.7038	18.8	166 -	178	1	---	K.KGQLQYDAMTSSR.M + Oxidation (M)
1500.7400	1499.7327	1499.7038	19.3	166 -	178	1	9	K.KGQLQYDAMTSSR.M + Oxidation (M)
1787.8407	1786.8334	1786.8737	-22.56	20 -	34	1	---	K.WLDVIDQGNEDDKIK.A
1990.9373	1989.9300	1989.9176	6.26	150 -	165	1	---	K.EQSLESEPVRMTMFYK.K + Oxidation (M)
2102.9954	2101.9881	2102.0176	-14.03	150 -	166	2	---	K.EQSLESEPVRMTMFYKK.G
2286.0986	2285.0913	2285.0643	11.8	160 -	178	2	---	R.MTMFYKKGQLQYDAMTSSR.M
2501.2207	2500.2134	2500.2705	-22.84	145 -	165	2	---	K.VAVIKEQSLESEPVRMTMFYK.K + Oxidation (M)

No match to: 716.3353, 718.2895, 724.2175, 726.3070, 728.2833, 729.4336, 730.2399, 732.2518, 734.4341, 743.3130, 745.3617, 747.4360, 753.3469, 756.3524, 758.3462, 759.3632, 768.3517, 779.4633, 781.3725, 789.3640, 794.3674, 796.3701, 799.2854, 801.4280, 805.4417, 807.4076, 810.3293, 812.3207, 818.3782, 828.4415, 829.4508, 830.4513, 834.3805, 842.5101, 849.3834, 852.4368, 857.4457, 860.4262, 868.4412, 870.4462, 873.4370, 877.4166, 880.4280, 882.4470, 883.4816, 887.4295, 889.4857, 891.4872, 894.4923, 898.4610, 900.5174, 905.4763, 907.4251, 912.5086, 914.4942, 920.4296, 922.4855, 928.4565, 929.4484, 933.4327, 936.4976, 938.5062, 944.4982, 946.4672, 949.5244, 950.5168, 957.5792, 961.5109, 965.5151, 967.4963, 972.5023, 974.4913, 976.5255, 978.5352, 981.5225, 983.6221, 986.5463, 988.4922, 993.5123, 995.4512, 998.5833, 1000.5594, 1006.5120, 1007.5640, 1021.5762, 1024.5542, 1031.5300, 1034.5703, 1037.5466, 1041.5637, 1043.5297, 1044.5375, 1045.5594, 1046.6058, 1054.5287, 1056.5575, 1057.6187, 1057.6200, 1068.5790, 1074.6162, 1076.6190, 1082.6056, 1105.5938, 1110.5851, 1113.5701, 1118.5687, 1120.6050, 1122.5892, 1123.6047, 1126.6241, 1128.5618, 1130.5756, 1135.5951, 1137.5647, 1143.5592, 1145.6373, 1148.6318, 1149.6230, 1150.6226, 1156.5652, 1157.5676, 1159.5890, 1160.5966, 1169.5873, 1177.6421, 1178.6371, 1180.6443, 1182.6539, 1184.6431, 1185.6815, 1193.6090, 1194.6001, 1209.6238, 1213.7180, 1216.7178, 1221.5953, 1227.6459, 1229.6874, 1234.6899, 1238.2251, 1239.6622, 1254.6993, 1255.6351, 1264.7139, 1265.1680, 1265.7196, 1269.6532, 1276.6729,

1278.6892, 1281.6375, 1285.6674, 1289.7029, 1293.6760, 1301.6362, 1303.6489, 1304.6685, 1306.6946, 1308.6797, 1314.7227, 1319.6770, 1320.6364, 1323.6815, 1326.6937, 1328.6799, 1333.7430, 1336.7301, 1337.6429, 1349.6729, 1351.6969, 1353.6891, 1358.6919, 1359.7013, 1361.7147, 1364.7118, 1365.6619, 1366.6965, 1375.6693, 1382.6771, 1383.7347, 1390.7089, 1393.8007, 1397.7449, 1403.7434, 1410.7849, 1416.7737, 1420.7114, 1423.7096, 1434.7847, 1436.7469, 1442.8257, 1445.7913, 1456.7555, 1457.7440, 1467.7560, 1470.7466, 1475.7615, 1479.8038, 1483.7579, 1487.7660, 1488.7366, 1493.7556, 1503.7648, 1504.7708, 1514.7063, 1516.7281, 1519.7705, 1522.7722, 1524.8040, 1526.7924, 1528.7079, 1530.7058, 1536.7252, 1537.7859, 1540.8043, 1543.7938, 1550.7543, 1551.7465, 1553.7668, 1555.7836, 1556.8011, 1562.8008, 1568.7620, 1572.7245, 1578.8058, 1580.8580, 1580.8600, 1584.8486, 1587.7675, 1608.8051, 1616.8165, 1621.7870, 1625.7886, 1628.7313, 1632.8120, 1635.7964, 1637.7974, 1642.7417, 1647.8439, 1650.8429, 1651.8369, 1655.8147, 1657.8483, 1658.8953, 1660.8379, 1662.8666, 1664.8490, 1666.7892, 1679.8091, 1680.8357, 1682.8409, 1696.8464, 1698.8180, 1701.8243, 1702.8386, 1707.8938, 1710.8904, 1714.7905, 1716.8219, 1717.9052, 1718.9094, 1721.8976, 1722.9003, 1724.9030, 1728.8840, 1730.8910, 1734.1617, 1738.8267, 1739.8165, 1741.8934, 1743.9005, 1747.9127, 1758.4807, 1758.8918, 1761.8734, 1765.8154, 1772.0012, 1774.9300, 1774.9319, 1779.9012, 1781.8585, 1783.8062, 1809.0583, 1810.0460, 1812.9126, 1829.8527, 1832.8584, 1833.8571, 1834.8650, 1836.8618, 1838.8677, 1845.8363, 1847.8600, 1848.8938, 1850.9128, 1851.8932, 1861.8531, 1862.9542, 1864.9258, 1880.9026, 1883.7704, 1887.8878, 1897.7863, 1902.9769, 1904.9276, 1908.8986, 1920.9131, 1923.9126, 1928.0443, 1932.0068, 1936.9510, 1938.0171, 1947.9314, 1958.9393, 1959.9655, 1960.9813, 1961.9463, 1967.1549, 1969.9951, 1973.9971, 1975.9755, 1976.9708, 1990.4971, 1991.4857, 1997.8479, 2001.8607, 2003.8768, 2004.8850, 2007.9500, 2007.9532, 2011.9381, 2012.8951, 2015.9694, 2017.9860, 2018.9740, 2020.1631, 2023.9482, 2026.9529, 2027.5033, 2029.9972, 2043.5356, 2044.9940, 2055.0205, 2057.0154, 2060.9878, 2066.9939, 2068.9661, 2082.9822, 2086.0254, 2088.1033, 2097.0371, 2098.4941, 2109.0095, 2112.9932, 2114.9707, 2122.9578, 2128.0444, 2130.9980, 2132.0200, 2136.9250, 2140.0063, 2143.0237, 2144.0559, 2145.0781, 2148.0483, 2153.0049, 2153.9973, 2174.0508, 2175.0435, 2178.0483, 2188.0610, 2191.0464, 2192.0427, 2199.1150, 2205.0615, 2211.1067, 2223.1533, 2225.1382, 2226.1472, 2239.1646, 2243.1448, 2246.9482, 2251.1108, 2255.1484, 2265.0837, 2271.1211, 2273.0735, 2277.0642, 2283.1606, 2285.1355, 2289.0901, 2291.0173, 2309.0349, 2328.9888, 2343.0608, 2352.9583, 2355.0383, 2366.9595, 2371.0325, 2392.1594, 2400.0532, 2424.1577, 2434.2429, 2438.2283, 2449.2180, 2450.2451, 2451.2480, 2465.1592, 2466.2458, 2467.2458, 2478.1377, 2523.2014, 2526.2585, 2540.2451, 2542.2578, 2545.2258, 2550.2373, 2561.6841, 2562.1826, 2562.6890, 2573.2495, 2576.1792, 2646.9758, 2659.2144, 2661.1409, 2662.0120, 2671.1924, 2681.2705, 2682.2888, 2685.3098, 2689.3059, 2695.1064, 2696.2695, 2699.2324, 2703.2529, 2705.2944, 2712.2937, 2717.2903, 2720.2839, 2721.2937, 2726.2935, 2727.2986, 2728.3079, 2739.2837, 2743.2729, 2744.2207, 2745.2256, 2746.2031, 2748.2295, 2755.2700, 2760.2590, 2763.1829, 2771.2400, 2778.1865, 2785.2969, 2786.2561, 2787.2524, 2790.2317, 2792.2166, 2800.3093, 2801.2932, 2803.2415, 2804.2429, 2807.2869, 2808.2227, 2812.2244, 2813.2373, 2817.2632, 2820.2441, 2824.2598, 2828.2454, 2833.2544, 2843.2900, 2844.3398, 2847.3630, 2851.3313, 2864.3188, 2890.3293, 2896.4324, 2908.3105, 2910.3325, 2911.3594, 2912.4265, 2925.3323, 2927.4434, 3023.3674, 3025.3865, 3026.3452, 3039.3838, 3042.3542, 3070.3745, 3071.3933, 3086.3984, 3087.3970, 3093.4294, 3101.3831, 3103.4006, 3104.4041, 3110.4143, 3127.4749, 3141.4126, 3143.5066, 3144.5298, 3159.5210, 3219.4854, 3236.5574, 3266.6021, 3280.5635, 3321.6216, 3344.4895, 3347.5515, 3348.5020, 3380.5210, 3396.5195, 3412.5801, 3427.6099, 3428.5874, 3444.5840, 3462.6301, 3478.6123, 3484.6497, 3489.7246, 3505.6919, 3510.5859, 3521.6392, 3552.6614, 3553.6028, 3554.5630, 3558.6460, 3573.6545, 3589.6523, 3608.6155, 3624.6685, 3630.5806, 3641.7090, 3651.7944, 3667.7266, 3683.6980, 3691.7681, 3707.7200, 3714.5493, 3715.6206, 3719.6914, 3720.7048, 3723.7041, 3735.7100, 3751.7539, 3830.8091, 3869.7393, 3876.6707, 3885.6946

27. Q0II95 **Mass:** 50922 **Score:** 17 **Expect:** 4.8e+002 **Matches:** 22

Inositol hexakisphosphate kinase 1 OS=Bos taurus GN=IP6K1 PE=2 SV=1

Observed	Mr(expt)	Mr(calc)	ppm	Start	End	Miss	Ions	Peptide
753.3469	752.3396	752.3487	-11.99	195	200	1	---	R.MRSESK.D + Oxidation (M)
781.3725	780.3652	780.3622	3.88	354	359	1	---	R.SEMRMK.H
1056.5575	1055.5502	1055.5294	19.7	187	194	1	---	R.CHKQQLSR.M
1349.6729	1348.6656	1348.6881	-16.66	190	200	2	---	K.QQLSRMRSESK.D
1359.7013	1358.6940	1358.6659	20.7	187	196	2	---	R.CHKQQLSRMR.S + Oxidation (M)
1365.6619	1364.6546	1364.6830	-20.80	190	200	2	---	K.QQLSRMRSESK.D + Oxidation (M)
1423.7096	1422.7023	1422.7076	-3.72	120	132	2	---	R.RSLHRSGSGSDHK.E
1436.7469	1435.7396	1435.7089	21.4	247	259	1	---	R.KCEQSTSATLGVR.V
1528.7079	1527.7006	1527.7099	-6.10	346	357	2	---	R.SESYLDRRSEMR.M
1556.8011	1555.7938	1555.7606	21.4	397	409	1	---	R.MIDFAHSTFKGFR.D
1572.7245	1571.7172	1571.7555	-24.35	397	409	1	---	R.MIDFAHSTFKGFR.D + Oxidation (M)
1851.8932	1850.8859	1850.9091	-12.51	244	259	2	---	R.QMRKCEQSTSATLGVR.V
1936.9510	1935.9437	1935.9400	1.91	330	345	1	---	R.FYSSSLLVIYDGKECR.S
2003.8768	2002.8695	2002.8659	1.80	2	18	1	---	M.CVCQTMEVGQYGKNASR.A + Oxidation (M)
2015.9694	2014.9621	2014.9856	-11.65	421	437	0	---	R.GYVFGLENLISIMEQMR.D + Oxidation (M)
2097.0371	2096.0298	2096.0578	-13.36	325	342	1	---	R.QASYRFYSSSLLVIYDGK.E
2285.1355	2284.1282	2284.1434	-6.63	133	153	2	---	K.EEKASLPLETSESPQEAKSPK.V
2526.2585	2525.2512	2525.2268	9.69	19	41	1	---	R.AGDRGVLLEPFIHQVGGHSSMMR.Y + 2 Oxidation (M)
2542.2578	2541.2505	2541.2322	7.22	325	345	2	---	R.QASYRFYSSSLLVIYDGKECR.S
2728.3079	2727.3006	2727.2835	6.29	125	150	2	---	R.SGSGSDHKEEKASLPLETSESPQEAK.S
2925.3323	2924.3250	2924.3974	-24.76	151	177	1	---	K.SPKVELHSHSDVPFQMLDGNSGLSSEK.I
3236.5574	3235.5501	3235.4914	18.1	410	437	1	---	R.DDPTVHDGPDRGYVFGLENLISIMEQMR.D + 2 Oxidation (M)

No match to: 716.3353, 718.2895, 724.2175, 726.3070, 728.2833, 729.4336, 730.2399, 732.2518, 734.4341, 743.3130, 745.3617, 747.4360, 756.3524, 758.3462, 759.3632, 768.3517, 779.4633, 789.3640, 794.3674, 796.3701, 799.2854, 801.4280, 805.4417, 807.4076, 810.3293, 812.3207, 818.3782, 820.3700, 828.4415, 829.4508, 830.4513, 834.3805, 842.5101, 849.3834, 852.4368, 857.4457, 860.4262, 868.4412, 870.4462, 873.4370, 877.4166, 880.4280, 882.4470, 883.4816, 887.4295, 889.4857, 891.4872, 894.4923, 898.4610, 900.5174, 905.4763, 907.4251, 912.5086, 914.4942, 920.4296, 922.4855, 928.4565, 929.4484, 933.4327, 936.4976, 938.5062, 944.4982, 946.4672, 949.5244, 950.5168, 957.5792, 961.5109, 965.5151, 967.4963, 972.5023, 974.4913, 976.5255, 978.5352, 981.5225, 983.6221, 986.5463, 988.4922, 993.5123, 995.4512, 998.5833, 1000.5594, 1006.5120, 1007.5640, 1021.5762, 1024.5542, 1031.5300, 1034.5703, 1037.5466, 1041.5637, 1043.5297, 1044.5375, 1045.5594, 1046.6058, 1054.5287, 1057.6187, 1057.6200, 1068.5790, 1074.6162, 1076.6190, 1082.6056, 1105.5938, 1110.5851, 1113.5701, 1118.5687, 1120.6050, 1122.5892, 1123.6047, 1126.6241, 1128.5618, 1130.5756, 1135.5951, 1137.5647, 1143.5592, 1145.6373, 1148.6318, 1149.6230, 1150.6226, 1156.5652, 1157.5676, 1159.5890, 1160.5966, 1169.5873, 1177.6421, 1178.6371, 1180.6443, 1182.6539, 1184.6431, 1185.6815, 1193.6090, 1194.6001, 1209.6238, 1213.7180, 1216.7178, 1221.5953, 1227.6459, 1229.6874, 1234.6899, 1238.2251, 1239.6622, 1254.6993, 1255.6351, 1264.7139, 1265.1680, 1265.7196, 1269.6532, 1276.6729, 1278.6892, 1281.6375, 1285.6674, 1289.7029, 1293.6760, 1301.6362, 1303.6489, 1304.6685, 1306.6946, 1308.6797, 1314.7227, 1319.6770, 1320.6364, 1323.6815, 1326.6937, 1328.6799, 1333.7430, 1336.7301, 1337.6429, 1351.6969, 1353.6891, 1356.6255, 1358.6919, 1361.7147, 1364.7118, 1366.6965, 1372.6228, 1375.6693, 1382.6771, 1383.7347, 1390.7089, 1393.8007, 1397.7449, 1403.7434, 1410.7849, 1416.7737, 1420.7114, 1434.7847, 1442.8257, 1445.7913, 1456.7555, 1457.7440, 1467.7560, 1470.7466, 1475.7615, 1479.8038, 1483.7579, 1484.7480, 1487.7660, 1488.7366, 1493.7556, 1500.7393, 1500.7400, 1503.7648, 1504.7708, 1514.7063, 1516.7281, 1519.7705, 1522.7722, 1524.8040, 1526.7924, 1530.7058, 1536.7252, 1537.7859, 1540.8043, 1543.7938, 1550.7543, 1551.7465, 1553.7668, 1555.7836, 1562.8008, 1568.7620, 1578.8058, 1580.8580, 1580.8600, 1584.8486, 1587.7675, 1608.8051, 1616.8165, 1621.7870, 1625.7886, 1628.7313, 1632.8120, 1635.7964, 1637.7974, 1642.7417, 1647.8439, 1650.8429, 1651.8369, 1655.8147, 1657.8483, 1658.8953, 1660.8379, 1662.8666, 1664.8490, 1666.7892, 1679.8091, 1680.8357, 1682.8409, 1696.8464, 1698.8180, 1701.8243, 1702.8386, 1707.8938, 1710.8904, 1714.7905, 1716.8219, 1717.9052, 1718.9094, 1721.8976, 1722.9003, 1724.9030, 1728.8840, 1730.8910, 1734.1617, 1738.8267, 1739.8165, 1741.8934, 1743.9005, 1747.9127, 1758.4807, 1758.8918, 1761.8734, 1765.8154, 1772.0012, 1774.9300, 1774.9319, 1779.9012, 1781.8585, 1783.8062, 1787.8407, 1809.0583, 1810.0460, 1812.9126, 1829.8527, 1832.8584, 1833.8571, 1834.8650, 1836.8618, 1838.8677, 1845.8363, 1847.8600, 1848.8938, 1850.9128, 1861.8531, 1862.9542, 1864.9258, 1880.9026, 1883.7704, 1887.8878, 1897.7863, 1902.9769, 1904.9276, 1908.8986, 1920.9131, 1923.9126, 1928.0443, 1932.0068, 1938.0171, 1947.9314, 1958.9393, 1959.9655, 1960.9813, 1961.9463, 1967.1549, 1969.9951, 1973.9971, 1975.9755, 1976.9708, 1990.4971, 1990.9373, 1991.4857, 1997.8479, 2001.8607, 2004.8850, 2007.9500, 2007.9532, 2011.9381, 2012.8951, 2017.9860, 2018.9740, 2020.1631, 2023.9482, 2026.9529, 2027.5033, 2029.9972, 2043.5356, 2044.9940, 2055.0205, 2057.0154, 2060.9878, 2066.9939, 2068.9661, 2082.9822, 2086.0254, 2088.1033, 2098.4941, 2102.9954, 2109.0095, 2112.9932, 2114.9707, 2122.9578, 2128.0444, 2130.9980, 2132.0200, 2136.9250, 2140.0063, 2143.0237, 2144.0559, 2145.0781, 2148.0483, 2153.0049, 2153.9973, 2174.0508, 2175.0435, 2178.0483, 2188.0610, 2191.0464, 2192.0427, 2199.1150, 2205.0615, 2211.1067, 2223.1533, 2225.1382, 2226.1472, 2239.1646, 2243.1448, 2246.9482, 2251.1108, 2255.1484, 2265.0837, 2271.1211, 2273.0735, 2277.0642, 2283.1606, 2286.0986, 2289.0901, 2291.0173, 2309.0349, 2328.9888, 2343.0608, 2352.9583, 2355.0383, 2366.9595, 2371.0325, 2392.1594, 2400.0532, 2424.1577, 2434.2429, 2438.2283, 2449.2180, 2450.2451, 2451.2480, 2465.1592, 2466.2458, 2467.2458, 2478.1377, 2501.2207, 2523.2014, 2540.2451, 2545.2258, 2550.2373, 2561.6841, 2562.1826, 2562.6890, 2573.2495, 2576.1792, 2646.9758, 2659.2144, 2661.1409, 2662.0120, 2671.1924, 2681.2705, 2682.2888, 2685.3098, 2689.3059, 2695.1064, 2696.2695, 2699.2324, 2703.2529, 2705.2944, 2712.2937, 2717.2903, 2720.2839, 2721.2937, 2726.2935, 2727.2986, 2739.2837, 2743.2729, 2744.2207,

2745.2256, 2746.2031, 2748.2295, 2755.2700, 2760.2590, 2763.1829, 2771.2400, 2778.1865, 2785.2969, 2786.2561, 2787.2524, 2790.2317, 2792.2166, 2800.3093, 2801.2932, 2803.2415, 2804.2429, 2807.2869, 2808.2227, 2812.2244, 2813.2373, 2817.2632, 2820.2441, 2824.2598, 2828.2454, 2833.2544, 2843.2900, 2844.3398, 2847.3630, 2851.3313, 2864.3188, 2890.3293, 2896.4324, 2908.3105, 2910.3325, 2911.3594, 2912.4265, 2927.4434, 3023.3674, 3025.3865, 3026.3452, 3039.3838, 3042.3542, 3070.3745, 3071.3933, 3086.3984, 3087.3970, 3093.4294, 3101.3831, 3103.4006, 3104.4041, 3110.4143, 3127.4749, 3141.4126, 3143.5066, 3144.5298, 3159.5210, 3219.4854, 3266.6021, 3280.5635, 3321.6216, 3344.4895, 3347.5515, 3348.5020, 3380.5210, 3396.5195, 3412.5801, 3427.6099, 3428.5874, 3444.5840, 3462.6301, 3478.6123, 3484.6497, 3489.7246, 3505.6919, 3510.5859, 3521.6392, 3552.6614, 3553.6028, 3554.5630, 3558.6460, 3573.6545, 3589.6523, 3608.6155, 3624.6685, 3630.5806, 3641.7090, 3651.7944, 3667.7266, 3683.6980, 3691.7681, 3707.7200, 3714.5493, 3715.6206, 3719.6914, 3720.7048, 3723.7041, 3735.7100, 3751.7539, 3830.8091, 3869.7393, 3876.6707, 3885.6946

28. F6QWT9 **Mass:** 13281 **Score:** 17 **Expect:** 4.8e+002 **Matches:** 5

Uncharacterized protein OS=Bos taurus GN=FAM229A PE=4 SV=1

Observed	Mr(expt)	Mr(calc)	ppm	Start	End	Miss	Ions	Peptide
1445.7913	1444.7840	1444.7746	6.52	31 -	46	1	---	R.ARAAASSLGLASASGR.A
2847.3630	2846.3557	2846.3046	18.0	100 -	124	0	---	R.CPGCHCLTLLPMPIDVYLAMGGSPR.A + 2 Oxidation (M)
3039.3838	3038.3765	3038.4451	-22.56	1 -	30	1	---	-.MQPSPSTPGPGRAADTCPAPPGPERPPAAR.A + Oxidation (M)
3266.6021	3265.5948	3265.5833	3.53	1 -	32	2	---	-.MQPSPSTPGPGRAADTCPAPPGPERPPAARAR.A + Oxidation (M)
3651.7944	3650.7871	3650.7965	-2.56	64 -	98	0	---	R.FPIEAGDSPGLAAAPESQDSPEPVATEHNPVRPLR.R

No match to: 716.3353, 718.2895, 724.2175, 726.3070, 728.2833, 729.4336, 730.2399, 732.2518, 734.4341, 743.3130, 745.3617, 747.4360, 753.3469, 756.3524, 758.3462, 759.3632, 768.3517, 779.4633, 781.3725, 789.3640, 794.3674, 796.3701, 799.2854, 801.4280, 805.4417, 807.4076, 810.3293, 812.3207, 818.3782, 820.3700, 828.4415, 829.4508, 830.4513, 834.3805, 842.5101, 849.3834, 852.4368, 857.4457, 860.4262, 868.4412, 870.4462, 873.4370, 877.4166, 880.4280, 882.4470, 883.4816, 887.4295, 889.4857, 891.4872, 894.4923, 898.4610, 900.5174, 905.4763, 907.4251, 912.5086, 914.4942, 920.4296, 922.4855, 928.4565, 929.4484, 933.4327, 936.4976, 938.5062, 944.4982, 946.4672, 949.5244, 950.5168, 957.5792, 961.5109, 965.5151, 967.4963, 972.5023, 974.4913, 976.5255, 978.5352, 981.5225, 983.6221, 986.5463, 988.4922, 993.5123, 995.4512, 998.5833, 1000.5594, 1006.5120, 1007.5640, 1021.5762, 1024.5542, 1031.5300, 1034.5703, 1037.5466, 1041.5637, 1043.5297, 1044.5375, 1045.5594, 1046.6058, 1054.5287, 1056.5575, 1057.6187, 1057.6200, 1068.5790, 1074.6162, 1076.6190, 1082.6056, 1105.5938, 1110.5851, 1113.5701, 1118.5687, 1120.6050, 1122.5892, 1123.6047, 1126.6241, 1128.5618, 1130.5756, 1135.5951, 1137.5647, 1143.5592, 1145.6373, 1148.6318, 1149.6230, 1150.6226, 1156.5652, 1157.5676, 1159.5890, 1160.5966, 1169.5873, 1177.6421, 1178.6371, 1180.6443, 1182.6539, 1184.6431, 1185.6815, 1193.6090, 1194.6001, 1209.6238, 1213.7180, 1216.7178, 1221.5953, 1227.6459, 1229.6874, 1234.6899, 1238.2251, 1239.6622, 1254.6993, 1255.6351, 1264.7139, 1265.1680, 1265.7196, 1269.6532, 1276.6729, 1278.6892, 1281.6375, 1285.6674, 1289.7029, 1293.6760, 1301.6362, 1303.6489, 1304.6685, 1306.6946, 1308.6797, 1314.7227, 1319.6770, 1320.6364, 1323.6815, 1326.6937, 1328.6799, 1333.7430, 1336.7301, 1337.6429, 1349.6729, 1351.6969, 1353.6891, 1356.6255, 1358.6919, 1359.7013, 1361.7147, 1364.7118, 1365.6619, 1366.6965, 1372.6228, 1375.6693, 1382.6771, 1383.7347, 1390.7089, 1393.8007, 1397.7449, 1403.7434, 1410.7849, 1416.7737, 1420.7114, 1423.7096, 1434.7847, 1436.7469, 1442.8257, 1456.7555, 1457.7440, 1467.7560, 1470.7466, 1475.7615, 1479.8038, 1483.7579, 1484.7480, 1487.7660, 1488.7366, 1493.7556, 1500.7393, 1500.7400, 1503.7648, 1504.7708, 1514.7063, 1516.7281, 1519.7705, 1522.7722, 1524.8040, 1526.7924, 1528.7079, 1530.7058, 1536.7252, 1537.7859, 1540.8043, 1543.7938, 1550.7543, 1551.7465, 1553.7668, 1555.7836, 1556.8011, 1562.8008, 1568.7620, 1572.7245, 1578.8058, 1580.8580, 1580.8600, 1584.8486, 1587.7675, 1608.8051, 1616.8165, 1621.7870, 1625.7886, 1628.7313, 1632.8120, 1635.7964, 1637.7974, 1642.7417, 1647.8439, 1650.8429, 1651.8369, 1655.8147, 1657.8483, 1658.8953, 1660.8379, 1662.8666, 1664.8490, 1666.7892, 1679.8091, 1680.8357, 1682.8409, 1696.8464, 1698.8180, 1701.8243, 1702.8386, 1707.8938, 1710.8904, 1714.7905, 1716.8219, 1717.9052, 1718.9094, 1721.8976, 1722.9003, 1724.9030, 1728.8840, 1730.8910, 1734.1617, 1738.8267, 1739.8165, 1741.8934, 1743.9005, 1747.9127, 1758.4807, 1758.8918, 1761.8734, 1765.8154, 1772.0012, 1774.9300, 1774.9319, 1779.9012, 1781.8585, 1783.8062, 1787.8407, 1809.0583, 1810.0460, 1812.9126, 1829.8527, 1832.8584, 1833.8571, 1834.8650, 1836.8618, 1838.8677, 1845.8363, 1847.8600, 1848.8938, 1850.9128, 1851.8932, 1861.8531, 1862.9542, 1864.9258, 1880.9026, 1883.7704, 1887.8878, 1897.7863, 1902.9769, 1904.9276, 1908.8986, 1920.9131, 1923.9126, 1928.0443, 1932.0068, 1936.9510, 1938.0171, 1947.9314, 1958.9393, 1959.9655, 1960.9813, 1961.9463, 1967.1549, 1969.9951, 1973.9971, 1975.9755, 1976.9708, 1990.4971, 1990.9373, 1991.4857, 1997.8479, 2001.8607, 2003.8768, 2004.8850, 2007.9500, 2007.9532, 2011.9381, 2012.8951, 2015.9694, 2017.9860, 2018.9740, 2020.1631, 2023.9482, 2026.9529, 2027.5033, 2029.9972, 2043.5356, 2044.9940, 2055.0205, 2057.0154, 2060.9878, 2066.9939, 2068.9661, 2082.9822, 2086.0254, 2088.1033, 2097.0371, 2098.4941, 2102.9954, 2109.0095, 2112.9932, 2114.9707, 2122.9578, 2128.0444, 2130.9980, 2132.0200, 2136.9250, 2140.0063, 2143.0237, 2144.0559, 2145.0781, 2148.0483, 2153.0049, 2153.9973, 2174.0508, 2175.0435, 2178.0483, 2188.0610, 2191.0464, 2192.0427, 2199.1150, 2205.0615, 2211.1067, 2223.1533, 2225.1382, 2226.1472, 2239.1646, 2243.1448, 2246.9482, 2251.1108, 2255.1484, 2265.0837, 2271.1211, 2273.0735, 2277.0642, 2283.1606, 2285.1355, 2286.0986, 2289.0901, 2291.0173, 2309.0349, 2328.9888, 2343.0608, 2352.9583, 2355.0383, 2366.9595, 2371.0325, 2392.1594, 2400.0532, 2424.1577, 2434.2429, 2438.2283, 2449.2180, 2450.2451, 2451.2480, 2465.1592, 2466.2458, 2467.2458, 2478.1377, 2501.2207, 2523.2014, 2526.2585, 2540.2451, 2542.2578, 2545.2258, 2550.2373, 2561.6841, 2562.1826, 2562.6890, 2573.2495, 2576.1792, 2646.9758, 2659.2144, 2661.1409, 2662.0120, 2671.1924, 2681.2705, 2682.2888, 2685.3098, 2689.3059, 2695.1064, 2696.2695, 2699.2324, 2703.2529, 2705.2944, 2712.2937, 2717.2903, 2720.2839, 2721.2937, 2726.2935, 2727.2986, 2728.3079, 2739.2837, 2743.2729, 2744.2207, 2745.2256, 2746.2031, 2748.2295, 2755.2700, 2760.2590, 2763.1829, 2771.2400, 2778.1865, 2785.2969, 2786.2561, 2787.2524, 2790.2317, 2792.2166, 2800.3093, 2801.2932, 2803.2415, 2804.2429, 2807.2869, 2808.2227, 2812.2244, 2813.2373, 2817.2632, 2820.2441, 2824.2598, 2828.2454, 2833.2544, 2843.2900, 2844.3398, 2851.3313, 2864.3188, 2890.3293, 2896.4324, 2908.3105, 2910.3325, 2911.3594, 2912.4265, 2925.3323, 2927.4434, 3023.3674, 3025.3865, 3026.3452, 3042.3542, 3070.3745, 3071.3933, 3086.3984, 3087.3970, 3093.4294, 3101.3831, 3103.4006, 3104.4041, 3110.4143, 3127.4749, 3141.4126, 3143.5066, 3144.5298, 3159.5210, 3219.4854, 3236.5574, 3280.5635, 3321.6216, 3344.4895, 3347.5515, 3348.5020, 3380.5210, 3396.5195, 3412.5801, 3427.6099, 3428.5874, 3444.5840, 3462.6301, 3478.6123, 3484.6497, 3489.7246, 3505.6919, 3510.5859, 3521.6392, 3552.6614, 3553.6028, 3554.5630, 3558.6460, 3573.6545, 3589.6523, 3608.6155, 3624.6685, 3630.5806, 3641.7090, 3667.7266, 3683.6980, 3691.7681, 3707.7200, 3714.5493, 3715.6206, 3719.6914, 3720.7048, 3723.7041, 3735.7100, 3751.7539, 3830.8091, 3869.7393, 3876.6707, 3885.6946

29. A4FV50 **Mass:** 40277 **Score:** 17 **Expect:** 4.8e+002 **Matches:** 17

MGC142792 protein OS=Bos taurus GN=MGC142792 PE=2 SV=1

Observed	Mr(expt)	Mr(calc)	ppm	Start	End	Miss	Ions	Peptide
1143.5592	1142.5519	1142.5655	-11.87	234 -	242	0	---	K.CEKPFLGHR.H
1145.6373	1144.6300	1144.6088	18.6	179 -	188	1	---	K.ELTADARELK.G
1326.6937	1325.6864	1325.6689	13.2	315 -	324	1	---	K.CYEKFPLELK.K
1543.7938	1542.7865	1542.7575	18.8	303 -	314	1	---	K.FVEFDMKPVCKK.C + Oxidation (M)
1657.8483	1656.8410	1656.8004	24.5	301 -	313	1	---	K.NKFVEFDMKPVCK.K + Oxidation (M)
1761.8734	1760.8661	1760.8226	24.7	186 -	199	1	---	R.ELKGELYCLPCHDK.M
1904.9276	1903.9203	1903.9118	4.47	124 -	138	2	---	K.NAGRHLCRPCHNREK.A
2140.0063	2138.9990	2139.0506	-24.13	216 -	233	1	---	R.VVNAMGKQWHVEHFVCAK.C
2355.0383	2354.0310	2354.0413	-4.35	2 -	24	0	---	M.LGVASGMTNSNMANALANASCER.C + Oxidation (M)
2371.0325	2370.0252	2370.0362	-4.63	2 -	24	0	---	M.LGVASGMTNSNMANALANASCER.C + 2 Oxidation (M)
2671.1924	2670.1851	2670.1730	4.53	2 -	26	1	---	M.LGVASGMTNSNMANALANASCERCR.G + Oxidation (M)
2786.2561	2785.2488	2785.2186	10.9	1 -	26	1	---	-.MLGVASGMTNSNMANALANASCERCR.G
3280.5635	3279.5562	3279.6005	-13.49	216 -	242	2	---	R.VVNAMGKQWHVEHFVCAKCEKPFLGHR.H + Oxidation (M)
3412.5801	3411.5728	3411.5540	5.52	2 -	34	2	---	M.LGVASGMTNSNMANALANASCERCRGGFAPAEK.I
3428.5874	3427.5801	3427.5489	9.10	2 -	34	2	---	M.LGVASGMTNSNMANALANASCERCRGGFAPAEK.I + Oxidation (M)
3444.5840	3443.5767	3443.5438	9.55	2 -	34	2	---	M.LGVASGMTNSNMANALANASCERCRGGFAPAEK.I + 2 Oxidation (M)
3510.5859	3509.5786	3509.5703	2.36	150 -	178	1	---	K.CHAIIDEQPLIFKNDPYHPDHFNCANCGK.E

No match to: 716.3353, 718.2895, 724.2175, 726.3070, 728.2833, 729.4336, 730.2399, 732.2518, 734.4341, 743.3130, 745.3617, 747.4360, 753.3469, 756.3524, 758.3462, 759.3632, 768.3517, 779.4633, 781.3725, 789.3640, 794.3674, 796.3701, 799.2854, 801.4280, 805.4417, 807.4076, 810.3293, 812.3207, 818.3782, 820.3700, 828.4415, 829.4508, 830.4513, 834.3805, 842.5101, 849.3834, 852.4368, 857.4457, 860.4262, 868.4412, 870.4462, 873.4370, 877.4166, 880.4280, 882.4470, 883.4816, 887.4295, 889.4857, 891.4872, 894.4923, 898.4610, 900.5174, 905.4763, 907.4251, 912.5086, 914.4942, 920.4296, 922.4855, 928.4565, 929.4484, 933.4327, 936.4976, 938.5062, 944.4982, 946.4672, 949.5244, 950.5168, 957.5792, 961.5109, 965.5151, 967.4963, 972.5023, 974.4913, 976.5255, 978.5352, 981.5225, 983.6221, 986.5463, 988.4922, 993.5123, 995.4512, 998.5833, 1000.5594, 1006.5120, 1007.5640, 1021.5762, 1024.5542, 1031.5300, 1034.5703, 1037.5466, 1041.5637, 1043.5297, 1044.5375, 1045.5594, 1046.6058, 1054.5287, 1056.5575, 1057.6187, 1057.6200, 1068.5790, 1074.6162, 1076.6190, 1082.6056, 1105.5938, 1110.5851, 1113.5701, 1118.5687, 1120.6050, 1122.5892, 1123.6047, 1126.6241, 1128.5618, 1130.5756, 1135.5951, 1137.5647, 1148.6318, 1149.6230, 1150.6226, 1156.5652, 1157.5676, 1159.5890, 1160.5966, 1169.5873, 1177.6421,

1178.6371, 1180.6443, 1182.6539, 1184.6431, 1185.6815, 1193.6090, 1194.6001, 1209.6238, 1213.7180, 1216.7178, 1221.5953, 1227.6459, 1229.6874, 1234.6899, 1238.2251, 1239.6622, 1254.6993, 1255.6351, 1264.7139, 1265.1680, 1265.7196, 1269.6532, 1276.6729, 1278.6892, 1281.6375, 1285.6674, 1289.7029, 1293.6760, 1301.6362, 1303.6489, 1304.6685, 1306.6946, 1308.6797, 1314.7227, 1319.6770, 1320.6364, 1323.6815, 1328.6799, 1333.7430, 1336.7301, 1337.6429, 1349.6729, 1351.6969, 1353.6891, 1356.6255, 1358.6919, 1359.7013, 1361.7147, 1364.7118, 1365.6619, 1366.6965, 1372.6228, 1375.6693, 1382.6771, 1383.7347, 1390.7089, 1393.8007, 1397.7449, 1403.7434, 1410.7849, 1416.7737, 1420.7114, 1423.7096, 1434.7847, 1436.7469, 1442.8257, 1445.7913, 1456.7555, 1457.7440, 1467.7560, 1470.7466, 1475.7615, 1479.8038, 1483.7579, 1484.7480, 1487.7660, 1488.7366, 1493.7556, 1500.7393, 1500.7400, 1503.7648, 1504.7708, 1514.7063, 1516.7281, 1519.7705, 1522.7722, 1524.8040, 1526.7924, 1528.7079, 1530.7058, 1536.7252, 1537.7859, 1540.8043, 1550.7543, 1551.7465, 1553.7668, 1555.7836, 1556.8011, 1562.8008, 1568.7620, 1572.7245, 1578.8058, 1580.8580, 1580.8600, 1584.8486, 1587.7675, 1608.8051, 1616.8165, 1621.7870, 1625.7886, 1628.7313, 1632.8120, 1635.7964, 1637.7974, 1642.7417, 1647.8439, 1650.8429, 1651.8369, 1655.8147, 1658.8953, 1660.8379, 1662.8666, 1664.8490, 1666.7892, 1679.8091, 1680.8357, 1682.8409, 1696.8464, 1698.8180, 1701.8243, 1702.8386, 1707.8938, 1710.8904, 1714.7905, 1716.8219, 1717.9052, 1718.9094, 1721.8976, 1722.9003, 1724.9030, 1728.8840, 1730.8910, 1734.1617, 1738.8267, 1739.8165, 1741.8934, 1743.9005, 1747.9127, 1758.4807, 1758.8918, 1765.8154, 1772.0012, 1774.9300, 1774.9319, 1779.9012, 1781.8585, 1783.8062, 1787.8407, 1809.0583, 1810.0460, 1812.9126, 1829.8527, 1832.8584, 1833.8571, 1834.8650, 1836.8618, 1838.8677, 1845.8363, 1847.8600, 1848.8938, 1850.9128, 1851.8932, 1861.8531, 1862.9542, 1864.9258, 1880.9026, 1883.7704, 1887.8878, 1897.7863, 1902.9769, 1908.8986, 1920.9131, 1923.9126, 1928.0443, 1932.0068, 1936.9510, 1938.0171, 1947.9314, 1958.9393, 1959.9655, 1960.9813, 1961.9463, 1967.1549, 1969.9951, 1973.9971, 1975.9755, 1976.9708, 1990.4971, 1990.9373, 1991.4857, 1997.8479, 2001.8607, 2003.8768, 2004.8850, 2007.9500, 2007.9532, 2011.9381, 2012.8951, 2015.9694, 2017.9860, 2018.9740, 2020.1631, 2023.9482, 2026.9529, 2027.5033, 2029.9972, 2043.5356, 2044.9940, 2055.0205, 2057.0154, 2060.9878, 2066.9939, 2068.9661, 2082.9822, 2086.0254, 2088.1033, 2097.0371, 2098.4941, 2102.9954, 2109.0095, 2112.9932, 2114.9707, 2122.9578, 2128.0444, 2130.9980, 2132.0200, 2136.9250, 2143.0237, 2144.0559, 2145.0781, 2148.0483, 2153.0049, 2153.9973, 2174.0508, 2175.0435, 2178.0483, 2188.0610, 2191.0464, 2192.0427, 2199.1150, 2205.0615, 2211.1067, 2223.1533, 2225.1382, 2226.1472, 2239.1646, 2243.1448, 2246.9482, 2251.1108, 2255.1484, 2265.0837, 2271.1211, 2273.0735, 2277.0642, 2283.1606, 2285.1355, 2286.0986, 2289.0901, 2291.0173, 2309.0349, 2328.9888, 2343.0608, 2352.9583, 2366.9595, 2392.1594, 2400.0532, 2424.1577, 2434.2429, 2438.2283, 2449.2180, 2450.2451, 2451.2480, 2465.1592, 2466.2458, 2467.2458, 2478.1377, 2501.2207, 2523.2014, 2526.2585, 2540.2451, 2542.2578, 2545.2258, 2550.2373, 2561.6841, 2562.1826, 2562.6890, 2573.2495, 2576.1792, 2646.9758, 2659.2144, 2661.1409, 2662.0120, 2681.2705, 2682.2888, 2685.3098, 2689.3059, 2695.1064, 2696.2695, 2699.2324, 2703.2529, 2705.2944, 2712.2937, 2717.2903, 2720.2839, 2721.2937, 2726.2935, 2727.2986, 2728.3079, 2739.2837, 2743.2729, 2744.2207, 2745.2256, 2746.2031, 2748.2295, 2755.2700, 2760.2590, 2763.1829, 2771.2400, 2778.1865, 2785.2969, 2787.2524, 2790.2317, 2792.2166, 2800.3093, 2801.2932, 2803.2415, 2804.2429, 2807.2869, 2808.2227, 2812.2244, 2813.2373, 2817.2632, 2820.2441, 2824.2598, 2828.2454, 2833.2544, 2843.2900, 2844.3398, 2847.3630, 2851.3313, 2864.3188, 2890.3293, 2896.4324, 2908.3105, 2910.3325, 2911.3594, 2912.4265, 2925.3323, 2927.4434, 3023.3674, 3025.3865, 3026.3452, 3039.3838, 3042.3542, 3070.3745, 3071.3933, 3086.3984, 3087.3970, 3093.4294, 3101.3831, 3103.4006, 3104.4041, 3110.4143, 3127.4749, 3141.4126, 3143.5066, 3144.5298, 3159.5210, 3219.4854, 3236.5574, 3266.6021, 3321.6216, 3344.4895, 3347.5515, 3348.5020, 3380.5210, 3396.5195, 3427.6099, 3462.6301, 3478.6123, 3484.6497, 3489.7246, 3505.6919, 3521.6392, 3552.6614, 3553.6028, 3554.5630, 3558.6460, 3573.6545, 3589.6523, 3608.6155, 3624.6685, 3630.5806, 3641.7090, 3651.7944, 3667.7266, 3683.6980, 3691.7681, 3707.7200, 3714.5493, 3715.6206, 3719.6914, 3720.7048, 3723.7041, 3735.7100, 3751.7539, 3830.8091, 3869.7393, 3876.6707, 3885.6946

30. G3MXX8 **Mass:** 36525 **Score:** 17 **Expect:** 4.9e+002 **Matches:** 13

Uncharacterized protein OS=Bos taurus GN=SCRT1 PE=4 SV=1

Observed	Mr(expt)	Mr(calc)	ppm	Start	End	Miss	Ions	Peptide
873.4370	872.4297	872.4100	22.5	180 -	190	0	---	R.AGPGAGSSGGR.H
889.4857	888.4784	888.4665	13.5	215 -	222	0	---	R.SLDSQLAR.R
1045.5594	1044.5521	1044.5676	-14.80	215 -	223	1	---	R.SLDSQLARR.C
1157.5676	1156.5603	1156.5448	13.5	293 -	302	0	---	R.AHMQTHSAFK.H
1364.7118	1363.7045	1363.6844	14.8	199 -	210	1	---	K.TYATSSNLSRHK.Q
1743.9005	1742.8932	1742.8521	23.6	159 -	179	2	---	R.GAGSGAGGRGGARAGAGTEAR.A
2029.9972	2028.9899	2028.9523	18.5	293 -	308	2	---	R.AHMQTHSAFKHFQCKR.C + Oxidation (M)
2188.0610	2187.0537	2187.0756	-9.99	284 -	302	2	---	K.AFADRSNLRAHMQTHSAFK.H
2523.2014	2522.1941	2522.1746	7.76	128 -	158	0	---	K.AANASAAAAPSAASAPAPDGDGGGGVGAGTR.G
2703.2529	2702.2456	2702.2554	-3.62	269 -	292	2	---	R.SHTGEKPFGCAHCGKAFADRSNLR.A
2820.2441	2819.2368	2819.3049	-24.16	224 -	247	1	---	R.CPTCGKVYVSMPAMAMHLLTHDLR.H + 2 Oxidation (M)
3093.4294	3092.4221	3092.3894	10.6	1 -	28	2	---	-.MPRSFPGQEGQTDTFSSADLESSYGRAR.S + Oxidation (M)
3321.6216	3320.6143	3320.5842	9.07	127 -	167	2	---	R.KAANASAAAAPSAASAPAPDGDGGGGVGAGTRGAGSGAGGR.G

No match to: 716.3353, 718.2895, 724.2175, 726.3070, 728.2833, 729.4336, 730.2399, 732.2518, 734.4341, 743.3130, 745.3617, 747.4360, 753.3469, 756.3524, 758.3462, 759.3632, 768.3517, 779.4633, 781.3725, 789.3640, 794.3674, 796.3701, 799.2854, 801.4280, 805.4417, 807.4076, 810.3293, 812.3207, 818.3782, 820.3700, 828.4415, 829.4508, 830.4513, 834.3805, 842.5101, 849.3834, 852.4368, 857.4457, 860.4262, 868.4412, 870.4462, 877.4166, 880.4280, 882.4470, 883.4816, 887.4295, 891.4872, 894.4923, 898.4610, 900.5174, 905.4763, 907.4251, 912.5086, 914.4942, 920.4296, 922.4855, 928.4565, 929.4484, 933.4327, 936.4976, 938.5062, 944.4982, 946.4672, 949.5244, 950.5168, 957.5792, 961.5109, 965.5151, 967.4963, 972.5023, 974.4913, 976.5255, 978.5352, 981.5225, 983.6221, 986.5463, 988.4922, 993.5123, 995.4512, 998.5833, 1000.5594, 1006.5120, 1007.5640, 1021.5762, 1024.5542, 1031.5300, 1034.5703, 1037.5466, 1041.5637, 1043.5297, 1044.5375, 1046.6058, 1054.5287, 1056.5575, 1057.6187, 1057.6200, 1068.5790, 1074.6162, 1076.6190, 1082.6056, 1105.5938, 1110.5851, 1113.5701, 1118.5687, 1120.6050, 1122.5892, 1123.6047, 1126.6241, 1128.5618, 1130.5756, 1135.5951, 1137.5647, 1143.5592, 1145.6373, 1148.6318, 1149.6230, 1150.6226, 1156.5652, 1159.5890, 1160.5966, 1169.5873, 1177.6421, 1178.6371, 1180.6443, 1182.6539, 1184.6431, 1185.6815, 1193.6090, 1194.6001, 1209.6238, 1213.7180, 1216.7178, 1221.5953, 1227.6459, 1229.6874, 1234.6899, 1238.2251, 1239.6622, 1254.6993, 1255.6351, 1264.7139, 1265.1680, 1265.7196, 1269.6532, 1276.6729, 1278.6892, 1281.6375, 1285.6674, 1289.7029, 1293.6760, 1301.6362, 1303.6489, 1304.6685, 1306.6946, 1308.6797, 1314.7227, 1319.6770, 1320.6364, 1323.6815, 1326.6937, 1328.6799, 1333.7430, 1336.7301, 1337.6429, 1349.6729, 1351.6969, 1353.6891, 1356.6255, 1358.6919, 1359.7013, 1361.7147, 1365.6619, 1366.6965, 1372.6228, 1375.6693, 1382.6771, 1383.7347, 1390.7089, 1393.8007, 1397.7449, 1403.7434, 1410.7849, 1416.7737, 1420.7114, 1423.7096, 1434.7847, 1436.7469, 1442.8257, 1445.7913, 1456.7555, 1457.7440, 1467.7560, 1470.7466, 1475.7615, 1479.8038, 1483.7579, 1484.7480, 1487.7660, 1488.7366, 1493.7556, 1500.7393, 1500.7400, 1503.7648, 1504.7708, 1514.7063, 1516.7281, 1519.7705, 1522.7722, 1524.8040, 1526.7924, 1528.7079, 1530.7058, 1536.7252, 1537.7859, 1540.8043, 1543.7938, 1550.7543, 1551.7465, 1553.7668, 1555.7836, 1556.8011, 1562.8008, 1568.7620, 1572.7245, 1578.8058, 1580.8580, 1580.8600, 1584.8486, 1587.7675, 1608.8051, 1616.8165, 1621.7870, 1625.7886, 1628.7313, 1632.8120, 1635.7964, 1637.7974, 1642.7417, 1647.8439, 1650.8429, 1651.8369, 1655.8147, 1657.8483, 1658.8953, 1660.8379, 1662.8666, 1664.8490, 1666.7892, 1679.8091, 1680.8357, 1682.8409, 1696.8464, 1698.8180, 1701.8243, 1702.8386, 1707.8938, 1710.8904, 1714.7905, 1716.8219, 1717.9052, 1718.9094, 1721.8976, 1722.9003, 1724.9030, 1728.8840, 1730.8910, 1734.1617, 1738.8267, 1739.8165, 1741.8934, 1747.9127, 1758.4807, 1758.8918, 1761.8734, 1765.8154, 1772.0012, 1774.9300, 1774.9319, 1779.9012, 1781.8585, 1783.8062, 1787.8407, 1809.0583, 1810.0460, 1812.9126, 1829.8527, 1832.8584, 1833.8571, 1834.8650, 1836.8618, 1838.8677, 1845.8363, 1847.8600, 1848.8938, 1850.9128, 1851.8932, 1861.8531, 1862.9542, 1864.9258, 1880.9026, 1883.7704, 1887.8878, 1897.7863, 1902.9769, 1904.9276, 1908.8986, 1920.9131, 1923.9126, 1928.0443, 1932.0068, 1936.9510, 1938.0171, 1947.9314, 1958.9393, 1959.9655, 1960.9813, 1961.9463, 1967.1549, 1969.9951, 1973.9971, 1975.9755, 1976.9708, 1990.4971, 1990.9373, 1991.4857, 1997.8479, 2001.8607, 2003.8768, 2004.8850, 2007.9500, 2007.9532, 2011.9381, 2012.8951, 2015.9694, 2017.9860, 2018.9740, 2020.1631, 2023.9482, 2026.9529, 2027.5033, 2043.5356, 2044.9940, 2055.0205, 2057.0154, 2060.9878, 2066.9939, 2068.9661, 2082.9822, 2086.0254, 2088.1033, 2097.0371, 2098.4941, 2102.9954, 2109.0095, 2112.9932, 2114.9707, 2122.9578, 2128.0444, 2130.9980, 2132.0200, 2136.9250, 2140.0063, 2143.0237, 2144.0559, 2145.0781, 2148.0483, 2153.0049, 2153.9973, 2174.0508, 2175.0435, 2178.0483, 2191.0464, 2192.0427, 2199.1150, 2205.0615, 2211.1067, 2223.1533, 2225.1382, 2226.1472, 2239.1646, 2243.1448, 2246.9482, 2251.1108, 2255.1484, 2265.0837, 2271.1211, 2273.0735, 2277.0642, 2283.1606, 2285.1355, 2286.0986, 2289.0901, 2291.0173, 2309.0349, 2328.9888, 2343.0608, 2352.9583, 2355.0383, 2366.9595, 2371.0325, 2392.1594, 2400.0532, 2424.1577, 2434.2429, 2438.2283, 2449.2180, 2450.2451, 2451.2480, 2465.1592, 2466.2458, 2467.2458, 2478.1377, 2501.2207, 2526.2585, 2540.2451, 2542.2578, 2545.2258, 2550.2373, 2561.6841, 2562.1826, 2562.6890, 2573.2495, 2576.1792, 2646.9758, 2659.2144, 2661.1409, 2662.0120, 2671.1924, 2681.2705, 2682.2888, 2685.3098, 2689.3059, 2695.1064, 2696.2695, 2699.2324, 2705.2944, 2712.2937, 2717.2903, 2720.2839, 2721.2937, 2726.2935, 2727.2986, 2728.3079, 2739.2837, 2743.2729, 2744.2207, 2745.2256, 2746.2031, 2748.2295, 2755.2700, 2760.2590, 2763.1829, 2771.2400, 2778.1865, 2785.2969, 2786.2561, 2787.2524, 2790.2317, 2792.2166, 2800.3093, 2801.2932, 2803.2415, 2804.2429, 2807.2869, 2808.2227, 2812.2244, 2813.2373, 2817.2632, 2824.2598, 2828.2454, 2833.2544, 2843.2900, 2844.3398, 2847.3630, 2851.3313, 2864.3188, 2890.3293, 2896.4324, 2908.3105, 2910.3325, 2911.3594, 2912.4265, 2925.3323, 2927.4434, 3023.3674, 3025.3865, 3026.3452, 3039.3838, 3042.3542, 3070.3745, 3071.3933, 3086.3984, 3087.3970, 3101.3831, 3103.4006, 3104.4041, 3110.4143, 3127.4749, 3141.4126, 3143.5066, 3144.5298, 3159.5210, 3219.4854, 3236.5574, 3266.6021, 3280.5635, 3344.4895, 3347.5515, 3348.5020, 3380.5210, 3396.5195, 3412.5801, 3427.6099, 3428.5874, 3444.5840, 3462.6301, 3478.6123, 3484.6497, 3489.7246, 3505.6919, 3510.5859, 3521.6392, 3552.6614, 3553.6028, 3554.5630, 3558.6460, 3573.6545, 3589.6523, 3608.6155, 3624.6685, 3630.5806, 3641.7090,

3651.7944, 3667.7266, 3683.6980, 3691.7681, 3707.7200, 3714.5493, 3715.6206, 3719.6914, 3720.7048, 3723.7041, 3735.7100, 3751.7539, 3830.8091, 3869.7393, 3876.6707, 3885.6946

31. G5E5D2 **Mass:** 21544 **Score:** 17 **Expect:** 4.9e+002 **Matches:** 14
Uncharacterized protein OS=Bos taurus GN=TNNI2 PE=4 SV=1

Observed	Mr(expt)	Mr(calc)	ppm	Start	End	Miss	Ions	Peptide
1137.5647	1136.5574	1136.5682	-9.46	115 -	124	1	---	R.VRMSADAMLK.A + Oxidation (M)
1293.6760	1292.6687	1292.6693	-0.43	114 -	124	2	---	R.RVRMSADAMLK.A + Oxidation (M)
1467.7560	1466.7487	1466.7473	1.00	117 -	130	1	---	R.MSADAMLKALLGSK.H + 2 Oxidation (M)
1484.7480	1483.7407	1483.7751	-23.20	131 -	142	2	---	K.HKVCMDLRANLK.Q
1488.7366	1487.7293	1487.7368	-5.05	157 -	168	2	---	R.DVGDWRKNIEEK.S
1500.7400	1499.7327	1499.7701	-24.90	131 -	142	2	---	K.HKVCMDLRANLK.Q + Oxidation (M)
1650.8429	1649.8356	1649.8083	16.6	92 -	104	1	---	K.ELEDMNQKLFDLR.G
1666.7892	1665.7819	1665.8032	-12.77	92 -	104	1	---	K.ELEDMNQKLFDLR.G + Oxidation (M)
1722.9003	1721.8930	1721.9168	-13.80	115 -	130	2	---	R.VRMSADAMLKALLGSK.H + 2 Oxidation (M)
1851.8932	1850.8859	1850.9196	-18.20	92 -	106	2	---	K.ELEDMNQKLFDLRGK.F + Oxidation (M)
2066.9939	2065.9866	2065.9990	-5.97	72 -	88	2	---	K.IDAAEEEKYDMEIRVQK.S
2082.9822	2081.9749	2081.9939	-9.11	72 -	88	2	---	K.IDAAEEEKYDMEIRVQK.S + Oxidation (M)
2289.0901	2288.0828	2288.1106	-12.15	67 -	85	2	---	R.QLHAKIDAAEEEKYDMEIR.V
3558.6460	3557.6387	3557.6966	-16.26	42 -	71	1	---	K.QNYLSEHCPPLHLPGSMSEVQELCRQLHAK.I

No match to: 716.3353, 718.2895, 724.2175, 726.3070, 728.2833, 729.4336, 730.2399, 732.2518, 734.4341, 743.3130, 745.3617, 747.4360, 753.3469, 756.3524, 758.3462, 759.3632, 768.3517, 779.4633, 781.3725, 789.3640, 794.3674, 796.3701, 799.2854, 801.4280, 805.4417, 807.4076, 810.3293, 812.3207, 818.3782, 820.3700, 828.4415, 829.4508, 830.4513, 834.3805, 842.5101, 849.3834, 852.4368, 857.4457, 860.4262, 868.4412, 870.4462, 873.4370, 877.4166, 880.4280, 882.4470, 883.4816, 887.4295, 889.4857, 891.4872, 894.4923, 898.4610, 900.5174, 905.4763, 907.4251, 912.5086, 914.4942, 920.4296, 922.4855, 928.4565, 929.4484, 933.4327, 936.4976, 938.5062, 944.4982, 946.4672, 949.5244, 950.5168, 957.5792, 961.5109, 965.5151, 967.4963, 972.5023, 974.4913, 976.5255, 978.5352, 981.5225, 983.6221, 986.5463, 988.4922, 993.5123, 995.4512, 998.5833, 1000.5594, 1006.5120, 1007.5640, 1021.5762, 1024.5542, 1031.5300, 1034.5703, 1037.5466, 1041.5637, 1043.5297, 1044.5375, 1045.5594, 1046.6058, 1054.5287, 1056.5575, 1057.6187, 1057.6200, 1068.5790, 1074.6162, 1076.6190, 1082.6056, 1105.5938, 1110.5851, 1113.5701, 1118.5687, 1120.6050, 1122.5892, 1123.6047, 1126.6241, 1128.5618, 1130.5756, 1135.5951, 1143.5592, 1145.6373, 1148.6318, 1149.6230, 1150.6226, 1156.5652, 1157.5676, 1159.5890, 1160.5966, 1169.5873, 1177.6421, 1178.6371, 1180.6443, 1182.6539, 1184.6431, 1185.6815, 1193.6090, 1194.6001, 1209.6238, 1213.7180, 1216.7178, 1221.5953, 1227.6459, 1229.6874, 1234.6899, 1238.2251, 1239.6622, 1254.6993, 1255.6351, 1264.7139, 1265.1680, 1265.7196, 1269.6532, 1276.6729, 1278.6892, 1281.6375, 1285.6674, 1289.7029, 1301.6362, 1303.6489, 1304.6685, 1306.6946, 1308.6797, 1314.7227, 1319.6770, 1320.6364, 1323.6815, 1326.6937, 1328.6799, 1333.7430, 1336.7301, 1337.6429, 1349.6729, 1351.6969, 1353.6891, 1356.6255, 1358.6919, 1359.7013, 1361.7147, 1364.7118, 1365.6619, 1366.6965, 1372.6228, 1375.6693, 1382.6771, 1383.7347, 1390.7089, 1393.8007, 1397.7449, 1403.7434, 1410.7849, 1416.7737, 1420.7114, 1423.7096, 1434.7847, 1436.7469, 1442.8257, 1445.7913, 1456.7555, 1457.7440, 1470.7466, 1475.7615, 1479.8038, 1483.7579, 1487.7660, 1493.7556, 1500.7393, 1503.7648, 1504.7708, 1514.7063, 1516.7281, 1519.7705, 1522.7722, 1524.8040, 1526.7924, 1528.7079, 1530.7058, 1536.7252, 1537.7859, 1540.8043, 1543.7938, 1550.7543, 1551.7465, 1553.7668, 1555.7836, 1556.8011, 1562.8008, 1568.7620, 1572.7245, 1578.8058, 1580.8580, 1580.8600, 1584.8486, 1587.7675, 1608.8051, 1616.8165, 1621.7870, 1625.7886, 1628.7313, 1632.8120, 1635.7964, 1637.7974, 1642.7417, 1647.8439, 1651.8369, 1655.8147, 1657.8483, 1658.8953, 1660.8379, 1662.8666, 1664.8490, 1679.8091, 1680.8357, 1682.8409, 1696.8464, 1698.8180, 1701.8243, 1702.8386, 1707.8938, 1710.8904, 1714.7905, 1716.8219, 1717.9052, 1718.9094, 1721.8976, 1724.9030, 1728.8840, 1730.8910, 1734.1617, 1738.8267, 1739.8165, 1741.8934, 1743.9005, 1747.9127, 1758.4807, 1758.8918, 1761.8734, 1765.8154, 1772.0012, 1774.9300, 1774.9319, 1779.9012, 1781.8585, 1783.8062, 1787.8407, 1809.0583, 1810.0460, 1812.9126, 1829.8527, 1832.8584, 1833.8571, 1834.8650, 1836.8618, 1838.8677, 1845.8363, 1847.8600, 1848.8938, 1850.9128, 1861.8531, 1862.9542, 1864.9258, 1880.9026, 1883.7704, 1887.8878, 1897.7863, 1902.9769, 1904.9276, 1908.8986, 1920.9131, 1923.9126, 1928.0443, 1932.0068, 1936.9510, 1938.0171, 1947.9314, 1958.9393, 1959.9655, 1960.9813, 1961.9463, 1967.1549, 1969.9951, 1973.9971, 1975.9755, 1976.9708, 1990.4971, 1990.9373, 1991.4857, 1997.8479, 2001.8607, 2003.8768, 2004.8850, 2007.9500, 2007.9532, 2011.9381, 2012.8951, 2015.9694, 2017.9860, 2018.9740, 2020.1631, 2023.9482, 2026.9529, 2027.5033, 2029.9972, 2043.5356, 2044.9940, 2055.0205, 2057.0154, 2060.9878, 2068.9661, 2086.0254, 2088.1033, 2097.0371, 2098.4941, 2102.9954, 2109.0095, 2112.9932, 2114.9707, 2122.9578, 2128.0444, 2130.9980, 2132.0200, 2136.9250, 2140.0063, 2143.0237, 2144.0559, 2145.0781, 2148.0483, 2153.0049, 2153.9973, 2174.0508, 2175.0435, 2178.0483, 2188.0610, 2191.0464, 2192.0427, 2199.1150, 2205.0615, 2211.1067, 2223.1533, 2225.1382, 2226.1472, 2239.1646, 2243.1448, 2246.9482, 2251.1108, 2255.1484, 2265.0837, 2271.1211, 2273.0735, 2277.0642, 2283.1606, 2285.1355, 2286.0986, 2291.0173, 2309.0349, 2328.9888, 2343.0608, 2352.9583, 2355.0383, 2366.9595, 2371.0325, 2392.1594, 2400.0532, 2424.1577, 2434.2429, 2438.2283, 2449.2180, 2450.2451, 2451.2480, 2465.1592, 2466.2458, 2467.2458, 2478.1377, 2501.2207, 2523.2014, 2526.2585, 2540.2451, 2542.2578, 2545.2258, 2550.2373, 2561.6841, 2562.1826, 2562.6890, 2573.2495, 2576.1792, 2646.9758, 2659.2144, 2661.1409, 2662.0120, 2671.1924, 2681.2705, 2682.2888, 2685.3098, 2689.3059, 2695.1064, 2696.2695, 2699.2324, 2703.2529, 2705.2944, 2712.2937, 2717.2903, 2720.2839, 2721.2937, 2726.2935, 2727.2986, 2728.3079, 2739.2837, 2743.2729, 2744.2207, 2745.2256, 2746.2031, 2748.2295, 2755.2700, 2760.2590, 2763.1829, 2771.2400, 2778.1865, 2785.2969, 2786.2561, 2787.2524, 2790.2317, 2792.2166, 2800.3093, 2801.2932, 2803.2415, 2804.2429, 2807.2869, 2808.2227, 2812.2244, 2813.2373, 2817.2632, 2820.2441, 2824.2598, 2828.2454, 2833.2544, 2843.2900, 2844.3398, 2847.3630, 2851.3313, 2864.3188, 2890.3293, 2896.4324, 2908.3105, 2910.3325, 2911.3594, 2912.4265, 2925.3323, 2927.4434, 3025.3865, 3026.3452, 3039.3838, 3042.3542, 3070.3745, 3071.3933, 3086.3984, 3087.3970, 3093.4294, 3101.3831, 3103.4006, 3104.4041, 3110.4143, 3127.4749, 3141.4126, 3143.5066, 3144.5298, 3159.5210, 3219.4854, 3236.5574, 3266.6021, 3280.5635, 3321.6216, 3344.4895, 3347.5515, 3348.5020, 3380.5210, 3396.5195, 3412.5801, 3427.6099, 3428.5874, 3444.5840, 3462.6301, 3478.6123, 3484.6497, 3489.7246, 3505.6919, 3510.5859, 3521.6392, 3552.6614, 3553.6028, 3554.5630, 3573.6545, 3589.6523, 3608.6155, 3624.6685, 3630.5806, 3641.7090, 3651.7944, 3667.7266, 3683.6980, 3691.7681, 3707.7200, 3714.5493, 3715.6206, 3719.6914, 3720.7048, 3723.7041, 3735.7100, 3751.7539, 3830.8091, 3869.7393, 3876.6707, 3885.6946

32. Q2YDE5 **Mass:** 22966 **Score:** 17 **Expect:** 5.1e+002 **Matches:** 13
Uncharacterized protein C14orf53 homolog OS=Bos taurus PE=2 SV=1

Observed	Mr(expt)	Mr(calc)	ppm	Start	End	Miss	Ions	Peptide
747.4360	746.4287	746.4109	23.9	68 -	73	0	---	R.SLQIMR.Q
860.4262	859.4189	859.4287	-11.41	28 -	34	0	---	K.ELNEDLK.L
880.4280	879.4207	879.4338	-14.87	21 -	27	0	---	K.LDDNYLK.E
1314.7227	1313.7154	1313.6939	16.4	135 -	146	2	---	K.NKTDLQDGKAPK.T
1504.7708	1503.7635	1503.7351	18.9	92 -	103	2	---	K.EAEEMKQNLERK.N
1608.8051	1607.7978	1607.7986	-0.45	106 -	117	2	---	K.MLRKEMEMLWNK.T
1714.7905	1713.7832	1713.8169	-19.66	2 -	17	1	---	M.TSASNSPESYLSSKTR.S
1721.8976	1720.8903	1720.8519	22.3	21 -	34	1	---	K.LDDNYLKELNEDLK.L
1845.8363	1844.8290	1844.8574	-15.38	1 -	17	1	---	-.MTSASNSPESYLSSKTR.S
1861.8531	1860.8458	1860.8523	-3.49	1 -	17	1	---	-.MTSASNSPESYLSSKTR.S + Oxidation (M)
2057.0154	2056.0081	2056.0000	3.93	118 -	134	1	---	K.TFNTEEFSDEEKVLQIK.N
2148.0483	2147.0410	2147.0164	11.5	1 -	20	2	---	-.MTSASNSPESYLSSKTRSSK.L
2812.2244	2811.2171	2811.2770	-21.30	173 -	198	1	---	R.LFLPPGKETEENGMGQVSGTSQHHSE.- + Oxidation (M)

No match to: 716.3353, 718.2895, 724.2175, 726.3070, 728.2833, 729.4336, 730.2399, 732.2518, 734.4341, 743.3130, 745.3617, 753.3469, 756.3524, 758.3462, 759.3632, 768.3517, 779.4633, 781.3725, 789.3640, 794.3674, 796.3701, 799.2854, 801.4280, 805.4417, 807.4076, 810.3293, 812.3207, 818.3782, 820.3700, 828.4415, 829.4508, 830.4513, 834.3805, 842.5101, 849.3834, 852.4368, 857.4457, 868.4412, 870.4462, 873.4370, 877.4166, 882.4470, 883.4816, 887.4295, 889.4857, 891.4872, 894.4923, 898.4610, 900.5174, 905.4763, 907.4251, 912.5086, 914.4942, 920.4296, 922.4855, 928.4565, 929.4484, 933.4327, 936.4976, 938.5062, 944.4982, 946.4672, 949.5244, 950.5168, 957.5792, 961.5109, 965.5151, 967.4963, 972.5023, 974.4913, 976.5255, 978.5352, 981.5225, 983.6221, 986.5463, 988.4922, 993.5123, 995.4512, 998.5833, 1000.5594, 1006.5120, 1007.5640, 1021.5762, 1024.5542, 1031.5300, 1034.5703, 1037.5466, 1041.5637, 1043.5297, 1044.5375, 1045.5594, 1046.6058, 1054.5287, 1056.5575, 1057.6187, 1057.6200, 1068.5790, 1074.6162, 1076.6190, 1082.6056, 1105.5938, 1110.5851, 1113.5701, 1118.5687, 1120.6050, 1122.5892, 1123.6047, 1126.6241, 1128.5618, 1130.5756, 1135.5951, 1137.5647, 1143.5592, 1145.6373, 1148.6318, 1149.6230, 1150.6226, 1156.5652, 1157.5676, 1159.5890, 1160.5966, 1169.5873, 1177.6421, 1178.6371, 1180.6443, 1182.6539, 1184.6431, 1185.6815, 1193.6090, 1194.6001, 1209.6238, 1213.7180, 1216.7178, 1221.5953, 1227.6459, 1229.6874,

1234.6899, 1238.2251, 1239.6622, 1254.6993, 1255.6351, 1264.7139, 1265.1680, 1265.7196, 1269.6532, 1276.6729, 1278.6892, 1281.6375, 1285.6674, 1289.7029, 1293.6760, 1301.6362, 1303.6489, 1304.6685, 1306.6946, 1308.6797, 1319.6770, 1320.6364, 1323.6815, 1326.6937, 1328.6799, 1333.7430, 1336.7301, 1337.6429, 1349.6729, 1351.6969, 1353.6891, 1356.6255, 1358.6919, 1359.7013, 1361.7147, 1364.7118, 1365.6619, 1366.6965, 1372.6228, 1375.6693, 1382.6771, 1383.7347, 1390.7089, 1393.8007, 1397.7449, 1403.7434, 1410.7849, 1416.7737, 1420.7114, 1423.7096, 1434.7847, 1436.7469, 1442.8257, 1445.7913, 1456.7555, 1457.7440, 1467.7560, 1470.7466, 1475.7615, 1479.8038, 1483.7579, 1484.7480, 1487.7660, 1488.7366, 1493.7556, 1500.7393, 1500.7400, 1503.7648, 1514.7063, 1516.7281, 1519.7705, 1522.7722, 1524.8040, 1526.7924, 1528.7079, 1530.7058, 1536.7252, 1537.7859, 1540.8043, 1543.7938, 1550.7543, 1551.7465, 1553.7668, 1555.7836, 1556.8011, 1562.8008, 1568.7620, 1572.7245, 1578.8058, 1580.8580, 1580.8600, 1584.8486, 1587.7675, 1616.8165, 1621.7870, 1625.7886, 1628.7313, 1632.8120, 1635.7964, 1637.7974, 1642.7417, 1647.8439, 1650.8429, 1651.8369, 1655.8147, 1657.8483, 1658.8953, 1660.8379, 1662.8666, 1664.8490, 1666.7892, 1679.8091, 1680.8357, 1682.8409, 1696.8464, 1698.8180, 1701.8243, 1702.8386, 1707.8938, 1710.8904, 1716.8219, 1717.9052, 1718.9094, 1722.9003, 1724.9030, 1728.8840, 1730.8910, 1734.1617, 1738.8267, 1739.8165, 1741.8934, 1743.9005, 1747.9127, 1758.4807, 1758.8918, 1761.8734, 1765.8154, 1772.0012, 1774.9300, 1774.9319, 1779.9012, 1781.8585, 1783.8062, 1787.8407, 1809.0583, 1810.0460, 1812.9126, 1829.8527, 1832.8584, 1833.8571, 1834.8650, 1836.8618, 1838.8677, 1847.8600, 1848.8938, 1850.9128, 1851.8932, 1862.9542, 1864.9258, 1880.9026, 1883.7704, 1887.8878, 1897.7863, 1902.9769, 1904.9276, 1908.8986, 1920.9131, 1923.9126, 1928.0443, 1932.0068, 1936.9510, 1938.0171, 1947.9314, 1958.9393, 1959.9655, 1960.9813, 1961.9463, 1967.1549, 1969.9951, 1973.9971, 1975.9755, 1976.9708, 1990.4971, 1990.9373, 1991.4857, 1997.8479, 2001.8607, 2003.8768, 2004.8850, 2007.9500, 2007.9532, 2011.9381, 2012.8951, 2015.9694, 2017.9860, 2018.9740, 2020.1631, 2023.9482, 2026.9529, 2027.5033, 2029.9972, 2043.5356, 2044.9940, 2055.0205, 2060.9878, 2066.9939, 2068.9661, 2082.9822, 2086.0254, 2088.1033, 2097.0371, 2098.4941, 2102.9954, 2109.0095, 2112.9932, 2114.9707, 2122.9578, 2128.0444, 2130.9980, 2132.0200, 2136.9250, 2140.0063, 2143.0237, 2144.0559, 2145.0781, 2153.0049, 2153.9973, 2174.0508, 2175.0435, 2178.0483, 2188.0610, 2191.0464, 2192.0427, 2199.1150, 2205.0615, 2211.1067, 2223.1533, 2225.1382, 2226.1472, 2239.1646, 2243.1448, 2246.9482, 2251.1108, 2255.1484, 2265.0837, 2271.1211, 2273.0735, 2277.0642, 2283.1606, 2285.1355, 2286.0986, 2289.0901, 2291.0173, 2309.0349, 2328.9888, 2343.0608, 2352.9583, 2355.0383, 2366.9595, 2371.0325, 2392.1594, 2400.0532, 2424.1577, 2434.2429, 2438.2283, 2449.2180, 2450.2451, 2451.2480, 2465.1592, 2466.2458, 2467.2458, 2478.1377, 2501.2207, 2523.2014, 2526.2585, 2540.2451, 2542.2578, 2545.2258, 2550.2373, 2561.6841, 2562.1826, 2562.6890, 2573.2495, 2576.1792, 2646.9758, 2659.2144, 2661.1409, 2662.0120, 2671.1924, 2681.2705, 2682.2888, 2685.3098, 2689.3059, 2695.1064, 2696.2695, 2699.2324, 2703.2529, 2705.2944, 2712.2937, 2717.2903, 2720.2839, 2721.2937, 2726.2935, 2727.2986, 2728.3079, 2739.2837, 2743.2729, 2744.2207, 2745.2256, 2746.2031, 2748.2295, 2755.2700, 2760.2590, 2763.1829, 2771.2400, 2778.1865, 2785.2969, 2786.2561, 2787.2524, 2790.2317, 2792.2166, 2800.3093, 2801.2932, 2803.2415, 2804.2429, 2807.2869, 2808.2227, 2813.2373, 2817.2632, 2820.2441, 2824.2598, 2828.2454, 2833.2544, 2843.2900, 2844.3398, 2847.3630, 2851.3313, 2864.3188, 2890.3293, 2896.4324, 2908.3105, 2910.3325, 2911.3594, 2912.4265, 2925.3323, 2927.4434, 3023.3674, 3025.3865, 3026.3452, 3039.3838, 3042.3542, 3070.3745, 3071.3933, 3086.3984, 3087.3970, 3093.4294, 3101.3831, 3103.4006, 3104.4041, 3110.4143, 3127.4749, 3141.4126, 3143.5066, 3144.5298, 3159.5210, 3219.4854, 3236.5574, 3266.6021, 3280.5635, 3321.6216, 3344.4895, 3347.5515, 3348.5020, 3380.5210, 3396.5195, 3412.5801, 3427.6099, 3428.5874, 3444.5840, 3462.6301, 3478.6123, 3484.6497, 3489.7246, 3505.6919, 3510.5859, 3521.6392, 3552.6614, 3553.6028, 3554.5630, 3558.6460, 3573.6545, 3589.6523, 3608.6155, 3624.6685, 3630.5806, 3641.7090, 3651.7944, 3667.7266, 3683.6980, 3691.7681, 3707.7200, 3714.5493, 3715.6206, 3719.6914, 3720.7048, 3723.7041, 3735.7100, 3751.7539, 3830.8091, 3869.7393, 3876.6707, 3885.6946

33. F1N7K1 **Mass:** 24749 **Score:** 17 **Expect:** 5.1e+002 **Matches:** 12
Ropporin-1-like protein OS=Bos taurus GN=ROPN1L PE=4 SV=1

Observed	Mr(expt)	Mr(calc)	ppm	Start		End	Miss	Ions	Peptide
972.5023	971.4950	971.5110	-16.47	103	-	110	0	---	K.NLCLPVEK.F
1301.6362	1300.6289	1300.6333	-3.35	113	-	123	0	---	R.ALLQLDPCEDK.I
1319.6770	1318.6697	1318.6373	24.6	59	-	69	1	---	K.DRIEMPMATQK.T
1349.6729	1348.6656	1348.6816	-11.83	163	-	173	0	---	R.IPFGTFSYVYR.Y
1456.7555	1455.7482	1455.7432	3.45	203	-	215	0	---	K.NGMIGLSDFFVLK.R + Oxidation (M)
1584.8486	1583.8413	1583.8382	2.00	202	-	215	1	---	R.KNGMIGLSDFFVLK.R + Oxidation (M)
1765.8154	1764.8081	1764.8465	-21.73	147	-	162	0	---	K.HLCEILTTDPEGGPAR.I
2097.0371	2096.0298	2096.0327	-1.37	33	-	51	0	---	R.TQPADVLQWSAGYFSALSR.G
2277.0642	2276.0569	2276.0895	-14.33	85	-	102	2	---	K.QCSHKEYVDLADLEQKWK.N
2540.2451	2539.2378	2539.3001	-24.51	124	-	146	1	---	K.IEWIKFLALGCSMLGGSLNTAMK.H
2689.3059	2688.2986	2688.3655	-24.89	2	-	24	0	---	M.PLPDTMFCAQQIHIPPELPDILK.Q + Oxidation (M)
3143.5066	3142.4993	3142.5305	-9.94	174	-	202	2	---	R.YLSGLDSDIPESDTEAYLSSLKENAAARK.N

No match to: 716.3353, 718.2895, 724.2175, 726.3070, 728.2833, 729.4336, 730.2399, 732.2518, 734.4341, 743.3130, 745.3617, 747.4360, 753.3469, 756.3524, 758.3462, 759.3632, 768.3517, 779.4633, 781.3725, 789.3640, 794.3674, 796.3701, 799.2854, 801.4280, 805.4417, 807.4076, 810.3293, 812.3207, 818.3782, 820.3700, 828.4415, 829.4508, 830.4513, 834.3805, 842.5101, 849.3834, 852.4368, 857.4457, 860.4262, 868.4412, 870.4462, 873.4370, 877.4166, 880.4280, 882.4470, 883.4816, 887.4295, 889.4857, 891.4872, 894.4923, 898.4610, 900.5174, 905.4763, 907.4251, 912.5086, 914.4942, 920.4296, 922.4855, 928.4565, 929.4484, 933.4327, 936.4976, 938.5062, 944.4982, 946.4672, 949.5244, 950.5168, 957.5792, 961.5109, 965.5151, 967.4963, 974.4913, 976.5255, 978.5352, 981.5225, 983.6221, 986.5463, 988.4922, 993.5123, 995.4512, 998.5833, 1000.5594, 1006.5120, 1007.5640, 1021.5762, 1024.5542, 1031.5300, 1034.5703, 1037.5466, 1041.5637, 1043.5297, 1044.5375, 1045.5594, 1046.6058, 1054.5287, 1056.5575, 1057.6187, 1057.6200, 1068.5790, 1074.6162, 1076.6190, 1082.6056, 1105.5938, 1110.5851, 1113.5701, 1118.5687, 1120.6050, 1122.5892, 1123.6047, 1126.6241, 1128.5618, 1130.5756, 1135.5951, 1137.5647, 1143.5592, 1145.6373, 1148.6318, 1149.6230, 1150.6226, 1156.5652, 1157.5676, 1159.5890, 1160.5966, 1169.5873, 1177.6421, 1178.6371, 1180.6443, 1182.6539, 1184.6431, 1185.6815, 1193.6090, 1194.6001, 1209.6238, 1213.7180, 1216.7178, 1221.5953, 1227.6459, 1229.6874, 1234.6899, 1238.2251, 1239.6622, 1254.6993, 1255.6351, 1264.7139, 1265.1680, 1265.7196, 1269.6532, 1276.6729, 1278.6892, 1281.6375, 1285.6674, 1289.7029, 1293.6760, 1303.6489, 1304.6685, 1306.6946, 1308.6797, 1314.7227, 1320.6364, 1323.6815, 1326.6937, 1328.6799, 1333.7430, 1336.7301, 1337.6429, 1351.6969, 1353.6891, 1356.6255, 1358.6919, 1359.7013, 1361.7147, 1364.7118, 1365.6619, 1366.6965, 1372.6228, 1375.6693, 1382.6771, 1383.7347, 1390.7089, 1393.8007, 1397.7449, 1403.7434, 1410.7849, 1416.7737, 1420.7114, 1423.7096, 1434.7847, 1436.7469, 1442.8257, 1445.7913, 1457.7440, 1467.7560, 1470.7466, 1475.7615, 1479.8038, 1483.7579, 1484.7480, 1487.7660, 1488.7366, 1493.7556, 1500.7393, 1500.7400, 1503.7648, 1504.7708, 1514.7063, 1516.7281, 1519.7705, 1522.7722, 1524.8040, 1526.7924, 1528.7079, 1530.7058, 1536.7252, 1537.7859, 1540.8043, 1543.7938, 1550.7543, 1551.7465, 1553.7668, 1555.7836, 1556.8011, 1562.8008, 1568.7620, 1572.7245, 1578.8058, 1580.8580, 1580.8600, 1587.7675, 1608.8051, 1616.8165, 1621.7870, 1625.7886, 1628.7313, 1632.8120, 1635.7964, 1637.7974, 1642.7417, 1647.8439, 1650.8429, 1651.8369, 1655.8147, 1657.8483, 1658.8953, 1660.8379, 1662.8666, 1664.8490, 1666.7892, 1679.8091, 1680.8357, 1682.8409, 1696.8464, 1698.8180, 1701.8243, 1702.8386, 1707.8938, 1710.8904, 1714.7905, 1716.8219, 1717.9052, 1718.9094, 1721.8976, 1722.9003, 1724.9030, 1728.8840, 1730.8910, 1734.1617, 1738.8267, 1739.8165, 1741.8934, 1743.9005, 1747.9127, 1758.4807, 1758.8918, 1761.8734, 1772.0012, 1774.9300, 1774.9319, 1779.9012, 1781.8585, 1783.8062, 1787.8407, 1809.0583, 1810.0460, 1812.9126, 1829.8527, 1832.8584, 1833.8571, 1834.8650, 1836.8618, 1838.8677, 1845.8363, 1847.8600, 1848.8938, 1850.9128, 1851.8932, 1861.8531, 1862.9542, 1864.9258, 1880.9026, 1883.7704, 1887.8878, 1897.7863, 1902.9769, 1904.9276, 1908.8986, 1920.9131, 1923.9126, 1928.0443, 1932.0068, 1936.9510, 1938.0171, 1947.9314, 1958.9393, 1959.9655, 1960.9813, 1961.9463, 1967.1549, 1969.9951, 1973.9971, 1975.9755, 1976.9708, 1990.4971, 1990.9373, 1991.4857, 1997.8479, 2001.8607, 2003.8768, 2004.8850, 2007.9500, 2007.9532, 2011.9381, 2012.8951, 2015.9694, 2017.9860, 2018.9740, 2020.1631, 2023.9482, 2026.9529, 2027.5033, 2029.9972, 2043.5356, 2044.9940, 2055.0205, 2057.0154, 2060.9878, 2066.9939, 2068.9661, 2082.9822, 2086.0254, 2088.1033, 2098.4941, 2102.9954, 2109.0095, 2112.9932, 2114.9707, 2122.9578, 2128.0444, 2130.9980, 2132.0200, 2136.9250, 2140.0063, 2143.0237, 2144.0559, 2145.0781, 2148.0483, 2153.0049, 2153.9973, 2174.0508, 2175.0435, 2178.0483, 2188.0610, 2191.0464, 2192.0427, 2199.1150, 2205.0615, 2211.1067, 2223.1533, 2225.1382, 2226.1472, 2239.1646, 2243.1448, 2246.9482, 2251.1108, 2255.1484, 2265.0837, 2271.1211, 2273.0735, 2283.1606, 2285.1355, 2286.0986, 2289.0901, 2291.0173, 2309.0349, 2328.9888, 2343.0608, 2352.9583, 2355.0383, 2366.9595, 2371.0325, 2392.1594, 2400.0532, 2424.1577, 2434.2429, 2438.2283, 2449.2180, 2450.2451, 2451.2480, 2465.1592, 2466.2458, 2467.2458, 2478.1377, 2501.2207, 2523.2014, 2526.2585, 2542.2578, 2545.2258, 2550.2373, 2561.6841, 2562.1826, 2562.6890, 2573.2495, 2576.1792, 2646.9758, 2659.2144, 2661.1409, 2662.0120, 2671.1924, 2681.2705, 2682.2888, 2685.3098, 2695.1064, 2696.2695, 2699.2324, 2703.2529, 2705.2944, 2712.2937, 2717.2903, 2720.2839, 2721.2937, 2726.2935, 2727.2986, 2728.3079, 2739.2837, 2743.2729, 2744.2207, 2745.2256, 2746.2031, 2748.2295, 2755.2700, 2760.2590, 2763.1829, 2771.2400, 2778.1865, 2785.2969, 2786.2561, 2787.2524, 2790.2317, 2792.2166, 2800.3093, 2801.2932, 2803.2415, 2804.2429, 2807.2869, 2808.2227, 2812.2244, 2813.2373, 2817.2632, 2820.2441, 2824.2598, 2828.2454, 2833.2544, 2843.2900, 2844.3398, 2847.3630, 2851.3313, 2864.3188, 2890.3293, 2896.4324, 2908.3105, 2910.3325, 2911.3594, 2912.4265, 2925.3323, 2927.4434, 3023.3674, 3025.3865, 3026.3452, 3039.3838, 3042.3542, 3070.3745, 3071.3933, 3086.3984, 3087.3970, 3093.4294, 3101.3831, 3103.4006, 3104.4041, 3110.4143, 3127.4749, 3141.4126, 3144.5298, 3159.5210, 3219.4854, 3236.5574, 3266.6021, 3280.5635, 3321.6216, 3344.4895, 3347.5515, 3348.5020, 3380.5210, 3396.5195, 3412.5801, 3427.6099, 3428.5874, 3444.5840, 3462.6301, 3478.6123, 3484.6497, 3489.7246, 3505.6919, 3510.5859, 3521.6392, 3552.6614, 3553.6028, 3554.5630, 3558.6460, 3573.6545, 3589.6523, 3608.6155, 3624.6685, 3630.5806, 3641.7090, 3651.7944, 3667.7266, 3683.6980, 3691.7681, 3707.7200, 3714.5493, 3715.6206, 3719.6914, 3720.7048, 3723.7041, 3735.7100, 3751.7539, 3830.8091, 3869.7393, 3876.6707, 3885.6946

34. F1N1Y9 **Mass:** 49406 **Score:** 17 **Expect:** 5.2e+002 **Matches:** 19

Zinc finger protein 2 OS=Bos taurus GN=ZNF2 PE=4 SV=2

Observed	Mr(expt)	Mr(calc)	ppm	Start	End	Miss	Ions	Peptide
950.5168	949.5096	949.4981	12.1	418 -	425	2	---	R.RYAKQGID.-
961.5109	960.5037	960.5253	-22.58	303 -	310	0	---	R.HQLIHTGR.K
1056.5575	1055.5502	1055.5764	-24.77	250 -	258	0	---	R.IHTGEKPFK.C
1110.5851	1109.5778	1109.6054	-24.83	410 -	418	1	---	K.SSVIQHQRR.Y
1113.5701	1112.5628	1112.5614	1.24	321 -	330	0	---	K.AFYGVSSLNR.H
1126.6241	1125.6168	1125.5938	20.5	382 -	389	2	---	R.CRLTRHQR.V
1130.5756	1129.5683	1129.5741	-5.08	331 -	340	1	---	R.HQKAHAGEPR.Y
1658.8953	1657.8880	1657.9012	-7.97	405 -	418	2	---	K.VFSSKSSVIQHQRR.Y
1920.9131	1919.9058	1919.8618	22.9	259 -	274	2	---	K.CSECGKAFFDRSSLTR.H
2225.1382	2224.1309	2224.1250	2.68	321 -	340	2	---	K.AFYGVSSLNRHQKAHAGEPR.Y
2239.1646	2238.1573	2238.1718	-6.47	30 -	47	2	---	R.LVPTQRDLYKEVMLENYK.S
2255.1484	2254.1411	2254.1667	-11.35	30 -	47	2	---	R.LVPTQRDLYKEVMLENYK.S + Oxidation (M)
2466.2458	2465.2385	2465.2696	-12.61	139 -	159	2	---	K.ESLTVEACKKPPSLEESLRHR.A
2526.2585	2525.2512	2525.2380	5.24	384 -	404	2	---	R.LTRHQRVHTGEKPFECSVCGK.V
2745.2256	2744.2183	2744.2507	-11.80	270 -	292	2	---	R.SSLTRHQRIHTGESPYECNQCGK.A
2911.3594	2910.3521	2910.3752	-7.94	278 -	302	2	---	R.IHTGESPYECNQCGKAFSQKSILTR.H
3280.5635	3279.5562	3279.5619	-1.73	2 -	29	2	---	M.AAVSPTRCQDPVTFEDVAVVFTDEEWRR.L
3321.6216	3320.6143	3320.5931	6.39	303 -	330	2	---	R.HQLIHTGRKPYECNECGKAFYGVSSLNR.H
3427.6099	3426.6026	3426.5973	1.55	1 -	29	2	---	-.MAAVSPTRCQDPVTFEDVAVVFTDEEWRR.L + Oxidation (M)

No match to: 716.3353, 718.2895, 724.2175, 726.3070, 728.2833, 729.4336, 730.2399, 732.2518, 734.4341, 743.3130, 745.3617, 747.4360, 753.3469, 756.3524, 758.3462, 759.3632, 768.3517, 779.4633, 781.3725, 789.3640, 794.3674, 796.3701, 799.2854, 801.4280, 805.4417, 807.4076, 810.3293, 812.3207, 818.3782, 820.3700, 828.4415, 829.4508, 830.4513, 834.3805, 842.5101, 849.3834, 852.4368, 857.4457, 860.4262, 868.4412, 870.4462, 873.4370, 877.4166, 880.4280, 882.4470, 883.4816, 887.4295, 889.4857, 891.4872, 894.4923, 898.4610, 900.5174, 905.4763, 907.4251, 912.5086, 914.4942, 920.4296, 922.4855, 928.4565, 929.4484, 933.4327, 936.4976, 938.5062, 944.4982, 946.4672, 949.5244, 957.5792, 965.5151, 967.4963, 972.5023, 974.4913, 976.5255, 978.5352, 981.5225, 983.6221, 986.5463, 988.4922, 993.5123, 995.4512, 998.5833, 1000.5594, 1006.5120, 1007.5640, 1021.5762, 1024.5542, 1031.5300, 1034.5703, 1037.5466, 1041.5637, 1043.5297, 1044.5375, 1045.5594, 1046.6058, 1054.5287, 1057.6187, 1057.6200, 1068.5790, 1074.6162, 1076.6190, 1082.6056, 1105.5938, 1118.5687, 1120.6050, 1122.5892, 1123.6047, 1128.5618, 1135.5951, 1137.5647, 1143.5592, 1145.6373, 1148.6318, 1149.6230, 1150.6226, 1156.5652, 1157.5676, 1159.5890, 1160.5966, 1169.5873, 1177.6421, 1178.6371, 1180.6443, 1182.6539, 1184.6431, 1185.6815, 1193.6090, 1194.6001, 1209.6238, 1213.7180, 1216.7178, 1221.5953, 1227.6459, 1229.6874, 1234.6899, 1238.2251, 1239.6622, 1254.6993, 1255.6351, 1264.7139, 1265.1680, 1265.7196, 1269.6532, 1276.6729, 1278.6892, 1281.6375, 1285.6674, 1289.7029, 1293.6760, 1301.6362, 1303.6489, 1304.6685, 1306.6946, 1308.6797, 1314.7227, 1319.6770, 1320.6364, 1323.6815, 1326.6937, 1328.6799, 1333.7430, 1336.7301, 1337.6429, 1349.6729, 1351.6969, 1353.6891, 1356.6255, 1358.6919, 1359.7013, 1361.7147, 1364.7118, 1365.6619, 1366.6965, 1372.6228, 1375.6693, 1382.6771, 1383.7347, 1390.7089, 1393.8007, 1397.7449, 1403.7434, 1410.7849, 1416.7737, 1420.7114, 1423.7096, 1434.7847, 1436.7469, 1442.8257, 1445.7913, 1456.7555, 1457.7440, 1467.7560, 1470.7466, 1475.7615, 1479.8038, 1483.7579, 1484.7480, 1487.7660, 1488.7366, 1493.7556, 1500.7393, 1500.7400, 1503.7648, 1504.7708, 1514.7063, 1516.7281, 1519.7705, 1522.7722, 1524.8040, 1526.7924, 1528.7079, 1530.7058, 1536.7252, 1537.7859, 1540.8043, 1543.7938, 1550.7543, 1551.7465, 1553.7668, 1555.7836, 1556.8011, 1562.8008, 1568.7620, 1572.7245, 1578.8058, 1580.8580, 1580.8600, 1584.8486, 1587.7675, 1608.8051, 1616.8165, 1621.7870, 1625.7886, 1628.7313, 1632.8120, 1635.7964, 1637.7974, 1642.7417, 1647.8439, 1650.8429, 1651.8369, 1655.8147, 1657.8483, 1660.8379, 1662.8666, 1664.8490, 1666.7892, 1679.8091, 1680.8357, 1682.8409, 1696.8464, 1698.8180, 1701.8243, 1702.8386, 1707.8938, 1710.8904, 1714.7905, 1716.8219, 1717.9052, 1718.9094, 1721.8976, 1722.9003, 1724.9030, 1728.8840, 1730.8910, 1734.1617, 1738.8267, 1739.8165, 1741.8934, 1743.9005, 1747.9127, 1758.4807, 1758.8918, 1761.8734, 1765.8154, 1772.0012, 1774.9300, 1774.9319, 1779.9012, 1781.8585, 1783.8062, 1787.8407, 1809.0583, 1810.0460, 1812.9126, 1829.8527, 1832.8584, 1833.8571, 1834.8650, 1836.8618, 1838.8677, 1845.8363, 1847.8600, 1848.8938, 1850.9128, 1851.8932, 1861.8531, 1862.9542, 1864.9258, 1880.9026, 1883.7704, 1887.8878, 1897.7863, 1902.9769, 1904.9276, 1908.8986, 1923.9126, 1928.0443, 1932.0068, 1936.9510, 1938.0171, 1947.9314, 1958.9393, 1959.9655, 1960.9813, 1961.9463, 1967.1549, 1969.9951, 1973.9971, 1975.9755, 1976.9708, 1990.4971, 1990.9373, 1991.4857, 1997.8479, 2001.8607, 2003.8768, 2004.8850, 2007.9500, 2007.9532, 2011.9381, 2012.8951, 2015.9694, 2017.9860, 2018.9740, 2020.1631, 2023.9482, 2026.9529, 2027.5033, 2029.9972, 2043.5356, 2044.9940, 2055.0205, 2057.0154, 2060.9878, 2066.9939, 2068.9661, 2082.9822, 2086.0254, 2088.1033, 2097.0371, 2098.4941, 2102.9954, 2109.0095, 2112.9932, 2114.9707, 2122.9578, 2128.0444, 2130.9980, 2132.0200, 2136.9250, 2140.0063, 2143.0237, 2144.0559, 2145.0781, 2148.0483, 2153.0049, 2153.9973, 2174.0508, 2175.0435, 2178.0483, 2188.0610, 2191.0464, 2192.0427, 2199.1150, 2205.0615, 2211.1067, 2223.1533, 2226.1472, 2243.1448, 2246.9482, 2251.1108, 2265.0837, 2271.1211, 2273.0735, 2277.0642, 2283.1606, 2285.1355, 2286.0986, 2289.0901, 2291.0173, 2309.0349, 2328.9888, 2343.0608, 2352.9583, 2355.0383, 2366.9595, 2371.0325, 2392.1594, 2400.0532, 2424.1577, 2434.2429, 2438.2283, 2449.2180, 2450.2451, 2451.2480, 2465.1592, 2467.2458, 2478.1377, 2501.2207, 2523.2014, 2540.2451, 2542.2578, 2545.2258, 2550.2373, 2561.6841, 2562.1826, 2562.6890, 2573.2495, 2576.1792, 2646.9758, 2659.2144, 2661.1409, 2662.0120, 2671.1924, 2681.2705, 2682.2888, 2685.3098, 2689.3059, 2695.1064, 2696.2695, 2699.2324, 2703.2529, 2705.2944, 2712.2937, 2717.2903, 2720.2839, 2721.2937, 2726.2935, 2727.2986, 2728.3079, 2739.2837, 2743.2729, 2744.2207, 2746.2031, 2748.2295, 2755.2700, 2760.2590, 2763.1829, 2771.2400, 2778.1865, 2785.2969, 2786.2561, 2787.2524, 2790.2317, 2792.2166, 2800.3093, 2801.2932, 2803.2415, 2804.2429, 2807.2869, 2808.2227, 2812.2244, 2813.2373, 2817.2632, 2820.2441, 2824.2598, 2828.2454, 2833.2544, 2843.2900, 2844.3398, 2847.3630, 2851.3313, 2864.3188, 2890.3293, 2896.4324, 2908.3105, 2910.3325, 2912.4265, 2925.3323, 2927.4434, 3023.3674, 3025.3865, 3026.3452, 3039.3838, 3042.3542, 3070.3745, 3071.3933, 3086.3984, 3087.3970, 3093.4294, 3101.3831, 3103.4006, 3104.4041, 3110.4143, 3127.4749, 3141.4126, 3143.5066, 3144.5298, 3159.5210, 3219.4854, 3236.5574, 3266.6021, 3344.4895, 3347.5515, 3348.5020, 3380.5210, 3396.5195, 3412.5801, 3428.5874, 3444.5840, 3462.6301, 3478.6123, 3484.6497, 3489.7246, 3505.6919, 3510.5859, 3521.6392, 3552.6614, 3553.6028, 3554.5630, 3558.6460, 3573.6545, 3589.6523, 3608.6155, 3624.6685, 3630.5806, 3641.7090, 3651.7944, 3667.7266, 3683.6980, 3691.7681, 3707.7200, 3714.5493, 3715.6206, 3719.6914, 3720.7048, 3723.7041, 3735.7100, 3751.7539, 3830.8091, 3869.7393, 3876.6707, 3885.6946

35. P79136 **Mass:** 34176 **Score:** 17 **Expect:** 5.2e+002 **Matches:** 14

F-actin-capping protein subunit beta OS=Bos taurus GN=CAPZB PE=1 SV=1

Observed	Mr(expt)	Mr(calc)	ppm	Start	End	Miss	Ions	Peptide
1006.5120	1005.5047	1005.4992	5.52	189 -	197	1	---	K.SSGRTAHYK.L
1057.6187	1056.6114	1056.5927	17.7	289 -	297	1	---	K.NDLVEALKR.K
1057.6200	1056.6127	1056.5927	18.9	289 -	297	1	7	K.NDLVEALKR.K
1110.5851	1109.5778	1109.5717	5.53	274 -	283	1	---	R.SVQTFADKSK.Q
1185.6815	1184.6742	1184.6877	-11.36	289 -	298	2	---	K.NDLVEALKRK.Q
1337.6429	1336.6356	1336.6405	-3.66	211 -	224	0	---	K.SGSGTMNLGGSLTR.Q
1568.7620	1567.7547	1567.7267	17.9	125 -	137	0	---	K.LEVEANNAFDQYR.D
1662.8666	1661.8593	1661.8737	-8.64	267 -	281	1	---	K.DIVNGLRSVQTFADK.S
1696.8464	1695.8391	1695.8216	10.3	124 -	137	1	---	R.KLEVEANNAFDQYR.D
1787.8407	1786.8334	1786.8672	-18.90	108 -	123	1	---	K.YDPPLEDGAMPSARLR.K
1932.0068	1930.9995	1930.9571	22.0	108 -	124	2	---	K.YDPPLEDGAMPSARLRK.L + Oxidation (M)
2055.0205	2054.0132	2054.0619	-23.69	172 -	188	2	---	K.KIKGCWDSIHVVEVQEK.S
2745.2256	2744.2183	2744.2381	-7.22	229 -	252	1	---	K.DETVSDCSPHIANIGRLVEDMENK.I + Oxidation (M)
2911.3594	2910.3521	2910.2879	22.1	96 -	121	2	---	R.DGDSYRSPWSNKYDPPLEDGAMPSAR.L

No match to: 716.3353, 718.2895, 724.2175, 726.3070, 728.2833, 729.4336, 730.2399, 732.2518, 734.4341, 743.3130, 745.3617, 747.4360, 753.3469, 756.3524, 758.3462, 759.3632, 768.3517, 779.4633, 781.3725, 789.3640, 794.3674, 796.3701, 799.2854, 801.4280, 805.4417, 807.4076, 810.3293, 812.3207, 818.3782, 820.3700, 828.4415, 829.4508, 830.4513, 834.3805, 842.5101, 849.3834, 852.4368, 857.4457, 860.4262, 868.4412, 870.4462, 873.4370, 877.4166, 880.4280, 882.4470, 883.4816, 887.4295, 889.4857, 891.4872, 894.4923, 898.4610, 900.5174, 905.4763, 907.4251, 912.5086, 914.4942, 920.4296, 922.4855, 928.4565, 929.4484, 933.4327, 936.4976, 938.5062, 944.4982, 946.4672, 949.5244, 950.5168, 957.5792, 961.5109, 965.5151, 967.4963, 972.5023, 974.4913, 976.5255, 978.5352, 981.5225, 983.6221, 986.5463, 988.4922, 993.5123, 995.4512, 998.5833, 1000.5594, 1007.5640, 1021.5762, 1024.5542, 1031.5300, 1034.5703,

1037.5466, 1041.5637, 1043.5297, 1044.5375, 1045.5594, 1046.6058, 1054.5287, 1056.5575, 1068.5790, 1074.6162, 1076.6190, 1082.6056, 1105.5938, 1113.5701, 1118.5687, 1120.6050, 1122.5892, 1123.6047, 1126.6241, 1128.5618, 1130.5756, 1135.5951, 1137.5647, 1143.5592, 1145.6373, 1148.6318, 1149.6230, 1150.6226, 1156.5652, 1157.5676, 1159.5890, 1160.5966, 1169.5873, 1177.6421, 1178.6371, 1180.6443, 1182.6539, 1184.6431, 1193.6090, 1194.6001, 1209.6238, 1213.7180, 1216.7178, 1221.5953, 1227.6459, 1229.6874, 1234.6899, 1238.2251, 1239.6622, 1254.6993, 1255.6351, 1264.7139, 1265.1680, 1265.7196, 1269.6532, 1276.6729, 1278.6892, 1281.6375, 1285.6674, 1289.7029, 1293.6760, 1301.6362, 1303.6489, 1304.6685, 1306.6946, 1308.6797, 1314.7227, 1319.6770, 1320.6364, 1323.6815, 1326.6937, 1328.6799, 1333.7430, 1336.7301, 1349.6729, 1351.6969, 1353.6891, 1356.6255, 1358.6919, 1359.7013, 1361.7147, 1364.7118, 1365.6619, 1366.6965, 1372.6228, 1375.6693, 1382.6771, 1383.7347, 1390.7089, 1393.8007, 1397.7449, 1403.7434, 1410.7849, 1416.7737, 1420.7114, 1423.7096, 1434.7847, 1436.7469, 1442.8257, 1445.7913, 1456.7555, 1457.7440, 1467.7560, 1470.7466, 1475.7615, 1479.8038, 1483.7579, 1484.7480, 1487.7660, 1488.7366, 1493.7556, 1500.7393, 1500.7400, 1503.7648, 1504.7708, 1514.7063, 1516.7281, 1519.7705, 1522.7722, 1524.8040, 1526.7924, 1528.7079, 1530.7058, 1536.7252, 1537.7859, 1540.8043, 1543.7938, 1550.7543, 1551.7465, 1553.7668, 1555.7836, 1556.8011, 1562.8008, 1572.7245, 1578.8058, 1580.8580, 1580.8600, 1584.8486, 1587.7675, 1608.8051, 1616.8165, 1621.7870, 1625.7886, 1628.7313, 1632.8120, 1635.7964, 1637.7974, 1642.7417, 1647.8439, 1650.8429, 1651.8369, 1655.8147, 1657.8483, 1658.8953, 1660.8379, 1664.8490, 1666.7892, 1679.8091, 1680.8357, 1682.8409, 1698.8180, 1701.8243, 1702.8386, 1707.8938, 1710.8904, 1714.7905, 1716.8219, 1717.9052, 1718.9094, 1721.8976, 1722.9003, 1724.9030, 1728.8840, 1730.8910, 1734.1617, 1738.8267, 1739.8165, 1741.8934, 1743.9005, 1747.9127, 1758.4807, 1758.8918, 1761.8734, 1765.8154, 1772.0012, 1774.9300, 1774.9319, 1779.9012, 1781.8585, 1783.8062, 1809.0583, 1810.0460, 1812.9126, 1829.8527, 1832.8584, 1833.8571, 1834.8650, 1836.8618, 1838.8677, 1845.8363, 1847.8600, 1848.8938, 1850.9128, 1851.8932, 1861.8531, 1862.9542, 1864.9258, 1880.9026, 1883.7704, 1887.8878, 1897.7863, 1902.9769, 1904.9276, 1908.8986, 1920.9131, 1923.9126, 1928.0443, 1936.9510, 1938.0171, 1947.9314, 1958.9393, 1959.9655, 1960.9813, 1961.9463, 1967.1549, 1969.9951, 1973.9971, 1975.9755, 1976.9708, 1990.4971, 1990.9373, 1991.4857, 1997.8479, 2001.8607, 2003.8768, 2004.8850, 2007.9500, 2007.9532, 2011.9381, 2012.8951, 2015.9694, 2017.9860, 2018.9740, 2020.1631, 2023.9482, 2026.9529, 2027.5033, 2029.9972, 2043.5356, 2044.9940, 2057.0154, 2060.9878, 2066.9939, 2068.9661, 2082.9822, 2086.0254, 2088.1033, 2097.0371, 2098.4941, 2102.9954, 2109.0095, 2112.9932, 2114.9707, 2122.9578, 2128.0444, 2130.9980, 2132.0200, 2136.9250, 2140.0063, 2143.0237, 2144.0559, 2145.0781, 2148.0483, 2153.0049, 2153.9973, 2174.0508, 2175.0435, 2178.0483, 2188.0610, 2191.0464, 2192.0427, 2199.1150, 2205.0615, 2211.1067, 2223.1533, 2225.1382, 2226.1472, 2239.1646, 2243.1448, 2246.9482, 2251.1108, 2255.1484, 2265.0837, 2271.1211, 2273.0735, 2277.0642, 2283.1606, 2285.1355, 2286.0986, 2289.0901, 2291.0173, 2309.0349, 2328.9888, 2343.0608, 2352.9583, 2355.0383, 2366.9595, 2371.0325, 2392.1594, 2400.0532, 2424.1577, 2434.2429, 2438.2283, 2449.2180, 2450.2451, 2451.2480, 2465.1592, 2466.2458, 2467.2458, 2478.1377, 2501.2207, 2523.2014, 2526.2585, 2540.2451, 2542.2578, 2545.2258, 2550.2373, 2561.6841, 2562.1826, 2562.6890, 2573.2495, 2576.1792, 2646.9758, 2659.2144, 2661.1409, 2662.0120, 2671.1924, 2681.2705, 2682.2888, 2685.3098, 2689.3059, 2695.1064, 2696.2695, 2699.2324, 2703.2529, 2705.2944, 2712.2937, 2717.2903, 2720.2839, 2721.2937, 2726.2935, 2727.2986, 2728.3079, 2739.2837, 2743.2729, 2744.2207, 2746.2031, 2748.2295, 2755.2700, 2760.2590, 2763.1829, 2771.2400, 2778.1865, 2785.2969, 2786.2561, 2787.2524, 2790.2317, 2792.2166, 2800.3093, 2801.2932, 2803.2415, 2804.2429, 2807.2869, 2808.2227, 2812.2244, 2813.2373, 2817.2632, 2820.2441, 2824.2598, 2828.2454, 2833.2544, 2843.2900, 2844.3398, 2847.3630, 2851.3313, 2864.3188, 2890.3293, 2896.4324, 2908.3105, 2910.3325, 2912.4265, 2925.3323, 2927.4434, 3023.3674, 3025.3865, 3026.3452, 3039.3838, 3042.3542, 3070.3745, 3071.3933, 3086.3984, 3087.3970, 3093.4294, 3101.3831, 3103.4006, 3104.4041, 3110.4143, 3127.4749, 3141.4126, 3143.5066, 3144.5298, 3159.5210, 3219.4854, 3236.5574, 3266.6021, 3280.5635, 3321.6216, 3344.4895, 3347.5515, 3348.5020, 3380.5210, 3396.5195, 3412.5801, 3427.6099, 3428.5874, 3444.5840, 3462.6301, 3478.6123, 3484.6497, 3489.7246, 3505.6919, 3510.5859, 3521.6392, 3552.6614, 3553.6028, 3554.5630, 3558.6460, 3573.6545, 3589.6523, 3608.6155, 3624.6685, 3630.5806, 3641.7090, 3651.7944, 3667.7266, 3683.6980, 3691.7681, 3707.7200, 3714.5493, 3715.6206, 3719.6914, 3720.7048, 3723.7041, 3735.7100, 3751.7539, 3830.8091, 3869.7393, 3876.6707, 3885.6946

36. Q0VC77 **Mass:** 19543 **Score:** 17 **Expect:** 5.3e+002 **Matches:** 9

CCDC49 protein OS=Bos taurus GN=CCDC49 PE=2 SV=1

Observed	Mr(expt)	Mr(calc)	ppm	Start		End	Miss	Ions	Peptide
805.4417	804.4344	804.4355	-1.27	144	-	149	1	---	R.VHHEKR.W
920.4296	919.4223	919.4433	-22.86	1	-	9	0	---	-.MGGGDLNLK.K + Oxidation (M)
1024.5542	1023.5469	1023.5250	21.4	11	-	18	0	---	K.SWHPQTLR.N
1185.6815	1184.6742	1184.6513	19.4	37	-	45	1	---	K.IEELQRELR.E
1851.8932	1850.8859	1850.8945	-4.61	49	-	64	2	---	R.AREEMQRYAEDVGAVK.K
1883.7704	1882.7631	1882.8057	-22.60	150	-	164	1	---	R.WAGRSTSWSQDCWEK.Y
2153.9973	2152.9900	2153.0034	-6.20	66	-	83	2	---	K.KEEKLDWMYQGPGGMVNR.D + Oxidation (M)
3236.5574	3235.5501	3235.5853	-10.87	104	-	135	1	---	K.EAGCSSETGLLPGSIFAPSGANSLLDMASKIR.E
3484.6497	3483.6424	3483.6207	6.23	100	-	133	1	---	K.MEEKEAGCSSETGLLPGSIFAPSGANSLLDMASK.I

No match to: 716.3353, 718.2895, 724.2175, 726.3070, 728.2833, 729.4336, 730.2399, 732.2518, 734.4341, 743.3130, 745.3617, 747.4360, 753.3469, 756.3524, 758.3462, 759.3632, 768.3517, 779.4633, 781.3725, 789.3640, 794.3674, 796.3701, 799.2854, 801.4280, 807.4076, 810.3293, 812.3207, 818.3782, 820.3700, 828.4415, 829.4508, 830.4513, 834.3805, 842.5101, 849.3834, 852.4368, 857.4457, 860.4262, 868.4412, 870.4462, 873.4370, 877.4166, 880.4280, 882.4470, 883.4816, 887.4295, 889.4857, 891.4872, 894.4923, 898.4610, 900.5174, 905.4763, 907.4251, 912.5086, 914.4942, 922.4855, 928.4565, 929.4484, 933.4327, 936.4976, 938.5062, 944.4982, 946.4672, 949.5244, 950.5168, 957.5792, 961.5109, 965.5151, 967.4963, 972.5023, 974.4913, 976.5255, 978.5352, 981.5225, 983.6221, 986.5463, 988.4922, 993.5123, 995.4512, 998.5833, 1000.5594, 1006.5120, 1007.5640, 1021.5762, 1031.5300, 1034.5703, 1037.5466, 1041.5637, 1043.5297, 1044.5375, 1045.5594, 1046.6058, 1054.5287, 1056.5575, 1057.6187, 1057.6200, 1068.5790, 1074.6162, 1076.6190, 1082.6056, 1105.5938, 1110.5851, 1113.5701, 1118.5687, 1120.6050, 1122.5892, 1123.6047, 1126.6241, 1128.5618, 1130.5756, 1135.5951, 1137.5647, 1143.5592, 1145.6373, 1148.6318, 1149.6230, 1150.6226, 1156.5652, 1157.5676, 1159.5890, 1160.5966, 1169.5873, 1177.6421, 1178.6371, 1180.6443, 1182.6539, 1184.6431, 1193.6090, 1194.6001, 1209.6238, 1213.7180, 1216.7178, 1221.5953, 1227.6459, 1229.6874, 1234.6899, 1238.2251, 1239.6622, 1254.6993, 1255.6351, 1264.7139, 1265.1680, 1265.7196, 1269.6532, 1276.6729, 1278.6892, 1281.6375, 1285.6674, 1289.7029, 1293.6760, 1301.6362, 1303.6489, 1304.6685, 1306.6946, 1308.6797, 1314.7227, 1319.6770, 1320.6364, 1323.6815, 1326.6937, 1328.6799, 1333.7430, 1336.7301, 1337.6429, 1349.6729, 1351.6969, 1353.6891, 1356.6255, 1358.6919, 1359.7013, 1361.7147, 1364.7118, 1365.6619, 1366.6965, 1372.6228, 1375.6693, 1382.6771, 1383.7347, 1390.7089, 1393.8007, 1397.7449, 1403.7434, 1410.7849, 1416.7737, 1420.7114, 1423.7096, 1434.7847, 1436.7469, 1442.8257, 1445.7913, 1456.7555, 1457.7440, 1467.7560, 1470.7466, 1475.7615, 1479.8038, 1483.7579, 1484.7480, 1487.7660, 1488.7366, 1493.7556, 1500.7393, 1500.7400, 1503.7648, 1504.7708, 1514.7063, 1516.7281, 1519.7705, 1522.7722, 1524.8040, 1526.7924, 1528.7079, 1530.7058, 1536.7252, 1537.7859, 1540.8043, 1543.7938, 1550.7543, 1551.7465, 1553.7668, 1555.7836, 1556.8011, 1562.8008, 1568.7620, 1572.7245, 1578.8058, 1580.8580, 1580.8600, 1584.8486, 1587.7675, 1608.8051, 1616.8165, 1621.7870, 1625.7886, 1628.7313, 1632.8120, 1635.7964, 1637.7974, 1642.7417, 1647.8439, 1650.8429, 1651.8369, 1655.8147, 1657.8483, 1658.8953, 1660.8379, 1662.8666, 1664.8490, 1666.7892, 1679.8091, 1680.8357, 1682.8409, 1696.8464, 1698.8180, 1701.8243, 1702.8386, 1707.8938, 1710.8904, 1714.7905, 1716.8219, 1717.9052, 1718.9094, 1721.8976, 1722.9003, 1724.9030, 1728.8840, 1730.8910, 1734.1617, 1738.8267, 1739.8165, 1741.8934, 1743.9005, 1747.9127, 1758.4807, 1758.8918, 1761.8734, 1765.8154, 1772.0012, 1774.9300, 1774.9319, 1779.9012, 1781.8585, 1783.8062, 1787.8407, 1809.0583, 1810.0460, 1812.9126, 1829.8527, 1832.8584, 1833.8571, 1834.8650, 1836.8618, 1838.8677, 1845.8363, 1847.8600, 1848.8938, 1850.9128, 1861.8531, 1862.9542, 1864.9258, 1880.9026, 1887.8878, 1897.7863, 1902.9769, 1904.9276, 1908.8986, 1920.9131, 1923.9126, 1928.0443, 1932.0068, 1936.9510, 1938.0171, 1947.9314, 1958.9393, 1959.9655, 1960.9813, 1961.9463, 1967.1549, 1969.9951, 1973.9971, 1975.9755, 1976.9708, 1990.4971, 1990.9373, 1991.4857, 1997.8479, 2001.8607, 2003.8768, 2004.8850, 2007.9500, 2007.9532, 2011.9381, 2012.8951, 2015.9694, 2017.9860, 2018.9740, 2020.1631, 2023.9482, 2026.9529, 2027.5033, 2029.9972, 2043.5356, 2044.9940, 2055.0205, 2057.0154, 2060.9878, 2066.9939, 2068.9661, 2082.9822, 2086.0254, 2088.1033, 2097.0371, 2098.4941, 2102.9954, 2109.0095, 2112.9932, 2114.9707, 2122.9578, 2128.0444, 2130.9980, 2132.0200, 2136.9250, 2140.0063, 2143.0237, 2144.0559, 2145.0781, 2148.0483, 2153.0049, 2174.0508, 2175.0435, 2178.0483, 2188.0610, 2191.0464, 2192.0427, 2199.1150, 2205.0615, 2211.1067, 2223.1533, 2225.1382, 2226.1472, 2239.1646, 2243.1448, 2246.9482, 2251.1108, 2255.1484, 2265.0837, 2271.1211, 2273.0735, 2277.0642, 2283.1606, 2285.1355, 2286.0986, 2289.0901, 2291.0173, 2309.0349, 2328.9888, 2343.0608, 2352.9583, 2355.0383, 2366.9595, 2371.0325, 2392.1594, 2400.0532, 2424.1577, 2434.2429, 2438.2283, 2449.2180, 2450.2451, 2451.2480, 2465.1592, 2466.2458, 2467.2458, 2478.1377, 2501.2207, 2523.2014, 2526.2585, 2540.2451, 2542.2578, 2545.2258, 2550.2373, 2561.6841, 2562.1826, 2562.6890, 2573.2495, 2576.1792, 2646.9758, 2659.2144, 2661.1409, 2662.0120, 2671.1924, 2681.2705, 2682.2888, 2685.3098, 2689.3059, 2695.1064, 2696.2695, 2699.2324, 2703.2529, 2705.2944, 2712.2937, 2717.2903, 2720.2839, 2721.2937, 2726.2935, 2727.2986, 2728.3079, 2739.2837, 2743.2729, 2744.2207, 2745.2256, 2746.2031, 2748.2295, 2755.2700, 2760.2590, 2763.1829, 2771.2400, 2778.1865, 2785.2969, 2786.2561, 2787.2524, 2790.2317, 2792.2166, 2800.3093, 2801.2932, 2803.2415, 2804.2429, 2807.2869, 2808.2227, 2812.2244, 2813.2373, 2817.2632, 2820.2441, 2824.2598, 2828.2454, 2833.2544, 2843.2900, 2844.3398, 2847.3630, 2851.3313, 2864.3188, 2890.3293, 2896.4324, 2908.3105, 2910.3325, 2911.3594, 2912.4265, 2925.3323, 2927.4434, 3023.3674, 3025.3865, 3026.3452, 3039.3838, 3042.3542, 3070.3745, 3071.3933, 3086.3984, 3087.3970, 3093.4294, 3101.3831, 3103.4006, 3104.4041, 3110.4143, 3127.4749, 3141.4126, 3143.5066, 3144.5298, 3159.5210, 3219.4854, 3266.6021, 3280.5635, 3321.6216, 3344.4895, 3347.5515, 3348.5020, 3380.5210, 3396.5195, 3412.5801, 3427.6099, 3428.5874, 3444.5840, 3462.6301, 3478.6123, 3489.7246, 3505.6919, 3510.5859, 3521.6392, 3552.6614, 3553.6028, 3554.5630, 3558.6460, 3573.6545, 3589.6523, 3608.6155, 3624.6685, 3630.5806, 3641.7090, 3651.7944, 3667.7266, 3683.6980, 3691.7681, 3707.7200, 3714.5493, 3715.6206, 3719.6914, 3720.7048, 3723.7041, 3735.7100, 3751.7539, 3830.8091, 3869.7393, 3876.6707, 3885.6946

37. F1MHL1 **Mass:** 40868 **Score:** 17 **Expect:** 5.3e+002 **Matches:** 19

Hsp90 co-chaperone Cdc37 OS=Bos taurus GN=CDC37 PE=1 SV=2

Observed	Mr(expt)	Mr(calc)	ppm	Start	End	Miss	Ions	Peptide
716.3353	715.3281	715.3177	14.5	205 -	210	0	---	R.SFYGDK.H
805.4417	804.4344	804.4163	22.5	71 -	76	1	---	K.LEEMRK.K
949.5244	948.5171	948.5062	11.5	71 -	77	2	---	K.LEEMRKK.E + Oxidation (M)
1043.5297	1042.5224	1042.5335	-10.62	119 -	126	1	---	K.TFVEKYEK.Q
1056.5575	1055.5502	1055.5723	-20.94	53 -	61	0	---	R.LQAEAQQLR.K
1145.6373	1144.6300	1144.6175	10.9	127 -	135	1	---	K.QIKHFGMLR.R + Oxidation (M)
1180.6443	1179.6370	1179.6302	5.82	224 -	233	0	---	R.RPGFHPWVGK.I
1194.6001	1193.5928	1193.5863	5.49	4 -	12	1	---	R.VERMEQFQK.E
1308.6797	1307.6724	1307.6874	-11.42	117 -	126	2	---	K.HKTFVEKYEK.Q
1358.6919	1357.6846	1357.7089	-17.86	35 -	47	1	---	K.LKELEVAEGEGGK.A
1375.6693	1374.6620	1374.6602	1.34	306 -	317	0	---	R.CIDSGLWVPNSK.S
1467.7560	1466.7487	1466.7187	20.4	4 -	14	2	---	R.VERMEQFQKEK.E + Oxidation (M)
1550.7543	1549.7470	1549.7446	1.55	78 -	90	1	---	K.EKSMPWNVDTLSK.D + Oxidation (M)
1662.8666	1661.8593	1661.8447	8.82	77 -	90	2	---	K.KEKSMPWNVDTLSK.D
1765.8154	1764.8081	1764.8100	-1.09	63 -	75	2	---	K.EERSWEQKLEEMR.K + Oxidation (M)
2068.9661	2067.9588	2067.9935	-16.78	78 -	95	2	---	K.EKSMPWNVDTLSKDGFSK.S
2192.0427	2191.0354	2191.0128	10.3	318 -	339	1	---	K.SSEAKDGEEAGTGDPLLEATSK.S
2277.0642	2276.0569	2276.1008	-19.28	262 -	279	1	---	K.ELHMTEQLSLHFFTTRDR.G + Oxidation (M)
2685.3098	2684.3025	2684.3500	-17.67	239 -	261	2	---	R.ERLPTPVFWPGEFHGLYSPGDRK.E

No match to: 718.2895, 724.2175, 726.3070, 728.2833, 729.4336, 730.2399, 732.2518, 734.4341, 743.3130, 745.3617, 747.4360, 753.3469, 756.3524, 758.3462, 759.3632, 768.3517, 779.4633, 781.3725, 789.3640, 794.3674, 796.3701, 799.2854, 801.4280, 807.4076, 810.3293, 812.3207, 818.3782, 820.3700, 828.4415, 829.4508, 830.4513, 834.3805, 842.5101, 849.3834, 852.4368, 857.4457, 860.4262, 868.4412, 870.4462, 873.4370, 877.4166, 880.4280, 882.4470, 883.4816, 887.4295, 889.4857, 891.4872, 894.4923, 898.4610, 900.5174, 905.4763, 907.4251, 912.5086, 914.4942, 920.4296, 922.4855, 928.4565, 929.4484, 933.4327, 936.4976, 938.5062, 944.4982, 946.4672, 950.5168, 957.5792, 961.5109, 965.5151, 967.4963, 972.5023, 974.4913, 976.5255, 978.5352, 981.5225, 983.6221, 986.5463, 988.4922, 993.5123, 995.4512, 998.5833, 1000.5594, 1006.5120, 1007.5640, 1021.5762, 1024.5542, 1031.5300, 1034.5703, 1037.5466, 1041.5637, 1044.5375, 1045.5594, 1046.6058, 1054.5287, 1057.6187, 1057.6200, 1068.5790, 1074.6162, 1076.6190, 1082.6056, 1105.5938, 1110.5851, 1113.5701, 1118.5687, 1120.6050, 1122.5892, 1123.6047, 1126.6241, 1128.5618, 1130.5756, 1135.5951, 1137.5647, 1143.5592, 1148.6318, 1149.6230, 1150.6226, 1156.5652, 1157.5676, 1159.5890, 1160.5966, 1169.5873, 1177.6421, 1178.6371, 1182.6539, 1184.6431, 1185.6815, 1193.6090, 1209.6238, 1213.7180, 1216.7178, 1221.5953, 1227.6459, 1229.6874, 1234.6899, 1238.2251, 1239.6622, 1254.6993, 1255.6351, 1264.7139, 1265.1680, 1265.7196, 1269.6532, 1276.6729, 1278.6892, 1281.6375, 1285.6674, 1289.7029, 1293.6760, 1301.6362, 1303.6489, 1304.6685, 1306.6946, 1314.7227, 1319.6770, 1320.6364, 1323.6815, 1326.6937, 1328.6799, 1333.7430, 1336.7301, 1337.6429, 1349.6729, 1351.6969, 1353.6891, 1356.6255, 1359.7013, 1361.7147, 1364.7118, 1365.6619, 1366.6965, 1372.6228, 1382.6771, 1383.7347, 1390.7089, 1393.8007, 1397.7449, 1403.7434, 1410.7849, 1416.7737, 1420.7114, 1423.7096, 1434.7847, 1436.7469, 1442.8257, 1445.7913, 1456.7555, 1457.7440, 1470.7466, 1475.7615, 1479.8038, 1483.7579, 1484.7480, 1487.7660, 1488.7366, 1493.7556, 1500.7393, 1500.7400, 1503.7648, 1504.7708, 1514.7063, 1516.7281, 1519.7705, 1522.7722, 1524.8040, 1526.7924, 1528.7079, 1530.7058, 1536.7252, 1537.7859, 1540.8043, 1543.7938, 1551.7465, 1553.7668, 1555.7836, 1556.8011, 1562.8008, 1568.7620, 1572.7245, 1578.8058, 1580.8580, 1580.8600, 1584.8486, 1587.7675, 1608.8051, 1616.8165, 1621.7870, 1625.7886, 1628.7313, 1632.8120, 1635.7964, 1637.7974, 1642.7417, 1647.8439, 1650.8429, 1651.8369, 1655.8147, 1657.8483, 1658.8953, 1660.8379, 1664.8490, 1666.7892, 1679.8091, 1680.8357, 1682.8409, 1696.8464, 1698.8180, 1701.8243, 1702.8386, 1707.8938, 1710.8904, 1714.7905, 1716.8219, 1717.9052, 1718.9094, 1721.8976, 1722.9003, 1724.9030, 1728.8840, 1730.8910, 1734.1617, 1738.8267, 1739.8165, 1741.8934, 1743.9005, 1747.9127, 1758.4807, 1758.8918, 1761.8734, 1772.0012, 1774.9300, 1774.9319, 1779.9012, 1781.8585, 1783.8062, 1787.8407, 1809.0583, 1810.0460, 1812.9126, 1829.8527, 1832.8584, 1833.8571, 1834.8650, 1836.8618, 1838.8677, 1845.8363, 1847.8600, 1848.8938, 1850.9128, 1851.8932, 1861.8531, 1862.9542, 1864.9258, 1880.9026, 1883.7704, 1887.8878, 1897.7863, 1902.9769, 1904.9276, 1908.8986, 1920.9131, 1923.9126, 1928.0443, 1932.0068, 1936.9510, 1938.0171, 1947.9314, 1958.9393, 1959.9655, 1960.9813, 1961.9463, 1967.1549, 1969.9951, 1973.9971, 1975.9755, 1976.9708, 1990.4971, 1990.9373, 1991.4857, 1997.8479, 2001.8607, 2003.8768, 2004.8850, 2007.9500, 2007.9532, 2011.9381, 2012.8951, 2015.9694, 2017.9860, 2018.9740, 2020.1631, 2023.9482, 2026.9529, 2027.5033, 2029.9972, 2043.5356, 2044.9940, 2055.0205, 2057.0154, 2060.9878, 2066.9939, 2082.9822, 2086.0254, 2088.1033, 2097.0371, 2098.4941, 2102.9954, 2109.0095, 2112.9932, 2114.9707, 2122.9578, 2128.0444, 2130.9980, 2132.0200, 2136.9250, 2140.0063, 2143.0237, 2144.0559, 2145.0781, 2148.0483, 2153.0049, 2153.9973, 2174.0508, 2175.0435, 2178.0483, 2188.0610, 2191.0464, 2199.1150, 2205.0615, 2211.1067, 2223.1533, 2225.1382, 2226.1472, 2239.1646, 2243.1448, 2246.9482, 2251.1108, 2255.1484, 2265.0837, 2271.1211, 2273.0735, 2283.1606, 2285.1355, 2286.0986, 2289.0901, 2291.0173, 2309.0349, 2328.9888, 2343.0608, 2352.9583, 2355.0383, 2366.9595, 2371.0325, 2392.1594, 2400.0532, 2424.1577, 2434.2429, 2438.2283, 2449.2180, 2450.2451, 2451.2480, 2465.1592, 2466.2458, 2467.2458, 2478.1377, 2501.2207, 2523.2014, 2526.2585, 2540.2451, 2542.2578, 2545.2258, 2550.2373, 2561.6841, 2562.1826, 2562.6890, 2573.2495, 2576.1792, 2646.9758, 2659.2144, 2661.1409, 2662.0120, 2671.1924, 2681.2705, 2682.2888, 2689.3059, 2695.1064, 2696.2695, 2699.2324, 2703.2529, 2705.2944, 2712.2937, 2717.2903, 2720.2839, 2721.2937, 2726.2935, 2727.2986, 2728.3079, 2739.2837, 2743.2729, 2744.2207, 2745.2256, 2746.2031, 2748.2295, 2755.2700, 2760.2590, 2763.1829, 2771.2400, 2778.1865, 2785.2969, 2786.2561, 2787.2524, 2790.2317, 2792.2166, 2800.3093, 2801.2932, 2803.2415, 2804.2429, 2807.2869, 2808.2227, 2812.2244, 2813.2373, 2817.2632, 2820.2441, 2824.2598, 2828.2454, 2833.2544, 2843.2900, 2844.3398, 2847.3630, 2851.3313, 2864.3188, 2890.3293, 2896.4324, 2908.3105, 2910.3325, 2911.3594, 2912.4265, 2925.3323, 2927.4434, 3023.3674, 3025.3865, 3026.3452, 3039.3838, 3042.3542, 3070.3745, 3071.3933, 3086.3984, 3087.3970, 3093.4294, 3101.3831, 3103.4006, 3104.4041, 3110.4143, 3127.4749, 3141.4126, 3143.5066, 3144.5298, 3159.5210, 3219.4854, 3236.5574, 3266.6021, 3280.5635, 3321.6216, 3344.4895, 3347.5515, 3348.5020, 3380.5210, 3396.5195, 3412.5801, 3427.6099, 3428.5874, 3444.5840, 3462.6301, 3478.6123, 3484.6497, 3489.7246, 3505.6919, 3510.5859, 3521.6392, 3552.6614, 3553.6028, 3554.5630, 3558.6460, 3573.6545, 3589.6523, 3608.6155, 3624.6685, 3630.5806, 3641.7090, 3651.7944, 3667.7266, 3683.6980, 3691.7681, 3707.7200, 3714.5493, 3715.6206, 3719.6914, 3720.7048, 3723.7041, 3735.7100, 3751.7539, 3830.8091, 3869.7393, 3876.6707, 3885.6946

38. F1MQW7 **Mass:** 14077 **Score:** 16 **Expect:** 5.4e+002 **Matches:** 6

Uncharacterized protein OS=Bos taurus GN=C15H11orf97 PE=4 SV=2

Observed	Mr(expt)	Mr(calc)	ppm	Start	End	Miss	Ions	Peptide
1221.5953	1220.5880	1220.6012	-10.79	51 -	59	1	---	K.KFLYCEPHK.R
1526.7924	1525.7851	1525.7736	7.54	3 -	17	0	---	R.EEEAAAVVTVPQAGR.D
1682.8409	1681.8336	1681.8747	-24.44	2 -	17	1	---	M.REEEAAAVVTVPQAGR.D
2760.2590	2759.2517	2759.2682	-5.96	18 -	46	1	---	R.DGEQPGPPAGLGCAAVRGEPGGGGPQESR.K
3159.5210	3158.5137	3158.5415	-8.79	3 -	34	1	---	R.EEEAAAVVTVPQAGRDGEQPGPPAGLGCAAVR.G
3462.6301	3461.6228	3461.6780	-15.93	1 -	34	2	---	-.MREEEAAAVVTVPQAGRDGEQPGPPAGLGCAAVR.G + Oxidation (M)

No match to: 716.3353, 718.2895, 724.2175, 726.3070, 728.2833, 729.4336, 730.2399, 732.2518, 734.4341, 743.3130, 745.3617, 747.4360, 753.3469, 756.3524, 758.3462, 759.3632, 768.3517, 779.4633, 781.3725, 789.3640, 794.3674, 796.3701, 799.2854, 801.4280, 805.4417, 807.4076, 810.3293, 812.3207, 818.3782, 820.3700, 828.4415, 829.4508, 830.4513, 834.3805, 842.5101, 849.3834, 852.4368, 857.4457, 860.4262, 868.4412, 870.4462, 873.4370, 877.4166, 880.4280, 882.4470, 883.4816, 887.4295, 889.4857, 891.4872, 894.4923, 898.4610, 900.5174, 905.4763, 907.4251, 912.5086, 914.4942, 920.4296, 922.4855, 928.4565, 929.4484, 933.4327, 936.4976, 938.5062, 944.4982, 946.4672, 949.5244, 950.5168, 957.5792, 961.5109, 965.5151, 967.4963, 972.5023, 974.4913, 976.5255, 978.5352, 981.5225, 983.6221, 986.5463, 988.4922, 993.5123, 995.4512, 998.5833, 1000.5594, 1006.5120, 1007.5640, 1021.5762, 1024.5542, 1031.5300, 1034.5703, 1037.5466, 1041.5637, 1043.5297, 1044.5375, 1045.5594, 1046.6058, 1054.5287, 1056.5575, 1057.6187, 1057.6200, 1068.5790, 1074.6162, 1076.6190, 1082.6056, 1105.5938, 1110.5851, 1113.5701, 1118.5687, 1120.6050, 1122.5892, 1123.6047, 1126.6241, 1128.5618, 1130.5756, 1135.5951, 1137.5647, 1143.5592, 1145.6373, 1148.6318, 1149.6230, 1150.6226, 1156.5652, 1157.5676, 1159.5890, 1160.5966, 1169.5873, 1177.6421, 1178.6371, 1180.6443, 1182.6539, 1184.6431, 1185.6815, 1193.6090, 1194.6001, 1209.6238, 1213.7180, 1216.7178, 1227.6459, 1229.6874, 1234.6899, 1238.2251, 1239.6622, 1254.6993, 1255.6351, 1264.7139, 1265.1680, 1265.7196, 1269.6532, 1276.6729, 1278.6892, 1281.6375, 1285.6674, 1289.7029, 1293.6760, 1301.6362, 1303.6489, 1304.6685, 1306.6946, 1308.6797, 1314.7227, 1319.6770, 1320.6364, 1323.6815, 1326.6937, 1328.6799, 1333.7430, 1336.7301, 1337.6429, 1349.6729, 1351.6969, 1353.6891, 1356.6255, 1358.6919, 1359.7013, 1361.7147, 1364.7118, 1365.6619, 1366.6965, 1372.6228, 1375.6693, 1382.6771, 1383.7347, 1390.7089, 1393.8007, 1397.7449, 1403.7434, 1410.7849, 1416.7737, 1420.7114, 1423.7096, 1434.7847, 1436.7469, 1442.8257, 1445.7913, 1456.7555, 1457.7440, 1467.7560,

1470.7466, 1475.7615, 1479.8038, 1483.7579, 1484.7480, 1487.7660, 1488.7366, 1493.7556, 1500.7393, 1500.7400, 1503.7648, 1504.7708, 1514.7063, 1516.7281, 1519.7705, 1522.7722, 1524.8040, 1528.7079, 1530.7058, 1536.7252, 1537.7859, 1540.8043, 1543.7938, 1550.7543, 1551.7465, 1553.7668, 1555.7836, 1556.8011, 1562.8008, 1568.7620, 1572.7245, 1578.8058, 1580.8580, 1580.8600, 1584.8486, 1587.7675, 1608.8051, 1616.8165, 1621.7870, 1625.7886, 1628.7313, 1632.8120, 1635.7964, 1637.7974, 1642.7417, 1647.8439, 1650.8429, 1651.8369, 1655.8147, 1657.8483, 1658.8953, 1660.8379, 1662.8666, 1664.8490, 1666.7892, 1679.8091, 1680.8357, 1696.8464, 1698.8180, 1701.8243, 1702.8386, 1707.8938, 1710.8904, 1714.7905, 1716.8219, 1717.9052, 1718.9094, 1721.8976, 1722.9003, 1724.9030, 1728.8840, 1730.8910, 1734.1617, 1738.8267, 1739.8165, 1741.8934, 1743.9005, 1747.9127, 1758.4807, 1758.8918, 1761.8734, 1765.8154, 1772.0012, 1774.9300, 1774.9319, 1779.9012, 1781.8585, 1783.8062, 1787.8407, 1809.0583, 1810.0460, 1812.9126, 1829.8527, 1832.8584, 1833.8571, 1834.8650, 1836.8618, 1838.8677, 1845.8363, 1847.8600, 1848.8938, 1850.9128, 1851.8932, 1861.8531, 1862.9542, 1864.9258, 1880.9026, 1883.7704, 1887.8878, 1897.7863, 1902.9769, 1904.9276, 1908.8986, 1920.9131, 1923.9126, 1928.0443, 1932.0068, 1936.9510, 1938.0171, 1947.9314, 1958.9393, 1959.9655, 1960.9813, 1961.9463, 1967.1549, 1969.9951, 1973.9971, 1975.9755, 1976.9708, 1990.4971, 1990.9373, 1991.4857, 1997.8479, 2001.8607, 2003.8768, 2004.8850, 2007.9500, 2007.9532, 2011.9381, 2012.8951, 2015.9694, 2017.9860, 2018.9740, 2020.1631, 2023.9482, 2026.9529, 2027.5033, 2029.9972, 2043.5356, 2044.9940, 2055.0205, 2057.0154, 2060.9878, 2066.9939, 2068.9661, 2082.9822, 2086.0254, 2088.1033, 2097.0371, 2098.4941, 2102.9954, 2109.0095, 2112.9932, 2114.9707, 2122.9578, 2128.0444, 2130.9980, 2132.0200, 2136.9250, 2140.0063, 2143.0237, 2144.0559, 2145.0781, 2148.0483, 2153.0049, 2153.9973, 2174.0508, 2175.0435, 2178.0483, 2188.0610, 2191.0464, 2192.0427, 2199.1150, 2205.0615, 2211.1067, 2223.1533, 2225.1382, 2226.1472, 2239.1646, 2243.1448, 2246.9482, 2251.1108, 2255.1484, 2265.0837, 2271.1211, 2273.0735, 2277.0642, 2283.1606, 2285.1355, 2286.0986, 2289.0901, 2291.0173, 2309.0349, 2328.9888, 2343.0608, 2352.9583, 2355.0383, 2366.9595, 2371.0325, 2392.1594, 2400.0532, 2424.1577, 2434.2429, 2438.2283, 2449.2180, 2450.2451, 2451.2480, 2465.1592, 2466.2458, 2467.2458, 2478.1377, 2501.2207, 2523.2014, 2526.2585, 2540.2451, 2542.2578, 2545.2258, 2550.2373, 2561.6841, 2562.1826, 2562.6890, 2573.2495, 2576.1792, 2646.9758, 2659.2144, 2661.1409, 2662.0120, 2671.1924, 2681.2705, 2682.2888, 2685.3098, 2689.3059, 2695.1064, 2696.2695, 2699.2324, 2703.2529, 2705.2944, 2712.2937, 2717.2903, 2720.2839, 2721.2937, 2726.2935, 2727.2986, 2728.3079, 2739.2837, 2743.2729, 2744.2207, 2745.2256, 2746.2031, 2748.2295, 2755.2700, 2763.1829, 2771.2400, 2778.1865, 2785.2969, 2786.2561, 2787.2524, 2790.2317, 2792.2166, 2800.3093, 2801.2932, 2803.2415, 2804.2429, 2807.2869, 2808.2227, 2812.2244, 2813.2373, 2817.2632, 2820.2441, 2824.2598, 2828.2454, 2833.2544, 2843.2900, 2844.3398, 2847.3630, 2851.3313, 2864.3188, 2890.3293, 2896.4324, 2908.3105, 2910.3325, 2911.3594, 2912.4265, 2925.3323, 2927.4434, 3023.3674, 3025.3865, 3026.3452, 3039.3838, 3042.3542, 3070.3745, 3071.3933, 3086.3984, 3087.3970, 3093.4294, 3101.3831, 3103.4006, 3104.4041, 3110.4143, 3127.4749, 3141.4126, 3143.5066, 3144.5298, 3219.4854, 3236.5574, 3266.6021, 3280.5635, 3321.6216, 3344.4895, 3347.5515, 3348.5020, 3380.5210, 3396.5195, 3412.5801, 3427.6099, 3428.5874, 3444.5840, 3478.6123, 3484.6497, 3489.7246, 3505.6919, 3510.5859, 3521.6392, 3552.6614, 3553.6028, 3554.5630, 3558.6460, 3573.6545, 3589.6523, 3608.6155, 3624.6685, 3630.5806, 3641.7090, 3651.7944, 3667.7266, 3683.6980, 3691.7681, 3707.7200, 3714.5493, 3715.6206, 3719.6914, 3720.7048, 3723.7041, 3735.7100, 3751.7539, 3830.8091, 3869.7393, 3876.6707, 3885.6946

39. E1B9E7 **Mass:** 17216 **Score:** 16 **Expect:** 5.4e+002 **Matches:** 10

Uncharacterized protein OS=Bos taurus GN=LOC783279 PE=4 SV=2

Observed	Mr(expt)	Mr(calc)	ppm	Start	End	Miss	Ions	Peptide
905.4763	904.4690	904.4510	19.9	50 -	56	0	---	K.MPICLQK.K + Oxidation (M)
1150.6226	1149.6153	1149.5924	19.9	1 -	10	2	---	-.MGSRTLKDSR.L
1372.6228	1371.6155	1371.6313	-11.54	97 -	107	1	---	R.DCSQRVDHLSR.N
1457.7440	1456.7367	1456.7133	16.1	79 -	89	1	---	K.DNFNMYLQKLR.V + Oxidation (M)
2003.8768	2002.8695	2002.8408	14.3	120 -	138	0	---	K.GNCQGDSGAPMVCTIHGSR.R
2174.0508	2173.0435	2173.0408	1.24	102 -	119	1	---	R.VDHLSRNMLCAWQELGTK.G + Oxidation (M)
2255.1484	2254.1411	2254.1389	0.97	90 -	107	2	---	R.VVQITWRDCSQRVDHLSR.N
2720.2839	2719.2766	2719.2378	14.3	120 -	144	2	---	K.GNCQGDSGAPMVCTIHGSRRLFQVG.- + Oxidation (M)
2804.2429	2803.2356	2803.2952	-21.26	97 -	119	2	---	R.DCSQRVDHLSRNMLCAWQELGTK.G
2820.2441	2819.2368	2819.2901	-18.91	97 -	119	2	---	R.DCSQRVDHLSRNMLCAWQELGTK.G + Oxidation (M)

No match to: 716.3353, 718.2895, 724.2175, 726.3070, 728.2833, 729.4336, 730.2399, 732.2518, 734.4341, 743.3130, 745.3617, 747.4360, 753.3469, 756.3524, 758.3462, 759.3632, 768.3517, 779.4633, 781.3725, 789.3640, 794.3674, 796.3701, 799.2854, 801.4280, 805.4417, 807.4076, 810.3293, 812.3207, 818.3782, 820.3700, 828.4415, 829.4508, 830.4513, 834.3805, 842.5101, 849.3834, 852.4368, 857.4457, 860.4262, 868.4412, 870.4462, 873.4370, 877.4166, 880.4280, 882.4470, 883.4816, 887.4295, 889.4857, 891.4872, 894.4923, 898.4610, 900.5174, 907.4251, 912.5086, 914.4942, 920.4296, 922.4855, 928.4565, 929.4484, 933.4327, 936.4976, 938.5062, 944.4982, 946.4672, 949.5244, 950.5168, 957.5792, 961.5109, 965.5151, 967.4963, 972.5023, 974.4913, 976.5255, 978.5352, 981.5225, 983.6221, 986.5463, 988.4922, 993.5123, 995.4512, 998.5833, 1000.5594, 1006.5120, 1007.5640, 1021.5762, 1024.5542, 1031.5300, 1034.5703, 1037.5466, 1041.5637, 1043.5297, 1044.5375, 1045.5594, 1046.6058, 1054.5287, 1056.5575, 1057.6187, 1057.6200, 1068.5790, 1074.6162, 1076.6190, 1082.6056, 1105.5938, 1110.5851, 1113.5701, 1118.5687, 1120.6050, 1122.5892, 1123.6047, 1126.6241, 1128.5618, 1130.5756, 1135.5951, 1137.5647, 1143.5592, 1145.6373, 1148.6318, 1149.6230, 1156.5652, 1157.5676, 1159.5890, 1160.5966, 1169.5873, 1177.6421, 1178.6371, 1180.6443, 1182.6539, 1184.6431, 1185.6815, 1193.6090, 1194.6001, 1209.6238, 1213.7180, 1216.7178, 1221.5953, 1227.6459, 1229.6874, 1234.6899, 1238.2251, 1239.6622, 1254.6993, 1255.6351, 1264.7139, 1265.1680, 1265.7196, 1269.6532, 1276.6729, 1278.6892, 1281.6375, 1285.6674, 1289.7029, 1293.6760, 1301.6362, 1303.6489, 1304.6685, 1306.6946, 1308.6797, 1314.7227, 1319.6770, 1320.6364, 1323.6815, 1326.6937, 1328.6799, 1333.7430, 1336.7301, 1337.6429, 1349.6729, 1351.6969, 1353.6891, 1356.6255, 1358.6919, 1359.7013, 1361.7147, 1364.7118, 1365.6619, 1366.6965, 1375.6693, 1382.6771, 1383.7347, 1390.7089, 1393.8007, 1397.7449, 1403.7434, 1410.7849, 1416.7737, 1420.7114, 1423.7096, 1434.7847, 1436.7469, 1442.8257, 1445.7913, 1456.7555, 1467.7560, 1470.7466, 1475.7615, 1479.8038, 1483.7579, 1484.7480, 1487.7660, 1488.7366, 1493.7556, 1500.7393, 1500.7400, 1503.7648, 1504.7708, 1514.7063, 1516.7281, 1519.7705, 1522.7722, 1524.8040, 1526.7924, 1528.7079, 1530.7058, 1536.7252, 1537.7859, 1540.8043, 1543.7938, 1550.7543, 1551.7465, 1553.7668, 1555.7836, 1556.8011, 1562.8008, 1568.7620, 1572.7245, 1578.8058, 1580.8580, 1580.8600, 1584.8486, 1587.7675, 1608.8051, 1616.8165, 1621.7870, 1625.7886, 1628.7313, 1632.8120, 1635.7964, 1637.7974, 1642.7417, 1647.8439, 1650.8429, 1651.8369, 1655.8147, 1657.8483, 1658.8953, 1660.8379, 1662.8666, 1664.8490, 1666.7892, 1679.8091, 1680.8357, 1682.8409, 1696.8464, 1698.8180, 1701.8243, 1702.8386, 1707.8938, 1710.8904, 1714.7905, 1716.8219, 1717.9052, 1718.9094, 1721.8976, 1722.9003, 1724.9030, 1728.8840, 1730.8910, 1734.1617, 1738.8267, 1739.8165, 1741.8934, 1743.9005, 1747.9127, 1758.4807, 1758.8918, 1761.8734, 1765.8154, 1772.0012, 1774.9300, 1774.9319, 1779.9012, 1781.8585, 1783.8062, 1787.8407, 1809.0583, 1810.0460, 1812.9126, 1829.8527, 1832.8584, 1833.8571, 1834.8650, 1836.8618, 1838.8677, 1845.8363, 1847.8600, 1848.8938, 1850.9128, 1851.8932, 1861.8531, 1862.9542, 1864.9258, 1880.9026, 1883.7704, 1887.8878, 1897.7863, 1902.9769, 1904.9276, 1908.8986, 1920.9131, 1923.9126, 1928.0443, 1932.0068, 1936.9510, 1938.0171, 1947.9314, 1958.9393, 1959.9655, 1960.9813, 1961.9463, 1967.1549, 1969.9951, 1973.9971, 1975.9755, 1976.9708, 1990.4971, 1990.9373, 1991.4857, 1997.8479, 2001.8607, 2004.8850, 2007.9500, 2007.9532, 2011.9381, 2012.8951, 2015.9694, 2017.9860, 2018.9740, 2020.1631, 2023.9482, 2026.9529, 2027.5033, 2029.9972, 2043.5356, 2044.9940, 2055.0205, 2057.0154, 2060.9878, 2066.9939, 2068.9661, 2082.9822, 2086.0254, 2088.1033, 2097.0371, 2098.4941, 2102.9954, 2109.0095, 2112.9932, 2114.9707, 2122.9578, 2128.0444, 2130.9980, 2132.0200, 2136.9250, 2140.0063, 2143.0237, 2144.0559, 2145.0781, 2148.0483, 2153.0049, 2153.9973, 2175.0435, 2178.0483, 2188.0610, 2191.0464, 2192.0427, 2199.1150, 2205.0615, 2211.1067, 2223.1533, 2225.1382, 2226.1472, 2239.1646, 2243.1448, 2246.9482, 2251.1108, 2265.0837, 2271.1211, 2273.0735, 2277.0642, 2283.1606, 2285.1355, 2286.0986, 2289.0901, 2291.0173, 2309.0349, 2328.9888, 2343.0608, 2352.9583, 2355.0383, 2366.9595, 2371.0325, 2392.1594, 2400.0532, 2424.1577, 2434.2429, 2438.2283, 2449.2180, 2450.2451, 2451.2480, 2465.1592, 2466.2458, 2467.2458, 2478.1377, 2501.2207, 2523.2014, 2526.2585, 2540.2451, 2542.2578, 2545.2258, 2550.2373, 2561.6841, 2562.1826, 2562.6890, 2573.2495, 2576.1792, 2646.9758, 2659.2144, 2661.1409, 2662.0120, 2671.1924, 2681.2705, 2682.2888, 2685.3098, 2689.3059, 2695.1064, 2696.2695, 2699.2324, 2703.2529, 2705.2944, 2712.2937, 2717.2903, 2721.2937, 2726.2935, 2727.2986, 2728.3079, 2739.2837, 2743.2729, 2744.2207, 2745.2256, 2746.2031, 2748.2295, 2755.2700, 2760.2590, 2763.1829, 2771.2400, 2778.1865, 2785.2969, 2786.2561, 2787.2524, 2790.2317, 2792.2166, 2800.3093, 2801.2932, 2803.2415, 2807.2869, 2808.2227, 2812.2244, 2813.2373, 2817.2632, 2824.2598, 2828.2454, 2833.2544, 2843.2900, 2844.3398, 2847.3630, 2851.3313, 2864.3188, 2890.3293, 2896.4324, 2908.3105, 2910.3325, 2911.3594, 2912.4265, 2925.3323, 2927.4434, 3023.3674, 3025.3865, 3026.3452, 3039.3838, 3042.3542, 3070.3745, 3071.3933, 3086.3984, 3087.3970, 3093.4294, 3101.3831, 3103.4006, 3104.4041, 3110.4143, 3127.4749, 3141.4126, 3143.5066, 3144.5298, 3159.5210, 3219.4854, 3236.5574, 3266.6021, 3280.5635, 3321.6216, 3344.4895, 3347.5515, 3348.5020, 3380.5210, 3396.5195, 3412.5801, 3427.6099, 3428.5874, 3444.5840, 3462.6301, 3478.6123, 3484.6497, 3489.7246, 3505.6919, 3510.5859, 3521.6392, 3552.6614, 3553.6028, 3554.5630, 3558.6460, 3573.6545, 3589.6523, 3608.6155, 3624.6685, 3630.5806, 3641.7090, 3651.7944, 3667.7266, 3683.6980, 3691.7681, 3707.7200, 3714.5493, 3715.6206, 3719.6914, 3720.7048, 3723.7041, 3735.7100, 3751.7539, 3830.8091, 3869.7393, 3876.6707, 3885.6946

40. E1BA70 **Mass:** 34295 **Score:** 16 **Expect:** 5.7e+002 **Matches:** 11

Uncharacterized protein OS=Bos taurus PE=4 SV=1

Observed	Mr(expt)	Mr(calc)	ppm	Start	End	Miss	Ions	Peptide
1514.7063	1513.6990	1513.7269	-18.40	76 -	88	0	---	R.MPEPCQIPVTNTK.T
1530.7058	1529.6985	1529.7218	-15.22	76 -	88	0	---	R.MPEPCQIPVTNTK.T + Oxidation (M)

1608.8051	1607.7978	1607.8090	-6.93	178	191	0	---	R.NQIVHEVMALPADR.W + Oxidation (M)
1833.8571	1832.8498	1832.8913	-22.64	74	88	1	---	K.FRMPEPCQIPVTNTK.T + Oxidation (M)
2289.0901	2288.0828	2288.1082	-11.09	254	273	0	---	R.ALFPCQEPPVAMSTWQATVR.A
2576.1792	2575.1719	2575.1382	13.1	105	129	2	---	K.GEKDTSGKDGNHDNQEQASGISSSK.Y
2681.2705	2680.2632	2680.2664	-1.18	230	253	0	---	R.SVTWTSDQSGRPCVYTVGSPINNR.A
3104.4041	3103.3968	3103.3396	18.5	130	155	0	---	K.YCCDTGNHGSEEFLLVLDCCDLSVLK.V
3236.5574	3235.5501	3235.5642	-4.35	274	304	0	---	R.AAASFVVLMSGENSAKPTQLQEGTVHVFCAS.-
3347.5515	3346.5442	3346.6036	-17.75	76	104	2	---	R.MPEPCQIPVTNTKTFSSKIGYNDFVICGK.G + Oxidation (M)
3641.7090	3640.7017	3640.6266	20.6	57	88	2	---	R.SSSTEVACPSESNEACKFRMPEPCQIPVTNTK.T

No match to: 716.3353, 718.2895, 724.2175, 726.3070, 728.2833, 729.4336, 730.2399, 732.2518, 734.4341, 743.3130, 745.3617, 747.4360, 753.3469, 756.3524, 758.3462, 759.3632, 768.3517, 779.4633, 781.3725, 789.3640, 794.3674, 796.3701, 799.2854, 801.4280, 805.4417, 807.4076, 810.3293, 812.3207, 818.3782, 820.3700, 828.4415, 829.4508, 830.4513, 834.3805, 842.5101, 849.3834, 852.4368, 857.4457, 860.4262, 868.4412, 870.4462, 873.4370, 877.4166, 880.4280, 882.4470, 883.4816, 887.4295, 889.4857, 891.4872, 894.4923, 898.4610, 900.5174, 905.4763, 907.4251, 912.5086, 914.4942, 920.4296, 922.4855, 928.4565, 929.4484, 933.4327, 936.4976, 938.5062, 944.4982, 946.4672, 949.5244, 950.5168, 957.5792, 961.5109, 965.5151, 967.4963, 972.5023, 974.4913, 976.5255, 978.5352, 981.5225, 983.6221, 986.5463, 988.4922, 993.5123, 995.4512, 998.5833, 1000.5594, 1006.5120, 1007.5640, 1021.5762, 1024.5542, 1031.5300, 1034.5703, 1037.5466, 1041.5637, 1043.5297, 1044.5375, 1045.5594, 1046.6058, 1054.5287, 1056.5575, 1057.6187, 1057.6200, 1068.5790, 1074.6162, 1076.6190, 1082.6056, 1105.5938, 1110.5851, 1113.5701, 1118.5687, 1120.6050, 1122.5892, 1123.6047, 1126.6241, 1128.5618, 1130.5756, 1135.5951, 1137.5647, 1143.5592, 1145.6373, 1148.6318, 1149.6230, 1150.6226, 1156.5652, 1157.5676, 1159.5890, 1160.5966, 1169.5873, 1177.6421, 1178.6371, 1180.6443, 1182.6539, 1184.6431, 1185.6815, 1193.6090, 1194.6001, 1209.6238, 1213.7180, 1216.7178, 1221.5953, 1227.6459, 1229.6874, 1234.6899, 1238.2251, 1239.6622, 1254.6993, 1255.6351, 1264.7139, 1265.1680, 1265.7196, 1269.6532, 1276.6729, 1278.6892, 1281.6375, 1285.6674, 1289.7029, 1293.6760, 1301.6362, 1303.6489, 1304.6685, 1306.6946, 1308.6797, 1314.7227, 1319.6770, 1320.6364, 1323.6815, 1326.6937, 1328.6799, 1333.7430, 1336.7301, 1337.6429, 1349.6729, 1351.6969, 1353.6891, 1356.6255, 1358.6919, 1359.7013, 1361.7147, 1364.7118, 1365.6619, 1366.6965, 1372.6228, 1375.6693, 1382.6771, 1383.7347, 1390.7089, 1393.8007, 1397.7449, 1403.7434, 1410.7849, 1416.7737, 1420.7114, 1423.7096, 1434.7847, 1436.7469, 1442.8257, 1445.7913, 1456.7555, 1457.7440, 1467.7560, 1470.7466, 1475.7615, 1479.8038, 1483.7579, 1484.7480, 1487.7660, 1488.7366, 1493.7556, 1500.7393, 1500.7400, 1503.7648, 1504.7708, 1516.7281, 1519.7705, 1522.7722, 1524.8040, 1526.7924, 1528.7079, 1536.7252, 1537.7859, 1540.8043, 1543.7938, 1550.7543, 1551.7465, 1553.7668, 1555.7836, 1556.8011, 1562.8008, 1568.7620, 1572.7245, 1578.8058, 1580.8580, 1580.8600, 1584.8486, 1587.7675, 1616.8165, 1621.7870, 1625.7886, 1628.7313, 1632.8120, 1635.7964, 1637.7974, 1642.7417, 1647.8439, 1650.8429, 1651.8369, 1655.8147, 1657.8483, 1658.8953, 1660.8379, 1662.8666, 1664.8490, 1666.7892, 1679.8091, 1680.8357, 1682.8409, 1696.8464, 1698.8180, 1701.8243, 1702.8386, 1707.8938, 1710.8904, 1714.7905, 1716.8219, 1717.9052, 1718.9094, 1721.8976, 1722.9003, 1724.9030, 1728.8840, 1730.8910, 1734.1617, 1738.8267, 1739.8165, 1741.8934, 1743.9005, 1747.9127, 1758.4807, 1758.8918, 1761.8734, 1765.8154, 1772.0012, 1774.9300, 1774.9319, 1779.9012, 1781.8585, 1783.8062, 1787.8407, 1809.0583, 1810.0460, 1812.9126, 1829.8527, 1832.8584, 1834.8650, 1836.8618, 1838.8677, 1845.8363, 1847.8600, 1848.8938, 1850.9128, 1851.8932, 1861.8531, 1862.9542, 1864.9258, 1880.9026, 1883.7704, 1887.8878, 1897.7863, 1902.9769, 1904.9276, 1908.8986, 1920.9131, 1923.9126, 1928.0443, 1932.0068, 1936.9510, 1938.0171, 1947.9314, 1958.9393, 1959.9655, 1960.9813, 1961.9463, 1967.1549, 1969.9951, 1973.9971, 1975.9755, 1976.9708, 1990.4971, 1990.9373, 1991.4857, 1997.8479, 2001.8607, 2003.8768, 2004.8850, 2007.9500, 2007.9532, 2011.9381, 2012.8951, 2015.9694, 2017.9860, 2018.9740, 2020.1631, 2023.9482, 2026.9529, 2027.5033, 2029.9972, 2043.5356, 2044.9940, 2055.0205, 2057.0154, 2060.9878, 2066.9939, 2068.9661, 2082.9822, 2086.0254, 2088.1033, 2097.0371, 2098.4941, 2102.9954, 2109.0095, 2112.9932, 2114.9707, 2122.9578, 2128.0444, 2130.9980, 2132.0200, 2136.9250, 2140.0063, 2143.0237, 2144.0559, 2145.0781, 2148.0483, 2153.0049, 2153.9973, 2174.0508, 2175.0435, 2178.0483, 2188.0610, 2191.0464, 2192.0427, 2199.1150, 2205.0615, 2211.1067, 2223.1533, 2225.1382, 2226.1472, 2239.1646, 2243.1448, 2246.9482, 2251.1108, 2255.1484, 2265.0837, 2271.1211, 2273.0735, 2277.0642, 2283.1606, 2285.1355, 2286.0986, 2291.0173, 2309.0349, 2328.9888, 2343.0608, 2352.9583, 2355.0383, 2366.9595, 2371.0325, 2392.1594, 2400.0532, 2424.1577, 2434.2429, 2438.2283, 2449.2180, 2450.2451, 2451.2480, 2465.1592, 2466.2458, 2467.2458, 2478.1377, 2501.2207, 2523.2014, 2526.2585, 2540.2451, 2542.2578, 2545.2258, 2550.2373, 2561.6841, 2562.1826, 2562.6890, 2573.2495, 2646.9758, 2659.2144, 2661.1409, 2662.0120, 2671.1924, 2682.2888, 2685.3098, 2689.3059, 2695.1064, 2696.2695, 2699.2324, 2703.2529, 2705.2944, 2712.2937, 2717.2903, 2720.2839, 2721.2937, 2726.2935, 2727.2986, 2728.3079, 2739.2837, 2743.2729, 2744.2207, 2745.2256, 2746.2031, 2748.2295, 2755.2700, 2760.2590, 2763.1829, 2771.2400, 2778.1865, 2785.2969, 2786.2561, 2787.2524, 2790.2317, 2792.2166, 2800.3093, 2801.2932, 2803.2415, 2804.2429, 2807.2869, 2808.2227, 2812.2244, 2813.2373, 2817.2632, 2820.2441, 2824.2598, 2828.2454, 2833.2544, 2843.2900, 2844.3398, 2847.3630, 2851.3313, 2864.3188, 2890.3293, 2896.4324, 2908.3105, 2910.3325, 2911.3594, 2912.4265, 2925.3323, 2927.4434, 3023.3674, 3025.3865, 3026.3452, 3039.3838, 3042.3542, 3070.3745, 3071.3933, 3086.3984, 3087.3970, 3093.4294, 3101.3831, 3103.4006, 3110.4143, 3127.4749, 3141.4126, 3143.5066, 3144.5298, 3159.5210, 3219.4854, 3266.6021, 3280.5635, 3321.6216, 3344.4895, 3348.5020, 3380.5210, 3396.5195, 3412.5801, 3427.6099, 3428.5874, 3444.5840, 3462.6301, 3478.6123, 3484.6497, 3489.7246, 3505.6919, 3510.5859, 3521.6392, 3552.6614, 3553.6028, 3554.5630, 3558.6460, 3573.6545, 3589.6523, 3608.6155, 3624.6685, 3630.5806, 3651.7944, 3667.7266, 3683.6980, 3691.7681, 3707.7200, 3714.5493, 3715.6206, 3719.6914, 3720.7048, 3723.7041, 3735.7100, 3751.7539, 3830.8091, 3869.7393, 3876.6707, 3885.6946

41. F1MR52 **Mass:** 116761 **Score:** 16 **Expect:** 5.7e+002 **Matches:** 43

Uncharacterized protein OS=Bos taurus PE=4 SV=2

Observed	Mr(expt)	Mr(calc)	ppm	Start	End	Miss	Ions	Peptide
972.5023	971.4950	971.4858	9.41	400	408	0	---	R.ASGLTQHMK.T
988.4922	987.4849	987.4808	4.21	400	408	0	---	R.ASGLTQHMK.T + Oxidation (M)
1044.5375	1043.5302	1043.5400	-9.36	780	788	0	---	K.THTGEKPFK.C
1056.5575	1055.5502	1055.5764	-24.77	808	816	0	---	K.IHTGEKPFK.C
1336.7301	1335.7228	1335.6895	24.9	378	389	1	---	R.HLQSHTGEKTAK.C
1382.6771	1381.6698	1381.6482	15.6	134	144	2	---	R.DQPYKCKVCGK.H
1410.7849	1409.7776	1409.7555	15.7	879	890	0	---	K.TFTTSSYLIIHK.R
1434.7847	1433.7774	1433.7813	-2.69	238	249	1	---	K.HMKIHTGEKPLK.C + Oxidation (M)
1483.7579	1482.7506	1482.7732	-15.21	947	958	2	---	K.YFRAHTGQKTFK.C
1484.7480	1483.7407	1483.7354	3.59	350	361	1	---	R.HMRTHTGEKPYK.C
1493.7556	1492.7483	1492.7457	1.79	396	408	1	---	K.TFTRASGLTQHMK.T + Oxidation (M)
1500.7393	1499.7320	1499.7303	1.14	350	361	1	---	R.HMRTHTGEKPYK.C + Oxidation (M)
1500.7400	1499.7327	1499.7303	1.61	350	361	1	---	R.HMRTHTGEKPYK.C + Oxidation (M)
1522.7722	1521.7649	1521.7610	2.61	739	751	1	---	K.TFTRSSGLIEHMK.T + Oxidation (M)
1524.8040	1523.7967	1523.7766	13.2	480	492	1	---	K.TFTTSLSVTMHKR.R + Oxidation (M)
1526.7924	1525.7851	1525.7677	11.4	851	863	0	---	K.AFANSSYLTIHFR.T
1537.7859	1536.7786	1536.7831	-2.91	795	807	2	---	K.TFTRSSGLTRHMK.I + Oxidation (M)
1555.7836	1554.7763	1554.7388	24.1	986	997	0	---	K.CTEVNTWELLYK.-
1616.8165	1615.8092	1615.8140	-2.98	651	663	2	---	K.ECGKTFRLSSYLR.V
1664.8490	1663.8417	1663.8828	-24.69	480	493	2	---	K.TFTTSLSVTMHKRR.H
1682.8409	1681.8336	1681.8477	-8.39	943	955	2	---	K.HFYKYFRAHTGQK.T
1739.8165	1738.8092	1738.8421	-18.88	378	392	2	---	R.HLQSHTGEKTAKCDK.C
1765.8154	1764.8081	1764.7924	8.93	780	794	1	---	K.THTGEKPFKCDTCGK.T
1781.8585	1780.8512	1780.8930	-23.47	208	222	0	---	R.LIEHMNTHTGEKPFK.C
1838.8677	1837.8604	1837.8451	8.34	158	172	2	---	K.THTGEQLYKCKECGK.A
1887.8878	1886.8805	1886.9210	-21.45	434	448	2	---	K.HFRTHTGEKPFKCDK.C
1960.9813	1959.9740	1960.0126	-19.69	5	23	0	---	R.ASIQSASLQAHVPTHTANK.H
2029.9972	2028.9899	2029.0051	-7.48	400	417	1	---	R.ASGLTQHMKTHTGEKPYK.C + Oxidation (M)
2097.0371	2096.0298	2096.0287	0.56	24	41	2	---	K.HYIYEDSGKVSDATLSRR.Q
2112.9932	2111.9859	2111.9477	18.1	590	607	2	---	K.CYRCEDSGKVSTASLSHR.Q
2140.0063	2138.9990	2138.9626	17.0	612	629	1	---	K.THTGEKPFQCDTCGKAFR.F
2174.0508	2173.0435	2173.0231	9.40	805	822	2	---	R.HMKIHTGEKPFKCDTCGK.A
2251.1108	2250.1035	2250.1447	-18.29	368	386	2	---	K.GFGFSFFLRRHLQSHTGEK.T

2271.1211	2270.1138	2270.0573	24.9	780 - 798	2	---	K.THTGEKPFKCDTCGKTFTR.S
2286.0986	2285.0913	2285.1409	-21.69	182 - 200	2	---	R.VHSQIHTGIKIYKCQECGK.T
2573.2495	2572.2422	2572.3009	-22.80	909 - 930	2	---	K.RFANSSPFAIHKCIHTGEIPYK.C
2748.2295	2747.2222	2747.2829	-22.10	743 - 766	2	---	R.SSGLIEHMKTHTGEKPFKCDTCGK.T
2847.3630	2846.3557	2846.3956	-14.01	808 - 832	2	---	K.IHTGEKPFKCDTCGKAFASSSHLIR.H
3025.3865	3024.3792	3024.4434	-21.21	297 - 323	2	---	R.TPTGEKPFKCDTCGKTFVQSSGLSQHK.K
3280.5635	3279.5562	3279.5223	10.3	195 - 222	2	---	K.CQECGKTFTGSSRLIEHMNTHTGEKPFK.C
3347.5515	3346.5442	3346.4958	14.5	705 - 732	2	---	K.CEECGKDFKHSLPFNIHMGTHTGEQPYK.C
3735.7100	3734.7027	3734.7790	-20.42	238 - 268	2	---	K.HMKIHTGEKPLKCDICGTTFTQTSYLTQHMR.T + Oxidation (M)
3751.7539	3750.7466	3750.7739	-7.27	238 - 268	2	---	K.HMKIHTGEKPLKCDICGTTFTQTSYLTQHMR.T + 2 Oxidation (M)

No match to: 716.3353, 718.2895, 724.2175, 726.3070, 728.2833, 729.4336, 730.2399, 732.2518, 734.4341, 743.3130, 745.3617, 747.4360, 753.3469, 756.3524, 758.3462, 759.3632, 768.3517, 779.4633, 781.3725, 789.3640, 794.3674, 796.3701, 799.2854, 801.4280, 805.4417, 807.4076, 810.3293, 812.3207, 818.3782, 820.3700, 828.4415, 829.4508, 830.4513, 834.3805, 842.5101, 849.3834, 852.4368, 857.4457, 860.4262, 868.4412, 870.4462, 873.4370, 877.4166, 880.4280, 882.4470, 883.4816, 887.4295, 889.4857, 891.4872, 894.4923, 898.4610, 900.5174, 905.4763, 907.4251, 912.5086, 914.4942, 920.4296, 922.4855, 928.4565, 929.4484, 933.4327, 936.4976, 938.5062, 944.4982, 946.4672, 949.5244, 950.5168, 957.5792, 961.5109, 965.5151, 967.4963, 974.4913, 976.5255, 978.5352, 981.5225, 983.6221, 986.5463, 993.5123, 995.4512, 998.5833, 1000.5594, 1006.5120, 1007.5640, 1021.5762, 1024.5542, 1031.5300, 1034.5703, 1037.5466, 1041.5637, 1043.5297, 1045.5594, 1046.6058, 1054.5287, 1057.6187, 1057.6200, 1068.5790, 1074.6162, 1076.6190, 1082.6056, 1105.5938, 1110.5851, 1113.5701, 1118.5687, 1120.6050, 1122.5892, 1123.6047, 1126.6241, 1128.5618, 1130.5756, 1135.5951, 1137.5647, 1143.5592, 1145.6373, 1148.6318, 1149.6230, 1150.6226, 1156.5652, 1157.5676, 1159.5890, 1160.5966, 1169.5873, 1177.6421, 1178.6371, 1180.6443, 1182.6539, 1184.6431, 1185.6815, 1193.6090, 1194.6001, 1209.6238, 1213.7180, 1216.7178, 1221.5953, 1227.6459, 1229.6874, 1234.6899, 1238.2251, 1239.6622, 1254.6993, 1255.6351, 1264.7139, 1265.1680, 1265.7196, 1269.6532, 1276.6729, 1278.6892, 1281.6375, 1285.6674, 1289.7029, 1293.6760, 1301.6362, 1303.6489, 1304.6685, 1306.6946, 1308.6797, 1314.7227, 1319.6770, 1320.6364, 1323.6815, 1326.6937, 1328.6799, 1333.7430, 1337.6429, 1349.6729, 1351.6969, 1353.6891, 1356.6255, 1358.6919, 1359.7013, 1361.7147, 1364.7118, 1365.6619, 1366.6965, 1372.6228, 1375.6693, 1383.7347, 1390.7089, 1393.8007, 1397.7449, 1403.7434, 1416.7737, 1420.7114, 1423.7096, 1436.7469, 1442.8257, 1445.7913, 1456.7555, 1457.7440, 1467.7560, 1470.7466, 1475.7615, 1479.8038, 1487.7660, 1488.7366, 1503.7648, 1504.7708, 1514.7063, 1516.7281, 1519.7705, 1528.7079, 1530.7058, 1536.7252, 1540.8043, 1543.7938, 1550.7543, 1551.7465, 1553.7668, 1556.8011, 1562.8008, 1568.7620, 1572.7245, 1578.8058, 1580.8580, 1580.8600, 1584.8486, 1587.7675, 1608.8051, 1621.7870, 1625.7886, 1628.7313, 1632.8120, 1635.7964, 1637.7974, 1642.7417, 1647.8439, 1650.8429, 1651.8369, 1655.8147, 1657.8483, 1658.8953, 1660.8379, 1662.8666, 1666.7892, 1679.8091, 1680.8357, 1696.8464, 1698.8180, 1701.8243, 1702.8386, 1707.8938, 1710.8904, 1714.7905, 1716.8219, 1717.9052, 1718.9094, 1721.8976, 1722.9003, 1724.9030, 1728.8840, 1730.8910, 1734.1617, 1738.8267, 1741.8934, 1743.9005, 1747.9127, 1758.4807, 1758.8918, 1761.8734, 1772.0012, 1774.9300, 1774.9319, 1779.9012, 1783.8062, 1787.8407, 1809.0583, 1810.0460, 1812.9126, 1829.8527, 1832.8584, 1833.8571, 1834.8650, 1836.8618, 1845.8363, 1847.8600, 1848.8938, 1850.9128, 1851.8932, 1861.8531, 1862.9542, 1864.9258, 1880.9026, 1883.7704, 1897.7863, 1902.9769, 1904.9276, 1908.8986, 1920.9131, 1923.9126, 1928.0443, 1932.0068, 1936.9510, 1938.0171, 1947.9314, 1958.9393, 1959.9655, 1961.9463, 1967.1549, 1969.9951, 1973.9971, 1975.9755, 1976.9708, 1990.4971, 1990.9373, 1991.4857, 1997.8479, 2001.8607, 2003.8768, 2004.8850, 2007.9500, 2007.9532, 2011.9381, 2012.8951, 2015.9694, 2017.9860, 2018.9740, 2020.1631, 2023.9482, 2026.9529, 2027.5033, 2043.5356, 2044.9940, 2055.0205, 2057.0154, 2060.9878, 2066.9939, 2068.9661, 2082.9822, 2086.0254, 2088.1033, 2098.4941, 2102.9954, 2109.0095, 2114.9707, 2122.9578, 2128.0444, 2130.9980, 2132.0200, 2136.9250, 2143.0237, 2144.0559, 2145.0781, 2148.0483, 2153.0049, 2153.9973, 2175.0435, 2178.0483, 2188.0610, 2191.0464, 2192.0427, 2199.1150, 2205.0615, 2211.1067, 2223.1533, 2225.1382, 2226.1472, 2239.1646, 2243.1448, 2246.9482, 2255.1484, 2265.0837, 2273.0735, 2277.0642, 2283.1606, 2285.1355, 2289.0901, 2291.0173, 2309.0349, 2328.9888, 2343.0608, 2352.9583, 2355.0383, 2366.9595, 2371.0325, 2392.1594, 2400.0532, 2424.1577, 2434.2429, 2438.2283, 2449.2180, 2450.2451, 2451.2480, 2465.1592, 2466.2458, 2467.2458, 2478.1377, 2501.2207, 2523.2014, 2526.2585, 2540.2451, 2542.2578, 2545.2258, 2550.2373, 2561.6841, 2562.1826, 2562.6890, 2576.1792, 2646.9758, 2659.2144, 2661.1409, 2662.0120, 2671.1924, 2681.2705, 2682.2888, 2685.3098, 2689.3059, 2695.1064, 2696.2695, 2699.2324, 2703.2529, 2705.2944, 2712.2937, 2717.2903, 2720.2839, 2721.2937, 2726.2935, 2727.2986, 2728.3079, 2739.2837, 2743.2729, 2744.2207, 2745.2256, 2746.2031, 2755.2700, 2760.2590, 2763.1829, 2771.2400, 2778.1865, 2785.2969, 2786.2561, 2787.2524, 2790.2317, 2792.2166, 2800.3093, 2801.2932, 2803.2415, 2804.2429, 2807.2869, 2808.2227, 2812.2244, 2813.2373, 2817.2632, 2820.2441, 2824.2598, 2828.2454, 2833.2544, 2843.2900, 2844.3398, 2851.3313, 2864.3188, 2890.3293, 2896.4324, 2908.3105, 2910.3325, 2911.3594, 2912.4265, 2925.3323, 2927.4434, 3023.3674, 3026.3452, 3039.3838, 3042.3542, 3070.3745, 3071.3933, 3086.3984, 3087.3970, 3093.4294, 3101.3831, 3103.4006, 3104.4041, 3110.4143, 3127.4749, 3141.4126, 3143.5066, 3144.5298, 3159.5210, 3219.4854, 3236.5574, 3266.6021, 3321.6216, 3344.4895, 3348.5020, 3380.5210, 3396.5195, 3412.5801, 3427.6099, 3428.5874, 3444.5840, 3462.6301, 3478.6123, 3484.6497, 3489.7246, 3505.6919, 3510.5859, 3521.6392, 3552.6614, 3553.6028, 3554.5630, 3558.6460, 3573.6545, 3589.6523, 3608.6155, 3624.6685, 3630.5806, 3641.7090, 3651.7944, 3667.7266, 3683.6980, 3691.7681, 3707.7200, 3714.5493, 3715.6206, 3719.6914, 3720.7048, 3723.7041, 3830.8091, 3869.7393, 3876.6707, 3885.6946

42. E1B705 **Mass:** 50364 **Score:** 16 **Expect:** 5.8e+002 **Matches:** 18

Uncharacterized protein OS=Bos taurus GN=RASSF9 PE=4 SV=2

Observed	Mr(expt)	Mr(calc)	ppm	Start - End	Miss	Ions	Peptide
734.4341	733.4269	733.4156	15.3	325 - 331	1	---	K.SMKAGLK.I
920.4296	919.4223	919.4433	-22.84	255 - 261	0	---	R.QMLEDLR.E + Oxidation (M)
1082.6056	1081.5983	1081.6244	-24.08	151 - 159	2	---	K.TLPPDKQKR.I
1145.6373	1144.6300	1144.6339	-3.40	283 - 292	1	---	K.LSAEIEKEVK.S
1351.6969	1350.6896	1350.6642	18.8	140 - 150	0	---	K.LWELSPANYMK.T
1403.7434	1402.7361	1402.7489	-9.14	203 - 213	2	---	K.RMKELDLEIEK.C
1470.7466	1469.7393	1469.7435	-2.87	342 - 353	1	---	K.EIKYSDSLLQMK.A + Oxidation (M)
1761.8734	1760.8661	1760.8556	5.98	102 - 116	0	---	K.AWGDEQPNMQFVLVK.A
1833.8571	1832.8498	1832.8905	-22.18	384 - 400	0	---	K.ESEVPTSNGVVSPFTQR.I
1969.9951	1968.9878	1968.9905	-1.34	262 - 277	1	---	R.ESNGIVQLEERLTYYR.K
2018.9740	2017.9667	2018.0069	-19.91	382 - 400	1	---	R.GKESEVPTSNGVVSPFTQR.I
2145.0781	2144.0708	2144.0572	6.37	206 - 222	2	---	K.ELDLEIEKCEAKFHLDR.V
2175.0435	2174.0362	2174.0637	-12.64	255 - 272	1	---	R.QMLEDLRESNGIVQLEER.L + Oxidation (M)
2205.0615	2204.0542	2204.1088	-24.78	99 - 116	1	---	K.LWKAWGDEQPNMQFVLVK.A + Oxidation (M)
2696.2695	2695.2622	2695.2357	9.85	17 - 38	2	---	R.SPTKEMDSEEKEIVVWVCQEEK.I + Oxidation (M)
2790.2317	2789.2244	2789.2490	-8.81	223 - 246	0	---	R.VENNGENYVQDAYLMPSFSEVEQK.L
3735.7100	3734.7027	3734.6378	17.4	401 - 435	0	---	R.IFNMYTNDTDSDTGISSNHSQDSETTAGDVVLLST.-
3876.6707	3875.6634	3875.7056	-10.89	293 - 327	2	---	K.SVCMEINEDAEGAAASELESSNLESVKCDLEKSMK.A + Oxidation (M)

No match to: 716.3353, 718.2895, 724.2175, 726.3070, 728.2833, 729.4336, 730.2399, 732.2518, 743.3130, 745.3617, 747.4360, 753.3469, 756.3524, 758.3462, 759.3632, 768.3517, 779.4633, 781.3725, 789.3640, 794.3674, 796.3701, 799.2854, 801.4280, 805.4417, 807.4076, 810.3293, 812.3207, 818.3782, 820.3700, 828.4415, 829.4508, 830.4513, 834.3805, 842.5101, 849.3834, 852.4368, 857.4457, 860.4262, 868.4412, 870.4462, 873.4370, 877.4166, 880.4280, 882.4470, 883.4816, 887.4295, 889.4857, 891.4872, 894.4923, 898.4610, 900.5174, 905.4763, 907.4251, 912.5086, 914.4942, 922.4855, 928.4565, 929.4484, 933.4327, 936.4976, 938.5062, 944.4982, 946.4672, 949.5244, 950.5168, 957.5792, 961.5109, 965.5151, 967.4963, 972.5023, 974.4913, 976.5255, 978.5352, 981.5225, 983.6221, 986.5463, 988.4922, 993.5123, 995.4512, 998.5833, 1000.5594, 1006.5120, 1007.5640, 1021.5762, 1024.5542, 1031.5300, 1034.5703, 1037.5466, 1041.5637, 1043.5297, 1044.5375, 1045.5594, 1046.6058, 1054.5287, 1056.5575, 1057.6187, 1057.6200, 1068.5790, 1074.6162, 1076.6190, 1105.5938, 1110.5851, 1113.5701, 1118.5687, 1120.6050, 1122.5892, 1123.6047, 1126.6241, 1128.5618, 1130.5756, 1135.5951, 1137.5647, 1143.5592, 1148.6318, 1149.6230, 1150.6226, 1156.5652, 1157.5676, 1159.5890, 1160.5966, 1169.5873, 1177.6421, 1178.6371, 1180.6443, 1182.6539, 1184.6431, 1185.6815, 1193.6090, 1194.6001, 1209.6238, 1213.7180, 1216.7178, 1221.5953, 1227.6459, 1229.6874, 1234.6899, 1238.2251, 1239.6622, 1254.6993, 1255.6351, 1264.7139, 1265.1680, 1265.7196, 1269.6532, 1276.6729, 1278.6892, 1281.6375, 1285.6674, 1289.7029, 1293.6760, 1301.6362, 1303.6489, 1304.6685, 1306.6946, 1308.6797, 1314.7227, 1319.6770, 1320.6364, 1323.6815, 1326.6937, 1328.6799, 1333.7430, 1336.7301, 1337.6429, 1349.6729, 1353.6891, 1356.6255, 1358.6919, 1359.7013, 1361.7147, 1364.7118, 1365.6619, 1366.6965, 1372.6228, 1375.6693, 1382.6771, 1383.7347, 1390.7089, 1393.8007, 1397.7449, 1410.7849, 1416.7737, 1420.7114, 1423.7096, 1434.7847, 1436.7469, 1442.8257, 1445.7913, 1456.7555, 1457.7440, 1467.7560, 1475.7615, 1479.8038, 1483.7579, 1484.7480, 1487.7660, 1488.7366, 1493.7556, 1500.7393, 1500.7400, 1503.7648, 1504.7708, 1514.7063, 1516.7281, 1519.7705, 1522.7722, 1524.8040, 1526.7924, 1528.7079, 1530.7058, 1536.7252, 1537.7859, 1540.8043, 1543.7938, 1550.7543, 1551.7465, 1553.7668, 1555.7836, 1556.8011, 1562.8008, 1568.7620, 1572.7245, 1578.8058, 1580.8580, 1580.8600, 1584.8486, 1587.7675, 1608.8051, 1616.8165, 1621.7870, 1625.7886, 1628.7313,

1632.8120, 1635.7964, 1637.7974, 1642.7417, 1647.8439, 1650.8429, 1651.8369, 1655.8147, 1657.8483, 1658.8953, 1660.8379, 1662.8666, 1664.8490, 1666.7892, 1679.8091, 1680.8357, 1682.8409, 1696.8464, 1698.8180, 1701.8243, 1702.8386, 1707.8938, 1710.8904, 1714.7905, 1716.8219, 1717.9052, 1718.9094, 1721.8976, 1722.9003, 1724.9030, 1728.8840, 1730.8910, 1734.1617, 1738.8267, 1739.8165, 1741.8934, 1743.9005, 1747.9127, 1758.4807, 1758.8918, 1765.8154, 1772.0012, 1774.9300, 1774.9319, 1779.9012, 1781.8585, 1783.8062, 1787.8407, 1809.0583, 1810.0460, 1812.9126, 1829.8527, 1832.8584, 1834.8650, 1836.8618, 1838.8677, 1845.8363, 1847.8600, 1848.8938, 1850.9128, 1851.8932, 1861.8531, 1862.9542, 1864.9258, 1880.9026, 1883.7704, 1887.8878, 1897.7863, 1902.9769, 1904.9276, 1908.8986, 1920.9131, 1923.9126, 1928.0443, 1932.0068, 1936.9510, 1938.0171, 1947.9314, 1958.9393, 1959.9655, 1960.9813, 1961.9463, 1967.1549, 1973.9971, 1975.9755, 1976.9708, 1990.4971, 1990.9373, 1991.4857, 1997.8479, 2001.8607, 2003.8768, 2004.8850, 2007.9500, 2007.9532, 2011.9381, 2012.8951, 2015.9694, 2017.9860, 2020.1631, 2023.9482, 2026.9529, 2027.5033, 2029.9972, 2043.5356, 2044.9940, 2055.0205, 2057.0154, 2060.9878, 2066.9939, 2068.9661, 2082.9822, 2086.0254, 2088.1033, 2097.0371, 2098.4941, 2102.9954, 2109.0095, 2112.9932, 2114.9707, 2122.9578, 2128.0444, 2130.9980, 2132.0200, 2136.9250, 2140.0063, 2143.0237, 2144.0559, 2148.0483, 2153.0049, 2153.9973, 2174.0508, 2178.0483, 2188.0610, 2191.0464, 2192.0427, 2199.1150, 2211.1067, 2223.1533, 2225.1382, 2226.1472, 2239.1646, 2243.1448, 2246.9482, 2251.1108, 2255.1484, 2265.0837, 2271.1211, 2273.0735, 2277.0642, 2283.1606, 2285.1355, 2286.0986, 2289.0901, 2291.0173, 2309.0349, 2328.9888, 2343.0608, 2352.9583, 2355.0383, 2366.9595, 2371.0325, 2392.1594, 2400.0532, 2424.1577, 2434.2429, 2438.2283, 2449.2180, 2450.2451, 2451.2480, 2465.1592, 2466.2458, 2467.2458, 2478.1377, 2501.2207, 2523.2014, 2526.2585, 2540.2451, 2542.2578, 2545.2258, 2550.2373, 2561.6841, 2562.1826, 2562.6890, 2573.2495, 2576.1792, 2646.9758, 2659.2144, 2661.1409, 2662.0120, 2671.1924, 2681.2705, 2682.2888, 2685.3098, 2689.3059, 2695.1064, 2699.2324, 2703.2529, 2705.2944, 2712.2937, 2717.2903, 2720.2839, 2721.2937, 2726.2935, 2727.2986, 2728.3079, 2739.2837, 2743.2729, 2744.2207, 2745.2256, 2746.2031, 2748.2295, 2755.2700, 2760.2590, 2763.1829, 2771.2400, 2778.1865, 2785.2969, 2786.2561, 2787.2524, 2792.2166, 2800.3093, 2801.2932, 2803.2415, 2804.2429, 2807.2869, 2808.2227, 2812.2244, 2813.2373, 2817.2632, 2820.2441, 2824.2598, 2828.2454, 2833.2544, 2843.2900, 2844.3398, 2847.3630, 2851.3313, 2864.3188, 2890.3293, 2896.4324, 2908.3105, 2910.3325, 2911.3594, 2912.4265, 2925.3323, 2927.4434, 3023.3674, 3025.3865, 3026.3452, 3039.3838, 3042.3542, 3070.3745, 3071.3933, 3086.3984, 3087.3970, 3093.4294, 3101.3831, 3103.4006, 3104.4041, 3110.4143, 3127.4749, 3141.4126, 3143.5066, 3144.5298, 3159.5210, 3219.4854, 3236.5574, 3266.6021, 3280.5635, 3321.6216, 3344.4895, 3347.5515, 3348.5020, 3380.5210, 3396.5195, 3412.5801, 3427.6099, 3428.5874, 3444.5840, 3462.6301, 3478.6123, 3484.6497, 3489.7246, 3505.6919, 3510.5859, 3521.6392, 3552.6614, 3553.6028, 3554.5630, 3558.6460, 3573.6545, 3589.6523, 3608.6155, 3624.6685, 3630.5806, 3641.7090, 3651.7944, 3667.7266, 3683.6980, 3691.7681, 3707.7200, 3714.5493, 3715.6206, 3719.6914, 3720.7048, 3723.7041, 3751.7539, 3830.8091, 3869.7393, 3885.6946

43. E1BDB7 **Mass:** 30920 **Score:** 16 **Expect:** 5.8e+002 **Matches:** 12

Uncharacterized protein OS=Bos taurus GN=CEBPE PE=4 SV=1

Observed	Mr(expt)	Mr(calc)	ppm	Start		End	Miss	Ions	Peptide
729.4336	728.4263	728.4405	-19.50	209	-	213	2	---	R.LRRER.N
998.5833	997.5760	997.5556	20.5	117	-	125	1	---	R.AVAVKEEPR.G
1057.6187	1056.6114	1056.6152	-3.56	214	-	222	2	---	R.NNIAVRKSR.D
1057.6200	1056.6127	1056.6152	-2.33	214	-	222	2	8	R.NNIAVRKSR.D
1194.6001	1193.5928	1193.6040	-9.38	199	-	208	1	---	K.AVNKDSLEYR.L
1484.7480	1483.7407	1483.7127	18.9	122	-	135	2	---	K.EEPRGPEGSRGASR.S
1568.7620	1567.7547	1567.7664	-7.45	236	-	247	1	---	K.VLEYMTENERLR.N + Oxidation (M)
2140.0063	2138.9990	2139.0517	-24.65	229	-	245	1	---	R.ILETQQKVLEYMTENER.L + Oxidation (M)
2771.2400	2770.2327	2770.2228	3.60	1	-	24	1	---	-.MSHGTYYECEPRAGQQPLEFSGAR.A
2787.2524	2786.2451	2786.2177	9.85	1	-	24	1	---	-.MSHGTYYECEPRAGQQPLEFSGAR.A + Oxidation (M)
3505.6919	3504.6846	3504.7031	-5.26	136	-	167	0	---	R.SSYNPLQYQVAHCGQTAMHLPPGLASPSQPLR.V
3521.6392	3520.6319	3520.6980	-18.76	136	-	167	0	---	R.SSYNPLQYQVAHCGQTAMHLPPGLASPSQPLR.V + Oxidation (M)

No match to: 716.3353, 718.2895, 724.2175, 726.3070, 728.2833, 730.2399, 732.2518, 734.4341, 743.3130, 745.3617, 747.4360, 753.3469, 756.3524, 758.3462, 759.3632, 768.3517, 779.4633, 781.3725, 789.3640, 794.3674, 796.3701, 799.2854, 801.4280, 805.4417, 807.4076, 810.3293, 812.3207, 818.3782, 820.3700, 828.4415, 829.4508, 830.4513, 834.3805, 842.5101, 849.3834, 852.4368, 857.4457, 860.4262, 868.4412, 870.4462, 873.4370, 877.4166, 880.4280, 882.4470, 883.4816, 887.4295, 889.4857, 891.4872, 894.4923, 898.4610, 900.5174, 905.4763, 907.4251, 912.5086, 914.4942, 920.4296, 922.4855, 928.4565, 929.4484, 933.4327, 936.4976, 938.5062, 944.4982, 946.4672, 949.5244, 950.5168, 957.5792, 961.5109, 965.5151, 967.4963, 972.5023, 974.4913, 976.5255, 978.5352, 981.5225, 983.6221, 986.5463, 988.4922, 993.5123, 995.4512, 1000.5594, 1006.5120, 1007.5640, 1021.5762, 1024.5542, 1031.5300, 1034.5703, 1037.5466, 1041.5637, 1043.5297, 1044.5375, 1045.5594, 1046.6058, 1054.5287, 1056.5575, 1068.5790, 1074.6162, 1076.6190, 1082.6056, 1105.5938, 1110.5851, 1113.5701, 1118.5687, 1120.6050, 1122.5892, 1123.6047, 1126.6241, 1128.5618, 1130.5756, 1135.5951, 1137.5647, 1143.5592, 1145.6373, 1148.6318, 1149.6230, 1150.6226, 1156.5652, 1157.5676, 1159.5890, 1160.5966, 1169.5873, 1177.6421, 1178.6371, 1180.6443, 1182.6539, 1184.6431, 1185.6815, 1193.6090, 1209.6238, 1213.7180, 1216.7178, 1221.5953, 1227.6459, 1229.6874, 1234.6899, 1238.2251, 1239.6622, 1254.6993, 1255.6351, 1264.7139, 1265.1680, 1265.7196, 1269.6532, 1276.6729, 1278.6892, 1281.6375, 1285.6674, 1289.7029, 1293.6760, 1301.6362, 1303.6489, 1304.6685, 1306.6946, 1308.6797, 1314.7227, 1319.6770, 1320.6364, 1323.6815, 1326.6937, 1328.6799, 1333.7430, 1336.7301, 1337.6429, 1349.6729, 1351.6969, 1353.6891, 1356.6255, 1358.6919, 1359.7013, 1361.7147, 1364.7118, 1365.6619, 1366.6965, 1372.6228, 1375.6693, 1382.6771, 1383.7347, 1390.7089, 1393.8007, 1397.7449, 1403.7434, 1410.7849, 1416.7737, 1420.7114, 1423.7096, 1434.7847, 1436.7469, 1442.8257, 1445.7913, 1456.7555, 1457.7440, 1467.7560, 1470.7466, 1475.7615, 1479.8038, 1483.7579, 1487.7660, 1488.7366, 1493.7556, 1500.7393, 1500.7400, 1503.7648, 1504.7708, 1514.7063, 1516.7281, 1519.7705, 1522.7722, 1524.8040, 1526.7924, 1528.7079, 1530.7058, 1536.7252, 1537.7859, 1540.8043, 1543.7938, 1550.7543, 1551.7465, 1553.7668, 1555.7836, 1556.8011, 1562.8008, 1572.7245, 1578.8058, 1580.8580, 1580.8600, 1584.8486, 1587.7675, 1608.8051, 1616.8165, 1621.7870, 1625.7886, 1628.7313, 1632.8120, 1635.7964, 1637.7974, 1642.7417, 1647.8439, 1650.8429, 1651.8369, 1655.8147, 1657.8483, 1658.8953, 1660.8379, 1662.8666, 1664.8490, 1666.7892, 1679.8091, 1680.8357, 1682.8409, 1696.8464, 1698.8180, 1701.8243, 1702.8386, 1707.8938, 1710.8904, 1714.7905, 1716.8219, 1717.9052, 1718.9094, 1721.8976, 1722.9003, 1724.9030, 1728.8840, 1730.8910, 1734.1617, 1738.8267, 1739.8165, 1741.8934, 1743.9005, 1747.9127, 1758.4807, 1758.8918, 1761.8734, 1765.8154, 1772.0012, 1774.9300, 1774.9319, 1779.9012, 1781.8585, 1783.8062, 1787.8407, 1809.0583, 1810.0460, 1812.9126, 1829.8527, 1832.8584, 1833.8571, 1834.8650, 1836.8618, 1838.8677, 1845.8363, 1847.8600, 1848.8938, 1850.9128, 1851.8932, 1861.8531, 1862.9542, 1864.9258, 1880.9026, 1883.7704, 1887.8878, 1897.7863, 1902.9769, 1904.9276, 1908.8986, 1920.9131, 1923.9126, 1928.0443, 1932.0068, 1936.9510, 1938.0171, 1947.9314, 1958.9393, 1959.9655, 1960.9813, 1961.9463, 1967.1549, 1969.9951, 1973.9971, 1975.9755, 1976.9708, 1990.4971, 1990.9373, 1991.4857, 1997.8479, 2001.8607, 2003.8768, 2004.8850, 2007.9500, 2007.9532, 2011.9381, 2012.8951, 2015.9694, 2017.9860, 2018.9740, 2020.1631, 2023.9482, 2026.9529, 2027.5033, 2029.9972, 2043.5356, 2044.9940, 2055.0205, 2057.0154, 2060.9878, 2066.9939, 2068.9661, 2082.9822, 2086.0254, 2088.1033, 2097.0371, 2098.4941, 2102.9954, 2109.0095, 2112.9932, 2114.9707, 2122.9578, 2128.0444, 2130.9980, 2132.0200, 2136.9250, 2143.0237, 2144.0559, 2145.0781, 2148.0483, 2153.0049, 2153.9973, 2174.0508, 2175.0435, 2178.0483, 2188.0610, 2191.0464, 2192.0427, 2199.1150, 2205.0615, 2211.1067, 2223.1533, 2225.1382, 2226.1472, 2239.1646, 2243.1448, 2246.9482, 2251.1108, 2255.1484, 2265.0837, 2271.1211, 2273.0735, 2277.0642, 2283.1606, 2285.1355, 2286.0986, 2289.0901, 2291.0173, 2309.0349, 2328.9888, 2343.0608, 2352.9583, 2355.0383, 2366.9595, 2371.0325, 2392.1594, 2400.0532, 2424.1577, 2434.2429, 2438.2283, 2449.2180, 2450.2451, 2451.2480, 2465.1592, 2466.2458, 2467.2458, 2478.1377, 2501.2207, 2523.2014, 2526.2585, 2540.2451, 2542.2578, 2545.2258, 2550.2373, 2561.6841, 2562.1826, 2562.6890, 2573.2495, 2576.1792, 2646.9758, 2659.2144, 2661.1409, 2662.0120, 2671.1924, 2681.2705, 2682.2888, 2685.3098, 2689.3059, 2695.1064, 2696.2695, 2699.2324, 2703.2529, 2705.2944, 2712.2937, 2717.2903, 2720.2839, 2721.2937, 2726.2935, 2727.2986, 2728.3079, 2739.2837, 2743.2729, 2744.2207, 2745.2256, 2746.2031, 2748.2295, 2755.2700, 2760.2590, 2763.1829, 2778.1865, 2785.2969, 2786.2561, 2790.2317, 2792.2166, 2800.3093, 2801.2932, 2803.2415, 2804.2429, 2807.2869, 2808.2227, 2812.2244, 2813.2373, 2817.2632, 2820.2441, 2824.2598, 2828.2454, 2833.2544, 2843.2900, 2844.3398, 2847.3630, 2851.3313, 2864.3188, 2890.3293, 2896.4324, 2908.3105, 2910.3325, 2911.3594, 2912.4265, 2925.3323, 2927.4434, 3023.3674, 3025.3865, 3026.3452, 3039.3838, 3042.3542, 3070.3745, 3071.3933, 3086.3984, 3087.3970, 3093.4294, 3101.3831, 3103.4006, 3104.4041, 3110.4143, 3127.4749, 3141.4126, 3143.5066, 3144.5298, 3159.5210, 3219.4854, 3236.5574, 3266.6021, 3280.5635, 3321.6216, 3344.4895, 3347.5515, 3348.5020, 3380.5210, 3396.5195, 3412.5801, 3427.6099, 3428.5874, 3444.5840, 3462.6301, 3478.6123, 3484.6497, 3489.7246, 3510.5859, 3552.6614, 3553.6028, 3554.5630, 3558.6460, 3573.6545, 3589.6523, 3608.6155, 3624.6685, 3630.5806, 3641.7090, 3651.7944, 3667.7266, 3683.6980, 3691.7681, 3707.7200, 3714.5493, 3715.6206, 3719.6914, 3720.7048, 3723.7041, 3735.7100, 3751.7539, 3830.8091, 3869.7393, 3876.6707, 3885.6946

44. Q0P584 **Mass:** 23768 **Score:** 16 **Expect:** 5.9e+002 **Matches:** 11

Uncharacterized protein OS=Bos taurus GN=ZMAT2 PE=2 SV=1

Observed	Mr(expt)	Mr(calc)	ppm	Start		End	Miss	Ions	Peptide
936.4976	935.4903	935.4746	16.9	132	-	138	2	---	K.KMEEKQK.D + Oxidation (M)
1074.6162	1073.6089	1073.6193	-9.66	117	-	125	2	---	R.STLDQVKKR.F
1229.6874	1228.6801	1228.6676	10.2	47	-	55	2	---	R.ELLRHRDYK.V
1320.6364	1319.6291	1319.6391	-7.52	146	-	155	2	---	R.MKELREEEEK.A

1553.7668	1552.7595	1552.7409	12.0	16 -	27	2	---	K.WDKDEYEKLAEK.R
1555.7836	1554.7763	1554.7573	12.3	1 -	14	2	---	-.MASGSGTKNLDFRR.K + Oxidation (M)
2813.2373	2812.2300	2812.1732	20.2	171 -	196	0	---	R.AEEDLTFEEDDEMAAVMGFSGFGSTK.K
3023.3674	3022.3601	3022.3909	-10.17	65 -	90	1	---	K.TIVITKTTPQSEMGGYYCNVCDCVVK.D
3039.3838	3038.3765	3038.3858	-3.05	65 -	90	1	---	K.TIVITKTTPQSEMGGYYCNVCDCVVK.D + Oxidation (M)
3141.4126	3140.4053	3140.3703	11.2	169 -	196	2	---	K.RRAEEDLTFEEDDEMAAVMGFSGFGSTK.K + Oxidation (M)
3321.6216	3320.6143	3320.5914	6.92	62 -	90	2	---	K.LGKTIVITKTTPQSEMGGYYCNVCDCVVK.D

No match to: 716.3353, 718.2895, 724.2175, 726.3070, 728.2833, 729.4336, 730.2399, 732.2518, 734.4341, 743.3130, 745.3617, 747.4360, 753.3469, 756.3524, 758.3462, 759.3632, 768.3517, 779.4633, 781.3725, 789.3640, 794.3674, 796.3701, 799.2854, 801.4280, 805.4417, 807.4076, 810.3293, 812.3207, 818.3782, 820.3700, 828.4415, 829.4508, 830.4513, 834.3805, 842.5101, 849.3834, 852.4368, 857.4457, 860.4262, 868.4412, 870.4462, 873.4370, 877.4166, 880.4280, 882.4470, 883.4816, 887.4295, 889.4857, 891.4872, 894.4923, 898.4610, 900.5174, 905.4763, 907.4251, 912.5086, 914.4942, 920.4296, 922.4855, 928.4565, 929.4484, 933.4327, 938.5062, 944.4982, 946.4672, 949.5244, 950.5168, 957.5792, 961.5109, 965.5151, 967.4963, 972.5023, 974.4913, 976.5255, 978.5352, 981.5225, 983.6221, 986.5463, 988.4922, 993.5123, 995.4512, 998.5833, 1000.5594, 1006.5120, 1007.5640, 1021.5762, 1024.5542, 1031.5300, 1034.5703, 1037.5466, 1041.5637, 1043.5297, 1044.5375, 1045.5594, 1046.6058, 1054.5287, 1056.5575, 1057.6187, 1057.6200, 1068.5790, 1076.6190, 1082.6056, 1105.5938, 1110.5851, 1113.5701, 1118.5687, 1120.6050, 1122.5892, 1123.6047, 1126.6241, 1128.5618, 1130.5756, 1135.5951, 1137.5647, 1143.5592, 1145.6373, 1148.6318, 1149.6230, 1150.6226, 1156.5652, 1157.5676, 1159.5890, 1160.5966, 1169.5873, 1177.6421, 1178.6371, 1180.6443, 1182.6539, 1184.6431, 1185.6815, 1193.6090, 1194.6001, 1209.6238, 1213.7180, 1216.7178, 1221.5953, 1227.6459, 1234.6899, 1238.2251, 1239.6622, 1254.6993, 1255.6351, 1264.7139, 1265.1680, 1265.7196, 1269.6532, 1276.6729, 1278.6892, 1281.6375, 1285.6674, 1289.7029, 1293.6760, 1301.6362, 1303.6489, 1304.6685, 1306.6946, 1308.6797, 1314.7227, 1319.6770, 1323.6815, 1326.6937, 1328.6799, 1333.7430, 1336.7301, 1337.6429, 1349.6729, 1351.6969, 1353.6891, 1356.6255, 1358.6919, 1359.7013, 1361.7147, 1364.7118, 1365.6619, 1366.6965, 1372.6228, 1375.6693, 1382.6771, 1383.7347, 1390.7089, 1393.8007, 1397.7449, 1403.7434, 1410.7849, 1416.7737, 1420.7114, 1423.7096, 1434.7847, 1436.7469, 1442.8257, 1445.7913, 1456.7555, 1457.7440, 1467.7560, 1470.7466, 1475.7615, 1479.8038, 1483.7579, 1484.7480, 1487.7660, 1488.7366, 1493.7556, 1500.7393, 1500.7400, 1503.7648, 1504.7708, 1514.7063, 1516.7281, 1519.7705, 1522.7722, 1524.8040, 1526.7924, 1528.7079, 1530.7058, 1536.7252, 1537.7859, 1540.8043, 1543.7938, 1550.7543, 1551.7465, 1556.8011, 1562.8008, 1568.7620, 1572.7245, 1578.8058, 1580.8580, 1580.8600, 1584.8486, 1587.7675, 1608.8051, 1616.8165, 1621.7870, 1625.7886, 1628.7313, 1632.8120, 1635.7964, 1637.7974, 1642.7417, 1647.8439, 1650.8429, 1651.8369, 1655.8147, 1657.8483, 1658.8953, 1660.8379, 1662.8666, 1664.8490, 1666.7892, 1679.8091, 1680.8357, 1682.8409, 1696.8464, 1698.8180, 1701.8243, 1702.8386, 1707.8938, 1710.8904, 1714.7905, 1716.8219, 1717.9052, 1718.9094, 1721.8976, 1722.9003, 1724.9030, 1728.8840, 1730.8910, 1734.1617, 1738.8267, 1739.8165, 1741.8934, 1743.9005, 1747.9127, 1758.4807, 1758.8918, 1761.8734, 1765.8154, 1772.0012, 1774.9300, 1774.9319, 1779.9012, 1781.8585, 1783.8062, 1787.8407, 1809.0583, 1810.0460, 1812.9126, 1829.8527, 1832.8584, 1833.8571, 1834.8650, 1836.8618, 1838.8677, 1845.8363, 1847.8600, 1848.8938, 1850.9128, 1851.8932, 1861.8531, 1862.9542, 1864.9258, 1880.9026, 1883.7704, 1887.8878, 1897.7863, 1902.9769, 1904.9276, 1908.8986, 1920.9131, 1923.9126, 1928.0443, 1932.0068, 1936.9510, 1938.0171, 1947.9314, 1958.9393, 1959.9655, 1960.9813, 1961.9463, 1967.1549, 1969.9951, 1973.9971, 1975.9755, 1976.9708, 1990.4971, 1990.9373, 1991.4857, 1997.8479, 2001.8607, 2003.8768, 2004.8850, 2007.9500, 2007.9532, 2011.9381, 2012.8951, 2015.9694, 2017.9860, 2018.9740, 2020.1631, 2023.9482, 2026.9529, 2027.5033, 2029.9972, 2043.5356, 2044.9940, 2055.0205, 2057.0154, 2060.9878, 2066.9939, 2068.9661, 2082.9822, 2086.0254, 2088.1033, 2097.0371, 2098.4941, 2102.9954, 2109.0095, 2112.9932, 2114.9707, 2122.9578, 2128.0444, 2130.9980, 2132.0200, 2136.9250, 2140.0063, 2143.0237, 2144.0559, 2145.0781, 2148.0483, 2153.0049, 2153.9973, 2174.0508, 2175.0435, 2178.0483, 2188.0610, 2191.0464, 2192.0427, 2199.1150, 2205.0615, 2211.1067, 2223.1533, 2225.1382, 2226.1472, 2239.1646, 2243.1448, 2246.9482, 2251.1108, 2255.1484, 2265.0837, 2271.1211, 2273.0735, 2277.0642, 2283.1606, 2285.1355, 2286.0986, 2289.0901, 2291.0173, 2309.0349, 2328.9888, 2343.0608, 2352.9583, 2355.0383, 2366.9595, 2371.0325, 2392.1594, 2400.0532, 2424.1577, 2434.2429, 2438.2283, 2449.2180, 2450.2451, 2451.2480, 2465.1592, 2466.2458, 2467.2458, 2478.1377, 2501.2207, 2523.2014, 2526.2585, 2540.2451, 2542.2578, 2545.2258, 2550.2373, 2561.6841, 2562.1826, 2562.6890, 2573.2495, 2576.1792, 2646.9758, 2659.2144, 2661.1409, 2662.0120, 2671.1924, 2681.2705, 2682.2888, 2685.3098, 2689.3059, 2695.1064, 2696.2695, 2699.2324, 2703.2529, 2705.2944, 2712.2937, 2717.2903, 2720.2839, 2721.2937, 2726.2935, 2727.2986, 2728.3079, 2739.2837, 2743.2729, 2744.2207, 2745.2256, 2746.2031, 2748.2295, 2755.2700, 2760.2590, 2763.1829, 2771.2400, 2778.1865, 2785.2969, 2786.2561, 2787.2524, 2790.2317, 2792.2166, 2800.3093, 2801.2932, 2803.2415, 2804.2429, 2807.2869, 2808.2227, 2812.2244, 2817.2632, 2820.2441, 2824.2598, 2828.2454, 2833.2544, 2843.2900, 2844.3398, 2847.3630, 2851.3313, 2864.3188, 2890.3293, 2896.4324, 2908.3105, 2910.3325, 2911.3594, 2912.4265, 2925.3323, 2927.4434, 3025.3865, 3026.3452, 3042.3542, 3070.3745, 3071.3933, 3086.3984, 3087.3970, 3093.4294, 3101.3831, 3103.4006, 3104.4041, 3110.4143, 3127.4749, 3143.5066, 3144.5298, 3159.5210, 3219.4854, 3236.5574, 3266.6021, 3280.5635, 3344.4895, 3347.5515, 3348.5020, 3380.5210, 3396.5195, 3412.5801, 3427.6099, 3428.5874, 3444.5840, 3462.6301, 3478.6123, 3484.6497, 3489.7246, 3505.6919, 3510.5859, 3521.6392, 3552.6614, 3553.6028, 3554.5630, 3558.6460, 3573.6545, 3589.6523, 3608.6155, 3624.6685, 3630.5806, 3641.7090, 3651.7944, 3667.7266, 3683.6980, 3691.7681, 3707.7200, 3714.5493, 3715.6206, 3719.6914, 3720.7048, 3723.7041, 3735.7100, 3751.7539, 3830.8091, 3869.7393, 3876.6707, 3885.6946

45. F1MH60 **Mass:** 51585 **Score:** 16 **Expect:** 6.1e+002 **Matches:** 18

Uncharacterized protein OS=Bos taurus GN=SPRED1 PE=4 SV=2

Observed	Mr(expt)	Mr(calc)	ppm	Start	End	Miss	Ions	Peptide
852.4368	851.4295	851.4137	18.5	207 -	213	0	---	R.SIEYGQR.Q
1122.5892	1121.5819	1121.5647	15.4	69 -	77	0	---	K.MVVLECMLK.K
1365.6619	1364.6546	1364.6866	-23.42	67 -	77	1	---	R.DKMVVLECMLK.K
1493.7556	1492.7483	1492.7815	-22.25	67 -	78	2	---	R.DKMVVLECMLKK.D
1637.7974	1636.7901	1636.7569	20.3	256 -	267	2	---	R.RYADYRHPDMWK.N
1650.8429	1649.8356	1649.8667	-18.82	65 -	77	2	---	R.LRDKMVVLECMLK.K + Oxidation (M)
1783.8062	1782.7989	1782.8272	-15.86	273 -	288	1	---	R.DDTDSSVQFSKPDSKK.S
1923.9126	1922.9053	1922.9520	-24.26	207 -	223	2	---	R.SIEYGQRQMPKESGGLK.S + Oxidation (M)
1997.8479	1996.8406	1996.8268	6.92	334 -	348	1	---	R.CVYCQERFNHEENGR.G
2057.0154	2056.0081	2055.9796	13.9	189 -	206	0	---	R.SQANQIMFSHPGLDIQDR.S
2109.0095	2108.0022	2107.9534	23.2	257 -	272	2	---	R.YADYRHPDMWKNDLER.D
2703.2529	2702.2456	2702.2388	2.53	1 -	24	2	---	-.MSEETATSDNDNSYARVRAVVMTR.D
2890.3293	2889.3220	2889.3828	-21.02	189 -	213	1	---	R.SQANQIMFSHPGLDIQDRSIEYGQR.Q
2896.4324	2895.4251	2895.4259	-0.28	19 -	46	1	---	R.AVVMTRDDSSGGWLPLGGSGLSCVTVFK.V
2912.4265	2911.4192	2911.4209	-0.56	19 -	46	1	---	R.AVVMTRDDSSGGWLPLGGSGLSCVTVFK.V + Oxidation (M)
3093.4294	3092.4221	3092.3782	14.2	262 -	287	2	---	R.HPDMWKNDLERDDTDSSVQFSKPDSK.K + Oxidation (M)
3144.5298	3143.5225	3143.5768	-17.25	399 -	423	1	---	K.FCLRWLALVALSFIVPCMCCYVPLR.M
3869.7393	3868.7320	3868.7432	-2.90	119 -	154	1	---	R.AIEDISQGCPSFKNEAEGTEDNLQAAEEDTSSSLVK.D

No match to: 716.3353, 718.2895, 724.2175, 726.3070, 728.2833, 729.4336, 730.2399, 732.2518, 734.4341, 743.3130, 745.3617, 747.4360, 753.3469, 756.3524, 758.3462, 759.3632, 768.3517, 779.4633, 781.3725, 789.3640, 794.3674, 796.3701, 799.2854, 801.4280, 805.4417, 807.4076, 810.3293, 812.3207, 818.3782, 820.3700, 828.4415, 829.4508, 830.4513, 834.3805, 842.5101, 849.3834, 857.4457, 860.4262, 868.4412, 870.4462, 873.4370, 877.4166, 880.4280, 882.4470, 883.4816, 887.4295, 889.4857, 891.4872, 894.4923, 898.4610, 900.5174, 905.4763, 907.4251, 912.5086, 914.4942, 920.4296, 922.4855, 928.4565, 929.4484, 933.4327, 936.4976, 938.5062, 944.4982, 946.4672, 949.5244, 950.5168, 957.5792, 961.5109, 965.5151, 967.4963, 972.5023, 974.4913, 976.5255, 978.5352, 981.5225, 983.6221, 986.5463, 988.4922, 993.5123, 995.4512, 998.5833, 1000.5594, 1006.5120, 1007.5640, 1021.5762, 1024.5542, 1031.5300, 1034.5703, 1037.5466, 1041.5637, 1043.5297, 1044.5375, 1045.5594, 1046.6058, 1054.5287, 1056.5575, 1057.6187, 1057.6200, 1068.5790, 1074.6162, 1076.6190, 1082.6056, 1105.5938, 1110.5851, 1113.5701, 1118.5687, 1120.6050, 1123.6047, 1126.6241, 1128.5618, 1130.5756, 1135.5951, 1137.5647, 1143.5592, 1145.6373, 1148.6318, 1149.6230, 1150.6226, 1156.5652, 1157.5676, 1159.5890, 1160.5966, 1169.5873, 1177.6421, 1178.6371, 1180.6443, 1182.6539, 1184.6431, 1185.6815, 1193.6090, 1194.6001, 1209.6238, 1213.7180, 1216.7178, 1221.5953, 1227.6459, 1229.6874, 1234.6899, 1238.2251, 1239.6622, 1254.6993, 1255.6351, 1264.7139, 1265.1680, 1265.7196, 1269.6532, 1276.6729, 1278.6892, 1281.6375, 1285.6674, 1289.7029, 1293.6760, 1301.6362, 1303.6489, 1304.6685, 1306.6946, 1308.6797, 1314.7227, 1319.6770, 1320.6364, 1323.6815, 1326.6937, 1328.6799, 1333.7430, 1336.7301, 1337.6429, 1349.6729, 1351.6969, 1353.6891, 1356.6255, 1358.6919, 1359.7013, 1361.7147, 1364.7118, 1366.6965, 1372.6228, 1375.6693, 1382.6771, 1383.7347, 1390.7089, 1393.8007, 1397.7449, 1403.7434, 1410.7849, 1416.7737, 1420.7114, 1423.7096, 1434.7847, 1436.7469, 1442.8257, 1445.7913, 1456.7555, 1457.7440, 1467.7560, 1470.7466, 1475.7615, 1479.8038, 1483.7579, 1484.7480, 1487.7660, 1488.7366, 1500.7393, 1500.7400, 1503.7648, 1504.7708, 1514.7063, 1516.7281, 1519.7705, 1522.7722, 1524.8040, 1526.7924, 1528.7079, 1530.7058, 1536.7252, 1537.7859, 1540.8043, 1543.7938, 1550.7543, 1551.7465, 1553.7668, 1555.7836, 1556.8011, 1562.8008, 1568.7620, 1572.7245, 1578.8058, 1580.8580, 1580.8600, 1584.8486, 1587.7675, 1608.8051, 1616.8165, 1621.7870, 1625.7886, 1628.7313, 1632.8120, 1635.7964, 1642.7417, 1647.8439, 1651.8369, 1655.8147, 1657.8483, 1658.8953, 1660.8379,

1662.8666, 1664.8490, 1666.7892, 1679.8091, 1680.8357, 1682.8409, 1696.8464, 1698.8180, 1701.8243, 1702.8386, 1707.8938, 1710.8904, 1714.7905, 1716.8219, 1717.9052, 1718.9094, 1721.8976, 1722.9003, 1724.9030, 1728.8840, 1730.8910, 1734.1617, 1738.8267, 1739.8165, 1741.8934, 1743.9005, 1747.9127, 1758.4807, 1758.8918, 1761.8734, 1765.8154, 1772.0012, 1774.9300, 1774.9319, 1779.9012, 1781.8585, 1787.8407, 1809.0583, 1810.0460, 1812.9126, 1829.8527, 1832.8584, 1833.8571, 1834.8650, 1836.8618, 1838.8677, 1845.8363, 1847.8600, 1848.8938, 1850.9128, 1851.8932, 1861.8531, 1862.9542, 1864.9258, 1880.9026, 1883.7704, 1887.8878, 1897.7863, 1902.9769, 1904.9276, 1908.8986, 1920.9131, 1928.0443, 1932.0068, 1936.9510, 1938.0171, 1947.9314, 1958.9393, 1959.9655, 1960.9813, 1961.9463, 1967.1549, 1969.9951, 1973.9971, 1975.9755, 1976.9708, 1990.4971, 1990.9373, 1991.4857, 2001.8607, 2003.8768, 2004.8850, 2007.9500, 2007.9532, 2011.9381, 2012.8951, 2015.9694, 2017.9860, 2018.9740, 2020.1631, 2023.9482, 2026.9529, 2027.5033, 2029.9972, 2043.5356, 2044.9940, 2055.0205, 2060.9878, 2066.9939, 2068.9661, 2082.9822, 2086.0254, 2088.1033, 2097.0371, 2098.4941, 2102.9954, 2112.9932, 2114.9707, 2122.9578, 2128.0444, 2130.9980, 2132.0200, 2136.9250, 2140.0063, 2143.0237, 2144.0559, 2145.0781, 2148.0483, 2153.0049, 2153.9973, 2174.0508, 2175.0435, 2178.0483, 2188.0610, 2191.0464, 2192.0427, 2199.1150, 2205.0615, 2211.1067, 2223.1533, 2225.1382, 2226.1472, 2239.1646, 2243.1448, 2246.9482, 2251.1108, 2255.1484, 2265.0837, 2271.1211, 2273.0735, 2277.0642, 2283.1606, 2285.1355, 2286.0986, 2289.0901, 2291.0173, 2309.0349, 2328.9888, 2343.0608, 2352.9583, 2355.0383, 2366.9595, 2371.0325, 2392.1594, 2400.0532, 2424.1577, 2434.2429, 2438.2283, 2449.2180, 2450.2451, 2451.2480, 2465.1592, 2466.2458, 2467.2458, 2478.1377, 2501.2207, 2523.2014, 2526.2585, 2540.2451, 2542.2578, 2545.2258, 2550.2373, 2561.6841, 2562.1826, 2562.6890, 2573.2495, 2576.1792, 2646.9758, 2659.2144, 2661.1409, 2662.0120, 2671.1924, 2681.2705, 2682.2888, 2685.3098, 2689.3059, 2695.1064, 2696.2695, 2699.2324, 2705.2944, 2712.2937, 2717.2903, 2720.2839, 2721.2937, 2726.2935, 2727.2986, 2728.3079, 2739.2837, 2743.2729, 2744.2207, 2745.2256, 2746.2031, 2748.2295, 2755.2700, 2760.2590, 2763.1829, 2771.2400, 2778.1865, 2785.2969, 2786.2561, 2787.2524, 2790.2317, 2792.2166, 2800.3093, 2801.2932, 2803.2415, 2804.2429, 2807.2869, 2808.2227, 2812.2244, 2813.2373, 2817.2632, 2820.2441, 2824.2598, 2828.2454, 2833.2544, 2843.2900, 2844.3398, 2847.3630, 2851.3313, 2864.3188, 2908.3105, 2910.3325, 2911.3594, 2925.3323, 2927.4434, 3023.3674, 3025.3865, 3026.3452, 3039.3838, 3042.3542, 3070.3745, 3071.3933, 3086.3984, 3087.3970, 3101.3831, 3103.4006, 3104.4041, 3110.4143, 3127.4749, 3141.4126, 3143.5066, 3159.5210, 3219.4854, 3236.5574, 3266.6021, 3280.5635, 3321.6216, 3344.4895, 3347.5515, 3348.5020, 3380.5210, 3396.5195, 3412.5801, 3427.6099, 3428.5874, 3444.5840, 3462.6301, 3478.6123, 3484.6497, 3489.7246, 3505.6919, 3510.5859, 3521.6392, 3552.6614, 3553.6028, 3554.5630, 3558.6460, 3573.6545, 3589.6523, 3608.6155, 3624.6685, 3630.5806, 3641.7090, 3651.7944, 3667.7266, 3683.6980, 3691.7681, 3707.7200, 3714.5493, 3715.6206, 3719.6914, 3720.7048, 3723.7041, 3735.7100, 3751.7539, 3830.8091, 3876.6707, 3885.6946

46. Q3ZCH0 **Mass:** 73981 **Score:** 16 **Expect:** 6.2e+002 **Matches:** 30

Stress-70 protein, mitochondrial OS=Bos taurus GN=HSPA9 PE=2 SV=1

Observed	Mr(expt)	Mr(calc)	ppm	Start	End	Miss	Ions	Peptide
900.5174	899.5101	899.5229	-14.17	285 -	291	1	---	R.HIVKEFK.R
1045.5594	1044.5521	1044.5314	19.9	647 -	654	1	---	K.LFEMAYKK.M + Oxidation (M)
1118.5687	1117.5614	1117.5662	-4.28	301 -	309	1	---	K.DNMALQRVR.E + Oxidation (M)
1148.6318	1147.6245	1147.6131	9.92	1 -	11	1	---	-.MISASRAAVSR.F
1149.6230	1148.6157	1148.6223	-5.72	611 -	619	2	---	K.LKEEISKMR.E + Oxidation (M)
1285.6674	1284.6601	1284.6786	-14.36	74 -	85	1	---	K.QAKVLENAEGAR.T
1349.6729	1348.6656	1348.6510	10.8	128 -	138	1	---	R.YDDPEVQKDIK.N
1361.7147	1360.7074	1360.7211	-10.08	12 -	25	1	---	R.FVGTAASRGPTAAR.H
1568.7620	1567.7547	1567.7631	-5.32	108 -	121	0	---	R.QAVTNPNNTFYATK.R
1608.8051	1607.7978	1607.7613	22.7	174 -	187	1	---	K.MKETAENYLGHTAK.N + Oxidation (M)
1635.7964	1634.7891	1634.7796	5.82	647 -	659	2	---	K.LFEMAYKKMASER.E + 2 Oxidation (M)
1662.8666	1661.8593	1661.8559	2.04	392 -	405	1	---	R.MPKVQQTVQDLFGR.A + Oxidation (M)
1724.9030	1723.8957	1723.8642	18.3	108 -	122	1	---	R.QAVTNPNNTFYATKR.L
1730.8910	1729.8837	1729.8958	-7.00	620 -	634	2	---	R.ELLARKDSETGENIR.Q
1862.9542	1861.9469	1861.9316	8.23	292 -	307	2	---	K.RETGVDLTKDNMALQR.V + Oxidation (M)
1936.9510	1935.9437	1935.9902	-23.99	468 -	485	1	---	K.KSQVFSTAADGQTQVEIK.V
1938.0171	1937.0098	1937.0265	-8.60	1 -	19	2	---	-.MISASRAAVSRFVGTAASR.G
2088.1033	2087.0960	2087.0971	-0.51	536 -	555	2	---	K.DKGTGREQQIVIQSSGGLSK.D
2109.0095	2108.0022	2108.0300	-13.18	26 -	43	1	---	R.HQDGWNGLSHEAFRIVSR.R
2265.0837	2264.0764	2264.1311	-24.16	26 -	44	2	---	R.HQDGWNGLSHEAFRIVSRR.D
2286.0986	2285.0913	2285.1321	-17.86	576 -	595	2	---	K.KERVEAVNMAEGIIHDTETK.M + Oxidation (M)
2434.2429	2433.2356	2433.2057	12.3	542 -	563	1	---	R.EQQIVIQSSGGLSKDDIENMVK.N + Oxidation (M)
2438.2283	2437.2210	2437.1829	15.7	369 -	391	1	---	K.AMQDAEVSKSDIGEVILVGGMTR.M + 2 Oxidation (M)
3127.4749	3126.4676	3126.5325	-20.77	44 -	73	2	---	R.RDYASEAIKGAVVGIDLGTTNSCVAVMEGK.Q + Oxidation (M)
3159.5210	3158.5137	3158.5780	-20.35	160 -	187	2	---	K.LYSPSQIGAFVLMKMKETAENYLGHTAK.N + 2 Oxidation (M)
3236.5574	3235.5501	3235.5887	-11.92	362 -	391	2	---	R.TIAPCQKAMQDAEVSKSDIGEVILVGGMTR.M + 2 Oxidation (M)
3651.7944	3650.7871	3650.7742	3.53	317 -	348	2	---	K.CELSSSVQTDINLPYLTMDASGPKHLNMKLTR.A + 2 Oxidation (M)
3691.7681	3690.7608	3690.6851	20.5	579 -	610	2	---	R.VEAVNMAEGIIHDTETKMEEFKDQLPADECNK.L
3707.7200	3706.7127	3706.6800	8.82	579 -	610	2	---	R.VEAVNMAEGIIHDTETKMEEFKDQLPADECNK.L + Oxidation (M)
3723.7041	3722.6968	3722.6750	5.87	579 -	610	2	---	R.VEAVNMAEGIIHDTETKMEEFKDQLPADECNK.L + 2 Oxidation (M)

No match to: 716.3353, 718.2895, 724.2175, 726.3070, 728.2833, 729.4336, 730.2399, 732.2518, 734.4341, 743.3130, 745.3617, 747.4360, 753.3469, 756.3524, 758.3462, 759.3632, 768.3517, 779.4633, 781.3725, 789.3640, 794.3674, 796.3701, 799.2854, 801.4280, 805.4417, 807.4076, 810.3293, 812.3207, 818.3782, 820.3700, 828.4415, 829.4508, 830.4513, 834.3805, 842.5101, 849.3834, 852.4368, 857.4457, 860.4262, 868.4412, 870.4462, 873.4370, 877.4166, 880.4280, 882.4470, 883.4816, 887.4295, 889.4857, 891.4872, 894.4923, 898.4610, 905.4763, 907.4251, 912.5086, 914.4942, 920.4296, 922.4855, 928.4565, 929.4484, 933.4327, 936.4976, 938.5062, 944.4982, 946.4672, 949.5244, 950.5168, 957.5792, 961.5109, 965.5151, 967.4963, 972.5023, 974.4913, 976.5255, 978.5352, 981.5225, 983.6221, 986.5463, 988.4922, 993.5123, 995.4512, 998.5833, 1000.5594, 1006.5120, 1007.5640, 1021.5762, 1024.5542, 1031.5300, 1034.5703, 1037.5466, 1041.5637, 1043.5297, 1044.5375, 1046.6058, 1054.5287, 1056.5575, 1057.6187, 1057.6200, 1068.5790, 1074.6162, 1076.6190, 1082.6056, 1105.5938, 1110.5851, 1113.5701, 1120.6050, 1122.5892, 1123.6047, 1126.6241, 1128.5618, 1130.5756, 1135.5951, 1137.5647, 1143.5592, 1145.6373, 1150.6226, 1156.5652, 1157.5676, 1159.5890, 1160.5966, 1169.5873, 1177.6421, 1178.6371, 1180.6443, 1182.6539, 1184.6431, 1185.6815, 1193.6090, 1194.6001, 1209.6238, 1213.7180, 1216.7178, 1221.5953, 1227.6459, 1229.6874, 1234.6899, 1238.2251, 1239.6622, 1254.6993, 1255.6351, 1264.7139, 1265.1680, 1265.7196, 1269.6532, 1276.6729, 1278.6892, 1281.6375, 1289.7029, 1293.6760, 1301.6362, 1303.6489, 1304.6685, 1306.6946, 1308.6797, 1314.7227, 1319.6770, 1320.6364, 1323.6815, 1326.6937, 1328.6799, 1333.7430, 1336.7301, 1337.6429, 1351.6969, 1353.6891, 1356.6255, 1358.6919, 1359.7013, 1364.7118, 1365.6619, 1366.6965, 1372.6228, 1375.6693, 1382.6771, 1383.7347, 1390.7089, 1393.8007, 1397.7449, 1403.7434, 1410.7849, 1416.7737, 1420.7114, 1423.7096, 1434.7847, 1436.7469, 1442.8257, 1445.7913, 1456.7555, 1457.7440, 1467.7560, 1470.7466, 1475.7615, 1479.8038, 1483.7579, 1484.7480, 1487.7660, 1488.7366, 1493.7556, 1500.7393, 1500.7400, 1503.7648, 1504.7708, 1514.7063, 1516.7281, 1519.7705, 1522.7722, 1524.8040, 1526.7924, 1528.7079, 1530.7058, 1536.7252, 1537.7859, 1540.8043, 1543.7938, 1550.7543, 1551.7465, 1553.7668, 1555.7836, 1556.8011, 1562.8008, 1572.7245, 1578.8058, 1580.8580, 1580.8600, 1584.8486, 1587.7675, 1616.8165, 1621.7870, 1625.7886, 1628.7313, 1632.8120, 1637.7974, 1642.7417, 1647.8439, 1650.8429, 1651.8369, 1655.8147, 1657.8483, 1658.8953, 1660.8379, 1664.8490, 1666.7892, 1679.8091, 1680.8357, 1682.8409, 1696.8464, 1698.8180, 1701.8243, 1702.8386, 1707.8938, 1710.8904, 1714.7905, 1716.8219, 1717.9052, 1718.9094, 1721.8976, 1722.9003, 1728.8840, 1734.1617, 1738.8267, 1739.8165, 1741.8934, 1743.9005, 1747.9127, 1758.4807, 1758.8918, 1761.8734, 1765.8154, 1772.0012, 1774.9300, 1774.9319, 1779.9012, 1781.8585, 1783.8062, 1787.8407, 1809.0583, 1810.0460, 1812.9126, 1829.8527, 1832.8584, 1833.8571, 1834.8650, 1836.8618, 1838.8677, 1845.8363, 1847.8600, 1848.8938, 1850.9128, 1851.8932, 1861.8531, 1864.9258, 1880.9026, 1883.7704, 1887.8878, 1897.7863, 1902.9769, 1904.9276, 1908.8986, 1920.9131, 1923.9126, 1928.0443, 1932.0068, 1947.9314, 1958.9393, 1959.9655, 1960.9813, 1961.9463, 1967.1549, 1969.9951, 1973.9971, 1975.9755, 1976.9708, 1990.4971, 1990.9373, 1991.4857, 1997.8479, 2001.8607, 2003.8768, 2004.8850, 2007.9500, 2007.9532, 2011.9381, 2012.8951, 2015.9694, 2017.9860, 2018.9740, 2020.1631, 2023.9482, 2026.9529, 2027.5033, 2029.9972, 2043.5356, 2044.9940, 2055.0205, 2057.0154, 2060.9878, 2066.9939, 2068.9661, 2082.9822, 2086.0254, 2097.0371, 2098.4941, 2102.9954, 2112.9932, 2114.9707, 2122.9578, 2128.0444, 2130.9980, 2132.0200, 2136.9250, 2140.0063, 2143.0237, 2144.0559, 2145.0781, 2148.0483, 2153.0049, 2153.9973, 2174.0508, 2175.0435, 2178.0483, 2188.0610, 2191.0464, 2192.0427, 2199.1150, 2205.0615, 2211.1067, 2223.1533, 2225.1382, 2226.1472, 2239.1646, 2243.1448, 2246.9482, 2251.1108, 2255.1484, 2271.1211, 2273.0735, 2277.0642, 2283.1606, 2285.1355, 2289.0901, 2291.0173, 2309.0349, 2328.9888, 2343.0608, 2352.9583, 2355.0383, 2366.9595, 2371.0325, 2392.1594, 2400.0532, 2424.1577, 2449.2180, 2450.2451, 2451.2480, 2465.1592, 2466.2458, 2467.2458, 2478.1377, 2501.2207, 2523.2014, 2526.2585, 2540.2451, 2542.2578, 2545.2258, 2550.2373, 2561.6841, 2562.1826, 2562.6890, 2573.2495, 2576.1792, 2646.9758, 2659.2144, 2661.1409, 2662.0120, 2671.1924, 2681.2705, 2682.2888, 2685.3098, 2689.3059, 2695.1064, 2696.2695, 2699.2324, 2703.2529, 2705.2944, 2712.2937,

2717.2903, 2720.2839, 2721.2937, 2726.2935, 2727.2986, 2728.3079, 2739.2837, 2743.2729, 2744.2207, 2745.2256, 2746.2031, 2748.2295, 2755.2700, 2760.2590, 2763.1829, 2771.2400, 2778.1865, 2785.2969, 2786.2561, 2787.2524, 2790.2317, 2792.2166, 2800.3093, 2801.2932, 2803.2415, 2804.2429, 2807.2869, 2808.2227, 2812.2244, 2813.2373, 2817.2632, 2820.2441, 2824.2598, 2828.2454, 2833.2544, 2843.2900, 2844.3398, 2847.3630, 2851.3313, 2864.3188, 2890.3293, 2896.4324, 2908.3105, 2910.3325, 2911.3594, 2912.4265, 2925.3323, 2927.4434, 3023.3674, 3025.3865, 3026.3452, 3039.3838, 3042.3542, 3070.3745, 3071.3933, 3086.3984, 3087.3970, 3093.4294, 3101.3831, 3103.4006, 3104.4041, 3110.4143, 3141.4126, 3143.5066, 3144.5298, 3219.4854, 3266.6021, 3280.5635, 3321.6216, 3344.4895, 3347.5515, 3348.5020, 3380.5210, 3396.5195, 3412.5801, 3427.6099, 3428.5874, 3444.5840, 3462.6301, 3478.6123, 3484.6497, 3489.7246, 3505.6919, 3510.5859, 3521.6392, 3552.6614, 3553.6028, 3554.5630, 3558.6460, 3573.6545, 3589.6523, 3608.6155, 3624.6685, 3630.5806, 3641.7090, 3667.7266, 3683.6980, 3714.5493, 3715.6206, 3719.6914, 3720.7048, 3735.7100, 3751.7539, 3830.8091, 3869.7393, 3876.6707, 3885.6946

47. Q0P571 **Mass:** 19114 **Score:** 16 **Expect:** 6.2e+002 **Matches:** 9

Myosin regulatory light chain 2, skeletal muscle isoform OS=Bos taurus GN=MYLPF PE=2 SV=1

Observed	Mr(expt)	Mr(calc)	ppm	Start	End	Miss	Ions	Peptide
880.4280	879.4207	879.4338	-14.87	132 -	138	0	---	R.FSQEEIK.N
1319.6770	1318.6697	1318.6405	22.2	94 -	106	0	---	K.GADPEDVITGAFK.V
1390.7089	1389.7016	1389.6711	22.0	156 -	167	0	---	K.NICYVITHGDAK.D
1550.7543	1549.7470	1549.7480	-0.61	61 -	73	1	---	R.LNVKNEELDAMMK.E + Oxidation (M)
1851.8932	1850.8859	1850.9309	-24.28	49 -	64	2	---	K.EDLRDTFAAMGRLNVK.N + Oxidation (M)
1880.9026	1879.8953	1879.8563	20.7	139 -	155	0	---	K.NMWAAFPPDVGGNVDYK.N
2465.1592	2464.1519	2464.1217	12.3	10 -	32	0	---	R.AAAEGGSSSVFSMFDQTQIQEFK.E
3110.4143	3109.4070	3109.4082	-0.39	65 -	91	1	---	K.NEELDAMMKEASGPINFTVFLNMFGEK.L + 3 Oxidation (M)
3624.6685	3623.6612	3623.6450	4.49	139 -	170	2	---	K.NMWAAFPPDVGGNVDYKNICYVITHGDAKDQE.-

No match to: 716.3353, 718.2895, 724.2175, 726.3070, 728.2833, 729.4336, 730.2399, 732.2518, 734.4341, 743.3130, 745.3617, 747.4360, 753.3469, 756.3524, 758.3462, 759.3632, 768.3517, 779.4633, 781.3725, 789.3640, 794.3674, 796.3701, 799.2854, 801.4280, 805.4417, 807.4076, 810.3293, 812.3207, 818.3782, 820.3700, 828.4415, 829.4508, 830.4513, 834.3805, 842.5101, 849.3834, 852.4368, 857.4457, 860.4262, 868.4412, 870.4462, 873.4370, 877.4166, 882.4470, 883.4816, 887.4295, 889.4857, 891.4872, 894.4923, 898.4610, 900.5174, 905.4763, 907.4251, 912.5086, 914.4942, 920.4296, 922.4855, 928.4565, 929.4484, 933.4327, 936.4976, 938.5062, 944.4982, 946.4672, 949.5244, 950.5168, 957.5792, 961.5109, 965.5151, 967.4963, 972.5023, 974.4913, 976.5255, 978.5352, 981.5225, 983.6221, 986.5463, 988.4922, 993.5123, 995.4512, 998.5833, 1000.5594, 1006.5120, 1007.5640, 1021.5762, 1024.5542, 1031.5300, 1034.5703, 1037.5466, 1041.5637, 1043.5297, 1044.5375, 1045.5594, 1046.6058, 1054.5287, 1056.5575, 1057.6187, 1057.6200, 1068.5790, 1074.6162, 1076.6190, 1082.6056, 1105.5938, 1110.5851, 1113.5701, 1118.5687, 1120.6050, 1122.5892, 1123.6047, 1126.6241, 1128.5618, 1130.5756, 1135.5951, 1137.5647, 1143.5592, 1145.6373, 1148.6318, 1149.6230, 1150.6226, 1156.5652, 1157.5676, 1159.5890, 1160.5966, 1169.5873, 1177.6421, 1178.6371, 1180.6443, 1182.6539, 1184.6431, 1185.6815, 1193.6090, 1194.6001, 1209.6238, 1213.7180, 1216.7178, 1221.5953, 1227.6459, 1229.6874, 1234.6899, 1238.2251, 1239.6622, 1254.6993, 1255.6351, 1264.7139, 1265.1680, 1265.7196, 1269.6532, 1276.6729, 1278.6892, 1281.6375, 1285.6674, 1289.7029, 1293.6760, 1301.6362, 1303.6489, 1304.6685, 1306.6946, 1308.6797, 1314.7227, 1320.6364, 1323.6815, 1326.6937, 1328.6799, 1333.7430, 1336.7301, 1337.6429, 1349.6729, 1351.6969, 1353.6891, 1356.6255, 1358.6919, 1359.7013, 1361.7147, 1364.7118, 1365.6619, 1366.6965, 1372.6228, 1375.6693, 1382.6771, 1383.7347, 1393.8007, 1397.7449, 1403.7434, 1410.7849, 1416.7737, 1420.7114, 1423.7096, 1434.7847, 1436.7469, 1442.8257, 1445.7913, 1456.7555, 1457.7440, 1467.7560, 1470.7466, 1475.7615, 1479.8038, 1483.7579, 1484.7480, 1487.7660, 1488.7366, 1493.7556, 1500.7393, 1500.7400, 1503.7648, 1504.7708, 1514.7063, 1516.7281, 1519.7705, 1522.7722, 1524.8040, 1526.7924, 1528.7079, 1530.7058, 1536.7252, 1537.7859, 1540.8043, 1543.7938, 1551.7465, 1553.7668, 1555.7836, 1556.8011, 1562.8008, 1568.7620, 1572.7245, 1578.8058, 1580.8580, 1580.8600, 1584.8486, 1587.7675, 1608.8051, 1616.8165, 1621.7870, 1625.7886, 1628.7313, 1632.8120, 1635.7964, 1637.7974, 1642.7417, 1647.8439, 1650.8429, 1651.8369, 1655.8147, 1657.8483, 1658.8953, 1660.8379, 1662.8666, 1664.8490, 1666.7892, 1679.8091, 1680.8357, 1682.8409, 1696.8464, 1698.8180, 1701.8243, 1702.8386, 1707.8938, 1710.8904, 1714.7905, 1716.8219, 1717.9052, 1718.9094, 1721.8976, 1722.9003, 1724.9030, 1728.8840, 1730.8910, 1734.1617, 1738.8267, 1739.8165, 1741.8934, 1743.9005, 1747.9127, 1758.4807, 1758.8918, 1761.8734, 1765.8154, 1772.0012, 1774.9300, 1774.9319, 1779.9012, 1781.8585, 1783.8062, 1787.8407, 1809.0583, 1810.0460, 1812.9126, 1829.8527, 1832.8584, 1833.8571, 1834.8650, 1836.8618, 1838.8677, 1845.8363, 1847.8600, 1848.8938, 1850.9128, 1861.8531, 1862.9542, 1864.9258, 1883.7704, 1887.8878, 1897.7863, 1902.9769, 1904.9276, 1908.8986, 1920.9131, 1923.9126, 1928.0443, 1932.0068, 1936.9510, 1938.0171, 1947.9314, 1958.9393, 1959.9655, 1960.9813, 1961.9463, 1967.1549, 1969.9951, 1973.9971, 1975.9755, 1976.9708, 1990.4971, 1990.9373, 1991.4857, 1997.8479, 2001.8607, 2003.8768, 2004.8850, 2007.9500, 2007.9532, 2011.9381, 2012.8951, 2015.9694, 2017.9860, 2018.9740, 2020.1631, 2023.9482, 2026.9529, 2027.5033, 2029.9972, 2043.5356, 2044.9940, 2055.0205, 2057.0154, 2060.9878, 2066.9939, 2068.9661, 2082.9822, 2086.0254, 2088.1033, 2097.0371, 2098.4941, 2102.9954, 2109.0095, 2112.9932, 2114.9707, 2122.9578, 2128.0444, 2130.9980, 2132.0200, 2136.9250, 2140.0063, 2143.0237, 2144.0559, 2145.0781, 2148.0483, 2153.0049, 2153.9973, 2174.0508, 2175.0435, 2178.0483, 2188.0610, 2191.0464, 2192.0427, 2199.1150, 2205.0615, 2211.1067, 2223.1533, 2225.1382, 2226.1472, 2239.1646, 2243.1448, 2246.9482, 2251.1108, 2255.1484, 2265.0837, 2271.1211, 2273.0735, 2277.0642, 2283.1606, 2285.1355, 2286.0986, 2289.0901, 2291.0173, 2309.0349, 2328.9888, 2343.0608, 2352.9583, 2355.0383, 2366.9595, 2371.0325, 2392.1594, 2400.0532, 2424.1577, 2434.2429, 2438.2283, 2449.2180, 2450.2451, 2451.2480, 2466.2458, 2467.2458, 2478.1377, 2501.2207, 2523.2014, 2526.2585, 2540.2451, 2542.2578, 2545.2258, 2550.2373, 2561.6841, 2562.1826, 2562.6890, 2573.2495, 2576.1792, 2646.9758, 2659.2144, 2661.1409, 2662.0120, 2671.1924, 2681.2705, 2682.2888, 2685.3098, 2689.3059, 2695.1064, 2696.2695, 2699.2324, 2703.2529, 2705.2944, 2712.2937, 2717.2903, 2720.2839, 2721.2937, 2726.2935, 2727.2986, 2728.3079, 2739.2837, 2743.2729, 2744.2207, 2745.2256, 2746.2031, 2748.2295, 2755.2700, 2760.2590, 2763.1829, 2771.2400, 2778.1865, 2785.2969, 2786.2561, 2787.2524, 2790.2317, 2792.2166, 2800.3093, 2801.2932, 2803.2415, 2804.2429, 2807.2869, 2808.2227, 2812.2244, 2813.2373, 2817.2632, 2820.2441, 2824.2598, 2828.2454, 2833.2544, 2843.2900, 2844.3398, 2847.3630, 2851.3313, 2864.3188, 2890.3293, 2896.4324, 2908.3105, 2910.3325, 2911.3594, 2912.4265, 2925.3323, 2927.4434, 3023.3674, 3025.3865, 3026.3452, 3039.3838, 3042.3542, 3070.3745, 3071.3933, 3086.3984, 3087.3970, 3093.4294, 3101.3831, 3103.4006, 3104.4041, 3127.4749, 3141.4126, 3143.5066, 3144.5298, 3159.5210, 3219.4854, 3236.5574, 3266.6021, 3280.5635, 3321.6216, 3344.4895, 3347.5515, 3348.5020, 3380.5210, 3396.5195, 3412.5801, 3427.6099, 3428.5874, 3444.5840, 3462.6301, 3478.6123, 3484.6497, 3489.7246, 3505.6919, 3510.5859, 3521.6392, 3552.6614, 3553.6028, 3554.5630, 3558.6460, 3573.6545, 3589.6523, 3608.6155, 3630.5806, 3641.7090, 3651.7944, 3667.7266, 3683.6980, 3691.7681, 3707.7200, 3714.5493, 3715.6206, 3719.6914, 3720.7048, 3723.7041, 3735.7100, 3751.7539, 3830.8091, 3869.7393, 3876.6707, 3885.6946

48. G3N0L1 **Mass:** 8718 **Score:** 16 **Expect:** 6.2e+002 **Matches:** 7

Uncharacterized protein OS=Bos taurus GN=FGF12 PE=3 SV=1

Observed	Mr(expt)	Mr(calc)	ppm	Start	End	Miss	Ions	Peptide
1057.6187	1056.6114	1056.5927	17.7	1 -	9	1	---	-.SRLSEPQLK.G
1057.6200	1056.6127	1056.5927	18.9	1 -	9	1	---	-.SRLSEPQLK.G
1761.8734	1760.8661	1760.8920	-14.69	10 -	24	0	---	K.GIVTMVFSQQGYFLR.M + Oxidation (M)
1880.9026	1879.8953	1879.8774	9.53	61 -	77	0	---	K.ASLYVAMNGEGYLYSSR.S
2896.4324	2895.4251	2895.3936	10.9	10 -	35	1	---	K.GIVTMVFSQQGYFLRMYPDGAVDGTK.D + Oxidation (M)
2912.4265	2911.4192	2911.3885	10.6	10 -	35	1	---	K.GIVTMVFSQQGYFLRMYPDGAVDGTK.D + 2 Oxidation (M)
3691.7681	3690.7608	3690.8426	-22.16	3 -	35	2	---	R.LSEPQLKGIVTMVFSQQGYFLRMYPDGAVDGTK.D + Oxidation (M)

No match to: 716.3353, 718.2895, 724.2175, 726.3070, 728.2833, 729.4336, 730.2399, 732.2518, 734.4341, 743.3130, 745.3617, 747.4360, 753.3469, 756.3524, 758.3462, 759.3632, 768.3517, 779.4633, 781.3725, 789.3640, 794.3674, 796.3701, 799.2854, 801.4280, 805.4417, 807.4076, 810.3293, 812.3207, 818.3782, 820.3700, 828.4415, 829.4508, 830.4513, 834.3805, 842.5101, 849.3834, 852.4368, 857.4457, 860.4262, 868.4412, 870.4462, 873.4370, 877.4166, 880.4280, 882.4470, 883.4816, 887.4295, 889.4857, 891.4872, 894.4923, 898.4610, 900.5174, 905.4763, 907.4251, 912.5086, 914.4942, 920.4296, 922.4855, 928.4565, 929.4484, 933.4327, 936.4976, 938.5062, 944.4982, 946.4672, 949.5244, 950.5168, 957.5792, 961.5109, 965.5151, 967.4963, 972.5023, 974.4913, 976.5255, 978.5352, 981.5225, 983.6221, 986.5463, 988.4922, 993.5123, 995.4512, 998.5833, 1000.5594, 1006.5120, 1007.5640, 1021.5762, 1024.5542, 1031.5300, 1034.5703, 1037.5466, 1041.5637, 1043.5297, 1044.5375, 1045.5594, 1046.6058, 1054.5287, 1056.5575, 1068.5790, 1074.6162, 1076.6190, 1082.6056, 1105.5938, 1110.5851, 1113.5701, 1118.5687, 1120.6050, 1122.5892, 1123.6047, 1126.6241, 1128.5618, 1130.5756, 1135.5951, 1137.5647, 1143.5592, 1145.6373, 1148.6318, 1149.6230, 1150.6226, 1156.5652, 1157.5676, 1159.5890, 1160.5966, 1169.5873, 1177.6421, 1178.6371, 1180.6443, 1182.6539, 1184.6431, 1185.6815, 1193.6090, 1194.6001, 1209.6238, 1213.7180, 1216.7178, 1221.5953, 1227.6459, 1229.6874, 1234.6899, 1238.2251, 1239.6622, 1254.6993, 1255.6351, 1264.7139, 1265.1680, 1265.7196, 1269.6532, 1276.6729, 1278.6892, 1281.6375, 1285.6674, 1289.7029, 1293.6760, 1301.6362, 1303.6489, 1304.6685, 1306.6946, 1308.6797, 1314.7227, 1319.6770, 1320.6364, 1323.6815, 1326.6937, 1328.6799, 1333.7430, 1336.7301, 1337.6429, 1349.6729, 1351.6969, 1353.6891, 1356.6255, 1358.6919, 1359.7013, 1361.7147, 1364.7118, 1365.6619, 1366.6965, 1372.6228, 1375.6693, 1382.6771, 1383.7347, 1390.7089, 1393.8007, 1397.7449, 1403.7434, 1410.7849, 1416.7737, 1420.7114, 1423.7096, 1434.7847, 1436.7469, 1442.8257, 1445.7913, 1456.7555, 1457.7440, 1467.7560, 1470.7466, 1475.7615, 1479.8038, 1483.7579, 1484.7480, 1487.7660, 1488.7366, 1493.7556, 1500.7393, 1500.7400, 1503.7648, 1504.7708, 1514.7063,

1516.7281, 1519.7705, 1522.7722, 1524.8040, 1526.7924, 1528.7079, 1530.7058, 1536.7252, 1537.7859, 1540.8043, 1543.7938, 1550.7543, 1551.7465, 1553.7668, 1555.7836, 1556.8011, 1562.8008, 1568.7620, 1572.7245, 1578.8058, 1580.8580, 1580.8600, 1584.8486, 1587.7675, 1608.8051, 1616.8165, 1621.7870, 1625.7886, 1628.7313, 1632.8120, 1635.7964, 1637.7974, 1642.7417, 1647.8439, 1650.8429, 1651.8369, 1655.8147, 1657.8483, 1658.8953, 1660.8379, 1662.8666, 1664.8490, 1666.7892, 1679.8091, 1680.8357, 1682.8409, 1696.8464, 1698.8180, 1701.8243, 1702.8386, 1707.8938, 1710.8904, 1714.7905, 1716.8219, 1717.9052, 1718.9094, 1721.8976, 1722.9003, 1724.9030, 1728.8840, 1730.8910, 1734.1617, 1738.8267, 1739.8165, 1741.8934, 1743.9005, 1747.9127, 1758.4807, 1758.8918, 1765.8154, 1772.0012, 1774.9300, 1774.9319, 1779.9012, 1781.8585, 1783.8062, 1787.8407, 1809.0583, 1810.0460, 1812.9126, 1829.8527, 1832.8584, 1833.8571, 1834.8650, 1836.8618, 1838.8677, 1845.8363, 1847.8600, 1848.8938, 1850.9128, 1851.8932, 1861.8531, 1862.9542, 1864.9258, 1883.7704, 1887.8878, 1897.7863, 1902.9769, 1904.9276, 1908.8986, 1920.9131, 1923.9126, 1928.0443, 1932.0068, 1936.9510, 1938.0171, 1947.9314, 1958.9393, 1959.9655, 1960.9813, 1961.9463, 1967.1549, 1969.9951, 1973.9971, 1975.9755, 1976.9708, 1990.4971, 1990.9373, 1991.4857, 1997.8479, 2001.8607, 2003.8768, 2004.8850, 2007.9500, 2007.9532, 2011.9381, 2012.8951, 2015.9694, 2017.9860, 2018.9740, 2020.1631, 2023.9482, 2026.9529, 2027.5033, 2029.9972, 2043.5356, 2044.9940, 2055.0205, 2057.0154, 2060.9878, 2066.9939, 2068.9661, 2082.9822, 2086.0254, 2088.1033, 2097.0371, 2098.4941, 2102.9954, 2109.0095, 2112.9932, 2114.9707, 2122.9578, 2128.0444, 2130.9980, 2132.0200, 2136.9250, 2140.0063, 2143.0237, 2144.0559, 2145.0781, 2148.0483, 2153.0049, 2153.9973, 2174.0508, 2175.0435, 2178.0483, 2188.0610, 2191.0464, 2192.0427, 2199.1150, 2205.0615, 2211.1067, 2223.1533, 2225.1382, 2226.1472, 2239.1646, 2243.1448, 2246.9482, 2251.1108, 2255.1484, 2265.0837, 2271.1211, 2273.0735, 2277.0642, 2283.1606, 2285.1355, 2286.0986, 2289.0901, 2291.0173, 2309.0349, 2328.9888, 2343.0608, 2352.9583, 2355.0383, 2366.9595, 2371.0325, 2392.1594, 2400.0532, 2424.1577, 2434.2429, 2438.2283, 2449.2180, 2450.2451, 2451.2480, 2465.1592, 2466.2458, 2467.2458, 2478.1377, 2501.2207, 2523.2014, 2526.2585, 2540.2451, 2542.2578, 2545.2258, 2550.2373, 2561.6841, 2562.1826, 2562.6890, 2573.2495, 2576.1792, 2646.9758, 2659.2144, 2661.1409, 2662.0120, 2671.1924, 2681.2705, 2682.2888, 2685.3098, 2689.3059, 2695.1064, 2696.2695, 2699.2324, 2703.2529, 2705.2944, 2712.2937, 2717.2903, 2720.2839, 2721.2937, 2726.2935, 2727.2986, 2728.3079, 2739.2837, 2743.2729, 2744.2207, 2745.2256, 2746.2031, 2748.2295, 2755.2700, 2760.2590, 2763.1829, 2771.2400, 2778.1865, 2785.2969, 2786.2561, 2787.2524, 2790.2317, 2792.2166, 2800.3093, 2801.2932, 2803.2415, 2804.2429, 2807.2869, 2808.2227, 2812.2244, 2813.2373, 2817.2632, 2820.2441, 2824.2598, 2828.2454, 2833.2544, 2843.2900, 2844.3398, 2847.3630, 2851.3313, 2864.3188, 2890.3293, 2908.3105, 2910.3325, 2911.3594, 2925.3323, 2927.4434, 3023.3674, 3025.3865, 3026.3452, 3039.3838, 3042.3542, 3070.3745, 3071.3933, 3086.3984, 3087.3970, 3093.4294, 3101.3831, 3103.4006, 3104.4041, 3110.4143, 3127.4749, 3141.4126, 3143.5066, 3144.5298, 3159.5210, 3219.4854, 3236.5574, 3266.6021, 3280.5635, 3321.6216, 3344.4895, 3347.5515, 3348.5020, 3380.5210, 3396.5195, 3412.5801, 3427.6099, 3428.5874, 3444.5840, 3462.6301, 3478.6123, 3484.6497, 3489.7246, 3505.6919, 3510.5859, 3521.6392, 3552.6614, 3553.6028, 3554.5630, 3558.6460, 3573.6545, 3589.6523, 3608.6155, 3624.6685, 3630.5806, 3641.7090, 3651.7944, 3667.7266, 3683.6980, 3707.7200, 3714.5493, 3715.6206, 3719.6914, 3720.7048, 3723.7041, 3735.7100, 3751.7539, 3830.8091, 3869.7393, 3876.6707, 3885.6946

49. G3MYV8 **Mass:** 6868 **Score:** 16 **Expect:** 6.4e+002 **Matches:** 6

Uncharacterized protein OS=Bos taurus PE=4 SV=1

Observed	Mr(expt)	Mr(calc)	ppm	Start	End	Miss	Ions	Peptide
1006.5120	1005.5047	1005.5138	-9.01	1 -	8	2	---	-.MTRGNQRK.L + Oxidation (M)
1031.5300	1030.5227	1030.5155	6.96	27 -	36	1	---	R.RDDGLSAAAR.K
1105.5938	1104.5865	1104.6073	-18.84	9 -	17	2	---	K.LARQKNMTK.Q + Oxidation (M)
1137.5647	1136.5574	1136.5496	6.92	14 -	23	1	---	K.NMTKQSDSVK.G
1159.5890	1158.5817	1158.6105	-24.84	27 -	37	2	---	R.RDDGLSAAARK.Q
1390.7089	1389.7016	1389.7034	-1.29	37 -	47	2	---	R.KQRDSEIMQQK.Q

No match to: 716.3353, 718.2895, 724.2175, 726.3070, 728.2833, 729.4336, 730.2399, 732.2518, 734.4341, 743.3130, 745.3617, 747.4360, 753.3469, 756.3524, 758.3462, 759.3632, 768.3517, 779.4633, 781.3725, 789.3640, 794.3674, 796.3701, 799.2854, 801.4280, 805.4417, 807.4076, 810.3293, 812.3207, 818.3782, 820.3700, 828.4415, 829.4508, 830.4513, 834.3805, 842.5101, 849.3834, 852.4368, 857.4457, 860.4262, 868.4412, 870.4462, 873.4370, 877.4166, 880.4280, 882.4470, 883.4816, 887.4295, 889.4857, 891.4872, 894.4923, 898.4610, 900.5174, 905.4763, 907.4251, 912.5086, 914.4942, 920.4296, 922.4855, 928.4565, 929.4484, 933.4327, 936.4976, 938.5062, 944.4982, 946.4672, 949.5244, 950.5168, 957.5792, 961.5109, 965.5151, 967.4963, 972.5023, 974.4913, 976.5255, 978.5352, 981.5225, 983.6221, 986.5463, 988.4922, 993.5123, 995.4512, 998.5833, 1000.5594, 1007.5640, 1021.5762, 1024.5542, 1034.5703, 1037.5466, 1041.5637, 1043.5297, 1044.5375, 1045.5594, 1046.6058, 1054.5287, 1056.5575, 1057.6187, 1057.6200, 1068.5790, 1074.6162, 1076.6190, 1082.6056, 1110.5851, 1113.5701, 1118.5687, 1120.6050, 1122.5892, 1123.6047, 1126.6241, 1128.5618, 1130.5756, 1135.5951, 1143.5592, 1145.6373, 1148.6318, 1149.6230, 1150.6226, 1156.5652, 1157.5676, 1160.5966, 1169.5873, 1177.6421, 1178.6371, 1180.6443, 1182.6539, 1184.6431, 1185.6815, 1193.6090, 1194.6001, 1209.6238, 1213.7180, 1216.7178, 1221.5953, 1227.6459, 1229.6874, 1234.6899, 1238.2251, 1239.6622, 1254.6993, 1255.6351, 1264.7139, 1265.1680, 1265.7196, 1269.6532, 1276.6729, 1278.6892, 1281.6375, 1285.6674, 1289.7029, 1293.6760, 1301.6362, 1303.6489, 1304.6685, 1306.6946, 1308.6797, 1314.7227, 1319.6770, 1320.6364, 1323.6815, 1326.6937, 1328.6799, 1333.7430, 1336.7301, 1337.6429, 1349.6729, 1351.6969, 1353.6891, 1356.6255, 1358.6919, 1359.7013, 1361.7147, 1364.7118, 1365.6619, 1366.6965, 1372.6228, 1375.6693, 1382.6771, 1383.7347, 1393.8007, 1397.7449, 1403.7434, 1410.7849, 1416.7737, 1420.7114, 1423.7096, 1434.7847, 1436.7469, 1442.8257, 1445.7913, 1456.7555, 1457.7440, 1467.7560, 1470.7466, 1475.7615, 1479.8038, 1483.7579, 1484.7480, 1487.7660, 1488.7366, 1493.7556, 1500.7393, 1500.7400, 1503.7648, 1504.7708, 1514.7063, 1516.7281, 1519.7705, 1522.7722, 1524.8040, 1526.7924, 1528.7079, 1530.7058, 1536.7252, 1537.7859, 1540.8043, 1543.7938, 1550.7543, 1551.7465, 1553.7668, 1555.7836, 1556.8011, 1562.8008, 1568.7620, 1572.7245, 1578.8058, 1580.8580, 1580.8600, 1584.8486, 1587.7675, 1608.8051, 1616.8165, 1621.7870, 1625.7886, 1628.7313, 1632.8120, 1635.7964, 1637.7974, 1642.7417, 1647.8439, 1650.8429, 1651.8369, 1655.8147, 1657.8483, 1658.8953, 1660.8379, 1662.8666, 1664.8490, 1666.7892, 1679.8091, 1680.8357, 1682.8409, 1696.8464, 1698.8180, 1701.8243, 1702.8386, 1707.8938, 1710.8904, 1714.7905, 1716.8219, 1717.9052, 1718.9094, 1721.8976, 1722.9003, 1724.9030, 1728.8840, 1730.8910, 1734.1617, 1738.8267, 1739.8165, 1741.8934, 1743.9005, 1747.9127, 1758.4807, 1758.8918, 1761.8734, 1765.8154, 1772.0012, 1774.9300, 1774.9319, 1779.9012, 1781.8585, 1783.8062, 1787.8407, 1809.0583, 1810.0460, 1812.9126, 1829.8527, 1832.8584, 1833.8571, 1834.8650, 1836.8618, 1838.8677, 1845.8363, 1847.8600, 1848.8938, 1850.9128, 1851.8932, 1861.8531, 1862.9542, 1864.9258, 1880.9026, 1883.7704, 1887.8878, 1897.7863, 1902.9769, 1904.9276, 1908.8986, 1920.9131, 1923.9126, 1928.0443, 1932.0068, 1936.9510, 1938.0171, 1947.9314, 1958.9393, 1959.9655, 1960.9813, 1961.9463, 1967.1549, 1969.9951, 1973.9971, 1975.9755, 1976.9708, 1990.4971, 1990.9373, 1991.4857, 1997.8479, 2001.8607, 2003.8768, 2004.8850, 2007.9500, 2007.9532, 2011.9381, 2012.8951, 2015.9694, 2017.9860, 2018.9740, 2020.1631, 2023.9482, 2026.9529, 2027.5033, 2029.9972, 2043.5356, 2044.9940, 2055.0205, 2057.0154, 2060.9878, 2066.9939, 2068.9661, 2082.9822, 2086.0254, 2088.1033, 2097.0371, 2098.4941, 2102.9954, 2109.0095, 2112.9932, 2114.9707, 2122.9578, 2128.0444, 2130.9980, 2132.0200, 2136.9250, 2140.0063, 2143.0237, 2144.0559, 2145.0781, 2148.0483, 2153.0049, 2153.9973, 2174.0508, 2175.0435, 2178.0483, 2188.0610, 2191.0464, 2192.0427, 2199.1150, 2205.0615, 2211.1067, 2223.1533, 2225.1382, 2226.1472, 2239.1646, 2243.1448, 2246.9482, 2251.1108, 2255.1484, 2265.0837, 2271.1211, 2273.0735, 2277.0642, 2283.1606, 2285.1355, 2286.0986, 2289.0901, 2291.0173, 2309.0349, 2328.9888, 2343.0608, 2352.9583, 2355.0383, 2366.9595, 2371.0325, 2392.1594, 2400.0532, 2424.1577, 2434.2429, 2438.2283, 2449.2180, 2450.2451, 2451.2480, 2465.1592, 2466.2458, 2467.2458, 2478.1377, 2501.2207, 2523.2014, 2526.2585, 2540.2451, 2542.2578, 2545.2258, 2550.2373, 2561.6841, 2562.1826, 2562.6890, 2573.2495, 2576.1792, 2646.9758, 2659.2144, 2661.1409, 2662.0120, 2671.1924, 2681.2705, 2682.2888, 2685.3098, 2689.3059, 2695.1064, 2696.2695, 2699.2324, 2703.2529, 2705.2944, 2712.2937, 2717.2903, 2720.2839, 2721.2937, 2726.2935, 2727.2986, 2728.3079, 2739.2837, 2743.2729, 2744.2207, 2745.2256, 2746.2031, 2748.2295, 2755.2700, 2760.2590, 2763.1829, 2771.2400, 2778.1865, 2785.2969, 2786.2561, 2787.2524, 2790.2317, 2792.2166, 2800.3093, 2801.2932, 2803.2415, 2804.2429, 2807.2869, 2808.2227, 2812.2244, 2813.2373, 2817.2632, 2820.2441, 2824.2598, 2828.2454, 2833.2544, 2843.2900, 2844.3398, 2847.3630, 2851.3313, 2864.3188, 2890.3293, 2896.4324, 2908.3105, 2910.3325, 2911.3594, 2912.4265, 2925.3323, 2927.4434, 3023.3674, 3025.3865, 3026.3452, 3039.3838, 3042.3542, 3070.3745, 3071.3933, 3086.3984, 3087.3970, 3093.4294, 3101.3831, 3103.4006, 3104.4041, 3110.4143, 3127.4749, 3141.4126, 3143.5066, 3144.5298, 3159.5210, 3219.4854, 3236.5574, 3266.6021, 3280.5635, 3321.6216, 3344.4895, 3347.5515, 3348.5020, 3380.5210, 3396.5195, 3412.5801, 3427.6099, 3428.5874, 3444.5840, 3462.6301, 3478.6123, 3484.6497, 3489.7246, 3505.6919, 3510.5859, 3521.6392, 3552.6614, 3553.6028, 3554.5630, 3558.6460, 3573.6545, 3589.6523, 3608.6155, 3624.6685, 3630.5806, 3641.7090, 3651.7944, 3667.7266, 3683.6980, 3691.7681, 3707.7200, 3714.5493, 3715.6206, 3719.6914, 3720.7048, 3723.7041, 3735.7100, 3751.7539, 3830.8091, 3869.7393, 3876.6707, 3885.6946

50. A2VDP3 **Mass:** 30659 **Score:** 16 **Expect:** 6.4e+002 **Matches:** 13

RASD family, member 2 OS=Bos taurus GN=RASD2 PE=2 SV=1

Observed	Mr(expt)	Mr(calc)	ppm	Start	End	Miss	Ions	Peptide
729.4336	728.4263	728.4432	-23.21	115 -	120	0	---	K.QILEVK.S
978.5352	977.5279	977.5328	-4.94	121 -	128	2	---	K.SCLKNKTK.E
1118.5687	1117.5614	1117.5476	12.4	254 -	262	2	---	R.ESQARERDK.C
1303.6489	1302.6416	1302.6503	-6.64	192 -	202	0	---	K.LPHEMSPALHR.K + Oxidation (M)
1660.8379	1659.8306	1659.8324	-1.06	127 -	141	1	---	K.TKEAAELPMVICGNK.N
1679.8091	1678.8018	1678.8382	-21.67	2 -	17	1	---	M.MKTLSSGNCALSVPAK.N + Oxidation (M)
1902.9769	1901.9696	1901.9703	-0.33	125 -	141	2	---	K.NKTKEAAELPMVICGNK.N

1908.8986	1907.8913	1907.8797	6.07	176 - 191	0	---	K.NTNVDEMFYVLFSMAK.L
2068.9661	2067.9588	2067.9645	-2.76	175 - 191	1	---	K.KNTNVDEMFYVLFSMAK.L + 2 Oxidation (M)
2550.2373	2549.2300	2549.1791	20.0	64 - 85	1	---	R.GDMYQLDILDTSGNHPFPAMRR.L + Oxidation (M)
2685.3098	2684.3025	2684.2640	14.4	150 - 174	0	---	R.QVPTTEAELLVSGDGNCAYFEVSAK.K
3039.3838	3038.3765	3038.4379	-20.18	59 - 84	1	---	K.VYNIRGDMYQLDILDTSGNHPFPAMR.R + Oxidation (M)
3321.6216	3320.6143	3320.6144	-0.03	176 - 203	2	---	K.NTNVDEMFYVLFSMAKLPHEMSPALHRK.I + Oxidation (M)

No match to: 716.3353, 718.2895, 724.2175, 726.3070, 728.2833, 730.2399, 732.2518, 734.4341, 743.3130, 745.3617, 747.4360, 753.3469, 756.3524, 758.3462, 759.3632, 768.3517, 779.4633, 781.3725, 789.3640, 794.3674, 796.3701, 799.2854, 801.4280, 805.4417, 807.4076, 810.3293, 812.3207, 818.3782, 820.3700, 828.4415, 829.4508, 830.4513, 834.3805, 842.5101, 849.3834, 852.4368, 857.4457, 860.4262, 868.4412, 870.4462, 873.4370, 877.4166, 880.4280, 882.4470, 883.4816, 887.4295, 889.4857, 891.4872, 894.4923, 898.4610, 900.5174, 905.4763, 907.4251, 912.5086, 914.4942, 920.4296, 922.4855, 928.4565, 929.4484, 933.4327, 936.4976, 938.5062, 944.4982, 946.4672, 949.5244, 950.5168, 957.5792, 961.5109, 965.5151, 967.4963, 972.5023, 974.4913, 976.5255, 981.5225, 983.6221, 986.5463, 988.4922, 993.5123, 995.4512, 998.5833, 1000.5594, 1006.5120, 1007.5640, 1021.5762, 1024.5542, 1031.5300, 1034.5703, 1037.5466, 1041.5637, 1043.5297, 1044.5375, 1045.5594, 1046.6058, 1054.5287, 1056.5575, 1057.6187, 1057.6200, 1068.5790, 1074.6162, 1076.6190, 1082.6056, 1105.5938, 1110.5851, 1113.5701, 1120.6050, 1122.5892, 1123.6047, 1126.6241, 1128.5618, 1130.5756, 1135.5951, 1137.5647, 1143.5592, 1145.6373, 1148.6318, 1149.6230, 1150.6226, 1156.5652, 1157.5676, 1159.5890, 1160.5966, 1169.5873, 1177.6421, 1178.6371, 1180.6443, 1182.6539, 1184.6431, 1185.6815, 1193.6090, 1194.6001, 1209.6238, 1213.7180, 1216.7178, 1221.5953, 1227.6459, 1229.6874, 1234.6899, 1238.2251, 1239.6622, 1254.6993, 1255.6351, 1264.7139, 1265.1680, 1265.7196, 1269.6532, 1276.6729, 1278.6892, 1281.6375, 1285.6674, 1289.7029, 1293.6760, 1301.6362, 1304.6685, 1306.6946, 1308.6797, 1314.7227, 1319.6770, 1320.6364, 1323.6815, 1326.6937, 1328.6799, 1333.7430, 1336.7301, 1337.6429, 1349.6729, 1351.6969, 1353.6891, 1356.6255, 1358.6919, 1359.7013, 1361.7147, 1364.7118, 1365.6619, 1366.6965, 1372.6228, 1375.6693, 1382.6771, 1383.7347, 1390.7089, 1393.8007, 1397.7449, 1403.7434, 1410.7849, 1416.7737, 1420.7114, 1423.7096, 1434.7847, 1436.7469, 1442.8257, 1445.7913, 1456.7555, 1457.7440, 1467.7560, 1470.7466, 1475.7615, 1479.8038, 1483.7579, 1484.7480, 1487.7660, 1488.7366, 1493.7556, 1500.7393, 1500.7400, 1503.7648, 1504.7708, 1514.7063, 1516.7281, 1519.7705, 1522.7722, 1524.8040, 1526.7924, 1528.7079, 1530.7058, 1536.7252, 1537.7859, 1540.8043, 1543.7938, 1550.7543, 1551.7465, 1553.7668, 1555.7836, 1556.8011, 1562.8008, 1568.7620, 1572.7245, 1578.8058, 1580.8580, 1580.8600, 1584.8486, 1587.7675, 1608.8051, 1616.8165, 1621.7870, 1625.7886, 1628.7313, 1632.8120, 1635.7964, 1637.7974, 1642.7417, 1647.8439, 1650.8429, 1651.8369, 1655.8147, 1657.8483, 1658.8953, 1662.8666, 1664.8490, 1666.7892, 1680.8357, 1682.8409, 1696.8464, 1698.8180, 1701.8243, 1702.8386, 1707.8938, 1710.8904, 1714.7905, 1716.8219, 1717.9052, 1718.9094, 1721.8976, 1722.9003, 1724.9030, 1728.8840, 1730.8910, 1734.1617, 1738.8267, 1739.8165, 1741.8934, 1743.9005, 1747.9127, 1758.4807, 1758.8918, 1761.8734, 1765.8154, 1772.0012, 1774.9300, 1774.9319, 1779.9012, 1781.8585, 1783.8062, 1787.8407, 1809.0583, 1810.0460, 1812.9126, 1829.8527, 1832.8584, 1833.8571, 1834.8650, 1836.8618, 1838.8677, 1845.8363, 1847.8600, 1848.8938, 1850.9128, 1851.8932, 1861.8531, 1862.9542, 1864.9258, 1880.9026, 1883.7704, 1887.8878, 1897.7863, 1904.9276, 1920.9131, 1923.9126, 1928.0443, 1932.0068, 1936.9510, 1938.0171, 1947.9314, 1958.9393, 1959.9655, 1960.9813, 1961.9463, 1967.1549, 1969.9951, 1973.9971, 1975.9755, 1976.9708, 1990.4971, 1990.9373, 1991.4857, 1997.8479, 2001.8607, 2003.8768, 2004.8850, 2007.9500, 2007.9532, 2011.9381, 2012.8951, 2015.9694, 2017.9860, 2018.9740, 2020.1631, 2023.9482, 2026.9529, 2027.5033, 2029.9972, 2043.5356, 2044.9940, 2055.0205, 2057.0154, 2060.9878, 2066.9939, 2082.9822, 2086.0254, 2088.1033, 2097.0371, 2098.4941, 2102.9954, 2109.0095, 2112.9932, 2114.9707, 2122.9578, 2128.0444, 2130.9980, 2132.0200, 2136.9250, 2140.0063, 2143.0237, 2144.0559, 2145.0781, 2148.0483, 2153.0049, 2153.9973, 2174.0508, 2175.0435, 2178.0483, 2188.0610, 2191.0464, 2192.0427, 2199.1150, 2205.0615, 2211.1067, 2223.1533, 2225.1382, 2226.1472, 2239.1646, 2243.1448, 2246.9482, 2251.1108, 2255.1484, 2265.0837, 2271.1211, 2273.0735, 2277.0642, 2283.1606, 2285.1355, 2286.0986, 2289.0901, 2291.0173, 2309.0349, 2328.9888, 2343.0608, 2352.9583, 2355.0383, 2366.9595, 2371.0325, 2392.1594, 2400.0532, 2424.1577, 2434.2429, 2438.2283, 2449.2180, 2450.2451, 2451.2480, 2465.1592, 2466.2458, 2467.2458, 2478.1377, 2501.2207, 2523.2014, 2526.2585, 2540.2451, 2542.2578, 2545.2258, 2561.6841, 2562.1826, 2562.6890, 2573.2495, 2576.1792, 2646.9758, 2659.2144, 2661.1409, 2662.0120, 2671.1924, 2681.2705, 2682.2888, 2689.3059, 2695.1064, 2696.2695, 2699.2324, 2703.2529, 2705.2944, 2712.2937, 2717.2903, 2720.2839, 2721.2937, 2726.2935, 2727.2986, 2728.3079, 2739.2837, 2743.2729, 2744.2207, 2745.2256, 2746.2031, 2748.2295, 2755.2700, 2760.2590, 2763.1829, 2771.2400, 2778.1865, 2785.2969, 2786.2561, 2787.2524, 2790.2317, 2792.2166, 2800.3093, 2801.2932, 2803.2415, 2804.2429, 2807.2869, 2808.2227, 2812.2244, 2813.2373, 2817.2632, 2820.2441, 2824.2598, 2828.2454, 2833.2544, 2843.2900, 2844.3398, 2847.3630, 2851.3313, 2864.3188, 2890.3293, 2896.4324, 2908.3105, 2910.3325, 2911.3594, 2912.4265, 2925.3323, 2927.4434, 3023.3674, 3025.3865, 3026.3452, 3042.3542, 3070.3745, 3071.3933, 3086.3984, 3087.3970, 3093.4294, 3101.3831, 3103.4006, 3104.4041, 3110.4143, 3127.4749, 3141.4126, 3143.5066, 3144.5298, 3159.5210, 3219.4854, 3236.5574, 3266.6021, 3280.5635, 3344.4895, 3347.5515, 3348.5020, 3380.5210, 3396.5195, 3412.5801, 3427.6099, 3428.5874, 3444.5840, 3462.6301, 3478.6123, 3484.6497, 3489.7246, 3505.6919, 3510.5859, 3521.6392, 3552.6614, 3553.6028, 3554.5630, 3558.6460, 3573.6545, 3589.6523, 3608.6155, 3624.6685, 3630.5806, 3641.7090, 3651.7944, 3667.7266, 3683.6980, 3691.7681, 3707.7200, 3714.5493, 3715.6206, 3719.6914, 3720.7048, 3723.7041, 3735.7100, 3751.7539, 3830.8091, 3869.7393, 3876.6707, 3885.6946

Search Parameters

Type of search : MS/MS Ion Search
Enzyme : Trypsin
Fixed modifications : Carbamidomethyl (C)
Variable modifications : Oxidation (M)
Mass values : Monoisotopic
Protein Mass : Unrestricted
Peptide Mass Tolerance : ± 25 ppm
Fragment Mass Tolerance: ± 0.5 Da
Max Missed Cleavages : 2
Instrument type : MALDI-TOF-TOF
Query1 (716.3353,1+): <no title>
Query2 (718.2895,1+): <no title>
Query3 (724.2175,1+): <no title>
Query4 (726.3070,1+): <no title>
Query5 (728.2833,1+): <no title>
Query6 (729.4336,1+): <no title>
Query7 (730.2399,1+): <no title>
Query8 (732.2518,1+): <no title>
Query9 (734.4341,1+): <no title>
Query10 (743.3130,1+): <no title>
Query11 (745.3617,1+): <no title>
Query12 (747.4360,1+): <no title>
Query13 (753.3469,1+): <no title>
Query14 (756.3524,1+): <no title>
Query15 (758.3462,1+): <no title>
Query16 (759.3632,1+): <no title>
Query17 (768.3517,1+): <no title>
Query18 (779.4633,1+): <no title>
Query19 (781.3725,1+): <no title>
Query20 (789.3640,1+): <no title>
Query21 (794.3674,1+): <no title>
Query22 (796.3701,1+): <no title>
Query23 (799.2854,1+): <no title>
Query24 (801.4280,1+): <no title>
Query25 (805.4417,1+): <no title>
Query26 (807.4076,1+): <no title>
Query27 (810.3293,1+): <no title>
Query28 (812.3207,1+): <no title>
Query29 (818.3782,1+): <no title>
Query30 (820.3700,1+): <no title>
Query31 (828.4415,1+): <no title>
Query32 (829.4508,1+): <no title>

Query33 (830.4513,1+): <no title>
Query34 (834.3805,1+): <no title>
Query35 (842.5101,1+): <no title>
Query36 (849.3834,1+): <no title>
Query37 (852.4368,1+): <no title>
Query38 (857.4457,1+): <no title>
Query39 (860.4262,1+): <no title>
Query40 (868.4412,1+): <no title>
Query41 (870.4462,1+): <no title>
Query42 (873.4370,1+): <no title>
Query43 (877.4166,1+): <no title>
Query44 (880.4280,1+): <no title>
Query45 (882.4470,1+): <no title>
Query46 (883.4816,1+): <no title>
Query47 (887.4295,1+): <no title>
Query48 (889.4857,1+): <no title>
Query49 (891.4872,1+): <no title>
Query50 (894.4923,1+): <no title>
Query51 (898.4610,1+): <no title>
Query52 (900.5174,1+): <no title>
Query53 (905.4763,1+): <no title>
Query54 (907.4251,1+): <no title>
Query55 (912.5086,1+): <no title>
Query56 (914.4942,1+): <no title>
Query57 (920.4296,1+): <no title>
Query58 (922.4855,1+): <no title>
Query59 (928.4565,1+): <no title>
Query60 (929.4484,1+): <no title>
Query61 (933.4327,1+): <no title>
Query62 (936.4976,1+): <no title>
Query63 (938.5062,1+): <no title>
Query64 (944.4982,1+): <no title>
Query65 (946.4672,1+): <no title>
Query66 (949.5244,1+): <no title>
Query67 (950.5168,1+): <no title>
Query68 (957.5792,1+): <no title>
Query69 (961.5109,1+): <no title>
Query70 (965.5151,1+): <no title>
Query71 (967.4963,1+): <no title>
Query72 (972.5023,1+): <no title>
Query73 (974.4913,1+): <no title>
Query74 (976.5255,1+): <no title>
Query75 (978.5352,1+): <no title>
Query76 (981.5225,1+): <no title>
Query77 (983.6221,1+): <no title>
Query78 (986.5463,1+): <no title>
Query79 (988.4922,1+): <no title>
Query80 (993.5123,1+): <no title>
Query81 (995.4512,1+): <no title>
Query82 (998.5833,1+): <no title>
Query83 (1000.5594,1+): <no title>
Query84 (1006.5120,1+): <no title>
Query85 (1007.5640,1+): <no title>
Query86 (1021.5762,1+): <no title>
Query87 (1024.5542,1+): <no title>
Query88 (1031.5300,1+): <no title>
Query89 (1034.5703,1+): <no title>
Query90 (1037.5466,1+): <no title>
Query91 (1041.5637,1+): <no title>
Query92 (1043.5297,1+): <no title>
Query93 (1044.5375,1+): <no title>
Query94 (1045.5594,1+): <no title>
Query95 (1046.6058,1+): <no title>
Query96 (1054.5287,1+): <no title>
Query97 (1056.5575,1+): <no title>
Query98 (1057.6187,1+): <no title>
Query99 (1057.6200,1+): Label: F23, Spot_Id: 221886, Peak_List_Id: 435408, MSMS Job_Run_Id: 30377, Comment:
Query100 (1068.5790,1+): <no title>
Query101 (1074.6162,1+): <no title>
Query102 (1076.6190,1+): <no title>
Query103 (1082.6056,1+): <no title>
Query104 (1105.5938,1+): <no title>
Query105 (1110.5851,1+): <no title>
Query106 (1113.5701,1+): <no title>
Query107 (1118.5687,1+): <no title>
Query108 (1120.6050,1+): <no title>
Query109 (1122.5892,1+): <no title>
Query110 (1123.6047,1+): <no title>
Query111 (1126.6241,1+): <no title>
Query112 (1128.5618,1+): <no title>
Query113 (1130.5756,1+): <no title>
Query114 (1135.5951,1+): <no title>
Query115 (1137.5647,1+): <no title>
Query116 (1143.5592,1+): <no title>
Query117 (1145.6373,1+): <no title>
Query118 (1148.6318,1+): <no title>
Query119 (1149.6230,1+): <no title>
Query120 (1150.6226,1+): <no title>
Query121 (1156.5652,1+): <no title>
Query122 (1157.5676,1+): <no title>
Query123 (1159.5890,1+): <no title>
Query124 (1160.5966,1+): <no title>
Query125 (1169.5873,1+): <no title>
Query126 (1177.6421,1+): <no title>
Query127 (1178.6371,1+): <no title>
Query128 (1180.6443,1+): <no title>
Query129 (1182.6539,1+): <no title>
Query130 (1184.6431,1+): <no title>
Query131 (1185.6815,1+): <no title>
Query132 (1193.6090,1+): <no title>

```
Query133 (1194.6001,1+): <no title>
Query134 (1209.6238,1+): <no title>
Query135 (1213.7180,1+): <no title>
Query136 (1216.7178,1+): <no title>
Query137 (1221.5953,1+): <no title>
Query138 (1227.6459,1+): <no title>
Query139 (1229.6874,1+): <no title>
Query140 (1234.6899,1+): <no title>
Query141 (1238.2251,1+): <no title>
Query142 (1239.6622,1+): <no title>
Query143 (1254.6993,1+): <no title>
Query144 (1255.6351,1+): <no title>
Query145 (1264.7139,1+): <no title>
Query146 (1265.1680,1+): <no title>
Query147 (1265.7196,1+): <no title>
Query148 (1269.6532,1+): <no title>
Query149 (1276.6729,1+): <no title>
Query150 (1278.6892,1+): <no title>
Query151 (1281.6375,1+): <no title>
Query152 (1285.6674,1+): <no title>
Query153 (1289.7029,1+): <no title>
Query154 (1293.6760,1+): <no title>
Query155 (1301.6362,1+): <no title>
Query156 (1303.6489,1+): <no title>
Query157 (1304.6685,1+): <no title>
Query158 (1306.6946,1+): <no title>
Query159 (1308.6797,1+): <no title>
Query160 (1314.7227,1+): <no title>
Query161 (1319.6770,1+): <no title>
Query162 (1320.6364,1+): <no title>
Query163 (1323.6815,1+): <no title>
Query164 (1326.6937,1+): <no title>
Query165 (1328.6799,1+): <no title>
Query166 (1333.7430,1+): <no title>
Query167 (1336.7301,1+): <no title>
Query168 (1337.6429,1+): <no title>
Query169 (1349.6729,1+): <no title>
Query170 (1351.6969,1+): <no title>
Query171 (1353.6891,1+): <no title>
Query172 (1356.6255,1+): <no title>
Query173 (1358.6919,1+): <no title>
Query174 (1359.7013,1+): <no title>
Query175 (1361.7147,1+): <no title>
Query176 (1364.7118,1+): <no title>
Query177 (1365.6619,1+): <no title>
Query178 (1366.6965,1+): <no title>
Query179 (1372.6228,1+): <no title>
Query180 (1375.6693,1+): <no title>
Query181 (1382.6771,1+): <no title>
Query182 (1383.7347,1+): <no title>
Query183 (1390.7089,1+): <no title>
Query184 (1393.8007,1+): <no title>
Query185 (1397.7449,1+): <no title>
Query186 (1403.7434,1+): <no title>
Query187 (1410.7849,1+): <no title>
Query188 (1416.7737,1+): <no title>
Query189 (1420.7114,1+): <no title>
Query190 (1423.7096,1+): <no title>
Query191 (1434.7847,1+): <no title>
Query192 (1436.7469,1+): <no title>
Query193 (1442.8257,1+): <no title>
Query194 (1445.7913,1+): <no title>
Query195 (1456.7555,1+): <no title>
Query196 (1457.7440,1+): <no title>
Query197 (1467.7560,1+): <no title>
Query198 (1470.7466,1+): <no title>
Query199 (1475.7615,1+): <no title>
Query200 (1479.8038,1+): <no title>
Query201 (1483.7579,1+): <no title>
Query202 (1484.7480,1+): <no title>
Query203 (1487.7660,1+): <no title>
Query204 (1488.7366,1+): <no title>
Query205 (1493.7556,1+): <no title>
Query206 (1500.7393,1+): <no title>
Query207 (1500.7400,1+): Label: F23, Spot_Id: 221886, Peak_List_Id: 435407, MSMS Job_Run_Id: 30377, Comment:
Query208 (1503.7648,1+): <no title>
Query209 (1504.7708,1+): <no title>
Query210 (1514.7063,1+): <no title>
Query211 (1516.7281,1+): <no title>
Query212 (1519.7705,1+): <no title>
Query213 (1522.7722,1+): <no title>
Query214 (1524.8040,1+): <no title>
Query215 (1526.7924,1+): <no title>
Query216 (1528.7079,1+): <no title>
Query217 (1530.7058,1+): <no title>
Query218 (1536.7252,1+): <no title>
Query219 (1537.7859,1+): <no title>
Query220 (1540.8043,1+): <no title>
Query221 (1543.7938,1+): <no title>
Query222 (1550.7543,1+): <no title>
Query223 (1551.7465,1+): <no title>
Query224 (1553.7668,1+): <no title>
Query225 (1555.7836,1+): <no title>
Query226 (1556.8011,1+): <no title>
Query227 (1562.8008,1+): <no title>
Query228 (1568.7620,1+): <no title>
Query229 (1572.7245,1+): <no title>
Query230 (1578.8058,1+): <no title>
Query231 (1580.8580,1+): <no title>
Query232 (1580.8600,1+): Label: F23, Spot_Id: 221886, Peak_List_Id: 435406, MSMS Job_Run_Id: 30377, Comment:
```

```
Query233 (1584.8486,1+): <no title>
Query234 (1587.7675,1+): <no title>
Query235 (1608.8051,1+): <no title>
Query236 (1616.8165,1+): <no title>
Query237 (1621.7870,1+): <no title>
Query238 (1625.7886,1+): <no title>
Query239 (1628.7313,1+): <no title>
Query240 (1632.8120,1+): <no title>
Query241 (1635.7964,1+): <no title>
Query242 (1637.7974,1+): <no title>
Query243 (1642.7417,1+): <no title>
Query244 (1647.8439,1+): <no title>
Query245 (1650.8429,1+): <no title>
Query246 (1651.8369,1+): <no title>
Query247 (1655.8147,1+): <no title>
Query248 (1657.8483,1+): <no title>
Query249 (1658.8953,1+): <no title>
Query250 (1660.8379,1+): <no title>
Query251 (1662.8666,1+): <no title>
Query252 (1664.8490,1+): <no title>
Query253 (1666.7892,1+): <no title>
Query254 (1679.8091,1+): <no title>
Query255 (1680.8357,1+): <no title>
Query256 (1682.8409,1+): <no title>
Query257 (1696.8464,1+): <no title>
Query258 (1698.8180,1+): <no title>
Query259 (1701.8243,1+): <no title>
Query260 (1702.8386,1+): <no title>
Query261 (1707.8938,1+): <no title>
Query262 (1710.8904,1+): <no title>
Query263 (1714.7905,1+): <no title>
Query264 (1716.8219,1+): <no title>
Query265 (1717.9052,1+): <no title>
Query266 (1718.9094,1+): <no title>
Query267 (1721.8976,1+): <no title>
Query268 (1722.9003,1+): <no title>
Query269 (1724.9030,1+): <no title>
Query270 (1728.8840,1+): <no title>
Query271 (1730.8910,1+): <no title>
Query272 (1734.1617,1+): <no title>
Query273 (1738.8267,1+): <no title>
Query274 (1739.8165,1+): <no title>
Query275 (1741.8934,1+): <no title>
Query276 (1743.9005,1+): <no title>
Query277 (1747.9127,1+): <no title>
Query278 (1758.4807,1+): <no title>
Query279 (1758.8918,1+): <no title>
Query280 (1761.8734,1+): <no title>
Query281 (1765.8154,1+): <no title>
Query282 (1772.0012,1+): <no title>
Query283 (1774.9300,1+): Label: F23, Spot_Id: 221886, Peak_List_Id: 435514, MSMS Job_Run_Id: 30377, Comment:
Query284 (1774.9319,1+): <no title>
Query285 (1779.9012,1+): <no title>
Query286 (1781.8585,1+): <no title>
Query287 (1783.8062,1+): <no title>
Query288 (1787.8407,1+): <no title>
Query289 (1809.0583,1+): <no title>
Query290 (1810.0460,1+): <no title>
Query291 (1812.9126,1+): <no title>
Query292 (1829.8527,1+): <no title>
Query293 (1832.8584,1+): <no title>
Query294 (1833.8571,1+): <no title>
Query295 (1834.8650,1+): <no title>
Query296 (1836.8618,1+): <no title>
Query297 (1838.8677,1+): <no title>
Query298 (1845.8363,1+): <no title>
Query299 (1847.8600,1+): <no title>
Query300 (1848.8938,1+): <no title>
Query301 (1850.9128,1+): <no title>
Query302 (1851.8932,1+): <no title>
Query303 (1861.8531,1+): <no title>
Query304 (1862.9542,1+): <no title>
Query305 (1864.9258,1+): <no title>
Query306 (1880.9026,1+): <no title>
Query307 (1883.7704,1+): <no title>
Query308 (1887.8878,1+): <no title>
Query309 (1897.7863,1+): <no title>
Query310 (1902.9769,1+): <no title>
Query311 (1904.9276,1+): <no title>
Query312 (1908.8986,1+): <no title>
Query313 (1920.9131,1+): <no title>
Query314 (1923.9126,1+): <no title>
Query315 (1928.0443,1+): <no title>
Query316 (1932.0068,1+): <no title>
Query317 (1936.9510,1+): <no title>
Query318 (1938.0171,1+): <no title>
Query319 (1947.9314,1+): <no title>
Query320 (1958.9393,1+): <no title>
Query321 (1959.9655,1+): <no title>
Query322 (1960.9813,1+): <no title>
Query323 (1961.9463,1+): <no title>
Query324 (1967.1549,1+): <no title>
Query325 (1969.9951,1+): <no title>
Query326 (1973.9971,1+): <no title>
Query327 (1975.9755,1+): <no title>
Query328 (1976.9708,1+): <no title>
Query329 (1990.4971,1+): <no title>
Query330 (1990.9373,1+): <no title>
Query331 (1991.4857,1+): <no title>
Query332 (1997.8479,1+): <no title>
```

```
Query333 (2001.8607,1+): <no title>
Query334 (2003.8768,1+): <no title>
Query335 (2004.8850,1+): <no title>
Query336 (2007.9500,1+): Label: F23, Spot_Id: 221886, Peak_List_Id: 435405, MSMS Job_Run_Id: 30377, Comment:
Query337 (2007.9532,1+): <no title>
Query338 (2011.9381,1+): <no title>
Query339 (2012.8951,1+): <no title>
Query340 (2015.9694,1+): <no title>
Query341 (2017.9860,1+): <no title>
Query342 (2018.9740,1+): <no title>
Query343 (2020.1631,1+): <no title>
Query344 (2023.9482,1+): <no title>
Query345 (2026.9529,1+): <no title>
Query346 (2027.5033,1+): <no title>
Query347 (2029.9972,1+): <no title>
Query348 (2043.5356,1+): <no title>
Query349 (2044.9940,1+): <no title>
Query350 (2055.0205,1+): <no title>
Query351 (2057.0154,1+): <no title>
Query352 (2060.9878,1+): <no title>
Query353 (2066.9939,1+): <no title>
Query354 (2068.9661,1+): <no title>
Query355 (2082.9822,1+): <no title>
Query356 (2086.0254,1+): <no title>
Query357 (2088.1033,1+): <no title>
Query358 (2097.0371,1+): <no title>
Query359 (2098.4941,1+): <no title>
Query360 (2102.9954,1+): <no title>
Query361 (2109.0095,1+): <no title>
Query362 (2112.9932,1+): <no title>
Query363 (2114.9707,1+): <no title>
Query364 (2122.9578,1+): <no title>
Query365 (2128.0444,1+): <no title>
Query366 (2130.9980,1+): <no title>
Query367 (2132.0200,1+): <no title>
Query368 (2136.9250,1+): <no title>
Query369 (2140.0063,1+): <no title>
Query370 (2143.0237,1+): <no title>
Query371 (2144.0559,1+): <no title>
Query372 (2145.0781,1+): <no title>
Query373 (2148.0483,1+): <no title>
Query374 (2153.0049,1+): <no title>
Query375 (2153.9973,1+): <no title>
Query376 (2174.0508,1+): <no title>
Query377 (2175.0435,1+): <no title>
Query378 (2178.0483,1+): <no title>
Query379 (2188.0610,1+): <no title>
Query380 (2191.0464,1+): <no title>
Query381 (2192.0427,1+): <no title>
Query382 (2199.1150,1+): <no title>
Query383 (2205.0615,1+): <no title>
Query384 (2211.1067,1+): <no title>
Query385 (2223.1533,1+): <no title>
Query386 (2225.1382,1+): <no title>
Query387 (2226.1472,1+): <no title>
Query388 (2239.1646,1+): <no title>
Query389 (2243.1448,1+): <no title>
Query390 (2246.9482,1+): <no title>
Query391 (2251.1108,1+): <no title>
Query392 (2255.1484,1+): <no title>
Query393 (2265.0837,1+): <no title>
Query394 (2271.1211,1+): <no title>
Query395 (2273.0735,1+): <no title>
Query396 (2277.0642,1+): <no title>
Query397 (2283.1606,1+): <no title>
Query398 (2285.1355,1+): <no title>
Query399 (2286.0986,1+): <no title>
Query400 (2289.0901,1+): <no title>
Query401 (2291.0173,1+): <no title>
Query402 (2309.0349,1+): <no title>
Query403 (2328.9888,1+): <no title>
Query404 (2343.0608,1+): <no title>
Query405 (2352.9583,1+): <no title>
Query406 (2355.0383,1+): <no title>
Query407 (2366.9595,1+): <no title>
Query408 (2371.0325,1+): <no title>
Query409 (2392.1594,1+): <no title>
Query410 (2400.0532,1+): <no title>
Query411 (2424.1577,1+): <no title>
Query412 (2434.2429,1+): <no title>
Query413 (2438.2283,1+): <no title>
Query414 (2449.2180,1+): <no title>
Query415 (2450.2451,1+): <no title>
Query416 (2451.2480,1+): <no title>
Query417 (2465.1592,1+): <no title>
Query418 (2466.2458,1+): <no title>
Query419 (2467.2458,1+): <no title>
Query420 (2478.1377,1+): <no title>
Query421 (2501.2207,1+): <no title>
Query422 (2523.2014,1+): <no title>
Query423 (2526.2585,1+): <no title>
Query424 (2540.2451,1+): <no title>
Query425 (2542.2578,1+): <no title>
Query426 (2545.2258,1+): <no title>
Query427 (2550.2373,1+): <no title>
Query428 (2561.6841,1+): <no title>
Query429 (2562.1826,1+): <no title>
Query430 (2562.6890,1+): <no title>
Query431 (2573.2495,1+): <no title>
Query432 (2576.1792,1+): <no title>
```

```
Query433 (2646.9758,1+): <no title>
Query434 (2659.2144,1+): <no title>
Query435 (2661.1409,1+): <no title>
Query436 (2662.0120,1+): <no title>
Query437 (2671.1924,1+): <no title>
Query438 (2681.2705,1+): <no title>
Query439 (2682.2888,1+): <no title>
Query440 (2685.3098,1+): <no title>
Query441 (2689.3059,1+): <no title>
Query442 (2695.1064,1+): <no title>
Query443 (2696.2695,1+): <no title>
Query444 (2699.2324,1+): <no title>
Query445 (2703.2529,1+): <no title>
Query446 (2705.2944,1+): <no title>
Query447 (2712.2937,1+): <no title>
Query448 (2717.2903,1+): <no title>
Query449 (2720.2839,1+): <no title>
Query450 (2721.2937,1+): <no title>
Query451 (2726.2935,1+): <no title>
Query452 (2727.2986,1+): <no title>
Query453 (2728.3079,1+): <no title>
Query454 (2739.2837,1+): <no title>
Query455 (2743.2729,1+): <no title>
Query456 (2744.2207,1+): <no title>
Query457 (2745.2256,1+): <no title>
Query458 (2746.2031,1+): <no title>
Query459 (2748.2295,1+): <no title>
Query460 (2755.2700,1+): <no title>
Query461 (2760.2590,1+): <no title>
Query462 (2763.1829,1+): <no title>
Query463 (2771.2400,1+): <no title>
Query464 (2778.1865,1+): <no title>
Query465 (2785.2969,1+): <no title>
Query466 (2786.2561,1+): <no title>
Query467 (2787.2524,1+): <no title>
Query468 (2790.2317,1+): <no title>
Query469 (2792.2166,1+): <no title>
Query470 (2800.3093,1+): <no title>
Query471 (2801.2932,1+): <no title>
Query472 (2803.2415,1+): <no title>
Query473 (2804.2429,1+): <no title>
Query474 (2807.2869,1+): <no title>
Query475 (2808.2227,1+): <no title>
Query476 (2812.2244,1+): <no title>
Query477 (2813.2373,1+): <no title>
Query478 (2817.2632,1+): <no title>
Query479 (2820.2441,1+): <no title>
Query480 (2824.2598,1+): <no title>
Query481 (2828.2454,1+): <no title>
Query482 (2833.2544,1+): <no title>
Query483 (2843.2900,1+): <no title>
Query484 (2844.3398,1+): <no title>
Query485 (2847.3630,1+): <no title>
Query486 (2851.3313,1+): <no title>
Query487 (2864.3188,1+): <no title>
Query488 (2890.3293,1+): <no title>
Query489 (2896.4324,1+): <no title>
Query490 (2908.3105,1+): <no title>
Query491 (2910.3325,1+): <no title>
Query492 (2911.3594,1+): <no title>
Query493 (2912.4265,1+): <no title>
Query494 (2925.3323,1+): <no title>
Query495 (2927.4434,1+): <no title>
Query496 (3023.3674,1+): <no title>
Query497 (3025.3865,1+): <no title>
Query498 (3026.3452,1+): <no title>
Query499 (3039.3838,1+): <no title>
Query500 (3042.3542,1+): <no title>
Query501 (3070.3745,1+): <no title>
Query502 (3071.3933,1+): <no title>
Query503 (3086.3984,1+): <no title>
Query504 (3087.3970,1+): <no title>
Query505 (3093.4294,1+): <no title>
Query506 (3101.3831,1+): <no title>
Query507 (3103.4006,1+): <no title>
Query508 (3104.4041,1+): <no title>
Query509 (3110.4143,1+): <no title>
Query510 (3127.4749,1+): <no title>
Query511 (3141.4126,1+): <no title>
Query512 (3143.5066,1+): <no title>
Query513 (3144.5298,1+): <no title>
Query514 (3159.5210,1+): <no title>
Query515 (3219.4854,1+): <no title>
Query516 (3236.5574,1+): <no title>
Query517 (3266.6021,1+): <no title>
Query518 (3280.5635,1+): <no title>
Query519 (3321.6216,1+): <no title>
Query520 (3344.4895,1+): <no title>
Query521 (3347.5515,1+): <no title>
Query522 (3348.5020,1+): <no title>
Query523 (3380.5210,1+): <no title>
Query524 (3396.5195,1+): <no title>
Query525 (3412.5801,1+): <no title>
Query526 (3427.6099,1+): <no title>
Query527 (3428.5874,1+): <no title>
Query528 (3444.5840,1+): <no title>
Query529 (3462.6301,1+): <no title>
Query530 (3478.6123,1+): <no title>
Query531 (3484.6497,1+): <no title>
Query532 (3489.7246,1+): <no title>
```

```
Query533 (3505.6919,1+): <no title>
Query534 (3510.5859,1+): <no title>
Query535 (3521.6392,1+): <no title>
Query536 (3552.6614,1+): <no title>
Query537 (3553.6028,1+): <no title>
Query538 (3554.5630,1+): <no title>
Query539 (3558.6460,1+): <no title>
Query540 (3573.6545,1+): <no title>
Query541 (3589.6523,1+): <no title>
Query542 (3608.6155,1+): <no title>
Query543 (3624.6685,1+): <no title>
Query544 (3630.5806,1+): <no title>
Query545 (3641.7090,1+): <no title>
Query546 (3651.7944,1+): <no title>
Query547 (3667.7266,1+): <no title>
Query548 (3683.6980,1+): <no title>
Query549 (3691.7681,1+): <no title>
Query550 (3707.7200,1+): <no title>
Query551 (3714.5493,1+): <no title>
Query552 (3715.6206,1+): <no title>
Query553 (3719.6914,1+): <no title>
Query554 (3720.7048,1+): <no title>
Query555 (3723.7041,1+): <no title>
Query556 (3735.7100,1+): <no title>
Query557 (3751.7539,1+): <no title>
Query558 (3830.8091,1+): <no title>
Query559 (3869.7393,1+): <no title>
Query560 (3876.6707,1+): <no title>
Query561 (3885.6946,1+): <no title>
```

Mascot: http://www.matrixscience.com/

MATRIX SCIENCE Mascot Search Results

```
User          : 2w_(F19@11273_12Mar2012)
Email         : proteomics@ipatimup.pt
Search title  : Project: Proteomica, Spot Set: Proteomica\12Jan2012, Label: F19, Spot Id: 221882, Peak List Id: 435347, MS Job Run Id: 30
MS data file  : C:\Documents and Settings\Administrator\Desktop\xu\xu\xu\Proteomica\12Jan2012\ppw_F19_146796935501.txt
Database      : OrganismSpecie Bos_taurus_Reference_Proteome_2016_06 (24214 sequences; 12839866 residues)
Timestamp     : 8 Jul 2016 at 10:03:05 GMT
Warning       : A Peptide summary report will usually give a much clearer picture of MS/MS search results.
Top Score     : 91 for F1MSR8, Collagen alpha-1(II) chain OS=Bos taurus GN=COL2A1 PE=4 SV=2
```

Mascot Score Histogram

Protein score is -10*Log(P), where P is the probability that the observed match is a random event.
Protein scores greater than 56 are significant (p<0.05).
Protein scores are derived from ions scores as a non-probabilistic basis for ranking protein hits.

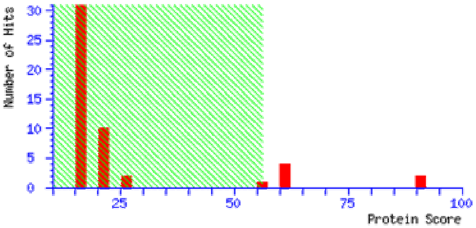

Protein Summary Report

Format As | Protein Summary (deprecated) | Help

Significance threshold p< 0.05 | Max. number of hits 50

Preferred taxonomy | All entries

Re-Search All | Search Unmatched

Index

	Accession	Mass	Score	Description
1.	F1MSR8	134858	91	Collagen alpha-1(II) chain OS=Bos taurus GN=COL2A1 PE=4 SV=2
2.	P02459	142825	91	Collagen alpha-1(II) chain OS=Bos taurus GN=COL2A1 PE=1 SV=4
3.	F1MYE4	41319	63	Chondroadherin OS=Bos taurus GN=CHAD PE=4 SV=2
4.	P19879	34530	62	Mimecan OS=Bos taurus GN=OGN PE=1 SV=2
5.	A5D9E8	34517	62	Mimecan OS=Bos taurus GN=OGN PE=1 SV=1
6.	G3N088	40915	61	Mimecan OS=Bos taurus GN=OGN PE=1 SV=1
7.	Q27972	41373	58	Chondroadherin OS=Bos taurus GN=CHAD PE=1 SV=1
8.	F1MYS7	68834	25	Uncharacterized protein OS=Bos taurus GN=EIF4B PE=1 SV=1
9.	E1BND5	218183	24	Uncharacterized protein OS=Bos taurus GN=NCOA6 PE=4 SV=1
10.	A7Z060	81637	22	RNF219 protein OS=Bos taurus GN=RNF219 PE=2 SV=1
11.	G5E523	28080	20	Uncharacterized protein OS=Bos taurus GN=STAU2 PE=4 SV=1
12.	A6QR15	32975	20	LOC535277 protein OS=Bos taurus GN=LOC535277 PE=1 SV=1
13.	G3N219	3783	20	Uncharacterized protein OS=Bos taurus PE=4 SV=1
14.	G3X6N6	52918	19	Uncharacterized protein OS=Bos taurus PE=4 SV=1
15.	Q5E9D0	38604	19	Eukaryotic translation initiation factor 2 subunit 2 OS=Bos taurus GN=EIF2S2 PE=2 SV=1
16.	A0A140T862	38576	19	Eukaryotic translation initiation factor 2 subunit 2 OS=Bos taurus GN=EIF2S2 PE=1 SV=1
17.	E1BDF0	81113	19	Uncharacterized protein OS=Bos taurus GN=NEXN PE=4 SV=2
18.	Q2TBK0	39253	19	Uncharacterized protein C6orf163 homolog OS=Bos taurus PE=2 SV=1
19.	Q2TBN3	19797	19	Centrin-2 OS=Bos taurus GN=CETN2 PE=2 SV=1
20.	G3MWY8	9886	19	Coiled-coil domain-containing protein 167 OS=Bos taurus GN=CCDC167 PE=4 SV=1
21.	Q3MHQ4	30565	19	Zinc finger protein SNAI2 OS=Bos taurus GN=SNAI2 PE=2 SV=1
22.	F1MMB7	28878	18	Uncharacterized protein OS=Bos taurus GN=TP53TG5 PE=4 SV=1
23.	F1N6K9	95221	18	Uncharacterized protein OS=Bos taurus PE=4 SV=2
24.	Q32KL9	28308	18	B-cell receptor-associated protein 29 OS=Bos taurus GN=BCAP29 PE=2 SV=1
25.	G3N097	6896	18	Uncharacterized protein OS=Bos taurus GN=SERF2 PE=4 SV=1
26.	F1MB56	24689	18	Uncharacterized protein OS=Bos taurus GN=TEX35 PE=4 SV=1
27.	G3MYV8	6868	18	Uncharacterized protein OS=Bos taurus PE=4 SV=1
28.	F1N3R9	25138	18	Uncharacterized protein OS=Bos taurus GN=TMCO5A PE=4 SV=1
29.	F1MN93	91564	18	Uncharacterized protein OS=Bos taurus GN=TOP1 PE=4 SV=1
30.	Q3SX42	23992	18	Charged multivesicular body protein 2b OS=Bos taurus GN=CHMP2B PE=2 SV=1
31.	F1MGT0	24053	18	Meiotic nuclear division protein 1 homolog OS=Bos taurus GN=MND1 PE=4 SV=1
32.	Q32L19	24083	18	Meiotic nuclear division protein 1 homolog OS=Bos taurus GN=MND1 PE=2 SV=1
33.	O62827	21064	18	ADM OS=Bos taurus GN=ADM PE=2 SV=2
34.	Q05B49	24773	18	UPF0711 protein C18orf21 homolog OS=Bos taurus PE=2 SV=1
35.	Q8MJN0	20622	18	FUN14 domain-containing protein 2 OS=Bos taurus GN=FUNDC2 PE=2 SV=1
36.	Q32LM7	28555	18	Coiled-coil domain-containing protein 152 OS=Bos taurus GN=CCDC152 PE=2 SV=2
37.	F1MJA8	28543	18	Coiled-coil domain-containing protein 152 OS=Bos taurus GN=CCDC152 PE=4 SV=2
38.	A5D7A0	26902	18	EF-hand domain-containing protein D2 OS=Bos taurus GN=EFHD2 PE=2 SV=1
39.	G3N365	141959	17	Structural maintenance of chromosomes protein OS=Bos taurus GN=SMC3 PE=3 SV=1
40.	E1BMK2	25092	17	Uncharacterized protein OS=Bos taurus PE=4 SV=2
41.	A7MBI0	50969	17	Protein kinase C and casein kinase substrate in neurons protein 1 OS=Bos taurus GN=PACSIN1 PE=2 SV=1
42.	G3N0Z2	40114	17	Uncharacterized protein OS=Bos taurus PE=3 SV=1
43.	Q0II81	14868	17	Regulator of G-protein signaling 13 OS=Bos taurus GN=RGS13 PE=2 SV=1
44.	P35246	37724	16	Pulmonary surfactant-associated protein D OS=Bos taurus GN=SFTPD PE=1 SV=2
45.	F1MWQ0	17662	16	Complexin-3 OS=Bos taurus GN=CPLX3 PE=4 SV=1
46.	Q0VC09	44563	16	RIB43A-like with coiled-coils protein 1 OS=Bos taurus GN=RIBC1 PE=2 SV=1
47.	E1BL95	154749	16	Uncharacterized protein OS=Bos taurus GN=CENPJ PE=4 SV=1
48.	P31976	68832	16	Ezrin OS=Bos taurus GN=EZR PE=1 SV=2

49. [Q2YDN4] 58176 16 Coiled-coil domain-containing protein 105 OS=Bos taurus GN=CCDC105 PE=2 SV=1
50. [G3MZ80] 7701 16 Uncharacterized protein OS=Bos taurus PE=4 SV=1

Results List

1. F1MSR8 **Mass:** 134858 **Score:** 91 **Expect:** 1.9e-005 **Matches:** 40
Collagen alpha-1(II) chain OS=Bos taurus GN=COL2A1 PE=4 SV=2

Observed	Mr(expt)	Mr(calc)	ppm	Start	End	Miss	Ions	Peptide
807.3893	806.3820	806.3711	13.5	1266	1271	0	---	K.NWWSSK.S
812.3985	811.3912	811.3898	1.75	1379	1384	0	---	K.TMIEYR.S
828.3904	827.3831	827.3847	-1.95	1379	1384	0	---	K.TMIEYR.S + Oxidation (M)
871.4945	870.4872	870.4851	2.44	1360	1366	0	---	R.FTYTVLK.D
912.4497	911.4424	911.4283	15.4	1038	1046	0	---	R.GMPGPQGPR.G + Oxidation (M)
935.4698	934.4625	934.4661	-3.82	1265	1271	1	---	K.KNWWSSK.S
969.4571	968.4498	968.4538	-4.13	1219	1225	0	---	K.LCHPEWK.S
1003.4687	1002.4614	1002.4553	6.12	1367	1375	1	---	K.DGCTKHTGK.W
1026.4922	1025.4849	1025.5003	-14.95	861	871	1	---	K.GARGDSGPPGR.A
1114.5396	1113.5323	1113.5414	-8.18	540	551	1	---	K.GANGEPGKAGEK.G
1155.6113	1154.6040	1154.5754	24.8	1379	1387	1	---	K.TMIEYRSQK.T
1173.6259	1172.6186	1172.6149	3.16	1192	1201	0	---	K.SLNNQIESLR.S
1183.6123	1182.6050	1182.5855	16.5	1376	1384	1	---	K.WGKTMIEYR.S
1196.6102	1195.6029	1195.5768	21.9	1038	1049	1	---	R.GMPGPQGPRGDK.G
1226.5948	1225.5875	1225.5939	-5.18	1181	1191	0	---	R.QHDAEVDATLK.S
1325.6637	1324.6564	1324.6598	-2.53	1216	1225	1	---	R.DLKLCHPEWK.S
1374.6412	1373.6339	1373.6510	-12.45	1367	1378	2	---	K.DGCTKHTGKWGK.T
1427.7872	1426.7799	1426.7780	1.37	1341	1353	0	---	K.ALLIQGSNDVEIR.A
1432.7133	1431.7060	1431.7068	-0.55	1360	1371	1	---	R.FTYTVLKDGCTK.H
1478.7439	1477.7366	1477.7412	-3.11	1326	1339	0	---	K.NSIAYLDEAAGNLK.K
1485.7720	1484.7647	1484.7623	1.61	1354	1366	1	---	R.AEGNSRFTYTVLK.D
1555.8816	1554.8743	1554.8729	0.90	1340	1353	1	---	K.KALLIQGSNDVEIR.A
1606.8384	1605.8311	1605.8362	-3.15	1326	1340	1	---	K.NSIAYLDEAAGNLKK.A
1622.8137	1621.8064	1621.8035	1.81	1372	1384	2	---	K.HTGKWGKTMIEYR.S + Oxidation (M)
1750.8356	1749.8283	1749.8356	-4.14	1311	1325	0	---	R.LLSTEGSQNITYHCK.N
1757.8322	1756.8249	1756.8605	-20.25	633	650	1	---	R.GFPGERGSPGSQGLQGAR.G
1786.9000	1785.8927	1785.8969	-2.34	1192	1207	1	76	K.SLNNQIESLRSPEGSR.K
1786.9017	1785.8944	1785.8969	-1.38	1192	1207	1	---	K.SLNNQIESLRSPEGSR.K
1914.9995	1913.9922	1913.9919	0.19	1192	1208	2	---	K.SLNNQIESLRSPEGSRK.N
2007.9894	2006.9821	2006.9341	23.9	1016	1037	1	---	K.QGDRGEAGAQGPMGPAGPAGAR.G
2042.0640	2041.0567	2041.0552	0.75	1341	1359	1	---	K.ALLIQGSNDVEIRAEGNSR.F
2047.0167	2046.0094	2045.9840	12.4	1354	1371	2	---	R.AEGNSRFTYTVLKDGCTK.H
2070.8835	2069.8762	2069.8823	-2.92	1226	1243	0	---	K.SGDYWIDPNQGCTLDAMK.V
2086.8828	2085.8755	2085.8772	-0.80	1226	1243	0	---	K.SGDYWIDPNQGCTLDAMK.V + Oxidation (M)
2098.9976	2097.9903	2097.9498	19.3	1038	1058	2	---	R.GMPGPQGPRGDKGETGEAGER.G + Oxidation (M)
2170.1467	2169.1394	2169.1501	-4.94	1340	1359	2	---	K.KALLIQGSNDVEIRAEGNSR.F
2400.0652	2399.0579	2399.0596	-0.70	1244	1264	0	---	K.VFCNMETGETCVYPNPASVPK.K
2416.0752	2415.0679	2415.0545	5.55	1244	1264	0	---	K.VFCNMETGETCVYPNPASVPK.K + Oxidation (M)
2452.0632	2451.0559	2451.1085	-21.44	231	257	1	---	K.GEAGAPGVKGESGSPGENGSPGPMGPR.G + Oxidation (M)
2528.1580	2527.1507	2527.1546	-1.52	1244	1265	1	---	K.VFCNMETGETCVYPNPASVPKK.N

No match to: 713.4334, 719.2388, 724.3009, 726.2899, 732.2586, 735.3865, 745.4124, 756.4687, 764.4056, 765.3908, 767.3679, 768.3909, 772.3955, 795.4186, 802.4548, 803.4370, 805.4229, 806.4631, 809.4480, 811.4104, 816.4408, 820.4244, 822.3905, 823.3846, 824.3756, 834.4043, 836.4589, 837.3853, 839.3696, 840.3839, 842.5085, 845.4100, 845.4123, 851.3975, 852.4056, 856.4893, 866.3914, 867.3724, 870.5402, 878.4625, 880.4403, 886.3815, 888.4233, 890.4479, 893.9562, 894.4579, 897.0289, 897.4412, 902.4597, 910.4410, 913.5118, 916.4905, 918.5391, 925.4747, 928.5064, 930.5048, 933.4619, 939.4758, 947.4628, 949.4591, 951.4703, 954.4674, 957.4657, 963.4676, 965.4761, 967.4568, 972.4793, 972.4800, 975.4650, 977.4630, 978.4976, 981.4565, 983.4664, 985.4842, 991.4688, 993.5331, 997.4298, 999.4453, 1001.4488, 1007.5668, 1018.5200, 1018.5212, 1023.4777, 1027.4972, 1030.5151, 1031.3850, 1033.5419, 1035.5352, 1036.5372, 1039.5402, 1042.5383, 1045.5654, 1056.5488, 1060.5400, 1060.5442, 1065.5425, 1086.5115, 1106.5372, 1111.5582, 1113.5546, 1115.5605, 1118.5529, 1122.5363, 1128.5775, 1129.6014, 1130.5935, 1140.5485, 1144.5709, 1156.5720, 1157.5952, 1158.6154, 1159.6273, 1165.5817, 1172.6301, 1179.6063, 1180.6165, 1182.6090, 1185.6317, 1187.6431, 1193.6096, 1195.6060, 1206.6770, 1209.1387, 1209.5848, 1210.1014, 1215.6627, 1216.6494, 1222.5994, 1229.6246, 1231.6174, 1234.6317, 1235.5846, 1240.6298, 1244.6324, 1248.6119, 1250.6144, 1268.6498, 1271.6696, 1273.6653, 1275.6644, 1291.6425, 1295.7233, 1300.6062, 1301.6205, 1307.6858, 1315.6570, 1317.6521, 1319.6852, 1323.6517, 1328.6809, 1329.6672, 1331.6763, 1332.6743, 1334.6592, 1335.6937, 1336.6715, 1337.6724, 1338.6471, 1342.6680, 1345.6760, 1346.6880, 1349.6991, 1354.6907, 1355.7155, 1357.7034, 1360.6882, 1361.6670, 1365.6913, 1368.6920, 1375.6798, 1376.6804, 1377.6794, 1382.6647, 1387.6444, 1410.7505, 1411.7208, 1421.7177, 1431.7961, 1434.7288, 1446.7256, 1450.7079, 1473.8054, 1475.7748, 1477.7524, 1486.7664, 1493.7539, 1494.7423, 1505.8649, 1523.7841, 1524.7848, 1537.8021, 1549.7388, 1558.8474, 1576.7987, 1579.7926, 1584.7897, 1588.8136, 1590.3611, 1604.8417, 1605.8098, 1615.8826, 1616.8605, 1628.8260, 1629.8358, 1630.8466, 1633.9790, 1635.7943, 1644.8046, 1647.8785, 1649.8463, 1651.8245, 1659.8241, 1662.7909, 1668.7810, 1679.9736, 1681.8214, 1683.8121, 1684.8204, 1706.8616, 1707.7838, 1716.8571, 1725.8353, 1728.8242, 1734.8152, 1740.8768, 1742.9031, 1746.1659, 1751.8787, 1753.8948, 1756.8905, 1763.8188, 1765.7690, 1767.8036, 1768.9027, 1769.4475, 1770.4261, 1774.9091, 1783.8499, 1791.7335, 1808.8868, 1824.8674, 1840.8225, 1844.8907, 1848.8322, 1850.8301, 1851.8488, 1867.9503, 1880.9277, 1887.9734, 1908.8838, 1919.0038, 1925.9418, 1928.0103, 1931.9463, 1953.9353, 1975.9609, 1992.9642, 1995.9020, 2011.8845, 2013.8983, 2024.8591, 2025.6234, 2036.5195, 2036.9486, 2047.9691, 2051.9792, 2052.9924, 2058.9963, 2060.0469, 2064.9900, 2068.8982, 2074.8914, 2083.9907, 2084.8987, 2092.8821, 2095.9941, 2100.9160, 2102.8872, 2106.9011, 2108.8835, 2109.9175, 2114.9180, 2116.9160, 2118.8835, 2119.8833, 2125.0176, 2132.8694, 2136.9180, 2211.1045, 2225.1191, 2273.0496, 2277.0137, 2307.9941, 2322.9980, 2341.0366, 2343.0271, 2352.0571, 2355.0459, 2359.0532, 2366.0813, 2368.0544, 2369.0710, 2382.0264, 2382.0349, 2383.6099, 2383.9600, 2390.0403, 2396.0342, 2398.0576, 2406.0737, 2411.0076, 2414.0813, 2417.0732, 2420.0718, 2422.0408, 2423.0466, 2428.0898, 2430.0828, 2431.0845, 2433.0779, 2436.0508, 2438.0457, 2439.0261, 2442.0618, 2449.0347, 2460.0254, 2461.9956, 2463.9858, 2510.1277, 2542.1509, 2545.1558, 2547.1531, 2552.1038, 2565.1543, 2585.1111, 2601.0889, 2705.2192, 2717.0693, 3129.3176, 3208.1736, 3223.2710, 3290.4131, 3291.4248, 3312.2756, 3451.3933, 3453.4365

2. P02459 **Mass:** 142825 **Score:** 91 **Expect:** 2e-005 **Matches:** 41
Collagen alpha-1(II) chain OS=Bos taurus GN=COL2A1 PE=1 SV=4

Observed	Mr(expt)	Mr(calc)	ppm	Start	End	Miss	Ions	Peptide
807.3893	806.3820	806.3711	13.5	1335	1340	0	---	K.NWWSSK.S
812.3985	811.3912	811.3898	1.75	1448	1453	0	---	K.TMIEYR.S
828.3904	827.3831	827.3847	-1.95	1448	1453	0	---	K.TMIEYR.S + Oxidation (M)
871.4945	870.4872	870.4851	2.44	1429	1435	0	---	R.FTYTVLK.D
912.4497	911.4424	911.4283	15.4	1107	1115	0	---	R.GMPGPQGPR.G + Oxidation (M)
935.4698	934.4625	934.4661	-3.82	1334	1340	1	---	K.KNWWSSK.S
969.4571	968.4498	968.4538	-4.13	1288	1294	0	---	K.LCHPEWK.S
1003.4687	1002.4614	1002.4553	6.12	1436	1444	1	---	K.DGCTKHTGK.W
1026.4922	1025.4849	1025.5003	-14.95	930	940	1	---	K.GARGDSGPPGR.A

1114.5396	1113.5323	1113.5414	-8.18	609	620	1	---	K.GANGEPGKAGEK.G
1155.6113	1154.6040	1154.5754	24.8	1448	1456	1	---	K.TMIEYRSQK.T
1173.6259	1172.6186	1172.6149	3.16	1261	1270	0	---	K.SLNNQIESLR.S
1183.6123	1182.6050	1182.5855	16.5	1445	1453	1	---	K.WGKTMIEYR.S
1196.6102	1195.6029	1195.5768	21.9	1107	1118	1	---	R.GMPGPQGPRGDK.G
1226.5948	1225.5875	1225.5939	-5.18	1250	1260	0	---	R.QHDAEVDATLK.S
1325.6637	1324.6564	1324.6598	-2.53	1285	1294	1	---	R.DLKLCHPEWK.S
1374.6412	1373.6339	1373.6510	-12.45	1436	1447	2	---	K.DGCTKHTGKWGK.T
1427.7872	1426.7799	1426.7780	1.37	1410	1422	0	---	K.ALLIQGSNDVEIR.A
1432.7133	1431.7060	1431.7068	-0.55	1429	1440	1	---	R.FTYTVLKDGCTK.H
1478.7439	1477.7366	1477.7412	-3.11	1395	1408	0	---	K.NSIAYLDEAAGNLK.K
1485.7720	1484.7647	1484.7623	1.61	1423	1435	1	---	R.AEGNSRFTYTVLK.D
1555.8816	1554.8743	1554.8729	0.90	1409	1422	1	---	K.KALLIQGSNDVEIR.A
1606.8384	1605.8311	1605.8362	-3.15	1395	1409	1	---	K.NSIAYLDEAAGNLKK.A
1622.8137	1621.8064	1621.8035	1.81	1441	1453	2	---	K.HTGKWGKTMIEYR.S + Oxidation (M)
1750.8356	1749.8283	1749.8356	-4.14	1380	1394	0	---	R.LLSTEGSQNITYHCK.N
1757.8322	1756.8249	1756.8605	-20.25	702	719	1	---	R.GFPGERGSPGSQGLQGAR.G
1786.9000	1785.8927	1785.8969	-2.34	1261	1276	1	76	K.SLNNQIESLRSPEGSR.K
1786.9017	1785.8944	1785.8969	-1.38	1261	1276	1	---	K.SLNNQIESLRSPEGSR.K
1914.9995	1913.9922	1913.9919	0.19	1261	1277	2	---	K.SLNNQIESLRSPEGSRK.N
2007.9894	2006.9821	2006.9341	23.9	1085	1106	1	---	K.QGDRGEAGAQGPMGPAGPAGAR.G
2042.0640	2041.0567	2041.0552	0.75	1410	1428	1	---	K.ALLIQGSNDVEIRAEGNSR.F
2047.0167	2046.0094	2045.9840	12.4	1423	1440	2	---	R.AEGNSRFTYTVLKDGCTK.H
2070.8835	2069.8762	2069.8823	-2.92	1295	1312	0	---	K.SGDYWIDPNQGCTLDAMK.V
2086.8828	2085.8755	2085.8772	-0.80	1295	1312	0	---	K.SGDYWIDPNQGCTLDAMK.V + Oxidation (M)
2098.9976	2097.9903	2097.9498	19.3	1107	1127	2	---	R.GMPGPQGPRGDKGETGEAGER.G + Oxidation (M)
2170.1467	2169.1394	2169.1501	-4.94	1409	1428	2	---	K.KALLIQGSNDVEIRAEGNSR.F
2400.0652	2399.0579	2399.0596	-0.70	1313	1333	0	---	K.VFCNMETGETCVYPNPASVPK.K
2416.0752	2415.0679	2415.0545	5.55	1313	1333	0	---	K.VFCNMETGETCVYPNPASVPK.K + Oxidation (M)
2417.0732	2416.0659	2416.0089	23.6	54	73	0	---	R.ICVCDTGTVLCDDIICEDMK.D
2452.0632	2451.0559	2451.1085	-21.44	300	326	1	---	K.GEAGAPGVKGESGSPGENGSPGPMGPR.G + Oxidation (M)
2528.1580	2527.1507	2527.1546	-1.52	1313	1334	1	---	K.VFCNMETGETCVYPNPASVPKK.N

No match to: 713.4334, 719.2388, 724.3009, 726.2899, 732.2586, 735.3865, 745.4124, 756.4687, 764.4056, 765.3908, 767.3679, 768.3909, 772.3955, 795.4186, 802.4548, 803.4370, 805.4229, 806.4631, 809.4480, 811.4104, 816.4408, 820.4244, 822.3905, 823.3846, 824.3756, 834.4043, 836.4589, 837.3853, 839.3696, 840.3839, 842.5085, 845.4100, 845.4123, 851.3975, 852.4056, 856.4893, 866.3914, 867.3724, 870.5402, 878.4625, 880.4403, 886.3815, 888.4233, 890.4479, 893.9562, 894.4579, 897.0289, 897.4412, 902.4597, 910.4410, 913.5118, 916.4905, 918.5391, 925.4747, 928.5064, 930.5048, 933.4619, 939.4758, 947.4628, 949.4591, 951.4703, 954.4674, 957.4657, 963.4676, 965.4761, 967.4568, 972.4793, 972.4800, 975.4650, 977.4630, 978.4976, 981.4565, 983.4664, 985.4842, 991.4688, 993.5331, 997.4298, 999.4453, 1001.4488, 1007.5668, 1018.5200, 1018.5212, 1023.4777, 1027.4972, 1030.5151, 1031.3850, 1033.5419, 1035.5352, 1036.5372, 1039.5402, 1042.5383, 1045.5654, 1056.5488, 1060.5400, 1060.5442, 1065.5425, 1086.5115, 1106.5372, 1111.5582, 1113.5546, 1115.5605, 1118.5529, 1122.5363, 1128.5775, 1129.6014, 1130.5935, 1140.5485, 1144.5709, 1156.5720, 1157.5952, 1158.6154, 1159.6273, 1165.5817, 1172.6301, 1179.6063, 1180.6165, 1182.6090, 1185.6317, 1187.6431, 1193.6096, 1195.6060, 1206.6770, 1209.1387, 1209.5848, 1210.1014, 1215.6627, 1216.6494, 1222.5994, 1229.6246, 1231.6174, 1234.6317, 1235.5846, 1240.6298, 1244.6324, 1248.6119, 1250.6144, 1268.6498, 1271.6696, 1273.6653, 1275.6644, 1291.6425, 1295.7233, 1300.6062, 1301.6205, 1307.6858, 1315.6570, 1317.6521, 1319.6852, 1323.6517, 1328.6809, 1329.6672, 1331.6763, 1332.6743, 1334.6592, 1335.6937, 1336.6715, 1337.6724, 1338.6471, 1342.6680, 1345.6760, 1346.6880, 1349.6991, 1354.6907, 1355.7155, 1357.7034, 1360.6882, 1361.6670, 1365.6913, 1368.6920, 1375.6798, 1376.6804, 1377.6794, 1382.6647, 1387.6444, 1410.7505, 1411.7208, 1421.7177, 1431.7961, 1434.7288, 1446.7256, 1450.7079, 1473.8054, 1475.7748, 1477.7524, 1486.7664, 1493.7539, 1494.7423, 1505.8649, 1523.7841, 1524.7848, 1537.8021, 1549.7388, 1558.8474, 1576.7987, 1579.7926, 1584.7897, 1588.8136, 1590.3611, 1604.8417, 1605.8098, 1615.8826, 1616.8605, 1628.8260, 1629.8358, 1630.8466, 1633.9790, 1635.7943, 1644.8046, 1647.8785, 1649.8463, 1651.8245, 1659.8241, 1662.7909, 1668.7810, 1679.9736, 1681.8214, 1683.8121, 1684.8204, 1706.8616, 1707.7838, 1716.8571, 1725.8353, 1728.8242, 1734.8152, 1740.8768, 1742.9031, 1746.1659, 1751.8787, 1753.8948, 1756.8905, 1763.8188, 1765.7690, 1767.8036, 1768.9027, 1769.4475, 1770.4261, 1774.9091, 1783.8499, 1791.7335, 1808.8868, 1824.8674, 1840.8225, 1844.8907, 1848.8322, 1850.8301, 1851.8488, 1867.9503, 1880.9277, 1887.9734, 1908.8838, 1919.0038, 1925.9418, 1928.0103, 1931.9463, 1953.9353, 1975.9609, 1992.9642, 1995.9020, 2011.8845, 2013.8983, 2024.8591, 2025.6234, 2036.5195, 2036.9486, 2047.9691, 2051.9792, 2052.9924, 2058.9963, 2060.0469, 2064.9900, 2068.8982, 2074.8914, 2083.9907, 2084.8987, 2092.8821, 2095.9941, 2100.9160, 2102.8872, 2106.9011, 2108.8835, 2109.9175, 2114.9180, 2116.9160, 2118.8835, 2119.8833, 2125.0176, 2132.8694, 2136.9180, 2211.1045, 2225.1191, 2273.0496, 2277.0137, 2307.9941, 2322.9980, 2341.0366, 2343.0271, 2352.0571, 2355.0459, 2359.0532, 2366.0813, 2368.0544, 2369.0710, 2376.0264, 2382.0349, 2383.6099, 2383.9600, 2390.0403, 2396.0342, 2398.0576, 2406.0737, 2411.0076, 2414.0813, 2420.0718, 2422.0408, 2423.0466, 2428.0898, 2430.0828, 2431.0845, 2433.0779, 2436.0508, 2438.0457, 2439.0261, 2442.0618, 2449.0347, 2460.0254, 2461.9956, 2463.9858, 2510.1277, 2542.1509, 2545.1558, 2547.1531, 2552.1038, 2565.1543, 2585.1111, 2601.0889, 2705.2192, 2717.0693, 3129.3176, 3208.1736, 3223.2710, 3290.4131, 3291.4248, 3312.2756, 3451.3933, 3453.4365

3. F1MYE4 **Mass:** 41319 **Score:** 63 **Expect:** 0.012 **Matches:** 20

Chondroadherin OS=Bos taurus GN=CHAD PE=4 SV=2

Observed	Mr(expt)	Mr(calc)	ppm	Start	End	Miss	Ions	Peptide
756.4687	755.4614	755.4653	-5.21	57	62	0	---	K.LLNLQR.N
765.3908	764.3835	764.3817	2.40	166	173	0	---	R.SGAFQGAK.D
802.4548	801.4475	801.4497	-2.74	315	320	1	---	R.RWLEAK.T
820.4244	819.4171	819.4239	-8.28	91	98	0	---	R.EVAAGAFR.G
837.3853	836.3780	836.3633	17.6	306	311	0	---	K.CTCQLR.G
913.5118	912.5045	912.5042	0.32	334	340	1	---	K.FRGQHIR.D
993.5331	992.5258	992.5226	3.26	347	354	2	---	R.GCKFPTKR.S
1018.5200	1017.5127	1017.5104	2.28	274	281	0	35	K.HVHLENNR.L
1018.5212	1017.5139	1017.5104	3.46	274	281	0	---	K.HVHLENNR.L
1315.6570	1314.6497	1314.6429	5.19	336	346	1	---	R.GQHIRDTDAFR.G
1319.6852	1318.6779	1318.6710	5.22	306	315	2	---	K.CTCQLRGLRR.W
1338.6471	1337.6398	1337.6364	2.55	235	246	0	---	K.SIPDNAFQSFGR.Y
1349.6991	1348.6918	1348.6888	2.27	63	74	0	---	R.NNFPVLAANSFR.A
1355.7155	1354.7082	1354.7133	-3.74	261	273	0	---	K.FSDGAFLGVTTLK.H
1361.6670	1360.6597	1360.6405	14.1	321	333	0	---	K.TSRPDATCASPAK.F
1365.6913	1364.6840	1364.6936	-6.99	207	219	0	---	R.NQLSSYPSAALSK.L
1505.8649	1504.8576	1504.8613	-2.44	222	234	1	---	R.VVEELKLSHNPLK.S
1647.8785	1646.8712	1646.8780	-4.12	102	114	0	---	K.QLIYLYLSHNDIR.V
1751.8787	1750.8714	1750.8777	-3.60	247	260	0	---	R.YLETLWLDNTNLEK.F
2060.0469	2059.0396	2059.0374	1.07	202	219	1	---	K.FYLDRNQLSSYPSAALSK.L

No match to: 713.4334, 719.2388, 724.3009, 726.2899, 732.2586, 735.3865, 745.4124, 764.4056, 767.3679, 768.3909, 772.3955, 795.4186, 803.4370, 805.4229, 806.4631, 807.3893, 809.4480, 811.4104, 812.3985, 816.4408, 822.3905, 823.3846, 824.3756, 828.3904, 834.4043, 836.4589, 839.3696, 840.3839, 842.5085, 845.4100, 845.4123, 851.3975, 852.4056, 856.4893, 866.3914, 867.3724, 870.5402, 871.4945, 878.4625, 880.4403, 886.3815, 888.4233, 890.4479, 893.9562, 894.4579, 897.0289, 897.4412, 902.4597, 910.4410, 912.4497, 916.4905, 918.5391, 925.4747, 928.5064, 930.5048, 933.4619, 935.4698, 939.4758, 947.4628, 949.4591, 951.4703, 954.4674, 957.4657,

963.4676, 965.4761, 967.4568, 969.4571, 972.4793, 972.4800, 975.4650, 977.4630, 978.4976, 981.4565, 983.4664, 985.4842, 991.4688, 997.4298, 999.4453, 1001.4488, 1003.4687, 1007.5668, 1023.4777, 1026.4922, 1027.4972, 1030.5151, 1031.3850, 1033.5419, 1035.5352, 1036.5372, 1039.5402, 1042.5383, 1045.5654, 1056.5488, 1060.5400, 1060.5442, 1065.5425, 1086.5115, 1106.5372, 1111.5582, 1113.5546, 1114.5396, 1115.5605, 1118.5529, 1122.5363, 1128.5775, 1129.6014, 1130.5935, 1140.5485, 1144.5709, 1155.6113, 1156.5720, 1157.5952, 1158.6154, 1159.6273, 1165.5817, 1172.6301, 1173.6259, 1179.6063, 1180.6165, 1182.6090, 1183.6123, 1185.6317, 1187.6431, 1193.6096, 1195.6060, 1196.6102, 1206.6770, 1209.1387, 1209.5848, 1210.1014, 1215.6627, 1216.6494, 1222.5994, 1226.5948, 1229.6246, 1231.6174, 1234.6317, 1235.5846, 1240.6298, 1244.6324, 1248.6119, 1250.6144, 1268.6498, 1271.6696, 1273.6653, 1275.6644, 1291.6425, 1295.7233, 1300.6062, 1301.6205, 1307.6858, 1317.6521, 1323.6517, 1325.6637, 1328.6809, 1329.6672, 1331.6763, 1332.6743, 1334.6592, 1335.6937, 1336.6715, 1337.6724, 1342.6680, 1345.6760, 1346.6880, 1354.6907, 1357.7034, 1360.6882, 1368.6920, 1374.6412, 1375.6798, 1376.6804, 1377.6794, 1382.6647, 1387.6444, 1410.7505, 1411.7208, 1421.7177, 1427.7872, 1431.7961, 1432.7133, 1434.7288, 1446.7256, 1450.7079, 1473.8054, 1475.7748, 1477.7524, 1478.7439, 1485.7720, 1486.7664, 1493.7539, 1494.7423, 1523.7841, 1524.7848, 1537.8021, 1549.7388, 1555.8816, 1558.8474, 1576.7987, 1579.7926, 1584.7897, 1588.8136, 1590.3611, 1604.8417, 1605.8098, 1606.8384, 1615.8826, 1616.8605, 1622.8137, 1628.8260, 1629.8358, 1630.8466, 1633.9790, 1635.7943, 1644.8046, 1649.8463, 1651.8245, 1659.8241, 1662.7909, 1668.7810, 1679.9736, 1681.8214, 1683.8121, 1684.8204, 1706.8616, 1707.7838, 1716.8571, 1725.8353, 1728.8242, 1734.8152, 1740.8768, 1742.9031, 1746.1659, 1750.8356, 1753.8948, 1756.8905, 1757.8322, 1763.8188, 1765.7690, 1767.8036, 1768.9027, 1769.4475, 1770.4261, 1774.9091, 1783.8499, 1786.9000, 1786.9017, 1791.7335, 1808.8868, 1824.8674, 1840.8225, 1844.8907, 1848.8322, 1850.8301, 1851.8488, 1867.9503, 1880.9277, 1887.9734, 1908.8838, 1914.9995, 1919.0038, 1925.9418, 1928.0103, 1931.9463, 1953.9353, 1975.9609, 1992.9642, 1995.9020, 2007.9894, 2011.8845, 2013.8983, 2024.8591, 2025.6234, 2036.5195, 2036.9486, 2042.0640, 2047.0167, 2047.9691, 2051.9792, 2052.9924, 2058.9963, 2064.9900, 2068.8982, 2070.8835, 2074.8914, 2083.9907, 2084.8987, 2086.8828, 2092.8821, 2095.9941, 2098.9976, 2100.9160, 2102.8872, 2106.9011, 2108.8835, 2109.9175, 2114.9180, 2116.9160, 2118.8835, 2119.8833, 2125.0176, 2132.8694, 2136.9180, 2170.1467, 2211.1045, 2225.1191, 2273.0496, 2277.0137, 2307.9941, 2322.9980, 2341.0366, 2343.0271, 2352.0571, 2355.0459, 2359.0532, 2366.0813, 2368.0544, 2369.0710, 2376.0264, 2382.0349, 2383.6099, 2383.9600, 2390.0403, 2396.0342, 2398.0576, 2400.0652, 2406.0737, 2411.0076, 2414.0813, 2416.0752, 2417.0732, 2420.0718, 2422.0408, 2423.0466, 2428.0898, 2430.0828, 2431.0845, 2433.0779, 2436.0508, 2438.0457, 2439.0261, 2442.0618, 2449.0347, 2452.0632, 2460.0254, 2461.9956, 2463.9858, 2510.1277, 2528.1580, 2542.1509, 2545.1558, 2547.1531, 2552.1038, 2565.1543, 2585.1111, 2601.0889, 2705.2192, 2717.0693, 3129.3176, 3208.1736, 3223.2710, 3290.4131, 3291.4248, 3312.2756, 3451.3933, 3453.4365

4. P19879 **Mass:** 34530 **Score:** 62 **Expect:** 0.015 **Matches:** 15

Mimecan OS=Bos taurus GN=OGN PE=1 SV=2

Observed	Mr(expt)	Mr(calc)	ppm	Start	End	Miss	Ions	Peptide
806.4631	805.4558	805.4698	-17.35	190	196	0	---	K.LTLFNAK.Y
918.5391	917.5318	917.5334	-1.80	292	299	1	---	K.RLPIGSYI.-
930.5048	929.4975	929.4930	4.81	266	272	1	---	R.DRIEEIR.L
939.4758	938.4686	938.4457	24.3	258	265	0	---	K.ANDTSYIR.D
972.4793	971.4720	971.4712	0.85	121	128	0	---	K.ESAYLYAR.F
972.4800	971.4727	971.4712	1.56	121	128	0	8	K.ESAYLYAR.F
1060.5400	1059.5327	1059.5349	-2.05	139	147	0	36	K.DFADIPNLR.R
1060.5442	1059.5369	1059.5349	1.91	139	147	0	---	K.DFADIPNLR.R
1115.5605	1114.5532	1114.5593	-5.49	283	291	0	---	K.HPNSFICLK.R
1216.6494	1215.6421	1215.6360	5.04	139	148	1	---	K.DFADIPNLRR.L
1271.6696	1270.6623	1270.6604	1.48	283	292	1	---	K.HPNSFICLKR.L
1361.6670	1360.6597	1360.6775	-13.07	121	131	1	---	K.ESAYLYARFNK.I
1473.8054	1472.7981	1472.7987	-0.40	135	147	1	---	K.LTAKDFADIPNLR.R
1679.9736	1678.9663	1678.9617	2.74	268	282	1	---	R.IEEIRLEGNPVILGK.H
2705.2192	2704.2119	2704.1704	15.3	29	50	0	---	R.IIYDYGTDNLEETFFSQDYEDK.Y

No match to: 713.4334, 719.2388, 724.3009, 726.2899, 732.2586, 735.3865, 745.4124, 756.4687, 764.4056, 765.3908, 767.3679, 768.3909, 772.3955, 795.4186, 802.4548, 803.4370, 805.4229, 807.3893, 809.4480, 811.4104, 812.3985, 816.4408, 820.4244, 822.3905, 823.3846, 824.3756, 828.3904, 834.4043, 836.4589, 837.3853, 839.3696, 840.3839, 842.5085, 845.4100, 845.4123, 851.3975, 852.4056, 856.4893, 866.3914, 867.3724, 870.5402, 871.4945, 878.4625, 880.4403, 886.3815, 888.4233, 890.4479, 893.9562, 894.4579, 897.0289, 897.4412, 902.4597, 910.4410, 912.4497, 913.5118, 916.4905, 925.4747, 928.5064, 933.4619, 935.4698, 947.4628, 949.4591, 951.4703, 954.4674, 957.4657, 963.4676, 965.4761, 967.4568, 969.4571, 975.4650, 977.4630, 978.4976, 981.4565, 983.4664, 985.4842, 991.4688, 993.5331, 997.4298, 999.4453, 1001.4488, 1003.4687, 1007.5668, 1018.5200, 1018.5212, 1023.4777, 1026.4922, 1027.4972, 1030.5151, 1031.3850, 1033.5419, 1035.5352, 1036.5372, 1039.5402, 1042.5383, 1045.5654, 1056.5488, 1065.5425, 1086.5115, 1106.5372, 1111.5582, 1113.5546, 1114.5396, 1118.5529, 1122.5363, 1128.5775, 1129.6014, 1130.5935, 1140.5485, 1144.5709, 1155.6113, 1156.5720, 1157.5952, 1158.6154, 1159.6273, 1165.5817, 1172.6301, 1173.6259, 1179.6063, 1180.6165, 1182.6090, 1183.6123, 1185.6317, 1187.6431, 1193.6096, 1195.6060, 1196.6102, 1206.6770, 1209.1387, 1209.5848, 1210.1014, 1215.6627, 1222.5994, 1226.5948, 1229.6246, 1231.6174, 1234.6317, 1235.5846, 1240.6298, 1244.6324, 1248.6119, 1250.6144, 1268.6498, 1273.6653, 1275.6644, 1291.6425, 1295.7233, 1300.6062, 1301.6205, 1307.6858, 1315.6570, 1317.6521, 1319.6852, 1323.6517, 1325.6637, 1328.6809, 1329.6672, 1331.6763, 1332.6743, 1334.6592, 1335.6937, 1336.6715, 1337.6724, 1338.6471, 1342.6680, 1345.6760, 1346.6880, 1349.6991, 1354.6907, 1355.7155, 1357.7034, 1360.6882, 1365.6913, 1368.6920, 1374.6412, 1375.6798, 1376.6804, 1377.6794, 1382.6647, 1387.6444, 1410.7505, 1411.7208, 1421.7177, 1427.7872, 1431.7961, 1432.7133, 1434.7288, 1446.7256, 1450.7079, 1475.7748, 1477.7524, 1478.7439, 1485.7720, 1486.7664, 1493.7539, 1494.7423, 1505.8649, 1523.7841, 1524.7848, 1537.8021, 1549.7388, 1555.8816, 1558.8474, 1576.7987, 1579.7926, 1584.7897, 1588.8136, 1590.3611, 1604.8417, 1605.8098, 1606.8384, 1615.8826, 1616.8605, 1622.8137, 1628.8260, 1629.8358, 1630.8466, 1633.9790, 1635.7943, 1644.8046, 1647.8785, 1649.8463, 1651.8245, 1659.8241, 1662.7909, 1668.7810, 1681.8214, 1683.8121, 1684.8204, 1706.8616, 1707.7838, 1716.8571, 1725.8353, 1728.8242, 1734.8152, 1740.8768, 1742.9031, 1746.1659, 1750.8356, 1751.8787, 1753.8948, 1756.8905, 1757.8322, 1763.8188, 1765.7690, 1767.8036, 1768.9027, 1769.4475, 1770.4261, 1774.9091, 1783.8499, 1786.9000, 1786.9017, 1791.7335, 1808.8868, 1824.8674, 1840.8225, 1844.8907, 1848.8322, 1850.8301, 1851.8488, 1867.9503, 1880.9277, 1887.9734, 1908.8838, 1914.9995, 1919.0038, 1925.9418, 1928.0103, 1931.9463, 1953.9353, 1975.9609, 1992.9642, 1995.9020, 2007.9894, 2011.8845, 2013.8983, 2024.8591, 2025.6234, 2036.5195, 2036.9486, 2042.0640, 2047.0167, 2047.9691, 2051.9792, 2052.9924, 2058.9963, 2060.0469, 2064.9900, 2068.8982, 2070.8835, 2074.8914, 2083.9907, 2084.8987, 2086.8828, 2092.8821, 2095.9941, 2098.9976, 2100.9160, 2102.8872, 2106.9011, 2108.8835, 2109.9175, 2114.9180, 2116.9160, 2118.8835, 2119.8833, 2125.0176, 2132.8694, 2136.9180, 2170.1467, 2211.1045, 2225.1191, 2273.0496, 2277.0137, 2307.9941, 2322.9980, 2341.0366, 2343.0271, 2352.0571, 2355.0459, 2359.0532, 2366.0813, 2368.0544, 2369.0710, 2376.0264, 2382.0349, 2383.6099, 2383.9600, 2390.0403, 2396.0342, 2398.0576, 2400.0652, 2406.0737, 2411.0076, 2414.0813, 2416.0752, 2417.0732, 2420.0718, 2422.0408, 2423.0466, 2428.0898, 2430.0828, 2431.0845, 2433.0779, 2436.0508, 2438.0457, 2439.0261, 2442.0618, 2449.0347, 2452.0632, 2460.0254, 2461.9956, 2463.9858, 2510.1277, 2528.1580, 2542.1509, 2545.1558, 2547.1531, 2552.1038, 2565.1543, 2585.1111, 2601.0889, 2717.0693, 3129.3176, 3208.1736, 3223.2710, 3290.4131, 3291.4248, 3312.2756, 3451.3933, 3453.4365

5. A5D9E8 **Mass:** 34517 **Score:** 62 **Expect:** 0.015 **Matches:** 15

Mimecan OS=Bos taurus GN=OGN PE=1 SV=1

Observed	Mr(expt)	Mr(calc)	ppm	Start	End	Miss	Ions	Peptide
806.4631	805.4558	805.4698	-17.35	190	196	0	---	K.LTLFNAK.Y
918.5391	917.5318	917.5334	-1.80	292	299	1	---	K.RLPIGSYI.-
930.5048	929.4975	929.4930	4.81	266	272	1	---	R.DRIEEIR.L
939.4758	938.4686	938.4457	24.3	258	265	0	---	K.ANDTSYIR.D
972.4793	971.4720	971.4712	0.85	121	128	0	---	K.ESAYLYAR.F
972.4800	971.4727	971.4712	1.56	121	128	0	8	K.ESAYLYAR.F
1060.5400	1059.5327	1059.5349	-2.05	139	147	0	36	K.DFADIPNLR.R
1060.5442	1059.5369	1059.5349	1.91	139	147	0	---	K.DFADIPNLR.R
1115.5605	1114.5532	1114.5593	-5.49	283	291	0	---	K.HPNSFICLK.R
1216.6494	1215.6421	1215.6360	5.04	139	148	1	---	K.DFADIPNLRR.L
1271.6696	1270.6623	1270.6604	1.48	283	292	1	---	K.HPNSFICLKR.L
1361.6670	1360.6597	1360.6775	-13.07	121	131	1	---	K.ESAYLYARFNK.I
1473.8054	1472.7981	1472.7987	-0.40	135	147	1	---	K.LTAKDFADIPNLR.R
1679.9736	1678.9663	1678.9617	2.74	268	282	1	---	R.IEEIRLEGNPVILGK.H

2705.2192	2704.2119	2704.1704	15.3	29 - 50	0	---	R.IIYDYGTDNLEETFFSQDYEDK.Y

No match to: 713.4334, 719.2388, 724.3009, 726.2899, 732.2586, 735.3865, 745.4124, 756.4687, 764.4056, 765.3908, 767.3679, 768.3909, 772.3955, 795.4186, 802.4548, 803.4370, 805.4229, 807.3893, 809.4480, 811.4104, 812.3985, 816.4408, 820.4244, 822.3905, 823.3846, 824.3756, 828.3904, 834.4043, 836.4589, 837.3853, 839.3696, 840.3839, 842.5085, 845.4100, 845.4123, 851.3975, 852.4056, 856.4893, 866.3914, 867.3724, 870.5402, 871.4945, 878.4625, 880.4403, 886.3815, 888.4233, 890.4479, 893.9562, 894.4579, 897.0289, 897.4412, 902.4597, 910.4410, 912.4497, 913.5118, 916.4905, 925.4747, 928.5064, 933.4619, 935.4698, 947.4628, 949.4591, 951.4703, 954.4674, 957.4657, 963.4676, 965.4761, 967.4568, 969.4571, 975.4650, 977.4630, 978.4976, 981.4565, 983.4664, 985.4842, 991.4688, 993.5331, 997.4298, 999.4453, 1001.4488, 1003.4687, 1007.5668, 1018.5200, 1018.5212, 1023.4777, 1026.4922, 1027.4972, 1030.5151, 1031.3850, 1033.5419, 1035.5352, 1036.5372, 1039.5402, 1042.5383, 1045.5654, 1056.5488, 1065.5425, 1086.5115, 1106.5372, 1111.5582, 1113.5546, 1114.5396, 1118.5529, 1122.5363, 1128.5775, 1129.6014, 1130.5935, 1140.5485, 1144.5709, 1155.6113, 1156.5720, 1157.5952, 1158.6154, 1159.6273, 1165.5817, 1172.6301, 1173.6259, 1179.6063, 1180.6165, 1182.6090, 1183.6123, 1185.6317, 1187.6431, 1193.6096, 1195.6060, 1196.6102, 1206.6770, 1209.1387, 1209.5848, 1210.1014, 1215.6627, 1222.5994, 1226.5948, 1229.6246, 1231.6174, 1234.6317, 1235.5846, 1240.6298, 1244.6324, 1248.6119, 1250.6144, 1268.6498, 1273.6653, 1275.6644, 1291.6425, 1295.7233, 1300.6062, 1301.6205, 1307.6858, 1315.6570, 1317.6521, 1319.6852, 1323.6517, 1325.6637, 1328.6809, 1329.6672, 1331.6763, 1332.6743, 1334.6592, 1335.6937, 1336.6715, 1337.6724, 1338.6471, 1342.6680, 1345.6760, 1346.6880, 1349.6991, 1354.6907, 1355.7155, 1357.7034, 1360.6882, 1365.6913, 1368.6920, 1374.6412, 1375.6798, 1376.6804, 1377.6794, 1382.6647, 1387.6444, 1410.7505, 1411.7208, 1421.7177, 1427.7872, 1431.7961, 1432.7133, 1434.7288, 1446.7256, 1450.7079, 1475.7748, 1477.7524, 1478.7439, 1485.7720, 1486.7664, 1493.7539, 1494.7423, 1505.8649, 1523.7841, 1524.7848, 1537.8021, 1549.7388, 1555.8816, 1558.8474, 1576.7987, 1579.7926, 1584.7897, 1588.8136, 1590.3611, 1604.8417, 1605.8098, 1606.8384, 1615.8826, 1616.8605, 1622.8137, 1628.8260, 1629.8358, 1630.8466, 1633.9790, 1635.7943, 1644.8046, 1647.8785, 1649.8463, 1651.8245, 1659.8241, 1662.7909, 1668.7810, 1681.8214, 1683.8121, 1684.8204, 1706.8616, 1707.7838, 1716.8571, 1725.8353, 1728.8242, 1734.8152, 1740.8768, 1742.9031, 1746.1659, 1750.8356, 1751.8787, 1753.8948, 1756.8905, 1757.8322, 1763.8188, 1765.7690, 1767.8036, 1768.9027, 1769.4475, 1770.4261, 1774.9091, 1783.8499, 1786.9000, 1786.9017, 1791.7335, 1808.8868, 1824.8674, 1840.8225, 1844.8907, 1848.8322, 1850.8301, 1851.8488, 1867.9503, 1880.9277, 1887.9734, 1908.8838, 1914.9995, 1919.0038, 1925.9418, 1928.0103, 1931.9463, 1953.9353, 1975.9609, 1992.9642, 1995.9020, 2007.9894, 2011.8845, 2013.8983, 2024.8591, 2025.6234, 2036.5195, 2036.9486, 2042.0640, 2047.0167, 2047.9691, 2051.9792, 2052.9924, 2058.9963, 2060.0469, 2064.9900, 2068.8982, 2070.8835, 2074.8914, 2083.9907, 2084.8987, 2086.8828, 2092.8821, 2095.9941, 2098.9976, 2100.9160, 2102.8872, 2106.9011, 2108.8835, 2109.9175, 2114.9180, 2116.9160, 2118.8835, 2119.8833, 2125.0176, 2132.8694, 2136.9180, 2170.1467, 2211.1045, 2225.1191, 2273.0496, 2277.0137, 2307.9941, 2322.9980, 2341.0366, 2343.0271, 2352.0571, 2355.0459, 2359.0532, 2366.0813, 2368.0544, 2369.0710, 2376.0264, 2382.0349, 2383.6099, 2383.9600, 2390.0403, 2396.0342, 2398.0576, 2400.0652, 2406.0737, 2411.0076, 2414.0813, 2416.0752, 2417.0732, 2420.0718, 2422.0408, 2423.0466, 2428.0898, 2430.0828, 2431.0845, 2433.0779, 2436.0508, 2438.0457, 2439.0261, 2442.0618, 2449.0347, 2452.0632, 2460.0254, 2461.9956, 2463.9858, 2510.1277, 2528.1580, 2542.1509, 2545.1558, 2547.1531, 2552.1038, 2565.1543, 2585.1111, 2601.0889, 2717.0693, 3129.3176, 3208.1736, 3223.2710, 3290.4131, 3291.4248, 3312.2756, 3451.3933, 3453.4365

6. G3N088 **Mass:** 40915 **Score:** 61 **Expect:** 0.021 **Matches:** 16

Mimecan OS=Bos taurus GN=OGN PE=1 SV=1

Observed	Mr(expt)	Mr(calc)	ppm	Start	End	Miss	Ions	Peptide
735.3865	734.3792	734.3963	-23.27	5	10	0	---	K.ISTTWK.L
806.4631	805.4558	805.4698	-17.35	247	253	0	---	K.LTLFNAK.Y
918.5391	917.5318	917.5334	-1.80	349	356	1	---	K.RLPIGSYI.-
930.5048	929.4975	929.4930	4.81	323	329	1	---	R.DRIEEIR.L
939.4758	938.4686	938.4457	24.3	315	322	0	---	K.ANDTSYIR.D
972.4793	971.4720	971.4712	0.85	178	185	0	---	K.ESAYLYAR.F
972.4800	971.4727	971.4712	1.56	178	185	0	8	K.ESAYLYAR.F
1060.5400	1059.5327	1059.5349	-2.05	196	204	0	36	K.DFADIPNLR.R
1060.5442	1059.5369	1059.5349	1.91	196	204	0	---	K.DFADIPNLR.R
1115.5605	1114.5532	1114.5593	-5.49	340	348	0	---	K.HPNSFICLK.R
1216.6494	1215.6421	1215.6360	5.04	196	205	1	---	K.DFADIPNLRR.L
1271.6696	1270.6623	1270.6604	1.48	340	349	1	---	K.HPNSFICLKR.L
1361.6670	1360.6597	1360.6775	-13.07	178	188	1	---	K.ESAYLYARFNK.I
1473.8054	1472.7981	1472.7987	-0.40	192	204	1	---	K.LTAKDFADIPNLR.R
1679.9736	1678.9663	1678.9617	2.74	325	339	1	---	R.IEEIRLEGNPVILGK.H
2705.2192	2704.2119	2704.1704	15.3	86	107	0	---	R.IIYDYGTDNLEETFFSQDYEDK.Y

No match to: 713.4334, 719.2388, 724.3009, 726.2899, 732.2586, 745.4124, 756.4687, 764.4056, 765.3908, 767.3679, 768.3909, 772.3955, 795.4186, 802.4548, 803.4370, 805.4229, 807.3893, 809.4480, 811.4104, 812.3985, 816.4408, 820.4244, 822.3905, 823.3846, 824.3756, 828.3904, 834.4043, 836.4589, 837.3853, 839.3696, 840.3839, 842.5085, 845.4100, 845.4123, 851.3975, 852.4056, 856.4893, 866.3914, 867.3724, 870.5402, 871.4945, 878.4625, 880.4403, 886.3815, 888.4233, 890.4479, 893.9562, 894.4579, 897.0289, 897.4412, 902.4597, 910.4410, 912.4497, 913.5118, 916.4905, 925.4747, 928.5064, 933.4619, 935.4698, 947.4628, 949.4591, 951.4703, 954.4674, 957.4657, 963.4676, 965.4761, 967.4568, 969.4571, 975.4650, 977.4630, 978.4976, 981.4565, 983.4664, 985.4842, 991.4688, 993.5331, 997.4298, 999.4453, 1001.4488, 1003.4687, 1007.5668, 1018.5200, 1018.5212, 1023.4777, 1026.4922, 1027.4972, 1030.5151, 1031.3850, 1033.5419, 1035.5352, 1036.5372, 1039.5402, 1042.5383, 1045.5654, 1056.5488, 1065.5425, 1086.5115, 1106.5372, 1111.5582, 1113.5546, 1114.5396, 1118.5529, 1122.5363, 1128.5775, 1129.6014, 1130.5935, 1140.5485, 1144.5709, 1155.6113, 1156.5720, 1157.5952, 1158.6154, 1159.6273, 1165.5817, 1172.6301, 1173.6259, 1179.6063, 1180.6165, 1182.6090, 1183.6123, 1185.6317, 1187.6431, 1193.6096, 1195.6060, 1196.6102, 1206.6770, 1209.1387, 1209.5848, 1210.1014, 1215.6627, 1222.5994, 1226.5948, 1229.6246, 1231.6174, 1234.6317, 1235.5846, 1240.6298, 1244.6324, 1248.6119, 1250.6144, 1268.6498, 1273.6653, 1275.6644, 1291.6425, 1295.7233, 1300.6062, 1301.6205, 1307.6858, 1315.6570, 1317.6521, 1319.6852, 1323.6517, 1325.6637, 1328.6809, 1329.6672, 1331.6763, 1332.6743, 1334.6592, 1335.6937, 1336.6715, 1337.6724, 1338.6471, 1342.6680, 1345.6760, 1346.6880, 1349.6991, 1354.6907, 1355.7155, 1357.7034, 1360.6882, 1365.6913, 1368.6920, 1374.6412, 1375.6798, 1376.6804, 1377.6794, 1382.6647, 1387.6444, 1410.7505, 1411.7208, 1421.7177, 1427.7872, 1431.7961, 1432.7133, 1434.7288, 1446.7256, 1450.7079, 1475.7748, 1477.7524, 1478.7439, 1485.7720, 1486.7664, 1493.7539, 1494.7423, 1505.8649, 1523.7841, 1524.7848, 1537.8021, 1549.7388, 1555.8816, 1558.8474, 1576.7987, 1579.7926, 1584.7897, 1588.8136, 1590.3611, 1604.8417, 1605.8098, 1606.8384, 1615.8826, 1616.8605, 1622.8137, 1628.8260, 1629.8358, 1630.8466, 1633.9790, 1635.7943, 1644.8046, 1647.8785, 1649.8463, 1651.8245, 1659.8241, 1662.7909, 1668.7810, 1681.8214, 1683.8121, 1684.8204, 1706.8616, 1707.7838, 1716.8571, 1725.8353, 1728.8242, 1734.8152, 1740.8768, 1742.9031, 1746.1659, 1750.8356, 1751.8787, 1753.8948, 1756.8905, 1757.8322, 1763.8188, 1765.7690, 1767.8036, 1768.9027, 1769.4475, 1770.4261, 1774.9091, 1783.8499, 1786.9000, 1786.9017, 1791.7335, 1808.8868, 1824.8674, 1840.8225, 1844.8907, 1848.8322, 1850.8301, 1851.8488, 1867.9503, 1880.9277, 1887.9734, 1908.8838, 1914.9995, 1919.0038, 1925.9418, 1928.0103, 1931.9463, 1953.9353, 1975.9609, 1992.9642, 1995.9020, 2007.9894, 2011.8845, 2013.8983, 2024.8591, 2025.6234, 2036.5195, 2036.9486, 2042.0640, 2047.0167, 2047.9691, 2051.9792, 2052.9924, 2058.9963, 2060.0469, 2064.9900, 2068.8982, 2070.8835, 2074.8914, 2083.9907, 2084.8987, 2086.8828, 2092.8821, 2095.9941, 2098.9976, 2100.9160, 2102.8872, 2106.9011, 2108.8835, 2109.9175, 2114.9180, 2116.9160, 2118.8835, 2119.8833, 2125.0176, 2132.8694, 2136.9180, 2170.1467, 2211.1045, 2225.1191, 2273.0496, 2277.0137, 2307.9941, 2322.9980, 2341.0366, 2343.0271, 2352.0571, 2355.0459, 2359.0532, 2366.0813, 2368.0544, 2369.0710, 2376.0264, 2382.0349, 2383.6099, 2383.9600, 2390.0403, 2396.0342, 2398.0576, 2400.0652, 2406.0737, 2411.0076, 2414.0813, 2416.0752, 2417.0732, 2420.0718, 2422.0408, 2423.0466, 2428.0898, 2430.0828, 2431.0845, 2433.0779, 2436.0508, 2438.0457, 2439.0261, 2442.0618, 2449.0347, 2452.0632, 2460.0254, 2461.9956, 2463.9858, 2510.1277, 2528.1580, 2542.1509, 2545.1558, 2547.1531, 2552.1038, 2565.1543, 2585.1111, 2601.0889, 2717.0693, 3129.3176, 3208.1736, 3223.2710, 3290.4131, 3291.4248, 3312.2756, 3451.3933, 3453.4365

7. Q27972 **Mass:** 41373 **Score:** 58 **Expect:** 0.039 **Matches:** 19

Chondroadherin OS=Bos taurus GN=CHAD PE=1 SV=1

Observed	Mr(expt)	Mr(calc)	ppm	Start	End	Miss	Ions	Peptide
756.4687	755.4614	755.4653	-5.21	57	62	0	---	K.LLNLQR.N
765.3908	764.3835	764.3817	2.40	166	173	0	---	R.SGAFQGAK.D
802.4548	801.4475	801.4497	-2.74	315	320	1	---	R.RWLEAK.T
820.4244	819.4171	819.4239	-8.28	91	98	0	---	R.EVAAGAFR.G
837.3853	836.3780	836.3633	17.6	306	311	0	---	K.CTCQLR.G
913.5118	912.5045	912.5042	0.32	334	340	1	---	K.FRGQHIR.D
993.5331	992.5258	992.5226	3.26	347	354	2	---	R.GCKFPTKR.S

1018.5200	1017.5127	1017.5104	2.28	274 - 281	0	35	K.HVHLENNR.L
1018.5212	1017.5139	1017.5104	3.46	274 - 281	0	---	K.HVHLENNR.L
1315.6570	1314.6497	1314.6429	5.19	336 - 346	1	---	R.GQHIRDTDAFR.G
1319.6852	1318.6779	1318.6710	5.22	306 - 315	2	---	K.CTCQLRGLRR.W
1338.6471	1337.6398	1337.6364	2.55	235 - 246	0	---	K.SIPDNAFQSFGR.Y
1355.7155	1354.7082	1354.7133	-3.74	261 - 273	0	---	K.FSDGAFLGVTTLK.H
1361.6670	1360.6597	1360.6405	14.1	321 - 333	0	---	K.TSRPDATCASPAK.F
1365.6913	1364.6840	1364.6936	-6.99	207 - 219	0	---	R.NQLSSYPSAALSK.L
1505.8649	1504.8576	1504.8613	-2.44	222 - 234	1	---	R.VVEELKLSHNPLK.S
1647.8785	1646.8712	1646.8780	-4.12	102 - 114	0	---	K.QLIYLYLSHNDIR.V
1751.8787	1750.8714	1750.8777	-3.60	247 - 260	0	---	R.YLETLWLDNTNLEK.F
2060.0469	2059.0396	2059.0374	1.07	202 - 219	1	---	K.FYLDRNQLSSYPSAALSK.L

No match to: 713.4334, 719.2388, 724.3009, 726.2899, 732.2586, 735.3865, 745.4124, 764.4056, 767.3679, 768.3909, 772.3955, 795.4186, 803.4370, 805.4229, 806.4631, 807.3893, 809.4480, 811.4104, 812.3985, 816.4408, 822.3905, 823.3846, 824.3756, 828.3904, 834.4043, 836.4589, 839.3696, 840.3839, 842.5085, 845.4100, 845.4123, 851.3975, 852.4056, 856.4893, 866.3914, 867.3724, 870.5402, 871.4945, 878.4625, 880.4403, 886.3815, 888.4233, 890.4479, 893.9562, 894.4579, 897.0289, 897.4412, 902.4597, 910.4410, 912.4497, 916.4905, 918.5391, 925.4747, 928.5064, 930.5048, 933.4619, 935.4698, 939.4758, 947.4628, 949.4591, 951.4703, 954.4674, 957.4657, 963.4676, 965.4761, 967.4568, 969.4571, 972.4793, 972.4800, 975.4650, 977.4630, 978.4976, 981.4565, 983.4664, 985.4842, 991.4688, 997.4298, 999.4453, 1001.4488, 1003.4687, 1007.5668, 1023.4777, 1026.4922, 1027.4972, 1030.5151, 1031.3850, 1033.5419, 1035.5352, 1036.5372, 1039.5402, 1042.5383, 1045.5654, 1056.5488, 1060.5400, 1060.5442, 1065.5425, 1086.5115, 1106.5372, 1111.5582, 1113.5546, 1114.5396, 1115.5605, 1118.5529, 1122.5363, 1128.5775, 1129.6014, 1130.5935, 1140.5485, 1144.5709, 1155.6113, 1156.5720, 1157.5952, 1158.6154, 1159.6273, 1165.5817, 1172.6301, 1173.6259, 1179.6063, 1180.6165, 1182.6090, 1183.6123, 1185.6317, 1187.6431, 1193.6096, 1195.6060, 1196.6102, 1206.6770, 1209.1387, 1209.5848, 1210.1014, 1215.6627, 1216.6494, 1222.5994, 1226.5948, 1229.6246, 1231.6174, 1234.6317, 1235.5846, 1240.6298, 1244.6324, 1248.6119, 1250.6144, 1268.6498, 1271.6696, 1273.6653, 1275.6644, 1291.6425, 1295.7233, 1300.6062, 1301.6205, 1307.6858, 1317.6521, 1323.6517, 1325.6637, 1328.6809, 1329.6672, 1331.6763, 1332.6743, 1334.6592, 1335.6937, 1336.6715, 1337.6724, 1342.6680, 1345.6760, 1346.6880, 1349.6991, 1354.6907, 1357.7034, 1360.6882, 1368.6920, 1374.6412, 1375.6798, 1376.6804, 1377.6794, 1382.6647, 1387.6444, 1410.7505, 1411.7208, 1421.7177, 1427.7872, 1431.7961, 1432.7133, 1434.7288, 1446.7256, 1450.7079, 1473.8054, 1475.7748, 1477.7524, 1478.7439, 1485.7720, 1486.7664, 1493.7539, 1494.7423, 1523.7841, 1524.7848, 1537.8021, 1549.7388, 1555.8816, 1558.8474, 1576.7987, 1579.7926, 1584.7897, 1588.8136, 1590.3611, 1604.8417, 1605.8098, 1606.8384, 1615.8826, 1616.8605, 1622.8137, 1628.8260, 1629.8358, 1630.8466, 1633.9790, 1635.7943, 1644.8046, 1649.8463, 1651.8245, 1659.8241, 1662.7909, 1668.7810, 1679.9736, 1681.8214, 1683.8121, 1684.8204, 1706.8616, 1707.7838, 1716.8571, 1725.8353, 1728.8242, 1734.8152, 1740.8768, 1742.9031, 1746.1659, 1750.8356, 1753.8948, 1756.8905, 1757.8322, 1763.8188, 1765.7690, 1767.8036, 1768.9027, 1769.4475, 1770.4261, 1774.9091, 1783.8499, 1786.9000, 1786.9017, 1791.7335, 1808.8868, 1824.8674, 1840.8225, 1844.8907, 1848.8322, 1850.8301, 1851.8488, 1867.9503, 1880.9277, 1887.9734, 1908.8838, 1914.9995, 1919.0038, 1925.9418, 1928.0103, 1931.9463, 1953.9353, 1975.9609, 1992.9642, 1995.9020, 2007.9894, 2011.8845, 2013.8983, 2024.8591, 2025.6234, 2036.5195, 2036.9486, 2042.0640, 2047.0167, 2047.9691, 2051.9792, 2052.9924, 2058.9963, 2064.9900, 2068.8982, 2070.8835, 2074.8914, 2083.9907, 2084.8987, 2086.8828, 2092.8821, 2095.9941, 2098.9976, 2100.9160, 2102.8872, 2106.9011, 2108.8835, 2109.9175, 2114.9180, 2116.9160, 2118.8835, 2119.8833, 2125.0176, 2132.8694, 2136.9180, 2170.1467, 2211.1045, 2225.1191, 2273.0496, 2277.0137, 2307.9941, 2322.9980, 2341.0366, 2343.0271, 2352.0571, 2355.0459, 2359.0532, 2366.0813, 2368.0544, 2369.0710, 2376.0264, 2382.0349, 2383.6099, 2383.9600, 2390.0403, 2396.0342, 2398.0576, 2400.0652, 2406.0737, 2411.0076, 2414.0813, 2416.0752, 2417.0732, 2420.0718, 2422.0408, 2423.0466, 2428.0898, 2430.0828, 2431.0845, 2433.0779, 2436.0508, 2438.0457, 2439.0261, 2442.0618, 2449.0347, 2452.0632, 2460.0254, 2461.9956, 2463.9858, 2510.1277, 2528.1580, 2542.1509, 2545.1558, 2547.1531, 2552.1038, 2565.1543, 2585.1111, 2601.0889, 2705.2192, 2717.0693, 3129.3176, 3208.1736, 3223.2710, 3290.4131, 3291.4248, 3312.2756, 3451.3933, 3453.4365

8. F1MYS7 **Mass:** 68834 **Score:** 25 **Expect:** 84 **Matches:** 16

Uncharacterized protein OS=Bos taurus GN=EIF4B PE=1 SV=1

Observed	Mr(expt)	Mr(calc)	ppm	Start - End	Miss	Ions	Peptide
713.4334	712.4261	712.4092	23.7	399 - 403	1	---	R.RPRER.H
828.3904	827.3831	827.3773	6.99	129 - 135	0	---	R.EPSNPER.L
836.4589	835.4516	835.4626	-13.17	473 - 480	0	---	K.VMPAPPPK.E
839.3696	838.3623	838.3569	6.39	287 - 292	1	---	R.RDDDYR.G
845.4100	844.4027	844.3926	12.0	579 - 586	0	15	K.AEENPASK.F
845.4123	844.4050	844.3926	14.7	579 - 586	0	---	K.AEENPASK.F
852.4056	851.3984	851.3886	11.5	180 - 186	1	---	R.DDRSFGR.D
930.5048	929.4975	929.4930	4.81	383 - 389	1	---	K.EQEKLQR.Q
1165.5817	1164.5744	1164.6000	-21.93	324 - 333	1	---	R.DDRGPPQRPK.L
1342.6680	1341.6607	1341.6888	-20.95	512 - 524	0	---	K.VVPAQLSEEGSAR.K
1357.7034	1356.6961	1356.6997	-2.66	166 - 177	1	---	R.IRVDVADQAQDK.D
1579.7926	1578.7853	1578.8228	-23.75	473 - 486	1	---	K.VMPAPPPKENAWVK.R + Oxidation (M)
1628.8260	1627.8187	1627.8278	-5.57	166 - 179	2	---	R.IRVDVADQAQDKDR.D
2013.8983	2012.8910	2012.9049	-6.89	538 - 557	2	---	K.GQSGSSSRGPGDGGNKDHWK.E
2352.0571	2351.0498	2351.0567	-2.92	195 - 214	1	---	K.TDTDWRARPAADSFDDYPPR.R
2390.0403	2389.0330	2389.0742	-17.23	488 - 511	1	---	R.SSNPPARSQSSDTEQQSPTSGGGK.V

No match to: 719.2388, 724.3009, 726.2899, 732.2586, 735.3865, 745.4124, 756.4687, 764.4056, 765.3908, 767.3679, 768.3909, 772.3955, 795.4186, 802.4548, 803.4370, 805.4229, 806.4631, 807.3893, 809.4480, 811.4104, 812.3985, 816.4408, 820.4244, 822.3905, 823.3846, 824.3756, 834.4043, 837.3853, 840.3839, 842.5085, 851.3975, 856.4893, 866.3914, 867.3724, 870.5402, 871.4945, 878.4625, 880.4403, 886.3815, 888.4233, 890.4479, 893.9562, 894.4579, 897.0289, 897.4412, 902.4597, 910.4410, 912.4497, 913.5118, 916.4905, 918.5391, 925.4747, 928.5064, 933.4619, 935.4698, 939.4758, 947.4628, 949.4591, 951.4703, 954.4674, 957.4657, 963.4676, 965.4761, 967.4568, 969.4571, 972.4793, 972.4800, 975.4650, 977.4630, 978.4976, 981.4565, 983.4664, 985.4842, 991.4688, 993.5331, 997.4298, 999.4453, 1001.4488, 1003.4687, 1007.5668, 1018.5200, 1018.5212, 1023.4777, 1026.4922, 1027.4972, 1030.5151, 1031.3850, 1033.5419, 1035.5352, 1036.5372, 1039.5402, 1042.5383, 1045.5654, 1056.5488, 1060.5400, 1060.5442, 1065.5425, 1086.5115, 1106.5372, 1111.5582, 1113.5546, 1114.5396, 1115.5605, 1118.5529, 1122.5363, 1128.5775, 1129.6014, 1130.5935, 1140.5485, 1144.5709, 1155.6113, 1156.5720, 1157.5952, 1158.6154, 1159.6273, 1172.6301, 1173.6259, 1179.6063, 1180.6165, 1182.6090, 1183.6123, 1185.6317, 1187.6431, 1193.6096, 1195.6060, 1196.6102, 1206.6770, 1209.1387, 1209.5848, 1210.1014, 1215.6627, 1216.6494, 1222.5994, 1226.5948, 1229.6246, 1231.6174, 1234.6317, 1235.5846, 1240.6298, 1244.6324, 1248.6119, 1250.6144, 1268.6498, 1271.6696, 1273.6653, 1275.6644, 1291.6425, 1295.7233, 1300.6062, 1301.6205, 1307.6858, 1315.6570, 1317.6521, 1319.6852, 1323.6517, 1325.6637, 1328.6809, 1329.6672, 1331.6763, 1332.6743, 1334.6592, 1335.6937, 1336.6715, 1337.6724, 1338.6471, 1345.6760, 1346.6880, 1349.6991, 1354.6907, 1355.7155, 1360.6882, 1361.6670, 1365.6913, 1368.6920, 1374.6412, 1375.6798, 1376.6804, 1377.6794, 1382.6647, 1387.6444, 1410.7505, 1411.7208, 1421.7177, 1427.7872, 1431.7961, 1432.7133, 1434.7288, 1446.7256, 1450.7079, 1473.8054, 1475.7748, 1477.7524, 1478.7439, 1485.7720, 1486.7664, 1493.7539, 1494.7423, 1505.8649, 1523.7841, 1524.7848, 1537.8021, 1549.7388, 1555.8816, 1558.8474, 1576.7987, 1584.7897, 1588.8136, 1590.3611, 1604.8417, 1605.8098, 1606.8384, 1615.8826, 1616.8605, 1622.8137, 1629.8358, 1630.8466, 1633.9790, 1635.7943, 1644.8046, 1647.8785, 1649.8463, 1651.8245, 1659.8241, 1662.7909, 1668.7810, 1679.9736, 1681.8214, 1683.8121, 1684.8204, 1706.8616, 1707.7838, 1716.8571, 1725.8353, 1728.8242, 1734.8152, 1740.8768, 1742.9031, 1746.1659, 1750.8356, 1751.8787, 1753.8948, 1756.8905, 1757.8322, 1763.8188, 1765.7690, 1767.8036, 1768.9027, 1769.4475, 1770.4261, 1774.9091, 1783.8499, 1786.9000, 1786.9017, 1791.7335, 1808.8868, 1824.8674, 1840.8225, 1844.8907, 1848.8322, 1850.8301, 1851.8488, 1867.9503, 1880.9277, 1887.9734, 1908.8838, 1914.9995, 1919.0038, 1925.9418, 1928.0103, 1931.9463, 1953.9353, 1975.9609, 1992.9642, 1995.9020, 2007.9894, 2011.8845, 2024.8591, 2025.6234, 2036.5195, 2036.9486, 2042.0640, 2047.0167, 2047.9691, 2051.9792, 2052.9924, 2058.9963, 2060.0469, 2064.9900, 2068.8982, 2070.8835, 2074.8914, 2083.9907, 2084.8987, 2086.8828, 2092.8821, 2095.9941, 2098.9976, 2100.9160, 2102.8872, 2106.9011, 2108.8835, 2109.9175, 2114.9180, 2116.9160, 2118.8835, 2119.8833, 2125.0176, 2132.8694, 2136.9180, 2170.1467, 2211.1045, 2225.1191, 2273.0496, 2277.0137, 2307.9941, 2322.9980, 2341.0366, 2343.0271, 2355.0459, 2359.0532, 2366.0813, 2368.0544, 2369.0710, 2376.0264, 2382.0349, 2383.6099, 2383.9600, 2396.0342, 2398.0576, 2400.0652, 2406.0737, 2411.0076, 2414.0813, 2416.0752, 2417.0732, 2420.0718, 2422.0408, 2423.0466, 2428.0898, 2430.0828, 2431.0845, 2433.0779, 2436.0508, 2438.0457, 2439.0261, 2442.0618, 2449.0347, 2452.0632, 2460.0254, 2461.9956, 2463.9858, 2510.1277, 2528.1580, 2542.1509, 2545.1558, 2547.1531, 2552.1038, 2565.1543, 2585.1111, 2601.0889, 2705.2192, 2717.0693, 3129.3176, 3208.1736, 3223.2710, 3290.4131, 3291.4248, 3312.2756, 3451.3933, 3453.4365

9. E1BND5 **Mass:** 218183 **Score:** 24 **Expect:** 1e+002 **Matches:** 12
Uncharacterized protein OS=Bos taurus GN=NCOA6 PE=4 SV=1

Observed	Mr(expt)	Mr(calc)	ppm	Start		End	Miss	Ions	Peptide
805.4229	804.4156	804.4090	8.21	1321	-	1328	1	---	R.SSPGSSRK.T
983.4664	982.4591	982.4832	-24.54	1047	-	1055	0	---	R.GPLNPDSQR.M
1128.5775	1127.5702	1127.5683	1.71	900	-	909	0	---	K.QNNTNANKPK.K
1165.5817	1164.5744	1164.5735	0.83	2026	-	2037	0	---	R.SSRPASASSSTK.V
1172.6301	1171.6228	1171.5945	24.1	1329	-	1339	1	---	K.TTPSPGRQNSK.A
1328.6809	1327.6736	1327.6983	-18.61	1517	-	1529	0	---	K.ASVIPSLQDPSSK.E
1365.6913	1364.6840	1364.6725	8.48	54	-	64	2	---	K.GNIDDKDFKWK.L
1786.9000	1785.8927	1785.9221	-16.44	1294	-	1311	1	24	K.LDSVVVNSGKQSNSGAPK.R
1786.9017	1785.8944	1785.9221	-15.49	1294	-	1311	1	---	K.LDSVVVNSGKQSNSGAPK.R
1931.9463	1930.9390	1930.9717	-16.92	1083	-	1099	2	---	K.QRMPMPVNTPLGSTSRK.M + 2 Oxidation (M)
1953.9353	1952.9280	1952.9700	-21.48	1432	-	1448	1	---	K.AVPPQEVKMVVPEDQCK.K
2211.1045	2210.0972	2210.0424	24.8	2004	-	2023	2	---	R.RNSRTDEPAVASESVENGHR.K

No match to: 713.4334, 719.2388, 724.3009, 726.2899, 732.2586, 735.3865, 745.4124, 756.4687, 764.4056, 765.3908, 767.3679, 768.3909, 772.3955, 795.4186, 802.4548, 803.4370, 806.4631, 807.3893, 809.4480, 811.4104, 812.3985, 816.4408, 820.4244, 822.3905, 823.3846, 824.3756, 828.3904, 834.4043, 836.4589, 837.3853, 839.3696, 840.3839, 842.5085, 845.4100, 845.4123, 851.3975, 852.4056, 856.4893, 866.3914, 867.3724, 870.5402, 871.4945, 878.4625, 880.4403, 886.3815, 888.4233, 890.4479, 893.9562, 894.4579, 897.0289, 897.4412, 902.4597, 910.4410, 912.4497, 913.5118, 916.4905, 918.5391, 925.4747, 928.5064, 930.5048, 933.4619, 935.4698, 939.4758, 947.4628, 949.4591, 951.4703, 954.4674, 957.4657, 963.4676, 965.4761, 967.4568, 969.4571, 972.4793, 972.4800, 975.4650, 977.4630, 978.4976, 981.4565, 985.4842, 991.4688, 993.5331, 997.4298, 999.4453, 1001.4488, 1003.4687, 1007.5668, 1018.5200, 1018.5212, 1023.4777, 1026.4922, 1027.4972, 1030.5151, 1031.3850, 1033.5419, 1035.5352, 1036.5372, 1039.5402, 1042.5383, 1045.5654, 1056.5488, 1060.5400, 1060.5442, 1065.5425, 1086.5115, 1106.5372, 1111.5582, 1113.5546, 1114.5396, 1115.5605, 1118.5529, 1122.5363, 1129.6014, 1130.5935, 1140.5485, 1144.5709, 1155.6113, 1156.5720, 1157.5952, 1158.6154, 1159.6273, 1173.6259, 1179.6063, 1180.6165, 1182.6090, 1183.6123, 1185.6317, 1187.6431, 1193.6096, 1195.6060, 1196.6102, 1206.6770, 1209.1387, 1209.5848, 1210.1014, 1215.6627, 1216.6494, 1222.5994, 1226.5948, 1229.6246, 1231.6174, 1234.6317, 1235.5846, 1240.6298, 1244.6324, 1248.6119, 1250.6144, 1268.6498, 1271.6696, 1273.6653, 1275.6644, 1291.6425, 1295.7233, 1300.6062, 1301.6205, 1307.6858, 1315.6570, 1317.6521, 1319.6852, 1323.6517, 1325.6637, 1329.6672, 1331.6763, 1332.6743, 1334.6592, 1335.6937, 1336.6715, 1337.6724, 1338.6471, 1342.6680, 1345.6760, 1346.6880, 1349.6991, 1354.6907, 1355.7155, 1357.7034, 1360.6882, 1361.6670, 1368.6920, 1374.6412, 1375.6798, 1376.6804, 1377.6794, 1382.6647, 1387.6444, 1410.7505, 1411.7208, 1421.7177, 1427.7872, 1431.7961, 1432.7133, 1434.7288, 1446.7256, 1450.7079, 1473.8054, 1475.7748, 1477.7524, 1478.7439, 1485.7720, 1486.7664, 1493.7539, 1494.7423, 1505.8649, 1523.7841, 1524.7848, 1537.8021, 1549.7388, 1555.8816, 1558.8474, 1576.7987, 1579.7926, 1584.7897, 1588.8136, 1590.3611, 1604.8417, 1605.8098, 1606.8384, 1615.8826, 1616.8605, 1622.8137, 1628.8260, 1629.8358, 1630.8466, 1633.9790, 1635.7943, 1644.8046, 1647.8785, 1649.8463, 1651.8245, 1659.8241, 1662.7909, 1668.7810, 1679.9736, 1681.8214, 1683.8121, 1684.8204, 1706.8616, 1707.7838, 1716.8571, 1725.8353, 1728.8242, 1734.8152, 1740.8768, 1742.9031, 1746.1659, 1750.8356, 1751.8787, 1753.8948, 1756.8905, 1757.8322, 1763.8188, 1765.7690, 1767.8036, 1768.9027, 1769.4475, 1770.4261, 1774.9091, 1783.8499, 1791.7335, 1808.8868, 1824.8674, 1840.8225, 1844.8907, 1848.8322, 1850.8301, 1851.8488, 1867.9503, 1880.9277, 1887.9734, 1908.8838, 1914.9995, 1919.0038, 1925.9418, 1928.0103, 1975.9609, 1992.9642, 1995.9020, 2007.9894, 2011.8845, 2013.8983, 2024.8591, 2025.6234, 2036.5195, 2036.9486, 2042.0640, 2047.0167, 2047.9691, 2051.9792, 2052.9924, 2058.9963, 2060.0469, 2064.9900, 2068.8982, 2070.8835, 2074.8914, 2083.9907, 2084.8987, 2086.8828, 2092.8821, 2095.9941, 2098.9976, 2100.9160, 2102.8872, 2106.9011, 2108.8835, 2109.9175, 2114.9180, 2116.9160, 2118.8835, 2119.8833, 2125.0176, 2132.8694, 2136.9180, 2170.1467, 2225.1191, 2273.0496, 2277.0137, 2307.9941, 2322.9980, 2341.0366, 2343.0271, 2352.0571, 2355.0459, 2359.0532, 2366.0813, 2368.0544, 2369.0710, 2376.0264, 2382.0349, 2383.6099, 2383.9600, 2390.0403, 2396.0342, 2398.0576, 2400.0652, 2406.0737, 2411.0076, 2414.0813, 2416.0752, 2417.0732, 2420.0718, 2422.0408, 2423.0466, 2428.0898, 2430.0828, 2431.0845, 2433.0779, 2436.0508, 2438.0457, 2439.0261, 2442.0618, 2449.0347, 2452.0632, 2460.0254, 2461.9956, 2463.9858, 2510.1277, 2528.1580, 2542.1509, 2545.1558, 2547.1531, 2552.1038, 2565.1543, 2585.1111, 2601.0889, 2705.2192, 2717.0693, 3129.3176, 3208.1736, 3223.2710, 3290.4131, 3291.4248, 3312.2756, 3451.3933, 3453.4365

10. A7Z060 **Mass:** 81637 **Score:** 22 **Expect:** 1.4e+002 **Matches:** 21
RNF219 protein OS=Bos taurus GN=RNF219 PE=2 SV=1

Observed	Mr(expt)	Mr(calc)	ppm	Start		End	Miss	Ions	Peptide
807.3893	806.3820	806.3923	-12.72	653	-	658	0	---	R.LFEDQR.F
823.3846	822.3774	822.3872	-11.91	226	-	231	0	---	K.VEQYER.E
845.4100	844.4027	844.4151	-14.67	293	-	300	1	12	K.NQGDGARK.Q
845.4123	844.4050	844.4151	-11.96	293	-	300	1	---	K.NQGDGARK.Q
916.4905	915.4832	915.4886	-5.90	693	-	700	1	---	K.NVNQSAKR.K
967.4568	966.4495	966.4658	-16.84	166	-	173	0	---	R.TANEIYEK.V
1158.6154	1157.6081	1157.6265	-15.86	691	-	700	2	---	R.NKNVNQSAKR.K
1315.6570	1314.6497	1314.6819	-24.51	152	-	162	1	---	K.IDPETVAEWKK.R
1323.6517	1322.6444	1322.6215	17.4	226	-	235	1	---	K.VEQYERETNR.L
1382.6647	1381.6574	1381.6772	-14.31	502	-	512	1	---	R.LNSLRSFEMNR.T + Oxidation (M)
1605.8098	1604.8025	1604.8369	-21.44	702	-	717	2	---	K.IQSSLSNASPSKATKS.-
1768.9027	1767.8954	1767.9155	-11.37	217	-	231	1	---	R.FTVAALQSKVEQYER.E
1851.8488	1850.8415	1850.8833	-22.55	544	-	560	1	---	K.SPCNNGFKSLDLDGLSK.S
1880.9277	1879.9204	1879.8846	19.0	327	-	341	2	---	K.TEPNCSKNQDLYQKR.V
2036.9486	2035.9413	2035.9779	-17.95	265	-	283	2	---	K.EAMNAICQRALSTDGKGSK.G
2052.9924	2051.9851	2051.9728	6.01	265	-	283	2	---	K.EAMNAICQRALSTDGKGSK.G + Oxidation (M)
2086.8828	2085.8755	2085.9160	-19.43	561	-	577	1	---	K.SSQCSEFLEETDKLEER.T
2136.9180	2135.9107	2135.9603	-23.19	342	-	359	0	---	R.VETMLTVTDTSMDTYLER.E + 2 Oxidation (M)
2355.0459	2354.0386	2354.0808	-17.93	321	-	340	2	---	R.QEGTSKTEPNCSKNQDLYQK.R
2510.1277	2509.1204	2509.1213	-0.35	507	-	528	2	---	R.SFEMNRTRTSSEASMDAAYLDK.I
2542.1509	2541.1436	2541.1111	12.8	507	-	528	2	---	R.SFEMNRTRTSSEASMDAAYLDK.I + 2 Oxidation (M)

No match to: 713.4334, 719.2388, 724.3009, 726.2899, 732.2586, 735.3865, 745.4124, 756.4687, 764.4056, 765.3908, 767.3679, 768.3909, 772.3955, 795.4186, 802.4548, 803.4370, 805.4229, 806.4631, 809.4480, 811.4104, 812.3985, 816.4408, 820.4244, 822.3905, 824.3756, 828.3904, 834.4043, 836.4589, 837.3853, 839.3696, 840.3839, 842.5085, 851.3975, 852.4056, 856.4893, 866.3914, 867.3724, 870.5402, 871.4945, 878.4625, 880.4403, 886.3815, 888.4233, 890.4479, 893.9562, 894.4579, 897.0289, 897.4412, 902.4597, 910.4410, 912.4497, 913.5118, 918.5391, 925.4747, 928.5064, 930.5048, 933.4619, 935.4698, 939.4758, 947.4628, 949.4591, 951.4703, 954.4674, 957.4657, 963.4676, 965.4761, 969.4571, 972.4793, 972.4800, 975.4650, 977.4630, 978.4976, 981.4565, 983.4664, 985.4842, 991.4688, 993.5331, 997.4298, 999.4453, 1001.4488, 1003.4687, 1007.5668, 1018.5200, 1018.5212, 1023.4777, 1026.4922, 1027.4972, 1030.5151, 1031.3850, 1033.5419, 1035.5352, 1036.5372, 1039.5402, 1042.5383, 1045.5654, 1056.5488, 1060.5400, 1060.5442, 1065.5425, 1086.5115, 1106.5372, 1111.5582, 1113.5546, 1114.5396, 1115.5605, 1118.5529, 1122.5363, 1128.5775, 1129.6014, 1130.5935, 1140.5485, 1144.5709, 1155.6113, 1156.5720, 1157.5952, 1159.6273, 1165.5817, 1172.6301, 1173.6259, 1179.6063, 1180.6165, 1182.6090, 1183.6123, 1185.6317, 1187.6431, 1193.6096, 1195.6060, 1196.6102, 1206.6770, 1209.1387, 1209.5848, 1210.1014, 1215.6627, 1216.6494, 1222.5994, 1226.5948, 1229.6246, 1231.6174, 1234.6317, 1235.5846, 1240.6298, 1244.6324, 1248.6119, 1250.6144, 1268.6498, 1271.6696, 1273.6653, 1275.6644, 1291.6425, 1295.7233, 1300.6062, 1301.6205, 1307.6858, 1317.6521, 1319.6852, 1325.6637, 1328.6809, 1329.6672, 1331.6763, 1332.6743, 1334.6592, 1335.6937, 1336.6715, 1337.6724, 1338.6471, 1342.6680, 1345.6760, 1346.6880, 1349.6991, 1354.6907, 1355.7155, 1357.7034, 1360.6882, 1361.6670, 1365.6913, 1368.6920, 1374.6412, 1375.6798, 1376.6804, 1377.6794, 1387.6444, 1410.7505, 1411.7208, 1421.7177, 1427.7872, 1431.7961, 1432.7133, 1434.7288, 1446.7256, 1450.7079, 1473.8054, 1475.7748, 1477.7524, 1478.7439, 1485.7720, 1486.7664, 1493.7539, 1494.7423, 1505.8649, 1523.7841, 1524.7848, 1537.8021, 1549.7388, 1555.8816, 1558.8474, 1576.7987, 1579.7926, 1584.7897, 1588.8136, 1590.3611, 1604.8417, 1606.8384, 1615.8826, 1616.8605, 1622.8137, 1628.8260, 1629.8358, 1630.8466, 1633.9790, 1635.7943, 1644.8046, 1647.8785, 1649.8463, 1651.8245, 1659.8241, 1662.7909, 1668.7810, 1679.9736, 1681.8214, 1683.8121, 1684.8204, 1706.8616, 1707.7838, 1716.8571, 1725.8353, 1728.8242, 1734.8152, 1740.8768, 1742.9031, 1746.1659, 1750.8356, 1751.8787, 1753.8948, 1756.8905, 1757.8322, 1763.8188, 1765.7690, 1767.8036, 1769.4475, 1770.4261, 1774.9091, 1783.8499, 1786.9000, 1786.9017, 1791.7335, 1808.8868,

1824.8674, 1840.8225, 1844.8907, 1848.8322, 1850.8301, 1867.9503, 1887.9734, 1908.8838, 1914.9995, 1919.0038, 1925.9418, 1928.0103, 1931.9463, 1953.9353, 1975.9609, 1992.9642, 1995.9020, 2007.9894, 2011.8845, 2013.8983, 2024.8591, 2025.6234, 2036.5195, 2042.0640, 2047.0167, 2047.9691, 2051.9792, 2058.9963, 2060.0469, 2064.9900, 2068.8982, 2070.8835, 2074.8914, 2083.9907, 2084.8987, 2092.8821, 2095.9941, 2098.9976, 2100.9160, 2102.8872, 2106.9011, 2108.8835, 2109.9175, 2114.9180, 2116.9160, 2118.8835, 2119.8833, 2125.0176, 2132.8694, 2170.1467, 2211.1045, 2225.1191, 2273.0496, 2277.0137, 2307.9941, 2322.9980, 2341.0366, 2343.0271, 2352.0571, 2359.0532, 2366.0813, 2368.0544, 2369.0710, 2376.0264, 2382.0349, 2383.6099, 2383.9600, 2390.0403, 2396.0342, 2398.0576, 2400.0652, 2406.0737, 2411.0076, 2414.0813, 2416.0752, 2417.0732, 2420.0718, 2422.0408, 2423.0466, 2428.0898, 2430.0828, 2431.0845, 2433.0779, 2436.0508, 2438.0457, 2439.0261, 2442.0618, 2449.0347, 2452.0632, 2460.0254, 2461.9956, 2463.9858, 2528.1580, 2545.1558, 2547.1531, 2552.1038, 2565.1543, 2585.1111, 2601.0889, 2705.2192, 2717.0693, 3129.3176, 3208.1736, 3223.2710, 3290.4131, 3291.4248, 3312.2756, 3451.3933, 3453.4365

11. G5E523 **Mass:** 28080 **Score:** 20 **Expect:** 2.5e+002 **Matches:** 13

Uncharacterized protein OS=Bos taurus GN=STAU2 PE=4 SV=1

Observed	Mr(expt)	Mr(calc)	ppm	Start		End	Miss	Ions	Peptide
880.4403	879.4330	879.4524	-22.05	203	-	209	0	---	R.EFVMQVK.V
1036.5372	1035.5299	1035.5535	-22.79	202	-	209	1	---	R.REFVMQVK.V
1056.5488	1055.5415	1055.5335	7.64	1	-	8	1	---	-.RYHCPVPK.I
1113.5546	1112.5473	1112.5509	-3.19	29	-	38	1	---	R.QAARHNAAMK.A + Oxidation (M)
1179.6063	1178.5990	1178.6005	-1.29	84	-	93	0	---	R.NMPVSFEVIK.E + Oxidation (M)
1319.6852	1318.6779	1318.7067	-21.84	83	-	93	1	---	K.RNMPVSFEVIK.E
1335.6937	1334.6864	1334.7016	-11.40	83	-	93	1	---	K.RNMPVSFEVIK.E + Oxidation (M)
1354.6907	1353.6834	1353.7047	-15.75	27	-	38	2	---	K.TRQAARHNAAMK.A
1421.7177	1420.7104	1420.7384	-19.68	228	-	240	0	---	K.NAAEAMLLQLGYK.A
1477.7524	1476.7451	1476.7460	-0.58	185	-	196	1	---	K.EKEPDYVLLSER.G
1605.8098	1604.8025	1604.8409	-23.94	184	-	196	2	---	K.KEKEPDYVLLSER.G
1919.0038	1917.9965	1917.9618	18.1	185	-	200	2	---	K.EKEPDYVLLSERGMPR.R
2058.9963	2057.9890	2057.9914	-1.17	84	-	101	1	---	R.NMPVSFEVIKESGPPHMK.S + 2 Oxidation (M)

No match to: 713.4334, 719.2388, 724.3009, 726.2899, 732.2586, 735.3865, 745.4124, 756.4687, 764.4056, 765.3908, 767.3679, 768.3909, 772.3955, 795.4186, 802.4548, 803.4370, 805.4229, 806.4631, 807.3893, 809.4480, 811.4104, 812.3985, 816.4408, 820.4244, 822.3905, 823.3846, 824.3756, 828.3904, 834.4043, 836.4589, 837.3853, 839.3696, 840.3839, 842.5085, 845.4100, 845.4123, 851.3975, 852.4056, 856.4893, 866.3914, 867.3724, 870.5402, 871.4945, 878.4625, 886.3815, 888.4233, 890.4479, 893.9562, 894.4579, 897.0289, 897.4412, 902.4597, 910.4410, 912.4497, 913.5118, 916.4905, 918.5391, 925.4747, 928.5064, 930.5048, 933.4619, 935.4698, 939.4758, 947.4628, 949.4591, 951.4703, 954.4674, 957.4657, 963.4676, 965.4761, 967.4568, 969.4571, 972.4793, 972.4800, 975.4650, 977.4630, 978.4976, 981.4565, 983.4664, 985.4842, 991.4688, 993.5331, 997.4298, 999.4453, 1001.4488, 1003.4687, 1007.5668, 1018.5200, 1018.5212, 1023.4777, 1026.4922, 1027.4972, 1030.5151, 1031.3850, 1033.5419, 1035.5352, 1039.5402, 1042.5383, 1045.5654, 1060.5400, 1060.5442, 1065.5425, 1086.5115, 1106.5372, 1111.5582, 1114.5396, 1115.5605, 1118.5529, 1122.5363, 1128.5775, 1129.6014, 1130.5935, 1140.5485, 1144.5709, 1155.6113, 1156.5720, 1157.5952, 1158.6154, 1159.6273, 1165.5817, 1172.6301, 1173.6259, 1180.6165, 1182.6090, 1183.6123, 1185.6317, 1187.6431, 1193.6096, 1195.6060, 1196.6102, 1206.6770, 1209.1387, 1209.5848, 1210.1014, 1215.6627, 1216.6494, 1222.5994, 1226.5948, 1229.6246, 1231.6174, 1234.6317, 1235.5846, 1240.6298, 1244.6324, 1248.6119, 1250.6144, 1268.6498, 1271.6696, 1273.6653, 1275.6644, 1291.6425, 1295.7233, 1300.6062, 1301.6205, 1307.6858, 1315.6570, 1317.6521, 1323.6517, 1325.6637, 1328.6809, 1329.6672, 1331.6763, 1332.6743, 1334.6592, 1336.6715, 1337.6724, 1338.6471, 1342.6680, 1345.6760, 1346.6880, 1349.6991, 1355.7155, 1357.7034, 1360.6882, 1361.6670, 1365.6913, 1368.6920, 1374.6412, 1375.6798, 1376.6804, 1377.6794, 1382.6647, 1387.6444, 1410.7505, 1411.7208, 1427.7872, 1431.7961, 1432.7133, 1434.7288, 1446.7256, 1450.7079, 1473.8054, 1475.7748, 1478.7439, 1485.7720, 1486.7664, 1493.7539, 1494.7423, 1505.8649, 1523.7841, 1524.7848, 1537.8021, 1549.7388, 1555.8816, 1558.8474, 1576.7987, 1579.7926, 1584.7897, 1588.8136, 1590.3611, 1604.8417, 1606.8384, 1615.8826, 1616.8605, 1622.8137, 1628.8260, 1629.8358, 1630.8466, 1633.9790, 1635.7943, 1644.8046, 1647.8785, 1649.8463, 1651.8245, 1659.8241, 1662.7909, 1668.7810, 1679.9736, 1681.8214, 1683.8121, 1684.8204, 1706.8616, 1707.7838, 1716.8571, 1725.8353, 1728.8242, 1734.8152, 1740.8768, 1742.9031, 1746.1659, 1750.8356, 1751.8787, 1753.8948, 1756.8905, 1757.8322, 1763.8188, 1765.7690, 1767.8036, 1768.9027, 1769.4475, 1770.4261, 1774.9091, 1783.8499, 1786.9000, 1786.9017, 1791.7335, 1808.8868, 1824.8674, 1840.8225, 1844.8907, 1848.8322, 1850.8301, 1851.8488, 1867.9503, 1880.9277, 1887.9734, 1908.8838, 1914.9995, 1925.9418, 1928.0103, 1931.9463, 1953.9353, 1975.9609, 1992.9642, 1995.9020, 2007.9894, 2011.8845, 2013.8983, 2024.8591, 2025.6234, 2036.5195, 2036.9486, 2042.0640, 2047.0167, 2047.9691, 2051.9792, 2052.9924, 2060.0469, 2064.9900, 2068.8982, 2070.8835, 2074.8914, 2083.9907, 2084.8987, 2086.8828, 2092.8821, 2095.9941, 2098.9976, 2100.9160, 2102.8872, 2106.9011, 2108.8835, 2109.9175, 2114.9180, 2116.9160, 2118.8835, 2119.8833, 2125.0176, 2132.8694, 2136.9180, 2170.1467, 2211.1045, 2225.1191, 2273.0496, 2277.0137, 2307.9941, 2322.9980, 2341.0366, 2343.0271, 2352.0571, 2355.0459, 2359.0532, 2366.0813, 2368.0544, 2369.0710, 2376.0264, 2382.0349, 2383.6099, 2383.9600, 2390.0403, 2396.0342, 2398.0576, 2400.0652, 2406.0737, 2411.0076, 2414.0813, 2416.0752, 2417.0732, 2420.0718, 2422.0408, 2423.0466, 2428.0898, 2430.0828, 2431.0845, 2433.0779, 2436.0508, 2438.0457, 2439.0261, 2442.0618, 2449.0347, 2452.0632, 2460.0254, 2461.9956, 2463.9858, 2510.1277, 2528.1580, 2542.1509, 2545.1558, 2547.1531, 2552.1038, 2565.1543, 2585.1111, 2601.0889, 2705.2192, 2717.0693, 3129.3176, 3208.1736, 3223.2710, 3290.4131, 3291.4248, 3312.2756, 3451.3933, 3453.4365

12. A6QR15 **Mass:** 32975 **Score:** 20 **Expect:** 2.5e+002 **Matches:** 14

LOC535277 protein OS=Bos taurus GN=LOC535277 PE=1 SV=1

Observed	Mr(expt)	Mr(calc)	ppm	Start		End	Miss	Ions	Peptide
802.4548	801.4475	801.4344	16.3	199	-	205	0	---	K.NVTNNLK.S
834.4043	833.3970	833.4130	-19.20	206	-	213	0	---	K.SLEAASEK.Y
866.3914	865.3841	865.3851	-1.11	134	-	140	1	---	R.AMKDEEK.M + Oxidation (M)
894.4579	893.4507	893.4606	-11.16	162	-	168	1	---	K.YEEVARK.L
1165.5817	1164.5744	1164.5518	19.4	141	-	149	0	---	K.MEIQEMQLK.E + Oxidation (M)
1268.6498	1267.6425	1267.6156	21.2	150	-	160	1	---	K.EAKHIAEEADR.K
1332.6743	1331.6670	1331.6820	-11.24	249	-	259	1	---	K.LEKTIDDLEEK.L
1431.7961	1430.7888	1430.7616	19.0	66	-	77	2	---	K.DAQEKLELTEKK.A
1473.8054	1472.7981	1472.7722	17.6	252	-	264	1	---	K.TIDDLEEKLAQAK.E
1477.7524	1476.7451	1476.7316	9.17	141	-	152	1	---	K.MEIQEMQLKEAK.H
1493.7539	1492.7466	1492.7265	13.5	141	-	152	1	---	K.MEIQEMQLKEAK.H + Oxidation (M)
1928.0103	1927.0030	1927.0374	-17.83	102	-	118	2	---	R.AQERLATALQKLEEAEK.A
1931.9463	1930.9390	1930.9232	8.22	13	-	29	2	---	K.LDKENAIDRAEQAESDK.K
1953.9353	1952.9280	1952.9578	-15.25	50	-	65	2	---	K.LKETEDELDKYSENLK.D

No match to: 713.4334, 719.2388, 724.3009, 726.2899, 732.2586, 735.3865, 745.4124, 756.4687, 764.4056, 765.3908, 767.3679, 768.3909, 772.3955, 795.4186, 803.4370, 805.4229, 806.4631, 807.3893, 809.4480, 811.4104, 812.3985, 816.4408, 820.4244, 822.3905, 823.3846, 824.3756, 828.3904, 836.4589, 837.3853, 839.3696, 840.3839, 842.5085, 845.4100, 845.4123, 851.3975, 852.4056, 856.4893, 867.3724, 870.5402, 871.4945, 878.4625, 880.4403, 886.3815, 888.4233, 890.4479, 893.9562, 897.0289, 897.4412, 902.4597, 910.4410, 912.4497, 913.5118, 916.4905, 918.5391, 925.4747, 928.5064, 930.5048, 933.4619, 935.4698, 939.4758, 947.4628, 949.4591, 951.4703, 954.4674, 957.4657, 963.4676, 965.4761, 967.4568, 969.4571, 972.4793, 972.4800, 975.4650, 977.4630, 978.4976, 981.4565, 983.4664, 985.4842, 991.4688, 993.5331, 997.4298, 999.4453, 1001.4488, 1003.4687, 1007.5668, 1018.5200, 1018.5212, 1023.4777, 1026.4922, 1027.4972, 1030.5151, 1031.3850, 1033.5419, 1035.5352, 1036.5372, 1039.5402, 1042.5383, 1045.5654, 1056.5488, 1060.5400, 1060.5442, 1065.5425, 1086.5115, 1106.5372, 1111.5582, 1113.5546, 1114.5396, 1115.5605, 1118.5529, 1122.5363, 1128.5775, 1129.6014, 1130.5935, 1140.5485, 1144.5709, 1155.6113, 1156.5720, 1157.5952, 1158.6154, 1159.6273, 1172.6301, 1173.6259, 1179.6063, 1180.6165, 1182.6090, 1183.6123, 1185.6317, 1187.6431, 1193.6096, 1195.6060, 1196.6102, 1206.6770, 1209.1387, 1209.5848, 1210.1014, 1215.6627, 1216.6494, 1222.5994, 1226.5948, 1229.6246, 1231.6174, 1234.6317, 1235.5846, 1240.6298, 1244.6324, 1248.6119, 1250.6144, 1271.6696, 1273.6653, 1275.6644, 1291.6425, 1295.7233, 1300.6062, 1301.6205, 1307.6858, 1315.6570, 1317.6521, 1319.6852, 1323.6517, 1325.6637, 1328.6809, 1329.6672, 1331.6763, 1334.6592, 1335.6937, 1336.6715, 1337.6724, 1338.6471, 1342.6680, 1345.6760, 1346.6880, 1349.6991, 1354.6907, 1355.7155, 1357.7034, 1360.6882, 1361.6670, 1365.6913, 1368.6920, 1374.6412, 1375.6798, 1376.6804, 1377.6794, 1382.6647, 1387.6444, 1410.7505, 1411.7208, 1421.7177, 1427.7872, 1432.7133, 1434.7288, 1446.7256, 1450.7079, 1475.7748, 1478.7439, 1485.7720, 1486.7664, 1494.7423, 1505.8649, 1523.7841, 1524.7848, 1537.8021, 1549.7388, 1555.8816, 1558.8474, 1576.7987, 1579.7926, 1584.7897, 1588.8136, 1590.3611, 1604.8417, 1605.8098, 1606.8384, 1615.8826, 1616.8605, 1622.8137, 1628.8260, 1629.8358, 1630.8466, 1633.9790, 1635.7943,

1644.8046, 1647.8785, 1649.8463, 1651.8245, 1659.8241, 1662.7909, 1668.7810, 1679.9736, 1681.8214, 1683.8121, 1684.8204, 1706.8616, 1707.7838, 1716.8571, 1725.8353, 1728.8242, 1734.8152, 1740.8768, 1742.9031, 1746.1659, 1750.8356, 1751.8787, 1753.8948, 1756.8905, 1757.8322, 1763.8188, 1765.7690, 1767.8036, 1768.9027, 1769.4475, 1770.4261, 1774.9091, 1783.8499, 1786.9000, 1786.9017, 1791.7335, 1808.8868, 1824.8674, 1840.8225, 1844.8907, 1848.8322, 1850.8301, 1851.8488, 1867.9503, 1880.9277, 1887.9734, 1908.8838, 1914.9995, 1919.0038, 1925.9418, 1975.9609, 1992.9642, 1995.9020, 2007.9894, 2011.8845, 2013.8983, 2024.8591, 2025.6234, 2036.5195, 2036.9486, 2042.0640, 2047.0167, 2047.9691, 2051.9792, 2052.9924, 2058.9963, 2060.0469, 2064.9900, 2068.8982, 2070.8835, 2074.8914, 2083.9907, 2084.8987, 2086.8828, 2092.8821, 2095.9941, 2098.9976, 2100.9160, 2102.8872, 2106.9011, 2108.8835, 2109.9175, 2114.9180, 2116.9160, 2118.8835, 2119.8833, 2125.0176, 2132.8694, 2136.9180, 2170.1467, 2211.1045, 2225.1191, 2273.0496, 2277.0137, 2307.9941, 2322.9980, 2341.0366, 2343.0271, 2352.0571, 2355.0459, 2359.0532, 2366.0813, 2368.0544, 2369.0710, 2376.0264, 2382.0349, 2383.6099, 2383.9600, 2390.0403, 2396.0342, 2398.0576, 2400.0652, 2406.0737, 2411.0076, 2414.0813, 2416.0752, 2417.0732, 2420.0718, 2422.0408, 2423.0466, 2428.0898, 2430.0828, 2431.0845, 2433.0779, 2436.0508, 2438.0457, 2439.0261, 2442.0618, 2449.0347, 2452.0632, 2460.0254, 2461.9956, 2463.9858, 2510.1277, 2528.1580, 2542.1509, 2545.1558, 2547.1531, 2552.1038, 2565.1543, 2585.1111, 2601.0889, 2705.2192, 2717.0693, 3129.3176, 3208.1736, 3223.2710, 3290.4131, 3291.4248, 3312.2756, 3451.3933, 3453.4365

13. G3N219 **Mass:** 3783 **Score:** 20 **Expect:** 2.7e+002 **Matches:** 4

Uncharacterized protein OS=Bos taurus PE=4 SV=1

Observed	Mr(expt)	Mr(calc)	ppm	Start	End	Miss	Ions	Peptide
935.4698	934.4625	934.4654	-3.11	24 -	31	1	---	K.LRNACSSK.E
1410.7505	1409.7432	1409.7370	4.38	14 -	25	1	---	K.SLQMVTATMKLR.N + 2 Oxidation (M)
1421.7177	1420.7104	1420.6802	21.2	24 -	35	2	---	K.LRNACSSKEMPL.- + Oxidation (M)
2042.0640	2041.0567	2041.0118	22.0	14 -	31	2	---	K.SLQMVTATMKLRNACSSK.E + Oxidation (M)

No match to: 713.4334, 719.2388, 724.3009, 726.2899, 732.2586, 735.3865, 745.4124, 756.4687, 764.4056, 765.3908, 767.3679, 768.3909, 772.3955, 795.4186, 802.4548, 803.4370, 805.4229, 806.4631, 807.3893, 809.4480, 811.4104, 812.3985, 816.4408, 820.4244, 822.3905, 823.3846, 824.3756, 828.3904, 834.4043, 836.4589, 837.3853, 839.3696, 840.3839, 842.5085, 845.4100, 845.4123, 851.3975, 852.4056, 856.4893, 866.3914, 867.3724, 870.5402, 871.4945, 878.4625, 880.4403, 886.3815, 888.4233, 890.4479, 893.9562, 894.4579, 897.0289, 897.4412, 902.4597, 910.4410, 912.4497, 913.5118, 916.4905, 918.5391, 925.4747, 928.5064, 930.5048, 933.4619, 939.4758, 947.4628, 949.4591, 951.4703, 954.4674, 957.4657, 963.4676, 965.4761, 967.4568, 969.4571, 972.4793, 972.4800, 975.4650, 977.4630, 978.4976, 981.4565, 983.4664, 985.4842, 991.4688, 993.5331, 997.4298, 999.4453, 1001.4488, 1003.4687, 1007.5668, 1018.5200, 1018.5212, 1023.4777, 1026.4922, 1027.4972, 1030.5151, 1031.3850, 1033.5419, 1035.5352, 1036.5372, 1039.5402, 1042.5383, 1045.5654, 1056.5488, 1060.5400, 1060.5442, 1065.5425, 1086.5115, 1106.5372, 1111.5582, 1113.5546, 1114.5396, 1115.5605, 1118.5529, 1122.5363, 1128.5775, 1129.6014, 1130.5935, 1140.5485, 1144.5709, 1155.6113, 1156.5720, 1157.5952, 1158.6154, 1159.6273, 1165.5817, 1172.6301, 1173.6259, 1179.6063, 1180.6165, 1182.6090, 1183.6123, 1185.6317, 1187.6431, 1193.6096, 1195.6060, 1196.6102, 1206.6770, 1209.1387, 1209.5848, 1210.1014, 1215.6627, 1216.6494, 1222.5994, 1226.5948, 1229.6246, 1231.6174, 1234.6317, 1235.5846, 1240.6298, 1244.6324, 1248.6119, 1250.6144, 1268.6498, 1271.6696, 1273.6653, 1275.6644, 1291.6425, 1295.7233, 1300.6062, 1301.6205, 1307.6858, 1315.6570, 1317.6521, 1319.6852, 1323.6517, 1325.6637, 1328.6809, 1329.6672, 1331.6763, 1332.6743, 1334.6592, 1335.6937, 1336.6715, 1337.6724, 1338.6471, 1342.6680, 1345.6760, 1346.6880, 1349.6991, 1354.6907, 1355.7155, 1357.7034, 1360.6882, 1361.6670, 1365.6913, 1368.6920, 1374.6412, 1375.6798, 1376.6804, 1377.6794, 1382.6647, 1387.6444, 1411.7208, 1427.7872, 1431.7961, 1432.7133, 1434.7288, 1446.7256, 1450.7079, 1473.8054, 1475.7748, 1477.7524, 1478.7439, 1485.7720, 1486.7664, 1493.7539, 1494.7423, 1505.8649, 1523.7841, 1524.7848, 1537.8021, 1549.7388, 1555.8816, 1558.8474, 1576.7987, 1579.7926, 1584.7897, 1588.8136, 1590.3611, 1604.8417, 1605.8098, 1606.8384, 1615.8826, 1616.8605, 1622.8137, 1628.8260, 1629.8358, 1630.8466, 1633.9790, 1635.7943, 1644.8046, 1647.8785, 1649.8463, 1651.8245, 1659.8241, 1662.7909, 1668.7810, 1679.9736, 1681.8214, 1683.8121, 1684.8204, 1706.8616, 1707.7838, 1716.8571, 1725.8353, 1728.8242, 1734.8152, 1740.8768, 1742.9031, 1746.1659, 1750.8356, 1751.8787, 1753.8948, 1756.8905, 1757.8322, 1763.8188, 1765.7690, 1767.8036, 1768.9027, 1769.4475, 1770.4261, 1774.9091, 1783.8499, 1786.9000, 1786.9017, 1791.7335, 1808.8868, 1824.8674, 1840.8225, 1844.8907, 1848.8322, 1850.8301, 1851.8488, 1867.9503, 1880.9277, 1887.9734, 1908.8838, 1914.9995, 1919.0038, 1925.9418, 1928.0103, 1931.9463, 1953.9353, 1975.9609, 1992.9642, 1995.9020, 2007.9894, 2011.8845, 2013.8983, 2024.8591, 2025.6234, 2036.5195, 2036.9486, 2047.0167, 2047.9691, 2051.9792, 2052.9924, 2058.9963, 2060.0469, 2064.9900, 2068.8982, 2070.8835, 2074.8914, 2083.9907, 2084.8987, 2086.8828, 2092.8821, 2095.9941, 2098.9976, 2100.9160, 2102.8872, 2106.9011, 2108.8835, 2109.9175, 2114.9180, 2116.9160, 2118.8835, 2119.8833, 2125.0176, 2132.8694, 2136.9180, 2170.1467, 2211.1045, 2225.1191, 2273.0496, 2277.0137, 2307.9941, 2322.9980, 2341.0366, 2343.0271, 2352.0571, 2355.0459, 2359.0532, 2366.0813, 2368.0544, 2369.0710, 2376.0264, 2382.0349, 2383.6099, 2383.9600, 2390.0403, 2396.0342, 2398.0576, 2400.0652, 2406.0737, 2411.0076, 2414.0813, 2416.0752, 2417.0732, 2420.0718, 2422.0408, 2423.0466, 2428.0898, 2430.0828, 2431.0845, 2433.0779, 2436.0508, 2438.0457, 2439.0261, 2442.0618, 2449.0347, 2452.0632, 2460.0254, 2461.9956, 2463.9858, 2510.1277, 2528.1580, 2542.1509, 2545.1558, 2547.1531, 2552.1038, 2565.1543, 2585.1111, 2601.0889, 2705.2192, 2717.0693, 3129.3176, 3208.1736, 3223.2710, 3290.4131, 3291.4248, 3312.2756, 3451.3933, 3453.4365

14. G3X6N6 **Mass:** 52918 **Score:** 19 **Expect:** 2.8e+002 **Matches:** 18

Uncharacterized protein OS=Bos taurus PE=4 SV=1

Observed	Mr(expt)	Mr(calc)	ppm	Start	End	Miss	Ions	Peptide
1036.5372	1035.5299	1035.5349	-4.80	366 -	374	0	---	K.VFNQNSSLK.G
1060.5400	1059.5327	1059.5349	-2.05	216 -	224	0	---	R.THTGEKPYK.C
1060.5442	1059.5369	1059.5349	1.91	216 -	224	0	---	R.THTGEKPYK.C
1122.5363	1121.5290	1121.5353	-5.58	323 -	331	1	---	K.SHTEEKTYK.C
1156.5720	1155.5647	1155.5706	-5.11	231 -	240	1	---	K.AFSRCTSLSK.H
1216.6494	1215.6421	1215.6360	5.04	215 -	224	1	---	K.RTHTGEKPYK.C
1387.6444	1386.6371	1386.6536	-11.91	85 -	95	1	---	R.MAFNQAKMFQR.F + Oxidation (M)
1411.7208	1410.7135	1410.7078	4.06	348 -	359	0	---	R.HSMIHTGEKPFK.C
1421.7177	1420.7104	1420.7245	-9.91	231 -	242	2	---	K.AFSRCTSLSKHK.R
1494.7423	1493.7350	1493.7370	-1.34	131 -	142	0	---	R.QQEMILMPSVYR.K
1622.8137	1621.8064	1621.8320	-15.76	131 -	143	1	---	R.QQEMILMPSVYRK.H
1644.8046	1643.7973	1643.7903	4.26	319 -	331	2	---	K.QHEKSHTEEKTYK.C
1651.8245	1650.8172	1650.8114	3.53	58 -	74	0	---	K.GSQPWAIASAAPGHSSK.D
1683.8121	1682.8048	1682.8385	-19.99	144 -	158	0	---	K.HISPAMAMGHSLIYR.V
1931.9463	1930.9390	1930.9506	-5.98	215 -	230	2	---	K.RTHTGEKPYKCPLCGK.A
2307.9941	2306.9868	2306.9692	7.66	375 -	393	1	---	K.GHERTHTGEKPCECHQCGK.A
2398.0576	2397.0503	2396.9974	22.1	263 -	281	2	---	K.EHEKAHRGENGYECHQCEK.V
2428.0898	2427.0825	2427.0631	8.02	379 -	398	1	---	R.THTGEKPCECHQCGKAFPQR.R

No match to: 713.4334, 719.2388, 724.3009, 726.2899, 732.2586, 735.3865, 745.4124, 756.4687, 764.4056, 765.3908, 767.3679, 768.3909, 772.3955, 795.4186, 802.4548, 803.4370, 805.4229, 806.4631, 807.3893, 809.4480, 811.4104, 812.3985, 816.4408, 820.4244, 822.3905, 823.3846, 824.3756, 828.3904, 834.4043, 836.4589, 837.3853, 839.3696, 840.3839, 842.5085, 845.4100, 845.4123, 851.3975, 852.4056, 856.4893, 866.3914, 867.3724, 870.5402, 871.4945, 878.4625, 880.4403, 886.3815, 888.4233, 890.4479, 893.9562, 894.4579, 897.0289, 897.4412, 902.4597, 910.4410, 912.4497, 913.5118, 916.4905, 918.5391, 925.4747, 928.5064, 930.5048, 933.4619, 935.4698, 939.4758, 947.4628, 949.4591, 951.4703, 954.4674, 957.4657, 963.4676, 965.4761, 967.4568, 969.4571, 972.4793, 972.4800, 975.4650, 977.4630, 978.4976, 981.4565, 983.4664, 985.4842, 991.4688, 993.5331, 997.4298, 999.4453, 1001.4488, 1003.4687, 1007.5668, 1018.5200, 1018.5212, 1023.4777, 1026.4922, 1027.4972, 1030.5151, 1031.3850, 1033.5419, 1035.5352, 1039.5402, 1042.5383, 1045.5654, 1056.5488, 1065.5425, 1086.5115, 1106.5372, 1111.5582, 1113.5546, 1114.5396, 1115.5605, 1118.5529, 1128.5775, 1129.6014, 1130.5935, 1140.5485, 1144.5709, 1155.6113, 1157.5952, 1158.6154, 1159.6273, 1165.5817, 1172.6301, 1173.6259, 1179.6063, 1180.6165, 1182.6090, 1183.6123, 1185.6317, 1187.6431, 1193.6096, 1195.6060, 1196.6102, 1206.6770, 1209.1387, 1209.5848, 1210.1014, 1215.6627, 1222.5994, 1226.5948, 1229.6246, 1231.6174, 1234.6317, 1235.5846, 1240.6298, 1244.6324, 1248.6119, 1250.6144, 1268.6498, 1271.6696, 1273.6653, 1275.6644, 1291.6425, 1295.7233, 1300.6062, 1301.6205, 1307.6858, 1315.6570, 1317.6521, 1319.6852, 1323.6517, 1325.6637, 1328.6809, 1329.6672, 1331.6763, 1332.6743, 1334.6592, 1335.6937, 1336.6715, 1337.6724, 1338.6471, 1342.6680, 1345.6760, 1346.6880, 1349.6991, 1354.6907, 1355.7155, 1357.7034, 1360.6882, 1361.6670, 1365.6913, 1368.6920, 1374.6412, 1375.6798, 1376.6804, 1377.6794, 1382.6647, 1410.7505, 1427.7872, 1431.7961, 1432.7133, 1434.7288, 1446.7256, 1450.7079, 1473.8054, 1475.7748, 1477.7524, 1478.7439, 1485.7720, 1486.7664, 1493.7539, 1505.8649, 1523.7841, 1524.7848, 1537.8021, 1549.7388, 1555.8816, 1558.8474, 1576.7987, 1579.7926, 1584.7897, 1588.8136, 1590.3611, 1604.8417, 1605.8098, 1606.8384, 1615.8826, 1616.8605, 1628.8260, 1629.8358, 1630.8466, 1633.9790, 1635.7943, 1647.8785, 1649.8463, 1659.8241, 1662.7909, 1668.7810, 1679.9736, 1681.8214, 1684.8204, 1706.8616, 1707.7838, 1716.8571, 1725.8353, 1728.8242, 1734.8152, 1740.8768, 1742.9031, 1746.1659, 1750.8356, 1751.8787, 1753.8948, 1756.8905, 1757.8322, 1763.8188, 1765.7690,

1767.8036, 1768.9027, 1769.4475, 1770.4261, 1774.9091, 1783.8499, 1786.9000, 1786.9017, 1791.7335, 1808.8868, 1824.8674, 1840.8225, 1844.8907, 1848.8322, 1850.8301, 1851.8488, 1867.9503, 1880.9277, 1887.9734, 1908.8838, 1914.9995, 1919.0038, 1925.9418, 1928.0103, 1953.9353, 1975.9609, 1992.9642, 1995.9020, 2007.9894, 2011.8845, 2013.8983, 2024.8591, 2025.6234, 2036.5195, 2036.9486, 2042.0640, 2047.0167, 2047.9691, 2051.9792, 2052.9924, 2058.9963, 2060.0469, 2064.9900, 2068.8982, 2070.8835, 2074.8914, 2083.9907, 2084.8987, 2086.8828, 2092.8821, 2095.9941, 2098.9976, 2100.9160, 2102.8872, 2106.9011, 2108.8835, 2109.9175, 2114.9180, 2116.9160, 2118.8835, 2119.8833, 2125.0176, 2132.8694, 2136.9180, 2170.1467, 2211.1045, 2225.1191, 2273.0496, 2277.0137, 2322.9980, 2341.0366, 2343.0271, 2352.0571, 2355.0459, 2359.0532, 2366.0813, 2368.0544, 2369.0710, 2376.0264, 2382.0349, 2383.6099, 2383.9600, 2390.0403, 2396.0342, 2400.0652, 2406.0737, 2411.0076, 2414.0813, 2416.0752, 2417.0732, 2420.0718, 2422.0408, 2423.0466, 2430.0828, 2431.0845, 2433.0779, 2436.0508, 2438.0457, 2439.0261, 2442.0618, 2449.0347, 2452.0632, 2460.0254, 2461.9956, 2463.9858, 2510.1277, 2528.1580, 2542.1509, 2545.1558, 2547.1531, 2552.1038, 2565.1543, 2585.1111, 2601.0889, 2705.2192, 2717.0693, 3129.3176, 3208.1736, 3223.2710, 3290.4131, 3291.4248, 3312.2756, 3451.3933, 3453.4365

15. Q5E9D0 **Mass:** 38604 **Score:** 19 **Expect:** 3e+002 **Matches:** 13
Eukaryotic translation initiation factor 2 subunit 2 OS=Bos taurus GN=EIF2S2 PE=2 SV=1

Observed	Mr(expt)	Mr(calc)	ppm	Start	End	Miss	Ions	Peptide
764.4056	763.3984	763.3898	11.2	309 -	315	0	---	R.CSVASIK.T
795.4186	794.4114	794.4109	0.60	183 -	188	0	---	R.VFNIMR.E + Oxidation (M)
871.4945	870.4872	870.4923	-5.81	266 -	272	0	---	K.QIENVLR.R
1036.5372	1035.5299	1035.5535	-22.78	183 -	190	1	---	R.VFNIMREK.N
1244.6324	1243.6251	1243.6343	-7.36	191 -	201	2	---	K.NPDMVAGEKRK.F
1273.6653	1272.6580	1272.6674	-7.35	286 -	296	1	---	R.SPDTILQKDTR.L
1558.8474	1557.8401	1557.8739	-21.70	262 -	273	2	---	R.FQQKQIENVLRR.Y
1615.8826	1614.8753	1614.8954	-12.43	260 -	272	2	---	K.GRFQQKQIENVLR.R
1844.8907	1843.8834	1843.8518	17.1	1 -	16	2	---	-.MSGDEMIFDPTMSKKK.K
2352.0571	2351.0498	2351.0176	13.7	133 -	152	1	---	K.FPDEDEVLEKDEALEDEDSK.K
2359.0532	2358.0459	2358.0919	-19.49	297 -	315	1	---	R.LYFLQCETCHSRCSVASIK.T
2510.1277	2509.1204	2509.1166	1.52	21 -	42	0	---	K.KPFMLDEEGDAQTEETQPSETK.E
3291.4248	3290.4175	3290.4752	-17.53	154 -	182	1	---	K.DDGISFSNQTGPAWAGSERDYTYEELLNR.V

No match to: 713.4334, 719.2388, 724.3009, 726.2899, 732.2586, 735.3865, 745.4124, 756.4687, 765.3908, 767.3679, 768.3909, 772.3955, 802.4548, 803.4370, 805.4229, 806.4631, 807.3893, 809.4480, 811.4104, 812.3985, 816.4408, 820.4244, 822.3905, 823.3846, 824.3756, 828.3904, 834.4043, 836.4589, 837.3853, 839.3696, 840.3839, 842.5085, 845.4100, 845.4123, 851.3975, 852.4056, 856.4893, 866.3914, 867.3724, 870.5402, 878.4625, 880.4403, 886.3815, 888.4233, 890.4479, 893.9562, 894.4579, 897.0289, 897.4412, 902.4597, 910.4410, 912.4497, 913.5118, 916.4905, 918.5391, 925.4747, 928.5064, 930.5048, 933.4619, 935.4698, 939.4758, 947.4628, 949.4591, 951.4703, 954.4674, 957.4657, 963.4676, 965.4761, 967.4568, 969.4571, 972.4793, 972.4800, 975.4650, 977.4630, 978.4976, 981.4565, 983.4664, 985.4842, 991.4688, 993.5331, 997.4298, 999.4453, 1001.4488, 1003.4687, 1007.5668, 1018.5200, 1018.5212, 1023.4777, 1026.4922, 1027.4972, 1030.5151, 1031.3850, 1033.5419, 1035.5352, 1039.5402, 1042.5383, 1045.5654, 1056.5488, 1060.5400, 1060.5442, 1065.5425, 1086.5115, 1106.5372, 1111.5582, 1113.5546, 1114.5396, 1115.5605, 1118.5529, 1122.5363, 1128.5775, 1129.6014, 1130.5935, 1140.5485, 1144.5709, 1155.6113, 1156.5720, 1157.5952, 1158.6154, 1159.6273, 1165.5817, 1172.6301, 1173.6259, 1179.6063, 1180.6165, 1182.6090, 1183.6123, 1185.6317, 1187.6431, 1193.6096, 1195.6060, 1196.6102, 1206.6770, 1209.1387, 1209.5848, 1210.1014, 1215.6627, 1216.6494, 1222.5994, 1226.5948, 1229.6246, 1231.6174, 1234.6317, 1235.5846, 1240.6298, 1248.6119, 1250.6144, 1268.6498, 1271.6696, 1275.6644, 1291.6425, 1295.7233, 1300.6062, 1301.6205, 1307.6858, 1315.6570, 1317.6521, 1319.6852, 1323.6517, 1325.6637, 1328.6809, 1329.6672, 1331.6763, 1332.6743, 1334.6592, 1335.6937, 1336.6715, 1337.6724, 1338.6471, 1342.6680, 1345.6760, 1346.6880, 1349.6991, 1354.6907, 1355.7155, 1357.7034, 1360.6882, 1361.6670, 1365.6913, 1368.6920, 1374.6412, 1375.6798, 1376.6804, 1377.6794, 1382.6647, 1387.6444, 1410.7505, 1411.7208, 1421.7177, 1427.7872, 1431.7961, 1432.7133, 1434.7288, 1446.7256, 1450.7079, 1473.8054, 1475.7748, 1477.7524, 1478.7439, 1485.7720, 1486.7664, 1493.7539, 1494.7423, 1505.8649, 1523.7841, 1524.7848, 1537.8021, 1549.7388, 1555.8816, 1576.7987, 1579.7926, 1584.7897, 1588.8136, 1590.3611, 1604.8417, 1605.8098, 1606.8384, 1616.8605, 1622.8137, 1628.8260, 1629.8358, 1630.8466, 1633.9790, 1635.7943, 1644.8046, 1647.8785, 1649.8463, 1651.8245, 1659.8241, 1662.7909, 1668.7810, 1679.9736, 1681.8214, 1683.8121, 1684.8204, 1706.8616, 1707.7838, 1716.8571, 1725.8353, 1728.8242, 1734.8152, 1740.8768, 1742.9031, 1746.1659, 1750.8356, 1751.8787, 1753.8948, 1756.8905, 1757.8322, 1763.8188, 1765.7690, 1767.8036, 1768.9027, 1769.4475, 1770.4261, 1774.9091, 1783.8499, 1786.9000, 1786.9017, 1791.7335, 1808.8868, 1824.8674, 1840.8225, 1848.8322, 1850.8301, 1851.8488, 1867.9503, 1880.9277, 1887.9734, 1908.8838, 1914.9995, 1919.0038, 1925.9418, 1928.0103, 1931.9463, 1953.9353, 1975.9609, 1992.9642, 1995.9020, 2007.9894, 2011.8845, 2013.8983, 2024.8591, 2025.6234, 2036.5195, 2036.9486, 2042.0640, 2047.0167, 2047.9691, 2051.9792, 2052.9924, 2058.9963, 2060.0469, 2064.9900, 2068.8982, 2070.8835, 2074.8914, 2083.9907, 2084.8987, 2086.8828, 2092.8821, 2095.9941, 2098.9976, 2100.9160, 2102.8872, 2106.9011, 2108.8835, 2109.9175, 2114.9180, 2116.9160, 2118.8835, 2119.8833, 2125.0176, 2132.8694, 2136.9180, 2170.1467, 2211.1045, 2225.1191, 2273.0496, 2277.0137, 2307.9941, 2322.9980, 2341.0366, 2343.0271, 2355.0459, 2366.0813, 2368.0544, 2369.0710, 2376.0264, 2382.0349, 2383.6099, 2383.9600, 2390.0403, 2396.0342, 2398.0576, 2400.0652, 2406.0737, 2411.0076, 2414.0813, 2416.0752, 2417.0732, 2420.0718, 2422.0408, 2423.0466, 2428.0898, 2430.0828, 2431.0845, 2433.0779, 2436.0508, 2438.0457, 2439.0261, 2442.0618, 2449.0347, 2452.0632, 2460.0254, 2461.9956, 2463.9858, 2528.1580, 2542.1509, 2545.1558, 2547.1531, 2552.1038, 2565.1543, 2585.1111, 2601.0889, 2705.2192, 2717.0693, 3129.3176, 3208.1736, 3223.2710, 3290.4131, 3312.2756, 3451.3933, 3453.4365

16. A0A140T862 **Mass:** 38576 **Score:** 19 **Expect:** 3e+002 **Matches:** 13
Eukaryotic translation initiation factor 2 subunit 2 OS=Bos taurus GN=EIF2S2 PE=1 SV=1

Observed	Mr(expt)	Mr(calc)	ppm	Start	End	Miss	Ions	Peptide
764.4056	763.3984	763.3898	11.2	309 -	315	0	---	R.CSVASIK.T
795.4186	794.4114	794.4109	0.60	183 -	188	0	---	R.VFNIMR.E + Oxidation (M)
871.4945	870.4872	870.4923	-5.81	266 -	272	0	---	K.QIENVLR.R
1036.5372	1035.5299	1035.5535	-22.78	183 -	190	1	---	R.VFNIMREK.N
1244.6324	1243.6251	1243.6343	-7.36	191 -	201	2	---	K.NPDMVAGEKRK.F
1273.6653	1272.6580	1272.6674	-7.35	286 -	296	1	---	R.SPDTILQKDTR.L
1558.8474	1557.8401	1557.8739	-21.70	262 -	273	2	---	R.FQQKQIENVLRR.Y
1615.8826	1614.8753	1614.8954	-12.43	260 -	272	2	---	K.GRFQQKQIENVLR.R
1844.8907	1843.8834	1843.8518	17.1	1 -	16	2	---	-.MSGDEMIFDPTMSKKK.K
2352.0571	2351.0498	2351.0176	13.7	133 -	152	1	---	K.FPDEDEVLEKDEALEDEDSK.K
2359.0532	2358.0459	2358.0919	-19.49	297 -	315	1	---	R.LYFLQCETCHSRCSVASIK.T
2510.1277	2509.1204	2509.1166	1.52	21 -	42	0	---	K.KPFMLDEEGDAQTEETQPSETK.E
3291.4248	3290.4175	3290.4752	-17.53	154 -	182	1	---	K.DDGISFSNQTGPAWAGSERDYTYEELLNR.V

No match to: 713.4334, 719.2388, 724.3009, 726.2899, 732.2586, 735.3865, 745.4124, 756.4687, 765.3908, 767.3679, 768.3909, 772.3955, 802.4548, 803.4370, 805.4229, 806.4631, 807.3893, 809.4480, 811.4104, 812.3985, 816.4408, 820.4244, 822.3905, 823.3846, 824.3756, 828.3904, 834.4043, 836.4589, 837.3853, 839.3696, 840.3839, 842.5085, 845.4100, 845.4123, 851.3975, 852.4056, 856.4893, 866.3914, 867.3724, 870.5402, 878.4625, 880.4403, 886.3815, 888.4233, 890.4479, 893.9562, 894.4579, 897.0289, 897.4412, 902.4597, 910.4410, 912.4497, 913.5118, 916.4905, 918.5391, 925.4747, 928.5064, 930.5048, 933.4619, 935.4698, 939.4758, 947.4628, 949.4591, 951.4703, 954.4674, 957.4657, 963.4676, 965.4761, 967.4568, 969.4571, 972.4793, 972.4800, 975.4650, 977.4630, 978.4976, 981.4565, 983.4664, 985.4842, 991.4688, 993.5331, 997.4298, 999.4453, 1001.4488, 1003.4687, 1007.5668, 1018.5200, 1018.5212, 1023.4777, 1026.4922, 1027.4972, 1030.5151, 1031.3850, 1033.5419, 1035.5352, 1039.5402, 1042.5383, 1045.5654, 1056.5488, 1060.5400, 1060.5442, 1065.5425, 1086.5115, 1106.5372, 1111.5582, 1113.5546, 1114.5396, 1115.5605, 1118.5529, 1122.5363, 1128.5775, 1129.6014, 1130.5935, 1140.5485, 1144.5709, 1155.6113, 1156.5720, 1157.5952, 1158.6154, 1159.6273, 1165.5817, 1172.6301, 1173.6259, 1179.6063, 1180.6165, 1182.6090, 1183.6123, 1185.6317, 1187.6431, 1193.6096, 1195.6060, 1196.6102, 1206.6770, 1209.1387, 1209.5848, 1210.1014, 1215.6627, 1216.6494, 1222.5994, 1226.5948, 1229.6246, 1231.6174, 1234.6317, 1235.5846, 1240.6298, 1248.6119, 1250.6144, 1268.6498, 1271.6696, 1275.6644, 1291.6425, 1295.7233, 1300.6062, 1301.6205, 1307.6858, 1315.6570, 1317.6521, 1319.6852, 1323.6517, 1325.6637, 1328.6809, 1329.6672, 1331.6763, 1332.6743, 1334.6592, 1335.6937, 1336.6715, 1337.6724, 1338.6471, 1342.6680, 1345.6760, 1346.6880, 1349.6991, 1354.6907, 1355.7155, 1357.7034, 1360.6882, 1361.6670, 1365.6913, 1368.6920, 1374.6412, 1375.6798, 1376.6804, 1377.6794, 1382.6647, 1387.6444, 1410.7505, 1411.7208, 1421.7177, 1427.7872, 1431.7961, 1432.7133, 1434.7288, 1446.7256, 1450.7079, 1473.8054, 1475.7748, 1477.7524, 1478.7439, 1485.7720, 1486.7664, 1493.7539, 1494.7423, 1505.8649, 1523.7841, 1524.7848, 1537.8021, 1549.7388, 1555.8816, 1576.7987, 1579.7926, 1584.7897, 1588.8136, 1590.3611, 1604.8417, 1605.8098, 1606.8384, 1616.8605, 1622.8137, 1628.8260, 1629.8358,

1630.8466, 1633.9790, 1635.7943, 1644.8046, 1647.8785, 1649.8463, 1651.8245, 1659.8241, 1662.7909, 1668.7810, 1679.9736, 1681.8214, 1683.8121, 1684.8204, 1706.8616, 1707.7838, 1716.8571, 1725.8353, 1728.8242, 1734.8152, 1740.8768, 1742.9031, 1746.1659, 1750.8356, 1751.8787, 1753.8948, 1756.8905, 1757.8322, 1763.8188, 1765.7690, 1767.8036, 1768.9027, 1769.4475, 1770.4261, 1774.9091, 1783.8499, 1786.9000, 1786.9017, 1791.7335, 1808.8868, 1824.8674, 1840.8225, 1848.8322, 1850.8301, 1851.8488, 1867.9503, 1880.9277, 1887.9734, 1908.8838, 1914.9995, 1919.0038, 1925.9418, 1928.0103, 1931.9463, 1953.9353, 1975.9609, 1992.9642, 1995.9020, 2007.9894, 2011.8845, 2013.8983, 2024.8591, 2025.6234, 2036.5195, 2036.9486, 2042.0640, 2047.0167, 2047.9691, 2051.9792, 2052.9924, 2058.9963, 2060.0469, 2064.9900, 2068.8982, 2070.8835, 2074.8914, 2083.9907, 2084.8987, 2086.8828, 2092.8821, 2095.9941, 2098.9976, 2100.9160, 2102.8872, 2106.9011, 2108.8835, 2109.9175, 2114.9180, 2116.9160, 2118.8835, 2119.8833, 2125.0176, 2132.8694, 2136.9180, 2170.1467, 2211.1045, 2225.1191, 2273.0496, 2277.0137, 2307.9941, 2322.9980, 2341.0366, 2343.0271, 2355.0459, 2366.0813, 2368.0544, 2369.0710, 2376.0264, 2382.0349, 2383.6099, 2383.9600, 2390.0403, 2396.0342, 2398.0576, 2400.0652, 2406.0737, 2411.0076, 2414.0813, 2416.0752, 2417.0732, 2420.0718, 2422.0408, 2423.0466, 2428.0898, 2430.0828, 2431.0845, 2433.0779, 2436.0508, 2438.0457, 2439.0261, 2442.0618, 2449.0347, 2452.0632, 2460.0254, 2461.9956, 2463.9858, 2528.1580, 2542.1509, 2545.1558, 2547.1531, 2552.1038, 2565.1543, 2585.1111, 2601.0889, 2705.2192, 2717.0693, 3129.3176, 3208.1736, 3223.2710, 3290.4131, 3312.2756, 3451.3933, 3453.4365

17. E1BDF0 **Mass:** 81113 **Score:** 19 **Expect:** 3e+002 **Matches:** 24

Uncharacterized protein OS=Bos taurus GN=NEXN PE=4 SV=2

Observed	Mr(expt)	Mr(calc)	ppm	Start	End	Miss	Ions	Peptide
802.4548	801.4475	801.4344	16.3	531 -	536	1	---	R.RIEEQK.L
803.4370	802.4297	802.4184	14.1	466 -	471	1	---	K.KIEEER.A
816.4408	815.4335	815.4474	-17.02	46 -	51	2	---	R.NQRRSR.D
820.4244	819.4171	819.4351	-21.97	343 -	349	1	---	K.AFAEARR.S
836.4589	835.4516	835.4552	-4.29	58 -	63	1	---	R.KEQYIR.E
839.3696	838.3623	838.3821	-23.58	204 -	209	1	---	K.YEEDKR.I
851.3975	850.3902	850.3855	5.64	1 -	7	0	---	-.MNDISQK.A + Oxidation (M)
878.4625	877.4552	877.4406	16.7	280 -	286	1	---	R.AFEEARR.Q
902.4597	901.4524	901.4617	-10.33	467 -	473	1	---	K.IEEERAR.R
910.4410	909.4338	909.4443	-11.62	202 -	208	1	---	R.VKYEEDK.R
918.5391	917.5318	917.5447	-14.04	304 -	311	1	---	K.GYRPGKLK.L
939.4758	938.4686	938.4531	16.4	104 -	111	1	---	K.GKFAEMEK.Q
949.4591	948.4518	948.4665	-15.50	406 -	412	0	---	K.QEFEQLR.Q
975.4650	974.4578	974.4417	16.5	525 -	531	1	---	R.EEEEQRR.I
993.5331	992.5258	992.5039	22.1	59 -	65	1	---	K.EQYIRER.E
1036.5372	1035.5299	1035.5237	6.06	249 -	256	0	---	K.LTFEELER.Q
1056.5488	1055.5415	1055.5359	5.30	393 -	400	2	---	R.TEEERKHK.L
1172.6301	1171.6228	1171.6448	-18.79	363 -	373	0	---	K.TISQESLIPGK.L
1195.6060	1194.5987	1194.5880	8.95	200 -	208	2	---	K.ERVKYEEDK.R
1307.6858	1306.6785	1306.6517	20.5	187 -	196	2	---	K.KNFEDIEKER.E
1376.6804	1375.6731	1375.6514	15.8	520 -	530	2	---	R.MAKAREEEEQR.R
1579.7926	1578.7853	1578.7711	8.98	401 -	412	1	---	K.LEMEKQEFEQLR.Q
1844.8907	1843.8834	1843.9250	-22.56	399 -	412	2	---	K.HKLEMEKQEFEQLR.Q
2211.1045	2210.0972	2210.0446	23.8	221 -	239	2	---	K.EAKCLSLVMDDELESEAKK.E + Oxidation (M)

No match to: 713.4334, 719.2388, 724.3009, 726.2899, 732.2586, 735.3865, 745.4124, 756.4687, 764.4056, 765.3908, 767.3679, 768.3909, 772.3955, 795.4186, 805.4229, 806.4631, 807.3893, 809.4480, 811.4104, 812.3985, 822.3905, 823.3846, 824.3756, 828.3904, 834.4043, 837.3853, 840.3839, 842.5085, 845.4100, 845.4123, 852.4056, 856.4893, 866.3914, 867.3724, 870.5402, 871.4945, 880.4403, 886.3815, 888.4233, 890.4479, 893.9562, 894.4579, 897.0289, 897.4412, 912.4497, 913.5118, 916.4905, 925.4747, 928.5064, 930.5048, 933.4619, 935.4698, 947.4628, 951.4703, 954.4674, 957.4657, 963.4676, 965.4761, 967.4568, 969.4571, 972.4793, 972.4800, 977.4630, 978.4976, 981.4565, 983.4664, 985.4842, 991.4688, 997.4298, 999.4453, 1001.4488, 1003.4687, 1007.5668, 1018.5200, 1018.5212, 1023.4777, 1026.4922, 1027.4972, 1030.5151, 1031.3850, 1033.5419, 1035.5352, 1039.5402, 1042.5383, 1045.5654, 1060.5400, 1060.5442, 1065.5425, 1086.5115, 1106.5372, 1111.5582, 1113.5546, 1114.5396, 1115.5605, 1118.5529, 1122.5363, 1128.5775, 1129.6014, 1130.5935, 1140.5485, 1144.5709, 1155.6113, 1156.5720, 1157.5952, 1158.6154, 1159.6273, 1165.5817, 1173.6259, 1179.6063, 1180.6165, 1182.6090, 1183.6123, 1185.6317, 1187.6431, 1193.6096, 1196.6102, 1206.6770, 1209.1387, 1209.5848, 1210.1014, 1215.6627, 1216.6494, 1222.5994, 1226.5948, 1229.6246, 1231.6174, 1234.6317, 1235.5846, 1240.6298, 1244.6324, 1248.6119, 1250.6144, 1268.6498, 1271.6696, 1273.6653, 1275.6644, 1291.6425, 1295.7233, 1300.6062, 1301.6205, 1315.6570, 1317.6521, 1319.6852, 1323.6517, 1325.6637, 1328.6809, 1329.6672, 1331.6763, 1332.6743, 1334.6592, 1335.6937, 1336.6715, 1337.6724, 1338.6471, 1342.6680, 1345.6760, 1346.6880, 1349.6991, 1354.6907, 1355.7155, 1357.7034, 1360.6882, 1361.6670, 1365.6913, 1368.6920, 1374.6412, 1375.6798, 1377.6794, 1382.6647, 1387.6444, 1410.7505, 1411.7208, 1421.7177, 1427.7872, 1431.7961, 1432.7133, 1434.7288, 1446.7256, 1450.7079, 1473.8054, 1475.7748, 1477.7524, 1478.7439, 1485.7720, 1486.7664, 1493.7539, 1494.7423, 1505.8649, 1523.7841, 1524.7848, 1537.8021, 1549.7388, 1555.8816, 1558.8474, 1576.7987, 1584.7897, 1588.8136, 1590.3611, 1604.8417, 1605.8098, 1606.8384, 1615.8826, 1616.8605, 1622.8137, 1628.8260, 1629.8358, 1630.8466, 1633.9790, 1635.7943, 1644.8046, 1647.8785, 1649.8463, 1651.8245, 1659.8241, 1662.7909, 1668.7810, 1679.9736, 1681.8214, 1683.8121, 1684.8204, 1706.8616, 1707.7838, 1716.8571, 1725.8353, 1728.8242, 1734.8152, 1740.8768, 1742.9031, 1746.1659, 1750.8356, 1751.8787, 1753.8948, 1756.8905, 1757.8322, 1763.8188, 1765.7690, 1767.8036, 1768.9027, 1769.4475, 1770.4261, 1774.9091, 1783.8499, 1786.9000, 1786.9017, 1791.7335, 1808.8868, 1824.8674, 1840.8225, 1848.8322, 1850.8301, 1851.8488, 1867.9503, 1880.9277, 1887.9734, 1908.8838, 1914.9995, 1919.0038, 1925.9418, 1928.0103, 1931.9463, 1953.9353, 1975.9609, 1992.9642, 1995.9020, 2007.9894, 2011.8845, 2013.8983, 2024.8591, 2025.6234, 2036.5195, 2036.9486, 2042.0640, 2047.0167, 2047.9691, 2051.9792, 2052.9924, 2058.9963, 2060.0469, 2064.9900, 2068.8982, 2070.8835, 2074.8914, 2083.9907, 2084.8987, 2086.8828, 2092.8821, 2095.9941, 2098.9976, 2100.9160, 2102.8872, 2106.9011, 2108.8835, 2109.9175, 2114.9180, 2116.9160, 2118.8835, 2119.8833, 2125.0176, 2132.8694, 2136.9180, 2170.1467, 2225.1191, 2273.0496, 2277.0137, 2307.9941, 2322.9980, 2341.0366, 2343.0271, 2352.0571, 2355.0459, 2359.0532, 2366.0813, 2368.0544, 2369.0710, 2376.0264, 2382.0349, 2383.6099, 2383.9600, 2390.0403, 2396.0342, 2398.0576, 2400.0652, 2406.0737, 2411.0076, 2414.0813, 2416.0752, 2417.0732, 2420.0718, 2422.0408, 2423.0466, 2428.0898, 2430.0828, 2431.0845, 2433.0779, 2436.0508, 2438.0457, 2439.0261, 2442.0618, 2449.0347, 2452.0632, 2460.0254, 2461.9956, 2463.9858, 2510.1277, 2528.1580, 2542.1509, 2545.1558, 2547.1531, 2552.1038, 2565.1543, 2585.1111, 2601.0889, 2705.2192, 2717.0693, 3129.3176, 3208.1736, 3223.2710, 3290.4131, 3291.4248, 3312.2756, 3451.3933, 3453.4365

18. Q2TBK0 **Mass:** 39253 **Score:** 19 **Expect:** 3e+002 **Matches:** 14

Uncharacterized protein C6orf163 homolog OS=Bos taurus PE=2 SV=1

Observed	Mr(expt)	Mr(calc)	ppm	Start	End	Miss	Ions	Peptide
765.3908	764.3835	764.3738	12.7	247 -	253	0	---	K.LGSVMDK.L + Oxidation (M)
852.4056	851.3984	851.4171	-21.99	118 -	124	1	---	K.EMATRTK.M + Oxidation (M)
1030.5151	1029.5078	1029.5090	-1.19	130 -	137	0	---	K.NLEEELQR.E
1206.6770	1205.6697	1205.6438	21.5	115 -	124	2	---	K.ELKEMATRTK.M
1215.6627	1214.6554	1214.6295	21.3	71 -	81	0	---	R.IAQAEADIWAK.A
1332.6743	1331.6670	1331.6503	12.5	174 -	184	0	---	R.LMAQEEIQSQR.R
1616.8605	1615.8532	1615.8464	4.24	174 -	186	2	---	R.LMAQEEIQSQRRK.A
1662.7909	1661.7836	1661.7800	2.20	150 -	161	2	---	R.IQRIMMECHREK.V + 2 Oxidation (M)
1683.8121	1682.8048	1682.8410	-21.47	125 -	137	1	---	K.MQLHKNLEEELQR.E + Oxidation (M)
1753.8948	1752.8875	1752.8716	9.09	88 -	102	2	---	R.QAVKKALEEANDMYK.M + Oxidation (M)
1867.9503	1866.9430	1866.9067	19.5	187 -	203	1	---	K.AMEELMSSGVTVVKDQK.K + Oxidation (M)
1953.9353	1952.9280	1952.9479	-10.20	276 -	291	1	---	K.DFLEEELQETRAAFQK.Y
2047.0167	2046.0094	2045.9912	8.91	170 -	185	2	---	R.EQERLMAQEEIQSQRR.K + Oxidation (M)
2277.0137	2276.0064	2275.9959	4.63	1 -	18	1	---	-.MIRNPNYTNFVCCAVCNK.I + Oxidation (M)

No match to: 713.4334, 719.2388, 724.3009, 726.2899, 732.2586, 735.3865, 745.4124, 756.4687, 764.4056, 767.3679, 768.3909, 772.3955, 795.4186, 802.4548, 803.4370, 805.4229, 806.4631, 807.3893, 809.4480, 811.4104, 812.3985, 816.4408, 820.4244, 822.3905, 823.3846, 824.3756, 828.3904, 834.4043, 836.4589, 837.3853, 839.3696, 840.3839, 842.5085, 845.4100, 845.4123, 851.3975, 856.4893,

866.3914, 867.3724, 870.5402, 871.4945, 878.4625, 880.4403, 886.3815, 888.4233, 890.4479, 893.9562, 894.4579, 897.0289, 897.4412, 902.4597, 910.4410, 912.4497, 913.5118, 916.4905, 918.5391, 925.4747, 928.5064, 930.5048, 933.4619, 935.4698, 939.4758, 947.4628, 949.4591, 951.4703, 954.4674, 957.4657, 963.4676, 965.4761, 967.4568, 969.4571, 972.4793, 972.4800, 975.4650, 977.4630, 978.4976, 981.4565, 983.4664, 985.4842, 991.4688, 993.5331, 997.4298, 999.4453, 1001.4488, 1003.4687, 1007.5668, 1018.5200, 1018.5212, 1023.4777, 1026.4922, 1027.4972, 1031.3850, 1033.5419, 1035.5352, 1036.5372, 1039.5402, 1042.5383, 1045.5654, 1056.5488, 1060.5400, 1060.5442, 1065.5425, 1086.5115, 1106.5372, 1111.5582, 1113.5546, 1114.5396, 1115.5605, 1118.5529, 1122.5363, 1128.5775, 1129.6014, 1130.5935, 1140.5485, 1144.5709, 1155.6113, 1156.5720, 1157.5952, 1158.6154, 1159.6273, 1165.5817, 1172.6301, 1173.6259, 1179.6063, 1180.6165, 1182.6090, 1183.6123, 1185.6317, 1187.6431, 1193.6096, 1195.6060, 1196.6102, 1209.1387, 1209.5848, 1210.1014, 1216.6494, 1222.5994, 1226.5948, 1229.6246, 1231.6174, 1234.6317, 1235.5846, 1240.6298, 1244.6324, 1248.6119, 1250.6144, 1268.6498, 1271.6696, 1273.6653, 1275.6644, 1291.6425, 1295.7233, 1300.6062, 1301.6205, 1307.6858, 1315.6570, 1317.6521, 1319.6852, 1323.6517, 1325.6637, 1328.6809, 1329.6672, 1331.6763, 1334.6592, 1335.6937, 1336.6715, 1337.6724, 1338.6471, 1342.6680, 1345.6760, 1346.6880, 1349.6991, 1354.6907, 1355.7155, 1357.7034, 1360.6882, 1361.6670, 1365.6913, 1368.6920, 1374.6412, 1375.6798, 1376.6804, 1377.6794, 1382.6647, 1387.6444, 1410.7505, 1411.7208, 1421.7177, 1427.7872, 1431.7961, 1432.7133, 1434.7288, 1446.7256, 1450.7079, 1473.8054, 1475.7748, 1477.7524, 1478.7439, 1485.7720, 1486.7664, 1493.7539, 1494.7423, 1505.8649, 1523.7841, 1524.7848, 1537.8021, 1549.7388, 1555.8816, 1558.8474, 1576.7987, 1579.7926, 1584.7897, 1588.8136, 1590.3611, 1604.8417, 1605.8098, 1606.8384, 1615.8826, 1622.8137, 1628.8260, 1629.8358, 1630.8466, 1633.9790, 1635.7943, 1644.8046, 1647.8785, 1649.8463, 1651.8245, 1659.8241, 1668.7810, 1679.9736, 1681.8214, 1684.8204, 1706.8616, 1707.7838, 1716.8571, 1725.8353, 1728.8242, 1734.8152, 1740.8768, 1742.9031, 1746.1659, 1750.8356, 1751.8787, 1756.8905, 1757.8322, 1763.8188, 1765.7690, 1767.8036, 1768.9027, 1769.4475, 1770.4261, 1774.9091, 1783.8499, 1786.9000, 1786.9017, 1791.7335, 1808.8868, 1824.8674, 1840.8225, 1844.8907, 1848.8322, 1850.8301, 1851.8488, 1880.9277, 1887.9734, 1908.8838, 1914.9995, 1919.0038, 1925.9418, 1928.0103, 1931.9463, 1975.9609, 1992.9642, 1995.9020, 2007.9894, 2011.8845, 2013.8983, 2024.8591, 2025.6234, 2036.5195, 2036.9486, 2042.0640, 2047.9691, 2051.9792, 2052.9924, 2058.9963, 2060.0469, 2064.9900, 2068.8982, 2070.8835, 2074.8914, 2083.9907, 2084.8987, 2086.8828, 2092.8821, 2095.9941, 2098.9976, 2100.9160, 2102.8872, 2106.9011, 2108.8835, 2109.9175, 2114.9180, 2116.9160, 2118.8835, 2119.8833, 2125.0176, 2132.8694, 2136.9180, 2170.1467, 2211.1045, 2225.1191, 2273.0496, 2307.9941, 2322.9980, 2341.0366, 2343.0271, 2352.0571, 2355.0459, 2359.0532, 2366.0813, 2368.0544, 2369.0710, 2376.0264, 2382.0349, 2383.6099, 2383.9600, 2390.0403, 2396.0342, 2398.0576, 2400.0652, 2406.0737, 2411.0076, 2414.0813, 2416.0752, 2417.0732, 2420.0718, 2422.0408, 2423.0466, 2428.0898, 2430.0828, 2431.0845, 2433.0779, 2436.0508, 2438.0457, 2439.0261, 2442.0618, 2449.0347, 2452.0632, 2460.0254, 2461.9956, 2463.9858, 2510.1277, 2528.1580, 2542.1509, 2545.1558, 2547.1531, 2552.1038, 2565.1543, 2585.1111, 2601.0889, 2705.2192, 2717.0693, 3129.3176, 3208.1736, 3223.2710, 3290.4131, 3291.4248, 3312.2756, 3451.3933, 3453.4365

19. Q2TBN3 **Mass:** 19797 **Score:** 19 **Expect:** 3.1e+002 **Matches:** 12

Centrin-2 OS=Bos taurus GN=CETN2 PE=2 SV=1

Observed	Mr(expt)	Mr(calc)	ppm	Start	End	Miss	Ions	Peptide
851.3975	850.3902	850.4106	-23.94	72 -	78	0	---	K.MISEIDK.E + Oxidation (M)
1234.6317	1233.6244	1233.6540	-23.94	55 -	65	1	---	K.VAMRALGFEPK.K + Oxidation (M)
1323.6517	1322.6444	1322.6388	4.28	72 -	83	1	---	K.MISEIDKEGTGK.M + Oxidation (M)
1432.7133	1431.7060	1431.6915	10.1	19 -	30	0	---	R.MSPKPELTEEQK.Q + Oxidation (M)
1478.7439	1477.7366	1477.7697	-22.41	67 -	78	2	---	K.EEIKKMISEIDK.E + Oxidation (M)
1588.8136	1587.8063	1587.7926	8.63	18 -	30	1	---	K.RMSPKPELTEEQK.Q + Oxidation (M)
1716.8571	1715.8498	1715.8876	-22.00	17 -	30	2	---	R.KRMSPKPELTEEQK.Q + Oxidation (M)
2007.9894	2006.9821	2006.9731	4.49	152 -	168	2	---	R.DGDGEVNEQEFLRIMKK.T
2036.9486	2035.9413	2035.9417	-0.18	84 -	100	1	---	K.MNFSDFLTVMTQKMSEK.D
2052.9924	2051.9851	2051.9366	23.6	84 -	100	1	---	K.MNFSDFLTVMTQKMSEK.D + Oxidation (M)
2068.8982	2067.8909	2067.9315	-19.64	84 -	100	1	---	K.MNFSDFLTVMTQKMSEK.D + 2 Oxidation (M)
2084.8987	2083.8914	2083.9265	-16.81	84 -	100	1	---	K.MNFSDFLTVMTQKMSEK.D + 3 Oxidation (M)

No match to: 713.4334, 719.2388, 724.3009, 726.2899, 732.2586, 735.3865, 745.4124, 756.4687, 764.4056, 765.3908, 767.3679, 768.3909, 772.3955, 795.4186, 802.4548, 803.4370, 805.4229, 806.4631, 807.3893, 809.4480, 811.4104, 812.3985, 816.4408, 820.4244, 822.3905, 823.3846, 824.3756, 828.3904, 834.4043, 836.4589, 837.3853, 839.3696, 840.3839, 842.5085, 845.4100, 845.4123, 852.4056, 856.4893, 866.3914, 867.3724, 870.5402, 871.4945, 878.4625, 880.4403, 886.3815, 888.4233, 890.4479, 893.9562, 894.4579, 897.0289, 897.4412, 902.4597, 910.4410, 912.4497, 913.5118, 916.4905, 918.5391, 925.4747, 928.5064, 930.5048, 933.4619, 935.4698, 939.4758, 947.4628, 949.4591, 951.4703, 954.4674, 957.4657, 963.4676, 965.4761, 967.4568, 969.4571, 972.4793, 972.4800, 975.4650, 977.4630, 978.4976, 981.4565, 983.4664, 985.4842, 991.4688, 993.5331, 997.4298, 999.4453, 1001.4488, 1003.4687, 1007.5668, 1018.5200, 1018.5212, 1023.4777, 1026.4922, 1027.4972, 1030.5151, 1031.3850, 1033.5419, 1035.5352, 1036.5372, 1039.5402, 1042.5383, 1045.5654, 1056.5488, 1060.5400, 1060.5442, 1065.5425, 1086.5115, 1106.5372, 1111.5582, 1113.5546, 1114.5396, 1115.5605, 1118.5529, 1122.5363, 1128.5775, 1129.6014, 1130.5935, 1140.5485, 1144.5709, 1155.6113, 1156.5720, 1157.5952, 1158.6154, 1159.6273, 1165.5817, 1172.6301, 1173.6259, 1179.6063, 1180.6165, 1182.6090, 1183.6123, 1185.6317, 1187.6431, 1193.6096, 1195.6060, 1196.6102, 1206.6770, 1209.1387, 1209.5848, 1210.1014, 1215.6627, 1216.6494, 1222.5994, 1226.5948, 1229.6246, 1231.6174, 1235.5846, 1240.6298, 1244.6324, 1248.6119, 1250.6144, 1268.6498, 1271.6696, 1273.6653, 1275.6644, 1291.6425, 1295.7233, 1300.6062, 1301.6205, 1307.6858, 1315.6570, 1317.6521, 1319.6852, 1325.6637, 1328.6809, 1329.6672, 1331.6763, 1332.6743, 1334.6592, 1335.6937, 1336.6715, 1337.6724, 1338.6471, 1342.6680, 1345.6760, 1346.6880, 1349.6991, 1354.6907, 1355.7155, 1357.7034, 1360.6882, 1361.6670, 1365.6913, 1368.6920, 1374.6412, 1375.6798, 1376.6804, 1377.6794, 1382.6647, 1387.6444, 1410.7505, 1411.7208, 1421.7177, 1427.7872, 1431.7961, 1434.7288, 1446.7256, 1450.7079, 1473.8054, 1475.7748, 1477.7524, 1485.7720, 1486.7664, 1493.7539, 1494.7423, 1505.8649, 1523.7841, 1524.7848, 1537.8021, 1549.7388, 1555.8816, 1558.8474, 1576.7987, 1579.7926, 1584.7897, 1590.3611, 1604.8417, 1605.8098, 1606.8384, 1615.8826, 1616.8605, 1622.8137, 1628.8260, 1629.8358, 1630.8466, 1633.9790, 1635.7943, 1644.8046, 1647.8785, 1649.8463, 1651.8245, 1659.8241, 1662.7909, 1668.7810, 1679.9736, 1681.8214, 1683.8121, 1684.8204, 1706.8616, 1707.7838, 1725.8353, 1728.8242, 1734.8152, 1740.8768, 1742.9031, 1746.1659, 1750.8356, 1751.8787, 1753.8948, 1756.8905, 1757.8322, 1763.8188, 1765.7690, 1767.8036, 1768.9027, 1769.4475, 1770.4261, 1774.9091, 1783.8499, 1786.9000, 1786.9017, 1791.7335, 1808.8868, 1824.8674, 1840.8225, 1844.8907, 1848.8322, 1850.8301, 1851.8488, 1867.9503, 1880.9277, 1887.9734, 1908.8838, 1914.9995, 1919.0038, 1925.9418, 1928.0103, 1931.9463, 1953.9353, 1975.9609, 1992.9642, 1995.9020, 2011.8845, 2013.8983, 2024.8591, 2025.6234, 2036.5195, 2042.0640, 2047.0167, 2047.9691, 2051.9792, 2058.9963, 2060.0469, 2064.9900, 2070.8835, 2074.8914, 2083.9907, 2086.8828, 2092.8821, 2095.9941, 2098.9976, 2100.9160, 2102.8872, 2106.9011, 2108.8835, 2109.9175, 2114.9180, 2116.9160, 2118.8835, 2119.8833, 2125.0176, 2132.8694, 2136.9180, 2170.1467, 2211.1045, 2225.1191, 2273.0496, 2277.0137, 2307.9941, 2322.9980, 2341.0366, 2343.0271, 2352.0571, 2355.0459, 2359.0532, 2366.0813, 2368.0544, 2369.0710, 2376.0264, 2382.0349, 2383.6099, 2383.9600, 2390.0403, 2396.0342, 2398.0576, 2400.0652, 2406.0737, 2411.0076, 2414.0813, 2416.0752, 2417.0732, 2420.0718, 2422.0408, 2423.0466, 2428.0898, 2430.0828, 2431.0845, 2433.0779, 2436.0508, 2438.0457, 2439.0261, 2442.0618, 2449.0347, 2452.0632, 2460.0254, 2461.9956, 2463.9858, 2510.1277, 2528.1580, 2542.1509, 2545.1558, 2547.1531, 2552.1038, 2565.1543, 2585.1111, 2601.0889, 2705.2192, 2717.0693, 3129.3176, 3208.1736, 3223.2710, 3290.4131, 3291.4248, 3312.2756, 3451.3933, 3453.4365

20. G3MWY8 **Mass:** 9886 **Score:** 19 **Expect:** 3.3e+002 **Matches:** 7

Coiled-coil domain-containing protein 167 OS=Bos taurus GN=CCDC167 PE=4 SV=1

Observed	Mr(expt)	Mr(calc)	ppm	Start	End	Miss	Ions	Peptide
802.4548	801.4475	801.4596	-15.10	2 -	8	1	---	M.IDGLEKK.L
805.4229	804.4156	804.4051	13.0	1 -	7	0	---	-.MIDGLEK.K
1376.6804	1375.6731	1375.6878	-10.65	23 -	34	1	---	R.LCGVELSSEARR.S
1387.6444	1386.6371	1386.6449	-5.59	41 -	52	1	---	K.SSLMNKASNYEK.E + Oxidation (M)
1628.8260	1627.8187	1627.7875	19.2	39 -	52	2	---	K.EKSSLMNKASNYEK.E
1644.8046	1643.7973	1643.7824	9.06	39 -	52	2	---	K.EKSSLMNKASNYEK.E + Oxidation (M)
1757.8322	1756.8249	1756.8665	-23.66	41 -	55	2	---	K.SSLMNKASNYEKELK.L + Oxidation (M)

No match to: 713.4334, 719.2388, 724.3009, 726.2899, 732.2586, 735.3865, 745.4124, 756.4687, 764.4056, 765.3908, 767.3679, 768.3909, 772.3955, 795.4186, 803.4370, 806.4631, 807.3893, 809.4480, 811.4104, 812.3985, 816.4408, 820.4244, 822.3905, 823.3846, 824.3756, 828.3904, 834.4043, 836.4589, 837.3853, 839.3696, 840.3839, 842.5085, 845.4100, 845.4123, 851.3975, 852.4056, 856.4893, 866.3914, 867.3724, 870.5402, 871.4945, 878.4625, 880.4403, 886.3815, 888.4233, 890.4479, 893.9562, 894.4579, 897.0289, 897.4412, 902.4597, 910.4410, 912.4497, 913.5118, 916.4905, 918.5391, 925.4747, 928.5064, 930.5048, 933.4619, 935.4698, 939.4758, 947.4628, 949.4591, 951.4703, 954.4674, 957.4657, 963.4676, 965.4761, 967.4568, 969.4571, 972.4793, 972.4800, 975.4650, 977.4630, 978.4976, 981.4565, 983.4664, 985.4842, 991.4688, 993.5331, 997.4298, 999.4453, 1001.4488, 1003.4687, 1007.5668, 1018.5200, 1018.5212, 1023.4777, 1026.4922, 1027.4972, 1030.5151, 1031.3850, 1033.5419, 1035.5352, 1036.5372, 1039.5402, 1042.5383, 1045.5654, 1056.5488, 1060.5400, 1060.5442, 1065.5425, 1086.5115, 1106.5372, 1111.5582, 1113.5546, 1114.5396, 1115.5605, 1118.5529, 1122.5363, 1128.5775,

1129.6014, 1130.5935, 1140.5485, 1144.5709, 1155.6113, 1156.5720, 1157.5952, 1158.6154, 1159.6273, 1165.5817, 1172.6301, 1173.6259, 1179.6063, 1180.6165, 1182.6090, 1183.6123, 1185.6317, 1187.6431, 1193.6096, 1195.6060, 1196.6102, 1206.6770, 1209.1387, 1209.5848, 1210.1014, 1215.6627, 1216.6494, 1222.5994, 1226.5948, 1229.6246, 1231.6174, 1234.6317, 1235.5846, 1240.6298, 1244.6324, 1248.6119, 1250.6144, 1268.6498, 1271.6696, 1273.6653, 1275.6644, 1291.6425, 1295.7233, 1300.6062, 1301.6205, 1307.6858, 1315.6570, 1317.6521, 1319.6852, 1323.6517, 1325.6637, 1328.6809, 1329.6672, 1331.6763, 1332.6743, 1334.6592, 1335.6937, 1336.6715, 1337.6724, 1338.6471, 1342.6680, 1345.6760, 1346.6880, 1349.6991, 1354.6907, 1355.7155, 1357.7034, 1360.6882, 1361.6670, 1365.6913, 1368.6920, 1374.6412, 1375.6798, 1377.6794, 1382.6647, 1410.7505, 1411.7208, 1421.7177, 1427.7872, 1431.7961, 1432.7133, 1434.7288, 1446.7256, 1450.7079, 1473.8054, 1475.7748, 1477.7524, 1478.7439, 1485.7720, 1486.7664, 1493.7539, 1494.7423, 1505.8649, 1523.7841, 1524.7848, 1537.8021, 1549.7388, 1555.8816, 1558.8474, 1576.7987, 1579.7926, 1584.7897, 1588.8136, 1590.3611, 1604.8417, 1605.8098, 1606.8384, 1615.8826, 1616.8605, 1622.8137, 1629.8358, 1630.8466, 1633.9790, 1635.7943, 1647.8785, 1649.8463, 1651.8245, 1659.8241, 1662.7909, 1668.7810, 1679.9736, 1681.8214, 1683.8121, 1684.8204, 1706.8616, 1707.7838, 1716.8571, 1725.8353, 1728.8242, 1734.8152, 1740.8768, 1742.9031, 1746.1659, 1750.8356, 1751.8787, 1753.8948, 1756.8905, 1763.8188, 1765.7690, 1767.8036, 1768.9027, 1769.4475, 1770.4261, 1774.9091, 1783.8499, 1786.9000, 1786.9017, 1791.7335, 1808.8868, 1824.8674, 1840.8225, 1844.8907, 1848.8322, 1850.8301, 1851.8488, 1867.9503, 1880.9277, 1887.9734, 1908.8838, 1914.9995, 1919.0038, 1925.9418, 1928.0103, 1931.9463, 1953.9353, 1975.9609, 1992.9642, 1995.9020, 2007.9894, 2011.8845, 2013.8983, 2024.8591, 2025.6234, 2036.5195, 2036.9486, 2042.0640, 2047.0167, 2047.9691, 2051.9792, 2052.9924, 2058.9963, 2060.0469, 2064.9900, 2068.8982, 2070.8835, 2074.8914, 2083.9907, 2084.8987, 2086.8828, 2092.8821, 2095.9941, 2098.9976, 2100.9160, 2102.8872, 2106.9011, 2108.8835, 2109.9175, 2114.9180, 2116.9160, 2118.8835, 2119.8833, 2125.0176, 2132.8694, 2136.9180, 2170.1467, 2211.1045, 2225.1191, 2273.0496, 2277.0137, 2307.9941, 2322.9980, 2341.0366, 2343.0271, 2352.0571, 2355.0459, 2359.0532, 2366.0813, 2368.0544, 2369.0710, 2376.0264, 2382.0349, 2383.6099, 2383.9600, 2390.0403, 2396.0342, 2398.0576, 2400.0652, 2406.0737, 2411.0076, 2414.0813, 2416.0752, 2417.0732, 2420.0718, 2422.0408, 2423.0466, 2428.0898, 2430.0828, 2431.0845, 2433.0779, 2436.0508, 2438.0457, 2439.0261, 2442.0618, 2449.0347, 2452.0632, 2460.0254, 2461.9956, 2463.9858, 2510.1277, 2528.1580, 2542.1509, 2545.1558, 2547.1531, 2552.1038, 2565.1543, 2585.1111, 2601.0889, 2705.2192, 2717.0693, 3129.3176, 3208.1736, 3223.2710, 3290.4131, 3291.4248, 3312.2756, 3451.3933, 3453.4365

21. Q3MHQ4 **Mass:** 30565 **Score:** 19 **Expect:** 3.3e+002 **Matches:** 14

Zinc finger protein SNAI2 OS=Bos taurus GN=SNAI2 PE=2 SV=1

Observed	Mr(expt)	Mr(calc)	ppm	Start	End	Miss	Ions	Peptide
972.4793	971.4720	971.4495	23.2	245 -	252	1	---	K.SCSKTFSR.M
972.4800	971.4727	971.4495	23.9	245 -	252	1	---	K.SCSKTFSR.M
993.5331	992.5258	992.5477	-22.07	1 -	8	1	---	-.MPRSFLVK.K + Oxidation (M)
1060.5400	1059.5327	1059.5237	8.53	136 -	145	0	---	K.TYSTFSGLGK.H
1060.5442	1059.5369	1059.5237	12.5	136 -	145	0	---	K.TYSTFSGLGK.H
1115.5605	1114.5532	1114.5264	24.1	180 -	188	0	---	R.THTLPCVCK.I
1209.5848	1208.5775	1208.6037	-21.64	117 -	127	0	---	K.LSDPHAIEAEK.F
1325.6637	1324.6564	1324.6776	-15.95	136 -	147	1	---	K.TYSTFSGLGKHK.Q
1605.8098	1604.8025	1604.8383	-22.28	226 -	239	1	---	R.SNLRAHLQTHSDVK.K
1668.7810	1667.7737	1667.8058	-19.25	176 -	188	1	---	K.MHIRTHTLPCVCK.I + Oxidation (M)
1681.8214	1680.8141	1680.8440	-17.75	245 -	258	2	---	K.SCSKTFSRMSLLHK.H
1851.8488	1850.8415	1850.8516	-5.43	148 -	162	2	---	K.QLHCDAQSRKSFSCK.Y
2098.9976	2097.9903	2098.0339	-20.79	163 -	179	2	---	K.YCDKEYVSLGALKMHIR.T + Oxidation (M)
2125.0176	2124.0103	2123.9769	15.8	128 -	145	1	---	K.FQCNLCNKTYSTFSGLGK.H

No match to: 713.4334, 719.2388, 724.3009, 726.2899, 732.2586, 735.3865, 745.4124, 756.4687, 764.4056, 765.3908, 767.3679, 768.3909, 772.3955, 795.4186, 802.4548, 803.4370, 805.4229, 806.4631, 807.3893, 809.4480, 811.4104, 812.3985, 816.4408, 820.4244, 822.3905, 823.3846, 824.3756, 828.3904, 834.4043, 836.4589, 837.3853, 839.3696, 840.3839, 842.5085, 845.4100, 845.4123, 851.3975, 852.4056, 856.4893, 866.3914, 867.3724, 870.5402, 871.4945, 878.4625, 880.4403, 886.3815, 888.4233, 890.4479, 893.9562, 894.4579, 897.0289, 897.4412, 902.4597, 910.4410, 912.4497, 913.5118, 916.4905, 918.5391, 925.4747, 928.5064, 930.5048, 933.4619, 935.4698, 939.4758, 947.4628, 949.4591, 951.4703, 954.4674, 957.4657, 963.4676, 965.4761, 967.4568, 969.4571, 975.4650, 977.4630, 978.4976, 981.4565, 983.4664, 985.4842, 991.4688, 997.4298, 999.4453, 1001.4488, 1003.4687, 1007.5668, 1018.5200, 1018.5212, 1023.4777, 1026.4922, 1027.4972, 1030.5151, 1031.3850, 1033.5419, 1035.5352, 1036.5372, 1039.5402, 1042.5383, 1045.5654, 1056.5488, 1065.5425, 1086.5115, 1106.5372, 1111.5582, 1113.5546, 1114.5396, 1118.5529, 1122.5363, 1128.5775, 1129.6014, 1130.5935, 1140.5485, 1144.5709, 1155.6113, 1156.5720, 1157.5952, 1158.6154, 1159.6273, 1165.5817, 1172.6301, 1173.6259, 1179.6063, 1180.6165, 1182.6090, 1183.6123, 1185.6317, 1187.6431, 1193.6096, 1195.6060, 1196.6102, 1206.6770, 1209.1387, 1210.1014, 1215.6627, 1216.6494, 1222.5994, 1226.5948, 1229.6246, 1231.6174, 1234.6317, 1235.5846, 1240.6298, 1244.6324, 1248.6119, 1250.6144, 1268.6498, 1271.6696, 1273.6653, 1275.6644, 1291.6425, 1295.7233, 1300.6062, 1301.6205, 1307.6858, 1315.6570, 1317.6521, 1319.6852, 1323.6517, 1328.6809, 1329.6672, 1331.6763, 1332.6743, 1334.6592, 1335.6937, 1336.6715, 1337.6724, 1338.6471, 1342.6680, 1345.6760, 1346.6880, 1349.6991, 1354.6907, 1355.7155, 1357.7034, 1360.6882, 1361.6670, 1365.6913, 1368.6920, 1374.6412, 1375.6798, 1376.6804, 1377.6794, 1382.6647, 1387.6444, 1410.7505, 1411.7208, 1421.7177, 1427.7872, 1431.7961, 1432.7133, 1434.7288, 1446.7256, 1450.7079, 1473.8054, 1475.7748, 1477.7524, 1478.7439, 1485.7720, 1486.7664, 1493.7539, 1494.7423, 1505.8649, 1523.7841, 1524.7848, 1537.8021, 1549.7388, 1555.8816, 1558.8474, 1576.7987, 1579.7926, 1584.7897, 1588.8136, 1590.3611, 1604.8417, 1606.8384, 1615.8826, 1616.8605, 1622.8137, 1628.8260, 1629.8358, 1630.8466, 1633.9790, 1635.7943, 1644.8046, 1647.8785, 1649.8463, 1651.8245, 1659.8241, 1662.7909, 1679.9736, 1683.8121, 1684.8204, 1706.8616, 1707.7838, 1716.8571, 1725.8353, 1728.8242, 1734.8152, 1740.8768, 1742.9031, 1746.1659, 1750.8356, 1751.8787, 1753.8948, 1756.8905, 1757.8322, 1763.8188, 1765.7690, 1767.8036, 1768.9027, 1769.4475, 1770.4261, 1774.9091, 1783.8499, 1786.9000, 1786.9017, 1791.7335, 1808.8868, 1824.8674, 1840.8225, 1844.8907, 1848.8322, 1850.8301, 1867.9503, 1880.9277, 1887.9734, 1908.8838, 1914.9995, 1919.0038, 1925.9418, 1928.0103, 1931.9463, 1953.9353, 1975.9609, 1992.9642, 1995.9020, 2007.9894, 2011.8845, 2013.8983, 2024.8591, 2025.6234, 2036.5195, 2036.9486, 2042.0640, 2047.0167, 2047.9691, 2051.9792, 2052.9924, 2058.9963, 2060.0469, 2064.9900, 2068.8982, 2070.8835, 2074.8914, 2083.9907, 2084.8987, 2086.8828, 2092.8821, 2095.9941, 2100.9160, 2102.8872, 2106.9011, 2108.8835, 2109.9175, 2114.9180, 2116.9160, 2118.8835, 2119.8833, 2132.8694, 2136.9180, 2170.1467, 2211.1045, 2225.1191, 2273.0496, 2277.0137, 2307.9941, 2322.9980, 2341.0366, 2343.0271, 2352.0571, 2355.0459, 2359.0532, 2366.0813, 2368.0544, 2369.0710, 2376.0264, 2382.0349, 2383.6099, 2383.9600, 2390.0403, 2396.0342, 2398.0576, 2400.0652, 2406.0737, 2411.0076, 2414.0813, 2416.0752, 2417.0732, 2420.0718, 2422.0408, 2423.0466, 2428.0898, 2430.0828, 2431.0845, 2433.0779, 2436.0508, 2438.0457, 2439.0261, 2442.0618, 2449.0347, 2452.0632, 2460.0254, 2461.9956, 2463.9858, 2510.1277, 2528.1580, 2542.1509, 2545.1558, 2547.1531, 2552.1038, 2565.1543, 2585.1111, 2601.0889, 2705.2192, 2717.0693, 3129.3176, 3208.1736, 3223.2710, 3290.4131, 3291.4248, 3312.2756, 3451.3933, 3453.4365

22. F1MMB7 **Mass:** 28878 **Score:** 18 **Expect:** 3.4e+002 **Matches:** 11

Uncharacterized protein OS=Bos taurus GN=TP53TG5 PE=4 SV=1

Observed	Mr(expt)	Mr(calc)	ppm	Start	End	Miss	Ions	Peptide
902.4597	901.4524	901.4327	21.8	132 -	139	0	---	R.AQEAMPQK.E
925.4747	924.4674	924.4777	-11.19	153 -	161	0	---	R.GHGLTTSPR.A
1113.5546	1112.5473	1112.5397	6.88	179 -	187	1	---	K.THQDRTPMK.D
1165.5817	1164.5744	1164.5645	8.56	224 -	231	2	---	R.WVKRCCTR.F
1328.6809	1327.6736	1327.6918	-13.69	184 -	194	2	---	R.TPMKDVKQPER.A
1337.6724	1336.6651	1336.6856	-15.35	208 -	218	1	---	R.VHLPGPRVMCR.S + Oxidation (M)
1880.9277	1879.9204	1879.9462	-13.69	17 -	32	2	---	K.MQAKEPEDKIHQPVSK.V + Oxidation (M)
1925.9418	1924.9345	1924.9796	-23.39	162 -	178	1	---	R.AQAQEPPTGDPRVVFWK.T
2013.8983	2012.8910	2012.8997	-4.29	99 -	115	1	---	K.ELQEEAGCSYQKLDSEK.L
2376.0264	2375.0191	2375.0531	-14.29	228 -	246	1	---	R.CCTRFCSASLETPMVHLYK.V + Oxidation (M)
2420.0718	2419.0645	2419.0631	0.58	132 -	152	1	---	R.AQEAMPQKEEQMEPEAPTTSR.G + 2 Oxidation (M)

No match to: 713.4334, 719.2388, 724.3009, 726.2899, 732.2586, 735.3865, 745.4124, 756.4687, 764.4056, 765.3908, 767.3679, 768.3909, 772.3955, 795.4186, 802.4548, 803.4370, 805.4229, 806.4631, 807.3893, 809.4480, 811.4104, 812.3985, 816.4408, 820.4244, 822.3905, 823.3846, 824.3756, 828.3904, 834.4043, 836.4589, 837.3853, 839.3696, 840.3839, 842.5085, 845.4100, 845.4123, 851.3975, 852.4056, 856.4893, 866.3914, 867.3724, 870.5402, 871.4945, 878.4625, 880.4403, 886.3815, 888.4233, 890.4479, 893.9562, 894.4579, 897.0289, 897.4412, 910.4410, 912.4497, 913.5118, 916.4905, 918.5391, 928.5064, 930.5048, 933.4619, 935.4698, 939.4758, 947.4628, 949.4591, 951.4703, 954.4674, 957.4657, 963.4676, 965.4761, 967.4568, 969.4571, 972.4793, 972.4800, 975.4650, 977.4630, 978.4976, 981.4565, 983.4664, 985.4842, 991.4688, 993.5331, 997.4298, 999.4453, 1001.4488, 1003.4687, 1007.5668, 1018.5200, 1018.5212,

1023.4777, 1026.4922, 1027.4972, 1030.5151, 1031.3850, 1033.5419, 1035.5352, 1036.5372, 1039.5402, 1042.5383, 1045.5654, 1056.5488, 1060.5400, 1060.5442, 1065.5425, 1086.5115, 1106.5372, 1111.5582, 1114.5396, 1115.5605, 1128.5775, 1129.6014, 1130.5935, 1140.5485, 1144.5709, 1155.6113, 1156.5720, 1157.5952, 1158.6154, 1159.6273, 1172.6301, 1173.6259, 1179.6063, 1180.6165, 1182.6090, 1183.6123, 1185.6317, 1187.6431, 1193.6096, 1195.6060, 1196.6102, 1206.6770, 1209.1387, 1209.5848, 1210.1014, 1215.6627, 1216.6494, 1222.5994, 1226.5948, 1229.6246, 1231.6174, 1234.6317, 1235.5846, 1240.6298, 1244.6324, 1248.6119, 1250.6144, 1268.6498, 1271.6696, 1273.6653, 1275.6644, 1291.6425, 1295.7233, 1300.6062, 1301.6205, 1307.6858, 1315.6570, 1317.6521, 1319.6852, 1323.6517, 1325.6637, 1329.6672, 1331.6763, 1332.6743, 1334.6592, 1335.6937, 1336.6715, 1338.6471, 1342.6680, 1345.6760, 1346.6880, 1349.6991, 1354.6907, 1355.7155, 1357.7034, 1360.6882, 1361.6670, 1365.6913, 1368.6920, 1374.6412, 1375.6798, 1376.6804, 1377.6794, 1382.6647, 1387.6444, 1410.7505, 1411.7208, 1421.7177, 1427.7872, 1431.7961, 1432.7133, 1434.7288, 1446.7256, 1450.7079, 1473.8054, 1475.7748, 1477.7524, 1478.7439, 1485.7720, 1486.7664, 1493.7539, 1494.7423, 1505.8649, 1523.7841, 1524.7848, 1537.8021, 1549.7388, 1555.8816, 1558.8474, 1576.7987, 1579.7926, 1584.7897, 1588.8136, 1590.3611, 1604.8417, 1605.8098, 1606.8384, 1615.8826, 1616.8605, 1622.8137, 1628.8260, 1629.8358, 1630.8466, 1633.9790, 1635.7943, 1644.8046, 1647.8785, 1649.8463, 1651.8245, 1659.8241, 1662.7909, 1668.7810, 1679.9736, 1681.8214, 1683.8121, 1684.8204, 1706.8616, 1707.7838, 1716.8571, 1725.8353, 1728.8242, 1734.8152, 1740.8768, 1742.9031, 1746.1659, 1750.8356, 1751.8787, 1753.8948, 1756.8905, 1757.8322, 1763.8188, 1765.7690, 1767.8036, 1768.9027, 1769.4475, 1770.4261, 1774.9091, 1783.8499, 1786.9000, 1786.9017, 1791.7335, 1808.8868, 1824.8674, 1840.8225, 1844.8907, 1848.8322, 1850.8301, 1851.8488, 1867.9503, 1887.9734, 1908.8838, 1914.9995, 1919.0038, 1928.0103, 1931.9463, 1953.9353, 1975.9609, 1992.9642, 1995.9020, 2007.9894, 2011.8845, 2024.8591, 2025.6234, 2036.5195, 2036.9486, 2042.0640, 2047.0167, 2047.9691, 2051.9792, 2052.9924, 2058.9963, 2060.0469, 2064.9900, 2068.8982, 2070.8835, 2074.8914, 2083.9907, 2084.8987, 2086.8828, 2092.8821, 2095.9941, 2098.9976, 2100.9160, 2102.8872, 2106.9011, 2108.8835, 2109.9175, 2114.9180, 2116.9160, 2118.8835, 2119.8833, 2125.0176, 2132.8694, 2136.9180, 2170.1467, 2211.1045, 2225.1191, 2273.0496, 2277.0137, 2307.9941, 2322.9980, 2341.0366, 2343.0271, 2352.0571, 2355.0459, 2359.0532, 2366.0813, 2368.0544, 2369.0710, 2382.0349, 2383.6099, 2383.9600, 2390.0403, 2396.0342, 2398.0576, 2400.0652, 2406.0737, 2411.0076, 2414.0813, 2416.0752, 2417.0732, 2422.0408, 2423.0466, 2428.0898, 2430.0828, 2431.0845, 2433.0779, 2436.0508, 2438.0457, 2439.0261, 2442.0618, 2449.0347, 2452.0632, 2460.0254, 2461.9956, 2463.9858, 2510.1277, 2528.1580, 2542.1509, 2545.1558, 2547.1531, 2552.1038, 2565.1543, 2585.1111, 2601.0889, 2705.2192, 2717.0693, 3129.3176, 3208.1736, 3223.2710, 3290.4131, 3291.4248, 3312.2756, 3451.3933, 3453.4365

23. F1N6K9 **Mass:** 95221 **Score:** 18 **Expect:** 3.5e+002 **Matches:** 27

Uncharacterized protein OS=Bos taurus PE=4 SV=2

Observed	Mr(expt)	Mr(calc)	ppm	Start	End	Miss	Ions	Peptide
807.3893	806.3820	806.3956	-16.88	583	589	0	---	K.NTMLNSK.L
823.3846	822.3774	822.3905	-16.01	583	589	0	---	K.NTMLNSK.L + Oxidation (M)
834.4043	833.3970	833.4130	-19.22	562	568	0	---	R.NEETLTK.T
897.4412	896.4339	896.4239	11.1	463	469	1	---	K.EDQYSKK.V
902.4597	901.4524	901.4366	17.5	440	446	1	---	K.QEGEQRR.N
983.4664	982.4591	982.4429	16.4	447	454	0	---	R.NADMLYEK.I
999.4453	998.4380	998.4379	0.17	447	454	0	---	R.NADMLYEK.I + Oxidation (M)
1030.5151	1029.5078	1029.5317	-23.23	1	9	1	---	-.MKIFGFGSK.K + Oxidation (M)
1155.6113	1154.6040	1154.5859	15.7	541	549	0	---	K.YFEDIEIVK.G
1158.6154	1157.6081	1157.6267	-16.04	1	10	2	---	-.MKIFGFGSKK.G + Oxidation (M)
1165.5817	1164.5744	1164.5710	2.98	622	630	1	---	K.RDMELAFQR.A
1173.6259	1172.6186	1172.6203	-1.43	28	36	1	---	R.RIHFQPGYR.I
1185.6317	1184.6244	1184.6513	-22.69	775	784	1	---	K.QGLVLEERNK.E
1268.6498	1267.6425	1267.6230	15.4	447	456	1	---	R.NADMLYEKIR.E + Oxidation (M)
1271.6696	1270.6623	1270.6339	22.3	197	207	1	---	K.EANIHAVDKMK.R + Oxidation (M)
1332.6743	1331.6670	1331.6364	23.0	66	76	2	---	K.NGLNDRDKMNR.T
1411.7208	1410.7135	1410.7401	-18.86	197	208	2	---	K.EANIHAVDKMKR.N
1421.7177	1420.7104	1420.7245	-9.90	633	643	2	---	R.DERLHLHDKMK.S
1434.7288	1433.7215	1433.7370	-10.82	683	693	1	---	R.EKILMLECVQR.D + Oxidation (M)
1576.7987	1575.7914	1575.8038	-7.87	583	595	2	---	K.NTMLNSKLENEKR.L
1588.8136	1587.8063	1587.8018	2.82	24	36	2	---	R.DSGRRIHFQPGYR.I
1615.8826	1614.8753	1614.9127	-23.13	469	481	2	---	K.KVEMKQQLELTLR.A
1668.7810	1667.7737	1667.7322	24.9	607	621	0	---	R.LAAASHDHDQCQTSK.R
1786.9000	1785.8927	1785.8969	-2.35	10	27	2	6	K.KGESPLGSSISPGRDSGR.R
1786.9017	1785.8944	1785.8969	-1.40	10	27	2	---	K.KGESPLGSSISPGRDSGR.R
1824.8674	1823.8601	1823.8333	14.7	607	622	1	---	R.LAAASHDHDQCQTSKR.D
1919.0038	1917.9965	1917.9982	-0.89	751	766	1	---	K.EKTVISIQDQFQPMVR.E

No match to: 713.4334, 719.2388, 724.3009, 726.2899, 732.2586, 735.3865, 745.4124, 756.4687, 764.4056, 765.3908, 767.3679, 768.3909, 772.3955, 795.4186, 802.4548, 803.4370, 805.4229, 806.4631, 809.4480, 811.4104, 812.3985, 816.4408, 820.4244, 822.3905, 824.3756, 828.3904, 836.4589, 837.3853, 839.3696, 840.3839, 842.5085, 845.4100, 845.4123, 851.3975, 852.4056, 856.4893, 866.3914, 867.3724, 870.5402, 871.4945, 878.4625, 880.4403, 886.3815, 888.4233, 890.4479, 893.9562, 894.4579, 897.0289, 910.4410, 912.4497, 913.5118, 916.4905, 918.5391, 925.4747, 928.5064, 930.5048, 933.4619, 935.4698, 939.4758, 947.4628, 949.4591, 951.4703, 954.4674, 957.4657, 963.4676, 965.4761, 967.4568, 969.4571, 972.4793, 972.4800, 975.4650, 977.4630, 978.4976, 981.4565, 985.4842, 991.4688, 993.5331, 997.4298, 1001.4488, 1003.4687, 1007.5668, 1018.5200, 1018.5212, 1023.4777, 1026.4922, 1027.4972, 1031.3850, 1033.5419, 1035.5352, 1036.5372, 1039.5402, 1042.5383, 1045.5654, 1056.5488, 1060.5400, 1060.5442, 1065.5425, 1086.5115, 1106.5372, 1111.5582, 1113.5546, 1114.5396, 1115.5605, 1118.5529, 1122.5363, 1128.5775, 1129.6014, 1130.5935, 1140.5485, 1144.5709, 1156.5720, 1157.5952, 1159.6273, 1172.6301, 1179.6063, 1180.6165, 1182.6090, 1183.6123, 1187.6431, 1193.6096, 1195.6060, 1196.6102, 1206.6770, 1209.1387, 1209.5848, 1210.1014, 1215.6627, 1216.6494, 1222.5994, 1226.5948, 1229.6246, 1231.6174, 1234.6317, 1235.5846, 1240.6298, 1244.6324, 1248.6119, 1250.6144, 1273.6653, 1275.6644, 1291.6425, 1295.7233, 1300.6062, 1301.6205, 1307.6858, 1315.6570, 1317.6521, 1319.6852, 1323.6517, 1325.6637, 1328.6809, 1329.6672, 1331.6763, 1334.6592, 1335.6937, 1336.6715, 1337.6724, 1338.6471, 1342.6680, 1345.6760, 1346.6880, 1349.6991, 1354.6907, 1355.7155, 1357.7034, 1360.6882, 1361.6670, 1365.6913, 1368.6920, 1374.6412, 1375.6798, 1376.6804, 1377.6794, 1382.6647, 1387.6444, 1410.7505, 1427.7872, 1431.7961, 1432.7133, 1446.7256, 1450.7079, 1473.8054, 1475.7748, 1477.7524, 1478.7439, 1485.7720, 1486.7664, 1493.7539, 1494.7423, 1505.8649, 1523.7841, 1524.7848, 1537.8021, 1549.7388, 1555.8816, 1558.8474, 1579.7926, 1584.7897, 1590.3611, 1604.8417, 1605.8098, 1606.8384, 1616.8605, 1622.8137, 1628.8260, 1629.8358, 1630.8466, 1633.9790, 1635.7943, 1644.8046, 1647.8785, 1649.8463, 1651.8245, 1659.8241, 1662.7909, 1679.9736, 1681.8214, 1683.8121, 1684.8204, 1706.8616, 1707.7838, 1716.8571, 1725.8353, 1728.8242, 1734.8152, 1740.8768, 1742.9031, 1746.1659, 1750.8356, 1751.8787, 1753.8948, 1756.8905, 1757.8322, 1763.8188, 1765.7690, 1767.8036, 1768.9027, 1769.4475, 1770.4261, 1774.9091, 1783.8499, 1791.7335, 1808.8868, 1840.8225, 1844.8907, 1848.8322, 1850.8301, 1851.8488, 1867.9503, 1880.9277, 1887.9734, 1908.8838, 1914.9995, 1925.9418, 1928.0103, 1931.9463, 1953.9353, 1975.9609, 1992.9642, 1995.9020, 2007.9894, 2011.8845, 2013.8983, 2024.8591, 2025.6234, 2036.5195, 2036.9486, 2042.0640, 2047.0167, 2047.9691, 2051.9792, 2052.9924, 2058.9963, 2060.0469, 2064.9900, 2068.8982, 2070.8835, 2074.8914, 2083.9907, 2084.8987, 2086.8828, 2092.8821, 2095.9941, 2098.9976, 2100.9160, 2102.8872, 2106.9011, 2108.8835, 2109.9175, 2114.9180, 2116.9160, 2118.8835, 2119.8833, 2125.0176, 2132.8694, 2136.9180, 2170.1467, 2211.1045, 2225.1191, 2273.0496, 2277.0137, 2307.9941, 2322.9980, 2341.0366, 2343.0271, 2352.0571, 2355.0459, 2359.0532, 2366.0813, 2368.0544, 2369.0710, 2376.0264, 2382.0349, 2383.6099, 2383.9600, 2390.0403, 2396.0342, 2398.0576, 2400.0652, 2406.0737, 2411.0076, 2414.0813, 2416.0752, 2417.0732, 2420.0718, 2422.0408, 2423.0466, 2428.0898, 2430.0828, 2431.0845, 2433.0779, 2436.0508, 2438.0457, 2439.0261, 2442.0618, 2449.0347, 2452.0632, 2460.0254, 2461.9956, 2463.9858, 2510.1277, 2528.1580, 2542.1509, 2545.1558, 2547.1531, 2552.1038, 2565.1543, 2585.1111, 2601.0889, 2705.2192, 2717.0693, 3129.3176, 3208.1736, 3223.2710, 3290.4131, 3291.4248, 3312.2756, 3451.3933, 3453.4365

24. Q32KL9 **Mass:** 28308 **Score:** 18 **Expect:** 3.8e+002 **Matches:** 12

B-cell receptor-associated protein 29 OS=Bos taurus GN=BCAP29 PE=2 SV=1

Observed	Mr(expt)	Mr(calc)	ppm	Start	End	Miss	Ions	Peptide
806.4631	805.4558	805.4559	-0.08	97	102	1	---	K.LFRSQR.N
890.4479	889.4407	889.4392	1.60	180	186	1	---	K.LEEDKEK.L
910.4410	909.4338	909.4556	-23.97	215	221	1	---	R.LSKEYDR.L
978.4976	977.4903	977.4964	-6.24	210	217	1	---	K.MQSERLSK.E

1018.5200	1017.5127	1017.5342	-21.10	179 - 186	2	---		K.KLEEDKEK.L
1018.5212	1017.5139	1017.5342	-19.92	179 - 186	2	---		K.KLEEDKEK.L
1065.5425	1064.5352	1064.5138	20.1	73 - 81	0	---		K.YSSTHTIEK.S
1193.6096	1192.6023	1192.6088	-5.41	72 - 81	1	---		R.KYSSTHTIEK.S
1195.6060	1194.5987	1194.5703	23.8	151 - 159	1	---		K.FMEENERLK.R
1323.6517	1322.6444	1322.6652	-15.72	150 - 159	2	---		K.KFMEENERLK.R
2095.9941	2094.9868	2095.0269	-19.12	82 - 99	1	---		K.SSASRPAAYEHTQMKLFR.S + Oxidation (M)
2369.0710	2368.0637	2368.1185	-23.11	194 - 214	2	---		K.ASDALSKAQNDVMIMKMQSER.L + Oxidation (M)

No match to: 713.4334, 719.2388, 724.3009, 726.2899, 732.2586, 735.3865, 745.4124, 756.4687, 764.4056, 765.3908, 767.3679, 768.3909, 772.3955, 795.4186, 802.4548, 803.4370, 805.4229, 807.3893, 809.4480, 811.4104, 812.3985, 816.4408, 820.4244, 822.3905, 823.3846, 824.3756, 828.3904, 834.4043, 836.4589, 837.3853, 839.3696, 840.3839, 842.5085, 845.4100, 845.4123, 851.3975, 852.4056, 856.4893, 866.3914, 867.3724, 870.5402, 871.4945, 878.4625, 880.4403, 886.3815, 888.4233, 893.9562, 894.4579, 897.0289, 897.4412, 902.4597, 912.4497, 913.5118, 916.4905, 918.5391, 925.4747, 928.5064, 930.5048, 933.4619, 935.4698, 939.4758, 947.4628, 949.4591, 951.4703, 954.4674, 957.4657, 963.4676, 965.4761, 967.4568, 969.4571, 972.4793, 972.4800, 975.4650, 977.4630, 981.4565, 983.4664, 985.4842, 991.4688, 993.5331, 997.4298, 999.4453, 1001.4488, 1003.4687, 1007.5668, 1023.4777, 1026.4922, 1027.4972, 1030.5151, 1031.3850, 1033.5419, 1035.5352, 1036.5372, 1039.5402, 1042.5383, 1045.5654, 1056.5488, 1060.5400, 1060.5442, 1086.5115, 1106.5372, 1111.5582, 1113.5546, 1114.5396, 1115.5605, 1118.5529, 1122.5363, 1128.5775, 1129.6014, 1130.5935, 1140.5485, 1144.5709, 1155.6113, 1156.5720, 1157.5952, 1158.6154, 1159.6273, 1165.5817, 1172.6301, 1173.6259, 1179.6063, 1180.6165, 1182.6090, 1183.6123, 1185.6317, 1187.6431, 1196.6102, 1206.6770, 1209.1387, 1209.5848, 1210.1014, 1215.6627, 1216.6494, 1222.5994, 1226.5948, 1229.6246, 1231.6174, 1234.6317, 1235.5846, 1240.6298, 1244.6324, 1248.6119, 1250.6144, 1268.6498, 1271.6696, 1273.6653, 1275.6644, 1291.6425, 1295.7233, 1300.6062, 1301.6205, 1307.6858, 1315.6570, 1317.6521, 1319.6852, 1325.6637, 1328.6809, 1329.6672, 1331.6763, 1332.6743, 1334.6592, 1335.6937, 1336.6715, 1337.6724, 1338.6471, 1342.6680, 1345.6760, 1346.6880, 1349.6991, 1354.6907, 1355.7155, 1357.7034, 1360.6882, 1361.6670, 1365.6913, 1368.6920, 1374.6412, 1375.6798, 1376.6804, 1377.6794, 1382.6647, 1387.6444, 1410.7505, 1411.7208, 1421.7177, 1427.7872, 1431.7961, 1432.7133, 1434.7288, 1446.7256, 1450.7079, 1473.8054, 1475.7748, 1477.7524, 1478.7439, 1485.7720, 1486.7664, 1493.7539, 1494.7423, 1505.8649, 1523.7841, 1524.7848, 1537.8021, 1549.7388, 1555.8816, 1558.8474, 1576.7987, 1579.7926, 1584.7897, 1588.8136, 1590.3611, 1604.8417, 1605.8098, 1606.8384, 1615.8826, 1616.8605, 1622.8137, 1628.8260, 1629.8358, 1630.8466, 1633.9790, 1635.7943, 1644.8046, 1647.8785, 1649.8463, 1651.8245, 1659.8241, 1662.7909, 1668.7810, 1679.9736, 1681.8214, 1683.8121, 1684.8204, 1706.8616, 1707.7838, 1716.8571, 1725.8353, 1728.8242, 1734.8152, 1740.8768, 1742.9031, 1746.1659, 1750.8356, 1751.8787, 1753.8948, 1756.8905, 1757.8322, 1763.8188, 1765.7690, 1767.8036, 1768.9027, 1769.4475, 1770.4261, 1774.9091, 1783.8499, 1786.9000, 1786.9017, 1791.7335, 1808.8868, 1824.8674, 1840.8225, 1844.8907, 1848.8322, 1850.8301, 1851.8488, 1867.9503, 1880.9277, 1887.9734, 1908.8838, 1914.9995, 1919.0038, 1925.9418, 1928.0103, 1931.9463, 1953.9353, 1975.9609, 1992.9642, 1995.9020, 2007.9894, 2011.8845, 2013.8983, 2024.8591, 2025.6234, 2036.5195, 2036.9486, 2042.0640, 2047.0167, 2047.9691, 2051.9792, 2052.9924, 2058.9963, 2060.0469, 2064.9900, 2068.8982, 2070.8835, 2074.8914, 2083.9907, 2084.8987, 2086.8828, 2092.8821, 2098.9976, 2100.9160, 2102.8872, 2106.9011, 2108.8835, 2109.9175, 2114.9180, 2116.9160, 2118.8835, 2119.8833, 2125.0176, 2132.8694, 2136.9180, 2170.1467, 2211.1045, 2225.1191, 2273.0496, 2277.0137, 2307.9941, 2322.9980, 2341.0366, 2343.0271, 2352.0571, 2355.0459, 2359.0532, 2366.0813, 2368.0544, 2376.0264, 2382.0349, 2383.6099, 2383.9600, 2390.0403, 2396.0342, 2398.0576, 2400.0652, 2406.0737, 2411.0076, 2414.0813, 2416.0752, 2417.0732, 2420.0718, 2422.0408, 2423.0466, 2428.0898, 2430.0828, 2431.0845, 2433.0779, 2436.0508, 2438.0457, 2439.0261, 2442.0618, 2449.0347, 2452.0632, 2460.0254, 2461.9956, 2463.9858, 2510.1277, 2528.1580, 2542.1509, 2545.1558, 2547.1531, 2552.1038, 2565.1543, 2585.1111, 2601.0889, 2705.2192, 2717.0693, 3129.3176, 3208.1736, 3223.2710, 3290.4131, 3291.4248, 3312.2756, 3451.3933, 3453.4365

25. G3N097 **Mass:** 6896 **Score:** 18 **Expect:** 3.8e+002 **Matches:** 6

Uncharacterized protein OS=Bos taurus GN=SERF2 PE=4 SV=1

Observed	Mr(expt)	Mr(calc)	ppm	Start - End	Miss	Ions	Peptide
1159.6273	1158.6200	1158.6105	8.22	27 - 37	2	---	R.RDDGLSAAARK.Q
1180.6165	1179.6092	1179.5918	14.8	14 - 23	2	---	K.NMKKQSDSVK.G + Oxidation (M)
1187.6431	1186.6358	1186.6167	16.2	26 - 36	2	---	K.RRDDGLSAAAR.K
1234.6317	1233.6244	1233.6023	17.9	40 - 49	1	---	R.DSEIMQQKQK.K
1250.6144	1249.6071	1249.5972	7.92	40 - 49	1	---	R.DSEIMQQKQK.K + Oxidation (M)
1331.6763	1330.6690	1330.6888	-14.84	1 - 11	2	---	-.MTRGNQRELAR.Q

No match to: 713.4334, 719.2388, 724.3009, 726.2899, 732.2586, 735.3865, 745.4124, 756.4687, 764.4056, 765.3908, 767.3679, 768.3909, 772.3955, 795.4186, 802.4548, 803.4370, 805.4229, 806.4631, 807.3893, 809.4480, 811.4104, 812.3985, 816.4408, 820.4244, 822.3905, 823.3846, 824.3756, 828.3904, 834.4043, 836.4589, 837.3853, 839.3696, 840.3839, 842.5085, 845.4100, 845.4123, 851.3975, 852.4056, 856.4893, 866.3914, 867.3724, 870.5402, 871.4945, 878.4625, 880.4403, 886.3815, 888.4233, 890.4479, 893.9562, 894.4579, 897.0289, 897.4412, 902.4597, 910.4410, 912.4497, 913.5118, 916.4905, 918.5391, 925.4747, 928.5064, 930.5048, 933.4619, 935.4698, 939.4758, 947.4628, 949.4591, 951.4703, 954.4674, 957.4657, 963.4676, 965.4761, 967.4568, 969.4571, 972.4793, 972.4800, 975.4650, 977.4630, 978.4976, 981.4565, 983.4664, 985.4842, 991.4688, 993.5331, 997.4298, 999.4453, 1001.4488, 1003.4687, 1007.5668, 1018.5200, 1018.5212, 1023.4777, 1026.4922, 1027.4972, 1030.5151, 1031.3850, 1033.5419, 1035.5352, 1036.5372, 1039.5402, 1042.5383, 1045.5654, 1056.5488, 1060.5400, 1060.5442, 1065.5425, 1086.5115, 1106.5372, 1111.5582, 1113.5546, 1114.5396, 1115.5605, 1118.5529, 1122.5363, 1128.5775, 1129.6014, 1130.5935, 1140.5485, 1144.5709, 1155.6113, 1156.5720, 1157.5952, 1158.6154, 1165.5817, 1172.6301, 1173.6259, 1179.6063, 1182.6090, 1183.6123, 1185.6317, 1193.6096, 1195.6060, 1196.6102, 1206.6770, 1209.1387, 1209.5848, 1210.1014, 1215.6627, 1216.6494, 1222.5994, 1226.5948, 1229.6246, 1231.6174, 1235.5846, 1240.6298, 1244.6324, 1248.6119, 1268.6498, 1271.6696, 1273.6653, 1275.6644, 1291.6425, 1295.7233, 1300.6062, 1301.6205, 1307.6858, 1315.6570, 1317.6521, 1319.6852, 1323.6517, 1325.6637, 1328.6809, 1329.6672, 1332.6743, 1334.6592, 1335.6937, 1336.6715, 1337.6724, 1338.6471, 1342.6680, 1345.6760, 1346.6880, 1349.6991, 1354.6907, 1355.7155, 1357.7034, 1360.6882, 1361.6670, 1365.6913, 1368.6920, 1374.6412, 1375.6798, 1376.6804, 1377.6794, 1382.6647, 1387.6444, 1410.7505, 1411.7208, 1421.7177, 1427.7872, 1431.7961, 1432.7133, 1434.7288, 1446.7256, 1450.7079, 1473.8054, 1475.7748, 1477.7524, 1478.7439, 1485.7720, 1486.7664, 1493.7539, 1494.7423, 1505.8649, 1523.7841, 1524.7848, 1537.8021, 1549.7388, 1555.8816, 1558.8474, 1576.7987, 1579.7926, 1584.7897, 1588.8136, 1590.3611, 1604.8417, 1605.8098, 1606.8384, 1615.8826, 1616.8605, 1622.8137, 1628.8260, 1629.8358, 1630.8466, 1633.9790, 1635.7943, 1644.8046, 1647.8785, 1649.8463, 1651.8245, 1659.8241, 1662.7909, 1668.7810, 1679.9736, 1681.8214, 1683.8121, 1684.8204, 1706.8616, 1707.7838, 1716.8571, 1725.8353, 1728.8242, 1734.8152, 1740.8768, 1742.9031, 1746.1659, 1750.8356, 1751.8787, 1753.8948, 1756.8905, 1757.8322, 1763.8188, 1765.7690, 1767.8036, 1768.9027, 1769.4475, 1770.4261, 1774.9091, 1783.8499, 1786.9000, 1786.9017, 1791.7335, 1808.8868, 1824.8674, 1840.8225, 1844.8907, 1848.8322, 1850.8301, 1851.8488, 1867.9503, 1880.9277, 1887.9734, 1908.8838, 1914.9995, 1919.0038, 1925.9418, 1928.0103, 1931.9463, 1953.9353, 1975.9609, 1992.9642, 1995.9020, 2007.9894, 2011.8845, 2013.8983, 2024.8591, 2025.6234, 2036.5195, 2036.9486, 2042.0640, 2047.0167, 2047.9691, 2051.9792, 2052.9924, 2058.9963, 2060.0469, 2064.9900, 2068.8982, 2070.8835, 2074.8914, 2083.9907, 2084.8987, 2086.8828, 2092.8821, 2095.9941, 2098.9976, 2100.9160, 2102.8872, 2106.9011, 2108.8835, 2109.9175, 2114.9180, 2116.9160, 2118.8835, 2119.8833, 2125.0176, 2132.8694, 2136.9180, 2170.1467, 2211.1045, 2225.1191, 2273.0496, 2277.0137, 2307.9941, 2322.9980, 2341.0366, 2343.0271, 2352.0571, 2355.0459, 2359.0532, 2366.0813, 2368.0544, 2369.0710, 2376.0264, 2382.0349, 2383.6099, 2383.9600, 2390.0403, 2396.0342, 2398.0576, 2400.0652, 2406.0737, 2411.0076, 2414.0813, 2416.0752, 2417.0732, 2420.0718, 2422.0408, 2423.0466, 2428.0898, 2430.0828, 2431.0845, 2433.0779, 2436.0508, 2438.0457, 2439.0261, 2442.0618, 2449.0347, 2452.0632, 2460.0254, 2461.9956, 2463.9858, 2510.1277, 2528.1580, 2542.1509, 2545.1558, 2547.1531, 2552.1038, 2565.1543, 2585.1111, 2601.0889, 2705.2192, 2717.0693, 3129.3176, 3208.1736, 3223.2710, 3290.4131, 3291.4248, 3312.2756, 3451.3933, 3453.4365

26. F1MB56 **Mass:** 24689 **Score:** 18 **Expect:** 3.8e+002 **Matches:** 12

Uncharacterized protein OS=Bos taurus GN=TEX35 PE=4 SV=1

Observed	Mr(expt)	Mr(calc)	ppm	Start - End	Miss	Ions	Peptide
756.4687	755.4614	755.4653	-5.21	5 - 10	2	---	K.RVEPKK.T
1045.5654	1044.5581	1044.5750	-16.12	1 - 9	2	---	-.MSAKRVEPK.K
1159.6273	1158.6200	1158.6244	-3.78	58 - 66	2	---	R.EVREELKEK.M
1215.6627	1214.6554	1214.6805	-20.64	122 - 131	1	---	K.MQQELRLIGK.T
1421.7177	1420.7104	1420.7119	-1.03	61 - 71	2	---	R.EELKEKMEEIK.Q + Oxidation (M)
1606.8384	1605.8311	1605.8106	12.8	67 - 79	2	---	K.MEEIKQIKDVMDK.D
1616.8605	1615.8532	1615.8570	-2.31	39 - 53	1	---	K.QEGPFTKPGGTKELK.N
1622.8137	1621.8064	1621.8055	0.57	67 - 79	2	---	K.MEEIKQIKDVMDK.D + Oxidation (M)
1684.8204	1683.8131	1683.8212	-4.77	84 - 96	1	---	K.LQEFVEIMKEMQK.D + 2 Oxidation (M)
2036.9486	2035.9413	2035.9918	-24.79	97 - 113	2	---	K.DMDEKMDVLINIQKNSK.F + Oxidation (M)

2052.9924	2051.9851	2051.9867	-0.77	97	113	2	---	K.DMDEKMDVLINIQKNSK.F + 2 Oxidation (M)
2376.0264	2375.0191	2374.9981	8.87	161	179	1	---	R.QQPKDPMDPLHQCDSCFEK.C + Oxidation (M)

No match to: 713.4334, 719.2388, 724.3009, 726.2899, 732.2586, 735.3865, 745.4124, 764.4056, 765.3908, 767.3679, 768.3909,
772.3955, 795.4186, 802.4548, 803.4370, 805.4229, 806.4631, 807.3893, 809.4480, 811.4104, 812.3985, 816.4408, 820.4244, 822.3905,
823.3846, 824.3756, 828.3904, 834.4043, 836.4589, 837.3853, 839.3696, 840.3839, 842.5085, 845.4100, 845.4123, 851.3975, 852.4056,
856.4893, 866.3914, 867.3724, 870.5402, 871.4945, 878.4625, 880.4403, 886.3815, 888.4233, 890.4479, 893.9562, 894.4579, 897.0289,
897.4412, 902.4597, 910.4410, 912.4497, 913.5118, 916.4905, 918.5391, 925.4747, 928.5064, 930.5048, 933.4619, 935.4698, 939.4758,
947.4628, 949.4591, 951.4703, 954.4674, 957.4657, 963.4676, 965.4761, 967.4568, 969.4571, 972.4793, 972.4800, 975.4650, 977.4630,
978.4976, 981.4565, 983.4664, 985.4842, 991.4688, 993.5331, 997.4298, 999.4453, 1001.4488, 1003.4687, 1007.5668, 1018.5200,
1018.5212, 1023.4777, 1026.4922, 1027.4972, 1030.5151, 1031.3850, 1033.5419, 1035.5352, 1036.5372, 1039.5402, 1042.5383, 1056.5488,
1060.5400, 1060.5442, 1065.5425, 1086.5115, 1106.5372, 1111.5582, 1113.5546, 1114.5396, 1115.5605, 1118.5529, 1122.5363, 1128.5775,
1129.6014, 1130.5935, 1140.5485, 1144.5709, 1155.6113, 1156.5720, 1157.5952, 1158.6154, 1165.5817, 1172.6301, 1173.6259, 1179.6063,
1180.6165, 1182.6090, 1183.6123, 1185.6317, 1187.6431, 1193.6096, 1195.6060, 1196.6102, 1206.6770, 1209.1387, 1209.5848, 1210.1014,
1216.6494, 1222.5994, 1226.5948, 1229.6246, 1231.6174, 1234.6317, 1235.5846, 1240.6298, 1244.6324, 1248.6119, 1250.6144, 1268.6498,
1271.6696, 1273.6653, 1275.6644, 1291.6425, 1295.7233, 1300.6062, 1301.6205, 1307.6858, 1315.6570, 1317.6521, 1319.6852, 1323.6517,
1325.6637, 1328.6809, 1329.6672, 1331.6763, 1332.6743, 1334.6592, 1335.6937, 1336.6715, 1337.6724, 1338.6471, 1342.6680, 1345.6760,
1346.6880, 1349.6991, 1354.6907, 1355.7155, 1357.7034, 1360.6882, 1361.6670, 1365.6913, 1368.6920, 1374.6412, 1375.6798, 1376.6804,
1377.6794, 1382.6647, 1387.6444, 1410.7505, 1411.7208, 1427.7872, 1431.7961, 1432.7133, 1434.7288, 1446.7256, 1450.7079, 1473.8054,
1475.7748, 1477.7524, 1478.7439, 1485.7720, 1486.7664, 1493.7539, 1494.7423, 1505.8649, 1523.7841, 1524.7848, 1537.8021, 1549.7388,
1555.8816, 1558.8474, 1576.7987, 1579.7926, 1584.7897, 1588.8136, 1590.3611, 1604.8417, 1605.8098, 1615.8826, 1628.8260, 1629.8358,
1630.8466, 1633.9790, 1635.7943, 1644.8046, 1647.8785, 1649.8463, 1651.8245, 1659.8241, 1662.7909, 1668.7810, 1679.9736, 1681.8214,
1683.8121, 1706.8616, 1707.7838, 1716.8571, 1725.8353, 1728.8242, 1734.8152, 1740.8768, 1742.9031, 1746.1659, 1750.8356, 1751.8787,
1753.8948, 1756.8905, 1757.8322, 1763.8188, 1765.7690, 1767.8036, 1768.9027, 1769.4475, 1770.4261, 1774.9091, 1783.8499, 1786.9000,
1786.9017, 1791.7335, 1808.8868, 1824.8674, 1840.8225, 1844.8907, 1848.8322, 1850.8301, 1851.8488, 1867.9503, 1880.9277, 1887.9734,
1908.8838, 1914.9995, 1919.0038, 1925.9418, 1928.0103, 1931.9463, 1953.9353, 1975.9609, 1992.9642, 1995.9020, 2007.9894, 2011.8845,
2013.8983, 2024.8591, 2025.6234, 2036.5195, 2042.0640, 2047.0167, 2047.9691, 2051.9792, 2058.9963, 2060.0469, 2064.9900, 2068.8982,
2070.8835, 2074.8914, 2083.9907, 2084.8987, 2086.8828, 2092.8821, 2095.9941, 2098.9976, 2100.9160, 2102.8872, 2106.9011, 2108.8835,
2109.9175, 2114.9180, 2116.9160, 2118.8835, 2119.8833, 2125.0176, 2132.8694, 2136.9180, 2170.1467, 2211.1045, 2225.1191, 2273.0496,
2277.0137, 2307.9941, 2322.9980, 2341.0366, 2343.0271, 2352.0571, 2355.0459, 2359.0532, 2366.0813, 2368.0544, 2369.0710, 2382.0349,
2383.6099, 2383.9600, 2390.0403, 2396.0342, 2398.0576, 2400.0652, 2406.0737, 2411.0076, 2414.0813, 2416.0752, 2417.0732, 2420.0718,
2422.0408, 2423.0466, 2428.0898, 2430.0828, 2431.0845, 2433.0779, 2436.0508, 2438.0457, 2439.0261, 2442.0618, 2449.0347, 2452.0632,
2460.0254, 2461.9956, 2463.9858, 2510.1277, 2528.1580, 2542.1509, 2545.1558, 2547.1531, 2552.1038, 2565.1543, 2585.1111, 2601.0889,
2705.2192, 2717.0693, 3129.3176, 3208.1736, 3223.2710, 3290.4131, 3291.4248, 3312.2756, 3451.3933, 3453.4365

27. G3MYV8 **Mass:** 6868 **Score:** 18 **Expect:** 3.8e+002 **Matches:** 6

Uncharacterized protein OS=Bos taurus PE=4 SV=1

Observed	Mr(expt)	Mr(calc)	ppm	Start	End	Miss	Ions	Peptide
765.3908	764.3835	764.3851	-1.99	12	17	1	---	R.QKNMTK.Q + Oxidation (M)
1159.6273	1158.6200	1158.6105	8.22	27	37	2	---	R.RDDGLSAAARK.Q
1187.6431	1186.6358	1186.6167	16.2	26	36	2	---	K.RRDDGLSAAAR.K
1234.6317	1233.6244	1233.6023	17.9	40	49	1	---	R.DSEIMQQKQK.K
1250.6144	1249.6071	1249.5972	7.92	40	49	1	---	R.DSEIMQQKQK.K + Oxidation (M)
1338.6471	1337.6398	1337.6609	-15.76	14	25	2	---	K.NMTKQSDSVKGK.R + Oxidation (M)

No match to: 713.4334, 719.2388, 724.3009, 726.2899, 732.2586, 735.3865, 745.4124, 756.4687, 764.4056, 767.3679, 768.3909,
772.3955, 795.4186, 802.4548, 803.4370, 805.4229, 806.4631, 807.3893, 809.4480, 811.4104, 812.3985, 816.4408, 820.4244, 822.3905,
823.3846, 824.3756, 828.3904, 834.4043, 836.4589, 837.3853, 839.3696, 840.3839, 842.5085, 845.4100, 845.4123, 851.3975, 852.4056,
856.4893, 866.3914, 867.3724, 870.5402, 871.4945, 878.4625, 880.4403, 886.3815, 888.4233, 890.4479, 893.9562, 894.4579, 897.0289,
897.4412, 902.4597, 910.4410, 912.4497, 913.5118, 916.4905, 918.5391, 925.4747, 928.5064, 930.5048, 933.4619, 935.4698, 939.4758,
947.4628, 949.4591, 951.4703, 954.4674, 957.4657, 963.4676, 965.4761, 967.4568, 969.4571, 972.4793, 972.4800, 975.4650, 977.4630,
978.4976, 981.4565, 983.4664, 985.4842, 991.4688, 993.5331, 997.4298, 999.4453, 1001.4488, 1003.4687, 1007.5668, 1018.5200,
1018.5212, 1023.4777, 1026.4922, 1027.4972, 1030.5151, 1031.3850, 1033.5419, 1035.5352, 1036.5372, 1039.5402, 1042.5383, 1045.5654,
1056.5488, 1060.5400, 1060.5442, 1065.5425, 1086.5115, 1106.5372, 1111.5582, 1113.5546, 1114.5396, 1115.5605, 1118.5529, 1122.5363,
1128.5775, 1129.6014, 1130.5935, 1140.5485, 1144.5709, 1155.6113, 1156.5720, 1157.5952, 1158.6154, 1165.5817, 1172.6301, 1173.6259,
1179.6063, 1180.6165, 1182.6090, 1183.6123, 1185.6317, 1193.6096, 1195.6060, 1196.6102, 1206.6770, 1209.1387, 1209.5848, 1210.1014,
1215.6627, 1216.6494, 1222.5994, 1226.5948, 1229.6246, 1231.6174, 1235.5846, 1240.6298, 1244.6324, 1248.6119, 1268.6498, 1271.6696,
1273.6653, 1275.6644, 1291.6425, 1295.7233, 1300.6062, 1301.6205, 1307.6858, 1315.6570, 1317.6521, 1319.6852, 1323.6517, 1325.6637,
1328.6809, 1329.6672, 1331.6763, 1332.6743, 1334.6592, 1335.6937, 1336.6715, 1337.6724, 1342.6680, 1345.6760, 1346.6880, 1349.6991,
1354.6907, 1355.7155, 1357.7034, 1360.6882, 1361.6670, 1365.6913, 1368.6920, 1374.6412, 1375.6798, 1376.6804, 1377.6794, 1382.6647,
1387.6444, 1410.7505, 1411.7208, 1421.7177, 1427.7872, 1431.7961, 1432.7133, 1434.7288, 1446.7256, 1450.7079, 1473.8054, 1475.7748,
1477.7524, 1478.7439, 1485.7720, 1486.7664, 1493.7539, 1494.7423, 1505.8649, 1523.7841, 1524.7848, 1537.8021, 1549.7388, 1555.8816,
1558.8474, 1576.7987, 1579.7926, 1584.7897, 1588.8136, 1590.3611, 1604.8417, 1605.8098, 1606.8384, 1615.8826, 1616.8605, 1622.8137,
1628.8260, 1629.8358, 1630.8466, 1633.9790, 1635.7943, 1644.8046, 1647.8785, 1649.8463, 1651.8245, 1659.8241, 1662.7909, 1668.7810,
1679.9736, 1681.8214, 1683.8121, 1684.8204, 1706.8616, 1707.7838, 1716.8571, 1725.8353, 1728.8242, 1734.8152, 1740.8768, 1742.9031,
1746.1659, 1750.8356, 1751.8787, 1753.8948, 1756.8905, 1757.8322, 1763.8188, 1765.7690, 1767.8036, 1768.9027, 1769.4475, 1770.4261,
1774.9091, 1783.8499, 1786.9000, 1786.9017, 1791.7335, 1808.8868, 1824.8674, 1840.8225, 1844.8907, 1848.8322, 1850.8301, 1851.8488,
1867.9503, 1880.9277, 1887.9734, 1908.8838, 1914.9995, 1919.0038, 1925.9418, 1928.0103, 1931.9463, 1953.9353, 1975.9609, 1992.9642,
1995.9020, 2007.9894, 2011.8845, 2013.8983, 2024.8591, 2025.6234, 2036.5195, 2036.9486, 2042.0640, 2047.0167, 2047.9691, 2051.9792,
2052.9924, 2058.9963, 2060.0469, 2064.9900, 2068.8982, 2070.8835, 2074.8914, 2083.9907, 2084.8987, 2086.8828, 2092.8821, 2095.9941,
2098.9976, 2100.9160, 2102.8872, 2106.9011, 2108.8835, 2109.9175, 2114.9180, 2116.9160, 2118.8835, 2119.8833, 2125.0176, 2132.8694,
2136.9180, 2170.1467, 2211.1045, 2225.1191, 2273.0496, 2277.0137, 2307.9941, 2322.9980, 2341.0366, 2343.0271, 2352.0571, 2355.0459,
2359.0532, 2366.0813, 2368.0544, 2369.0710, 2376.0264, 2382.0349, 2383.6099, 2383.9600, 2390.0403, 2396.0342, 2398.0576, 2400.0652,
2406.0737, 2411.0076, 2414.0813, 2416.0752, 2417.0732, 2420.0718, 2422.0408, 2423.0466, 2428.0898, 2430.0828, 2431.0845, 2433.0779,
2436.0508, 2438.0457, 2439.0261, 2442.0618, 2449.0347, 2452.0632, 2460.0254, 2461.9956, 2463.9858, 2510.1277, 2528.1580, 2542.1509,
2545.1558, 2547.1531, 2552.1038, 2565.1543, 2585.1111, 2601.0889, 2705.2192, 2717.0693, 3129.3176, 3208.1736, 3223.2710, 3290.4131,
3291.4248, 3312.2756, 3451.3933, 3453.4365

28. F1N3R9 **Mass:** 25138 **Score:** 18 **Expect:** 3.8e+002 **Matches:** 10

Uncharacterized protein OS=Bos taurus GN=TMCO5A PE=4 SV=1

Observed	Mr(expt)	Mr(calc)	ppm	Start	End	Miss	Ions	Peptide
806.4631	805.4558	805.4480	9.70	197	202	2	---	R.EVMKRK.R + Oxidation (M)
997.4298	996.4225	996.4182	4.28	75	82	0	---	K.ENTTTMER.E + Oxidation (M)
1295.7233	1294.7160	1294.6993	12.9	46	55	2	---	K.IHKKEDEIQR.L
1334.6592	1333.6519	1333.6296	16.8	20	30	0	---	K.NINSLNMDLER.D + Oxidation (M)
1446.7256	1445.7183	1445.7296	-7.81	19	30	1	---	K.KNINSLNMDLER.D
1549.7388	1548.7315	1548.7566	-16.18	118	130	2	---	K.SQKATKCEQDNLK.G
1558.8474	1557.8401	1557.8297	6.70	1	13	2	---	-.MQGPRSEKVEILR.L + Oxidation (M)
1848.8322	1847.8249	1847.8618	-19.94	20	34	1	---	K.NINSLNMDLERDMQR.I
1992.9642	1991.9569	1991.9516	2.65	19	34	2	---	K.KNINSLNMDLERDMQR.I + Oxidation (M)
2438.0457	2437.0384	2437.0914	-21.74	65	84	2	---	R.DLAEDEELEKENTTTMERER.A

No match to: 713.4334, 719.2388, 724.3009, 726.2899, 732.2586, 735.3865, 745.4124, 756.4687, 764.4056, 765.3908, 767.3679,
768.3909, 772.3955, 795.4186, 802.4548, 803.4370, 805.4229, 807.3893, 809.4480, 811.4104, 812.3985, 816.4408, 820.4244, 822.3905,
823.3846, 824.3756, 828.3904, 834.4043, 836.4589, 837.3853, 839.3696, 840.3839, 842.5085, 845.4100, 845.4123, 851.3975, 852.4056,
856.4893, 866.3914, 867.3724, 870.5402, 871.4945, 878.4625, 880.4403, 886.3815, 888.4233, 890.4479, 893.9562, 894.4579, 897.0289,
897.4412, 902.4597, 910.4410, 912.4497, 913.5118, 916.4905, 918.5391, 925.4747, 928.5064, 930.5048, 933.4619, 935.4698, 939.4758,
947.4628, 949.4591, 951.4703, 954.4674, 957.4657, 963.4676, 965.4761, 967.4568, 969.4571, 972.4793, 972.4800, 975.4650, 977.4630,

978.4976, 981.4565, 983.4664, 985.4842, 991.4688, 993.5331, 999.4453, 1001.4488, 1003.4687, 1007.5668, 1018.5200, 1018.5212, 1023.4777, 1026.4922, 1027.4972, 1030.5151, 1031.3850, 1033.5419, 1035.5352, 1036.5372, 1039.5402, 1042.5383, 1045.5654, 1056.5488, 1060.5400, 1060.5442, 1065.5425, 1086.5115, 1106.5372, 1111.5582, 1113.5546, 1114.5396, 1115.5605, 1118.5529, 1122.5363, 1128.5775, 1129.6014, 1130.5935, 1140.5485, 1144.5709, 1155.6113, 1156.5720, 1157.5952, 1158.6154, 1159.6273, 1165.5817, 1172.6301, 1173.6259, 1179.6063, 1180.6165, 1182.6090, 1183.6123, 1185.6317, 1187.6431, 1193.6096, 1195.6060, 1196.6102, 1206.6770, 1209.1387, 1209.5848, 1210.1014, 1215.6627, 1216.6494, 1222.5994, 1226.5948, 1229.6246, 1231.6174, 1234.6317, 1235.5846, 1240.6298, 1244.6324, 1248.6119, 1250.6144, 1268.6498, 1271.6696, 1273.6653, 1275.6644, 1291.6425, 1300.6062, 1301.6205, 1307.6858, 1315.6570, 1317.6521, 1319.6852, 1323.6517, 1325.6637, 1328.6809, 1329.6672, 1331.6763, 1332.6743, 1335.6937, 1336.6715, 1337.6724, 1338.6471, 1342.6680, 1345.6760, 1346.6880, 1349.6991, 1354.6907, 1355.7155, 1357.7034, 1360.6882, 1361.6670, 1365.6913, 1368.6920, 1374.6412, 1375.6798, 1376.6804, 1377.6794, 1382.6647, 1387.6444, 1410.7505, 1411.7208, 1421.7177, 1427.7872, 1431.7961, 1432.7133, 1434.7288, 1450.7079, 1473.8054, 1475.7748, 1477.7524, 1478.7439, 1485.7720, 1486.7664, 1493.7539, 1494.7423, 1505.8649, 1523.7841, 1524.7848, 1537.8021, 1555.8816, 1576.7987, 1579.7926, 1584.7897, 1588.8136, 1590.3611, 1604.8417, 1605.8098, 1606.8384, 1615.8826, 1616.8605, 1622.8137, 1628.8260, 1629.8358, 1630.8466, 1633.9790, 1635.7943, 1644.8046, 1647.8785, 1649.8463, 1651.8245, 1659.8241, 1662.7909, 1668.7810, 1679.9736, 1681.8214, 1683.8121, 1684.8204, 1706.8616, 1707.7838, 1716.8571, 1725.8353, 1728.8242, 1734.8152, 1740.8768, 1742.9031, 1746.1659, 1750.8356, 1751.8787, 1753.8948, 1756.8905, 1757.8322, 1763.8188, 1765.7690, 1767.8036, 1768.9027, 1769.4475, 1770.4261, 1774.9091, 1783.8499, 1786.9000, 1786.9017, 1791.7335, 1808.8868, 1824.8674, 1840.8225, 1844.8907, 1850.8301, 1851.8488, 1867.9503, 1880.9277, 1887.9734, 1908.8838, 1914.9995, 1919.0038, 1925.9418, 1928.0103, 1931.9463, 1953.9353, 1975.9609, 1995.9020, 2007.9894, 2011.8845, 2013.8983, 2024.8591, 2025.6234, 2036.5195, 2036.9486, 2042.0640, 2047.0167, 2047.9691, 2051.9792, 2052.9924, 2058.9963, 2060.0469, 2064.9900, 2068.8982, 2070.8835, 2074.8914, 2083.9907, 2084.8987, 2086.8828, 2092.8821, 2095.9941, 2098.9976, 2100.9160, 2102.8872, 2106.9011, 2108.8835, 2109.9175, 2114.9180, 2116.9160, 2118.8835, 2119.8833, 2125.0176, 2132.8694, 2136.9180, 2170.1467, 2211.1045, 2225.1191, 2273.0496, 2277.0137, 2307.9941, 2322.9980, 2341.0366, 2343.0271, 2352.0571, 2355.0459, 2359.0532, 2366.0813, 2368.0544, 2369.0710, 2376.0264, 2382.0349, 2383.6099, 2383.9600, 2390.0403, 2396.0342, 2398.0576, 2400.0652, 2406.0737, 2411.0076, 2414.0813, 2416.0752, 2417.0732, 2420.0718, 2422.0408, 2423.0466, 2428.0898, 2430.0828, 2431.0845, 2433.0779, 2436.0508, 2439.0261, 2442.0618, 2449.0347, 2452.0632, 2460.0254, 2461.9956, 2463.9858, 2510.1277, 2528.1580, 2542.1509, 2545.1558, 2547.1531, 2552.1038, 2565.1543, 2585.1111, 2601.0889, 2705.2192, 2717.0693, 3129.3176, 3208.1736, 3223.2710, 3290.4131, 3291.4248, 3312.2756, 3451.3933, 3453.4365

29. F1MN93 **Mass:** 91564 **Score:** 18 **Expect:** 3.8e+002 **Matches:** 29

Uncharacterized protein OS=Bos taurus GN=TOP1 PE=4 SV=1

Observed	Mr(expt)	Mr(calc)	ppm	Start	End	Miss	Ions	Peptide
735.3865	734.3792	734.3931	-18.95	374	379	1	---	K.MGMLKR.R
795.4186	794.4114	794.4035	9.93	448	453	1	---	K.YETARR.L
803.4370	802.4297	802.4297	0.06	315	321	1	---	K.AQTEARK.Q
806.4631	805.4558	805.4558	-0.06	35	40	2	---	R.HKEHKK.D
842.5085	841.5012	841.4909	12.3	133	139	0	---	K.EDIKPLK.R
890.4479	889.4407	889.4294	12.7	441	447	1	---	K.GEKDWQK.Y
897.4412	896.4339	896.4464	-13.97	407	413	1	---	K.EVRHDNK.V
918.5391	917.5318	917.5117	21.9	456	462	2	---	K.KCVDKIR.N
928.5064	927.4991	927.5178	-20.18	359	366	0	---	K.IEPPGLFR.G
930.5048	929.4975	929.4930	4.81	659	666	1	---	K.KEQLADAR.R
978.4976	977.4903	977.5043	-14.29	18	25	1	---	R.LNDSHKHK.D
1128.5775	1127.5702	1127.5580	10.9	369	378	1	---	R.GNHPKMGMLK.R + Oxidation (M)
1144.5709	1143.5636	1143.5529	9.40	369	378	1	---	R.GNHPKMGMLK.R + 2 Oxidation (M)
1231.6174	1230.6101	1230.6357	-20.76	109	119	1	---	K.ENGFSSPPRIK.D
1268.6498	1267.6425	1267.6641	-17.05	369	379	2	---	R.GNHPKMGMLKR.R
1295.7233	1294.7160	1294.7033	9.80	541	550	2	---	R.YYNKVPVEKR.V
1325.6637	1324.6564	1324.6856	-22.03	367	378	2	---	R.GRGNHPKMGMLK.R
1355.7155	1354.7082	1354.6915	12.4	580	591	0	---	K.HLQDLMEGLTAK.V
1357.7034	1356.6961	1356.6754	15.2	367	378	2	---	R.GRGNHPKMGMLK.R + 2 Oxidation (M)
1365.6913	1364.6840	1364.6792	3.56	647	658	1	---	K.SMMNLQSKIDAK.K
1375.6798	1374.6725	1374.6891	-12.09	106	117	2	---	K.KEKENGFSSPPR.I
1432.7133	1431.7060	1431.7101	-2.88	381	392	0	---	R.IMPEDIIINCSK.D
1493.7539	1492.7466	1492.7741	-18.42	647	659	2	---	K.SMMNLQSKIDAKK.E
1537.8021	1536.7948	1536.8188	-15.58	253	266	1	---	K.LSPKAEEVATFFAK.M
1588.8136	1587.8063	1587.8112	-3.10	380	392	1	---	R.RIMPEDIIINCSK.D
1604.8417	1603.8344	1603.8062	17.6	380	392	1	---	R.RIMPEDIIINCSK.D + Oxidation (M)
1753.8948	1752.8875	1752.9087	-12.06	228	243	0	---	K.GPVFAPPYEPLPENVK.F
1840.8225	1839.8152	1839.8136	0.88	2	17	0	---	M.SGDHLHNDSQIEADFR.L
1919.0038	1917.9965	1917.9652	16.3	380	395	2	---	R.RIMPEDIIINCSKDAK.V + Oxidation (M)

No match to: 713.4334, 719.2388, 724.3009, 726.2899, 732.2586, 745.4124, 756.4687, 764.4056, 765.3908, 767.3679, 768.3909, 772.3955, 802.4548, 805.4229, 807.3893, 809.4480, 811.4104, 812.3985, 816.4408, 820.4244, 822.3905, 823.3846, 824.3756, 828.3904, 834.4043, 836.4589, 837.3853, 839.3696, 840.3839, 845.4100, 845.4123, 851.3975, 852.4056, 856.4893, 866.3914, 867.3724, 870.5402, 871.4945, 878.4625, 880.4403, 886.3815, 888.4233, 893.9562, 894.4579, 897.0289, 902.4597, 910.4410, 912.4497, 913.5118, 916.4905, 925.4747, 933.4619, 935.4698, 939.4758, 947.4628, 949.4591, 951.4703, 954.4674, 957.4657, 963.4676, 965.4761, 967.4568, 969.4571, 972.4793, 972.4800, 975.4650, 977.4630, 981.4565, 983.4664, 985.4842, 991.4688, 993.5331, 997.4298, 999.4453, 1001.4488, 1003.4687, 1007.5668, 1018.5200, 1018.5212, 1023.4777, 1026.4922, 1027.4972, 1030.5151, 1031.3850, 1033.5419, 1035.5352, 1036.5372, 1039.5402, 1042.5383, 1045.5654, 1056.5488, 1060.5400, 1060.5442, 1065.5425, 1086.5115, 1106.5372, 1111.5582, 1113.5546, 1114.5396, 1115.5605, 1118.5529, 1122.5363, 1129.6014, 1130.5935, 1140.5485, 1155.6113, 1156.5720, 1157.5952, 1158.6154, 1159.6273, 1165.5817, 1172.6301, 1173.6259, 1179.6063, 1180.6165, 1182.6090, 1183.6123, 1185.6317, 1187.6431, 1193.6096, 1195.6060, 1196.6102, 1206.6770, 1209.1387, 1209.5848, 1210.1014, 1215.6627, 1216.6494, 1222.5994, 1226.5948, 1229.6246, 1234.6317, 1235.5846, 1240.6298, 1244.6324, 1248.6119, 1250.6144, 1271.6696, 1273.6653, 1275.6644, 1291.6425, 1300.6062, 1301.6205, 1307.6858, 1315.6570, 1317.6521, 1319.6852, 1323.6517, 1328.6809, 1329.6672, 1331.6763, 1332.6743, 1334.6592, 1335.6937, 1336.6715, 1337.6724, 1338.6471, 1342.6680, 1345.6760, 1346.6880, 1349.6991, 1354.6907, 1360.6882, 1361.6670, 1368.6920, 1374.6412, 1376.6804, 1377.6794, 1382.6647, 1387.6444, 1410.7505, 1411.7208, 1421.7177, 1427.7872, 1431.7961, 1434.7288, 1446.7256, 1450.7079, 1473.8054, 1475.7748, 1477.7524, 1478.7439, 1485.7720, 1486.7664, 1494.7423, 1505.8649, 1523.7841, 1524.7848, 1549.7388, 1555.8816, 1558.8474, 1576.7987, 1579.7926, 1584.7897, 1590.3611, 1605.8098, 1606.8384, 1615.8826, 1616.8605, 1622.8137, 1628.8260, 1629.8358, 1630.8466, 1633.9790, 1635.7943, 1644.8046, 1647.8785, 1649.8463, 1651.8245, 1659.8241, 1662.7909, 1668.7810, 1679.9736, 1681.8214, 1683.8121, 1684.8204, 1706.8616, 1707.7838, 1716.8571, 1725.8353, 1728.8242, 1734.8152, 1740.8768, 1742.9031, 1746.1659, 1750.8356, 1751.8787, 1756.8905, 1757.8322, 1763.8188, 1765.7690, 1767.8036, 1768.9027, 1769.4475, 1770.4261, 1774.9091, 1783.8499, 1786.9000, 1786.9017, 1791.7335, 1808.8868, 1824.8674, 1844.8907, 1848.8322, 1850.8301, 1851.8488, 1867.9503, 1880.9277, 1887.9734, 1908.8838, 1914.9995, 1925.9418, 1928.0103, 1931.9463, 1953.9353, 1975.9609, 1992.9642, 1995.9020, 2007.9894, 2011.8845, 2013.8983, 2024.8591, 2025.6234, 2036.5195, 2036.9486, 2042.0640, 2047.0167, 2047.9691, 2051.9792, 2052.9924, 2058.9963, 2060.0469, 2064.9900, 2068.8982, 2070.8835, 2074.8914, 2083.9907, 2084.8987, 2086.8828, 2092.8821, 2095.9941, 2098.9976, 2100.9160, 2102.8872, 2106.9011, 2108.8835, 2109.9175, 2114.9180, 2116.9160, 2118.8835, 2119.8833, 2125.0176, 2132.8694, 2136.9180, 2170.1467, 2211.1045, 2225.1191, 2273.0496, 2277.0137, 2307.9941, 2322.9980, 2341.0366, 2343.0271, 2352.0571, 2355.0459, 2359.0532, 2366.0813, 2368.0544, 2369.0710, 2376.0264, 2382.0349, 2383.6099, 2383.9600, 2390.0403, 2396.0342, 2398.0576, 2400.0652, 2406.0737, 2411.0076, 2414.0813, 2416.0752, 2417.0732, 2420.0718, 2422.0408, 2423.0466, 2428.0898, 2430.0828, 2431.0845, 2433.0779, 2436.0508, 2438.0457, 2439.0261, 2442.0618, 2449.0347, 2452.0632, 2460.0254, 2461.9956, 2463.9858, 2510.1277, 2528.1580, 2542.1509, 2545.1558, 2547.1531, 2552.1038, 2565.1543, 2585.1111, 2601.0889, 2705.2192, 2717.0693, 3129.3176, 3208.1736, 3223.2710, 3290.4131, 3291.4248, 3312.2756, 3451.3933, 3453.4365

30. Q3SX42 **Mass:** 23992 **Score:** 18 **Expect:** 3.9e+002 **Matches:** 10

Charged multivesicular body protein 2b OS=Bos taurus GN=CHMP2B PE=2 SV=1

Observed	Mr(expt)	Mr(calc)	ppm	Start	End	Miss	Ions	Peptide
802.4548	801.4475	801.4344	16.3	31	37	1	---	R.DRAALEK.Q

807.3893	806.3820	806.3956	-16.90	108	114	0	---	K.TMQAVNK.K + Oxidation (M)
837.3853	836.3780	836.3884	-12.43	91	97	0	---	K.VMNSQMK.M
916.4905	915.4832	915.5025	-21.08	33	40	1	---	R.AALEKQEK.Q
947.4628	946.4556	946.4654	-10.43	52	59	1	---	K.IGNKEACR.V
1187.6431	1186.6358	1186.6306	4.44	31	40	2	---	R.DRAALEKQEK.Q
1740.8768	1739.8695	1739.8368	18.8	98	114	1	---	K.MAGAMSTTAKTMQAVNK.K
1848.8322	1847.8249	1847.8427	-9.64	82	97	1	---	K.VTSMSTQTKVMNSQMK.M + 3 Oxidation (M)
1850.8301	1849.8228	1849.8042	10.1	91	107	1	---	K.VMNSQMKMAGAMSTTAK.T + 4 Oxidation (M)
2036.9486	2035.9413	2035.9909	-24.36	187	205	1	---	R.SLPSASTSKSTISDEEIER.Q

No match to: 713.4334, 719.2388, 724.3009, 726.2899, 732.2586, 735.3865, 745.4124, 756.4687, 764.4056, 765.3908, 767.3679, 768.3909, 772.3955, 795.4186, 803.4370, 805.4229, 806.4631, 809.4480, 811.4104, 812.3985, 816.4408, 820.4244, 822.3905, 823.3846, 824.3756, 828.3904, 834.4043, 836.4589, 839.3696, 840.3839, 842.5085, 845.4100, 845.4123, 851.3975, 852.4056, 856.4893, 866.3914, 867.3724, 870.5402, 871.4945, 878.4625, 880.4403, 886.3815, 888.4233, 890.4479, 893.9562, 894.4579, 897.0289, 897.4412, 902.4597, 910.4410, 912.4497, 913.5118, 918.5391, 925.4747, 928.5064, 930.5048, 933.4619, 935.4698, 939.4758, 949.4591, 951.4703, 954.4674, 957.4657, 963.4676, 965.4761, 967.4568, 969.4571, 972.4793, 972.4800, 975.4650, 977.4630, 978.4976, 981.4565, 983.4664, 985.4842, 991.4688, 993.5331, 997.4298, 999.4453, 1001.4488, 1003.4687, 1007.5668, 1018.5200, 1018.5212, 1023.4777, 1026.4922, 1027.4972, 1030.5151, 1031.3850, 1033.5419, 1035.5352, 1036.5372, 1039.5402, 1042.5383, 1045.5654, 1056.5488, 1060.5400, 1060.5442, 1065.5425, 1086.5115, 1106.5372, 1111.5582, 1113.5546, 1114.5396, 1115.5605, 1118.5529, 1122.5363, 1128.5775, 1129.6014, 1130.5935, 1140.5485, 1144.5709, 1155.6113, 1156.5720, 1157.5952, 1158.6154, 1159.6273, 1165.5817, 1172.6301, 1173.6259, 1179.6063, 1180.6165, 1182.6090, 1183.6123, 1185.6317, 1193.6096, 1195.6060, 1196.6102, 1206.6770, 1209.1387, 1209.5848, 1210.1014, 1215.6627, 1216.6494, 1222.5994, 1226.5948, 1229.6246, 1231.6174, 1234.6317, 1235.5846, 1240.6298, 1244.6324, 1248.6119, 1250.6144, 1268.6498, 1271.6696, 1273.6653, 1275.6644, 1291.6425, 1295.7233, 1300.6062, 1301.6205, 1307.6858, 1315.6570, 1317.6521, 1319.6852, 1323.6517, 1325.6637, 1328.6809, 1329.6672, 1331.6763, 1332.6743, 1334.6592, 1335.6937, 1336.6715, 1337.6724, 1338.6471, 1342.6680, 1345.6760, 1346.6880, 1349.6991, 1354.6907, 1355.7155, 1357.7034, 1360.6882, 1361.6670, 1365.6913, 1368.6920, 1374.6412, 1375.6798, 1376.6804, 1377.6794, 1382.6647, 1387.6444, 1410.7505, 1411.7208, 1421.7177, 1427.7872, 1431.7961, 1432.7133, 1434.7288, 1446.7256, 1450.7079, 1473.8054, 1475.7748, 1477.7524, 1478.7439, 1485.7720, 1486.7664, 1493.7539, 1494.7423, 1505.8649, 1523.7841, 1524.7848, 1537.8021, 1549.7388, 1555.8816, 1558.8474, 1576.7987, 1579.7926, 1584.7897, 1588.8136, 1590.3611, 1604.8417, 1605.8098, 1606.8384, 1615.8826, 1616.8605, 1622.8137, 1628.8260, 1629.8358, 1630.8466, 1633.9790, 1635.7943, 1644.8046, 1647.8785, 1649.8463, 1651.8245, 1659.8241, 1662.7909, 1668.7810, 1679.9736, 1681.8214, 1683.8121, 1684.8204, 1706.8616, 1707.7838, 1716.8571, 1725.8353, 1728.8242, 1734.8152, 1742.9031, 1746.1659, 1750.8356, 1751.8787, 1753.8948, 1756.8905, 1757.8322, 1763.8188, 1765.7690, 1767.8036, 1768.9027, 1769.4475, 1770.4261, 1774.9091, 1783.8499, 1786.9000, 1786.9017, 1791.7335, 1808.8868, 1824.8674, 1840.8225, 1844.8907, 1851.8488, 1867.9503, 1880.9277, 1887.9734, 1908.8838, 1914.9995, 1919.0038, 1925.9418, 1928.0103, 1931.9463, 1953.9353, 1975.9609, 1992.9642, 1995.9020, 2007.9894, 2011.8845, 2013.8983, 2024.8591, 2025.6234, 2036.5195, 2042.0640, 2047.0167, 2047.9691, 2051.9792, 2052.9924, 2058.9963, 2060.0469, 2064.9900, 2068.8982, 2070.8835, 2074.8914, 2083.9907, 2084.8987, 2086.8828, 2092.8821, 2095.9941, 2098.9976, 2100.9160, 2102.8872, 2106.9011, 2108.8835, 2109.9175, 2114.9180, 2116.9160, 2118.8835, 2119.8833, 2125.0176, 2132.8694, 2136.9180, 2170.1467, 2211.1045, 2225.1191, 2273.0496, 2277.0137, 2307.9941, 2322.9980, 2341.0366, 2343.0271, 2352.0571, 2355.0459, 2359.0532, 2366.0813, 2368.0544, 2369.0710, 2376.0264, 2382.0349, 2383.6099, 2383.9600, 2390.0403, 2396.0342, 2398.0576, 2400.0652, 2406.0737, 2411.0076, 2414.0813, 2416.0752, 2417.0732, 2420.0718, 2422.0408, 2423.0466, 2428.0898, 2430.0828, 2431.0845, 2433.0779, 2436.0508, 2438.0457, 2439.0261, 2442.0618, 2449.0347, 2452.0632, 2460.0254, 2461.9956, 2463.9858, 2510.1277, 2528.1580, 2542.1509, 2545.1558, 2547.1531, 2552.1038, 2565.1543, 2585.1111, 2601.0889, 2705.2192, 2717.0693, 3129.3176, 3208.1736, 3223.2710, 3290.4131, 3291.4248, 3312.2756, 3451.3933, 3453.4365

31. F1MGT0 **Mass:** 24053 **Score:** 18 **Expect:** 3.9e+002 **Matches:** 10

Meiotic nuclear division protein 1 homolog OS=Bos taurus GN=MND1 PE=4 SV=1

Observed	Mr(expt)	Mr(calc)	ppm	Start	End	Miss	Ions	Peptide
806.4631	805.4558	805.4368	23.6	41	48	0	---	K.GITAMSVK.E
894.4579	893.4507	893.4606	-11.16	139	145	1	---	K.AEVEKYR.E
916.4905	915.4832	915.4774	6.37	132	138	1	---	R.DQREQLK.A
1226.5948	1225.5875	1225.5979	-8.45	184	193	1	---	K.FGFEENKIDK.N
1268.6498	1267.6425	1267.6118	24.2	25	34	1	---	K.DVFQLKDMEK.I + Oxidation (M)
1354.6907	1353.6834	1353.6928	-6.96	183	193	2	---	R.KFGFEENKIDK.N
1579.7926	1578.7853	1578.8194	-21.61	169	181	1	---	R.WTDNIFAIKSWAK.R
1647.8785	1646.8712	1646.8774	-3.72	121	134	2	---	R.TMLAKELSSLRDQR.E
1953.9353	1952.9280	1952.9264	0.85	16	30	1	---	R.MMEIFYETKDVFQLK.D + 2 Oxidation (M)
2211.1045	2210.0972	2210.0752	9.99	14	30	2	---	R.TRMMEIFYETKDVFQLK.D + 2 Oxidation (M)

No match to: 713.4334, 719.2388, 724.3009, 726.2899, 732.2586, 735.3865, 745.4124, 756.4687, 764.4056, 765.3908, 767.3679, 768.3909, 772.3955, 795.4186, 802.4548, 803.4370, 805.4229, 807.3893, 809.4480, 811.4104, 812.3985, 816.4408, 820.4244, 822.3905, 823.3846, 824.3756, 828.3904, 834.4043, 836.4589, 837.3853, 839.3696, 840.3839, 842.5085, 845.4100, 845.4123, 851.3975, 852.4056, 856.4893, 866.3914, 867.3724, 870.5402, 871.4945, 878.4625, 880.4403, 886.3815, 888.4233, 890.4479, 893.9562, 897.0289, 897.4412, 902.4597, 910.4410, 912.4497, 913.5118, 918.5391, 925.4747, 928.5064, 930.5048, 933.4619, 935.4698, 939.4758, 947.4628, 949.4591, 951.4703, 954.4674, 957.4657, 963.4676, 965.4761, 967.4568, 969.4571, 972.4793, 972.4800, 975.4650, 977.4630, 978.4976, 981.4565, 983.4664, 985.4842, 991.4688, 993.5331, 997.4298, 999.4453, 1001.4488, 1003.4687, 1007.5668, 1018.5200, 1018.5212, 1023.4777, 1026.4922, 1027.4972, 1030.5151, 1031.3850, 1033.5419, 1035.5352, 1036.5372, 1039.5402, 1042.5383, 1045.5654, 1056.5488, 1060.5400, 1060.5442, 1065.5425, 1086.5115, 1106.5372, 1111.5582, 1113.5546, 1114.5396, 1115.5605, 1118.5529, 1122.5363, 1128.5775, 1129.6014, 1130.5935, 1140.5485, 1144.5709, 1155.6113, 1156.5720, 1157.5952, 1158.6154, 1159.6273, 1165.5817, 1172.6301, 1173.6259, 1179.6063, 1180.6165, 1182.6090, 1183.6123, 1185.6317, 1187.6431, 1193.6096, 1195.6060, 1196.6102, 1206.6770, 1209.1387, 1209.5848, 1210.1014, 1215.6627, 1216.6494, 1222.5994, 1229.6246, 1231.6174, 1234.6317, 1235.5846, 1240.6298, 1244.6324, 1248.6119, 1250.6144, 1271.6696, 1273.6653, 1275.6644, 1291.6425, 1295.7233, 1300.6062, 1301.6205, 1307.6858, 1315.6570, 1317.6521, 1319.6852, 1323.6517, 1325.6637, 1328.6809, 1329.6672, 1331.6763, 1332.6743, 1334.6592, 1335.6937, 1336.6715, 1337.6724, 1338.6471, 1342.6680, 1345.6760, 1346.6880, 1349.6991, 1355.7155, 1357.7034, 1360.6882, 1361.6670, 1365.6913, 1368.6920, 1374.6412, 1375.6798, 1376.6804, 1377.6794, 1382.6647, 1387.6444, 1410.7505, 1411.7208, 1421.7177, 1427.7872, 1431.7961, 1432.7133, 1434.7288, 1446.7256, 1450.7079, 1473.8054, 1475.7748, 1477.7524, 1478.7439, 1485.7720, 1486.7664, 1493.7539, 1494.7423, 1505.8649, 1523.7841, 1524.7848, 1537.8021, 1549.7388, 1555.8816, 1558.8474, 1576.7987, 1584.7897, 1588.8136, 1590.3611, 1604.8417, 1605.8098, 1606.8384, 1615.8826, 1616.8605, 1622.8137, 1628.8260, 1629.8358, 1630.8466, 1633.9790, 1635.7943, 1644.8046, 1649.8463, 1651.8245, 1659.8241, 1662.7909, 1668.7810, 1679.9736, 1681.8214, 1683.8121, 1684.8204, 1706.8616, 1707.7838, 1716.8571, 1725.8353, 1728.8242, 1734.8152, 1740.8768, 1742.9031, 1746.1659, 1750.8356, 1751.8787, 1753.8948, 1756.8905, 1757.8322, 1763.8188, 1765.7690, 1767.8036, 1768.9027, 1769.4475, 1770.4261, 1774.9091, 1783.8499, 1786.9000, 1786.9017, 1791.7335, 1808.8868, 1824.8674, 1840.8225, 1844.8907, 1848.8322, 1850.8301, 1851.8488, 1867.9503, 1880.9277, 1887.9734, 1908.8838, 1914.9995, 1919.0038, 1925.9418, 1928.0103, 1931.9463, 1975.9609, 1992.9642, 1995.9020, 2007.9894, 2011.8845, 2013.8983, 2024.8591, 2025.6234, 2036.5195, 2036.9486, 2042.0640, 2047.0167, 2047.9691, 2051.9792, 2052.9924, 2058.9963, 2060.0469, 2064.9900, 2068.8982, 2070.8835, 2074.8914, 2083.9907, 2084.8987, 2086.8828, 2092.8821, 2095.9941, 2098.9976, 2100.9160, 2102.8872, 2106.9011, 2108.8835, 2109.9175, 2114.9180, 2116.9160, 2118.8835, 2119.8833, 2125.0176, 2132.8694, 2136.9180, 2170.1467, 2225.1191, 2273.0496, 2277.0137, 2307.9941, 2322.9980, 2341.0366, 2343.0271, 2352.0571, 2355.0459, 2359.0532, 2366.0813, 2368.0544, 2369.0710, 2376.0264, 2382.0349, 2383.6099, 2383.9600, 2390.0403, 2396.0342, 2398.0576, 2400.0652, 2406.0737, 2411.0076, 2414.0813, 2416.0752, 2417.0732, 2420.0718, 2422.0408, 2423.0466, 2428.0898, 2430.0828, 2431.0845, 2433.0779, 2436.0508, 2438.0457, 2439.0261, 2442.0618, 2449.0347, 2452.0632, 2460.0254, 2461.9956, 2463.9858, 2510.1277, 2528.1580, 2542.1509, 2545.1558, 2547.1531, 2552.1038, 2565.1543, 2585.1111, 2601.0889, 2705.2192, 2717.0693, 3129.3176, 3208.1736, 3223.2710, 3290.4131, 3291.4248, 3312.2756, 3451.3933, 3453.4365

32. Q32L19 **Mass:** 24083 **Score:** 18 **Expect:** 3.9e+002 **Matches:** 10

Meiotic nuclear division protein 1 homolog OS=Bos taurus GN=MND1 PE=2 SV=1

Observed	Mr(expt)	Mr(calc)	ppm	Start	End	Miss	Ions	Peptide
836.4589	835.4516	835.4473	5.08	41	48	0	---	K.GITTMSVK.E
894.4579	893.4507	893.4606	-11.16	139	145	1	---	K.AEVEKYR.E
916.4905	915.4832	915.4774	6.37	132	138	1	---	R.DQREQLK.A
1226.5948	1225.5875	1225.5979	-8.45	184	193	1	---	K.FGFEENKIDK.N
1268.6498	1267.6425	1267.6118	24.2	25	34	1	---	K.DVFQLKDMEK.I + Oxidation (M)

1354.6907	1353.6834	1353.6928	-6.96	183 - 193	2	---	R.KFGFEENKIDK.N
1579.7926	1578.7853	1578.8194	-21.61	169 - 181	1	---	R.WTDNIFAIKSWAK.R
1647.8785	1646.8712	1646.8774	-3.72	121 - 134	2	---	R.TMLAKELSSLRDQR.E
1953.9353	1952.9280	1952.9264	0.85	16 - 30	1	---	R.MMEIFYETKDVFQLK.D + 2 Oxidation (M)
2211.1045	2210.0972	2210.0752	9.99	14 - 30	2	---	R.TRMMEIFYETKDVFQLK.D + 2 Oxidation (M)

No match to: 713.4334, 719.2388, 724.3009, 726.2899, 732.2586, 735.3865, 745.4124, 756.4687, 764.4056, 765.3908, 767.3679, 768.3909, 772.3955, 795.4186, 802.4548, 803.4370, 805.4229, 806.4631, 807.3893, 809.4480, 811.4104, 812.3985, 816.4408, 820.4244, 822.3905, 823.3846, 824.3756, 828.3904, 834.4043, 837.3853, 839.3696, 840.3839, 842.5085, 845.4100, 845.4123, 851.3975, 852.4056, 856.4893, 866.3914, 867.3724, 870.5402, 871.4945, 878.4625, 880.4403, 886.3815, 888.4233, 890.4479, 893.9562, 897.0289, 897.4412, 902.4597, 910.4410, 912.4497, 913.5118, 918.5391, 925.4747, 928.5064, 930.5048, 933.4619, 935.4698, 939.4758, 947.4628, 949.4591, 951.4703, 954.4674, 957.4657, 963.4676, 965.4761, 967.4568, 969.4571, 972.4793, 972.4800, 975.4650, 977.4630, 978.4976, 981.4565, 983.4664, 985.4842, 991.4688, 993.5331, 997.4298, 999.4453, 1001.4488, 1003.4687, 1007.5668, 1018.5200, 1018.5212, 1023.4777, 1026.4922, 1027.4972, 1030.5151, 1031.3850, 1033.5419, 1035.5352, 1036.5372, 1039.5402, 1042.5383, 1045.5654, 1056.5488, 1060.5400, 1060.5442, 1065.5425, 1086.5115, 1106.5372, 1111.5582, 1113.5546, 1114.5396, 1115.5605, 1118.5529, 1122.5363, 1128.5775, 1129.6014, 1130.5935, 1140.5485, 1144.5709, 1155.6113, 1156.5720, 1157.5952, 1158.6154, 1159.6273, 1165.5817, 1172.6301, 1173.6259, 1179.6063, 1180.6165, 1182.6090, 1183.6123, 1185.6317, 1187.6431, 1193.6096, 1195.6060, 1196.6102, 1206.6770, 1209.1387, 1209.5848, 1210.1014, 1215.6627, 1216.6494, 1222.5994, 1229.6246, 1231.6174, 1234.6317, 1235.5846, 1240.6298, 1244.6324, 1248.6119, 1250.6144, 1271.6696, 1273.6653, 1275.6644, 1291.6425, 1295.7233, 1300.6062, 1301.6205, 1307.6858, 1315.6570, 1317.6521, 1319.6852, 1323.6517, 1325.6637, 1328.6809, 1329.6672, 1331.6763, 1332.6743, 1334.6592, 1335.6937, 1336.6715, 1337.6724, 1338.6471, 1342.6680, 1345.6760, 1346.6880, 1349.6991, 1355.7155, 1357.7034, 1360.6882, 1361.6670, 1365.6913, 1368.6920, 1374.6412, 1375.6798, 1376.6804, 1377.6794, 1382.6647, 1387.6444, 1410.7505, 1411.7208, 1421.7177, 1427.7872, 1431.7961, 1432.7133, 1434.7288, 1446.7256, 1450.7079, 1473.8054, 1475.7748, 1477.7524, 1478.7439, 1485.7720, 1486.7664, 1493.7539, 1494.7423, 1505.8649, 1523.7841, 1524.7848, 1537.8021, 1549.7388, 1555.8816, 1558.8474, 1576.7987, 1584.7897, 1588.8136, 1590.3611, 1604.8417, 1605.8098, 1606.8384, 1615.8826, 1616.8605, 1622.8137, 1628.8260, 1629.8358, 1630.8466, 1633.9790, 1635.7943, 1644.8046, 1649.8463, 1651.8245, 1659.8241, 1662.7909, 1668.7810, 1679.9736, 1681.8214, 1683.8121, 1684.8204, 1706.8616, 1707.7838, 1716.8571, 1725.8353, 1728.8242, 1734.8152, 1740.8768, 1746.1659, 1750.8356, 1751.8787, 1753.8948, 1756.8905, 1757.8322, 1763.8188, 1765.7690, 1767.8036, 1768.9027, 1769.4475, 1770.4261, 1774.9091, 1783.8499, 1786.9000, 1786.9017, 1791.7335, 1808.8868, 1824.8674, 1840.8225, 1844.8907, 1848.8322, 1850.8301, 1851.8488, 1867.9503, 1880.9277, 1887.9734, 1908.8838, 1914.9995, 1919.0038, 1925.9418, 1928.0103, 1931.9463, 1975.9609, 1992.9642, 1995.9020, 2007.9894, 2011.8845, 2013.8983, 2024.8591, 2025.6234, 2036.5195, 2036.9486, 2042.0640, 2047.0167, 2047.9691, 2051.9792, 2052.9924, 2058.9963, 2060.0469, 2064.9900, 2068.8982, 2070.8835, 2074.8914, 2083.9907, 2084.8987, 2086.8828, 2092.8821, 2095.9941, 2098.9976, 2100.9160, 2102.8872, 2106.9011, 2108.8835, 2109.9175, 2114.9180, 2116.9160, 2118.8835, 2119.8833, 2125.0176, 2132.8694, 2136.9180, 2170.1467, 2225.1191, 2273.0496, 2277.0137, 2307.9941, 2322.9980, 2341.0366, 2343.0271, 2352.0571, 2355.0459, 2359.0532, 2366.0813, 2368.0544, 2369.0710, 2376.0264, 2382.0349, 2383.6099, 2383.9600, 2390.0403, 2396.0342, 2398.0576, 2400.0652, 2406.0737, 2411.0076, 2414.0813, 2416.0752, 2417.0732, 2420.0718, 2422.0408, 2423.0466, 2428.0898, 2430.0828, 2431.0845, 2433.0779, 2436.0508, 2438.0457, 2439.0261, 2442.0618, 2449.0347, 2452.0632, 2460.0254, 2461.9956, 2463.9858, 2510.1277, 2528.1580, 2542.1509, 2545.1558, 2547.1531, 2552.1038, 2565.1543, 2585.1111, 2601.0889, 2705.2192, 2717.0693, 3129.3176, 3208.1736, 3223.2710, 3290.4131, 3291.4248, 3312.2756, 3451.3933, 3453.4365

33. O62827 **Mass:** 21064 **Score:** 18 **Expect:** 4e+002 **Matches:** 10
ADM OS=Bos taurus GN=ADM PE=2 SV=2

Observed	Mr(expt)	Mr(calc)	ppm	Start - End	Miss	Ions	Peptide
845.4100	844.4027	844.4039	-1.37	131 - 138	1	5	K.DKDGSAPR.S
845.4123	844.4050	844.4039	1.34	131 - 138	1	---	K.DKDGSAPR.S
871.4945	870.4872	870.4671	23.1	171 - 178	1	---	K.LRGAPDSR.V
1033.5419	1032.5346	1032.5465	-11.47	141 - 149	1	---	K.ISPQGYGRR.R
1060.5400	1059.5327	1059.5309	1.75	131 - 140	2	---	K.DKDGSAPRSK.I
1060.5442	1059.5369	1059.5309	5.71	131 - 140	2	---	K.DKDGSAPRSK.I
1086.5115	1085.5042	1085.5101	-5.44	79 - 89	0	---	R.SPQASSPDAAR.I
1355.7155	1354.7082	1354.6953	9.54	79 - 91	1	---	R.SPQASSPDAARIR.V
1751.8787	1750.8714	1750.8737	-1.31	45 - 60	1	---	R.ELRESSSYPTGLADVK.A
1783.8499	1782.8426	1782.8584	-8.84	97 - 111	1	---	R.QSLNNFQGLRSFGCR.F

No match to: 713.4334, 719.2388, 724.3009, 726.2899, 732.2586, 735.3865, 745.4124, 756.4687, 764.4056, 765.3908, 767.3679, 768.3909, 772.3955, 795.4186, 802.4548, 803.4370, 805.4229, 806.4631, 807.3893, 809.4480, 811.4104, 812.3985, 816.4408, 820.4244, 822.3905, 823.3846, 824.3756, 828.3904, 834.4043, 836.4589, 837.3853, 839.3696, 840.3839, 842.5085, 851.3975, 852.4056, 856.4893, 866.3914, 867.3724, 870.5402, 878.4625, 880.4403, 886.3815, 888.4233, 890.4479, 893.9562, 894.4579, 897.0289, 897.4412, 902.4597, 910.4410, 912.4497, 913.5118, 916.4905, 918.5391, 925.4747, 928.5064, 930.5048, 933.4619, 935.4698, 939.4758, 947.4628, 949.4591, 951.4703, 954.4674, 957.4657, 963.4676, 965.4761, 967.4568, 969.4571, 972.4793, 972.4800, 975.4650, 977.4630, 978.4976, 981.4565, 983.4664, 985.4842, 991.4688, 993.5331, 997.4298, 999.4453, 1001.4488, 1003.4687, 1007.5668, 1018.5200, 1018.5212, 1023.4777, 1026.4922, 1027.4972, 1030.5151, 1031.3850, 1035.5352, 1036.5372, 1039.5402, 1042.5383, 1045.5654, 1056.5488, 1065.5425, 1106.5372, 1111.5582, 1113.5546, 1114.5396, 1115.5605, 1118.5529, 1122.5363, 1128.5775, 1129.6014, 1130.5935, 1140.5485, 1144.5709, 1155.6113, 1156.5720, 1157.5952, 1158.6154, 1159.6273, 1165.5817, 1172.6301, 1173.6259, 1179.6063, 1180.6165, 1182.6090, 1183.6123, 1185.6317, 1187.6431, 1193.6096, 1195.6060, 1196.6102, 1206.6770, 1209.1387, 1209.5848, 1210.1014, 1215.6627, 1216.6494, 1222.5994, 1226.5948, 1229.6246, 1231.6174, 1234.6317, 1235.5846, 1240.6298, 1244.6324, 1248.6119, 1250.6144, 1268.6498, 1271.6696, 1273.6653, 1275.6644, 1291.6425, 1295.7233, 1300.6062, 1301.6205, 1307.6858, 1315.6570, 1317.6521, 1319.6852, 1323.6517, 1325.6637, 1328.6809, 1329.6672, 1331.6763, 1332.6743, 1334.6592, 1335.6937, 1336.6715, 1337.6724, 1338.6471, 1342.6680, 1345.6760, 1346.6880, 1349.6991, 1354.6907, 1357.7034, 1360.6882, 1361.6670, 1365.6913, 1368.6920, 1374.6412, 1375.6798, 1376.6804, 1377.6794, 1382.6647, 1387.6444, 1410.7505, 1411.7208, 1421.7177, 1427.7872, 1431.7961, 1432.7133, 1434.7288, 1446.7256, 1450.7079, 1473.8054, 1475.7748, 1477.7524, 1478.7439, 1485.7720, 1486.7664, 1493.7539, 1494.7423, 1505.8649, 1523.7841, 1524.7848, 1537.8021, 1549.7388, 1555.8816, 1558.8474, 1576.7987, 1579.7926, 1584.7897, 1588.8136, 1590.3611, 1604.8417, 1605.8098, 1606.8384, 1615.8826, 1616.8605, 1622.8137, 1628.8260, 1629.8358, 1630.8466, 1633.9790, 1635.7943, 1644.8046, 1647.8785, 1649.8463, 1651.8245, 1659.8241, 1662.7909, 1668.7810, 1679.9736, 1681.8214, 1683.8121, 1684.8204, 1706.8616, 1707.7838, 1716.8571, 1725.8353, 1728.8242, 1734.8152, 1740.8768, 1742.9031, 1746.1659, 1750.8356, 1753.8948, 1756.8905, 1757.8322, 1763.8188, 1765.7690, 1767.8036, 1768.9027, 1769.4475, 1770.4261, 1774.9091, 1786.9000, 1786.9017, 1791.7335, 1808.8868, 1824.8674, 1840.8225, 1844.8907, 1848.8322, 1850.8301, 1851.8488, 1867.9503, 1880.9277, 1887.9734, 1908.8838, 1914.9995, 1919.0038, 1925.9418, 1928.0103, 1931.9463, 1953.9353, 1975.9609, 1992.9642, 1995.9020, 2007.9894, 2011.8845, 2013.8983, 2024.8591, 2025.6234, 2036.5195, 2036.9486, 2042.0640, 2047.0167, 2047.9691, 2051.9792, 2052.9924, 2058.9963, 2060.0469, 2064.9900, 2068.8982, 2070.8835, 2074.8914, 2083.9907, 2084.8987, 2086.8828, 2092.8821, 2095.9941, 2098.9976, 2100.9160, 2102.8872, 2106.9011, 2108.8835, 2109.9175, 2114.9180, 2116.9160, 2118.8835, 2119.8833, 2125.0176, 2132.8694, 2136.9180, 2170.1467, 2211.1045, 2225.1191, 2273.0496, 2277.0137, 2307.9941, 2322.9980, 2341.0366, 2343.0271, 2352.0571, 2355.0459, 2359.0532, 2366.0813, 2368.0544, 2369.0710, 2376.0264, 2382.0349, 2383.6099, 2383.9600, 2390.0403, 2396.0342, 2398.0576, 2400.0652, 2406.0737, 2411.0076, 2414.0813, 2416.0752, 2417.0732, 2420.0718, 2422.0408, 2423.0466, 2428.0898, 2430.0828, 2431.0845, 2433.0779, 2436.0508, 2438.0457, 2439.0261, 2442.0618, 2449.0347, 2452.0632, 2460.0254, 2461.9956, 2463.9858, 2510.1277, 2528.1580, 2542.1509, 2545.1558, 2547.1531, 2552.1038, 2565.1543, 2585.1111, 2601.0889, 2705.2192, 2717.0693, 3129.3176, 3208.1736, 3223.2710, 3290.4131, 3291.4248, 3312.2756, 3451.3933, 3453.4365

34. Q05B49 **Mass:** 24773 **Score:** 18 **Expect:** 4.1e+002 **Matches:** 10
UPF0711 protein C18orf21 homolog OS=Bos taurus PE=2 SV=1

Observed	Mr(expt)	Mr(calc)	ppm	Start - End	Miss	Ions	Peptide
806.4631	805.4558	805.4559	-0.10	110 - 116	1	---	R.TVKHHGK.S
871.4945	870.4872	870.5035	-18.70	72 - 78	1	---	K.LLNREAR.N
878.4625	877.4552	877.4440	12.8	106 - 112	1	---	K.TCNRTVK.H
1035.5352	1034.5279	1034.5331	-5.01	203 - 210	2	---	K.KNPKMDFR.N
1229.6246	1228.6173	1228.5910	21.4	207 - 216	1	---	K.MDFRNFLSSL.-
1337.6724	1336.6651	1336.6782	-9.81	106 - 116	2	---	K.TCNRTVKHHGK.S
1584.7897	1583.7824	1583.7766	3.68	204 - 216	2	---	K.NPKMDFRNFLSSL.- + Oxidation (M)
1647.8785	1646.8712	1646.8351	21.9	1 - 13	2	---	-.MRQKHYLEAAAWK.L + Oxidation (M)
2225.1191	2224.1118	2224.1158	-1.78	160 - 180	1	---	K.SPALIFRTPTSGQSTSICSSK.N

2449.0347 2448.0274 2448.0872 -24.43 37 - 56 1 --- K.STFEGTCPYCCQLLVQDKSR.V

No match to: 713.4334, 719.2388, 724.3009, 726.2899, 732.2586, 735.3865, 745.4124, 756.4687, 764.4056, 765.3908, 767.3679, 768.3909, 772.3955, 795.4186, 802.4548, 803.4370, 805.4229, 807.3893, 809.4480, 811.4104, 812.3985, 816.4408, 820.4244, 822.3905, 823.3846, 824.3756, 828.3904, 834.4043, 836.4589, 837.3853, 839.3696, 840.3839, 842.5085, 845.4100, 845.4123, 851.3975, 852.4056, 856.4893, 866.3914, 867.3724, 870.5402, 880.4403, 886.3815, 888.4233, 890.4479, 893.9562, 894.4579, 897.0289, 897.4412, 902.4597, 910.4410, 912.4497, 913.5118, 916.4905, 918.5391, 925.4747, 928.5064, 930.5048, 933.4619, 935.4698, 939.4758, 947.4628, 949.4591, 951.4703, 954.4674, 957.4657, 963.4676, 965.4761, 967.4568, 969.4571, 972.4793, 972.4800, 975.4650, 977.4630, 978.4976, 981.4565, 983.4664, 985.4842, 991.4688, 993.5331, 997.4298, 999.4453, 1001.4488, 1003.4687, 1007.5668, 1018.5200, 1018.5212, 1023.4777, 1026.4922, 1027.4972, 1030.5151, 1031.3850, 1033.5419, 1036.5372, 1039.5402, 1042.5383, 1045.5654, 1056.5488, 1060.5400, 1060.5442, 1065.5425, 1086.5115, 1106.5372, 1111.5582, 1113.5546, 1114.5396, 1115.5605, 1118.5529, 1122.5363, 1128.5775, 1129.6014, 1130.5935, 1140.5485, 1144.5709, 1155.6113, 1156.5720, 1157.5952, 1158.6154, 1159.6273, 1165.5817, 1172.6301, 1173.6259, 1179.6063, 1180.6165, 1182.6090, 1183.6123, 1185.6317, 1187.6431, 1193.6096, 1195.6060, 1196.6102, 1206.6770, 1209.1387, 1209.5848, 1210.1014, 1215.6627, 1216.6494, 1222.5994, 1226.5948, 1231.6174, 1234.6317, 1235.5846, 1240.6298, 1244.6324, 1248.6119, 1250.6144, 1268.6498, 1271.6696, 1273.6653, 1275.6644, 1291.6425, 1295.7233, 1300.6062, 1301.6205, 1307.6858, 1315.6570, 1317.6521, 1319.6852, 1323.6517, 1325.6637, 1328.6809, 1329.6672, 1331.6763, 1332.6743, 1334.6592, 1335.6937, 1336.6715, 1338.6471, 1342.6680, 1345.6760, 1346.6880, 1349.6991, 1354.6907, 1355.7155, 1357.7034, 1360.6882, 1361.6670, 1365.6913, 1368.6920, 1374.6412, 1375.6798, 1376.6804, 1377.6794, 1382.6647, 1387.6444, 1410.7505, 1411.7208, 1421.7177, 1427.7872, 1431.7961, 1432.7133, 1434.7288, 1446.7256, 1450.7079, 1473.8054, 1475.7748, 1477.7524, 1478.7439, 1485.7720, 1486.7664, 1493.7539, 1494.7423, 1505.8649, 1523.7841, 1524.7848, 1537.8021, 1549.7388, 1555.8816, 1558.8474, 1576.7987, 1579.7926, 1588.8136, 1590.3611, 1604.8417, 1605.8098, 1606.8384, 1615.8826, 1616.8605, 1622.8137, 1628.8260, 1629.8358, 1630.8466, 1633.9790, 1635.7943, 1644.8046, 1649.8463, 1651.8245, 1659.8241, 1662.7909, 1668.7810, 1679.9736, 1681.8214, 1683.8121, 1684.8204, 1706.8616, 1707.7838, 1716.8571, 1725.8353, 1728.8242, 1734.8152, 1740.8768, 1742.9031, 1746.1659, 1750.8356, 1751.8787, 1753.8948, 1756.8905, 1757.8322, 1763.8188, 1765.7690, 1767.8036, 1768.9027, 1769.4475, 1770.4261, 1774.9091, 1783.8499, 1786.9000, 1786.9017, 1791.7335, 1808.8868, 1824.8674, 1840.8225, 1844.8907, 1848.8322, 1850.8301, 1851.8488, 1867.9503, 1880.9277, 1887.9734, 1908.8838, 1914.9995, 1919.0038, 1925.9418, 1928.0103, 1931.9463, 1953.9353, 1975.9609, 1992.9642, 1995.9020, 2007.9894, 2011.8845, 2013.8983, 2024.8591, 2025.6234, 2036.5195, 2036.9486, 2042.0640, 2047.0167, 2047.9691, 2051.9792, 2052.9924, 2058.9963, 2060.0469, 2064.9900, 2068.8982, 2070.8835, 2074.8914, 2083.9907, 2084.8987, 2086.8828, 2092.8821, 2095.9941, 2098.9976, 2100.9160, 2102.8872, 2106.9011, 2108.8835, 2109.9175, 2114.9180, 2116.9160, 2118.8835, 2119.8833, 2125.0176, 2132.8694, 2136.9180, 2170.1467, 2211.1045, 2273.0496, 2277.0137, 2307.9941, 2322.9980, 2341.0366, 2343.0271, 2352.0571, 2355.0459, 2359.0532, 2366.0813, 2368.0544, 2369.0710, 2376.0264, 2382.0349, 2383.6099, 2383.9600, 2390.0403, 2396.0342, 2398.0576, 2400.0652, 2406.0737, 2411.0076, 2414.0813, 2416.0752, 2417.0732, 2420.0718, 2422.0408, 2423.0466, 2428.0898, 2430.0828, 2431.0845, 2433.0779, 2436.0508, 2438.0457, 2439.0261, 2442.0618, 2452.0632, 2460.0254, 2461.9956, 2463.9858, 2510.1277, 2528.1580, 2542.1509, 2545.1558, 2547.1531, 2552.1038, 2565.1543, 2585.1111, 2601.0889, 2705.2192, 2717.0693, 3129.3176, 3208.1736, 3223.2710, 3290.4131, 3291.4248, 3312.2756, 3451.3933, 3453.4365

35. Q8MJN0 **Mass:** 20622 **Score:** 18 **Expect:** 4.1e+002 **Matches:** 10

FUN14 domain-containing protein 2 OS=Bos taurus GN=FUNDC2 PE=2 SV=1

Observed	Mr(expt)	Mr(calc)	ppm	Start		End	Miss	Ions	Peptide
765.3908	764.3835	764.3738	12.7	136	-	141	1	---	R.VEKDMK.K + Oxidation (M)
772.3955	771.3882	771.3816	8.55	64	-	68	0	---	K.QPWWR.T
852.4056	851.3984	851.3807	20.7	1	-	7	0	---	-.METSTQR.T
1128.5775	1127.5702	1127.5432	24.0	20	-	30	1	---	R.HSASSRGEAAR.V
1183.6123	1182.6050	1182.6105	-4.65	8	-	19	0	---	R.TGSHLAQTAAAR.H
1767.8036	1766.7963	1766.7781	10.3	35	-	51	0	---	R.DELAEMAAASQGNFEGK.F
1786.9000	1785.8927	1785.9124	-11.03	173	-	190	0	---	K.NVLVTGGFFGGFLLGMAS.-
1786.9017	1785.8944	1785.9124	-10.07	173	-	190	0	---	K.NVLVTGGFFGGFLLGMAS.-
1808.8868	1807.8795	1807.9037	-13.39	8	-	25	1	---	R.TGSHLAQTAAARHSASSR.G
1914.9995	1913.9922	1914.0074	-7.91	172	-	190	1	---	K.KNVLVTGGFFGGFLLGMAS.-

No match to: 713.4334, 719.2388, 724.3009, 726.2899, 732.2586, 735.3865, 745.4124, 756.4687, 764.4056, 767.3679, 768.3909, 795.4186, 802.4548, 803.4370, 805.4229, 806.4631, 807.3893, 809.4480, 811.4104, 812.3985, 816.4408, 820.4244, 822.3905, 823.3846, 824.3756, 828.3904, 834.4043, 836.4589, 837.3853, 839.3696, 840.3839, 842.5085, 845.4100, 845.4123, 851.3975, 856.4893, 866.3914, 867.3724, 870.5402, 871.4945, 878.4625, 880.4403, 886.3815, 888.4233, 890.4479, 893.9562, 894.4579, 897.0289, 897.4412, 902.4597, 910.4410, 912.4497, 913.5118, 916.4905, 918.5391, 925.4747, 928.5064, 930.5048, 933.4619, 935.4698, 939.4758, 947.4628, 949.4591, 951.4703, 954.4674, 957.4657, 963.4676, 965.4761, 967.4568, 969.4571, 972.4793, 972.4800, 975.4650, 977.4630, 978.4976, 981.4565, 983.4664, 985.4842, 991.4688, 993.5331, 997.4298, 999.4453, 1001.4488, 1003.4687, 1007.5668, 1018.5200, 1018.5212, 1023.4777, 1026.4922, 1027.4972, 1030.5151, 1031.3850, 1033.5419, 1035.5352, 1036.5372, 1039.5402, 1042.5383, 1045.5654, 1056.5488, 1060.5400, 1060.5442, 1065.5425, 1086.5115, 1106.5372, 1111.5582, 1113.5546, 1114.5396, 1115.5605, 1118.5529, 1122.5363, 1129.6014, 1130.5935, 1140.5485, 1144.5709, 1155.6113, 1156.5720, 1157.5952, 1158.6154, 1159.6273, 1165.5817, 1172.6301, 1173.6259, 1179.6063, 1180.6165, 1182.6090, 1185.6317, 1187.6431, 1193.6096, 1195.6060, 1196.6102, 1206.6770, 1209.1387, 1209.5848, 1210.1014, 1215.6627, 1216.6494, 1222.5994, 1226.5948, 1229.6246, 1231.6174, 1234.6317, 1235.5846, 1240.6298, 1244.6324, 1248.6119, 1250.6144, 1268.6498, 1271.6696, 1273.6653, 1275.6644, 1291.6425, 1295.7233, 1300.6062, 1301.6205, 1307.6858, 1315.6570, 1317.6521, 1319.6852, 1323.6517, 1325.6637, 1328.6809, 1329.6672, 1331.6763, 1332.6743, 1334.6592, 1335.6937, 1336.6715, 1337.6724, 1338.6471, 1342.6680, 1345.6760, 1346.6880, 1349.6991, 1354.6907, 1355.7155, 1357.7034, 1360.6882, 1361.6670, 1365.6913, 1368.6920, 1374.6412, 1375.6798, 1376.6804, 1377.6794, 1382.6647, 1387.6444, 1410.7505, 1411.7208, 1421.7177, 1427.7872, 1431.7961, 1432.7133, 1434.7288, 1446.7256, 1450.7079, 1473.8054, 1475.7748, 1477.7524, 1478.7439, 1485.7720, 1486.7664, 1493.7539, 1494.7423, 1505.8649, 1523.7841, 1524.7848, 1537.8021, 1549.7388, 1555.8816, 1558.8474, 1576.7987, 1579.7926, 1584.7897, 1588.8136, 1590.3611, 1604.8417, 1605.8098, 1606.8384, 1615.8826, 1616.8605, 1622.8137, 1628.8260, 1629.8358, 1630.8466, 1633.9790, 1635.7943, 1644.8046, 1647.8785, 1649.8463, 1651.8245, 1659.8241, 1662.7909, 1668.7810, 1679.9736, 1681.8214, 1683.8121, 1684.8204, 1706.8616, 1707.7838, 1716.8571, 1725.8353, 1728.8242, 1734.8152, 1740.8768, 1742.9031, 1746.1659, 1750.8356, 1751.8787, 1753.8948, 1756.8905, 1757.8322, 1763.8188, 1765.7690, 1768.9027, 1769.4475, 1770.4261, 1774.9091, 1783.8499, 1791.7335, 1824.8674, 1840.8225, 1844.8907, 1848.8322, 1850.8301, 1851.8488, 1867.9503, 1880.9277, 1887.9734, 1908.8838, 1919.0038, 1925.9418, 1928.0103, 1931.9463, 1953.9353, 1975.9609, 1992.9642, 1995.9020, 2007.9894, 2011.8845, 2013.8983, 2024.8591, 2025.6234, 2036.5195, 2036.9486, 2042.0640, 2047.0167, 2047.9691, 2051.9792, 2052.9924, 2058.9963, 2060.0469, 2064.9900, 2068.8982, 2070.8835, 2074.8914, 2083.9907, 2084.8987, 2086.8828, 2092.8821, 2095.9941, 2098.9976, 2100.9160, 2102.8872, 2106.9011, 2108.8835, 2109.9175, 2114.9180, 2116.9160, 2118.8835, 2119.8833, 2125.0176, 2132.8694, 2136.9180, 2170.1467, 2211.1045, 2225.1191, 2273.0496, 2277.0137, 2307.9941, 2322.9980, 2341.0366, 2343.0271, 2352.0571, 2355.0459, 2359.0532, 2366.0813, 2368.0544, 2369.0710, 2376.0264, 2382.0349, 2383.6099, 2383.9600, 2390.0403, 2396.0342, 2398.0576, 2400.0652, 2406.0737, 2411.0076, 2414.0813, 2416.0752, 2417.0732, 2420.0718, 2422.0408, 2423.0466, 2428.0898, 2430.0828, 2431.0845, 2433.0779, 2436.0508, 2438.0457, 2439.0261, 2442.0618, 2449.0347, 2452.0632, 2460.0254, 2461.9956, 2463.9858, 2510.1277, 2528.1580, 2542.1509, 2545.1558, 2547.1531, 2552.1038, 2565.1543, 2585.1111, 2601.0889, 2705.2192, 2717.0693, 3129.3176, 3208.1736, 3223.2710, 3290.4131, 3291.4248, 3312.2756, 3451.3933, 3453.4365

36. Q32LM7 **Mass:** 28555 **Score:** 18 **Expect:** 4.2e+002 **Matches:** 12

Coiled-coil domain-containing protein 152 OS=Bos taurus GN=CCDC152 PE=2 SV=2

Observed	Mr(expt)	Mr(calc)	ppm	Start		End	Miss	Ions	Peptide
767.3679	766.3606	766.3531	9.86	31	-	37	0	---	K.MIESSGK.N + Oxidation (M)
802.4548	801.4475	801.4457	2.26	146	-	151	1	---	K.LRTQER.E
1035.5352	1034.5279	1034.5066	20.6	152	-	159	1	---	R.EKQNEMIK.L + Oxidation (M)
1130.5935	1129.5862	1129.5840	2.00	209	-	217	1	---	R.NTVRDLEQR.L
1180.6165	1179.6092	1179.5992	8.54	54	-	63	1	---	R.VMQTKEVAMK.Q + Oxidation (M)
1196.6102	1195.6029	1195.5941	7.41	54	-	63	1	---	R.VMQTKEVAMK.Q + 2 Oxidation (M)
1349.6991	1348.6918	1348.6955	-2.73	48	-	58	1	---	K.ANCLLRVMQTK.E + Oxidation (M)
1524.7848	1523.7775	1523.8017	-15.88	108	-	119	2	---	K.IEKTKLYQDMQK.K
1549.7388	1548.7315	1548.7566	-16.17	148	-	159	2	---	R.TQEREKQNEMIK.L + Oxidation (M)
1659.8241	1658.8168	1658.8410	-14.55	59	-	72	2	---	K.EVAMKQGENEQLKR.N
1706.8616	1705.8543	1705.8709	-9.70	154	-	167	1	---	K.QNEMIKLQLEFDAK.L
2051.9792	2050.9719	2050.9775	-2.74	2	-	19	2	---	M.NQNSEGCMKKISSVNLDK.L

No match to: 713.4334, 719.2388, 724.3009, 726.2899, 732.2586, 735.3865, 745.4124, 756.4687, 764.4056, 765.3908, 768.3909, 772.3955, 795.4186, 803.4370, 805.4229, 806.4631, 807.3893, 809.4480, 811.4104, 812.3985, 816.4408, 820.4244, 822.3905, 823.3846,

824.3756, 828.3904, 834.4043, 836.4589, 837.3853, 839.3696, 840.3839, 842.5085, 845.4100, 845.4123, 851.3975, 852.4056, 856.4893, 866.3914, 867.3724, 870.5402, 871.4945, 878.4625, 880.4403, 886.3815, 888.4233, 890.4479, 893.9562, 894.4579, 897.0289, 897.4412, 902.4597, 910.4410, 912.4497, 913.5118, 916.4905, 918.5391, 925.4747, 928.5064, 930.5048, 933.4619, 935.4698, 939.4758, 947.4628, 949.4591, 951.4703, 954.4674, 957.4657, 963.4676, 965.4761, 967.4568, 969.4571, 972.4793, 972.4800, 975.4650, 977.4630, 978.4976, 981.4565, 983.4664, 985.4842, 991.4688, 993.5331, 997.4298, 999.4453, 1001.4488, 1003.4687, 1007.5668, 1018.5200, 1018.5212, 1023.4777, 1026.4922, 1027.4972, 1030.5151, 1031.3850, 1033.5419, 1036.5372, 1039.5402, 1042.5383, 1045.5654, 1056.5488, 1060.5400, 1060.5442, 1065.5425, 1086.5115, 1106.5372, 1111.5582, 1113.5546, 1114.5396, 1115.5605, 1118.5529, 1122.5363, 1128.5775, 1129.6014, 1140.5485, 1144.5709, 1155.6113, 1156.5720, 1157.5952, 1158.6154, 1159.6273, 1165.5817, 1172.6301, 1173.6259, 1179.6063, 1182.6090, 1183.6123, 1185.6317, 1187.6431, 1193.6096, 1195.6060, 1206.6770, 1209.1387, 1209.5848, 1210.1014, 1215.6627, 1216.6494, 1222.5994, 1226.5948, 1229.6246, 1231.6174, 1234.6317, 1235.5846, 1240.6298, 1244.6324, 1248.6119, 1250.6144, 1268.6498, 1271.6696, 1273.6653, 1275.6644, 1291.6425, 1295.7233, 1300.6062, 1301.6205, 1307.6858, 1315.6570, 1317.6521, 1319.6852, 1323.6517, 1325.6637, 1328.6809, 1329.6672, 1331.6763, 1332.6743, 1334.6592, 1335.6937, 1336.6715, 1337.6724, 1338.6471, 1342.6680, 1345.6760, 1346.6880, 1354.6907, 1355.7155, 1357.7034, 1360.6882, 1361.6670, 1365.6913, 1368.6920, 1374.6412, 1375.6798, 1376.6804, 1377.6794, 1382.6647, 1387.6444, 1410.7505, 1411.7208, 1421.7177, 1427.7872, 1431.7961, 1432.7133, 1434.7288, 1446.7256, 1450.7079, 1473.8054, 1475.7748, 1477.7524, 1478.7439, 1485.7720, 1486.7664, 1493.7539, 1494.7423, 1505.8649, 1523.7841, 1537.8021, 1555.8816, 1558.8474, 1576.7987, 1579.7926, 1584.7897, 1588.8136, 1590.3611, 1604.8417, 1605.8098, 1606.8384, 1615.8826, 1616.8605, 1622.8137, 1628.8260, 1629.8358, 1630.8466, 1633.9790, 1635.7943, 1644.8046, 1647.8785, 1649.8463, 1651.8245, 1662.7909, 1668.7810, 1679.9736, 1681.8214, 1683.8121, 1684.8204, 1707.7838, 1716.8571, 1725.8353, 1728.8242, 1734.8152, 1740.8768, 1742.9031, 1746.1659, 1750.8356, 1751.8787, 1753.8948, 1756.8905, 1757.8322, 1763.8188, 1765.7690, 1767.8036, 1768.9027, 1769.4475, 1770.4261, 1774.9091, 1783.8499, 1786.9000, 1786.9017, 1791.7335, 1808.8868, 1824.8674, 1840.8225, 1844.8907, 1848.8322, 1850.8301, 1851.8488, 1867.9503, 1880.9277, 1887.9734, 1908.8838, 1914.9995, 1919.0038, 1925.9418, 1928.0103, 1931.9463, 1953.9353, 1975.9609, 1992.9642, 1995.9020, 2007.9894, 2011.8845, 2013.8983, 2024.8591, 2025.6234, 2036.5195, 2036.9486, 2042.0640, 2047.0167, 2047.9691, 2052.9924, 2058.9963, 2060.0469, 2064.9900, 2068.8982, 2070.8835, 2074.8914, 2083.9907, 2084.8987, 2086.8828, 2092.8821, 2095.9941, 2098.9976, 2100.9160, 2102.8872, 2106.9011, 2108.8835, 2109.9175, 2114.9180, 2116.9160, 2118.8835, 2119.8833, 2125.0176, 2132.8694, 2136.9180, 2170.1467, 2211.1045, 2225.1191, 2273.0496, 2277.0137, 2307.9941, 2322.9980, 2341.0366, 2343.0271, 2352.0571, 2355.0459, 2359.0532, 2366.0813, 2368.0544, 2369.0710, 2376.0264, 2382.0349, 2383.6099, 2383.9600, 2390.0403, 2396.0342, 2398.0576, 2400.0652, 2406.0737, 2411.0076, 2414.0813, 2416.0752, 2417.0732, 2420.0718, 2422.0408, 2423.0466, 2428.0898, 2430.0828, 2431.0845, 2433.0779, 2436.0508, 2438.0457, 2439.0261, 2442.0618, 2449.0347, 2452.0632, 2460.0254, 2461.9956, 2463.9858, 2510.1277, 2528.1580, 2542.1509, 2545.1558, 2547.1531, 2552.1038, 2565.1543, 2585.1111, 2601.0889, 2705.2192, 2717.0693, 3129.3176, 3208.1736, 3223.2710, 3290.4131, 3291.4248, 3312.2756, 3451.3933, 3453.4365

37. F1MJA8 **Mass:** 28543 **Score:** 18 **Expect:** 4.2e+002 **Matches:** 12

Coiled-coil domain-containing protein 152 OS=Bos taurus GN=CCDC152 PE=4 SV=2

Observed	Mr(expt)	Mr(calc)	ppm	Start		End	Miss	Ions	Peptide
767.3679	766.3606	766.3531	9.86	31	-	37	0	---	K.MIESSGK.N + Oxidation (M)
802.4548	801.4475	801.4457	2.26	146	-	151	1	---	K.LRTQER.E
1035.5352	1034.5279	1034.5066	20.6	152	-	159	1	---	R.EKQNEMIK.L + Oxidation (M)
1130.5935	1129.5862	1129.5840	2.00	209	-	217	1	---	R.NTVRDLEQR.L
1180.6165	1179.6092	1179.5992	8.54	54	-	63	1	---	R.VMQTKEVAMK.Q + Oxidation (M)
1196.6102	1195.6029	1195.5941	7.41	54	-	63	1	---	R.VMQTKEVAMK.Q + 2 Oxidation (M)
1349.6991	1348.6918	1348.6955	-2.73	48	-	58	1	---	K.ANCLLRVMQTK.E + Oxidation (M)
1524.7848	1523.7775	1523.8017	-15.88	108	-	119	2	---	K.IEKTKLYQDMQK.K
1549.7388	1548.7315	1548.7566	-16.17	148	-	159	2	---	R.TQEREKQNEMIK.L + Oxidation (M)
1659.8241	1658.8168	1658.8410	-14.55	59	-	72	2	---	K.EVAMKQGENEQLKR.N
1706.8616	1705.8543	1705.8709	-9.70	154	-	167	1	---	K.QNEMIKLQLEFDAK.L
2051.9792	2050.9719	2050.9775	-2.74	2	-	19	2	---	M.NQNSEGCMKKISSVNLDK.L

No match to: 713.4334, 719.2388, 724.3009, 726.2899, 732.2586, 735.3865, 745.4124, 756.4687, 764.4056, 765.3908, 768.3909, 772.3955, 795.4186, 803.4370, 805.4229, 806.4631, 807.3893, 809.4480, 811.4104, 812.3985, 816.4408, 820.4244, 822.3905, 823.3846, 824.3756, 828.3904, 834.4043, 836.4589, 837.3853, 839.3696, 840.3839, 842.5085, 845.4100, 845.4123, 851.3975, 852.4056, 856.4893, 866.3914, 867.3724, 870.5402, 871.4945, 878.4625, 880.4403, 886.3815, 888.4233, 890.4479, 893.9562, 894.4579, 897.0289, 897.4412, 902.4597, 910.4410, 912.4497, 913.5118, 916.4905, 918.5391, 925.4747, 928.5064, 930.5048, 933.4619, 935.4698, 939.4758, 947.4628, 949.4591, 951.4703, 954.4674, 957.4657, 963.4676, 965.4761, 967.4568, 969.4571, 972.4793, 972.4800, 975.4650, 977.4630, 978.4976, 981.4565, 983.4664, 985.4842, 991.4688, 993.5331, 997.4298, 999.4453, 1001.4488, 1003.4687, 1007.5668, 1018.5200, 1018.5212, 1023.4777, 1026.4922, 1027.4972, 1030.5151, 1031.3850, 1033.5419, 1036.5372, 1039.5402, 1042.5383, 1045.5654, 1056.5488, 1060.5400, 1060.5442, 1065.5425, 1086.5115, 1106.5372, 1111.5582, 1113.5546, 1114.5396, 1115.5605, 1118.5529, 1122.5363, 1128.5775, 1129.6014, 1140.5485, 1144.5709, 1155.6113, 1156.5720, 1157.5952, 1158.6154, 1159.6273, 1165.5817, 1172.6301, 1173.6259, 1179.6063, 1182.6090, 1183.6123, 1185.6317, 1187.6431, 1193.6096, 1195.6060, 1206.6770, 1209.1387, 1209.5848, 1210.1014, 1215.6627, 1216.6494, 1222.5994, 1226.5948, 1229.6246, 1231.6174, 1234.6317, 1235.5846, 1240.6298, 1244.6324, 1248.6119, 1250.6144, 1268.6498, 1271.6696, 1273.6653, 1275.6644, 1291.6425, 1295.7233, 1300.6062, 1301.6205, 1307.6858, 1315.6570, 1317.6521, 1319.6852, 1323.6517, 1325.6637, 1328.6809, 1329.6672, 1331.6763, 1332.6743, 1334.6592, 1335.6937, 1336.6715, 1337.6724, 1338.6471, 1342.6680, 1345.6760, 1346.6880, 1354.6907, 1355.7155, 1357.7034, 1360.6882, 1361.6670, 1365.6913, 1368.6920, 1374.6412, 1375.6798, 1376.6804, 1377.6794, 1382.6647, 1387.6444, 1410.7505, 1411.7208, 1421.7177, 1427.7872, 1431.7961, 1432.7133, 1434.7288, 1446.7256, 1450.7079, 1473.8054, 1475.7748, 1477.7524, 1478.7439, 1485.7720, 1486.7664, 1493.7539, 1494.7423, 1505.8649, 1523.7841, 1537.8021, 1555.8816, 1558.8474, 1576.7987, 1579.7926, 1584.7897, 1588.8136, 1590.3611, 1604.8417, 1605.8098, 1606.8384, 1615.8826, 1616.8605, 1622.8137, 1628.8260, 1629.8358, 1630.8466, 1633.9790, 1635.7943, 1644.8046, 1647.8785, 1649.8463, 1651.8245, 1662.7909, 1668.7810, 1679.9736, 1681.8214, 1683.8121, 1684.8204, 1707.7838, 1716.8571, 1725.8353, 1728.8242, 1734.8152, 1740.8768, 1742.9031, 1746.1659, 1750.8356, 1751.8787, 1753.8948, 1756.8905, 1757.8322, 1763.8188, 1765.7690, 1767.8036, 1768.9027, 1769.4475, 1770.4261, 1774.9091, 1783.8499, 1786.9000, 1786.9017, 1791.7335, 1808.8868, 1824.8674, 1840.8225, 1844.8907, 1848.8322, 1850.8301, 1851.8488, 1867.9503, 1880.9277, 1887.9734, 1908.8838, 1914.9995, 1919.0038, 1925.9418, 1928.0103, 1931.9463, 1953.9353, 1975.9609, 1992.9642, 1995.9020, 2007.9894, 2011.8845, 2013.8983, 2024.8591, 2025.6234, 2036.5195, 2036.9486, 2042.0640, 2047.0167, 2047.9691, 2052.9924, 2058.9963, 2060.0469, 2064.9900, 2068.8982, 2070.8835, 2074.8914, 2083.9907, 2084.8987, 2086.8828, 2092.8821, 2095.9941, 2098.9976, 2100.9160, 2102.8872, 2106.9011, 2108.8835, 2109.9175, 2114.9180, 2116.9160, 2118.8835, 2119.8833, 2125.0176, 2132.8694, 2136.9180, 2170.1467, 2211.1045, 2225.1191, 2273.0496, 2277.0137, 2307.9941, 2322.9980, 2341.0366, 2343.0271, 2352.0571, 2355.0459, 2359.0532, 2366.0813, 2368.0544, 2369.0710, 2376.0264, 2382.0349, 2383.6099, 2383.9600, 2390.0403, 2396.0342, 2398.0576, 2400.0652, 2406.0737, 2411.0076, 2414.0813, 2416.0752, 2417.0732, 2420.0718, 2422.0408, 2423.0466, 2428.0898, 2430.0828, 2431.0845, 2433.0779, 2436.0508, 2438.0457, 2439.0261, 2442.0618, 2449.0347, 2452.0632, 2460.0254, 2461.9956, 2463.9858, 2510.1277, 2528.1580, 2542.1509, 2545.1558, 2547.1531, 2552.1038, 2565.1543, 2585.1111, 2601.0889, 2705.2192, 2717.0693, 3129.3176, 3208.1736, 3223.2710, 3290.4131, 3291.4248, 3312.2756, 3451.3933, 3453.4365

38. A5D7A0 **Mass:** 26902 **Score:** 18 **Expect:** 4.3e+002 **Matches:** 14

EF-hand domain-containing protein D2 OS=Bos taurus GN=EFHD2 PE=2 SV=1

Observed	Mr(expt)	Mr(calc)	ppm	Start		End	Miss	Ions	Peptide
834.4043	833.3970	833.4130	-19.22	2	-	9	0	---	M.ATDELASK.L
965.4761	964.4689	964.4535	15.9	1	-	9	0	---	-.MATDELASK.L
981.4565	980.4493	980.4484	0.84	1	-	9	0	---	-.MATDELASK.L + Oxidation (M)
1035.5352	1034.5279	1034.5430	-14.57	94	-	101	2	---	R.KQIKDMEK.M + Oxidation (M)
1144.5709	1143.5636	1143.5601	3.12	81	-	89	0	---	R.VFNPYTEFK.E
1180.6165	1179.6092	1179.5845	20.9	111	-	120	0	---	R.DGFIDLMELK.L
1196.6102	1195.6029	1195.5795	19.6	111	-	120	0	---	R.DGFIDLMELK.L + Oxidation (M)
1329.6672	1328.6599	1328.6468	9.86	95	-	104	2	---	K.QIKDMEKMFK.E + 2 Oxidation (M)
1337.6724	1336.6651	1336.6656	-0.38	1	-	12	1	---	-.MATDELASKLSR.R + Oxidation (M)
1477.7524	1476.7451	1476.7718	-18.08	1	-	13	2	---	-.MATDELASKLSRR.L
1493.7539	1492.7466	1492.7667	-13.47	1	-	13	2	---	-.MATDELASKLSRR.L + Oxidation (M)
1716.8571	1715.8498	1715.8301	11.5	194	-	208	1	---	K.SFFEAKVQAMNVSSR.F + Oxidation (M)
1844.8907	1843.8834	1843.8770	3.51	111	-	125	1	---	R.DGFIDLMELKLMMEK.L + 2 Oxidation (M)
1880.9277	1879.9204	1879.9612	-21.71	64	-	80	2	---	R.RADLNQGIGEPQSPSRR.V

No match to: 713.4334, 719.2388, 724.3009, 726.2899, 732.2586, 735.3865, 745.4124, 756.4687, 764.4056, 765.3908, 767.3679, 768.3909, 772.3955, 795.4186, 802.4548, 803.4370, 805.4229, 806.4631, 807.3893, 809.4480, 811.4104, 812.3985, 816.4408, 820.4244, 822.3905, 823.3846, 824.3756, 828.3904, 836.4589, 837.3853, 839.3696, 840.3839, 842.5085, 845.4100, 845.4123, 851.3975, 852.4056, 856.4893, 866.3914, 867.3724, 870.5402, 871.4945, 878.4625, 880.4403, 886.3815, 888.4233, 890.4479, 893.9562, 894.4579, 897.0289, 897.4412, 902.4597, 910.4410, 912.4497, 913.5118, 916.4905, 918.5391, 925.4747, 928.5064, 930.5048, 933.4619, 935.4698, 939.4758, 947.4628, 949.4591, 951.4703, 954.4674, 957.4657, 963.4676, 967.4568, 969.4571, 972.4793, 972.4800, 975.4650, 977.4630, 978.4976, 983.4664, 985.4842, 991.4688, 993.5331, 997.4298, 999.4453, 1001.4488, 1003.4687, 1007.5668, 1018.5200, 1018.5212, 1023.4777, 1026.4922, 1027.4972, 1030.5151, 1031.3850, 1033.5419, 1036.5372, 1039.5402, 1042.5383, 1045.5654, 1056.5488, 1060.5400, 1060.5442, 1065.5425, 1086.5115, 1106.5372, 1111.5582, 1113.5546, 1114.5396, 1115.5605, 1118.5529, 1122.5363, 1128.5775, 1129.6014, 1130.5935, 1140.5485, 1155.6113, 1156.5720, 1157.5952, 1158.6154, 1159.6273, 1165.5817, 1172.6301, 1173.6259, 1179.6063, 1182.6090, 1183.6123, 1185.6317, 1187.6431, 1193.6096, 1195.6060, 1206.6770, 1209.1387, 1209.5848, 1210.1014, 1215.6627, 1216.6494, 1222.5994, 1226.5948, 1229.6246, 1231.6174, 1234.6317, 1235.5846, 1240.6298, 1244.6324, 1248.6119, 1250.6144, 1268.6498, 1271.6696, 1273.6653, 1275.6644, 1291.6425, 1295.7233, 1300.6062, 1301.6205, 1307.6858, 1315.6570, 1317.6521, 1319.6852, 1323.6517, 1325.6637, 1328.6809, 1331.6763, 1332.6743, 1334.6592, 1335.6937, 1336.6715, 1338.6471, 1342.6680, 1345.6760, 1346.6880, 1349.6991, 1354.6907, 1355.7155, 1357.7034, 1360.6882, 1361.6670, 1365.6913, 1368.6920, 1374.6412, 1375.6798, 1376.6804, 1377.6794, 1382.6647, 1387.6444, 1410.7505, 1411.7208, 1421.7177, 1427.7872, 1431.7961, 1432.7133, 1434.7288, 1446.7256, 1450.7079, 1473.8054, 1475.7748, 1478.7439, 1485.7720, 1486.7664, 1494.7423, 1505.8649, 1523.7841, 1524.7848, 1537.8021, 1549.7388, 1555.8816, 1558.8474, 1576.7987, 1579.7926, 1584.7897, 1588.8136, 1590.3611, 1604.8417, 1605.8098, 1606.8384, 1615.8826, 1616.8605, 1622.8137, 1628.8260, 1629.8358, 1630.8466, 1633.9790, 1635.7943, 1644.8046, 1647.8785, 1649.8463, 1651.8245, 1659.8241, 1662.7909, 1668.7810, 1679.9736, 1681.8214, 1683.8121, 1684.8204, 1706.8616, 1707.7838, 1725.8353, 1728.8242, 1734.8152, 1740.8768, 1742.9031, 1746.1659, 1750.8356, 1751.8787, 1753.8948, 1756.8905, 1757.8322, 1763.8188, 1765.7690, 1767.8036, 1768.9027, 1769.4475, 1770.4261, 1774.9091, 1783.8499, 1786.9000, 1786.9017, 1791.7335, 1808.8868, 1824.8674, 1840.8225, 1848.8322, 1850.8301, 1851.8488, 1867.9503, 1887.9734, 1908.8838, 1914.9995, 1919.0038, 1925.9418, 1928.0103, 1931.9463, 1953.9353, 1975.9609, 1992.9642, 1995.9020, 2007.9894, 2011.8845, 2013.8983, 2024.8591, 2025.6234, 2036.5195, 2036.9486, 2042.0640, 2047.0167, 2047.9691, 2051.9792, 2052.9924, 2058.9963, 2060.0469, 2064.9900, 2068.8982, 2070.8835, 2074.8914, 2083.9907, 2084.8987, 2086.8828, 2092.8821, 2095.9941, 2098.9976, 2100.9160, 2102.8872, 2106.9011, 2108.8835, 2109.9175, 2114.9180, 2116.9160, 2118.8835, 2119.8833, 2125.0176, 2132.8694, 2136.9180, 2170.1467, 2211.1045, 2225.1191, 2273.0496, 2277.0137, 2307.9941, 2322.9980, 2341.0366, 2343.0271, 2352.0571, 2355.0459, 2359.0532, 2366.0813, 2368.0544, 2369.0710, 2376.0264, 2382.0349, 2383.6099, 2383.9600, 2390.0403, 2396.0342, 2398.0576, 2400.0652, 2406.0737, 2411.0076, 2414.0813, 2416.0752, 2417.0732, 2420.0718, 2422.0408, 2423.0466, 2428.0898, 2430.0828, 2431.0845, 2433.0779, 2436.0508, 2438.0457, 2439.0261, 2442.0618, 2449.0347, 2452.0632, 2460.0254, 2461.9956, 2463.9858, 2510.1277, 2528.1580, 2542.1509, 2545.1558, 2547.1531, 2552.1038, 2565.1543, 2585.1111, 2601.0889, 2705.2192, 2717.0693, 3129.3176, 3208.1736, 3223.2710, 3290.4131, 3291.4248, 3312.2756, 3451.3933, 3453.4365

39. G3N365 **Mass:** 141959 **Score:** 17 **Expect:** 4.6e+002 **Matches:** 39

Structural maintenance of chromosomes protein OS=Bos taurus GN=SMC3 PE=3 SV=1

Observed	Mr(expt)	Mr(calc)	ppm	Start	End	Miss	Ions	Peptide
806.4631	805.4558	805.4731	-21.54	1053 -	1059	1	---	K.ATLVMKK.G + Oxidation (M)
820.4244	819.4171	819.4273	-12.40	873 -	879	1	---	R.VKDTMAR.S
824.3756	823.3683	823.3568	14.0	937 -	942	1	---	K.EECMKK.I
851.3975	850.3902	850.4007	-12.29	904 -	909	1	---	R.WKNMEK.E + Oxidation (M)
852.4056	851.3984	851.3959	2.84	900 -	905	1	---	K.SMERWK.N + Oxidation (M)
894.4579	893.4507	893.4680	-19.45	572 -	578	0	---	K.ILMEFNK.M
916.4905	915.4832	915.4774	6.37	365 -	372	0	---	R.LAQATQER.T
918.5391	917.5318	917.5368	-5.47	1069 -	1076	2	---	R.MKEKGVVK.E
935.4698	934.4625	934.4793	-17.99	281 -	288	1	---	K.ISAMKEEK.E
939.4758	938.4686	938.4610	8.05	615 -	621	1	---	R.YNPRFDK.A
951.4703	950.4631	950.4742	-11.76	281 -	288	1	---	K.ISAMKEEK.E + Oxidation (M)
1056.5488	1055.5415	1055.5287	12.1	107 -	114	1	---	K.DQYFLDKK.M
1158.6154	1157.6081	1157.6346	-22.83	1185 -	1193	2	---	K.FYGVKFRNK.V
1165.5817	1164.5744	1164.5775	-2.60	428 -	436	1	---	K.EKNLEQYNK.L
1172.6301	1171.6228	1171.6084	12.3	341 -	350	1	---	K.QKELAETEPK.F
1180.6165	1179.6092	1179.6169	-6.49	279 -	288	2	---	K.TKISAMKEEK.E + Oxidation (M)
1235.5846	1234.5773	1234.5976	-16.41	1060 -	1070	1	---	K.GDVEGSQLRMK.E + Oxidation (M)
1250.6144	1249.6071	1249.6302	-18.49	828 -	837	0	---	R.VETYLNENLR.K
1301.6205	1300.6132	1300.6259	-9.71	475 -	486	0	---	R.EENAEQQALAAK.R
1334.6592	1333.6519	1333.6812	-21.96	896 -	905	2	---	K.ELQKSMERWK.N
1335.6937	1334.6864	1334.7162	-22.33	925 -	935	2	---	K.MTNRQGMLLKK.K + Oxidation (M)
1338.6471	1337.6398	1337.6220	13.3	900 -	909	2	---	K.SMERWKNMEK.E
1345.6760	1344.6687	1344.6772	-6.32	684 -	695	1	---	R.KAEEELGELEAK.L
1434.7288	1433.7215	1433.6933	19.7	265 -	275	2	---	R.DKMEDIERQVR.E + Oxidation (M)
1485.7720	1484.7647	1484.7471	11.9	840 -	851	0	---	R.LDQVEQELNELR.E
1523.7841	1522.7768	1522.7562	13.5	747 -	758	2	---	R.QQSEKTFMPKQR.S + Oxidation (M)
1588.8136	1587.8063	1587.8039	1.55	144 -	157	1	---	K.INQMATAPDSQRLK.L + Oxidation (M)
1616.8605	1615.8532	1615.8569	-2.30	943 -	956	1	---	K.IRELGSLPQEAFEK.Y
1644.8046	1643.7973	1643.8049	-4.63	141 -	155	1	---	K.QGKINQMATAPDSQR.L
1706.8616	1705.8543	1705.8855	-18.26	921 -	934	2	---	K.ELEKMTNRQGMLLK.K + Oxidation (M)
1734.8152	1733.8079	1733.8319	-13.83	880 -	895	1	---	R.SEDLDNSIDKTEAGIK.E
1740.8768	1739.8695	1739.8301	22.7	208 -	220	2	---	K.EELAQYQKWDKMR.R + Oxidation (M)
1742.9031	1741.8958	1741.9071	-6.45	289 -	302	2	---	K.EQLSAERQEQIKQR.T
1786.9000	1785.8927	1785.9010	-4.61	373 -	388	2	---	R.TDLYAKQGRGSQFTSK.E
1786.9017	1785.8944	1785.9010	-3.66	373 -	388	2	---	R.TDLYAKQGRGSQFTSK.E
1844.8907	1843.8834	1843.9275	-23.91	286 -	300	2	---	K.EEKEQLSAERQEQIK.Q
1995.9020	1994.8947	1994.8826	6.06	645 -	661	0	---	R.AFTMDCITLEGDQVSHR.G + Oxidation (M)
2042.0640	2041.0567	2041.0440	6.25	437 -	453	2	---	K.LDQDLNEVKARVEELDR.K
2060.0469	2059.0396	2059.0116	13.6	757 -	774	1	---	K.QRSLQSLEASLHAMESTR.E + Oxidation (M)

No match to: 713.4334, 719.2388, 724.3009, 726.2899, 732.2586, 735.3865, 745.4124, 756.4687, 764.4056, 765.3908, 767.3679, 768.3909, 772.3955, 795.4186, 802.4548, 803.4370, 805.4229, 807.3893, 809.4480, 811.4104, 812.3985, 816.4408, 822.3905, 823.3846, 828.3904, 834.4043, 836.4589, 837.3853, 839.3696, 840.3839, 842.5085, 845.4100, 845.4123, 856.4893, 866.3914, 867.3724, 870.5402, 871.4945, 878.4625, 880.4403, 886.3815, 888.4233, 890.4479, 893.9562, 897.0289, 897.4412, 902.4597, 910.4410, 912.4497, 913.5118, 925.4747, 928.5064, 930.5048, 933.4619, 947.4628, 949.4591, 954.4674, 957.4657, 963.4676, 965.4761, 967.4568, 969.4571, 972.4793, 972.4800, 975.4650, 977.4630, 978.4976, 981.4565, 983.4664, 985.4842, 991.4688, 993.5331, 997.4298, 999.4453, 1001.4488, 1003.4687, 1007.5668, 1018.5200, 1018.5212, 1023.4777, 1026.4922, 1027.4972, 1030.5151, 1031.3850, 1033.5419, 1035.5352, 1036.5372, 1039.5402, 1042.5383, 1045.5654, 1060.5400, 1060.5442, 1065.5425, 1086.5115, 1106.5372, 1111.5582, 1113.5546, 1114.5396, 1115.5605, 1118.5529, 1122.5363, 1128.5775, 1129.6014, 1130.5935, 1140.5485, 1144.5709, 1155.6113, 1156.5720, 1157.5952, 1159.6273, 1173.6259, 1179.6063, 1182.6090, 1183.6123, 1185.6317, 1187.6431, 1193.6096, 1195.6060, 1196.6102, 1206.6770, 1209.1387, 1209.5848, 1210.1014, 1215.6627, 1216.6494, 1222.5994, 1226.5948, 1229.6246, 1231.6174, 1234.6317, 1240.6298, 1244.6324, 1248.6119, 1268.6498, 1271.6696, 1273.6653, 1275.6644, 1291.6425, 1295.7233, 1300.6062, 1307.6858, 1315.6570, 1317.6521, 1319.6852, 1323.6517, 1325.6637, 1328.6809, 1329.6672, 1331.6763, 1332.6743, 1336.6715, 1337.6724, 1342.6680, 1346.6880, 1349.6991, 1354.6907, 1355.7155, 1357.7034, 1360.6882, 1361.6670, 1365.6913, 1368.6920, 1374.6412, 1375.6798, 1376.6804, 1377.6794, 1382.6647, 1387.6444, 1410.7505, 1411.7208, 1421.7177, 1427.7872, 1431.7961, 1432.7133, 1446.7256, 1450.7079, 1473.8054, 1475.7748, 1477.7524, 1478.7439, 1486.7664, 1493.7539, 1494.7423, 1505.8649, 1524.7848, 1537.8021, 1549.7388, 1555.8816, 1558.8474, 1576.7987, 1579.7926, 1584.7897, 1590.3611, 1604.8417, 1605.8098, 1606.8384, 1615.8826, 1622.8137, 1628.8260, 1629.8358, 1630.8466, 1633.9790, 1635.7943, 1647.8785, 1649.8463, 1651.8245, 1659.8241, 1662.7909, 1668.7810, 1679.9736, 1681.8214, 1683.8121, 1684.8204, 1707.7838, 1716.8571, 1725.8353, 1728.8242, 1746.1659, 1750.8356, 1751.8787, 1753.8948, 1756.8905, 1757.8322, 1763.8188, 1765.7690, 1767.8036, 1768.9027, 1769.4475, 1770.4261, 1774.9091, 1783.8499, 1791.7335,

1808.8868, 1824.8674, 1840.8225, 1848.8322, 1850.8301, 1851.8488, 1867.9503, 1880.9277, 1887.9734, 1908.8838, 1914.9995, 1919.0038, 1925.9418, 1928.0103, 1931.9463, 1953.9353, 1975.9609, 1992.9642, 2007.9894, 2011.8845, 2013.8983, 2024.8591, 2025.6234, 2036.5195, 2036.9486, 2047.0167, 2047.9691, 2051.9792, 2052.9924, 2058.9963, 2064.9900, 2068.8982, 2070.8835, 2074.8914, 2083.9907, 2084.8987, 2086.8828, 2092.8821, 2095.9941, 2098.9976, 2100.9160, 2102.8872, 2106.9011, 2108.8835, 2109.9175, 2114.9180, 2116.9160, 2118.8835, 2119.8833, 2125.0176, 2132.8694, 2136.9180, 2170.1467, 2211.1045, 2225.1191, 2273.0496, 2277.0137, 2307.9941, 2322.9980, 2341.0366, 2343.0271, 2352.0571, 2355.0459, 2359.0532, 2366.0813, 2368.0544, 2369.0710, 2376.0264, 2382.0349, 2383.6099, 2383.9600, 2390.0403, 2396.0342, 2398.0576, 2400.0652, 2406.0737, 2411.0076, 2414.0813, 2416.0752, 2417.0732, 2420.0718, 2422.0408, 2423.0466, 2428.0898, 2430.0828, 2431.0845, 2433.0779, 2436.0508, 2438.0457, 2439.0261, 2442.0618, 2449.0347, 2452.0632, 2460.0254, 2461.9956, 2463.9858, 2510.1277, 2528.1580, 2542.1509, 2545.1558, 2547.1531, 2552.1038, 2565.1543, 2585.1111, 2601.0889, 2705.2192, 2717.0693, 3129.3176, 3208.1736, 3223.2710, 3290.4131, 3291.4248, 3312.2756, 3451.3933, 3453.4365

40. E1BMK2 **Mass:** 25092 **Score:** 17 **Expect:** 4.7e+002 **Matches:** 10

Uncharacterized protein OS=Bos taurus PE=4 SV=2

Observed	Mr(expt)	Mr(calc)	ppm	Start		End	Miss	Ions	Peptide
916.4905	915.4832	915.4814	1.97	89	-	96	1	---	K.FKDPNAPK.R
1036.5372	1035.5299	1035.5461	-15.63	66	-	73	2	---	K.VDKARYER.E
1128.5775	1127.5702	1127.5611	8.11	155	-	163	1	---	K.YEKDIAAYR.A
1129.6014	1128.5941	1128.6073	-11.72	1	-	10	2	---	-.MGKGDPKKPR.G + Oxidation (M)
1182.6090	1181.6017	1181.5750	22.6	56	-	65	2	---	K.EKGKFEDMAK.V
1325.6637	1324.6564	1324.6445	8.99	60	-	70	2	---	K.FEDMAKVDKAR.Y + Oxidation (M)
1537.8021	1536.7948	1536.7858	5.90	74	-	86	2	---	R.EMKTYIPPKGETK.K + Oxidation (M)
1681.8214	1680.8141	1680.7752	23.1	11	-	24	1	---	R.GKMSSYAFFVQTCR.E
2125.0176	2124.0103	2123.9694	19.3	31	-	48	2	---	K.HPDASVNFSEFSKKCSER.W
2136.9180	2135.9107	2135.9405	-13.92	13	-	29	1	---	K.MSSYAFFVQTCREEHTK.K + Oxidation (M)

No match to: 713.4334, 719.2388, 724.3009, 726.2899, 732.2586, 735.3865, 745.4124, 756.4687, 764.4056, 765.3908, 767.3679, 768.3909, 772.3955, 795.4186, 802.4548, 803.4370, 805.4229, 806.4631, 807.3893, 809.4480, 811.4104, 812.3985, 816.4408, 820.4244, 822.3905, 823.3846, 824.3756, 828.3904, 834.4043, 836.4589, 837.3853, 839.3696, 840.3839, 842.5085, 845.4100, 845.4123, 851.3975, 852.4056, 856.4893, 866.3914, 867.3724, 870.5402, 871.4945, 878.4625, 880.4403, 886.3815, 888.4233, 890.4479, 893.9562, 894.4579, 897.0289, 897.4412, 902.4597, 910.4410, 912.4497, 913.5118, 918.5391, 925.4747, 928.5064, 930.5048, 933.4619, 935.4698, 939.4758, 947.4628, 949.4591, 951.4703, 954.4674, 957.4657, 963.4676, 965.4761, 967.4568, 969.4571, 972.4793, 972.4800, 975.4650, 977.4630, 978.4976, 981.4565, 983.4664, 985.4842, 991.4688, 993.5331, 997.4298, 999.4453, 1001.4488, 1003.4687, 1007.5668, 1018.5200, 1018.5212, 1023.4777, 1026.4922, 1027.4972, 1030.5151, 1031.3850, 1033.5419, 1035.5352, 1039.5402, 1042.5383, 1045.5654, 1056.5488, 1060.5400, 1060.5442, 1065.5425, 1086.5115, 1106.5372, 1111.5582, 1113.5546, 1114.5396, 1115.5605, 1118.5529, 1122.5363, 1130.5935, 1140.5485, 1144.5709, 1155.6113, 1156.5720, 1157.5952, 1158.6154, 1159.6273, 1165.5817, 1172.6301, 1173.6259, 1179.6063, 1180.6165, 1183.6123, 1185.6317, 1187.6431, 1193.6096, 1195.6060, 1196.6102, 1206.6770, 1209.1387, 1209.5848, 1210.1014, 1215.6627, 1216.6494, 1222.5994, 1226.5948, 1229.6246, 1231.6174, 1234.6317, 1235.5846, 1240.6298, 1244.6324, 1248.6119, 1250.6144, 1268.6498, 1271.6696, 1273.6653, 1275.6644, 1291.6425, 1295.7233, 1300.6062, 1301.6205, 1307.6858, 1315.6570, 1317.6521, 1319.6852, 1323.6517, 1328.6809, 1329.6672, 1331.6763, 1332.6743, 1334.6592, 1335.6937, 1336.6715, 1337.6724, 1338.6471, 1342.6680, 1345.6760, 1346.6880, 1349.6991, 1354.6907, 1355.7155, 1357.7034, 1360.6882, 1361.6670, 1365.6913, 1368.6920, 1374.6412, 1375.6798, 1376.6804, 1377.6794, 1382.6647, 1387.6444, 1410.7505, 1411.7208, 1421.7177, 1427.7872, 1431.7961, 1432.7133, 1434.7288, 1446.7256, 1450.7079, 1473.8054, 1475.7748, 1477.7524, 1478.7439, 1485.7720, 1486.7664, 1493.7539, 1494.7423, 1505.8649, 1523.7841, 1524.7848, 1549.7388, 1555.8816, 1558.8474, 1576.7987, 1579.7926, 1584.7897, 1588.8136, 1590.3611, 1604.8417, 1605.8098, 1606.8384, 1615.8826, 1616.8605, 1622.8137, 1628.8260, 1629.8358, 1630.8466, 1633.9790, 1635.7943, 1644.8046, 1647.8785, 1649.8463, 1651.8245, 1659.8241, 1662.7909, 1668.7810, 1679.9736, 1683.8121, 1684.8204, 1706.8616, 1707.7838, 1716.8571, 1725.8353, 1728.8242, 1734.8152, 1740.8768, 1742.9031, 1746.1659, 1750.8356, 1751.8787, 1753.8948, 1756.8905, 1757.8322, 1763.8188, 1765.7690, 1767.8036, 1768.9027, 1769.4475, 1770.4261, 1774.9091, 1783.8499, 1786.9000, 1786.9017, 1791.7335, 1808.8868, 1824.8674, 1840.8225, 1844.8907, 1848.8322, 1850.8301, 1851.8488, 1867.9503, 1880.9277, 1887.9734, 1908.8838, 1914.9995, 1919.0038, 1925.9418, 1928.0103, 1931.9463, 1953.9353, 1975.9609, 1992.9642, 1995.9020, 2007.9894, 2011.8845, 2013.8983, 2024.8591, 2025.6234, 2036.5195, 2036.9486, 2042.0640, 2047.0167, 2047.9691, 2051.9792, 2052.9924, 2058.9963, 2060.0469, 2064.9900, 2068.8982, 2070.8835, 2074.8914, 2083.9907, 2084.8987, 2086.8828, 2092.8821, 2095.9941, 2098.9976, 2100.9160, 2102.8872, 2106.9011, 2108.8835, 2109.9175, 2114.9180, 2116.9160, 2118.8835, 2119.8833, 2132.8694, 2170.1467, 2211.1045, 2225.1191, 2273.0496, 2277.0137, 2307.9941, 2322.9980, 2341.0366, 2343.0271, 2352.0571, 2355.0459, 2359.0532, 2366.0813, 2368.0544, 2369.0710, 2376.0264, 2382.0349, 2383.6099, 2383.9600, 2390.0403, 2396.0342, 2398.0576, 2400.0652, 2406.0737, 2411.0076, 2414.0813, 2416.0752, 2417.0732, 2420.0718, 2422.0408, 2423.0466, 2428.0898, 2430.0828, 2431.0845, 2433.0779, 2436.0508, 2438.0457, 2439.0261, 2442.0618, 2449.0347, 2452.0632, 2460.0254, 2461.9956, 2463.9858, 2510.1277, 2528.1580, 2542.1509, 2545.1558, 2547.1531, 2552.1038, 2565.1543, 2585.1111, 2601.0889, 2705.2192, 2717.0693, 3129.3176, 3208.1736, 3223.2710, 3290.4131, 3291.4248, 3312.2756, 3451.3933, 3453.4365

41. A7MBI0 **Mass:** 50969 **Score:** 17 **Expect:** 4.9e+002 **Matches:** 17

Protein kinase C and casein kinase substrate in neurons protein 1 OS=Bos taurus GN=PACSIN1 PE=2 SV=1

Observed	Mr(expt)	Mr(calc)	ppm	Start		End	Miss	Ions	Peptide
745.4124	744.4051	744.4018	4.51	212	-	218	0	---	K.VLDDVGK.T
802.4548	801.4475	801.4497	-2.76	113	-	118	1	---	K.VKNWQK.D
925.4747	924.4674	924.4552	13.2	205	-	211	1	---	K.TQEKYEK.V
935.4698	934.4625	934.4793	-17.99	150	-	157	1	---	K.MKELEAAK.K + Oxidation (M)
951.4703	950.4631	950.4457	18.2	134	-	141	1	---	K.EAEDGFRK.A
1165.5817	1164.5744	1164.6026	-24.23	209	-	218	1	---	K.YEKVLDDVGK.T
1248.6119	1247.6046	1247.5968	6.26	159	-	168	1	---	K.AYHLACKEEK.L
1376.6804	1375.6731	1375.6918	-13.56	158	-	168	2	---	K.KAYHLACKEEK.L
1450.7079	1449.7006	1449.7364	-24.70	55	-	66	1	---	K.AYAQQLTDWAKR.W
1523.7841	1522.7768	1522.7627	9.27	200	-	211	2	---	K.QDVQKTQEKYEK.V
1651.8245	1650.8172	1650.8465	-17.70	205	-	218	2	---	K.TQEKYEKVLDDVGK.T
1768.9027	1767.8954	1767.8614	19.3	119	-	133	2	---	K.DAYHKQIMGGFKETK.E + Oxidation (M)
2007.9894	2006.9821	2006.9579	12.1	174	-	190	2	---	R.EMNSKTEQSVTPEQQKK.L + Oxidation (M)
2092.8821	2091.8748	2091.9167	-20.03	408	-	425	1	---	K.AGDELTKLGEEDEQGWCR.G
2390.0403	2389.0330	2389.0685	-14.86	31	-	49	2	---	K.RIDDGHRLCNDLMSCVQER.A + Oxidation (M)
2417.0732	2416.0659	2416.1046	-15.99	32	-	51	2	---	R.IDDGHRLCNDLMSCVQERAK.I
2433.0779	2432.0706	2432.0995	-11.87	32	-	51	2	---	R.IDDGHRLCNDLMSCVQERAK.I + Oxidation (M)

No match to: 713.4334, 719.2388, 724.3009, 726.2899, 732.2586, 735.3865, 756.4687, 764.4056, 765.3908, 767.3679, 768.3909, 772.3955, 795.4186, 803.4370, 805.4229, 806.4631, 807.3893, 809.4480, 811.4104, 812.3985, 816.4408, 820.4244, 822.3905, 823.3846, 824.3756, 828.3904, 834.4043, 836.4589, 837.3853, 839.3696, 840.3839, 842.5085, 845.4100, 845.4123, 851.3975, 852.4056, 856.4893, 866.3914, 867.3724, 870.5402, 871.4945, 878.4625, 880.4403, 886.3815, 888.4233, 890.4479, 893.9562, 894.4579, 897.0289, 897.4412, 902.4597, 910.4410, 912.4497, 913.5118, 916.4905, 918.5391, 928.5064, 930.5048, 933.4619, 939.4758, 947.4628, 949.4591, 954.4674, 957.4657, 963.4676, 965.4761, 967.4568, 969.4571, 972.4793, 972.4800, 975.4650, 977.4630, 978.4976, 981.4565, 983.4664, 985.4842, 991.4688, 993.5331, 997.4298, 999.4453, 1001.4488, 1003.4687, 1007.5668, 1018.5200, 1018.5212, 1023.4777, 1026.4922, 1027.4972, 1030.5151, 1031.3850, 1033.5419, 1035.5352, 1036.5372, 1039.5402, 1042.5383, 1045.5654, 1056.5488, 1060.5400, 1060.5442, 1065.5425, 1086.5115, 1106.5372, 1111.5582, 1113.5546, 1114.5396, 1115.5605, 1118.5529, 1122.5363, 1128.5775, 1129.6014, 1130.5935, 1140.5485, 1144.5709, 1155.6113, 1156.5720, 1157.5952, 1158.6154, 1159.6273, 1172.6301, 1173.6259, 1179.6063, 1180.6165, 1182.6090, 1183.6123, 1185.6317, 1187.6431, 1193.6096, 1195.6060, 1196.6102, 1206.6770, 1209.1387, 1209.5848, 1210.1014, 1215.6627, 1216.6494, 1222.5994, 1226.5948, 1229.6246, 1231.6174, 1234.6317, 1235.5846, 1240.6298, 1244.6324, 1250.6144, 1268.6498, 1271.6696, 1273.6653, 1275.6644, 1291.6425, 1295.7233, 1300.6062, 1301.6205, 1307.6858, 1315.6570, 1317.6521, 1319.6852, 1323.6517, 1325.6637, 1328.6809, 1329.6672, 1331.6763, 1332.6743, 1334.6592, 1335.6937, 1336.6715, 1337.6724, 1338.6471, 1342.6680, 1345.6760, 1346.6880, 1349.6991, 1354.6907, 1355.7155, 1357.7034, 1360.6882, 1361.6670, 1365.6913, 1368.6920, 1374.6412, 1375.6798, 1377.6794, 1382.6647, 1387.6444, 1410.7505, 1411.7208, 1421.7177, 1427.7872, 1431.7961, 1432.7133, 1434.7288, 1446.7256, 1473.8054, 1475.7748, 1477.7524, 1478.7439, 1485.7720, 1486.7664, 1493.7539, 1494.7423, 1505.8649, 1524.7848, 1537.8021, 1549.7388, 1555.8816, 1558.8474, 1576.7987, 1579.7926, 1584.7897, 1588.8136, 1590.3611, 1604.8417, 1605.8098, 1606.8384, 1615.8826, 1616.8605, 1622.8137, 1628.8260, 1629.8358, 1630.8466, 1633.9790,

1635.7943, 1644.8046, 1647.8785, 1649.8463, 1659.8241, 1662.7909, 1668.7810, 1679.9736, 1681.8214, 1683.8121, 1684.8204, 1706.8616, 1707.7838, 1716.8571, 1725.8353, 1728.8242, 1734.8152, 1740.8768, 1742.9031, 1746.1659, 1750.8356, 1751.8787, 1753.8948, 1756.8905, 1757.8322, 1763.8188, 1765.7690, 1767.8036, 1769.4475, 1770.4261, 1774.9091, 1783.8499, 1786.9000, 1786.9017, 1791.7335, 1808.8868, 1824.8674, 1840.8225, 1844.8907, 1848.8322, 1850.8301, 1851.8488, 1867.9503, 1880.9277, 1887.9734, 1908.8838, 1914.9995, 1919.0038, 1925.9418, 1928.0103, 1931.9463, 1953.9353, 1975.9609, 1992.9642, 1995.9020, 2011.8845, 2013.8983, 2024.8591, 2025.6234, 2036.5195, 2036.9486, 2042.0640, 2047.0167, 2047.9691, 2051.9792, 2052.9924, 2058.9963, 2060.0469, 2064.9900, 2068.8982, 2070.8835, 2074.8914, 2083.9907, 2084.8987, 2086.8828, 2095.9941, 2098.9976, 2100.9160, 2102.8872, 2106.9011, 2108.8835, 2109.9175, 2114.9180, 2116.9160, 2118.8835, 2119.8833, 2125.0176, 2132.8694, 2136.9180, 2170.1467, 2211.1045, 2225.1191, 2273.0496, 2277.0137, 2307.9941, 2322.9980, 2341.0366, 2343.0271, 2352.0571, 2355.0459, 2359.0532, 2366.0813, 2368.0544, 2369.0710, 2376.0264, 2382.0349, 2383.6099, 2383.9600, 2396.0342, 2398.0576, 2400.0652, 2406.0737, 2411.0076, 2414.0813, 2416.0752, 2420.0718, 2422.0408, 2423.0466, 2428.0898, 2430.0828, 2431.0845, 2436.0508, 2438.0457, 2439.0261, 2442.0618, 2449.0347, 2452.0632, 2460.0254, 2461.9956, 2463.9858, 2510.1277, 2528.1580, 2542.1509, 2545.1558, 2547.1531, 2552.1038, 2565.1543, 2585.1111, 2601.0889, 2705.2192, 2717.0693, 3129.3176, 3208.1736, 3223.2710, 3290.4131, 3291.4248, 3312.2756, 3451.3933, 3453.4365

42. G3N0Z2 **Mass:** 40114 **Score:** 17 **Expect:** 5.1e+002 **Matches:** 4
Uncharacterized protein OS=Bos taurus PE=3 SV=1

Observed	Mr(expt)	Mr(calc)	ppm	Start	End	Miss	Ions	Peptide
1222.5994	1221.5921	1221.6210	-23.60	199 -	209	0	---	R.VNSSMLMLVGR.D + Oxidation (M)
1786.9000	1785.8927	1785.9301	-20.94	180 -	195	1	19	K.KQGAISDPVFSFYLSK.K
1786.9017	1785.8944	1785.9301	-19.99	181 -	196	1	---	K.QGAISDPVFSFYLSKK.C
1914.9995	1913.9922	1914.0251	-17.17	180 -	196	2	---	K.KQGAISDPVFSFYLSKK.C

No match to: 713.4334, 719.2388, 724.3009, 726.2899, 732.2586, 735.3865, 745.4124, 756.4687, 764.4056, 765.3908, 767.3679, 768.3909, 772.3955, 795.4186, 802.4548, 803.4370, 805.4229, 806.4631, 807.3893, 809.4480, 811.4104, 812.3985, 816.4408, 820.4244, 822.3905, 823.3846, 824.3756, 828.3904, 834.4043, 836.4589, 837.3853, 839.3696, 840.3839, 842.5085, 845.4100, 845.4123, 851.3975, 852.4056, 856.4893, 866.3914, 867.3724, 870.5402, 871.4945, 878.4625, 880.4403, 886.3815, 888.4233, 890.4479, 893.9562, 894.4579, 897.0289, 897.4412, 902.4597, 910.4410, 912.4497, 913.5118, 916.4905, 918.5391, 925.4747, 928.5064, 930.5048, 933.4619, 935.4698, 939.4758, 947.4628, 949.4591, 951.4703, 954.4674, 957.4657, 963.4676, 965.4761, 967.4568, 969.4571, 972.4793, 972.4800, 975.4650, 977.4630, 978.4976, 981.4565, 983.4664, 985.4842, 991.4688, 993.5331, 997.4298, 999.4453, 1001.4488, 1003.4687, 1007.5668, 1018.5200, 1018.5212, 1023.4777, 1026.4922, 1027.4972, 1030.5151, 1031.3850, 1033.5419, 1035.5352, 1036.5372, 1039.5402, 1042.5383, 1045.5654, 1056.5488, 1060.5400, 1060.5442, 1065.5425, 1086.5115, 1106.5372, 1111.5582, 1113.5546, 1114.5396, 1115.5605, 1118.5529, 1122.5363, 1128.5775, 1129.6014, 1130.5935, 1140.5485, 1144.5709, 1155.6113, 1156.5720, 1157.5952, 1158.6154, 1159.6273, 1165.5817, 1172.6301, 1173.6259, 1179.6063, 1180.6165, 1182.6090, 1183.6123, 1185.6317, 1187.6431, 1193.6096, 1195.6060, 1196.6102, 1206.6770, 1209.1387, 1209.5848, 1210.1014, 1215.6627, 1216.6494, 1226.5948, 1229.6246, 1231.6174, 1234.6317, 1235.5846, 1240.6298, 1244.6324, 1248.6119, 1250.6144, 1268.6498, 1271.6696, 1273.6653, 1275.6644, 1291.6425, 1295.7233, 1300.6062, 1301.6205, 1307.6858, 1315.6570, 1317.6521, 1319.6852, 1323.6517, 1325.6637, 1328.6809, 1329.6672, 1331.6763, 1332.6743, 1334.6592, 1335.6937, 1336.6715, 1337.6724, 1338.6471, 1342.6680, 1345.6760, 1346.6880, 1349.6991, 1354.6907, 1355.7155, 1357.7034, 1360.6882, 1361.6670, 1365.6913, 1368.6920, 1374.6412, 1375.6798, 1376.6804, 1377.6794, 1382.6647, 1387.6444, 1410.7505, 1411.7208, 1421.7177, 1427.7872, 1431.7961, 1432.7133, 1434.7288, 1446.7256, 1450.7079, 1473.8054, 1475.7748, 1477.7524, 1478.7439, 1485.7720, 1486.7664, 1493.7539, 1494.7423, 1505.8649, 1523.7841, 1524.7848, 1537.8021, 1549.7388, 1555.8816, 1558.8474, 1576.7987, 1579.7926, 1584.7897, 1588.8136, 1590.3611, 1604.8417, 1605.8098, 1606.8384, 1615.8826, 1616.8605, 1622.8137, 1628.8260, 1629.8358, 1630.8466, 1633.9790, 1635.7943, 1644.8046, 1647.8785, 1649.8463, 1651.8245, 1659.8241, 1662.7909, 1668.7810, 1679.9736, 1681.8214, 1683.8121, 1684.8204, 1706.8616, 1707.7838, 1716.8571, 1725.8353, 1728.8242, 1734.8152, 1740.8768, 1742.9031, 1746.1659, 1750.8356, 1751.8787, 1753.8948, 1756.8905, 1757.8322, 1763.8188, 1765.7690, 1767.8036, 1768.9027, 1769.4475, 1770.4261, 1774.9091, 1783.8499, 1791.7335, 1808.8868, 1824.8674, 1840.8225, 1844.8907, 1848.8322, 1850.8301, 1851.8488, 1867.9503, 1880.9277, 1887.9734, 1908.8838, 1919.0038, 1925.9418, 1928.0103, 1931.9463, 1953.9353, 1975.9609, 1992.9642, 1995.9020, 2007.9894, 2011.8845, 2013.8983, 2024.8591, 2025.6234, 2036.5195, 2036.9486, 2042.0640, 2047.0167, 2047.9691, 2051.9792, 2052.9924, 2058.9963, 2060.0469, 2064.9900, 2068.8982, 2070.8835, 2074.8914, 2083.9907, 2084.8987, 2086.8828, 2092.8821, 2095.9941, 2098.9976, 2100.9160, 2102.8872, 2106.9011, 2108.8835, 2109.9175, 2114.9180, 2116.9160, 2118.8835, 2119.8833, 2125.0176, 2132.8694, 2136.9180, 2170.1467, 2211.1045, 2225.1191, 2273.0496, 2277.0137, 2307.9941, 2322.9980, 2341.0366, 2343.0271, 2352.0571, 2355.0459, 2359.0532, 2366.0813, 2368.0544, 2369.0710, 2376.0264, 2382.0349, 2383.6099, 2383.9600, 2390.0403, 2396.0342, 2398.0576, 2400.0652, 2406.0737, 2411.0076, 2414.0813, 2416.0752, 2417.0732, 2420.0718, 2422.0408, 2423.0466, 2428.0898, 2430.0828, 2431.0845, 2433.0779, 2436.0508, 2438.0457, 2439.0261, 2442.0618, 2449.0347, 2452.0632, 2460.0254, 2461.9956, 2463.9858, 2510.1277, 2528.1580, 2542.1509, 2545.1558, 2547.1531, 2552.1038, 2565.1543, 2585.1111, 2601.0889, 2705.2192, 2717.0693, 3129.3176, 3208.1736, 3223.2710, 3290.4131, 3291.4248, 3312.2756, 3451.3933, 3453.4365

43. Q0II81 **Mass:** 14868 **Score:** 17 **Expect:** 5.2e+002 **Matches:** 8
Regulator of G-protein signaling 13 OS=Bos taurus GN=RGS13 PE=2 SV=1

Observed	Mr(expt)	Mr(calc)	ppm	Start	End	Miss	Ions	Peptide
1155.6113	1154.6040	1154.6005	3.04	109 -	117	1	---	K.SEMYQKLLK.T + Oxidation (M)
1173.6259	1172.6186	1172.5900	24.5	106 -	114	1	---	R.FLKSEMYQK.L
1179.6063	1178.5990	1178.5792	16.8	39 -	48	1	---	K.TASQWSRTSR.A
1215.6627	1214.6554	1214.6619	-5.30	115 -	125	1	---	K.LLKTIQANNS.-
1307.6858	1306.6785	1306.6742	3.33	38 -	48	2	---	K.KTASQWSRTSR.A
1728.8242	1727.8169	1727.7759	23.7	93 -	105	1	---	K.IVYMHMERDSYPR.F + 2 Oxidation (M)
2417.0732	2416.0659	2416.0827	-6.96	20 -	38	2	---	K.TEHSDENIKFWMACETYKK.T
2433.0779	2432.0706	2432.0777	-2.89	20 -	38	2	---	K.TEHSDENIKFWMACETYKK.T + Oxidation (M)

No match to: 713.4334, 719.2388, 724.3009, 726.2899, 732.2586, 735.3865, 745.4124, 756.4687, 764.4056, 765.3908, 767.3679, 768.3909, 772.3955, 795.4186, 802.4548, 803.4370, 805.4229, 806.4631, 807.3893, 809.4480, 811.4104, 812.3985, 816.4408, 820.4244, 822.3905, 823.3846, 824.3756, 828.3904, 834.4043, 836.4589, 837.3853, 839.3696, 840.3839, 842.5085, 845.4100, 845.4123, 851.3975, 852.4056, 856.4893, 866.3914, 867.3724, 870.5402, 871.4945, 878.4625, 880.4403, 886.3815, 888.4233, 890.4479, 893.9562, 894.4579, 897.0289, 897.4412, 902.4597, 910.4410, 912.4497, 913.5118, 916.4905, 918.5391, 925.4747, 928.5064, 930.5048, 933.4619, 935.4698, 939.4758, 947.4628, 949.4591, 951.4703, 954.4674, 957.4657, 963.4676, 965.4761, 967.4568, 969.4571, 972.4793, 972.4800, 975.4650, 977.4630, 978.4976, 981.4565, 983.4664, 985.4842, 991.4688, 993.5331, 997.4298, 999.4453, 1001.4488, 1003.4687, 1007.5668, 1018.5200, 1018.5212, 1023.4777, 1026.4922, 1027.4972, 1030.5151, 1031.3850, 1033.5419, 1035.5352, 1036.5372, 1039.5402, 1042.5383, 1045.5654, 1056.5488, 1060.5400, 1060.5442, 1065.5425, 1086.5115, 1106.5372, 1111.5582, 1113.5546, 1114.5396, 1115.5605, 1118.5529, 1122.5363, 1128.5775, 1129.6014, 1130.5935, 1140.5485, 1144.5709, 1156.5720, 1157.5952, 1158.6154, 1159.6273, 1165.5817, 1172.6301, 1180.6165, 1182.6090, 1183.6123, 1185.6317, 1187.6431, 1193.6096, 1195.6060, 1196.6102, 1206.6770, 1209.1387, 1209.5848, 1210.1014, 1216.6494, 1222.5994, 1226.5948, 1229.6246, 1231.6174, 1234.6317, 1235.5846, 1240.6298, 1244.6324, 1248.6119, 1250.6144, 1268.6498, 1271.6696, 1273.6653, 1275.6644, 1291.6425, 1295.7233, 1300.6062, 1301.6205, 1315.6570, 1317.6521, 1319.6852, 1323.6517, 1325.6637, 1328.6809, 1329.6672, 1331.6763, 1332.6743, 1334.6592, 1335.6937, 1336.6715, 1337.6724, 1338.6471, 1342.6680, 1345.6760, 1346.6880, 1349.6991, 1354.6907, 1355.7155, 1357.7034, 1360.6882, 1361.6670, 1365.6913, 1368.6920, 1374.6412, 1375.6798, 1376.6804, 1377.6794, 1382.6647, 1387.6444, 1410.7505, 1411.7208, 1421.7177, 1427.7872, 1431.7961, 1432.7133, 1434.7288, 1446.7256, 1450.7079, 1473.8054, 1475.7748, 1477.7524, 1478.7439, 1485.7720, 1486.7664, 1493.7539, 1494.7423, 1505.8649, 1523.7841, 1524.7848, 1537.8021, 1549.7388, 1555.8816, 1558.8474, 1576.7987, 1579.7926, 1584.7897, 1588.8136, 1590.3611, 1604.8417, 1605.8098, 1606.8384, 1615.8826, 1616.8605, 1622.8137, 1628.8260, 1629.8358, 1630.8466, 1633.9790, 1635.7943, 1644.8046, 1647.8785, 1649.8463, 1651.8245, 1659.8241, 1662.7909, 1668.7810, 1679.9736, 1681.8214, 1683.8121, 1684.8204, 1706.8616, 1707.7838, 1716.8571, 1725.8353, 1734.8152, 1740.8768, 1742.9031, 1746.1659, 1750.8356, 1751.8787, 1753.8948, 1756.8905, 1757.8322, 1763.8188, 1765.7690, 1767.8036, 1768.9027, 1769.4475, 1770.4261, 1774.9091, 1783.8499, 1786.9000, 1786.9017, 1791.7335, 1808.8868, 1824.8674, 1840.8225, 1844.8907, 1848.8322, 1850.8301, 1851.8488, 1867.9503, 1880.9277, 1887.9734, 1908.8838, 1914.9995, 1919.0038, 1925.9418, 1928.0103, 1931.9463, 1953.9353, 1975.9609, 1992.9642, 1995.9020, 2007.9894, 2011.8845, 2013.8983, 2024.8591, 2025.6234, 2036.5195, 2036.9486, 2042.0640, 2047.0167, 2047.9691, 2051.9792, 2052.9924, 2058.9963, 2060.0469, 2064.9900, 2068.8982, 2070.8835, 2074.8914, 2083.9907, 2084.8987, 2086.8828, 2092.8821, 2095.9941, 2098.9976, 2100.9160, 2102.8872, 2106.9011, 2108.8835, 2109.9175, 2114.9180, 2116.9160, 2118.8835, 2119.8833, 2125.0176, 2132.8694, 2136.9180, 2170.1467, 2211.1045, 2225.1191, 2273.0496, 2277.0137, 2307.9941, 2322.9980, 2341.0366, 2343.0271, 2352.0571, 2355.0459, 2359.0532, 2366.0813, 2368.0544, 2369.0710, 2376.0264, 2382.0349, 2383.6099, 2383.9600, 2390.0403, 2396.0342, 2398.0576, 2400.0652, 2406.0737, 2411.0076, 2414.0813, 2416.0752, 2420.0718, 2422.0408, 2423.0466, 2428.0898, 2430.0828, 2431.0845, 2436.0508, 2438.0457, 2439.0261, 2442.0618, 2449.0347, 2452.0632, 2460.0254, 2461.9956, 2463.9858, 2510.1277, 2528.1580, 2542.1509, 2545.1558, 2547.1531, 2552.1038, 2565.1543, 2585.1111, 2601.0889, 2705.2192, 2717.0693, 3129.3176, 3208.1736, 3223.2710, 3290.4131, 3291.4248, 3312.2756, 3451.3933, 3453.4365

44. P35246 **Mass:** 37724 **Score:** 16 **Expect:** 5.4e+002 **Matches:** 14

Pulmonary surfactant-associated protein D OS=Bos taurus GN=SFTPD PE=1 SV=2

Observed	Mr(expt)	Mr(calc)	ppm	Start	End	Miss	Ions	Peptide
735.3865	734.3792	734.3633	21.7	18 -	24	0	---	R.SLGAEMK.I
772.3955	771.3882	771.3875	0.96	57 -	63	1	---	R.EGPRGEK.G
965.4761	964.4689	964.4726	-3.92	163 -	173	0	---	R.GAPGEPGAPGR.A
967.4568	966.4495	966.4519	-2.49	64 -	74	0	---	K.GDPGSPGPAGR.A
1033.5419	1032.5346	1032.5538	-18.62	244 -	252	1	---	K.KAMLFPNGR.S
1114.5396	1113.5323	1113.5275	4.31	51 -	60	2	---	R.DGRDGREGPR.G
1215.6627	1214.6554	1214.6255	24.6	211 -	222	0	---	K.GESGLAEVNALR.Q
1226.5948	1225.5875	1225.5761	9.29	123 -	135	0	---	K.QGSMGPPGTPGPK.G + Oxidation (M)
1354.6907	1353.6834	1353.6962	-9.44	18 -	29	1	---	R.SLGAEMKIYSQK.T
1421.7177	1420.7104	1420.7133	-2.00	245 -	257	1	---	K.AMLFPNGRSVGEK.I + Oxidation (M)
1493.7539	1492.7466	1492.7746	-18.77	193 -	207	2	---	R.GPPGLKGDRGTPGER.G
1588.8136	1587.8063	1587.8118	-3.42	174 -	192	0	---	R.AGAPGPAGAIGPQGPSGAR.G
2052.9924	2051.9851	2051.9557	14.3	354 -	369	2	---	K.WNDKVCGEQRLVICEF.-
2277.0137	2276.0064	2276.0058	0.28	30 -	50	0	---	K.TMANACTLVMCSPPEDGLPGR.D

No match to: 713.4334, 719.2388, 724.3009, 726.2899, 732.2586, 745.4124, 756.4687, 764.4056, 765.3908, 767.3679, 768.3909, 795.4186, 802.4548, 803.4370, 805.4229, 806.4631, 807.3893, 809.4480, 811.4104, 812.3985, 816.4408, 820.4244, 822.3905, 823.3846, 824.3756, 828.3904, 834.4043, 836.4589, 837.3853, 839.3696, 840.3839, 842.5085, 845.4100, 845.4123, 851.3975, 852.4056, 856.4893, 866.3914, 867.3724, 870.5402, 871.4945, 878.4625, 880.4403, 886.3815, 888.4233, 890.4479, 893.9562, 894.4579, 897.0289, 897.4412, 902.4597, 910.4410, 912.4497, 913.5118, 916.4905, 918.5391, 925.4747, 928.5064, 930.5048, 933.4619, 935.4698, 939.4758, 947.4628, 949.4591, 951.4703, 954.4674, 957.4657, 963.4676, 969.4571, 972.4793, 972.4800, 975.4650, 977.4630, 978.4976, 981.4565, 983.4664, 985.4842, 991.4688, 993.5331, 997.4298, 999.4453, 1001.4488, 1003.4687, 1007.5668, 1018.5200, 1018.5212, 1023.4777, 1026.4922, 1027.4972, 1030.5151, 1031.3850, 1035.5352, 1036.5372, 1039.5402, 1042.5383, 1045.5654, 1056.5488, 1060.5400, 1060.5442, 1065.5425, 1086.5115, 1106.5372, 1111.5582, 1113.5546, 1115.5605, 1118.5529, 1122.5363, 1128.5775, 1129.6014, 1130.5935, 1140.5485, 1144.5709, 1155.6113, 1156.5720, 1157.5952, 1158.6154, 1159.6273, 1165.5817, 1172.6301, 1173.6259, 1179.6063, 1180.6165, 1182.6090, 1183.6123, 1185.6317, 1187.6431, 1193.6096, 1195.6060, 1196.6102, 1206.6770, 1209.1387, 1209.5848, 1210.1014, 1216.6494, 1222.5994, 1229.6246, 1231.6174, 1234.6317, 1235.5846, 1240.6298, 1244.6324, 1248.6119, 1250.6144, 1268.6498, 1271.6696, 1273.6653, 1275.6644, 1291.6425, 1295.7233, 1300.6062, 1301.6205, 1307.6858, 1315.6570, 1317.6521, 1319.6852, 1323.6517, 1325.6637, 1328.6809, 1329.6672, 1331.6763, 1332.6743, 1334.6592, 1335.6937, 1336.6715, 1337.6724, 1338.6471, 1342.6680, 1345.6760, 1346.6880, 1349.6991, 1355.7155, 1357.7034, 1360.6882, 1361.6670, 1365.6913, 1368.6920, 1374.6412, 1375.6798, 1376.6804, 1377.6794, 1382.6647, 1387.6444, 1410.7505, 1411.7208, 1427.7872, 1431.7961, 1432.7133, 1434.7288, 1446.7256, 1450.7079, 1473.8054, 1475.7748, 1477.7524, 1478.7439, 1485.7720, 1486.7664, 1494.7423, 1505.8649, 1523.7841, 1524.7848, 1537.8021, 1549.7388, 1555.8816, 1558.8474, 1576.7987, 1579.7926, 1584.7897, 1590.3611, 1604.8417, 1605.8098, 1606.8384, 1615.8826, 1616.8605, 1622.8137, 1628.8260, 1629.8358, 1630.8466, 1633.9790, 1635.7943, 1644.8046, 1647.8785, 1649.8463, 1651.8245, 1659.8241, 1662.7909, 1668.7810, 1679.9736, 1681.8214, 1683.8121, 1684.8204, 1706.8616, 1707.7838, 1716.8571, 1725.8353, 1728.8242, 1734.8152, 1740.8768, 1742.9031, 1746.1659, 1750.8356, 1751.8787, 1753.8948, 1756.8905, 1757.8322, 1763.8188, 1765.7690, 1767.8036, 1768.9027, 1769.4475, 1770.4261, 1774.9091, 1783.8499, 1786.9000, 1786.9017, 1791.7335, 1808.8868, 1824.8674, 1840.8225, 1844.8907, 1848.8322, 1850.8301, 1851.8488, 1867.9503, 1880.9277, 1887.9734, 1908.8838, 1914.9995, 1919.0038, 1925.9418, 1928.0103, 1931.9463, 1953.9353, 1975.9609, 1992.9642, 1995.9020, 2007.9894, 2011.8845, 2013.8983, 2024.8591, 2025.6234, 2036.5195, 2036.9486, 2042.0640, 2047.0167, 2047.9691, 2051.9792, 2058.9963, 2060.0469, 2064.9900, 2068.8982, 2070.8835, 2074.8914, 2083.9907, 2084.8987, 2086.8828, 2092.8821, 2095.9941, 2098.9976, 2100.9160, 2102.8872, 2106.9011, 2108.8835, 2109.9175, 2114.9180, 2116.9160, 2118.8835, 2119.8833, 2125.0176, 2132.8694, 2136.9180, 2170.1467, 2211.1045, 2225.1191, 2273.0496, 2307.9941, 2322.9980, 2341.0366, 2343.0271, 2352.0571, 2355.0459, 2359.0532, 2366.0813, 2368.0544, 2369.0710, 2376.0264, 2382.0349, 2383.6099, 2383.9600, 2390.0403, 2396.0342, 2398.0576, 2400.0652, 2406.0737, 2411.0076, 2414.0813, 2416.0752, 2417.0732, 2420.0718, 2422.0408, 2423.0466, 2428.0898, 2430.0828, 2431.0845, 2433.0779, 2436.0508, 2438.0457, 2439.0261, 2442.0618, 2449.0347, 2452.0632, 2460.0254, 2461.9956, 2463.9858, 2510.1277, 2528.1580, 2542.1509, 2545.1558, 2547.1531, 2552.1038, 2565.1543, 2585.1111, 2601.0889, 2705.2192, 2717.0693, 3129.3176, 3208.1736, 3223.2710, 3290.4131, 3291.4248, 3312.2756, 3451.3933, 3453.4365

45. F1MWQ0 **Mass:** 17662 **Score:** 16 **Expect:** 5.4e+002 **Matches:** 8

Complexin-3 OS=Bos taurus GN=CPLX3 PE=4 SV=1

Observed	Mr(expt)	Mr(calc)	ppm	Start	End	Miss	Ions	Peptide
745.4124	744.4051	744.4017	4.55	50 -	55	0	---	K.QLVEEK.M
993.5331	992.5258	992.5039	22.0	59 -	66	1	---	R.DAQFTQRK.A
1023.4777	1022.4704	1022.4451	24.8	32 -	41	0	---	K.SAAEAQGMSR.E + Oxidation (M)
1349.6991	1348.6918	1348.6847	5.25	59 -	69	2	---	R.DAQFTQRKAER.A
1475.7748	1474.7675	1474.7627	3.26	141 -	154	1	---	K.AQSTLGDLKQSAEK.C
1588.8136	1587.8063	1587.7823	15.1	1 -	14	1	---	-.MAFMVKTMVGGQLK.N + 3 Oxidation (M)
1995.9020	1994.8947	1994.8626	16.1	104 -	119	1	---	R.ELAKMIEEDTEEEEER.A + Oxidation (M)
2007.9894	2006.9821	2006.9843	-1.11	50 -	65	2	---	K.QLVEEKMERDAQFTQR.K

No match to: 713.4334, 719.2388, 724.3009, 726.2899, 732.2586, 735.3865, 756.4687, 764.4056, 765.3908, 767.3679, 768.3909, 772.3955, 795.4186, 802.4548, 803.4370, 805.4229, 806.4631, 807.3893, 809.4480, 811.4104, 812.3985, 816.4408, 820.4244, 822.3905, 823.3846, 824.3756, 828.3904, 834.4043, 836.4589, 837.3853, 839.3696, 840.3839, 842.5085, 845.4100, 845.4123, 851.3975, 852.4056, 856.4893, 866.3914, 867.3724, 870.5402, 871.4945, 878.4625, 880.4403, 886.3815, 888.4233, 890.4479, 893.9562, 894.4579, 897.0289, 897.4412, 902.4597, 910.4410, 912.4497, 913.5118, 916.4905, 918.5391, 925.4747, 928.5064, 930.5048, 933.4619, 935.4698, 939.4758, 947.4628, 949.4591, 951.4703, 954.4674, 957.4657, 963.4676, 965.4761, 967.4568, 969.4571, 972.4793, 972.4800, 975.4650, 977.4630, 978.4976, 981.4565, 983.4664, 985.4842, 991.4688, 997.4298, 999.4453, 1001.4488, 1003.4687, 1007.5668, 1018.5200, 1018.5212, 1026.4922, 1027.4972, 1030.5151, 1031.3850, 1033.5419, 1035.5352, 1036.5372, 1039.5402, 1042.5383, 1045.5654, 1056.5488, 1060.5400, 1060.5442, 1065.5425, 1086.5115, 1106.5372, 1111.5582, 1113.5546, 1114.5396, 1115.5605, 1118.5529, 1122.5363, 1128.5775, 1129.6014, 1130.5935, 1140.5485, 1144.5709, 1155.6113, 1156.5720, 1157.5952, 1158.6154, 1159.6273, 1165.5817, 1172.6301, 1173.6259, 1179.6063, 1180.6165, 1182.6090, 1183.6123, 1185.6317, 1187.6431, 1193.6096, 1195.6060, 1196.6102, 1206.6770, 1209.1387, 1209.5848, 1210.1014, 1215.6627, 1216.6494, 1222.5994, 1226.5948, 1229.6246, 1231.6174, 1234.6317, 1235.5846, 1240.6298, 1244.6324, 1248.6119, 1250.6144, 1268.6498, 1271.6696, 1273.6653, 1275.6644, 1291.6425, 1295.7233, 1300.6062, 1301.6205, 1307.6858, 1315.6570, 1317.6521, 1319.6852, 1323.6517, 1325.6637, 1328.6809, 1329.6672, 1331.6763, 1332.6743, 1334.6592, 1335.6937, 1336.6715, 1337.6724, 1338.6471, 1342.6680, 1345.6760, 1346.6880, 1354.6907, 1355.7155, 1357.7034, 1360.6882, 1361.6670, 1365.6913, 1368.6920, 1374.6412, 1375.6798, 1376.6804, 1377.6794, 1382.6647, 1387.6444, 1410.7505, 1411.7208, 1421.7177, 1427.7872, 1431.7961, 1432.7133, 1434.7288, 1446.7256, 1450.7079, 1473.8054, 1477.7524, 1478.7439, 1485.7720, 1486.7664, 1493.7539, 1494.7423, 1505.8649, 1523.7841, 1524.7848, 1537.8021, 1549.7388, 1555.8816, 1558.8474, 1576.7987, 1579.7926, 1584.7897, 1590.3611, 1604.8417, 1605.8098, 1606.8384, 1615.8826, 1616.8605, 1622.8137, 1628.8260, 1629.8358, 1630.8466, 1633.9790, 1635.7943, 1644.8046, 1647.8785, 1649.8463, 1651.8245, 1659.8241, 1662.7909, 1668.7810, 1679.9736, 1681.8214, 1683.8121, 1684.8204, 1706.8616, 1707.7838, 1716.8571, 1725.8353, 1728.8242, 1734.8152, 1740.8768, 1742.9031, 1746.1659, 1750.8356, 1751.8787, 1753.8948, 1756.8905, 1757.8322, 1763.8188, 1765.7690, 1767.8036, 1768.9027, 1769.4475, 1770.4261, 1774.9091, 1783.8499, 1786.9000, 1786.9017, 1791.7335, 1808.8868, 1824.8674, 1840.8225, 1844.8907, 1848.8322, 1850.8301, 1851.8488, 1867.9503, 1880.9277, 1887.9734, 1908.8838, 1914.9995, 1919.0038, 1925.9418, 1928.0103, 1931.9463, 1953.9353, 1975.9609, 1992.9642, 2011.8845, 2013.8983, 2024.8591, 2025.6234, 2036.5195, 2036.9486, 2042.0640, 2047.0167, 2047.9691, 2051.9792, 2052.9924, 2058.9963, 2060.0469, 2064.9900, 2068.8982, 2070.8835, 2074.8914, 2083.9907, 2084.8987, 2086.8828, 2092.8821, 2095.9941, 2098.9976, 2100.9160, 2102.8872, 2106.9011, 2108.8835, 2109.9175, 2114.9180, 2116.9160, 2118.8835, 2119.8833, 2125.0176, 2132.8694, 2136.9180, 2170.1467, 2211.1045, 2225.1191, 2273.0496, 2277.0137, 2307.9941, 2322.9980, 2341.0366, 2343.0271, 2352.0571, 2355.0459, 2359.0532, 2366.0813, 2368.0544, 2369.0710, 2376.0264, 2382.0349, 2383.6099, 2383.9600, 2390.0403, 2396.0342, 2398.0576, 2400.0652, 2406.0737, 2411.0076, 2414.0813, 2416.0752, 2417.0732, 2420.0718, 2422.0408, 2423.0466, 2428.0898, 2430.0828, 2431.0845, 2433.0779, 2436.0508, 2438.0457, 2439.0261, 2442.0618, 2449.0347, 2452.0632, 2460.0254, 2461.9956, 2463.9858, 2510.1277, 2528.1580, 2542.1509, 2545.1558, 2547.1531, 2552.1038, 2565.1543, 2585.1111, 2601.0889, 2705.2192, 2717.0693, 3129.3176, 3208.1736, 3223.2710, 3290.4131, 3291.4248, 3312.2756, 3451.3933, 3453.4365

46. Q0VC09 **Mass:** 44563 **Score:** 16 **Expect:** 5.4e+002 **Matches:** 15

RIB43A-like with coiled-coils protein 1 OS=Bos taurus GN=RIBC1 PE=2 SV=1

Observed	Mr(expt)	Mr(calc)	ppm	Start	End	Miss	Ions	Peptide
965.4761	964.4689	964.4841	-15.75	269	275	0	---	R.VLPYCWK.G
1045.5654	1044.5581	1044.5424	15.0	83	90	2	---	K.EQAERTRR.L
1128.5775	1127.5702	1127.5683	1.69	167	175	0	---	R.QLQEQQQAR.V
1179.6063	1178.5990	1178.5787	17.2	208	219	0	---	R.IAMMAATASANK.A
1195.6060	1194.5987	1194.5736	21.0	208	219	0	---	R.IAMMAATASANK.A + Oxidation (M)
1275.6644	1274.6571	1274.6401	13.4	276	286	1	---	K.GMTPEQRATIR.K + Oxidation (M)
1323.6517	1322.6444	1322.6327	8.86	225	235	0	---	K.LAEQQGQEHQR.Q
1334.6592	1333.6519	1333.6846	-24.49	190	201	1	---	R.LAMDMRAAQLAK.L + Oxidation (M)
1375.6798	1374.6725	1374.6925	-14.53	196	207	1	---	R.AAQLAKLEESCR.I
1767.8036	1766.7963	1766.7835	7.27	109	121	0	---	R.CELDFWNSNQLWR.E
1953.9353	1952.9280	1952.9117	8.34	202	219	1	---	K.LEESCRIAMMAATASANK.A
2058.9963	2057.9890	2058.0164	-13.30	35	52	1	---	R.TRVMGVDVEALNNQVEER.K
2095.9941	2094.9868	2095.0368	-23.83	73	89	2	---	R.YDLVAQMLEKEQAERTR.R + Oxidation (M)
2125.0176	2124.0103	2124.0125	-1.04	190	207	2	---	R.LAMDMRAAQLAKLEESCR.I + 2 Oxidation (M)
2442.0618	2441.0545	2441.0443	4.18	176	195	1	---	R.VDENCADMLNDQLRLAMDMR.A + 2 Oxidation (M)

No match to: 713.4334, 719.2388, 724.3009, 726.2899, 732.2586, 735.3865, 745.4124, 756.4687, 764.4056, 765.3908, 767.3679, 768.3909, 772.3955, 795.4186, 802.4548, 803.4370, 805.4229, 806.4631, 807.3893, 809.4480, 811.4104, 812.3985, 816.4408, 820.4244, 822.3905, 823.3846, 824.3756, 828.3904, 834.4043, 836.4589, 837.3853, 839.3696, 840.3839, 842.5085, 845.4100, 845.4123, 851.3975, 852.4056, 856.4893, 866.3914, 867.3724, 870.5402, 871.4945, 878.4625, 880.4403, 886.3815, 888.4233, 890.4479, 893.9562, 894.4579, 897.0289, 897.4412, 902.4597, 910.4410, 912.4497, 913.5118, 916.4905, 918.5391, 925.4747, 928.5064, 930.5048, 933.4619, 935.4698, 939.4758, 947.4628, 949.4591, 951.4703, 954.4674, 957.4657, 963.4676, 967.4568, 969.4571, 972.4793, 972.4800, 975.4650, 977.4630, 978.4976, 981.4565, 983.4664, 985.4842, 991.4688, 993.5331, 997.4298, 999.4453, 1001.4488, 1003.4687, 1007.5668, 1018.5200, 1018.5212, 1023.4777, 1026.4922, 1027.4972, 1030.5151, 1031.3850, 1033.5419, 1035.5352, 1036.5372, 1039.5402, 1042.5383, 1056.5488, 1060.5400, 1060.5442, 1065.5425, 1086.5115, 1106.5372, 1111.5582, 1113.5546, 1114.5396, 1115.5605, 1118.5529, 1122.5363, 1129.6014, 1130.5935, 1140.5485, 1144.5709, 1155.6113, 1156.5720, 1157.5952, 1158.6154, 1159.6273, 1165.5817, 1172.6301, 1173.6259, 1180.6165, 1182.6090, 1183.6123, 1185.6317, 1187.6431, 1193.6096, 1196.6102, 1206.6770, 1209.1387, 1209.5848, 1210.1014, 1215.6627, 1216.6494, 1222.5994, 1226.5948, 1229.6246, 1231.6174, 1234.6317, 1235.5846, 1240.6298, 1244.6324, 1248.6119, 1250.6144, 1268.6498, 1271.6696, 1273.6653, 1291.6425, 1295.7233, 1300.6062, 1301.6205, 1307.6858, 1315.6570, 1317.6521, 1319.6852, 1325.6637, 1328.6809, 1329.6672, 1331.6763, 1332.6743, 1335.6937, 1336.6715, 1337.6724, 1338.6471, 1342.6680, 1345.6760, 1346.6880, 1349.6991, 1354.6907, 1355.7155, 1357.7034, 1360.6882, 1361.6670, 1365.6913, 1368.6920, 1374.6412, 1376.6804, 1377.6794, 1382.6647, 1387.6444, 1410.7505, 1411.7208, 1421.7177, 1427.7872, 1431.7961, 1432.7133, 1434.7288, 1446.7256, 1450.7079, 1473.8054, 1475.7748, 1477.7524, 1478.7439, 1485.7720, 1486.7664, 1493.7539, 1494.7423, 1505.8649, 1523.7841, 1524.7848, 1537.8021, 1549.7388, 1555.8816, 1558.8474, 1576.7987, 1579.7926, 1584.7897, 1588.8136, 1590.3611, 1604.8417, 1605.8098, 1606.8384, 1615.8826, 1616.8605, 1622.8137, 1628.8260, 1629.8358, 1630.8466, 1633.9790, 1635.7943, 1644.8046, 1647.8785, 1649.8463, 1651.8245, 1659.8241, 1662.7909, 1668.7810, 1679.9736, 1681.8214, 1683.8121, 1684.8204, 1706.8616, 1707.7838, 1716.8571, 1725.8353, 1728.8242, 1734.8152, 1740.8768, 1742.9031, 1746.1659, 1750.8356, 1751.8787, 1753.8948, 1756.8905, 1757.8322, 1763.8188, 1765.7690, 1768.9027, 1769.4475, 1770.4261, 1774.9091, 1783.8499, 1786.9000, 1786.9017, 1791.7335, 1808.8868, 1824.8674, 1840.8225, 1844.8907, 1848.8322, 1850.8301, 1851.8488, 1867.9503, 1880.9277, 1887.9734, 1908.8838, 1914.9995, 1919.0038, 1925.9418, 1928.0103, 1931.9463, 1975.9609, 1992.9642, 1995.9020, 2007.9894, 2011.8845, 2013.8983, 2024.8591, 2025.6234, 2036.5195, 2036.9486, 2042.0640, 2047.0167, 2047.9691, 2051.9792, 2052.9924, 2060.0469, 2064.9900, 2068.8982, 2070.8835, 2074.8914, 2083.9907, 2084.8987, 2086.8828, 2092.8821, 2098.9976, 2100.9160, 2102.8872, 2106.9011, 2108.8835, 2109.9175, 2114.9180, 2116.9160, 2118.8835, 2119.8833, 2132.8694, 2136.9180, 2170.1467, 2211.1045, 2225.1191, 2273.0496, 2277.0137, 2307.9941, 2322.9980, 2341.0366, 2343.0271, 2352.0571, 2355.0459, 2359.0532, 2366.0813, 2368.0544, 2369.0710, 2376.0264, 2382.0349, 2383.6099, 2383.9600, 2390.0403, 2396.0342, 2398.0576, 2400.0652, 2406.0737, 2411.0076, 2414.0813, 2416.0752, 2417.0732, 2420.0718, 2422.0408, 2423.0466, 2428.0898, 2430.0828, 2431.0845, 2433.0779, 2436.0508, 2438.0457, 2439.0261, 2449.0347, 2452.0632, 2460.0254, 2461.9956, 2463.9858, 2510.1277, 2528.1580, 2542.1509, 2545.1558, 2547.1531, 2552.1038, 2565.1543, 2585.1111, 2601.0889, 2705.2192, 2717.0693, 3129.3176, 3208.1736, 3223.2710, 3290.4131, 3291.4248, 3312.2756, 3451.3933, 3453.4365

47. E1BL95 **Mass:** 154749 **Score:** 16 **Expect:** 5.4e+002 **Matches:** 40

Uncharacterized protein OS=Bos taurus GN=CENPJ PE=4 SV=1

Observed	Mr(expt)	Mr(calc)	ppm	Start	End	Miss	Ions	Peptide
735.3865	734.3792	734.3963	-23.27	996	1001	1	---	R.TFPDKK.E
795.4186	794.4114	794.4286	-21.74	388	394	1	---	R.FTNAKSK.F
803.4370	802.4297	802.4184	14.1	932	939	0	---	K.AENASLAK.L
816.4408	815.4335	815.4250	10.5	1288	1294	1	---	R.VQRDGNK.I
820.4244	819.4171	819.4199	-3.36	784	790	1	---	K.LSDSSRR.H
910.4410	909.4338	909.4127	23.2	483	489	1	---	K.KCDGQFR.E
916.4905	915.4832	915.4886	-5.90	885	892	2	---	K.SKSKSHSR.S
947.4628	946.4556	946.4760	-21.57	80	87	0	---	K.LEPNSPYK.L
981.4565	980.4493	980.4419	7.47	1075	1083	0	---	R.MGQCVTASK.K
997.4298	996.4225	996.4256	-3.15	838	845	0	---	K.SEMCVLDK.T + Oxidation (M)
1033.5419	1032.5346	1032.5273	7.05	1050	1057	1	---	R.EEIKVMER.F
1115.5605	1114.5532	1114.5692	-14.36	291	300	0	---	K.GEGILPCLEK.M
1129.6014	1128.5941	1128.6040	-8.73	1304	1312	1	---	R.ELHTAQFKR.R
1172.6301	1171.6228	1171.6197	2.70	351	359	1	---	R.LEELERQQK.Q
1187.6431	1186.6358	1186.6598	-20.17	135	144	1	---	K.EVPYKDPLVK.K
1216.6494	1215.6421	1215.6598	-14.53	920	929	0	---	K.IIELETEIEK.F
1231.6174	1230.6101	1230.6356	-20.73	563	571	2	---	K.KLENWEREK.E
1235.5846	1234.5773	1234.5863	-7.31	1342	1352	1	---	K.DKDGNVLMDTK.L
1319.6852	1318.6779	1318.7106	-24.75	381	392	2	---	K.RGEGLARFTNAK.S
1323.6517	1322.6444	1322.6574	-9.81	838	848	1	---	K.SEMCVLDKTIK.R
1331.6763	1330.6690	1330.6841	-11.32	757	767	2	---	K.RRDVDLDLSDK.D
1336.6715	1335.6642	1335.6969	-24.44	1050	1059	2	---	R.EEIKVMERFR.L
1338.6471	1337.6398	1337.6649	-18.74	969	978	2	---	R.IEEFKKEEMR.K
1354.6907	1353.6834	1353.6598	17.4	969	978	2	---	R.IEEFKKEEMR.K + Oxidation (M)
1360.6882	1359.6809	1359.6782	1.99	564	573	2	---	K.LENWEREKEK.E
1432.7133	1431.7060	1431.6954	7.44	1129	1142	1	---	R.DLGSSEKGQTASPR.E
1434.7288	1433.7215	1433.7514	-20.84	953	964	2	---	R.KEIADFEQQKAK.E
1473.8054	1472.7981	1472.7973	0.54	918	929	1	---	R.EKIIELETEIEK.F
1486.7664	1485.7591	1485.7899	-20.74	1011	1022	2	---	K.RQVSDLQEDLKR.K
1494.7423	1493.7350	1493.7218	8.86	1340	1352	2	---	R.MKDKDGNVLMDTK.L
1584.7897	1583.7824	1583.7566	16.3	768	781	0	---	K.DYSSDDSLIIESLK.N
1659.8241	1658.8168	1658.7828	20.5	1143	1156	0	---	R.ELPEPVNFPDPDYK.E
1851.8488	1850.8415	1850.8258	8.52	663	679	0	---	R.DSVFSPGASWAPSCEVR.R
1887.9734	1886.9661	1886.9711	-2.63	1288	1303	2	---	R.VQRDGNKIIEFNNGQR.E
1931.9463	1930.9390	1930.8955	22.5	232	247	1	---	K.KHPSNILSSEQNDFCR.T
2051.9792	2050.9719	2050.9744	-1.21	866	884	0	---	R.GPSPPPTSGLMMEFFPSLK.S + 2 Oxidation (M)
2058.9963	2057.9890	2058.0242	-17.11	1111	1128	2	---	K.NYLPTQGNPSQRSKSEPR.D
2170.1467	2169.1394	2169.1641	-11.37	1004	1021	2	---	R.EEIQVLKRQVSDLQEDLK.R

2542.1509	2541.1436	2541.1626	-7.48	629 -	652	1	---	K.TATLGSVSQHNQNEDPGRAQCGSK.A
2565.1543	2564.1470	2564.2064	-23.15	279 -	300	2	---	K.NDLKEENDNYSKGEGILPCLEK.M

No match to: 713.4334, 719.2388, 724.3009, 726.2899, 732.2586, 745.4124, 756.4687, 764.4056, 765.3908, 767.3679, 768.3909, 772.3955, 802.4548, 805.4229, 806.4631, 807.3893, 809.4480, 811.4104, 812.3985, 822.3905, 823.3846, 824.3756, 828.3904, 834.4043, 836.4589, 837.3853, 839.3696, 840.3839, 842.5085, 845.4100, 845.4123, 851.3975, 852.4056, 856.4893, 866.3914, 867.3724, 870.5402, 871.4945, 878.4625, 880.4403, 886.3815, 888.4233, 890.4479, 893.9562, 894.4579, 897.0289, 897.4412, 902.4597, 912.4497, 913.5118, 918.5391, 925.4747, 928.5064, 930.5048, 933.4619, 935.4698, 939.4758, 949.4591, 951.4703, 954.4674, 957.4657, 963.4676, 965.4761, 967.4568, 969.4571, 972.4793, 972.4800, 975.4650, 977.4630, 978.4976, 983.4664, 985.4842, 991.4688, 993.5331, 999.4453, 1001.4488, 1003.4687, 1007.5668, 1018.5200, 1018.5212, 1023.4777, 1026.4922, 1027.4972, 1030.5151, 1031.3850, 1035.5352, 1036.5372, 1039.5402, 1042.5383, 1045.5654, 1056.5488, 1060.5400, 1060.5442, 1065.5425, 1086.5115, 1106.5372, 1111.5582, 1113.5546, 1114.5396, 1118.5529, 1122.5363, 1128.5775, 1130.5935, 1140.5485, 1144.5709, 1155.6113, 1156.5720, 1157.5952, 1158.6154, 1159.6273, 1165.5817, 1173.6259, 1179.6063, 1180.6165, 1182.6090, 1183.6123, 1185.6317, 1193.6096, 1195.6060, 1196.6102, 1206.6770, 1209.1387, 1209.5848, 1210.1014, 1215.6627, 1222.5994, 1226.5948, 1229.6246, 1234.6317, 1240.6298, 1244.6324, 1248.6119, 1250.6144, 1268.6498, 1271.6696, 1273.6653, 1275.6644, 1291.6425, 1295.7233, 1300.6062, 1301.6205, 1307.6858, 1315.6570, 1317.6521, 1325.6637, 1328.6809, 1329.6672, 1332.6743, 1334.6592, 1335.6937, 1337.6724, 1342.6680, 1345.6760, 1346.6880, 1349.6991, 1355.7155, 1357.7034, 1361.6670, 1365.6913, 1368.6920, 1374.6412, 1375.6798, 1376.6804, 1377.6794, 1382.6647, 1387.6444, 1410.7505, 1411.7208, 1421.7177, 1427.7872, 1431.7961, 1446.7256, 1450.7079, 1475.7748, 1477.7524, 1478.7439, 1485.7720, 1493.7539, 1505.8649, 1523.7841, 1524.7848, 1537.8021, 1549.7388, 1555.8816, 1558.8474, 1576.7987, 1579.7926, 1588.8136, 1590.3611, 1604.8417, 1605.8098, 1606.8384, 1615.8826, 1616.8605, 1622.8137, 1628.8260, 1629.8358, 1630.8466, 1633.9790, 1635.7943, 1644.8046, 1647.8785, 1649.8463, 1651.8245, 1662.7909, 1668.7810, 1679.9736, 1681.8214, 1683.8121, 1684.8204, 1706.8616, 1707.7838, 1716.8571, 1725.8353, 1728.8242, 1734.8152, 1740.8768, 1742.9031, 1746.1659, 1750.8356, 1751.8787, 1753.8948, 1756.8905, 1757.8322, 1763.8188, 1765.7690, 1767.8036, 1768.9027, 1769.4475, 1770.4261, 1774.9091, 1783.8499, 1786.9000, 1786.9017, 1791.7335, 1808.8868, 1824.8674, 1840.8225, 1844.8907, 1848.8322, 1850.8301, 1867.9503, 1880.9277, 1908.8838, 1914.9995, 1919.0038, 1925.9418, 1928.0103, 1953.9353, 1975.9609, 1992.9642, 1995.9020, 2007.9894, 2011.8845, 2013.8983, 2024.8591, 2025.6234, 2036.5195, 2036.9486, 2042.0640, 2047.0167, 2047.9691, 2052.9924, 2060.0469, 2064.9900, 2068.8982, 2070.8835, 2074.8914, 2083.9907, 2084.8987, 2086.8828, 2092.8821, 2095.9941, 2098.9976, 2100.9160, 2102.8872, 2106.9011, 2108.8835, 2109.9175, 2114.9180, 2116.9160, 2118.8835, 2119.8833, 2125.0176, 2132.8694, 2136.9180, 2211.1045, 2225.1191, 2273.0496, 2277.0137, 2307.9941, 2322.9980, 2341.0366, 2343.0271, 2352.0571, 2355.0459, 2359.0532, 2366.0813, 2368.0544, 2369.0710, 2376.0264, 2382.0349, 2383.6099, 2383.9600, 2390.0403, 2396.0342, 2398.0576, 2400.0652, 2406.0737, 2411.0076, 2414.0813, 2416.0752, 2417.0732, 2420.0718, 2422.0408, 2423.0466, 2428.0898, 2430.0828, 2431.0845, 2433.0779, 2436.0508, 2438.0457, 2439.0261, 2442.0618, 2449.0347, 2452.0632, 2460.0254, 2461.9956, 2463.9858, 2510.1277, 2528.1580, 2545.1558, 2547.1531, 2552.1038, 2585.1111, 2601.0889, 2705.2192, 2717.0693, 3129.3176, 3208.1736, 3223.2710, 3290.4131, 3291.4248, 3312.2756, 3451.3933, 3453.4365

48. P31976 **Mass:** 68832 **Score:** 16 **Expect:** 5.5e+002 **Matches:** 21

Ezrin OS=Bos taurus GN=EZR PE=1 SV=2

Observed	Mr(expt)	Mr(calc)	ppm	Start	End	Miss	Ions	Peptide
726.2899	725.2826	725.2836	-1.44	338 -	342	0	---	K.EQMMR.E + 2 Oxidation (M)
803.4370	802.4297	802.4184	14.1	375 -	380	1	---	K.LEEERK.R
816.4408	815.4335	815.4389	-6.56	231 -	237	1	---	K.DDKLTPK.I
852.4056	851.3984	851.4137	-18.03	144 -	151	0	---	K.AGYLGSER.L
890.4479	889.4407	889.4505	-11.04	331 -	337	1	---	R.ETVEREK.E
894.4579	893.4507	893.4719	-23.75	555 -	561	2	---	R.QGRDKYK.T
967.4568	966.4495	966.4263	24.1	338 -	344	1	---	K.EQMMREK.E + Oxidation (M)
1159.6273	1158.6200	1158.6357	-13.49	321 -	329	2	---	R.QQLETEKKR.R
1182.6090	1181.6017	1181.5869	12.5	264 -	273	0	---	K.APDFVFYAPR.L
1250.6144	1249.6071	1249.6091	-1.59	134 -	143	1	---	K.FGDYNKELHK.A
1332.6743	1331.6670	1331.6755	-6.36	296 -	306	0	---	R.KPDTIEVQQMK.A + Oxidation (M)
1346.6880	1345.6807	1345.6837	-2.21	508 -	518	2	---	R.NEEKRITEAEK.N
1477.7524	1476.7451	1476.7606	-10.48	525 -	537	0	---	R.QLMTLTSELSQAR.D
1493.7539	1492.7466	1492.7555	-5.96	525 -	537	0	---	R.QLMTLTSELSQAR.D + Oxidation (M)
1604.8417	1603.8344	1603.8405	-3.81	172 -	184	1	---	R.IQVWHAEHRGMLK.D
1628.8260	1627.8187	1627.8529	-20.99	399 -	412	2	---	R.AKEELERQAADQIK.S
1681.8214	1680.8141	1680.8206	-3.85	413 -	427	0	---	K.SQEQLATELAEYTAK.I
1742.9031	1741.8958	1741.9145	-10.71	296 -	310	1	---	R.KPDTIEVQQMKAQAR.E
1850.8301	1849.8228	1849.8602	-20.18	543 -	557	1	---	R.THNDIIHNENMRQGR.D + Oxidation (M)
1914.9995	1913.9922	1914.0105	-9.55	295 -	310	2	---	R.RKPDTIEVQQMKAQAR.E + Oxidation (M)
2083.9907	2082.9834	2083.0123	-13.85	134 -	151	2	---	K.FGDYNKELHKAGYLGSER.L

No match to: 713.4334, 719.2388, 724.3009, 732.2586, 735.3865, 745.4124, 756.4687, 764.4056, 765.3908, 767.3679, 768.3909, 772.3955, 795.4186, 802.4548, 805.4229, 806.4631, 807.3893, 809.4480, 811.4104, 812.3985, 820.4244, 822.3905, 823.3846, 824.3756, 828.3904, 834.4043, 836.4589, 837.3853, 839.3696, 840.3839, 842.5085, 845.4100, 845.4123, 851.3975, 856.4893, 866.3914, 867.3724, 870.5402, 871.4945, 878.4625, 880.4403, 886.3815, 888.4233, 893.9562, 897.0289, 897.4412, 902.4597, 910.4410, 912.4497, 913.5118, 916.4905, 918.5391, 925.4747, 928.5064, 930.5048, 933.4619, 935.4698, 939.4758, 947.4628, 949.4591, 951.4703, 954.4674, 957.4657, 963.4676, 965.4761, 969.4571, 972.4793, 972.4800, 975.4650, 977.4630, 978.4976, 981.4565, 983.4664, 985.4842, 991.4688, 993.5331, 997.4298, 999.4453, 1001.4488, 1003.4687, 1007.5668, 1018.5200, 1018.5212, 1023.4777, 1026.4922, 1027.4972, 1030.5151, 1031.3850, 1033.5419, 1035.5352, 1036.5372, 1039.5402, 1042.5383, 1045.5654, 1056.5488, 1060.5400, 1060.5442, 1065.5425, 1086.5115, 1106.5372, 1111.5582, 1113.5546, 1114.5396, 1115.5605, 1118.5529, 1122.5363, 1128.5775, 1129.6014, 1130.5935, 1140.5485, 1144.5709, 1155.6113, 1156.5720, 1157.5952, 1158.6154, 1165.5817, 1172.6301, 1173.6259, 1179.6063, 1180.6165, 1183.6123, 1185.6317, 1187.6431, 1193.6096, 1195.6060, 1196.6102, 1206.6770, 1209.1387, 1209.5848, 1210.1014, 1215.6627, 1216.6494, 1222.5994, 1226.5948, 1229.6246, 1231.6174, 1234.6317, 1235.5846, 1240.6298, 1244.6324, 1248.6119, 1268.6498, 1271.6696, 1273.6653, 1275.6644, 1291.6425, 1295.7233, 1300.6062, 1301.6205, 1307.6858, 1315.6570, 1317.6521, 1319.6852, 1323.6517, 1325.6637, 1328.6809, 1329.6672, 1331.6763, 1334.6592, 1335.6937, 1336.6715, 1337.6724, 1338.6471, 1342.6680, 1345.6760, 1349.6991, 1354.6907, 1355.7155, 1357.7034, 1360.6882, 1361.6670, 1365.6913, 1368.6920, 1374.6412, 1375.6798, 1376.6804, 1377.6794, 1382.6647, 1387.6444, 1410.7505, 1411.7208, 1421.7177, 1427.7872, 1431.7961, 1432.7133, 1434.7288, 1446.7256, 1450.7079, 1473.8054, 1475.7748, 1478.7439, 1485.7720, 1486.7664, 1494.7423, 1505.8649, 1523.7841, 1524.7848, 1537.8021, 1549.7388, 1555.8816, 1558.8474, 1576.7987, 1579.7926, 1584.7897, 1588.8136, 1590.3611, 1605.8098, 1606.8384, 1615.8826, 1616.8605, 1622.8137, 1629.8358, 1630.8466, 1633.9790, 1635.7943, 1644.8046, 1647.8785, 1649.8463, 1651.8245, 1659.8241, 1662.7909, 1668.7810, 1679.9736, 1683.8121, 1684.8204, 1706.8616, 1707.7838, 1716.8571, 1725.8353, 1728.8242, 1734.8152, 1740.8768, 1746.1659, 1750.8356, 1751.8787, 1753.8948, 1756.8905, 1757.8322, 1763.8188, 1765.7690, 1767.8036, 1768.9027, 1769.4475, 1770.4261, 1774.9091, 1783.8499, 1786.9000, 1786.9017, 1791.7335, 1808.8868, 1824.8674, 1840.8225, 1844.8907, 1848.8322, 1851.8488, 1867.9503, 1880.9277, 1887.9734, 1908.8838, 1919.0038, 1925.9418, 1928.0103, 1931.9463, 1953.9353, 1975.9609, 1992.9642, 1995.9020, 2007.9894, 2011.8845, 2013.8983, 2024.8591, 2025.6234, 2036.5195, 2036.9486, 2042.0640, 2047.0167, 2047.9691, 2051.9792, 2052.9924, 2058.9963, 2060.0469, 2064.9900, 2068.8982, 2070.8835, 2074.8914, 2084.8987, 2086.8828, 2092.8821, 2095.9941, 2098.9976, 2100.9160, 2102.8872, 2106.9011, 2108.8835, 2109.9175, 2114.9180, 2116.9160, 2118.8835, 2119.8833, 2125.0176, 2132.8694, 2136.9180, 2170.1467, 2211.1045, 2225.1191, 2273.0496, 2277.0137, 2307.9941, 2322.9980, 2341.0366, 2343.0271, 2352.0571, 2355.0459, 2359.0532, 2366.0813, 2368.0544, 2369.0710, 2376.0264, 2382.0349, 2383.6099, 2383.9600, 2390.0403, 2396.0342, 2398.0576, 2400.0652, 2406.0737, 2411.0076, 2414.0813, 2416.0752, 2417.0732, 2420.0718, 2422.0408, 2423.0466, 2428.0898, 2430.0828, 2431.0845, 2433.0779, 2436.0508, 2438.0457, 2439.0261, 2442.0618, 2449.0347, 2452.0632, 2460.0254, 2461.9956, 2463.9858, 2510.1277, 2528.1580, 2542.1509, 2545.1558, 2547.1531, 2552.1038, 2565.1543, 2585.1111, 2601.0889, 2705.2192, 2717.0693, 3129.3176, 3208.1736, 3223.2710, 3290.4131, 3291.4248, 3312.2756, 3451.3933, 3453.4365

49. Q2YDN4 **Mass:** 58176 **Score:** 16 **Expect:** 5.5e+002 **Matches:** 20

Coiled-coil domain-containing protein 105 OS=Bos taurus GN=CCDC105 PE=2 SV=1

Observed	Mr(expt)	Mr(calc)	ppm	Start	End	Miss	Ions	Peptide
880.4403	879.4330	879.4371	-4.66	204 -	210	1	---	K.EELKSMK.R + Oxidation (M)
981.4565	980.4493	980.4307	19.0	212 -	219	1	---	R.KMEADMEK.S
997.4298	996.4225	996.4256	-3.12	212 -	219	1	---	R.KMEADMEK.S + Oxidation (M)

1035.5352	1034.5279	1034.5430	-14.57	340 - 347	1	---	K.MRETLELK.D + Oxidation (M)
1036.5372	1035.5299	1035.5382	-8.03	204 - 211	2	---	K.EELKSMKR.K + Oxidation (M)
1106.5372	1105.5299	1105.5437	-12.49	432 - 440	0	---	K.NLDELLSMR.K + Oxidation (M)
1179.6063	1178.5990	1178.5900	7.67	332 - 341	1	---	R.VCASLAQKMR.E + Oxidation (M)
1183.6123	1182.6050	1182.5856	16.5	443 - 451	1	---	K.LTWSFNCKK.I
1234.6317	1233.6244	1233.6387	-11.56	432 - 441	1	---	K.NLDELLSMRK.K + Oxidation (M)
1319.6852	1318.6779	1318.6849	-5.32	348 - 358	1	---	K.DRLNMTLGLMR.G
1331.6763	1330.6690	1330.6953	-19.74	159 - 169	2	---	R.EATDRRLSEVR.K
1335.6937	1334.6864	1334.6799	4.92	348 - 358	1	---	K.DRLNMTLGLMR.G + Oxidation (M)
1494.7423	1493.7350	1493.7105	16.4	213 - 225	1	---	K.MEADMEKSEALLK.T
1579.7926	1578.7853	1578.7599	16.1	195 - 207	1	---	K.ADSMLTWEKEELK.S
1622.8137	1621.8064	1621.8055	0.59	212 - 225	2	---	R.KMEADMEKSEALLK.T
1628.8260	1627.8187	1627.8392	-12.57	367 - 379	1	---	K.FNQEMYITRGLIK.G + Oxidation (M)
1662.7909	1661.7836	1661.7930	-5.65	309 - 322	1	---	K.DTLLDMAKNEEDIR.E
1756.8905	1755.8832	1755.8838	-0.36	400 - 414	1	---	R.MYQRHVGTQLPEAAR.L
1840.8225	1839.8152	1839.8560	-22.17	188 - 203	1	---	K.SETISDKADSMLTWEK.E
1925.9418	1924.9345	1924.9274	3.71	195 - 210	2	---	K.ADSMLTWEKEELKSMK.R

No match to: 713.4334, 719.2388, 724.3009, 726.2899, 732.2586, 735.3865, 745.4124, 756.4687, 764.4056, 765.3908, 767.3679, 768.3909, 772.3955, 795.4186, 802.4548, 803.4370, 805.4229, 806.4631, 807.3893, 809.4480, 811.4104, 812.3985, 816.4408, 820.4244, 822.3905, 823.3846, 824.3756, 828.3904, 834.4043, 836.4589, 837.3853, 839.3696, 840.3839, 842.5085, 845.4100, 845.4123, 851.3975, 852.4056, 856.4893, 866.3914, 867.3724, 870.5402, 871.4945, 878.4625, 886.3815, 888.4233, 890.4479, 893.9562, 894.4579, 897.0289, 897.4412, 902.4597, 910.4410, 912.4497, 913.5118, 916.4905, 918.5391, 925.4747, 928.5064, 930.5048, 933.4619, 935.4698, 939.4758, 947.4628, 949.4591, 951.4703, 954.4674, 957.4657, 963.4676, 965.4761, 967.4568, 969.4571, 972.4793, 972.4800, 975.4650, 977.4630, 978.4976, 983.4664, 985.4842, 991.4688, 993.5331, 999.4453, 1001.4488, 1003.4687, 1007.5668, 1018.5200, 1018.5212, 1023.4777, 1026.4922, 1027.4972, 1030.5151, 1031.3850, 1033.5419, 1039.5402, 1042.5383, 1045.5654, 1056.5488, 1060.5400, 1060.5442, 1065.5425, 1086.5115, 1111.5582, 1113.5546, 1114.5396, 1115.5605, 1118.5529, 1122.5363, 1128.5775, 1129.6014, 1130.5935, 1140.5485, 1144.5709, 1155.6113, 1156.5720, 1157.5952, 1158.6154, 1159.6273, 1165.5817, 1172.6301, 1173.6259, 1180.6165, 1182.6090, 1185.6317, 1187.6431, 1193.6096, 1195.6060, 1196.6102, 1206.6770, 1209.1387, 1209.5848, 1210.1014, 1215.6627, 1216.6494, 1222.5994, 1226.5948, 1229.6246, 1231.6174, 1235.5846, 1240.6298, 1244.6324, 1248.6119, 1250.6144, 1268.6498, 1271.6696, 1273.6653, 1275.6644, 1291.6425, 1295.7233, 1300.6062, 1301.6205, 1307.6858, 1315.6570, 1317.6521, 1323.6517, 1325.6637, 1328.6809, 1329.6672, 1332.6743, 1334.6592, 1336.6715, 1337.6724, 1338.6471, 1342.6680, 1345.6760, 1346.6880, 1349.6991, 1354.6907, 1355.7155, 1357.7034, 1360.6882, 1361.6670, 1365.6913, 1368.6920, 1374.6412, 1375.6798, 1376.6804, 1377.6794, 1382.6647, 1387.6444, 1410.7505, 1411.7208, 1421.7177, 1427.7872, 1431.7961, 1432.7133, 1434.7288, 1446.7256, 1450.7079, 1473.8054, 1475.7748, 1477.7524, 1478.7439, 1485.7720, 1486.7664, 1493.7539, 1505.8649, 1523.7841, 1524.7848, 1537.8021, 1549.7388, 1555.8816, 1558.8474, 1576.7987, 1584.7897, 1588.8136, 1590.3611, 1604.8417, 1605.8098, 1606.8384, 1615.8826, 1616.8605, 1629.8358, 1630.8466, 1633.9790, 1635.7943, 1644.8046, 1647.8785, 1649.8463, 1651.8245, 1659.8241, 1668.7810, 1679.9736, 1681.8214, 1683.8121, 1684.8204, 1706.8616, 1707.7838, 1716.8571, 1725.8353, 1728.8242, 1734.8152, 1740.8768, 1742.9031, 1746.1659, 1750.8356, 1751.8787, 1753.8948, 1757.8322, 1763.8188, 1765.7690, 1767.8036, 1768.9027, 1769.4475, 1770.4261, 1774.9091, 1783.8499, 1786.9000, 1786.9017, 1791.7335, 1808.8868, 1824.8674, 1844.8907, 1848.8322, 1850.8301, 1851.8488, 1867.9503, 1880.9277, 1887.9734, 1908.8838, 1914.9995, 1919.0038, 1928.0103, 1931.9463, 1953.9353, 1975.9609, 1992.9642, 1995.9020, 2007.9894, 2011.8845, 2013.8983, 2024.8591, 2025.6234, 2036.5195, 2036.9486, 2042.0640, 2047.0167, 2047.9691, 2051.9792, 2052.9924, 2058.9963, 2060.0469, 2064.9900, 2068.8982, 2070.8835, 2074.8914, 2083.9907, 2084.8987, 2086.8828, 2092.8821, 2095.9941, 2098.9976, 2100.9160, 2102.8872, 2106.9011, 2108.8835, 2109.9175, 2114.9180, 2116.9160, 2118.8835, 2119.8833, 2125.0176, 2132.8694, 2136.9180, 2170.1467, 2211.1045, 2225.1191, 2273.0496, 2277.0137, 2307.9941, 2322.9980, 2341.0366, 2343.0271, 2352.0571, 2355.0459, 2359.0532, 2366.0813, 2368.0544, 2369.0710, 2376.0264, 2382.0349, 2383.6099, 2383.9600, 2390.0403, 2396.0342, 2398.0576, 2400.0652, 2406.0737, 2411.0076, 2414.0813, 2416.0752, 2417.0732, 2420.0718, 2422.0408, 2423.0466, 2428.0898, 2430.0828, 2431.0845, 2433.0779, 2436.0508, 2438.0457, 2439.0261, 2442.0618, 2449.0347, 2452.0632, 2460.0254, 2461.9956, 2463.9858, 2510.1277, 2528.1580, 2542.1509, 2545.1558, 2547.1531, 2552.1038, 2565.1543, 2585.1111, 2601.0889, 2705.2192, 2717.0693, 3129.3176, 3208.1736, 3223.2710, 3290.4131, 3291.4248, 3312.2756, 3451.3933, 3453.4365

50. G3MZ80 **Mass:** 7701 **Score:** 16 **Expect:** 5.7e+002 **Matches:** 7

Uncharacterized protein OS=Bos taurus PE=4 SV=1

Observed	Mr(expt)	Mr(calc)	ppm	Start	End	Miss	Ions	Peptide
949.4591	948.4518	948.4698	-19.06	32 -	39	1	---	R.EKVADTMR.Q
965.4761	964.4689	964.4648	4.25	32 -	39	1	---	R.EKVADTMR.Q + Oxidation (M)
1033.5419	1032.5346	1032.5274	7.04	40 -	48	0	---	R.QMEALQLGK.Q + Oxidation (M)
1216.6494	1215.6421	1215.6434	-1.05	49 -	58	1	---	K.QGMHLKIFDK.I
1473.8054	1472.7981	1472.8173	-13.05	49 -	60	2	---	K.QGMHLKIFDKIK.Y + Oxidation (M)
1706.8616	1705.8543	1705.8491	3.06	34 -	48	1	---	K.VADTMRQMEALQLGK.Q + Oxidation (M)
1757.8322	1756.8249	1756.8340	-5.16	18 -	33	1	---	R.QQQAETPTAADQGREK.V

No match to: 713.4334, 719.2388, 724.3009, 726.2899, 732.2586, 735.3865, 745.4124, 756.4687, 764.4056, 765.3908, 767.3679, 768.3909, 772.3955, 795.4186, 802.4548, 803.4370, 805.4229, 806.4631, 807.3893, 809.4480, 811.4104, 812.3985, 816.4408, 820.4244, 822.3905, 823.3846, 824.3756, 828.3904, 834.4043, 836.4589, 837.3853, 839.3696, 840.3839, 842.5085, 845.4100, 845.4123, 851.3975, 852.4056, 856.4893, 866.3914, 867.3724, 870.5402, 871.4945, 878.4625, 880.4403, 886.3815, 888.4233, 890.4479, 893.9562, 894.4579, 897.0289, 897.4412, 902.4597, 910.4410, 912.4497, 913.5118, 916.4905, 918.5391, 925.4747, 928.5064, 930.5048, 933.4619, 935.4698, 939.4758, 947.4628, 951.4703, 954.4674, 957.4657, 963.4676, 967.4568, 969.4571, 972.4793, 972.4800, 975.4650, 977.4630, 978.4976, 981.4565, 983.4664, 985.4842, 991.4688, 993.5331, 997.4298, 999.4453, 1001.4488, 1003.4687, 1007.5668, 1018.5200, 1018.5212, 1023.4777, 1026.4922, 1027.4972, 1030.5151, 1031.3850, 1035.5352, 1036.5372, 1039.5402, 1042.5383, 1045.5654, 1056.5488, 1060.5400, 1060.5442, 1065.5425, 1086.5115, 1106.5372, 1111.5582, 1113.5546, 1114.5396, 1115.5605, 1118.5529, 1122.5363, 1128.5775, 1129.6014, 1130.5935, 1140.5485, 1144.5709, 1155.6113, 1156.5720, 1157.5952, 1158.6154, 1159.6273, 1165.5817, 1172.6301, 1173.6259, 1179.6063, 1180.6165, 1182.6090, 1183.6123, 1185.6317, 1187.6431, 1193.6096, 1195.6060, 1196.6102, 1206.6770, 1209.1387, 1209.5848, 1210.1014, 1215.6627, 1222.5994, 1226.5948, 1229.6246, 1231.6174, 1234.6317, 1235.5846, 1240.6298, 1244.6324, 1248.6119, 1250.6144, 1268.6498, 1271.6696, 1273.6653, 1275.6644, 1291.6425, 1295.7233, 1300.6062, 1301.6205, 1307.6858, 1315.6570, 1317.6521, 1319.6852, 1323.6517, 1325.6637, 1328.6809, 1329.6672, 1331.6763, 1332.6743, 1334.6592, 1335.6937, 1336.6715, 1337.6724, 1338.6471, 1342.6680, 1345.6760, 1346.6880, 1349.6991, 1354.6907, 1355.7155, 1357.7034, 1360.6882, 1361.6670, 1365.6913, 1368.6920, 1374.6412, 1375.6798, 1376.6804, 1377.6794, 1382.6647, 1387.6444, 1410.7505, 1411.7208, 1421.7177, 1427.7872, 1431.7961, 1432.7133, 1434.7288, 1446.7256, 1450.7079, 1475.7748, 1477.7524, 1478.7439, 1485.7720, 1486.7664, 1493.7539, 1494.7423, 1505.8649, 1523.7841, 1524.7848, 1537.8021, 1549.7388, 1555.8816, 1558.8474, 1576.7987, 1579.7926, 1584.7897, 1588.8136, 1590.3611, 1604.8417, 1605.8098, 1606.8384, 1615.8826, 1616.8605, 1622.8137, 1628.8260, 1629.8358, 1630.8466, 1633.9790, 1635.7943, 1644.8046, 1647.8785, 1649.8463, 1651.8245, 1659.8241, 1662.7909, 1668.7810, 1679.9736, 1681.8214, 1683.8121, 1684.8204, 1707.7838, 1716.8571, 1725.8353, 1728.8242, 1734.8152, 1740.8768, 1742.9031, 1746.1659, 1750.8356, 1751.8787, 1753.8948, 1756.8905, 1763.8188, 1765.7690, 1767.8036, 1768.9027, 1769.4475, 1770.4261, 1774.9091, 1783.8499, 1786.9000, 1786.9017, 1791.7335, 1808.8868, 1824.8674, 1840.8225, 1844.8907, 1848.8322, 1850.8301, 1851.8488, 1867.9503, 1880.9277, 1887.9734, 1908.8838, 1914.9995, 1919.0038, 1925.9418, 1928.0103, 1931.9463, 1953.9353, 1975.9609, 1992.9642, 1995.9020, 2007.9894, 2011.8845, 2013.8983, 2024.8591, 2025.6234, 2036.5195, 2036.9486, 2042.0640, 2047.0167, 2047.9691, 2051.9792, 2052.9924, 2058.9963, 2060.0469, 2064.9900, 2068.8982, 2070.8835, 2074.8914, 2083.9907, 2084.8987, 2086.8828, 2092.8821, 2095.9941, 2098.9976, 2100.9160, 2102.8872, 2106.9011, 2108.8835, 2109.9175, 2114.9180, 2116.9160, 2118.8835, 2119.8833, 2125.0176, 2132.8694, 2136.9180, 2170.1467, 2211.1045, 2225.1191, 2273.0496, 2277.0137, 2307.9941, 2322.9980, 2341.0366, 2343.0271, 2352.0571, 2355.0459, 2359.0532, 2366.0813, 2368.0544, 2369.0710, 2376.0264, 2382.0349, 2383.6099, 2383.9600, 2390.0403, 2396.0342, 2398.0576, 2400.0652, 2406.0737, 2411.0076, 2414.0813, 2416.0752, 2417.0732, 2420.0718, 2422.0408, 2423.0466, 2428.0898, 2430.0828, 2431.0845, 2433.0779, 2436.0508, 2438.0457, 2439.0261, 2442.0618, 2449.0347, 2452.0632, 2460.0254, 2461.9956, 2463.9858, 2510.1277, 2528.1580, 2542.1509, 2545.1558, 2547.1531, 2552.1038, 2565.1543, 2585.1111, 2601.0889, 2705.2192, 2717.0693, 3129.3176, 3208.1736, 3223.2710, 3290.4131, 3291.4248, 3312.2756, 3451.3933, 3453.4365

Search Parameters

```
Type of search         : MS/MS Ion Search
Enzyme                 : Trypsin
Fixed modifications    : Carbamidomethyl (C)
Variable modifications : Oxidation (M)
Mass values            : Monoisotopic
Protein Mass           : Unrestricted
Peptide Mass Tolerance : ± 25 ppm
Fragment Mass Tolerance: ± 0.5 Da
Max Missed Cleavages   : 2
Instrument type        : MALDI-TOF-TOF
   Query1 (713.4334,1+): <no title>
   Query2 (719.2388,1+): <no title>
   Query3 (724.3009,1+): <no title>
   Query4 (726.2899,1+): <no title>
   Query5 (732.2586,1+): <no title>
   Query6 (735.3865,1+): <no title>
   Query7 (745.4124,1+): <no title>
   Query8 (756.4687,1+): <no title>
   Query9 (764.4056,1+): <no title>
  Query10 (765.3908,1+): <no title>
  Query11 (767.3679,1+): <no title>
  Query12 (768.3909,1+): <no title>
  Query13 (772.3955,1+): <no title>
  Query14 (795.4186,1+): <no title>
  Query15 (802.4548,1+): <no title>
  Query16 (803.4370,1+): <no title>
  Query17 (805.4229,1+): <no title>
  Query18 (806.4631,1+): <no title>
  Query19 (807.3893,1+): <no title>
  Query20 (809.4480,1+): <no title>
  Query21 (811.4104,1+): <no title>
  Query22 (812.3985,1+): <no title>
  Query23 (816.4408,1+): <no title>
  Query24 (820.4244,1+): <no title>
  Query25 (822.3905,1+): <no title>
  Query26 (823.3846,1+): <no title>
  Query27 (824.3756,1+): <no title>
  Query28 (828.3904,1+): <no title>
  Query29 (834.4043,1+): <no title>
  Query30 (836.4589,1+): <no title>
  Query31 (837.3853,1+): <no title>
  Query32 (839.3696,1+): <no title>
  Query33 (840.3839,1+): <no title>
  Query34 (842.5085,1+): <no title>
  Query35 (845.4100,1+): Label: F19, Spot_Id: 221882, Peak_List_Id: 435513, MSMS Job_Run_Id: 30377, Comment:
  Query36 (845.4123,1+): <no title>
  Query37 (851.3975,1+): <no title>
  Query38 (852.4056,1+): <no title>
  Query39 (856.4893,1+): <no title>
  Query40 (866.3914,1+): <no title>
  Query41 (867.3724,1+): <no title>
  Query42 (870.5402,1+): <no title>
  Query43 (871.4945,1+): <no title>
  Query44 (878.4625,1+): <no title>
  Query45 (880.4403,1+): <no title>
  Query46 (886.3815,1+): <no title>
  Query47 (888.4233,1+): <no title>
  Query48 (890.4479,1+): <no title>
  Query49 (893.9562,1+): <no title>
  Query50 (894.4579,1+): <no title>
  Query51 (897.0289,1+): <no title>
  Query52 (897.4412,1+): <no title>
  Query53 (902.4597,1+): <no title>
  Query54 (910.4410,1+): <no title>
  Query55 (912.4497,1+): <no title>
  Query56 (913.5118,1+): <no title>
  Query57 (916.4905,1+): <no title>
  Query58 (918.5391,1+): <no title>
  Query59 (925.4747,1+): <no title>
  Query60 (928.5064,1+): <no title>
  Query61 (930.5048,1+): <no title>
  Query62 (933.4619,1+): <no title>
  Query63 (935.4698,1+): <no title>
  Query64 (939.4758,1+): <no title>
  Query65 (947.4628,1+): <no title>
  Query66 (949.4591,1+): <no title>
  Query67 (951.4703,1+): <no title>
  Query68 (954.4674,1+): <no title>
  Query69 (957.4657,1+): <no title>
  Query70 (963.4676,1+): <no title>
  Query71 (965.4761,1+): <no title>
  Query72 (967.4568,1+): <no title>
  Query73 (969.4571,1+): <no title>
  Query74 (972.4793,1+): <no title>
  Query75 (972.4800,1+): Label: F19, Spot_Id: 221882, Peak_List_Id: 435404, MSMS Job_Run_Id: 30377, Comment:
  Query76 (975.4650,1+): <no title>
  Query77 (977.4630,1+): <no title>
  Query78 (978.4976,1+): <no title>
  Query79 (981.4565,1+): <no title>
  Query80 (983.4664,1+): <no title>
  Query81 (985.4842,1+): <no title>
  Query82 (991.4688,1+): <no title>
  Query83 (993.5331,1+): <no title>
  Query84 (997.4298,1+): <no title>
  Query85 (999.4453,1+): <no title>
 Query86 (1001.4488,1+): <no title>
 Query87 (1003.4687,1+): <no title>
 Query88 (1007.5668,1+): <no title>
```

```
 Query89 (1018.5200,1+): Label: F19, Spot_Id: 221882, Peak_List_Id: 435402, MSMS Job_Run_Id: 30377, Comment:
 Query90 (1018.5212,1+): <no title>
 Query91 (1023.4777,1+): <no title>
 Query92 (1026.4922,1+): <no title>
 Query93 (1027.4972,1+): <no title>
 Query94 (1030.5151,1+): <no title>
 Query95 (1031.3850,1+): <no title>
 Query96 (1033.5419,1+): <no title>
 Query97 (1035.5352,1+): <no title>
 Query98 (1036.5372,1+): <no title>
 Query99 (1039.5402,1+): <no title>
Query100 (1042.5383,1+): <no title>
Query101 (1045.5654,1+): <no title>
Query102 (1056.5488,1+): <no title>
Query103 (1060.5400,1+): Label: F19, Spot_Id: 221882, Peak_List_Id: 435403, MSMS Job_Run_Id: 30377, Comment:
Query104 (1060.5442,1+): <no title>
Query105 (1065.5425,1+): <no title>
Query106 (1086.5115,1+): <no title>
Query107 (1106.5372,1+): <no title>
Query108 (1111.5582,1+): <no title>
Query109 (1113.5546,1+): <no title>
Query110 (1114.5396,1+): <no title>
Query111 (1115.5605,1+): <no title>
Query112 (1118.5529,1+): <no title>
Query113 (1122.5363,1+): <no title>
Query114 (1128.5775,1+): <no title>
Query115 (1129.6014,1+): <no title>
Query116 (1130.5935,1+): <no title>
Query117 (1140.5485,1+): <no title>
Query118 (1144.5709,1+): <no title>
Query119 (1155.6113,1+): <no title>
Query120 (1156.5720,1+): <no title>
Query121 (1157.5952,1+): <no title>
Query122 (1158.6154,1+): <no title>
Query123 (1159.6273,1+): <no title>
Query124 (1165.5817,1+): <no title>
Query125 (1172.6301,1+): <no title>
Query126 (1173.6259,1+): <no title>
Query127 (1179.6063,1+): <no title>
Query128 (1180.6165,1+): <no title>
Query129 (1182.6090,1+): <no title>
Query130 (1183.6123,1+): <no title>
Query131 (1185.6317,1+): <no title>
Query132 (1187.6431,1+): <no title>
Query133 (1193.6096,1+): <no title>
Query134 (1195.6060,1+): <no title>
Query135 (1196.6102,1+): <no title>
Query136 (1206.6770,1+): <no title>
Query137 (1209.1387,1+): <no title>
Query138 (1209.5848,1+): <no title>
Query139 (1210.1014,1+): <no title>
Query140 (1215.6627,1+): <no title>
Query141 (1216.6494,1+): <no title>
Query142 (1222.5994,1+): <no title>
Query143 (1226.5948,1+): <no title>
Query144 (1229.6246,1+): <no title>
Query145 (1231.6174,1+): <no title>
Query146 (1234.6317,1+): <no title>
Query147 (1235.5846,1+): <no title>
Query148 (1240.6298,1+): <no title>
Query149 (1244.6324,1+): <no title>
Query150 (1248.6119,1+): <no title>
Query151 (1250.6144,1+): <no title>
Query152 (1268.6498,1+): <no title>
Query153 (1271.6696,1+): <no title>
Query154 (1273.6653,1+): <no title>
Query155 (1275.6644,1+): <no title>
Query156 (1291.6425,1+): <no title>
Query157 (1295.7233,1+): <no title>
Query158 (1300.6062,1+): <no title>
Query159 (1301.6205,1+): <no title>
Query160 (1307.6858,1+): <no title>
Query161 (1315.6570,1+): <no title>
Query162 (1317.6521,1+): <no title>
Query163 (1319.6852,1+): <no title>
Query164 (1323.6517,1+): <no title>
Query165 (1325.6637,1+): <no title>
Query166 (1328.6809,1+): <no title>
Query167 (1329.6672,1+): <no title>
Query168 (1331.6763,1+): <no title>
Query169 (1332.6743,1+): <no title>
Query170 (1334.6592,1+): <no title>
Query171 (1335.6937,1+): <no title>
Query172 (1336.6715,1+): <no title>
Query173 (1337.6724,1+): <no title>
Query174 (1338.6471,1+): <no title>
Query175 (1342.6680,1+): <no title>
Query176 (1345.6760,1+): <no title>
Query177 (1346.6880,1+): <no title>
Query178 (1349.6991,1+): <no title>
Query179 (1354.6907,1+): <no title>
Query180 (1355.7155,1+): <no title>
Query181 (1357.7034,1+): <no title>
Query182 (1360.6882,1+): <no title>
Query183 (1361.6670,1+): <no title>
Query184 (1365.6913,1+): <no title>
Query185 (1368.6920,1+): <no title>
Query186 (1374.6412,1+): <no title>
Query187 (1375.6798,1+): <no title>
Query188 (1376.6804,1+): <no title>
```

```
Query189 (1377.6794,1+): <no title>
Query190 (1382.6647,1+): <no title>
Query191 (1387.6444,1+): <no title>
Query192 (1410.7505,1+): <no title>
Query193 (1411.7208,1+): <no title>
Query194 (1421.7177,1+): <no title>
Query195 (1427.7872,1+): <no title>
Query196 (1431.7961,1+): <no title>
Query197 (1432.7133,1+): <no title>
Query198 (1434.7288,1+): <no title>
Query199 (1446.7256,1+): <no title>
Query200 (1450.7079,1+): <no title>
Query201 (1473.8054,1+): <no title>
Query202 (1475.7748,1+): <no title>
Query203 (1477.7524,1+): <no title>
Query204 (1478.7439,1+): <no title>
Query205 (1485.7720,1+): <no title>
Query206 (1486.7664,1+): <no title>
Query207 (1493.7539,1+): <no title>
Query208 (1494.7423,1+): <no title>
Query209 (1505.8649,1+): <no title>
Query210 (1523.7841,1+): <no title>
Query211 (1524.7848,1+): <no title>
Query212 (1537.8021,1+): <no title>
Query213 (1549.7388,1+): <no title>
Query214 (1555.8816,1+): <no title>
Query215 (1558.8474,1+): <no title>
Query216 (1576.7987,1+): <no title>
Query217 (1579.7926,1+): <no title>
Query218 (1584.7897,1+): <no title>
Query219 (1588.8136,1+): <no title>
Query220 (1590.3611,1+): <no title>
Query221 (1604.8417,1+): <no title>
Query222 (1605.8098,1+): <no title>
Query223 (1606.8384,1+): <no title>
Query224 (1615.8826,1+): <no title>
Query225 (1616.8605,1+): <no title>
Query226 (1622.8137,1+): <no title>
Query227 (1628.8260,1+): <no title>
Query228 (1629.8358,1+): <no title>
Query229 (1630.8466,1+): <no title>
Query230 (1633.9790,1+): <no title>
Query231 (1635.7943,1+): <no title>
Query232 (1644.8046,1+): <no title>
Query233 (1647.8785,1+): <no title>
Query234 (1649.8463,1+): <no title>
Query235 (1651.8245,1+): <no title>
Query236 (1659.8241,1+): <no title>
Query237 (1662.7909,1+): <no title>
Query238 (1668.7810,1+): <no title>
Query239 (1679.9736,1+): <no title>
Query240 (1681.8214,1+): <no title>
Query241 (1683.8121,1+): <no title>
Query242 (1684.8204,1+): <no title>
Query243 (1706.8616,1+): <no title>
Query244 (1707.7838,1+): <no title>
Query245 (1716.8571,1+): <no title>
Query246 (1725.8353,1+): <no title>
Query247 (1728.8242,1+): <no title>
Query248 (1734.8152,1+): <no title>
Query249 (1740.8768,1+): <no title>
Query250 (1742.9031,1+): <no title>
Query251 (1746.1659,1+): <no title>
Query252 (1750.8356,1+): <no title>
Query253 (1751.8787,1+): <no title>
Query254 (1753.8948,1+): <no title>
Query255 (1756.8905,1+): <no title>
Query256 (1757.8322,1+): <no title>
Query257 (1763.8188,1+): <no title>
Query258 (1765.7690,1+): <no title>
Query259 (1767.8036,1+): <no title>
Query260 (1768.9027,1+): <no title>
Query261 (1769.4475,1+): <no title>
Query262 (1770.4261,1+): <no title>
Query263 (1774.9091,1+): <no title>
Query264 (1783.8499,1+): <no title>
Query265 (1786.9000,1+): Label: F19, Spot_Id: 221882, Peak_List_Id: 435401, MSMS Job_Run_Id: 30377, Comment:
Query266 (1786.9017,1+): <no title>
Query267 (1791.7335,1+): <no title>
Query268 (1808.8868,1+): <no title>
Query269 (1824.8674,1+): <no title>
Query270 (1840.8225,1+): <no title>
Query271 (1844.8907,1+): <no title>
Query272 (1848.8322,1+): <no title>
Query273 (1850.8301,1+): <no title>
Query274 (1851.8488,1+): <no title>
Query275 (1867.9503,1+): <no title>
Query276 (1880.9277,1+): <no title>
Query277 (1887.9734,1+): <no title>
Query278 (1908.8838,1+): <no title>
Query279 (1914.9995,1+): <no title>
Query280 (1919.0038,1+): <no title>
Query281 (1925.9418,1+): <no title>
Query282 (1928.0103,1+): <no title>
Query283 (1931.9463,1+): <no title>
Query284 (1953.9353,1+): <no title>
Query285 (1975.9609,1+): <no title>
Query286 (1992.9642,1+): <no title>
Query287 (1995.9020,1+): <no title>
Query288 (2007.9894,1+): <no title>
```

```
Query289 (2011.8845,1+): <no title>
Query290 (2013.8983,1+): <no title>
Query291 (2024.8591,1+): <no title>
Query292 (2025.6234,1+): <no title>
Query293 (2036.5195,1+): <no title>
Query294 (2036.9486,1+): <no title>
Query295 (2042.0640,1+): <no title>
Query296 (2047.0167,1+): <no title>
Query297 (2047.9691,1+): <no title>
Query298 (2051.9792,1+): <no title>
Query299 (2052.9924,1+): <no title>
Query300 (2058.9963,1+): <no title>
Query301 (2060.0469,1+): <no title>
Query302 (2064.9900,1+): <no title>
Query303 (2068.8982,1+): <no title>
Query304 (2070.8835,1+): <no title>
Query305 (2074.8914,1+): <no title>
Query306 (2083.9907,1+): <no title>
Query307 (2084.8987,1+): <no title>
Query308 (2086.8828,1+): <no title>
Query309 (2092.8821,1+): <no title>
Query310 (2095.9941,1+): <no title>
Query311 (2098.9976,1+): <no title>
Query312 (2100.9160,1+): <no title>
Query313 (2102.8872,1+): <no title>
Query314 (2106.9011,1+): <no title>
Query315 (2108.8835,1+): <no title>
Query316 (2109.9175,1+): <no title>
Query317 (2114.9180,1+): <no title>
Query318 (2116.9160,1+): <no title>
Query319 (2118.8835,1+): <no title>
Query320 (2119.8833,1+): <no title>
Query321 (2125.0176,1+): <no title>
Query322 (2132.8694,1+): <no title>
Query323 (2136.9180,1+): <no title>
Query324 (2170.1467,1+): <no title>
Query325 (2211.1045,1+): <no title>
Query326 (2225.1191,1+): <no title>
Query327 (2273.0496,1+): <no title>
Query328 (2277.0137,1+): <no title>
Query329 (2307.9941,1+): <no title>
Query330 (2322.9980,1+): <no title>
Query331 (2341.0366,1+): <no title>
Query332 (2343.0271,1+): <no title>
Query333 (2352.0571,1+): <no title>
Query334 (2355.0459,1+): <no title>
Query335 (2359.0532,1+): <no title>
Query336 (2366.0813,1+): <no title>
Query337 (2368.0544,1+): <no title>
Query338 (2369.0710,1+): <no title>
Query339 (2376.0264,1+): <no title>
Query340 (2382.0349,1+): <no title>
Query341 (2383.6099,1+): <no title>
Query342 (2383.9600,1+): <no title>
Query343 (2390.0403,1+): <no title>
Query344 (2396.0342,1+): <no title>
Query345 (2398.0576,1+): <no title>
Query346 (2400.0652,1+): <no title>
Query347 (2406.0737,1+): <no title>
Query348 (2411.0076,1+): <no title>
Query349 (2414.0813,1+): <no title>
Query350 (2416.0752,1+): <no title>
Query351 (2417.0732,1+): <no title>
Query352 (2420.0718,1+): <no title>
Query353 (2422.0408,1+): <no title>
Query354 (2423.0466,1+): <no title>
Query355 (2428.0898,1+): <no title>
Query356 (2430.0828,1+): <no title>
Query357 (2431.0845,1+): <no title>
Query358 (2433.0779,1+): <no title>
Query359 (2436.0508,1+): <no title>
Query360 (2438.0457,1+): <no title>
Query361 (2439.0261,1+): <no title>
Query362 (2442.0618,1+): <no title>
Query363 (2449.0347,1+): <no title>
Query364 (2452.0632,1+): <no title>
Query365 (2460.0254,1+): <no title>
Query366 (2461.9956,1+): <no title>
Query367 (2463.9858,1+): <no title>
Query368 (2510.1277,1+): <no title>
Query369 (2528.1580,1+): <no title>
Query370 (2542.1509,1+): <no title>
Query371 (2545.1558,1+): <no title>
Query372 (2547.1531,1+): <no title>
Query373 (2552.1038,1+): <no title>
Query374 (2565.1543,1+): <no title>
Query375 (2585.1111,1+): <no title>
Query376 (2601.0889,1+): <no title>
Query377 (2705.2192,1+): <no title>
Query378 (2717.0693,1+): <no title>
Query379 (3129.3176,1+): <no title>
Query380 (3208.1736,1+): <no title>
Query381 (3223.2710,1+): <no title>
Query382 (3290.4131,1+): <no title>
Query383 (3291.4248,1+): <no title>
Query384 (3312.2756,1+): <no title>
Query385 (3451.3933,1+): <no title>
Query386 (3453.4365,1+): <no title>
```

**Mascot:** http://www.matrixscience.com/

Mascot Search Results

**User**	: 3w_(F17@11273_12Mar2012)
**Email**	: proteomics@ipatimup.pt
**Search title**	: Project: Proteomica, Spot Set: Proteomica\12Jan2012, Label: F17, Spot Id: 221880, Peak List Id: 435346, MS Job Run Id: 30
**MS data file**	: C:\Documents and Settings\Administrator\Desktop\xu\xu\xu\Proteomica\12Jan2012\ppw_F17_146796935400.txt
**Database**	: OrganismSpecie Bos_taurus_Reference_Proteome_2016_06 (24214 sequences; 12839866 residues)
**Timestamp**	: 8 Jul 2016 at 09:59:43 GMT
**Warning**	: A Peptide summary report will usually give a much clearer picture of MS/MS search results.
**Top Score**	: 269 for F1MYE4, Chondroadherin OS=Bos taurus GN=CHAD PE=4 SV=2

Mascot Score Histogram

Protein score is -10*Log(P), where P is the probability that the observed match is a random event.
Protein scores greater than 56 are significant (p<0.05).
Protein scores are derived from ions scores as a non-probabilistic basis for ranking protein hits.

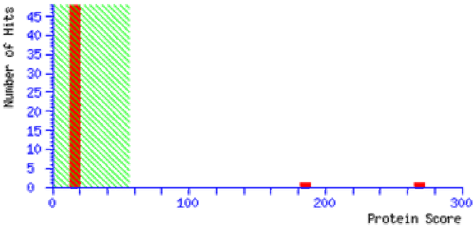

Protein Summary Report

Format As | Protein Summary (deprecated) | Help

Significance threshold p< 0.05 | Max. number of hits 50

Preferred taxonomy | All entries

Re-Search All | Search Unmatched

Index

	Accession	Mass	Score	Description
1.	F1MYE4	41319	269	Chondroadherin OS=Bos taurus GN=CHAD PE=4 SV=2
2.	Q27972	41373	183	Chondroadherin OS=Bos taurus GN=CHAD PE=1 SV=1
3.	E1BFX3	11338	20	Ribosomal protein L37 OS=Bos taurus GN=RPL37 PE=3 SV=1
4.	Q1PSA0	42952	20	Uncharacterized protein OS=Bos taurus GN=ZAR1 PE=2 SV=1
5.	E1B7W1	108879	20	Uncharacterized protein OS=Bos taurus GN=THRAP3 PE=4 SV=1
6.	G3X6N6	52918	20	Uncharacterized protein OS=Bos taurus PE=4 SV=1
7.	Q02368	16615	20	NADH dehydrogenase [ubiquinone] 1 beta subcomplex subunit 7 OS=Bos taurus GN=NDUFB7 PE=1 SV=2
8.	F1N1U9	64259	20	Uncharacterized protein OS=Bos taurus PE=4 SV=2
9.	F1N7L8	63126	20	Uncharacterized protein OS=Bos taurus GN=BBOF1 PE=4 SV=1
10.	G3N2J6	36179	19	Uncharacterized protein OS=Bos taurus GN=TNIP3 PE=4 SV=1
11.	Q1PSA4	20552	19	Uncharacterized protein OS=Bos taurus GN=ZAR1 PE=4 SV=1
12.	Q17Q91	10021	19	Coiled-coil-helix-coiled-coil-helix domain-containing protein 7 OS=Bos taurus GN=CHCHD7 PE=3 SV=1
13.	Q0IIC8	17012	19	Phospholipase A(2) OS=Bos taurus GN=PLA2G2A PE=2 SV=1
14.	Q5KR49	32732	19	Tropomyosin alpha-1 chain OS=Bos taurus GN=TPM1 PE=2 SV=1
15.	G1K1U4	51632	19	DNA polymerase delta subunit 3 OS=Bos taurus GN=POLD3 PE=4 SV=2
16.	Q5KR47	32856	19	Tropomyosin alpha-3 chain OS=Bos taurus GN=TPM3 PE=2 SV=1
17.	Q2T9X5	23537	19	Uncharacterized protein C7orf61 homolog OS=Bos taurus PE=2 SV=1
18.	A6QPI2	49923	19	GAS7 protein OS=Bos taurus GN=GAS7 PE=2 SV=1
19.	Q2TBU3	28638	19	Golgi SNAP receptor complex member 1 OS=Bos taurus GN=GOSR1 PE=2 SV=1
20.	Q2TBN3	19797	18	Centrin-2 OS=Bos taurus GN=CETN2 PE=2 SV=1
21.	F1MMV5	66566	18	Nuclear speckle-splicing regulatory protein 1 OS=Bos taurus GN=NSRP1 PE=4 SV=2
22.	Q2KIC0	66733	18	Nuclear speckle splicing regulatory protein 1 OS=Bos taurus GN=NSRP1 PE=2 SV=1
23.	E1BDW0	49794	18	Uncharacterized protein OS=Bos taurus GN=ALS2CR12 PE=4 SV=1
24.	G3N3V8	69281	18	Uncharacterized protein OS=Bos taurus GN=C3orf67 PE=4 SV=1
25.	F1MNR4	58473	18	Uncharacterized protein OS=Bos taurus GN=C3orf67 PE=4 SV=2
26.	Q6YFP9	10868	18	Cytochrome c oxidase subunit 6B2 OS=Bos taurus GN=COX6B2 PE=2 SV=1
27.	P11019	26180	18	V-type proton ATPase subunit E 1 OS=Bos taurus GN=ATP6V1E1 PE=2 SV=1
28.	F1MLG8	9765	18	Uncharacterized protein OS=Bos taurus GN=SMIM4 PE=4 SV=2
29.	E1BEC7	19673	18	Mimitin, mitochondrial OS=Bos taurus GN=NDUFAF2 PE=4 SV=2
30.	A6QLN3	7445	18	FXYD2 protein OS=Bos taurus GN=FXYD2 PE=2 SV=1
31.	F1MIT2	31839	18	Transcription elongation factor A protein 1 OS=Bos taurus GN=TCEA1 PE=4 SV=1
32.	E1BLN1	117436	18	Uncharacterized protein OS=Bos taurus GN=SYCP1 PE=4 SV=1
33.	Q3ZBL3	49458	17	Inositol hexakisphosphate kinase 2 OS=Bos taurus GN=IP6K2 PE=2 SV=1
34.	Q56JZ9	16931	17	Glia maturation factor gamma OS=Bos taurus GN=GMFG PE=2 SV=1
35.	F1MQU4	106272	17	Uncharacterized protein OS=Bos taurus GN=BCLAF1 PE=4 SV=2
36.	G3MYV8	6868	17	Uncharacterized protein OS=Bos taurus PE=4 SV=1
37.	E1B8A3	13661	17	Uncharacterized protein OS=Bos taurus GN=LOC100298655 PE=4 SV=1
38.	G3MWK4	30649	17	Uncharacterized protein OS=Bos taurus GN=SRSF12 PE=4 SV=1
39.	G3N0W0	3134	17	Uncharacterized protein OS=Bos taurus PE=4 SV=1
40.	Q2KHY2	12884	17	Vesicle-associated membrane protein 5 OS=Bos taurus GN=VAMP5 PE=3 SV=1
41.	E1BPQ7	23626	17	Uncharacterized protein OS=Bos taurus GN=LNP1 PE=4 SV=1
42.	Q5EA89	36065	17	Protein FAM76A OS=Bos taurus GN=FAM76A PE=2 SV=1
43.	Q9N287	23425	17	DnaJ homolog subfamily C member 12 OS=Bos taurus GN=DNAJC12 PE=2 SV=1
44.	F1MJH0	128067	17	Uncharacterized protein OS=Bos taurus GN=SPINK5 PE=4 SV=2
45.	F1MJI7	69096	17	Uncharacterized protein OS=Bos taurus GN=LMNB2 PE=3 SV=1
46.	G3MZ80	7701	17	Uncharacterized protein OS=Bos taurus PE=4 SV=1
47.	A5D7M3	56128	16	Growth arrest-specific protein 8 OS=Bos taurus GN=GAS8 PE=2 SV=1
48.	F1MYR3	56242	16	Growth arrest-specific protein 8 OS=Bos taurus GN=GAS8 PE=4 SV=1

49. E1BF46	243942	16	Uncharacterized protein OS=Bos taurus GN=ZNF407 PE=4 SV=2
50. F1MYI2	58389	16	Uncharacterized protein OS=Bos taurus GN=HNRNPM PE=1 SV=2

Results List

1. F1MYE4 **Mass:** 41319 **Score:** 269 **Expect:** 3e-023 **Matches:** 29

Chondroadherin OS=Bos taurus GN=CHAD PE=4 SV=2

Observed	Mr(expt)	Mr(calc)	ppm	Start	End	Miss	Ions	Peptide
756.4777	755.4704	755.4653	6.75	57 -	62	0	---	K.LLNLQR.N
765.3959	764.3887	764.3817	9.11	166 -	173	0	---	R.SGAFQGAK.D
802.4609	801.4537	801.4497	4.96	315 -	320	1	---	R.RWLEAK.T
820.4252	819.4180	819.4239	-7.24	91 -	98	0	---	R.EVAAGAFR.G
837.3794	836.3721	836.3633	10.5	306 -	311	0	---	K.CTCQLR.G
913.5080	912.5007	912.5042	-3.82	334 -	340	1	---	K.FRGQHIR.D
913.5100	912.5027	912.5042	-1.63	334 -	340	1	28	K.FRGQHIR.D
985.6071	984.5998	984.5968	3.14	220 -	227	1	---	K.LRVVEELK.L
993.5267	992.5195	992.5226	-3.14	347 -	354	2	---	R.GCKFPTKR.S
1018.5130	1017.5057	1017.5104	-4.60	274 -	281	0	---	K.HVHLENNR.L
1069.4846	1068.4773	1068.4659	10.7	341 -	349	1	---	R.DTDAFRGCK.F
1149.5912	1148.5839	1148.5938	-8.61	166 -	176	1	---	R.SGAFQGAKDLR.W
1163.6145	1162.6072	1162.6095	-1.92	163 -	173	1	---	R.ELRSGAFQGAK.D
1315.6364	1314.6291	1314.6429	-10.48	336 -	346	1	---	R.GQHIRDTDAFR.G
1338.6300	1337.6227	1337.6364	-10.23	235 -	246	0	---	K.SIPDNAFQSFGR.Y
1338.6300	1337.6227	1337.6364	-10.23	235 -	246	0	67	K.SIPDNAFQSFGR.Y
1349.6800	1348.6727	1348.6888	-11.89	63 -	74	0	80	R.NNFPVLAANSFR.A
1349.6827	1348.6754	1348.6888	-9.89	63 -	74	0	---	R.NNFPVLAANSFR.A
1355.6964	1354.6891	1354.7133	-17.84	261 -	273	0	---	K.FSDGAFLGVTTLK.H
1361.6373	1360.6300	1360.6405	-7.70	321 -	333	0	---	K.TSRPDATCASPAK.F
1365.6752	1364.6679	1364.6936	-18.78	207 -	219	0	---	R.NQLSSYPSAALSK.L
1505.8511	1504.8438	1504.8613	-11.61	222 -	234	1	---	R.VVEELKLSHNPLK.S
1647.8730	1646.8657	1646.8780	-7.46	102 -	114	0	---	K.QLIYLYLSHNDIR.V
1751.8657	1750.8584	1750.8777	-11.03	247 -	260	0	---	R.YLETLWLDNTNLEK.F
1775.0302	1774.0229	1774.0465	-13.27	220 -	234	2	---	K.LRVVEELKLSHNPLK.S
1916.9882	1915.9809	1915.9873	-3.31	75 -	90	0	---	R.AMPNLVSLHLQHCQIR.E
1932.9813	1931.9740	1931.9822	-4.23	75 -	90	0	---	R.AMPNLVSLHLQHCQIR.E + Oxidation (M)
2060.0400	2059.0327	2059.0374	-2.28	202 -	219	1	44	K.FYLDRNQLSSYPSAALSK.L
2060.0405	2059.0332	2059.0374	-2.04	202 -	219	1	---	K.FYLDRNQLSSYPSAALSK.L

No match to: 713.4400, 713.4415, 724.3446, 728.2467, 730.2754, 732.2383, 734.2729, 735.3820, 736.3320, 740.2330, 743.2514, 748.2947, 750.2650, 764.3574, 768.3784, 772.4155, 774.3952, 778.4551, 780.3343, 782.3223, 783.3115, 787.2390, 791.2662, 805.3347, 806.4342, 807.3921, 808.4378, 812.4002, 814.4128, 828.3922, 830.3668, 834.3623, 840.3857, 842.5100, 845.4155, 851.3846, 856.5103, 865.3634, 869.4703, 870.5254, 871.4965, 872.6990, 888.3988, 891.4706, 893.9500, 894.4534, 897.0277, 905.4637, 911.4314, 912.4475, 918.5267, 925.4804, 927.4970, 930.5015, 933.4662, 935.4675, 943.4757, 951.4646, 952.4799, 957.4673, 961.4901, 962.4866, 967.4440, 969.4496, 972.4781, 974.5051, 975.4521, 977.4406, 978.4772, 979.4924, 983.4706, 991.4533, 995.5177, 999.4602, 1001.4565, 1001.5621, 1003.5043, 1007.5638, 1012.5004, 1014.5331, 1022.5398, 1023.5525, 1028.5991, 1030.5259, 1035.5314, 1036.5333, 1037.5145, 1040.5127, 1042.5243, 1045.5603, 1060.5398, 1075.5515, 1092.6041, 1106.5313, 1109.5087, 1111.5457, 1113.5419, 1115.5394, 1118.5659, 1121.5419, 1122.5701, 1131.5602, 1138.5428, 1155.5768, 1157.5907, 1158.6328, 1173.6122, 1177.6130, 1178.6060, 1179.5918, 1180.6001, 1181.6368, 1183.6030, 1187.6376, 1192.6014, 1193.5928, 1195.5924, 1199.6785, 1206.6066, 1215.6306, 1216.6482, 1226.5839, 1227.6051, 1231.6115, 1232.6251, 1233.6230, 1234.6500, 1235.6155, 1248.6078, 1250.6483, 1254.6526, 1255.6036, 1256.6123, 1268.6208, 1271.6423, 1276.6179, 1277.6350, 1289.6449, 1292.6322, 1294.6362, 1295.6366, 1300.5895, 1303.6643, 1305.6523, 1307.6600, 1308.6443, 1310.6051, 1320.6128, 1321.6134, 1323.6320, 1325.6456, 1331.6674, 1332.6602, 1333.2076, 1334.6744, 1336.6710, 1337.6279, 1345.6146, 1347.6653, 1354.6820, 1359.7053, 1360.6173, 1371.6718, 1375.6670, 1377.6776, 1382.6367, 1383.6619, 1387.6700, 1393.6727, 1400.6235, 1409.7107, 1411.6742, 1427.7725, 1431.7605, 1432.6987, 1434.7190, 1449.7479, 1466.6953, 1473.7905, 1475.7498, 1478.7303, 1479.7468, 1485.7531, 1486.7472, 1493.7345, 1495.7502, 1500.7242, 1503.7690, 1509.6786, 1520.7347, 1522.7239, 1523.7567, 1525.7864, 1527.8068, 1532.7472, 1537.7886, 1539.7711, 1549.7833, 1555.8657, 1558.8440, 1559.8573, 1568.7592, 1570.7814, 1578.7899, 1606.8213, 1615.8425, 1619.7972, 1621.7831, 1628.8156, 1630.8202, 1631.8217, 1639.9263, 1644.8083, 1649.8650, 1650.8523, 1658.8206, 1659.8125, 1661.8284, 1663.7981, 1665.8070, 1669.8174, 1677.8353, 1679.9403, 1680.8076, 1682.7780, 1696.8055, 1697.7845, 1698.7950, 1707.7931, 1709.8029, 1710.8878, 1712.9166, 1716.8563, 1725.8188, 1727.8683, 1730.8236, 1731.8221, 1742.8798, 1746.1482, 1749.8406, 1756.8782, 1757.8225, 1763.8152, 1765.7922, 1766.8105, 1768.8596, 1769.4275, 1770.4087, 1773.8615, 1774.8795, 1783.8599, 1785.8483, 1786.8871, 1790.8822, 1792.7894, 1801.9315, 1804.9026, 1808.8663, 1824.8685, 1830.8608, 1849.8231, 1851.8430, 1867.9100, 1868.9441, 1870.9304, 1880.9020, 1884.9191, 1908.8989, 1914.9821, 1920.9862, 1930.9827, 1934.9822, 1947.0288, 1958.9543, 1994.8763, 2011.9012, 2013.8700, 2020.9592, 2025.5920, 2042.0568, 2047.0238, 2047.9805, 2049.9497, 2052.9905, 2053.9851, 2058.9988, 2065.0427, 2066.0137, 2066.9895, 2067.9763, 2068.9128, 2070.8792, 2074.8860, 2082.0293, 2084.8977, 2086.8872, 2087.8835, 2090.8708, 2092.8674, 2093.8711, 2096.9836, 2097.9961, 2100.8787, 2101.8999, 2103.9092, 2107.8730, 2119.9924, 2122.9397, 2136.9133, 2211.1047, 2217.9368, 2225.1243, 2232.0229, 2233.0840, 2238.1050, 2250.0259, 2257.9934, 2262.9368, 2263.9700, 2273.0264, 2277.9500, 2283.1741, 2290.9570, 2307.9631, 2321.9558, 2323.9670, 2324.9824, 2335.9763, 2339.9456, 2343.0320, 2349.9844, 2352.0308, 2355.0315, 2358.0854, 2368.0815, 2369.0588, 2378.0020, 2379.0002, 2380.0007, 2381.9963, 2383.0444, 2383.9822, 2393.0020, 2394.0181, 2398.0447, 2400.0771, 2405.0879, 2414.1050, 2417.0933, 2421.0769, 2422.0593, 2426.0476, 2428.0725, 2430.0947, 2432.0962, 2435.0254, 2438.0706, 2444.0901, 2451.0278, 2459.9802, 2461.0254, 2501.2520, 2528.1902, 2543.2092, 2564.1965, 2705.2144, 2717.1655, 2834.2925, 2888.2483, 2904.4053, 3130.4312, 3291.4875, 3312.3765, 3451.6833, 3454.6270

2. Q27972 **Mass:** 41373 **Score:** 183 **Expect:** 1.2e-014 **Matches:** 27

Chondroadherin OS=Bos taurus GN=CHAD PE=1 SV=1

Observed	Mr(expt)	Mr(calc)	ppm	Start	End	Miss	Ions	Peptide
756.4777	755.4704	755.4653	6.75	57 -	62	0	---	K.LLNLQR.N
765.3959	764.3887	764.3817	9.11	166 -	173	0	---	R.SGAFQGAK.D
802.4609	801.4537	801.4497	4.96	315 -	320	1	---	R.RWLEAK.T
820.4252	819.4180	819.4239	-7.24	91 -	98	0	---	R.EVAAGAFR.G
837.3794	836.3721	836.3633	10.5	306 -	311	0	---	K.CTCQLR.G
913.5080	912.5007	912.5042	-3.82	334 -	340	1	---	K.FRGQHIR.D
913.5100	912.5027	912.5042	-1.63	334 -	340	1	28	K.FRGQHIR.D
985.6071	984.5998	984.5968	3.14	220 -	227	1	---	K.LRVVEELK.L
993.5267	992.5195	992.5226	-3.14	347 -	354	2	---	R.GCKFPTKR.S
1018.5130	1017.5057	1017.5104	-4.60	274 -	281	0	---	K.HVHLENNR.L
1069.4846	1068.4773	1068.4659	10.7	341 -	349	1	---	R.DTDAFRGCK.F
1149.5912	1148.5839	1148.5938	-8.61	166 -	176	1	---	R.SGAFQGAKDLR.W
1163.6145	1162.6072	1162.6095	-1.92	163 -	173	1	---	R.ELRSGAFQGAK.D
1315.6364	1314.6291	1314.6429	-10.48	336 -	346	1	---	R.GQHIRDTDAFR.G
1338.6300	1337.6227	1337.6364	-10.23	235 -	246	0	---	K.SIPDNAFQSFGR.Y
1338.6300	1337.6227	1337.6364	-10.23	235 -	246	0	67	K.SIPDNAFQSFGR.Y
1355.6964	1354.6891	1354.7133	-17.84	261 -	273	0	---	K.FSDGAFLGVTTLK.H
1361.6373	1360.6300	1360.6405	-7.70	321 -	333	0	---	K.TSRPDATCASPAK.F

1365.6752	1364.6679	1364.6936	-18.78	207 -	219	0	---	R.NQLSSYPSAALSK.L
1505.8511	1504.8438	1504.8613	-11.61	222 -	234	1	---	R.VVEELKLSHNPLK.S
1647.8730	1646.8657	1646.8780	-7.46	102 -	114	0	---	K.QLIYLYLSHNDIR.V
1751.8657	1750.8584	1750.8777	-11.03	247 -	260	0	---	R.YLETLWLDNTNLEK.F
1775.0302	1774.0229	1774.0465	-13.27	220 -	234	2	---	K.LRVVEELKLSHNPLK.S
1916.9882	1915.9809	1915.9873	-3.31	75 -	90	0	---	R.AMPNLVSLHLQHCQIR.E
1932.9813	1931.9740	1931.9822	-4.23	75 -	90	0	---	R.AMPNLVSLHLQHCQIR.E + Oxidation (M)
2060.0400	2059.0327	2059.0374	-2.28	202 -	219	1	44	K.FYLDRNQLSSYPSAALSK.L
2060.0405	2059.0332	2059.0374	-2.04	202 -	219	1	---	K.FYLDRNQLSSYPSAALSK.L

No match to: 713.4400, 713.4415, 724.3446, 728.2467, 730.2754, 732.2383, 734.2729, 735.3820, 736.3320, 740.2330, 743.2514, 748.2947, 750.2650, 764.3574, 768.3784, 772.4155, 774.3952, 778.4551, 780.3343, 782.3223, 783.3115, 787.2390, 791.2662, 805.3347, 806.4342, 807.3921, 808.4378, 812.4002, 814.4128, 828.3922, 830.3668, 834.3623, 840.3857, 842.5100, 845.4155, 851.3846, 856.5103, 865.3634, 869.4703, 870.5254, 871.4965, 872.6990, 888.3988, 891.4706, 893.9500, 894.4534, 897.0277, 905.4637, 911.4314, 912.4475, 918.5267, 925.4804, 927.4970, 930.5015, 933.4662, 935.4675, 943.4757, 951.4646, 952.4799, 957.4673, 961.4901, 962.4866, 967.4440, 969.4496, 972.4781, 974.5051, 975.4521, 977.4406, 978.4772, 979.4924, 983.4706, 991.4533, 995.5177, 999.4602, 1001.4565, 1001.5621, 1003.5043, 1007.5638, 1012.5004, 1014.5331, 1022.5398, 1023.5525, 1028.5991, 1030.5259, 1035.5314, 1036.5333, 1037.5145, 1040.5127, 1042.5243, 1045.5603, 1060.5398, 1075.5515, 1092.6041, 1106.5313, 1109.5087, 1111.5457, 1113.5419, 1115.5394, 1118.5659, 1121.5419, 1122.5701, 1131.5602, 1138.5428, 1155.5768, 1157.5907, 1158.6328, 1173.6122, 1177.6130, 1178.6060, 1179.5918, 1180.6001, 1181.6368, 1183.6030, 1187.6376, 1192.6014, 1193.5928, 1195.5924, 1199.6785, 1206.6066, 1215.6306, 1216.6482, 1226.5839, 1227.6051, 1231.6115, 1232.6251, 1233.6230, 1234.6500, 1235.6155, 1248.6078, 1250.6483, 1254.6526, 1255.6036, 1256.6123, 1268.6208, 1271.6423, 1276.6179, 1277.6350, 1289.6449, 1292.6322, 1294.6362, 1295.6366, 1300.5895, 1303.6643, 1305.6523, 1307.6600, 1308.6443, 1310.6051, 1320.6128, 1321.6134, 1323.6320, 1325.6456, 1331.6674, 1332.6602, 1333.2076, 1334.6744, 1336.6710, 1337.6279, 1345.6146, 1347.6653, 1349.6800, 1349.6827, 1354.6820, 1359.7053, 1360.6173, 1371.6718, 1375.6670, 1377.6776, 1382.6367, 1383.6619, 1387.6700, 1393.6727, 1400.6235, 1409.7107, 1411.6742, 1427.7725, 1431.7605, 1432.6987, 1434.7190, 1449.7479, 1466.6953, 1473.7905, 1475.7498, 1478.7303, 1479.7468, 1485.7531, 1486.7472, 1493.7345, 1495.7502, 1500.7242, 1503.7690, 1509.6786, 1520.7347, 1522.7239, 1523.7567, 1525.7864, 1527.8068, 1532.7472, 1537.7886, 1539.7711, 1549.7833, 1555.8657, 1558.8440, 1559.8573, 1568.7592, 1570.7814, 1578.7899, 1606.8213, 1615.8425, 1619.7972, 1621.7831, 1628.8156, 1630.8202, 1631.8217, 1639.9263, 1644.8083, 1649.8650, 1650.8523, 1658.8206, 1659.8125, 1661.8284, 1663.7981, 1665.8070, 1669.8174, 1677.8353, 1679.9403, 1680.8076, 1682.7780, 1696.8055, 1697.7845, 1698.7950, 1707.7931, 1709.8029, 1710.8878, 1712.9166, 1716.8563, 1725.8188, 1727.8683, 1730.8236, 1731.8221, 1742.8798, 1746.1482, 1749.8406, 1756.8782, 1757.8225, 1763.8152, 1765.7922, 1766.8105, 1768.8596, 1769.4275, 1770.4087, 1773.8615, 1774.8795, 1783.8599, 1785.8483, 1786.8871, 1790.8822, 1792.7894, 1801.9315, 1804.9026, 1808.8663, 1824.8685, 1830.8608, 1849.8231, 1851.8430, 1867.9100, 1868.9441, 1870.9304, 1880.9020, 1884.9191, 1908.8989, 1914.9821, 1920.9862, 1930.9827, 1934.9822, 1947.0288, 1958.9543, 1994.8763, 2011.9012, 2013.8700, 2020.9592, 2025.5920, 2042.0568, 2047.0238, 2047.9805, 2049.9497, 2052.9905, 2053.9851, 2058.9988, 2065.0427, 2066.0137, 2066.9895, 2067.9763, 2068.9128, 2070.8792, 2074.8860, 2082.0293, 2084.8977, 2086.8872, 2087.8835, 2090.8708, 2092.8674, 2093.8711, 2096.9836, 2097.9961, 2100.8787, 2101.8999, 2103.9092, 2107.8730, 2119.9924, 2122.9397, 2136.9133, 2211.1047, 2217.9368, 2225.1243, 2232.0229, 2233.0840, 2238.1050, 2250.0259, 2257.9934, 2262.9368, 2263.9700, 2273.0264, 2277.9500, 2283.1741, 2290.9570, 2307.9631, 2321.9558, 2323.9670, 2324.9824, 2335.9763, 2339.9456, 2343.0320, 2349.9844, 2352.0308, 2355.0315, 2358.0854, 2368.0815, 2369.0588, 2378.0020, 2379.0002, 2380.0007, 2381.9963, 2383.0444, 2383.9822, 2393.0020, 2394.0181, 2398.0447, 2400.0771, 2405.0879, 2414.1050, 2417.0933, 2421.0769, 2422.0593, 2426.0476, 2428.0725, 2430.0947, 2432.0962, 2435.0254, 2438.0706, 2444.0901, 2451.0278, 2459.9802, 2461.0254, 2501.2520, 2528.1902, 2543.2092, 2564.1965, 2705.2144, 2717.1655, 2834.2925, 2888.2483, 2904.4053, 3130.4312, 3291.4875, 3312.3765, 3451.6833, 3454.6270

3\. E1BFX3 **Mass:** 11338 **Score:** 20 **Expect:** 2.4e+002 **Matches:** 7

Ribosomal protein L37 OS=Bos taurus GN=RPL37 PE=3 SV=1

Observed	Mr(expt)	Mr(calc)	ppm	Start	End	Miss	Ions	Peptide
768.3784	767.3711	767.3602	14.2	47 -	52	0	---	K.YNWSAK.A
995.5177	994.5104	994.5308	-20.51	4 -	12	2	---	K.GTSSFGKRR.N
1037.5145	1036.5072	1036.5124	-4.97	37 -	44	2	---	K.CDYPAKRK.R
1149.5912	1148.5839	1148.5833	0.57	56 -	65	2	---	R.RNTTGTGRMR.H
1215.6306	1214.6233	1214.6078	12.8	1 -	11	2	---	-.MTKGTSSFGKR.R + Oxidation (M)
1292.6322	1291.6249	1291.6343	-7.25	26 -	36	1	---	K.AYHLQKSTCGK.C
2096.9836	2095.9763	2096.0156	-18.75	15 -	31	2	---	K.THTLCHRCGSKAYHLQK.S

No match to: 713.4400, 713.4415, 724.3446, 728.2467, 730.2754, 732.2383, 734.2729, 735.3820, 736.3320, 740.2330, 743.2514, 748.2947, 750.2650, 756.4777, 764.3574, 765.3959, 772.4155, 774.3952, 778.4551, 780.3343, 782.3223, 783.3115, 787.2390, 791.2662, 802.4609, 805.3347, 806.4342, 807.3921, 808.4378, 812.4002, 814.4128, 820.4252, 828.3922, 830.3668, 834.3623, 837.3794, 840.3857, 842.5100, 845.4155, 851.3846, 856.5103, 865.3634, 869.4703, 870.5254, 871.4965, 872.6990, 888.3988, 891.4706, 893.9500, 894.4534, 897.0277, 905.4637, 911.4314, 912.4475, 913.5080, 913.5100, 918.5267, 925.4804, 927.4970, 930.5015, 933.4662, 935.4675, 943.4757, 951.4646, 952.4799, 957.4673, 961.4901, 962.4866, 967.4440, 969.4496, 972.4781, 974.5051, 975.4521, 977.4406, 978.4772, 979.4924, 983.4706, 985.6071, 991.4533, 993.5267, 999.4602, 1001.4565, 1001.5621, 1003.5043, 1007.5638, 1012.5004, 1014.5331, 1018.5130, 1022.5398, 1023.5525, 1028.5991, 1030.5259, 1035.5314, 1036.5333, 1040.5127, 1042.5243, 1045.5603, 1060.5398, 1069.4846, 1075.5515, 1092.6041, 1106.5313, 1109.5087, 1111.5457, 1113.5419, 1115.5394, 1118.5659, 1121.5419, 1122.5701, 1131.5602, 1138.5428, 1155.5768, 1157.5907, 1158.6328, 1163.6145, 1173.6122, 1177.6130, 1178.6060, 1179.5918, 1180.6001, 1181.6368, 1183.6030, 1187.6376, 1192.6014, 1193.5928, 1195.5924, 1199.6785, 1206.6066, 1216.6482, 1226.5839, 1227.6051, 1231.6115, 1232.6251, 1233.6230, 1234.6500, 1235.6155, 1248.6078, 1250.6483, 1254.6526, 1255.6036, 1256.6123, 1268.6208, 1271.6423, 1276.6179, 1277.6350, 1289.6449, 1294.6362, 1295.6366, 1300.5895, 1303.6643, 1305.6523, 1307.6600, 1308.6443, 1310.6051, 1315.6364, 1320.6128, 1321.6134, 1323.6320, 1325.6456, 1331.6674, 1332.6602, 1333.2076, 1334.6744, 1336.6710, 1337.6279, 1338.6300, 1338.6300, 1345.6146, 1347.6653, 1349.6800, 1349.6827, 1354.6820, 1355.6964, 1359.7053, 1360.6173, 1361.6373, 1365.6752, 1371.6718, 1375.6670, 1377.6776, 1382.6367, 1383.6619, 1387.6700, 1393.6727, 1400.6235, 1409.7107, 1411.6742, 1427.7725, 1431.7605, 1432.6987, 1434.7190, 1449.7479, 1466.6953, 1473.7905, 1475.7498, 1478.7303, 1479.7468, 1485.7531, 1486.7472, 1493.7345, 1495.7502, 1500.7242, 1503.7690, 1505.8511, 1509.6786, 1520.7347, 1522.7239, 1523.7567, 1525.7864, 1527.8068, 1532.7472, 1537.7886, 1539.7711, 1549.7833, 1555.8657, 1558.8440, 1559.8573, 1568.7592, 1570.7814, 1578.7899, 1606.8213, 1615.8425, 1619.7972, 1621.7831, 1628.8156, 1630.8202, 1631.8217, 1639.9263, 1644.8083, 1647.8730, 1649.8650, 1650.8523, 1658.8206, 1659.8125, 1661.8284, 1663.7981, 1665.8070, 1669.8174, 1677.8353, 1679.9403, 1680.8076, 1682.7780, 1696.8055, 1697.7845, 1698.7950, 1707.7931, 1709.8029, 1710.8878, 1712.9166, 1716.8563, 1725.8188, 1727.8683, 1730.8236, 1731.8221, 1742.8798, 1746.1482, 1749.8406, 1751.8657, 1756.8782, 1757.8225, 1763.8152, 1765.7922, 1766.8105, 1768.8596, 1769.4275, 1770.4087, 1773.8615, 1774.8795, 1775.0302, 1783.8599, 1785.8483, 1786.8871, 1790.8822, 1792.7894, 1801.9315, 1804.9026, 1808.8663, 1824.8685, 1830.8608, 1849.8231, 1851.8430, 1867.9100, 1868.9441, 1870.9304, 1880.9020, 1884.9191, 1908.8989, 1914.9821, 1916.9882, 1920.9862, 1930.9827, 1932.9813, 1934.9822, 1947.0288, 1958.9543, 1994.8763, 2011.9012, 2013.8700, 2020.9592, 2025.5920, 2042.0568, 2047.0238, 2047.9805, 2049.9497, 2052.9905, 2053.9851, 2058.9988, 2060.0400, 2060.0405, 2065.0427, 2066.0137, 2066.9895, 2067.9763, 2068.9128, 2070.8792, 2074.8860, 2082.0293, 2084.8977, 2086.8872, 2087.8835, 2090.8708, 2092.8674, 2093.8711, 2097.9961, 2100.8787, 2101.8999, 2103.9092, 2107.8730, 2119.9924, 2122.9397, 2136.9133, 2211.1047, 2217.9368, 2225.1243, 2232.0229, 2233.0840, 2238.1050, 2250.0259, 2257.9934, 2262.9368, 2263.9700, 2273.0264, 2277.9500, 2283.1741, 2290.9570, 2307.9631, 2321.9558, 2323.9670, 2324.9824, 2335.9763, 2339.9456, 2343.0320, 2349.9844, 2352.0308, 2355.0315, 2358.0854, 2368.0815, 2369.0588, 2378.0020, 2379.0002, 2380.0007, 2381.9963, 2383.0444, 2383.9822, 2393.0020, 2394.0181, 2398.0447, 2400.0771, 2405.0879, 2414.1050, 2417.0933, 2421.0769, 2422.0593, 2426.0476, 2428.0725, 2430.0947, 2432.0962, 2435.0254, 2438.0706, 2444.0901, 2451.0278, 2459.9802, 2461.0254, 2501.2520, 2528.1902, 2543.2092, 2564.1965, 2705.2144, 2717.1655, 2834.2925, 2888.2483, 2904.4053, 3130.4312, 3291.4875, 3312.3765, 3451.6833, 3454.6270

4\. Q1PSA0 **Mass:** 42952 **Score:** 20 **Expect:** 2.5e+002 **Matches:** 16

Uncharacterized protein OS=Bos taurus GN=ZAR1 PE=2 SV=1

Observed	Mr(expt)	Mr(calc)	ppm	Start	End	Miss	Ions	Peptide
1035.5314	1034.5241	1034.5219	2.18	24 -	33	0	---	R.CLYPAAAAAK.G
1069.4846	1068.4773	1068.4836	-5.87	156 -	165	0	---	R.GPEQDSPPSR.A
1271.6423	1270.6350	1270.6266	6.65	258 -	269	1	---	R.VPDTAGERSSPR.S
1276.6179	1275.6106	1275.6354	-19.40	111 -	121	1	---	R.DVSVQCSLGRR.T
1294.6362	1293.6289	1293.6473	-14.18	357 -	366	1	---	K.RPHRQDLCGR.C
1347.6653	1346.6580	1346.6289	21.6	371 -	381	1	---	K.RLSCDSTFSFK.Y

1355.6964	1354.6891	1354.6953	-4.56	266 - 277	2	---	R.SSPRSPQPSKER.L
1393.6727	1392.6654	1392.6746	-6.57	156 - 168	1	---	R.GPEQDSPPSRAPR.R
1532.7472	1531.7399	1531.7453	-3.52	369 - 381	2	---	K.GKRLSCDSTFSFK.Y
1549.7833	1548.7760	1548.7757	0.22	156 - 169	2	---	R.GPEQDSPPSRAPRR.V
1680.8076	1679.8003	1679.7912	5.42	313 - 324	2	---	K.VYYKQFCRTCQK.S
1870.9304	1869.9231	1869.9493	-13.98	352 - 366	2	---	R.HVDPKRPHRQDLCGR.C
1930.9827	1929.9754	1929.9769	-0.76	211 - 229	2	---	R.GPPPARPRGPEEGDGSARK.V
2383.0444	2382.0371	2382.0549	-7.45	331 - 349	2	---	R.VEDITCQNCKQTRCSCPVK.L
2432.0962	2431.0889	2431.1009	-4.91	325 - 343	2	---	K.SYNPYRVEDITCQNCKQTR.C
2564.1965	2563.1892	2563.1254	24.9	321 - 340	2	---	R.TCQKSYNPYRVEDITCQNCK.Q

No match to: 713.4400, 713.4415, 724.3446, 728.2467, 730.2754, 732.2383, 734.2729, 735.3820, 736.3320, 740.2330, 743.2514, 748.2947, 750.2650, 756.4777, 764.3574, 765.3959, 768.3784, 772.4155, 774.3952, 778.4551, 780.3343, 782.3223, 783.3115, 787.2390, 791.2662, 802.4609, 805.3347, 806.4342, 807.3921, 808.4378, 812.4002, 814.4128, 820.4252, 828.3922, 830.3668, 834.3623, 837.3794, 840.3857, 842.5100, 845.4155, 851.3846, 856.5103, 865.3634, 869.4703, 870.5254, 871.4965, 872.6990, 888.3988, 891.4706, 893.9500, 894.4534, 897.0277, 905.4637, 911.4314, 912.4475, 913.5080, 913.5100, 918.5267, 925.4804, 927.4970, 930.5015, 933.4662, 935.4675, 943.4757, 951.4646, 952.4799, 957.4673, 961.4901, 962.4866, 967.4440, 969.4496, 972.4781, 974.5051, 975.4521, 977.4406, 978.4772, 979.4924, 983.4706, 985.6071, 991.4533, 993.5267, 995.5177, 999.4602, 1001.4565, 1001.5621, 1003.5043, 1007.5638, 1012.5004, 1014.5331, 1018.5130, 1022.5398, 1023.5525, 1028.5991, 1030.5259, 1036.5333, 1037.5145, 1040.5127, 1042.5243, 1045.5603, 1060.5398, 1075.5515, 1092.6041, 1106.5313, 1109.5087, 1111.5457, 1113.5419, 1115.5394, 1118.5659, 1121.5419, 1122.5701, 1131.5602, 1138.5428, 1149.5912, 1155.5768, 1157.5907, 1158.6328, 1163.6145, 1173.6122, 1177.6130, 1178.6060, 1179.5918, 1180.6001, 1181.6368, 1183.6030, 1187.6376, 1192.6014, 1193.5928, 1195.5924, 1199.6785, 1206.6066, 1215.6306, 1216.6482, 1226.5839, 1227.6051, 1231.6115, 1232.6251, 1233.6230, 1234.6500, 1235.6155, 1248.6078, 1250.6483, 1254.6526, 1255.6036, 1256.6123, 1268.6208, 1277.6350, 1289.6449, 1292.6322, 1295.6366, 1300.5895, 1303.6643, 1305.6523, 1307.6600, 1308.6443, 1310.6051, 1315.6364, 1320.6128, 1321.6134, 1323.6320, 1325.6456, 1331.6674, 1332.6602, 1333.2076, 1334.6744, 1336.6710, 1337.6279, 1338.6300, 1338.6300, 1345.6146, 1349.6800, 1349.6827, 1354.6820, 1359.7053, 1360.6173, 1361.6373, 1365.6752, 1371.6718, 1375.6670, 1377.6776, 1382.6367, 1383.6619, 1387.6700, 1400.6235, 1409.7107, 1411.6742, 1427.7725, 1431.7605, 1432.6987, 1434.7190, 1449.7479, 1466.6953, 1473.7905, 1475.7498, 1478.7303, 1479.7468, 1485.7531, 1486.7472, 1493.7345, 1495.7502, 1500.7242, 1503.7690, 1505.8511, 1509.6786, 1520.7347, 1522.7239, 1523.7567, 1525.7864, 1527.8068, 1537.7886, 1539.7711, 1555.8657, 1558.8440, 1559.8573, 1568.7592, 1570.7814, 1578.7899, 1606.8213, 1615.8425, 1619.7972, 1621.7831, 1628.8156, 1630.8202, 1631.8217, 1639.9263, 1644.8083, 1647.8730, 1649.8650, 1650.8523, 1658.8206, 1659.8125, 1661.8284, 1663.7981, 1665.8070, 1669.8174, 1677.8353, 1679.9403, 1682.7780, 1696.8055, 1697.7845, 1698.7950, 1707.7931, 1709.8029, 1710.8878, 1712.9166, 1716.8563, 1725.8188, 1727.8683, 1730.8236, 1731.8221, 1742.8798, 1746.1482, 1749.8406, 1751.8657, 1756.8782, 1757.8225, 1763.8152, 1765.7922, 1766.8105, 1768.8596, 1769.4275, 1770.4087, 1773.8615, 1774.8795, 1775.0302, 1783.8599, 1785.8483, 1786.8871, 1790.8822, 1792.7894, 1801.9315, 1804.9026, 1808.8663, 1824.8685, 1830.8608, 1849.8231, 1851.8430, 1867.9100, 1868.9441, 1880.9020, 1884.9191, 1908.8989, 1914.9821, 1916.9882, 1920.9862, 1932.9813, 1934.9822, 1947.0288, 1958.9543, 1994.8763, 2011.9012, 2013.8700, 2020.9592, 2025.5920, 2042.0568, 2047.0238, 2047.9805, 2049.9497, 2052.9905, 2053.9851, 2058.9988, 2060.0400, 2060.0405, 2065.0427, 2066.0137, 2066.9895, 2067.9763, 2068.9128, 2070.8792, 2074.8860, 2082.0293, 2084.8977, 2086.8872, 2087.8835, 2090.8708, 2092.8674, 2093.8711, 2096.9836, 2097.9961, 2100.8787, 2101.8999, 2103.9092, 2107.8730, 2119.9924, 2122.9397, 2136.9133, 2211.1047, 2217.9368, 2225.1243, 2232.0229, 2233.0840, 2238.1050, 2250.0259, 2257.9934, 2262.9368, 2263.9700, 2273.0264, 2277.9500, 2283.1741, 2290.9570, 2307.9631, 2321.9558, 2323.9670, 2324.9824, 2335.9763, 2339.9456, 2343.0320, 2349.9844, 2352.0308, 2355.0315, 2358.0854, 2368.0815, 2369.0588, 2378.0020, 2379.0002, 2380.0007, 2381.9963, 2383.9822, 2393.0020, 2394.0181, 2398.0447, 2400.0771, 2405.0879, 2414.1050, 2417.0933, 2421.0769, 2422.0593, 2426.0476, 2428.0725, 2430.0947, 2435.0254, 2438.0706, 2444.0901, 2451.0278, 2459.9802, 2461.0254, 2501.2520, 2528.1902, 2543.2092, 2705.2144, 2717.1655, 2834.2925, 2888.2483, 2904.4053, 3130.4312, 3291.4875, 3312.3765, 3451.6833, 3454.6270

5. E1B7W1 **Mass:** 108879 **Score:** 20 **Expect:** 2.5e+002 **Matches:** 34

Uncharacterized protein OS=Bos taurus GN=THRAP3 PE=4 SV=1

Observed	Mr(expt)	Mr(calc)	ppm	Start - End	Miss	Ions	Peptide
764.3574	763.3501	763.3323	23.4	423 - 428	0	---	K.MADFHK.E + Oxidation (M)
808.4378	807.4305	807.4160	18.0	371 - 376	2	---	K.EKMKEK.G + Oxidation (M)
828.3922	827.3849	827.3998	-18.03	511 - 517	1	---	K.RSESGHR.G
933.4662	932.4590	932.4716	-13.50	702 - 709	1	---	K.SPREPGYK.A
935.4675	934.4602	934.4468	14.3	741 - 748	1	---	K.SRESVDSR.D
967.4440	966.4367	966.4308	6.14	109 - 115	0	---	R.SNWQNYR.Q
978.4772	977.4700	977.4818	-12.10	705 - 713	1	---	R.EPGYKAEGK.Y
979.4924	978.4852	978.4955	-10.56	9 - 17	2	---	K.SGSRSSRSR.S
993.5267	992.5195	992.5111	8.39	13 - 21	2	---	R.SSRSRSASR.S
1035.5314	1034.5241	1034.5397	-15.02	683 - 691	0	---	R.IDISPSTFR.K
1042.5243	1041.5170	1041.5291	-11.55	910 - 917	2	---	R.GRGRFMFR.K + Oxidation (M)
1157.5907	1156.5834	1156.5697	11.9	507 - 517	2	---	R.GGSKRSESGHR.G
1158.6328	1157.6255	1157.6040	18.6	757 - 766	2	---	R.SAEKTEKTHK.G
1163.6145	1162.6072	1162.6346	-23.56	683 - 692	1	---	R.IDISPSTFRK.H
1183.6030	1182.5957	1182.6006	-4.14	63 - 71	1	---	R.NHPRVYQNR.D
1216.6482	1215.6409	1215.6288	9.97	473 - 483	0	---	K.WEGLVYAPPGK.E
1235.6155	1234.6082	1234.5829	20.5	335 - 347	0	---	K.EEAAAPGGAAYTK.R
1277.6350	1276.6277	1276.6173	8.13	72 - 81	2	---	R.DFRGHNRGYR.R
1305.6523	1304.6450	1304.6725	-21.04	818 - 829	1	---	R.LGTKDFVGPSER.G
1331.6674	1330.6601	1330.6703	-7.68	914 - 924	2	---	R.FMFRKSSTSPK.W + Oxidation (M)
1334.6744	1333.6671	1333.6474	14.8	360 - 370	2	---	K.DKEQKQTNTDK.E
1361.6373	1360.6300	1360.5967	24.5	743 - 754	1	---	R.ESVDSRDSSHSR.E
1371.6718	1370.6645	1370.6942	-21.68	918 - 929	2	---	R.KSSTSPKWAHDK.F
1377.6776	1376.6703	1376.6619	6.12	693 - 704	1	---	K.HGLAHDEMKSPR.E
1393.6727	1392.6654	1392.6568	6.18	693 - 704	1	---	K.HGLAHDEMKSPR.E + Oxidation (M)
1473.7905	1472.7832	1472.7663	11.5	473 - 485	1	---	K.WEGLVYAPPGKEK.Q
1485.7531	1484.7458	1484.7484	-1.74	511 - 523	2	---	K.RSESGHRGFVPEK.N
1669.8174	1668.8101	1668.7757	20.6	109 - 121	1	---	R.SNWQNYRQAYSPR.R
1725.8188	1724.8115	1724.8370	-14.75	391 - 405	1	---	K.SDSFAPKTDTEKPFR.G
1765.7922	1764.7849	1764.8206	-20.22	869 - 882	2	---	R.EEEWDPEYTPKSKK.Y
1808.8663	1807.8590	1807.8409	10.1	157 - 173	2	---	R.SSSSRSSSNHSRVESSK.R
1880.9020	1879.8947	1879.8588	19.1	867 - 881	2	---	R.SREEEWDPEYTPKSK.K
2273.0264	2272.0191	2271.9965	9.96	849 - 868	2	---	R.GNYSGNNNNSSNNDFQKRSR.E
2380.0007	2378.9934	2378.9873	2.55	796 - 815	1	---	K.AEEYTEETEEREESTTGFDK.S

No match to: 713.4400, 713.4415, 724.3446, 728.2467, 730.2754, 732.2383, 734.2729, 735.3820, 736.3320, 740.2330, 743.2514, 748.2947, 750.2650, 756.4777, 765.3959, 768.3784, 772.4155, 774.3952, 778.4551, 780.3343, 782.3223, 783.3115, 787.2390, 791.2662, 802.4609, 805.3347, 806.4342, 807.3921, 812.4002, 814.4128, 820.4252, 830.3668, 834.3623, 837.3794, 840.3857, 842.5100, 845.4155, 851.3846, 856.5103, 865.3634, 869.4703, 870.5254, 871.4965, 872.6990, 888.3988, 891.4706, 893.9500, 894.4534, 897.0277, 905.4637, 911.4314, 912.4475, 913.5080, 913.5100, 918.5267, 925.4804, 927.4970, 930.5015, 943.4757, 951.4646, 952.4799, 957.4673, 961.4901, 962.4866, 969.4496, 972.4781, 974.5051, 975.4521, 977.4406, 983.4706, 985.6071, 991.4533, 995.5177, 999.4602, 1001.4565, 1001.5621, 1003.5043, 1007.5638, 1012.5004, 1014.5331, 1018.5130, 1022.5398, 1023.5525, 1028.5991, 1030.5259, 1036.5333, 1037.5145, 1040.5127, 1045.5603, 1060.5398, 1069.4846, 1075.5515, 1092.6041, 1106.5313, 1109.5087, 1111.5457, 1113.5419, 1115.5394, 1118.5659, 1121.5419, 1122.5701, 1131.5602, 1138.5428, 1149.5912, 1155.5768, 1173.6122, 1177.6130, 1178.6060, 1179.5918, 1180.6001, 1181.6368, 1187.6376, 1192.6014, 1193.5928, 1195.5924, 1199.6785, 1206.6066, 1215.6306, 1226.5839, 1227.6051, 1231.6115, 1232.6251, 1233.6230, 1234.6500, 1248.6078, 1250.6483, 1254.6526, 1255.6036, 1256.6123, 1268.6208, 1271.6423, 1276.6179, 1289.6449, 1292.6322, 1294.6362, 1295.6366, 1300.5895, 1303.6643, 1307.6600, 1308.6443, 1310.6051, 1315.6364, 1320.6128, 1321.6134, 1323.6320, 1325.6456, 1332.6602, 1333.2076,

1336.6710, 1337.6279, 1338.6300, 1338.6300, 1345.6146, 1347.6653, 1349.6800, 1349.6827, 1354.6820, 1355.6964, 1359.7053, 1360.6173, 1365.6752, 1375.6670, 1382.6367, 1383.6619, 1387.6700, 1400.6235, 1409.7107, 1411.6742, 1427.7725, 1431.7605, 1432.6987, 1434.7190, 1449.7479, 1466.6953, 1475.7498, 1478.7303, 1479.7468, 1486.7472, 1493.7345, 1495.7502, 1500.7242, 1503.7690, 1505.8511, 1509.6786, 1520.7347, 1522.7239, 1523.7567, 1525.7864, 1527.8068, 1532.7472, 1537.7886, 1539.7711, 1549.7833, 1555.8657, 1558.8440, 1559.8573, 1568.7592, 1570.7814, 1578.7899, 1606.8213, 1615.8425, 1619.7972, 1621.7831, 1628.8156, 1630.8202, 1631.8217, 1639.9263, 1644.8083, 1647.8730, 1649.8650, 1650.8523, 1658.8206, 1659.8125, 1661.8284, 1663.7981, 1665.8070, 1677.8353, 1679.9403, 1680.8076, 1682.7780, 1696.8055, 1697.7845, 1698.7950, 1707.7931, 1709.8029, 1710.8878, 1712.9166, 1716.8563, 1727.8683, 1730.8236, 1731.8221, 1742.8798, 1746.1482, 1749.8406, 1751.8657, 1756.8782, 1757.8225, 1763.8152, 1766.8105, 1768.8596, 1769.4275, 1770.4087, 1773.8615, 1774.8795, 1775.0302, 1783.8599, 1785.8483, 1786.8871, 1790.8822, 1792.7894, 1801.9315, 1804.9026, 1824.8685, 1830.8608, 1849.8231, 1851.8430, 1867.9100, 1868.9441, 1870.9304, 1884.9191, 1908.8989, 1914.9821, 1916.9882, 1920.9862, 1930.9827, 1932.9813, 1934.9822, 1947.0288, 1958.9543, 1994.8763, 2011.9012, 2013.8700, 2020.9592, 2025.5920, 2042.0568, 2047.0238, 2047.9805, 2049.9497, 2052.9905, 2053.9851, 2058.9988, 2060.0400, 2060.0405, 2065.0427, 2066.0137, 2066.9895, 2067.9763, 2068.9128, 2070.8792, 2074.8860, 2082.0293, 2084.8977, 2086.8872, 2087.8835, 2090.8708, 2092.8674, 2093.8711, 2096.9836, 2097.9961, 2100.8787, 2101.8999, 2103.9092, 2107.8730, 2119.9924, 2122.9397, 2136.9133, 2211.1047, 2217.9368, 2225.1243, 2232.0229, 2233.0840, 2238.1050, 2250.0259, 2257.9934, 2262.9368, 2263.9700, 2277.9500, 2283.1741, 2290.9570, 2307.9631, 2321.9558, 2323.9670, 2324.9824, 2335.9763, 2339.9456, 2343.0320, 2349.9844, 2352.0308, 2355.0315, 2358.0854, 2368.0815, 2369.0588, 2378.0020, 2379.0002, 2381.9963, 2383.0444, 2383.9822, 2393.0020, 2394.0181, 2398.0447, 2400.0771, 2405.0879, 2414.1050, 2417.0933, 2421.0769, 2422.0593, 2426.0476, 2428.0725, 2430.0947, 2432.0962, 2435.0254, 2438.0706, 2444.0901, 2451.0278, 2459.9802, 2461.0254, 2501.2520, 2528.1902, 2543.2092, 2564.1965, 2705.2144, 2717.1655, 2834.2925, 2888.2483, 2904.4053, 3130.4312, 3291.4875, 3312.3765, 3451.6833, 3454.6270

6. G3X6N6 **Mass:** 52918 **Score:** 20 **Expect:** 2.5e+002 **Matches:** 19

Uncharacterized protein OS=Bos taurus PE=4 SV=1

Observed	Mr(expt)	Mr(calc)	ppm	Start	End	Miss	Ions	Peptide
977.4406	976.4333	976.4284	5.00	49 -	57	0	---	K.SDGLCPSNK.G
1001.5621	1000.5548	1000.5454	9.42	170 -	177	1	---	R.IHTEKAFR.N
1036.5333	1035.5260	1035.5349	-8.57	366 -	374	0	---	K.VFNQNSSLK.G
1040.5127	1039.5054	1039.4934	11.6	178 -	186	0	---	R.NYSNLSSQK.K
1060.5398	1059.5325	1059.5349	-2.24	216 -	224	0	---	R.THTGEKPYK.C
1122.5701	1121.5628	1121.5353	24.6	323 -	331	1	---	K.SHTEEKTYK.C
1178.6060	1177.5987	1177.5840	12.5	166 -	174	1	---	K.EHERIHTEK.A
1195.5924	1194.5851	1194.5638	17.9	225 -	234	1	---	K.CPLCGKAFSR.C
1216.6482	1215.6409	1215.6360	4.05	215 -	224	1	---	K.RTHTGEKPYK.C
1310.6051	1309.5978	1309.5696	21.6	329 -	337	1	---	K.TYKCHQCWK.V
1371.6718	1370.6645	1370.6587	4.23	85 -	95	1	---	R.MAFNQAKMFQR.F
1387.6700	1386.6627	1386.6536	6.55	85 -	95	1	---	R.MAFNQAKMFQR.F + Oxidation (M)
1503.7690	1502.7617	1502.7664	-3.08	255 -	266	2	---	R.LCGRFSLKEHEK.A
1644.8083	1643.8010	1643.7903	6.52	319 -	331	2	---	K.QHEKSHTEEKTYK.C
1804.9026	1803.8953	1803.8648	16.9	407 -	421	0	---	K.IHTGEKPYLCLECGK.F
1930.9827	1929.9754	1929.9917	-8.43	187 -	202	2	---	K.KIHTGEKPFKCQLCGK.V
2307.9631	2306.9558	2306.9692	-5.78	375 -	393	1	---	K.GHERTHTGEKPCECHQCGK.A
2398.0447	2397.0374	2396.9974	16.7	263 -	281	2	---	K.EHEKAHRGENGYECHQCEK.V
2428.0725	2427.0652	2427.0631	0.89	379 -	398	1	---	R.THTGEKPCECHQCGKAFPQR.R

No match to: 713.4400, 713.4415, 724.3446, 728.2467, 730.2754, 732.2383, 734.2729, 735.3820, 736.3320, 740.2330, 743.2514, 748.2947, 750.2650, 756.4777, 764.3574, 765.3959, 768.3784, 772.4155, 774.3952, 778.4551, 780.3343, 782.3223, 783.3115, 787.2390, 791.2662, 802.4609, 805.3347, 806.4342, 807.3921, 808.4378, 812.4002, 814.4128, 820.4252, 828.3922, 830.3668, 834.3623, 837.3794, 840.3857, 842.5100, 845.4155, 851.3846, 856.5103, 865.3634, 869.4703, 870.5254, 871.4965, 872.6990, 888.3988, 891.4706, 893.9500, 894.4534, 897.0277, 905.4637, 911.4314, 912.4475, 913.5080, 913.5100, 918.5267, 925.4804, 927.4970, 930.5015, 933.4662, 935.4675, 943.4757, 951.4646, 952.4799, 957.4673, 961.4901, 962.4866, 967.4440, 969.4496, 972.4781, 974.5051, 975.4521, 978.4772, 979.4924, 983.4706, 985.6071, 991.4533, 993.5267, 995.5177, 999.4602, 1001.4565, 1003.5043, 1007.5638, 1012.5004, 1014.5331, 1018.5130, 1022.5398, 1023.5525, 1028.5991, 1030.5259, 1035.5314, 1037.5145, 1042.5243, 1045.5603, 1069.4846, 1075.5515, 1092.6041, 1106.5313, 1109.5087, 1111.5457, 1113.5419, 1115.5394, 1118.5659, 1121.5419, 1131.5602, 1138.5428, 1149.5912, 1155.5768, 1157.5907, 1158.6328, 1163.6145, 1173.6122, 1177.6130, 1179.5918, 1180.6001, 1181.6368, 1183.6030, 1187.6376, 1192.6014, 1193.5928, 1199.6785, 1206.6066, 1215.6306, 1226.5839, 1227.6051, 1231.6115, 1232.6251, 1233.6230, 1234.6500, 1235.6155, 1248.6078, 1250.6483, 1254.6526, 1255.6036, 1256.6123, 1268.6208, 1271.6423, 1276.6179, 1277.6350, 1289.6449, 1292.6322, 1294.6362, 1295.6366, 1300.5895, 1303.6643, 1305.6523, 1307.6600, 1308.6443, 1315.6364, 1320.6128, 1321.6134, 1323.6320, 1325.6456, 1331.6674, 1332.6602, 1333.2076, 1334.6744, 1336.6710, 1337.6279, 1338.6300, 1338.6300, 1345.6146, 1347.6653, 1349.6800, 1349.6827, 1354.6820, 1355.6964, 1359.7053, 1360.6173, 1361.6373, 1365.6752, 1375.6670, 1377.6776, 1382.6367, 1383.6619, 1393.6727, 1400.6235, 1409.7107, 1411.6742, 1427.7725, 1431.7605, 1432.6987, 1434.7190, 1449.7479, 1466.6953, 1473.7905, 1475.7498, 1478.7303, 1479.7468, 1485.7531, 1486.7472, 1493.7345, 1495.7502, 1500.7242, 1505.8511, 1509.6786, 1520.7347, 1522.7239, 1523.7567, 1525.7864, 1527.8068, 1532.7472, 1537.7886, 1539.7711, 1549.7833, 1555.8657, 1558.8440, 1559.8573, 1568.7592, 1570.7814, 1578.7899, 1606.8213, 1615.8425, 1619.7972, 1621.7831, 1628.8156, 1630.8202, 1631.8217, 1639.9263, 1647.8730, 1649.8650, 1650.8523, 1658.8206, 1659.8125, 1661.8284, 1663.7981, 1665.8070, 1669.8174, 1677.8353, 1679.9403, 1680.8076, 1682.7780, 1696.8055, 1697.7845, 1698.7950, 1707.7931, 1709.8029, 1710.8878, 1712.9166, 1716.8563, 1725.8188, 1727.8683, 1730.8236, 1731.8221, 1742.8798, 1746.1482, 1749.8406, 1751.8657, 1756.8782, 1757.8225, 1763.8152, 1765.7922, 1766.8105, 1768.8596, 1769.4275, 1770.4087, 1773.8615, 1774.8795, 1775.0302, 1783.8599, 1785.8483, 1786.8871, 1790.8822, 1792.7894, 1801.9315, 1808.8663, 1824.8685, 1830.8608, 1849.8231, 1851.8430, 1867.9100, 1868.9441, 1870.9304, 1880.9020, 1884.9191, 1908.8989, 1914.9821, 1916.9882, 1920.9862, 1932.9813, 1934.9822, 1947.0288, 1958.9543, 1994.8763, 2011.9012, 2013.8700, 2020.9592, 2025.5920, 2042.0568, 2047.0238, 2047.9805, 2049.9497, 2052.9905, 2053.9851, 2058.9988, 2060.0400, 2060.0405, 2065.0427, 2066.0137, 2066.9895, 2067.9763, 2068.9128, 2070.8792, 2074.8860, 2082.0293, 2084.8977, 2086.8872, 2087.8835, 2090.8708, 2092.8674, 2093.8711, 2096.9836, 2097.9961, 2100.8787, 2101.8999, 2103.9092, 2107.8730, 2119.9924, 2122.9397, 2136.9133, 2211.1047, 2217.9368, 2225.1243, 2232.0229, 2233.0840, 2238.1050, 2250.0259, 2257.9934, 2262.9368, 2263.9700, 2273.0264, 2277.9500, 2283.1741, 2290.9570, 2321.9558, 2323.9670, 2324.9824, 2335.9763, 2339.9456, 2343.0320, 2349.9844, 2352.0308, 2355.0315, 2358.0854, 2368.0815, 2369.0588, 2378.0020, 2379.0002, 2380.0007, 2381.9963, 2383.0444, 2383.9822, 2393.0020, 2394.0181, 2400.0771, 2405.0879, 2414.1050, 2417.0933, 2421.0769, 2422.0593, 2426.0476, 2430.0947, 2432.0962, 2435.0254, 2438.0706, 2444.0901, 2451.0278, 2459.9802, 2461.0254, 2501.2520, 2528.1902, 2543.2092, 2564.1965, 2705.2144, 2717.1655, 2834.2925, 2888.2483, 2904.4053, 3130.4312, 3291.4875, 3312.3765, 3451.6833, 3454.6270

7. Q02368 **Mass:** 16615 **Score:** 20 **Expect:** 2.5e+002 **Matches:** 8

NADH dehydrogenase [ubiquinone] 1 beta subcomplex subunit 7 OS=Bos taurus GN=NDUFB7 PE=1 SV=2

Observed	Mr(expt)	Mr(calc)	ppm	Start	End	Miss	Ions	Peptide
927.4970	926.4897	926.4868	3.13	1 -	8	1	---	-.MGAHLARR.Y + Oxidation (M)
995.5177	994.5104	994.5018	8.67	98 -	104	2	---	K.RMKEFER.E
1233.6230	1232.6157	1232.5931	18.3	115 -	124	2	---	R.REQREADMAK.G
1354.6820	1353.6747	1353.6499	18.3	71 -	81	1	---	K.RDSFPNFLACK.H
1697.7845	1696.7772	1696.7919	-8.67	22 -	35	1	---	R.MPTFPPDYGFPERK.E + Oxidation (M)
2232.0229	2231.0156	2231.0674	-23.22	38 -	56	0	---	R.EMVATQQEMNDAQLVLQQR.D
2283.1741	2282.1668	2282.1212	20.0	116 -	137	2	---	R.EQREADMAKGLGPGEVAPEVAL.- + Oxidation (M)
2358.0854	2357.0781	2357.0396	16.4	82 -	98	2	---	K.HERHDWDYCEHLDYVKR.M

No match to: 713.4400, 713.4415, 724.3446, 728.2467, 730.2754, 732.2383, 734.2729, 735.3820, 736.3320, 740.2330, 743.2514, 748.2947, 750.2650, 756.4777, 764.3574, 765.3959, 768.3784, 772.4155, 774.3952, 778.4551, 780.3343, 782.3223, 783.3115, 787.2390, 791.2662, 802.4609, 805.3347, 806.4342, 807.3921, 808.4378, 812.4002, 814.4128, 820.4252, 828.3922, 830.3668, 834.3623, 837.3794, 840.3857, 842.5100, 845.4155, 851.3846, 856.5103, 865.3634, 869.4703, 870.5254, 871.4965, 872.6990, 888.3988, 891.4706, 893.9500, 894.4534, 897.0277, 905.4637, 911.4314, 912.4475, 913.5080, 913.5100, 918.5267, 925.4804, 930.5015, 933.4662, 935.4675, 943.4757, 951.4646, 952.4799, 957.4673, 961.4901, 962.4866, 967.4440, 969.4496, 972.4781, 974.5051, 975.4521, 977.4406, 978.4772, 979.4924, 983.4706, 985.6071, 991.4533, 993.5267, 999.4602, 1001.4565, 1001.5621, 1003.5043, 1007.5638, 1012.5004, 1014.5331, 1018.5130, 1022.5398, 1023.5525, 1028.5991, 1030.5259, 1035.5314, 1036.5333, 1037.5145, 1040.5127, 1042.5243, 1045.5603, 1060.5398, 1069.4846,

1075.5515, 1092.6041, 1106.5313, 1109.5087, 1111.5457, 1113.5419, 1115.5394, 1118.5659, 1121.5419, 1122.5701, 1131.5602, 1138.5428, 1149.5912, 1155.5768, 1157.5907, 1158.6328, 1163.6145, 1173.6122, 1177.6130, 1178.6060, 1179.5918, 1180.6001, 1181.6368, 1183.6030, 1187.6376, 1192.6014, 1193.5928, 1195.5924, 1199.6785, 1206.6066, 1215.6306, 1216.6482, 1226.5839, 1227.6051, 1231.6115, 1232.6251, 1234.6500, 1235.6155, 1248.6078, 1250.6483, 1254.6526, 1255.6036, 1256.6123, 1268.6208, 1271.6423, 1276.6179, 1277.6350, 1289.6449, 1292.6322, 1294.6362, 1295.6366, 1300.5895, 1303.6643, 1305.6523, 1307.6600, 1308.6443, 1310.6051, 1315.6364, 1320.6128, 1321.6134, 1323.6320, 1325.6456, 1331.6674, 1332.6602, 1333.2076, 1334.6744, 1336.6710, 1337.6279, 1338.6300, 1338.6300, 1345.6146, 1347.6653, 1349.6800, 1349.6827, 1355.6964, 1359.7053, 1360.6173, 1361.6373, 1365.6752, 1371.6718, 1375.6670, 1377.6776, 1382.6367, 1383.6619, 1387.6700, 1393.6727, 1400.6235, 1409.7107, 1411.6742, 1427.7725, 1431.7605, 1432.6987, 1434.7190, 1449.7479, 1466.6953, 1473.7905, 1475.7498, 1478.7303, 1479.7468, 1485.7531, 1486.7472, 1493.7345, 1495.7502, 1500.7242, 1503.7690, 1505.8511, 1509.6786, 1520.7347, 1522.7239, 1523.7567, 1525.7864, 1527.8068, 1532.7472, 1537.7886, 1539.7711, 1549.7833, 1555.8657, 1558.8440, 1559.8573, 1568.7592, 1570.7814, 1578.7899, 1606.8213, 1615.8425, 1619.7972, 1621.7831, 1628.8156, 1630.8202, 1631.8217, 1639.9263, 1644.8083, 1647.8730, 1649.8650, 1650.8523, 1658.8206, 1659.8125, 1661.8284, 1663.7981, 1665.8070, 1669.8174, 1677.8353, 1679.9403, 1680.8076, 1682.7780, 1696.8055, 1698.7950, 1707.7931, 1709.8029, 1710.8878, 1712.9166, 1716.8563, 1725.8188, 1727.8683, 1730.8236, 1731.8221, 1742.8798, 1746.1482, 1749.8406, 1751.8657, 1756.8782, 1757.8225, 1763.8152, 1765.7922, 1766.8105, 1768.8596, 1769.4275, 1770.4087, 1773.8615, 1774.8795, 1775.0302, 1783.8599, 1785.8483, 1786.8871, 1790.8822, 1792.7894, 1801.9315, 1804.9026, 1808.8663, 1824.8685, 1830.8608, 1849.8231, 1851.8430, 1867.9100, 1868.9441, 1870.9304, 1880.9020, 1884.9191, 1908.8989, 1914.9821, 1916.9882, 1920.9862, 1930.9827, 1932.9813, 1934.9822, 1947.0288, 1958.9543, 1994.8763, 2011.9012, 2013.8700, 2020.9592, 2025.5920, 2042.0568, 2047.0238, 2047.9805, 2049.9497, 2052.9905, 2053.9851, 2058.9988, 2060.0400, 2060.0405, 2065.0427, 2066.0137, 2066.9895, 2067.9763, 2068.9128, 2070.8792, 2074.8860, 2082.0293, 2084.8977, 2086.8872, 2087.8835, 2090.8708, 2092.8674, 2093.8711, 2096.9836, 2097.9961, 2100.8787, 2101.8999, 2103.9092, 2107.8730, 2119.9924, 2122.9397, 2136.9133, 2211.1047, 2217.9368, 2225.1243, 2233.0840, 2238.1050, 2250.0259, 2257.9934, 2262.9368, 2263.9700, 2273.0264, 2277.9500, 2290.9570, 2307.9631, 2321.9558, 2323.9670, 2324.9824, 2335.9763, 2339.9456, 2343.0320, 2349.9844, 2352.0308, 2355.0315, 2368.0815, 2369.0588, 2378.0020, 2379.0002, 2380.0007, 2381.9963, 2383.0444, 2383.9822, 2393.0020, 2394.0181, 2398.0447, 2400.0771, 2405.0879, 2414.1050, 2417.0933, 2421.0769, 2422.0593, 2426.0476, 2428.0725, 2430.0947, 2432.0962, 2435.0254, 2438.0706, 2444.0901, 2451.0278, 2459.9802, 2461.0254, 2501.2520, 2528.1902, 2543.2092, 2564.1965, 2705.2144, 2717.1655, 2834.2925, 2888.2483, 2904.4053, 3130.4312, 3291.4875, 3312.3765, 3451.6833, 3454.6270

8. F1N1U9 **Mass:** 64259 **Score:** 20 **Expect:** 2.5e+002 **Matches:** 20

Uncharacterized protein OS=Bos taurus PE=4 SV=2

Observed	Mr(expt)	Mr(calc)	ppm	Start	End	Miss	Ions	Peptide
969.4496	968.4423	968.4577	-15.85	456 -	463	0	---	K.SHFSAHQR.I
979.4924	978.4852	978.4995	-14.69	272 -	279	0	---	K.THLSTHQR.I
1018.5130	1017.5057	1017.4920	13.5	96 -	103	0	---	R.FTNAFYQK.L
1060.5398	1059.5325	1059.5574	-23.44	160 -	167	0	---	K.EHLIQHQR.T
1113.5419	1112.5346	1112.5107	21.5	44 -	52	0	---	K.TATLFNCMR.T
1192.6014	1191.5941	1191.5918	1.99	24 -	33	1	---	K.RLLTGEESCK.D
1216.6482	1215.6409	1215.6546	-11.26	414 -	422	1	---	R.NLICNIWRK.T
1332.6602	1331.6529	1331.6517	0.93	516 -	526	1	---	K.VHHTRMHTGEK.N
1432.6987	1431.6914	1431.7153	-16.71	328 -	339	2	---	K.HCAHQKTHTGKK.L
1486.7472	1485.7399	1485.7688	-19.44	211 -	223	1	---	K.TFADNSVLKAHQR.I
1500.7242	1499.7169	1499.7225	-3.70	44 -	56	1	---	K.TATLFNCMRTGTK.E
1539.7711	1538.7638	1538.7365	17.8	112 -	123	2	---	R.THKEEKFYQSDK.Y
1697.7845	1696.7772	1696.7726	2.71	25 -	38	1	---	R.LLTGEESCKDNEFR.E
1707.7931	1706.7858	1706.7869	-0.60	196 -	210	1	---	R.THMGVNLYKCNEGGK.T
1773.8615	1772.8542	1772.8549	-0.40	44 -	58	2	---	K.TATLFNCMRTGTKEK.C + Oxidation (M)
1804.9026	1803.8953	1803.8978	-1.35	258 -	271	2	---	K.LYKCNEFQKTFAQK.T
2233.0840	2232.0767	2232.0382	17.3	220 -	237	1	---	K.AHQRIHTGEKPYECSEYK.K
2307.9631	2306.9558	2306.9606	-2.07	280 -	298	0	---	R.INTGEKPYECCECGTFSQK.S
2421.0769	2420.0696	2420.0307	16.1	34 -	52	2	---	K.DNEFRENCDKTATLFNCMR.T
3130.4312	3129.4239	3129.4971	-23.40	57 -	83	2	---	K.EKCFDLNECALSVKNLSQSSAHTVHQK.T

No match to: 713.4400, 713.4415, 724.3446, 728.2467, 730.2754, 732.2383, 734.2729, 735.3820, 736.3320, 740.2330, 743.2514, 748.2947, 750.2650, 756.4777, 764.3574, 765.3959, 768.3784, 772.4155, 774.3952, 778.4551, 780.3343, 782.3223, 783.3115, 787.2390, 791.2662, 802.4609, 805.3347, 806.4342, 807.3921, 808.4378, 812.4002, 814.4128, 820.4252, 828.3922, 830.3668, 834.3623, 837.3794, 840.3857, 842.5100, 845.4155, 851.3846, 856.5103, 865.3634, 869.4703, 870.5254, 871.4965, 872.6990, 888.3988, 891.4706, 893.9500, 894.4534, 897.0277, 905.4637, 911.4314, 912.4475, 913.5080, 913.5100, 918.5267, 925.4804, 927.4970, 930.5015, 933.4662, 935.4675, 943.4757, 951.4646, 952.4799, 957.4673, 961.4901, 962.4866, 967.4440, 972.4781, 974.5051, 975.4521, 977.4406, 978.4772, 983.4706, 985.6071, 991.4533, 993.5267, 995.5177, 999.4602, 1001.4565, 1001.5621, 1003.5043, 1007.5638, 1012.5004, 1014.5331, 1022.5398, 1023.5525, 1028.5991, 1030.5259, 1035.5314, 1036.5333, 1037.5145, 1040.5127, 1042.5243, 1045.5603, 1069.4846, 1075.5515, 1092.6041, 1106.5313, 1109.5087, 1111.5457, 1115.5394, 1118.5659, 1121.5419, 1122.5701, 1131.5602, 1138.5428, 1149.5912, 1155.5768, 1157.5907, 1158.6328, 1163.6145, 1173.6122, 1177.6130, 1178.6060, 1179.5918, 1180.6001, 1181.6368, 1183.6030, 1187.6376, 1193.5928, 1195.5924, 1199.6785, 1206.6066, 1215.6306, 1226.5839, 1227.6051, 1231.6115, 1232.6251, 1233.6230, 1234.6500, 1235.6155, 1248.6078, 1250.6483, 1254.6526, 1255.6036, 1256.6123, 1268.6208, 1271.6423, 1276.6179, 1277.6350, 1289.6449, 1292.6322, 1294.6362, 1295.6366, 1300.5895, 1303.6643, 1305.6523, 1307.6600, 1308.6443, 1310.6051, 1315.6364, 1320.6128, 1321.6134, 1323.6320, 1325.6456, 1331.6674, 1333.2076, 1334.6744, 1336.6710, 1337.6279, 1338.6300, 1338.6300, 1345.6146, 1347.6653, 1349.6800, 1349.6827, 1354.6820, 1355.6964, 1359.7053, 1360.6173, 1361.6373, 1365.6752, 1371.6718, 1375.6670, 1377.6776, 1382.6367, 1383.6619, 1387.6700, 1393.6727, 1400.6235, 1409.7107, 1411.6742, 1427.7725, 1431.7605, 1434.7190, 1449.7479, 1466.6953, 1473.7905, 1475.7498, 1478.7303, 1479.7468, 1485.7531, 1493.7345, 1495.7502, 1503.7690, 1505.8511, 1509.6786, 1520.7347, 1522.7239, 1523.7567, 1525.7864, 1527.8068, 1532.7472, 1537.7886, 1549.7833, 1555.8657, 1558.8440, 1559.8573, 1568.7592, 1570.7814, 1578.7899, 1606.8213, 1615.8425, 1619.7972, 1621.7831, 1628.8156, 1630.8202, 1631.8217, 1639.9263, 1644.8083, 1647.8730, 1649.8650, 1650.8523, 1658.8206, 1659.8125, 1661.8284, 1663.7981, 1665.8070, 1669.8174, 1677.8353, 1679.9403, 1680.8076, 1682.7780, 1696.8055, 1698.7950, 1709.8029, 1710.8878, 1712.9166, 1716.8563, 1725.8188, 1727.8683, 1730.8236, 1731.8221, 1742.8798, 1746.1482, 1749.8406, 1751.8657, 1756.8782, 1757.8225, 1763.8152, 1765.7922, 1766.8105, 1768.8596, 1769.4275, 1770.4087, 1774.8795, 1775.0302, 1783.8599, 1785.8483, 1786.8871, 1790.8822, 1792.7894, 1801.9315, 1808.8663, 1824.8685, 1830.8608, 1849.8231, 1851.8430, 1867.9100, 1868.9441, 1870.9304, 1880.9020, 1884.9191, 1908.8989, 1914.9821, 1916.9882, 1920.9862, 1930.9827, 1932.9813, 1934.9822, 1947.0288, 1958.9543, 1994.8763, 2011.9012, 2013.8700, 2020.9592, 2025.5920, 2042.0568, 2047.0238, 2047.9805, 2049.9497, 2052.9905, 2053.9851, 2058.9988, 2060.0400, 2060.0405, 2065.0427, 2066.0137, 2066.9895, 2067.9763, 2068.9128, 2070.8792, 2074.8860, 2082.0293, 2084.8977, 2086.8872, 2087.8835, 2090.8708, 2092.8674, 2093.8711, 2096.9836, 2097.9961, 2100.8787, 2101.8999, 2103.9092, 2107.8730, 2119.9924, 2122.9397, 2136.9133, 2211.1047, 2217.9368, 2225.1243, 2232.0229, 2238.1050, 2250.0259, 2257.9934, 2262.9368, 2263.9700, 2273.0264, 2277.9500, 2283.1741, 2290.9570, 2321.9558, 2323.9670, 2324.9824, 2335.9763, 2339.9456, 2343.0320, 2349.9844, 2352.0308, 2355.0315, 2358.0854, 2368.0815, 2369.0588, 2378.0020, 2379.0002, 2380.0007, 2381.9963, 2383.0444, 2383.9822, 2393.0020, 2394.0181, 2398.0447, 2400.0771, 2405.0879, 2414.1050, 2417.0933, 2422.0593, 2426.0476, 2428.0725, 2430.0947, 2432.0962, 2435.0254, 2438.0706, 2444.0901, 2451.0278, 2459.9802, 2461.0254, 2501.2520, 2528.1902, 2543.2092, 2564.1965, 2705.2144, 2717.1655, 2834.2925, 2888.2483, 2904.4053, 3291.4875, 3312.3765, 3451.6833, 3454.6270

9. F1N7L8 **Mass:** 63126 **Score:** 20 **Expect:** 2.6e+002 **Matches:** 22

Uncharacterized protein OS=Bos taurus GN=BBOF1 PE=4 SV=1

Observed	Mr(expt)	Mr(calc)	ppm	Start	End	Miss	Ions	Peptide
774.3952	773.3879	773.3919	-5.15	248 -	254	0	---	K.EADALQK.N
802.4609	801.4537	801.4708	-21.39	121 -	126	2	---	K.EKLERK.F
806.4342	805.4269	805.4116	19.0	347 -	352	1	---	K.EMNRIK.K + Oxidation (M)
820.4252	819.4180	819.4061	14.4	463 -	469	0	---	K.MNGFVPR.K
918.5267	917.5195	917.5116	8.53	347 -	353	2	---	K.EMNRIKK.L
952.4799	951.4726	951.4848	-12.79	407 -	413	1	---	R.MEYPKIR.T + Oxidation (M)
974.5051	973.4978	973.5080	-10.48	30 -	37	0	---	K.LEESVLER.A
978.4772	977.4700	977.4535	16.8	399 -	406	1	---	K.MREACVGR.M
1001.5621	1000.5548	1000.5739	-19.08	337 -	344	0	---	K.LQQLLQMK.D

1118.5659	1117.5586	1117.5615	-2.54	114 - 122	2	---	K.EKAQEEKEK.L
1177.6130	1176.6057	1176.6060	-0.24	144 - 153	0	---	K.EIGMIQTELK.T + Oxidation (M)
1183.6030	1182.5957	1182.5954	0.24	83 - 92	0	---	K.DTMSVLNYLK.K
1195.5924	1194.5851	1194.6105	-21.25	57 - 65	2	---	R.IEYRDTSRR.L
1216.6482	1215.6409	1215.6360	4.07	38 - 48	1	---	R.AKANASLWEAR.L
1231.6115	1230.6042	1230.6204	-13.14	248 - 258	1	---	K.EADALQKNSQK.L
1359.7053	1358.6980	1358.6677	22.3	94 - 104	1	---	K.QDQEKDNLIEK.L
1479.7468	1478.7395	1478.7473	-5.25	298 - 310	0	---	K.VVSLENALVCMTK.E + Oxidation (M)
1495.7502	1494.7429	1494.7249	12.1	69 - 79	2	---	R.CNEELKRQQYK.M
1790.8822	1789.8749	1789.9178	-23.97	337 - 350	2	---	K.LQQLLQMKDKEMNR.I + Oxidation (M)
1808.8663	1807.8590	1807.8563	1.49	127 - 141	0	---	K.FTMQVSELEGQFHQK.A
1824.8685	1823.8612	1823.8512	5.47	127 - 141	0	---	K.FTMQVSELEGQFHQK.A + Oxidation (M)
2394.0181	2393.0108	2393.0554	-18.61	414 - 434	1	---	R.TFDGREHSTNSVNQDLMEADK.W

No match to: 713.4400, 713.4415, 724.3446, 728.2467, 730.2754, 732.2383, 734.2729, 735.3820, 736.3320, 740.2330, 743.2514, 748.2947, 750.2650, 756.4777, 764.3574, 765.3959, 768.3784, 772.4155, 778.4551, 780.3343, 782.3223, 783.3115, 787.2390, 791.2662, 805.3347, 807.3921, 808.4378, 812.4002, 814.4128, 828.3922, 830.3668, 834.3623, 837.3794, 840.3857, 842.5100, 845.4155, 851.3846, 856.5103, 865.3634, 869.4703, 870.5254, 871.4965, 872.6990, 888.3988, 891.4706, 893.9500, 894.4534, 897.0277, 905.4637, 911.4314, 912.4475, 913.5080, 913.5100, 925.4804, 927.4970, 930.5015, 933.4662, 935.4675, 943.4757, 951.4646, 957.4673, 961.4901, 962.4866, 967.4440, 969.4496, 972.4781, 975.4521, 977.4406, 979.4924, 983.4706, 985.6071, 991.4533, 993.5267, 995.5177, 999.4602, 1001.4565, 1003.5043, 1007.5638, 1012.5004, 1014.5331, 1018.5130, 1022.5398, 1023.5525, 1028.5991, 1030.5259, 1035.5314, 1036.5333, 1037.5145, 1040.5127, 1042.5243, 1045.5603, 1060.5398, 1069.4846, 1075.5515, 1092.6041, 1106.5313, 1109.5087, 1111.5457, 1113.5419, 1115.5394, 1121.5419, 1122.5701, 1131.5602, 1138.5428, 1149.5912, 1155.5768, 1157.5907, 1158.6328, 1163.6145, 1173.6122, 1178.6060, 1179.5918, 1180.6001, 1181.6368, 1187.6376, 1192.6014, 1193.5928, 1199.6785, 1206.6066, 1215.6306, 1226.5839, 1227.6051, 1232.6251, 1233.6230, 1234.6500, 1235.6155, 1248.6078, 1250.6483, 1254.6526, 1255.6036, 1256.6123, 1268.6208, 1271.6423, 1276.6179, 1277.6350, 1289.6449, 1292.6322, 1294.6362, 1295.6366, 1300.5895, 1303.6643, 1305.6523, 1307.6600, 1308.6443, 1310.6051, 1315.6364, 1320.6128, 1321.6134, 1323.6320, 1325.6456, 1331.6674, 1332.6602, 1333.2076, 1334.6744, 1336.6710, 1337.6279, 1338.6300, 1338.6300, 1345.6146, 1347.6653, 1349.6800, 1349.6827, 1354.6820, 1355.6964, 1360.6173, 1361.6373, 1365.6752, 1371.6718, 1375.6670, 1377.6776, 1382.6367, 1383.6619, 1387.6700, 1393.6727, 1400.6235, 1409.7107, 1411.6742, 1427.7725, 1431.7605, 1432.6987, 1434.7190, 1449.7479, 1466.6953, 1473.7905, 1475.7498, 1478.7303, 1485.7531, 1486.7472, 1493.7345, 1500.7242, 1503.7690, 1505.8511, 1509.6786, 1520.7347, 1522.7239, 1523.7567, 1525.7864, 1527.8068, 1532.7472, 1537.7886, 1539.7711, 1549.7833, 1555.8657, 1558.8440, 1559.8573, 1568.7592, 1570.7814, 1578.7899, 1606.8213, 1615.8425, 1619.7972, 1621.7831, 1628.8156, 1630.8202, 1631.8217, 1639.9263, 1644.8083, 1647.8730, 1649.8650, 1650.8523, 1658.8206, 1659.8125, 1661.8284, 1663.7981, 1665.8070, 1669.8174, 1677.8353, 1679.9403, 1680.8076, 1682.7780, 1696.8055, 1697.7845, 1698.7950, 1707.7931, 1709.8029, 1710.8878, 1712.9166, 1716.8563, 1725.8188, 1727.8683, 1730.8236, 1731.8221, 1742.8798, 1746.1482, 1749.8406, 1751.8657, 1756.8782, 1757.8225, 1763.8152, 1765.7922, 1766.8105, 1768.8596, 1769.4275, 1770.4087, 1773.8615, 1774.8795, 1775.0302, 1783.8599, 1785.8483, 1786.8871, 1792.7894, 1801.9315, 1804.9026, 1830.8608, 1849.8231, 1851.8430, 1867.9100, 1868.9441, 1870.9304, 1880.9020, 1884.9191, 1908.8989, 1914.9821, 1916.9882, 1920.9862, 1930.9827, 1932.9813, 1934.9822, 1947.0288, 1958.9543, 1994.8763, 2011.9012, 2013.8700, 2020.9592, 2025.5920, 2042.0568, 2047.0238, 2047.9805, 2049.9497, 2052.9905, 2053.9851, 2058.9988, 2060.0400, 2060.0405, 2065.0427, 2066.0137, 2066.9895, 2067.9763, 2068.9128, 2070.8792, 2074.8860, 2082.0293, 2084.8977, 2086.8872, 2087.8835, 2090.8708, 2092.8674, 2093.8711, 2096.9836, 2097.9961, 2100.8787, 2101.8999, 2103.9092, 2107.8730, 2119.9924, 2122.9397, 2136.9133, 2211.1047, 2217.9368, 2225.1243, 2232.0229, 2233.0840, 2238.1050, 2250.0259, 2257.9934, 2262.9368, 2263.9700, 2273.0264, 2277.9500, 2283.1741, 2290.9570, 2307.9631, 2321.9558, 2323.9670, 2324.9824, 2335.9763, 2339.9456, 2343.0320, 2349.9844, 2352.0308, 2355.0315, 2358.0854, 2368.0815, 2369.0588, 2378.0020, 2379.0002, 2380.0007, 2381.9963, 2383.0444, 2383.9822, 2393.0020, 2398.0447, 2400.0771, 2405.0879, 2414.1050, 2417.0933, 2421.0769, 2422.0593, 2426.0476, 2428.0725, 2430.0947, 2432.0962, 2435.0254, 2438.0706, 2444.0901, 2451.0278, 2459.9802, 2461.0254, 2501.2520, 2528.1902, 2543.2092, 2564.1965, 2705.2144, 2717.1655, 2834.2925, 2888.2483, 2904.4053, 3130.4312, 3291.4875, 3312.3765, 3451.6833, 3454.6270

10. G3N2J6 **Mass:** 36179 **Score:** 19 **Expect:** 2.8e+002 **Matches:** 13

Uncharacterized protein OS=Bos taurus GN=TNIP3 PE=4 SV=1

Observed	Mr(expt)	Mr(calc)	ppm	Start - End	Miss	Ions	Peptide
782.3223	781.3150	781.3098	6.59	221 - 226	0	---	K.ACQMEK.E + Oxidation (M)
905.4637	904.4564	904.4614	-5.50	55 - 62	1	---	K.SKLDSAER.F
1183.6030	1182.5957	1182.6066	-9.23	41 - 49	2	---	R.SMKELYERK.V
1268.6208	1267.6135	1267.6408	-21.51	97 - 107	0	---	K.ESLNGELHELK.I
1449.7479	1448.7406	1448.7115	20.1	215 - 226	1	---	R.LNSQIKACQMEK.E
1525.7864	1524.7791	1524.7783	0.52	95 - 107	1	---	K.EKESLNGELHELK.I
1801.9315	1800.9242	1800.9080	9.01	251 - 266	0	---	R.DPYLLMDPGAVQNLQK.H
1868.9441	1867.9368	1867.9111	13.8	77 - 91	2	---	R.ESFPPHCRTSEPLRR.A
1914.9821	1913.9748	1913.9806	-3.04	199 - 214	0	---	K.EELQQIIQTSQSQLNR.L
2058.9988	2057.9915	2057.9775	6.83	117 - 132	2	---	K.NALMNHKKQHYECEIK.R + Oxidation (M)
2321.9558	2320.9485	2320.9643	-6.81	157 - 174	2	---	K.SEMECSREEMRTEMEVLK.Q + 3 Oxidation (M)
2417.0933	2416.0860	2416.0873	-0.52	267 - 286	0	---	K.HPPDDQWYAPDQFPPDVQHK.A
2834.2925	2833.2852	2833.3150	-10.50	164 - 185	2	---	R.EEMRTEMEVLKQQVQIYEEDFK.K + 2 Oxidation (M)

No match to: 713.4400, 713.4415, 724.3446, 728.2467, 730.2754, 732.2383, 734.2729, 735.3820, 736.3320, 740.2330, 743.2514, 748.2947, 750.2650, 756.4777, 764.3574, 765.3959, 768.3784, 772.4155, 774.3952, 778.4551, 780.3343, 783.3115, 787.2390, 791.2662, 802.4609, 805.3347, 806.4342, 807.3921, 808.4378, 812.4002, 814.4128, 820.4252, 828.3922, 830.3668, 834.3623, 837.3794, 840.3857, 842.5100, 845.4155, 851.3846, 856.5103, 865.3634, 869.4703, 870.5254, 871.4965, 872.6990, 888.3988, 891.4706, 893.9500, 894.4534, 897.0277, 911.4314, 912.4475, 913.5080, 913.5100, 918.5267, 925.4804, 927.4970, 930.5015, 933.4662, 935.4675, 943.4757, 951.4646, 952.4799, 957.4673, 961.4901, 962.4866, 967.4440, 969.4496, 972.4781, 974.5051, 975.4521, 977.4406, 978.4772, 979.4924, 983.4706, 985.6071, 991.4533, 993.5267, 995.5177, 999.4602, 1001.4565, 1001.5621, 1003.5043, 1007.5638, 1012.5004, 1014.5331, 1018.5130, 1022.5398, 1023.5525, 1028.5991, 1030.5259, 1035.5314, 1036.5333, 1037.5145, 1040.5127, 1042.5243, 1045.5603, 1060.5398, 1069.4846, 1075.5515, 1092.6041, 1106.5313, 1109.5087, 1111.5457, 1113.5419, 1115.5394, 1118.5659, 1121.5419, 1122.5701, 1131.5602, 1138.5428, 1149.5912, 1155.5768, 1157.5907, 1158.6328, 1163.6145, 1173.6122, 1177.6130, 1178.6060, 1179.5918, 1180.6001, 1181.6368, 1187.6376, 1192.6014, 1193.5928, 1195.5924, 1199.6785, 1206.6066, 1215.6306, 1216.6482, 1226.5839, 1227.6051, 1231.6115, 1232.6251, 1233.6230, 1234.6500, 1235.6155, 1248.6078, 1250.6483, 1254.6526, 1255.6036, 1256.6123, 1271.6423, 1276.6179, 1277.6350, 1289.6449, 1292.6322, 1294.6362, 1295.6366, 1300.5895, 1303.6643, 1305.6523, 1307.6600, 1308.6443, 1310.6051, 1315.6364, 1320.6128, 1321.6134, 1323.6320, 1325.6456, 1331.6674, 1332.6602, 1333.2076, 1334.6744, 1336.6710, 1337.6279, 1338.6300, 1338.6300, 1345.6146, 1347.6653, 1349.6800, 1349.6827, 1354.6820, 1355.6964, 1359.7053, 1360.6173, 1361.6373, 1365.6752, 1371.6718, 1375.6670, 1377.6776, 1382.6367, 1383.6619, 1387.6700, 1393.6727, 1400.6235, 1409.7107, 1411.6742, 1427.7725, 1431.7605, 1432.6987, 1434.7190, 1466.6953, 1473.7905, 1475.7498, 1478.7303, 1479.7468, 1485.7531, 1486.7472, 1493.7345, 1495.7502, 1500.7242, 1503.7690, 1505.8511, 1509.6786, 1520.7347, 1522.7239, 1523.7567, 1527.8068, 1532.7472, 1537.7886, 1539.7711, 1549.7833, 1555.8657, 1558.8440, 1559.8573, 1568.7592, 1570.7814, 1578.7899, 1606.8213, 1615.8425, 1619.7972, 1621.7831, 1628.8156, 1630.8202, 1631.8217, 1639.9263, 1644.8083, 1647.8730, 1649.8650, 1650.8523, 1658.8206, 1659.8125, 1661.8284, 1663.7981, 1665.8070, 1669.8174, 1677.8353, 1679.9403, 1680.8076, 1682.7780, 1696.8055, 1697.7845, 1698.7950, 1707.7931, 1709.8029, 1710.8878, 1712.9166, 1716.8563, 1725.8188, 1727.8683, 1730.8236, 1731.8221, 1742.8798, 1746.1482, 1749.8406, 1751.8657, 1756.8782, 1757.8225, 1763.8152, 1765.7922, 1766.8105, 1768.8596, 1769.4275, 1770.4087, 1773.8615, 1774.8795, 1775.0302, 1783.8599, 1785.8483, 1786.8871, 1790.8822, 1792.7894, 1804.9026, 1808.8663, 1824.8685, 1830.8608, 1849.8231, 1851.8430, 1867.9100, 1870.9304, 1880.9020, 1884.9191, 1908.8989, 1916.9882, 1920.9862, 1930.9827, 1932.9813, 1934.9822, 1947.0288, 1958.9543, 1994.8763, 2011.9012, 2013.8700, 2020.9592, 2025.5920, 2042.0568, 2047.0238, 2047.9805, 2049.9497, 2052.9905, 2053.9851, 2060.0400, 2060.0405, 2065.0427, 2066.0137, 2066.9895, 2067.9763, 2068.9128, 2070.8792, 2074.8860, 2082.0293, 2084.8977, 2086.8872, 2087.8835, 2090.8708, 2092.8674, 2093.8711, 2096.9836, 2097.9961, 2100.8787, 2101.8999, 2103.9092, 2107.8730, 2119.9924, 2122.9397, 2136.9133, 2211.1047, 2217.9368, 2225.1243, 2232.0229, 2233.0840, 2238.1050, 2250.0259, 2257.9934, 2262.9368, 2263.9700, 2273.0264, 2277.9500, 2283.1741, 2290.9570, 2307.9631, 2323.9670, 2324.9824, 2335.9763, 2339.9456, 2343.0320, 2349.9844, 2352.0308, 2355.0315, 2358.0854, 2368.0815, 2369.0588, 2378.0020, 2379.0002, 2380.0007, 2381.9963, 2383.0444, 2383.9822, 2393.0020, 2394.0181, 2398.0447, 2400.0771, 2405.0879, 2414.1050, 2421.0769, 2422.0593, 2426.0476, 2428.0725, 2430.0947, 2432.0962, 2435.0254, 2438.0706, 2444.0901, 2451.0278, 2459.9802, 2461.0254, 2501.2520, 2528.1902, 2543.2092, 2564.1965, 2705.2144, 2717.1655, 2888.2483, 2904.4053, 3130.4312, 3291.4875, 3312.3765, 3451.6833, 3454.6270

11. Q1PSA4 **Mass:** 20552 **Score:** 19 **Expect:** 2.8e+002 **Matches:** 9

Uncharacterized protein OS=Bos taurus GN=ZAR1 PE=4 SV=1

Observed	Mr(expt)	Mr(calc)	ppm	Start	End	Miss	Ions	Peptide
1035.5314	1034.5241	1034.5219	2.18	24 -	33	0	---	R.CLYPAAAAAK.G
1294.6362	1293.6289	1293.6473	-14.18	143 -	152	1	---	K.RPHRQDLCGR.C
1347.6653	1346.6580	1346.6289	21.6	157 -	167	1	---	K.RLSCDSTFSFK.Y
1532.7472	1531.7399	1531.7453	-3.52	155 -	167	2	---	K.GKRLSCDSTFSFK.Y
1680.8076	1679.8003	1679.7912	5.42	99 -	110	2	---	K.VYYKQFCRTCQK.S
1870.9304	1869.9231	1869.9493	-13.98	138 -	152	2	---	R.HVDPKRPHRQDLCGR.C
2383.0444	2382.0371	2382.0549	-7.45	117 -	135	2	---	R.VEDITCQNCKQTRCSCPVK.L
2432.0962	2431.0889	2431.1009	-4.91	111 -	129	2	---	K.SYNPYRVEDITCQNCKQTR.C
2564.1965	2563.1892	2563.1254	24.9	107 -	126	2	---	R.TCQKSYNPYRVEDITCQNCK.Q

No match to: 713.4400, 713.4415, 724.3446, 728.2467, 730.2754, 732.2383, 734.2729, 735.3820, 736.3320, 740.2330, 743.2514, 748.2947, 750.2650, 756.4777, 764.3574, 765.3959, 768.3784, 772.4155, 774.3952, 778.4551, 780.3343, 782.3223, 783.3115, 787.2390, 791.2662, 802.4609, 805.3347, 806.4342, 807.3921, 808.4378, 812.4002, 814.4128, 820.4252, 828.3922, 830.3668, 834.3623, 837.3794, 840.3857, 842.5100, 845.4155, 851.3846, 856.5103, 865.3634, 869.4703, 870.5254, 871.4965, 872.6990, 888.3988, 891.4706, 893.9500, 894.4534, 897.0277, 905.4637, 911.4314, 912.4475, 913.5080, 913.5100, 918.5267, 925.4804, 927.4970, 930.5015, 933.4662, 935.4675, 943.4757, 951.4646, 952.4799, 957.4673, 961.4901, 962.4866, 967.4440, 969.4496, 972.4781, 974.5051, 975.4521, 977.4406, 978.4772, 979.4924, 983.4706, 985.6071, 991.4533, 993.5267, 995.5177, 999.4602, 1001.4565, 1001.5621, 1003.5043, 1007.5638, 1012.5004, 1014.5331, 1018.5130, 1022.5398, 1023.5525, 1028.5991, 1030.5259, 1036.5333, 1037.5145, 1040.5127, 1042.5243, 1045.5603, 1060.5398, 1069.4846, 1075.5515, 1092.6041, 1106.5313, 1109.5087, 1111.5457, 1113.5419, 1115.5394, 1118.5659, 1121.5419, 1122.5701, 1131.5602, 1138.5428, 1149.5912, 1155.5768, 1157.5907, 1158.6328, 1163.6145, 1173.6122, 1177.6130, 1178.6060, 1179.5918, 1180.6001, 1181.6368, 1183.6030, 1187.6376, 1192.6014, 1193.5928, 1195.5924, 1199.6785, 1206.6066, 1215.6306, 1216.6482, 1226.5839, 1227.6051, 1231.6115, 1232.6251, 1233.6230, 1234.6500, 1235.6155, 1248.6078, 1250.6483, 1254.6526, 1255.6036, 1256.6123, 1268.6208, 1271.6423, 1276.6179, 1277.6350, 1289.6449, 1292.6322, 1295.6366, 1300.5895, 1303.6643, 1305.6523, 1307.6600, 1308.6443, 1310.6051, 1315.6364, 1320.6128, 1321.6134, 1323.6320, 1325.6456, 1331.6674, 1332.6602, 1333.2076, 1334.6744, 1336.6710, 1337.6279, 1338.6300, 1338.6300, 1345.6146, 1349.6800, 1349.6827, 1354.6820, 1355.6964, 1359.7053, 1360.6173, 1361.6373, 1365.6752, 1371.6718, 1375.6670, 1377.6776, 1382.6367, 1383.6619, 1387.6700, 1393.6727, 1400.6235, 1409.7107, 1411.6742, 1427.7725, 1431.7605, 1432.6987, 1434.7190, 1449.7479, 1466.6953, 1473.7905, 1475.7498, 1478.7303, 1479.7468, 1485.7531, 1486.7472, 1493.7345, 1495.7502, 1500.7242, 1503.7690, 1505.8511, 1509.6786, 1520.7347, 1522.7239, 1523.7567, 1525.7864, 1527.8068, 1537.7886, 1539.7711, 1549.7833, 1555.8657, 1558.8440, 1559.8573, 1568.7592, 1570.7814, 1578.7899, 1606.8213, 1615.8425, 1619.7972, 1621.7831, 1628.8156, 1630.8202, 1631.8217, 1639.9263, 1644.8083, 1647.8730, 1649.8650, 1650.8523, 1658.8206, 1659.8125, 1661.8284, 1663.7981, 1665.8070, 1669.8174, 1677.8353, 1679.9403, 1682.7780, 1696.8055, 1697.7845, 1698.7950, 1707.7931, 1709.8029, 1710.8878, 1712.9166, 1716.8563, 1725.8188, 1727.8683, 1730.8236, 1731.8221, 1742.8798, 1746.1482, 1749.8406, 1751.8657, 1756.8782, 1757.8225, 1763.8152, 1765.7922, 1766.8105, 1768.8596, 1769.4275, 1770.4087, 1773.8615, 1774.8795, 1775.0302, 1783.8599, 1785.8483, 1786.8871, 1790.8822, 1792.7894, 1801.9315, 1804.9026, 1808.8663, 1824.8685, 1830.8608, 1849.8231, 1851.8430, 1867.9100, 1868.9441, 1880.9020, 1884.9191, 1908.8989, 1914.9821, 1916.9882, 1920.9862, 1930.9827, 1932.9813, 1934.9822, 1947.0288, 1958.9543, 1994.8763, 2011.9012, 2013.8700, 2020.9592, 2025.5920, 2042.0568, 2047.0238, 2047.9805, 2049.9497, 2052.9905, 2053.9851, 2058.9988, 2060.0400, 2060.0405, 2065.0427, 2066.0137, 2066.9895, 2067.9763, 2068.9128, 2070.8792, 2074.8860, 2082.0293, 2084.8977, 2086.8872, 2087.8835, 2090.8708, 2092.8674, 2093.8711, 2096.9836, 2097.9961, 2100.8787, 2101.8999, 2103.9092, 2107.8730, 2119.9924, 2122.9397, 2136.9133, 2211.1047, 2217.9368, 2225.1243, 2232.0229, 2233.0840, 2238.1050, 2250.0259, 2257.9934, 2262.9368, 2263.9700, 2273.0264, 2277.9500, 2283.1741, 2290.9570, 2307.9631, 2321.9558, 2323.9670, 2324.9824, 2335.9763, 2339.9456, 2343.0320, 2349.9844, 2352.0308, 2355.0315, 2358.0854, 2368.0815, 2369.0588, 2378.0020, 2379.0002, 2380.0007, 2381.9963, 2383.9822, 2393.0020, 2394.0181, 2398.0447, 2400.0771, 2405.0879, 2414.1050, 2417.0933, 2421.0769, 2422.0593, 2426.0476, 2428.0725, 2430.0947, 2435.0254, 2438.0706, 2444.0901, 2451.0278, 2459.9802, 2461.0254, 2501.2520, 2528.1902, 2543.2092, 2705.2144, 2717.1655, 2834.2925, 2888.2483, 2904.4053, 3130.4312, 3291.4875, 3312.3765, 3451.6833, 3454.6270

12. Q17Q91 **Mass:** 10021 **Score:** 19 **Expect:** 2.9e+002 **Matches:** 7

Coiled-coil-helix-coiled-coil-helix domain-containing protein 7 OS=Bos taurus GN=CHCHD7 PE=3 SV=1

Observed	Mr(expt)	Mr(calc)	ppm	Start	End	Miss	Ions	Peptide
1028.5991	1027.5918	1027.6073	-15.05	2 -	9	2	---	M.PMVTRRLR.D
1180.6001	1179.5928	1179.5859	5.88	50 -	58	0	---	K.FWNSVMIQR.R
1292.6322	1291.6249	1291.6482	-18.03	74 -	85	1	---	R.DEILGGLGKMPY.-
1308.6443	1307.6370	1307.6431	-4.67	74 -	85	1	---	R.DEILGGLGKMPY.- + Oxidation (M)
1336.6710	1335.6637	1335.6870	-17.42	50 -	59	1	---	K.FWNSVMIQRR.Q
1495.7502	1494.7429	1494.7613	-12.28	60 -	73	0	---	R.QNGVKPPMPTAAER.D
2888.2483	2887.2410	2887.1695	24.8	10 -	34	1	---	R.DPDVNPCLSESDASTRCMAENNYDK.E

No match to: 713.4400, 713.4415, 724.3446, 728.2467, 730.2754, 732.2383, 734.2729, 735.3820, 736.3320, 740.2330, 743.2514, 748.2947, 750.2650, 756.4777, 764.3574, 765.3959, 768.3784, 772.4155, 774.3952, 778.4551, 780.3343, 782.3223, 783.3115, 787.2390, 791.2662, 802.4609, 805.3347, 806.4342, 807.3921, 808.4378, 812.4002, 814.4128, 820.4252, 828.3922, 830.3668, 834.3623, 837.3794, 840.3857, 842.5100, 845.4155, 851.3846, 856.5103, 865.3634, 869.4703, 870.5254, 871.4965, 872.6990, 888.3988, 891.4706, 893.9500, 894.4534, 897.0277, 905.4637, 911.4314, 912.4475, 913.5080, 913.5100, 918.5267, 925.4804, 927.4970, 930.5015, 933.4662, 935.4675, 943.4757, 951.4646, 952.4799, 957.4673, 961.4901, 962.4866, 967.4440, 969.4496, 972.4781, 974.5051, 975.4521, 977.4406, 978.4772, 979.4924, 983.4706, 985.6071, 991.4533, 993.5267, 995.5177, 999.4602, 1001.4565, 1001.5621, 1003.5043, 1007.5638, 1012.5004, 1014.5331, 1018.5130, 1022.5398, 1023.5525, 1030.5259, 1035.5314, 1036.5333, 1037.5145, 1040.5127, 1042.5243, 1045.5603, 1060.5398, 1069.4846, 1075.5515, 1092.6041, 1106.5313, 1109.5087, 1111.5457, 1113.5419, 1115.5394, 1118.5659, 1121.5419, 1122.5701, 1131.5602, 1138.5428, 1149.5912, 1155.5768, 1157.5907, 1158.6328, 1163.6145, 1173.6122, 1177.6130, 1178.6060, 1179.5918, 1181.6368, 1183.6030, 1187.6376, 1192.6014, 1193.5928, 1195.5924, 1199.6785, 1206.6066, 1215.6306, 1216.6482, 1226.5839, 1227.6051, 1231.6115, 1232.6251, 1233.6230, 1234.6500, 1235.6155, 1248.6078, 1250.6483, 1254.6526, 1255.6036, 1256.6123, 1268.6208, 1271.6423, 1276.6179, 1277.6350, 1289.6449, 1294.6362, 1295.6366, 1300.5895, 1303.6643, 1305.6523, 1307.6600, 1310.6051, 1315.6364, 1320.6128, 1321.6134, 1323.6320, 1325.6456, 1331.6674, 1332.6602, 1333.2076, 1334.6744, 1337.6279, 1338.6300, 1338.6300, 1345.6146, 1347.6653, 1349.6800, 1349.6827, 1354.6820, 1355.6964, 1359.7053, 1360.6173, 1361.6373, 1365.6752, 1371.6718, 1375.6670, 1377.6776, 1382.6367, 1383.6619, 1387.6700, 1393.6727, 1400.6235, 1409.7107, 1411.6742, 1427.7725, 1431.7605, 1432.6987, 1434.7190, 1449.7479, 1466.6953, 1473.7905, 1475.7498, 1478.7303, 1479.7468, 1485.7531, 1486.7472, 1493.7345, 1500.7242, 1503.7690, 1505.8511, 1509.6786, 1520.7347, 1522.7239, 1523.7567, 1525.7864, 1527.8068, 1532.7472, 1537.7886, 1539.7711, 1549.7833, 1555.8657, 1558.8440, 1559.8573, 1568.7592, 1570.7814, 1578.7899, 1606.8213, 1615.8425, 1619.7972, 1621.7831, 1628.8156, 1630.8202, 1631.8217, 1639.9263, 1644.8083, 1647.8730, 1649.8650, 1650.8523, 1658.8206, 1659.8125, 1661.8284, 1663.7981, 1665.8070, 1669.8174, 1677.8353, 1679.9403, 1680.8076, 1682.7780, 1696.8055, 1697.7845, 1698.7950, 1707.7931, 1709.8029, 1710.8878, 1712.9166, 1716.8563, 1725.8188, 1727.8683, 1730.8236, 1731.8221, 1742.8798, 1746.1482, 1749.8406, 1751.8657, 1756.8782, 1757.8225, 1763.8152, 1765.7922, 1766.8105, 1768.8596, 1769.4275, 1770.4087, 1773.8615, 1774.8795, 1775.0302, 1783.8599, 1785.8483, 1786.8871, 1790.8822, 1792.7894, 1801.9315, 1804.9026, 1808.8663, 1824.8685, 1830.8608, 1849.8231, 1851.8430, 1867.9100, 1868.9441, 1870.9304, 1880.9020, 1884.9191, 1908.8989, 1914.9821, 1916.9882, 1920.9862, 1930.9827, 1932.9813, 1934.9822, 1947.0288, 1958.9543, 1994.8763, 2011.9012, 2013.8700, 2020.9592, 2025.5920, 2042.0568, 2047.0238, 2047.9805, 2049.9497, 2052.9905, 2053.9851, 2058.9988, 2060.0400, 2060.0405, 2065.0427, 2066.0137, 2066.9895, 2067.9763, 2068.9128, 2070.8792, 2074.8860, 2082.0293, 2084.8977, 2086.8872, 2087.8835, 2090.8708, 2092.8674, 2093.8711, 2096.9836, 2097.9961, 2100.8787, 2101.8999, 2103.9092, 2107.8730, 2119.9924, 2122.9397, 2136.9133, 2211.1047, 2217.9368, 2225.1243, 2232.0229, 2233.0840, 2238.1050, 2250.0259, 2257.9934, 2262.9368, 2263.9700, 2273.0264, 2277.9500, 2283.1741, 2290.9570, 2307.9631, 2321.9558, 2323.9670, 2324.9824, 2335.9763, 2339.9456, 2343.0320, 2349.9844, 2352.0308, 2355.0315, 2358.0854, 2368.0815, 2369.0588, 2378.0020, 2379.0002, 2380.0007, 2381.9963, 2383.0444, 2383.9822, 2393.0020, 2394.0181, 2398.0447, 2400.0771, 2405.0879, 2414.1050, 2417.0933, 2421.0769, 2422.0593, 2426.0476, 2428.0725, 2430.0947, 2432.0962, 2435.0254, 2438.0706, 2444.0901, 2451.0278, 2459.9802, 2461.0254, 2501.2520, 2528.1902, 2543.2092, 2564.1965, 2705.2144, 2717.1655, 2834.2925, 2904.4053, 3130.4312, 3291.4875, 3312.3765, 3451.6833, 3454.6270

13. Q0IIC8 **Mass:** 17012 **Score:** 19 **Expect:** 3e+002 **Matches:** 8

Phospholipase A(2) OS=Bos taurus GN=PLA2G2A PE=2 SV=1

Observed	Mr(expt)	Mr(calc)	ppm	Start	End	Miss	Ions	Peptide
807.3921	806.3848	806.3705	17.8	134 -	140	0	---	R.NSLCTGR.A

1023.5525	1022.5452	1022.5509	-5.51	121 - 128	2	---	R.NQKTYNKK.L
1037.5145	1036.5072	1036.5236	-15.83	74 - 82	1	---	K.LKHHGCGTK.F
1178.6060	1177.5987	1177.6026	-3.28	114 - 123	1	---	K.AALCFARNQK.T
1520.7347	1519.7274	1519.6984	19.1	93 - 105	1	---	R.GQITCADQGVCRR.I
1658.8206	1657.8133	1657.8358	-13.58	128 - 140	2	---	K.KLQYYRNSLCTGR.A
2058.9988	2057.9915	2058.0330	-20.15	76 - 92	2	---	K.HHGCGTKFLNYNVSIRR.G
2834.2925	2833.2852	2833.3276	-14.95	31 - 57	2	---	K.HTTGKGALLSYSAYGCYCGVSGRGSPK.D

No match to: 713.4400, 713.4415, 724.3446, 728.2467, 730.2754, 732.2383, 734.2729, 735.3820, 736.3320, 740.2330, 743.2514, 748.2947, 750.2650, 756.4777, 764.3574, 765.3959, 768.3784, 772.4155, 774.3952, 778.4551, 780.3343, 782.3223, 783.3115, 787.2390, 791.2662, 802.4609, 805.3347, 806.4342, 808.4378, 812.4002, 814.4128, 820.4252, 828.3922, 830.3668, 834.3623, 837.3794, 840.3857, 842.5100, 845.4155, 851.3846, 856.5103, 865.3634, 869.4703, 870.5254, 871.4965, 872.6990, 888.3988, 891.4706, 893.9500, 894.4534, 897.0277, 905.4637, 911.4314, 912.4475, 913.5080, 913.5100, 918.5267, 925.4804, 927.4970, 930.5015, 933.4662, 935.4675, 943.4757, 951.4646, 952.4799, 957.4673, 961.4901, 962.4866, 967.4440, 969.4496, 972.4781, 974.5051, 975.4521, 977.4406, 978.4772, 979.4924, 983.4706, 985.6071, 991.4533, 993.5267, 995.5177, 999.4602, 1001.4565, 1001.5621, 1003.5043, 1007.5638, 1012.5004, 1014.5331, 1018.5130, 1022.5398, 1028.5991, 1030.5259, 1035.5314, 1036.5333, 1040.5127, 1042.5243, 1045.5603, 1060.5398, 1069.4846, 1075.5515, 1092.6041, 1106.5313, 1109.5087, 1111.5457, 1113.5419, 1115.5394, 1118.5659, 1121.5419, 1122.5701, 1131.5602, 1138.5428, 1149.5912, 1155.5768, 1157.5907, 1158.6328, 1163.6145, 1173.6122, 1177.6130, 1179.5918, 1180.6001, 1181.6368, 1183.6030, 1187.6376, 1192.6014, 1193.5928, 1195.5924, 1199.6785, 1206.6066, 1215.6306, 1216.6482, 1226.5839, 1227.6051, 1231.6115, 1232.6251, 1233.6230, 1234.6500, 1235.6155, 1248.6078, 1250.6483, 1254.6526, 1255.6036, 1256.6123, 1268.6208, 1271.6423, 1276.6179, 1277.6350, 1289.6449, 1292.6322, 1294.6362, 1295.6366, 1300.5895, 1303.6643, 1305.6523, 1307.6600, 1308.6443, 1310.6051, 1315.6364, 1320.6128, 1321.6134, 1323.6320, 1325.6456, 1331.6674, 1332.6602, 1333.2076, 1334.6744, 1336.6710, 1337.6279, 1338.6300, 1338.6300, 1345.6146, 1347.6653, 1349.6800, 1349.6827, 1354.6820, 1355.6964, 1359.7053, 1360.6173, 1361.6373, 1365.6752, 1371.6718, 1375.6670, 1377.6776, 1382.6367, 1383.6619, 1387.6700, 1393.6727, 1400.6235, 1409.7107, 1411.6742, 1427.7725, 1431.7605, 1432.6987, 1434.7190, 1449.7479, 1466.6953, 1473.7905, 1475.7498, 1478.7303, 1479.7468, 1485.7531, 1486.7472, 1493.7345, 1495.7502, 1500.7242, 1503.7690, 1505.8511, 1509.6786, 1522.7239, 1523.7567, 1525.7864, 1527.8068, 1532.7472, 1537.7886, 1539.7711, 1549.7833, 1555.8657, 1558.8440, 1559.8573, 1568.7592, 1570.7814, 1578.7899, 1606.8213, 1615.8425, 1619.7972, 1621.7831, 1628.8156, 1630.8202, 1631.8217, 1639.9263, 1644.8083, 1647.8730, 1649.8650, 1650.8523, 1659.8125, 1661.8284, 1663.7981, 1665.8070, 1669.8174, 1677.8353, 1679.9403, 1680.8076, 1682.7780, 1696.8055, 1697.7845, 1698.7950, 1707.7931, 1709.8029, 1710.8878, 1712.9166, 1716.8563, 1725.8188, 1727.8683, 1730.8236, 1731.8221, 1742.8798, 1746.1482, 1749.8406, 1751.8657, 1756.8782, 1757.8225, 1763.8152, 1765.7922, 1766.8105, 1768.8596, 1769.4275, 1770.4087, 1773.8615, 1774.8795, 1775.0302, 1783.8599, 1785.8483, 1786.8871, 1790.8822, 1792.7894, 1801.9315, 1804.9026, 1808.8663, 1824.8685, 1830.8608, 1849.8231, 1851.8430, 1867.9100, 1868.9441, 1870.9304, 1880.9020, 1884.9191, 1908.8989, 1914.9821, 1916.9882, 1920.9862, 1930.9827, 1932.9813, 1934.9822, 1947.0288, 1958.9543, 1994.8763, 2011.9012, 2013.8700, 2020.9592, 2025.5920, 2042.0568, 2047.0238, 2047.9805, 2049.9497, 2052.9905, 2053.9851, 2060.0400, 2060.0405, 2065.0427, 2066.0137, 2066.9895, 2067.9763, 2068.9128, 2070.8792, 2074.8860, 2082.0293, 2084.8977, 2086.8872, 2087.8835, 2090.8708, 2092.8674, 2093.8711, 2096.9836, 2097.9961, 2100.8787, 2101.8999, 2103.9092, 2107.8730, 2119.9924, 2122.9397, 2136.9133, 2211.1047, 2217.9368, 2225.1243, 2232.0229, 2233.0840, 2238.1050, 2250.0259, 2257.9934, 2262.9368, 2263.9700, 2273.0264, 2277.9500, 2283.1741, 2290.9570, 2307.9631, 2321.9558, 2323.9670, 2324.9824, 2335.9763, 2339.9456, 2343.0320, 2349.9844, 2352.0308, 2355.0315, 2358.0854, 2368.0815, 2369.0588, 2378.0020, 2379.0002, 2380.0007, 2381.9963, 2383.0444, 2383.9822, 2393.0020, 2394.0181, 2398.0447, 2400.0771, 2405.0879, 2414.1050, 2417.0933, 2421.0769, 2422.0593, 2426.0476, 2428.0725, 2430.0947, 2432.0962, 2435.0254, 2438.0706, 2444.0901, 2451.0278, 2459.9802, 2461.0254, 2501.2520, 2528.1902, 2543.2092, 2564.1965, 2705.2144, 2717.1655, 2888.2483, 2904.4053, 3130.4312, 3291.4875, 3312.3765, 3451.6833, 3454.6270

14. Q5KR49 **Mass:** 32732 **Score:** 19 **Expect:** 3.1e+002 **Matches:** 14

Tropomyosin alpha-1 chain OS=Bos taurus GN=TPM1 PE=2 SV=1

Observed	Mr(expt)	Mr(calc)	ppm	Start - End	Miss	Ions	Peptide
894.4534	893.4461	893.4606	-16.29	162 - 168	1	---	K.YEEVARK.L
1022.5398	1021.5325	1021.5300	2.47	8 - 15	1	---	K.MQMLKLDK.E + Oxidation (M)
1109.5087	1108.5014	1108.4819	17.7	119 - 128	1	---	K.AADESERGMK.V + Oxidation (M)
1308.6443	1307.6370	1307.6106	20.2	234 - 244	1	---	K.EAETRAEFAER.S
1332.6602	1331.6529	1331.6317	15.9	78 - 90	0	---	K.ATDAEADVASLNR.R
1431.7605	1430.7532	1430.7365	11.7	129 - 140	2	---	K.VIESRAQKDEEK.M
1475.7498	1474.7425	1474.7701	-18.69	141 - 152	1	---	K.MEIQEIQLKEAK.H + Oxidation (M)
1549.7833	1548.7760	1548.7896	-8.74	232 - 244	2	---	K.LKEAETRAEFAER.S
1677.8353	1676.8280	1676.8151	7.68	119 - 133	2	---	K.AADESERGMKVIESR.A
1727.8683	1726.8610	1726.8849	-13.85	92 - 105	1	---	R.IQLVEEELDRAQER.L
1808.8663	1807.8590	1807.8257	18.4	113 - 128	2	---	K.LEEAEKAADESERGMK.V + Oxidation (M)
1908.8989	1907.8916	1907.9364	-23.45	249 - 264	1	---	K.LEKSIDDLEDELYAQK.L
2049.9497	2048.9424	2048.9724	-14.64	267 - 284	1	---	K.YKAISEELDHALNDMTSI.-
2066.0137	2065.0064	2064.9673	18.9	267 - 284	1	---	K.YKAISEELDHALNDMTSI.- + Oxidation (M)

No match to: 713.4400, 713.4415, 724.3446, 728.2467, 730.2754, 732.2383, 734.2729, 735.3820, 736.3320, 740.2330, 743.2514, 748.2947, 750.2650, 756.4777, 764.3574, 765.3959, 768.3784, 772.4155, 774.3952, 778.4551, 780.3343, 782.3223, 783.3115, 787.2390, 791.2662, 802.4609, 805.3347, 806.4342, 807.3921, 808.4378, 812.4002, 814.4128, 820.4252, 828.3922, 830.3668, 834.3623, 837.3794, 840.3857, 842.5100, 845.4155, 851.3846, 856.5103, 865.3634, 869.4703, 870.5254, 871.4965, 872.6990, 888.3988, 891.4706, 893.9500, 897.0277, 905.4637, 911.4314, 912.4475, 913.5080, 913.5100, 918.5267, 925.4804, 927.4970, 930.5015, 933.4662, 935.4675, 943.4757, 951.4646, 952.4799, 957.4673, 961.4901, 962.4866, 967.4440, 969.4496, 972.4781, 974.5051, 975.4521, 977.4406, 978.4772, 979.4924, 983.4706, 985.6071, 991.4533, 993.5267, 995.5177, 999.4602, 1001.4565, 1001.5621, 1003.5043, 1007.5638, 1012.5004, 1014.5331, 1018.5130, 1023.5525, 1028.5991, 1030.5259, 1035.5314, 1036.5333, 1037.5145, 1040.5127, 1042.5243, 1045.5603, 1060.5398, 1069.4846, 1075.5515, 1092.6041, 1106.5313, 1111.5457, 1113.5419, 1115.5394, 1118.5659, 1121.5419, 1122.5701, 1131.5602, 1138.5428, 1149.5912, 1155.5768, 1157.5907, 1158.6328, 1163.6145, 1173.6122, 1177.6130, 1178.6060, 1179.5918, 1180.6001, 1181.6368, 1183.6030, 1187.6376, 1192.6014, 1193.5928, 1195.5924, 1199.6785, 1206.6066, 1215.6306, 1216.6482, 1226.5839, 1227.6051, 1231.6115, 1232.6251, 1233.6230, 1234.6500, 1235.6155, 1248.6078, 1250.6483, 1254.6526, 1255.6036, 1256.6123, 1268.6208, 1271.6423, 1276.6179, 1277.6350, 1289.6449, 1292.6322, 1294.6362, 1295.6366, 1300.5895, 1303.6643, 1305.6523, 1307.6600, 1310.6051, 1315.6364, 1320.6128, 1321.6134, 1323.6320, 1325.6456, 1331.6674, 1333.2076, 1334.6744, 1336.6710, 1337.6279, 1338.6300, 1338.6300, 1345.6146, 1347.6653, 1349.6800, 1349.6827, 1354.6820, 1355.6964, 1359.7053, 1360.6173, 1361.6373, 1365.6752, 1371.6718, 1375.6670, 1377.6776, 1382.6367, 1383.6619, 1387.6700, 1393.6727, 1400.6235, 1409.7107, 1411.6742, 1427.7725, 1432.6987, 1434.7190, 1449.7479, 1466.6953, 1473.7905, 1478.7303, 1479.7468, 1485.7531, 1486.7472, 1493.7345, 1495.7502, 1500.7242, 1503.7690, 1505.8511, 1509.6786, 1520.7347, 1522.7239, 1523.7567, 1525.7864, 1527.8068, 1532.7472, 1537.7886, 1539.7711, 1555.8657, 1558.8440, 1559.8573, 1568.7592, 1570.7814, 1578.7899, 1606.8213, 1615.8425, 1619.7972, 1621.7831, 1628.8156, 1630.8202, 1631.8217, 1639.9263, 1644.8083, 1647.8730, 1649.8650, 1650.8523, 1658.8206, 1659.8125, 1661.8284, 1663.7981, 1665.8070, 1669.8174, 1679.9403, 1680.8076, 1682.7780, 1696.8055, 1697.7845, 1698.7950, 1707.7931, 1709.8029, 1710.8878, 1712.9166, 1716.8563, 1725.8188, 1730.8236, 1731.8221, 1742.8798, 1746.1482, 1749.8406, 1751.8657, 1756.8782, 1757.8225, 1763.8152, 1765.7922, 1766.8105, 1768.8596, 1769.4275, 1770.4087, 1773.8615, 1774.8795, 1775.0302, 1783.8599, 1785.8483, 1786.8871, 1790.8822, 1792.7894, 1801.9315, 1804.9026, 1824.8685, 1830.8608, 1849.8231, 1851.8430, 1867.9100, 1868.9441, 1870.9304, 1880.9020, 1884.9191, 1914.9821, 1916.9882, 1920.9862, 1930.9827, 1932.9813, 1934.9822, 1947.0288, 1958.9543, 1994.8763, 2011.9012, 2013.8700, 2020.9592, 2025.5920, 2042.0568, 2047.0238, 2047.9805, 2052.9905, 2053.9851, 2058.9988, 2060.0400, 2060.0405, 2065.0427, 2066.9895, 2067.9763, 2068.9128, 2070.8792, 2074.8860, 2082.0293, 2084.8977, 2086.8872, 2087.8835, 2090.8708, 2092.8674, 2093.8711, 2096.9836, 2097.9961, 2100.8787, 2101.8999, 2103.9092, 2107.8730, 2119.9924, 2122.9397, 2136.9133, 2211.1047, 2217.9368, 2225.1243, 2232.0229, 2233.0840, 2238.1050, 2250.0259, 2257.9934, 2262.9368, 2263.9700, 2273.0264, 2277.9500, 2283.1741, 2290.9570, 2307.9631, 2321.9558, 2323.9670, 2324.9824, 2335.9763, 2339.9456, 2343.0320, 2349.9844, 2352.0308, 2355.0315, 2358.0854, 2368.0815, 2369.0588, 2378.0020, 2379.0002, 2380.0007, 2381.9963, 2383.0444, 2383.9822, 2393.0020, 2394.0181, 2398.0447, 2400.0771, 2405.0879, 2414.1050, 2417.0933, 2421.0769, 2422.0593, 2426.0476, 2428.0725, 2430.0947, 2432.0962, 2435.0254, 2438.0706, 2444.0901, 2451.0278, 2459.9802, 2461.0254, 2501.2520, 2528.1902, 2543.2092, 2564.1965, 2705.2144, 2717.1655, 2834.2925, 2888.2483, 2904.4053, 3130.4312, 3291.4875, 3312.3765, 3451.6833, 3454.6270

15. G1K1U4 **Mass:** 51632 **Score:** 19 **Expect:** 3.2e+002 **Matches:** 19

DNA polymerase delta subunit 3 OS=Bos taurus GN=POLD3 PE=4 SV=2

Observed	Mr(expt)	Mr(calc)	ppm	Start - End	Miss	Ions	Peptide

806.4342	805.4269	805.4181	10.9	209	215	1	---	K.ETKTEAK.E
807.3921	806.3848	806.3956	-13.39	292	297	1	---	R.MQQKEK.K + Oxidation (M)
957.4673	956.4600	956.4460	14.7	190	198	0	---	K.GIMGMFASK.A + Oxidation (M)
993.5267	992.5195	992.5179	1.61	363	371	0	---	K.TEPEPPPVK.G
1001.5621	1000.5548	1000.5665	-11.68	283	291	2	---	K.SSKKAEPVR.M
1003.5043	1002.4970	1002.5094	-12.33	199	208	1	---	K.AASKAQDANK.E
1045.5603	1044.5530	1044.5750	-21.02	440	450	2	---	K.GPKKGTAAMGK.A
1118.5659	1117.5586	1117.5437	13.3	216	226	0	---	K.EVMNASSVGPK.A
1323.6320	1322.6247	1322.6024	16.9	301	311	1	---	R.KQMELSDDETK.E
1359.7053	1358.6980	1358.6976	0.31	287	297	2	---	K.AEPVRMQQKEK.K + Oxidation (M)
1479.7468	1478.7395	1478.7035	24.4	300	311	2	---	R.RKQMELSDDETK.E
1615.8425	1614.8352	1614.8518	-10.28	25	38	0	---	K.WLSYTLGVHVNQAK.Q
1680.8076	1679.8003	1679.8002	0.05	363	378	1	---	K.TEPEPPPVKGSDGENK.R
1756.8782	1755.8709	1755.8284	24.2	183	198	1	---	K.QSSQQPKGIMGMFASK.A + 2 Oxidation (M)
1916.9882	1915.9809	1915.9673	7.11	212	230	2	---	K.TEAKEVMNASSVGPKAPGK.G + Oxidation (M)
1932.9813	1931.9740	1931.9986	-12.71	241	257	2	---	K.AAMNKLKVNLDSEQEVK.E + Oxidation (M)
2082.0293	2081.0220	2081.0397	-8.52	183	202	2	---	K.QSSQQPKGIMGMFASKAASK.A
2087.8835	2086.8762	2086.9034	-13.04	302	318	2	---	K.QMELSDDETKETENMKK.K + 2 Oxidation (M)
2097.9961	2096.9888	2097.0347	-21.86	183	202	2	---	K.QSSQQPKGIMGMFASKAASK.A + Oxidation (M)

No match to: 713.4400, 713.4415, 724.3446, 728.2467, 730.2754, 732.2383, 734.2729, 735.3820, 736.3320, 740.2330, 743.2514, 748.2947, 750.2650, 756.4777, 764.3574, 765.3959, 768.3784, 772.4155, 774.3952, 778.4551, 780.3343, 782.3223, 783.3115, 787.2390, 791.2662, 802.4609, 805.3347, 808.4378, 812.4002, 814.4128, 820.4252, 828.3922, 830.3668, 834.3623, 837.3794, 840.3857, 842.5100, 845.4155, 851.3846, 856.5103, 865.3634, 869.4703, 870.5254, 871.4965, 872.6990, 888.3988, 891.4706, 893.9500, 894.4534, 897.0277, 905.4637, 911.4314, 912.4475, 913.5080, 913.5100, 918.5267, 925.4804, 927.4970, 930.5015, 933.4662, 935.4675, 943.4757, 951.4646, 952.4799, 961.4901, 962.4866, 967.4440, 969.4496, 972.4781, 974.5051, 975.4521, 977.4406, 978.4772, 979.4924, 983.4706, 985.6071, 991.4533, 995.5177, 999.4602, 1001.4565, 1007.5638, 1012.5004, 1014.5331, 1018.5130, 1022.5398, 1023.5525, 1028.5991, 1030.5259, 1035.5314, 1036.5333, 1037.5145, 1040.5127, 1042.5243, 1060.5398, 1069.4846, 1075.5515, 1092.6041, 1106.5313, 1109.5087, 1111.5457, 1113.5419, 1115.5394, 1121.5419, 1122.5701, 1131.5602, 1138.5428, 1149.5912, 1155.5768, 1157.5907, 1158.6328, 1163.6145, 1173.6122, 1177.6130, 1178.6060, 1179.5918, 1180.6001, 1181.6368, 1183.6030, 1187.6376, 1192.6014, 1193.5928, 1195.5924, 1199.6785, 1206.6066, 1215.6306, 1216.6482, 1226.5839, 1227.6051, 1231.6115, 1232.6251, 1233.6230, 1234.6500, 1235.6155, 1248.6078, 1250.6483, 1254.6526, 1255.6036, 1256.6123, 1268.6208, 1271.6423, 1276.6179, 1277.6350, 1289.6449, 1292.6322, 1294.6362, 1295.6366, 1300.5895, 1303.6643, 1305.6523, 1307.6600, 1308.6443, 1310.6051, 1315.6364, 1320.6128, 1321.6134, 1325.6456, 1331.6674, 1332.6602, 1333.2076, 1334.6744, 1336.6710, 1337.6279, 1338.6300, 1338.6300, 1345.6146, 1347.6653, 1349.6800, 1349.6827, 1354.6820, 1355.6964, 1360.6173, 1361.6373, 1365.6752, 1371.6718, 1375.6670, 1377.6776, 1382.6367, 1383.6619, 1387.6700, 1393.6727, 1400.6235, 1409.7107, 1411.6742, 1427.7725, 1431.7605, 1432.6987, 1434.7190, 1449.7479, 1466.6953, 1473.7905, 1475.7498, 1478.7303, 1485.7531, 1486.7472, 1493.7345, 1495.7502, 1500.7242, 1503.7690, 1505.8511, 1509.6786, 1520.7347, 1522.7239, 1523.7567, 1525.7864, 1527.8068, 1532.7472, 1537.7886, 1539.7711, 1549.7833, 1555.8657, 1558.8440, 1559.8573, 1568.7592, 1570.7814, 1578.7899, 1606.8213, 1619.7972, 1621.7831, 1628.8156, 1630.8202, 1631.8217, 1639.9263, 1644.8083, 1647.8730, 1649.8650, 1650.8523, 1658.8206, 1659.8125, 1661.8284, 1663.7981, 1665.8070, 1669.8174, 1677.8353, 1679.9403, 1682.7780, 1696.8055, 1697.7845, 1698.7950, 1707.7931, 1709.8029, 1710.8878, 1712.9166, 1716.8563, 1725.8188, 1727.8683, 1730.8236, 1731.8221, 1742.8798, 1746.1482, 1749.8406, 1751.8657, 1757.8225, 1763.8152, 1765.7922, 1766.8105, 1768.8596, 1769.4275, 1770.4087, 1773.8615, 1774.8795, 1775.0302, 1783.8599, 1785.8483, 1786.8871, 1790.8822, 1792.7894, 1801.9315, 1804.9026, 1808.8663, 1824.8685, 1830.8608, 1849.8231, 1851.8430, 1867.9100, 1868.9441, 1870.9304, 1880.9020, 1884.9191, 1908.8989, 1914.9821, 1920.9862, 1930.9827, 1934.9822, 1947.0288, 1958.9543, 1994.8763, 2011.9012, 2013.8700, 2020.9592, 2025.5920, 2042.0568, 2047.0238, 2047.9805, 2049.9497, 2052.9905, 2053.9851, 2058.9988, 2060.0400, 2060.0405, 2065.0427, 2066.0137, 2066.9895, 2067.9763, 2068.9128, 2070.8792, 2074.8860, 2084.8977, 2086.8872, 2090.8708, 2092.8674, 2093.8711, 2096.9836, 2100.8787, 2101.8999, 2103.9092, 2107.8730, 2119.9924, 2122.9397, 2136.9133, 2211.1047, 2217.9368, 2225.1243, 2232.0229, 2233.0840, 2238.1050, 2250.0259, 2257.9934, 2262.9368, 2263.9700, 2273.0264, 2277.9500, 2283.1741, 2290.9570, 2307.9631, 2321.9558, 2323.9670, 2324.9824, 2335.9763, 2339.9456, 2343.0320, 2349.9844, 2352.0308, 2355.0315, 2358.0854, 2368.0815, 2369.0588, 2378.0020, 2379.0002, 2380.0007, 2381.9963, 2383.0444, 2383.9822, 2393.0020, 2394.0181, 2398.0447, 2400.0771, 2405.0879, 2414.1050, 2417.0933, 2421.0769, 2422.0593, 2426.0476, 2428.0725, 2430.0947, 2432.0962, 2435.0254, 2438.0706, 2444.0901, 2451.0278, 2459.9802, 2461.0254, 2501.2520, 2528.1902, 2543.2092, 2564.1965, 2705.2144, 2717.1655, 2834.2925, 2888.2483, 2904.4053, 3130.4312, 3291.4875, 3312.3765, 3451.6833, 3454.6270

16. Q5KR47 **Mass:** 32856 **Score:** 19 **Expect:** 3.2e+002 **Matches:** 14

Tropomyosin alpha-3 chain OS=Bos taurus GN=TPM3 PE=2 SV=1

Observed	Mr(expt)	Mr(calc)	ppm	Start	End	Miss	Ions	Peptide
802.4609	801.4537	801.4344	24.0	199	205	0	---	K.NVTNNLK.S
894.4534	893.4461	893.4606	-16.29	162	168	1	---	K.YEEVARK.L
962.4866	961.4793	961.5015	-23.07	126	133	1	---	R.GMKVIENR.A + Oxidation (M)
1022.5398	1021.5325	1021.5300	2.47	8	15	1	---	K.MQMLKLDK.E + Oxidation (M)
1109.5087	1108.5014	1108.4819	17.7	119	128	1	---	K.AADESERGMK.V + Oxidation (M)
1268.6208	1267.6135	1267.6156	-1.66	150	160	1	---	K.EAKHIAEEADR.K
1308.6443	1307.6370	1307.6106	20.2	234	244	1	---	K.EAETRAEFAER.S
1475.7498	1474.7425	1474.7701	-18.69	141	152	1	---	K.MELQEIQLKEAK.H + Oxidation (M)
1549.7833	1548.7760	1548.7896	-8.74	232	244	2	---	K.LKEAETRAEFAER.S
1727.8683	1726.8610	1726.8849	-13.85	92	105	1	---	R.IQLVEEELDRAQER.L
1808.8663	1807.8590	1807.8257	18.4	113	128	2	---	K.LEEAEKAADESERGMK.V + Oxidation (M)
2049.9497	2048.9424	2048.9724	-14.64	267	284	1	---	K.YKAISEELDHALNDMTSI.-
2066.0137	2065.0064	2064.9673	18.9	267	284	1	---	K.YKAISEELDHALNDMTSI.- + Oxidation (M)
2119.9924	2118.9851	2119.0215	-17.16	31	48	2	---	K.QAEERSKQLEDELAAMQK.K + Oxidation (M)

No match to: 713.4400, 713.4415, 724.3446, 728.2467, 730.2754, 732.2383, 734.2729, 735.3820, 736.3320, 740.2330, 743.2514, 748.2947, 750.2650, 756.4777, 764.3574, 765.3959, 768.3784, 772.4155, 774.3952, 778.4551, 780.3343, 782.3223, 783.3115, 787.2390, 791.2662, 805.3347, 806.4342, 807.3921, 808.4378, 812.4002, 814.4128, 820.4252, 828.3922, 830.3668, 834.3623, 837.3794, 840.3857, 842.5100, 845.4155, 851.3846, 856.5103, 865.3634, 869.4703, 870.5254, 871.4965, 872.6990, 888.3988, 891.4706, 893.9500, 897.0277, 905.4637, 911.4314, 912.4475, 913.5080, 913.5100, 918.5267, 925.4804, 927.4970, 930.5015, 933.4662, 935.4675, 943.4757, 951.4646, 952.4799, 957.4673, 961.4901, 967.4440, 969.4496, 972.4781, 974.5051, 975.4521, 977.4406, 978.4772, 979.4924, 983.4706, 985.6071, 991.4533, 993.5267, 995.5177, 999.4602, 1001.4565, 1001.5621, 1003.5043, 1007.5638, 1012.5004, 1014.5331, 1018.5130, 1023.5525, 1028.5991, 1030.5259, 1035.5314, 1036.5333, 1037.5145, 1040.5127, 1042.5243, 1045.5603, 1060.5398, 1069.4846, 1075.5515, 1092.6041, 1106.5313, 1111.5457, 1113.5419, 1115.5394, 1118.5659, 1121.5419, 1122.5701, 1131.5602, 1138.5428, 1149.5912, 1155.5768, 1157.5907, 1158.6328, 1163.6145, 1173.6122, 1177.6130, 1178.6060, 1179.5918, 1180.6001, 1181.6368, 1183.6030, 1187.6376, 1192.6014, 1193.5928, 1195.5924, 1199.6785, 1206.6066, 1215.6306, 1216.6482, 1226.5839, 1227.6051, 1231.6115, 1232.6251, 1233.6230, 1234.6500, 1235.6155, 1248.6078, 1250.6483, 1254.6526, 1255.6036, 1256.6123, 1271.6423, 1276.6179, 1277.6350, 1289.6449, 1292.6322, 1294.6362, 1295.6366, 1300.5895, 1303.6643, 1305.6523, 1307.6600, 1310.6051, 1315.6364, 1320.6128, 1321.6134, 1323.6320, 1325.6456, 1331.6674, 1332.6602, 1333.2076, 1334.6744, 1336.6710, 1337.6279, 1338.6300, 1338.6300, 1345.6146, 1347.6653, 1349.6800, 1349.6827, 1354.6820, 1355.6964, 1359.7053, 1360.6173, 1361.6373, 1365.6752, 1371.6718, 1375.6670, 1377.6776, 1382.6367, 1383.6619, 1387.6700, 1393.6727, 1400.6235, 1409.7107, 1411.6742, 1427.7725, 1431.7605, 1432.6987, 1434.7190, 1449.7479, 1466.6953, 1473.7905, 1478.7303, 1479.7468, 1485.7531, 1486.7472, 1493.7345, 1495.7502, 1500.7242, 1503.7690, 1505.8511, 1509.6786, 1520.7347, 1522.7239, 1523.7567, 1525.7864, 1527.8068, 1532.7472, 1537.7886, 1539.7711, 1555.8657, 1558.8440, 1559.8573, 1568.7592, 1570.7814, 1578.7899, 1606.8213, 1615.8425, 1619.7972, 1621.7831, 1628.8156, 1630.8202, 1631.8217, 1639.9263, 1644.8083, 1647.8730, 1649.8650, 1650.8523, 1658.8206, 1659.8125, 1661.8284, 1663.7981, 1665.8070, 1669.8174, 1677.8353, 1679.9403, 1680.8076, 1682.7780, 1696.8055, 1697.7845, 1698.7950, 1707.7931, 1709.8029, 1710.8878, 1712.9166, 1716.8563, 1725.8188, 1730.8236, 1731.8221, 1742.8798, 1746.1482, 1749.8406, 1751.8657, 1756.8782, 1757.8225, 1763.8152, 1765.7922, 1766.8105, 1768.8596, 1769.4275, 1770.4087, 1773.8615, 1774.8795, 1775.0302, 1783.8599, 1785.8483, 1786.8871, 1790.8822, 1792.7894, 1801.9315, 1804.9026, 1824.8685, 1830.8608, 1849.8231, 1851.8430, 1867.9100, 1868.9441, 1870.9304, 1880.9020, 1884.9191, 1908.8989, 1914.9821, 1916.9882, 1920.9862, 1930.9827, 1932.9813, 1934.9822, 1947.0288, 1958.9543, 1994.8763, 2011.9012, 2013.8700, 2020.9592, 2025.5920, 2042.0568, 2047.0238, 2047.9805, 2052.9905, 2053.9851, 2058.9988, 2060.0400, 2060.0405, 2065.0427,

2066.9895, 2067.9763, 2068.9128, 2070.8792, 2074.8860, 2082.0293, 2084.8977, 2086.8872, 2087.8835, 2090.8708, 2092.8674, 2093.8711, 2096.9836, 2097.9961, 2100.8787, 2101.8999, 2103.9092, 2107.8730, 2122.9397, 2136.9133, 2211.1047, 2217.9368, 2225.1243, 2232.0229, 2233.0840, 2238.1050, 2250.0259, 2257.9934, 2262.9368, 2263.9700, 2273.0264, 2277.9500, 2283.1741, 2290.9570, 2307.9631, 2321.9558, 2323.9670, 2324.9824, 2335.9763, 2339.9456, 2343.0320, 2349.9844, 2352.0308, 2355.0315, 2358.0854, 2368.0815, 2369.0588, 2378.0020, 2379.0002, 2380.0007, 2381.9963, 2383.0444, 2383.9822, 2393.0020, 2394.0181, 2398.0447, 2400.0771, 2405.0879, 2414.1050, 2417.0933, 2421.0769, 2422.0593, 2426.0476, 2428.0725, 2430.0947, 2432.0962, 2435.0254, 2438.0706, 2444.0901, 2451.0278, 2459.9802, 2461.0254, 2501.2520, 2528.1902, 2543.2092, 2564.1965, 2705.2144, 2717.1655, 2834.2925, 2888.2483, 2904.4053, 3130.4312, 3291.4875, 3312.3765, 3451.6833, 3454.6270

17. Q2T9X5 **Mass:** 23537 **Score:** 19 **Expect:** 3.3e+002 **Matches:** 12

Uncharacterized protein C7orf61 homolog OS=Bos taurus PE=2 SV=1

Observed	Mr(expt)	Mr(calc)	ppm	Start	End	Miss	Ions	Peptide
870.5254	869.5182	869.5195	-1.54	166 -	172	1	---	K.QLRQVAR.R
1177.6130	1176.6057	1176.6233	-14.92	18 -	25	1	---	R.WVFFWKHK.A
1276.6179	1275.6106	1275.6129	-1.78	28 -	38	1	---	K.SVIMDHTDSKK.N + Oxidation (M)
1331.6674	1330.6601	1330.6551	3.78	26 -	37	1	---	K.AKSVIMDHTDSK.K
1334.6744	1333.6671	1333.6911	-18.00	61 -	72	2	---	K.EAKVSKMDVSPK.V + Oxidation (M)
1347.6653	1346.6580	1346.6500	5.95	26 -	37	1	---	K.AKSVIMDHTDSK.K + Oxidation (M)
1359.7053	1358.6980	1358.6976	0.32	143 -	154	1	---	R.SPLESREAAMLR.E
1375.6670	1374.6597	1374.6925	-23.85	143 -	154	1	---	R.SPLESREAAMLR.E + Oxidation (M)
1475.7498	1474.7425	1474.7450	-1.66	26 -	38	2	---	K.AKSVIMDHTDSKK.N + Oxidation (M)
1932.9813	1931.9740	1931.9272	24.2	184 -	201	1	---	R.AHMGPQPDPAQGLKQDAR.S + Oxidation (M)
2225.1243	2224.1170	2224.1117	2.38	128 -	148	2	---	K.SSVSSTSVASQMKTRSPLESR.E
2335.9763	2334.9690	2335.0105	-17.78	108 -	127	0	---	K.SVSMLMASALQSGWQMCSWK.S + 3 Oxidation (M)

No match to: 713.4400, 713.4415, 724.3446, 728.2467, 730.2754, 732.2383, 734.2729, 735.3820, 736.3320, 740.2330, 743.2514, 748.2947, 750.2650, 756.4777, 764.3574, 765.3959, 768.3784, 772.4155, 774.3952, 778.4551, 780.3343, 782.3223, 783.3115, 787.2390, 791.2662, 802.4609, 805.3347, 806.4342, 807.3921, 808.4378, 812.4002, 814.4128, 820.4252, 828.3922, 830.3668, 834.3623, 837.3794, 840.3857, 842.5100, 845.4155, 851.3846, 856.5103, 865.3634, 869.4703, 871.4965, 872.6990, 888.3988, 891.4706, 893.9500, 894.4534, 897.0277, 905.4637, 911.4314, 912.4475, 913.5080, 913.5100, 918.5267, 925.4804, 927.4970, 930.5015, 933.4662, 935.4675, 943.4757, 951.4646, 952.4799, 957.4673, 961.4901, 962.4866, 967.4440, 969.4496, 972.4781, 974.5051, 975.4521, 977.4406, 978.4772, 979.4924, 983.4706, 985.6071, 991.4533, 993.5267, 995.5177, 999.4602, 1001.4565, 1001.5621, 1003.5043, 1007.5638, 1012.5004, 1014.5331, 1018.5130, 1022.5398, 1023.5525, 1028.5991, 1030.5259, 1035.5314, 1036.5333, 1037.5145, 1040.5127, 1042.5243, 1045.5603, 1060.5398, 1069.4846, 1075.5515, 1092.6041, 1106.5313, 1109.5087, 1111.5457, 1113.5419, 1115.5394, 1118.5659, 1121.5419, 1122.5701, 1131.5602, 1138.5428, 1149.5912, 1155.5768, 1157.5907, 1158.6328, 1163.6145, 1173.6122, 1178.6060, 1179.5918, 1180.6001, 1181.6368, 1183.6030, 1187.6376, 1192.6014, 1193.5928, 1195.5924, 1199.6785, 1206.6066, 1215.6306, 1216.6482, 1226.5839, 1227.6051, 1231.6115, 1232.6251, 1233.6230, 1234.6500, 1235.6155, 1248.6078, 1250.6483, 1254.6526, 1255.6036, 1256.6123, 1268.6208, 1271.6423, 1277.6350, 1289.6449, 1292.6322, 1294.6362, 1295.6366, 1300.5895, 1303.6643, 1305.6523, 1307.6600, 1308.6443, 1310.6051, 1315.6364, 1320.6128, 1321.6134, 1323.6320, 1325.6456, 1332.6602, 1333.2076, 1336.6710, 1337.6279, 1338.6300, 1338.6300, 1345.6146, 1349.6800, 1349.6827, 1354.6820, 1355.6964, 1360.6173, 1361.6373, 1365.6752, 1371.6718, 1377.6776, 1382.6367, 1383.6619, 1387.6700, 1393.6727, 1400.6235, 1409.7107, 1411.6742, 1427.7725, 1431.7605, 1432.6987, 1434.7190, 1449.7479, 1466.6953, 1473.7905, 1478.7303, 1479.7468, 1485.7531, 1486.7472, 1493.7345, 1495.7502, 1500.7242, 1503.7690, 1505.8511, 1509.6786, 1520.7347, 1522.7239, 1523.7567, 1525.7864, 1527.8068, 1532.7472, 1537.7886, 1539.7711, 1549.7833, 1555.8657, 1558.8440, 1559.8573, 1568.7592, 1570.7814, 1578.7899, 1606.8213, 1615.8425, 1619.7972, 1621.7831, 1628.8156, 1630.8202, 1631.8217, 1639.9263, 1644.8083, 1647.8730, 1649.8650, 1650.8523, 1658.8206, 1659.8125, 1661.8284, 1663.7981, 1665.8070, 1669.8174, 1677.8353, 1679.9403, 1680.8076, 1682.7780, 1696.8055, 1697.7845, 1698.7950, 1707.7931, 1709.8029, 1710.8878, 1712.9166, 1716.8563, 1725.8188, 1727.8683, 1730.8236, 1731.8221, 1742.8798, 1746.1482, 1749.8406, 1751.8657, 1756.8782, 1757.8225, 1763.8152, 1765.7922, 1766.8105, 1768.8596, 1769.4275, 1770.4087, 1773.8615, 1774.8795, 1775.0302, 1783.8599, 1785.8483, 1786.8871, 1790.8822, 1792.7894, 1801.9315, 1804.9026, 1808.8663, 1824.8685, 1830.8608, 1849.8231, 1851.8430, 1867.9100, 1868.9441, 1870.9304, 1880.9020, 1884.9191, 1908.8989, 1914.9821, 1916.9882, 1920.9862, 1930.9827, 1934.9822, 1947.0288, 1958.9543, 1994.8763, 2011.9012, 2013.8700, 2020.9592, 2025.5920, 2042.0568, 2047.0238, 2047.9805, 2049.9497, 2052.9905, 2053.9851, 2058.9988, 2060.0400, 2060.0405, 2065.0427, 2066.0137, 2066.9895, 2067.9763, 2068.9128, 2070.8792, 2074.8860, 2082.0293, 2084.8977, 2086.8872, 2087.8835, 2090.8708, 2092.8674, 2093.8711, 2096.9836, 2097.9961, 2100.8787, 2101.8999, 2103.9092, 2107.8730, 2119.9924, 2122.9397, 2136.9133, 2211.1047, 2217.9368, 2232.0229, 2233.0840, 2238.1050, 2250.0259, 2257.9934, 2262.9368, 2263.9700, 2273.0264, 2277.9500, 2283.1741, 2290.9570, 2307.9631, 2321.9558, 2323.9670, 2324.9824, 2339.9456, 2343.0320, 2349.9844, 2352.0308, 2355.0315, 2358.0854, 2368.0815, 2369.0588, 2378.0020, 2379.0002, 2380.0007, 2381.9963, 2383.0444, 2383.9822, 2393.0020, 2394.0181, 2398.0447, 2400.0771, 2405.0879, 2414.1050, 2417.0933, 2421.0769, 2422.0593, 2426.0476, 2428.0725, 2430.0947, 2432.0962, 2435.0254, 2438.0706, 2444.0901, 2451.0278, 2459.9802, 2461.0254, 2501.2520, 2528.1902, 2543.2092, 2564.1965, 2705.2144, 2717.1655, 2834.2925, 2888.2483, 2904.4053, 3130.4312, 3291.4875, 3312.3765, 3451.6833, 3454.6270

18. A6QPI2 **Mass:** 49923 **Score:** 19 **Expect:** 3.3e+002 **Matches:** 19

GAS7 protein OS=Bos taurus GN=GAS7 PE=2 SV=1

Observed	Mr(expt)	Mr(calc)	ppm	Start	End	Miss	Ions	Peptide
735.3820	734.3747	734.3745	0.34	185 -	190	1	---	K.GKQMQK.E + Oxidation (M)
764.3574	763.3501	763.3460	5.38	30 -	37	0	---	R.TDSAAGSR.R
911.4314	910.4241	910.4218	2.52	191 -	197	0	---	K.EMSEFIR.E
935.4675	934.4602	934.4542	6.42	302 -	308	1	---	R.QRDLEMK.T + Oxidation (M)
1001.5621	1000.5548	1000.5777	-22.91	296 -	303	2	---	R.KALTERQR.D
1028.5991	1027.5918	1027.5927	-0.83	60 -	68	1	---	R.LLSFHRAGK.A
1109.5087	1108.5014	1108.4819	17.6	331 -	340	0	---	K.STQAGDDLMR.C + Oxidation (M)
1307.6600	1306.6527	1306.6768	-18.44	202 -	212	1	---	K.IEEEYAKNLAK.L
1325.6456	1324.6383	1324.6081	22.8	341 -	351	0	---	R.CVDLYNQAQSK.W
1377.6776	1376.6703	1376.6830	-9.23	329 -	340	2	---	R.RKSTQAGDDLMR.C
1393.6727	1392.6654	1392.6779	-8.99	329 -	340	2	---	R.RKSTQAGDDLMR.C + Oxidation (M)
1475.7498	1474.7425	1474.7701	-18.70	304 -	315	1	---	R.DLEMKTQQLEIK.L
1493.7345	1492.7272	1492.6987	19.1	1 -	14	2	---	-.MARGCASPRASSSR.C
1509.6786	1508.6713	1508.6936	-14.77	1 -	14	2	---	-.MARGCASPRASSSR.C + Oxidation (M)
1615.8425	1614.8352	1614.8188	10.2	251 -	263	0	---	K.LHSEVEKPLMNFR.E + Oxidation (M)
1783.8599	1782.8526	1782.8498	1.57	352 -	365	0	---	K.WFEEMVTTTLELER.L
1908.8989	1907.8916	1907.8771	7.63	39 -	53	1	---	R.ATCSCWRSLEWSPLR.R
2400.0771	2399.0698	2399.0845	-6.14	331 -	351	1	---	K.STQAGDDLMRCVDLYNQAQSK.W
2528.1902	2527.1829	2527.1795	1.35	330 -	351	2	---	R.KSTQAGDDLMRCVDLYNQAQSK.W

No match to: 713.4400, 713.4415, 724.3446, 728.2467, 730.2754, 732.2383, 734.2729, 736.3320, 740.2330, 743.2514, 748.2947, 750.2650, 756.4777, 765.3959, 768.3784, 772.4155, 774.3952, 778.4551, 780.3343, 782.3223, 783.3115, 787.2390, 791.2662, 802.4609, 805.3347, 806.4342, 807.3921, 808.4378, 812.4002, 814.4128, 820.4252, 828.3922, 830.3668, 834.3623, 837.3794, 840.3857, 842.5100, 845.4155, 851.3846, 856.5103, 865.3634, 869.4703, 870.5254, 871.4965, 872.6990, 888.3988, 891.4706, 893.9500, 894.4534, 897.0277, 905.4637, 912.4475, 913.5080, 913.5100, 918.5267, 925.4804, 927.4970, 930.5015, 933.4662, 943.4757, 951.4646, 952.4799, 957.4673, 961.4901, 962.4866, 967.4440, 969.4496, 972.4781, 974.5051, 975.4521, 977.4406, 978.4772, 979.4924, 983.4706, 985.6071, 991.4533, 993.5267, 995.5177, 999.4602, 1001.4565, 1003.5043, 1007.5638, 1012.5004, 1014.5331, 1018.5130, 1022.5398, 1023.5525, 1030.5259, 1035.5314, 1036.5333, 1037.5145, 1040.5127, 1042.5243, 1045.5603, 1060.5398, 1069.4846, 1075.5515, 1092.6041, 1106.5313, 1111.5457, 1113.5419, 1115.5394, 1118.5659, 1121.5419, 1122.5701, 1131.5602, 1138.5428, 1149.5912, 1155.5768, 1157.5907, 1158.6328, 1163.6145, 1173.6122, 1177.6130, 1178.6060, 1179.5918, 1180.6001, 1181.6368, 1183.6030, 1187.6376, 1192.6014, 1193.5928, 1195.5924, 1199.6785, 1206.6066, 1215.6306, 1216.6482, 1226.5839, 1227.6051, 1231.6115, 1232.6251, 1233.6230, 1234.6500, 1235.6155, 1248.6078, 1250.6483, 1254.6526, 1255.6036, 1256.6123, 1268.6208, 1271.6423, 1276.6179, 1277.6350, 1289.6449, 1292.6322, 1294.6362, 1295.6366, 1300.5895, 1303.6643, 1305.6523, 1308.6443, 1310.6051, 1315.6364, 1320.6128, 1321.6134, 1323.6320, 1331.6674, 1332.6602, 1333.2076, 1334.6744,

1336.6710, 1337.6279, 1338.6300, 1338.6300, 1345.6146, 1347.6653, 1349.6800, 1349.6827, 1354.6820, 1355.6964, 1359.7053, 1360.6173, 1361.6373, 1365.6752, 1371.6718, 1375.6670, 1382.6367, 1383.6619, 1387.6700, 1400.6235, 1409.7107, 1411.6742, 1427.7725, 1431.7605, 1432.6987, 1434.7190, 1449.7479, 1466.6953, 1473.7905, 1478.7303, 1479.7468, 1485.7531, 1486.7472, 1495.7502, 1500.7242, 1503.7690, 1505.8511, 1520.7347, 1522.7239, 1523.7567, 1525.7864, 1527.8068, 1532.7472, 1537.7886, 1539.7711, 1549.7833, 1555.8657, 1558.8440, 1559.8573, 1568.7592, 1570.7814, 1578.7899, 1606.8213, 1619.7972, 1621.7831, 1628.8156, 1630.8202, 1631.8217, 1639.9263, 1644.8083, 1647.8730, 1649.8650, 1650.8523, 1658.8206, 1659.8125, 1661.8284, 1663.7981, 1665.8070, 1669.8174, 1677.8353, 1679.9403, 1680.8076, 1682.7780, 1696.8055, 1697.7845, 1698.7950, 1707.7931, 1709.8029, 1710.8878, 1712.9166, 1716.8563, 1725.8188, 1727.8683, 1730.8236, 1731.8221, 1742.8798, 1746.1482, 1749.8406, 1751.8657, 1756.8782, 1757.8225, 1763.8152, 1765.7922, 1766.8105, 1768.8596, 1769.4275, 1770.4087, 1773.8615, 1774.8795, 1775.0302, 1785.8483, 1786.8871, 1790.8822, 1792.7894, 1801.9315, 1804.9026, 1808.8663, 1824.8685, 1830.8608, 1849.8231, 1851.8430, 1867.9100, 1868.9441, 1870.9304, 1880.9020, 1884.9191, 1914.9821, 1916.9882, 1920.9862, 1930.9827, 1932.9813, 1934.9822, 1947.0288, 1958.9543, 1994.8763, 2011.9012, 2013.8700, 2020.9592, 2025.5920, 2042.0568, 2047.0238, 2047.9805, 2049.9497, 2052.9905, 2053.9851, 2058.9988, 2060.0400, 2060.0405, 2065.0427, 2066.0137, 2066.9895, 2067.9763, 2068.9128, 2070.8792, 2074.8860, 2082.0293, 2084.8977, 2086.8872, 2087.8835, 2090.8708, 2092.8674, 2093.8711, 2096.9836, 2097.9961, 2100.8787, 2101.8999, 2103.9092, 2107.8730, 2119.9924, 2122.9397, 2136.9133, 2211.1047, 2217.9368, 2225.1243, 2232.0229, 2233.0840, 2238.1050, 2250.0259, 2257.9934, 2262.9368, 2263.9700, 2273.0264, 2277.9500, 2283.1741, 2290.9570, 2307.9631, 2321.9558, 2323.9670, 2324.9824, 2335.9763, 2339.9456, 2343.0320, 2349.9844, 2352.0308, 2355.0315, 2358.0854, 2368.0815, 2369.0588, 2378.0020, 2379.0002, 2380.0007, 2381.9963, 2383.0444, 2383.9822, 2393.0020, 2394.0181, 2398.0447, 2405.0879, 2414.1050, 2417.0933, 2421.0769, 2422.0593, 2426.0476, 2428.0725, 2430.0947, 2432.0962, 2435.0254, 2438.0706, 2444.0901, 2451.0278, 2459.9802, 2461.0254, 2501.2520, 2543.2092, 2564.1965, 2705.2144, 2717.1655, 2834.2925, 2888.2483, 2904.4053, 3130.4312, 3291.4875, 3312.3765, 3451.6833, 3454.6270

19. Q2TBU3 **Mass:** 28638 **Score:** 19 **Expect:** 3.3e+002 **Matches:** 13

Golgi SNAP receptor complex member 1 OS=Bos taurus GN=GOSR1 PE=2 SV=1

Observed	Mr(expt)	Mr(calc)	ppm	Start	End	Miss	Ions	Peptide
774.3952	773.3879	773.3892	-1.70	44 -	49	2	---	R.DGRRDR.Y
865.3634	864.3561	864.3759	-22.93	189 -	195	0	---	K.ENMTSQR.G
905.4637	904.4564	904.4436	14.1	136 -	143	0	---	R.ENLMGSVR.K
1007.5638	1006.5565	1006.5481	8.37	196 -	204	1	---	R.GMLKSIQSK.M + Oxidation (M)
1206.6066	1205.5993	1205.5822	14.2	134 -	143	1	---	R.ERENLMGSVR.K + Oxidation (M)
1294.6362	1293.6289	1293.6169	9.28	189 -	199	1	---	K.ENMTSQRGMLK.S
1310.6051	1309.5978	1309.6118	-10.70	189 -	199	1	---	K.ENMTSQRGMLK.S + Oxidation (M)
1334.6744	1333.6671	1333.6772	-7.56	134 -	144	2	---	R.ERENLMGSVRK.D + Oxidation (M)
1382.6367	1381.6294	1381.6262	2.32	2 -	13	0	---	M.AAGTSNYWEDLR.K
1537.7886	1536.7813	1536.8049	-15.31	160 -	171	1	---	R.TELFLKEHDHLR.N
1768.8596	1767.8523	1767.8825	-17.06	136 -	150	2	---	R.ENLMGSVRKDIESYK.S
1774.8795	1773.8722	1773.8686	2.05	113 -	126	1	---	R.DILQDYTHEFHKTK.A
2011.9012	2010.8939	2010.9243	-15.09	48 -	65	1	---	R.DRYSSDTTPLLNGSSQDR.M

No match to: 713.4400, 713.4415, 724.3446, 728.2467, 730.2754, 732.2383, 734.2729, 735.3820, 736.3320, 740.2330, 743.2514, 748.2947, 750.2650, 756.4777, 764.3574, 765.3959, 768.3784, 772.4155, 778.4551, 780.3343, 782.3223, 783.3115, 787.2390, 791.2662, 802.4609, 805.3347, 806.4342, 807.3921, 808.4378, 812.4002, 814.4128, 820.4252, 828.3922, 830.3668, 834.3623, 837.3794, 840.3857, 842.5100, 845.4155, 851.3846, 856.5103, 869.4703, 870.5254, 871.4965, 872.6990, 888.3988, 891.4706, 893.9500, 894.4534, 897.0277, 911.4314, 912.4475, 913.5080, 913.5100, 918.5267, 925.4804, 927.4970, 930.5015, 933.4662, 935.4675, 943.4757, 951.4646, 952.4799, 957.4673, 961.4901, 962.4866, 967.4440, 969.4496, 972.4781, 974.5051, 975.4521, 977.4406, 978.4772, 979.4924, 983.4706, 985.6071, 991.4533, 993.5267, 995.5177, 999.4602, 1001.4565, 1001.5621, 1003.5043, 1012.5004, 1014.5331, 1018.5130, 1022.5398, 1023.5525, 1028.5991, 1030.5259, 1035.5314, 1036.5333, 1037.5145, 1040.5127, 1042.5243, 1045.5603, 1060.5398, 1069.4846, 1075.5515, 1092.6041, 1106.5313, 1109.5087, 1111.5457, 1113.5419, 1115.5394, 1118.5659, 1121.5419, 1122.5701, 1131.5602, 1138.5428, 1149.5912, 1155.5768, 1157.5907, 1158.6328, 1163.6145, 1173.6122, 1177.6130, 1178.6060, 1179.5918, 1180.6001, 1181.6368, 1183.6030, 1187.6376, 1192.6014, 1193.5928, 1195.5924, 1199.6785, 1215.6306, 1216.6482, 1226.5839, 1227.6051, 1231.6115, 1232.6251, 1233.6230, 1234.6500, 1235.6155, 1248.6078, 1250.6483, 1254.6526, 1255.6036, 1256.6123, 1268.6208, 1271.6423, 1276.6179, 1277.6350, 1289.6449, 1292.6322, 1295.6366, 1300.5895, 1303.6643, 1305.6523, 1307.6600, 1308.6443, 1315.6364, 1320.6128, 1321.6134, 1323.6320, 1325.6456, 1331.6674, 1332.6602, 1333.2076, 1336.6710, 1337.6279, 1338.6300, 1338.6300, 1345.6146, 1347.6653, 1349.6800, 1349.6827, 1354.6820, 1355.6964, 1359.7053, 1360.6173, 1361.6373, 1365.6752, 1371.6718, 1375.6670, 1377.6776, 1383.6619, 1387.6700, 1393.6727, 1400.6235, 1409.7107, 1411.6742, 1427.7725, 1431.7605, 1432.6987, 1434.7190, 1449.7479, 1466.6953, 1473.7905, 1475.7498, 1478.7303, 1479.7468, 1485.7531, 1486.7472, 1493.7345, 1495.7502, 1500.7242, 1503.7690, 1505.8511, 1509.6786, 1520.7347, 1522.7239, 1523.7567, 1525.7864, 1527.8068, 1532.7472, 1539.7711, 1549.7833, 1555.8657, 1558.8440, 1559.8573, 1568.7592, 1570.7814, 1578.7899, 1606.8213, 1615.8425, 1619.7972, 1621.7831, 1628.8156, 1630.8202, 1631.8217, 1639.9263, 1644.8083, 1647.8730, 1649.8650, 1650.8523, 1658.8206, 1659.8125, 1661.8284, 1663.7981, 1665.8070, 1669.8174, 1677.8353, 1679.9403, 1680.8076, 1682.7780, 1696.8055, 1697.7845, 1698.7950, 1707.7931, 1709.8029, 1710.8878, 1712.9166, 1716.8563, 1725.8188, 1727.8683, 1730.8236, 1731.8221, 1742.8798, 1746.1482, 1749.8406, 1751.8657, 1756.8782, 1757.8225, 1763.8152, 1765.7922, 1766.8105, 1769.4275, 1770.4087, 1773.8615, 1775.0302, 1783.8599, 1785.8483, 1786.8871, 1790.8822, 1792.7894, 1801.9315, 1804.9026, 1808.8663, 1824.8685, 1830.8608, 1849.8231, 1851.8430, 1867.9100, 1868.9441, 1870.9304, 1880.9020, 1884.9191, 1908.8989, 1914.9821, 1916.9882, 1920.9862, 1930.9827, 1932.9813, 1934.9822, 1947.0288, 1958.9543, 1994.8763, 2013.8700, 2020.9592, 2025.5920, 2042.0568, 2047.0238, 2047.9805, 2049.9497, 2052.9905, 2053.9851, 2058.9988, 2060.0400, 2060.0405, 2065.0427, 2066.0137, 2066.9895, 2067.9763, 2068.9128, 2070.8792, 2074.8860, 2082.0293, 2084.8977, 2086.8872, 2087.8835, 2090.8708, 2092.8674, 2093.8711, 2096.9836, 2097.9961, 2100.8787, 2101.8999, 2103.9092, 2107.8730, 2119.9924, 2122.9397, 2136.9133, 2211.1047, 2217.9368, 2225.1243, 2232.0229, 2233.0840, 2238.1050, 2250.0259, 2257.9934, 2262.9368, 2263.9700, 2273.0264, 2277.9500, 2283.1741, 2290.9570, 2307.9631, 2321.9558, 2323.9670, 2324.9824, 2335.9763, 2339.9456, 2343.0320, 2349.9844, 2352.0308, 2355.0315, 2358.0854, 2368.0815, 2369.0588, 2378.0020, 2379.0002, 2380.0007, 2381.9963, 2383.0444, 2383.9822, 2393.0020, 2394.0181, 2398.0447, 2400.0771, 2405.0879, 2414.1050, 2417.0933, 2421.0769, 2422.0593, 2426.0476, 2428.0725, 2430.0947, 2432.0962, 2435.0254, 2438.0706, 2444.0901, 2451.0278, 2459.9802, 2461.0254, 2501.2520, 2528.1902, 2543.2092, 2564.1965, 2705.2144, 2717.1655, 2834.2925, 2888.2483, 2904.4053, 3130.4312, 3291.4875, 3312.3765, 3451.6833, 3454.6270

20. Q2TBN3 **Mass:** 19797 **Score:** 18 **Expect:** 3.4e+002 **Matches:** 12

Centrin-2 OS=Bos taurus GN=CETN2 PE=2 SV=1

Observed	Mr(expt)	Mr(calc)	ppm	Start	End	Miss	Ions	Peptide
979.4924	978.4852	978.5056	-20.85	71 -	78	1	---	K.KMISEIDK.E + Oxidation (M)
1234.6500	1233.6427	1233.6540	-9.11	55 -	65	1	---	K.VAMRALGFEPK.K + Oxidation (M)
1307.6600	1306.6527	1306.6438	6.80	72 -	83	1	---	K.MISEIDKEGTGK.M
1323.6320	1322.6247	1322.6388	-10.61	72 -	83	1	---	K.MISEIDKEGTGK.M + Oxidation (M)
1432.6987	1431.6914	1431.6915	-0.06	19 -	30	0	---	R.MSPKPELTEEQK.Q + Oxidation (M)
1716.8563	1715.8490	1715.8876	-22.47	17 -	30	2	---	R.KRMSPKPELTEEQK.Q + Oxidation (M)
1785.8483	1784.8410	1784.8661	-14.07	1 -	16	2	---	-.MASNFKKANMASTTQR.K
1958.9543	1957.9470	1957.9778	-15.74	19 -	34	1	---	R.MSPKPELTEEQKQEIR.E + Oxidation (M)
2049.9497	2048.9424	2048.9547	-6.00	79 -	96	1	---	K.EGTGKMNFSDFLTVMTQK.M + Oxidation (M)
2052.9905	2051.9832	2051.9366	22.7	84 -	100	1	---	K.MNFSDFLTVMTQKMSEK.D + Oxidation (M)
2068.9128	2067.9055	2067.9315	-12.58	84 -	100	1	---	K.MNFSDFLTVMTQKMSEK.D + 2 Oxidation (M)
2084.8977	2083.8904	2083.9265	-17.29	84 -	100	1	---	K.MNFSDFLTVMTQKMSEK.D + 3 Oxidation (M)

No match to: 713.4400, 713.4415, 724.3446, 728.2467, 730.2754, 732.2383, 734.2729, 735.3820, 736.3320, 740.2330, 743.2514, 748.2947, 750.2650, 756.4777, 764.3574, 765.3959, 768.3784, 772.4155, 774.3952, 778.4551, 780.3343, 782.3223, 783.3115, 787.2390, 791.2662, 802.4609, 805.3347, 806.4342, 807.3921, 808.4378, 812.4002, 814.4128, 820.4252, 828.3922, 830.3668, 834.3623, 837.3794, 840.3857, 842.5100, 845.4155, 851.3846, 856.5103, 865.3634, 869.4703, 870.5254, 871.4965, 872.6990, 888.3988, 891.4706, 893.9500, 894.4534, 897.0277, 905.4637, 911.4314, 912.4475, 913.5080, 913.5100, 918.5267, 925.4804, 927.4970, 930.5015, 933.4662, 935.4675, 943.4757, 951.4646, 952.4799, 957.4673, 961.4901, 962.4866, 967.4440, 969.4496, 972.4781, 974.5051, 975.4521, 977.4406, 978.4772, 983.4706, 985.6071, 991.4533, 993.5267, 995.5177, 999.4602, 1001.4565, 1001.5621, 1003.5043, 1007.5638, 1012.5004, 1014.5331, 1018.5130, 1022.5398, 1023.5525, 1028.5991, 1030.5259, 1035.5314, 1036.5333, 1037.5145, 1040.5127, 1042.5243, 1045.5603, 1060.5398, 1069.4846, 1075.5515, 1092.6041, 1106.5313, 1109.5087, 1111.5457, 1113.5419, 1115.5394, 1118.5659, 1121.5419, 1122.5701, 1131.5602,

1138.5428, 1149.5912, 1155.5768, 1157.5907, 1158.6328, 1163.6145, 1173.6122, 1177.6130, 1178.6060, 1179.5918, 1180.6001, 1181.6368, 1183.6030, 1187.6376, 1192.6014, 1193.5928, 1195.5924, 1199.6785, 1206.6066, 1215.6306, 1216.6482, 1226.5839, 1227.6051, 1231.6115, 1232.6251, 1233.6230, 1235.6155, 1248.6078, 1250.6483, 1254.6526, 1255.6036, 1256.6123, 1268.6208, 1271.6423, 1276.6179, 1277.6350, 1289.6449, 1292.6322, 1294.6362, 1295.6366, 1300.5895, 1303.6643, 1305.6523, 1308.6443, 1310.6051, 1315.6364, 1320.6128, 1321.6134, 1325.6456, 1331.6674, 1332.6602, 1333.2076, 1334.6744, 1336.6710, 1337.6279, 1338.6300, 1338.6300, 1345.6146, 1347.6653, 1349.6800, 1349.6827, 1354.6820, 1355.6964, 1359.7053, 1360.6173, 1361.6373, 1365.6752, 1371.6718, 1375.6670, 1377.6776, 1382.6367, 1383.6619, 1387.6700, 1393.6727, 1400.6235, 1409.7107, 1411.6742, 1427.7725, 1431.7605, 1434.7190, 1449.7479, 1466.6953, 1473.7905, 1475.7498, 1478.7303, 1479.7468, 1485.7531, 1486.7472, 1493.7345, 1495.7502, 1500.7242, 1503.7690, 1505.8511, 1509.6786, 1520.7347, 1522.7239, 1523.7567, 1525.7864, 1527.8068, 1532.7472, 1537.7886, 1539.7711, 1549.7833, 1555.8657, 1558.8440, 1559.8573, 1568.7592, 1570.7814, 1578.7899, 1606.8213, 1615.8425, 1619.7972, 1621.7831, 1628.8156, 1630.8202, 1631.8217, 1639.9263, 1644.8083, 1647.8730, 1649.8650, 1650.8523, 1658.8206, 1659.8125, 1661.8284, 1663.7981, 1665.8070, 1669.8174, 1677.8353, 1679.9403, 1680.8076, 1682.7780, 1696.8055, 1697.7845, 1698.7950, 1707.7931, 1709.8029, 1710.8878, 1712.9166, 1725.8188, 1727.8683, 1730.8236, 1731.8221, 1742.8798, 1746.1482, 1749.8406, 1751.8657, 1756.8782, 1757.8225, 1763.8152, 1765.7922, 1766.8105, 1768.8596, 1769.4275, 1770.4087, 1773.8615, 1774.8795, 1775.0302, 1783.8599, 1786.8871, 1790.8822, 1792.7894, 1801.9315, 1804.9026, 1808.8663, 1824.8685, 1830.8608, 1849.8231, 1851.8430, 1867.9100, 1868.9441, 1870.9304, 1880.9020, 1884.9191, 1908.8989, 1914.9821, 1916.9882, 1920.9862, 1930.9827, 1932.9813, 1934.9822, 1947.0288, 1994.8763, 2011.9012, 2013.8700, 2020.9592, 2025.5920, 2042.0568, 2047.0238, 2047.9805, 2053.9851, 2058.9988, 2060.0400, 2060.0405, 2065.0427, 2066.0137, 2066.9895, 2067.9763, 2070.8792, 2074.8860, 2082.0293, 2086.8872, 2087.8835, 2090.8708, 2092.8674, 2093.8711, 2096.9836, 2097.9961, 2100.8787, 2101.8999, 2103.9092, 2107.8730, 2119.9924, 2122.9397, 2136.9133, 2211.1047, 2217.9368, 2225.1243, 2232.0229, 2233.0840, 2238.1050, 2250.0259, 2257.9934, 2262.9368, 2263.9700, 2273.0264, 2277.9500, 2283.1741, 2290.9570, 2307.9631, 2321.9558, 2323.9670, 2324.9824, 2335.9763, 2339.9456, 2343.0320, 2349.9844, 2352.0308, 2355.0315, 2358.0854, 2368.0815, 2369.0588, 2378.0020, 2379.0002, 2380.0007, 2381.9963, 2383.0444, 2383.9822, 2393.0020, 2394.0181, 2398.0447, 2400.0771, 2405.0879, 2414.1050, 2417.0933, 2421.0769, 2422.0593, 2426.0476, 2428.0725, 2430.0947, 2432.0962, 2435.0254, 2438.0706, 2444.0901, 2451.0278, 2459.9802, 2461.0254, 2501.2520, 2528.1902, 2543.2092, 2564.1965, 2705.2144, 2717.1655, 2834.2925, 2888.2483, 2904.4053, 3130.4312, 3291.4875, 3312.3765, 3451.6833, 3454.6270

21. F1MMV5 **Mass:** 66566 **Score:** 18 **Expect:** 3.4e+002 **Matches:** 22

Nuclear speckle-splicing regulatory protein 1 OS=Bos taurus GN=NSRP1 PE=4 SV=2

Observed	Mr(expt)	Mr(calc)	ppm	Start	End	Miss	Ions	Peptide
807.3921	806.3848	806.3896	-5.90	343 -	348	1	---	R.DSHRHR.E
828.3922	827.3849	827.4038	-22.90	354 -	359	1	---	R.DSHWKR.H
837.3794	836.3721	836.3698	2.79	130 -	135	1	---	R.EREMEK.G + Oxidation (M)
869.4703	868.4631	868.4627	0.39	310 -	316	2	---	R.SRGHEKR.E
927.4970	926.4897	926.4682	23.2	340 -	346	2	---	K.EKRDSHR.H
1001.4565	1000.4492	1000.4396	9.61	403 -	410	1	---	R.HNEKGCEK.E
1111.5457	1110.5384	1110.5161	20.1	472 -	480	2	---	R.ASKMRDMEK.D + Oxidation (M)
1115.5394	1114.5321	1114.5519	-17.77	372 -	379	2	---	R.EPDREWKR.E
1181.6368	1180.6295	1180.6061	19.9	360 -	368	2	---	R.HEEERLRGR.D
1234.6500	1233.6427	1233.6135	23.7	127 -	135	2	---	K.IQREREMEK.G + Oxidation (M)
1295.6366	1294.6293	1294.6564	-20.92	537 -	546	2	---	R.DRYLARQMAR.I + Oxidation (M)
1303.6643	1302.6570	1302.6415	11.9	271 -	282	1	---	R.EKGTETLSSHSK.H
1345.6146	1344.6073	1344.6058	1.13	428 -	437	2	---	R.YENSDKYRDR.E
1349.6800	1348.6727	1348.6847	-8.91	464 -	474	2	---	K.DRFVNQERASK.M
1349.6827	1348.6754	1348.6847	-6.91	464 -	474	2	---	K.DRFVNQERASK.M
1365.6752	1364.6679	1364.6983	-22.24	466 -	476	2	---	R.FVNQERASKMR.D
1434.7190	1433.7117	1433.7110	0.51	438 -	449	2	---	R.EKREVSVESSER.N
1520.7347	1519.7274	1519.7339	-4.24	526 -	538	2	---	K.RSNEETVTSARDR.Y
1786.8871	1785.8798	1785.8857	-3.26	483 -	499	1	---	K.ERNPEKPSSSEASLGAK.H
1867.9100	1866.9027	1866.9184	-8.38	527 -	542	2	---	R.SNEETVTSARDRYLAR.Q
1914.9821	1913.9748	1913.9568	9.40	273 -	289	2	---	K.GTETLSSHSKHHRNPAR.S
2398.0447	2397.0374	2397.0330	1.86	317 -	335	1	---	R.EDEHQERPAREQDSYHTDR.D

No match to: 713.4400, 713.4415, 724.3446, 728.2467, 730.2754, 732.2383, 734.2729, 735.3820, 736.3320, 740.2330, 743.2514, 748.2947, 750.2650, 756.4777, 764.3574, 765.3959, 768.3784, 772.4155, 774.3952, 778.4551, 780.3343, 782.3223, 783.3115, 787.2390, 791.2662, 802.4609, 805.3347, 806.4342, 808.4378, 812.4002, 814.4128, 820.4252, 830.3668, 834.3623, 840.3857, 842.5100, 845.4155, 851.3846, 856.5103, 865.3634, 870.5254, 871.4965, 872.6990, 888.3988, 891.4706, 893.9500, 894.4534, 897.0277, 905.4637, 911.4314, 912.4475, 913.5080, 913.5100, 918.5267, 925.4804, 930.5015, 933.4662, 935.4675, 943.4757, 951.4646, 952.4799, 957.4673, 961.4901, 962.4866, 967.4440, 969.4496, 972.4781, 974.5051, 975.4521, 977.4406, 978.4772, 979.4924, 983.4706, 985.6071, 991.4533, 993.5267, 995.5177, 999.4602, 1001.5621, 1003.5043, 1007.5638, 1012.5004, 1014.5331, 1018.5130, 1022.5398, 1023.5525, 1028.5991, 1030.5259, 1035.5314, 1036.5333, 1037.5145, 1040.5127, 1042.5243, 1045.5603, 1060.5398, 1069.4846, 1075.5515, 1092.6041, 1106.5313, 1109.5087, 1113.5419, 1118.5659, 1121.5419, 1122.5701, 1131.5602, 1138.5428, 1149.5912, 1155.5768, 1157.5907, 1158.6328, 1163.6145, 1173.6122, 1177.6130, 1178.6060, 1179.5918, 1180.6001, 1183.6030, 1187.6376, 1192.6014, 1193.5928, 1195.5924, 1199.6785, 1206.6066, 1215.6306, 1216.6482, 1226.5839, 1227.6051, 1231.6115, 1232.6251, 1233.6230, 1235.6155, 1248.6078, 1250.6483, 1254.6526, 1255.6036, 1256.6123, 1268.6208, 1271.6423, 1276.6179, 1277.6350, 1289.6449, 1292.6322, 1294.6362, 1300.5895, 1305.6523, 1307.6600, 1308.6443, 1310.6051, 1315.6364, 1320.6128, 1321.6134, 1323.6320, 1325.6456, 1331.6674, 1332.6602, 1333.2076, 1334.6744, 1336.6710, 1337.6279, 1338.6300, 1338.6300, 1347.6653, 1354.6820, 1355.6964, 1359.7053, 1360.6173, 1361.6373, 1371.6718, 1375.6670, 1377.6776, 1382.6367, 1383.6619, 1387.6700, 1393.6727, 1400.6235, 1409.7107, 1411.6742, 1427.7725, 1431.7605, 1432.6987, 1449.7479, 1466.6953, 1473.7905, 1475.7498, 1478.7303, 1479.7468, 1485.7531, 1486.7472, 1493.7345, 1495.7502, 1500.7242, 1503.7690, 1505.8511, 1509.6786, 1522.7239, 1523.7567, 1525.7864, 1527.8068, 1532.7472, 1537.7886, 1539.7711, 1549.7833, 1555.8657, 1558.8440, 1559.8573, 1568.7592, 1570.7814, 1578.7899, 1606.8213, 1615.8425, 1619.7972, 1621.7831, 1628.8156, 1630.8202, 1631.8217, 1639.9263, 1644.8083, 1647.8730, 1649.8650, 1650.8523, 1658.8206, 1659.8125, 1661.8284, 1663.7981, 1665.8070, 1669.8174, 1677.8353, 1679.9403, 1680.8076, 1682.7780, 1696.8055, 1697.7845, 1698.7950, 1707.7931, 1709.8029, 1710.8878, 1712.9166, 1716.8563, 1725.8188, 1727.8683, 1730.8236, 1731.8221, 1742.8798, 1746.1482, 1749.8406, 1751.8657, 1756.8782, 1757.8225, 1763.8152, 1765.7922, 1766.8105, 1768.8596, 1769.4275, 1770.4087, 1773.8615, 1774.8795, 1775.0302, 1783.8599, 1785.8483, 1790.8822, 1792.7894, 1801.9315, 1804.9026, 1808.8663, 1824.8685, 1830.8608, 1849.8231, 1851.8430, 1868.9441, 1870.9304, 1880.9020, 1884.9191, 1908.8989, 1916.9882, 1920.9862, 1930.9827, 1932.9813, 1934.9822, 1947.0288, 1958.9543, 1994.8763, 2011.9012, 2013.8700, 2020.9592, 2025.5920, 2042.0568, 2047.0238, 2047.9805, 2049.9497, 2052.9905, 2053.9851, 2058.9988, 2060.0400, 2060.0405, 2065.0427, 2066.0137, 2066.9895, 2067.9763, 2068.9128, 2070.8792, 2074.8860, 2082.0293, 2084.8977, 2086.8872, 2087.8835, 2090.8708, 2092.8674, 2093.8711, 2096.9836, 2097.9961, 2100.8787, 2101.8999, 2103.9092, 2107.8730, 2119.9924, 2122.9397, 2136.9133, 2211.1047, 2217.9368, 2225.1243, 2232.0229, 2233.0840, 2238.1050, 2250.0259, 2257.9934, 2262.9368, 2263.9700, 2273.0264, 2277.9500, 2283.1741, 2290.9570, 2307.9631, 2321.9558, 2323.9670, 2324.9824, 2335.9763, 2339.9456, 2343.0320, 2349.9844, 2352.0308, 2355.0315, 2358.0854, 2368.0815, 2369.0588, 2378.0020, 2379.0002, 2380.0007, 2381.9963, 2383.0444, 2383.9822, 2393.0020, 2394.0181, 2400.0771, 2405.0879, 2414.1050, 2417.0933, 2421.0769, 2422.0593, 2426.0476, 2428.0725, 2430.0947, 2432.0962, 2435.0254, 2438.0706, 2444.0901, 2451.0278, 2459.9802, 2461.0254, 2501.2520, 2528.1902, 2543.2092, 2564.1965, 2705.2144, 2717.1655, 2834.2925, 2888.2483, 2904.4053, 3130.4312, 3291.4875, 3312.3765, 3451.6833, 3454.6270

22. Q2KIC0 **Mass:** 66733 **Score:** 18 **Expect:** 3.4e+002 **Matches:** 22

Nuclear speckle splicing regulatory protein 1 OS=Bos taurus GN=NSRP1 PE=2 SV=1

Observed	Mr(expt)	Mr(calc)	ppm	Start	End	Miss	Ions	Peptide
807.3921	806.3848	806.3896	-5.90	343 -	348	1	---	R.DSHRHR.E
828.3922	827.3849	827.4038	-22.90	354 -	359	1	---	R.DSHWKR.H
837.3794	836.3721	836.3698	2.79	130 -	135	1	---	R.EREMEK.G + Oxidation (M)
869.4703	868.4631	868.4627	0.39	310 -	316	2	---	R.SRGHEKR.E
927.4970	926.4897	926.4682	23.2	340 -	346	2	---	K.EKRDSHR.H
1001.4565	1000.4492	1000.4396	9.61	404 -	411	1	---	R.HNEKGCEK.E
1003.5043	1002.4970	1002.4995	-2.47	374 -	380	2	---	R.NDREWKR.E
1111.5457	1110.5384	1110.5161	20.1	473 -	481	2	---	R.ASKMRDMEK.D + Oxidation (M)

1181.6368	1180.6295	1180.6061	19.9	360 - 368	2	---	R.HEEERLRGR.D
1234.6500	1233.6427	1233.6135	23.7	127 - 135	2	---	K.IQREREMEK.G + Oxidation (M)
1295.6366	1294.6293	1294.6564	-20.92	538 - 547	2	---	R.DRYLARQMAR.I + Oxidation (M)
1303.6643	1302.6570	1302.6415	11.9	271 - 282	1	---	R.EKGTETLSSHSK.H
1345.6146	1344.6073	1344.6058	1.13	429 - 438	2	---	R.YENSDKYRDR.E
1349.6800	1348.6727	1348.6847	-8.91	465 - 475	2	---	K.DRFVNQERASK.M
1349.6827	1348.6754	1348.6847	-6.91	465 - 475	2	---	K.DRFVNQERASK.M
1365.6752	1364.6679	1364.6983	-22.24	467 - 477	2	---	R.FVNQERASKMR.D
1434.7190	1433.7117	1433.7110	0.51	439 - 450	2	---	R.EKREVSVESSER.N
1520.7347	1519.7274	1519.7339	-4.24	527 - 539	2	---	K.RSNEETVTSARDR.Y
1786.8871	1785.8798	1785.8857	-3.26	484 - 500	1	---	K.ERNPEKPSSSEASLGAK.H
1867.9100	1866.9027	1866.9184	-8.38	528 - 543	2	---	R.SNEETVTSARDRYLAR.Q
1914.9821	1913.9748	1913.9568	9.40	273 - 289	2	---	K.GTETLSSHSKHHRNPAR.S
2398.0447	2397.0374	2397.0330	1.86	317 - 335	1	---	R.EDEHQERPAREQDSYHTDR.D

No match to: 713.4400, 713.4415, 724.3446, 728.2467, 730.2754, 732.2383, 734.2729, 735.3820, 736.3320, 740.2330, 743.2514, 748.2947, 750.2650, 756.4777, 764.3574, 765.3959, 768.3784, 772.4155, 774.3952, 778.4551, 780.3343, 782.3223, 783.3115, 787.2390, 791.2662, 802.4609, 805.3347, 806.4342, 808.4378, 812.4002, 814.4128, 820.4252, 830.3668, 834.3623, 840.3857, 842.5100, 845.4155, 851.3846, 856.5103, 865.3634, 870.5254, 871.4965, 872.6990, 888.3988, 891.4706, 893.9500, 894.4534, 897.0277, 905.4637, 911.4314, 912.4475, 913.5080, 913.5100, 918.5267, 925.4804, 930.5015, 933.4662, 935.4675, 943.4757, 951.4646, 952.4799, 957.4673, 961.4901, 962.4866, 967.4440, 969.4496, 972.4781, 974.5051, 975.4521, 977.4406, 978.4772, 979.4924, 983.4706, 985.6071, 991.4533, 993.5267, 995.5177, 999.4602, 1001.5621, 1007.5638, 1012.5004, 1014.5331, 1018.5130, 1022.5398, 1023.5525, 1028.5991, 1030.5259, 1035.5314, 1036.5333, 1037.5145, 1040.5127, 1042.5243, 1045.5603, 1060.5398, 1069.4846, 1075.5515, 1092.6041, 1106.5313, 1109.5087, 1113.5419, 1115.5394, 1118.5659, 1121.5419, 1122.5701, 1131.5602, 1138.5428, 1149.5912, 1155.5768, 1157.5907, 1158.6328, 1163.6145, 1173.6122, 1177.6130, 1178.6060, 1179.5918, 1180.6001, 1183.6030, 1187.6376, 1192.6014, 1193.5928, 1195.5924, 1199.6785, 1206.6066, 1215.6306, 1216.6482, 1226.5839, 1227.6051, 1231.6115, 1232.6251, 1233.6230, 1235.6155, 1248.6078, 1250.6483, 1254.6526, 1255.6036, 1256.6123, 1268.6208, 1271.6423, 1276.6179, 1277.6350, 1289.6449, 1292.6322, 1294.6362, 1300.5895, 1305.6523, 1307.6600, 1308.6443, 1310.6051, 1315.6364, 1320.6128, 1321.6134, 1323.6320, 1325.6456, 1331.6674, 1332.6602, 1333.2076, 1334.6744, 1336.6710, 1337.6279, 1338.6300, 1338.6300, 1347.6653, 1354.6820, 1355.6964, 1359.7053, 1360.6173, 1361.6373, 1371.6718, 1375.6670, 1377.6776, 1382.6367, 1383.6619, 1387.6700, 1393.6727, 1400.6235, 1409.7107, 1411.6742, 1427.7725, 1431.7605, 1432.6987, 1449.7479, 1466.6953, 1473.7905, 1475.7498, 1478.7303, 1479.7468, 1485.7531, 1486.7472, 1493.7345, 1495.7502, 1500.7242, 1503.7690, 1505.8511, 1509.6786, 1522.7239, 1523.7567, 1525.7864, 1527.8068, 1532.7472, 1537.7886, 1539.7711, 1549.7833, 1555.8657, 1558.8440, 1559.8573, 1568.7592, 1570.7814, 1578.7899, 1606.8213, 1615.8425, 1619.7972, 1621.7831, 1628.8156, 1630.8202, 1631.8217, 1639.9263, 1644.8083, 1647.8730, 1649.8650, 1650.8523, 1658.8206, 1659.8125, 1661.8284, 1663.7981, 1665.8070, 1669.8174, 1677.8353, 1679.9403, 1680.8076, 1682.7780, 1696.8055, 1697.7845, 1698.7950, 1707.7931, 1709.8029, 1710.8878, 1712.9166, 1716.8563, 1725.8188, 1727.8683, 1730.8236, 1731.8221, 1742.8798, 1746.1482, 1749.8406, 1751.8657, 1756.8782, 1757.8225, 1763.8152, 1765.7922, 1766.8105, 1768.8596, 1769.4275, 1770.4087, 1773.8615, 1774.8795, 1775.0302, 1783.8599, 1785.8483, 1790.8822, 1792.7894, 1801.9315, 1804.9026, 1808.8663, 1824.8685, 1830.8608, 1849.8231, 1851.8430, 1868.9441, 1870.9304, 1880.9020, 1884.9191, 1908.8989, 1916.9882, 1920.9862, 1930.9827, 1932.9813, 1934.9822, 1947.0288, 1958.9543, 1994.8763, 2011.9012, 2013.8700, 2020.9592, 2025.5920, 2042.0568, 2047.0238, 2047.9805, 2049.9497, 2052.9905, 2053.9851, 2058.9988, 2060.0400, 2060.0405, 2065.0427, 2066.0137, 2066.9895, 2067.9763, 2068.9128, 2070.8792, 2074.8860, 2082.0293, 2084.8977, 2086.8872, 2087.8835, 2090.8708, 2092.8674, 2093.8711, 2096.9836, 2097.9961, 2100.8787, 2101.8999, 2103.9092, 2107.8730, 2119.9924, 2122.9397, 2136.9133, 2211.1047, 2217.9368, 2225.1243, 2232.0229, 2233.0840, 2238.1050, 2250.0259, 2257.9934, 2262.9368, 2263.9700, 2273.0264, 2277.9500, 2283.1741, 2290.9570, 2307.9631, 2321.9558, 2323.9670, 2324.9824, 2335.9763, 2339.9456, 2343.0320, 2349.9844, 2352.0308, 2355.0315, 2358.0854, 2368.0815, 2369.0588, 2378.0020, 2379.0002, 2380.0007, 2381.9963, 2383.0444, 2383.9822, 2393.0020, 2394.0181, 2400.0771, 2405.0879, 2414.1050, 2417.0933, 2421.0769, 2422.0593, 2426.0476, 2428.0725, 2430.0947, 2432.0962, 2435.0254, 2438.0706, 2444.0901, 2451.0278, 2459.9802, 2461.0254, 2501.2520, 2528.1902, 2543.2092, 2564.1965, 2705.2144, 2717.1655, 2834.2925, 2888.2483, 2904.4053, 3130.4312, 3291.4875, 3312.3765, 3451.6833, 3454.6270

23. E1BDW0 **Mass:** 49794 **Score:** 18 **Expect:** 3.4e+002 **Matches:** 17

Uncharacterized protein OS=Bos taurus GN=ALS2CR12 PE=4 SV=1

Observed	Mr(expt)	Mr(calc)	ppm	Start - End	Miss	Ions	Peptide
782.3223	781.3150	781.3098	6.59	164 - 169	0	---	R.CAEMQK.N + Oxidation (M)
918.5267	917.5195	917.4970	24.4	240 - 246	2	---	K.KKAEWEK.D
1106.5313	1105.5240	1105.5437	-17.83	155 - 163	0	---	K.LLSNEMDLR.C + Oxidation (M)
1115.5394	1114.5321	1114.5255	5.99	110 - 119	1	---	K.KHSESEDVGK.V
1181.6368	1180.6295	1180.6200	8.07	294 - 302	1	---	K.EELLKQHER.D
1295.6366	1294.6293	1294.6041	19.5	267 - 276	1	---	K.FELESEEERK.K
1332.6602	1331.6529	1331.6755	-16.92	202 - 213	1	---	K.LAAQDKLEEMGK.E
1432.6987	1431.6914	1431.6994	-5.54	242 - 252	2	---	K.AEWEKDEKLER.E
1473.7905	1472.7832	1472.8198	-24.85	303 - 314	2	---	R.DTLQLEELRKTK.E
1527.8068	1526.7995	1526.7875	7.89	64 - 76	2	---	K.MKVHLGKQEESNK.L
1570.7814	1569.7741	1569.8111	-23.53	111 - 124	2	---	K.HSESEDVGKVKISR.T
1665.8070	1664.7997	1664.8369	-22.34	263 - 275	2	---	K.ITRKFELESEEER.K
1696.8055	1695.7982	1695.8315	-19.61	177 - 191	0	---	R.ELEEAHAAQLSELEK.N
1768.8596	1767.8523	1767.8712	-10.70	202 - 216	2	---	K.LAAQDKLEEMGKEYK.Y + Oxidation (M)
1783.8599	1782.8526	1782.8458	3.82	95 - 109	1	---	R.EQISVTLGDEMFDRK.K + Oxidation (M)
2053.9851	2052.9778	2052.9898	-5.85	93 - 109	2	---	R.NREQISVTLGDEMFDRK.K + Oxidation (M)
2211.1047	2210.0974	2210.1113	-6.29	133 - 150	1	---	K.EQISELTVIIEQMNRDHR.S

No match to: 713.4400, 713.4415, 724.3446, 728.2467, 730.2754, 732.2383, 734.2729, 735.3820, 736.3320, 740.2330, 743.2514, 748.2947, 750.2650, 756.4777, 764.3574, 765.3959, 768.3784, 772.4155, 774.3952, 778.4551, 780.3343, 783.3115, 787.2390, 791.2662, 802.4609, 805.3347, 806.4342, 807.3921, 808.4378, 812.4002, 814.4128, 820.4252, 828.3922, 830.3668, 834.3623, 837.3794, 840.3857, 842.5100, 845.4155, 851.3846, 856.5103, 865.3634, 869.4703, 870.5254, 871.4965, 872.6990, 888.3988, 891.4706, 893.9500, 894.4534, 897.0277, 905.4637, 911.4314, 912.4475, 913.5080, 913.5100, 925.4804, 927.4970, 930.5015, 933.4662, 935.4675, 943.4757, 951.4646, 952.4799, 957.4673, 961.4901, 962.4866, 967.4440, 969.4496, 972.4781, 974.5051, 975.4521, 977.4406, 978.4772, 979.4924, 983.4706, 985.6071, 991.4533, 993.5267, 995.5177, 999.4602, 1001.4565, 1001.5621, 1003.5043, 1007.5638, 1012.5004, 1014.5331, 1018.5130, 1022.5398, 1023.5525, 1028.5991, 1030.5259, 1035.5314, 1036.5333, 1037.5145, 1040.5127, 1042.5243, 1045.5603, 1060.5398, 1069.4846, 1075.5515, 1092.6041, 1109.5087, 1111.5457, 1113.5419, 1118.5659, 1121.5419, 1122.5701, 1131.5602, 1138.5428, 1149.5912, 1155.5768, 1157.5907, 1158.6328, 1163.6145, 1173.6122, 1177.6130, 1178.6060, 1179.5918, 1180.6001, 1183.6030, 1187.6376, 1192.6014, 1193.5928, 1195.5924, 1199.6785, 1206.6066, 1215.6306, 1216.6482, 1226.5839, 1227.6051, 1231.6115, 1232.6251, 1233.6230, 1234.6500, 1235.6155, 1248.6078, 1250.6483, 1254.6526, 1255.6036, 1256.6123, 1268.6208, 1271.6423, 1276.6179, 1277.6350, 1289.6449, 1292.6322, 1294.6362, 1300.5895, 1303.6643, 1305.6523, 1307.6600, 1308.6443, 1310.6051, 1315.6364, 1320.6128, 1321.6134, 1323.6320, 1325.6456, 1331.6674, 1333.2076, 1334.6744, 1336.6710, 1337.6279, 1338.6300, 1338.6300, 1345.6146, 1347.6653, 1349.6800, 1349.6827, 1354.6820, 1355.6964, 1359.7053, 1360.6173, 1361.6373, 1365.6752, 1371.6718, 1375.6670, 1377.6776, 1382.6367, 1383.6619, 1387.6700, 1393.6727, 1400.6235, 1409.7107, 1411.6742, 1427.7725, 1431.7605, 1434.7190, 1449.7479, 1466.6953, 1475.7498, 1478.7303, 1479.7468, 1485.7531, 1486.7472, 1493.7345, 1495.7502, 1500.7242, 1503.7690, 1505.8511, 1509.6786, 1520.7347, 1522.7239, 1523.7567, 1525.7864, 1532.7472, 1537.7886, 1539.7711, 1549.7833, 1555.8657, 1558.8440, 1559.8573, 1568.7592, 1578.7899, 1606.8213, 1615.8425, 1619.7972, 1621.7831, 1628.8156, 1630.8202, 1631.8217, 1639.9263, 1644.8083, 1647.8730, 1649.8650, 1650.8523, 1658.8206, 1659.8125, 1661.8284, 1663.7981, 1669.8174, 1677.8353, 1679.9403, 1680.8076, 1682.7780, 1697.7845, 1698.7950, 1707.7931, 1709.8029, 1710.8878, 1712.9166, 1716.8563, 1725.8188, 1727.8683, 1730.8236, 1731.8221, 1742.8798, 1746.1482, 1749.8406, 1751.8657, 1756.8782, 1757.8225, 1763.8152, 1765.7922, 1766.8105, 1769.4275, 1770.4087, 1773.8615, 1774.8795, 1775.0302, 1785.8483, 1786.8871, 1790.8822, 1792.7894, 1801.9315, 1804.9026, 1808.8663, 1824.8685, 1830.8608, 1849.8231, 1851.8430, 1867.9100, 1868.9441, 1870.9304, 1880.9020, 1884.9191, 1908.8989, 1914.9821, 1916.9882, 1920.9862, 1930.9827, 1932.9813, 1934.9822, 1947.0288, 1958.9543, 1994.8763, 2011.9012, 2013.8700, 2020.9592, 2025.5920, 2042.0568, 2047.0238, 2047.9805, 2049.9497, 2052.9905, 2058.9988, 2060.0400, 2060.0405, 2065.0427, 2066.0137, 2066.9895, 2067.9763, 2068.9128, 2070.8792, 2074.8860, 2082.0293, 2084.8977, 2086.8872, 2087.8835, 2090.8708, 2092.8674, 2093.8711, 2096.9836, 2097.9961, 2100.8787, 2101.8999, 2103.9092, 2107.8730, 2119.9924, 2122.9397, 2136.9133, 2217.9368, 2225.1243, 2232.0229, 2233.0840, 2238.1050, 2250.0259,

2257.9934, 2262.9368, 2263.9700, 2273.0264, 2277.9500, 2283.1741, 2290.9570, 2307.9631, 2321.9558, 2323.9670, 2324.9824, 2335.9763, 2339.9456, 2343.0320, 2349.9844, 2352.0308, 2355.0315, 2358.0854, 2368.0815, 2369.0588, 2378.0020, 2379.0002, 2380.0007, 2381.9963, 2383.0444, 2383.9822, 2393.0020, 2394.0181, 2398.0447, 2400.0771, 2405.0879, 2414.1050, 2417.0933, 2421.0769, 2422.0593, 2426.0476, 2428.0725, 2430.0947, 2432.0962, 2435.0254, 2438.0706, 2444.0901, 2451.0278, 2459.9802, 2461.0254, 2501.2520, 2528.1902, 2543.2092, 2564.1965, 2705.2144, 2717.1655, 2834.2925, 2888.2483, 2904.4053, 3130.4312, 3291.4875, 3312.3765, 3451.6833, 3454.6270

24. G3N3V8 **Mass:** 69281 **Score:** 18 **Expect:** 3.7e+002 **Matches:** 12

Uncharacterized protein OS=Bos taurus GN=C3orf67 PE=4 SV=1

Observed	Mr(expt)	Mr(calc)	ppm	Start	End	Miss	Ions	Peptide
735.3820	734.3747	734.3745	0.36	437	443	1	---	K.AAKCASK.E
807.3921	806.3848	806.3770	9.67	384	390	0	---	K.EDTSVTR.S
979.4924	978.4852	978.4917	-6.64	120	128	1	---	R.GTVSMRNSK.H
995.5177	994.5104	994.4866	24.0	120	128	1	---	R.GTVSMRNSK.H + Oxidation (M)
1069.4846	1068.4773	1068.4546	21.3	611	620	0	---	K.EMNPPSPSNP.-
1292.6322	1291.6249	1291.6554	-23.61	267	278	0	---	K.AATVLTAMSQQR.A + Oxidation (M)
1349.6800	1348.6727	1348.6517	15.6	152	162	1	18	R.NNMRLSNETVR.S + Oxidation (M)
1349.6827	1348.6754	1348.6517	17.6	152	162	1	---	R.NNMRLSNETVR.S + Oxidation (M)
1409.7107	1408.7034	1408.7310	-19.60	502	514	0	---	K.DLPTHQVSASINK.T
1679.9403	1678.9330	1678.9049	16.7	141	155	2	---	K.VLGPPPLSGRRNNMR.L + Oxidation (M)
1727.8683	1726.8610	1726.8209	23.2	126	140	1	---	R.NSKHQDVCHIAFGSK.V
2211.1047	2210.0974	2210.0525	20.3	538	556	0	---	R.NYQPSEISESELQMLASLR.R + Oxidation (M)

No match to: 713.4400, 713.4415, 724.3446, 728.2467, 730.2754, 732.2383, 734.2729, 736.3320, 740.2330, 743.2514, 748.2947, 750.2650, 756.4777, 764.3574, 765.3959, 768.3784, 772.4155, 774.3952, 778.4551, 780.3343, 782.3223, 783.3115, 787.2390, 791.2662, 802.4609, 805.3347, 806.4342, 808.4378, 812.4002, 814.4128, 820.4252, 828.3922, 830.3668, 834.3623, 837.3794, 840.3857, 842.5100, 845.4155, 851.3846, 856.5103, 865.3634, 869.4703, 870.5254, 871.4965, 872.6990, 888.3988, 891.4706, 893.9500, 894.4534, 897.0277, 905.4637, 911.4314, 912.4475, 913.5080, 913.5100, 918.5267, 925.4804, 927.4970, 930.5015, 933.4662, 935.4675, 943.4757, 951.4646, 952.4799, 957.4673, 961.4901, 962.4866, 967.4440, 969.4496, 972.4781, 974.5051, 975.4521, 977.4406, 978.4772, 983.4706, 985.6071, 991.4533, 993.5267, 999.4602, 1001.4565, 1001.5621, 1003.5043, 1007.5638, 1012.5004, 1014.5331, 1018.5130, 1022.5398, 1023.5525, 1028.5991, 1030.5259, 1035.5314, 1036.5333, 1037.5145, 1040.5127, 1042.5243, 1045.5603, 1060.5398, 1075.5515, 1092.6041, 1106.5313, 1109.5087, 1111.5457, 1113.5419, 1115.5394, 1118.5659, 1121.5419, 1122.5701, 1131.5602, 1138.5428, 1149.5912, 1155.5768, 1157.5907, 1158.6328, 1163.6145, 1173.6122, 1177.6130, 1178.6060, 1179.5918, 1180.6001, 1181.6368, 1183.6030, 1187.6376, 1192.6014, 1193.5928, 1195.5924, 1199.6785, 1206.6066, 1215.6306, 1216.6482, 1226.5839, 1227.6051, 1231.6115, 1232.6251, 1233.6230, 1234.6500, 1235.6155, 1248.6078, 1250.6483, 1254.6526, 1255.6036, 1256.6123, 1268.6208, 1271.6423, 1276.6179, 1277.6350, 1289.6449, 1294.6362, 1295.6366, 1300.5895, 1303.6643, 1305.6523, 1307.6600, 1308.6443, 1310.6051, 1315.6364, 1320.6128, 1321.6134, 1323.6320, 1325.6456, 1331.6674, 1332.6602, 1333.2076, 1334.6744, 1336.6710, 1337.6279, 1338.6300, 1338.6300, 1345.6146, 1347.6653, 1354.6820, 1355.6964, 1359.7053, 1360.6173, 1361.6373, 1365.6752, 1371.6718, 1375.6670, 1377.6776, 1382.6367, 1383.6619, 1387.6700, 1393.6727, 1400.6235, 1411.6742, 1427.7725, 1431.7605, 1432.6987, 1434.7190, 1449.7479, 1466.6953, 1473.7905, 1475.7498, 1478.7303, 1479.7468, 1485.7531, 1486.7472, 1493.7345, 1495.7502, 1500.7242, 1503.7690, 1505.8511, 1509.6786, 1520.7347, 1522.7239, 1523.7567, 1525.7864, 1527.8068, 1532.7472, 1537.7886, 1539.7711, 1549.7833, 1555.8657, 1558.8440, 1559.8573, 1568.7592, 1570.7814, 1578.7899, 1606.8213, 1615.8425, 1619.7972, 1621.7831, 1628.8156, 1630.8202, 1631.8217, 1639.9263, 1644.8083, 1647.8730, 1649.8650, 1650.8523, 1658.8206, 1659.8125, 1661.8284, 1663.7981, 1665.8070, 1669.8174, 1677.8353, 1680.8076, 1682.7780, 1696.8055, 1697.7845, 1698.7950, 1707.7931, 1709.8029, 1710.8878, 1712.9166, 1716.8563, 1725.8188, 1730.8236, 1731.8221, 1742.8798, 1746.1482, 1749.8406, 1751.8657, 1756.8782, 1757.8225, 1763.8152, 1765.7922, 1766.8105, 1768.8596, 1769.4275, 1770.4087, 1773.8615, 1774.8795, 1775.0302, 1783.8599, 1785.8483, 1786.8871, 1790.8822, 1792.7894, 1801.9315, 1804.9026, 1808.8663, 1824.8685, 1830.8608, 1849.8231, 1851.8430, 1867.9100, 1868.9441, 1870.9304, 1880.9020, 1884.9191, 1908.8989, 1914.9821, 1916.9882, 1920.9862, 1930.9827, 1932.9813, 1934.9822, 1947.0288, 1958.9543, 1994.8763, 2011.9012, 2013.8700, 2020.9592, 2025.5920, 2042.0568, 2047.0238, 2047.9805, 2049.9497, 2052.9905, 2053.9851, 2058.9988, 2060.0400, 2060.0405, 2065.0427, 2066.0137, 2066.9895, 2067.9763, 2068.9128, 2070.8792, 2074.8860, 2082.0293, 2084.8977, 2086.8872, 2087.8835, 2090.8708, 2092.8674, 2093.8711, 2096.9836, 2097.9961, 2100.8787, 2101.8999, 2103.9092, 2107.8730, 2119.9924, 2122.9397, 2136.9133, 2217.9368, 2225.1243, 2232.0229, 2233.0840, 2238.1050, 2250.0259, 2257.9934, 2262.9368, 2263.9700, 2273.0264, 2277.9500, 2283.1741, 2290.9570, 2307.9631, 2321.9558, 2323.9670, 2324.9824, 2335.9763, 2339.9456, 2343.0320, 2349.9844, 2352.0308, 2355.0315, 2358.0854, 2368.0815, 2369.0588, 2378.0020, 2379.0002, 2380.0007, 2381.9963, 2383.0444, 2383.9822, 2393.0020, 2394.0181, 2398.0447, 2400.0771, 2405.0879, 2414.1050, 2417.0933, 2421.0769, 2422.0593, 2426.0476, 2428.0725, 2430.0947, 2432.0962, 2435.0254, 2438.0706, 2444.0901, 2451.0278, 2459.9802, 2461.0254, 2501.2520, 2528.1902, 2543.2092, 2564.1965, 2705.2144, 2717.1655, 2834.2925, 2888.2483, 2904.4053, 3130.4312, 3291.4875, 3312.3765, 3451.6833, 3454.6270

25. F1MNR4 **Mass:** 58473 **Score:** 18 **Expect:** 3.7e+002 **Matches:** 11

Uncharacterized protein OS=Bos taurus GN=C3orf67 PE=4 SV=2

Observed	Mr(expt)	Mr(calc)	ppm	Start	End	Miss	Ions	Peptide
735.3820	734.3747	734.3671	10.4	265	270	1	---	K.STSRER.T
807.3921	806.3848	806.3770	9.67	406	412	0	---	K.EDTSVTR.S
979.4924	978.4852	978.4917	-6.64	143	151	1	---	R.GTVSMRNSK.H
995.5177	994.5104	994.4866	24.0	143	151	1	---	R.GTVSMRNSK.H + Oxidation (M)
1069.4846	1068.4773	1068.4546	21.3	508	517	0	---	K.EMNPPSPSNP.-
1292.6322	1291.6249	1291.6554	-23.61	289	300	0	---	K.AATVLTAMSQQR.A + Oxidation (M)
1349.6800	1348.6727	1348.6517	15.6	175	185	1	18	R.NNMRLSNETVR.S + Oxidation (M)
1349.6827	1348.6754	1348.6517	17.6	175	185	1	---	R.NNMRLSNETVR.S + Oxidation (M)
1679.9403	1678.9330	1678.9049	16.7	164	178	2	---	K.VLGPPPLSGRRNNMR.L + Oxidation (M)
1727.8683	1726.8610	1726.8209	23.2	149	163	1	---	R.NSKHQDVCHIAFGSK.V
2211.1047	2210.0974	2210.0525	20.3	435	453	0	---	R.NYQPSEISESELQMLASLR.R + Oxidation (M)

No match to: 713.4400, 713.4415, 724.3446, 728.2467, 730.2754, 732.2383, 734.2729, 736.3320, 740.2330, 743.2514, 748.2947, 750.2650, 756.4777, 764.3574, 765.3959, 768.3784, 772.4155, 774.3952, 778.4551, 780.3343, 782.3223, 783.3115, 787.2390, 791.2662, 802.4609, 805.3347, 806.4342, 808.4378, 812.4002, 814.4128, 820.4252, 828.3922, 830.3668, 834.3623, 837.3794, 840.3857, 842.5100, 845.4155, 851.3846, 856.5103, 865.3634, 869.4703, 870.5254, 871.4965, 872.6990, 888.3988, 891.4706, 893.9500, 894.4534, 897.0277, 905.4637, 911.4314, 912.4475, 913.5080, 913.5100, 918.5267, 925.4804, 927.4970, 930.5015, 933.4662, 935.4675, 943.4757, 951.4646, 952.4799, 957.4673, 961.4901, 962.4866, 967.4440, 969.4496, 972.4781, 974.5051, 975.4521, 977.4406, 978.4772, 983.4706, 985.6071, 991.4533, 993.5267, 999.4602, 1001.4565, 1001.5621, 1003.5043, 1007.5638, 1012.5004, 1014.5331, 1018.5130, 1022.5398, 1023.5525, 1028.5991, 1030.5259, 1035.5314, 1036.5333, 1037.5145, 1040.5127, 1042.5243, 1045.5603, 1060.5398, 1075.5515, 1092.6041, 1106.5313, 1109.5087, 1111.5457, 1113.5419, 1115.5394, 1118.5659, 1121.5419, 1122.5701, 1131.5602, 1138.5428, 1149.5912, 1155.5768, 1157.5907, 1158.6328, 1163.6145, 1173.6122, 1177.6130, 1178.6060, 1179.5918, 1180.6001, 1181.6368, 1183.6030, 1187.6376, 1192.6014, 1193.5928, 1195.5924, 1199.6785, 1206.6066, 1215.6306, 1216.6482, 1226.5839, 1227.6051, 1231.6115, 1232.6251, 1233.6230, 1234.6500, 1235.6155, 1248.6078, 1250.6483, 1254.6526, 1255.6036, 1256.6123, 1268.6208, 1271.6423, 1276.6179, 1277.6350, 1289.6449, 1294.6362, 1295.6366, 1300.5895, 1303.6643, 1305.6523, 1307.6600, 1308.6443, 1310.6051, 1315.6364, 1320.6128, 1321.6134, 1323.6320, 1325.6456, 1331.6674, 1332.6602, 1333.2076, 1334.6744, 1336.6710, 1337.6279, 1338.6300, 1338.6300, 1345.6146, 1347.6653, 1354.6820, 1355.6964, 1359.7053, 1360.6173, 1361.6373, 1365.6752, 1371.6718, 1375.6670, 1377.6776, 1382.6367, 1383.6619, 1387.6700, 1393.6727, 1400.6235, 1409.7107, 1411.6742, 1427.7725, 1431.7605, 1432.6987, 1434.7190, 1449.7479, 1466.6953, 1473.7905, 1475.7498, 1478.7303, 1479.7468, 1485.7531, 1486.7472, 1493.7345, 1495.7502, 1500.7242, 1503.7690, 1505.8511, 1509.6786, 1520.7347, 1522.7239, 1523.7567, 1525.7864, 1527.8068, 1532.7472, 1537.7886, 1539.7711, 1549.7833, 1555.8657, 1558.8440, 1559.8573, 1568.7592, 1570.7814, 1578.7899, 1606.8213, 1615.8425, 1619.7972, 1621.7831, 1628.8156, 1630.8202, 1631.8217, 1639.9263, 1644.8083, 1647.8730, 1649.8650, 1650.8523, 1658.8206, 1659.8125, 1661.8284, 1663.7981, 1665.8070, 1669.8174, 1677.8353, 1680.8076, 1682.7780, 1696.8055, 1697.7845, 1698.7950, 1707.7931, 1709.8029, 1710.8878, 1712.9166, 1716.8563, 1725.8188, 1730.8236, 1731.8221, 1742.8798, 1746.1482, 1749.8406, 1751.8657, 1756.8782, 1757.8225, 1763.8152, 1765.7922, 1766.8105, 1768.8596, 1769.4275, 1770.4087, 1773.8615, 1774.8795, 1775.0302, 1783.8599, 1785.8483, 1786.8871, 1790.8822, 1792.7894, 1801.9315, 1804.9026, 1808.8663, 1824.8685, 1830.8608, 1849.8231, 1851.8430, 1867.9100, 1868.9441, 1870.9304, 1880.9020, 1884.9191, 1908.8989, 1914.9821, 1916.9882, 1920.9862, 1930.9827, 1932.9813, 1934.9822, 1947.0288, 1958.9543, 1994.8763, 2011.9012, 2013.8700, 2020.9592, 2025.5920, 2042.0568, 2047.0238, 2047.9805, 2049.9497, 2052.9905, 2053.9851, 2058.9988, 2060.0400, 2060.0405, 2065.0427, 2066.0137, 2066.9895, 2067.9763, 2068.9128, 2070.8792, 2074.8860, 2082.0293, 2084.8977, 2086.8872, 2087.8835,

2090.8708, 2092.8674, 2093.8711, 2096.9836, 2097.9961, 2100.8787, 2101.8999, 2103.9092, 2107.8730, 2119.9924, 2122.9397, 2136.9133, 2217.9368, 2225.1243, 2232.0229, 2233.0840, 2238.1050, 2250.0259, 2257.9934, 2262.9368, 2263.9700, 2273.0264, 2277.9500, 2283.1741, 2290.9570, 2307.9631, 2321.9558, 2323.9670, 2324.9824, 2335.9763, 2339.9456, 2343.0320, 2349.9844, 2352.0308, 2355.0315, 2358.0854, 2368.0815, 2369.0588, 2378.0020, 2379.0002, 2380.0007, 2381.9963, 2383.0444, 2383.9822, 2393.0020, 2394.0181, 2398.0447, 2400.0771, 2405.0879, 2414.1050, 2417.0933, 2421.0769, 2422.0593, 2426.0476, 2428.0725, 2430.0947, 2432.0962, 2435.0254, 2438.0706, 2444.0901, 2451.0278, 2459.9802, 2461.0254, 2501.2520, 2528.1902, 2543.2092, 2564.1965, 2705.2144, 2717.1655, 2834.2925, 2888.2483, 2904.4053, 3130.4312, 3291.4875, 3312.3765, 3451.6833, 3454.6270

26. Q6YFP9 **Mass:** 10868 **Score:** 18 **Expect:** 3.7e+002 **Matches:** 6

Cytochrome c oxidase subunit 6B2 OS=Bos taurus GN=COX6B2 PE=2 SV=1

Observed	Mr(expt)	Mr(calc)	ppm	Start		End	Miss	Ions	Peptide
808.4378	807.4305	807.4127	22.1	81	-	88	1	---	K.DGTFAGKI.-
1193.5928	1192.5855	1192.6088	-19.51	77	-	87	1	---	K.EQIKDGTFAGK.I
1475.7498	1474.7425	1474.7094	22.4	1	-	13	1	---	-.MLGAECPKPCKGK.W
1644.8083	1643.8010	1643.8242	-14.12	62	-	74	0	---	R.VYHSLCPISWVQR.W
2097.9961	2096.9888	2097.0181	-13.94	14	-	30	1	---	K.WPTPPFDPRFPNQNQTR.N
2283.1741	2282.1668	2282.1345	14.2	12	-	30	2	---	K.GKWPTPPFDPRFPNQNQTR.N

No match to: 713.4400, 713.4415, 724.3446, 728.2467, 730.2754, 732.2383, 734.2729, 735.3820, 736.3320, 740.2330, 743.2514, 748.2947, 750.2650, 756.4777, 764.3574, 765.3959, 768.3784, 772.4155, 774.3952, 778.4551, 780.3343, 782.3223, 783.3115, 787.2390, 791.2662, 802.4609, 805.3347, 806.4342, 807.3921, 812.4002, 814.4128, 820.4252, 828.3922, 830.3668, 834.3623, 837.3794, 840.3857, 842.5100, 845.4155, 851.3846, 856.5103, 865.3634, 869.4703, 870.5254, 871.4965, 872.6990, 888.3988, 891.4706, 893.9500, 894.4534, 897.0277, 905.4637, 911.4314, 912.4475, 913.5080, 913.5100, 918.5267, 925.4804, 927.4970, 930.5015, 933.4662, 935.4675, 943.4757, 951.4646, 952.4799, 957.4673, 961.4901, 962.4866, 967.4440, 969.4496, 972.4781, 974.5051, 975.4521, 977.4406, 978.4772, 979.4924, 983.4706, 985.6071, 991.4533, 993.5267, 995.5177, 999.4602, 1001.4565, 1001.5621, 1003.5043, 1007.5638, 1012.5004, 1014.5331, 1018.5130, 1022.5398, 1023.5525, 1028.5991, 1030.5259, 1035.5314, 1036.5333, 1037.5145, 1040.5127, 1042.5243, 1045.5603, 1060.5398, 1069.4846, 1075.5515, 1092.6041, 1106.5313, 1109.5087, 1111.5457, 1113.5419, 1115.5394, 1118.5659, 1121.5419, 1122.5701, 1131.5602, 1138.5428, 1149.5912, 1155.5768, 1157.5907, 1158.6328, 1163.6145, 1173.6122, 1177.6130, 1178.6060, 1179.5918, 1180.6001, 1181.6368, 1183.6030, 1187.6376, 1192.6014, 1195.5924, 1199.6785, 1206.6066, 1215.6306, 1216.6482, 1226.5839, 1227.6051, 1231.6115, 1232.6251, 1233.6230, 1234.6500, 1235.6155, 1248.6078, 1250.6483, 1254.6526, 1255.6036, 1256.6123, 1268.6208, 1271.6423, 1276.6179, 1277.6350, 1289.6449, 1292.6322, 1294.6362, 1295.6366, 1300.5895, 1303.6643, 1305.6523, 1307.6600, 1308.6443, 1310.6051, 1315.6364, 1320.6128, 1321.6134, 1323.6320, 1325.6456, 1331.6674, 1332.6602, 1333.2076, 1334.6744, 1336.6710, 1337.6279, 1338.6300, 1338.6300, 1345.6146, 1347.6653, 1349.6800, 1349.6827, 1354.6820, 1355.6964, 1359.7053, 1360.6173, 1361.6373, 1365.6752, 1371.6718, 1375.6670, 1377.6776, 1382.6367, 1383.6619, 1387.6700, 1393.6727, 1400.6235, 1409.7107, 1411.6742, 1427.7725, 1431.7605, 1432.6987, 1434.7190, 1449.7479, 1466.6953, 1473.7905, 1478.7303, 1479.7468, 1485.7531, 1486.7472, 1493.7345, 1495.7502, 1500.7242, 1503.7690, 1505.8511, 1509.6786, 1520.7347, 1522.7239, 1523.7567, 1525.7864, 1527.8068, 1532.7472, 1537.7886, 1539.7711, 1549.7833, 1555.8657, 1558.8440, 1559.8573, 1568.7592, 1570.7814, 1578.7899, 1606.8213, 1615.8425, 1619.7972, 1621.7831, 1628.8156, 1630.8202, 1631.8217, 1639.9263, 1647.8730, 1649.8650, 1650.8523, 1658.8206, 1659.8125, 1661.8284, 1663.7981, 1665.8070, 1669.8174, 1677.8353, 1679.9403, 1680.8076, 1682.7780, 1696.8055, 1697.7845, 1698.7950, 1707.7931, 1709.8029, 1710.8878, 1712.9166, 1716.8563, 1725.8188, 1727.8683, 1730.8236, 1731.8221, 1742.8798, 1746.1482, 1749.8406, 1751.8657, 1756.8782, 1757.8225, 1763.8152, 1765.7922, 1766.8105, 1768.8596, 1769.4275, 1770.4087, 1773.8615, 1774.8795, 1775.0302, 1783.8599, 1785.8483, 1786.8871, 1790.8822, 1792.7894, 1801.9315, 1804.9026, 1808.8663, 1824.8685, 1830.8608, 1849.8231, 1851.8430, 1867.9100, 1868.9441, 1870.9304, 1880.9020, 1884.9191, 1908.8989, 1914.9821, 1916.9882, 1920.9862, 1930.9827, 1932.9813, 1934.9822, 1947.0288, 1958.9543, 1994.8763, 2011.9012, 2013.8700, 2020.9592, 2025.5920, 2042.0568, 2047.0238, 2047.9805, 2049.9497, 2052.9905, 2053.9851, 2058.9988, 2060.0400, 2060.0405, 2065.0427, 2066.0137, 2066.9895, 2067.9763, 2068.9128, 2070.8792, 2074.8860, 2082.0293, 2084.8977, 2086.8872, 2087.8835, 2090.8708, 2092.8674, 2093.8711, 2096.9836, 2100.8787, 2101.8999, 2103.9092, 2107.8730, 2119.9924, 2122.9397, 2136.9133, 2211.1047, 2217.9368, 2225.1243, 2232.0229, 2233.0840, 2238.1050, 2250.0259, 2257.9934, 2262.9368, 2263.9700, 2273.0264, 2277.9500, 2290.9570, 2307.9631, 2321.9558, 2323.9670, 2324.9824, 2335.9763, 2339.9456, 2343.0320, 2349.9844, 2352.0308, 2355.0315, 2358.0854, 2368.0815, 2369.0588, 2378.0020, 2379.0002, 2380.0007, 2381.9963, 2383.0444, 2383.9822, 2393.0020, 2394.0181, 2398.0447, 2400.0771, 2405.0879, 2414.1050, 2417.0933, 2421.0769, 2422.0593, 2426.0476, 2428.0725, 2430.0947, 2432.0962, 2435.0254, 2438.0706, 2444.0901, 2451.0278, 2459.9802, 2461.0254, 2501.2520, 2528.1902, 2543.2092, 2564.1965, 2705.2144, 2717.1655, 2834.2925, 2888.2483, 2904.4053, 3130.4312, 3291.4875, 3312.3765, 3451.6833, 3454.6270

27. P11019 **Mass:** 26180 **Score:** 18 **Expect:** 3.8e+002 **Matches:** 15

V-type proton ATPase subunit E 1 OS=Bos taurus GN=ATP6V1E1 PE=2 SV=1

Observed	Mr(expt)	Mr(calc)	ppm	Start		End	Miss	Ions	Peptide
905.4637	904.4564	904.4614	-5.52	192	-	199	0	---	K.VSNTLESR.L
975.4521	974.4449	974.4419	3.07	53	-	59	0	---	K.IMEYYEK.K
985.6071	984.5998	984.6080	-8.29	45	-	52	1	---	R.LVQTQRLK.I
991.4533	990.4460	990.4368	9.26	53	-	59	0	---	K.IMEYYEK.K + Oxidation (M)
1158.6328	1157.6255	1157.6404	-12.85	61	-	69	2	---	K.EKQIEQQKK.I
1216.6482	1215.6409	1215.6209	16.5	51	-	59	1	---	R.LKIMEYYEK.K
1232.6251	1231.6178	1231.6158	1.63	51	-	59	1	---	R.LKIMEYYEK.K + Oxidation (M)
1305.6523	1304.6450	1304.6329	9.30	70	-	80	0	---	K.IQMSNLMNQAR.L
1321.6134	1320.6061	1320.6278	-16.42	70	-	80	0	---	K.IQMSNLMNQAR.L + Oxidation (M)
1337.6279	1336.6206	1336.6227	-1.57	70	-	80	0	---	K.IQMSNLMNQAR.L + 2 Oxidation (M)
1359.7053	1358.6980	1358.6929	3.77	88	-	99	0	---	R.DDLITDLLNEAK.Q
1449.7479	1448.7406	1448.7228	12.3	69	-	80	1	---	K.KIQMSNLMNQAR.L + Oxidation (M)
1578.7899	1577.7826	1577.8017	-12.12	70	-	82	1	---	K.IQMSNLMNQARLK.V + 2 Oxidation (M)
1947.0288	1946.0215	1946.0553	-17.37	70	-	85	2	---	K.IQMSNLMNQARLKVLR.A + 2 Oxidation (M)
2047.0238	2046.0165	2046.0494	-16.06	34	-	50	2	---	K.AEEEFNIEKGRLVQTQR.L

No match to: 713.4400, 713.4415, 724.3446, 728.2467, 730.2754, 732.2383, 734.2729, 735.3820, 736.3320, 740.2330, 743.2514, 748.2947, 750.2650, 756.4777, 764.3574, 765.3959, 768.3784, 772.4155, 774.3952, 778.4551, 780.3343, 782.3223, 783.3115, 787.2390, 791.2662, 802.4609, 805.3347, 806.4342, 807.3921, 808.4378, 812.4002, 814.4128, 820.4252, 828.3922, 830.3668, 834.3623, 837.3794, 840.3857, 842.5100, 845.4155, 851.3846, 856.5103, 865.3634, 869.4703, 870.5254, 871.4965, 872.6990, 888.3988, 891.4706, 893.9500, 894.4534, 897.0277, 911.4314, 912.4475, 913.5080, 913.5100, 918.5267, 925.4804, 927.4970, 930.5015, 933.4662, 935.4675, 943.4757, 951.4646, 952.4799, 957.4673, 961.4901, 962.4866, 967.4440, 969.4496, 972.4781, 974.5051, 977.4406, 978.4772, 979.4924, 983.4706, 993.5267, 995.5177, 999.4602, 1001.4565, 1001.5621, 1003.5043, 1007.5638, 1012.5004, 1014.5331, 1018.5130, 1022.5398, 1023.5525, 1028.5991, 1030.5259, 1035.5314, 1036.5333, 1037.5145, 1040.5127, 1042.5243, 1045.5603, 1060.5398, 1069.4846, 1075.5515, 1092.6041, 1106.5313, 1109.5087, 1111.5457, 1113.5419, 1115.5394, 1118.5659, 1121.5419, 1122.5701, 1131.5602, 1138.5428, 1149.5912, 1155.5768, 1157.5907, 1163.6145, 1173.6122, 1177.6130, 1178.6060, 1179.5918, 1180.6001, 1181.6368, 1183.6030, 1187.6376, 1192.6014, 1193.5928, 1195.5924, 1199.6785, 1206.6066, 1215.6306, 1226.5839, 1227.6051, 1231.6115, 1233.6230, 1234.6500, 1235.6155, 1248.6078, 1250.6483, 1254.6526, 1255.6036, 1256.6123, 1268.6208, 1271.6423, 1276.6179, 1277.6350, 1289.6449, 1292.6322, 1294.6362, 1295.6366, 1300.5895, 1303.6643, 1307.6600, 1308.6443, 1310.6051, 1315.6364, 1320.6128, 1323.6320, 1325.6456, 1331.6674, 1332.6602, 1333.2076, 1334.6744, 1336.6710, 1338.6300, 1338.6300, 1345.6146, 1347.6653, 1349.6800, 1349.6827, 1354.6820, 1355.6964, 1360.6173, 1361.6373, 1365.6752, 1371.6718, 1375.6670, 1377.6776, 1382.6367, 1383.6619, 1387.6700, 1393.6727, 1400.6235, 1409.7107, 1411.6742, 1427.7725, 1431.7605, 1432.6987, 1434.7190, 1466.6953, 1473.7905, 1475.7498, 1478.7303, 1479.7468, 1485.7531, 1486.7472, 1493.7345, 1495.7502, 1500.7242, 1503.7690, 1505.8511, 1509.6786, 1520.7347, 1522.7239, 1523.7567, 1525.7864, 1527.8068, 1532.7472, 1537.7886, 1539.7711, 1549.7833, 1555.8657, 1558.8440, 1559.8573, 1568.7592, 1570.7814, 1606.8213, 1615.8425, 1619.7972, 1621.7831, 1628.8156, 1630.8202, 1631.8217, 1639.9263, 1644.8083, 1647.8730, 1649.8650, 1650.8523, 1658.8206, 1659.8125, 1661.8284, 1663.7981, 1665.8070, 1669.8174, 1677.8353, 1679.9403, 1680.8076, 1682.7780, 1696.8055, 1697.7845, 1698.7950, 1707.7931, 1709.8029, 1710.8878, 1712.9166, 1716.8563, 1725.8188, 1727.8683, 1730.8236, 1731.8221, 1742.8798, 1746.1482, 1749.8406, 1751.8657, 1756.8782, 1757.8225, 1763.8152, 1765.7922, 1766.8105, 1768.8596, 1769.4275, 1770.4087, 1773.8615, 1774.8795, 1775.0302, 1783.8599, 1785.8483, 1786.8871, 1790.8822, 1792.7894, 1801.9315, 1804.9026, 1808.8663, 1824.8685, 1830.8608, 1849.8231, 1851.8430, 1867.9100, 1868.9441, 1870.9304, 1880.9020, 1884.9191, 1908.8989, 1914.9821, 1916.9882, 1920.9862, 1930.9827, 1932.9813, 1934.9822, 1958.9543, 1994.8763, 2011.9012, 2013.8700, 2020.9592, 2025.5920, 2042.0568, 2047.9805, 2049.9497, 2052.9905, 2053.9851, 2058.9988, 2060.0400, 2060.0405, 2065.0427, 2066.0137, 2066.9895, 2067.9763, 2068.9128, 2070.8792, 2074.8860, 2082.0293, 2084.8977, 2086.8872, 2087.8835, 2090.8708, 2092.8674, 2093.8711, 2096.9836, 2097.9961,

2100.8787, 2101.8999, 2103.9092, 2107.8730, 2119.9924, 2122.9397, 2136.9133, 2211.1047, 2217.9368, 2225.1243, 2232.0229, 2233.0840, 2238.1050, 2250.0259, 2257.9934, 2262.9368, 2263.9700, 2273.0264, 2277.9500, 2283.1741, 2290.9570, 2307.9631, 2321.9558, 2323.9670, 2324.9824, 2335.9763, 2339.9456, 2343.0320, 2349.9844, 2352.0308, 2355.0315, 2358.0854, 2368.0815, 2369.0588, 2378.0020, 2379.0002, 2380.0007, 2381.9963, 2383.0444, 2383.9822, 2393.0020, 2394.0181, 2398.0447, 2400.0771, 2405.0879, 2414.1050, 2417.0933, 2421.0769, 2422.0593, 2426.0476, 2428.0725, 2430.0947, 2432.0962, 2435.0254, 2438.0706, 2444.0901, 2451.0278, 2459.9802, 2461.0254, 2501.2520, 2528.1902, 2543.2092, 2564.1965, 2705.2144, 2717.1655, 2834.2925, 2888.2483, 2904.4053, 3130.4312, 3291.4875, 3312.3765, 3451.6833, 3454.6270

28. F1MLG8 **Mass:** 9765 **Score:** 18 **Expect:** 3.8e+002 **Matches:** 6
Uncharacterized protein OS=Bos taurus GN=SMIM4 PE=4 SV=2

Observed	Mr(expt)	Mr(calc)	ppm	Start	End	Miss	Ions	Peptide
925.4804	924.4731	924.4930	-21.53	18 -	24	1	---	K.QRFGVYR.F
1037.5145	1036.5072	1036.5050	2.17	58 -	65	1	---	K.ASERQYQR.R
1193.5928	1192.5855	1192.6061	-17.24	58 -	66	2	---	K.ASERQYQRR.L
1354.6820	1353.6747	1353.6976	-16.88	14 -	24	2	---	R.MPGKQRFGVYR.F + Oxidation (M)
1532.7472	1531.7399	1531.7419	-1.32	45 -	56	1	---	R.VGQETFYDVYRR.K
1631.8217	1630.8144	1630.8104	2.49	43 -	55	1	---	K.VRVGQETFYDVYR.R

No match to: 713.4400, 713.4415, 724.3446, 728.2467, 730.2754, 732.2383, 734.2729, 735.3820, 736.3320, 740.2330, 743.2514, 748.2947, 750.2650, 756.4777, 764.3574, 765.3959, 768.3784, 772.4155, 774.3952, 778.4551, 780.3343, 782.3223, 783.3115, 787.2390, 791.2662, 802.4609, 805.3347, 806.4342, 807.3921, 808.4378, 812.4002, 814.4128, 820.4252, 828.3922, 830.3668, 834.3623, 837.3794, 840.3857, 842.5100, 845.4155, 851.3846, 856.5103, 865.3634, 869.4703, 870.5254, 871.4965, 872.6990, 888.3988, 891.4706, 893.9500, 894.4534, 897.0277, 905.4637, 911.4314, 912.4475, 913.5080, 913.5100, 918.5267, 927.4970, 930.5015, 933.4662, 935.4675, 943.4757, 951.4646, 952.4799, 957.4673, 961.4901, 962.4866, 967.4440, 969.4496, 972.4781, 974.5051, 975.4521, 977.4406, 978.4772, 979.4924, 983.4706, 985.6071, 991.4533, 993.5267, 995.5177, 999.4602, 1001.4565, 1001.5621, 1003.5043, 1007.5638, 1012.5004, 1014.5331, 1018.5130, 1022.5398, 1023.5525, 1028.5991, 1030.5259, 1035.5314, 1036.5333, 1040.5127, 1042.5243, 1045.5603, 1060.5398, 1069.4846, 1075.5515, 1092.6041, 1106.5313, 1109.5087, 1111.5457, 1113.5419, 1115.5394, 1118.5659, 1121.5419, 1122.5701, 1131.5602, 1138.5428, 1149.5912, 1155.5768, 1157.5907, 1158.6328, 1163.6145, 1173.6122, 1177.6130, 1178.6060, 1179.5918, 1180.6001, 1181.6368, 1183.6030, 1187.6376, 1192.6014, 1195.5924, 1199.6785, 1206.6066, 1215.6306, 1216.6482, 1226.5839, 1227.6051, 1231.6115, 1232.6251, 1233.6230, 1234.6500, 1235.6155, 1248.6078, 1250.6483, 1254.6526, 1255.6036, 1256.6123, 1268.6208, 1271.6423, 1276.6179, 1277.6350, 1289.6449, 1292.6322, 1294.6362, 1295.6366, 1300.5895, 1303.6643, 1305.6523, 1307.6600, 1308.6443, 1310.6051, 1315.6364, 1320.6128, 1321.6134, 1323.6320, 1325.6456, 1331.6674, 1332.6602, 1333.2076, 1334.6744, 1336.6710, 1337.6279, 1338.6300, 1338.6300, 1345.6146, 1347.6653, 1349.6800, 1349.6827, 1355.6964, 1359.7053, 1360.6173, 1361.6373, 1365.6752, 1371.6718, 1375.6670, 1377.6776, 1382.6367, 1383.6619, 1387.6700, 1393.6727, 1400.6235, 1409.7107, 1411.6742, 1427.7725, 1431.7605, 1432.6987, 1434.7190, 1449.7479, 1466.6953, 1473.7905, 1475.7498, 1478.7303, 1479.7468, 1485.7531, 1486.7472, 1493.7345, 1495.7502, 1500.7242, 1503.7690, 1505.8511, 1509.6786, 1520.7347, 1522.7239, 1523.7567, 1525.7864, 1527.8068, 1537.7886, 1539.7711, 1549.7833, 1555.8657, 1558.8440, 1559.8573, 1568.7592, 1570.7814, 1578.7899, 1606.8213, 1615.8425, 1619.7972, 1621.7831, 1628.8156, 1630.8202, 1639.9263, 1644.8083, 1647.8730, 1649.8650, 1650.8523, 1658.8206, 1659.8125, 1661.8284, 1663.7981, 1665.8070, 1669.8174, 1677.8353, 1679.9403, 1680.8076, 1682.7780, 1696.8055, 1697.7845, 1698.7950, 1707.7931, 1709.8029, 1710.8878, 1712.9166, 1716.8563, 1725.8188, 1727.8683, 1730.8236, 1731.8221, 1742.8798, 1746.1482, 1749.8406, 1751.8657, 1756.8782, 1757.8225, 1763.8152, 1765.7922, 1766.8105, 1768.8596, 1769.4275, 1770.4087, 1773.8615, 1774.8795, 1775.0302, 1783.8599, 1785.8483, 1786.8871, 1790.8822, 1792.7894, 1801.9315, 1804.9026, 1808.8663, 1824.8685, 1830.8608, 1849.8231, 1851.8430, 1867.9100, 1868.9441, 1870.9304, 1880.9020, 1884.9191, 1908.8989, 1914.9821, 1916.9882, 1920.9862, 1930.9827, 1932.9813, 1934.9822, 1947.0288, 1958.9543, 1994.8763, 2011.9012, 2013.8700, 2020.9592, 2025.5920, 2042.0568, 2047.0238, 2047.9805, 2049.9497, 2052.9905, 2053.9851, 2058.9988, 2060.0400, 2060.0405, 2065.0427, 2066.0137, 2066.9895, 2067.9763, 2068.9128, 2070.8792, 2074.8860, 2082.0293, 2084.8977, 2086.8872, 2087.8835, 2090.8708, 2092.8674, 2093.8711, 2096.9836, 2097.9961, 2100.8787, 2101.8999, 2103.9092, 2107.8730, 2119.9924, 2122.9397, 2136.9133, 2211.1047, 2217.9368, 2225.1243, 2232.0229, 2233.0840, 2238.1050, 2250.0259, 2257.9934, 2262.9368, 2263.9700, 2273.0264, 2277.9500, 2283.1741, 2290.9570, 2307.9631, 2321.9558, 2323.9670, 2324.9824, 2335.9763, 2339.9456, 2343.0320, 2349.9844, 2352.0308, 2355.0315, 2358.0854, 2368.0815, 2369.0588, 2378.0020, 2379.0002, 2380.0007, 2381.9963, 2383.0444, 2383.9822, 2393.0020, 2394.0181, 2398.0447, 2400.0771, 2405.0879, 2414.1050, 2417.0933, 2421.0769, 2422.0593, 2426.0476, 2428.0725, 2430.0947, 2432.0962, 2435.0254, 2438.0706, 2444.0901, 2451.0278, 2459.9802, 2461.0254, 2501.2520, 2528.1902, 2543.2092, 2564.1965, 2705.2144, 2717.1655, 2834.2925, 2888.2483, 2904.4053, 3130.4312, 3291.4875, 3312.3765, 3451.6833, 3454.6270

29. E1BEC7 **Mass:** 19673 **Score:** 18 **Expect:** 3.9e+002 **Matches:** 10
Mimitin, mitochondrial OS=Bos taurus GN=NDUFAF2 PE=4 SV=2

Observed	Mr(expt)	Mr(calc)	ppm	Start	End	Miss	Ions	Peptide
764.3574	763.3501	763.3435	8.66	1 -	6	0	---	-.MGWSQR.L
780.3343	779.3271	779.3385	-14.60	1 -	6	0	---	-.MGWSQR.L + Oxidation (M)
1109.5087	1108.5014	1108.5005	0.84	94 -	101	1	---	K.CREEIQMK.S + Oxidation (M)
1138.5428	1137.5355	1137.5382	-2.37	32 -	39	0	---	K.YYYIPEYK.N
1320.6128	1319.6055	1319.6101	-3.47	80 -	90	0	---	R.QTPPTMEEIMK.N + Oxidation (M)
1449.7479	1448.7406	1448.7711	-21.02	1 -	12	1	---	-.MGWSQRLFGVIR.R
1559.8573	1558.8500	1558.8315	11.9	14 -	27	2	---	R.ALSKEVKEQVGTDR.F
1606.8213	1605.8140	1605.8111	1.83	18 -	31	2	---	K.EVKEQVGTDRFGNK.Y
1707.7931	1706.7858	1706.7855	0.19	80 -	93	1	---	R.QTPPTMEEIMKNEK.C + 2 Oxidation (M)
1932.9813	1931.9740	1931.9444	15.3	78 -	93	2	---	K.TRQTPPTMEEIMKNEK.C

No match to: 713.4400, 713.4415, 724.3446, 728.2467, 730.2754, 732.2383, 734.2729, 735.3820, 736.3320, 740.2330, 743.2514, 748.2947, 750.2650, 756.4777, 765.3959, 768.3784, 772.4155, 774.3952, 778.4551, 782.3223, 783.3115, 787.2390, 791.2662, 802.4609, 805.3347, 806.4342, 807.3921, 808.4378, 812.4002, 814.4128, 820.4252, 828.3922, 830.3668, 834.3623, 837.3794, 840.3857, 842.5100, 845.4155, 851.3846, 856.5103, 865.3634, 869.4703, 870.5254, 871.4965, 872.6990, 888.3988, 891.4706, 893.9500, 894.4534, 897.0277, 905.4637, 911.4314, 912.4475, 913.5080, 913.5100, 918.5267, 925.4804, 927.4970, 930.5015, 933.4662, 935.4675, 943.4757, 951.4646, 952.4799, 957.4673, 961.4901, 962.4866, 967.4440, 969.4496, 972.4781, 974.5051, 975.4521, 977.4406, 978.4772, 979.4924, 983.4706, 985.6071, 991.4533, 993.5267, 995.5177, 999.4602, 1001.4565, 1001.5621, 1003.5043, 1007.5638, 1012.5004, 1014.5331, 1018.5130, 1022.5398, 1023.5525, 1028.5991, 1030.5259, 1035.5314, 1036.5333, 1037.5145, 1040.5127, 1042.5243, 1045.5603, 1060.5398, 1069.4846, 1075.5515, 1092.6041, 1106.5313, 1111.5457, 1113.5419, 1115.5394, 1118.5659, 1121.5419, 1122.5701, 1131.5602, 1149.5912, 1155.5768, 1157.5907, 1158.6328, 1163.6145, 1173.6122, 1177.6130, 1178.6060, 1179.5918, 1180.6001, 1181.6368, 1183.6030, 1187.6376, 1192.6014, 1193.5928, 1195.5924, 1199.6785, 1206.6066, 1215.6306, 1216.6482, 1226.5839, 1227.6051, 1231.6115, 1232.6251, 1233.6230, 1234.6500, 1235.6155, 1248.6078, 1250.6483, 1254.6526, 1255.6036, 1256.6123, 1268.6208, 1271.6423, 1276.6179, 1277.6350, 1289.6449, 1292.6322, 1294.6362, 1295.6366, 1300.5895, 1303.6643, 1305.6523, 1307.6600, 1308.6443, 1310.6051, 1315.6364, 1321.6134, 1323.6320, 1325.6456, 1331.6674, 1332.6602, 1333.2076, 1334.6744, 1336.6710, 1337.6279, 1338.6300, 1338.6300, 1345.6146, 1347.6653, 1349.6800, 1349.6827, 1354.6820, 1355.6964, 1359.7053, 1360.6173, 1361.6373, 1365.6752, 1371.6718, 1375.6670, 1377.6776, 1382.6367, 1383.6619, 1387.6700, 1393.6727, 1400.6235, 1409.7107, 1411.6742, 1427.7725, 1431.7605, 1432.6987, 1434.7190, 1466.6953, 1473.7905, 1475.7498, 1478.7303, 1479.7468, 1485.7531, 1486.7472, 1493.7345, 1495.7502, 1500.7242, 1503.7690, 1505.8511, 1509.6786, 1520.7347, 1522.7239, 1523.7567, 1525.7864, 1527.8068, 1532.7472, 1537.7886, 1539.7711, 1549.7833, 1555.8657, 1558.8440, 1568.7592, 1570.7814, 1578.7899, 1615.8425, 1619.7972, 1621.7831, 1628.8156, 1630.8202, 1631.8217, 1639.9263, 1644.8083, 1647.8730, 1649.8650, 1650.8523, 1658.8206, 1659.8125, 1661.8284, 1663.7981, 1665.8070, 1669.8174, 1677.8353, 1679.9403, 1680.8076, 1682.7780, 1696.8055, 1697.7845, 1698.7950, 1709.8029, 1710.8878, 1712.9166, 1716.8563, 1725.8188, 1727.8683, 1730.8236, 1731.8221, 1742.8798, 1746.1482, 1749.8406, 1751.8657, 1756.8782, 1757.8225, 1763.8152, 1765.7922, 1766.8105, 1768.8596, 1769.4275, 1770.4087, 1773.8615, 1774.8795, 1775.0302, 1783.8599, 1785.8483, 1786.8871, 1790.8822, 1792.7894, 1801.9315, 1804.9026, 1808.8663, 1824.8685, 1830.8608, 1849.8231, 1851.8430, 1867.9100, 1868.9441, 1870.9304, 1880.9020, 1884.9191, 1908.8989, 1914.9821, 1916.9882, 1920.9862, 1930.9827, 1934.9822, 1947.0288, 1958.9543, 1994.8763, 2011.9012, 2013.8700, 2020.9592, 2025.5920, 2042.0568, 2047.0238, 2047.9805, 2049.9497, 2052.9905, 2053.9851, 2058.9988, 2060.0400, 2060.0405, 2065.0427, 2066.0137, 2066.9895, 2067.9763, 2068.9128, 2070.8792, 2074.8860, 2082.0293, 2084.8977, 2086.8872, 2087.8835, 2090.8708, 2092.8674, 2093.8711, 2096.9836, 2097.9961, 2100.8787, 2101.8999, 2103.9092, 2107.8730, 2119.9924, 2122.9397, 2136.9133, 2211.1047, 2217.9368, 2225.1243, 2232.0229, 2233.0840, 2238.1050, 2250.0259, 2257.9934, 2262.9368, 2263.9700, 2273.0264, 2277.9500, 2283.1741, 2290.9570, 2307.9631, 2321.9558, 2323.9670, 2324.9824, 2335.9763, 2339.9456, 2343.0320, 2349.9844, 2352.0308, 2355.0315, 2358.0854, 2368.0815, 2369.0588, 2378.0020, 2379.0002, 2380.0007, 2381.9963, 2383.0444, 2383.9822, 2393.0020, 2394.0181, 2398.0447, 2400.0771, 2405.0879, 2414.1050, 2417.0933, 2421.0769, 2422.0593, 2426.0476, 2428.0725, 2430.0947, 2432.0962, 2435.0254, 2438.0706,

2444.0901, 2451.0278, 2459.9802, 2461.0254, 2501.2520, 2528.1902, 2543.2092, 2564.1965, 2705.2144, 2717.1655, 2834.2925, 2888.2483, 2904.4053, 3130.4312, 3291.4875, 3312.3765, 3451.6833, 3454.6270

30. A6QLN3 **Mass:** 7445 **Score:** 18 **Expect:** 4.1e+002 **Matches:** 6

FXYD2 protein OS=Bos taurus GN=FXYD2 PE=2 SV=1

Observed	Mr(expt)	Mr(calc)	ppm	Start	End	Miss	Ions	Peptide
894.4534	893.4461	893.4654	-21.58	48 -	54	2	---	R.FRCGAKR.Q
1012.5004	1011.4931	1011.5145	-21.08	50 -	57	2	---	R.CGAKRQHR.Q
1178.6060	1177.5987	1177.5880	9.11	2 -	11	1	---	M.DRWYLGGSPK.E
1192.6014	1191.5941	1191.5996	-4.62	55 -	64	1	---	R.QHRQIPEDGL.-
1325.6456	1324.6383	1324.6234	11.3	1 -	11	1	---	-.MDRWYLGGSPK.E + Oxidation (M)
3130.4312	3129.4239	3129.3814	13.6	1 -	25	2	---	-.MDRWYLGGSPKENEDPFYYDYETVR.N

No match to: 713.4400, 713.4415, 724.3446, 728.2467, 730.2754, 732.2383, 734.2729, 735.3820, 736.3320, 740.2330, 743.2514, 748.2947, 750.2650, 756.4777, 764.3574, 765.3959, 768.3784, 772.4155, 774.3952, 778.4551, 780.3343, 782.3223, 783.3115, 787.2390, 791.2662, 802.4609, 805.3347, 806.4342, 807.3921, 808.4378, 812.4002, 814.4128, 820.4252, 828.3922, 830.3668, 834.3623, 837.3794, 840.3857, 842.5100, 845.4155, 851.3846, 856.5103, 865.3634, 869.4703, 870.5254, 871.4965, 872.6990, 888.3988, 891.4706, 893.9500, 897.0277, 905.4637, 911.4314, 912.4475, 913.5080, 913.5100, 918.5267, 925.4804, 927.4970, 930.5015, 933.4662, 935.4675, 943.4757, 951.4646, 952.4799, 957.4673, 961.4901, 962.4866, 967.4440, 969.4496, 972.4781, 974.5051, 975.4521, 977.4406, 978.4772, 979.4924, 983.4706, 985.6071, 991.4533, 993.5267, 995.5177, 999.4602, 1001.4565, 1001.5621, 1003.5043, 1007.5638, 1014.5331, 1018.5130, 1022.5398, 1023.5525, 1028.5991, 1030.5259, 1035.5314, 1036.5333, 1037.5145, 1040.5127, 1042.5243, 1045.5603, 1060.5398, 1069.4846, 1075.5515, 1092.6041, 1106.5313, 1109.5087, 1111.5457, 1113.5419, 1115.5394, 1118.5659, 1121.5419, 1122.5701, 1131.5602, 1138.5428, 1149.5912, 1155.5768, 1157.5907, 1158.6328, 1163.6145, 1173.6122, 1177.6130, 1179.5918, 1180.6001, 1181.6368, 1183.6030, 1187.6376, 1193.5928, 1195.5924, 1199.6785, 1206.6066, 1215.6306, 1216.6482, 1226.5839, 1227.6051, 1231.6115, 1232.6251, 1233.6230, 1234.6500, 1235.6155, 1248.6078, 1250.6483, 1254.6526, 1255.6036, 1256.6123, 1268.6208, 1271.6423, 1276.6179, 1277.6350, 1289.6449, 1292.6322, 1294.6362, 1295.6366, 1300.5895, 1303.6643, 1305.6523, 1307.6600, 1308.6443, 1310.6051, 1315.6364, 1320.6128, 1321.6134, 1323.6320, 1331.6674, 1332.6602, 1333.2076, 1334.6744, 1336.6710, 1337.6279, 1338.6300, 1338.6300, 1345.6146, 1347.6653, 1349.6800, 1349.6827, 1354.6820, 1355.6964, 1359.7053, 1360.6173, 1361.6373, 1365.6752, 1371.6718, 1375.6670, 1377.6776, 1382.6367, 1383.6619, 1387.6700, 1393.6727, 1400.6235, 1409.7107, 1411.6742, 1427.7725, 1431.7605, 1432.6987, 1434.7190, 1449.7479, 1466.6953, 1473.7905, 1475.7498, 1478.7303, 1479.7468, 1485.7531, 1486.7472, 1493.7345, 1495.7502, 1500.7242, 1503.7690, 1505.8511, 1509.6786, 1520.7347, 1522.7239, 1523.7567, 1525.7864, 1527.8068, 1532.7472, 1537.7886, 1539.7711, 1549.7833, 1555.8657, 1558.8440, 1559.8573, 1568.7592, 1570.7814, 1578.7899, 1606.8213, 1615.8425, 1619.7972, 1621.7831, 1628.8156, 1630.8202, 1631.8217, 1639.9263, 1644.8083, 1647.8730, 1649.8650, 1650.8523, 1658.8206, 1659.8125, 1661.8284, 1663.7981, 1665.8070, 1669.8174, 1677.8353, 1679.9403, 1680.8076, 1682.7780, 1696.8055, 1697.7845, 1698.7950, 1707.7931, 1709.8029, 1710.8878, 1712.9166, 1716.8563, 1725.8188, 1727.8683, 1730.8236, 1731.8221, 1742.8798, 1746.1482, 1749.8406, 1751.8657, 1756.8782, 1757.8225, 1763.8152, 1765.7922, 1766.8105, 1768.8596, 1769.4275, 1770.4087, 1773.8615, 1774.8795, 1775.0302, 1783.8599, 1785.8483, 1786.8871, 1790.8822, 1792.7894, 1801.9315, 1804.9026, 1808.8663, 1824.8685, 1830.8608, 1849.8231, 1851.8430, 1867.9100, 1868.9441, 1870.9304, 1880.9020, 1884.9191, 1908.8989, 1914.9821, 1916.9882, 1920.9862, 1930.9827, 1932.9813, 1934.9822, 1947.0288, 1958.9543, 1994.8763, 2011.9012, 2013.8700, 2020.9592, 2025.5920, 2042.0568, 2047.0238, 2047.9805, 2049.9497, 2052.9905, 2053.9851, 2058.9988, 2060.0400, 2060.0405, 2065.0427, 2066.0137, 2066.9895, 2067.9763, 2068.9128, 2070.8792, 2074.8860, 2082.0293, 2084.8977, 2086.8872, 2087.8835, 2090.8708, 2092.8674, 2093.8711, 2096.9836, 2097.9961, 2100.8787, 2101.8999, 2103.9092, 2107.8730, 2119.9924, 2122.9397, 2136.9133, 2211.1047, 2217.9368, 2225.1243, 2232.0229, 2233.0840, 2238.1050, 2250.0259, 2257.9934, 2262.9368, 2263.9700, 2273.0264, 2277.9500, 2283.1741, 2290.9570, 2307.9631, 2321.9558, 2323.9670, 2324.9824, 2335.9763, 2339.9456, 2343.0320, 2349.9844, 2352.0308, 2355.0315, 2358.0854, 2368.0815, 2369.0588, 2378.0020, 2379.0002, 2380.0007, 2381.9963, 2383.0444, 2383.9822, 2393.0020, 2394.0181, 2398.0447, 2400.0771, 2405.0879, 2414.1050, 2417.0933, 2421.0769, 2422.0593, 2426.0476, 2428.0725, 2430.0947, 2432.0962, 2435.0254, 2438.0706, 2444.0901, 2451.0278, 2459.9802, 2461.0254, 2501.2520, 2528.1902, 2543.2092, 2564.1965, 2705.2144, 2717.1655, 2834.2925, 2888.2483, 2904.4053, 3291.4875, 3312.3765, 3451.6833, 3454.6270

31. F1MIT2 **Mass:** 31839 **Score:** 18 **Expect:** 4.2e+002 **Matches:** 13

Transcription elongation factor A protein 1 OS=Bos taurus GN=TCEA1 PE=4 SV=1

Observed	Mr(expt)	Mr(calc)	ppm	Start	End	Miss	Ions	Peptide
927.4970	926.4897	926.4933	-3.90	179 -	186	1	---	K.DAKNPNLR.K
1206.6066	1205.5993	1205.6009	-1.27	123 -	132	1	---	K.CREMLAAALR.T + Oxidation (M)
1307.6600	1306.6527	1306.6252	21.0	35 -	46	0	---	K.QSTDEEVTSLAK.S
1375.6670	1374.6597	1374.6375	16.1	71 -	83	0	---	K.DTAVTSQNSPEAR.E
1431.7605	1430.7532	1430.7850	-22.18	121 -	132	2	---	R.LKCREMLAAALR.T
1503.7690	1502.7617	1502.7325	19.5	70 -	83	1	---	K.KDTAVTSQNSPEAR.E
1631.8217	1630.8144	1630.8274	-7.98	69 -	83	2	---	K.KKDTAVTSQNSPEAR.E
1661.8284	1660.8211	1660.8155	3.36	55 -	69	2	---	K.LLDGPSTDKDSEEKK.K
1757.8225	1756.8152	1756.8016	7.73	97 -	111	1	---	K.DETNARDTYVSSFPR.A
2086.8872	2085.8799	2085.8554	11.7	259 -	276	0	---	R.SADEPMTTFVVCNECGNR.W
2087.8835	2086.8762	2086.8821	-2.82	84 -	102	2	---	R.EESSSSGNMSSRKDETNAR.D + Oxidation (M)
2398.0447	2397.0374	2397.0876	-20.91	226 -	246	2	---	R.EHQMAKTGGTQTDLFTCGKCK.K
2414.1050	2413.0977	2413.0825	6.32	226 -	246	2	---	R.EHQMAKTGGTQTDLFTCGKCK.K + Oxidation (M)

No match to: 713.4400, 713.4415, 724.3446, 728.2467, 730.2754, 732.2383, 734.2729, 735.3820, 736.3320, 740.2330, 743.2514, 748.2947, 750.2650, 756.4777, 764.3574, 765.3959, 768.3784, 772.4155, 774.3952, 778.4551, 780.3343, 782.3223, 783.3115, 787.2390, 791.2662, 802.4609, 805.3347, 806.4342, 807.3921, 808.4378, 812.4002, 814.4128, 820.4252, 828.3922, 830.3668, 834.3623, 837.3794, 840.3857, 842.5100, 845.4155, 851.3846, 856.5103, 865.3634, 869.4703, 870.5254, 871.4965, 872.6990, 888.3988, 891.4706, 893.9500, 894.4534, 897.0277, 905.4637, 911.4314, 912.4475, 913.5080, 913.5100, 918.5267, 925.4804, 930.5015, 933.4662, 935.4675, 943.4757, 951.4646, 952.4799, 957.4673, 961.4901, 962.4866, 967.4440, 969.4496, 972.4781, 974.5051, 975.4521, 977.4406, 978.4772, 979.4924, 983.4706, 985.6071, 991.4533, 993.5267, 995.5177, 999.4602, 1001.4565, 1001.5621, 1003.5043, 1007.5638, 1012.5004, 1014.5331, 1018.5130, 1022.5398, 1023.5525, 1028.5991, 1030.5259, 1035.5314, 1036.5333, 1037.5145, 1040.5127, 1042.5243, 1045.5603, 1060.5398, 1069.4846, 1075.5515, 1092.6041, 1106.5313, 1109.5087, 1111.5457, 1113.5419, 1115.5394, 1118.5659, 1121.5419, 1122.5701, 1131.5602, 1138.5428, 1149.5912, 1155.5768, 1157.5907, 1158.6328, 1163.6145, 1173.6122, 1177.6130, 1178.6060, 1179.5918, 1180.6001, 1181.6368, 1183.6030, 1187.6376, 1192.6014, 1193.5928, 1195.5924, 1199.6785, 1215.6306, 1216.6482, 1226.5839, 1227.6051, 1231.6115, 1232.6251, 1233.6230, 1234.6500, 1235.6155, 1248.6078, 1250.6483, 1254.6526, 1255.6036, 1256.6123, 1268.6208, 1271.6423, 1276.6179, 1277.6350, 1289.6449, 1292.6322, 1294.6362, 1295.6366, 1300.5895, 1303.6643, 1305.6523, 1308.6443, 1310.6051, 1315.6364, 1320.6128, 1321.6134, 1323.6320, 1325.6456, 1331.6674, 1332.6602, 1333.2076, 1334.6744, 1336.6710, 1337.6279, 1338.6300, 1338.6300, 1345.6146, 1347.6653, 1349.6800, 1349.6827, 1354.6820, 1355.6964, 1359.7053, 1360.6173, 1361.6373, 1365.6752, 1371.6718, 1377.6776, 1382.6367, 1383.6619, 1387.6700, 1393.6727, 1400.6235, 1409.7107, 1411.6742, 1427.7725, 1432.6987, 1434.7190, 1449.7479, 1466.6953, 1473.7905, 1475.7498, 1478.7303, 1479.7468, 1485.7531, 1486.7472, 1493.7345, 1495.7502, 1500.7242, 1505.8511, 1509.6786, 1520.7347, 1522.7239, 1523.7567, 1525.7864, 1527.8068, 1532.7472, 1537.7886, 1539.7711, 1549.7833, 1555.8657, 1558.8440, 1559.8573, 1568.7592, 1570.7814, 1578.7899, 1606.8213, 1615.8425, 1619.7972, 1621.7831, 1628.8156, 1630.8202, 1639.9263, 1644.8083, 1647.8730, 1649.8650, 1650.8523, 1658.8206, 1659.8125, 1663.7981, 1665.8070, 1669.8174, 1677.8353, 1679.9403, 1680.8076, 1682.7780, 1696.8055, 1697.7845, 1698.7950, 1707.7931, 1709.8029, 1710.8878, 1712.9166, 1716.8563, 1725.8188, 1727.8683, 1730.8236, 1731.8221, 1742.8798, 1746.1482, 1749.8406, 1751.8657, 1756.8782, 1763.8152, 1765.7922, 1766.8105, 1768.8596, 1769.4275, 1770.4087, 1773.8615, 1774.8795, 1775.0302, 1783.8599, 1785.8483, 1786.8871, 1790.8822, 1792.7894, 1801.9315, 1804.9026, 1808.8663, 1824.8685, 1830.8608, 1849.8231, 1851.8430, 1867.9100, 1868.9441, 1870.9304, 1880.9020, 1884.9191, 1908.8989, 1914.9821, 1916.9882, 1920.9862, 1930.9827, 1932.9813, 1934.9822, 1947.0288, 1958.9543, 1994.8763, 2011.9012, 2013.8700, 2020.9592, 2025.5920, 2042.0568, 2047.0238, 2047.9805, 2049.9497, 2052.9905, 2053.9851, 2058.9988, 2060.0400, 2060.0405, 2065.0427, 2066.0137, 2066.9895, 2067.9763, 2068.9128, 2070.8792, 2074.8860, 2082.0293, 2084.8977, 2090.8708, 2092.8674, 2093.8711, 2096.9836, 2097.9961, 2100.8787, 2101.8999, 2103.9092, 2107.8730, 2119.9924, 2122.9397, 2136.9133, 2211.1047, 2217.9368, 2225.1243, 2232.0229, 2233.0840, 2238.1050, 2250.0259, 2257.9934, 2262.9368, 2263.9700, 2273.0264, 2277.9500, 2283.1741, 2290.9570, 2307.9631, 2321.9558, 2323.9670, 2324.9824, 2335.9763, 2339.9456, 2343.0320, 2349.9844, 2352.0308, 2355.0315, 2358.0854, 2368.0815, 2369.0588, 2378.0020, 2379.0002, 2380.0007, 2381.9963, 2383.0444, 2383.9822, 2393.0020, 2394.0181, 2400.0771, 2405.0879, 2417.0933, 2421.0769, 2422.0593, 2426.0476, 2428.0725, 2430.0947, 2432.0962, 2435.0254, 2438.0706, 2444.0901, 2451.0278, 2459.9802, 2461.0254, 2501.2520, 2528.1902, 2543.2092, 2564.1965, 2705.2144, 2717.1655, 2834.2925, 2888.2483, 2904.4053, 3130.4312, 3291.4875, 3312.3765, 3451.6833, 3454.6270

32. E1BLN1 **Mass:** 117436 **Score:** 18 **Expect:** 4.2e+002 **Matches:** 34

Uncharacterized protein OS=Bos taurus GN=SYCP1 PE=4 SV=1

Observed	Mr(expt)	Mr(calc)	ppm	Start	End	Miss	Ions	Peptide
933.4662	932.4590	932.4749	-17.10	556	562	1	---	R.QEERMLK.Q
985.6071	984.5998	984.6080	-8.25	140	147	2	---	R.KIIEAQRK.A
1001.5621	1000.5548	1000.5440	10.8	669	677	0	---	K.LEIELEGAK.Q
1030.5259	1029.5186	1029.5203	-1.61	132	139	1	---	K.ENKLQENR.K
1045.5603	1044.5530	1044.5790	-24.86	238	245	1	---	R.LEMHFKLK.E
1060.5398	1059.5325	1059.5560	-22.14	795	802	2	---	R.EEKEELKR.E
1069.4846	1068.4773	1068.4645	12.0	417	425	0	---	K.SSELEEMTK.L + Oxidation (M)
1158.6328	1157.6255	1157.6152	8.89	132	140	2	---	K.ENKLQENRK.I
1173.6122	1172.6049	1172.6149	-8.52	172	181	1	---	K.DLIKENNATR.H
1192.6014	1191.5941	1191.6169	-19.11	803	812	1	---	R.EMKENTVTLK.G
1199.6785	1198.6712	1198.6921	-17.43	710	720	1	---	K.VVADEAVKLQK.E
1231.6115	1230.6042	1230.5952	7.30	546	555	1	---	K.QQEDINNSKR.Q
1234.6500	1233.6427	1233.6605	-14.38	453	462	1	---	K.QFEKIAEELK.E
1276.6179	1275.6106	1275.6418	-24.48	594	604	2	---	K.SKLDKSEENAR.S
1277.6350	1276.6277	1276.6299	-1.70	637	646	0	---	K.FIEELQQENK.A
1294.6362	1293.6289	1293.6486	-15.19	417	427	1	---	K.SSELEEMTKLK.N
1295.6366	1294.6293	1294.6266	2.14	847	858	1	---	R.SFPSANQSRSSK.D
1305.6523	1304.6450	1304.6394	4.30	770	780	0	---	K.ASLETELCNLR.N
1334.6744	1333.6671	1333.6548	9.26	954	966	0	---	K.TPSSLTTPGSMQK.S
1359.7053	1358.6980	1358.6902	5.76	545	555	2	---	K.KQQEDINNSKR.Q
1431.7605	1430.7532	1430.7261	18.9	730	741	1	---	K.IAEMVALMEKHK.H + 2 Oxidation (M)
1478.7303	1477.7230	1477.7446	-14.63	953	966	1	---	K.KTPSSLTTPGSMQK.S + Oxidation (M)
1495.7502	1494.7429	1494.7752	-21.58	283	294	1	---	K.MKELTFLLEESR.N
1650.8523	1649.8450	1649.8294	9.48	417	430	2	---	K.SSELEEMTKLKNNK.E
1663.7981	1662.7908	1662.7883	1.55	599	612	1	---	K.SEENARSIECEVIK.K
1669.8174	1668.8101	1668.8318	-13.00	789	801	2	---	K.HLETEREEKEELK.R
1725.8188	1724.8115	1724.8443	-19.01	238	250	2	---	R.LEMHFKLKEDYEK.I + Oxidation (M)
1773.8615	1772.8542	1772.8401	7.97	546	559	2	---	K.QQEDINNSKRQEER.M
1804.9026	1803.8953	1803.9400	-24.79	950	966	2	---	R.TPKKTPSSLTTPGSMQK.S + Oxidation (M)
1947.0288	1946.0215	1946.0619	-20.73	383	398	2	---	K.TTICNLKKLLTTEQQR.L
2058.9988	2057.9915	2058.0051	-6.62	318	334	1	---	K.QDHLTSELEDIKMSLQR.S + Oxidation (M)
2060.0400	2059.0327	2059.0797	-22.80	391	407	2	---	K.LLTTEQQRLEKSEDELK.I
2060.0405	2059.0332	2059.0797	-22.55	391	407	2	---	K.LLTTEQQRLEKSEDELK.I
2066.9895	2065.9822	2066.0136	-15.19	954	972	2	---	K.TPSSLTTPGSMQKSETMRK.T

No match to: 713.4400, 713.4415, 724.3446, 728.2467, 730.2754, 732.2383, 734.2729, 735.3820, 736.3320, 740.2330, 743.2514, 748.2947, 750.2650, 756.4777, 764.3574, 765.3959, 768.3784, 772.4155, 774.3952, 778.4551, 780.3343, 782.3223, 783.3115, 787.2390, 791.2662, 802.4609, 805.3347, 806.4342, 807.3921, 808.4378, 812.4002, 814.4128, 820.4252, 828.3922, 830.3668, 834.3623, 837.3794, 840.3857, 842.5100, 845.4155, 851.3846, 856.5103, 865.3634, 869.4703, 870.5254, 871.4965, 872.6990, 888.3988, 891.4706, 893.9500, 894.4534, 897.0277, 905.4637, 911.4314, 912.4475, 913.5080, 913.5100, 918.5267, 925.4804, 927.4970, 930.5015, 935.4675, 943.4757, 951.4646, 952.4799, 957.4673, 961.4901, 962.4866, 967.4440, 969.4496, 972.4781, 974.5051, 975.4521, 977.4406, 978.4772, 979.4924, 983.4706, 991.4533, 993.5267, 995.5177, 999.4602, 1001.4565, 1003.5043, 1007.5638, 1012.5004, 1014.5331, 1018.5130, 1022.5398, 1023.5525, 1028.5991, 1035.5314, 1036.5333, 1037.5145, 1040.5127, 1042.5243, 1075.5515, 1092.6041, 1106.5313, 1109.5087, 1111.5457, 1113.5419, 1115.5394, 1118.5659, 1121.5419, 1122.5701, 1131.5602, 1138.5428, 1149.5912, 1155.5768, 1157.5907, 1163.6145, 1177.6130, 1178.6060, 1179.5918, 1180.6001, 1181.6368, 1183.6030, 1187.6376, 1193.5928, 1195.5924, 1206.6066, 1215.6306, 1216.6482, 1226.5839, 1227.6051, 1232.6251, 1233.6230, 1235.6155, 1248.6078, 1250.6483, 1254.6526, 1255.6036, 1256.6123, 1268.6208, 1271.6423, 1289.6449, 1292.6322, 1300.5895, 1303.6643, 1307.6600, 1308.6443, 1310.6051, 1315.6364, 1320.6128, 1321.6134, 1323.6320, 1325.6456, 1331.6674, 1332.6602, 1333.2076, 1336.6710, 1337.6279, 1338.6300, 1338.6300, 1345.6146, 1347.6653, 1349.6800, 1349.6827, 1354.6820, 1355.6964, 1360.6173, 1361.6373, 1365.6752, 1371.6718, 1375.6670, 1377.6776, 1382.6367, 1383.6619, 1387.6700, 1393.6727, 1400.6235, 1409.7107, 1411.6742, 1427.7725, 1432.6987, 1434.7190, 1449.7479, 1466.6953, 1473.7905, 1475.7498, 1479.7468, 1485.7531, 1486.7472, 1493.7345, 1500.7242, 1503.7690, 1505.8511, 1509.6786, 1520.7347, 1522.7239, 1523.7567, 1525.7864, 1527.8068, 1532.7472, 1537.7886, 1539.7711, 1549.7833, 1555.8657, 1558.8440, 1559.8573, 1568.7592, 1570.7814, 1578.7899, 1606.8213, 1615.8425, 1619.7972, 1621.7831, 1628.8156, 1630.8202, 1631.8217, 1639.9263, 1644.8083, 1647.8730, 1649.8650, 1658.8206, 1659.8125, 1661.8284, 1665.8070, 1677.8353, 1679.9403, 1680.8076, 1682.7780, 1696.8055, 1697.7845, 1698.7950, 1707.7931, 1709.8029, 1710.8878, 1712.9166, 1716.8563, 1727.8683, 1730.8236, 1731.8221, 1742.8798, 1746.1482, 1749.8406, 1751.8657, 1756.8782, 1757.8225, 1763.8152, 1765.7922, 1766.8105, 1768.8596, 1769.4275, 1770.4087, 1774.8795, 1775.0302, 1783.8599, 1785.8483, 1786.8871, 1790.8822, 1792.7894, 1801.9315, 1808.8663, 1824.8685, 1830.8608, 1849.8231, 1851.8430, 1867.9100, 1868.9441, 1870.9304, 1880.9020, 1884.9191, 1908.8989, 1914.9821, 1916.9882, 1920.9862, 1930.9827, 1932.9813, 1934.9822, 1958.9543, 1994.8763, 2011.9012, 2013.8700, 2020.9592, 2025.5920, 2042.0568, 2047.0238, 2047.9805, 2049.9497, 2052.9905, 2053.9851, 2065.0427, 2066.0137, 2067.9763, 2068.9128, 2070.8792, 2074.8860, 2082.0293, 2084.8977, 2086.8872, 2087.8835, 2090.8708, 2092.8674, 2093.8711, 2096.9836, 2097.9961, 2100.8787, 2101.8999, 2103.9092, 2107.8730, 2119.9924, 2122.9397, 2136.9133, 2211.1047, 2217.9368, 2225.1243, 2232.0229, 2233.0840, 2238.1050, 2250.0259, 2257.9934, 2262.9368, 2263.9700, 2273.0264, 2277.9500, 2283.1741, 2290.9570, 2307.9631, 2321.9558, 2323.9670, 2324.9824, 2335.9763, 2339.9456, 2343.0320, 2349.9844, 2352.0308, 2355.0315, 2358.0854, 2368.0815, 2369.0588, 2378.0020, 2379.0002, 2380.0007, 2381.9963, 2383.0444, 2383.9822, 2393.0020, 2394.0181, 2398.0447, 2400.0771, 2405.0879, 2414.1050, 2417.0933, 2421.0769, 2422.0593, 2426.0476, 2428.0725, 2430.0947, 2432.0962, 2435.0254, 2438.0706, 2444.0901, 2451.0278, 2459.9802, 2461.0254, 2501.2520, 2528.1902, 2543.2092, 2564.1965, 2705.2144, 2717.1655, 2834.2925, 2888.2483, 2904.4053, 3130.4312, 3291.4875, 3312.3765, 3451.6833, 3454.6270

33. Q3ZBL3 **Mass:** 49458 **Score:** 17 **Expect:** 4.5e+002 **Matches:** 17

Inositol hexakisphosphate kinase 2 OS=Bos taurus GN=IP6K2 PE=2 SV=1

Observed	Mr(expt)	Mr(calc)	ppm	Start	End	Miss	Ions	Peptide
724.3446	723.3373	723.3374	-0.09	1	6	0	---	-.MSPAFR.A + Oxidation (M)
778.4551	777.4478	777.4385	12.0	279	285	0	---	K.LSVQGFK.E
891.4706	890.4634	890.4610	2.64	126	132	1	---	K.TSKDWVR.Q
978.4772	977.4700	977.4930	-23.62	118	125	0	---	K.HHVLETDK.T
1305.6523	1304.6450	1304.6507	-4.35	243	254	0	---	K.CQQSTSAVIGVR.V
1365.6752	1364.6679	1364.6626	3.93	286	296	0	---	K.EALFQFFHNGR.Y
1375.6670	1374.6597	1374.6714	-8.50	2	13	1	---	M.SPAFRAMDVEPR.D
1522.7239	1521.7166	1521.7068	6.45	1	13	1	---	-.MSPAFRAMDVEPR.D + Oxidation (M)
1749.8406	1748.8333	1748.8338	-0.26	1	15	2	---	-.MSPAFRAMDVEPRDK.G
1765.7922	1764.7849	1764.8287	-24.81	1	15	2	---	-.MSPAFRAMDVEPRDK.G + Oxidation (M)
1768.8596	1767.8523	1767.8136	21.9	226	241	1	---	R.QHGDDASEEKAANQIR.K
1801.9315	1800.9242	1800.9410	-9.31	140	153	2	---	K.VKSHKLEEEFEWLK.K
2232.0229	2231.0156	2231.0383	-10.14	62	80	1	---	K.FTPQYKGVVSVSFFEEDEDR.N
2400.0771	2399.0698	2399.1198	-20.81	174	191	2	---	K.HYNPWSMKCHQQQLQRMK.E
2432.0962	2431.0889	2431.1096	-8.50	174	191	2	---	K.HYNPWSMKCHQQQLQRMK.E + 2 Oxidation (M)
2543.2092	2542.2019	2542.2314	-11.61	48	67	2	---	R.EHQFYETLPAEMRKFTPQYK.G
3451.6833	3450.6760	3450.5975	22.8	243	273	1	---	K.CQQSTSAVIGVRVCGMQVYQAGSGQLMFMNK.Y + Oxidation (M)

No match to: 713.4400, 713.4415, 728.2467, 730.2754, 732.2383, 734.2729, 735.3820, 736.3320, 740.2330, 743.2514, 748.2947, 750.2650, 756.4777, 764.3574, 765.3959, 768.3784, 772.4155, 774.3952, 780.3343, 782.3223, 783.3115, 787.2390, 791.2662, 802.4609, 805.3347, 806.4342, 807.3921, 808.4378, 812.4002, 814.4128, 820.4252, 828.3922, 830.3668, 834.3623, 837.3794, 840.3857, 842.5100, 845.4155, 851.3846, 856.5103, 865.3634, 869.4703, 870.5254, 871.4965, 872.6990, 888.3988, 893.9500, 894.4534, 897.0277, 905.4637, 911.4314, 912.4475, 913.5080, 913.5100, 918.5267, 925.4804, 927.4970, 930.5015, 933.4662, 935.4675, 943.4757, 951.4646, 952.4799, 957.4673, 961.4901, 962.4866, 967.4440, 969.4496, 972.4781, 974.5051, 975.4521, 977.4406, 979.4924, 983.4706, 985.6071, 991.4533, 993.5267, 995.5177, 999.4602, 1001.4565, 1001.5621, 1003.5043, 1007.5638, 1012.5004, 1014.5331, 1018.5130, 1022.5398, 1023.5525, 1028.5991, 1030.5259, 1035.5314, 1036.5333, 1037.5145, 1040.5127, 1042.5243, 1045.5603, 1060.5398, 1069.4846, 1075.5515, 1092.6041, 1106.5313, 1109.5087, 1111.5457, 1113.5419, 1115.5394, 1118.5659, 1121.5419, 1122.5701, 1131.5602, 1138.5428, 1149.5912, 1155.5768, 1157.5907, 1158.6328, 1163.6145, 1173.6122, 1177.6130, 1178.6060, 1179.5918, 1180.6001, 1181.6368, 1183.6030, 1187.6376, 1192.6014, 1193.5928, 1195.5924, 1199.6785, 1206.6066, 1215.6306, 1216.6482, 1226.5839, 1227.6051, 1231.6115, 1232.6251, 1233.6230, 1234.6500, 1235.6155, 1248.6078, 1250.6483, 1254.6526, 1255.6036, 1256.6123, 1268.6208, 1271.6423, 1276.6179, 1277.6350, 1289.6449, 1292.6322, 1294.6362, 1295.6366, 1300.5895, 1303.6643, 1307.6600, 1308.6443, 1310.6051, 1315.6364, 1320.6128, 1321.6134, 1323.6320, 1325.6456, 1331.6674, 1332.6602, 1333.2076, 1334.6744, 1336.6710, 1337.6279, 1338.6300, 1338.6300, 1345.6146, 1347.6653, 1349.6800, 1349.6827, 1354.6820, 1355.6964, 1359.7053, 1360.6173, 1361.6373, 1371.6718, 1377.6776, 1382.6367, 1383.6619, 1387.6700, 1393.6727, 1400.6235, 1409.7107, 1411.6742, 1427.7725, 1431.7605, 1432.6987, 1434.7190, 1449.7479, 1466.6953, 1473.7905, 1475.7498, 1478.7303, 1479.7468, 1485.7531, 1486.7472, 1493.7345, 1495.7502, 1500.7242, 1503.7690, 1505.8511, 1509.6786, 1520.7347, 1523.7567, 1525.7864, 1527.8068, 1532.7472, 1537.7886, 1539.7711, 1549.7833, 1555.8657, 1558.8440, 1559.8573, 1568.7592, 1570.7814, 1578.7899, 1606.8213, 1615.8425, 1619.7972, 1621.7831, 1628.8156, 1630.8202, 1631.8217, 1639.9263, 1644.8083, 1647.8730, 1649.8650, 1650.8523, 1658.8206, 1659.8125, 1661.8284, 1663.7981, 1665.8070, 1669.8174, 1677.8353, 1679.9403, 1680.8076, 1682.7780, 1696.8055, 1697.7845, 1698.7950, 1707.7931, 1709.8029, 1710.8878, 1712.9166, 1716.8563, 1725.8188, 1727.8683, 1730.8236, 1731.8221, 1742.8798, 1746.1482, 1751.8657, 1756.8782, 1757.8225, 1763.8152, 1766.8105, 1769.4275, 1770.4087, 1773.8615, 1774.8795, 1775.0302, 1783.8599, 1785.8483, 1786.8871, 1790.8822, 1792.7894, 1804.9026, 1808.8663, 1824.8685, 1830.8608, 1849.8231, 1851.8430, 1867.9100, 1868.9441, 1870.9304, 1880.9020, 1884.9191, 1908.8989, 1914.9821, 1916.9882, 1920.9862, 1930.9827, 1932.9813, 1934.9822, 1947.0288, 1958.9543, 1994.8763, 2011.9012, 2013.8700, 2020.9592, 2025.5920, 2042.0568, 2047.0238, 2047.9805, 2049.9497, 2052.9905, 2053.9851, 2058.9988, 2060.0400, 2060.0405, 2065.0427, 2066.0137, 2066.9895, 2067.9763, 2068.9128, 2070.8792, 2074.8860, 2082.0293, 2084.8977, 2086.8872, 2087.8835, 2090.8708, 2092.8674, 2093.8711, 2096.9836, 2097.9961, 2100.8787, 2101.8999, 2103.9092, 2107.8730, 2119.9924, 2122.9397, 2136.9133, 2211.1047, 2217.9368, 2225.1243, 2233.0840, 2238.1050, 2250.0259, 2257.9934, 2262.9368, 2263.9700, 2273.0264, 2277.9500, 2283.1741, 2290.9570, 2307.9631, 2321.9558, 2323.9670, 2324.9824, 2335.9763, 2339.9456, 2343.0320, 2349.9844, 2352.0308, 2355.0315, 2358.0854, 2368.0815, 2369.0588, 2378.0020, 2379.0002, 2380.0007, 2381.9963, 2383.0444, 2383.9822, 2393.0020, 2394.0181, 2398.0447, 2405.0879, 2414.1050, 2417.0933, 2421.0769, 2422.0593, 2426.0476, 2428.0725, 2430.0947, 2435.0254, 2438.0706, 2444.0901, 2451.0278, 2459.9802, 2461.0254, 2501.2520, 2528.1902, 2564.1965, 2705.2144, 2717.1655, 2834.2925, 2888.2483, 2904.4053, 3130.4312, 3291.4875, 3312.3765, 3454.6270

34. Q56JZ9 **Mass:** 16931 **Score:** 17 **Expect:** 4.6e+002 **Matches:** 8

Glia maturation factor gamma OS=Bos taurus GN=GMFG PE=2 SV=1

Observed	Mr(expt)	Mr(calc)	ppm	Start	End	Miss	Ions	Peptide
774.3952	773.3879	773.3741	17.8	59 -	64	0	---	K.MELPER.Q
905.4637	904.4564	904.4695	-14.43	68 -	74	0	---	R.FVVYSYK.Y
1155.5768	1154.5695	1154.5866	-14.78	59 -	67	1	---	K.MELPERQPR.F
1235.6155	1234.6082	1234.6227	-11.73	25 -	35	1	---	R.KETDNAAIVMK.V + Oxidation (M)
1292.6322	1291.6249	1291.6296	-3.61	125 -	135	0	---	R.TTDDLTEAWLK.E
1449.7479	1448.7406	1448.7181	15.6	26 -	38	1	---	K.ETDNAAIVMKVDK.D + Oxidation (M)
1549.7833	1548.7760	1548.7671	5.74	125 -	137	1	---	R.TTDDLTEAWLKEK.L
2042.0568	2041.0495	2041.0455	1.98	59 -	74	2	---	K.MELPERQPRFVVYSYK.Y

No match to: 713.4400, 713.4415, 724.3446, 728.2467, 730.2754, 732.2383, 734.2729, 735.3820, 736.3320, 740.2330, 743.2514, 748.2947, 750.2650, 756.4777, 764.3574, 765.3959, 768.3784, 772.4155, 778.4551, 780.3343, 782.3223, 783.3115, 787.2390, 791.2662, 802.4609, 805.3347, 806.4342, 807.3921, 808.4378, 812.4002, 814.4128, 820.4252, 828.3922, 830.3668, 834.3623, 837.3794, 840.3857, 842.5100, 845.4155, 851.3846, 856.5103, 865.3634, 869.4703, 870.5254, 871.4965, 872.6990, 888.3988, 891.4706, 893.9500, 894.4534, 897.0277, 911.4314, 912.4475, 913.5080, 913.5100, 918.5267, 925.4804, 927.4970, 930.5015, 933.4662, 935.4675, 943.4757, 951.4646, 952.4799, 957.4673, 961.4901, 962.4866, 967.4440, 969.4496, 972.4781, 974.5051, 975.4521, 977.4406, 978.4772, 979.4924, 983.4706, 985.6071, 991.4533, 993.5267, 995.5177, 999.4602, 1001.4565, 1001.5621, 1003.5043, 1007.5638, 1012.5004, 1014.5331, 1018.5130, 1022.5398, 1023.5525, 1028.5991, 1030.5259, 1035.5314, 1036.5333, 1037.5145, 1040.5127, 1042.5243, 1045.5603, 1060.5398, 1069.4846, 1075.5515, 1092.6041, 1106.5313, 1109.5087, 1111.5457, 1113.5419, 1115.5394, 1118.5659, 1121.5419, 1122.5701, 1131.5602, 1138.5428, 1149.5912, 1157.5907, 1158.6328, 1163.6145, 1173.6122, 1177.6130, 1178.6060, 1179.5918, 1180.6001, 1181.6368, 1183.6030, 1187.6376, 1192.6014, 1193.5928, 1195.5924, 1199.6785, 1206.6066, 1215.6306, 1216.6482, 1226.5839, 1227.6051, 1231.6115, 1232.6251, 1233.6230, 1234.6500, 1248.6078, 1250.6483, 1254.6526, 1255.6036, 1256.6123, 1268.6208, 1271.6423, 1276.6179, 1277.6350, 1289.6449, 1294.6362, 1295.6366, 1300.5895, 1303.6643, 1305.6523, 1307.6600, 1308.6443, 1310.6051, 1315.6364, 1320.6128, 1321.6134, 1323.6320, 1325.6456, 1331.6674, 1332.6602, 1333.2076, 1334.6744, 1336.6710, 1337.6279, 1338.6300, 1338.6300, 1345.6146, 1347.6653, 1349.6800, 1349.6827, 1354.6820, 1355.6964, 1359.7053, 1360.6173, 1361.6373, 1365.6752, 1371.6718, 1375.6670, 1377.6776, 1382.6367, 1383.6619, 1387.6700, 1393.6727, 1400.6235, 1409.7107, 1411.6742, 1427.7725, 1431.7605, 1432.6987, 1434.7190, 1466.6953, 1473.7905, 1475.7498, 1478.7303, 1479.7468, 1485.7531, 1486.7472, 1493.7345, 1495.7502, 1500.7242, 1503.7690, 1505.8511, 1509.6786, 1520.7347, 1522.7239, 1523.7567, 1525.7864, 1527.8068, 1532.7472, 1537.7886, 1539.7711, 1555.8657, 1558.8440, 1559.8573, 1568.7592, 1570.7814, 1578.7899, 1606.8213, 1615.8425, 1619.7972, 1621.7831, 1628.8156, 1630.8202, 1631.8217, 1639.9263, 1644.8083, 1647.8730, 1649.8650, 1650.8523, 1658.8206, 1659.8125, 1661.8284, 1663.7981, 1665.8070, 1669.8174, 1677.8353, 1679.9403, 1680.8076, 1682.7780, 1696.8055, 1697.7845, 1698.7950, 1707.7931, 1709.8029, 1710.8878, 1712.9166, 1716.8563, 1725.8188, 1727.8683, 1730.8236, 1731.8221, 1742.8798, 1746.1482, 1749.8406, 1751.8657, 1756.8782, 1757.8225, 1763.8152, 1765.7922, 1766.8105, 1768.8596, 1769.4275, 1770.4087, 1773.8615, 1774.8795, 1775.0302, 1783.8599, 1785.8483, 1786.8871, 1790.8822, 1792.7894, 1801.9315, 1804.9026, 1808.8663, 1824.8685, 1830.8608, 1849.8231, 1851.8430, 1867.9100, 1868.9441, 1870.9304, 1880.9020, 1884.9191, 1908.8989, 1914.9821, 1916.9882, 1920.9862, 1930.9827, 1932.9813, 1934.9822, 1947.0288, 1958.9543, 1994.8763, 2011.9012, 2013.8700, 2020.9592, 2025.5920, 2047.0238, 2047.9805, 2049.9497, 2052.9905, 2053.9851, 2058.9988, 2060.0400, 2060.0405, 2065.0427, 2066.0137, 2066.9895, 2067.9763, 2068.9128, 2070.8792, 2074.8860, 2082.0293, 2084.8977, 2086.8872, 2087.8835, 2090.8708, 2092.8674, 2093.8711, 2096.9836, 2097.9961, 2100.8787, 2101.8999, 2103.9092, 2107.8730, 2119.9924, 2122.9397, 2136.9133, 2211.1047, 2217.9368, 2225.1243, 2232.0229, 2233.0840, 2238.1050, 2250.0259, 2257.9934, 2262.9368, 2263.9700, 2273.0264, 2277.9500, 2283.1741, 2290.9570, 2307.9631, 2321.9558, 2323.9670, 2324.9824, 2335.9763, 2339.9456, 2343.0320, 2349.9844, 2352.0308, 2355.0315, 2358.0854, 2368.0815, 2369.0588, 2378.0020, 2379.0002, 2380.0007, 2381.9963, 2383.0444, 2383.9822, 2393.0020, 2394.0181, 2398.0447, 2400.0771, 2405.0879, 2414.1050, 2417.0933, 2421.0769, 2422.0593, 2426.0476, 2428.0725, 2430.0947, 2432.0962, 2435.0254, 2438.0706, 2444.0901, 2451.0278, 2459.9802, 2461.0254, 2501.2520, 2528.1902, 2543.2092, 2564.1965, 2705.2144, 2717.1655, 2834.2925, 2888.2483, 2904.4053, 3130.4312, 3291.4875, 3312.3765, 3451.6833, 3454.6270

35. F1MQU4 **Mass:** 106272 **Score:** 17 **Expect:** 4.7e+002 **Matches:** 30

Uncharacterized protein OS=Bos taurus GN=BCLAF1 PE=4 SV=2

Observed	Mr(expt)	Mr(calc)	ppm	Start	End	Miss	Ions	Peptide
807.3921	806.3848	806.3817	3.86	1 -	7	1	---	-.MGRSNSR.S
837.3794	836.3721	836.3889	-20.05	60 -	65	1	---	R.DYRNNR.G
845.4155	844.4083	844.3967	13.7	729 -	734	1	---	K.DYKEYK.S
894.4534	893.4461	893.4315	16.3	17 -	24	1	---	R.SQSSSRSR.S
905.4637	904.4564	904.4614	-5.52	160 -	167	1	---	R.GSQEKQTK.K
933.4662	932.4590	932.4464	13.5	422 -	429	0	---	K.NFATTSHR.N
972.4781	971.4708	971.4784	-7.85	738 -	745	2	---	K.DDSKHKSR.E
1075.5515	1074.5442	1074.5305	12.8	677 -	685	2	---	K.EENQKGDKK.L
1149.5912	1148.5839	1148.6010	-14.88	119 -	128	2	---	R.SVSSQRSRSR.S
1155.5768	1154.5695	1154.5767	-6.22	66 -	74	1	---	R.GMRRPYGYR.G
1173.6122	1172.6049	1172.6037	1.04	492 -	501	1	---	K.KETQSPEQVK.S
1193.5928	1192.5855	1192.5908	-4.45	135 -	145	2	---	R.SSRSPRSSSSR.S
1232.6251	1231.6178	1231.6017	13.1	4 -	14	2	---	R.SNSRSHSSRSK.S
1233.6230	1232.6157	1232.6295	-11.20	685 -	694	2	---	K.KLRCDSADLR.H

1308.6443	1307.6370	1307.6622	-19.26	844 -	853	2	---	K.SKKYFLHDDR.D
1310.6051	1309.5978	1309.5746	17.7	752 -	765	0	---	R.SSSSSASPSSPSSR.E
1325.6456	1324.6383	1324.6524	-10.62	786 -	797	0	---	K.EYSGFAGVSRPR.G
1331.6674	1330.6601	1330.6557	3.29	507 -	517	0	---	K.DLFDYSPPLHK.N
1336.6710	1335.6637	1335.6895	-19.29	518 -	528	2	---	K.NLDAREKSTFR.E
1361.6373	1360.6300	1360.6378	-5.72	1 -	12	2	---	-.MGRSNSRSHSSR.S
1409.7107	1408.7034	1408.6834	14.2	430 -	441	2	---	R.NTEEEGPKYKSK.V
1478.7303	1477.7230	1477.7525	-19.93	523 -	534	2	---	R.EKSTFREESPLR.I
1539.7711	1538.7638	1538.7637	0.11	63 -	74	2	---	R.NNRGMRRPYGYR.G
1716.8563	1715.8490	1715.8869	-22.07	88 -	100	2	---	R.YHRGGYRPVWNRR.H
1757.8225	1756.8152	1756.8591	-24.99	141 -	157	2	---	R.SSSSRSSSPYSKSPVSK.R
1765.7922	1764.7849	1764.8206	-20.22	833 -	846	2	---	K.EEEWDPEYTPKSKK.Y
1916.9882	1915.9809	1916.0043	-12.22	502 -	517	2	---	K.SEKLKDLFDYSPPLHK.N
2273.0264	2272.0191	2271.9920	11.9	369 -	387	1	---	K.GRAEGEWEDQEVLDYFSDK.E
2461.0254	2460.0181	2460.0605	-17.21	371 -	391	1	---	R.AEGEWEDQEVLDYFSDKESGK.Q
2717.1655	2716.1582	2716.2140	-20.54	371 -	393	2	---	R.AEGEWEDQEVLDYFSDKESGKQK.F

No match to: 713.4400, 713.4415, 724.3446, 728.2467, 730.2754, 732.2383, 734.2729, 735.3820, 736.3320, 740.2330, 743.2514, 748.2947, 750.2650, 756.4777, 764.3574, 765.3959, 768.3784, 772.4155, 774.3952, 778.4551, 780.3343, 782.3223, 783.3115, 787.2390, 791.2662, 802.4609, 805.3347, 806.4342, 808.4378, 812.4002, 814.4128, 820.4252, 828.3922, 830.3668, 834.3623, 840.3857, 842.5100, 851.3846, 856.5103, 865.3634, 869.4703, 870.5254, 871.4965, 872.6990, 888.3988, 891.4706, 893.9500, 897.0277, 911.4314, 912.4475, 913.5080, 913.5100, 918.5267, 925.4804, 927.4970, 930.5015, 935.4675, 943.4757, 951.4646, 952.4799, 957.4673, 961.4901, 962.4866, 967.4440, 969.4496, 974.5051, 975.4521, 977.4406, 978.4772, 979.4924, 983.4706, 985.6071, 991.4533, 993.5267, 995.5177, 999.4602, 1001.4565, 1001.5621, 1003.5043, 1007.5638, 1012.5004, 1014.5331, 1018.5130, 1022.5398, 1023.5525, 1028.5991, 1030.5259, 1035.5314, 1036.5333, 1037.5145, 1040.5127, 1042.5243, 1045.5603, 1060.5398, 1069.4846, 1092.6041, 1106.5313, 1109.5087, 1111.5457, 1113.5419, 1115.5394, 1118.5659, 1121.5419, 1122.5701, 1131.5602, 1138.5428, 1157.5907, 1158.6328, 1163.6145, 1177.6130, 1178.6060, 1179.5918, 1180.6001, 1181.6368, 1183.6030, 1187.6376, 1192.6014, 1195.5924, 1199.6785, 1206.6066, 1215.6306, 1216.6482, 1226.5839, 1227.6051, 1231.6115, 1234.6500, 1235.6155, 1248.6078, 1250.6483, 1254.6526, 1255.6036, 1256.6123, 1268.6208, 1271.6423, 1276.6179, 1277.6350, 1289.6449, 1292.6322, 1294.6362, 1295.6366, 1300.5895, 1303.6643, 1305.6523, 1307.6600, 1315.6364, 1320.6128, 1321.6134, 1323.6320, 1332.6602, 1333.2076, 1334.6744, 1337.6279, 1338.6300, 1338.6300, 1345.6146, 1347.6653, 1349.6800, 1349.6827, 1354.6820, 1355.6964, 1359.7053, 1360.6173, 1365.6752, 1371.6718, 1375.6670, 1377.6776, 1382.6367, 1383.6619, 1387.6700, 1393.6727, 1400.6235, 1411.6742, 1427.7725, 1431.7605, 1432.6987, 1434.7190, 1449.7479, 1466.6953, 1473.7905, 1475.7498, 1479.7468, 1485.7531, 1486.7472, 1493.7345, 1495.7502, 1500.7242, 1503.7690, 1505.8511, 1509.6786, 1520.7347, 1522.7239, 1523.7567, 1525.7864, 1527.8068, 1532.7472, 1537.7886, 1549.7833, 1555.8657, 1558.8440, 1559.8573, 1568.7592, 1570.7814, 1578.7899, 1606.8213, 1615.8425, 1619.7972, 1621.7831, 1628.8156, 1630.8202, 1631.8217, 1639.9263, 1644.8083, 1647.8730, 1649.8650, 1650.8523, 1658.8206, 1659.8125, 1661.8284, 1663.7981, 1665.8070, 1669.8174, 1677.8353, 1679.9403, 1680.8076, 1682.7780, 1696.8055, 1697.7845, 1698.7950, 1707.7931, 1709.8029, 1710.8878, 1712.9166, 1725.8188, 1727.8683, 1730.8236, 1731.8221, 1742.8798, 1746.1482, 1749.8406, 1751.8657, 1756.8782, 1763.8152, 1766.8105, 1768.8596, 1769.4275, 1770.4087, 1773.8615, 1774.8795, 1775.0302, 1783.8599, 1785.8483, 1786.8871, 1790.8822, 1792.7894, 1801.9315, 1804.9026, 1808.8663, 1824.8685, 1830.8608, 1849.8231, 1851.8430, 1867.9100, 1868.9441, 1870.9304, 1880.9020, 1884.9191, 1908.8989, 1914.9821, 1920.9862, 1930.9827, 1932.9813, 1934.9822, 1947.0288, 1958.9543, 1994.8763, 2011.9012, 2013.8700, 2020.9592, 2025.5920, 2042.0568, 2047.0238, 2047.9805, 2049.9497, 2052.9905, 2053.9851, 2058.9988, 2060.0400, 2060.0405, 2065.0427, 2066.0137, 2066.9895, 2067.9763, 2068.9128, 2070.8792, 2074.8860, 2082.0293, 2084.8977, 2086.8872, 2087.8835, 2090.8708, 2092.8674, 2093.8711, 2096.9836, 2097.9961, 2100.8787, 2101.8999, 2103.9092, 2107.8730, 2119.9924, 2122.9397, 2136.9133, 2211.1047, 2217.9368, 2225.1243, 2232.0229, 2233.0840, 2238.1050, 2250.0259, 2257.9934, 2262.9368, 2263.9700, 2277.9500, 2283.1741, 2290.9570, 2307.9631, 2321.9558, 2323.9670, 2324.9824, 2335.9763, 2339.9456, 2343.0320, 2349.9844, 2352.0308, 2355.0315, 2358.0854, 2368.0815, 2369.0588, 2378.0020, 2379.0002, 2380.0007, 2381.9963, 2383.0444, 2383.9822, 2393.0020, 2394.0181, 2398.0447, 2400.0771, 2405.0879, 2414.1050, 2417.0933, 2421.0769, 2422.0593, 2426.0476, 2428.0725, 2430.0947, 2432.0962, 2435.0254, 2438.0706, 2444.0901, 2451.0278, 2459.9802, 2501.2520, 2528.1902, 2543.2092, 2564.1965, 2705.2144, 2834.2925, 2888.2483, 2904.4053, 3130.4312, 3291.4875, 3312.3765, 3451.6833, 3454.6270

36. G3MYV8 **Mass:** 6868 **Score:** 17 **Expect:** 4.7e+002 **Matches:** 5

Uncharacterized protein OS=Bos taurus PE=4 SV=1

Observed	Mr(expt)	Mr(calc)	ppm	Start	End	Miss	Ions	Peptide
765.3959	764.3887	764.3851	4.72	12 -	17	1	---	R.QKNMTK.Q + Oxidation (M)
978.4772	977.4700	977.4488	21.7	40 -	47	0	---	R.DSEIMQQK.Q
1003.5043	1002.4970	1002.5094	-12.34	28 -	37	1	---	R.DDGLSAAARK.Q
1187.6376	1186.6303	1186.6167	11.5	26 -	36	2	---	K.RRDDGLSAAAR.K
1409.7107	1408.7034	1408.6980	3.84	12 -	23	2	---	R.QKNMTKQSDSVK.G + Oxidation (M)

No match to: 713.4400, 713.4415, 724.3446, 728.2467, 730.2754, 732.2383, 734.2729, 735.3820, 736.3320, 740.2330, 743.2514, 748.2947, 750.2650, 756.4777, 764.3574, 768.3784, 772.4155, 774.3952, 778.4551, 780.3343, 782.3223, 783.3115, 787.2390, 791.2662, 802.4609, 805.3347, 806.4342, 807.3921, 808.4378, 812.4002, 814.4128, 820.4252, 828.3922, 830.3668, 834.3623, 837.3794, 840.3857, 842.5100, 845.4155, 851.3846, 856.5103, 865.3634, 869.4703, 870.5254, 871.4965, 872.6990, 888.3988, 891.4706, 893.9500, 894.4534, 897.0277, 905.4637, 911.4314, 912.4475, 913.5080, 913.5100, 918.5267, 925.4804, 927.4970, 930.5015, 933.4662, 935.4675, 943.4757, 951.4646, 952.4799, 957.4673, 961.4901, 962.4866, 967.4440, 969.4496, 972.4781, 974.5051, 975.4521, 977.4406, 979.4924, 983.4706, 985.6071, 991.4533, 993.5267, 995.5177, 999.4602, 1001.4565, 1001.5621, 1007.5638, 1012.5004, 1014.5331, 1018.5130, 1022.5398, 1023.5525, 1028.5991, 1030.5259, 1035.5314, 1036.5333, 1037.5145, 1040.5127, 1042.5243, 1045.5603, 1060.5398, 1069.4846, 1075.5515, 1092.6041, 1106.5313, 1109.5087, 1111.5457, 1113.5419, 1115.5394, 1118.5659, 1121.5419, 1122.5701, 1131.5602, 1138.5428, 1149.5912, 1155.5768, 1157.5907, 1158.6328, 1163.6145, 1173.6122, 1177.6130, 1178.6060, 1179.5918, 1180.6001, 1181.6368, 1183.6030, 1192.6014, 1193.5928, 1195.5924, 1199.6785, 1206.6066, 1215.6306, 1216.6482, 1226.5839, 1227.6051, 1231.6115, 1232.6251, 1233.6230, 1234.6500, 1235.6155, 1248.6078, 1250.6483, 1254.6526, 1255.6036, 1256.6123, 1268.6208, 1271.6423, 1276.6179, 1277.6350, 1289.6449, 1292.6322, 1294.6362, 1295.6366, 1300.5895, 1303.6643, 1305.6523, 1307.6600, 1308.6443, 1310.6051, 1315.6364, 1320.6128, 1321.6134, 1323.6320, 1325.6456, 1331.6674, 1332.6602, 1333.2076, 1334.6744, 1336.6710, 1337.6279, 1338.6300, 1338.6300, 1345.6146, 1347.6653, 1349.6800, 1349.6827, 1354.6820, 1355.6964, 1359.7053, 1360.6173, 1361.6373, 1365.6752, 1371.6718, 1375.6670, 1377.6776, 1382.6367, 1383.6619, 1387.6700, 1393.6727, 1400.6235, 1411.6742, 1427.7725, 1431.7605, 1432.6987, 1434.7190, 1449.7479, 1466.6953, 1473.7905, 1475.7498, 1478.7303, 1479.7468, 1485.7531, 1486.7472, 1493.7345, 1495.7502, 1500.7242, 1503.7690, 1505.8511, 1509.6786, 1520.7347, 1522.7239, 1523.7567, 1525.7864, 1527.8068, 1532.7472, 1537.7886, 1539.7711, 1549.7833, 1555.8657, 1558.8440, 1559.8573, 1568.7592, 1570.7814, 1578.7899, 1606.8213, 1615.8425, 1619.7972, 1621.7831, 1628.8156, 1630.8202, 1631.8217, 1639.9263, 1644.8083, 1647.8730, 1649.8650, 1650.8523, 1658.8206, 1659.8125, 1661.8284, 1663.7981, 1665.8070, 1669.8174, 1677.8353, 1679.9403, 1680.8076, 1682.7780, 1696.8055, 1697.7845, 1698.7950, 1707.7931, 1709.8029, 1710.8878, 1712.9166, 1716.8563, 1725.8188, 1727.8683, 1730.8236, 1731.8221, 1742.8798, 1746.1482, 1749.8406, 1751.8657, 1756.8782, 1757.8225, 1763.8152, 1765.7922, 1766.8105, 1768.8596, 1769.4275, 1770.4087, 1773.8615, 1774.8795, 1775.0302, 1783.8599, 1785.8483, 1786.8871, 1790.8822, 1792.7894, 1801.9315, 1804.9026, 1808.8663, 1824.8685, 1830.8608, 1849.8231, 1851.8430, 1867.9100, 1868.9441, 1870.9304, 1880.9020, 1884.9191, 1908.8989, 1914.9821, 1916.9882, 1920.9862, 1930.9827, 1932.9813, 1934.9822, 1947.0288, 1958.9543, 1994.8763, 2011.9012, 2013.8700, 2020.9592, 2025.5920, 2042.0568, 2047.0238, 2047.9805, 2049.9497, 2052.9905, 2053.9851, 2058.9988, 2060.0400, 2060.0405, 2065.0427, 2066.0137, 2066.9895, 2067.9763, 2068.9128, 2070.8792, 2074.8860, 2082.0293, 2084.8977, 2086.8872, 2087.8835, 2090.8708, 2092.8674, 2093.8711, 2096.9836, 2097.9961, 2100.8787, 2101.8999, 2103.9092, 2107.8730, 2119.9924, 2122.9397, 2136.9133, 2211.1047, 2217.9368, 2225.1243, 2232.0229, 2233.0840, 2238.1050, 2250.0259, 2257.9934, 2262.9368, 2263.9700, 2273.0264, 2277.9500, 2283.1741, 2290.9570, 2307.9631, 2321.9558, 2323.9670, 2324.9824, 2335.9763, 2339.9456, 2343.0320, 2349.9844, 2352.0308, 2355.0315, 2358.0854, 2368.0815, 2369.0588, 2378.0020, 2379.0002, 2380.0007, 2381.9963, 2383.0444, 2383.9822, 2393.0020, 2394.0181, 2398.0447, 2400.0771, 2405.0879, 2414.1050, 2417.0933, 2421.0769, 2422.0593, 2426.0476, 2428.0725, 2430.0947, 2432.0962, 2435.0254, 2438.0706, 2444.0901, 2451.0278, 2459.9802, 2461.0254, 2501.2520, 2528.1902, 2543.2092, 2564.1965, 2705.2144, 2717.1655, 2834.2925, 2888.2483, 2904.4053, 3130.4312, 3291.4875, 3312.3765, 3451.6833, 3454.6270

37. E1B8A3 **Mass:** 13661 **Score:** 17 **Expect:** 4.7e+002 **Matches:** 7

Uncharacterized protein OS=Bos taurus GN=LOC100298655 PE=4 SV=1

Observed	Mr(expt)	Mr(calc)	ppm	Start	End	Miss	Ions	Peptide
756.4777	755.4704	755.4766	-8.14	4 -	9	1	---	K.VTRKPR.Q
911.4314	910.4241	910.4113	14.1	40 -	46	1	---	K.ARNMTMR.V + 2 Oxidation (M)

979.4924	978.4852	978.5069	-22.24	14 -	21	1	---	R.VPMRFASR.M + Oxidation (M)
985.6071	984.5998	984.6192	-19.69	2 -	9	2	---	M.TKVTRKPR.Q
1045.5603	1044.5530	1044.5611	-7.70	10 -	17	2	---	R.QSRRVPMR.F + Oxidation (M)
1250.6483	1249.6410	1249.6383	2.16	36 -	46	2	---	R.GSVKARNMTMR.V
1409.7107	1408.7034	1408.7067	-2.35	14 -	25	2	---	R.VPMRFASRMNGK.K + Oxidation (M)

No match to: 713.4400, 713.4415, 724.3446, 728.2467, 730.2754, 732.2383, 734.2729, 735.3820, 736.3320, 740.2330, 743.2514,
748.2947, 750.2650, 764.3574, 765.3959, 768.3784, 772.4155, 774.3952, 778.4551, 780.3343, 782.3223, 783.3115, 787.2390, 791.2662,
802.4609, 805.3347, 806.4342, 807.3921, 808.4378, 812.4002, 814.4128, 820.4252, 828.3922, 830.3668, 834.3623, 837.3794, 840.3857,
842.5100, 845.4155, 851.3846, 856.5103, 865.3634, 869.4703, 870.5254, 871.4965, 872.6990, 888.3988, 891.4706, 893.9500, 894.4534,
897.0277, 905.4637, 912.4475, 913.5080, 913.5100, 918.5267, 925.4804, 927.4970, 930.5015, 933.4662, 935.4675, 943.4757, 951.4646,
952.4799, 957.4673, 961.4901, 962.4866, 967.4440, 969.4496, 972.4781, 974.5051, 975.4521, 977.4406, 978.4772, 983.4706, 991.4533,
993.5267, 995.5177, 999.4602, 1001.4565, 1001.5621, 1003.5043, 1007.5638, 1012.5004, 1014.5331, 1018.5130, 1022.5398, 1023.5525,
1028.5991, 1030.5259, 1035.5314, 1036.5333, 1037.5145, 1040.5127, 1042.5243, 1060.5398, 1069.4846, 1075.5515, 1092.6041, 1106.5313,
1109.5087, 1111.5457, 1113.5419, 1115.5394, 1118.5659, 1121.5419, 1122.5701, 1131.5602, 1138.5428, 1149.5912, 1155.5768, 1157.5907,
1158.6328, 1163.6145, 1173.6122, 1177.6130, 1178.6060, 1179.5918, 1180.6001, 1181.6368, 1183.6030, 1187.6376, 1192.6014, 1193.5928,
1195.5924, 1199.6785, 1206.6066, 1215.6306, 1216.6482, 1226.5839, 1227.6051, 1231.6115, 1232.6251, 1233.6230, 1234.6500, 1235.6155,
1248.6078, 1254.6526, 1255.6036, 1256.6123, 1268.6208, 1271.6423, 1276.6179, 1277.6350, 1289.6449, 1292.6322, 1294.6362, 1295.6366,
1300.5895, 1303.6643, 1305.6523, 1307.6600, 1308.6443, 1310.6051, 1315.6364, 1320.6128, 1321.6134, 1323.6320, 1325.6456, 1331.6674,
1332.6602, 1333.2076, 1334.6744, 1336.6710, 1337.6279, 1338.6300, 1338.6300, 1345.6146, 1347.6653, 1349.6800, 1349.6827, 1354.6820,
1355.6964, 1359.7053, 1360.6173, 1361.6373, 1365.6752, 1371.6718, 1375.6670, 1377.6776, 1382.6367, 1383.6619, 1387.6700, 1393.6727,
1400.6235, 1411.6742, 1427.7725, 1431.7605, 1432.6987, 1434.7190, 1449.7479, 1466.6953, 1473.7905, 1475.7498, 1478.7303, 1479.7468,
1485.7531, 1486.7472, 1493.7345, 1495.7502, 1500.7242, 1503.7690, 1505.8511, 1509.6786, 1520.7347, 1522.7239, 1523.7567, 1525.7864,
1527.8068, 1532.7472, 1537.7886, 1539.7711, 1549.7833, 1555.8657, 1558.8440, 1559.8573, 1568.7592, 1570.7814, 1578.7899, 1606.8213,
1615.8425, 1619.7972, 1621.7831, 1628.8156, 1630.8202, 1631.8217, 1639.9263, 1644.8083, 1647.8730, 1649.8650, 1650.8523, 1658.8206,
1659.8125, 1661.8284, 1663.7981, 1665.8070, 1669.8174, 1677.8353, 1679.9403, 1680.8076, 1682.7780, 1696.8055, 1697.7845, 1698.7950,
1707.7931, 1709.8029, 1710.8878, 1712.9166, 1716.8563, 1725.8188, 1727.8683, 1730.8236, 1731.8221, 1742.8798, 1746.1482, 1749.8406,
1751.8657, 1756.8782, 1757.8225, 1763.8152, 1765.7922, 1766.8105, 1768.8596, 1769.4275, 1770.4087, 1773.8615, 1774.8795, 1775.0302,
1783.8599, 1785.8483, 1786.8871, 1790.8822, 1792.7894, 1801.9315, 1804.9026, 1808.8663, 1824.8685, 1830.8608, 1849.8231, 1851.8430,
1867.9100, 1868.9441, 1870.9304, 1880.9020, 1884.9191, 1908.8989, 1914.9821, 1916.9882, 1920.9862, 1930.9827, 1932.9813, 1934.9822,
1947.0288, 1958.9543, 1994.8763, 2011.9012, 2013.8700, 2020.9592, 2025.5920, 2042.0568, 2047.0238, 2047.9805, 2049.9497, 2052.9905,
2053.9851, 2058.9988, 2060.0400, 2060.0405, 2065.0427, 2066.0137, 2066.9895, 2067.9763, 2068.9128, 2070.8792, 2074.8860, 2082.0293,
2084.8977, 2086.8872, 2087.8835, 2090.8708, 2092.8674, 2093.8711, 2096.9836, 2097.9961, 2100.8787, 2101.8999, 2103.9092, 2107.8730,
2119.9924, 2122.9397, 2136.9133, 2211.1047, 2217.9368, 2225.1243, 2232.0229, 2233.0840, 2238.1050, 2250.0259, 2257.9934, 2262.9368,
2263.9700, 2273.0264, 2277.9500, 2283.1741, 2290.9570, 2307.9631, 2321.9558, 2323.9670, 2324.9824, 2335.9763, 2339.9456, 2343.0320,
2349.9844, 2352.0308, 2355.0315, 2358.0854, 2368.0815, 2369.0588, 2378.0020, 2379.0002, 2380.0007, 2381.9963, 2383.0444, 2383.9822,
2393.0020, 2394.0181, 2398.0447, 2400.0771, 2405.0879, 2414.1050, 2417.0933, 2421.0769, 2422.0593, 2426.0476, 2428.0725, 2430.0947,
2432.0962, 2435.0254, 2438.0706, 2444.0901, 2451.0278, 2459.9802, 2461.0254, 2501.2520, 2528.1902, 2543.2092, 2564.1965, 2705.2144,
2717.1655, 2834.2925, 2888.2483, 2904.4053, 3130.4312, 3291.4875, 3312.3765, 3451.6833, 3454.6270

38. G3MWK4 **Mass:** 30649 **Score:** 17 **Expect:** 4.8e+002 **Matches:** 12

Uncharacterized protein OS=Bos taurus GN=SRSF12 PE=4 SV=1

Observed	Mr(expt)	Mr(calc)	ppm	Start	End	Miss	Ions	Peptide
951.4646	950.4573	950.4781	-21.88	203 -	211	1	---	K.QTSSGTKSR.S
961.4901	960.4828	960.4849	-2.22	172 -	179	2	---	R.RNSGSRER.S
1030.5259	1029.5186	1029.5428	-23.45	169 -	177	2	---	R.TPRRNSGSR.E
1177.6130	1176.6057	1176.5921	11.6	92 -	101	2	---	K.TPGQMKSKER.H + Oxidation (M)
1179.5918	1178.5845	1178.5905	-5.03	120 -	129	2	---	R.TRSRSSSWGR.N
1192.6014	1191.5941	1191.5857	7.08	122 -	131	2	---	R.SRSSSWGRNR.R
1233.6230	1232.6157	1232.6361	-16.50	191 -	202	1	---	K.SIGKSQSSSPQK.Q
1295.6366	1294.6293	1294.6530	-18.32	142 -	151	2	---	R.HRRFSYSQSK.S
1393.6727	1392.6654	1392.6871	-15.60	243 -	253	2	---	K.HSHFRSHSRSR.S
1649.8650	1648.8577	1648.8295	17.1	238 -	251	2	---	K.AQTAKHSHFRSHSR.S
1697.7845	1696.7772	1696.8090	-18.73	223 -	237	2	---	R.SPCKSPKAYTNSETK.A
1930.9827	1929.9754	1929.9877	-6.34	33 -	48	0	---	R.YGPIVDVYIPLDFYTR.R

No match to: 713.4400, 713.4415, 724.3446, 728.2467, 730.2754, 732.2383, 734.2729, 735.3820, 736.3320, 740.2330, 743.2514,
748.2947, 750.2650, 756.4777, 764.3574, 765.3959, 768.3784, 772.4155, 774.3952, 778.4551, 780.3343, 782.3223, 783.3115, 787.2390,
791.2662, 802.4609, 805.3347, 806.4342, 807.3921, 808.4378, 812.4002, 814.4128, 820.4252, 828.3922, 830.3668, 834.3623, 837.3794,
840.3857, 842.5100, 845.4155, 851.3846, 856.5103, 865.3634, 869.4703, 870.5254, 871.4965, 872.6990, 888.3988, 891.4706, 893.9500,
894.4534, 897.0277, 905.4637, 911.4314, 912.4475, 913.5080, 913.5100, 918.5267, 925.4804, 927.4970, 930.5015, 933.4662, 935.4675,
943.4757, 952.4799, 957.4673, 962.4866, 967.4440, 969.4496, 972.4781, 974.5051, 975.4521, 977.4406, 978.4772, 979.4924, 983.4706,
985.6071, 991.4533, 993.5267, 995.5177, 999.4602, 1001.4565, 1001.5621, 1003.5043, 1007.5638, 1012.5004, 1014.5331, 1018.5130,
1022.5398, 1023.5525, 1028.5991, 1035.5314, 1036.5333, 1037.5145, 1040.5127, 1042.5243, 1045.5603, 1060.5398, 1069.4846, 1075.5515,
1092.6041, 1106.5313, 1109.5087, 1111.5457, 1113.5419, 1115.5394, 1118.5659, 1121.5419, 1122.5701, 1131.5602, 1138.5428, 1149.5912,
1155.5768, 1157.5907, 1158.6328, 1163.6145, 1173.6122, 1178.6060, 1180.6001, 1181.6368, 1183.6030, 1187.6376, 1193.5928, 1195.5924,
1199.6785, 1206.6066, 1215.6306, 1216.6482, 1226.5839, 1227.6051, 1231.6115, 1232.6251, 1234.6500, 1235.6155, 1248.6078, 1250.6483,
1254.6526, 1255.6036, 1256.6123, 1268.6208, 1271.6423, 1276.6179, 1277.6350, 1289.6449, 1292.6322, 1294.6362, 1300.5895, 1303.6643,
1305.6523, 1307.6600, 1308.6443, 1310.6051, 1315.6364, 1320.6128, 1321.6134, 1323.6320, 1325.6456, 1331.6674, 1332.6602, 1333.2076,
1334.6744, 1336.6710, 1337.6279, 1338.6300, 1338.6300, 1345.6146, 1347.6653, 1349.6800, 1349.6827, 1354.6820, 1355.6964, 1359.7053,
1360.6173, 1361.6373, 1365.6752, 1371.6718, 1375.6670, 1377.6776, 1382.6367, 1383.6619, 1387.6700, 1400.6235, 1409.7107, 1411.6742,
1427.7725, 1431.7605, 1432.6987, 1434.7190, 1449.7479, 1466.6953, 1473.7905, 1475.7498, 1478.7303, 1479.7468, 1485.7531, 1486.7472,
1493.7345, 1495.7502, 1500.7242, 1503.7690, 1505.8511, 1509.6786, 1520.7347, 1522.7239, 1523.7567, 1525.7864, 1527.8068, 1532.7472,
1537.7886, 1539.7711, 1549.7833, 1555.8657, 1558.8440, 1559.8573, 1568.7592, 1570.7814, 1578.7899, 1606.8213, 1615.8425, 1619.7972,
1621.7831, 1628.8156, 1630.8202, 1631.8217, 1639.9263, 1644.8083, 1647.8730, 1650.8523, 1658.8206, 1659.8125, 1661.8284, 1663.7981,
1665.8070, 1669.8174, 1677.8353, 1679.9403, 1680.8076, 1682.7780, 1696.8055, 1698.7950, 1707.7931, 1709.8029, 1710.8878, 1712.9166,
1716.8563, 1725.8188, 1727.8683, 1730.8236, 1731.8221, 1742.8798, 1746.1482, 1749.8406, 1751.8657, 1756.8782, 1757.8225, 1763.8152,
1765.7922, 1766.8105, 1768.8596, 1769.4275, 1770.4087, 1773.8615, 1774.8795, 1775.0302, 1783.8599, 1785.8483, 1786.8871, 1790.8822,
1792.7894, 1801.9315, 1804.9026, 1808.8663, 1824.8685, 1830.8608, 1849.8231, 1851.8430, 1867.9100, 1868.9441, 1870.9304, 1880.9020,
1884.9191, 1908.8989, 1914.9821, 1916.9882, 1920.9862, 1932.9813, 1934.9822, 1947.0288, 1958.9543, 1994.8763, 2011.9012, 2013.8700,
2020.9592, 2025.5920, 2042.0568, 2047.0238, 2047.9805, 2049.9497, 2052.9905, 2053.9851, 2058.9988, 2060.0400, 2060.0405, 2065.0427,
2066.0137, 2066.9895, 2067.9763, 2068.9128, 2070.8792, 2074.8860, 2082.0293, 2084.8977, 2086.8872, 2087.8835, 2090.8708, 2092.8674,
2093.8711, 2096.9836, 2097.9961, 2100.8787, 2101.8999, 2103.9092, 2107.8730, 2119.9924, 2122.9397, 2136.9133, 2211.1047, 2217.9368,
2225.1243, 2232.0229, 2233.0840, 2238.1050, 2250.0259, 2257.9934, 2262.9368, 2263.9700, 2273.0264, 2277.9500, 2283.1741, 2290.9570,
2307.9631, 2321.9558, 2323.9670, 2324.9824, 2335.9763, 2339.9456, 2343.0320, 2349.9844, 2352.0308, 2355.0315, 2358.0854, 2368.0815,
2369.0588, 2378.0020, 2379.0002, 2380.0007, 2381.9963, 2383.0444, 2383.9822, 2393.0020, 2394.0181, 2398.0447, 2400.0771, 2405.0879,
2414.1050, 2417.0933, 2421.0769, 2422.0593, 2426.0476, 2428.0725, 2430.0947, 2432.0962, 2435.0254, 2438.0706, 2444.0901, 2451.0278,
2459.9802, 2461.0254, 2501.2520, 2528.1902, 2543.2092, 2564.1965, 2705.2144, 2717.1655, 2834.2925, 2888.2483, 2904.4053, 3130.4312,
3291.4875, 3312.3765, 3451.6833, 3454.6270

39. G3N0W0 **Mass:** 3134 **Score:** 17 **Expect:** 4.9e+002 **Matches:** 4

Uncharacterized protein OS=Bos taurus PE=4 SV=1

Observed	Mr(expt)	Mr(calc)	ppm	Start	End	Miss	Ions	Peptide
765.3959	764.3887	764.3970	-10.87	20 -	25	1	---	R.WGRTVF.-
2119.9924	2118.9851	2118.9617	11.0	1 -	17	0	---	-.MLWFCYGYTMGTLVFSK.E + Oxidation (M)
2405.0879	2404.0806	2404.1054	-10.32	1 -	19	1	---	-.MLWFCYGYTMGTLVFSKER.W + Oxidation (M)
2421.0769	2420.0696	2420.1003	-12.69	1 -	19	1	---	-.MLWFCYGYTMGTLVFSKER.W + 2 Oxidation (M)
[truncated: 92,674 more chars]
