# Supplementary material for: Matrisome Profiling During Intervertebral Disc Development And Ageing
Source: Sci Rep. 2017 Sep 14;7:11629. doi: 10.1038/s41598-017-11960-0 (PMC5599645; doi:10.1038/s41598-017-11960-0)
Supplement: Supplementary file 4 — Supplementary Data 4 [file 41598_2017_11960_MOESM4_ESM.pdf]

MATRIX SCIENCE Mascot Search Results

User	: 1F_(L1@52381_05Apr2017)
Email	: proteomics@i3s.up.pt
Search title	: Project: Proteomics, Spot Set: Proteomics\05Apr2017, Label: L1, Spot Id: 226996, Peak List Id: 447667, MS Job Run Id: 316
MS data file	: C:\Documents and Settings\Administrator\Desktop\xu\29JUNHO\Proteomics\05Apr2017\pmf_L1_149875820400.txt
Database	: OrganismSpecie Bos_taurus_Reference_Proteome_2016_06 (24214 sequences; 12839866 residues)
Timestamp	: 29 Jun 2017 at 17:59:09 GMT
Top Score	: 161 for Mixture 1, F1MRZ5 + E1BB91

Mascot Score Histogram

Protein score is -10*Log(P), where P is the probability that the observed match is a random event.
Protein scores greater than 56 are significant (p<0.05).

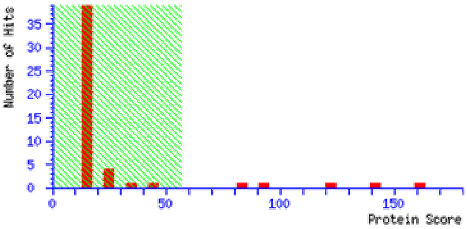

Protein Summary Report

Format As Protein Summary [Help]

Significance threshold p< 0.05 Max. number of hits 50

Preferred taxonomy All entries

Re-Search All Search Unmatched

Index

	Accession	Mass	Score	Description
1.	Mixture 1		161	F1MRZ5 + E1BB91
2.	Mixture 2		142	F1MRZ6 + E1BB91
3.	F1MRZ5	196677	124	Uncharacterized protein OS=Bos taurus GN=TNC PE=4 SV=1
4.	F1MRZ6	250106	97	Uncharacterized protein OS=Bos taurus GN=TNC PE=4 SV=2
5.	E1BB91	343908	79	Uncharacterized protein OS=Bos taurus GN=COL6A3 PE=1 SV=1
6.	P21809	41963	46	Biglycan OS=Bos taurus GN=BGN PE=1 SV=3
7.	G3N3E4	185893	33	Uncharacterized protein OS=Bos taurus GN=COL6A3 PE=1 SV=1
8.	G3N126	135082	27	Uncharacterized protein OS=Bos taurus GN=COL6A3 PE=1 SV=1
9.	Q17R06	24530	23	Ras-related protein Rab-21 OS=Bos taurus GN=RAB21 PE=2 SV=1
10.	F1MPP1	23962	21	Ras-related protein Rab-21 OS=Bos taurus GN=RAB21 PE=4 SV=2
11.	E1BMT4	22224	20	Uncharacterized protein OS=Bos taurus GN=LOC618985 PE=3 SV=2
12.	Q58CY3	41067	18	Chromosome 20 open reading frame 4 OS=Bos taurus GN=C20orf4 PE=2 SV=1
13.	Q32L59	41560	18	Transmembrane and coiled-coil domain-containing protein 5B OS=Bos taurus GN=TMCO5B PE=2 SV=1
14.	F1MWR4	41530	18	Transmembrane and coiled-coil domain-containing protein 5B OS=Bos taurus GN=TMCO5B PE=4 SV=1
15.	F1MQ25	19349	18	39S ribosomal protein L49, mitochondrial OS=Bos taurus GN=MRPL49 PE=4 SV=2
16.	Q2KIA2	14368	18	Multifunctional methyltransferase subunit TRM112-like protein OS=Bos taurus GN=TRMT112 PE=2 SV=1
17.	P79244	11299	18	60S ribosomal protein L37 OS=Bos taurus GN=RPL37 PE=3 SV=4
18.	E1BFX3	11338	18	Ribosomal protein L37 OS=Bos taurus GN=RPL37 PE=3 SV=1
19.	A6H782	57102	18	Tektin-3 OS=Bos taurus GN=TEKT3 PE=2 SV=1
20.	Q2KIN6	19801	17	Protein Mpv17 OS=Bos taurus GN=MPV17 PE=2 SV=1
21.	P02526	21482	17	Gamma-crystallin B OS=Bos taurus GN=CRYGB PE=1 SV=2
22.	A8YXZ5	11449	17	Uncharacterized protein C8orf59 homolog OS=Bos taurus PE=3 SV=2
23.	Q0P568	26340	17	Ubiquitin fusion degradation 1 like OS=Bos taurus GN=UFD1L PE=2 SV=1
24.	Q5EAC0	34118	17	Solute carrier family 25 member 38 OS=Bos taurus GN=SLC25A38 PE=2 SV=2
25.	F1MUP9	43041	17	Uncharacterized protein OS=Bos taurus GN=VAT1 PE=4 SV=2
26.	P82292	13660	17	Spermadhesin Z13 OS=Bos taurus PE=1 SV=1
27.	Q2TBX4	51945	17	Heat shock 70 kDa protein 13 OS=Bos taurus GN=HSPA13 PE=2 SV=1
28.	P01966	15175	16	Hemoglobin subunit alpha OS=Bos taurus GN=HBA PE=1 SV=2
29.	G3MZZ4	7599	16	Uncharacterized protein OS=Bos taurus PE=4 SV=1
30.	F1MGJ7	31607	16	Uncharacterized protein OS=Bos taurus GN=AUH PE=1 SV=2
31.	G3N1R0	18422	16	Major histocompatibility complex class I-related gene protein OS=Bos taurus GN=MR1 PE=3 SV=1
32.	F1MCS0	15398	16	Uncharacterized protein OS=Bos taurus PE=4 SV=2
33.	F1N0K7	15524	16	Uncharacterized protein OS=Bos taurus PE=4 SV=1
34.	G3X8G8	36269	16	Olfactory receptor OS=Bos taurus GN=LOC509526 PE=3 SV=1
35.	A6QLP2	67758	16	Adenosylhomocysteinase 3 OS=Bos taurus GN=AHCYL2 PE=1 SV=1
36.	Q2KIL1	49974	16	Uncharacterized protein C1orf141 homolog OS=Bos taurus PE=2 SV=1
37.	F1MNH1	49975	16	Uncharacterized protein OS=Bos taurus GN=C1orf141 PE=4 SV=2
38.	P08220	54381	16	Gamma-aminobutyric acid receptor subunit beta-1 OS=Bos taurus GN=GABRB1 PE=1 SV=1
39.	A1A4P3	13522	16	Purkinje cell protein 2 OS=Bos taurus GN=PCP2 PE=2 SV=1
40.	Q5EA89	36065	15	Protein FAM76A OS=Bos taurus GN=FAM76A PE=2 SV=1
41.	Q5EA43	36620	15	Sideroflexin-2 OS=Bos taurus GN=SFXN2 PE=2 SV=2
42.	F1MY80	33505	15	Uncharacterized protein OS=Bos taurus GN=HOXC10 PE=4 SV=2
43.	A0A140T855	36964	15	Sideroflexin OS=Bos taurus GN=SFXN2 PE=3 SV=1
44.	F1MU12	53494	15	Keratin, type II cytoskeletal 8 OS=Bos taurus GN=KRT8 PE=1 SV=1
45.	G3N2V0	18032	15	Ras-related protein Rab-5A OS=Bos taurus GN=RAB5A PE=4 SV=1
46.	G3MWH3	76263	15	Uncharacterized protein OS=Bos taurus GN=PIK3CB PE=3 SV=1
47.	E1BDP9	81899	15	Uncharacterized protein OS=Bos taurus GN=CEP63 PE=4 SV=1
48.	E1BKL0	21110	15	Uncharacterized protein OS=Bos taurus PE=4 SV=2
49.	Q2KIB6	22324	15	Protein lin-7 homolog B OS=Bos taurus GN=LIN7B PE=2 SV=1
50.	E1BAN2	60255	15	Katanin p60 ATPase-containing subunit A-like 2 OS=Bos taurus GN=KATNAL2 PE=3 SV=2

Results List

1. **Mixture 1** **Total score:** **161** **Expect:** 1.9e-012 **Matches:** 145

Components: 1. F1MRZ5 Uncharacterized protein OS=Bos taurus GN=TNC PE=4 SV=1
2. E1BB91 Uncharacterized protein OS=Bos taurus GN=COL6A3 PE=1 SV=1

Observed	Mr(expt)	Mr(calc)	ppm	Start	End	Miss	Comp	Peptide
710.3746	709.3673	709.3581	12.9	2505	2510	0	2	R.SGFLMR.K
729.4379	728.4306	728.4181	17.2	701	707	0	1	K.SIPVSAR.V
734.4639	733.4567	733.4487	10.9	1593	1598	0	2	R.LVFTVR.E
745.4249	744.4176	744.4130	6.18	1028	1033	0	1	K.EVQLTR.D
747.4562	746.4490	746.4439	6.75	1774	1780	0	2	K.VFAVGVR.N
753.3594	752.3522	752.3453	9.14	918	924	0	1	K.AAADSYR.I
756.3831	755.3758	755.3715	5.72	1012	1016	1	1	K.FDRYR.L
768.4982	767.4910	767.4766	18.7	104	109	1	1	R.INIPRR.A
771.3934	770.3861	770.3752	14.2	1687	1692	0	1	K.GAFWYK.N
805.4591	804.4518	804.4494	3.06	1520	1526	0	2	K.ALEFVAR.N
823.4842	822.4769	822.4824	-6.66	1060	1066	1	1	R.HKSKPAR.V
889.5049	888.4977	888.4930	5.29	1731	1737	0	1	K.LRPSNFR.N
922.4780	921.4708	921.4668	4.28	763	769	0	1	R.RPETTYR.Q
938.4989	937.4916	937.4869	5.03	1034	1041	0	1	R.DTTSFVLR.G
958.4880	957.4807	957.4767	4.18	1602	1609	0	2	R.DLPSIEER.M
968.4982	967.4910	967.5127	-22.49	2848	2855	0	2	K.NLVQFGYK.Q
973.5403	972.5331	972.5352	-2.23	1119	1128	0	2	R.NILVGSAGSR.I
979.3778	978.3705	978.3648	5.90	501	508	0	1	R.CPGDCSQR.G
979.5679	978.5607	978.5723	-11.88	1722	1729	1	2	K.VGLEHLRR.N
983.6275	982.6202	982.6175	2.78	1977	1985	0	2	R.ALILVGLER.V
988.3948	987.3875	987.3651	22.7	439	446	0	1	R.CPSDCHGR.G
993.5026	992.4953	992.4927	2.66	1654	1661	0	2	R.DSFQEVLR.F
1017.5743	1016.5670	1016.5655	1.54	1412	1420	1	1	R.IFAEKGPQK.S
1031.6372	1030.6299	1030.6288	1.12	1771	1780	1	2	R.GVKVFAVGVR.N
1036.5481	1035.5408	1035.5349	5.71	1068	1076	0	2	R.VAVVQYSDR.T
1051.5096	1050.5023	1050.4982	3.93	1428	1436	0	1	K.FTTDLDSPR.D
1056.5107	1055.5034	1055.4784	23.7	1576	1583	1	1	K.NGREDFYR.N
1113.5466	1112.5393	1112.5363	2.72	1730	1739	0	2	R.NHFVPEAGSR.L
1123.6021	1122.5948	1122.5934	1.26	2000	2008	0	2	R.GFTYNRPLR.L
1143.4449	1142.4376	1142.4346	2.66	563	572	0	1	R.CPGDCHGQGR.C
1153.6440	1152.6367	1152.6251	10.1	2042	2053	1	2	R.GDRGPIGSIGPK.G
1172.6813	1171.6740	1171.6713	2.29	1362	1372	0	2	K.QFGVAPLTIAR.N
1178.6198	1177.6125	1177.6091	2.89	1609	1618	0	1	K.ITAQGQYELR.V
1187.7026	1186.6953	1186.6935	1.58	1323	1332	1	2	R.NIFKRPLGSR.I
1194.5691	1193.5618	1193.5612	0.56	155	164	0	1	R.VDTRPFCSGR.G
1207.5975	1206.5902	1206.5881	1.78	887	897	0	1	K.ETFTTGLDAPR.N
1215.6563	1214.6490	1214.6731	-19.83	1496	1506	1	2	K.SQASVLDAIRR.L
1216.4995	1215.4922	1215.4873	4.03	376	384	1	1	R.RCPSDCHER.G
1255.6042	1254.5969	1254.5973	-1.87	535	545	0	2	R.NNLFTEAAGYR.A
1265.7039	1264.6966	1264.6928	3.02	2512	2522	0	2	K.VAVFFSNKPTR.A
1269.6589	1268.6516	1268.6374	11.2	1729	1739	1	2	R.RNHFVPEAGSR.L
1273.6492	1272.6419	1272.6422	-0.21	1966	1976	1	2	R.ASEELRQEGVR.A
1278.6917	1277.6844	1277.6840	0.33	760	769	1	1	K.SLRRPETTYR.Q
1286.7411	1285.7338	1285.7394	-4.36	966	977	0	2	K.QSGVVPFILQAK.N
1288.7273	1287.7200	1287.7074	9.77	708	719	0	1	R.VATYLPTPEGLK.F
1299.5450	1298.5377	1298.5357	1.57	562	572	1	1	R.RCPGDCHGQGR.C
1304.6617	1303.6544	1303.6554	-0.78	1876	1887	0	2	K.ISCSGSQLPTVR.V
1317.7113	1316.7040	1316.7048	-0.59	1206	1216	1	2	R.VTQLSREELSR.L
1351.6887	1350.6814	1350.6819	-0.39	681	691	0	1	R.ELEPGVEYFIR.V
1356.7275	1355.7202	1355.7157	3.30	1145	1157	1	2	R.SGDDVRGPSVVLR.R
1371.7595	1370.7522	1370.7518	0.33	1194	1205	0	2	R.QLGTIQQVISER.V
1375.7335	1374.7262	1374.7256	0.49	1310	1322	0	2	R.QVNVGGALEYVAR.N
1390.6962	1389.6889	1389.6888	0.08	1599	1609	1	2	R.EFRDLPSIEER.M
1393.7622	1392.7549	1392.7878	-23.58	2511	2522	1	2	R.KVAVFFSNKPTR.A
1413.6547	1412.6474	1412.6507	-2.29	1719	1730	0	1	K.GHEHSIQFAEMK.L
1420.7949	1419.7876	1419.7874	0.14	1043	1054	1	2	R.SGFPLLKEFVQR.V
1440.7788	1439.7715	1439.7733	-1.20	1055	1067	1	2	R.VVESLDVGPDRVR.V
1450.8590	1449.8517	1449.8555	-2.60	1003	1015	0	2	K.IGDLQPQIVNLLK.S
1456.7791	1455.7718	1455.7722	-0.24	1920	1932	0	2	R.AQHPYVLTADTLK.L
1467.7502	1466.7429	1466.7517	-6.01	914	926	0	2	K.ALNLGYALDYAQR.Y
1470.7725	1469.7652	1469.7726	-5.00	863	875	0	2	K.VIDELDVKPEGTR.V
1512.8181	1511.8108	1511.8168	-3.99	1145	1158	2	2	R.SGDDVRGPSVVLRR.G
1513.7562	1512.7489	1512.7460	1.90	1259	1271	0	2	R.LVDYLDVGFDTTR.V
1562.7806	1561.7733	1561.7736	-0.19	902	914	0	1	R.ISQTDNSITLEWR.N
1580.8347	1579.8274	1579.8318	-2.79	2169	2185	1	2	R.GAKGETGDIGPVGLPGR.D
1590.7754	1589.7681	1589.7654	1.72	3101	3114	2	2	K.ADICKLPKDGGTCR.E
1616.7943	1615.7870	1615.7876	-0.33	2429	2442	0	2	K.ILDDLTIAESNCPR.G
1628.8575	1627.8502	1627.8464	2.36	2954	2968	1	2	R.SMATRSEAPRPQAVK.L
1635.7671	1634.7598	1634.7570	1.74	1756	1770	0	2	K.SVEDAQEASMALTQR.G
1647.8516	1646.8443	1646.8450	-0.41	1986	1999	0	2	R.VANLEQLMQLEFGR.G
1657.9042	1656.8969	1656.8988	-1.12	1514	1527	1	1	K.TIFTTSGVLYRFPR.D
1663.8501	1662.8428	1662.8399	1.75	1986	1999	0	2	R.VANLEQLMQLEFGR.G + Oxidation (M)
1664.8555	1663.8482	1663.8893	-24.71	1028	1041	1	1	K.EVQLTRDTTSFVLR.G
1696.8273	1695.8200	1695.8216	-0.95	927	942	0	1	K.YAPISGGDHAEVEVPR.S
1707.9615	1706.9542	1706.9355	10.9	1638	1652	1	2	K.KADIVFLLDGSINFR.R
1717.8761	1716.8688	1716.8287	23.4	110	125	1	1	R.ACGCAAAPDVKELLSR.L
1718.8779	1717.8706	1717.8747	-2.39	901	914	1	1	R.RISQTDNSITLEWR.N
1721.9366	1720.9293	1720.9512	-12.72	1537	1552	0	2	R.IEDGVPQHLVLFLGGK.S
1741.9938	1740.9865	1740.9887	-1.23	1740	1755	1	2	R.LDQRVPQIAFVITGGK.S

1747.9058	1746.8985	1746.9039	-3.10	1042 - 1057	0	1	R.GLEPGQEYSILLTAEK.G
1755.8904	1754.8831	1754.8839	-0.45	518 - 534	0	2	K.ALDGSALYTGSALDFVR.N
1758.9028	1757.8955	1757.8988	-1.88	1382 - 1396	0	2	K.ISLSPEYVFSVNTFR.E
1784.0254	1783.0181	1783.0204	-1.25	1217 - 1233	1	2	R.LQASVTPLTTPVVSSKR.D
1862.9503	1861.9430	1861.9422	0.47	2534 - 2550	0	2	K.LSDAGITPLFLTSQEDR.Q
1870.7574	1869.7501	1869.7404	5.21	138 - 154	0	1	R.EQCTSGAGCCLQSAEGR.V
1883.7222	1882.7149	1882.7145	0.21	593 - 608	0	1	R.SCPNDCSNWGQCVSGR.C
1938.0079	1937.0006	1937.0007	-0.02	925 - 942	1	1	R.IKYAPISGGDHAEVEVPR.S
1961.0325	1960.0252	1960.0265	-0.66	1042 - 1059	1	1	R.GLEPGQEYSILLTAEKGR.H
1969.9990	1968.9917	1968.9905	0.60	1948 - 1965	0	2	K.VVIHFTDGVDGDLADLQR.A
1979.0790	1978.0717	1978.0748	-1.56	891 - 906	1	2	R.FDQHQNKPEILNLVKR.M
1981.0576	1980.0503	1980.0357	7.40	1476 - 1492	0	2	R.IGVLQFSNDVFPEFQLK.T
2008.9901	2007.9828	2007.9672	7.78	1687 - 1702	2	1	K.GAFWYKNCHRVNLMGR.Y
2060.9788	2059.9715	2059.9348	17.8	1623 - 1641	1	1	R.DHGESAHAVYDKFSVGDAR.T
2067.1584	2066.1511	2066.1524	-0.63	1098 - 1118	0	2	R.GLTLLGGPAPNTGAALEFVLR.N
2086.1133	2085.1060	2085.0994	3.18	993 - 1011	0	1	R.ISDPTETSLTLVWQTPVAK.F
2108.9963	2107.9890	2107.9844	2.18	878 - 897	1	1	R.ADMSSNPAKETFTTGLDAPR.N
2112.9805	2111.9732	2111.9694	1.79	1646 - 1665	1	1	R.LKVEGYSGTAGDSMAYHNGR.S
2137.1189	2136.1116	2136.1103	0.63	858 - 875	1	2	R.DFLYKVIDELDVKPEGTR.V
2180.1812	2179.1739	2179.1750	-0.47	1532 - 1552	1	2	K.SAGSRIEDGVPQHLVLFLGGK.S
2241.1355	2240.1282	2240.1114	7.53	1234 - 1253	0	2	R.DVVFLIDGSQSAGPEFQYIR.T
2245.8403	2244.8330	2244.8293	1.66	392 - 410	0	1	R.CECNDGFTGADCGELQCPR.D
2265.1016	2264.0943	2264.0855	3.89	877 - 897	2	1	R.RADMSSNPAKETFTTGLDAPR.N
2273.0654	2272.0581	2272.0509	3.18	85 - 103	0	1	R.ESFQEHTVDGENQIVFTHR.I
2281.1055	2280.0982	2280.0804	7.80	877 - 897	2	1	R.RADMSSNPAKETFTTGLDAPR.N + Oxidation (M)
2318.1824	2317.1751	2317.1703	2.10	1437 - 1456	0	1	R.DFTATEVQSETALLTWRPPR.A
2328.9529	2327.9456	2327.9392	2.76	542 - 561	0	1	R.CVDGQCVCLEGFTGPDCAQR.R
2347.8682	2346.8609	2346.8246	15.5	356 - 375	0	1	R.CDEGQCVCDEGFAGPDCSER.R
2352.9265	2351.9192	2351.9141	2.19	573 - 592	0	1	R.CVDGQCVCHEGFTGPDCAQR.S
2363.8860	2362.8787	2362.8672	4.90	418 - 437	0	1	R.CVNGQCVCDEGYTGEDCGQR.R
2371.0420	2370.0347	2370.0686	-14.28	1666 - 1686	1	1	R.SFSTFDKDTDSAITNCALSYK.G
2389.2756	2388.2683	2388.2649	1.42	1287 - 1310	1	1	R.VTYVPIAGGAPSAVTVDGTKTQTR.L
2397.2231	2396.2158	2396.2125	1.40	1233 - 1253	1	2	K.RDVVFLIDGSQSAGPEFQYIR.T
2406.2913	2405.2840	2405.2855	-0.63	1920 - 1939	2	2	R.AQHPYVLTADTLKLYQNKFR.Q
2431.2300	2430.2227	2430.2107	4.94	1587 - 1608	1	1	K.AYAAGFGDLKEEFWLGLDTLSK.I
2467.2122	2466.2049	2466.1850	8.10	1835 - 1857	0	2	K.ACNLDVILGFDGSGDQNVFVAQK.G
2474.2263	2473.2190	2473.2060	5.27	1077 - 1097	0	2	R.TRPEFYLNSYMDQQSVVGAIR.G
2477.2573	2476.2500	2476.2598	-3.95	670 - 691	1	1	R.VPGDQTSATIRELEPGVEYFIR.V
2485.0691	2484.0618	2484.0403	8.66	542 - 562	1	1	R.CVDGQCVCLEGFTGPDCAQRR.C
2499.1719	2498.1646	2498.2150	-20.16	927 - 949	1	1	K.YAPISGGDHAEVEVPRSQQTTTR.T
2519.9934	2518.9861	2518.9683	7.09	418 - 438	1	1	R.CVNGQCVCDEGYTGEDCGQRR.C
2541.2542	2540.2469	2540.2257	8.35	649 - 669	0	1	R.VTEYLIVYTPTHEDGLEMQFR.V
2542.2739	2541.2666	2541.2598	2.67	1389 - 1411	0	1	R.TVSGNTVEYALTNLEPATEYTLR.I
2580.0227	2579.0154	2578.9968	7.23	480 - 500	1	1	R.CVNGMCVCDDAYTGEDCRELR.C
2683.4189	2682.4116	2682.3905	7.89	1382 - 1404	1	2	K.ISLSPEYVFSVNTFRELPSLEQK.L
2695.0366	2694.0293	2694.0138	5.75	516 - 539	0	1	R.CVCEDGFAGPDCADLACPGNCHGR.G
2799.3379	2798.3306	2798.3392	-3.08	58 - 84	1	1	K.LPVGSQCSVDLESASGEKDLAAPSEPR.E
2813.4517	2812.4444	2812.4283	5.73	1373 - 1396	1	2	R.NVDQEELVKISLSPEYVFSVNTFR.E
2833.1284	2832.1211	2832.0779	15.3	387 - 410	1	1	R.CVDGRCECNDGFTGADCGELQCPR.D
2842.5049	2841.4976	2841.4581	13.9	1566 - 1592	2	2	K.SAGIASLGVGDRNIDRTELQTITSDPR.L
2846.4216	2845.4143	2845.4644	-17.59	1744 - 1770	1	2	R.VPQIAFVITGGKSVEDAQEASMALTQR.G
2961.2437	2960.2364	2960.1993	12.5	268 - 291	1	1	R.CVCQEGFAGEDCREPLCLHNCHGR.G
3029.4883	3028.4810	3028.4488	10.6	1260 - 1286	0	1	K.EIIFSDITENAASVSWMAPTTQVESFR.V
3161.1531	3160.1458	3160.1158	9.51	454 - 479	0	1	R.CECQPGFQGDDCGEMSCPHDCHQHGR.C
3177.1650	3176.1577	3176.1107	14.8	454 - 479	0	1	R.CECQPGFQGDDCGEMSCPHDCHQHGR.C + Oxidation (M)
3237.6042	3236.5969	3236.6565	-18.40	1383 - 1411	1	1	R.VPEITRTVSGNTVEYALTNLEPATEYTLR.I
3248.6902	3247.6829	3247.7200	-11.43	740 - 769	0	2	R.IQDHVPQLLLLLTAGQSEDSYLQAANALAR.A
3360.7197	3359.7124	3359.7302	-5.30	2999 - 3027	0	2	K.LHWERPEPPSPYLYNLTVTSAHDQAVVLK.Q
3411.8193	3410.8120	3410.7583	15.8	1272 - 1301	2	2	R.VAVIQFSDDPRVEFLLNVHSSKDEVQNAVR.R
3435.7471	3434.7398	3434.6841	16.2	847 - 876	0	1	R.TSIDLTHEENQYSIGNLKPDTEYEVALISR.R
3568.9385	3567.9312	3567.8944	10.3	3028 - 3059	2	2	K.QNLTVTDRVIGGLLPGQTYHVTVTCSLRSQVR.A
3625.7847	3624.7774	3624.7810	-0.99	722 - 751	2	1	K.SIKETSVEVEWDPLDIAFETWEVIFRNMNK.D
3645.8701	3644.8628	3644.7861	21.0	1888 - 1919	1	2	R.VSVVALTPSGPVEAFDFAEYQPELFEKFQNMR.A
3722.5232	3721.5159	3721.4307	22.9	231 - 262	0	1	K.CVDGACVCFEGYSGLDCSQETCPVPCSEEHGR.C
3733.5112	3732.5039	3732.4552	13.1	323 - 355	1	1	R.GRCLNGTCSCDEGFTGEDCGQLACPHACHGHGR.C
4074.1699	4073.1626	4073.0804	20.2	950 - 989	2	1	R.TTLTGLRPGTEYGIGVSAVKGDKESDPATINAATELDTPK.D

No match to: 706.2868, 707.2966, 708.3044, 713.3099, 714.3496, 717.4172, 722.4243, 735.4285, 738.4069, 744.3897, 758.3950, 779.4738, 783.3916, 818.4537, 832.3158, 834.3223, 840.4870, 842.5089, 876.2845, 878.2925, 883.4848, 891.4897, 923.5152, 929.4479, 949.5408, 962.3525, 967.4243, 969.5316, 974.4454, 976.4499, 978.5390, 985.6091, 986.5857, 1007.5763, 1021.5607, 1034.5828, 1046.6013, 1057.6161, 1074.6067, 1087.5623, 1126.4208, 1157.4840, 1216.7017, 1248.6692, 1254.6897, 1257.6483, 1264.7133, 1284.6440, 1301.5977, 1314.7190, 1321.7212, 1338.6622, 1349.6757, 1354.7229, 1358.7100, 1382.5935, 1383.7274, 1423.7593, 1457.7606, 1488.7212, 1494.7396, 1500.7236, 1528.6954, 1539.7452, 1556.7537, 1570.7695, 1574.7894, 1578.7858, 1592.7883, 1628.7009, 1631.8130, 1642.7155, 1650.8444, 1693.0090, 1706.8668, 1715.8461, 1730.8695, 1771.0238, 1774.9207, 1787.8574, 1895.7236, 1897.7325, 1909.7473, 1911.7430, 1913.7366, 1925.7604, 1974.8882, 2027.1294, 2144.0579, 2223.1646, 2239.1501, 2255.1479, 2335.9097, 2342.9673, 2356.9395, 2361.8997, 2366.9265, 2380.9539, 2414.2468, 2478.2725, 2503.0352, 2518.0303, 2534.0215, 2558.3401, 2594.0615, 2631.4961, 2678.0144, 2701.2097, 2709.0559, 2717.3198, 2723.0757, 2770.4492, 2787.3171, 2817.2305, 2872.3596, 2944.3171, 2975.3135, 2989.3062, 3004.4270, 3059.4963, 3081.6570, 3086.5718, 3147.5710, 3157.4875, 3185.5222, 3254.5940, 3331.5864, 3335.7378, 3347.5420, 3351.6597, 3428.6296, 3457.6697, 3471.7146, 3475.7085, 3489.7715, 3503.8445, 3528.3743, 3542.4243, 3552.7373, 3603.9502, 3640.7600, 3662.8821, 3691.0452, 3727.5442, 3731.5757, 3736.5735, 3741.5391, 3746.5410, 3748.5002, 3751.5356, 3755.5571, 3756.5549, 3763.5049, 3767.5706, 3778.5320, 3884.9326, 3955.6738, 4149.0591, 4163.0181, 4238.0752, 4300.3838, 4382.1450, 4397.2036, 4441.5176, 4453.2886, 4458.5176, 4464.4150, 4495.2642, 4511.1577, 4692.4893

2. **Mixture 2** **Total score:** **142** **Expect:** 1.5e-010 **Matches:** 147

Components: 1. F1MRZ6 Uncharacterized protein OS=Bos taurus GN=TNC PE=4 SV=2
2. E1BB91 Uncharacterized protein OS=Bos taurus GN=COL6A3 PE=1 SV=1

Observed	Mr(expt)	Mr(calc)	ppm	Start - End	Miss	Comp	Peptide
710.3746	709.3673	709.3581	12.9	2505 - 2510	0	2	R.SGFLMR.K
729.4379	728.4306	728.4181	17.2	731 - 737	0	1	K.SIPVSAR.V

734.4639	733.4567	733.4487	10.9	1593 - 1598	0	2	R.LVFTVR.E
745.4249	744.4176	744.4130	6.18	1058 - 1063	0	1	K.EVQLTR.D
747.4562	746.4490	746.4439	6.75	1774 - 1780	0	2	K.VFAVGVR.N
753.3594	752.3522	752.3453	9.14	948 - 954	0	1	K.AAADSYR.I
756.3831	755.3758	755.3715	5.72	1042 - 1046	1	1	K.FDRYR.L
768.4982	767.4910	767.4766	18.7	134 - 139	1	1	R.INIPRR.A
771.3934	770.3861	770.3752	14.2	2172 - 2177	0	1	K.GAFWYK.N
805.4591	804.4518	804.4494	3.06	1520 - 1526	0	2	K.ALEFVAR.N
823.4842	822.4769	822.4824	-6.66	1090 - 1096	1	1	R.HKSKPAR.V
889.5049	888.4977	888.4930	5.29	2216 - 2222	0	1	K.LRPSNFR.N
922.4780	921.4708	921.4668	4.28	793 - 799	0	1	R.RPETTYR.Q
938.4989	937.4916	937.4869	5.03	1064 - 1071	0	1	R.DTTSFVLR.G
958.4880	957.4807	957.4767	4.18	1602 - 1609	0	2	R.DLPSIEER.M
968.4982	967.4910	967.5127	-22.49	2848 - 2855	0	2	K.NLVQFGYK.Q
973.5403	972.5331	972.5352	-2.23	1119 - 1128	0	2	R.NILVGSAGSR.I
979.3778	978.3705	978.3648	5.90	531 - 538	0	1	R.CPGDCSQR.G
979.5679	978.5607	978.5723	-11.88	1722 - 1729	1	2	K.VGLEHLRR.N
983.6275	982.6202	982.6175	2.78	1977 - 1985	0	2	R.ALILVGLER.V
988.3948	987.3875	987.3651	22.7	469 - 476	0	1	R.CPSDCHGR.G
993.5026	992.4953	992.4927	2.66	1654 - 1661	0	2	R.DSFQEVLR.F
1017.5743	1016.5670	1016.5655	1.54	1897 - 1905	1	1	R.IFAEKGPQK.S
1031.6372	1030.6299	1030.6288	1.12	1771 - 1780	1	2	R.GVKVFAVGVR.N
1036.5481	1035.5408	1035.5349	5.71	1068 - 1076	0	2	R.VAVVQYSDR.T
1051.5096	1050.5023	1050.4982	3.93	1913 - 1921	0	1	K.FTTDLDSPR.D
1056.5107	1055.5034	1055.4784	23.7	2061 - 2068	1	1	K.NGREDFYR.N
1113.5466	1112.5393	1112.5363	2.72	1730 - 1739	0	2	R.NHFVPEAGSR.L
1123.6021	1122.5948	1122.5934	1.26	2000 - 2008	0	2	R.GFTYNRPLR.L
1143.4449	1142.4376	1142.4346	2.66	593 - 602	0	1	R.CPGDCHGQGR.C
1153.6440	1152.6367	1152.6251	10.1	2042 - 2053	1	2	R.GDRGPIGSIGPK.G
1172.6813	1171.6740	1171.6713	2.29	1362 - 1372	0	2	K.QFGVAPLTIAR.N
1178.6198	1177.6125	1177.6091	2.89	2094 - 2103	0	1	K.ITAQGQYELR.V
1187.7026	1186.6953	1186.6935	1.58	1323 - 1332	1	2	R.NIFKRPLGSR.I
1194.5691	1193.5618	1193.5612	0.56	185 - 194	0	1	R.VDTRPFCSGR.G
1207.5975	1206.5902	1206.5881	1.78	917 - 927	0	1	K.ETFTTGLDAPR.N
1215.6563	1214.6490	1214.6731	-19.83	1496 - 1506	1	2	K.SQASVLDAIRR.L
1216.4995	1215.4922	1215.4873	4.03	406 - 414	1	1	R.RCPSDCHER.G
1255.6042	1254.5969	1254.5993	-1.87	535 - 545	0	2	R.NNLFTEAAGYR.A
1265.7039	1264.6966	1264.6928	3.02	2512 - 2522	0	2	K.VAVFFSNKPTR.A
1269.6589	1268.6516	1268.6374	11.2	1729 - 1739	1	2	R.RNHFVPEAGSR.L
1273.6492	1272.6419	1272.6422	-0.21	1966 - 1976	1	2	R.ASEELRQEGVR.A
1278.6917	1277.6844	1277.6840	0.33	790 - 799	1	1	K.SLRRPETTYR.Q
1286.7411	1285.7338	1285.7394	-4.36	966 - 977	0	2	K.QSGVVPFILQAK.N
1288.7273	1287.7200	1287.7074	9.77	738 - 749	0	1	R.VATYLPTPEGLK.F
1299.5450	1298.5377	1298.5357	1.57	592 - 602	1	1	R.RCPGDCHGQGR.C
1304.6617	1303.6544	1303.6554	-0.78	1876 - 1887	0	2	K.ISCSGSQLPTVR.V
1317.7113	1316.7040	1316.7048	-0.59	1206 - 1216	1	2	R.VTQLSREELSR.L
1351.6887	1350.6814	1350.6819	-0.39	711 - 721	0	1	R.ELEPGVEYFIR.V
1356.7275	1355.7202	1355.7157	3.30	1145 - 1157	1	2	R.SGDDVRGPSVVLR.R
1371.7595	1370.7522	1370.7518	0.33	1194 - 1205	0	2	R.QLGTIQQVISER.V
1375.7335	1374.7262	1374.7256	0.49	1310 - 1322	0	2	R.QVNVGGALEYVAR.N
1390.6962	1389.6889	1389.6888	0.08	1599 - 1609	1	2	R.EFRDLPSIEER.M
1393.7622	1392.7549	1392.7878	-23.58	2511 - 2522	1	2	R.KVAVFFSNKPTR.A
1413.6547	1412.6474	1412.6507	-2.29	2204 - 2215	0	1	K.GHEHSIQFAEMK.L
1420.7949	1419.7876	1419.7874	0.14	1043 - 1054	1	2	R.SGFPLLKEFVQR.V
1440.7788	1439.7715	1439.7733	-1.20	1055 - 1067	1	2	R.VVESLDVGPDRVR.V
1450.8590	1449.8517	1449.8555	-2.60	1003 - 1015	0	2	K.IGDLQPQIVNLLK.S
1456.7791	1455.7718	1455.7722	-0.24	1920 - 1932	0	2	R.AQHPYVLTADTLK.L
1467.7502	1466.7429	1466.7517	-6.01	914 - 926	0	2	K.ALNLGYALDYAQR.Y
1470.7725	1469.7652	1469.7726	-5.00	863 - 875	0	2	K.VIDELDVKPEGTR.V
1512.8181	1511.8108	1511.8168	-3.99	1145 - 1158	2	2	R.SGDDVRGPSVVLRR.G
1513.7562	1512.7489	1512.7460	1.90	1259 - 1271	0	2	R.LVDYLDVGFDTTR.V
1562.7806	1561.7733	1561.7736	-0.19	932 - 944	0	1	R.ISQTDNSITLEWR.N
1578.7858	1577.7785	1577.8161	-23.84	1453 - 1467	1	1	R.GYRTPVLSAEASTAR.E
1580.8347	1579.8274	1579.8318	-2.79	2169 - 2185	1	2	R.GAKGETGDIGPVGLPGR.D
1590.7754	1589.7681	1589.7654	1.72	3101 - 3114	2	2	K.ADICKLPKDGGTCR.E
1616.7943	1615.7870	1615.7876	-0.33	2429 - 2442	0	2	K.ILDDLTIAESNCPR.G
1628.8575	1627.8502	1627.8464	2.36	2954 - 2968	1	2	R.SMATRSEAPRPQAVK.L
1635.7671	1634.7598	1634.7570	1.74	1756 - 1770	0	2	K.SVEDAQEASMALTQR.G
1647.8516	1646.8443	1646.8450	-0.41	1986 - 1999	0	2	R.VANLEQLMQLEFGR.G
1657.9042	1656.8969	1656.8988	-1.12	1999 - 2012	1	1	K.TIFTTSGVLYRFPR.D
1663.8501	1662.8428	1662.8399	1.75	1986 - 1999	0	2	R.VANLEQLMQLEFGR.G + Oxidation (M)
1664.8555	1663.8482	1663.8893	-24.71	1058 - 1071	1	1	K.EVQLTRDTTSFVLR.G
1696.8273	1695.8200	1695.8216	-0.95	957 - 972	0	1	K.YAPISGGDHAEVEVPR.S
1707.9615	1706.9542	1706.9355	10.9	1638 - 1652	1	2	K.KADIVFLLDGSINFR.R
1717.8761	1716.8688	1716.8287	23.4	140 - 155	1	1	R.ACGCAAAPDVKELLSR.L
1718.8779	1717.8706	1717.8747	-2.39	931 - 944	1	1	R.RISQTDNSITLEWR.N
1721.9366	1720.9293	1720.9512	-12.72	1537 - 1552	0	2	R.IEDGVPQHLVLFLGGK.S
1741.9938	1740.9865	1740.9887	-1.23	1740 - 1755	1	2	R.LDQRVPQIAFVITGGK.S
1747.9058	1746.8985	1746.9039	-3.10	1072 - 1087	0	1	R.GLEPGQEYSILLTAEK.G
1755.8904	1754.8831	1754.8839	-0.45	518 - 534	0	2	K.ALDGSALYTGSALDFVR.N
1758.9028	1757.8955	1757.8988	-1.88	1382 - 1396	0	2	K.ISLSPEYVFSVNTFR.E
1784.0254	1783.0181	1783.0204	-1.25	1217 - 1233	1	2	R.LQASVTPLTTPVVSSKR.D
1862.9503	1861.9430	1861.9422	0.47	2534 - 2550	0	2	K.LSDAGITPLFLTSQEDR.Q
1870.7574	1869.7501	1869.7404	5.21	168 - 184	0	1	R.EQCTSGAGCCLQSAEGR.V
1883.7222	1882.7149	1882.7145	0.21	623 - 638	0	1	R.SCPNDCSNWGQCVSGR.C

1938.0079	1937.0006	1937.0007	-0.02	955 -	972	1	1	R.IKYAPISGGDHAEVEVPR.S
1961.0325	1960.0252	1960.0265	-0.66	1072 -	1089	1	1	R.GLEPGQEYSILLTAEKGR.H
1969.9990	1968.9917	1968.9905	0.60	1948 -	1965	0	2	K.VVIHFTDGVDGDLADLQR.A
1979.0790	1978.0717	1978.0748	-1.56	891 -	906	1	2	R.FDQHQNKPEILNLVKR.M
1981.0576	1980.0503	1980.0357	7.40	1476 -	1492	0	2	R.IGVLQFSNDVFPEFQLK.T
2008.9901	2007.9828	2007.9672	7.78	2172 -	2187	2	1	K.GAFWYKNCHRVNLMGR.Y
2060.9788	2059.9715	2059.9348	17.8	2108 -	2126	1	1	R.DHGESAHAVYDKFSVGDAR.T
2067.1584	2066.1511	2066.1524	-0.63	1098 -	1118	0	2	R.GLTLLGGPAPNTGAALEFVLR.N
2086.1133	2085.1060	2085.0994	3.18	1023 -	1041	0	1	R.ISDPTETSLTLVWQTPVAK.F
2108.9963	2107.9890	2107.9844	2.18	908 -	927	1	1	R.ADMSSNPAKETFTTGLDAPR.N
2112.9805	2111.9732	2111.9694	1.79	2131 -	2150	1	1	R.LKVEGYSGTAGDSMAYHNGR.S
2137.1189	2136.1116	2136.1103	0.63	858 -	875	1	2	R.DFLYKVIDELDVKPEGTR.V
2180.1812	2179.1739	2179.1750	-0.47	1532 -	1552	1	2	K.SAGSRIEDGVPQHLVLFLGGK.S
2241.1355	2240.1282	2240.1114	7.53	1234 -	1253	0	2	R.DVVFLIDGSQSAGPEFQYIR.T
2245.8403	2244.8330	2244.8293	1.66	422 -	440	0	1	R.CECNDGFTGADCGELQCPR.D
2265.1016	2264.0943	2264.0855	3.89	907 -	927	2	1	R.RADMSSNPAKETFTTGLDAPR.N
2273.0654	2272.0581	2272.0509	3.18	115 -	133	0	1	R.ESFQEHTVDGENQIVFTHR.I
2281.1055	2280.0982	2280.0804	7.80	907 -	927	2	1	R.RADMSSNPAKETFTTGLDAPR.N + Oxidation (M)
2318.1824	2317.1751	2317.1703	2.10	1922 -	1941	0	1	R.DFTATEVQSETALLTWRPPR.A
2328.9529	2327.9456	2327.9392	2.76	572 -	591	0	1	R.CVDGQCVCLEGFTGPDCAQR.R
2347.8682	2346.8609	2346.8246	15.5	386 -	405	0	1	R.CDEGQCVCDEGFAGPDCSER.R
2352.9265	2351.9192	2351.9141	2.19	603 -	622	0	1	R.CVDGQCVCHEGFTGPDCAQR.S
2363.8860	2362.8787	2362.8672	4.90	448 -	467	0	1	R.CVNGQCVCDEGYTGEDCGQR.R
2371.0420	2370.0347	2370.0686	-14.28	2151 -	2171	1	1	R.SFSTFDKDTDSAITNCALSYK.G
2389.2756	2388.2683	2388.2649	1.42	1772 -	1795	1	1	R.VTYVPIAGGAPSAVTVDGTKTQTR.L
2397.2231	2396.2158	2396.2125	1.40	1233 -	1253	1	2	K.RDVVFLIDGSQSAGPEFQYIR.T
2406.2913	2405.2840	2405.2855	-0.63	1920 -	1939	2	2	R.AQHPYVLTADTLKLYQNKFR.Q
2431.2300	2430.2227	2430.2107	4.94	2072 -	2093	1	1	K.AYAAGFGDLKEEFWLGLDTLSK.I
2467.2122	2466.2049	2466.1850	8.10	1835 -	1857	0	2	K.ACNLDVILGFDGSGDQNVFVAQK.G
2474.2263	2473.2190	2473.2060	5.27	1077 -	1097	0	2	R.TRPEFYLNSYMDQQSVVGAIR.G
2477.2573	2476.2500	2476.2598	-3.95	700 -	721	1	1	R.VPGDQTSATIRELEPGVEYFIR.V
2485.0691	2484.0618	2484.0403	8.66	572 -	592	1	1	R.CVDGQCVCLEGFTGPDCAQRR.C
2499.1719	2498.1646	2498.2150	-20.16	957 -	979	1	1	K.YAPISGGDHAEVEVPRSQTTTR.T
2519.9934	2518.9861	2518.9683	7.09	448 -	468	1	1	R.CVNGQCVCDEGYTGEDCGQRR.C
2541.2542	2540.2469	2540.2257	8.35	679 -	699	0	1	R.VTEYLIVYTPTHEDGLEMQFR.V
2542.2739	2541.2666	2541.2598	2.67	1874 -	1896	0	1	R.TVSGNTVEYALTNLEPATEYTLR.I
2580.0227	2579.0154	2578.9968	7.23	510 -	530	1	1	R.CVNGMCVCDDAYTGEDCRELR.C
2683.4189	2682.4116	2682.3905	7.89	1382 -	1404	1	2	K.ISLSPEYVFSVNTFRELPSLEQK.L
2695.0366	2694.0293	2694.0138	5.75	546 -	569	0	1	R.CVCEDGFAGPDCADLACPGNCHGR.G
2799.3379	2798.3306	2798.3392	-3.08	88 -	114	1	1	K.LPVGSQCSVDLESASGEKDLAAPSEPR.E
2813.4517	2812.4444	2812.4283	5.73	1373 -	1396	1	2	R.NVDQEELVKISLSPEYVFSVNTFR.E
2833.1284	2832.1211	2832.0779	15.3	417 -	440	1	1	R.CVDGRCECNDGFTGADCGELQCPR.D
2842.5049	2841.4976	2841.4581	13.9	1566 -	1592	2	2	K.SAGIASLGVGDRNIDRTELQTITSDPR.L
2846.4216	2845.4143	2845.4644	-17.59	1744 -	1770	1	2	R.VPQIAFVITGGKSVEDAQEASMALTQR.G
2961.2437	2960.2364	2960.1993	12.5	298 -	321	1	1	R.CVCQEGFAGEDCREPLCLHNCHGR.G
3029.4883	3028.4810	3028.4488	10.6	1745 -	1771	0	1	K.EIIFSDITENAASVSWMAPTTQVESFR.V
3081.6570	3080.6497	3080.5891	19.7	1320 -	1347	1	1	K.IRETDQPQEVHSLTVPGSQHSVEIPGLK.A
3161.1531	3160.1458	3160.1158	9.51	484 -	509	0	1	R.CECQPGFQGDDCGEMSCPHDCHQHGR.C
3177.1650	3176.1577	3176.1107	14.8	484 -	509	0	1	R.CECQPGFQGDDCGEMSCPHDCHQHGR.C + Oxidation (M)
3237.6042	3236.5969	3236.6565	-18.40	1868 -	1896	1	1	R.VPEITRTVSGNTVEYALTNLEPATEYTLR.I
3248.6902	3247.6829	3247.7200	-11.43	740 -	769	0	2	R.IQDHVPQLLLLLTAGQSEDSYLQAANALAR.A
3360.7197	3359.7124	3359.7302	-5.30	2999 -	3027	0	2	K.LHWERPEPPSPYLYNLTVTSAHDQAVVLK.Q
3411.8193	3410.8120	3410.7583	15.8	1272 -	1301	2	2	R.VAVIQFSDDPRVEFLLNVHSSKDEVQNAVR.R
3435.7471	3434.7398	3434.6841	16.2	877 -	906	0	1	R.TSIDLTHEENQYSIGNLKPDTEYEVALISR.R
3568.9385	3567.9312	3567.9049	7.37	1316 -	1347	2	1	R.FVIKIRETDQPQEVHSLTVPGSQHSVEIPGLK.A
3625.7847	3624.7774	3624.7810	-0.99	752 -	781	2	1	K.SIKETSVEVEWDPLDIAFETWEVIFRNMNK.D
3645.8701	3644.8628	3644.7861	21.0	1888 -	1919	1	2	R.VSVVALTPSGPVEAFDFAEYQPELFEKFQNMR.A
3722.5232	3721.5159	3721.4307	22.9	261 -	292	0	1	K.CVDGACVCFEGYSGLDCSQETCPVPCSEEHGR.C
3733.5112	3732.5039	3732.4552	13.1	353 -	385	1	1	R.GRCLNGTCSCDEGFTGEDCGQLACPHACHGHGR.C
4074.1699	4073.1626	4073.0804	20.2	980 -	1019	2	1	R.TTLTGLRPGTEYGIGVSAVKGDKESDPATINAATELDTPK.D

No match to: 706.2868, 707.2966, 708.3044, 713.3099, 714.3496, 717.4172, 722.4243, 735.4285, 738.4069, 744.3897, 758.3950, 779.4738, 783.3916, 818.4537, 832.3158, 834.3223, 840.4870, 842.5089, 876.2845, 878.2925, 883.4848, 891.4897, 923.5152, 929.4479, 949.5408, 962.3525, 967.4243, 969.5316, 974.4454, 976.4499, 978.5390, 985.6091, 986.5857, 1007.5763, 1021.5607, 1034.5828, 1046.6013, 1057.6161, 1074.6067, 1087.5623, 1126.4208, 1157.4840, 1216.7017, 1248.6692, 1254.6897, 1257.6483, 1264.7133, 1284.6440, 1301.5977, 1314.7190, 1321.7212, 1338.6622, 1349.6757, 1354.7229, 1358.7100, 1382.5935, 1383.7274, 1423.7593, 1457.7606, 1488.7212, 1494.7396, 1500.7236, 1528.6954, 1539.7452, 1556.7537, 1570.7695, 1574.7894, 1592.7883, 1628.7009, 1631.8130, 1642.7155, 1650.8444, 1693.0090, 1706.8668, 1715.8461, 1730.8695, 1771.0238, 1774.9207, 1787.8574, 1895.7236, 1897.7325, 1909.7473, 1911.7430, 1913.7366, 1925.7604, 1974.8882, 2027.1294, 2144.0579, 2223.1646, 2239.1501, 2255.1479, 2335.9097, 2342.9673, 2356.9395, 2361.8997, 2366.9265, 2380.9539, 2414.2468, 2478.2725, 2503.0352, 2518.0303, 2534.0215, 2558.3401, 2594.0615, 2631.4961, 2678.0144, 2701.2097, 2709.0559, 2717.3198, 2723.0757, 2770.4492, 2787.3171, 2817.2305, 2872.3596, 2944.3171, 2975.3135, 2989.3062, 3004.4270, 3059.4963, 3086.5718, 3147.5710, 3157.4875, 3185.5222, 3254.5940, 3331.5864, 3335.7378, 3347.5420, 3351.6597, 3428.6296, 3457.6697, 3471.7146, 3475.7085, 3489.7715, 3503.8445, 3528.3743, 3542.4243, 3552.7373, 3603.9502, 3640.7600, 3662.8821, 3691.0452, 3727.5442, 3731.5757, 3736.5735, 3741.5391, 3746.5410, 3748.5002, 3751.5356, 3755.5571, 3756.5549, 3763.5049, 3767.5706, 3778.5320, 3884.9326, 3955.6738, 4149.0591, 4163.0181, 4238.0752, 4300.3838, 4382.1450, 4397.2036, 4441.5176, 4453.2886, 4458.5176, 4464.4150, 4495.2642, 4511.1577, 4692.4893

3. F1MRZ5 **Mass:** 196677 **Score:** **124** **Expect:** 9.6e-009 **Matches:** 73

Uncharacterized protein OS=Bos taurus GN=TNC PE=4 SV=1

Observed	Mr(expt)	Mr(calc)	ppm	Start	End	Miss	Peptide
729.4379	728.4306	728.4181	17.2	701 -	707	0	K.SIPVSAR.V
745.4249	744.4176	744.4130	6.18	1028 -	1033	0	K.EVQLTR.D
753.3594	752.3522	752.3453	9.14	918 -	924	0	K.AAADSYR.I
756.3831	755.3758	755.3715	5.72	1012 -	1016	1	K.FDRYR.L
768.4982	767.4910	767.4766	18.7	104 -	109	1	R.INIPRR.A
771.3934	770.3861	770.3752	14.2	1687 -	1692	0	K.GAFWYK.N
823.4842	822.4769	822.4824	-6.66	1060 -	1066	1	R.HKSKPAR.V
889.5049	888.4977	888.4930	5.29	1731 -	1737	0	K.LRPSNFR.N
922.4780	921.4708	921.4668	4.28	763 -	769	0	R.RPETTYR.Q

938.4989	937.4916	937.4869	5.03	1034 - 1041	0	R.DTTSFVLR.G
979.3778	978.3705	978.3648	5.90	501 - 508	0	R.CPGDCSQR.G
988.3948	987.3875	987.3651	22.7	439 - 446	0	R.CPSDCHGR.G
1017.5743	1016.5670	1016.5655	1.54	1412 - 1420	1	R.IFAEKGPQK.S
1051.5096	1050.5023	1050.4982	3.93	1428 - 1436	0	K.FTTDLDSPR.D
1056.5107	1055.5034	1055.4784	23.7	1576 - 1583	1	K.NGREDFYR.N
1143.4449	1142.4376	1142.4346	2.66	563 - 572	0	R.CPGDCHGQGR.C
1178.6198	1177.6125	1177.6091	2.89	1609 - 1618	0	K.ITAQGQYELR.V
1194.5691	1193.5618	1193.5612	0.56	155 - 164	0	R.VDTRPFCSGR.G
1207.5975	1206.5902	1206.5881	1.78	887 - 897	0	K.ETFTTGLDAPR.N
1216.4995	1215.4922	1215.4873	4.03	376 - 384	1	R.RCPSDCHER.G
1278.6917	1277.6844	1277.6840	0.33	760 - 769	1	K.SLRRPETTYR.Q
1288.7273	1287.7200	1287.7074	9.77	708 - 719	0	R.VATYLPTPEGLK.F
1299.5450	1298.5377	1298.5357	1.57	562 - 572	1	R.RCPGDCHGQGR.C
1351.6887	1350.6814	1350.6819	-0.39	681 - 691	0	R.ELEPGVEYFIR.V
1413.6547	1412.6474	1412.6507	-2.29	1719 - 1730	0	K.GHEHSIQFAEMK.L
1562.7806	1561.7733	1561.7736	-0.19	902 - 914	0	R.ISQTDNSITLEWR.N
1657.9042	1656.8969	1656.8988	-1.12	1514 - 1527	1	K.TIFTTSGVLYRFPR.D
1664.8555	1663.8482	1663.8893	-24.71	1028 - 1041	1	K.EVQLTRDTTSFVLR.G
1696.8273	1695.8200	1695.8216	-0.95	927 - 942	0	K.YAPISGGDHAEVEVPR.S
1717.8761	1716.8688	1716.8287	23.4	110 - 125	1	R.ACGCAAAPDVKELLSR.L
1718.8779	1717.8706	1717.8747	-2.39	901 - 914	1	R.RISQTDNSITLEWR.N
1747.9058	1746.8985	1746.9039	-3.10	1042 - 1057	0	R.GLEPGQEYSILLTAEK.G
1870.7574	1869.7501	1869.7404	5.21	138 - 154	0	R.EQCTSGAGCCLQSAEGR.V
1883.7222	1882.7149	1882.7145	0.21	593 - 608	0	R.SCPNDCSNWGQCVSGR.C
1938.0079	1937.0006	1937.0007	-0.02	925 - 942	1	R.IKYAPISGGDHAEVEVPR.S
1961.0325	1960.0252	1960.0265	-0.66	1042 - 1059	1	R.GLEPGQEYSILLTAEKGR.H
2008.9901	2007.9828	2007.9672	7.78	1687 - 1702	2	K.GAFWYKNCHRVNLMGR.Y
2060.9788	2059.9715	2059.9348	17.8	1623 - 1641	1	R.DHGESAHAVYDKFSVGDAR.T
2086.1133	2085.1060	2085.0994	3.18	993 - 1011	0	R.ISDPTETSLTLVWQTPVAK.F
2108.9963	2107.9890	2107.9844	2.18	878 - 897	1	R.ADMSSNPAKETFTTGLDAPR.N
2112.9805	2111.9732	2111.9694	1.79	1646 - 1665	1	R.LKVEGYSGTAGDSMAYHNGR.S
2245.8403	2244.8330	2244.8293	1.66	392 - 410	0	R.CECNDGFTGADCGELQCPR.D
2265.1016	2264.0943	2264.0855	3.89	877 - 897	2	R.RADMSSNPAKETFTTGLDAPR.N
2273.0654	2272.0581	2272.0509	3.18	85 - 103	0	R.ESFQEHTVDGENQIVFTHR.I
2281.1055	2280.0982	2280.0804	7.80	877 - 897	2	R.RADMSSNPAKETFTTGLDAPR.N + Oxidation (M)
2318.1824	2317.1751	2317.1703	2.10	1437 - 1456	0	R.DFTATEVQSETALLTWRPPR.A
2328.9529	2327.9456	2327.9392	2.76	542 - 561	0	R.CVDGQCVCLEGFTGPDCAQR.R
2347.8682	2346.8609	2346.8246	15.5	356 - 375	0	R.CDEGQCVCDEGFAGPDCSER.R
2352.9265	2351.9192	2351.9141	2.19	573 - 592	0	R.CVDGQCVCHEGFTGPDCAQR.S
2363.8860	2362.8787	2362.8672	4.90	418 - 437	0	R.CVNGQCVCDEGYTGEDCGQR.R
2371.0420	2370.0347	2370.0686	-14.28	1666 - 1686	1	R.SFSTFDKDTDSAITNCALSYK.G
2389.2756	2388.2683	2388.2649	1.42	1287 - 1310	1	R.VTYVPIAGGAPSAVTVDGTKTQTR.L
2431.2300	2430.2227	2430.2107	4.94	1587 - 1608	1	K.AYAAGFGDLKEEFWLGLDTLSK.I
2477.2573	2476.2500	2476.2598	-3.95	670 - 691	1	R.VPGDQTSATIRELEPGVEYFIR.V
2485.0691	2484.0618	2484.0403	8.66	542 - 562	1	R.CVDGQCVCLEGFTGPDCAQRR.C
2499.1719	2498.1646	2498.2150	-20.16	927 - 949	1	K.YAPISGGDHAEVEVPRSQQTTTR.T
2519.9934	2518.9861	2518.9683	7.09	418 - 438	1	R.CVNGQCVCDEGYTGEDCGQRR.C
2541.2542	2540.2469	2540.2257	8.35	649 - 669	0	R.VTEYLIVYTPTHEDGLEMQFR.V
2542.2739	2541.2666	2541.2598	2.67	1389 - 1411	0	R.TVSGNTVEYALTNLEPATEYTLR.I
2580.0227	2579.0154	2578.9968	7.23	480 - 500	1	R.CVNGMCVCDDAYTGEDCRELR.C
2695.0366	2694.0293	2694.0138	5.75	516 - 539	0	R.CVCEDGFAGPDCADLACPGNCHGR.G
2799.3379	2798.3306	2798.3392	-3.08	58 - 84	1	K.LPVGSQCSVDLESASGEKDLAAPSEPR.E
2833.1284	2832.1211	2832.0779	15.3	387 - 410	1	R.CVDGRCECNDGFTGADCGELQCPR.D
2961.2437	2960.2364	2960.1993	12.5	268 - 291	1	R.CVCQEGFAGEDCREPLCLHNCHGR.G
3029.4883	3028.4810	3028.4488	10.6	1260 - 1286	0	K.EIIFSDITENAASVSWMAPTTQVESFR.V
3161.1531	3160.1458	3160.1158	9.51	454 - 479	0	R.CECQPGFQGDDCGEMSCPHDCHQHGR.C
3177.1650	3176.1577	3176.1107	14.8	454 - 479	0	R.CECQPGFQGDDCGEMSCPHDCHQHGR.C + Oxidation (M)
3237.6042	3236.5969	3236.6565	-18.40	1383 - 1411	1	R.VPEITRTVSGNTVEYALTNLEPATEYTLR.I
3435.7471	3434.7398	3434.6841	16.2	847 - 876	0	R.TSIDLTHEENQYSIGNLKPDTEYEVALISR.R
3625.7847	3624.7774	3624.7810	-0.99	722 - 751	2	K.SIKETSVEVEWDPLDIAFETWEVIFRNMNK.D
3722.5232	3721.5159	3721.4307	22.9	231 - 262	0	K.CVDGACVCFEGYSGLDCSQETCPVPCSEEHGR.C
3733.5112	3732.5039	3732.4552	13.1	323 - 355	1	R.GRCLNGTCSCDEGFTGEDCGQLACPHACHGHGR.C
4074.1699	4073.1626	4073.0804	20.2	950 - 989	2	R.TTLTGLRPGTEYGIGVSAVKGDKESDPATINAATELDTPK.D

No match to: 706.2868, 707.2966, 708.3044, 710.3746, 713.3099, 714.3496, 717.4172, 722.4243, 734.4639, 735.4285, 738.4069, 744.3897, 747.4562, 758.3950, 779.4738, 783.3916, 805.4591, 818.4537, 832.3158, 834.3223, 840.4870, 842.5089, 876.2845, 878.2925, 883.4848, 891.4897, 923.5152, 929.4479, 949.5408, 958.4880, 962.3525, 967.4243, 968.4982, 969.5316, 973.5403, 974.4454, 976.4499, 978.5390, 979.5679, 983.6275, 985.6091, 986.5857, 993.5026, 1007.5763, 1021.5607, 1031.6372, 1034.5828, 1036.5481, 1046.6013, 1057.6161, 1074.6067, 1087.5623, 1113.5466, 1123.6021, 1126.4208, 1153.6440, 1157.4840, 1172.6813, 1187.7026, 1215.6563, 1216.7017, 1248.6692, 1254.6897, 1255.6042, 1257.6483, 1264.7133, 1265.7039, 1269.6589, 1273.6492, 1284.6440, 1286.7411, 1301.5977, 1304.6617, 1314.7190, 1317.7113, 1321.7212, 1338.6622, 1349.6757, 1354.7229, 1356.7275, 1358.7100, 1371.7595, 1375.7335, 1382.5935, 1383.7274, 1390.6962, 1393.7622, 1420.7949, 1423.7593, 1440.7788, 1450.8590, 1456.7791, 1457.7606, 1467.7502, 1470.7725, 1488.7212, 1494.7396, 1500.7236, 1512.8181, 1513.7562, 1528.6954, 1539.7452, 1556.7537, 1570.7695, 1574.7894, 1578.7858, 1580.8347, 1590.7754, 1592.7883, 1616.7943, 1628.7009, 1628.8575, 1631.8130, 1635.7671, 1642.7155, 1647.8516, 1650.8444, 1663.8501, 1693.0090, 1706.8668, 1707.9615, 1715.8461, 1721.9366, 1730.8695, 1741.9938, 1755.8904, 1758.9028, 1771.0238, 1774.9207, 1784.0254, 1787.8574, 1862.9503, 1895.7236, 1897.7325, 1909.7473, 1911.7430, 1913.7366, 1969.9990, 1974.8882, 1979.0790, 1981.0576, 2027.1294, 2067.1584, 2137.1189, 2144.0579, 2180.1812, 2223.1646, 2239.1501, 2241.1355, 2255.1479, 2335.9097, 2342.9673, 2356.9395, 2361.8997, 2366.9265, 2380.9539, 2397.2231, 2406.2913, 2414.2468, 2467.2122, 2474.2263, 2478.2725, 2503.0352, 2518.0303, 2534.0215, 2558.3401, 2594.0615, 2631.4961, 2678.0144, 2683.4189, 2701.2097, 2709.0559, 2717.3198, 2723.0757, 2770.4492, 2787.3171, 2813.4517, 2817.2305, 2842.5049, 2846.4216, 2872.3596, 2944.3171, 2975.3135, 2989.3062, 3004.4270, 3059.4963, 3081.6570, 3086.5718, 3147.5710, 3157.4875, 3185.5222, 3248.6902, 3254.5940, 3331.5864, 3335.7378, 3347.5420, 3351.6597, 3360.7197, 3411.8193, 3428.6296, 3457.6697, 3471.7146, 3475.7085, 3489.7715, 3503.8445, 3528.3743, 3542.4243, 3552.7373, 3568.9385, 3603.9502, 3640.7600, 3645.8701, 3662.8821, 3691.0452, 3727.5442, 3731.5757, 3736.5735, 3741.5391, 3746.5410, 3748.5002, 3751.5356, 3755.5571, 3756.5549, 3763.5049, 3767.5706, 3778.5320, 3884.9326, 3955.6738, 4149.0591, 4163.0181, 4238.0752, 4300.3838, 4382.1450, 4397.2036, 4441.5176, 4453.2886, 4458.5176, 4464.4150, 4495.2642, 4511.1577, 4692.4893

4. F1MRZ6 **Mass:** 250106 **Score:** 97 **Expect:** 5.2e-006 **Matches:** 76
Uncharacterized protein OS=Bos taurus GN=TNC PE=4 SV=2

Observed	Mr(expt)	Mr(calc)	ppm	Start	End	Miss	Peptide
729.4379	728.4306	728.4181	17.2	731 -	737	0	K.SIPVSAR.V
745.4249	744.4176	744.4130	6.18	1058 -	1063	0	K.EVQLTR.D
753.3594	752.3522	752.3453	9.14	948 -	954	0	K.AAADSYR.I
756.3831	755.3758	755.3715	5.72	1042 -	1046	1	K.FDRYR.L
768.4982	767.4910	767.4766	18.7	134 -	139	1	R.INIPRR.A
771.3934	770.3861	770.3752	14.2	2172 -	2177	0	K.GAFWYK.N
823.4842	822.4769	822.4824	-6.66	1090 -	1096	1	R.HKSKPAR.V
889.5049	888.4977	888.4930	5.29	2216 -	2222	0	K.LRPSNFR.N
922.4780	921.4708	921.4668	4.28	793 -	799	0	R.RPETTYR.Q
938.4989	937.4916	937.4869	5.03	1064 -	1071	0	R.DTTSFVLR.G
979.3778	978.3705	978.3648	5.90	531 -	538	0	R.CPGDCSQR.G
988.3948	987.3875	987.3651	22.7	469 -	476	0	R.CPSDCHGR.G
1017.5743	1016.5670	1016.5655	1.54	1897 -	1905	1	R.IFAEKGPQK.S
1051.5096	1050.5023	1050.4982	3.93	1913 -	1921	0	K.FTTDLDSPR.D
1056.5107	1055.5034	1055.4784	23.7	2061 -	2068	1	K.NGREDFYR.N
1143.4449	1142.4376	1142.4346	2.66	593 -	602	0	R.CPGDCHGQGR.C
1178.6198	1177.6125	1177.6091	2.89	2094 -	2103	0	K.ITAQGQYELR.V
1194.5691	1193.5618	1193.5612	0.56	185 -	194	0	R.VDTRPFCSGR.G
1207.5975	1206.5902	1206.5881	1.78	917 -	927	0	K.ETFTTGLDAPR.N
1216.4995	1215.4922	1215.4873	4.03	406 -	414	1	R.RCPSDCHER.G
1278.6917	1277.6844	1277.6840	0.33	790 -	799	1	K.SLRRPETTYR.Q
1288.7273	1287.7200	1287.7074	9.77	738 -	749	0	R.VATYLPTPEGLK.F
1299.5450	1298.5377	1298.5357	1.57	592 -	602	1	R.RCPGDCHGQGR.C
1351.6887	1350.6814	1350.6819	-0.39	711 -	721	0	R.ELEPGVEYFIR.V
1413.6547	1412.6474	1412.6507	-2.29	2204 -	2215	0	K.GHEHSIQFAEMK.L
1562.7806	1561.7733	1561.7736	-0.19	932 -	944	0	R.ISQTDNSITLEWR.N
1578.7858	1577.7785	1577.8161	-23.84	1453 -	1467	1	R.GYRTPVLSAEASTAR.E
1657.9042	1656.8969	1656.8988	-1.12	1999 -	2012	1	K.TIFTTSGVLYRFPR.D
1664.8555	1663.8482	1663.8893	-24.71	1058 -	1071	1	K.EVQLTRDTTSFVLR.G
1696.8273	1695.8200	1695.8216	-0.95	957 -	972	0	K.YAPISGGDHAEVEVPR.S
1717.8761	1716.8688	1716.8287	23.4	140 -	155	1	R.ACGCAAAPDVKELLSR.L
1718.8779	1717.8706	1717.8747	-2.39	931 -	944	1	R.RISQTDNSITLEWR.N
1747.9058	1746.8985	1746.9039	-3.10	1072 -	1087	0	R.GLEPGQEYSILLTAEK.G
1870.7574	1869.7501	1869.7404	5.21	168 -	184	0	R.EQCTSGAGCCLQSAEGR.V
1883.7222	1882.7149	1882.7145	0.21	623 -	638	0	R.SCPNDCSNWGQCVSGR.C
1938.0079	1937.0006	1937.0007	-0.02	955 -	972	1	R.IKYAPISGGDHAEVEVPR.S
1961.0325	1960.0252	1960.0265	-0.66	1072 -	1089	1	R.GLEPGQEYSILLTAEKGR.H
2008.9901	2007.9828	2007.9672	7.78	2172 -	2187	2	K.GAFWYKNCHRVNLMGR.Y
2060.9788	2059.9715	2059.9348	17.8	2108 -	2126	1	R.DHGESAHAVYDKFSVGDAR.T
2086.1133	2085.1060	2085.0994	3.18	1023 -	1041	0	R.ISDPTETSLTLVWQTPVAK.F
2108.9963	2107.9890	2107.9844	2.18	908 -	927	1	R.ADMSSNPAKETFTTGLDAPR.N
2112.9805	2111.9732	2111.9694	1.79	2131 -	2150	1	R.LKVEGYSGTAGDSMAYHNGR.S
2245.8403	2244.8330	2244.8293	1.66	422 -	440	0	R.CECNDGFTGADCGELQCPR.D
2265.1016	2264.0943	2264.0855	3.89	907 -	927	2	R.RADMSSNPAKETFTTGLDAPR.N
2273.0654	2272.0581	2272.0509	3.18	115 -	133	0	R.ESFQEHTVDGENQIVFTHR.I
2281.1055	2280.0982	2280.0804	7.80	907 -	927	2	R.RADMSSNPAKETFTTGLDAPR.N + Oxidation (M)
2318.1824	2317.1751	2317.1703	2.10	1922 -	1941	0	R.DFTATEVQSETALLTWRPPR.A
2328.9529	2327.9456	2327.9392	2.76	572 -	591	0	R.CVDGQCVCLEGFTGPDCAQR.R
2347.8682	2346.8609	2346.8246	15.5	386 -	405	0	R.CDEGQCVCDEGFAGPDCSER.R
2352.9265	2351.9192	2351.9141	2.19	603 -	622	0	R.CVDGQCVCHEGFTGPDCAQR.S
2363.8860	2362.8787	2362.8672	4.90	448 -	467	0	R.CVNGQCVCDEGYTGEDCGQR.R
2371.0420	2370.0347	2370.0686	-14.28	2151 -	2171	1	R.SFSTFDKDTDSAITNCALSYK.G
2389.2756	2388.2683	2388.2649	1.42	1772 -	1795	1	R.VTYVPIAGGAPSAVTVDGTKTQTR.L
2431.2300	2430.2227	2430.2107	4.94	2072 -	2093	1	K.AYAAGFGDLKEEFWLGLDTLSK.I
2477.2573	2476.2500	2476.2598	-3.95	700 -	721	1	R.VPGDQTSATIRELEPGVEYFIR.V
2485.0691	2484.0618	2484.0403	8.66	572 -	592	1	R.CVDGQCVCLEGFTGPDCAQRR.C
2499.1719	2498.1646	2498.2150	-20.16	957 -	979	1	K.YAPISGGDHAEVEVPRSQQTTTR.T
2519.9934	2518.9861	2518.9683	7.09	448 -	468	1	R.CVNGQCVCDEGYTGEDCGQRR.C
2541.2542	2540.2469	2540.2257	8.35	679 -	699	0	R.VTEYLIVYTPTHEDGLEMQFR.V
2542.2739	2541.2666	2541.2598	2.67	1874 -	1896	0	R.TVSGNTVEYALTNLEPATEYTLR.I
2580.0227	2579.0154	2578.9968	7.23	510 -	530	1	R.CVNGMCVCDDAYTGEDCRELR.C
2695.0366	2694.0293	2694.0138	5.75	546 -	569	0	R.CVCEDGFAGPDCADLACPGNCHGR.G
2799.3379	2798.3306	2798.3392	-3.08	88 -	114	1	K.LPVGSQCSVDLESASGEKDLAAPSEPR.E
2833.1284	2832.1211	2832.0779	15.3	417 -	440	1	R.CVDGRCECNDGFTGADCGELQCPR.D
2961.2437	2960.2364	2960.1993	12.5	298 -	321	1	R.CVCQEGFAGEDCREPLCLHNCHGR.G
3029.4883	3028.4810	3028.4488	10.6	1745 -	1771	0	K.EIIFSDITENAASVSWMAPTTQVESFR.V
3081.6570	3080.6497	3080.5891	19.7	1320 -	1347	1	K.IRETDQPQEVHSLTVPGSQHSVEIPGLK.A
3161.1531	3160.1458	3160.1158	9.51	484 -	509	0	R.CECQPGFQGDDCGEMSCPHDCHQHGR.C
3177.1650	3176.1577	3176.1107	14.8	484 -	509	0	R.CECQPGFQGDDCGEMSCPHDCHQHGR.C + Oxidation (M)
3237.6042	3236.5969	3236.6565	-18.40	1868 -	1896	1	R.VPEITRTVSGNTVEYALTNLEPATEYTLR.I
3435.7471	3434.7398	3434.6841	16.2	877 -	906	0	R.TSIDLTHEENQYSIGNLKPDTEYEVALISR.R
3568.9385	3567.9312	3567.9049	7.37	1316 -	1347	2	R.FVIKIRETDQPQEVHSLTVPGSQHSVEIPGLK.A
3625.7847	3624.7774	3624.7810	-0.99	752 -	781	2	K.SIKETSVEVEWDPLDIAFETWEVIFRNMNK.D
3722.5232	3721.5159	3721.4307	22.9	261 -	292	0	K.CVDGACVCFEGYSGLDCSQETCPVPCSEEHGR.C
3733.5112	3732.5039	3732.4552	13.1	353 -	385	1	R.GRCLNGTCSCDEGFTGEDCGQLACPHACHGHGR.C
4074.1699	4073.1626	4073.0804	20.2	980 -	1019	2	R.TTLTGLRPGTEYGIGVSAVKGDKESDPATINAATELDTPK.D

No match to: 706.2868, 707.2966, 708.3044, 710.3746, 713.3099, 714.3496, 717.4172, 722.4243, 734.4639, 735.4285, 738.4069, 744.3897, 747.4562, 758.3950, 779.4738, 783.3916, 805.4591, 818.4537, 832.3158, 834.3223, 840.4870, 842.5089, 876.2845, 878.2925, 883.4848, 891.4897, 923.5152, 929.4479, 949.5408, 958.4880, 962.3525, 967.4243, 968.4982, 969.5316, 973.5403, 974.4454, 976.4499, 978.5390, 979.5679, 983.6275, 985.6091, 986.5857, 993.5026, 1007.5763, 1021.5607, 1031.6372, 1034.5828, 1036.5481, 1046.6013, 1057.6161, 1074.6067, 1087.5623, 1113.5466, 1123.6021, 1126.4208, 1153.6440, 1157.4840, 1172.6813, 1187.7026, 1215.6563, 1216.7017, 1248.6692, 1254.6897, 1255.6042, 1257.6483, 1264.7133, 1265.7039, 1269.6589, 1273.6492, 1284.6440, 1286.7411, 1301.5977, 1304.6617, 1314.7190, 1317.7113, 1321.7212, 1338.6622, 1349.6757, 1354.7229, 1356.7275, 1358.7100, 1371.7595, 1375.7335, 1382.5935, 1383.7274, 1390.6962, 1393.7622, 1420.7949, 1423.7593, 1440.7788, 1450.8590, 1456.7791, 1457.7606, 1467.7502, 1470.7725, 1488.7212, 1494.7396, 1500.7236, 1512.8181, 1513.7562, 1528.6954, 1539.7452, 1556.7537, 1570.7695, 1574.7894, 1580.8347, 1590.7754, 1592.7883, 1616.7943, 1628.7009, 1628.8575, 1631.8130, 1635.7671, 1642.7155, 1647.8516, 1650.8444, 1663.8501, 1693.0090, 1706.8668, 1707.9615, 1715.8461,

1721.9366, 1730.8695, 1741.9938, 1755.8904, 1758.9028, 1771.0238, 1774.9207, 1784.0254, 1787.8574, 1862.9503, 1895.7236, 1897.7325, 1909.7473, 1911.7430, 1913.7366, 1925.7604, 1969.9990, 1974.8882, 1979.0790, 1981.0576, 2027.1294, 2067.1584, 2137.1189, 2144.0579, 2180.1812, 2223.1646, 2239.1501, 2241.1355, 2255.1479, 2335.9097, 2342.9673, 2356.9395, 2361.8997, 2366.9265, 2380.9539, 2397.2231, 2406.2913, 2414.2468, 2467.2122, 2474.2263, 2478.2725, 2503.0352, 2518.0303, 2534.0215, 2558.3401, 2594.0615, 2631.4961, 2678.0144, 2683.4189, 2701.2097, 2709.0559, 2717.3198, 2723.0757, 2770.4492, 2787.3171, 2813.4517, 2817.2305, 2842.5049, 2846.4216, 2872.3596, 2944.3171, 2975.3135, 2989.3062, 3004.4270, 3059.4963, 3086.5718, 3147.5710, 3157.4875, 3185.5222, 3248.6902, 3254.5940, 3331.5864, 3335.7378, 3347.5420, 3351.6597, 3360.7197, 3411.8193, 3428.6296, 3457.6697, 3471.7146, 3475.7085, 3489.7715, 3503.8445, 3528.3743, 3542.4243, 3552.7373, 3603.9502, 3640.7600, 3645.8701, 3662.8821, 3691.0452, 3727.5442, 3731.5757, 3736.5735, 3741.5391, 3746.5410, 3748.5002, 3751.5356, 3755.5571, 3756.5549, 3763.5049, 3767.5706, 3778.5320, 3884.9326, 3955.6738, 4149.0591, 4163.0181, 4238.0752, 4300.3838, 4382.1450, 4397.2036, 4441.5176, 4453.2886, 4458.5176, 4464.4150, 4495.2642, 4511.1577, 4692.4893

5. E1BB91 **Mass:** 343908 **Score:** 79 **Expect:** 0.00032 **Matches:** 82

Uncharacterized protein OS=Bos taurus GN=COL6A3 PE=1 SV=1

Observed	Mr(expt)	Mr(calc)	ppm	Start	End	Miss	Peptide
710.3746	709.3673	709.3581	12.9	2505	2510	0	R.SGFLMR.K
734.4639	733.4567	733.4487	10.9	1593	1598	0	R.LVFTVR.E
747.4562	746.4490	746.4439	6.75	1774	1780	0	K.VFAVGVR.N
805.4591	804.4518	804.4494	3.06	1520	1526	0	K.ALEFVAR.N
823.4842	822.4769	822.4712	6.98	1722	1728	0	K.VGLEHLR.R
958.4880	957.4807	957.4767	4.18	1602	1609	0	R.DLPSIEER.M
968.4982	967.4910	967.5127	-22.49	2848	2855	0	K.NLVQFGYK.Q
973.5403	972.5331	972.5352	-2.23	1119	1128	0	R.NILVGSAGSR.I
979.5679	978.5607	978.5723	-11.88	1722	1729	1	K.VGLEHLRR.N
983.6275	982.6202	982.6175	2.78	1977	1985	0	R.ALILVGLER.V
993.5026	992.4953	992.4927	2.66	1654	1661	0	R.DSFQEVLR.F
1031.6372	1030.6299	1030.6288	1.12	1771	1780	1	R.GVKVFAVGVR.N
1036.5481	1035.5408	1035.5349	5.71	1068	1076	0	R.VAVVQYSDR.T
1056.5107	1055.5034	1055.4884	14.3	2186	2197	0	R.DGVSGGPGEPGK.S
1113.5466	1112.5393	1112.5363	2.72	1730	1739	0	R.NHFVPEAGSR.L
1123.6021	1122.5948	1122.5934	1.26	2000	2008	0	R.GFTYNRPLR.L
1153.6440	1152.6367	1152.6251	10.1	2042	2053	1	R.GDRGPIGSIGPK.G
1172.6813	1171.6740	1171.6713	2.29	1362	1372	0	K.QFGVAPLTIAR.N
1178.6198	1177.6125	1177.5979	12.4	876	886	0	R.VAVAQFSDDVK.V
1187.7026	1186.6953	1186.6935	1.58	1323	1332	1	R.NIFKRPLGSR.I
1194.5691	1193.5618	1193.5353	22.2	2357	2368	0	K.GDPGYPGPSGYK.G
1215.6563	1214.6490	1214.6731	-19.83	1496	1506	1	K.SQASVLDAIRR.L
1255.6042	1254.5969	1254.5993	-1.87	535	545	0	R.NNLFTEAAGYR.A
1265.7039	1264.6966	1264.6928	3.02	2512	2522	0	K.VAVFFSNKPTR.A
1269.6589	1268.6516	1268.6374	11.2	1729	1739	1	R.RNHFVPEAGSR.L
1273.6492	1272.6419	1272.6422	-0.21	1966	1976	1	R.ASEELRQEGVR.A
1286.7411	1285.7338	1285.7394	-4.36	966	977	0	K.QSGVVPFILQAK.N
1304.6617	1303.6544	1303.6554	-0.78	1876	1887	0	K.ISCSGSQLPTVR.V
1317.7113	1316.7040	1316.7048	-0.59	1206	1216	1	R.VTQLSREELSR.L
1356.7275	1355.7202	1355.7157	3.30	1145	1157	1	R.SGDDVRGPSVVLR.R
1371.7595	1370.7522	1370.7518	0.33	1194	1205	0	R.QLGTIQQVISER.V
1375.7335	1374.7262	1374.7256	0.49	1310	1322	0	R.QVNVGGALEYVAR.N
1390.6962	1389.6889	1389.6888	0.08	1599	1609	1	R.EFRDLPSIEER.M
1393.7622	1392.7549	1392.7878	-23.58	2511	2522	1	R.KVAVFFSNKPTR.A
1420.7949	1419.7876	1419.7874	0.14	1043	1054	1	R.SGFPLLKEFVQR.V
1440.7788	1439.7715	1439.7733	-1.20	1055	1067	1	R.VVESLDVGPDRVR.V
1450.8590	1449.8517	1449.8555	-2.60	1003	1015	0	K.IGDLQPQIVNLLK.S
1456.7791	1455.7718	1455.7722	-0.24	1920	1932	0	R.AQHPYVLTADTLK.L
1467.7502	1466.7429	1466.7517	-6.01	914	926	0	K.ALNLGYALDYAQR.Y
1470.7725	1469.7652	1469.7726	-5.00	863	875	0	K.VIDELDVKPEGTR.V
1512.8181	1511.8108	1511.8168	-3.99	1145	1158	2	R.SGDDVRGPSVVLRR.G
1513.7562	1512.7489	1512.7460	1.90	1259	1271	0	R.LVDYLDVGFDTTR.V
1580.8347	1579.8274	1579.8318	-2.79	2169	2185	1	R.GAKGETGDIGPVGLPGR.D
1590.7754	1589.7681	1589.7654	1.72	3101	3114	2	K.ADICKLPKDGGTCR.E
1616.7943	1615.7870	1615.7876	-0.33	2429	2442	0	K.ILDDLTIAESNCPR.G
1628.8575	1627.8502	1627.8464	2.36	2954	2968	1	R.SMATRSEAPRPQAVK.L
1635.7671	1634.7598	1634.7570	1.74	1756	1770	0	K.SVEDAQEASMALTQR.G
1647.8516	1646.8443	1646.8450	-0.41	1986	1999	0	R.VANLEQLMQLEFGR.G
1657.9042	1656.8969	1656.9174	-12.36	2765	2778	2	K.CKGYFFVILGIGRK.V
1663.8501	1662.8428	1662.8399	1.75	1986	1999	0	R.VANLEQLMQLEFGR.G + Oxidation (M)
1707.9615	1706.9542	1706.9355	10.9	1638	1652	1	K.KADIVFLLDGSINFR.R
1718.8779	1717.8706	1717.8999	-17.04	1774	1789	1	K.VFAVGVRNIDSEEVGK.I
1721.9366	1720.9293	1720.9512	-12.72	1537	1552	0	R.IEDGVPQHLVLFLGGK.S
1741.9938	1740.9865	1740.9887	-1.23	1740	1755	1	R.LDQRVPQIAFVITGGK.S
1747.9058	1746.8985	1746.8761	12.8	2274	2291	2	K.GEPGDPGPKGSIGNRGPR.G
1755.8904	1754.8831	1754.8839	-0.45	518	534	0	K.ALDGSALYTGSALDFVR.N
1758.9028	1757.8955	1757.8988	-1.88	1382	1396	0	K.ISLSPEYVFSVNTFR.E
1784.0254	1783.0181	1783.0204	-1.25	1217	1233	1	R.LQASVTPLTTPVVSSKR.D
1862.9503	1861.9430	1861.9422	0.47	2534	2550	0	K.LSDAGITPLFLTSQEDR.Q
1969.9990	1968.9917	1968.9905	0.60	1948	1965	0	K.VVIHFTDGVDGDLADLQR.A
1979.0790	1978.0717	1978.0748	-1.56	891	906	1	R.FDQHQNKPEILNLVKR.M
1981.0576	1980.0503	1980.0357	7.40	1476	1492	0	R.IGVLQFSNDVFPEFQLK.T
2008.9901	2007.9828	2007.9861	-1.64	1781	1799	1	R.NIDSEEVGKIASNSATAFR.V
2067.1584	2066.1511	2066.1524	-0.63	1098	1118	0	R.GLTLLGGPAPNTGAALEFVLR.N
2137.1189	2136.1116	2136.1103	0.63	858	875	1	R.DFLYKVIDELDVKPEGTR.V
2180.1812	2179.1739	2179.1750	-0.47	1532	1552	1	K.SAGSRIEDGVPQHLVLFLGGK.S
2241.1355	2240.1282	2240.1114	7.53	1234	1253	0	R.DVVFLIDGSQSAGPEFQYIR.T
2397.2231	2396.2158	2396.2125	1.40	1233	1253	1	K.RDVVFLIDGSQSAGPEFQYIR.T
2406.2913	2405.2840	2405.2855	-0.63	1920	1939	2	R.AQHPYVLTADTLKLYQNKFR.Q
2467.2122	2466.2049	2466.1850	8.10	1835	1857	0	K.ACNLDVILGFDGSGDQNVFVAQK.G
2474.2263	2473.2190	2473.2060	5.27	1077	1097	0	R.TRPEFYLNSYMDQQSVVGAIR.G
2683.4189	2682.4116	2682.3905	7.89	1382	1404	1	K.ISLSPEYVFSVNTFRELPSLEQK.L
2799.3379	2798.3306	2798.3122	6.57	2825	2847	2	K.DAFYLSPDIRKQCDWFQADQPAK.N

2813.4517	2812.4444	2812.4283	5.73	1373	- 1396	1	R.NVDQEELVKISLSPEYVFSVNTFR.E
2842.5049	2841.4976	2841.4581	13.9	1566	- 1592	2	K.SAGIASLGVGDRNIDRTELQTITSDPR.L
2846.4216	2845.4143	2845.4644	-17.59	1744	- 1770	1	R.VPQIAFVITGGKSVEDAQEASMALTQR.G
2961.2437	2960.2364	2960.2031	11.3	3120	- 3143	1	K.WYYDSVTENCARFWYGGCGGNENR.F
3248.6902	3247.6829	3247.7200	-11.43	740	- 769	0	R.IQDHVPQLLLLLTAGQSEDSYLQAANALAR.A
3360.7197	3359.7124	3359.7302	-5.30	2999	- 3027	0	K.LHWERPEPPSPYLYNLTVTSAHDQAVVLK.Q
3411.8193	3410.8120	3410.7583	15.8	1272	- 1301	2	R.VAVIQFSDDPRVEFLLNVHSSKDEVQNAVR.R
3568.9385	3567.9312	3567.8944	10.3	3028	- 3059	2	K.QNLTVTDRVIGGLLPGQTYHVTVTCSLRSQVR.A
3645.8701	3644.8628	3644.7861	21.0	1888	- 1919	1	R.VSVVALTPSGPVEAFDFAEYQPELFEKFQNMR.A

No match to: 706.2868, 707.2966, 708.3044, 713.3099, 714.3496, 717.4172, 722.4243, 729.4379, 735.4285, 738.4069, 744.3897, 745.4249, 753.3594, 756.3831, 758.3950, 768.4982, 771.3934, 779.4738, 783.3916, 818.4537, 832.3158, 834.3223, 840.4870, 842.5089, 876.2845, 878.2925, 883.4848, 889.5049, 891.4897, 922.4780, 923.5152, 929.4479, 938.4989, 949.5408, 962.3525, 967.4243, 969.5316, 974.4454, 976.4499, 978.5390, 979.3778, 985.6091, 986.5857, 988.3948, 1007.5763, 1017.5743, 1021.5607, 1034.5828, 1046.6013, 1051.5096, 1057.6161, 1074.6067, 1087.5623, 1126.4208, 1143.4449, 1157.4840, 1207.5975, 1216.4995, 1216.7017, 1248.6692, 1254.6897, 1257.6483, 1264.7133, 1278.6917, 1284.6440, 1288.7273, 1299.5450, 1301.5977, 1314.7190, 1321.7212, 1338.6622, 1349.6757, 1351.6887, 1354.7229, 1358.7100, 1382.5935, 1383.7274, 1413.6547, 1423.7593, 1457.7606, 1488.7212, 1494.7396, 1500.7236, 1528.6954, 1539.7452, 1556.7537, 1562.7806, 1570.7695, 1574.7894, 1578.7858, 1592.7883, 1628.7009, 1631.8130, 1642.7155, 1650.8444, 1664.8555, 1693.0090, 1696.8273, 1706.8668, 1715.8461, 1717.8761, 1730.8695, 1771.0238, 1774.9207, 1787.8574, 1870.7574, 1883.7222, 1895.7236, 1897.7325, 1909.7473, 1911.7430, 1913.7366, 1925.7604, 1938.0079, 1961.0325, 1974.8882, 2027.1294, 2060.9788, 2086.1133, 2108.9963, 2112.9805, 2144.0579, 2223.1646, 2239.1501, 2245.8403, 2255.1479, 2265.1016, 2273.0654, 2281.1055, 2318.1824, 2328.9529, 2335.9097, 2342.9673, 2347.8682, 2352.9265, 2356.9395, 2361.8997, 2363.8860, 2366.9265, 2371.0420, 2380.9539, 2389.2756, 2414.2468, 2431.2300, 2477.2573, 2478.2725, 2485.0691, 2499.1719, 2503.0352, 2518.0303, 2519.9934, 2534.0215, 2541.2542, 2542.2739, 2558.3401, 2580.0227, 2594.0615, 2631.4961, 2678.0144, 2695.0366, 2701.2097, 2709.0559, 2717.3198, 2723.0757, 2770.4492, 2787.3171, 2817.2305, 2833.1284, 2872.3596, 2944.3171, 2975.3135, 2989.3062, 3004.4270, 3029.4883, 3059.4963, 3081.6570, 3086.5718, 3147.5710, 3157.4875, 3161.1531, 3177.1650, 3185.5222, 3237.6042, 3254.5940, 3331.5864, 3335.7378, 3347.5420, 3351.6597, 3428.6296, 3435.7471, 3457.6697, 3471.7146, 3475.7085, 3489.7715, 3503.8445, 3528.3743, 3542.4243, 3552.7373, 3603.9502, 3625.7847, 3640.7600, 3662.8821, 3691.0452, 3722.5232, 3727.5442, 3731.5757, 3733.5112, 3736.5735, 3741.5391, 3746.5410, 3748.5002, 3751.5356, 3755.5571, 3756.5549, 3763.5049, 3767.5706, 3778.5320, 3884.9326, 3955.6738, 4074.1699, 4149.0591, 4163.0181, 4238.0752, 4300.3838, 4382.1450, 4397.2036, 4441.5176, 4453.2886, 4458.5176, 4464.4150, 4495.2642, 4511.1577, 4692.4893

6. P21809 **Mass:** 41963 **Score:** 46 **Expect:** 0.58 **Matches:** 16
Biglycan OS=Bos taurus GN=BGN PE=1 SV=3

Observed	Mr(expt)	Mr(calc)	ppm	Start	End	Miss	Peptide
735.4285	734.4212	734.4075	18.6	178	- 184	0	K.GVFSGLR.N
923.5152	922.5079	922.5097	-1.90	167	- 173	1	R.IHDNRIR.K
1007.5763	1006.5690	1006.5672	1.81	259	- 267	0	R.LGLGHNQIR.M
1187.7026	1186.6953	1186.7186	-19.61	134	- 143	2	K.AFSPLRKLQK.L
1494.7396	1493.7323	1493.7337	-0.94	316	- 329	0	K.VGVNDFCPVGFGVK.R
1650.8444	1649.8371	1649.8348	1.38	316	- 330	1	K.VGVNDFCPVGFGVKR.A
1787.8574	1786.8501	1786.8849	-19.49	227	- 241	0	K.DLPETLNELHLDHNK.I
2027.1294	2026.1221	2026.1211	0.51	149	- 166	0	K.NHLVEIPPNLPSSLVELR.I
2397.2231	2396.2158	2396.2699	-22.58	221	- 241	1	K.LTGIPKDLPETLNELHLDHNK.I
2414.2468	2413.2395	2413.2336	2.45	88	- 108	1	K.EISPDTTLLDLQNNDISELRK.D
2541.2542	2540.2469	2540.3057	-23.12	268	- 289	1	R.MIENGSLSFLPTLRELHLDNNK.L
2631.4961	2630.4888	2630.4795	3.54	144	- 166	1	K.LYISKNHLVEIPPNLPSSLVELR.I
2846.4216	2845.4143	2845.3864	9.82	331	- 354	0	R.AYYNGISLFNNPVPYWEVQPATFR.C
3081.6570	3080.6497	3080.6142	11.5	227	- 252	1	K.DLPETLNELHLDHNKIQAIELEDLLR.Y
3457.6697	3456.6624	3456.5900	20.9	185	- 215	1	R.NMNCIEMGGNPLENSGFEPGAFDGLKLNYLR.I
3691.0452	3690.0379	3689.9992	10.5	221	- 252	2	K.LTGIPKDLPETLNELHLDHNKIQAIELEDLLR.Y

No match to: 706.2868, 707.2966, 708.3044, 710.3746, 713.3099, 714.3496, 717.4172, 722.4243, 729.4379, 734.4639, 738.4069, 744.3897, 745.4249, 747.4562, 753.3594, 756.3831, 758.3950, 768.4982, 771.3934, 779.4738, 783.3916, 805.4591, 818.4537, 823.4842, 832.3158, 834.3223, 840.4870, 842.5089, 876.2845, 878.2925, 883.4848, 889.5049, 891.4897, 922.4780, 929.4479, 938.4989, 949.5408, 958.4880, 962.3525, 967.4243, 968.4982, 969.5316, 973.5403, 974.4454, 976.4499, 978.5390, 979.3778, 979.5679, 983.6275, 985.6091, 986.5857, 988.3948, 993.5026, 1017.5743, 1021.5607, 1031.6372, 1034.5828, 1036.5481, 1046.6013, 1051.5096, 1056.5107, 1057.6161, 1074.6067, 1087.5623, 1113.5466, 1123.6021, 1126.4208, 1143.4449, 1153.6440, 1157.4840, 1172.6813, 1178.6198, 1194.5691, 1207.5975, 1215.6563, 1216.4995, 1216.7017, 1248.6692, 1254.6897, 1255.6042, 1257.6483, 1264.7133, 1265.7039, 1269.6589, 1273.6492, 1278.6917, 1284.6440, 1286.7411, 1288.7273, 1299.5450, 1301.5977, 1304.6617, 1314.7190, 1317.7113, 1321.7212, 1338.6622, 1349.6757, 1351.6887, 1354.7229, 1356.7275, 1358.7100, 1371.7595, 1375.7335, 1382.5935, 1383.7274, 1390.6962, 1393.7622, 1413.6547, 1420.7949, 1423.7593, 1440.7788, 1450.8590, 1456.7791, 1457.7606, 1467.7502, 1470.7725, 1488.7212, 1500.7236, 1512.8181, 1513.7562, 1528.6954, 1539.7452, 1556.7537, 1562.7806, 1570.7695, 1574.7894, 1578.7858, 1580.8347, 1590.7754, 1592.7883, 1616.7943, 1628.7009, 1628.8575, 1631.8130, 1635.7671, 1642.7155, 1647.8516, 1657.9042, 1663.8501, 1664.8555, 1693.0090, 1696.8273, 1706.8668, 1707.9615, 1715.8461, 1717.8761, 1718.8779, 1721.9366, 1730.8695, 1741.9938, 1747.9058, 1755.8904, 1758.9028, 1771.0238, 1774.9207, 1784.0254, 1862.9503, 1870.7574, 1883.7222, 1895.7236, 1897.7325, 1909.7473, 1911.7430, 1913.7366, 1925.7604, 1938.0079, 1961.0325, 1969.9990, 1974.8882, 1979.0790, 1981.0576, 2008.9901, 2060.9788, 2067.1584, 2086.1133, 2108.9963, 2112.9805, 2137.1189, 2144.0579, 2180.1812, 2223.1646, 2239.1501, 2241.1355, 2245.8403, 2255.1479, 2265.1016, 2273.0654, 2281.1055, 2318.1824, 2328.9529, 2335.9097, 2342.9673, 2347.8682, 2352.9265, 2356.9395, 2361.8997, 2363.8860, 2366.9265, 2371.0420, 2380.9539, 2389.2756, 2406.2913, 2431.2300, 2467.2122, 2474.2263, 2477.2573, 2478.2725, 2485.0691, 2499.1719, 2503.0352, 2518.0303, 2519.9934, 2534.0215, 2542.2739, 2558.3401, 2580.0227, 2594.0615, 2678.0144, 2683.4189, 2695.0366, 2701.2097, 2709.0559, 2717.3198, 2723.0757, 2770.4492, 2787.3171, 2799.3379, 2813.4517, 2817.2305, 2833.1284, 2842.5049, 2872.3596, 2944.3171, 2961.2437, 2975.3135, 2989.3062, 3004.4270, 3029.4883, 3059.4963, 3086.5718, 3147.5710, 3157.4875, 3161.1531, 3177.1650, 3185.5222, 3237.6042, 3248.6902, 3254.5940, 3331.5864, 3335.7378, 3347.5420, 3351.6597, 3360.7197, 3411.8193, 3428.6296, 3435.7471, 3471.7146, 3475.7085, 3489.7715, 3503.8445, 3528.3743, 3542.4243, 3552.7373, 3568.9385, 3603.9502, 3625.7847, 3640.7600, 3645.8701, 3662.8821, 3722.5232, 3727.5442, 3731.5757, 3733.5112, 3736.5735, 3741.5391, 3746.5410, 3748.5002, 3751.5356, 3755.5571, 3756.5549, 3763.5049, 3767.5706, 3778.5320, 3884.9326, 3955.6738, 4074.1699, 4149.0591, 4163.0181, 4238.0752, 4300.3838, 4382.1450, 4397.2036, 4441.5176, 4453.2886, 4458.5176, 4464.4150, 4495.2642, 4511.1577, 4692.4893

7. G3N3E4 **Mass:** 185893 **Score:** 33 **Expect:** 11 **Matches:** 44
Uncharacterized protein OS=Bos taurus GN=COL6A3 PE=1 SV=1

Observed	Mr(expt)	Mr(calc)	ppm	Start	End	Miss	Peptide
734.4639	733.4567	733.4487	10.9	1589	- 1594	0	R.LVFTVR.E
805.4591	804.4518	804.4494	3.06	1516	- 1522	0	K.ALEFVAR.N
958.4880	957.4807	957.4767	4.18	1598	- 1605	0	R.DLPSIEER.M
973.5403	972.5331	972.5352	-2.23	1115	- 1124	0	R.NILVGSAGSR.I
993.5026	992.4953	992.4927	2.66	1650	- 1657	0	R.DSFQEVLR.F
1036.5481	1035.5408	1035.5349	5.71	1064	- 1072	0	R.VAVVQYSDR.T
1172.6813	1171.6740	1171.6713	2.29	1358	- 1368	0	K.QFGVAPLTIAR.N
1178.6198	1177.6125	1177.5979	12.4	874	- 884	0	R.VAVAQFSDDVK.V
1187.7026	1186.6953	1186.6935	1.58	1319	- 1328	1	R.NIFKRPLGSR.I
1215.6563	1214.6490	1214.6731	-19.83	1492	- 1502	1	K.SQASVLDAIRR.L
1255.6042	1254.5969	1254.5993	-1.87	533	- 543	0	R.NNLFTEAAGYR.A
1286.7411	1285.7338	1285.7394	-4.36	964	- 975	0	K.QSGVVPFILQAK.N
1317.7113	1316.7040	1316.7048	-0.59	1202	- 1212	1	R.VTQLSREELSR.L
1356.7275	1355.7202	1355.7157	3.30	1141	- 1153	1	R.SGDDVRGPSVVLR.R

1371.7595	1370.7522	1370.7518	0.33	1190 -	1201	0	R.QLGTIQQVISER.V
1375.7335	1374.7262	1374.7256	0.49	1306 -	1318	0	R.QVNVGGALEYVAR.N
1390.6962	1389.6889	1389.6888	0.08	1595 -	1605	1	R.EFRDLPSIEER.M
1420.7949	1419.7876	1419.7874	0.14	1039 -	1050	1	R.SGFPLLKEFVQR.V
1440.7788	1439.7715	1439.7733	-1.20	1051 -	1063	1	R.VVESLDVGPDRVR.V
1450.8590	1449.8517	1449.8555	-2.60	1001 -	1013	0	K.IGDLQPQIVNLLK.S
1456.7791	1455.7718	1455.7874	-10.71	1516 -	1527	1	K.ALEFVARNYFVK.S
1467.7502	1466.7429	1466.7517	-6.01	912 -	924	0	K.ALNLGYALDYAQR.Y
1470.7725	1469.7652	1469.7726	-5.00	861 -	873	0	K.VIDELDVKPEGTR.V
1512.8181	1511.8108	1511.8168	-3.99	1141 -	1154	2	R.SGDDVRGPSVVLRR.G
1513.7562	1512.7489	1512.7460	1.90	1255 -	1267	0	R.LVDYLDVGFDTTR.V
1707.9615	1706.9542	1706.9355	10.9	1634 -	1648	1	K.KADIVFLLDGSINFR.R
1721.9366	1720.9293	1720.9512	-12.72	1533 -	1548	0	R.IEDGVPQHLVLFLGGK.S
1755.8904	1754.8831	1754.8839	-0.45	516 -	532	0	K.ALDGSALYTGSALDFVR.N
1758.9028	1757.8955	1757.8988	-1.88	1378 -	1392	0	K.ISLSPEYVFSVNTFR.E
1784.0254	1783.0181	1783.0204	-1.25	1213 -	1229	1	R.LQASVTPLTTPVVSSKR.D
1979.0790	1978.0717	1978.0748	-1.56	889 -	904	1	R.FDQHQNKPEILNLVKR.M
1981.0576	1980.0503	1980.0357	7.40	1472 -	1488	0	R.IGVLQFSNDVFPEFQLK.T
2067.1584	2066.1511	2066.1524	-0.63	1094 -	1114	0	R.GLTLLGGPAPNTGAALEFVLR.N
2137.1189	2136.1116	2136.1103	0.63	856 -	873	1	R.DFLYKVIDELDVKPEGTR.V
2180.1812	2179.1739	2179.1750	-0.47	1528 -	1548	1	K.SAGSRIEDGVPQHLVLFLGGK.S
2241.1355	2240.1282	2240.1114	7.53	1230 -	1249	0	R.DVVFLIDGSQSAGPEFQYIR.T
2397.2231	2396.2158	2396.2125	1.40	1229 -	1249	1	K.RDVVFLIDGSQSAGPEFQYIR.T
2467.2122	2466.2049	2466.1809	9.74	97 -	119	1	R.SKQEVLSHVSNMSYIGGSNQTGK.G + Oxidation (M)
2474.2263	2473.2190	2473.2060	5.27	1073 -	1093	0	R.TRPEFYLNSYMDQQSVVGAIR.G
2683.4189	2682.4116	2682.3905	7.89	1378 -	1400	1	K.ISLSPEYVFSVNTFRELPSLEQK.L
2813.4517	2812.4444	2812.4283	5.73	1369 -	1392	1	R.NVDQEELVKISLSPEYVFSVNTFR.E
2842.5049	2841.4976	2841.4581	13.9	1562 -	1588	2	K.SAGIASLGVGDRNIDRTELQTITSDPR.L
3248.6902	3247.6829	3247.7200	-11.43	738 -	767	0	R.IQDHVPQLLLLLTAGQSEDSYLQAANALAR.A
3411.8193	3410.8120	3410.7583	15.8	1268 -	1297	2	R.VAVIQFSDDPRVEFLLNVHSSKDEVQNAVR.R

No match to: 706.2868, 707.2966, 708.3044, 710.3746, 713.3099, 714.3496, 717.4172, 722.4243, 729.4379, 735.4285, 738.4069, 744.3897, 745.4249, 747.4562, 753.3594, 756.3831, 758.3950, 768.4982, 771.3934, 779.4738, 783.3916, 818.4537, 823.4842, 832.3158, 834.3223, 840.4870, 842.5089, 876.2845, 878.2925, 883.4848, 889.5049, 891.4897, 922.4780, 923.5152, 929.4479, 938.4989, 949.5408, 962.3525, 967.4243, 968.4982, 969.5316, 974.4454, 976.4499, 978.5390, 979.3778, 979.5679, 983.6275, 985.6091, 986.5857, 988.3948, 1007.5763, 1017.5743, 1021.5607, 1031.6372, 1034.5828, 1046.6013, 1051.5096, 1056.5107, 1057.6161, 1074.6067, 1087.5623, 1113.5466, 1123.6021, 1126.4208, 1143.4449, 1153.6440, 1157.4840, 1194.5691, 1207.5975, 1216.4995, 1216.7017, 1248.6692, 1254.6897, 1257.6483, 1264.7133, 1265.7039, 1269.6589, 1273.6492, 1278.6917, 1284.6440, 1288.7273, 1299.5450, 1301.5977, 1304.6617, 1314.7190, 1321.7212, 1338.6622, 1349.6757, 1351.6887, 1354.7229, 1358.7100, 1382.5935, 1383.7274, 1393.7622, 1413.6547, 1423.7593, 1457.7606, 1488.7212, 1494.7396, 1500.7236, 1528.6954, 1539.7452, 1556.7537, 1562.7806, 1570.7695, 1574.7894, 1578.7858, 1580.8347, 1590.7754, 1592.7883, 1616.7943, 1628.7009, 1628.8575, 1631.8130, 1635.7671, 1642.7155, 1647.8516, 1650.8444, 1657.9042, 1663.8501, 1664.8555, 1693.0090, 1696.8273, 1706.8668, 1715.8461, 1717.8761, 1718.8779, 1730.8695, 1741.9938, 1747.9058, 1771.0238, 1774.9207, 1787.8574, 1862.9503, 1870.7574, 1883.7222, 1895.7236, 1897.7325, 1909.7473, 1911.7430, 1913.7366, 1925.7604, 1938.0079, 1961.0325, 1969.9990, 1974.8882, 2008.9901, 2027.1294, 2060.9788, 2086.1133, 2108.9963, 2112.9805, 2144.0579, 2223.1646, 2239.1501, 2245.8403, 2255.1479, 2265.1016, 2273.0654, 2281.1055, 2318.1824, 2328.9529, 2335.9097, 2342.9673, 2347.8682, 2352.9265, 2356.9395, 2361.8997, 2363.8860, 2366.9265, 2371.0420, 2380.9539, 2389.2756, 2406.2913, 2414.2468, 2431.2300, 2477.2573, 2478.2725, 2485.0691, 2499.1719, 2503.0352, 2518.0303, 2519.9934, 2534.0215, 2541.2542, 2542.2739, 2558.3401, 2580.0227, 2594.0615, 2631.4961, 2678.0144, 2695.0366, 2701.2097, 2709.0559, 2717.3198, 2723.0757, 2770.4492, 2787.3171, 2799.3379, 2817.2305, 2833.1284, 2846.4216, 2872.3596, 2944.3171, 2961.2437, 2975.3135, 2989.3062, 3004.4270, 3029.4883, 3059.4963, 3081.6570, 3086.5718, 3147.5710, 3157.4875, 3161.1531, 3177.1650, 3185.5222, 3237.6042, 3254.5940, 3331.5864, 3335.7378, 3347.5420, 3351.6597, 3360.7197, 3428.6296, 3435.7471, 3457.6697, 3471.7146, 3475.7085, 3489.7715, 3503.8445, 3528.3743, 3542.4243, 3552.7373, 3568.9385, 3603.9502, 3625.7847, 3640.7600, 3645.8701, 3662.8821, 3691.0452, 3722.5232, 3727.5442, 3731.5757, 3733.5112, 3736.5735, 3741.5391, 3746.5410, 3748.5002, 3751.5356, 3755.5571, 3756.5549, 3763.5049, 3767.5706, 3778.5320, 3884.9326, 3955.6738, 4074.1699, 4149.0591, 4163.0181, 4238.0752, 4300.3838, 4382.1450, 4397.2036, 4441.5176, 4453.2886, 4458.5176, 4464.4150, 4495.2642, 4511.1577, 4692.4893

8. G3N126 **Mass:** 135082 **Score:** 27 **Expect:** 48 **Matches:** 32

Uncharacterized protein OS=Bos taurus GN=COL6A3 PE=1 SV=1

Observed	Mr(expt)	Mr(calc)	ppm	Start	End	Miss	Peptide
973.5403	972.5331	972.5352	-2.23	916 -	925	0	R.NILVGSAGSR.I
1036.5481	1035.5408	1035.5349	5.71	865 -	873	0	R.VAVVQYSDR.T
1172.6813	1171.6740	1171.6713	2.29	1159 -	1169	0	K.QFGVAPLTIAR.N
1178.6198	1177.6125	1177.5979	12.4	673 -	683	0	R.VAVAQFSDDVK.V
1187.7026	1186.6953	1186.6935	1.58	1120 -	1129	1	R.NIFKRPLGSR.I
1255.6042	1254.5969	1254.5993	-1.87	332 -	342	0	R.NNLFTEAAGYR.A
1286.7411	1285.7338	1285.7394	-4.36	763 -	774	0	K.QSGVVPFILQAK.N
1317.7113	1316.7040	1316.7048	-0.59	1003 -	1013	1	R.VTQLSREELSR.L
1356.7275	1355.7202	1355.7157	3.30	942 -	954	1	R.SGDDVRGPSVVLR.R
1371.7595	1370.7522	1370.7518	0.33	991 -	1002	0	R.QLGTIQQVISER.V
1375.7335	1374.7262	1374.7256	0.49	1107 -	1119	0	R.QVNVGGALEYVAR.N
1420.7949	1419.7876	1419.7874	0.14	840 -	851	1	R.SGFPLLKEFVQR.V
1440.7788	1439.7715	1439.7733	-1.20	852 -	864	1	R.VVESLDVGPDRVR.V
1450.8590	1449.8517	1449.8555	-2.60	800 -	812	0	K.IGDLQPQIVNLLK.S
1467.7502	1466.7429	1466.7517	-6.01	711 -	723	0	K.ALNLGYALDYAQR.Y
1470.7725	1469.7652	1469.7726	-5.00	660 -	672	0	K.VIDELDVKPEGTR.V
1512.8181	1511.8108	1511.8168	-3.99	942 -	955	2	R.SGDDVRGPSVVLRR.G
1513.7562	1512.7489	1512.7460	1.90	1056 -	1068	0	R.LVDYLDVGFDTTR.V
1707.9615	1706.9542	1706.9931	-22.75	926 -	941	0	R.IAEGVPQLLIVLTADR.S
1755.8904	1754.8831	1754.8839	-0.45	315 -	331	0	K.ALDGSALYTGSALDFVR.N
1758.9028	1757.8955	1757.8988	-1.88	1179 -	1193	0	K.ISLSPEYVFSVNTFR.E
1784.0254	1783.0181	1783.0204	-1.25	1014 -	1030	1	R.LQASVTPLTTPVVSSKR.D
1979.0790	1978.0717	1978.0748	-1.56	688 -	703	1	R.FDQHQNKPEILNLVKR.M
2067.1584	2066.1511	2066.1524	-0.63	895 -	915	0	R.GLTLLGGPAPNTGAALEFVLR.N
2137.1189	2136.1116	2136.1103	0.63	655 -	672	1	R.DFLYKVIDELDVKPEGTR.V
2241.1355	2240.1282	2240.1114	7.53	1031 -	1050	0	R.DVVFLIDGSQSAGPEFQYIR.T
2397.2231	2396.2158	2396.2125	1.40	1030 -	1050	1	K.RDVVFLIDGSQSAGPEFQYIR.T
2474.2263	2473.2190	2473.2060	5.27	874 -	894	0	R.TRPEFYLNSYMDQQSVVGAIR.G
2683.4189	2682.4116	2682.3905	7.89	1179 -	1201	1	K.ISLSPEYVFSVNTFRELPSLEQK.L
2813.4517	2812.4444	2812.4283	5.73	1170 -	1193	1	R.NVDQEELVKISLSPEYVFSVNTFR.E
3248.6902	3247.6829	3247.7200	-11.43	537 -	566	0	R.IQDHVPQLLLLLTAGQSEDSYLQAANALAR.A
3411.8193	3410.8120	3410.7583	15.8	1069 -	1098	2	R.VAVIQFSDDPRVEFLLNVHSSKDEVQNAVR.R

No match to: 706.2868, 707.2966, 708.3044, 710.3746, 713.3099, 714.3496, 717.4172, 722.4243, 729.4379, 734.4639, 735.4285, 738.4069, 744.3897, 745.4249, 747.4562, 753.3594, 756.3831, 758.3950, 768.4982, 771.3934, 779.4738, 783.3916, 805.4591, 818.4537, 823.4842, 832.3158, 834.3223, 840.4870, 842.5089, 876.2845, 878.2925, 883.4848, 889.5049, 891.4897, 922.4780, 923.5152, 929.4479, 938.4989, 949.5408, 958.4880, 962.3525, 967.4243, 968.4982, 969.5316, 974.4454, 976.4499, 978.5390, 979.3778, 979.5679, 983.6275, 985.6091, 986.5857, 988.3948, 993.5026, 1007.5763, 1017.5743, 1021.5607, 1031.6372, 1034.5828, 1046.6013, 1051.5096, 1056.5107, 1057.6161, 1074.6067, 1087.5623, 1113.5466, 1123.6021, 1126.4208, 1143.4449, 1153.6440, 1157.4840, 1194.5691, 1207.5975, 1215.6563, 1216.4995, 1216.7017, 1248.6692, 1254.6897, 1257.6483, 1264.7133, 1265.7039, 1269.6589, 1273.6492, 1278.6917, 1284.6440, 1288.7273, 1299.5450, 1301.5977, 1304.6617, 1314.7190, 1321.7212, 1338.6622, 1349.6757, 1351.6887, 1354.7229, 1358.7100, 1382.5935, 1383.7274, 1390.6962, 1393.7622, 1413.6547, 1423.7593, 1456.7791, 1457.7606, 1488.7212, 1494.7396, 1500.7236, 1528.6954, 1539.7452, 1556.7537, 1562.7806, 1570.7695, 1574.7894, 1578.7858, 1580.8347, 1590.7754, 1592.7883, 1616.7943, 1628.7009, 1628.8575, 1631.8130, 1635.7671, 1642.7155, 1647.8516, 1650.8444, 1657.9042, 1663.8501, 1664.8555, 1693.0090, 1696.8273, 1706.8668, 1715.8461, 1717.8761, 1718.8779, 1721.9366, 1730.8695, 1741.9938, 1747.9058, 1771.0238, 1774.9207, 1787.8574, 1862.9503, 1870.7574, 1883.7222, 1895.7236, 1897.7325, 1909.7473, 1911.7430, 1913.7366, 1925.7604, 1938.0079, 1961.0325, 1969.9990, 1974.8882, 1981.0576, 2008.9901, 2027.1294, 2060.9788, 2086.1133, 2108.9963, 2112.9805, 2144.0579, 2180.1812, 2223.1646, 2239.1501, 2245.8403, 2255.1479, 2265.1016, 2273.0654, 2281.1055, 2318.1824, 2328.9529, 2335.9097, 2342.9673, 2347.8682, 2352.9265, 2356.9395, 2361.8997, 2363.8860, 2366.9265, 2371.0420, 2380.9539, 2389.2756, 2406.2913, 2414.2468, 2431.2300, 2467.2122, 2477.2573, 2478.2725, 2485.0691, 2499.1719, 2503.0352, 2518.0303, 2519.9934, 2534.0215, 2541.2542, 2542.2739, 2558.3401, 2580.0227, 2594.0615, 2631.4961, 2678.0144, 2695.0366, 2701.2097, 2709.0559, 2717.3198, 2723.0757, 2770.4492, 2787.3171, 2799.3379, 2817.2305, 2833.1284, 2842.5049, 2846.4216, 2872.3596, 2944.3171, 2961.2437, 2975.3135, 2989.3062, 3004.4270, 3029.4883, 3059.4963, 3081.6570, 3086.5718, 3147.5710, 3157.4875, 3161.1531, 3177.1650, 3185.5222, 3237.6042, 3254.5940, 3331.5864, 3335.7378, 3347.5420, 3351.6597, 3360.7197, 3428.6296, 3435.7471, 3457.6697, 3471.7146, 3475.7085, 3489.7715, 3503.8445, 3528.3743, 3542.4243, 3552.7373, 3568.9385, 3603.9502, 3625.7847, 3640.7600, 3645.8701, 3662.8821, 3691.0452, 3722.5232, 3727.5442, 3731.5757, 3733.5112, 3736.5735, 3741.5391, 3746.5410, 3748.5002, 3751.5356, 3755.5571, 3756.5549, 3763.5049, 3767.5706, 3778.5320, 3884.9326, 3955.6738, 4074.1699, 4149.0591, 4163.0181, 4238.0752, 4300.3838, 4382.1450, 4397.2036, 4441.5176, 4453.2886, 4458.5176, 4464.4150, 4495.2642, 4511.1577, 4692.4893

9. Q17R06 **Mass:** 24530 **Score:** 23 **Expect:** 1.2e+002 **Matches:** 9

Ras-related protein Rab-21 OS=Bos taurus GN=RAB21 PE=2 SV=1

Observed	Mr(expt)	Mr(calc)	ppm	Start	End	Miss	Peptide
713.3099	712.3027	712.2850	24.8	36 -	40	0	R.YCENK.F
1356.7275	1355.7202	1355.7018	13.6	187 -	200	2	R.AKGNGSSQPGAARR.G
1390.6962	1389.6889	1389.6922	-2.35	177 -	188	1	R.MIETAQVDERAK.G
1562.7806	1561.7733	1561.7882	-9.53	176 -	188	2	K.RMIETAQVDERAK.G + Oxidation (M)
1628.8575	1627.8502	1627.8430	4.42	64 -	77	1	K.RVNLAIWDTAGQER.F
1706.8668	1705.8595	1705.8709	-6.66	162 -	175	1	K.QNKGIEELFLDLCK.R
1862.9503	1861.9430	1861.9720	-15.55	162 -	176	2	K.QNKGIEELFLDLCKR.M
2846.4216	2845.4143	2845.4776	-22.23	64 -	87	2	K.RVNLAIWDTAGQERFHALGPIYYR.D
3347.5420	3346.5347	3346.6146	-23.87	78 -	106	1	R.FHALGPIYYRDSNGAILVYDITDEDSFQK.V

No match to: 706.2868, 707.2966, 708.3044, 710.3746, 714.3496, 717.4172, 722.4243, 729.4379, 734.4639, 735.4285, 738.4069, 744.3897, 745.4249, 747.4562, 753.3594, 756.3831, 758.3950, 768.4982, 771.3934, 779.4738, 783.3916, 805.4591, 818.4537, 823.4842, 832.3158, 834.3223, 840.4870, 842.5089, 876.2845, 878.2925, 883.4848, 889.5049, 891.4897, 922.4780, 923.5152, 929.4479, 938.4989, 949.5408, 958.4880, 962.3525, 967.4243, 968.4982, 969.5316, 973.5403, 974.4454, 976.4499, 978.5390, 979.3778, 979.5679, 983.6275, 985.6091, 986.5857, 988.3948, 993.5026, 1007.5763, 1017.5743, 1021.5607, 1031.6372, 1034.5828, 1036.5481, 1046.6013, 1051.5096, 1056.5107, 1057.6161, 1074.6067, 1087.5623, 1113.5466, 1123.6021, 1126.4208, 1143.4449, 1153.6440, 1157.4840, 1172.6813, 1178.6198, 1187.7026, 1194.5691, 1207.5975, 1215.6563, 1216.4995, 1216.7017, 1248.6692, 1254.6897, 1255.6042, 1257.6483, 1264.7133, 1265.7039, 1269.6589, 1273.6492, 1278.6917, 1284.6440, 1286.7411, 1288.7273, 1299.5450, 1301.5977, 1304.6617, 1314.7190, 1317.7113, 1321.7212, 1338.6622, 1349.6757, 1351.6887, 1354.7229, 1358.7100, 1371.7595, 1375.7335, 1382.5935, 1383.7274, 1393.7622, 1413.6547, 1420.7949, 1423.7593, 1440.7788, 1450.8590, 1456.7791, 1457.7606, 1467.7502, 1470.7725, 1488.7212, 1494.7396, 1500.7236, 1512.8181, 1513.7562, 1528.6954, 1539.7452, 1556.7537, 1570.7695, 1574.7894, 1578.7858, 1580.8347, 1590.7754, 1592.7883, 1616.7943, 1628.7009, 1631.8130, 1635.7671, 1642.7155, 1647.8516, 1650.8444, 1657.9042, 1663.8501, 1664.8555, 1693.0090, 1696.8273, 1707.9615, 1715.8461, 1717.8761, 1718.8779, 1721.9366, 1730.8695, 1741.9938, 1747.9058, 1755.8904, 1758.9028, 1771.0238, 1774.9207, 1784.0254, 1787.8574, 1870.7574, 1883.7222, 1895.7236, 1897.7325, 1909.7473, 1911.7430, 1913.7366, 1925.7604, 1938.0079, 1961.0325, 1969.9990, 1974.8882, 1979.0790, 1981.0576, 2008.9901, 2027.1294, 2060.9788, 2067.1584, 2086.1133, 2108.9963, 2112.9805, 2137.1189, 2144.0579, 2180.1812, 2223.1646, 2239.1501, 2241.1355, 2245.8403, 2255.1479, 2265.1016, 2273.0654, 2281.1055, 2318.1824, 2328.9529, 2335.9097, 2342.9673, 2347.8682, 2352.9265, 2356.9395, 2361.8997, 2363.8860, 2366.9265, 2371.0420, 2380.9539, 2389.2756, 2397.2231, 2406.2913, 2414.2468, 2431.2300, 2467.2122, 2474.2263, 2477.2573, 2478.2725, 2485.0691, 2499.1719, 2503.0352, 2518.0303, 2519.9934, 2534.0215, 2541.2542, 2542.2739, 2558.3401, 2580.0227, 2594.0615, 2631.4961, 2678.0144, 2683.4189, 2695.0366, 2701.2097, 2709.0559, 2717.3198, 2723.0757, 2770.4492, 2787.3171, 2799.3379, 2813.4517, 2817.2305, 2833.1284, 2842.5049, 2872.3596, 2944.3171, 2961.2437, 2975.3135, 2989.3062, 3004.4270, 3029.4883, 3059.4963, 3081.6570, 3086.5718, 3147.5710, 3157.4875, 3161.1531, 3177.1650, 3185.5222, 3237.6042, 3248.6902, 3254.5940, 3331.5864, 3335.7378, 3351.6597, 3360.7197, 3411.8193, 3428.6296, 3435.7471, 3457.6697, 3471.7146, 3475.7085, 3489.7715, 3503.8445, 3528.3743, 3542.4243, 3552.7373, 3568.9385, 3603.9502, 3625.7847, 3640.7600, 3645.8701, 3662.8821, 3691.0452, 3722.5232, 3727.5442, 3731.5757, 3733.5112, 3736.5735, 3741.5391, 3746.5410, 3748.5002, 3751.5356, 3755.5571, 3756.5549, 3763.5049, 3767.5706, 3778.5320, 3884.9326, 3955.6738, 4074.1699, 4149.0591, 4163.0181, 4238.0752, 4300.3838, 4382.1450, 4397.2036, 4441.5176, 4453.2886, 4458.5176, 4464.4150, 4495.2642, 4511.1577, 4692.4893

10. F1MPP1 **Mass:** 23962 **Score:** 21 **Expect:** 1.8e+002 **Matches:** 8

Ras-related protein Rab-21 OS=Bos taurus GN=RAB21 PE=4 SV=2

Observed	Mr(expt)	Mr(calc)	ppm	Start	End	Miss	Peptide
1356.7275	1355.7202	1355.7018	13.6	179 -	192	2	R.AKGNGSSQPGAARR.G
1390.6962	1389.6889	1389.6922	-2.35	169 -	180	1	R.MIETAQVDERAK.G
1562.7806	1561.7733	1561.7882	-9.53	168 -	180	2	K.RMIETAQVDERAK.G + Oxidation (M)
1628.8575	1627.8502	1627.8430	4.42	56 -	69	1	K.RVNLAIWDTAGQER.F
1706.8668	1705.8595	1705.8709	-6.66	154 -	167	1	K.QNKGIEELFLDLCK.R
1862.9503	1861.9430	1861.9720	-15.55	154 -	168	2	K.QNKGIEELFLDLCKR.M
2846.4216	2845.4143	2845.4776	-22.23	56 -	79	2	K.RVNLAIWDTAGQERFHALGPIYYR.D
3347.5420	3346.5347	3346.6146	-23.87	70 -	98	1	R.FHALGPIYYRDSNGAILVYDITDEDSFQK.V

No match to: 706.2868, 707.2966, 708.3044, 710.3746, 713.3099, 714.3496, 717.4172, 722.4243, 729.4379, 734.4639, 735.4285, 738.4069, 744.3897, 745.4249, 747.4562, 753.3594, 756.3831, 758.3950, 768.4982, 771.3934, 779.4738, 783.3916, 805.4591, 818.4537, 823.4842, 832.3158, 834.3223, 840.4870, 842.5089, 876.2845, 878.2925, 883.4848, 889.5049, 891.4897, 922.4780, 923.5152, 929.4479, 938.4989, 949.5408, 958.4880, 962.3525, 967.4243, 968.4982, 969.5316, 973.5403, 974.4454, 976.4499, 978.5390, 979.3778, 979.5679, 983.6275, 985.6091, 986.5857, 988.3948, 993.5026, 1007.5763, 1017.5743, 1021.5607, 1031.6372, 1034.5828, 1036.5481, 1046.6013, 1051.5096, 1056.5107, 1057.6161, 1074.6067, 1087.5623, 1113.5466, 1123.6021, 1126.4208, 1143.4449, 1153.6440, 1157.4840, 1172.6813, 1178.6198, 1187.7026, 1194.5691, 1207.5975, 1215.6563, 1216.4995, 1216.7017, 1248.6692, 1254.6897, 1255.6042, 1257.6483, 1264.7133, 1265.7039, 1269.6589, 1273.6492, 1278.6917, 1284.6440, 1286.7411, 1288.7273, 1299.5450, 1301.5977, 1304.6617, 1314.7190, 1317.7113, 1321.7212, 1338.6622, 1349.6757, 1351.6887, 1354.7229, 1358.7100, 1371.7595, 1375.7335, 1382.5935, 1383.7274, 1393.7622, 1413.6547, 1420.7949, 1423.7593, 1440.7788, 1450.8590, 1456.7791, 1457.7606, 1467.7502, 1470.7725, 1488.7212, 1494.7396, 1500.7236, 1512.8181, 1513.7562, 1528.6954, 1539.7452, 1556.7537, 1570.7695, 1574.7894, 1578.7858, 1580.8347, 1590.7754, 1592.7883, 1616.7943, 1628.7009, 1631.8130, 1635.7671, 1642.7155, 1647.8516, 1650.8444, 1657.9042, 1663.8501, 1664.8555, 1693.0090, 1696.8273, 1707.9615, 1715.8461, 1717.8761, 1718.8779, 1721.9366, 1730.8695, 1741.9938, 1747.9058, 1755.8904, 1758.9028, 1771.0238, 1774.9207, 1784.0254, 1787.8574, 1870.7574, 1883.7222, 1895.7236, 1897.7325, 1909.7473, 1911.7430, 1913.7366, 1925.7604, 1938.0079, 1961.0325, 1969.9990, 1974.8882, 1979.0790, 1981.0576, 2008.9901, 2027.1294, 2060.9788, 2067.1584, 2086.1133, 2108.9963, 2112.9805, 2137.1189, 2144.0579, 2180.1812, 2223.1646, 2239.1501, 2241.1355, 2245.8403, 2255.1479, 2265.1016, 2273.0654, 2281.1055, 2318.1824, 2328.9529, 2335.9097, 2342.9673, 2347.8682, 2352.9265, 2356.9395, 2361.8997, 2363.8860, 2366.9265, 2371.0420, 2380.9539, 2389.2756, 2397.2231, 2406.2913, 2414.2468, 2431.2300, 2467.2122, 2474.2263, 2477.2573, 2478.2725, 2485.0691, 2499.1719, 2503.0352, 2518.0303, 2519.9934, 2534.0215, 2541.2542, 2542.2739, 2558.3401, 2580.0227, 2594.0615, 2631.4961, 2678.0144, 2683.4189, 2695.0366, 2701.2097, 2709.0559, 2717.3198, 2723.0757, 2770.4492, 2787.3171, 2799.3379, 2813.4517, 2817.2305, 2833.1284, 2842.5049, 2872.3596, 2944.3171, 2961.2437, 2975.3135, 2989.3062, 3004.4270, 3029.4883, 3059.4963, 3081.6570, 3086.5718, 3147.5710, 3157.4875, 3161.1531, 3177.1650, 3185.5222, 3237.6042, 3248.6902,

3254.5940, 3331.5864, 3335.7378, 3351.6597, 3360.7197, 3411.8193, 3428.6296, 3435.7471, 3457.6697, 3471.7146, 3475.7085, 3489.7715, 3503.8445, 3528.3743, 3542.4243, 3552.7373, 3568.9385, 3603.9502, 3625.7847, 3640.7600, 3645.8701, 3662.8821, 3691.0452, 3722.5232, 3727.5442, 3731.5757, 3733.5112, 3736.5735, 3741.5391, 3746.5410, 3748.5002, 3751.5356, 3755.5571, 3756.5549, 3763.5049, 3767.5706, 3778.5320, 3884.9326, 3955.6738, 4074.1699, 4149.0591, 4163.0181, 4238.0752, 4300.3838, 4382.1450, 4397.2036, 4441.5176, 4453.2886, 4458.5176, 4464.4150, 4495.2642, 4511.1577, 4692.4893

11. E1BMT4 **Mass:** 22224 **Score:** 20 **Expect:** 2.3e+002 **Matches:** 5

Uncharacterized protein OS=Bos taurus GN=LOC618985 PE=3 SV=2

Observed	Mr(expt)	Mr(calc)	ppm	Start	End	Miss	Peptide
1194.5691	1193.5618	1193.5888	-22.58	174	184	0	R.SLSSSTSLQER.L
1278.6917	1277.6844	1277.6663	14.2	36	45	1	R.QNLRFLGQMR.R + Oxidation (M)
1590.7754	1589.7681	1589.8049	-23.14	96	109	0	R.SSAAWDTTLLEQLR.T
3457.6697	3456.6624	3456.7017	-11.36	158	186	2	K.EYSDCAWEIVIVEIMRSLSSSTSLQERLR.M
4074.1699	4073.1626	4073.1554	1.77	2	39	1	M.AFVLSLLMALVLVSYSSGGSLGCDLSQNHVLVGRQNLR.F

No match to: 706.2868, 707.2966, 708.3044, 710.3746, 713.3099, 714.3496, 717.4172, 722.4243, 729.4379, 734.4639, 735.4285, 738.4069, 744.3897, 745.4249, 747.4562, 753.3594, 756.3831, 758.3950, 768.4982, 771.3934, 779.4738, 783.3916, 805.4591, 818.4537, 823.4842, 832.3158, 834.3223, 840.4870, 842.5089, 876.2845, 878.2925, 883.4848, 889.5049, 891.4897, 922.4780, 923.5152, 929.4479, 938.4989, 949.5408, 958.4880, 962.3525, 967.4243, 968.4982, 969.5316, 973.5403, 974.4454, 976.4499, 978.5390, 979.3778, 979.5679, 983.6275, 985.6091, 986.5857, 988.3948, 993.5026, 1007.5763, 1017.5743, 1021.5607, 1031.6372, 1034.5828, 1036.5481, 1046.6013, 1051.5096, 1056.5107, 1057.6161, 1074.6067, 1087.5623, 1113.5466, 1123.6021, 1126.4208, 1143.4449, 1153.6440, 1157.4840, 1172.6813, 1178.6198, 1187.7026, 1207.5975, 1215.6563, 1216.4995, 1216.7017, 1248.6692, 1254.6897, 1255.6042, 1257.6483, 1264.7133, 1265.7039, 1269.6589, 1273.6492, 1284.6440, 1286.7411, 1288.7273, 1299.5450, 1301.5977, 1304.6617, 1314.7190, 1317.7113, 1321.7212, 1338.6622, 1349.6757, 1351.6887, 1354.7229, 1356.7275, 1358.7100, 1371.7595, 1375.7335, 1382.5935, 1383.7274, 1390.6962, 1393.7622, 1413.6547, 1420.7949, 1423.7593, 1440.7788, 1450.8590, 1456.7791, 1457.7606, 1467.7502, 1470.7725, 1488.7212, 1494.7396, 1500.7236, 1512.8181, 1513.7562, 1528.6954, 1539.7452, 1556.7537, 1562.7806, 1570.7695, 1574.7894, 1578.7858, 1580.8347, 1592.7883, 1616.7943, 1628.7009, 1628.8575, 1631.8130, 1635.7671, 1642.7155, 1647.8516, 1650.8444, 1657.9042, 1663.8501, 1664.8555, 1693.0090, 1696.8273, 1706.8668, 1707.9615, 1715.8461, 1717.8761, 1718.8779, 1721.9366, 1730.8695, 1741.9938, 1747.9058, 1755.8904, 1758.9028, 1771.0238, 1774.9207, 1784.0254, 1787.8574, 1862.9503, 1870.7574, 1883.7222, 1895.7236, 1897.7325, 1909.7473, 1911.7430, 1913.7366, 1925.7604, 1938.0079, 1961.0325, 1969.9990, 1974.8882, 1979.0790, 1981.0576, 2008.9901, 2027.1294, 2060.9788, 2067.1584, 2086.1133, 2108.9963, 2112.9805, 2137.1189, 2144.0579, 2180.1812, 2223.1646, 2239.1501, 2241.1355, 2245.8403, 2255.1479, 2265.1016, 2273.0654, 2281.1055, 2318.1824, 2328.9529, 2335.9097, 2342.9673, 2347.8682, 2352.9265, 2356.9395, 2361.8997, 2363.8860, 2366.9265, 2371.0420, 2380.9539, 2389.2756, 2397.2231, 2406.2913, 2414.2468, 2431.2300, 2467.2122, 2474.2263, 2477.2573, 2478.2725, 2485.0691, 2499.1719, 2503.0352, 2518.0303, 2519.9934, 2534.0215, 2541.2542, 2542.2739, 2558.3401, 2580.0227, 2594.0615, 2631.4961, 2678.0144, 2683.4189, 2695.0366, 2701.2097, 2709.0559, 2717.3198, 2723.0757, 2770.4492, 2787.3171, 2799.3379, 2813.4517, 2817.2305, 2833.1284, 2842.5049, 2846.4216, 2872.3596, 2944.3171, 2961.2437, 2975.3135, 2989.3062, 3004.4270, 3029.4883, 3059.4963, 3081.6570, 3086.5718, 3147.5710, 3157.4875, 3161.1531, 3177.1650, 3185.5222, 3237.6042, 3248.6902, 3254.5940, 3331.5864, 3335.7378, 3347.5420, 3351.6597, 3360.7197, 3411.8193, 3428.6296, 3435.7471, 3471.7146, 3475.7085, 3489.7715, 3503.8445, 3528.3743, 3542.4243, 3552.7373, 3568.9385, 3603.9502, 3625.7847, 3640.7600, 3645.8701, 3662.8821, 3691.0452, 3722.5232, 3727.5442, 3731.5757, 3733.5112, 3736.5735, 3741.5391, 3746.5410, 3748.5002, 3751.5356, 3755.5571, 3756.5549, 3763.5049, 3767.5706, 3778.5320, 3884.9326, 3955.6738, 4149.0591, 4163.0181, 4238.0752, 4300.3838, 4382.1450, 4397.2036, 4441.5176, 4453.2886, 4458.5176, 4464.4150, 4495.2642, 4511.1577, 4692.4893

12. Q58CY3 **Mass:** 41067 **Score:** 18 **Expect:** 3.4e+002 **Matches:** 10

Chromosome 20 open reading frame 4 OS=Bos taurus GN=C20orf4 PE=2 SV=1

Observed	Mr(expt)	Mr(calc)	ppm	Start	End	Miss	Peptide
1057.6161	1056.6088	1056.6226	-13.03	251	258	1	K.RLLNLLCR.S
1187.7026	1186.6953	1186.6670	23.9	312	322	1	R.SVAVDATLRQK.A
1269.6589	1268.6516	1268.6336	14.2	50	59	0	R.MGFFLNLQQR.G + Oxidation (M)
1420.7949	1419.7876	1419.7695	12.8	144	155	2	R.HTKDRVGQNLPR.C
1456.7791	1455.7718	1455.7946	-15.66	321	332	2	R.QKAERFQAHLTK.K
1488.7212	1487.7139	1487.6973	11.2	149	161	1	R.VGQNLPRCGAECK.S
1628.7009	1627.6936	1627.7083	-8.99	156	169	1	R.CGAECKSYQEGLAR.L
1774.9207	1773.9134	1773.9195	-3.45	162	176	1	K.SYQEGLARLPEMKPR.A
2474.2263	2473.2190	2473.1736	18.4	1	21	2	-.MPKGTEFGIDYNSWEVGPKFR.G + Oxidation (M)
2842.5049	2841.4976	2841.4629	12.2	123	146	2	K.LQPESRQICAFSEVLPVLSMRHTK.D + Oxidation (M)

No match to: 706.2868, 707.2966, 708.3044, 710.3746, 713.3099, 714.3496, 717.4172, 722.4243, 729.4379, 734.4639, 735.4285, 738.4069, 744.3897, 745.4249, 747.4562, 753.3594, 756.3831, 758.3950, 768.4982, 771.3934, 779.4738, 783.3916, 805.4591, 818.4537, 823.4842, 832.3158, 834.3223, 840.4870, 842.5089, 876.2845, 878.2925, 883.4848, 889.5049, 891.4897, 922.4780, 923.5152, 929.4479, 938.4989, 949.5408, 958.4880, 962.3525, 967.4243, 968.4982, 969.5316, 973.5403, 974.4454, 976.4499, 978.5390, 979.3778, 979.5679, 983.6275, 985.6091, 986.5857, 988.3948, 993.5026, 1007.5763, 1017.5743, 1021.5607, 1031.6372, 1034.5828, 1036.5481, 1046.6013, 1051.5096, 1056.5107, 1074.6067, 1087.5623, 1113.5466, 1123.6021, 1126.4208, 1143.4449, 1153.6440, 1157.4840, 1172.6813, 1178.6198, 1194.5691, 1207.5975, 1215.6563, 1216.4995, 1216.7017, 1248.6692, 1254.6897, 1255.6042, 1257.6483, 1264.7133, 1265.7039, 1273.6492, 1278.6917, 1284.6440, 1286.7411, 1288.7273, 1299.5450, 1301.5977, 1304.6617, 1314.7190, 1317.7113, 1321.7212, 1338.6622, 1349.6757, 1351.6887, 1354.7229, 1356.7275, 1358.7100, 1371.7595, 1375.7335, 1382.5935, 1383.7274, 1390.6962, 1393.7622, 1413.6547, 1423.7593, 1440.7788, 1450.8590, 1457.7606, 1467.7502, 1470.7725, 1494.7396, 1500.7236, 1512.8181, 1513.7562, 1528.6954, 1539.7452, 1556.7537, 1562.7806, 1570.7695, 1574.7894, 1578.7858, 1580.8347, 1590.7754, 1592.7883, 1616.7943, 1628.8575, 1631.8130, 1635.7671, 1642.7155, 1647.8516, 1650.8444, 1657.9042, 1663.8501, 1664.8555, 1693.0090, 1696.8273, 1706.8668, 1707.9615, 1715.8461, 1717.8761, 1718.8779, 1721.9366, 1730.8695, 1741.9938, 1747.9058, 1755.8904, 1758.9028, 1771.0238, 1784.0254, 1787.8574, 1862.9503, 1870.7574, 1883.7222, 1895.7236, 1897.7325, 1909.7473, 1911.7430, 1913.7366, 1925.7604, 1938.0079, 1961.0325, 1969.9990, 1974.8882, 1979.0790, 1981.0576, 2008.9901, 2027.1294, 2060.9788, 2067.1584, 2086.1133, 2108.9963, 2112.9805, 2137.1189, 2144.0579, 2180.1812, 2223.1646, 2239.1501, 2241.1355, 2245.8403, 2255.1479, 2265.1016, 2273.0654, 2281.1055, 2318.1824, 2328.9529, 2335.9097, 2342.9673, 2347.8682, 2352.9265, 2356.9395, 2361.8997, 2363.8860, 2366.9265, 2371.0420, 2380.9539, 2389.2756, 2397.2231, 2406.2913, 2414.2468, 2431.2300, 2467.2122, 2477.2573, 2478.2725, 2485.0691, 2499.1719, 2503.0352, 2518.0303, 2519.9934, 2534.0215, 2541.2542, 2542.2739, 2558.3401, 2580.0227, 2594.0615, 2631.4961, 2678.0144, 2683.4189, 2695.0366, 2701.2097, 2709.0559, 2717.3198, 2723.0757, 2770.4492, 2787.3171, 2799.3379, 2813.4517, 2817.2305, 2833.1284, 2846.4216, 2872.3596, 2944.3171, 2961.2437, 2975.3135, 2989.3062, 3004.4270, 3029.4883, 3059.4963, 3081.6570, 3086.5718, 3147.5710, 3157.4875, 3161.1531, 3177.1650, 3185.5222, 3237.6042, 3248.6902, 3254.5940, 3331.5864, 3335.7378, 3347.5420, 3351.6597, 3360.7197, 3411.8193, 3428.6296, 3435.7471, 3457.6697, 3471.7146, 3475.7085, 3489.7715, 3503.8445, 3528.3743, 3542.4243, 3552.7373, 3568.9385, 3603.9502, 3625.7847, 3640.7600, 3645.8701, 3662.8821, 3691.0452, 3722.5232, 3727.5442, 3731.5757, 3733.5112, 3736.5735, 3741.5391, 3746.5410, 3748.5002, 3751.5356, 3755.5571, 3756.5549, 3763.5049, 3767.5706, 3778.5320, 3884.9326, 3955.6738, 4074.1699, 4149.0591, 4163.0181, 4238.0752, 4300.3838, 4382.1450, 4397.2036, 4441.5176, 4453.2886, 4458.5176, 4464.4150, 4495.2642, 4511.1577, 4692.4893

13. Q32L59 **Mass:** 41560 **Score:** 18 **Expect:** 3.4e+002 **Matches:** 10

Transmembrane and coiled-coil domain-containing protein 5B OS=Bos taurus GN=TMCO5B PE=2 SV=1

Observed	Mr(expt)	Mr(calc)	ppm	Start	End	Miss	Peptide
891.4897	890.4824	890.4783	4.65	194	200	0	K.EMLLLEK.E + Oxidation (M)
949.5408	948.5335	948.5392	-6.05	181	187	1	K.KYQEILR.Q
1288.7273	1287.7200	1287.6922	21.6	93	103	1	R.DLELETAKLEK.N
1304.6617	1303.6544	1303.6806	-20.05	42	52	1	R.MDEANQVLLKK.I + Oxidation (M)
1664.8555	1663.8482	1663.8524	-2.52	188	200	2	R.QMEKEKEMLLLEK.E + Oxidation (M)
1938.0079	1937.0006	1937.0175	-8.70	287	300	1	R.HFRYFIFVVMIFFR.L + Oxidation (M)
2541.2542	2540.2469	2540.2653	-7.22	207	230	1	R.SSIQSAKTRPGADCGSDHELLIAK.F
2717.3198	2716.3125	2716.3701	-21.21	249	273	0	K.AQNNATQIVKPGSTLVETIQSNMEK.T + Oxidation (M)
3029.4883	3028.4810	3028.5135	-10.71	26	51	2	K.QNLDSLNSDLEKDLQRMDEANQVLLK.K
3086.5718	3085.5645	3085.5066	18.8	157	181	2	K.ELVKIESDYQSVYQLCEDQAHYIKK.Y

No match to: 706.2868, 707.2966, 708.3044, 710.3746, 713.3099, 714.3496, 717.4172, 722.4243, 729.4379, 734.4639, 735.4285, 738.4069, 744.3897, 745.4249, 747.4562, 753.3594, 756.3831, 758.3950, 768.4982, 771.3934, 779.4738, 783.3916, 805.4591, 818.4537, 823.4842, 832.3158, 834.3223, 840.4870, 842.5089, 876.2845, 878.2925, 883.4848, 889.5049, 922.4780, 923.5152, 929.4479, 938.4989, 958.4880, 962.3525, 967.4243, 968.4982, 969.5316, 973.5403, 974.4454, 976.4499, 978.5390, 979.3778, 979.5679, 983.6275, 985.6091, 986.5857, 988.3948, 993.5026, 1007.5763, 1017.5743, 1021.5607, 1031.6372, 1034.5828, 1036.5481, 1046.6013, 1051.5096, 1056.5107, 1057.6161, 1074.6067, 1087.5623, 1113.5466, 1123.6021, 1126.4208, 1143.4449, 1153.6440, 1157.4840, 1172.6813, 1178.6198, 1187.7026, 1194.5691, 1207.5975, 1215.6563, 1216.4995, 1216.7017, 1248.6692, 1254.6897, 1255.6042, 1257.6483, 1264.7133, 1265.7039, 1269.6589, 1273.6492, 1278.6917, 1284.6440, 1286.7411, 1299.5450, 1301.5977, 1314.7190, 1317.7113, 1321.7212, 1338.6622, 1349.6757, 1351.6887, 1354.7229, 1356.7275, 1358.7100, 1371.7595, 1375.7335, 1382.5935, 1383.7274, 1390.6962, 1393.7622, 1413.6547, 1420.7949, 1423.7593, 1440.7788, 1450.8590, 1456.7791, 1457.7606, 1467.7502, 1470.7725, 1488.7212, 1494.7396, 1500.7236, 1512.8181, 1513.7562, 1528.6954, 1539.7452, 1556.7537, 1562.7806, 1570.7695, 1574.7894, 1578.7858, 1580.8347, 1590.7754, 1592.7883, 1616.7943, 1628.7009, 1628.8575, 1631.8130, 1635.7671, 1642.7155, 1647.8516, 1650.8444, 1657.9042, 1663.8501, 1693.0090, 1696.8273, 1706.8668, 1707.9615, 1715.8461, 1717.8761, 1718.8779, 1721.9366, 1730.8695, 1741.9938, 1747.9058, 1755.8904, 1758.9028, 1771.0238, 1774.9207, 1784.0254, 1787.8574, 1862.9503, 1870.7574, 1883.7222, 1895.7236, 1897.7325, 1909.7473, 1911.7430, 1913.7366, 1925.7604, 1961.0325, 1969.9990, 1974.8882, 1979.0790, 1981.0576, 2008.9901, 2027.1294, 2060.9788, 2067.1584, 2086.1133, 2108.9963, 2112.9805, 2137.1189, 2144.0579, 2180.1812, 2223.1646, 2239.1501, 2241.1355, 2245.8403, 2255.1479, 2265.1016, 2273.0654, 2281.1055, 2318.1824, 2328.9529, 2335.9097, 2342.9673, 2347.8682, 2352.9265, 2356.9395, 2361.8997, 2363.8860, 2366.9265, 2371.0420, 2380.9539, 2389.2756, 2397.2231, 2406.2913, 2414.2468, 2431.2300, 2467.2122, 2474.2263, 2477.2573, 2478.2725, 2485.0691, 2499.1719, 2503.0352, 2518.0303, 2519.9934, 2534.0215, 2542.2739, 2558.3401, 2580.0227, 2594.0615, 2631.4961, 2678.0144, 2683.4189, 2695.0366, 2701.2097, 2709.0559, 2723.0757, 2770.4492, 2787.3171, 2799.3379, 2813.4517, 2817.2305, 2833.1284, 2842.5049, 2846.4216, 2872.3596, 2944.3171, 2961.2437, 2975.3135, 2989.3062, 3004.4270, 3059.4963, 3081.6570, 3147.5710, 3157.4875, 3161.1531, 3177.1650, 3185.5222, 3237.6042, 3248.6902, 3254.5940, 3331.5864, 3335.7378, 3347.5420, 3351.6597, 3360.7197, 3411.8193, 3428.6296, 3435.7471, 3457.6697, 3471.7146, 3475.7085, 3489.7715, 3503.8445, 3528.3743, 3542.4243, 3552.7373, 3568.9385, 3603.9502, 3625.7847, 3640.7600, 3645.8701, 3662.8821, 3691.0452, 3722.5232, 3727.5442, 3731.5757, 3733.5112, 3736.5735, 3741.5391, 3746.5410, 3748.5002, 3751.5356, 3755.5571, 3756.5549, 3763.5049, 3767.5706, 3778.5320, 3884.9326, 3955.6738, 4074.1699, 4149.0591, 4163.0181, 4238.0752, 4300.3838, 4382.1450, 4397.2036, 4441.5176, 4453.2886, 4458.5176, 4464.4150, 4495.2642, 4511.1577, 4692.4893

14. F1MWR4 **Mass:** 41530 **Score:** 18 **Expect:** 3.4e+002 **Matches:** 10

Transmembrane and coiled-coil domain-containing protein 5B OS=Bos taurus GN=TMCO5B PE=4 SV=1

Observed	Mr(expt)	Mr(calc)	ppm	Start		End	Miss	Peptide
891.4897	890.4824	890.4783	4.65	194	-	200	0	K.EMLLLEK.E + Oxidation (M)
949.5408	948.5335	948.5392	-6.05	181	-	187	1	K.KYQEILR.Q
1288.7273	1287.7200	1287.6922	21.6	93	-	103	1	R.DLELETAKLEK.N
1304.6617	1303.6544	1303.6806	-20.05	42	-	52	1	R.MDEANQVLLKK.I + Oxidation (M)
1664.8555	1663.8482	1663.8524	-2.52	188	-	200	2	R.QMEKEKEMLLLEK.E + Oxidation (M)
1938.0079	1937.0006	1937.0175	-8.70	287	-	300	1	R.HFRYFIFVVMIFFR.L + Oxidation (M)
2541.2542	2540.2469	2540.2653	-7.22	207	-	230	1	R.SSIQSAKTRPGADCGSDHELLIAK.F
2717.3198	2716.3125	2716.3701	-21.21	249	-	273	0	K.AQNNATQIVKPGSTLVETIQSNMEK.T + Oxidation (M)
3029.4883	3028.4810	3028.5135	-10.71	26	-	51	2	K.QNLDSLNSDLEKDLQRMDEANQVLLK.K
3086.5718	3085.5645	3085.5066	18.8	157	-	181	2	K.ELVKIESDYQSVYQLCEDQAHYIKK.Y

No match to: 706.2868, 707.2966, 708.3044, 710.3746, 713.3099, 714.3496, 717.4172, 722.4243, 729.4379, 734.4639, 735.4285, 738.4069, 744.3897, 745.4249, 747.4562, 753.3594, 756.3831, 758.3950, 768.4982, 771.3934, 779.4738, 783.3916, 805.4591, 818.4537, 823.4842, 832.3158, 834.3223, 840.4870, 842.5089, 876.2845, 878.2925, 883.4848, 889.5049, 922.4780, 923.5152, 929.4479, 938.4989, 958.4880, 962.3525, 967.4243, 968.4982, 969.5316, 973.5403, 974.4454, 976.4499, 978.5390, 979.3778, 979.5679, 983.6275, 985.6091, 986.5857, 988.3948, 993.5026, 1007.5763, 1017.5743, 1021.5607, 1031.6372, 1034.5828, 1036.5481, 1046.6013, 1051.5096, 1056.5107, 1057.6161, 1074.6067, 1087.5623, 1113.5466, 1123.6021, 1126.4208, 1143.4449, 1153.6440, 1157.4840, 1172.6813, 1178.6198, 1187.7026, 1194.5691, 1207.5975, 1215.6563, 1216.4995, 1216.7017, 1248.6692, 1254.6897, 1255.6042, 1257.6483, 1264.7133, 1265.7039, 1269.6589, 1273.6492, 1278.6917, 1284.6440, 1286.7411, 1299.5450, 1301.5977, 1314.7190, 1317.7113, 1321.7212, 1338.6622, 1349.6757, 1351.6887, 1354.7229, 1356.7275, 1358.7100, 1371.7595, 1375.7335, 1382.5935, 1383.7274, 1390.6962, 1393.7622, 1413.6547, 1420.7949, 1423.7593, 1440.7788, 1450.8590, 1456.7791, 1457.7606, 1467.7502, 1470.7725, 1488.7212, 1494.7396, 1500.7236, 1512.8181, 1513.7562, 1528.6954, 1539.7452, 1556.7537, 1562.7806, 1570.7695, 1574.7894, 1578.7858, 1580.8347, 1590.7754, 1592.7883, 1616.7943, 1628.7009, 1628.8575, 1631.8130, 1635.7671, 1642.7155, 1647.8516, 1650.8444, 1657.9042, 1663.8501, 1693.0090, 1696.8273, 1706.8668, 1707.9615, 1715.8461, 1717.8761, 1718.8779, 1721.9366, 1730.8695, 1741.9938, 1747.9058, 1755.8904, 1758.9028, 1771.0238, 1774.9207, 1784.0254, 1787.8574, 1862.9503, 1870.7574, 1883.7222, 1895.7236, 1897.7325, 1909.7473, 1911.7430, 1913.7366, 1925.7604, 1961.0325, 1969.9990, 1974.8882, 1979.0790, 1981.0576, 2008.9901, 2027.1294, 2060.9788, 2067.1584, 2086.1133, 2108.9963, 2112.9805, 2137.1189, 2144.0579, 2180.1812, 2223.1646, 2239.1501, 2241.1355, 2245.8403, 2255.1479, 2265.1016, 2273.0654, 2281.1055, 2318.1824, 2328.9529, 2335.9097, 2342.9673, 2347.8682, 2352.9265, 2356.9395, 2361.8997, 2363.8860, 2366.9265, 2371.0420, 2380.9539, 2389.2756, 2397.2231, 2406.2913, 2414.2468, 2431.2300, 2467.2122, 2474.2263, 2477.2573, 2478.2725, 2485.0691, 2499.1719, 2503.0352, 2518.0303, 2519.9934, 2534.0215, 2542.2739, 2558.3401, 2580.0227, 2594.0615, 2631.4961, 2678.0144, 2683.4189, 2695.0366, 2701.2097, 2709.0559, 2723.0757, 2770.4492, 2787.3171, 2799.3379, 2813.4517, 2817.2305, 2833.1284, 2842.5049, 2846.4216, 2872.3596, 2944.3171, 2961.2437, 2975.3135, 2989.3062, 3004.4270, 3059.4963, 3081.6570, 3147.5710, 3157.4875, 3161.1531, 3177.1650, 3185.5222, 3237.6042, 3248.6902, 3254.5940, 3331.5864, 3335.7378, 3347.5420, 3351.6597, 3360.7197, 3411.8193, 3428.6296, 3435.7471, 3457.6697, 3471.7146, 3475.7085, 3489.7715, 3503.8445, 3528.3743, 3542.4243, 3552.7373, 3568.9385, 3603.9502, 3625.7847, 3640.7600, 3645.8701, 3662.8821, 3691.0452, 3722.5232, 3727.5442, 3731.5757, 3733.5112, 3736.5735, 3741.5391, 3746.5410, 3748.5002, 3751.5356, 3755.5571, 3756.5549, 3763.5049, 3767.5706, 3778.5320, 3884.9326, 3955.6738, 4074.1699, 4149.0591, 4163.0181, 4238.0752, 4300.3838, 4382.1450, 4397.2036, 4441.5176, 4453.2886, 4458.5176, 4464.4150, 4495.2642, 4511.1577, 4692.4893

15. F1MQ25 **Mass:** 19349 **Score:** 18 **Expect:** 3.8e+002 **Matches:** 7

39S ribosomal protein L49, mitochondrial OS=Bos taurus GN=MRPL49 PE=4 SV=2

Observed	Mr(expt)	Mr(calc)	ppm	Start		End	Miss	Peptide
783.3916	782.3843	782.3745	12.6	1	-	7	0	-.MAAATFR.F + Oxidation (M)
891.4897	890.4824	890.5008	-20.61	106	-	112	1	R.QMTVIRK.V + Oxidation (M)
1248.6692	1247.6619	1247.6662	-3.46	149	-	158	1	R.IKGYFDHQLK.A
1286.7411	1285.7338	1285.7030	24.0	112	-	122	1	R.KVEGDIWALQK.D
1470.7725	1469.7652	1469.7779	-8.64	77	-	88	1	R.DPPPNLPYFVRR.S
1747.9058	1746.8985	1746.9093	-6.18	151	-	164	1	K.GYFDHQLKAWLLEK.G
2842.5049	2841.4976	2841.5124	-5.20	123	-	148	1	K.DVEDFLSPLLGKTPITQVNEVTGTLR.I

No match to: 706.2868, 707.2966, 708.3044, 710.3746, 713.3099, 714.3496, 717.4172, 722.4243, 729.4379, 734.4639, 735.4285, 738.4069, 744.3897, 745.4249, 747.4562, 753.3594, 756.3831, 758.3950, 768.4982, 771.3934, 779.4738, 805.4591, 818.4537, 823.4842, 832.3158, 834.3223, 840.4870, 842.5089, 876.2845, 878.2925, 883.4848, 889.5049, 922.4780, 923.5152, 929.4479, 938.4989, 949.5408, 958.4880, 962.3525, 967.4243, 968.4982, 969.5316, 973.5403, 974.4454, 976.4499, 978.5390, 979.3778, 979.5679, 983.6275, 985.6091, 986.5857, 988.3948, 993.5026, 1007.5763, 1017.5743, 1021.5607, 1031.6372, 1034.5828, 1036.5481, 1046.6013, 1051.5096, 1056.5107, 1057.6161, 1074.6067, 1087.5623, 1113.5466, 1123.6021, 1126.4208, 1143.4449, 1153.6440, 1157.4840, 1172.6813, 1178.6198, 1187.7026, 1194.5691, 1207.5975, 1215.6563, 1216.4995, 1216.7017, 1254.6897, 1255.6042, 1257.6483, 1264.7133, 1265.7039, 1269.6589, 1273.6492, 1278.6917, 1284.6440, 1288.7273, 1299.5450, 1301.5977, 1304.6617, 1314.7190, 1317.7113, 1321.7212, 1338.6622, 1349.6757, 1351.6887, 1354.7229, 1356.7275, 1358.7100, 1371.7595, 1375.7335, 1382.5935, 1383.7274, 1390.6962, 1393.7622, 1413.6547, 1420.7949, 1423.7593, 1440.7788, 1450.8590, 1456.7791, 1457.7606, 1467.7502, 1488.7212, 1494.7396, 1500.7236, 1512.8181, 1513.7562, 1528.6954, 1539.7452, 1556.7537, 1562.7806, 1570.7695, 1574.7894, 1578.7858, 1580.8347, 1590.7754, 1592.7883, 1616.7943, 1628.7009, 1628.8575, 1631.8130, 1635.7671, 1642.7155, 1647.8516, 1650.8444, 1657.9042, 1663.8501, 1664.8555, 1693.0090, 1696.8273, 1706.8668, 1707.9615, 1715.8461, 1717.8761, 1718.8779, 1721.9366, 1730.8695, 1741.9938, 1755.8904, 1758.9028, 1771.0238, 1774.9207, 1784.0254, 1787.8574, 1862.9503, 1870.7574, 1883.7222, 1895.7236, 1897.7325, 1909.7473, 1911.7430, 1913.7366, 1925.7604, 1938.0079, 1961.0325, 1969.9990, 1974.8882, 1979.0790, 1981.0576, 2008.9901, 2027.1294, 2060.9788, 2067.1584, 2086.1133, 2108.9963, 2112.9805, 2137.1189, 2144.0579, 2180.1812, 2223.1646, 2239.1501, 2241.1355, 2245.8403, 2255.1479, 2265.1016, 2273.0654, 2281.1055, 2318.1824, 2328.9529, 2335.9097, 2342.9673, 2347.8682, 2352.9265, 2356.9395, 2361.8997, 2363.8860, 2366.9265, 2371.0420, 2380.9539, 2389.2756, 2397.2231, 2406.2913, 2414.2468, 2431.2300, 2467.2122, 2474.2263, 2477.2573, 2478.2725, 2485.0691, 2499.1719, 2503.0352, 2518.0303, 2519.9934, 2534.0215, 2541.2542, 2542.2739, 2558.3401, 2580.0227, 2594.0615, 2631.4961, 2678.0144, 2683.4189, 2695.0366, 2701.2097, 2709.0559, 2717.3198, 2723.0757,

2770.4492, 2787.3171, 2799.3379, 2813.4517, 2817.2305, 2833.1284, 2846.4216, 2872.3596, 2944.3171, 2961.2437, 2975.3135, 2989.3062, 3004.4270, 3029.4883, 3059.4963, 3081.6570, 3086.5718, 3147.5710, 3157.4875, 3161.1531, 3177.1650, 3185.5222, 3237.6042, 3248.6902, 3254.5940, 3331.5864, 3335.7378, 3347.5420, 3351.6597, 3360.7197, 3411.8193, 3428.6296, 3435.7471, 3457.6697, 3471.7146, 3475.7085, 3489.7715, 3503.8445, 3528.3743, 3542.4243, 3552.7373, 3568.9385, 3603.9502, 3625.7847, 3640.7600, 3645.8701, 3662.8821, 3691.0452, 3722.5232, 3727.5442, 3731.5757, 3733.5112, 3736.5735, 3741.5391, 3746.5410, 3748.5002, 3751.5356, 3755.5571, 3756.5549, 3763.5049, 3767.5706, 3778.5320, 3884.9326, 3955.6738, 4074.1699, 4149.0591, 4163.0181, 4238.0752, 4300.3838, 4382.1450, 4397.2036, 4441.5176, 4453.2886, 4458.5176, 4464.4150, 4495.2642, 4511.1577, 4692.4893

16. Q2KIA2 **Mass:** 14368 **Score:** 18 **Expect:** 3.8e+002 **Matches:** 4
Multifunctional methyltransferase subunit TRM112-like protein OS=Bos taurus GN=TRMT112 PE=2 SV=1

Observed	Mr(expt)	Mr(calc)	ppm	Start	End	Miss	Peptide
2842.5049	2841.4976	2841.5211	-8.26	25 -	48	2	R.LQATEVRINPVEFNPDFIVRMIPK.V + Oxidation (M)
3248.6902	3247.6829	3247.6482	10.7	84 -	111	1	K.MHHVLLEVEVLEGTLQCPESGRVFPISR.G + Oxidation (M)
3360.7197	3359.7124	3359.7482	-10.66	83 -	111	2	R.KMHHVLLEVEVLEGTLQCPESGRVFPISR.G
4464.4150	4463.4077	4463.3099	21.9	45 -	82	2	R.MIPKVEWAALLEAADHLHLIQVPKEPIQGYEHNEEFLR.K

No match to: 706.2868, 707.2966, 708.3044, 710.3746, 713.3099, 714.3496, 717.4172, 722.4243, 729.4379, 734.4639, 735.4285, 738.4069, 744.3897, 745.4249, 747.4562, 753.3594, 756.3831, 758.3950, 768.4982, 771.3934, 779.4738, 783.3916, 805.4591, 818.4537, 823.4842, 832.3158, 834.3223, 840.4870, 842.5089, 876.2845, 878.2925, 883.4848, 889.5049, 891.4897, 922.4780, 923.5152, 929.4479, 938.4989, 949.5408, 958.4880, 962.3525, 967.4243, 968.4982, 969.5316, 973.5403, 974.4454, 976.4499, 978.5390, 979.3778, 979.5679, 983.6275, 985.6091, 986.5857, 988.3948, 993.5026, 1007.5763, 1017.5743, 1021.5607, 1031.6372, 1034.5828, 1036.5481, 1046.6013, 1051.5096, 1056.5107, 1057.6161, 1074.6067, 1087.5623, 1113.5466, 1123.6021, 1126.4208, 1143.4449, 1153.6440, 1157.4840, 1172.6813, 1178.6198, 1187.7026, 1194.5691, 1207.5975, 1215.6563, 1216.4995, 1216.7017, 1248.6692, 1254.6897, 1255.6042, 1257.6483, 1264.7133, 1265.7039, 1269.6589, 1273.6492, 1278.6917, 1284.6440, 1286.7411, 1288.7273, 1299.5450, 1301.5977, 1304.6617, 1314.7190, 1317.7113, 1321.7212, 1338.6622, 1349.6757, 1351.6887, 1354.7229, 1356.7275, 1358.7100, 1371.7595, 1375.7335, 1382.5935, 1383.7274, 1390.6962, 1393.7622, 1413.6547, 1420.7949, 1423.7593, 1440.7788, 1450.8590, 1456.7791, 1457.7606, 1467.7502, 1470.7725, 1488.7212, 1494.7396, 1500.7236, 1512.8181, 1513.7562, 1528.6954, 1539.7452, 1556.7537, 1562.7806, 1570.7695, 1574.7894, 1578.7858, 1580.8347, 1590.7754, 1592.7883, 1616.7943, 1628.7009, 1628.8575, 1631.8130, 1635.7671, 1642.7155, 1647.8516, 1650.8444, 1657.9042, 1663.8501, 1664.8555, 1693.0090, 1696.8273, 1706.8668, 1707.9615, 1715.8461, 1717.8761, 1718.8779, 1721.9366, 1730.8695, 1741.9938, 1747.9058, 1755.8904, 1758.9028, 1771.0238, 1774.9207, 1784.0254, 1787.8574, 1862.9503, 1870.7574, 1883.7222, 1895.7236, 1897.7325, 1909.7473, 1911.7430, 1913.7366, 1925.7604, 1938.0079, 1961.0325, 1969.9990, 1974.8882, 1979.0790, 1981.0576, 2008.9901, 2027.1294, 2060.9788, 2067.1584, 2086.1133, 2108.9963, 2112.9805, 2137.1189, 2144.0579, 2180.1812, 2223.1646, 2239.1501, 2241.1355, 2245.8403, 2255.1479, 2265.1016, 2273.0654, 2281.1055, 2318.1824, 2328.9529, 2335.9097, 2342.9673, 2347.8682, 2352.9265, 2356.9395, 2361.8997, 2363.8860, 2366.9265, 2371.0420, 2380.9539, 2389.2756, 2397.2231, 2406.2913, 2414.2468, 2431.2300, 2467.2122, 2474.2263, 2477.2573, 2478.2725, 2485.0691, 2499.1719, 2503.0352, 2518.0303, 2519.9934, 2534.0215, 2541.2542, 2542.2739, 2558.3401, 2580.0227, 2594.0615, 2631.4961, 2678.0144, 2683.4189, 2695.0366, 2701.2097, 2709.0559, 2717.3198, 2723.0757, 2770.4492, 2787.3171, 2799.3379, 2813.4517, 2817.2305, 2833.1284, 2846.4216, 2872.3596, 2944.3171, 2961.2437, 2975.3135, 2989.3062, 3004.4270, 3029.4883, 3059.4963, 3081.6570, 3086.5718, 3147.5710, 3157.4875, 3161.1531, 3177.1650, 3185.5222, 3237.6042, 3254.5940, 3331.5864, 3335.7378, 3347.5420, 3351.6597, 3411.8193, 3428.6296, 3435.7471, 3457.6697, 3471.7146, 3475.7085, 3489.7715, 3503.8445, 3528.3743, 3542.4243, 3552.7373, 3568.9385, 3603.9502, 3625.7847, 3640.7600, 3645.8701, 3662.8821, 3691.0452, 3722.5232, 3727.5442, 3731.5757, 3733.5112, 3736.5735, 3741.5391, 3746.5410, 3748.5002, 3751.5356, 3755.5571, 3756.5549, 3763.5049, 3767.5706, 3778.5320, 3884.9326, 3955.6738, 4074.1699, 4149.0591, 4163.0181, 4238.0752, 4300.3838, 4382.1450, 4397.2036, 4441.5176, 4453.2886, 4458.5176, 4495.2642, 4511.1577, 4692.4893

17. P79244 **Mass:** 11299 **Score:** 18 **Expect:** 3.9e+002 **Matches:** 5
60S ribosomal protein L37 OS=Bos taurus GN=RPL37 PE=3 SV=4

Observed	Mr(expt)	Mr(calc)	ppm	Start	End	Miss	Peptide
993.5026	992.4953	992.4822	13.3	57 -	65	1	R.NTTGTGRMR.H
1123.6021	1122.5948	1122.5934	1.28	47 -	55	2	K.YNWSAKAKR.R
1354.7229	1353.7156	1353.7153	0.22	76 -	87	1	R.HGFREGTTPKPK.R
1371.7595	1370.7522	1370.7201	23.4	57 -	68	2	R.NTTGTGRMRHLK.I
1657.9042	1656.8969	1656.8849	7.28	74 -	87	2	R.FRHGFREGTTPKPK.R

No match to: 706.2868, 707.2966, 708.3044, 710.3746, 713.3099, 714.3496, 717.4172, 722.4243, 729.4379, 734.4639, 735.4285, 738.4069, 744.3897, 745.4249, 747.4562, 753.3594, 756.3831, 758.3950, 768.4982, 771.3934, 779.4738, 783.3916, 805.4591, 818.4537, 823.4842, 832.3158, 834.3223, 840.4870, 842.5089, 876.2845, 878.2925, 883.4848, 889.5049, 891.4897, 922.4780, 923.5152, 929.4479, 938.4989, 949.5408, 958.4880, 962.3525, 967.4243, 968.4982, 969.5316, 973.5403, 974.4454, 976.4499, 978.5390, 979.3778, 979.5679, 983.6275, 985.6091, 986.5857, 988.3948, 1007.5763, 1017.5743, 1021.5607, 1031.6372, 1034.5828, 1036.5481, 1046.6013, 1051.5096, 1056.5107, 1057.6161, 1074.6067, 1087.5623, 1113.5466, 1126.4208, 1143.4449, 1153.6440, 1157.4840, 1172.6813, 1178.6198, 1187.7026, 1194.5691, 1207.5975, 1215.6563, 1216.4995, 1216.7017, 1248.6692, 1254.6897, 1255.6042, 1257.6483, 1264.7133, 1265.7039, 1269.6589, 1273.6492, 1278.6917, 1284.6440, 1286.7411, 1288.7273, 1299.5450, 1301.5977, 1304.6617, 1314.7190, 1317.7113, 1321.7212, 1338.6622, 1349.6757, 1351.6887, 1356.7275, 1358.7100, 1375.7335, 1382.5935, 1383.7274, 1390.6962, 1393.7622, 1413.6547, 1420.7949, 1423.7593, 1440.7788, 1450.8590, 1456.7791, 1457.7606, 1467.7502, 1470.7725, 1488.7212, 1494.7396, 1500.7236, 1512.8181, 1513.7562, 1528.6954, 1539.7452, 1556.7537, 1562.7806, 1570.7695, 1574.7894, 1578.7858, 1580.8347, 1590.7754, 1592.7883, 1616.7943, 1628.7009, 1628.8575, 1631.8130, 1635.7671, 1642.7155, 1647.8516, 1650.8444, 1663.8501, 1664.8555, 1693.0090, 1696.8273, 1706.8668, 1707.9615, 1715.8461, 1717.8761, 1718.8779, 1721.9366, 1730.8695, 1741.9938, 1747.9058, 1755.8904, 1758.9028, 1771.0238, 1774.9207, 1784.0254, 1787.8574, 1862.9503, 1870.7574, 1883.7222, 1895.7236, 1897.7325, 1909.7473, 1911.7430, 1913.7366, 1925.7604, 1938.0079, 1961.0325, 1969.9990, 1974.8882, 1979.0790, 1981.0576, 2008.9901, 2027.1294, 2060.9788, 2067.1584, 2086.1133, 2108.9963, 2112.9805, 2137.1189, 2144.0579, 2180.1812, 2223.1646, 2239.1501, 2241.1355, 2245.8403, 2255.1479, 2265.1016, 2273.0654, 2281.1055, 2318.1824, 2328.9529, 2335.9097, 2342.9673, 2347.8682, 2352.9265, 2356.9395, 2361.8997, 2363.8860, 2366.9265, 2371.0420, 2380.9539, 2389.2756, 2397.2231, 2406.2913, 2414.2468, 2431.2300, 2467.2122, 2474.2263, 2477.2573, 2478.2725, 2485.0691, 2499.1719, 2503.0352, 2518.0303, 2519.9934, 2534.0215, 2541.2542, 2542.2739, 2558.3401, 2580.0227, 2594.0615, 2631.4961, 2678.0144, 2683.4189, 2695.0366, 2701.2097, 2709.0559, 2717.3198, 2723.0757, 2770.4492, 2787.3171, 2799.3379, 2813.4517, 2817.2305, 2833.1284, 2842.5049, 2846.4216, 2872.3596, 2944.3171, 2961.2437, 2975.3135, 2989.3062, 3004.4270, 3029.4883, 3059.4963, 3081.6570, 3086.5718, 3147.5710, 3157.4875, 3161.1531, 3177.1650, 3185.5222, 3237.6042, 3248.6902, 3254.5940, 3331.5864, 3335.7378, 3347.5420, 3351.6597, 3360.7197, 3411.8193, 3428.6296, 3435.7471, 3457.6697, 3471.7146, 3475.7085, 3489.7715, 3503.8445, 3528.3743, 3542.4243, 3552.7373, 3568.9385, 3603.9502, 3625.7847, 3640.7600, 3645.8701, 3662.8821, 3691.0452, 3722.5232, 3727.5442, 3731.5757, 3733.5112, 3736.5735, 3741.5391, 3746.5410, 3748.5002, 3751.5356, 3755.5571, 3756.5549, 3763.5049, 3767.5706, 3778.5320, 3884.9326, 3955.6738, 4074.1699, 4149.0591, 4163.0181, 4238.0752, 4300.3838, 4382.1450, 4397.2036, 4441.5176, 4453.2886, 4458.5176, 4464.4150, 4495.2642, 4511.1577, 4692.4893

18. E1BFX3 **Mass:** 11338 **Score:** 18 **Expect:** 3.9e+002 **Matches:** 5
Ribosomal protein L37 OS=Bos taurus GN=RPL37 PE=3 SV=1

Observed	Mr(expt)	Mr(calc)	ppm	Start	End	Miss	Peptide
993.5026	992.4953	992.4822	13.3	57 -	65	1	R.NTTGTGRMR.H
1123.6021	1122.5948	1122.5934	1.28	47 -	55	2	K.YNWSAKAKR.R
1354.7229	1353.7156	1353.7153	0.22	76 -	87	1	R.HGFREGTTPKPK.R
1371.7595	1370.7522	1370.7201	23.4	57 -	68	2	R.NTTGTGRMRHLK.I
1657.9042	1656.8969	1656.8849	7.28	74 -	87	2	R.FRHGFREGTTPKPK.R

No match to: 706.2868, 707.2966, 708.3044, 710.3746, 713.3099, 714.3496, 717.4172, 722.4243, 729.4379, 734.4639, 735.4285, 738.4069, 744.3897, 745.4249, 747.4562, 753.3594, 756.3831, 758.3950, 768.4982, 771.3934, 779.4738, 783.3916, 805.4591, 818.4537, 823.4842, 832.3158, 834.3223, 840.4870, 842.5089, 876.2845, 878.2925, 883.4848, 889.5049, 891.4897, 922.4780, 923.5152, 929.4479, 938.4989, 949.5408, 958.4880, 962.3525, 967.4243, 968.4982, 969.5316, 973.5403, 974.4454, 976.4499, 978.5390, 979.3778, 979.5679, 983.6275, 985.6091, 986.5857, 988.3948, 1007.5763, 1017.5743, 1021.5607, 1031.6372, 1034.5828, 1036.5481, 1046.6013, 1051.5096, 1056.5107, 1057.6161, 1074.6067, 1087.5623, 1113.5466, 1126.4208, 1143.4449, 1153.6440, 1157.4840, 1172.6813, 1178.6198, 1187.7026, 1194.5691, 1207.5975, 1215.6563, 1216.4995, 1216.7017, 1248.6692, 1254.6897, 1255.6042, 1257.6483, 1264.7133, 1265.7039, 1269.6589, 1273.6492, 1278.6917, 1284.6440, 1286.7411, 1288.7273, 1299.5450, 1301.5977, 1304.6617, 1314.7190, 1317.7113, 1321.7212, 1338.6622, 1349.6757, 1351.6887, 1356.7275, 1358.7100, 1375.7335, 1382.5935, 1383.7274, 1390.6962, 1393.7622, 1413.6547, 1420.7949, 1423.7593, 1440.7788, 1450.8590, 1456.7791, 1457.7606, 1467.7502, 1470.7725, 1488.7212, 1494.7396, 1500.7236, 1512.8181, 1513.7562, 1528.6954, 1539.7452, 1556.7537, 1562.7806, 1570.7695, 1574.7894, 1578.7858, 1580.8347, 1590.7754, 1592.7883, 1616.7943, 1628.7009, 1628.8575,

1631.8130, 1635.7671, 1642.7155, 1647.8516, 1650.8444, 1663.8501, 1664.8555, 1693.0090, 1696.8273, 1706.8668, 1707.9615, 1715.8461, 1717.8761, 1718.8779, 1721.9366, 1730.8695, 1741.9938, 1747.9058, 1755.8904, 1758.9028, 1771.0238, 1774.9207, 1784.0254, 1787.8574, 1862.9503, 1870.7574, 1883.7222, 1895.7236, 1897.7325, 1909.7473, 1911.7430, 1913.7366, 1925.7604, 1938.0079, 1961.0325, 1969.9990, 1974.8882, 1979.0790, 1981.0576, 2008.9901, 2027.1294, 2060.9788, 2067.1584, 2086.1133, 2108.9963, 2112.9805, 2137.1189, 2144.0579, 2180.1812, 2223.1646, 2239.1501, 2241.1355, 2245.8403, 2255.1479, 2265.1016, 2273.0654, 2281.1055, 2318.1824, 2328.9529, 2335.9097, 2342.9673, 2347.8682, 2352.9265, 2356.9395, 2361.8997, 2363.8860, 2366.9265, 2371.0420, 2380.9539, 2389.2756, 2397.2231, 2406.2913, 2414.2468, 2431.2300, 2467.2122, 2474.2263, 2477.2573, 2478.2725, 2485.0691, 2499.1719, 2503.0352, 2518.0303, 2519.9934, 2534.0215, 2541.2542, 2542.2739, 2558.3401, 2580.0227, 2594.0615, 2631.4961, 2678.0144, 2683.4189, 2695.0366, 2701.2097, 2709.0559, 2717.3198, 2723.0757, 2770.4492, 2787.3171, 2799.3379, 2813.4517, 2817.2305, 2833.1284, 2842.5049, 2846.4216, 2872.3596, 2944.3171, 2961.2437, 2975.3135, 2989.3062, 3004.4270, 3029.4883, 3059.4963, 3081.6570, 3086.5718, 3147.5710, 3157.4875, 3161.1531, 3177.1650, 3185.5222, 3237.6042, 3248.6902, 3254.5940, 3331.5864, 3335.7378, 3347.5420, 3351.6597, 3360.7197, 3411.8193, 3428.6296, 3435.7471, 3457.6697, 3471.7146, 3475.7085, 3489.7715, 3503.8445, 3528.3743, 3542.4243, 3552.7373, 3568.9385, 3603.9502, 3625.7847, 3640.7600, 3645.8701, 3662.8821, 3691.0452, 3722.5232, 3727.5442, 3731.5757, 3733.5112, 3736.5735, 3741.5391, 3746.5410, 3748.5002, 3751.5356, 3755.5571, 3756.5549, 3763.5049, 3767.5706, 3778.5320, 3884.9326, 3955.6738, 4074.1699, 4149.0591, 4163.0181, 4238.0752, 4300.3838, 4382.1450, 4397.2036, 4441.5176, 4453.2886, 4458.5176, 4464.4150, 4495.2642, 4511.1577, 4692.4893

19. A6H782 **Mass:** 57102 **Score:** 18 **Expect:** 4e+002 **Matches:** 12

Tektin-3 OS=Bos taurus GN=TEKT3 PE=2 SV=1

Observed	Mr(expt)	Mr(calc)	ppm	Start	End	Miss	Peptide
738.4069	737.3996	737.4072	-10.32	88 -	93	0	R.TTLFTR.Y
993.5026	992.4953	992.4927	2.66	303 -	310	0	K.FTDDNILR.S
1087.5623	1086.5550	1086.5782	-21.29	395 -	403	1	K.VAQTRLDER.T
1314.7190	1313.7117	1313.6986	9.98	404 -	413	1	R.TRRPNIELCR.D
1383.7274	1382.7201	1382.7017	13.3	479 -	490	1	R.SFPSTLRLVGFC.-
1420.7949	1419.7876	1419.7834	2.98	127 -	137	2	R.LIQDKYQQTRK.T
1556.7537	1555.7464	1555.7664	-12.86	205 -	217	1	K.RMGIDLVHDEVEK.E + Oxidation (M)
1787.8574	1786.8501	1786.8930	-24.01	406 -	419	1	R.RPNIELCRDMAQLR.L + Oxidation (M)
2008.9901	2007.9828	2007.9973	-7.23	303 -	320	2	K.FTDDNILRSQSERAASAK.L
2770.4492	2769.4419	2769.3751	24.1	218 -	239	2	K.ELLTEVDTILCCQERMKLYLDK.A
3640.7600	3639.7527	3639.7277	6.87	271 -	302	2	K.CHHLRNTSDGVSYFHGVERVDATVSVPESWAK.F
4511.1577	4510.1504	4510.1705	-4.46	35 -	71	2	R.DRFPHYNLTHSLSLPWRPSTYYKAASNWPTLDPYCTR.S

No match to: 706.2868, 707.2966, 708.3044, 710.3746, 713.3099, 714.3496, 717.4172, 722.4243, 729.4379, 734.4639, 735.4285, 744.3897, 745.4249, 747.4562, 753.3594, 756.3831, 758.3950, 768.4982, 771.3934, 779.4738, 783.3916, 805.4591, 818.4537, 823.4842, 832.3158, 834.3223, 840.4870, 842.5089, 876.2845, 878.2925, 883.4848, 889.5049, 891.4897, 922.4780, 923.5152, 929.4479, 938.4989, 949.5408, 958.4880, 962.3525, 967.4243, 968.4982, 969.5316, 973.5403, 974.4454, 976.4499, 978.5390, 979.3778, 979.5679, 983.6275, 985.6091, 986.5857, 988.3948, 1007.5763, 1017.5743, 1021.5607, 1031.6372, 1034.5828, 1036.5481, 1046.6013, 1051.5096, 1056.5107, 1057.6161, 1074.6067, 1113.5466, 1123.6021, 1126.4208, 1143.4449, 1153.6440, 1157.4840, 1172.6813, 1178.6198, 1187.7026, 1194.5691, 1207.5975, 1215.6563, 1216.4995, 1216.7017, 1248.6692, 1254.6897, 1255.6042, 1257.6483, 1264.7133, 1265.7039, 1269.6589, 1273.6492, 1278.6917, 1284.6440, 1286.7411, 1288.7273, 1299.5450, 1301.5977, 1304.6617, 1317.7113, 1321.7212, 1338.6622, 1349.6757, 1351.6887, 1354.7229, 1356.7275, 1358.7100, 1371.7595, 1375.7335, 1382.5935, 1390.6962, 1393.7622, 1413.6547, 1423.7593, 1440.7788, 1450.8590, 1456.7791, 1457.7606, 1467.7502, 1470.7725, 1488.7212, 1494.7396, 1500.7236, 1512.8181, 1513.7562, 1528.6954, 1539.7452, 1562.7806, 1570.7695, 1574.7894, 1578.7858, 1580.8347, 1590.7754, 1592.7883, 1616.7943, 1628.7009, 1628.8575, 1631.8130, 1635.7671, 1642.7155, 1647.8516, 1650.8444, 1657.9042, 1663.8501, 1664.8555, 1693.0090, 1696.8273, 1706.8668, 1707.9615, 1715.8461, 1717.8761, 1718.8779, 1721.9366, 1730.8695, 1741.9938, 1747.9058, 1755.8904, 1758.9028, 1771.0238, 1774.9207, 1784.0254, 1862.9503, 1870.7574, 1883.7222, 1895.7236, 1897.7325, 1909.7473, 1911.7430, 1913.7366, 1925.7604, 1938.0079, 1961.0325, 1969.9990, 1974.8882, 1979.0790, 1981.0576, 2027.1294, 2060.9788, 2067.1584, 2086.1133, 2108.9963, 2112.9805, 2137.1189, 2144.0579, 2180.1812, 2223.1646, 2239.1501, 2241.1355, 2245.8403, 2255.1479, 2265.1016, 2273.0654, 2281.1055, 2318.1824, 2328.9529, 2335.9097, 2342.9673, 2347.8682, 2352.9265, 2356.9395, 2361.8997, 2363.8860, 2366.9265, 2371.0420, 2380.9539, 2389.2756, 2397.2231, 2406.2913, 2414.2468, 2431.2300, 2467.2122, 2474.2263, 2477.2573, 2478.2725, 2485.0691, 2499.1719, 2503.0352, 2518.0303, 2519.9934, 2534.0215, 2541.2542, 2542.2739, 2558.3401, 2580.0227, 2594.0615, 2631.4961, 2678.0144, 2683.4189, 2695.0366, 2701.2097, 2709.0559, 2717.3198, 2723.0757, 2787.3171, 2799.3379, 2813.4517, 2817.2305, 2833.1284, 2842.5049, 2846.4216, 2872.3596, 2944.3171, 2961.2437, 2975.3135, 2989.3062, 3004.4270, 3029.4883, 3059.4963, 3081.6570, 3086.5718, 3147.5710, 3157.4875, 3161.1531, 3177.1650, 3185.5222, 3237.6042, 3248.6902, 3254.5940, 3331.5864, 3335.7378, 3347.5420, 3351.6597, 3360.7197, 3411.8193, 3428.6296, 3435.7471, 3457.6697, 3471.7146, 3475.7085, 3489.7715, 3503.8445, 3528.3743, 3542.4243, 3552.7373, 3568.9385, 3603.9502, 3625.7847, 3645.8701, 3662.8821, 3691.0452, 3722.5232, 3727.5442, 3731.5757, 3733.5112, 3736.5735, 3741.5391, 3746.5410, 3748.5002, 3751.5356, 3755.5571, 3756.5549, 3763.5049, 3767.5706, 3778.5320, 3884.9326, 3955.6738, 4074.1699, 4149.0591, 4163.0181, 4238.0752, 4300.3838, 4382.1450, 4397.2036, 4441.5176, 4453.2886, 4458.5176, 4464.4150, 4495.2642, 4692.4893

20. Q2KIN6 **Mass:** 19801 **Score:** 17 **Expect:** 4.5e+002 **Matches:** 4

Protein Mpv17 OS=Bos taurus GN=MPV17 PE=2 SV=1

Observed	Mr(expt)	Mr(calc)	ppm	Start	End	Miss	Peptide
729.4379	728.4306	728.4432	-17.36	76 -	82	0	R.LIPGTTK.V
923.5152	922.5079	922.5025	5.91	10 -	17	0	R.ALTAHPWK.V
3503.8445	3502.8372	3502.8791	-11.95	18 -	50	2	K.VQVLTAGSLMGLGDVISQQLVERRGLQAHQAGR.T + Oxidation (M)
3645.8701	3644.8628	3644.8457	4.70	42 -	75	2	R.GLQAHQAGRTLTMASLGCGFVGPVVGGWYRVLDR.L + Oxidation (M)

No match to: 706.2868, 707.2966, 708.3044, 710.3746, 713.3099, 714.3496, 717.4172, 722.4243, 734.4639, 735.4285, 738.4069, 744.3897, 745.4249, 747.4562, 753.3594, 756.3831, 758.3950, 768.4982, 771.3934, 779.4738, 783.3916, 805.4591, 818.4537, 823.4842, 832.3158, 834.3223, 840.4870, 842.5089, 876.2845, 878.2925, 883.4848, 889.5049, 891.4897, 922.4780, 929.4479, 938.4989, 949.5408, 958.4880, 962.3525, 967.4243, 968.4982, 969.5316, 973.5403, 974.4454, 976.4499, 978.5390, 979.3778, 979.5679, 983.6275, 985.6091, 986.5857, 988.3948, 993.5026, 1007.5763, 1017.5743, 1021.5607, 1031.6372, 1034.5828, 1036.5481, 1046.6013, 1051.5096, 1056.5107, 1057.6161, 1074.6067, 1087.5623, 1113.5466, 1123.6021, 1126.4208, 1143.4449, 1153.6440, 1157.4840, 1172.6813, 1178.6198, 1187.7026, 1194.5691, 1207.5975, 1215.6563, 1216.4995, 1216.7017, 1248.6692, 1254.6897, 1255.6042, 1257.6483, 1264.7133, 1265.7039, 1269.6589, 1273.6492, 1278.6917, 1284.6440, 1286.7411, 1288.7273, 1299.5450, 1301.5977, 1304.6617, 1314.7190, 1317.7113, 1321.7212, 1338.6622, 1349.6757, 1351.6887, 1354.7229, 1356.7275, 1358.7100, 1371.7595, 1375.7335, 1382.5935, 1383.7274, 1390.6962, 1393.7622, 1413.6547, 1420.7949, 1423.7593, 1440.7788, 1450.8590, 1456.7791, 1457.7606, 1467.7502, 1470.7725, 1488.7212, 1494.7396, 1500.7236, 1512.8181, 1513.7562, 1528.6954, 1539.7452, 1556.7537, 1562.7806, 1570.7695, 1574.7894, 1578.7858, 1580.8347, 1590.7754, 1592.7883, 1616.7943, 1628.7009, 1628.8575, 1631.8130, 1635.7671, 1642.7155, 1647.8516, 1650.8444, 1657.9042, 1663.8501, 1664.8555, 1693.0090, 1696.8273, 1706.8668, 1707.9615, 1715.8461, 1717.8761, 1718.8779, 1721.9366, 1730.8695, 1741.9938, 1747.9058, 1755.8904, 1758.9028, 1771.0238, 1774.9207, 1784.0254, 1787.8574, 1862.9503, 1870.7574, 1883.7222, 1895.7236, 1897.7325, 1909.7473, 1911.7430, 1913.7366, 1925.7604, 1938.0079, 1961.0325, 1969.9990, 1974.8882, 1979.0790, 1981.0576, 2008.9901, 2027.1294, 2060.9788, 2067.1584, 2086.1133, 2108.9963, 2112.9805, 2137.1189, 2144.0579, 2180.1812, 2223.1646, 2239.1501, 2241.1355, 2245.8403, 2255.1479, 2265.1016, 2273.0654, 2281.1055, 2318.1824, 2328.9529, 2335.9097, 2342.9673, 2347.8682, 2352.9265, 2356.9395, 2361.8997, 2363.8860, 2366.9265, 2371.0420, 2380.9539, 2389.2756, 2397.2231, 2406.2913, 2414.2468, 2431.2300, 2467.2122, 2474.2263, 2477.2573, 2478.2725, 2485.0691, 2499.1719, 2503.0352, 2518.0303, 2519.9934, 2534.0215, 2541.2542, 2542.2739, 2558.3401, 2580.0227, 2594.0615, 2631.4961, 2678.0144, 2683.4189, 2695.0366, 2701.2097, 2709.0559, 2717.3198, 2723.0757, 2770.4492, 2787.3171, 2799.3379, 2813.4517, 2817.2305, 2833.1284, 2842.5049, 2846.4216, 2872.3596, 2944.3171, 2961.2437, 2975.3135, 2989.3062, 3004.4270, 3029.4883, 3059.4963, 3081.6570, 3086.5718, 3147.5710, 3157.4875, 3161.1531, 3177.1650, 3185.5222, 3237.6042, 3248.6902, 3254.5940, 3331.5864, 3335.7378, 3347.5420, 3351.6597, 3360.7197, 3411.8193, 3428.6296, 3435.7471, 3457.6697, 3471.7146, 3475.7085, 3489.7715, 3528.3743, 3542.4243, 3552.7373, 3568.9385, 3603.9502, 3625.7847, 3640.7600, 3662.8821, 3691.0452, 3722.5232, 3727.5442, 3731.5757, 3733.5112, 3736.5735, 3741.5391, 3746.5410, 3748.5002, 3751.5356, 3755.5571, 3756.5549, 3763.5049, 3767.5706, 3778.5320, 3884.9326, 3955.6738, 4074.1699, 4149.0591, 4163.0181, 4238.0752, 4300.3838, 4382.1450, 4397.2036, 4441.5176, 4453.2886, 4458.5176, 4464.4150, 4495.2642, 4511.1577, 4692.4893

21. P02526 **Mass:** 21482 **Score:** 17 **Expect:** 4.5e+002 **Matches:** 7

Gamma-crystallin B OS=Bos taurus GN=CRYGB PE=1 SV=2

Observed	Mr(expt)	Mr(calc)	ppm	Start	End	Miss	Peptide

1113.5466	1112.5393	1112.5251	12.8	93 -	100	1	R.IYERDDFR.G
1358.7100	1357.7027	1357.6813	15.8	165 -	175	2	K.VGSLRRVMDFY.- + Oxidation (M)
1456.7791	1455.7718	1455.7769	-3.48	81 -	92	1	R.LIPQHTGTFRMR.I
1696.8273	1695.8200	1695.8403	-11.93	155 -	169	1	R.YLDWGAMNAKVGSLR.R + Oxidation (M)
2709.0559	2708.0486	2708.0843	-13.16	11 -	32	0	R.GFQGHCYECSSDCPNLQPYFSR.C
2975.3135	2974.3062	2974.3755	-23.30	38 -	60	1	R.VDSGCWMLYERPNYQGHQYFLRR.G
4495.2642	4494.2569	4494.2695	-2.80	117 -	153	2	R.FHLTEVHSLNVLEGSWVLYEMPSYRGRQYLLRPGEYR.R

No match to: 706.2868, 707.2966, 708.3044, 710.3746, 713.3099, 714.3496, 717.4172, 722.4243, 729.4379, 734.4639, 735.4285, 738.4069, 744.3897, 745.4249, 747.4562, 753.3594, 756.3831, 758.3950, 768.4982, 771.3934, 779.4738, 783.3916, 805.4591, 818.4537, 823.4842, 832.3158, 834.3223, 840.4870, 842.5089, 876.2845, 878.2925, 883.4848, 889.5049, 891.4897, 922.4780, 923.5152, 929.4479, 938.4989, 949.5408, 958.4880, 962.3525, 967.4243, 968.4982, 969.5316, 973.5403, 974.4454, 976.4499, 978.5390, 979.3778, 979.5679, 983.6275, 985.6091, 986.5857, 988.3948, 993.5026, 1007.5763, 1017.5743, 1021.5607, 1031.6372, 1034.5828, 1036.5481, 1046.6013, 1051.5096, 1056.5107, 1057.6161, 1074.6067, 1087.5623, 1123.6021, 1126.4208, 1143.4449, 1153.6440, 1157.4840, 1172.6813, 1178.6198, 1187.7026, 1194.5691, 1207.5975, 1215.6563, 1216.4995, 1216.7017, 1248.6692, 1254.6897, 1255.6042, 1257.6483, 1264.7133, 1265.7039, 1269.6589, 1273.6492, 1278.6917, 1284.6440, 1286.7411, 1288.7273, 1299.5450, 1301.5977, 1304.6617, 1314.7190, 1317.7113, 1321.7212, 1338.6622, 1349.6757, 1351.6887, 1354.7229, 1356.7275, 1371.7595, 1375.7335, 1382.5935, 1383.7274, 1390.6962, 1393.7622, 1413.6547, 1420.7949, 1423.7593, 1440.7788, 1450.8590, 1457.7606, 1467.7502, 1470.7725, 1488.7212, 1494.7396, 1500.7236, 1512.8181, 1513.7562, 1528.6954, 1539.7452, 1556.7537, 1562.7806, 1570.7695, 1574.7894, 1578.7858, 1580.8347, 1590.7754, 1592.7883, 1616.7943, 1628.7009, 1628.8575, 1631.8130, 1635.7671, 1642.7155, 1647.8516, 1650.8444, 1657.9042, 1663.8501, 1664.8555, 1693.0090, 1706.8668, 1707.9615, 1715.8461, 1717.8761, 1718.8779, 1721.9366, 1730.8695, 1741.9938, 1747.9058, 1755.8904, 1758.9028, 1771.0238, 1774.9207, 1784.0254, 1787.8574, 1862.9503, 1870.7574, 1883.7222, 1895.7236, 1897.7325, 1909.7473, 1911.7430, 1913.7366, 1925.7604, 1938.0079, 1961.0325, 1969.9990, 1974.8882, 1979.0790, 1981.0576, 2008.9901, 2027.1294, 2060.9788, 2067.1584, 2086.1133, 2108.9963, 2112.9805, 2137.1189, 2144.0579, 2180.1812, 2223.1646, 2239.1501, 2241.1355, 2245.8403, 2255.1479, 2265.1016, 2273.0654, 2281.1055, 2318.1824, 2328.9529, 2335.9097, 2342.9673, 2347.8682, 2352.9265, 2356.9395, 2361.8997, 2363.8860, 2366.9265, 2371.0420, 2380.9539, 2389.2756, 2397.2231, 2406.2913, 2414.2468, 2431.2300, 2467.2122, 2474.2263, 2477.2573, 2478.2725, 2485.0691, 2499.1719, 2503.0352, 2518.0303, 2519.9934, 2534.0215, 2541.2542, 2542.2739, 2558.3401, 2580.0227, 2594.0615, 2631.4961, 2678.0144, 2683.4189, 2695.0366, 2701.2097, 2717.3198, 2723.0757, 2770.4492, 2787.3171, 2799.3379, 2813.4517, 2817.2305, 2833.1284, 2842.5049, 2846.4216, 2872.3596, 2944.3171, 2961.2437, 2989.3062, 3004.4270, 3029.4883, 3059.4963, 3081.6570, 3086.5718, 3147.5710, 3157.4875, 3161.1531, 3177.1650, 3185.5222, 3237.6042, 3248.6902, 3254.5940, 3331.5864, 3335.7378, 3347.5420, 3351.6597, 3360.7197, 3411.8193, 3428.6296, 3435.7471, 3457.6697, 3471.7146, 3475.7085, 3489.7715, 3503.8445, 3528.3743, 3542.4243, 3552.7373, 3568.9385, 3603.9502, 3625.7847, 3640.7600, 3645.8701, 3662.8821, 3691.0452, 3722.5232, 3727.5442, 3731.5757, 3733.5112, 3736.5735, 3741.5391, 3746.5410, 3748.5002, 3751.5356, 3755.5571, 3756.5549, 3763.5049, 3767.5706, 3778.5320, 3884.9326, 3955.6738, 4074.1699, 4149.0591, 4163.0181, 4238.0752, 4300.3838, 4382.1450, 4397.2036, 4441.5176, 4453.2886, 4458.5176, 4464.4150, 4511.1577, 4692.4893

22. A8YXZ5 **Mass:** 11449 **Score:** 17 **Expect:** 4.7e+002 **Matches:** 4

Uncharacterized protein C8orf59 homolog OS=Bos taurus PE=3 SV=2

Observed	Mr(expt)	Mr(calc)	ppm	Start	End	Miss	Peptide
729.4379	728.4306	728.4293	1.77	47 -	52	1	K.VNRVNK.A
805.4591	804.4518	804.4494	3.04	53 -	59	0	K.AFVNVQK.E
883.4848	882.4775	882.4923	-16.74	76 -	82	0	K.ELIPQQR.H
1375.7335	1374.7262	1374.6925	24.5	39 -	49	1	K.INIMNEEKVNR.V + Oxidation (M)

No match to: 706.2868, 707.2966, 708.3044, 710.3746, 713.3099, 714.3496, 717.4172, 722.4243, 734.4639, 735.4285, 738.4069, 744.3897, 745.4249, 747.4562, 753.3594, 756.3831, 758.3950, 768.4982, 771.3934, 779.4738, 783.3916, 818.4537, 823.4842, 832.3158, 834.3223, 840.4870, 842.5089, 876.2845, 878.2925, 889.5049, 891.4897, 922.4780, 923.5152, 929.4479, 938.4989, 949.5408, 958.4880, 962.3525, 967.4243, 968.4982, 969.5316, 973.5403, 974.4454, 976.4499, 978.5390, 979.3778, 979.5679, 983.6275, 985.6091, 986.5857, 988.3948, 993.5026, 1007.5763, 1017.5743, 1021.5607, 1031.6372, 1034.5828, 1036.5481, 1046.6013, 1051.5096, 1056.5107, 1057.6161, 1074.6067, 1087.5623, 1113.5466, 1123.6021, 1126.4208, 1143.4449, 1153.6440, 1157.4840, 1172.6813, 1178.6198, 1187.7026, 1194.5691, 1207.5975, 1215.6563, 1216.4995, 1216.7017, 1248.6692, 1254.6897, 1255.6042, 1257.6483, 1264.7133, 1265.7039, 1269.6589, 1273.6492, 1278.6917, 1284.6440, 1286.7411, 1288.7273, 1299.5450, 1301.5977, 1304.6617, 1314.7190, 1317.7113, 1321.7212, 1338.6622, 1349.6757, 1351.6887, 1354.7229, 1356.7275, 1358.7100, 1371.7595, 1382.5935, 1383.7274, 1390.6962, 1393.7622, 1413.6547, 1420.7949, 1423.7593, 1440.7788, 1450.8590, 1456.7791, 1457.7606, 1467.7502, 1470.7725, 1488.7212, 1494.7396, 1500.7236, 1512.8181, 1513.7562, 1528.6954, 1539.7452, 1556.7537, 1562.7806, 1570.7695, 1574.7894, 1578.7858, 1580.8347, 1590.7754, 1592.7883, 1616.7943, 1628.7009, 1628.8575, 1631.8130, 1635.7671, 1642.7155, 1647.8516, 1650.8444, 1657.9042, 1663.8501, 1664.8555, 1693.0090, 1696.8273, 1706.8668, 1707.9615, 1715.8461, 1717.8761, 1718.8779, 1721.9366, 1730.8695, 1741.9938, 1747.9058, 1755.8904, 1758.9028, 1771.0238, 1774.9207, 1784.0254, 1787.8574, 1862.9503, 1870.7574, 1883.7222, 1895.7236, 1897.7325, 1909.7473, 1911.7430, 1913.7366, 1925.7604, 1938.0079, 1961.0325, 1969.9990, 1974.8882, 1979.0790, 1981.0576, 2008.9901, 2027.1294, 2060.9788, 2067.1584, 2086.1133, 2108.9963, 2112.9805, 2137.1189, 2144.0579, 2180.1812, 2223.1646, 2239.1501, 2241.1355, 2245.8403, 2255.1479, 2265.1016, 2273.0654, 2281.1055, 2318.1824, 2328.9529, 2335.9097, 2342.9673, 2347.8682, 2352.9265, 2356.9395, 2361.8997, 2363.8860, 2366.9265, 2371.0420, 2380.9539, 2389.2756, 2397.2231, 2406.2913, 2414.2468, 2431.2300, 2467.2122, 2474.2263, 2477.2573, 2478.2725, 2485.0691, 2499.1719, 2503.0352, 2518.0303, 2519.9934, 2534.0215, 2541.2542, 2542.2739, 2558.3401, 2580.0227, 2594.0615, 2631.4961, 2678.0144, 2683.4189, 2695.0366, 2701.2097, 2709.0559, 2717.3198, 2723.0757, 2770.4492, 2787.3171, 2799.3379, 2813.4517, 2817.2305, 2833.1284, 2842.5049, 2846.4216, 2872.3596, 2944.3171, 2961.2437, 2975.3135, 2989.3062, 3004.4270, 3029.4883, 3059.4963, 3081.6570, 3086.5718, 3147.5710, 3157.4875, 3161.1531, 3177.1650, 3185.5222, 3237.6042, 3248.6902, 3254.5940, 3331.5864, 3335.7378, 3347.5420, 3351.6597, 3360.7197, 3411.8193, 3428.6296, 3435.7471, 3457.6697, 3471.7146, 3475.7085, 3489.7715, 3503.8445, 3528.3743, 3542.4243, 3552.7373, 3568.9385, 3603.9502, 3625.7847, 3640.7600, 3645.8701, 3662.8821, 3691.0452, 3722.5232, 3727.5442, 3731.5757, 3733.5112, 3736.5735, 3741.5391, 3746.5410, 3748.5002, 3751.5356, 3755.5571, 3756.5549, 3763.5049, 3767.5706, 3778.5320, 3884.9326, 3955.6738, 4074.1699, 4149.0591, 4163.0181, 4238.0752, 4300.3838, 4382.1450, 4397.2036, 4441.5176, 4453.2886, 4458.5176, 4464.4150, 4495.2642, 4511.1577, 4692.4893

23. Q0P568 **Mass:** 26340 **Score:** 17 **Expect:** 4.7e+002 **Matches:** 7

Ubiquitin fusion degradation 1 like OS=Bos taurus GN=UFD1L PE=2 SV=1

Observed	Mr(expt)	Mr(calc)	ppm	Start	End	Miss	Peptide
1440.7788	1439.7715	1439.7806	-6.31	46 -	58	0	K.IIMPPSALDQLSR.L
1456.7791	1455.7718	1455.7755	-2.54	46 -	58	0	K.IIMPPSALDQLSR.L + Oxidation (M)
2241.1355	2240.1282	2240.1834	-24.65	38 -	58	2	R.SDVEKGGKIIMPPSALDQLSR.L
2273.0654	2272.0581	2272.0392	8.34	179 -	198	0	K.AVSIIECDMNVDFDAPLGYK.E + Oxidation (M)
2717.3198	2716.3125	2716.3530	-14.91	148 -	170	1	R.NFACLTTGDVIAINYNEKIYELR.V
3185.5222	3184.5149	3184.5130	0.59	171 -	198	1	R.VMETKPDKAVSIIECDMNVDFDAPLGYK.E
3645.8701	3644.8628	3644.8218	11.3	148 -	178	2	R.NFACLTTGDVIAINYNEKIYELRVMETKPDK.A

No match to: 706.2868, 707.2966, 708.3044, 710.3746, 713.3099, 714.3496, 717.4172, 722.4243, 729.4379, 734.4639, 735.4285, 738.4069, 744.3897, 745.4249, 747.4562, 753.3594, 756.3831, 758.3950, 768.4982, 771.3934, 779.4738, 783.3916, 805.4591, 818.4537, 823.4842, 832.3158, 834.3223, 840.4870, 842.5089, 876.2845, 878.2925, 883.4848, 889.5049, 891.4897, 922.4780, 923.5152, 929.4479, 938.4989, 949.5408, 958.4880, 962.3525, 967.4243, 968.4982, 969.5316, 973.5403, 974.4454, 976.4499, 978.5390, 979.3778, 979.5679, 983.6275, 985.6091, 986.5857, 988.3948, 993.5026, 1007.5763, 1017.5743, 1021.5607, 1031.6372, 1034.5828, 1036.5481, 1046.6013, 1051.5096, 1056.5107, 1057.6161, 1074.6067, 1087.5623, 1113.5466, 1123.6021, 1126.4208, 1143.4449, 1153.6440, 1157.4840, 1172.6813, 1178.6198, 1187.7026, 1194.5691, 1207.5975, 1215.6563, 1216.4995, 1216.7017, 1248.6692, 1254.6897, 1255.6042, 1257.6483, 1264.7133, 1265.7039, 1269.6589, 1273.6492, 1278.6917, 1284.6440, 1286.7411, 1288.7273, 1299.5450, 1301.5977, 1304.6617, 1314.7190, 1317.7113, 1321.7212, 1338.6622, 1349.6757, 1351.6887, 1354.7229, 1356.7275, 1358.7100, 1371.7595, 1375.7335, 1382.5935, 1383.7274, 1390.6962, 1393.7622, 1413.6547, 1420.7949, 1423.7593, 1450.8590, 1457.7606, 1467.7502, 1470.7725, 1488.7212, 1494.7396, 1500.7236, 1512.8181, 1513.7562, 1528.6954, 1539.7452, 1556.7537, 1562.7806, 1570.7695, 1574.7894, 1578.7858, 1580.8347, 1590.7754, 1592.7883, 1616.7943, 1628.7009, 1628.8575, 1631.8130, 1635.7671, 1642.7155, 1647.8516, 1650.8444, 1657.9042, 1663.8501, 1664.8555, 1693.0090, 1696.8273, 1706.8668, 1707.9615, 1715.8461, 1717.8761, 1718.8779, 1721.9366, 1730.8695, 1741.9938, 1747.9058, 1755.8904, 1758.9028, 1771.0238, 1774.9207, 1784.0254, 1787.8574, 1862.9503, 1870.7574, 1883.7222, 1895.7236, 1897.7325, 1909.7473, 1911.7430, 1913.7366, 1925.7604, 1938.0079, 1961.0325, 1969.9990, 1974.8882, 1979.0790, 1981.0576, 2008.9901, 2027.1294, 2060.9788, 2067.1584, 2086.1133, 2108.9963, 2112.9805, 2137.1189, 2144.0579, 2180.1812, 2223.1646, 2239.1501, 2245.8403, 2255.1479, 2265.1016, 2281.1055, 2318.1824, 2328.9529, 2335.9097, 2342.9673, 2347.8682, 2352.9265, 2356.9395, 2361.8997, 2363.8860, 2366.9265, 2371.0420, 2380.9539, 2389.2756, 2397.2231,

2406.2913, 2414.2468, 2431.2300, 2467.2122, 2474.2263, 2477.2573, 2478.2725, 2485.0691, 2499.1719, 2503.0352, 2518.0303, 2519.9934, 2534.0215, 2541.2542, 2542.2739, 2558.3401, 2580.0227, 2594.0615, 2631.4961, 2678.0144, 2683.4189, 2695.0366, 2701.2097, 2709.0559, 2723.0757, 2770.4492, 2787.3171, 2799.3379, 2813.4517, 2817.2305, 2833.1284, 2842.5049, 2846.4216, 2872.3596, 2944.3171, 2961.2437, 2975.3135, 2989.3062, 3004.4270, 3029.4883, 3059.4963, 3081.6570, 3086.5718, 3147.5710, 3157.4875, 3161.1531, 3177.1650, 3237.6042, 3248.6902, 3254.5940, 3331.5864, 3335.7378, 3347.5420, 3351.6597, 3360.7197, 3411.8193, 3428.6296, 3435.7471, 3457.6697, 3471.7146, 3475.7085, 3489.7715, 3503.8445, 3528.3743, 3542.4243, 3552.7373, 3568.9385, 3603.9502, 3625.7847, 3640.7600, 3662.8821, 3691.0452, 3722.5232, 3727.5442, 3731.5757, 3733.5112, 3736.5735, 3741.5391, 3746.5410, 3748.5002, 3751.5356, 3755.5571, 3756.5549, 3763.5049, 3767.5706, 3778.5320, 3884.9326, 3955.6738, 4074.1699, 4149.0591, 4163.0181, 4238.0752, 4300.3838, 4382.1450, 4397.2036, 4441.5176, 4453.2886, 4458.5176, 4464.4150, 4495.2642, 4511.1577, 4692.4893

24. Q5EAC0 **Mass:** 34118 **Score:** 17 **Expect:** 5.1e+002 **Matches:** 6

Solute carrier family 25 member 38 OS=Bos taurus GN=SLC25A38 PE=2 SV=2

Observed	Mr(expt)	Mr(calc)	ppm	Start	End	Miss	Peptide
1286.7411	1285.7338	1285.7316	1.77	19 -	29	0	R.VETLMLQPVIK.A + Oxidation (M)
1467.7502	1466.7429	1466.7163	18.2	245 -	255	1	K.THMQLFPMKFR.W + 2 Oxidation (M)
1707.9615	1706.9542	1706.9288	14.9	53 -	67	2	K.TRLQTLQPSAHGSRR.V
1961.0325	1960.0252	1960.0602	-17.87	2 -	18	1	M.IQKSRPALLQHQDVGDR.V
2541.2542	2540.2469	2540.3019	-21.63	30 -	52	0	K.AFLCGSISGTCSTLLFQPLDLLK.T
4074.1699	4073.1626	4073.1190	10.7	30 -	66	2	K.AFLCGSISGTCSTLLFQPLDLLKTRLQTLQPSAHGSR.R

No match to: 706.2868, 707.2966, 708.3044, 710.3746, 713.3099, 714.3496, 717.4172, 722.4243, 729.4379, 734.4639, 735.4285, 738.4069, 744.3897, 745.4249, 747.4562, 753.3594, 756.3831, 758.3950, 768.4982, 771.3934, 779.4738, 783.3916, 805.4591, 818.4537, 823.4842, 832.3158, 834.3223, 840.4870, 842.5089, 876.2845, 878.2925, 883.4848, 889.5049, 891.4897, 922.4780, 923.5152, 929.4479, 938.4989, 949.5408, 958.4880, 962.3525, 967.4243, 968.4982, 969.5316, 973.5403, 974.4454, 976.4499, 978.5390, 979.3778, 979.5679, 983.6275, 985.6091, 986.5857, 988.3948, 993.5026, 1007.5763, 1017.5743, 1021.5607, 1031.6372, 1034.5828, 1036.5481, 1046.6013, 1051.5096, 1056.5107, 1057.6161, 1074.6067, 1087.5623, 1113.5466, 1123.6021, 1126.4208, 1143.4449, 1153.6440, 1157.4840, 1172.6813, 1178.6198, 1187.7026, 1194.5691, 1207.5975, 1215.6563, 1216.4995, 1216.7017, 1248.6692, 1254.6897, 1255.6042, 1257.6483, 1264.7133, 1265.7039, 1269.6589, 1273.6492, 1278.6917, 1284.6440, 1288.7273, 1299.5450, 1301.5977, 1304.6617, 1314.7190, 1317.7113, 1321.7212, 1338.6622, 1349.6757, 1351.6887, 1354.7229, 1356.7275, 1358.7100, 1371.7595, 1375.7335, 1382.5935, 1383.7274, 1390.6962, 1393.7622, 1413.6547, 1420.7949, 1423.7593, 1440.7788, 1450.8590, 1456.7791, 1457.7606, 1470.7725, 1488.7212, 1494.7396, 1500.7236, 1512.8181, 1513.7562, 1528.6954, 1539.7452, 1556.7537, 1562.7806, 1570.7695, 1574.7894, 1578.7858, 1580.8347, 1590.7754, 1592.7883, 1616.7943, 1628.7009, 1628.8575, 1631.8130, 1635.7671, 1642.7155, 1647.8516, 1650.8444, 1657.9042, 1663.8501, 1664.8555, 1693.0090, 1696.8273, 1706.8668, 1715.8461, 1717.8761, 1718.8779, 1721.9366, 1730.8695, 1741.9938, 1747.9058, 1755.8904, 1758.9028, 1771.0238, 1774.9207, 1784.0254, 1787.8574, 1862.9503, 1870.7574, 1883.7222, 1895.7236, 1897.7325, 1909.7473, 1911.7430, 1913.7366, 1925.7604, 1938.0079, 1969.9990, 1974.8882, 1979.0790, 1981.0576, 2008.9901, 2027.1294, 2060.9788, 2067.1584, 2086.1133, 2108.9963, 2112.9805, 2137.1189, 2144.0579, 2180.1812, 2223.1646, 2239.1501, 2241.1355, 2245.8403, 2255.1479, 2265.1016, 2273.0654, 2281.1055, 2318.1824, 2328.9529, 2335.9097, 2342.9673, 2347.8682, 2352.9265, 2356.9395, 2361.8997, 2363.8860, 2366.9265, 2371.0420, 2380.9539, 2389.2756, 2397.2231, 2406.2913, 2414.2468, 2431.2300, 2467.2122, 2474.2263, 2477.2573, 2478.2725, 2485.0691, 2499.1719, 2503.0352, 2518.0303, 2519.9934, 2534.0215, 2542.2739, 2558.3401, 2580.0227, 2594.0615, 2631.4961, 2678.0144, 2683.4189, 2695.0366, 2701.2097, 2709.0559, 2717.3198, 2723.0757, 2770.4492, 2787.3171, 2799.3379, 2813.4517, 2817.2305, 2833.1284, 2842.5049, 2846.4216, 2872.3596, 2944.3171, 2961.2437, 2975.3135, 2989.3062, 3004.4270, 3029.4883, 3059.4963, 3081.6570, 3086.5718, 3147.5710, 3157.4875, 3161.1531, 3177.1650, 3185.5222, 3237.6042, 3248.6902, 3254.5940, 3331.5864, 3335.7378, 3347.5420, 3351.6597, 3360.7197, 3411.8193, 3428.6296, 3435.7471, 3457.6697, 3471.7146, 3475.7085, 3489.7715, 3503.8445, 3528.3743, 3542.4243, 3552.7373, 3568.9385, 3603.9502, 3625.7847, 3640.7600, 3645.8701, 3662.8821, 3691.0452, 3722.5232, 3727.5442, 3731.5757, 3733.5112, 3736.5735, 3741.5391, 3746.5410, 3748.5002, 3751.5356, 3755.5571, 3756.5549, 3763.5049, 3767.5706, 3778.5320, 3884.9326, 3955.6738, 4149.0591, 4163.0181, 4238.0752, 4300.3838, 4382.1450, 4397.2036, 4441.5176, 4453.2886, 4458.5176, 4464.4150, 4495.2642, 4511.1577, 4692.4893

25. F1MUP9 **Mass:** 43041 **Score:** 17 **Expect:** 5.1e+002 **Matches:** 8

Uncharacterized protein OS=Bos taurus GN=VAT1 PE=4 SV=2

Observed	Mr(expt)	Mr(calc)	ppm	Start	End	Miss	Peptide
1187.7026	1186.6953	1186.6856	8.19	139 -	148	1	K.IGDRVMVLIR.S + Oxidation (M)
1338.6622	1337.6549	1337.6220	24.6	94 -	105	0	K.ACGLNFADLMAR.Q
1574.7894	1573.7821	1573.7658	10.4	265 -	280	0	K.GVDIVMDPLGGSDTAK.G
1590.7754	1589.7681	1589.7607	4.66	265 -	280	0	K.GVDIVMDPLGGSDTAK.G + Oxidation (M)
2389.2756	2388.2683	2388.3133	-18.85	291 -	312	2	K.VVTYGMANLLTGPKRNLMALAR.T
2474.2263	2473.2190	2473.1761	17.3	240 -	260	1	K.ENGVTHPIDYHTTDYVDEIKK.I
3086.5718	3085.5645	3085.6396	-24.32	305 -	330	2	K.RNLMALARTWWNQFSVTALQLLPANR.A + Oxidation (M)
3435.7471	3434.7398	3434.7980	-16.95	106 -	138	2	R.QGLYDRLPQLPVTPGMEGAGVVIAVGEGVNDRK.I

No match to: 706.2868, 707.2966, 708.3044, 710.3746, 713.3099, 714.3496, 717.4172, 722.4243, 729.4379, 734.4639, 735.4285, 738.4069, 744.3897, 745.4249, 747.4562, 753.3594, 756.3831, 758.3950, 768.4982, 771.3934, 779.4738, 783.3916, 805.4591, 818.4537, 823.4842, 832.3158, 834.3223, 840.4870, 842.5089, 876.2845, 878.2925, 883.4848, 889.5049, 891.4897, 922.4780, 923.5152, 929.4479, 938.4989, 949.5408, 958.4880, 962.3525, 967.4243, 968.4982, 969.5316, 973.5403, 974.4454, 976.4499, 978.5390, 979.3778, 979.5679, 983.6275, 985.6091, 986.5857, 988.3948, 993.5026, 1007.5763, 1017.5743, 1021.5607, 1031.6372, 1034.5828, 1036.5481, 1046.6013, 1051.5096, 1056.5107, 1057.6161, 1074.6067, 1087.5623, 1113.5466, 1123.6021, 1126.4208, 1143.4449, 1153.6440, 1157.4840, 1172.6813, 1178.6198, 1194.5691, 1207.5975, 1215.6563, 1216.4995, 1216.7017, 1248.6692, 1254.6897, 1255.6042, 1257.6483, 1264.7133, 1265.7039, 1269.6589, 1273.6492, 1278.6917, 1284.6440, 1286.7411, 1288.7273, 1299.5450, 1301.5977, 1304.6617, 1314.7190, 1317.7113, 1321.7212, 1349.6757, 1351.6887, 1354.7229, 1356.7275, 1358.7100, 1371.7595, 1375.7335, 1382.5935, 1383.7274, 1390.6962, 1393.7622, 1413.6547, 1420.7949, 1423.7593, 1440.7788, 1450.8590, 1456.7791, 1457.7606, 1467.7502, 1470.7725, 1488.7212, 1494.7396, 1500.7236, 1512.8181, 1513.7562, 1528.6954, 1539.7452, 1556.7537, 1562.7806, 1570.7695, 1578.7858, 1580.8347, 1592.7883, 1616.7943, 1628.7009, 1628.8575, 1631.8130, 1635.7671, 1642.7155, 1647.8516, 1650.8444, 1657.9042, 1663.8501, 1664.8555, 1693.0090, 1696.8273, 1706.8668, 1707.9615, 1715.8461, 1717.8761, 1718.8779, 1721.9366, 1730.8695, 1741.9938, 1747.9058, 1755.8904, 1758.9028, 1771.0238, 1774.9207, 1784.0254, 1787.8574, 1862.9503, 1870.7574, 1883.7222, 1895.7236, 1897.7325, 1909.7473, 1911.7430, 1913.7366, 1925.7604, 1938.0079, 1961.0325, 1969.9990, 1974.8882, 1979.0790, 1981.0576, 2008.9901, 2027.1294, 2060.9788, 2067.1584, 2086.1133, 2108.9963, 2112.9805, 2137.1189, 2144.0579, 2180.1812, 2223.1646, 2239.1501, 2241.1355, 2245.8403, 2255.1479, 2265.1016, 2273.0654, 2281.1055, 2318.1824, 2328.9529, 2335.9097, 2342.9673, 2347.8682, 2352.9265, 2356.9395, 2361.8997, 2363.8860, 2366.9265, 2371.0420, 2380.9539, 2397.2231, 2406.2913, 2414.2468, 2431.2300, 2467.2122, 2477.2573, 2478.2725, 2485.0691, 2499.1719, 2503.0352, 2518.0303, 2519.9934, 2534.0215, 2541.2542, 2542.2739, 2558.3401, 2580.0227, 2594.0615, 2631.4961, 2678.0144, 2683.4189, 2695.0366, 2701.2097, 2709.0559, 2717.3198, 2723.0757, 2770.4492, 2787.3171, 2799.3379, 2813.4517, 2817.2305, 2833.1284, 2842.5049, 2846.4216, 2872.3596, 2944.3171, 2961.2437, 2975.3135, 2989.3062, 3004.4270, 3029.4883, 3059.4963, 3081.6570, 3147.5710, 3157.4875, 3161.1531, 3177.1650, 3185.5222, 3237.6042, 3248.6902, 3254.5940, 3331.5864, 3335.7378, 3347.5420, 3351.6597, 3360.7197, 3411.8193, 3428.6296, 3457.6697, 3471.7146, 3475.7085, 3489.7715, 3503.8445, 3528.3743, 3542.4243, 3552.7373, 3568.9385, 3603.9502, 3625.7847, 3640.7600, 3645.8701, 3662.8821, 3691.0452, 3722.5232, 3727.5442, 3731.5757, 3733.5112, 3736.5735, 3741.5391, 3746.5410, 3748.5002, 3751.5356, 3755.5571, 3756.5549, 3763.5049, 3767.5706, 3778.5320, 3884.9326, 3955.6738, 4074.1699, 4149.0591, 4163.0181, 4238.0752, 4300.3838, 4382.1450, 4397.2036, 4441.5176, 4453.2886, 4458.5176, 4464.4150, 4495.2642, 4511.1577, 4692.4893

26. P82292 **Mass:** 13660 **Score:** 17 **Expect:** 5.2e+002 **Matches:** 3

Spermadhesin Z13 OS=Bos taurus PE=1 SV=1

Observed	Mr(expt)	Mr(calc)	ppm	Start	End	Miss	Peptide
2067.1584	2066.1511	2066.1578	-3.22	100 -	116	2	R.KPNHPAPDFFLIFRRVL.-
3435.7471	3434.7398	3434.7099	8.70	47 -	76	2	R.ILLTVRDVHENCNKESLEIIEGPPESSNSR.K
3640.7600	3639.7527	3639.8058	-14.60	1 -	32	2	-.DSTDGLLVKDKYLCGDLYGEEYGVIFPYLGLK.T

No match to: 706.2868, 707.2966, 708.3044, 710.3746, 713.3099, 714.3496, 717.4172, 722.4243, 729.4379, 734.4639, 735.4285, 738.4069, 744.3897, 745.4249, 747.4562, 753.3594, 756.3831, 758.3950, 768.4982, 771.3934, 779.4738, 783.3916, 805.4591, 818.4537, 823.4842, 832.3158, 834.3223, 840.4870, 842.5089, 876.2845, 878.2925, 883.4848, 889.5049, 891.4897, 922.4780, 923.5152, 929.4479, 938.4989, 949.5408, 958.4880, 962.3525, 967.4243, 968.4982, 969.5316, 973.5403, 974.4454, 976.4499, 978.5390, 979.3778, 979.5679, 983.6275, 985.6091, 986.5857, 988.3948, 993.5026, 1007.5763, 1017.5743, 1021.5607, 1031.6372, 1034.5828, 1036.5481, 1046.6013,

1051.5096, 1056.5107, 1057.6161, 1074.6067, 1087.5623, 1113.5466, 1123.6021, 1126.4208, 1143.4449, 1153.6440, 1157.4840, 1172.6813, 1178.6198, 1187.7026, 1194.5691, 1207.5975, 1215.6563, 1216.4995, 1216.7017, 1248.6692, 1254.6897, 1255.6042, 1257.6483, 1264.7133, 1265.7039, 1269.6589, 1273.6492, 1278.6917, 1284.6440, 1286.7411, 1288.7273, 1299.5450, 1301.5977, 1304.6617, 1314.7190, 1317.7113, 1321.7212, 1338.6622, 1349.6757, 1351.6887, 1354.7229, 1356.7275, 1358.7100, 1371.7595, 1375.7335, 1382.5935, 1383.7274, 1390.6962, 1393.7622, 1413.6547, 1420.7949, 1423.7593, 1440.7788, 1450.8590, 1456.7791, 1457.7606, 1467.7502, 1470.7725, 1488.7212, 1494.7396, 1500.7236, 1512.8181, 1513.7562, 1528.6954, 1539.7452, 1556.7537, 1562.7806, 1570.7695, 1574.7894, 1578.7858, 1580.8347, 1590.7754, 1592.7883, 1616.7943, 1628.7009, 1628.8575, 1631.8130, 1635.7671, 1642.7155, 1647.8516, 1650.8444, 1657.9042, 1663.8501, 1664.8555, 1693.0090, 1696.8273, 1706.8668, 1707.9615, 1715.8461, 1717.8761, 1718.8779, 1721.9366, 1730.8695, 1741.9938, 1747.9058, 1755.8904, 1758.9028, 1771.0238, 1774.9207, 1784.0254, 1787.8574, 1862.9503, 1870.7574, 1883.7222, 1895.7236, 1897.7325, 1909.7473, 1911.7430, 1913.7366, 1925.7604, 1938.0079, 1961.0325, 1969.9990, 1974.8882, 1979.0790, 1981.0576, 2008.9901, 2027.1294, 2060.9788, 2086.1133, 2108.9963, 2112.9805, 2137.1189, 2144.0579, 2180.1812, 2223.1646, 2239.1501, 2241.1355, 2245.8403, 2255.1479, 2265.1016, 2273.0654, 2281.1055, 2318.1824, 2328.9529, 2335.9097, 2342.9673, 2347.8682, 2352.9265, 2356.9395, 2361.8997, 2363.8860, 2366.9265, 2371.0420, 2380.9539, 2389.2756, 2397.2231, 2406.2913, 2414.2468, 2431.2300, 2467.2122, 2474.2263, 2477.2573, 2478.2725, 2485.0691, 2499.1719, 2503.0352, 2518.0303, 2519.9934, 2534.0215, 2541.2542, 2542.2739, 2558.3401, 2580.0227, 2594.0615, 2631.4961, 2678.0144, 2683.4189, 2695.0366, 2701.2097, 2709.0559, 2717.3198, 2723.0757, 2770.4492, 2787.3171, 2799.3379, 2813.4517, 2817.2305, 2833.1284, 2842.5049, 2846.4216, 2872.3596, 2944.3171, 2961.2437, 2975.3135, 2989.3062, 3004.4270, 3029.4883, 3059.4963, 3081.6570, 3086.5718, 3147.5710, 3157.4875, 3161.1531, 3177.1650, 3185.5222, 3237.6042, 3248.6902, 3254.5940, 3331.5864, 3335.7378, 3347.5420, 3351.6597, 3360.7197, 3411.8193, 3428.6296, 3457.6697, 3471.7146, 3475.7085, 3489.7715, 3503.8445, 3528.3743, 3542.4243, 3552.7373, 3568.9385, 3603.9502, 3625.7847, 3645.8701, 3662.8821, 3691.0452, 3722.5232, 3727.5442, 3731.5757, 3733.5112, 3736.5735, 3741.5391, 3746.5410, 3748.5002, 3751.5356, 3755.5571, 3756.5549, 3763.5049, 3767.5706, 3778.5320, 3884.9326, 3955.6738, 4074.1699, 4149.0591, 4163.0181, 4238.0752, 4300.3838, 4382.1450, 4397.2036, 4441.5176, 4453.2886, 4458.5176, 4464.4150, 4495.2642, 4511.1577, 4692.4893

27. Q2TBX4 **Mass:** 51945 **Score:** 17 **Expect:** 5.2e+002 **Matches:** 7

Heat shock 70 kDa protein 13 OS=Bos taurus GN=HSPA13 PE=2 SV=1

Observed	Mr(expt)	Mr(calc)	ppm	Start	End	Miss	Peptide
1578.7858	1577.7785	1577.7685	6.33	354 -	367	0	K.NGEGQVLFETEISR.K
1706.8668	1705.8595	1705.8635	-2.33	354 -	368	1	K.NGEGQVLFETEISRK.L
2542.2739	2541.2666	2541.2744	-3.08	297 -	318	0	K.LNLTLHETAQMSVLLTVEENDR.K + Oxidation (M)
3086.5718	3085.5645	3085.5642	0.12	127 -	154	1	K.NGMVEFSVTSNETITVSPEYVGSRLLLK.L + Oxidation (M)
3248.6902	3247.6829	3247.6620	6.43	155 -	183	2	K.LKEMAEEYLGMPVANAVISVPAEFDLKQR.N
3471.7146	3470.7073	3470.7749	-19.46	290 -	319	2	R.QAVEMVKLNLTLHETAQMSVLLTVEENDRK.G + 2 Oxidation (M)
4458.5176	4457.5103	4457.4508	13.3	197 -	238	2	K.ILRVINEPTAAAMAYGLHKAEVFHVLVIDLGGGTLDVSLLNK.Q

No match to: 706.2868, 707.2966, 708.3044, 710.3746, 713.3099, 714.3496, 717.4172, 722.4243, 729.4379, 734.4639, 735.4285, 738.4069, 744.3897, 745.4249, 747.4562, 753.3594, 756.3831, 758.3950, 768.4982, 771.3934, 779.4738, 783.3916, 805.4591, 818.4537, 823.4842, 832.3158, 834.3223, 840.4870, 842.5089, 876.2845, 878.2925, 883.4848, 889.5049, 891.4897, 922.4780, 923.5152, 929.4479, 938.4989, 949.5408, 958.4880, 962.3525, 967.4243, 968.4982, 969.5316, 973.5403, 974.4454, 976.4499, 978.5390, 979.3778, 979.5679, 983.6275, 985.6091, 986.5857, 988.3948, 993.5026, 1007.5763, 1017.5743, 1021.5607, 1031.6372, 1034.5828, 1036.5481, 1046.6013, 1051.5096, 1056.5107, 1057.6161, 1074.6067, 1087.5623, 1113.5466, 1123.6021, 1126.4208, 1143.4449, 1153.6440, 1157.4840, 1172.6813, 1178.6198, 1187.7026, 1194.5691, 1207.5975, 1215.6563, 1216.4995, 1216.7017, 1248.6692, 1254.6897, 1255.6042, 1257.6483, 1264.7133, 1265.7039, 1269.6589, 1273.6492, 1278.6917, 1284.6440, 1286.7411, 1288.7273, 1299.5450, 1301.5977, 1304.6617, 1314.7190, 1317.7113, 1321.7212, 1338.6622, 1349.6757, 1351.6887, 1354.7229, 1356.7275, 1358.7100, 1371.7595, 1375.7335, 1382.5935, 1383.7274, 1390.6962, 1393.7622, 1413.6547, 1420.7949, 1423.7593, 1440.7788, 1450.8590, 1456.7791, 1457.7606, 1467.7502, 1470.7725, 1488.7212, 1494.7396, 1500.7236, 1512.8181, 1513.7562, 1528.6954, 1539.7452, 1556.7537, 1562.7806, 1570.7695, 1574.7894, 1580.8347, 1590.7754, 1592.7883, 1616.7943, 1628.7009, 1628.8575, 1631.8130, 1635.7671, 1642.7155, 1647.8516, 1650.8444, 1657.9042, 1663.8501, 1664.8555, 1693.0090, 1696.8273, 1707.9615, 1715.8461, 1717.8761, 1718.8779, 1721.9366, 1730.8695, 1741.9938, 1747.9058, 1755.8904, 1758.9028, 1771.0238, 1774.9207, 1784.0254, 1787.8574, 1862.9503, 1870.7574, 1883.7222, 1895.7236, 1897.7325, 1909.7473, 1911.7430, 1913.7366, 1925.7604, 1938.0079, 1961.0325, 1969.9990, 1974.8882, 1979.0790, 1981.0576, 2008.9901, 2027.1294, 2060.9788, 2067.1584, 2086.1133, 2108.9963, 2112.9805, 2137.1189, 2144.0579, 2180.1812, 2223.1646, 2239.1501, 2241.1355, 2245.8403, 2255.1479, 2265.1016, 2273.0654, 2281.1055, 2318.1824, 2328.9529, 2335.9097, 2342.9673, 2347.8682, 2352.9265, 2356.9395, 2361.8997, 2363.8860, 2366.9265, 2371.0420, 2380.9539, 2389.2756, 2397.2231, 2406.2913, 2414.2468, 2431.2300, 2467.2122, 2474.2263, 2477.2573, 2478.2725, 2485.0691, 2499.1719, 2503.0352, 2518.0303, 2519.9934, 2534.0215, 2541.2542, 2558.3401, 2580.0227, 2594.0615, 2631.4961, 2678.0144, 2683.4189, 2695.0366, 2701.2097, 2709.0559, 2717.3198, 2723.0757, 2770.4492, 2787.3171, 2799.3379, 2813.4517, 2817.2305, 2833.1284, 2842.5049, 2846.4216, 2872.3596, 2944.3171, 2961.2437, 2975.3135, 2989.3062, 3004.4270, 3029.4883, 3059.4963, 3081.6570, 3147.5710, 3157.4875, 3161.1531, 3177.1650, 3185.5222, 3237.6042, 3254.5940, 3331.5864, 3335.7378, 3347.5420, 3351.6597, 3360.7197, 3411.8193, 3428.6296, 3435.7471, 3457.6697, 3475.7085, 3489.7715, 3503.8445, 3528.3743, 3542.4243, 3552.7373, 3568.9385, 3603.9502, 3625.7847, 3640.7600, 3645.8701, 3662.8821, 3691.0452, 3722.5232, 3727.5442, 3731.5757, 3733.5112, 3736.5735, 3741.5391, 3746.5410, 3748.5002, 3751.5356, 3755.5571, 3756.5549, 3763.5049, 3767.5706, 3778.5320, 3884.9326, 3955.6738, 4074.1699, 4149.0591, 4163.0181, 4238.0752, 4300.3838, 4382.1450, 4397.2036, 4441.5176, 4453.2886, 4464.4150, 4495.2642, 4511.1577, 4692.4893

28. P01966 **Mass:** 15175 **Score:** 16 **Expect:** 5.4e+002 **Matches:** 5

Hemoglobin subunit alpha OS=Bos taurus GN=HBA PE=1 SV=2

Observed	Mr(expt)	Mr(calc)	ppm	Start	End	Miss	Peptide
818.4537	817.4464	817.4334	15.9	94 -	100	0	R.VDPVNFK.L
1087.5623	1086.5550	1086.5420	12.0	33 -	41	0	R.MFLSFPTTK.T + Oxidation (M)
1248.6692	1247.6619	1247.6544	6.06	1 -	12	1	-.MVLSAADKGNVK.A + Oxidation (M)
3435.7471	3434.7398	3434.7946	-15.96	70 -	100	2	K.AVEHLDDLPGALSELSDLHAHKLRVDPVNFK.L
4397.2036	4396.1963	4396.1375	13.4	18 -	57	2	K.VGGHAAEYGAEALERMFLSFPTTKTYFPHFDLSHGSAQVK.G

No match to: 706.2868, 707.2966, 708.3044, 710.3746, 713.3099, 714.3496, 717.4172, 722.4243, 729.4379, 734.4639, 735.4285, 738.4069, 744.3897, 745.4249, 747.4562, 753.3594, 756.3831, 758.3950, 768.4982, 771.3934, 779.4738, 783.3916, 805.4591, 823.4842, 832.3158, 834.3223, 840.4870, 842.5089, 876.2845, 878.2925, 883.4848, 889.5049, 891.4897, 922.4780, 923.5152, 929.4479, 938.4989, 949.5408, 958.4880, 962.3525, 967.4243, 968.4982, 969.5316, 973.5403, 974.4454, 976.4499, 978.5390, 979.3778, 979.5679, 983.6275, 985.6091, 986.5857, 988.3948, 993.5026, 1007.5763, 1017.5743, 1021.5607, 1031.6372, 1034.5828, 1036.5481, 1046.6013, 1051.5096, 1056.5107, 1057.6161, 1074.6067, 1113.5466, 1123.6021, 1126.4208, 1143.4449, 1153.6440, 1157.4840, 1172.6813, 1178.6198, 1187.7026, 1194.5691, 1207.5975, 1215.6563, 1216.4995, 1216.7017, 1254.6897, 1255.6042, 1257.6483, 1264.7133, 1265.7039, 1269.6589, 1273.6492, 1278.6917, 1284.6440, 1286.7411, 1288.7273, 1299.5450, 1301.5977, 1304.6617, 1314.7190, 1317.7113, 1321.7212, 1338.6622, 1349.6757, 1351.6887, 1354.7229, 1356.7275, 1358.7100, 1371.7595, 1375.7335, 1382.5935, 1383.7274, 1390.6962, 1393.7622, 1413.6547, 1420.7949, 1423.7593, 1440.7788, 1450.8590, 1456.7791, 1457.7606, 1467.7502, 1470.7725, 1488.7212, 1494.7396, 1500.7236, 1512.8181, 1513.7562, 1528.6954, 1539.7452, 1556.7537, 1562.7806, 1570.7695, 1574.7894, 1578.7858, 1580.8347, 1590.7754, 1592.7883, 1616.7943, 1628.7009, 1628.8575, 1631.8130, 1635.7671, 1642.7155, 1647.8516, 1650.8444, 1657.9042, 1663.8501, 1664.8555, 1693.0090, 1696.8273, 1706.8668, 1707.9615, 1715.8461, 1717.8761, 1718.8779, 1721.9366, 1730.8695, 1741.9938, 1747.9058, 1755.8904, 1758.9028, 1771.0238, 1774.9207, 1784.0254, 1787.8574, 1862.9503, 1870.7574, 1883.7222, 1895.7236, 1897.7325, 1909.7473, 1911.7430, 1913.7366, 1925.7604, 1938.0079, 1961.0325, 1969.9990, 1974.8882, 1979.0790, 1981.0576, 2008.9901, 2027.1294, 2060.9788, 2067.1584, 2086.1133, 2108.9963, 2112.9805, 2137.1189, 2144.0579, 2180.1812, 2223.1646, 2239.1501, 2241.1355, 2245.8403, 2255.1479, 2265.1016, 2273.0654, 2281.1055, 2318.1824, 2328.9529, 2335.9097, 2342.9673, 2347.8682, 2352.9265, 2356.9395, 2361.8997, 2363.8860, 2366.9265, 2371.0420, 2380.9539, 2389.2756, 2397.2231, 2406.2913, 2414.2468, 2431.2300, 2467.2122, 2474.2263, 2477.2573, 2478.2725, 2485.0691, 2499.1719, 2503.0352, 2518.0303, 2519.9934, 2534.0215, 2541.2542, 2542.2739, 2558.3401, 2580.0227, 2594.0615, 2631.4961, 2678.0144, 2683.4189, 2695.0366, 2701.2097, 2709.0559, 2717.3198, 2723.0757, 2770.4492, 2787.3171, 2799.3379, 2813.4517, 2817.2305, 2833.1284, 2842.5049, 2846.4216, 2872.3596, 2944.3171, 2961.2437, 2975.3135, 2989.3062, 3004.4270, 3029.4883, 3059.4963, 3081.6570, 3086.5718, 3147.5710, 3157.4875, 3161.1531, 3177.1650, 3185.5222, 3237.6042, 3248.6902, 3254.5940, 3331.5864, 3335.7378, 3347.5420, 3351.6597, 3360.7197, 3411.8193, 3428.6296, 3457.6697, 3471.7146, 3475.7085, 3489.7715, 3503.8445, 3528.3743, 3542.4243, 3552.7373, 3568.9385, 3603.9502, 3625.7847, 3640.7600, 3645.8701, 3662.8821, 3691.0452, 3722.5232, 3727.5442, 3731.5757, 3733.5112, 3736.5735, 3741.5391, 3746.5410, 3748.5002, 3751.5356, 3755.5571, 3756.5549, 3763.5049, 3767.5706, 3778.5320, 3884.9326, 3955.6738, 4074.1699, 4149.0591, 4163.0181, 4238.0752, 4300.3838, 4382.1450, 4441.5176, 4453.2886, 4458.5176, 4464.4150, 4495.2642, 4511.1577, 4692.4893

29. G3MZZ4 **Mass:** 7599 **Score:** 16 **Expect:** 5.8e+002 **Matches:** 4

Uncharacterized protein OS=Bos taurus PE=4 SV=1

Observed	Mr(expt)	Mr(calc)	ppm	Start		End	Miss	Peptide
805.4591	804.4518	804.4341	22.0	55	-	61	1	R.ESKNISK.E
1301.5977	1300.5904	1300.5717	14.4	2	-	12	0	K.MEEGHLENAQK.S + Oxidation (M)
1413.6547	1412.6474	1412.6718	-17.23	1	-	12	1	-.KMEEGHLENAQK.S
2008.9901	2007.9828	2007.9612	10.8	13	-	30	1	K.SFETTVGYFGMKPKSGEK.E + Oxidation (M)

No match to: 706.2868, 707.2966, 708.3044, 710.3746, 713.3099, 714.3496, 717.4172, 722.4243, 729.4379, 734.4639, 735.4285, 738.4069, 744.3897, 745.4249, 747.4562, 753.3594, 756.3831, 758.3950, 768.4982, 771.3934, 779.4738, 783.3916, 818.4537, 823.4842, 832.3158, 834.3223, 840.4870, 842.5089, 876.2845, 878.2925, 883.4848, 889.5049, 891.4897, 922.4780, 923.5152, 929.4479, 938.4989, 949.5408, 958.4880, 962.3525, 967.4243, 968.4982, 969.5316, 973.5403, 974.4454, 976.4499, 978.5390, 979.3778, 979.5679, 983.6275, 985.6091, 986.5857, 988.3948, 993.5026, 1007.5763, 1017.5743, 1021.5607, 1031.6372, 1034.5828, 1036.5481, 1046.6013, 1051.5096, 1056.5107, 1057.6161, 1074.6067, 1087.5623, 1113.5466, 1123.6021, 1126.4208, 1143.4449, 1153.6440, 1157.4840, 1172.6813, 1178.6198, 1187.7026, 1194.5691, 1207.5975, 1215.6563, 1216.4995, 1216.7017, 1248.6692, 1254.6897, 1255.6042, 1257.6483, 1264.7133, 1265.7039, 1269.6589, 1273.6492, 1278.6917, 1284.6440, 1286.7411, 1288.7273, 1299.5450, 1304.6617, 1314.7190, 1317.7113, 1321.7212, 1338.6622, 1349.6757, 1351.6887, 1354.7229, 1356.7275, 1358.7100, 1371.7595, 1375.7335, 1382.5935, 1383.7274, 1390.6962, 1393.7622, 1420.7949, 1423.7593, 1440.7788, 1450.8590, 1456.7791, 1457.7606, 1467.7502, 1470.7725, 1488.7212, 1494.7396, 1500.7236, 1512.8181, 1513.7562, 1528.6954, 1539.7452, 1556.7537, 1562.7806, 1570.7695, 1574.7894, 1578.7858, 1580.8347, 1590.7754, 1592.7883, 1616.7943, 1628.7009, 1628.8575, 1631.8130, 1635.7671, 1642.7155, 1647.8516, 1650.8444, 1657.9042, 1663.8501, 1664.8555, 1693.0090, 1696.8273, 1706.8668, 1707.9615, 1715.8461, 1717.8761, 1718.8779, 1721.9366, 1730.8695, 1741.9938, 1747.9058, 1755.8904, 1758.9028, 1771.0238, 1774.9207, 1784.0254, 1787.8574, 1862.9503, 1870.7574, 1883.7222, 1895.7236, 1897.7325, 1909.7473, 1911.7430, 1913.7366, 1925.7604, 1938.0079, 1961.0325, 1969.9990, 1974.8882, 1979.0790, 1981.0576, 2027.1294, 2060.9788, 2067.1584, 2086.1133, 2108.9963, 2112.9805, 2137.1189, 2144.0579, 2180.1812, 2223.1646, 2239.1501, 2241.1355, 2245.8403, 2255.1479, 2265.1016, 2273.0654, 2281.1055, 2318.1824, 2328.9529, 2335.9097, 2342.9673, 2347.8682, 2352.9265, 2356.9395, 2361.8997, 2363.8860, 2366.9265, 2371.0420, 2380.9539, 2389.2756, 2397.2231, 2406.2913, 2414.2468, 2431.2300, 2467.2122, 2474.2263, 2477.2573, 2478.2725, 2485.0691, 2499.1719, 2503.0352, 2518.0303, 2519.9934, 2534.0215, 2541.2542, 2542.2739, 2558.3401, 2580.0227, 2594.0615, 2631.4961, 2678.0144, 2683.4189, 2695.0366, 2701.2097, 2709.0559, 2717.3198, 2723.0757, 2770.4492, 2787.3171, 2799.3379, 2813.4517, 2817.2305, 2833.1284, 2842.5049, 2846.4216, 2872.3596, 2944.3171, 2961.2437, 2975.3135, 2989.3062, 3004.4270, 3029.4883, 3059.4963, 3081.6570, 3086.5718, 3147.5710, 3157.4875, 3161.1531, 3177.1650, 3185.5222, 3237.6042, 3248.6902, 3254.5940, 3331.5864, 3335.7378, 3347.5420, 3351.6597, 3360.7197, 3411.8193, 3428.6296, 3435.7471, 3457.6697, 3471.7146, 3475.7085, 3489.7715, 3503.8445, 3528.3743, 3542.4243, 3552.7373, 3568.9385, 3603.9502, 3625.7847, 3640.7600, 3645.8701, 3662.8821, 3691.0452, 3722.5232, 3727.5442, 3731.5757, 3733.5112, 3736.5735, 3741.5391, 3746.5410, 3748.5002, 3751.5356, 3755.5571, 3756.5549, 3763.5049, 3767.5706, 3778.5320, 3884.9326, 3955.6738, 4074.1699, 4149.0591, 4163.0181, 4238.0752, 4300.3838, 4382.1450, 4397.2036, 4441.5176, 4453.2886, 4458.5176, 4464.4150, 4495.2642, 4511.1577, 4692.4893

30. F1MGJ7 **Mass:** 31607 **Score:** 16 **Expect:** 5.8e+002 **Matches:** 8

Uncharacterized protein OS=Bos taurus GN=AUH PE=1 SV=2

Observed	Mr(expt)	Mr(calc)	ppm	Start		End	Miss	Peptide
753.3594	752.3522	752.3705	-24.31	65	-	71	0	R.GYSAEVK.T
1074.6067	1073.5994	1073.6080	-8.03	113	-	122	2	R.AVDALKSDKK.V
1539.7452	1538.7379	1538.7763	-24.92	148	-	161	1	K.MSSSEVGPFVSKIR.A + Oxidation (M)
1663.8501	1662.8428	1662.8229	12.0	22	-	35	2	R.LVAACCTRLGARCR.L
1758.9028	1757.8955	1757.9094	-7.89	1	-	19	0	-.MAVAASAAPGALGTLQASR.A + Oxidation (M)
2108.9963	2107.9890	2108.0286	-18.79	60	-	77	2	K.AAWQRGYSAEVKTEDELR.V
2770.4492	2769.4419	2769.4490	-2.56	2	-	29	2	M.AVAASAAPGALGTLQASRARLVAACCTR.L
3435.7471	3434.7398	3434.7061	9.81	240	-	270	1	K.LAINQGMEVDLVTGLAIEEACYAQTIPTKDR.L + Oxidation (M)

No match to: 706.2868, 707.2966, 708.3044, 710.3746, 713.3099, 714.3496, 717.4172, 722.4243, 729.4379, 734.4639, 735.4285, 738.4069, 744.3897, 745.4249, 747.4562, 756.3831, 758.3950, 768.4982, 771.3934, 779.4738, 783.3916, 805.4591, 818.4537, 823.4842, 832.3158, 834.3223, 840.4870, 842.5089, 876.2845, 878.2925, 883.4848, 889.5049, 891.4897, 922.4780, 923.5152, 929.4479, 938.4989, 949.5408, 958.4880, 962.3525, 967.4243, 968.4982, 969.5316, 973.5403, 974.4454, 976.4499, 978.5390, 979.3778, 979.5679, 983.6275, 985.6091, 986.5857, 988.3948, 993.5026, 1007.5763, 1017.5743, 1021.5607, 1031.6372, 1034.5828, 1036.5481, 1046.6013, 1051.5096, 1056.5107, 1057.6161, 1087.5623, 1113.5466, 1123.6021, 1126.4208, 1143.4449, 1153.6440, 1157.4840, 1172.6813, 1178.6198, 1187.7026, 1194.5691, 1207.5975, 1215.6563, 1216.4995, 1216.7017, 1248.6692, 1254.6897, 1255.6042, 1257.6483, 1264.7133, 1265.7039, 1269.6589, 1273.6492, 1278.6917, 1284.6440, 1286.7411, 1288.7273, 1299.5450, 1301.5977, 1304.6617, 1314.7190, 1317.7113, 1321.7212, 1338.6622, 1349.6757, 1351.6887, 1354.7229, 1356.7275, 1358.7100, 1371.7595, 1375.7335, 1382.5935, 1383.7274, 1390.6962, 1393.7622, 1413.6547, 1420.7949, 1423.7593, 1440.7788, 1450.8590, 1456.7791, 1457.7606, 1467.7502, 1470.7725, 1488.7212, 1494.7396, 1500.7236, 1512.8181, 1513.7562, 1528.6954, 1556.7537, 1562.7806, 1570.7695, 1574.7894, 1578.7858, 1580.8347, 1590.7754, 1592.7883, 1616.7943, 1628.7009, 1628.8575, 1631.8130, 1635.7671, 1642.7155, 1647.8516, 1650.8444, 1657.9042, 1664.8555, 1693.0090, 1696.8273, 1706.8668, 1707.9615, 1715.8461, 1717.8761, 1718.8779, 1721.9366, 1730.8695, 1741.9938, 1747.9058, 1755.8904, 1771.0238, 1774.9207, 1787.8574, 1784.0254, 1862.9503, 1870.7574, 1883.7222, 1895.7236, 1897.7325, 1909.7473, 1911.7430, 1913.7366, 1925.7604, 1938.0079, 1961.0325, 1969.9990, 1974.8882, 1979.0790, 1981.0576, 2008.9901, 2027.1294, 2060.9788, 2067.1584, 2086.1133, 2112.9805, 2137.1189, 2144.0579, 2180.1812, 2223.1646, 2239.1501, 2241.1355, 2245.8403, 2255.1479, 2265.1016, 2273.0654, 2281.1055, 2318.1824, 2328.9529, 2335.9097, 2342.9673, 2347.8682, 2352.9265, 2356.9395, 2361.8997, 2363.8860, 2366.9265, 2371.0420, 2380.9539, 2389.2756, 2397.2231, 2406.2913, 2414.2468, 2431.2300, 2467.2122, 2474.2263, 2477.2573, 2478.2725, 2485.0691, 2499.1719, 2503.0352, 2518.0303, 2519.9934, 2534.0215, 2541.2542, 2542.2739, 2558.3401, 2580.0227, 2594.0615, 2631.4961, 2678.0144, 2683.4189, 2695.0366, 2701.2097, 2709.0559, 2717.3198, 2723.0757, 2787.3171, 2799.3379, 2813.4517, 2817.2305, 2833.1284, 2842.5049, 2846.4216, 2872.3596, 2944.3171, 2961.2437, 2975.3135, 2989.3062, 3004.4270, 3029.4883, 3059.4963, 3081.6570, 3086.5718, 3147.5710, 3157.4875, 3161.1531, 3177.1650, 3185.5222, 3237.6042, 3248.6902, 3254.5940, 3331.5864, 3335.7378, 3347.5420, 3351.6597, 3360.7197, 3411.8193, 3428.6296, 3457.6697, 3471.7146, 3475.7085, 3489.7715, 3503.8445, 3528.3743, 3542.4243, 3552.7373, 3568.9385, 3603.9502, 3625.7847, 3640.7600, 3645.8701, 3662.8821, 3691.0452, 3722.5232, 3727.5442, 3731.5757, 3733.5112, 3736.5735, 3741.5391, 3746.5410, 3748.5002, 3751.5356, 3755.5571, 3756.5549, 3763.5049, 3767.5706, 3778.5320, 3884.9326, 3955.6738, 4074.1699, 4149.0591, 4163.0181, 4238.0752, 4300.3838, 4382.1450, 4397.2036, 4441.5176, 4453.2886, 4458.5176, 4464.4150, 4495.2642, 4511.1577, 4692.4893

31. G3N1R0 **Mass:** 18422 **Score:** 16 **Expect:** 5.8e+002 **Matches:** 4

Major histocompatibility complex class I-related gene protein OS=Bos taurus GN=MR1 PE=3 SV=1

Observed	Mr(expt)	Mr(calc)	ppm	Start		End	Miss	Peptide
756.3831	755.3758	755.3854	-12.70	99	-	104	0	R.FLEYGK.D
1590.7754	1589.7681	1589.7548	8.40	86	-	97	0	K.NWLEEECIAWLK.R
3081.6570	3080.6497	3080.6411	2.80	1	-	25	2	-.MMLLLPLIIVLMMKLSDARFHTYQR.M + 3 Oxidation (M)
3457.6697	3456.6624	3456.6258	10.6	26	-	55	0	R.MIGCELLEDGSITGFLQYAYDGQDFLIFNK.D

No match to: 706.2868, 707.2966, 708.3044, 710.3746, 713.3099, 714.3496, 717.4172, 722.4243, 729.4379, 734.4639, 735.4285, 738.4069, 744.3897, 745.4249, 747.4562, 753.3594, 758.3950, 768.4982, 771.3934, 779.4738, 783.3916, 805.4591, 818.4537, 823.4842, 832.3158, 834.3223, 840.4870, 842.5089, 876.2845, 878.2925, 883.4848, 889.5049, 891.4897, 922.4780, 923.5152, 929.4479, 938.4989, 949.5408, 958.4880, 962.3525, 967.4243, 968.4982, 969.5316, 973.5403, 974.4454, 976.4499, 978.5390, 979.3778, 979.5679, 983.6275, 985.6091, 986.5857, 988.3948, 993.5026, 1007.5763, 1017.5743, 1021.5607, 1031.6372, 1034.5828, 1036.5481, 1046.6013, 1051.5096, 1056.5107, 1057.6161, 1074.6067, 1087.5623, 1113.5466, 1123.6021, 1126.4208, 1143.4449, 1153.6440, 1157.4840, 1172.6813, 1178.6198, 1187.7026, 1194.5691, 1207.5975, 1215.6563, 1216.4995, 1216.7017, 1248.6692, 1254.6897, 1255.6042, 1257.6483, 1264.7133, 1265.7039, 1269.6589, 1273.6492, 1278.6917, 1284.6440, 1286.7411, 1288.7273, 1299.5450, 1301.5977, 1304.6617, 1314.7190, 1317.7113, 1321.7212, 1338.6622, 1349.6757, 1351.6887, 1354.7229, 1356.7275, 1358.7100, 1371.7595, 1375.7335, 1382.5935, 1383.7274, 1390.6962, 1393.7622, 1413.6547, 1420.7949, 1423.7593, 1440.7788, 1450.8590, 1456.7791, 1457.7606, 1467.7502, 1470.7725, 1488.7212, 1494.7396, 1500.7236, 1512.8181, 1513.7562, 1528.6954, 1539.7452, 1556.7537, 1562.7806, 1570.7695, 1574.7894, 1578.7858, 1580.8347, 1592.7883, 1616.7943, 1628.7009, 1628.8575, 1631.8130, 1635.7671, 1642.7155, 1647.8516, 1650.8444, 1657.9042, 1663.8501, 1664.8555, 1693.0090, 1696.8273, 1706.8668, 1707.9615, 1715.8461, 1717.8761, 1718.8779, 1721.9366, 1730.8695, 1741.9938, 1747.9058, 1755.8904, 1758.9028, 1771.0238, 1774.9207, 1784.0254, 1787.8574, 1862.9503, 1870.7574, 1883.7222, 1895.7236, 1897.7325, 1909.7473, 1911.7430, 1913.7366, 1925.7604, 1938.0079, 1961.0325, 1969.9990, 1974.8882, 1979.0790, 1981.0576, 2008.9901, 2027.1294, 2060.9788, 2067.1584, 2086.1133, 2108.9963, 2112.9805, 2137.1189, 2144.0579, 2180.1812, 2223.1646, 2239.1501, 2241.1355, 2245.8403, 2255.1479, 2265.1016, 2273.0654, 2281.1055, 2318.1824, 2328.9529, 2335.9097, 2342.9673, 2347.8682, 2352.9265, 2356.9395, 2361.8997, 2363.8860, 2366.9265, 2371.0420, 2380.9539, 2389.2756, 2397.2231, 2406.2913, 2414.2468, 2431.2300, 2467.2122, 2474.2263, 2477.2573, 2478.2725, 2485.0691, 2499.1719, 2503.0352, 2518.0303, 2519.9934, 2534.0215, 2541.2542, 2542.2739, 2558.3401, 2580.0227, 2594.0615, 2631.4961, 2678.0144, 2683.4189, 2695.0366,

2701.2097, 2709.0559, 2717.3198, 2723.0757, 2770.4492, 2787.3171, 2799.3379, 2813.4517, 2817.2305, 2833.1284, 2842.5049, 2846.4216, 2872.3596, 2944.3171, 2961.2437, 2975.3135, 2989.3062, 3004.4270, 3029.4883, 3059.4963, 3086.5718, 3147.5710, 3157.4875, 3161.1531, 3177.1650, 3185.5222, 3237.6042, 3248.6902, 3254.5940, 3331.5864, 3335.7378, 3347.5420, 3351.6597, 3360.7197, 3411.8193, 3428.6296, 3435.7471, 3471.7146, 3475.7085, 3489.7715, 3503.8445, 3528.3743, 3542.4243, 3552.7373, 3568.9385, 3603.9502, 3625.7847, 3640.7600, 3645.8701, 3662.8821, 3691.0452, 3722.5232, 3727.5442, 3731.5757, 3733.5112, 3736.5735, 3741.5391, 3746.5410, 3748.5002, 3751.5356, 3755.5571, 3756.5549, 3763.5049, 3767.5706, 3778.5320, 3884.9326, 3955.6738, 4074.1699, 4149.0591, 4163.0181, 4238.0752, 4300.3838, 4382.1450, 4397.2036, 4441.5176, 4453.2886, 4458.5176, 4464.4150, 4495.2642, 4511.1577, 4692.4893

32. F1MCS0 **Mass:** 15398 **Score:** 16 **Expect:** 5.8e+002 **Matches:** 6
Uncharacterized protein OS=Bos taurus PE=4 SV=2

Observed	Mr(expt)	Mr(calc)	ppm	Start	End	Miss	Peptide
717.4172	716.4099	716.4068	4.27	1 -	6	1	-.IDDKVK.D
1248.6692	1247.6619	1247.6544	6.06	1 -	10	2	-.IDDKVKDIMR.L + Oxidation (M)
1513.7562	1512.7489	1512.7646	-10.38	120 -	131	0	K.QLVDMLNNYFIK.E + Oxidation (M)
1741.9938	1740.9865	1740.9484	21.9	70 -	84	0	K.FKPVPQIIMELPADK.Q + Oxidation (M)
2478.2725	2477.2652	2477.2658	-0.24	98 -	119	1	R.NPSWTSVEQLTMLVMGSKTLQK.Q
4458.5176	4457.5103	4457.4550	12.4	23 -	69	1	K.VAFKYPLLGGLVSGTVATVGGLVGGLPGIVFGGAVGGLLGSWVMNGK.F + Oxidation (M)

No match to: 706.2868, 707.2966, 708.3044, 710.3746, 713.3099, 714.3496, 722.4243, 729.4379, 734.4639, 735.4285, 738.4069, 744.3897, 745.4249, 747.4562, 753.3594, 756.3831, 758.3950, 768.4982, 771.3934, 779.4738, 783.3916, 805.4591, 818.4537, 823.4842, 832.3158, 834.3223, 840.4870, 842.5089, 876.2845, 878.2925, 883.4848, 889.5049, 891.4897, 922.4780, 923.5152, 929.4479, 938.4989, 949.5408, 958.4880, 962.3525, 967.4243, 968.4982, 969.5316, 973.5403, 974.4454, 976.4499, 978.5390, 979.3778, 979.5679, 983.6275, 985.6091, 986.5857, 988.3948, 993.5026, 1007.5763, 1017.5743, 1021.5607, 1031.6372, 1034.5828, 1036.5481, 1046.6013, 1051.5096, 1056.5107, 1057.6161, 1074.6067, 1087.5623, 1113.5466, 1123.6021, 1126.4208, 1143.4449, 1153.6440, 1157.4840, 1172.6813, 1178.6198, 1187.7026, 1194.5691, 1207.5975, 1215.6563, 1216.4995, 1216.7017, 1254.6897, 1255.6042, 1257.6483, 1265.7039, 1269.6589, 1273.6492, 1278.6917, 1284.6440, 1286.7411, 1288.7273, 1299.5450, 1301.5977, 1304.6617, 1314.7190, 1317.7113, 1321.7212, 1338.6622, 1349.6757, 1351.6887, 1354.7229, 1356.7275, 1358.7100, 1371.7595, 1375.7335, 1382.5935, 1383.7274, 1390.6962, 1393.7622, 1413.6547, 1420.7949, 1423.7593, 1440.7788, 1450.8590, 1456.7791, 1457.7606, 1467.7502, 1470.7725, 1488.7212, 1494.7396, 1500.7236, 1512.8181, 1528.6954, 1539.7452, 1556.7537, 1562.7806, 1570.7695, 1574.7894, 1578.7858, 1580.8347, 1590.7754, 1592.7883, 1616.7943, 1628.7009, 1628.8575, 1631.8130, 1635.7671, 1642.7155, 1647.8516, 1650.8444, 1657.9042, 1663.8501, 1664.8555, 1693.0090, 1696.8273, 1706.8668, 1707.9615, 1715.8461, 1717.8761, 1718.8779, 1721.9366, 1730.8695, 1747.9058, 1755.8904, 1758.9028, 1771.0238, 1774.9207, 1784.0254, 1787.8574, 1862.9503, 1870.7574, 1883.7222, 1895.7236, 1897.7325, 1909.7473, 1911.7430, 1913.7366, 1925.7604, 1938.0079, 1961.0325, 1969.9990, 1974.8882, 1979.0790, 1981.0576, 2008.9901, 2027.1294, 2060.9788, 2067.1584, 2086.1133, 2108.9963, 2112.9805, 2137.1189, 2144.0579, 2180.1812, 2223.1646, 2239.1501, 2241.1355, 2245.8403, 2255.1479, 2265.1016, 2273.0654, 2281.1055, 2318.1824, 2328.9529, 2335.9097, 2342.9673, 2347.8682, 2352.9265, 2356.9395, 2361.8997, 2363.8860, 2366.9265, 2371.0420, 2380.9539, 2389.2756, 2397.2231, 2406.2913, 2414.2468, 2431.2300, 2467.2122, 2474.2263, 2477.2573, 2485.0691, 2499.1719, 2503.0352, 2518.0303, 2519.9934, 2534.0215, 2541.2542, 2542.2739, 2558.3401, 2580.0227, 2594.0615, 2631.4961, 2678.0144, 2683.4189, 2695.0366, 2701.2097, 2709.0559, 2717.3198, 2723.0757, 2770.4492, 2787.3171, 2799.3379, 2813.4517, 2817.2305, 2833.1284, 2842.5049, 2846.4216, 2872.3596, 2944.3171, 2961.2437, 2975.3135, 2989.3062, 3004.4270, 3029.4883, 3059.4963, 3081.6570, 3086.5718, 3147.5710, 3157.4875, 3161.1531, 3177.1650, 3185.5222, 3237.6042, 3248.6902, 3254.5940, 3331.5864, 3335.7378, 3347.5420, 3351.6597, 3360.7197, 3411.8193, 3428.6296, 3435.7471, 3457.6697, 3471.7146, 3475.7085, 3489.7715, 3503.8445, 3528.3743, 3542.4243, 3552.7373, 3568.9385, 3603.9502, 3625.7847, 3640.7600, 3645.8701, 3662.8821, 3691.0452, 3722.5232, 3727.5442, 3731.5757, 3733.5112, 3736.5735, 3741.5391, 3746.5410, 3748.5002, 3751.5356, 3755.5571, 3756.5549, 3763.5049, 3767.5706, 3778.5320, 3884.9326, 3955.6738, 4074.1699, 4149.0591, 4163.0181, 4238.0752, 4300.3838, 4382.1450, 4397.2036, 4441.5176, 4453.2886, 4464.4150, 4495.2642, 4511.1577, 4692.4893

33. F1N0K7 **Mass:** 15524 **Score:** 16 **Expect:** 5.9e+002 **Matches:** 6
Uncharacterized protein OS=Bos taurus PE=4 SV=1

Observed	Mr(expt)	Mr(calc)	ppm	Start	End	Miss	Peptide
717.4172	716.4099	716.4068	4.27	1 -	6	1	-.IDDKVK.D
1248.6692	1247.6619	1247.6544	6.06	1 -	10	2	-.IDDKVKDIMR.L + Oxidation (M)
1513.7562	1512.7489	1512.7646	-10.38	120 -	131	0	K.QLVDMLNNYFIK.E + Oxidation (M)
1741.9938	1740.9865	1740.9484	21.9	70 -	84	0	K.FKPVPQIIMELPADK.Q + Oxidation (M)
2478.2725	2477.2652	2477.2658	-0.24	98 -	119	1	R.NPSWTSVEQLTMLVMGSKTLQK.Q
4458.5176	4457.5103	4457.4550	12.4	23 -	69	1	K.VAFKYPLLGGLVSGTVATVGGLVGGLPGIVFGGAVGGLLGSWVMNGK.F + Oxidation (M)

No match to: 706.2868, 707.2966, 708.3044, 710.3746, 713.3099, 714.3496, 722.4243, 729.4379, 734.4639, 735.4285, 738.4069, 744.3897, 745.4249, 747.4562, 753.3594, 756.3831, 758.3950, 768.4982, 771.3934, 779.4738, 783.3916, 805.4591, 818.4537, 823.4842, 832.3158, 834.3223, 840.4870, 842.5089, 876.2845, 878.2925, 883.4848, 889.5049, 891.4897, 922.4780, 923.5152, 929.4479, 938.4989, 949.5408, 958.4880, 962.3525, 967.4243, 968.4982, 969.5316, 973.5403, 974.4454, 976.4499, 978.5390, 979.3778, 979.5679, 983.6275, 985.6091, 986.5857, 988.3948, 993.5026, 1007.5763, 1017.5743, 1021.5607, 1031.6372, 1034.5828, 1036.5481, 1046.6013, 1051.5096, 1056.5107, 1057.6161, 1074.6067, 1087.5623, 1113.5466, 1123.6021, 1126.4208, 1143.4449, 1153.6440, 1157.4840, 1172.6813, 1178.6198, 1187.7026, 1194.5691, 1207.5975, 1215.6563, 1216.4995, 1216.7017, 1254.6897, 1255.6042, 1257.6483, 1264.7133, 1265.7039, 1269.6589, 1273.6492, 1278.6917, 1284.6440, 1286.7411, 1288.7273, 1299.5450, 1301.5977, 1304.6617, 1314.7190, 1317.7113, 1321.7212, 1338.6622, 1349.6757, 1351.6887, 1354.7229, 1356.7275, 1358.7100, 1371.7595, 1375.7335, 1382.5935, 1383.7274, 1390.6962, 1393.7622, 1413.6547, 1420.7949, 1423.7593, 1440.7788, 1450.8590, 1456.7791, 1457.7606, 1467.7502, 1470.7725, 1488.7212, 1494.7396, 1500.7236, 1512.8181, 1528.6954, 1539.7452, 1556.7537, 1562.7806, 1570.7695, 1574.7894, 1578.7858, 1580.8347, 1590.7754, 1592.7883, 1616.7943, 1628.7009, 1628.8575, 1631.8130, 1635.7671, 1642.7155, 1647.8516, 1650.8444, 1657.9042, 1663.8501, 1664.8555, 1693.0090, 1696.8273, 1706.8668, 1707.9615, 1715.8461, 1717.8761, 1718.8779, 1721.9366, 1730.8695, 1747.9058, 1755.8904, 1758.9028, 1771.0238, 1774.9207, 1784.0254, 1787.8574, 1862.9503, 1870.7574, 1883.7222, 1895.7236, 1897.7325, 1909.7473, 1911.7430, 1913.7366, 1925.7604, 1938.0079, 1961.0325, 1969.9990, 1974.8882, 1979.0790, 1981.0576, 2008.9901, 2027.1294, 2060.9788, 2067.1584, 2086.1133, 2108.9963, 2112.9805, 2137.1189, 2144.0579, 2180.1812, 2223.1646, 2239.1501, 2241.1355, 2245.8403, 2255.1479, 2265.1016, 2273.0654, 2281.1055, 2318.1824, 2328.9529, 2335.9097, 2342.9673, 2347.8682, 2352.9265, 2356.9395, 2361.8997, 2363.8860, 2366.9265, 2371.0420, 2380.9539, 2389.2756, 2397.2231, 2406.2913, 2414.2468, 2431.2300, 2467.2122, 2474.2263, 2477.2573, 2485.0691, 2499.1719, 2503.0352, 2518.0303, 2519.9934, 2534.0215, 2541.2542, 2542.2739, 2558.3401, 2580.0227, 2594.0615, 2631.4961, 2678.0144, 2683.4189, 2695.0366, 2701.2097, 2709.0559, 2717.3198, 2723.0757, 2770.4492, 2787.3171, 2799.3379, 2813.4517, 2817.2305, 2833.1284, 2842.5049, 2846.4216, 2872.3596, 2944.3171, 2961.2437, 2975.3135, 2989.3062, 3004.4270, 3029.4883, 3059.4963, 3081.6570, 3086.5718, 3147.5710, 3157.4875, 3161.1531, 3177.1650, 3185.5222, 3237.6042, 3248.6902, 3254.5940, 3331.5864, 3335.7378, 3347.5420, 3351.6597, 3360.7197, 3411.8193, 3428.6296, 3435.7471, 3457.6697, 3471.7146, 3475.7085, 3489.7715, 3503.8445, 3528.3743, 3542.4243, 3552.7373, 3568.9385, 3603.9502, 3625.7847, 3640.7600, 3645.8701, 3662.8821, 3691.0452, 3722.5232, 3727.5442, 3731.5757, 3733.5112, 3736.5735, 3741.5391, 3746.5410, 3748.5002, 3751.5356, 3755.5571, 3756.5549, 3763.5049, 3767.5706, 3778.5320, 3884.9326, 3955.6738, 4074.1699, 4149.0591, 4163.0181, 4238.0752, 4300.3838, 4382.1450, 4397.2036, 4441.5176, 4453.2886, 4464.4150, 4495.2642, 4511.1577, 4692.4893

34. G3X8G8 **Mass:** 36269 **Score:** 16 **Expect:** 6.1e+002 **Matches:** 8
Olfactory receptor OS=Bos taurus GN=LOC509526 PE=3 SV=1

Observed	Mr(expt)	Mr(calc)	ppm	Start	End	Miss	Peptide
1215.6563	1214.6490	1214.6190	24.8	6 -	15	1	R.LMQPEARTNR.T
3029.4883	3028.4810	3028.4398	13.6	245 -	270	1	K.KAFSTCGSHLTVVCLFYGTGIFNYMR.L
3157.4875	3156.4802	3156.5348	-17.28	244 -	270	2	R.KKAFSTCGSHLTVVCLFYGTGIFNYMR.L
3335.7378	3334.7305	3334.7280	0.74	64 -	93	0	K.LHTPMYFFLGNLSVLDIGCITVTVPAMLGR.L
3351.6597	3350.6524	3350.7230	-21.05	64 -	93	0	K.LHTPMYFFLGNLSVLDIGCITVTVPAMLGR.L + Oxidation (M)
3489.7715	3488.7642	3488.8238	-17.09	282 -	312	1	K.GVGVFNTIINPMLNPLIYSLRNSDVQGSLWR.V + Oxidation (M)
3884.9326	3883.9253	3883.8711	14.0	99 -	131	1	K.RTVPYAGCLSQLFFFHLLAGMDCFLLTAMAYDR.F
4495.2642	4494.2569	4494.2149	9.34	94 -	131	2	R.LLSHKRTVPYAGCLSQLFFFHLLAGMDCFLLTAMAYDR.F + 2 Oxidation (M)

No match to: 706.2868, 707.2966, 708.3044, 710.3746, 713.3099, 714.3496, 717.4172, 722.4243, 729.4379, 734.4639, 735.4285, 738.4069, 744.3897, 745.4249, 747.4562, 753.3594, 756.3831, 758.3950, 768.4982, 771.3934, 779.4738, 783.3916, 805.4591, 818.4537, 823.4842, 832.3158, 834.3223, 840.4870, 842.5089, 876.2845, 878.2925, 883.4848, 889.5049, 891.4897, 922.4780, 923.5152, 929.4479,

938.4989, 949.5408, 958.4880, 962.3525, 967.4243, 968.4982, 969.5316, 973.5403, 974.4454, 976.4499, 978.5390, 979.3778, 979.5679, 983.6275, 985.6091, 986.5857, 988.3948, 993.5026, 1007.5763, 1017.5743, 1021.5607, 1031.6372, 1034.5828, 1036.5481, 1046.6013, 1051.5096, 1056.5107, 1057.6161, 1074.6067, 1087.5623, 1113.5466, 1123.6021, 1126.4208, 1143.4449, 1153.6440, 1157.4840, 1172.6813, 1178.6198, 1187.7026, 1194.5691, 1207.5975, 1216.4995, 1216.7017, 1248.6692, 1254.6897, 1255.6042, 1257.6483, 1264.7133, 1265.7039, 1269.6589, 1273.6492, 1278.6917, 1284.6440, 1286.7411, 1288.7273, 1299.5450, 1301.5977, 1304.6617, 1314.7190, 1317.7113, 1321.7212, 1338.6622, 1349.6757, 1351.6887, 1354.7229, 1356.7275, 1358.7100, 1371.7595, 1375.7335, 1382.5935, 1383.7274, 1390.6962, 1393.7622, 1413.6547, 1420.7949, 1423.7593, 1440.7788, 1450.8590, 1456.7791, 1457.7606, 1467.7502, 1470.7725, 1488.7212, 1494.7396, 1500.7236, 1512.8181, 1513.7562, 1528.6954, 1539.7452, 1556.7537, 1562.7806, 1570.7695, 1574.7894, 1578.7858, 1590.7754, 1592.7883, 1616.7943, 1628.7009, 1628.8575, 1631.8130, 1635.7671, 1642.7155, 1647.8516, 1650.8444, 1657.9042, 1663.8501, 1664.8555, 1693.0090, 1696.8273, 1706.8668, 1707.9615, 1715.8461, 1717.8761, 1718.8779, 1721.9366, 1730.8695, 1741.9938, 1747.9058, 1755.8904, 1758.9028, 1771.0238, 1774.9207, 1784.0254, 1787.8574, 1862.9503, 1870.7574, 1883.7222, 1895.7236, 1897.7325, 1909.7473, 1911.7430, 1913.7366, 1925.7604, 1938.0079, 1961.0325, 1969.9990, 1974.8882, 1979.0790, 1981.0576, 2008.9901, 2027.1294, 2060.9788, 2067.1584, 2086.1133, 2108.9963, 2112.9805, 2137.1189, 2144.0579, 2180.1812, 2223.1646, 2239.1501, 2241.1355, 2245.8403, 2255.1479, 2265.1016, 2273.0654, 2281.1055, 2318.1824, 2328.9529, 2335.9097, 2342.9673, 2347.8682, 2352.9265, 2356.9395, 2361.8997, 2363.8860, 2366.9265, 2371.0420, 2380.9539, 2389.2756, 2397.2231, 2406.2913, 2414.2468, 2431.2300, 2467.2122, 2474.2263, 2477.2573, 2478.2725, 2485.0691, 2499.1719, 2503.0352, 2518.0303, 2519.9934, 2534.0215, 2541.2542, 2542.2739, 2558.3401, 2580.0227, 2594.0615, 2631.4961, 2678.0144, 2683.4189, 2695.0366, 2701.2097, 2709.0559, 2717.3198, 2723.0757, 2770.4492, 2787.3171, 2799.3379, 2813.4517, 2817.2305, 2833.1284, 2842.5049, 2846.4216, 2872.3596, 2944.3171, 2961.2437, 2975.3135, 2989.3062, 3004.4270, 3059.4963, 3081.6570, 3086.5718, 3147.5710, 3161.1531, 3177.1650, 3185.5222, 3237.6042, 3248.6902, 3254.5940, 3331.5864, 3347.5420, 3360.7197, 3411.8193, 3428.6296, 3435.7471, 3457.6697, 3471.7146, 3475.7085, 3503.8445, 3528.3743, 3542.4243, 3552.7373, 3568.9385, 3603.9502, 3625.7847, 3640.7600, 3645.8701, 3662.8821, 3691.0452, 3722.5232, 3727.5442, 3731.5757, 3733.5112, 3736.5735, 3741.5391, 3746.5410, 3748.5002, 3751.5356, 3755.5571, 3756.5549, 3763.5049, 3767.5706, 3778.5320, 3955.6738, 4074.1699, 4149.0591, 4163.0181, 4238.0752, 4300.3838, 4382.1450, 4397.2036, 4441.5176, 4453.2886, 4458.5176, 4464.4150, 4511.1577, 4692.4893

35. A6QLP2 **Mass:** 67758 **Score:** 16 **Expect:** 6.2e+002 **Matches:** 11

Adenosylhomocysteinase 3 OS=Bos taurus GN=AHCYL2 PE=1 SV=1

Observed	Mr(expt)	Mr(calc)	ppm	Start	End	Miss	Peptide
983.6275	982.6202	982.6148	5.51	135	142	2	K.RPTKIGRR.S
1007.5763	1006.5690	1006.5811	-12.00	343	351	1	R.LYQLSKAGK.L
1248.6692	1247.6619	1247.6544	6.06	1	13	0	-.MSVQVVSAAAAAK.V + Oxidation (M)
1255.6042	1254.5969	1254.6145	-14.03	601	610	0	K.NGPFKPNYYR.Y
1513.7562	1512.7489	1512.7831	-22.58	459	470	2	K.NVVTREHLDRMK.N + Oxidation (M)
1590.7754	1589.7681	1589.7784	-6.48	88	99	2	K.RSDPHHQHQRHR.D
1969.9990	1968.9917	1968.9476	22.4	182	198	2	K.GNSDFCVKNIKQAEFGR.R
2027.1294	2026.1221	2026.1132	4.39	1	20	1	-.MSVQVVSAAAAAKVPEVELK.D
3086.5718	3085.5645	3085.5007	20.7	255	282	0	R.WAACNIYSTLNEVAAALAESGFPVFAWK.G
3157.4875	3156.4802	3156.5480	-21.47	413	440	1	K.AMGSIVYVTEIDPICALQACMDGFRLVK.L
3662.8821	3661.8748	3661.7974	21.1	569	600	1	K.MDEYVASLHLPTFDAHLTELTDEQAKYLGLNK.N

No match to: 706.2868, 707.2966, 708.3044, 710.3746, 713.3099, 714.3496, 717.4172, 722.4243, 729.4379, 734.4639, 735.4285, 738.4069, 744.3897, 745.4249, 747.4562, 753.3594, 756.3831, 758.3950, 768.4982, 771.3934, 779.4738, 783.3916, 805.4591, 818.4537, 823.4842, 832.3158, 834.3223, 840.4870, 842.5089, 876.2845, 878.2925, 883.4848, 889.5049, 891.4897, 922.4780, 923.5152, 929.4479, 938.4989, 949.5408, 958.4880, 962.3525, 967.4243, 968.4982, 969.5316, 973.5403, 974.4454, 976.4499, 978.5390, 979.3778, 979.5679, 985.6091, 986.5857, 988.3948, 993.5026, 1017.5743, 1021.5607, 1031.6372, 1034.5828, 1036.5481, 1046.6013, 1051.5096, 1056.5107, 1057.6161, 1074.6067, 1087.5623, 1113.5466, 1123.6021, 1126.4208, 1143.4449, 1153.6440, 1157.4840, 1172.6813, 1178.6198, 1187.7026, 1194.5691, 1207.5975, 1215.6563, 1216.4995, 1216.7017, 1254.6897, 1257.6483, 1264.7133, 1265.7039, 1269.6589, 1273.6492, 1278.6917, 1284.6440, 1286.7411, 1288.7273, 1299.5450, 1301.5977, 1304.6617, 1314.7190, 1317.7113, 1321.7212, 1338.6622, 1349.6757, 1351.6887, 1354.7229, 1356.7275, 1358.7100, 1371.7595, 1375.7335, 1382.5935, 1383.7274, 1390.6962, 1393.7622, 1413.6547, 1420.7949, 1423.7593, 1440.7788, 1450.8590, 1456.7791, 1457.7606, 1467.7502, 1470.7725, 1488.7212, 1494.7396, 1500.7236, 1512.8181, 1528.6954, 1539.7452, 1556.7537, 1562.7806, 1570.7695, 1574.7894, 1578.7858, 1580.8347, 1592.7883, 1616.7943, 1628.7009, 1628.8575, 1631.8130, 1635.7671, 1642.7155, 1647.8516, 1650.8444, 1657.9042, 1663.8501, 1664.8555, 1693.0090, 1696.8273, 1706.8668, 1707.9615, 1715.8461, 1717.8761, 1718.8779, 1721.9366, 1730.8695, 1741.9938, 1747.9058, 1755.8904, 1758.9028, 1771.0238, 1774.9207, 1784.0254, 1787.8574, 1862.9503, 1870.7574, 1883.7222, 1895.7236, 1897.7325, 1909.7473, 1911.7430, 1913.7366, 1925.7604, 1938.0079, 1961.0325, 1974.8882, 1979.0790, 1981.0576, 2008.9901, 2060.9788, 2067.1584, 2086.1133, 2108.9963, 2112.9805, 2137.1189, 2144.0579, 2180.1812, 2223.1646, 2239.1501, 2241.1355, 2245.8403, 2255.1479, 2265.1016, 2273.0654, 2281.1055, 2318.1824, 2328.9529, 2335.9097, 2342.9673, 2347.8682, 2352.9265, 2356.9395, 2361.8997, 2363.8860, 2366.9265, 2371.0420, 2380.9539, 2389.2756, 2397.2231, 2406.2913, 2414.2468, 2431.2300, 2467.2122, 2474.2263, 2477.2573, 2478.2725, 2485.0691, 2499.1719, 2503.0352, 2518.0303, 2519.9934, 2534.0215, 2541.2542, 2542.2739, 2558.3401, 2580.0227, 2594.0615, 2631.4961, 2678.0144, 2683.4189, 2695.0366, 2701.2097, 2709.0559, 2717.3198, 2723.0757, 2770.4492, 2787.3171, 2799.3379, 2813.4517, 2817.2305, 2833.1284, 2842.5049, 2846.4216, 2872.3596, 2944.3171, 2961.2437, 2975.3135, 2989.3062, 3004.4270, 3029.4883, 3059.4963, 3081.6570, 3147.5710, 3161.1531, 3177.1650, 3185.5222, 3237.6042, 3248.6902, 3254.5940, 3335.7378, 3347.5420, 3351.6597, 3360.7197, 3411.8193, 3428.6296, 3435.7471, 3457.6697, 3471.7146, 3475.7085, 3489.7715, 3503.8445, 3528.3743, 3542.4243, 3552.7373, 3568.9385, 3603.9502, 3625.7847, 3640.7600, 3645.8701, 3691.0452, 3722.5232, 3727.5442, 3731.5757, 3733.5112, 3736.5735, 3741.5391, 3746.5410, 3748.5002, 3751.5356, 3755.5571, 3756.5549, 3763.5049, 3767.5706, 3778.5320, 3884.9326, 3955.6738, 4074.1699, 4149.0591, 4163.0181, 4238.0752, 4300.3838, 4382.1450, 4397.2036, 4441.5176, 4453.2886, 4458.5176, 4464.4150, 4495.2642, 4511.1577, 4692.4893

36. Q2KIL1 **Mass:** 49974 **Score:** 16 **Expect:** 6.2e+002 **Matches:** 11

Uncharacterized protein C1orf141 homolog OS=Bos taurus PE=2 SV=1

Observed	Mr(expt)	Mr(calc)	ppm	Start	End	Miss	Peptide
756.3831	755.3758	755.3609	19.7	154	158	1	R.HQCRR.T
1207.5975	1206.5902	1206.5849	4.42	284	293	1	R.NCEMLKSVAR.D
1393.7622	1392.7549	1392.7626	-5.50	124	134	2	K.SRSIRSFHYLK.D
1467.7502	1466.7429	1466.7591	-11.06	79	90	2	R.YVSFKNMPEPKK.S
1590.7754	1589.7681	1589.8018	-21.16	277	289	1	R.SNVVLQRNCEMLK.S
1663.8501	1662.8428	1662.8617	-11.36	347	360	1	K.LYNFSQTFSSLTKK.F
2112.9805	2111.9732	2111.9946	-10.13	159	177	0	R.TLCSTVFSSVPSNQSHAYK.K
2241.1355	2240.1282	2240.0896	17.3	159	178	1	R.TLCSTVFSSVPSNQSHAYKK.E
2397.2231	2396.2158	2396.1907	10.5	158	178	2	R.RTLCSTVFSSVPSNQSHAYKK.E
2467.2122	2466.2049	2466.1923	5.12	238	258	0	K.NDTNPIMFHEAGYVQMLLLTK.N + 2 Oxidation (M)
2770.4492	2769.4419	2769.3836	21.1	383	406	1	K.MFSTVKPVSEFSASPFKYYSKPSR.N

No match to: 706.2868, 707.2966, 708.3044, 710.3746, 713.3099, 714.3496, 717.4172, 722.4243, 729.4379, 734.4639, 735.4285, 738.4069, 744.3897, 745.4249, 747.4562, 753.3594, 758.3950, 768.4982, 771.3934, 779.4738, 783.3916, 805.4591, 818.4537, 823.4842, 832.3158, 834.3223, 840.4870, 842.5089, 876.2845, 878.2925, 883.4848, 889.5049, 891.4897, 922.4780, 923.5152, 929.4479, 938.4989, 949.5408, 958.4880, 962.3525, 967.4243, 968.4982, 969.5316, 973.5403, 974.4454, 976.4499, 978.5390, 979.3778, 979.5679, 983.6275, 985.6091, 986.5857, 988.3948, 993.5026, 1007.5763, 1017.5743, 1021.5607, 1031.6372, 1034.5828, 1036.5481, 1046.6013, 1051.5096, 1056.5107, 1057.6161, 1074.6067, 1087.5623, 1113.5466, 1123.6021, 1126.4208, 1143.4449, 1153.6440, 1157.4840, 1172.6813, 1178.6198, 1187.7026, 1194.5691, 1215.6563, 1216.4995, 1216.7017, 1248.6692, 1254.6897, 1255.6042, 1257.6483, 1264.7133, 1265.7039, 1269.6589, 1273.6492, 1278.6917, 1284.6440, 1286.7411, 1288.7273, 1299.5450, 1301.5977, 1304.6617, 1314.7190, 1317.7113, 1321.7212, 1338.6622, 1349.6757, 1351.6887, 1354.7229, 1356.7275, 1358.7100, 1371.7595, 1375.7335, 1382.5935, 1383.7274, 1390.6962, 1413.6547, 1420.7949, 1423.7593, 1440.7788, 1450.8590, 1456.7791, 1457.7606, 1470.7725, 1488.7212, 1494.7396, 1500.7236, 1512.8181, 1513.7562, 1528.6954, 1539.7452, 1556.7537, 1562.7806, 1570.7695, 1574.7894, 1578.7858, 1580.8347, 1592.7883, 1616.7943, 1628.7009, 1628.8575, 1631.8130, 1635.7671, 1642.7155, 1647.8516, 1650.8444, 1657.9042, 1664.8555, 1693.0090, 1696.8273, 1706.8668, 1707.9615, 1715.8461, 1717.8761, 1718.8779, 1721.9366, 1730.8695, 1741.9938, 1747.9058, 1755.8904, 1758.9028, 1771.0238, 1774.9207, 1784.0254, 1787.8574, 1862.9503, 1870.7574, 1883.7222, 1895.7236, 1897.7325, 1909.7473, 1911.7430, 1913.7366, 1925.7604, 1938.0079, 1961.0325, 1969.9990, 1974.8882, 1979.0790, 1981.0576, 2008.9901, 2027.1294, 2060.9788, 2067.1584, 2086.1133, 2108.9963, 2137.1189, 2144.0579, 2180.1812, 2223.1646, 2239.1501, 2245.8403, 2255.1479, 2265.1016, 2273.0654, 2281.1055, 2318.1824, 2328.9529, 2335.9097, 2342.9673, 2347.8682, 2352.9265,

2356.9395, 2361.8997, 2363.8860, 2366.9265, 2371.0420, 2380.9539, 2389.2756, 2406.2913, 2414.2468, 2431.2300, 2474.2263, 2477.2573, 2478.2725, 2485.0691, 2499.1719, 2503.0352, 2518.0303, 2519.9934, 2534.0215, 2541.2542, 2542.2739, 2558.3401, 2580.0227, 2594.0615, 2631.4961, 2678.0144, 2683.4189, 2695.0366, 2701.2097, 2709.0559, 2717.3198, 2723.0757, 2787.3171, 2799.3379, 2813.4517, 2817.2305, 2833.1284, 2842.5049, 2846.4216, 2872.3596, 2944.3171, 2961.2437, 2975.3135, 2989.3062, 3004.4270, 3029.4883, 3059.4963, 3081.6570, 3086.5718, 3147.5710, 3157.4875, 3161.1531, 3177.1650, 3185.5222, 3237.6042, 3248.6902, 3254.5940, 3331.5864, 3335.7378, 3347.5420, 3351.6597, 3360.7197, 3411.8193, 3428.6296, 3435.7471, 3457.6697, 3471.7146, 3475.7085, 3489.7715, 3503.8445, 3528.3743, 3542.4243, 3552.7373, 3568.9385, 3603.9502, 3625.7847, 3640.7600, 3645.8701, 3662.8821, 3691.0452, 3722.5232, 3727.5442, 3731.5757, 3733.5112, 3736.5735, 3741.5391, 3746.5410, 3748.5002, 3751.5356, 3755.5571, 3756.5549, 3763.5049, 3767.5706, 3778.5320, 3884.9326, 3955.6738, 4074.1699, 4149.0591, 4163.0181, 4238.0752, 4300.3838, 4382.1450, 4397.2036, 4441.5176, 4453.2886, 4458.5176, 4464.4150, 4495.2642, 4511.1577, 4692.4893

37. F1MNH1 **Mass:** 49975 **Score:** 16 **Expect:** 6.2e+002 **Matches:** 11

Uncharacterized protein OS=Bos taurus GN=C1orf141 PE=4 SV=2

Observed	Mr(expt)	Mr(calc)	ppm	Start	End	Miss	Peptide
756.3831	755.3758	755.3609	19.7	154	158	1	R.HQCRR.T
1207.5975	1206.5902	1206.5849	4.42	284	293	1	R.NCEMLKSVAR.D
1393.7622	1392.7549	1392.7626	-5.50	124	134	2	K.SRSIRSFHYLK.D
1467.7502	1466.7429	1466.7591	-11.06	79	90	2	R.YVSFKNMPEPKK.S
1590.7754	1589.7681	1589.8018	-21.16	277	289	1	R.SNVVLQRNCEMLK.S
1663.8501	1662.8428	1662.8617	-11.36	347	360	1	K.LYNFSQTFSSLTKK.F
2112.9805	2111.9732	2111.9946	-10.13	159	177	0	R.TLCSTVFSSVPSNQSHAYK.K
2241.1355	2240.1282	2240.0896	17.3	159	178	1	R.TLCSTVFSSVPSNQSHAYKK.E
2397.2231	2396.2158	2396.1907	10.5	158	178	2	R.RTLCSTVFSSVPSNQSHAYKK.E
2467.2122	2466.2049	2466.1923	5.12	238	258	0	K.NDTNPIMFHEAGYVQMLLLTK.N + 2 Oxidation (M)
2770.4492	2769.4419	2769.3836	21.1	383	406	1	K.MFSTVKPVSEFSASPFKYYSKPSR.N

No match to: 706.2868, 707.2966, 708.3044, 710.3746, 713.3099, 714.3496, 717.4172, 722.4243, 729.4379, 734.4639, 735.4285, 738.4069, 744.3897, 745.4249, 747.4562, 753.3594, 758.3950, 768.4982, 771.3934, 779.4738, 783.3916, 805.4591, 818.4537, 823.4842, 832.3158, 834.3223, 840.4870, 842.5089, 876.2845, 878.2925, 883.4848, 889.5049, 891.4897, 922.4780, 923.5152, 929.4479, 938.4989, 949.5408, 958.4880, 962.3525, 967.4243, 968.4982, 969.5316, 973.5403, 974.4454, 976.4499, 978.5390, 979.3778, 979.5679, 983.6275, 985.6091, 986.5857, 988.3948, 993.5026, 1007.5763, 1017.5743, 1021.5607, 1031.6372, 1034.5828, 1036.5481, 1046.6013, 1051.5096, 1056.5107, 1057.6161, 1074.6067, 1087.5623, 1113.5466, 1123.6021, 1126.4208, 1143.4449, 1153.6440, 1157.4840, 1172.6813, 1178.6198, 1187.7026, 1194.5691, 1215.6563, 1216.4995, 1216.7017, 1248.6692, 1254.6897, 1255.6042, 1257.6483, 1264.7133, 1265.7039, 1269.6589, 1273.6492, 1278.6917, 1284.6440, 1286.7411, 1288.7273, 1299.5450, 1301.5977, 1304.6617, 1314.7190, 1317.7113, 1321.7212, 1338.6622, 1349.6757, 1351.6887, 1354.7229, 1356.7275, 1358.7100, 1371.7595, 1375.7335, 1382.5935, 1383.7274, 1390.6962, 1413.6547, 1420.7949, 1423.7593, 1440.7788, 1450.8590, 1456.7791, 1457.7606, 1470.7725, 1488.7212, 1494.7396, 1500.7236, 1512.8181, 1513.7562, 1528.6954, 1539.7452, 1556.7537, 1562.7806, 1570.7695, 1574.7894, 1578.7858, 1580.8347, 1592.7883, 1616.7943, 1628.7009, 1628.8575, 1631.8130, 1635.7671, 1642.7155, 1647.8516, 1650.8444, 1657.9042, 1664.8555, 1693.0090, 1696.8273, 1706.8668, 1707.9615, 1715.8461, 1717.8761, 1718.8779, 1721.9366, 1730.8695, 1741.9938, 1747.9058, 1755.8904, 1758.9028, 1771.0238, 1774.9207, 1784.0254, 1787.8574, 1862.9503, 1870.7574, 1883.7222, 1895.7236, 1897.7325, 1909.7473, 1911.7430, 1913.7366, 1925.7604, 1938.0079, 1961.0325, 1969.9990, 1974.8882, 1979.0790, 1981.0576, 2008.9901, 2027.1294, 2060.9788, 2067.1584, 2086.1133, 2108.9963, 2137.1189, 2144.0579, 2180.1812, 2223.1646, 2239.1501, 2245.8403, 2255.1479, 2265.1016, 2273.0654, 2281.1055, 2318.1824, 2328.9529, 2335.9097, 2342.9673, 2347.8682, 2352.9265, 2356.9395, 2361.8997, 2363.8860, 2366.9265, 2371.0420, 2380.9539, 2389.2756, 2406.2913, 2414.2468, 2431.2300, 2474.2263, 2477.2573, 2478.2725, 2485.0691, 2499.1719, 2503.0352, 2518.0303, 2519.9934, 2534.0215, 2541.2542, 2542.2739, 2558.3401, 2580.0227, 2594.0615, 2631.4961, 2678.0144, 2683.4189, 2695.0366, 2701.2097, 2709.0559, 2717.3198, 2723.0757, 2787.3171, 2799.3379, 2813.4517, 2817.2305, 2833.1284, 2842.5049, 2846.4216, 2872.3596, 2944.3171, 2961.2437, 2975.3135, 2989.3062, 3004.4270, 3029.4883, 3059.4963, 3081.6570, 3086.5718, 3147.5710, 3157.4875, 3161.1531, 3177.1650, 3185.5222, 3237.6042, 3248.6902, 3254.5940, 3331.5864, 3335.7378, 3347.5420, 3351.6597, 3360.7197, 3411.8193, 3428.6296, 3435.7471, 3457.6697, 3471.7146, 3475.7085, 3489.7715, 3503.8445, 3528.3743, 3542.4243, 3552.7373, 3568.9385, 3603.9502, 3625.7847, 3640.7600, 3645.8701, 3662.8821, 3691.0452, 3722.5232, 3727.5442, 3731.5757, 3733.5112, 3736.5735, 3741.5391, 3746.5410, 3748.5002, 3751.5356, 3755.5571, 3756.5549, 3763.5049, 3767.5706, 3778.5320, 3884.9326, 3955.6738, 4074.1699, 4149.0591, 4163.0181, 4238.0752, 4300.3838, 4382.1450, 4397.2036, 4441.5176, 4453.2886, 4458.5176, 4464.4150, 4495.2642, 4511.1577, 4692.4893

38. P08220 **Mass:** 54381 **Score:** 16 **Expect:** 6.4e+002 **Matches:** 11

Gamma-aminobutyric acid receptor subunit beta-1 OS=Bos taurus GN=GABRB1 PE=1 SV=1

Observed	Mr(expt)	Mr(calc)	ppm	Start	End	Miss	Peptide
722.4243	721.4170	721.4123	6.55	233	238	0	R.LSLSFR.L
973.5403	972.5331	972.5393	-6.40	129	137	0	K.SFVHGVTVK.N
1187.7026	1186.6953	1186.6961	-0.68	295	304	1	R.ETLPKIPYVK.A
1304.6617	1303.6544	1303.6857	-24.02	417	428	2	R.ALDRHGAHSKGR.I
1371.7595	1370.7522	1370.7783	-19.00	128	139	2	K.KSFVHGVTVKNR.M
1539.7452	1538.7379	1538.7367	0.78	155	167	1	R.ITTTAACMMDLRR.Y
1628.8575	1627.8502	1627.8141	22.2	52	66	0	R.LRPDFGGPPVDVGMR.I + Oxidation (M)
1758.9028	1757.8955	1757.8948	0.41	440	454	1	K.IPDLTDVNSIDKWSR.M
2027.1294	2026.1221	2026.0894	16.1	138	154	2	K.NRMIRLHPDGTVLYGLR.I + Oxidation (M)
3457.6697	3456.6624	3456.5928	20.2	67	95	1	R.IDVASIDMVSEVNMDYTLTMYFQQSWKDK.R
4397.2036	4396.1963	4396.1397	12.9	8	46	2	R.ESLGLLSFPVMIAMVCCAHSANEPSNMSYVKETVDRLLK.G

No match to: 706.2868, 707.2966, 708.3044, 710.3746, 713.3099, 714.3496, 717.4172, 729.4379, 734.4639, 735.4285, 738.4069, 744.3897, 745.4249, 747.4562, 753.3594, 756.3831, 758.3950, 768.4982, 771.3934, 779.4738, 783.3916, 805.4591, 818.4537, 823.4842, 832.3158, 834.3223, 840.4870, 842.5089, 876.2845, 878.2925, 883.4848, 889.5049, 891.4897, 922.4780, 923.5152, 929.4479, 938.4989, 949.5408, 958.4880, 962.3525, 967.4243, 968.4982, 969.5316, 974.4454, 976.4499, 978.5390, 979.3778, 979.5679, 983.6275, 985.6091, 986.5857, 988.3948, 993.5026, 1007.5763, 1017.5743, 1021.5607, 1031.6372, 1034.5828, 1036.5481, 1046.6013, 1051.5096, 1056.5107, 1057.6161, 1074.6067, 1087.5623, 1113.5466, 1123.6021, 1126.4208, 1143.4449, 1153.6440, 1157.4840, 1172.6813, 1178.6198, 1194.5691, 1207.5975, 1215.6563, 1216.4995, 1216.7017, 1248.6692, 1254.6897, 1255.6042, 1257.6483, 1264.7133, 1265.7039, 1269.6589, 1273.6492, 1278.6917, 1284.6440, 1286.7411, 1288.7273, 1299.5450, 1301.5977, 1314.7190, 1317.7113, 1321.7212, 1338.6622, 1349.6757, 1351.6887, 1354.7229, 1356.7275, 1358.7100, 1375.7335, 1382.5935, 1383.7274, 1390.6962, 1393.7622, 1413.6547, 1420.7949, 1423.7593, 1440.7788, 1450.8590, 1456.7791, 1457.7606, 1467.7502, 1470.7725, 1488.7212, 1494.7396, 1500.7236, 1512.8181, 1513.7562, 1528.6954, 1556.7537, 1562.7806, 1570.7695, 1574.7894, 1578.7858, 1580.8347, 1590.7754, 1592.7883, 1616.7943, 1628.7009, 1631.8130, 1635.7671, 1642.7155, 1647.8516, 1650.8444, 1657.9042, 1663.8501, 1664.8555, 1693.0090, 1696.8273, 1706.8668, 1707.9615, 1715.8461, 1717.8761, 1718.8779, 1721.9366, 1730.8695, 1741.9938, 1747.9058, 1755.8904, 1771.0238, 1774.9207, 1784.0254, 1787.8574, 1862.9503, 1870.7574, 1883.7222, 1895.7236, 1897.7325, 1909.7473, 1911.7430, 1913.7366, 1925.7604, 1938.0079, 1961.0325, 1969.9990, 1974.8882, 1979.0790, 1981.0576, 2008.9901, 2060.9788, 2067.1584, 2086.1133, 2108.9963, 2112.9805, 2137.1189, 2144.0579, 2180.1812, 2223.1646, 2239.1501, 2241.1355, 2245.8403, 2255.1479, 2265.1016, 2273.0654, 2281.1055, 2318.1824, 2328.9529, 2335.9097, 2342.9673, 2347.8682, 2352.9265, 2356.9395, 2361.8997, 2363.8860, 2366.9265, 2371.0420, 2380.9539, 2389.2756, 2397.2231, 2406.2913, 2414.2468, 2431.2300, 2467.2122, 2474.2263, 2477.2573, 2478.2725, 2485.0691, 2499.1719, 2503.0352, 2518.0303, 2519.9934, 2534.0215, 2541.2542, 2542.2739, 2558.3401, 2580.0227, 2594.0615, 2631.4961, 2678.0144, 2683.4189, 2695.0366, 2701.2097, 2709.0559, 2717.3198, 2723.0757, 2770.4492, 2787.3171, 2799.3379, 2813.4517, 2817.2305, 2833.1284, 2842.5049, 2846.4216, 2872.3596, 2944.3171, 2961.2437, 2975.3135, 2989.3062, 3004.4270, 3029.4883, 3059.4963, 3081.6570, 3086.5718, 3147.5710, 3157.4875, 3161.1531, 3177.1650, 3185.5222, 3237.6042, 3248.6902, 3254.5940, 3331.5864, 3335.7378, 3347.5420, 3351.6597, 3360.7197, 3411.8193, 3428.6296, 3435.7471, 3471.7146, 3475.7085, 3489.7715, 3503.8445, 3528.3743, 3542.4243, 3552.7373, 3568.9385, 3603.9502, 3625.7847, 3640.7600, 3645.8701, 3662.8821, 3691.0452, 3722.5232, 3727.5442, 3731.5757, 3733.5112, 3736.5735, 3741.5391, 3746.5410, 3748.5002, 3751.5356, 3755.5571, 3756.5549, 3763.5049, 3767.5706, 3778.5320, 3884.9326, 3955.6738, 4074.1699, 4149.0591, 4163.0181, 4238.0752, 4300.3838, 4382.1450, 4441.5176, 4453.2886, 4458.5176, 4464.4150, 4495.2642, 4511.1577, 4692.4893

39. A1A4P3 **Mass:** 13522 **Score:** 16 **Expect:** 6.7e+002 **Matches:** 4

Purkinje cell protein 2 OS=Bos taurus GN=PCP2 PE=2 SV=1

Observed	Mr(expt)	Mr(calc)	ppm	Start		End	Miss	Peptide
708.3044	707.2972	707.2908	8.97	22	-	26	0	R.MEEQR.C + Oxidation (M)
2180.1812	2179.1739	2179.1637	4.69	94	-	114	0	R.AGTLSPQPLLTPQDPAALSFR.R
2683.4189	2682.4116	2682.3800	11.8	68	-	92	2	R.MDDQRVTVSALPGFQPLGPKDGVQK.R
3471.7146	3470.7073	3470.7906	-24.00	94	-	126	2	R.AGTLSPQPLLTPQDPAALSFRRNSSPQPQTQAP.-

No match to: 706.2868, 707.2966, 710.3746, 713.3099, 714.3496, 717.4172, 722.4243, 729.4379, 734.4639, 735.4285, 738.4069, 744.3897, 745.4249, 747.4562, 753.3594, 756.3831, 758.3950, 768.4982, 771.3934, 779.4738, 783.3916, 805.4591, 818.4537, 823.4842, 832.3158, 834.3223, 840.4870, 842.5089, 876.2845, 878.2925, 883.4848, 889.5049, 891.4897, 922.4780, 923.5152, 929.4479, 938.4989, 949.5408, 958.4880, 962.3525, 967.4243, 968.4982, 969.5316, 973.5403, 974.4454, 976.4499, 978.5390, 979.3778, 979.5679, 983.6275, 985.6091, 986.5857, 988.3948, 993.5026, 1007.5763, 1017.5743, 1021.5607, 1031.6372, 1034.5828, 1036.5481, 1046.6013, 1051.5096, 1056.5107, 1057.6161, 1074.6067, 1087.5623, 1113.5466, 1123.6021, 1126.4208, 1143.4449, 1153.6440, 1157.4840, 1172.6813, 1178.6198, 1187.7026, 1194.5691, 1207.5975, 1215.6563, 1216.4995, 1216.7017, 1248.6692, 1254.6897, 1255.6042, 1257.6483, 1264.7133, 1265.7039, 1269.6589, 1273.6492, 1278.6917, 1284.6440, 1286.7411, 1288.7273, 1299.5450, 1301.5977, 1304.6617, 1314.7190, 1317.7113, 1321.7212, 1338.6622, 1349.6757, 1351.6887, 1354.7229, 1356.7275, 1358.7100, 1371.7595, 1375.7335, 1382.5935, 1383.7274, 1390.6962, 1393.7622, 1413.6547, 1420.7949, 1423.7593, 1440.7788, 1450.8590, 1456.7791, 1457.7606, 1467.7502, 1470.7725, 1488.7212, 1494.7396, 1500.7236, 1512.8181, 1513.7562, 1528.6954, 1539.7452, 1556.7537, 1562.7806, 1570.7695, 1574.7894, 1578.7858, 1580.8347, 1590.7754, 1592.7883, 1616.7943, 1628.7009, 1628.8575, 1631.8130, 1635.7671, 1642.7155, 1647.8516, 1650.8444, 1657.9042, 1663.8501, 1664.8555, 1693.0090, 1696.8273, 1706.8668, 1707.9615, 1715.8461, 1717.8761, 1718.8779, 1721.9366, 1730.8695, 1741.9938, 1747.9058, 1755.8904, 1758.9028, 1771.0238, 1774.9207, 1784.0254, 1787.8574, 1862.9503, 1870.7574, 1883.7222, 1895.7236, 1897.7325, 1909.7473, 1911.7430, 1913.7366, 1925.7604, 1938.0079, 1961.0325, 1969.9990, 1974.8882, 1979.0790, 1981.0576, 2008.9901, 2027.1294, 2060.9788, 2067.1584, 2086.1133, 2108.9963, 2112.9805, 2137.1189, 2144.0579, 2223.1646, 2239.1501, 2241.1355, 2245.8403, 2255.1479, 2265.1016, 2273.0654, 2281.1055, 2318.1824, 2328.9529, 2335.9097, 2342.9673, 2347.8682, 2352.9265, 2356.9395, 2361.8997, 2363.8860, 2366.9265, 2371.0420, 2380.9539, 2389.2756, 2397.2231, 2406.2913, 2414.2468, 2431.2300, 2467.2122, 2474.2263, 2477.2573, 2478.2725, 2485.0691, 2499.1719, 2503.0352, 2518.0303, 2519.9934, 2534.0215, 2541.2542, 2542.2739, 2558.3401, 2580.0227, 2594.0615, 2631.4961, 2678.0144, 2695.0366, 2701.2097, 2709.0559, 2717.3198, 2723.0757, 2770.4492, 2787.3171, 2799.3379, 2813.4517, 2817.2305, 2833.1284, 2842.5049, 2846.4216, 2872.3596, 2944.3171, 2961.2437, 2975.3135, 2989.3062, 3004.4270, 3029.4883, 3059.4963, 3081.6570, 3086.5718, 3147.5710, 3157.4875, 3161.1531, 3177.1650, 3185.5222, 3237.6042, 3248.6902, 3254.5940, 3331.5864, 3335.7378, 3347.5420, 3351.6597, 3360.7197, 3411.8193, 3428.6296, 3435.7471, 3457.6697, 3475.7085, 3489.7715, 3503.8445, 3528.3743, 3542.4243, 3552.7373, 3568.9385, 3603.9502, 3625.7847, 3640.7600, 3645.8701, 3662.8821, 3691.0452, 3722.5232, 3727.5442, 3731.5757, 3733.5112, 3736.5735, 3741.5391, 3746.5410, 3748.5002, 3751.5356, 3755.5571, 3756.5549, 3763.5049, 3767.5706, 3778.5320, 3884.9326, 3955.6738, 4074.1699, 4149.0591, 4163.0181, 4238.0752, 4300.3838, 4382.1450, 4397.2036, 4441.5176, 4453.2886, 4458.5176, 4464.4150, 4495.2642, 4511.1577, 4692.4893

40. Q5EA89 **Mass:** 36065 **Score:** 15 **Expect:** 7e+002 **Matches:** 7

Protein FAM76A OS=Bos taurus GN=FAM76A PE=2 SV=1

Observed	Mr(expt)	Mr(calc)	ppm	Start		End	Miss	Peptide
1031.6372	1030.6299	1030.6134	16.0	288	-	297	2	K.QAAALSKSKK.S
1314.7190	1313.7117	1313.6800	24.2	141	-	151	2	K.EQRKHLSSSSR.A
1513.7562	1512.7489	1512.7792	-20.03	231	-	242	1	K.MLHQKDQMILEK.E
1590.7754	1589.7681	1589.8022	-21.44	144	-	157	2	R.KHLSSSSRASHHEK.E
1657.9042	1656.8969	1656.8691	16.8	230	-	242	2	K.KMLHQKDQMILEK.E + Oxidation (M)
2842.5049	2841.4976	2841.4418	19.6	14	-	37	2	R.FPFEALSQGQQLCKECRIAHPVVK.C
3435.7471	3434.7398	3434.6882	15.0	191	-	222	0	K.FESITTNGDSFSPDLALDSPGTDHFVIIAQLK.E

No match to: 706.2868, 707.2966, 708.3044, 710.3746, 713.3099, 714.3496, 717.4172, 722.4243, 729.4379, 734.4639, 735.4285, 738.4069, 744.3897, 745.4249, 747.4562, 753.3594, 756.3831, 758.3950, 768.4982, 771.3934, 779.4738, 783.3916, 805.4591, 818.4537, 823.4842, 832.3158, 834.3223, 840.4870, 842.5089, 876.2845, 878.2925, 883.4848, 889.5049, 891.4897, 922.4780, 923.5152, 929.4479, 938.4989, 949.5408, 958.4880, 962.3525, 967.4243, 968.4982, 969.5316, 973.5403, 974.4454, 976.4499, 978.5390, 979.3778, 979.5679, 983.6275, 985.6091, 986.5857, 988.3948, 993.5026, 1007.5763, 1017.5743, 1021.5607, 1034.5828, 1036.5481, 1046.6013, 1051.5096, 1056.5107, 1057.6161, 1074.6067, 1087.5623, 1113.5466, 1123.6021, 1126.4208, 1143.4449, 1153.6440, 1157.4840, 1172.6813, 1178.6198, 1187.7026, 1194.5691, 1207.5975, 1215.6563, 1216.4995, 1216.7017, 1248.6692, 1254.6897, 1255.6042, 1257.6483, 1264.7133, 1265.7039, 1269.6589, 1273.6492, 1278.6917, 1284.6440, 1286.7411, 1288.7273, 1299.5450, 1301.5977, 1304.6617, 1317.7113, 1321.7212, 1338.6622, 1349.6757, 1351.6887, 1354.7229, 1356.7275, 1358.7100, 1371.7595, 1375.7335, 1382.5935, 1383.7274, 1390.6962, 1393.7622, 1413.6547, 1420.7949, 1423.7593, 1440.7788, 1450.8590, 1456.7791, 1457.7606, 1467.7502, 1470.7725, 1488.7212, 1494.7396, 1500.7236, 1512.8181, 1528.6954, 1539.7452, 1556.7537, 1562.7806, 1570.7695, 1574.7894, 1578.7858, 1580.8347, 1592.7883, 1616.7943, 1628.7009, 1628.8575, 1631.8130, 1635.7671, 1642.7155, 1647.8516, 1650.8444, 1663.8501, 1664.8555, 1693.0090, 1696.8273, 1706.8668, 1707.9615, 1715.8461, 1717.8761, 1718.8779, 1721.9366, 1730.8695, 1741.9938, 1747.9058, 1755.8904, 1758.9028, 1771.0238, 1774.9207, 1784.0254, 1787.8574, 1862.9503, 1870.7574, 1883.7222, 1895.7236, 1897.7325, 1909.7473, 1911.7430, 1913.7366, 1925.7604, 1938.0079, 1961.0325, 1969.9990, 1974.8882, 1979.0790, 1981.0576, 2008.9901, 2027.1294, 2060.9788, 2067.1584, 2086.1133, 2108.9963, 2112.9805, 2137.1189, 2144.0579, 2180.1812, 2223.1646, 2239.1501, 2241.1355, 2245.8403, 2255.1479, 2265.1016, 2273.0654, 2281.1055, 2318.1824, 2328.9529, 2335.9097, 2342.9673, 2347.8682, 2352.9265, 2356.9395, 2361.8997, 2363.8860, 2366.9265, 2371.0420, 2380.9539, 2389.2756, 2397.2231, 2406.2913, 2414.2468, 2431.2300, 2467.2122, 2474.2263, 2477.2573, 2478.2725, 2485.0691, 2499.1719, 2503.0352, 2518.0303, 2519.9934, 2534.0215, 2541.2542, 2542.2739, 2558.3401, 2580.0227, 2594.0615, 2631.4961, 2678.0144, 2683.4189, 2695.0366, 2701.2097, 2709.0559, 2717.3198, 2723.0757, 2770.4492, 2787.3171, 2799.3379, 2813.4517, 2817.2305, 2833.1284, 2846.4216, 2872.3596, 2944.3171, 2961.2437, 2975.3135, 2989.3062, 3004.4270, 3029.4883, 3059.4963, 3081.6570, 3086.5718, 3147.5710, 3157.4875, 3161.1531, 3177.1650, 3185.5222, 3237.6042, 3248.6902, 3254.5940, 3331.5864, 3335.7378, 3347.5420, 3351.6597, 3360.7197, 3411.8193, 3428.6296, 3457.6697, 3471.7146, 3475.7085, 3489.7715, 3503.8445, 3528.3743, 3542.4243, 3552.7373, 3568.9385, 3603.9502, 3625.7847, 3640.7600, 3645.8701, 3662.8821, 3691.0452, 3722.5232, 3727.5442, 3731.5757, 3733.5112, 3736.5735, 3741.5391, 3746.5410, 3748.5002, 3751.5356, 3755.5571, 3756.5549, 3763.5049, 3767.5706, 3778.5320, 3884.9326, 3955.6738, 4074.1699, 4149.0591, 4163.0181, 4238.0752, 4300.3838, 4382.1450, 4397.2036, 4441.5176, 4453.2886, 4458.5176, 4464.4150, 4495.2642, 4511.1577, 4692.4893

41. Q5EA43 **Mass:** 36620 **Score:** 15 **Expect:** 7e+002 **Matches:** 7

Sideroflexin-2 OS=Bos taurus GN=SFXN2 PE=2 SV=2

Observed	Mr(expt)	Mr(calc)	ppm	Start		End	Miss	Peptide
1590.7754	1589.7681	1589.7872	-11.97	42	-	54	1	R.ELDWAKVMVEQSR.M
1718.8779	1717.8706	1717.8940	-13.61	309	-	322	2	K.YREPVPHVYFNKGL.-
1747.9058	1746.8985	1746.9205	-12.61	307	-	320	2	K.AKYREPVPHVYFNK.G
2683.4189	2682.4116	2682.3918	7.38	26	-	47	2	K.HFFNITDPRTVLVPERELDWAK.V
3457.6697	3456.6624	3456.7415	-22.87	136	-	169	1	R.NAASPTSVRQMAVSYITATTAVATAVGMNMLTK.R
3489.7715	3488.7642	3488.7313	9.43	136	-	169	1	R.NAASPTSVRQMAVSYITATTAVATAVGMNMLTK.R + 2 Oxidation (M)
3645.8701	3644.8628	3644.8324	8.34	136	-	170	2	R.NAASPTSVRQMAVSYITATTAVATAVGMNMLTKR.A + 2 Oxidation (M)

No match to: 706.2868, 707.2966, 708.3044, 710.3746, 713.3099, 714.3496, 717.4172, 722.4243, 729.4379, 734.4639, 735.4285, 738.4069, 744.3897, 745.4249, 747.4562, 753.3594, 756.3831, 758.3950, 768.4982, 771.3934, 779.4738, 783.3916, 805.4591, 818.4537, 823.4842, 832.3158, 834.3223, 840.4870, 842.5089, 876.2845, 878.2925, 883.4848, 889.5049, 891.4897, 922.4780, 923.5152, 929.4479, 938.4989, 949.5408, 958.4880, 962.3525, 967.4243, 968.4982, 969.5316, 973.5403, 974.4454, 976.4499, 978.5390, 979.3778, 979.5679, 983.6275, 985.6091, 986.5857, 988.3948, 993.5026, 1007.5763, 1017.5743, 1021.5607, 1031.6372, 1034.5828, 1036.5481, 1046.6013, 1051.5096, 1056.5107, 1057.6161, 1074.6067, 1087.5623, 1113.5466, 1123.6021, 1126.4208, 1143.4449, 1153.6440, 1157.4840, 1172.6813, 1178.6198, 1187.7026, 1194.5691, 1207.5975, 1215.6563, 1216.4995, 1216.7017, 1248.6692, 1254.6897, 1255.6042, 1257.6483, 1264.7133, 1265.7039, 1269.6589, 1273.6492, 1278.6917, 1284.6440, 1286.7411, 1288.7273, 1299.5450, 1301.5977, 1304.6617, 1314.7190, 1317.7113, 1321.7212, 1338.6622, 1349.6757, 1351.6887, 1354.7229, 1356.7275, 1358.7100, 1371.7595, 1375.7335, 1382.5935, 1383.7274, 1390.6962, 1393.7622, 1413.6547, 1420.7949, 1423.7593, 1440.7788, 1450.8590, 1456.7791, 1457.7606, 1467.7502, 1470.7725, 1488.7212, 1494.7396, 1500.7236, 1512.8181, 1513.7562, 1528.6954, 1539.7452, 1556.7537, 1562.7806, 1570.7695, 1574.7894, 1578.7858, 1580.8347, 1592.7883, 1616.7943, 1628.7009, 1628.8575, 1631.8130, 1635.7671, 1642.7155, 1647.8516, 1650.8444, 1657.9042, 1663.8501, 1664.8555, 1693.0090, 1696.8273, 1706.8668, 1707.9615, 1715.8461, 1717.8761, 1721.9366, 1730.8695, 1741.9938, 1755.8904, 1758.9028, 1771.0238, 1774.9207, 1784.0254, 1787.8574, 1862.9503, 1870.7574, 1883.7222, 1895.7236, 1897.7325, 1909.7473, 1911.7430, 1913.7366, 1925.7604, 1938.0079, 1961.0325, 1969.9990, 1974.8882, 1979.0790, 1981.0576, 2008.9901, 2027.1294, 2060.9788, 2067.1584, 2086.1133, 2108.9963, 2112.9805,

2137.1189, 2144.0579, 2180.1812, 2223.1646, 2239.1501, 2241.1355, 2245.8403, 2255.1479, 2265.1016, 2273.0654, 2281.1055, 2318.1824, 2328.9529, 2335.9097, 2342.9673, 2347.8682, 2352.9265, 2356.9395, 2361.8997, 2363.8860, 2366.9265, 2371.0420, 2380.9539, 2389.2756, 2397.2231, 2406.2913, 2414.2468, 2431.2300, 2467.2122, 2474.2263, 2477.2573, 2478.2725, 2485.0691, 2499.1719, 2503.0352, 2518.0303, 2519.9934, 2534.0215, 2541.2542, 2542.2739, 2558.3401, 2580.0227, 2594.0615, 2631.4961, 2678.0144, 2695.0366, 2701.2097, 2709.0559, 2717.3198, 2723.0757, 2770.4492, 2787.3171, 2799.3379, 2813.4517, 2817.2305, 2833.1284, 2842.5049, 2846.4216, 2872.3596, 2944.3171, 2961.2437, 2975.3135, 2989.3062, 3004.4270, 3029.4883, 3059.4963, 3081.6570, 3086.5718, 3147.5710, 3157.4875, 3161.1531, 3177.1650, 3185.5222, 3237.6042, 3248.6902, 3254.5940, 3331.5864, 3335.7378, 3347.5420, 3351.6597, 3360.7197, 3411.8193, 3428.6296, 3435.7471, 3471.7146, 3475.7085, 3503.8445, 3528.3743, 3542.4243, 3552.7373, 3568.9385, 3603.9502, 3625.7847, 3640.7600, 3662.8821, 3691.0452, 3722.5232, 3727.5442, 3731.5757, 3733.5112, 3736.5735, 3741.5391, 3746.5410, 3748.5002, 3751.5356, 3755.5571, 3756.5549, 3763.5049, 3767.5706, 3778.5320, 3884.9326, 3955.6738, 4074.1699, 4149.0591, 4163.0181, 4238.0752, 4300.3838, 4382.1450, 4397.2036, 4441.5176, 4453.2886, 4458.5176, 4464.4150, 4495.2642, 4511.1577, 4692.4893

42. F1MY80 **Mass:** 33505 **Score:** 15 **Expect:** 7.1e+002 **Matches:** 7
Uncharacterized protein OS=Bos taurus GN=HOXC10 PE=4 SV=2

Observed	Mr(expt)	Mr(calc)	ppm	Start	End	Miss	Peptide
805.4591	804.4518	804.4640	-15.07	275	280	2	K.LKKMNR.E + Oxidation (M)
1187.7026	1186.6953	1186.6670	23.9	256	265	1	K.TINLTDRQVK.I
1278.6917	1277.6844	1277.6815	2.29	266	274	2	K.IWFQNRRMK.L
1349.6757	1348.6684	1348.6485	14.7	238	247	0	K.EFLFNMYLTR.E + Oxidation (M)
1774.9207	1773.9134	1773.8984	8.46	238	250	2	K.EFLFNMYLTRERR.L
2241.1355	2240.1282	2240.0920	16.2	156	176	1	K.SDSQTPSPNEIKTEQNLAGPK.G
3471.7146	3470.7073	3470.6565	14.7	72	102	1	R.AKSGPEAALYSHPLQESCLGEHEVPVPSYYR.A

No match to: 706.2868, 707.2966, 708.3044, 710.3746, 713.3099, 714.3496, 717.4172, 722.4243, 729.4379, 734.4639, 735.4285, 738.4069, 744.3897, 745.4249, 747.4562, 753.3594, 756.3831, 758.3950, 768.4982, 771.3934, 779.4738, 783.3916, 818.4537, 823.4842, 832.3158, 834.3223, 840.4870, 842.5089, 876.2845, 878.2925, 883.4848, 889.5049, 891.4897, 922.4780, 923.5152, 929.4479, 938.4989, 949.5408, 958.4880, 962.3525, 967.4243, 968.4982, 969.5316, 973.5403, 974.4454, 976.4499, 978.5390, 979.3778, 979.5679, 983.6275, 985.6091, 986.5857, 988.3948, 993.5026, 1007.5763, 1017.5743, 1021.5607, 1031.6372, 1034.5828, 1036.5481, 1046.6013, 1051.5096, 1056.5107, 1057.6161, 1074.6067, 1087.5623, 1113.5466, 1123.6021, 1126.4208, 1143.4449, 1153.6440, 1157.4840, 1172.6813, 1178.6198, 1194.5691, 1207.5975, 1215.6563, 1216.4995, 1216.7017, 1248.6692, 1254.6897, 1255.6042, 1257.6483, 1264.7133, 1265.7039, 1269.6589, 1273.6492, 1284.6440, 1286.7411, 1288.7273, 1299.5450, 1301.5977, 1304.6617, 1314.7190, 1317.7113, 1321.7212, 1338.6622, 1351.6887, 1354.7229, 1356.7275, 1358.7100, 1371.7595, 1375.7335, 1382.5935, 1383.7274, 1390.6962, 1393.7622, 1413.6547, 1420.7949, 1423.7593, 1440.7788, 1450.8590, 1456.7791, 1457.7606, 1467.7502, 1470.7725, 1488.7212, 1494.7396, 1500.7236, 1512.8181, 1513.7562, 1528.6954, 1539.7452, 1556.7537, 1562.7806, 1570.7695, 1574.7894, 1578.7858, 1580.8347, 1590.7754, 1592.7883, 1616.7943, 1628.7009, 1628.8575, 1631.8130, 1635.7671, 1642.7155, 1647.8516, 1650.8444, 1657.9042, 1663.8501, 1664.8555, 1693.0090, 1696.8273, 1706.8668, 1707.9615, 1715.8461, 1717.8761, 1718.8779, 1721.9366, 1730.8695, 1741.9938, 1747.9058, 1755.8904, 1758.9028, 1771.0238, 1784.0254, 1787.8574, 1862.9503, 1870.7574, 1883.7222, 1895.7236, 1897.7325, 1909.7473, 1911.7430, 1913.7366, 1925.7604, 1938.0079, 1961.0325, 1969.9990, 1974.8882, 1979.0790, 1981.0576, 2008.9901, 2027.1294, 2060.9788, 2067.1584, 2086.1133, 2108.9963, 2112.9805, 2137.1189, 2144.0579, 2180.1812, 2223.1646, 2239.1501, 2245.8403, 2255.1479, 2265.1016, 2273.0654, 2281.1055, 2318.1824, 2328.9529, 2335.9097, 2342.9673, 2347.8682, 2352.9265, 2356.9395, 2361.8997, 2363.8860, 2366.9265, 2371.0420, 2380.9539, 2389.2756, 2397.2231, 2406.2913, 2414.2468, 2431.2300, 2467.2122, 2474.2263, 2477.2573, 2478.2725, 2485.0691, 2499.1719, 2503.0352, 2518.0303, 2519.9934, 2534.0215, 2541.2542, 2542.2739, 2558.3401, 2580.0227, 2594.0615, 2631.4961, 2678.0144, 2683.4189, 2695.0366, 2701.2097, 2709.0559, 2717.3198, 2723.0757, 2770.4492, 2787.3171, 2799.3379, 2813.4517, 2817.2305, 2833.1284, 2842.5049, 2846.4216, 2872.3596, 2944.3171, 2961.2437, 2975.3135, 2989.3062, 3004.4270, 3029.4883, 3059.4963, 3081.6570, 3086.5718, 3147.5710, 3157.4875, 3161.1531, 3177.1650, 3185.5222, 3237.6042, 3248.6902, 3254.5940, 3331.5864, 3335.7378, 3347.5420, 3351.6597, 3360.7197, 3411.8193, 3428.6296, 3435.7471, 3457.6697, 3475.7085, 3489.7715, 3503.8445, 3528.3743, 3542.4243, 3552.7373, 3568.9385, 3603.9502, 3625.7847, 3640.7600, 3645.8701, 3662.8821, 3691.0452, 3722.5232, 3727.5442, 3731.5757, 3733.5112, 3736.5735, 3741.5391, 3746.5410, 3748.5002, 3751.5356, 3755.5571, 3756.5549, 3763.5049, 3767.5706, 3778.5320, 3884.9326, 3955.6738, 4074.1699, 4149.0591, 4163.0181, 4238.0752, 4300.3838, 4382.1450, 4397.2036, 4441.5176, 4453.2886, 4458.5176, 4464.4150, 4495.2642, 4511.1577, 4692.4893

43. A0A140T855 **Mass:** 36964 **Score:** 15 **Expect:** 7.1e+002 **Matches:** 7
Sideroflexin OS=Bos taurus GN=SFXN2 PE=3 SV=1

Observed	Mr(expt)	Mr(calc)	ppm	Start	End	Miss	Peptide
1590.7754	1589.7681	1589.7872	-11.97	45	57	1	R.ELDWAKVMVEQSR.M
1718.8779	1717.8706	1717.8940	-13.61	312	325	2	K.YREPVPHVYFNKGL.-
1747.9058	1746.8985	1746.9205	-12.61	310	323	2	K.AKYREPVPHVYFNK.G
2683.4189	2682.4116	2682.3918	7.38	29	50	2	K.HFFNITDPRTVLVPERELDWAK.V
3457.6697	3456.6624	3456.7415	-22.87	139	172	1	R.NAASPTSVRQMAVSYITATTAVATAVGMNMLTK.R
3489.7715	3488.7642	3488.7313	9.43	139	172	1	R.NAASPTSVRQMAVSYITATTAVATAVGMNMLTK.R + 2 Oxidation (M)
3645.8701	3644.8628	3644.8324	8.34	139	173	2	R.NAASPTSVRQMAVSYITATTAVATAVGMNMLTKR.A + 2 Oxidation (M)

No match to: 706.2868, 707.2966, 708.3044, 710.3746, 713.3099, 714.3496, 717.4172, 722.4243, 729.4379, 734.4639, 735.4285, 738.4069, 744.3897, 745.4249, 747.4562, 753.3594, 756.3831, 758.3950, 768.4982, 771.3934, 779.4738, 783.3916, 805.4591, 818.4537, 823.4842, 832.3158, 834.3223, 840.4870, 842.5089, 876.2845, 878.2925, 883.4848, 889.5049, 891.4897, 922.4780, 923.5152, 929.4479, 938.4989, 949.5408, 958.4880, 962.3525, 967.4243, 968.4982, 969.5316, 973.5403, 974.4454, 976.4499, 978.5390, 979.3778, 979.5679, 983.6275, 985.6091, 986.5857, 988.3948, 993.5026, 1007.5763, 1017.5743, 1021.5607, 1031.6372, 1034.5828, 1036.5481, 1046.6013, 1051.5096, 1056.5107, 1057.6161, 1074.6067, 1087.5623, 1113.5466, 1123.6021, 1126.4208, 1143.4449, 1153.6440, 1157.4840, 1172.6813, 1178.6198, 1187.7026, 1194.5691, 1207.5975, 1215.6563, 1216.4995, 1216.7017, 1248.6692, 1254.6897, 1255.6042, 1257.6483, 1264.7133, 1265.7039, 1269.6589, 1273.6492, 1278.6917, 1284.6440, 1286.7411, 1288.7273, 1299.5450, 1301.5977, 1304.6617, 1314.7190, 1317.7113, 1321.7212, 1338.6622, 1349.6757, 1351.6887, 1354.7229, 1356.7275, 1358.7100, 1371.7595, 1375.7335, 1382.5935, 1383.7274, 1390.6962, 1393.7622, 1413.6547, 1420.7949, 1423.7593, 1440.7788, 1450.8590, 1456.7791, 1457.7606, 1467.7502, 1470.7725, 1488.7212, 1494.7396, 1500.7236, 1512.8181, 1513.7562, 1528.6954, 1539.7452, 1556.7537, 1562.7806, 1570.7695, 1574.7894, 1578.7858, 1580.8347, 1592.7883, 1616.7943, 1628.7009, 1628.8575, 1631.8130, 1635.7671, 1642.7155, 1647.8516, 1650.8444, 1657.9042, 1663.8501, 1664.8555, 1693.0090, 1696.8273, 1706.8668, 1707.9615, 1715.8461, 1717.8761, 1721.9366, 1730.8695, 1741.9938, 1755.8904, 1758.9028, 1771.0238, 1774.9207, 1784.0254, 1787.8574, 1862.9503, 1870.7574, 1883.7222, 1895.7236, 1897.7325, 1909.7473, 1911.7430, 1913.7366, 1925.7604, 1938.0079, 1961.0325, 1969.9990, 1974.8882, 1979.0790, 1981.0576, 2008.9901, 2027.1294, 2060.9788, 2067.1584, 2086.1133, 2108.9963, 2112.9805, 2137.1189, 2144.0579, 2180.1812, 2223.1646, 2239.1501, 2241.1355, 2245.8403, 2255.1479, 2265.1016, 2273.0654, 2281.1055, 2318.1824, 2328.9529, 2335.9097, 2342.9673, 2347.8682, 2352.9265, 2356.9395, 2361.8997, 2363.8860, 2366.9265, 2371.0420, 2380.9539, 2389.2756, 2397.2231, 2406.2913, 2414.2468, 2431.2300, 2467.2122, 2474.2263, 2477.2573, 2478.2725, 2485.0691, 2499.1719, 2503.0352, 2518.0303, 2519.9934, 2534.0215, 2541.2542, 2542.2739, 2558.3401, 2580.0227, 2594.0615, 2631.4961, 2678.0144, 2695.0366, 2701.2097, 2709.0559, 2717.3198, 2723.0757, 2770.4492, 2787.3171, 2799.3379, 2813.4517, 2817.2305, 2833.1284, 2842.5049, 2846.4216, 2872.3596, 2944.3171, 2961.2437, 2975.3135, 2989.3062, 3004.4270, 3029.4883, 3059.4963, 3081.6570, 3086.5718, 3147.5710, 3157.4875, 3161.1531, 3177.1650, 3185.5222, 3237.6042, 3248.6902, 3254.5940, 3331.5864, 3335.7378, 3347.5420, 3351.6597, 3360.7197, 3411.8193, 3428.6296, 3435.7471, 3471.7146, 3475.7085, 3503.8445, 3528.3743, 3542.4243, 3552.7373, 3568.9385, 3603.9502, 3625.7847, 3640.7600, 3662.8821, 3691.0452, 3722.5232, 3727.5442, 3731.5757, 3733.5112, 3736.5735, 3741.5391, 3746.5410, 3748.5002, 3751.5356, 3755.5571, 3756.5549, 3763.5049, 3767.5706, 3778.5320, 3884.9326, 3955.6738, 4074.1699, 4149.0591, 4163.0181, 4238.0752, 4300.3838, 4382.1450, 4397.2036, 4441.5176, 4453.2886, 4458.5176, 4464.4150, 4495.2642, 4511.1577, 4692.4893

44. F1MU12 **Mass:** 53494 **Score:** 15 **Expect:** 7.1e+002 **Matches:** 16
Keratin, type II cytoskeletal 8 OS=Bos taurus GN=KRT8 PE=1 SV=1

Observed	Mr(expt)	Mr(calc)	ppm	Start	End	Miss	Peptide
818.4537	817.4464	817.4294	20.8	461	467	1	K.IETRDGK.L
978.5390	977.5317	977.5328	-1.09	1	8	1	-.MSIRVTQK.S + Oxidation (M)
1021.5607	1020.5534	1020.5637	-10.10	96	103	2	R.TQEKKIMK.S + Oxidation (M)
1215.6563	1214.6490	1214.6619	-10.57	156	165	1	R.RQLETLAQEK.L
1338.6622	1337.6549	1337.6537	0.92	194	204	0	R.TDMENEFVIIK.K
1356.7275	1355.7202	1355.7231	-2.12	1	11	2	-.MSIRVTQKSYK.V + Oxidation (M)

1494.7396	1493.7323	1493.7548	-15.04	193 -	204	1	K.RTDMENEFVIIK.K
1721.9366	1720.9293	1720.9584	-16.89	116 -	129	2	K.VRHLEQQNKVLETK.W
1981.0576	1980.0503	1980.0462	2.07	101 -	117	2	K.IMKSVNQNLATFTDKVR.H + Oxidation (M)
2414.2468	2413.2395	2413.2199	8.15	380 -	399	1	R.EYQELMNVKLALDVEIATYR.K + Oxidation (M)
2474.2263	2473.2190	2473.2594	-16.33	312 -	332	2	K.TEISEMNRNINRLQAEIEGLK.G + Oxidation (M)
2542.2739	2541.2666	2541.3148	-18.96	380 -	400	2	R.EYQELMNVKLALDVEIATYRK.L + Oxidation (M)
3335.7378	3334.7305	3334.6901	12.1	49 -	84	0	R.GGLGTGMSMAGSYGGAPGLGGITAVTVNQSLLSPLK.L + Oxidation (M)
3351.6597	3350.6524	3350.6851	-9.74	49 -	84	0	R.GGLGTGMSMAGSYGGAPGLGGITAVTVNQSLLSPLK.L + 2 Oxidation (M)
4495.2642	4494.2569	4494.1894	15.0	233 -	271	2	R.QLYEEEIREMQSQISDTSVVLSMDNNRNLDLDGIIAEVK.A
4511.1577	4510.1504	4510.1843	-7.51	233 -	271	2	R.QLYEEEIREMQSQISDTSVVLSMDNNRNLDLDGIIAEVK.A + Oxidation (M)

No match to: 706.2868, 707.2966, 708.3044, 710.3746, 713.3099, 714.3496, 717.4172, 722.4243, 729.4379, 734.4639, 735.4285, 738.4069, 744.3897, 745.4249, 747.4562, 753.3594, 756.3831, 758.3950, 768.4982, 771.3934, 779.4738, 783.3916, 805.4591, 823.4842, 832.3158, 834.3223, 840.4870, 842.5089, 876.2845, 878.2925, 883.4848, 889.5049, 891.4897, 922.4780, 923.5152, 929.4479, 938.4989, 949.5408, 958.4880, 962.3525, 967.4243, 968.4982, 969.5316, 973.5403, 974.4454, 976.4499, 979.3778, 979.5679, 983.6275, 985.6091, 986.5857, 988.3948, 993.5026, 1007.5763, 1017.5743, 1031.6372, 1034.5828, 1036.5481, 1046.6013, 1051.5096, 1056.5107, 1057.6161, 1074.6067, 1087.5623, 1113.5466, 1123.6021, 1126.4208, 1143.4449, 1153.6440, 1157.4840, 1172.6813, 1178.6198, 1187.7026, 1194.5691, 1207.5975, 1216.4995, 1216.7017, 1248.6692, 1254.6897, 1255.6042, 1257.6483, 1264.7133, 1265.7039, 1269.6589, 1273.6492, 1278.6917, 1284.6440, 1286.7411, 1288.7273, 1299.5450, 1301.5977, 1304.6617, 1314.7190, 1317.7113, 1321.7212, 1349.6757, 1351.6887, 1354.7229, 1358.7100, 1371.7595, 1375.7335, 1382.5935, 1383.7274, 1390.6962, 1393.7622, 1413.6547, 1420.7949, 1423.7593, 1440.7788, 1450.8590, 1456.7791, 1457.7606, 1467.7502, 1470.7725, 1488.7212, 1500.7236, 1512.8181, 1513.7562, 1528.6954, 1539.7452, 1556.7537, 1562.7806, 1570.7695, 1574.7894, 1578.7858, 1580.8347, 1590.7754, 1592.7883, 1616.7943, 1628.7009, 1628.8575, 1631.8130, 1635.7671, 1642.7155, 1647.8516, 1650.8444, 1657.9042, 1663.8501, 1664.8555, 1693.0090, 1696.8273, 1706.8668, 1707.9615, 1715.8461, 1717.8761, 1718.8779, 1730.8695, 1741.9938, 1747.9058, 1755.8904, 1758.9028, 1771.0238, 1774.9207, 1784.0254, 1787.8574, 1862.9503, 1870.7574, 1883.7222, 1895.7236, 1897.7325, 1909.7473, 1911.7430, 1913.7366, 1925.7604, 1938.0079, 1961.0325, 1969.9990, 1974.8882, 1979.0790, 2008.9901, 2027.1294, 2060.9788, 2067.1584, 2086.1133, 2108.9963, 2112.9805, 2137.1189, 2144.0579, 2180.1812, 2223.1646, 2239.1501, 2241.1355, 2245.8403, 2255.1479, 2265.1016, 2273.0654, 2281.1055, 2318.1824, 2328.9529, 2335.9097, 2342.9673, 2347.8682, 2352.9265, 2356.9395, 2361.8997, 2363.8860, 2366.9265, 2371.0420, 2380.9539, 2389.2756, 2397.2231, 2406.2913, 2431.2300, 2467.2122, 2477.2573, 2478.2725, 2485.0691, 2499.1719, 2503.0352, 2518.0303, 2519.9934, 2534.0215, 2541.2542, 2558.3401, 2580.0227, 2594.0615, 2631.4961, 2678.0144, 2683.4189, 2695.0366, 2701.2097, 2709.0559, 2717.3198, 2723.0757, 2770.4492, 2787.3171, 2799.3379, 2813.4517, 2817.2305, 2833.1284, 2842.5049, 2846.4216, 2872.3596, 2944.3171, 2961.2437, 2975.3135, 2989.3062, 3004.4270, 3029.4883, 3059.4963, 3081.6570, 3086.5718, 3147.5710, 3157.4875, 3161.1531, 3177.1650, 3185.5222, 3237.6042, 3248.6902, 3254.5940, 3331.5864, 3347.5420, 3360.7197, 3411.8193, 3428.6296, 3435.7471, 3457.6697, 3471.7146, 3475.7085, 3489.7715, 3503.8445, 3528.3743, 3542.4243, 3552.7373, 3568.9385, 3603.9502, 3625.7847, 3640.7600, 3645.8701, 3662.8821, 3691.0452, 3722.5232, 3727.5442, 3731.5757, 3733.5112, 3736.5735, 3741.5391, 3746.5410, 3748.5002, 3751.5356, 3755.5571, 3756.5549, 3763.5049, 3767.5706, 3778.5320, 3884.9326, 3955.6738, 4074.1699, 4149.0591, 4163.0181, 4238.0752, 4300.3838, 4382.1450, 4397.2036, 4441.5176, 4453.2886, 4458.5176, 4464.4150, 4692.4893

45. G3N2V0 **Mass:** 18032 **Score:** 15 **Expect:** 7.3e+002 **Matches:** 4

Ras-related protein Rab-5A OS=Bos taurus GN=RAB5A PE=4 SV=1

Observed	Mr(expt)	Mr(calc)	ppm	Start	End	Miss	Peptide
745.4249	744.4176	744.4282	-14.30	57 -	62	1	R.AKNWVK.E
2180.1812	2179.1739	2179.2073	-15.30	67 -	87	2	R.QASPNIVIALSGNKADLANKR.A
3351.6597	3350.6524	3350.6030	14.8	28 -	56	1	R.YHSLAPMYYRGAQAAIVVYDITNEESFAR.A + Oxidation (M)
4397.2036	4396.1963	4396.0933	23.4	1 -	37	2	-.AAFLTQTVCLDDTTVKFEIWDTAGQERYHSLAPMYYR.G

No match to: 706.2868, 707.2966, 708.3044, 710.3746, 713.3099, 714.3496, 717.4172, 722.4243, 729.4379, 734.4639, 735.4285, 738.4069, 744.3897, 745.4249, 747.4562, 753.3594, 756.3831, 758.3950, 768.4982, 771.3934, 779.4738, 783.3916, 805.4591, 818.4537, 823.4842, 832.3158, 834.3223, 840.4870, 842.5089, 876.2845, 878.2925, 883.4848, 889.5049, 891.4897, 922.4780, 923.5152, 929.4479, 938.4989, 949.5408, 958.4880, 962.3525, 967.4243, 968.4982, 969.5316, 973.5403, 974.4454, 976.4499, 978.5390, 979.3778, 979.5679, 983.6275, 985.6091, 986.5857, 988.3948, 993.5026, 1007.5763, 1017.5743, 1021.5607, 1031.6372, 1034.5828, 1036.5481, 1046.6013, 1051.5096, 1056.5107, 1057.6161, 1074.6067, 1087.5623, 1113.5466, 1123.6021, 1126.4208, 1143.4449, 1153.6440, 1157.4840, 1172.6813, 1178.6198, 1187.7026, 1194.5691, 1207.5975, 1215.6563, 1216.4995, 1216.7017, 1248.6692, 1254.6897, 1255.6042, 1257.6483, 1264.7133, 1265.7039, 1269.6589, 1273.6492, 1278.6917, 1284.6440, 1286.7411, 1288.7273, 1299.5450, 1301.5977, 1304.6617, 1314.7190, 1317.7113, 1321.7212, 1338.6622, 1349.6757, 1351.6887, 1354.7229, 1356.7275, 1358.7100, 1371.7595, 1375.7335, 1382.5935, 1383.7274, 1390.6962, 1393.7622, 1413.6547, 1420.7949, 1423.7593, 1440.7788, 1450.8590, 1456.7791, 1457.7606, 1467.7502, 1470.7725, 1488.7212, 1494.7396, 1500.7236, 1512.8181, 1513.7562, 1528.6954, 1539.7452, 1556.7537, 1562.7806, 1570.7695, 1574.7894, 1578.7858, 1580.8347, 1590.7754, 1592.7883, 1616.7943, 1628.7009, 1628.8575, 1631.8130, 1635.7671, 1642.7155, 1647.8516, 1650.8444, 1657.9042, 1663.8501, 1664.8555, 1693.0090, 1696.8273, 1706.8668, 1707.9615, 1715.8461, 1717.8761, 1718.8779, 1721.9366, 1730.8695, 1741.9938, 1747.9058, 1755.8904, 1758.9028, 1771.0238, 1774.9207, 1784.0254, 1787.8574, 1862.9503, 1870.7574, 1883.7222, 1895.7236, 1897.7325, 1909.7473, 1911.7430, 1913.7366, 1925.7604, 1938.0079, 1961.0325, 1969.9990, 1974.8882, 1979.0790, 1981.0576, 2008.9901, 2027.1294, 2060.9788, 2067.1584, 2086.1133, 2108.9963, 2112.9805, 2137.1189, 2144.0579, 2223.1646, 2239.1501, 2241.1355, 2245.8403, 2255.1479, 2265.1016, 2273.0654, 2281.1055, 2318.1824, 2328.9529, 2335.9097, 2342.9673, 2347.8682, 2352.9265, 2356.9395, 2361.8997, 2363.8860, 2366.9265, 2371.0420, 2380.9539, 2389.2756, 2397.2231, 2406.2913, 2414.2468, 2431.2300, 2467.2122, 2474.2263, 2477.2573, 2478.2725, 2485.0691, 2499.1719, 2503.0352, 2518.0303, 2519.9934, 2534.0215, 2541.2542, 2542.2739, 2558.3401, 2580.0227, 2594.0615, 2631.4961, 2678.0144, 2683.4189, 2695.0366, 2701.2097, 2709.0559, 2717.3198, 2723.0757, 2770.4492, 2787.3171, 2799.3379, 2813.4517, 2817.2305, 2833.1284, 2842.5049, 2846.4216, 2872.3596, 2944.3171, 2961.2437, 2975.3135, 2989.3062, 3004.4270, 3029.4883, 3059.4963, 3081.6570, 3086.5718, 3147.5710, 3157.4875, 3161.1531, 3177.1650, 3185.5222, 3237.6042, 3248.6902, 3254.5940, 3331.5864, 3335.7378, 3347.5420, 3360.7197, 3411.8193, 3428.6296, 3435.7471, 3457.6697, 3471.7146, 3475.7085, 3489.7715, 3503.8445, 3528.3743, 3542.4243, 3552.7373, 3568.9385, 3603.9502, 3625.7847, 3640.7600, 3645.8701, 3662.8821, 3691.0452, 3722.5232, 3727.5442, 3731.5757, 3733.5112, 3736.5735, 3741.5391, 3746.5410, 3748.5002, 3751.5356, 3755.5571, 3756.5549, 3763.5049, 3767.5706, 3778.5320, 3884.9326, 3955.6738, 4074.1699, 4149.0591, 4163.0181, 4238.0752, 4300.3838, 4382.1450, 4441.5176, 4453.2886, 4458.5176, 4464.4150, 4495.2642, 4511.1577, 4692.4893

46. G3MWH3 **Mass:** 76263 **Score:** 15 **Expect:** 7.5e+002 **Matches:** 13

Uncharacterized protein OS=Bos taurus GN=PIK3CB PE=3 SV=1

Observed	Mr(expt)	Mr(calc)	ppm	Start	End	Miss	Peptide
993.5026	992.4953	992.4961	-0.72	20 -	28	1	K.STKTMNPSK.Y
1087.5623	1086.5550	1086.5492	5.39	508 -	516	1	R.HSDNIMVKK.T + Oxidation (M)
1113.5466	1112.5393	1112.5549	-14.03	644 -	652	0	K.VNWMAHTVR.K
1248.6692	1247.6619	1247.6366	20.3	399 -	408	0	R.QDMLTLQMLR.L
1257.6483	1256.6410	1256.6448	-3.01	644 -	653	1	K.VNWMAHTVRK.D + Oxidation (M)
1338.6622	1337.6549	1337.6761	-15.87	23 -	33	1	K.TMNPSKYQTIR.K
2060.9788	2059.9715	2060.0043	-15.93	99 -	114	2	K.FPENKKQPYYYPPFDK.S
2180.1812	2179.1739	2179.1203	24.6	399 -	415	1	R.QDMLTLQMLRLMDLLWK.E + 2 Oxidation (M)
2241.1355	2240.1282	2240.1089	8.64	35 -	53	1	K.SGKVHYPVAWVNTMVFDFK.G + Oxidation (M)
2318.1824	2317.1751	2317.1421	14.3	363 -	380	2	K.CYMDSKMKPLWLVYNNK.V
2683.4189	2682.4116	2682.4244	-4.75	370 -	392	1	K.MKPLWLVYNNKVFGEDSVGVIFK.N
3086.5718	3085.5645	3085.5478	5.43	267 -	294	1	R.SEVHIPAVSVQFGVILEAYCRGSVGHMK.A + Oxidation (M)
4074.1699	4073.1626	4073.1320	7.51	435 -	473	1	R.SGLIEVVSTSETIADIQLNSSNVAAAAAFNKDALLNWLK.E

No match to: 706.2868, 707.2966, 708.3044, 710.3746, 713.3099, 714.3496, 717.4172, 722.4243, 729.4379, 734.4639, 735.4285, 738.4069, 744.3897, 745.4249, 747.4562, 753.3594, 756.3831, 758.3950, 768.4982, 771.3934, 779.4738, 783.3916, 805.4591, 818.4537, 823.4842, 832.3158, 834.3223, 840.4870, 842.5089, 876.2845, 878.2925, 883.4848, 889.5049, 891.4897, 922.4780, 923.5152, 929.4479, 938.4989, 949.5408, 958.4880, 962.3525, 967.4243, 968.4982, 969.5316, 973.5403, 974.4454, 976.4499, 978.5390, 979.3778, 979.5679, 983.6275, 985.6091, 986.5857, 988.3948, 1007.5763, 1017.5743, 1021.5607, 1031.6372, 1034.5828, 1036.5481, 1046.6013, 1051.5096, 1056.5107, 1057.6161, 1074.6067, 1123.6021, 1126.4208, 1143.4449, 1153.6440, 1157.4840, 1172.6813, 1178.6198, 1187.7026, 1194.5691, 1207.5975, 1215.6563, 1216.4995, 1216.7017, 1254.6897, 1255.6042, 1264.7133, 1265.7039, 1269.6589, 1273.6492, 1278.6917, 1284.6440, 1286.7411, 1288.7273, 1299.5450, 1301.5977, 1304.6617, 1314.7190, 1317.7113, 1321.7212, 1349.6757, 1351.6887, 1354.7229, 1356.7275,

1358.7100, 1371.7595, 1375.7335, 1382.5935, 1383.7274, 1390.6962, 1393.7622, 1413.6547, 1420.7949, 1423.7593, 1440.7788, 1450.8590, 1456.7791, 1457.7606, 1467.7502, 1470.7725, 1488.7212, 1494.7396, 1500.7236, 1512.8181, 1513.7562, 1528.6954, 1539.7452, 1556.7537, 1562.7806, 1570.7695, 1574.7894, 1578.7858, 1580.8347, 1590.7754, 1592.7883, 1616.7943, 1628.7009, 1628.8575, 1631.8130, 1635.7671, 1642.7155, 1647.8516, 1650.8444, 1657.9042, 1663.8501, 1664.8555, 1693.0090, 1696.8273, 1706.8668, 1707.9615, 1715.8461, 1717.8761, 1718.8779, 1721.9366, 1730.8695, 1741.9938, 1747.9058, 1755.8904, 1758.9028, 1771.0238, 1774.9207, 1784.0254, 1787.8574, 1862.9503, 1870.7574, 1883.7222, 1895.7236, 1897.7325, 1909.7473, 1911.7430, 1913.7366, 1925.7604, 1938.0079, 1961.0325, 1969.9990, 1974.8882, 1979.0790, 1981.0576, 2008.9901, 2027.1294, 2067.1584, 2086.1133, 2108.9963, 2112.9805, 2137.1189, 2144.0579, 2223.1646, 2239.1501, 2245.8403, 2255.1479, 2265.1016, 2273.0654, 2281.1055, 2328.9529, 2335.9097, 2342.9673, 2347.8682, 2352.9265, 2356.9395, 2361.8997, 2363.8860, 2366.9265, 2371.0420, 2380.9539, 2389.2756, 2397.2231, 2406.2913, 2414.2468, 2431.2300, 2467.2122, 2474.2263, 2477.2573, 2478.2725, 2485.0691, 2499.1719, 2503.0352, 2518.0303, 2519.9934, 2534.0215, 2541.2542, 2542.2739, 2558.3401, 2580.0227, 2594.0615, 2631.4961, 2678.0144, 2695.0366, 2701.2097, 2709.0559, 2717.3198, 2723.0757, 2770.4492, 2787.3171, 2799.3379, 2813.4517, 2817.2305, 2833.1284, 2842.5049, 2846.4216, 2872.3596, 2944.3171, 2961.2437, 2975.3135, 2989.3062, 3004.4270, 3029.4883, 3059.4963, 3081.6570, 3147.5710, 3157.4875, 3161.1531, 3177.1650, 3185.5222, 3237.6042, 3248.6902, 3254.5940, 3331.5864, 3335.7378, 3347.5420, 3351.6597, 3360.7197, 3411.8193, 3428.6296, 3435.7471, 3457.6697, 3471.7146, 3475.7085, 3489.7715, 3503.8445, 3528.3743, 3542.4243, 3552.7373, 3568.9385, 3603.9502, 3625.7847, 3640.7600, 3645.8701, 3662.8821, 3691.0452, 3722.5232, 3727.5442, 3731.5757, 3733.5112, 3736.5735, 3741.5391, 3746.5410, 3748.5002, 3751.5356, 3755.5571, 3756.5549, 3763.5049, 3767.5706, 3778.5320, 3884.9326, 3955.6738, 4149.0591, 4163.0181, 4238.0752, 4300.3838, 4382.1450, 4397.2036, 4441.5176, 4453.2886, 4458.5176, 4464.4150, 4495.2642, 4511.1577, 4692.4893

47. E1BDP9 **Mass:** 81899 **Score:** 15 **Expect:** 7.7e+002 **Matches:** 17

Uncharacterized protein OS=Bos taurus GN=CEP63 PE=4 SV=1

Observed	Mr(expt)	Mr(calc)	ppm	Start		End	Miss	Peptide
735.4285	734.4212	734.4327	-15.64	700	-	705	0	K.QFTVLK.-
883.4848	882.4775	882.4559	24.5	103	-	109	0	K.LHEELSR.L
891.4897	890.4824	890.4722	11.4	676	-	682	0	R.SHHILER.L
923.5152	922.5079	922.5236	-16.97	110	-	116	2	R.LKRSYEK.L
985.6091	984.6019	984.6080	-6.21	444	-	451	2	K.RLKAEIQK.A
993.5026	992.4953	992.4961	-0.72	415	-	422	0	K.MEISQLTR.E + Oxidation (M)
1273.6492	1272.6419	1272.6496	-6.02	1	-	11	0	-.MEALLEGIQNR.G
1286.7411	1285.7338	1285.7241	7.56	445	-	455	2	R.LKAEIQKAEEK.A
1317.7113	1316.7040	1316.6837	15.5	151	-	160	2	R.QKSLDWEKQR.L
1706.8668	1705.8595	1705.8999	-23.64	137	-	150	2	R.SEIERLTGKIEEFR.Q
1747.9058	1746.8985	1746.8821	9.38	257	-	270	2	K.ESEKMLEVLQEEKR.G
1981.0576	1980.0503	1980.0172	16.7	475	-	491	1	R.HLSEMVMKLELGLHEAK.E + Oxidation (M)
2467.2122	2466.2049	2466.1982	2.75	353	-	374	1	R.LEGSLESVSATCKQLSQELMEK.Y
2477.2573	2476.2500	2476.2930	-17.34	471	-	491	2	K.LENRHLSEMVMKLELGLHEAK.E
3029.4883	3028.4810	3028.5209	-13.16	397	-	422	2	K.KQILQAEQSYSSVLEGMKMEISQLTR.E + 2 Oxidation (M)
3147.5710	3146.5637	3146.5951	-9.98	509	-	535	2	K.LVSENQQLQKDLMDTKGQLEISTQICK.K
3157.4875	3156.4802	3156.5042	-7.60	12	-	39	1	R.GHDGGFLTSCEAELQELMKQIDIMVAHK.K

No match to: 706.2868, 707.2966, 708.3044, 710.3746, 713.3099, 714.3496, 717.4172, 722.4243, 729.4379, 734.4639, 738.4069, 744.3897, 745.4249, 747.4562, 753.3594, 756.3831, 758.3950, 768.4982, 771.3934, 779.4738, 783.3916, 805.4591, 818.4537, 823.4842, 832.3158, 834.3223, 840.4870, 842.5089, 876.2845, 878.2925, 889.5049, 922.4780, 929.4479, 938.4989, 949.5408, 958.4880, 962.3525, 967.4243, 968.4982, 969.5316, 973.5403, 974.4454, 976.4499, 978.5390, 979.3778, 979.5679, 983.6275, 986.5857, 988.3948, 1007.5763, 1017.5743, 1021.5607, 1031.6372, 1034.5828, 1036.5481, 1046.6013, 1051.5096, 1056.5107, 1057.6161, 1074.6067, 1087.5623, 1113.5466, 1123.6021, 1126.4208, 1143.4449, 1153.6440, 1157.4840, 1172.6813, 1178.6198, 1187.7026, 1194.5691, 1207.5975, 1215.6563, 1216.4995, 1216.7017, 1248.6692, 1254.6897, 1255.6042, 1257.6483, 1264.7133, 1265.7039, 1269.6589, 1278.6917, 1284.6440, 1288.7273, 1299.5450, 1301.5977, 1304.6617, 1314.7190, 1321.7212, 1338.6622, 1349.6757, 1351.6887, 1354.7229, 1356.7275, 1358.7100, 1371.7595, 1375.7335, 1382.5935, 1383.7274, 1390.6962, 1393.7622, 1413.6547, 1420.7949, 1423.7593, 1440.7788, 1450.8590, 1456.7791, 1457.7606, 1467.7502, 1470.7725, 1488.7212, 1494.7396, 1500.7236, 1512.8181, 1513.7562, 1528.6954, 1539.7452, 1556.7537, 1562.7806, 1570.7695, 1574.7894, 1578.7858, 1580.8347, 1590.7754, 1592.7883, 1616.7943, 1628.7009, 1628.8575, 1631.8130, 1635.7671, 1642.7155, 1647.8516, 1650.8444, 1657.9042, 1663.8501, 1664.8555, 1693.0090, 1696.8273, 1707.9615, 1715.8461, 1717.8761, 1718.8779, 1721.9366, 1730.8695, 1741.9938, 1755.8904, 1758.9028, 1771.0238, 1774.9207, 1784.0254, 1787.8574, 1862.9503, 1870.7574, 1883.7222, 1895.7236, 1897.7325, 1909.7473, 1911.7430, 1913.7366, 1925.7604, 1938.0079, 1961.0325, 1969.9990, 1974.8882, 1979.0790, 2008.9901, 2027.1294, 2060.9788, 2067.1584, 2086.1133, 2108.9963, 2112.9805, 2137.1189, 2144.0579, 2180.1812, 2223.1646, 2239.1501, 2241.1355, 2245.8403, 2255.1479, 2265.1016, 2273.0654, 2281.1055, 2318.1824, 2328.9529, 2335.9097, 2342.9673, 2347.8682, 2352.9265, 2356.9395, 2361.8997, 2363.8860, 2366.9265, 2371.0420, 2380.9539, 2389.2756, 2397.2231, 2406.2913, 2414.2468, 2431.2300, 2474.2263, 2478.2725, 2485.0691, 2499.1719, 2503.0352, 2518.0303, 2519.9934, 2534.0215, 2541.2542, 2542.2739, 2558.3401, 2580.0227, 2594.0615, 2631.4961, 2678.0144, 2683.4189, 2695.0366, 2701.2097, 2709.0559, 2717.3198, 2723.0757, 2770.4492, 2787.3171, 2799.3379, 2813.4517, 2817.2305, 2833.1284, 2842.5049, 2846.4216, 2872.3596, 2944.3171, 2961.2437, 2975.3135, 2989.3062, 3004.4270, 3059.4963, 3081.6570, 3086.5718, 3161.1531, 3177.1650, 3185.5222, 3237.6042, 3248.6902, 3254.5940, 3331.5864, 3335.7378, 3347.5420, 3351.6597, 3360.7197, 3411.8193, 3428.6296, 3435.7471, 3457.6697, 3471.7146, 3475.7085, 3489.7715, 3503.8445, 3528.3743, 3542.4243, 3552.7373, 3568.9385, 3603.9502, 3625.7847, 3640.7600, 3645.8701, 3662.8821, 3691.0452, 3722.5232, 3727.5442, 3731.5757, 3733.5112, 3736.5735, 3741.5391, 3746.5410, 3748.5002, 3751.5356, 3755.5571, 3756.5549, 3763.5049, 3767.5706, 3778.5320, 3884.9326, 3955.6738, 4074.1699, 4149.0591, 4163.0181, 4238.0752, 4300.3838, 4382.1450, 4397.2036, 4441.5176, 4453.2886, 4458.5176, 4464.4150, 4495.2642, 4511.1577, 4692.4893

48. E1BKL0 **Mass:** 21110 **Score:** 15 **Expect:** 7.7e+002 **Matches:** 4

Uncharacterized protein OS=Bos taurus PE=4 SV=2

Observed	Mr(expt)	Mr(calc)	ppm	Start		End	Miss	Peptide
2108.9963	2107.9890	2108.0287	-18.81	27	-	44	0	K.VFHQNDAHLSEVQELQSK.R
2255.1479	2254.1406	2254.1198	9.23	127	-	145	2	R.DMVICVSTGRSFRTQQELK.Q
2265.1016	2264.0943	2264.1298	-15.66	27	-	45	1	K.VFHQNDAHLSEVQELQSKR.N
3640.7600	3639.7527	3639.7991	-12.75	13	-	44	1	R.GLGIETGENFLLCKVFHQNDAHLSEVQELQSK.R

No match to: 706.2868, 707.2966, 708.3044, 710.3746, 713.3099, 714.3496, 717.4172, 722.4243, 729.4379, 734.4639, 735.4285, 738.4069, 744.3897, 745.4249, 747.4562, 753.3594, 756.3831, 758.3950, 768.4982, 771.3934, 779.4738, 783.3916, 805.4591, 818.4537, 823.4842, 832.3158, 834.3223, 840.4870, 842.5089, 876.2845, 878.2925, 883.4848, 889.5049, 891.4897, 922.4780, 923.5152, 929.4479, 938.4989, 949.5408, 958.4880, 962.3525, 967.4243, 968.4982, 969.5316, 973.5403, 974.4454, 976.4499, 978.5390, 979.3778, 979.5679, 983.6275, 985.6091, 986.5857, 988.3948, 993.5026, 1007.5763, 1017.5743, 1021.5607, 1031.6372, 1034.5828, 1036.5481, 1046.6013, 1051.5096, 1056.5107, 1057.6161, 1074.6067, 1087.5623, 1113.5466, 1123.6021, 1126.4208, 1143.4449, 1153.6440, 1157.4840, 1172.6813, 1178.6198, 1187.7026, 1194.5691, 1207.5975, 1215.6563, 1216.4995, 1216.7017, 1248.6692, 1254.6897, 1255.6042, 1257.6483, 1264.7133, 1265.7039, 1269.6589, 1273.6492, 1278.6917, 1284.6440, 1286.7411, 1288.7273, 1299.5450, 1301.5977, 1304.6617, 1317.7113, 1314.7190, 1321.7212, 1338.6622, 1349.6757, 1351.6887, 1354.7229, 1356.7275, 1358.7100, 1371.7595, 1375.7335, 1382.5935, 1383.7274, 1390.6962, 1393.7622, 1413.6547, 1420.7949, 1423.7593, 1440.7788, 1450.8590, 1456.7791, 1457.7606, 1467.7502, 1470.7725, 1488.7212, 1494.7396, 1500.7236, 1512.8181, 1513.7562, 1528.6954, 1539.7452, 1556.7537, 1562.7806, 1570.7695, 1574.7894, 1578.7858, 1580.8347, 1590.7754, 1592.7883, 1616.7943, 1628.7009, 1628.8575, 1631.8130, 1635.7671, 1642.7155, 1647.8516, 1650.8444, 1657.9042, 1663.8501, 1664.8555, 1693.0090, 1696.8273, 1706.8668, 1707.9615, 1715.8461, 1717.8761, 1718.8779, 1721.9366, 1730.8695, 1741.9938, 1747.9058, 1755.8904, 1758.9028, 1771.0238, 1774.9207, 1784.0254, 1787.8574, 1862.9503, 1870.7574, 1883.7222, 1895.7236, 1897.7325, 1909.7473, 1911.7430, 1913.7366, 1925.7604, 1938.0079, 1961.0325, 1969.9990, 1974.8882, 1979.0790, 1981.0576, 2008.9901, 2027.1294, 2060.9788, 2067.1584, 2086.1133, 2112.9805, 2137.1189, 2144.0579, 2180.1812, 2223.1646, 2239.1501, 2241.1355, 2245.8403, 2273.0654, 2281.1055, 2318.1824, 2328.9529, 2335.9097, 2342.9673, 2347.8682, 2352.9265, 2356.9395, 2361.8997, 2363.8860, 2366.9265, 2371.0420, 2380.9539, 2389.2756, 2397.2231, 2406.2913, 2414.2468, 2431.2300, 2467.2122, 2474.2263, 2477.2573, 2478.2725, 2485.0691, 2499.1719, 2503.0352, 2518.0303, 2519.9934, 2534.0215, 2541.2542, 2542.2739, 2558.3401, 2580.0227, 2594.0615, 2631.4961, 2678.0144, 2683.4189, 2695.0366, 2701.2097, 2709.0559, 2717.3198, 2723.0757, 2770.4492, 2787.3171, 2799.3379, 2813.4517, 2817.2305, 2833.1284, 2842.5049, 2846.4216, 2872.3596, 2944.3171, 2961.2437, 2975.3135, 2989.3062, 3004.4270, 3029.4883, 3059.4963, 3081.6570, 3086.5718, 3147.5710, 3157.4875, 3161.1531, 3177.1650, 3185.5222, 3237.6042, 3248.6902, 3254.5940, 3331.5864, 3335.7378, 3347.5420, 3351.6597, 3360.7197, 3411.8193, 3428.6296, 3435.7471, 3457.6697, 3471.7146, 3475.7085, 3489.7715, 3503.8445, 3528.3743, 3542.4243, 3552.7373, 3568.9385, 3603.9502, 3625.7847, 3645.8701, 3662.8821, 3691.0452, 3722.5232, 3727.5442, 3731.5757, 3733.5112, 3736.5735, 3741.5391, 3746.5410, 3748.5002, 3751.5356,

3755.5571, 3756.5549, 3763.5049, 3767.5706, 3778.5320, 3884.9326, 3955.6738, 4074.1699, 4149.0591, 4163.0181, 4238.0752, 4300.3838, 4382.1450, 4397.2036, 4441.5176, 4453.2886, 4458.5176, 4464.4150, 4495.2642, 4511.1577, 4692.4893

49. Q2KIB6 **Mass:** 22324 **Score:** 15 **Expect:** 7.7e+002 **Matches:** 7

Protein lin-7 homolog B OS=Bos taurus GN=LIN7B PE=2 SV=1

Observed	Mr(expt)	Mr(calc)	ppm	Start		End	Miss	Peptide
710.3746	709.3673	709.3581	12.9	185	-	189	1	R.FEKMR.S
753.3594	752.3522	752.3639	-15.65	46	-	51	0	R.FCSAIR.E
883.4848	882.4775	882.4923	-16.77	122	-	130	0	R.VIPGGVADR.H
1314.7190	1313.7117	1313.7013	7.94	1	-	12	0	-.MAALVEPLGLER.E + Oxidation (M)
1338.6622	1337.6549	1337.6286	19.7	99	-	111	0	K.TDEGLGFNIMGGK.E
1961.0325	1960.0252	1960.0564	-15.90	169	-	184	2	K.LVVRYTPRVLEEMEAR.F
2542.2739	2541.2666	2541.2169	19.6	99	-	121	1	K.TDEGLGFNIMGGKEQNSPIYISR.V + Oxidation (M)

No match to: 706.2868, 707.2966, 708.3044, 713.3099, 714.3496, 717.4172, 722.4243, 729.4379, 734.4639, 735.4285, 738.4069, 744.3897, 745.4249, 747.4562, 756.3831, 758.3950, 768.4982, 771.3934, 779.4738, 783.3916, 805.4591, 818.4537, 823.4842, 832.3158, 834.3223, 840.4870, 842.5089, 876.2845, 878.2925, 889.5049, 891.4897, 922.4780, 923.5152, 929.4479, 938.4989, 949.5408, 958.4880, 962.3525, 967.4243, 968.4982, 969.5316, 973.5403, 974.4454, 976.4499, 978.5390, 979.3778, 979.5679, 983.6275, 985.6091, 986.5857, 988.3948, 993.5026, 1007.5763, 1017.5743, 1021.5607, 1031.6372, 1034.5828, 1036.5481, 1046.6013, 1051.5096, 1056.5107, 1057.6161, 1074.6067, 1087.5623, 1113.5466, 1123.6021, 1126.4208, 1143.4449, 1153.6440, 1157.4840, 1172.6813, 1178.6198, 1187.7026, 1194.5691, 1207.5975, 1215.6563, 1216.4995, 1216.7017, 1248.6692, 1254.6897, 1255.6042, 1257.6483, 1264.7133, 1265.7039, 1269.6589, 1273.6492, 1278.6917, 1284.6440, 1286.7411, 1288.7273, 1299.5450, 1301.5977, 1304.6617, 1317.7113, 1321.7212, 1349.6757, 1351.6887, 1354.7229, 1356.7275, 1358.7100, 1371.7595, 1375.7335, 1382.5935, 1383.7274, 1390.6962, 1393.7622, 1413.6547, 1420.7949, 1423.7593, 1440.7788, 1450.8590, 1456.7791, 1457.7606, 1467.7502, 1470.7725, 1488.7212, 1494.7396, 1500.7236, 1512.8181, 1513.7562, 1528.6954, 1539.7452, 1556.7537, 1562.7806, 1570.7695, 1574.7894, 1578.7858, 1580.8347, 1590.7754, 1592.7883, 1616.7943, 1628.7009, 1628.8575, 1631.8130, 1635.7671, 1642.7155, 1647.8516, 1650.8444, 1657.9042, 1663.8501, 1664.8555, 1693.0090, 1696.8273, 1706.8668, 1707.9615, 1715.8461, 1717.8761, 1718.8779, 1721.9366, 1730.8695, 1741.9938, 1747.9058, 1755.8904, 1758.9028, 1771.0238, 1774.9207, 1784.0254, 1787.8574, 1862.9503, 1870.7574, 1883.7222, 1895.7236, 1897.7325, 1909.7473, 1911.7430, 1913.7366, 1925.7604, 1938.0079, 1969.9990, 1974.8882, 1979.0790, 1981.0576, 2008.9901, 2027.1294, 2060.9788, 2067.1584, 2086.1133, 2108.9963, 2112.9805, 2137.1189, 2144.0579, 2180.1812, 2223.1646, 2239.1501, 2241.1355, 2245.8403, 2255.1479, 2265.1016, 2273.0654, 2281.1055, 2318.1824, 2328.9529, 2335.9097, 2342.9673, 2347.8682, 2352.9265, 2356.9395, 2361.8997, 2363.8860, 2366.9265, 2371.0420, 2380.9539, 2389.2756, 2397.2231, 2406.2913, 2414.2468, 2431.2300, 2467.2122, 2474.2263, 2477.2573, 2478.2725, 2485.0691, 2499.1719, 2503.0352, 2518.0303, 2519.9934, 2534.0215, 2541.2542, 2558.3401, 2580.0227, 2594.0615, 2631.4961, 2678.0144, 2683.4189, 2695.0366, 2701.2097, 2709.0559, 2717.3198, 2723.0757, 2770.4492, 2787.3171, 2799.3379, 2813.4517, 2817.2305, 2833.1284, 2842.5049, 2846.4216, 2872.3596, 2944.3171, 2961.2437, 2975.3135, 2989.3062, 3004.4270, 3029.4883, 3059.4963, 3081.6570, 3086.5718, 3147.5710, 3157.4875, 3161.1531, 3177.1650, 3185.5222, 3237.6042, 3248.6902, 3254.5940, 3331.5864, 3335.7378, 3347.5420, 3351.6597, 3360.7197, 3411.8193, 3428.6296, 3435.7471, 3457.6697, 3471.7146, 3475.7085, 3489.7715, 3503.8445, 3528.3743, 3542.4243, 3552.7373, 3568.9385, 3603.9502, 3625.7847, 3640.7600, 3645.8701, 3662.8821, 3691.0452, 3722.5232, 3727.5442, 3731.5757, 3733.5112, 3736.5735, 3741.5391, 3746.5410, 3748.5002, 3751.5356, 3755.5571, 3756.5549, 3763.5049, 3767.5706, 3778.5320, 3884.9326, 3955.6738, 4074.1699, 4149.0591, 4163.0181, 4238.0752, 4300.3838, 4382.1450, 4397.2036, 4441.5176, 4453.2886, 4458.5176, 4464.4150, 4495.2642, 4511.1577, 4692.4893

50. E1BAN2 **Mass:** 60255 **Score:** 15 **Expect:** 7.8e+002 **Matches:** 9

Katanin p60 ATPase-containing subunit A-like 2 OS=Bos taurus GN=KATNAL2 PE=3 SV=2

Observed	Mr(expt)	Mr(calc)	ppm	Start		End	Miss	Peptide
1057.6161	1056.6088	1056.5862	21.4	470	-	478	1	R.EAAMRPVRK.I
1172.6813	1171.6740	1171.6601	11.9	285	-	296	0	K.GLLLYGPPGTGK.T
1215.6563	1214.6490	1214.6295	16.1	249	-	259	0	K.WNDIIGLDAAK.Q
1457.7606	1456.7533	1456.7755	-15.21	466	-	477	1	K.LVCREAAMRPVR.K
1562.7806	1561.7733	1561.7823	-5.78	425	-	437	0	R.EAMIHHWLPAVSR.S + Oxidation (M)
3086.5718	3085.5645	3085.6335	-22.35	24	-	50	0	K.NLLILILHYLTQEGYIDAANALEQETK.M
3248.6902	3247.6829	3247.7353	-16.12	231	-	259	2	R.ELAAVVSRDIYLHNPNIKWNDIIGLDAAK.Q
3435.7471	3434.7398	3434.8020	-18.11	330	-	358	2	K.LVRVLFELARYHAPSTIFLDELESVMSQR.G + Oxidation (M)
3471.7146	3470.7073	3470.6525	15.8	340	-	371	1	R.YHAPSTIFLDELESVMSQRGTAPGGEHEGSLR.M

No match to: 706.2868, 707.2966, 708.3044, 710.3746, 713.3099, 714.3496, 717.4172, 722.4243, 729.4379, 734.4639, 735.4285, 738.4069, 744.3897, 745.4249, 747.4562, 753.3594, 756.3831, 758.3950, 768.4982, 771.3934, 779.4738, 783.3916, 805.4591, 818.4537, 823.4842, 832.3158, 834.3223, 840.4870, 842.5089, 876.2845, 878.2925, 883.4848, 889.5049, 891.4897, 922.4780, 923.5152, 929.4479, 938.4989, 949.5408, 958.4880, 962.3525, 967.4243, 968.4982, 969.5316, 973.5403, 974.4454, 976.4499, 978.5390, 979.3778, 979.5679, 983.6275, 985.6091, 986.5857, 988.3948, 993.5026, 1007.5763, 1017.5743, 1021.5607, 1031.6372, 1034.5828, 1036.5481, 1046.6013, 1051.5096, 1056.5107, 1074.6067, 1087.5623, 1113.5466, 1123.6021, 1126.4208, 1143.4449, 1153.6440, 1157.4840, 1178.6198, 1187.7026, 1194.5691, 1207.5975, 1216.4995, 1216.7017, 1248.6692, 1254.6897, 1255.6042, 1257.6483, 1264.7133, 1265.7039, 1269.6589, 1273.6492, 1278.6917, 1284.6440, 1286.7411, 1288.7273, 1299.5450, 1301.5977, 1304.6617, 1314.7190, 1317.7113, 1321.7212, 1338.6622, 1349.6757, 1351.6887, 1354.7229, 1356.7275, 1358.7100, 1371.7595, 1375.7335, 1382.5935, 1383.7274, 1390.6962, 1393.7622, 1413.6547, 1420.7949, 1423.7593, 1440.7788, 1450.8590, 1456.7791, 1467.7502, 1470.7725, 1488.7212, 1494.7396, 1500.7236, 1512.8181, 1513.7562, 1528.6954, 1539.7452, 1556.7537, 1570.7695, 1574.7894, 1578.7858, 1580.8347, 1590.7754, 1592.7883, 1616.7943, 1628.7009, 1628.8575, 1631.8130, 1635.7671, 1642.7155, 1647.8516, 1650.8444, 1657.9042, 1663.8501, 1664.8555, 1693.0090, 1696.8273, 1706.8668, 1707.9615, 1715.8461, 1717.8761, 1718.8779, 1721.9366, 1730.8695, 1741.9938, 1747.9058, 1755.8904, 1758.9028, 1771.0238, 1774.9207, 1784.0254, 1787.8574, 1862.9503, 1870.7574, 1883.7222, 1895.7236, 1897.7325, 1909.7473, 1911.7430, 1913.7366, 1925.7604, 1938.0079, 1961.0325, 1969.9990, 1974.8882, 1979.0790, 1981.0576, 2008.9901, 2027.1294, 2060.9788, 2067.1584, 2086.1133, 2108.9963, 2112.9805, 2137.1189, 2144.0579, 2180.1812, 2223.1646, 2239.1501, 2241.1355, 2245.8403, 2255.1479, 2265.1016, 2273.0654, 2281.1055, 2318.1824, 2328.9529, 2335.9097, 2342.9673, 2347.8682, 2352.9265, 2356.9395, 2361.8997, 2363.8860, 2366.9265, 2371.0420, 2380.9539, 2389.2756, 2397.2231, 2406.2913, 2414.2468, 2431.2300, 2467.2122, 2474.2263, 2477.2573, 2478.2725, 2485.0691, 2499.1719, 2503.0352, 2518.0303, 2519.9934, 2534.0215, 2541.2542, 2542.2739, 2558.3401, 2580.0227, 2594.0615, 2631.4961, 2678.0144, 2683.4189, 2695.0366, 2701.2097, 2709.0559, 2717.3198, 2723.0757, 2770.4492, 2787.3171, 2799.3379, 2813.4517, 2817.2305, 2833.1284, 2842.5049, 2846.4216, 2872.3596, 2944.3171, 2961.2437, 2975.3135, 2989.3062, 3004.4270, 3029.4883, 3059.4963, 3081.6570, 3147.5710, 3157.4875, 3161.1531, 3177.1650, 3185.5222, 3237.6042, 3254.5940, 3331.5864, 3335.7378, 3347.5420, 3351.6597, 3360.7197, 3411.8193, 3428.6296, 3457.6697, 3475.7085, 3489.7715, 3503.8445, 3528.3743, 3542.4243, 3552.7373, 3568.9385, 3603.9502, 3625.7847, 3640.7600, 3645.8701, 3662.8821, 3691.0452, 3722.5232, 3727.5442, 3731.5757, 3733.5112, 3736.5735, 3741.5391, 3746.5410, 3748.5002, 3751.5356, 3755.5571, 3756.5549, 3763.5049, 3767.5706, 3778.5320, 3884.9326, 3955.6738, 4074.1699, 4149.0591, 4163.0181, 4238.0752, 4300.3838, 4382.1450, 4397.2036, 4441.5176, 4453.2886, 4458.5176, 4464.4150, 4495.2642, 4511.1577, 4692.4893

Search Parameters

Type of search : Peptide Mass Fingerprint
Enzyme : Trypsin
Fixed modifications : Carbamidomethyl (C)
Variable modifications : Oxidation (M)
Mass values : Monoisotopic
Protein Mass : Unrestricted
Peptide Mass Tolerance : ± 25 ppm
Peptide Charge State : 1+
Max Missed Cleavages : 2
Number of queries : 315
Selected for scoring : 160

**Mascot:** http://www.matrixscience.com/

MATRIX SCIENCE Mascot Search Results

```
User          : 2F_(L2@52381_05Apr2017)
Email         : proteomics@i3s.up.pt
Search title  : Project: Proteomics, Spot Set: Proteomics\05Apr2017, Label: L2, Spot Id: 227007, Peak List Id: 447668, MS Job Run Id: 316
MS data file  : C:\Documents and Settings\Administrator\Desktop\xu\29JUNHO\Proteomics\05Apr2017\pmf_L2_149875820511.txt
Database      : OrganismSpecie Bos_taurus_Reference_Proteome_2016_06 (24214 sequences; 12839866 residues)
Timestamp     : 29 Jun 2017 at 18:03:10 GMT
Top Score     : 59 for F1MKG2, Uncharacterized protein OS=Bos taurus GN=COL6A2 PE=1 SV=2
```

Mascot Score Histogram

Protein score is -10*Log(P), where P is the probability that the observed match is a random event.
Protein scores greater than 56 are significant (p<0.05).

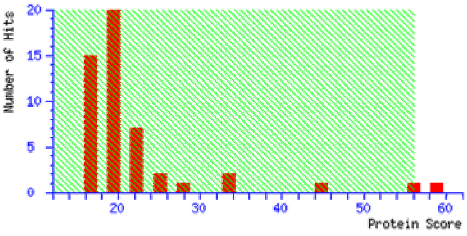

Protein Summary Report

Format As: Protein Summary | Help

Significance threshold p< 0.05 | Max. number of hits 50

Preferred taxonomy: All entries

Re-Search All | Search Unmatched

Index

	Accession	Mass	Score	Description
1.	F1MKG2	110451	59	Uncharacterized protein OS=Bos taurus GN=COL6A2 PE=1 SV=2
2.	E1BI98	109744	57	Uncharacterized protein OS=Bos taurus GN=COL6A1 PE=1 SV=1
3.	Q28024	8166	45	Guanine nucleotide-binding protein G(I)/G(S)/G(O) subunit gamma-12 OS=Bos taurus GN=GNG12 PE=1 SV=2
4.	Q1JQB0	98215	34	Collagen, type VI, alpha 2 OS=Bos taurus GN=COL6A2 PE=1 SV=1
5.	G3MXU3	25162	33	Uncharacterized protein OS=Bos taurus GN=COL6A2 PE=4 SV=1
6.	F1MNG6	140022	26	Uncharacterized protein OS=Bos taurus GN=PPIP5K2 PE=3 SV=2
7.	P21809	41963	26	Biglycan OS=Bos taurus GN=BGN PE=1 SV=3
8.	F1N290	52538	24	Uncharacterized protein OS=Bos taurus PE=4 SV=1
9.	E1BIL2	57570	22	Uncharacterized protein OS=Bos taurus PE=3 SV=2
10.	E1BFZ6	58545	22	Uncharacterized protein OS=Bos taurus GN=FAM126A PE=4 SV=2
11.	Q0P578	24511	22	Estrogen receptor binding site associated, antigen, 9 OS=Bos taurus GN=EBAG9 PE=2 SV=1
12.	F1MDD0	13795	22	Uncharacterized protein OS=Bos taurus GN=PSMG4 PE=4 SV=2
13.	E1BMG0	50437	21	Uncharacterized protein OS=Bos taurus PE=4 SV=2
14.	E1BAL3	14666	21	Uncharacterized protein OS=Bos taurus PE=4 SV=1
15.	A0JN40	89660	21	Kinesin-like protein KIF3C OS=Bos taurus GN=KIF3C PE=2 SV=1
16.	Q0VCX5	49228	21	Eukaryotic peptide chain release factor subunit 1 OS=Bos taurus GN=ETF1 PE=2 SV=3
17.	Q3B7N2	103486	20	Alpha-actinin-1 OS=Bos taurus GN=ACTN1 PE=2 SV=1
18.	P08239	40611	20	Guanine nucleotide-binding protein G(o) subunit alpha OS=Bos taurus GN=GNAO1 PE=1 SV=4
19.	Q5E9I6	20645	20	ADP-ribosylation factor 3 OS=Bos taurus GN=ARF3 PE=2 SV=3
20.	Q5BIS3	9033	20	CDC42 small effector protein 1 OS=Bos taurus GN=CDC42SE1 PE=3 SV=1
21.	G3N2Z1	8956	20	Uncharacterized protein OS=Bos taurus GN=SMIM1 PE=4 SV=1
22.	A2VDZ9	27369	20	Vesicle-associated membrane protein-associated protein B OS=Bos taurus GN=VAPB PE=2 SV=1
23.	F1N091	43136	20	Uncharacterized protein OS=Bos taurus GN=CCDC6 PE=4 SV=1
24.	F1MQD7	47708	20	Uncharacterized protein OS=Bos taurus GN=CIR1 PE=4 SV=2
25.	A4FV58	53257	19	Cytosolic Fe-S cluster assembly factor NARFL OS=Bos taurus GN=NARFL PE=2 SV=2
26.	M5FMT8	53257	19	Cytosolic Fe-S cluster assembly factor NARFL OS=Bos taurus GN=NARFL PE=3 SV=1
27.	A6H762	22782	19	C3H1orf149 protein OS=Bos taurus GN=C3H1orf149 PE=2 SV=1
28.	G3MXC7	12858	19	Uncharacterized protein OS=Bos taurus PE=4 SV=1
29.	Q2TBR9	15151	19	Uncharacterized protein C12orf31 homolog OS=Bos taurus PE=2 SV=1
30.	F1MP35	62777	19	Uncharacterized protein OS=Bos taurus GN=ZNF783 PE=4 SV=2
31.	Q32KZ1	30885	19	Translation initiation factor IF-3, mitochondrial OS=Bos taurus GN=MTIF3 PE=2 SV=1
32.	Q3SZK8	39864	18	Na(+)/H(+) exchange regulatory cofactor NHE-RF1 OS=Bos taurus GN=SLC9A3R1 PE=2 SV=1
33.	Q3ZC37	68867	18	TNFAIP3 interacting protein 1 OS=Bos taurus GN=TNIP1 PE=2 SV=1
34.	Q08DV5	63716	18	E3 ubiquitin-protein ligase RNF220 OS=Bos taurus GN=RNF220 PE=2 SV=1
35.	F1MYL0	63667	18	E3 ubiquitin-protein ligase RNF220 OS=Bos taurus GN=RNF220 PE=4 SV=2
36.	Q3T0J9	65538	18	Guanine nucleotide-binding protein-like 3-like protein OS=Bos taurus GN=GNL3L PE=2 SV=1
37.	G8JKZ5	31228	18	Guanine nucleotide-binding protein G(o) subunit alpha OS=Bos taurus GN=GNAO1 PE=4 SV=1
38.	G3N315	15541	18	Uncharacterized protein OS=Bos taurus PE=4 SV=1
39.	F1MPK4	44043	17	3-hydroxyisobutyryl-CoA hydrolase, mitochondrial OS=Bos taurus GN=HIBCH PE=3 SV=2
40.	F1MRS8	14805	17	Uncharacterized protein OS=Bos taurus GN=FABP9 PE=3 SV=2
41.	F1MN77	21893	17	Uncharacterized protein OS=Bos taurus GN=TFDP2 PE=3 SV=1
42.	Q3T169	26842	17	40S ribosomal protein S3 OS=Bos taurus GN=RPS3 PE=2 SV=1
43.	E1BGJ9	68502	17	Uncharacterized protein OS=Bos taurus GN=CTIF PE=4 SV=2
44.	Q32PB8	9220	17	40S ribosomal protein S21 OS=Bos taurus GN=RPS21 PE=3 SV=1
45.	E1BI02	43291	17	Fibromodulin OS=Bos taurus GN=FMOD PE=4 SV=1
46.	P13605	43410	17	Fibromodulin OS=Bos taurus GN=FMOD PE=1 SV=2
47.	F1MRI8	35973	17	Uncharacterized protein OS=Bos taurus PE=4 SV=1
48.	E1BEI2	25560	17	Uncharacterized protein OS=Bos taurus GN=SPCS2 PE=1 SV=1
49.	G3X7L3	66157	17	Uncharacterized protein OS=Bos taurus GN=DENND6B PE=4 SV=1
50.	F1MM31	177649	17	Uncharacterized protein OS=Bos taurus GN=NCKAP5 PE=4 SV=2

Results List

1. F1MKG2 **Mass:** 110451 **Score:** 59 **Expect:** 0.031 **Matches:** 34

Uncharacterized protein OS=Bos taurus GN=COL6A2 PE=1 SV=2

Observed	Mr(expt)	Mr(calc)	ppm	Start	End	Miss	Peptide
829.4349	828.4277	828.4453	-21.34	204 -	210	0	K.LNEQGLR.D
830.4365	829.4292	829.4406	-13.67	188 -	194	1	R.AREEGIR.L
865.4302	864.4230	864.4341	-12.91	863 -	869	0	R.FVEEVSR.R
972.4810	971.4737	971.4825	-9.04	852 -	859	0	R.LGEQNFHK.V
998.5886	997.5813	997.5960	-14.79	195 -	203	0	R.LFAVPPNLK.L
1021.5605	1020.5532	1020.5352	17.6	863 -	870	1	R.FVEEVSRR.L
1024.5408	1023.5335	1023.5349	-1.34	129 -	137	0	K.SLQSISSFR.R
1043.5085	1042.5012	1042.5043	-2.97	876 -	884	1	R.KDDDPLNAR.V
1057.6062	1056.5989	1056.6080	-8.61	885 -	894	0	R.VALLQFGGPR.E
1074.6005	1073.5932	1073.5982	-4.61	644 -	652	0	K.NFVINVVNR.L
1145.6267	1144.6194	1144.6241	-4.04	996 -	1006	0	K.ISLGDPAAVFR.E
1177.6389	1176.6316	1176.6363	-4.01	862 -	870	2	R.RFVEEVSRR.L
1180.6364	1179.6291	1179.6360	-5.83	129 -	138	1	K.SLQSISSFRR.G
1227.6558	1226.6485	1226.6520	-2.83	852 -	861	1	R.LGEQNFHKVR.R
1328.6610	1327.6537	1327.6520	1.27	211 -	221	0	R.DIANTPHELYR.N
1365.6167	1364.6094	1364.6433	-24.81	743 -	753	1	R.HDPRDDDLNLR.A
1530.6902	1529.6829	1529.6787	2.77	1009 -	1021	0	K.DYDSLAQPGFFDR.F
1706.8651	1705.8578	1705.8384	11.4	453 -	470	1	K.GDPGPEGPRGLAGEVGNK.G
1774.9175	1773.9102	1773.8983	6.74	381 -	398	2	K.GDPGRPGRRGPPGENGAK.G
1787.8247	1786.8174	1786.8162	0.67	1007 -	1021	1	R.EKDYDSLAQPGFFDR.F
1809.0355	1808.0282	1808.0308	-1.44	195 -	210	1	R.LFAVPPNLKLNEQGLR.D
1932.0079	1931.0006	1930.9973	1.70	474 -	493	2	K.GDRGLPGPRGPQGTVGEPGK.Q
2032.0186	2031.0113	2031.0610	-24.46	477 -	497	2	R.GLPGPRGPQGTVGEPGKQGSR.G
2139.0918	2138.0845	2138.0868	-1.08	204 -	221	1	K.LNEQGLRDIANTPHELYR.N
2179.0405	2178.0332	2178.0382	-2.29	104 -	123	0	R.YGGLHFSDLVEVFSPPGSDR.A
2270.1790	2269.1717	2269.2166	-19.77	974 -	995	1	R.KQNVVPTVVAVGSDVDTDVLSK.I
2284.1729	2283.1656	2283.1682	-1.13	160 -	182	0	K.GVVNFAVVITDGHVTGSPCGGIK.L
2423.2490	2422.2417	2422.2353	2.64	733 -	753	2	R.VFAVVITDGRHDPRDDDLNLR.A
2558.3389	2557.3316	2557.3401	-3.33	918 -	941	0	R.YLNSFSHVGTGIVQAINQVVQGAR.A
2713.3413	2712.3340	2712.3184	5.76	104 -	128	1	R.YGGLHFSDLVEVFSPPGSDRASFTK.S
2846.3865	2845.3792	2845.3601	6.72	245 -	269	2	K.VMKHEAYGECYKVSCLEIPGPPGPK.G
3118.6643	3117.6570	3117.6723	-4.90	195 -	221	2	R.LFAVPPNLKLNEQGLRDIANTPHELYR.N
3269.6123	3268.6050	3268.5888	4.96	668 -	696	1	R.VGVVQYSHEGTFEAIQLDDERIDSLSSFK.E
3696.7678	3695.7605	3695.8319	-19.31	668 -	700	2	R.VGVVQYSHEGTFEAIQLDDERIDSLSSFKEAVK.N

No match to: 710.3558, 715.3582, 722.4182, 729.4472, 734.4399, 735.4044, 737.4075, 747.4354, 756.3666, 759.4210, 772.4085, 779.4621, 800.3990, 801.4063, 805.4392, 818.4569, 823.4658, 828.4204, 832.2986, 834.3055, 842.4619, 856.3810, 870.4144, 873.3766, 880.4450, 883.4655, 887.4088, 889.4736, 891.4725, 898.4407, 900.5031, 912.4936, 923.5015, 925.4745, 929.4329, 938.4833, 957.5629, 961.4780, 967.4121, 970.4429, 974.4342, 978.5226, 983.6148, 988.4271, 993.4903, 995.3998, 1006.4926, 1007.5641, 1032.5048, 1034.5706, 1036.5566, 1044.5150, 1046.5917, 1056.5394, 1087.5505, 1113.5382, 1122.5513, 1123.5905, 1130.5541, 1143.4395, 1178.6249, 1184.6396, 1194.5638, 1198.6896, 1216.7015, 1243.6030, 1248.6648, 1254.6876, 1255.5952, 1264.7084, 1269.6405, 1273.6479, 1278.6892, 1281.6271, 1289.6858, 1301.6105, 1304.6399, 1312.7454, 1325.6327, 1326.6665, 1336.7188, 1337.6541, 1338.6735, 1349.6659, 1351.6846, 1356.6124, 1361.6910, 1372.5992, 1382.5925, 1383.7246, 1390.6946, 1420.7117, 1423.6814, 1445.7860, 1456.7703, 1457.7407, 1467.7371, 1470.7511, 1488.7211, 1494.7380, 1497.8768, 1500.7181, 1503.7421, 1513.7554, 1519.7505, 1528.6958, 1540.7820, 1544.8356, 1556.8394, 1562.7800, 1563.7925, 1572.7056, 1578.7816, 1580.8422, 1599.9109, 1616.7953, 1621.7692, 1628.7004, 1628.8589, 1635.7825, 1642.7168, 1647.8525, 1650.8430, 1657.9056, 1666.7905, 1696.8221, 1707.9131, 1717.9056, 1722.9384, 1730.8652, 1738.7958, 1741.9946, 1755.8914, 1758.9082, 1771.0306, 1862.9480, 1883.7240, 1928.0236, 1938.0043, 1940.0157, 1953.8590, 1959.9622, 1961.0220, 1969.9949, 1981.0421, 1990.9496, 1997.8253, 2007.9401, 2011.8949, 2015.9641, 2023.9448, 2027.1273, 2044.0441, 2045.9841, 2060.9731, 2067.1531, 2086.0945, 2088.1196, 2108.9956, 2112.9929, 2114.9592, 2144.0564, 2160.0481, 2174.1157, 2199.1233, 2211.0984, 2229.1216, 2239.1421, 2242.1345, 2245.8398, 2251.1340, 2255.1416, 2265.0903, 2273.0667, 2287.1509, 2293.1445, 2328.9482, 2343.1899, 2352.9153, 2354.9983, 2356.0093, 2364.8684, 2414.2334, 2450.2375, 2466.2253, 2468.2173, 2475.2114, 2486.1069, 2519.9941, 2541.2319, 2542.2505, 2575.1797, 2646.9695, 2660.9880, 2675.0371, 2695.0181, 2702.2866, 2705.2827, 2717.3323, 2718.2998, 2721.2900, 2744.2466, 2763.3267, 2770.3513, 2785.3567, 2787.2734, 2788.2341, 2792.2034, 2798.2407, 2801.2722, 2803.2302, 2808.2080, 2817.2480, 2820.2319, 2833.2026, 2843.3906, 2847.3792, 2870.3926, 2873.3279, 2889.3057, 2892.4070, 2896.4392, 2912.4277, 2915.4314, 2919.4495, 2961.2297, 3071.3845, 3076.4626, 3081.6108, 3087.3884, 3101.4309, 3143.5120, 3159.5193, 3173.5476, 3184.4751, 3236.5454, 3248.6077, 3253.6089, 3265.5996, 3285.5964, 3331.5325, 3334.6331, 3335.6592, 3347.4841, 3397.6499, 3411.6399, 3412.5662, 3427.6182, 3428.5718, 3435.6516, 3444.5942, 3457.6045, 3463.5820, 3473.5864, 3487.6257, 3491.6506, 3503.7678, 3509.5947, 3521.6812, 3552.5681, 3574.6165, 3590.6309, 3624.6504, 3636.7607, 3640.6731, 3646.7363, 3651.7256, 3665.7231, 3669.7129, 3692.6494, 3707.6807, 3714.6301, 3719.6519, 3722.5593, 3723.6516, 3735.6885, 3751.6423, 3830.7644, 3937.7585, 3941.8464, 3942.8328, 3955.8433, 4092.8938, 4147.8613, 4162.9160, 4254.9053, 4272.9224, 4290.9102, 4306.9575, 4309.8506, 4323.8926, 4397.9277, 4415.8896, 4449.1777, 4455.1221, 4459.2368, 4494.0420, 4501.0767, 4510.0269, 4525.1040, 4530.0112, 4839.1138, 4846.0161

2. E1BI98 **Mass:** 109744 **Score:** 57 **Expect:** 0.044 **Matches:** 39

Uncharacterized protein OS=Bos taurus GN=COL6A1 PE=1 SV=1

Observed	Mr(expt)	Mr(calc)	ppm	Start	End	Miss	Peptide
801.4063	800.3990	800.4140	-18.83	957 -	963	0	K.AVQEAQR.A
887.4088	886.4015	886.4145	-14.60	864 -	871	0	R.TDPGQDVR.V
957.5629	956.5556	956.5655	-10.33	723 -	731	0	R.IALVITDGR.S
978.5226	977.5154	977.5043	11.3	976 -	983	0	R.QVNEPHVR.V
1006.4926	1005.4853	1005.4879	-2.61	69 -	76	0	R.FIDNLNDR.Y
1021.5605	1020.5532	1020.5716	-18.01	714 -	722	0	R.LLPPTPNNR.I
1032.5048	1031.4975	1031.5070	-9.15	106 -	114	1	R.MPSGRDELK.S
1046.5917	1045.5844	1045.5920	-7.26	1005 -	1013	0	R.VPSYQALLR.G
1056.5394	1055.5321	1055.5400	-7.47	1014 -	1022	0	R.GVFYQTVSR.K
1178.6249	1177.6176	1177.6428	-21.41	431 -	442	2	R.GPRGTPGARGPR.G
1184.6396	1183.6323	1183.6350	-2.23	1014 -	1023	1	R.GVFYQTVSRK.V
1216.7015	1215.6942	1215.6976	-2.75	964 -	975	0	R.AGVEIFAVVVGR.Q
1243.6030	1242.5957	1242.5993	-2.84	925 -	935	1	R.FYREASSNAAK.K
1255.5952	1254.5879	1254.5881	-0.11	990 -	1000	0	K.AAEYDVVFGER.H
1264.7084	1263.7011	1263.7047	-2.86	712 -	722	1	R.SRLLPPTPNNR.I
1289.6858	1288.6785	1288.6775	0.77	200 -	210	0	R.LSIIATDHTYR.R
1445.7860	1444.7787	1444.7786	0.06	200 -	211	1	R.LSIIATDHTYRR.N
1488.7211	1487.7138	1487.7157	-1.26	69 -	79	1	R.FIDNLNDRYYR.C
1544.8356	1543.8283	1543.8318	-2.26	723 -	736	1	R.IALVITDGRSDTQR.D
1562.7800	1561.7727	1561.7849	-7.78	858 -	871	1	R.FLTASRTDPGQDVR.V

1580.8422	1579.8349	1579.8358	-0.58	186 -	199	0	K.VFSVAITPDHLEPR.L
1599.9109	1598.9036	1598.9045	-0.55	1001 -	1013	1	R.HLFRVPSYQALLR.G
1722.9384	1721.9311	1721.9312	-0.03	136 -	151	1	K.GLEELLVGGSHLKENK.Y
1774.9175	1773.9102	1773.9122	-1.11	872 -	887	0	R.VAVVQYSGTGQQRPER.A
1932.0079	1931.0006	1931.0000	0.33	938 -	956	0	R.LLLFSDGNSQGATPAAIEK.A
1997.8253	1996.8180	1996.8190	-0.47	241 -	256	0	K.NNVEQVCCSFECQPAR.G
2015.9641	2014.9568	2014.9571	-0.14	694 -	711	0	K.LQWMGGGTFTGEALQYTR.S
2032.0186	2031.0113	2031.0497	-18.92	854 -	871	2	R.LAERFLTASRTDPGQDVR.V
2088.1196	2087.1123	2087.1011	5.38	937 -	956	1	K.RLLLFSDGNSQGATPAAIEK.A
2144.0564	2143.0491	2143.0521	-1.37	693 -	711	1	K.KLQWMGGGTFTGEALQYTR.S
2160.0481	2159.0408	2159.0470	-2.85	693 -	711	1	K.KLQWMGGGTFTGEALQYTR.S + Oxidation (M)
2199.1233	2198.1160	2198.1120	1.84	83 -	101	0	R.NLVWNAGALHYSDEVEIIR.G
2713.3413	2712.3340	2712.3469	-4.76	28 -	51	0	R.AIAFQDCPVDLFFVLDTSESVALR.L
3159.5193	3158.5120	3158.5230	-3.48	152 -	180	1	K.YLVVVTDGHPLEGYKEPCGGLEDAVNEAK.H
3269.6123	3268.6050	3268.5645	12.4	572 -	602	1	K.GYRGPEGPQGPPGHVGPPGPDECEILDIIMK.M
3285.5964	3284.5891	3284.5594	9.04	572 -	602	1	K.GYRGPEGPQGPPGHVGPPGPDECEILDIIMK.M + Oxidation (M)
3411.6399	3410.6326	3410.6412	-2.52	211 -	240	2	R.RNFTAADWGQSRDAEEVISQTIDTITDMIK.N
3427.6182	3426.6109	3426.6189	-2.33	443 -	478	2	R.GDPGEAGPQGDQGREGPVGVPGDPGEAGPIGPKGYR.G
3941.8464	3940.8391	3940.8612	-5.60	814 -	849	1	K.CPDYSCPITFSSPADITILLDGSASVGSHNFDITKR.F

No match to: 710.3558, 715.3582, 722.4182, 729.4472, 734.4399, 735.4044, 737.4075, 747.4354, 756.3666, 759.4210, 772.4085, 779.4621, 800.3990, 805.4392, 818.4569, 823.4658, 828.4204, 829.4349, 830.4365, 832.2986, 834.3055, 842.4619, 856.3810, 865.4302, 870.4144, 873.3766, 880.4450, 883.4655, 889.4736, 891.4725, 898.4407, 900.5031, 912.4936, 923.5015, 925.4745, 929.4329, 938.4833, 961.4780, 967.4121, 970.4429, 972.4810, 974.4342, 983.6148, 988.4271, 993.4903, 995.3998, 998.5886, 1007.5641, 1024.5408, 1034.5706, 1036.5566, 1043.5085, 1044.5150, 1057.6062, 1074.6005, 1087.5505, 1113.5382, 1122.5513, 1123.5905, 1130.5541, 1143.4395, 1145.6267, 1177.6389, 1180.6364, 1194.5638, 1198.6896, 1227.6558, 1248.6648, 1254.6876, 1269.6405, 1273.6479, 1278.6892, 1281.6271, 1301.6105, 1304.6399, 1312.7454, 1325.6327, 1326.6665, 1328.6610, 1336.7188, 1337.6541, 1338.6735, 1349.6659, 1351.6846, 1356.6124, 1361.6910, 1365.6167, 1372.5992, 1382.5925, 1383.7246, 1390.6946, 1420.7117, 1423.6814, 1456.7703, 1457.7407, 1467.7371, 1470.7511, 1494.7380, 1497.8768, 1500.7181, 1503.7421, 1513.7554, 1519.7505, 1528.6958, 1530.6902, 1540.7820, 1556.8394, 1563.7925, 1572.7056, 1578.7816, 1616.7953, 1621.7692, 1628.7004, 1628.8589, 1635.7825, 1642.7168, 1647.8525, 1650.8430, 1657.9056, 1666.7905, 1696.8221, 1706.8651, 1707.9131, 1717.9056, 1730.8652, 1738.7958, 1741.9946, 1755.8914, 1758.9082, 1771.0306, 1787.8247, 1809.0355, 1862.9480, 1883.7240, 1928.0236, 1938.0043, 1940.0157, 1953.8590, 1959.9622, 1961.0220, 1969.9949, 1981.0421, 1990.9496, 2007.9401, 2011.8949, 2023.9448, 2027.1273, 2044.0441, 2045.9841, 2060.9731, 2067.1531, 2086.0945, 2108.9956, 2112.9929, 2114.9592, 2139.0918, 2174.1157, 2179.0405, 2211.0984, 2229.1216, 2239.1421, 2242.1345, 2245.8398, 2251.1340, 2255.1416, 2265.0903, 2270.1790, 2273.0667, 2284.1729, 2287.1509, 2293.1445, 2328.9482, 2343.1899, 2352.9153, 2354.9983, 2356.0093, 2364.8684, 2414.2334, 2423.2490, 2450.2375, 2466.2253, 2468.2173, 2475.2114, 2486.1069, 2519.9941, 2541.2319, 2542.2505, 2558.3389, 2575.1797, 2646.9695, 2660.9880, 2675.0371, 2695.0181, 2702.2866, 2705.2827, 2717.3323, 2718.2998, 2721.2900, 2744.2466, 2763.3267, 2770.3513, 2785.3567, 2787.2734, 2788.2341, 2792.2034, 2798.2407, 2801.2722, 2803.2302, 2808.2080, 2817.2480, 2820.2319, 2833.2026, 2843.3906, 2846.3865, 2847.3792, 2870.3926, 2873.3279, 2889.3057, 2892.4070, 2896.4392, 2912.4277, 2915.4314, 2919.4495, 2961.2297, 3071.3845, 3076.4626, 3081.6108, 3087.3884, 3101.4309, 3118.6643, 3143.5120, 3173.5476, 3184.4751, 3236.5454, 3248.6077, 3253.6089, 3265.5996, 3331.5325, 3334.6331, 3335.6592, 3347.4841, 3397.6499, 3412.5662, 3428.5718, 3435.6516, 3444.5942, 3457.6045, 3463.5820, 3473.5864, 3487.6257, 3491.6506, 3503.7678, 3509.5947, 3521.6812, 3552.5681, 3574.6165, 3590.6309, 3624.6504, 3636.7607, 3640.6731, 3646.7363, 3651.7256, 3665.7231, 3669.7129, 3692.6494, 3696.7678, 3707.6807, 3714.6301, 3719.6519, 3722.5593, 3723.6516, 3735.6885, 3751.6423, 3830.7644, 3937.7585, 3942.8328, 3955.8433, 4092.8938, 4147.8613, 4162.9160, 4254.9053, 4272.9224, 4290.9102, 4306.9575, 4309.8506, 4323.8926, 4397.9277, 4415.8896, 4449.1777, 4455.1221, 4459.2368, 4494.0420, 4501.0767, 4510.0269, 4525.1040, 4530.0112, 4839.1138, 4846.0161

3. Q28024 **Mass:** 8166 **Score:** 45 **Expect:** 0.78 **Matches:** 10

Guanine nucleotide-binding protein G(I)/G(S)/G(O) subunit gamma-12 OS=Bos taurus GN=GNG12 PE=1 SV=2

Observed	Mr(expt)	Mr(calc)	ppm	Start	End	Miss	Peptide
735.4044	734.3971	734.3997	-3.42	67 -	72	1	K.KTCTIL.-
978.5226	977.5154	977.5216	-6.34	65 -	72	2	K.DKKTCTIL.-
1953.8590	1952.8517	1952.8720	-10.39	32 -	48	1	K.VSKASADLMSYCEEHAR.N
2015.9641	2014.9568	2015.0034	-23.09	49 -	66	1	R.NDPLLMGIPTSENPFKDK.K
2032.0186	2031.0113	2030.9983	6.42	49 -	66	1	R.NDPLLMGIPTSENPFKDK.K + Oxidation (M)
2144.0564	2143.0491	2143.0983	-22.95	49 -	67	2	R.NDPLLMGIPTSENPFKDKK.T
2160.0481	2159.0408	2159.0932	-24.27	49 -	67	2	R.NDPLLMGIPTSENPFKDKK.T + Oxidation (M)
2211.0984	2210.0911	2210.0459	20.4	30 -	48	2	R.IKVSKASADLMSYCEEHAR.N + Oxidation (M)
3636.7607	3635.7534	3635.6694	23.1	35 -	66	2	K.ASADLMSYCEEHARNDPLLMGIPTSENPFKDK.K
3707.6807	3706.6734	3706.7429	-18.74	32 -	64	2	K.VSKASADLMSYCEEHARNDPLLMGIPTSENPFK.D

No match to: 710.3558, 715.3582, 722.4182, 729.4472, 734.4399, 737.4075, 747.4354, 756.3666, 759.4210, 772.4085, 779.4621, 800.3990, 801.4063, 805.4392, 818.4569, 823.4658, 828.4204, 829.4349, 830.4365, 832.2986, 834.3055, 842.4619, 856.3810, 865.4302, 870.4144, 873.3766, 880.4450, 883.4655, 887.4088, 889.4736, 891.4725, 898.4407, 900.5031, 912.4936, 923.5015, 925.4745, 929.4329, 938.4833, 957.5629, 961.4780, 967.4121, 970.4429, 972.4810, 974.4342, 983.6148, 988.4271, 993.4903, 995.3998, 998.5886, 1006.4926, 1007.5641, 1021.5605, 1024.5408, 1032.5048, 1034.5706, 1036.5566, 1043.5085, 1044.5150, 1046.5917, 1056.5394, 1057.6062, 1074.6005, 1087.5505, 1113.5382, 1122.5513, 1123.5905, 1130.5541, 1143.4395, 1145.6267, 1177.6389, 1178.6249, 1180.6364, 1184.6396, 1194.5638, 1198.6896, 1216.7015, 1227.6558, 1243.6030, 1248.6648, 1254.6876, 1255.5952, 1264.7084, 1269.6405, 1273.6479, 1278.6892, 1281.6271, 1289.6858, 1301.6105, 1304.6399, 1312.7454, 1325.6327, 1326.6665, 1328.6610, 1336.7188, 1337.6541, 1338.6735, 1349.6659, 1351.6846, 1356.6124, 1361.6910, 1365.6167, 1372.5992, 1382.5925, 1383.7246, 1390.6946, 1420.7117, 1423.6814, 1445.7860, 1456.7703, 1457.7407, 1467.7371, 1470.7511, 1488.7211, 1494.7380, 1497.8768, 1500.7181, 1503.7421, 1513.7554, 1519.7505, 1528.6958, 1530.6902, 1540.7820, 1544.8356, 1556.8394, 1562.7800, 1563.7925, 1572.7056, 1578.7816, 1580.8422, 1599.9109, 1616.7953, 1621.7692, 1628.7004, 1628.8589, 1635.7825, 1642.7168, 1647.8525, 1650.8430, 1657.9056, 1666.7905, 1696.8221, 1706.8651, 1707.9131, 1717.9056, 1722.9384, 1730.8652, 1738.7958, 1741.9946, 1755.8914, 1758.9082, 1771.0306, 1774.9175, 1787.8247, 1809.0355, 1862.9480, 1883.7240, 1928.0236, 1932.0079, 1938.0043, 1940.0157, 1959.9622, 1961.0220, 1969.9949, 1981.0421, 1990.9496, 1997.8253, 2007.9401, 2011.8949, 2023.9448, 2027.1273, 2044.0441, 2045.9841, 2060.9731, 2067.1531, 2086.0945, 2088.1196, 2108.9956, 2112.9929, 2114.9592, 2139.0918, 2174.1157, 2179.0405, 2199.1233, 2229.1216, 2239.1421, 2242.1345, 2245.8398, 2251.1340, 2255.1416, 2265.0903, 2270.1790, 2273.0667, 2284.1729, 2287.1509, 2293.1445, 2328.9482, 2343.1899, 2352.9153, 2354.9983, 2356.0093, 2364.8684, 2414.2334, 2423.2490, 2450.2375, 2466.2253, 2468.2173, 2475.2114, 2486.1069, 2519.9941, 2541.2319, 2542.2505, 2558.3389, 2575.1797, 2646.9695, 2660.9880, 2675.0371, 2695.0181, 2702.2866, 2705.2827, 2713.3413, 2717.3323, 2718.2998, 2721.2900, 2744.2466, 2763.3267, 2770.3513, 2785.3567, 2787.2734, 2788.2341, 2792.2034, 2798.2407, 2801.2722, 2803.2302, 2808.2080, 2817.2480, 2820.2319, 2833.2026, 2843.3906, 2846.3865, 2847.3792, 2870.3926, 2873.3279, 2889.3057, 2892.4070, 2896.4392, 2912.4277, 2915.4314, 2919.4495, 2961.2297, 3071.3845, 3076.4626, 3081.6108, 3087.3884, 3101.4309, 3118.6643, 3143.5120, 3159.5193, 3173.5476, 3184.4751, 3236.5454, 3248.6077, 3253.6089, 3265.5996, 3269.6123, 3285.5964, 3331.5325, 3334.6331, 3335.6592, 3347.4841, 3397.6499, 3411.6399, 3412.5662, 3427.6182, 3428.5718, 3435.6516, 3444.5942, 3457.6045, 3463.5820, 3473.5864, 3487.6257, 3491.6506, 3503.7678, 3509.5947, 3521.6812, 3552.5681, 3574.6165, 3590.6309, 3624.6504, 3640.6731, 3646.7363, 3651.7256, 3665.7231, 3669.7129, 3692.6494, 3696.7678, 3714.6301, 3719.6519, 3722.5593, 3723.6516, 3735.6885, 3751.6423, 3830.7644, 3937.7585, 3941.8464, 3942.8328, 3955.8433, 4092.8938, 4147.8613, 4162.9160, 4254.9053, 4272.9224, 4290.9102, 4306.9575, 4309.8506, 4323.8926, 4397.9277, 4415.8896, 4449.1777, 4455.1221, 4459.2368, 4494.0420, 4501.0767, 4510.0269, 4525.1040, 4530.0112, 4839.1138, 4846.0161

4. Q1JQB0 **Mass:** 98215 **Score:** 34 **Expect:** 8.8 **Matches:** 24

Collagen, type VI, alpha 2 OS=Bos taurus GN=COL6A2 PE=1 SV=1

Observed	Mr(expt)	Mr(calc)	ppm	Start	End	Miss	Peptide
829.4349	828.4277	828.4453	-21.34	195 -	201	0	K.LNEQGLR.D
830.4365	829.4292	829.4406	-13.67	179 -	185	1	R.AREEGIR.L
993.4903	992.4830	992.5039	-21.06	880 -	888	1	R.AKFATGNER.Q

998.5886	997.5813	997.5960	-14.79	186 -	194	0	R.LFAVPPNLK.L
1024.5408	1023.5335	1023.5349	-1.34	120 -	128	0	K.SLQSISSFR.R
1074.6005	1073.5932	1073.5982	-4.61	635 -	643	0	K.NFVINVVNR.L
1180.6364	1179.6291	1179.6360	-5.83	120 -	129	1	K.SLQSISSFRR.G
1328.6610	1327.6537	1327.6520	1.27	202 -	212	0	R.DIANTPHELYR.N
1365.6167	1364.6094	1364.6433	-24.81	734 -	744	1	R.HDPRDDDLNLR.A
1530.6902	1529.6829	1529.6643	12.2	236 -	247	1	K.VMKHEAYGECYK.V + Oxidation (M)
1706.8651	1705.8578	1705.8384	11.4	444 -	461	1	K.GDPGPEGPRGLAGEVGNK.G
1774.9175	1773.9102	1773.8983	6.74	372 -	389	2	K.GDPGRPGRRGPPGENGAK.G
1809.0355	1808.0282	1808.0308	-1.44	186 -	201	1	R.LFAVPPNLKLNEQGLR.D
1932.0079	1931.0006	1930.9973	1.70	465 -	484	2	K.GDRGLPGPRGPQGTVGEPGK.Q
2032.0186	2031.0113	2031.0610	-24.46	468 -	488	2	R.GLPGPRGPQGTVGEPGKQGSR.G
2139.0918	2138.0845	2138.0868	-1.08	195 -	212	1	K.LNEQGLRDIANTPHELYR.N
2179.0405	2178.0332	2178.0382	-2.29	95 -	114	0	R.YGGLHFSDLVEVFSPPGSDR.A
2284.1729	2283.1656	2283.1682	-1.13	151 -	173	0	K.GVVNFAVVITDGHVTGSPCGGIK.L
2423.2490	2422.2417	2422.2353	2.64	724 -	744	2	R.VFAVVITDGRHDPRDDDLNLR.A
2713.3413	2712.3340	2712.3184	5.76	95 -	119	1	R.YGGLHFSDLVEVFSPPGSDRASFTK.S
2846.3865	2845.3792	2845.3601	6.72	236 -	260	2	K.VMKHEAYGECYKVSCLEIPGPPGPK.G
3118.6643	3117.6570	3117.6723	-4.90	186 -	212	2	R.LFAVPPNLKLNEQGLRDIANTPHELYR.N
3269.6123	3268.6050	3268.5888	4.96	659 -	687	1	R.VGVVQYSHEGTFEAIQLDDERIDSLSSFK.E
3696.7678	3695.7605	3695.8319	-19.31	659 -	691	2	R.VGVVQYSHEGTFEAIQLDDERIDSLSSFKEAVK.N

No match to: 710.3558, 715.3582, 722.4182, 729.4472, 734.4399, 735.4044, 737.4075, 747.4354, 756.3666, 759.4210, 772.4085, 779.4621, 800.3990, 801.4063, 805.4392, 818.4569, 823.4658, 828.4204, 832.2986, 834.3055, 842.4619, 856.3810, 865.4302, 870.4144, 873.3766, 880.4450, 883.4655, 887.4088, 889.4736, 891.4725, 898.4407, 900.5031, 912.4936, 923.5015, 925.4745, 929.4329, 938.4833, 957.5629, 961.4780, 967.4121, 970.4429, 972.4810, 974.4342, 978.5226, 983.6148, 988.4271, 995.3998, 1006.4926, 1007.5641, 1021.5605, 1032.5048, 1034.5706, 1036.5566, 1043.5085, 1044.5150, 1046.5917, 1056.5394, 1057.6062, 1087.5505, 1113.5382, 1122.5513, 1123.5905, 1130.5541, 1143.4395, 1145.6267, 1177.6389, 1178.6249, 1184.6396, 1194.5638, 1198.6896, 1216.7015, 1227.6558, 1243.6030, 1248.6648, 1254.6876, 1255.5952, 1264.7084, 1269.6405, 1273.6479, 1278.6892, 1281.6271, 1289.6858, 1301.6105, 1304.6399, 1312.7454, 1325.6327, 1326.6665, 1336.7188, 1337.6541, 1338.6735, 1349.6659, 1351.6846, 1356.6124, 1361.6910, 1372.5992, 1382.5925, 1383.7246, 1390.6946, 1420.7117, 1423.6814, 1445.7860, 1456.7703, 1457.7407, 1467.7371, 1470.7511, 1488.7211, 1494.7380, 1497.8768, 1500.7181, 1503.7421, 1513.7554, 1519.7505, 1528.6958, 1540.7820, 1544.8356, 1556.8394, 1562.7800, 1563.7925, 1572.7056, 1578.7816, 1580.8422, 1599.9109, 1616.7953, 1621.7692, 1628.7004, 1628.8589, 1635.7825, 1642.7168, 1647.8525, 1650.8430, 1657.9056, 1666.7905, 1696.8221, 1707.9131, 1717.9056, 1722.9384, 1730.8652, 1738.7958, 1741.9946, 1755.8914, 1758.9082, 1771.0306, 1787.8247, 1862.9480, 1883.7240, 1928.0236, 1938.0043, 1940.0157, 1953.8590, 1959.9622, 1961.0220, 1969.9949, 1981.0421, 1990.9496, 1997.8253, 2007.9401, 2011.8949, 2015.9641, 2023.9448, 2027.1273, 2044.0441, 2045.9841, 2060.9731, 2067.1531, 2086.0945, 2088.1196, 2108.9956, 2112.9929, 2114.9592, 2144.0564, 2160.0481, 2174.1157, 2199.1233, 2211.0984, 2229.1216, 2239.1421, 2242.1345, 2245.8398, 2251.1340, 2255.1416, 2265.0903, 2270.1790, 2273.0667, 2287.1509, 2293.1445, 2328.9482, 2343.1899, 2352.9153, 2354.9983, 2356.0093, 2364.8684, 2414.2334, 2450.2375, 2466.2253, 2468.2173, 2475.2114, 2486.1069, 2519.9941, 2541.2319, 2542.2505, 2558.3389, 2575.1797, 2646.9695, 2660.9880, 2675.0371, 2695.0181, 2702.2866, 2705.2827, 2717.3323, 2718.2998, 2721.2900, 2744.2466, 2763.3267, 2770.3513, 2785.3567, 2787.2734, 2788.2341, 2792.2034, 2798.2407, 2801.2722, 2803.2302, 2808.2080, 2817.2480, 2820.2319, 2833.2026, 2843.3906, 2847.3792, 2870.3926, 2873.3279, 2889.3057, 2892.4070, 2896.4392, 2912.4277, 2915.4314, 2919.4495, 2961.2297, 3071.3845, 3076.4626, 3081.6108, 3087.3884, 3101.4309, 3143.5120, 3159.5193, 3173.5476, 3184.4751, 3236.5454, 3248.6077, 3253.6089, 3265.5996, 3285.5964, 3331.5325, 3334.6331, 3335.6592, 3347.4841, 3397.6499, 3411.6399, 3412.5662, 3427.6182, 3428.5718, 3435.6516, 3444.5942, 3457.6045, 3463.5820, 3473.5864, 3487.6257, 3491.6506, 3503.7678, 3509.5947, 3521.6812, 3552.5681, 3574.6165, 3590.6309, 3624.6504, 3636.7607, 3640.6731, 3646.7363, 3651.7256, 3665.7231, 3669.7129, 3692.6494, 3707.6807, 3714.6301, 3719.6519, 3722.5593, 3723.6516, 3735.6885, 3751.6423, 3830.7644, 3937.7585, 3941.8464, 3942.8328, 3955.8433, 4092.8938, 4147.8613, 4162.9160, 4254.9053, 4272.9224, 4290.9102, 4306.9575, 4309.8506, 4323.8926, 4397.9277, 4415.8896, 4449.1777, 4455.1221, 4459.2368, 4494.0420, 4501.0767, 4510.0269, 4525.1040, 4530.0112, 4839.1138, 4846.0161

5. G3MXU3 **Mass:** 25162 **Score:** 33 **Expect:** 12 **Matches:** 12

Uncharacterized protein OS=Bos taurus GN=COL6A2 PE=4 SV=1

Observed	Mr(expt)	Mr(calc)	ppm	Start	End	Miss	Peptide
865.4302	864.4230	864.4341	-12.91	65 -	71	0	R.FVEEVSR.R
972.4810	971.4737	971.4825	-9.04	54 -	61	0	R.LGEQNFHK.V
1021.5605	1020.5532	1020.5352	17.6	65 -	72	1	R.FVEEVSRR.L
1043.5085	1042.5012	1042.5043	-2.97	78 -	86	1	R.KDDDPLNAR.V
1057.6062	1056.5989	1056.6080	-8.61	87 -	96	0	R.VALLQFGGPR.E
1145.6267	1144.6194	1144.6241	-4.04	198 -	208	0	K.ISLGDPAAVFR.E
1177.6389	1176.6316	1176.6363	-4.01	64 -	72	2	R.RFVEEVSRR.L
1227.6558	1226.6485	1226.6520	-2.83	54 -	63	1	R.LGEQNFHKVR.R
1530.6902	1529.6829	1529.6787	2.77	211 -	223	0	K.DYDSLAQPGFFDR.F
1787.8247	1786.8174	1786.8162	0.67	209 -	223	1	R.EKDYDSLAQPGFFDR.F
2270.1790	2269.1717	2269.2166	-19.77	176 -	197	1	R.KQNVVPTVVAVGSDVDTDVLSK.I
2558.3389	2557.3316	2557.3401	-3.33	120 -	143	0	R.YLNSFSHVGTGIVQAINQVVQGAR.A

No match to: 710.3558, 715.3582, 722.4182, 729.4472, 734.4399, 735.4044, 737.4075, 747.4354, 756.3666, 759.4210, 772.4085, 779.4621, 800.3990, 801.4063, 805.4392, 818.4569, 823.4658, 828.4204, 829.4349, 830.4365, 832.2986, 834.3055, 842.4619, 856.3810, 870.4144, 873.3766, 880.4450, 883.4655, 887.4088, 889.4736, 891.4725, 898.4407, 900.5031, 912.4936, 923.5015, 925.4745, 929.4329, 938.4833, 957.5629, 961.4780, 967.4121, 970.4429, 974.4342, 978.5226, 983.6148, 988.4271, 993.4903, 995.3998, 998.5886, 1006.4926, 1007.5641, 1024.5408, 1032.5048, 1034.5706, 1036.5566, 1044.5150, 1046.5917, 1056.5394, 1074.6005, 1087.5505, 1113.5382, 1122.5513, 1123.5905, 1130.5541, 1143.4395, 1178.6249, 1180.6364, 1184.6396, 1194.5638, 1198.6896, 1216.7015, 1243.6030, 1248.6648, 1254.6876, 1255.5952, 1264.7084, 1269.6405, 1273.6479, 1278.6892, 1281.6271, 1289.6858, 1301.6105, 1304.6399, 1312.7454, 1325.6327, 1326.6665, 1328.6610, 1336.7188, 1337.6541, 1338.6735, 1349.6659, 1351.6846, 1356.6124, 1361.6910, 1365.6167, 1372.5992, 1382.5925, 1383.7246, 1390.6946, 1420.7117, 1423.6814, 1445.7860, 1456.7703, 1457.7407, 1467.7371, 1470.7511, 1488.7211, 1494.7380, 1497.8768, 1500.7181, 1503.7421, 1513.7554, 1519.7505, 1528.6958, 1540.7820, 1544.8356, 1556.8394, 1562.7800, 1563.7925, 1572.7056, 1578.7816, 1580.8422, 1599.9109, 1616.7953, 1621.7692, 1628.7004, 1628.8589, 1635.7825, 1642.7168, 1647.8525, 1650.8430, 1657.9056, 1666.7905, 1696.8221, 1706.8651, 1707.9131, 1717.9056, 1722.9384, 1730.8652, 1738.7958, 1741.9946, 1755.8914, 1758.9082, 1771.0306, 1774.9175, 1809.0355, 1862.9480, 1883.7240, 1928.0236, 1932.0079, 1938.0043, 1940.0157, 1953.8590, 1959.9622, 1961.0220, 1969.9949, 1981.0421, 1990.9496, 1997.8253, 2007.9401, 2011.8949, 2015.9641, 2023.9448, 2027.1273, 2032.0186, 2044.0441, 2045.9841, 2060.9731, 2067.1531, 2086.0945, 2088.1196, 2108.9956, 2112.9929, 2114.9592, 2139.0918, 2144.0564, 2160.0481, 2174.1157, 2179.0405, 2199.1233, 2211.0984, 2229.1216, 2239.1421, 2242.1345, 2245.8398, 2251.1340, 2255.1416, 2265.0903, 2273.0667, 2284.1729, 2287.1509, 2293.1445, 2328.9482, 2343.1899, 2352.9153, 2354.9983, 2356.0093, 2364.8684, 2414.2334, 2423.2490, 2450.2375, 2466.2253, 2468.2173, 2475.2114, 2486.1069, 2519.9941, 2541.2319, 2542.2505, 2575.1797, 2646.9695, 2660.9880, 2675.0371, 2695.0181, 2702.2866, 2705.2827, 2713.3413, 2717.3323, 2718.2998, 2721.2900, 2744.2466, 2763.3267, 2770.3513, 2785.3567, 2787.2734, 2788.2341, 2792.2034, 2798.2407, 2801.2722, 2803.2302, 2808.2080, 2817.2480, 2820.2319, 2833.2026, 2843.3906, 2846.3865, 2847.3792, 2870.3926, 2873.3279, 2889.3057, 2892.4070, 2896.4392, 2912.4277, 2915.4314, 2919.4495, 2961.2297, 3071.3845, 3076.4626, 3081.6108, 3087.3884, 3101.4309, 3118.6643, 3143.5120, 3159.5193, 3173.5476, 3184.4751, 3236.5454, 3248.6077, 3253.6089, 3265.5996, 3269.6123, 3285.5964, 3331.5325, 3334.6331, 3335.6592, 3347.4841, 3397.6499, 3411.6399, 3412.5662, 3427.6182, 3428.5718, 3435.6516, 3444.5942, 3457.6045, 3463.5820, 3473.5864, 3487.6257, 3491.6506, 3503.7678, 3509.5947, 3521.6812, 3552.5681, 3574.6165, 3590.6309, 3624.6504, 3636.7607, 3640.6731, 3646.7363, 3651.7256, 3665.7231, 3669.7129, 3692.6494, 3696.7678, 3707.6807, 3714.6301, 3719.6519, 3722.5593, 3723.6516, 3735.6885, 3751.6423, 3830.7644, 3937.7585, 3941.8464, 3942.8328, 3955.8433, 4092.8938, 4147.8613, 4162.9160, 4254.9053, 4272.9224, 4290.9102, 4306.9575, 4309.8506, 4323.8926, 4397.9277, 4415.8896, 4449.1777, 4455.1221, 4459.2368, 4494.0420, 4501.0767, 4510.0269, 4525.1040, 4530.0112, 4839.1138, 4846.0161

6. F1MNG6 **Mass:** 140022 **Score:** 26 **Expect:** 54 **Matches:** 29

Uncharacterized protein OS=Bos taurus GN=PPIP5K2 PE=3 SV=2

Observed	Mr(expt)	Mr(calc)	ppm	Start	End	Miss	Peptide
737.4075	736.4002	736.3942	8.17	303	308	0	K.VCLAFK.Q
1043.5085	1042.5012	1042.5196	-17.62	517	526	0	K.WGGELTPAGR.V
1180.6364	1179.6291	1179.6288	0.29	710	718	2	R.WSKLEKDFK.T
1184.6396	1183.6323	1183.6561	-20.06	435	444	0	K.QLQEVLDIAR.Q
1269.6405	1268.6332	1268.6626	-23.12	558	567	1	R.LHSTYRHDLK.I
1338.6735	1337.6662	1337.6768	-7.92	410	419	1	R.HQKFFDLFEK.C
1445.7860	1444.7787	1444.8071	-19.66	61	72	2	K.SKSKPMKEILER.I
1513.7554	1512.7481	1512.7494	-0.82	748	759	1	K.LEDTMELYRLSK.A + Oxidation (M)
1628.8589	1627.8516	1627.8642	-7.70	1010	1024	2	R.RRSGEQITSSPVSPK.S
1932.0079	1931.0006	1930.9636	19.2	1127	1144	0	K.QVDEFLASIASPSSEVPR.K
2108.9956	2107.9883	2108.0361	-22.64	124	140	0	R.NPFVINDLNMQYLIQDR.R + Oxidation (M)
2179.0405	2178.0332	2178.0678	-15.88	911	929	2	K.NLPSGYGYRPASRENEGRR.A
2251.1340	2250.1267	2250.1467	-8.86	465	483	1	K.LEQLKTVLEMYGHFSGINR.K + Oxidation (M)
2265.0903	2264.0830	2264.1372	-23.91	124	141	1	R.NPFVINDLNMQYLIQDRR.E + Oxidation (M)
2423.2490	2422.2417	2422.2526	-4.49	664	684	1	K.NPVKTCDTVYSLIQSLTSQIR.H
2450.2375	2449.2302	2449.2787	-19.81	463	483	2	K.SKLEQLKTVLEMYGHFSGINR.K
2466.2253	2465.2180	2465.2737	-22.56	463	483	2	K.SKLEQLKTVLEMYGHFSGINR.K + Oxidation (M)
3071.3845	3070.3772	3070.4454	-22.21	2	28	2	M.SNEIKMSEAPRFFVGPEDTEINAGNYR.H
3087.3884	3086.3811	3086.4403	-19.18	2	28	2	M.SNEIKMSEAPRFFVGPEDTEINAGNYR.H + Oxidation (M)
3265.5996	3264.5923	3264.5392	16.3	859	885	0	R.AMDYLNVVNELNYMTQIVIMLYEDPNK.D + 2 Oxidation (M)
3269.6123	3268.6050	3268.6245	-5.95	435	462	1	K.QLQEVLDIARQLLMELGQNNDSEIEENK.S
3285.5964	3284.5891	3284.6194	-9.21	435	462	1	K.QLQEVLDIARQLLMELGQNNDSEIEENK.S + Oxidation (M)
3428.5718	3427.5645	3427.6428	-22.83	729	756	2	K.IPDIYDCIKYDVQHNGSLKLEDTMELYR.L
3444.5942	3443.5869	3443.6377	-14.75	729	756	2	K.IPDIYDCIKYDVQHNGSLKLEDTMELYR.L + Oxidation (M)
3509.5947	3508.5874	3508.6165	-8.29	976	1008	1	K.MATNEEESPLSVSSPEGTGTWLHYTSGVGTGRR.R + Oxidation (M)
3665.7231	3664.7158	3664.7176	-0.49	976	1009	2	K.MATNEEESPLSVSSPEGTGTWLHYTSGVGTGRRR.R + Oxidation (M)
3696.7678	3695.7605	3695.8249	-17.41	1094	1126	1	K.KLTSSGCIDGFELYSMVPSICPLETLHNALSLK.Q + Oxidation (M)
4290.9102	4289.9029	4289.8111	21.4	13	48	1	R.FFVGPEDTEINAGNYRHFFHHADEDEEEDESPPER.Q
4449.1777	4448.1704	4448.2237	-11.97	586	626	2	K.GLLALEGELTPILVQMVKSANMNGLLDSDSDSLSSCQQRVK.A + 2 Oxidation (M)

No match to: 710.3558, 715.3582, 722.4182, 729.4472, 734.4399, 735.4044, 747.4354, 756.3666, 759.4210, 772.4085, 779.4621, 800.3990, 801.4063, 805.4392, 818.4569, 823.4658, 828.4204, 829.4349, 830.4365, 832.2986, 834.3055, 842.4619, 856.3810, 865.4302, 870.4144, 873.3766, 880.4450, 883.4655, 887.4088, 889.4736, 891.4725, 898.4407, 900.5031, 912.4936, 923.5015, 925.4745, 929.4329, 938.4833, 957.5629, 961.4780, 967.4121, 970.4429, 972.4810, 974.4342, 978.5226, 983.6148, 988.4271, 993.4903, 995.3998, 998.5886, 1006.4926, 1007.5641, 1021.5605, 1024.5408, 1032.5048, 1034.5706, 1036.5566, 1044.5150, 1046.5917, 1056.5394, 1057.6062, 1074.6005, 1087.5505, 1113.5382, 1122.5513, 1123.5905, 1130.5541, 1143.4395, 1145.6267, 1177.6389, 1178.6249, 1194.5638, 1198.6896, 1216.7015, 1227.6558, 1243.6030, 1248.6648, 1254.6876, 1255.5952, 1264.7084, 1273.6479, 1278.6892, 1281.6271, 1289.6858, 1301.6105, 1304.6399, 1312.7454, 1325.6327, 1326.6665, 1328.6610, 1336.7188, 1337.6541, 1349.6659, 1351.6846, 1356.6124, 1361.6910, 1365.6167, 1372.5992, 1382.5925, 1383.7246, 1390.6946, 1420.7117, 1423.6814, 1456.7703, 1457.7407, 1467.7371, 1470.7511, 1488.7211, 1494.7380, 1497.8768, 1500.7181, 1503.7421, 1519.7505, 1528.6958, 1530.6902, 1540.7820, 1544.8356, 1556.8394, 1562.7800, 1563.7925, 1572.7056, 1578.7816, 1580.8422, 1599.9109, 1616.7953, 1621.7692, 1628.7004, 1635.7825, 1642.7168, 1647.8525, 1650.8430, 1657.9056, 1666.7905, 1696.8221, 1706.8651, 1707.9131, 1717.9056, 1722.9384, 1730.8652, 1738.7958, 1741.9946, 1755.8914, 1758.9082, 1771.0306, 1774.9175, 1787.8247, 1809.0355, 1862.9480, 1883.7240, 1928.0236, 1938.0043, 1940.0157, 1953.8590, 1959.9622, 1961.0220, 1969.9949, 1981.0421, 1990.9496, 1997.8253, 2007.9401, 2011.8949, 2015.9641, 2023.9448, 2027.1273, 2032.0186, 2044.0441, 2045.9841, 2060.9731, 2067.1531, 2086.0945, 2088.1196, 2112.9929, 2114.9592, 2139.0918, 2144.0564, 2160.0481, 2174.1157, 2199.1233, 2211.0984, 2229.1216, 2239.1421, 2242.1345, 2245.8398, 2255.1416, 2270.1790, 2273.0667, 2284.1729, 2287.1509, 2293.1445, 2328.9482, 2343.1899, 2352.9153, 2354.9983, 2356.0093, 2364.8684, 2414.2334, 2468.2173, 2475.2114, 2486.1069, 2519.9941, 2541.2319, 2542.2505, 2558.3389, 2575.1797, 2646.9695, 2660.9880, 2675.0371, 2695.0181, 2702.2866, 2705.2827, 2713.3413, 2717.3323, 2718.2998, 2721.2900, 2744.2466, 2763.3267, 2770.3513, 2785.3567, 2787.2734, 2788.2341, 2792.2034, 2798.2407, 2801.2722, 2803.2302, 2808.2080, 2817.2480, 2820.2319, 2833.2026, 2843.3906, 2846.3865, 2847.3792, 2870.3926, 2873.3279, 2889.3057, 2892.4070, 2896.4392, 2912.4277, 2915.4314, 2919.4495, 2961.2297, 3076.4626, 3081.6108, 3101.4309, 3118.6643, 3143.5120, 3159.5193, 3173.5476, 3184.4751, 3236.5454, 3248.6077, 3253.6089, 3331.5325, 3334.6331, 3335.6592, 3347.4841, 3397.6499, 3411.6399, 3412.5662, 3427.6182, 3435.6516, 3457.6045, 3463.5820, 3473.5864, 3487.6257, 3491.6506, 3503.7678, 3521.6812, 3552.5681, 3574.6165, 3590.6309, 3624.6504, 3636.7607, 3640.6731, 3646.7363, 3651.7256, 3669.7129, 3692.6494, 3707.6807, 3714.6301, 3719.6519, 3722.5593, 3723.6516, 3735.6885, 3751.6423, 3830.7644, 3937.7585, 3941.8464, 3942.8328, 3955.8433, 4092.8938, 4147.8613, 4162.9160, 4254.9053, 4272.9224, 4306.9575, 4309.8506, 4323.8926, 4397.9277, 4415.8896, 4455.1221, 4459.2368, 4494.0420, 4501.0767, 4510.0269, 4525.1040, 4530.0112, 4839.1138, 4846.0161

7. P21809 **Mass:** 41963 **Score:** 26 **Expect:** 61 **Matches:** 14

Biglycan OS=Bos taurus GN=BGN PE=1 SV=3

Observed	Mr(expt)	Mr(calc)	ppm	Start	End	Miss	Peptide
735.4044	734.3971	734.4075	-14.14	178	184	0	K.GVFSGLR.N
923.5015	922.4943	922.5097	-16.72	167	173	1	R.IHDNRIR.K
1007.5641	1006.5568	1006.5672	-10.31	259	267	0	R.LGLGHNQIR.M
1312.7454	1311.7381	1311.7398	-1.26	242	252	0	K.IQAIELEDLLR.Y
1494.7380	1493.7307	1493.7337	-2.02	316	329	0	K.VGVNDFCPVGFGVK.R
1650.8430	1649.8357	1649.8348	0.54	316	330	1	K.VGVNDFCPVGFGVKR.A
2027.1273	2026.1200	2026.1211	-0.53	149	166	0	K.NHLVEIPPNLPSSLVELR.I
2414.2334	2413.2261	2413.2336	-3.10	88	108	1	K.EISPDTTLLDLQNNDISELRK.D
2798.2407	2797.2334	2797.2146	6.74	185	210	0	R.NMNCIEMGGNPLENSGFEPGAFDGLK.L
2846.3865	2845.3792	2845.3864	-2.52	331	354	0	R.AYYNGISLFNNPVPYWEVQPATFR.C
2919.4495	2918.4422	2918.4509	-2.97	88	112	2	K.EISPDTTLLDLQNNDISELRKDDFK.G
3081.6108	3080.6035	3080.6142	-3.45	227	252	1	K.DLPETLNELHLDHNKIQAIELEDLLR.Y
3457.6045	3456.5972	3456.5900	2.08	185	215	1	R.NMNCIEMGGNPLENSGFEPGAFDGLKLNYLR.I
3473.5864	3472.5791	3472.5850	-1.68	185	215	1	R.NMNCIEMGGNPLENSGFEPGAFDGLKLNYLR.I + Oxidation (M)

No match to: 710.3558, 715.3582, 722.4182, 729.4472, 734.4399, 737.4075, 747.4354, 756.3666, 759.4210, 772.4085, 779.4621, 800.3990, 801.4063, 805.4392, 818.4569, 823.4658, 828.4204, 829.4349, 830.4365, 832.2986, 834.3055, 842.4619, 856.3810, 865.4302, 870.4144, 873.3766, 880.4450, 883.4655, 887.4088, 889.4736, 891.4725, 898.4407, 900.5031, 912.4936, 925.4745, 929.4329, 938.4833, 957.5629, 961.4780, 967.4121, 970.4429, 972.4810, 974.4342, 978.5226, 983.6148, 988.4271, 993.4903, 995.3998, 998.5886, 1006.4926, 1021.5605, 1024.5408, 1032.5048, 1034.5706, 1036.5566, 1043.5085, 1044.5150, 1046.5917, 1056.5394, 1057.6062, 1074.6005, 1087.5505, 1113.5382, 1122.5513, 1123.5905, 1130.5541, 1143.4395, 1145.6267, 1177.6389, 1178.6249, 1180.6364, 1184.6396, 1194.5638, 1198.6896, 1216.7015, 1227.6558, 1243.6030, 1248.6648, 1254.6876, 1255.5952, 1264.7084, 1269.6405, 1273.6479, 1278.6892, 1281.6271, 1289.6858, 1301.6105, 1304.6399, 1325.6327, 1326.6665, 1328.6610, 1336.7188, 1337.6541, 1338.6735, 1349.6659, 1351.6846, 1356.6124, 1361.6910, 1365.6167, 1372.5992, 1382.5925, 1383.7246, 1390.6946, 1420.7117, 1423.6814, 1445.7860, 1456.7703, 1457.7407, 1467.7371, 1470.7511, 1488.7211, 1497.8768, 1500.7181, 1503.7421, 1513.7554, 1519.7505, 1528.6958, 1530.6902, 1540.7820, 1544.8356, 1556.8394, 1562.7800, 1563.7925, 1572.7056, 1578.7816, 1580.8422, 1599.9109, 1616.7953, 1621.7692, 1628.7004, 1628.8589, 1635.7825, 1642.7168, 1647.8525, 1657.9056, 1666.7905, 1696.8221, 1706.8651, 1707.9131, 1717.9056, 1722.9384, 1730.8652, 1738.7958, 1741.9946, 1755.8914, 1758.9082, 1771.0306, 1774.9175, 1787.8247, 1809.0355, 1862.9480, 1883.7240, 1928.0236, 1932.0079, 1938.0043, 1940.0157, 1953.8590, 1959.9622, 1961.0220, 1969.9949, 1981.0421, 1990.9496, 1997.8253, 2007.9401, 2011.8949, 2015.9641, 2023.9448, 2032.0186, 2044.0441, 2045.9841, 2060.9731, 2067.1531, 2086.0945, 2088.1196, 2108.9956, 2112.9929, 2114.9592, 2139.0918, 2144.0564, 2160.0481, 2174.1157, 2179.0405, 2199.1233, 2211.0984, 2229.1216, 2239.1421, 2242.1345, 2245.8398, 2251.1340, 2255.1416, 2265.0903, 2270.1790, 2273.0667, 2284.1729, 2287.1509, 2293.1445, 2328.9482, 2343.1899, 2352.9153, 2354.9983, 2356.0093, 2364.8684, 2423.2490, 2450.2375, 2466.2253, 2468.2173, 2475.2114, 2486.1069, 2519.9941, 2541.2319, 2542.2505, 2558.3389, 2575.1797, 2646.9695, 2660.9880, 2675.0371, 2695.0181, 2702.2866,

2705.2827, 2713.3413, 2717.3323, 2718.2998, 2721.2900, 2744.2466, 2763.3267, 2770.3513, 2785.3567, 2787.2734, 2788.2341, 2792.2034, 2801.2722, 2803.2302, 2808.2080, 2817.2480, 2820.2319, 2833.2026, 2843.3906, 2847.3792, 2870.3926, 2873.3279, 2889.3057, 2892.4070, 2896.4392, 2912.4277, 2915.4314, 2961.2297, 3071.3845, 3076.4626, 3087.3884, 3101.4309, 3118.6643, 3143.5120, 3159.5193, 3173.5476, 3184.4751, 3236.5454, 3248.6077, 3253.6089, 3265.5996, 3269.6123, 3285.5964, 3331.5325, 3334.6331, 3335.6592, 3347.4841, 3397.6499, 3411.6399, 3412.5662, 3427.6182, 3428.5718, 3435.6516, 3444.5942, 3463.5820, 3487.6257, 3491.6506, 3503.7678, 3509.5947, 3521.6812, 3552.5681, 3574.6165, 3590.6309, 3624.6504, 3636.7607, 3640.6731, 3646.7363, 3651.7256, 3665.7231, 3669.7129, 3692.6494, 3696.7678, 3707.6807, 3714.6301, 3719.6519, 3722.5593, 3723.6516, 3735.6885, 3751.6423, 3830.7644, 3937.7585, 3941.8464, 3942.8328, 3955.8433, 4092.8938, 4147.8613, 4162.9160, 4254.9053, 4272.9224, 4290.9102, 4306.9575, 4309.8506, 4323.8926, 4397.9277, 4415.8896, 4449.1777, 4455.1221, 4459.2368, 4494.0420, 4501.0767, 4510.0269, 4525.1040, 4530.0112, 4839.1138, 4846.0161

8. F1N290 **Mass:** 52538 **Score:** 24 **Expect:** 88 **Matches:** 11

Uncharacterized protein OS=Bos taurus PE=4 SV=1

Observed	Mr(expt)	Mr(calc)	ppm	Start	End	Miss	Peptide
735.4044	734.3971	734.4075	-14.14	10 -	15	0	K.NVTFVR.K
923.5015	922.4943	922.5124	-19.63	21 -	29	0	K.APLPPAETK.C
1006.4926	1005.4853	1005.4614	23.8	432 -	440	0	K.DEAENTSLK.S
1500.7181	1499.7108	1499.7442	-22.29	180 -	191	0	K.CEFDPLHTQLLK.D
1717.9056	1716.8983	1716.9158	-10.21	279 -	293	2	K.QYISNTLPSDAPKKR.R
2843.3906	2842.3833	2842.3728	3.70	212 -	235	2	R.ELYAIDVSKESCQISQNLDLMKDK.E + Oxidation (M)
3269.6123	3268.6050	3268.5903	4.49	294 -	323	1	R.RAPLPPMPGSQSSPQDPAHIQERPASCMVK.S
3285.5964	3284.5891	3284.5853	1.18	294 -	323	1	R.RAPLPPMPGSQSSPQDPAHIQERPASCMVK.S + Oxidation (M)
3457.6045	3456.5972	3456.6813	-24.32	293 -	323	2	K.RRAPLPPMPGSQSSPQDPAHIQERPASCMVK.S + 2 Oxidation (M)
3942.8328	3941.8255	3941.7856	10.1	21 -	56	2	K.APLPPAETKCLDASSVDDSVESSAFIMDQKENMTDK.D + Oxidation (M)
4839.1138	4838.1065	4838.2222	-23.90	295 -	339	1	R.APLPPMPGSQSSPQDPAHIQERPASCMVKSLSVDETEESSYGEGR.V

No match to: 710.3558, 715.3582, 722.4182, 729.4472, 734.4399, 737.4075, 747.4354, 756.3666, 759.4210, 772.4085, 779.4621, 800.3990, 801.4063, 805.4392, 818.4569, 823.4658, 828.4204, 829.4349, 830.4365, 832.2986, 834.3055, 842.4619, 856.3810, 865.4302, 870.4144, 873.3766, 880.4450, 883.4655, 887.4088, 889.4736, 891.4725, 898.4407, 900.5031, 912.4936, 925.4745, 929.4329, 938.4833, 957.5629, 961.4780, 967.4121, 970.4429, 972.4810, 974.4342, 978.5226, 983.6148, 988.4271, 993.4903, 995.3998, 998.5886, 1007.5641, 1021.5605, 1024.5408, 1032.5048, 1034.5706, 1036.5566, 1043.5085, 1044.5150, 1046.5917, 1056.5394, 1057.6062, 1074.6005, 1087.5505, 1113.5382, 1122.5513, 1123.5905, 1130.5541, 1143.4395, 1145.6267, 1177.6389, 1178.6249, 1180.6364, 1184.6396, 1194.5638, 1198.6896, 1216.7015, 1227.6558, 1243.6030, 1248.6648, 1254.6876, 1255.5952, 1264.7084, 1269.6405, 1273.6479, 1278.6892, 1281.6271, 1289.6858, 1301.6105, 1304.6399, 1312.7454, 1325.6327, 1326.6665, 1328.6610, 1336.7188, 1337.6541, 1338.6735, 1349.6659, 1351.6846, 1356.6124, 1361.6910, 1365.6167, 1372.5992, 1382.5925, 1383.7246, 1390.6946, 1420.7117, 1423.6814, 1445.7860, 1456.7703, 1457.7407, 1467.7371, 1470.7511, 1488.7211, 1494.7380, 1497.8768, 1503.7421, 1513.7554, 1519.7505, 1528.6958, 1530.6902, 1540.7820, 1544.8356, 1556.8394, 1562.7800, 1563.7925, 1572.7056, 1578.7816, 1580.8422, 1599.9109, 1616.7953, 1621.7692, 1628.7004, 1628.8589, 1635.7825, 1642.7168, 1647.8525, 1650.8430, 1657.9056, 1666.7905, 1696.8221, 1706.8651, 1707.9131, 1722.9384, 1730.8652, 1738.7958, 1741.9946, 1755.8914, 1758.9082, 1771.0306, 1774.9175, 1787.8247, 1809.0355, 1862.9480, 1883.7240, 1928.0236, 1932.0079, 1938.0043, 1940.0157, 1953.8590, 1959.9622, 1961.0220, 1969.9949, 1981.0421, 1990.9496, 1997.8253, 2007.9401, 2011.8949, 2015.9641, 2023.9448, 2027.1273, 2032.0186, 2044.0441, 2045.9841, 2060.9731, 2067.1531, 2086.0945, 2088.1196, 2108.9956, 2112.9929, 2114.9592, 2139.0918, 2144.0564, 2160.0481, 2174.1157, 2179.0405, 2199.1233, 2211.0984, 2229.1216, 2239.1421, 2242.1345, 2245.8398, 2251.1340, 2255.1416, 2265.0903, 2270.1790, 2273.0667, 2284.1729, 2287.1509, 2293.1445, 2328.9482, 2343.1899, 2352.9153, 2354.9983, 2356.0093, 2364.8684, 2414.2334, 2423.2490, 2450.2375, 2466.2253, 2468.2173, 2475.2114, 2486.1069, 2519.9941, 2541.2319, 2542.2505, 2558.3389, 2575.1797, 2646.9695, 2660.9880, 2675.0371, 2695.0181, 2702.2866, 2705.2827, 2713.3413, 2717.3323, 2718.2998, 2721.2900, 2744.2466, 2763.3267, 2770.3513, 2785.3567, 2787.2734, 2788.2341, 2792.2034, 2798.2407, 2801.2722, 2803.2302, 2808.2080, 2817.2480, 2820.2319, 2833.2026, 2846.3865, 2847.3792, 2870.3926, 2873.3279, 2889.3057, 2892.4070, 2896.4392, 2912.4277, 2915.4314, 2919.4495, 2961.2297, 3071.3845, 3076.4626, 3081.6108, 3087.3884, 3101.4309, 3118.6643, 3143.5120, 3159.5193, 3173.5476, 3184.4751, 3236.5454, 3248.6077, 3253.6089, 3265.5996, 3331.5325, 3334.6331, 3335.6592, 3347.4841, 3397.6499, 3411.6399, 3412.5662, 3427.6182, 3428.5718, 3435.6516, 3444.5942, 3463.5820, 3473.5864, 3487.6257, 3491.6506, 3503.7678, 3509.5947, 3521.6812, 3552.5681, 3574.6165, 3590.6309, 3624.6504, 3636.7607, 3640.6731, 3646.7363, 3651.7256, 3665.7231, 3669.7129, 3692.6494, 3696.7678, 3707.6807, 3714.6301, 3719.6519, 3722.5593, 3723.6516, 3735.6885, 3751.6423, 3830.7644, 3937.7585, 3941.8464, 3955.8433, 4092.8938, 4147.8613, 4162.9160, 4254.9053, 4272.9224, 4290.9102, 4306.9575, 4309.8506, 4323.8926, 4397.9277, 4415.8896, 4449.1777, 4455.1221, 4459.2368, 4494.0420, 4501.0767, 4510.0269, 4525.1040, 4530.0112, 4846.0161

9. E1BIL2 **Mass:** 57570 **Score:** 22 **Expect:** 1.5e+002 **Matches:** 10

Uncharacterized protein OS=Bos taurus PE=3 SV=2

Observed	Mr(expt)	Mr(calc)	ppm	Start	End	Miss	Peptide
957.5629	956.5556	956.5654	-10.30	442 -	449	1	K.EDLARLLK.E
1383.7246	1382.7173	1382.6864	22.4	379 -	389	1	K.SIKTEIMEFNR.M + Oxidation (M)
1503.7421	1502.7348	1502.7226	8.12	15 -	29	0	R.QGFSGHSAVVSGQSR.V
1696.8221	1695.8148	1695.8389	-14.20	274 -	288	1	K.DIDAAYMTKVELEAK.V
2255.1416	2254.1343	2254.1627	-12.60	361 -	381	1	K.IQLGELQTTAGMHGDDLKSIK.T
2343.1899	2342.1826	2342.1828	-0.06	262 -	282	2	R.TAAENDFVVLKKDIDAAYMTK.V
2847.3792	2846.3719	2846.3076	22.6	41 -	71	1	R.SGGGGAGSAACALMGGGFGSQSLYNLGGNKK.I + Oxidation (M)
3081.6108	3080.6035	3080.5607	13.9	210 -	235	2	R.NLEPFFENYISSLRAFLDGLHVEKDK.L
3248.6077	3247.6004	3247.6030	-0.80	349 -	378	2	R.SKAEAEALYQSKIQLGELQTTAGMHGDDLK.S + Oxidation (M)
4449.1777	4448.1704	4448.2765	-23.86	114 -	159	0	R.AGGFGGGVGGFGGPGGFPGGIQEVTVNQSLLQPLNVEIDPQIGQVK.A

No match to: 710.3558, 715.3582, 722.4182, 729.4472, 734.4399, 735.4044, 737.4075, 747.4354, 756.3666, 759.4210, 772.4085, 779.4621, 800.3990, 801.4063, 805.4392, 818.4569, 823.4658, 828.4204, 829.4349, 830.4365, 832.2986, 834.3055, 842.4619, 856.3810, 865.4302, 870.4144, 873.3766, 880.4450, 883.4655, 887.4088, 889.4736, 891.4725, 898.4407, 900.5031, 912.4936, 923.5015, 925.4745, 929.4329, 938.4833, 961.4780, 967.4121, 970.4429, 972.4810, 974.4342, 978.5226, 983.6148, 988.4271, 993.4903, 995.3998, 998.5886, 1006.4926, 1007.5641, 1021.5605, 1024.5408, 1032.5048, 1034.5706, 1036.5566, 1043.5085, 1044.5150, 1046.5917, 1056.5394, 1057.6062, 1074.6005, 1087.5505, 1113.5382, 1122.5513, 1123.5905, 1130.5541, 1143.4395, 1145.6267, 1177.6389, 1178.6249, 1180.6364, 1184.6396, 1194.5638, 1198.6896, 1216.7015, 1227.6558, 1243.6030, 1248.6648, 1254.6876, 1255.5952, 1264.7084, 1269.6405, 1273.6479, 1278.6892, 1281.6271, 1289.6858, 1301.6105, 1304.6399, 1312.7454, 1325.6327, 1326.6665, 1328.6610, 1336.7188, 1337.6541, 1338.6735, 1349.6659, 1351.6846, 1356.6124, 1361.6910, 1365.6167, 1372.5992, 1382.5925, 1390.6946, 1420.7117, 1423.6814, 1445.7860, 1456.7703, 1457.7407, 1467.7371, 1470.7511, 1488.7211, 1494.7380, 1497.8768, 1500.7181, 1513.7554, 1519.7505, 1528.6958, 1530.6902, 1540.7820, 1544.8356, 1556.8394, 1562.7800, 1563.7925, 1572.7056, 1578.7816, 1580.8422, 1599.9109, 1616.7953, 1621.7692, 1628.7004, 1628.8589, 1635.7825, 1642.7168, 1647.8525, 1650.8430, 1657.9056, 1666.7905, 1706.8651, 1707.9131, 1717.9056, 1722.9384, 1730.8652, 1738.7958, 1741.9946, 1755.8914, 1758.9082, 1771.0306, 1774.9175, 1787.8247, 1809.0355, 1862.9480, 1883.7240, 1928.0236, 1932.0079, 1938.0043, 1940.0157, 1953.8590, 1959.9622, 1961.0220, 1969.9949, 1981.0421, 1990.9496, 1997.8253, 2007.9401, 2011.8949, 2015.9641, 2023.9448, 2027.1273, 2032.0186, 2044.0441, 2045.9841, 2060.9731, 2067.1531, 2086.0945, 2088.1196, 2108.9956, 2112.9929, 2114.9592, 2139.0918, 2144.0564, 2160.0481, 2174.1157, 2179.0405, 2199.1233, 2211.0984, 2229.1216, 2239.1421, 2242.1345, 2245.8398, 2251.1340, 2265.0903, 2270.1790, 2273.0667, 2284.1729, 2287.1509, 2293.1445, 2328.9482, 2352.9153, 2354.9983, 2356.0093, 2364.8684, 2414.2334, 2423.2490, 2450.2375, 2466.2253, 2468.2173, 2475.2114, 2486.1069, 2519.9941, 2541.2319, 2542.2505, 2558.3389, 2575.1797, 2646.9695, 2660.9880, 2675.0371, 2695.0181, 2702.2866, 2705.2827, 2713.3413, 2717.3323, 2718.2998, 2721.2900, 2744.2466, 2763.3267, 2770.3513, 2785.3567, 2787.2734, 2788.2341, 2792.2034, 2798.2407, 2801.2722, 2803.2302, 2808.2080, 2817.2480, 2820.2319, 2833.2026, 2843.3906, 2846.3865, 2870.3926, 2873.3279, 2889.3057, 2892.4070, 2896.4392, 2912.4277, 2915.4314, 2919.4495, 2961.2297, 3071.3845, 3076.4626, 3087.3884, 3101.4309, 3118.6643, 3143.5120, 3159.5193, 3173.5476, 3184.4751, 3236.5454, 3253.6089, 3265.5996, 3269.6123, 3285.5964, 3331.5325, 3334.6331, 3335.6592, 3347.4841, 3397.6499, 3411.6399, 3412.5662, 3427.6182, 3428.5718, 3435.6516, 3444.5942, 3457.6045, 3463.5820, 3473.5864, 3487.6257, 3491.6506, 3503.7678, 3509.5947, 3521.6812, 3552.5681, 3574.6165, 3590.6309, 3624.6504, 3636.7607, 3640.6731, 3646.7363, 3651.7256, 3665.7231, 3669.7129, 3692.6494, 3696.7678, 3707.6807, 3714.6301, 3719.6519, 3722.5593, 3723.6516, 3735.6885, 3751.6423, 3830.7644, 3937.7585, 3941.8464, 3942.8328, 3955.8433, 4092.8938, 4147.8613, 4162.9160, 4254.9053, 4272.9224, 4290.9102, 4306.9575, 4309.8506, 4323.8926, 4397.9277, 4415.8896, 4455.1221, 4459.2368, 4494.0420, 4501.0767, 4510.0269, 4525.1040, 4530.0112, 4839.1138, 4846.0161

10. E1BFZ6 **Mass:** 58545 **Score:** 22 **Expect:** 1.6e+002 **Matches:** 13

Uncharacterized protein OS=Bos taurus GN=FAM126A PE=4 SV=2

Observed	Mr(expt)	Mr(calc)	ppm	Start	End	Miss	Peptide
978.5226	977.5154	977.5182	-2.87	259 -	266	0	K.ALDDIIYR.A
1046.5917	1045.5844	1045.5992	-14.16	375 -	383	2	K.TSIGKTQRR.S
1328.6610	1327.6537	1327.6779	-18.20	315 -	326	1	R.NAVTSMSIRGHR.W
1390.6946	1389.6873	1389.6922	-3.52	300 -	311	0	R.CIQVEITPTSSR.I
1580.8422	1579.8349	1579.8279	4.42	491 -	503	1	K.LIYTSERTELPMK.H
2045.9841	2044.9768	2044.9816	-2.32	1 -	17	1	-.MFTSEKGVVEEWLSEFK.T
2060.9731	2059.9658	2059.9884	-10.97	481 -	497	1	R.FSACSLQEEKLIYTSER.T
2414.2334	2413.2261	2413.2504	-10.06	74 -	93	0	R.FTLQFLPELIWCYLAVSASR.N
2787.2734	2786.2661	2786.2679	-0.62	453 -	480	0	K.SFEQVSGVSVPHNPSSAVGCGAGTDANR.F
2912.4277	2911.4204	2911.4054	5.18	355 -	382	2	R.AASSTSQSGLSNISQNCSNKTSIGKTQR.R
3412.5662	3411.5589	3411.5677	-2.57	327 -	354	2	R.WKRHENTELTGQEELMEISEVDEGFYSR.A
3428.5718	3427.5645	3427.5626	0.55	327 -	354	2	R.WKRHENTELTGQEELMEISEVDEGFYSR.A + Oxidation (M)
3830.7644	3829.7571	3829.8484	-23.84	225 -	258	1	K.GISSRVPVSSGFMVQMLTGIYFAFYNGEWDLAQK.A + 2 Oxidation (M)

No match to: 710.3558, 715.3582, 722.4182, 729.4472, 734.4399, 735.4044, 737.4075, 747.4354, 756.3666, 759.4210, 772.4085, 779.4621, 800.3990, 801.4063, 805.4392, 818.4569, 823.4658, 828.4204, 829.4349, 830.4365, 832.2986, 834.3055, 842.4619, 856.3810, 865.4302, 870.4144, 873.3766, 880.4450, 883.4655, 887.4088, 889.4736, 891.4725, 898.4407, 900.5031, 912.4936, 923.5015, 925.4745, 929.4329, 938.4833, 957.5629, 961.4780, 967.4121, 970.4429, 972.4810, 974.4342, 983.6148, 988.4271, 993.4903, 995.3998, 998.5886, 1006.4926, 1007.5641, 1021.5605, 1024.5408, 1032.5048, 1034.5706, 1036.5566, 1043.5085, 1044.5150, 1056.5394, 1057.6062, 1074.6005, 1087.5505, 1113.5382, 1122.5513, 1123.5905, 1130.5541, 1143.4395, 1145.6267, 1177.6389, 1178.6249, 1180.6364, 1184.6396, 1194.5638, 1198.6896, 1216.7015, 1227.6558, 1243.6030, 1248.6648, 1254.6876, 1255.5952, 1264.7084, 1269.6405, 1273.6479, 1278.6892, 1281.6271, 1289.6858, 1301.6105, 1304.6399, 1312.7454, 1325.6327, 1326.6665, 1336.7188, 1337.6541, 1338.6735, 1349.6659, 1351.6846, 1356.6124, 1361.6910, 1365.6167, 1372.5992, 1382.5925, 1383.7246, 1420.7117, 1423.6814, 1445.7860, 1456.7703, 1457.7407, 1467.7371, 1470.7511, 1488.7211, 1494.7380, 1497.8768, 1500.7181, 1503.7421, 1513.7554, 1519.7505, 1528.6958, 1530.6902, 1540.7820, 1544.8356, 1556.8394, 1562.7800, 1563.7925, 1572.7056, 1578.7816, 1599.9109, 1616.7953, 1621.7692, 1628.7004, 1628.8589, 1635.7825, 1642.7168, 1647.8525, 1650.8430, 1657.9056, 1666.7905, 1696.8221, 1706.8651, 1707.9131, 1717.9056, 1722.9384, 1730.8652, 1738.7958, 1741.9946, 1755.8914, 1758.9082, 1771.0306, 1774.9175, 1787.8247, 1809.0355, 1862.9480, 1883.7240, 1928.0236, 1932.0079, 1938.0043, 1940.0157, 1953.8590, 1959.9622, 1961.0220, 1969.9949, 1981.0421, 1990.9496, 1997.8253, 2007.9401, 2011.8949, 2015.9641, 2023.9448, 2027.1273, 2032.0186, 2044.0441, 2067.1531, 2086.0945, 2088.1196, 2108.9956, 2112.9929, 2114.9592, 2139.0918, 2144.0564, 2160.0481, 2174.1157, 2179.0405, 2199.1233, 2211.0984, 2229.1216, 2239.1421, 2242.1345, 2245.8398, 2251.1340, 2255.1416, 2265.0903, 2270.1790, 2273.0667, 2284.1729, 2287.1509, 2293.1445, 2328.9482, 2343.1899, 2352.9153, 2354.9983, 2356.0093, 2364.8684, 2423.2490, 2450.2375, 2466.2253, 2468.2173, 2475.2114, 2486.1069, 2519.9941, 2541.2319, 2542.2505, 2558.3389, 2575.1797, 2646.9695, 2660.9880, 2675.0371, 2695.0181, 2702.2866, 2705.2827, 2713.3413, 2717.3323, 2718.2998, 2721.2900, 2744.2466, 2763.3267, 2770.3513, 2785.3567, 2788.2341, 2792.2034, 2798.2407, 2801.2722, 2803.2302, 2808.2080, 2817.2480, 2820.2319, 2833.2026, 2843.3906, 2846.3865, 2847.3792, 2870.3926, 2873.3279, 2889.3057, 2892.4070, 2896.4392, 2915.4314, 2919.4495, 2961.2297, 3071.3845, 3076.4626, 3081.6108, 3087.3884, 3101.4309, 3118.6643, 3143.5120, 3159.5193, 3173.5476, 3184.4751, 3236.5454, 3248.6077, 3253.6089, 3265.5996, 3269.6123, 3285.5964, 3331.5325, 3334.6331, 3335.6592, 3347.4841, 3397.6499, 3411.6399, 3427.6182, 3435.6516, 3444.5942, 3457.6045, 3463.5820, 3473.5864, 3487.6257, 3491.6506, 3503.7678, 3509.5947, 3521.6812, 3552.5681, 3574.6165, 3590.6309, 3624.6504, 3636.7607, 3640.6731, 3646.7363, 3651.7256, 3665.7231, 3669.7129, 3692.6494, 3696.7678, 3707.6807, 3714.6301, 3719.6519, 3722.5593, 3723.6516, 3735.6885, 3751.6423, 3937.7585, 3941.8464, 3942.8328, 3955.8433, 4092.8938, 4147.8613, 4162.9160, 4254.9053, 4272.9224, 4290.9102, 4306.9575, 4309.8506, 4323.8926, 4397.9277, 4415.8896, 4449.1777, 4455.1221, 4459.2368, 4494.0420, 4501.0767, 4510.0269, 4525.1040, 4530.0112, 4839.1138, 4846.0161

11. Q0P578 **Mass:** 24511 **Score:** 22 **Expect:** 1.6e+002 **Matches:** 7

Estrogen receptor binding site associated, antigen, 9 OS=Bos taurus GN=EBAG9 PE=2 SV=1

Observed	Mr(expt)	Mr(calc)	ppm	Start	End	Miss	Peptide
735.4044	734.3971	734.4075	-14.12	2 -	7	0	M.AITQFR.L
830.4365	829.4292	829.4406	-13.67	185 -	191	1	R.AAEQQRK.K
891.4725	890.4653	890.4644	1.01	196 -	202	1	K.EAQRLMK.K + Oxidation (M)
1034.5706	1033.5633	1033.5590	4.21	200 -	207	2	R.LMKKEQNK.I + Oxidation (M)
1074.6005	1073.5932	1073.5876	5.22	25 -	33	2	R.LICRSGRGR.K
1254.6876	1253.6803	1253.6954	-12.05	1 -	10	1	-.MAITQFRLFK.V
4839.1138	4838.1065	4838.2107	-21.54	54 -	96	1	K.QTDVEEWTSWDEDAPTSVKIEGGNGNVATQQNALEQLEPDYFK.D

No match to: 710.3558, 715.3582, 722.4182, 729.4472, 734.4399, 737.4075, 747.4354, 756.3666, 759.4210, 772.4085, 779.4621, 800.3990, 801.4063, 805.4392, 818.4569, 823.4658, 828.4204, 829.4349, 832.2986, 834.3055, 842.4619, 856.3810, 865.4302, 870.4144, 873.3766, 880.4450, 883.4655, 887.4088, 889.4736, 898.4407, 900.5031, 912.4936, 923.5015, 925.4745, 929.4329, 938.4833, 957.5629, 961.4780, 967.4121, 970.4429, 972.4810, 974.4342, 978.5226, 983.6148, 988.4271, 993.4903, 995.3998, 998.5886, 1006.4926, 1007.5641, 1021.5605, 1024.5408, 1032.5048, 1036.5566, 1043.5085, 1044.5150, 1046.5917, 1056.5394, 1057.6062, 1087.5505, 1113.5382, 1122.5513, 1123.5905, 1130.5541, 1143.4395, 1145.6267, 1177.6389, 1178.6249, 1180.6364, 1184.6396, 1194.5638, 1198.6896, 1216.7015, 1227.6558, 1243.6030, 1248.6648, 1255.5952, 1264.7084, 1269.6405, 1273.6479, 1278.6892, 1281.6271, 1289.6858, 1301.6105, 1304.6399, 1312.7454, 1325.6327, 1326.6665, 1328.6610, 1336.7188, 1337.6541, 1338.6735, 1349.6659, 1351.6846, 1356.6124, 1361.6910, 1365.6167, 1372.5992, 1382.5925, 1383.7246, 1390.6946, 1420.7117, 1423.6814, 1445.7860, 1456.7703, 1457.7407, 1467.7371, 1470.7511, 1488.7211, 1494.7380, 1497.8768, 1500.7181, 1503.7421, 1513.7554, 1519.7505, 1528.6958, 1530.6902, 1540.7820, 1544.8356, 1556.8394, 1562.7800, 1563.7925, 1572.7056, 1578.7816, 1580.8422, 1599.9109, 1616.7953, 1621.7692, 1628.7004, 1628.8589, 1635.7825, 1642.7168, 1647.8525, 1650.8430, 1657.9056, 1666.7905, 1696.8221, 1706.8651, 1707.9131, 1717.9056, 1722.9384, 1730.8652, 1738.7958, 1741.9946, 1755.8914, 1758.9082, 1771.0306, 1774.9175, 1787.8247, 1809.0355, 1862.9480, 1883.7240, 1928.0236, 1932.0079, 1938.0043, 1940.0157, 1953.8590, 1959.9622, 1961.0220, 1969.9949, 1981.0421, 1990.9496, 1997.8253, 2007.9401, 2011.8949, 2015.9641, 2023.9448, 2027.1273, 2032.0186, 2044.0441, 2045.9841, 2060.9731, 2067.1531, 2086.0945, 2088.1196, 2108.9956, 2112.9929, 2114.9592, 2139.0918, 2144.0564, 2160.0481, 2174.1157, 2179.0405, 2199.1233, 2211.0984, 2229.1216, 2239.1421, 2242.1345, 2245.8398, 2251.1340, 2255.1416, 2265.0903, 2270.1790, 2273.0667, 2284.1729, 2287.1509, 2293.1445, 2328.9482, 2343.1899, 2352.9153, 2354.9983, 2356.0093, 2364.8684, 2414.2334, 2423.2490, 2450.2375, 2466.2253, 2468.2173, 2475.2114, 2486.1069, 2519.9941, 2541.2319, 2542.2505, 2558.3389, 2575.1797, 2646.9695, 2660.9880, 2675.0371, 2695.0181, 2702.2866, 2705.2827, 2713.3413, 2717.3323, 2718.2998, 2721.2900, 2744.2466, 2763.3267, 2770.3513, 2785.3567, 2787.2734, 2788.2341, 2792.2034, 2798.2407, 2801.2722, 2803.2302, 2808.2080, 2817.2480, 2820.2319, 2833.2026, 2843.3906, 2846.3865, 2847.3792, 2870.3926, 2873.3279, 2889.3057, 2892.4070, 2896.4392, 2912.4277, 2915.4314, 2919.4495, 2961.2297, 3071.3845, 3076.4626, 3081.6108, 3087.3884, 3101.4309, 3118.6643, 3143.5120, 3159.5193, 3173.5476, 3184.4751, 3236.5454, 3248.6077, 3253.6089, 3265.5996, 3269.6123, 3285.5964, 3331.5325, 3334.6331, 3335.6592, 3347.4841, 3397.6499, 3411.6399, 3412.5662, 3427.6182, 3428.5718, 3435.6516, 3444.5942, 3457.6045, 3463.5820, 3473.5864, 3487.6257, 3491.6506, 3503.7678, 3509.5947, 3521.6812, 3552.5681, 3574.6165, 3590.6309, 3624.6504, 3636.7607, 3640.6731, 3646.7363, 3651.7256, 3665.7231, 3669.7129, 3692.6494, 3696.7678, 3707.6807, 3714.6301, 3719.6519, 3722.5593, 3723.6516, 3735.6885, 3751.6423, 3830.7644, 3937.7585, 3941.8464, 3942.8328, 3955.8433, 4092.8938, 4147.8613, 4162.9160, 4254.9053, 4272.9224, 4290.9102, 4306.9575, 4309.8506, 4323.8926, 4397.9277, 4415.8896, 4449.1777, 4455.1221, 4459.2368, 4494.0420, 4501.0767, 4510.0269, 4525.1040, 4530.0112, 4846.0161

12. F1MDD0 **Mass:** 13795 **Score:** 22 **Expect:** 1.6e+002 **Matches:** 4

Uncharacterized protein OS=Bos taurus GN=PSMG4 PE=4 SV=2

Observed	Mr(expt)	Mr(calc)	ppm	Start	End	Miss	Peptide
1255.5952	1254.5879	1254.5591	23.0	114 -	123	1	K.EEMQAFPEKF.-
1349.6659	1348.6586	1348.6696	-8.17	112 -	122	1	R.IKEEMQAFPEK.F
3081.6108	3080.6035	3080.5812	7.25	58 -	86	2	R.YDSIPLSTALLGDTSDTMSTGLAQRLARK.T
3503.7678	3502.7605	3502.7429	5.03	2 -	32	1	M.ERPAAAGGGDVSLHNFSARLWEQLVHFHVMR.L + Oxidation (M)

No match to: 710.3558, 715.3582, 722.4182, 729.4472, 734.4399, 735.4044, 737.4075, 747.4354, 756.3666, 759.4210, 772.4085, 779.4621, 800.3990, 801.4063, 805.4392, 818.4569, 823.4658, 828.4204, 829.4349, 830.4365, 832.2986, 834.3055, 842.4619, 856.3810, 865.4302, 870.4144, 873.3766, 880.4450, 883.4655, 887.4088, 889.4736, 891.4725, 898.4407, 900.5031, 912.4936, 923.5015, 925.4745, 929.4329, 938.4833, 957.5629, 961.4780, 967.4121, 970.4429, 972.4810, 974.4342, 978.5226, 983.6148, 988.4271, 993.4903, 995.3998, 998.5886, 1006.4926, 1007.5641, 1021.5605, 1024.5408, 1032.5048, 1034.5706, 1036.5566, 1043.5085, 1044.5150, 1046.5917, 1056.5394,

1057.6062, 1074.6005, 1087.5505, 1113.5382, 1122.5513, 1123.5905, 1130.5541, 1143.4395, 1145.6267, 1177.6389, 1178.6249, 1180.6364, 1184.6396, 1194.5638, 1198.6896, 1216.7015, 1227.6558, 1243.6030, 1248.6648, 1254.6876, 1264.7084, 1269.6405, 1273.6479, 1278.6892, 1281.6271, 1289.6858, 1301.6105, 1304.6399, 1312.7454, 1325.6327, 1326.6665, 1328.6610, 1336.7188, 1337.6541, 1338.6735, 1351.6846, 1356.6124, 1361.6910, 1365.6167, 1372.5992, 1382.5925, 1383.7246, 1390.6946, 1420.7117, 1423.6814, 1445.7860, 1456.7703, 1457.7407, 1467.7371, 1470.7511, 1488.7211, 1494.7380, 1497.8768, 1500.7181, 1503.7421, 1513.7554, 1519.7505, 1528.6958, 1530.6902, 1540.7820, 1544.8356, 1556.8394, 1562.7800, 1563.7925, 1572.7056, 1578.7816, 1580.8422, 1599.9109, 1616.7953, 1621.7692, 1628.7004, 1628.8589, 1635.7825, 1642.7168, 1647.8525, 1650.8430, 1657.9056, 1666.7905, 1696.8221, 1706.8651, 1707.9131, 1717.9056, 1722.9384, 1730.8652, 1738.7958, 1741.9946, 1755.8914, 1758.9082, 1771.0306, 1774.9175, 1787.8247, 1809.0355, 1862.9480, 1883.7240, 1928.0236, 1932.0079, 1938.0043, 1940.0157, 1953.8590, 1959.9622, 1961.0220, 1969.9949, 1981.0421, 1990.9496, 1997.8253, 2007.9401, 2011.8949, 2015.9641, 2023.9448, 2027.1273, 2032.0186, 2044.0441, 2045.9841, 2060.9731, 2067.1531, 2086.0945, 2088.1196, 2108.9956, 2112.9929, 2114.9592, 2139.0918, 2144.0564, 2160.0481, 2174.1157, 2179.0405, 2199.1233, 2211.0984, 2229.1216, 2239.1421, 2242.1345, 2245.8398, 2251.1340, 2255.1416, 2265.0903, 2270.1790, 2273.0667, 2284.1729, 2287.1509, 2293.1445, 2328.9482, 2343.1899, 2352.9153, 2354.9983, 2356.0093, 2364.8684, 2414.2334, 2423.2490, 2450.2375, 2466.2253, 2468.2173, 2475.2114, 2486.1069, 2519.9941, 2541.2319, 2542.2505, 2558.3389, 2575.1797, 2646.9695, 2660.9880, 2675.0371, 2695.0181, 2702.2866, 2705.2827, 2713.3413, 2717.3323, 2718.2998, 2721.2900, 2744.2466, 2763.3267, 2770.3513, 2785.3567, 2787.2734, 2788.2341, 2792.2034, 2798.2407, 2801.2722, 2803.2302, 2808.2080, 2817.2480, 2820.2319, 2833.2026, 2843.3906, 2846.3865, 2847.3792, 2870.3926, 2873.3279, 2889.3057, 2892.4070, 2896.4392, 2912.4277, 2915.4314, 2919.4495, 2961.2297, 3071.3845, 3076.4626, 3087.3884, 3101.4309, 3118.6643, 3143.5120, 3159.5193, 3173.5476, 3184.4751, 3236.5454, 3248.6077, 3253.6089, 3265.5996, 3269.6123, 3285.5964, 3331.5325, 3334.6331, 3335.6592, 3347.4841, 3397.6499, 3411.6399, 3412.5662, 3427.6182, 3428.5718, 3435.6516, 3444.5942, 3457.6045, 3463.5820, 3473.5864, 3487.6257, 3491.6506, 3509.5947, 3521.6812, 3552.5681, 3574.6165, 3590.6309, 3624.6504, 3636.7607, 3640.6731, 3646.7363, 3651.7256, 3665.7231, 3669.7129, 3692.6494, 3696.7678, 3707.6807, 3714.6301, 3719.6519, 3722.5593, 3723.6516, 3735.6885, 3751.6423, 3830.7644, 3937.7585, 3941.8464, 3942.8328, 3955.8433, 4092.8938, 4147.8613, 4162.9160, 4254.9053, 4272.9224, 4290.9102, 4306.9575, 4309.8506, 4323.8926, 4397.9277, 4415.8896, 4449.1777, 4455.1221, 4459.2368, 4494.0420, 4501.0767, 4510.0269, 4525.1040, 4530.0112, 4839.1138, 4846.0161

13. E1BMG0 **Mass:** 50437 **Score:** 21 **Expect:** 1.8e+002 **Matches:** 12

Uncharacterized protein OS=Bos taurus PE=4 SV=2

Observed	Mr(expt)	Mr(calc)	ppm	Start	End	Miss	Peptide
961.4780	960.4708	960.4512	20.3	10	19	0	K.GDEALSAGNK.D
1361.6910	1360.6837	1360.7099	-19.25	43	54	2	R.SVSFTKKGDHQK.Y
1787.8247	1786.8174	1786.8481	-17.17	367	380	1	K.DCEKYIQLEMTLTK.G + Oxidation (M)
2015.9641	2014.9568	2014.9306	13.0	123	139	0	K.NPFIVSDLYQMLDSDSR.T + Oxidation (M)
2179.0405	2178.0332	2178.0501	-7.75	340	357	1	K.GDYPQARNHQQVSCLLHR.T
2273.0667	2272.0594	2272.0794	-8.79	123	141	1	K.NPFIVSDLYQMLDSDSRTR.T + Oxidation (M)
2541.2319	2540.2246	2540.2693	-17.60	142	163	0	R.TLLMDPTYQELIGQHTTGHLTR.A + Oxidation (M)
2721.2900	2720.2827	2720.2184	23.7	250	272	1	R.QSQHQDPTNMTHMTNQGARYFTK.D
2912.4277	2911.4204	2911.4074	4.49	413	437	1	K.QVADSHHGYTVLQNHQHNSLEDVKR.-
3248.6077	3247.6004	3247.6374	-11.37	285	312	2	K.AFEEQLNNHQQIAKPYTQIGRAYSKEGK.Y
3331.5325	3330.5252	3330.4854	11.9	240	268	1	K.TNFDSALNVRQSQHQDPTNMTHMTNQGAR.Y + 2 Oxidation (M)
3427.6182	3426.6109	3426.5906	5.94	239	268	2	K.KTNFDSALNVRQSQHQDPTNMTHMTNQGAR.Y

No match to: 710.3558, 715.3582, 722.4182, 729.4472, 734.4399, 735.4044, 737.4075, 747.4354, 756.3666, 759.4210, 772.4085, 779.4621, 800.3990, 801.4063, 805.4392, 818.4569, 823.4658, 828.4204, 829.4349, 830.4365, 832.2986, 834.3055, 842.4619, 856.3810, 865.4302, 870.4144, 873.3766, 880.4450, 883.4655, 887.4088, 889.4736, 891.4725, 898.4407, 900.5031, 912.4936, 923.5015, 925.4745, 929.4329, 938.4833, 957.5629, 967.4121, 970.4429, 972.4810, 974.4342, 978.5226, 983.6148, 988.4271, 993.4903, 995.3998, 998.5886, 1006.4926, 1007.5641, 1021.5605, 1024.5408, 1032.5048, 1034.5706, 1036.5566, 1043.5085, 1044.5150, 1046.5917, 1056.5394, 1057.6062, 1074.6005, 1087.5505, 1113.5382, 1122.5513, 1123.5905, 1130.5541, 1143.4395, 1145.6267, 1177.6389, 1178.6249, 1180.6364, 1184.6396, 1194.5638, 1198.6896, 1216.7015, 1227.6558, 1243.6030, 1248.6648, 1254.6876, 1255.5952, 1264.7084, 1269.6405, 1273.6479, 1278.6892, 1281.6271, 1289.6858, 1301.6105, 1304.6399, 1312.7454, 1325.6327, 1326.6665, 1328.6610, 1336.7188, 1337.6541, 1338.6735, 1349.6659, 1351.6846, 1356.6124, 1365.6167, 1372.5992, 1382.5925, 1383.7246, 1390.6946, 1420.7117, 1423.6814, 1445.7860, 1456.7703, 1457.7407, 1467.7371, 1470.7511, 1488.7211, 1494.7380, 1497.8768, 1500.7181, 1503.7421, 1513.7554, 1519.7505, 1528.6958, 1530.6902, 1540.7820, 1544.8356, 1556.8394, 1562.7800, 1563.7925, 1572.7056, 1578.7816, 1580.8422, 1599.9109, 1616.7953, 1621.7692, 1628.7004, 1628.8589, 1635.7825, 1642.7168, 1647.8525, 1650.8430, 1657.9056, 1666.7905, 1696.8221, 1706.8651, 1707.9131, 1717.9056, 1722.9384, 1730.8652, 1738.7958, 1741.9946, 1755.8914, 1758.9082, 1771.0306, 1774.9175, 1809.0355, 1862.9480, 1883.7240, 1928.0236, 1932.0079, 1938.0043, 1940.0157, 1953.8590, 1959.9622, 1961.0220, 1969.9949, 1981.0421, 1990.9496, 1997.8253, 2007.9401, 2011.8949, 2023.9448, 2027.1273, 2032.0186, 2044.0441, 2045.9841, 2060.9731, 2067.1531, 2086.0945, 2088.1196, 2108.9956, 2112.9929, 2114.9592, 2139.0918, 2144.0564, 2160.0481, 2174.1157, 2199.1233, 2211.0984, 2229.1216, 2239.1421, 2242.1345, 2245.8398, 2251.1340, 2255.1416, 2265.0903, 2270.1790, 2284.1729, 2287.1509, 2293.1445, 2328.9482, 2343.1899, 2352.9153, 2354.9983, 2356.0093, 2364.8684, 2414.2334, 2423.2490, 2450.2375, 2466.2253, 2468.2173, 2475.2114, 2486.1069, 2519.9941, 2542.2505, 2558.3389, 2575.1797, 2646.9695, 2660.9880, 2675.0371, 2695.0181, 2702.2866, 2705.2827, 2713.3413, 2717.3323, 2718.2998, 2744.2466, 2763.3267, 2770.3513, 2785.3567, 2787.2734, 2788.2341, 2792.2034, 2798.2407, 2801.2722, 2803.2302, 2808.2080, 2817.2480, 2820.2319, 2833.2026, 2843.3906, 2846.3865, 2847.3792, 2870.3926, 2873.3279, 2889.3057, 2892.4070, 2896.4392, 2915.4314, 2919.4495, 2961.2297, 3071.3845, 3076.4626, 3081.6108, 3087.3884, 3101.4309, 3118.6643, 3143.5120, 3159.5193, 3173.5476, 3184.4751, 3236.5454, 3253.6089, 3265.5996, 3269.6123, 3285.5964, 3334.6331, 3335.6592, 3347.4841, 3397.6499, 3411.6399, 3412.5662, 3428.5718, 3435.6516, 3444.5942, 3457.6045, 3463.5820, 3473.5864, 3487.6257, 3491.6506, 3503.7678, 3509.5947, 3521.6812, 3552.5681, 3574.6165, 3590.6309, 3624.6504, 3636.7607, 3640.6731, 3646.7363, 3651.7256, 3665.7231, 3669.7129, 3692.6494, 3696.7678, 3707.6807, 3714.6301, 3719.6519, 3722.5593, 3723.6516, 3735.6885, 3751.6423, 3830.7644, 3937.7585, 3941.8464, 3942.8328, 3955.8433, 4092.8938, 4147.8613, 4162.9160, 4254.9053, 4272.9224, 4290.9102, 4306.9575, 4309.8506, 4323.8926, 4397.9277, 4415.8896, 4449.1777, 4455.1221, 4459.2368, 4494.0420, 4501.0767, 4510.0269, 4525.1040, 4530.0112, 4839.1138, 4846.0161

14. E1BAL3 **Mass:** 14666 **Score:** 21 **Expect:** 1.8e+002 **Matches:** 5

Uncharacterized protein OS=Bos taurus PE=4 SV=1

Observed	Mr(expt)	Mr(calc)	ppm	Start	End	Miss	Peptide
1666.7905	1665.7832	1665.7958	-7.56	73	87	1	R.SSEPGDVLDRYSVSR.S
2284.1729	2283.1656	2283.1607	2.15	83	102	2	R.YSVSRSNTENFPLTLKSANR.S
2721.2900	2720.2827	2720.2224	22.2	34	55	1	R.RGQSTTLTCAQDLGHNYMYWYR.Q
2847.3792	2846.3719	2846.3368	12.4	103	127	0	R.SQTSVYFCASSYSTVLHGHLLSMQK.S + Oxidation (M)
3457.6045	3456.5972	3456.5728	7.07	35	63	1	R.GQSTTLTCAQDLGHNYMYWYRQDLGHGLR.L + Oxidation (M)

No match to: 710.3558, 715.3582, 722.4182, 729.4472, 734.4399, 735.4044, 737.4075, 747.4354, 756.3666, 759.4210, 772.4085, 779.4621, 800.3990, 801.4063, 805.4392, 818.4569, 823.4658, 828.4204, 829.4349, 830.4365, 832.2986, 834.3055, 842.4619, 856.3810, 865.4302, 870.4144, 873.3766, 880.4450, 883.4655, 887.4088, 889.4736, 891.4725, 898.4407, 900.5031, 912.4936, 923.5015, 925.4745, 929.4329, 938.4833, 957.5629, 961.4780, 967.4121, 970.4429, 972.4810, 974.4342, 978.5226, 983.6148, 988.4271, 993.4903, 995.3998, 998.5886, 1006.4926, 1007.5641, 1021.5605, 1024.5408, 1032.5048, 1034.5706, 1036.5566, 1043.5085, 1044.5150, 1046.5917, 1056.5394, 1057.6062, 1074.6005, 1087.5505, 1113.5382, 1122.5513, 1123.5905, 1130.5541, 1143.4395, 1145.6267, 1177.6389, 1178.6249, 1180.6364, 1184.6396, 1194.5638, 1198.6896, 1216.7015, 1227.6558, 1243.6030, 1248.6648, 1254.6876, 1255.5952, 1264.7084, 1269.6405, 1273.6479, 1278.6892, 1281.6271, 1289.6858, 1301.6105, 1304.6399, 1312.7454, 1325.6327, 1326.6665, 1328.6610, 1336.7188, 1337.6541, 1338.6735, 1349.6659, 1351.6846, 1356.6124, 1361.6910, 1365.6167, 1372.5992, 1382.5925, 1383.7246, 1390.6946, 1420.7117, 1423.6814, 1445.7860, 1456.7703, 1457.7407, 1467.7371, 1470.7511, 1488.7211, 1494.7380, 1497.8768, 1500.7181, 1503.7421, 1513.7554, 1519.7505, 1528.6958, 1530.6902, 1540.7820, 1544.8356, 1556.8394, 1562.7800, 1563.7925, 1572.7056, 1578.7816, 1580.8422, 1599.9109, 1616.7953, 1621.7692, 1628.7004, 1628.8589, 1635.7825, 1642.7168, 1647.8525, 1650.8430, 1657.9056, 1696.8221, 1706.8651, 1707.9131, 1717.9056, 1722.9384, 1730.8652, 1738.7958, 1741.9946, 1755.8914, 1758.9082, 1771.0306, 1774.9175, 1787.8247, 1809.0355, 1862.9480, 1883.7240, 1928.0236, 1932.0079, 1938.0043, 1940.0157, 1953.8590, 1959.9622, 1961.0220, 1969.9949, 1981.0421, 1990.9496, 1997.8253, 2007.9401, 2011.8949, 2015.9641, 2023.9448, 2027.1273, 2032.0186, 2044.0441, 2045.9841, 2060.9731, 2067.1531, 2086.0945, 2088.1196, 2108.9956, 2112.9929, 2114.9592, 2139.0918, 2144.0564, 2160.0481, 2174.1157, 2179.0405, 2199.1233, 2211.0984, 2229.1216, 2239.1421, 2242.1345, 2245.8398, 2251.1340, 2255.1416, 2265.0903, 2270.1790, 2273.0667, 2287.1509, 2293.1445, 2328.9482, 2343.1899, 2352.9153, 2354.9983, 2356.0093, 2364.8684, 2414.2334, 2423.2490, 2450.2375, 2466.2253, 2468.2173, 2475.2114, 2486.1069, 2519.9941, 2541.2319, 2542.2505, 2558.3389, 2575.1797, 2646.9695, 2660.9880, 2675.0371, 2695.0181, 2702.2866, 2705.2827, 2713.3413, 2717.3323, 2718.2998, 2744.2466, 2763.3267, 2770.3513, 2785.3567, 2787.2734, 2788.2341, 2792.2034, 2798.2407, 2801.2722, 2803.2302, 2808.2080, 2817.2480, 2820.2319, 2833.2026, 2843.3906, 2846.3865, 2870.3926, 2873.3279, 2889.3057, 2892.4070, 2896.4392, 2912.4277, 2915.4314, 2919.4495, 2961.2297, 3071.3845,

3076.4626, 3081.6108, 3087.3884, 3101.4309, 3118.6643, 3143.5120, 3159.5193, 3173.5476, 3184.4751, 3236.5454, 3248.6077, 3253.6089, 3265.5996, 3269.6123, 3285.5964, 3331.5325, 3334.6331, 3335.6592, 3347.4841, 3397.6499, 3411.6399, 3412.5662, 3427.6182, 3428.5718, 3435.6516, 3444.5942, 3463.5820, 3473.5864, 3487.6257, 3491.6506, 3503.7678, 3509.5947, 3521.6812, 3552.5681, 3574.6165, 3590.6309, 3624.6504, 3636.7607, 3640.6731, 3646.7363, 3651.7256, 3665.7231, 3669.7129, 3692.6494, 3696.7678, 3707.6807, 3714.6301, 3719.6519, 3722.5593, 3723.6516, 3735.6885, 3751.6423, 3830.7644, 3937.7585, 3941.8464, 3942.8328, 3955.8433, 4092.8938, 4147.8613, 4162.9160, 4254.9053, 4272.9224, 4290.9102, 4306.9575, 4309.8506, 4323.8926, 4397.9277, 4415.8896, 4449.1777, 4455.1221, 4459.2368, 4494.0420, 4501.0767, 4510.0269, 4525.1040, 4530.0112, 4839.1138, 4846.0161

15. A0JN40 **Mass:** 89660 **Score:** 21 **Expect:** 2e+002 **Matches:** 18

Kinesin-like protein KIF3C OS=Bos taurus GN=KIF3C PE=2 SV=1

Observed	Mr(expt)	Mr(calc)	ppm	Start	End	Miss	Peptide
756.3666	755.3593	755.3748	-20.54	734 -	739	0	R.ALHMER.L
978.5226	977.5154	977.5294	-14.38	675 -	683	0	K.RPTSAVGYK.R
1264.7084	1263.7011	1263.6856	12.3	1 -	12	2	-.MASKTKASEALK.V
1513.7554	1512.7481	1512.7460	1.42	143 -	154	0	R.ASYLEIYQEEIR.D
1540.7820	1539.7747	1539.7464	18.4	190 -	202	0	K.EIEHVMNLGNQTR.A
1556.8394	1555.8321	1555.8318	0.22	242 -	255	1	K.LNLVDLAGSERQNK.A
1647.8525	1646.8452	1646.8086	22.2	216 -	229	0	R.SHAIFVITVECSER.G
2044.0441	2043.0368	2043.0254	5.57	684 -	701	1	K.RPISQYARVAMAMGSHPR.Y + Oxidation (M)
2086.0945	2085.0872	2085.0450	20.2	610 -	626	2	R.VRQDLEEAQNEQTRELK.L
2144.0564	2143.0491	2143.0514	-1.04	525 -	541	2	R.NIMDHTNEQQKMLELKR.Q + Oxidation (M)
2160.0481	2159.0408	2159.0463	-2.53	525 -	541	2	R.NIMDHTNEQQKMLELKR.Q + 2 Oxidation (M)
2896.4392	2895.4319	2895.3716	20.9	190 -	215	1	K.EIEHVMNLGNQTRAVGSTHMNEVSSR.S
2912.4277	2911.4204	2911.3665	18.5	190 -	215	1	K.EIEHVMNLGNQTRAVGSTHMNEVSSR.S + Oxidation (M)
3253.6089	3252.6016	3252.5728	8.87	187 -	215	2	K.NVKEIEHVMNLGNQTRAVGSTHMNEVSSR.S + Oxidation (M)
3269.6123	3268.6050	3268.5677	11.4	187 -	215	2	K.NVKEIEHVMNLGNQTRAVGSTHMNEVSSR.S + 2 Oxidation (M)
3646.7363	3645.7290	3645.7952	-18.14	70 -	103	0	K.QADLYDETVRPLVDSVLQGFNGTVFAYGQTGTGK.T
4449.1777	4448.1704	4448.2665	-21.60	118 -	154	2	R.GVIPNAFEHIFTHISRSQNQQYLVRASYLEIYQEEIR.D
4525.1040	4524.0967	4524.1696	-16.12	190 -	229	2	K.EIEHVMNLGNQTRAVGSTHMNEVSSRSHAIFVITVECSER.G

No match to: 710.3558, 715.3582, 722.4182, 729.4472, 734.4399, 735.4044, 737.4075, 747.4354, 759.4210, 772.4085, 779.4621, 800.3990, 801.4063, 805.4392, 818.4569, 823.4658, 828.4204, 829.4349, 830.4365, 832.2986, 834.3055, 842.4619, 856.3810, 865.4302, 870.4144, 873.3766, 880.4450, 883.4655, 887.4088, 889.4736, 891.4725, 898.4407, 900.5031, 912.4936, 923.5015, 925.4745, 929.4329, 938.4833, 957.5629, 961.4780, 967.4121, 970.4429, 972.4810, 974.4342, 983.6148, 988.4271, 993.4903, 995.3998, 998.5886, 1006.4926, 1007.5641, 1021.5605, 1024.5408, 1032.5048, 1034.5706, 1036.5566, 1043.5085, 1044.5150, 1046.5917, 1056.5394, 1057.6062, 1074.6005, 1087.5505, 1113.5382, 1122.5513, 1123.5905, 1130.5541, 1143.4395, 1145.6267, 1177.6389, 1178.6249, 1180.6364, 1184.6396, 1194.5638, 1198.6896, 1216.7015, 1227.6558, 1243.6030, 1248.6648, 1254.6876, 1255.5952, 1269.6405, 1273.6479, 1278.6892, 1281.6271, 1289.6858, 1301.6105, 1304.6399, 1312.7454, 1325.6327, 1326.6665, 1328.6610, 1336.7188, 1337.6541, 1338.6735, 1349.6659, 1351.6846, 1356.6124, 1361.6910, 1365.6167, 1372.5992, 1382.5925, 1383.7246, 1390.6946, 1420.7117, 1423.6814, 1445.7860, 1456.7703, 1457.7407, 1467.7371, 1470.7511, 1488.7211, 1494.7380, 1497.8768, 1500.7181, 1503.7421, 1519.7505, 1528.6958, 1530.6902, 1544.8356, 1562.7800, 1563.7925, 1572.7056, 1578.7816, 1580.8422, 1599.9109, 1616.7953, 1621.7692, 1628.7004, 1628.8589, 1635.7825, 1642.7168, 1650.8430, 1657.9056, 1666.7905, 1696.8221, 1706.8651, 1707.9131, 1717.9056, 1722.9384, 1730.8652, 1738.7958, 1741.9946, 1755.8914, 1758.9082, 1771.0306, 1774.9175, 1787.8247, 1809.0355, 1862.9480, 1883.7240, 1928.0236, 1932.0079, 1938.0043, 1940.0157, 1953.8590, 1959.9622, 1961.0220, 1969.9949, 1981.0421, 1990.9496, 1997.8253, 2007.9401, 2011.8949, 2015.9641, 2023.9448, 2027.1273, 2032.0186, 2045.9841, 2060.9731, 2067.1531, 2088.1196, 2108.9956, 2112.9929, 2114.9592, 2139.0918, 2174.1157, 2179.0405, 2199.1233, 2211.0984, 2229.1216, 2239.1421, 2242.1345, 2245.8398, 2251.1340, 2255.1416, 2265.0903, 2270.1790, 2273.0667, 2284.1729, 2287.1509, 2293.1445, 2328.9482, 2343.1899, 2352.9153, 2354.9983, 2356.0093, 2364.8684, 2414.2334, 2423.2490, 2450.2375, 2466.2253, 2468.2173, 2475.2114, 2486.1069, 2519.9941, 2541.2319, 2542.2505, 2558.3389, 2575.1797, 2646.9695, 2660.9880, 2675.0371, 2695.0181, 2702.2866, 2705.2827, 2713.3413, 2717.3323, 2718.2998, 2721.2900, 2744.2466, 2763.3267, 2770.3513, 2785.3567, 2787.2734, 2788.2341, 2792.2034, 2798.2407, 2801.2722, 2803.2302, 2808.2080, 2817.2480, 2820.2319, 2833.2026, 2843.3906, 2846.3865, 2847.3792, 2870.3926, 2873.3279, 2889.3057, 2892.4070, 2915.4314, 2919.4495, 2961.2297, 3071.3845, 3076.4626, 3081.6108, 3087.3884, 3101.4309, 3118.6643, 3143.5120, 3159.5193, 3173.5476, 3184.4751, 3236.5454, 3248.6077, 3265.5996, 3285.5964, 3331.5325, 3334.6331, 3335.6592, 3347.4841, 3397.6499, 3411.6399, 3412.5662, 3427.6182, 3428.5718, 3435.6516, 3444.5942, 3457.6045, 3463.5820, 3473.5864, 3487.6257, 3491.6506, 3503.7678, 3509.5947, 3521.6812, 3552.5681, 3574.6165, 3590.6309, 3624.6504, 3636.7607, 3640.6731, 3651.7256, 3665.7231, 3669.7129, 3692.6494, 3696.7678, 3707.6807, 3714.6301, 3719.6519, 3722.5593, 3723.6516, 3735.6885, 3751.6423, 3830.7644, 3937.7585, 3941.8464, 3942.8328, 3955.8433, 4092.8938, 4147.8613, 4162.9160, 4254.9053, 4272.9224, 4290.9102, 4306.9575, 4309.8506, 4323.8926, 4397.9277, 4415.8896, 4455.1221, 4459.2368, 4494.0420, 4501.0767, 4510.0269, 4530.0112, 4839.1138, 4846.0161

16. Q0VCX5 **Mass:** 49228 **Score:** 21 **Expect:** 2.1e+002 **Matches:** 9

Eukaryotic peptide chain release factor subunit 1 OS=Bos taurus GN=ETF1 PE=2 SV=3

Observed	Mr(expt)	Mr(calc)	ppm	Start	End	Miss	Peptide
1647.8525	1646.8452	1646.8152	18.3	131 -	145	0	K.FHTEALTALLSDDSK.F
1928.0236	1927.0163	1926.9799	18.9	2 -	18	2	M.ADDPSAADRNVEIWKIK.K
1938.0043	1936.9970	1937.0040	-3.61	48 -	65	2	R.VAKMLADEFGTASNIKSR.V
2027.1273	2026.1200	2026.0881	15.8	29 -	47	1	R.GNGTSMISLIIPPKDQISR.V
2032.0186	2031.0113	2031.0347	-11.51	88 -	106	0	K.VPPNGLVVYCGTIVTEEGK.E
2896.4392	2895.4319	2895.4502	-6.30	253 -	279	0	K.LVDISYGGENGFNQAIELSTEVLSNVK.F
2912.4277	2911.4204	2911.4789	-20.10	107 -	130	2	K.EKKVNIDFEPFKPINTSLYLCDNK.F
3509.5947	3508.5874	3508.6245	-10.57	355 -	383	1	K.SHFTDKETGQEHELIESMPLLEWFANNYK.K + Oxidation (M)
3751.6423	3750.6350	3750.6573	-5.95	405 -	437	2	K.GFGGIGGILRYRVDFQGMEYQGGDDEFFDLDDY.-

No match to: 710.3558, 715.3582, 722.4182, 729.4472, 734.4399, 735.4044, 737.4075, 747.4354, 756.3666, 759.4210, 772.4085, 779.4621, 800.3990, 801.4063, 805.4392, 818.4569, 823.4658, 828.4204, 829.4349, 830.4365, 832.2986, 834.3055, 842.4619, 856.3810, 865.4302, 870.4144, 873.3766, 880.4450, 883.4655, 887.4088, 889.4736, 891.4725, 898.4407, 900.5031, 912.4936, 923.5015, 925.4745, 929.4329, 938.4833, 957.5629, 961.4780, 967.4121, 970.4429, 972.4810, 974.4342, 978.5226, 983.6148, 988.4271, 993.4903, 995.3998, 998.5886, 1006.4926, 1007.5641, 1021.5605, 1024.5408, 1032.5048, 1034.5706, 1036.5566, 1043.5085, 1044.5150, 1046.5917, 1056.5394, 1057.6062, 1074.6005, 1087.5505, 1113.5382, 1122.5513, 1123.5905, 1130.5541, 1143.4395, 1145.6267, 1177.6389, 1178.6249, 1180.6364, 1184.6396, 1194.5638, 1198.6896, 1216.7015, 1227.6558, 1243.6030, 1248.6648, 1254.6876, 1255.5952, 1264.7084, 1269.6405, 1273.6479, 1278.6892, 1281.6271, 1289.6858, 1301.6105, 1304.6399, 1312.7454, 1325.6327, 1326.6665, 1328.6610, 1336.7188, 1337.6541, 1338.6735, 1349.6659, 1351.6846, 1356.6124, 1361.6910, 1365.6167, 1372.5992, 1382.5925, 1383.7246, 1390.6946, 1420.7117, 1423.6814, 1445.7860, 1456.7703, 1457.7407, 1467.7371, 1470.7511, 1488.7211, 1494.7380, 1497.8768, 1500.7181, 1503.7421, 1513.7554, 1519.7505, 1528.6958, 1530.6902, 1540.7820, 1544.8356, 1556.8394, 1562.7800, 1563.7925, 1572.7056, 1578.7816, 1580.8422, 1599.9109, 1616.7953, 1621.7692, 1628.7004, 1628.8589, 1635.7825, 1642.7168, 1650.8430, 1657.9056, 1666.7905, 1696.8221, 1706.8651, 1707.9131, 1717.9056, 1722.9384, 1730.8652, 1738.7958, 1741.9946, 1755.8914, 1758.9082, 1771.0306, 1774.9175, 1787.8247, 1809.0355, 1862.9480, 1883.7240, 1932.0079, 1940.0157, 1953.8590, 1959.9622, 1961.0220, 1969.9949, 1981.0421, 1990.9496, 1997.8253, 2007.9401, 2011.8949, 2015.9641, 2023.9448, 2044.0441, 2045.9841, 2060.9731, 2067.1531, 2086.0945, 2088.1196, 2108.9956, 2112.9929, 2114.9592, 2139.0918, 2144.0564, 2160.0481, 2174.1157, 2179.0405, 2199.1233, 2211.0984, 2229.1216, 2239.1421, 2242.1345, 2245.8398, 2251.1340, 2255.1416, 2265.0903, 2270.1790, 2273.0667, 2284.1729, 2287.1509, 2293.1445, 2328.9482, 2343.1899, 2352.9153, 2354.9983, 2356.0093, 2364.8684, 2414.2334, 2423.2490, 2450.2375, 2466.2253, 2468.2173, 2475.2114, 2486.1069, 2519.9941, 2541.2319, 2542.2505, 2558.3389, 2575.1797, 2646.9695, 2660.9880, 2675.0371, 2695.0181, 2702.2866, 2705.2827, 2713.3413, 2717.3323, 2718.2998, 2721.2900, 2744.2466, 2763.3267, 2770.3513, 2785.3567, 2787.2734, 2788.2341, 2792.2034, 2798.2407, 2801.2722, 2803.2302, 2808.2080, 2817.2480, 2820.2319, 2833.2026, 2843.3906, 2846.3865, 2847.3792, 2870.3926, 2873.3279, 2889.3057, 2892.4070, 2915.4314, 2919.4495, 2961.2297, 3071.3845, 3076.4626, 3081.6108, 3087.3884, 3101.4309, 3118.6643, 3143.5120, 3159.5193, 3173.5476, 3184.4751, 3236.5454, 3248.6077, 3253.6089, 3265.5996, 3269.6123, 3285.5964, 3331.5325, 3334.6331, 3335.6592, 3347.4841, 3397.6499, 3411.6399, 3412.5662, 3427.6182, 3428.5718, 3435.6516, 3444.5942, 3457.6045, 3463.5820, 3473.5864, 3487.6257, 3491.6506, 3503.7678, 3521.6812, 3552.5681, 3574.6165, 3590.6309, 3624.6504, 3636.7607, 3640.6731, 3646.7363, 3651.7256, 3665.7231, 3669.7129, 3692.6494, 3696.7678, 3707.6807, 3714.6301, 3719.6519, 3722.5593, 3723.6516, 3735.6885,

3830.7644, 3937.7585, 3941.8464, 3942.8328, 3955.8433, 4092.8938, 4147.8613, 4162.9160, 4254.9053, 4272.9224, 4290.9102, 4306.9575, 4309.8506, 4323.8926, 4397.9277, 4415.8896, 4449.1777, 4455.1221, 4459.2368, 4494.0420, 4501.0767, 4510.0269, 4525.1040, 4530.0112, 4839.1138, 4846.0161

17. Q3B7N2 **Mass:** 103486 **Score:** 20 **Expect:** 2.3e+002 **Matches:** 25
Alpha-actinin-1 OS=Bos taurus GN=ACTN1 PE=2 SV=1

Observed	Mr(expt)	Mr(calc)	ppm	Start	End	Miss	Peptide
830.4365	829.4292	829.4181	13.4	556 -	563	0	K.ATLPDADK.E
891.4725	890.4653	890.4644	1.01	414 -	420	1	K.EAMLRQK.D + Oxidation (M)
1113.5382	1112.5309	1112.5251	5.28	313 -	320	1	K.LEDFRDYR.R
1122.5513	1121.5440	1121.5717	-24.65	841 -	849	0	K.NYITVDELR.R
1184.6396	1183.6323	1183.6462	-11.69	292 -	300	1	R.RTIPWLENR.A
1227.6558	1226.6485	1226.6407	6.34	186 -	195	0	R.HRPELIDYGK.L
1269.6405	1268.6332	1268.6262	5.57	313 -	321	2	K.LEDFRDYRR.L
1278.6892	1277.6819	1277.6728	7.16	841 -	850	1	K.NYITVDELRR.E
1301.6105	1300.6032	1300.5982	3.84	705 -	714	0	K.HTNYTMEHIR.V
1325.6327	1324.6254	1324.6483	-17.30	614 -	624	1	R.RDQALTEEHAR.Q
1470.7511	1469.7438	1469.7263	11.9	401 -	413	1	R.QKASIHEAWTDGK.E
1730.8652	1729.8579	1729.8862	-16.34	804 -	818	0	R.LGVVTFQAFIDFMSR.E
1862.9480	1861.9407	1861.9872	-24.98	148 -	162	2	K.EGLLLWCQRKTAPYK.N
2007.9401	2006.9328	2006.9560	-11.56	237 -	254	0	K.AIMTYVSSFYHAFSGAQK.A
2023.9448	2022.9375	2022.9509	-6.63	237 -	254	0	K.AIMTYVSSFYHAFSGAQK.A + Oxidation (M)
2045.9841	2044.9768	2044.9458	15.1	301 -	317	1	R.APENTMHAMQQKLEDFR.D
2067.1531	2066.1458	2066.1921	-22.41	65 -	82	1	K.LMLLLEVISGERLAKPER.G
2721.2900	2720.2827	2720.3017	-6.96	237 -	261	1	K.AIMTYVSSFYHAFSGAQKAETAANR.I
3071.3845	3070.3772	3070.3464	10.0	864 -	892	0	R.MAPYTGPDAVPGALDYMSFSTALYGESDL.- + 2 Oxidation (M)
3248.6077	3247.6004	3247.6070	-2.03	265 -	291	1	K.VLAVNQENEQLMEDYEKLASDLLEWIR.R
3269.6123	3268.6050	3268.5968	2.51	650 -	676	2	K.MEEIGRISIEMHGTLEDQLNHLRQYEK.S
3285.5964	3284.5891	3284.5917	-0.80	650 -	676	2	K.MEEIGRISIEMHGTLEDQLNHLRQYEK.S + Oxidation (M)
3457.6045	3456.5972	3456.6218	-7.11	804 -	834	1	R.LGVVTFQAFIDFMSRETADTDTADQVMASFK.I + Oxidation (M)
3473.5864	3472.5791	3472.6167	-10.82	804 -	834	1	R.LGVVTFQAFIDFMSRETADTDTADQVMASFK.I + 2 Oxidation (M)
4494.0420	4493.0347	4493.1267	-20.48	221 -	261	2	K.MLDAEDIVGTARPDEKAIMTYVSSFYHAFSGAQKAETAANR.I + 2 Oxidation (M)

No match to: 710.3558, 715.3582, 722.4182, 729.4472, 734.4399, 735.4044, 737.4075, 747.4354, 756.3666, 759.4210, 772.4085, 779.4621, 800.3990, 801.4063, 805.4392, 818.4569, 823.4658, 828.4204, 829.4349, 832.2986, 834.3055, 842.4619, 856.3810, 865.4302, 870.4144, 873.3766, 880.4450, 883.4655, 887.4088, 889.4736, 898.4407, 900.5031, 912.4936, 923.5015, 925.4745, 929.4329, 938.4833, 957.5629, 961.4780, 967.4121, 970.4429, 972.4810, 974.4342, 978.5226, 983.6148, 988.4271, 993.4903, 995.3998, 998.5886, 1006.4926, 1007.5641, 1021.5605, 1024.5408, 1032.5048, 1034.5706, 1036.5566, 1043.5085, 1044.5150, 1046.5917, 1056.5394, 1057.6062, 1074.6005, 1087.5505, 1123.5905, 1130.5541, 1143.4395, 1145.6267, 1177.6389, 1178.6249, 1180.6364, 1194.5638, 1198.6896, 1216.7015, 1243.6030, 1248.6648, 1254.6876, 1255.5952, 1264.7084, 1273.6479, 1281.6271, 1289.6858, 1304.6399, 1312.7454, 1326.6665, 1328.6610, 1336.7188, 1337.6541, 1338.6735, 1349.6659, 1351.6846, 1356.6124, 1361.6910, 1365.6167, 1372.5992, 1382.5925, 1383.7246, 1390.6946, 1420.7117, 1423.6814, 1445.7860, 1456.7703, 1457.7407, 1467.7371, 1488.7211, 1494.7380, 1497.8768, 1500.7181, 1503.7421, 1513.7554, 1519.7505, 1528.6958, 1530.6902, 1540.7820, 1544.8356, 1556.8394, 1562.7800, 1563.7925, 1572.7056, 1578.7816, 1580.8422, 1599.9109, 1616.7953, 1621.7692, 1628.7004, 1628.8589, 1635.7825, 1642.7168, 1647.8525, 1650.8430, 1657.9056, 1666.7905, 1696.8221, 1706.8651, 1707.9131, 1717.9056, 1722.9384, 1738.7958, 1741.9946, 1755.8914, 1758.9082, 1771.0306, 1774.9175, 1787.8247, 1809.0355, 1883.7240, 1928.0236, 1932.0079, 1938.0043, 1940.0157, 1953.8590, 1959.9622, 1961.0220, 1969.9949, 1981.0421, 1990.9496, 1997.8253, 2011.8949, 2015.9641, 2027.1273, 2032.0186, 2044.0441, 2060.9731, 2086.0945, 2088.1196, 2108.9956, 2112.9929, 2114.9592, 2139.0918, 2144.0564, 2160.0481, 2174.1157, 2179.0405, 2199.1233, 2211.0984, 2229.1216, 2239.1421, 2242.1345, 2245.8398, 2251.1340, 2255.1416, 2265.0903, 2270.1790, 2273.0667, 2284.1729, 2287.1509, 2293.1445, 2328.9482, 2343.1899, 2352.9153, 2354.9983, 2356.0093, 2364.8684, 2414.2334, 2423.2490, 2450.2375, 2466.2253, 2468.2173, 2475.2114, 2486.1069, 2519.9941, 2541.2319, 2542.2505, 2558.3389, 2575.1797, 2646.9695, 2660.9880, 2675.0371, 2695.0181, 2702.2866, 2705.2827, 2713.3413, 2717.3323, 2718.2998, 2744.2466, 2763.3267, 2770.3513, 2785.3567, 2787.2734, 2788.2341, 2792.2034, 2798.2407, 2801.2722, 2803.2302, 2808.2080, 2817.2480, 2820.2319, 2833.2026, 2843.3906, 2846.3865, 2847.3792, 2870.3926, 2873.3279, 2889.3057, 2892.4070, 2896.4392, 2912.4277, 2915.4314, 2919.4495, 2961.2297, 3076.4626, 3081.6108, 3087.3884, 3101.4309, 3118.6643, 3143.5120, 3159.5193, 3173.5476, 3184.4751, 3236.5454, 3253.6089, 3265.5996, 3331.5325, 3334.6331, 3335.6592, 3347.4841, 3397.6499, 3411.6399, 3412.5662, 3427.6182, 3428.5718, 3435.6516, 3444.5942, 3463.5820, 3487.6257, 3491.6506, 3503.7678, 3509.5947, 3521.6812, 3552.5681, 3574.6165, 3590.6309, 3624.6504, 3636.7607, 3640.6731, 3646.7363, 3651.7256, 3665.7231, 3669.7129, 3692.6494, 3696.7678, 3707.6807, 3714.6301, 3719.6519, 3722.5593, 3723.6516, 3735.6885, 3751.6423, 3830.7644, 3937.7585, 3941.8464, 3942.8328, 3955.8433, 4092.8938, 4147.8613, 4162.9160, 4254.9053, 4272.9224, 4290.9102, 4306.9575, 4309.8506, 4323.8926, 4397.9277, 4415.8896, 4449.1777, 4455.1221, 4459.2368, 4501.0767, 4510.0269, 4525.1040, 4530.0112, 4839.1138, 4846.0161

18. P08239 **Mass:** 40611 **Score:** 20 **Expect:** 2.3e+002 **Matches:** 12
Guanine nucleotide-binding protein G(o) subunit alpha OS=Bos taurus GN=GNAO1 PE=1 SV=4

Observed	Mr(expt)	Mr(calc)	ppm	Start	End	Miss	Peptide
891.4725	890.4653	890.4610	4.74	199 -	206	0	R.LFDVGGQR.S
1044.5150	1043.5077	1043.4924	14.7	155 -	162	0	K.YYLDSLDR.I
1057.6062	1056.5989	1056.6179	-17.95	36 -	46	0	K.LLLLGAGESGK.S
1145.6267	1144.6194	1144.6088	9.31	22 -	32	1	K.NLKEDGISAAK.D
1494.7380	1493.7307	1493.7330	-1.55	101 -	113	2	R.KADAKMVCDVVSR.M + Oxidation (M)
1562.7800	1561.7727	1561.7518	13.4	2 -	15	1	M.GCTLSAEERAALER.S
2174.1157	2173.1084	2173.1491	-18.72	163 -	181	2	R.IGAADYQPTEQDILRTRVK.T
2873.3279	2872.3206	2872.3198	0.28	131 -	154	2	R.LWGDSGIQECFNRSREYQLNDSAK.Y
3331.5325	3330.5252	3330.5063	5.70	102 -	130	2	K.ADAKMVCDVVSRMEDTEPFSPELLSAMMR.L + Oxidation (M)
3347.4841	3346.4768	3346.5012	-7.28	102 -	130	2	K.ADAKMVCDVVSRMEDTEPFSPELLSAMMR.L + 2 Oxidation (M)
4290.9102	4289.9029	4289.9854	-19.22	318 -	354	1	K.EIYCHMTCATDTNNIQVVFDAVTDIIIANNLRGCGLY.- + Oxidation (M)
4525.1040	4524.0967	4523.9987	21.7	106 -	143	2	K.MVCDVVSRMEDTEPFSPELLSAMMRLWGDSGIQECFNR.S + 2 Oxidation (M)

No match to: 710.3558, 715.3582, 722.4182, 729.4472, 734.4399, 735.4044, 737.4075, 747.4354, 756.3666, 759.4210, 772.4085, 779.4621, 800.3990, 801.4063, 805.4392, 818.4569, 823.4658, 828.4204, 829.4349, 830.4365, 832.2986, 834.3055, 842.4619, 856.3810, 865.4302, 870.4144, 873.3766, 880.4450, 883.4655, 887.4088, 889.4736, 898.4407, 900.5031, 912.4936, 923.5015, 925.4745, 929.4329, 938.4833, 957.5629, 961.4780, 967.4121, 970.4429, 972.4810, 974.4342, 978.5226, 983.6148, 988.4271, 993.4903, 995.3998, 998.5886, 1006.4926, 1007.5641, 1021.5605, 1024.5408, 1032.5048, 1034.5706, 1036.5566, 1043.5085, 1046.5917, 1056.5394, 1074.6005, 1087.5505, 1113.5382, 1122.5513, 1123.5905, 1130.5541, 1143.4395, 1177.6389, 1178.6249, 1180.6364, 1184.6396, 1194.5638, 1198.6896, 1216.7015, 1227.6558, 1243.6030, 1248.6648, 1254.6876, 1255.5952, 1264.7084, 1269.6405, 1273.6479, 1278.6892, 1281.6271, 1289.6858, 1301.6105, 1304.6399, 1312.7454, 1325.6327, 1326.6665, 1328.6610, 1336.7188, 1337.6541, 1338.6735, 1349.6659, 1351.6846, 1356.6124, 1361.6910, 1365.6167, 1372.5992, 1382.5925, 1383.7246, 1390.6946, 1420.7117, 1423.6814, 1445.7860, 1456.7703, 1457.7407, 1467.7371, 1470.7511, 1488.7211, 1497.8768, 1500.7181, 1503.7421, 1513.7554, 1519.7505, 1528.6958, 1530.6902, 1540.7820, 1544.8356, 1556.8394, 1563.7925, 1572.7056, 1578.7816, 1580.8422, 1599.9109, 1616.7953, 1621.7692, 1628.7004, 1628.8589, 1635.7825, 1642.7168, 1647.8525, 1650.8430, 1657.9056, 1666.7905, 1696.8221, 1706.8651, 1707.9131, 1717.9056, 1722.9384, 1730.8652, 1738.7958, 1741.9946, 1755.8914, 1758.9082, 1771.0306, 1774.9175, 1787.8247, 1809.0355, 1862.9480, 1883.7240, 1928.0236, 1932.0079, 1938.0043, 1940.0157, 1953.8590, 1959.9622, 1961.0220, 1969.9949, 1981.0421, 1990.9496, 1997.8253, 2007.9401, 2011.8949, 2015.9641, 2023.9448, 2027.1273, 2032.0186, 2044.0441, 2045.9841, 2060.9731, 2067.1531, 2086.0945, 2088.1196, 2108.9956, 2112.9929, 2114.9592, 2139.0918, 2144.0564, 2160.0481, 2179.0405, 2199.1233, 2211.0984, 2229.1216, 2239.1421, 2242.1345, 2245.8398, 2251.1340, 2255.1416, 2265.0903, 2270.1790, 2273.0667, 2284.1729, 2287.1509, 2293.1445, 2328.9482, 2343.1899, 2352.9153, 2354.9983, 2356.0093, 2364.8684, 2414.2334, 2423.2490, 2450.2375, 2466.2253, 2468.2173, 2475.2114, 2486.1069, 2519.9941, 2541.2319, 2542.2505, 2558.3389, 2575.1797, 2646.9695, 2660.9880, 2675.0371, 2695.0181,

2702.2866, 2705.2827, 2713.3413, 2717.3323, 2718.2998, 2721.2900, 2744.2466, 2763.3267, 2770.3513, 2785.3567, 2787.2734, 2788.2341, 2792.2034, 2798.2407, 2801.2722, 2803.2302, 2808.2080, 2817.2480, 2820.2319, 2833.2026, 2843.3906, 2846.3865, 2847.3792, 2870.3926, 2889.3057, 2892.4070, 2896.4392, 2912.4277, 2915.4314, 2919.4495, 2961.2297, 3071.3845, 3076.4626, 3081.6108, 3087.3884, 3101.4309, 3118.6643, 3143.5120, 3159.5193, 3173.5476, 3184.4751, 3236.5454, 3248.6077, 3253.6089, 3265.5996, 3269.6123, 3285.5964, 3334.6331, 3335.6592, 3397.6499, 3411.6399, 3412.5662, 3427.6182, 3428.5718, 3435.6516, 3444.5942, 3457.6045, 3463.5820, 3473.5864, 3487.6257, 3491.6506, 3503.7678, 3509.5947, 3521.6812, 3552.5681, 3574.6165, 3590.6309, 3624.6504, 3636.7607, 3640.6731, 3646.7363, 3651.7256, 3665.7231, 3669.7129, 3692.6494, 3696.7678, 3707.6807, 3714.6301, 3719.6519, 3722.5593, 3723.6516, 3735.6885, 3751.6423, 3830.7644, 3937.7585, 3941.8464, 3942.8328, 3955.8433, 4092.8938, 4147.8613, 4162.9160, 4254.9053, 4272.9224, 4306.9575, 4309.8506, 4323.8926, 4397.9277, 4415.8896, 4449.1777, 4455.1221, 4459.2368, 4494.0420, 4501.0767, 4510.0269, 4530.0112, 4839.1138, 4846.0161

19. Q5E9I6 **Mass:** 20645 **Score:** 20 **Expect:** 2.3e+002 **Matches:** 6

ADP-ribosylation factor 3 OS=Bos taurus GN=ARF3 PE=2 SV=3

Observed	Mr(expt)	Mr(calc)	ppm	Start	End	Miss	Peptide
1647.8525	1646.8452	1646.8847	-24.00	16 -	30	2	K.KEMRILMVGLDAAGK.T + Oxidation (M)
1666.7905	1665.7832	1665.7702	7.84	105 -	117	1	R.EELMRMLAEDELR.D + 2 Oxidation (M)
2423.2490	2422.2417	2422.2274	5.91	128 -	149	1	K.QDLPNAMNAAEITDKLGLHSLR.H + Oxidation (M)
2717.3323	2716.3250	2716.3741	-18.08	118 -	142	1	R.DAVLLVFANKQDLPNAMNAAEITDK.L + Oxidation (M)
3087.3884	3086.3811	3086.4444	-20.49	152 -	178	0	R.NWYIQATCATSGDGLYEGLDWLANQLK.N
3457.6045	3456.5972	3456.6772	-23.14	152 -	181	2	R.NWYIQATCATSGDGLYEGLDWLANQLKNKK.-

No match to: 710.3558, 715.3582, 722.4182, 729.4472, 734.4399, 735.4044, 737.4075, 747.4354, 756.3666, 759.4210, 772.4085, 779.4621, 800.3990, 801.4063, 805.4392, 818.4569, 823.4658, 828.4204, 829.4349, 830.4365, 832.2986, 834.3055, 842.4619, 856.3810, 865.4302, 870.4144, 873.3766, 880.4450, 883.4655, 887.4088, 889.4736, 891.4725, 898.4407, 900.5031, 912.4936, 923.5015, 925.4745, 929.4329, 938.4833, 957.5629, 961.4780, 967.4121, 970.4429, 972.4810, 974.4342, 978.5226, 983.6148, 988.4271, 993.4903, 995.3998, 998.5886, 1006.4926, 1007.5641, 1021.5605, 1024.5408, 1032.5048, 1034.5706, 1036.5566, 1043.5085, 1044.5150, 1046.5917, 1056.5394, 1057.6062, 1074.6005, 1087.5505, 1113.5382, 1122.5513, 1123.5905, 1130.5541, 1143.4395, 1145.6267, 1177.6389, 1178.6249, 1180.6364, 1184.6396, 1194.5638, 1198.6896, 1216.7015, 1227.6558, 1243.6030, 1248.6648, 1254.6876, 1255.5952, 1264.7084, 1269.6405, 1273.6479, 1278.6892, 1281.6271, 1289.6858, 1301.6105, 1304.6399, 1312.7454, 1325.6327, 1326.6665, 1328.6610, 1336.7188, 1337.6541, 1338.6735, 1349.6659, 1351.6846, 1356.6124, 1361.6910, 1365.6167, 1372.5992, 1382.5925, 1383.7246, 1390.6946, 1420.7117, 1423.6814, 1445.7860, 1456.7703, 1457.7407, 1467.7371, 1470.7511, 1488.7211, 1494.7380, 1497.8768, 1500.7181, 1503.7421, 1513.7554, 1519.7505, 1528.6958, 1530.6902, 1540.7820, 1544.8356, 1556.8394, 1562.7800, 1563.7925, 1572.7056, 1578.7816, 1580.8422, 1599.9109, 1616.7953, 1621.7692, 1628.7004, 1628.8589, 1635.7825, 1642.7168, 1650.8430, 1657.9056, 1696.8221, 1706.8651, 1707.9131, 1717.9056, 1722.9384, 1730.8652, 1738.7958, 1741.9946, 1755.8914, 1758.9082, 1771.0306, 1774.9175, 1787.8247, 1809.0355, 1862.9480, 1883.7240, 1928.0236, 1932.0079, 1938.0043, 1940.0157, 1953.8590, 1959.9622, 1961.0220, 1969.9949, 1981.0421, 1990.9496, 1997.8253, 2007.9401, 2011.8949, 2015.9641, 2023.9448, 2027.1273, 2032.0186, 2044.0441, 2045.9841, 2060.9731, 2067.1531, 2086.0945, 2088.1196, 2108.9956, 2112.9929, 2114.9592, 2139.0918, 2144.0564, 2160.0481, 2174.1157, 2179.0405, 2199.1233, 2211.0984, 2229.1216, 2239.1421, 2242.1345, 2245.8398, 2251.1340, 2255.1416, 2265.0903, 2270.1790, 2273.0667, 2284.1729, 2287.1509, 2293.1445, 2328.9482, 2343.1899, 2352.9153, 2354.9983, 2356.0093, 2364.8684, 2414.2334, 2450.2375, 2466.2253, 2468.2173, 2475.2114, 2486.1069, 2519.9941, 2541.2319, 2542.2505, 2558.3389, 2575.1797, 2646.9695, 2660.9880, 2675.0371, 2695.0181, 2702.2866, 2705.2827, 2713.3413, 2718.2998, 2721.2900, 2744.2466, 2763.3267, 2770.3513, 2785.3567, 2787.2734, 2788.2341, 2792.2034, 2798.2407, 2801.2722, 2803.2302, 2808.2080, 2817.2480, 2820.2319, 2833.2026, 2843.3906, 2846.3865, 2847.3792, 2870.3926, 2873.3279, 2889.3057, 2892.4070, 2896.4392, 2912.4277, 2915.4314, 2919.4495, 2961.2297, 3071.3845, 3076.4626, 3081.6108, 3101.4309, 3118.6643, 3143.5120, 3159.5193, 3173.5476, 3184.4751, 3236.5454, 3248.6077, 3253.6089, 3265.5996, 3269.6123, 3285.5964, 3331.5325, 3334.6331, 3335.6592, 3347.4841, 3397.6499, 3411.6399, 3412.5662, 3427.6182, 3428.5718, 3435.6516, 3444.5942, 3463.5820, 3473.5864, 3487.6257, 3491.6506, 3503.7678, 3509.5947, 3521.6812, 3552.5681, 3574.6165, 3590.6309, 3624.6504, 3636.7607, 3640.6731, 3646.7363, 3651.7256, 3665.7231, 3669.7129, 3692.6494, 3696.7678, 3707.6807, 3714.6301, 3719.6519, 3722.5593, 3723.6516, 3735.6885, 3751.6423, 3830.7644, 3937.7585, 3941.8464, 3942.8328, 3955.8433, 4092.8938, 4147.8613, 4162.9160, 4254.9053, 4272.9224, 4290.9102, 4306.9575, 4309.8506, 4323.8926, 4397.9277, 4415.8896, 4449.1777, 4455.1221, 4459.2368, 4494.0420, 4501.0767, 4510.0269, 4525.1040, 4530.0112, 4839.1138, 4846.0161

20. Q5BIS3 **Mass:** 9033 **Score:** 20 **Expect:** 2.4e+002 **Matches:** 3

CDC42 small effector protein 1 OS=Bos taurus GN=CDC42SE1 PE=3 SV=1

Observed	Mr(expt)	Mr(calc)	ppm	Start	End	Miss	Peptide
2229.1216	2228.1143	2228.0871	12.2	2 -	19	1	M.SEFWHKLGCCVVEKPQPK.K
3937.7585	3936.7512	3936.7573	-1.55	28 -	64	0	R.TMIGEPMNFVHLTHIGSGEMGAGDGLAMTGAVQEQMR.S + 4 Oxidation (M)
4494.0420	4493.0347	4493.0655	-6.84	24 -	64	2	R.RIDRTMIGEPMNFVHLTHIGSGEMGAGDGLAMTGAVQEQMR.S + 5 Oxidation (M)

No match to: 710.3558, 715.3582, 722.4182, 729.4472, 734.4399, 735.4044, 737.4075, 747.4354, 756.3666, 759.4210, 772.4085, 779.4621, 800.3990, 801.4063, 805.4392, 818.4569, 823.4658, 828.4204, 829.4349, 830.4365, 832.2986, 834.3055, 842.4619, 856.3810, 865.4302, 870.4144, 873.3766, 880.4450, 883.4655, 887.4088, 889.4736, 891.4725, 898.4407, 900.5031, 912.4936, 923.5015, 925.4745, 929.4329, 938.4833, 957.5629, 961.4780, 967.4121, 970.4429, 972.4810, 974.4342, 978.5226, 983.6148, 988.4271, 993.4903, 995.3998, 998.5886, 1006.4926, 1007.5641, 1021.5605, 1024.5408, 1032.5048, 1034.5706, 1036.5566, 1043.5085, 1044.5150, 1046.5917, 1056.5394, 1057.6062, 1074.6005, 1087.5505, 1113.5382, 1122.5513, 1123.5905, 1130.5541, 1143.4395, 1145.6267, 1177.6389, 1178.6249, 1180.6364, 1184.6396, 1194.5638, 1198.6896, 1216.7015, 1227.6558, 1243.6030, 1248.6648, 1254.6876, 1255.5952, 1264.7084, 1269.6405, 1273.6479, 1278.6892, 1281.6271, 1289.6858, 1301.6105, 1304.6399, 1312.7454, 1325.6327, 1326.6665, 1328.6610, 1336.7188, 1337.6541, 1338.6735, 1349.6659, 1351.6846, 1356.6124, 1361.6910, 1365.6167, 1372.5992, 1382.5925, 1383.7246, 1390.6946, 1420.7117, 1423.6814, 1445.7860, 1456.7703, 1457.7407, 1467.7371, 1470.7511, 1488.7211, 1494.7380, 1497.8768, 1500.7181, 1503.7421, 1513.7554, 1519.7505, 1528.6958, 1530.6902, 1540.7820, 1544.8356, 1556.8394, 1562.7800, 1563.7925, 1572.7056, 1578.7816, 1580.8422, 1599.9109, 1616.7953, 1621.7692, 1628.7004, 1628.8589, 1635.7825, 1642.7168, 1647.8525, 1650.8430, 1657.9056, 1666.7905, 1696.8221, 1706.8651, 1707.9131, 1717.9056, 1722.9384, 1730.8652, 1738.7958, 1741.9946, 1755.8914, 1758.9082, 1771.0306, 1774.9175, 1787.8247, 1809.0355, 1862.9480, 1883.7240, 1928.0236, 1932.0079, 1938.0043, 1940.0157, 1953.8590, 1959.9622, 1961.0220, 1969.9949, 1981.0421, 1990.9496, 1997.8253, 2007.9401, 2011.8949, 2015.9641, 2023.9448, 2027.1273, 2032.0186, 2044.0441, 2045.9841, 2060.9731, 2067.1531, 2086.0945, 2088.1196, 2108.9956, 2112.9929, 2114.9592, 2139.0918, 2144.0564, 2160.0481, 2174.1157, 2179.0405, 2199.1233, 2211.0984, 2239.1421, 2242.1345, 2245.8398, 2251.1340, 2255.1416, 2265.0903, 2270.1790, 2273.0667, 2284.1729, 2287.1509, 2293.1445, 2328.9482, 2343.1899, 2352.9153, 2354.9983, 2356.0093, 2364.8684, 2414.2334, 2423.2490, 2450.2375, 2466.2253, 2468.2173, 2475.2114, 2486.1069, 2519.9941, 2541.2319, 2542.2505, 2558.3389, 2575.1797, 2646.9695, 2660.9880, 2675.0371, 2695.0181, 2702.2866, 2705.2827, 2713.3413, 2717.3323, 2718.2998, 2721.2900, 2744.2466, 2763.3267, 2770.3513, 2785.3567, 2787.2734, 2788.2341, 2792.2034, 2798.2407, 2801.2722, 2803.2302, 2808.2080, 2817.2480, 2820.2319, 2833.2026, 2843.3906, 2846.3865, 2847.3792, 2870.3926, 2873.3279, 2889.3057, 2892.4070, 2896.4392, 2912.4277, 2915.4314, 2919.4495, 2961.2297, 3071.3845, 3076.4626, 3081.6108, 3087.3884, 3101.4309, 3118.6643, 3143.5120, 3159.5193, 3173.5476, 3184.4751, 3236.5454, 3248.6077, 3253.6089, 3265.5996, 3269.6123, 3285.5964, 3331.5325, 3334.6331, 3335.6592, 3347.4841, 3397.6499, 3411.6399, 3412.5662, 3427.6182, 3428.5718, 3435.6516, 3444.5942, 3457.6045, 3463.5820, 3473.5864, 3487.6257, 3491.6506, 3503.7678, 3509.5947, 3521.6812, 3552.5681, 3574.6165, 3590.6309, 3624.6504, 3636.7607, 3640.6731, 3646.7363, 3651.7256, 3665.7231, 3669.7129, 3692.6494, 3696.7678, 3707.6807, 3714.6301, 3719.6519, 3722.5593, 3723.6516, 3735.6885, 3751.6423, 3830.7644, 3941.8464, 3942.8328, 3955.8433, 4092.8938, 4147.8613, 4162.9160, 4254.9053, 4272.9224, 4290.9102, 4306.9575, 4309.8506, 4323.8926, 4397.9277, 4415.8896, 4449.1777, 4455.1221, 4459.2368, 4501.0767, 4510.0269, 4525.1040, 4530.0112, 4839.1138, 4846.0161

21. G3N2Z1 **Mass:** 8956 **Score:** 20 **Expect:** 2.4e+002 **Matches:** 3

Uncharacterized protein OS=Bos taurus GN=SMIM1 PE=4 SV=1

Observed	Mr(expt)	Mr(calc)	ppm	Start	End	Miss	Peptide
1177.6389	1176.6316	1176.6359	-3.60	43 -	53	1	K.LCSGKLGIAMK.V
2792.2034	2791.1961	2791.2222	-9.36	14 -	38	0	R.WENSHPDEVHVASGPSTEEASGWQR.V
3640.6731	3639.6658	3639.6727	-1.88	11 -	42	2	R.YSRWENSHPDEVHVASGPSTEEASGWQRVSQK.L

No match to: 710.3558, 715.3582, 722.4182, 729.4472, 734.4399, 735.4044, 737.4075, 747.4354, 756.3666, 759.4210, 772.4085, 779.4621, 800.3990, 801.4063, 805.4392, 818.4569, 823.4658, 828.4204, 829.4349, 830.4365, 832.2986, 834.3055, 842.4619, 856.3810, 865.4302, 870.4144, 873.3766, 880.4450, 883.4655, 887.4088, 889.4736, 891.4725, 898.4407, 900.5031, 912.4936, 923.5015, 925.4745, 929.4329, 938.4833, 957.5629, 961.4780, 967.4121, 970.4429, 972.4810, 974.4342, 978.5226, 983.6148, 988.4271, 993.4903, 995.3998, 998.5886, 1006.4926, 1007.5641, 1021.5605, 1024.5408, 1032.5048, 1034.5706, 1036.5566, 1043.5085, 1044.5150, 1046.5917, 1056.5394, 1057.6062, 1074.6005, 1087.5505, 1113.5382, 1122.5513, 1123.5905, 1130.5541, 1143.4395, 1145.6267, 1178.6249, 1180.6364, 1184.6396, 1194.5638, 1198.6896, 1216.7015, 1227.6558, 1243.6030, 1248.6648, 1254.6876, 1255.5952, 1264.7084, 1269.6405, 1273.6479, 1278.6892,

1281.6271, 1289.6858, 1301.6105, 1304.6399, 1312.7454, 1325.6327, 1326.6665, 1328.6610, 1336.7188, 1337.6541, 1338.6735, 1349.6659, 1351.6846, 1356.6124, 1361.6910, 1365.6167, 1372.5992, 1382.5925, 1383.7246, 1390.6946, 1420.7117, 1423.6814, 1445.7860, 1456.7703, 1457.7407, 1467.7371, 1470.7511, 1488.7211, 1494.7380, 1497.8768, 1500.7181, 1503.7421, 1513.7554, 1519.7505, 1528.6958, 1530.6902, 1540.7820, 1544.8356, 1556.8394, 1562.7800, 1563.7925, 1572.7056, 1578.7816, 1580.8422, 1599.9109, 1616.7953, 1621.7692, 1628.7004, 1628.8589, 1635.7825, 1642.7168, 1647.8525, 1650.8430, 1657.9056, 1666.7905, 1696.8221, 1706.8651, 1707.9131, 1717.9056, 1722.9384, 1730.8652, 1738.7958, 1741.9946, 1755.8914, 1758.9082, 1771.0306, 1774.9175, 1787.8247, 1809.0355, 1862.9480, 1883.7240, 1928.0236, 1932.0079, 1938.0043, 1940.0157, 1953.8590, 1959.9622, 1961.0220, 1969.9949, 1981.0421, 1990.9496, 1997.8253, 2007.9401, 2011.8949, 2015.9641, 2023.9448, 2027.1273, 2032.0186, 2044.0441, 2045.9841, 2060.9731, 2067.1531, 2086.0945, 2088.1196, 2108.9956, 2112.9929, 2114.9592, 2139.0918, 2144.0564, 2160.0481, 2174.1157, 2179.0405, 2199.1233, 2211.0984, 2229.1216, 2239.1421, 2242.1345, 2245.8398, 2251.1340, 2255.1416, 2265.0903, 2270.1790, 2273.0667, 2284.1729, 2287.1509, 2293.1445, 2328.9482, 2343.1899, 2352.9153, 2354.9983, 2356.0093, 2364.8684, 2414.2334, 2423.2490, 2450.2375, 2466.2253, 2468.2173, 2475.2114, 2486.1069, 2519.9941, 2541.2319, 2542.2505, 2558.3389, 2575.1797, 2646.9695, 2660.9880, 2675.0371, 2695.0181, 2702.2866, 2705.2827, 2713.3413, 2717.3323, 2718.2998, 2721.2900, 2744.2466, 2763.3267, 2770.3513, 2785.3567, 2787.2734, 2788.2341, 2798.2407, 2801.2722, 2803.2302, 2808.2080, 2817.2480, 2820.2319, 2833.2026, 2843.3906, 2846.3865, 2847.3792, 2870.3926, 2873.3279, 2889.3057, 2892.4070, 2896.4392, 2915.4314, 2919.4495, 2961.2297, 3071.3845, 3076.4626, 3081.6108, 3087.3884, 3101.4309, 3118.6643, 3143.5120, 3159.5193, 3173.5476, 3184.4751, 3236.5454, 3248.6077, 3253.6089, 3265.5996, 3269.6123, 3285.5964, 3331.5325, 3334.6331, 3335.6592, 3347.4841, 3397.6499, 3411.6399, 3412.5662, 3427.6182, 3428.5718, 3435.6516, 3444.5942, 3457.6045, 3463.5820, 3473.5864, 3487.6257, 3491.6506, 3503.7678, 3509.5947, 3521.6812, 3552.5681, 3574.6165, 3590.6309, 3624.6504, 3636.7607, 3646.7363, 3651.7256, 3665.7231, 3669.7129, 3692.6494, 3696.7678, 3707.6807, 3714.6301, 3719.6519, 3722.5593, 3723.6516, 3735.6885, 3751.6423, 3830.7644, 3937.7585, 3941.8464, 3942.8328, 3955.8433, 4092.8938, 4147.8613, 4162.9160, 4254.9053, 4272.9224, 4290.9102, 4306.9575, 4309.8506, 4323.8926, 4397.9277, 4415.8896, 4449.1777, 4455.1221, 4459.2368, 4494.0420, 4501.0767, 4510.0269, 4525.1040, 4530.0112, 4839.1138, 4846.0161

22. A2VDZ9 **Mass:** 27369 **Score:** 20 **Expect:** 2.5e+002 **Matches:** 10

Vesicle-associated membrane protein-associated protein B OS=Bos taurus GN=VAPB PE=2 SV=1

Observed	Mr(expt)	Mr(calc)	ppm	Start	End	Miss	Peptide
772.4085	771.4012	771.3875	17.7	32 -	38	0	K.LGNPTDR.N
967.4121	966.4048	966.4263	-22.18	169 -	175	1	K.VMEECKR.L + Oxidation (M)
1420.7117	1419.7044	1419.6929	8.13	32 -	43	1	K.LGNPTDRNVCFK.V
1488.7211	1487.7138	1487.7038	6.73	201 -	216	0	K.TAQSNSPAPASAMAGK.E
1616.7953	1615.7880	1615.7988	-6.65	200 -	216	1	R.KTAQSNSPAPASAMAGK.E
1621.7692	1620.7619	1620.7743	-7.65	185 -	197	2	R.EENKQFKEEDGLR.M
1628.8589	1627.8516	1627.8641	-7.69	176 -	188	2	R.LQSEVQRLREENK.Q
1647.8525	1646.8452	1646.8563	-6.70	32 -	45	2	K.LGNPTDRNVCFKVK.T
2801.2722	2800.2649	2800.3023	-13.34	84 -	107	2	K.SKHKFMVQSMFAPTDTSDMEAVWK.E
2817.2480	2816.2407	2816.2972	-20.05	84 -	107	2	K.SKHKFMVQSMFAPTDTSDMEAVWK.E + Oxidation (M)

No match to: 710.3558, 715.3582, 722.4182, 729.4472, 734.4399, 735.4044, 737.4075, 747.4354, 756.3666, 759.4210, 779.4621, 800.3990, 801.4063, 805.4392, 818.4569, 823.4658, 828.4204, 829.4349, 830.4365, 832.2986, 834.3055, 842.4619, 856.3810, 865.4302, 870.4144, 873.3766, 880.4450, 883.4655, 887.4088, 889.4736, 891.4725, 898.4407, 900.5031, 912.4936, 923.5015, 925.4745, 929.4329, 938.4833, 957.5629, 961.4780, 970.4429, 972.4810, 974.4342, 978.5226, 983.6148, 988.4271, 993.4903, 995.3998, 998.5886, 1006.4926, 1007.5641, 1021.5605, 1024.5408, 1032.5048, 1034.5706, 1036.5566, 1043.5085, 1044.5150, 1046.5917, 1056.5394, 1057.6062, 1074.6005, 1087.5505, 1113.5382, 1122.5513, 1123.5905, 1130.5541, 1143.4395, 1145.6267, 1177.6389, 1178.6249, 1180.6364, 1184.6396, 1194.5638, 1198.6896, 1216.7015, 1227.6558, 1243.6030, 1248.6648, 1254.6876, 1255.5952, 1264.7084, 1269.6405, 1273.6479, 1278.6892, 1281.6271, 1289.6858, 1301.6105, 1304.6399, 1312.7454, 1325.6327, 1326.6665, 1328.6610, 1336.7188, 1337.6541, 1338.6735, 1349.6659, 1351.6846, 1356.6124, 1361.6910, 1365.6167, 1372.5992, 1382.5925, 1383.7246, 1390.6946, 1423.6814, 1445.7860, 1456.7703, 1457.7407, 1467.7371, 1470.7511, 1494.7380, 1497.8768, 1500.7181, 1503.7421, 1513.7554, 1519.7505, 1528.6958, 1530.6902, 1540.7820, 1544.8356, 1556.8394, 1562.7800, 1563.7925, 1572.7056, 1578.7816, 1580.8422, 1599.9109, 1628.7004, 1635.7825, 1642.7168, 1650.8430, 1657.9056, 1666.7905, 1696.8221, 1706.8651, 1707.9131, 1717.9056, 1722.9384, 1730.8652, 1738.7958, 1741.9946, 1755.8914, 1758.9082, 1771.0306, 1774.9175, 1787.8247, 1809.0355, 1862.9480, 1883.7240, 1928.0236, 1932.0079, 1938.0043, 1940.0157, 1953.8590, 1959.9622, 1961.0220, 1969.9949, 1981.0421, 1990.9496, 1997.8253, 2007.9401, 2011.8949, 2015.9641, 2023.9448, 2027.1273, 2032.0186, 2044.0441, 2045.9841, 2060.9731, 2067.1531, 2086.0945, 2088.1196, 2108.9956, 2112.9929, 2114.9592, 2139.0918, 2144.0564, 2160.0481, 2174.1157, 2179.0405, 2199.1233, 2211.0984, 2229.1216, 2239.1421, 2242.1345, 2245.8398, 2251.1340, 2255.1416, 2265.0903, 2270.1790, 2273.0667, 2284.1729, 2287.1509, 2293.1445, 2328.9482, 2343.1899, 2352.9153, 2354.9983, 2356.0093, 2364.8684, 2414.2334, 2423.2490, 2450.2375, 2466.2253, 2468.2173, 2475.2114, 2486.1069, 2519.9941, 2541.2319, 2542.2505, 2558.3389, 2575.1797, 2646.9695, 2660.9880, 2675.0371, 2695.0181, 2702.2866, 2705.2827, 2713.3413, 2717.3323, 2718.2998, 2721.2900, 2744.2466, 2763.3267, 2770.3513, 2785.3567, 2787.2734, 2788.2341, 2792.2034, 2798.2407, 2803.2302, 2808.2080, 2820.2319, 2833.2026, 2843.3906, 2846.3865, 2847.3792, 2870.3926, 2873.3279, 2889.3057, 2892.4070, 2896.4392, 2912.4277, 2915.4314, 2919.4495, 2961.2297, 3071.3845, 3076.4626, 3081.6108, 3087.3884, 3101.4309, 3118.6643, 3143.5120, 3159.5193, 3173.5476, 3184.4751, 3236.5454, 3248.6077, 3253.6089, 3265.5996, 3269.6123, 3285.5964, 3331.5325, 3334.6331, 3335.6592, 3347.4841, 3397.6499, 3411.6399, 3412.5662, 3427.6182, 3428.5718, 3435.6516, 3444.5942, 3457.6045, 3463.5820, 3473.5864, 3487.6257, 3491.6506, 3503.7678, 3509.5947, 3521.6812, 3552.5681, 3574.6165, 3590.6309, 3624.6504, 3636.7607, 3640.6731, 3646.7363, 3651.7256, 3665.7231, 3669.7129, 3692.6494, 3696.7678, 3707.6807, 3714.6301, 3719.6519, 3722.5593, 3723.6516, 3735.6885, 3751.6423, 3830.7644, 3937.7585, 3941.8464, 3942.8328, 3955.8433, 4092.8938, 4147.8613, 4162.9160, 4254.9053, 4272.9224, 4290.9102, 4306.9575, 4309.8506, 4323.8926, 4397.9277, 4415.8896, 4449.1777, 4455.1221, 4459.2368, 4494.0420, 4501.0767, 4510.0269, 4525.1040, 4530.0112, 4839.1138, 4846.0161

23. F1N091 **Mass:** 43136 **Score:** 20 **Expect:** 2.5e+002 **Matches:** 10

Uncharacterized protein OS=Bos taurus GN=CCDC6 PE=4 SV=1

Observed	Mr(expt)	Mr(calc)	ppm	Start	End	Miss	Peptide
957.5629	956.5556	956.5654	-10.30	19 -	26	1	K.IQALQKEK.E
961.4780	960.4708	960.4811	-10.73	207 -	213	1	K.LQREMER.R
1034.5706	1033.5633	1033.5556	7.46	163 -	170	1	R.FLKNEVER.L
1349.6659	1348.6586	1348.6339	18.3	210 -	219	2	R.EMERREALCR.Q
1365.6167	1364.6094	1364.6289	-14.24	210 -	219	2	R.EMERREALCR.Q + Oxidation (M)
1494.7380	1493.7307	1493.7361	-3.63	36 -	47	1	K.EEEFLTNELSRK.L
2542.2505	2541.2432	2541.2598	-6.52	27 -	47	2	K.ETLAVNYEKEEEFLTNELSRK.L
2558.3389	2557.3316	2557.2706	23.8	174 -	194	2	K.QLRAAQLQHSEKMAQYLEEER.H
2912.4277	2911.4204	2911.3514	23.7	134 -	159	1	K.LDQPVSAPPSPRDISMEIDSPENMMR.H
3334.6331	3333.6258	3333.5904	10.6	134 -	162	2	K.LDQPVSAPPSPRDISMEIDSPENMMRHIR.F + Oxidation (M)

No match to: 710.3558, 715.3582, 722.4182, 729.4472, 734.4399, 735.4044, 737.4075, 747.4354, 756.3666, 759.4210, 772.4085, 779.4621, 800.3990, 801.4063, 805.4392, 818.4569, 823.4658, 828.4204, 829.4349, 830.4365, 832.2986, 834.3055, 842.4619, 856.3810, 865.4302, 870.4144, 873.3766, 880.4450, 883.4655, 887.4088, 889.4736, 891.4725, 898.4407, 900.5031, 912.4936, 923.5015, 925.4745, 929.4329, 938.4833, 967.4121, 970.4429, 972.4810, 974.4342, 978.5226, 983.6148, 988.4271, 993.4903, 995.3998, 998.5886, 1006.4926, 1007.5641, 1021.5605, 1024.5408, 1032.5048, 1036.5566, 1043.5085, 1044.5150, 1046.5917, 1056.5394, 1057.6062, 1074.6005, 1087.5505, 1113.5382, 1122.5513, 1123.5905, 1130.5541, 1143.4395, 1145.6267, 1177.6389, 1178.6249, 1180.6364, 1184.6396, 1194.5638, 1198.6896, 1216.7015, 1227.6558, 1243.6030, 1248.6648, 1254.6876, 1255.5952, 1264.7084, 1269.6405, 1273.6479, 1278.6892, 1281.6271, 1289.6858, 1301.6105, 1304.6399, 1312.7454, 1325.6327, 1326.6665, 1328.6610, 1336.7188, 1337.6541, 1338.6735, 1351.6846, 1356.6124, 1361.6910, 1372.5992, 1382.5925, 1383.7246, 1390.6946, 1420.7117, 1423.6814, 1445.7860, 1456.7703, 1457.7407, 1467.7371, 1470.7511, 1488.7211, 1497.8768, 1500.7181, 1503.7421, 1513.7554, 1519.7505, 1528.6958, 1530.6902, 1540.7820, 1544.8356, 1556.8394, 1562.7800, 1563.7925, 1572.7056, 1578.7816, 1580.8422, 1599.9109, 1616.7953, 1621.7692, 1628.7004, 1628.8589, 1635.7825, 1642.7168, 1647.8525, 1650.8430, 1657.9056, 1666.7905, 1696.8221, 1706.8651, 1707.9131, 1717.9056, 1722.9384, 1730.8652, 1738.7958, 1741.9946, 1755.8914, 1758.9082, 1771.0306, 1774.9175, 1787.8247, 1809.0355, 1862.9480, 1883.7240, 1928.0236, 1932.0079, 1938.0043, 1940.0157, 1953.8590, 1959.9622, 1961.0220, 1969.9949, 1981.0421, 1990.9496, 1997.8253, 2007.9401, 2011.8949, 2015.9641, 2023.9448, 2027.1273, 2032.0186, 2044.0441, 2045.9841, 2060.9731, 2067.1531, 2086.0945, 2088.1196, 2108.9956, 2112.9929, 2114.9592, 2139.0918, 2144.0564, 2160.0481, 2174.1157, 2179.0405, 2199.1233, 2211.0984, 2229.1216, 2239.1421, 2242.1345, 2245.8398, 2251.1340, 2255.1416, 2265.0903, 2270.1790, 2273.0667, 2284.1729, 2287.1509, 2293.1445, 2328.9482, 2343.1899, 2352.9153, 2354.9983, 2356.0093, 2364.8684, 2414.2334, 2423.2490, 2450.2375, 2466.2253, 2468.2173, 2475.2114, 2486.1069, 2519.9941, 2541.2319, 2575.1797, 2646.9695, 2660.9880, 2675.0371, 2695.0181, 2702.2866,

2705.2827, 2713.3413, 2717.3323, 2718.2998, 2721.2900, 2744.2466, 2763.3267, 2770.3513, 2785.3567, 2787.2734, 2788.2341, 2792.2034, 2798.2407, 2801.2722, 2803.2302, 2808.2080, 2817.2480, 2820.2319, 2833.2026, 2843.3906, 2846.3865, 2847.3792, 2870.3926, 2873.3279, 2889.3057, 2892.4070, 2896.4392, 2915.4314, 2919.4495, 2961.2297, 3071.3845, 3076.4626, 3081.6108, 3087.3884, 3101.4309, 3118.6643, 3143.5120, 3159.5193, 3173.5476, 3184.4751, 3236.5454, 3248.6077, 3253.6089, 3265.5996, 3269.6123, 3285.5964, 3331.5325, 3335.6592, 3347.4841, 3397.6499, 3411.6399, 3412.5662, 3427.6182, 3428.5718, 3435.6516, 3444.5942, 3457.6045, 3463.5820, 3473.5864, 3487.6257, 3491.6506, 3503.7678, 3509.5947, 3521.6812, 3552.5681, 3574.6165, 3590.6309, 3624.6504, 3636.7607, 3640.6731, 3646.7363, 3651.7256, 3665.7231, 3669.7129, 3692.6494, 3696.7678, 3707.6807, 3714.6301, 3719.6519, 3722.5593, 3723.6516, 3735.6885, 3751.6423, 3830.7644, 3937.7585, 3941.8464, 3942.8328, 3955.8433, 4092.8938, 4147.8613, 4162.9160, 4254.9053, 4272.9224, 4290.9102, 4306.9575, 4309.8506, 4323.8926, 4397.9277, 4415.8896, 4449.1777, 4455.1221, 4459.2368, 4494.0420, 4501.0767, 4510.0269, 4525.1040, 4530.0112, 4839.1138, 4846.0161

24. F1MQD7 **Mass:** 47708 **Score:** 20 **Expect:** 2.5e+002 **Matches:** 6
Uncharacterized protein OS=Bos taurus GN=CIR1 PE=4 SV=2

Observed	Mr(expt)	Mr(calc)	ppm	Start	End	Miss	Peptide
801.4063	800.3990	800.3817	21.6	13 -	19	0	K.DFHPASK.S
1325.6327	1324.6254	1324.6333	-5.92	39 -	48	0	K.QEELMQQYLK.E + Oxidation (M)
1513.7554	1512.7481	1512.7282	13.1	25 -	36	1	K.VWMAEQKISYDK.K + Oxidation (M)
3696.7678	3695.7605	3695.7690	-2.28	192 -	224	2	R.NLTANDPSQEYVASEGEEDPEVEFLKSLTTKQK.Q
4449.1777	4448.1704	4448.1199	11.4	145 -	186	2	R.ECPLFGLSGINASSVPTDGSGPSMHPSELIAEMRNSGFALKR.N + 2 Oxidation (M)
4525.1040	4524.0967	4524.0897	1.56	137 -	178	1	K.WGHVNTDRECPLFGLSGINASSVPTDGSGPSMHPSELIAEMR.N + Oxidation (M)

No match to: 710.3558, 715.3582, 722.4182, 729.4472, 734.4399, 735.4044, 737.4075, 747.4354, 756.3666, 759.4210, 772.4085, 779.4621, 800.3990, 805.4392, 818.4569, 823.4658, 828.4204, 829.4349, 830.4365, 832.2986, 834.3055, 842.4619, 856.3810, 865.4302, 870.4144, 873.3766, 880.4450, 883.4655, 887.4088, 889.4736, 891.4725, 898.4407, 900.5031, 912.4936, 923.5015, 925.4745, 929.4329, 938.4833, 957.5629, 961.4780, 967.4121, 970.4429, 972.4810, 974.4342, 978.5226, 983.6148, 988.4271, 993.4903, 995.3998, 998.5886, 1006.4926, 1007.5641, 1021.5605, 1024.5408, 1032.5048, 1034.5706, 1036.5566, 1043.5085, 1044.5150, 1046.5917, 1056.5394, 1057.6062, 1074.6005, 1087.5505, 1113.5382, 1122.5513, 1123.5905, 1130.5541, 1143.4395, 1145.6267, 1177.6389, 1178.6249, 1180.6364, 1184.6396, 1194.5638, 1198.6896, 1216.7015, 1227.6558, 1243.6030, 1248.6648, 1254.6876, 1255.5952, 1264.7084, 1269.6405, 1273.6479, 1278.6892, 1281.6271, 1289.6858, 1301.6105, 1304.6399, 1312.7454, 1326.6665, 1328.6610, 1336.7188, 1337.6541, 1338.6735, 1349.6659, 1351.6846, 1356.6124, 1361.6910, 1365.6167, 1372.5992, 1382.5925, 1383.7246, 1390.6946, 1420.7117, 1423.6814, 1445.7860, 1456.7703, 1457.7407, 1467.7371, 1470.7511, 1488.7211, 1494.7380, 1497.8768, 1500.7181, 1503.7421, 1519.7505, 1528.6958, 1530.6902, 1540.7820, 1544.8356, 1556.8394, 1562.7800, 1563.7925, 1572.7056, 1578.7816, 1580.8422, 1599.9109, 1616.7953, 1621.7692, 1628.7004, 1628.8589, 1635.7825, 1642.7168, 1647.8525, 1650.8430, 1657.9056, 1666.7905, 1696.8221, 1706.8651, 1707.9131, 1717.9056, 1722.9384, 1730.8652, 1738.7958, 1741.9946, 1755.8914, 1758.9082, 1771.0306, 1774.9175, 1787.8247, 1809.0355, 1862.9480, 1883.7240, 1928.0236, 1932.0079, 1938.0043, 1940.0157, 1953.8590, 1959.9622, 1961.0220, 1969.9949, 1981.0421, 1990.9496, 1997.8253, 2007.9401, 2011.8949, 2015.9641, 2023.9448, 2027.1273, 2032.0186, 2044.0441, 2045.9841, 2060.9731, 2067.1531, 2086.0945, 2088.1196, 2108.9956, 2112.9929, 2114.9592, 2139.0918, 2144.0564, 2160.0481, 2174.1157, 2179.0405, 2199.1233, 2211.0984, 2229.1216, 2239.1421, 2242.1345, 2245.8398, 2251.1340, 2255.1416, 2265.0903, 2270.1790, 2273.0667, 2284.1729, 2287.1509, 2293.1445, 2328.9482, 2343.1899, 2352.9153, 2354.9983, 2356.0093, 2364.8684, 2414.2334, 2423.2490, 2450.2375, 2466.2253, 2468.2173, 2475.2114, 2486.1069, 2519.9941, 2541.2319, 2542.2505, 2558.3389, 2575.1797, 2646.9695, 2660.9880, 2675.0371, 2695.0181, 2702.2866, 2705.2827, 2713.3413, 2717.3323, 2718.2998, 2721.2900, 2744.2466, 2763.3267, 2770.3513, 2785.3567, 2787.2734, 2788.2341, 2792.2034, 2798.2407, 2801.2722, 2803.2302, 2808.2080, 2817.2480, 2820.2319, 2833.2026, 2843.3906, 2846.3865, 2847.3792, 2870.3926, 2873.3279, 2889.3057, 2892.4070, 2896.4392, 2912.4277, 2915.4314, 2919.4495, 2961.2297, 3071.3845, 3076.4626, 3081.6108, 3087.3884, 3101.4309, 3118.6643, 3143.5120, 3159.5193, 3173.5476, 3184.4751, 3236.5454, 3248.6077, 3253.6089, 3265.5996, 3269.6123, 3285.5964, 3331.5325, 3334.6331, 3335.6592, 3347.4841, 3397.6499, 3411.6399, 3412.5662, 3427.6182, 3428.5718, 3435.6516, 3444.5942, 3457.6045, 3463.5820, 3473.5864, 3487.6257, 3491.6506, 3503.7678, 3509.5947, 3521.6812, 3552.5681, 3574.6165, 3590.6309, 3624.6504, 3636.7607, 3640.6731, 3646.7363, 3651.7256, 3665.7231, 3669.7129, 3692.6494, 3707.6807, 3714.6301, 3719.6519, 3722.5593, 3723.6516, 3735.6885, 3751.6423, 3830.7644, 3937.7585, 3941.8464, 3942.8328, 3955.8433, 4092.8938, 4147.8613, 4162.9160, 4254.9053, 4272.9224, 4290.9102, 4306.9575, 4309.8506, 4323.8926, 4397.9277, 4415.8896, 4455.1221, 4459.2368, 4494.0420, 4501.0767, 4510.0269, 4530.0112, 4839.1138, 4846.0161

25. A4FV58 **Mass:** 53257 **Score:** 19 **Expect:** 2.9e+002 **Matches:** 8
Cytosolic Fe-S cluster assembly factor NARFL OS=Bos taurus GN=NARFL PE=2 SV=2

Observed	Mr(expt)	Mr(calc)	ppm	Start	End	Miss	Peptide
715.3582	714.3509	714.3371	19.4	408 -	414	0	K.APDMPGK.E
729.4472	728.4399	728.4545	-19.94	96 -	102	1	R.KVLGANK.T
2558.3389	2557.3316	2557.2714	23.6	146 -	167	1	K.IGAHYVFDTAFSRNFSLLESQR.E
3248.6077	3247.6004	3247.5680	9.97	30 -	59	2	K.VDRRPGSGVAKIHIEDDGSYFQVSQDGGMK.K
3411.6399	3410.6326	3410.6201	3.66	251 -	279	1	K.LEASRPDFFSQEHQTRDVDCVITTGEVFK.L
3435.6516	3434.6443	3434.6850	-11.85	2 -	32	1	M.ASPFSGALQLTDLDDFIAPSQDCIKPMKVDR.R
3503.7678	3502.7605	3502.7480	3.56	423 -	453	2	R.LYGLVRTEAPEDAPGIQELYERWLQGAGSER.A
4272.9224	4271.9151	4272.0047	-20.96	385 -	422	2	R.CPYHYVEVMACPAGCLNGGGQLKAPDMPGKELLQQVER.L

No match to: 710.3558, 722.4182, 734.4399, 735.4044, 737.4075, 747.4354, 756.3666, 759.4210, 772.4085, 779.4621, 800.3990, 801.4063, 805.4392, 818.4569, 823.4658, 828.4204, 829.4349, 830.4365, 832.2986, 834.3055, 842.4619, 856.3810, 865.4302, 870.4144, 873.3766, 880.4450, 883.4655, 887.4088, 889.4736, 891.4725, 898.4407, 900.5031, 912.4936, 923.5015, 925.4745, 929.4329, 938.4833, 957.5629, 961.4780, 967.4121, 970.4429, 972.4810, 974.4342, 978.5226, 983.6148, 988.4271, 993.4903, 995.3998, 998.5886, 1006.4926, 1007.5641, 1021.5605, 1024.5408, 1032.5048, 1034.5706, 1036.5566, 1043.5085, 1044.5150, 1046.5917, 1056.5394, 1057.6062, 1074.6005, 1087.5505, 1113.5382, 1122.5513, 1123.5905, 1130.5541, 1143.4395, 1145.6267, 1177.6389, 1178.6249, 1180.6364, 1184.6396, 1194.5638, 1198.6896, 1216.7015, 1227.6558, 1243.6030, 1248.6648, 1254.6876, 1255.5952, 1264.7084, 1269.6405, 1273.6479, 1278.6892, 1281.6271, 1289.6858, 1301.6105, 1304.6399, 1312.7454, 1325.6327, 1326.6665, 1328.6610, 1336.7188, 1337.6541, 1338.6735, 1349.6659, 1351.6846, 1356.6124, 1361.6910, 1365.6167, 1372.5992, 1382.5925, 1383.7246, 1390.6946, 1420.7117, 1423.6814, 1445.7860, 1456.7703, 1457.7407, 1467.7371, 1470.7511, 1488.7211, 1494.7380, 1497.8768, 1500.7181, 1503.7421, 1513.7554, 1519.7505, 1528.6958, 1530.6902, 1540.7820, 1544.8356, 1556.8394, 1562.7800, 1563.7925, 1572.7056, 1578.7816, 1580.8422, 1599.9109, 1616.7953, 1621.7692, 1628.7004, 1628.8589, 1635.7825, 1642.7168, 1647.8525, 1650.8430, 1657.9056, 1666.7905, 1696.8221, 1706.8651, 1707.9131, 1717.9056, 1722.9384, 1730.8652, 1738.7958, 1741.9946, 1755.8914, 1758.9082, 1771.0306, 1774.9175, 1787.8247, 1809.0355, 1862.9480, 1883.7240, 1928.0236, 1932.0079, 1938.0043, 1940.0157, 1953.8590, 1959.9622, 1961.0220, 1969.9949, 1981.0421, 1990.9496, 1997.8253, 2007.9401, 2011.8949, 2015.9641, 2023.9448, 2027.1273, 2032.0186, 2044.0441, 2045.9841, 2060.9731, 2067.1531, 2086.0945, 2088.1196, 2108.9956, 2112.9929, 2114.9592, 2139.0918, 2144.0564, 2160.0481, 2174.1157, 2179.0405, 2199.1233, 2211.0984, 2229.1216, 2239.1421, 2242.1345, 2245.8398, 2251.1340, 2255.1416, 2265.0903, 2270.1790, 2273.0667, 2284.1729, 2287.1509, 2293.1445, 2328.9482, 2343.1899, 2352.9153, 2354.9983, 2356.0093, 2364.8684, 2414.2334, 2423.2490, 2450.2375, 2466.2253, 2468.2173, 2475.2114, 2486.1069, 2519.9941, 2541.2319, 2542.2505, 2575.1797, 2646.9695, 2660.9880, 2675.0371, 2695.0181, 2702.2866, 2705.2827, 2713.3413, 2717.3323, 2718.2998, 2721.2900, 2744.2466, 2763.3267, 2770.3513, 2785.3567, 2787.2734, 2788.2341, 2792.2034, 2798.2407, 2801.2722, 2803.2302, 2808.2080, 2817.2480, 2820.2319, 2833.2026, 2843.3906, 2846.3865, 2847.3792, 2870.3926, 2873.3279, 2889.3057, 2892.4070, 2896.4392, 2912.4277, 2915.4314, 2919.4495, 2961.2297, 3071.3845, 3076.4626, 3081.6108, 3087.3884, 3101.4309, 3118.6643, 3143.5120, 3159.5193, 3173.5476, 3184.4751, 3236.5454, 3253.6089, 3265.5996, 3269.6123, 3285.5964, 3331.5325, 3334.6331, 3335.6592, 3347.4841, 3397.6499, 3412.5662, 3427.6182, 3428.5718, 3444.5942, 3457.6045, 3463.5820, 3473.5864, 3487.6257, 3491.6506, 3509.5947, 3521.6812, 3552.5681, 3574.6165, 3590.6309, 3624.6504, 3636.7607, 3640.6731, 3646.7363, 3651.7256, 3665.7231, 3669.7129, 3692.6494, 3696.7678, 3707.6807, 3714.6301, 3719.6519, 3722.5593, 3723.6516, 3735.6885, 3751.6423, 3830.7644, 3937.7585, 3941.8464, 3942.8328, 3955.8433, 4092.8938, 4147.8613, 4162.9160, 4254.9053, 4290.9102, 4306.9575, 4309.8506, 4323.8926, 4397.9277, 4415.8896, 4449.1777, 4455.1221, 4459.2368, 4494.0420, 4501.0767, 4510.0269, 4525.1040, 4530.0112, 4839.1138, 4846.0161

26. M5FMT8 **Mass:** 53257 **Score:** 19 **Expect:** 2.9e+002 **Matches:** 8
Cytosolic Fe-S cluster assembly factor NARFL OS=Bos taurus GN=NARFL PE=3 SV=1

Observed	Mr(expt)	Mr(calc)	ppm	Start	End	Miss	Peptide
715.3582	714.3509	714.3371	19.4	408 -	414	0	K.APDMPGK.E
729.4472	728.4399	728.4545	-19.94	96 -	102	1	R.KVLGANK.T
2558.3389	2557.3316	2557.2714	23.6	146 -	167	1	K.IGAHYVFDTAFSRNFSLLESQR.E

3248.6077	3247.6004	3247.5680	9.97	30 -	59	2	K.VDRRPGSGVAKIHIEDDGSYFQVSQDGGMK.K
3411.6399	3410.6326	3410.6201	3.66	251 -	279	1	K.LEASRPDFFSQEHQTRDVDCVITTGEVFK.L
3435.6516	3434.6443	3434.6850	-11.85	2 -	32	1	M.ASPFSGALQLTDLDDFIAPSQDCIKPMKVDR.R
3503.7678	3502.7605	3502.7480	3.56	423 -	453	2	R.LYGLVRTEAPEDAPGIQELYERWLQGAGSER.A
4272.9224	4271.9151	4272.0047	-20.96	385 -	422	2	R.CPYHYVEVMACPAGCLNGGGQLKAPDMPGKELLQQVER.L

No match to: 710.3558, 722.4182, 734.4399, 735.4044, 737.4075, 747.4354, 756.3666, 759.4210, 772.4085, 779.4621, 800.3990, 801.4063, 805.4392, 818.4569, 823.4658, 828.4204, 829.4349, 830.4365, 832.2986, 834.3055, 842.4619, 856.3810, 865.4302, 870.4144, 873.3766, 880.4450, 883.4655, 887.4088, 889.4736, 891.4725, 898.4407, 900.5031, 912.4936, 923.5015, 925.4745, 929.4329, 938.4833, 957.5629, 961.4780, 967.4121, 970.4429, 972.4810, 974.4342, 978.5226, 983.6148, 988.4271, 993.4903, 995.3998, 998.5886, 1006.4926, 1007.5641, 1021.5605, 1024.5408, 1032.5048, 1034.5706, 1036.5566, 1043.5085, 1044.5150, 1046.5917, 1056.5394, 1057.6062, 1074.6005, 1087.5505, 1113.5382, 1122.5513, 1123.5905, 1130.5541, 1143.4395, 1145.6267, 1177.6389, 1178.6249, 1180.6364, 1184.6396, 1194.5638, 1198.6896, 1216.7015, 1227.6558, 1243.6030, 1248.6648, 1254.6876, 1255.5952, 1264.7084, 1269.6405, 1273.6479, 1278.6892, 1281.6271, 1289.6858, 1301.6105, 1304.6399, 1312.7454, 1325.6327, 1326.6665, 1328.6610, 1336.7188, 1337.6541, 1338.6735, 1349.6659, 1351.6846, 1356.6124, 1361.6910, 1365.6167, 1372.5992, 1382.5925, 1383.7246, 1390.6946, 1420.7117, 1423.6814, 1445.7860, 1456.7703, 1457.7407, 1467.7371, 1470.7511, 1488.7211, 1494.7380, 1497.8768, 1500.7181, 1503.7421, 1513.7554, 1519.7505, 1528.6958, 1530.6902, 1540.7820, 1544.8356, 1556.8394, 1562.7800, 1563.7925, 1572.7056, 1578.7816, 1580.8422, 1599.9109, 1616.7953, 1621.7692, 1628.7004, 1628.8589, 1635.7825, 1642.7168, 1647.8525, 1650.8430, 1657.9056, 1666.7905, 1696.8221, 1706.8651, 1707.9131, 1717.9056, 1722.9384, 1730.8652, 1738.7958, 1741.9946, 1755.8914, 1758.9082, 1771.0306, 1774.9175, 1787.8247, 1809.0355, 1862.9480, 1883.7240, 1928.0236, 1932.0079, 1938.0043, 1940.0157, 1953.8590, 1959.9622, 1961.0220, 1969.9949, 1981.0421, 1990.9496, 1997.8253, 2007.9401, 2011.8949, 2015.9641, 2023.9448, 2027.1273, 2032.0186, 2044.0441, 2045.9841, 2060.9731, 2067.1531, 2086.0945, 2088.1196, 2108.9956, 2112.9929, 2114.9592, 2139.0918, 2144.0564, 2160.0481, 2174.1157, 2179.0405, 2199.1233, 2211.0984, 2229.1216, 2239.1421, 2242.1345, 2245.8398, 2251.1340, 2255.1416, 2265.0903, 2270.1790, 2273.0667, 2284.1729, 2287.1509, 2293.1445, 2328.9482, 2343.1899, 2352.9153, 2354.9983, 2356.0093, 2364.8684, 2414.2334, 2423.2490, 2450.2375, 2466.2253, 2468.2173, 2475.2114, 2486.1069, 2519.9941, 2541.2319, 2542.2505, 2575.1797, 2646.9695, 2660.9880, 2675.0371, 2695.0181, 2702.2866, 2705.2827, 2713.3413, 2717.3323, 2718.2998, 2721.2900, 2744.2466, 2763.3267, 2770.3513, 2785.3567, 2787.2734, 2788.2341, 2792.2034, 2798.2407, 2801.2722, 2803.2302, 2808.2080, 2817.2480, 2820.2319, 2833.2026, 2843.3906, 2846.3865, 2847.3792, 2870.3926, 2873.3279, 2889.3057, 2892.4070, 2896.4392, 2912.4277, 2915.4314, 2919.4495, 2961.2297, 3071.3845, 3076.4626, 3081.6108, 3087.3884, 3101.4309, 3118.6643, 3143.5120, 3159.5193, 3173.5476, 3184.4751, 3236.5454, 3253.6089, 3265.5996, 3269.6123, 3285.5964, 3331.5325, 3334.6331, 3335.6592, 3347.4841, 3397.6499, 3412.5662, 3427.6182, 3428.5718, 3444.5942, 3457.6045, 3463.5820, 3473.5864, 3487.6257, 3491.6506, 3509.5947, 3521.6812, 3552.5681, 3574.6165, 3590.6309, 3624.6504, 3636.7607, 3640.6731, 3646.7363, 3651.7256, 3665.7231, 3669.7129, 3692.6494, 3696.7678, 3707.6807, 3714.6301, 3719.6519, 3722.5593, 3723.6516, 3735.6885, 3751.6423, 3830.7644, 3937.7585, 3941.8464, 3942.8328, 3955.8433, 4092.8938, 4147.8613, 4162.9160, 4254.9053, 4290.9102, 4306.9575, 4309.8506, 4323.8926, 4397.9277, 4415.8896, 4449.1777, 4455.1221, 4459.2368, 4494.0420, 4501.0767, 4510.0269, 4525.1040, 4530.0112, 4839.1138, 4846.0161

27. A6H762 **Mass:** 22782 **Score:** 19 **Expect:** 3.1e+002 **Matches:** 5
C3H1orf149 protein OS=Bos taurus GN=C3H1orf149 PE=2 SV=1

Observed	Mr(expt)	Mr(calc)	ppm	Start	End	Miss	Peptide
830.4365	829.4292	829.4267	3.11	75 -	80	2	K.NDRRNR.K
957.5629	956.5556	956.5654	-10.30	18 -	25	1	R.ELAELVKR.K
1254.6876	1253.6803	1253.6768	2.82	82 -	91	2	K.FKEAERLFSK.S
2542.2505	2541.2432	2541.1958	18.7	175 -	195	2	K.NRHSPSGMFDYDFEIDLKLNK.K + Oxidation (M)
4494.0420	4493.0347	4492.9763	13.0	114 -	154	1	K.REPGSGTESDTSPDFHNQENEPNQEDPEDLDGSVQGVKPQK.A

No match to: 710.3558, 715.3582, 722.4182, 729.4472, 734.4399, 735.4044, 737.4075, 747.4354, 756.3666, 759.4210, 772.4085, 779.4621, 800.3990, 801.4063, 805.4392, 818.4569, 823.4658, 828.4204, 829.4349, 832.2986, 834.3055, 842.4619, 856.3810, 865.4302, 870.4144, 873.3766, 880.4450, 883.4655, 887.4088, 889.4736, 891.4725, 898.4407, 900.5031, 912.4936, 923.5015, 925.4745, 929.4329, 938.4833, 961.4780, 967.4121, 970.4429, 972.4810, 974.4342, 978.5226, 983.6148, 988.4271, 993.4903, 995.3998, 998.5886, 1006.4926, 1007.5641, 1021.5605, 1024.5408, 1032.5048, 1034.5706, 1036.5566, 1043.5085, 1044.5150, 1046.5917, 1056.5394, 1057.6062, 1074.6005, 1087.5505, 1113.5382, 1122.5513, 1123.5905, 1130.5541, 1143.4395, 1145.6267, 1177.6389, 1178.6249, 1180.6364, 1184.6396, 1194.5638, 1198.6896, 1216.7015, 1227.6558, 1243.6030, 1248.6648, 1255.5952, 1264.7084, 1269.6405, 1273.6479, 1278.6892, 1281.6271, 1289.6858, 1301.6105, 1304.6399, 1312.7454, 1325.6327, 1326.6665, 1328.6610, 1336.7188, 1337.6541, 1338.6735, 1349.6659, 1351.6846, 1356.6124, 1361.6910, 1365.6167, 1372.5992, 1382.5925, 1383.7246, 1390.6946, 1420.7117, 1423.6814, 1445.7860, 1456.7703, 1457.7407, 1467.7371, 1470.7511, 1488.7211, 1494.7380, 1497.8768, 1500.7181, 1503.7421, 1513.7554, 1519.7505, 1528.6958, 1530.6902, 1540.7820, 1544.8356, 1556.8394, 1562.7800, 1563.7925, 1572.7056, 1578.7816, 1580.8422, 1599.9109, 1616.7953, 1621.7692, 1628.7004, 1628.8589, 1635.7825, 1642.7168, 1647.8525, 1650.8430, 1657.9056, 1666.7905, 1696.8221, 1706.8651, 1707.9131, 1717.9056, 1722.9384, 1730.8652, 1738.7958, 1741.9946, 1755.8914, 1758.9082, 1771.0306, 1774.9175, 1787.8247, 1809.0355, 1862.9480, 1883.7240, 1928.0236, 1932.0079, 1938.0043, 1940.0157, 1953.8590, 1959.9622, 1961.0220, 1969.9949, 1981.0421, 1990.9496, 1997.8253, 2007.9401, 2011.8949, 2015.9641, 2023.9448, 2027.1273, 2032.0186, 2044.0441, 2045.9841, 2060.9731, 2067.1531, 2086.0945, 2088.1196, 2108.9956, 2112.9929, 2114.9592, 2139.0918, 2144.0564, 2160.0481, 2174.1157, 2179.0405, 2199.1233, 2211.0984, 2229.1216, 2239.1421, 2242.1345, 2245.8398, 2251.1340, 2255.1416, 2265.0903, 2270.1790, 2273.0667, 2284.1729, 2287.1509, 2293.1445, 2328.9482, 2343.1899, 2352.9153, 2354.9983, 2356.0093, 2364.8684, 2414.2334, 2423.2490, 2450.2375, 2466.2253, 2468.2173, 2475.2114, 2486.1069, 2519.9941, 2541.2319, 2558.3389, 2575.1797, 2646.9695, 2660.9880, 2675.0371, 2695.0181, 2702.2866, 2705.2827, 2713.3413, 2717.3323, 2718.2998, 2721.2900, 2744.2466, 2763.3267, 2770.3513, 2785.3567, 2787.2734, 2788.2341, 2792.2034, 2798.2407, 2801.2722, 2803.2302, 2808.2080, 2817.2480, 2820.2319, 2833.2026, 2843.3906, 2846.3865, 2847.3792, 2870.3926, 2873.3279, 2889.3057, 2892.4070, 2896.4392, 2912.4277, 2915.4314, 2919.4495, 2961.2297, 3071.3845, 3076.4626, 3081.6108, 3087.3884, 3101.4309, 3118.6643, 3143.5120, 3159.5193, 3173.5476, 3184.4751, 3236.5454, 3248.6077, 3253.6089, 3265.5996, 3269.6123, 3285.5964, 3331.5325, 3334.6331, 3335.6592, 3347.4841, 3397.6499, 3411.6399, 3412.5662, 3427.6182, 3428.5718, 3435.6516, 3444.5942, 3457.6045, 3463.5820, 3473.5864, 3487.6257, 3491.6506, 3503.7678, 3509.5947, 3521.6812, 3552.5681, 3574.6165, 3590.6309, 3624.6504, 3636.7607, 3640.6731, 3646.7363, 3651.7256, 3665.7231, 3669.7129, 3692.6494, 3696.7678, 3707.6807, 3714.6301, 3719.6519, 3722.5593, 3723.6516, 3735.6885, 3751.6423, 3830.7644, 3937.7585, 3941.8464, 3942.8328, 3955.8433, 4092.8938, 4147.8613, 4162.9160, 4254.9053, 4272.9224, 4290.9102, 4306.9575, 4309.8506, 4323.8926, 4397.9277, 4415.8896, 4449.1777, 4455.1221, 4459.2368, 4501.0767, 4510.0269, 4525.1040, 4530.0112, 4839.1138, 4846.0161

28. G3MXC7 **Mass:** 12858 **Score:** 19 **Expect:** 3.1e+002 **Matches:** 3
Uncharacterized protein OS=Bos taurus PE=4 SV=1

Observed	Mr(expt)	Mr(calc)	ppm	Start	End	Miss	Peptide
779.4621	778.4548	778.4701	-19.61	29 -	34	1	K.YLIKSR.E
3411.6399	3410.6326	3410.5612	20.9	83 -	113	1	R.FSGKQFSDFHSELNLSSLELTDSAAYLCASS.-
4147.8613	4146.8540	4146.9282	-17.90	42 -	76	2	R.CSPESGHRSVYWYQQALGQGPQFLVQYYNGKENEK.G

No match to: 710.3558, 715.3582, 722.4182, 729.4472, 734.4399, 735.4044, 737.4075, 747.4354, 756.3666, 759.4210, 772.4085, 800.3990, 801.4063, 805.4392, 818.4569, 823.4658, 828.4204, 829.4349, 830.4365, 832.2986, 834.3055, 842.4619, 856.3810, 865.4302, 870.4144, 873.3766, 880.4450, 883.4655, 887.4088, 889.4736, 891.4725, 898.4407, 900.5031, 912.4936, 923.5015, 925.4745, 929.4329, 938.4833, 957.5629, 961.4780, 967.4121, 970.4429, 972.4810, 974.4342, 978.5226, 983.6148, 988.4271, 993.4903, 995.3998, 998.5886, 1006.4926, 1007.5641, 1021.5605, 1024.5408, 1032.5048, 1034.5706, 1036.5566, 1043.5085, 1044.5150, 1046.5917, 1056.5394, 1057.6062, 1074.6005, 1087.5505, 1113.5382, 1122.5513, 1123.5905, 1130.5541, 1143.4395, 1145.6267, 1177.6389, 1178.6249, 1180.6364, 1184.6396, 1194.5638, 1198.6896, 1216.7015, 1227.6558, 1243.6030, 1248.6648, 1254.6876, 1255.5952, 1264.7084, 1269.6405, 1273.6479, 1278.6892, 1281.6271, 1289.6858, 1301.6105, 1304.6399, 1312.7454, 1325.6327, 1326.6665, 1328.6610, 1336.7188, 1337.6541, 1338.6735, 1349.6659, 1351.6846, 1356.6124, 1361.6910, 1365.6167, 1372.5992, 1382.5925, 1383.7246, 1390.6946, 1420.7117, 1423.6814, 1445.7860, 1456.7703, 1457.7407, 1467.7371, 1470.7511, 1488.7211, 1494.7380, 1497.8768, 1500.7181, 1503.7421, 1513.7554, 1519.7505, 1528.6958, 1530.6902, 1540.7820, 1544.8356, 1556.8394, 1562.7800, 1563.7925, 1572.7056, 1578.7816, 1580.8422, 1599.9109, 1616.7953, 1621.7692, 1628.7004, 1628.8589, 1635.7825, 1642.7168, 1647.8525, 1650.8430, 1657.9056, 1666.7905, 1696.8221, 1706.8651, 1707.9131, 1717.9056, 1722.9384, 1730.8652, 1738.7958, 1741.9946, 1755.8914, 1758.9082, 1771.0306, 1774.9175, 1787.8247, 1809.0355, 1862.9480, 1883.7240, 1928.0236, 1932.0079, 1938.0043, 1940.0157, 1953.8590, 1959.9622, 1961.0220, 1969.9949, 1981.0421, 1990.9496, 1997.8253, 2007.9401, 2011.8949, 2015.9641, 2023.9448, 2027.1273, 2032.0186, 2044.0441, 2045.9841, 2060.9731, 2067.1531, 2086.0945, 2088.1196, 2108.9956, 2112.9929, 2114.9592, 2139.0918, 2144.0564, 2160.0481, 2174.1157, 2179.0405, 2199.1233, 2211.0984, 2229.1216, 2239.1421, 2242.1345, 2245.8398, 2251.1340, 2255.1416, 2265.0903, 2270.1790, 2273.0667, 2284.1729, 2287.1509, 2293.1445, 2328.9482, 2343.1899, 2352.9153, 2354.9983, 2356.0093, 2364.8684, 2414.2334, 2423.2490, 2450.2375, 2466.2253, 2468.2173, 2475.2114, 2486.1069, 2519.9941, 2541.2319, 2542.2505, 2558.3389, 2575.1797, 2646.9695, 2660.9880, 2675.0371, 2695.0181, 2702.2866, 2705.2827, 2713.3413, 2717.3323, 2718.2998, 2721.2900,

2744.2466, 2763.3267, 2770.3513, 2785.3567, 2787.2734, 2788.2341, 2792.2034, 2798.2407, 2801.2722, 2803.2302, 2808.2080, 2817.2480, 2820.2319, 2833.2026, 2843.3906, 2846.3865, 2847.3792, 2870.3926, 2873.3279, 2889.3057, 2892.4070, 2896.4392, 2912.4277, 2915.4314, 2919.4495, 2961.2297, 3071.3845, 3076.4626, 3081.6108, 3087.3884, 3101.4309, 3118.6643, 3143.5120, 3159.5193, 3173.5476, 3184.4751, 3236.5454, 3248.6077, 3253.6089, 3265.5996, 3269.6123, 3285.5964, 3331.5325, 3334.6331, 3335.6592, 3347.4841, 3397.6499, 3412.5662, 3427.6182, 3428.5718, 3435.6516, 3444.5942, 3457.6045, 3463.5820, 3473.5864, 3487.6257, 3491.6506, 3503.7678, 3509.5947, 3521.6812, 3552.5681, 3574.6165, 3590.6309, 3624.6504, 3636.7607, 3640.6731, 3646.7363, 3651.7256, 3665.7231, 3669.7129, 3692.6494, 3696.7678, 3707.6807, 3714.6301, 3719.6519, 3722.5593, 3723.6516, 3735.6885, 3751.6423, 3830.7644, 3937.7585, 3941.8464, 3942.8328, 3955.8433, 4092.8938, 4162.9160, 4254.9053, 4272.9224, 4290.9102, 4306.9575, 4309.8506, 4323.8926, 4397.9277, 4415.8896, 4449.1777, 4455.1221, 4459.2368, 4494.0420, 4501.0767, 4510.0269, 4525.1040, 4530.0112, 4839.1138, 4846.0161

29. Q2TBR9 **Mass:** 15151 **Score:** 19 **Expect:** 3.2e+002 **Matches:** 4

Uncharacterized protein C12orf31 homolog OS=Bos taurus PE=2 SV=1

Observed	Mr(expt)	Mr(calc)	ppm	Start	End	Miss	Peptide
2015.9641	2014.9568	2014.9683	-5.71	83 -	98	0	K.TLLDQHGQYPIWMNQR.Q + Oxidation (M)
2144.0564	2143.0491	2143.0633	-6.60	82 -	98	1	K.KTLLDQHGQYPIWMNQR.Q + Oxidation (M)
2199.1233	2198.1160	2198.1504	-15.65	35 -	54	1	K.IDGDVLMKDVQEIATVVEPK.H
2284.1729	2283.1656	2283.1331	14.3	83 -	100	1	K.TLLDQHGQYPIWMNQRQR.K

No match to: 710.3558, 715.3582, 722.4182, 729.4472, 734.4399, 735.4044, 737.4075, 747.4354, 756.3666, 759.4210, 772.4085, 779.4621, 800.3990, 801.4063, 805.4392, 818.4569, 823.4658, 828.4204, 829.4349, 830.4365, 832.2986, 834.3055, 842.4619, 856.3810, 865.4302, 870.4144, 873.3766, 880.4450, 883.4655, 887.4088, 889.4736, 891.4725, 898.4407, 900.5031, 912.4936, 923.5015, 925.4745, 929.4329, 938.4833, 957.5629, 961.4780, 967.4121, 970.4429, 972.4810, 974.4342, 978.5226, 983.6148, 988.4271, 993.4903, 995.3998, 998.5886, 1006.4926, 1007.5641, 1021.5605, 1024.5408, 1032.5048, 1034.5706, 1036.5566, 1043.5085, 1044.5150, 1046.5917, 1056.5394, 1057.6062, 1074.6005, 1087.5505, 1113.5382, 1122.5513, 1123.5905, 1130.5541, 1143.4395, 1145.6267, 1177.6389, 1178.6249, 1180.6364, 1184.6396, 1194.5638, 1198.6896, 1216.7015, 1227.6558, 1243.6030, 1248.6648, 1254.6876, 1255.5952, 1264.7084, 1269.6405, 1273.6479, 1278.6892, 1281.6271, 1289.6858, 1301.6105, 1304.6399, 1312.7454, 1325.6327, 1326.6665, 1328.6610, 1336.7188, 1337.6541, 1338.6735, 1349.6659, 1351.6846, 1356.6124, 1361.6910, 1365.6167, 1372.5992, 1382.5925, 1383.7246, 1390.6946, 1420.7117, 1423.6814, 1445.7860, 1456.7703, 1457.7407, 1467.7371, 1470.7511, 1488.7211, 1494.7380, 1497.8768, 1500.7181, 1503.7421, 1513.7554, 1519.7505, 1528.6958, 1530.6902, 1540.7820, 1544.8356, 1556.8394, 1562.7800, 1563.7925, 1572.7056, 1578.7816, 1580.8422, 1599.9109, 1616.7953, 1621.7692, 1628.7004, 1628.8589, 1635.7825, 1642.7168, 1647.8525, 1650.8430, 1657.9056, 1666.7905, 1696.8221, 1706.8651, 1707.9131, 1717.9056, 1722.9384, 1730.8652, 1738.7958, 1741.9946, 1755.8914, 1758.9082, 1771.0306, 1774.9175, 1787.8247, 1809.0355, 1862.9480, 1883.7240, 1928.0236, 1932.0079, 1938.0043, 1940.0157, 1953.8590, 1959.9622, 1961.0220, 1969.9949, 1981.0421, 1990.9496, 1997.8253, 2007.9401, 2011.8949, 2023.9448, 2027.1273, 2032.0186, 2044.0441, 2045.9841, 2060.9731, 2067.1531, 2086.0945, 2088.1196, 2108.9956, 2112.9929, 2114.9592, 2139.0918, 2160.0481, 2174.1157, 2179.0405, 2211.0984, 2229.1216, 2239.1421, 2242.1345, 2245.8398, 2251.1340, 2255.1416, 2265.0903, 2270.1790, 2273.0667, 2287.1509, 2293.1445, 2328.9482, 2343.1899, 2352.9153, 2354.9983, 2356.0093, 2364.8684, 2414.2334, 2423.2490, 2450.2375, 2466.2253, 2468.2173, 2475.2114, 2486.1069, 2519.9941, 2541.2319, 2542.2505, 2558.3389, 2575.1797, 2646.9695, 2660.9880, 2675.0371, 2695.0181, 2702.2866, 2705.2827, 2713.3413, 2717.3323, 2718.2998, 2721.2900, 2744.2466, 2763.3267, 2770.3513, 2785.3567, 2787.2734, 2788.2341, 2792.2034, 2798.2407, 2801.2722, 2803.2302, 2808.2080, 2817.2480, 2820.2319, 2833.2026, 2843.3906, 2846.3865, 2847.3792, 2870.3926, 2873.3279, 2889.3057, 2892.4070, 2896.4392, 2912.4277, 2915.4314, 2919.4495, 2961.2297, 3071.3845, 3076.4626, 3081.6108, 3087.3884, 3101.4309, 3118.6643, 3143.5120, 3159.5193, 3173.5476, 3184.4751, 3236.5454, 3248.6077, 3253.6089, 3265.5996, 3269.6123, 3285.5964, 3331.5325, 3334.6331, 3335.6592, 3347.4841, 3397.6499, 3411.6399, 3412.5662, 3427.6182, 3428.5718, 3435.6516, 3444.5942, 3457.6045, 3463.5820, 3473.5864, 3487.6257, 3491.6506, 3503.7678, 3509.5947, 3521.6812, 3552.5681, 3574.6165, 3590.6309, 3624.6504, 3636.7607, 3640.6731, 3646.7363, 3651.7256, 3665.7231, 3669.7129, 3692.6494, 3696.7678, 3707.6807, 3714.6301, 3719.6519, 3722.5593, 3723.6516, 3735.6885, 3751.6423, 3830.7644, 3937.7585, 3941.8464, 3942.8328, 3955.8433, 4092.8938, 4147.8613, 4162.9160, 4254.9053, 4272.9224, 4290.9102, 4306.9575, 4309.8506, 4323.8926, 4397.9277, 4415.8896, 4449.1777, 4455.1221, 4459.2368, 4494.0420, 4501.0767, 4510.0269, 4525.1040, 4530.0112, 4839.1138, 4846.0161

30. F1MP35 **Mass:** 62777 **Score:** 19 **Expect:** 3.3e+002 **Matches:** 10

Uncharacterized protein OS=Bos taurus GN=ZNF783 PE=4 SV=2

Observed	Mr(expt)	Mr(calc)	ppm	Start	End	Miss	Peptide
978.5226	977.5154	977.4964	19.4	24 -	31	1	K.KVDSCLTR.L
1264.7084	1263.7011	1263.6836	13.8	509 -	518	1	K.AFRRPSDLFR.H
1351.6846	1350.6773	1350.6819	-3.41	114 -	123	1	K.LDEWQKELYK.H
1457.7407	1456.7334	1456.7019	21.7	411 -	425	1	R.AGDSRSTGAGTQPPR.V
1928.0236	1927.0163	1926.9952	10.9	258 -	277	1	R.KGSSLPPFHPGSGFGGGTLK.R
2114.9592	2113.9519	2113.9786	-12.62	467 -	484	2	R.GGSPTGCSECLRFFQQRK.S
2199.1233	2198.1160	2198.1345	-8.41	259 -	280	2	K.GSSLPPFHPGSGFGGGTLKRSR.G
2414.2334	2413.2261	2413.2199	2.59	128 -	148	0	R.GNYEMLVSLDYAISKPDILTR.M + Oxidation (M)
2846.3865	2845.3792	2845.3990	-6.94	128 -	151	1	R.GNYEMLVSLDYAISKPDILTRMER.G + 2 Oxidation (M)
2896.4392	2895.4319	2895.3756	19.4	196 -	222	1	R.KTLQMGSHQPALPGTSCPAWGQVSEGR.R + Oxidation (M)

No match to: 710.3558, 715.3582, 722.4182, 729.4472, 734.4399, 735.4044, 737.4075, 747.4354, 756.3666, 759.4210, 772.4085, 779.4621, 800.3990, 801.4063, 805.4392, 818.4569, 823.4658, 828.4204, 829.4349, 830.4365, 832.2986, 834.3055, 842.4619, 856.3810, 865.4302, 870.4144, 873.3766, 880.4450, 883.4655, 887.4088, 889.4736, 891.4725, 898.4407, 900.5031, 912.4936, 923.5015, 925.4745, 929.4329, 938.4833, 957.5629, 961.4780, 967.4121, 970.4429, 972.4810, 974.4342, 983.6148, 988.4271, 993.4903, 995.3998, 998.5886, 1006.4926, 1007.5641, 1021.5605, 1024.5408, 1032.5048, 1034.5706, 1036.5566, 1043.5085, 1044.5150, 1046.5917, 1056.5394, 1057.6062, 1074.6005, 1087.5505, 1113.5382, 1122.5513, 1123.5905, 1130.5541, 1143.4395, 1145.6267, 1177.6389, 1178.6249, 1180.6364, 1184.6396, 1194.5638, 1198.6896, 1216.7015, 1227.6558, 1243.6030, 1248.6648, 1254.6876, 1255.5952, 1269.6405, 1273.6479, 1278.6892, 1281.6271, 1289.6858, 1301.6105, 1304.6399, 1312.7454, 1325.6327, 1326.6665, 1328.6610, 1336.7188, 1337.6541, 1338.6735, 1349.6659, 1356.6124, 1361.6910, 1365.6167, 1372.5992, 1382.5925, 1383.7246, 1390.6946, 1420.7117, 1423.6814, 1445.7860, 1456.7703, 1467.7371, 1470.7511, 1488.7211, 1494.7380, 1497.8768, 1500.7181, 1503.7421, 1513.7554, 1519.7505, 1528.6958, 1530.6902, 1540.7820, 1544.8356, 1556.8394, 1562.7800, 1563.7925, 1572.7056, 1578.7816, 1580.8422, 1599.9109, 1616.7953, 1621.7692, 1628.7004, 1628.8589, 1635.7825, 1642.7168, 1647.8525, 1650.8430, 1657.9056, 1666.7905, 1696.8221, 1706.8651, 1707.9131, 1717.9056, 1722.9384, 1730.8652, 1738.7958, 1741.9946, 1755.8914, 1758.9082, 1771.0306, 1774.9175, 1787.8247, 1809.0355, 1862.9480, 1883.7240, 1932.0079, 1938.0043, 1940.0157, 1953.8590, 1959.9622, 1961.0220, 1969.9949, 1981.0421, 1990.9496, 1997.8253, 2007.9401, 2011.8949, 2015.9641, 2023.9448, 2027.1273, 2032.0186, 2044.0441, 2045.9841, 2060.9731, 2067.1531, 2086.0945, 2088.1196, 2108.9956, 2112.9929, 2139.0918, 2144.0564, 2160.0481, 2174.1157, 2179.0405, 2211.0984, 2229.1216, 2239.1421, 2242.1345, 2245.8398, 2251.1340, 2255.1416, 2265.0903, 2270.1790, 2273.0667, 2284.1729, 2287.1509, 2293.1445, 2328.9482, 2343.1899, 2352.9153, 2354.9983, 2356.0093, 2364.8684, 2423.2490, 2450.2375, 2466.2253, 2468.2173, 2475.2114, 2486.1069, 2519.9941, 2541.2319, 2542.2505, 2558.3389, 2575.1797, 2646.9695, 2660.9880, 2675.0371, 2695.0181, 2702.2866, 2705.2827, 2713.3413, 2717.3323, 2718.2998, 2721.2900, 2744.2466, 2763.3267, 2770.3513, 2785.3567, 2787.2734, 2788.2341, 2792.2034, 2798.2407, 2801.2722, 2803.2302, 2808.2080, 2817.2480, 2820.2319, 2833.2026, 2843.3906, 2847.3792, 2870.3926, 2873.3279, 2889.3057, 2892.4070, 2912.4277, 2915.4314, 2919.4495, 2961.2297, 3071.3845, 3076.4626, 3081.6108, 3087.3884, 3101.4309, 3118.6643, 3143.5120, 3159.5193, 3173.5476, 3184.4751, 3236.5454, 3248.6077, 3253.6089, 3265.5996, 3269.6123, 3285.5964, 3331.5325, 3334.6331, 3335.6592, 3347.4841, 3397.6499, 3411.6399, 3412.5662, 3427.6182, 3428.5718, 3435.6516, 3444.5942, 3457.6045, 3463.5820, 3473.5864, 3487.6257, 3491.6506, 3503.7678, 3509.5947, 3521.6812, 3552.5681, 3574.6165, 3590.6309, 3624.6504, 3636.7607, 3640.6731, 3646.7363, 3651.7256, 3665.7231, 3669.7129, 3692.6494, 3696.7678, 3707.6807, 3714.6301, 3719.6519, 3722.5593, 3723.6516, 3735.6885, 3751.6423, 3830.7644, 3937.7585, 3941.8464, 3942.8328, 3955.8433, 4092.8938, 4147.8613, 4162.9160, 4254.9053, 4272.9224, 4290.9102, 4306.9575, 4309.8506, 4323.8926, 4397.9277, 4415.8896, 4449.1777, 4455.1221, 4459.2368, 4494.0420, 4501.0767, 4510.0269, 4525.1040, 4530.0112, 4839.1138, 4846.0161

31. Q32KZ1 **Mass:** 30885 **Score:** 19 **Expect:** 3.3e+002 **Matches:** 9

Translation initiation factor IF-3, mitochondrial OS=Bos taurus GN=MTIF3 PE=2 SV=1

Observed	Mr(expt)	Mr(calc)	ppm	Start	End	Miss	Peptide
883.4655	882.4582	882.4671	-10.17	248 -	255	1	K.AAPDTPRR.D
1289.6858	1288.6785	1288.6995	-16.31	230 -	240	0	K.AVMCVLRPLSK.K + Oxidation (M)
1556.8394	1555.8321	1555.8616	-18.97	98 -	110	2	R.ANVIRLMAERDLR.L
1707.9131	1706.9058	1706.8700	21.0	66 -	80	2	K.NETAFSSVGRKINER.I

3071.3845	3070.3772	3070.4456	-22.28	201 - 226	1	K.MEEMCNRIVQTMSGIATFSSRPQPIR.G + 2 Oxidation (M)
3081.6108	3080.6035	3080.6142	-3.47	144 - 172	2	R.AAPKPGPTLTKELTFSSNIGQHDLDTKSK.Q
3087.3884	3086.3811	3086.4406	-19.26	201 - 226	1	K.MEEMCNRIVQTMSGIATFSSRPQPIR.G + 3 Oxidation (M)
3942.8328	3941.8255	3941.8016	6.08	193 - 226	2	K.SADEPEDKMEEMCNRIVQTMSGIATFSSRPQPIR.G + 2 Oxidation (M)
3955.8433	3954.8360	3954.9244	-22.35	28 - 62	1	K.YILQGPAPTQQPPRPSCLIHAKAFSTEDTQDEMTK.K

No match to: 710.3558, 715.3582, 722.4182, 729.4472, 734.4399, 735.4044, 737.4075, 747.4354, 756.3666, 759.4210, 772.4085, 779.4621, 800.3990, 801.4063, 805.4392, 818.4569, 823.4658, 828.4204, 829.4349, 830.4365, 832.2986, 834.3055, 842.4619, 856.3810, 865.4302, 870.4144, 873.3766, 880.4450, 887.4088, 889.4736, 891.4725, 898.4407, 900.5031, 912.4936, 923.5015, 925.4745, 929.4329, 938.4833, 957.5629, 961.4780, 967.4121, 970.4429, 972.4810, 974.4342, 978.5226, 983.6148, 988.4271, 993.4903, 995.3998, 998.5886, 1006.4926, 1007.5641, 1021.5605, 1024.5408, 1032.5048, 1034.5706, 1036.5566, 1043.5085, 1044.5150, 1046.5917, 1056.5394, 1057.6062, 1074.6005, 1087.5505, 1113.5382, 1122.5513, 1123.5905, 1130.5541, 1143.4395, 1145.6267, 1177.6389, 1178.6249, 1180.6364, 1184.6396, 1194.5638, 1198.6896, 1216.7015, 1227.6558, 1243.6030, 1248.6648, 1254.6876, 1255.5952, 1264.7084, 1269.6405, 1273.6479, 1278.6892, 1281.6271, 1301.6105, 1304.6399, 1312.7454, 1325.6327, 1326.6665, 1328.6610, 1336.7188, 1337.6541, 1338.6735, 1349.6659, 1351.6846, 1356.6124, 1361.6910, 1365.6167, 1372.5992, 1382.5925, 1383.7246, 1390.6946, 1420.7117, 1423.6814, 1445.7860, 1456.7703, 1457.7407, 1467.7371, 1470.7511, 1488.7211, 1494.7380, 1497.8768, 1500.7181, 1503.7421, 1513.7554, 1519.7505, 1528.6958, 1530.6902, 1540.7820, 1544.8356, 1562.7800, 1563.7925, 1572.7056, 1578.7816, 1580.8422, 1599.9109, 1616.7953, 1621.7692, 1628.7004, 1628.8589, 1635.7825, 1642.7168, 1647.8525, 1650.8430, 1657.9056, 1666.7905, 1696.8221, 1706.8651, 1717.9056, 1722.9384, 1730.8652, 1738.7958, 1741.9946, 1755.8914, 1758.9082, 1771.0306, 1774.9175, 1787.8247, 1809.0355, 1862.9480, 1883.7240, 1928.0236, 1932.0079, 1938.0043, 1940.0157, 1953.8590, 1959.9622, 1961.0220, 1969.9949, 1981.0421, 1990.9496, 1997.8253, 2007.9401, 2011.8949, 2015.9641, 2023.9448, 2027.1273, 2032.0186, 2044.0441, 2045.9841, 2060.9731, 2067.1531, 2086.0945, 2088.1196, 2108.9956, 2112.9929, 2114.9592, 2139.0918, 2144.0564, 2160.0481, 2174.1157, 2179.0405, 2199.1233, 2211.0984, 2229.1216, 2239.1421, 2242.1345, 2245.8398, 2251.1340, 2255.1416, 2265.0903, 2270.1790, 2273.0667, 2284.1729, 2287.1509, 2293.1445, 2328.9482, 2343.1899, 2352.9153, 2354.9983, 2356.0093, 2364.8684, 2414.2334, 2423.2490, 2450.2375, 2466.2253, 2468.2173, 2475.2114, 2486.1069, 2519.9941, 2541.2319, 2542.2505, 2558.3389, 2575.1797, 2646.9695, 2660.9880, 2675.0371, 2695.0181, 2702.2866, 2705.2827, 2713.3413, 2717.3323, 2718.2998, 2721.2900, 2744.2466, 2763.3267, 2770.3513, 2785.3567, 2787.2734, 2788.2341, 2792.2034, 2798.2407, 2801.2722, 2803.2302, 2808.2080, 2817.2480, 2820.2319, 2833.2026, 2843.3906, 2846.3865, 2847.3792, 2870.3926, 2873.3279, 2889.3057, 2892.4070, 2896.4392, 2912.4277, 2915.4314, 2919.4495, 2961.2297, 3076.4626, 3101.4309, 3118.6643, 3143.5120, 3159.5193, 3173.5476, 3184.4751, 3236.5454, 3248.6077, 3253.6089, 3265.5996, 3269.6123, 3285.5964, 3331.5325, 3334.6331, 3335.6592, 3347.4841, 3397.6499, 3411.6399, 3412.5662, 3427.6182, 3428.5718, 3435.6516, 3444.5942, 3457.6045, 3463.5820, 3473.5864, 3487.6257, 3491.6506, 3503.7678, 3509.5947, 3521.6812, 3552.5681, 3574.6165, 3590.6309, 3624.6504, 3636.7607, 3640.6731, 3646.7363, 3651.7256, 3665.7231, 3669.7129, 3692.6494, 3696.7678, 3707.6807, 3714.6301, 3719.6519, 3722.5593, 3723.6516, 3735.6885, 3751.6423, 3830.7644, 3937.7585, 3941.8464, 4092.8938, 4147.8613, 4162.9160, 4254.9053, 4272.9224, 4290.9102, 4306.9575, 4309.8506, 4323.8926, 4397.9277, 4415.8896, 4449.1777, 4455.1221, 4459.2368, 4494.0420, 4501.0767, 4510.0269, 4525.1040, 4530.0112, 4839.1138, 4846.0161

32. Q3SZK8 **Mass:** 39864 **Score:** 18 **Expect:** 3.4e+002 **Matches:** 9

Na(+)/H(+) exchange regulatory cofactor NHE-RF1 OS=Bos taurus GN=SLC9A3R1 PE=2 SV=1

Observed	Mr(expt)	Mr(calc)	ppm	Start End	Miss	Peptide
735.4044	734.3971	734.4075	-14.12	35 - 40	0	K.VGQYIR.L
1043.5085	1042.5012	1042.4971	3.94	242 - 249	1	R.ETDEFFKK.C
1289.6858	1288.6785	1288.6744	3.23	157 - 166	1	R.ELRPRLCAMK.K + Oxidation (M)
1445.7860	1444.7787	1444.7755	2.25	156 - 166	2	R.RELRPRLCAMK.K + Oxidation (M)
1519.7505	1518.7432	1518.7063	24.3	131 - 146	0	R.GAGGENEPPAAAPEPR.E
1928.0236	1927.0163	1927.0527	-18.87	33 - 50	2	K.GKVGQYIRLVEPGSPAEK.S
2239.1421	2238.1348	2238.1168	8.04	229 - 248	2	K.AGGDEAKLLVVDRETDEFFK.K
2912.4277	2911.4204	2911.4298	-3.23	313 - 341	1	K.KQDSTAPSSTSSSSSDPVLDFSISLAVAK.E
3937.7585	3936.7512	3936.8296	-19.90	108 - 146	2	R.EEMLRAQEGPGQAEPPAAAAEERGAGGENEPPAAAPEPR.E

No match to: 710.3558, 715.3582, 722.4182, 729.4472, 734.4399, 737.4075, 747.4354, 756.3666, 759.4210, 772.4085, 779.4621, 800.3990, 801.4063, 805.4392, 818.4569, 823.4658, 828.4204, 829.4349, 830.4365, 832.2986, 834.3055, 842.4619, 856.3810, 865.4302, 870.4144, 873.3766, 880.4450, 883.4655, 887.4088, 889.4736, 891.4725, 898.4407, 900.5031, 912.4936, 923.5015, 925.4745, 929.4329, 938.4833, 957.5629, 961.4780, 967.4121, 970.4429, 972.4810, 974.4342, 978.5226, 983.6148, 988.4271, 993.4903, 995.3998, 998.5886, 1006.4926, 1007.5641, 1021.5605, 1024.5408, 1032.5048, 1034.5706, 1036.5566, 1044.5150, 1046.5917, 1056.5394, 1057.6062, 1074.6005, 1087.5505, 1113.5382, 1122.5513, 1123.5905, 1130.5541, 1143.4395, 1145.6267, 1177.6389, 1178.6249, 1180.6364, 1184.6396, 1194.5638, 1198.6896, 1216.7015, 1227.6558, 1243.6030, 1248.6648, 1254.6876, 1255.5952, 1264.7084, 1269.6405, 1273.6479, 1278.6892, 1281.6271, 1301.6105, 1304.6399, 1312.7454, 1325.6327, 1326.6665, 1328.6610, 1336.7188, 1337.6541, 1338.6735, 1349.6659, 1351.6846, 1356.6124, 1361.6910, 1365.6167, 1372.5992, 1382.5925, 1383.7246, 1390.6946, 1420.7117, 1423.6814, 1456.7703, 1457.7407, 1467.7371, 1470.7511, 1488.7211, 1494.7380, 1497.8768, 1500.7181, 1503.7421, 1513.7554, 1528.6958, 1530.6902, 1540.7820, 1544.8356, 1556.8394, 1562.7800, 1563.7925, 1572.7056, 1578.7816, 1580.8422, 1599.9109, 1616.7953, 1621.7692, 1628.7004, 1628.8589, 1635.7825, 1642.7168, 1647.8525, 1650.8430, 1657.9056, 1666.7905, 1696.8221, 1706.8651, 1707.9131, 1717.9056, 1722.9384, 1730.8652, 1738.7958, 1741.9946, 1755.8914, 1758.9082, 1771.0306, 1774.9175, 1787.8247, 1809.0355, 1862.9480, 1883.7240, 1932.0079, 1938.0043, 1940.0157, 1953.8590, 1959.9622, 1961.0220, 1969.9949, 1981.0421, 1990.9496, 1997.8253, 2007.9401, 2011.8949, 2015.9641, 2023.9448, 2027.1273, 2032.0186, 2044.0441, 2045.9841, 2060.9731, 2067.1531, 2086.0945, 2088.1196, 2108.9956, 2112.9929, 2114.9592, 2139.0918, 2144.0564, 2160.0481, 2174.1157, 2179.0405, 2199.1233, 2211.0984, 2229.1216, 2242.1345, 2245.8398, 2251.1340, 2255.1416, 2265.0903, 2270.1790, 2273.0667, 2284.1729, 2287.1509, 2293.1445, 2328.9482, 2343.1899, 2352.9153, 2354.9983, 2356.0093, 2364.8684, 2414.2334, 2423.2490, 2450.2375, 2466.2253, 2468.2173, 2475.2114, 2486.1069, 2519.9941, 2541.2319, 2542.2505, 2558.3389, 2575.1797, 2646.9695, 2660.9880, 2675.0371, 2695.0181, 2702.2866, 2705.2827, 2713.3413, 2717.3323, 2718.2998, 2721.2900, 2744.2466, 2763.3267, 2770.3513, 2785.3567, 2787.2734, 2788.2341, 2792.2034, 2798.2407, 2801.2722, 2803.2302, 2808.2080, 2817.2480, 2820.2319, 2833.2026, 2843.3906, 2846.3865, 2847.3792, 2870.3926, 2873.3279, 2889.3057, 2892.4070, 2896.4392, 2915.4314, 2919.4495, 2961.2297, 3071.3845, 3076.4626, 3081.6108, 3087.3884, 3101.4309, 3118.6643, 3143.5120, 3159.5193, 3173.5476, 3184.4751, 3236.5454, 3248.6077, 3253.6089, 3265.5996, 3269.6123, 3285.5964, 3331.5325, 3334.6331, 3335.6592, 3347.4841, 3397.6499, 3411.6399, 3412.5662, 3427.6182, 3428.5718, 3435.6516, 3444.5942, 3457.6045, 3463.5820, 3473.5864, 3487.6257, 3491.6506, 3503.7678, 3509.5947, 3521.6812, 3552.5681, 3574.6165, 3590.6309, 3624.6504, 3636.7607, 3640.6731, 3646.7363, 3651.7256, 3665.7231, 3669.7129, 3692.6494, 3696.7678, 3707.6807, 3714.6301, 3719.6519, 3722.5593, 3723.6516, 3735.6885, 3751.6423, 3830.7644, 3941.8464, 3942.8328, 3955.8433, 4092.8938, 4147.8613, 4162.9160, 4254.9053, 4272.9224, 4290.9102, 4306.9575, 4309.8506, 4323.8926, 4397.9277, 4415.8896, 4449.1777, 4455.1221, 4459.2368, 4494.0420, 4501.0767, 4510.0269, 4525.1040, 4530.0112, 4839.1138, 4846.0161

33. Q3ZC37 **Mass:** 68867 **Score:** 18 **Expect:** 3.5e+002 **Matches:** 13

TNFAIP3 interacting protein 1 OS=Bos taurus GN=TNIP1 PE=2 SV=1

Observed	Mr(expt)	Mr(calc)	ppm	Start End	Miss	Peptide
880.4450	879.4377	879.4272	11.9	188 - 194	0	R.MALEFNR.L
898.4407	897.4334	897.4556	-24.71	382 - 389	0	R.EHLTAEAK.E
1046.5917	1045.5844	1045.5767	7.37	218 - 226	2	R.KENEALKSK.L
1057.6062	1056.5989	1056.6040	-4.76	516 - 524	2	K.AKDALRQQK.R
1628.8589	1627.8516	1627.8278	14.7	227 - 240	2	K.LDKGLEQRDQAAER.L
1738.7958	1737.7885	1737.7913	-1.59	44 - 58	0	K.MLGELLEESQMEASR.L + Oxidation (M)
1774.9175	1773.9102	1773.9043	3.33	251 - 268	1	K.LLMSSSKDGASGRPGSPK.M
1928.0236	1927.0163	1926.9986	9.22	592 - 609	1	R.LNTPGGCPVEGYVIRAPK.-
2007.9401	2006.9328	2006.9765	-21.74	44 - 60	1	K.MLGELLEESQMEASRLR.Q + Oxidation (M)
2023.9448	2022.9375	2022.9714	-16.73	44 - 60	1	K.MLGELLEESQMEASRLR.Q + 2 Oxidation (M)
2284.1729	2283.1656	2283.2223	-24.81	496 - 515	2	K.LQAQVTLSHAQLKAFKDEEK.A
2414.2334	2413.2261	2413.2365	-4.29	571 - 591	1	R.YPPPPTAMEHPPPLPNSRLFR.L
2423.2490	2422.2417	2422.1984	17.9	144 - 164	0	R.NTMELGPLPQEDSTLMLHLQR.L

No match to: 710.3558, 715.3582, 722.4182, 729.4472, 734.4399, 735.4044, 737.4075, 747.4354, 756.3666, 759.4210, 772.4085, 779.4621, 800.3990, 801.4063, 805.4392, 818.4569, 823.4658, 828.4204, 829.4349, 830.4365, 832.2986, 834.3055, 842.4619, 856.3810, 865.4302, 870.4144, 873.3766, 883.4655, 887.4088, 889.4736, 891.4725, 900.5031, 912.4936, 923.5015, 925.4745, 929.4329, 938.4833,

957.5629, 961.4780, 967.4121, 970.4429, 972.4810, 974.4342, 978.5226, 983.6148, 988.4271, 993.4903, 995.3998, 998.5886, 1006.4926, 1007.5641, 1021.5605, 1024.5408, 1032.5048, 1034.5706, 1036.5566, 1043.5085, 1044.5150, 1056.5394, 1074.6005, 1087.5505, 1113.5382, 1122.5513, 1123.5905, 1130.5541, 1143.4395, 1145.6267, 1177.6389, 1178.6249, 1180.6364, 1184.6396, 1194.5638, 1198.6896, 1216.7015, 1227.6558, 1243.6030, 1248.6648, 1254.6876, 1255.5952, 1264.7084, 1269.6405, 1273.6479, 1278.6892, 1281.6271, 1289.6858, 1301.6105, 1304.6399, 1312.7454, 1325.6327, 1326.6665, 1328.6610, 1336.7188, 1337.6541, 1338.6735, 1349.6659, 1351.6846, 1356.6124, 1361.6910, 1365.6167, 1372.5992, 1382.5925, 1383.7246, 1390.6946, 1420.7117, 1423.6814, 1445.7860, 1456.7703, 1457.7407, 1467.7371, 1470.7511, 1488.7211, 1494.7380, 1497.8768, 1500.7181, 1503.7421, 1513.7554, 1519.7505, 1528.6958, 1530.6902, 1540.7820, 1544.8356, 1556.8394, 1562.7800, 1563.7925, 1572.7056, 1578.7816, 1580.8422, 1599.9109, 1616.7953, 1621.7692, 1628.7004, 1635.7825, 1642.7168, 1647.8525, 1650.8430, 1657.9056, 1666.7905, 1696.8221, 1706.8651, 1707.9131, 1717.9056, 1722.9384, 1730.8652, 1741.9946, 1755.8914, 1758.9082, 1771.0306, 1787.8247, 1809.0355, 1862.9480, 1883.7240, 1932.0079, 1938.0043, 1940.0157, 1953.8590, 1959.9622, 1961.0220, 1969.9949, 1981.0421, 1990.9496, 1997.8253, 2011.8949, 2015.9641, 2027.1273, 2032.0186, 2044.0441, 2045.9841, 2060.9731, 2067.1531, 2086.0945, 2088.1196, 2108.9956, 2112.9929, 2114.9592, 2139.0918, 2144.0564, 2160.0481, 2174.1157, 2179.0405, 2199.1233, 2211.0984, 2229.1216, 2239.1421, 2242.1345, 2245.8398, 2251.1340, 2255.1416, 2265.0903, 2270.1790, 2273.0667, 2287.1509, 2293.1445, 2328.9482, 2343.1899, 2352.9153, 2354.9983, 2356.0093, 2364.8684, 2450.2375, 2466.2253, 2468.2173, 2475.2114, 2486.1069, 2519.9941, 2541.2319, 2542.2505, 2558.3389, 2575.1797, 2646.9695, 2660.9880, 2675.0371, 2695.0181, 2702.2866, 2705.2827, 2713.3413, 2717.3323, 2718.2998, 2721.2900, 2744.2466, 2763.3267, 2770.3513, 2785.3567, 2787.2734, 2788.2341, 2792.2034, 2798.2407, 2801.2722, 2803.2302, 2808.2080, 2817.2480, 2820.2319, 2833.2026, 2843.3906, 2846.3865, 2847.3792, 2870.3926, 2873.3279, 2889.3057, 2892.4070, 2896.4392, 2912.4277, 2915.4314, 2919.4495, 2961.2297, 3071.3845, 3076.4626, 3081.6108, 3087.3884, 3101.4309, 3118.6643, 3143.5120, 3159.5193, 3173.5476, 3184.4751, 3236.5454, 3248.6077, 3253.6089, 3265.5996, 3269.6123, 3285.5964, 3331.5325, 3334.6331, 3335.6592, 3347.4841, 3397.6499, 3411.6399, 3412.5662, 3427.6182, 3428.5718, 3435.6516, 3444.5942, 3457.6045, 3463.5820, 3473.5864, 3487.6257, 3491.6506, 3503.7678, 3509.5947, 3521.6812, 3552.5681, 3574.6165, 3590.6309, 3624.6504, 3636.7607, 3640.6731, 3646.7363, 3651.7256, 3665.7231, 3669.7129, 3692.6494, 3696.7678, 3707.6807, 3714.6301, 3719.6519, 3722.5593, 3723.6516, 3735.6885, 3751.6423, 3830.7644, 3937.7585, 3941.8464, 3942.8328, 3955.8433, 4092.8938, 4147.8613, 4162.9160, 4254.9053, 4272.9224, 4290.9102, 4306.9575, 4309.8506, 4323.8926, 4397.9277, 4415.8896, 4449.1777, 4455.1221, 4459.2368, 4494.0420, 4501.0767, 4510.0269, 4525.1040, 4530.0112, 4839.1138, 4846.0161

34. Q08DV5 **Mass:** 63716 **Score:** 18 **Expect:** 3.8e+002 **Matches:** 12

E3 ubiquitin-protein ligase RNF220 OS=Bos taurus GN=RNF220 PE=2 SV=1

Observed	Mr(expt)	Mr(calc)	ppm	Start	End	Miss	Peptide
887.4088	886.4015	886.4218	-22.91	260	268	0	K.DAMAPGTPK.S
1046.5917	1045.5844	1045.6066	-21.21	315	323	2	R.LNARIGKMK.R + Oxidation (M)
1177.6389	1176.6316	1176.6503	-15.85	186	196	0	K.IFAVSGLISDR.E
1365.6167	1364.6094	1364.5891	14.9	144	154	0	K.NCHDTESPHLR.F
1928.0236	1927.0163	1927.0163	-0.00	433	451	1	R.GAVLNGGPPSTRITPEFSK.W
2211.0984	2210.0911	2210.0954	-1.93	477	496	1	K.NSDIEKITEDSAVTTFEALK.A
2817.2480	2816.2407	2816.2051	12.6	452	476	2	K.WASDEMPSTSNGESSKQEAMQKTCK.N
2833.2026	2832.1953	2832.2000	-1.66	452	476	2	K.WASDEMPSTSNGESSKQEAMQKTCK.N + Oxidation (M)
3253.6089	3252.6016	3252.6158	-4.36	6	36	1	R.AAFKMENSSYLPNPLASPALMVLASTAEASR.D + Oxidation (M)
3269.6123	3268.6050	3268.6107	-1.75	6	36	1	R.AAFKMENSSYLPNPLASPALMVLASTAEASR.D + 2 Oxidation (M)
4290.9102	4289.9029	4289.8494	12.5	509	541	2	R.GDRYKCLICMDSYSMPLTSIQCWHVHCEECWLR.T
4306.9575	4305.9502	4305.8444	24.6	509	541	2	R.GDRYKCLICMDSYSMPLTSIQCWHVHCEECWLR.T + Oxidation (M)

No match to: 710.3558, 715.3582, 722.4182, 729.4472, 734.4399, 735.4044, 737.4075, 747.4354, 756.3666, 759.4210, 772.4085, 779.4621, 800.3990, 801.4063, 805.4392, 818.4569, 823.4658, 828.4204, 829.4349, 830.4365, 832.2986, 834.3055, 842.4619, 856.3810, 865.4302, 870.4144, 873.3766, 880.4450, 883.4655, 889.4736, 891.4725, 898.4407, 900.5031, 912.4936, 923.5015, 925.4745, 929.4329, 938.4833, 957.5629, 961.4780, 967.4121, 970.4429, 972.4810, 974.4342, 978.5226, 983.6148, 988.4271, 993.4903, 995.3998, 998.5886, 1006.4926, 1007.5641, 1021.5605, 1024.5408, 1032.5048, 1034.5706, 1036.5566, 1043.5085, 1044.5150, 1056.5394, 1057.6062, 1074.6005, 1087.5505, 1113.5382, 1122.5513, 1123.5905, 1130.5541, 1143.4395, 1145.6267, 1178.6249, 1180.6364, 1184.6396, 1194.5638, 1198.6896, 1216.7015, 1227.6558, 1243.6030, 1248.6648, 1254.6876, 1255.5952, 1264.7084, 1269.6405, 1273.6479, 1278.6892, 1281.6271, 1289.6858, 1301.6105, 1304.6399, 1312.7454, 1325.6327, 1326.6665, 1328.6610, 1336.7188, 1337.6541, 1338.6735, 1349.6659, 1351.6846, 1356.6124, 1361.6910, 1372.5992, 1382.5925, 1383.7246, 1390.6946, 1420.7117, 1423.6814, 1445.7860, 1456.7703, 1457.7407, 1467.7371, 1470.7511, 1488.7211, 1494.7380, 1497.8768, 1500.7181, 1503.7421, 1513.7554, 1519.7505, 1528.6958, 1530.6902, 1540.7820, 1544.8356, 1556.8394, 1562.7800, 1563.7925, 1572.7056, 1578.7816, 1580.8422, 1599.9109, 1616.7953, 1621.7692, 1628.7004, 1628.8589, 1635.7825, 1642.7168, 1647.8525, 1650.8430, 1657.9056, 1666.7905, 1696.8221, 1706.8651, 1707.9131, 1717.9056, 1722.9384, 1730.8652, 1738.7958, 1741.9946, 1755.8914, 1758.9082, 1771.0306, 1774.9175, 1787.8247, 1809.0355, 1862.9480, 1883.7240, 1932.0079, 1938.0043, 1940.0157, 1953.8590, 1959.9622, 1961.0220, 1969.9949, 1981.0421, 1990.9496, 1997.8253, 2007.9401, 2011.8949, 2015.9641, 2023.9448, 2027.1273, 2032.0186, 2044.0441, 2045.9841, 2060.9731, 2067.1531, 2086.0945, 2088.1196, 2108.9956, 2112.9929, 2114.9592, 2139.0918, 2144.0564, 2160.0481, 2174.1157, 2179.0405, 2199.1233, 2229.1216, 2239.1421, 2242.1345, 2245.8398, 2251.1340, 2255.1416, 2265.0903, 2270.1790, 2273.0667, 2284.1729, 2287.1509, 2293.1445, 2328.9482, 2343.1899, 2352.9153, 2354.9983, 2356.0093, 2364.8684, 2414.2334, 2423.2490, 2450.2375, 2466.2253, 2468.2173, 2475.2114, 2486.1069, 2519.9941, 2541.2319, 2542.2505, 2558.3389, 2575.1797, 2646.9695, 2660.9880, 2675.0371, 2695.0181, 2702.2866, 2705.2827, 2713.3413, 2717.3323, 2718.2998, 2721.2900, 2744.2466, 2763.3267, 2770.3513, 2785.3567, 2787.2734, 2788.2341, 2792.2034, 2798.2407, 2801.2722, 2803.2302, 2808.2080, 2820.2319, 2843.3906, 2846.3865, 2847.3792, 2870.3926, 2873.3279, 2889.3057, 2892.4070, 2896.4392, 2912.4277, 2915.4314, 2919.4495, 2961.2297, 3071.3845, 3076.4626, 3081.6108, 3087.3884, 3101.4309, 3118.6643, 3143.5120, 3159.5193, 3173.5476, 3184.4751, 3236.5454, 3248.6077, 3265.5996, 3285.5964, 3331.5325, 3334.6331, 3335.6592, 3347.4841, 3397.6499, 3411.6399, 3412.5662, 3427.6182, 3428.5718, 3435.6516, 3444.5942, 3457.6045, 3463.5820, 3473.5864, 3487.6257, 3491.6506, 3503.7678, 3509.5947, 3521.6812, 3552.5681, 3574.6165, 3590.6309, 3624.6504, 3636.7607, 3640.6731, 3646.7363, 3651.7256, 3665.7231, 3669.7129, 3692.6494, 3696.7678, 3707.6807, 3714.6301, 3719.6519, 3722.5593, 3723.6516, 3735.6885, 3751.6423, 3830.7644, 3937.7585, 3941.8464, 3942.8328, 3955.8433, 4092.8938, 4147.8613, 4162.9160, 4254.9053, 4272.9224, 4309.8506, 4323.8926, 4397.9277, 4415.8896, 4449.1777, 4455.1221, 4459.2368, 4494.0420, 4501.0767, 4510.0269, 4525.1040, 4530.0112, 4839.1138, 4846.0161

35. F1MYL0 **Mass:** 63667 **Score:** 18 **Expect:** 3.8e+002 **Matches:** 12

E3 ubiquitin-protein ligase RNF220 OS=Bos taurus GN=RNF220 PE=4 SV=2

Observed	Mr(expt)	Mr(calc)	ppm	Start	End	Miss	Peptide
887.4088	886.4015	886.4218	-22.91	260	268	0	K.DAMAPGTPK.S
1046.5917	1045.5844	1045.6066	-21.21	315	323	2	R.LNARIGKMK.R + Oxidation (M)
1177.6389	1176.6316	1176.6503	-15.85	186	196	0	K.IFAVSGLISDR.E
1365.6167	1364.6094	1364.5891	14.9	144	154	0	K.NCHDTESPHLR.F
1928.0236	1927.0163	1927.0163	-0.00	433	451	1	R.GAVLNGGPPSTRITPEFSK.W
2211.0984	2210.0911	2210.0954	-1.93	477	496	1	K.NSDIEKITEDSAVTTFEALK.A
2817.2480	2816.2407	2816.2051	12.6	452	476	2	K.WASDEMPSTSNGESSKQEAMQKTCK.N
2833.2026	2832.1953	2832.2000	-1.66	452	476	2	K.WASDEMPSTSNGESSKQEAMQKTCK.N + Oxidation (M)
3253.6089	3252.6016	3252.6158	-4.36	6	36	1	R.AAFKMENSSYLPNPLASPALMVLASTAEASR.D + Oxidation (M)
3269.6123	3268.6050	3268.6107	-1.75	6	36	1	R.AAFKMENSSYLPNPLASPALMVLASTAEASR.D + 2 Oxidation (M)
4290.9102	4289.9029	4289.8494	12.5	509	541	2	R.GDRYKCLICMDSYSMPLTSIQCWHVHCEECWLR.T
4306.9575	4305.9502	4305.8444	24.6	509	541	2	R.GDRYKCLICMDSYSMPLTSIQCWHVHCEECWLR.T + Oxidation (M)

No match to: 710.3558, 715.3582, 722.4182, 729.4472, 734.4399, 735.4044, 737.4075, 747.4354, 756.3666, 759.4210, 772.4085, 779.4621, 800.3990, 801.4063, 805.4392, 818.4569, 823.4658, 828.4204, 829.4349, 830.4365, 832.2986, 834.3055, 842.4619, 856.3810, 865.4302, 870.4144, 873.3766, 880.4450, 883.4655, 889.4736, 891.4725, 898.4407, 900.5031, 912.4936, 923.5015, 925.4745, 929.4329, 938.4833, 957.5629, 961.4780, 967.4121, 970.4429, 972.4810, 974.4342, 978.5226, 983.6148, 988.4271, 993.4903, 995.3998, 998.5886, 1006.4926, 1007.5641, 1021.5605, 1024.5408, 1032.5048, 1034.5706, 1036.5566, 1043.5085, 1044.5150, 1056.5394, 1057.6062, 1074.6005, 1087.5505, 1113.5382, 1122.5513, 1123.5905, 1130.5541, 1143.4395, 1145.6267, 1178.6249, 1180.6364, 1184.6396, 1194.5638, 1198.6896, 1216.7015, 1227.6558, 1243.6030, 1248.6648, 1254.6876, 1255.5952, 1264.7084, 1269.6405, 1273.6479, 1278.6892, 1281.6271, 1289.6858, 1301.6105, 1304.6399, 1312.7454, 1325.6327, 1326.6665, 1328.6610, 1336.7188, 1337.6541, 1338.6735, 1349.6659, 1351.6846, 1356.6124, 1361.6910, 1372.5992, 1382.5925, 1383.7246, 1390.6946, 1420.7117, 1423.6814, 1445.7860, 1456.7703, 1457.7407, 1467.7371, 1470.7511, 1488.7211, 1494.7380, 1497.8768, 1500.7181, 1503.7421, 1513.7554, 1519.7505, 1528.6958, 1530.6902, 1540.7820, 1544.8356, 1556.8394, 1562.7800, 1563.7925, 1572.7056, 1578.7816, 1580.8422, 1599.9109, 1616.7953, 1621.7692, 1628.7004, 1628.8589, 1635.7825, 1642.7168, 1647.8525, 1650.8430, 1657.9056, 1666.7905, 1696.8221, 1706.8651, 1707.9131, 1717.9056, 1722.9384, 1730.8652, 1738.7958, 1741.9946,

1755.8914, 1758.9082, 1771.0306, 1774.9175, 1787.8247, 1809.0355, 1862.9480, 1883.7240, 1932.0079, 1938.0043, 1940.0157, 1953.8590, 1959.9622, 1961.0220, 1969.9949, 1981.0421, 1990.9496, 1997.8253, 2007.9401, 2011.8949, 2015.9641, 2023.9448, 2027.1273, 2032.0186, 2044.0441, 2045.9841, 2060.9731, 2067.1531, 2086.0945, 2088.1196, 2108.9956, 2112.9929, 2114.9592, 2139.0918, 2144.0564, 2160.0481, 2174.1157, 2179.0405, 2199.1233, 2229.1216, 2239.1421, 2242.1345, 2245.8398, 2251.1340, 2255.1416, 2265.0903, 2270.1790, 2273.0667, 2284.1729, 2287.1509, 2293.1445, 2328.9482, 2343.1899, 2352.9153, 2354.9983, 2356.0093, 2364.8684, 2414.2334, 2423.2490, 2450.2375, 2466.2253, 2468.2173, 2475.2114, 2486.1069, 2519.9941, 2541.2319, 2542.2505, 2558.3389, 2575.1797, 2646.9695, 2660.9880, 2675.0371, 2695.0181, 2702.2866, 2705.2827, 2713.3413, 2717.3323, 2718.2998, 2721.2900, 2744.2466, 2763.3267, 2770.3513, 2785.3567, 2787.2734, 2788.2341, 2792.2034, 2798.2407, 2801.2722, 2803.2302, 2808.2080, 2820.2319, 2843.3906, 2846.3865, 2847.3792, 2870.3926, 2873.3279, 2889.3057, 2892.4070, 2896.4392, 2912.4277, 2915.4314, 2919.4495, 2961.2297, 3071.3845, 3076.4626, 3081.6108, 3087.3884, 3101.4309, 3118.6643, 3143.5120, 3159.5193, 3173.5476, 3184.4751, 3236.5454, 3248.6077, 3265.5996, 3285.5964, 3331.5325, 3334.6331, 3335.6592, 3347.4841, 3397.6499, 3411.6399, 3412.5662, 3427.6182, 3428.5718, 3435.6516, 3444.5942, 3457.6045, 3463.5820, 3473.5864, 3487.6257, 3491.6506, 3503.7678, 3509.5947, 3521.6812, 3552.5681, 3574.6165, 3590.6309, 3624.6504, 3636.7607, 3640.6731, 3646.7363, 3651.7256, 3665.7231, 3669.7129, 3692.6494, 3696.7678, 3707.6807, 3714.6301, 3719.6519, 3722.5593, 3723.6516, 3735.6885, 3751.6423, 3830.7644, 3937.7585, 3941.8464, 3942.8328, 3955.8433, 4092.8938, 4147.8613, 4162.9160, 4254.9053, 4272.9224, 4309.8506, 4323.8926, 4397.9277, 4415.8896, 4449.1777, 4455.1221, 4459.2368, 4494.0420, 4501.0767, 4510.0269, 4525.1040, 4530.0112, 4839.1138, 4846.0161

36. Q3T0J9 **Mass:** 65538 **Score:** 18 **Expect:** 3.9e+002 **Matches:** 13

Guanine nucleotide-binding protein-like 3-like protein OS=Bos taurus GN=GNL3L PE=2 SV=1

Observed	Mr(expt)	Mr(calc)	ppm	Start	End	Miss	Peptide
710.3558	709.3486	709.3615	-18.22	1 -	5	1	-.MMKLR.H + 2 Oxidation (M)
830.4365	829.4292	829.4406	-13.67	62 -	68	1	R.EKQQAAR.E
891.4725	890.4653	890.4756	-11.62	55 -	61	2	K.KRVGEMR.E + Oxidation (M)
898.4407	897.4334	897.4491	-17.46	314 -	320	0	R.NCIHVQK.L
998.5886	997.5813	997.6032	-21.99	20 -	28	2	K.KPAKQNGKK.A
1046.5917	1045.5844	1045.5702	13.6	10 -	19	2	K.KPGKGSKGCK.K
1145.6267	1144.6194	1144.5911	24.8	270 -	281	0	R.ACSVGAVPGVTK.F
1544.8356	1543.8283	1543.8253	1.96	267 -	281	2	K.RSRACSVGAVPGVTK.F
2475.2114	2474.2041	2474.1696	14.0	476 -	496	2	K.IGDLTGYCTNPNRHQMGWAKR.N
2542.2505	2541.2432	2541.2720	-11.31	268 -	290	2	R.SRACSVGAVPGVTKFMQEVYLDK.F
2801.2722	2800.2649	2800.2379	9.67	548 -	575	2	R.ADKLASKLSDSMMSALDLSGNADDSAGD.- + Oxidation (M)
2817.2480	2816.2407	2816.2328	2.82	548 -	575	2	R.ADKLASKLSDSMMSALDLSGNADDSAGD.- + 2 Oxidation (M)
4839.1138	4838.1065	4838.0160	18.7	413 -	454	0	K.EMTEVFDIEDTEQANEDTMECLATGESDELLGDMDPLEMEIK.W + Oxidation (M)

No match to: 715.3582, 722.4182, 729.4472, 734.4399, 735.4044, 737.4075, 747.4354, 756.3666, 759.4210, 772.4085, 779.4621, 800.3990, 801.4063, 805.4392, 818.4569, 823.4658, 828.4204, 829.4349, 832.2986, 834.3055, 842.4619, 856.3810, 865.4302, 870.4144, 873.3766, 880.4450, 883.4655, 887.4088, 889.4736, 900.5031, 912.4936, 923.5015, 925.4745, 929.4329, 938.4833, 957.5629, 961.4780, 967.4121, 970.4429, 972.4810, 974.4342, 978.5226, 983.6148, 988.4271, 993.4903, 995.3998, 1006.4926, 1007.5641, 1021.5605, 1024.5408, 1032.5048, 1034.5706, 1036.5566, 1043.5085, 1044.5150, 1056.5394, 1057.6062, 1074.6005, 1087.5505, 1113.5382, 1122.5513, 1123.5905, 1130.5541, 1143.4395, 1177.6389, 1178.6249, 1180.6364, 1184.6396, 1194.5638, 1198.6896, 1216.7015, 1227.6558, 1243.6030, 1248.6648, 1254.6876, 1255.5952, 1264.7084, 1269.6405, 1273.6479, 1278.6892, 1281.6271, 1289.6858, 1301.6105, 1304.6399, 1312.7454, 1325.6327, 1326.6665, 1328.6610, 1336.7188, 1337.6541, 1338.6735, 1349.6659, 1351.6846, 1356.6124, 1361.6910, 1365.6167, 1372.5992, 1382.5925, 1383.7246, 1390.6946, 1420.7117, 1423.6814, 1445.7860, 1456.7703, 1457.7407, 1467.7371, 1470.7511, 1488.7211, 1494.7380, 1497.8768, 1500.7181, 1503.7421, 1513.7554, 1519.7505, 1528.6958, 1530.6902, 1540.7820, 1556.8394, 1562.7800, 1563.7925, 1572.7056, 1578.7816, 1580.8422, 1599.9109, 1616.7953, 1621.7692, 1628.7004, 1628.8589, 1635.7825, 1642.7168, 1647.8525, 1650.8430, 1657.9056, 1666.7905, 1696.8221, 1706.8651, 1707.9131, 1717.9056, 1722.9384, 1730.8652, 1738.7958, 1741.9946, 1755.8914, 1758.9082, 1771.0306, 1774.9175, 1787.8247, 1809.0355, 1862.9480, 1883.7240, 1928.0236, 1932.0079, 1938.0043, 1940.0157, 1953.8590, 1959.9622, 1961.0220, 1969.9949, 1981.0421, 1990.9496, 1997.8253, 2007.9401, 2011.8949, 2015.9641, 2023.9448, 2027.1273, 2032.0186, 2044.0441, 2045.9841, 2060.9731, 2067.1531, 2086.0945, 2088.1196, 2108.9956, 2112.9929, 2114.9592, 2139.0918, 2144.0564, 2160.0481, 2174.1157, 2179.0405, 2199.1233, 2211.0984, 2229.1216, 2239.1421, 2242.1345, 2245.8398, 2251.1340, 2255.1416, 2265.0903, 2270.1790, 2273.0667, 2284.1729, 2287.1509, 2293.1445, 2328.9482, 2343.1899, 2352.9153, 2354.9983, 2356.0093, 2364.8684, 2414.2334, 2423.2490, 2450.2375, 2466.2253, 2468.2173, 2486.1069, 2519.9941, 2541.2319, 2558.3389, 2575.1797, 2646.9695, 2660.9880, 2675.0371, 2695.0181, 2702.2866, 2705.2827, 2713.3413, 2717.3323, 2718.2998, 2721.2900, 2744.2466, 2763.3267, 2770.3513, 2785.3567, 2787.2734, 2788.2341, 2792.2034, 2798.2407, 2803.2302, 2808.2080, 2820.2319, 2833.2026, 2843.3906, 2846.3865, 2847.3792, 2870.3926, 2873.3279, 2889.3057, 2892.4070, 2896.4392, 2912.4277, 2915.4314, 2919.4495, 2961.2297, 3071.3845, 3076.4626, 3081.6108, 3087.3884, 3101.4309, 3118.6643, 3143.5120, 3159.5193, 3173.5476, 3184.4751, 3236.5454, 3248.6077, 3253.6089, 3265.5996, 3269.6123, 3285.5964, 3331.5325, 3334.6331, 3335.6592, 3347.4841, 3397.6499, 3411.6399, 3412.5662, 3427.6182, 3428.5718, 3435.6516, 3444.5942, 3457.6045, 3463.5820, 3473.5864, 3487.6257, 3491.6506, 3503.7678, 3509.5947, 3521.6812, 3552.5681, 3574.6165, 3590.6309, 3624.6504, 3636.7607, 3640.6731, 3646.7363, 3651.7256, 3665.7231, 3669.7129, 3692.6494, 3696.7678, 3707.6807, 3714.6301, 3719.6519, 3722.5593, 3723.6516, 3735.6885, 3751.6423, 3830.7644, 3937.7585, 3941.8464, 3942.8328, 3955.8433, 4092.8938, 4147.8613, 4162.9160, 4254.9053, 4272.9224, 4290.9102, 4306.9575, 4309.8506, 4323.8926, 4397.9277, 4415.8896, 4449.1777, 4455.1221, 4459.2368, 4494.0420, 4501.0767, 4510.0269, 4525.1040, 4530.0112, 4846.0161

37. G8JKZ5 **Mass:** 31228 **Score:** 18 **Expect:** 4.1e+002 **Matches:** 9

Guanine nucleotide-binding protein G(o) subunit alpha OS=Bos taurus GN=GNAO1 PE=4 SV=1

Observed	Mr(expt)	Mr(calc)	ppm	Start	End	Miss	Peptide
891.4725	890.4653	890.4610	4.74	112 -	119	0	R.LFDVGGQR.S
1044.5150	1043.5077	1043.4924	14.7	68 -	75	0	K.YYLDSLDR.I
1494.7380	1493.7307	1493.7330	-1.55	14 -	26	2	R.KADAKMVCDVVSR.M + Oxidation (M)
2174.1157	2173.1084	2173.1491	-18.72	76 -	94	2	R.IGAADYQPTEQDILRTRVK.T
2873.3279	2872.3206	2872.3198	0.28	44 -	67	2	R.LWGDSGIQECFNRSREYQLNDSAK.Y
3331.5325	3330.5252	3330.5063	5.70	15 -	43	2	K.ADAKMVCDVVSRMEDTEPFSPELLSAMMR.L + Oxidation (M)
3347.4841	3346.4768	3346.5012	-7.28	15 -	43	2	K.ADAKMVCDVVSRMEDTEPFSPELLSAMMR.L + 2 Oxidation (M)
4290.9102	4289.9029	4289.9854	-19.22	231 -	267	1	K.EIYCHMTCATDTNNIQVVFDAVTDIIIANNLRGCGLY.- + Oxidation (M)
4525.1040	4524.0967	4523.9987	21.7	19 -	56	2	K.MVCDVVSRMEDTEPFSPELLSAMMRLWGDSGIQECFNR.S + 2 Oxidation (M)

No match to: 710.3558, 715.3582, 722.4182, 729.4472, 734.4399, 735.4044, 737.4075, 747.4354, 756.3666, 759.4210, 772.4085, 779.4621, 800.3990, 801.4063, 805.4392, 818.4569, 823.4658, 828.4204, 829.4349, 830.4365, 832.2986, 834.3055, 842.4619, 856.3810, 865.4302, 870.4144, 873.3766, 880.4450, 883.4655, 887.4088, 889.4736, 898.4407, 900.5031, 912.4936, 923.5015, 925.4745, 929.4329, 938.4833, 957.5629, 961.4780, 967.4121, 970.4429, 972.4810, 974.4342, 978.5226, 983.6148, 988.4271, 993.4903, 995.3998, 998.5886, 1006.4926, 1007.5641, 1021.5605, 1024.5408, 1032.5048, 1034.5706, 1036.5566, 1043.5085, 1046.5917, 1056.5394, 1057.6062, 1074.6005, 1087.5505, 1113.5382, 1122.5513, 1123.5905, 1130.5541, 1143.4395, 1145.6267, 1177.6389, 1178.6249, 1180.6364, 1184.6396, 1194.5638, 1198.6896, 1216.7015, 1227.6558, 1243.6030, 1248.6648, 1254.6876, 1255.5952, 1264.7084, 1269.6405, 1273.6479, 1278.6892, 1281.6271, 1289.6858, 1301.6105, 1304.6399, 1312.7454, 1325.6327, 1326.6665, 1328.6610, 1336.7188, 1337.6541, 1338.6735, 1349.6659, 1351.6846, 1356.6124, 1361.6910, 1365.6167, 1372.5992, 1382.5925, 1383.7246, 1390.6946, 1420.7117, 1423.6814, 1445.7860, 1456.7703, 1457.7407, 1467.7371, 1470.7511, 1488.7211, 1497.8768, 1500.7181, 1503.7421, 1513.7554, 1519.7505, 1528.6958, 1530.6902, 1540.7820, 1544.8356, 1556.8394, 1562.7800, 1563.7925, 1572.7056, 1578.7816, 1580.8422, 1599.9109, 1616.7953, 1621.7692, 1628.7004, 1628.8589, 1635.7825, 1642.7168, 1647.8525, 1650.8430, 1657.9056, 1666.7905, 1696.8221, 1706.8651, 1707.9131, 1717.9056, 1722.9384, 1730.8652, 1738.7958, 1741.9946, 1755.8914, 1758.9082, 1771.0306, 1774.9175, 1787.8247, 1809.0355, 1862.9480, 1883.7240, 1928.0236, 1932.0079, 1938.0043, 1940.0157, 1953.8590, 1959.9622, 1961.0220, 1969.9949, 1981.0421, 1990.9496, 1997.8253, 2007.9401, 2011.8949, 2015.9641, 2023.9448, 2027.1273, 2032.0186, 2044.0441, 2045.9841, 2060.9731, 2067.1531, 2086.0945, 2088.1196, 2108.9956, 2112.9929, 2114.9592, 2139.0918, 2144.0564, 2160.0481, 2179.0405, 2199.1233, 2211.0984, 2229.1216, 2239.1421, 2242.1345, 2245.8398, 2251.1340, 2255.1416, 2265.0903, 2270.1790, 2273.0667, 2284.1729, 2287.1509, 2293.1445, 2328.9482, 2343.1899, 2352.9153, 2354.9983, 2356.0093, 2364.8684, 2414.2334, 2423.2490, 2450.2375, 2466.2253, 2468.2173, 2475.2114, 2486.1069, 2519.9941, 2541.2319, 2542.2505, 2558.3389, 2575.1797, 2646.9695, 2660.9880, 2675.0371, 2695.0181, 2702.2866, 2705.2827, 2713.3413, 2717.3323, 2718.2998, 2721.2900, 2744.2466, 2763.3267, 2770.3513, 2785.3567, 2787.2734, 2788.2341, 2792.2034, 2798.2407, 2801.2722, 2803.2302, 2808.2080, 2817.2480, 2820.2319, 2833.2026, 2843.3906, 2846.3865, 2847.3792, 2870.3926, 2889.3057, 2892.4070, 2896.4392, 2912.4277, 2915.4314, 2919.4495, 2961.2297, 3071.3845, 3076.4626, 3081.6108, 3087.3884, 3101.4309, 3118.6643, 3143.5120, 3159.5193, 3173.5476, 3184.4751, 3236.5454, 3248.6077, 3253.6089, 3265.5996, 3269.6123, 3285.5964, 3334.6331, 3335.6592, 3397.6499, 3411.6399, 3412.5662, 3427.6182, 3428.5718, 3435.6516, 3444.5942, 3457.6045,

3463.5820, 3473.5864, 3487.6257, 3491.6506, 3503.7678, 3509.5947, 3521.6812, 3552.5681, 3574.6165, 3590.6309, 3624.6504, 3636.7607, 3640.6731, 3646.7363, 3651.7256, 3665.7231, 3669.7129, 3692.6494, 3696.7678, 3707.6807, 3714.6301, 3719.6519, 3722.5593, 3723.6516, 3735.6885, 3751.6423, 3830.7644, 3937.7585, 3941.8464, 3942.8328, 3955.8433, 4092.8938, 4147.8613, 4162.9160, 4254.9053, 4272.9224, 4306.9575, 4309.8506, 4323.8926, 4397.9277, 4415.8896, 4449.1777, 4455.1221, 4459.2368, 4494.0420, 4501.0767, 4510.0269, 4530.0112, 4839.1138, 4846.0161

38. G3N315 **Mass:** 15541 **Score:** 18 **Expect:** 4.3e+002 **Matches:** 5

Uncharacterized protein OS=Bos taurus PE=4 SV=1

Observed	Mr(expt)	Mr(calc)	ppm	Start	End	Miss	Peptide
830.4365	829.4292	829.4294	-0.17	24	32	0	K.VGSTPNGAK.D
978.5226	977.5154	977.5038	11.8	33	40	1	K.DCLKAIMK.R
1248.6648	1247.6575	1247.6874	-23.93	76	86	1	R.DFATEVRAVIK.N
1528.6958	1527.6885	1527.6915	-1.97	99	110	0	K.SLMVEWSEEFQK.D + Oxidation (M)
3159.5193	3158.5120	3158.5668	-17.33	99	125	2	K.SLMVEWSEEFQKDPQFSLISATIKSMK.E

No match to: 710.3558, 715.3582, 722.4182, 729.4472, 734.4399, 735.4044, 737.4075, 747.4354, 756.3666, 759.4210, 772.4085, 779.4621, 800.3990, 801.4063, 805.4392, 818.4569, 823.4658, 828.4204, 829.4349, 832.2986, 834.3055, 842.4619, 856.3810, 865.4302, 870.4144, 873.3766, 880.4450, 883.4655, 887.4088, 889.4736, 891.4725, 898.4407, 900.5031, 912.4936, 923.5015, 925.4745, 929.4329, 938.4833, 957.5629, 961.4780, 967.4121, 970.4429, 972.4810, 974.4342, 983.6148, 988.4271, 993.4903, 995.3998, 998.5886, 1006.4926, 1007.5641, 1021.5605, 1024.5408, 1032.5048, 1034.5706, 1036.5566, 1043.5085, 1044.5150, 1046.5917, 1056.5394, 1057.6062, 1074.6005, 1087.5505, 1113.5382, 1122.5513, 1123.5905, 1130.5541, 1143.4395, 1145.6267, 1177.6389, 1178.6249, 1180.6364, 1184.6396, 1194.5638, 1198.6896, 1216.7015, 1227.6558, 1243.6030, 1254.6876, 1255.5952, 1264.7084, 1269.6405, 1273.6479, 1278.6892, 1281.6271, 1289.6858, 1301.6105, 1304.6399, 1312.7454, 1325.6327, 1326.6665, 1328.6610, 1336.7188, 1337.6541, 1338.6735, 1349.6659, 1351.6846, 1356.6124, 1361.6910, 1365.6167, 1372.5992, 1382.5925, 1383.7246, 1390.6946, 1420.7117, 1423.6814, 1445.7860, 1456.7703, 1457.7407, 1467.7371, 1470.7511, 1488.7211, 1494.7380, 1497.8768, 1500.7181, 1503.7421, 1513.7554, 1519.7505, 1530.6902, 1540.7820, 1544.8356, 1556.8394, 1562.7800, 1563.7925, 1572.7056, 1578.7816, 1580.8422, 1599.9109, 1616.7953, 1621.7692, 1628.7004, 1628.8589, 1635.7825, 1642.7168, 1647.8525, 1650.8430, 1657.9056, 1666.7905, 1696.8221, 1706.8651, 1707.9131, 1717.9056, 1722.9384, 1730.8652, 1738.7958, 1741.9946, 1755.8914, 1758.9082, 1771.0306, 1774.9175, 1787.8247, 1809.0355, 1862.9480, 1883.7240, 1928.0236, 1932.0079, 1938.0043, 1940.0157, 1953.8590, 1959.9622, 1961.0220, 1969.9949, 1981.0421, 1990.9496, 1997.8253, 2007.9401, 2011.8949, 2015.9641, 2023.9448, 2027.1273, 2032.0186, 2044.0441, 2045.9841, 2060.9731, 2067.1531, 2086.0945, 2088.1196, 2108.9956, 2112.9929, 2114.9592, 2139.0918, 2144.0564, 2160.0481, 2174.1157, 2179.0405, 2199.1233, 2211.0984, 2229.1216, 2239.1421, 2242.1345, 2245.8398, 2251.1340, 2255.1416, 2265.0903, 2270.1790, 2273.0667, 2284.1729, 2287.1509, 2293.1445, 2328.9482, 2343.1899, 2352.9153, 2354.9983, 2356.0093, 2364.8684, 2414.2334, 2423.2490, 2450.2375, 2466.2253, 2468.2173, 2475.2114, 2486.1069, 2519.9941, 2541.2319, 2542.2505, 2558.3389, 2575.1797, 2646.9695, 2660.9880, 2675.0371, 2695.0181, 2702.2866, 2705.2827, 2713.3413, 2717.3323, 2718.2998, 2721.2900, 2744.2466, 2763.3267, 2770.3513, 2785.3567, 2787.2734, 2788.2341, 2792.2034, 2798.2407, 2801.2722, 2803.2302, 2808.2080, 2817.2480, 2820.2319, 2833.2026, 2843.3906, 2846.3865, 2847.3792, 2870.3926, 2873.3279, 2889.3057, 2892.4070, 2896.4392, 2912.4277, 2915.4314, 2919.4495, 2961.2297, 3071.3845, 3076.4626, 3081.6108, 3087.3884, 3101.4309, 3118.6643, 3143.5120, 3173.5476, 3184.4751, 3236.5454, 3248.6077, 3253.6089, 3265.5996, 3269.6123, 3285.5964, 3331.5325, 3334.6331, 3335.6592, 3347.4841, 3397.6499, 3411.6399, 3412.5662, 3427.6182, 3428.5718, 3435.6516, 3444.5942, 3457.6045, 3463.5820, 3473.5864, 3487.6257, 3491.6506, 3503.7678, 3509.5947, 3521.6812, 3552.5681, 3574.6165, 3590.6309, 3624.6504, 3636.7607, 3640.6731, 3646.7363, 3651.7256, 3665.7231, 3669.7129, 3692.6494, 3696.7678, 3707.6807, 3714.6301, 3719.6519, 3722.5593, 3723.6516, 3735.6885, 3751.6423, 3830.7644, 3937.7585, 3941.8464, 3942.8328, 3955.8433, 4092.8938, 4147.8613, 4162.9160, 4254.9053, 4272.9224, 4290.9102, 4306.9575, 4309.8506, 4323.8926, 4397.9277, 4415.8896, 4449.1777, 4455.1221, 4459.2368, 4494.0420, 4501.0767, 4510.0269, 4525.1040, 4530.0112, 4839.1138, 4846.0161

39. F1MPK4 **Mass:** 44043 **Score:** 17 **Expect:** 4.4e+002 **Matches:** 9

3-hydroxyisobutyryl-CoA hydrolase, mitochondrial OS=Bos taurus GN=HIBCH PE=3 SV=2

Observed	Mr(expt)	Mr(calc)	ppm	Start	End	Miss	Peptide
830.4365	829.4292	829.4446	-18.53	69	75	0	K.AYALHQK.W
938.4833	937.4761	937.4691	7.40	114	120	0	K.MLQDLFR.E + Oxidation (M)
1628.8589	1627.8516	1627.8565	-2.97	56	68	1	K.MKLESILLDMLYK.A + 2 Oxidation (M)
1717.9056	1716.8983	1716.8682	17.5	239	253	1	K.ENIADVLETYHAKSK.T
2060.9731	2059.9658	2059.9884	-10.98	252	268	1	K.SKTDQDKPFILEEHMDK.I
2139.0918	2138.0845	2138.1075	-10.75	303	321	2	K.MSPTSLKITLRQLMEGSSK.T + 2 Oxidation (M)
2179.0405	2178.0332	2178.0600	-12.28	94	113	2	K.AFCAGGDIRALSEARNTNQK.M
2541.2319	2540.2246	2540.2774	-20.77	164	187	0	K.SVFAMPETAIGLFPDVGGGYFLPR.L
3248.6077	3247.6004	3247.5576	13.2	322	348	2	K.TLPEVLIMEYRLSQACMKGHDFHEGVR.A + 2 Oxidation (M)

No match to: 710.3558, 715.3582, 722.4182, 729.4472, 734.4399, 735.4044, 737.4075, 747.4354, 756.3666, 759.4210, 772.4085, 779.4621, 800.3990, 801.4063, 805.4392, 818.4569, 823.4658, 828.4204, 829.4349, 832.2986, 834.3055, 842.4619, 856.3810, 865.4302, 870.4144, 873.3766, 880.4450, 883.4655, 887.4088, 889.4736, 891.4725, 898.4407, 900.5031, 912.4936, 923.5015, 925.4745, 929.4329, 957.5629, 961.4780, 967.4121, 970.4429, 972.4810, 974.4342, 978.5226, 983.6148, 988.4271, 993.4903, 995.3998, 998.5886, 1006.4926, 1007.5641, 1021.5605, 1024.5408, 1032.5048, 1034.5706, 1036.5566, 1043.5085, 1044.5150, 1046.5917, 1056.5394, 1057.6062, 1074.6005, 1087.5505, 1113.5382, 1122.5513, 1123.5905, 1130.5541, 1143.4395, 1145.6267, 1177.6389, 1178.6249, 1180.6364, 1184.6396, 1194.5638, 1198.6896, 1216.7015, 1227.6558, 1243.6030, 1248.6648, 1254.6876, 1255.5952, 1264.7084, 1269.6405, 1273.6479, 1278.6892, 1281.6271, 1289.6858, 1301.6105, 1304.6399, 1312.7454, 1325.6327, 1326.6665, 1328.6610, 1336.7188, 1337.6541, 1338.6735, 1349.6659, 1351.6846, 1356.6124, 1361.6910, 1365.6167, 1372.5992, 1382.5925, 1383.7246, 1390.6946, 1420.7117, 1423.6814, 1445.7860, 1456.7703, 1457.7407, 1467.7371, 1470.7511, 1488.7211, 1494.7380, 1497.8768, 1500.7181, 1503.7421, 1513.7554, 1519.7505, 1528.6958, 1530.6902, 1540.7820, 1544.8356, 1556.8394, 1562.7800, 1563.7925, 1572.7056, 1578.7816, 1580.8422, 1599.9109, 1616.7953, 1621.7692, 1628.7004, 1635.7825, 1642.7168, 1647.8525, 1650.8430, 1657.9056, 1666.7905, 1696.8221, 1706.8651, 1707.9131, 1722.9384, 1730.8652, 1738.7958, 1741.9946, 1755.8914, 1758.9082, 1771.0306, 1774.9175, 1787.8247, 1809.0355, 1862.9480, 1883.7240, 1928.0236, 1932.0079, 1938.0043, 1940.0157, 1953.8590, 1959.9622, 1961.0220, 1969.9949, 1981.0421, 1990.9496, 1997.8253, 2007.9401, 2011.8949, 2015.9641, 2023.9448, 2027.1273, 2032.0186, 2044.0441, 2045.9841, 2067.1531, 2086.0945, 2088.1196, 2108.9956, 2112.9929, 2114.9592, 2144.0564, 2160.0481, 2174.1157, 2199.1233, 2211.0984, 2229.1216, 2239.1421, 2242.1345, 2245.8398, 2251.1340, 2255.1416, 2265.0903, 2270.1790, 2273.0667, 2284.1729, 2287.1509, 2293.1445, 2328.9482, 2343.1899, 2352.9153, 2354.9983, 2356.0093, 2364.8684, 2414.2334, 2423.2490, 2450.2375, 2466.2253, 2468.2173, 2475.2114, 2486.1069, 2519.9941, 2542.2505, 2558.3389, 2575.1797, 2646.9695, 2660.9880, 2675.0371, 2695.0181, 2702.2866, 2705.2827, 2713.3413, 2717.3323, 2718.2998, 2721.2900, 2744.2466, 2763.3267, 2770.3513, 2785.3567, 2787.2734, 2788.2341, 2792.2034, 2798.2407, 2801.2722, 2803.2302, 2808.2080, 2817.2480, 2820.2319, 2833.2026, 2843.3906, 2846.3865, 2847.3792, 2870.3926, 2873.3279, 2889.3057, 2892.4070, 2896.4392, 2912.4277, 2915.4314, 2919.4495, 2961.2297, 3071.3845, 3076.4626, 3081.6108, 3087.3884, 3101.4309, 3118.6643, 3143.5120, 3159.5193, 3173.5476, 3184.4751, 3236.5454, 3253.6089, 3265.5996, 3269.6123, 3285.5964, 3331.5325, 3334.6331, 3335.6592, 3347.4841, 3397.6499, 3411.6399, 3412.5662, 3427.6182, 3428.5718, 3435.6516, 3444.5942, 3457.6045, 3463.5820, 3473.5864, 3487.6257, 3491.6506, 3503.7678, 3509.5947, 3521.6812, 3552.5681, 3574.6165, 3590.6309, 3624.6504, 3636.7607, 3640.6731, 3646.7363, 3651.7256, 3665.7231, 3669.7129, 3692.6494, 3696.7678, 3707.6807, 3714.6301, 3719.6519, 3722.5593, 3723.6516, 3735.6885, 3751.6423, 3830.7644, 3937.7585, 3941.8464, 3942.8328, 3955.8433, 4092.8938, 4147.8613, 4162.9160, 4254.9053, 4272.9224, 4290.9102, 4306.9575, 4309.8506, 4323.8926, 4397.9277, 4415.8896, 4449.1777, 4455.1221, 4459.2368, 4494.0420, 4501.0767, 4510.0269, 4525.1040, 4530.0112, 4839.1138, 4846.0161

40. F1MRS8 **Mass:** 14805 **Score:** 17 **Expect:** 4.4e+002 **Matches:** 5

Uncharacterized protein OS=Bos taurus GN=FABP9 PE=3 SV=2

Observed	Mr(expt)	Mr(calc)	ppm	Start	End	Miss	Peptide
747.4354	746.4281	746.4286	-0.66	102	107	1	K.ETTIKR.K
1530.6902	1529.6829	1529.7184	-23.19	11	22	1	K.LVSRENFDEYMK.E
1666.7905	1665.7832	1665.7846	-0.81	67	80	1	K.LGEEFDETTIDNRK.V
1928.0236	1927.0163	1927.0414	-13.04	50	66	2	K.ISIKTQSAFKNSEISFK.L
2199.1233	2198.1160	2198.0785	17.1	114	132	1	K.MLVEYIMNNVVSTGVCEKL.-

No match to: 710.3558, 715.3582, 722.4182, 729.4472, 734.4399, 735.4044, 737.4075, 756.3666, 759.4210, 772.4085, 779.4621, 800.3990, 801.4063, 805.4392, 818.4569, 823.4658, 828.4204, 829.4349, 830.4365, 832.2986, 834.3055, 842.4619, 856.3810, 865.4302, 870.4144, 873.3766, 880.4450, 883.4655, 887.4088, 889.4736, 891.4725, 898.4407, 900.5031, 912.4936, 923.5015, 925.4745, 929.4329,

938.4833, 957.5629, 961.4780, 967.4121, 970.4429, 972.4810, 974.4342, 978.5226, 983.6148, 988.4271, 993.4903, 995.3998, 998.5886, 1006.4926, 1007.5641, 1021.5605, 1024.5408, 1032.5048, 1034.5706, 1036.5566, 1043.5085, 1044.5150, 1046.5917, 1056.5394, 1057.6062, 1074.6005, 1087.5505, 1113.5382, 1122.5513, 1123.5905, 1130.5541, 1143.4395, 1145.6267, 1177.6389, 1178.6249, 1180.6364, 1184.6396, 1194.5638, 1198.6896, 1216.7015, 1227.6558, 1243.6030, 1248.6648, 1254.6876, 1255.5952, 1264.7084, 1269.6405, 1273.6479, 1278.6892, 1281.6271, 1289.6858, 1301.6105, 1304.6399, 1312.7454, 1325.6327, 1326.6665, 1328.6610, 1336.7188, 1337.6541, 1338.6735, 1349.6659, 1351.6846, 1356.6124, 1361.6910, 1365.6167, 1372.5992, 1382.5925, 1383.7246, 1390.6946, 1420.7117, 1423.6814, 1445.7860, 1456.7703, 1457.7407, 1467.7371, 1470.7511, 1488.7211, 1494.7380, 1497.8768, 1500.7181, 1503.7421, 1513.7554, 1519.7505, 1528.6958, 1540.7820, 1544.8356, 1556.8394, 1562.7800, 1563.7925, 1572.7056, 1578.7816, 1580.8422, 1599.9109, 1616.7953, 1628.7004, 1628.8589, 1635.7825, 1642.7168, 1647.8525, 1650.8430, 1657.9056, 1696.8221, 1706.8651, 1707.9131, 1717.9056, 1722.9384, 1730.8652, 1738.7958, 1741.9946, 1755.8914, 1758.9082, 1771.0306, 1774.9175, 1787.8247, 1809.0355, 1862.9480, 1883.7240, 1932.0079, 1938.0043, 1940.0157, 1953.8590, 1959.9622, 1961.0220, 1969.9949, 1981.0421, 1990.9496, 1997.8253, 2007.9401, 2011.8949, 2015.9641, 2023.9448, 2027.1273, 2032.0186, 2044.0441, 2045.9841, 2060.9731, 2067.1531, 2086.0945, 2088.1196, 2108.9956, 2112.9929, 2114.9592, 2139.0918, 2144.0564, 2160.0481, 2174.1157, 2179.0405, 2211.0984, 2229.1216, 2239.1421, 2242.1345, 2245.8398, 2251.1340, 2255.1416, 2265.0903, 2270.1790, 2273.0667, 2284.1729, 2287.1509, 2293.1445, 2328.9482, 2343.1899, 2352.9153, 2354.9983, 2356.0093, 2364.8684, 2414.2334, 2423.2490, 2450.2375, 2466.2253, 2468.2173, 2475.2114, 2486.1069, 2519.9941, 2541.2319, 2542.2505, 2558.3389, 2575.1797, 2646.9695, 2660.9880, 2675.0371, 2695.0181, 2702.2866, 2705.2827, 2713.3413, 2717.3323, 2718.2998, 2721.2900, 2744.2466, 2763.3267, 2770.3513, 2785.3567, 2787.2734, 2788.2341, 2792.2034, 2798.2407, 2801.2722, 2803.2302, 2808.2080, 2817.2480, 2820.2319, 2833.2026, 2843.3906, 2846.3865, 2847.3792, 2870.3926, 2873.3279, 2889.3057, 2892.4070, 2896.4392, 2912.4277, 2915.4314, 2919.4495, 2961.2297, 3071.3845, 3076.4626, 3081.6108, 3087.3884, 3101.4309, 3118.6643, 3143.5120, 3159.5193, 3173.5476, 3184.4751, 3236.5454, 3248.6077, 3253.6089, 3265.5996, 3269.6123, 3285.5964, 3331.5325, 3334.6331, 3335.6592, 3347.4841, 3397.6499, 3411.6399, 3412.5662, 3427.6182, 3428.5718, 3435.6516, 3444.5942, 3457.6045, 3463.5820, 3473.5864, 3487.6257, 3491.6506, 3503.7678, 3509.5947, 3521.6812, 3552.5681, 3574.6165, 3590.6309, 3624.6504, 3636.7607, 3640.6731, 3646.7363, 3651.7256, 3665.7231, 3669.7129, 3692.6494, 3696.7678, 3707.6807, 3714.6301, 3719.6519, 3722.5593, 3723.6516, 3735.6885, 3751.6423, 3830.7644, 3937.7585, 3941.8464, 3942.8328, 3955.8433, 4092.8938, 4147.8613, 4162.9160, 4254.9053, 4272.9224, 4290.9102, 4306.9575, 4309.8506, 4323.8926, 4397.9277, 4415.8896, 4449.1777, 4455.1221, 4459.2368, 4494.0420, 4501.0767, 4510.0269, 4525.1040, 4530.0112, 4839.1138, 4846.0161

41. F1MN77 **Mass:** 21893 **Score:** 17 **Expect:** 4.4e+002 **Matches:** 5

Uncharacterized protein OS=Bos taurus GN=TFDP2 PE=3 SV=1

Observed	Mr(expt)	Mr(calc)	ppm	Start	End	Miss	Peptide
805.4392	804.4319	804.4494	-21.77	187	193	0	R.FQKPGTK.K
1046.5917	1045.5844	1045.5702	13.6	78	85	2	K.VCEKVQRK.G
1530.6902	1529.6829	1529.6998	-11.02	47	59	1	R.AREFIDSDFSESK.R
2139.0918	2138.0845	2138.1340	-23.13	122	139	2	R.RRVYDALNVLMAMNIISK.E + 2 Oxidation (M)
4449.1777	4448.1704	4448.2798	-24.60	2	44	1	M.IISTPQRLTSSGSVLIGSPYTPAPAMVTQTHIAEAAGWVPGDR.K + Oxidation (M)

No match to: 710.3558, 715.3582, 722.4182, 729.4472, 734.4399, 735.4044, 737.4075, 747.4354, 756.3666, 759.4210, 772.4085, 779.4621, 800.3990, 801.4063, 818.4569, 823.4658, 828.4204, 829.4349, 830.4365, 832.2986, 834.3055, 842.4619, 856.3810, 865.4302, 870.4144, 873.3766, 880.4450, 883.4655, 887.4088, 889.4736, 891.4725, 898.4407, 900.5031, 912.4936, 923.5015, 925.4745, 929.4329, 938.4833, 957.5629, 961.4780, 967.4121, 970.4429, 972.4810, 974.4342, 978.5226, 983.6148, 988.4271, 993.4903, 995.3998, 998.5886, 1006.4926, 1007.5641, 1021.5605, 1024.5408, 1032.5048, 1034.5706, 1036.5566, 1043.5085, 1044.5150, 1056.5394, 1057.6062, 1074.6005, 1087.5505, 1113.5382, 1122.5513, 1123.5905, 1130.5541, 1143.4395, 1145.6267, 1177.6389, 1178.6249, 1180.6364, 1184.6396, 1194.5638, 1198.6896, 1216.7015, 1227.6558, 1243.6030, 1248.6648, 1254.6876, 1255.5952, 1264.7084, 1269.6405, 1273.6479, 1278.6892, 1281.6271, 1289.6858, 1301.6105, 1304.6399, 1312.7454, 1325.6327, 1326.6665, 1328.6610, 1336.7188, 1337.6541, 1338.6735, 1349.6659, 1351.6846, 1356.6124, 1361.6910, 1365.6167, 1372.5992, 1382.5925, 1383.7246, 1390.6946, 1420.7117, 1423.6814, 1445.7860, 1456.7703, 1457.7407, 1467.7371, 1470.7511, 1488.7211, 1494.7380, 1497.8768, 1500.7181, 1503.7421, 1513.7554, 1519.7505, 1528.6958, 1540.7820, 1544.8356, 1556.8394, 1562.7800, 1563.7925, 1572.7056, 1578.7816, 1580.8422, 1599.9109, 1616.7953, 1621.7692, 1628.7004, 1628.8589, 1635.7825, 1642.7168, 1647.8525, 1650.8430, 1657.9056, 1666.7905, 1696.8221, 1706.8651, 1707.9131, 1717.9056, 1722.9384, 1730.8652, 1738.7958, 1741.9946, 1755.8914, 1758.9082, 1771.0306, 1774.9175, 1787.8247, 1809.0355, 1862.9480, 1883.7240, 1928.0236, 1932.0079, 1938.0043, 1940.0157, 1953.8590, 1959.9622, 1961.0220, 1969.9949, 1981.0421, 1990.9496, 1997.8253, 2007.9401, 2011.8949, 2015.9641, 2023.9448, 2027.1273, 2032.0186, 2044.0441, 2045.9841, 2060.9731, 2067.1531, 2086.0945, 2088.1196, 2108.9956, 2112.9929, 2114.9592, 2144.0564, 2160.0481, 2174.1157, 2179.0405, 2199.1233, 2211.0984, 2229.1216, 2239.1421, 2242.1345, 2245.8398, 2251.1340, 2255.1416, 2265.0903, 2270.1790, 2273.0667, 2284.1729, 2287.1509, 2293.1445, 2328.9482, 2343.1899, 2352.9153, 2354.9983, 2356.0093, 2364.8684, 2414.2334, 2423.2490, 2450.2375, 2466.2253, 2468.2173, 2475.2114, 2486.1069, 2519.9941, 2541.2319, 2542.2505, 2558.3389, 2575.1797, 2646.9695, 2660.9880, 2675.0371, 2695.0181, 2702.2866, 2705.2827, 2713.3413, 2717.3323, 2718.2998, 2721.2900, 2744.2466, 2763.3267, 2770.3513, 2785.3567, 2787.2734, 2788.2341, 2792.2034, 2798.2407, 2801.2722, 2803.2302, 2808.2080, 2817.2480, 2820.2319, 2833.2026, 2843.3906, 2846.3865, 2847.3792, 2870.3926, 2873.3279, 2889.3057, 2892.4070, 2896.4392, 2912.4277, 2915.4314, 2919.4495, 2961.2297, 3071.3845, 3076.4626, 3081.6108, 3087.3884, 3101.4309, 3118.6643, 3143.5120, 3159.5193, 3173.5476, 3184.4751, 3236.5454, 3248.6077, 3253.6089, 3265.5996, 3269.6123, 3285.5964, 3331.5325, 3334.6331, 3335.6592, 3347.4841, 3397.6499, 3411.6399, 3412.5662, 3427.6182, 3428.5718, 3435.6516, 3444.5942, 3457.6045, 3463.5820, 3473.5864, 3487.6257, 3491.6506, 3503.7678, 3509.5947, 3521.6812, 3552.5681, 3574.6165, 3590.6309, 3624.6504, 3636.7607, 3640.6731, 3646.7363, 3651.7256, 3665.7231, 3669.7129, 3692.6494, 3696.7678, 3707.6807, 3714.6301, 3719.6519, 3722.5593, 3723.6516, 3735.6885, 3751.6423, 3830.7644, 3937.7585, 3941.8464, 3942.8328, 3955.8433, 4092.8938, 4147.8613, 4162.9160, 4254.9053, 4272.9224, 4290.9102, 4306.9575, 4309.8506, 4323.8926, 4397.9277, 4415.8896, 4455.1221, 4459.2368, 4494.0420, 4501.0767, 4510.0269, 4525.1040, 4530.0112, 4839.1138, 4846.0161

42. Q3T169 **Mass:** 26842 **Score:** 17 **Expect:** 4.4e+002 **Matches:** 8

40S ribosomal protein S3 OS=Bos taurus GN=RPS3 PE=2 SV=1

Observed	Mr(expt)	Mr(calc)	ppm	Start	End	Miss	Peptide
898.4407	897.4334	897.4266	7.58	125	132	0	R.FIMESGAK.G + Oxidation (M)
1423.6814	1422.6741	1422.6627	8.05	28	40	0	R.ELAEDGYSGVEVR.V
1470.7511	1469.7438	1469.7613	-11.91	215	227	0	K.DEILPTTPISEQK.G
1544.8356	1543.8283	1543.8253	1.96	133	146	2	K.GCEVVVSGKLRGQR.A
1969.9949	1968.9876	1969.0309	-21.98	11	27	1	K.FVADGIFKAELNEFLTR.E
2468.2173	2467.2100	2467.1842	10.5	152	173	0	K.FVDGLMIHSGDPVNYYVDTAVR.H
2846.3865	2845.3792	2845.3415	13.3	149	173	1	K.SMKFVDGLMIHSGDPVNYYVDTAVR.H + 2 Oxidation (M)
3269.6123	3268.6050	3268.6550	-15.29	77	106	2	R.FGFPEGSVELYAEKVATRGLCAIAQAESLR.Y

No match to: 710.3558, 715.3582, 722.4182, 729.4472, 734.4399, 735.4044, 737.4075, 747.4354, 756.3666, 759.4210, 772.4085, 779.4621, 800.3990, 801.4063, 805.4392, 818.4569, 823.4658, 828.4204, 829.4349, 830.4365, 832.2986, 834.3055, 842.4619, 856.3810, 865.4302, 870.4144, 873.3766, 880.4450, 883.4655, 887.4088, 889.4736, 891.4725, 900.5031, 912.4936, 923.5015, 925.4745, 929.4329, 938.4833, 957.5629, 961.4780, 967.4121, 970.4429, 972.4810, 974.4342, 978.5226, 983.6148, 988.4271, 993.4903, 995.3998, 998.5886, 1006.4926, 1007.5641, 1021.5605, 1024.5408, 1032.5048, 1034.5706, 1036.5566, 1043.5085, 1044.5150, 1046.5917, 1056.5394, 1057.6062, 1074.6005, 1087.5505, 1113.5382, 1122.5513, 1123.5905, 1130.5541, 1143.4395, 1145.6267, 1177.6389, 1178.6249, 1180.6364, 1184.6396, 1194.5638, 1198.6896, 1216.7015, 1227.6558, 1243.6030, 1248.6648, 1254.6876, 1255.5952, 1264.7084, 1269.6405, 1273.6479, 1278.6892, 1281.6271, 1289.6858, 1301.6105, 1304.6399, 1312.7454, 1325.6327, 1326.6665, 1328.6610, 1336.7188, 1337.6541, 1338.6735, 1349.6659, 1351.6846, 1356.6124, 1361.6910, 1365.6167, 1372.5992, 1382.5925, 1383.7246, 1390.6946, 1420.7117, 1445.7860, 1456.7703, 1457.7407, 1467.7371, 1488.7211, 1494.7380, 1497.8768, 1500.7181, 1503.7421, 1513.7554, 1519.7505, 1528.6958, 1530.6902, 1540.7820, 1556.8394, 1562.7800, 1563.7925, 1572.7056, 1578.7816, 1580.8422, 1599.9109, 1616.7953, 1621.7692, 1628.7004, 1628.8589, 1635.7825, 1642.7168, 1647.8525, 1650.8430, 1657.9056, 1666.7905, 1696.8221, 1706.8651, 1707.9131, 1717.9056, 1722.9384, 1730.8652, 1738.7958, 1741.9946, 1755.8914, 1758.9082, 1771.0306, 1774.9175, 1787.8247, 1809.0355, 1862.9480, 1883.7240, 1928.0236, 1932.0079, 1938.0043, 1940.0157, 1953.8590, 1959.9622, 1961.0220, 1981.0421, 1990.9496, 1997.8253, 2007.9401, 2011.8949, 2015.9641, 2023.9448, 2027.1273, 2032.0186, 2044.0441, 2045.9841, 2060.9731, 2067.1531, 2086.0945, 2088.1196, 2108.9956, 2112.9929, 2114.9592, 2139.0918, 2144.0564, 2160.0481, 2174.1157, 2179.0405, 2199.1233, 2211.0984, 2229.1216, 2239.1421, 2242.1345, 2245.8398, 2251.1340, 2255.1416, 2265.0903, 2270.1790, 2273.0667, 2284.1729, 2287.1509, 2293.1445, 2328.9482, 2343.1899, 2352.9153, 2354.9983, 2356.0093, 2364.8684, 2414.2334, 2423.2490, 2450.2375, 2466.2253, 2475.2114, 2486.1069, 2519.9941, 2541.2319, 2542.2505, 2558.3389, 2575.1797, 2646.9695, 2660.9880, 2675.0371, 2695.0181, 2702.2866, 2705.2827, 2713.3413, 2717.3323, 2718.2998, 2721.2900, 2744.2466, 2763.3267, 2770.3513, 2785.3567, 2787.2734, 2788.2341, 2792.2034, 2798.2407, 2801.2722, 2803.2302, 2808.2080, 2817.2480, 2820.2319, 2833.2026, 2843.3906, 2847.3792, 2870.3926, 2873.3279, 2889.3057, 2892.4070, 2896.4392, 2912.4277, 2915.4314, 2919.4495, 2961.2297, 3071.3845, 3076.4626, 3081.6108, 3087.3884, 3101.4309, 3118.6643, 3143.5120, 3159.5193, 3173.5476, 3184.4751, 3236.5454, 3248.6077, 3253.6089, 3265.5996, 3285.5964, 3331.5325,

3334.6331, 3335.6592, 3347.4841, 3397.6499, 3411.6399, 3412.5662, 3427.6182, 3428.5718, 3435.6516, 3444.5942, 3457.6045, 3463.5820, 3473.5864, 3487.6257, 3491.6506, 3503.7678, 3509.5947, 3521.6812, 3552.5681, 3574.6165, 3590.6309, 3624.6504, 3636.7607, 3640.6731, 3646.7363, 3651.7256, 3665.7231, 3669.7129, 3692.6494, 3696.7678, 3707.6807, 3714.6301, 3719.6519, 3722.5593, 3723.6516, 3735.6885, 3751.6423, 3830.7644, 3937.7585, 3941.8464, 3942.8328, 3955.8433, 4092.8938, 4147.8613, 4162.9160, 4254.9053, 4272.9224, 4290.9102, 4306.9575, 4309.8506, 4323.8926, 4397.9277, 4415.8896, 4449.1777, 4455.1221, 4459.2368, 4494.0420, 4501.0767, 4510.0269, 4525.1040, 4530.0112, 4839.1138, 4846.0161

43. E1BGJ9 **Mass:** 68502 **Score:** 17 **Expect:** 4.5e+002 **Matches:** 14

Uncharacterized protein OS=Bos taurus GN=CTIF PE=4 SV=2

Observed	Mr(expt)	Mr(calc)	ppm	Start	End	Miss	Peptide
829.4349	828.4277	828.4341	-7.78	329 -	335	0	K.DSIIPER.G
1180.6364	1179.6291	1179.6248	3.69	420 -	429	1	R.TIYQKAVSDR.S
1328.6610	1327.6537	1327.6732	-14.65	272 -	283	1	R.NAKETVPTESPR.L
1356.6124	1355.6051	1355.5883	12.4	558 -	568	0	K.MLCPSESMLTR.S + 2 Oxidation (M)
1494.7380	1493.7307	1493.7581	-18.36	376 -	387	1	K.MDKLIEILNSMR.N + 2 Oxidation (M)
1628.7004	1627.6931	1627.6743	11.5	1 -	17	0	-.MENSSAASASSEAGSSR.S
1969.9949	1968.9876	1968.9952	-3.85	133 -	149	2	R.HTPKQPLPHIDREGCGK.G
2044.0441	2043.0368	2043.0380	-0.57	538 -	555	0	R.LLEEQLPEMMAELLASAR.D
2255.1416	2254.1343	2254.1239	4.64	494 -	512	1	R.SSTGEPFRVLVCPIYTCLR.E
2287.1509	2286.1436	2286.1599	-7.11	538 -	557	1	R.LLEEQLPEMMAELLASARDK.M
2423.2490	2422.2417	2422.1832	24.2	376 -	396	2	K.MDKLIEILNSMRNNSTDVDAK.L + Oxidation (M)
2466.2253	2465.2180	2465.2157	0.95	430 -	451	2	R.SFAFTAAKLCDKMALFMVEGTK.F
3640.6731	3639.6658	3639.7326	-18.34	538 -	568	2	R.LLEEQLPEMMAELLASARDKMLCPSESMLTR.S + 3 Oxidation (M)
4092.8938	4091.8865	4091.9510	-15.75	468 -	501	2	R.EELQQQDVERWLGFITFLCEVFGTMRSSTGEPFR.V

No match to: 710.3558, 715.3582, 722.4182, 729.4472, 734.4399, 735.4044, 737.4075, 747.4354, 756.3666, 759.4210, 772.4085, 779.4621, 800.3990, 801.4063, 805.4392, 818.4569, 823.4658, 828.4204, 830.4365, 832.2986, 834.3055, 842.4619, 856.3810, 865.4302, 870.4144, 873.3766, 880.4450, 883.4655, 887.4088, 889.4736, 891.4725, 898.4407, 900.5031, 912.4936, 923.5015, 925.4745, 929.4329, 938.4833, 957.5629, 961.4780, 967.4121, 970.4429, 972.4810, 974.4342, 978.5226, 983.6148, 988.4271, 993.4903, 995.3998, 998.5886, 1006.4926, 1007.5641, 1021.5605, 1024.5408, 1032.5048, 1034.5706, 1036.5566, 1043.5085, 1044.5150, 1046.5917, 1056.5394, 1057.6062, 1074.6005, 1087.5505, 1113.5382, 1122.5513, 1123.5905, 1130.5541, 1143.4395, 1145.6267, 1177.6389, 1178.6249, 1184.6396, 1194.5638, 1198.6896, 1216.7015, 1227.6558, 1243.6030, 1248.6648, 1254.6876, 1255.5952, 1264.7084, 1269.6405, 1273.6479, 1278.6892, 1281.6271, 1289.6858, 1301.6105, 1304.6399, 1312.7454, 1325.6327, 1326.6665, 1336.7188, 1337.6541, 1338.6735, 1349.6659, 1351.6846, 1361.6910, 1365.6167, 1372.5992, 1382.5925, 1383.7246, 1390.6946, 1420.7117, 1423.6814, 1445.7860, 1456.7703, 1457.7407, 1467.7371, 1470.7511, 1488.7211, 1497.8768, 1500.7181, 1503.7421, 1513.7554, 1519.7505, 1528.6958, 1530.6902, 1540.7820, 1544.8356, 1556.8394, 1562.7800, 1563.7925, 1572.7056, 1578.7816, 1580.8422, 1599.9109, 1616.7953, 1621.7692, 1628.8589, 1635.7825, 1642.7168, 1647.8525, 1650.8430, 1657.9056, 1666.7905, 1696.8221, 1706.8651, 1707.9131, 1717.9056, 1722.9384, 1730.8652, 1738.7958, 1741.9946, 1755.8914, 1758.9082, 1771.0306, 1774.9175, 1787.8247, 1809.0355, 1862.9480, 1883.7240, 1928.0236, 1932.0079, 1938.0043, 1940.0157, 1953.8590, 1959.9622, 1961.0220, 1981.0421, 1990.9496, 1997.8253, 2007.9401, 2011.8949, 2015.9641, 2023.9448, 2027.1273, 2032.0186, 2045.9841, 2060.9731, 2067.1531, 2086.0945, 2088.1196, 2108.9956, 2112.9929, 2114.9592, 2139.0918, 2144.0564, 2160.0481, 2174.1157, 2179.0405, 2199.1233, 2211.0984, 2229.1216, 2239.1421, 2242.1345, 2245.8398, 2251.1340, 2265.0903, 2270.1790, 2273.0667, 2284.1729, 2293.1445, 2328.9482, 2343.1899, 2352.9153, 2354.9983, 2356.0093, 2364.8684, 2414.2334, 2450.2375, 2468.2173, 2475.2114, 2486.1069, 2519.9941, 2541.2319, 2542.2505, 2558.3389, 2575.1797, 2646.9695, 2660.9880, 2675.0371, 2695.0181, 2702.2866, 2705.2827, 2713.3413, 2717.3323, 2718.2998, 2721.2900, 2744.2466, 2763.3267, 2770.3513, 2785.3567, 2787.2734, 2788.2341, 2792.2034, 2798.2407, 2801.2722, 2803.2302, 2808.2080, 2817.2480, 2820.2319, 2833.2026, 2843.3906, 2846.3865, 2847.3792, 2870.3926, 2873.3279, 2889.3057, 2892.4070, 2896.4392, 2912.4277, 2915.4314, 2919.4495, 2961.2297, 3071.3845, 3076.4626, 3081.6108, 3087.3884, 3101.4309, 3118.6643, 3143.5120, 3159.5193, 3173.5476, 3184.4751, 3236.5454, 3248.6077, 3253.6089, 3265.5996, 3269.6123, 3285.5964, 3331.5325, 3334.6331, 3335.6592, 3347.4841, 3397.6499, 3411.6399, 3412.5662, 3427.6182, 3428.5718, 3435.6516, 3444.5942, 3457.6045, 3463.5820, 3473.5864, 3487.6257, 3491.6506, 3503.7678, 3509.5947, 3521.6812, 3552.5681, 3574.6165, 3590.6309, 3624.6504, 3636.7607, 3646.7363, 3651.7256, 3665.7231, 3669.7129, 3692.6494, 3696.7678, 3707.6807, 3714.6301, 3719.6519, 3722.5593, 3723.6516, 3735.6885, 3751.6423, 3830.7644, 3937.7585, 3941.8464, 3942.8328, 3955.8433, 4147.8613, 4162.9160, 4254.9053, 4272.9224, 4290.9102, 4306.9575, 4309.8506, 4323.8926, 4397.9277, 4415.8896, 4449.1777, 4455.1221, 4459.2368, 4494.0420, 4501.0767, 4510.0269, 4525.1040, 4530.0112, 4839.1138, 4846.0161

44. Q32PB8 **Mass:** 9220 **Score:** 17 **Expect:** 4.6e+002 **Matches:** 5

40S ribosomal protein S21 OS=Bos taurus GN=RPS21 PE=3 SV=1

Observed	Mr(expt)	Mr(calc)	ppm	Start	End	Miss	Peptide
1024.5408	1023.5335	1023.5171	16.0	52 -	60	0	K.TYAICGAIR.R
1180.6364	1179.6291	1179.6182	9.23	52 -	61	1	K.TYAICGAIRR.M
1572.7056	1571.6983	1571.7250	-16.95	28 -	41	0	K.DHASIQMNVAEVDK.V + Oxidation (M)
1969.9949	1968.9876	1968.9687	9.60	28 -	45	1	K.DHASIQMNVAEVDKVTGR.F
2284.1729	2283.1656	2283.1100	24.4	52 -	71	2	K.TYAICGAIRRMGESDDSILR.L

No match to: 710.3558, 715.3582, 722.4182, 729.4472, 734.4399, 735.4044, 737.4075, 747.4354, 756.3666, 759.4210, 772.4085, 779.4621, 800.3990, 801.4063, 805.4392, 818.4569, 823.4658, 828.4204, 829.4349, 830.4365, 832.2986, 834.3055, 842.4619, 856.3810, 865.4302, 870.4144, 873.3766, 880.4450, 883.4655, 887.4088, 889.4736, 891.4725, 898.4407, 900.5031, 912.4936, 923.5015, 925.4745, 929.4329, 938.4833, 957.5629, 961.4780, 967.4121, 970.4429, 972.4810, 974.4342, 978.5226, 983.6148, 988.4271, 993.4903, 995.3998, 998.5886, 1006.4926, 1007.5641, 1021.5605, 1032.5048, 1034.5706, 1036.5566, 1043.5085, 1044.5150, 1046.5917, 1056.5394, 1057.6062, 1074.6005, 1087.5505, 1113.5382, 1122.5513, 1123.5905, 1130.5541, 1143.4395, 1145.6267, 1177.6389, 1178.6249, 1184.6396, 1194.5638, 1198.6896, 1216.7015, 1227.6558, 1243.6030, 1248.6648, 1254.6876, 1255.5952, 1264.7084, 1269.6405, 1273.6479, 1278.6892, 1281.6271, 1289.6858, 1301.6105, 1304.6399, 1312.7454, 1325.6327, 1326.6665, 1328.6610, 1336.7188, 1337.6541, 1338.6735, 1349.6659, 1351.6846, 1356.6124, 1361.6910, 1365.6167, 1372.5992, 1382.5925, 1383.7246, 1390.6946, 1420.7117, 1423.6814, 1445.7860, 1456.7703, 1457.7407, 1467.7371, 1470.7511, 1488.7211, 1494.7380, 1497.8768, 1500.7181, 1503.7421, 1513.7554, 1519.7505, 1528.6958, 1530.6902, 1540.7820, 1544.8356, 1556.8394, 1562.7800, 1563.7925, 1578.7816, 1580.8422, 1599.9109, 1616.7953, 1621.7692, 1628.7004, 1628.8589, 1635.7825, 1642.7168, 1647.8525, 1650.8430, 1657.9056, 1666.7905, 1696.8221, 1706.8651, 1707.9131, 1717.9056, 1722.9384, 1730.8652, 1738.7958, 1741.9946, 1755.8914, 1758.9082, 1771.0306, 1774.9175, 1787.8247, 1809.0355, 1862.9480, 1883.7240, 1928.0236, 1932.0079, 1938.0043, 1940.0157, 1953.8590, 1959.9622, 1961.0220, 1981.0421, 1990.9496, 1997.8253, 2007.9401, 2011.8949, 2015.9641, 2023.9448, 2027.1273, 2032.0186, 2044.0441, 2045.9841, 2060.9731, 2067.1531, 2086.0945, 2088.1196, 2108.9956, 2112.9929, 2114.9592, 2139.0918, 2144.0564, 2160.0481, 2174.1157, 2179.0405, 2199.1233, 2211.0984, 2229.1216, 2239.1421, 2242.1345, 2245.8398, 2251.1340, 2255.1416, 2265.0903, 2270.1790, 2273.0667, 2287.1509, 2293.1445, 2328.9482, 2343.1899, 2352.9153, 2354.9983, 2356.0093, 2364.8684, 2414.2334, 2423.2490, 2450.2375, 2466.2253, 2468.2173, 2475.2114, 2486.1069, 2519.9941, 2541.2319, 2542.2505, 2558.3389, 2575.1797, 2646.9695, 2660.9880, 2675.0371, 2695.0181, 2702.2866, 2705.2827, 2713.3413, 2717.3323, 2718.2998, 2721.2900, 2744.2466, 2763.3267, 2770.3513, 2785.3567, 2787.2734, 2788.2341, 2792.2034, 2798.2407, 2801.2722, 2803.2302, 2808.2080, 2817.2480, 2820.2319, 2833.2026, 2843.3906, 2846.3865, 2847.3792, 2870.3926, 2873.3279, 2889.3057, 2892.4070, 2896.4392, 2912.4277, 2915.4314, 2919.4495, 2961.2297, 3071.3845, 3076.4626, 3081.6108, 3087.3884, 3101.4309, 3118.6643, 3143.5120, 3159.5193, 3173.5476, 3184.4751, 3236.5454, 3248.6077, 3253.6089, 3265.5996, 3269.6123, 3285.5964, 3331.5325, 3334.6331, 3335.6592, 3347.4841, 3397.6499, 3411.6399, 3412.5662, 3427.6182, 3428.5718, 3435.6516, 3444.5942, 3457.6045, 3463.5820, 3473.5864, 3487.6257, 3491.6506, 3503.7678, 3509.5947, 3521.6812, 3552.5681, 3574.6165, 3590.6309, 3624.6504, 3636.7607, 3640.6731, 3646.7363, 3651.7256, 3665.7231, 3669.7129, 3692.6494, 3696.7678, 3707.6807, 3714.6301, 3719.6519, 3722.5593, 3723.6516, 3735.6885, 3751.6423, 3830.7644, 3937.7585, 3941.8464, 3942.8328, 3955.8433, 4092.8938, 4147.8613, 4162.9160, 4254.9053, 4272.9224, 4290.9102, 4306.9575, 4309.8506, 4323.8926, 4397.9277, 4415.8896, 4449.1777, 4455.1221, 4459.2368, 4494.0420, 4501.0767, 4510.0269, 4525.1040, 4530.0112, 4839.1138, 4846.0161

45. E1BI02 **Mass:** 43291 **Score:** 17 **Expect:** 4.7e+002 **Matches:** 10

Fibromodulin OS=Bos taurus GN=FMOD PE=4 SV=1

Observed	Mr(expt)	Mr(calc)	ppm	Start	End	Miss	Peptide
779.4621	778.4548	778.4701	-19.63	169 -	175	0	R.IPSPLPR.S
978.5226	977.5154	977.5334	-18.50	98 -	105	0	K.YLPFVPSR.M

1301.6105	1300.6032	1300.6268	-18.09	357 - 368	0	R.SAMPADAPLCLR.L
1361.6910	1360.6837	1360.6847	-0.75	179 - 189	0	R.ELHLDHNQISR.V
1457.7407	1456.7334	1456.7279	3.82	356 - 368	1	K.RSAMPADAPLCLR.L
1717.9056	1716.8983	1716.9019	-2.10	176 - 189	1	R.SLRELHLDHNQISR.V
1928.0236	1927.0163	1927.0163	0.01	307 - 323	0	K.IPPVSTNLENLYLQGNR.I
2646.9695	2645.9622	2645.9702	-3.02	74 - 94	0	R.DCPQECDCPPNFPTAMYCDNR.N
3334.6331	3333.6258	3333.6670	-12.34	236 - 264	1	R.KVPDGLPSALEQLYLEHNNVFSVPDSYFR.G
4459.2368	4458.2295	4458.1954	7.65	106 - 144	1	R.MKYVYFQNNQISSIQEGVFDNATGLLWIALHGNQITSDK.V + Oxidation (M)

No match to: 710.3558, 715.3582, 722.4182, 729.4472, 734.4399, 735.4044, 737.4075, 747.4354, 756.3666, 759.4210, 772.4085, 800.3990, 801.4063, 805.4392, 818.4569, 823.4658, 828.4204, 829.4349, 830.4365, 832.2986, 834.3055, 842.4619, 856.3810, 865.4302, 870.4144, 873.3766, 880.4450, 883.4655, 887.4088, 889.4736, 891.4725, 898.4407, 900.5031, 912.4936, 923.5015, 925.4745, 929.4329, 938.4833, 957.5629, 961.4780, 967.4121, 970.4429, 972.4810, 974.4342, 983.6148, 988.4271, 993.4903, 995.3998, 998.5886, 1006.4926, 1007.5641, 1021.5605, 1024.5408, 1032.5048, 1034.5706, 1036.5566, 1043.5085, 1044.5150, 1046.5917, 1056.5394, 1057.6062, 1074.6005, 1087.5505, 1113.5382, 1122.5513, 1123.5905, 1130.5541, 1143.4395, 1145.6267, 1177.6389, 1178.6249, 1180.6364, 1184.6396, 1194.5638, 1198.6896, 1216.7015, 1227.6558, 1243.6030, 1248.6648, 1254.6876, 1255.5952, 1264.7084, 1269.6405, 1273.6479, 1278.6892, 1281.6271, 1289.6858, 1304.6399, 1312.7454, 1325.6327, 1326.6665, 1328.6610, 1336.7188, 1337.6541, 1338.6735, 1349.6659, 1351.6846, 1356.6124, 1365.6167, 1372.5992, 1382.5925, 1383.7246, 1390.6946, 1420.7117, 1423.6814, 1445.7860, 1456.7703, 1467.7371, 1470.7511, 1488.7211, 1494.7380, 1497.8768, 1500.7181, 1503.7421, 1513.7554, 1519.7505, 1528.6958, 1530.6902, 1540.7820, 1544.8356, 1556.8394, 1562.7800, 1563.7925, 1572.7056, 1578.7816, 1580.8422, 1599.9109, 1616.7953, 1621.7692, 1628.7004, 1628.8589, 1635.7825, 1642.7168, 1647.8525, 1650.8430, 1657.9056, 1666.7905, 1696.8221, 1706.8651, 1707.9131, 1722.9384, 1730.8652, 1738.7958, 1741.9946, 1755.8914, 1758.9082, 1771.0306, 1774.9175, 1787.8247, 1809.0355, 1862.9480, 1883.7240, 1932.0079, 1938.0043, 1940.0157, 1953.8590, 1959.9622, 1961.0220, 1969.9949, 1981.0421, 1990.9496, 1997.8253, 2007.9401, 2011.8949, 2015.9641, 2023.9448, 2027.1273, 2032.0186, 2044.0441, 2045.9841, 2060.9731, 2067.1531, 2086.0945, 2088.1196, 2108.9956, 2112.9929, 2114.9592, 2139.0918, 2144.0564, 2160.0481, 2174.1157, 2179.0405, 2199.1233, 2211.0984, 2229.1216, 2239.1421, 2242.1345, 2245.8398, 2251.1340, 2255.1416, 2265.0903, 2270.1790, 2273.0667, 2284.1729, 2287.1509, 2293.1445, 2328.9482, 2343.1899, 2352.9153, 2354.9983, 2356.0093, 2364.8684, 2414.2334, 2423.2490, 2450.2375, 2466.2253, 2468.2173, 2475.2114, 2486.1069, 2519.9941, 2541.2319, 2542.2505, 2558.3389, 2575.1797, 2660.9880, 2675.0371, 2695.0181, 2702.2866, 2705.2827, 2713.3413, 2717.3323, 2718.2998, 2721.2900, 2744.2466, 2763.3267, 2770.3513, 2785.3567, 2787.2734, 2788.2341, 2792.2034, 2798.2407, 2801.2722, 2803.2302, 2808.2080, 2817.2480, 2820.2319, 2833.2026, 2843.3906, 2846.3865, 2847.3792, 2870.3926, 2873.3279, 2889.3057, 2892.4070, 2896.4392, 2912.4277, 2915.4314, 2919.4495, 2961.2297, 3071.3845, 3076.4626, 3081.6108, 3087.3884, 3101.4309, 3118.6643, 3143.5120, 3159.5193, 3173.5476, 3184.4751, 3236.5454, 3248.6077, 3253.6089, 3265.5996, 3269.6123, 3285.5964, 3331.5325, 3335.6592, 3347.4841, 3397.6499, 3411.6399, 3412.5662, 3427.6182, 3428.5718, 3435.6516, 3444.5942, 3457.6045, 3463.5820, 3473.5864, 3487.6257, 3491.6506, 3503.7678, 3509.5947, 3521.6812, 3552.5681, 3574.6165, 3590.6309, 3624.6504, 3636.7607, 3640.6731, 3646.7363, 3651.7256, 3665.7231, 3669.7129, 3692.6494, 3696.7678, 3707.6807, 3714.6301, 3719.6519, 3722.5593, 3723.6516, 3735.6885, 3751.6423, 3830.7644, 3937.7585, 3941.8464, 3942.8328, 3955.8433, 4092.8938, 4147.8613, 4162.9160, 4254.9053, 4272.9224, 4290.9102, 4306.9575, 4309.8506, 4323.8926, 4397.9277, 4415.8896, 4449.1777, 4455.1221, 4494.0420, 4501.0767, 4510.0269, 4525.1040, 4530.0112, 4839.1138, 4846.0161

46. P13605 **Mass:** 43410 **Score:** 17 **Expect:** 4.7e+002 **Matches:** 10
Fibromodulin OS=Bos taurus GN=FMOD PE=1 SV=2

Observed	Mr(expt)	Mr(calc)	ppm	Start End	Miss	Peptide
779.4621	778.4548	778.4701	-19.63	170 - 176	0	R.IPSPLPR.S
978.5226	977.5154	977.5334	-18.50	99 - 106	0	K.YLPFVPSR.M
1301.6105	1300.6032	1300.6268	-18.09	358 - 369	0	R.SAMPADAPLCLR.L
1361.6910	1360.6837	1360.6847	-0.75	180 - 190	0	R.ELHLDHNQISR.V
1457.7407	1456.7334	1456.7279	3.82	357 - 369	1	K.RSAMPADAPLCLR.L
1717.9056	1716.8983	1716.9019	-2.10	177 - 190	1	R.SLRELHLDHNQISR.V
1928.0236	1927.0163	1927.0163	0.01	308 - 324	0	K.IPPVSTNLENLYLQGNR.I
2646.9695	2645.9622	2645.9702	-3.02	75 - 95	0	R.DCPQECDCPPNFPTAMYCDNR.N
3334.6331	3333.6258	3333.6670	-12.34	237 - 265	1	R.KVPDGLPSALEQLYLEHNNVFSVPDSYFR.G
4459.2368	4458.2295	4458.1954	7.65	107 - 145	1	R.MKYVYFQNNQISSIQEGVFDNATGLLWIALHGNQITSDK.V + Oxidation (M)

No match to: 710.3558, 715.3582, 722.4182, 729.4472, 734.4399, 735.4044, 737.4075, 747.4354, 756.3666, 759.4210, 772.4085, 800.3990, 801.4063, 805.4392, 818.4569, 823.4658, 828.4204, 829.4349, 830.4365, 832.2986, 834.3055, 842.4619, 856.3810, 865.4302, 870.4144, 873.3766, 880.4450, 883.4655, 887.4088, 889.4736, 891.4725, 898.4407, 900.5031, 912.4936, 923.5015, 925.4745, 929.4329, 938.4833, 957.5629, 961.4780, 967.4121, 970.4429, 972.4810, 974.4342, 983.6148, 988.4271, 993.4903, 995.3998, 998.5886, 1006.4926, 1007.5641, 1021.5605, 1024.5408, 1032.5048, 1034.5706, 1036.5566, 1043.5085, 1044.5150, 1046.5917, 1056.5394, 1057.6062, 1074.6005, 1087.5505, 1113.5382, 1122.5513, 1123.5905, 1130.5541, 1143.4395, 1145.6267, 1177.6389, 1178.6249, 1180.6364, 1184.6396, 1194.5638, 1198.6896, 1216.7015, 1227.6558, 1243.6030, 1248.6648, 1254.6876, 1255.5952, 1264.7084, 1269.6405, 1273.6479, 1278.6892, 1281.6271, 1289.6858, 1304.6399, 1312.7454, 1325.6327, 1326.6665, 1328.6610, 1336.7188, 1337.6541, 1338.6735, 1349.6659, 1351.6846, 1356.6124, 1365.6167, 1372.5992, 1382.5925, 1383.7246, 1390.6946, 1420.7117, 1423.6814, 1445.7860, 1456.7703, 1467.7371, 1470.7511, 1488.7211, 1494.7380, 1497.8768, 1500.7181, 1503.7421, 1513.7554, 1519.7505, 1528.6958, 1530.6902, 1540.7820, 1544.8356, 1556.8394, 1562.7800, 1563.7925, 1572.7056, 1578.7816, 1580.8422, 1599.9109, 1616.7953, 1621.7692, 1628.7004, 1628.8589, 1635.7825, 1642.7168, 1647.8525, 1650.8430, 1657.9056, 1666.7905, 1696.8221, 1706.8651, 1707.9131, 1722.9384, 1730.8652, 1738.7958, 1741.9946, 1755.8914, 1758.9082, 1771.0306, 1774.9175, 1787.8247, 1809.0355, 1862.9480, 1883.7240, 1932.0079, 1938.0043, 1940.0157, 1953.8590, 1959.9622, 1961.0220, 1969.9949, 1981.0421, 1990.9496, 1997.8253, 2007.9401, 2011.8949, 2015.9641, 2023.9448, 2027.1273, 2032.0186, 2044.0441, 2045.9841, 2060.9731, 2067.1531, 2086.0945, 2088.1196, 2108.9956, 2112.9929, 2114.9592, 2139.0918, 2144.0564, 2160.0481, 2174.1157, 2179.0405, 2199.1233, 2211.0984, 2229.1216, 2239.1421, 2242.1345, 2245.8398, 2251.1340, 2255.1416, 2265.0903, 2270.1790, 2273.0667, 2284.1729, 2287.1509, 2293.1445, 2328.9482, 2343.1899, 2352.9153, 2354.9983, 2356.0093, 2364.8684, 2414.2334, 2423.2490, 2450.2375, 2466.2253, 2468.2173, 2475.2114, 2486.1069, 2519.9941, 2541.2319, 2542.2505, 2558.3389, 2575.1797, 2660.9880, 2675.0371, 2695.0181, 2702.2866, 2705.2827, 2713.3413, 2717.3323, 2718.2998, 2721.2900, 2744.2466, 2763.3267, 2770.3513, 2785.3567, 2787.2734, 2788.2341, 2792.2034, 2798.2407, 2801.2722, 2803.2302, 2808.2080, 2817.2480, 2820.2319, 2833.2026, 2843.3906, 2846.3865, 2847.3792, 2870.3926, 2873.3279, 2889.3057, 2892.4070, 2896.4392, 2912.4277, 2915.4314, 2919.4495, 2961.2297, 3071.3845, 3076.4626, 3081.6108, 3087.3884, 3101.4309, 3118.6643, 3143.5120, 3159.5193, 3173.5476, 3184.4751, 3236.5454, 3248.6077, 3253.6089, 3265.5996, 3269.6123, 3285.5964, 3331.5325, 3335.6592, 3347.4841, 3397.6499, 3411.6399, 3412.5662, 3427.6182, 3428.5718, 3435.6516, 3444.5942, 3457.6045, 3463.5820, 3473.5864, 3487.6257, 3491.6506, 3503.7678, 3509.5947, 3521.6812, 3552.5681, 3574.6165, 3590.6309, 3624.6504, 3636.7607, 3640.6731, 3646.7363, 3651.7256, 3665.7231, 3669.7129, 3692.6494, 3696.7678, 3707.6807, 3714.6301, 3719.6519, 3722.5593, 3723.6516, 3735.6885, 3751.6423, 3830.7644, 3937.7585, 3941.8464, 3942.8328, 3955.8433, 4092.8938, 4147.8613, 4162.9160, 4254.9053, 4272.9224, 4290.9102, 4306.9575, 4309.8506, 4323.8926, 4397.9277, 4415.8896, 4449.1777, 4455.1221, 4494.0420, 4501.0767, 4510.0269, 4525.1040, 4530.0112, 4839.1138, 4846.0161

47. F1MRI8 **Mass:** 35973 **Score:** 17 **Expect:** 4.8e+002 **Matches:** 7
Uncharacterized protein OS=Bos taurus PE=4 SV=1

Observed	Mr(expt)	Mr(calc)	ppm	Start End	Miss	Peptide
1556.8394	1555.8321	1555.8585	-16.94	9 - 20	1	K.NQIQMIFLFKFK.M
1809.0355	1808.0282	1807.9944	18.7	114 - 128	2	K.KLSKWVPHELTTNQK.I
1969.9949	1968.9876	1969.0017	-7.16	25 - 43	1	K.AVETTHNINNAFGPGTAKK.R
2044.0441	2043.0368	2043.0848	-23.47	75 - 93	1	K.DQLRAIIEADLTTTGEVAK.E
2060.9731	2059.9658	2060.0145	-23.60	3 - 18	1	R.EMMLDKNQIQMIFLFK.F + 2 Oxidation (M)
2713.3413	2712.3340	2712.3983	-23.71	177 - 201	2	K.KGHGSAAGLIHYSFLNPRETITSEK.Y
3719.6519	3718.6446	3718.7341	-24.07	263 - 292	1	K.HLNNFLQGKHFHNQQEAEYAFQEFVESWNK.L

No match to: 710.3558, 715.3582, 722.4182, 729.4472, 734.4399, 735.4044, 737.4075, 747.4354, 756.3666, 759.4210, 772.4085, 779.4621, 800.3990, 801.4063, 805.4392, 818.4569, 823.4658, 828.4204, 829.4349, 830.4365, 832.2986, 834.3055, 842.4619, 856.3810, 865.4302, 870.4144, 873.3766, 880.4450, 883.4655, 887.4088, 889.4736, 891.4725, 898.4407, 900.5031, 912.4936, 923.5015, 925.4745, 929.4329, 938.4833, 957.5629, 961.4780, 967.4121, 970.4429, 972.4810, 974.4342, 978.5226, 983.6148, 988.4271, 993.4903, 995.3998, 998.5886, 1006.4926, 1007.5641, 1021.5605, 1024.5408, 1032.5048, 1034.5706, 1036.5566, 1043.5085, 1044.5150, 1046.5917, 1056.5394,

1057.6062, 1074.6005, 1087.5505, 1113.5382, 1122.5513, 1123.5905, 1130.5541, 1143.4395, 1145.6267, 1177.6389, 1178.6249, 1180.6364, 1184.6396, 1194.5638, 1198.6896, 1216.7015, 1227.6558, 1243.6030, 1248.6648, 1254.6876, 1255.5952, 1264.7084, 1269.6405, 1273.6479, 1278.6892, 1281.6271, 1289.6858, 1301.6105, 1304.6399, 1312.7454, 1325.6327, 1326.6665, 1328.6610, 1336.7188, 1337.6541, 1338.6735, 1349.6659, 1351.6846, 1356.6124, 1361.6910, 1365.6167, 1372.5992, 1382.5925, 1383.7246, 1390.6946, 1420.7117, 1423.6814, 1445.7860, 1456.7703, 1457.7407, 1467.7371, 1470.7511, 1488.7211, 1494.7380, 1497.8768, 1500.7181, 1503.7421, 1513.7554, 1519.7505, 1528.6958, 1530.6902, 1540.7820, 1544.8356, 1562.7800, 1563.7925, 1572.7056, 1578.7816, 1580.8422, 1599.9109, 1616.7953, 1621.7692, 1628.7004, 1628.8589, 1635.7825, 1642.7168, 1647.8525, 1650.8430, 1657.9056, 1666.7905, 1696.8221, 1706.8651, 1707.9131, 1717.9056, 1722.9384, 1730.8652, 1738.7958, 1741.9946, 1755.8914, 1758.9082, 1771.0306, 1774.9175, 1787.8247, 1862.9480, 1883.7240, 1928.0236, 1932.0079, 1938.0043, 1940.0157, 1953.8590, 1959.9622, 1961.0220, 1981.0421, 1990.9496, 1997.8253, 2007.9401, 2011.8949, 2015.9641, 2023.9448, 2027.1273, 2032.0186, 2045.9841, 2067.1531, 2086.0945, 2088.1196, 2108.9956, 2112.9929, 2114.9592, 2139.0918, 2144.0564, 2160.0481, 2174.1157, 2179.0405, 2199.1233, 2211.0984, 2229.1216, 2239.1421, 2242.1345, 2245.8398, 2251.1340, 2255.1416, 2265.0903, 2270.1790, 2273.0667, 2284.1729, 2287.1509, 2293.1445, 2328.9482, 2343.1899, 2352.9153, 2354.9983, 2356.0093, 2364.8684, 2414.2334, 2423.2490, 2450.2375, 2466.2253, 2468.2173, 2475.2114, 2486.1069, 2519.9941, 2541.2319, 2542.2505, 2558.3389, 2575.1797, 2646.9695, 2660.9880, 2675.0371, 2695.0181, 2702.2866, 2705.2827, 2717.3323, 2718.2998, 2721.2900, 2744.2466, 2763.3267, 2770.3513, 2785.3567, 2787.2734, 2788.2341, 2792.2034, 2798.2407, 2801.2722, 2803.2302, 2808.2080, 2817.2480, 2820.2319, 2833.2026, 2843.3906, 2846.3865, 2847.3792, 2870.3926, 2873.3279, 2889.3057, 2892.4070, 2896.4392, 2912.4277, 2915.4314, 2919.4495, 2961.2297, 3071.3845, 3076.4626, 3081.6108, 3087.3884, 3101.4309, 3118.6643, 3143.5120, 3159.5193, 3173.5476, 3184.4751, 3236.5454, 3248.6077, 3253.6089, 3265.5996, 3269.6123, 3285.5964, 3331.5325, 3334.6331, 3335.6592, 3347.4841, 3397.6499, 3411.6399, 3412.5662, 3427.6182, 3428.5718, 3435.6516, 3444.5942, 3457.6045, 3463.5820, 3473.5864, 3487.6257, 3491.6506, 3503.7678, 3509.5947, 3521.6812, 3552.5681, 3574.6165, 3590.6309, 3624.6504, 3636.7607, 3640.6731, 3646.7363, 3651.7256, 3665.7231, 3669.7129, 3692.6494, 3696.7678, 3707.6807, 3714.6301, 3722.5593, 3723.6516, 3735.6885, 3751.6423, 3830.7644, 3937.7585, 3941.8464, 3942.8328, 3955.8433, 4092.8938, 4147.8613, 4162.9160, 4254.9053, 4272.9224, 4290.9102, 4306.9575, 4309.8506, 4323.8926, 4397.9277, 4415.8896, 4449.1777, 4455.1221, 4459.2368, 4494.0420, 4501.0767, 4510.0269, 4525.1040, 4530.0112, 4839.1138, 4846.0161

48. E1BEI2 **Mass:** 25560 **Score:** 17 **Expect:** 4.9e+002 **Matches:** 5
Uncharacterized protein OS=Bos taurus GN=SPCS2 PE=1 SV=1

Observed	Mr(expt)	Mr(calc)	ppm	Start	End	Miss	Peptide
1123.5905	1122.5832	1122.5921	-7.87	190	199	1	R.EAEFTKSIAK.F
1741.9946	1740.9873	1740.9774	5.70	33	47	2	R.SGLLDKWKIDDKPVK.I
3236.5454	3235.5381	3235.5244	4.24	200	227	1	K.FFDHSGTLVMDAYEPEISRLHDSLATER.K
3334.6331	3333.6258	3333.5863	11.8	190	218	2	R.EAEFTKSIAKFFDHSGTLVMDAYEPEISR.L + Oxidation (M)
3651.7256	3650.7183	3650.7675	-13.47	196	227	2	K.SIAKFFDHSGTLVMDAYEPEISRLHDSLATER.K + Oxidation (M)

No match to: 710.3558, 715.3582, 722.4182, 729.4472, 734.4399, 735.4044, 737.4075, 747.4354, 756.3666, 759.4210, 772.4085, 779.4621, 800.3990, 801.4063, 805.4392, 818.4569, 823.4658, 828.4204, 829.4349, 830.4365, 832.2986, 834.3055, 842.4619, 856.3810, 865.4302, 870.4144, 873.3766, 880.4450, 883.4655, 887.4088, 889.4736, 891.4725, 898.4407, 900.5031, 912.4936, 923.5015, 925.4745, 929.4329, 938.4833, 957.5629, 961.4780, 967.4121, 970.4429, 972.4810, 974.4342, 978.5226, 983.6148, 988.4271, 993.4903, 995.3998, 998.5886, 1006.4926, 1007.5641, 1021.5605, 1024.5408, 1032.5048, 1034.5706, 1036.5566, 1043.5085, 1044.5150, 1046.5917, 1056.5394, 1057.6062, 1074.6005, 1087.5505, 1113.5382, 1122.5513, 1130.5541, 1143.4395, 1145.6267, 1177.6389, 1178.6249, 1180.6364, 1184.6396, 1194.5638, 1198.6896, 1216.7015, 1227.6558, 1243.6030, 1248.6648, 1254.6876, 1255.5952, 1264.7084, 1269.6405, 1273.6479, 1278.6892, 1281.6271, 1289.6858, 1301.6105, 1304.6399, 1312.7454, 1325.6327, 1326.6665, 1328.6610, 1336.7188, 1337.6541, 1338.6735, 1349.6659, 1351.6846, 1356.6124, 1361.6910, 1365.6167, 1372.5992, 1382.5925, 1383.7246, 1390.6946, 1420.7117, 1423.6814, 1445.7860, 1456.7703, 1457.7407, 1467.7371, 1470.7511, 1488.7211, 1494.7380, 1497.8768, 1500.7181, 1503.7421, 1513.7554, 1519.7505, 1528.6958, 1530.6902, 1540.7820, 1544.8356, 1556.8394, 1562.7800, 1563.7925, 1572.7056, 1578.7816, 1580.8422, 1599.9109, 1616.7953, 1621.7692, 1628.7004, 1628.8589, 1635.7825, 1642.7168, 1647.8525, 1650.8430, 1657.9056, 1666.7905, 1696.8221, 1706.8651, 1707.9131, 1717.9056, 1722.9384, 1730.8652, 1738.7958, 1755.8914, 1758.9082, 1771.0306, 1774.9175, 1787.8247, 1809.0355, 1862.9480, 1883.7240, 1928.0236, 1932.0079, 1938.0043, 1940.0157, 1953.8590, 1959.9622, 1961.0220, 1969.9949, 1981.0421, 1990.9496, 1997.8253, 2007.9401, 2011.8949, 2015.9641, 2023.9448, 2027.1273, 2032.0186, 2044.0441, 2045.9841, 2060.9731, 2067.1531, 2086.0945, 2088.1196, 2108.9956, 2112.9929, 2114.9592, 2139.0918, 2144.0564, 2160.0481, 2174.1157, 2179.0405, 2199.1233, 2211.0984, 2229.1216, 2239.1421, 2242.1345, 2245.8398, 2251.1340, 2255.1416, 2265.0903, 2270.1790, 2273.0667, 2284.1729, 2287.1509, 2293.1445, 2328.9482, 2343.1899, 2352.9153, 2354.9983, 2356.0093, 2364.8684, 2414.2334, 2423.2490, 2450.2375, 2466.2253, 2468.2173, 2475.2114, 2486.1069, 2519.9941, 2541.2319, 2542.2505, 2558.3389, 2575.1797, 2646.9695, 2660.9880, 2675.0371, 2695.0181, 2702.2866, 2705.2827, 2713.3413, 2717.3323, 2718.2998, 2721.2900, 2744.2466, 2763.3267, 2770.3513, 2785.3567, 2787.2734, 2788.2341, 2792.2034, 2798.2407, 2801.2722, 2803.2302, 2808.2080, 2817.2480, 2820.2319, 2833.2026, 2843.3906, 2846.3865, 2847.3792, 2870.3926, 2873.3279, 2889.3057, 2892.4070, 2896.4392, 2912.4277, 2915.4314, 2919.4495, 2961.2297, 3071.3845, 3076.4626, 3081.6108, 3087.3884, 3101.4309, 3118.6643, 3143.5120, 3159.5193, 3173.5476, 3184.4751, 3248.6077, 3253.6089, 3265.5996, 3269.6123, 3285.5964, 3331.5325, 3335.6592, 3347.4841, 3397.6499, 3411.6399, 3412.5662, 3427.6182, 3428.5718, 3435.6516, 3444.5942, 3457.6045, 3463.5820, 3473.5864, 3487.6257, 3491.6506, 3503.7678, 3509.5947, 3521.6812, 3552.5681, 3574.6165, 3590.6309, 3624.6504, 3636.7607, 3640.6731, 3646.7363, 3665.7231, 3669.7129, 3692.6494, 3696.7678, 3707.6807, 3714.6301, 3719.6519, 3722.5593, 3723.6516, 3735.6885, 3751.6423, 3830.7644, 3937.7585, 3941.8464, 3942.8328, 3955.8433, 4092.8938, 4147.8613, 4162.9160, 4254.9053, 4272.9224, 4290.9102, 4306.9575, 4309.8506, 4323.8926, 4397.9277, 4415.8896, 4449.1777, 4455.1221, 4459.2368, 4494.0420, 4501.0767, 4510.0269, 4525.1040, 4530.0112, 4839.1138, 4846.0161

49. G3X7L3 **Mass:** 66157 **Score:** 17 **Expect:** 5.2e+002 **Matches:** 11
Uncharacterized protein OS=Bos taurus GN=DENND6B PE=4 SV=1

Observed	Mr(expt)	Mr(calc)	ppm	Start	End	Miss	Peptide
1046.5917	1045.5844	1045.5880	-3.42	133	141	2	R.QVKDSSVKR.G
1273.6479	1272.6406	1272.6384	1.76	346	357	1	R.VGEPKMSGDLPK.Q + Oxidation (M)
1628.8589	1627.8516	1627.8603	-5.35	346	360	2	R.VGEPKMSGDLPKQVK.L + Oxidation (M)
2060.9731	2059.9658	2059.9970	-15.13	101	120	1	R.CPGHAGEKPGDRGAPASLQR.E
2284.1729	2283.1656	2283.2184	-23.13	560	580	1	R.LYIDTAIGSLPKDLQAVLCPP.-
2343.1899	2342.1826	2342.2131	-13.02	445	463	1	K.TPPQIRPFRQDDFLQSLER.S
2542.2505	2541.2432	2541.2607	-6.86	504	524	1	K.LEALHLEAICEANIEIWMKDK.S + Oxidation (M)
3143.5120	3142.5047	3142.5500	-14.42	499	524	2	R.EMTQKLEALHLEAICEANIEIWMKDK.S
3159.5193	3158.5120	3158.5450	-10.43	499	524	2	R.EMTQKLEALHLEAICEANIEIWMKDK.S + Oxidation (M)
3590.6309	3589.6236	3589.7062	-23.00	101	132	2	R.CPGHAGEKPGDRGAPASLQREPAHYFGYVYFR.Q
3723.6516	3722.6443	3722.6777	-8.97	69	100	2	K.SSICYLSFPDSHSGCLGDTQFSFRIRQCGGQR.C

No match to: 710.3558, 715.3582, 722.4182, 729.4472, 734.4399, 735.4044, 737.4075, 747.4354, 756.3666, 759.4210, 772.4085, 779.4621, 800.3990, 801.4063, 805.4392, 818.4569, 823.4658, 828.4204, 829.4349, 830.4365, 832.2986, 834.3055, 842.4619, 856.3810, 865.4302, 870.4144, 873.3766, 880.4450, 883.4655, 887.4088, 889.4736, 891.4725, 898.4407, 900.5031, 912.4936, 923.5015, 925.4745, 929.4329, 938.4833, 957.5629, 961.4780, 967.4121, 970.4429, 972.4810, 974.4342, 978.5226, 983.6148, 988.4271, 993.4903, 995.3998, 998.5886, 1006.4926, 1007.5641, 1021.5605, 1024.5408, 1032.5048, 1034.5706, 1036.5566, 1043.5085, 1044.5150, 1056.5394, 1057.6062, 1074.6005, 1087.5505, 1113.5382, 1122.5513, 1123.5905, 1130.5541, 1143.4395, 1145.6267, 1177.6389, 1178.6249, 1180.6364, 1184.6396, 1194.5638, 1198.6896, 1216.7015, 1227.6558, 1243.6030, 1248.6648, 1254.6876, 1255.5952, 1264.7084, 1269.6405, 1278.6892, 1281.6271, 1289.6858, 1301.6105, 1304.6399, 1312.7454, 1325.6327, 1326.6665, 1328.6610, 1336.7188, 1337.6541, 1338.6735, 1349.6659, 1351.6846, 1356.6124, 1361.6910, 1365.6167, 1372.5992, 1382.5925, 1383.7246, 1390.6946, 1420.7117, 1423.6814, 1445.7860, 1456.7703, 1457.7407, 1467.7371, 1470.7511, 1488.7211, 1494.7380, 1497.8768, 1500.7181, 1503.7421, 1513.7554, 1519.7505, 1528.6958, 1530.6902, 1540.7820, 1544.8356, 1556.8394, 1562.7800, 1563.7925, 1572.7056, 1578.7816, 1580.8422, 1599.9109, 1616.7953, 1621.7692, 1628.7004, 1635.7825, 1642.7168, 1647.8525, 1650.8430, 1657.9056, 1666.7905, 1696.8221, 1706.8651, 1707.9131, 1717.9056, 1722.9384, 1730.8652, 1738.7958, 1741.9946, 1755.8914, 1758.9082, 1771.0306, 1774.9175, 1787.8247, 1809.0355, 1862.9480, 1883.7240, 1928.0236, 1932.0079, 1938.0043, 1940.0157, 1953.8590, 1959.9622, 1961.0220, 1969.9949, 1981.0421, 1990.9496, 1997.8253, 2007.9401, 2011.8949, 2015.9641, 2023.9448, 2027.1273, 2032.0186, 2044.0441, 2045.9841, 2067.1531, 2086.0945, 2088.1196, 2108.9956, 2112.9929, 2114.9592, 2139.0918, 2144.0564, 2160.0481, 2174.1157, 2179.0405, 2199.1233, 2211.0984, 2229.1216, 2239.1421, 2242.1345, 2245.8398, 2251.1340, 2255.1416, 2265.0903, 2270.1790, 2273.0667, 2287.1509, 2293.1445, 2328.9482, 2352.9153, 2354.9983, 2356.0093, 2364.8684, 2414.2334, 2423.2490, 2450.2375, 2466.2253, 2468.2173, 2475.2114, 2486.1069, 2519.9941, 2541.2319, 2558.3389, 2575.1797, 2646.9695, 2660.9880, 2675.0371, 2695.0181, 2702.2866, 2705.2827, 2713.3413, 2717.3323, 2718.2998, 2721.2900, 2744.2466, 2763.3267, 2770.3513, 2785.3567, 2787.2734, 2788.2341, 2792.2034, 2798.2407, 2801.2722, 2803.2302, 2808.2080, 2817.2480, 2820.2319, 2833.2026, 2843.3906, 2846.3865, 2847.3792, 2870.3926, 2873.3279, 2889.3057, 2892.4070, 2896.4392, 2912.4277, 2915.4314, 2919.4495, 2961.2297, 3071.3845, 3076.4626, 3081.6108, 3087.3884,

3101.4309, 3118.6643, 3173.5476, 3184.4751, 3236.5454, 3248.6077, 3253.6089, 3265.5996, 3269.6123, 3285.5964, 3331.5325, 3334.6331, 3335.6592, 3347.4841, 3397.6499, 3411.6399, 3412.5662, 3427.6182, 3428.5718, 3435.6516, 3444.5942, 3457.6045, 3463.5820, 3473.5864, 3487.6257, 3491.6506, 3503.7678, 3509.5947, 3521.6812, 3552.5681, 3574.6165, 3624.6504, 3636.7607, 3640.6731, 3646.7363, 3651.7256, 3665.7231, 3669.7129, 3692.6494, 3696.7678, 3707.6807, 3714.6301, 3719.6519, 3722.5593, 3735.6885, 3751.6423, 3830.7644, 3937.7585, 3941.8464, 3942.8328, 3955.8433, 4092.8938, 4147.8613, 4162.9160, 4254.9053, 4272.9224, 4290.9102, 4306.9575, 4309.8506, 4323.8926, 4397.9277, 4415.8896, 4449.1777, 4455.1221, 4459.2368, 4494.0420, 4501.0767, 4510.0269, 4525.1040, 4530.0112, 4839.1138, 4846.0161

50. F1MM31 **Mass:** 177649 **Score:** 17 **Expect:** 5.3e+002 **Matches:** 22

Uncharacterized protein OS=Bos taurus GN=NCKAP5 PE=4 SV=2

Observed	Mr(expt)	Mr(calc)	ppm	Start	End	Miss	Peptide
957.5629	956.5556	956.5403	16.0	813 -	821	2	K.GVSPGRKEK.L
978.5226	977.5154	977.5142	1.23	1169 -	1177	1	R.TSKLETSGR.Y
1198.6896	1197.6823	1197.6830	-0.53	533 -	543	2	K.IPTRGKSSPQK.S
1255.5952	1254.5879	1254.5914	-2.80	1342 -	1351	0	K.DTFMTELLNR.V + Oxidation (M)
1304.6399	1303.6326	1303.6381	-4.22	98 -	107	2	K.RQSSQNWDKR.L
1497.8768	1496.8695	1496.8675	1.38	885 -	898	2	K.SPQLLRKSSTVPGK.Q
1503.7421	1502.7348	1502.7478	-8.61	1158 -	1171	1	R.TPHPSSFTASRTSK.L
1628.8589	1627.8516	1627.8206	19.1	1313 -	1326	1	R.NNPKTPPDIYDQVK.F
1774.9175	1773.9102	1773.9333	-13.01	1028 -	1043	1	R.TQIITNTAERGNSLTR.Q
2239.1421	2238.1348	2238.0917	19.3	1004 -	1025	0	R.SQSFSAHSGEKPPTPPTEGLGK.V
2255.1416	2254.1343	2254.1302	1.83	1078 -	1100	1	R.QGSLGSTGSSSSQHGSPSKLPLR.I
2414.2334	2413.2261	2413.1869	16.3	548 -	571	1	R.EPEASLMMPSAGSVTLEKSPAPGK.L
2450.2375	2449.2302	2449.1867	17.8	1371 -	1394	1	R.SVLKGSSQGSCLTSSSLSTQGNHK.K
2466.2253	2465.2180	2465.2299	-4.81	1002 -	1025	1	K.ARSQSFSAHSGEKPPTPPTEGLGK.V
2542.2505	2541.2432	2541.3048	-24.23	774 -	797	1	R.NAPRAPPHQTLGTTQNNTGLQTPK.N
2558.3389	2557.3316	2557.3098	8.55	108 -	131	1	R.LSIDSSLPSGFASPMDELPPTRIK.E
2843.3906	2842.3833	2842.4508	-23.73	610 -	633	2	R.RDWVQSSKNQMLASQLLSRPPTER.S + Oxidation (M)
2873.3279	2872.3206	2872.3565	-12.48	271 -	295	2	K.LTGCASNCPLEQKLCPGMQGPRVQR.E + Oxidation (M)
3509.5947	3508.5874	3508.6133	-7.38	254 -	283	2	K.HCSEVTSSVYSRERPEKLTGCASNCPLEQK.L
3521.6812	3520.6739	3520.6674	1.85	1283 -	1312	2	K.KKPELQCEMENELHRDIELADGPDSGLQNR.N
3707.6807	3706.6734	3706.6329	10.9	65 -	97	2	R.SSYSSSELSLSSTCSEYSSSSSYTWHDGKNLRK.R
3942.8328	3941.8255	3941.8735	-12.18	7 -	42	1	R.ILDLSSGDLLSDVEGSQSLTHSRTDVEMHGCQLNTK.A

No match to: 710.3558, 715.3582, 722.4182, 729.4472, 734.4399, 735.4044, 737.4075, 747.4354, 756.3666, 759.4210, 772.4085, 779.4621, 800.3990, 801.4063, 805.4392, 818.4569, 823.4658, 828.4204, 829.4349, 830.4365, 832.2986, 834.3055, 842.4619, 856.3810, 865.4302, 870.4144, 873.3766, 880.4450, 883.4655, 887.4088, 889.4736, 891.4725, 898.4407, 900.5031, 912.4936, 923.5015, 925.4745, 929.4329, 938.4833, 961.4780, 967.4121, 970.4429, 972.4810, 974.4342, 983.6148, 988.4271, 993.4903, 995.3998, 998.5886, 1006.4926, 1007.5641, 1021.5605, 1024.5408, 1032.5048, 1034.5706, 1036.5566, 1043.5085, 1044.5150, 1046.5917, 1056.5394, 1057.6062, 1074.6005, 1087.5505, 1113.5382, 1122.5513, 1123.5905, 1130.5541, 1143.4395, 1145.6267, 1177.6389, 1178.6249, 1180.6364, 1184.6396, 1194.5638, 1216.7015, 1227.6558, 1243.6030, 1248.6648, 1254.6876, 1264.7084, 1269.6405, 1273.6479, 1278.6892, 1281.6271, 1289.6858, 1301.6105, 1312.7454, 1325.6327, 1326.6665, 1328.6610, 1336.7188, 1337.6541, 1338.6735, 1349.6659, 1351.6846, 1356.6124, 1361.6910, 1365.6167, 1372.5992, 1382.5925, 1383.7246, 1390.6946, 1420.7117, 1423.6814, 1445.7860, 1456.7703, 1457.7407, 1467.7371, 1470.7511, 1488.7211, 1494.7380, 1500.7181, 1513.7554, 1519.7505, 1528.6958, 1530.6902, 1540.7820, 1544.8356, 1556.8394, 1562.7800, 1563.7925, 1572.7056, 1578.7816, 1580.8422, 1599.9109, 1616.7953, 1621.7692, 1628.7004, 1635.7825, 1642.7168, 1647.8525, 1650.8430, 1657.9056, 1666.7905, 1696.8221, 1706.8651, 1707.9131, 1717.9056, 1722.9384, 1730.8652, 1738.7958, 1741.9946, 1755.8914, 1758.9082, 1771.0306, 1787.8247, 1809.0355, 1862.9480, 1883.7240, 1928.0236, 1932.0079, 1938.0043, 1940.0157, 1953.8590, 1959.9622, 1961.0220, 1969.9949, 1981.0421, 1990.9496, 1997.8253, 2007.9401, 2011.8949, 2015.9641, 2023.9448, 2027.1273, 2032.0186, 2044.0441, 2045.9841, 2060.9731, 2067.1531, 2086.0945, 2088.1196, 2108.9956, 2112.9929, 2114.9592, 2139.0918, 2144.0564, 2160.0481, 2174.1157, 2179.0405, 2199.1233, 2211.0984, 2229.1216, 2242.1345, 2245.8398, 2251.1340, 2265.0903, 2270.1790, 2273.0667, 2284.1729, 2287.1509, 2293.1445, 2328.9482, 2343.1899, 2352.9153, 2354.9983, 2356.0093, 2364.8684, 2423.2490, 2468.2173, 2475.2114, 2486.1069, 2519.9941, 2541.2319, 2575.1797, 2646.9695, 2660.9880, 2675.0371, 2695.0181, 2702.2866, 2705.2827, 2713.3413, 2717.3323, 2718.2998, 2721.2900, 2744.2466, 2763.3267, 2770.3513, 2785.3567, 2787.2734, 2788.2341, 2792.2034, 2798.2407, 2801.2722, 2803.2302, 2808.2080, 2817.2480, 2820.2319, 2833.2026, 2846.3865, 2847.3792, 2870.3926, 2889.3057, 2892.4070, 2896.4392, 2912.4277, 2915.4314, 2919.4495, 2961.2297, 3071.3845, 3076.4626, 3081.6108, 3087.3884, 3101.4309, 3118.6643, 3143.5120, 3159.5193, 3173.5476, 3184.4751, 3236.5454, 3248.6077, 3253.6089, 3265.5996, 3269.6123, 3285.5964, 3331.5325, 3334.6331, 3335.6592, 3347.4841, 3397.6499, 3411.6399, 3412.5662, 3427.6182, 3428.5718, 3435.6516, 3444.5942, 3457.6045, 3463.5820, 3473.5864, 3487.6257, 3491.6506, 3503.7678, 3552.5681, 3574.6165, 3590.6309, 3624.6504, 3636.7607, 3640.6731, 3646.7363, 3651.7256, 3665.7231, 3669.7129, 3692.6494, 3696.7678, 3714.6301, 3719.6519, 3722.5593, 3723.6516, 3735.6885, 3751.6423, 3830.7644, 3937.7585, 3941.8464, 3955.8433, 4092.8938, 4147.8613, 4162.9160, 4254.9053, 4272.9224, 4290.9102, 4306.9575, 4309.8506, 4323.8926, 4397.9277, 4415.8896, 4449.1777, 4455.1221, 4459.2368, 4494.0420, 4501.0767, 4510.0269, 4525.1040, 4530.0112, 4839.1138, 4846.0161

Search Parameters

**Type of search**	: **Peptide Mass Fingerprint**
**Enzyme**	: **Trypsin**
**Fixed modifications**	: **Carbamidomethyl (C)**
**Variable modifications**	: **Oxidation (M)**
**Mass values**	: **Monoisotopic**
**Protein Mass**	: **Unrestricted**
**Peptide Mass Tolerance**	: **± 25 ppm**
**Peptide Charge State**	: **1+**
**Max Missed Cleavages**	: **2**
**Number of queries**	: **337**
**Selected for scoring**	: **136**

Mascot: http://www.matrixscience.com/

MATRIX SCIENCE Mascot Search Results

```
User           : 3F_(L3@52381_05Apr2017)
Email          : proteomics@i3s.up.pt
Search title   : Project: Proteomics, Spot Set: Proteomics\05Apr2017, Label: L3, Spot Id: 227013, Peak List Id: 447669, MS Job Run Id: 316
MS data file   : C:\Documents and Settings\Administrator\Desktop\xu\29JUNHO\Proteomics\05Apr2017\pmf_L3_149875820512.txt
Database       : OrganismSpecie Bos_taurus_Reference_Proteome_2016_06 (24214 sequences; 12839866 residues)
Timestamp      : 29 Jun 2017 at 18:04:02 GMT
Top Score      : 110 for Mixture 1, P21809 + P13605
```

Mascot Score Histogram

Protein score is -10*Log(P), where P is the probability that the observed match is a random event.
Protein scores greater than 56 are significant (p<0.05).

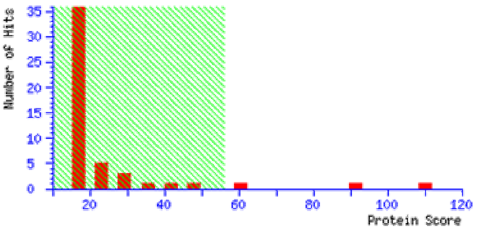

Protein Summary Report

Format As | Protein Summary | Help

Significance threshold p< 0.05 | Max. number of hits 50

Preferred taxonomy All entries

Re-Search All | Search Unmatched

Index

	Accession	Mass	Score	Description
1.	Mixture 1		110	P21809 + P13605
2.	P21809	41963	94	Biglycan OS=Bos taurus GN=BGN PE=1 SV=3
3.	P13605	43410	62	Fibromodulin OS=Bos taurus GN=FMOD PE=1 SV=2
4.	E1BI02	43291	51	Fibromodulin OS=Bos taurus GN=FMOD PE=4 SV=1
5.	P35445	84876	40	Cartilage oligomeric matrix protein OS=Bos taurus GN=COMP PE=1 SV=2
6.	F1MTZ9	84415	36	Cartilage oligomeric matrix protein OS=Bos taurus GN=COMP PE=1 SV=2
7.	P13608	248150	28	Aggrecan core protein OS=Bos taurus GN=ACAN PE=1 SV=3
8.	F1N367	244177	27	Aggrecan core protein OS=Bos taurus GN=ACAN PE=4 SV=2
9.	F1N368	248513	26	Aggrecan core protein OS=Bos taurus GN=ACAN PE=4 SV=2
10.	F1N024	7899	26	Uncharacterized protein OS=Bos taurus GN=FAM19A4 PE=4 SV=1
11.	Q95118	43638	22	Cytokine receptor common subunit gamma OS=Bos taurus GN=IL2RG PE=2 SV=1
12.	F1MX63	44102	21	Prolargin OS=Bos taurus GN=PRELP PE=4 SV=1
13.	Q3SWY9	24956	21	Ras-related protein Rab-28 OS=Bos taurus GN=RAB28 PE=2 SV=1
14.	Q1JPF1	21780	20	NFKB inhibitor interacting Ras-like 2 OS=Bos taurus GN=NKIRAS2 PE=2 SV=1
15.	Q32KZ1	30885	20	Translation initiation factor IF-3, mitochondrial OS=Bos taurus GN=MTIF3 PE=2 SV=1
16.	Q2YDP6	24765	20	Testis-expressed sequence 35 protein OS=Bos taurus GN=Tex35 PE=2 SV=1
17.	F1N1Z4	19257	19	Uncharacterized protein OS=Bos taurus GN=VWA3B PE=4 SV=1
18.	F1MTZ7	34297	19	Glycine N-acyltransferase OS=Bos taurus GN=GLYAT PE=1 SV=2
19.	E1BLR1	58429	19	Uncharacterized protein OS=Bos taurus GN=SPATA2 PE=4 SV=1
20.	Q3ZC17	10037	19	Myelin-associated oligodendrocyte basic protein OS=Bos taurus GN=MOBP PE=2 SV=1
21.	A8E4Q5	35464	19	GAS2 protein OS=Bos taurus GN=GAS2 PE=2 SV=1
22.	Q2NKR1	10554	19	Mitochondrial import inner membrane translocase subunit Tim10 OS=Bos taurus GN=TIMM10 PE=3 SV=1
23.	F1MY76	78375	19	cGMP-dependent protein kinase OS=Bos taurus GN=PRKG1 PE=3 SV=2
24.	G3X7B5	54428	18	RAC-alpha serine/threonine-protein kinase OS=Bos taurus GN=AKT1 PE=4 SV=1
25.	F1N148	15972	18	39S ribosomal protein L54, mitochondrial OS=Bos taurus GN=MRPL54 PE=4 SV=1
26.	A6QLD1	111322	18	EXOC4 protein OS=Bos taurus GN=EXOC4 PE=2 SV=1
27.	G3X8G9	52451	18	Uncharacterized protein OS=Bos taurus PE=3 SV=1
28.	E1BM95	60596	18	Uncharacterized protein OS=Bos taurus GN=SCLT1 PE=4 SV=2
29.	F1MDD0	13795	18	Uncharacterized protein OS=Bos taurus GN=PSMG4 PE=4 SV=2
30.	Q2KJH4	66900	18	WD repeat-containing protein 1 OS=Bos taurus GN=WDR1 PE=2 SV=3
31.	F1MTP5	66919	18	WD repeat-containing protein 1 OS=Bos taurus GN=WDR1 PE=4 SV=2
32.	E1BJY5	27252	18	Uncharacterized protein OS=Bos taurus GN=RNF125 PE=4 SV=1
33.	G3X7H8	43831	18	Uncharacterized protein OS=Bos taurus GN=LOC782894 PE=3 SV=1
34.	Q3T016	13891	17	TSSK6-activating co-chaperone protein OS=Bos taurus GN=TSACC PE=2 SV=1
35.	F1MFH1	13894	17	TSSK6-activating co-chaperone protein OS=Bos taurus GN=TSACC PE=4 SV=1
36.	Q0VC60	56014	17	Dihydrouridine synthase 2-like, SMM1 homolog (S. cerevisiae) OS=Bos taurus GN=DUS2L PE=2 SV=1
37.	P00516	76997	17	cGMP-dependent protein kinase 1 OS=Bos taurus GN=PRKG1 PE=1 SV=2
38.	G5E6K4	12963	17	Uncharacterized protein OS=Bos taurus GN=ATXN7 PE=4 SV=1
39.	Q0VC96	22800	17	Thymocyte nuclear protein 1 OS=Bos taurus GN=THYN1 PE=2 SV=1
40.	M0QVY0	61166	17	Uncharacterized protein OS=Bos taurus GN=KRT6A PE=3 SV=1
41.	Q32LF3	24094	17	Similar to type 1 protein phosphatase inhibitor OS=Bos taurus GN=LOC520057 PE=2 SV=1
42.	F1MYI4	12478	17	Uncharacterized protein OS=Bos taurus GN=IQCJ PE=4 SV=2
43.	E1BPX5	48955	17	Uncharacterized protein OS=Bos taurus GN=DYX1C1 PE=4 SV=2
44.	F6QNC4	81939	17	Uncharacterized protein OS=Bos taurus GN=PCCA PE=4 SV=1
45.	Q3T071	23858	17	Translation machinery-associated protein 16 OS=Bos taurus GN=TMA16 PE=2 SV=1
46.	F1MUI3	23858	17	Translation machinery-associated protein 16 OS=Bos taurus GN=TMA16 PE=4 SV=1
47.	F1MCM7	143110	17	Uncharacterized protein OS=Bos taurus GN=KIAA1683 PE=4 SV=2
48.	F1MWM1	34838	17	Uncharacterized protein OS=Bos taurus GN=MLIP PE=4 SV=1
49.	Q58CY3	41067	17	Chromosome 20 open reading frame 4 OS=Bos taurus GN=C20orf4 PE=2 SV=1
50.	E1BDB4	55638	17	Uncharacterized protein OS=Bos taurus GN=CCDC7 PE=4 SV=1

Results List

1. **Mixture 1** **Total score:** **110** **Expect:** 2.4e-007 **Matches:** 43

Components: 1. P21809 Biglycan OS=Bos taurus GN=BGN PE=1 SV=3
2. P13605 Fibromodulin OS=Bos taurus GN=FMOD PE=1 SV=2

Observed	Mr(expt)	Mr(calc)	ppm	Start	End	Miss	Comp	Peptide
735.4166	734.4094	734.4075	2.48	178	184	0	1	K.GVFSGLR.N
779.4761	778.4689	778.4701	-1.60	170	176	0	2	R.IPSPLPR.S
818.4884	817.4811	817.4810	0.15	134	140	1	1	K.AFSPLRK.L
823.4870	822.4797	822.4824	-3.24	154	159	1	2	K.LRHLER.L
829.4492	828.4419	828.4494	-9.02	253	258	1	1	R.YSKLYR.L
909.5356	908.5283	908.5331	-5.29	293	301	0	1	R.VPAGLPDLK.L
923.5190	922.5118	922.5097	2.27	167	173	1	1	R.IHDNRIR.K
944.5178	943.5105	943.5087	1.93	350	357	1	2	R.LDGNEIKR.S
978.5416	977.5344	977.5334	0.93	99	106	0	2	K.YLPFVPSR.M
1007.5735	1006.5662	1006.5672	-0.97	259	267	0	1	R.LGLGHNQIR.M
1118.5896	1117.5823	1117.5802	1.94	74	83	0	1	R.VVQCSDLGLK.A
1301.6294	1300.6221	1300.6268	-3.56	358	369	0	2	R.SAMPADAPLCLR.L
1312.7516	1311.7443	1311.7398	3.47	242	252	0	1	K.IQAIELEDLLR.Y
1317.6367	1316.6294	1316.6217	5.89	358	369	0	2	R.SAMPADAPLCLR.L + Oxidation (M)
1338.7152	1337.7079	1337.7051	2.09	282	292	1	1	R.ELHLDNNKLSR.V
1361.6908	1360.6835	1360.6847	-0.89	180	190	0	2	R.ELHLDHNQISR.V
1457.7417	1456.7344	1456.7279	4.51	357	369	1	2	K.RSAMPADAPLCLR.L
1494.7421	1493.7348	1493.7337	0.73	316	329	0	1	K.VGVNDFCPVGFGVK.R
1556.8414	1555.8341	1555.8722	-24.47	224	236	0	2	R.SLILLDLSYNHLR.K
1650.8427	1649.8354	1649.8348	0.35	316	330	1	1	K.VGVNDFCPVGFGVKR.A
1717.9019	1716.8946	1716.9019	-4.26	177	190	1	2	R.SLRELHLDHNQISR.V
1787.8689	1786.8616	1786.8849	-13.05	227	241	0	1	K.DLPETLNELHLDHNK.I
1928.0237	1927.0164	1927.0163	0.07	308	324	0	2	K.IPPVSTNLENLYLQGNR.I
2027.1273	2026.1200	2026.1211	-0.53	149	166	0	1	K.NHLVEIPPNLPSSLVELR.I
2286.1472	2285.1399	2285.1387	0.55	88	107	0	1	K.EISPDTTLLDLQNNDISELR.K
2324.0913	2323.0840	2323.0865	-1.05	325	344	0	2	R.INEFSISSFCTVVDVMNFSK.L
2340.1006	2339.0933	2339.0814	5.10	325	344	0	2	R.INEFSISSFCTVVDVMNFSK.L + Oxidation (M)
2414.2415	2413.2342	2413.2336	0.25	88	108	1	1	K.EISPDTTLLDLQNNDISELRK.D
2646.9749	2645.9676	2645.9702	-0.98	75	95	0	2	R.DCPQECDCPPNFPTAMYCDNR.N
2798.2268	2797.2195	2797.2146	1.77	185	210	0	1	R.NMNCIEMGGNPLENSGFEPGAFDGLK.L
2809.4873	2808.4800	2808.4869	-2.43	84	108	2	1	K.AVPKEISPDTTLLDLQNNDISELRK.D
2814.2390	2813.2317	2813.2095	7.90	185	210	0	1	R.NMNCIEMGGNPLENSGFEPGAFDGLK.L + Oxidation (M)
2846.3894	2845.3821	2845.3864	-1.50	331	354	0	1	R.AYYNGISLFNNPVPYWEVQPATFR.C
2919.4480	2918.4407	2918.4509	-3.48	88	112	2	1	K.EISPDTTLLDLQNNDISELRKDDFK.G
3002.4827	3001.4754	3001.4875	-4.02	330	354	1	1	K.RAYYNGISLFNNPVPYWEVQPATFR.C
3081.6121	3080.6048	3080.6142	-3.03	227	252	1	1	K.DLPETLNELHLDHNKIQAIELEDLLR.Y
3334.6743	3333.6670	3333.6670	0.01	237	265	1	2	R.KVPDGLPSALEQLYLEHNNVFSVPDSYFR.G
3457.6028	3456.5955	3456.5900	1.59	185	215	1	1	R.NMNCIEMGGNPLENSGFEPGAFDGLKLNYLR.I
3489.6655	3488.6582	3488.5799	22.5	185	215	1	1	R.NMNCIEMGGNPLENSGFEPGAFDGLKLNYLR.I + 2 Oxidation (M)
4174.0122	4173.0049	4172.9870	4.29	178	215	2	1	K.GVFSGLRNMNCIEMGGNPLENSGFEPGAFDGLKLNYLR.I
4459.3091	4458.3018	4458.1954	23.9	107	145	1	2	R.MKYVYFQNNQISSIQEGVFDNATGLLWIALHGNQITSDK.V + Oxidation (M)
4679.2988	4678.2915	4678.3197	-6.03	180	220	1	2	R.ELHLDHNQISRVPNNALEGLENLTALYLHHNEIQEVGSSMK.G + Oxidation (M)
4844.0703	4843.0630	4843.0463	3.46	33	74	0	2	R.NQQSTYDDPYDPYPYEPYEPYPYGGEEGPAYAYGSPPQPEPR.D

No match to: 706.3109, 710.3683, 712.2512, 712.4277, 717.4089, 719.3850, 722.4312, 729.4136, 734.4397, 747.4490, 750.4445, 756.3809, 759.4347, 772.4293, 783.3987, 799.3317, 831.4715, 832.3093, 834.3143, 842.4991, 856.3599, 862.4179, 870.4754, 873.3850, 876.2836, 880.4593, 883.4774, 887.4115, 889.4967, 890.4962, 891.4855, 895.5654, 900.5347, 925.4849, 927.5414, 929.4692, 936.4928, 938.4988, 945.5171, 949.5491, 955.5369, 957.5576, 967.4212, 972.4985, 974.4442, 976.4541, 977.5377, 981.5450, 983.6256, 988.4442, 993.5175, 1015.4863, 1021.5621, 1025.5615, 1031.5157, 1034.5802, 1044.5248, 1046.5699, 1051.5020, 1056.5302, 1057.5824, 1074.5834, 1087.5496, 1099.6482, 1113.5366, 1120.5878, 1122.5590, 1133.6019, 1145.6392, 1168.6099, 1178.6198, 1180.6312, 1185.6841, 1187.6882, 1194.5767, 1216.7070, 1221.6462, 1248.6903, 1254.6893, 1255.6096, 1264.6997, 1269.6152, 1273.6505, 1281.6354, 1292.7681, 1314.7264, 1315.6790, 1322.6362, 1325.6277, 1336.7238, 1349.6917, 1351.6910, 1352.6709, 1365.5895, 1368.6443, 1372.6029, 1382.5973, 1383.7275, 1390.7019, 1397.7428, 1412.6068, 1414.6772, 1420.7469, 1442.8217, 1454.7571, 1467.7429, 1485.7374, 1486.7495, 1488.7402, 1497.8790, 1500.7224, 1513.7649, 1517.8065, 1518.8060, 1520.7325, 1527.7305, 1528.7021, 1531.6841, 1537.8251, 1539.7679, 1555.7635, 1562.7877, 1567.7642, 1570.7750, 1571.8055, 1578.7964, 1580.8280, 1581.8616, 1583.7979, 1585.7704, 1592.7926, 1610.6801, 1616.7987, 1624.7253, 1628.7031, 1628.8617, 1642.7180, 1647.8546, 1649.8544, 1657.9053, 1663.8506, 1664.8582, 1666.8584, 1680.8226, 1683.8226, 1696.8158, 1698.7761, 1701.9166, 1706.8654, 1707.8828, 1710.8179, 1711.7795, 1714.7788, 1715.8093, 1726.8021, 1728.7821, 1730.8467, 1739.6987, 1747.9039, 1753.7146, 1755.7096, 1758.9032, 1771.0311, 1774.9202, 1783.6785, 1797.6923, 1809.0245, 1811.7025, 1816.9102, 1836.8322, 1838.8318, 1850.8512, 1862.9421, 1866.8452, 1937.9972, 1953.8524, 1961.0308, 1965.8595, 1970.0011, 1982.0062, 1984.0415, 1995.8663, 2007.9470, 2061.0056, 2067.0483, 2086.1091, 2099.1079, 2109.0042, 2136.8455, 2144.0537, 2150.8601, 2173.1655, 2179.0540, 2239.1436, 2251.1506, 2255.1462, 2265.0896, 2270.0999, 2274.0774, 2285.1733, 2289.0583, 2293.1331, 2301.2546, 2303.0488, 2343.1824, 2344.1963, 2352.9204, 2356.0154, 2359.1130, 2375.0210, 2389.0332, 2467.2080, 2525.2712, 2541.2361, 2542.2598, 2660.9883, 2675.0051, 2679.0034, 2689.0183, 2692.0198, 2693.0125, 2695.0129, 2705.2800, 2717.3184, 2763.3374, 2771.4116, 2787.2793, 2792.2427, 2795.3037, 2803.2610, 2813.3884, 2819.2676, 2872.3164, 2876.3669, 2877.3923, 2884.3479, 2887.3311, 2888.3215, 2891.4258, 2901.3557, 3029.4448, 3036.5105, 3053.4414, 3067.4578, 3071.4341, 3126.7246, 3151.3789, 3169.6074, 3184.4443, 3192.2090, 3232.5308, 3237.5469, 3248.6238, 3264.5508, 3305.5381, 3316.7190, 3321.5339, 3330.5715, 3339.6362, 3346.5017, 3350.5159, 3396.6165, 3412.6321, 3428.5723, 3435.6877, 3471.6187, 3476.5935, 3503.7874, 3624.7163, 3639.7131, 3645.7556, 3651.6982, 3692.0066, 3721.6226, 3990.8337, 4074.9656, 4093.8804, 4147.9131, 4153.9287, 4160.9331, 4177.8853, 4253.9565, 4416.9512, 4452.1763, 4466.1865

2. P21809 **Mass:** 41963 **Score:** **94** **Expect:** 9.6e-006 **Matches:** 25

Biglycan OS=Bos taurus GN=BGN PE=1 SV=3

Observed	Mr(expt)	Mr(calc)	ppm	Start	End	Miss	Peptide
735.4166	734.4094	734.4075	2.48	178	184	0	K.GVFSGLR.N
818.4884	817.4811	817.4810	0.15	134	140	1	K.AFSPLRK.L
829.4492	828.4419	828.4494	-9.02	253	258	1	R.YSKLYR.L
909.5356	908.5283	908.5331	-5.29	293	301	0	R.VPAGLPDLK.L
923.5190	922.5118	922.5097	2.27	167	173	1	R.IHDNRIR.K
1007.5735	1006.5662	1006.5672	-0.97	259	267	0	R.LGLGHNQIR.M
1118.5896	1117.5823	1117.5802	1.94	74	83	0	R.VVQCSDLGLK.A
1312.7516	1311.7443	1311.7398	3.47	242	252	0	K.IQAIELEDLLR.Y
1338.7152	1337.7079	1337.7051	2.09	282	292	1	R.ELHLDNNKLSR.V
1494.7421	1493.7348	1493.7337	0.73	316	329	0	K.VGVNDFCPVGFGVK.R
1650.8427	1649.8354	1649.8348	0.35	316	330	1	K.VGVNDFCPVGFGVKR.A
1787.8689	1786.8616	1786.8849	-13.05	227	241	0	K.DLPETLNELHLDHNK.I

2027.1273	2026.1200	2026.1211	-0.53	149	- 166	0	K.NHLVEIPPNLPSSLVELR.I
2286.1472	2285.1399	2285.1387	0.55	88	- 107	0	K.EISPDTTLLDLQNNDISELR.K
2414.2415	2413.2342	2413.2336	0.25	88	- 108	1	K.EISPDTTLLDLQNNDISELRK.D
2798.2268	2797.2195	2797.2146	1.77	185	- 210	0	R.NMNCIEMGGNPLENSGFEPGAFDGLK.L
2809.4873	2808.4800	2808.4869	-2.43	84	- 108	2	K.AVPKEISPDTTLLDLQNNDISELRK.D
2814.2390	2813.2317	2813.2095	7.90	185	- 210	0	R.NMNCIEMGGNPLENSGFEPGAFDGLK.L + Oxidation (M)
2846.3894	2845.3821	2845.3864	-1.50	331	- 354	0	R.AYYNGISLFNNPVPYWEVQPATFR.C
2919.4480	2918.4407	2918.4509	-3.48	88	- 112	2	K.EISPDTTLLDLQNNDISELRKDDFK.G
3002.4827	3001.4754	3001.4875	-4.02	330	- 354	1	K.RAYYNGISLFNNPVPYWEVQPATFR.C
3081.6121	3080.6048	3080.6142	-3.03	227	- 252	1	K.DLPETLNELHLDHNKIQAIELEDLLR.Y
3457.6028	3456.5955	3456.5900	1.59	185	- 215	1	R.NMNCIEMGGNPLENSGFEPGAFDGLKLNYLR.I
3489.6655	3488.6582	3488.5799	22.5	185	- 215	1	R.NMNCIEMGGNPLENSGFEPGAFDGLKLNYLR.I + 2 Oxidation (M)
4174.0122	4173.0049	4172.9870	4.29	178	- 215	2	K.GVFSGLRNMNCIEMGGNPLENSGFEPGAFDGLKLNYLR.I

No match to: 706.3109, 710.3683, 712.2512, 712.4277, 717.4089, 719.3850, 722.4312, 729.4136, 734.4397, 747.4490, 750.4445, 756.3809, 759.4347, 772.4293, 779.4761, 783.3987, 799.3317, 823.4870, 831.4715, 832.3093, 834.3143, 842.4991, 856.3599, 862.4179, 870.4754, 873.3850, 876.2836, 880.4593, 883.4774, 887.4115, 889.4967, 890.4962, 891.4855, 895.5654, 900.5347, 925.4849, 927.5414, 929.4692, 936.4928, 938.4988, 944.5178, 945.5171, 949.5491, 955.5369, 957.5576, 967.4212, 972.4985, 974.4442, 976.4541, 977.5377, 978.5416, 981.5450, 983.6256, 988.4442, 993.5175, 1015.4863, 1021.5621, 1025.5615, 1031.5157, 1034.5802, 1044.5248, 1046.5699, 1051.5020, 1056.5302, 1057.5824, 1074.5834, 1087.5496, 1099.6482, 1113.5366, 1120.5878, 1122.5590, 1133.6019, 1145.6392, 1168.6099, 1178.6198, 1180.6312, 1185.6841, 1187.6882, 1194.5767, 1216.7070, 1221.6462, 1248.6903, 1254.6893, 1255.6096, 1264.6997, 1269.6152, 1273.6505, 1281.6354, 1292.7681, 1301.6294, 1314.7264, 1315.6790, 1317.6367, 1322.6362, 1325.6277, 1336.7238, 1349.6917, 1351.6910, 1352.6709, 1361.6908, 1365.5895, 1368.6443, 1372.6029, 1382.5973, 1383.7275, 1390.7019, 1397.7428, 1412.6068, 1414.6772, 1420.7469, 1442.8217, 1454.7571, 1457.7417, 1467.7429, 1485.7374, 1486.7495, 1488.7402, 1497.8790, 1500.7224, 1513.7649, 1517.8065, 1518.8060, 1520.7325, 1527.7305, 1528.7021, 1531.6841, 1537.8251, 1539.7679, 1555.7635, 1556.8414, 1562.7877, 1567.7642, 1570.7750, 1571.8055, 1578.7964, 1580.8280, 1581.8616, 1583.7979, 1585.7704, 1592.7926, 1610.6801, 1616.7987, 1624.7253, 1628.7031, 1628.8617, 1642.7180, 1647.8546, 1649.8544, 1657.9053, 1663.8506, 1664.8582, 1666.8584, 1680.8226, 1683.8226, 1696.8158, 1698.7761, 1701.9166, 1706.8654, 1707.8828, 1710.8179, 1711.7795, 1714.7788, 1715.8093, 1717.9019, 1726.8021, 1728.7821, 1730.8467, 1739.6987, 1747.9039, 1753.7146, 1755.7096, 1758.9032, 1771.0311, 1774.9202, 1783.6785, 1797.6923, 1809.0245, 1811.7025, 1816.9102, 1836.8322, 1838.8318, 1850.8512, 1862.9421, 1866.8452, 1928.0237, 1937.9972, 1953.8524, 1961.0308, 1965.8595, 1970.0011, 1982.0062, 1984.0415, 1995.8663, 2007.9470, 2061.0056, 2067.0483, 2086.1091, 2099.1079, 2109.0042, 2136.8455, 2144.0537, 2150.8601, 2173.1655, 2179.0540, 2239.1436, 2251.1506, 2255.1462, 2265.0896, 2270.0999, 2274.0774, 2285.1733, 2289.0583, 2293.1331, 2301.2546, 2303.0488, 2324.0913, 2340.1006, 2343.1824, 2344.1963, 2352.9204, 2356.0154, 2359.1130, 2375.0210, 2389.0332, 2467.2080, 2525.2712, 2541.2361, 2542.2598, 2646.9749, 2660.9883, 2675.0051, 2679.0034, 2689.0183, 2692.0198, 2693.0125, 2695.0129, 2705.2800, 2717.3184, 2763.3374, 2771.4116, 2787.2793, 2792.2427, 2795.3037, 2803.2610, 2813.3884, 2819.2676, 2872.3164, 2876.3669, 2877.3923, 2884.3479, 2887.3311, 2888.3215, 2891.4258, 2901.3557, 3029.4448, 3036.5105, 3053.4414, 3067.4578, 3071.4341, 3126.7246, 3151.3789, 3169.6074, 3184.4443, 3192.2090, 3232.5308, 3237.5469, 3248.6238, 3264.5508, 3305.5381, 3316.7190, 3321.5339, 3330.5715, 3334.6743, 3339.6362, 3346.5017, 3350.5159, 3396.6165, 3412.6321, 3428.5723, 3435.6877, 3471.6187, 3476.5935, 3503.7874, 3624.7163, 3639.7131, 3645.7556, 3651.6982, 3692.0066, 3721.6226, 3990.8337, 4074.9656, 4093.8804, 4147.9131, 4153.9287, 4160.9331, 4177.8853, 4253.9565, 4416.9512, 4452.1763, 4459.3091, 4466.1865, 4679.2988, 4844.0703

3. P13605 **Mass:** 43410 **Score:** **62** **Expect:** 0.017 **Matches:** 18

Fibromodulin OS=Bos taurus GN=FMOD PE=1 SV=2

Observed	Mr(expt)	Mr(calc)	ppm	Start	End	Miss	Peptide
779.4761	778.4689	778.4701	-1.60	170	- 176	0	R.IPSPLPR.S
823.4870	822.4797	822.4824	-3.24	154	- 159	1	K.LRHLER.L
944.5178	943.5105	943.5087	1.93	350	- 357	1	R.LDGNEIKR.S
978.5416	977.5344	977.5334	0.93	99	- 106	0	K.YLPFVPSR.M
1301.6294	1300.6221	1300.6268	-3.56	358	- 369	0	R.SAMPADAPLCLR.L
1317.6367	1316.6294	1316.6217	5.89	358	- 369	0	R.SAMPADAPLCLR.L + Oxidation (M)
1361.6908	1360.6835	1360.6847	-0.89	180	- 190	0	K.ELHLDHNQISR.V
1457.7417	1456.7344	1456.7279	4.51	357	- 369	1	K.RSAMPADAPLCLR.L
1556.8414	1555.8341	1555.8722	-24.47	224	- 236	0	R.SLILLDLSYNHLR.K
1717.9019	1716.8946	1716.9019	-4.26	177	- 190	1	R.SLRELHLDHNQISR.V
1928.0237	1927.0164	1927.0163	0.07	308	- 324	0	K.IPPVSTNLENLYLQGNR.I
2324.0913	2323.0840	2323.0865	-1.05	325	- 344	0	R.INEFSISSFCTVVDVMNFSK.L
2340.1006	2339.0933	2339.0814	5.10	325	- 344	0	R.INEFSISSFCTVVDVMNFSK.L + Oxidation (M)
2646.9749	2645.9676	2645.9702	-0.98	75	- 95	0	R.DCPQECDCPPNFPTAMYCDNR.N
3334.6743	3333.6670	3333.6670	0.01	237	- 265	1	R.KVPDGLPSALEQLYLEHNNVFSVPDSYFR.G
4459.3091	4458.3018	4458.1954	23.9	107	- 145	1	R.MKYVYFQNNQISSIQEGVFDNATGLLWIALHGNQITSDK.V + Oxidation (M)
4679.2988	4678.2915	4678.3197	-6.03	180	- 220	1	R.ELHLDHNQISRVPNNALEGLENLTALYLHHNEIQEVGSSMK.G + Oxidation (M)
4844.0703	4843.0630	4843.0463	3.46	33	- 74	0	R.NQQSTYDDPYDPYPYEPYEPYPYGGEEGPAYAYGSPPQPEPR.D

No match to: 706.3109, 710.3683, 712.2512, 712.4277, 717.4089, 719.3850, 722.4312, 729.4136, 734.4397, 735.4166, 747.4490, 750.4445, 756.3809, 759.4347, 772.4293, 783.3987, 799.3317, 818.4884, 829.4492, 831.4715, 832.3093, 834.3143, 842.4991, 856.3599, 862.4179, 870.4754, 873.3850, 876.2836, 880.4593, 883.4774, 887.4115, 889.4967, 890.4962, 891.4855, 895.5654, 900.5347, 909.5356, 923.5190, 925.4849, 927.5414, 929.4692, 936.4928, 938.4988, 945.5171, 949.5491, 955.5369, 957.5576, 967.4212, 972.4985, 974.4442, 976.4541, 977.5377, 981.5450, 983.6256, 988.4442, 993.5175, 1007.5735, 1015.4863, 1021.5621, 1025.5615, 1031.5157, 1034.5802, 1044.5248, 1046.5699, 1051.5020, 1056.5302, 1057.5824, 1074.5834, 1087.5496, 1099.6482, 1113.5366, 1118.5896, 1120.5878, 1122.5590, 1133.6019, 1145.6392, 1168.6099, 1178.6198, 1180.6312, 1185.6841, 1187.6882, 1194.5767, 1216.7070, 1221.6462, 1248.6903, 1254.6893, 1255.6096, 1264.6997, 1269.6152, 1273.6505, 1281.6354, 1292.7681, 1312.7516, 1314.7264, 1315.6790, 1322.6362, 1325.6277, 1336.7238, 1338.7152, 1349.6917, 1351.6910, 1352.6709, 1365.5895, 1368.6443, 1372.6029, 1382.5973, 1383.7275, 1390.7019, 1397.7428, 1412.6068, 1414.6772, 1420.7469, 1442.8217, 1454.7571, 1467.7429, 1485.7374, 1486.7495, 1488.7402, 1494.7421, 1497.8790, 1500.7224, 1513.7649, 1517.8065, 1518.8060, 1520.7325, 1527.7305, 1528.7021, 1531.6841, 1537.8251, 1539.7679, 1555.7635, 1562.7877, 1567.7642, 1570.7750, 1571.8055, 1578.7964, 1580.8280, 1581.8616, 1583.7979, 1585.7704, 1592.7926, 1610.6801, 1616.7987, 1624.7253, 1628.7031, 1628.8617, 1642.7180, 1647.8546, 1649.8544, 1650.8427, 1657.9053, 1663.8506, 1664.8582, 1666.8584, 1680.8226, 1683.8226, 1696.8158, 1698.7761, 1701.9166, 1706.8654, 1707.8828, 1710.8179, 1711.7795, 1714.7788, 1715.8093, 1726.8021, 1728.7821, 1730.8467, 1739.6987, 1747.9039, 1753.7146, 1755.7096, 1758.9032, 1771.0311, 1774.9202, 1783.6785, 1787.8689, 1797.6923, 1809.0245, 1811.7025, 1816.9102, 1836.8322, 1838.8318, 1850.8512, 1862.9421, 1866.8452, 1937.9972, 1953.8524, 1961.0308, 1965.8595, 1970.0011, 1982.0062, 1984.0415, 1995.8663, 2007.9470, 2027.1273, 2061.0056, 2067.0483, 2086.1091, 2099.1079, 2109.0042, 2136.8455, 2144.0537, 2150.8601, 2173.1655, 2179.0540, 2239.1436, 2251.1506, 2255.1462, 2265.0896, 2270.0999, 2274.0774, 2285.1733, 2286.1472, 2289.0583, 2293.1331, 2301.2546, 2303.0488, 2343.1824, 2344.1963, 2352.9204, 2356.0154, 2359.1130, 2375.0210, 2389.0332, 2414.2415, 2467.2080, 2525.2712, 2541.2361, 2542.2598, 2660.9883, 2675.0051, 2679.0034, 2689.0183, 2692.0198, 2693.0125, 2695.0129, 2705.2800, 2717.3184, 2763.3374, 2771.4116, 2787.2793, 2792.2427, 2795.3037, 2798.2268, 2803.2610, 2809.4873, 2813.3884, 2814.2390, 2819.2676, 2846.3894, 2872.3164, 2876.3669, 2877.3923, 2884.3479, 2887.3311, 2888.3215, 2891.4258, 2901.3557, 2919.4480, 3002.4827, 3029.4448, 3036.5105, 3053.4414, 3067.4578, 3071.4341, 3081.6121, 3126.7246, 3151.3789, 3169.6074, 3184.4443, 3192.2090, 3232.5308, 3237.5469, 3248.6238, 3264.5508, 3305.5381, 3316.7190, 3321.5339, 3330.5715, 3339.6362, 3346.5017, 3350.5159, 3396.6165, 3412.6321, 3428.5723, 3435.6877, 3457.6028, 3471.6187, 3476.5935, 3489.6655, 3503.7874, 3624.7163, 3639.7131, 3645.7556, 3651.6982, 3692.0066, 3721.6226, 3990.8337, 4074.9656, 4093.8804, 4147.9131, 4153.9287, 4160.9331, 4174.0122, 4177.8853, 4253.9565, 4416.9512, 4452.1763, 4466.1865

4. E1BI02 **Mass:** 43291 **Score:** 51 **Expect:** 0.19 **Matches:** 17

Fibromodulin OS=Bos taurus GN=FMOD PE=4 SV=1

Observed	Mr(expt)	Mr(calc)	ppm	Start	End	Miss	Peptide
779.4761	778.4689	778.4701	-1.60	169	- 175	0	R.IPSPLPR.S
823.4870	822.4797	822.4824	-3.24	153	- 158	1	K.LRHLER.L

944.5178	943.5105	943.5087	1.93	349	356	1	R.LDGNEIKR.S
978.5416	977.5344	977.5334	0.93	98	105	0	K.YLPFVPSR.M
1301.6294	1300.6221	1300.6268	-3.56	357	368	0	R.SAMPADAPLCLR.L
1317.6367	1316.6294	1316.6217	5.89	357	368	0	R.SAMPADAPLCLR.L + Oxidation (M)
1361.6908	1360.6835	1360.6847	-0.89	179	189	0	R.ELHLDHNQISR.V
1457.7417	1456.7344	1456.7279	4.51	356	368	1	K.RSAMPADAPLCLR.L
1556.8414	1555.8341	1555.8722	-24.47	223	235	0	R.SLILLDLSYNHLR.K
1717.9019	1716.8946	1716.9019	-4.26	176	189	1	R.SLRELHLDHNQISR.V
1928.0237	1927.0164	1927.0163	0.07	307	323	0	K.IPPVSTNLENLYLQGNR.I
2324.0913	2323.0840	2323.0865	-1.05	324	343	0	R.INEFSISSFCTVVDVMNFSK.L
2340.1006	2339.0933	2339.0814	5.10	324	343	0	R.INEFSISSFCTVVDVMNFSK.L + Oxidation (M)
2646.9749	2645.9676	2645.9702	-0.98	74	94	0	R.DCPQECDCPPNFPTAMYCDNR.N
3334.6743	3333.6670	3333.6670	0.01	236	264	1	R.KVPDGLPSALEQLYLEHNNVFSVPDSYFR.G
4459.3091	4458.3018	4458.1954	23.9	106	144	1	R.MKYVYFQNNQISSIQEGVFDNATGLLWIALHGNQITSDK.V + Oxidation (M)
4679.2988	4678.2915	4678.3197	-6.03	179	219	1	R.ELHLDHNQISRVPNNALEGLENLTALYLHHNEIQEVGSSMK.G + Oxidation (M)

No match to: 706.3109, 710.3683, 712.2512, 712.4277, 717.4089, 719.3850, 722.4312, 729.4136, 734.4397, 735.4166, 747.4490, 750.4445, 756.3809, 759.4347, 772.4293, 783.3987, 799.3317, 818.4884, 829.4492, 831.4715, 832.3093, 834.3143, 842.4991, 856.3599, 862.4179, 870.4754, 873.3850, 876.2836, 880.4593, 883.4774, 887.4115, 889.4967, 890.4962, 891.4855, 895.5654, 900.5347, 909.5356, 923.5190, 925.4849, 927.5414, 929.4692, 936.4928, 938.4988, 945.5171, 949.5491, 955.5369, 957.5576, 967.4212, 972.4985, 974.4442, 976.4541, 977.5377, 981.5450, 983.6256, 988.4442, 993.5175, 1007.5735, 1015.4863, 1021.5621, 1025.5615, 1031.5157, 1034.5802, 1044.5248, 1046.5699, 1051.5020, 1056.5302, 1057.5824, 1074.5834, 1087.5496, 1099.6482, 1113.5366, 1118.5896, 1120.5878, 1122.5590, 1133.6019, 1145.6392, 1168.6099, 1178.6198, 1180.6312, 1185.6841, 1187.6882, 1194.5767, 1216.7070, 1221.6462, 1248.6903, 1254.6893, 1255.6096, 1264.6997, 1269.6152, 1273.6505, 1281.6354, 1292.7681, 1312.7516, 1314.7264, 1315.6790, 1322.6362, 1325.6277, 1336.7238, 1338.7152, 1349.6917, 1351.6910, 1352.6709, 1365.5895, 1368.6443, 1372.6029, 1382.5973, 1383.7275, 1390.7019, 1397.7428, 1412.6068, 1414.6772, 1420.7469, 1442.8217, 1454.7571, 1467.7429, 1485.7374, 1486.7495, 1488.7402, 1494.7421, 1497.8790, 1500.7224, 1513.7649, 1517.8065, 1518.8060, 1520.7325, 1527.7305, 1528.7021, 1531.6841, 1537.8251, 1539.7679, 1555.7635, 1562.7877, 1567.7642, 1570.7750, 1571.8055, 1578.7964, 1580.8280, 1581.8616, 1583.7979, 1585.7704, 1592.7926, 1610.6801, 1616.7987, 1624.7253, 1628.7031, 1628.8617, 1642.7180, 1647.8546, 1649.8544, 1650.8427, 1657.9053, 1663.8506, 1664.8582, 1666.8584, 1680.8226, 1683.8226, 1696.8158, 1698.7761, 1701.9166, 1706.8654, 1707.8828, 1710.8179, 1711.7795, 1714.7788, 1715.8093, 1726.8021, 1728.7821, 1730.8467, 1739.6987, 1747.9039, 1753.7146, 1755.7096, 1758.9032, 1771.0311, 1774.9202, 1783.6785, 1787.8689, 1797.6923, 1809.0245, 1811.7025, 1816.9102, 1836.8322, 1838.8318, 1850.8512, 1862.9421, 1866.8452, 1937.9972, 1953.8524, 1961.0308, 1965.8595, 1970.0011, 1982.0062, 1984.0415, 1995.8663, 2007.9470, 2027.1273, 2061.0056, 2067.0483, 2086.1091, 2099.1079, 2109.0042, 2136.8455, 2144.0537, 2150.8601, 2173.1655, 2179.0540, 2239.1436, 2251.1506, 2255.1462, 2265.0896, 2270.0999, 2274.0774, 2285.1733, 2286.1472, 2289.0583, 2293.1331, 2301.2546, 2303.0488, 2343.1824, 2344.1963, 2352.9204, 2356.0154, 2359.1130, 2375.0210, 2389.0332, 2414.2415, 2467.2080, 2525.2712, 2541.2361, 2542.2598, 2660.9883, 2675.0051, 2679.0034, 2689.0183, 2692.0198, 2693.0125, 2695.0129, 2705.2800, 2717.3184, 2763.3374, 2771.4116, 2787.2793, 2792.2427, 2795.3037, 2798.2268, 2803.2610, 2809.4873, 2813.3884, 2814.2390, 2819.2676, 2846.3894, 2872.3164, 2876.3669, 2877.3923, 2884.3479, 2887.3311, 2888.3215, 2891.4258, 2901.3557, 2919.4480, 3002.4827, 3029.4448, 3036.5105, 3053.4414, 3067.4578, 3071.4341, 3081.6121, 3126.7246, 3151.3789, 3169.6074, 3184.4443, 3192.2090, 3232.5308, 3237.5469, 3248.6238, 3264.5508, 3305.5381, 3316.7190, 3321.5339, 3330.5715, 3339.6362, 3346.5017, 3350.5159, 3396.6165, 3412.6321, 3428.5723, 3435.6877, 3457.6028, 3471.6187, 3476.5935, 3489.6655, 3503.7874, 3624.7163, 3639.7131, 3645.7556, 3651.6982, 3692.0066, 3721.6226, 3990.8337, 4074.9656, 4093.8804, 4147.9131, 4153.9287, 4160.9331, 4174.0122, 4177.8853, 4253.9565, 4416.9512, 4452.1763, 4466.1865, 4844.0703

5. P35445 **Mass:** 84876 **Score:** 40 **Expect:** 2.3 **Matches:** 27

Cartilage oligomeric matrix protein OS=Bos taurus GN=COMP PE=1 SV=2

Observed	Mr(expt)	Mr(calc)	ppm	Start	End	Miss	Peptide
712.4277	711.4204	711.4252	-6.78	222	226	1	R.RRPQR.F
750.4445	749.4372	749.4323	6.48	58	63	0	K.EITFLK.N
862.4179	861.4106	861.4014	10.7	240	246	0	K.ADCVLER.D
927.5414	926.5341	926.5338	0.36	665	671	1	R.LLWKDPR.N
1031.5157	1030.5084	1030.5043	3.97	641	650	0	K.SSTGPGEQLR.N
1051.5020	1050.4947	1050.4917	2.91	141	149	0	R.CINTSPGFR.C
1486.7495	1485.7422	1485.7423	-0.06	37	49	0	R.ELQETNAALQDVR.D
1520.7325	1519.7252	1519.7155	6.43	268	280	1	R.DTDLDGFPDEKLR.C
1555.7635	1554.7562	1554.7539	1.49	651	664	0	R.NALWHTGDTASQVR.L
1610.6801	1609.6728	1609.6678	3.10	740	752	0	R.CNDTIPEDYEAQR.L
1698.7761	1697.7688	1697.7620	4.01	615	627	0	K.QMEQTYWQANPFR.A
1711.7795	1710.7722	1710.7995	-15.96	381	395	1	R.NPVDNCPKVPNSDQK.D
1714.7788	1713.7715	1713.7569	8.52	615	627	0	K.QMEQTYWQANPFR.A + Oxidation (M)
1739.6987	1738.6914	1738.6895	1.09	64	78	0	K.NTVMECDACGMQPAR.T
1755.7096	1754.7023	1754.6844	10.2	64	78	0	K.NTVMECDACGMQPAR.T + Oxidation (M)
1783.6785	1782.6712	1782.6686	1.47	337	351	1	R.NTDGDKWGDACDNCR.S
1836.8322	1835.8249	1835.8230	1.07	251	267	0	R.SCVCAVGWAGNGLICGR.D
1984.0415	1983.0342	1983.0385	-2.15	37	53	1	R.ELQETNAALQDVRDLLR.Q
2067.0483	2066.0410	2066.0408	0.13	721	737	0	R.LGVFCFSQENIIWANLR.Y
2270.0999	2269.0926	2269.0936	-0.45	698	717	0	R.FYEGPELVADSNVILDTTMR.G
2286.1472	2285.1399	2285.0886	22.5	698	717	0	R.FYEGPELVADSNVILDTTMR.G + Oxidation (M)
2289.0583	2288.0510	2288.0354	6.81	150	171	0	R.CEACPPGFSGPTHEGVGLAFAK.A
2375.0210	2374.0137	2374.0141	-0.14	227	246	1	R.FCPDGTPSPCHEKADCVLER.D
2525.2712	2524.2639	2524.2632	0.30	696	717	1	R.VRFYEGPELVADSNVILDTTMR.G
2541.2361	2540.2288	2540.2581	-11.52	696	717	1	R.VRFYEGPELVADSNVILDTTMR.G + Oxidation (M)
3053.4414	3052.4341	3052.4318	0.77	82	109	0	K.LTVRPLSQCSPGFCFPGVACTETANGAR.C
3321.5339	3320.5266	3320.5166	3.03	141	171	1	R.CINTSPGFRCEACPPGFSGPTHEGVGLAFAK.A

No match to: 706.3109, 710.3683, 712.2512, 717.4089, 719.3850, 722.4312, 729.4136, 734.4397, 735.4166, 747.4490, 756.3809, 759.4347, 772.4293, 779.4761, 783.3987, 799.3317, 818.4884, 823.4870, 829.4492, 831.4715, 832.3093, 834.3143, 842.4991, 856.3599, 870.4754, 873.3850, 876.2836, 880.4593, 883.4774, 887.4115, 889.4967, 890.4962, 891.4855, 895.5654, 900.5347, 909.5356, 923.5190, 925.4849, 929.4692, 936.4928, 938.4988, 944.5178, 945.5171, 949.5491, 955.5369, 957.5576, 967.4212, 972.4985, 974.4442, 976.4541, 977.5377, 978.5416, 981.5450, 983.6256, 988.4442, 993.5175, 1007.5735, 1015.4863, 1021.5621, 1025.5615, 1034.5802, 1044.5248, 1046.5699, 1056.5302, 1057.5824, 1074.5834, 1087.5496, 1099.6482, 1113.5366, 1118.5896, 1120.5878, 1122.5590, 1133.6019, 1145.6392, 1168.6099, 1178.6198, 1180.6312, 1185.6841, 1187.6882, 1194.5767, 1216.7070, 1221.6462, 1248.6903, 1254.6893, 1255.6096, 1264.6997, 1269.6152, 1273.6505, 1281.6354, 1292.7681, 1301.6294, 1312.7516, 1314.7264, 1315.6790, 1317.6367, 1322.6362, 1325.6277, 1336.7238, 1338.7152, 1349.6917, 1351.6910, 1352.6709, 1361.6908, 1365.5895, 1368.6443, 1372.6029, 1382.5973, 1383.7275, 1390.7019, 1397.7428, 1412.6068, 1414.6772, 1420.7469, 1442.8217, 1454.7571, 1457.7417, 1467.7429, 1485.7374, 1488.7402, 1494.7421, 1497.8790, 1500.7224, 1513.7649, 1517.8065, 1518.8060, 1527.7305, 1528.7021, 1531.6841, 1537.8251, 1539.7679, 1556.8414, 1562.7877, 1567.7642, 1570.7750, 1571.8055, 1578.7964, 1580.8280, 1581.8616, 1583.7979, 1585.7704, 1592.7926, 1616.7987, 1624.7253, 1628.7031, 1628.8617, 1642.7180, 1647.8546, 1649.8544, 1650.8427, 1657.9053, 1663.8506, 1664.8582, 1666.8584, 1680.8226, 1683.8226, 1696.8158, 1701.9166, 1706.8654, 1707.8828, 1710.8179, 1715.8093, 1717.9019, 1726.8021, 1728.7821, 1730.8467, 1747.9039, 1753.7146, 1758.9032, 1771.0311, 1774.9202, 1787.8689, 1797.6923, 1809.0245, 1811.7025, 1816.9102, 1838.8318, 1850.8512, 1862.9421, 1866.8452, 1928.0237, 1937.9972, 1953.8524, 1961.0308, 1965.8595, 1970.0011, 1982.0062, 1995.8663, 2007.9470, 2027.1273, 2061.0056, 2086.1091, 2099.1079, 2109.0042, 2136.8455, 2144.0537, 2150.8601, 2173.1655, 2179.0540, 2239.1436, 2251.1506, 2255.1462, 2265.0896, 2274.0774, 2285.1733, 2293.1331, 2301.2546, 2303.0488, 2324.0913, 2340.1006, 2343.1824, 2344.1963, 2352.9204, 2356.0154, 2359.1130, 2389.0332, 2414.2415, 2467.2080, 2542.2598, 2646.9749, 2660.9883, 2675.0051, 2679.0034, 2689.0183, 2692.0198, 2693.0125, 2695.0129, 2705.2800, 2717.3184, 2763.3374, 2771.4116, 2787.2793, 2792.2427, 2795.3037, 2798.2268, 2803.2610, 2809.4873, 2813.3884, 2814.2390, 2819.2676, 2846.3894, 2872.3164, 2876.3669,

2877.3923, 2884.3479, 2887.3311, 2888.3215, 2891.4258, 2901.3557, 2919.4480, 3002.4827, 3029.4448, 3036.5105, 3067.4578, 3071.4341, 3081.6121, 3126.7246, 3151.3789, 3169.6074, 3184.4443, 3192.2090, 3232.5308, 3237.5469, 3248.6238, 3264.5508, 3305.5381, 3316.7190, 3330.5715, 3334.6743, 3339.6362, 3346.5017, 3350.5159, 3396.6165, 3412.6321, 3428.5723, 3435.6877, 3457.6028, 3471.6187, 3476.5935, 3489.6655, 3503.7874, 3624.7163, 3639.7131, 3645.7556, 3651.6982, 3692.0066, 3721.6226, 3990.8337, 4074.9656, 4093.8804, 4147.9131, 4153.9287, 4160.9331, 4174.0122, 4177.8853, 4253.9565, 4416.9512, 4452.1763, 4459.3091, 4466.1865, 4679.2988, 4844.0703

6. F1MTZ9 **Mass:** 84415 **Score:** 36 **Expect:** 6.7 **Matches:** 24

Cartilage oligomeric matrix protein OS=Bos taurus GN=COMP PE=1 SV=2

Observed	Mr(expt)	Mr(calc)	ppm	Start	End	Miss	Peptide
712.4277	711.4204	711.4252	-6.78	222	226	1	R.RRPQR.F
750.4445	749.4372	749.4323	6.48	58	63	0	K.EITFLK.N
927.5414	926.5341	926.5338	0.36	664	670	1	R.LLWKDPR.N
1031.5157	1030.5084	1030.5043	3.97	640	649	0	K.SSTGPGEQLR.N
1051.5020	1050.4947	1050.4917	2.91	141	149	0	R.CINTSPGFR.C
1486.7495	1485.7422	1485.7423	-0.06	37	49	0	R.ELQETNAALQDVR.D
1520.7325	1519.7252	1519.7155	6.43	267	279	1	R.DTDLDGFPDEKLR.C
1555.7635	1554.7562	1554.7539	1.49	650	663	0	R.NALWHTGDTASQVR.L
1610.6801	1609.6728	1609.6678	3.10	739	751	0	R.CNDTIPEDYEAQR.L
1698.7761	1697.7688	1697.7620	4.01	614	626	0	K.QMEQTYWQANPFR.A
1711.7795	1710.7722	1710.7995	-15.96	380	394	1	R.NPVDNCPKVPNSDQK.D
1714.7788	1713.7715	1713.7569	8.52	614	626	0	K.QMEQTYWQANPFR.A + Oxidation (M)
1739.6987	1738.6914	1738.6895	1.09	64	78	0	K.NTVMECDACGMQPAR.T
1755.7096	1754.7023	1754.6844	10.2	64	78	0	K.NTVMECDACGMQPAR.T + Oxidation (M)
1836.8322	1835.8249	1835.8230	1.07	250	266	0	R.SCVCAVGWAGNGLICGR.D
1984.0415	1983.0342	1983.0385	-2.15	37	53	1	R.ELQETNAALQDVRDLLR.Q
2067.0483	2066.0410	2066.0408	0.13	720	736	0	R.LGVFCFSQENIIWANLR.Y
2270.0999	2269.0926	2269.0936	-0.45	697	716	0	R.FYEGPELVADSNVILDTTMR.G
2286.1472	2285.1399	2285.0886	22.5	697	716	0	R.FYEGPELVADSNVILDTTMR.G + Oxidation (M)
2289.0583	2288.0510	2288.0354	6.81	150	171	0	R.CEACPPGFSGPTHEGVGLAFAK.A
2525.2712	2524.2639	2524.2632	0.30	695	716	1	R.VRFYEGPELVADSNVILDTTMR.G
2541.2361	2540.2288	2540.2581	-11.52	695	716	1	R.VRFYEGPELVADSNVILDTTMR.G + Oxidation (M)
3053.4414	3052.4341	3052.4318	0.77	82	109	0	K.LTVRPLSQCSPGFCFPGVACTETANGAR.C
3321.5339	3320.5266	3320.5166	3.03	141	171	1	R.CINTSPGFRCEACPPGFSGPTHEGVGLAFAK.A

No match to: 706.3109, 710.3683, 712.2512, 717.4089, 719.3850, 722.4312, 729.4136, 734.4397, 735.4166, 747.4490, 756.3809, 759.4347, 772.4293, 779.4761, 783.3987, 799.3317, 818.4884, 823.4870, 829.4492, 831.4715, 832.3093, 834.3143, 842.4991, 856.3599, 862.4179, 870.4754, 873.3850, 876.2836, 880.4593, 883.4774, 887.4115, 889.4967, 890.4962, 891.4855, 895.5654, 900.5347, 909.5356, 923.5190, 925.4849, 929.4692, 936.4928, 938.4988, 944.5178, 945.5171, 949.5491, 955.5369, 957.5576, 967.4212, 972.4985, 974.4442, 976.4541, 977.5377, 978.5416, 981.5450, 983.6256, 988.4442, 993.5175, 1007.5735, 1015.4863, 1021.5621, 1025.5615, 1034.5802, 1044.5248, 1046.5699, 1056.5302, 1057.5824, 1074.5834, 1087.5496, 1099.6482, 1113.5366, 1118.5896, 1120.5878, 1122.5590, 1133.6019, 1145.6392, 1168.6099, 1178.6198, 1180.6312, 1185.6841, 1187.6882, 1194.5767, 1216.7070, 1221.6462, 1248.6903, 1254.6893, 1255.6096, 1264.6997, 1269.6152, 1273.6505, 1281.6354, 1292.7681, 1301.6294, 1312.7516, 1314.7264, 1315.6790, 1317.6367, 1322.6362, 1325.6277, 1336.7238, 1338.7152, 1349.6917, 1351.6910, 1352.6709, 1361.6908, 1365.5895, 1368.6443, 1372.6029, 1382.5973, 1383.7275, 1390.7019, 1397.7428, 1412.6068, 1414.6772, 1420.7469, 1442.8217, 1454.7571, 1457.7417, 1467.7429, 1485.7374, 1488.7402, 1494.7421, 1497.8790, 1500.7224, 1513.7649, 1517.8065, 1518.8060, 1527.7305, 1528.7021, 1531.6841, 1537.8251, 1539.7679, 1556.8414, 1562.7877, 1567.7642, 1570.7750, 1571.8055, 1578.7964, 1580.8280, 1581.8616, 1583.7979, 1585.7704, 1592.7926, 1616.7987, 1624.7253, 1628.7031, 1628.8617, 1642.7180, 1647.8546, 1649.8544, 1650.8427, 1657.9053, 1663.8506, 1664.8582, 1666.8584, 1680.8226, 1683.8226, 1696.8158, 1701.9166, 1706.8654, 1707.8828, 1710.8179, 1715.8093, 1717.9019, 1726.8021, 1728.7821, 1730.8467, 1747.9039, 1753.7146, 1758.9032, 1771.0311, 1774.9202, 1783.6785, 1787.8689, 1797.6923, 1809.0245, 1811.7025, 1816.9102, 1838.8318, 1850.8512, 1862.9421, 1866.8452, 1928.0237, 1937.9972, 1953.8524, 1961.0308, 1965.8595, 1970.0011, 1982.0062, 1995.8663, 2007.9470, 2027.1273, 2061.0056, 2086.1091, 2099.1079, 2109.0042, 2136.8455, 2144.0537, 2150.8601, 2173.1655, 2179.0540, 2239.1436, 2251.1506, 2255.1462, 2265.0896, 2274.0774, 2285.1733, 2293.1331, 2301.2546, 2303.0488, 2324.0913, 2340.1006, 2343.1824, 2344.1963, 2352.9204, 2356.0154, 2359.1130, 2375.0210, 2389.0332, 2414.2415, 2467.2080, 2542.2598, 2646.9749, 2660.9883, 2675.0051, 2679.0034, 2689.0183, 2692.0198, 2693.0125, 2695.0129, 2705.2800, 2717.3184, 2763.3374, 2771.4116, 2787.2793, 2792.2427, 2795.3037, 2798.2268, 2803.2610, 2809.4873, 2813.3884, 2814.2390, 2819.2676, 2846.3894, 2872.3164, 2876.3669, 2877.3923, 2884.3479, 2887.3311, 2888.3215, 2891.4258, 2901.3557, 2919.4480, 3002.4827, 3029.4448, 3036.5105, 3067.4578, 3071.4341, 3081.6121, 3126.7246, 3151.3789, 3169.6074, 3184.4443, 3192.2090, 3232.5308, 3237.5469, 3248.6238, 3264.5508, 3305.5381, 3316.7190, 3330.5715, 3334.6743, 3339.6362, 3346.5017, 3350.5159, 3396.6165, 3412.6321, 3428.5723, 3435.6877, 3457.6028, 3471.6187, 3476.5935, 3489.6655, 3503.7874, 3624.7163, 3639.7131, 3645.7556, 3651.6982, 3692.0066, 3721.6226, 3990.8337, 4074.9656, 4093.8804, 4147.9131, 4153.9287, 4160.9331, 4174.0122, 4177.8853, 4253.9565, 4416.9512, 4452.1763, 4459.3091, 4466.1865, 4679.2988, 4844.0703

7. P13608 **Mass:** 248150 **Score:** 28 **Expect:** 38 **Matches:** 39

Aggrecan core protein OS=Bos taurus GN=ACAN PE=1 SV=3

Observed	Mr(expt)	Mr(calc)	ppm	Start	End	Miss	Peptide
831.4715	830.4642	830.4650	-0.97	545	551	0	R.YPIVSPR.T
883.4774	882.4701	882.4712	-1.17	211	217	0	R.YPIHTPR.E
891.4855	890.4782	890.4763	2.16	152	158	0	K.GIVFHYR.A
949.5491	948.5419	948.5392	2.75	306	313	1	R.SVRYPISK.A
967.4212	966.4139	966.4130	0.93	2161	2167	0	K.FQGHCYR.H
974.4442	973.4369	973.4341	2.91	678	684	0	R.HHAFCFR.G
977.5377	976.5304	976.5341	-3.79	2300	2307	0	R.YEINALVR.Y
1087.5496	1086.5423	1086.5233	17.5	498	506	0	R.YSLTFEEAK.Q
1168.6099	1167.6026	1167.5924	8.74	585	594	0	R.LEGEVFFATR.L
1185.6841	1184.6768	1184.6765	0.29	83	93	0	K.EVVLLVATEGR.V
1248.6903	1247.6830	1247.6622	16.7	2298	2307	1	K.DRYEINALVR.Y
1325.6277	1324.6204	1324.6445	-18.20	2335	2345	1	R.ITCTDPATYKR.R
1368.6443	1367.6370	1367.6292	5.71	631	642	0	K.CYAGWLADGSLR.Y
1372.6029	1371.5956	1371.5877	5.75	264	274	0	K.FTFQEAANECR.R
1382.5973	1381.5900	1381.5833	4.85	2324	2334	0	R.CQPSGHWEEPR.I
1383.7275	1382.7202	1382.7201	0.09	314	326	0	K.ARPNCGGNLLGVR.T
1442.8217	1441.8144	1441.8140	0.28	81	93	1	K.EKEVVLLVATEGR.V
1457.7417	1456.7344	1456.7310	2.33	159	170	1	R.AISTRYTLDFDR.A
1528.7021	1527.6948	1527.6888	3.92	264	275	1	K.FTFQEAANECRR.L
1578.7964	1577.7891	1577.7620	17.2	2277	2291	0	K.GTVACGEPPVVEHAR.I
1628.7031	1627.6958	1627.6937	1.32	218	231	1	R.EGCYGDKDEFPGVR.T
1628.8617	1627.8544	1627.8583	-2.39	483	497	0	R.LPGGVVFHYRPGSSR.Y
1706.8654	1705.8581	1705.8570	0.66	2276	2291	1	K.KGTVACGEPPVVEHAR.I
1715.8093	1714.8020	1714.8348	-19.13	498	511	1	R.YSLTFEEAKQACLR.T
1771.0311	1770.0238	1770.0251	-0.70	78	93	2	R.ISKEKEVVLLVATEGR.V
2144.0537	2143.0464	2143.0289	8.16	133	151	0	R.CEVMHGIEDSQATLEVVVK.G
2340.1006	2339.0933	2339.0529	17.3	566	584	0	R.TYGVRPPSETYDVYCYVDR.L

2343.1824	2342.1751	2342.1940	-8.06	104 -	124	0	K.VTLPNYPAIPSDATLEIQNMR.S
2872.3164	2871.3091	2871.3142	-1.78	280 -	305	0	R.LATTGQLYLAWQGGMDMCSAGWLADR.S
2888.3215	2887.3142	2887.3092	1.75	280 -	305	0	R.LATTGQLYLAWQGGMDMCSAGWLADR.S + Oxidation (M)
3002.4827	3001.4754	3001.4485	8.97	125 -	151	1	R.SNDSGILRCEVMHGIEDSQATLEVVVK.G + Oxidation (M)
3248.6238	3247.6165	3247.6183	-0.54	96 -	124	1	R.VNSAYQDKVTLPNYPAIPSDATLEIQNMR.S
3264.5508	3263.5435	3263.6132	-21.35	96 -	124	1	R.VNSAYQDKVTLPNYPAIPSDATLEIQNMR.S + Oxidation (M)
3457.6028	3456.5955	3456.6489	-15.45	275 -	305	2	R.RLGARLATTGQLYLAWQGGMDMCSAGWLADR.S + 2 Oxidation (M)
3489.6655	3488.6582	3488.6347	6.74	566 -	594	1	R.TYGVRPPSETYDVYCYVDRLEGEVFFATR.L
3503.7874	3502.7801	3502.7878	-2.19	94 -	124	2	R.VRVNSAYQDKVTLPNYPAIPSDATLEIQNMR.S
3639.7131	3638.7058	3638.6947	3.05	512 -	544	0	R.TGAIIASPEQLQAAYEAGYEQCDAGWLQDQTVR.Y
4147.9131	4146.9058	4146.9164	-2.54	174 -	210	0	R.ACLQNSAIIATPEQLQAAYEDGFHQCDAGWLADQTVR.Y
4452.1763	4451.1690	4451.1492	4.46	512 -	551	1	R.TGAIIASPEQLQAAYEAGYEQCDAGWLQDQTVRYPIVSPR.T

No match to: 706.3109, 710.3683, 712.2512, 712.4277, 717.4089, 719.3850, 722.4312, 729.4136, 734.4397, 735.4166, 747.4490, 750.4445, 756.3809, 759.4347, 772.4293, 779.4761, 783.3987, 799.3317, 818.4884, 823.4870, 829.4492, 832.3093, 834.3143, 842.4991, 856.3599, 862.4179, 870.4754, 873.3850, 876.2836, 880.4593, 887.4115, 889.4967, 890.4962, 895.5654, 900.5347, 909.5356, 923.5190, 925.4849, 927.5414, 929.4692, 936.4928, 938.4988, 944.5178, 945.5171, 955.5369, 957.5576, 972.4985, 976.4541, 978.5416, 981.5450, 983.6256, 988.4442, 993.5175, 1007.5735, 1015.4863, 1021.5621, 1025.5615, 1031.5157, 1034.5802, 1044.5248, 1046.5699, 1051.5020, 1056.5302, 1057.5824, 1074.5834, 1099.6482, 1113.5366, 1118.5896, 1120.5878, 1122.5590, 1133.6019, 1145.6392, 1178.6198, 1180.6312, 1187.6882, 1194.5767, 1216.7070, 1221.6462, 1254.6893, 1255.6096, 1264.6997, 1269.6152, 1273.6505, 1281.6354, 1292.7681, 1301.6294, 1312.7516, 1314.7264, 1315.6790, 1317.6367, 1322.6362, 1336.7238, 1338.7152, 1349.6917, 1351.6910, 1352.6709, 1361.6908, 1365.5895, 1390.7019, 1397.7428, 1412.6068, 1414.6772, 1420.7469, 1454.7571, 1467.7429, 1485.7374, 1486.7495, 1488.7402, 1494.7421, 1497.8790, 1500.7224, 1513.7649, 1517.8065, 1518.8060, 1520.7325, 1527.7305, 1531.6841, 1537.8251, 1539.7679, 1555.7635, 1556.8414, 1562.7877, 1567.7642, 1570.7750, 1571.8055, 1580.8280, 1581.8616, 1583.7979, 1585.7704, 1592.7926, 1610.6801, 1616.7987, 1624.7253, 1642.7180, 1647.8546, 1649.8544, 1650.8427, 1657.9053, 1663.8506, 1664.8582, 1666.8584, 1680.8226, 1683.8226, 1696.8158, 1698.7761, 1701.9166, 1707.8828, 1710.8179, 1711.7795, 1714.7788, 1717.9019, 1726.8021, 1728.7821, 1730.8467, 1739.6987, 1747.9039, 1753.7146, 1755.7096, 1758.9032, 1774.9202, 1783.6785, 1787.8689, 1797.6923, 1809.0245, 1811.7025, 1816.9102, 1836.8322, 1838.8318, 1850.8512, 1862.9421, 1866.8452, 1928.0237, 1937.9972, 1953.8524, 1961.0308, 1965.8595, 1970.0011, 1982.0062, 1984.0415, 1995.8663, 2007.9470, 2027.1273, 2061.0056, 2067.0483, 2086.1091, 2099.1079, 2109.0042, 2136.8455, 2150.8601, 2173.1655, 2179.0540, 2239.1436, 2251.1506, 2255.1462, 2265.0896, 2270.0999, 2274.0774, 2285.1733, 2286.1472, 2289.0583, 2293.1331, 2301.2546, 2303.0488, 2324.0913, 2344.1963, 2352.9204, 2356.0154, 2359.1130, 2375.0210, 2389.0332, 2414.2415, 2467.2080, 2525.2712, 2541.2361, 2542.2598, 2646.9749, 2660.9883, 2675.0051, 2679.0034, 2689.0183, 2692.0198, 2693.0125, 2695.0129, 2705.2800, 2717.3184, 2763.3374, 2771.4116, 2787.2793, 2792.2427, 2795.3037, 2798.2268, 2803.2610, 2809.4873, 2813.3884, 2814.2390, 2819.2676, 2846.3894, 2876.3669, 2877.3923, 2884.3479, 2887.3311, 2891.4258, 2901.3557, 2919.4480, 3029.4448, 3036.5105, 3053.4414, 3067.4578, 3071.4341, 3081.6121, 3126.7246, 3151.3789, 3169.6074, 3184.4443, 3192.2090, 3232.5308, 3237.5469, 3305.5381, 3316.7190, 3321.5339, 3330.5715, 3334.6743, 3339.6362, 3346.5017, 3350.5159, 3396.6165, 3412.6321, 3428.5723, 3435.6877, 3471.6187, 3476.5935, 3624.7163, 3645.7556, 3651.6982, 3692.0066, 3721.6226, 3990.8337, 4074.9656, 4093.8804, 4153.9287, 4160.9331, 4174.0122, 4177.8853, 4253.9565, 4416.9512, 4459.3091, 4466.1865, 4679.2988, 4844.0703

8. F1N367 **Mass:** 244177 **Score:** 27 **Expect:** 46 **Matches:** 40

Aggrecan core protein OS=Bos taurus GN=ACAN PE=4 SV=2

Observed	Mr(expt)	Mr(calc)	ppm	Start	End	Miss	Peptide
722.4312	721.4239	721.4235	0.59	2282 -	2287	0	R.HVPTIR.C
831.4715	830.4642	830.4650	-0.97	545 -	551	0	R.YPIVSPR.T
883.4774	882.4701	882.4712	-1.17	211 -	217	0	R.YPIHTPR.E
891.4855	890.4782	890.4763	2.16	152 -	158	0	K.GIVFHYR.A
949.5491	948.5419	948.5392	2.75	306 -	313	1	R.SVRYPISK.A
967.4212	966.4139	966.4130	0.93	2125 -	2131	0	K.FQGHCYR.H
974.4442	973.4369	973.4341	2.91	678 -	684	0	R.HHAFCFR.G
977.5377	976.5304	976.5341	-3.79	2264 -	2271	0	R.YEINALVR.Y
1087.5496	1086.5423	1086.5233	17.5	498 -	506	0	R.YSLTFEEAK.Q
1168.6099	1167.6026	1167.5924	8.74	585 -	594	0	R.LEGEVFFATR.L
1185.6841	1184.6768	1184.6765	0.29	83 -	93	0	K.EVVLLVATEGR.V
1248.6903	1247.6830	1247.6622	16.7	2262 -	2271	1	K.DRYEINALVR.Y
1325.6277	1324.6204	1324.6445	-18.20	2299 -	2309	1	R.ITCTDPATYKR.R
1368.6443	1367.6370	1367.6292	5.71	631 -	642	0	K.CYAGWLADGSLR.Y
1372.6029	1371.5956	1371.5877	5.75	264 -	274	0	K.FTFQEAANECR.R
1382.5973	1381.5900	1381.5833	4.85	2288 -	2298	0	R.CQPSGHWEEPR.I
1383.7275	1382.7202	1382.7201	0.09	314 -	326	0	K.ARPNCGGNLLGVR.T
1442.8217	1441.8144	1441.8140	0.28	81 -	93	1	K.EKEVVLLVATEGR.V
1457.7417	1456.7344	1456.7310	2.33	159 -	170	1	R.AISTRYTLDFDR.A
1528.7021	1527.6948	1527.6888	3.92	264 -	275	1	K.FTFQEAANECRR.L
1578.7964	1577.7891	1577.7620	17.2	2241 -	2255	0	K.GTVACGEPPVVEHAR.I
1628.7031	1627.6958	1627.6937	1.32	218 -	231	1	K.EGCYGDKDEFPGVR.T
1628.8617	1627.8544	1627.8583	-2.39	483 -	497	0	R.LPGGVVFHYRPGSSR.Y
1706.8654	1705.8581	1705.8570	0.66	2240 -	2255	1	K.KGTVACGEPPVVEHAR.I
1715.8093	1714.8020	1714.8348	-19.13	498 -	511	1	R.YSLTFEEAKQACLR.T
1771.0311	1770.0238	1770.0251	-0.70	78 -	93	2	R.ISKEKEVVLLVATEGR.V
2144.0537	2143.0464	2143.0289	8.16	133 -	151	0	R.CEVMHGIEDSQATLEVVVK.G
2340.1006	2339.0933	2339.0529	17.3	566 -	584	0	R.TYGVRPPSETYDVYCYVDR.L
2343.1824	2342.1751	2342.1940	-8.06	104 -	124	0	K.VTLPNYPAIPSDATLEIQNMR.S
2872.3164	2871.3091	2871.3142	-1.78	280 -	305	0	R.LATTGQLYLAWQGGMDMCSAGWLADR.S
2888.3215	2887.3142	2887.3092	1.75	280 -	305	0	R.LATTGQLYLAWQGGMDMCSAGWLADR.S + Oxidation (M)
3036.5105	3035.5032	3035.4328	23.2	125 -	151	1	R.SNDSGIYRCEVMHGIEDSQATLEVVVK.G
3248.6238	3247.6165	3247.6183	-0.54	96 -	124	1	R.VNSAYQDKVTLPNYPAIPSDATLEIQNMR.S
3264.5508	3263.5435	3263.6132	-21.35	96 -	124	1	R.VNSAYQDKVTLPNYPAIPSDATLEIQNMR.S + Oxidation (M)
3457.6028	3456.5955	3456.6489	-15.45	275 -	305	2	R.RLGARLATTGQLYLAWQGGMDMCSAGWLADR.S + 2 Oxidation (M)
3489.6655	3488.6582	3488.6347	6.74	566 -	594	1	R.TYGVRPPSETYDVYCYVDRLEGEVFFATR.L
3503.7874	3502.7801	3502.7878	-2.19	94 -	124	2	R.VRVNSAYQDKVTLPNYPAIPSDATLEIQNMR.S
3639.7131	3638.7058	3638.6947	3.05	512 -	544	0	R.TGAIIASPEQLQAAYEAGYEQCDAGWLQDQTVR.Y
4147.9131	4146.9058	4146.9164	-2.54	174 -	210	0	R.ACLQNSAIIATPEQLQAAYEDGFHQCDAGWLADQTVR.Y
4452.1763	4451.1690	4451.1492	4.46	512 -	551	1	R.TGAIIASPEQLQAAYEAGYEQCDAGWLQDQTVRYPIVSPR.T

No match to: 706.3109, 710.3683, 712.2512, 712.4277, 717.4089, 719.3850, 729.4136, 734.4397, 735.4166, 747.4490, 750.4445, 756.3809, 759.4347, 772.4293, 779.4761, 783.3987, 799.3317, 818.4884, 823.4870, 829.4492, 832.3093, 834.3143, 842.4991, 856.3599, 862.4179, 870.4754, 873.3850, 876.2836, 880.4593, 887.4115, 889.4967, 890.4962, 895.5654, 900.5347, 909.5356, 923.5190, 925.4849, 927.5414, 929.4692, 936.4928, 938.4988, 944.5178, 945.5171, 955.5369, 957.5576, 972.4985, 976.4541, 978.5416, 981.5450, 983.6256, 988.4442, 993.5175, 1007.5735, 1015.4863, 1021.5621, 1025.5615, 1031.5157, 1034.5802, 1044.5248, 1046.5699, 1051.5020, 1056.5302, 1057.5824, 1074.5834, 1099.6482, 1113.5366, 1118.5896, 1120.5878, 1122.5590, 1133.6019, 1145.6392, 1178.6198, 1180.6312, 1187.6882, 1194.5767, 1216.7070, 1221.6462, 1254.6893, 1255.6096, 1264.6997, 1269.6152, 1273.6505, 1281.6354, 1292.7681, 1301.6294, 1312.7516, 1314.7264, 1315.6790, 1317.6367, 1322.6362, 1336.7238, 1338.7152, 1349.6917, 1351.6910, 1352.6709, 1361.6908, 1365.5895, 1390.7019, 1397.7428, 1412.6068, 1414.6772, 1420.7469, 1454.7571, 1467.7429, 1485.7374, 1486.7495, 1488.7402, 1494.7421, 1497.8790, 1500.7224, 1513.7649, 1517.8065, 1518.8060, 1520.7325, 1527.7305, 1531.6841, 1537.8251, 1539.7679, 1555.7635, 1556.8414, 1562.7877, 1567.7642,

1570.7750, 1571.8055, 1580.8280, 1581.8616, 1583.7979, 1585.7704, 1592.7926, 1610.6801, 1616.7987, 1624.7253, 1642.7180, 1647.8546, 1649.8544, 1650.8427, 1657.9053, 1663.8506, 1664.8582, 1666.8584, 1680.8226, 1683.8226, 1696.8158, 1698.7761, 1701.9166, 1707.8828, 1710.8179, 1711.7795, 1714.7788, 1717.9019, 1726.8021, 1728.7821, 1730.8467, 1739.6987, 1747.9039, 1753.7146, 1755.7096, 1758.9032, 1774.9202, 1783.6785, 1787.8689, 1797.6923, 1809.0245, 1811.7025, 1816.9102, 1836.8322, 1838.8318, 1850.8512, 1862.9421, 1866.8452, 1928.0237, 1937.9972, 1953.8524, 1961.0308, 1965.8595, 1970.0011, 1982.0062, 1984.0415, 1995.8663, 2007.9470, 2027.1273, 2061.0056, 2067.0483, 2086.1091, 2099.1079, 2109.0042, 2136.8455, 2150.8601, 2173.1655, 2179.0540, 2239.1436, 2251.1506, 2255.1462, 2265.0896, 2270.0999, 2274.0774, 2285.1733, 2286.1472, 2289.0583, 2293.1331, 2301.2546, 2303.0488, 2324.0913, 2344.1963, 2352.9204, 2356.0154, 2359.1130, 2375.0210, 2389.0332, 2414.2415, 2467.2080, 2525.2712, 2541.2361, 2542.2598, 2646.9749, 2660.9883, 2675.0051, 2679.0034, 2689.0183, 2692.0198, 2693.0125, 2695.0129, 2705.2800, 2717.3184, 2763.3374, 2771.4116, 2787.2793, 2792.2427, 2795.3037, 2798.2268, 2803.2610, 2809.4873, 2813.3884, 2814.2390, 2819.2676, 2846.3894, 2876.3669, 2877.3923, 2884.3479, 2887.3311, 2891.4258, 2901.3557, 2919.4480, 3002.4827, 3029.4448, 3053.4414, 3067.4578, 3071.4341, 3081.6121, 3126.7246, 3151.3789, 3169.6074, 3184.4443, 3192.2090, 3232.5308, 3237.5469, 3305.5381, 3316.7190, 3321.5339, 3330.5715, 3334.6743, 3339.6362, 3346.5017, 3350.5159, 3396.6165, 3412.6321, 3428.5723, 3435.6877, 3471.6187, 3476.5935, 3624.7163, 3645.7556, 3651.6982, 3692.0066, 3721.6226, 3990.8337, 4074.9656, 4093.8804, 4153.9287, 4160.9331, 4174.0122, 4177.8853, 4253.9565, 4416.9512, 4459.3091, 4466.1865, 4679.2988, 4844.0703

9. F1N368 **Mass:** 248513 **Score:** 26 **Expect:** 55 **Matches:** 40

Aggrecan core protein OS=Bos taurus GN=ACAN PE=4 SV=2

Observed	Mr(expt)	Mr(calc)	ppm	Start	End	Miss	Peptide
722.4312	721.4239	721.4235	0.59	2320	2325	0	R.HVPTIR.C
831.4715	830.4642	830.4650	-0.97	545	551	0	R.YPIVSPR.T
883.4774	882.4701	882.4712	-1.17	211	217	0	R.YPIHTPR.E
891.4855	890.4782	890.4763	2.16	152	158	0	K.GIVFHYR.A
949.5491	948.5419	948.5392	2.75	306	313	1	R.SVRYPISK.A
967.4212	966.4139	966.4130	0.93	2163	2169	0	K.FQGHCYR.H
974.4442	973.4369	973.4341	2.91	678	684	0	R.HHAFCFR.G
977.5377	976.5304	976.5341	-3.79	2302	2309	0	R.YEINALVR.Y
1087.5496	1086.5423	1086.5233	17.5	498	506	0	R.YSLTFEEAK.Q
1168.6099	1167.6026	1167.5924	8.74	585	594	0	R.LEGEVFFATR.L
1185.6841	1184.6768	1184.6765	0.29	83	93	0	K.EVVLLVATEGR.V
1248.6903	1247.6830	1247.6622	16.7	2300	2309	1	K.DRYEINALVR.Y
1325.6277	1324.6204	1324.6445	-18.20	2337	2347	1	R.ITCTDPATYKR.R
1368.6443	1367.6370	1367.6292	5.71	631	642	0	K.CYAGWLADGSLR.Y
1372.6029	1371.5956	1371.5877	5.75	264	274	0	K.FTFQEAANECR.R
1382.5973	1381.5900	1381.5833	4.85	2326	2336	0	R.CQPSGHWEEPR.I
1383.7275	1382.7202	1382.7201	0.09	314	326	0	K.ARPNCGGNLLGVR.T
1442.8217	1441.8144	1441.8140	0.28	81	93	1	K.EKEVVLLVATEGR.V
1457.7417	1456.7344	1456.7310	2.33	159	170	1	R.AISTRYTLDFDR.A
1528.7021	1527.6948	1527.6888	3.92	264	275	1	K.FTFQEAANECRR.L
1578.7964	1577.7891	1577.7620	17.2	2279	2293	0	K.GTVACGEPPVVEHAR.I
1628.7031	1627.6958	1627.6937	1.32	218	231	1	R.EGCYGDKDEFPGVR.T
1628.8617	1627.8544	1627.8583	-2.39	483	497	0	R.LPGGVVFHYRPGSSR.Y
1706.8654	1705.8581	1705.8570	0.66	2278	2293	1	K.KGTVACGEPPVVEHAR.I
1715.8093	1714.8020	1714.8348	-19.13	498	511	1	R.YSLTFEEAKQACLR.T
1771.0311	1770.0238	1770.0251	-0.70	78	93	2	R.ISKEKEVVLLVATEGR.V
2144.0537	2143.0464	2143.0289	8.16	133	151	0	R.CEVMHGIEDSQATLEVVVK.G
2340.1006	2339.0933	2339.0529	17.3	566	584	0	R.TYGVRPPSETYDVYCYVDR.L
2343.1824	2342.1751	2342.1940	-8.06	104	124	0	K.VTLPNYPAIPSDATLEIQNMR.S
2872.3164	2871.3091	2871.3142	-1.78	280	305	0	R.LATTGQLYLAWQGGMDMCSAGWLADR.S
2888.3215	2887.3142	2887.3092	1.75	280	305	0	R.LATTGQLYLAWQGGMDMCSAGWLADR.S + Oxidation (M)
3036.5105	3035.5032	3035.4328	23.2	125	151	1	R.SNDSGIYRCEVMHGIEDSQATLEVVVK.G
3248.6238	3247.6165	3247.6183	-0.54	96	124	1	R.VNSAYQDKVTLPNYPAIPSDATLEIQNMR.S
3264.5508	3263.5435	3263.6132	-21.35	96	124	1	R.VNSAYQDKVTLPNYPAIPSDATLEIQNMR.S + Oxidation (M)
3457.6028	3456.5955	3456.6489	-15.45	275	305	2	R.RLGARLATTGQLYLAWQGGMDMCSAGWLADR.S + 2 Oxidation (M)
3489.6655	3488.6582	3488.6347	6.74	566	594	1	R.TYGVRPPSETYDVYCYVDRLEGEVFFATR.L
3503.7874	3502.7801	3502.7878	-2.19	94	124	2	R.VRVNSAYQDKVTLPNYPAIPSDATLEIQNMR.S
3639.7131	3638.7058	3638.6947	3.05	512	544	0	R.TGAIIASPEQLQAAYEAGYEQCDAGWLQDQTVR.Y
4147.9131	4146.9058	4146.9164	-2.54	174	210	0	R.ACLQNSAIIATPEQLQAAYEDGFHQCDAGWLADQTVR.Y
4452.1763	4451.1690	4451.1492	4.46	512	551	1	R.TGAIIASPEQLQAAYEAGYEQCDAGWLQDQTVRYPIVSPR.T

No match to: 706.3109, 710.3683, 712.2512, 712.4277, 717.4089, 719.3850, 729.4136, 734.4397, 735.4166, 747.4490, 750.4445, 756.3809, 759.4347, 772.4293, 779.4761, 783.3987, 799.3317, 818.4884, 823.4870, 829.4492, 832.3093, 834.3143, 842.4991, 856.3599, 862.4179, 870.4754, 873.3850, 876.2836, 880.4593, 887.4115, 889.4967, 890.4962, 895.5654, 900.5347, 909.5356, 923.5190, 925.4849, 927.5414, 929.4692, 936.4928, 938.4988, 944.5178, 945.5171, 955.5369, 957.5576, 972.4985, 976.4541, 978.5416, 981.5450, 983.6256, 988.4442, 993.5175, 1007.5735, 1015.4863, 1021.5621, 1025.5615, 1031.5157, 1034.5802, 1044.5248, 1046.5699, 1051.5020, 1056.5302, 1057.5824, 1074.5834, 1099.6482, 1113.5366, 1118.5896, 1120.5878, 1122.5590, 1133.6019, 1145.6392, 1178.6198, 1180.6312, 1187.6882, 1194.5767, 1216.7070, 1221.6462, 1254.6893, 1255.6096, 1264.6997, 1269.6152, 1273.6505, 1281.6354, 1292.7681, 1301.6294, 1312.7516, 1314.7264, 1315.6790, 1317.6367, 1322.6362, 1336.7238, 1338.7152, 1349.6917, 1351.6910, 1352.6709, 1361.6908, 1365.5895, 1390.7019, 1397.7428, 1412.6068, 1414.6772, 1420.7469, 1454.7571, 1467.7429, 1485.7374, 1486.7495, 1488.7402, 1494.7421, 1497.8790, 1500.7224, 1513.7649, 1517.8065, 1518.8060, 1520.7325, 1527.7305, 1531.6841, 1537.8251, 1539.7679, 1555.7635, 1556.8414, 1562.7877, 1567.7642, 1570.7750, 1571.8055, 1580.8280, 1581.8616, 1583.7979, 1585.7704, 1592.7926, 1610.6801, 1616.7987, 1624.7253, 1642.7180, 1647.8546, 1649.8544, 1650.8427, 1657.9053, 1663.8506, 1664.8582, 1666.8584, 1680.8226, 1683.8226, 1696.8158, 1698.7761, 1701.9166, 1707.8828, 1710.8179, 1711.7795, 1714.7788, 1717.9019, 1726.8021, 1728.7821, 1730.8467, 1739.6987, 1747.9039, 1753.7146, 1755.7096, 1758.9032, 1774.9202, 1783.6785, 1787.8689, 1797.6923, 1809.0245, 1811.7025, 1816.9102, 1836.8322, 1838.8318, 1850.8512, 1862.9421, 1866.8452, 1928.0237, 1937.9972, 1953.8524, 1961.0308, 1965.8595, 1970.0011, 1982.0062, 1984.0415, 1995.8663, 2007.9470, 2027.1273, 2061.0056, 2067.0483, 2086.1091, 2099.1079, 2109.0042, 2136.8455, 2150.8601, 2173.1655, 2179.0540, 2239.1436, 2251.1506, 2255.1462, 2265.0896, 2270.0999, 2274.0774, 2285.1733, 2286.1472, 2289.0583, 2293.1331, 2301.2546, 2303.0488, 2324.0913, 2344.1963, 2352.9204, 2356.0154, 2359.1130, 2375.0210, 2389.0332, 2414.2415, 2467.2080, 2525.2712, 2541.2361, 2542.2598, 2646.9749, 2660.9883, 2675.0051, 2679.0034, 2689.0183, 2692.0198, 2693.0125, 2695.0129, 2705.2800, 2717.3184, 2763.3374, 2771.4116, 2787.2793, 2792.2427, 2795.3037, 2798.2268, 2803.2610, 2809.4873, 2813.3884, 2814.2390, 2819.2676, 2846.3894, 2876.3669, 2877.3923, 2884.3479, 2887.3311, 2891.4258, 2901.3557, 2919.4480, 3002.4827, 3029.4448, 3053.4414, 3067.4578, 3071.4341, 3081.6121, 3126.7246, 3151.3789, 3169.6074, 3184.4443, 3192.2090, 3232.5308, 3237.5469, 3305.5381, 3316.7190, 3321.5339, 3330.5715, 3334.6743, 3339.6362, 3346.5017, 3350.5159, 3396.6165, 3412.6321, 3428.5723, 3435.6877, 3471.6187, 3476.5935, 3624.7163, 3645.7556, 3651.6982, 3692.0066, 3721.6226, 3990.8337, 4074.9656, 4093.8804, 4153.9287, 4160.9331, 4174.0122, 4177.8853, 4253.9565, 4416.9512, 4459.3091, 4466.1865, 4679.2988, 4844.0703

10. F1N024 **Mass:** 7899 **Score:** 26 **Expect:** 68 **Matches:** 5

Uncharacterized protein OS=Bos taurus GN=FAM19A4 PE=4 SV=1

Observed	Mr(expt)	Mr(calc)	ppm	Start	End	Miss	Peptide
1255.6096	1254.6023	1254.6139	-9.24	19	29	0	K.QGTCEVVAVHR.C
2251.1506	2250.1433	2250.1189	10.9	10	29	1	R.GHTGHHQIKQGTCEVVAVHR.C
2813.3884	2812.3811	2812.3181	22.4	10	33	2	R.GHTGHHQIKQGTCEVVAVHRCCNK.N
3248.6238	3247.6165	3247.5952	6.56	1	29	2	-.MSASSQHLRGHTGHHQIKQGTCEVVAVHR.C
3264.5508	3263.5435	3263.5901	-14.28	1	29	2	-.MSASSQHLRGHTGHHQIKQGTCEVVAVHR.C + Oxidation (M)

No match to: 706.3109, 710.3683, 712.2512, 712.4277, 717.4089, 719.3850, 722.4312, 729.4136, 734.4397, 735.4166, 747.4490, 750.4445, 756.3809, 759.4347, 772.4293, 779.4761, 783.3987, 799.3317, 818.4884, 823.4870, 829.4492, 831.4715, 832.3093, 834.3143, 842.4991, 856.3599, 862.4179, 870.4754, 873.3850, 876.2836, 880.4593, 883.4774, 887.4115, 889.4967, 890.4962, 891.4855, 895.5654, 900.5347, 909.5356, 923.5190, 925.4849, 927.5414, 929.4692, 936.4928, 938.4988, 944.5178, 945.5171, 949.5491, 955.5369, 957.5576, 967.4212, 972.4985, 974.4442, 976.4541, 977.5377, 978.5416, 981.5450, 983.6256, 988.4442, 993.5175, 1007.5735, 1015.4863, 1021.5621, 1025.5615, 1031.5157, 1034.5802, 1044.5248, 1046.5699, 1051.5020, 1056.5302, 1057.5824, 1074.5834, 1087.5496, 1099.6482, 1113.5366, 1118.5896, 1120.5878, 1122.5590, 1133.6019, 1145.6392, 1168.6099, 1178.6198, 1180.6312, 1185.6841, 1187.6882, 1194.5767, 1216.7070, 1221.6462, 1248.6903, 1254.6893, 1264.6997, 1269.6152, 1273.6505, 1281.6354, 1292.7681, 1301.6294, 1312.7516, 1314.7264, 1315.6790, 1317.6367, 1322.6362, 1325.6277, 1336.7238, 1338.7152, 1349.6917, 1351.6910, 1352.6709, 1361.6908, 1365.5895, 1368.6443, 1372.6029, 1382.5973, 1383.7275, 1390.7019, 1397.7428, 1412.6068, 1414.6772, 1420.7469, 1442.8217, 1454.7571, 1457.7417, 1467.7429, 1485.7374, 1486.7495, 1488.7402, 1494.7421, 1497.8790, 1500.7224, 1513.7649, 1517.8065, 1518.8060, 1520.7325, 1527.7305, 1528.7021, 1531.6841, 1537.8251, 1539.7679, 1555.7635, 1556.8414, 1562.7877, 1567.7642, 1570.7750, 1571.8055, 1578.7964, 1580.8280, 1581.8616, 1583.7979, 1585.7704, 1592.7926, 1610.6801, 1616.7987, 1624.7253, 1628.7031, 1628.8617, 1642.7180, 1647.8546, 1649.8544, 1650.8427, 1657.9053, 1663.8506, 1664.8582, 1666.8584, 1680.8226, 1683.8226, 1696.8158, 1698.7761, 1701.9166, 1706.8654, 1707.8828, 1710.8179, 1711.7795, 1714.7788, 1715.8093, 1717.9019, 1726.8021, 1728.7821, 1730.8467, 1739.6987, 1747.9039, 1753.7146, 1755.7096, 1758.9032, 1771.0311, 1774.9202, 1783.6785, 1787.8689, 1797.6923, 1809.0245, 1811.7025, 1816.9102, 1836.8322, 1838.8318, 1850.8512, 1862.9421, 1866.8452, 1928.0237, 1937.9972, 1953.8524, 1961.0308, 1965.8595, 1970.0011, 1982.0062, 1984.0415, 1995.8663, 2007.9470, 2027.1273, 2061.0056, 2067.0483, 2086.1091, 2099.1079, 2109.0042, 2136.8455, 2144.0537, 2150.8601, 2173.1655, 2179.0540, 2239.1436, 2255.1462, 2265.0896, 2270.0999, 2274.0774, 2285.1733, 2286.1472, 2289.0583, 2293.1331, 2301.2546, 2303.0488, 2324.0913, 2340.1006, 2343.1824, 2344.1963, 2352.9204, 2356.0154, 2359.1130, 2375.0210, 2389.0332, 2414.2415, 2467.2080, 2525.2712, 2541.2361, 2542.2598, 2646.9749, 2660.9883, 2675.0051, 2679.0034, 2689.0183, 2692.0198, 2693.0125, 2695.0129, 2705.2800, 2717.3184, 2763.3374, 2771.4116, 2787.2793, 2792.2427, 2795.3037, 2798.2268, 2803.2610, 2809.4873, 2814.2390, 2819.2676, 2846.3894, 2872.3164, 2876.3669, 2877.3923, 2884.3479, 2887.3311, 2888.3215, 2891.4258, 2901.3557, 2919.4480, 3002.4827, 3029.4448, 3036.5105, 3053.4414, 3067.4578, 3071.4341, 3081.6121, 3126.7246, 3151.3789, 3169.6074, 3184.4443, 3192.2090, 3232.5308, 3237.5469, 3305.5381, 3316.7190, 3321.5339, 3330.5715, 3334.6743, 3339.6362, 3346.5017, 3350.5159, 3396.6165, 3412.6321, 3428.5723, 3435.6877, 3457.6028, 3471.6187, 3476.5935, 3489.6655, 3503.7874, 3624.7163, 3639.7131, 3645.7556, 3651.6982, 3692.0066, 3721.6226, 3990.8337, 4074.9656, 4093.8804, 4147.9131, 4153.9287, 4160.9331, 4174.0122, 4177.8853, 4253.9565, 4416.9512, 4452.1763, 4459.3091, 4466.1865, 4679.2988, 4844.0703

11. Q95118 **Mass:** 43638 **Score:** 22 **Expect:** 1.6e+002 **Matches:** 7

Cytokine receptor common subunit gamma OS=Bos taurus GN=IL2RG PE=2 SV=1

Observed	Mr(expt)	Mr(calc)	ppm	Start	End	Miss	Peptide
1315.6790	1314.6717	1314.6568	11.3	213	224	0	R.HSFSLPSVDAQK.L
2251.1506	2250.1433	2250.1909	-21.16	213	231	2	R.HSFSLPSVDAQKLYTFRVR.S
2541.2361	2540.2288	2540.2044	9.61	203	224	1	R.SWTEQSVDHRHSFSLPSVDAQK.L
3002.4827	3001.4754	3001.4215	18.0	106	130	2	K.LQECGHYLFSEGITSGCWFGKKEIR.L
3321.5339	3320.5266	3320.4846	12.7	232	259	1	R.SRYNPLCGSAQHWSDWSYPIHWGSNTSK.E
4174.0122	4173.0049	4172.9183	20.8	342	379	1	R.LCHVSEIPPKGGEGPGGSPCSQHSPYWAPPCYTLKPEP.-
4459.3091	4458.3018	4458.2677	7.66	260	298	0	K.ENIENPENPSLFALEAVLIPLGSMGLIVSLICVYCWLER.T

No match to: 706.3109, 710.3683, 712.2512, 712.4277, 717.4089, 719.3850, 722.4312, 729.4136, 734.4397, 735.4166, 747.4490, 750.4445, 756.3809, 759.4347, 772.4293, 779.4761, 783.3987, 799.3317, 818.4884, 823.4870, 829.4492, 831.4715, 832.3093, 834.3143, 842.4991, 856.3599, 862.4179, 870.4754, 873.3850, 876.2836, 880.4593, 883.4774, 887.4115, 889.4967, 890.4962, 891.4855, 895.5654, 900.5347, 909.5356, 923.5190, 925.4849, 927.5414, 929.4692, 936.4928, 938.4988, 944.5178, 945.5171, 949.5491, 955.5369, 957.5576, 967.4212, 972.4985, 974.4442, 976.4541, 977.5377, 978.5416, 981.5450, 983.6256, 988.4442, 993.5175, 1007.5735, 1015.4863, 1021.5621, 1025.5615, 1031.5157, 1034.5802, 1044.5248, 1046.5699, 1051.5020, 1056.5302, 1057.5824, 1074.5834, 1087.5496, 1099.6482, 1113.5366, 1118.5896, 1120.5878, 1122.5590, 1133.6019, 1145.6392, 1168.6099, 1178.6198, 1180.6312, 1185.6841, 1187.6882, 1194.5767, 1216.7070, 1221.6462, 1248.6903, 1254.6893, 1255.6096, 1264.6997, 1269.6152, 1273.6505, 1281.6354, 1292.7681, 1301.6294, 1312.7516, 1314.7264, 1317.6367, 1322.6362, 1325.6277, 1336.7238, 1338.7152, 1349.6917, 1351.6910, 1352.6709, 1361.6908, 1365.5895, 1368.6443, 1372.6029, 1382.5973, 1383.7275, 1390.7019, 1397.7428, 1412.6068, 1414.6772, 1420.7469, 1442.8217, 1454.7571, 1457.7417, 1467.7429, 1485.7374, 1486.7495, 1488.7402, 1494.7421, 1497.8790, 1500.7224, 1513.7649, 1517.8065, 1518.8060, 1520.7325, 1527.7305, 1528.7021, 1531.6841, 1537.8251, 1539.7679, 1555.7635, 1556.8414, 1562.7877, 1567.7642, 1570.7750, 1571.8055, 1578.7964, 1580.8280, 1581.8616, 1583.7979, 1585.7704, 1592.7926, 1610.6801, 1616.7987, 1624.7253, 1628.7031, 1628.8617, 1642.7180, 1647.8546, 1649.8544, 1650.8427, 1657.9053, 1663.8506, 1664.8582, 1666.8584, 1680.8226, 1683.8226, 1696.8158, 1698.7761, 1701.9166, 1706.8654, 1707.8828, 1710.8179, 1711.7795, 1714.7788, 1715.8093, 1717.9019, 1726.8021, 1728.7821, 1730.8467, 1739.6987, 1747.9039, 1753.7146, 1755.7096, 1758.9032, 1771.0311, 1774.9202, 1783.6785, 1787.8689, 1797.6923, 1809.0245, 1811.7025, 1816.9102, 1836.8322, 1838.8318, 1850.8512, 1862.9421, 1866.8452, 1928.0237, 1937.9972, 1953.8524, 1961.0308, 1965.8595, 1970.0011, 1982.0062, 1984.0415, 1995.8663, 2007.9470, 2027.1273, 2061.0056, 2067.0483, 2086.1091, 2099.1079, 2109.0042, 2136.8455, 2144.0537, 2150.8601, 2173.1655, 2179.0540, 2239.1436, 2255.1462, 2265.0896, 2270.0999, 2274.0774, 2285.1733, 2286.1472, 2289.0583, 2293.1331, 2301.2546, 2303.0488, 2324.0913, 2340.1006, 2343.1824, 2344.1963, 2352.9204, 2356.0154, 2359.1130, 2375.0210, 2389.0332, 2414.2415, 2467.2080, 2525.2712, 2542.2598, 2646.9749, 2660.9883, 2675.0051, 2679.0034, 2689.0183, 2692.0198, 2693.0125, 2695.0129, 2705.2800, 2717.3184, 2763.3374, 2771.4116, 2787.2793, 2792.2427, 2795.3037, 2798.2268, 2803.2610, 2809.4873, 2813.3884, 2814.2390, 2819.2676, 2846.3894, 2872.3164, 2876.3669, 2877.3923, 2884.3479, 2887.3311, 2888.3215, 2891.4258, 2901.3557, 2919.4480, 3029.4448, 3036.5105, 3053.4414, 3067.4578, 3071.4341, 3081.6121, 3126.7246, 3151.3789, 3169.6074, 3184.4443, 3192.2090, 3232.5308, 3237.5469, 3248.6238, 3264.5508, 3305.5381, 3316.7190, 3330.5715, 3334.6743, 3339.6362, 3346.5017, 3350.5159, 3396.6165, 3412.6321, 3428.5723, 3435.6877, 3457.6028, 3471.6187, 3476.5935, 3489.6655, 3503.7874, 3624.7163, 3639.7131, 3645.7556, 3651.6982, 3692.0066, 3721.6226, 3990.8337, 4074.9656, 4093.8804, 4147.9131, 4153.9287, 4160.9331, 4177.8853, 4253.9565, 4416.9512, 4452.1763, 4466.1865, 4679.2988, 4844.0703

12. F1MX63 **Mass:** 44102 **Score:** 21 **Expect:** 2e+002 **Matches:** 12

Prolargin OS=Bos taurus GN=PRELP PE=4 SV=1

Observed	Mr(expt)	Mr(calc)	ppm	Start	End	Miss	Peptide
772.4293	771.4220	771.4239	-2.45	171	176	0	R.NLEQLR.L
895.5654	894.5581	894.5651	-7.81	95	102	1	R.KVPVIPSR.I
945.5171	944.5098	944.5039	6.24	177	184	0	R.LSQNQISR.I
1044.5248	1043.5175	1043.5148	2.60	129	136	0	R.WINLDNNR.I
1122.5590	1121.5517	1121.5465	4.65	272	280	0	R.LNYNQLSDR.G
1336.7238	1335.7165	1335.7146	1.42	159	170	0	K.NQLEEVPAALPR.N
1517.8065	1516.7992	1516.7998	-0.35	272	284	1	R.LNYNQLSDRGLPK.N
1537.8251	1536.8178	1536.8194	-1.05	221	233	0	K.NLMQLNLAHNTLR.K
1809.0245	1808.0172	1808.0157	0.82	144	158	1	R.VLEKLPSLVFLYMEK.N
2136.8455	2135.8382	2135.8387	-0.22	75	91	0	R.ECYCPPDFPSALYCDSR.N
2293.1331	2292.1258	2292.1469	-9.18	356	374	0	R.LDGNYLKPPIPLDLMMCFR.L
3126.7246	3125.7173	3125.7198	-0.80	144	170	2	R.VLEKLPSLVFLYMEKNQLEEVPAALPR.N

No match to: 706.3109, 710.3683, 712.2512, 712.4277, 717.4089, 719.3850, 722.4312, 729.4136, 734.4397, 735.4166, 747.4490, 750.4445, 756.3809, 759.4347, 779.4761, 783.3987, 799.3317, 818.4884, 823.4870, 829.4492, 831.4715, 832.3093, 834.3143, 842.4991, 856.3599, 862.4179, 870.4754, 873.3850, 876.2836, 880.4593, 883.4774, 887.4115, 889.4967, 890.4962, 891.4855, 900.5347, 909.5356, 923.5190, 925.4849, 927.5414, 929.4692, 936.4928, 938.4988, 944.5178, 949.5491, 955.5369, 957.5576, 967.4212, 972.4985, 974.4442, 976.4541, 977.5377, 978.5416, 981.5450, 983.6256, 988.4442, 993.5175, 1007.5735, 1015.4863, 1021.5621, 1025.5615, 1031.5157, 1034.5802, 1046.5699, 1051.5020, 1056.5302, 1057.5824, 1074.5834, 1087.5496, 1099.6482, 1113.5366, 1118.5896, 1120.5878, 1133.6019, 1145.6392, 1168.6099, 1178.6198, 1180.6312, 1185.6841, 1187.6882, 1194.5767, 1216.7070, 1221.6462, 1248.6903, 1254.6893, 1255.6096, 1264.6997, 1269.6152, 1273.6505, 1281.6354, 1292.7681, 1301.6294, 1312.7516, 1314.7264, 1315.6790, 1317.6367, 1322.6362, 1325.6277, 1338.7152, 1349.6917, 1351.6910, 1352.6709, 1361.6908, 1365.5895, 1368.6443, 1372.6029, 1382.5973, 1383.7275, 1390.7019, 1397.7428, 1412.6068, 1414.6772, 1420.7469, 1442.8217, 1454.7571, 1457.7417, 1467.7429, 1485.7374, 1486.7495, 1488.7402, 1494.7421, 1497.8790, 1500.7224, 1513.7649, 1518.8060, 1520.7325, 1527.7305, 1528.7021, 1531.6841, 1539.7679, 1555.7635, 1556.8414, 1562.7877, 1567.7642, 1570.7750, 1571.8055, 1578.7964, 1580.8280, 1581.8616, 1583.7979, 1585.7704, 1592.7926, 1610.6801, 1616.7987, 1624.7253, 1628.7031, 1628.8617, 1642.7180, 1647.8546, 1649.8544, 1650.8427, 1657.9053, 1663.8506, 1664.8582, 1666.8584, 1680.8226, 1683.8226, 1696.8158, 1698.7761, 1701.9166, 1706.8654, 1707.8828, 1710.8179, 1711.7795, 1714.7788, 1715.8093, 1717.9019, 1726.8021, 1728.7821, 1730.8467,

1739.6987, 1747.9039, 1753.7146, 1755.7096, 1758.9032, 1771.0311, 1774.9202, 1783.6785, 1787.8689, 1797.6923, 1811.7025, 1816.9102, 1836.8322, 1838.8318, 1850.8512, 1862.9421, 1866.8452, 1928.0237, 1937.9972, 1953.8524, 1961.0308, 1965.8595, 1970.0011, 1982.0062, 1984.0415, 1995.8663, 2007.9470, 2027.1273, 2061.0056, 2067.0483, 2086.1091, 2099.1079, 2109.0042, 2144.0537, 2150.8601, 2173.1655, 2179.0540, 2239.1436, 2251.1506, 2255.1462, 2265.0896, 2270.0999, 2274.0774, 2285.1733, 2286.1472, 2289.0583, 2301.2546, 2303.0488, 2324.0913, 2340.1006, 2343.1824, 2344.1963, 2352.9204, 2356.0154, 2359.1130, 2375.0210, 2389.0332, 2414.2415, 2467.2080, 2525.2712, 2541.2361, 2542.2598, 2646.9749, 2660.9883, 2675.0051, 2679.0034, 2689.0183, 2692.0198, 2693.0125, 2695.0129, 2705.2800, 2717.3184, 2763.3374, 2771.4116, 2787.2793, 2792.2427, 2795.3037, 2798.2268, 2803.2610, 2809.4873, 2813.3884, 2814.2390, 2819.2676, 2846.3894, 2872.3164, 2876.3669, 2877.3923, 2884.3479, 2887.3311, 2888.3215, 2891.4258, 2901.3557, 2919.4480, 3002.4827, 3029.4448, 3036.5105, 3053.4414, 3067.4578, 3071.4341, 3081.6121, 3151.3789, 3169.6074, 3184.4443, 3192.2090, 3232.5308, 3237.5469, 3248.6238, 3264.5508, 3305.5381, 3316.7190, 3321.5339, 3330.5715, 3334.6743, 3339.6362, 3346.5017, 3350.5159, 3396.6165, 3412.6321, 3428.5723, 3435.6877, 3457.6028, 3471.6187, 3476.5935, 3489.6655, 3503.7874, 3624.7163, 3639.7131, 3645.7556, 3651.6982, 3692.0066, 3721.6226, 3990.8337, 4074.9656, 4093.8804, 4147.9131, 4153.9287, 4160.9331, 4174.0122, 4177.8853, 4253.9565, 4416.9512, 4452.1763, 4459.3091, 4466.1865, 4679.2988, 4844.0703

13. Q3SWY9 **Mass:** 24956 **Score:** 21 **Expect:** 2.1e+002 **Matches:** 7

Ras-related protein Rab-28 OS=Bos taurus GN=RAB28 PE=2 SV=1

Observed	Mr(expt)	Mr(calc)	ppm	Start	End	Miss	Peptide
929.4692	928.4620	928.4436	19.7	131	137	0	K.IDLEHMR.T + Oxidation (M)
1301.6294	1300.6221	1300.6122	7.63	162	172	0	K.TGDSVFLCFQK.V
1315.6790	1314.6717	1314.6891	-13.24	182	192	1	K.LNKAEIEQSQR.V
1628.8617	1627.8544	1627.8722	-10.93	40	52	1	K.QYKQTIGLDFFLR.R
1862.9421	1861.9348	1861.9356	-0.42	193	208	1	R.VVKADIVNYNQEPMSR.T
1928.0237	1927.0164	1927.0262	-5.07	113	130	1	K.KVSEESETQPLVALVGNK.I
3126.7246	3125.7173	3125.6907	8.51	53	81	2	R.RITLPGSLNVTLQVWDIGGQTIGGKMLDK.Y + Oxidation (M)

No match to: 706.3109, 710.3683, 712.2512, 712.4277, 717.4089, 719.3850, 722.4312, 729.4136, 734.4397, 735.4166, 747.4490, 750.4445, 756.3809, 759.4347, 772.4293, 779.4761, 783.3987, 799.3317, 818.4884, 823.4870, 829.4492, 831.4715, 832.3093, 834.3143, 842.4991, 856.3599, 862.4179, 870.4754, 873.3850, 876.2836, 880.4593, 883.4774, 887.4115, 889.4967, 890.4962, 891.4855, 895.5654, 900.5347, 909.5356, 923.5190, 925.4849, 927.5414, 936.4928, 938.4988, 944.5178, 945.5171, 949.5491, 955.5369, 957.5576, 967.4212, 972.4985, 974.4442, 976.4541, 977.5377, 978.5416, 981.5450, 983.6256, 988.4442, 993.5175, 1007.5735, 1015.4863, 1021.5621, 1025.5615, 1031.5157, 1034.5802, 1044.5248, 1046.5699, 1051.5020, 1056.5302, 1057.5824, 1074.5834, 1087.5496, 1099.6482, 1113.5366, 1118.5896, 1120.5878, 1122.5590, 1133.6019, 1145.6392, 1168.6099, 1178.6198, 1180.6312, 1185.6841, 1187.6882, 1194.5767, 1216.7070, 1221.6462, 1248.6903, 1254.6893, 1255.6096, 1264.6997, 1269.6152, 1273.6505, 1281.6354, 1292.7681, 1312.7516, 1314.7264, 1317.6367, 1322.6362, 1325.6277, 1336.7238, 1338.7152, 1349.6917, 1351.6910, 1352.6709, 1361.6908, 1365.5895, 1368.6443, 1372.6029, 1382.5973, 1383.7275, 1390.7019, 1397.7428, 1412.6068, 1414.6772, 1420.7469, 1442.8217, 1454.7571, 1457.7417, 1467.7429, 1485.7374, 1486.7495, 1488.7402, 1494.7421, 1497.8790, 1500.7224, 1513.7649, 1517.8065, 1518.8060, 1520.7325, 1527.7305, 1528.7021, 1531.6841, 1537.8251, 1539.7679, 1555.7635, 1556.8414, 1562.7877, 1567.7642, 1570.7750, 1571.8055, 1578.7964, 1580.8280, 1581.8616, 1583.7979, 1585.7704, 1592.7926, 1610.6801, 1616.7987, 1624.7253, 1628.7031, 1642.7180, 1647.8546, 1649.8544, 1650.8427, 1657.9053, 1663.8506, 1664.8582, 1666.8584, 1680.8226, 1683.8226, 1696.8158, 1698.7761, 1701.9166, 1706.8654, 1707.8828, 1710.8179, 1711.7795, 1714.7788, 1715.8093, 1717.9019, 1726.8021, 1728.7821, 1730.8467, 1739.6987, 1747.9039, 1753.7146, 1755.7096, 1758.9032, 1771.0311, 1774.9202, 1783.6785, 1787.8689, 1797.6923, 1809.0245, 1811.7025, 1816.9102, 1836.8322, 1838.8318, 1850.8512, 1866.8452, 1937.9972, 1953.8524, 1961.0308, 1965.8595, 1970.0011, 1982.0062, 1984.0415, 1995.8663, 2007.9470, 2027.1273, 2061.0056, 2067.0483, 2086.1091, 2099.1079, 2109.0042, 2136.8455, 2144.0537, 2150.8601, 2173.1655, 2179.0540, 2239.1436, 2251.1506, 2255.1462, 2265.0896, 2270.0999, 2274.0774, 2285.1733, 2286.1472, 2289.0583, 2293.1331, 2301.2546, 2303.0488, 2324.0913, 2340.1006, 2343.1824, 2344.1963, 2352.9204, 2356.0154, 2359.1130, 2375.0210, 2389.0332, 2414.2415, 2467.2080, 2525.2712, 2541.2361, 2542.2598, 2646.9749, 2660.9883, 2675.0051, 2679.0034, 2689.0183, 2692.0198, 2693.0125, 2695.0129, 2705.2800, 2717.3184, 2763.3374, 2771.4116, 2787.2793, 2792.2427, 2795.3037, 2798.2268, 2803.2610, 2809.4873, 2813.3884, 2814.2390, 2819.2676, 2846.3894, 2872.3164, 2876.3669, 2877.3923, 2884.3479, 2887.3311, 2888.3215, 2891.4258, 2901.3557, 2919.4480, 3002.4827, 3029.4448, 3036.5105, 3053.4414, 3067.4578, 3071.4341, 3081.6121, 3151.3789, 3169.6074, 3184.4443, 3192.2090, 3232.5308, 3237.5469, 3248.6238, 3264.5508, 3305.5381, 3316.7190, 3321.5339, 3330.5715, 3334.6743, 3339.6362, 3346.5017, 3350.5159, 3396.6165, 3412.6321, 3428.5723, 3435.6877, 3457.6028, 3471.6187, 3476.5935, 3489.6655, 3503.7874, 3624.7163, 3639.7131, 3645.7556, 3651.6982, 3692.0066, 3721.6226, 3990.8337, 4074.9656, 4093.8804, 4147.9131, 4153.9287, 4160.9331, 4174.0122, 4177.8853, 4253.9565, 4416.9512, 4452.1763, 4459.3091, 4466.1865, 4679.2988, 4844.0703

14. Q1JPF1 **Mass:** 21780 **Score:** 20 **Expect:** 2.3e+002 **Matches:** 9

NFKB inhibitor interacting Ras-like 2 OS=Bos taurus GN=NKIRAS2 PE=2 SV=1

Observed	Mr(expt)	Mr(calc)	ppm	Start	End	Miss	Peptide
719.3850	718.3777	718.3861	-11.64	103	108	1	K.EIDKSK.D
1074.5834	1073.5761	1073.5506	23.8	146	154	0	K.LWEVSVADR.R
1248.6903	1247.6830	1247.6874	-3.48	92	101	1	R.ESFQRVELLK.K
1442.8217	1441.8144	1441.8504	-24.96	109	121	2	K.DKKEVTIVVLGNK.C
1539.7679	1538.7606	1538.7954	-22.58	55	66	2	R.EQVRFYDTRGLR.D
1663.8506	1662.8433	1662.8546	-6.76	2	17	2	M.GKSCKVVVCGQASVGK.T
2814.2390	2813.2317	2813.2174	5.09	74	96	1	R.HCFSCTDGYVLVYSTDSRESFQR.V
3396.6165	3395.6092	3395.5915	5.23	74	101	2	R.HCFSCTDGYVLVYSTDSRESFQRVELLK.K
4679.2988	4678.2915	4678.3185	-5.76	18	58	2	K.TSILEQLLYGNHVVGSEMIETQEDIYVGSIETDRGVREQVR.F + Oxidation (M)

No match to: 706.3109, 710.3683, 712.2512, 712.4277, 717.4089, 722.4312, 729.4136, 734.4397, 735.4166, 747.4490, 750.4445, 756.3809, 759.4347, 772.4293, 779.4761, 783.3987, 799.3317, 818.4884, 823.4870, 829.4492, 831.4715, 832.3093, 834.3143, 842.4991, 856.3599, 862.4179, 870.4754, 873.3850, 876.2836, 880.4593, 883.4774, 887.4115, 889.4967, 890.4962, 891.4855, 895.5654, 900.5347, 909.5356, 923.5190, 925.4849, 927.5414, 929.4692, 936.4928, 938.4988, 944.5178, 945.5171, 949.5491, 955.5369, 957.5576, 967.4212, 972.4985, 974.4442, 976.4541, 977.5377, 978.5416, 981.5450, 983.6256, 988.4442, 993.5175, 1007.5735, 1015.4863, 1021.5621, 1025.5615, 1031.5157, 1034.5802, 1044.5248, 1046.5699, 1051.5020, 1056.5302, 1057.5824, 1087.5496, 1099.6482, 1113.5366, 1118.5896, 1120.5878, 1122.5590, 1133.6019, 1145.6392, 1168.6099, 1178.6198, 1180.6312, 1185.6841, 1187.6882, 1194.5767, 1216.7070, 1221.6462, 1254.6893, 1255.6096, 1264.6997, 1269.6152, 1273.6505, 1281.6354, 1292.7681, 1301.6294, 1312.7516, 1314.7264, 1315.6790, 1317.6367, 1322.6362, 1325.6277, 1336.7238, 1338.7152, 1349.6917, 1351.6910, 1352.6709, 1361.6908, 1365.5895, 1368.6443, 1372.6029, 1382.5973, 1383.7275, 1390.7019, 1397.7428, 1412.6068, 1414.6772, 1420.7469, 1454.7571, 1457.7417, 1467.7429, 1485.7374, 1486.7495, 1488.7402, 1494.7421, 1497.8790, 1500.7224, 1513.7649, 1517.8065, 1518.8060, 1520.7325, 1527.7305, 1528.7021, 1531.6841, 1537.8251, 1555.7635, 1556.8414, 1562.7877, 1567.7642, 1570.7750, 1571.8055, 1578.7964, 1580.8280, 1581.8616, 1583.7979, 1585.7704, 1592.7926, 1610.6801, 1616.7987, 1624.7253, 1628.7031, 1628.8617, 1642.7180, 1647.8546, 1649.8544, 1650.8427, 1657.9053, 1664.8582, 1666.8584, 1680.8226, 1683.8226, 1696.8158, 1698.7761, 1701.9166, 1706.8654, 1707.8828, 1710.8179, 1711.7795, 1714.7788, 1715.8093, 1717.9019, 1726.8021, 1728.7821, 1730.8467, 1739.6987, 1747.9039, 1753.7146, 1755.7096, 1758.9032, 1771.0311, 1774.9202, 1783.6785, 1787.8689, 1797.6923, 1809.0245, 1811.7025, 1816.9102, 1836.8322, 1838.8318, 1850.8512, 1862.9421, 1866.8452, 1928.0237, 1937.9972, 1953.8524, 1961.0308, 1965.8595, 1970.0011, 1982.0062, 1984.0415, 1995.8663, 2007.9470, 2027.1273, 2061.0056, 2067.0483, 2086.1091, 2099.1079, 2109.0042, 2136.8455, 2144.0537, 2150.8601, 2173.1655, 2179.0540, 2239.1436, 2251.1506, 2255.1462, 2265.0896, 2270.0999, 2274.0774, 2285.1733, 2286.1472, 2289.0583, 2293.1331, 2301.2546, 2303.0488, 2324.0913, 2340.1006, 2343.1824, 2344.1963, 2352.9204, 2356.0154, 2359.1130, 2375.0210, 2389.0332, 2414.2415, 2467.2080, 2525.2712, 2541.2361, 2542.2598, 2646.9749, 2660.9883, 2675.0051, 2679.0034, 2689.0183, 2692.0198, 2693.0125, 2695.0129, 2705.2800, 2717.3184, 2763.3374, 2771.4116, 2787.2793, 2792.2427, 2795.3037, 2798.2268, 2803.2610, 2809.4873, 2813.3884, 2819.2676, 2846.3894, 2872.3164, 2876.3669, 2877.3923, 2884.3479, 2887.3311, 2888.3215, 2891.4258, 2901.3557, 2919.4480, 3002.4827, 3029.4448, 3036.5105, 3053.4414, 3067.4578, 3071.4341, 3081.6121, 3126.7246, 3151.3789, 3169.6074, 3184.4443, 3192.2090, 3232.5308, 3237.5469, 3248.6238, 3264.5508, 3305.5381, 3316.7190, 3321.5339, 3330.5715, 3334.6743, 3339.6362, 3346.5017, 3350.5159, 3412.6321, 3428.5723, 3435.6877, 3457.6028, 3471.6187, 3476.5935, 3489.6655, 3503.7874, 3624.7163, 3639.7131, 3645.7556, 3651.6982, 3692.0066, 3721.6226, 3990.8337, 4074.9656, 4093.8804, 4147.9131, 4153.9287, 4160.9331, 4174.0122, 4177.8853, 4253.9565, 4416.9512, 4452.1763, 4459.3091, 4466.1865, 4844.0703

15. Q32KZ1 **Mass:** 30885 **Score:** 20 **Expect:** 2.5e+002 **Matches:** 7

Translation initiation factor IF-3, mitochondrial OS=Bos taurus GN=MTIF3 PE=2 SV=1

Observed	Mr(expt)	Mr(calc)	ppm	Start	End	Miss	Peptide

883.4774	882.4701	882.4671	3.40	248 - 255	1	K.AAPDTPRR.D
1556.8414	1555.8341	1555.8616	-17.68	98 - 110	2	R.ANVIRLMAERDLR.L
1707.8828	1706.8755	1706.8700	3.26	66 - 80	2	K.NETAFSSVGRKINER.I
1710.8179	1709.8106	1709.8445	-19.81	248 - 264	2	K.AAPDTPRRDALNGGDGK.D
3071.4341	3070.4268	3070.4456	-6.13	201 - 226	1	K.MEEMCNRIVQTMSGIATFSSRPQPIR.G + 2 Oxidation (M)
3081.6121	3080.6048	3080.6142	-3.05	144 - 172	2	R.AAPKPGPTLTKELTFSSNIGQHDLDTKSK.Q
3169.6074	3168.6001	3168.6033	-0.99	81 - 107	2	R.IIHVLDEQGNDLGHMHRANVIRLMAER.D + 2 Oxidation (M)

No match to: 706.3109, 710.3683, 712.2512, 712.4277, 717.4089, 719.3850, 722.4312, 729.4136, 734.4397, 735.4166, 747.4490, 750.4445, 756.3809, 759.4347, 772.4293, 779.4761, 783.3987, 799.3317, 818.4884, 823.4870, 829.4492, 831.4715, 832.3093, 834.3143, 842.4991, 856.3599, 862.4179, 870.4754, 873.3850, 876.2836, 880.4593, 887.4115, 889.4967, 890.4962, 891.4855, 895.5654, 900.5347, 909.5356, 923.5190, 925.4849, 927.5414, 929.4692, 936.4928, 938.4988, 944.5178, 945.5171, 949.5491, 955.5369, 957.5576, 967.4212, 972.4985, 974.4442, 976.4541, 977.5377, 978.5416, 981.5450, 983.6256, 988.4442, 993.5175, 1007.5735, 1015.4863, 1021.5621, 1025.5615, 1031.5157, 1034.5802, 1044.5248, 1046.5699, 1051.5020, 1056.5302, 1057.5824, 1074.5834, 1087.5496, 1099.6482, 1113.5366, 1118.5896, 1120.5878, 1122.5590, 1133.6019, 1145.6392, 1168.6099, 1178.6198, 1180.6312, 1185.6841, 1187.6882, 1194.5767, 1216.7070, 1221.6462, 1248.6903, 1254.6893, 1255.6096, 1264.6997, 1269.6152, 1273.6505, 1281.6354, 1292.7681, 1301.6294, 1312.7516, 1314.7264, 1315.6790, 1317.6367, 1322.6362, 1325.6277, 1336.7238, 1338.7152, 1349.6917, 1351.6910, 1352.6709, 1361.6908, 1365.5895, 1368.6443, 1372.6029, 1382.5973, 1383.7275, 1390.7019, 1397.7428, 1412.6068, 1414.6772, 1420.7469, 1442.8217, 1454.7571, 1457.7417, 1467.7429, 1485.7374, 1486.7495, 1488.7402, 1494.7421, 1497.8790, 1500.7224, 1513.7649, 1517.8065, 1518.8060, 1520.7325, 1527.7305, 1528.7021, 1531.6841, 1537.8251, 1539.7679, 1555.7635, 1562.7877, 1567.7642, 1570.7750, 1571.8055, 1578.7964, 1580.8280, 1581.8616, 1583.7979, 1585.7704, 1592.7926, 1610.6801, 1616.7987, 1624.7253, 1628.7031, 1628.8617, 1642.7180, 1647.8546, 1649.8544, 1650.8427, 1657.9053, 1663.8506, 1664.8582, 1666.8584, 1680.8226, 1683.8226, 1696.8158, 1698.7761, 1701.9166, 1706.8654, 1711.7795, 1714.7788, 1715.8093, 1717.9019, 1726.8021, 1728.7821, 1730.8467, 1739.6987, 1747.9039, 1753.7146, 1755.7096, 1758.9032, 1771.0311, 1774.9202, 1783.6785, 1787.8689, 1797.6923, 1809.0245, 1811.7025, 1816.9102, 1836.8322, 1838.8318, 1850.8512, 1862.9421, 1866.8452, 1928.0237, 1937.9972, 1953.8524, 1961.0308, 1965.8595, 1970.0011, 1982.0062, 1984.0415, 1995.8663, 2007.9470, 2027.1273, 2061.0056, 2067.0483, 2086.1091, 2099.1079, 2109.0042, 2136.8455, 2144.0537, 2150.8601, 2173.1655, 2179.0540, 2239.1436, 2251.1506, 2255.1462, 2265.0896, 2270.0999, 2274.0774, 2285.1733, 2286.1472, 2289.0583, 2293.1331, 2301.2546, 2303.0488, 2324.0913, 2340.1006, 2343.1824, 2344.1963, 2352.9204, 2356.0154, 2359.1130, 2375.0210, 2389.0332, 2414.2415, 2467.2080, 2525.2712, 2541.2361, 2542.2598, 2646.9749, 2660.9883, 2675.0051, 2679.0034, 2689.0183, 2692.0198, 2693.0125, 2695.0129, 2705.2800, 2717.3184, 2763.3374, 2771.4116, 2787.2793, 2792.2427, 2795.3037, 2798.2268, 2803.2610, 2809.4873, 2813.3884, 2814.2390, 2819.2676, 2846.3894, 2872.3164, 2876.3669, 2877.3923, 2884.3479, 2887.3311, 2888.3215, 2891.4258, 2901.3557, 2919.4480, 3002.4827, 3029.4448, 3036.5105, 3053.4414, 3067.4578, 3126.7246, 3151.3789, 3184.4443, 3192.2090, 3232.5308, 3237.5469, 3248.6238, 3264.5508, 3305.5381, 3316.7190, 3321.5339, 3330.5715, 3334.6743, 3339.6362, 3346.5017, 3350.5159, 3396.6165, 3412.6321, 3428.5723, 3435.6877, 3457.6028, 3471.6187, 3476.5935, 3489.6655, 3503.7874, 3624.7163, 3639.7131, 3645.7556, 3651.6982, 3692.0066, 3721.6226, 3990.8337, 4074.9656, 4093.8804, 4147.9131, 4153.9287, 4160.9331, 4174.0122, 4177.8853, 4253.9565, 4416.9512, 4452.1763, 4459.3091, 4466.1865, 4679.2988, 4844.0703

16. Q2YDP6 **Mass:** 24765 **Score:** 20 **Expect:** 2.7e+002 **Matches:** 7
Testis-expressed sequence 35 protein OS=Bos taurus GN=Tex35 PE=2 SV=1

Observed	Mr(expt)	Mr(calc)	ppm	Start - End	Miss	Peptide
1031.5157	1030.5084	1030.5229	-14.09	157 - 164	1	K.MVERQQPK.D + Oxidation (M)
1034.5802	1033.5729	1033.5477	24.4	67 - 74	1	K.MEEIKQIK.D + Oxidation (M)
1118.5896	1117.5823	1117.5914	-8.08	119 - 127	1	R.GLKMQQELR.L + Oxidation (M)
1269.6152	1268.6079	1268.6183	-8.19	143 - 155	0	K.MDGAGGAPLSLHK.K + Oxidation (M)
1397.7428	1396.7355	1396.7133	15.9	143 - 156	1	K.MDGAGGAPLSLHKK.M + Oxidation (M)
2067.0483	2066.0410	2066.0758	-16.82	19 - 35	1	R.AVCLELKPEPIKTYDYK.G
2303.0488	2302.0415	2302.0531	-5.02	84 - 101	2	K.LQEFVEIMKEMQKDMDEK.M + 2 Oxidation (M)

No match to: 706.3109, 710.3683, 712.2512, 712.4277, 717.4089, 719.3850, 722.4312, 729.4136, 734.4397, 735.4166, 747.4490, 750.4445, 756.3809, 759.4347, 772.4293, 779.4761, 783.3987, 799.3317, 818.4884, 823.4870, 829.4492, 831.4715, 832.3093, 834.3143, 842.4991, 856.3599, 862.4179, 870.4754, 873.3850, 876.2836, 880.4593, 883.4774, 887.4115, 889.4967, 890.4962, 891.4855, 895.5654, 900.5347, 909.5356, 923.5190, 925.4849, 927.5414, 929.4692, 936.4928, 938.4988, 944.5178, 945.5171, 949.5491, 955.5369, 957.5576, 967.4212, 972.4985, 974.4442, 976.4541, 977.5377, 978.5416, 981.5450, 983.6256, 988.4442, 993.5175, 1007.5735, 1015.4863, 1021.5621, 1025.5615, 1044.5248, 1046.5699, 1051.5020, 1056.5302, 1057.5824, 1074.5834, 1087.5496, 1099.6482, 1113.5366, 1120.5878, 1122.5590, 1133.6019, 1145.6392, 1168.6099, 1178.6198, 1180.6312, 1185.6841, 1187.6882, 1194.5767, 1216.7070, 1221.6462, 1248.6903, 1254.6893, 1255.6096, 1264.6997, 1273.6505, 1281.6354, 1292.7681, 1301.6294, 1312.7516, 1314.7264, 1315.6790, 1317.6367, 1322.6362, 1325.6277, 1336.7238, 1338.7152, 1349.6917, 1351.6910, 1352.6709, 1361.6908, 1365.5895, 1368.6443, 1372.6029, 1382.5973, 1383.7275, 1390.7019, 1412.6068, 1414.6772, 1420.7469, 1442.8217, 1454.7571, 1457.7417, 1467.7429, 1485.7374, 1486.7495, 1488.7402, 1494.7421, 1497.8790, 1500.7224, 1513.7649, 1517.8065, 1518.8060, 1520.7325, 1527.7305, 1528.7021, 1531.6841, 1537.8251, 1539.7679, 1555.7635, 1556.8414, 1562.7877, 1567.7642, 1570.7750, 1571.8055, 1578.7964, 1580.8280, 1581.8616, 1583.7979, 1585.7704, 1592.7926, 1610.6801, 1616.7987, 1624.7253, 1628.7031, 1628.8617, 1642.7180, 1647.8546, 1649.8544, 1650.8427, 1657.9053, 1663.8506, 1664.8582, 1666.8584, 1680.8226, 1683.8226, 1696.8158, 1698.7761, 1701.9166, 1706.8654, 1707.8828, 1710.8179, 1711.7795, 1714.7788, 1715.8093, 1717.9019, 1726.8021, 1728.7821, 1730.8467, 1739.6987, 1747.9039, 1753.7146, 1755.7096, 1758.9032, 1771.0311, 1774.9202, 1783.6785, 1787.8689, 1797.6923, 1809.0245, 1811.7025, 1816.9102, 1836.8322, 1838.8318, 1850.8512, 1862.9421, 1866.8452, 1928.0237, 1937.9972, 1953.8524, 1961.0308, 1965.8595, 1970.0011, 1982.0062, 1984.0415, 1995.8663, 2007.9470, 2027.1273, 2061.0056, 2086.1091, 2099.1079, 2109.0042, 2136.8455, 2144.0537, 2150.8601, 2173.1655, 2179.0540, 2239.1436, 2251.1506, 2255.1462, 2265.0896, 2270.0999, 2274.0774, 2285.1733, 2286.1472, 2289.0583, 2293.1331, 2301.2546, 2324.0913, 2340.1006, 2343.1824, 2344.1963, 2352.9204, 2356.0154, 2359.1130, 2375.0210, 2389.0332, 2414.2415, 2467.2080, 2525.2712, 2541.2361, 2542.2598, 2646.9749, 2660.9883, 2675.0051, 2679.0034, 2689.0183, 2692.0198, 2693.0125, 2695.0129, 2705.2800, 2717.3184, 2763.3374, 2771.4116, 2787.2793, 2792.2427, 2795.3037, 2798.2268, 2803.2610, 2809.4873, 2813.3884, 2814.2390, 2819.2676, 2846.3894, 2872.3164, 2876.3669, 2877.3923, 2884.3479, 2887.3311, 2888.3215, 2891.4258, 2901.3557, 2919.4480, 3002.4827, 3029.4448, 3036.5105, 3053.4414, 3067.4578, 3071.4341, 3081.6121, 3126.7246, 3151.3789, 3169.6074, 3184.4443, 3192.2090, 3232.5308, 3237.5469, 3248.6238, 3264.5508, 3305.5381, 3316.7190, 3321.5339, 3330.5715, 3334.6743, 3339.6362, 3346.5017, 3350.5159, 3396.6165, 3412.6321, 3428.5723, 3435.6877, 3457.6028, 3471.6187, 3476.5935, 3489.6655, 3503.7874, 3624.7163, 3639.7131, 3645.7556, 3651.6982, 3692.0066, 3721.6226, 3990.8337, 4074.9656, 4093.8804, 4147.9131, 4153.9287, 4160.9331, 4174.0122, 4177.8853, 4253.9565, 4416.9512, 4452.1763, 4459.3091, 4466.1865, 4679.2988, 4844.0703

17. F1N1Z4 **Mass:** 19257 **Score:** 19 **Expect:** 2.9e+002 **Matches:** 7
Uncharacterized protein OS=Bos taurus GN=VWA3B PE=4 SV=1

Observed	Mr(expt)	Mr(calc)	ppm	Start - End	Miss	Peptide
856.3599	855.3526	855.3401	14.7	1 - 7	0	-.MAMQGMR.S + 2 Oxidation (M)
944.5178	943.5105	943.4909	20.8	71 - 78	0	K.LDANKPMR.Y
1216.7070	1215.6997	1215.6757	19.7	69 - 78	2	K.KKLDANKPMR.Y + Oxidation (M)
1663.8506	1662.8433	1662.8577	-8.62	128 - 141	2	K.KENYVGKALEEQQK.I
1683.8226	1682.8153	1682.8008	8.66	114 - 127	0	K.MYVQQAMELQEAVK.K + Oxidation (M)
1698.7761	1697.7688	1697.7654	2.03	153 - 165	1	K.NWITQRPEGYCKM.- + Oxidation (M)
2414.2415	2413.2342	2413.2926	-24.20	93 - 113	1	R.LNWPISLKELSMLENEILAGK.M + Oxidation (M)

No match to: 706.3109, 710.3683, 712.2512, 712.4277, 717.4089, 719.3850, 722.4312, 729.4136, 734.4397, 735.4166, 747.4490, 750.4445, 756.3809, 759.4347, 772.4293, 779.4761, 783.3987, 799.3317, 818.4884, 823.4870, 829.4492, 831.4715, 832.3093, 834.3143, 842.4991, 862.4179, 870.4754, 873.3850, 876.2836, 880.4593, 883.4774, 887.4115, 889.4967, 890.4962, 891.4855, 895.5654, 900.5347, 909.5356, 923.5190, 925.4849, 927.5414, 929.4692, 936.4928, 938.4988, 945.5171, 949.5491, 955.5369, 957.5576, 967.4212, 972.4985, 974.4442, 976.4541, 977.5377, 978.5416, 981.5450, 983.6256, 988.4442, 993.5175, 1007.5735, 1015.4863, 1021.5621, 1025.5615, 1031.5157, 1034.5802, 1044.5248, 1046.5699, 1051.5020, 1056.5302, 1057.5824, 1074.5834, 1087.5496, 1099.6482, 1113.5366, 1118.5896, 1120.5878, 1122.5590, 1133.6019, 1145.6392, 1168.6099, 1178.6198, 1180.6312, 1185.6841, 1187.6882, 1194.5767, 1221.6462, 1248.6903, 1254.6893, 1255.6096, 1264.6997, 1269.6152, 1273.6505, 1281.6354, 1292.7681, 1301.6294, 1312.7516, 1314.7264, 1315.6790, 1317.6367, 1322.6362, 1325.6277, 1336.7238, 1338.7152, 1349.6917, 1351.6910, 1352.6709, 1361.6908, 1365.5895, 1368.6443, 1372.6029, 1382.5973, 1383.7275, 1390.7019, 1397.7428, 1412.6068, 1414.6772, 1420.7469, 1442.8217, 1454.7571, 1457.7417, 1467.7429, 1485.7374, 1486.7495, 1488.7402, 1494.7421, 1497.8790, 1500.7224, 1513.7649, 1517.8065, 1518.8060, 1520.7325, 1527.7305, 1528.7021, 1531.6841, 1537.8251, 1539.7679, 1555.7635, 1556.8414, 1562.7877, 1567.7642, 1570.7750, 1571.8055, 1578.7964, 1580.8280, 1581.8616, 1583.7979, 1585.7704,

1592.7926, 1610.6801, 1616.7987, 1624.7253, 1628.7031, 1628.8617, 1642.7180, 1647.8546, 1649.8544, 1650.8427, 1657.9053, 1664.8582,
1666.8584, 1680.8226, 1696.8158, 1701.9166, 1706.8654, 1707.8828, 1710.8179, 1711.7795, 1714.7788, 1715.8093, 1717.9019, 1726.8021,
1728.7821, 1730.8467, 1739.6987, 1747.9039, 1753.7146, 1755.7096, 1758.9032, 1771.0311, 1774.9202, 1783.6785, 1787.8689, 1797.6923,
1809.0245, 1811.7025, 1816.9102, 1836.8322, 1838.8318, 1850.8512, 1862.9421, 1866.8452, 1928.0237, 1937.9972, 1953.8524, 1961.0308,
1965.8595, 1970.0011, 1982.0062, 1984.0415, 1995.8663, 2007.9470, 2027.1273, 2061.0056, 2067.0483, 2086.1091, 2099.1079, 2109.0042,
2136.8455, 2144.0537, 2150.8601, 2173.1655, 2179.0540, 2239.1436, 2251.1506, 2255.1462, 2265.0896, 2270.0999, 2274.0774, 2285.1733,
2286.1472, 2289.0583, 2293.1331, 2301.2546, 2303.0488, 2324.0913, 2340.1006, 2343.1824, 2344.1963, 2352.9204, 2356.0154, 2359.1130,
2375.0210, 2389.0332, 2467.2080, 2525.2712, 2541.2361, 2542.2598, 2646.9749, 2660.9883, 2675.0051, 2679.0034, 2689.0183, 2692.0198,
2693.0125, 2695.0129, 2705.2800, 2717.3184, 2763.3374, 2771.4116, 2787.2793, 2792.2427, 2795.3037, 2798.2268, 2803.2610, 2809.4873,
2813.3884, 2814.2390, 2819.2676, 2846.3894, 2872.3164, 2876.3669, 2877.3923, 2884.3479, 2887.3311, 2888.3215, 2891.4258, 2901.3557,
2919.4480, 3002.4827, 3029.4448, 3036.5105, 3053.4414, 3067.4578, 3071.4341, 3081.6121, 3126.7246, 3151.3789, 3169.6074, 3184.4443,
3192.2090, 3232.5308, 3237.5469, 3248.6238, 3264.5508, 3305.5381, 3316.7190, 3321.5339, 3330.5715, 3334.6743, 3339.6362, 3346.5017,
3350.5159, 3396.6165, 3412.6321, 3428.5723, 3435.6877, 3457.6028, 3471.6187, 3476.5935, 3489.6655, 3503.7874, 3624.7163, 3639.7131,
3645.7556, 3651.6982, 3692.0066, 3721.6226, 3990.8337, 4074.9656, 4093.8804, 4147.9131, 4153.9287, 4160.9331, 4174.0122, 4177.8853,
4253.9565, 4416.9512, 4452.1763, 4459.3091, 4466.1865, 4679.2988, 4844.0703

18. F1MTZ7 **Mass:** 34297 **Score:** 19 **Expect:** 2.9e+002 **Matches:** 7

Glycine N-acyltransferase OS=Bos taurus GN=GLYAT PE=1 SV=2

Observed	Mr(expt)	Mr(calc)	ppm	Start	End	Miss	Peptide
1513.7649	1512.7576	1512.7857	-18.58	129 -	141	1	R.SKNILYMASETIK.E + Oxidation (M)
2144.0537	2143.0464	2143.0997	-24.85	257 -	274	2	K.RGFPVYSHVDPKNQIMQK.M
2763.3374	2762.3301	2762.3342	-1.49	21 -	44	1	K.SLPMSLKVYGTVMHMNHGNPFNLK.A + 3 Oxidation (M)
2891.4258	2890.4185	2890.4292	-3.69	20 -	44	2	R.KSLPMSLKVYGTVMHMNHGNPFNLK.A + 3 Oxidation (M)
3428.5723	3427.5650	3427.5206	13.0	269 -	296	1	K.NQIMQKMSQSLNHVPMPSDWNQWNCEPL.- + Oxidation (M)
3457.6028	3456.5955	3456.5611	9.96	200 -	229	0	R.CIQSFPNFCLLGPEGTPVSWSLMDQTGEMR.M
3489.6655	3488.6582	3488.7299	-20.56	162 -	192	2	K.AIDPEMFKLSSVDPSHAAVVNRFWLFGGNER.S

No match to: 706.3109, 710.3683, 712.2512, 712.4277, 717.4089, 719.3850, 722.4312, 729.4136, 734.4397, 735.4166, 747.4490,
750.4445, 756.3809, 759.4347, 772.4293, 779.4761, 783.3987, 799.3317, 818.4884, 823.4870, 829.4492, 831.4715, 832.3093, 834.3143,
842.4991, 856.3599, 862.4179, 870.4754, 873.3850, 876.2836, 880.4593, 883.4774, 887.4115, 889.4967, 890.4962, 891.4855, 895.5654,
900.5347, 909.5356, 923.5190, 925.4849, 927.5414, 929.4692, 936.4928, 938.4988, 944.5178, 945.5171, 949.5491, 955.5369, 957.5576,
967.4212, 972.4985, 974.4442, 976.4541, 977.5377, 978.5416, 981.5450, 983.6256, 988.4442, 993.5175, 1007.5735, 1015.4863,
1021.5621, 1025.5615, 1031.5157, 1034.5802, 1044.5248, 1046.5699, 1051.5020, 1056.5302, 1057.5824, 1074.5834, 1087.5496, 1099.6482,
1113.5366, 1118.5896, 1120.5878, 1122.5590, 1133.6019, 1145.6392, 1168.6099, 1178.6198, 1180.6312, 1185.6841, 1187.6882, 1194.5767,
1216.7070, 1221.6462, 1248.6903, 1254.6893, 1255.6096, 1264.6997, 1269.6152, 1273.6505, 1281.6354, 1292.7681, 1301.6294, 1312.7516,
1314.7264, 1315.6790, 1317.6367, 1322.6362, 1325.6277, 1336.7238, 1338.7152, 1349.6917, 1351.6910, 1352.6709, 1361.6908, 1365.5895,
1368.6443, 1372.6029, 1382.5973, 1383.7275, 1390.7019, 1397.7428, 1412.6068, 1414.6772, 1420.7469, 1442.8217, 1454.7571, 1457.7417,
1467.7429, 1485.7374, 1486.7495, 1488.7402, 1494.7421, 1497.8790, 1500.7224, 1517.8065, 1518.8060, 1520.7325, 1527.7305, 1528.7021,
1531.6841, 1537.8251, 1539.7679, 1555.7635, 1556.8414, 1562.7877, 1567.7642, 1570.7750, 1571.8055, 1578.7964, 1580.8280, 1581.8616,
1583.7979, 1585.7704, 1592.7926, 1610.6801, 1616.7987, 1624.7253, 1628.7031, 1628.8617, 1642.7180, 1647.8546, 1649.8544, 1650.8427,
1657.9053, 1663.8506, 1664.8582, 1666.8584, 1680.8226, 1683.8226, 1696.8158, 1698.7761, 1701.9166, 1706.8654, 1707.8828, 1710.8179,
1711.7795, 1714.7788, 1715.8093, 1717.9019, 1726.8021, 1728.7821, 1730.8467, 1739.6987, 1747.9039, 1753.7146, 1755.7096, 1758.9032,
1771.0311, 1774.9202, 1783.6785, 1787.8689, 1797.6923, 1809.0245, 1811.7025, 1816.9102, 1836.8322, 1838.8318, 1850.8512, 1862.9421,
1866.8452, 1928.0237, 1937.9972, 1953.8524, 1961.0308, 1965.8595, 1970.0011, 1982.0062, 1984.0415, 1995.8663, 2007.9470, 2027.1273,
2061.0056, 2067.0483, 2086.1091, 2099.1079, 2109.0042, 2136.8455, 2150.8601, 2173.1655, 2179.0540, 2239.1436, 2251.1506, 2255.1462,
2265.0896, 2270.0999, 2274.0774, 2285.1733, 2286.1472, 2289.0583, 2293.1331, 2301.2546, 2303.0488, 2324.0913, 2340.1006, 2343.1824,
2344.1963, 2352.9204, 2356.0154, 2359.1130, 2375.0210, 2389.0332, 2414.2415, 2467.2080, 2525.2712, 2541.2361, 2542.2598, 2646.9749,
2660.9883, 2675.0051, 2679.0034, 2689.0183, 2692.0198, 2693.0125, 2695.0129, 2705.2800, 2717.3184, 2771.4116, 2787.2793, 2792.2427,
2795.3037, 2798.2268, 2803.2610, 2809.4873, 2813.3884, 2814.2390, 2819.2676, 2846.3894, 2872.3164, 2876.3669, 2877.3923, 2884.3479,
2887.3311, 2888.3215, 2901.3557, 2919.4480, 3002.4827, 3029.4448, 3036.5105, 3053.4414, 3067.4578, 3071.4341, 3081.6121, 3126.7246,
3151.3789, 3169.6074, 3184.4443, 3192.2090, 3232.5308, 3237.5469, 3248.6238, 3264.5508, 3305.5381, 3316.7190, 3321.5339, 3330.5715,
3334.6743, 3339.6362, 3346.5017, 3350.5159, 3396.6165, 3412.6321, 3435.6877, 3471.6187, 3476.5935, 3503.7874, 3624.7163, 3639.7131,
3645.7556, 3651.6982, 3692.0066, 3721.6226, 3990.8337, 4074.9656, 4093.8804, 4147.9131, 4153.9287, 4160.9331, 4174.0122, 4177.8853,
4253.9565, 4416.9512, 4452.1763, 4459.3091, 4466.1865, 4679.2988, 4844.0703

19. E1BLR1 **Mass:** 58429 **Score:** 19 **Expect:** 3.1e+002 **Matches:** 13

Uncharacterized protein OS=Bos taurus GN=SPATA2 PE=4 SV=1

Observed	Mr(expt)	Mr(calc)	ppm	Start	End	Miss	Peptide
1051.5020	1050.4947	1050.4838	10.4	1 -	10	0	-.MGKPSSMDAK.Y
1118.5896	1117.5823	1117.5979	-13.91	367 -	377	1	R.STPAKESALSK.C
1194.5767	1193.5694	1193.5690	0.37	357 -	366	1	R.NDAHPGYHKR.S
1666.8584	1665.8511	1665.8322	11.4	180 -	193	2	K.DKGYTELDVVSERR.G
1862.9421	1861.9348	1861.9145	10.9	493 -	507	1	K.SELHKFMPNNQLNYK.S
2286.1472	2285.1399	2285.1047	15.4	82 -	100	0	R.CAFSVLETMGINLFLCPWK.K
2414.2415	2413.2342	2413.1996	14.3	82 -	101	1	R.CAFSVLETMGINLFLCPWKK.E
2541.2361	2540.2288	2540.1819	18.5	321 -	343	1	R.GPYLPGSDDVDLYTDAEPRAAYR.R
2846.3894	2845.3821	2845.4118	-10.41	82 -	104	2	R.CAFSVLETMGINLFLCPWKKEFR.S
2884.3479	2883.3406	2883.3095	10.8	367 -	392	2	R.STPAKESALSKCQNCGLSCSAALCQR.C
3081.6121	3080.6048	3080.5290	24.6	493 -	517	2	K.SELHKFMPNNQLNYKSTQFSHLVYR.-
3237.5469	3236.5396	3236.5329	2.07	166 -	192	2	K.VECEQMLEIHSQVKDKGYTELDVVSER.R + Oxidation (M)
3248.6238	3247.6165	3247.6872	-21.77	128 -	155	2	R.AILSYMGYMPELGTAYRLKELVETLQVK.M + 2 Oxidation (M)

No match to: 706.3109, 710.3683, 712.2512, 712.4277, 717.4089, 719.3850, 722.4312, 729.4136, 734.4397, 735.4166, 747.4490,
750.4445, 756.3809, 759.4347, 772.4293, 779.4761, 783.3987, 799.3317, 818.4884, 823.4870, 829.4492, 831.4715, 832.3093, 834.3143,
842.4991, 856.3599, 862.4179, 870.4754, 873.3850, 876.2836, 880.4593, 883.4774, 887.4115, 889.4967, 890.4962, 891.4855, 895.5654,
900.5347, 909.5356, 923.5190, 925.4849, 927.5414, 929.4692, 936.4928, 938.4988, 944.5178, 945.5171, 949.5491, 955.5369, 957.5576,
967.4212, 972.4985, 974.4442, 976.4541, 977.5377, 978.5416, 981.5450, 983.6256, 988.4442, 993.5175, 1007.5735, 1015.4863,
1021.5621, 1025.5615, 1031.5157, 1034.5802, 1044.5248, 1046.5699, 1056.5302, 1057.5824, 1074.5834, 1087.5496, 1099.6482, 1113.5366,
1120.5878, 1122.5590, 1133.6019, 1145.6392, 1168.6099, 1178.6198, 1180.6312, 1185.6841, 1187.6882, 1216.7070, 1221.6462, 1248.6903,
1254.6893, 1255.6096, 1264.6997, 1269.6152, 1273.6505, 1281.6354, 1292.7681, 1301.6294, 1312.7516, 1314.7264, 1315.6790, 1317.6367,
1322.6362, 1325.6277, 1336.7238, 1338.7152, 1349.6917, 1351.6910, 1352.6709, 1361.6908, 1365.5895, 1368.6443, 1372.6029, 1382.5973,
1383.7275, 1390.7019, 1397.7428, 1412.6068, 1414.6772, 1420.7469, 1442.8217, 1454.7571, 1457.7417, 1467.7429, 1485.7374, 1486.7495,
1488.7402, 1494.7421, 1497.8790, 1500.7224, 1513.7649, 1517.8065, 1518.8060, 1520.7325, 1527.7305, 1528.7021, 1531.6841, 1537.8251,
1539.7679, 1555.7635, 1556.8414, 1562.7877, 1567.7642, 1570.7750, 1571.8055, 1578.7964, 1580.8280, 1581.8616, 1583.7979, 1585.7704,
1592.7926, 1610.6801, 1616.7987, 1624.7253, 1628.7031, 1628.8617, 1642.7180, 1647.8546, 1649.8544, 1650.8427, 1657.9053, 1663.8506,
1664.8582, 1680.8226, 1683.8226, 1696.8158, 1698.7761, 1701.9166, 1706.8654, 1707.8828, 1710.8179, 1711.7795, 1714.7788, 1715.8093,
1717.9019, 1726.8021, 1728.7821, 1730.8467, 1739.6987, 1747.9039, 1753.7146, 1755.7096, 1758.9032, 1771.0311, 1774.9202, 1783.6785,
1787.8689, 1797.6923, 1809.0245, 1811.7025, 1816.9102, 1836.8322, 1838.8318, 1850.8512, 1866.8452, 1928.0237, 1937.9972, 1953.8524,
1961.0308, 1965.8595, 1970.0011, 1982.0062, 1984.0415, 1995.8663, 2007.9470, 2027.1273, 2061.0056, 2067.0483, 2086.1091, 2099.1079,
2109.0042, 2136.8455, 2144.0537, 2150.8601, 2173.1655, 2179.0540, 2239.1436, 2251.1506, 2255.1462, 2265.0896, 2270.0999, 2274.0774,
2285.1733, 2289.0583, 2293.1331, 2301.2546, 2303.0488, 2324.0913, 2340.1006, 2343.1824, 2344.1963, 2352.9204, 2356.0154, 2359.1130,
2375.0210, 2389.0332, 2467.2080, 2525.2712, 2542.2598, 2646.9749, 2660.9883, 2675.0051, 2679.0034, 2689.0183, 2692.0198, 2693.0125,
2695.0129, 2705.2800, 2717.3184, 2763.3374, 2771.4116, 2787.2793, 2792.2427, 2795.3037, 2798.2268, 2803.2610, 2809.4873, 2813.3884,
2814.2390, 2819.2676, 2872.3164, 2876.3669, 2877.3923, 2887.3311, 2888.3215, 2891.4258, 2901.3557, 2919.4480, 3002.4827, 3029.4448,
3036.5105, 3053.4414, 3067.4578, 3071.4341, 3126.7246, 3151.3789, 3169.6074, 3184.4443, 3192.2090, 3232.5308, 3264.5508, 3305.5381,
3316.7190, 3321.5339, 3330.5715, 3334.6743, 3339.6362, 3346.5017, 3350.5159, 3396.6165, 3412.6321, 3428.5723, 3435.6877, 3457.6028,
3471.6187, 3476.5935, 3489.6655, 3503.7874, 3624.7163, 3639.7131, 3645.7556, 3651.6982, 3692.0066, 3721.6226, 3990.8337, 4074.9656,

4093.8804, 4147.9131, 4153.9287, 4160.9331, 4174.0122, 4177.8853, 4253.9565, 4416.9512, 4452.1763, 4459.3091, 4466.1865, 4679.2988, 4844.0703

20. Q3ZC17 **Mass:** 10037 **Score:** 19 **Expect:** 3.1e+002 **Matches:** 5

Myelin-associated oligodendrocyte basic protein OS=Bos taurus GN=MOBP PE=2 SV=1

Observed	Mr(expt)	Mr(calc)	ppm	Start	End	Miss	Peptide
717.4089	716.4017	716.4181	-22.89	12 -	17	1	R.LSKNQK.F
759.4347	758.4274	758.4286	-1.61	38 -	43	1	R.EIVDRK.Y
983.6256	982.6183	982.6036	15.0	72 -	79	2	K.RKTKPTPR.K
1755.7096	1754.7023	1754.7175	-8.62	57 -	69	1	K.EEDWICCACQKTR.L
3053.4414	3052.4341	3052.4688	-11.35	12 -	36	2	R.LSKNQKFSEHFSIHCCPPFTFLNSK.R

No match to: 706.3109, 710.3683, 712.2512, 712.4277, 719.3850, 722.4312, 729.4136, 734.4397, 735.4166, 747.4490, 750.4445, 756.3809, 772.4293, 779.4761, 783.3987, 799.3317, 818.4884, 823.4870, 829.4492, 831.4715, 832.3093, 834.3143, 842.4991, 856.3599, 862.4179, 870.4754, 873.3850, 876.2836, 880.4593, 883.4774, 887.4115, 889.4967, 890.4962, 891.4855, 895.5654, 900.5347, 909.5356, 923.5190, 925.4849, 927.5414, 929.4692, 936.4928, 938.4988, 944.5178, 945.5171, 949.5491, 955.5369, 957.5576, 967.4212, 972.4985, 974.4442, 976.4541, 977.5377, 978.5416, 981.5450, 988.4442, 993.5175, 1007.5735, 1015.4863, 1021.5621, 1025.5615, 1031.5157, 1034.5802, 1044.5248, 1046.5699, 1051.5020, 1056.5302, 1057.5824, 1074.5834, 1087.5496, 1099.6482, 1113.5366, 1118.5896, 1120.5878, 1122.5590, 1133.6019, 1145.6392, 1168.6099, 1178.6198, 1180.6312, 1185.6841, 1187.6882, 1194.5767, 1216.7070, 1221.6462, 1248.6903, 1254.6893, 1255.6096, 1264.6997, 1269.6152, 1273.6505, 1281.6354, 1292.7681, 1301.6294, 1312.7516, 1314.7264, 1315.6790, 1317.6367, 1322.6362, 1325.6277, 1336.7238, 1338.7152, 1349.6917, 1351.6910, 1352.6709, 1361.6908, 1365.5895, 1368.6443, 1372.6029, 1382.5973, 1383.7275, 1390.7019, 1397.7428, 1412.6068, 1414.6772, 1420.7469, 1442.8217, 1454.7571, 1457.7417, 1467.7429, 1485.7374, 1486.7495, 1488.7402, 1494.7421, 1497.8790, 1500.7224, 1513.7649, 1517.8065, 1518.8060, 1520.7325, 1527.7305, 1528.7021, 1531.6841, 1537.8251, 1539.7679, 1555.7635, 1556.8414, 1562.7877, 1567.7642, 1570.7750, 1571.8055, 1578.7964, 1580.8280, 1581.8616, 1583.7979, 1585.7704, 1592.7926, 1610.6801, 1616.7987, 1624.7253, 1628.7031, 1628.8617, 1642.7180, 1647.8546, 1649.8544, 1650.8427, 1657.9053, 1663.8506, 1664.8582, 1666.8584, 1680.8226, 1683.8226, 1696.8158, 1698.7761, 1701.9166, 1706.8654, 1707.8828, 1710.8179, 1711.7795, 1714.7788, 1715.8093, 1717.9019, 1726.8021, 1728.7821, 1730.8467, 1739.6987, 1747.9039, 1753.7146, 1758.9032, 1771.0311, 1774.9202, 1783.6785, 1787.8689, 1797.6923, 1809.0245, 1811.7025, 1816.9102, 1836.8322, 1838.8318, 1850.8512, 1862.9421, 1866.8452, 1928.0237, 1937.9972, 1953.8524, 1961.0308, 1965.8595, 1970.0011, 1982.0062, 1984.0415, 1995.8663, 2007.9470, 2027.1273, 2061.0056, 2067.0483, 2086.1091, 2099.1079, 2109.0042, 2136.8455, 2144.0537, 2150.8601, 2173.1655, 2179.0540, 2239.1436, 2251.1506, 2255.1462, 2265.0896, 2270.0999, 2274.0774, 2285.1733, 2286.1472, 2289.0583, 2293.1331, 2301.2546, 2303.0488, 2324.0913, 2340.1006, 2343.1824, 2344.1963, 2352.9204, 2356.0154, 2359.1130, 2375.0210, 2389.0332, 2414.2415, 2467.2080, 2525.2712, 2541.2361, 2542.2598, 2646.9749, 2660.9883, 2675.0051, 2679.0034, 2689.0183, 2692.0198, 2693.0125, 2695.0129, 2705.2800, 2717.3184, 2763.3374, 2771.4116, 2787.2793, 2792.2427, 2795.3037, 2798.2268, 2803.2610, 2809.4873, 2813.3884, 2814.2390, 2819.2676, 2846.3894, 2872.3164, 2876.3669, 2877.3923, 2884.3479, 2887.3311, 2888.3215, 2891.4258, 2901.3557, 2919.4480, 3002.4827, 3029.4448, 3036.5105, 3067.4578, 3071.4341, 3081.6121, 3126.7246, 3151.3789, 3169.6074, 3184.4443, 3192.2090, 3232.5308, 3237.5469, 3248.6238, 3264.5508, 3305.5381, 3316.7190, 3321.5339, 3330.5715, 3334.6743, 3339.6362, 3346.5017, 3350.5159, 3396.6165, 3412.6321, 3428.5723, 3435.6877, 3457.6028, 3471.6187, 3476.5935, 3489.6655, 3503.7874, 3624.7163, 3639.7131, 3645.7556, 3651.6982, 3692.0066, 3721.6226, 3990.8337, 4074.9656, 4093.8804, 4147.9131, 4153.9287, 4160.9331, 4174.0122, 4177.8853, 4253.9565, 4416.9512, 4452.1763, 4459.3091, 4466.1865, 4679.2988, 4844.0703

21. A8E4Q5 **Mass:** 35464 **Score:** 19 **Expect:** 3.2e+002 **Matches:** 11

GAS2 protein OS=Bos taurus GN=GAS2 PE=2 SV=1

Observed	Mr(expt)	Mr(calc)	ppm	Start	End	Miss	Peptide
1145.6392	1144.6319	1144.6088	20.2	277 -	287	1	R.VDGKTSPVQSK.S
1178.6198	1177.6125	1177.5947	15.1	1 -	10	1	-.MCTALSPKVR.S + Oxidation (M)
1264.6997	1263.6924	1263.6692	18.4	242 -	251	1	R.MLHNKHVMVR.V
1361.6908	1360.6835	1360.6843	-0.56	144 -	154	0	R.EVCLCLLELGR.I
1442.8217	1441.8144	1441.8180	-2.51	159 -	171	1	R.YGVEPPGLIKLEK.E
1570.7750	1569.7677	1569.7834	-10.00	221 -	232	2	K.FCVERLSQGRYR.V
1715.8093	1714.8020	1714.7872	8.64	293 -	307	0	K.DMNPDNYLVVSASYK.A
2109.0042	2107.9969	2107.9489	22.8	209 -	225	2	R.ISEDPPCKCPSKFCVER.L
2542.2598	2541.2525	2541.2533	-0.32	122 -	143	1	R.DLGVDETCLFESEGLVLHKQPR.E
2891.4258	2890.4185	2890.4371	-6.42	252 -	276	2	R.VGGGWETFAGYLLKHDPCRMLQISR.V
3053.4414	3052.4341	3052.4647	-10.02	11 -	37	1	R.SGPGLSDMHQYSQWLASRHEANLLPMK.E

No match to: 706.3109, 710.3683, 712.2512, 712.4277, 717.4089, 719.3850, 722.4312, 729.4136, 734.4397, 735.4166, 747.4490, 750.4445, 756.3809, 759.4347, 772.4293, 779.4761, 783.3987, 799.3317, 818.4884, 823.4870, 829.4492, 831.4715, 832.3093, 834.3143, 842.4991, 856.3599, 862.4179, 870.4754, 873.3850, 876.2836, 880.4593, 883.4774, 887.4115, 889.4967, 890.4962, 891.4855, 895.5654, 900.5347, 909.5356, 923.5190, 925.4849, 927.5414, 929.4692, 936.4928, 938.4988, 944.5178, 945.5171, 949.5491, 955.5369, 957.5576, 967.4212, 972.4985, 974.4442, 976.4541, 977.5377, 978.5416, 981.5450, 983.6256, 988.4442, 993.5175, 1007.5735, 1015.4863, 1021.5621, 1025.5615, 1031.5157, 1034.5802, 1044.5248, 1046.5699, 1051.5020, 1056.5302, 1057.5824, 1074.5834, 1087.5496, 1099.6482, 1113.5366, 1118.5896, 1120.5878, 1122.5590, 1133.6019, 1168.6099, 1180.6312, 1185.6841, 1187.6882, 1194.5767, 1216.7070, 1221.6462, 1248.6903, 1254.6893, 1255.6096, 1269.6152, 1273.6505, 1281.6354, 1292.7681, 1301.6294, 1312.7516, 1314.7264, 1315.6790, 1317.6367, 1322.6362, 1325.6277, 1336.7238, 1338.7152, 1349.6917, 1351.6910, 1352.6709, 1365.5895, 1368.6443, 1372.6029, 1382.5973, 1383.7275, 1390.7019, 1397.7428, 1412.6068, 1414.6772, 1420.7469, 1454.7571, 1457.7417, 1467.7429, 1485.7374, 1486.7495, 1488.7402, 1494.7421, 1497.8790, 1500.7224, 1513.7649, 1517.8065, 1518.8060, 1520.7325, 1527.7305, 1528.7021, 1531.6841, 1537.8251, 1539.7679, 1555.7635, 1556.8414, 1562.7877, 1567.7642, 1571.8055, 1578.7964, 1580.8280, 1581.8616, 1583.7979, 1585.7704, 1592.7926, 1610.6801, 1616.7987, 1624.7253, 1628.7031, 1628.8617, 1642.7180, 1647.8546, 1649.8544, 1650.8427, 1657.9053, 1663.8506, 1664.8582, 1666.8584, 1680.8226, 1683.8226, 1696.8158, 1698.7761, 1701.9166, 1706.8654, 1707.8828, 1710.8179, 1711.7795, 1714.7788, 1717.9019, 1726.8021, 1728.7821, 1730.8467, 1739.6987, 1747.9039, 1753.7146, 1755.7096, 1758.9032, 1771.0311, 1774.9202, 1783.6785, 1787.8689, 1797.6923, 1809.0245, 1811.7025, 1816.9102, 1836.8322, 1838.8318, 1850.8512, 1862.9421, 1866.8452, 1928.0237, 1937.9972, 1953.8524, 1961.0308, 1965.8595, 1970.0011, 1982.0062, 1984.0415, 1995.8663, 2007.9470, 2027.1273, 2061.0056, 2067.0483, 2086.1091, 2099.1079, 2136.8455, 2144.0537, 2150.8601, 2173.1655, 2179.0540, 2239.1436, 2251.1506, 2255.1462, 2265.0896, 2270.0999, 2274.0774, 2285.1733, 2286.1472, 2289.0583, 2293.1331, 2301.2546, 2303.0488, 2324.0913, 2340.1006, 2343.1824, 2344.1963, 2352.9204, 2356.0154, 2359.1130, 2375.0210, 2389.0332, 2414.2415, 2467.2080, 2525.2712, 2541.2361, 2646.9749, 2660.9883, 2675.0051, 2679.0034, 2689.0183, 2692.0198, 2693.0125, 2695.0129, 2705.2800, 2717.3184, 2763.3374, 2771.4116, 2787.2793, 2792.2427, 2795.3037, 2798.2268, 2803.2610, 2809.4873, 2813.3884, 2814.2390, 2819.2676, 2846.3894, 2872.3164, 2876.3669, 2877.3923, 2884.3479, 2887.3311, 2888.3215, 2901.3557, 2919.4480, 3002.4827, 3029.4448, 3036.5105, 3067.4578, 3071.4341, 3081.6121, 3126.7246, 3151.3789, 3169.6074, 3184.4443, 3192.2090, 3232.5308, 3237.5469, 3248.6238, 3264.5508, 3305.5381, 3316.7190, 3321.5339, 3330.5715, 3334.6743, 3339.6362, 3346.5017, 3350.5159, 3396.6165, 3412.6321, 3428.5723, 3435.6877, 3457.6028, 3471.6187, 3476.5935, 3489.6655, 3503.7874, 3624.7163, 3639.7131, 3645.7556, 3651.6982, 3692.0066, 3721.6226, 3990.8337, 4074.9656, 4093.8804, 4147.9131, 4153.9287, 4160.9331, 4174.0122, 4177.8853, 4253.9565, 4416.9512, 4452.1763, 4459.3091, 4466.1865, 4679.2988, 4844.0703

22. Q2NKR1 **Mass:** 10554 **Score:** 19 **Expect:** 3.3e+002 **Matches:** 5

Mitochondrial import inner membrane translocase subunit Tim10 OS=Bos taurus GN=TIMM10 PE=3 SV=1

Observed	Mr(expt)	Mr(calc)	ppm	Start	End	Miss	Peptide
929.4692	928.4620	928.4726	-11.50	82 -	90	1	K.RAQQSSGPV.-
1592.7926	1591.7853	1591.7512	21.5	40 -	53	1	K.EAELSKGESVCLDR.C
1710.8179	1709.8106	1709.8215	-6.38	68 -	81	1	K.KLTELSMQDEELMK.R + Oxidation (M)
1726.8021	1725.7948	1725.8165	-12.53	68 -	81	1	K.KLTELSMQDEELMK.R + 2 Oxidation (M)
3184.4443	3183.4370	3183.4391	-0.66	6 -	32	2	R.AQQLAAELEVEMMADMYNRMTSACHRK.E

No match to: 706.3109, 710.3683, 712.2512, 712.4277, 717.4089, 719.3850, 722.4312, 729.4136, 734.4397, 735.4166, 747.4490, 750.4445, 756.3809, 759.4347, 772.4293, 779.4761, 783.3987, 799.3317, 818.4884, 823.4870, 829.4492, 831.4715, 832.3093, 834.3143, 842.4991, 856.3599, 862.4179, 870.4754, 873.3850, 876.2836, 880.4593, 883.4774, 887.4115, 889.4967, 890.4962, 891.4855, 895.5654, 900.5347, 909.5356, 923.5190, 925.4849, 927.5414, 936.4928, 938.4988, 944.5178, 945.5171, 949.5491, 955.5369, 957.5576, 967.4212, 972.4985, 974.4442, 976.4541, 977.5377, 978.5416, 981.5450, 983.6256, 988.4442, 993.5175, 1007.5735, 1015.4863, 1021.5621,

1025.5615, 1031.5157, 1034.5802, 1044.5248, 1046.5699, 1051.5020, 1056.5302, 1057.5824, 1074.5834, 1087.5496, 1099.6482, 1113.5366, 1118.5896, 1120.5878, 1122.5590, 1133.6019, 1145.6392, 1168.6099, 1178.6198, 1180.6312, 1185.6841, 1187.6882, 1194.5767, 1216.7070, 1221.6462, 1248.6903, 1254.6893, 1255.6096, 1264.6997, 1269.6152, 1273.6505, 1281.6354, 1292.7681, 1301.6294, 1312.7516, 1314.7264, 1315.6790, 1317.6367, 1322.6362, 1325.6277, 1336.7238, 1338.7152, 1349.6917, 1351.6910, 1352.6709, 1361.6908, 1365.5895, 1368.6443, 1372.6029, 1382.5973, 1383.7275, 1390.7019, 1397.7428, 1412.6068, 1414.6772, 1420.7469, 1442.8217, 1454.7571, 1457.7417, 1467.7429, 1485.7374, 1486.7495, 1488.7402, 1494.7421, 1497.8790, 1500.7224, 1513.7649, 1517.8065, 1518.8060, 1520.7325, 1527.7305, 1528.7021, 1531.6841, 1537.8251, 1539.7679, 1555.7635, 1556.8414, 1562.7877, 1567.7642, 1570.7750, 1571.8055, 1578.7964, 1580.8280, 1581.8616, 1583.7979, 1585.7704, 1610.6801, 1616.7987, 1624.7253, 1628.7031, 1628.8617, 1642.7180, 1647.8546, 1649.8544, 1650.8427, 1657.9053, 1663.8506, 1664.8582, 1666.8584, 1680.8226, 1683.8226, 1696.8158, 1698.7761, 1701.9166, 1706.8654, 1707.8828, 1711.7795, 1714.7788, 1715.8093, 1717.9019, 1728.7821, 1730.8467, 1739.6987, 1747.9039, 1753.7146, 1755.7096, 1758.9032, 1771.0311, 1774.9202, 1783.6785, 1787.8689, 1797.6923, 1809.0245, 1811.7025, 1816.9102, 1836.8322, 1838.8318, 1850.8512, 1862.9421, 1866.8452, 1928.0237, 1937.9972, 1953.8524, 1961.0308, 1965.8595, 1970.0011, 1982.0062, 1984.0415, 1995.8663, 2007.9470, 2027.1273, 2061.0056, 2067.0483, 2086.1091, 2099.1079, 2109.0042, 2136.8455, 2144.0537, 2150.8601, 2173.1655, 2179.0540, 2239.1436, 2251.1506, 2255.1462, 2265.0896, 2270.0999, 2274.0774, 2285.1733, 2286.1472, 2289.0583, 2293.1331, 2301.2546, 2303.0488, 2324.0913, 2340.1006, 2343.1824, 2344.1963, 2352.9204, 2356.0154, 2359.1130, 2375.0210, 2389.0332, 2414.2415, 2467.2080, 2525.2712, 2541.2361, 2542.2598, 2646.9749, 2660.9883, 2675.0051, 2679.0034, 2689.0183, 2692.0198, 2693.0125, 2695.0129, 2705.2800, 2717.3184, 2763.3374, 2771.4116, 2787.2793, 2792.2427, 2795.3037, 2798.2268, 2803.2610, 2809.4873, 2813.3884, 2814.2390, 2819.2676, 2846.3894, 2872.3164, 2876.3669, 2877.3923, 2884.3479, 2887.3311, 2888.3215, 2891.4258, 2901.3557, 2919.4480, 3002.4827, 3029.4448, 3036.5105, 3053.4414, 3067.4578, 3071.4341, 3081.6121, 3126.7246, 3151.3789, 3169.6074, 3192.2090, 3232.5308, 3237.5469, 3248.6238, 3264.5508, 3305.5381, 3316.7190, 3321.5339, 3330.5715, 3334.6743, 3339.6362, 3346.5017, 3350.5159, 3396.6165, 3412.6321, 3428.5723, 3435.6877, 3457.6028, 3471.6187, 3476.5935, 3489.6655, 3503.7874, 3624.7163, 3639.7131, 3645.7556, 3651.6982, 3692.0066, 3721.6226, 3990.8337, 4074.9656, 4093.8804, 4147.9131, 4153.9287, 4160.9331, 4174.0122, 4177.8853, 4253.9565, 4416.9512, 4452.1763, 4459.3091, 4466.1865, 4679.2988, 4844.0703

23. F1MY76 **Mass:** 78375 **Score:** 19 **Expect:** 3.3e+002 **Matches:** 16

cGMP-dependent protein kinase OS=Bos taurus GN=PRKG1 PE=3 SV=2

Observed	Mr(expt)	Mr(calc)	ppm	Start	End	Miss	Peptide
1178.6198	1177.6125	1177.6091	2.91	42 -	50	1	K.LQNELDKYR.S
1315.6790	1314.6717	1314.6972	-19.39	511 -	522	1	R.GYAKLVDFGFAK.K
1555.7635	1554.7562	1554.7864	-19.43	180 -	192	0	K.VFGELAILYNCTR.T
1664.8582	1663.8509	1663.8491	1.11	107 -	120	1	K.DLIKEAILDNDFMK.N
1680.8226	1679.8153	1679.8440	-17.07	107 -	120	1	K.DLIKEAILDNDFMK.N + Oxidation (M)
1747.9039	1746.8966	1746.9152	-10.61	6 -	19	1	R.DLQYALQEKIEELR.Q
1758.9032	1757.8959	1757.8627	18.9	210 -	223	1	R.QCFQTIMMRTGLIK.H + 2 Oxidation (M)
1816.9102	1815.9029	1815.9003	1.45	286 -	301	1	R.EDSPNEDPVFLRTLGK.G
1928.0237	1927.0164	1926.9898	13.8	20 -	35	1	R.QRDALIDELELELDQK.D
2239.1436	2238.1363	2238.1031	14.9	530 -	548	0	K.TWTFCGTPEYVAPEIILNK.G
2293.1331	2292.1258	2292.1784	-22.93	317 -	337	1	R.TANVIAAEAVTCLVIDRDSFK.H
2344.1963	2343.1890	2343.1706	7.86	338 -	359	2	K.HLIGGLDDVSNKAYEDAEAKAK.Y
2813.3884	2812.3811	2812.4074	-9.35	168 -	192	2	K.EGVKLCTMGPGKVFGELAILYNCTR.T
3334.6743	3333.6670	3333.7034	-10.90	358 -	388	2	K.AKYEAEAAFFANLKLSDFNIIDTLGVGGFGR.V
3503.7874	3502.7801	3502.7515	8.18	302 -	333	2	K.GDWFGEKALQGEDVRTANVIAAEAVTCLVIDR.D
4174.0122	4173.0049	4173.0360	-7.43	107 -	141	2	K.DLIKEAILDNDFMKNLELSQIQEIVDCMYPVEYGK.D

No match to: 706.3109, 710.3683, 712.2512, 712.4277, 717.4089, 719.3850, 722.4312, 729.4136, 734.4397, 735.4166, 747.4490, 750.4445, 756.3809, 759.4347, 772.4293, 779.4761, 783.3987, 799.3317, 818.4884, 823.4870, 829.4492, 831.4715, 832.3093, 834.3143, 842.4991, 856.3599, 862.4179, 870.4754, 873.3850, 876.2836, 880.4593, 883.4774, 887.4115, 889.4967, 890.4962, 891.4855, 895.5654, 900.5347, 909.5356, 923.5190, 925.4849, 927.5414, 929.4692, 936.4928, 938.4988, 944.5178, 945.5171, 949.5491, 955.5369, 957.5576, 967.4212, 972.4985, 974.4442, 976.4541, 977.5377, 978.5416, 981.5450, 983.6256, 988.4442, 993.5175, 1007.5735, 1015.4863, 1021.5621, 1025.5615, 1031.5157, 1034.5802, 1044.5248, 1046.5699, 1051.5020, 1056.5302, 1057.5824, 1074.5834, 1087.5496, 1099.6482, 1113.5366, 1118.5896, 1120.5878, 1122.5590, 1133.6019, 1145.6392, 1168.6099, 1180.6312, 1185.6841, 1187.6882, 1194.5767, 1216.7070, 1221.6462, 1248.6903, 1254.6893, 1255.6096, 1264.6997, 1269.6152, 1273.6505, 1281.6354, 1292.7681, 1301.6294, 1312.7516, 1314.7264, 1317.6367, 1322.6362, 1325.6277, 1336.7238, 1338.7152, 1349.6917, 1351.6910, 1352.6709, 1361.6908, 1365.5895, 1368.6443, 1372.6029, 1382.5973, 1383.7275, 1390.7019, 1397.7428, 1412.6068, 1414.6772, 1420.7469, 1442.8217, 1454.7571, 1457.7417, 1467.7429, 1485.7374, 1486.7495, 1488.7402, 1494.7421, 1497.8790, 1500.7224, 1513.7649, 1517.8065, 1518.8060, 1520.7325, 1527.7305, 1528.7021, 1531.6841, 1537.8251, 1539.7679, 1556.8414, 1562.7877, 1567.7642, 1570.7750, 1571.8055, 1578.7964, 1580.8280, 1581.8616, 1583.7979, 1585.7704, 1592.7926, 1610.6801, 1616.7987, 1624.7253, 1628.7031, 1628.8617, 1642.7180, 1647.8546, 1649.8544, 1650.8427, 1657.9053, 1663.8506, 1666.8584, 1683.8226, 1696.8158, 1698.7761, 1701.9166, 1706.8654, 1707.8828, 1710.8179, 1711.7795, 1714.7788, 1715.8093, 1717.9019, 1726.8021, 1728.7821, 1730.8467, 1739.6987, 1753.7146, 1755.7096, 1771.0311, 1774.9202, 1783.6785, 1787.8689, 1797.6923, 1809.0245, 1811.7025, 1836.8322, 1838.8318, 1850.8512, 1862.9421, 1866.8452, 1937.9972, 1953.8524, 1961.0308, 1965.8595, 1970.0011, 1982.0062, 1984.0415, 1995.8663, 2007.9470, 2027.1273, 2061.0056, 2067.0483, 2086.1091, 2099.1079, 2109.0042, 2136.8455, 2144.0537, 2150.8601, 2173.1655, 2179.0540, 2251.1506, 2255.1462, 2265.0896, 2270.0999, 2274.0774, 2285.1733, 2286.1472, 2289.0583, 2301.2546, 2303.0488, 2324.0913, 2340.1006, 2343.1824, 2352.9204, 2356.0154, 2359.1130, 2375.0210, 2389.0332, 2414.2415, 2467.2080, 2525.2712, 2541.2361, 2542.2598, 2646.9749, 2660.9883, 2675.0051, 2679.0034, 2689.0183, 2692.0198, 2693.0125, 2695.0129, 2705.2800, 2717.3184, 2763.3374, 2771.4116, 2787.2793, 2792.2427, 2795.3037, 2798.2268, 2803.2610, 2809.4873, 2814.2390, 2819.2676, 2846.3894, 2872.3164, 2876.3669, 2877.3923, 2884.3479, 2887.3311, 2888.3215, 2891.4258, 2901.3557, 2919.4480, 3002.4827, 3029.4448, 3036.5105, 3053.4414, 3067.4578, 3071.4341, 3081.6121, 3126.7246, 3151.3789, 3169.6074, 3184.4443, 3192.2090, 3232.5308, 3237.5469, 3248.6238, 3264.5508, 3305.5381, 3316.7190, 3321.5339, 3330.5715, 3339.6362, 3346.5017, 3350.5159, 3396.6165, 3412.6321, 3428.5723, 3435.6877, 3457.6028, 3471.6187, 3476.5935, 3489.6655, 3624.7163, 3639.7131, 3645.7556, 3651.6982, 3692.0066, 3721.6226, 3990.8337, 4074.9656, 4093.8804, 4147.9131, 4153.9287, 4160.9331, 4177.8853, 4253.9565, 4416.9512, 4452.1763, 4459.3091, 4466.1865, 4679.2988, 4844.0703

24. G3X7B5 **Mass:** 54428 **Score:** 18 **Expect:** 3.4e+002 **Matches:** 10

RAC-alpha serine/threonine-protein kinase OS=Bos taurus GN=AKT1 PE=4 SV=1

Observed	Mr(expt)	Mr(calc)	ppm	Start	End	Miss	Peptide
889.4967	888.4894	888.4739	17.5	1 -	8	0	-.MNDVAIVK.E
925.4849	924.4776	924.4930	-16.64	9 -	15	1	K.EGWLHKR.G
1145.6392	1144.6319	1144.6240	6.89	360 -	369	1	R.FPRTLSPEAK.S
1317.6367	1316.6294	1316.6547	-19.17	169 -	179	2	K.EKATGRYYAMK.I
1457.7417	1456.7344	1456.7270	5.09	379 -	392	2	K.DPKQRLGGGSEDAK.E
1961.0308	1960.0235	1959.9955	14.3	207 -	222	1	R.HPFLTALKYSFQTHDR.L
1982.0062	1980.9989	1980.9977	0.62	190 -	206	1	K.DEVAHTLTENRVLQNSR.H
3457.6028	3456.5955	3456.5409	15.8	112 -	142	2	K.RQEEETMDFRSGSPGENSGAEEMEVSLAKPK.H + 2 Oxidation (M)
3651.6982	3650.6909	3650.7344	-11.92	122 -	154	2	R.SGSPGENSGAEEMEVSLAKPKHRVTMNEFEYLK.L
4174.0122	4173.0049	4173.0477	-10.25	77 -	111	2	R.CLQWTTVIERTFHVETPEEREEWTTAIQTVADGLK.R

No match to: 706.3109, 710.3683, 712.2512, 712.4277, 717.4089, 719.3850, 722.4312, 729.4136, 734.4397, 735.4166, 747.4490, 750.4445, 756.3809, 759.4347, 772.4293, 779.4761, 783.3987, 799.3317, 818.4884, 823.4870, 829.4492, 831.4715, 832.3093, 834.3143, 842.4991, 856.3599, 862.4179, 870.4754, 873.3850, 876.2836, 880.4593, 883.4774, 887.4115, 890.4962, 891.4855, 895.5654, 900.5347, 909.5356, 923.5190, 927.5414, 929.4692, 936.4928, 938.4988, 944.5178, 945.5171, 949.5491, 955.5369, 957.5576, 967.4212, 972.4985, 974.4442, 976.4541, 977.5377, 978.5416, 981.5450, 983.6256, 988.4442, 993.5175, 1007.5735, 1015.4863, 1021.5621, 1025.5615, 1031.5157, 1034.5802, 1044.5248, 1046.5699, 1051.5020, 1056.5302, 1057.5824, 1074.5834, 1087.5496, 1099.6482, 1113.5366, 1118.5896, 1120.5878, 1122.5590, 1133.6019, 1168.6099, 1178.6198, 1180.6312, 1185.6841, 1187.6882, 1194.5767, 1216.7070, 1221.6462, 1248.6903, 1254.6893, 1255.6096, 1264.6997, 1269.6152, 1273.6505, 1281.6354, 1292.7681, 1301.6294, 1312.7516, 1314.7264, 1315.6790, 1322.6362, 1325.6277, 1336.7238, 1338.7152, 1349.6917, 1351.6910, 1352.6709, 1361.6908, 1365.5895, 1368.6443, 1372.6029, 1382.5973, 1383.7275, 1390.7019, 1397.7428, 1412.6068, 1414.6772, 1420.7469, 1442.8217, 1454.7571, 1467.7429, 1485.7374, 1486.7495, 1488.7402, 1494.7421, 1497.8790, 1500.7224, 1513.7649, 1517.8065, 1518.8060, 1520.7325, 1527.7305, 1528.7021, 1531.6841, 1537.8251, 1539.7679, 1555.7635, 1556.8414, 1562.7877, 1567.7642, 1570.7750, 1571.8055, 1578.7964, 1580.8280, 1581.8616, 1583.7979, 1585.7704, 1592.7926, 1610.6801,

1616.7987, 1624.7253, 1628.7031, 1628.8617, 1642.7180, 1647.8546, 1649.8544, 1650.8427, 1657.9053, 1663.8506, 1664.8582, 1666.8584, 1680.8226, 1683.8226, 1696.8158, 1698.7761, 1701.9166, 1706.8654, 1707.8828, 1710.8179, 1711.7795, 1714.7788, 1715.8093, 1717.9019, 1726.8021, 1728.7821, 1730.8467, 1739.6987, 1747.9039, 1753.7146, 1755.7096, 1758.9032, 1771.0311, 1774.9202, 1783.6785, 1787.8689, 1797.6923, 1809.0245, 1811.7025, 1816.9102, 1836.8322, 1838.8318, 1850.8512, 1862.9421, 1866.8452, 1928.0237, 1937.9972, 1953.8524, 1965.8595, 1970.0011, 1984.0415, 1995.8663, 2007.9470, 2027.1273, 2061.0056, 2067.0483, 2086.1091, 2099.1079, 2109.0042, 2136.8455, 2144.0537, 2150.8601, 2173.1655, 2179.0540, 2239.1436, 2251.1506, 2255.1462, 2265.0896, 2270.0999, 2274.0774, 2285.1733, 2286.1472, 2289.0583, 2293.1331, 2301.2546, 2303.0488, 2324.0913, 2340.1006, 2343.1824, 2344.1963, 2352.9204, 2356.0154, 2359.1130, 2375.0210, 2389.0332, 2414.2415, 2467.2080, 2525.2712, 2541.2361, 2542.2598, 2646.9749, 2660.9883, 2675.0051, 2679.0034, 2689.0183, 2692.0198, 2693.0125, 2695.0129, 2705.2800, 2717.3184, 2763.3374, 2771.4116, 2787.2793, 2792.2427, 2795.3037, 2798.2268, 2803.2610, 2809.4873, 2813.3884, 2814.2390, 2819.2676, 2846.3894, 2872.3164, 2876.3669, 2877.3923, 2884.3479, 2887.3311, 2888.3215, 2891.4258, 2901.3557, 2919.4480, 3002.4827, 3029.4448, 3036.5105, 3053.4414, 3067.4578, 3071.4341, 3081.6121, 3126.7246, 3151.3789, 3169.6074, 3184.4443, 3192.2090, 3232.5308, 3237.5469, 3248.6238, 3264.5508, 3305.5381, 3316.7190, 3321.5339, 3330.5715, 3334.6743, 3339.6362, 3346.5017, 3350.5159, 3396.6165, 3412.6321, 3428.5723, 3435.6877, 3471.6187, 3476.5935, 3489.6655, 3503.7874, 3624.7163, 3639.7131, 3645.7556, 3692.0066, 3721.6226, 3990.8337, 4074.9656, 4093.8804, 4147.9131, 4153.9287, 4160.9331, 4177.8853, 4253.9565, 4416.9512, 4452.1763, 4459.3091, 4466.1865, 4679.2988, 4844.0703

25. F1N148 **Mass:** 15972 **Score:** 18 **Expect:** 3.5e+002 **Matches:** 6
39S ribosomal protein L54, mitochondrial OS=Bos taurus GN=MRPL54 PE=4 SV=1

Observed	Mr(expt)	Mr(calc)	ppm	Start	End	Miss	Peptide
717.4089	716.4017	716.4181	-22.89	132 -	137	1	R.LSKNQK.F
1221.6462	1220.6389	1220.6414	-2.06	5 -	15	1	R.RLFGATGSWAR.W
1650.8427	1649.8354	1649.8572	-13.22	1 -	15	2	-.MAARRLFGATGSWAR.W
1666.8584	1665.8511	1665.8522	-0.62	1 -	15	2	-.MAARRLFGATGSWAR.W + Oxidation (M)
3002.4827	3001.4754	3001.4532	7.42	80 -	105	0	K.EGQDVVLKPDSEYPEWLFQMNVGPPK.K
3330.5715	3329.5642	3329.6272	-18.91	49 -	79	1	K.GAAVGETLKDPEVCTDPVQLTTHAMGVNIYK.E + Oxidation (M)

No match to: 706.3109, 710.3683, 712.2512, 712.4277, 719.3850, 722.4312, 729.4136, 734.4397, 735.4166, 747.4490, 750.4445, 756.3809, 759.4347, 772.4293, 779.4761, 783.3987, 799.3317, 818.4884, 823.4870, 829.4492, 831.4715, 832.3093, 834.3143, 842.4991, 856.3599, 862.4179, 870.4754, 873.3850, 876.2836, 880.4593, 883.4774, 887.4115, 889.4967, 890.4962, 891.4855, 895.5654, 900.5347, 909.5356, 923.5190, 925.4849, 927.5414, 929.4692, 936.4928, 938.4988, 944.5178, 945.5171, 949.5491, 955.5369, 957.5576, 967.4212, 972.4985, 974.4442, 976.4541, 977.5377, 978.5416, 981.5450, 983.6256, 988.4442, 993.5175, 1007.5735, 1015.4863, 1021.5621, 1025.5615, 1031.5157, 1034.5802, 1044.5248, 1046.5699, 1051.5020, 1056.5302, 1057.5824, 1074.5834, 1087.5496, 1099.6482, 1113.5366, 1118.5896, 1120.5878, 1122.5590, 1133.6019, 1145.6392, 1168.6099, 1178.6198, 1180.6312, 1185.6841, 1187.6882, 1194.5767, 1216.7070, 1248.6903, 1254.6893, 1255.6096, 1264.6997, 1269.6152, 1273.6505, 1281.6354, 1292.7681, 1301.6294, 1312.7516, 1314.7264, 1315.6790, 1317.6367, 1322.6362, 1325.6277, 1336.7238, 1338.7152, 1349.6917, 1351.6910, 1352.6709, 1361.6908, 1365.5895, 1368.6443, 1372.6029, 1382.5973, 1383.7275, 1390.7019, 1397.7428, 1412.6068, 1414.6772, 1420.7469, 1442.8217, 1454.7571, 1457.7417, 1467.7429, 1485.7374, 1486.7495, 1488.7402, 1494.7421, 1497.8790, 1500.7224, 1513.7649, 1517.8065, 1518.8060, 1520.7325, 1527.7305, 1528.7021, 1531.6841, 1537.8251, 1539.7679, 1555.7635, 1556.8414, 1562.7877, 1567.7642, 1570.7750, 1571.8055, 1578.7964, 1580.8280, 1581.8616, 1583.7979, 1585.7704, 1592.7926, 1610.6801, 1616.7987, 1624.7253, 1628.7031, 1628.8617, 1642.7180, 1647.8546, 1649.8544, 1657.9053, 1663.8506, 1664.8582, 1680.8226, 1683.8226, 1696.8158, 1698.7761, 1701.9166, 1706.8654, 1707.8828, 1710.8179, 1711.7795, 1714.7788, 1715.8093, 1717.9019, 1726.8021, 1728.7821, 1730.8467, 1739.6987, 1747.9039, 1753.7146, 1755.7096, 1758.9032, 1771.0311, 1774.9202, 1783.6785, 1787.8689, 1797.6923, 1809.0245, 1811.7025, 1816.9102, 1836.8322, 1838.8318, 1850.8512, 1862.9421, 1866.8452, 1928.0237, 1937.9972, 1953.8524, 1961.0308, 1965.8595, 1970.0011, 1982.0062, 1984.0415, 1995.8663, 2007.9470, 2027.1273, 2061.0056, 2067.0483, 2086.1091, 2099.1079, 2109.0042, 2136.8455, 2144.0537, 2150.8601, 2173.1655, 2179.0540, 2239.1436, 2251.1506, 2255.1462, 2265.0896, 2270.0999, 2274.0774, 2285.1733, 2286.1472, 2289.0583, 2293.1331, 2301.2546, 2303.0488, 2324.0913, 2340.1006, 2343.1824, 2344.1963, 2352.9204, 2356.0154, 2359.1130, 2375.0210, 2389.0332, 2414.2415, 2467.2080, 2525.2712, 2541.2361, 2542.2598, 2646.9749, 2660.9883, 2675.0051, 2679.0034, 2689.0183, 2692.0198, 2693.0125, 2695.0129, 2705.2800, 2717.3184, 2763.3374, 2771.4116, 2787.2793, 2792.2427, 2795.3037, 2798.2268, 2803.2610, 2809.4873, 2813.3884, 2814.2390, 2819.2676, 2846.3894, 2872.3164, 2876.3669, 2877.3923, 2884.3479, 2887.3311, 2888.3215, 2891.4258, 2901.3557, 2919.4480, 3029.4448, 3036.5105, 3053.4414, 3067.4578, 3071.4341, 3081.6121, 3126.7246, 3151.3789, 3169.6074, 3184.4443, 3192.2090, 3232.5308, 3237.5469, 3248.6238, 3264.5508, 3305.5381, 3316.7190, 3321.5339, 3334.6743, 3339.6362, 3346.5017, 3350.5159, 3396.6165, 3412.6321, 3428.5723, 3435.6877, 3457.6028, 3471.6187, 3476.5935, 3489.6655, 3503.7874, 3624.7163, 3639.7131, 3645.7556, 3651.6982, 3692.0066, 3721.6226, 3990.8337, 4074.9656, 4093.8804, 4147.9131, 4153.9287, 4160.9331, 4174.0122, 4177.8853, 4253.9565, 4416.9512, 4452.1763, 4459.3091, 4466.1865, 4679.2988, 4844.0703

26. A6QLD1 **Mass:** 111322 **Score:** 18 **Expect:** 3.6e+002 **Matches:** 21
EXOC4 protein OS=Bos taurus GN=EXOC4 PE=2 SV=1

Observed	Mr(expt)	Mr(calc)	ppm	Start	End	Miss	Peptide
759.4347	758.4274	758.4174	13.2	304 -	309	0	R.LEQELK.Q
818.4884	817.4811	817.4657	18.8	965 -	971	2	K.QATKDKK.I
889.4967	888.4894	888.5029	-15.11	862 -	869	1	R.RISESGIK.K
945.5171	944.5098	944.5178	-8.50	258 -	265	0	K.EISLQDIK.E
1044.5248	1043.5175	1043.5361	-17.83	527 -	534	0	R.EFLTMYIK.N
1074.5834	1073.5761	1073.5717	4.15	643 -	651	1	K.DDDISRLLK.S
1122.5590	1121.5517	1121.5465	4.66	45 -	53	1	K.GRLEEAYER.C
1185.6841	1184.6768	1184.6626	12.0	208 -	217	2	R.VVQQNKEKGR.M
1255.6096	1254.6023	1254.5955	5.47	380 -	389	0	K.LYDMADVWVK.I + Oxidation (M)
1273.6505	1272.6432	1272.6561	-10.14	683 -	694	0	K.ESEVLIGNLGDK.L
1486.7495	1485.7422	1485.7544	-8.20	102 -	112	2	K.MLLHCKRDELR.K + Oxidation (M)
1562.7877	1561.7804	1561.7592	13.6	136 -	148	1	K.QVPQKLEQCMASK.H + Oxidation (M)
1696.8158	1695.8085	1695.8216	-7.72	669 -	682	2	K.REEEEDFIRAAFGK.E
1747.9039	1746.8966	1746.9152	-10.63	678 -	694	1	R.AAFGKESEVLIGNLGDK.L
2061.0056	2059.9983	2060.0295	-15.14	652 -	668	2	K.SLPNWMNMAQPKQLRSK.R + 2 Oxidation (M)
2255.1462	2254.1389	2254.0912	21.2	445 -	463	2	K.FESSSHAISMSAYLREQRR.E
2795.3037	2794.2964	2794.3661	-24.93	670 -	694	2	R.EEEEDFIRAAFGKESEVLIGNLGDK.L
2809.4873	2808.4800	2808.4228	20.4	874 -	897	1	R.NIFVLQQNLTNITMSREADLDFAR.Q
3248.6238	3247.6165	3247.6421	-7.87	258 -	285	1	K.EISLQDIKEDLELDPEENSTLFMGILIK.G + Oxidation (M)
3651.6982	3650.6909	3650.7450	-14.82	890 -	920	1	R.EADLDFARQYYEMLYNTADELLNLVVDQGVK.Y + Oxidation (M)
4466.1865	4465.1792	4465.2516	-16.21	350 -	389	1	K.FNAVATAHSVVLGYLQDTVVTPLSQQEDVKLYDMADVWVK.I + Oxidation (M)

No match to: 706.3109, 710.3683, 712.2512, 712.4277, 717.4089, 719.3850, 722.4312, 729.4136, 734.4397, 735.4166, 747.4490, 750.4445, 756.3809, 772.4293, 779.4761, 783.3987, 799.3317, 823.4870, 829.4492, 831.4715, 832.3093, 834.3143, 842.4991, 856.3599, 862.4179, 870.4754, 873.3850, 876.2836, 880.4593, 883.4774, 887.4115, 890.4962, 891.4855, 895.5654, 900.5347, 909.5356, 923.5190, 925.4849, 927.5414, 929.4692, 936.4928, 938.4988, 944.5178, 949.5491, 955.5369, 957.5576, 967.4212, 972.4985, 974.4442, 976.4541, 977.5377, 978.5416, 981.5450, 983.6256, 988.4442, 993.5175, 1007.5735, 1015.4863, 1021.5621, 1025.5615, 1031.5157, 1034.5802, 1046.5699, 1051.5020, 1056.5302, 1057.5824, 1087.5496, 1099.6482, 1113.5366, 1118.5896, 1120.5878, 1133.6019, 1145.6392, 1168.6099, 1178.6198, 1180.6312, 1187.6882, 1194.5767, 1216.7070, 1221.6462, 1248.6903, 1254.6893, 1264.6997, 1269.6152, 1281.6354, 1292.7681, 1301.6294, 1312.7516, 1314.7264, 1315.6790, 1317.6367, 1322.6362, 1325.6277, 1336.7238, 1338.7152, 1349.6917, 1351.6910, 1352.6709, 1361.6908, 1365.5895, 1368.6443, 1372.6029, 1382.5973, 1383.7275, 1390.7019, 1397.7428, 1412.6068, 1414.6772, 1420.7469, 1442.8217, 1454.7571, 1457.7417, 1467.7429, 1485.7374, 1488.7402, 1494.7421, 1497.8790, 1500.7224, 1513.7649, 1517.8065, 1518.8060, 1520.7325, 1527.7305, 1528.7021, 1531.6841, 1537.8251, 1539.7679, 1555.7635, 1556.8414, 1567.7642, 1570.7750, 1571.8055, 1578.7964, 1580.8280, 1581.8616, 1583.7979, 1585.7704, 1592.7926, 1610.6801, 1616.7987, 1624.7253, 1628.7031, 1628.8617, 1642.7180, 1647.8546, 1649.8544, 1650.8427, 1657.9053, 1663.8506, 1664.8582, 1666.8584, 1680.8226, 1683.8226, 1698.7761, 1701.9166, 1706.8654, 1707.8828, 1710.8179, 1711.7795, 1714.7788, 1715.8093, 1717.9019, 1726.8021, 1728.7821, 1730.8467, 1739.6987, 1753.7146, 1755.7096, 1758.9032, 1771.0311, 1774.9202, 1783.6785, 1787.8689, 1797.6923, 1809.0245, 1811.7025, 1816.9102, 1836.8322, 1838.8318, 1850.8512, 1862.9421, 1866.8452, 1928.0237, 1937.9972, 1953.8524, 1961.0308, 1965.8595, 1970.0011, 1982.0062, 1984.0415, 1995.8663, 2007.9470, 2027.1273, 2067.0483, 2086.1091, 2099.1079, 2109.0042, 2136.8455, 2144.0537, 2150.8601, 2173.1655, 2179.0540, 2239.1436, 2251.1506, 2265.0896, 2270.0999,

2274.0774, 2285.1733, 2286.1472, 2289.0583, 2293.1331, 2301.2546, 2303.0488, 2324.0913, 2340.1006, 2343.1824, 2344.1963, 2352.9204, 2356.0154, 2359.1130, 2375.0210, 2389.0332, 2414.2415, 2467.2080, 2525.2712, 2541.2361, 2542.2598, 2646.9749, 2660.9883, 2675.0051, 2679.0034, 2689.0183, 2692.0198, 2693.0125, 2695.0129, 2705.2800, 2717.3184, 2763.3374, 2771.4116, 2787.2793, 2792.2427, 2798.2268, 2803.2610, 2813.3884, 2814.2390, 2819.2676, 2846.3894, 2872.3164, 2876.3669, 2877.3923, 2884.3479, 2887.3311, 2888.3215, 2891.4258, 2901.3557, 2919.4480, 3002.4827, 3029.4448, 3036.5105, 3053.4414, 3067.4578, 3071.4341, 3081.6121, 3126.7246, 3151.3789, 3169.6074, 3184.4443, 3192.2090, 3232.5308, 3237.5469, 3264.5508, 3305.5381, 3316.7190, 3321.5339, 3330.5715, 3334.6743, 3339.6362, 3346.5017, 3350.5159, 3396.6165, 3412.6321, 3428.5723, 3435.6877, 3457.6028, 3471.6187, 3476.5935, 3489.6655, 3503.7874, 3624.7163, 3639.7131, 3645.7556, 3692.0066, 3721.6226, 3990.8337, 4074.9656, 4093.8804, 4147.9131, 4153.9287, 4160.9331, 4174.0122, 4177.8853, 4253.9565, 4416.9512, 4452.1763, 4459.3091, 4679.2988, 4844.0703

27. G3X8G9 **Mass:** 52451 **Score:** 18 **Expect:** 3.6e+002 **Matches:** 11

Uncharacterized protein OS=Bos taurus PE=3 SV=1

Observed	Mr(expt)	Mr(calc)	ppm	Start	End	Miss	Peptide
978.5416	977.5344	977.5328	1.60	1 -	8	1	-.MSIRVTQK.S + Oxidation (M)
1057.5824	1056.5751	1056.5716	3.33	120 -	127	0	K.WNLLQQQK.T
1281.6354	1280.6281	1280.6473	-14.98	12 -	23	1	K.VSTSAPRSFSSR.S
1397.7428	1396.7355	1396.7350	0.34	94 -	105	1	K.TLNNKFASFIDK.V
1486.7495	1485.7422	1485.7358	4.35	302 -	313	1	K.TEIPEMNRNINR.L
2285.1733	2284.1660	2284.1957	-12.99	350 -	369	2	K.LAELEAALRNAKQDMAQQLR.E + Oxidation (M)
2286.1472	2285.1399	2285.0858	23.7	128 -	146	1	K.TAQSNIDNMFESYINNLRR.Q
2414.2415	2413.2342	2413.1835	21.0	370 -	389	1	R.EYQELMNDKLALDVEIATYR.K
2542.2598	2541.2525	2541.2784	-10.19	370 -	390	2	R.EYQELMNDKLALDVEIATYRK.L
3264.5508	3263.5435	3263.5292	4.39	196 -	222	2	K.DVDEAYMNKVELESRLEGLTDEINFYR.Q + Oxidation (M)
4253.9565	4252.9492	4252.9893	-9.41	391 -	430	2	K.LLEGEESRLESGMQNMSIHTKTTSGYAGGLTSSYGTPGFK.Y + 2 Oxidation (M)

No match to: 706.3109, 710.3683, 712.2512, 712.4277, 717.4089, 719.3850, 722.4312, 729.4136, 734.4397, 735.4166, 747.4490, 750.4445, 756.3809, 759.4347, 772.4293, 779.4761, 783.3987, 799.3317, 818.4884, 823.4870, 829.4492, 831.4715, 832.3093, 834.3143, 842.4991, 856.3599, 862.4179, 870.4754, 873.3850, 876.2836, 880.4593, 883.4774, 887.4115, 889.4967, 890.4962, 891.4855, 895.5654, 900.5347, 909.5356, 923.5190, 925.4849, 927.5414, 929.4692, 936.4928, 938.4988, 944.5178, 945.5171, 949.5491, 955.5369, 957.5576, 967.4212, 972.4985, 974.4442, 976.4541, 977.5377, 981.5450, 983.6256, 988.4442, 993.5175, 1007.5735, 1015.4863, 1021.5621, 1025.5615, 1031.5157, 1034.5802, 1044.5248, 1046.5699, 1051.5020, 1056.5302, 1074.5834, 1087.5496, 1099.6482, 1113.5366, 1118.5896, 1120.5878, 1122.5590, 1133.6019, 1145.6392, 1168.6099, 1178.6198, 1180.6312, 1185.6841, 1187.6882, 1194.5767, 1216.7070, 1221.6462, 1248.6903, 1254.6893, 1255.6096, 1264.6997, 1269.6152, 1273.6505, 1292.7681, 1301.6294, 1312.7516, 1314.7264, 1315.6790, 1317.6367, 1322.6362, 1325.6277, 1336.7238, 1338.7152, 1349.6917, 1351.6910, 1352.6709, 1361.6908, 1365.5895, 1368.6443, 1372.6029, 1382.5973, 1383.7275, 1390.7019, 1412.6068, 1414.6772, 1420.7469, 1442.8217, 1454.7571, 1457.7417, 1467.7429, 1485.7374, 1488.7402, 1494.7421, 1497.8790, 1500.7224, 1513.7649, 1517.8065, 1518.8060, 1520.7325, 1527.7305, 1528.7021, 1531.6841, 1537.8251, 1539.7679, 1555.7635, 1556.8414, 1562.7877, 1567.7642, 1570.7750, 1571.8055, 1578.7964, 1580.8280, 1581.8616, 1583.7979, 1585.7704, 1592.7926, 1610.6801, 1616.7987, 1624.7253, 1628.7031, 1628.8617, 1642.7180, 1647.8546, 1649.8544, 1650.8427, 1657.9053, 1663.8506, 1664.8582, 1666.8584, 1680.8226, 1683.8226, 1696.8158, 1698.7761, 1701.9166, 1706.8654, 1707.8828, 1710.8179, 1711.7795, 1714.7788, 1715.8093, 1717.9019, 1726.8021, 1728.7821, 1730.8467, 1739.6987, 1747.9039, 1753.7146, 1755.7096, 1758.9032, 1771.0311, 1774.9202, 1783.6785, 1787.8689, 1797.6923, 1809.0245, 1811.7025, 1816.9102, 1836.8322, 1838.8318, 1850.8512, 1862.9421, 1866.8452, 1928.0237, 1937.9972, 1953.8524, 1961.0308, 1965.8595, 1970.0011, 1982.0062, 1984.0415, 1995.8663, 2007.9470, 2027.1273, 2061.0056, 2067.0483, 2086.1091, 2099.1079, 2109.0042, 2136.8455, 2144.0537, 2150.8601, 2173.1655, 2179.0540, 2239.1436, 2251.1506, 2255.1462, 2265.0896, 2270.0999, 2274.0774, 2289.0583, 2293.1331, 2301.2546, 2303.0488, 2324.0913, 2340.1006, 2343.1824, 2344.1963, 2352.9204, 2356.0154, 2359.1130, 2375.0210, 2389.0332, 2467.2080, 2525.2712, 2541.2361, 2646.9749, 2660.9883, 2675.0051, 2679.0034, 2689.0183, 2692.0198, 2693.0125, 2695.0129, 2705.2800, 2717.3184, 2763.3374, 2771.4116, 2787.2793, 2792.2427, 2795.3037, 2798.2268, 2803.2610, 2809.4873, 2813.3884, 2814.2390, 2819.2676, 2846.3894, 2872.3164, 2876.3669, 2877.3923, 2884.3479, 2887.3311, 2888.3215, 2891.4258, 2901.3557, 2919.4480, 3002.4827, 3029.4448, 3036.5105, 3053.4414, 3067.4578, 3071.4341, 3081.6121, 3126.7246, 3151.3789, 3169.6074, 3184.4443, 3192.2090, 3232.5308, 3237.5469, 3248.6238, 3305.5381, 3316.7190, 3321.5339, 3330.5715, 3334.6743, 3339.6362, 3346.5017, 3350.5159, 3396.6165, 3412.6321, 3428.5723, 3435.6877, 3457.6028, 3471.6187, 3476.5935, 3489.6655, 3503.7874, 3624.7163, 3639.7131, 3645.7556, 3651.6982, 3692.0066, 3721.6226, 3990.8337, 4074.9656, 4093.8804, 4147.9131, 4153.9287, 4160.9331, 4174.0122, 4177.8853, 4416.9512, 4452.1763, 4459.3091, 4466.1865, 4679.2988, 4844.0703

28. E1BM95 **Mass:** 60596 **Score:** 18 **Expect:** 3.8e+002 **Matches:** 15

Uncharacterized protein OS=Bos taurus GN=SCLT1 PE=4 SV=2

Observed	Mr(expt)	Mr(calc)	ppm	Start	End	Miss	Peptide
706.3109	705.3036	705.2929	15.2	176 -	181	0	R.EEASDR.R
862.4179	861.4106	861.3940	19.3	176 -	182	1	R.EEASDRR.L
944.5178	943.5105	943.4974	13.8	254 -	261	0	K.NQALLEEK.Q
977.5377	976.5304	976.5454	-15.29	233 -	240	1	K.YEAIVRAR.N
1046.5699	1045.5626	1045.5767	-13.50	503 -	511	1	K.KLTESAEIR.I
1051.5020	1050.4947	1050.5094	-13.96	344 -	351	1	R.INENDYRK.L
1074.5834	1073.5761	1073.5604	14.6	329 -	337	1	K.KAVEEELEK.I
1314.7264	1313.7191	1313.7303	-8.51	197 -	208	0	R.LSVAVQEATQIR.A
1361.6908	1360.6835	1360.7020	-13.58	262 -	272	2	K.QKQEAIEEMKK.T
1520.7325	1519.7252	1519.7188	4.24	220 -	232	1	R.ALQAKCSELEDEK.Y
1562.7877	1561.7804	1561.8174	-23.66	449 -	461	1	K.LKISTMEHEFSIK.E
1578.7964	1577.7891	1577.8123	-14.69	449 -	461	1	K.LKISTMEHEFSIK.E + Oxidation (M)
2705.2800	2704.2727	2704.3238	-18.90	410 -	431	2	R.ENCGFVSEQRLKLQQENEQLQK.Q
2809.4873	2808.4800	2808.4592	7.42	67 -	89	2	K.LQKLYQEHMTEAQIHVLESQKQK.D
3639.7131	3638.7058	3638.7556	-13.67	294 -	323	2	R.KQYNIQISQLTEELSALQMECAEKQDQIER.A + Oxidation (M)

No match to: 710.3683, 712.2512, 712.4277, 717.4089, 719.3850, 722.4312, 729.4136, 734.4397, 735.4166, 747.4490, 750.4445, 756.3809, 759.4347, 772.4293, 779.4761, 783.3987, 799.3317, 818.4884, 823.4870, 829.4492, 831.4715, 832.3093, 834.3143, 842.4991, 856.3599, 870.4754, 873.3850, 876.2836, 880.4593, 883.4774, 887.4115, 889.4967, 890.4962, 891.4855, 895.5654, 900.5347, 909.5356, 923.5190, 925.4849, 927.5414, 929.4692, 936.4928, 938.4988, 945.5171, 949.5491, 955.5369, 957.5576, 967.4212, 972.4985, 974.4442, 976.4541, 978.5416, 981.5450, 983.6256, 988.4442, 993.5175, 1007.5735, 1015.4863, 1021.5621, 1025.5615, 1031.5157, 1034.5802, 1044.5248, 1056.5302, 1057.5824, 1087.5496, 1099.6482, 1113.5366, 1118.5896, 1120.5878, 1122.5590, 1133.6019, 1145.6392, 1168.6099, 1178.6198, 1180.6312, 1185.6841, 1187.6882, 1194.5767, 1216.7070, 1221.6462, 1248.6903, 1254.6893, 1255.6096, 1264.6997, 1269.6152, 1273.6505, 1281.6354, 1292.7681, 1301.6294, 1312.7516, 1315.6790, 1317.6367, 1322.6362, 1325.6277, 1336.7238, 1338.7152, 1349.6917, 1351.6910, 1352.6709, 1365.5895, 1368.6443, 1372.6029, 1382.5973, 1383.7275, 1390.7019, 1397.7428, 1412.6068, 1414.6772, 1420.7469, 1442.8217, 1454.7571, 1457.7417, 1467.7429, 1485.7374, 1486.7495, 1488.7402, 1494.7421, 1497.8790, 1500.7224, 1513.7649, 1517.8065, 1518.8060, 1527.7305, 1528.7021, 1531.6841, 1537.8251, 1539.7679, 1555.7635, 1556.8414, 1567.7642, 1570.7750, 1571.8055, 1580.8280, 1581.8616, 1583.7979, 1585.7704, 1592.7926, 1610.6801, 1616.7987, 1624.7253, 1628.7031, 1628.8617, 1642.7180, 1647.8546, 1649.8544, 1650.8427, 1657.9053, 1663.8506, 1664.8582, 1666.8584, 1680.8226, 1683.8226, 1696.8158, 1698.7761, 1701.9166, 1706.8654, 1707.8828, 1710.8179, 1711.7795, 1714.7788, 1715.8093, 1717.9019, 1726.8021, 1728.7821, 1730.8467, 1739.6987, 1747.9039, 1753.7146, 1755.7096, 1758.9032, 1771.0311, 1774.9202, 1783.6785, 1787.8689, 1797.6923, 1809.0245, 1811.7025, 1816.9102, 1836.8322, 1838.8318, 1850.8512, 1862.9421, 1866.8452, 1928.0237, 1937.9972, 1953.8524, 1961.0308, 1965.8595, 1970.0011, 1982.0062, 1984.0415, 1995.8663, 2007.9470, 2027.1273, 2061.0056, 2067.0483, 2086.1091, 2099.1079, 2109.0042, 2136.8455, 2144.0537, 2150.8601, 2173.1655, 2179.0540, 2239.1436, 2251.1506, 2255.1462, 2265.0896, 2270.0999, 2274.0774, 2285.1733, 2286.1472, 2289.0583, 2293.1331, 2301.2546, 2303.0488, 2324.0913, 2340.1006, 2343.1824, 2344.1963, 2352.9204, 2356.0154, 2359.1130, 2375.0210, 2389.0332, 2414.2415, 2467.2080, 2525.2712, 2541.2361, 2542.2598, 2646.9749, 2660.9883, 2675.0051, 2679.0034, 2689.0183, 2692.0198, 2693.0125, 2695.0129, 2717.3184, 2763.3374, 2771.4116, 2787.2793, 2792.2427, 2795.3037, 2798.2268, 2803.2610, 2813.3884, 2814.2390, 2819.2676, 2846.3894, 2872.3164, 2876.3669, 2877.3923, 2884.3479, 2887.3311, 2888.3215, 2891.4258, 2901.3557, 2919.4480, 3002.4827, 3029.4448, 3036.5105, 3053.4414, 3067.4578, 3071.4341, 3081.6121, 3126.7246, 3151.3789, 3169.6074, 3184.4443, 3192.2090, 3232.5308, 3237.5469, 3248.6238, 3264.5508, 3305.5381, 3316.7190, 3321.5339, 3330.5715, 3334.6743, 3339.6362, 3346.5017, 3350.5159, 3396.6165, 3412.6321, 3428.5723, 3435.6877, 3457.6028, 3471.6187,

3476.5935, 3489.6655, 3503.7874, 3624.7163, 3645.7556, 3651.6982, 3692.0066, 3721.6226, 3990.8337, 4074.9656, 4093.8804, 4147.9131, 4153.9287, 4160.9331, 4174.0122, 4177.8853, 4253.9565, 4416.9512, 4452.1763, 4459.3091, 4466.1865, 4679.2988, 4844.0703

29. F1MDD0 **Mass:** 13795 **Score:** 18 **Expect:** 4e+002 **Matches:** 5
Uncharacterized protein OS=Bos taurus GN=PSMG4 PE=4 SV=2

Observed	Mr(expt)	Mr(calc)	ppm	Start	End	Miss	Peptide
1051.5020	1050.4947	1050.4950	-0.29	49 -	57	0	R.NLAVAMCTR.Y + Oxidation (M)
1349.6917	1348.6844	1348.6696	11.0	112 -	122	1	R.IKEEMQAFPEK.F
3081.6121	3080.6048	3080.5812	7.67	58 -	86	2	R.YDSIPLSTALLGDTSDTMSTGLAQRLARK.T
3503.7874	3502.7801	3502.7429	10.6	2 -	32	1	M.ERPAAAGGGDVSLHNFSARLWEQLVHFHVMR.L + Oxidation (M)
3645.7556	3644.7483	3644.7484	-0.03	49 -	82	1	R.NLAVAMCTRYDSIPLSTALLGDTSDTMSTGLAQR.L + Oxidation (M)

No match to: 706.3109, 710.3683, 712.2512, 712.4277, 717.4089, 719.3850, 722.4312, 729.4136, 734.4397, 735.4166, 747.4490, 750.4445, 756.3809, 759.4347, 772.4293, 779.4761, 783.3987, 799.3317, 818.4884, 823.4870, 829.4492, 831.4715, 832.3093, 834.3143, 842.4991, 856.3599, 862.4179, 870.4754, 873.3850, 876.2836, 880.4593, 883.4774, 887.4115, 889.4967, 890.4962, 891.4855, 895.5654, 900.5347, 909.5356, 923.5190, 925.4849, 927.5414, 929.4692, 936.4928, 938.4988, 944.5178, 945.5171, 949.5491, 955.5369, 957.5576, 967.4212, 972.4985, 974.4442, 976.4541, 977.5377, 978.5416, 981.5450, 983.6256, 988.4442, 993.5175, 1007.5735, 1015.4863, 1021.5621, 1025.5615, 1031.5157, 1034.5802, 1044.5248, 1046.5699, 1056.5302, 1057.5824, 1074.5834, 1087.5496, 1099.6482, 1113.5366, 1118.5896, 1120.5878, 1122.5590, 1133.6019, 1145.6392, 1168.6099, 1178.6198, 1180.6312, 1185.6841, 1187.6882, 1194.5767, 1216.7070, 1221.6462, 1248.6903, 1254.6893, 1255.6096, 1264.6997, 1269.6152, 1273.6505, 1281.6354, 1292.7681, 1301.6294, 1312.7516, 1314.7264, 1315.6790, 1317.6367, 1322.6362, 1325.6277, 1336.7238, 1338.7152, 1351.6910, 1352.6709, 1361.6908, 1365.5895, 1368.6443, 1372.6029, 1382.5973, 1383.7275, 1390.7019, 1397.7428, 1412.6068, 1414.6772, 1420.7469, 1442.8217, 1454.7571, 1457.7417, 1467.7429, 1485.7374, 1486.7495, 1488.7402, 1494.7421, 1497.8790, 1500.7224, 1513.7649, 1517.8065, 1518.8060, 1520.7325, 1527.7305, 1528.7021, 1531.6841, 1537.8251, 1539.7679, 1555.7635, 1556.8414, 1562.7877, 1567.7642, 1570.7750, 1571.8055, 1578.7964, 1580.8280, 1581.8616, 1583.7979, 1585.7704, 1592.7926, 1610.6801, 1616.7987, 1624.7253, 1628.7031, 1628.8617, 1642.7180, 1647.8546, 1649.8544, 1650.8427, 1657.9053, 1663.8506, 1664.8582, 1666.8584, 1680.8226, 1683.8226, 1696.8158, 1698.7761, 1701.9166, 1706.8654, 1707.8828, 1710.8179, 1711.7795, 1714.7788, 1715.8093, 1717.9019, 1726.8021, 1728.7821, 1730.8467, 1739.6987, 1747.9039, 1753.7146, 1755.7096, 1758.9032, 1771.0311, 1774.9202, 1783.6785, 1787.8689, 1797.6923, 1809.0245, 1811.7025, 1816.9102, 1836.8322, 1838.8318, 1850.8512, 1862.9421, 1866.8452, 1928.0237, 1937.9972, 1953.8524, 1961.0308, 1965.8595, 1970.0011, 1982.0062, 1984.0415, 1995.8663, 2007.9470, 2027.1273, 2061.0056, 2067.0483, 2086.1091, 2099.1079, 2109.0042, 2136.8455, 2144.0537, 2150.8601, 2173.1655, 2179.0540, 2239.1436, 2251.1506, 2255.1462, 2265.0896, 2270.0999, 2274.0774, 2285.1733, 2286.1472, 2289.0583, 2293.1331, 2301.2546, 2303.0488, 2324.0913, 2340.1006, 2343.1824, 2344.1963, 2352.9204, 2356.0154, 2359.1130, 2375.0210, 2389.0332, 2414.2415, 2467.2080, 2525.2712, 2541.2361, 2542.2598, 2646.9749, 2660.9883, 2675.0051, 2679.0034, 2689.0183, 2692.0198, 2693.0125, 2695.0129, 2705.2800, 2717.3184, 2763.3374, 2771.4116, 2787.2793, 2792.2427, 2795.3037, 2798.2268, 2803.2610, 2809.4873, 2813.3884, 2814.2390, 2819.2676, 2846.3894, 2872.3164, 2876.3669, 2877.3923, 2884.3479, 2887.3311, 2888.3215, 2891.4258, 2901.3557, 2919.4480, 3002.4827, 3029.4448, 3036.5105, 3053.4414, 3067.4578, 3071.4341, 3126.7246, 3151.3789, 3169.6074, 3184.4443, 3192.2090, 3232.5308, 3237.5469, 3248.6238, 3264.5508, 3305.5381, 3316.7190, 3321.5339, 3330.5715, 3334.6743, 3339.6362, 3346.5017, 3350.5159, 3396.6165, 3412.6321, 3428.5723, 3435.6877, 3457.6028, 3471.6187, 3476.5935, 3489.6655, 3624.7163, 3639.7131, 3651.6982, 3692.0066, 3721.6226, 3990.8337, 4074.9656, 4093.8804, 4147.9131, 4153.9287, 4160.9331, 4174.0122, 4177.8853, 4253.9565, 4416.9512, 4452.1763, 4459.3091, 4466.1865, 4679.2988, 4844.0703

30. Q2KJH4 **Mass:** 66900 **Score:** 18 **Expect:** 4.1e+002 **Matches:** 9
WD repeat-containing protein 1 OS=Bos taurus GN=WDR1 PE=2 SV=3

Observed	Mr(expt)	Mr(calc)	ppm	Start	End	Miss	Peptide
1057.5824	1056.5751	1056.5927	-16.68	117 -	126	1	R.IAVVGEGREK.F
1145.6392	1144.6319	1144.6241	6.88	8 -	17	0	K.VFASLPQVER.G
1537.8251	1536.8178	1536.8399	-14.37	471 -	484	1	R.LYSILGTTLKDEGK.L
1567.7642	1566.7569	1566.7362	13.3	191 -	203	1	R.FVNCVRFSPDGNR.F
2891.4258	2890.4185	2890.4171	0.49	204 -	231	2	R.FATASADGQIFIYDGKTGEKVCALGGSK.A
2901.3557	2900.3484	2900.3941	-15.73	232 -	259	1	K.AHDGGIYAISWSPDSTHLLSASGDKTSK.I
3503.7874	3502.7801	3502.7706	2.73	570 -	600	1	K.IQDAHRLHHVSSLAWLDEHTLVTTSHDASVK.E
3990.8337	3989.8264	3989.8558	-7.36	371 -	405	2	R.MTVDEHGQLVSCSMDDTVRYTNLTLRDYSGQGVVK.L + Oxidation (M)
4459.3091	4458.3018	4458.2166	19.1	438 -	480	1	K.CFSIDNPGYEPEVVAVHPGGETVAVGGADGNVRLYSILGTTLK.D

No match to: 706.3109, 710.3683, 712.2512, 712.4277, 717.4089, 719.3850, 722.4312, 729.4136, 734.4397, 735.4166, 747.4490, 750.4445, 756.3809, 759.4347, 772.4293, 779.4761, 783.3987, 799.3317, 818.4884, 823.4870, 829.4492, 831.4715, 832.3093, 834.3143, 842.4991, 856.3599, 862.4179, 870.4754, 873.3850, 876.2836, 880.4593, 883.4774, 887.4115, 889.4967, 890.4962, 891.4855, 895.5654, 900.5347, 909.5356, 923.5190, 925.4849, 927.5414, 929.4692, 936.4928, 938.4988, 944.5178, 945.5171, 949.5491, 955.5369, 957.5576, 967.4212, 972.4985, 974.4442, 976.4541, 977.5377, 978.5416, 981.5450, 983.6256, 988.4442, 993.5175, 1007.5735, 1015.4863, 1021.5621, 1025.5615, 1031.5157, 1034.5802, 1044.5248, 1046.5699, 1051.5020, 1056.5302, 1074.5834, 1087.5496, 1099.6482, 1113.5366, 1118.5896, 1120.5878, 1122.5590, 1133.6019, 1168.6099, 1178.6198, 1180.6312, 1185.6841, 1187.6882, 1194.5767, 1216.7070, 1221.6462, 1248.6903, 1254.6893, 1255.6096, 1264.6997, 1269.6152, 1273.6505, 1281.6354, 1292.7681, 1301.6294, 1312.7516, 1314.7264, 1315.6790, 1317.6367, 1322.6362, 1325.6277, 1336.7238, 1338.7152, 1349.6917, 1351.6910, 1352.6709, 1361.6908, 1365.5895, 1368.6443, 1372.6029, 1382.5973, 1383.7275, 1390.7019, 1397.7428, 1412.6068, 1414.6772, 1420.7469, 1442.8217, 1454.7571, 1457.7417, 1467.7429, 1485.7374, 1486.7495, 1488.7402, 1494.7421, 1497.8790, 1500.7224, 1513.7649, 1517.8065, 1518.8060, 1520.7325, 1527.7305, 1528.7021, 1531.6841, 1539.7679, 1555.7635, 1556.8414, 1562.7877, 1570.7750, 1571.8055, 1578.7964, 1580.8280, 1581.8616, 1583.7979, 1585.7704, 1592.7926, 1610.6801, 1616.7987, 1624.7253, 1628.7031, 1628.8617, 1642.7180, 1647.8546, 1649.8544, 1650.8427, 1657.9053, 1663.8506, 1664.8582, 1666.8584, 1680.8226, 1683.8226, 1696.8158, 1698.7761, 1701.9166, 1706.8654, 1707.8828, 1710.8179, 1711.7795, 1714.7788, 1715.8093, 1717.9019, 1726.8021, 1728.7821, 1730.8467, 1739.6987, 1747.9039, 1753.7146, 1755.7096, 1758.9032, 1771.0311, 1774.9202, 1783.6785, 1787.8689, 1797.6923, 1809.0245, 1811.7025, 1816.9102, 1836.8322, 1838.8318, 1850.8512, 1862.9421, 1866.8452, 1928.0237, 1937.9972, 1953.8524, 1961.0308, 1965.8595, 1970.0011, 1982.0062, 1984.0415, 1995.8663, 2007.9470, 2027.1273, 2061.0056, 2067.0483, 2086.1091, 2099.1079, 2109.0042, 2136.8455, 2144.0537, 2150.8601, 2173.1655, 2179.0540, 2239.1436, 2251.1506, 2255.1462, 2265.0896, 2270.0999, 2274.0774, 2285.1733, 2286.1472, 2289.0583, 2293.1331, 2301.2546, 2303.0488, 2324.0913, 2340.1006, 2343.1824, 2344.1963, 2352.9204, 2356.0154, 2359.1130, 2375.0210, 2389.0332, 2414.2415, 2467.2080, 2525.2712, 2541.2361, 2542.2598, 2646.9749, 2660.9883, 2675.0051, 2679.0034, 2689.0183, 2692.0198, 2693.0125, 2695.0129, 2705.2800, 2717.3184, 2763.3374, 2771.4116, 2787.2793, 2792.2427, 2795.3037, 2798.2268, 2803.2610, 2809.4873, 2813.3884, 2814.2390, 2819.2676, 2846.3894, 2872.3164, 2876.3669, 2877.3923, 2884.3479, 2887.3311, 2888.3215, 2919.4480, 3002.4827, 3029.4448, 3036.5105, 3053.4414, 3067.4578, 3071.4341, 3081.6121, 3126.7246, 3151.3789, 3169.6074, 3184.4443, 3192.2090, 3232.5308, 3237.5469, 3248.6238, 3264.5508, 3305.5381, 3316.7190, 3321.5339, 3330.5715, 3334.6743, 3339.6362, 3346.5017, 3350.5159, 3396.6165, 3412.6321, 3428.5723, 3435.6877, 3457.6028, 3471.6187, 3476.5935, 3489.6655, 3624.7163, 3639.7131, 3645.7556, 3651.6982, 3692.0066, 3721.6226, 4074.9656, 4093.8804, 4147.9131, 4153.9287, 4160.9331, 4174.0122, 4177.8853, 4253.9565, 4416.9512, 4452.1763, 4466.1865, 4679.2988, 4844.0703

31. F1MTP5 **Mass:** 66919 **Score:** 18 **Expect:** 4.1e+002 **Matches:** 8
WD repeat-containing protein 1 OS=Bos taurus GN=WDR1 PE=4 SV=2

Observed	Mr(expt)	Mr(calc)	ppm	Start	End	Miss	Peptide
1057.5824	1056.5751	1056.5927	-16.68	117 -	126	1	R.IAVVGEGREK.F
1145.6392	1144.6319	1144.6241	6.88	8 -	17	0	K.VFASLPQVER.G
1537.8251	1536.8178	1536.8399	-14.37	471 -	484	1	R.LYSILGTTLKDEGK.L
1567.7642	1566.7569	1566.7362	13.3	191 -	203	1	R.FVNCVRFSPDGNR.F
2891.4258	2890.4185	2890.4171	0.49	204 -	231	2	R.FATASADGQIFIYDGKTGEKVCALGGSK.A
3503.7874	3502.7801	3502.7706	2.73	570 -	600	1	K.IQDAHRLHHVSSLAWLDEHTLVTTSHDASVK.E
3990.8337	3989.8264	3989.8558	-7.36	371 -	405	2	R.MTVDEHGQLVSCSMDDTVRYTNLTLRDYSGQGVVK.L + Oxidation (M)
4459.3091	4458.3018	4458.2166	19.1	438 -	480	1	K.CFSIDNPGYEPEVVAVHPGGETVAVGGADGNVRLYSILGTTLK.D

No match to: 706.3109, 710.3683, 712.2512, 712.4277, 717.4089, 719.3850, 722.4312, 729.4136, 734.4397, 735.4166, 747.4490, 750.4445, 756.3809, 759.4347, 772.4293, 779.4761, 783.3987, 799.3317, 818.4884, 823.4870, 829.4492, 831.4715, 832.3093, 834.3143, 842.4991, 856.3599, 862.4179, 870.4754, 873.3850, 876.2836, 880.4593, 883.4774, 887.4115, 889.4967, 890.4962, 891.4855, 895.5654, 900.5347, 909.5356, 923.5190, 925.4849, 927.5414, 929.4692, 936.4928, 938.4988, 944.5178, 945.5171, 949.5491, 955.5369, 957.5576,

967.4212, 972.4985, 974.4442, 976.4541, 977.5377, 978.5416, 981.5450, 983.6256, 988.4442, 993.5175, 1007.5735, 1015.4863, 1021.5621, 1025.5615, 1031.5157, 1034.5802, 1044.5248, 1046.5699, 1051.5020, 1056.5302, 1074.5834, 1087.5496, 1099.6482, 1113.5366, 1118.5896, 1120.5878, 1122.5590, 1133.6019, 1168.6099, 1178.6198, 1180.6312, 1185.6841, 1187.6882, 1194.5767, 1216.7070, 1221.6462, 1248.6903, 1254.6893, 1255.6096, 1264.6997, 1269.6152, 1273.6505, 1281.6354, 1292.7681, 1301.6294, 1312.7516, 1314.7264, 1315.6790, 1317.6367, 1322.6362, 1325.6277, 1336.7238, 1338.7152, 1349.6917, 1351.6910, 1352.6709, 1361.6908, 1365.5895, 1368.6443, 1372.6029, 1382.5973, 1383.7275, 1390.7019, 1397.7428, 1412.6068, 1414.6772, 1420.7469, 1442.8217, 1454.7571, 1457.7417, 1467.7429, 1485.7374, 1486.7495, 1488.7402, 1494.7421, 1497.8790, 1500.7224, 1513.7649, 1517.8065, 1518.8060, 1520.7325, 1527.7305, 1528.7021, 1531.6841, 1539.7679, 1555.7635, 1556.8414, 1562.7877, 1570.7750, 1571.8055, 1578.7964, 1580.8280, 1581.8616, 1583.7979, 1585.7704, 1592.7926, 1610.6801, 1616.7987, 1624.7253, 1628.7031, 1628.8617, 1642.7180, 1647.8546, 1649.8544, 1650.8427, 1657.9053, 1663.8506, 1664.8582, 1666.8584, 1680.8226, 1683.8226, 1696.8158, 1698.7761, 1701.9166, 1706.8654, 1707.8828, 1710.8179, 1711.7795, 1714.7788, 1715.8093, 1717.9019, 1726.8021, 1728.7821, 1730.8467, 1739.6987, 1747.9039, 1753.7146, 1755.7096, 1758.9032, 1771.0311, 1774.9202, 1783.6785, 1787.8689, 1797.6923, 1809.0245, 1811.7025, 1816.9102, 1836.8322, 1838.8318, 1850.8512, 1862.9421, 1866.8452, 1928.0237, 1937.9972, 1953.8524, 1961.0308, 1965.8595, 1970.0011, 1982.0062, 1984.0415, 1995.8663, 2007.9470, 2027.1273, 2061.0056, 2067.0483, 2086.1091, 2099.1079, 2109.0042, 2136.8455, 2144.0537, 2150.8601, 2173.1655, 2179.0540, 2239.1436, 2251.1506, 2255.1462, 2265.0896, 2270.0999, 2274.0774, 2285.1733, 2286.1472, 2289.0583, 2293.1331, 2301.2546, 2303.0488, 2324.0913, 2340.1006, 2343.1824, 2344.1963, 2352.9204, 2356.0154, 2359.1130, 2375.0210, 2389.0332, 2414.2415, 2467.2080, 2525.2712, 2541.2361, 2542.2598, 2646.9749, 2660.9883, 2675.0051, 2679.0034, 2689.0183, 2692.0198, 2693.0125, 2695.0129, 2705.2800, 2717.3184, 2763.3374, 2771.4116, 2787.2793, 2792.2427, 2795.3037, 2798.2268, 2803.2610, 2809.4873, 2813.3884, 2814.2390, 2819.2676, 2846.3894, 2872.3164, 2876.3669, 2877.3923, 2884.3479, 2887.3311, 2888.3215, 2901.3557, 2919.4480, 3002.4827, 3029.4448, 3036.5105, 3053.4414, 3067.4578, 3071.4341, 3081.6121, 3126.7246, 3151.3789, 3169.6074, 3184.4443, 3192.2090, 3232.5308, 3237.5469, 3248.6238, 3264.5508, 3305.5381, 3316.7190, 3321.5339, 3330.5715, 3334.6743, 3339.6362, 3346.5017, 3350.5159, 3396.6165, 3412.6321, 3428.5723, 3435.6877, 3457.6028, 3471.6187, 3476.5935, 3489.6655, 3624.7163, 3639.7131, 3645.7556, 3651.6982, 3692.0066, 3721.6226, 4074.9656, 4093.8804, 4147.9131, 4153.9287, 4160.9331, 4174.0122, 4177.8853, 4253.9565, 4416.9512, 4452.1763, 4466.1865, 4679.2988, 4844.0703

32. E1BJY5 **Mass:** 27252 **Score:** 18 **Expect:** 4.3e+002 **Matches:** 7

Uncharacterized protein OS=Bos taurus GN=RNF125 PE=4 SV=1

Observed	Mr(expt)	Mr(calc)	ppm	Start	End	Miss	Peptide
1520.7325	1519.7252	1519.7565	-20.60	114 -	125	2	R.AHTRTCQKYIDK.Y
1774.9202	1773.9129	1773.8857	15.3	2 -	20	1	M.GSVLSSDSGKSAPASATPR.A
1984.0415	1983.0342	1982.9949	19.8	122 -	138	1	K.YIDKYGPLQELGETTTR.C
2763.3374	2762.3301	2762.3157	5.24	26 -	49	0	R.GDPELPVTSFDCSVCLEVLHHPVR.T
2872.3164	2871.3091	2871.2520	19.9	95 -	117	2	K.SEFRNCTECDTLVCLSEMRAHTR.T
2888.3215	2887.3142	2887.2470	23.3	95 -	117	2	K.SEFRNCTECDTLVCLSEMRAHTR.T + Oxidation (M)
2919.4480	2918.4407	2918.4168	8.21	25 -	49	1	R.RGDPELPVTSFDCSVCLEVLHHPVR.T

No match to: 706.3109, 710.3683, 712.2512, 712.4277, 717.4089, 719.3850, 722.4312, 729.4136, 734.4397, 735.4166, 747.4490, 750.4445, 756.3809, 759.4347, 772.4293, 779.4761, 783.3987, 799.3317, 818.4884, 823.4870, 829.4492, 831.4715, 832.3093, 834.3143, 842.4991, 856.3599, 862.4179, 870.4754, 873.3850, 876.2836, 880.4593, 883.4774, 887.4115, 889.4967, 890.4962, 891.4855, 895.5654, 900.5347, 909.5356, 923.5190, 925.4849, 927.5414, 929.4692, 936.4928, 938.4988, 944.5178, 945.5171, 949.5491, 955.5369, 957.5576, 967.4212, 972.4985, 974.4442, 976.4541, 977.5377, 978.5416, 981.5450, 983.6256, 988.4442, 993.5175, 1007.5735, 1015.4863, 1021.5621, 1025.5615, 1031.5157, 1034.5802, 1044.5248, 1046.5699, 1051.5020, 1056.5302, 1057.5824, 1074.5834, 1087.5496, 1099.6482, 1113.5366, 1118.5896, 1120.5878, 1122.5590, 1133.6019, 1145.6392, 1168.6099, 1178.6198, 1180.6312, 1185.6841, 1187.6882, 1194.5767, 1216.7070, 1221.6462, 1248.6903, 1254.6893, 1255.6096, 1264.6997, 1269.6152, 1273.6505, 1281.6354, 1292.7681, 1301.6294, 1312.7516, 1314.7264, 1315.6790, 1317.6367, 1322.6362, 1325.6277, 1336.7238, 1338.7152, 1349.6917, 1351.6910, 1352.6709, 1361.6908, 1365.5895, 1368.6443, 1372.6029, 1382.5973, 1383.7275, 1390.7019, 1397.7428, 1412.6068, 1414.6772, 1420.7469, 1442.8217, 1454.7571, 1457.7417, 1467.7429, 1485.7374, 1486.7495, 1488.7402, 1494.7421, 1497.8790, 1500.7224, 1513.7649, 1517.8065, 1518.8060, 1527.7305, 1528.7021, 1531.6841, 1537.8251, 1539.7679, 1555.7635, 1556.8414, 1562.7877, 1567.7642, 1570.7750, 1571.8055, 1578.7964, 1580.8280, 1581.8616, 1583.7979, 1585.7704, 1592.7926, 1610.6801, 1616.7987, 1624.7253, 1628.7031, 1628.8617, 1642.7180, 1647.8546, 1649.8544, 1650.8427, 1657.9053, 1663.8506, 1664.8582, 1666.8584, 1680.8226, 1683.8226, 1696.8158, 1698.7761, 1701.9166, 1706.8654, 1707.8828, 1710.8179, 1711.7795, 1714.7788, 1715.8093, 1717.9019, 1726.8021, 1728.7821, 1730.8467, 1739.6987, 1747.9039, 1753.7146, 1755.7096, 1758.9032, 1771.0311, 1783.6785, 1787.8689, 1797.6923, 1809.0245, 1811.7025, 1816.9102, 1836.8322, 1838.8318, 1850.8512, 1862.9421, 1866.8452, 1928.0237, 1937.9972, 1953.8524, 1961.0308, 1965.8595, 1970.0011, 1982.0062, 1995.8663, 2007.9470, 2027.1273, 2061.0056, 2067.0483, 2086.1091, 2099.1079, 2109.0042, 2136.8455, 2144.0537, 2150.8601, 2173.1655, 2179.0540, 2239.1436, 2251.1506, 2255.1462, 2265.0896, 2270.0999, 2274.0774, 2285.1733, 2286.1472, 2289.0583, 2293.1331, 2301.2546, 2303.0488, 2324.0913, 2340.1006, 2343.1824, 2344.1963, 2352.9204, 2356.0154, 2359.1130, 2375.0210, 2389.0332, 2414.2415, 2467.2080, 2525.2712, 2541.2361, 2542.2598, 2646.9749, 2660.9883, 2675.0051, 2679.0034, 2689.0183, 2692.0198, 2693.0125, 2695.0129, 2705.2800, 2717.3184, 2771.4116, 2787.2793, 2792.2427, 2795.3037, 2798.2268, 2803.2610, 2809.4873, 2813.3884, 2814.2390, 2819.2676, 2846.3894, 2876.3669, 2877.3923, 2884.3479, 2887.3311, 2891.4258, 2901.3557, 3002.4827, 3029.4448, 3036.5105, 3053.4414, 3067.4578, 3071.4341, 3081.6121, 3126.7246, 3151.3789, 3169.6074, 3184.4443, 3192.2090, 3232.5308, 3237.5469, 3248.6238, 3264.5508, 3305.5381, 3316.7190, 3321.5339, 3330.5715, 3334.6743, 3339.6362, 3346.5017, 3350.5159, 3396.6165, 3412.6321, 3428.5723, 3435.6877, 3457.6028, 3471.6187, 3476.5935, 3489.6655, 3503.7874, 3624.7163, 3639.7131, 3645.7556, 3651.6982, 3692.0066, 3721.6226, 3990.8337, 4074.9656, 4093.8804, 4147.9131, 4153.9287, 4160.9331, 4174.0122, 4177.8853, 4253.9565, 4416.9512, 4452.1763, 4459.3091, 4466.1865, 4679.2988, 4844.0703

33. G3X7H8 **Mass:** 43831 **Score:** 18 **Expect:** 4.3e+002 **Matches:** 10

Uncharacterized protein OS=Bos taurus GN=LOC782894 PE=3 SV=1

Observed	Mr(expt)	Mr(calc)	ppm	Start	End	Miss	Peptide
1537.8251	1536.8178	1536.7905	17.8	193 -	204	1	K.NVQKPVQMMFRK.S + 2 Oxidation (M)
1624.7253	1623.7180	1623.7351	-10.54	100 -	112	1	K.TYNFLSSFKDSCR.K
1650.8427	1649.8354	1649.8314	2.43	172 -	184	2	K.GNWDKQFYKVHTK.E
1663.8506	1662.8433	1662.8828	-23.76	2 -	17	0	M.DALSEANGTFALTLLK.K
1680.8226	1679.8153	1679.8559	-24.15	95 -	108	1	R.LFGEKTYNFLSSFK.D
2303.0488	2302.0415	2302.0397	0.80	62 -	82	0	K.SSGGGEDVHQGFQNLLCEVNR.T
2846.3894	2845.3821	2845.3945	-4.35	355 -	378	0	R.FCANRPFLFFIQHSSTGAILFCGR.F
3067.4578	3066.4505	3066.4362	4.69	293 -	320	1	R.DLGMTDGFNMARADFIGLSCQPGLHLSK.V + Oxidation (M)
3321.5339	3320.5266	3320.5956	-20.78	62 -	90	2	K.SSGGGEDVHQGFQNLLCEVNRTDTRYLLR.T
3692.0066	3690.9993	3690.9551	12.0	209 -	239	1	K.MTYIAEICTQILVLPYVGQELNMVILLPRER.T + Oxidation (M)

No match to: 706.3109, 710.3683, 712.2512, 712.4277, 717.4089, 719.3850, 722.4312, 729.4136, 734.4397, 735.4166, 747.4490, 750.4445, 756.3809, 759.4347, 772.4293, 779.4761, 783.3987, 799.3317, 818.4884, 823.4870, 829.4492, 831.4715, 832.3093, 834.3143, 842.4991, 856.3599, 862.4179, 870.4754, 873.3850, 876.2836, 880.4593, 883.4774, 887.4115, 889.4967, 890.4962, 891.4855, 895.5654, 900.5347, 909.5356, 923.5190, 925.4849, 927.5414, 929.4692, 936.4928, 938.4988, 944.5178, 945.5171, 949.5491, 955.5369, 957.5576, 967.4212, 972.4985, 974.4442, 976.4541, 977.5377, 978.5416, 981.5450, 983.6256, 988.4442, 993.5175, 1007.5735, 1015.4863, 1021.5621, 1025.5615, 1031.5157, 1034.5802, 1044.5248, 1046.5699, 1051.5020, 1056.5302, 1057.5824, 1074.5834, 1087.5496, 1099.6482, 1113.5366, 1118.5896, 1120.5878, 1122.5590, 1133.6019, 1145.6392, 1168.6099, 1178.6198, 1180.6312, 1185.6841, 1187.6882, 1194.5767, 1216.7070, 1221.6462, 1248.6903, 1254.6893, 1255.6096, 1264.6997, 1269.6152, 1273.6505, 1281.6354, 1292.7681, 1301.6294, 1312.7516, 1314.7264, 1315.6790, 1317.6367, 1322.6362, 1325.6277, 1336.7238, 1338.7152, 1349.6917, 1351.6910, 1352.6709, 1361.6908, 1365.5895, 1368.6443, 1372.6029, 1382.5973, 1383.7275, 1390.7019, 1397.7428, 1412.6068, 1414.6772, 1420.7469, 1442.8217, 1454.7571, 1457.7417, 1467.7429, 1485.7374, 1486.7495, 1488.7402, 1494.7421, 1497.8790, 1500.7224, 1513.7649, 1517.8065, 1518.8060, 1520.7325, 1527.7305, 1528.7021, 1531.6841, 1539.7679, 1555.7635, 1556.8414, 1562.7877, 1567.7642, 1570.7750, 1571.8055, 1578.7964, 1580.8280, 1581.8616, 1583.7979, 1585.7704, 1592.7926, 1610.6801, 1616.7987, 1628.7031, 1628.8617, 1642.7180, 1647.8546, 1649.8544, 1657.9053, 1664.8582, 1666.8584, 1683.8226, 1696.8158, 1698.7761, 1701.9166, 1706.8654, 1707.8828, 1710.8179, 1711.7795, 1714.7788, 1715.8093, 1717.9019, 1726.8021, 1728.7821, 1730.8467, 1739.6987, 1747.9039, 1753.7146, 1755.7096, 1758.9032, 1771.0311, 1774.9202, 1783.6785, 1787.8689, 1797.6923, 1809.0245, 1811.7025, 1816.9102, 1836.8322, 1838.8318, 1850.8512, 1862.9421, 1866.8452, 1928.0237, 1937.9972, 1953.8524, 1961.0308, 1965.8595, 1970.0011, 1982.0062, 1984.0415, 1995.8663, 2007.9470, 2027.1273, 2061.0056, 2067.0483, 2086.1091, 2099.1079, 2109.0042, 2136.8455, 2144.0537, 2150.8601, 2173.1655, 2179.0540, 2239.1436, 2251.1506, 2255.1462, 2265.0896, 2270.0999, 2274.0774, 2285.1733, 2286.1472, 2289.0583, 2293.1331, 2301.2546, 2324.0913, 2340.1006, 2343.1824, 2344.1963, 2352.9204, 2356.0154, 2359.1130, 2375.0210, 2389.0332, 2414.2415, 2467.2080, 2525.2712, 2541.2361, 2542.2598, 2646.9749, 2660.9883, 2675.0051, 2679.0034, 2689.0183, 2692.0198, 2693.0125, 2695.0129, 2705.2800, 2717.3184, 2763.3374, 2771.4116, 2787.2793, 2792.2427, 2795.3037, 2798.2268, 2803.2610,

2809.4873, 2813.3884, 2814.2390, 2819.2676, 2872.3164, 2876.3669, 2877.3923, 2884.3479, 2887.3311, 2888.3215, 2891.4258, 2901.3557, 2919.4480, 3002.4827, 3029.4448, 3036.5105, 3053.4414, 3071.4341, 3081.6121, 3126.7246, 3151.3789, 3169.6074, 3184.4443, 3192.2090, 3232.5308, 3237.5469, 3248.6238, 3264.5508, 3305.5381, 3316.7190, 3330.5715, 3334.6743, 3339.6362, 3346.5017, 3350.5159, 3396.6165, 3412.6321, 3428.5723, 3435.6877, 3457.6028, 3471.6187, 3476.5935, 3489.6655, 3503.7874, 3624.7163, 3639.7131, 3645.7556, 3651.6982, 3721.6226, 3990.8337, 4074.9656, 4093.8804, 4147.9131, 4153.9287, 4160.9331, 4174.0122, 4177.8853, 4253.9565, 4416.9512, 4452.1763, 4459.3091, 4466.1865, 4679.2988, 4844.0703

34. Q3T016 **Mass:** 13891 **Score:** 17 **Expect:** 4.4e+002 **Matches:** 6

TSSK6-activating co-chaperone protein OS=Bos taurus GN=TSACC PE=2 SV=1

Observed	Mr(expt)	Mr(calc)	ppm	Start	End	Miss	Peptide
944.5178	943.5105	943.5087	1.93	105 -	113	0	K.NSSLPALSR.N
1178.6198	1177.6125	1177.5840	24.2	2 -	11	0	M.EQLPSHPTNR.R
1315.6790	1314.6717	1314.6891	-13.24	102 -	113	1	K.ENKNSSLPALSR.N
1325.6277	1324.6204	1324.6194	0.80	1 -	11	0	-.MEQLPSHPTNR.R + Oxidation (M)
1664.8582	1663.8509	1663.8576	-4.02	1 -	14	2	-.MEQLPSHPTNRRAK.E
1680.8226	1679.8153	1679.8525	-22.15	1 -	14	2	-.MEQLPSHPTNRRAK.E + Oxidation (M)

No match to: 706.3109, 710.3683, 712.2512, 712.4277, 717.4089, 719.3850, 722.4312, 729.4136, 734.4397, 735.4166, 747.4490, 750.4445, 756.3809, 759.4347, 772.4293, 779.4761, 783.3987, 799.3317, 818.4884, 823.4870, 829.4492, 831.4715, 832.3093, 834.3143, 842.4991, 856.3599, 862.4179, 870.4754, 873.3850, 876.2836, 880.4593, 883.4774, 887.4115, 889.4967, 890.4962, 891.4855, 895.5654, 900.5347, 909.5356, 923.5190, 925.4849, 927.5414, 929.4692, 936.4928, 938.4988, 945.5171, 949.5491, 955.5369, 957.5576, 967.4212, 972.4985, 974.4442, 976.4541, 977.5377, 978.5416, 981.5450, 983.6256, 988.4442, 993.5175, 1007.5735, 1015.4863, 1021.5621, 1025.5615, 1031.5157, 1034.5802, 1044.5248, 1046.5699, 1051.5020, 1056.5302, 1057.5824, 1074.5834, 1087.5496, 1099.6482, 1113.5366, 1118.5896, 1120.5878, 1122.5590, 1133.6019, 1145.6392, 1168.6099, 1180.6312, 1185.6841, 1187.6882, 1194.5767, 1216.7070, 1221.6462, 1248.6903, 1254.6893, 1255.6096, 1264.6997, 1269.6152, 1273.6505, 1281.6354, 1292.7681, 1301.6294, 1312.7516, 1314.7264, 1317.6367, 1322.6362, 1336.7238, 1338.7152, 1349.6917, 1351.6910, 1352.6709, 1361.6908, 1365.5895, 1368.6443, 1372.6029, 1382.5973, 1383.7275, 1390.7019, 1397.7428, 1412.6068, 1414.6772, 1420.7469, 1442.8217, 1454.7571, 1457.7417, 1467.7429, 1485.7374, 1486.7495, 1488.7402, 1494.7421, 1497.8790, 1500.7224, 1513.7649, 1517.8065, 1518.8060, 1520.7325, 1527.7305, 1528.7021, 1531.6841, 1537.8251, 1539.7679, 1555.7635, 1556.8414, 1562.7877, 1567.7642, 1570.7750, 1571.8055, 1578.7964, 1580.8280, 1581.8616, 1583.7979, 1585.7704, 1592.7926, 1610.6801, 1616.7987, 1624.7253, 1628.7031, 1628.8617, 1642.7180, 1647.8546, 1649.8544, 1650.8427, 1657.9053, 1663.8506, 1666.8584, 1683.8226, 1696.8158, 1698.7761, 1701.9166, 1706.8654, 1707.8828, 1710.8179, 1711.7795, 1714.7788, 1715.8093, 1717.9019, 1726.8021, 1728.7821, 1730.8467, 1739.6987, 1747.9039, 1753.7146, 1755.7096, 1758.9032, 1771.0311, 1774.9202, 1783.6785, 1787.8689, 1797.6923, 1809.0245, 1811.7025, 1816.9102, 1836.8322, 1838.8318, 1850.8512, 1862.9421, 1866.8452, 1928.0237, 1937.9972, 1953.8524, 1961.0308, 1965.8595, 1970.0011, 1982.0062, 1984.0415, 1995.8663, 2007.9470, 2027.1273, 2061.0056, 2067.0483, 2086.1091, 2099.1079, 2109.0042, 2136.8455, 2144.0537, 2150.8601, 2173.1655, 2179.0540, 2239.1436, 2251.1506, 2255.1462, 2265.0896, 2270.0999, 2274.0774, 2285.1733, 2286.1472, 2289.0583, 2293.1331, 2301.2546, 2303.0488, 2324.0913, 2340.1006, 2343.1824, 2344.1963, 2352.9204, 2356.0154, 2359.1130, 2375.0210, 2389.0332, 2414.2415, 2467.2080, 2525.2712, 2541.2361, 2542.2598, 2646.9749, 2660.9883, 2675.0051, 2679.0034, 2689.0183, 2692.0198, 2693.0125, 2695.0129, 2705.2800, 2717.3184, 2763.3374, 2771.4116, 2787.2793, 2792.2427, 2795.3037, 2798.2268, 2803.2610, 2809.4873, 2813.3884, 2814.2390, 2819.2676, 2846.3894, 2872.3164, 2876.3669, 2877.3923, 2884.3479, 2887.3311, 2888.3215, 2891.4258, 2901.3557, 2919.4480, 3002.4827, 3029.4448, 3036.5105, 3053.4414, 3067.4578, 3071.4341, 3081.6121, 3126.7246, 3151.3789, 3169.6074, 3184.4443, 3192.2090, 3232.5308, 3237.5469, 3248.6238, 3264.5508, 3305.5381, 3316.7190, 3321.5339, 3330.5715, 3334.6743, 3339.6362, 3346.5017, 3350.5159, 3396.6165, 3412.6321, 3428.5723, 3435.6877, 3457.6028, 3471.6187, 3476.5935, 3489.6655, 3503.7874, 3624.7163, 3639.7131, 3645.7556, 3651.6982, 3692.0066, 3721.6226, 3990.8337, 4074.9656, 4093.8804, 4147.9131, 4153.9287, 4160.9331, 4174.0122, 4177.8853, 4253.9565, 4416.9512, 4452.1763, 4459.3091, 4466.1865, 4679.2988, 4844.0703

35. F1MFH1 **Mass:** 13894 **Score:** 17 **Expect:** 4.4e+002 **Matches:** 6

TSSK6-activating co-chaperone protein OS=Bos taurus GN=TSACC PE=4 SV=1

Observed	Mr(expt)	Mr(calc)	ppm	Start	End	Miss	Peptide
944.5178	943.5105	943.5087	1.93	105 -	113	0	K.NSSLPALSR.N
1178.6198	1177.6125	1177.5840	24.2	2 -	11	0	M.EQLPSHPTNR.R
1315.6790	1314.6717	1314.6891	-13.24	102 -	113	1	K.ENKNSSLPALSR.N
1325.6277	1324.6204	1324.6194	0.80	1 -	11	0	-.MEQLPSHPTNR.R + Oxidation (M)
1664.8582	1663.8509	1663.8576	-4.02	1 -	14	2	-.MEQLPSHPTNRRAK.E
1680.8226	1679.8153	1679.8525	-22.15	1 -	14	2	-.MEQLPSHPTNRRAK.E + Oxidation (M)

No match to: 706.3109, 710.3683, 712.2512, 712.4277, 717.4089, 719.3850, 722.4312, 729.4136, 734.4397, 735.4166, 747.4490, 750.4445, 756.3809, 759.4347, 772.4293, 779.4761, 783.3987, 799.3317, 818.4884, 823.4870, 829.4492, 831.4715, 832.3093, 834.3143, 842.4991, 856.3599, 862.4179, 870.4754, 873.3850, 876.2836, 880.4593, 883.4774, 887.4115, 889.4967, 890.4962, 891.4855, 895.5654, 900.5347, 909.5356, 923.5190, 925.4849, 927.5414, 929.4692, 936.4928, 938.4988, 945.5171, 949.5491, 955.5369, 957.5576, 967.4212, 972.4985, 974.4442, 976.4541, 977.5377, 978.5416, 981.5450, 983.6256, 988.4442, 993.5175, 1007.5735, 1015.4863, 1021.5621, 1025.5615, 1031.5157, 1034.5802, 1044.5248, 1046.5699, 1051.5020, 1056.5302, 1057.5824, 1074.5834, 1087.5496, 1099.6482, 1113.5366, 1118.5896, 1120.5878, 1122.5590, 1133.6019, 1145.6392, 1168.6099, 1180.6312, 1185.6841, 1187.6882, 1194.5767, 1216.7070, 1221.6462, 1248.6903, 1254.6893, 1255.6096, 1264.6997, 1269.6152, 1273.6505, 1281.6354, 1292.7681, 1301.6294, 1312.7516, 1314.7264, 1317.6367, 1322.6362, 1336.7238, 1338.7152, 1349.6917, 1351.6910, 1352.6709, 1361.6908, 1365.5895, 1368.6443, 1372.6029, 1382.5973, 1383.7275, 1390.7019, 1397.7428, 1412.6068, 1414.6772, 1420.7469, 1442.8217, 1454.7571, 1457.7417, 1467.7429, 1485.7374, 1486.7495, 1488.7402, 1494.7421, 1497.8790, 1500.7224, 1513.7649, 1517.8065, 1518.8060, 1520.7325, 1527.7305, 1528.7021, 1531.6841, 1537.8251, 1539.7679, 1555.7635, 1556.8414, 1562.7877, 1567.7642, 1570.7750, 1571.8055, 1578.7964, 1580.8280, 1581.8616, 1583.7979, 1585.7704, 1592.7926, 1610.6801, 1616.7987, 1624.7253, 1628.7031, 1628.8617, 1642.7180, 1647.8546, 1649.8544, 1650.8427, 1657.9053, 1663.8506, 1666.8584, 1683.8226, 1696.8158, 1698.7761, 1701.9166, 1706.8654, 1707.8828, 1710.8179, 1711.7795, 1714.7788, 1715.8093, 1717.9019, 1726.8021, 1728.7821, 1730.8467, 1739.6987, 1747.9039, 1753.7146, 1755.7096, 1758.9032, 1771.0311, 1774.9202, 1783.6785, 1787.8689, 1797.6923, 1809.0245, 1811.7025, 1816.9102, 1836.8322, 1838.8318, 1850.8512, 1862.9421, 1866.8452, 1928.0237, 1937.9972, 1953.8524, 1961.0308, 1965.8595, 1970.0011, 1982.0062, 1984.0415, 1995.8663, 2007.9470, 2027.1273, 2061.0056, 2067.0483, 2086.1091, 2099.1079, 2109.0042, 2136.8455, 2144.0537, 2150.8601, 2173.1655, 2179.0540, 2239.1436, 2251.1506, 2255.1462, 2265.0896, 2270.0999, 2274.0774, 2285.1733, 2286.1472, 2289.0583, 2293.1331, 2301.2546, 2303.0488, 2324.0913, 2340.1006, 2343.1824, 2344.1963, 2352.9204, 2356.0154, 2359.1130, 2375.0210, 2389.0332, 2414.2415, 2467.2080, 2525.2712, 2541.2361, 2542.2598, 2646.9749, 2660.9883, 2675.0051, 2679.0034, 2689.0183, 2692.0198, 2693.0125, 2695.0129, 2705.2800, 2717.3184, 2763.3374, 2771.4116, 2787.2793, 2792.2427, 2795.3037, 2798.2268, 2803.2610, 2809.4873, 2813.3884, 2814.2390, 2819.2676, 2846.3894, 2872.3164, 2876.3669, 2877.3923, 2884.3479, 2887.3311, 2888.3215, 2891.4258, 2901.3557, 2919.4480, 3002.4827, 3029.4448, 3036.5105, 3053.4414, 3067.4578, 3071.4341, 3081.6121, 3126.7246, 3151.3789, 3169.6074, 3184.4443, 3192.2090, 3232.5308, 3237.5469, 3248.6238, 3264.5508, 3305.5381, 3316.7190, 3321.5339, 3330.5715, 3334.6743, 3339.6362, 3346.5017, 3350.5159, 3396.6165, 3412.6321, 3428.5723, 3435.6877, 3457.6028, 3471.6187, 3476.5935, 3489.6655, 3503.7874, 3624.7163, 3639.7131, 3645.7556, 3651.6982, 3692.0066, 3721.6226, 3990.8337, 4074.9656, 4093.8804, 4147.9131, 4153.9287, 4160.9331, 4174.0122, 4177.8853, 4253.9565, 4416.9512, 4452.1763, 4459.3091, 4466.1865, 4679.2988, 4844.0703

36. Q0VC60 **Mass:** 56014 **Score:** 17 **Expect:** 4.4e+002 **Matches:** 13

Dihydrouridine synthase 2-like, SMM1 homolog (S. cerevisiae) OS=Bos taurus GN=DUS2L PE=2 SV=1

Observed	Mr(expt)	Mr(calc)	ppm	Start	End	Miss	Peptide
880.4593	879.4521	879.4636	-13.15	355 -	361	1	K.MAIKFDR.R
1074.5834	1073.5761	1073.5764	-0.26	147 -	155	1	K.GTRRPVTCK.I
1255.6096	1254.6023	1254.6244	-17.62	410 -	419	1	K.YQSTLWDKSK.K
1314.7264	1313.7191	1313.7125	5.02	421 -	432	0	K.LAEQAAAIVCLR.S
1368.6443	1367.6370	1367.6429	-4.33	453 -	464	1	K.REALDQDPGDPR.T
1383.7275	1382.7202	1382.7194	0.60	410 -	420	2	K.YQSTLWDKSKK.L
1442.8217	1441.8144	1441.8075	4.82	420 -	432	1	K.KLAEQAAAIVCLR.S
1657.9053	1656.8980	1656.9345	-21.99	418 -	432	2	K.SKKLAEQAAAIVCLR.S
2255.1462	2254.1389	2254.1328	2.71	334 -	354	1	K.LLARSPEEAEEPAEDTSGIIK.M
3036.5105	3035.5032	3035.5056	-0.78	96 -	123	2	R.ALAVARLVENDVAGIDVNMGCPKEYSTK.G + Oxidation (M)

3126.7246	3125.7173	3125.6664	16.3	2 - 28	2	M.TVNNLSLCYHNKLILAPMVRVGTLPMR.L + Oxidation (M)
3651.6982	3650.6909	3650.7266	-9.78	102 - 136	2	R.LVENDVAGIDVNMGCPKEYSTKGGMGAALLSDPDK.I
4452.1763	4451.1690	4451.2655	-21.68	202 - 243	2	K.AIAETLSIPVIANGGSHDHIQGYLDIEDFRRATAASSVMVAR.A

No match to: 706.3109, 710.3683, 712.2512, 712.4277, 717.4089, 719.3850, 722.4312, 729.4136, 734.4397, 735.4166, 747.4490, 750.4445, 756.3809, 759.4347, 772.4293, 779.4761, 783.3987, 799.3317, 818.4884, 823.4870, 829.4492, 831.4715, 832.3093, 834.3143, 842.4991, 856.3599, 862.4179, 870.4754, 873.3850, 876.2836, 883.4774, 887.4115, 889.4967, 890.4962, 891.4855, 895.5654, 900.5347, 909.5356, 923.5190, 925.4849, 927.5414, 929.4692, 936.4928, 938.4988, 944.5178, 945.5171, 949.5491, 955.5369, 957.5576, 967.4212, 972.4985, 974.4442, 976.4541, 977.5377, 978.5416, 981.5450, 983.6256, 988.4442, 993.5175, 1007.5735, 1015.4863, 1021.5621, 1025.5615, 1031.5157, 1034.5802, 1044.5248, 1046.5699, 1051.5020, 1056.5302, 1057.5824, 1087.5496, 1099.6482, 1113.5366, 1118.5896, 1120.5878, 1122.5590, 1133.6019, 1145.6392, 1168.6099, 1178.6198, 1180.6312, 1185.6841, 1187.6882, 1194.5767, 1216.7070, 1221.6462, 1248.6903, 1254.6893, 1264.6997, 1269.6152, 1273.6505, 1281.6354, 1292.7681, 1301.6294, 1312.7516, 1315.6790, 1317.6367, 1322.6362, 1325.6277, 1336.7238, 1338.7152, 1349.6917, 1351.6910, 1352.6709, 1361.6908, 1365.5895, 1372.6029, 1382.5973, 1390.7019, 1397.7428, 1412.6068, 1414.6772, 1420.7469, 1454.7571, 1457.7417, 1467.7429, 1485.7374, 1486.7495, 1488.7402, 1494.7421, 1497.8790, 1500.7224, 1513.7649, 1517.8065, 1518.8060, 1520.7325, 1527.7305, 1528.7021, 1531.6841, 1537.8251, 1539.7679, 1555.7635, 1556.8414, 1562.7877, 1567.7642, 1570.7750, 1571.8055, 1578.7964, 1580.8280, 1581.8616, 1583.7979, 1585.7704, 1592.7926, 1610.6801, 1616.7987, 1624.7253, 1628.7031, 1628.8617, 1642.7180, 1647.8546, 1649.8544, 1650.8427, 1663.8506, 1664.8582, 1666.8584, 1680.8226, 1683.8226, 1696.8158, 1698.7761, 1701.9166, 1706.8654, 1707.8828, 1710.8179, 1711.7795, 1714.7788, 1715.8093, 1717.9019, 1726.8021, 1728.7821, 1730.8467, 1739.6987, 1747.9039, 1753.7146, 1755.7096, 1758.9032, 1771.0311, 1774.9202, 1783.6785, 1787.8689, 1797.6923, 1809.0245, 1811.7025, 1816.9102, 1836.8322, 1838.8318, 1850.8512, 1862.9421, 1866.8452, 1928.0237, 1937.9972, 1953.8524, 1961.0308, 1965.8595, 1970.0011, 1982.0062, 1984.0415, 1995.8663, 2007.9470, 2027.1273, 2061.0056, 2067.0483, 2086.1091, 2099.1079, 2109.0042, 2136.8455, 2144.0537, 2150.8601, 2173.1655, 2179.0540, 2239.1436, 2251.1506, 2265.0896, 2270.0999, 2274.0774, 2285.1733, 2286.1472, 2289.0583, 2293.1331, 2301.2546, 2303.0488, 2324.0913, 2340.1006, 2343.1824, 2344.1963, 2352.9204, 2356.0154, 2359.1130, 2375.0210, 2389.0332, 2414.2415, 2467.2080, 2525.2712, 2541.2361, 2542.2598, 2646.9749, 2660.9883, 2675.0051, 2679.0034, 2689.0183, 2692.0198, 2693.0125, 2695.0129, 2705.2800, 2717.3184, 2763.3374, 2771.4116, 2787.2793, 2792.2427, 2795.3037, 2798.2268, 2803.2610, 2809.4873, 2813.3884, 2814.2390, 2819.2676, 2846.3894, 2872.3164, 2876.3669, 2877.3923, 2884.3479, 2887.3311, 2888.3215, 2891.4258, 2901.3557, 2919.4480, 3002.4827, 3029.4448, 3053.4414, 3067.4578, 3071.4341, 3081.6121, 3151.3789, 3169.6074, 3184.4443, 3192.2090, 3232.5308, 3237.5469, 3248.6238, 3264.5508, 3305.5381, 3316.7190, 3321.5339, 3330.5715, 3334.6743, 3339.6362, 3346.5017, 3350.5159, 3396.6165, 3412.6321, 3428.5723, 3435.6877, 3457.6028, 3471.6187, 3476.5935, 3489.6655, 3503.7874, 3624.7163, 3639.7131, 3645.7556, 3692.0066, 3721.6226, 3990.8337, 4074.9656, 4093.8804, 4147.9131, 4153.9287, 4160.9331, 4174.0122, 4177.8853, 4253.9565, 4416.9512, 4459.3091, 4466.1865, 4679.2988, 4844.0703

37. P00516 **Mass:** 76997 **Score:** 17 **Expect:** 4.5e+002 **Matches:** 14
cGMP-dependent protein kinase 1 OS=Bos taurus GN=PRKG1 PE=1 SV=2

Observed	Mr(expt)	Mr(calc)	ppm	Start End	Miss	Peptide
1315.6790	1314.6717	1314.6972	-19.39	496 - 507	1	R.GYAKLVDFGFAK.K
1555.7635	1554.7562	1554.7864	-19.43	165 - 177	0	K.VFGELAILYNCTR.T
1647.8546	1646.8473	1646.8563	-5.44	43 - 57	0	K.CQSVLPVPSTHIGPR.T
1664.8582	1663.8509	1663.8491	1.11	92 - 105	1	K.DLIKEAILDNDFMK.N
1680.8226	1679.8153	1679.8440	-17.07	92 - 105	1	K.DLIKEAILDNDFMK.N + Oxidation (M)
1758.9032	1757.8959	1757.8627	18.9	195 - 208	1	R.QCFQTIMMRTGLIK.H + 2 Oxidation (M)
1816.9102	1815.9029	1815.9003	1.45	271 - 286	1	R.EDSPNEDPVFLRTLGK.G
2239.1436	2238.1363	2238.1031	14.9	515 - 533	0	K.TWTFCGTPEYVAPEIILNK.G
2293.1331	2292.1258	2292.1784	-22.93	302 - 322	1	R.TANVIAAEAVTCLVIDRDSFK.H
2344.1963	2343.1890	2343.1706	7.86	323 - 344	2	K.HLIGGLDDVSNKAYEDAEAKAK.Y
2813.3884	2812.3811	2812.4074	-9.35	153 - 177	2	K.EGVKLCTMGPGKVFGELAILYNCTR.T
3334.6743	3333.6670	3333.7034	-10.90	343 - 373	2	K.AKYEAEAAFFANLKLSDFNIIDTLGVGGFGR.V
3503.7874	3502.7801	3502.7515	8.18	287 - 318	2	K.GDWFGEKALQGEDVRTANVIAAEAVTCLVIDR.D
4174.0122	4173.0049	4173.0360	-7.43	92 - 126	2	K.DLIKEAILDNDFMKNLELSQIQEIVDCMYPVEYGK.D

No match to: 706.3109, 710.3683, 712.2512, 712.4277, 717.4089, 719.3850, 722.4312, 729.4136, 734.4397, 735.4166, 747.4490, 750.4445, 756.3809, 759.4347, 772.4293, 779.4761, 783.3987, 799.3317, 818.4884, 823.4870, 829.4492, 831.4715, 832.3093, 834.3143, 842.4991, 856.3599, 862.4179, 870.4754, 873.3850, 876.2836, 880.4593, 883.4774, 887.4115, 889.4967, 890.4962, 891.4855, 895.5654, 900.5347, 909.5356, 923.5190, 925.4849, 927.5414, 929.4692, 936.4928, 938.4988, 944.5178, 945.5171, 949.5491, 955.5369, 957.5576, 967.4212, 972.4985, 974.4442, 976.4541, 977.5377, 978.5416, 981.5450, 983.6256, 988.4442, 993.5175, 1007.5735, 1015.4863, 1021.5621, 1025.5615, 1031.5157, 1034.5802, 1044.5248, 1046.5699, 1051.5020, 1056.5302, 1057.5824, 1074.5834, 1087.5496, 1099.6482, 1113.5366, 1118.5896, 1120.5878, 1122.5590, 1133.6019, 1145.6392, 1168.6099, 1178.6198, 1180.6312, 1185.6841, 1187.6882, 1194.5767, 1216.7070, 1221.6462, 1248.6903, 1254.6893, 1255.6096, 1264.6997, 1269.6152, 1273.6505, 1281.6354, 1292.7681, 1301.6294, 1312.7516, 1314.7264, 1317.6367, 1322.6362, 1325.6277, 1336.7238, 1338.7152, 1349.6917, 1351.6910, 1352.6709, 1361.6908, 1365.5895, 1368.6443, 1372.6029, 1382.5973, 1383.7275, 1390.7019, 1397.7428, 1412.6068, 1414.6772, 1420.7469, 1442.8217, 1454.7571, 1457.7417, 1467.7429, 1485.7374, 1486.7495, 1488.7402, 1494.7421, 1497.8790, 1500.7224, 1513.7649, 1517.8065, 1518.8060, 1520.7325, 1527.7305, 1528.7021, 1531.6841, 1537.8251, 1539.7679, 1556.8414, 1562.7877, 1567.7642, 1570.7750, 1571.8055, 1578.7964, 1580.8280, 1581.8616, 1583.7979, 1585.7704, 1592.7926, 1610.6801, 1616.7987, 1624.7253, 1628.7031, 1628.8617, 1642.7180, 1649.8544, 1650.8427, 1657.9053, 1663.8506, 1666.8584, 1683.8226, 1696.8158, 1698.7761, 1701.9166, 1706.8654, 1707.8828, 1710.8179, 1711.7795, 1714.7788, 1715.8093, 1717.9019, 1726.8021, 1728.7821, 1730.8467, 1739.6987, 1747.9039, 1753.7146, 1755.7096, 1771.0311, 1774.9202, 1783.6785, 1787.8689, 1797.6923, 1809.0245, 1811.7025, 1836.8322, 1838.8318, 1850.8512, 1862.9421, 1866.8452, 1928.0237, 1937.9972, 1953.8524, 1961.0308, 1965.8595, 1970.0011, 1982.0062, 1984.0415, 1995.8663, 2007.9470, 2027.1273, 2061.0056, 2067.0483, 2086.1091, 2099.1079, 2109.0042, 2136.8455, 2144.0537, 2150.8601, 2173.1655, 2179.0540, 2251.1506, 2255.1462, 2265.0896, 2270.0999, 2274.0774, 2285.1733, 2286.1472, 2289.0583, 2301.2546, 2303.0488, 2324.0913, 2340.1006, 2343.1824, 2352.9204, 2356.0154, 2359.1130, 2375.0210, 2389.0332, 2414.2415, 2467.2080, 2525.2712, 2541.2361, 2542.2598, 2646.9749, 2660.9883, 2675.0051, 2679.0034, 2689.0183, 2692.0198, 2693.0125, 2695.0129, 2705.2800, 2717.3184, 2763.3374, 2771.4116, 2787.2793, 2792.2427, 2795.3037, 2798.2268, 2803.2610, 2809.4873, 2814.2390, 2819.2676, 2846.3894, 2872.3164, 2876.3669, 2877.3923, 2884.3479, 2887.3311, 2888.3215, 2891.4258, 2901.3557, 2919.4480, 3002.4827, 3036.5105, 3053.4414, 3067.4578, 3071.4341, 3081.6121, 3126.7246, 3151.3789, 3169.6074, 3184.4443, 3192.2090, 3232.5308, 3237.5469, 3248.6238, 3264.5508, 3305.5381, 3316.7190, 3321.5339, 3330.5715, 3339.6362, 3346.5017, 3350.5159, 3396.6165, 3412.6321, 3428.5723, 3435.6877, 3457.6028, 3471.6187, 3476.5935, 3489.6655, 3624.7163, 3639.7131, 3645.7556, 3651.6982, 3692.0066, 3721.6226, 3990.8337, 4074.9656, 4093.8804, 4147.9131, 4153.9287, 4160.9331, 4177.8853, 4253.9565, 4416.9512, 4452.1763, 4459.3091, 4466.1865, 4679.2988, 4844.0703

38. G5E6K4 **Mass:** 12963 **Score:** 17 **Expect:** 4.5e+002 **Matches:** 7
Uncharacterized protein OS=Bos taurus GN=ATXN7 PE=4 SV=1

Observed	Mr(expt)	Mr(calc)	ppm	Start End	Miss	Peptide
1122.5590	1121.5517	1121.5321	17.5	113 - 121	1	K.NREVMGLCK.E + Oxidation (M)
1457.7417	1456.7344	1456.7018	22.4	2 - 14	2	M.SERAADDVRGEPR.R
1567.7642	1566.7569	1566.7646	-4.93	109 - 121	2	K.EFGKNREVMGLCK.E
1583.7979	1582.7906	1582.7596	19.6	109 - 121	2	K.EFGKNREVMGLCK.E + Oxidation (M)
2343.1824	2342.1751	2342.1284	19.9	48 - 72	0	R.LRPEDGGPGAASTSAAAAMATVGER.R
2359.1130	2358.1057	2358.1234	-7.48	48 - 72	0	R.LRPEDGGPGAASTSAAAAMATVGER.R + Oxidation (M)
2414.2415	2413.2342	2413.2323	0.82	16 - 40	1	R.AAAAAGGAAAAAARQQQQPPQPQR.Q

No match to: 706.3109, 710.3683, 712.2512, 712.4277, 717.4089, 719.3850, 722.4312, 729.4136, 734.4397, 735.4166, 747.4490, 750.4445, 756.3809, 759.4347, 772.4293, 779.4761, 783.3987, 799.3317, 818.4884, 823.4870, 829.4492, 831.4715, 832.3093, 834.3143, 842.4991, 856.3599, 862.4179, 870.4754, 873.3850, 876.2836, 880.4593, 883.4774, 887.4115, 889.4967, 890.4962, 891.4855, 895.5654, 900.5347, 909.5356, 923.5190, 925.4849, 927.5414, 929.4692, 936.4928, 938.4988, 944.5178, 945.5171, 949.5491, 955.5369, 957.5576, 967.4212, 972.4985, 974.4442, 976.4541, 977.5377, 978.5416, 981.5450, 983.6256, 988.4442, 993.5175, 1007.5735, 1015.4863, 1021.5621, 1025.5615, 1031.5157, 1034.5802, 1044.5248, 1046.5699, 1051.5020, 1056.5302, 1057.5824, 1074.5834, 1087.5496, 1099.6482, 1113.5366, 1118.5896, 1120.5878, 1133.6019, 1145.6392, 1168.6099, 1178.6198, 1180.6312, 1185.6841, 1187.6882, 1194.5767, 1216.7070, 1221.6462, 1248.6903, 1254.6893, 1255.6096, 1264.6997, 1269.6152, 1273.6505, 1281.6354, 1292.7681, 1301.6294, 1312.7516, 1314.7264, 1315.6790, 1317.6367, 1322.6362, 1325.6277, 1336.7238, 1338.7152, 1349.6917, 1351.6910, 1352.6709, 1361.6908, 1365.5895, 1368.6443,

1372.6029, 1382.5973, 1383.7275, 1390.7019, 1397.7428, 1412.6068, 1414.6772, 1420.7469, 1442.8217, 1454.7571, 1467.7429, 1485.7374, 1486.7495, 1488.7402, 1494.7421, 1497.8790, 1500.7224, 1513.7649, 1517.8065, 1518.8060, 1520.7325, 1527.7305, 1528.7021, 1531.6841, 1537.8251, 1539.7679, 1555.7635, 1556.8414, 1562.7877, 1570.7750, 1571.8055, 1578.7964, 1580.8280, 1581.8616, 1585.7704, 1592.7926, 1610.6801, 1616.7987, 1624.7253, 1628.7031, 1628.8617, 1642.7180, 1647.8546, 1649.8544, 1650.8427, 1657.9053, 1663.8506, 1664.8582, 1666.8584, 1680.8226, 1683.8226, 1696.8158, 1698.7761, 1701.9166, 1706.8654, 1707.8828, 1710.8179, 1711.7795, 1714.7788, 1715.8093, 1717.9019, 1726.8021, 1728.7821, 1730.8467, 1739.6987, 1747.9039, 1753.7146, 1755.7096, 1758.9032, 1771.0311, 1774.9202, 1783.6785, 1787.8689, 1797.6923, 1809.0245, 1811.7025, 1816.9102, 1836.8322, 1838.8318, 1850.8512, 1862.9421, 1866.8452, 1928.0237, 1937.9972, 1953.8524, 1961.0308, 1965.8595, 1970.0011, 1982.0062, 1984.0415, 1995.8663, 2007.9470, 2027.1273, 2061.0056, 2067.0483, 2086.1091, 2099.1079, 2109.0042, 2136.8455, 2144.0537, 2150.8601, 2173.1655, 2179.0540, 2239.1436, 2251.1506, 2255.1462, 2265.0896, 2270.0999, 2274.0774, 2285.1733, 2286.1472, 2289.0583, 2293.1331, 2301.2546, 2303.0488, 2324.0913, 2340.1006, 2344.1963, 2352.9204, 2356.0154, 2375.0210, 2389.0332, 2467.2080, 2525.2712, 2541.2361, 2542.2598, 2646.9749, 2660.9883, 2675.0051, 2679.0034, 2689.0183, 2692.0198, 2693.0125, 2695.0129, 2705.2800, 2717.3184, 2763.3374, 2771.4116, 2787.2793, 2792.2427, 2795.3037, 2798.2268, 2803.2610, 2809.4873, 2813.3884, 2814.2390, 2819.2676, 2846.3894, 2872.3164, 2876.3669, 2877.3923, 2884.3479, 2887.3311, 2888.3215, 2891.4258, 2901.3557, 2919.4480, 3002.4827, 3029.4448, 3036.5105, 3053.4414, 3067.4578, 3071.4341, 3081.6121, 3126.7246, 3151.3789, 3169.6074, 3184.4443, 3192.2090, 3232.5308, 3237.5469, 3248.6238, 3264.5508, 3305.5381, 3316.7190, 3321.5339, 3330.5715, 3334.6743, 3339.6362, 3346.5017, 3350.5159, 3396.6165, 3412.6321, 3428.5723, 3435.6877, 3457.6028, 3471.6187, 3476.5935, 3489.6655, 3503.7874, 3624.7163, 3639.7131, 3645.7556, 3651.6982, 3692.0066, 3721.6226, 3990.8337, 4074.9656, 4093.8804, 4147.9131, 4153.9287, 4160.9331, 4174.0122, 4177.8853, 4253.9565, 4416.9512, 4452.1763, 4459.3091, 4466.1865, 4679.2988, 4844.0703

39. Q0VC96 **Mass:** 22800 **Score:** 17 **Expect:** 4.5e+002 **Matches:** 9

Thymocyte nuclear protein 1 OS=Bos taurus GN=THYN1 PE=2 SV=1

Observed	Mr(expt)	Mr(calc)	ppm	Start	End	Miss	Peptide
944.5178	943.5105	943.5087	1.93	15 -	22	2	K.GPEEKRTK.T
1178.6198	1177.6125	1177.6026	8.43	156 -	164	1	K.WSMRLSVQR.L + Oxidation (M)
1254.6893	1253.6820	1253.6775	3.63	4 -	14	2	R.ARRRPAGPDMK.G
1539.7679	1538.7606	1538.7888	-18.31	93 -	104	2	R.NYQARNFLRAMR.L
1555.7635	1554.7562	1554.7837	-17.69	93 -	104	2	R.NYQARNFLRAMR.L + Oxidation (M)
1567.7642	1566.7569	1566.7936	-23.42	7 -	20	2	R.RPAGPDMKGPEEKR.T
1583.7979	1582.7906	1582.7885	1.31	7 -	20	2	R.RPAGPDMKGPEEKR.T + Oxidation (M)
1701.9166	1700.9093	1700.8805	16.9	25 -	40	1	K.NLGRASAQVENSSLQK.T
3071.4341	3070.4268	3070.4441	-5.61	41 -	67	2	K.TPVSEDSGEALSSYWLMKSEPESRLEK.G + Oxidation (M)

No match to: 706.3109, 710.3683, 712.2512, 712.4277, 717.4089, 719.3850, 722.4312, 729.4136, 734.4397, 735.4166, 747.4490, 750.4445, 756.3809, 759.4347, 772.4293, 779.4761, 783.3987, 799.3317, 818.4884, 823.4870, 829.4492, 831.4715, 832.3093, 834.3143, 842.4991, 856.3599, 862.4179, 870.4754, 873.3850, 876.2836, 880.4593, 883.4774, 887.4115, 889.4967, 890.4962, 891.4855, 895.5654, 900.5347, 909.5356, 923.5190, 925.4849, 927.5414, 929.4692, 936.4928, 938.4988, 945.5171, 949.5491, 955.5369, 957.5576, 967.4212, 972.4985, 974.4442, 976.4541, 977.5377, 978.5416, 981.5450, 983.6256, 988.4442, 993.5175, 1007.5735, 1015.4863, 1021.5621, 1025.5615, 1031.5157, 1034.5802, 1044.5248, 1046.5699, 1051.5020, 1056.5302, 1057.5824, 1074.5834, 1087.5496, 1099.6482, 1113.5366, 1118.5896, 1120.5878, 1122.5590, 1133.6019, 1145.6392, 1168.6099, 1180.6312, 1185.6841, 1187.6882, 1194.5767, 1216.7070, 1221.6462, 1248.6903, 1255.6096, 1264.6997, 1269.6152, 1273.6505, 1281.6354, 1292.7681, 1301.6294, 1312.7516, 1314.7264, 1315.6790, 1317.6367, 1322.6362, 1325.6277, 1336.7238, 1338.7152, 1349.6917, 1351.6910, 1352.6709, 1361.6908, 1365.5895, 1368.6443, 1372.6029, 1382.5973, 1383.7275, 1390.7019, 1397.7428, 1412.6068, 1414.6772, 1420.7469, 1442.8217, 1454.7571, 1457.7417, 1467.7429, 1485.7374, 1486.7495, 1488.7402, 1494.7421, 1497.8790, 1500.7224, 1513.7649, 1517.8065, 1518.8060, 1520.7325, 1527.7305, 1528.7021, 1531.6841, 1537.8251, 1556.8414, 1562.7877, 1570.7750, 1571.8055, 1578.7964, 1580.8280, 1581.8616, 1585.7704, 1592.7926, 1610.6801, 1616.7987, 1624.7253, 1628.7031, 1628.8617, 1642.7180, 1647.8546, 1649.8544, 1650.8427, 1657.9053, 1663.8506, 1664.8582, 1666.8584, 1680.8226, 1683.8226, 1696.8158, 1698.7761, 1706.8654, 1707.8828, 1710.8179, 1711.7795, 1714.7788, 1715.8093, 1717.9019, 1726.8021, 1728.7821, 1730.8467, 1739.6987, 1747.9039, 1753.7146, 1755.7096, 1758.9032, 1771.0311, 1774.9202, 1783.6785, 1787.8689, 1797.6923, 1809.0245, 1811.7025, 1816.9102, 1836.8322, 1838.8318, 1850.8512, 1862.9421, 1866.8452, 1928.0237, 1937.9972, 1953.8524, 1961.0308, 1965.8595, 1970.0011, 1982.0062, 1984.0415, 1995.8663, 2007.9470, 2027.1273, 2061.0056, 2067.0483, 2086.1091, 2099.1079, 2109.0042, 2136.8455, 2144.0537, 2150.8601, 2173.1655, 2179.0540, 2239.1436, 2251.1506, 2255.1462, 2265.0896, 2270.0999, 2274.0774, 2285.1733, 2286.1472, 2289.0583, 2293.1331, 2301.2546, 2303.0488, 2324.0913, 2340.1006, 2343.1824, 2344.1963, 2352.9204, 2356.0154, 2359.1130, 2375.0210, 2389.0332, 2414.2415, 2467.2080, 2525.2712, 2541.2361, 2542.2598, 2646.9749, 2660.9883, 2675.0051, 2679.0034, 2689.0183, 2692.0198, 2693.0125, 2695.0129, 2705.2800, 2717.3184, 2763.3374, 2771.4116, 2787.2793, 2792.2427, 2795.3037, 2798.2268, 2803.2610, 2809.4873, 2813.3884, 2814.2390, 2819.2676, 2846.3894, 2872.3164, 2876.3669, 2877.3923, 2884.3479, 2887.3311, 2888.3215, 2891.4258, 2901.3557, 2919.4480, 3002.4827, 3029.4448, 3036.5105, 3053.4414, 3067.4578, 3081.6121, 3126.7246, 3151.3789, 3169.6074, 3184.4443, 3192.2090, 3232.5308, 3237.5469, 3248.6238, 3264.5508, 3305.5381, 3316.7190, 3321.5339, 3330.5715, 3334.6743, 3339.6362, 3346.5017, 3350.5159, 3396.6165, 3412.6321, 3428.5723, 3435.6877, 3457.6028, 3471.6187, 3476.5935, 3489.6655, 3503.7874, 3624.7163, 3639.7131, 3645.7556, 3651.6982, 3692.0066, 3721.6226, 3990.8337, 4074.9656, 4093.8804, 4147.9131, 4153.9287, 4160.9331, 4174.0122, 4177.8853, 4253.9565, 4416.9512, 4452.1763, 4459.3091, 4466.1865, 4679.2988, 4844.0703

40. M0QVY0 **Mass:** 61166 **Score:** 17 **Expect:** 4.6e+002 **Matches:** 15

Uncharacterized protein OS=Bos taurus GN=KRT6A PE=3 SV=1

Observed	Mr(expt)	Mr(calc)	ppm	Start	End	Miss	Peptide
880.4593	879.4521	879.4563	-4.75	15 -	23	1	R.KGFSAGSAR.V
945.5171	944.5098	944.5039	6.24	233 -	240	1	R.GRLDSELR.G
955.5369	954.5297	954.5134	17.0	393 -	400	1	R.SEIDHVKK.Q
1187.6882	1186.6809	1186.6669	11.8	416 -	426	2	R.GELALKDARSK.L
1314.7264	1313.7191	1313.7190	0.06	427 -	438	1	K.LADLEDALQKAK.Q
1397.7428	1396.7355	1396.7350	0.34	169 -	180	1	K.TLNNKFASFIDK.V
1420.7469	1419.7396	1419.7041	25.0	16 -	29	1	K.GFSAGSARVPGVCR.S
1710.8179	1709.8106	1709.8294	-10.99	273 -	287	1	K.DVDAAYMNKVELQAK.V + Oxidation (M)
1937.9972	1936.9899	1936.9901	-0.11	24 -	41	2	R.VPGVCRSGFSSVSLSRSR.G
1970.0011	1968.9938	1969.0421	-24.53	174 -	189	2	K.FASFIDKVRFLEQQNK.V
2303.0488	2302.0415	2302.0423	-0.34	241 -	259	2	R.GMQDTVEDFKNKYEDEINK.R
2414.2415	2413.2342	2413.2199	5.95	447 -	466	1	K.EYQELMNVKLALDVEIATYR.K + Oxidation (M)
2542.2598	2541.2525	2541.3148	-24.51	447 -	467	2	K.EYQELMNVKLALDVEIATYRK.L + Oxidation (M)
2717.3184	2716.3111	2716.3100	0.41	30 -	58	2	R.SGFSSVSLSRSRGSGGLAGVCGGAGFGSR.S
3081.6121	3080.6048	3080.5488	18.2	273 -	299	2	K.DVDAAYMNKVELQAKVDALTDEINFLR.T

No match to: 706.3109, 710.3683, 712.2512, 712.4277, 717.4089, 719.3850, 722.4312, 729.4136, 734.4397, 735.4166, 747.4490, 750.4445, 756.3809, 759.4347, 772.4293, 779.4761, 783.3987, 799.3317, 818.4884, 823.4870, 829.4492, 831.4715, 832.3093, 834.3143, 842.4991, 856.3599, 862.4179, 870.4754, 873.3850, 876.2836, 883.4774, 887.4115, 889.4967, 890.4962, 891.4855, 895.5654, 900.5347, 909.5356, 923.5190, 925.4849, 927.5414, 929.4692, 936.4928, 938.4988, 944.5178, 949.5491, 957.5576, 967.4212, 972.4985, 974.4442, 976.4541, 977.5377, 978.5416, 981.5450, 983.6256, 988.4442, 993.5175, 1007.5735, 1015.4863, 1021.5621, 1025.5615, 1031.5157, 1034.5802, 1044.5248, 1046.5699, 1051.5020, 1056.5302, 1057.5824, 1074.5834, 1087.5496, 1099.6482, 1113.5366, 1118.5896, 1120.5878, 1122.5590, 1133.6019, 1145.6392, 1168.6099, 1178.6198, 1180.6312, 1185.6841, 1194.5767, 1216.7070, 1221.6462, 1248.6903, 1254.6893, 1255.6096, 1264.6997, 1269.6152, 1273.6505, 1281.6354, 1292.7681, 1301.6294, 1312.7516, 1315.6790, 1317.6367, 1322.6362, 1325.6277, 1336.7238, 1338.7152, 1349.6917, 1351.6910, 1352.6709, 1361.6908, 1365.5895, 1368.6443, 1372.6029, 1382.5973, 1383.7275, 1390.7019, 1412.6068, 1414.6772, 1442.8217, 1454.7571, 1457.7417, 1467.7429, 1485.7374, 1486.7495, 1488.7402, 1494.7421, 1497.8790, 1500.7224, 1513.7649, 1517.8065, 1518.8060, 1520.7325, 1527.7305, 1528.7021, 1531.6841, 1537.8251, 1539.7679, 1555.7635, 1556.8414, 1562.7877, 1567.7642, 1570.7750, 1571.8055, 1578.7964, 1580.8280, 1581.8616, 1583.7979, 1585.7704, 1592.7926, 1610.6801, 1616.7987, 1624.7253, 1628.7031, 1628.8617, 1642.7180, 1647.8546, 1649.8544, 1650.8427, 1657.9053, 1663.8506, 1664.8582, 1666.8584, 1680.8226, 1683.8226, 1696.8158, 1698.7761, 1701.9166, 1706.8654, 1707.8828, 1711.7795, 1714.7788, 1715.8093, 1717.9019, 1726.8021, 1728.7821, 1730.8467, 1739.6987, 1747.9039, 1753.7146, 1755.7096, 1758.9032, 1771.0311, 1774.9202, 1783.6785, 1787.8689, 1797.6923, 1809.0245, 1811.7025, 1816.9102, 1836.8322, 1838.8318, 1850.8512, 1862.9421, 1866.8452, 1928.0237, 1953.8524, 1961.0308, 1965.8595, 1982.0062, 1984.0415, 1995.8663, 2007.9470, 2027.1273, 2061.0056, 2067.0483, 2086.1091, 2099.1079, 2109.0042, 2136.8455, 2144.0537, 2150.8601, 2173.1655, 2179.0540, 2239.1436, 2251.1506, 2255.1462, 2265.0896, 2270.0999, 2274.0774, 2285.1733, 2286.1472, 2289.0583, 2293.1331, 2301.2546,

2324.0913, 2340.1006, 2343.1824, 2344.1963, 2352.9204, 2356.0154, 2359.1130, 2375.0210, 2389.0332, 2467.2080, 2525.2712, 2541.2361, 2646.9749, 2660.9883, 2675.0051, 2679.0034, 2689.0183, 2692.0198, 2693.0125, 2695.0129, 2705.2800, 2763.3374, 2771.4116, 2787.2793, 2792.2427, 2795.3037, 2798.2268, 2803.2610, 2809.4873, 2813.3884, 2814.2390, 2819.2676, 2846.3894, 2872.3164, 2876.3669, 2877.3923, 2884.3479, 2887.3311, 2888.3215, 2891.4258, 2901.3557, 2919.4480, 3002.4827, 3029.4448, 3036.5105, 3053.4414, 3067.4578, 3071.4341, 3126.7246, 3151.3789, 3169.6074, 3184.4443, 3192.2090, 3232.5308, 3237.5469, 3248.6238, 3264.5508, 3305.5381, 3316.7190, 3321.5339, 3330.5715, 3334.6743, 3339.6362, 3346.5017, 3350.5159, 3396.6165, 3412.6321, 3428.5723, 3435.6877, 3457.6028, 3471.6187, 3476.5935, 3489.6655, 3503.7874, 3624.7163, 3639.7131, 3645.7556, 3651.6982, 3692.0066, 3721.6226, 3990.8337, 4074.9656, 4093.8804, 4147.9131, 4153.9287, 4160.9331, 4174.0122, 4177.8853, 4253.9565, 4416.9512, 4452.1763, 4459.3091, 4466.1865, 4679.2988, 4844.0703

41. Q32LF3 **Mass:** 24094 **Score:** 17 **Expect:** 4.7e+002 **Matches:** 10

Similar to type 1 protein phosphatase inhibitor OS=Bos taurus GN=LOC520057 PE=2 SV=1

Observed	Mr(expt)	Mr(calc)	ppm	Start	End	Miss	Peptide
834.3143	833.3071	833.3266	-23.40	62 -	67	0	R.DYDFMK.M + Oxidation (M)
1336.7238	1335.7165	1335.6929	17.7	1 -	12	1	-.MSASTSSRRPIK.G + Oxidation (M)
1494.7421	1493.7348	1493.7110	15.9	100 -	113	0	R.LAATHTSDSSYSVR.D
1578.7964	1577.7891	1577.7896	-0.33	85 -	99	1	R.SAESEATALDSLTKR.L
1650.8427	1649.8354	1649.8121	14.1	99 -	113	1	K.RLAATHTSDSSYSVR.D
2467.2080	2466.2007	2466.2575	-23.02	19 -	43	2	K.GSTASSVAGSAQQSGGPLQEVHRKK.S
2787.2793	2786.2720	2786.3219	-17.92	99 -	124	2	K.RLAATHTSDSSYSVRDPELDGAHSSK.I
3071.4341	3070.4268	3070.4130	4.49	44 -	67	2	K.SQKWDESNILATYRPEYRDYDFMK.M + Oxidation (M)
3305.5381	3304.5308	3304.5517	-6.33	68 -	98	1	K.MNEPSTPQFGPQNLGERSAESEATALDSLTK.R
3321.5339	3320.5266	3320.5466	-6.03	68 -	98	1	K.MNEPSTPQFGPQNLGERSAESEATALDSLTK.R + Oxidation (M)

No match to: 706.3109, 710.3683, 712.2512, 712.4277, 717.4089, 719.3850, 722.4312, 729.4136, 734.4397, 735.4166, 747.4490, 750.4445, 756.3809, 759.4347, 772.4293, 779.4761, 783.3987, 799.3317, 818.4884, 823.4870, 829.4492, 831.4715, 832.3093, 842.4991, 856.3599, 862.4179, 870.4754, 873.3850, 876.2836, 880.4593, 883.4774, 887.4115, 889.4967, 890.4962, 891.4855, 895.5654, 900.5347, 909.5356, 923.5190, 925.4849, 927.5414, 929.4692, 936.4928, 938.4988, 944.5178, 945.5171, 949.5491, 955.5369, 957.5576, 967.4212, 972.4985, 974.4442, 976.4541, 977.5377, 978.5416, 981.5450, 983.6256, 988.4442, 993.5175, 1007.5735, 1015.4863, 1021.5621, 1025.5615, 1031.5157, 1034.5802, 1044.5248, 1046.5699, 1051.5020, 1056.5302, 1057.5824, 1074.5834, 1087.5496, 1099.6482, 1113.5366, 1118.5896, 1120.5878, 1122.5590, 1133.6019, 1145.6392, 1168.6099, 1178.6198, 1180.6312, 1185.6841, 1187.6882, 1194.5767, 1216.7070, 1221.6462, 1248.6903, 1254.6893, 1255.6096, 1264.6997, 1269.6152, 1273.6505, 1281.6354, 1292.7681, 1301.6294, 1312.7516, 1314.7264, 1315.6790, 1317.6367, 1322.6362, 1325.6277, 1338.7152, 1349.6917, 1351.6910, 1352.6709, 1361.6908, 1365.5895, 1368.6443, 1372.6029, 1382.5973, 1383.7275, 1390.7019, 1397.7428, 1412.6068, 1414.6772, 1420.7469, 1442.8217, 1454.7571, 1457.7417, 1467.7429, 1485.7374, 1486.7495, 1488.7402, 1497.8790, 1500.7224, 1513.7649, 1517.8065, 1518.8060, 1520.7325, 1527.7305, 1528.7021, 1531.6841, 1537.8251, 1539.7679, 1555.7635, 1556.8414, 1562.7877, 1567.7642, 1570.7750, 1571.8055, 1580.8280, 1581.8616, 1583.7979, 1585.7704, 1592.7926, 1610.6801, 1616.7987, 1624.7253, 1628.7031, 1628.8617, 1642.7180, 1647.8546, 1649.8544, 1657.9053, 1663.8506, 1664.8582, 1666.8584, 1680.8226, 1683.8226, 1696.8158, 1698.7761, 1701.9166, 1706.8654, 1707.8828, 1710.8179, 1711.7795, 1714.7788, 1715.8093, 1717.9019, 1726.8021, 1728.7821, 1730.8467, 1739.6987, 1747.9039, 1753.7146, 1755.7096, 1758.9032, 1771.0311, 1774.9202, 1783.6785, 1787.8689, 1797.6923, 1809.0245, 1811.7025, 1816.9102, 1836.8322, 1838.8318, 1850.8512, 1862.9421, 1866.8452, 1928.0237, 1937.9972, 1953.8524, 1961.0308, 1965.8595, 1970.0011, 1982.0062, 1984.0415, 1995.8663, 2007.9470, 2027.1273, 2061.0056, 2067.0483, 2086.1091, 2099.1079, 2109.0042, 2136.8455, 2144.0537, 2150.8601, 2173.1655, 2179.0540, 2239.1436, 2251.1506, 2255.1462, 2265.0896, 2270.0999, 2274.0774, 2285.1733, 2286.1472, 2289.0583, 2293.1331, 2301.2546, 2303.0488, 2324.0913, 2340.1006, 2343.1824, 2344.1963, 2352.9204, 2356.0154, 2359.1130, 2375.0210, 2389.0332, 2414.2415, 2525.2712, 2541.2361, 2542.2598, 2646.9749, 2660.9883, 2675.0051, 2679.0034, 2689.0183, 2692.0198, 2693.0125, 2695.0129, 2705.2800, 2717.3184, 2763.3374, 2771.4116, 2792.2427, 2795.3037, 2798.2268, 2803.2610, 2809.4873, 2813.3884, 2814.2390, 2819.2676, 2846.3894, 2872.3164, 2876.3669, 2877.3923, 2884.3479, 2887.3311, 2888.3215, 2891.4258, 2901.3557, 2919.4480, 3002.4827, 3029.4448, 3036.5105, 3053.4414, 3067.4578, 3081.6121, 3126.7246, 3151.3789, 3169.6074, 3184.4443, 3192.2090, 3232.5308, 3237.5469, 3248.6238, 3264.5508, 3316.7190, 3330.5715, 3334.6743, 3339.6362, 3346.5017, 3350.5159, 3396.6165, 3412.6321, 3428.5723, 3435.6877, 3457.6028, 3471.6187, 3476.5935, 3489.6655, 3503.7874, 3624.7163, 3639.7131, 3645.7556, 3651.6982, 3692.0066, 3721.6226, 3990.8337, 4074.9656, 4093.8804, 4147.9131, 4153.9287, 4160.9331, 4174.0122, 4177.8853, 4253.9565, 4416.9512, 4452.1763, 4459.3091, 4466.1865, 4679.2988, 4844.0703

42. F1MYI4 **Mass:** 12478 **Score:** 17 **Expect:** 4.7e+002 **Matches:** 5

Uncharacterized protein OS=Bos taurus GN=IQCJ PE=4 SV=2

Observed	Mr(expt)	Mr(calc)	ppm	Start	End	Miss	Peptide
712.4277	711.4204	711.4279	-10.55	9 -	14	0	R.LQNPLK.Q
880.4593	879.4521	879.4338	20.8	1 -	7	0	-.FSQEELK.R
883.4774	882.4701	882.4559	16.1	64 -	70	1	R.QEPPEKR.S
1087.5496	1086.5423	1086.5193	21.2	71 -	81	0	R.SPSPPSVSSDK.L
2813.3884	2812.3811	2812.3813	-0.08	15 -	38	1	K.QVNDGKYLLENHQLAMDVENNIQK.C

No match to: 706.3109, 710.3683, 712.2512, 717.4089, 719.3850, 722.4312, 729.4136, 734.4397, 735.4166, 747.4490, 750.4445, 756.3809, 759.4347, 772.4293, 779.4761, 783.3987, 799.3317, 818.4884, 823.4870, 829.4492, 831.4715, 832.3093, 834.3143, 842.4991, 856.3599, 862.4179, 870.4754, 873.3850, 876.2836, 887.4115, 889.4967, 890.4962, 891.4855, 895.5654, 900.5347, 909.5356, 923.5190, 925.4849, 927.5414, 929.4692, 936.4928, 938.4988, 944.5178, 945.5171, 949.5491, 955.5369, 957.5576, 967.4212, 972.4985, 974.4442, 976.4541, 977.5377, 978.5416, 981.5450, 983.6256, 988.4442, 993.5175, 1007.5735, 1015.4863, 1021.5621, 1025.5615, 1031.5157, 1034.5802, 1044.5248, 1046.5699, 1051.5020, 1056.5302, 1057.5824, 1074.5834, 1099.6482, 1113.5366, 1118.5896, 1120.5878, 1122.5590, 1133.6019, 1145.6392, 1168.6099, 1178.6198, 1180.6312, 1185.6841, 1187.6882, 1194.5767, 1216.7070, 1221.6462, 1248.6903, 1254.6893, 1255.6096, 1264.6997, 1269.6152, 1273.6505, 1281.6354, 1292.7681, 1301.6294, 1312.7516, 1314.7264, 1315.6790, 1317.6367, 1322.6362, 1325.6277, 1336.7238, 1338.7152, 1349.6917, 1351.6910, 1352.6709, 1361.6908, 1365.5895, 1368.6443, 1372.6029, 1382.5973, 1383.7275, 1390.7019, 1397.7428, 1412.6068, 1414.6772, 1420.7469, 1442.8217, 1454.7571, 1457.7417, 1467.7429, 1485.7374, 1486.7495, 1488.7402, 1494.7421, 1497.8790, 1500.7224, 1513.7649, 1517.8065, 1518.8060, 1520.7325, 1527.7305, 1528.7021, 1531.6841, 1537.8251, 1539.7679, 1555.7635, 1556.8414, 1562.7877, 1567.7642, 1570.7750, 1571.8055, 1578.7964, 1580.8280, 1581.8616, 1583.7979, 1585.7704, 1592.7926, 1610.6801, 1616.7987, 1624.7253, 1628.7031, 1628.8617, 1642.7180, 1647.8546, 1649.8544, 1650.8427, 1657.9053, 1663.8506, 1664.8582, 1666.8584, 1680.8226, 1683.8226, 1696.8158, 1698.7761, 1701.9166, 1706.8654, 1707.8828, 1710.8179, 1711.7795, 1714.7788, 1715.8093, 1717.9019, 1726.8021, 1728.7821, 1730.8467, 1739.6987, 1747.9039, 1753.7146, 1755.7096, 1758.9032, 1771.0311, 1774.9202, 1783.6785, 1787.8689, 1797.6923, 1809.0245, 1811.7025, 1816.9102, 1836.8322, 1838.8318, 1850.8512, 1862.9421, 1866.8452, 1928.0237, 1937.9972, 1953.8524, 1961.0308, 1965.8595, 1970.0011, 1982.0062, 1984.0415, 1995.8663, 2007.9470, 2027.1273, 2061.0056, 2067.0483, 2086.1091, 2099.1079, 2109.0042, 2136.8455, 2144.0537, 2150.8601, 2173.1655, 2179.0540, 2239.1436, 2251.1506, 2255.1462, 2265.0896, 2270.0999, 2274.0774, 2285.1733, 2286.1472, 2289.0583, 2293.1331, 2301.2546, 2303.0488, 2324.0913, 2340.1006, 2343.1824, 2352.9204, 2344.1963, 2356.0154, 2359.1130, 2375.0210, 2389.0332, 2414.2415, 2467.2080, 2525.2712, 2541.2361, 2542.2598, 2646.9749, 2660.9883, 2675.0051, 2679.0034, 2689.0183, 2692.0198, 2693.0125, 2695.0129, 2705.2800, 2717.3184, 2763.3374, 2771.4116, 2787.2793, 2792.2427, 2795.3037, 2798.2268, 2803.2610, 2809.4873, 2814.2390, 2819.2676, 2846.3894, 2872.3164, 2876.3669, 2877.3923, 2884.3479, 2887.3311, 2888.3215, 2891.4258, 2901.3557, 2919.4480, 3002.4827, 3029.4448, 3036.5105, 3053.4414, 3067.4578, 3071.4341, 3081.6121, 3126.7246, 3151.3789, 3169.6074, 3184.4443, 3192.2090, 3232.5308, 3237.5469, 3248.6238, 3264.5508, 3305.5381, 3316.7190, 3321.5339, 3330.5715, 3334.6743, 3339.6362, 3346.5017, 3350.5159, 3396.6165, 3412.6321, 3428.5723, 3435.6877, 3457.6028, 3471.6187, 3476.5935, 3489.6655, 3503.7874, 3624.7163, 3639.7131, 3645.7556, 3651.6982, 3692.0066, 3721.6226, 3990.8337, 4074.9656, 4093.8804, 4147.9131, 4153.9287, 4160.9331, 4174.0122, 4177.8853, 4253.9565, 4416.9512, 4452.1763, 4459.3091, 4466.1865, 4679.2988, 4844.0703

43. E1BPX5 **Mass:** 48955 **Score:** 17 **Expect:** 4.7e+002 **Matches:** 15

Uncharacterized protein OS=Bos taurus GN=DYX1C1 PE=4 SV=2

Observed	Mr(expt)	Mr(calc)	ppm	Start	End	Miss	Peptide
717.4089	716.4017	716.4068	-7.19	168 -	173	1	R.KAEELK.I
759.4347	758.4274	758.4399	-16.42	208 -	214	1	K.NLATKGR.N
891.4855	890.4782	890.4895	-12.70	142 -	148	2	R.KKIEDMK.E
927.5414	926.5341	926.5232	11.8	366 -	372	2	R.MKAHIRR.G + Oxidation (M)
944.5178	943.5105	943.5087	1.93	103 -	110	0	K.SILQAQER.A

1015.4863	1014.4790	1014.4878	-8.66	128 - 135	0	K.YTLNVMMK.I + Oxidation (M)
1031.5157	1030.5084	1030.4827	24.9	128 - 135	0	K.YTLNVMMK.I + 2 Oxidation (M)
1099.6482	1098.6409	1098.6397	1.15	169 - 177	1	K.AEELKIIQR.E
1185.6841	1184.6768	1184.6513	21.5	401 - 410	1	K.IVQNDAEKIR.N
1301.6294	1300.6221	1300.6299	-5.97	285 - 294	1	K.EEEKNPEWLK.E
1531.6841	1530.6768	1530.7058	-18.93	124 - 135	1	R.EDQKYTLNVMMK.I + 2 Oxidation (M)
1570.7750	1569.7677	1569.7998	-20.45	395 - 408	1	K.IDPSNKIVQNDAEK.I
1581.8616	1580.8543	1580.8311	14.7	213 - 225	2	K.GRNSENIFFEKLK.E
1664.8582	1663.8509	1663.8893	-23.06	350 - 365	0	K.ALEILTPPVADNANAR.M
3081.6121	3080.6048	3080.5315	23.8	246 - 271	2	R.VFPTALRESQVAEEEEWLHKQAEAQR.A

No match to: 706.3109, 710.3683, 712.2512, 712.4277, 719.3850, 722.4312, 729.4136, 734.4397, 735.4166, 747.4490, 750.4445, 756.3809, 772.4293, 779.4761, 783.3987, 799.3317, 818.4884, 823.4870, 829.4492, 831.4715, 832.3093, 834.3143, 842.4991, 856.3599, 862.4179, 870.4754, 873.3850, 876.2836, 880.4593, 883.4774, 887.4115, 889.4967, 890.4962, 895.5654, 900.5347, 909.5356, 923.5190, 925.4849, 929.4692, 936.4928, 938.4988, 945.5171, 949.5491, 955.5369, 957.5576, 967.4212, 972.4985, 974.4442, 976.4541, 977.5377, 978.5416, 981.5450, 983.6256, 988.4442, 993.5175, 1007.5735, 1021.5621, 1025.5615, 1034.5802, 1044.5248, 1046.5699, 1051.5020, 1056.5302, 1057.5824, 1074.5834, 1087.5496, 1113.5366, 1118.5896, 1120.5878, 1122.5590, 1133.6019, 1145.6392, 1168.6099, 1178.6198, 1180.6312, 1187.6882, 1194.5767, 1216.7070, 1221.6462, 1248.6903, 1254.6893, 1255.6096, 1264.6997, 1269.6152, 1273.6505, 1281.6354, 1292.7681, 1312.7516, 1314.7264, 1315.6790, 1317.6367, 1322.6362, 1325.6277, 1336.7238, 1338.7152, 1349.6917, 1351.6910, 1352.6709, 1361.6908, 1365.5895, 1368.6443, 1372.6029, 1382.5973, 1383.7275, 1390.7019, 1397.7428, 1412.6068, 1414.6772, 1420.7469, 1442.8217, 1454.7571, 1457.7417, 1467.7429, 1485.7374, 1486.7495, 1488.7402, 1494.7421, 1497.8790, 1500.7224, 1513.7649, 1517.8065, 1518.8060, 1520.7325, 1527.7305, 1528.7021, 1537.8251, 1539.7679, 1555.7635, 1556.8414, 1562.7877, 1567.7642, 1571.8055, 1578.7964, 1580.8280, 1583.7979, 1585.7704, 1592.7926, 1610.6801, 1616.7987, 1624.7253, 1628.7031, 1628.8617, 1642.7180, 1647.8546, 1649.8544, 1650.8427, 1657.9053, 1663.8506, 1666.8584, 1680.8226, 1683.8226, 1696.8158, 1698.7761, 1701.9166, 1706.8654, 1707.8828, 1710.8179, 1711.7795, 1714.7788, 1715.8093, 1717.9019, 1726.8021, 1728.7821, 1730.8467, 1739.6987, 1747.9039, 1753.7146, 1755.7096, 1758.9032, 1771.0311, 1774.9202, 1783.6785, 1787.8689, 1797.6923, 1809.0245, 1811.7025, 1816.9102, 1836.8322, 1838.8318, 1850.8512, 1862.9421, 1866.8452, 1928.0237, 1937.9972, 1953.8524, 1961.0308, 1965.8595, 1970.0011, 1982.0062, 1984.0415, 1995.8663, 2007.9470, 2027.1273, 2061.0056, 2067.0483, 2086.1091, 2099.1079, 2109.0042, 2136.8455, 2144.0537, 2150.8601, 2173.1655, 2179.0540, 2239.1436, 2251.1506, 2255.1462, 2265.0896, 2270.0999, 2274.0774, 2285.1733, 2286.1472, 2289.0583, 2293.1331, 2301.2546, 2303.0488, 2324.0913, 2340.1006, 2343.1824, 2344.1963, 2352.9204, 2356.0154, 2359.1130, 2375.0210, 2389.0332, 2414.2415, 2467.2080, 2525.2712, 2541.2361, 2542.2598, 2646.9749, 2660.9883, 2675.0051, 2679.0034, 2689.0183, 2692.0198, 2693.0125, 2695.0129, 2705.2800, 2717.3184, 2763.3374, 2771.4116, 2787.2793, 2792.2427, 2795.3037, 2798.2268, 2803.2610, 2809.4873, 2813.3884, 2814.2390, 2819.2676, 2846.3894, 2872.3164, 2876.3669, 2877.3923, 2884.3479, 2887.3311, 2888.3215, 2891.4258, 2901.3557, 2919.4480, 3002.4827, 3029.4448, 3036.5105, 3053.4414, 3067.4578, 3071.4341, 3126.7246, 3151.3789, 3169.6074, 3184.4443, 3192.2090, 3232.5308, 3237.5469, 3248.6238, 3264.5508, 3305.5381, 3316.7190, 3321.5339, 3330.5715, 3334.6743, 3339.6362, 3346.5017, 3350.5159, 3396.6165, 3412.6321, 3428.5723, 3435.6877, 3457.6028, 3471.6187, 3476.5935, 3489.6655, 3503.7874, 3624.7163, 3639.7131, 3645.7556, 3651.6982, 3692.0066, 3721.6226, 3990.8337, 4074.9656, 4093.8804, 4147.9131, 4153.9287, 4160.9331, 4174.0122, 4177.8853, 4253.9565, 4416.9512, 4452.1763, 4459.3091, 4466.1865, 4679.2988, 4844.0703

44. F6QNC4 **Mass:** 81939 **Score:** 17 **Expect:** 4.8e+002 **Matches:** 17

Uncharacterized protein OS=Bos taurus GN=PCCA PE=4 SV=1

Observed	Mr(expt)	Mr(calc)	ppm	Start	End	Miss	Peptide
717.4089	716.4017	716.4181	-22.89	460	- 465	1	R.TEALKR.M
870.4754	869.4681	869.4872	-21.88	379	- 385	1	K.GYPLRHK.Q
967.4212	966.4139	966.4263	-12.77	708	- 716	0	K.MQNSMTAGK.T
981.5450	980.5378	980.5147	23.5	81	- 88	2	K.TCKKMGIK.T + Oxidation (M)
1099.6482	1098.6409	1098.6484	-6.81	20	- 27	2	R.RWPKLLMR.S
1269.6152	1268.6079	1268.5853	17.8	708	- 719	1	K.MQNSMTAGKTGK.V + Oxidation (M)
1420.7469	1419.7396	1419.7259	9.67	2	- 16	0	M.AGFWTGAAALATAGR.R
1467.7429	1466.7356	1466.7187	11.5	465	- 476	1	K.RMEDALDSYVIR.G
1567.7642	1566.7569	1566.7613	-2.79	1	- 16	0	-.MAGFWTGAAALATAGR.R + Oxidation (M)
1650.8427	1649.8354	1649.8447	-5.61	211	- 227	1	R.EIGYPVMIKASAGGGGK.G + Oxidation (M)
1664.8582	1663.8509	1663.8530	-1.23	89	- 104	0	K.TVAIHSDVDASSVHVK.M
1707.8828	1706.8755	1706.8675	4.71	1	- 17	1	-.MAGFWTGAAALATAGRR.G
1730.8467	1729.8394	1729.8345	2.84	633	- 648	0	R.EAGGNMSIQFLGTVYK.V + Oxidation (M)
1953.8524	1952.8451	1952.8799	-17.81	228	- 243	2	K.GMRIAWDDEETRDGFR.F
1970.0011	1968.9938	1969.0091	-7.77	633	- 650	1	R.EAGGNMSIQFLGTVYKVR.I
3503.7874	3502.7801	3502.7885	-2.39	400	- 430	2	R.VYAEDPYKSFGLPSVGRLSQYEEPIHLPGVR.V
3639.7131	3638.7058	3638.7743	-18.81	85	- 119	2	K.MGIKTVAIHSDVDASSVHVKMADEAVCVGPAPTSK.S + 2 Oxidation (M)

No match to: 706.3109, 710.3683, 712.2512, 712.4277, 719.3850, 722.4312, 729.4136, 734.4397, 735.4166, 747.4490, 750.4445, 756.3809, 759.4347, 772.4293, 779.4761, 783.3987, 799.3317, 818.4884, 823.4870, 829.4492, 831.4715, 832.3093, 834.3143, 842.4991, 856.3599, 862.4179, 873.3850, 876.2836, 880.4593, 883.4774, 887.4115, 889.4967, 890.4962, 891.4855, 895.5654, 900.5347, 909.5356, 923.5190, 925.4849, 927.5414, 929.4692, 936.4928, 938.4988, 944.5178, 945.5171, 949.5491, 955.5369, 957.5576, 972.4985, 974.4442, 976.4541, 977.5377, 978.5416, 983.6256, 988.4442, 993.5175, 1007.5735, 1015.4863, 1021.5621, 1025.5615, 1031.5157, 1034.5802, 1044.5248, 1046.5699, 1051.5020, 1056.5302, 1057.5824, 1074.5834, 1087.5496, 1113.5366, 1118.5896, 1120.5878, 1122.5590, 1133.6019, 1145.6392, 1168.6099, 1178.6198, 1180.6312, 1185.6841, 1187.6882, 1194.5767, 1216.7070, 1221.6462, 1248.6903, 1254.6893, 1255.6096, 1264.6997, 1273.6505, 1281.6354, 1292.7681, 1301.6294, 1312.7516, 1314.7264, 1315.6790, 1317.6367, 1322.6362, 1325.6277, 1336.7238, 1338.7152, 1349.6917, 1351.6910, 1352.6709, 1361.6908, 1365.5895, 1368.6443, 1372.6029, 1382.5973, 1383.7275, 1390.7019, 1397.7428, 1412.6068, 1414.6772, 1442.8217, 1454.7571, 1457.7417, 1485.7374, 1486.7495, 1488.7402, 1494.7421, 1497.8790, 1500.7224, 1513.7649, 1517.8065, 1518.8060, 1520.7325, 1527.7305, 1528.7021, 1531.6841, 1537.8251, 1539.7679, 1555.7635, 1556.8414, 1562.7877, 1570.7750, 1571.8055, 1578.7964, 1580.8280, 1581.8616, 1583.7979, 1585.7704, 1592.7926, 1610.6801, 1616.7987, 1624.7253, 1628.7031, 1628.8617, 1642.7180, 1647.8546, 1649.8544, 1657.9053, 1663.8506, 1666.8584, 1680.8226, 1683.8226, 1696.8158, 1698.7761, 1701.9166, 1706.8654, 1710.8179, 1711.7795, 1714.7788, 1715.8093, 1717.9019, 1726.8021, 1728.7821, 1739.6987, 1747.9039, 1753.7146, 1755.7096, 1758.9032, 1771.0311, 1774.9202, 1783.6785, 1787.8689, 1797.6923, 1809.0245, 1811.7025, 1816.9102, 1836.8322, 1838.8318, 1850.8512, 1862.9421, 1866.8452, 1928.0237, 1937.9972, 1961.0308, 1965.8595, 1982.0062, 1984.0415, 1995.8663, 2007.9470, 2027.1273, 2061.0056, 2067.0483, 2086.1091, 2099.1079, 2109.0042, 2136.8455, 2144.0537, 2150.8601, 2173.1655, 2179.0540, 2239.1436, 2251.1506, 2255.1462, 2265.0896, 2270.0999, 2274.0774, 2285.1733, 2286.1472, 2289.0583, 2293.1331, 2301.2546, 2303.0488, 2324.0913, 2340.1006, 2343.1824, 2344.1963, 2352.9204, 2356.0154, 2359.1130, 2375.0210, 2389.0332, 2414.2415, 2467.2080, 2525.2712, 2541.2361, 2542.2598, 2646.9749, 2660.9883, 2675.0051, 2679.0034, 2689.0183, 2692.0198, 2693.0125, 2695.0129, 2705.2800, 2717.3184, 2763.3374, 2771.4116, 2787.2793, 2792.2427, 2795.3037, 2798.2268, 2803.2610, 2809.4873, 2813.3884, 2814.2390, 2819.2676, 2846.3894, 2872.3164, 2876.3669, 2877.3923, 2884.3479, 2887.3311, 2888.3215, 2891.4258, 2901.3557, 2919.4480, 3002.4827, 3029.4448, 3036.5105, 3053.4414, 3067.4578, 3071.4341, 3081.6121, 3126.7246, 3151.3789, 3169.6074, 3184.4443, 3192.2090, 3232.5308, 3237.5469, 3248.6238, 3264.5508, 3305.5381, 3316.7190, 3321.5339, 3330.5715, 3334.6743, 3339.6362, 3346.5017, 3350.5159, 3396.6165, 3412.6321, 3428.5723, 3435.6877, 3457.6028, 3471.6187, 3476.5935, 3489.6655, 3624.7163, 3645.7556, 3651.6982, 3692.0066, 3721.6226, 3990.8337, 4074.9656, 4093.8804, 4147.9131, 4153.9287, 4160.9331, 4174.0122, 4177.8853, 4253.9565, 4416.9512, 4452.1763, 4459.3091, 4466.1865, 4679.2988, 4844.0703

45. Q3T071 **Mass:** 23858 **Score:** 17 **Expect:** 4.8e+002 **Matches:** 7

Translation machinery-associated protein 16 OS=Bos taurus GN=TMA16 PE=2 SV=1

Observed	Mr(expt)	Mr(calc)	ppm	Start	End	Miss	Peptide
1056.5302	1055.5229	1055.5155	7.02	102	- 109	2	R.QGRRHCSR.E
1074.5834	1073.5761	1073.5539	20.7	161	- 170	1	K.VCASDAVPKK.C
1361.6908	1360.6835	1360.6955	-8.81	158	- 169	2	K.MRKVCASDAVPK.K
1497.8790	1496.8717	1496.8674	2.88	42	- 54	2	K.NEKALRLNLIGEK.L
1701.9166	1700.9093	1700.9196	-6.05	174	- 189	1	K.KAVTTIDGDLGELELK.D
1707.8828	1706.8755	1706.8522	13.7	72	- 84	2	K.RDACELIERYLNR.F

3489.6655	3488.6582	3488.7364	-22.42	123 - 151	2	R.QYQAHGLEIPDILNADNLKTFREWDFDLK.K

No match to: 706.3109, 710.3683, 712.2512, 712.4277, 717.4089, 719.3850, 722.4312, 729.4136, 734.4397, 735.4166, 747.4490, 750.4445, 756.3809, 759.4347, 772.4293, 779.4761, 783.3987, 799.3317, 818.4884, 823.4870, 829.4492, 831.4715, 832.3093, 834.3143, 842.4991, 856.3599, 862.4179, 870.4754, 873.3850, 876.2836, 880.4593, 883.4774, 887.4115, 889.4967, 890.4962, 891.4855, 895.5654, 900.5347, 909.5356, 923.5190, 925.4849, 927.5414, 929.4692, 936.4928, 938.4988, 944.5178, 945.5171, 949.5491, 955.5369, 957.5576, 967.4212, 972.4985, 974.4442, 976.4541, 977.5377, 978.5416, 981.5450, 983.6256, 988.4442, 993.5175, 1007.5735, 1015.4863, 1021.5621, 1025.5615, 1031.5157, 1034.5802, 1044.5248, 1046.5699, 1051.5020, 1057.5824, 1087.5496, 1099.6482, 1113.5366, 1118.5896, 1120.5878, 1122.5590, 1133.6019, 1145.6392, 1168.6099, 1178.6198, 1180.6312, 1185.6841, 1187.6882, 1194.5767, 1216.7070, 1221.6462, 1248.6903, 1254.6893, 1255.6096, 1264.6997, 1269.6152, 1273.6505, 1281.6354, 1292.7681, 1301.6294, 1312.7516, 1314.7264, 1315.6790, 1317.6367, 1322.6362, 1325.6277, 1336.7238, 1338.7152, 1349.6917, 1351.6910, 1352.6709, 1365.5895, 1368.6443, 1372.6029, 1382.5973, 1383.7275, 1390.7019, 1397.7428, 1412.6068, 1414.6772, 1420.7469, 1442.8217, 1454.7571, 1457.7417, 1467.7429, 1485.7374, 1486.7495, 1488.7402, 1494.7421, 1500.7224, 1513.7649, 1517.8065, 1518.8060, 1520.7325, 1527.7305, 1528.7021, 1531.6841, 1537.8251, 1539.7679, 1555.7635, 1556.8414, 1562.7877, 1567.7642, 1570.7750, 1571.8055, 1578.7964, 1580.8280, 1581.8616, 1583.7979, 1585.7704, 1592.7926, 1610.6801, 1616.7987, 1624.7253, 1628.7031, 1628.8617, 1642.7180, 1647.8546, 1649.8544, 1650.8427, 1657.9053, 1663.8506, 1664.8582, 1666.8584, 1680.8226, 1683.8226, 1696.8158, 1698.7761, 1706.8654, 1710.8179, 1711.7795, 1714.7788, 1715.8093, 1717.9019, 1726.8021, 1728.7821, 1730.8467, 1739.6987, 1747.9039, 1753.7146, 1755.7096, 1758.9032, 1771.0311, 1774.9202, 1783.6785, 1787.8689, 1797.6923, 1809.0245, 1811.7025, 1816.9102, 1836.8322, 1838.8318, 1850.8512, 1862.9421, 1866.8452, 1928.0237, 1937.9972, 1953.8524, 1961.0308, 1965.8595, 1970.0011, 1982.0062, 1984.0415, 1995.8663, 2007.9470, 2027.1273, 2061.0056, 2067.0483, 2086.1091, 2099.1079, 2109.0042, 2136.8455, 2144.0537, 2150.8601, 2173.1655, 2179.0540, 2239.1436, 2251.1506, 2255.1462, 2265.0896, 2270.0999, 2274.0774, 2285.1733, 2286.1472, 2289.0583, 2293.1331, 2301.2546, 2303.0488, 2324.0913, 2340.1006, 2343.1824, 2344.1963, 2352.9204, 2356.0154, 2359.1130, 2375.0210, 2389.0332, 2414.2415, 2467.2080, 2525.2712, 2541.2361, 2542.2598, 2646.9749, 2660.9883, 2675.0051, 2679.0034, 2689.0183, 2692.0198, 2693.0125, 2695.0129, 2705.2800, 2717.3184, 2763.3374, 2771.4116, 2787.2793, 2792.2427, 2795.3037, 2798.2268, 2803.2610, 2809.4873, 2813.3884, 2814.2390, 2819.2676, 2846.3894, 2872.3164, 2876.3669, 2877.3923, 2884.3479, 2887.3311, 2888.3215, 2891.4258, 2901.3557, 2919.4480, 3002.4827, 3029.4448, 3036.5105, 3053.4414, 3067.4578, 3071.4341, 3081.6121, 3126.7246, 3151.3789, 3169.6074, 3184.4443, 3192.2090, 3232.5308, 3237.5469, 3248.6238, 3264.5508, 3305.5381, 3316.7190, 3321.5339, 3330.5715, 3334.6743, 3339.6362, 3346.5017, 3350.5159, 3396.6165, 3412.6321, 3428.5723, 3435.6877, 3457.6028, 3471.6187, 3476.5935, 3503.7874, 3624.7163, 3639.7131, 3645.7556, 3651.6982, 3692.0066, 3721.6226, 3990.8337, 4074.9656, 4093.8804, 4147.9131, 4153.9287, 4160.9331, 4174.0122, 4177.8853, 4253.9565, 4416.9512, 4452.1763, 4459.3091, 4466.1865, 4679.2988, 4844.0703

46. F1MUI3 **Mass:** 23858 **Score:** 17 **Expect:** 4.8e+002 **Matches:** 7
Translation machinery-associated protein 16 OS=Bos taurus GN=TMA16 PE=4 SV=1

Observed	Mr(expt)	Mr(calc)	ppm	Start End	Miss	Peptide
1056.5302	1055.5229	1055.5155	7.02	102 - 109	2	R.QGRRHCSR.E
1074.5834	1073.5761	1073.5539	20.7	161 - 170	1	K.VCASDAVPKK.C
1361.6908	1360.6835	1360.6955	-8.81	158 - 169	2	K.MRKVCASDAVPK.K
1497.8790	1496.8717	1496.8674	2.88	42 - 54	2	K.NEKALRLNLIGEK.L
1701.9166	1700.9093	1700.9196	-6.05	174 - 189	1	K.KAVTTIDGDLGELELK.D
1707.8828	1706.8755	1706.8522	13.7	72 - 84	2	K.RDACELIERYLNR.F
3489.6655	3488.6582	3488.7364	-22.42	123 - 151	2	R.QYQAHGLEIPDILNADNLKTFREWDFDLK.K

No match to: 706.3109, 710.3683, 712.2512, 712.4277, 717.4089, 719.3850, 722.4312, 729.4136, 734.4397, 735.4166, 747.4490, 750.4445, 756.3809, 759.4347, 772.4293, 779.4761, 783.3987, 799.3317, 818.4884, 823.4870, 829.4492, 831.4715, 832.3093, 834.3143, 842.4991, 856.3599, 862.4179, 870.4754, 873.3850, 876.2836, 880.4593, 883.4774, 887.4115, 889.4967, 890.4962, 891.4855, 895.5654, 900.5347, 909.5356, 923.5190, 925.4849, 927.5414, 929.4692, 936.4928, 938.4988, 944.5178, 945.5171, 949.5491, 955.5369, 957.5576, 967.4212, 972.4985, 974.4442, 976.4541, 977.5377, 978.5416, 981.5450, 983.6256, 988.4442, 993.5175, 1007.5735, 1015.4863, 1021.5621, 1025.5615, 1031.5157, 1034.5802, 1044.5248, 1046.5699, 1051.5020, 1057.5824, 1087.5496, 1099.6482, 1113.5366, 1118.5896, 1120.5878, 1122.5590, 1133.6019, 1145.6392, 1168.6099, 1178.6198, 1180.6312, 1185.6841, 1187.6882, 1194.5767, 1216.7070, 1221.6462, 1248.6903, 1254.6893, 1255.6096, 1264.6997, 1269.6152, 1273.6505, 1281.6354, 1292.7681, 1301.6294, 1312.7516, 1314.7264, 1315.6790, 1317.6367, 1322.6362, 1325.6277, 1336.7238, 1338.7152, 1349.6917, 1351.6910, 1352.6709, 1365.5895, 1368.6443, 1372.6029, 1382.5973, 1383.7275, 1390.7019, 1397.7428, 1412.6068, 1414.6772, 1420.7469, 1442.8217, 1454.7571, 1457.7417, 1467.7429, 1485.7374, 1486.7495, 1488.7402, 1494.7421, 1500.7224, 1513.7649, 1517.8065, 1518.8060, 1520.7325, 1527.7305, 1528.7021, 1531.6841, 1537.8251, 1539.7679, 1555.7635, 1556.8414, 1562.7877, 1567.7642, 1570.7750, 1571.8055, 1578.7964, 1580.8280, 1581.8616, 1583.7979, 1585.7704, 1592.7926, 1610.6801, 1616.7987, 1624.7253, 1628.7031, 1628.8617, 1642.7180, 1647.8546, 1649.8544, 1650.8427, 1657.9053, 1663.8506, 1664.8582, 1666.8584, 1680.8226, 1683.8226, 1696.8158, 1698.7761, 1706.8654, 1710.8179, 1711.7795, 1714.7788, 1715.8093, 1717.9019, 1726.8021, 1728.7821, 1730.8467, 1739.6987, 1747.9039, 1753.7146, 1755.7096, 1758.9032, 1771.0311, 1774.9202, 1783.6785, 1787.8689, 1797.6923, 1809.0245, 1811.7025, 1816.9102, 1836.8322, 1838.8318, 1850.8512, 1862.9421, 1866.8452, 1928.0237, 1937.9972, 1953.8524, 1961.0308, 1965.8595, 1970.0011, 1982.0062, 1984.0415, 1995.8663, 2007.9470, 2027.1273, 2061.0056, 2067.0483, 2086.1091, 2099.1079, 2109.0042, 2136.8455, 2144.0537, 2150.8601, 2173.1655, 2179.0540, 2239.1436, 2251.1506, 2255.1462, 2265.0896, 2270.0999, 2274.0774, 2285.1733, 2286.1472, 2289.0583, 2293.1331, 2301.2546, 2303.0488, 2324.0913, 2340.1006, 2343.1824, 2344.1963, 2352.9204, 2356.0154, 2359.1130, 2375.0210, 2389.0332, 2414.2415, 2467.2080, 2525.2712, 2541.2361, 2542.2598, 2646.9749, 2660.9883, 2675.0051, 2679.0034, 2689.0183, 2692.0198, 2693.0125, 2695.0129, 2705.2800, 2717.3184, 2763.3374, 2771.4116, 2787.2793, 2792.2427, 2795.3037, 2798.2268, 2803.2610, 2809.4873, 2813.3884, 2814.2390, 2819.2676, 2846.3894, 2872.3164, 2876.3669, 2877.3923, 2884.3479, 2887.3311, 2888.3215, 2891.4258, 2901.3557, 2919.4480, 3002.4827, 3029.4448, 3036.5105, 3053.4414, 3067.4578, 3071.4341, 3081.6121, 3126.7246, 3151.3789, 3169.6074, 3184.4443, 3192.2090, 3232.5308, 3237.5469, 3248.6238, 3264.5508, 3305.5381, 3316.7190, 3321.5339, 3330.5715, 3334.6743, 3339.6362, 3346.5017, 3350.5159, 3396.6165, 3412.6321, 3428.5723, 3435.6877, 3457.6028, 3471.6187, 3476.5935, 3503.7874, 3624.7163, 3639.7131, 3645.7556, 3651.6982, 3692.0066, 3721.6226, 3990.8337, 4074.9656, 4093.8804, 4147.9131, 4153.9287, 4160.9331, 4174.0122, 4177.8853, 4253.9565, 4416.9512, 4452.1763, 4459.3091, 4466.1865, 4679.2988, 4844.0703

47. F1MCM7 **Mass:** 143110 **Score:** 17 **Expect:** 4.8e+002 **Matches:** 21
Uncharacterized protein OS=Bos taurus GN=KIAA1683 PE=4 SV=2

Observed	Mr(expt)	Mr(calc)	ppm	Start End	Miss	Peptide
747.4490	746.4417	746.4360	7.60	496 - 502	0	R.LAAMITK.T
831.4715	830.4642	830.4610	3.88	561 - 567	1	R.QATREVK.V
1074.5834	1073.5761	1073.5618	13.4	1270 - 1279	0	K.AATAIQSAWR.G
1248.6903	1247.6830	1247.6730	8.07	121 - 131	1	R.QKLISQMMAAK.A
1264.6997	1263.6924	1263.6679	19.4	121 - 131	1	R.QKLISQMMAAK.A + Oxidation (M)
1314.7264	1313.7191	1313.7276	-6.45	553 - 564	2	K.ARATVNARQATR.E
1315.6790	1314.6717	1314.6667	3.83	63 - 73	1	K.ETLLEQPDKDK.T
1336.7238	1335.7165	1335.7221	-4.15	319 - 331	0	K.MPPQTGPAPVITK.T
1349.6917	1348.6844	1348.6888	-3.23	162 - 172	0	R.DIPYHPPQQVR.F
1486.7495	1485.7422	1485.7398	1.62	1024 - 1038	0	R.GSLSAAWGPSLGPMR.L
1494.7421	1493.7348	1493.7040	20.6	412 - 424	0	K.VPLQSCLAAMMNK.T + 2 Oxidation (M)
1520.7325	1519.7252	1519.7374	-8.04	93 - 105	0	R.NVLVDEMDIMVSR.A
1862.9421	1861.9348	1861.9435	-4.65	158 - 172	1	K.VEERDIPYHPPQQVR.F
1953.8524	1952.8451	1952.8465	-0.68	1006 - 1023	0	K.TMGSGMTVMPAPVEVDCR.G + Oxidation (M)
1961.0308	1960.0235	1960.0498	-13.42	123 - 139	2	K.LISQMMAAKAIQASWRR.F
2809.4873	2808.4800	2808.4262	19.2	82 - 105	2	R.LRAVVESQAFRNVLVDEMDIMVSR.A + 2 Oxidation (M)
2846.3894	2845.3821	2845.3669	5.37	453 - 478	1	K.IPPQMCPPATATKTPLQSCLAAMMSK.I + Oxidation (M)
3067.4578	3066.4505	3066.5201	-22.70	93 - 118	2	R.NVLVDEMDIMVSRAATLIQACWRGYR.L
3990.8337	3989.8264	3989.8930	-16.69	1006 - 1043	2	K.TMGSGMTVMPAPVEVDCRGSLSAAWGPSLGPMRLQPSK.G + 2 Oxidation (M)
4074.9656	4073.9583	4073.9290	7.19	1044 - 1084	2	K.GPEDTAMSGGQAPGGAWEPAGGTVPWDVVGSKAAVDPRQPR.E + Oxidation (M)
4452.1763	4451.1690	4451.2548	-19.26	412 - 452	2	K.VPLQSCLAAMMNKTLPQPCPMSTVTITKAPPQVYPQGPVGK.I + Oxidation (M)

No match to: 706.3109, 710.3683, 712.2512, 712.4277, 717.4089, 719.3850, 722.4312, 729.4136, 734.4397, 735.4166, 750.4445, 756.3809, 759.4347, 772.4293, 779.4761, 783.3987, 799.3317, 818.4884, 823.4870, 829.4492, 832.3093, 834.3143, 842.4991, 856.3599, 862.4179, 870.4754, 873.3850, 876.2836, 880.4593, 883.4774, 887.4115, 889.4967, 890.4962, 891.4855, 895.5654, 900.5347, 909.5356,

923.5190, 925.4849, 927.5414, 929.4692, 936.4928, 938.4988, 944.5178, 945.5171, 949.5491, 955.5369, 957.5576, 967.4212, 972.4985, 974.4442, 976.4541, 977.5377, 978.5416, 981.5450, 983.6256, 988.4442, 993.5175, 1007.5735, 1015.4863, 1021.5621, 1025.5615, 1031.5157, 1034.5802, 1044.5248, 1046.5699, 1051.5020, 1056.5302, 1057.5824, 1087.5496, 1099.6482, 1113.5366, 1118.5896, 1120.5878, 1122.5590, 1133.6019, 1145.6392, 1168.6099, 1178.6198, 1180.6312, 1185.6841, 1187.6882, 1194.5767, 1216.7070, 1221.6462, 1254.6893, 1255.6096, 1269.6152, 1273.6505, 1281.6354, 1292.7681, 1301.6294, 1312.7516, 1317.6367, 1322.6362, 1325.6277, 1338.7152, 1351.6910, 1352.6709, 1361.6908, 1365.5895, 1368.6443, 1372.6029, 1382.5973, 1383.7275, 1390.7019, 1397.7428, 1412.6068, 1414.6772, 1420.7469, 1442.8217, 1454.7571, 1457.7417, 1467.7429, 1485.7374, 1488.7402, 1497.8790, 1500.7224, 1513.7649, 1517.8065, 1518.8060, 1527.7305, 1528.7021, 1531.6841, 1537.8251, 1539.7679, 1555.7635, 1556.8414, 1562.7877, 1567.7642, 1570.7750, 1571.8055, 1578.7964, 1580.8280, 1581.8616, 1583.7979, 1585.7704, 1592.7926, 1610.6801, 1616.7987, 1624.7253, 1628.7031, 1628.8617, 1642.7180, 1647.8546, 1649.8544, 1650.8427, 1657.9053, 1663.8506, 1664.8582, 1666.8584, 1680.8226, 1683.8226, 1696.8158, 1698.7761, 1701.9166, 1706.8654, 1707.8828, 1710.8179, 1711.7795, 1714.7788, 1715.8093, 1717.9019, 1726.8021, 1728.7821, 1730.8467, 1739.6987, 1747.9039, 1753.7146, 1755.7096, 1758.9032, 1771.0311, 1774.9202, 1783.6785, 1787.8689, 1797.6923, 1809.0245, 1811.7025, 1816.9102, 1836.8322, 1838.8318, 1850.8512, 1866.8452, 1928.0237, 1937.9972, 1965.8595, 1970.0011, 1982.0062, 1984.0415, 1995.8663, 2007.9470, 2027.1273, 2061.0056, 2067.0483, 2086.1091, 2099.1079, 2109.0042, 2136.8455, 2144.0537, 2150.8601, 2173.1655, 2179.0540, 2239.1436, 2251.1506, 2255.1462, 2265.0896, 2270.0999, 2274.0774, 2285.1733, 2286.1472, 2289.0583, 2293.1331, 2301.2546, 2303.0488, 2324.0913, 2340.1006, 2343.1824, 2344.1963, 2352.9204, 2356.0154, 2359.1130, 2375.0210, 2389.0332, 2414.2415, 2467.2080, 2525.2712, 2541.2361, 2542.2598, 2646.9749, 2660.9883, 2675.0051, 2679.0034, 2689.0183, 2692.0198, 2693.0125, 2695.0129, 2705.2800, 2717.3184, 2763.3374, 2771.4116, 2787.2793, 2792.2427, 2795.3037, 2798.2268, 2803.2610, 2813.3884, 2814.2390, 2819.2676, 2872.3164, 2876.3669, 2877.3923, 2884.3479, 2887.3311, 2888.3215, 2891.4258, 2901.3557, 2919.4480, 3002.4827, 3029.4448, 3036.5105, 3053.4414, 3071.4341, 3081.6121, 3126.7246, 3151.3789, 3169.6074, 3184.4443, 3192.2090, 3232.5308, 3237.5469, 3248.6238, 3264.5508, 3305.5381, 3316.7190, 3321.5339, 3330.5715, 3334.6743, 3339.6362, 3346.5017, 3350.5159, 3396.6165, 3412.6321, 3428.5723, 3435.6877, 3457.6028, 3471.6187, 3476.5935, 3489.6655, 3503.7874, 3624.7163, 3639.7131, 3645.7556, 3651.6982, 3692.0066, 3721.6226, 4093.8804, 4147.9131, 4153.9287, 4160.9331, 4174.0122, 4177.8853, 4253.9565, 4416.9512, 4459.3091, 4466.1865, 4679.2988, 4844.0703

48. F1MWM1 **Mass:** 34838 **Score:** 17 **Expect:** 4.9e+002 **Matches:** 8

Uncharacterized protein OS=Bos taurus GN=MLIP PE=4 SV=1

Observed	Mr(expt)	Mr(calc)	ppm	Start	End	Miss	Peptide
862.4179	861.4106	861.4304	-22.97	53	60	1	K.ASQGRESK.E
1663.8506	1662.8433	1662.8399	2.05	61	74	1	K.EKKPQGMQQSDLFK.A
1696.8158	1695.8085	1695.8428	-20.19	150	165	1	R.HGQALDEPAKTDSISK.D
2414.2415	2413.2342	2413.2047	12.3	160	181	1	K.TDSISKDSTLDPPLEMPTTLPR.A
3321.5339	3320.5266	3320.6068	-24.15	129	159	1	R.GADLQAPSHPERPQAMASQQRHGQALDEPAK.T
3412.6321	3411.6248	3411.6293	-1.30	63	92	1	K.KPQGMQQSDLFKAEYVFIVDSEGEEEVPGR.K
3428.5723	3427.5650	3427.6242	-17.26	63	92	1	K.KPQGMQQSDLFKAEYVFIVDSEGEEEVPGR.K + Oxidation (M)
3489.6655	3488.6582	3488.6888	-8.78	236	265	0	K.YLEDNSDFFSEQDVPAPPKPVSLHPLYQTR.L

No match to: 706.3109, 710.3683, 712.2512, 712.4277, 717.4089, 719.3850, 722.4312, 729.4136, 734.4397, 735.4166, 747.4490, 750.4445, 756.3809, 759.4347, 772.4293, 779.4761, 783.3987, 799.3317, 818.4884, 823.4870, 829.4492, 831.4715, 832.3093, 834.3143, 842.4991, 856.3599, 870.4754, 873.3850, 876.2836, 880.4593, 883.4774, 887.4115, 889.4967, 890.4962, 891.4855, 895.5654, 900.5347, 909.5356, 923.5190, 925.4849, 927.5414, 929.4692, 936.4928, 938.4988, 944.5178, 945.5171, 949.5491, 955.5369, 957.5576, 967.4212, 972.4985, 974.4442, 976.4541, 977.5377, 978.5416, 981.5450, 983.6256, 988.4442, 993.5175, 1007.5735, 1015.4863, 1021.5621, 1025.5615, 1031.5157, 1034.5802, 1044.5248, 1046.5699, 1051.5020, 1056.5302, 1057.5824, 1074.5834, 1087.5496, 1099.6482, 1113.5366, 1118.5896, 1120.5878, 1122.5590, 1133.6019, 1145.6392, 1168.6099, 1178.6198, 1180.6312, 1185.6841, 1187.6882, 1194.5767, 1216.7070, 1221.6462, 1248.6903, 1254.6893, 1255.6096, 1264.6997, 1269.6152, 1273.6505, 1281.6354, 1292.7681, 1301.6294, 1312.7516, 1314.7264, 1315.6790, 1317.6367, 1322.6362, 1325.6277, 1336.7238, 1338.7152, 1349.6917, 1351.6910, 1352.6709, 1361.6908, 1365.5895, 1368.6443, 1372.6029, 1382.5973, 1383.7275, 1390.7019, 1397.7428, 1412.6068, 1414.6772, 1420.7469, 1442.8217, 1454.7571, 1457.7417, 1467.7429, 1485.7374, 1486.7495, 1488.7402, 1494.7421, 1497.8790, 1500.7224, 1513.7649, 1517.8065, 1518.8060, 1520.7325, 1527.7305, 1528.7021, 1531.6841, 1537.8251, 1539.7679, 1555.7635, 1556.8414, 1562.7877, 1567.7642, 1570.7750, 1571.8055, 1578.7964, 1580.8280, 1581.8616, 1583.7979, 1585.7704, 1592.7926, 1610.6801, 1616.7987, 1624.7253, 1628.7031, 1628.8617, 1642.7180, 1647.8546, 1649.8544, 1650.8427, 1657.9053, 1664.8582, 1666.8584, 1680.8226, 1683.8226, 1698.7761, 1701.9166, 1706.8654, 1707.8828, 1710.8179, 1711.7795, 1714.7788, 1715.8093, 1717.9019, 1726.8021, 1728.7821, 1730.8467, 1739.6987, 1747.9039, 1753.7146, 1755.7096, 1758.9032, 1771.0311, 1774.9202, 1783.6785, 1787.8689, 1797.6923, 1809.0245, 1811.7025, 1816.9102, 1836.8322, 1838.8318, 1850.8512, 1862.9421, 1866.8452, 1928.0237, 1937.9972, 1953.8524, 1961.0308, 1965.8595, 1970.0011, 1982.0062, 1984.0415, 1995.8663, 2007.9470, 2027.1273, 2061.0056, 2067.0483, 2086.1091, 2099.1079, 2109.0042, 2136.8455, 2144.0537, 2150.8601, 2173.1655, 2179.0540, 2239.1436, 2251.1506, 2255.1462, 2265.0896, 2270.0999, 2274.0774, 2285.1733, 2286.1472, 2289.0583, 2293.1331, 2301.2546, 2303.0488, 2324.0913, 2340.1006, 2343.1824, 2344.1963, 2352.9204, 2356.0154, 2359.1130, 2375.0210, 2389.0332, 2467.2080, 2525.2712, 2541.2361, 2542.2598, 2646.9749, 2660.9883, 2675.0051, 2679.0034, 2689.0183, 2692.0198, 2693.0125, 2695.0129, 2705.2800, 2717.3184, 2763.3374, 2771.4116, 2787.2793, 2792.2427, 2795.3037, 2798.2268, 2803.2610, 2809.4873, 2813.3884, 2814.2390, 2819.2676, 2846.3894, 2872.3164, 2876.3669, 2877.3923, 2884.3479, 2887.3311, 2888.3215, 2891.4258, 2901.3557, 2919.4480, 3002.4827, 3029.4448, 3036.5105, 3053.4414, 3067.4578, 3071.4341, 3081.6121, 3126.7246, 3151.3789, 3169.6074, 3184.4443, 3192.2090, 3232.5308, 3237.5469, 3248.6238, 3264.5508, 3305.5381, 3316.7190, 3330.5715, 3334.6743, 3339.6362, 3346.5017, 3350.5159, 3396.6165, 3435.6877, 3457.6028, 3471.6187, 3476.5935, 3503.7874, 3624.7163, 3639.7131, 3645.7556, 3651.6982, 3692.0066, 3721.6226, 3990.8337, 4074.9656, 4093.8804, 4147.9131, 4153.9287, 4160.9331, 4174.0122, 4177.8853, 4253.9565, 4416.9512, 4452.1763, 4459.3091, 4466.1865, 4679.2988, 4844.0703

49. Q58CY3 **Mass:** 41067 **Score:** 17 **Expect:** 4.9e+002 **Matches:** 9

Chromosome 20 open reading frame 4 OS=Bos taurus GN=C20orf4 PE=2 SV=1

Observed	Mr(expt)	Mr(calc)	ppm	Start	End	Miss	Peptide
870.4754	869.4681	869.4793	-12.83	170	176	0	R.LPEMKPR.A
1187.6882	1186.6809	1186.6670	11.8	312	322	1	R.SVAVDATLRQK.A
1269.6152	1268.6079	1268.6336	-20.22	50	59	0	R.MGFFLNLQQR.G + Oxidation (M)
1420.7469	1419.7396	1419.7695	-21.03	144	155	2	R.HTKDRVGQNLPR.C
1488.7402	1487.7329	1487.6973	23.9	149	161	1	R.VGQNLPRCGAECK.S
1628.7031	1627.6958	1627.7083	-7.64	156	169	1	R.CGAECKSYQEGLAR.L
1774.9202	1773.9129	1773.9195	-3.73	162	176	1	K.SYQEGLARLPEMKPR.A
4466.1865	4465.1792	4465.2297	-11.31	177	217	2	R.AGTEIRFSELPTQMFPAGATPAEITRHSMDLSYALETVLSK.Q
4679.2988	4678.2915	4678.2537	8.09	66	106	2	R.WDAAREEVDLSPAPEAEVEAMRANLQELDQFLGPYPYTTLK.K + Oxidation (M)

No match to: 706.3109, 710.3683, 712.2512, 712.4277, 717.4089, 719.3850, 722.4312, 729.4136, 734.4397, 735.4166, 747.4490, 750.4445, 756.3809, 759.4347, 772.4293, 779.4761, 783.3987, 799.3317, 818.4884, 823.4870, 829.4492, 831.4715, 832.3093, 834.3143, 842.4991, 856.3599, 862.4179, 873.3850, 876.2836, 880.4593, 883.4774, 887.4115, 889.4967, 890.4962, 891.4855, 895.5654, 900.5347, 909.5356, 923.5190, 925.4849, 927.5414, 929.4692, 936.4928, 938.4988, 944.5178, 945.5171, 949.5491, 955.5369, 957.5576, 967.4212, 972.4985, 974.4442, 976.4541, 977.5377, 978.5416, 981.5450, 983.6256, 988.4442, 993.5175, 1007.5735, 1015.4863, 1021.5621, 1025.5615, 1031.5157, 1034.5802, 1044.5248, 1046.5699, 1051.5020, 1056.5302, 1057.5824, 1074.5834, 1087.5496, 1099.6482, 1113.5366, 1118.5896, 1120.5878, 1122.5590, 1133.6019, 1145.6392, 1168.6099, 1178.6198, 1180.6312, 1185.6841, 1194.5767, 1216.7070, 1221.6462, 1248.6903, 1254.6893, 1255.6096, 1264.6997, 1273.6505, 1281.6354, 1292.7681, 1301.6294, 1312.7516, 1314.7264, 1315.6790, 1317.6367, 1322.6362, 1325.6277, 1336.7238, 1338.7152, 1349.6917, 1351.6910, 1352.6709, 1361.6908, 1365.5895, 1368.6443, 1372.6029, 1382.5973, 1383.7275, 1390.7019, 1397.7428, 1412.6068, 1414.6772, 1442.8217, 1454.7571, 1457.7417, 1467.7429, 1485.7374, 1486.7495, 1494.7421, 1497.8790, 1500.7224, 1513.7649, 1517.8065, 1518.8060, 1520.7325, 1527.7305, 1528.7021, 1531.6841, 1537.8251, 1539.7679, 1555.7635, 1556.8414, 1562.7877, 1567.7642, 1570.7750, 1571.8055, 1578.7964, 1580.8280, 1581.8616, 1583.7979, 1585.7704, 1592.7926, 1610.6801, 1616.7987, 1624.7253, 1628.8617, 1642.7180, 1647.8546, 1649.8544, 1650.8427, 1657.9053, 1663.8506, 1664.8582, 1666.8584, 1680.8226, 1683.8226, 1696.8158, 1698.7761, 1701.9166, 1706.8654, 1707.8828, 1710.8179, 1711.7795, 1714.7788, 1715.8093, 1717.9019, 1726.8021, 1728.7821, 1730.8467, 1739.6987, 1747.9039, 1753.7146, 1755.7096, 1758.9032, 1771.0311, 1783.6785, 1787.8689, 1797.6923, 1809.0245, 1811.7025, 1816.9102, 1836.8322, 1838.8318, 1850.8512, 1862.9421, 1866.8452, 1928.0237, 1937.9972, 1953.8524, 1961.0308, 1965.8595, 1970.0011, 1982.0062, 1984.0415, 1995.8663, 2007.9470, 2027.1273, 2061.0056, 2067.0483, 2086.1091, 2099.1079, 2109.0042, 2136.8455, 2144.0537, 2150.8601, 2173.1655, 2179.0540, 2239.1436, 2251.1506, 2255.1462, 2265.0896, 2270.0999, 2274.0774, 2285.1733, 2286.1472, 2289.0583, 2293.1331, 2301.2546, 2303.0488, 2324.0913, 2340.1006, 2343.1824, 2344.1963, 2352.9204, 2356.0154, 2359.1130, 2375.0210, 2389.0332, 2414.2415, 2467.2080, 2525.2712, 2541.2361, 2542.2598, 2646.9749, 2660.9883, 2675.0051, 2679.0034, 2689.0183, 2692.0198, 2693.0125, 2695.0129, 2705.2800, 2717.3184, 2763.3374, 2771.4116, 2787.2793, 2792.2427, 2795.3037, 2798.2268, 2803.2610, 2809.4873,

2813.3884, 2814.2390, 2819.2676, 2846.3894, 2872.3164, 2876.3669, 2877.3923, 2884.3479, 2887.3311, 2888.3215, 2891.4258, 2901.3557, 2919.4480, 3002.4827, 3029.4448, 3036.5105, 3053.4414, 3067.4578, 3071.4341, 3081.6121, 3126.7246, 3151.3789, 3169.6074, 3184.4443, 3192.2090, 3232.5308, 3237.5469, 3248.6238, 3264.5508, 3305.5381, 3316.7190, 3321.5339, 3330.5715, 3334.6743, 3339.6362, 3346.5017, 3350.5159, 3396.6165, 3412.6321, 3428.5723, 3435.6877, 3457.6028, 3471.6187, 3476.5935, 3489.6655, 3503.7874, 3624.7163, 3639.7131, 3645.7556, 3651.6982, 3692.0066, 3721.6226, 3990.8337, 4074.9656, 4093.8804, 4147.9131, 4153.9287, 4160.9331, 4174.0122, 4177.8853, 4253.9565, 4416.9512, 4452.1763, 4459.3091, 4844.0703

50. E1BDB4 **Mass:** 55638 **Score:** 17 **Expect:** 4.9e+002 **Matches:** 19

Uncharacterized protein OS=Bos taurus GN=CCDC7 PE=4 SV=1

Observed	Mr(expt)	Mr(calc)	ppm	Start	End	Miss	Peptide
712.2512	711.2439	711.2316	17.3	483 -	488	0	R.NSNMCS.-
735.4166	734.4094	734.3996	13.2	344 -	349	1	K.VMKTEK.K
750.4445	749.4372	749.4469	-13.00	341 -	346	2	R.TKKVMK.T + Oxidation (M)
1021.5621	1020.5548	1020.5564	-1.50	334 -	342	2	K.TKATSERTK.K
1034.5802	1033.5729	1033.5808	-7.59	244 -	251	1	K.QKIEEFIK.T
1133.6019	1132.5946	1132.5798	13.1	319 -	327	0	K.LMLENELQK.L + Oxidation (M)
1180.6312	1179.6239	1179.6499	-22.02	419 -	428	2	K.EFLKSEAKTK.T
1361.6908	1360.6835	1360.7099	-19.36	282 -	292	1	K.IFENQANKLER.A
1442.8217	1441.8144	1441.8327	-12.65	43 -	54	2	K.VVRDKLEPMVLK.S + Oxidation (M)
1580.8280	1579.8207	1579.8358	-9.54	406 -	418	1	R.AKQQLAYFLNQEK.E
1585.7704	1584.7631	1584.7665	-2.13	423 -	437	2	K.SEAKTKTTTEMGTGK.L + Oxidation (M)
1666.8584	1665.8511	1665.8209	18.1	442 -	455	1	K.GEDSKYILLENETR.K
1707.8828	1706.8755	1706.8331	24.9	290 -	303	1	K.LERAMNEQLMLEAK.Y + 2 Oxidation (M)
1961.0308	1960.0235	1959.9875	18.4	467 -	482	2	K.TNDKITYPQVRNNQNR.N
2798.2268	2797.2195	2797.2752	-19.92	165 -	188	1	R.QVNQMEEISKDQTFSEAEFPTPDK.T
2809.4873	2808.4800	2808.5459	-23.45	48 -	73	2	K.LEPMVLKSPPTGESIVRYALPIPSSK.T
2814.2390	2813.2317	2813.2702	-13.66	165 -	188	1	R.QVNQMEEISKDQTFSEAEFPTPDK.T + Oxidation (M)
3071.4341	3070.4268	3070.4838	-18.56	293 -	318	1	R.AMNEQLMLEAKYTQIQSDLQVLSEEK.L + 2 Oxidation (M)
3330.5715	3329.5642	3329.6185	-16.29	252 -	281	0	K.THSTEEITDVSVTEPLETSPVTNQLNTMLK.I + Oxidation (M)

No match to: 706.3109, 710.3683, 712.4277, 717.4089, 719.3850, 722.4312, 729.4136, 734.4397, 747.4490, 756.3809, 759.4347, 772.4293, 779.4761, 783.3987, 799.3317, 818.4884, 823.4870, 829.4492, 831.4715, 832.3093, 834.3143, 842.4991, 856.3599, 862.4179, 870.4754, 873.3850, 876.2836, 880.4593, 883.4774, 887.4115, 889.4967, 890.4962, 891.4855, 895.5654, 900.5347, 909.5356, 923.5190, 925.4849, 927.5414, 929.4692, 936.4928, 938.4988, 944.5178, 945.5171, 949.5491, 955.5369, 957.5576, 967.4212, 972.4985, 974.4442, 976.4541, 977.5377, 978.5416, 981.5450, 983.6256, 988.4442, 993.5175, 1007.5735, 1015.4863, 1025.5615, 1031.5157, 1044.5248, 1046.5699, 1051.5020, 1056.5302, 1057.5824, 1074.5834, 1087.5496, 1099.6482, 1113.5366, 1118.5896, 1120.5878, 1122.5590, 1145.6392, 1168.6099, 1178.6198, 1185.6841, 1187.6882, 1194.5767, 1216.7070, 1221.6462, 1248.6903, 1254.6893, 1255.6096, 1264.6997, 1269.6152, 1273.6505, 1281.6354, 1292.7681, 1301.6294, 1312.7516, 1314.7264, 1315.6790, 1317.6367, 1322.6362, 1325.6277, 1336.7238, 1338.7152, 1349.6917, 1351.6910, 1352.6709, 1365.5895, 1368.6443, 1372.6029, 1382.5973, 1383.7275, 1390.7019, 1397.7428, 1412.6068, 1414.6772, 1420.7469, 1454.7571, 1457.7417, 1467.7429, 1485.7374, 1486.7495, 1488.7402, 1494.7421, 1497.8790, 1500.7224, 1513.7649, 1517.8065, 1518.8060, 1520.7325, 1527.7305, 1528.7021, 1531.6841, 1537.8251, 1539.7679, 1555.7635, 1556.8414, 1562.7877, 1567.7642, 1570.7750, 1571.8055, 1578.7964, 1581.8616, 1583.7979, 1592.7926, 1610.6801, 1616.7987, 1624.7253, 1628.7031, 1628.8617, 1642.7180, 1647.8546, 1649.8544, 1650.8427, 1657.9053, 1663.8506, 1664.8582, 1680.8226, 1683.8226, 1696.8158, 1698.7761, 1701.9166, 1706.8654, 1710.8179, 1711.7795, 1714.7788, 1715.8093, 1717.9019, 1726.8021, 1728.7821, 1730.8467, 1739.6987, 1747.9039, 1753.7146, 1755.7096, 1758.9032, 1771.0311, 1774.9202, 1783.6785, 1787.8689, 1797.6923, 1809.0245, 1811.7025, 1816.9102, 1836.8322, 1838.8318, 1850.8512, 1862.9421, 1866.8452, 1928.0237, 1937.9972, 1953.8524, 1965.8595, 1970.0011, 1982.0062, 1984.0415, 1995.8663, 2007.9470, 2027.1273, 2061.0056, 2067.0483, 2086.1091, 2099.1079, 2109.0042, 2136.8455, 2144.0537, 2150.8601, 2173.1655, 2179.0540, 2239.1436, 2251.1506, 2255.1462, 2265.0896, 2270.0999, 2274.0774, 2285.1733, 2286.1472, 2289.0583, 2293.1331, 2301.2546, 2303.0488, 2324.0913, 2340.1006, 2343.1824, 2344.1963, 2352.9204, 2356.0154, 2359.1130, 2375.0210, 2389.0332, 2414.2415, 2467.2080, 2525.2712, 2541.2361, 2542.2598, 2646.9749, 2660.9883, 2675.0051, 2679.0034, 2689.0183, 2692.0198, 2693.0125, 2695.0129, 2705.2800, 2717.3184, 2763.3374, 2771.4116, 2787.2793, 2792.2427, 2795.3037, 2803.2610, 2813.3884, 2819.2676, 2846.3894, 2872.3164, 2876.3669, 2877.3923, 2884.3479, 2887.3311, 2888.3215, 2891.4258, 2901.3557, 2919.4480, 3002.4827, 3029.4448, 3036.5105, 3053.4414, 3067.4578, 3081.6121, 3126.7246, 3151.3789, 3169.6074, 3184.4443, 3192.2090, 3232.5308, 3237.5469, 3248.6238, 3264.5508, 3305.5381, 3316.7190, 3321.5339, 3334.6743, 3339.6362, 3346.5017, 3350.5159, 3396.6165, 3412.6321, 3428.5723, 3435.6877, 3457.6028, 3471.6187, 3476.5935, 3489.6655, 3503.7874, 3624.7163, 3639.7131, 3645.7556, 3651.6982, 3692.0066, 3721.6226, 3990.8337, 4074.9656, 4093.8804, 4147.9131, 4153.9287, 4160.9331, 4174.0122, 4177.8853, 4253.9565, 4416.9512, 4452.1763, 4459.3091, 4466.1865, 4679.2988, 4844.0703

Search Parameters

Type of search : Peptide Mass Fingerprint
Enzyme : Trypsin
Fixed modifications : Carbamidomethyl (C)
Variable modifications : Oxidation (M)
Mass values : Monoisotopic
Protein Mass : Unrestricted
Peptide Mass Tolerance : ± 25 ppm
Peptide Charge State : 1+
Max Missed Cleavages : 2
Number of queries : 328
Selected for scoring : 198

Mascot: http://www.matrixscience.com/

MATRIX SCIENCE Mascot Search Results

```
User          : 4F_(L4@52381_05Apr2017)
Email         : proteomics@i3s.up.pt
Search title  : Project: Proteomics, Spot Set: Proteomics\05Apr2017, Label: L4, Spot Id: 227014, Peak List Id: 447670, MS Job Run Id: 316
MS data file  : C:\Documents and Settings\Administrator\Desktop\xu\29JUNHO\Proteomics\05Apr2017\pmf_L4_149875820513.txt
Database      : OrganismSpecie Bos_taurus_Reference_Proteome_2016_06 (24214 sequences; 12839866 residues)
Timestamp     : 29 Jun 2017 at 18:05:08 GMT
Top Score     : 91 for E1BI02, Fibromodulin OS=Bos taurus GN=FMOD PE=4 SV=1
```

Mascot Score Histogram

Protein score is -10*Log(P), where P is the probability that the observed match is a random event.
Protein scores greater than 56 are significant (p<0.05).

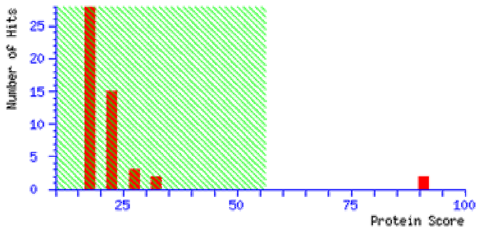

Protein Summary Report

Format As | Protein Summary | Help

Significance threshold p< 0.05 | Max. number of hits 50

Preferred taxonomy | All entries

Re-Search All | Search Unmatched

Index

	Accession	Mass	Score	Description
1.	E1BI02	43291	91	Fibromodulin OS=Bos taurus GN=FMOD PE=4 SV=1
2.	P13605	43410	91	Fibromodulin OS=Bos taurus GN=FMOD PE=1 SV=2
3.	E1BET7	49322	31	Uncharacterized protein OS=Bos taurus GN=SPRYD3 PE=4 SV=2
4.	P21809	41963	30	Biglycan OS=Bos taurus GN=BGN PE=1 SV=3
5.	G5E6K4	12963	29	Uncharacterized protein OS=Bos taurus GN=ATXN7 PE=4 SV=1
6.	A6QLB7	51512	26	Adenylyl cyclase-associated protein OS=Bos taurus GN=CAP1 PE=1 SV=1
7.	Q3SYV4	51583	26	Adenylyl cyclase-associated protein 1 OS=Bos taurus GN=CAP1 PE=2 SV=3
8.	Q3SWY9	24956	24	Ras-related protein Rab-28 OS=Bos taurus GN=RAB28 PE=2 SV=1
9.	F1MP35	62777	24	Uncharacterized protein OS=Bos taurus GN=ZNF783 PE=4 SV=2
10.	Q0VCQ3	32810	23	MAX interactor 1 OS=Bos taurus GN=MXI1 PE=2 SV=1
11.	Q3T063	31473	22	Nicotinate-nucleotide pyrophosphorylase [carboxylating] OS=Bos taurus GN=QPRT PE=2 SV=1
12.	F1MHB8	31507	22	Nicotinate-nucleotide pyrophosphorylase [carboxylating] OS=Bos taurus GN=QPRT PE=3 SV=1
13.	P56965	31554	22	N(G),N(G)-dimethylarginine dimethylaminohydrolase 1 OS=Bos taurus GN=DDAH1 PE=1 SV=3
14.	F1MVI1	62511	21	Cleavage stimulation factor subunit 2 OS=Bos taurus GN=CSTF2 PE=4 SV=1
15.	Q8HXM1	60513	21	Cleavage stimulation factor subunit 2 OS=Bos taurus GN=CSTF2 PE=2 SV=1
16.	Q27979	35414	21	11-cis retinol dehydrogenase OS=Bos taurus GN=RDH5 PE=1 SV=1
17.	A0A0A0MP97	34740	21	11-cis retinol dehydrogenase OS=Bos taurus GN=RDH5 PE=3 SV=1
18.	E1BFB9	61308	21	Protein FAM114A2 OS=Bos taurus GN=FAM114A1 PE=4 SV=1
19.	F1MMK0	12474	20	Catechol O-methyltransferase OS=Bos taurus GN=COMT PE=4 SV=2
20.	A4FUG5	57651	20	BIN2 protein OS=Bos taurus GN=BIN2 PE=2 SV=1
21.	E1BP99	28979	20	Uncharacterized protein OS=Bos taurus GN=APOBEC1 PE=4 SV=1
22.	F6RN30	69980	20	Uncharacterized protein OS=Bos taurus GN=GGA1 PE=4 SV=1
23.	Q3T171	12273	20	60S ribosomal protein L36 OS=Bos taurus GN=RPL36 PE=3 SV=3
24.	G3N2X2	12242	20	60S ribosomal protein L36 OS=Bos taurus PE=3 SV=1
25.	A6QP80	45891	20	LOC532995 protein OS=Bos taurus GN=LOC532995 PE=2 SV=1
26.	Q28055	12345	20	cAMP-regulated phosphoprotein 19 OS=Bos taurus GN=ARPP19 PE=1 SV=2
27.	G1K177	68709	20	Cleavage stimulation factor subunit 2 OS=Bos taurus GN=CSTF2 PE=4 SV=2
28.	Q2HJ73	43607	20	3-hydroxyisobutyryl-CoA hydrolase, mitochondrial OS=Bos taurus GN=HIBCH PE=2 SV=1
29.	E1B8F8	42403	20	Uncharacterized protein OS=Bos taurus PE=4 SV=1
30.	F1MPK4	44043	20	3-hydroxyisobutyryl-CoA hydrolase, mitochondrial OS=Bos taurus GN=HIBCH PE=3 SV=2
31.	F1MYN2	136237	19	Uncharacterized protein OS=Bos taurus GN=CGN PE=4 SV=2
32.	F1N052	122565	19	Uncharacterized protein OS=Bos taurus GN=SMARCA5 PE=1 SV=2
33.	F1MHZ5	32519	19	Uncharacterized protein OS=Bos taurus GN=EPHA4 PE=4 SV=1
34.	F1MV63	78027	19	Uncharacterized protein OS=Bos taurus GN=MACC1 PE=4 SV=1
35.	A6QQS9	110367	18	NBR1 protein OS=Bos taurus GN=NBR1 PE=2 SV=1
36.	A7MB88	93244	18	TMEM63A protein OS=Bos taurus GN=TMEM63A PE=2 SV=1
37.	Q3B8S0	14393	18	NHP2-like protein 1 OS=Bos taurus GN=SNU13 PE=2 SV=3
38.	E1BE67	14027	18	Uncharacterized protein OS=Bos taurus PE=4 SV=2
39.	E1BLR1	58429	18	Uncharacterized protein OS=Bos taurus GN=SPATA2 PE=4 SV=1
40.	A1L579	53486	18	Oviduct-specific glycoprotein OS=Bos taurus GN=OVGP1 PE=2 SV=1
41.	F1N6X9	54088	18	Uncharacterized protein OS=Bos taurus GN=LOC100141215 PE=4 SV=2
42.	G3N2A1	46196	18	Uncharacterized protein OS=Bos taurus GN=CCDC96 PE=4 SV=1
43.	P79134	76202	18	Annexin A6 OS=Bos taurus GN=ANXA6 PE=1 SV=2
44.	F1MZH9	51716	18	Probable proline dehydrogenase 2 OS=Bos taurus GN=PRODH2 PE=4 SV=2
45.	A7E321	51226	18	PNMA-like protein 1 OS=Bos taurus GN=PNMAL1 PE=2 SV=1
46.	Q9GKN8	44054	18	Prolargin OS=Bos taurus GN=PRELP PE=2 SV=1
47.	Q08DB0	44179	18	Centrosomal protein of 44 kDa OS=Bos taurus GN=CEP44 PE=2 SV=1
48.	F1N402	44161	18	Centrosomal protein of 44 kDa OS=Bos taurus GN=CEP44 PE=4 SV=2
49.	Q58DS5	23184	18	Ras-related protein Rab-13 OS=Bos taurus GN=RAB13 PE=1 SV=1
50.	F1MIN9	51015	17	Uncharacterized protein OS=Bos taurus GN=ZNRF4 PE=4 SV=1

Results List

1. E1BI02 **Mass:** 43291 **Score:** 91 **Expect:** 2e-005 **Matches:** 18

Fibromodulin OS=Bos taurus GN=FMOD PE=4 SV=1

Observed	Mr(expt)	Mr(calc)	ppm	Start	End	Miss	Peptide
749.4561	748.4488	748.4595	-14.40	149 -	154	1	K.VFSKLR.H
779.4789	778.4716	778.4701	1.92	169 -	175	0	R.IPSPLPR.S
823.4901	822.4828	822.4824	0.54	153 -	158	1	K.LRHLER.L
944.5204	943.5132	943.5087	4.78	349 -	356	1	R.LDGNEIKR.S
978.5431	977.5358	977.5334	2.42	98 -	105	0	K.YLPFVPSR.M
1301.6338	1300.6265	1300.6268	-0.18	357 -	368	0	R.SAMPADAPLCLR.L
1317.6354	1316.6281	1316.6217	4.90	357 -	368	0	R.SAMPADAPLCLR.L + Oxidation (M)
1333.7570	1332.7497	1332.7554	-4.25	95 -	105	1	R.NLKYLPFVPSR.M
1361.6875	1360.6802	1360.6847	-3.32	179 -	189	0	R.ELHLDHNQISR.V
1457.7394	1456.7321	1456.7279	2.93	356 -	368	1	K.RSAMPADAPLCLR.L
1556.8799	1555.8726	1555.8722	0.28	223 -	235	0	R.SLILLDLSYNHLR.K
1717.9056	1716.8983	1716.9019	-2.10	176 -	189	1	R.SLRELHLDHNQISR.V
1928.0238	1927.0165	1927.0163	0.12	307 -	323	0	K.IPPVSTNLENLYLQGNR.I
2324.0981	2323.0908	2323.0865	1.87	324 -	343	0	R.INEFSISSFCTVVDVMNFSK.L
2340.0979	2339.0906	2339.0814	3.95	324 -	343	0	R.INEFSISSFCTVVDVMNFSK.L + Oxidation (M)
2646.9875	2645.9802	2645.9702	3.78	74 -	94	0	R.DCPQECDCPPNFPTAMYCDNR.N
3206.6179	3205.6106	3205.5720	12.0	237 -	264	0	K.VPDGLPSALEQLYLEHNNVFSVPDSYFR.G
3334.6953	3333.6880	3333.6670	6.31	236 -	264	1	R.KVPDGLPSALEQLYLEHNNVFSVPDSYFR.G

No match to: 706.3098, 710.3790, 712.2532, 714.3089, 717.3984, 719.4317, 720.4312, 722.4346, 735.4181, 756.4000, 759.4391, 772.4356, 774.4578, 778.4783, 818.4724, 831.4754, 832.3123, 834.3210, 842.5065, 844.4971, 870.5016, 871.4923, 873.3899, 876.2862, 880.4520, 883.4794, 891.4870, 895.5731, 900.5293, 909.5164, 913.4845, 918.5409, 923.5157, 925.4807, 927.5140, 929.4449, 942.5470, 945.5183, 949.5508, 950.5472, 967.4262, 972.4835, 974.4452, 976.4556, 977.4954, 988.4708, 1007.5730, 1016.5761, 1026.5627, 1031.5581, 1034.5808, 1044.5240, 1051.5095, 1056.5281, 1057.5575, 1066.5067, 1074.5374, 1087.5460, 1103.5287, 1118.5885, 1122.5553, 1148.5439, 1168.5985, 1178.6211, 1185.6898, 1244.6118, 1248.6686, 1253.6313, 1255.6216, 1312.7423, 1313.7227, 1314.7188, 1315.6721, 1320.6331, 1322.6371, 1325.6360, 1327.6959, 1336.7218, 1338.7179, 1339.7092, 1343.6960, 1349.6917, 1350.6987, 1351.6946, 1372.5978, 1381.7642, 1382.5950, 1383.7253, 1397.7454, 1439.7549, 1442.8190, 1463.7950, 1488.7245, 1494.7383, 1497.8735, 1504.7484, 1513.7760, 1517.8062, 1520.7523, 1528.6951, 1537.8245, 1539.8104, 1549.8242, 1552.8073, 1553.8669, 1555.8348, 1567.7986, 1570.7749, 1578.7872, 1592.7849, 1628.7002, 1628.8571, 1638.8861, 1642.7155, 1647.8503, 1650.8458, 1664.8571, 1680.8226, 1696.8146, 1698.7845, 1699.8228, 1705.8712, 1707.8536, 1715.8536, 1730.8564, 1739.6990, 1771.0344, 1786.9199, 1809.0262, 1816.9354, 1820.8855, 1836.8507, 1907.9603, 1909.9886, 1940.0148, 1953.8596, 1961.0111, 1965.8679, 1974.9255, 1982.0082, 1983.9866, 1985.8589, 1995.9136, 2027.1289, 2067.1079, 2110.0598, 2136.8484, 2144.0432, 2150.8660, 2164.8794, 2211.0857, 2239.1533, 2251.1509, 2255.1836, 2270.1113, 2273.0972, 2274.0605, 2286.1492, 2289.0857, 2290.0530, 2293.1558, 2309.1570, 2338.1033, 2343.1770, 2352.9316, 2375.0454, 2383.9480, 2414.2512, 2499.2480, 2542.2678, 2589.9707, 2600.0022, 2603.9897, 2613.9939, 2627.9993, 2644.0112, 2657.4248, 2659.0405, 2661.0017, 2665.0042, 2674.0601, 2675.0151, 2678.0159, 2689.0330, 2692.0283, 2695.0315, 2703.0417, 2706.0959, 2763.3606, 2798.2788, 2815.3203, 2846.4011, 2872.3391, 2876.3679, 2884.3535, 2888.3455, 2891.4131, 2892.4519, 2919.4543, 3002.3042, 3012.4717, 3016.2314, 3020.2483, 3030.3025, 3033.2446, 3053.4917, 3081.6296, 3126.7493, 3171.7131, 3184.4602, 3207.6111, 3248.6445, 3254.6370, 3265.6309, 3300.7825, 3317.7288, 3321.6318, 3347.5437, 3429.5991, 3457.6255, 3471.6121, 3489.7363, 3503.8608, 3518.5715, 3639.7969, 4148.0293, 4162.0039, 4453.2720, 4844.2046

2. P13605 **Mass:** 43410 **Score:** 91 **Expect:** 2.1e-005 **Matches:** 18

Fibromodulin OS=Bos taurus GN=FMOD PE=1 SV=2

Observed	Mr(expt)	Mr(calc)	ppm	Start	End	Miss	Peptide
749.4561	748.4488	748.4595	-14.40	150 -	155	1	K.VFSKLR.H
779.4789	778.4716	778.4701	1.92	170 -	176	0	R.IPSPLPR.S
823.4901	822.4828	822.4824	0.54	154 -	159	1	K.LRHLER.L
944.5204	943.5132	943.5087	4.78	350 -	357	1	R.LDGNEIKR.S
978.5431	977.5358	977.5334	2.42	99 -	106	0	K.YLPFVPSR.M
1301.6338	1300.6265	1300.6268	-0.18	358 -	369	0	R.SAMPADAPLCLR.L
1317.6354	1316.6281	1316.6217	4.90	358 -	369	0	R.SAMPADAPLCLR.L + Oxidation (M)
1333.7570	1332.7497	1332.7554	-4.25	96 -	106	1	R.NLKYLPFVPSR.M
1361.6875	1360.6802	1360.6847	-3.32	180 -	190	0	R.ELHLDHNQISR.V
1457.7394	1456.7321	1456.7279	2.93	357 -	369	1	K.RSAMPADAPLCLR.L
1556.8799	1555.8726	1555.8722	0.28	224 -	236	0	R.SLILLDLSYNHLR.K
1717.9056	1716.8983	1716.9019	-2.10	177 -	190	1	R.SLRELHLDHNQISR.V
1928.0238	1927.0165	1927.0163	0.12	308 -	324	0	K.IPPVSTNLENLYLQGNR.I
2324.0981	2323.0908	2323.0865	1.87	325 -	344	0	R.INEFSISSFCTVVDVMNFSK.L
2340.0979	2339.0906	2339.0814	3.95	325 -	344	0	R.INEFSISSFCTVVDVMNFSK.L + Oxidation (M)
2646.9875	2645.9802	2645.9702	3.78	75 -	95	0	R.DCPQECDCPPNFPTAMYCDNR.N
3206.6179	3205.6106	3205.5720	12.0	238 -	265	0	K.VPDGLPSALEQLYLEHNNVFSVPDSYFR.G
3334.6953	3333.6880	3333.6670	6.31	237 -	265	1	R.KVPDGLPSALEQLYLEHNNVFSVPDSYFR.G

No match to: 706.3098, 710.3790, 712.2532, 714.3089, 717.3984, 719.4317, 720.4312, 722.4346, 735.4181, 756.4000, 759.4391, 772.4356, 774.4578, 778.4783, 818.4724, 831.4754, 832.3123, 834.3210, 842.5065, 844.4971, 870.5016, 871.4923, 873.3899, 876.2862, 880.4520, 883.4794, 891.4870, 895.5731, 900.5293, 909.5164, 913.4845, 918.5409, 923.5157, 925.4807, 927.5140, 929.4449, 942.5470, 945.5183, 949.5508, 950.5472, 967.4262, 972.4835, 974.4452, 976.4556, 977.4954, 988.4708, 1007.5730, 1016.5761, 1026.5627, 1031.5581, 1034.5808, 1044.5240, 1051.5095, 1056.5281, 1057.5575, 1066.5067, 1074.5374, 1087.5460, 1103.5287, 1118.5885, 1122.5553, 1148.5439, 1168.5985, 1178.6211, 1185.6898, 1244.6118, 1248.6686, 1253.6313, 1255.6216, 1312.7423, 1313.7227, 1314.7188, 1315.6721, 1320.6331, 1322.6371, 1325.6360, 1327.6959, 1336.7218, 1338.7179, 1339.7092, 1343.6960, 1349.6917, 1350.6987, 1351.6946, 1372.5978, 1381.7642, 1382.5950, 1383.7253, 1397.7454, 1439.7549, 1442.8190, 1463.7950, 1488.7245, 1494.7383, 1497.8735, 1504.7484, 1513.7760, 1517.8062, 1520.7523, 1528.6951, 1537.8245, 1539.8104, 1549.8242, 1552.8073, 1553.8669, 1555.8348, 1567.7986, 1570.7749, 1578.7872, 1592.7849, 1628.7002, 1628.8571, 1638.8861, 1642.7155, 1647.8503, 1650.8458, 1664.8571, 1680.8226, 1696.8146, 1698.7845, 1699.8228, 1705.8712, 1707.8536, 1715.8536, 1730.8564, 1739.6990, 1771.0344, 1786.9199, 1809.0262, 1816.9354, 1820.8855, 1836.8507, 1907.9603, 1909.9886, 1940.0148, 1953.8596, 1961.0111, 1965.8679, 1974.9255, 1982.0082, 1983.9866, 1985.8589, 1995.9136, 2027.1289, 2067.1079, 2110.0598, 2136.8484, 2144.0432, 2150.8660, 2164.8794, 2211.0857, 2239.1533, 2251.1509, 2255.1836, 2270.1113, 2273.0972, 2274.0605, 2286.1492, 2289.0857, 2290.0530, 2293.1558, 2309.1570, 2338.1033, 2343.1770, 2352.9316, 2375.0454, 2383.9480, 2414.2512, 2499.2480, 2542.2678, 2589.9707, 2600.0022, 2603.9897, 2613.9939, 2627.9993, 2644.0112, 2657.4248, 2659.0405, 2661.0017, 2665.0042, 2674.0601, 2675.0151, 2678.0159, 2689.0330, 2692.0283, 2695.0315, 2703.0417, 2706.0959, 2763.3606, 2798.2788, 2815.3203, 2846.4011, 2872.3391, 2876.3679, 2884.3535, 2888.3455, 2891.4131, 2892.4519, 2919.4543, 3002.3042, 3012.4717, 3016.2314, 3020.2483, 3030.3025, 3033.2446, 3053.4917, 3081.6296, 3126.7493, 3171.7131, 3184.4602, 3207.6111, 3248.6445, 3254.6370, 3265.6309, 3300.7825, 3317.7288, 3321.6318, 3347.5437, 3429.5991, 3457.6255, 3471.6121, 3489.7363, 3503.8608, 3518.5715, 3639.7969, 4148.0293, 4162.0039, 4453.2720, 4844.2046

3. E1BET7 **Mass:** 49322 **Score:** 31 **Expect:** 18 **Matches:** 6

Uncharacterized protein OS=Bos taurus GN=SPRYD3 PE=4 SV=2

Observed	Mr(expt)	Mr(calc)	ppm	Start	End	Miss	Peptide
735.4181	734.4108	734.3970	18.9	1 -	5	2	-.MRRTR.R + Oxidation (M)
891.4870	890.4797	890.4875	-8.72	24 -	29	1	R.FLNWRR.R
978.5431	977.5358	977.5406	-4.95	130 -	138	2	R.AKGRQFGSK.C
1034.5808	1033.5735	1033.5781	-4.41	125 -	133	2	K.LYNGRAKGR.Q

1313.7227	1312.7154	1312.7463	-23.51	239 - 251	1	K.GKSIVDVGLAQAR.R
2846.4011	2845.3938	2845.3449	17.2	170 - 196	0	R.VGSTIMPMSPDGLFPAVGMHSLGEEVR.L + 2 Oxidation (M)

No match to: 706.3098, 710.3790, 712.2532, 714.3089, 717.3984, 719.4317, 720.4312, 722.4346, 749.4561, 756.4000, 759.4391, 772.4356, 774.4578, 778.4783, 779.4789, 818.4724, 823.4901, 831.4754, 832.3123, 834.3210, 842.5065, 844.4971, 870.5016, 871.4923, 873.3899, 876.2862, 880.4520, 883.4794, 895.5731, 900.5293, 909.5164, 913.4845, 918.5409, 923.5157, 925.4807, 927.5140, 929.4449, 942.5470, 944.5204, 945.5183, 949.5508, 950.5472, 967.4262, 972.4835, 974.4452, 976.4556, 977.4954, 988.4708, 1007.5730, 1016.5761, 1026.5627, 1031.5581, 1044.5240, 1051.5095, 1056.5281, 1057.5575, 1066.5067, 1074.5374, 1087.5460, 1103.5287, 1118.5885, 1122.5553, 1148.5439, 1168.5985, 1178.6211, 1185.6898, 1244.6118, 1248.6686, 1253.6313, 1255.6216, 1301.6338, 1312.7423, 1314.7188, 1315.6721, 1317.6354, 1320.6331, 1322.6371, 1325.6360, 1327.6959, 1333.7570, 1336.7218, 1338.7179, 1339.7092, 1343.6960, 1349.6917, 1350.6987, 1351.6946, 1361.6875, 1372.5978, 1381.7642, 1382.5950, 1383.7253, 1397.7454, 1439.7549, 1442.8190, 1457.7394, 1463.7950, 1488.7245, 1494.7383, 1497.8735, 1504.7484, 1513.7760, 1517.8062, 1520.7523, 1528.6951, 1537.8245, 1539.8104, 1549.8242, 1552.8073, 1553.8669, 1555.8348, 1556.8799, 1567.7986, 1570.7749, 1578.7872, 1592.7849, 1628.7002, 1628.8571, 1638.8861, 1642.7155, 1647.8503, 1650.8458, 1664.8571, 1680.8226, 1696.8146, 1698.7845, 1699.8228, 1705.8712, 1707.8536, 1715.8536, 1717.9056, 1730.8564, 1739.6990, 1771.0344, 1786.9199, 1809.0262, 1816.9354, 1820.8855, 1836.8507, 1907.9603, 1909.9886, 1928.0238, 1940.0148, 1953.8596, 1961.0111, 1965.8679, 1974.9255, 1982.0082, 1983.9866, 1985.8589, 1995.9136, 2027.1289, 2067.1079, 2110.0598, 2136.8484, 2144.0432, 2150.8660, 2164.8794, 2211.0857, 2239.1533, 2251.1509, 2255.1836, 2270.1113, 2273.0972, 2274.0605, 2286.1492, 2289.0857, 2290.0530, 2293.1558, 2309.1570, 2324.0981, 2338.1033, 2340.0979, 2343.1770, 2352.9316, 2375.0454, 2383.9480, 2414.2512, 2499.2480, 2542.2678, 2589.9707, 2600.0022, 2603.9897, 2613.9939, 2627.9993, 2644.0112, 2646.9875, 2657.4248, 2659.0405, 2661.0017, 2665.0042, 2674.0601, 2675.0151, 2678.0159, 2689.0330, 2692.0283, 2695.0315, 2703.0417, 2706.0959, 2763.3606, 2798.2788, 2815.3203, 2872.3391, 2876.3679, 2884.3535, 2888.3455, 2891.4131, 2892.4519, 2919.4543, 3002.3042, 3012.4717, 3016.2314, 3020.2483, 3030.3025, 3033.2446, 3053.4917, 3081.6296, 3126.7493, 3171.7131, 3184.4602, 3206.6179, 3207.6111, 3248.6445, 3254.6370, 3265.6309, 3300.7825, 3317.7288, 3321.6318, 3334.6953, 3347.5437, 3429.5991, 3457.6255, 3471.6121, 3489.7363, 3503.8608, 3518.5715, 3639.7969, 4148.0293, 4162.0039, 4453.2720, 4844.2046

4. P21809 **Mass:** 41963 **Score:** 30 **Expect:** 26 **Matches:** 17

Biglycan OS=Bos taurus GN=BGN PE=1 SV=3

Observed	Mr(expt)	Mr(calc)	ppm	Start - End	Miss	Peptide
735.4181	734.4108	734.4075	4.47	178 - 184	0	K.GVFSGLR.N
818.4724	817.4651	817.4810	-19.41	134 - 140	1	K.AFSPLRK.L
923.5157	922.5084	922.5097	-1.37	167 - 173	1	R.IHDNRIR.K
1007.5730	1006.5657	1006.5672	-1.47	259 - 267	0	R.LGLGHNQIR.M
1118.5885	1117.5812	1117.5802	0.96	74 - 83	0	R.VVQCSDLGLK.A
1312.7423	1311.7350	1311.7398	-3.62	242 - 252	0	K.IQAIELEDLLR.Y
1338.7179	1337.7106	1337.7051	4.11	282 - 292	1	R.ELHLDNNKLSR.V
1494.7383	1493.7310	1493.7337	-1.81	316 - 329	0	K.VGVNDFCPVGFGVK.R
1650.8458	1649.8385	1649.8348	2.23	316 - 330	1	K.VGVNDFCPVGFGVKR.A
2027.1289	2026.1216	2026.1211	0.26	149 - 166	0	K.NHLVEIPPNLPSSLVELR.I
2286.1492	2285.1419	2285.1387	1.43	88 - 107	0	K.EISPDTTLLDLQNNDISELR.K
2414.2512	2413.2439	2413.2336	4.27	88 - 108	1	K.EISPDTTLLDLQNNDISELRK.D
2798.2788	2797.2715	2797.2146	20.4	185 - 210	0	R.NMNCIEMGGNPLENSGFEPGAFDGLK.L
2846.4011	2845.3938	2845.3864	2.62	331 - 354	0	R.AYYNGISLFNNPVPYWEVQPATFR.C
2919.4543	2918.4470	2918.4509	-1.32	88 - 112	2	K.EISPDTTLLDLQNNDISELRKDDFK.G
3081.6296	3080.6223	3080.6142	2.65	227 - 252	1	K.DLPETLNELHLDHNKIQAIELEDLLR.Y
3457.6255	3456.6182	3456.5900	8.15	185 - 215	1	R.NMNCIEMGGNPLENSGFEPGAFDGLKLNYLR.I

No match to: 706.3098, 710.3790, 712.2532, 714.3089, 717.3984, 719.4317, 720.4312, 722.4346, 749.4561, 756.4000, 759.4391, 772.4356, 774.4578, 778.4783, 779.4789, 823.4901, 831.4754, 832.3123, 834.3210, 842.5065, 844.4971, 870.5016, 871.4923, 873.3899, 876.2862, 880.4520, 883.4794, 891.4870, 895.5731, 900.5293, 909.5164, 913.4845, 918.5409, 925.4807, 927.5140, 929.4449, 942.5470, 944.5204, 945.5183, 949.5508, 950.5472, 967.4262, 972.4835, 974.4452, 976.4556, 977.4954, 978.5431, 988.4708, 1016.5761, 1026.5627, 1031.5581, 1034.5808, 1044.5240, 1051.5095, 1056.5281, 1057.5575, 1066.5067, 1074.5374, 1087.5460, 1103.5287, 1122.5553, 1148.5439, 1168.5985, 1178.6211, 1185.6898, 1244.6118, 1248.6686, 1253.6313, 1255.6216, 1301.6338, 1313.7227, 1314.7188, 1315.6721, 1317.6354, 1320.6331, 1322.6371, 1325.6360, 1327.6959, 1333.7570, 1336.7218, 1339.7092, 1343.6960, 1349.6917, 1350.6987, 1351.6946, 1361.6875, 1372.5978, 1381.7642, 1382.5950, 1383.7253, 1397.7454, 1439.7549, 1442.8190, 1457.7394, 1463.7950, 1488.7245, 1497.8735, 1504.7484, 1513.7760, 1517.8062, 1520.7523, 1528.6951, 1537.8245, 1539.8104, 1549.8242, 1552.8073, 1553.8669, 1555.8348, 1556.8799, 1567.7986, 1570.7749, 1578.7872, 1592.7849, 1628.7002, 1628.8571, 1638.8861, 1642.7155, 1647.8503, 1664.8571, 1680.8226, 1696.8146, 1698.7845, 1699.8228, 1705.8712, 1707.8536, 1715.8536, 1717.9056, 1730.8564, 1739.6990, 1771.0344, 1786.9199, 1809.0262, 1816.9354, 1820.8855, 1836.8507, 1907.9603, 1909.9886, 1928.0238, 1940.0148, 1953.8596, 1961.0111, 1965.8679, 1974.9255, 1982.0082, 1983.9866, 1985.8589, 1995.9136, 2067.1079, 2110.0598, 2136.8484, 2144.0432, 2150.8660, 2164.8794, 2211.0857, 2239.1533, 2251.1509, 2255.1836, 2270.1113, 2273.0972, 2274.0605, 2289.0857, 2290.0530, 2293.1558, 2309.1570, 2324.0981, 2338.1033, 2340.0979, 2343.1770, 2352.9316, 2375.0454, 2383.9480, 2499.2480, 2542.2678, 2589.9707, 2600.0022, 2603.9897, 2613.9939, 2627.9993, 2644.0112, 2646.9875, 2657.4248, 2659.0405, 2661.0017, 2665.0042, 2674.0601, 2675.0151, 2678.0159, 2689.0330, 2692.0283, 2695.0315, 2703.0417, 2706.0959, 2763.3606, 2815.3203, 2872.3391, 2876.3679, 2884.3535, 2888.3455, 2891.4131, 2892.4519, 3002.3042, 3012.4717, 3016.2314, 3020.2483, 3030.3025, 3033.2446, 3053.4917, 3126.7493, 3171.7131, 3184.4602, 3206.6179, 3207.6111, 3248.6445, 3254.6370, 3265.6309, 3300.7825, 3317.7288, 3321.6318, 3334.6953, 3347.5437, 3429.5991, 3471.6121, 3489.7363, 3503.8608, 3518.5715, 3639.7969, 4148.0293, 4162.0039, 4453.2720, 4844.2046

5. G5E6K4 **Mass:** 12963 **Score:** 29 **Expect:** 30 **Matches:** 6

Uncharacterized protein OS=Bos taurus GN=ATXN7 PE=4 SV=1

Observed	Mr(expt)	Mr(calc)	ppm	Start - End	Miss	Peptide
1122.5553	1121.5480	1121.5321	14.2	113 - 121	1	K.NREVMGLCK.E + Oxidation (M)
1457.7394	1456.7321	1456.7018	20.8	2 - 14	2	M.SERAADDVRGEPR.R
1567.7986	1566.7913	1566.7646	17.0	109 - 121	2	K.EFGKNREVMGLCK.E
2343.1770	2342.1697	2342.1284	17.6	48 - 72	0	R.LRPEDGGPGAASTSAAAAMATVGER.R
2414.2512	2413.2439	2413.2323	4.84	16 - 40	1	R.AAAAAGGAAAAAARQQQQQPPQPQR.Q
2499.2480	2498.2407	2498.2296	4.47	47 - 72	1	R.RLRPEDGGPGAASTSAAAAMATVGER.R

No match to: 706.3098, 710.3790, 712.2532, 714.3089, 717.3984, 719.4317, 720.4312, 722.4346, 735.4181, 749.4561, 756.4000, 759.4391, 772.4356, 774.4578, 778.4783, 779.4789, 818.4724, 823.4901, 831.4754, 832.3123, 834.3210, 842.5065, 844.4971, 870.5016, 871.4923, 873.3899, 876.2862, 880.4520, 883.4794, 891.4870, 895.5731, 900.5293, 909.5164, 913.4845, 918.5409, 923.5157, 925.4807, 927.5140, 929.4449, 942.5470, 944.5204, 945.5183, 949.5508, 950.5472, 967.4262, 972.4835, 974.4452, 976.4556, 977.4954, 978.5431, 988.4708, 1007.5730, 1016.5761, 1026.5627, 1031.5581, 1034.5808, 1044.5240, 1051.5095, 1056.5281, 1057.5575, 1066.5067, 1074.5374, 1087.5460, 1103.5287, 1118.5885, 1148.5439, 1168.5985, 1178.6211, 1185.6898, 1244.6118, 1248.6686, 1253.6313, 1255.6216, 1301.6338, 1312.7423, 1313.7227, 1314.7188, 1315.6721, 1317.6354, 1320.6331, 1322.6371, 1325.6360, 1327.6959, 1333.7570, 1336.7218, 1338.7179, 1339.7092, 1343.6960, 1349.6917, 1350.6987, 1351.6946, 1361.6875, 1372.5978, 1381.7642, 1382.5950, 1383.7253, 1397.7454, 1439.7549, 1442.8190, 1463.7950, 1488.7245, 1494.7383, 1497.8735, 1504.7484, 1513.7760, 1517.8062, 1520.7523, 1528.6951, 1537.8245, 1539.8104, 1549.8242, 1552.8073, 1553.8669, 1555.8348, 1556.8799, 1570.7749, 1578.7872, 1592.7849, 1628.7002, 1628.8571, 1638.8861, 1642.7155, 1647.8503, 1650.8458, 1664.8571, 1680.8226, 1696.8146, 1698.7845, 1699.8228, 1705.8712, 1707.8536, 1715.8536, 1717.9056, 1730.8564, 1739.6990, 1771.0344, 1786.9199, 1809.0262, 1816.9354, 1820.8855, 1836.8507, 1907.9603, 1909.9886, 1928.0238, 1940.0148, 1953.8596, 1961.0111, 1965.8679, 1974.9255, 1982.0082, 1983.9866, 1985.8589, 1995.9136, 2027.1289, 2067.1079, 2110.0598, 2136.8484, 2144.0432, 2150.8660, 2164.8794, 2211.0857, 2239.1533, 2251.1509, 2255.1836, 2270.1113, 2273.0972, 2274.0605, 2286.1492, 2289.0857, 2290.0530, 2293.1558, 2309.1570, 2324.0981, 2338.1033, 2340.0979, 2352.9316, 2375.0454, 2383.9480, 2542.2678, 2589.9707, 2600.0022, 2603.9897, 2613.9939, 2627.9993, 2644.0112, 2646.9875, 2657.4248, 2659.0405, 2661.0017, 2665.0042, 2674.0601, 2675.0151, 2678.0159, 2689.0330, 2692.0283, 2695.0315, 2703.0417, 2706.0959, 2763.3606, 2798.2788, 2815.3203, 2846.4011, 2872.3391, 2876.3679, 2884.3535, 2888.3455, 2891.4131, 2892.4519, 2919.4543, 3002.3042, 3012.4717, 3016.2314, 3020.2483, 3030.3025, 3033.2446, 3053.4917, 3081.6296, 3126.7493, 3171.7131, 3184.4602, 3206.6179, 3207.6111, 3248.6445, 3254.6370, 3265.6309, 3300.7825, 3317.7288, 3321.6318, 3334.6953, 3347.5437, 3429.5991, 3457.6255, 3471.6121, 3489.7363, 3503.8608, 3518.5715, 3639.7969, 4148.0293, 4162.0039, 4453.2720, 4844.2046

6. A6QLB7 **Mass:** 51512 **Score:** 26 **Expect:** 58 **Matches:** 7

Adenylyl cyclase-associated protein OS=Bos taurus GN=CAP1 PE=1 SV=1

Observed	Mr(expt)	Mr(calc)	ppm	Start	End	Miss	Peptide
779.4789	778.4716	778.4589	16.4	167	172	1	R.VLKEYK.D
1122.5553	1121.5480	1121.5652	-15.27	71	80	0	K.HAEMVHTGLK.L
1517.8062	1516.7989	1516.7780	13.8	2	13	2	M.ADMRNLVERLER.V + Oxidation (M)
1664.8571	1663.8498	1663.8134	21.9	1	13	2	-.MADMRNLVERLER.V + 2 Oxidation (M)
1928.0238	1927.0165	1927.0163	0.12	254	271	0	R.SALFAQINQGESITHALK.H
2293.1558	2292.1485	2292.1896	-17.92	60	80	2	K.ISKEIGGDVQKHAEMVHTGLK.L + Oxidation (M)
3248.6445	3247.6372	3247.6217	4.78	401	429	2	K.VPTISINKTDGCHVYLSKNSLDCEIVSAK.S

No match to: 706.3098, 710.3790, 712.2532, 714.3089, 717.3984, 719.4317, 720.4312, 722.4346, 735.4181, 749.4561, 756.4000, 759.4391, 772.4356, 774.4578, 778.4783, 818.4724, 823.4901, 831.4754, 832.3123, 834.3210, 842.5065, 844.4971, 870.5016, 871.4923, 873.3899, 876.2862, 880.4520, 883.4794, 891.4870, 895.5731, 900.5293, 909.5164, 913.4845, 918.5409, 923.5157, 925.4807, 927.5140, 929.4449, 942.5470, 944.5204, 945.5183, 949.5508, 950.5472, 967.4262, 972.4835, 974.4452, 976.4556, 977.4954, 978.5431, 988.4708, 1007.5730, 1016.5761, 1026.5627, 1031.5581, 1034.5808, 1044.5240, 1051.5095, 1056.5281, 1057.5575, 1066.5067, 1074.5374, 1087.5460, 1103.5287, 1118.5885, 1148.5439, 1168.5985, 1178.6211, 1185.6898, 1244.6118, 1248.6686, 1253.6313, 1255.6216, 1301.6338, 1312.7423, 1313.7227, 1314.7188, 1315.6721, 1317.6354, 1320.6331, 1322.6371, 1325.6360, 1327.6959, 1333.7570, 1336.7218, 1338.7179, 1339.7092, 1343.6960, 1349.6917, 1350.6987, 1351.6946, 1361.6875, 1372.5978, 1381.7642, 1382.5950, 1383.7253, 1397.7454, 1439.7549, 1442.8190, 1457.7394, 1463.7950, 1488.7245, 1494.7383, 1497.8735, 1504.7484, 1513.7760, 1520.7523, 1528.6951, 1537.8245, 1539.8104, 1549.8242, 1552.8073, 1553.8669, 1555.8348, 1556.8799, 1567.7986, 1570.7749, 1578.7872, 1592.7849, 1628.7002, 1628.8571, 1638.8861, 1642.7155, 1647.8503, 1650.8458, 1680.8226, 1696.8146, 1698.7845, 1699.8228, 1705.8712, 1707.8536, 1715.8536, 1717.9056, 1730.8564, 1739.6990, 1771.0344, 1786.9199, 1809.0262, 1816.9354, 1820.8855, 1836.8507, 1907.9603, 1909.9886, 1940.0148, 1953.8596, 1961.0111, 1965.8679, 1974.9255, 1982.0082, 1983.9866, 1985.8589, 1995.9136, 2027.1289, 2067.1079, 2110.0598, 2136.8484, 2144.0432, 2150.8660, 2164.8794, 2211.0857, 2239.1533, 2251.1509, 2255.1836, 2270.1113, 2273.0972, 2274.0605, 2286.1492, 2289.0857, 2290.0530, 2309.1570, 2324.0981, 2338.1033, 2340.0979, 2343.1770, 2352.9316, 2375.0454, 2383.9480, 2414.2512, 2499.2480, 2542.2678, 2589.9707, 2600.0022, 2603.9897, 2613.9939, 2627.9993, 2644.0112, 2646.9875, 2657.4248, 2659.0405, 2661.0017, 2665.0042, 2674.0601, 2675.0151, 2678.0159, 2689.0330, 2692.0283, 2695.0315, 2703.0417, 2706.0959, 2763.3606, 2798.2788, 2815.3203, 2846.4011, 2872.3391, 2876.3679, 2884.3535, 2888.3455, 2891.4131, 2892.4519, 2919.4543, 3002.3042, 3012.4717, 3016.2314, 3020.2483, 3030.3025, 3033.2446, 3053.4917, 3081.6296, 3126.7493, 3171.7131, 3184.4602, 3206.6179, 3207.6111, 3254.6370, 3265.6309, 3300.7825, 3317.7288, 3321.6318, 3334.6953, 3347.5437, 3429.5991, 3457.6255, 3471.6121, 3489.7363, 3503.8608, 3518.5715, 3639.7969, 4148.0293, 4162.0039, 4453.2720, 4844.2046

7. Q3SYV4 **Mass:** 51583 **Score:** 26 **Expect:** 58 **Matches:** 7

Adenylyl cyclase-associated protein 1 OS=Bos taurus GN=CAP1 PE=2 SV=3

Observed	Mr(expt)	Mr(calc)	ppm	Start	End	Miss	Peptide
779.4789	778.4716	778.4589	16.4	168	173	1	R.VLKEYK.D
1122.5553	1121.5480	1121.5652	-15.27	72	81	0	K.HAEMVHTGLK.L
1517.8062	1516.7989	1516.7780	13.8	2	13	2	M.ADMRNLVERLER.V + Oxidation (M)
1664.8571	1663.8498	1663.8134	21.9	1	13	2	-.MADMRNLVERLER.V + 2 Oxidation (M)
1928.0238	1927.0165	1927.0163	0.12	255	272	0	R.SALFAQINQGESITHALK.H
2293.1558	2292.1485	2292.1896	-17.92	61	81	2	K.ISKEIGGDVQKHAEMVHTGLK.L + Oxidation (M)
3248.6445	3247.6372	3247.6217	4.78	402	430	2	K.VPTISINKTDGCHVYLSKNSLDCEIVSAK.S

No match to: 706.3098, 710.3790, 712.2532, 714.3089, 717.3984, 719.4317, 720.4312, 722.4346, 735.4181, 749.4561, 756.4000, 759.4391, 772.4356, 774.4578, 778.4783, 818.4724, 823.4901, 831.4754, 832.3123, 834.3210, 842.5065, 844.4971, 870.5016, 871.4923, 873.3899, 876.2862, 880.4520, 883.4794, 891.4870, 895.5731, 900.5293, 909.5164, 913.4845, 918.5409, 923.5157, 925.4807, 927.5140, 929.4449, 942.5470, 944.5204, 945.5183, 949.5508, 950.5472, 967.4262, 972.4835, 974.4452, 976.4556, 977.4954, 978.5431, 988.4708, 1007.5730, 1016.5761, 1026.5627, 1031.5581, 1034.5808, 1044.5240, 1051.5095, 1056.5281, 1057.5575, 1066.5067, 1074.5374, 1087.5460, 1103.5287, 1118.5885, 1148.5439, 1168.5985, 1178.6211, 1185.6898, 1244.6118, 1248.6686, 1253.6313, 1255.6216, 1301.6338, 1312.7423, 1313.7227, 1314.7188, 1315.6721, 1317.6354, 1320.6331, 1322.6371, 1325.6360, 1327.6959, 1333.7570, 1336.7218, 1338.7179, 1339.7092, 1343.6960, 1349.6917, 1350.6987, 1351.6946, 1361.6875, 1372.5978, 1381.7642, 1382.5950, 1383.7253, 1397.7454, 1439.7549, 1442.8190, 1457.7394, 1463.7950, 1488.7245, 1494.7383, 1497.8735, 1504.7484, 1513.7760, 1520.7523, 1528.6951, 1537.8245, 1539.8104, 1549.8242, 1552.8073, 1553.8669, 1555.8348, 1556.8799, 1567.7986, 1570.7749, 1578.7872, 1592.7849, 1628.7002, 1628.8571, 1638.8861, 1642.7155, 1647.8503, 1650.8458, 1680.8226, 1696.8146, 1698.7845, 1699.8228, 1705.8712, 1707.8536, 1715.8536, 1717.9056, 1730.8564, 1739.6990, 1771.0344, 1786.9199, 1809.0262, 1816.9354, 1820.8855, 1836.8507, 1907.9603, 1909.9886, 1940.0148, 1953.8596, 1961.0111, 1965.8679, 1974.9255, 1982.0082, 1983.9866, 1985.8589, 1995.9136, 2027.1289, 2067.1079, 2110.0598, 2136.8484, 2144.0432, 2150.8660, 2164.8794, 2211.0857, 2239.1533, 2251.1509, 2255.1836, 2270.1113, 2273.0972, 2274.0605, 2286.1492, 2289.0857, 2290.0530, 2309.1570, 2324.0981, 2338.1033, 2340.0979, 2343.1770, 2352.9316, 2375.0454, 2383.9480, 2414.2512, 2499.2480, 2542.2678, 2589.9707, 2600.0022, 2603.9897, 2613.9939, 2627.9993, 2644.0112, 2646.9875, 2657.4248, 2659.0405, 2661.0017, 2665.0042, 2674.0601, 2675.0151, 2678.0159, 2689.0330, 2692.0283, 2695.0315, 2703.0417, 2706.0959, 2763.3606, 2798.2788, 2815.3203, 2846.4011, 2872.3391, 2876.3679, 2884.3535, 2888.3455, 2891.4131, 2892.4519, 2919.4543, 3002.3042, 3012.4717, 3016.2314, 3020.2483, 3030.3025, 3033.2446, 3053.4917, 3081.6296, 3126.7493, 3171.7131, 3184.4602, 3206.6179, 3207.6111, 3254.6370, 3265.6309, 3300.7825, 3317.7288, 3321.6318, 3334.6953, 3347.5437, 3429.5991, 3457.6255, 3471.6121, 3489.7363, 3503.8608, 3518.5715, 3639.7969, 4148.0293, 4162.0039, 4453.2720, 4844.2046

8. Q3SWY9 **Mass:** 24956 **Score:** 24 **Expect:** 90 **Matches:** 6

Ras-related protein Rab-28 OS=Bos taurus GN=RAB28 PE=2 SV=1

Observed	Mr(expt)	Mr(calc)	ppm	Start	End	Miss	Peptide
929.4449	928.4376	928.4436	-6.48	131	137	0	K.IDLEHMR.T + Oxidation (M)
1301.6338	1300.6265	1300.6122	11.0	162	172	0	K.TGDSVFLCFQK.V
1315.6721	1314.6648	1314.6891	-18.49	182	192	1	K.LNKAEIEQSQR.V
1628.8571	1627.8498	1627.8722	-13.76	40	52	1	K.QYKQTIGLDFFLR.R
1928.0238	1927.0165	1927.0262	-5.02	113	130	1	K.KVSEESETQPLVALVGNK.I
3126.7493	3125.7420	3125.6907	16.4	53	81	2	R.RITLPGSLNVTLQVWDIGGQTIGGKMLDK.Y + Oxidation (M)

No match to: 706.3098, 710.3790, 712.2532, 714.3089, 717.3984, 719.4317, 720.4312, 722.4346, 735.4181, 749.4561, 756.4000, 759.4391, 772.4356, 774.4578, 778.4783, 779.4789, 818.4724, 823.4901, 831.4754, 832.3123, 834.3210, 842.5065, 844.4971, 870.5016, 871.4923, 873.3899, 876.2862, 880.4520, 883.4794, 891.4870, 895.5731, 900.5293, 909.5164, 913.4845, 918.5409, 923.5157, 925.4807, 927.5140, 942.5470, 944.5204, 945.5183, 949.5508, 950.5472, 967.4262, 972.4835, 974.4452, 976.4556, 977.4954, 978.5431, 988.4708, 1007.5730, 1016.5761, 1026.5627, 1031.5581, 1034.5808, 1044.5240, 1051.5095, 1056.5281, 1057.5575, 1066.5067, 1074.5374, 1087.5460, 1103.5287, 1118.5885, 1122.5553, 1148.5439, 1168.5985, 1178.6211, 1185.6898, 1244.6118, 1248.6686, 1253.6313, 1255.6216, 1312.7423, 1313.7227, 1314.7188, 1317.6354, 1320.6331, 1322.6371, 1325.6360, 1327.6959, 1333.7570, 1336.7218, 1338.7179, 1339.7092, 1343.6960, 1349.6917, 1350.6987, 1351.6946, 1361.6875, 1372.5978, 1381.7642, 1382.5950, 1383.7253, 1397.7454, 1439.7549, 1442.8190, 1457.7394, 1463.7950, 1488.7245, 1494.7383, 1497.8735, 1504.7484, 1513.7760, 1517.8062, 1520.7523, 1528.6951, 1537.8245, 1539.8104, 1549.8242, 1552.8073, 1553.8669, 1555.8348, 1556.8799, 1567.7986, 1570.7749, 1578.7872, 1592.7849, 1628.7002, 1638.8861, 1642.7155, 1647.8503, 1650.8458, 1664.8571, 1680.8226, 1696.8146, 1698.7845, 1699.8228, 1705.8712, 1707.8536, 1715.8536, 1717.9056, 1730.8564, 1739.6990, 1771.0344, 1786.9199, 1809.0262, 1816.9354, 1820.8855, 1836.8507, 1907.9603, 1909.9886, 1940.0148, 1953.8596, 1961.0111, 1965.8679, 1974.9255, 1982.0082, 1983.9866, 1985.8589, 1995.9136, 2027.1289, 2067.1079, 2110.0598, 2136.8484, 2144.0432, 2150.8660, 2164.8794, 2211.0857, 2239.1533, 2251.1509, 2255.1836, 2270.1113, 2273.0972, 2274.0605, 2286.1492, 2289.0857, 2290.0530, 2293.1558, 2309.1570, 2324.0981, 2338.1033, 2340.0979, 2343.1770, 2352.9316, 2375.0454, 2383.9480, 2414.2512, 2499.2480, 2542.2678, 2589.9707, 2600.0022, 2603.9897, 2613.9939, 2627.9993, 2644.0112, 2646.9875, 2657.4248, 2659.0405, 2661.0017, 2665.0042, 2674.0601, 2675.0151, 2678.0159, 2689.0330, 2692.0283, 2695.0315, 2703.0417, 2706.0959, 2763.3606, 2798.2788, 2815.3203, 2846.4011, 2872.3391, 2876.3679, 2884.3535, 2888.3455, 2891.4131, 2892.4519, 2919.4543, 3002.3042, 3012.4717, 3016.2314, 3020.2483, 3030.3025, 3033.2446, 3053.4917, 3081.6296, 3171.7131, 3184.4602, 3206.6179, 3207.6111, 3248.6445, 3254.6370, 3265.6309, 3300.7825, 3317.7288, 3321.6318, 3334.6953, 3347.5437, 3429.5991, 3457.6255, 3471.6121, 3489.7363, 3503.8608, 3518.5715, 3639.7969, 4148.0293, 4162.0039, 4453.2720, 4844.2046

9. F1MP35 **Mass:** 62777 **Score:** 24 **Expect:** 1e+002 **Matches:** 10

Uncharacterized protein OS=Bos taurus GN=ZNF783 PE=4 SV=2

Observed	Mr(expt)	Mr(calc)	ppm	Start	End	Miss	Peptide

Observed	Mr(expt)	Mr(calc)	ppm	Start	End	Miss	Peptide
1026.5627	1025.5554	1025.5440	11.1	383	392	1	R.AACGIPKGPR.S
1351.6946	1350.6873	1350.6819	3.99	114	123	1	K.LDEWQKELYK.H
1457.7394	1456.7321	1456.7019	20.8	411	425	1	R.AGDSRSTGAGTQPPR.V
1549.8242	1548.8169	1548.8022	9.53	519	530	1	R.HQRIHTGERPYR.C
1567.7986	1566.7913	1566.7586	20.9	541	553	0	R.NHHLAVHMQTHAR.G + Oxidation (M)
1928.0238	1927.0165	1926.9952	11.1	258	277	1	R.KGSSLPPFHPGSGFGGGTLK.R
2239.1533	2238.1460	2238.1981	-23.27	446	463	2	R.LKINLTIHQRSHVEEEHR.E
2414.2512	2413.2439	2413.2199	9.96	128	148	0	R.GNYEMLVSLDYAISKPDILTR.M + Oxidation (M)
2798.2788	2797.2715	2797.3085	-13.21	531	553	2	R.CPQCGRAFNRNHHLAVHMQTHAR.G
2846.4011	2845.3938	2845.3990	-1.81	128	151	1	R.GNYEMLVSLDYAISKPDILTRMER.G + 2 Oxidation (M)

No match to: 706.3098, 710.3790, 712.2532, 714.3089, 717.3984, 719.4317, 720.4312, 722.4346, 735.4181, 749.4561, 756.4000, 759.4391, 772.4356, 774.4578, 778.4783, 779.4789, 818.4724, 823.4901, 831.4754, 832.3123, 834.3210, 842.5065, 844.4971, 870.5016, 871.4923, 873.3899, 876.2862, 880.4520, 883.4794, 891.4870, 895.5731, 900.5293, 909.5164, 913.4845, 918.5409, 923.5157, 925.4807, 927.5140, 929.4449, 942.5470, 944.5204, 945.5183, 949.5508, 950.5472, 967.4262, 972.4835, 974.4452, 976.4556, 977.4954, 978.5431, 988.4708, 1007.5730, 1016.5761, 1031.5581, 1034.5808, 1044.5240, 1051.5095, 1056.5281, 1057.5575, 1066.5067, 1074.5374, 1087.5460, 1103.5287, 1118.5885, 1122.5553, 1148.5439, 1168.5985, 1178.6211, 1185.6898, 1244.6118, 1248.6686, 1253.6313, 1255.6216, 1301.6338, 1312.7423, 1313.7227, 1314.7188, 1315.6721, 1317.6354, 1320.6331, 1322.6371, 1325.6360, 1327.6959, 1333.7570, 1336.7218, 1338.7179, 1339.7092, 1343.6960, 1349.6917, 1350.6987, 1361.6875, 1372.5978, 1381.7642, 1382.5950, 1383.7253, 1397.7454, 1439.7549, 1442.8190, 1463.7950, 1488.7245, 1494.7383, 1497.8735, 1504.7484, 1513.7760, 1517.8062, 1520.7523, 1528.6951, 1537.8245, 1539.8104, 1552.8073, 1553.8669, 1555.8348, 1556.8799, 1570.7749, 1578.7872, 1592.7849, 1628.7002, 1628.8571, 1638.8861, 1642.7155, 1647.8503, 1650.8458, 1664.8571, 1680.8226, 1696.8146, 1698.7845, 1699.8228, 1705.8712, 1707.8536, 1715.8536, 1717.9056, 1730.8564, 1739.6990, 1771.0344, 1786.9199, 1809.0262, 1816.9354, 1820.8855, 1836.8507, 1907.9603, 1909.9886, 1940.0148, 1953.8596, 1961.0111, 1965.8679, 1974.9255, 1982.0082, 1983.9866, 1985.8589, 1995.9136, 2027.1289, 2067.1079, 2110.0598, 2136.8484, 2144.0432, 2150.8660, 2164.8794, 2211.0857, 2251.1509, 2255.1836, 2270.1113, 2273.0972, 2274.0605, 2286.1492, 2289.0857, 2290.0530, 2293.1558, 2309.1570, 2324.0981, 2338.1033, 2340.0979, 2343.1770, 2352.9316, 2375.0454, 2383.9480, 2499.2480, 2542.2678, 2589.9707, 2600.0022, 2603.9897, 2613.9939, 2627.9993, 2644.0112, 2646.9875, 2657.4248, 2659.0405, 2661.0017, 2665.0042, 2674.0601, 2675.0151, 2678.0159, 2689.0330, 2692.0283, 2695.0315, 2703.0417, 2706.0959, 2763.3606, 2815.3203, 2872.3391, 2876.3679, 2884.3535, 2888.3455, 2891.4131, 2892.4519, 2919.4543, 3002.3042, 3012.4717, 3016.2314, 3020.2483, 3030.3025, 3033.2446, 3053.4917, 3081.6296, 3126.7493, 3171.7131, 3184.4602, 3206.6179, 3207.6111, 3248.6445, 3254.6370, 3265.6309, 3300.7825, 3317.7288, 3321.6318, 3334.6953, 3347.5437, 3429.5991, 3457.6255, 3471.6121, 3489.7363, 3503.8608, 3518.5715, 3639.7969, 4148.0293, 4162.0039, 4453.2720, 4844.2046

10. Q0VCQ3 **Mass:** 32810 **Score:** 23 **Expect:** 1.3e+002 **Matches:** 5

MAX interactor 1 OS=Bos taurus GN=MXI1 PE=2 SV=1

Observed	Mr(expt)	Mr(calc)	ppm	Start	End	Miss	Peptide
1253.6313	1252.6240	1252.6160	6.42	189	198	0	K.SQHQLENLER.E
1383.7253	1382.7180	1382.7014	12.0	133	143	2	R.STHNELEKNRR.A
1457.7394	1456.7321	1456.6980	23.4	207	218	0	R.LEQLQGPQEMER.I
3206.6179	3205.6106	3205.5826	8.75	10	42	1	K.EARCEGAGLAPAAPPAVTAPQPPAQPEEPAGAK.L
3334.6953	3333.6880	3333.6775	3.15	9	42	2	R.KEARCEGAGLAPAAPPAVTAPQPPAQPEEPAGAK.L

No match to: 706.3098, 710.3790, 712.2532, 714.3089, 717.3984, 719.4317, 720.4312, 722.4346, 735.4181, 749.4561, 756.4000, 759.4391, 772.4356, 774.4578, 778.4783, 779.4789, 818.4724, 823.4901, 831.4754, 832.3123, 834.3210, 842.5065, 844.4971, 870.5016, 871.4923, 873.3899, 876.2862, 880.4520, 883.4794, 891.4870, 895.5731, 900.5293, 909.5164, 913.4845, 918.5409, 923.5157, 925.4807, 927.5140, 929.4449, 942.5470, 944.5204, 945.5183, 949.5508, 950.5472, 967.4262, 972.4835, 974.4452, 976.4556, 977.4954, 978.5431, 988.4708, 1007.5730, 1016.5761, 1026.5627, 1031.5581, 1034.5808, 1044.5240, 1051.5095, 1056.5281, 1057.5575, 1066.5067, 1074.5374, 1087.5460, 1103.5287, 1118.5885, 1122.5553, 1148.5439, 1168.5985, 1178.6211, 1185.6898, 1244.6118, 1248.6686, 1255.6216, 1301.6338, 1312.7423, 1313.7227, 1314.7188, 1315.6721, 1317.6354, 1320.6331, 1322.6371, 1325.6360, 1327.6959, 1333.7570, 1336.7218, 1338.7179, 1339.7092, 1343.6960, 1349.6917, 1350.6987, 1351.6946, 1361.6875, 1372.5978, 1381.7642, 1382.5950, 1397.7454, 1439.7549, 1442.8190, 1463.7950, 1488.7245, 1494.7383, 1497.8735, 1504.7484, 1513.7760, 1517.8062, 1520.7523, 1528.6951, 1537.8245, 1539.8104, 1549.8242, 1552.8073, 1553.8669, 1555.8348, 1556.8799, 1567.7986, 1570.7749, 1578.7872, 1592.7849, 1628.7002, 1628.8571, 1638.8861, 1642.7155, 1647.8503, 1650.8458, 1664.8571, 1680.8226, 1696.8146, 1698.7845, 1699.8228, 1705.8712, 1707.8536, 1715.8536, 1717.9056, 1730.8564, 1739.6990, 1771.0344, 1786.9199, 1809.0262, 1816.9354, 1820.8855, 1836.8507, 1907.9603, 1909.9886, 1928.0238, 1940.0148, 1953.8596, 1961.0111, 1965.8679, 1974.9255, 1982.0082, 1983.9866, 1985.8589, 1995.9136, 2027.1289, 2067.1079, 2110.0598, 2136.8484, 2144.0432, 2150.8660, 2164.8794, 2211.0857, 2239.1533, 2251.1509, 2255.1836, 2270.1113, 2273.0972, 2274.0605, 2286.1492, 2289.0857, 2290.0530, 2293.1558, 2309.1570, 2324.0981, 2338.1033, 2340.0979, 2343.1770, 2352.9316, 2375.0454, 2383.9480, 2414.2512, 2499.2480, 2542.2678, 2589.9707, 2600.0022, 2603.9897, 2613.9939, 2627.9993, 2644.0112, 2646.9875, 2657.4248, 2659.0405, 2661.0017, 2665.0042, 2674.0601, 2675.0151, 2678.0159, 2689.0330, 2692.0283, 2695.0315, 2703.0417, 2706.0959, 2763.3606, 2798.2788, 2815.3203, 2846.4011, 2872.3391, 2876.3679, 2884.3535, 2888.3455, 2891.4131, 2892.4519, 2919.4543, 3002.3042, 3012.4717, 3016.2314, 3020.2483, 3030.3025, 3033.2446, 3053.4917, 3081.6296, 3126.7493, 3171.7131, 3184.4602, 3207.6111, 3248.6445, 3254.6370, 3265.6309, 3300.7825, 3317.7288, 3321.6318, 3347.5437, 3429.5991, 3457.6255, 3471.6121, 3489.7363, 3503.8608, 3518.5715, 3639.7969, 4148.0293, 4162.0039, 4453.2720, 4844.2046

11. Q3T063 **Mass:** 31473 **Score:** 22 **Expect:** 1.4e+002 **Matches:** 4

Nicotinate-nucleotide pyrophosphorylase [carboxylating] OS=Bos taurus GN=QPRT PE=2 SV=1

Observed	Mr(expt)	Mr(calc)	ppm	Start	End	Miss	Peptide
735.4181	734.4108	734.3963	19.8	191	197	0	R.AADFALK.V
891.4870	890.4797	890.4974	-19.82	190	197	1	R.RAADFALK.V
1552.8073	1551.8000	1551.8304	-19.55	172	186	2	K.DNHVMAAGGVKKAVR.A
2846.4011	2845.3938	2845.3712	7.96	111	139	2	R.CSGVASMAAAAVETARGTGWAGHVAGTRK.T + Oxidation (M)

No match to: 706.3098, 710.3790, 712.2532, 714.3089, 717.3984, 719.4317, 720.4312, 722.4346, 749.4561, 756.4000, 759.4391, 772.4356, 774.4578, 778.4783, 779.4789, 818.4724, 823.4901, 831.4754, 832.3123, 834.3210, 842.5065, 844.4971, 870.5016, 871.4923, 873.3899, 876.2862, 880.4520, 883.4794, 895.5731, 900.5293, 909.5164, 913.4845, 918.5409, 923.5157, 925.4807, 927.5140, 929.4449, 942.5470, 944.5204, 945.5183, 949.5508, 950.5472, 967.4262, 972.4835, 974.4452, 976.4556, 977.4954, 978.5431, 988.4708, 1007.5730, 1016.5761, 1026.5627, 1031.5581, 1034.5808, 1044.5240, 1051.5095, 1056.5281, 1057.5575, 1066.5067, 1074.5374, 1087.5460, 1103.5287, 1118.5885, 1122.5553, 1148.5439, 1168.5985, 1178.6211, 1185.6898, 1244.6118, 1248.6686, 1253.6313, 1255.6216, 1301.6338, 1312.7423, 1313.7227, 1314.7188, 1315.6721, 1317.6354, 1320.6331, 1322.6371, 1325.6360, 1327.6959, 1333.7570, 1336.7218, 1338.7179, 1339.7092, 1343.6960, 1349.6917, 1350.6987, 1351.6946, 1361.6875, 1372.5978, 1381.7642, 1382.5950, 1383.7253, 1397.7454, 1439.7549, 1442.8190, 1457.7394, 1463.7950, 1488.7245, 1494.7383, 1497.8735, 1504.7484, 1513.7760, 1517.8062, 1520.7523, 1528.6951, 1537.8245, 1539.8104, 1549.8242, 1553.8669, 1555.8348, 1556.8799, 1567.7986, 1570.7749, 1578.7872, 1592.7849, 1628.7002, 1628.8571, 1638.8861, 1642.7155, 1647.8503, 1650.8458, 1664.8571, 1680.8226, 1696.8146, 1698.7845, 1699.8228, 1705.8712, 1707.8536, 1715.8536, 1717.9056, 1730.8564, 1739.6990, 1771.0344, 1786.9199, 1809.0262, 1816.9354, 1820.8855, 1836.8507, 1907.9603, 1909.9886, 1928.0238, 1940.0148, 1953.8596, 1961.0111, 1965.8679, 1974.9255, 1982.0082, 1983.9866, 1985.8589, 1995.9136, 2027.1289, 2067.1079, 2110.0598, 2136.8484, 2144.0432, 2150.8660, 2164.8794, 2211.0857, 2239.1533, 2251.1509, 2255.1836, 2270.1113, 2273.0972, 2274.0605, 2286.1492, 2289.0857, 2290.0530, 2293.1558, 2309.1570, 2324.0981, 2338.1033, 2340.0979, 2343.1770, 2352.9316, 2375.0454, 2383.9480, 2414.2512, 2499.2480, 2542.2678, 2589.9707, 2600.0022, 2603.9897, 2613.9939, 2627.9993, 2644.0112, 2646.9875, 2657.4248, 2659.0405, 2661.0017, 2665.0042, 2674.0601, 2675.0151, 2678.0159, 2689.0330, 2692.0283, 2695.0315, 2703.0417, 2706.0959, 2763.3606, 2798.2788, 2815.3203, 2872.3391, 2876.3679, 2884.3535, 2888.3455, 2891.4131, 2892.4519, 2919.4543, 3002.3042, 3012.4717, 3016.2314, 3020.2483, 3030.3025, 3033.2446, 3053.4917, 3081.6296, 3126.7493, 3171.7131, 3184.4602, 3206.6179, 3207.6111, 3248.6445, 3254.6370, 3265.6309, 3300.7825, 3317.7288, 3321.6318, 3334.6953, 3347.5437, 3429.5991, 3457.6255, 3471.6121, 3489.7363, 3503.8608, 3518.5715, 3639.7969, 4148.0293, 4162.0039, 4453.2720, 4844.2046

12. F1MHB8 **Mass:** 31507 **Score:** 22 **Expect:** 1.4e+002 **Matches:** 4

Nicotinate-nucleotide pyrophosphorylase [carboxylating] OS=Bos taurus GN=QPRT PE=3 SV=1

Observed	Mr(expt)	Mr(calc)	ppm	Start	End	Miss	Peptide
735.4181	734.4108	734.3963	19.8	191	197	0	R.AADFALK.V
891.4870	890.4797	890.4974	-19.82	190	197	1	R.RAADFALK.V

1552.8073	1551.8000	1551.8304	-19.55	172 - 186	2	K.DNHVMAAGGVKKAVR.A
2846.4011	2845.3938	2845.3712	7.96	111 - 139	2	R.CSGVASMAAAAVETARGTGWAGHVAGTRK.T + Oxidation (M)

No match to: 706.3098, 710.3790, 712.2532, 714.3089, 717.3984, 719.4317, 720.4312, 722.4346, 749.4561, 756.4000, 759.4391, 772.4356, 774.4578, 778.4783, 779.4789, 818.4724, 823.4901, 831.4754, 832.3123, 834.3210, 842.5065, 844.4971, 870.5016, 871.4923, 873.3899, 876.2862, 880.4520, 883.4794, 895.5731, 900.5293, 909.5164, 913.4845, 918.5409, 923.5157, 925.4807, 927.5140, 929.4449, 942.5470, 944.5204, 945.5183, 949.5508, 950.5472, 967.4262, 972.4835, 974.4452, 976.4556, 977.4954, 978.5431, 988.4708, 1007.5730, 1016.5761, 1026.5627, 1031.5581, 1034.5808, 1044.5240, 1051.5095, 1056.5281, 1057.5575, 1066.5067, 1074.5374, 1087.5460, 1103.5287, 1118.5885, 1122.5553, 1148.5439, 1168.5985, 1178.6211, 1185.6898, 1244.6118, 1248.6686, 1253.6313, 1255.6216, 1301.6338, 1312.7423, 1313.7227, 1314.7188, 1315.6721, 1317.6354, 1320.6331, 1322.6371, 1325.6360, 1327.6959, 1333.7570, 1336.7218, 1338.7179, 1339.7092, 1343.6960, 1349.6917, 1350.6987, 1351.6946, 1361.6875, 1372.5978, 1381.7642, 1382.5950, 1383.7253, 1397.7454, 1439.7549, 1442.8190, 1457.7394, 1463.7950, 1488.7245, 1494.7383, 1497.8735, 1504.7484, 1513.7760, 1517.8062, 1520.7523, 1528.6951, 1537.8245, 1539.8104, 1549.8242, 1553.8669, 1555.8348, 1556.8799, 1567.7986, 1570.7749, 1578.7872, 1592.7849, 1628.7002, 1628.8571, 1638.8861, 1642.7155, 1647.8503, 1650.8458, 1664.8571, 1680.8226, 1696.8146, 1698.7845, 1699.8228, 1705.8712, 1707.8536, 1715.8536, 1717.9056, 1730.8564, 1739.6990, 1771.0344, 1786.9199, 1809.0262, 1816.9354, 1820.8855, 1836.8507, 1907.9603, 1909.9886, 1928.0238, 1940.0148, 1953.8596, 1961.0111, 1965.8679, 1974.9255, 1982.0082, 1983.9866, 1985.8589, 1995.9136, 2027.1289, 2067.1079, 2110.0598, 2136.8484, 2144.0432, 2150.8660, 2164.8794, 2211.0857, 2239.1533, 2251.1509, 2255.1836, 2270.1113, 2273.0972, 2274.0605, 2286.1492, 2289.0857, 2290.0530, 2293.1558, 2309.1570, 2324.0981, 2338.1033, 2340.0979, 2343.1770, 2352.9316, 2375.0454, 2383.9480, 2414.2512, 2499.2480, 2542.2678, 2589.9707, 2600.0022, 2603.9897, 2613.9939, 2627.9993, 2644.0112, 2646.9875, 2657.4248, 2659.0405, 2661.0017, 2665.0042, 2674.0601, 2675.0151, 2678.0159, 2689.0330, 2692.0283, 2695.0315, 2703.0417, 2706.0959, 2763.3606, 2798.2788, 2815.3203, 2872.3391, 2876.3679, 2884.3535, 2888.3455, 2891.4131, 2892.4519, 2919.4543, 3002.3042, 3012.4717, 3016.2314, 3020.2483, 3030.3025, 3033.2446, 3053.4917, 3081.6296, 3126.7493, 3171.7131, 3184.4602, 3206.6179, 3207.6111, 3248.6445, 3254.6370, 3265.6309, 3300.7825, 3317.7288, 3321.6318, 3334.6953, 3347.5437, 3429.5991, 3457.6255, 3471.6121, 3489.7363, 3503.8608, 3518.5715, 3639.7969, 4148.0293, 4162.0039, 4453.2720, 4844.2046

13. P56965 **Mass:** 31554 **Score:** 22 **Expect:** 1.6e+002 **Matches:** 6

N(G),N(G)-dimethylarginine dimethylaminohydrolase 1 OS=Bos taurus GN=DDAH1 PE=1 SV=3

Observed	Mr(expt)	Mr(calc)	ppm	Start - End	Miss	Peptide
779.4789	778.4716	778.4589	16.4	248 - 253	1	K.VYEKLK.D
831.4754	830.4681	830.4875	-23.33	231 - 237	0	K.GHVLLHR.T
1244.6118	1243.6045	1243.6132	-6.96	1 - 12	0	-.MASLGHPATFGR.A
1457.7394	1456.7321	1456.7279	2.93	197 - 208	1	K.ALKIMQQMSDHR.Y
3300.7825	3299.7752	3299.7673	2.40	1 - 31	2	-.MASLGHPATFGRATHVVVRALPESLAQQALR.R + Oxidation (M)
3334.6953	3333.6880	3333.6762	3.54	107 - 136	2	K.EALEKLQLNIVEMKDENATLDGGDVLFTGR.E + Oxidation (M)

No match to: 706.3098, 710.3790, 712.2532, 714.3089, 717.3984, 719.4317, 720.4312, 722.4346, 735.4181, 749.4561, 756.4000, 759.4391, 772.4356, 774.4578, 778.4783, 818.4724, 823.4901, 832.3123, 834.3210, 842.5065, 844.4971, 870.5016, 871.4923, 873.3899, 876.2862, 880.4520, 883.4794, 891.4870, 895.5731, 900.5293, 909.5164, 913.4845, 918.5409, 923.5157, 925.4807, 927.5140, 929.4449, 942.5470, 944.5204, 945.5183, 949.5508, 950.5472, 967.4262, 972.4835, 974.4452, 976.4556, 977.4954, 978.5431, 988.4708, 1007.5730, 1016.5761, 1026.5627, 1031.5581, 1034.5808, 1044.5240, 1051.5095, 1056.5281, 1057.5575, 1066.5067, 1074.5374, 1087.5460, 1103.5287, 1118.5885, 1122.5553, 1148.5439, 1168.5985, 1178.6211, 1185.6898, 1248.6686, 1253.6313, 1255.6216, 1301.6338, 1312.7423, 1313.7227, 1314.7188, 1315.6721, 1317.6354, 1320.6331, 1322.6371, 1325.6360, 1327.6959, 1333.7570, 1336.7218, 1338.7179, 1339.7092, 1343.6960, 1349.6917, 1350.6987, 1351.6946, 1361.6875, 1372.5978, 1381.7642, 1382.5950, 1383.7253, 1397.7454, 1439.7549, 1442.8190, 1463.7950, 1488.7245, 1494.7383, 1497.8735, 1504.7484, 1513.7760, 1517.8062, 1520.7523, 1528.6951, 1537.8245, 1539.8104, 1549.8242, 1552.8073, 1553.8669, 1555.8348, 1556.8799, 1567.7986, 1570.7749, 1578.7872, 1592.7849, 1628.7002, 1628.8571, 1638.8861, 1642.7155, 1647.8503, 1650.8458, 1664.8571, 1680.8226, 1696.8146, 1698.7845, 1699.8228, 1705.8712, 1707.8536, 1715.8536, 1717.9056, 1730.8564, 1739.6990, 1771.0344, 1786.9199, 1809.0262, 1816.9354, 1820.8855, 1836.8507, 1907.9603, 1909.9886, 1928.0238, 1940.0148, 1953.8596, 1961.0111, 1965.8679, 1974.9255, 1982.0082, 1983.9866, 1985.8589, 1995.9136, 2027.1289, 2067.1079, 2110.0598, 2136.8484, 2144.0432, 2150.8660, 2164.8794, 2211.0857, 2239.1533, 2251.1509, 2255.1836, 2270.1113, 2273.0972, 2274.0605, 2286.1492, 2289.0857, 2290.0530, 2293.1558, 2309.1570, 2324.0981, 2338.1033, 2340.0979, 2343.1770, 2352.9316, 2375.0454, 2383.9480, 2414.2512, 2499.2480, 2542.2678, 2589.9707, 2600.0022, 2603.9897, 2613.9939, 2627.9993, 2644.0112, 2646.9875, 2657.4248, 2659.0405, 2661.0017, 2665.0042, 2674.0601, 2675.0151, 2678.0159, 2689.0330, 2692.0283, 2695.0315, 2703.0417, 2706.0959, 2763.3606, 2798.2788, 2815.3203, 2846.4011, 2872.3391, 2876.3679, 2884.3535, 2888.3455, 2891.4131, 2892.4519, 2919.4543, 3002.3042, 3012.4717, 3016.2314, 3020.2483, 3030.3025, 3033.2446, 3053.4917, 3081.6296, 3126.7493, 3171.7131, 3184.4602, 3206.6179, 3207.6111, 3248.6445, 3254.6370, 3265.6309, 3317.7288, 3321.6318, 3347.5437, 3429.5991, 3457.6255, 3471.6121, 3489.7363, 3503.8608, 3518.5715, 3639.7969, 4148.0293, 4162.0039, 4453.2720, 4844.2046

14. F1MVI1 **Mass:** 62511 **Score:** 21 **Expect:** 1.8e+002 **Matches:** 9

Cleavage stimulation factor subunit 2 OS=Bos taurus GN=CSTF2 PE=4 SV=1

Observed	Mr(expt)	Mr(calc)	ppm	Start - End	Miss	Peptide
1057.5575	1056.5502	1056.5676	-16.43	439 - 448	1	R.GLDARGLEAR.A
1301.6338	1300.6265	1300.6194	5.50	149 - 159	0	K.LCVQNSPQEAR.N
1349.6917	1348.6844	1348.6960	-8.58	416 - 428	2	R.GPPLDGRGGRDPR.G
1457.7394	1456.7321	1456.7205	8.01	426 - 438	2	R.DPRGIDARAMEAR.G
2110.0598	2109.0525	2109.0308	10.3	131 - 148	1	K.AVASLPPEQMFELMKQMK.L + 2 Oxidation (M)
2273.0972	2272.0899	2272.0709	8.39	365 - 385	0	R.GYLGPPHQGPPMHHVPGHDSR.G
2289.0857	2288.0784	2288.0658	5.53	365 - 385	0	R.GYLGPPHQGPPMHHVPGHDSR.G + Oxidation (M)
2324.0981	2323.0908	2323.1049	-6.07	491 - 514	0	R.GPMPSGIQGPSPINMGAVGPQGSR.Q + 2 Oxidation (M)
2414.2512	2413.2439	2413.2973	-22.14	160 - 180	0	R.NMLLQNPQLAYALLQAQVVMR.I

No match to: 706.3098, 710.3790, 712.2532, 714.3089, 717.3984, 719.4317, 720.4312, 722.4346, 735.4181, 749.4561, 756.4000, 759.4391, 772.4356, 774.4578, 778.4783, 779.4789, 818.4724, 823.4901, 831.4754, 832.3123, 834.3210, 842.5065, 844.4971, 870.5016, 871.4923, 873.3899, 876.2862, 880.4520, 883.4794, 891.4870, 895.5731, 900.5293, 909.5164, 913.4845, 918.5409, 923.5157, 925.4807, 927.5140, 929.4449, 942.5470, 944.5204, 945.5183, 949.5508, 950.5472, 967.4262, 972.4835, 974.4452, 976.4556, 977.4954, 978.5431, 988.4708, 1007.5730, 1016.5761, 1026.5627, 1031.5581, 1034.5808, 1044.5240, 1051.5095, 1056.5281, 1066.5067, 1074.5374, 1087.5460, 1103.5287, 1118.5885, 1122.5553, 1148.5439, 1168.5985, 1178.6211, 1185.6898, 1244.6118, 1248.6686, 1253.6313, 1255.6216, 1312.7423, 1313.7227, 1314.7188, 1315.6721, 1317.6354, 1320.6331, 1322.6371, 1325.6360, 1327.6959, 1333.7570, 1336.7218, 1338.7179, 1339.7092, 1343.6960, 1350.6987, 1351.6946, 1361.6875, 1372.5978, 1381.7642, 1382.5950, 1383.7253, 1397.7454, 1439.7549, 1442.8190, 1463.7950, 1488.7245, 1494.7383, 1497.8735, 1504.7484, 1513.7760, 1517.8062, 1520.7523, 1528.6951, 1537.8245, 1539.8104, 1549.8242, 1552.8073, 1553.8669, 1555.8348, 1556.8799, 1567.7986, 1570.7749, 1578.7872, 1592.7849, 1628.7002, 1628.8571, 1638.8861, 1642.7155, 1647.8503, 1650.8458, 1664.8571, 1680.8226, 1696.8146, 1698.7845, 1699.8228, 1705.8712, 1707.8536, 1715.8536, 1717.9056, 1730.8564, 1739.6990, 1771.0344, 1786.9199, 1809.0262, 1816.9354, 1820.8855, 1836.8507, 1907.9603, 1909.9886, 1928.0238, 1940.0148, 1953.8596, 1961.0111, 1965.8679, 1974.9255, 1982.0082, 1983.9866, 1985.8589, 1995.9136, 2027.1289, 2067.1079, 2136.8484, 2144.0432, 2150.8660, 2164.8794, 2211.0857, 2239.1533, 2251.1509, 2255.1836, 2270.1113, 2274.0605, 2286.1492, 2290.0530, 2293.1558, 2309.1570, 2338.1033, 2340.0979, 2343.1770, 2352.9316, 2375.0454, 2383.9480, 2499.2480, 2542.2678, 2589.9707, 2600.0022, 2603.9897, 2613.9939, 2627.9993, 2644.0112, 2646.9875, 2657.4248, 2659.0405, 2661.0017, 2665.0042, 2674.0601, 2675.0151, 2678.0159, 2689.0330, 2692.0283, 2695.0315, 2703.0417, 2706.0959, 2763.3606, 2798.2788, 2815.3203, 2846.4011, 2872.3391, 2876.3679, 2884.3535, 2888.3455, 2891.4131, 2892.4519, 2919.4543, 3002.3042, 3012.4717, 3016.2314, 3020.2483, 3030.3025, 3033.2446, 3053.4917, 3081.6296, 3126.7493, 3171.7131, 3184.4602, 3206.6179, 3207.6111, 3248.6445, 3254.6370, 3265.6309, 3300.7825, 3317.7288, 3321.6318, 3334.6953, 3347.5437, 3429.5991, 3457.6255, 3471.6121, 3489.7363, 3503.8608, 3518.5715, 3639.7969, 4148.0293, 4162.0039, 4453.2720, 4844.2046

15. Q8HXM1 **Mass:** 60513 **Score:** 21 **Expect:** 1.8e+002 **Matches:** 9

Cleavage stimulation factor subunit 2 OS=Bos taurus GN=CSTF2 PE=2 SV=1

Observed	Mr(expt)	Mr(calc)	ppm	Start - End	Miss	Peptide
1057.5575	1056.5502	1056.5676	-16.43	419 - 428	1	R.GLDARGLEAR.A
1301.6338	1300.6265	1300.6194	5.50	149 - 159	0	K.LCVQNSPQEAR.N
1349.6917	1348.6844	1348.6960	-8.58	396 - 408	2	R.GPPLDGRGGRDPR.G
1457.7394	1456.7321	1456.7205	8.01	406 - 418	2	R.DPRGIDARAMEAR.G

2110.0598	2109.0525	2109.0308	10.3	131 - 148	1	K.AVASLPPEQMFELMKQMK.L + 2 Oxidation (M)
2273.0972	2272.0899	2272.0709	8.39	345 - 365	0	R.GYLGPPHQGPPMHHVPGHDSR.G
2289.0857	2288.0784	2288.0658	5.53	345 - 365	0	R.GYLGPPHQGPPMHHVPGHDSR.G + Oxidation (M)
2324.0981	2323.0908	2323.1049	-6.07	471 - 494	0	R.GPMPSGIQGPSPINMGAVGPQGSR.Q + 2 Oxidation (M)
2414.2512	2413.2439	2413.2973	-22.14	160 - 180	0	R.NMLLQNPQLAYALLQAQVVMR.I

No match to: 706.3098, 710.3790, 712.2532, 714.3089, 717.3984, 719.4317, 720.4312, 722.4346, 735.4181, 749.4561, 756.4000, 759.4391, 772.4356, 774.4578, 778.4783, 779.4789, 818.4724, 823.4901, 831.4754, 832.3123, 834.3210, 842.5065, 844.4971, 870.5016, 871.4923, 873.3899, 876.2862, 880.4520, 883.4794, 891.4870, 895.5731, 900.5293, 909.5164, 913.4845, 918.5409, 923.5157, 925.4807, 927.5140, 929.4449, 942.5470, 944.5204, 945.5183, 949.5508, 950.5472, 967.4262, 972.4835, 974.4452, 976.4556, 977.4954, 978.5431, 988.4708, 1007.5730, 1016.5761, 1026.5627, 1031.5581, 1034.5808, 1044.5240, 1051.5095, 1056.5281, 1066.5067, 1074.5374, 1087.5460, 1103.5287, 1118.5885, 1122.5553, 1148.5439, 1168.5985, 1178.6211, 1185.6898, 1244.6118, 1248.6686, 1253.6313, 1255.6216, 1312.7423, 1313.7227, 1314.7188, 1315.6721, 1317.6354, 1320.6331, 1322.6371, 1325.6360, 1327.6959, 1333.7570, 1336.7218, 1338.7179, 1339.7092, 1343.6960, 1350.6987, 1351.6946, 1361.6875, 1372.5978, 1381.7642, 1382.5950, 1383.7253, 1397.7454, 1439.7549, 1442.8190, 1463.7950, 1488.7245, 1494.7383, 1497.8735, 1504.7484, 1513.7760, 1517.8062, 1520.7523, 1528.6951, 1537.8245, 1539.8104, 1549.8242, 1552.8073, 1553.8669, 1555.8348, 1556.8799, 1567.7986, 1570.7749, 1578.7872, 1592.7849, 1628.7002, 1628.8571, 1638.8861, 1642.7155, 1647.8503, 1650.8458, 1664.8571, 1680.8226, 1696.8146, 1698.7845, 1699.8228, 1705.8712, 1707.8536, 1715.8536, 1717.9056, 1730.8564, 1739.6990, 1771.0344, 1786.9199, 1809.0262, 1816.9354, 1820.8855, 1836.8507, 1907.9603, 1909.9886, 1928.0238, 1940.0148, 1953.8596, 1961.0111, 1965.8679, 1974.9255, 1982.0082, 1983.9866, 1985.8589, 1995.9136, 2027.1289, 2067.1079, 2136.8484, 2144.0432, 2150.8660, 2164.8794, 2211.0857, 2239.1533, 2251.1509, 2255.1836, 2270.1113, 2274.0605, 2286.1492, 2290.0530, 2293.1558, 2309.1570, 2338.1033, 2340.0979, 2343.1770, 2352.9316, 2375.0454, 2383.9480, 2499.2480, 2542.2678, 2589.9707, 2600.0022, 2603.9897, 2613.9939, 2627.9993, 2644.0112, 2646.9875, 2657.4248, 2659.0405, 2661.0017, 2665.0042, 2674.0601, 2675.0151, 2678.0159, 2689.0330, 2692.0283, 2695.0315, 2703.0417, 2706.0959, 2763.3606, 2798.2788, 2815.3203, 2846.4011, 2872.3391, 2876.3679, 2884.3535, 2888.3455, 2891.4131, 2892.4519, 2919.4543, 3002.3042, 3012.4717, 3016.2314, 3020.2483, 3030.3025, 3033.2446, 3053.4917, 3081.6296, 3126.7493, 3171.7131, 3184.4602, 3206.6179, 3207.6111, 3248.6445, 3254.6370, 3265.6309, 3300.7825, 3317.7288, 3321.6318, 3334.6953, 3347.5437, 3429.5991, 3457.6255, 3471.6121, 3489.7363, 3503.8608, 3518.5715, 3639.7969, 4148.0293, 4162.0039, 4453.2720, 4844.2046

16. Q27979 **Mass:** 35414 **Score:** 21 **Expect:** 1.9e+002 **Matches:** 7

11-cis retinol dehydrogenase OS=Bos taurus GN=RDH5 PE=1 SV=1

Observed	Mr(expt)	Mr(calc)	ppm	Start - End	Miss	Peptide
891.4870	890.4797	890.4722	8.40	48 - 54	1	R.LDQRGFR.V
1016.5761	1015.5688	1015.5563	12.3	191 - 199	1	R.RDVAPFGVR.V
1397.7454	1396.7381	1396.7099	20.2	180 - 191	1	K.FGLEAFSDSLRR.D
1570.7749	1569.7676	1569.7800	-7.91	276 - 288	2	R.HPRTRYSPGWDAK.L
1717.9056	1716.8983	1716.8954	1.69	267 - 280	2	R.CLEHALTARHPRTR.Y
2239.1533	2238.1460	2238.1546	-3.81	180 - 199	2	K.FGLEAFSDSLRRDVAPFGVR.V
2846.4011	2845.3938	2845.3707	8.13	252 - 275	2	R.IMNMICDPDLAKVSRCLEHALTAR.H + 2 Oxidation (M)

No match to: 706.3098, 710.3790, 712.2532, 714.3089, 717.3984, 719.4317, 720.4312, 722.4346, 735.4181, 749.4561, 756.4000, 759.4391, 772.4356, 774.4578, 778.4783, 779.4789, 818.4724, 823.4901, 831.4754, 832.3123, 834.3210, 842.5065, 844.4971, 870.5016, 871.4923, 873.3899, 876.2862, 880.4520, 883.4794, 895.5731, 900.5293, 909.5164, 913.4845, 918.5409, 923.5157, 925.4807, 927.5140, 929.4449, 942.5470, 944.5204, 945.5183, 949.5508, 950.5472, 967.4262, 972.4835, 974.4452, 976.4556, 977.4954, 978.5431, 988.4708, 1007.5730, 1026.5627, 1031.5581, 1034.5808, 1044.5240, 1051.5095, 1056.5281, 1057.5575, 1066.5067, 1074.5374, 1087.5460, 1103.5287, 1118.5885, 1122.5553, 1148.5439, 1168.5985, 1178.6211, 1185.6898, 1244.6118, 1248.6686, 1253.6313, 1255.6216, 1301.6338, 1312.7423, 1313.7227, 1314.7188, 1315.6721, 1317.6354, 1320.6331, 1322.6371, 1325.6360, 1327.6959, 1333.7570, 1336.7218, 1338.7179, 1339.7092, 1343.6960, 1349.6917, 1350.6987, 1351.6946, 1361.6875, 1372.5978, 1381.7642, 1382.5950, 1383.7253, 1439.7549, 1442.8190, 1457.7394, 1463.7950, 1488.7245, 1494.7383, 1497.8735, 1504.7484, 1513.7760, 1517.8062, 1520.7523, 1528.6951, 1537.8245, 1539.8104, 1549.8242, 1552.8073, 1553.8669, 1555.8348, 1556.8799, 1567.7986, 1578.7872, 1592.7849, 1628.7002, 1628.8571, 1638.8861, 1642.7155, 1647.8503, 1650.8458, 1664.8571, 1680.8226, 1696.8146, 1698.7845, 1699.8228, 1705.8712, 1707.8536, 1715.8536, 1730.8564, 1739.6990, 1771.0344, 1786.9199, 1809.0262, 1816.9354, 1820.8855, 1836.8507, 1907.9603, 1909.9886, 1928.0238, 1940.0148, 1953.8596, 1961.0111, 1965.8679, 1974.9255, 1982.0082, 1983.9866, 1985.8589, 1995.9136, 2027.1289, 2067.1079, 2110.0598, 2136.8484, 2144.0432, 2150.8660, 2164.8794, 2211.0857, 2251.1509, 2255.1836, 2270.1113, 2273.0972, 2274.0605, 2286.1492, 2289.0857, 2290.0530, 2293.1558, 2309.1570, 2324.0981, 2338.1033, 2340.0979, 2343.1770, 2352.9316, 2375.0454, 2383.9480, 2414.2512, 2499.2480, 2542.2678, 2589.9707, 2600.0022, 2603.9897, 2613.9939, 2627.9993, 2644.0112, 2646.9875, 2657.4248, 2659.0405, 2661.0017, 2665.0042, 2674.0601, 2675.0151, 2678.0159, 2689.0330, 2692.0283, 2695.0315, 2703.0417, 2706.0959, 2763.3606, 2798.2788, 2815.3203, 2872.3391, 2876.3679, 2884.3535, 2888.3455, 2891.4131, 2892.4519, 2919.4543, 3002.3042, 3012.4717, 3016.2314, 3020.2483, 3030.3025, 3033.2446, 3053.4917, 3081.6296, 3126.7493, 3171.7131, 3184.4602, 3206.6179, 3207.6111, 3248.6445, 3254.6370, 3265.6309, 3300.7825, 3317.7288, 3321.6318, 3334.6953, 3347.5437, 3429.5991, 3457.6255, 3471.6121, 3489.7363, 3503.8608, 3518.5715, 3639.7969, 4148.0293, 4162.0039, 4453.2720, 4844.2046

17. A0A0A0MP97 **Mass:** 34740 **Score:** 21 **Expect:** 1.9e+002 **Matches:** 7

11-cis retinol dehydrogenase OS=Bos taurus GN=RDH5 PE=3 SV=1

Observed	Mr(expt)	Mr(calc)	ppm	Start - End	Miss	Peptide
891.4870	890.4797	890.4722	8.40	48 - 54	1	R.LDQRGFR.V
1016.5761	1015.5688	1015.5563	12.3	191 - 199	1	R.RDVAPFGVR.V
1397.7454	1396.7381	1396.7099	20.2	180 - 191	1	K.FGLEAFSDSLRR.D
1570.7749	1569.7676	1569.7800	-7.91	276 - 288	2	R.HPRTRYSPGWDAK.L
1717.9056	1716.8983	1716.8954	1.69	267 - 280	2	R.CLEHALTARHPRTR.Y
2239.1533	2238.1460	2238.1546	-3.81	180 - 199	2	K.FGLEAFSDSLRRDVAPFGVR.V
2846.4011	2845.3938	2845.3707	8.13	252 - 275	2	R.IMNMICDPDLAKVSRCLEHALTAR.H + 2 Oxidation (M)

No match to: 706.3098, 710.3790, 712.2532, 714.3089, 717.3984, 719.4317, 720.4312, 722.4346, 735.4181, 749.4561, 756.4000, 759.4391, 772.4356, 774.4578, 778.4783, 779.4789, 818.4724, 823.4901, 831.4754, 832.3123, 834.3210, 842.5065, 844.4971, 870.5016, 871.4923, 873.3899, 876.2862, 880.4520, 883.4794, 895.5731, 900.5293, 909.5164, 913.4845, 918.5409, 923.5157, 925.4807, 927.5140, 929.4449, 942.5470, 944.5204, 945.5183, 949.5508, 950.5472, 967.4262, 972.4835, 974.4452, 976.4556, 977.4954, 978.5431, 988.4708, 1007.5730, 1026.5627, 1031.5581, 1034.5808, 1044.5240, 1051.5095, 1056.5281, 1057.5575, 1066.5067, 1074.5374, 1087.5460, 1103.5287, 1118.5885, 1122.5553, 1148.5439, 1168.5985, 1178.6211, 1185.6898, 1244.6118, 1248.6686, 1253.6313, 1255.6216, 1301.6338, 1312.7423, 1313.7227, 1314.7188, 1315.6721, 1317.6354, 1320.6331, 1322.6371, 1325.6360, 1327.6959, 1333.7570, 1336.7218, 1338.7179, 1339.7092, 1343.6960, 1349.6917, 1350.6987, 1351.6946, 1361.6875, 1372.5978, 1381.7642, 1382.5950, 1383.7253, 1439.7549, 1442.8190, 1457.7394, 1463.7950, 1488.7245, 1494.7383, 1497.8735, 1504.7484, 1513.7760, 1517.8062, 1520.7523, 1528.6951, 1537.8245, 1539.8104, 1549.8242, 1552.8073, 1553.8669, 1555.8348, 1556.8799, 1567.7986, 1578.7872, 1592.7849, 1628.7002, 1628.8571, 1638.8861, 1642.7155, 1647.8503, 1650.8458, 1664.8571, 1680.8226, 1696.8146, 1698.7845, 1699.8228, 1705.8712, 1707.8536, 1715.8536, 1730.8564, 1739.6990, 1771.0344, 1786.9199, 1809.0262, 1816.9354, 1820.8855, 1836.8507, 1907.9603, 1909.9886, 1928.0238, 1940.0148, 1953.8596, 1961.0111, 1965.8679, 1974.9255, 1982.0082, 1983.9866, 1985.8589, 1995.9136, 2027.1289, 2067.1079, 2110.0598, 2136.8484, 2144.0432, 2150.8660, 2164.8794, 2211.0857, 2251.1509, 2255.1836, 2270.1113, 2273.0972, 2274.0605, 2286.1492, 2289.0857, 2290.0530, 2293.1558, 2309.1570, 2324.0981, 2338.1033, 2340.0979, 2343.1770, 2352.9316, 2375.0454, 2383.9480, 2414.2512, 2499.2480, 2542.2678, 2589.9707, 2600.0022, 2603.9897, 2613.9939, 2627.9993, 2644.0112, 2646.9875, 2657.4248, 2659.0405, 2661.0017, 2665.0042, 2674.0601, 2675.0151, 2678.0159, 2689.0330, 2692.0283, 2695.0315, 2703.0417, 2706.0959, 2763.3606, 2798.2788, 2815.3203, 2872.3391, 2876.3679, 2884.3535, 2888.3455, 2891.4131, 2892.4519, 2919.4543, 3002.3042, 3012.4717, 3016.2314, 3020.2483, 3030.3025, 3033.2446, 3053.4917, 3081.6296, 3126.7493, 3171.7131, 3184.4602, 3206.6179, 3207.6111, 3248.6445, 3254.6370, 3265.6309, 3300.7825, 3317.7288, 3321.6318, 3334.6953, 3347.5437, 3429.5991, 3457.6255, 3471.6121, 3489.7363, 3503.8608, 3518.5715, 3639.7969, 4148.0293, 4162.0039, 4453.2720, 4844.2046

18. E1BFB9 **Mass:** 61308 **Score:** 21 **Expect:** 2e+002 **Matches:** 9

Protein FAM114A2 OS=Bos taurus GN=FAM114A1 PE=4 SV=1

Observed	Mr(expt)	Mr(calc)	ppm	Start - End	Miss	Peptide
1034.5808	1033.5735	1033.5702	3.20	251 - 258	2	K.RTKTLMER.T
1313.7227	1312.7154	1312.7211	-4.34	275 - 285	1	K.QRLAQQLTAER.T
1504.7484	1503.7411	1503.7715	-20.21	207 - 221	0	R.GMLSAITNAVQNTGK.S

1520.7523	1519.7450	1519.7664	-14.09	207 - 221	0	R.GMLSAITNAVQNTGK.S + Oxidation (M)
1664.8571	1663.8498	1663.8749	-15.09	254 - 267	1	K.TLMERTVSLSQMLR.E
1909.9886	1908.9813	1909.0125	-16.32	252 - 267	2	R.TKTLMERTVSLSQMLR.E + Oxidation (M)
2499.2480	2498.2407	2498.2574	-6.67	436 - 457	1	K.TNKTVEEVYMLSIESLAEVTAR.C + Oxidation (M)
2846.4011	2845.3938	2845.4571	-22.25	363 - 387	1	K.EEFASMLTELLFELHVAATPDKLNK.A
4844.2046	4843.1973	4843.2227	-5.24	29 - 73	2	K.DAEVHCDSATISNELTPANPRRDVHENEAVQGAETADTGLAEEPK.D

No match to: 706.3098, 710.3790, 712.2532, 714.3089, 717.3984, 719.4317, 720.4312, 722.4346, 735.4181, 749.4561, 756.4000, 759.4391, 772.4356, 774.4578, 778.4783, 779.4789, 818.4724, 823.4901, 831.4754, 832.3123, 834.3210, 842.5065, 844.4971, 870.5016, 871.4923, 873.3899, 876.2862, 880.4520, 883.4794, 891.4870, 895.5731, 900.5293, 909.5164, 913.4845, 918.5409, 923.5157, 925.4807, 927.5140, 929.4449, 942.5470, 944.5204, 945.5183, 949.5508, 950.5472, 967.4262, 972.4835, 974.4452, 976.4556, 977.4954, 978.5431, 988.4708, 1007.5730, 1016.5761, 1026.5627, 1031.5581, 1044.5240, 1051.5095, 1056.5281, 1057.5575, 1066.5067, 1074.5374, 1087.5460, 1103.5287, 1118.5885, 1122.5553, 1148.5439, 1168.5985, 1178.6211, 1185.6898, 1244.6118, 1248.6686, 1253.6313, 1255.6216, 1301.6338, 1312.7423, 1314.7188, 1315.6721, 1317.6354, 1320.6331, 1322.6371, 1325.6360, 1327.6959, 1333.7570, 1336.7218, 1338.7179, 1339.7092, 1343.6960, 1349.6917, 1350.6987, 1351.6946, 1361.6875, 1372.5978, 1381.7642, 1382.5950, 1383.7253, 1397.7454, 1439.7549, 1442.8190, 1457.7394, 1463.7950, 1488.7245, 1494.7383, 1497.8735, 1513.7760, 1517.8062, 1528.6951, 1537.8245, 1539.8104, 1549.8242, 1552.8073, 1553.8669, 1555.8348, 1556.8799, 1567.7986, 1570.7749, 1578.7872, 1592.7849, 1628.7002, 1628.8571, 1638.8861, 1642.7155, 1647.8503, 1650.8458, 1680.8226, 1696.8146, 1698.7845, 1699.8228, 1705.8712, 1707.8536, 1715.8536, 1717.9056, 1730.8564, 1739.6990, 1771.0344, 1786.9199, 1809.0262, 1816.9354, 1820.8855, 1836.8507, 1907.9603, 1928.0238, 1940.0148, 1953.8596, 1961.0111, 1965.8679, 1974.9255, 1982.0082, 1983.9866, 1985.8589, 1995.9136, 2027.1289, 2067.1079, 2110.0598, 2136.8484, 2144.0432, 2150.8660, 2164.8794, 2211.0857, 2239.1533, 2251.1509, 2255.1836, 2270.1113, 2273.0972, 2274.0605, 2286.1492, 2289.0857, 2290.0530, 2293.1558, 2309.1570, 2324.0981, 2338.1033, 2340.0979, 2343.1770, 2352.9316, 2375.0454, 2383.9480, 2414.2512, 2542.2678, 2589.9707, 2600.0022, 2603.9897, 2613.9939, 2627.9993, 2644.0112, 2646.9875, 2657.4248, 2659.0405, 2661.0017, 2665.0042, 2674.0601, 2675.0151, 2678.0159, 2689.0330, 2692.0283, 2695.0315, 2703.0417, 2706.0959, 2763.3606, 2798.2788, 2815.3203, 2872.3391, 2876.3679, 2884.3535, 2888.3455, 2891.4131, 2892.4519, 2919.4543, 3002.3042, 3012.4717, 3016.2314, 3020.2483, 3030.3025, 3033.2446, 3053.4917, 3081.6296, 3126.7493, 3171.7131, 3184.4602, 3206.6179, 3207.6111, 3248.6445, 3254.6370, 3265.6309, 3300.7825, 3317.7288, 3321.6318, 3334.6953, 3347.5437, 3429.5991, 3457.6255, 3471.6121, 3489.7363, 3503.8608, 3518.5715, 3639.7969, 4148.0293, 4162.0039, 4453.2720

19. F1MMK0 **Mass:** 12474 **Score:** 20 **Expect:** 2.2e+002 **Matches:** 2

Catechol O-methyltransferase OS=Bos taurus GN=COMT PE=4 SV=2

Observed	Mr(expt)	Mr(calc)	ppm	Start - End	Miss	Peptide
1122.5553	1121.5480	1121.5678	-17.65	101 - 109	0	R.YLPDMLLLE.- + Oxidation (M)
3334.6953	3333.6880	3333.7325	-13.35	10 - 39	2	R.EQQPSVLLELGAYCGYSAVRMARLLLPGAR.L + Oxidation (M)

No match to: 706.3098, 710.3790, 712.2532, 714.3089, 717.3984, 719.4317, 720.4312, 722.4346, 735.4181, 749.4561, 756.4000, 759.4391, 772.4356, 774.4578, 778.4783, 779.4789, 818.4724, 823.4901, 831.4754, 832.3123, 834.3210, 842.5065, 844.4971, 870.5016, 871.4923, 873.3899, 876.2862, 880.4520, 883.4794, 891.4870, 895.5731, 900.5293, 909.5164, 913.4845, 918.5409, 923.5157, 925.4807, 927.5140, 929.4449, 942.5470, 944.5204, 945.5183, 949.5508, 950.5472, 967.4262, 972.4835, 974.4452, 976.4556, 977.4954, 978.5431, 988.4708, 1007.5730, 1016.5761, 1026.5627, 1031.5581, 1034.5808, 1044.5240, 1051.5095, 1056.5281, 1057.5575, 1066.5067, 1074.5374, 1087.5460, 1103.5287, 1118.5885, 1148.5439, 1168.5985, 1178.6211, 1185.6898, 1244.6118, 1248.6686, 1253.6313, 1255.6216, 1301.6338, 1312.7423, 1313.7227, 1314.7188, 1315.6721, 1317.6354, 1320.6331, 1322.6371, 1325.6360, 1327.6959, 1333.7570, 1336.7218, 1338.7179, 1339.7092, 1343.6960, 1349.6917, 1350.6987, 1351.6946, 1361.6875, 1372.5978, 1381.7642, 1382.5950, 1383.7253, 1397.7454, 1439.7549, 1442.8190, 1457.7394, 1463.7950, 1488.7245, 1494.7383, 1497.8735, 1504.7484, 1513.7760, 1517.8062, 1520.7523, 1528.6951, 1537.8245, 1539.8104, 1549.8242, 1552.8073, 1553.8669, 1555.8348, 1556.8799, 1567.7986, 1570.7749, 1578.7872, 1592.7849, 1628.7002, 1628.8571, 1638.8861, 1642.7155, 1647.8503, 1650.8458, 1664.8571, 1680.8226, 1696.8146, 1698.7845, 1699.8228, 1705.8712, 1707.8536, 1715.8536, 1717.9056, 1730.8564, 1739.6990, 1771.0344, 1786.9199, 1809.0262, 1816.9354, 1820.8855, 1836.8507, 1907.9603, 1909.9886, 1928.0238, 1940.0148, 1953.8596, 1961.0111, 1965.8679, 1974.9255, 1982.0082, 1983.9866, 1985.8589, 1995.9136, 2027.1289, 2067.1079, 2110.0598, 2136.8484, 2144.0432, 2150.8660, 2164.8794, 2211.0857, 2239.1533, 2251.1509, 2255.1836, 2270.1113, 2273.0972, 2274.0605, 2286.1492, 2289.0857, 2290.0530, 2293.1558, 2309.1570, 2324.0981, 2338.1033, 2340.0979, 2343.1770, 2352.9316, 2375.0454, 2383.9480, 2414.2512, 2499.2480, 2542.2678, 2589.9707, 2600.0022, 2603.9897, 2613.9939, 2627.9993, 2644.0112, 2646.9875, 2657.4248, 2659.0405, 2661.0017, 2665.0042, 2674.0601, 2675.0151, 2678.0159, 2689.0330, 2692.0283, 2695.0315, 2703.0417, 2706.0959, 2763.3606, 2798.2788, 2815.3203, 2846.4011, 2872.3391, 2876.3679, 2884.3535, 2888.3455, 2891.4131, 2892.4519, 2919.4543, 3002.3042, 3012.4717, 3016.2314, 3020.2483, 3030.3025, 3033.2446, 3053.4917, 3081.6296, 3126.7493, 3171.7131, 3184.4602, 3206.6179, 3207.6111, 3248.6445, 3254.6370, 3265.6309, 3300.7825, 3317.7288, 3321.6318, 3347.5437, 3429.5991, 3457.6255, 3471.6121, 3489.7363, 3503.8608, 3518.5715, 3639.7969, 4148.0293, 4162.0039, 4453.2720, 4844.2046

20. A4FUG5 **Mass:** 57651 **Score:** 20 **Expect:** 2.2e+002 **Matches:** 9

BIN2 protein OS=Bos taurus GN=BIN2 PE=2 SV=1

Observed	Mr(expt)	Mr(calc)	ppm	Start - End	Miss	Peptide
779.4789	778.4716	778.4589	16.4	60 - 65	1	K.LYKDLK.N
977.4954	976.4881	976.4825	5.69	35 - 42	1	K.TVETKDER.F
1066.5067	1065.4994	1065.4826	15.8	393 - 402	0	R.TSSEGSEELK.K
1333.7570	1332.7497	1332.7514	-1.23	21 - 31	2	K.FSRAQEKVLQK.L
1383.7253	1382.7180	1382.6929	18.2	81 - 92	0	R.VSETLQEIYSSK.W
1539.8104	1538.8031	1538.7940	5.93	80 - 92	1	K.RVSETLQEIYSSK.W
1556.8799	1555.8726	1555.8722	0.26	238 - 250	2	K.LEKQHSDKVFVVK.G
1592.7849	1591.7776	1591.8028	-15.82	66 - 79	1	K.NFLSAVKVMHESSK.R + Oxidation (M)
1717.9056	1716.8983	1716.8868	6.69	227 - 240	1	K.LNHNLYEVMSKLEK.Q

No match to: 706.3098, 710.3790, 712.2532, 714.3089, 717.3984, 719.4317, 720.4312, 722.4346, 735.4181, 749.4561, 756.4000, 759.4391, 772.4356, 774.4578, 778.4783, 818.4724, 823.4901, 831.4754, 832.3123, 834.3210, 842.5065, 844.4971, 870.5016, 871.4923, 873.3899, 876.2862, 880.4520, 883.4794, 891.4870, 895.5731, 900.5293, 909.5164, 913.4845, 918.5409, 923.5157, 925.4807, 927.5140, 929.4449, 942.5470, 944.5204, 945.5183, 949.5508, 950.5472, 967.4262, 972.4835, 974.4452, 976.4556, 978.5431, 988.4708, 1007.5730, 1016.5761, 1026.5627, 1031.5581, 1034.5808, 1044.5240, 1051.5095, 1056.5281, 1057.5575, 1074.5374, 1087.5460, 1103.5287, 1118.5885, 1122.5553, 1148.5439, 1168.5985, 1178.6211, 1185.6898, 1244.6118, 1248.6686, 1253.6313, 1255.6216, 1301.6338, 1312.7423, 1313.7227, 1314.7188, 1315.6721, 1317.6354, 1320.6331, 1322.6371, 1325.6360, 1327.6959, 1336.7218, 1338.7179, 1339.7092, 1343.6960, 1349.6917, 1350.6987, 1351.6946, 1361.6875, 1372.5978, 1381.7642, 1382.5950, 1397.7454, 1439.7549, 1442.8190, 1457.7394, 1463.7950, 1488.7245, 1494.7383, 1497.8735, 1504.7484, 1513.7760, 1517.8062, 1520.7523, 1528.6951, 1537.8245, 1549.8242, 1552.8073, 1553.8669, 1555.8348, 1567.7986, 1570.7749, 1578.7872, 1628.7002, 1628.8571, 1638.8861, 1642.7155, 1647.8503, 1650.8458, 1664.8571, 1680.8226, 1696.8146, 1698.7845, 1699.8228, 1705.8712, 1707.8536, 1715.8536, 1730.8564, 1739.6990, 1771.0344, 1786.9199, 1809.0262, 1816.9354, 1820.8855, 1836.8507, 1907.9603, 1909.9886, 1928.0238, 1940.0148, 1953.8596, 1961.0111, 1965.8679, 1974.9255, 1982.0082, 1983.9866, 1985.8589, 1995.9136, 2027.1289, 2067.1079, 2110.0598, 2136.8484, 2144.0432, 2150.8660, 2164.8794, 2211.0857, 2239.1533, 2251.1509, 2255.1836, 2270.1113, 2273.0972, 2274.0605, 2286.1492, 2289.0857, 2290.0530, 2293.1558, 2309.1570, 2324.0981, 2338.1033, 2340.0979, 2343.1770, 2352.9316, 2375.0454, 2383.9480, 2414.2512, 2499.2480, 2542.2678, 2589.9707, 2600.0022, 2603.9897, 2613.9939, 2627.9993, 2644.0112, 2646.9875, 2657.4248, 2659.0405, 2661.0017, 2665.0042, 2674.0601, 2675.0151, 2678.0159, 2689.0330, 2692.0283, 2695.0315, 2703.0417, 2706.0959, 2763.3606, 2798.2788, 2815.3203, 2846.4011, 2872.3391, 2876.3679, 2884.3535, 2888.3455, 2891.4131, 2892.4519, 2919.4543, 3002.3042, 3012.4717, 3016.2314, 3020.2483, 3030.3025, 3033.2446, 3053.4917, 3081.6296, 3126.7493, 3171.7131, 3184.4602, 3206.6179, 3207.6111, 3248.6445, 3254.6370, 3265.6309, 3300.7825, 3317.7288, 3321.6318, 3334.6953, 3347.5437, 3429.5991, 3457.6255, 3471.6121, 3489.7363, 3503.8608, 3518.5715, 3639.7969, 4148.0293, 4162.0039, 4453.2720, 4844.2046

21. E1BP99 **Mass:** 28979 **Score:** 20 **Expect:** 2.2e+002 **Matches:** 5

Uncharacterized protein OS=Bos taurus GN=APOBEC1 PE=4 SV=1

Observed	Mr(expt)	Mr(calc)	ppm	Start - End	Miss	Peptide
1301.6338	1300.6265	1300.5983	21.7	119 - 128	0	R.LFQHMDPQNR.Q + Oxidation (M)
1336.7218	1335.7145	1335.7411	-19.92	198 - 207	1	R.RYQNQLIVFR.L
1696.8146	1695.8073	1695.8475	-23.67	1 - 16	2	-.MASDRGPPAGDPTLRR.R
1698.7845	1697.7772	1697.7726	2.74	18 - 30	0	R.IEPWEFEFSFDPR.K

2027.1289	2026.1216	2026.1000	10.7	102 - 118	0	R.EFLNQHPNVTLVIYIAR.L

No match to: 706.3098, 710.3790, 712.2532, 714.3089, 717.3984, 719.4317, 720.4312, 722.4346, 735.4181, 749.4561, 756.4000, 759.4391, 772.4356, 774.4578, 778.4783, 779.4789, 818.4724, 823.4901, 831.4754, 832.3123, 834.3210, 842.5065, 844.4971, 870.5016, 871.4923, 873.3899, 876.2862, 880.4520, 883.4794, 891.4870, 895.5731, 900.5293, 909.5164, 913.4845, 918.5409, 923.5157, 925.4807, 927.5140, 929.4449, 942.5470, 944.5204, 945.5183, 949.5508, 950.5472, 967.4262, 972.4835, 974.4452, 976.4556, 977.4954, 978.5431, 988.4708, 1007.5730, 1016.5761, 1026.5627, 1031.5581, 1034.5808, 1044.5240, 1051.5095, 1056.5281, 1057.5575, 1066.5067, 1074.5374, 1087.5460, 1103.5287, 1118.5885, 1122.5553, 1148.5439, 1168.5985, 1178.6211, 1185.6898, 1244.6118, 1248.6686, 1253.6313, 1255.6216, 1312.7423, 1313.7227, 1314.7188, 1315.6721, 1317.6354, 1320.6331, 1322.6371, 1325.6360, 1327.6959, 1333.7570, 1338.7179, 1339.7092, 1343.6960, 1349.6917, 1350.6987, 1351.6946, 1361.6875, 1372.5978, 1381.7642, 1382.5950, 1383.7253, 1397.7454, 1439.7549, 1442.8190, 1457.7394, 1463.7950, 1488.7245, 1494.7383, 1497.8735, 1504.7484, 1513.7760, 1517.8062, 1520.7523, 1528.6951, 1537.8245, 1539.8104, 1549.8242, 1552.8073, 1553.8669, 1555.8348, 1556.8799, 1567.7986, 1570.7749, 1578.7872, 1592.7849, 1628.7002, 1628.8571, 1638.8861, 1642.7155, 1647.8503, 1650.8458, 1664.8571, 1680.8226, 1699.8228, 1705.8712, 1707.8536, 1715.8536, 1717.9056, 1730.8564, 1739.6990, 1771.0344, 1786.9199, 1809.0262, 1816.9354, 1820.8855, 1836.8507, 1907.9603, 1909.9886, 1928.0238, 1940.0148, 1953.8596, 1961.0111, 1965.8679, 1974.9255, 1982.0082, 1983.9866, 1985.8589, 1995.9136, 2067.1079, 2110.0598, 2136.8484, 2144.0432, 2150.8660, 2164.8794, 2211.0857, 2239.1533, 2251.1509, 2255.1836, 2270.1113, 2273.0972, 2274.0605, 2286.1492, 2289.0857, 2290.0530, 2293.1558, 2309.1570, 2324.0981, 2338.1033, 2340.0979, 2343.1770, 2352.9316, 2375.0454, 2383.9480, 2414.2512, 2499.2480, 2542.2678, 2589.9707, 2600.0022, 2603.9897, 2613.9939, 2627.9993, 2644.0112, 2646.9875, 2657.4248, 2659.0405, 2661.0017, 2665.0042, 2674.0601, 2675.0151, 2678.0159, 2689.0330, 2692.0283, 2695.0315, 2703.0417, 2706.0959, 2763.3606, 2798.2788, 2815.3203, 2846.4011, 2872.3391, 2876.3679, 2884.3535, 2888.3455, 2891.4131, 2892.4519, 2919.4543, 3002.3042, 3012.4717, 3016.2314, 3020.2483, 3030.3025, 3033.2446, 3053.4917, 3081.6296, 3126.7493, 3171.7131, 3184.4602, 3206.6179, 3207.6111, 3248.6445, 3254.6370, 3265.6309, 3300.7825, 3317.7288, 3321.6318, 3334.6953, 3347.5437, 3429.5991, 3457.6255, 3471.6121, 3489.7363, 3503.8608, 3518.5715, 3639.7969, 4148.0293, 4162.0039, 4453.2720, 4844.2046

22. F6RN30 **Mass:** 69980 **Score:** 20 **Expect:** 2.4e+002 **Matches:** 5

Uncharacterized protein OS=Bos taurus GN=GGA1 PE=4 SV=1

Observed	Mr(expt)	Mr(calc)	ppm	Start	End	Miss	Peptide
735.4181	734.4108	734.3996	15.2	208 -	213	1	R.MEKISK.R
891.4870	890.4797	890.5007	-23.59	208 -	214	2	R.MEKISKR.A
1122.5553	1121.5480	1121.5240	21.4	166 -	174	0	K.NVIFEDEEK.S
2027.1289	2026.1216	2026.1133	4.12	533 -	551	0	R.SDVLVVVVSMLSTAPQPIR.N + Oxidation (M)
3265.6309	3264.6236	3264.5689	16.8	227 -	255	2	K.LLTEMVMNHSQGGAAARSSEDLMKELYQR.C

No match to: 706.3098, 710.3790, 712.2532, 714.3089, 717.3984, 719.4317, 720.4312, 722.4346, 749.4561, 756.4000, 759.4391, 772.4356, 774.4578, 778.4783, 779.4789, 818.4724, 823.4901, 831.4754, 832.3123, 834.3210, 842.5065, 844.4971, 870.5016, 871.4923, 873.3899, 876.2862, 880.4520, 883.4794, 895.5731, 900.5293, 909.5164, 913.4845, 918.5409, 923.5157, 925.4807, 927.5140, 929.4449, 942.5470, 944.5204, 945.5183, 949.5508, 950.5472, 967.4262, 972.4835, 974.4452, 976.4556, 977.4954, 978.5431, 988.4708, 1007.5730, 1016.5761, 1026.5627, 1031.5581, 1034.5808, 1044.5240, 1051.5095, 1056.5281, 1057.5575, 1066.5067, 1074.5374, 1087.5460, 1103.5287, 1118.5885, 1148.5439, 1168.5985, 1178.6211, 1185.6898, 1244.6118, 1248.6686, 1253.6313, 1255.6216, 1301.6338, 1312.7423, 1313.7227, 1314.7188, 1315.6721, 1317.6354, 1320.6331, 1322.6371, 1325.6360, 1327.6959, 1333.7570, 1336.7218, 1338.7179, 1339.7092, 1343.6960, 1349.6917, 1350.6987, 1351.6946, 1361.6875, 1372.5978, 1381.7642, 1382.5950, 1383.7253, 1397.7454, 1439.7549, 1442.8190, 1457.7394, 1463.7950, 1488.7245, 1494.7383, 1497.8735, 1504.7484, 1513.7760, 1517.8062, 1520.7523, 1528.6951, 1537.8245, 1539.8104, 1549.8242, 1552.8073, 1553.8669, 1555.8348, 1556.8799, 1567.7986, 1570.7749, 1578.7872, 1592.7849, 1628.7002, 1628.8571, 1638.8861, 1642.7155, 1647.8503, 1650.8458, 1664.8571, 1680.8226, 1696.8146, 1698.7845, 1699.8228, 1705.8712, 1707.8536, 1715.8536, 1717.9056, 1730.8564, 1739.6990, 1771.0344, 1786.9199, 1809.0262, 1816.9354, 1820.8855, 1836.8507, 1907.9603, 1909.9886, 1928.0238, 1940.0148, 1953.8596, 1961.0111, 1965.8679, 1974.9255, 1982.0082, 1983.9866, 1985.8589, 1995.9136, 2067.1079, 2110.0598, 2136.8484, 2144.0432, 2150.8660, 2164.8794, 2211.0857, 2239.1533, 2251.1509, 2255.1836, 2270.1113, 2273.0972, 2274.0605, 2286.1492, 2289.0857, 2290.0530, 2293.1558, 2309.1570, 2324.0981, 2338.1033, 2340.0979, 2343.1770, 2352.9316, 2375.0454, 2383.9480, 2414.2512, 2499.2480, 2542.2678, 2589.9707, 2600.0022, 2603.9897, 2613.9939, 2627.9993, 2644.0112, 2646.9875, 2657.4248, 2659.0405, 2661.0017, 2665.0042, 2674.0601, 2675.0151, 2678.0159, 2689.0330, 2692.0283, 2695.0315, 2703.0417, 2706.0959, 2763.3606, 2798.2788, 2815.3203, 2846.4011, 2872.3391, 2876.3679, 2884.3535, 2888.3455, 2891.4131, 2892.4519, 2919.4543, 3002.3042, 3012.4717, 3016.2314, 3020.2483, 3030.3025, 3033.2446, 3053.4917, 3081.6296, 3126.7493, 3171.7131, 3184.4602, 3206.6179, 3207.6111, 3248.6445, 3254.6370, 3300.7825, 3317.7288, 3321.6318, 3334.6953, 3347.5437, 3429.5991, 3457.6255, 3471.6121, 3489.7363, 3503.8608, 3518.5715, 3639.7969, 4148.0293, 4162.0039, 4453.2720, 4844.2046

23. Q3T171 **Mass:** 12273 **Score:** 20 **Expect:** 2.5e+002 **Matches:** 4

60S ribosomal protein L36 OS=Bos taurus GN=RPL36 PE=3 SV=3

Observed	Mr(expt)	Mr(calc)	ppm	Start	End	Miss	Peptide
1034.5808	1033.5735	1033.5841	-10.27	57 -	65	1	R.AMELLKVSK.D + Oxidation (M)
1314.7188	1313.7115	1313.6914	15.3	5 -	16	1	R.YPMAVGLNKGHK.V
1463.7950	1462.7877	1462.7788	6.08	1 -	13	1	-.MALRYPMAVGLNK.G
1717.9056	1716.8983	1716.9192	-12.15	88 -	103	2	R.EELSNVLAAMRKAAAK.K + Oxidation (M)

No match to: 706.3098, 710.3790, 712.2532, 714.3089, 717.3984, 719.4317, 720.4312, 722.4346, 735.4181, 749.4561, 756.4000, 759.4391, 772.4356, 774.4578, 778.4783, 779.4789, 818.4724, 823.4901, 831.4754, 832.3123, 834.3210, 842.5065, 844.4971, 870.5016, 871.4923, 873.3899, 876.2862, 880.4520, 883.4794, 891.4870, 895.5731, 900.5293, 909.5164, 913.4845, 918.5409, 923.5157, 925.4807, 927.5140, 929.4449, 942.5470, 944.5204, 945.5183, 949.5508, 950.5472, 967.4262, 972.4835, 974.4452, 976.4556, 977.4954, 978.5431, 988.4708, 1007.5730, 1016.5761, 1026.5627, 1031.5581, 1044.5240, 1051.5095, 1056.5281, 1057.5575, 1066.5067, 1074.5374, 1087.5460, 1103.5287, 1118.5885, 1122.5553, 1148.5439, 1168.5985, 1178.6211, 1185.6898, 1244.6118, 1248.6686, 1253.6313, 1255.6216, 1301.6338, 1312.7423, 1313.7227, 1315.6721, 1317.6354, 1320.6331, 1322.6371, 1325.6360, 1327.6959, 1333.7570, 1336.7218, 1338.7179, 1339.7092, 1343.6960, 1349.6917, 1350.6987, 1351.6946, 1361.6875, 1372.5978, 1381.7642, 1382.5950, 1383.7253, 1397.7454, 1439.7549, 1442.8190, 1457.7394, 1488.7245, 1494.7383, 1497.8735, 1504.7484, 1513.7760, 1517.8062, 1520.7523, 1528.6951, 1537.8245, 1539.8104, 1549.8242, 1552.8073, 1553.8669, 1555.8348, 1556.8799, 1567.7986, 1570.7749, 1578.7872, 1592.7849, 1628.7002, 1628.8571, 1638.8861, 1642.7155, 1647.8503, 1650.8458, 1664.8571, 1680.8226, 1696.8146, 1698.7845, 1699.8228, 1705.8712, 1707.8536, 1715.8536, 1730.8564, 1739.6990, 1771.0344, 1786.9199, 1809.0262, 1816.9354, 1820.8855, 1836.8507, 1907.9603, 1909.9886, 1928.0238, 1940.0148, 1953.8596, 1961.0111, 1965.8679, 1974.9255, 1982.0082, 1983.9866, 1985.8589, 1995.9136, 2027.1289, 2067.1079, 2110.0598, 2136.8484, 2144.0432, 2150.8660, 2164.8794, 2211.0857, 2239.1533, 2251.1509, 2255.1836, 2270.1113, 2273.0972, 2274.0605, 2286.1492, 2289.0857, 2290.0530, 2293.1558, 2309.1570, 2324.0981, 2338.1033, 2340.0979, 2343.1770, 2352.9316, 2375.0454, 2383.9480, 2414.2512, 2499.2480, 2542.2678, 2589.9707, 2600.0022, 2603.9897, 2613.9939, 2627.9993, 2644.0112, 2646.9875, 2657.4248, 2659.0405, 2661.0017, 2665.0042, 2674.0601, 2675.0151, 2678.0159, 2689.0330, 2692.0283, 2695.0315, 2703.0417, 2706.0959, 2763.3606, 2798.2788, 2815.3203, 2846.4011, 2872.3391, 2876.3679, 2884.3535, 2888.3455, 2891.4131, 2892.4519, 2919.4543, 3002.3042, 3012.4717, 3016.2314, 3020.2483, 3030.3025, 3033.2446, 3053.4917, 3081.6296, 3126.7493, 3171.7131, 3184.4602, 3206.6179, 3207.6111, 3248.6445, 3254.6370, 3265.6309, 3300.7825, 3317.7288, 3321.6318, 3334.6953, 3347.5437, 3429.5991, 3457.6255, 3471.6121, 3489.7363, 3503.8608, 3518.5715, 3639.7969, 4148.0293, 4162.0039, 4453.2720, 4844.2046

24. G3N2X2 **Mass:** 12242 **Score:** 20 **Expect:** 2.5e+002 **Matches:** 5

60S ribosomal protein L36 OS=Bos taurus PE=3 SV=1

Observed	Mr(expt)	Mr(calc)	ppm	Start	End	Miss	Peptide
1034.5808	1033.5735	1033.5841	-10.27	57 -	65	1	R.AMELLKVSK.D + Oxidation (M)
1349.6917	1348.6844	1348.6557	21.3	46 -	56	1	R.EVCGLAPYERR.A
1517.8062	1516.7989	1516.7667	21.2	86 -	98	1	R.EREELSNVLAAMR.K
1570.7749	1569.7676	1569.7980	-19.36	17 -	29	2	K.VTKNMGKPRHSCR.R
1717.9056	1716.8983	1716.9192	-12.15	88 -	103	2	R.EELSNVLAAMRKAAAK.K + Oxidation (M)

No match to: 706.3098, 710.3790, 712.2532, 714.3089, 717.3984, 719.4317, 720.4312, 722.4346, 735.4181, 749.4561, 756.4000, 759.4391, 772.4356, 774.4578, 778.4783, 779.4789, 818.4724, 823.4901, 831.4754, 832.3123, 834.3210, 842.5065, 844.4971, 870.5016, 871.4923, 873.3899, 876.2862, 880.4520, 883.4794, 891.4870, 895.5731, 900.5293, 909.5164, 913.4845, 918.5409, 923.5157, 925.4807, 927.5140, 929.4449, 942.5470, 944.5204, 945.5183, 949.5508, 950.5472, 967.4262, 972.4835, 974.4452, 976.4556, 977.4954, 978.5431, 988.4708, 1007.5730, 1016.5761, 1026.5627, 1031.5581, 1044.5240, 1051.5095, 1056.5281, 1057.5575, 1066.5067, 1074.5374, 1087.5460, 1103.5287, 1118.5885, 1122.5553, 1148.5439, 1168.5985, 1178.6211, 1185.6898, 1244.6118, 1248.6686, 1253.6313, 1255.6216, 1301.6338, 1312.7423, 1313.7227, 1314.7188, 1315.6721, 1317.6354, 1320.6331, 1322.6371, 1325.6360, 1327.6959, 1333.7570, 1336.7218, 1338.7179,

1339.7092, 1343.6960, 1350.6987, 1351.6946, 1361.6875, 1372.5978, 1381.7642, 1382.5950, 1383.7253, 1397.7454, 1439.7549, 1442.8190, 1457.7394, 1463.7950, 1488.7245, 1494.7383, 1497.8735, 1504.7484, 1513.7760, 1520.7523, 1528.6951, 1537.8245, 1539.8104, 1549.8242, 1552.8073, 1553.8669, 1555.8348, 1556.8799, 1567.7986, 1578.7872, 1592.7849, 1628.7002, 1628.8571, 1638.8861, 1642.7155, 1647.8503, 1650.8458, 1664.8571, 1680.8226, 1696.8146, 1698.7845, 1699.8228, 1705.8712, 1707.8536, 1715.8536, 1730.8564, 1739.6990, 1771.0344, 1786.9199, 1809.0262, 1816.9354, 1820.8855, 1836.8507, 1907.9603, 1909.9886, 1928.0238, 1940.0148, 1953.8596, 1961.0111, 1965.8679, 1974.9255, 1982.0082, 1983.9866, 1985.8589, 1995.9136, 2027.1289, 2067.1079, 2110.0598, 2136.8484, 2144.0432, 2150.8660, 2164.8794, 2211.0857, 2239.1533, 2251.1509, 2255.1836, 2270.1113, 2273.0972, 2274.0605, 2286.1492, 2289.0857, 2290.0530, 2293.1558, 2309.1570, 2324.0981, 2338.1033, 2340.0979, 2343.1770, 2352.9316, 2375.0454, 2383.9480, 2414.2512, 2499.2480, 2542.2678, 2589.9707, 2600.0022, 2603.9897, 2613.9939, 2627.9993, 2644.0112, 2646.9875, 2657.4248, 2659.0405, 2661.0017, 2665.0042, 2674.0601, 2675.0151, 2678.0159, 2689.0330, 2692.0283, 2695.0315, 2703.0417, 2706.0959, 2763.3606, 2798.2788, 2815.3203, 2846.4011, 2872.3391, 2876.3679, 2884.3535, 2888.3455, 2891.4131, 2892.4519, 2919.4543, 3002.3042, 3012.4717, 3016.2314, 3020.2483, 3030.3025, 3033.2446, 3053.4917, 3081.6296, 3126.7493, 3171.7131, 3184.4602, 3206.6179, 3207.6111, 3248.6445, 3254.6370, 3265.6309, 3300.7825, 3317.7288, 3321.6318, 3334.6953, 3347.5437, 3429.5991, 3457.6255, 3471.6121, 3489.7363, 3503.8608, 3518.5715, 3639.7969, 4148.0293, 4162.0039, 4453.2720, 4844.2046

25. A6QP80 **Mass:** 45891 **Score:** 20 **Expect:** 2.6e+002 **Matches:** 9

LOC532995 protein OS=Bos taurus GN=LOC532995 PE=2 SV=1

Observed	Mr(expt)	Mr(calc)	ppm	Start		End	Miss	Peptide
818.4724	817.4651	817.4731	-9.80	240	-	246	1	K.VMIPSKK.N + Oxidation (M)
1178.6211	1177.6138	1177.5873	22.5	96	-	105	2	K.KRSQMNPSSK.D + Oxidation (M)
1253.6313	1252.6240	1252.6485	-19.56	209	-	219	0	R.LESGAYTLLMR.G
1463.7950	1462.7877	1462.7609	18.3	2	-	13	1	M.TFYFTGIRSFPK.L
1705.8712	1704.8639	1704.8253	22.6	80	-	96	1	K.QVSADSSHMFAAGAAKK.R
1928.0238	1927.0165	1926.9986	9.32	152	-	169	1	K.CHSSVDVAKSVLAWVAAK.N
2110.0598	2109.0525	2109.0630	-4.95	355	-	372	1	K.TVESIHLSSEEYEFLKAK.I
2414.2512	2413.2439	2413.2576	-5.67	209	-	228	2	R.LESGAYTLLMRGLIYTDRWR.E
2846.4011	2845.3938	2845.4255	-11.12	137	-	160	2	K.EKANFENWIILQMVKCHSSVDVAK.S

No match to: 706.3098, 710.3790, 712.2532, 714.3089, 717.3984, 719.4317, 720.4312, 722.4346, 735.4181, 749.4561, 756.4000, 759.4391, 772.4356, 774.4578, 778.4783, 779.4789, 823.4901, 831.4754, 832.3123, 834.3210, 842.5065, 844.4971, 870.5016, 871.4923, 873.3899, 876.2862, 880.4520, 883.4794, 891.4870, 895.5731, 900.5293, 909.5164, 913.4845, 918.5409, 923.5157, 925.4807, 927.5140, 929.4449, 942.5470, 944.5204, 945.5183, 949.5508, 950.5472, 967.4262, 972.4835, 974.4452, 976.4556, 977.4954, 978.5431, 988.4708, 1007.5730, 1016.5761, 1026.5627, 1031.5581, 1034.5808, 1044.5240, 1051.5095, 1056.5281, 1057.5575, 1066.5067, 1074.5374, 1087.5460, 1103.5287, 1118.5885, 1122.5553, 1148.5439, 1168.5985, 1185.6898, 1244.6118, 1248.6686, 1255.6216, 1301.6338, 1312.7423, 1313.7227, 1314.7188, 1315.6721, 1317.6354, 1320.6331, 1322.6371, 1325.6360, 1327.6959, 1333.7570, 1336.7218, 1338.7179, 1339.7092, 1343.6960, 1349.6917, 1350.6987, 1351.6946, 1361.6875, 1372.5978, 1381.7642, 1382.5950, 1383.7253, 1397.7454, 1439.7549, 1442.8190, 1457.7394, 1488.7245, 1494.7383, 1497.8735, 1504.7484, 1513.7760, 1517.8062, 1520.7523, 1528.6951, 1537.8245, 1539.8104, 1549.8242, 1552.8073, 1553.8669, 1555.8348, 1556.8799, 1567.7986, 1570.7749, 1578.7872, 1592.7849, 1628.7002, 1628.8571, 1638.8861, 1642.7155, 1647.8503, 1650.8458, 1664.8571, 1680.8226, 1696.8146, 1698.7845, 1699.8228, 1707.8536, 1715.8536, 1717.9056, 1730.8564, 1739.6990, 1771.0344, 1786.9199, 1809.0262, 1816.9354, 1820.8855, 1836.8507, 1907.9603, 1909.9886, 1940.0148, 1953.8596, 1961.0111, 1965.8679, 1974.9255, 1982.0082, 1983.9866, 1985.8589, 1995.9136, 2027.1289, 2067.1079, 2136.8484, 2144.0432, 2150.8660, 2164.8794, 2211.0857, 2239.1533, 2251.1509, 2255.1836, 2270.1113, 2273.0972, 2274.0605, 2286.1492, 2289.0857, 2290.0530, 2293.1558, 2309.1570, 2324.0981, 2338.1033, 2340.0979, 2343.1770, 2352.9316, 2375.0454, 2383.9480, 2499.2480, 2542.2678, 2589.9707, 2600.0022, 2603.9897, 2613.9939, 2627.9993, 2644.0112, 2646.9875, 2657.4248, 2659.0405, 2661.0017, 2665.0042, 2674.0601, 2675.0151, 2678.0159, 2689.0330, 2692.0283, 2695.0315, 2703.0417, 2706.0959, 2763.3606, 2798.2788, 2815.3203, 2872.3391, 2876.3679, 2884.3535, 2888.3455, 2891.4131, 2892.4519, 2919.4543, 3002.3042, 3012.4717, 3016.2314, 3020.2483, 3030.3025, 3033.2446, 3053.4917, 3081.6296, 3126.7493, 3171.7131, 3184.4602, 3206.6179, 3207.6111, 3248.6445, 3254.6370, 3265.6309, 3300.7825, 3317.7288, 3321.6318, 3334.6953, 3347.5437, 3429.5991, 3457.6255, 3471.6121, 3489.7363, 3503.8608, 3518.5715, 3639.7969, 4148.0293, 4162.0039, 4453.2720, 4844.2046

26. Q28055 **Mass:** 12345 **Score:** 20 **Expect:** 2.7e+002 **Matches:** 5

cAMP-regulated phosphoprotein 19 OS=Bos taurus GN=ARPP19 PE=1 SV=2

Observed	Mr(expt)	Mr(calc)	ppm	Start		End	Miss	Peptide
719.4317	718.4244	718.4159	11.8	70	-	75	2	K.AKMKNK.Q
735.4181	734.4108	734.4109	-0.06	70	-	75	2	K.AKMKNK.Q + Oxidation (M)
1820.8855	1819.8782	1819.8509	15.0	16	-	31	2	K.EMEDKVTSPEKAEEAK.L
1836.8507	1835.8434	1835.8458	-1.31	16	-	31	2	K.EMEDKVTSPEKAEEAK.L + Oxidation (M)
2027.1289	2026.1216	2026.0861	17.6	34	-	51	2	K.ARYPHLGQKPGGSDFLRK.R

No match to: 706.3098, 710.3790, 712.2532, 714.3089, 717.3984, 720.4312, 722.4346, 749.4561, 756.4000, 759.4391, 772.4356, 774.4578, 778.4783, 779.4789, 818.4724, 823.4901, 831.4754, 832.3123, 834.3210, 842.5065, 844.4971, 870.5016, 871.4923, 873.3899, 876.2862, 880.4520, 883.4794, 891.4870, 895.5731, 900.5293, 909.5164, 913.4845, 918.5409, 923.5157, 925.4807, 927.5140, 929.4449, 942.5470, 944.5204, 945.5183, 949.5508, 950.5472, 967.4262, 972.4835, 974.4452, 976.4556, 977.4954, 978.5431, 988.4708, 1007.5730, 1016.5761, 1026.5627, 1031.5581, 1034.5808, 1044.5240, 1051.5095, 1056.5281, 1057.5575, 1066.5067, 1074.5374, 1087.5460, 1103.5287, 1118.5885, 1122.5553, 1148.5439, 1168.5985, 1178.6211, 1185.6898, 1244.6118, 1248.6686, 1253.6313, 1255.6216, 1301.6338, 1312.7423, 1313.7227, 1314.7188, 1315.6721, 1317.6354, 1320.6331, 1322.6371, 1325.6360, 1327.6959, 1333.7570, 1336.7218, 1338.7179, 1339.7092, 1343.6960, 1349.6917, 1350.6987, 1351.6946, 1361.6875, 1372.5978, 1381.7642, 1382.5950, 1383.7253, 1397.7454, 1439.7549, 1442.8190, 1457.7394, 1463.7950, 1488.7245, 1494.7383, 1497.8735, 1504.7484, 1513.7760, 1517.8062, 1520.7523, 1528.6951, 1537.8245, 1539.8104, 1549.8242, 1552.8073, 1553.8669, 1555.8348, 1556.8799, 1567.7986, 1570.7749, 1578.7872, 1592.7849, 1628.7002, 1628.8571, 1638.8861, 1642.7155, 1647.8503, 1650.8458, 1664.8571, 1680.8226, 1696.8146, 1698.7845, 1699.8228, 1705.8712, 1707.8536, 1715.8536, 1717.9056, 1730.8564, 1739.6990, 1771.0344, 1786.9199, 1809.0262, 1816.9354, 1907.9603, 1909.9886, 1928.0238, 1940.0148, 1953.8596, 1961.0111, 1965.8679, 1974.9255, 1982.0082, 1983.9866, 1985.8589, 1995.9136, 2067.1079, 2110.0598, 2136.8484, 2144.0432, 2150.8660, 2164.8794, 2211.0857, 2239.1533, 2251.1509, 2255.1836, 2270.1113, 2273.0972, 2274.0605, 2286.1492, 2289.0857, 2290.0530, 2293.1558, 2309.1570, 2324.0981, 2338.1033, 2340.0979, 2343.1770, 2352.9316, 2375.0454, 2383.9480, 2414.2512, 2499.2480, 2542.2678, 2589.9707, 2600.0022, 2603.9897, 2613.9939, 2627.9993, 2644.0112, 2646.9875, 2657.4248, 2659.0405, 2661.0017, 2665.0042, 2674.0601, 2675.0151, 2678.0159, 2689.0330, 2692.0283, 2695.0315, 2703.0417, 2706.0959, 2763.3606, 2798.2788, 2815.3203, 2846.4011, 2872.3391, 2876.3679, 2884.3535, 2888.3455, 2891.4131, 2892.4519, 2919.4543, 3002.3042, 3012.4717, 3016.2314, 3020.2483, 3030.3025, 3033.2446, 3053.4917, 3081.6296, 3126.7493, 3171.7131, 3184.4602, 3206.6179, 3207.6111, 3248.6445, 3254.6370, 3265.6309, 3300.7825, 3317.7288, 3321.6318, 3334.6953, 3347.5437, 3429.5991, 3457.6255, 3471.6121, 3489.7363, 3503.8608, 3518.5715, 3639.7969, 4148.0293, 4162.0039, 4453.2720, 4844.2046

27. G1K177 **Mass:** 68709 **Score:** 20 **Expect:** 2.7e+002 **Matches:** 9

Cleavage stimulation factor subunit 2 OS=Bos taurus GN=CSTF2 PE=4 SV=2

Observed	Mr(expt)	Mr(calc)	ppm	Start		End	Miss	Peptide
1057.5575	1056.5502	1056.5676	-16.43	421	-	430	1	R.GLDARGLEAR.A
1301.6338	1300.6265	1300.6194	5.50	149	-	159	0	K.LCVQNSPQEAR.N
1349.6917	1348.6844	1348.6960	-8.58	398	-	410	2	R.GPPLDGRGGRDPR.G
1457.7394	1456.7321	1456.7205	8.01	408	-	420	2	R.DPRGIDARAMEAR.G
2110.0598	2109.0525	2109.0308	10.3	131	-	148	1	K.AVASLPPEQMFELMKQMK.L + 2 Oxidation (M)
2273.0972	2272.0899	2272.0709	8.39	347	-	367	0	R.GYLGPPHQGPPMHHVPGHDSR.G
2289.0857	2288.0784	2288.0658	5.53	347	-	367	0	R.GYLGPPHQGPPMHHVPGHDSR.G + Oxidation (M)
2324.0981	2323.0908	2323.1049	-6.07	473	-	496	0	R.GPMPSGIQGPSPINMGAVGPQGSR.Q + 2 Oxidation (M)
2414.2512	2413.2439	2413.2973	-22.14	160	-	180	0	R.NMLLQNPQLAYALLQAQVVMR.I

No match to: 706.3098, 710.3790, 712.2532, 714.3089, 717.3984, 719.4317, 720.4312, 722.4346, 735.4181, 749.4561, 756.4000, 759.4391, 772.4356, 774.4578, 778.4783, 779.4789, 818.4724, 823.4901, 831.4754, 832.3123, 834.3210, 842.5065, 844.4971, 870.5016, 871.4923, 873.3899, 876.2862, 880.4520, 883.4794, 891.4870, 895.5731, 900.5293, 909.5164, 913.4845, 918.5409, 923.5157, 925.4807, 927.5140, 929.4449, 942.5470, 944.5204, 945.5183, 949.5508, 950.5472, 967.4262, 972.4835, 974.4452, 976.4556, 977.4954, 978.5431, 988.4708, 1007.5730, 1016.5761, 1026.5627, 1031.5581, 1034.5808, 1044.5240, 1051.5095, 1056.5281, 1066.5067, 1074.5374, 1087.5460, 1103.5287, 1118.5885, 1122.5553, 1148.5439, 1168.5985, 1178.6211, 1185.6898, 1244.6118, 1248.6686, 1253.6313, 1255.6216, 1312.7423,

1313.7227, 1314.7188, 1315.6721, 1317.6354, 1320.6331, 1322.6371, 1325.6360, 1327.6959, 1333.7570, 1336.7218, 1338.7179, 1339.7092, 1343.6960, 1350.6987, 1351.6946, 1361.6875, 1372.5978, 1381.7642, 1382.5950, 1383.7253, 1397.7454, 1439.7549, 1442.8190, 1463.7950, 1488.7245, 1494.7383, 1497.8735, 1504.7484, 1513.7760, 1517.8062, 1520.7523, 1528.6951, 1537.8245, 1539.8104, 1549.8242, 1552.8073, 1553.8669, 1555.8348, 1556.8799, 1567.7986, 1570.7749, 1578.7872, 1592.7849, 1628.7002, 1628.8571, 1638.8861, 1642.7155, 1647.8503, 1650.8458, 1664.8571, 1680.8226, 1696.8146, 1698.7845, 1699.8228, 1705.8712, 1707.8536, 1715.8536, 1717.9056, 1730.8564, 1739.6990, 1771.0344, 1786.9199, 1809.0262, 1816.9354, 1820.8855, 1836.8507, 1907.9603, 1909.9886, 1928.0238, 1940.0148, 1953.8596, 1961.0111, 1965.8679, 1974.9255, 1982.0082, 1983.9866, 1985.8589, 1995.9136, 2027.1289, 2067.1079, 2136.8484, 2144.0432, 2150.8660, 2164.8794, 2211.0857, 2239.1533, 2251.1509, 2255.1836, 2270.1113, 2274.0605, 2286.1492, 2290.0530, 2293.1558, 2309.1570, 2338.1033, 2340.0979, 2343.1770, 2352.9316, 2375.0454, 2383.9480, 2499.2480, 2542.2678, 2589.9707, 2600.0022, 2603.9897, 2613.9939, 2627.9993, 2644.0112, 2646.9875, 2657.4248, 2659.0405, 2661.0017, 2665.0042, 2674.0601, 2675.0151, 2678.0159, 2689.0330, 2692.0283, 2695.0315, 2703.0417, 2706.0959, 2763.3606, 2798.2788, 2815.3203, 2846.4011, 2872.3391, 2876.3679, 2884.3535, 2888.3455, 2891.4131, 2892.4519, 2919.4543, 3002.3042, 3012.4717, 3016.2314, 3020.2483, 3030.3025, 3033.2446, 3053.4917, 3081.6296, 3126.7493, 3171.7131, 3184.4602, 3206.6179, 3207.6111, 3248.6445, 3254.6370, 3265.6309, 3300.7825, 3317.7288, 3321.6318, 3334.6953, 3347.5437, 3429.5991, 3457.6255, 3471.6121, 3489.7363, 3503.8608, 3518.5715, 3639.7969, 4148.0293, 4162.0039, 4453.2720, 4844.2046

28. Q2HJ73 **Mass:** 43607 **Score:** 20 **Expect:** 2.7e+002 **Matches:** 4

3-hydroxyisobutyryl-CoA hydrolase, mitochondrial OS=Bos taurus GN=HIBCH PE=2 SV=1

Observed	Mr(expt)	Mr(calc)	ppm	Start		End	Miss	Peptide
891.4870	890.4797	890.4895	-11.00	301	-	308	1	K.KMSPTSLK.I
1313.7227	1312.7154	1312.7034	9.17	44	-	55	0	K.GCAGVITLNRPR.F
1717.9056	1716.8983	1716.8682	17.5	238	-	252	1	K.ENIADVLETYHAKSK.T
3248.6445	3247.6372	3247.5576	24.5	321	-	347	2	K.TLPEVLIMEYRLSQACMKGHDFHEGVR.A + 2 Oxidation (M)

No match to: 706.3098, 710.3790, 712.2532, 714.3089, 717.3984, 719.4317, 720.4312, 722.4346, 735.4181, 749.4561, 756.4000, 759.4391, 772.4356, 774.4578, 778.4783, 779.4789, 818.4724, 823.4901, 831.4754, 832.3123, 834.3210, 842.5065, 844.4971, 870.5016, 871.4923, 873.3899, 876.2862, 880.4520, 883.4794, 895.5731, 900.5293, 909.5164, 913.4845, 918.5409, 923.5157, 925.4807, 927.5140, 929.4449, 942.5470, 944.5204, 945.5183, 949.5508, 950.5472, 967.4262, 972.4835, 974.4452, 976.4556, 977.4954, 978.5431, 988.4708, 1007.5730, 1016.5761, 1026.5627, 1031.5581, 1034.5808, 1044.5240, 1051.5095, 1056.5281, 1057.5575, 1066.5067, 1074.5374, 1087.5460, 1103.5287, 1118.5885, 1122.5553, 1148.5439, 1168.5985, 1178.6211, 1185.6898, 1244.6118, 1248.6686, 1253.6313, 1255.6216, 1301.6338, 1312.7423, 1314.7188, 1315.6721, 1317.6354, 1320.6331, 1322.6371, 1325.6360, 1327.6959, 1333.7570, 1336.7218, 1338.7179, 1339.7092, 1343.6960, 1349.6917, 1350.6987, 1351.6946, 1361.6875, 1372.5978, 1381.7642, 1382.5950, 1383.7253, 1397.7454, 1439.7549, 1442.8190, 1457.7394, 1463.7950, 1488.7245, 1494.7383, 1497.8735, 1504.7484, 1513.7760, 1517.8062, 1520.7523, 1528.6951, 1537.8245, 1539.8104, 1549.8242, 1552.8073, 1553.8669, 1555.8348, 1556.8799, 1567.7986, 1570.7749, 1578.7872, 1592.7849, 1628.7002, 1628.8571, 1638.8861, 1642.7155, 1647.8503, 1650.8458, 1664.8571, 1680.8226, 1696.8146, 1698.7845, 1699.8228, 1705.8712, 1707.8536, 1715.8536, 1730.8564, 1739.6990, 1771.0344, 1786.9199, 1809.0262, 1816.9354, 1820.8855, 1836.8507, 1907.9603, 1909.9886, 1928.0238, 1940.0148, 1953.8596, 1961.0111, 1965.8679, 1974.9255, 1982.0082, 1983.9866, 1985.8589, 1995.9136, 2027.1289, 2067.1079, 2110.0598, 2136.8484, 2144.0432, 2150.8660, 2164.8794, 2211.0857, 2239.1533, 2251.1509, 2255.1836, 2270.1113, 2273.0972, 2274.0605, 2286.1492, 2289.0857, 2290.0530, 2293.1558, 2309.1570, 2324.0981, 2338.1033, 2340.0979, 2343.1770, 2352.9316, 2375.0454, 2383.9480, 2414.2512, 2499.2480, 2542.2678, 2589.9707, 2600.0022, 2603.9897, 2613.9939, 2627.9993, 2644.0112, 2646.9875, 2657.4248, 2659.0405, 2661.0017, 2665.0042, 2674.0601, 2675.0151, 2678.0159, 2689.0330, 2692.0283, 2695.0315, 2703.0417, 2706.0959, 2763.3606, 2798.2788, 2815.3203, 2846.4011, 2872.3391, 2876.3679, 2884.3535, 2888.3455, 2891.4131, 2892.4519, 2919.4543, 3002.3042, 3012.4717, 3016.2314, 3020.2483, 3030.3025, 3033.2446, 3053.4917, 3081.6296, 3126.7493, 3171.7131, 3184.4602, 3206.6179, 3207.6111, 3254.6370, 3265.6309, 3300.7825, 3317.7288, 3321.6318, 3334.6953, 3347.5437, 3429.5991, 3457.6255, 3471.6121, 3489.7363, 3503.8608, 3518.5715, 3639.7969, 4148.0293, 4162.0039, 4453.2720, 4844.2046

29. E1B8F8 **Mass:** 42403 **Score:** 20 **Expect:** 2.7e+002 **Matches:** 6

Uncharacterized protein OS=Bos taurus PE=4 SV=1

Observed	Mr(expt)	Mr(calc)	ppm	Start		End	Miss	Peptide
1343.6960	1342.6887	1342.6604	21.1	250	-	259	1	R.YFSSLFMHRR.I
1539.8104	1538.8031	1538.8178	-9.55	280	-	293	1	K.AHLQGHQRIHSGAK.T
1647.8503	1646.8430	1646.8529	-5.99	191	-	203	2	K.TFSTKTKLDQHWR.V
1717.9056	1716.8983	1716.8671	18.2	247	-	259	2	K.AFRYFSSLFMHRR.I
2150.8660	2149.8587	2149.8615	-1.30	208	-	223	1	R.ERPYECEECGRTYNCK.R
2324.0981	2323.0908	2323.0395	22.1	294	-	312	2	K.TFECSECGRCFSQKSVLTK.H

No match to: 706.3098, 710.3790, 712.2532, 714.3089, 717.3984, 719.4317, 720.4312, 722.4346, 735.4181, 749.4561, 756.4000, 759.4391, 772.4356, 774.4578, 778.4783, 779.4789, 818.4724, 823.4901, 831.4754, 832.3123, 834.3210, 842.5065, 844.4971, 870.5016, 871.4923, 873.3899, 876.2862, 880.4520, 883.4794, 891.4870, 895.5731, 900.5293, 909.5164, 913.4845, 918.5409, 923.5157, 925.4807, 927.5140, 929.4449, 942.5470, 944.5204, 945.5183, 949.5508, 950.5472, 967.4262, 972.4835, 974.4452, 976.4556, 977.4954, 978.5431, 988.4708, 1007.5730, 1016.5761, 1026.5627, 1031.5581, 1034.5808, 1044.5240, 1051.5095, 1056.5281, 1057.5575, 1066.5067, 1074.5374, 1087.5460, 1103.5287, 1118.5885, 1122.5553, 1148.5439, 1168.5985, 1178.6211, 1185.6898, 1244.6118, 1248.6686, 1253.6313, 1255.6216, 1301.6338, 1312.7423, 1313.7227, 1314.7188, 1315.6721, 1317.6354, 1320.6331, 1322.6371, 1325.6360, 1327.6959, 1333.7570, 1336.7218, 1338.7179, 1339.7092, 1349.6917, 1350.6987, 1351.6946, 1361.6875, 1372.5978, 1381.7642, 1382.5950, 1383.7253, 1397.7454, 1439.7549, 1442.8190, 1457.7394, 1463.7950, 1488.7245, 1494.7383, 1497.8735, 1504.7484, 1513.7760, 1517.8062, 1520.7523, 1528.6951, 1537.8245, 1549.8242, 1552.8073, 1553.8669, 1555.8348, 1556.8799, 1567.7986, 1570.7749, 1578.7872, 1592.7849, 1628.7002, 1628.8571, 1638.8861, 1642.7155, 1650.8458, 1664.8571, 1680.8226, 1696.8146, 1698.7845, 1699.8228, 1705.8712, 1707.8536, 1715.8536, 1730.8564, 1739.6990, 1771.0344, 1786.9199, 1809.0262, 1816.9354, 1820.8855, 1836.8507, 1907.9603, 1909.9886, 1928.0238, 1940.0148, 1953.8596, 1961.0111, 1965.8679, 1974.9255, 1982.0082, 1983.9866, 1985.8589, 1995.9136, 2027.1289, 2067.1079, 2110.0598, 2136.8484, 2144.0432, 2164.8794, 2211.0857, 2239.1533, 2251.1509, 2255.1836, 2270.1113, 2273.0972, 2274.0605, 2286.1492, 2289.0857, 2290.0530, 2293.1558, 2309.1570, 2338.1033, 2340.0979, 2343.1770, 2352.9316, 2375.0454, 2383.9480, 2414.2512, 2499.2480, 2542.2678, 2589.9707, 2600.0022, 2603.9897, 2613.9939, 2627.9993, 2644.0112, 2646.9875, 2657.4248, 2659.0405, 2661.0017, 2665.0042, 2674.0601, 2675.0151, 2678.0159, 2689.0330, 2692.0283, 2695.0315, 2703.0417, 2706.0959, 2763.3606, 2798.2788, 2815.3203, 2846.4011, 2872.3391, 2876.3679, 2884.3535, 2888.3455, 2891.4131, 2892.4519, 2919.4543, 3002.3042, 3012.4717, 3016.2314, 3020.2483, 3030.3025, 3033.2446, 3053.4917, 3081.6296, 3126.7493, 3171.7131, 3184.4602, 3206.6179, 3207.6111, 3248.6445, 3254.6370, 3265.6309, 3300.7825, 3317.7288, 3321.6318, 3334.6953, 3347.5437, 3429.5991, 3457.6255, 3471.6121, 3489.7363, 3503.8608, 3518.5715, 3639.7969, 4148.0293, 4162.0039, 4453.2720, 4844.2046

30. F1MPK4 **Mass:** 44043 **Score:** 20 **Expect:** 2.7e+002 **Matches:** 4

3-hydroxyisobutyryl-CoA hydrolase, mitochondrial OS=Bos taurus GN=HIBCH PE=3 SV=2

Observed	Mr(expt)	Mr(calc)	ppm	Start		End	Miss	Peptide
891.4870	890.4797	890.4895	-11.00	302	-	309	1	K.KMSPTSLK.I
1628.8571	1627.8498	1627.8565	-4.08	56	-	68	1	K.MKLESILLDMLYK.A + 2 Oxidation (M)
1717.9056	1716.8983	1716.8682	17.5	239	-	253	1	K.ENIADVLETYHAKSK.T
3248.6445	3247.6372	3247.5576	24.5	322	-	348	2	K.TLPEVLIMEYRLSQACMKGHDFHEGVR.A + 2 Oxidation (M)

No match to: 706.3098, 710.3790, 712.2532, 714.3089, 717.3984, 719.4317, 720.4312, 722.4346, 735.4181, 749.4561, 756.4000, 759.4391, 772.4356, 774.4578, 778.4783, 779.4789, 818.4724, 823.4901, 831.4754, 832.3123, 834.3210, 842.5065, 844.4971, 870.5016, 871.4923, 873.3899, 876.2862, 880.4520, 883.4794, 895.5731, 900.5293, 909.5164, 913.4845, 918.5409, 923.5157, 925.4807, 927.5140, 929.4449, 942.5470, 944.5204, 945.5183, 949.5508, 950.5472, 967.4262, 972.4835, 974.4452, 976.4556, 977.4954, 978.5431, 988.4708, 1007.5730, 1016.5761, 1026.5627, 1031.5581, 1034.5808, 1044.5240, 1051.5095, 1056.5281, 1057.5575, 1066.5067, 1074.5374, 1087.5460, 1103.5287, 1118.5885, 1122.5553, 1148.5439, 1168.5985, 1178.6211, 1185.6898, 1244.6118, 1248.6686, 1253.6313, 1255.6216, 1301.6338, 1312.7423, 1313.7227, 1314.7188, 1315.6721, 1317.6354, 1320.6331, 1322.6371, 1325.6360, 1327.6959, 1333.7570, 1336.7218, 1338.7179, 1339.7092, 1343.6960, 1349.6917, 1350.6987, 1351.6946, 1361.6875, 1372.5978, 1381.7642, 1382.5950, 1383.7253, 1397.7454, 1439.7549, 1442.8190, 1457.7394, 1463.7950, 1488.7245, 1494.7383, 1497.8735, 1504.7484, 1513.7760, 1517.8062, 1520.7523, 1528.6951, 1537.8245, 1539.8104, 1549.8242, 1552.8073, 1553.8669, 1555.8348, 1556.8799, 1567.7986, 1570.7749, 1578.7872, 1592.7849, 1628.7002, 1638.8861, 1642.7155, 1647.8503, 1650.8458, 1664.8571, 1680.8226, 1696.8146, 1698.7845, 1699.8228, 1705.8712, 1707.8536, 1715.8536, 1730.8564, 1739.6990, 1771.0344, 1786.9199, 1809.0262, 1816.9354, 1820.8855, 1836.8507, 1907.9603, 1909.9886, 1928.0238, 1940.0148, 1953.8596, 1961.0111, 1965.8679, 1974.9255, 1982.0082, 1983.9866, 1985.8589, 1995.9136, 2027.1289, 2067.1079, 2110.0598, 2136.8484, 2144.0432, 2150.8660, 2164.8794, 2211.0857, 2239.1533, 2251.1509, 2255.1836, 2270.1113, 2273.0972, 2274.0605, 2286.1492, 2289.0857, 2290.0530, 2293.1558, 2309.1570, 2324.0981, 2338.1033, 2340.0979, 2343.1770, 2352.9316, 2375.0454, 2383.9480, 2414.2512, 2499.2480, 2542.2678,

2589.9707, 2600.0022, 2603.9897, 2613.9939, 2627.9993, 2644.0112, 2646.9875, 2657.4248, 2659.0405, 2661.0017, 2665.0042, 2674.0601, 2675.0151, 2678.0159, 2689.0330, 2692.0283, 2695.0315, 2703.0417, 2706.0959, 2763.3606, 2798.2788, 2815.3203, 2846.4011, 2872.3391, 2876.3679, 2884.3535, 2888.3455, 2891.4131, 2892.4519, 2919.4543, 3002.3042, 3012.4717, 3016.2314, 3020.2483, 3030.3025, 3033.2446, 3053.4917, 3081.6296, 3126.7493, 3171.7131, 3184.4602, 3206.6179, 3207.6111, 3254.6370, 3265.6309, 3300.7825, 3317.7288, 3321.6318, 3334.6953, 3347.5437, 3429.5991, 3457.6255, 3471.6121, 3489.7363, 3503.8608, 3518.5715, 3639.7969, 4148.0293, 4162.0039, 4453.2720, 4844.2046

31. F1MYN2 **Mass:** 136237 **Score:** 19 **Expect:** 2.8e+002 **Matches:** 20

Uncharacterized protein OS=Bos taurus GN=CGN PE=4 SV=2

Observed	Mr(expt)	Mr(calc)	ppm	Start	End	Miss	Peptide
717.3984	716.3911	716.3817	13.2	222	227	0	R.ATLQER.E
900.5293	899.5220	899.5076	16.0	400	406	0	R.LQELLER.R
923.5157	922.5084	922.4872	23.0	48	56	0	R.ASTYGVAVR.V
1016.5761	1015.5688	1015.5662	2.61	369	376	2	K.LDEEVKKR.Q
1057.5575	1056.5502	1056.5676	-16.43	478	485	1	K.QRVEEQLR.L
1087.5460	1086.5387	1086.5418	-2.80	911	920	0	R.QALQASQADR.D
1383.7253	1382.7180	1382.7518	-24.39	377	388	1	R.QKLEPSQVGLER.Q
1442.8190	1441.8117	1441.7776	23.7	468	479	1	R.ELLEEVLEGKQR.V
1488.7245	1487.7172	1487.7467	-19.83	845	857	1	R.LNKELEQIGDDSK.Q
1556.8799	1555.8726	1555.8682	2.86	921	933	2	R.DTARLDKELLVQR.L
1647.8503	1646.8430	1646.8522	-5.58	974	987	2	K.STVELLTDRMNRGR.D
1717.9056	1716.8983	1716.9383	-23.30	41	56	2	R.RPAKDARASTYGVAVR.V
1816.9354	1815.9281	1815.9551	-14.86	895	910	2	K.TSGGLSRLQDETQRLR.Q
2067.1079	2066.1006	2066.1445	-21.26	333	352	0	K.VSLVLEQLQPLVMASPGSAK.V
2211.0857	2210.0784	2210.1291	-22.92	312	331	2	K.ATIYGILREGSSESETSVRR.K
2289.0857	2288.0784	2288.1205	-18.40	588	606	2	R.MEKDELEEELGEKIEALQR.E
2309.1570	2308.1497	2308.1052	19.3	1	21	1	-.MEQASTMAEPRGPVDHGVQIR.F
2324.0981	2323.0908	2323.1040	-5.67	710	728	2	R.TAVETTLRETQEENDEFRR.R
2891.4131	2890.4058	2890.3555	17.4	550	573	2	R.GLQKELEETSEETGHWQNMFQKNK.E
3248.6445	3247.6372	3247.5561	25.0	680	708	2	K.TLQQLRQDCEEASKAQMAAETEAAVLGQR.R + Oxidation (M)

No match to: 706.3098, 710.3790, 712.2532, 714.3089, 719.4317, 720.4312, 722.4346, 735.4181, 749.4561, 756.4000, 759.4391, 772.4356, 774.4578, 778.4783, 779.4789, 818.4724, 823.4901, 831.4754, 832.3123, 834.3210, 842.5065, 844.4971, 870.5016, 871.4923, 873.3899, 876.2862, 880.4520, 883.4794, 891.4870, 895.5731, 909.5164, 913.4845, 918.5409, 925.4807, 927.5140, 929.4449, 942.5470, 944.5204, 945.5183, 949.5508, 950.5472, 967.4262, 972.4835, 974.4452, 976.4556, 977.4954, 978.5431, 988.4708, 1007.5730, 1026.5627, 1031.5581, 1034.5808, 1044.5240, 1051.5095, 1056.5281, 1066.5067, 1074.5374, 1103.5287, 1118.5885, 1122.5553, 1148.5439, 1168.5985, 1178.6211, 1185.6898, 1244.6118, 1248.6686, 1253.6313, 1255.6216, 1301.6338, 1312.7423, 1313.7227, 1314.7188, 1315.6721, 1317.6354, 1320.6331, 1322.6371, 1325.6360, 1327.6959, 1333.7570, 1336.7218, 1338.7179, 1339.7092, 1343.6960, 1349.6917, 1350.6987, 1351.6946, 1361.6875, 1372.5978, 1381.7642, 1382.5950, 1397.7454, 1439.7549, 1457.7394, 1463.7950, 1494.7383, 1497.8735, 1504.7484, 1513.7760, 1517.8062, 1520.7523, 1528.6951, 1537.8245, 1539.8104, 1549.8242, 1552.8073, 1553.8669, 1555.8348, 1567.7986, 1570.7749, 1578.7872, 1592.7849, 1628.7002, 1628.8571, 1638.8861, 1642.7155, 1650.8458, 1664.8571, 1680.8226, 1696.8146, 1698.7845, 1699.8228, 1705.8712, 1707.8536, 1715.8536, 1730.8564, 1739.6990, 1771.0344, 1786.9199, 1809.0262, 1820.8855, 1836.8507, 1907.9603, 1909.9886, 1928.0238, 1940.0148, 1953.8596, 1961.0111, 1965.8679, 1974.9255, 1982.0082, 1983.9866, 1985.8589, 1995.9136, 2027.1289, 2110.0598, 2136.8484, 2144.0432, 2150.8660, 2164.8794, 2239.1533, 2251.1509, 2255.1836, 2270.1113, 2273.0972, 2274.0605, 2286.1492, 2290.0530, 2293.1558, 2338.1033, 2340.0979, 2343.1770, 2352.9316, 2375.0454, 2383.9480, 2414.2512, 2499.2480, 2542.2678, 2589.9707, 2600.0022, 2603.9897, 2613.9939, 2627.9993, 2644.0112, 2646.9875, 2657.4248, 2659.0405, 2661.0017, 2665.0042, 2674.0601, 2675.0151, 2678.0159, 2689.0330, 2692.0283, 2695.0315, 2703.0417, 2706.0959, 2763.3606, 2798.2788, 2815.3203, 2846.4011, 2872.3391, 2876.3679, 2884.3535, 2888.3455, 2892.4519, 2919.4543, 3002.3042, 3012.4717, 3016.2314, 3020.2483, 3030.3025, 3033.2446, 3053.4917, 3081.6296, 3126.7493, 3171.7131, 3184.4602, 3206.6179, 3207.6111, 3254.6370, 3265.6309, 3300.7825, 3317.7288, 3321.6318, 3334.6953, 3347.5437, 3429.5991, 3457.6255, 3471.6121, 3489.7363, 3503.8608, 3518.5715, 3639.7969, 4148.0293, 4162.0039, 4453.2720, 4844.2046

32. F1N052 **Mass:** 122565 **Score:** 19 **Expect:** 2.9e+002 **Matches:** 14

Uncharacterized protein OS=Bos taurus GN=SMARCA5 PE=1 SV=2

Observed	Mr(expt)	Mr(calc)	ppm	Start	End	Miss	Peptide
779.4789	778.4716	778.4589	16.3	412	418	0	K.IYVGLSK.M
834.3210	833.3137	833.3338	-24.08	1037	1044	0	K.MDGAPDGR.G + Oxidation (M)
1051.5095	1050.5022	1050.4838	17.6	648	655	0	K.DEMLQMIR.H + Oxidation (M)
1148.5439	1147.5366	1147.5179	16.3	891	899	0	R.CNELQDIEK.I
1255.6216	1254.6143	1254.6431	-22.90	422	430	1	R.EWYTRILMK.D + Oxidation (M)
1317.6354	1316.6281	1316.6513	-17.61	739	749	1	R.KANYAVDAYFR.E
1349.6917	1348.6844	1348.6843	0.12	645	655	1	K.IGKDEMLQMIR.H + Oxidation (M)
1383.7253	1382.7180	1382.7479	-21.62	212	223	0	K.TLQTISLLGYMK.H + Oxidation (M)
1457.7394	1456.7321	1456.7535	-14.67	133	144	1	K.QNLLSVGDYRHR.R
1664.8571	1663.8498	1663.8529	-1.86	129	142	2	K.KDEKQNLLSVGDYR.H
1786.9199	1785.9126	1785.9162	-1.98	739	753	2	R.KANYAVDAYFREALR.V
1928.0238	1927.0165	1926.9774	20.3	985	999	2	R.FDWFLKSRTAMELQR.R
1982.0082	1981.0009	1981.0381	-18.78	601	616	2	K.TVRVFRFITDNTVEER.I
2324.0981	2323.0908	2323.1127	-9.43	967	984	2	K.ENVYDELRQCIRNSPQFR.F

No match to: 706.3098, 710.3790, 712.2532, 714.3089, 717.3984, 719.4317, 720.4312, 722.4346, 735.4181, 749.4561, 756.4000, 759.4391, 772.4356, 774.4578, 778.4783, 818.4724, 823.4901, 831.4754, 832.3123, 842.5065, 844.4971, 870.5016, 871.4923, 873.3899, 876.2862, 880.4520, 883.4794, 891.4870, 895.5731, 900.5293, 909.5164, 913.4845, 918.5409, 923.5157, 925.4807, 927.5140, 929.4449, 942.5470, 944.5204, 945.5183, 949.5508, 950.5472, 967.4262, 972.4835, 974.4452, 976.4556, 977.4954, 978.5431, 988.4708, 1007.5730, 1016.5761, 1026.5627, 1031.5581, 1034.5808, 1044.5240, 1056.5281, 1057.5575, 1066.5067, 1074.5374, 1087.5460, 1103.5287, 1118.5885, 1122.5553, 1168.5985, 1178.6211, 1185.6898, 1244.6118, 1248.6686, 1253.6313, 1301.6338, 1312.7423, 1313.7227, 1314.7188, 1315.6721, 1320.6331, 1322.6371, 1325.6360, 1327.6959, 1333.7570, 1336.7218, 1338.7179, 1339.7092, 1343.6960, 1350.6987, 1351.6946, 1361.6875, 1372.5978, 1381.7642, 1382.5950, 1397.7454, 1439.7549, 1442.8190, 1463.7950, 1488.7245, 1494.7383, 1497.8735, 1504.7484, 1513.7760, 1517.8062, 1520.7523, 1528.6951, 1537.8245, 1539.8104, 1549.8242, 1552.8073, 1553.8669, 1555.8348, 1556.8799, 1567.7986, 1570.7749, 1578.7872, 1592.7849, 1628.7002, 1628.8571, 1638.8861, 1642.7155, 1647.8503, 1650.8458, 1680.8226, 1696.8146, 1698.7845, 1699.8228, 1705.8712, 1707.8536, 1715.8536, 1717.9056, 1730.8564, 1739.6990, 1771.0344, 1809.0262, 1816.9354, 1820.8855, 1836.8507, 1907.9603, 1909.9886, 1940.0148, 1953.8596, 1961.0111, 1965.8679, 1974.9255, 1983.9866, 1985.8589, 1995.9136, 2027.1289, 2067.1079, 2110.0598, 2136.8484, 2144.0432, 2150.8660, 2164.8794, 2211.0857, 2239.1533, 2251.1509, 2255.1836, 2270.1113, 2273.0972, 2274.0605, 2286.1492, 2289.0857, 2290.0530, 2293.1558, 2309.1570, 2338.1033, 2340.0979, 2343.1770, 2352.9316, 2375.0454, 2383.9480, 2414.2512, 2499.2480, 2542.2678, 2589.9707, 2600.0022, 2603.9897, 2613.9939, 2627.9993, 2644.0112, 2646.9875, 2657.4248, 2659.0405, 2661.0017, 2665.0042, 2674.0601, 2675.0151, 2678.0159, 2689.0330, 2692.0283, 2695.0315, 2703.0417, 2706.0959, 2763.3606, 2798.2788, 2815.3203, 2846.4011, 2872.3391, 2876.3679, 2884.3535, 2888.3455, 2891.4131, 2892.4519, 2919.4543, 3002.3042, 3012.4717, 3016.2314, 3020.2483, 3030.3025, 3033.2446, 3053.4917, 3081.6296, 3126.7493, 3171.7131, 3184.4602, 3206.6179, 3207.6111, 3248.6445, 3254.6370, 3265.6309, 3300.7825, 3317.7288, 3321.6318, 3334.6953, 3347.5437, 3429.5991, 3457.6255, 3471.6121, 3489.7363, 3503.8608, 3518.5715, 3639.7969, 4148.0293, 4162.0039, 4453.2720, 4844.2046

33. F1MHZ5 **Mass:** 32519 **Score:** 19 **Expect:** 3.2e+002 **Matches:** 5

Uncharacterized protein OS=Bos taurus GN=EPHA4 PE=4 SV=1

Observed	Mr(expt)	Mr(calc)	ppm	Start	End	Miss	Peptide
720.4312	719.4239	719.4078	22.3	134	138	1	K.ERFIR.E

2338.1033	2337.0960	2337.1522	-24.05	145 - 165	1	K.IDTIAADESFTQVDIGDRIMK.L
2846.4011	2845.3938	2845.3480	16.1	38 - 63	0	R.SVQGELGWIASPLEGGWEEVSIMDEK.N
3347.5437	3346.5364	3346.4628	22.0	241 - 269	1	K.DVPKMYCGADGEWLVPIGNCLCNAGHEER.N
4844.2046	4843.1973	4843.1611	7.49	241 - 281	2	K.DVPKMYCGADGEWLVPIGNCLCNAGHEERNGECQGLPWWLR.W

No match to: 706.3098, 710.3790, 712.2532, 714.3089, 717.3984, 719.4317, 722.4346, 735.4181, 749.4561, 756.4000, 759.4391, 772.4356, 774.4578, 778.4783, 779.4789, 818.4724, 823.4901, 831.4754, 832.3123, 834.3210, 842.5065, 844.4971, 870.5016, 871.4923, 873.3899, 876.2862, 880.4520, 883.4794, 891.4870, 895.5731, 900.5293, 909.5164, 913.4845, 918.5409, 923.5157, 925.4807, 927.5140, 929.4449, 942.5470, 944.5204, 945.5183, 949.5508, 950.5472, 967.4262, 972.4835, 974.4452, 976.4556, 977.4954, 978.5431, 988.4708, 1007.5730, 1016.5761, 1026.5627, 1031.5581, 1034.5808, 1044.5240, 1051.5095, 1056.5281, 1057.5575, 1066.5067, 1074.5374, 1087.5460, 1103.5287, 1118.5885, 1122.5553, 1148.5439, 1168.5985, 1178.6211, 1185.6898, 1244.6118, 1248.6686, 1253.6313, 1255.6216, 1301.6338, 1312.7423, 1313.7227, 1314.7188, 1315.6721, 1317.6354, 1320.6331, 1322.6371, 1325.6360, 1327.6959, 1333.7570, 1336.7218, 1338.7179, 1339.7092, 1343.6960, 1349.6917, 1350.6987, 1351.6946, 1361.6875, 1372.5978, 1381.7642, 1382.5950, 1383.7253, 1397.7454, 1439.7549, 1442.8190, 1457.7394, 1463.7950, 1488.7245, 1494.7383, 1497.8735, 1504.7484, 1513.7760, 1517.8062, 1520.7523, 1528.6951, 1537.8245, 1539.8104, 1549.8242, 1552.8073, 1553.8669, 1555.8348, 1556.8799, 1567.7986, 1570.7749, 1578.7872, 1592.7849, 1628.7002, 1628.8571, 1638.8861, 1642.7155, 1647.8503, 1650.8458, 1664.8571, 1680.8226, 1696.8146, 1698.7845, 1699.8228, 1705.8712, 1707.8536, 1715.8536, 1717.9056, 1730.8564, 1739.6990, 1771.0344, 1786.9199, 1809.0262, 1816.9354, 1820.8855, 1836.8507, 1907.9603, 1909.9886, 1928.0238, 1940.0148, 1953.8596, 1961.0111, 1965.8679, 1974.9255, 1982.0082, 1983.9866, 1985.8589, 1995.9136, 2027.1289, 2067.1079, 2110.0598, 2136.8484, 2144.0432, 2150.8660, 2164.8794, 2211.0857, 2239.1533, 2251.1509, 2255.1836, 2270.1113, 2273.0972, 2274.0605, 2286.1492, 2289.0857, 2290.0530, 2293.1558, 2309.1570, 2324.0981, 2340.0979, 2343.1770, 2352.9316, 2375.0454, 2383.9480, 2414.2512, 2499.2480, 2542.2678, 2589.9707, 2600.0022, 2603.9897, 2613.9939, 2627.9993, 2644.0112, 2646.9875, 2657.4248, 2659.0405, 2661.0017, 2665.0042, 2674.0601, 2675.0151, 2678.0159, 2689.0330, 2692.0283, 2695.0315, 2703.0417, 2706.0959, 2763.3606, 2798.2788, 2815.3203, 2872.3391, 2876.3679, 2884.3535, 2888.3455, 2891.4131, 2892.4519, 2919.4543, 3002.3042, 3012.4717, 3016.2314, 3020.2483, 3030.3025, 3033.2446, 3053.4917, 3081.6296, 3126.7493, 3171.7131, 3184.4602, 3206.6179, 3207.6111, 3248.6445, 3254.6370, 3265.6309, 3300.7825, 3317.7288, 3321.6318, 3334.6953, 3429.5991, 3457.6255, 3471.6121, 3489.7363, 3503.8608, 3518.5715, 3639.7969, 4148.0293, 4162.0039, 4453.2720

34. F1MV63 **Mass:** 78027 **Score:** 19 **Expect:** 3.3e+002 **Matches:** 4
Uncharacterized protein OS=Bos taurus GN=MACC1 PE=4 SV=1

Observed	Mr(expt)	Mr(calc)	ppm	Start - End	Miss	Peptide
1034.5808	1033.5735	1033.5808	-7.03	196 - 205	0	R.TISVGIYGPK.Y
1647.8503	1646.8430	1646.8298	8.06	633 - 646	1	R.DVLQDLQSALDRMK.N + Oxidation (M)
3334.6953	3333.6880	3333.7676	-23.87	444 - 472	2	R.VIAKEQVMSMSDNVFTTRNLLEQITLPLK.K + Oxidation (M)
4844.2046	4843.1973	4843.2323	-7.22	604 - 644	1	K.SGDLAVEMMWKPAYDFLYTWSAHYGNSYRDVLQDLQSALDR.M + 2 Oxidation (M)

No match to: 706.3098, 710.3790, 712.2532, 714.3089, 717.3984, 719.4317, 720.4312, 722.4346, 735.4181, 749.4561, 756.4000, 759.4391, 772.4356, 774.4578, 778.4783, 779.4789, 818.4724, 823.4901, 831.4754, 832.3123, 834.3210, 842.5065, 844.4971, 870.5016, 871.4923, 873.3899, 876.2862, 880.4520, 883.4794, 891.4870, 895.5731, 900.5293, 909.5164, 913.4845, 918.5409, 923.5157, 925.4807, 927.5140, 929.4449, 942.5470, 944.5204, 945.5183, 949.5508, 950.5472, 967.4262, 972.4835, 974.4452, 976.4556, 977.4954, 978.5431, 988.4708, 1007.5730, 1016.5761, 1026.5627, 1031.5581, 1044.5240, 1051.5095, 1056.5281, 1057.5575, 1066.5067, 1074.5374, 1087.5460, 1103.5287, 1118.5885, 1122.5553, 1148.5439, 1168.5985, 1178.6211, 1185.6898, 1244.6118, 1248.6686, 1253.6313, 1255.6216, 1301.6338, 1312.7423, 1313.7227, 1314.7188, 1315.6721, 1317.6354, 1320.6331, 1322.6371, 1325.6360, 1327.6959, 1333.7570, 1336.7218, 1338.7179, 1339.7092, 1343.6960, 1349.6917, 1350.6987, 1351.6946, 1361.6875, 1372.5978, 1381.7642, 1382.5950, 1383.7253, 1397.7454, 1439.7549, 1442.8190, 1457.7394, 1463.7950, 1488.7245, 1494.7383, 1497.8735, 1504.7484, 1513.7760, 1517.8062, 1520.7523, 1528.6951, 1537.8245, 1539.8104, 1549.8242, 1552.8073, 1553.8669, 1555.8348, 1556.8799, 1567.7986, 1570.7749, 1578.7872, 1592.7849, 1628.7002, 1628.8571, 1638.8861, 1642.7155, 1650.8458, 1664.8571, 1680.8226, 1696.8146, 1698.7845, 1699.8228, 1705.8712, 1707.8536, 1715.8536, 1717.9056, 1730.8564, 1739.6990, 1771.0344, 1786.9199, 1809.0262, 1816.9354, 1820.8855, 1836.8507, 1907.9603, 1909.9886, 1928.0238, 1940.0148, 1953.8596, 1961.0111, 1965.8679, 1974.9255, 1982.0082, 1983.9866, 1985.8589, 1995.9136, 2027.1289, 2067.1079, 2110.0598, 2136.8484, 2144.0432, 2150.8660, 2164.8794, 2211.0857, 2239.1533, 2251.1509, 2255.1836, 2270.1113, 2273.0972, 2274.0605, 2286.1492, 2289.0857, 2290.0530, 2293.1558, 2309.1570, 2324.0981, 2338.1033, 2340.0979, 2343.1770, 2352.9316, 2375.0454, 2383.9480, 2414.2512, 2499.2480, 2542.2678, 2589.9707, 2600.0022, 2603.9897, 2613.9939, 2627.9993, 2644.0112, 2646.9875, 2657.4248, 2659.0405, 2661.0017, 2665.0042, 2674.0601, 2675.0151, 2678.0159, 2689.0330, 2692.0283, 2695.0315, 2703.0417, 2706.0959, 2763.3606, 2798.2788, 2815.3203, 2846.4011, 2872.3391, 2876.3679, 2884.3535, 2888.3455, 2891.4131, 2892.4519, 2919.4543, 3002.3042, 3012.4717, 3016.2314, 3020.2483, 3030.3025, 3033.2446, 3053.4917, 3081.6296, 3126.7493, 3171.7131, 3184.4602, 3206.6179, 3207.6111, 3248.6445, 3254.6370, 3265.6309, 3300.7825, 3317.7288, 3321.6318, 3347.5437, 3429.5991, 3457.6255, 3471.6121, 3489.7363, 3503.8608, 3518.5715, 3639.7969, 4148.0293, 4162.0039, 4453.2720

35. A6QQS9 **Mass:** 110367 **Score:** 18 **Expect:** 3.4e+002 **Matches:** 10
NBR1 protein OS=Bos taurus GN=NBR1 PE=2 SV=1

Observed	Mr(expt)	Mr(calc)	ppm	Start - End	Miss	Peptide
759.4391	758.4318	758.4398	-10.60	307 - 312	2	R.AEKKQR.K
871.4923	870.4850	870.5035	-21.29	537 - 544	2	K.AKNAPSKR.E
891.4870	890.4797	890.4644	17.2	398 - 405	1	R.MKNTGNVK.W
900.5293	899.5220	899.5301	-8.93	303 - 309	2	K.QRLRAEK.K
978.5431	977.5358	977.5182	18.0	406 - 413	1	K.WSTDTKLK.F
1016.5761	1015.5688	1015.5550	13.6	617 - 626	0	K.ALPDSTVSVK.K
1556.8799	1555.8726	1555.8470	16.4	275 - 288	1	R.FSTPRLPAALEQAR.L
1638.8861	1637.8788	1637.8849	-3.70	871 - 885	1	R.EELRGPSGLVNSRPK.S
3053.4917	3052.4844	3052.5063	-7.16	676 - 702	1	R.AELQLYATEEQQPTVLPGFCSKESSLK.F
4844.2046	4843.1973	4843.2503	-10.93	181 - 222	0	K.LVLQHPSLGSCPSEVSMPVSEETLFLPENQFSWHIACSSCQR.S

No match to: 706.3098, 710.3790, 712.2532, 714.3089, 717.3984, 719.4317, 720.4312, 722.4346, 735.4181, 749.4561, 756.4000, 772.4356, 774.4578, 778.4783, 779.4789, 818.4724, 823.4901, 831.4754, 832.3123, 834.3210, 842.5065, 844.4971, 870.5016, 873.3899, 876.2862, 880.4520, 883.4794, 895.5731, 909.5164, 913.4845, 918.5409, 923.5157, 925.4807, 927.5140, 929.4449, 942.5470, 944.5204, 945.5183, 949.5508, 950.5472, 967.4262, 972.4835, 974.4452, 976.4556, 977.4954, 988.4708, 1007.5730, 1026.5627, 1031.5581, 1034.5808, 1044.5240, 1051.5095, 1056.5281, 1057.5575, 1066.5067, 1074.5374, 1087.5460, 1103.5287, 1118.5885, 1122.5553, 1148.5439, 1168.5985, 1178.6211, 1185.6898, 1244.6118, 1248.6686, 1253.6313, 1255.6216, 1301.6338, 1312.7423, 1313.7227, 1314.7188, 1315.6721, 1317.6354, 1320.6331, 1322.6371, 1325.6360, 1327.6959, 1333.7570, 1336.7218, 1338.7179, 1339.7092, 1343.6960, 1349.6917, 1350.6987, 1351.6946, 1361.6875, 1372.5978, 1381.7642, 1382.5950, 1383.7253, 1397.7454, 1439.7549, 1442.8190, 1457.7394, 1463.7950, 1488.7245, 1494.7383, 1497.8735, 1504.7484, 1513.7760, 1517.8062, 1520.7523, 1528.6951, 1537.8245, 1539.8104, 1549.8242, 1552.8073, 1553.8669, 1555.8348, 1567.7986, 1570.7749, 1578.7872, 1592.7849, 1628.7002, 1628.8571, 1642.7155, 1647.8503, 1650.8458, 1664.8571, 1680.8226, 1696.8146, 1698.7845, 1699.8228, 1705.8712, 1707.8536, 1715.8536, 1717.9056, 1730.8564, 1739.6990, 1771.0344, 1786.9199, 1809.0262, 1816.9354, 1820.8855, 1836.8507, 1907.9603, 1909.9886, 1928.0238, 1940.0148, 1953.8596, 1961.0111, 1965.8679, 1974.9255, 1982.0082, 1983.9866, 1985.8589, 1995.9136, 2027.1289, 2067.1079, 2110.0598, 2136.8484, 2144.0432, 2150.8660, 2164.8794, 2211.0857, 2239.1533, 2251.1509, 2255.1836, 2270.1113, 2273.0972, 2274.0605, 2286.1492, 2289.0857, 2290.0530, 2293.1558, 2309.1570, 2324.0981, 2338.1033, 2340.0979, 2343.1770, 2352.9316, 2375.0454, 2383.9480, 2414.2512, 2499.2480, 2542.2678, 2589.9707, 2600.0022, 2603.9897, 2613.9939, 2627.9993, 2644.0112, 2646.9875, 2657.4248, 2659.0405, 2661.0017, 2665.0042, 2674.0601, 2675.0151, 2678.0159, 2689.0330, 2692.0283, 2695.0315, 2703.0417, 2706.0959, 2763.3606, 2798.2788, 2815.3203, 2846.4011, 2872.3391, 2876.3679, 2884.3535, 2888.3455, 2891.4131, 2892.4519, 2919.4543, 3002.3042, 3012.4717, 3016.2314, 3020.2483, 3030.3025, 3033.2446, 3081.6296, 3126.7493, 3171.7131, 3184.4602, 3206.6179, 3207.6111, 3248.6445, 3254.6370, 3265.6309, 3300.7825, 3317.7288, 3321.6318, 3334.6953, 3347.5437, 3429.5991, 3457.6255, 3471.6121, 3489.7363, 3503.8608, 3518.5715, 3639.7969, 4148.0293, 4162.0039, 4453.2720

36. A7MB88 **Mass:** 93244 **Score:** 18 **Expect:** 3.4e+002 **Matches:** 8
TMEM63A protein OS=Bos taurus GN=TMEM63A PE=2 SV=1

Observed	Mr(expt)	Mr(calc)	ppm	Start - End	Miss	Peptide
1051.5095	1050.5022	1050.4843	17.1	378 - 387	0	K.GEPQPSSHGR.E
1253.6313	1252.6240	1252.6268	-2.18	584 - 594	2	R.MVMAKTAADRK.N + 2 Oxidation (M)
1336.7218	1335.7145	1335.6975	12.7	647 - 657	0	R.HNLYFAYLPAK.L

1539.8104	1538.8031	1538.7729	19.6	530 -	542	1	R.WLFDKTSSEASIR.L
1628.7002	1627.6929	1627.6905	1.48	365 -	377	1	K.DFNACKCQGLQCK.G
2324.0981	2323.0908	2323.0910	-0.06	371 -	390	2	K.CQGLQCKGEPQPSSHGRELR.I
2763.3606	2762.3533	2762.3659	-4.57	341 -	364	1	R.VQDQPLGMAFVTFQEKSMATYVLK.D + 2 Oxidation (M)
3334.6953	3333.6880	3333.6083	23.9	249 -	275	2	R.DAYPTCEVVEVQLCYDVAKLIYLCKER.K

No match to: 706.3098, 710.3790, 712.2532, 714.3089, 717.3984, 719.4317, 720.4312, 722.4346, 735.4181, 749.4561, 756.4000, 759.4391, 772.4356, 774.4578, 778.4783, 779.4789, 818.4724, 823.4901, 831.4754, 832.3123, 834.3210, 842.5065, 844.4971, 870.5016, 871.4923, 873.3899, 876.2862, 880.4520, 883.4794, 891.4870, 895.5731, 900.5293, 909.5164, 913.4845, 918.5409, 923.5157, 925.4807, 927.5140, 929.4449, 942.5470, 944.5204, 945.5183, 949.5508, 950.5472, 967.4262, 972.4835, 974.4452, 976.4556, 977.4954, 978.5431, 988.4708, 1007.5730, 1016.5761, 1026.5627, 1031.5581, 1034.5808, 1044.5240, 1056.5281, 1057.5575, 1066.5067, 1074.5374, 1087.5460, 1103.5287, 1118.5885, 1122.5553, 1148.5439, 1168.5985, 1178.6211, 1185.6898, 1244.6118, 1248.6686, 1255.6216, 1301.6338, 1312.7423, 1313.7227, 1314.7188, 1315.6721, 1317.6354, 1320.6331, 1322.6371, 1325.6360, 1327.6959, 1333.7570, 1338.7179, 1339.7092, 1343.6960, 1349.6917, 1350.6987, 1351.6946, 1361.6875, 1372.5978, 1381.7642, 1382.5950, 1383.7253, 1397.7454, 1439.7549, 1442.8190, 1457.7394, 1463.7950, 1488.7245, 1494.7383, 1497.8735, 1504.7484, 1513.7760, 1517.8062, 1520.7523, 1528.6951, 1537.8245, 1549.8242, 1552.8073, 1553.8669, 1555.8348, 1556.8799, 1567.7986, 1570.7749, 1578.7872, 1592.7849, 1628.8571, 1638.8861, 1642.7155, 1647.8503, 1650.8458, 1664.8571, 1680.8226, 1696.8146, 1698.7845, 1699.8228, 1705.8712, 1707.8536, 1715.8536, 1717.9056, 1730.8564, 1739.6990, 1771.0344, 1786.9199, 1809.0262, 1816.9354, 1820.8855, 1836.8507, 1907.9603, 1909.9886, 1928.0238, 1940.0148, 1953.8596, 1961.0111, 1965.8679, 1974.9255, 1982.0082, 1983.9866, 1985.8589, 1995.9136, 2027.1289, 2067.1079, 2110.0598, 2136.8484, 2144.0432, 2150.8660, 2164.8794, 2211.0857, 2239.1533, 2251.1509, 2255.1836, 2270.1113, 2273.0972, 2274.0605, 2286.1492, 2289.0857, 2290.0530, 2293.1558, 2309.1570, 2338.1033, 2340.0979, 2343.1770, 2352.9316, 2375.0454, 2383.9480, 2414.2512, 2499.2480, 2542.2678, 2589.9707, 2600.0022, 2603.9897, 2613.9939, 2627.9993, 2644.0112, 2646.9875, 2657.4248, 2659.0405, 2661.0017, 2665.0042, 2674.0601, 2675.0151, 2678.0159, 2689.0330, 2692.0283, 2695.0315, 2703.0417, 2706.0959, 2798.2788, 2815.3203, 2846.4011, 2872.3391, 2876.3679, 2884.3535, 2888.3455, 2891.4131, 2892.4519, 2919.4543, 3002.3042, 3012.4717, 3016.2314, 3020.2483, 3030.3025, 3033.2446, 3053.4917, 3081.6296, 3126.7493, 3171.7131, 3184.4602, 3206.6179, 3207.6111, 3248.6445, 3254.6370, 3265.6309, 3300.7825, 3317.7288, 3321.6318, 3347.5437, 3429.5991, 3457.6255, 3471.6121, 3489.7363, 3503.8608, 3518.5715, 3639.7969, 4148.0293, 4162.0039, 4453.2720, 4844.2046

37. Q3B8S0 **Mass:** 14393 **Score:** 18 **Expect:** 3.5e+002 **Matches:** 3

NHP2-like protein 1 OS=Bos taurus GN=SNU13 PE=2 SV=3

Observed	Mr(expt)	Mr(calc)	ppm	Start	End	Miss	Peptide
759.4391	758.4318	758.4399	-10.62	85 -	91	1	R.SKQALGR.A
1457.7394	1456.7321	1456.7634	-21.46	114 -	125	0	K.QQIQSIQQSIER.L
1717.9056	1716.8983	1716.9015	-1.85	92 -	107	0	R.ACGVSRPVIACSVTIK.E

No match to: 706.3098, 710.3790, 712.2532, 714.3089, 717.3984, 719.4317, 720.4312, 722.4346, 735.4181, 749.4561, 756.4000, 772.4356, 774.4578, 778.4783, 779.4789, 818.4724, 823.4901, 831.4754, 832.3123, 834.3210, 842.5065, 844.4971, 870.5016, 871.4923, 873.3899, 876.2862, 880.4520, 883.4794, 891.4870, 895.5731, 900.5293, 909.5164, 913.4845, 918.5409, 923.5157, 925.4807, 927.5140, 929.4449, 942.5470, 944.5204, 945.5183, 949.5508, 950.5472, 967.4262, 972.4835, 974.4452, 976.4556, 977.4954, 978.5431, 988.4708, 1007.5730, 1016.5761, 1026.5627, 1031.5581, 1034.5808, 1044.5240, 1051.5095, 1056.5281, 1057.5575, 1066.5067, 1074.5374, 1087.5460, 1103.5287, 1118.5885, 1122.5553, 1148.5439, 1168.5985, 1178.6211, 1185.6898, 1244.6118, 1248.6686, 1253.6313, 1255.6216, 1301.6338, 1312.7423, 1313.7227, 1314.7188, 1315.6721, 1317.6354, 1320.6331, 1322.6371, 1325.6360, 1327.6959, 1333.7570, 1336.7218, 1338.7179, 1339.7092, 1343.6960, 1349.6917, 1350.6987, 1351.6946, 1361.6875, 1372.5978, 1381.7642, 1382.5950, 1383.7253, 1397.7454, 1439.7549, 1442.8190, 1463.7950, 1488.7245, 1494.7383, 1497.8735, 1504.7484, 1513.7760, 1517.8062, 1520.7523, 1528.6951, 1537.8245, 1539.8104, 1549.8242, 1552.8073, 1553.8669, 1555.8348, 1556.8799, 1567.7986, 1570.7749, 1578.7872, 1592.7849, 1628.7002, 1628.8571, 1638.8861, 1642.7155, 1647.8503, 1650.8458, 1664.8571, 1680.8226, 1696.8146, 1698.7845, 1699.8228, 1705.8712, 1707.8536, 1715.8536, 1730.8564, 1739.6990, 1771.0344, 1786.9199, 1809.0262, 1816.9354, 1820.8855, 1836.8507, 1907.9603, 1909.9886, 1928.0238, 1940.0148, 1953.8596, 1961.0111, 1965.8679, 1974.9255, 1982.0082, 1983.9866, 1985.8589, 1995.9136, 2027.1289, 2067.1079, 2110.0598, 2136.8484, 2144.0432, 2150.8660, 2164.8794, 2211.0857, 2239.1533, 2251.1509, 2255.1836, 2270.1113, 2273.0972, 2274.0605, 2286.1492, 2289.0857, 2290.0530, 2293.1558, 2309.1570, 2324.0981, 2338.1033, 2340.0979, 2343.1770, 2352.9316, 2375.0454, 2383.9480, 2414.2512, 2499.2480, 2542.2678, 2589.9707, 2600.0022, 2603.9897, 2613.9939, 2627.9993, 2644.0112, 2646.9875, 2657.4248, 2659.0405, 2661.0017, 2665.0042, 2674.0601, 2675.0151, 2678.0159, 2689.0330, 2692.0283, 2695.0315, 2703.0417, 2706.0959, 2763.3606, 2798.2788, 2815.3203, 2846.4011, 2872.3391, 2876.3679, 2884.3535, 2888.3455, 2891.4131, 2892.4519, 2919.4543, 3002.3042, 3012.4717, 3016.2314, 3020.2483, 3030.3025, 3033.2446, 3053.4917, 3081.6296, 3126.7493, 3171.7131, 3184.4602, 3206.6179, 3207.6111, 3248.6445, 3254.6370, 3265.6309, 3300.7825, 3317.7288, 3321.6318, 3334.6953, 3347.5437, 3429.5991, 3457.6255, 3471.6121, 3489.7363, 3503.8608, 3518.5715, 3639.7969, 4148.0293, 4162.0039, 4453.2720, 4844.2046

38. E1BE67 **Mass:** 14027 **Score:** 18 **Expect:** 3.6e+002 **Matches:** 3

Uncharacterized protein OS=Bos taurus PE=4 SV=2

Observed	Mr(expt)	Mr(calc)	ppm	Start	End	Miss	Peptide
1122.5553	1121.5480	1121.5326	13.7	115 -	125	2	K.GEGAPAGHRRD.-
1350.6987	1349.6914	1349.6912	0.15	112 -	124	2	R.NTKGEGAPAGHRR.D
1556.8799	1555.8726	1555.8947	-14.18	99 -	111	2	K.LVSKHRAQVVYTR.N

No match to: 706.3098, 710.3790, 712.2532, 714.3089, 717.3984, 719.4317, 720.4312, 722.4346, 735.4181, 749.4561, 756.4000, 759.4391, 772.4356, 774.4578, 778.4783, 779.4789, 818.4724, 823.4901, 831.4754, 832.3123, 834.3210, 842.5065, 844.4971, 870.5016, 871.4923, 873.3899, 876.2862, 880.4520, 883.4794, 891.4870, 895.5731, 900.5293, 909.5164, 913.4845, 918.5409, 923.5157, 925.4807, 927.5140, 929.4449, 942.5470, 944.5204, 945.5183, 949.5508, 950.5472, 967.4262, 972.4835, 974.4452, 976.4556, 977.4954, 978.5431, 988.4708, 1007.5730, 1016.5761, 1026.5627, 1031.5581, 1034.5808, 1044.5240, 1051.5095, 1056.5281, 1057.5575, 1066.5067, 1074.5374, 1087.5460, 1103.5287, 1118.5885, 1148.5439, 1168.5985, 1178.6211, 1185.6898, 1244.6118, 1248.6686, 1253.6313, 1255.6216, 1301.6338, 1312.7423, 1313.7227, 1314.7188, 1315.6721, 1317.6354, 1320.6331, 1322.6371, 1325.6360, 1327.6959, 1333.7570, 1336.7218, 1338.7179, 1339.7092, 1343.6960, 1349.6917, 1351.6946, 1361.6875, 1372.5978, 1381.7642, 1382.5950, 1383.7253, 1397.7454, 1439.7549, 1442.8190, 1457.7394, 1463.7950, 1488.7245, 1494.7383, 1497.8735, 1504.7484, 1513.7760, 1517.8062, 1520.7523, 1528.6951, 1537.8245, 1539.8104, 1549.8242, 1552.8073, 1553.8669, 1555.8348, 1567.7986, 1570.7749, 1578.7872, 1592.7849, 1628.7002, 1628.8571, 1638.8861, 1642.7155, 1647.8503, 1650.8458, 1664.8571, 1680.8226, 1696.8146, 1698.7845, 1699.8228, 1705.8712, 1707.8536, 1715.8536, 1717.9056, 1730.8564, 1739.6990, 1771.0344, 1786.9199, 1809.0262, 1816.9354, 1820.8855, 1836.8507, 1907.9603, 1909.9886, 1928.0238, 1940.0148, 1953.8596, 1961.0111, 1965.8679, 1974.9255, 1982.0082, 1983.9866, 1985.8589, 1995.9136, 2027.1289, 2067.1079, 2110.0598, 2136.8484, 2144.0432, 2150.8660, 2164.8794, 2211.0857, 2239.1533, 2251.1509, 2255.1836, 2270.1113, 2273.0972, 2274.0605, 2286.1492, 2289.0857, 2290.0530, 2293.1558, 2309.1570, 2324.0981, 2338.1033, 2340.0979, 2343.1770, 2352.9316, 2375.0454, 2383.9480, 2414.2512, 2499.2480, 2542.2678, 2589.9707, 2600.0022, 2603.9897, 2613.9939, 2627.9993, 2644.0112, 2646.9875, 2657.4248, 2659.0405, 2661.0017, 2665.0042, 2674.0601, 2675.0151, 2678.0159, 2689.0330, 2692.0283, 2695.0315, 2703.0417, 2706.0959, 2763.3606, 2798.2788, 2815.3203, 2846.4011, 2872.3391, 2876.3679, 2884.3535, 2888.3455, 2891.4131, 2892.4519, 2919.4543, 3002.3042, 3012.4717, 3016.2314, 3020.2483, 3030.3025, 3033.2446, 3053.4917, 3081.6296, 3126.7493, 3171.7131, 3184.4602, 3206.6179, 3207.6111, 3248.6445, 3254.6370, 3265.6309, 3300.7825, 3317.7288, 3321.6318, 3334.6953, 3347.5437, 3429.5991, 3457.6255, 3471.6121, 3489.7363, 3503.8608, 3518.5715, 3639.7969, 4148.0293, 4162.0039, 4453.2720, 4844.2046

39. E1BLR1 **Mass:** 58429 **Score:** 18 **Expect:** 3.7e+002 **Matches:** 10

Uncharacterized protein OS=Bos taurus GN=SPATA2 PE=4 SV=1

Observed	Mr(expt)	Mr(calc)	ppm	Start	End	Miss	Peptide
977.4954	976.4881	976.4648	23.9	214 -	222	0	R.EPLSASMAR.V + Oxidation (M)
1051.5095	1050.5022	1050.4838	17.6	1 -	10	0	-.MGKPSSMDAK.Y
1118.5885	1117.5812	1117.5979	-14.89	367 -	377	1	R.STPAKESALSK.C
2286.1492	2285.1419	2285.1047	16.3	82 -	100	0	R.CAFSVLETMGINLFLCPWK.K
2414.2512	2413.2439	2413.1996	18.3	82 -	101	1	R.CAFSVLETMGINLFLCPWKK.E
2657.4248	2656.4175	2656.3735	16.6	345 -	366	2	R.QDALRPDVWLLRNDAHPGYHKR.S
2846.4011	2845.3938	2845.4118	-6.30	82 -	104	2	R.CAFSVLETMGINLFLCPWKKEFR.S
2884.3535	2883.3462	2883.3095	12.7	367 -	392	2	R.STPAKESALSKCQNCGLSCSAALCQR.C
3248.6445	3247.6372	3247.6872	-15.39	128 -	155	2	R.AILSYMGYMPELGTAYRLKELVETLQVK.M + 2 Oxidation (M)

3265.6309 3264.6236 3264.6410 -5.32 119 - 146 2 K.STLLEEDIRAILSYMGYMPELGTAYRLK.E + 2 Oxidation (M)

No match to: 706.3098, 710.3790, 712.2532, 714.3089, 717.3984, 719.4317, 720.4312, 722.4346, 735.4181, 749.4561, 756.4000, 759.4391, 772.4356, 774.4578, 778.4783, 779.4789, 818.4724, 823.4901, 831.4754, 832.3123, 834.3210, 842.5065, 844.4971, 870.5016, 871.4923, 873.3899, 876.2862, 880.4520, 883.4794, 891.4870, 895.5731, 900.5293, 909.5164, 913.4845, 918.5409, 923.5157, 925.4807, 927.5140, 929.4449, 942.5470, 944.5204, 945.5183, 949.5508, 950.5472, 967.4262, 972.4835, 974.4452, 976.4556, 978.5431, 988.4708, 1007.5730, 1016.5761, 1026.5627, 1031.5581, 1034.5808, 1044.5240, 1056.5281, 1057.5575, 1066.5067, 1074.5374, 1087.5460, 1103.5287, 1122.5553, 1148.5439, 1168.5985, 1178.6211, 1185.6898, 1244.6118, 1248.6686, 1253.6313, 1255.6216, 1301.6338, 1312.7423, 1313.7227, 1314.7188, 1315.6721, 1317.6354, 1320.6331, 1322.6371, 1325.6360, 1327.6959, 1333.7570, 1336.7218, 1338.7179, 1339.7092, 1343.6960, 1349.6917, 1350.6987, 1351.6946, 1361.6875, 1372.5978, 1381.7642, 1382.5950, 1383.7253, 1397.7454, 1439.7549, 1442.8190, 1457.7394, 1463.7950, 1488.7245, 1494.7383, 1497.8735, 1504.7484, 1513.7760, 1517.8062, 1520.7523, 1528.6951, 1537.8245, 1539.8104, 1549.8242, 1552.8073, 1553.8669, 1555.8348, 1556.8799, 1567.7986, 1570.7749, 1578.7872, 1592.7849, 1628.7002, 1628.8571, 1638.8861, 1642.7155, 1647.8503, 1650.8458, 1664.8571, 1680.8226, 1696.8146, 1698.7845, 1699.8228, 1705.8712, 1707.8536, 1715.8536, 1717.9056, 1730.8564, 1739.6990, 1771.0344, 1786.9199, 1809.0262, 1816.9354, 1820.8855, 1836.8507, 1907.9603, 1909.9886, 1928.0238, 1940.0148, 1953.8596, 1961.0111, 1965.8679, 1974.9255, 1982.0082, 1983.9866, 1985.8589, 1995.9136, 2027.1289, 2067.1079, 2110.0598, 2136.8484, 2144.0432, 2150.8660, 2164.8794, 2211.0857, 2239.1533, 2251.1509, 2255.1836, 2270.1113, 2273.0972, 2274.0605, 2289.0857, 2290.0530, 2293.1558, 2309.1570, 2324.0981, 2338.1033, 2340.0979, 2343.1770, 2352.9316, 2375.0454, 2383.9480, 2499.2480, 2542.2678, 2589.9707, 2600.0022, 2603.9897, 2613.9939, 2627.9993, 2644.0112, 2646.9875, 2659.0405, 2661.0017, 2665.0042, 2674.0601, 2675.0151, 2678.0159, 2689.0330, 2692.0283, 2695.0315, 2703.0417, 2706.0959, 2763.3606, 2798.2788, 2815.3203, 2872.3391, 2876.3679, 2888.3455, 2891.4131, 2892.4519, 2919.4543, 3002.3042, 3012.4717, 3016.2314, 3020.2483, 3030.3025, 3033.2446, 3053.4917, 3081.6296, 3126.7493, 3171.7131, 3184.4602, 3206.6179, 3207.6111, 3254.6370, 3300.7825, 3317.7288, 3321.6318, 3334.6953, 3347.5437, 3429.5991, 3457.6255, 3471.6121, 3489.7363, 3503.8608, 3518.5715, 3639.7969, 4148.0293, 4162.0039, 4453.2720, 4844.2046

40. A1L579 **Mass:** 53486 **Score:** 18 **Expect:** 3.8e+002 **Matches:** 9

Oviduct-specific glycoprotein OS=Bos taurus GN=OVGP1 PE=2 SV=1

Observed	Mr(expt)	Mr(calc)	ppm	Start	End	Miss	Peptide
1148.5439	1147.5366	1147.5366	0.06	346	355	0	R.IGPEMPTMTR.D + Oxidation (M)
1301.6338	1300.6265	1300.6200	4.98	335	345	0	K.FWFSTAVNSSR.I
1320.6331	1319.6258	1319.6180	5.93	47	57	0	R.FTTMLSTFSNR.E + Oxidation (M)
1556.8799	1555.8726	1555.8470	16.4	206	218	1	R.TFHLLKASQNELR.A
1699.8228	1698.8155	1698.8518	-21.36	252	264	1	K.RWINDQYVPYAFK.G
1961.0111	1960.0038	1960.0299	-13.31	378	395	2	K.KSETMTITPKGEIATPTR.T
2289.0857	2288.0784	2288.0758	1.14	230	247	1	K.YTKQAGFLAYYEICCFVR.R
2763.3606	2762.3533	2762.3585	-1.87	1	23	2	-.MSNNQIVPKDPQDEKILYPEFNK.L + Oxidation (M)
3334.6953	3333.6880	3333.6697	5.49	346	377	1	R.IGPEMPTMTRDLTTGLGILPPGGEAVATETHR.K + Oxidation (M)

No match to: 706.3098, 710.3790, 712.2532, 714.3089, 717.3984, 719.4317, 720.4312, 722.4346, 735.4181, 749.4561, 756.4000, 759.4391, 772.4356, 774.4578, 778.4783, 779.4789, 818.4724, 823.4901, 831.4754, 832.3123, 834.3210, 842.5065, 844.4971, 870.5016, 871.4923, 873.3899, 876.2862, 880.4520, 883.4794, 891.4870, 895.5731, 900.5293, 909.5164, 913.4845, 918.5409, 923.5157, 925.4807, 927.5140, 929.4449, 942.5470, 944.5204, 945.5183, 949.5508, 950.5472, 967.4262, 972.4835, 974.4452, 976.4556, 977.4954, 978.5431, 988.4708, 1007.5730, 1016.5761, 1026.5627, 1031.5581, 1034.5808, 1044.5240, 1051.5095, 1056.5281, 1057.5575, 1066.5067, 1074.5374, 1087.5460, 1103.5287, 1118.5885, 1122.5553, 1168.5985, 1178.6211, 1185.6898, 1244.6118, 1248.6686, 1253.6313, 1255.6216, 1312.7423, 1313.7227, 1314.7188, 1315.6721, 1317.6354, 1322.6371, 1325.6360, 1327.6959, 1333.7570, 1336.7218, 1338.7179, 1339.7092, 1343.6960, 1349.6917, 1350.6987, 1351.6946, 1361.6875, 1372.5978, 1381.7642, 1382.5950, 1383.7253, 1397.7454, 1439.7549, 1442.8190, 1457.7394, 1463.7950, 1488.7245, 1494.7383, 1497.8735, 1504.7484, 1513.7760, 1517.8062, 1520.7523, 1528.6951, 1537.8245, 1539.8104, 1549.8242, 1552.8073, 1553.8669, 1555.8348, 1567.7986, 1570.7749, 1578.7872, 1592.7849, 1628.7002, 1628.8571, 1638.8861, 1642.7155, 1647.8503, 1650.8458, 1664.8571, 1680.8226, 1696.8146, 1698.7845, 1705.8712, 1707.8536, 1715.8536, 1717.9056, 1730.8564, 1739.6990, 1771.0344, 1786.9199, 1809.0262, 1816.9354, 1820.8855, 1836.8507, 1907.9603, 1909.9886, 1928.0238, 1940.0148, 1953.8596, 1965.8679, 1974.9255, 1982.0082, 1983.9866, 1985.8589, 1995.9136, 2027.1289, 2067.1079, 2110.0598, 2136.8484, 2144.0432, 2150.8660, 2164.8794, 2211.0857, 2239.1533, 2251.1509, 2255.1836, 2270.1113, 2273.0972, 2274.0605, 2286.1492, 2290.0530, 2293.1558, 2309.1570, 2324.0981, 2338.1033, 2340.0979, 2343.1770, 2352.9316, 2375.0454, 2383.9480, 2414.2512, 2499.2480, 2542.2678, 2589.9707, 2600.0022, 2603.9897, 2613.9939, 2627.9993, 2644.0112, 2646.9875, 2657.4248, 2659.0405, 2661.0017, 2665.0042, 2674.0601, 2675.0151, 2678.0159, 2689.0330, 2692.0283, 2695.0315, 2703.0417, 2706.0959, 2798.2788, 2815.3203, 2846.4011, 2872.3391, 2876.3679, 2884.3535, 2888.3455, 2891.4131, 2892.4519, 2919.4543, 3002.3042, 3012.4717, 3016.2314, 3020.2483, 3030.3025, 3033.2446, 3053.4917, 3081.6296, 3126.7493, 3171.7131, 3184.4602, 3206.6179, 3207.6111, 3248.6445, 3254.6370, 3265.6309, 3300.7825, 3317.7288, 3321.6318, 3347.5437, 3429.5991, 3457.6255, 3471.6121, 3489.7363, 3503.8608, 3518.5715, 3639.7969, 4148.0293, 4162.0039, 4453.2720, 4844.2046

41. F1N6X9 **Mass:** 54088 **Score:** 18 **Expect:** 3.9e+002 **Matches:** 5

Uncharacterized protein OS=Bos taurus GN=LOC100141215 PE=4 SV=2

Observed	Mr(expt)	Mr(calc)	ppm	Start	End	Miss	Peptide
891.4870	890.4797	890.4644	17.3	410	416	1	R.RALEQMK.G + Oxidation (M)
1312.7423	1311.7350	1311.7047	23.1	437	446	2	R.GRRLDYITYR.S
1317.6354	1316.6281	1316.6448	-12.67	108	117	0	R.RPFCYQPPPR.C
2414.2512	2413.2439	2413.2437	0.08	297	317	1	R.RVVGFLHCTHLHAPSEDGLLR.C
3248.6445	3247.6372	3247.6461	-2.75	134	161	2	R.SFGFFSANLCLLPDGLARFNNLPHTQRR.A

No match to: 706.3098, 710.3790, 712.2532, 714.3089, 717.3984, 719.4317, 720.4312, 722.4346, 735.4181, 749.4561, 756.4000, 759.4391, 772.4356, 774.4578, 778.4783, 779.4789, 818.4724, 823.4901, 831.4754, 832.3123, 834.3210, 842.5065, 844.4971, 870.5016, 871.4923, 873.3899, 876.2862, 880.4520, 883.4794, 895.5731, 900.5293, 909.5164, 913.4845, 918.5409, 923.5157, 925.4807, 927.5140, 929.4449, 942.5470, 944.5204, 945.5183, 949.5508, 950.5472, 967.4262, 972.4835, 974.4452, 976.4556, 977.4954, 978.5431, 988.4708, 1007.5730, 1016.5761, 1026.5627, 1031.5581, 1034.5808, 1044.5240, 1051.5095, 1056.5281, 1057.5575, 1066.5067, 1074.5374, 1087.5460, 1103.5287, 1118.5885, 1122.5553, 1148.5439, 1168.5985, 1178.6211, 1185.6898, 1244.6118, 1248.6686, 1253.6313, 1255.6216, 1301.6338, 1313.7227, 1314.7188, 1315.6721, 1320.6331, 1322.6371, 1325.6360, 1327.6959, 1333.7570, 1336.7218, 1338.7179, 1339.7092, 1343.6960, 1349.6917, 1350.6987, 1351.6946, 1361.6875, 1372.5978, 1381.7642, 1382.5950, 1383.7253, 1397.7454, 1439.7549, 1442.8190, 1457.7394, 1463.7950, 1488.7245, 1494.7383, 1497.8735, 1504.7484, 1513.7760, 1517.8062, 1520.7523, 1528.6951, 1537.8245, 1539.8104, 1549.8242, 1552.8073, 1553.8669, 1555.8348, 1556.8799, 1567.7986, 1570.7749, 1578.7872, 1592.7849, 1628.7002, 1628.8571, 1638.8861, 1642.7155, 1647.8503, 1650.8458, 1664.8571, 1680.8226, 1696.8146, 1698.7845, 1699.8228, 1705.8712, 1707.8536, 1715.8536, 1717.9056, 1730.8564, 1739.6990, 1771.0344, 1786.9199, 1809.0262, 1816.9354, 1820.8855, 1836.8507, 1907.9603, 1909.9886, 1928.0238, 1940.0148, 1953.8596, 1961.0111, 1965.8679, 1974.9255, 1982.0082, 1983.9866, 1985.8589, 1995.9136, 2027.1289, 2067.1079, 2110.0598, 2136.8484, 2144.0432, 2150.8660, 2164.8794, 2211.0857, 2239.1533, 2251.1509, 2255.1836, 2270.1113, 2273.0972, 2274.0605, 2286.1492, 2289.0857, 2290.0530, 2293.1558, 2309.1570, 2324.0981, 2338.1033, 2340.0979, 2343.1770, 2352.9316, 2375.0454, 2383.9480, 2499.2480, 2542.2678, 2589.9707, 2600.0022, 2603.9897, 2613.9939, 2627.9993, 2644.0112, 2646.9875, 2657.4248, 2659.0405, 2661.0017, 2665.0042, 2674.0601, 2675.0151, 2678.0159, 2689.0330, 2692.0283, 2695.0315, 2703.0417, 2706.0959, 2763.3606, 2798.2788, 2815.3203, 2846.4011, 2872.3391, 2876.3679, 2884.3535, 2888.3455, 2891.4131, 2892.4519, 2919.4543, 3002.3042, 3012.4717, 3016.2314, 3020.2483, 3030.3025, 3033.2446, 3053.4917, 3081.6296, 3126.7493, 3171.7131, 3184.4602, 3206.6179, 3207.6111, 3254.6370, 3265.6309, 3300.7825, 3317.7288, 3321.6318, 3334.6953, 3347.5437, 3429.5991, 3457.6255, 3471.6121, 3489.7363, 3503.8608, 3518.5715, 3639.7969, 4148.0293, 4162.0039, 4453.2720, 4844.2046

42. G3N2A1 **Mass:** 46196 **Score:** 18 **Expect:** 3.9e+002 **Matches:** 6

Uncharacterized protein OS=Bos taurus GN=CCDC96 PE=4 SV=1

Observed	Mr(expt)	Mr(calc)	ppm	Start	End	Miss	Peptide
1122.5553	1121.5480	1121.5353	11.4	250	258	0	K.IENQTFNEK.V
1301.6338	1300.6265	1300.6510	-18.84	194	204	0	R.EVEQIQALEDK.K
1628.8571	1627.8498	1627.8165	20.5	3	17	2	R.EEKGAEPQALESGRK.R
2255.1836	2254.1763	2254.2011	-10.97	76	92	2	R.YQRYNIYLQHKIFEALR.K
2324.0981	2323.0908	2323.1345	-18.80	133	150	2	R.KQEADDLRWYHHELDQLK.L
3489.7363	3488.7290	3488.7973	-19.58	221	249	2	K.QSLVHFETRMRTQEDLTEGLLLIDFEQLK.I

No match to: 706.3098, 710.3790, 712.2532, 714.3089, 717.3984, 719.4317, 720.4312, 722.4346, 735.4181, 749.4561, 756.4000, 759.4391, 772.4356, 774.4578, 778.4783, 779.4789, 818.4724, 823.4901, 831.4754, 832.3123, 834.3210, 842.5065, 844.4971, 870.5016, 871.4923, 873.3899, 876.2862, 880.4520, 883.4794, 891.4870, 895.5731, 900.5293, 909.5164, 913.4845, 918.5409, 923.5157, 925.4807, 927.5140, 929.4449, 942.5470, 944.5204, 945.5183, 949.5508, 950.5472, 967.4262, 972.4835, 974.4452, 976.4556, 977.4954, 978.5431, 988.4708, 1007.5730, 1016.5761, 1026.5627, 1031.5581, 1034.5808, 1044.5240, 1051.5095, 1056.5281, 1057.5575, 1066.5067, 1074.5374, 1087.5460, 1103.5287, 1118.5885, 1148.5439, 1168.5985, 1178.6211, 1185.6898, 1244.6118, 1248.6686, 1253.6313, 1255.6216, 1312.7423, 1313.7227, 1314.7188, 1315.6721, 1317.6354, 1320.6331, 1322.6371, 1325.6360, 1327.6959, 1333.7570, 1336.7218, 1338.7179, 1339.7092, 1343.6960, 1349.6917, 1350.6987, 1351.6946, 1361.6875, 1372.5978, 1381.7642, 1382.5950, 1383.7253, 1397.7454, 1439.7549, 1442.8190, 1457.7394, 1463.7950, 1488.7245, 1494.7383, 1497.8735, 1504.7484, 1513.7760, 1517.8062, 1520.7523, 1528.6951, 1537.8245, 1539.8104, 1549.8242, 1552.8073, 1553.8669, 1555.8348, 1556.8799, 1567.7986, 1570.7749, 1578.7872, 1592.7849, 1628.7002, 1638.8861, 1642.7155, 1647.8503, 1650.8458, 1664.8571, 1680.8226, 1696.8146, 1698.7845, 1699.8228, 1705.8712, 1707.8536, 1715.8536, 1717.9056, 1730.8564, 1739.6990, 1771.0344, 1786.9199, 1809.0262, 1816.9354, 1820.8855, 1836.8507, 1907.9603, 1909.9886, 1928.0238, 1940.0148, 1953.8596, 1961.0111, 1965.8679, 1974.9255, 1982.0082, 1983.9866, 1985.8589, 1995.9136, 2027.1289, 2067.1079, 2110.0598, 2136.8484, 2144.0432, 2150.8660, 2164.8794, 2211.0857, 2239.1533, 2251.1509, 2270.1113, 2273.0972, 2274.0605, 2286.1492, 2289.0857, 2290.0530, 2293.1558, 2309.1570, 2338.1033, 2340.0979, 2343.1770, 2352.9316, 2375.0454, 2383.9480, 2414.2512, 2499.2480, 2542.2678, 2589.9707, 2600.0022, 2603.9897, 2613.9939, 2627.9993, 2644.0112, 2646.9875, 2657.4248, 2659.0405, 2661.0017, 2665.0042, 2674.0601, 2675.0151, 2678.0159, 2689.0330, 2692.0283, 2695.0315, 2703.0417, 2706.0959, 2763.3606, 2798.2788, 2815.3203, 2846.4011, 2872.3391, 2876.3679, 2884.3535, 2888.3455, 2891.4131, 2892.4519, 2919.4543, 3002.3042, 3012.4717, 3016.2314, 3020.2483, 3030.3025, 3033.2446, 3053.4917, 3081.6296, 3126.7493, 3171.7131, 3184.4602, 3206.6179, 3207.6111, 3248.6445, 3254.6370, 3265.6309, 3300.7825, 3317.7288, 3321.6318, 3334.6953, 3347.5437, 3429.5991, 3457.6255, 3471.6121, 3503.8608, 3518.5715, 3639.7969, 4148.0293, 4162.0039, 4453.2720, 4844.2046

43. P79134 **Mass:** 76202 **Score:** 18 **Expect:** 3.9e+002 **Matches:** 12
Annexin A6 OS=Bos taurus GN=ANXA6 PE=1 SV=2

Observed	Mr(expt)	Mr(calc)	ppm	Start	End	Miss	Peptide
710.3790	709.3718	709.3646	10.0	76	81	0	K.YELTGK.F
1026.5627	1025.5554	1025.5433	11.8	213	220	0	R.LVFDEYLK.T
1103.5287	1102.5214	1102.4973	21.8	547	554	0	R.FMMILCTR.S + 2 Oxidation (M)
1333.7570	1332.7497	1332.7184	23.5	271	281	1	R.DNTLIRIMVSR.S + Oxidation (M)
1463.7950	1462.7877	1462.7779	6.69	466	478	1	R.TNAEIQAINKAYK.E
1494.7383	1493.7310	1493.7184	8.44	569	580	1	K.MTNYDVEHTIKK.E + Oxidation (M)
1909.9886	1908.9813	1908.9516	15.6	403	418	2	R.QTFKSHFGRDLMADLK.S + Oxidation (M)
2027.1289	2026.1216	2026.0986	11.4	64	81	2	K.SLYGKDLIADLKYELTGK.F
2239.1533	2238.1460	2238.1500	-1.79	277	294	2	R.IMVSRSELDMLDIREIFR.T + Oxidation (M)
2255.1836	2254.1763	2254.1449	13.9	277	294	2	R.IMVSRSELDMLDIREIFR.T + 2 Oxidation (M)
2414.2512	2413.2439	2413.2100	14.1	561	579	2	R.RVFQEFIKMTNYDVEHTIK.K + Oxidation (M)
4844.2046	4843.1973	4843.2988	-20.95	167	208	2	R.EEDDVVSEDLVQQDVQDLYEAGELKWGTDEAQFIYILGNRSK.Q

No match to: 706.3098, 712.2532, 714.3089, 717.3984, 719.4317, 720.4312, 722.4346, 735.4181, 749.4561, 756.4000, 759.4391, 772.4356, 774.4578, 778.4783, 779.4789, 818.4724, 823.4901, 831.4754, 832.3123, 834.3210, 842.5065, 844.4971, 870.5016, 871.4923, 873.3899, 876.2862, 880.4520, 883.4794, 891.4870, 895.5731, 900.5293, 909.5164, 913.4845, 918.5409, 923.5157, 925.4807, 927.5140, 929.4449, 942.5470, 944.5204, 945.5183, 949.5508, 950.5472, 967.4262, 972.4835, 974.4452, 976.4556, 977.4954, 978.5431, 988.4708, 1007.5730, 1016.5761, 1031.5581, 1034.5808, 1044.5240, 1051.5095, 1056.5281, 1057.5575, 1066.5067, 1074.5374, 1087.5460, 1118.5885, 1122.5553, 1148.5439, 1168.5985, 1178.6211, 1185.6898, 1244.6118, 1248.6686, 1253.6313, 1255.6216, 1301.6338, 1312.7423, 1313.7227, 1314.7188, 1315.6721, 1317.6354, 1320.6331, 1322.6371, 1325.6360, 1327.6959, 1336.7218, 1338.7179, 1339.7092, 1343.6960, 1349.6917, 1350.6987, 1351.6946, 1361.6875, 1372.5978, 1381.7642, 1382.5950, 1383.7253, 1397.7454, 1439.7549, 1442.8190, 1457.7394, 1488.7245, 1497.8735, 1504.7484, 1513.7760, 1517.8062, 1520.7523, 1528.6951, 1537.8245, 1539.8104, 1549.8242, 1552.8073, 1553.8669, 1555.8348, 1556.8799, 1567.7986, 1570.7749, 1578.7872, 1592.7849, 1628.7002, 1628.8571, 1638.8861, 1642.7155, 1647.8503, 1650.8458, 1664.8571, 1680.8226, 1696.8146, 1698.7845, 1699.8228, 1705.8712, 1707.8536, 1715.8536, 1717.9056, 1730.8564, 1739.6990, 1771.0344, 1786.9199, 1809.0262, 1816.9354, 1820.8855, 1836.8507, 1907.9603, 1928.0238, 1940.0148, 1953.8596, 1961.0111, 1965.8679, 1974.9255, 1982.0082, 1983.9866, 1985.8589, 1995.9136, 2067.1079, 2110.0598, 2136.8484, 2144.0432, 2150.8660, 2164.8794, 2211.0857, 2251.1509, 2270.1113, 2273.0972, 2274.0605, 2286.1492, 2289.0857, 2290.0530, 2293.1558, 2309.1570, 2324.0981, 2338.1033, 2340.0979, 2343.1770, 2352.9316, 2375.0454, 2383.9480, 2499.2480, 2542.2678, 2589.9707, 2600.0022, 2603.9897, 2613.9939, 2627.9993, 2644.0112, 2646.9875, 2657.4248, 2659.0405, 2661.0017, 2665.0042, 2674.0601, 2675.0151, 2678.0159, 2689.0330, 2692.0283, 2695.0315, 2703.0417, 2706.0959, 2763.3606, 2798.2788, 2815.3203, 2846.4011, 2872.3391, 2876.3679, 2884.3535, 2888.3455, 2891.4131, 2892.4519, 2919.4543, 3002.3042, 3012.4717, 3016.2314, 3020.2483, 3030.3025, 3033.2446, 3053.4917, 3081.6296, 3126.7493, 3171.7131, 3184.4602, 3206.6179, 3207.6111, 3248.6445, 3254.6370, 3265.6309, 3300.7825, 3317.7288, 3321.6318, 3334.6953, 3347.5437, 3429.5991, 3457.6255, 3471.6121, 3489.7363, 3503.8608, 3518.5715, 3639.7969, 4148.0293, 4162.0039, 4453.2720

44. F1MZH9 **Mass:** 51716 **Score:** 18 **Expect:** 4e+002 **Matches:** 4
Probable proline dehydrogenase 2 OS=Bos taurus GN=PRODH2 PE=4 SV=2

Observed	Mr(expt)	Mr(calc)	ppm	Start	End	Miss	Peptide
1034.5808	1033.5735	1033.5920	-17.89	302	310	1	K.LVRGAYLDK.E
1442.8190	1441.8117	1441.8015	7.12	221	232	1	R.LHRVVQHAQAQR.V
1556.8799	1555.8726	1555.8430	19.0	439	450	2	R.EQELLSQELRRR.L
3334.6953	3333.6880	3333.7247	-11.00	161	189	2	K.MTALMSTRLCKQLTSWVLRPGDSLELSPK.R + Oxidation (M)

No match to: 706.3098, 710.3790, 712.2532, 714.3089, 717.3984, 719.4317, 720.4312, 722.4346, 735.4181, 749.4561, 756.4000, 759.4391, 772.4356, 774.4578, 778.4783, 779.4789, 818.4724, 823.4901, 831.4754, 832.3123, 834.3210, 842.5065, 844.4971, 870.5016, 871.4923, 873.3899, 876.2862, 880.4520, 883.4794, 891.4870, 895.5731, 900.5293, 909.5164, 913.4845, 918.5409, 923.5157, 925.4807, 927.5140, 929.4449, 942.5470, 944.5204, 945.5183, 949.5508, 950.5472, 967.4262, 972.4835, 974.4452, 976.4556, 977.4954, 978.5431, 988.4708, 1007.5730, 1016.5761, 1026.5627, 1031.5581, 1044.5240, 1051.5095, 1056.5281, 1057.5575, 1066.5067, 1074.5374, 1087.5460, 1103.5287, 1118.5885, 1122.5553, 1148.5439, 1168.5985, 1178.6211, 1185.6898, 1244.6118, 1248.6686, 1253.6313, 1255.6216, 1301.6338, 1312.7423, 1313.7227, 1314.7188, 1315.6721, 1317.6354, 1320.6331, 1322.6371, 1325.6360, 1327.6959, 1333.7570, 1336.7218, 1338.7179, 1339.7092, 1343.6960, 1349.6917, 1350.6987, 1351.6946, 1361.6875, 1372.5978, 1381.7642, 1382.5950, 1383.7253, 1397.7454, 1439.7549, 1457.7394, 1463.7950, 1488.7245, 1494.7383, 1497.8735, 1504.7484, 1513.7760, 1517.8062, 1520.7523, 1528.6951, 1537.8245, 1539.8104, 1549.8242, 1552.8073, 1553.8669, 1555.8348, 1567.7986, 1570.7749, 1578.7872, 1592.7849, 1628.7002, 1628.8571, 1638.8861, 1642.7155, 1647.8503, 1650.8458, 1664.8571, 1680.8226, 1696.8146, 1698.7845, 1699.8228, 1705.8712, 1707.8536, 1715.8536, 1717.9056, 1730.8564, 1739.6990, 1771.0344, 1786.9199, 1809.0262, 1816.9354, 1820.8855, 1836.8507, 1907.9603, 1909.9886, 1928.0238, 1940.0148, 1953.8596, 1961.0111, 1965.8679, 1974.9255, 1982.0082, 1983.9866, 1985.8589, 1995.9136, 2027.1289, 2067.1079, 2110.0598, 2136.8484, 2144.0432, 2150.8660, 2164.8794, 2211.0857, 2239.1533, 2251.1509, 2255.1836, 2270.1113, 2273.0972, 2274.0605, 2286.1492, 2289.0857, 2290.0530, 2293.1558, 2309.1570, 2324.0981, 2338.1033, 2340.0979, 2343.1770, 2352.9316, 2375.0454, 2383.9480, 2414.2512, 2499.2480, 2542.2678, 2589.9707, 2600.0022, 2603.9897, 2613.9939, 2627.9993, 2644.0112, 2646.9875, 2657.4248, 2659.0405, 2661.0017, 2665.0042, 2674.0601, 2675.0151, 2678.0159, 2689.0330, 2692.0283, 2695.0315, 2703.0417, 2706.0959, 2763.3606, 2798.2788, 2815.3203, 2846.4011, 2872.3391, 2876.3679, 2884.3535, 2888.3455, 2891.4131, 2892.4519, 2919.4543, 3002.3042, 3012.4717, 3016.2314, 3020.2483, 3030.3025, 3033.2446, 3053.4917, 3081.6296, 3126.7493, 3171.7131, 3184.4602, 3206.6179, 3207.6111, 3248.6445, 3254.6370, 3265.6309, 3300.7825, 3317.7288, 3321.6318, 3347.5437, 3429.5991, 3457.6255, 3471.6121, 3489.7363, 3503.8608, 3518.5715, 3639.7969, 4148.0293, 4162.0039, 4453.2720, 4844.2046

45. A7E321 **Mass:** 51226 **Score:** 18 **Expect:** 4e+002 **Matches:** 8
PNMA-like protein 1 OS=Bos taurus GN=PNMAL1 PE=2 SV=1

Observed	Mr(expt)	Mr(calc)	ppm	Start	End	Miss	Peptide
883.4794	882.4722	882.4599	13.8	250	256	0	K.QEPVPWK.K
1570.7749	1569.7676	1569.7432	15.6	343	356	1	K.AMGWVSAKSPAYMR.K + Oxidation (M)
1642.7155	1641.7082	1641.6949	8.11	1	13	0	-.MTMAANLLEDWCR.G + 2 Oxidation (M)
2290.0530	2289.0457	2289.0734	-12.08	257	278	2	K.KSQGSHSHSSASLKHPEADDGK.N
2798.2788	2797.2715	2797.2912	-7.02	433	457	2	R.SHLGCVNKWMEGEEQQGKAGAQEPK.W

2919.4543	2918.4470	2918.4159	10.7	312 - 340	1	K.FPSNLPHVAASRGVASESDQDGGLEGPPK.K
3334.6953	3333.6880	3333.7204	-9.72	107 - 135	2	K.NLNEFLDAEERTLEDVVHLLELSRASPPK.T
4844.2046	4843.1973	4843.2782	-16.70	384 - 432	2	K.KGPGSGQDAPDQKAPGVPQAHESPTSASQGPEAKPQSPLHASSGENDGR.S

No match to: 706.3098, 710.3790, 712.2532, 714.3089, 717.3984, 719.4317, 720.4312, 722.4346, 735.4181, 749.4561, 756.4000, 759.4391, 772.4356, 774.4578, 778.4783, 779.4789, 818.4724, 823.4901, 831.4754, 832.3123, 834.3210, 842.5065, 844.4971, 870.5016, 871.4923, 873.3899, 876.2862, 880.4520, 891.4870, 895.5731, 900.5293, 909.5164, 913.4845, 918.5409, 923.5157, 925.4807, 927.5140, 929.4449, 942.5470, 944.5204, 945.5183, 949.5508, 950.5472, 967.4262, 972.4835, 974.4452, 976.4556, 977.4954, 978.5431, 988.4708, 1007.5730, 1016.5761, 1026.5627, 1031.5581, 1034.5808, 1044.5240, 1051.5095, 1056.5281, 1057.5575, 1066.5067, 1074.5374, 1087.5460, 1103.5287, 1118.5885, 1122.5553, 1148.5439, 1168.5985, 1178.6211, 1185.6898, 1244.6118, 1248.6686, 1253.6313, 1255.6216, 1301.6338, 1312.7423, 1313.7227, 1314.7188, 1315.6721, 1317.6354, 1320.6331, 1322.6371, 1325.6360, 1327.6959, 1333.7570, 1336.7218, 1338.7179, 1339.7092, 1343.6960, 1349.6917, 1350.6987, 1351.6946, 1361.6875, 1372.5978, 1381.7642, 1382.5950, 1383.7253, 1397.7454, 1439.7549, 1442.8190, 1457.7394, 1463.7950, 1488.7245, 1494.7383, 1497.8735, 1504.7484, 1513.7760, 1517.8062, 1520.7523, 1528.6951, 1537.8245, 1539.8104, 1549.8242, 1552.8073, 1553.8669, 1555.8348, 1556.8799, 1567.7986, 1578.7872, 1592.7849, 1628.7002, 1628.8571, 1638.8861, 1647.8503, 1650.8458, 1664.8571, 1680.8226, 1696.8146, 1698.7845, 1699.8228, 1705.8712, 1707.8536, 1715.8536, 1717.9056, 1730.8564, 1739.6990, 1771.0344, 1786.9199, 1809.0262, 1816.9354, 1820.8855, 1836.8507, 1907.9603, 1909.9886, 1928.0238, 1940.0148, 1953.8596, 1961.0111, 1965.8679, 1974.9255, 1982.0082, 1983.9866, 1985.8589, 1995.9136, 2027.1289, 2067.1079, 2110.0598, 2136.8484, 2144.0432, 2150.8660, 2164.8794, 2211.0857, 2239.1533, 2251.1509, 2255.1836, 2270.1113, 2273.0972, 2274.0605, 2286.1492, 2289.0857, 2293.1558, 2309.1570, 2324.0981, 2338.1033, 2340.0979, 2343.1770, 2352.9316, 2375.0454, 2383.9480, 2414.2512, 2499.2480, 2542.2678, 2589.9707, 2600.0022, 2603.9897, 2613.9939, 2627.9993, 2644.0112, 2646.9875, 2657.4248, 2659.0405, 2661.0017, 2665.0042, 2674.0601, 2675.0151, 2678.0159, 2689.0330, 2692.0283, 2695.0315, 2703.0417, 2706.0959, 2763.3606, 2815.3203, 2846.4011, 2872.3391, 2876.3679, 2884.3535, 2888.3455, 2891.4131, 2892.4519, 3002.3042, 3012.4717, 3016.2314, 3020.2483, 3030.3025, 3033.2446, 3053.4917, 3081.6296, 3126.7493, 3171.7131, 3184.4602, 3206.6179, 3207.6111, 3248.6445, 3254.6370, 3265.6309, 3300.7825, 3317.7288, 3321.6318, 3347.5437, 3429.5991, 3457.6255, 3471.6121, 3489.7363, 3503.8608, 3518.5715, 3639.7969, 4148.0293, 4162.0039, 4453.2720

46. Q9GKN8 **Mass:** 44054 **Score:** 18 **Expect:** 4e+002 **Matches:** 16

Prolargin OS=Bos taurus GN=PRELP PE=2 SV=1

Observed	Mr(expt)	Mr(calc)	ppm	Start - End	Miss	Peptide
772.4356	771.4283	771.4239	5.70	171 - 176	0	R.NLEQLR.L
844.4971	843.4899	843.4854	5.23	185 - 192	0	R.IPPGVFSK.L
895.5731	894.5658	894.5651	0.79	95 - 102	1	R.KVPVIPSR.I
945.5183	944.5110	944.5039	7.53	177 - 184	0	R.LSQNQISR.I
1016.5761	1015.5688	1015.5662	2.59	302 - 311	0	R.ISSVPAISSR.L
1034.5808	1033.5735	1033.5709	2.55	263 - 271	0	K.GFPNLAFIR.L
1044.5240	1043.5167	1043.5148	1.83	129 - 136	0	R.WINLDNNR.I
1122.5553	1121.5480	1121.5465	1.35	272 - 280	0	R.LNYNQLSDR.G
1313.7227	1312.7154	1312.7000	11.8	129 - 138	1	R.WINLDNNRIR.K
1336.7218	1335.7145	1335.7146	-0.08	159 - 170	0	K.NQLEEVPAALPR.N
1517.8062	1516.7989	1516.7998	-0.55	272 - 284	1	R.LNYNQLSDRGLPK.N
1537.8245	1536.8172	1536.8194	-1.44	221 - 233	0	K.NLMQLNLAHNTLR.K
2136.8484	2135.8411	2135.8387	1.14	75 - 91	0	R.ECYCPPDFPSALYCDSR.N
2293.1558	2292.1485	2292.1469	0.72	356 - 374	0	R.LDGNYLKPPIPLDLMMCFR.L
2309.1570	2308.1497	2308.1418	3.44	356 - 374	0	R.LDGNYLKPPIPLDLMMCFR.L + Oxidation (M)
3171.7131	3170.7058	3170.6586	14.9	235 - 262	2	K.MPPKVPSAIHQLYLDSNRIEAIPSGYFK.G

No match to: 706.3098, 710.3790, 712.2532, 714.3089, 717.3984, 719.4317, 720.4312, 722.4346, 735.4181, 749.4561, 756.4000, 759.4391, 774.4578, 778.4783, 779.4789, 818.4724, 823.4901, 831.4754, 832.3123, 834.3210, 842.5065, 870.5016, 871.4923, 873.3899, 876.2862, 880.4520, 883.4794, 891.4870, 900.5293, 909.5164, 913.4845, 918.5409, 923.5157, 925.4807, 927.5140, 929.4449, 942.5470, 944.5204, 949.5508, 950.5472, 967.4262, 972.4835, 974.4452, 976.4556, 977.4954, 978.5431, 988.4708, 1007.5730, 1026.5627, 1031.5581, 1051.5095, 1056.5281, 1057.5575, 1066.5067, 1074.5374, 1087.5460, 1103.5287, 1118.5885, 1148.5439, 1168.5985, 1178.6211, 1185.6898, 1244.6118, 1248.6686, 1253.6313, 1255.6216, 1301.6338, 1312.7423, 1314.7188, 1315.6721, 1317.6354, 1320.6331, 1322.6371, 1325.6360, 1327.6959, 1333.7570, 1338.7179, 1339.7092, 1343.6960, 1349.6917, 1350.6987, 1351.6946, 1361.6875, 1372.5978, 1381.7642, 1382.5950, 1383.7253, 1397.7454, 1439.7549, 1442.8190, 1457.7394, 1463.7950, 1488.7245, 1494.7383, 1497.8735, 1504.7484, 1513.7760, 1520.7523, 1528.6951, 1539.8104, 1549.8242, 1552.8073, 1553.8669, 1555.8348, 1556.8799, 1567.7986, 1570.7749, 1578.7872, 1592.7849, 1628.7002, 1628.8571, 1638.8861, 1642.7155, 1647.8503, 1650.8458, 1664.8571, 1680.8226, 1696.8146, 1698.7845, 1699.8228, 1705.8712, 1707.8536, 1715.8536, 1717.9056, 1730.8564, 1739.6990, 1771.0344, 1786.9199, 1809.0262, 1816.9354, 1820.8855, 1836.8507, 1907.9603, 1909.9886, 1928.0238, 1940.0148, 1953.8596, 1961.0111, 1965.8679, 1974.9255, 1982.0082, 1983.9866, 1985.8589, 1995.9136, 2027.1289, 2067.1079, 2110.0598, 2144.0432, 2150.8660, 2164.8794, 2211.0857, 2239.1533, 2251.1509, 2255.1836, 2270.1113, 2273.0972, 2274.0605, 2286.1492, 2289.0857, 2290.0530, 2324.0981, 2338.1033, 2340.0979, 2343.1770, 2352.9316, 2375.0454, 2383.9480, 2414.2512, 2499.2480, 2542.2678, 2589.9707, 2600.0022, 2603.9897, 2613.9939, 2627.9993, 2644.0112, 2646.9875, 2657.4248, 2659.0405, 2661.0017, 2665.0042, 2674.0601, 2675.0151, 2678.0159, 2689.0330, 2692.0283, 2695.0315, 2703.0417, 2706.0959, 2763.3606, 2798.2788, 2815.3203, 2846.4011, 2872.3391, 2876.3679, 2884.3535, 2888.3455, 2891.4131, 2892.4519, 2919.4543, 3002.3042, 3012.4717, 3016.2314, 3020.2483, 3030.3025, 3033.2446, 3053.4917, 3081.6296, 3126.7493, 3184.4602, 3206.6179, 3207.6111, 3248.6445, 3254.6370, 3265.6309, 3300.7825, 3317.7288, 3321.6318, 3334.6953, 3347.5437, 3429.5991, 3457.6255, 3471.6121, 3489.7363, 3503.8608, 3518.5715, 3639.7969, 4148.0293, 4162.0039, 4453.2720, 4844.2046

47. Q08DB0 **Mass:** 44179 **Score:** 18 **Expect:** 4.2e+002 **Matches:** 7

Centrosomal protein of 44 kDa OS=Bos taurus GN=CEP44 PE=2 SV=1

Observed	Mr(expt)	Mr(calc)	ppm	Start - End	Miss	Peptide
749.4561	748.4488	748.4443	6.01	135 - 141	2	R.KKTSSAK.S
871.4923	870.4850	870.4923	-8.41	12 - 18	0	R.NLEQVLR.S
891.4870	890.4797	890.4644	17.2	1 - 8	1	-.MATGDLKR.S
1122.5553	1121.5480	1121.5638	-14.05	255 - 263	1	R.LESLEEKMK.G + Oxidation (M)
1338.7179	1337.7106	1337.6900	15.4	371 - 381	1	K.KMFEETAELLK.C
1383.7253	1382.7180	1382.7194	-1.00	69 - 79	1	K.NDLRFIDTVYK.L
1982.0082	1981.0009	1980.9860	7.55	248 - 263	2	K.LTCIESRLESLEEKMK.G + Oxidation (M)

No match to: 706.3098, 710.3790, 712.2532, 714.3089, 717.3984, 719.4317, 720.4312, 722.4346, 735.4181, 756.4000, 759.4391, 772.4356, 774.4578, 778.4783, 779.4789, 818.4724, 823.4901, 831.4754, 832.3123, 834.3210, 842.5065, 844.4971, 870.5016, 873.3899, 876.2862, 880.4520, 883.4794, 895.5731, 900.5293, 909.5164, 913.4845, 918.5409, 923.5157, 925.4807, 927.5140, 929.4449, 942.5470, 944.5204, 945.5183, 949.5508, 950.5472, 967.4262, 972.4835, 974.4452, 976.4556, 977.4954, 978.5431, 988.4708, 1007.5730, 1016.5761, 1026.5627, 1031.5581, 1034.5808, 1044.5240, 1051.5095, 1056.5281, 1057.5575, 1066.5067, 1074.5374, 1087.5460, 1103.5287, 1118.5885, 1148.5439, 1168.5985, 1178.6211, 1185.6898, 1244.6118, 1248.6686, 1253.6313, 1255.6216, 1301.6338, 1312.7423, 1313.7227, 1314.7188, 1315.6721, 1317.6354, 1320.6331, 1322.6371, 1325.6360, 1327.6959, 1333.7570, 1336.7218, 1339.7092, 1343.6960, 1349.6917, 1350.6987, 1351.6946, 1361.6875, 1372.5978, 1381.7642, 1382.5950, 1397.7454, 1439.7549, 1442.8190, 1457.7394, 1463.7950, 1488.7245, 1494.7383, 1497.8735, 1504.7484, 1513.7760, 1517.8062, 1520.7523, 1528.6951, 1537.8245, 1539.8104, 1549.8242, 1552.8073, 1553.8669, 1555.8348, 1556.8799, 1567.7986, 1570.7749, 1578.7872, 1592.7849, 1628.7002, 1628.8571, 1638.8861, 1642.7155, 1647.8503, 1650.8458, 1664.8571, 1680.8226, 1696.8146, 1698.7845, 1699.8228, 1705.8712, 1707.8536, 1715.8536, 1717.9056, 1730.8564, 1739.6990, 1771.0344, 1786.9199, 1809.0262, 1816.9354, 1820.8855, 1836.8507, 1907.9603, 1909.9886, 1928.0238, 1940.0148, 1953.8596, 1961.0111, 1965.8679, 1974.9255, 1983.9866, 1985.8589, 1995.9136, 2027.1289, 2067.1079, 2110.0598, 2136.8484, 2144.0432, 2150.8660, 2164.8794, 2211.0857, 2239.1533, 2251.1509, 2255.1836, 2270.1113, 2273.0972, 2274.0605, 2286.1492, 2289.0857, 2290.0530, 2293.1558, 2309.1570, 2324.0981, 2338.1033, 2340.0979, 2343.1770, 2352.9316, 2375.0454, 2383.9480, 2414.2512, 2499.2480, 2542.2678, 2589.9707, 2600.0022, 2603.9897, 2613.9939, 2627.9993, 2644.0112, 2646.9875, 2657.4248, 2659.0405, 2661.0017, 2665.0042, 2674.0601, 2675.0151, 2678.0159, 2689.0330, 2692.0283, 2695.0315, 2703.0417, 2706.0959, 2763.3606, 2798.2788, 2815.3203, 2846.4011, 2872.3391, 2876.3679, 2884.3535, 2888.3455, 2891.4131, 2892.4519, 2919.4543, 3002.3042, 3012.4717, 3016.2314, 3020.2483, 3030.3025, 3033.2446, 3053.4917, 3081.6296, 3126.7493, 3171.7131, 3184.4602, 3206.6179, 3207.6111, 3248.6445, 3254.6370, 3265.6309, 3300.7825, 3317.7288, 3321.6318, 3334.6953, 3347.5437, 3429.5991, 3457.6255, 3471.6121, 3489.7363, 3503.8608, 3518.5715, 3639.7969, 4148.0293, 4162.0039, 4453.2720, 4844.2046

48. F1N402 **Mass:** 44161 **Score:** 18 **Expect:** 4.2e+002 **Matches:** 7

Centrosomal protein of 44 kDa OS=Bos taurus GN=CEP44 PE=4 SV=2

Observed	Mr(expt)	Mr(calc)	ppm	Start	End	Miss	Peptide
749.4561	748.4488	748.4443	6.01	135 -	141	2	R.KKTSSAK.S
871.4923	870.4850	870.4923	-8.41	12 -	18	0	R.NLEQVLR.S
891.4870	890.4797	890.4644	17.2	1 -	8	1	-.MATGDLKR.S
1122.5553	1121.5480	1121.5638	-14.05	255 -	263	1	R.LESLEEKMK.G + Oxidation (M)
1338.7179	1337.7106	1337.6900	15.4	371 -	381	1	K.KMFEETAELLK.C
1383.7253	1382.7180	1382.7194	-1.00	69 -	79	1	K.NDLRFIDTVYK.L
1982.0082	1981.0009	1980.9860	7.55	248 -	263	2	K.LTCIESRLESLEEKMK.G + Oxidation (M)

No match to: 706.3098, 710.3790, 712.2532, 714.3089, 717.3984, 719.4317, 720.4312, 722.4346, 735.4181, 756.4000, 759.4391, 772.4356, 774.4578, 778.4783, 779.4789, 818.4724, 823.4901, 831.4754, 832.3123, 834.3210, 842.5065, 844.4971, 870.5016, 873.3899, 876.2862, 880.4520, 883.4794, 895.5731, 900.5293, 909.5164, 913.4845, 918.5409, 923.5157, 925.4807, 927.5140, 929.4449, 942.5470, 944.5204, 945.5183, 949.5508, 950.5472, 967.4262, 972.4835, 974.4452, 976.4556, 977.4954, 978.5431, 988.4708, 1007.5730, 1016.5761, 1026.5627, 1031.5581, 1034.5808, 1044.5240, 1051.5095, 1056.5281, 1057.5575, 1066.5067, 1074.5374, 1087.5460, 1103.5287, 1118.5885, 1148.5439, 1168.5985, 1178.6211, 1185.6898, 1244.6118, 1248.6686, 1253.6313, 1255.6216, 1301.6338, 1312.7423, 1313.7227, 1314.7188, 1315.6721, 1317.6354, 1320.6331, 1322.6371, 1325.6360, 1327.6959, 1333.7570, 1336.7218, 1339.7092, 1343.6960, 1349.6917, 1350.6987, 1351.6946, 1361.6875, 1372.5978, 1381.7642, 1382.5950, 1397.7454, 1439.7549, 1442.8190, 1457.7394, 1463.7950, 1488.7245, 1494.7383, 1497.8735, 1504.7484, 1513.7760, 1517.8062, 1520.7523, 1528.6951, 1537.8245, 1539.8104, 1549.8242, 1552.8073, 1553.8669, 1555.8348, 1556.8799, 1567.7986, 1570.7749, 1578.7872, 1592.7849, 1628.7002, 1628.8571, 1638.8861, 1642.7155, 1647.8503, 1650.8458, 1664.8571, 1680.8226, 1696.8146, 1698.7845, 1699.8228, 1705.8712, 1707.8536, 1715.8536, 1717.9056, 1730.8564, 1739.6990, 1771.0344, 1786.9199, 1809.0262, 1816.9354, 1820.8855, 1836.8507, 1907.9603, 1909.9886, 1928.0238, 1940.0148, 1953.8596, 1961.0111, 1965.8679, 1974.9255, 1983.9866, 1985.8589, 1995.9136, 2027.1289, 2067.1079, 2110.0598, 2136.8484, 2144.0432, 2150.8660, 2164.8794, 2211.0857, 2239.1533, 2251.1509, 2255.1836, 2270.1113, 2273.0972, 2274.0605, 2286.1492, 2289.0857, 2290.0530, 2293.1558, 2309.1570, 2324.0981, 2338.1033, 2340.0979, 2343.1770, 2352.9316, 2375.0454, 2383.9480, 2414.2512, 2499.2480, 2542.2678, 2589.9707, 2600.0022, 2603.9897, 2613.9939, 2627.9993, 2644.0112, 2646.9875, 2657.4248, 2659.0405, 2661.0017, 2665.0042, 2674.0601, 2675.0151, 2678.0159, 2689.0330, 2692.0283, 2695.0315, 2703.0417, 2706.0959, 2763.3606, 2798.2788, 2815.3203, 2846.4011, 2872.3391, 2876.3679, 2884.3535, 2888.3455, 2891.4131, 2892.4519, 2919.4543, 3002.3042, 3012.4717, 3016.2314, 3020.2483, 3030.3025, 3033.2446, 3053.4917, 3081.6296, 3126.7493, 3171.7131, 3184.4602, 3206.6179, 3207.6111, 3248.6445, 3254.6370, 3265.6309, 3300.7825, 3317.7288, 3321.6318, 3334.6953, 3347.5437, 3429.5991, 3457.6255, 3471.6121, 3489.7363, 3503.8608, 3518.5715, 3639.7969, 4148.0293, 4162.0039, 4453.2720, 4844.2046

49. Q58DS5 **Mass:** 23184 **Score:** 18 **Expect:** 4.2e+002 **Matches:** 4

Ras-related protein Rab-13 OS=Bos taurus GN=RAB13 PE=1 SV=1

Observed	Mr(expt)	Mr(calc)	ppm	Start	End	Miss	Peptide
945.5183	944.5110	944.4927	19.4	131 -	138	1	K.VQKEQADK.L
2324.0981	2323.0908	2323.0790	5.07	147 -	167	1	R.FFETSAKSSMNVDEAFSSLAR.D
2340.0979	2339.0906	2339.0740	7.12	147 -	167	1	R.FFETSAKSSMNVDEAFSSLAR.D + Oxidation (M)
3248.6445	3247.6372	3247.6149	6.86	28 -	55	2	R.FAEDNFNNTYISTIGIDFRIRTVDIEGK.K

No match to: 706.3098, 710.3790, 712.2532, 714.3089, 717.3984, 719.4317, 720.4312, 722.4346, 735.4181, 749.4561, 756.4000, 759.4391, 772.4356, 774.4578, 778.4783, 779.4789, 818.4724, 823.4901, 831.4754, 832.3123, 834.3210, 842.5065, 844.4971, 870.5016, 871.4923, 873.3899, 876.2862, 880.4520, 883.4794, 891.4870, 895.5731, 900.5293, 909.5164, 913.4845, 918.5409, 923.5157, 925.4807, 927.5140, 929.4449, 942.5470, 944.5204, 949.5508, 950.5472, 967.4262, 972.4835, 974.4452, 976.4556, 977.4954, 978.5431, 988.4708, 1007.5730, 1016.5761, 1026.5627, 1031.5581, 1034.5808, 1044.5240, 1051.5095, 1056.5281, 1057.5575, 1066.5067, 1074.5374, 1087.5460, 1103.5287, 1118.5885, 1122.5553, 1148.5439, 1168.5985, 1178.6211, 1185.6898, 1244.6118, 1248.6686, 1253.6313, 1255.6216, 1301.6338, 1312.7423, 1313.7227, 1314.7188, 1315.6721, 1317.6354, 1320.6331, 1322.6371, 1325.6360, 1327.6959, 1333.7570, 1336.7218, 1338.7179, 1339.7092, 1343.6960, 1349.6917, 1350.6987, 1351.6946, 1361.6875, 1372.5978, 1381.7642, 1382.5950, 1383.7253, 1397.7454, 1439.7549, 1442.8190, 1457.7394, 1463.7950, 1488.7245, 1494.7383, 1497.8735, 1504.7484, 1513.7760, 1517.8062, 1520.7523, 1528.6951, 1537.8245, 1539.8104, 1549.8242, 1552.8073, 1553.8669, 1555.8348, 1556.8799, 1567.7986, 1570.7749, 1578.7872, 1592.7849, 1628.7002, 1628.8571, 1638.8861, 1642.7155, 1647.8503, 1650.8458, 1664.8571, 1680.8226, 1696.8146, 1698.7845, 1699.8228, 1705.8712, 1707.8536, 1715.8536, 1717.9056, 1730.8564, 1739.6990, 1771.0344, 1786.9199, 1809.0262, 1816.9354, 1820.8855, 1836.8507, 1907.9603, 1909.9886, 1928.0238, 1940.0148, 1953.8596, 1961.0111, 1965.8679, 1974.9255, 1982.0082, 1983.9866, 1985.8589, 1995.9136, 2027.1289, 2067.1079, 2110.0598, 2136.8484, 2144.0432, 2150.8660, 2164.8794, 2211.0857, 2239.1533, 2251.1509, 2255.1836, 2270.1113, 2273.0972, 2274.0605, 2286.1492, 2289.0857, 2290.0530, 2293.1558, 2309.1570, 2338.1033, 2343.1770, 2352.9316, 2375.0454, 2383.9480, 2414.2512, 2499.2480, 2542.2678, 2589.9707, 2600.0022, 2603.9897, 2613.9939, 2627.9993, 2644.0112, 2646.9875, 2657.4248, 2659.0405, 2661.0017, 2665.0042, 2674.0601, 2675.0151, 2678.0159, 2689.0330, 2692.0283, 2695.0315, 2703.0417, 2706.0959, 2763.3606, 2798.2788, 2815.3203, 2846.4011, 2872.3391, 2876.3679, 2884.3535, 2888.3455, 2891.4131, 2892.4519, 2919.4543, 3002.3042, 3012.4717, 3016.2314, 3020.2483, 3030.3025, 3033.2446, 3053.4917, 3081.6296, 3126.7493, 3171.7131, 3184.4602, 3206.6179, 3207.6111, 3254.6370, 3265.6309, 3300.7825, 3317.7288, 3321.6318, 3334.6953, 3347.5437, 3429.5991, 3457.6255, 3471.6121, 3489.7363, 3503.8608, 3518.5715, 3639.7969, 4148.0293, 4162.0039, 4453.2720, 4844.2046

50. F1MIN9 **Mass:** 51015 **Score:** 17 **Expect:** 4.4e+002 **Matches:** 3

Uncharacterized protein OS=Bos taurus GN=ZNRF4 PE=4 SV=1

Observed	Mr(expt)	Mr(calc)	ppm	Start	End	Miss	Peptide
891.4870	890.4797	890.4644	17.2	261 -	267	1	R.VITRCDK.A
978.5431	977.5358	977.5407	-4.96	334 -	341	1	R.AQVRTFTR.C
2846.4011	2845.3938	2845.4287	-12.25	1 -	27	2	-.NRTMSQSLGASRTLGAPGLSVICQEGR.L

No match to: 706.3098, 710.3790, 712.2532, 714.3089, 717.3984, 719.4317, 720.4312, 722.4346, 735.4181, 749.4561, 756.4000, 759.4391, 772.4356, 774.4578, 778.4783, 779.4789, 818.4724, 823.4901, 831.4754, 832.3123, 834.3210, 842.5065, 844.4971, 870.5016, 871.4923, 873.3899, 876.2862, 880.4520, 883.4794, 895.5731, 900.5293, 909.5164, 913.4845, 918.5409, 923.5157, 925.4807, 927.5140, 929.4449, 942.5470, 944.5204, 945.5183, 949.5508, 950.5472, 967.4262, 972.4835, 974.4452, 976.4556, 977.4954, 988.4708, 1007.5730, 1016.5761, 1026.5627, 1031.5581, 1034.5808, 1044.5240, 1051.5095, 1056.5281, 1057.5575, 1066.5067, 1074.5374, 1087.5460, 1103.5287, 1118.5885, 1122.5553, 1148.5439, 1168.5985, 1178.6211, 1185.6898, 1244.6118, 1248.6686, 1253.6313, 1255.6216, 1301.6338, 1312.7423, 1313.7227, 1314.7188, 1315.6721, 1317.6354, 1320.6331, 1322.6371, 1325.6360, 1327.6959, 1333.7570, 1336.7218, 1338.7179, 1339.7092, 1343.6960, 1349.6917, 1350.6987, 1351.6946, 1361.6875, 1372.5978, 1381.7642, 1382.5950, 1383.7253, 1397.7454, 1439.7549, 1442.8190, 1457.7394, 1463.7950, 1488.7245, 1494.7383, 1497.8735, 1504.7484, 1513.7760, 1517.8062, 1520.7523, 1528.6951, 1537.8245, 1539.8104, 1549.8242, 1552.8073, 1553.8669, 1555.8348, 1556.8799, 1567.7986, 1570.7749, 1578.7872, 1592.7849, 1628.7002, 1628.8571, 1638.8861, 1642.7155, 1647.8503, 1650.8458, 1664.8571, 1680.8226, 1696.8146, 1698.7845, 1699.8228, 1705.8712, 1707.8536, 1715.8536, 1717.9056, 1730.8564, 1739.6990, 1771.0344, 1786.9199, 1809.0262, 1816.9354, 1820.8855, 1836.8507, 1907.9603, 1909.9886, 1928.0238, 1940.0148, 1953.8596, 1961.0111, 1965.8679, 1974.9255, 1982.0082, 1983.9866, 1985.8589, 1995.9136, 2027.1289, 2067.1079, 2110.0598, 2136.8484, 2144.0432, 2150.8660, 2164.8794, 2211.0857, 2239.1533, 2251.1509, 2255.1836, 2270.1113, 2273.0972, 2274.0605, 2286.1492, 2289.0857, 2290.0530, 2293.1558, 2309.1570, 2324.0981, 2338.1033, 2340.0979, 2343.1770, 2352.9316, 2375.0454, 2383.9480, 2414.2512, 2499.2480, 2542.2678, 2589.9707, 2600.0022, 2603.9897, 2613.9939, 2627.9993, 2644.0112, 2646.9875, 2657.4248, 2659.0405, 2661.0017, 2665.0042, 2674.0601, 2675.0151, 2678.0159, 2689.0330, 2692.0283, 2695.0315, 2703.0417, 2706.0959, 2763.3606, 2798.2788, 2815.3203, 2872.3391, 2876.3679, 2884.3535, 2888.3455, 2891.4131, 2892.4519, 2919.4543, 3002.3042, 3012.4717, 3016.2314, 3020.2483, 3030.3025, 3033.2446, 3053.4917, 3081.6296, 3126.7493, 3171.7131, 3184.4602, 3206.6179, 3207.6111, 3248.6445, 3254.6370, 3265.6309, 3300.7825, 3317.7288, 3321.6318, 3334.6953, 3347.5437, 3429.5991, 3457.6255, 3471.6121, 3489.7363, 3503.8608, 3518.5715, 3639.7969, 4148.0293, 4162.0039, 4453.2720, 4844.2046

Search Parameters

Type of search : Peptide Mass Fingerprint
Enzyme : Trypsin

```
Fixed modifications    : Carbamidomethyl (C)
Variable modifications : Oxidation (M)
Mass values            : Monoisotopic
Protein Mass           : Unrestricted
Peptide Mass Tolerance : ± 25 ppm
Peptide Charge State   : 1+
Max Missed Cleavages   : 2
Number of queries      : 247
Selected for scoring   : 25
```

Mascot: http://www.matrixscience.com/

MATRIX SCIENCE Mascot Search Results

```
User         : 5F_(L5@52381_05Apr2017)
Email        : proteomics@i3s.up.pt
Search title : Project: Proteomics, Spot Set: Proteomics\05Apr2017, Label: L5, Spot Id: 227015, Peak List Id: 447671, MS Job Run Id: 316
MS data file : C:\Documents and Settings\Administrator\Desktop\xu\29JUNHO\Proteomics\05Apr2017\pmf_L5_149875820514.txt
Database     : OrganismSpecie Bos_taurus_Reference_Proteome_2016_06 (24214 sequences; 12839866 residues)
Timestamp    : 29 Jun 2017 at 18:07:22 GMT
Top Score    : 124 for P13605, Fibromodulin OS=Bos taurus GN=FMOD PE=1 SV=2
```

Mascot Score Histogram

Protein score is -10*Log(P), where P is the probability that the observed match is a random event.
Protein scores greater than 56 are significant (p<0.05).

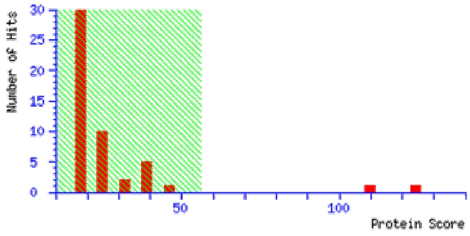

Protein Summary Report

Format As Protein Summary Help

Significance threshold p< 0.05 Max. number of hits 50

Preferred taxonomy All entries

Re-Search All Search Unmatched

Index

	Accession	Mass	Score	Description
1.	P13605	43410	124	Fibromodulin OS=Bos taurus GN=FMOD PE=1 SV=2
2.	E1BI02	43291	108	Fibromodulin OS=Bos taurus GN=FMOD PE=4 SV=1
3.	P55906	74988	46	Transforming growth factor-beta-induced protein ig-h3 OS=Bos taurus GN=TGFBI PE=1 SV=2
4.	F1N367	244177	42	Aggrecan core protein OS=Bos taurus GN=ACAN PE=4 SV=2
5.	F1N368	248513	42	Aggrecan core protein OS=Bos taurus GN=ACAN PE=4 SV=2
6.	F1MBS3	72834	41	Transforming growth factor-beta-induced protein ig-h3 OS=Bos taurus GN=TGFBI PE=4 SV=2
7.	P13608	248150	38	Aggrecan core protein OS=Bos taurus GN=ACAN PE=1 SV=3
8.	F1MX63	44102	36	Prolargin OS=Bos taurus GN=PRELP PE=4 SV=1
9.	Q9GKN8	44054	35	Prolargin OS=Bos taurus GN=PRELP PE=2 SV=1
10.	Q32KP0	36069	29	Spermatid-specific manchette-related protein 1 OS=Bos taurus GN=SMRP1 PE=2 SV=1
11.	A6QLB7	51512	28	Adenylyl cyclase-associated protein OS=Bos taurus GN=CAP1 PE=1 SV=1
12.	Q3SYV4	51583	28	Adenylyl cyclase-associated protein 1 OS=Bos taurus GN=CAP1 PE=2 SV=3
13.	E1BKL4	128535	24	Uncharacterized protein OS=Bos taurus GN=CILP2 PE=4 SV=1
14.	G3MXC7	12858	24	Uncharacterized protein OS=Bos taurus PE=4 SV=1
15.	F1MQI1	273592	22	Uncharacterized protein OS=Bos taurus GN=TLN2 PE=4 SV=2
16.	A6QR51	63694	22	CNOT6 protein OS=Bos taurus GN=CNOT6 PE=2 SV=1
17.	E1BP29	68878	22	Protein-serine/threonine kinase OS=Bos taurus GN=GRK6 PE=3 SV=2
18.	E1BNK9	20498	22	Uncharacterized protein OS=Bos taurus GN=FAM219B PE=4 SV=1
19.	Q5E9X7	46964	22	Inactive serine protease 35 OS=Bos taurus GN=PRSS35 PE=2 SV=2
20.	G3X761	54315	22	Uncharacterized protein OS=Bos taurus GN=PLD4 PE=4 SV=1
21.	Q58DQ8	29298	21	Putative uncharacterized protein FLJ21128 OS=Bos taurus GN=FLJ21128 PE=2 SV=1
22.	E1B9T7	83794	21	Uncharacterized protein OS=Bos taurus GN=SUZ12 PE=4 SV=1
23.	Q17QV0	38378	20	Poly(RC) binding protein 3 OS=Bos taurus GN=PCBP3 PE=2 SV=1
24.	G3MX30	89186	20	Uncharacterized protein OS=Bos taurus GN=OVCH1 PE=3 SV=1
25.	Q3SWY9	24956	20	Ras-related protein Rab-28 OS=Bos taurus GN=RAB28 PE=2 SV=1
26.	A5D7R8	26930	20	Thymidine kinase, cytosolic OS=Bos taurus GN=TK1 PE=2 SV=1
27.	Q32KR8	39412	20	[Protein ADP-ribosylarginine] hydrolase OS=Bos taurus GN=ADPRH PE=2 SV=1
28.	Q0VCL5	40078	19	High-mobility group 20A OS=Bos taurus GN=HMG20A PE=2 SV=1
29.	F1MVT5	36896	19	Uncharacterized protein OS=Bos taurus GN=SLAIN1 PE=4 SV=2
30.	E1B7E6	110359	19	Uncharacterized protein OS=Bos taurus GN=PIWIL2 PE=3 SV=2
31.	P56717	3809	19	Orexin-A OS=Bos taurus GN=HCRT PE=1 SV=1
32.	G1K212	46341	19	Dynein assembly factor with WDR repeat domains 1 OS=Bos taurus GN=DAW1 PE=4 SV=2
33.	Q0P593	46626	19	Dynein assembly factor with WDR repeat domains 1 OS=Bos taurus GN=DAW1 PE=2 SV=1
34.	F6Q0Z5	24053	19	Uncharacterized protein OS=Bos taurus GN=LRRC57 PE=4 SV=1
35.	G3N188	15089	19	Uncharacterized protein OS=Bos taurus PE=4 SV=1
36.	G3MWI9	34349	19	Uncharacterized protein OS=Bos taurus PE=4 SV=1
37.	F1MZL6	54511	18	V-type proton ATPase subunit H OS=Bos taurus GN=ATP6V1H PE=4 SV=1
38.	A6QPQ5	36919	18	39S ribosomal protein L1, mitochondrial OS=Bos taurus GN=MRPL1 PE=1 SV=1
39.	F6QNC4	81939	18	Uncharacterized protein OS=Bos taurus GN=PCCA PE=4 SV=1
40.	F1MDJ4	73301	18	Uncharacterized protein OS=Bos taurus GN=CCDC93 PE=4 SV=1
41.	G3MZ54	74700	18	Uncharacterized protein OS=Bos taurus GN=SNX9 PE=4 SV=1
42.	E1BPW2	128317	18	Uncharacterized protein OS=Bos taurus GN=ERC1 PE=4 SV=2
43.	P07514	34328	18	NADH-cytochrome b5 reductase 3 OS=Bos taurus GN=CYB5R3 PE=1 SV=3
44.	F1N7T1	34443	18	NADH-cytochrome b5 reductase OS=Bos taurus GN=CYB5R3 PE=1 SV=2
45.	G5E5P6	43934	18	Uncharacterized protein OS=Bos taurus GN=AGPAT3 PE=4 SV=1
46.	F1MVJ7	106871	18	Uncharacterized protein OS=Bos taurus GN=LOC104968473 PE=4 SV=2
47.	G3MX47	64111	18	Uncharacterized protein OS=Bos taurus GN=KLHDC7B PE=4 SV=1
48.	Q2M2T3	19496	18	Mitochondrial pyruvate carrier-like protein OS=Bos taurus PE=2 SV=1
49.	G3X6Y1	19510	18	Uncharacterized protein OS=Bos taurus GN=MPC1L PE=4 SV=1
50.	A2VDT4	31057	18	Putative transcription factor Ovo-like 1 OS=Bos taurus GN=OVOL1 PE=2 SV=1

Results List

1. P13605 **Mass:** 43410 **Score:** **124** **Expect:** 9.6e-009 **Matches:** 23

Fibromodulin OS=Bos taurus GN=FMOD PE=1 SV=2

Observed	Mr(expt)	Mr(calc)	ppm	Start	End	Miss	Peptide
779.4799	778.4726	778.4701	3.26	170 -	176	0	R.IPSPLPR.S
823.4933	822.4860	822.4824	4.40	154 -	159	1	K.LRHLER.L
944.5216	943.5143	943.5087	6.01	350 -	357	1	R.LDGNEIKR.S
978.5469	977.5396	977.5334	6.30	99 -	106	0	K.YLPFVPSR.M
1253.6409	1252.6336	1252.6638	-24.09	99 -	108	1	K.YLPFVPSRMK.Y + Oxidation (M)
1301.6381	1300.6308	1300.6268	3.13	358 -	369	0	R.SAMPADAPLCLR.L
1317.6417	1316.6344	1316.6217	9.68	358 -	369	0	R.SAMPADAPLCLR.L + Oxidation (M)
1333.7577	1332.7504	1332.7554	-3.72	96 -	106	1	R.NLKYLPFVPSR.M
1361.6941	1360.6868	1360.6847	1.53	180 -	190	0	R.ELHLDHNQISR.V
1457.7437	1456.7364	1456.7279	5.88	357 -	369	1	K.RSAMPADAPLCLR.L
1553.8821	1552.8748	1552.9049	-19.36	345 -	357	2	K.LQVLRLDGNEIKR.S
1556.8846	1555.8773	1555.8722	3.30	224 -	236	0	R.SLILLDLSYNHLR.K
1717.9054	1716.8981	1716.9019	-2.22	177 -	190	1	R.SLRELHLDHNQISR.V
1928.0238	1927.0165	1927.0163	0.12	308 -	324	0	K.IPPVSTNLENLYLQGNR.I
2324.0906	2323.0833	2323.0865	-1.36	325 -	344	0	R.INEFSISSFCTVVDVMNFSK.L
2340.1016	2339.0943	2339.0814	5.53	325 -	344	0	R.INEFSISSFCTVVDVMNFSK.L + Oxidation (M)
2646.9685	2645.9612	2645.9702	-3.40	75 -	95	0	R.DCPQECDCPPNFPTAMYCDNR.N
3002.1853	3001.1780	3001.1922	-4.71	75 -	98	1	R.DCPQECDCPPNFPTAMYCDNRNLK.Y
3206.5684	3205.5611	3205.5720	-3.40	238 -	265	0	K.VPDGLPSALEQLYLEHNNVFSVPDSYFR.G
3320.6321	3319.6248	3319.6506	-7.78	191 -	220	0	R.VPNNALEGLENLTALYLHHNEIQEVGSSMK.G
3334.6604	3333.6531	3333.6670	-4.16	237 -	265	1	R.KVPDGLPSALEQLYLEHNNVFSVPDSYFR.G
4233.1089	4232.1016	4232.0922	2.22	308 -	344	1	K.IPPVSTNLENLYLQGNRINEFSISSFCTVVDVMNFSK.L
4844.0430	4843.0357	4843.0463	-2.18	33 -	74	0	R.NQQSTYDDPYDPYPYEPYEPYPYGGEEGPAYAYGSPPQPEPR.D

No match to: 706.2851, 710.3807, 717.4042, 719.4268, 720.4332, 722.4348, 723.4337, 735.4208, 744.3633, 749.4401, 756.4190, 759.4415, 766.3942, 772.4352, 774.4582, 831.4758, 832.3166, 834.3207, 842.5047, 843.4840, 844.4902, 846.4869, 870.4950, 873.3982, 876.2891, 880.4586, 883.4816, 885.4853, 891.4897, 895.5765, 900.5383, 918.5458, 923.5204, 927.5097, 928.5096, 929.4465, 945.5218, 949.5538, 967.4249, 969.4326, 972.4861, 974.4482, 976.4711, 977.4844, 988.4647, 1007.5762, 1033.4938, 1034.5852, 1044.5280, 1056.5298, 1074.5454, 1087.5504, 1097.5125, 1103.5261, 1113.4890, 1119.5367, 1122.5620, 1145.6725, 1178.6276, 1185.6924, 1188.5890, 1220.6172, 1234.4895, 1248.6737, 1284.6428, 1313.7157, 1314.7207, 1315.6769, 1316.6554, 1325.6414, 1327.7157, 1336.7279, 1338.7021, 1349.7028, 1355.6765, 1358.7172, 1372.6039, 1381.7673, 1382.5994, 1383.7312, 1397.7480, 1442.8285, 1447.7806, 1461.8053, 1479.8021, 1487.7090, 1497.8783, 1517.8105, 1528.7001, 1532.7877, 1537.8292, 1539.8185, 1552.8179, 1562.8353, 1567.7686, 1578.7893, 1592.7920, 1593.8013, 1628.7037, 1628.8643, 1642.7200, 1650.8497, 1651.8568, 1667.8695, 1680.8239, 1687.9133, 1691.7732, 1706.8671, 1715.8597, 1724.8430, 1728.8602, 1730.8619, 1747.9176, 1771.0326, 1805.9197, 1809.0233, 1813.0017, 1820.9197, 1855.0103, 1887.9128, 1894.9668, 1910.9791, 1953.8596, 1965.8589, 1974.9517, 1981.9934, 1983.9583, 1995.8975, 2020.9868, 2022.0580, 2027.1263, 2044.1479, 2084.0510, 2110.0601, 2136.8467, 2144.0354, 2150.8611, 2155.0640, 2162.8757, 2164.8806, 2170.0522, 2174.1650, 2203.0303, 2217.0352, 2239.1282, 2251.1396, 2253.1372, 2255.2141, 2271.1794, 2274.0801, 2277.0820, 2286.1489, 2289.0703, 2293.1492, 2297.1624, 2309.1501, 2343.1846, 2384.2053, 2410.1479, 2414.2322, 2462.2893, 2464.2695, 2465.2659, 2529.2080, 2542.2549, 2645.0503, 2657.4148, 2660.0061, 2660.9807, 2675.0012, 2689.0129, 2693.0125, 2703.0303, 2706.0225, 2718.3938, 2763.3359, 2774.3179, 2780.3301, 2787.3474, 2826.4065, 2846.3816, 2872.3054, 2876.3391, 2884.3103, 2887.3201, 2889.3223, 2891.4019, 2895.4370, 2900.3384, 2915.4314, 2983.4602, 2995.5381, 2999.4780, 3016.2046, 3020.2017, 3030.2197, 3034.2266, 3044.2632, 3053.4902, 3064.5010, 3081.5806, 3126.7075, 3169.7002, 3234.5972, 3248.6172, 3264.5881, 3299.7673, 3316.7351, 3427.5977, 3439.7319, 3453.7163, 3457.6880, 3458.6204, 3473.6636, 3490.6494, 3503.7805, 3517.5906, 3529.7371, 3616.8076, 3639.6938, 3652.7173, 3704.8706, 3893.7073, 3959.0081, 3991.8828, 4147.9468, 4161.9458, 4177.9121, 4226.0356, 4252.8618, 4280.2700, 4452.1279, 4465.1328, 4679.1919

2. E1BI02 **Mass:** 43291 **Score:** **108** **Expect:** 3.8e-007 **Matches:** 22

Fibromodulin OS=Bos taurus GN=FMOD PE=4 SV=1

Observed	Mr(expt)	Mr(calc)	ppm	Start	End	Miss	Peptide
779.4799	778.4726	778.4701	3.26	169 -	175	0	R.IPSPLPR.S
823.4933	822.4860	822.4824	4.40	153 -	158	1	K.LRHLER.L
944.5216	943.5143	943.5087	6.01	349 -	356	1	R.LDGNEIKR.S
978.5469	977.5396	977.5334	6.30	98 -	105	0	K.YLPFVPSR.M
1253.6409	1252.6336	1252.6638	-24.09	98 -	107	1	K.YLPFVPSRMK.Y + Oxidation (M)
1301.6381	1300.6308	1300.6268	3.13	357 -	368	0	R.SAMPADAPLCLR.L
1317.6417	1316.6344	1316.6217	9.68	357 -	368	0	R.SAMPADAPLCLR.L + Oxidation (M)
1333.7577	1332.7504	1332.7554	-3.72	95 -	105	1	R.NLKYLPFVPSR.M
1361.6941	1360.6868	1360.6847	1.53	179 -	189	0	R.ELHLDHNQISR.V
1457.7437	1456.7364	1456.7279	5.88	356 -	368	1	K.RSAMPADAPLCLR.L
1553.8821	1552.8748	1552.9049	-19.36	344 -	356	2	K.LQVLRLDGNEIKR.S
1556.8846	1555.8773	1555.8722	3.30	223 -	235	0	R.SLILLDLSYNHLR.K
1717.9054	1716.8981	1716.9019	-2.22	176 -	189	1	R.SLRELHLDHNQISR.V
1928.0238	1927.0165	1927.0163	0.12	307 -	323	0	K.IPPVSTNLENLYLQGNR.I
2324.0906	2323.0833	2323.0865	-1.36	324 -	343	0	R.INEFSISSFCTVVDVMNFSK.L
2340.1016	2339.0943	2339.0814	5.53	324 -	343	0	R.INEFSISSFCTVVDVMNFSK.L + Oxidation (M)
2646.9685	2645.9612	2645.9702	-3.40	74 -	94	0	R.DCPQECDCPPNFPTAMYCDNR.N
3002.1853	3001.1780	3001.1922	-4.71	74 -	97	1	R.DCPQECDCPPNFPTAMYCDNRNLK.Y
3206.5684	3205.5611	3205.5720	-3.40	237 -	264	0	K.VPDGLPSALEQLYLEHNNVFSVPDSYFR.G
3320.6321	3319.6248	3319.6506	-7.78	190 -	219	0	R.VPNNALEGLENLTALYLHHNEIQEVGSSMK.G
3334.6604	3333.6531	3333.6670	-4.16	236 -	264	1	R.KVPDGLPSALEQLYLEHNNVFSVPDSYFR.G
4233.1089	4232.1016	4232.0922	2.22	307 -	343	1	K.IPPVSTNLENLYLQGNRINEFSISSFCTVVDVMNFSK.L

No match to: 706.2851, 710.3807, 717.4042, 719.4268, 720.4332, 722.4348, 723.4337, 735.4208, 744.3633, 749.4401, 756.4190, 759.4415, 766.3942, 772.4352, 774.4582, 831.4758, 832.3166, 834.3207, 842.5047, 843.4840, 844.4902, 846.4869, 870.4950, 873.3982, 876.2891, 880.4586, 883.4816, 885.4853, 891.4897, 895.5765, 900.5383, 918.5458, 923.5204, 927.5097, 928.5096, 929.4465, 945.5218, 949.5538, 967.4249, 969.4326, 972.4861, 974.4482, 976.4711, 977.4844, 988.4647, 1007.5762, 1033.4938, 1034.5852, 1044.5280, 1056.5298, 1074.5454, 1087.5504, 1097.5125, 1103.5261, 1113.4890, 1119.5367, 1122.5620, 1145.6725, 1178.6276, 1185.6924, 1188.5890, 1220.6172, 1234.4895, 1248.6737, 1284.6428, 1313.7157, 1314.7207, 1315.6769, 1316.6554, 1325.6414, 1327.7157, 1336.7279, 1338.7021, 1349.7028, 1355.6765, 1358.7172, 1372.6039, 1381.7673, 1382.5994, 1383.7312, 1397.7480, 1442.8285, 1447.7806, 1461.8053, 1479.8021, 1487.7090, 1497.8783, 1517.8105, 1528.7001, 1532.7877, 1537.8292, 1539.8185, 1552.8179, 1562.8353, 1567.7686, 1578.7893, 1592.7920, 1593.8013, 1628.7037, 1628.8643, 1642.7200, 1650.8497, 1651.8568, 1667.8695, 1680.8239, 1687.9133, 1691.7732, 1706.8671, 1715.8597, 1724.8430, 1728.8602, 1730.8619, 1747.9176, 1771.0326, 1805.9197, 1809.0233, 1813.0017, 1820.9197, 1855.0103, 1887.9128, 1894.9668, 1910.9791, 1953.8596, 1965.8589, 1974.9517, 1981.9934, 1983.9583, 1995.8975, 2020.9868, 2022.0580, 2027.1263, 2044.1479, 2084.0510, 2110.0601, 2136.8467, 2144.0354, 2150.8611, 2155.0640, 2162.8757, 2164.8806, 2170.0522, 2174.1650, 2203.0303, 2217.0352, 2239.1282, 2251.1396, 2253.1372, 2255.2141, 2271.1794, 2274.0801, 2277.0820, 2286.1489, 2289.0703, 2293.1492, 2297.1624, 2309.1501, 2343.1846, 2384.2053, 2410.1479, 2414.2322, 2462.2893, 2464.2695, 2465.2659, 2529.2080, 2542.2549, 2645.0503, 2657.4148, 2660.0061, 2660.9807, 2675.0012, 2689.0129, 2693.0125, 2703.0303, 2706.0225, 2718.3938, 2763.3359, 2774.3179, 2780.3301, 2787.3474, 2826.4065, 2846.3816, 2872.3054, 2876.3391, 2884.3103, 2887.3201, 2889.3223, 2891.4019, 2895.4370, 2900.3384, 2915.4314, 2983.4602, 2995.5381, 2999.4780,

3016.2046, 3020.2017, 3030.2197, 3034.2266, 3044.2632, 3053.4902, 3064.5010, 3081.5806, 3126.7075, 3169.7002, 3234.5972, 3248.6172, 3264.5881, 3299.7673, 3316.7351, 3427.5977, 3439.7319, 3453.7163, 3457.6880, 3458.6204, 3473.6636, 3490.6494, 3503.7805, 3517.5906, 3529.7371, 3616.8076, 3639.6938, 3652.7173, 3704.8706, 3893.7073, 3959.0081, 3991.8828, 4147.9468, 4161.9458, 4177.9121, 4226.0356, 4252.8618, 4280.2700, 4452.1279, 4465.1328, 4679.1919, 4844.0430

3. P55906 **Mass:** 74988 **Score:** 46 **Expect:** 0.64 **Matches:** 29

Transforming growth factor-beta-induced protein ig-h3 OS=Bos taurus GN=TGFBI PE=1 SV=2

Observed	Mr(expt)	Mr(calc)	ppm	Start	End	Miss	Peptide
766.3942	765.3870	765.3769	13.1	656	662	0	K.QASAFSR.A
846.4869	845.4796	845.4759	4.35	670	676	0	K.LAPVYQR.L
976.4711	975.4638	975.4709	-7.24	173	179	1	R.YHMVDRR.V
1103.5261	1102.5188	1102.5117	6.43	488	496	0	R.YGTLFTMDR.M
1119.5367	1118.5294	1118.5067	20.4	488	496	0	R.YGTLFTMDR.M + Oxidation (M)
1316.6554	1315.6481	1315.6343	10.5	486	496	1	R.GRYGTLFTMDR.M
1327.7157	1326.7084	1326.7044	3.02	28	38	0	R.SPYQLVLQHSR.L
1333.7577	1332.7504	1332.7475	2.17	410	421	0	R.LTLLAPMNSVFK.D
1355.6765	1354.6692	1354.6589	7.61	397	409	0	R.QAGLSSHLSGNER.L
1532.7877	1531.7804	1531.7812	-0.53	497	510	0	R.MLTPPMGTVMDVLK.G
1593.8013	1592.7940	1592.7947	-0.43	448	461	0	K.YLYHGQTLDTLGGR.K
1855.0103	1854.0030	1854.0000	1.66	571	588	0	K.YHVGDEILVSGGIGALVR.L
1887.9128	1886.9055	1886.8727	17.4	470	485	1	R.NSLCIENSCIAAHDKR.G
1974.9517	1973.9444	1973.9737	-14.82	497	514	1	R.MLTPPMGTVMDVLKGDNR.F
2022.0580	2021.0507	2021.0615	-5.35	515	533	0	R.FSMLVAAIQSAGLTETLNR.E
2044.1479	2043.1406	2043.1476	-3.43	296	313	1	R.ILGDPEALRDLLNNHILK.S
2084.0510	2083.0437	2083.0473	-1.74	378	396	0	K.TLFELAADSDVSTAIDLFR.Q
2110.0601	2109.0528	2109.0531	-0.13	534	552	0	R.EGVYTVFAPTNEAFQALPR.G
2155.0640	2154.0567	2154.0593	-1.19	643	662	1	R.GDELADSALEIFKQASAFSR.A
2203.0303	2202.0230	2201.9973	11.7	77	95	1	K.STVISYECCPGYEKVPGEK.G
2255.2141	2254.2068	2254.2144	-3.34	410	430	1	R.LTLLAPMNSVFKDGTPPINAR.T
2271.1794	2270.1721	2270.2093	-16.36	410	430	1	R.LTLLAPMNSVFKDGTPPINAR.T + Oxidation (M)
2464.2695	2463.2622	2463.2540	3.35	511	533	1	K.GDNRFSMLVAAIQSAGLTETLNR.E
2846.3816	2845.3743	2845.3999	-8.99	486	510	2	R.GRYGTLFTMDRMLTPPMGTVMDVLK.G + Oxidation (M)
3064.5010	3063.4937	3063.5400	-15.11	258	287	0	R.AAVAASGLNTLLEGDGQYTLLAPTNEAFEK.I
3081.5806	3080.5733	3080.5489	7.94	96	124	0	K.GCPAALPLSNLYETLGVVGATTTQLYTDR.T
3439.7319	3438.7246	3438.7341	-2.75	96	127	1	K.GCPAALPLSNLYETLGVVGATTTQLYTDRTEK.L
3458.6204	3457.6131	3457.6078	1.54	187	216	0	K.HGMALTSMYQNSNIQIHHYPNGIVTVNCAR.L + 2 Oxidation (M)
3959.0081	3958.0008	3958.0323	-7.96	258	295	1	R.AAVAASGLNTLLEGDGQYTLLAPTNEAFEKIPAETLNR.I

No match to: 706.2851, 710.3807, 717.4042, 719.4268, 720.4332, 722.4348, 723.4337, 735.4208, 744.3633, 749.4401, 756.4190, 759.4415, 772.4352, 774.4582, 779.4799, 823.4933, 831.4758, 832.3166, 834.3207, 842.5047, 843.4840, 844.4902, 870.4950, 873.3982, 876.2891, 880.4586, 883.4816, 885.4853, 891.4897, 895.5765, 900.5383, 918.5458, 923.5204, 927.5097, 928.5096, 929.4465, 944.5216, 945.5218, 949.5538, 967.4249, 969.4326, 972.4861, 974.4482, 977.4844, 978.5469, 988.4647, 1007.5762, 1033.4938, 1034.5852, 1044.5280, 1056.5298, 1074.5454, 1087.5504, 1097.5125, 1113.4890, 1122.5620, 1145.6725, 1178.6276, 1185.6924, 1188.5890, 1220.6172, 1234.4895, 1248.6737, 1253.6409, 1284.6428, 1301.6381, 1313.7157, 1314.7207, 1315.6769, 1317.6417, 1325.6414, 1336.7279, 1338.7021, 1349.7028, 1358.7172, 1361.6941, 1372.6039, 1381.7673, 1382.5994, 1383.7312, 1397.7480, 1442.8285, 1447.7806, 1457.7437, 1461.8053, 1479.8021, 1487.7090, 1497.8783, 1517.8105, 1528.7001, 1537.8292, 1539.8185, 1552.8179, 1553.8821, 1556.8846, 1562.8353, 1567.7686, 1578.7893, 1592.7920, 1628.7037, 1628.8643, 1642.7200, 1650.8497, 1651.8568, 1667.8695, 1680.8239, 1687.9133, 1691.7732, 1706.8671, 1715.8597, 1717.9054, 1724.8430, 1728.8602, 1730.8619, 1747.9176, 1771.0326, 1805.9197, 1809.0233, 1813.0017, 1820.9197, 1894.9668, 1910.9791, 1928.0238, 1953.8596, 1965.8589, 1981.9934, 1983.9583, 1995.8975, 2020.9868, 2027.1263, 2136.8467, 2144.0354, 2150.8611, 2162.8757, 2164.8806, 2170.0522, 2174.1650, 2217.0352, 2239.1282, 2251.1396, 2253.1372, 2274.0801, 2277.0820, 2286.1489, 2289.0703, 2293.1492, 2297.1624, 2309.1501, 2324.0906, 2340.1016, 2343.1846, 2384.2053, 2410.1479, 2414.2322, 2462.2893, 2465.2659, 2529.2080, 2542.2549, 2645.0503, 2646.9685, 2657.4148, 2660.0061, 2660.9807, 2675.0012, 2689.0129, 2693.0125, 2703.0303, 2706.0225, 2718.3938, 2763.3359, 2774.3179, 2780.3301, 2787.3474, 2826.4065, 2872.3054, 2876.3391, 2884.3103, 2887.3201, 2889.3223, 2891.4019, 2895.4370, 2900.3384, 2915.4314, 2983.4602, 2995.5381, 2999.4780, 3002.1853, 3016.2046, 3020.2017, 3030.2197, 3034.2266, 3044.2632, 3053.4902, 3126.7075, 3169.7002, 3206.5684, 3234.5972, 3248.6172, 3264.5881, 3299.7673, 3316.7351, 3320.6321, 3334.6604, 3427.5977, 3453.7163, 3457.6880, 3473.6636, 3490.6494, 3503.7805, 3517.5906, 3529.7371, 3616.8076, 3639.6938, 3652.7173, 3704.8706, 3893.7073, 3991.8828, 4147.9468, 4161.9458, 4177.9121, 4226.0356, 4233.1089, 4252.8618, 4280.2700, 4452.1279, 4465.1328, 4679.1919, 4844.0430

4. F1N367 **Mass:** 244177 **Score:** 42 **Expect:** 1.4 **Matches:** 37

Aggrecan core protein OS=Bos taurus GN=ACAN PE=4 SV=2

Observed	Mr(expt)	Mr(calc)	ppm	Start	End	Miss	Peptide
720.4332	719.4260	719.4330	-9.79	2256	2261	1	R.IFGQKK.D
722.4348	721.4275	721.4235	5.58	2282	2287	0	R.HVPTIR.C
831.4758	830.4685	830.4650	4.17	545	551	0	R.YPIVSPR.T
883.4816	882.4744	882.4712	3.60	211	217	0	R.YPIHTPR.E
891.4897	890.4824	890.4763	6.90	152	158	0	K.GIVFHYR.A
929.4465	928.4393	928.4290	11.0	164	170	0	R.YTLDFDR.A
949.5538	948.5466	948.5392	7.70	306	313	1	R.SVRYPISK.A
967.4249	966.4177	966.4130	4.79	2125	2131	0	K.FQGHCYR.H
974.4482	973.4410	973.4341	7.05	678	684	0	R.HHAFCFR.G
1087.5504	1086.5431	1086.5233	18.2	498	506	0	R.YSLTFEEAK.Q
1185.6924	1184.6851	1184.6765	7.29	83	93	0	K.EVVLLVATEGR.V
1248.6737	1247.6664	1247.6622	3.38	2262	2271	1	K.DRYEINALVR.Y
1284.6428	1283.6355	1283.6258	7.55	164	173	1	R.YTLDFDRAQR.A
1325.6414	1324.6341	1324.6445	-7.86	2299	2309	1	R.ITCTDPATYKR.R
1372.6039	1371.5966	1371.5877	6.48	264	274	0	K.FTFQEAANECR.R
1382.5994	1381.5921	1381.5833	6.37	2288	2298	0	R.CQPSGHWEEPR.I
1383.7312	1382.7239	1382.7201	2.77	314	326	0	K.ARPNCGGNLLGVR.T
1442.8285	1441.8212	1441.8140	4.99	81	93	1	K.EKEVVLLVATEGR.V
1457.7437	1456.7364	1456.7310	3.70	159	170	1	R.AISTRYTLDFDR.A
1528.7001	1527.6928	1527.6888	2.61	264	275	1	K.FTFQEAANECRR.L
1578.7893	1577.7820	1577.7620	12.7	2241	2255	0	K.GTVACGEPPVVEHAR.I
1628.7037	1627.6964	1627.6937	1.69	218	231	1	R.EGCYGDKDEFPGVR.T
1628.8643	1627.8570	1627.8583	-0.80	483	497	0	R.LPGGVVFHYRPGSSR.Y
1706.8671	1705.8598	1705.8570	1.66	2240	2255	1	K.KGTVACGEPPVVEHAR.I
1715.8597	1714.8524	1714.8348	10.3	498	511	1	R.YSLTFEEAKQACLR.T
1771.0326	1770.0253	1770.0251	0.14	78	93	2	R.ISKEKEVVLLVATEGR.V
2144.0354	2143.0281	2143.0289	-0.38	133	151	0	R.CEVMHGIEDSQATLEVVVK.G
2340.1016	2339.0943	2339.0529	17.7	566	584	0	R.TYGVRPPSETYDVYCYVDR.L
2343.1846	2342.1773	2342.1940	-7.12	104	124	0	K.VTLPNYPAIPSDATLEIQNMR.S

2872.3054	2871.2981	2871.3142	-5.61	280 -	305	0	R.LATTGQLYLAWQGGMDMCSAGWLADR.S
3248.6172	3247.6099	3247.6183	-2.57	96 -	124	1	R.VNSAYQDKVTLPNYPAIPSDATLEIQNMR.S
3264.5881	3263.5808	3263.6132	-9.92	96 -	124	1	R.VNSAYQDKVTLPNYPAIPSDATLEIQNMR.S + Oxidation (M)
3457.6880	3456.6807	3456.6489	9.20	275 -	305	2	R.RLGARLATTGQLYLAWQGGMDMCSAGWLADR.S + 2 Oxidation (M)
3503.7805	3502.7732	3502.7878	-4.16	94 -	124	2	R.VRVNSAYQDKVTLPNYPAIPSDATLEIQNMR.S
3639.6938	3638.6865	3638.6947	-2.25	512 -	544	0	R.TGAIIASPEQLQAAYEAGYEQCDAGWLQDQTVR.Y
4147.9468	4146.9395	4146.9164	5.58	174 -	210	0	R.ACLQNSAIIATPEQLQAAYEDGFHQCDAGWLADQTVR.Y
4452.1279	4451.1206	4451.1492	-6.42	512 -	551	1	R.TGAIIASPEQLQAAYEAGYEQCDAGWLQDQTVRYPIVSPR.T

No match to: 706.2851, 710.3807, 717.4042, 719.4268, 723.4337, 735.4208, 744.3633, 749.4401, 756.4190, 759.4415, 766.3942, 772.4352, 774.4582, 779.4799, 823.4933, 832.3166, 834.3207, 842.5047, 843.4840, 844.4902, 846.4869, 870.4950, 873.3982, 876.2891, 880.4586, 885.4853, 895.5765, 900.5383, 918.5458, 923.5204, 927.5097, 928.5096, 944.5216, 945.5218, 969.4326, 972.4861, 976.4711, 977.4844, 978.5469, 988.4647, 1007.5762, 1033.4938, 1034.5852, 1044.5280, 1056.5298, 1074.5454, 1097.5125, 1103.5261, 1113.4890, 1119.5367, 1122.5620, 1145.6725, 1178.6276, 1188.5890, 1220.6172, 1234.4895, 1253.6409, 1301.6381, 1313.7157, 1314.7207, 1315.6769, 1316.6554, 1317.6417, 1327.7157, 1333.7577, 1336.7279, 1338.7021, 1349.7028, 1355.6765, 1358.7172, 1361.6941, 1381.7673, 1397.7480, 1447.7806, 1461.8053, 1479.8021, 1487.7090, 1497.8783, 1517.8105, 1532.7877, 1537.8292, 1539.8185, 1552.8179, 1553.8821, 1556.8846, 1562.8353, 1567.7686, 1592.7920, 1593.8013, 1642.7200, 1650.8497, 1651.8568, 1667.8695, 1680.8239, 1687.9133, 1691.7732, 1717.9054, 1724.8430, 1728.8602, 1730.8619, 1747.9176, 1805.9197, 1809.0233, 1813.0017, 1820.9197, 1855.0103, 1887.9128, 1894.9668, 1910.9791, 1928.0238, 1953.8596, 1965.8589, 1974.9517, 1981.9934, 1983.9583, 1995.8975, 2020.9868, 2022.0580, 2027.1263, 2044.1479, 2084.0510, 2110.0601, 2136.8467, 2150.8611, 2155.0640, 2162.8757, 2164.8806, 2170.0522, 2174.1650, 2203.0303, 2217.0352, 2239.1282, 2251.1396, 2253.1372, 2255.2141, 2271.1794, 2274.0801, 2277.0820, 2286.1489, 2289.0703, 2293.1492, 2297.1624, 2309.1501, 2324.0906, 2384.2053, 2410.1479, 2414.2322, 2462.2893, 2464.2695, 2465.2659, 2529.2080, 2542.2549, 2645.0503, 2646.9685, 2657.4148, 2660.0061, 2660.9807, 2675.0012, 2689.0129, 2693.0125, 2703.0303, 2706.0225, 2718.3938, 2763.3359, 2774.3179, 2780.3301, 2787.3474, 2826.4065, 2846.3816, 2876.3391, 2884.3103, 2887.3201, 2889.3223, 2891.4019, 2895.4370, 2900.3384, 2915.4314, 2983.4602, 2995.5381, 2999.4780, 3002.1853, 3016.2046, 3020.2017, 3030.2197, 3034.2266, 3044.2632, 3053.4902, 3064.5010, 3081.5806, 3126.7075, 3169.7002, 3206.5684, 3234.5972, 3299.7673, 3316.7351, 3320.6321, 3334.6604, 3427.5977, 3439.7319, 3453.7163, 3458.6204, 3473.6636, 3490.6494, 3517.5906, 3529.7371, 3616.8076, 3652.7173, 3704.8706, 3893.7073, 3959.0081, 3991.8828, 4161.9458, 4177.9121, 4226.0356, 4233.1089, 4252.8618, 4280.2700, 4465.1328, 4679.1919, 4844.0430

5. F1N368 **Mass:** 248513 **Score:** 42 **Expect:** 1.7 **Matches:** 37

Aggrecan core protein OS=Bos taurus GN=ACAN PE=4 SV=2

Observed	Mr(expt)	Mr(calc)	ppm	Start	End	Miss	Peptide
720.4332	719.4260	719.4330	-9.79	2294 -	2299	1	R.IFGQKK.D
722.4348	721.4275	721.4235	5.58	2320 -	2325	0	R.HVPTIR.C
831.4758	830.4685	830.4650	4.17	545 -	551	0	R.YPIVSPR.T
883.4816	882.4744	882.4712	3.60	211 -	217	0	R.YPIHTPR.E
891.4897	890.4824	890.4763	6.90	152 -	158	0	K.GIVFHYR.A
929.4465	928.4393	928.4290	11.0	164 -	170	0	R.YTLDFDR.A
949.5538	948.5466	948.5392	7.70	306 -	313	1	R.SVRYPISK.A
967.4249	966.4177	966.4130	4.79	2163 -	2169	0	K.FQGHCYR.H
974.4482	973.4410	973.4341	7.05	678 -	684	0	R.HHAFCFR.G
1087.5504	1086.5431	1086.5233	18.2	498 -	506	0	R.YSLTFEEAK.Q
1185.6924	1184.6851	1184.6765	7.29	83 -	93	0	K.EVVLLVATEGR.V
1248.6737	1247.6664	1247.6622	3.38	2300 -	2309	1	K.DRYEINALVR.Y
1284.6428	1283.6355	1283.6258	7.55	164 -	173	1	R.YTLDFDRAQR.A
1325.6414	1324.6341	1324.6445	-7.86	2337 -	2347	1	R.ITCTDPATYKR.R
1372.6039	1371.5966	1371.5877	6.48	264 -	274	0	K.FTFQEAANECR.R
1382.5994	1381.5921	1381.5833	6.37	2326 -	2336	0	R.CQPSGHWEEPR.I
1383.7312	1382.7239	1382.7201	2.77	314 -	326	0	K.ARPNCGGNLLGVR.T
1442.8285	1441.8212	1441.8140	4.99	81 -	93	1	K.EKEVVLLVATEGR.V
1457.7437	1456.7364	1456.7310	3.70	159 -	170	1	K.AISTRYTLDFDR.A
1528.7001	1527.6928	1527.6888	2.61	264 -	275	1	K.FTFQEAANECRR.L
1578.7893	1577.7820	1577.7620	12.7	2279 -	2293	0	K.GTVACGEPPVVEHAR.I
1628.7037	1627.6964	1627.6937	1.69	218 -	231	1	R.EGCYGDKDEFPGVR.T
1628.8643	1627.8570	1627.8583	-0.80	483 -	497	0	R.LPGGVVFHYRPGSSR.Y
1706.8671	1705.8598	1705.8570	1.66	2278 -	2293	1	K.KGTVACGEPPVVEHAR.I
1715.8597	1714.8524	1714.8348	10.3	498 -	511	1	R.YSLTFEEAKQACLR.T
1771.0326	1770.0253	1770.0251	0.14	78 -	93	2	R.ISKEKEVVLLVATEGR.V
2144.0354	2143.0281	2143.0289	-0.38	133 -	151	0	R.CEVMHGIEDSQATLEVVVK.G
2340.1016	2339.0943	2339.0529	17.7	566 -	584	0	R.TYGVRPPSETYDVYCYVDR.L
2343.1846	2342.1773	2342.1940	-7.12	104 -	124	0	K.VTLPNYPAIPSDATLEIQNMR.S
2872.3054	2871.2981	2871.3142	-5.61	280 -	305	0	R.LATTGQLYLAWQGGMDMCSAGWLADR.S
3248.6172	3247.6099	3247.6183	-2.57	96 -	124	1	R.VNSAYQDKVTLPNYPAIPSDATLEIQNMR.S
3264.5881	3263.5808	3263.6132	-9.92	96 -	124	1	R.VNSAYQDKVTLPNYPAIPSDATLEIQNMR.S + Oxidation (M)
3457.6880	3456.6807	3456.6489	9.20	275 -	305	2	R.RLGARLATTGQLYLAWQGGMDMCSAGWLADR.S + 2 Oxidation (M)
3503.7805	3502.7732	3502.7878	-4.16	94 -	124	2	R.VRVNSAYQDKVTLPNYPAIPSDATLEIQNMR.S
3639.6938	3638.6865	3638.6947	-2.25	512 -	544	0	R.TGAIIASPEQLQAAYEAGYEQCDAGWLQDQTVR.Y
4147.9468	4146.9395	4146.9164	5.58	174 -	210	0	R.ACLQNSAIIATPEQLQAAYEDGFHQCDAGWLADQTVR.Y
4452.1279	4451.1206	4451.1492	-6.42	512 -	551	1	R.TGAIIASPEQLQAAYEAGYEQCDAGWLQDQTVRYPIVSPR.T

No match to: 706.2851, 710.3807, 717.4042, 719.4268, 723.4337, 735.4208, 744.3633, 749.4401, 756.4190, 759.4415, 766.3942, 772.4352, 774.4582, 779.4799, 823.4933, 832.3166, 834.3207, 842.5047, 843.4840, 844.4902, 846.4869, 870.4950, 873.3982, 876.2891, 880.4586, 885.4853, 895.5765, 900.5383, 918.5458, 923.5204, 927.5097, 928.5096, 944.5216, 945.5218, 969.4326, 972.4861, 976.4711, 977.4844, 978.5469, 988.4647, 1007.5762, 1033.4938, 1034.5852, 1044.5280, 1056.5298, 1074.5454, 1097.5125, 1103.5261, 1113.4890, 1119.5367, 1122.5620, 1145.6725, 1178.6276, 1188.5890, 1220.6172, 1234.4895, 1253.6409, 1301.6381, 1313.7157, 1314.7207, 1315.6769, 1316.6554, 1317.6417, 1327.7157, 1333.7577, 1336.7279, 1338.7021, 1349.7028, 1355.6765, 1358.7172, 1361.6941, 1381.7673, 1397.7480, 1447.7806, 1461.8053, 1479.8021, 1487.7090, 1497.8783, 1517.8105, 1532.7877, 1537.8292, 1539.8185, 1552.8179, 1553.8821, 1556.8846, 1562.8353, 1567.7686, 1592.7920, 1593.8013, 1642.7200, 1650.8497, 1651.8568, 1667.8695, 1680.8239, 1687.9133, 1691.7732, 1717.9054, 1724.8430, 1728.8602, 1730.8619, 1747.9176, 1805.9197, 1809.0233, 1813.0017, 1820.9197, 1855.0103, 1887.9128, 1894.9668, 1910.9791, 1928.0238, 1953.8596, 1965.8589, 1974.9517, 1981.9934, 1983.9583, 1995.8975, 2020.9868, 2022.0580, 2027.1263, 2044.1479, 2084.0510, 2110.0601, 2136.8467, 2150.8611, 2155.0640, 2162.8757, 2164.8806, 2170.0522, 2174.1650, 2203.0303, 2217.0352, 2239.1282, 2251.1396, 2253.1372, 2255.2141, 2271.1794, 2274.0801, 2277.0820, 2286.1489, 2289.0703, 2293.1492, 2297.1624, 2309.1501, 2324.0906, 2384.2053, 2410.1479, 2414.2322, 2462.2893, 2464.2695, 2465.2659, 2529.2080, 2542.2549, 2645.0503, 2646.9685, 2657.4148, 2660.0061, 2660.9807, 2675.0012, 2689.0129, 2693.0125, 2703.0303, 2706.0225, 2718.3938, 2763.3359, 2774.3179, 2780.3301, 2787.3474, 2826.4065, 2846.3816, 2876.3391, 2884.3103, 2887.3201, 2889.3223, 2891.4019, 2895.4370, 2900.3384, 2915.4314, 2983.4602, 2995.5381, 2999.4780, 3002.1853, 3016.2046, 3020.2017, 3030.2197, 3034.2266, 3044.2632, 3053.4902, 3064.5010, 3081.5806, 3126.7075, 3169.7002, 3206.5684, 3234.5972, 3299.7673, 3316.7351, 3320.6321, 3334.6604, 3427.5977, 3439.7319, 3453.7163, 3458.6204, 3473.6636, 3490.6494, 3517.5906, 3529.7371, 3616.8076, 3652.7173, 3704.8706, 3893.7073, 3959.0081, 3991.8828, 4161.9458, 4177.9121, 4226.0356, 4233.1089, 4252.8618, 4280.2700, 4465.1328, 4679.1919, 4844.0430

6. F1MBS3 **Mass:** 72834 **Score:** 41 **Expect:** 1.9 **Matches:** 28

Transforming growth factor-beta-induced protein ig-h3 OS=Bos taurus GN=TGFBI PE=4 SV=2

Observed	Mr(expt)	Mr(calc)	ppm	Start	End	Miss	Peptide

766.3942	765.3870	765.3769	13.1	634	640	0	K.QASAFSR.A
846.4869	845.4796	845.4759	4.35	648	654	0	K.LAPVYQR.L
976.4711	975.4638	975.4709	-7.24	151	157	1	R.YHMVDRR.V
1103.5261	1102.5188	1102.5117	6.43	466	474	0	R.YGTLFTMDR.M
1119.5367	1118.5294	1118.5067	20.4	466	474	0	R.YGTLFTMDR.M + Oxidation (M)
1316.6554	1315.6481	1315.6343	10.5	464	474	1	R.GRYGTLFTMDR.M
1333.7577	1332.7504	1332.7475	2.17	388	399	0	R.LTLLAPMNSVFK.D
1355.6765	1354.6692	1354.6589	7.61	375	387	0	R.QAGLSSHLSGNER.L
1532.7877	1531.7804	1531.7812	-0.53	475	488	0	R.MLTPPMGTVMDVLK.G
1593.8013	1592.7940	1592.7947	-0.43	426	439	0	K.YLYHGQTLDTLGGR.K
1855.0103	1854.0030	1854.0000	1.66	549	566	0	K.YHVGDEILVSGGIGALVR.L
1887.9128	1886.9055	1886.8727	17.4	448	463	1	R.NSLCIENSCIAAHDKR.G
1974.9517	1973.9444	1973.9737	-14.82	475	492	1	R.MLTPPMGTVMDVLKGDNR.F
2022.0580	2021.0507	2021.0615	-5.35	493	511	0	R.FSMLVAAIQSAGLTETLNR.E
2044.1479	2043.1406	2043.1476	-3.43	274	291	1	R.ILGDPEALRDLLNNHILK.S
2084.0510	2083.0437	2083.0473	-1.74	356	374	0	K.TLFELAADSDVSTAIDLFR.Q
2110.0601	2109.0528	2109.0531	-0.13	512	530	0	R.EGVYTVFAPTNEAFQALPR.G
2155.0640	2154.0567	2154.0593	-1.19	621	640	1	R.GDELADSALEIFKQASAFSR.A
2203.0303	2202.0230	2201.9973	11.7	55	73	1	K.STVISYECCPGYEKVPGEK.G
2255.2141	2254.2068	2254.2144	-3.34	388	408	1	R.LTLLAPMNSVFKDGTPPINAR.T
2271.1794	2270.1721	2270.2093	-16.36	388	408	1	R.LTLLAPMNSVFKDGTPPINAR.T + Oxidation (M)
2464.2695	2463.2622	2463.2540	3.35	489	511	1	K.GDNRFSMLVAAIQSAGLTETLNR.E
2846.3816	2845.3743	2845.3999	-8.99	464	488	2	R.GRYGTLFTMDRMLTPPMGTVMDVLK.G + Oxidation (M)
3064.5010	3063.4937	3063.5400	-15.11	236	265	0	R.AAVAASGLNTLLEGDGQYTLLAPTNEAFEK.I
3081.5806	3080.5733	3080.5489	7.94	74	102	0	K.GCPAALPLSNLYETLGVVGATTTQLYTDR.T
3439.7319	3438.7246	3438.7341	-2.75	74	105	1	K.GCPAALPLSNLYETLGVVGATTTQLYTDRTEK.L
3458.6204	3457.6131	3457.6078	1.54	165	194	0	K.HGMALTSMYQNSNIQIHHYPNGIVTVNCAR.L + 2 Oxidation (M)
3959.0081	3958.0008	3958.0323	-7.96	236	273	1	R.AAVAASGLNTLLEGDGQYTLLAPTNEAFEKIPAETLNR.I

No match to: 706.2851, 710.3807, 717.4042, 719.4268, 720.4332, 722.4348, 723.4337, 735.4208, 744.3633, 749.4401, 756.4190, 759.4415, 772.4352, 774.4582, 779.4799, 823.4933, 831.4758, 832.3166, 834.3207, 842.5047, 843.4840, 844.4902, 870.4950, 873.3982, 876.2891, 880.4586, 883.4816, 885.4853, 891.4897, 895.5765, 900.5383, 918.5458, 923.5204, 927.5097, 928.5096, 929.4465, 944.5216, 945.5218, 949.5538, 967.4249, 969.4326, 972.4861, 974.4482, 977.4844, 978.5469, 988.4647, 1007.5762, 1033.4938, 1034.5852, 1044.5280, 1056.5298, 1074.5454, 1087.5504, 1097.5125, 1113.4890, 1122.5620, 1145.6725, 1178.6276, 1185.6924, 1188.5890, 1220.6172, 1234.4895, 1248.6737, 1253.6409, 1284.6428, 1301.6381, 1313.7157, 1314.7207, 1315.6769, 1317.6417, 1325.6414, 1327.7157, 1336.7279, 1338.7021, 1349.7028, 1358.7172, 1361.6941, 1372.6039, 1381.7673, 1382.5994, 1383.7312, 1397.7480, 1442.8285, 1447.7806, 1457.7437, 1461.8053, 1479.8021, 1487.7090, 1497.8783, 1517.8105, 1528.7001, 1537.8292, 1539.8185, 1552.8179, 1553.8821, 1556.8846, 1562.8353, 1567.7686, 1578.7893, 1592.7920, 1628.7037, 1628.8643, 1642.7200, 1650.8497, 1651.8568, 1667.8695, 1680.8239, 1687.9133, 1691.7732, 1706.8671, 1715.8597, 1717.9054, 1724.8430, 1728.8602, 1730.8619, 1747.9176, 1771.0326, 1805.9197, 1809.0233, 1813.0017, 1820.9197, 1894.9668, 1910.9791, 1928.0238, 1953.8596, 1965.8589, 1981.9934, 1983.9583, 1995.8975, 2020.9868, 2027.1263, 2136.8467, 2144.0354, 2150.8611, 2162.8757, 2164.8806, 2170.0522, 2174.1650, 2217.0352, 2239.1282, 2251.1396, 2253.1372, 2274.0801, 2277.0820, 2286.1489, 2289.0703, 2293.1492, 2297.1624, 2309.1501, 2324.0906, 2340.1016, 2343.1846, 2384.2053, 2410.1479, 2414.2322, 2462.2893, 2465.2659, 2529.2080, 2542.2549, 2645.0503, 2646.9685, 2657.4148, 2660.0061, 2660.9807, 2675.0012, 2689.0129, 2693.0125, 2703.0303, 2706.0225, 2718.3938, 2763.3359, 2774.3179, 2780.3301, 2787.3474, 2826.4065, 2872.3054, 2876.3391, 2884.3103, 2887.3201, 2889.3223, 2891.4019, 2895.4370, 2900.3384, 2915.4314, 2983.4602, 2995.5381, 2999.4780, 3002.1853, 3016.2046, 3020.2017, 3030.2197, 3034.2266, 3044.2632, 3053.4902, 3126.7075, 3169.7002, 3206.5684, 3234.5972, 3248.6172, 3264.5881, 3299.7673, 3316.7351, 3320.6321, 3334.6604, 3427.5977, 3453.7163, 3457.6880, 3473.6636, 3490.6494, 3503.7805, 3517.5906, 3529.7371, 3616.8076, 3639.6938, 3652.7173, 3704.8706, 3893.7073, 3991.8828, 4147.9468, 4161.9458, 4177.9121, 4226.0356, 4233.1089, 4252.8618, 4280.2700, 4452.1279, 4465.1328, 4679.1919, 4844.0430

7. P13608 **Mass:** 248150 **Score:** 38 **Expect:** 3.8 **Matches:** 36

Aggrecan core protein OS=Bos taurus GN=ACAN PE=1 SV=3

Observed	Mr(expt)	Mr(calc)	ppm	Start	End	Miss	Peptide
720.4332	719.4260	719.4330	-9.79	2292	2297	1	R.IFGQKK.D
831.4758	830.4685	830.4650	4.17	545	551	0	R.YPIVSPR.T
883.4816	882.4744	882.4712	3.60	211	217	0	R.YPIHTPR.E
891.4897	890.4824	890.4763	6.90	152	158	0	K.GIVFHYR.A
929.4465	928.4393	928.4290	11.0	164	170	0	R.YTLDFDR.A
949.5538	948.5466	948.5392	7.70	306	313	1	R.SVRYPISK.A
967.4249	966.4177	966.4130	4.79	2161	2167	0	K.FQGHCYR.H
974.4482	973.4410	973.4341	7.05	678	684	0	R.HHAFCFR.G
1087.5504	1086.5431	1086.5233	18.2	498	506	0	R.YSLTFEEAK.Q
1185.6924	1184.6851	1184.6765	7.29	83	93	0	K.EVVLLVATEGR.V
1248.6737	1247.6664	1247.6622	3.38	2298	2307	1	K.DRYEINALVR.Y
1284.6428	1283.6355	1283.6258	7.55	164	173	1	R.YTLDFDRAQR.A
1325.6414	1324.6341	1324.6445	-7.86	2335	2345	1	R.ITCTDPATYKR.R
1372.6039	1371.5966	1371.5877	6.48	264	274	0	K.FTFQEAANECR.R
1382.5994	1381.5921	1381.5833	6.37	2324	2334	0	R.CQPSGHWEEPR.I
1383.7312	1382.7239	1382.7201	2.77	314	326	0	K.ARPNCGGNLLGVR.T
1442.8285	1441.8212	1441.8140	4.99	81	93	1	K.EKEVVLLVATEGR.V
1457.7437	1456.7364	1456.7310	3.70	159	170	1	R.AISTRYTLDFDR.A
1528.7001	1527.6928	1527.6888	2.61	264	275	1	K.FTFQEAANECRR.L
1578.7893	1577.7820	1577.7620	12.7	2277	2291	0	K.GTVACGEPPVVEHAR.I
1628.7037	1627.6964	1627.6937	1.69	218	231	1	R.EGCYGDKDEFPGVR.T
1628.8643	1627.8570	1627.8583	-0.80	483	497	0	R.LPGGVVFHYRPGSSR.Y
1706.8671	1705.8598	1705.8570	1.66	2276	2291	1	K.KGTVACGEPPVVEHAR.I
1715.8597	1714.8524	1714.8348	10.3	498	511	1	R.YSLTFEEAKQACLR.T
1771.0326	1770.0253	1770.0251	0.14	78	93	2	R.ISKEKEVVLLVATEGR.V
2144.0354	2143.0281	2143.0289	-0.38	133	151	0	R.CEVMHGIEDSQATLEVVVK.G
2340.1016	2339.0943	2339.0529	17.7	566	584	0	R.TYGVRPPSETYDVYCYVDR.L
2343.1846	2342.1773	2342.1940	-7.12	104	124	0	K.VTLPNYPAIPSDATLEIQNMR.S
2872.3054	2871.2981	2871.3142	-5.61	280	305	0	R.LATTGQLYLAWQGGMDMCSAGWLADR.S
3248.6172	3247.6099	3247.6183	-2.57	96	124	1	R.VNSAYQDKVTLPNYPAIPSDATLEIQNMR.S
3264.5881	3263.5808	3263.6132	-9.92	96	124	1	R.VNSAYQDKVTLPNYPAIPSDATLEIQNMR.S + Oxidation (M)
3457.6880	3456.6807	3456.6489	9.20	275	305	2	R.RLGARLATTGQLYLAWQGGMDMCSAGWLADR.S + 2 Oxidation (M)
3503.7805	3502.7732	3502.7878	-4.16	94	124	2	R.VRVNSAYQDKVTLPNYPAIPSDATLEIQNMR.S
3639.6938	3638.6865	3638.6947	-2.25	512	544	0	R.TGAIIASPEQLQAAYEAGYEQCDAGWLQDQTVR.Y
4147.9468	4146.9395	4146.9164	5.58	174	210	0	R.ACLQNSAIIATPEQLQAAYEDGFHQCDAGWLADQTVR.Y
4452.1279	4451.1206	4451.1492	-6.42	512	551	1	R.TGAIIASPEQLQAAYEAGYEQCDAGWLQDQTVRYPIVSPR.T

No match to: 706.2851, 710.3807, 717.4042, 719.4268, 722.4348, 723.4337, 735.4208, 744.3633, 749.4401, 756.4190, 759.4415, 766.3942, 772.4352, 774.4582, 779.4799, 823.4933, 832.3166, 834.3207, 842.5047, 843.4840, 844.4902, 846.4869, 870.4950, 873.3982, 876.2891, 880.4586, 885.4853, 895.5765, 900.5383, 918.5458, 923.5204, 927.5097, 928.5096, 944.5216, 945.5218, 969.4326, 972.4861, 976.4711, 977.4844, 978.5469, 988.4647, 1007.5762, 1033.4938, 1034.5852, 1044.5280, 1056.5298, 1074.5454, 1097.5125, 1103.5261, 1113.4890, 1119.5367, 1122.5620, 1145.6725, 1178.6276, 1188.5890, 1220.6172, 1234.4895, 1253.6409, 1301.6381, 1313.7157, 1314.7207, 1315.6769, 1316.6554, 1317.6417, 1327.7157, 1333.7577, 1336.7279, 1338.7021, 1349.7028, 1355.6765, 1358.7172, 1361.6941, 1381.7673, 1397.7480, 1447.7806, 1461.8053, 1479.8021, 1487.7090, 1497.8783, 1517.8105, 1532.7877, 1537.8292, 1539.8185, 1552.8179, 1553.8821, 1556.8846, 1562.8353, 1567.7686, 1592.7920, 1593.8013, 1642.7200, 1650.8497, 1651.8568, 1667.8695, 1680.8239, 1687.9133, 1691.7732, 1717.9054, 1724.8430, 1728.8602, 1730.8619, 1747.9176, 1805.9197, 1809.0233, 1813.0017, 1820.9197, 1855.0103, 1887.9128, 1894.9668, 1910.9791, 1928.0238, 1953.8596, 1965.8589, 1974.9517, 1981.9934, 1983.9583, 1995.8975, 2020.9868, 2022.0580, 2027.1263, 2044.1479, 2084.0510, 2110.0601, 2136.8467, 2150.8611, 2155.0640, 2162.8757, 2164.8806, 2170.0522, 2174.1650, 2203.0303, 2217.0352, 2239.1282, 2251.1396, 2253.1372, 2255.2141, 2271.1794, 2274.0801, 2277.0820, 2286.1489, 2289.0703, 2293.1492, 2297.1624, 2309.1501, 2324.0906, 2384.2053, 2410.1479, 2414.2322, 2462.2893, 2464.2695, 2465.2659, 2529.2080, 2542.2549, 2645.0503, 2646.9685, 2657.4148, 2660.0061, 2660.9807, 2675.0012, 2689.0129, 2693.0125, 2703.0303, 2706.0225, 2718.3938, 2763.3359, 2774.3179, 2780.3301, 2787.3474, 2826.4065, 2846.3816, 2876.3391, 2884.3103, 2887.3201, 2889.3223, 2891.4019, 2895.4370, 2900.3384, 2915.4314, 2983.4602, 2995.5381, 2999.4780, 3002.1853, 3016.2046, 3020.2017, 3030.2197, 3034.2266, 3044.2632, 3053.4902, 3064.5010, 3081.5806, 3126.7075, 3169.7002, 3206.5684, 3234.5972, 3299.7673, 3316.7351, 3320.6321, 3334.6604, 3427.5977, 3439.7319, 3453.7163, 3458.6204, 3473.6636, 3490.6494, 3517.5906, 3529.7371, 3616.8076, 3652.7173, 3704.8706, 3893.7073, 3959.0081, 3991.8828, 4161.9458, 4177.9121, 4226.0356, 4233.1089, 4252.8618, 4280.2700, 4465.1328, 4679.1919, 4844.0430

8. F1MX63 **Mass:** 44102 **Score:** 36 **Expect:** 5.4 **Matches:** 18

Prolargin OS=Bos taurus GN=PRELP PE=4 SV=1

Observed	Mr(expt)	Mr(calc)	ppm	Start	End	Miss	Peptide
772.4352	771.4280	771.4239	5.30	171 -	176	0	R.NLEQLR.L
844.4902	843.4829	843.4854	-3.02	185 -	192	0	R.IPPGVFSK.L
895.5765	894.5692	894.5651	4.61	95 -	102	1	R.KVPVIPSR.I
945.5218	944.5145	944.5039	11.2	177 -	184	0	R.LSQNQISR.I
1044.5280	1043.5207	1043.5148	5.66	129 -	136	0	R.WINLDNNR.I
1122.5620	1121.5547	1121.5465	7.32	272 -	280	0	R.LNYNQLSDR.G
1313.7157	1312.7084	1312.7000	6.43	129 -	138	1	R.WINLDNNRIR.K
1336.7279	1335.7206	1335.7146	4.49	159 -	170	0	K.NQLEEVPAALPR.N
1517.8105	1516.8032	1516.7998	2.28	272 -	284	1	R.LNYNQLSDRGLPK.N
1537.8292	1536.8219	1536.8194	1.61	221 -	233	0	K.NLMQLNLAHNTLR.K
1651.8568	1650.8495	1650.8617	-7.40	206 -	220	0	K.LSDGVFKPDTFQGLK.N
1809.0233	1808.0160	1808.0157	0.15	144 -	158	1	R.VLEKLPSLVFLYMEK.N
2136.8467	2135.8394	2135.8387	0.34	75 -	91	0	R.ECYCPPDFPSALYCDSR.N
2293.1492	2292.1419	2292.1469	-2.16	356 -	374	0	R.LDGNYLKPPIPLDLMMCFR.L
2309.1501	2308.1428	2308.1418	0.45	356 -	374	0	R.LDGNYLKPPIPLDLMMCFR.L + Oxidation (M)
2657.4148	2656.4075	2656.4298	-8.38	148 -	170	1	K.LPSLVFLYMEKNQLEEVPAALPR.N
2718.3938	2717.3865	2717.4177	-11.46	239 -	262	1	K.VPSAIHQLYLDSNRIEAIPSGYFK.S
3126.7075	3125.7002	3125.7198	-6.27	144 -	170	2	R.VLEKLPSLVFLYMEKNQLEEVPAALPR.N

No match to: 706.2851, 710.3807, 717.4042, 719.4268, 720.4332, 722.4348, 723.4337, 735.4208, 744.3633, 749.4401, 756.4190, 759.4415, 766.3942, 774.4582, 779.4799, 823.4933, 831.4758, 832.3166, 834.3207, 842.5047, 843.4840, 846.4869, 870.4950, 873.3982, 876.2891, 880.4586, 883.4816, 885.4853, 891.4897, 900.5383, 918.5458, 923.5204, 927.5097, 928.5096, 929.4465, 944.5216, 949.5538, 967.4249, 969.4326, 972.4861, 974.4482, 976.4711, 977.4844, 978.5469, 988.4647, 1007.5762, 1033.4938, 1034.5852, 1056.5298, 1074.5454, 1087.5504, 1097.5125, 1103.5261, 1113.4890, 1119.5367, 1145.6725, 1178.6276, 1185.6924, 1188.5890, 1220.6172, 1234.4895, 1248.6737, 1253.6409, 1284.6428, 1301.6381, 1314.7207, 1315.6769, 1316.6554, 1317.6417, 1325.6414, 1327.7157, 1333.7577, 1338.7021, 1349.7028, 1355.6765, 1358.7172, 1361.6941, 1372.6039, 1381.7673, 1382.5994, 1383.7312, 1397.7480, 1442.8285, 1447.7806, 1457.7437, 1461.8053, 1479.8021, 1487.7090, 1497.8783, 1528.7001, 1532.7877, 1539.8185, 1552.8179, 1553.8821, 1556.8846, 1562.8353, 1567.7686, 1578.7893, 1592.7920, 1593.8013, 1628.7037, 1628.8643, 1642.7200, 1650.8497, 1667.8695, 1680.8239, 1687.9133, 1691.7732, 1706.8671, 1715.8597, 1717.9054, 1724.8430, 1728.8602, 1730.8619, 1747.9176, 1771.0326, 1805.9197, 1813.0017, 1820.9197, 1855.0103, 1887.9128, 1894.9668, 1910.9791, 1928.0238, 1953.8596, 1965.8589, 1974.9517, 1981.9934, 1983.9583, 1995.8975, 2020.9868, 2022.0580, 2027.1263, 2044.1479, 2084.0510, 2110.0601, 2144.0354, 2150.8611, 2155.0640, 2162.8757, 2164.8806, 2170.0522, 2174.1650, 2203.0303, 2217.0352, 2239.1282, 2251.1396, 2253.1372, 2255.2141, 2271.1794, 2274.0801, 2277.0820, 2286.1489, 2289.0703, 2297.1624, 2324.0906, 2340.1016, 2343.1846, 2384.2053, 2410.1479, 2414.2322, 2462.2893, 2464.2695, 2465.2659, 2529.2080, 2542.2549, 2645.0503, 2646.9685, 2660.0061, 2660.9807, 2675.0012, 2689.0129, 2693.0125, 2703.0303, 2706.0225, 2763.3359, 2774.3179, 2780.3301, 2787.3474, 2826.4065, 2846.3816, 2872.3054, 2876.3391, 2884.3103, 2887.3201, 2889.3223, 2891.4019, 2895.4370, 2900.3384, 2915.4314, 2983.4602, 2995.5381, 2999.4780, 3002.1853, 3016.2046, 3020.2017, 3030.2197, 3034.2266, 3044.2632, 3053.4902, 3064.5010, 3081.5806, 3169.7002, 3206.5684, 3234.5972, 3248.6172, 3264.5881, 3299.7673, 3316.7351, 3320.6321, 3334.6604, 3427.5977, 3439.7319, 3453.7163, 3457.6880, 3458.6204, 3473.6636, 3490.6494, 3503.7805, 3517.5906, 3529.7371, 3616.8076, 3639.6938, 3652.7173, 3704.8706, 3893.7073, 3959.0081, 3991.8828, 4147.9468, 4161.9458, 4177.9121, 4226.0356, 4233.1089, 4252.8618, 4280.2700, 4452.1279, 4465.1328, 4679.1919, 4844.0430

9. Q9GKN8 **Mass:** 44054 **Score:** 35 **Expect:** 8.2 **Matches:** 16

Prolargin OS=Bos taurus GN=PRELP PE=2 SV=1

Observed	Mr(expt)	Mr(calc)	ppm	Start	End	Miss	Peptide
772.4352	771.4280	771.4239	5.30	171 -	176	0	R.NLEQLR.L
844.4902	843.4829	843.4854	-3.02	185 -	192	0	R.IPPGVFSK.L
895.5765	894.5692	894.5651	4.61	95 -	102	1	R.KVPVIPSR.I
945.5218	944.5145	944.5039	11.2	177 -	184	0	R.LSQNQISR.I
1034.5852	1033.5779	1033.5709	6.80	263 -	271	0	K.GFPNLAFIR.L
1044.5280	1043.5207	1043.5148	5.66	129 -	136	0	R.WINLDNNR.I
1122.5620	1121.5547	1121.5465	7.32	272 -	280	0	R.LNYNQLSDR.G
1313.7157	1312.7084	1312.7000	6.43	129 -	138	1	R.WINLDNNRIR.K
1336.7279	1335.7206	1335.7146	4.49	159 -	170	0	K.NQLEEVPAALPR.N
1517.8105	1516.8032	1516.7998	2.28	272 -	284	1	R.LNYNQLSDRGLPK.N
1537.8292	1536.8219	1536.8194	1.61	221 -	233	0	K.NLMQLNLAHNTLR.K
1651.8568	1650.8495	1650.8617	-7.40	206 -	220	0	K.LSDGVFKPDTFQGLK.N
2136.8467	2135.8394	2135.8387	0.34	75 -	91	0	R.ECYCPPDFPSALYCDSR.N
2293.1492	2292.1419	2292.1469	-2.16	356 -	374	0	R.LDGNYLKPPIPLDLMMCFR.L
2309.1501	2308.1428	2308.1418	0.45	356 -	374	0	R.LDGNYLKPPIPLDLMMCFR.L + Oxidation (M)
2718.3938	2717.3865	2717.4177	-11.46	239 -	262	1	K.VPSAIHQLYLDSNRIEAIPSGYFK.G

No match to: 706.2851, 710.3807, 717.4042, 719.4268, 720.4332, 722.4348, 723.4337, 735.4208, 744.3633, 749.4401, 756.4190, 759.4415, 766.3942, 774.4582, 779.4799, 823.4933, 831.4758, 832.3166, 834.3207, 842.5047, 843.4840, 846.4869, 870.4950, 873.3982, 876.2891, 880.4586, 883.4816, 885.4853, 891.4897, 900.5383, 918.5458, 923.5204, 927.5097, 928.5096, 929.4465, 944.5216, 949.5538, 967.4249, 969.4326, 972.4861, 974.4482, 976.4711, 977.4844, 978.5469, 988.4647, 1007.5762, 1033.4938, 1056.5298, 1074.5454, 1087.5504, 1097.5125, 1103.5261, 1113.4890, 1119.5367, 1145.6725, 1178.6276, 1185.6924, 1188.5890, 1220.6172, 1234.4895, 1248.6737, 1253.6409, 1284.6428, 1301.6381, 1314.7207, 1315.6769, 1316.6554, 1317.6417, 1325.6414, 1327.7157, 1333.7577, 1338.7021, 1349.7028, 1355.6765, 1358.7172, 1361.6941, 1372.6039, 1381.7673, 1382.5994, 1383.7312, 1397.7480, 1442.8285, 1447.7806, 1457.7437, 1461.8053, 1479.8021, 1487.7090, 1497.8783, 1528.7001, 1532.7877, 1539.8185, 1552.8179, 1553.8821, 1556.8846, 1562.8353, 1567.7686, 1578.7893, 1592.7920, 1593.8013, 1628.7037, 1628.8643, 1642.7200, 1650.8497, 1667.8695, 1680.8239, 1687.9133, 1691.7732, 1706.8671, 1715.8597, 1717.9054, 1724.8430, 1728.8602, 1730.8619, 1747.9176, 1771.0326, 1805.9197, 1809.0233, 1813.0017, 1820.9197, 1855.0103, 1887.9128, 1894.9668, 1910.9791, 1928.0238, 1953.8596, 1965.8589, 1974.9517, 1981.9934, 1983.9583, 1995.8975, 2020.9868, 2022.0580, 2027.1263, 2044.1479, 2084.0510, 2110.0601, 2144.0354, 2150.8611, 2155.0640, 2162.8757, 2164.8806, 2170.0522, 2174.1650, 2203.0303, 2217.0352,

2239.1282, 2251.1396, 2253.1372, 2255.2141, 2271.1794, 2274.0801, 2277.0820, 2286.1489, 2289.0703, 2297.1624, 2324.0906, 2340.1016, 2343.1846, 2384.2053, 2410.1479, 2414.2322, 2462.2893, 2464.2695, 2465.2659, 2529.2080, 2542.2549, 2645.0503, 2646.9685, 2657.4148, 2660.0061, 2660.9807, 2675.0012, 2689.0129, 2693.0125, 2703.0303, 2706.0225, 2763.3359, 2774.3179, 2780.3301, 2787.3474, 2826.4065, 2846.3816, 2872.3054, 2876.3391, 2884.3103, 2887.3201, 2889.3223, 2891.4019, 2895.4370, 2900.3384, 2915.4314, 2983.4602, 2995.5381, 2999.4780, 3002.1853, 3016.2046, 3020.2017, 3030.2197, 3034.2266, 3044.2632, 3053.4902, 3064.5010, 3081.5806, 3126.7075, 3169.7002, 3206.5684, 3234.5972, 3248.6172, 3264.5881, 3299.7673, 3316.7351, 3320.6321, 3334.6604, 3427.5977, 3439.7319, 3453.7163, 3457.6880, 3458.6204, 3473.6636, 3490.6494, 3503.7805, 3517.5906, 3529.7371, 3616.8076, 3639.6938, 3652.7173, 3704.8706, 3893.7073, 3959.0081, 3991.8828, 4147.9468, 4161.9458, 4177.9121, 4226.0356, 4233.1089, 4252.8618, 4280.2700, 4452.1279, 4465.1328, 4679.1919, 4844.0430

10. Q32KP0 **Mass:** 36069 **Score:** 29 **Expect:** 30 **Matches:** 7

Spermatid-specific manchette-related protein 1 OS=Bos taurus GN=SMRP1 PE=2 SV=1

Observed	Mr(expt)	Mr(calc)	ppm	Start	End	Miss	Peptide
928.5096	927.5024	927.5000	2.52	1	7	1	-.MFLFSRK.T
944.5216	943.5143	943.4949	20.5	1	7	1	-.MFLFSRK.T + Oxidation (M)
1248.6737	1247.6664	1247.6696	-2.56	128	137	1	K.CTTYLPRLPK.E
1327.7157	1326.7084	1326.7045	2.99	42	53	0	K.FVTPGLTHTAQR.H
1383.7312	1382.7239	1382.7380	-10.20	147	158	0	R.GMPLVYPPKPER.L
3503.7805	3502.7732	3502.8064	-9.48	147	176	2	R.GMPLVYPPKPERLNAYEREVVVNMLNSLSR.N + 2 Oxidation (M)
3616.8076	3615.8003	3615.8436	-11.97	165	196	2	R.EVVVNMLNSLSRNQPLPQITPRCGCVDPLPGR.L

No match to: 706.2851, 710.3807, 717.4042, 719.4268, 720.4332, 722.4348, 723.4337, 735.4208, 744.3633, 749.4401, 756.4190, 759.4415, 766.3942, 772.4352, 774.4582, 779.4799, 823.4933, 831.4758, 832.3166, 834.3207, 842.5047, 843.4840, 844.4902, 846.4869, 870.4950, 873.3982, 876.2891, 880.4586, 883.4816, 885.4853, 891.4897, 895.5765, 900.5383, 918.5458, 923.5204, 927.5097, 929.4465, 945.5218, 949.5538, 967.4249, 969.4326, 972.4861, 974.4482, 976.4711, 977.4844, 978.5469, 988.4647, 1007.5762, 1033.4938, 1034.5852, 1044.5280, 1056.5298, 1074.5454, 1087.5504, 1097.5125, 1103.5261, 1113.4890, 1119.5367, 1122.5620, 1145.6725, 1178.6276, 1185.6924, 1188.5890, 1220.6172, 1234.4895, 1253.6409, 1284.6428, 1301.6381, 1313.7157, 1314.7207, 1315.6769, 1316.6554, 1317.6417, 1325.6414, 1333.7577, 1336.7279, 1338.7021, 1349.7028, 1355.6765, 1358.7172, 1361.6941, 1372.6039, 1381.7673, 1382.5994, 1397.7480, 1442.8285, 1447.7806, 1457.7437, 1461.8053, 1479.8021, 1487.7090, 1497.8783, 1517.8105, 1528.7001, 1532.7877, 1537.8292, 1539.8185, 1552.8179, 1553.8821, 1556.8846, 1562.8353, 1567.7686, 1578.7893, 1592.7920, 1593.8013, 1628.7037, 1628.8643, 1642.7200, 1650.8497, 1651.8568, 1667.8695, 1680.8239, 1687.9133, 1691.7732, 1706.8671, 1715.8597, 1717.9054, 1724.8430, 1728.8602, 1730.8619, 1747.9176, 1771.0326, 1805.9197, 1809.0233, 1813.0017, 1820.9197, 1855.0103, 1887.9128, 1894.9668, 1910.9791, 1928.0238, 1953.8596, 1965.8589, 1974.9517, 1981.9934, 1983.9583, 1995.8975, 2020.9868, 2022.0580, 2027.1263, 2044.1479, 2084.0510, 2110.0601, 2136.8467, 2144.0354, 2150.8611, 2155.0640, 2162.8757, 2164.8806, 2170.0522, 2174.1650, 2203.0303, 2217.0352, 2239.1282, 2251.1396, 2253.1372, 2255.2141, 2271.1794, 2274.0801, 2277.0820, 2286.1489, 2289.0703, 2293.1492, 2297.1624, 2309.1501, 2324.0906, 2340.1016, 2343.1846, 2384.2053, 2410.1479, 2414.2322, 2462.2893, 2464.2695, 2465.2659, 2529.2080, 2542.2549, 2645.0503, 2646.9685, 2657.4148, 2660.0061, 2660.9807, 2675.0012, 2689.0129, 2693.0125, 2703.0303, 2706.0225, 2718.3938, 2763.3359, 2774.3179, 2780.3301, 2787.3474, 2826.4065, 2846.3816, 2872.3054, 2876.3391, 2884.3103, 2887.3201, 2889.3223, 2891.4019, 2895.4370, 2900.3384, 2915.4314, 2983.4602, 2995.5381, 2999.4780, 3002.1853, 3016.2046, 3020.2017, 3030.2197, 3034.2266, 3044.2632, 3053.4902, 3064.5010, 3081.5806, 3126.7075, 3169.7002, 3206.5684, 3234.5972, 3248.6172, 3264.5881, 3299.7673, 3316.7351, 3320.6321, 3334.6604, 3427.5977, 3439.7319, 3453.7163, 3457.6880, 3458.6204, 3473.6636, 3490.6494, 3517.5906, 3529.7371, 3639.6938, 3652.7173, 3704.8706, 3893.7073, 3959.0081, 3991.8828, 4147.9468, 4161.9458, 4177.9121, 4226.0356, 4233.1089, 4252.8618, 4280.2700, 4452.1279, 4465.1328, 4679.1919, 4844.0430

11. A6QLB7 **Mass:** 51512 **Score:** 28 **Expect:** 37 **Matches:** 9

Adenylyl cyclase-associated protein OS=Bos taurus GN=CAP1 PE=1 SV=1

Observed	Mr(expt)	Mr(calc)	ppm	Start	End	Miss	Peptide
779.4799	778.4726	778.4589	17.7	167	172	1	R.VLKEYK.D
1119.5367	1118.5294	1118.5502	-18.59	2	10	1	M.ADMRNLVER.L + Oxidation (M)
1122.5620	1121.5547	1121.5652	-9.30	71	80	0	K.HAEMVHTGLK.L
1517.8105	1516.8032	1516.7780	16.7	2	13	2	M.ADMRNLVERLER.V + Oxidation (M)
1928.0238	1927.0165	1927.0163	0.12	254	271	0	R.SALFAQINQGESITHALK.H
2084.0510	2083.0437	2083.0844	-19.52	81	99	1	K.LERALLVTASQCQQPAGNK.L
2293.1492	2292.1419	2292.1896	-20.80	60	80	2	K.ISKEIGGDVQKHAEMVHTGLK.L + Oxidation (M)
3126.7075	3125.7002	3125.6372	20.2	126	154	0	K.LFNHLSAVSESIQALGWVAMAPKPGPYVK.E + Oxidation (M)
3248.6172	3247.6099	3247.6217	-3.62	401	429	2	K.VPTISINKTDGCHVYLSKNSLDCEIVSAK.S

No match to: 706.2851, 710.3807, 717.4042, 719.4268, 720.4332, 722.4348, 723.4337, 735.4208, 744.3633, 749.4401, 756.4190, 759.4415, 766.3942, 772.4352, 774.4582, 823.4933, 831.4758, 832.3166, 834.3207, 842.5047, 843.4840, 844.4902, 846.4869, 870.4950, 873.3982, 876.2891, 880.4586, 883.4816, 885.4853, 891.4897, 895.5765, 900.5383, 918.5458, 923.5204, 927.5097, 928.5096, 929.4465, 944.5216, 945.5218, 949.5538, 967.4249, 969.4326, 972.4861, 974.4482, 976.4711, 977.4844, 978.5469, 988.4647, 1007.5762, 1033.4938, 1034.5852, 1044.5280, 1056.5298, 1074.5454, 1087.5504, 1097.5125, 1103.5261, 1113.4890, 1145.6725, 1178.6276, 1185.6924, 1188.5890, 1220.6172, 1234.4895, 1248.6737, 1253.6409, 1284.6428, 1301.6381, 1313.7157, 1314.7207, 1315.6769, 1316.6554, 1317.6417, 1325.6414, 1327.7157, 1333.7577, 1336.7279, 1338.7021, 1349.7028, 1355.6765, 1358.7172, 1361.6941, 1372.6039, 1381.7673, 1382.5994, 1383.7312, 1397.7480, 1442.8285, 1447.7806, 1457.7437, 1461.8053, 1479.8021, 1487.7090, 1497.8783, 1528.7001, 1532.7877, 1537.8292, 1539.8185, 1552.8179, 1553.8821, 1556.8846, 1562.8353, 1567.7686, 1578.7893, 1592.7920, 1593.8013, 1628.7037, 1628.8643, 1642.7200, 1650.8497, 1651.8568, 1667.8695, 1680.8239, 1687.9133, 1691.7732, 1706.8671, 1715.8597, 1717.9054, 1724.8430, 1728.8602, 1730.8619, 1747.9176, 1771.0326, 1805.9197, 1809.0233, 1813.0017, 1820.9197, 1855.0103, 1887.9128, 1894.9668, 1910.9791, 1953.8596, 1965.8589, 1974.9517, 1981.9934, 1983.9583, 1995.8975, 2020.9868, 2022.0580, 2027.1263, 2044.1479, 2110.0601, 2136.8467, 2144.0354, 2150.8611, 2155.0640, 2162.8757, 2164.8806, 2170.0522, 2174.1650, 2203.0303, 2217.0352, 2239.1282, 2251.1396, 2253.1372, 2255.2141, 2271.1794, 2274.0801, 2277.0820, 2286.1489, 2289.0703, 2297.1624, 2309.1501, 2324.0906, 2340.1016, 2343.1846, 2384.2053, 2410.1479, 2414.2322, 2462.2893, 2464.2695, 2465.2659, 2529.2080, 2542.2549, 2645.0503, 2646.9685, 2657.4148, 2660.0061, 2660.9807, 2675.0012, 2689.0129, 2693.0125, 2703.0303, 2706.0225, 2718.3938, 2763.3359, 2774.3179, 2780.3301, 2787.3474, 2826.4065, 2846.3816, 2872.3054, 2876.3391, 2884.3103, 2887.3201, 2889.3223, 2891.4019, 2895.4370, 2900.3384, 2915.4314, 2983.4602, 2995.5381, 2999.4780, 3002.1853, 3016.2046, 3020.2017, 3030.2197, 3034.2266, 3044.2632, 3053.4902, 3064.5010, 3081.5806, 3169.7002, 3206.5684, 3234.5972, 3264.5881, 3299.7673, 3316.7351, 3320.6321, 3334.6604, 3427.5977, 3439.7319, 3453.7163, 3457.6880, 3458.6204, 3473.6636, 3490.6494, 3503.7805, 3517.5906, 3529.7371, 3616.8076, 3639.6938, 3652.7173, 3704.8706, 3893.7073, 3959.0081, 3991.8828, 4147.9468, 4161.9458, 4177.9121, 4226.0356, 4233.1089, 4252.8618, 4280.2700, 4452.1279, 4465.1328, 4679.1919, 4844.0430

12. Q3SYV4 **Mass:** 51583 **Score:** 28 **Expect:** 37 **Matches:** 9

Adenylyl cyclase-associated protein 1 OS=Bos taurus GN=CAP1 PE=2 SV=3

Observed	Mr(expt)	Mr(calc)	ppm	Start	End	Miss	Peptide
779.4799	778.4726	778.4589	17.7	168	173	1	R.VLKEYK.D
1119.5367	1118.5294	1118.5502	-18.59	2	10	1	M.ADMRNLVER.L + Oxidation (M)
1122.5620	1121.5547	1121.5652	-9.30	72	81	0	K.HAEMVHTGLK.L
1517.8105	1516.8032	1516.7780	16.7	2	13	2	M.ADMRNLVERLER.V + Oxidation (M)
1928.0238	1927.0165	1927.0163	0.12	255	272	0	R.SALFAQINQGESITHALK.H
2084.0510	2083.0437	2083.0844	-19.52	82	100	1	K.LERALLVTASQCQQPAGNK.L
2293.1492	2292.1419	2292.1896	-20.80	61	81	2	K.ISKEIGGDVQKHAEMVHTGLK.L + Oxidation (M)
3126.7075	3125.7002	3125.6372	20.2	127	155	0	K.LFNHLSAVSESIQALGWVAMAPKPGPYVK.E + Oxidation (M)
3248.6172	3247.6099	3247.6217	-3.62	402	430	2	K.VPTISINKTDGCHVYLSKNSLDCEIVSAK.S

No match to: 706.2851, 710.3807, 717.4042, 719.4268, 720.4332, 722.4348, 723.4337, 735.4208, 744.3633, 749.4401, 756.4190, 759.4415, 766.3942, 772.4352, 774.4582, 823.4933, 831.4758, 832.3166, 834.3207, 842.5047, 843.4840, 844.4902, 846.4869, 870.4950, 873.3982, 876.2891, 880.4586, 883.4816, 885.4853, 891.4897, 895.5765, 900.5383, 918.5458, 923.5204, 927.5097, 928.5096, 929.4465, 944.5216, 945.5218, 949.5538, 967.4249, 969.4326, 972.4861, 974.4482, 976.4711, 977.4844, 978.5469, 988.4647, 1007.5762, 1033.4938, 1034.5852, 1044.5280, 1056.5298, 1074.5454, 1087.5504, 1097.5125, 1103.5261, 1113.4890, 1145.6725, 1178.6276, 1185.6924, 1188.5890, 1220.6172, 1234.4895, 1248.6737, 1253.6409, 1284.6428, 1301.6381, 1313.7157, 1314.7207, 1315.6769, 1316.6554, 1317.6417, 1325.6414, 1327.7157, 1333.7577, 1336.7279, 1338.7021, 1349.7028, 1355.6765, 1358.7172, 1361.6941, 1372.6039, 1381.7673, 1382.5994, 1383.7312,

1397.7480, 1442.8285, 1447.7806, 1457.7437, 1461.8053, 1479.8021, 1487.7090, 1497.8783, 1528.7001, 1532.7877, 1537.8292, 1539.8185, 1552.8179, 1553.8821, 1556.8846, 1562.8353, 1567.7686, 1578.7893, 1592.7920, 1593.8013, 1628.7037, 1628.8643, 1642.7200, 1650.8497, 1651.8568, 1667.8695, 1680.8239, 1687.9133, 1691.7732, 1706.8671, 1715.8597, 1717.9054, 1724.8430, 1728.8602, 1730.8619, 1747.9176, 1771.0326, 1805.9197, 1809.0233, 1813.0017, 1820.9197, 1855.0103, 1887.9128, 1894.9668, 1910.9791, 1953.8596, 1965.8589, 1974.9517, 1981.9934, 1983.9583, 1995.8975, 2020.9868, 2022.0580, 2027.1263, 2044.1479, 2110.0601, 2136.8467, 2144.0354, 2150.8611, 2155.0640, 2162.8757, 2164.8806, 2170.0522, 2174.1650, 2203.0303, 2217.0352, 2239.1282, 2251.1396, 2253.1372, 2255.2141, 2271.1794, 2274.0801, 2277.0820, 2286.1489, 2289.0703, 2297.1624, 2309.1501, 2324.0906, 2340.1016, 2343.1846, 2384.2053, 2410.1479, 2414.2322, 2462.2893, 2464.2695, 2465.2659, 2529.2080, 2542.2549, 2645.0503, 2646.9685, 2657.4148, 2660.0061, 2660.9807, 2675.0012, 2689.0129, 2693.0125, 2703.0303, 2706.0225, 2718.3938, 2763.3359, 2774.3179, 2780.3301, 2787.3474, 2826.4065, 2846.3816, 2872.3054, 2876.3391, 2884.3103, 2887.3201, 2889.3223, 2891.4019, 2895.4370, 2900.3384, 2915.4314, 2983.4602, 2995.5381, 2999.4780, 3002.1853, 3016.2046, 3020.2017, 3030.2197, 3034.2266, 3044.2632, 3053.4902, 3064.5010, 3081.5806, 3169.7002, 3206.5684, 3234.5972, 3264.5881, 3299.7673, 3316.7351, 3320.6321, 3334.6604, 3427.5977, 3439.7319, 3453.7163, 3457.6880, 3458.6204, 3473.6636, 3490.6494, 3503.7805, 3517.5906, 3529.7371, 3616.8076, 3639.6938, 3652.7173, 3704.8706, 3893.7073, 3959.0081, 3991.8828, 4147.9468, 4161.9458, 4177.9121, 4226.0356, 4233.1089, 4252.8618, 4280.2700, 4452.1279, 4465.1328, 4679.1919, 4844.0430

13. E1BKL4 **Mass:** 128535 **Score:** 24 **Expect:** 88 **Matches:** 25
Uncharacterized protein OS=Bos taurus GN=CILP2 PE=4 SV=1

Observed	Mr(expt)	Mr(calc)	ppm	Start	End	Miss	Peptide
717.4042	716.3969	716.3817	21.2	480 -	486	0	R.VVAADSR.E
772.4352	771.4280	771.4239	5.28	666 -	672	0	R.VAADQIR.M
843.4840	842.4767	842.4650	13.8	538 -	544	0	R.VLPFDPR.G
1113.4890	1112.4817	1112.4743	6.67	996 -	1004	0	K.CSGMLFDQR.Q
1145.6725	1144.6652	1144.6604	4.17	714 -	723	0	R.VFLVGNVEIR.E
1188.5890	1187.5817	1187.5645	14.5	640 -	649	0	R.TYGMFSVDLR.A
1220.6172	1219.6099	1219.6019	6.56	946 -	955	0	K.IQGPQEYMVR.S
1234.4895	1233.4822	1233.4721	8.23	1081 -	1091	0	R.CFDGSSDGFSR.E
1317.6417	1316.6344	1316.6659	-23.92	545 -	556	1	R.GAGVYHEVKAMR.K
1358.7172	1357.7099	1357.7102	-0.23	1107 -	1118	0	R.EPTAGRPSLFQR.L
1447.7806	1446.7733	1446.7653	5.54	944 -	955	1	K.VKIQGPQEYMVR.S
1461.8053	1460.7980	1460.7987	-0.48	1022 -	1034	0	R.VAVNGLLQDYLTR.H
1728.8602	1727.8529	1727.8519	0.61	903 -	916	1	R.VEADKYEYNVVPFR.E
1747.9176	1746.9103	1746.9199	-5.49	376 -	391	0	R.LTVLAPGQPACDPRPR.E
1887.9128	1886.9055	1886.9057	-0.10	884 -	899	1	R.SLRECQEAPVTASHFR.F
1910.9791	1909.9718	1909.9608	5.76	521 -	537	0	R.LVMTFVDPSGQFVDTVR.V
2020.9868	2019.9795	2019.9796	-0.04	978 -	995	1	R.SVRDPQRPSSSAACVEFK.C
2277.0820	2276.0747	2276.1186	-19.27	594 -	614	1	R.RADGQPYTGAVEAQVTFVDPR.N
2462.2893	2461.2820	2461.2866	-1.86	862 -	883	0	R.INLAKPRPGEPTEANGPVYPWR.S
2826.4065	2825.3992	2825.4269	-9.80	567 -	593	0	R.QSNTISLGEMGEEAPLGELIIPPGAFR.R
2995.5381	2994.5308	2994.5451	-4.76	494 -	520	1	R.ILLGREPIGFTSYQGDFTLEVAPSTQR.L
3334.6604	3333.6531	3333.7285	-22.62	650 -	681	2	R.AAGSAEQLQTGLVAVRVAADQIRMPGHMEALK.L + Oxidation (M)
3616.8076	3615.8003	3615.8209	-5.70	744 -	777	1	R.AYANDKFTPSEQVEGVVVTLVNLEPAPGFSANPR.A
3959.0081	3958.0008	3958.0119	-2.80	1095 -	1131	2	K.ADAGTAVTFQCREPTAGRPSLFQRLLESPSAALGDIR.R
4233.1089	4232.1016	4232.0089	21.9	392 -	430	2	R.EHLIKLPDDCGQPGRGPTYLDVGLCPDTLCPSPAGSSPR.C

No match to: 706.2851, 710.3807, 719.4268, 720.4332, 722.4348, 723.4337, 735.4208, 744.3633, 749.4401, 756.4190, 759.4415, 766.3942, 774.4582, 779.4799, 823.4933, 831.4758, 832.3166, 834.3207, 842.5047, 844.4902, 846.4869, 870.4950, 873.3982, 876.2891, 880.4586, 883.4816, 885.4853, 891.4897, 895.5765, 900.5383, 918.5458, 923.5204, 927.5097, 928.5096, 929.4465, 944.5216, 945.5218, 949.5538, 967.4249, 969.4326, 972.4861, 974.4482, 976.4711, 977.4844, 978.5469, 988.4647, 1007.5762, 1033.4938, 1034.5852, 1044.5280, 1056.5298, 1074.5454, 1087.5504, 1097.5125, 1103.5261, 1119.5367, 1122.5620, 1178.6276, 1185.6924, 1248.6737, 1253.6409, 1284.6428, 1301.6381, 1313.7157, 1314.7207, 1315.6769, 1316.6554, 1325.6414, 1327.7157, 1333.7577, 1336.7279, 1338.7021, 1349.7028, 1355.6765, 1361.6941, 1372.6039, 1381.7673, 1382.5994, 1383.7312, 1397.7480, 1442.8285, 1457.7437, 1479.8021, 1487.7090, 1497.8783, 1517.8105, 1528.7001, 1532.7877, 1537.8292, 1539.8185, 1552.8179, 1553.8821, 1556.8846, 1562.8353, 1567.7686, 1578.7893, 1592.7920, 1593.8013, 1628.7037, 1628.8643, 1642.7200, 1650.8497, 1651.8568, 1667.8695, 1680.8239, 1687.9133, 1691.7732, 1706.8671, 1715.8597, 1717.9054, 1724.8430, 1730.8619, 1771.0326, 1805.9197, 1809.0233, 1813.0017, 1820.9197, 1855.0103, 1894.9668, 1928.0238, 1953.8596, 1965.8589, 1974.9517, 1981.9934, 1983.9583, 1995.8975, 2022.0580, 2027.1263, 2044.1479, 2084.0510, 2110.0601, 2136.8467, 2144.0354, 2150.8611, 2155.0640, 2162.8757, 2164.8806, 2170.0522, 2174.1650, 2203.0303, 2217.0352, 2239.1282, 2251.1396, 2253.1372, 2255.2141, 2271.1794, 2274.0801, 2286.1489, 2289.0703, 2293.1492, 2297.1624, 2309.1501, 2324.0906, 2340.1016, 2343.1846, 2384.2053, 2410.1479, 2414.2322, 2464.2695, 2465.2659, 2529.2080, 2542.2549, 2645.0503, 2646.9685, 2657.4148, 2660.0061, 2660.9807, 2675.0012, 2689.0129, 2693.0125, 2703.0303, 2706.0225, 2718.3938, 2763.3359, 2774.3179, 2780.3301, 2787.3474, 2846.3816, 2872.3054, 2876.3391, 2884.3103, 2887.3201, 2889.3223, 2891.4019, 2895.4370, 2900.3384, 2915.4314, 2983.4602, 2999.4780, 3002.1853, 3016.2046, 3020.2017, 3030.2197, 3034.2266, 3044.2632, 3053.4902, 3064.5010, 3081.5806, 3126.7075, 3169.7002, 3206.5684, 3234.5972, 3248.6172, 3264.5881, 3299.7673, 3316.7351, 3320.6321, 3427.5977, 3439.7319, 3453.7163, 3457.6880, 3458.6204, 3473.6636, 3490.6494, 3503.7805, 3517.5906, 3529.7371, 3639.6938, 3652.7173, 3704.8706, 3893.7073, 3991.8828, 4147.9468, 4161.9458, 4177.9121, 4226.0356, 4252.8618, 4280.2700, 4452.1279, 4465.1328, 4679.1919, 4844.0430

14. G3MXC7 **Mass:** 12858 **Score:** 24 **Expect:** 92 **Matches:** 3
Uncharacterized protein OS=Bos taurus PE=4 SV=1

Observed	Mr(expt)	Mr(calc)	ppm	Start	End	Miss	Peptide
779.4799	778.4726	778.4701	3.28	29 -	34	1	K.YLIKSR.E
3126.7075	3125.7002	3125.6328	21.6	5 -	32	1	R.FLCCVTLCLLGAGLVDSGVTQTPKYLIK.S
4147.9468	4146.9395	4146.9282	2.72	42 -	76	2	R.CSPESGHRSVYWYQQALGQGPQFLVQYYNGKENEK.G

No match to: 706.2851, 710.3807, 717.4042, 719.4268, 720.4332, 722.4348, 723.4337, 735.4208, 744.3633, 749.4401, 756.4190, 759.4415, 766.3942, 772.4352, 774.4582, 823.4933, 831.4758, 832.3166, 834.3207, 842.5047, 843.4840, 844.4902, 846.4869, 870.4950, 873.3982, 876.2891, 880.4586, 883.4816, 885.4853, 891.4897, 895.5765, 900.5383, 918.5458, 923.5204, 927.5097, 928.5096, 929.4465, 944.5216, 945.5218, 949.5538, 967.4249, 969.4326, 972.4861, 974.4482, 976.4711, 977.4844, 978.5469, 988.4647, 1007.5762, 1033.4938, 1034.5852, 1044.5280, 1056.5298, 1074.5454, 1087.5504, 1097.5125, 1103.5261, 1113.4890, 1119.5367, 1122.5620, 1145.6725, 1178.6276, 1185.6924, 1188.5890, 1220.6172, 1234.4895, 1248.6737, 1253.6409, 1284.6428, 1301.6381, 1313.7157, 1314.7207, 1315.6769, 1316.6554, 1317.6417, 1325.6414, 1327.7157, 1333.7577, 1336.7279, 1338.7021, 1349.7028, 1355.6765, 1358.7172, 1361.6941, 1372.6039, 1381.7673, 1382.5994, 1383.7312, 1397.7480, 1442.8285, 1447.7806, 1457.7437, 1461.8053, 1479.8021, 1487.7090, 1497.8783, 1517.8105, 1528.7001, 1532.7877, 1537.8292, 1539.8185, 1552.8179, 1553.8821, 1556.8846, 1562.8353, 1567.7686, 1578.7893, 1592.7920, 1593.8013, 1628.7037, 1628.8643, 1642.7200, 1650.8497, 1651.8568, 1667.8695, 1680.8239, 1687.9133, 1691.7732, 1706.8671, 1715.8597, 1717.9054, 1724.8430, 1728.8602, 1730.8619, 1747.9176, 1771.0326, 1805.9197, 1809.0233, 1813.0017, 1820.9197, 1855.0103, 1887.9128, 1894.9668, 1910.9791, 1928.0238, 1953.8596, 1965.8589, 1974.9517, 1981.9934, 1983.9583, 1995.8975, 2020.9868, 2022.0580, 2027.1263, 2044.1479, 2084.0510, 2110.0601, 2136.8467, 2144.0354, 2150.8611, 2155.0640, 2162.8757, 2164.8806, 2170.0522, 2174.1650, 2203.0303, 2217.0352, 2239.1282, 2251.1396, 2253.1372, 2255.2141, 2271.1794, 2274.0801, 2277.0820, 2286.1489, 2289.0703, 2293.1492, 2297.1624, 2309.1501, 2324.0906, 2340.1016, 2343.1846, 2384.2053, 2410.1479, 2414.2322, 2462.2893, 2464.2695, 2465.2659, 2529.2080, 2542.2549, 2645.0503, 2646.9685, 2657.4148, 2660.0061, 2660.9807, 2675.0012, 2689.0129, 2693.0125, 2703.0303, 2706.0225, 2718.3938, 2763.3359, 2774.3179, 2780.3301, 2787.3474, 2826.4065, 2846.3816, 2872.3054, 2876.3391, 2884.3103, 2887.3201, 2889.3223, 2891.4019, 2895.4370, 2900.3384, 2915.4314, 2983.4602, 2995.5381, 2999.4780, 3002.1853, 3016.2046, 3020.2017, 3030.2197, 3034.2266, 3044.2632, 3053.4902, 3064.5010, 3081.5806, 3169.7002, 3206.5684, 3234.5972, 3248.6172, 3264.5881, 3299.7673, 3316.7351, 3320.6321, 3334.6604, 3427.5977, 3439.7319, 3453.7163, 3457.6880, 3458.6204, 3473.6636, 3490.6494, 3503.7805, 3517.5906, 3529.7371, 3616.8076, 3639.6938, 3652.7173, 3704.8706, 3893.7073, 3959.0081, 3991.8828, 4161.9458, 4177.9121, 4226.0356, 4233.1089, 4252.8618, 4280.2700, 4452.1279, 4465.1328, 4679.1919, 4844.0430

15. F1MQI1 **Mass:** 273592 **Score:** 22 **Expect:** 1.4e+002 **Matches:** 34

Uncharacterized protein OS=Bos taurus GN=TLN2 PE=4 SV=2

Observed	Mr(expt)	Mr(calc)	ppm	Start		End	Miss	Peptide
759.4415	758.4343	758.4174	22.2	1209	-	1215	0	K.DVDVALK.S
842.5047	841.4974	841.4770	24.3	1517	-	1524	1	K.TGNPVAKR.H
844.4902	843.4829	843.4675	18.3	275	-	281	2	K.QRGAEKR.I
846.4869	845.4796	845.4607	22.4	864	-	871	0	K.LLADSTAR.M
891.4897	890.4824	890.4974	-16.80	2473	-	2481	1	R.AAQKAAFGK.A
1034.5852	1033.5779	1033.5590	18.3	92	-	100	1	K.IRMLDGSVK.T + Oxidation (M)
1074.5454	1073.5381	1073.5215	15.4	69	-	76	0	R.TLDYYMLR.N
1248.6737	1247.6664	1247.6908	-19.50	332	-	342	1	R.LLGITKDSVMR.V + Oxidation (M)
1284.6428	1283.6355	1283.6591	-18.36	8	-	17	1	K.ICVRHCNVVK.T
1315.6769	1314.6696	1314.6489	15.7	1606	-	1616	0	K.TMLESSSYLIR.T + Oxidation (M)
1316.6554	1315.6481	1315.6732	-19.04	2198	-	2209	0	R.QEDVIATANLSR.K
1325.6414	1324.6341	1324.6367	-1.91	1863	-	1874	0	K.AIAVTAQEMMTK.S + 2 Oxidation (M)
1447.7806	1446.7733	1446.7566	11.6	1209	-	1222	1	K.DVDVALKSIGESSK.K
1532.7877	1531.7804	1531.7954	-9.79	2131	-	2144	2	K.TVKAVEDEATRGTR.A
1651.8568	1650.8495	1650.8876	-23.04	442	-	457	1	R.TGKVEHGSVALPAVMR.S
1667.8695	1666.8622	1666.8825	-12.15	442	-	457	1	R.TGKVEHGSVALPAVMR.S + Oxidation (M)
2027.1263	2026.1190	2026.1002	9.29	1	-	17	2	-.MVALSLKICVRHCNVVK.T
2144.0354	2143.0281	2143.0190	4.24	18	-	35	1	K.TMQFEPSTAVYDACRVIR.E
2155.0640	2154.0567	2154.0660	-4.32	2169	-	2188	2	K.TSSPEESIRMTKGITMATAK.A + Oxidation (M)
2239.1282	2238.1209	2238.1236	-1.19	101	-	120	1	K.TVMVDDSKTVGELLVTICSR.I + Oxidation (M)
2274.0801	2273.0728	2273.0668	2.66	1042	-	1062	0	K.AHEACGPMEIDSALSTVQTLK.S + Oxidation (M)
2277.0820	2276.0747	2276.0929	-7.98	282	-	300	2	R.IFQEHKNCGEMSEIEAKVK.Y
2293.1492	2292.1419	2292.0878	23.6	282	-	300	2	R.IFQEHKNCGEMSEIEAKVK.Y + Oxidation (M)
2340.1016	2339.0943	2339.1407	-19.82	165	-	183	2	K.AKLHTDDDLNWLDHSRTFR.E
2343.1846	2342.1773	2342.1583	8.12	1502	-	1523	2	K.HTSALCNACRIASSKTGNPVAK.R
2763.3359	2762.3286	2762.3327	-1.48	744	-	767	2	K.LVDRSVENCVRACQAATDDTELLK.Q
3030.2197	3029.2124	3029.2763	-21.08	800	-	826	0	R.YDQATDTIMCVTESIFSSMGDAGEMVR.Q + Oxidation (M)
3248.6172	3247.6099	3247.6805	-21.74	1186	-	1215	2	R.LAQVAKAVSHSLNNCVNCLPGQKDVDVALK.S
3299.7673	3298.7600	3298.7442	4.79	596	-	627	2	K.LLAALMDDEVGSGEDLLRAARTLAGAVSDLLK.A + Oxidation (M)
3320.6321	3319.6248	3319.6792	-16.38	1753	-	1781	1	K.ILDHQQMTVLDQTKTLAESALQMLYAAK.E + 2 Oxidation (M)
3427.5977	3426.5904	3426.6613	-20.68	412	-	441	2	R.FGLEGDEESTMLEESVSPKKSTILQQQFNR.T
3439.7319	3438.7246	3438.7273	-0.79	925	-	958	1	K.QAAAAATQTIAASQNAAVSNKNPAAQQQLVQSCK.A
3639.6938	3638.6865	3638.5997	23.9	794	-	826	1	R.GEPIGRYDQATDTIMCVTESIFSSMGDAGEMVR.Q + Oxidation (M)
4452.1279	4451.1206	4451.1268	-1.38	1088	-	1130	2	K.CAQDLGSTSKAVGSSMAQLLTCAAQGNEHYTGVAARETAQALK.T

No match to: 706.2851, 710.3807, 717.4042, 719.4268, 720.4332, 722.4348, 723.4337, 735.4208, 744.3633, 749.4401, 756.4190, 766.3942, 772.4352, 774.4582, 779.4799, 823.4933, 831.4758, 832.3166, 834.3207, 843.4840, 870.4950, 873.3982, 876.2891, 880.4586, 883.4816, 885.4853, 895.5765, 900.5383, 918.5458, 923.5204, 927.5097, 928.5096, 929.4465, 944.5216, 945.5218, 949.5538, 967.4249, 969.4326, 972.4861, 974.4482, 976.4711, 977.4844, 978.5469, 988.4647, 1007.5762, 1033.4938, 1044.5280, 1056.5298, 1087.5504, 1097.5125, 1103.5261, 1113.4890, 1119.5367, 1122.5620, 1145.6725, 1178.6276, 1185.6924, 1188.5890, 1220.6172, 1234.4895, 1253.6409, 1301.6381, 1313.7157, 1314.7207, 1317.6417, 1327.7157, 1333.7577, 1336.7279, 1338.7021, 1349.7028, 1355.6765, 1358.7172, 1361.6941, 1372.6039, 1381.7673, 1382.5994, 1383.7312, 1397.7480, 1442.8285, 1457.7437, 1461.8053, 1479.8021, 1487.7090, 1497.8783, 1517.8105, 1528.7001, 1537.8292, 1539.8185, 1552.8179, 1553.8821, 1556.8846, 1562.8353, 1567.7686, 1578.7893, 1592.7920, 1593.8013, 1628.7037, 1628.8643, 1642.7200, 1650.8497, 1680.8239, 1687.9133, 1691.7732, 1706.8671, 1715.8597, 1717.9054, 1724.8430, 1728.8602, 1730.8619, 1747.9176, 1771.0326, 1805.9197, 1809.0233, 1813.0017, 1820.9197, 1855.0103, 1887.9128, 1894.9668, 1910.9791, 1928.0238, 1953.8596, 1965.8589, 1974.9517, 1981.9934, 1983.9583, 1995.8975, 2020.9868, 2022.0580, 2044.1479, 2084.0510, 2110.0601, 2136.8467, 2150.8611, 2162.8757, 2164.8806, 2170.0522, 2174.1650, 2203.0303, 2217.0352, 2251.1396, 2253.1372, 2255.2141, 2271.1794, 2286.1489, 2289.0703, 2297.1624, 2309.1501, 2324.0906, 2384.2053, 2410.1479, 2414.2322, 2462.2893, 2464.2695, 2465.2659, 2529.2080, 2542.2549, 2645.0503, 2646.9685, 2657.4148, 2660.0061, 2660.9807, 2675.0012, 2689.0129, 2693.0125, 2703.0303, 2706.0225, 2718.3938, 2774.3179, 2780.3301, 2787.3474, 2826.4065, 2846.3816, 2872.3054, 2876.3391, 2884.3103, 2887.3201, 2889.3223, 2891.4019, 2895.4370, 2900.3384, 2915.4314, 2983.4602, 2995.5381, 2999.4780, 3002.1853, 3016.2046, 3020.2017, 3034.2266, 3044.2632, 3053.4902, 3064.5010, 3081.5806, 3126.7075, 3169.7002, 3206.5684, 3234.5972, 3264.5881, 3316.7351, 3334.6604, 3453.7163, 3457.6880, 3458.6204, 3473.6636, 3490.6494, 3503.7805, 3517.5906, 3529.7371, 3616.8076, 3652.7173, 3704.8706, 3893.7073, 3959.0081, 3991.8828, 4147.9468, 4161.9458, 4177.9121, 4226.0356, 4233.1089, 4252.8618, 4280.2700, 4465.1328, 4679.1919, 4844.0430

16. A6QR51 **Mass:** 63694 **Score:** 22 **Expect:** 1.6e+002 **Matches:** 10

CNOT6 protein OS=Bos taurus GN=CNOT6 PE=2 SV=1

Observed	Mr(expt)	Mr(calc)	ppm	Start		End	Miss	Peptide
873.3982	872.3909	872.4028	-13.64	6	-	12	0	K.YEPPDPR.R
1284.6428	1283.6355	1283.6510	-12.05	29	-	39	0	K.SHWVELEISGK.V
1301.6381	1300.6308	1300.6200	8.30	256	-	266	1	K.ERGYNGFFSPK.S
1355.6765	1354.6692	1354.6881	-13.92	2	-	12	2	M.PKEKYEPPDPR.R
1457.7437	1456.7364	1456.7306	4.01	373	-	384	0	K.LVQTMMFLSEVK.N + 2 Oxidation (M)
1539.8185	1538.8112	1538.8205	-6.04	29	-	41	1	K.SHWVELEISGKVR.S
1691.7732	1690.7659	1690.7542	6.95	14	-	28	1	R.MYTIMSSEEAANGKK.S + 2 Oxidation (M)
1771.0326	1770.0253	1770.0073	10.2	320	-	335	1	R.VMTKDNIGVAVLLELR.K
4233.1089	4232.1016	4232.1028	-0.27	298	-	335	2	K.HTVEFNQLAMANSEGSEAMLNRVMTKDNIGVAVLLELR.K + 2 Oxidation (M)
4452.1279	4451.1206	4451.1534	-7.37	164	-	200	2	R.IATEQPPPRSWIMLQEPDRTRPTALFSVMCYNVLCDK.Y + 2 Oxidation (M)

No match to: 706.2851, 710.3807, 717.4042, 719.4268, 720.4332, 722.4348, 723.4337, 735.4208, 744.3633, 749.4401, 756.4190, 759.4415, 766.3942, 772.4352, 774.4582, 779.4799, 823.4933, 831.4758, 832.3166, 834.3207, 842.5047, 843.4840, 844.4902, 846.4869, 870.4950, 876.2891, 880.4586, 883.4816, 885.4853, 891.4897, 895.5765, 900.5383, 918.5458, 923.5204, 927.5097, 928.5096, 929.4465, 944.5216, 945.5218, 949.5538, 967.4249, 969.4326, 972.4861, 974.4482, 976.4711, 977.4844, 978.5469, 988.4647, 1007.5762, 1033.4938, 1034.5852, 1044.5280, 1056.5298, 1074.5454, 1087.5504, 1097.5125, 1103.5261, 1113.4890, 1119.5367, 1122.5620, 1145.6725, 1178.6276, 1185.6924, 1188.5890, 1220.6172, 1234.4895, 1248.6737, 1253.6409, 1313.7157, 1314.7207, 1315.6769, 1316.6554, 1317.6417, 1325.6414, 1327.7157, 1333.7577, 1336.7279, 1338.7021, 1349.7028, 1358.7172, 1361.6941, 1372.6039, 1381.7673, 1382.5994, 1383.7312, 1397.7480, 1442.8285, 1447.7806, 1461.8053, 1479.8021, 1487.7090, 1497.8783, 1517.8105, 1528.7001, 1532.7877, 1537.8292, 1552.8179, 1553.8821, 1556.8846, 1562.8353, 1567.7686, 1578.7893, 1592.7920, 1593.8013, 1628.7037, 1628.8643, 1642.7200, 1650.8497, 1651.8568, 1667.8695, 1680.8239, 1687.9133, 1706.8671, 1715.8597, 1717.9054, 1724.8430, 1728.8602, 1730.8619, 1747.9176, 1805.9197, 1809.0233, 1813.0017, 1820.9197, 1855.0103, 1887.9128, 1894.9668, 1910.9791, 1928.0238, 1953.8596, 1965.8589, 1974.9517, 1981.9934, 1983.9583, 1995.8975, 2020.9868, 2022.0580, 2027.1263, 2044.1479, 2084.0510, 2110.0601, 2136.8467, 2144.0354, 2150.8611, 2155.0640, 2162.8757, 2164.8806, 2170.0522, 2174.1650, 2203.0303, 2217.0352, 2239.1282, 2251.1396, 2253.1372, 2255.2141, 2271.1794, 2274.0801, 2277.0820, 2286.1489, 2289.0703, 2293.1492, 2297.1624, 2309.1501, 2324.0906, 2340.1016, 2343.1846, 2384.2053, 2410.1479, 2414.2322, 2462.2893, 2464.2695, 2465.2659, 2529.2080, 2542.2549, 2645.0503, 2646.9685, 2657.4148, 2660.0061, 2660.9807, 2675.0012, 2689.0129, 2693.0125, 2703.0303, 2706.0225, 2718.3938, 2763.3359, 2774.3179, 2780.3301, 2787.3474, 2826.4065, 2846.3816, 2872.3054, 2876.3391, 2884.3103, 2887.3201, 2889.3223, 2891.4019, 2895.4370, 2900.3384, 2915.4314, 2983.4602, 2995.5381, 2999.4780, 3002.1853, 3016.2046, 3020.2017, 3030.2197, 3034.2266, 3044.2632, 3053.4902, 3064.5010, 3081.5806, 3126.7075, 3169.7002, 3206.5684, 3234.5972, 3248.6172, 3264.5881, 3299.7673, 3316.7351, 3320.6321, 3334.6604, 3427.5977, 3439.7319, 3453.7163, 3457.6880, 3458.6204, 3473.6636, 3490.6494, 3503.7805, 3517.5906, 3529.7371, 3616.8076, 3639.6938, 3652.7173, 3704.8706, 3893.7073, 3959.0081, 3991.8828, 4147.9468, 4161.9458, 4177.9121, 4226.0356, 4252.8618, 4280.2700, 4465.1328, 4679.1919, 4844.0430

17. E1BP29 **Mass:** 68878 **Score:** 22 **Expect:** 1.6e+002 **Matches:** 14
Protein-serine/threonine kinase OS=Bos taurus GN=GRK6 PE=3 SV=2

Observed	Mr(expt)	Mr(calc)	ppm	Start	End	Miss	Peptide
1122.5620	1121.5547	1121.5573	-2.29	420 -	428	0	R.SLCTQLLCK.D
1349.7028	1348.6955	1348.7061	-7.83	348 -	360	0	R.VGTVGYMAPEVVK.N
1381.7673	1380.7600	1380.7626	-1.87	184 -	194	2	K.NTFRQYRVLGK.G
1537.8292	1536.8219	1536.8161	3.79	179 -	190	2	R.QPVTKNTFRQYR.V
1562.8353	1561.8280	1561.8287	-0.41	346 -	360	1	K.GRVGTVGYMAPEVVK.N
1717.9054	1716.8981	1716.9080	-5.73	228 -	242	2	R.KGEAMALNEKQILEK.V + Oxidation (M)
2274.0801	2273.0728	2273.0834	-4.63	30 -	46	1	K.WRQMLQFPHISQCEELR.L + Oxidation (M)
2340.1016	2339.0943	2339.1467	-22.40	80 -	99	2	R.CIAFLDGVAEYEVTPDEKRK.A
2787.3474	2786.3401	2786.3511	-3.94	397 -	419	2	R.EEVERLVKEVPEEYSEHFSPQAR.S
3234.5972	3233.5899	3233.6438	-16.65	105 -	132	1	R.LMQNFLSHTGPDLIPEVPRQLVTNCAQR.L
3248.6172	3247.6099	3247.6005	2.89	402 -	428	2	R.LVKEVPEEYSEHFSPQARSLCTQLLCK.D
3264.5881	3263.5808	3263.5209	18.4	364 -	390	0	R.YTFSPDWWALGCLLYEMIAGQSPFQQR.K
3490.6494	3489.6421	3489.6996	-16.46	259 -	289	1	K.DALCLVLTLMNGGDLKFHIYHMGQAGFPEAR.A + Oxidation (M)
4452.1279	4451.1206	4451.0767	9.88	504 -	542	0	K.FATGSVPIPWQNEMVETECFQELNVFGLDGSVPPDLDWK.G

No match to: 706.2851, 710.3807, 717.4042, 719.4268, 720.4332, 722.4348, 723.4337, 735.4208, 744.3633, 749.4401, 756.4190, 759.4415, 766.3942, 772.4352, 774.4582, 779.4799, 823.4933, 831.4758, 832.3166, 834.3207, 842.5047, 843.4840, 844.4902, 846.4869, 870.4950, 873.3982, 876.2891, 880.4586, 883.4816, 885.4853, 891.4897, 895.5765, 900.5383, 918.5458, 923.5204, 927.5097, 928.5096, 929.4465, 944.5216, 945.5218, 949.5538, 967.4249, 969.4326, 972.4861, 974.4482, 976.4711, 977.4844, 978.5469, 988.4647, 1007.5762, 1033.4938, 1034.5852, 1044.5280, 1056.5298, 1074.5454, 1087.5504, 1097.5125, 1103.5261, 1113.4890, 1119.5367, 1145.6725, 1178.6276, 1185.6924, 1188.5890, 1220.6172, 1234.4895, 1248.6737, 1253.6409, 1284.6428, 1301.6381, 1313.7157, 1314.7207, 1315.6769, 1316.6554, 1317.6417, 1325.6414, 1327.7157, 1333.7577, 1336.7279, 1338.7021, 1355.6765, 1358.7172, 1361.6941, 1372.6039, 1382.5994, 1383.7312, 1397.7480, 1442.8285, 1447.7806, 1457.7437, 1461.8053, 1479.8021, 1487.7090, 1497.8783, 1517.8105, 1528.7001, 1532.7877, 1539.8185, 1552.8179, 1553.8821, 1556.8846, 1567.7686, 1578.7893, 1592.7920, 1593.8013, 1628.7037, 1628.8643, 1642.7200, 1650.8497, 1651.8568, 1667.8695, 1680.8239, 1687.9133, 1691.7732, 1706.8671, 1715.8597, 1724.8430, 1728.8602, 1730.8619, 1747.9176, 1771.0326, 1805.9197, 1809.0233, 1813.0017, 1820.9197, 1855.0103, 1887.9128, 1894.9668, 1910.9791, 1928.0238, 1953.8596, 1965.8589, 1974.9517, 1981.9934, 1983.9583, 1995.8975, 2020.9868, 2022.0580, 2027.1263, 2044.1479, 2084.0510, 2110.0601, 2136.8467, 2144.0354, 2150.8611, 2155.0640, 2162.8757, 2164.8806, 2170.0522, 2174.1650, 2203.0303, 2217.0352, 2239.1282, 2251.1396, 2253.1372, 2255.2141, 2271.1794, 2277.0820, 2286.1489, 2289.0703, 2293.1492, 2297.1624, 2309.1501, 2324.0906, 2343.1846, 2384.2053, 2410.1479, 2414.2322, 2462.2893, 2464.2695, 2465.2659, 2529.2080, 2542.2549, 2645.0503, 2646.9685, 2657.4148, 2660.0061, 2660.9807, 2675.0012, 2689.0129, 2693.0125, 2703.0303, 2706.0225, 2718.3938, 2763.3359, 2774.3179, 2780.3301, 2826.4065, 2846.3816, 2872.3054, 2876.3391, 2884.3103, 2887.3201, 2889.3223, 2891.4019, 2895.4370, 2900.3384, 2915.4314, 2983.4602, 2995.5381, 2999.4780, 3002.1853, 3016.2046, 3020.2017, 3030.2197, 3034.2266, 3044.2632, 3053.4902, 3064.5010, 3081.5806, 3126.7075, 3169.7002, 3206.5684, 3299.7673, 3316.7351, 3320.6321, 3334.6604, 3427.5977, 3439.7319, 3453.7163, 3457.6880, 3458.6204, 3473.6636, 3503.7805, 3517.5906, 3529.7371, 3616.8076, 3639.6938, 3652.7173, 3704.8706, 3893.7073, 3959.0081, 3991.8828, 4147.9468, 4161.9458, 4177.9121, 4226.0356, 4233.1089, 4252.8618, 4280.2700, 4465.1328, 4679.1919, 4844.0430

18. E1BNK9 **Mass:** 20498 **Score:** 22 **Expect:** 1.6e+002 **Matches:** 4
Uncharacterized protein OS=Bos taurus GN=FAM219B PE=4 SV=1

Observed	Mr(expt)	Mr(calc)	ppm	Start	End	Miss	Peptide
722.4348	721.4275	721.4123	21.2	96 -	101	1	R.SVKFNK.G
1457.7437	1456.7364	1456.7092	18.7	80 -	94	0	K.GMLGAAPNRPDSSGK.R
1928.0238	1927.0165	1927.0058	5.59	80 -	98	2	K.GMLGAAPNRPDSSGKRSVK.F
2884.3103	2883.3030	2883.2894	4.74	102 -	128	0	K.GYTALSQSPDENLVSLDSDSDGELESR.Y

No match to: 706.2851, 710.3807, 717.4042, 719.4268, 720.4332, 723.4337, 735.4208, 744.3633, 749.4401, 756.4190, 759.4415, 766.3942, 772.4352, 774.4582, 779.4799, 823.4933, 831.4758, 832.3166, 834.3207, 842.5047, 843.4840, 844.4902, 846.4869, 870.4950, 873.3982, 876.2891, 880.4586, 883.4816, 885.4853, 891.4897, 895.5765, 900.5383, 918.5458, 923.5204, 927.5097, 928.5096, 929.4465, 944.5216, 945.5218, 949.5538, 967.4249, 969.4326, 972.4861, 974.4482, 976.4711, 977.4844, 978.5469, 988.4647, 1007.5762, 1033.4938, 1034.5852, 1044.5280, 1056.5298, 1074.5454, 1087.5504, 1097.5125, 1103.5261, 1113.4890, 1119.5367, 1122.5620, 1145.6725, 1178.6276, 1185.6924, 1188.5890, 1220.6172, 1234.4895, 1248.6737, 1253.6409, 1284.6428, 1301.6381, 1313.7157, 1314.7207, 1315.6769, 1316.6554, 1317.6417, 1325.6414, 1327.7157, 1333.7577, 1336.7279, 1338.7021, 1349.7028, 1355.6765, 1358.7172, 1361.6941, 1381.7673, 1372.6039, 1382.5994, 1383.7312, 1397.7480, 1442.8285, 1447.7806, 1461.8053, 1479.8021, 1487.7090, 1497.8783, 1517.8105, 1528.7001, 1532.7877, 1537.8292, 1539.8185, 1552.8179, 1553.8821, 1556.8846, 1562.8353, 1567.7686, 1578.7893, 1592.7920, 1593.8013, 1628.7037, 1628.8643, 1642.7200, 1650.8497, 1651.8568, 1667.8695, 1680.8239, 1687.9133, 1691.7732, 1706.8671, 1715.8597, 1717.9054, 1724.8430, 1728.8602, 1730.8619, 1747.9176, 1771.0326, 1805.9197, 1809.0233, 1813.0017, 1820.9197, 1855.0103, 1887.9128, 1894.9668, 1910.9791, 1953.8596, 1965.8589, 1974.9517, 1981.9934, 1983.9583, 1995.8975, 2020.9868, 2022.0580, 2027.1263, 2044.1479, 2084.0510, 2110.0601, 2136.8467, 2144.0354, 2150.8611, 2155.0640, 2162.8757, 2164.8806, 2170.0522, 2174.1650, 2203.0303, 2217.0352, 2239.1282, 2251.1396, 2253.1372, 2255.2141, 2271.1794, 2274.0801, 2277.0820, 2286.1489, 2289.0703, 2293.1492, 2297.1624, 2309.1501, 2324.0906, 2340.1016, 2343.1846, 2384.2053, 2410.1479, 2414.2322, 2462.2893, 2464.2695, 2465.2659, 2529.2080, 2542.2549, 2645.0503, 2646.9685, 2657.4148, 2660.0061, 2660.9807, 2675.0012, 2689.0129, 2693.0125, 2703.0303, 2706.0225, 2718.3938, 2763.3359, 2774.3179, 2780.3301, 2787.3474, 2826.4065, 2846.3816, 2872.3054, 2876.3391, 2887.3201, 2889.3223, 2891.4019, 2895.4370, 2900.3384, 2915.4314, 2983.4602, 2995.5381, 2999.4780, 3002.1853, 3016.2046, 3020.2017, 3030.2197, 3034.2266, 3044.2632, 3053.4902, 3064.5010, 3081.5806, 3126.7075, 3169.7002, 3206.5684, 3234.5972, 3248.6172, 3264.5881, 3299.7673, 3316.7351, 3320.6321, 3334.6604, 3427.5977, 3439.7319, 3453.7163, 3457.6880, 3458.6204, 3473.6636, 3490.6494, 3503.7805, 3517.5906, 3529.7371, 3616.8076, 3639.6938, 3652.7173, 3704.8706, 3893.7073, 3959.0081, 3991.8828, 4147.9468, 4161.9458, 4177.9121, 4226.0356, 4233.1089, 4252.8618, 4280.2700, 4452.1279, 4465.1328, 4679.1919, 4844.0430

19. Q5E9X7 **Mass:** 46964 **Score:** 22 **Expect:** 1.6e+002 **Matches:** 8
Inactive serine protease 35 OS=Bos taurus GN=PRSS35 PE=2 SV=2

Observed	Mr(expt)	Mr(calc)	ppm	Start	End	Miss	Peptide
772.4352	771.4280	771.4239	5.30	355 -	360	2	K.EPDKKR.W
1253.6409	1252.6336	1252.6373	-2.95	289 -	299	0	K.YMELGVSPTIK.K + Oxidation (M)
1381.7673	1380.7600	1380.7323	20.1	289 -	300	1	K.YMELGVSPTIKK.L + Oxidation (M)
1628.8643	1627.8570	1627.8206	22.4	123 -	136	1	R.QVYGTDSRFSILDK.K
1820.9197	1819.9124	1819.8676	24.6	300 -	315	1	K.KLPGGMIHFSGFDQDR.A + Oxidation (M)
2995.5381	2994.5308	2994.5828	-17.36	364 -	389	2	R.KIIAIYSGHQWVDVHGVQKDYNVAVR.I
3439.7319	3438.7246	3438.7154	2.67	69 -	99	2	K.DLPAPSLSDLEDSLSYETVFENGSRTLTRVK.V
4147.9468	4146.9395	4147.0158	-18.40	2 -	36	2	M.GAMFFGLMLFTLGWTLIDGSESEQDFMWHVRKIPR.L + 2 Oxidation (M)

No match to: 706.2851, 710.3807, 717.4042, 719.4268, 720.4332, 722.4348, 723.4337, 735.4208, 744.3633, 749.4401, 756.4190, 759.4415, 766.3942, 774.4582, 779.4799, 823.4933, 831.4758, 832.3166, 834.3207, 842.5047, 843.4840, 844.4902, 846.4869, 870.4950, 873.3982, 876.2891, 880.4586, 883.4816, 885.4853, 891.4897, 895.5765, 900.5383, 918.5458, 923.5204, 927.5097, 928.5096, 929.4465, 944.5216, 945.5218, 949.5538, 967.4249, 969.4326, 972.4861, 974.4482, 976.4711, 977.4844, 978.5469, 988.4647, 1007.5762, 1033.4938, 1034.5852, 1044.5280, 1056.5298, 1074.5454, 1087.5504, 1097.5125, 1103.5261, 1113.4890, 1119.5367, 1122.5620, 1145.6725, 1178.6276, 1185.6924, 1188.5890, 1220.6172, 1234.4895, 1248.6737, 1284.6428, 1301.6381, 1313.7157, 1314.7207, 1315.6769, 1316.6554, 1317.6417, 1325.6414, 1327.7157, 1333.7577, 1336.7279, 1338.7021, 1349.7028, 1355.6765, 1358.7172, 1361.6941, 1372.6039, 1382.5994, 1383.7312, 1397.7480, 1442.8285, 1447.7806, 1457.7437, 1461.8053, 1479.8021, 1487.7090, 1497.8783, 1517.8105, 1528.7001, 1532.7877, 1537.8292, 1539.8185, 1552.8179, 1553.8821, 1556.8846, 1562.8353, 1567.7686, 1578.7893, 1592.7920, 1593.8013, 1628.7037, 1642.7200, 1650.8497, 1651.8568, 1667.8695, 1680.8239, 1687.9133, 1691.7732, 1706.8671, 1715.8597, 1717.9054, 1724.8430, 1728.8602, 1730.8619, 1747.9176, 1771.0326, 1805.9197, 1809.0233, 1813.0017, 1855.0103, 1887.9128, 1894.9668, 1910.9791, 1928.0238, 1953.8596, 1965.8589, 1974.9517, 1981.9934, 1983.9583, 1995.8975, 2020.9868, 2022.0580, 2027.1263, 2044.1479, 2084.0510, 2110.0601, 2136.8467, 2144.0354, 2150.8611, 2155.0640, 2162.8757, 2164.8806, 2170.0522, 2174.1650, 2203.0303, 2217.0352, 2239.1282, 2251.1396, 2253.1372, 2255.2141, 2271.1794, 2274.0801, 2277.0820, 2286.1489, 2289.0703, 2293.1492, 2297.1624, 2309.1501, 2324.0906, 2340.1016, 2343.1846, 2384.2053, 2410.1479, 2414.2322, 2462.2893, 2464.2695, 2465.2659, 2529.2080, 2542.2549, 2645.0503, 2646.9685, 2657.4148, 2660.0061, 2660.9807, 2675.0012,

2689.0129, 2693.0125, 2703.0303, 2706.0225, 2718.3938, 2763.3359, 2774.3179, 2780.3301, 2787.3474, 2826.4065, 2846.3816, 2872.3054, 2876.3391, 2884.3103, 2887.3201, 2889.3223, 2891.4019, 2895.4370, 2900.3384, 2915.4314, 2983.4602, 2999.4780, 3002.1853, 3016.2046, 3020.2017, 3030.2197, 3034.2266, 3044.2632, 3053.4902, 3064.5010, 3081.5806, 3126.7075, 3169.7002, 3206.5684, 3234.5972, 3248.6172, 3264.5881, 3299.7673, 3316.7351, 3320.6321, 3334.6604, 3427.5977, 3453.7163, 3457.6880, 3458.6204, 3473.6636, 3490.6494, 3503.7805, 3517.5906, 3529.7371, 3616.8076, 3639.6938, 3652.7173, 3704.8706, 3893.7073, 3959.0081, 3991.8828, 4161.9458, 4177.9121, 4226.0356, 4233.1089, 4252.8618, 4280.2700, 4452.1279, 4465.1328, 4679.1919, 4844.0430

20. G3X761 **Mass:** 54315 **Score:** 22 **Expect:** 1.6e+002 **Matches:** 7

Uncharacterized protein OS=Bos taurus GN=PLD4 PE=4 SV=1

Observed	Mr(expt)	Mr(calc)	ppm	Start		End	Miss	Peptide
1315.6769	1314.6696	1314.6965	-20.48	320	-	332	0	R.DLDALLAVMGAAR.E
1361.6941	1360.6868	1360.6656	15.6	381	-	393	0	R.LSNPAANVSMDVK.V + Oxidation (M)
2324.0906	2323.0833	2323.0957	-5.32	217	-	235	1	K.FWVVDGRHIYVGSANMDWR.S + Oxidation (M)
3439.7319	3438.7246	3438.6952	8.56	318	-	347	2	R.TRDLDALLAVMGAAREFLYASVMEYFPTTR.F + 2 Oxidation (M)
3453.7163	3452.7090	3452.7261	-4.94	320	-	349	2	R.DLDALLAVMGAAREFLYASVMEYFPTTRFR.H
3616.8076	3615.8003	3615.7192	22.4	415	-	447	1	K.FMVTEKAAYIGTSNWSEDYFSSTSGVGLVVSQR.A
4233.1089	4232.1016	4232.1834	-19.32	92	-	130	2	K.DSCRLVLVESIPQDLRSVAGSPAAQPLAQAWMQLLDAAR.E

No match to: 706.2851, 710.3807, 717.4042, 719.4268, 720.4332, 722.4348, 723.4337, 735.4208, 744.3633, 749.4401, 756.4190, 759.4415, 766.3942, 772.4352, 774.4582, 779.4799, 823.4933, 831.4758, 832.3166, 834.3207, 842.5047, 843.4840, 844.4902, 846.4869, 870.4950, 873.3982, 876.2891, 880.4586, 883.4816, 885.4853, 891.4897, 895.5765, 900.5383, 918.5458, 923.5204, 927.5097, 928.5096, 929.4465, 944.5216, 945.5218, 949.5538, 967.4249, 969.4326, 972.4861, 974.4482, 976.4711, 977.4844, 978.5469, 988.4647, 1007.5762, 1033.4938, 1034.5852, 1044.5280, 1056.5298, 1074.5454, 1087.5504, 1097.5125, 1103.5261, 1113.4890, 1119.5367, 1122.5620, 1145.6725, 1178.6276, 1185.6924, 1188.5890, 1220.6172, 1234.4895, 1248.6737, 1253.6409, 1284.6428, 1301.6381, 1313.7157, 1314.7207, 1316.6554, 1317.6417, 1325.6414, 1327.7157, 1333.7577, 1336.7279, 1338.7021, 1349.7028, 1355.6765, 1358.7172, 1372.6039, 1381.7673, 1382.5994, 1383.7312, 1397.7480, 1442.8285, 1447.7806, 1457.7437, 1461.8053, 1479.8021, 1487.7090, 1497.8783, 1517.8105, 1528.7001, 1532.7877, 1537.8292, 1539.8185, 1552.8179, 1553.8821, 1556.8846, 1562.8353, 1567.7686, 1578.7893, 1592.7920, 1593.8013, 1628.7037, 1628.8643, 1642.7200, 1650.8497, 1651.8568, 1667.8695, 1680.8239, 1687.9133, 1691.7732, 1706.8671, 1715.8597, 1717.9054, 1724.8430, 1728.8602, 1730.8619, 1747.9176, 1771.0326, 1805.9197, 1809.0233, 1813.0017, 1820.9197, 1855.0103, 1887.9128, 1894.9668, 1910.9791, 1928.0238, 1953.8596, 1965.8589, 1974.9517, 1981.9934, 1983.9583, 1995.8975, 2020.9868, 2022.0580, 2027.1263, 2044.1479, 2084.0510, 2110.0601, 2136.8467, 2144.0354, 2150.8611, 2155.0640, 2162.8757, 2164.8806, 2170.0522, 2174.1650, 2203.0303, 2217.0352, 2239.1282, 2251.1396, 2253.1372, 2255.2141, 2271.1794, 2274.0801, 2277.0820, 2286.1489, 2289.0703, 2293.1492, 2297.1624, 2309.1501, 2340.1016, 2343.1846, 2384.2053, 2410.1479, 2414.2322, 2462.2893, 2464.2695, 2465.2659, 2529.2080, 2542.2549, 2645.0503, 2646.9685, 2657.4148, 2660.0061, 2660.9807, 2675.0012, 2689.0129, 2693.0125, 2703.0303, 2706.0225, 2718.3938, 2763.3359, 2774.3179, 2780.3301, 2787.3474, 2826.4065, 2846.3816, 2872.3054, 2876.3391, 2884.3103, 2887.3201, 2889.3223, 2891.4019, 2895.4370, 2900.3384, 2915.4314, 2983.4602, 2995.5381, 2999.4780, 3002.1853, 3016.2046, 3020.2017, 3030.2197, 3034.2266, 3044.2632, 3053.4902, 3064.5010, 3081.5806, 3126.7075, 3169.7002, 3206.5684, 3234.5972, 3248.6172, 3264.5881, 3299.7673, 3316.7351, 3320.6321, 3334.6604, 3427.5977, 3457.6880, 3458.6204, 3473.6636, 3490.6494, 3503.7805, 3517.5906, 3529.7371, 3639.6938, 3652.7173, 3704.8706, 3893.7073, 3959.0081, 3991.8828, 4147.9468, 4161.9458, 4177.9121, 4226.0356, 4252.8618, 4280.2700, 4452.1279, 4465.1328, 4679.1919, 4844.0430

21. Q58DQ8 **Mass:** 29298 **Score:** 21 **Expect:** 2.1e+002 **Matches:** 5

Putative uncharacterized protein FLJ21128 OS=Bos taurus GN=FLJ21128 PE=2 SV=1

Observed	Mr(expt)	Mr(calc)	ppm	Start		End	Miss	Peptide
1220.6172	1219.6099	1219.6343	-19.97	177	-	187	2	K.SGLKNGQMKNK.D + Oxidation (M)
1562.8353	1561.8280	1561.7961	20.5	145	-	157	2	R.HTESLSQSKSFRR.R
1953.8596	1952.8523	1952.8608	-4.36	249	-	266	0	R.TSWSQSPLSTVGSQCPTM.-
2324.0906	2323.0833	2323.0863	-1.27	125	-	144	1	K.SQDVAISPQQQQCSKSYVDR.H
4147.9468	4146.9395	4147.0137	-17.89	227	-	266	2	R.GEVVPVPGASQTRGLLGTATMRTSWSQSPLSTVGSQCPTM.- + Oxidation (M)

No match to: 706.2851, 710.3807, 717.4042, 719.4268, 720.4332, 722.4348, 723.4337, 735.4208, 744.3633, 749.4401, 756.4190, 759.4415, 766.3942, 772.4352, 774.4582, 779.4799, 823.4933, 831.4758, 832.3166, 834.3207, 842.5047, 843.4840, 844.4902, 846.4869, 870.4950, 873.3982, 876.2891, 880.4586, 883.4816, 885.4853, 891.4897, 895.5765, 900.5383, 918.5458, 923.5204, 927.5097, 928.5096, 929.4465, 944.5216, 945.5218, 949.5538, 967.4249, 969.4326, 972.4861, 974.4482, 976.4711, 977.4844, 978.5469, 988.4647, 1007.5762, 1033.4938, 1034.5852, 1044.5280, 1056.5298, 1074.5454, 1087.5504, 1097.5125, 1103.5261, 1113.4890, 1119.5367, 1122.5620, 1145.6725, 1178.6276, 1185.6924, 1188.5890, 1234.4895, 1248.6737, 1253.6409, 1284.6428, 1301.6381, 1313.7157, 1314.7207, 1315.6769, 1316.6554, 1317.6417, 1325.6414, 1327.7157, 1333.7577, 1336.7279, 1338.7021, 1349.7028, 1355.6765, 1358.7172, 1361.6941, 1372.6039, 1381.7673, 1382.5994, 1383.7312, 1397.7480, 1442.8285, 1447.7806, 1457.7437, 1461.8053, 1479.8021, 1487.7090, 1497.8783, 1517.8105, 1528.7001, 1532.7877, 1537.8292, 1539.8185, 1552.8179, 1553.8821, 1556.8846, 1567.7686, 1578.7893, 1592.7920, 1593.8013, 1628.7037, 1628.8643, 1642.7200, 1650.8497, 1651.8568, 1667.8695, 1680.8239, 1687.9133, 1691.7732, 1706.8671, 1715.8597, 1717.9054, 1724.8430, 1728.8602, 1730.8619, 1747.9176, 1771.0326, 1805.9197, 1809.0233, 1813.0017, 1820.9197, 1855.0103, 1887.9128, 1894.9668, 1910.9791, 1928.0238, 1965.8589, 1974.9517, 1981.9934, 1983.9583, 1995.8975, 2020.9868, 2022.0580, 2027.1263, 2044.1479, 2084.0510, 2110.0601, 2136.8467, 2144.0354, 2150.8611, 2155.0640, 2162.8757, 2164.8806, 2170.0522, 2174.1650, 2203.0303, 2217.0352, 2239.1282, 2251.1396, 2253.1372, 2255.2141, 2271.1794, 2274.0801, 2277.0820, 2286.1489, 2289.0703, 2293.1492, 2297.1624, 2309.1501, 2340.1016, 2343.1846, 2384.2053, 2410.1479, 2414.2322, 2462.2893, 2464.2695, 2465.2659, 2529.2080, 2542.2549, 2645.0503, 2646.9685, 2657.4148, 2660.0061, 2660.9807, 2675.0012, 2689.0129, 2693.0125, 2703.0303, 2706.0225, 2718.3938, 2763.3359, 2774.3179, 2780.3301, 2787.3474, 2826.4065, 2846.3816, 2872.3054, 2876.3391, 2884.3103, 2887.3201, 2889.3223, 2891.4019, 2895.4370, 2900.3384, 2915.4314, 2983.4602, 2995.5381, 2999.4780, 3002.1853, 3016.2046, 3020.2017, 3030.2197, 3034.2266, 3044.2632, 3053.4902, 3064.5010, 3081.5806, 3126.7075, 3169.7002, 3206.5684, 3234.5972, 3248.6172, 3264.5881, 3299.7673, 3316.7351, 3320.6321, 3334.6604, 3427.5977, 3439.7319, 3453.7163, 3457.6880, 3458.6204, 3473.6636, 3490.6494, 3503.7805, 3517.5906, 3529.7371, 3616.8076, 3639.6938, 3652.7173, 3704.8706, 3893.7073, 3959.0081, 3991.8828, 4161.9458, 4177.9121, 4226.0356, 4233.1089, 4252.8618, 4280.2700, 4452.1279, 4465.1328, 4679.1919, 4844.0430

22. E1B9T7 **Mass:** 83794 **Score:** 21 **Expect:** 2.1e+002 **Matches:** 11

Uncharacterized protein OS=Bos taurus GN=SUZ12 PE=4 SV=1

Observed	Mr(expt)	Mr(calc)	ppm	Start		End	Miss	Peptide
885.4853	884.4780	884.4756	2.75	247	-	253	0	K.SYSLLFR.V
1327.7157	1326.7084	1326.6853	17.4	130	-	140	2	R.KTFKVDDMLSK.V + Oxidation (M)
1539.8185	1538.8112	1538.8014	6.37	131	-	143	2	K.TFKVDDMLSKVEK.M
1592.7920	1591.7847	1591.7783	4.03	428	-	439	0	R.IFYQFLYNNNTR.Q
1728.8602	1727.8529	1727.8777	-14.36	294	-	307	2	K.TFVAQMTVFDKNRR.L + Oxidation (M)
2846.3816	2845.3743	2845.3857	-4.01	76	-	98	0	K.MEHVQADHELFLQAFEKPTQIYR.F + Oxidation (M)
3206.5684	3205.5611	3205.5623	-0.37	625	-	651	2	K.HGFIADNQMNHACMLFVENYGQKIIKK.N
3503.7805	3502.7732	3502.7932	-5.70	76	-	103	2	K.MEHVQADHELFLQAFEKPTQIYRFLRTR.N
3991.8828	3990.8755	3990.8404	8.81	565	-	596	2	R.LYFHSDTCLPLRPQEMEVDSEDEKDPEWLREK.T
4147.9468	4146.9395	4147.0052	-15.85	617	-	650	2	K.LWNLHVMKHGFIADNQMNHACMLFVENYGQKIIK.K + 3 Oxidation (M)
4233.1089	4232.1016	4232.0039	23.1	613	-	647	2	K.EVMKLWNLHVMKHGFIADNQMNHACMLFVENYGQK.I

No match to: 706.2851, 710.3807, 717.4042, 719.4268, 720.4332, 722.4348, 723.4337, 735.4208, 744.3633, 749.4401, 756.4190, 759.4415, 766.3942, 772.4352, 774.4582, 779.4799, 823.4933, 831.4758, 832.3166, 834.3207, 842.5047, 843.4840, 844.4902, 846.4869, 870.4950, 873.3982, 876.2891, 880.4586, 883.4816, 891.4897, 895.5765, 900.5383, 918.5458, 923.5204, 927.5097, 928.5096, 929.4465, 944.5216, 945.5218, 949.5538, 967.4249, 969.4326, 972.4861, 974.4482, 976.4711, 977.4844, 978.5469, 988.4647, 1007.5762, 1033.4938, 1034.5852, 1044.5280, 1056.5298, 1074.5454, 1087.5504, 1097.5125, 1103.5261, 1113.4890, 1119.5367, 1122.5620, 1145.6725, 1178.6276, 1185.6924, 1188.5890, 1220.6172, 1234.4895, 1248.6737, 1253.6409, 1284.6428, 1301.6381, 1313.7157, 1314.7207, 1315.6769, 1316.6554, 1317.6417, 1325.6414, 1333.7577, 1336.7279, 1338.7021, 1349.7028, 1355.6765, 1358.7172, 1361.6941, 1372.6039, 1381.7673, 1382.5994, 1383.7312, 1397.7480, 1442.8285, 1447.7806, 1457.7437, 1461.8053, 1479.8021, 1487.7090, 1497.8783, 1517.8105, 1528.7001, 1532.7877, 1537.8292, 1552.8179, 1553.8821, 1556.8846, 1562.8353, 1567.7686, 1578.7893, 1593.8013, 1628.7037, 1628.8643, 1642.7200, 1650.8497, 1651.8568, 1667.8695, 1680.8239, 1687.9133, 1691.7732, 1706.8671, 1715.8597, 1717.9054, 1724.8430, 1730.8619, 1747.9176, 1771.0326, 1805.9197, 1809.0233, 1813.0017, 1820.9197, 1855.0103, 1887.9128, 1894.9668, 1910.9791, 1928.0238, 1953.8596, 1965.8589, 1974.9517,

1981.9934, 1983.9583, 1995.8975, 2020.9868, 2022.0580, 2027.1263, 2044.1479, 2084.0510, 2110.0601, 2136.8467, 2144.0354, 2150.8611, 2155.0640, 2162.8757, 2164.8806, 2170.0522, 2174.1650, 2203.0303, 2217.0352, 2239.1282, 2251.1396, 2253.1372, 2255.2141, 2271.1794, 2274.0801, 2277.0820, 2286.1489, 2289.0703, 2293.1492, 2297.1624, 2309.1501, 2324.0906, 2340.1016, 2343.1846, 2384.2053, 2410.1479, 2414.2322, 2462.2893, 2464.2695, 2465.2659, 2529.2080, 2542.2549, 2645.0503, 2646.9685, 2657.4148, 2660.0061, 2660.9807, 2675.0012, 2689.0129, 2693.0125, 2703.0303, 2706.0225, 2718.3938, 2763.3359, 2774.3179, 2780.3301, 2787.3474, 2826.4065, 2872.3054, 2876.3391, 2884.3103, 2887.3201, 2889.3223, 2891.4019, 2895.4370, 2900.3384, 2915.4314, 2983.4602, 2995.5381, 2999.4780, 3002.1853, 3016.2046, 3020.2017, 3030.2197, 3034.2266, 3044.2632, 3053.4902, 3064.5010, 3081.5806, 3126.7075, 3169.7002, 3234.5972, 3248.6172, 3264.5881, 3299.7673, 3316.7351, 3320.6321, 3334.6604, 3427.5977, 3439.7319, 3453.7163, 3457.6880, 3458.6204, 3473.6636, 3490.6494, 3517.5906, 3529.7371, 3616.8076, 3639.6938, 3652.7173, 3704.8706, 3893.7073, 3959.0081, 4161.9458, 4177.9121, 4226.0356, 4252.8618, 4280.2700, 4452.1279, 4465.1328, 4679.1919, 4844.0430

23. Q17QV0 **Mass:** 38378 **Score:** 20 **Expect:** 2.3e+002 **Matches:** 4
Poly(RC) binding protein 3 OS=Bos taurus GN=PCBP3 PE=2 SV=1

Observed	Mr(expt)	Mr(calc)	ppm	Start	End	Miss	Peptide
1497.8783	1496.8710	1496.8385	21.7	18 -	31	1	R.LLMHGKEVGSIIGK.K + Oxidation (M)
2763.3359	2762.3286	2762.3850	-20.42	234 -	258	0	K.LHQLAMQQTPFPPLGQTNPAFPGEK.L + Oxidation (M)
3959.0081	3958.0008	3958.0180	-4.33	79 -	115	1	K.FEEDIINSMSNSPATSKPPVTLRLVVPASQCGSLIGK.G + Oxidation (M)
4233.1089	4232.1016	4232.1859	-19.91	311 -	350	2	R.QMSGAQIKIANATEGSSERQITITGTPANISLAQYLINAR.L + Oxidation (M)

No match to: 706.2851, 710.3807, 717.4042, 719.4268, 720.4332, 722.4348, 723.4337, 735.4208, 744.3633, 749.4401, 756.4190, 759.4415, 766.3942, 772.4352, 774.4582, 779.4799, 823.4933, 831.4758, 832.3166, 834.3207, 842.5047, 843.4840, 844.4902, 846.4869, 870.4950, 873.3982, 876.2891, 880.4586, 883.4816, 885.4853, 891.4897, 895.5765, 900.5383, 918.5458, 923.5204, 927.5097, 928.5096, 929.4465, 944.5216, 945.5218, 949.5538, 967.4249, 969.4326, 972.4861, 974.4482, 976.4711, 977.4844, 978.5469, 988.4647, 1007.5762, 1033.4938, 1034.5852, 1044.5280, 1056.5298, 1074.5454, 1087.5504, 1097.5125, 1103.5261, 1113.4890, 1119.5367, 1122.5620, 1145.6725, 1178.6276, 1185.6924, 1188.5890, 1220.6172, 1234.4895, 1248.6737, 1253.6409, 1284.6428, 1301.6381, 1313.7157, 1314.7207, 1315.6769, 1316.6554, 1317.6417, 1325.6414, 1327.7157, 1333.7577, 1336.7279, 1338.7021, 1349.7028, 1355.6765, 1358.7172, 1361.6941, 1372.6039, 1381.7673, 1382.5994, 1383.7312, 1397.7480, 1442.8285, 1447.7806, 1457.7437, 1461.8053, 1479.8021, 1487.7090, 1517.8105, 1528.7001, 1532.7877, 1537.8292, 1539.8185, 1552.8179, 1553.8821, 1556.8846, 1562.8353, 1567.7686, 1578.7893, 1592.7920, 1593.8013, 1628.7037, 1628.8643, 1642.7200, 1650.8497, 1651.8568, 1667.8695, 1680.8239, 1687.9133, 1691.7732, 1706.8671, 1715.8597, 1717.9054, 1724.8430, 1728.8602, 1730.8619, 1747.9176, 1771.0326, 1805.9197, 1809.0233, 1813.0017, 1820.9197, 1855.0103, 1887.9128, 1894.9668, 1910.9791, 1928.0238, 1953.8596, 1965.8589, 1974.9517, 1981.9934, 1983.9583, 1995.8975, 2020.9868, 2022.0580, 2027.1263, 2044.1479, 2084.0510, 2110.0601, 2136.8467, 2144.0354, 2150.8611, 2155.0640, 2162.8757, 2164.8806, 2170.0522, 2174.1650, 2203.0303, 2217.0352, 2239.1282, 2251.1396, 2253.1372, 2255.2141, 2271.1794, 2274.0801, 2277.0820, 2286.1489, 2289.0703, 2293.1492, 2297.1624, 2309.1501, 2324.0906, 2340.1016, 2343.1846, 2384.2053, 2410.1479, 2414.2322, 2462.2893, 2464.2695, 2465.2659, 2529.2080, 2542.2549, 2645.0503, 2646.9685, 2657.4148, 2660.0061, 2660.9807, 2675.0012, 2689.0129, 2693.0125, 2703.0303, 2706.0225, 2718.3938, 2774.3179, 2780.3301, 2787.3474, 2826.4065, 2846.3816, 2872.3054, 2876.3391, 2884.3103, 2887.3201, 2889.3223, 2891.4019, 2895.4370, 2900.3384, 2915.4314, 2983.4602, 2995.5381, 2999.4780, 3002.1853, 3016.2046, 3020.2017, 3030.2197, 3034.2266, 3044.2632, 3053.4902, 3064.5010, 3081.5806, 3126.7075, 3169.7002, 3206.5684, 3234.5972, 3248.6172, 3264.5881, 3299.7673, 3316.7351, 3320.6321, 3334.6604, 3427.5977, 3439.7319, 3453.7163, 3457.6880, 3458.6204, 3473.6636, 3490.6494, 3503.7805, 3517.5906, 3529.7371, 3616.8076, 3639.6938, 3652.7173, 3704.8706, 3893.7073, 3991.8828, 4147.9468, 4161.9458, 4177.9121, 4226.0356, 4252.8618, 4280.2700, 4452.1279, 4465.1328, 4679.1919, 4844.0430

24. G3MX30 **Mass:** 89186 **Score:** 20 **Expect:** 2.4e+002 **Matches:** 10
Uncharacterized protein OS=Bos taurus GN=OVCH1 PE=3 SV=1

Observed	Mr(expt)	Mr(calc)	ppm	Start	End	Miss	Peptide
774.4582	773.4509	773.4548	-5.03	759 -	765	0	R.KPGVFAR.V
978.5469	977.5396	977.5480	-8.62	301 -	307	2	R.LTWRMKK.T + Oxidation (M)
1044.5280	1043.5207	1043.5287	-7.67	475 -	482	2	K.YFSEKKDK.K
2027.1263	2026.1190	2026.0710	23.7	422 -	438	0	R.TCLLVAILFGFHHLEEK.S
2044.1479	2043.1406	2043.1703	-14.53	334 -	350	1	K.SLLFCFSHSPLLILLRK.S
2891.4019	2890.3946	2890.3266	23.6	697 -	723	2	R.TYYSAHPGGISEKMICAGFAASGEKDK.C + Oxidation (M)
2995.5381	2994.5308	2994.4957	11.7	559 -	585	0	R.FLGNHQCGGAIINSIWILTAAHCVQSK.N
3427.5977	3426.5904	3426.6217	-9.14	142 -	170	2	K.ISETSEYSNILQEVEVPIMDDRRCGAMLR.G + Oxidation (M)
3439.7319	3438.7246	3438.7760	-14.94	452 -	479	1	K.QHLLNIILLIFSFPSCLYSTFMKYFSEK.K
3503.7805	3502.7732	3502.7919	-5.32	422 -	451	1	R.TCLLVAILFGFHHLEEKSNEYQNVTSNLVK.Q

No match to: 706.2851, 710.3807, 717.4042, 719.4268, 720.4332, 722.4348, 723.4337, 735.4208, 744.3633, 749.4401, 756.4190, 759.4415, 766.3942, 772.4352, 779.4799, 823.4933, 831.4758, 832.3166, 834.3207, 842.5047, 843.4840, 844.4902, 846.4869, 870.4950, 873.3982, 876.2891, 880.4586, 883.4816, 885.4853, 891.4897, 895.5765, 900.5383, 918.5458, 923.5204, 927.5097, 928.5096, 929.4465, 944.5216, 945.5218, 949.5538, 967.4249, 969.4326, 972.4861, 974.4482, 976.4711, 977.4844, 988.4647, 1007.5762, 1033.4938, 1034.5852, 1056.5298, 1074.5454, 1087.5504, 1097.5125, 1103.5261, 1113.4890, 1119.5367, 1122.5620, 1145.6725, 1178.6276, 1185.6924, 1188.5890, 1220.6172, 1234.4895, 1248.6737, 1253.6409, 1284.6428, 1301.6381, 1313.7157, 1314.7207, 1315.6769, 1316.6554, 1317.6417, 1325.6414, 1327.7157, 1333.7577, 1336.7279, 1338.7021, 1349.7028, 1355.6765, 1358.7172, 1361.6941, 1372.6039, 1381.7673, 1382.5994, 1383.7312, 1397.7480, 1442.8285, 1447.7806, 1457.7437, 1461.8053, 1479.8021, 1487.7090, 1497.8783, 1517.8105, 1528.7001, 1532.7877, 1537.8292, 1539.8185, 1552.8179, 1553.8821, 1556.8846, 1562.8353, 1567.7686, 1578.7893, 1592.7920, 1593.8013, 1628.7037, 1628.8643, 1642.7200, 1650.8497, 1651.8568, 1667.8695, 1680.8239, 1687.9133, 1691.7732, 1706.8671, 1715.8597, 1717.9054, 1724.8430, 1728.8602, 1730.8619, 1747.9176, 1771.0326, 1805.9197, 1809.0233, 1813.0017, 1820.9197, 1855.0103, 1887.9128, 1894.9668, 1910.9791, 1928.0238, 1953.8596, 1965.8589, 1974.9517, 1981.9934, 1983.9583, 1995.8975, 2020.9868, 2022.0580, 2084.0510, 2110.0601, 2136.8467, 2144.0354, 2150.8611, 2155.0640, 2162.8757, 2164.8806, 2170.0522, 2174.1650, 2203.0303, 2217.0352, 2239.1282, 2251.1396, 2253.1372, 2255.2141, 2271.1794, 2274.0801, 2277.0820, 2286.1489, 2289.0703, 2293.1492, 2297.1624, 2309.1501, 2324.0906, 2340.1016, 2343.1846, 2384.2053, 2410.1479, 2414.2322, 2462.2893, 2464.2695, 2465.2659, 2529.2080, 2542.2549, 2645.0503, 2646.9685, 2657.4148, 2660.0061, 2660.9807, 2675.0012, 2689.0129, 2693.0125, 2703.0303, 2706.0225, 2718.3938, 2763.3359, 2774.3179, 2780.3301, 2787.3474, 2826.4065, 2846.3816, 2872.3054, 2876.3391, 2884.3103, 2887.3201, 2889.3223, 2895.4370, 2900.3384, 2915.4314, 2983.4602, 2999.4780, 3002.1853, 3016.2046, 3020.2017, 3030.2197, 3034.2266, 3044.2632, 3053.4902, 3064.5010, 3081.5806, 3126.7075, 3169.7002, 3206.5684, 3234.5972, 3248.6172, 3264.5881, 3299.7673, 3316.7351, 3320.6321, 3334.6604, 3453.7163, 3457.6880, 3458.6204, 3473.6636, 3490.6494, 3517.5906, 3529.7371, 3616.8076, 3639.6938, 3652.7173, 3704.8706, 3893.7073, 3959.0081, 3991.8828, 4147.9468, 4161.9458, 4177.9121, 4226.0356, 4233.1089, 4252.8618, 4280.2700, 4452.1279, 4465.1328, 4679.1919, 4844.0430

25. Q3SWY9 **Mass:** 24956 **Score:** 20 **Expect:** 2.4e+002 **Matches:** 7
Ras-related protein Rab-28 OS=Bos taurus GN=RAB28 PE=2 SV=1

Observed	Mr(expt)	Mr(calc)	ppm	Start	End	Miss	Peptide
929.4465	928.4393	928.4436	-4.72	131 -	137	0	K.IDLEHMR.T + Oxidation (M)
1301.6381	1300.6308	1300.6122	14.3	162 -	172	0	K.TGDSVFLCFQK.V
1315.6769	1314.6696	1314.6891	-14.84	182 -	192	1	K.LNKAEIEQSQR.V
1628.8643	1627.8570	1627.8722	-9.34	40 -	52	1	K.QYKQTIGLDFFLR.R
1928.0238	1927.0165	1927.0262	-5.02	113 -	130	1	K.KVSEESETQPLVALVGNK.I
2217.0352	2216.0279	2216.0644	-16.46	196 -	214	1	K.ADIVNYNQEPMSRTVNPPR.S + Oxidation (M)
3126.7075	3125.7002	3125.6907	3.04	53 -	81	2	R.RITLPGSLNVTLQVWDIGGQTIGGKMLDK.Y + Oxidation (M)

No match to: 706.2851, 710.3807, 717.4042, 719.4268, 720.4332, 722.4348, 723.4337, 735.4208, 744.3633, 749.4401, 756.4190, 759.4415, 766.3942, 772.4352, 774.4582, 779.4799, 823.4933, 831.4758, 832.3166, 834.3207, 842.5047, 843.4840, 844.4902, 846.4869, 870.4950, 873.3982, 876.2891, 880.4586, 883.4816, 885.4853, 891.4897, 895.5765, 900.5383, 918.5458, 923.5204, 927.5097, 928.5096, 944.5216, 945.5218, 949.5538, 967.4249, 969.4326, 972.4861, 974.4482, 976.4711, 977.4844, 978.5469, 988.4647, 1007.5762, 1033.4938, 1034.5852, 1044.5280, 1056.5298, 1074.5454, 1087.5504, 1097.5125, 1103.5261, 1113.4890, 1119.5367, 1122.5620, 1145.6725, 1178.6276, 1185.6924, 1188.5890, 1220.6172, 1234.4895, 1248.6737, 1253.6409, 1284.6428, 1313.7157, 1314.7207, 1316.6554, 1317.6417, 1325.6414, 1327.7157, 1333.7577, 1336.7279, 1338.7021, 1349.7028, 1355.6765, 1358.7172, 1361.6941, 1372.6039, 1381.7673, 1382.5994, 1383.7312, 1397.7480, 1442.8285, 1447.7806, 1457.7437, 1461.8053, 1479.8021, 1487.7090, 1497.8783, 1517.8105, 1528.7001, 1532.7877, 1537.8292, 1539.8185, 1552.8179, 1553.8821, 1556.8846, 1562.8353, 1567.7686, 1578.7893, 1592.7920, 1593.8013, 1628.7037, 1642.7200, 1650.8497,

1651.8568, 1667.8695, 1680.8239, 1687.9133, 1691.7732, 1706.8671, 1715.8597, 1717.9054, 1724.8430, 1728.8602, 1730.8619, 1747.9176, 1771.0326, 1805.9197, 1809.0233, 1813.0017, 1820.9197, 1855.0103, 1887.9128, 1894.9668, 1910.9791, 1953.8596, 1965.8589, 1974.9517, 1981.9934, 1983.9583, 1995.8975, 2020.9868, 2022.0580, 2027.1263, 2044.1479, 2084.0510, 2110.0601, 2136.8467, 2144.0354, 2150.8611, 2155.0640, 2162.8757, 2164.8806, 2170.0522, 2174.1650, 2203.0303, 2239.1282, 2251.1396, 2253.1372, 2255.2141, 2271.1794, 2274.0801, 2277.0820, 2286.1489, 2289.0703, 2293.1492, 2297.1624, 2309.1501, 2324.0906, 2340.1016, 2343.1846, 2384.2053, 2410.1479, 2414.2322, 2462.2893, 2464.2695, 2465.2659, 2529.2080, 2542.2549, 2645.0503, 2646.9685, 2657.4148, 2660.0061, 2660.9807, 2675.0012, 2689.0129, 2693.0125, 2703.0303, 2706.0225, 2718.3938, 2763.3359, 2774.3179, 2780.3301, 2787.3474, 2826.4065, 2846.3816, 2872.3054, 2876.3391, 2884.3103, 2887.3201, 2889.3223, 2891.4019, 2895.4370, 2900.3384, 2915.4314, 2983.4602, 2995.5381, 2999.4780, 3002.1853, 3016.2046, 3020.2017, 3030.2197, 3034.2266, 3044.2632, 3053.4902, 3064.5010, 3081.5806, 3169.7002, 3206.5684, 3234.5972, 3248.6172, 3264.5881, 3299.7673, 3316.7351, 3320.6321, 3334.6604, 3427.5977, 3439.7319, 3453.7163, 3457.6880, 3458.6204, 3473.6636, 3490.6494, 3503.7805, 3517.5906, 3529.7371, 3616.8076, 3639.6938, 3652.7173, 3704.8706, 3893.7073, 3959.0081, 3991.8828, 4147.9468, 4161.9458, 4177.9121, 4226.0356, 4233.1089, 4252.8618, 4280.2700, 4452.1279, 4465.1328, 4679.1919, 4844.0430

26. A5D7R8 **Mass:** 26930 **Score:** 20 **Expect:** 2.6e+002 **Matches:** 6

Thymidine kinase, cytosolic OS=Bos taurus GN=TK1 PE=2 SV=1

Observed	Mr(expt)	Mr(calc)	ppm	Start	End	Miss	Peptide
883.4816	882.4744	882.4599	16.3	43 -	49	0	R.FQVAQYK.C
1747.9176	1746.9103	1746.9451	-19.90	17 -	32	1	K.TRGQIQVILGPMFSGK.S + Oxidation (M)
1771.0326	1770.0253	1770.0291	-2.14	132 -	148	0	K.AFGTILNLVPLAESVVK.L
1965.8589	1964.8516	1964.8794	-14.15	149 -	164	1	K.LTAVCMECFREAAYTK.R + Oxidation (M)
2465.2659	2464.2586	2464.2930	-13.96	17 -	38	2	K.TRGQIQVILGPMFSGKSTELMR.R + Oxidation (M)
3639.6938	3638.6865	3638.7544	-18.66	207 -	238	2	K.ENCPMTLGKPAEAPGVRKLFATHQIWQCSQAN.-

No match to: 706.2851, 710.3807, 717.4042, 719.4268, 720.4332, 722.4348, 723.4337, 735.4208, 744.3633, 749.4401, 756.4190, 759.4415, 766.3942, 772.4352, 774.4582, 779.4799, 823.4933, 831.4758, 832.3166, 834.3207, 842.5047, 843.4840, 844.4902, 846.4869, 870.4950, 873.3982, 876.2891, 880.4586, 885.4853, 891.4897, 895.5765, 900.5383, 918.5458, 923.5204, 927.5097, 928.5096, 929.4465, 944.5216, 945.5218, 949.5538, 967.4249, 969.4326, 972.4861, 974.4482, 976.4711, 977.4844, 978.5469, 988.4647, 1007.5762, 1033.4938, 1034.5852, 1044.5280, 1056.5298, 1074.5454, 1087.5504, 1097.5125, 1103.5261, 1113.4890, 1119.5367, 1122.5620, 1145.6725, 1178.6276, 1185.6924, 1188.5890, 1220.6172, 1234.4895, 1248.6737, 1253.6409, 1284.6428, 1301.6381, 1313.7157, 1314.7207, 1315.6769, 1316.6554, 1317.6417, 1325.6414, 1327.7157, 1333.7577, 1336.7279, 1338.7021, 1349.7028, 1355.6765, 1358.7172, 1361.6941, 1372.6039, 1381.7673, 1382.5994, 1383.7312, 1397.7480, 1442.8285, 1447.7806, 1457.7437, 1461.8053, 1479.8021, 1487.7090, 1497.8783, 1517.8105, 1528.7001, 1532.7877, 1537.8292, 1539.8185, 1552.8179, 1553.8821, 1556.8846, 1562.8353, 1567.7686, 1578.7893, 1592.7920, 1593.8013, 1628.7037, 1628.8643, 1642.7200, 1650.8497, 1651.8568, 1667.8695, 1680.8239, 1687.9133, 1691.7732, 1706.8671, 1715.8597, 1717.9054, 1724.8430, 1728.8602, 1730.8619, 1805.9197, 1809.0233, 1813.0017, 1820.9197, 1855.0103, 1887.9128, 1894.9668, 1910.9791, 1928.0238, 1953.8596, 1974.9517, 1981.9934, 1983.9583, 1995.8975, 2020.9868, 2022.0580, 2027.1263, 2044.1479, 2084.0510, 2110.0601, 2136.8467, 2144.0354, 2150.8611, 2155.0640, 2162.8757, 2164.8806, 2170.0522, 2174.1650, 2203.0303, 2217.0352, 2239.1282, 2251.1396, 2253.1372, 2255.2141, 2271.1794, 2274.0801, 2277.0820, 2286.1489, 2289.0703, 2293.1492, 2297.1624, 2309.1501, 2324.0906, 2340.1016, 2343.1846, 2384.2053, 2410.1479, 2414.2322, 2462.2893, 2464.2695, 2529.2080, 2542.2549, 2645.0503, 2646.9685, 2657.4148, 2660.0061, 2660.9807, 2675.0012, 2689.0129, 2693.0125, 2703.0303, 2706.0225, 2718.3938, 2763.3359, 2774.3179, 2780.3301, 2787.3474, 2826.4065, 2846.3816, 2872.3054, 2876.3391, 2884.3103, 2887.3201, 2889.3223, 2891.4019, 2895.4370, 2900.3384, 2915.4314, 2983.4602, 2995.5381, 2999.4780, 3002.1853, 3016.2046, 3020.2017, 3030.2197, 3034.2266, 3044.2632, 3053.4902, 3064.5010, 3081.5806, 3126.7075, 3169.7002, 3206.5684, 3234.5972, 3248.6172, 3264.5881, 3299.7673, 3316.7351, 3320.6321, 3334.6604, 3427.5977, 3439.7319, 3453.7163, 3457.6880, 3458.6204, 3473.6636, 3490.6494, 3503.7805, 3517.5906, 3529.7371, 3616.8076, 3652.7173, 3704.8706, 3893.7073, 3959.0081, 3991.8828, 4147.9468, 4161.9458, 4177.9121, 4226.0356, 4233.1089, 4252.8618, 4280.2700, 4452.1279, 4465.1328, 4679.1919, 4844.0430

27. Q32KR8 **Mass:** 39412 **Score:** 20 **Expect:** 2.7e+002 **Matches:** 5

[Protein ADP-ribosylarginine] hydrolase OS=Bos taurus GN=ADPRH PE=2 SV=1

Observed	Mr(expt)	Mr(calc)	ppm	Start	End	Miss	Peptide
722.4348	721.4275	721.4122	21.2	348 -	353	0	R.ALYSLR.-
944.5216	943.5143	943.5239	-10.18	238 -	245	1	K.LRGIWDGK.S
967.4249	966.4177	966.4085	9.46	1 -	8	0	-.MCGALMER.Y
3206.5684	3205.5611	3205.4934	21.1	210 -	234	0	K.YIVQSGFFVEQNLQHWSYFQDQWEK.Y
3334.6604	3333.6531	3333.5883	19.4	209 -	234	1	K.KYIVQSGFFVEQNLQHWSYFQDQWEK.Y

No match to: 706.2851, 710.3807, 717.4042, 719.4268, 720.4332, 723.4337, 735.4208, 744.3633, 749.4401, 756.4190, 759.4415, 766.3942, 772.4352, 774.4582, 779.4799, 823.4933, 831.4758, 832.3166, 834.3207, 842.5047, 843.4840, 844.4902, 846.4869, 870.4950, 873.3982, 876.2891, 880.4586, 883.4816, 885.4853, 891.4897, 895.5765, 900.5383, 918.5458, 923.5204, 927.5097, 928.5096, 929.4465, 945.5218, 949.5538, 969.4326, 972.4861, 974.4482, 976.4711, 977.4844, 978.5469, 988.4647, 1007.5762, 1033.4938, 1034.5852, 1044.5280, 1056.5298, 1074.5454, 1087.5504, 1097.5125, 1103.5261, 1113.4890, 1119.5367, 1122.5620, 1145.6725, 1178.6276, 1185.6924, 1188.5890, 1220.6172, 1234.4895, 1248.6737, 1253.6409, 1284.6428, 1301.6381, 1313.7157, 1314.7207, 1315.6769, 1316.6554, 1317.6417, 1325.6414, 1327.7157, 1333.7577, 1336.7279, 1338.7021, 1349.7028, 1355.6765, 1358.7172, 1361.6941, 1372.6039, 1381.7673, 1382.5994, 1383.7312, 1397.7480, 1442.8285, 1447.7806, 1457.7437, 1461.8053, 1479.8021, 1487.7090, 1497.8783, 1517.8105, 1528.7001, 1532.7877, 1537.8292, 1539.8185, 1552.8179, 1553.8821, 1556.8846, 1562.8353, 1567.7686, 1578.7893, 1592.7920, 1593.8013, 1628.7037, 1628.8643, 1642.7200, 1650.8497, 1651.8568, 1667.8695, 1680.8239, 1687.9133, 1691.7732, 1706.8671, 1715.8597, 1717.9054, 1724.8430, 1728.8602, 1730.8619, 1747.9176, 1771.0326, 1805.9197, 1809.0233, 1813.0017, 1820.9197, 1855.0103, 1887.9128, 1894.9668, 1910.9791, 1928.0238, 1953.8596, 1965.8589, 1974.9517, 1981.9934, 1983.9583, 1995.8975, 2020.9868, 2022.0580, 2027.1263, 2044.1479, 2084.0510, 2110.0601, 2136.8467, 2144.0354, 2150.8611, 2155.0640, 2162.8757, 2164.8806, 2170.0522, 2174.1650, 2203.0303, 2217.0352, 2239.1282, 2251.1396, 2253.1372, 2255.2141, 2271.1794, 2274.0801, 2277.0820, 2286.1489, 2289.0703, 2293.1492, 2297.1624, 2309.1501, 2324.0906, 2340.1016, 2343.1846, 2384.2053, 2410.1479, 2414.2322, 2462.2893, 2464.2695, 2465.2659, 2529.2080, 2542.2549, 2645.0503, 2646.9685, 2657.4148, 2660.0061, 2660.9807, 2675.0012, 2689.0129, 2693.0125, 2703.0303, 2706.0225, 2718.3938, 2763.3359, 2774.3179, 2780.3301, 2787.3474, 2826.4065, 2846.3816, 2872.3054, 2876.3391, 2884.3103, 2887.3201, 2889.3223, 2891.4019, 2895.4370, 2900.3384, 2915.4314, 2983.4602, 2995.5381, 2999.4780, 3002.1853, 3016.2046, 3020.2017, 3030.2197, 3034.2266, 3044.2632, 3053.4902, 3064.5010, 3081.5806, 3126.7075, 3169.7002, 3234.5972, 3248.6172, 3264.5881, 3299.7673, 3316.7351, 3320.6321, 3427.5977, 3439.7319, 3453.7163, 3457.6880, 3458.6204, 3473.6636, 3490.6494, 3503.7805, 3517.5906, 3529.7371, 3616.8076, 3639.6938, 3652.7173, 3704.8706, 3893.7073, 3959.0081, 3991.8828, 4147.9468, 4161.9458, 4177.9121, 4226.0356, 4233.1089, 4252.8618, 4280.2700, 4452.1279, 4465.1328, 4679.1919, 4844.0430

28. Q0VCL5 **Mass:** 40078 **Score:** 19 **Expect:** 2.8e+002 **Matches:** 8

High-mobility group 20A OS=Bos taurus GN=HMG20A PE=2 SV=1

Observed	Mr(expt)	Mr(calc)	ppm	Start	End	Miss	Peptide
900.5383	899.5310	899.5301	1.04	118 -	124	2	R.REQLRAK.R
1383.7312	1382.7239	1382.7088	10.9	250 -	261	1	R.NAALQKHVESMR.T
1628.8643	1627.8570	1627.8781	-12.94	262 -	275	1	R.TAVEKLEVDVIQER.S
1928.0238	1927.0165	1926.9945	11.4	250 -	266	2	R.NAALQKHVESMRTAVEK.L + Oxidation (M)
2155.0640	2154.0567	2154.0038	24.6	2 -	21	0	M.ETLMTSSTLPPLFADEDGSK.E + Oxidation (M)
2286.1489	2285.1416	2285.1626	-9.20	125 -	143	1	K.RPEVPFPEITRMLGNEWSK.L
2384.2053	2383.1980	2383.2165	-7.77	256 -	275	2	K.HVESMRTAVEKLEVDVIQER.S + Oxidation (M)
2995.5381	2994.5308	2994.5273	1.18	125 -	149	2	K.RPEVPFPEITRMLGNEWSKLPPEEK.Q + Oxidation (M)

No match to: 706.2851, 710.3807, 717.4042, 719.4268, 720.4332, 722.4348, 723.4337, 735.4208, 744.3633, 749.4401, 756.4190, 759.4415, 766.3942, 772.4352, 774.4582, 779.4799, 823.4933, 831.4758, 832.3166, 834.3207, 842.5047, 843.4840, 844.4902, 846.4869, 870.4950, 873.3982, 876.2891, 880.4586, 883.4816, 885.4853, 891.4897, 895.5765, 918.5458, 923.5204, 927.5097, 928.5096, 929.4465, 944.5216, 945.5218, 949.5538, 967.4249, 969.4326, 972.4861, 974.4482, 976.4711, 977.4844, 978.5469, 988.4647, 1007.5762, 1033.4938, 1034.5852, 1044.5280, 1056.5298, 1074.5454, 1087.5504, 1097.5125, 1103.5261, 1113.4890, 1119.5367, 1122.5620, 1145.6725, 1178.6276, 1185.6924, 1188.5890, 1220.6172, 1234.4895, 1248.6737, 1253.6409, 1284.6428, 1301.6381, 1313.7157, 1314.7207, 1315.6769, 1316.6554, 1317.6417, 1325.6414, 1327.7157, 1333.7577, 1336.7279, 1338.7021, 1349.7028, 1355.6765, 1358.7172, 1361.6941, 1372.6039, 1381.7673, 1382.5994, 1397.7480, 1442.8285, 1447.7806, 1457.7437, 1461.8053, 1479.8021, 1487.7090, 1497.8783, 1517.8105, 1528.7001, 1532.7877, 1537.8292, 1539.8185, 1552.8179, 1553.8821, 1556.8846, 1562.8353, 1567.7686, 1578.7893, 1592.7920, 1593.8013, 1628.7037, 1642.7200, 1650.8497, 1651.8568, 1667.8695, 1680.8239, 1687.9133, 1691.7732, 1706.8671, 1715.8597, 1717.9054, 1724.8430, 1728.8602, 1730.8619,

1747.9176, 1771.0326, 1805.9197, 1809.0233, 1813.0017, 1820.9197, 1855.0103, 1887.9128, 1894.9668, 1910.9791, 1953.8596, 1965.8589, 1974.9517, 1981.9934, 1983.9583, 1995.8975, 2020.9868, 2022.0580, 2027.1263, 2044.1479, 2084.0510, 2110.0601, 2136.8467, 2144.0354, 2150.8611, 2162.8757, 2164.8806, 2170.0522, 2174.1650, 2203.0303, 2217.0352, 2239.1282, 2251.1396, 2253.1372, 2255.2141, 2271.1794, 2274.0801, 2277.0820, 2289.0703, 2293.1492, 2297.1624, 2309.1501, 2324.0906, 2340.1016, 2343.1846, 2410.1479, 2414.2322, 2462.2893, 2464.2695, 2465.2659, 2529.2080, 2542.2549, 2645.0503, 2646.9685, 2657.4148, 2660.0061, 2660.9807, 2675.0012, 2689.0129, 2693.0125, 2703.0303, 2706.0225, 2718.3938, 2763.3359, 2774.3179, 2780.3301, 2787.3474, 2826.4065, 2846.3816, 2872.3054, 2876.3391, 2884.3103, 2887.3201, 2889.3223, 2891.4019, 2895.4370, 2900.3384, 2915.4314, 2983.4602, 2999.4780, 3002.1853, 3016.2046, 3020.2017, 3030.2197, 3034.2266, 3044.2632, 3053.4902, 3064.5010, 3081.5806, 3126.7075, 3169.7002, 3206.5684, 3234.5972, 3264.5881, 3299.7673, 3316.7351, 3320.6321, 3334.6604, 3427.5977, 3439.7319, 3453.7163, 3457.6880, 3458.6204, 3473.6636, 3490.6494, 3503.7805, 3517.5906, 3529.7371, 3616.8076, 3639.6938, 3652.7173, 3704.8706, 3893.7073, 3959.0081, 3991.8828, 4147.9468, 4161.9458, 4177.9121, 4226.0356, 4233.1089, 4252.8618, 4280.2700, 4452.1279, 4465.1328, 4679.1919, 4844.0430

29. F1MVT5 **Mass:** 36896 **Score:** 19 **Expect:** 2.9e+002 **Matches:** 4

Uncharacterized protein OS=Bos taurus GN=SLAIN1 PE=4 SV=2

Observed	Mr(expt)	Mr(calc)	ppm	Start	End	Miss	Peptide
944.5216	943.5143	943.5021	12.9	169 -	176	1	R.RSMPNLAR.M
1771.0326	1770.0253	1770.0040	12.1	311 -	326	0	R.SFLQPPKPLSSLSTLR.D
2763.3359	2762.3286	2762.3803	-18.72	169 -	194	2	R.RSMPNLARMPSTTTISSNGSSPVTVR.N + Oxidation (M)
4452.1279	4451.1206	4451.1194	0.28	170 -	212	2	R.SMPNLARMPSTTTISSNGSSPVTVRNSQSFDSSLHGAANGISR.I + 2 Oxidation (M)

No match to: 706.2851, 710.3807, 717.4042, 719.4268, 720.4332, 722.4348, 723.4337, 735.4208, 744.3633, 749.4401, 756.4190, 759.4415, 766.3942, 772.4352, 774.4582, 779.4799, 823.4933, 831.4758, 832.3166, 834.3207, 842.5047, 843.4840, 844.4902, 846.4869, 870.4950, 873.3982, 876.2891, 880.4586, 883.4816, 885.4853, 891.4897, 895.5765, 900.5383, 918.5458, 923.5204, 927.5097, 928.5096, 929.4465, 945.5218, 949.5538, 967.4249, 969.4326, 972.4861, 974.4482, 976.4711, 977.4844, 978.5469, 988.4647, 1007.5762, 1033.4938, 1034.5852, 1044.5280, 1056.5298, 1074.5454, 1087.5504, 1097.5125, 1103.5261, 1113.4890, 1119.5367, 1122.5620, 1145.6725, 1178.6276, 1185.6924, 1188.5890, 1220.6172, 1234.4895, 1248.6737, 1253.6409, 1284.6428, 1301.6381, 1313.7157, 1314.7207, 1315.6769, 1316.6554, 1317.6417, 1325.6414, 1327.7157, 1333.7577, 1336.7279, 1338.7021, 1349.7028, 1355.6765, 1358.7172, 1361.6941, 1372.6039, 1381.7673, 1382.5994, 1383.7312, 1397.7480, 1442.8285, 1447.7806, 1457.7437, 1461.8053, 1479.8021, 1487.7090, 1497.8783, 1517.8105, 1528.7001, 1532.7877, 1537.8292, 1539.8185, 1552.8179, 1553.8821, 1556.8846, 1562.8353, 1567.7686, 1578.7893, 1592.7920, 1593.8013, 1628.7037, 1628.8643, 1642.7200, 1650.8497, 1651.8568, 1667.8695, 1680.8239, 1687.9133, 1691.7732, 1706.8671, 1715.8597, 1717.9054, 1724.8430, 1728.8602, 1730.8619, 1747.9176, 1805.9197, 1809.0233, 1813.0017, 1820.9197, 1855.0103, 1887.9128, 1894.9668, 1910.9791, 1928.0238, 1953.8596, 1965.8589, 1974.9517, 1981.9934, 1983.9583, 1995.8975, 2020.9868, 2022.0580, 2027.1263, 2044.1479, 2084.0510, 2110.0601, 2136.8467, 2144.0354, 2150.8611, 2155.0640, 2162.8757, 2164.8806, 2170.0522, 2174.1650, 2203.0303, 2217.0352, 2239.1282, 2251.1396, 2253.1372, 2255.2141, 2271.1794, 2274.0801, 2277.0820, 2286.1489, 2289.0703, 2293.1492, 2297.1624, 2309.1501, 2324.0906, 2340.1016, 2343.1846, 2384.2053, 2410.1479, 2414.2322, 2462.2893, 2464.2695, 2465.2659, 2529.2080, 2542.2549, 2645.0503, 2646.9685, 2657.4148, 2660.0061, 2660.9807, 2675.0012, 2689.0129, 2693.0125, 2703.0303, 2706.0225, 2718.3938, 2774.3179, 2780.3301, 2787.3474, 2826.4065, 2846.3816, 2872.3054, 2876.3391, 2884.3103, 2887.3201, 2889.3223, 2891.4019, 2895.4370, 2900.3384, 2915.4314, 2983.4602, 2995.5381, 2999.4780, 3002.1853, 3016.2046, 3020.2017, 3030.2197, 3034.2266, 3044.2632, 3053.4902, 3064.5010, 3081.5806, 3126.7075, 3169.7002, 3206.5684, 3234.5972, 3248.6172, 3264.5881, 3299.7673, 3316.7351, 3320.6321, 3334.6604, 3427.5977, 3439.7319, 3453.7163, 3457.6880, 3458.6204, 3473.6636, 3490.6494, 3503.7805, 3517.5906, 3529.7371, 3616.8076, 3639.6938, 3652.7173, 3704.8706, 3893.7073, 3959.0081, 3991.8828, 4147.9468, 4161.9458, 4177.9121, 4226.0356, 4233.1089, 4252.8618, 4280.2700, 4465.1328, 4679.1919, 4844.0430

30. E1B7E6 **Mass:** 110359 **Score:** 19 **Expect:** 3e+002 **Matches:** 16

Uncharacterized protein OS=Bos taurus GN=PIWIL2 PE=3 SV=2

Observed	Mr(expt)	Mr(calc)	ppm	Start	End	Miss	Peptide
772.4352	771.4280	771.4239	5.26	699 -	705	0	R.TIGQPTR.L
923.5204	922.5132	922.5348	-23.48	88 -	95	1	K.REVPPLGR.G
949.5538	948.5466	948.5467	-0.13	843 -	850	0	K.MVVFVVQK.K
1178.6276	1177.6203	1177.6013	16.2	651 -	661	0	R.TIQSMLGVEGK.I + Oxidation (M)
1314.7207	1313.7134	1313.7191	-4.30	75 -	87	0	R.GLGLETASQTPLK.R
1628.8643	1627.8570	1627.8682	-6.87	365 -	379	1	R.RTDGGLFLLADVSHK.V
1771.0326	1770.0253	1769.9934	18.0	141 -	156	2	R.MLRRGTLDASLLPVGR.A + Oxidation (M)
1953.8596	1952.8523	1952.8972	-22.97	383 -	398	0	R.NDSVLDIMHTMYLQNK.E + 2 Oxidation (M)
2239.1282	2238.1209	2238.0867	15.3	919 -	935	1	R.LTFKLCHMYWNWPGTIR.V + Oxidation (M)
2884.3103	2883.3030	2883.3215	-6.40	894 -	918	0	R.QGCGIPTHYVCVLNTANLSPDHMQR.L + Oxidation (M)
2891.4019	2890.3946	2890.4233	-9.93	919 -	941	2	R.LTFKLCHMYWNWPGTIRVPAPCK.Y + Oxidation (M)
3248.6172	3247.6099	3247.6886	-24.22	767 -	793	2	K.WYSRVVFQMPHQEIVDSLKLCLVGSLK.K + Oxidation (M)
3334.6604	3333.6531	3333.6829	-8.93	338 -	365	2	R.NFYDPTSAMVLQQHRLQIWPGYAASIRR.T + Oxidation (M)
3439.7319	3438.7246	3438.7747	-14.56	651 -	681	2	R.TIQSMLGVEGKIQMVVCIIMGTRDDLYGAIK.K
3457.6880	3456.6807	3456.6112	20.1	380 -	407	2	K.VIRNDSVLDIMHTMYLQNKENFQDECTK.L + Oxidation (M)
3473.6636	3472.6563	3472.6061	14.5	380 -	407	2	K.VIRNDSVLDIMHTMYLQNKENFQDECTK.L + 2 Oxidation (M)

No match to: 706.2851, 710.3807, 717.4042, 719.4268, 720.4332, 722.4348, 723.4337, 735.4208, 744.3633, 749.4401, 756.4190, 759.4415, 766.3942, 774.4582, 779.4799, 823.4933, 831.4758, 832.3166, 834.3207, 842.5047, 843.4840, 844.4902, 846.4869, 870.4950, 873.3982, 876.2891, 880.4586, 883.4816, 885.4853, 891.4897, 895.5765, 900.5383, 918.5458, 927.5097, 928.5096, 929.4465, 944.5216, 945.5218, 967.4249, 969.4326, 972.4861, 974.4482, 976.4711, 977.4844, 978.5469, 988.4647, 1007.5762, 1033.4938, 1034.5852, 1044.5280, 1056.5298, 1074.5454, 1087.5504, 1097.5125, 1103.5261, 1113.4890, 1119.5367, 1122.5620, 1145.6725, 1185.6924, 1188.5890, 1220.6172, 1234.4895, 1248.6737, 1253.6409, 1284.6428, 1301.6381, 1313.7157, 1315.6769, 1316.6554, 1317.6417, 1325.6414, 1327.7157, 1333.7577, 1336.7279, 1338.7021, 1349.7028, 1355.6765, 1358.7172, 1361.6941, 1372.6039, 1381.7673, 1382.5994, 1383.7312, 1397.7480, 1442.8285, 1447.7806, 1457.7437, 1461.8053, 1479.8021, 1487.7090, 1497.8783, 1517.8105, 1528.7001, 1532.7877, 1537.8292, 1539.8185, 1552.8179, 1553.8821, 1556.8846, 1562.8353, 1567.7686, 1578.7893, 1592.7920, 1593.8013, 1628.7037, 1642.7200, 1650.8497, 1651.8568, 1667.8695, 1680.8239, 1687.9133, 1691.7732, 1706.8671, 1715.8597, 1717.9054, 1724.8430, 1728.8602, 1730.8619, 1747.9176, 1805.9197, 1809.0233, 1813.0017, 1820.9197, 1855.0103, 1887.9128, 1894.9668, 1910.9791, 1928.0238, 1965.8589, 1974.9517, 1981.9934, 1983.9583, 1995.8975, 2020.9868, 2022.0580, 2027.1263, 2044.1479, 2084.0510, 2110.0601, 2136.8467, 2144.0354, 2150.8611, 2155.0640, 2162.8757, 2164.8806, 2170.0522, 2174.1650, 2203.0303, 2217.0352, 2251.1396, 2253.1372, 2255.2141, 2271.1794, 2274.0801, 2277.0820, 2286.1489, 2289.0703, 2293.1492, 2297.1624, 2309.1501, 2324.0906, 2340.1016, 2343.1846, 2384.2053, 2410.1479, 2414.2322, 2462.2893, 2464.2695, 2465.2659, 2529.2080, 2542.2549, 2645.0503, 2646.9685, 2657.4148, 2660.0061, 2660.9807, 2675.0012, 2689.0129, 2693.0125, 2703.0303, 2706.0225, 2718.3938, 2763.3359, 2774.3179, 2780.3301, 2787.3474, 2826.4065, 2846.3816, 2872.3054, 2876.3391, 2887.3201, 2889.3223, 2895.4370, 2900.3384, 2915.4314, 2983.4602, 2995.5381, 2999.4780, 3002.1853, 3016.2046, 3020.2017, 3030.2197, 3034.2266, 3044.2632, 3053.4902, 3064.5010, 3081.5806, 3126.7075, 3169.7002, 3206.5684, 3234.5972, 3264.5881, 3299.7673, 3316.7351, 3320.6321, 3427.5977, 3453.7163, 3458.6204, 3490.6494, 3503.7805, 3517.5906, 3529.7371, 3616.8076, 3639.6938, 3652.7173, 3704.8706, 3893.7073, 3959.0081, 3991.8828, 4147.9468, 4161.9458, 4177.9121, 4226.0356, 4233.1089, 4252.8618, 4280.2700, 4452.1279, 4465.1328, 4679.1919, 4844.0430

31. P56717 **Mass:** 3809 **Score:** 19 **Expect:** 3e+002 **Matches:** 2

Orexin-A OS=Bos taurus GN=HCRT PE=1 SV=1

Observed	Mr(expt)	Mr(calc)	ppm	Start	End	Miss	Peptide
1301.6381	1300.6308	1300.6016	22.5	1 -	10	1	-.QPLPDCCRQK.T
1965.8589	1964.8516	1964.8438	4.01	1 -	15	2	-.QPLPDCCRQKTCSCR.L

No match to: 706.2851, 710.3807, 717.4042, 719.4268, 720.4332, 722.4348, 723.4337, 735.4208, 744.3633, 749.4401, 756.4190, 759.4415, 766.3942, 772.4352, 774.4582, 779.4799, 823.4933, 831.4758, 832.3166, 834.3207, 842.5047, 843.4840, 844.4902, 846.4869, 870.4950, 873.3982, 876.2891, 880.4586, 883.4816, 885.4853, 891.4897, 895.5765, 900.5383, 918.5458, 923.5204, 927.5097, 928.5096, 929.4465, 944.5216, 945.5218, 949.5538, 967.4249, 969.4326, 972.4861, 974.4482, 976.4711, 977.4844, 978.5469, 988.4647, 1007.5762, 1033.4938, 1034.5852, 1044.5280, 1056.5298, 1074.5454, 1087.5504, 1097.5125, 1103.5261, 1113.4890, 1119.5367, 1122.5620, 1145.6725, 1178.6276, 1185.6924, 1188.5890, 1220.6172, 1234.4895, 1248.6737, 1253.6409, 1284.6428, 1313.7157, 1314.7207, 1315.6769, 1316.6554, 1317.6417, 1325.6414, 1327.7157, 1333.7577, 1336.7279, 1338.7021, 1349.7028, 1355.6765, 1358.7172, 1361.6941, 1372.6039, 1381.7673, 1382.5994, 1383.7312, 1397.7480, 1442.8285, 1447.7806, 1457.7437, 1461.8053, 1479.8021, 1487.7090, 1497.8783, 1517.8105, 1528.7001,

1532.7877, 1537.8292, 1539.8185, 1552.8179, 1553.8821, 1556.8846, 1562.8353, 1567.7686, 1578.7893, 1592.7920, 1593.8013, 1628.7037, 1628.8643, 1642.7200, 1650.8497, 1651.8568, 1667.8695, 1680.8239, 1687.9133, 1691.7732, 1706.8671, 1715.8597, 1717.9054, 1724.8430, 1728.8602, 1730.8619, 1747.9176, 1771.0326, 1805.9197, 1809.0233, 1813.0017, 1820.9197, 1855.0103, 1887.9128, 1894.9668, 1910.9791, 1928.0238, 1953.8596, 1974.9517, 1981.9934, 1983.9583, 1995.8975, 2020.9868, 2022.0580, 2027.1263, 2044.1479, 2084.0510, 2110.0601, 2136.8467, 2144.0354, 2150.8611, 2155.0640, 2162.8757, 2164.8806, 2170.0522, 2174.1650, 2203.0303, 2217.0352, 2239.1282, 2251.1396, 2253.1372, 2255.2141, 2271.1794, 2274.0801, 2277.0820, 2286.1489, 2289.0703, 2293.1492, 2297.1624, 2309.1501, 2324.0906, 2340.1016, 2343.1846, 2384.2053, 2410.1479, 2414.2322, 2462.2893, 2464.2695, 2465.2659, 2529.2080, 2542.2549, 2645.0503, 2646.9685, 2657.4148, 2660.0061, 2660.9807, 2675.0012, 2689.0129, 2693.0125, 2703.0303, 2706.0225, 2718.3938, 2763.3359, 2774.3179, 2780.3301, 2787.3474, 2826.4065, 2846.3816, 2872.3054, 2876.3391, 2884.3103, 2887.3201, 2889.3223, 2891.4019, 2895.4370, 2900.3384, 2915.4314, 2983.4602, 2995.5381, 2999.4780, 3002.1853, 3016.2046, 3020.2017, 3030.2197, 3034.2266, 3044.2632, 3053.4902, 3064.5010, 3081.5806, 3126.7075, 3169.7002, 3206.5684, 3234.5972, 3248.6172, 3264.5881, 3299.7673, 3316.7351, 3320.6321, 3334.6604, 3427.5977, 3439.7319, 3453.7163, 3457.6880, 3458.6204, 3473.6636, 3490.6494, 3503.7805, 3517.5906, 3529.7371, 3616.8076, 3639.6938, 3652.7173, 3704.8706, 3893.7073, 3959.0081, 3991.8828, 4147.9468, 4161.9458, 4177.9121, 4226.0356, 4233.1089, 4252.8618, 4280.2700, 4452.1279, 4465.1328, 4679.1919, 4844.0430

32. G1K212 **Mass:** 46341 **Score:** 19 **Expect:** 3.1e+002 **Matches:** 6
Dynein assembly factor with WDR repeat domains 1 OS=Bos taurus GN=DAW1 PE=4 SV=2

Observed	Mr(expt)	Mr(calc)	ppm	Start	End	Miss	Peptide
717.4042	716.3969	716.3897	9.99	82 -	86	0	K.FYLFK.V
1715.8597	1714.8524	1714.8196	19.2	334 -	348	1	R.ECVTKLEGHEGEISK.I
2027.1263	2026.1190	2026.1548	-17.65	65 -	81	2	K.LLIQRLQNKLGQHSNHK.F
2340.1016	2339.0943	2339.0964	-0.90	115 -	134	1	R.TCKLWDTASGEELHTLEGHR.N
2462.2893	2461.2820	2461.2271	22.3	327 -	348	2	R.IFSAATRECVTKLEGHEGEISK.I
3616.8076	3615.8003	3615.7382	17.2	118 -	149	1	K.LWDTASGEELHTLEGHRNVVYAIAFNNPYGDK.I

No match to: 706.2851, 710.3807, 719.4268, 720.4332, 722.4348, 723.4337, 735.4208, 744.3633, 749.4401, 756.4190, 759.4415, 766.3942, 772.4352, 774.4582, 779.4799, 823.4933, 831.4758, 832.3166, 834.3207, 842.5047, 843.4840, 844.4902, 846.4869, 870.4950, 873.3982, 876.2891, 880.4586, 883.4816, 885.4853, 891.4897, 895.5765, 900.5383, 918.5458, 923.5204, 927.5097, 928.5096, 929.4465, 944.5216, 945.5218, 949.5538, 967.4249, 969.4326, 972.4861, 974.4482, 976.4711, 977.4844, 978.5469, 988.4647, 1007.5762, 1033.4938, 1034.5852, 1044.5280, 1056.5298, 1074.5454, 1087.5504, 1097.5125, 1103.5261, 1113.4890, 1119.5367, 1122.5620, 1145.6725, 1178.6276, 1185.6924, 1188.5890, 1220.6172, 1234.4895, 1248.6737, 1253.6409, 1284.6428, 1301.6381, 1313.7157, 1314.7207, 1315.6769, 1316.6554, 1317.6417, 1325.6414, 1327.7157, 1333.7577, 1336.7279, 1338.7021, 1349.7028, 1355.6765, 1358.7172, 1361.6941, 1372.6039, 1381.7673, 1382.5994, 1383.7312, 1397.7480, 1442.8285, 1447.7806, 1457.7437, 1461.8053, 1479.8021, 1487.7090, 1497.8783, 1517.8105, 1528.7001, 1532.7877, 1537.8292, 1539.8185, 1552.8179, 1553.8821, 1556.8846, 1562.8353, 1567.7686, 1578.7893, 1592.7920, 1593.8013, 1628.7037, 1628.8643, 1642.7200, 1650.8497, 1651.8568, 1667.8695, 1680.8239, 1687.9133, 1691.7732, 1706.8671, 1717.9054, 1724.8430, 1728.8602, 1730.8619, 1747.9176, 1771.0326, 1805.9197, 1809.0233, 1813.0017, 1820.9197, 1855.0103, 1887.9128, 1894.9668, 1910.9791, 1928.0238, 1953.8596, 1965.8589, 1974.9517, 1981.9934, 1983.9583, 1995.8975, 2020.9868, 2022.0580, 2044.1479, 2084.0510, 2110.0601, 2136.8467, 2144.0354, 2150.8611, 2155.0640, 2162.8757, 2164.8806, 2170.0522, 2174.1650, 2203.0303, 2217.0352, 2239.1282, 2251.1396, 2253.1372, 2255.2141, 2271.1794, 2274.0801, 2277.0820, 2286.1489, 2289.0703, 2293.1492, 2297.1624, 2309.1501, 2324.0906, 2343.1846, 2384.2053, 2410.1479, 2414.2322, 2464.2695, 2465.2659, 2529.2080, 2542.2549, 2645.0503, 2646.9685, 2657.4148, 2660.0061, 2660.9807, 2675.0012, 2689.0129, 2693.0125, 2703.0303, 2706.0225, 2718.3938, 2763.3359, 2774.3179, 2780.3301, 2787.3474, 2826.4065, 2846.3816, 2872.3054, 2876.3391, 2884.3103, 2887.3201, 2889.3223, 2891.4019, 2895.4370, 2900.3384, 2915.4314, 2983.4602, 2995.5381, 2999.4780, 3002.1853, 3016.2046, 3020.2017, 3030.2197, 3034.2266, 3044.2632, 3053.4902, 3064.5010, 3081.5806, 3126.7075, 3169.7002, 3206.5684, 3234.5972, 3248.6172, 3264.5881, 3299.7673, 3316.7351, 3320.6321, 3334.6604, 3427.5977, 3439.7319, 3453.7163, 3457.6880, 3458.6204, 3473.6636, 3490.6494, 3503.7805, 3517.5906, 3529.7371, 3639.6938, 3652.7173, 3704.8706, 3893.7073, 3959.0081, 3991.8828, 4147.9468, 4161.9458, 4177.9121, 4226.0356, 4233.1089, 4252.8618, 4280.2700, 4452.1279, 4465.1328, 4679.1919, 4844.0430

33. Q0P593 **Mass:** 46626 **Score:** 19 **Expect:** 3.2e+002 **Matches:** 6
Dynein assembly factor with WDR repeat domains 1 OS=Bos taurus GN=DAW1 PE=2 SV=1

Observed	Mr(expt)	Mr(calc)	ppm	Start	End	Miss	Peptide
717.4042	716.3969	716.3897	9.99	82 -	86	0	K.FYLFK.V
1715.8597	1714.8524	1714.8196	19.2	336 -	350	1	R.ECVTKLEGHEGEISK.I
2027.1263	2026.1190	2026.1548	-17.65	65 -	81	2	K.LLIQRLQNKLGQHSNHK.F
2340.1016	2339.0943	2339.0964	-0.90	115 -	134	1	R.TCKLWDTASGEELHTLEGHR.N
2462.2893	2461.2820	2461.2271	22.3	329 -	350	2	R.IFSAATRECVTKLEGHEGEISK.I
3616.8076	3615.8003	3615.7382	17.2	118 -	149	1	K.LWDTASGEELHTLEGHRNVVYAIAFNNPYGDK.I

No match to: 706.2851, 710.3807, 719.4268, 720.4332, 722.4348, 723.4337, 735.4208, 744.3633, 749.4401, 756.4190, 759.4415, 766.3942, 772.4352, 774.4582, 779.4799, 823.4933, 831.4758, 832.3166, 834.3207, 842.5047, 843.4840, 844.4902, 846.4869, 870.4950, 873.3982, 876.2891, 880.4586, 883.4816, 885.4853, 891.4897, 895.5765, 900.5383, 918.5458, 923.5204, 927.5097, 928.5096, 929.4465, 944.5216, 945.5218, 949.5538, 967.4249, 969.4326, 972.4861, 974.4482, 976.4711, 977.4844, 978.5469, 988.4647, 1007.5762, 1033.4938, 1034.5852, 1044.5280, 1056.5298, 1074.5454, 1087.5504, 1097.5125, 1103.5261, 1113.4890, 1119.5367, 1122.5620, 1145.6725, 1178.6276, 1185.6924, 1188.5890, 1220.6172, 1234.4895, 1248.6737, 1253.6409, 1284.6428, 1301.6381, 1313.7157, 1314.7207, 1315.6769, 1316.6554, 1317.6417, 1325.6414, 1327.7157, 1333.7577, 1336.7279, 1338.7021, 1349.7028, 1355.6765, 1358.7172, 1361.6941, 1372.6039, 1381.7673, 1382.5994, 1383.7312, 1397.7480, 1442.8285, 1447.7806, 1457.7437, 1461.8053, 1479.8021, 1487.7090, 1497.8783, 1517.8105, 1528.7001, 1532.7877, 1537.8292, 1539.8185, 1552.8179, 1553.8821, 1556.8846, 1562.8353, 1567.7686, 1578.7893, 1592.7920, 1593.8013, 1628.7037, 1628.8643, 1642.7200, 1650.8497, 1651.8568, 1667.8695, 1680.8239, 1687.9133, 1691.7732, 1706.8671, 1717.9054, 1724.8430, 1728.8602, 1730.8619, 1747.9176, 1771.0326, 1805.9197, 1809.0233, 1813.0017, 1820.9197, 1855.0103, 1887.9128, 1894.9668, 1910.9791, 1928.0238, 1953.8596, 1965.8589, 1974.9517, 1981.9934, 1983.9583, 1995.8975, 2020.9868, 2022.0580, 2044.1479, 2084.0510, 2110.0601, 2136.8467, 2144.0354, 2150.8611, 2155.0640, 2162.8757, 2164.8806, 2170.0522, 2174.1650, 2203.0303, 2217.0352, 2239.1282, 2251.1396, 2253.1372, 2255.2141, 2271.1794, 2274.0801, 2277.0820, 2286.1489, 2289.0703, 2293.1492, 2297.1624, 2309.1501, 2324.0906, 2343.1846, 2384.2053, 2410.1479, 2414.2322, 2464.2695, 2465.2659, 2529.2080, 2542.2549, 2645.0503, 2646.9685, 2657.4148, 2660.0061, 2660.9807, 2675.0012, 2689.0129, 2693.0125, 2703.0303, 2706.0225, 2718.3938, 2763.3359, 2774.3179, 2780.3301, 2787.3474, 2826.4065, 2846.3816, 2872.3054, 2876.3391, 2884.3103, 2887.3201, 2889.3223, 2891.4019, 2895.4370, 2900.3384, 2915.4314, 2983.4602, 2995.5381, 2999.4780, 3002.1853, 3016.2046, 3020.2017, 3030.2197, 3034.2266, 3044.2632, 3053.4902, 3064.5010, 3081.5806, 3126.7075, 3169.7002, 3206.5684, 3234.5972, 3248.6172, 3264.5881, 3299.7673, 3316.7351, 3320.6321, 3334.6604, 3427.5977, 3439.7319, 3453.7163, 3457.6880, 3458.6204, 3473.6636, 3490.6494, 3503.7805, 3517.5906, 3529.7371, 3639.6938, 3652.7173, 3704.8706, 3893.7073, 3959.0081, 3991.8828, 4147.9468, 4161.9458, 4177.9121, 4226.0356, 4233.1089, 4252.8618, 4280.2700, 4452.1279, 4465.1328, 4679.1919, 4844.0430

34. F6Q0Z5 **Mass:** 24053 **Score:** 19 **Expect:** 3.2e+002 **Matches:** 4
Uncharacterized protein OS=Bos taurus GN=LRRC57 PE=4 SV=1

Observed	Mr(expt)	Mr(calc)	ppm	Start	End	Miss	Peptide
883.4816	882.4744	882.4559	20.9	8 -	15	0	R.AHVETAQK.T
1248.6737	1247.6664	1247.6398	21.4	25 -	35	0	R.GLTEFPSELQK.L
1928.0238	1927.0165	1927.0276	-5.73	8 -	24	2	R.AHVETAQKTGVFQLKDR.G
2255.2141	2254.2068	2254.2182	-5.05	2 -	22	2	M.GNSALRAHVETAQKTGVFQLK.D

No match to: 706.2851, 710.3807, 717.4042, 719.4268, 720.4332, 722.4348, 723.4337, 735.4208, 744.3633, 749.4401, 756.4190, 759.4415, 766.3942, 772.4352, 774.4582, 779.4799, 823.4933, 831.4758, 832.3166, 834.3207, 842.5047, 843.4840, 844.4902, 846.4869, 870.4950, 873.3982, 876.2891, 880.4586, 885.4853, 891.4897, 895.5765, 900.5383, 918.5458, 923.5204, 927.5097, 928.5096, 929.4465, 944.5216, 945.5218, 949.5538, 967.4249, 969.4326, 972.4861, 974.4482, 976.4711, 977.4844, 978.5469, 988.4647, 1007.5762, 1033.4938, 1034.5852, 1044.5280, 1056.5298, 1074.5454, 1087.5504, 1097.5125, 1103.5261, 1113.4890, 1119.5367, 1122.5620, 1145.6725, 1178.6276, 1185.6924, 1188.5890, 1220.6172, 1234.4895, 1253.6409, 1284.6428, 1301.6381, 1313.7157, 1314.7207, 1315.6769, 1316.6554, 1317.6417, 1325.6414, 1327.7157, 1333.7577, 1336.7279, 1338.7021, 1349.7028, 1355.6765, 1358.7172, 1361.6941, 1372.6039, 1381.7673, 1382.5994, 1383.7312, 1397.7480, 1442.8285, 1447.7806, 1457.7437, 1461.8053, 1479.8021, 1487.7090, 1497.8783, 1517.8105, 1528.7001, 1532.7877, 1537.8292, 1539.8185, 1552.8179, 1553.8821, 1556.8846, 1562.8353, 1567.7686, 1578.7893, 1592.7920, 1593.8013, 1628.7037, 1628.8643, 1642.7200, 1650.8497, 1651.8568, 1667.8695, 1680.8239, 1687.9133, 1691.7732, 1706.8671, 1715.8597, 1717.9054, 1724.8430, 1728.8602, 1730.8619, 1747.9176, 1771.0326, 1805.9197, 1809.0233, 1813.0017, 1820.9197, 1855.0103, 1887.9128, 1894.9668, 1910.9791, 1953.8596,

1965.8589, 1974.9517, 1981.9934, 1983.9583, 1995.8975, 2020.9868, 2022.0580, 2027.1263, 2044.1479, 2084.0510, 2110.0601, 2136.8467, 2144.0354, 2150.8611, 2155.0640, 2162.8757, 2164.8806, 2170.0522, 2174.1650, 2203.0303, 2217.0352, 2239.1282, 2251.1396, 2253.1372, 2271.1794, 2274.0801, 2277.0820, 2286.1489, 2289.0703, 2293.1492, 2297.1624, 2309.1501, 2324.0906, 2340.1016, 2343.1846, 2384.2053, 2410.1479, 2414.2322, 2462.2893, 2464.2695, 2465.2659, 2529.2080, 2542.2549, 2645.0503, 2646.9685, 2657.4148, 2660.0061, 2660.9807, 2675.0012, 2689.0129, 2693.0125, 2703.0303, 2706.0225, 2718.3938, 2763.3359, 2774.3179, 2780.3301, 2787.3474, 2826.4065, 2846.3816, 2872.3054, 2876.3391, 2884.3103, 2887.3201, 2889.3223, 2891.4019, 2895.4370, 2900.3384, 2915.4314, 2983.4602, 2995.5381, 2999.4780, 3002.1853, 3016.2046, 3020.2017, 3030.2197, 3034.2266, 3044.2632, 3053.4902, 3064.5010, 3081.5806, 3126.7075, 3169.7002, 3206.5684, 3234.5972, 3248.6172, 3264.5881, 3299.7673, 3316.7351, 3320.6321, 3334.6604, 3427.5977, 3439.7319, 3453.7163, 3457.6880, 3458.6204, 3473.6636, 3490.6494, 3503.7805, 3517.5906, 3529.7371, 3616.8076, 3639.6938, 3652.7173, 3704.8706, 3893.7073, 3959.0081, 3991.8828, 4147.9468, 4161.9458, 4177.9121, 4226.0356, 4233.1089, 4252.8618, 4280.2700, 4452.1279, 4465.1328, 4679.1919, 4844.0430

35. G3N188 **Mass:** 15089 **Score:** 19 **Expect:** 3.3e+002 **Matches:** 3

Uncharacterized protein OS=Bos taurus PE=4 SV=1

Observed	Mr(expt)	Mr(calc)	ppm	Start	End	Miss	Peptide
1248.6737	1247.6664	1247.6833	-13.55	84	94	2	K.SRLSITKDNSK.S
2110.0601	2109.0528	2109.0565	-1.75	121	140	0	R.GSGFVPVVNVEPTTICPLSH.-
2846.3816	2845.3743	2845.4359	-21.62	58	85	2	R.QAPGKALEWLGDISSGGSTGYNPGLKSR.L

No match to: 706.2851, 710.3807, 717.4042, 719.4268, 720.4332, 722.4348, 723.4337, 735.4208, 744.3633, 749.4401, 756.4190, 759.4415, 766.3942, 772.4352, 774.4582, 779.4799, 823.4933, 831.4758, 832.3166, 834.3207, 842.5047, 843.4840, 844.4902, 846.4869, 870.4950, 873.3982, 876.2891, 880.4586, 883.4816, 885.4853, 891.4897, 895.5765, 900.5383, 918.5458, 923.5204, 927.5097, 928.5096, 929.4465, 944.5216, 945.5218, 949.5538, 967.4249, 969.4326, 972.4861, 974.4482, 976.4711, 977.4844, 978.5469, 988.4647, 1007.5762, 1033.4938, 1034.5852, 1044.5280, 1056.5298, 1074.5454, 1087.5504, 1097.5125, 1103.5261, 1113.4890, 1119.5367, 1122.5620, 1145.6725, 1178.6276, 1185.6924, 1188.5890, 1220.6172, 1234.4895, 1253.6409, 1284.6428, 1301.6381, 1313.7157, 1314.7207, 1315.6769, 1316.6554, 1317.6417, 1325.6414, 1327.7157, 1333.7577, 1336.7279, 1338.7021, 1349.7028, 1355.6765, 1358.7172, 1361.6941, 1372.6039, 1381.7673, 1382.5994, 1383.7312, 1397.7480, 1442.8285, 1447.7806, 1457.7437, 1461.8053, 1479.8021, 1487.7090, 1497.8783, 1517.8105, 1528.7001, 1532.7877, 1537.8292, 1539.8185, 1552.8179, 1553.8821, 1556.8846, 1562.8353, 1567.7686, 1578.7893, 1592.7920, 1593.8013, 1628.7037, 1628.8643, 1642.7200, 1650.8497, 1651.8568, 1667.8695, 1680.8239, 1687.9133, 1691.7732, 1706.8671, 1715.8597, 1717.9054, 1724.8430, 1728.8602, 1730.8619, 1747.9176, 1771.0326, 1805.9197, 1809.0233, 1813.0017, 1820.9197, 1855.0103, 1887.9128, 1894.9668, 1910.9791, 1928.0238, 1953.8596, 1965.8589, 1974.9517, 1981.9934, 1983.9583, 1995.8975, 2020.9868, 2022.0580, 2027.1263, 2044.1479, 2084.0510, 2136.8467, 2144.0354, 2150.8611, 2155.0640, 2162.8757, 2164.8806, 2170.0522, 2174.1650, 2203.0303, 2217.0352, 2239.1282, 2251.1396, 2253.1372, 2255.2141, 2271.1794, 2274.0801, 2277.0820, 2286.1489, 2289.0703, 2293.1492, 2297.1624, 2309.1501, 2324.0906, 2340.1016, 2343.1846, 2384.2053, 2410.1479, 2414.2322, 2462.2893, 2464.2695, 2465.2659, 2529.2080, 2542.2549, 2645.0503, 2646.9685, 2657.4148, 2660.0061, 2660.9807, 2675.0012, 2689.0129, 2693.0125, 2703.0303, 2706.0225, 2718.3938, 2763.3359, 2774.3179, 2780.3301, 2787.3474, 2826.4065, 2872.3054, 2876.3391, 2884.3103, 2887.3201, 2889.3223, 2891.4019, 2895.4370, 2900.3384, 2915.4314, 2983.4602, 2995.5381, 2999.4780, 3002.1853, 3016.2046, 3020.2017, 3030.2197, 3034.2266, 3044.2632, 3053.4902, 3064.5010, 3081.5806, 3126.7075, 3169.7002, 3206.5684, 3234.5972, 3248.6172, 3264.5881, 3299.7673, 3316.7351, 3320.6321, 3334.6604, 3427.5977, 3439.7319, 3453.7163, 3457.6880, 3458.6204, 3473.6636, 3490.6494, 3503.7805, 3517.5906, 3529.7371, 3616.8076, 3639.6938, 3652.7173, 3704.8706, 3893.7073, 3959.0081, 3991.8828, 4147.9468, 4161.9458, 4177.9121, 4226.0356, 4233.1089, 4252.8618, 4280.2700, 4452.1279, 4465.1328, 4679.1919, 4844.0430

36. G3MWI9 **Mass:** 34349 **Score:** 19 **Expect:** 3.3e+002 **Matches:** 5

Uncharacterized protein OS=Bos taurus PE=4 SV=1

Observed	Mr(expt)	Mr(calc)	ppm	Start	End	Miss	Peptide
772.4352	771.4280	771.4239	5.30	82	87	0	K.QNLELR.A
883.4816	882.4744	882.4672	8.15	103	111	0	R.SAAGSVHVR.R
1327.7157	1326.7084	1326.6853	17.4	126	138	0	K.MSPLPADTGITPK.H
1628.8643	1627.8570	1627.8715	-8.91	74	87	1	R.AQMDALVKQNLELR.A
2027.1263	2026.1190	2026.0993	9.74	71	87	2	R.ELRAQMDALVKQNLELR.A

No match to: 706.2851, 710.3807, 717.4042, 719.4268, 720.4332, 722.4348, 723.4337, 735.4208, 744.3633, 749.4401, 756.4190, 759.4415, 766.3942, 774.4582, 779.4799, 823.4933, 831.4758, 832.3166, 834.3207, 842.5047, 843.4840, 844.4902, 846.4869, 870.4950, 873.3982, 876.2891, 880.4586, 885.4853, 891.4897, 895.5765, 900.5383, 918.5458, 923.5204, 927.5097, 928.5096, 929.4465, 944.5216, 945.5218, 949.5538, 967.4249, 969.4326, 972.4861, 974.4482, 976.4711, 977.4844, 978.5469, 988.4647, 1007.5762, 1033.4938, 1034.5852, 1044.5280, 1056.5298, 1074.5454, 1087.5504, 1097.5125, 1103.5261, 1113.4890, 1119.5367, 1122.5620, 1145.6725, 1178.6276, 1185.6924, 1188.5890, 1220.6172, 1234.4895, 1248.6737, 1253.6409, 1284.6428, 1301.6381, 1313.7157, 1314.7207, 1315.6769, 1316.6554, 1317.6417, 1325.6414, 1333.7577, 1336.7279, 1338.7021, 1349.7028, 1355.6765, 1358.7172, 1361.6941, 1372.6039, 1381.7673, 1382.5994, 1383.7312, 1397.7480, 1442.8285, 1447.7806, 1457.7437, 1461.8053, 1479.8021, 1487.7090, 1497.8783, 1517.8105, 1528.7001, 1532.7877, 1537.8292, 1539.8185, 1552.8179, 1553.8821, 1556.8846, 1562.8353, 1567.7686, 1578.7893, 1592.7920, 1593.8013, 1628.7037, 1642.7200, 1650.8497, 1651.8568, 1667.8695, 1680.8239, 1687.9133, 1691.7732, 1706.8671, 1715.8597, 1717.9054, 1724.8430, 1728.8602, 1730.8619, 1747.9176, 1771.0326, 1805.9197, 1809.0233, 1813.0017, 1820.9197, 1855.0103, 1887.9128, 1894.9668, 1910.9791, 1928.0238, 1953.8596, 1965.8589, 1974.9517, 1981.9934, 1983.9583, 1995.8975, 2020.9868, 2022.0580, 2044.1479, 2084.0510, 2110.0601, 2136.8467, 2144.0354, 2150.8611, 2155.0640, 2162.8757, 2164.8806, 2170.0522, 2174.1650, 2203.0303, 2217.0352, 2239.1282, 2251.1396, 2253.1372, 2255.2141, 2271.1794, 2274.0801, 2277.0820, 2286.1489, 2289.0703, 2293.1492, 2297.1624, 2309.1501, 2324.0906, 2340.1016, 2343.1846, 2384.2053, 2410.1479, 2414.2322, 2462.2893, 2464.2695, 2465.2659, 2529.2080, 2542.2549, 2645.0503, 2646.9685, 2657.4148, 2660.0061, 2660.9807, 2675.0012, 2689.0129, 2693.0125, 2703.0303, 2706.0225, 2718.3938, 2763.3359, 2774.3179, 2780.3301, 2787.3474, 2826.4065, 2846.3816, 2872.3054, 2876.3391, 2884.3103, 2887.3201, 2889.3223, 2891.4019, 2895.4370, 2900.3384, 2915.4314, 2983.4602, 2995.5381, 2999.4780, 3002.1853, 3016.2046, 3020.2017, 3030.2197, 3034.2266, 3044.2632, 3053.4902, 3064.5010, 3081.5806, 3126.7075, 3169.7002, 3206.5684, 3234.5972, 3248.6172, 3264.5881, 3299.7673, 3316.7351, 3320.6321, 3334.6604, 3427.5977, 3439.7319, 3453.7163, 3457.6880, 3458.6204, 3473.6636, 3490.6494, 3503.7805, 3517.5906, 3529.7371, 3616.8076, 3639.6938, 3652.7173, 3704.8706, 3893.7073, 3959.0081, 3991.8828, 4147.9468, 4161.9458, 4177.9121, 4226.0356, 4233.1089, 4252.8618, 4280.2700, 4452.1279, 4465.1328, 4679.1919, 4844.0430

37. F1MZL6 **Mass:** 54511 **Score:** 18 **Expect:** 3.4e+002 **Matches:** 8

V-type proton ATPase subunit H OS=Bos taurus GN=ATP6V1H PE=4 SV=1

Observed	Mr(expt)	Mr(calc)	ppm	Start	End	Miss	Peptide
710.3807	709.3734	709.3581	21.6	53	57	1	R.FEMKR.S
843.4840	842.4767	842.4862	-11.26	409	416	0	R.VIEQLGGK.Q
1044.5280	1043.5207	1043.5359	-14.56	360	368	1	R.ENAARLNEK.N
2110.0601	2109.0528	2109.0023	23.9	198	216	0	R.FAWVEADGVNCIMGVLSNK.C
2763.3359	2762.3286	2762.3538	-9.11	319	342	1	K.FLLEKLGESVQDLSSFDEYSSELK.S
2787.3474	2786.3401	2786.3156	8.80	193	216	1	R.VNEYRFAWVEADGVNCIMGVLSNK.C + Oxidation (M)
3334.6604	3333.6531	3333.5904	18.8	170	197	1	K.TQLSSQSSQYVQCVAGCLQLMLRVNEYR.F + Oxidation (M)
3503.7805	3502.7732	3502.8572	-23.98	375	405	2	K.ILTKLLEVSDDPQVLAVAAHDVGEYVRHYPR.G

No match to: 706.2851, 717.4042, 719.4268, 720.4332, 722.4348, 723.4337, 735.4208, 744.3633, 749.4401, 756.4190, 759.4415, 766.3942, 772.4352, 774.4582, 779.4799, 823.4933, 831.4758, 832.3166, 834.3207, 842.5047, 844.4902, 846.4869, 870.4950, 873.3982, 876.2891, 880.4586, 883.4816, 885.4853, 891.4897, 895.5765, 900.5383, 918.5458, 923.5204, 927.5097, 928.5096, 929.4465, 944.5216, 945.5218, 949.5538, 967.4249, 969.4326, 972.4861, 974.4482, 976.4711, 977.4844, 978.5469, 988.4647, 1007.5762, 1033.4938, 1034.5852, 1056.5298, 1074.5454, 1087.5504, 1097.5125, 1103.5261, 1113.4890, 1119.5367, 1122.5620, 1145.6725, 1178.6276, 1185.6924, 1188.5890, 1220.6172, 1234.4895, 1248.6737, 1253.6409, 1284.6428, 1301.6381, 1313.7157, 1314.7207, 1315.6769, 1316.6554, 1317.6417, 1325.6414, 1327.7157, 1333.7577, 1336.7279, 1338.7021, 1349.7028, 1355.6765, 1358.7172, 1361.6941, 1372.6039, 1381.7673, 1382.5994, 1383.7312, 1397.7480, 1442.8285, 1447.7806, 1457.7437, 1461.8053, 1479.8021, 1487.7090, 1497.8783, 1517.8105, 1528.7001, 1532.7877, 1537.8292, 1539.8185, 1552.8179, 1553.8821, 1556.8846, 1562.8353, 1567.7686, 1578.7893, 1592.7920, 1593.8013, 1628.7037, 1628.8643, 1642.7200, 1650.8497, 1651.8568, 1667.8695, 1680.8239, 1687.9133, 1691.7732, 1706.8671, 1715.8597, 1717.9054, 1724.8430, 1728.8602, 1730.8619, 1747.9176, 1771.0326, 1805.9197, 1809.0233, 1813.0017, 1820.9197, 1855.0103, 1887.9128, 1894.9668, 1910.9791, 1928.0238, 1953.8596, 1965.8589, 1974.9517, 1981.9934, 1983.9583, 1995.8975, 2020.9868, 2022.0580, 2027.1263, 2044.1479, 2084.0510, 2136.8467, 2144.0354, 2150.8611, 2155.0640, 2162.8757, 2164.8806, 2170.0522, 2174.1650, 2203.0303, 2217.0352, 2239.1282, 2251.1396, 2253.1372, 2255.2141, 2271.1794, 2274.0801, 2277.0820, 2286.1489, 2289.0703, 2293.1492, 2297.1624, 2309.1501, 2324.0906, 2340.1016, 2343.1846, 2384.2053, 2410.1479, 2414.2322, 2462.2893, 2464.2695, 2465.2659, 2529.2080, 2542.2549, 2645.0503, 2646.9685, 2657.4148, 2660.0061,

2660.9807, 2675.0012, 2689.0129, 2693.0125, 2703.0303, 2706.0225, 2718.3938, 2774.3179, 2780.3301, 2826.4065, 2846.3816, 2872.3054, 2876.3391, 2884.3103, 2887.3201, 2889.3223, 2891.4019, 2895.4370, 2900.3384, 2915.4314, 2983.4602, 2995.5381, 2999.4780, 3002.1853, 3016.2046, 3020.2017, 3030.2197, 3034.2266, 3044.2632, 3053.4902, 3064.5010, 3081.5806, 3126.7075, 3169.7002, 3206.5684, 3234.5972, 3248.6172, 3264.5881, 3299.7673, 3316.7351, 3320.6321, 3427.5977, 3439.7319, 3453.7163, 3457.6880, 3458.6204, 3473.6636, 3490.6494, 3517.5906, 3529.7371, 3616.8076, 3639.6938, 3652.7173, 3704.8706, 3893.7073, 3959.0081, 3991.8828, 4147.9468, 4161.9458, 4177.9121, 4226.0356, 4233.1089, 4252.8618, 4280.2700, 4452.1279, 4465.1328, 4679.1919, 4844.0430

38. A6QPQ5 **Mass:** 36919 **Score:** 18 **Expect:** 3.4e+002 **Matches:** 6

39S ribosomal protein L1, mitochondrial OS=Bos taurus GN=MRPL1 PE=1 SV=1

Observed	Mr(expt)	Mr(calc)	ppm	Start	End	Miss	Peptide
880.4586	879.4513	879.4338	19.9	250	256	0	R.ENFLETK.I
1034.5852	1033.5779	1033.5655	12.0	297	306	0	R.SSTSEGLLLK.I
1336.7279	1335.7206	1335.7187	1.45	108	118	1	K.KFQVLDFTNPK.Q
1813.0017	1811.9944	1811.9516	23.6	307	322	2	K.IEPLLPKEGETKESDK.K
3503.7805	3502.7732	3502.7436	8.47	250	280	1	R.ENFLETKIATLDMPSDQIAANLQAVINEVCR.Q
4147.9468	4146.9395	4147.0201	-19.44	245	280	2	K.VDEERENFLETKIATLDMPSDQIAANLQAVINEVCR.Q + Oxidation (M)

No match to: 706.2851, 710.3807, 717.4042, 719.4268, 720.4332, 722.4348, 723.4337, 735.4208, 744.3633, 749.4401, 756.4190, 759.4415, 766.3942, 772.4352, 774.4582, 779.4799, 823.4933, 831.4758, 832.3166, 834.3207, 842.5047, 843.4840, 844.4902, 846.4869, 870.4950, 873.3982, 876.2891, 883.4816, 885.4853, 891.4897, 895.5765, 900.5383, 918.5458, 923.5204, 927.5097, 928.5096, 929.4465, 944.5216, 945.5218, 949.5538, 967.4249, 969.4326, 972.4861, 974.4482, 976.4711, 977.4844, 978.5469, 988.4647, 1007.5762, 1033.4938, 1044.5280, 1056.5298, 1074.5454, 1087.5504, 1097.5125, 1103.5261, 1113.4890, 1119.5367, 1122.5620, 1145.6725, 1178.6276, 1185.6924, 1188.5890, 1220.6172, 1234.4895, 1248.6737, 1253.6409, 1284.6428, 1301.6381, 1313.7157, 1314.7207, 1315.6769, 1316.6554, 1317.6417, 1325.6414, 1327.7157, 1333.7577, 1338.7021, 1349.7028, 1355.6765, 1358.7172, 1361.6941, 1372.6039, 1381.7673, 1382.5994, 1383.7312, 1397.7480, 1442.8285, 1447.7806, 1457.7437, 1461.8053, 1479.8021, 1487.7090, 1497.8783, 1517.8105, 1528.7001, 1532.7877, 1537.8292, 1539.8185, 1552.8179, 1553.8821, 1556.8846, 1562.8353, 1567.7686, 1578.7893, 1592.7920, 1593.8013, 1628.7037, 1628.8643, 1642.7200, 1650.8497, 1651.8568, 1667.8695, 1680.8239, 1687.9133, 1691.7732, 1706.8671, 1715.8597, 1717.9054, 1724.8430, 1728.8602, 1730.8619, 1747.9176, 1771.0326, 1805.9197, 1809.0233, 1820.9197, 1855.0103, 1887.9128, 1894.9668, 1910.9791, 1928.0238, 1953.8596, 1965.8589, 1974.9517, 1981.9934, 1983.9583, 1995.8975, 2020.9868, 2022.0580, 2027.1263, 2044.1479, 2084.0510, 2110.0601, 2136.8467, 2144.0354, 2150.8611, 2155.0640, 2162.8757, 2164.8806, 2170.0522, 2174.1650, 2203.0303, 2217.0352, 2239.1282, 2251.1396, 2253.1372, 2255.2141, 2271.1794, 2274.0801, 2277.0820, 2286.1489, 2289.0703, 2293.1492, 2297.1624, 2309.1501, 2324.0906, 2340.1016, 2343.1846, 2384.2053, 2410.1479, 2414.2322, 2462.2893, 2464.2695, 2465.2659, 2529.2080, 2542.2549, 2645.0503, 2646.9685, 2657.4148, 2660.0061, 2660.9807, 2675.0012, 2689.0129, 2693.0125, 2703.0303, 2706.0225, 2718.3938, 2763.3359, 2774.3179, 2780.3301, 2787.3474, 2826.4065, 2846.3816, 2872.3054, 2876.3391, 2884.3103, 2887.3201, 2889.3223, 2891.4019, 2895.4370, 2900.3384, 2915.4314, 2983.4602, 2995.5381, 2999.4780, 3002.1853, 3016.2046, 3020.2017, 3030.2197, 3034.2266, 3044.2632, 3053.4902, 3064.5010, 3081.5806, 3126.7075, 3169.7002, 3206.5684, 3234.5972, 3248.6172, 3264.5881, 3299.7673, 3316.7351, 3320.6321, 3334.6604, 3427.5977, 3439.7319, 3453.7163, 3457.6880, 3458.6204, 3473.6636, 3490.6494, 3517.5906, 3529.7371, 3616.8076, 3639.6938, 3652.7173, 3704.8706, 3893.7073, 3959.0081, 3991.8828, 4161.9458, 4177.9121, 4226.0356, 4233.1089, 4252.8618, 4280.2700, 4452.1279, 4465.1328, 4679.1919, 4844.0430

39. F6QNC4 **Mass:** 81939 **Score:** 18 **Expect:** 3.7e+002 **Matches:** 10

Uncharacterized protein OS=Bos taurus GN=PCCA PE=4 SV=1

Observed	Mr(expt)	Mr(calc)	ppm	Start	End	Miss	Peptide
870.4950	869.4877	869.4872	0.66	379	385	1	K.GYPLRHK.Q
967.4249	966.4177	966.4263	-8.92	708	716	0	K.MQNSMTAGK.T
1567.7686	1566.7613	1566.7613	0.02	1	16	0	-.MAGFWTGAAALATAGR.R + Oxidation (M)
1650.8497	1649.8424	1649.8447	-1.37	211	227	1	R.EIGYPVMIKASAGGGGK.G + Oxidation (M)
1730.8619	1729.8546	1729.8345	11.6	633	648	0	R.EAGGNMSIQFLGTVYK.V + Oxidation (M)
1953.8596	1952.8523	1952.8799	-14.12	228	243	2	K.GMRIAWDDEETRDGFR.F
2110.0601	2109.0528	2109.0889	-17.08	85	104	1	K.MGIKTVAIHSDVDASSVHVK.M + Oxidation (M)
3206.5684	3205.5611	3205.5489	3.81	431	459	1	R.VDSGIQPGSDISIYYDPMISKLITYGSDR.T + Oxidation (M)
3503.7805	3502.7732	3502.7885	-4.36	400	430	2	R.VYAEDPYKSFGLPSVGRLSQYEEPIHLPGVR.V
3639.6938	3638.6865	3638.7743	-24.12	85	119	2	K.MGIKTVAIHSDVDASSVHVKMADEAVCVGPAPTSK.S + 2 Oxidation (M)

No match to: 706.2851, 710.3807, 717.4042, 719.4268, 720.4332, 722.4348, 723.4337, 735.4208, 744.3633, 749.4401, 756.4190, 759.4415, 766.3942, 772.4352, 774.4582, 779.4799, 823.4933, 831.4758, 832.3166, 834.3207, 842.5047, 843.4840, 844.4902, 846.4869, 873.3982, 876.2891, 880.4586, 883.4816, 885.4853, 891.4897, 895.5765, 900.5383, 918.5458, 923.5204, 927.5097, 928.5096, 929.4465, 944.5216, 945.5218, 949.5538, 969.4326, 972.4861, 974.4482, 976.4711, 977.4844, 978.5469, 988.4647, 1007.5762, 1033.4938, 1034.5852, 1044.5280, 1056.5298, 1074.5454, 1087.5504, 1097.5125, 1103.5261, 1113.4890, 1119.5367, 1122.5620, 1145.6725, 1178.6276, 1185.6924, 1188.5890, 1220.6172, 1234.4895, 1248.6737, 1253.6409, 1284.6428, 1301.6381, 1313.7157, 1314.7207, 1315.6769, 1316.6554, 1317.6417, 1325.6414, 1327.7157, 1333.7577, 1336.7279, 1338.7021, 1349.7028, 1355.6765, 1358.7172, 1361.6941, 1372.6039, 1381.7673, 1382.5994, 1383.7312, 1397.7480, 1442.8285, 1447.7806, 1457.7437, 1461.8053, 1479.8021, 1487.7090, 1497.8783, 1517.8105, 1528.7001, 1532.7877, 1537.8292, 1539.8185, 1552.8179, 1553.8821, 1556.8846, 1562.8353, 1578.7893, 1592.7920, 1593.8013, 1628.7037, 1628.8643, 1642.7200, 1651.8568, 1667.8695, 1680.8239, 1687.9133, 1691.7732, 1706.8671, 1715.8597, 1717.9054, 1724.8430, 1728.8602, 1747.9176, 1771.0326, 1805.9197, 1809.0233, 1813.0017, 1820.9197, 1855.0103, 1887.9128, 1894.9668, 1910.9791, 1928.0238, 1965.8589, 1974.9517, 1981.9934, 1983.9583, 1995.8975, 2020.9868, 2022.0580, 2027.1263, 2044.1479, 2084.0510, 2136.8467, 2144.0354, 2150.8611, 2155.0640, 2162.8757, 2164.8806, 2170.0522, 2174.1650, 2203.0303, 2217.0352, 2239.1282, 2251.1396, 2253.1372, 2255.2141, 2271.1794, 2274.0801, 2277.0820, 2286.1489, 2289.0703, 2293.1492, 2297.1624, 2309.1501, 2324.0906, 2340.1016, 2343.1846, 2384.2053, 2410.1479, 2414.2322, 2462.2893, 2464.2695, 2465.2659, 2529.2080, 2542.2549, 2645.0503, 2646.9685, 2657.4148, 2660.0061, 2660.9807, 2675.0012, 2689.0129, 2693.0125, 2703.0303, 2706.0225, 2718.3938, 2763.3359, 2774.3179, 2780.3301, 2787.3474, 2826.4065, 2846.3816, 2872.3054, 2876.3391, 2884.3103, 2887.3201, 2889.3223, 2891.4019, 2895.4370, 2900.3384, 2915.4314, 2983.4602, 2995.5381, 2999.4780, 3002.1853, 3016.2046, 3020.2017, 3030.2197, 3034.2266, 3044.2632, 3053.4902, 3064.5010, 3081.5806, 3126.7075, 3169.7002, 3234.5972, 3248.6172, 3264.5881, 3299.7673, 3316.7351, 3320.6321, 3334.6604, 3427.5977, 3439.7319, 3453.7163, 3457.6880, 3458.6204, 3473.6636, 3490.6494, 3517.5906, 3529.7371, 3616.8076, 3652.7173, 3704.8706, 3893.7073, 3959.0081, 3991.8828, 4147.9468, 4161.9458, 4177.9121, 4226.0356, 4233.1089, 4252.8618, 4280.2700, 4452.1279, 4465.1328, 4679.1919, 4844.0430

40. F1MDJ4 **Mass:** 73301 **Score:** 18 **Expect:** 3.8e+002 **Matches:** 14

Uncharacterized protein OS=Bos taurus GN=CCDC93 PE=4 SV=1

Observed	Mr(expt)	Mr(calc)	ppm	Start	End	Miss	Peptide
756.4190	755.4117	755.4038	10.5	423	429	1	K.AERAPGR.D
774.4582	773.4509	773.4395	14.7	580	585	2	K.KKQENK.M
779.4799	778.4726	778.4589	17.7	529	534	1	K.KVYLEK.E
923.5204	922.5132	922.5348	-23.48	309	316	0	K.LGISQLHR.R
972.4861	971.4789	971.4825	-3.70	209	216	1	R.YGFSRQSK.T
977.4844	976.4772	976.4872	-10.29	582	588	2	K.QENKMRR.D + Oxidation (M)
978.5469	977.5396	977.5215	18.5	618	625	1	R.KNEMLLSK.V + Oxidation (M)
1087.5504	1086.5431	1086.5346	7.88	140	148	0	R.SYSISQFQK.T
1361.6941	1360.6868	1360.6696	12.6	457	466	2	R.YNMEKEKLYK.I + Oxidation (M)
1820.9197	1819.9124	1819.9284	-8.78	562	576	2	R.QMEQIVEGIKQSRMK.M + Oxidation (M)
2340.1016	2339.0943	2339.0877	2.84	6	26	1	K.GPEGQGLPEVETREDEEQNVK.L
2529.2080	2528.2007	2528.1417	23.3	140	160	1	R.SYSISQFQKTYSLPEDDDFMK.R
2872.3054	2871.2981	2871.3556	-20.02	1	26	2	-.MGLSKGPEGQGLPEVETREDEEQNVK.L + Oxidation (M)
4233.1089	4232.1016	4232.0994	0.53	535	571	2	K.EISLLNSIHENFSQAMASPASRDQFLRQMEQIVEGIK.Q + Oxidation (M)

No match to: 706.2851, 710.3807, 717.4042, 719.4268, 720.4332, 722.4348, 723.4337, 735.4208, 744.3633, 749.4401, 759.4415, 766.3942, 772.4352, 823.4933, 831.4758, 832.3166, 834.3207, 842.5047, 843.4840, 844.4902, 846.4869, 870.4950, 873.3982, 876.2891, 880.4586, 883.4816, 885.4853, 891.4897, 895.5765, 900.5383, 918.5458, 927.5097, 928.5096, 929.4465, 944.5216, 945.5218, 949.5538,

967.4249, 969.4326, 974.4482, 976.4711, 988.4647, 1007.5762, 1033.4938, 1034.5852, 1044.5280, 1056.5298, 1074.5454, 1097.5125, 1103.5261, 1113.4890, 1119.5367, 1122.5620, 1145.6725, 1178.6276, 1185.6924, 1188.5890, 1220.6172, 1234.4895, 1248.6737, 1253.6409, 1284.6428, 1301.6381, 1313.7157, 1314.7207, 1315.6769, 1316.6554, 1317.6417, 1325.6414, 1327.7157, 1333.7577, 1336.7279, 1338.7021, 1349.7028, 1355.6765, 1358.7172, 1372.6039, 1381.7673, 1382.5994, 1383.7312, 1397.7480, 1442.8285, 1447.7806, 1457.7437, 1461.8053, 1479.8021, 1487.7090, 1497.8783, 1517.8105, 1528.7001, 1532.7877, 1537.8292, 1539.8185, 1552.8179, 1553.8821, 1556.8846, 1562.8353, 1567.7686, 1578.7893, 1592.7920, 1593.8013, 1628.7037, 1628.8643, 1642.7200, 1650.8497, 1651.8568, 1667.8695, 1680.8239, 1687.9133, 1691.7732, 1706.8671, 1715.8597, 1717.9054, 1724.8430, 1728.8602, 1730.8619, 1747.9176, 1771.0326, 1805.9197, 1809.0233, 1813.0017, 1855.0103, 1887.9128, 1894.9668, 1910.9791, 1928.0238, 1953.8596, 1965.8589, 1974.9517, 1981.9934, 1983.9583, 1995.8975, 2020.9868, 2022.0580, 2027.1263, 2044.1479, 2084.0510, 2110.0601, 2136.8467, 2144.0354, 2150.8611, 2155.0640, 2162.8757, 2164.8806, 2170.0522, 2174.1650, 2203.0303, 2217.0352, 2239.1282, 2251.1396, 2253.1372, 2255.2141, 2271.1794, 2274.0801, 2277.0820, 2286.1489, 2289.0703, 2293.1492, 2297.1624, 2309.1501, 2324.0906, 2343.1846, 2384.2053, 2410.1479, 2414.2322, 2462.2893, 2464.2695, 2465.2659, 2542.2549, 2645.0503, 2646.9685, 2657.4148, 2660.0061, 2660.9807, 2675.0012, 2689.0129, 2693.0125, 2703.0303, 2706.0225, 2718.3938, 2763.3359, 2774.3179, 2780.3301, 2787.3474, 2826.4065, 2846.3816, 2876.3391, 2884.3103, 2887.3201, 2889.3223, 2891.4019, 2895.4370, 2900.3384, 2915.4314, 2983.4602, 2995.5381, 2999.4780, 3002.1853, 3016.2046, 3020.2017, 3030.2197, 3034.2266, 3044.2632, 3053.4902, 3064.5010, 3081.5806, 3126.7075, 3169.7002, 3206.5684, 3234.5972, 3248.6172, 3264.5881, 3299.7673, 3316.7351, 3320.6321, 3334.6604, 3427.5977, 3439.7319, 3453.7163, 3457.6880, 3458.6204, 3473.6636, 3490.6494, 3503.7805, 3517.5906, 3529.7371, 3616.8076, 3639.6938, 3652.7173, 3704.8706, 3893.7073, 3959.0081, 3991.8828, 4147.9468, 4161.9458, 4177.9121, 4226.0356, 4252.8618, 4280.2700, 4452.1279, 4465.1328, 4679.1919, 4844.0430

41. G3MZ54 **Mass:** 74700 **Score:** 18 **Expect:** 3.8e+002 **Matches:** 8

Uncharacterized protein OS=Bos taurus GN=SNX9 PE=4 SV=1

Observed	Mr(expt)	Mr(calc)	ppm	Start		End	Miss	Peptide
883.4816	882.4744	882.4633	12.5	325	-	332	1	K.GSKMYGLK.S
928.5096	927.5024	927.4960	6.84	491	-	498	1	K.RCTGPLPK.E
2044.1479	2043.1406	2043.0982	20.8	53	-	69	1	R.ALFPLLKWVYSEFFGAR.S
2293.1492	2292.1419	2292.1247	7.52	333	-	351	1	K.SYIEYQLTPTNTNRSVNHR.Y
2774.3179	2773.3106	2773.3779	-24.24	35	-	59	2	K.EEVDGGVPCSQEAEKAMRALFPLLK.W
3439.7319	3438.7246	3438.6992	7.38	295	-	324	2	K.EKIPIIVGDYGPMWVYPSSTFDCVVADPRK.G
3503.7805	3502.7732	3502.7462	7.70	433	-	463	2	R.KAEKDELVGVMIFSTMEPEAPDLDLVEIEQK.C
4452.1279	4451.1206	4451.0976	5.18	70	-	110	1	R.SQARVMYDFSAEPGNNELTVNEGEIITITNPDVGGGWLEGR.N + Oxidation (M)

No match to: 706.2851, 710.3807, 717.4042, 719.4268, 720.4332, 722.4348, 723.4337, 735.4208, 744.3633, 749.4401, 756.4190, 759.4415, 766.3942, 772.4352, 774.4582, 779.4799, 823.4933, 831.4758, 832.3166, 834.3207, 842.5047, 843.4840, 844.4902, 846.4869, 870.4950, 873.3982, 876.2891, 880.4586, 885.4853, 891.4897, 895.5765, 900.5383, 918.5458, 923.5204, 927.5097, 929.4465, 944.5216, 945.5218, 949.5538, 967.4249, 969.4326, 972.4861, 974.4482, 976.4711, 977.4844, 978.5469, 988.4647, 1007.5762, 1033.4938, 1034.5852, 1044.5280, 1056.5298, 1074.5454, 1087.5504, 1097.5125, 1103.5261, 1113.4890, 1119.5367, 1122.5620, 1145.6725, 1178.6276, 1185.6924, 1188.5890, 1220.6172, 1234.4895, 1248.6737, 1253.6409, 1284.6428, 1301.6381, 1313.7157, 1314.7207, 1315.6769, 1316.6554, 1317.6417, 1325.6414, 1327.7157, 1333.7577, 1336.7279, 1338.7021, 1349.7028, 1355.6765, 1358.7172, 1361.6941, 1372.6039, 1381.7673, 1382.5994, 1383.7312, 1397.7480, 1442.8285, 1447.7806, 1457.7437, 1461.8053, 1479.8021, 1487.7090, 1497.8783, 1517.8105, 1528.7001, 1532.7877, 1537.8292, 1539.8185, 1552.8179, 1553.8821, 1556.8846, 1562.8353, 1567.7686, 1578.7893, 1592.7920, 1593.8013, 1628.7037, 1628.8643, 1642.7200, 1650.8497, 1651.8568, 1667.8695, 1680.8239, 1687.9133, 1691.7732, 1706.8671, 1715.8597, 1717.9054, 1724.8430, 1728.8602, 1730.8619, 1747.9176, 1771.0326, 1805.9197, 1809.0233, 1813.0017, 1820.9197, 1855.0103, 1887.9128, 1894.9668, 1910.9791, 1928.0238, 1953.8596, 1965.8589, 1974.9517, 1981.9934, 1983.9583, 1995.8975, 2020.9868, 2022.0580, 2027.1263, 2084.0510, 2110.0601, 2136.8467, 2144.0354, 2150.8611, 2155.0640, 2162.8757, 2164.8806, 2170.0522, 2174.1650, 2203.0303, 2217.0352, 2239.1282, 2251.1396, 2253.1372, 2255.2141, 2271.1794, 2274.0801, 2277.0820, 2286.1489, 2289.0703, 2297.1624, 2309.1501, 2324.0906, 2340.1016, 2343.1846, 2384.2053, 2410.1479, 2414.2322, 2462.2893, 2464.2695, 2465.2659, 2529.2080, 2542.2549, 2645.0503, 2646.9685, 2657.4148, 2660.0061, 2660.9807, 2675.0012, 2689.0129, 2693.0125, 2703.0303, 2706.0225, 2718.3938, 2763.3359, 2780.3301, 2787.3474, 2826.4065, 2846.3816, 2872.3054, 2876.3391, 2884.3103, 2887.3201, 2889.3223, 2891.4019, 2895.4370, 2900.3384, 2915.4314, 2983.4602, 2995.5381, 2999.4780, 3002.1853, 3016.2046, 3020.2017, 3030.2197, 3034.2266, 3044.2632, 3053.4902, 3064.5010, 3081.5806, 3126.7075, 3169.7002, 3206.5684, 3234.5972, 3248.6172, 3264.5881, 3299.7673, 3316.7351, 3320.6321, 3334.6604, 3427.5977, 3453.7163, 3457.6880, 3458.6204, 3473.6636, 3490.6494, 3517.5906, 3529.7371, 3616.8076, 3639.6938, 3652.7173, 3704.8706, 3893.7073, 3959.0081, 3991.8828, 4147.9468, 4161.9458, 4177.9121, 4226.0356, 4233.1089, 4252.8618, 4280.2700, 4465.1328, 4679.1919, 4844.0430

42. E1BPW2 **Mass:** 128317 **Score:** 18 **Expect:** 3.8e+002 **Matches:** 24

Uncharacterized protein OS=Bos taurus GN=ERC1 PE=4 SV=2

Observed	Mr(expt)	Mr(calc)	ppm	Start		End	Miss	Peptide
717.4042	716.3969	716.4068	-13.89	170	-	175	1	R.KDVEVK.E
873.3982	872.3909	872.3923	-1.54	362	-	367	1	R.EEMHRR.F + Oxidation (M)
944.5216	943.5143	943.5199	-5.90	632	-	638	2	R.LKEQRDR.D
978.5469	977.5396	977.5631	-24.05	27	-	34	2	R.SPRLGHRR.T
1103.5261	1102.5188	1102.5115	6.62	634	-	641	2	K.EQRDRDER.E
1122.5620	1121.5547	1121.5611	-5.72	87	-	97	1	K.STMTLGRSGGR.L
1314.7207	1313.7134	1313.6939	14.9	680	-	692	1	K.EHASSLASSGLKK.D
1325.6414	1324.6341	1324.6622	-21.23	743	-	753	2	R.EIARYKDESSK.A
1397.7480	1396.7407	1396.7059	24.9	11	-	23	1	K.VEPSTQSPGRSPR.L
1487.7090	1486.7017	1486.7263	-16.53	840	-	851	1	K.DDRIEELEEALR.E
1497.8783	1496.8710	1496.8562	9.90	754	-	766	1	K.AQAEVDRLLEILK.E
1667.8695	1666.8622	1666.8726	-6.20	1029	-	1041	1	K.LYIRHLTALCHDR.D
1747.9176	1746.9103	1746.9185	-4.70	286	-	299	2	R.KTLEEMELRIETQK.Q
2044.1479	2043.1406	2043.1099	15.0	661	-	679	1	K.VSVLQGDLSEKEASLLDLK.E
2110.0601	2109.0528	2109.0273	12.1	1	-	20	2	-.MYGSARSVGKVEPSTQSPGR.S + Oxidation (M)
2155.0640	2154.0567	2154.0739	-7.97	553	-	571	2	K.GTQAGEIHDLKDMLDVKER.K
2174.1650	2173.1577	2173.1113	21.3	440	-	458	2	K.VEQLKEELSSKEAQGEELK.K
2286.1489	2285.1416	2285.1586	-7.43	335	-	353	1	R.RLAEAEMHVHHLESLLEQK.E + Oxidation (M)
2293.1492	2292.1419	2292.1453	-1.48	391	-	409	2	K.ISSMERGLRDLEEEIQMLK.S + Oxidation (M)
2297.1624	2296.1551	2296.1984	-18.84	923	-	942	1	K.HLEEVLEMKQEALLAAISEK.D + Oxidation (M)
2309.1501	2308.1428	2308.1402	1.12	391	-	409	2	K.ISSMERGLRDLEEEIQMLK.S + 2 Oxidation (M)
2657.4148	2656.4075	2656.3854	8.34	148	-	169	2	R.DNTIMDLQTQLKEVLRENDLLR.K
3529.7371	3528.7298	3528.7201	2.74	213	-	240	2	K.EQYRVVQEENQHMQMTIQALQDELRIQR.D + Oxidation (M)
4147.9468	4146.9395	4146.9925	-12.78	104	-	144	0	R.MTAMGSSPNIASSGVASDTIAFGEHHLPPVSMASTVPHSLR.Q

No match to: 706.2851, 710.3807, 719.4268, 720.4332, 722.4348, 723.4337, 735.4208, 744.3633, 749.4401, 756.4190, 759.4415, 766.3942, 772.4352, 774.4582, 779.4799, 823.4933, 831.4758, 832.3166, 834.3207, 842.5047, 843.4840, 844.4902, 846.4869, 870.4950, 876.2891, 880.4586, 883.4816, 885.4853, 891.4897, 895.5765, 900.5383, 918.5458, 923.5204, 927.5097, 928.5096, 929.4465, 945.5218, 949.5538, 967.4249, 969.4326, 972.4861, 974.4482, 976.4711, 977.4844, 988.4647, 1007.5762, 1033.4938, 1034.5852, 1044.5280, 1056.5298, 1074.5454, 1087.5504, 1097.5125, 1113.4890, 1119.5367, 1145.6725, 1178.6276, 1185.6924, 1188.5890, 1220.6172, 1234.4895, 1248.6737, 1253.6409, 1284.6428, 1301.6381, 1313.7157, 1315.6769, 1316.6554, 1317.6417, 1327.7157, 1333.7577, 1336.7279, 1338.7021, 1349.7028, 1355.6765, 1358.7172, 1361.6941, 1372.6039, 1381.7673, 1382.5994, 1383.7312, 1442.8285, 1447.7806, 1457.7437, 1461.8053, 1479.8021, 1517.8105, 1528.7001, 1532.7877, 1537.8292, 1539.8185, 1552.8179, 1553.8821, 1556.8846, 1562.8353, 1567.7686, 1578.7893, 1592.7920, 1593.8013, 1628.7037, 1628.8643, 1642.7200, 1650.8497, 1651.8568, 1680.8239, 1687.9133, 1691.7732, 1706.8671, 1715.8597, 1717.9054, 1724.8430, 1728.8602, 1730.8619, 1771.0326, 1805.9197, 1809.0233, 1813.0017, 1820.9197, 1855.0103, 1887.9128, 1894.9668, 1910.9791, 1928.0238, 1953.8596, 1965.8589, 1974.9517, 1981.9934, 1983.9583, 1995.8975, 2020.9868, 2022.0580, 2027.1263, 2084.0510, 2136.8467, 2144.0354, 2150.8611, 2162.8757, 2164.8806, 2170.0522, 2203.0303, 2217.0352, 2239.1282, 2251.1396, 2253.1372, 2255.2141, 2271.1794, 2274.0801, 2277.0820, 2289.0703, 2324.0906, 2340.1016, 2343.1846, 2384.2053, 2410.1479, 2414.2322, 2462.2893, 2464.2695, 2465.2659, 2529.2080, 2542.2549, 2645.0503, 2646.9685, 2660.0061, 2660.9807, 2675.0012, 2689.0129, 2693.0125, 2703.0303, 2706.0225,

2718.3938, 2763.3359, 2774.3179, 2780.3301, 2787.3474, 2826.4065, 2846.3816, 2872.3054, 2876.3391, 2884.3103, 2887.3201, 2889.3223, 2891.4019, 2895.4370, 2900.3384, 2915.4314, 2983.4602, 2995.5381, 2999.4780, 3002.1853, 3016.2046, 3020.2017, 3030.2197, 3034.2266, 3044.2632, 3053.4902, 3064.5010, 3081.5806, 3126.7075, 3169.7002, 3206.5684, 3234.5972, 3248.6172, 3264.5881, 3299.7673, 3316.7351, 3320.6321, 3334.6604, 3427.5977, 3439.7319, 3453.7163, 3457.6880, 3458.6204, 3473.6636, 3490.6494, 3503.7805, 3517.5906, 3616.8076, 3639.6938, 3652.7173, 3704.8706, 3893.7073, 3959.0081, 3991.8828, 4161.9458, 4177.9121, 4226.0356, 4233.1089, 4252.8618, 4280.2700, 4452.1279, 4465.1328, 4679.1919, 4844.0430

43. P07514 **Mass:** 34328 **Score:** 18 **Expect:** 3.8e+002 **Matches:** 5

NADH-cytochrome b5 reductase 3 OS=Bos taurus GN=CYB5R3 PE=1 SV=3

Observed	Mr(expt)	Mr(calc)	ppm	Start	End	Miss	Peptide
846.4869	845.4796	845.4759	4.35	157	163	0	K.FAIRPDK.K
1928.0238	1927.0165	1927.0414	-12.93	30	46	1	R.STPAITLENPDIKYPLR.L
2465.2659	2464.2586	2464.3186	-24.33	215	234	2	K.DILLRPELEELRNEHSARFK.L
3206.5684	3205.5611	3205.5788	-5.50	127	154	2	K.MSQYLESMKIGDTIEFRGPNGLLVYQGK.G + 2 Oxidation (M)
3616.8076	3615.8003	3615.7991	0.33	197	226	1	K.DPDDHTVCHLLFANQTEKDILLRPELEELR.N

No match to: 706.2851, 710.3807, 717.4042, 719.4268, 720.4332, 722.4348, 723.4337, 735.4208, 744.3633, 749.4401, 756.4190, 759.4415, 766.3942, 772.4352, 774.4582, 779.4799, 823.4933, 831.4758, 832.3166, 834.3207, 842.5047, 843.4840, 844.4902, 870.4950, 873.3982, 876.2891, 880.4586, 883.4816, 885.4853, 891.4897, 895.5765, 900.5383, 918.5458, 923.5204, 927.5097, 928.5096, 929.4465, 944.5216, 945.5218, 949.5538, 967.4249, 969.4326, 972.4861, 974.4482, 976.4711, 977.4844, 978.5469, 988.4647, 1007.5762, 1033.4938, 1034.5852, 1044.5280, 1056.5298, 1074.5454, 1087.5504, 1097.5125, 1103.5261, 1113.4890, 1119.5367, 1122.5620, 1145.6725, 1178.6276, 1185.6924, 1188.5890, 1220.6172, 1234.4895, 1248.6737, 1253.6409, 1284.6428, 1301.6381, 1313.7157, 1314.7207, 1315.6769, 1316.6554, 1317.6417, 1325.6414, 1327.7157, 1333.7577, 1336.7279, 1338.7021, 1349.7028, 1355.6765, 1358.7172, 1361.6941, 1372.6039, 1381.7673, 1382.5994, 1383.7312, 1397.7480, 1442.8285, 1447.7806, 1457.7437, 1461.8053, 1479.8021, 1487.7090, 1497.8783, 1517.8105, 1528.7001, 1532.7877, 1537.8292, 1539.8185, 1552.8179, 1553.8821, 1556.8846, 1562.8353, 1567.7686, 1578.7893, 1592.7920, 1593.8013, 1628.7037, 1628.8643, 1642.7200, 1650.8497, 1651.8568, 1667.8695, 1680.8239, 1687.9133, 1691.7732, 1706.8671, 1715.8597, 1717.9054, 1724.8430, 1728.8602, 1730.8619, 1747.9176, 1771.0326, 1805.9197, 1809.0233, 1813.0017, 1820.9197, 1855.0103, 1887.9128, 1894.9668, 1910.9791, 1953.8596, 1965.8589, 1974.9517, 1981.9934, 1983.9583, 1995.8975, 2020.9868, 2022.0580, 2027.1263, 2044.1479, 2084.0510, 2110.0601, 2136.8467, 2144.0354, 2150.8611, 2155.0640, 2162.8757, 2164.8806, 2170.0522, 2174.1650, 2203.0303, 2217.0352, 2239.1282, 2251.1396, 2253.1372, 2255.2141, 2271.1794, 2274.0801, 2277.0820, 2286.1489, 2289.0703, 2293.1492, 2297.1624, 2309.1501, 2324.0906, 2340.1016, 2343.1846, 2384.2053, 2410.1479, 2414.2322, 2462.2893, 2464.2695, 2529.2080, 2542.2549, 2645.0503, 2646.9685, 2657.4148, 2660.0061, 2660.9807, 2675.0012, 2689.0129, 2693.0125, 2703.0303, 2706.0225, 2718.3938, 2763.3359, 2774.3179, 2780.3301, 2787.3474, 2826.4065, 2846.3816, 2872.3054, 2876.3391, 2884.3103, 2887.3201, 2889.3223, 2891.4019, 2895.4370, 2900.3384, 2915.4314, 2983.4602, 2995.5381, 2999.4780, 3002.1853, 3016.2046, 3020.2017, 3030.2197, 3034.2266, 3044.2632, 3053.4902, 3064.5010, 3081.5806, 3126.7075, 3169.7002, 3234.5972, 3248.6172, 3264.5881, 3299.7673, 3316.7351, 3320.6321, 3334.6604, 3427.5977, 3439.7319, 3453.7163, 3457.6880, 3458.6204, 3473.6636, 3490.6494, 3503.7805, 3517.5906, 3529.7371, 3639.6938, 3652.7173, 3704.8706, 3893.7073, 3959.0081, 3991.8828, 4147.9468, 4161.9458, 4177.9121, 4226.0356, 4233.1089, 4252.8618, 4280.2700, 4452.1279, 4465.1328, 4679.1919, 4844.0430

44. F1N7T1 **Mass:** 34443 **Score:** 18 **Expect:** 3.9e+002 **Matches:** 5

NADH-cytochrome b5 reductase OS=Bos taurus GN=CYB5R3 PE=1 SV=2

Observed	Mr(expt)	Mr(calc)	ppm	Start	End	Miss	Peptide
846.4869	845.4796	845.4759	4.35	158	164	0	K.FAIRPDK.K
1928.0238	1927.0165	1927.0414	-12.93	31	47	1	R.STPAITLENPDIKYPLR.L
2465.2659	2464.2586	2464.3186	-24.33	216	235	2	K.DILLRPELEELRNEHSARFK.L
3206.5684	3205.5611	3205.5788	-5.50	128	155	2	K.MSQYLESMKIGDTIEFRGPNGLLVYQGK.G + 2 Oxidation (M)
3616.8076	3615.8003	3615.7991	0.33	198	227	1	K.DPDDHTVCHLLFANQTEKDILLRPELEELR.N

No match to: 706.2851, 710.3807, 717.4042, 719.4268, 720.4332, 722.4348, 723.4337, 735.4208, 744.3633, 749.4401, 756.4190, 759.4415, 766.3942, 772.4352, 774.4582, 779.4799, 823.4933, 831.4758, 832.3166, 834.3207, 842.5047, 843.4840, 844.4902, 870.4950, 873.3982, 876.2891, 880.4586, 883.4816, 885.4853, 891.4897, 895.5765, 900.5383, 918.5458, 923.5204, 927.5097, 928.5096, 929.4465, 944.5216, 945.5218, 949.5538, 967.4249, 969.4326, 972.4861, 974.4482, 976.4711, 977.4844, 978.5469, 988.4647, 1007.5762, 1033.4938, 1034.5852, 1044.5280, 1056.5298, 1074.5454, 1087.5504, 1097.5125, 1103.5261, 1113.4890, 1119.5367, 1122.5620, 1145.6725, 1178.6276, 1185.6924, 1188.5890, 1220.6172, 1234.4895, 1248.6737, 1253.6409, 1284.6428, 1301.6381, 1313.7157, 1314.7207, 1315.6769, 1316.6554, 1317.6417, 1325.6414, 1327.7157, 1333.7577, 1336.7279, 1338.7021, 1349.7028, 1355.6765, 1358.7172, 1361.6941, 1372.6039, 1381.7673, 1382.5994, 1383.7312, 1397.7480, 1442.8285, 1447.7806, 1457.7437, 1461.8053, 1479.8021, 1487.7090, 1497.8783, 1517.8105, 1528.7001, 1532.7877, 1537.8292, 1539.8185, 1552.8179, 1553.8821, 1556.8846, 1562.8353, 1567.7686, 1578.7893, 1592.7920, 1593.8013, 1628.7037, 1628.8643, 1642.7200, 1650.8497, 1651.8568, 1667.8695, 1680.8239, 1687.9133, 1691.7732, 1706.8671, 1715.8597, 1717.9054, 1724.8430, 1728.8602, 1730.8619, 1747.9176, 1771.0326, 1805.9197, 1809.0233, 1813.0017, 1820.9197, 1855.0103, 1887.9128, 1894.9668, 1910.9791, 1953.8596, 1965.8589, 1974.9517, 1981.9934, 1983.9583, 1995.8975, 2020.9868, 2022.0580, 2027.1263, 2044.1479, 2084.0510, 2110.0601, 2136.8467, 2144.0354, 2150.8611, 2155.0640, 2162.8757, 2164.8806, 2170.0522, 2174.1650, 2203.0303, 2217.0352, 2239.1282, 2251.1396, 2253.1372, 2255.2141, 2271.1794, 2274.0801, 2277.0820, 2286.1489, 2289.0703, 2293.1492, 2297.1624, 2309.1501, 2324.0906, 2340.1016, 2343.1846, 2384.2053, 2410.1479, 2414.2322, 2462.2893, 2464.2695, 2529.2080, 2542.2549, 2645.0503, 2646.9685, 2657.4148, 2660.0061, 2660.9807, 2675.0012, 2689.0129, 2693.0125, 2703.0303, 2706.0225, 2718.3938, 2763.3359, 2774.3179, 2780.3301, 2787.3474, 2826.4065, 2846.3816, 2872.3054, 2876.3391, 2884.3103, 2887.3201, 2889.3223, 2891.4019, 2895.4370, 2900.3384, 2915.4314, 2983.4602, 2995.5381, 2999.4780, 3002.1853, 3016.2046, 3020.2017, 3030.2197, 3034.2266, 3044.2632, 3053.4902, 3064.5010, 3081.5806, 3126.7075, 3169.7002, 3234.5972, 3248.6172, 3264.5881, 3299.7673, 3316.7351, 3320.6321, 3334.6604, 3427.5977, 3439.7319, 3453.7163, 3457.6880, 3458.6204, 3473.6636, 3490.6494, 3503.7805, 3517.5906, 3529.7371, 3639.6938, 3652.7173, 3704.8706, 3893.7073, 3959.0081, 3991.8828, 4147.9468, 4161.9458, 4177.9121, 4226.0356, 4233.1089, 4252.8618, 4280.2700, 4452.1279, 4465.1328, 4679.1919, 4844.0430

45. G5E5P6 **Mass:** 43934 **Score:** 18 **Expect:** 3.9e+002 **Matches:** 8

Uncharacterized protein OS=Bos taurus GN=AGPAT3 PE=4 SV=1

Observed	Mr(expt)	Mr(calc)	ppm	Start	End	Miss	Peptide
735.4208	734.4135	734.4187	-7.12	44	48	1	K.QLYRR.L
945.5218	944.5145	944.5192	-4.94	180	186	2	R.FTEKKHR.V
1358.7172	1357.7099	1357.7136	-2.70	184	195	2	K.KHRVSMEVAASK.G + Oxidation (M)
1855.0103	1854.0030	1853.9636	21.3	220	236	1	R.GTVTAVYDVTLNFRGNK.N
2255.2141	2254.2068	2254.1593	21.1	354	373	2	R.RLIGVTEIEKGSSYGNQEFK.K
2293.1492	2292.1419	2292.1790	-16.17	258	276	1	R.FPLEEIPLDEKEAAQWLHK.L
2343.1846	2342.1773	2342.1762	0.46	237	256	2	K.NPSLLGILYGKKYEADMCVR.R + Oxidation (M)
2846.3816	2845.3743	2845.4398	-23.02	269	291	2	K.EAAQWLHKLYQEKDALQEIYNQK.G

No match to: 706.2851, 710.3807, 717.4042, 719.4268, 720.4332, 722.4348, 723.4337, 744.3633, 749.4401, 756.4190, 759.4415, 766.3942, 772.4352, 774.4582, 779.4799, 823.4933, 831.4758, 832.3166, 834.3207, 842.5047, 843.4840, 844.4902, 846.4869, 870.4950, 873.3982, 876.2891, 880.4586, 883.4816, 885.4853, 891.4897, 895.5765, 900.5383, 918.5458, 923.5204, 927.5097, 928.5096, 929.4465, 944.5216, 949.5538, 967.4249, 969.4326, 972.4861, 974.4482, 976.4711, 977.4844, 978.5469, 988.4647, 1007.5762, 1033.4938, 1034.5852, 1044.5280, 1056.5298, 1074.5454, 1087.5504, 1097.5125, 1103.5261, 1113.4890, 1119.5367, 1122.5620, 1145.6725, 1178.6276, 1185.6924, 1188.5890, 1220.6172, 1234.4895, 1248.6737, 1253.6409, 1284.6428, 1301.6381, 1313.7157, 1314.7207, 1315.6769, 1316.6554, 1317.6417, 1325.6414, 1327.7157, 1333.7577, 1336.7279, 1338.7021, 1349.7028, 1355.6765, 1361.6941, 1372.6039, 1381.7673, 1382.5994, 1383.7312, 1397.7480, 1442.8285, 1447.7806, 1457.7437, 1461.8053, 1479.8021, 1487.7090, 1497.8783, 1517.8105, 1528.7001, 1532.7877, 1537.8292, 1539.8185, 1552.8179, 1553.8821, 1556.8846, 1562.8353, 1567.7686, 1578.7893, 1592.7920, 1593.8013, 1628.7037, 1628.8643, 1642.7200, 1650.8497, 1651.8568, 1667.8695, 1680.8239, 1687.9133, 1691.7732, 1706.8671, 1715.8597, 1717.9054, 1724.8430, 1728.8602, 1730.8619, 1747.9176, 1771.0326, 1805.9197, 1809.0233, 1813.0017, 1820.9197, 1887.9128, 1894.9668, 1910.9791, 1928.0238, 1953.8596, 1965.8589, 1974.9517, 1981.9934, 1983.9583, 1995.8975, 2020.9868, 2022.0580, 2027.1263, 2044.1479, 2084.0510, 2110.0601, 2136.8467, 2144.0354, 2150.8611, 2155.0640, 2162.8757, 2164.8806, 2170.0522, 2174.1650, 2203.0303, 2217.0352, 2239.1282, 2251.1396, 2253.1372, 2271.1794, 2274.0801, 2277.0820, 2286.1489, 2289.0703, 2297.1624, 2309.1501, 2324.0906, 2340.1016, 2384.2053, 2410.1479, 2414.2322, 2462.2893, 2464.2695, 2465.2659, 2529.2080, 2542.2549, 2645.0503, 2646.9685, 2657.4148, 2660.0061, 2660.9807, 2675.0012, 2689.0129, 2693.0125, 2703.0303, 2706.0225, 2718.3938, 2763.3359, 2774.3179, 2780.3301, 2787.3474, 2826.4065, 2872.3054, 2876.3391, 2884.3103, 2887.3201, 2889.3223, 2891.4019, 2895.4370, 2900.3384, 2915.4314, 2983.4602, 2995.5381, 2999.4780, 3002.1853, 3016.2046, 3020.2017,

3030.2197, 3034.2266, 3044.2632, 3053.4902, 3064.5010, 3081.5806, 3126.7075, 3169.7002, 3206.5684, 3234.5972, 3248.6172, 3264.5881, 3299.7673, 3316.7351, 3320.6321, 3334.6604, 3427.5977, 3439.7319, 3453.7163, 3457.6880, 3458.6204, 3473.6636, 3490.6494, 3503.7805, 3517.5906, 3529.7371, 3616.8076, 3639.6938, 3652.7173, 3704.8706, 3893.7073, 3959.0081, 3991.8828, 4147.9468, 4161.9458, 4177.9121, 4226.0356, 4233.1089, 4252.8618, 4280.2700, 4452.1279, 4465.1328, 4679.1919, 4844.0430

46. F1MVJ7 **Mass:** 106871 **Score:** 18 **Expect:** 3.9e+002 **Matches:** 11
Uncharacterized protein OS=Bos taurus GN=LOC104968473 PE=4 SV=2

Observed	Mr(expt)	Mr(calc)	ppm	Start	End	Miss	Peptide
944.5216	943.5143	943.5127	1.72	896	903	0	K.IHILNTFS.-
1122.5620	1121.5547	1121.5499	4.32	16	25	1	K.ESGMALSQKR.L + Oxidation (M)
1220.6172	1219.6099	1219.5842	21.1	371	379	1	K.LFKCHICDK.V
1301.6381	1300.6308	1300.6598	-22.25	1	10	1	-.CWPEEALKLR.K
1327.7157	1326.7084	1326.7156	-5.44	75	85	2	K.HLQPKGNSYKR.E
1338.7021	1337.6948	1337.7092	-10.72	893	903	1	K.HEKIHILNTFS.-
1894.9668	1893.9595	1893.9333	13.8	553	569	0	R.EQLAGHQSVHSGEKPYK.C
1910.9791	1909.9718	1909.9543	9.20	533	547	2	K.IHTGQKLFKCDICYK.V
2027.1263	2026.1190	2026.1072	5.85	609	625	2	K.STLLRNQRVHTGEKPYK.C
2340.1016	2339.0943	2339.1038	-4.07	617	636	2	R.VHTGEKPYKCDVCGKAFSEK.S
4844.0430	4843.0357	4843.1160	-16.57	86	124	2	R.ETFQTLMLGRPESCDIKHFHLWESQENTCDFECQGRDDK.Q

No match to: 706.2851, 710.3807, 717.4042, 719.4268, 720.4332, 722.4348, 723.4337, 735.4208, 744.3633, 749.4401, 756.4190, 759.4415, 766.3942, 772.4352, 774.4582, 779.4799, 823.4933, 831.4758, 832.3166, 834.3207, 842.5047, 843.4840, 844.4902, 846.4869, 870.4950, 873.3982, 876.2891, 880.4586, 883.4816, 885.4853, 891.4897, 895.5765, 900.5383, 918.5458, 923.5204, 927.5097, 928.5096, 929.4465, 945.5218, 949.5538, 967.4249, 969.4326, 972.4861, 974.4482, 976.4711, 977.4844, 978.5469, 988.4647, 1007.5762, 1033.4938, 1034.5852, 1044.5280, 1056.5298, 1074.5454, 1087.5504, 1097.5125, 1103.5261, 1113.4890, 1119.5367, 1145.6725, 1178.6276, 1185.6924, 1188.5890, 1234.4895, 1248.6737, 1253.6409, 1284.6428, 1313.7157, 1314.7207, 1315.6769, 1316.6554, 1317.6417, 1325.6414, 1333.7577, 1336.7279, 1349.7028, 1355.6765, 1358.7172, 1361.6941, 1372.6039, 1381.7673, 1382.5994, 1383.7312, 1397.7480, 1442.8285, 1447.7806, 1457.7437, 1461.8053, 1479.8021, 1487.7090, 1497.8783, 1517.8105, 1528.7001, 1532.7877, 1537.8292, 1539.8185, 1552.8179, 1553.8821, 1556.8846, 1562.8353, 1567.7686, 1578.7893, 1592.7920, 1593.8013, 1628.7037, 1628.8643, 1642.7200, 1650.8497, 1651.8568, 1667.8695, 1680.8239, 1687.9133, 1691.7732, 1706.8671, 1715.8597, 1717.9054, 1724.8430, 1728.8602, 1730.8619, 1747.9176, 1771.0326, 1805.9197, 1809.0233, 1813.0017, 1820.9197, 1855.0103, 1887.9128, 1928.0238, 1953.8596, 1965.8589, 1974.9517, 1981.9934, 1983.9583, 1995.8975, 2020.9868, 2022.0580, 2044.1479, 2084.0510, 2110.0601, 2136.8467, 2144.0354, 2150.8611, 2155.0640, 2162.8757, 2164.8806, 2170.0522, 2174.1650, 2203.0303, 2217.0352, 2239.1282, 2251.1396, 2253.1372, 2255.2141, 2271.1794, 2274.0801, 2277.0820, 2286.1489, 2289.0703, 2293.1492, 2297.1624, 2309.1501, 2324.0906, 2343.1846, 2384.2053, 2410.1479, 2414.2322, 2462.2893, 2464.2695, 2465.2659, 2529.2080, 2542.2549, 2645.0503, 2646.9685, 2657.4148, 2660.0061, 2660.9807, 2675.0012, 2689.0129, 2693.0125, 2703.0303, 2706.0225, 2718.3938, 2763.3359, 2774.3179, 2780.3301, 2787.3474, 2826.4065, 2846.3816, 2872.3054, 2876.3391, 2884.3103, 2887.3201, 2889.3223, 2891.4019, 2895.4370, 2900.3384, 2915.4314, 2983.4602, 2995.5381, 2999.4780, 3002.1853, 3016.2046, 3020.2017, 3030.2197, 3034.2266, 3044.2632, 3053.4902, 3064.5010, 3081.5806, 3126.7075, 3169.7002, 3206.5684, 3234.5972, 3248.6172, 3264.5881, 3299.7673, 3316.7351, 3320.6321, 3334.6604, 3427.5977, 3439.7319, 3453.7163, 3457.6880, 3458.6204, 3473.6636, 3490.6494, 3503.7805, 3517.5906, 3529.7371, 3616.8076, 3639.6938, 3652.7173, 3704.8706, 3893.7073, 3959.0081, 3991.8828, 4147.9468, 4161.9458, 4177.9121, 4226.0356, 4233.1089, 4252.8618, 4280.2700, 4452.1279, 4465.1328, 4679.1919

47. G3MX47 **Mass:** 64111 **Score:** 18 **Expect:** 4e+002 **Matches:** 4
Uncharacterized protein OS=Bos taurus GN=KLHDC7B PE=4 SV=1

Observed	Mr(expt)	Mr(calc)	ppm	Start	End	Miss	Peptide
1248.6737	1247.6664	1247.6622	3.36	131	141	1	R.QDPQPVPPSRK.R
3334.6604	3333.6531	3333.6083	13.4	391	419	1	R.AQLKLVALDGLLYAIGGECLYSMECYDPR.T + Oxidation (M)
4147.9468	4146.9395	4146.9853	-11.03	391	426	2	R.AQLKLVALDGLLYAIGGECLYSMECYDPRTDAWSPR.A + Oxidation (M)
4452.1279	4451.1206	4451.0835	8.35	49	93	2	R.HGAEDLATRAGPGGLEEAGARPQQSHTETEGAAAEGTPSGPRGDR.T

No match to: 706.2851, 710.3807, 717.4042, 719.4268, 720.4332, 722.4348, 723.4337, 735.4208, 744.3633, 749.4401, 756.4190, 759.4415, 766.3942, 772.4352, 774.4582, 779.4799, 823.4933, 831.4758, 832.3166, 834.3207, 842.5047, 843.4840, 844.4902, 846.4869, 870.4950, 873.3982, 876.2891, 880.4586, 883.4816, 885.4853, 891.4897, 895.5765, 900.5383, 918.5458, 923.5204, 927.5097, 928.5096, 929.4465, 944.5216, 945.5218, 949.5538, 967.4249, 969.4326, 972.4861, 974.4482, 976.4711, 977.4844, 978.5469, 988.4647, 1007.5762, 1033.4938, 1034.5852, 1044.5280, 1056.5298, 1074.5454, 1087.5504, 1097.5125, 1103.5261, 1113.4890, 1119.5367, 1122.5620, 1145.6725, 1178.6276, 1185.6924, 1188.5890, 1220.6172, 1234.4895, 1253.6409, 1284.6428, 1301.6381, 1313.7157, 1314.7207, 1315.6769, 1316.6554, 1317.6417, 1325.6414, 1327.7157, 1333.7577, 1336.7279, 1338.7021, 1349.7028, 1355.6765, 1358.7172, 1361.6941, 1372.6039, 1381.7673, 1382.5994, 1383.7312, 1397.7480, 1442.8285, 1447.7806, 1457.7437, 1461.8053, 1479.8021, 1487.7090, 1497.8783, 1517.8105, 1528.7001, 1532.7877, 1537.8292, 1539.8185, 1552.8179, 1553.8821, 1556.8846, 1562.8353, 1567.7686, 1578.7893, 1592.7920, 1593.8013, 1628.7037, 1628.8643, 1642.7200, 1650.8497, 1651.8568, 1667.8695, 1680.8239, 1687.9133, 1691.7732, 1706.8671, 1715.8597, 1717.9054, 1724.8430, 1728.8602, 1730.8619, 1747.9176, 1771.0326, 1805.9197, 1809.0233, 1813.0017, 1820.9197, 1855.0103, 1887.9128, 1894.9668, 1910.9791, 1928.0238, 1953.8596, 1965.8589, 1974.9517, 1981.9934, 1983.9583, 1995.8975, 2020.9868, 2022.0580, 2027.1263, 2044.1479, 2084.0510, 2110.0601, 2136.8467, 2144.0354, 2150.8611, 2155.0640, 2162.8757, 2164.8806, 2170.0522, 2174.1650, 2203.0303, 2217.0352, 2239.1282, 2251.1396, 2253.1372, 2255.2141, 2271.1794, 2274.0801, 2277.0820, 2286.1489, 2289.0703, 2293.1492, 2297.1624, 2309.1501, 2324.0906, 2340.1016, 2343.1846, 2384.2053, 2410.1479, 2414.2322, 2462.2893, 2464.2695, 2465.2659, 2529.2080, 2542.2549, 2645.0503, 2646.9685, 2657.4148, 2660.0061, 2660.9807, 2675.0012, 2689.0129, 2693.0125, 2703.0303, 2706.0225, 2718.3938, 2763.3359, 2774.3179, 2780.3301, 2787.3474, 2826.4065, 2846.3816, 2872.3054, 2876.3391, 2884.3103, 2887.3201, 2889.3223, 2891.4019, 2895.4370, 2900.3384, 2915.4314, 2983.4602, 2995.5381, 2999.4780, 3002.1853, 3016.2046, 3020.2017, 3030.2197, 3034.2266, 3044.2632, 3053.4902, 3064.5010, 3081.5806, 3126.7075, 3169.7002, 3206.5684, 3234.5972, 3248.6172, 3264.5881, 3299.7673, 3316.7351, 3320.6321, 3427.5977, 3439.7319, 3453.7163, 3457.6880, 3458.6204, 3473.6636, 3490.6494, 3503.7805, 3517.5906, 3529.7371, 3616.8076, 3639.6938, 3652.7173, 3704.8706, 3893.7073, 3959.0081, 3991.8828, 4161.9458, 4177.9121, 4226.0356, 4233.1089, 4252.8618, 4280.2700, 4465.1328, 4679.1919, 4844.0430

48. Q2M2T3 **Mass:** 19496 **Score:** 18 **Expect:** 4.1e+002 **Matches:** 4
Mitochondrial pyruvate carrier-like protein OS=Bos taurus PE=2 SV=1

Observed	Mr(expt)	Mr(calc)	ppm	Start	End	Miss	Peptide
1317.6417	1316.6344	1316.6507	-12.34	45	56	1	R.DMRASPDIISGR.M
1355.6765	1354.6692	1354.6915	-16.42	11	21	2	K.AMETVKTKEFR.D + Oxidation (M)
3334.6604	3333.6531	3333.6645	-3.41	71	99	2	R.FAYRVQPRNLLLMACHGTNIVAQSMQAGR.Y + 2 Oxidation (M)
3439.7319	3438.7246	3438.6720	15.3	19	47	2	K.EFRDYLASTHFWGPVANWGLPLAAFRDMR.A + Oxidation (M)

No match to: 706.2851, 710.3807, 717.4042, 719.4268, 720.4332, 722.4348, 723.4337, 735.4208, 744.3633, 749.4401, 756.4190, 759.4415, 766.3942, 772.4352, 774.4582, 779.4799, 823.4933, 831.4758, 832.3166, 834.3207, 842.5047, 843.4840, 844.4902, 846.4869, 870.4950, 873.3982, 876.2891, 880.4586, 883.4816, 885.4853, 891.4897, 895.5765, 900.5383, 918.5458, 923.5204, 927.5097, 928.5096, 929.4465, 944.5216, 945.5218, 949.5538, 967.4249, 969.4326, 972.4861, 974.4482, 976.4711, 977.4844, 978.5469, 988.4647, 1007.5762, 1033.4938, 1034.5852, 1044.5280, 1056.5298, 1074.5454, 1087.5504, 1097.5125, 1103.5261, 1113.4890, 1119.5367, 1122.5620, 1145.6725, 1178.6276, 1185.6924, 1188.5890, 1220.6172, 1234.4895, 1248.6737, 1253.6409, 1284.6428, 1301.6381, 1313.7157, 1314.7207, 1315.6769, 1316.6554, 1325.6414, 1327.7157, 1333.7577, 1336.7279, 1338.7021, 1349.7028, 1358.7172, 1361.6941, 1372.6039, 1381.7673, 1382.5994, 1383.7312, 1397.7480, 1442.8285, 1447.7806, 1457.7437, 1461.8053, 1479.8021, 1487.7090, 1497.8783, 1517.8105, 1528.7001, 1532.7877, 1537.8292, 1539.8185, 1552.8179, 1553.8821, 1556.8846, 1562.8353, 1567.7686, 1578.7893, 1592.7920, 1593.8013, 1628.7037, 1628.8643, 1642.7200, 1650.8497, 1651.8568, 1667.8695, 1680.8239, 1687.9133, 1691.7732, 1706.8671, 1715.8597, 1717.9054, 1724.8430, 1728.8602, 1730.8619, 1747.9176, 1771.0326, 1805.9197, 1809.0233, 1813.0017, 1820.9197, 1855.0103, 1887.9128, 1894.9668, 1910.9791, 1928.0238, 1953.8596, 1965.8589, 1974.9517, 1981.9934, 1983.9583, 1995.8975, 2020.9868, 2022.0580, 2027.1263, 2044.1479, 2084.0510, 2110.0601, 2136.8467, 2144.0354, 2150.8611, 2155.0640, 2162.8757, 2164.8806, 2170.0522, 2174.1650, 2203.0303, 2217.0352, 2239.1282, 2251.1396, 2253.1372, 2255.2141, 2271.1794, 2274.0801, 2277.0820, 2286.1489, 2289.0703, 2293.1492, 2297.1624, 2309.1501, 2324.0906, 2340.1016, 2343.1846, 2384.2053, 2410.1479, 2414.2322, 2462.2893, 2464.2695, 2465.2659, 2529.2080, 2542.2549, 2645.0503, 2646.9685, 2657.4148, 2660.0061, 2660.9807, 2675.0012, 2689.0129, 2693.0125, 2703.0303, 2706.0225, 2718.3938, 2763.3359, 2774.3179, 2780.3301, 2787.3474, 2826.4065, 2846.3816, 2872.3054, 2876.3391, 2884.3103, 2887.3201, 2889.3223, 2891.4019, 2895.4370, 2900.3384, 2915.4314, 2983.4602, 2995.5381, 2999.4780, 3002.1853, 3016.2046, 3020.2017, 3030.2197, 3034.2266, 3044.2632, 3053.4902, 3064.5010, 3081.5806, 3126.7075,

3169.7002, 3206.5684, 3234.5972, 3248.6172, 3264.5881, 3299.7673, 3316.7351, 3320.6321, 3427.5977, 3453.7163, 3457.6880, 3458.6204, 3473.6636, 3490.6494, 3503.7805, 3517.5906, 3529.7371, 3616.8076, 3639.6938, 3652.7173, 3704.8706, 3893.7073, 3959.0081, 3991.8828, 4147.9468, 4161.9458, 4177.9121, 4226.0356, 4233.1089, 4252.8618, 4280.2700, 4452.1279, 4465.1328, 4679.1919, 4844.0430

49. G3X6Y1 **Mass:** 19510 **Score:** 18 **Expect:** 4.1e+002 **Matches:** 4

Uncharacterized protein OS=Bos taurus GN=MPC1L PE=4 SV=1

Observed	Mr(expt)	Mr(calc)	ppm	Start	End	Miss	Peptide
1317.6417	1316.6344	1316.6507	-12.34	45 -	56	1	R.DMRASPDIISGR.M
1355.6765	1354.6692	1354.6915	-16.42	11 -	21	2	K.AMETVKTKEFR.D + Oxidation (M)
3334.6604	3333.6531	3333.6645	-3.41	71 -	99	2	R.FAYRVQPRNLLLMACHGTNIVAQSMQAGR.Y + 2 Oxidation (M)
3439.7319	3438.7246	3438.6720	15.3	19 -	47	2	K.EFRDYLASTHFWGPVANWGLPLAAFRDMR.A + Oxidation (M)

No match to: 706.2851, 710.3807, 717.4042, 719.4268, 720.4332, 722.4348, 723.4337, 735.4208, 744.3633, 749.4401, 756.4190, 759.4415, 766.3942, 772.4352, 774.4582, 779.4799, 823.4933, 831.4758, 832.3166, 834.3207, 842.5047, 843.4840, 844.4902, 846.4869, 870.4950, 873.3982, 876.2891, 880.4586, 883.4816, 885.4853, 891.4897, 895.5765, 900.5383, 918.5458, 923.5204, 927.5097, 928.5096, 929.4465, 944.5216, 945.5218, 949.5538, 967.4249, 969.4326, 972.4861, 974.4482, 976.4711, 977.4844, 978.5469, 988.4647, 1007.5762, 1033.4938, 1034.5852, 1044.5280, 1056.5298, 1074.5454, 1087.5504, 1097.5125, 1103.5261, 1113.4890, 1119.5367, 1122.5620, 1145.6725, 1178.6276, 1185.6924, 1188.5890, 1220.6172, 1234.4895, 1248.6737, 1253.6409, 1284.6428, 1301.6381, 1313.7157, 1314.7207, 1315.6769, 1316.6554, 1325.6414, 1327.7157, 1333.7577, 1336.7279, 1338.7021, 1349.7028, 1358.7172, 1361.6941, 1372.6039, 1381.7673, 1382.5994, 1383.7312, 1397.7480, 1442.8285, 1447.7806, 1457.7437, 1461.8053, 1479.8021, 1487.7090, 1497.8783, 1517.8105, 1528.7001, 1532.7877, 1537.8292, 1539.8185, 1552.8179, 1553.8821, 1556.8846, 1562.8353, 1567.7686, 1578.7893, 1592.7920, 1593.8013, 1628.7037, 1628.8643, 1642.7200, 1650.8497, 1651.8568, 1667.8695, 1680.8239, 1687.9133, 1691.7732, 1706.8671, 1715.8597, 1717.9054, 1724.8430, 1728.8602, 1730.8619, 1747.9176, 1771.0326, 1805.9197, 1809.0233, 1813.0017, 1820.9197, 1855.0103, 1887.9128, 1894.9668, 1910.9791, 1928.0238, 1953.8596, 1965.8589, 1974.9517, 1981.9934, 1983.9583, 1995.8975, 2020.9868, 2022.0580, 2027.1263, 2044.1479, 2084.0510, 2110.0601, 2136.8467, 2144.0354, 2150.8611, 2155.0640, 2162.8757, 2164.8806, 2170.0522, 2174.1650, 2203.0303, 2217.0352, 2239.1282, 2251.1396, 2253.1372, 2255.2141, 2271.1794, 2274.0801, 2277.0820, 2286.1489, 2289.0703, 2293.1492, 2297.1624, 2309.1501, 2324.0906, 2340.1016, 2343.1846, 2384.2053, 2410.1479, 2414.2322, 2462.2893, 2464.2695, 2465.2659, 2529.2080, 2542.2549, 2645.0503, 2646.9685, 2657.4148, 2660.0061, 2660.9807, 2675.0012, 2689.0129, 2693.0125, 2703.0303, 2706.0225, 2718.3938, 2763.3359, 2774.3179, 2780.3301, 2787.3474, 2826.4065, 2846.3816, 2872.3054, 2876.3391, 2884.3103, 2887.3201, 2889.3223, 2891.4019, 2895.4370, 2900.3384, 2915.4314, 2983.4602, 2995.5381, 2999.4780, 3002.1853, 3016.2046, 3020.2017, 3030.2197, 3034.2266, 3044.2632, 3053.4902, 3064.5010, 3081.5806, 3126.7075, 3169.7002, 3206.5684, 3234.5972, 3248.6172, 3264.5881, 3299.7673, 3316.7351, 3320.6321, 3427.5977, 3453.7163, 3457.6880, 3458.6204, 3473.6636, 3490.6494, 3503.7805, 3517.5906, 3529.7371, 3616.8076, 3639.6938, 3652.7173, 3704.8706, 3893.7073, 3959.0081, 3991.8828, 4147.9468, 4161.9458, 4177.9121, 4226.0356, 4233.1089, 4252.8618, 4280.2700, 4452.1279, 4465.1328, 4679.1919, 4844.0430

50. A2VDT4 **Mass:** 31057 **Score:** 18 **Expect:** 4.1e+002 **Matches:** 6

Putative transcription factor Ovo-like 1 OS=Bos taurus GN=OVOL1 PE=2 SV=1

Observed	Mr(expt)	Mr(calc)	ppm	Start	End	Miss	Peptide
744.3633	743.3560	743.3715	-20.81	97 -	102	0	R.DHGFLR.T
1248.6737	1247.6664	1247.6445	17.6	97 -	106	2	R.DHGFLRTKMK.V + Oxidation (M)
1315.6769	1314.6696	1314.6503	14.7	126 -	135	1	K.AFTYQRMLNR.H + Oxidation (M)
1537.8292	1536.8219	1536.8156	4.09	4 -	16	1	R.AFLVKKPCVSTCK.R
2340.1016	2339.0943	2339.0820	5.25	176 -	194	2	K.CSLCDKAFTQRCSLESHLK.K
3206.5684	3205.5611	3205.5132	14.9	67 -	96	1	R.NSSYSVTPGPCVVAQLPSEDMSRLAGPQSR.D + Oxidation (M)

No match to: 706.2851, 710.3807, 717.4042, 719.4268, 720.4332, 722.4348, 723.4337, 735.4208, 749.4401, 756.4190, 759.4415, 766.3942, 772.4352, 774.4582, 779.4799, 823.4933, 831.4758, 832.3166, 834.3207, 842.5047, 843.4840, 844.4902, 846.4869, 870.4950, 873.3982, 876.2891, 880.4586, 883.4816, 885.4853, 891.4897, 895.5765, 900.5383, 918.5458, 923.5204, 927.5097, 928.5096, 929.4465, 944.5216, 945.5218, 949.5538, 967.4249, 969.4326, 972.4861, 974.4482, 976.4711, 977.4844, 978.5469, 988.4647, 1007.5762, 1033.4938, 1034.5852, 1044.5280, 1056.5298, 1074.5454, 1087.5504, 1097.5125, 1103.5261, 1113.4890, 1119.5367, 1122.5620, 1145.6725, 1178.6276, 1185.6924, 1188.5890, 1220.6172, 1234.4895, 1253.6409, 1284.6428, 1301.6381, 1313.7157, 1314.7207, 1316.6554, 1317.6417, 1325.6414, 1327.7157, 1333.7577, 1336.7279, 1338.7021, 1349.7028, 1355.6765, 1358.7172, 1361.6941, 1372.6039, 1381.7673, 1382.5994, 1383.7312, 1397.7480, 1442.8285, 1447.7806, 1457.7437, 1461.8053, 1479.8021, 1487.7090, 1497.8783, 1517.8105, 1528.7001, 1532.7877, 1539.8185, 1552.8179, 1553.8821, 1556.8846, 1562.8353, 1567.7686, 1578.7893, 1592.7920, 1593.8013, 1628.7037, 1628.8643, 1642.7200, 1650.8497, 1651.8568, 1667.8695, 1680.8239, 1687.9133, 1691.7732, 1706.8671, 1715.8597, 1717.9054, 1724.8430, 1728.8602, 1730.8619, 1747.9176, 1771.0326, 1805.9197, 1809.0233, 1813.0017, 1820.9197, 1855.0103, 1887.9128, 1894.9668, 1910.9791, 1928.0238, 1953.8596, 1965.8589, 1974.9517, 1981.9934, 1983.9583, 1995.8975, 2020.9868, 2022.0580, 2027.1263, 2044.1479, 2084.0510, 2110.0601, 2136.8467, 2144.0354, 2150.8611, 2155.0640, 2162.8757, 2164.8806, 2170.0522, 2174.1650, 2203.0303, 2217.0352, 2239.1282, 2251.1396, 2253.1372, 2255.2141, 2271.1794, 2274.0801, 2277.0820, 2286.1489, 2289.0703, 2293.1492, 2297.1624, 2309.1501, 2324.0906, 2343.1846, 2384.2053, 2410.1479, 2414.2322, 2462.2893, 2464.2695, 2465.2659, 2529.2080, 2542.2549, 2645.0503, 2646.9685, 2657.4148, 2660.0061, 2660.9807, 2675.0012, 2689.0129, 2693.0125, 2703.0303, 2706.0225, 2718.3938, 2763.3359, 2774.3179, 2780.3301, 2787.3474, 2826.4065, 2846.3816, 2872.3054, 2876.3391, 2884.3103, 2887.3201, 2889.3223, 2891.4019, 2895.4370, 2900.3384, 2915.4314, 2983.4602, 2995.5381, 2999.4780, 3002.1853, 3016.2046, 3020.2017, 3030.2197, 3034.2266, 3044.2632, 3053.4902, 3064.5010, 3081.5806, 3126.7075, 3169.7002, 3234.5972, 3248.6172, 3264.5881, 3299.7673, 3316.7351, 3320.6321, 3334.6604, 3427.5977, 3439.7319, 3453.7163, 3457.6880, 3458.6204, 3473.6636, 3490.6494, 3503.7805, 3517.5906, 3529.7371, 3616.8076, 3639.6938, 3652.7173, 3704.8706, 3893.7073, 3959.0081, 3991.8828, 4147.9468, 4161.9458, 4177.9121, 4226.0356, 4233.1089, 4252.8618, 4280.2700, 4452.1279, 4465.1328, 4679.1919, 4844.0430

Search Parameters

**Type of search**	: **Peptide Mass Fingerprint**
**Enzyme**	: **Trypsin**
**Fixed modifications**	: **Carbamidomethyl (C)**
**Variable modifications**	: **Oxidation (M)**
**Mass values**	: **Monoisotopic**
**Protein Mass**	: **Unrestricted**
**Peptide Mass Tolerance**	: **± 25 ppm**
**Peptide Charge State**	: **1+**
**Max Missed Cleavages**	: **2**
**Number of queries**	: **257**
**Selected for scoring**	: **78**

Mascot: http://www.matrixscience.com/

MATRIX SCIENCE Mascot Search Results

```
User          : 6F_(L6@52381_05Apr2017)
Email         : proteomics@i3s.up.pt
Search title  : Project: Proteomics, Spot Set: Proteomics\05Apr2017, Label: L6, Spot Id: 227016, Peak List Id: 447672, MS Job Run Id: 316
MS data file  : C:\Documents and Settings\Administrator\Desktop\xu\29JUNHO\Proteomics\05Apr2017\pmf_L6_149875820515.txt
Database      : OrganismSpecie Bos_taurus_Reference_Proteome_2016_06 (24214 sequences; 12839866 residues)
Timestamp     : 29 Jun 2017 at 18:08:35 GMT
Top Score     : 76 for P55252, Hyaluronan and proteoglycan link protein 1 OS=Bos taurus GN=HAPLN1 PE=2 SV=1
```

Mascot Score Histogram

Protein score is -10*Log(P), where P is the probability that the observed match is a random event.
Protein scores greater than 56 are significant (p<0.05).

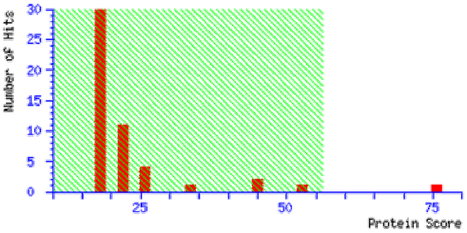

Protein Summary Report

Format As | Protein Summary | Help

Significance threshold p< 0.05 | Max. number of hits 50

Preferred taxonomy | All entries

Re-Search All | Search Unmatched

Index

	Accession	Mass	Score	Description
1.	P55252	40889	76	Hyaluronan and proteoglycan link protein 1 OS=Bos taurus GN=HAPLN1 PE=2 SV=1
2.	P63258	42108	52	Actin, cytoplasmic 2 OS=Bos taurus GN=ACTG1 PE=1 SV=1
3.	P60712	42052	46	Actin, cytoplasmic 1 OS=Bos taurus GN=ACTB PE=1 SV=1
4.	Q3T0P6	44908	43	Phosphoglycerate kinase 1 OS=Bos taurus GN=PGK1 PE=2 SV=3
5.	F1MRD0	42167	35	Actin, cytoplasmic 1 OS=Bos taurus GN=ACTB PE=3 SV=2
6.	F1MVK7	40714	27	Uncharacterized protein OS=Bos taurus PE=4 SV=2
7.	P19879	34530	26	Mimecan OS=Bos taurus GN=OGN PE=1 SV=2
8.	F1N367	244177	25	Aggrecan core protein OS=Bos taurus GN=ACAN PE=4 SV=2
9.	F1N368	248513	24	Aggrecan core protein OS=Bos taurus GN=ACAN PE=4 SV=2
10.	F1MFI7	57809	24	Uncharacterized protein OS=Bos taurus GN=SYBU PE=4 SV=2
11.	F1MSC3	264516	23	Uncharacterized protein OS=Bos taurus GN=ACACB PE=4 SV=2
12.	A5D9E8	34517	22	Mimecan OS=Bos taurus GN=OGN PE=1 SV=1
13.	F1N6I7	76622	22	Versican core protein OS=Bos taurus GN=VCAN PE=1 SV=2
14.	Q0VC60	56014	22	Dihydrouridine synthase 2-like, SMM1 homolog (S. cerevisiae) OS=Bos taurus GN=DUS2L PE=2 SV=1
15.	Q32LF3	24094	22	Similar to type 1 protein phosphatase inhibitor OS=Bos taurus GN=LOC520057 PE=2 SV=1
16.	F1MHQ4	14751	21	Fatty acid-binding protein, adipocyte OS=Bos taurus GN=FABP4 PE=1 SV=2
17.	E1BP29	68878	21	Protein-serine/threonine kinase OS=Bos taurus GN=GRK6 PE=3 SV=2
18.	Q32L62	51390	21	Hemogen OS=Bos taurus GN=HEMGN PE=2 SV=1
19.	G3N088	40915	21	Mimecan OS=Bos taurus GN=OGN PE=1 SV=1
20.	A8E4P9	20587	20	HAPLN1 protein OS=Bos taurus GN=HAPLN1 PE=2 SV=1
21.	G3N1F5	34506	20	Uncharacterized protein OS=Bos taurus PE=3 SV=1
22.	P13608	248150	20	Aggrecan core protein OS=Bos taurus GN=ACAN PE=1 SV=3
23.	Q3T0X8	57425	19	Na(+)/H(+) exchange regulatory cofactor NHE-RF3 OS=Bos taurus GN=PDZK1 PE=2 SV=1
24.	A5PK10	43166	19	TOM1L2 protein OS=Bos taurus GN=TOM1L2 PE=2 SV=1
25.	Q3SYU8	19979	19	Tumor protein D52-like 2 OS=Bos taurus GN=TPD52L2 PE=2 SV=1
26.	E1BKZ5	378904	19	Uncharacterized protein OS=Bos taurus GN=GOLGB1 PE=4 SV=2
27.	E1BA62	59054	19	Uncharacterized protein OS=Bos taurus GN=DNM3 PE=3 SV=2
28.	P67827	37771	19	Casein kinase I isoform alpha OS=Bos taurus GN=CSNK1A1 PE=1 SV=1
29.	Q32LI4	38763	19	CSNK1A1 protein OS=Bos taurus GN=CSNK1A1 PE=2 SV=1
30.	A6QP66	23698	19	Ras-related and estrogen-regulated growth inhibitor-like protein OS=Bos taurus GN=RERGL PE=2 SV=1
31.	E1BEI2	25560	19	Uncharacterized protein OS=Bos taurus GN=SPCS2 PE=1 SV=1
32.	F1MWE0	51365	19	Uncharacterized protein OS=Bos taurus GN=PSMC3 PE=3 SV=2
33.	A3KMV6	71197	19	HNRNPR protein OS=Bos taurus GN=HNRNPR PE=1 SV=1
34.	F1N5N0	72401	19	Uncharacterized protein OS=Bos taurus GN=TRAK1 PE=4 SV=2
35.	Q3SX14	80966	19	Gelsolin OS=Bos taurus GN=GSN PE=2 SV=1
36.	E1BEE0	55035	19	Uncharacterized protein OS=Bos taurus GN=TOM1L2 PE=4 SV=2
37.	Q2TBL4	60789	19	DNA-directed RNA polymerase III subunit RPC3 OS=Bos taurus GN=POLR3C PE=2 SV=1
38.	F1MUY3	60801	19	DNA-directed RNA polymerase III subunit RPC3 OS=Bos taurus GN=POLR3C PE=4 SV=1
39.	F1MDV8	53058	18	Septin-10 OS=Bos taurus GN=SEPT10 PE=3 SV=2
40.	E1BL71	84208	18	Uncharacterized protein OS=Bos taurus GN=TYW1 PE=4 SV=2
41.	Q28088	21431	18	Gamma-crystallin C OS=Bos taurus GN=CRYGC PE=1 SV=3
42.	Q58DL0	26401	18	Chromosome X open reading frame 23 OS=Bos taurus GN=CXorf23 PE=2 SV=1
43.	F1MU80	46943	18	Uncharacterized protein OS=Bos taurus GN=LOC520336 PE=4 SV=2
44.	F1N3G4	157905	18	Condensin complex subunit 1 OS=Bos taurus GN=LOC509171 PE=3 SV=2
45.	Q9TTS3	266788	18	Acetyl-CoA carboxylase 1 OS=Bos taurus GN=ACACA PE=2 SV=1
46.	F1MUT6	54881	18	Uncharacterized protein OS=Bos taurus GN=DDX19B PE=4 SV=1
47.	F1MV51	314440	18	Uncharacterized protein OS=Bos taurus GN=APC PE=4 SV=2
48.	G3N0V2	63341	18	Uncharacterized protein OS=Bos taurus GN=KRT1 PE=1 SV=1
49.	F1MHQ3	40861	18	Hexosyltransferase OS=Bos taurus PE=3 SV=1
50.	A5D7M3	56128	18	Growth arrest-specific protein 8 OS=Bos taurus GN=GAS8 PE=2 SV=1

Results List

1. P55252 **Mass:** 40889 **Score:** 76 **Expect:** 0.00067 **Matches:** 21

Hyaluronan and proteoglycan link protein 1 OS=Bos taurus GN=HAPLN1 PE=2 SV=1

Observed	Mr(expt)	Mr(calc)	ppm	Start	End	Miss	Peptide
736.4033	735.3960	735.3915	6.06	298	- 303	0	K.LLGYDR.C
888.4968	887.4896	887.4977	-9.19	316	- 322	0	R.YPISRPR.R
910.4130	909.4057	909.4093	-3.99	104	- 111	0	K.TYGGYHGR.V
929.4340	928.4267	928.4079	20.2	237	- 243	0	R.NYGFWDK.D
937.5071	936.4998	936.5069	-7.59	334	- 341	1	R.FVGFPDKK.H
976.4465	975.4392	975.4444	-5.30	325	- 333	0	R.CSPSEAAVR.F
1000.5594	999.5521	999.5600	-7.91	40	- 48	0	R.LLVEAEQAK.V
1019.5631	1018.5558	1018.5600	-4.10	289	- 297	0	K.VGQIFAAWK.L
1077.5199	1076.5126	1076.5113	1.20	344	- 351	0	K.LYGVYCFR.A
1132.5316	1131.5243	1131.5455	-18.69	324	- 333	1	R.RCSPSEAAVR.F
1181.6516	1180.6443	1180.6281	13.8	261	- 269	0	R.FYYLIHPTK.L
1254.6965	1253.6892	1253.6656	18.9	81	- 90	1	K.WTKLTSDYLK.E
1342.6847	1341.6774	1341.6652	9.11	342	- 351	1	K.HKLYGVYCFR.A
1370.6589	1369.6516	1369.6409	7.85	224	- 236	0	R.EPCGGQNTVPGVR.N
1386.7084	1385.7011	1385.7052	-2.92	66	- 78	1	R.DPTAFGSGTHKIR.I
1397.7515	1396.7442	1396.7252	13.6	104	- 115	1	K.TYGGYHGRVFLK.G
2211.0654	2210.0581	2210.0339	11.0	116	- 136	0	K.GGSDNDASLVITDLTLEDYGR.Y
2698.3696	2697.3623	2697.3497	4.66	112	- 136	1	R.VFLKGGSDNDASLVITDLTLEDYGR.Y
2707.3074	2706.3001	2706.2860	5.20	200	- 223	0	R.SGLDWCNAGWLSDGSVQYPITKPR.E
3254.6448	3253.6375	3253.5979	12.2	2	- 28	1	M.KSLLLLVLISFCWADHHSDNYTVDHDR.V
3601.6826	3600.6753	3600.6368	10.7	170	- 199	0	R.YNLNFHEAQQACLDQDAVIASFDQLYDAWR.S

No match to: 701.3809, 706.2637, 708.2758, 717.3943, 719.4086, 722.4171, 734.4380, 735.4089, 747.4274, 756.3826, 759.3994, 772.4160, 779.4522, 795.4493, 806.4573, 831.4625, 832.3028, 834.3088, 842.4966, 876.2747, 878.2808, 883.4713, 890.4931, 891.4778, 900.5316, 918.5359, 936.4846, 938.5070, 949.5414, 967.4175, 969.4572, 972.4759, 974.4473, 978.5344, 983.6115, 988.4531, 990.4647, 993.4956, 1007.5680, 1021.5624, 1031.5641, 1034.5791, 1036.5952, 1044.5217, 1046.5728, 1051.5135, 1056.5387, 1057.6144, 1060.5447, 1074.5956, 1087.5442, 1091.5437, 1113.5406, 1122.5580, 1123.5852, 1145.6403, 1161.6290, 1168.6013, 1171.5769, 1172.5704, 1178.6254, 1180.6395, 1185.6835, 1198.7111, 1216.6992, 1234.6646, 1248.6750, 1255.6359, 1273.6595, 1278.6974, 1281.6390, 1284.6533, 1301.6256, 1304.6489, 1314.7235, 1315.6904, 1325.6581, 1336.7290, 1338.6799, 1349.6997, 1351.6993, 1354.6641, 1361.6997, 1368.6772, 1372.6199, 1381.7728, 1382.6112, 1383.7378, 1388.7888, 1390.7301, 1425.7671, 1439.7701, 1442.8314, 1457.7517, 1467.7510, 1473.8167, 1488.7367, 1497.8859, 1500.7335, 1513.7731, 1516.7435, 1528.7101, 1556.8929, 1559.8439, 1562.8004, 1563.8088, 1578.7965, 1580.8445, 1592.8094, 1601.9139, 1616.8481, 1623.8562, 1627.7197, 1628.7129, 1628.8722, 1634.8071, 1638.8757, 1642.7318, 1645.7701, 1647.8682, 1650.8612, 1673.8375, 1679.9817, 1680.8936, 1681.8372, 1696.8403, 1706.8660, 1715.8601, 1717.9037, 1730.8719, 1742.0088, 1758.9255, 1769.0105, 1771.0138, 1774.9404, 1783.9508, 1786.9517, 1787.9071, 1790.9086, 1835.8361, 1837.8712, 1862.9679, 1928.0435, 1940.0333, 1954.0796, 1961.0397, 1970.0139, 1982.0297, 1984.0314, 2023.0551, 2027.1292, 2032.0228, 2061.0330, 2105.0796, 2144.0679, 2170.0872, 2179.0752, 2215.0876, 2239.1650, 2242.1501, 2246.2151, 2251.1675, 2252.1614, 2255.1719, 2273.1008, 2289.1030, 2293.1750, 2304.2087, 2316.2031, 2343.2134, 2354.0020, 2391.2788, 2475.2476, 2491.2402, 2507.1975, 2514.2664, 2542.2795, 2550.2063, 2558.3499, 2587.3516, 2605.2615, 2646.9883, 2661.0090, 2675.0190, 2681.3855, 2705.3142, 2714.3647, 2717.3394, 2719.3167, 2722.3188, 2763.3738, 2792.2446, 2826.3545, 2843.4670, 2846.4014, 2847.4082, 2872.3359, 2884.3538, 2887.3474, 2889.3481, 2892.4165, 2894.4639, 2896.4890, 2914.4551, 2987.5244, 2995.4431, 3004.5840, 3029.4812, 3054.4724, 3069.4849, 3072.4231, 3101.5515, 3126.7327, 3166.6106, 3171.6748, 3183.6196, 3199.6411, 3231.5630, 3236.5791, 3243.6301, 3245.6216, 3247.5737, 3248.6313, 3262.7324, 3267.7256, 3278.7546, 3321.5828, 3333.5762, 3334.6729, 3335.6787, 3338.7097, 3347.4907, 3395.6821, 3412.6426, 3428.5991, 3435.7024, 3457.6995, 3487.8022, 3490.6885, 3503.8049, 3534.6365, 3548.6917, 3551.6768, 3552.6902, 3616.7095, 3624.6824, 3639.7014, 3645.7827, 3651.7559, 3655.7354, 3667.7646, 3697.8025, 3716.7253, 3762.8396, 3929.9304, 4040.7656, 4057.9707, 4071.9524, 4092.8394, 4147.9063, 4160.9473, 4174.9575, 4193.9395, 4253.8755, 4415.9736, 4452.1865, 4465.1670, 4845.0845

2. P63258 **Mass:** 42108 **Score:** 52 **Expect:** 0.16 **Matches:** 19

Actin, cytoplasmic 2 OS=Bos taurus GN=ACTG1 PE=1 SV=1

Observed	Mr(expt)	Mr(calc)	ppm	Start	End	Miss	Peptide
976.4465	975.4392	975.4410	-1.86	19	- 28	0	K.AGFAGDDAPR.A
1132.5316	1131.5243	1131.5197	4.13	197	- 206	0	R.GYSFTTTAER.E
1161.6290	1160.6217	1160.6111	9.17	316	- 326	0	K.EITALAPSTMK.I
1171.5769	1170.5696	1170.5638	4.98	40	- 50	0	R.HQGVMVGMGQK.D
1198.7111	1197.7038	1197.6982	4.67	29	- 39	0	R.AVFPSIVGRPR.H
1623.8562	1622.8489	1622.8338	9.34	178	- 191	1	R.LDLAGRDLTDYLMK.I
1790.9086	1789.9013	1789.8846	9.33	239	- 254	0	K.SYELPDGQVITIGNER.F
1835.8361	1834.8288	1834.8441	-8.31	2	- 18	0	M.EEEIAALVIDNGSGMCK.A
1954.0796	1953.0723	1953.0571	7.80	96	- 113	0	R.VAPEEHPVLLTEAPLNPK.A
2215.0876	2214.0803	2214.0627	7.97	292	- 312	0	K.DLYANTVLSGGTTMYPGIADR.M
2242.1501	2241.1428	2241.1753	-14.50	192	- 210	2	K.ILTERGYSFTTTAEREIVR.D
2252.1614	2251.1541	2251.1882	-15.13	178	- 196	2	R.LDLAGRDLTDYLMKILTER.G + Oxidation (M)
2343.2134	2342.2061	2342.1576	20.7	291	- 312	1	R.KDLYANTVLSGGTTMYPGIADR.M
2507.1975	2506.1902	2506.1693	8.35	40	- 62	2	R.HQGVMVGMGQKDSYVGDEAQSKR.G
2550.2063	2549.1990	2549.1665	12.8	216	- 238	0	K.LCYVALDFEQEMATAASSSSLEK.S
3101.5515	3100.5442	3100.4958	15.6	285	- 312	2	K.CDVDIRKDLYANTVLSGGTTMYPGIADR.M
3183.6196	3182.6123	3182.6071	1.66	148	- 177	0	R.TTGIVMDSGDGVTHTVPIYEGYALPHAILR.L
3199.6411	3198.6338	3198.6020	9.96	148	- 177	0	R.TTGIVMDSGDGVTHTVPIYEGYALPHAILR.L + Oxidation (M)
3534.6365	3533.6292	3533.6240	1.47	255	- 284	1	R.FRCPEALFQPSFLGMESCGIHETTFNSIMK.C

No match to: 701.3809, 706.2637, 708.2758, 717.3943, 719.4086, 722.4171, 734.4380, 735.4089, 736.4033, 747.4274, 756.3826, 759.3994, 772.4160, 779.4522, 795.4493, 806.4573, 831.4625, 832.3028, 834.3088, 842.4966, 876.2747, 878.2808, 883.4713, 888.4968, 890.4931, 891.4778, 900.5316, 910.4130, 918.5359, 929.4340, 936.4846, 937.5071, 938.5070, 949.5414, 967.4175, 969.4572, 972.4759, 974.4473, 978.5344, 983.6115, 988.4531, 990.4647, 993.4956, 1000.5594, 1007.5680, 1019.5631, 1021.5624, 1031.5641, 1034.5791, 1036.5952, 1044.5217, 1046.5728, 1051.5135, 1056.5387, 1057.6144, 1060.5447, 1074.5956, 1077.5199, 1087.5442, 1091.5437, 1113.5406, 1122.5580, 1123.5852, 1145.6403, 1168.6013, 1172.5704, 1178.6254, 1180.6395, 1181.6516, 1185.6835, 1216.6992, 1234.6646, 1248.6750, 1254.6965, 1255.6359, 1273.6595, 1278.6974, 1281.6390, 1284.6533, 1301.6256, 1304.6489, 1314.7235, 1315.6904, 1325.6581, 1336.7290, 1338.6799, 1342.6847, 1349.6997, 1351.6993, 1354.6641, 1361.6997, 1368.6772, 1370.6589, 1372.6199, 1381.7728, 1382.6112, 1383.7378, 1386.7084, 1388.7888, 1390.7301, 1397.7515, 1425.7671, 1439.7701, 1442.8314, 1457.7517, 1467.7510, 1473.8167, 1488.7367, 1497.8859, 1500.7335, 1513.7731, 1516.7435, 1528.7101, 1556.8929, 1559.8439, 1562.8004, 1563.8088, 1578.7965, 1580.8445, 1592.8094, 1601.9139, 1616.8481, 1627.7197, 1628.7129, 1628.8722, 1634.8071, 1638.8757, 1642.7318, 1645.7701, 1647.8682, 1650.8612, 1673.8375, 1679.9817, 1680.8936, 1681.8372, 1696.8403, 1706.8660, 1715.8601, 1717.9037, 1730.8719, 1742.0088, 1758.9255, 1769.0105, 1771.0138, 1774.9404, 1783.9508, 1786.9517, 1787.9071, 1837.8712, 1862.9679, 1928.0435, 1940.0333, 1961.0397, 1970.0139, 1982.0297, 1984.0314, 2023.0551, 2027.1292, 2032.0228, 2061.0330, 2105.0796, 2144.0679, 2170.0872, 2179.0752, 2211.0654, 2239.1650, 2246.2151, 2251.1675, 2255.1719, 2273.1008, 2289.1030, 2293.1750, 2304.2087, 2316.2031, 2354.0020, 2391.2788, 2475.2476, 2491.2402, 2514.2664, 2542.2795, 2558.3499, 2587.3516, 2605.2615, 2646.9883, 2661.0090, 2675.0190, 2681.3855, 2698.3696, 2705.3142, 2707.3074, 2714.3647, 2717.3394, 2719.3167, 2722.3188, 2763.3738, 2792.2446, 2826.3545, 2843.4670, 2846.4014, 2847.4082, 2872.3359, 2884.3538, 2887.3474, 2889.3481, 2892.4165, 2894.4639, 2896.4890, 2914.4551, 2987.5244, 2995.4431, 3004.5840, 3029.4812, 3054.4724, 3069.4849, 3072.4231, 3126.7327, 3166.6106, 3171.6748, 3231.5630, 3236.5791, 3243.6301, 3245.6216, 3247.5737, 3248.6313, 3254.6448, 3262.7324, 3267.7256, 3278.7546, 3321.5828,

3333.5762, 3334.6729, 3335.6787, 3338.7097, 3347.4907, 3395.6821, 3412.6426, 3428.5991, 3435.7024, 3457.6995, 3487.8022, 3490.6885, 3503.8049, 3548.6917, 3551.6768, 3552.6902, 3601.6826, 3616.7095, 3624.6824, 3639.7014, 3645.7827, 3651.7559, 3655.7354, 3667.7646, 3697.8025, 3716.7253, 3762.8396, 3929.9304, 4040.7656, 4057.9707, 4071.9524, 4092.8394, 4147.9063, 4160.9473, 4174.9575, 4193.9395, 4253.8755, 4415.9736, 4452.1865, 4465.1670, 4845.0845

3. P60712 **Mass:** 42052 **Score:** 46 **Expect:** 0.59 **Matches:** 18

Actin, cytoplasmic 1 OS=Bos taurus GN=ACTB PE=1 SV=1

Observed	Mr(expt)	Mr(calc)	ppm	Start	End	Miss	Peptide
976.4465	975.4392	975.4410	-1.86	19	28	0	K.AGFAGDDAPR.A
1132.5316	1131.5243	1131.5197	4.13	197	206	0	R.GYSFTTTAER.E
1161.6290	1160.6217	1160.6111	9.17	316	326	0	K.EITALAPSTMK.I
1171.5769	1170.5696	1170.5638	4.98	40	50	0	R.HQGVMVGMGQK.D
1198.7111	1197.7038	1197.6982	4.67	29	39	0	R.AVFPSIVGRPR.H
1623.8562	1622.8489	1622.8338	9.34	178	191	1	R.LDLAGRDLTDYLMK.I
1790.9086	1789.9013	1789.8846	9.33	239	254	0	K.SYELPDGQVITIGNER.F
1954.0796	1953.0723	1953.0571	7.80	96	113	0	R.VAPEEHPVLLTEAPLNPK.A
2215.0876	2214.0803	2214.0627	7.97	292	312	0	K.DLYANTVLSGGTTMYPGIADR.M
2242.1501	2241.1428	2241.1753	-14.50	192	210	2	K.ILTERGYSFTTTAEREIVR.D
2252.1614	2251.1541	2251.1882	-15.13	178	196	2	R.LDLAGRDLTDYLMKILTER.G + Oxidation (M)
2343.2134	2342.2061	2342.1576	20.7	291	312	1	R.KDLYANTVLSGGTTMYPGIADR.M
2507.1975	2506.1902	2506.1693	8.35	40	62	2	R.HQGVMVGMGQKDSYVGDEAQSKR.G
2550.2063	2549.1990	2549.1665	12.8	216	238	0	K.LCYVALDFEQEMATAASSSSLEK.S
3101.5515	3100.5442	3100.4958	15.6	285	312	2	K.CDVDIRKDLYANTVLSGGTTMYPGIADR.M
3183.6196	3182.6123	3182.6071	1.66	148	177	0	R.TTGIVMDSGDGVTHTVPIYEGYALPHAILR.L
3199.6411	3198.6338	3198.6020	9.96	148	177	0	R.TTGIVMDSGDGVTHTVPIYEGYALPHAILR.L + Oxidation (M)
3534.6365	3533.6292	3533.6240	1.47	255	284	1	R.FRCPEALFQPSFLGMESCGIHETTFNSIMK.C

No match to: 701.3809, 706.2637, 708.2758, 717.3943, 719.4086, 722.4171, 734.4380, 735.4089, 736.4033, 747.4274, 756.3826, 759.3994, 772.4160, 779.4522, 795.4493, 806.4573, 831.4625, 832.3028, 834.3088, 842.4966, 876.2747, 878.2808, 883.4713, 888.4968, 890.4931, 891.4778, 900.5316, 910.4130, 918.5359, 929.4340, 936.4846, 937.5071, 938.5070, 949.5414, 967.4175, 969.4572, 972.4759, 974.4473, 978.5344, 983.6115, 988.4531, 990.4647, 993.4956, 1000.5594, 1007.5680, 1019.5631, 1021.5624, 1031.5641, 1034.5791, 1036.5952, 1044.5217, 1046.5728, 1051.5135, 1056.5387, 1057.6144, 1060.5447, 1074.5956, 1077.5199, 1087.5442, 1091.5437, 1113.5406, 1122.5580, 1123.5852, 1145.6403, 1168.6013, 1172.5704, 1178.6254, 1180.6395, 1181.6516, 1185.6835, 1216.6992, 1234.6646, 1248.6750, 1254.6965, 1255.6359, 1273.6595, 1278.6974, 1281.6390, 1284.6533, 1301.6256, 1304.6489, 1314.7235, 1315.6904, 1325.6581, 1336.7290, 1338.6799, 1342.6847, 1349.6997, 1351.6993, 1354.6641, 1361.6997, 1368.6772, 1370.6589, 1372.6199, 1381.7728, 1382.6112, 1383.7378, 1386.7084, 1388.7888, 1390.7301, 1397.7515, 1425.7671, 1439.7701, 1442.8314, 1457.7517, 1467.7510, 1473.8167, 1488.7367, 1497.8859, 1500.7335, 1513.7731, 1516.7435, 1528.7101, 1556.8929, 1559.8439, 1562.8004, 1563.8088, 1578.7965, 1580.8445, 1592.8094, 1601.9139, 1616.8481, 1627.7197, 1628.7129, 1628.8722, 1634.8071, 1638.8757, 1642.7318, 1645.7701, 1647.8682, 1650.8612, 1673.8375, 1679.9817, 1680.8936, 1681.8372, 1696.8403, 1706.8660, 1715.8601, 1717.9037, 1730.8719, 1742.0088, 1758.9255, 1769.0105, 1771.0138, 1774.9404, 1783.9508, 1786.9517, 1787.9071, 1835.8361, 1837.8712, 1862.9679, 1928.0435, 1940.0333, 1961.0397, 1970.0139, 1982.0297, 1984.0314, 2023.0551, 2027.1292, 2032.0228, 2061.0330, 2105.0796, 2144.0679, 2170.0872, 2179.0752, 2211.0654, 2239.1650, 2246.2151, 2251.1675, 2255.1719, 2273.1008, 2289.1030, 2293.1750, 2304.2087, 2316.2031, 2354.0020, 2391.2788, 2475.2476, 2491.2402, 2514.2664, 2542.2795, 2558.3499, 2587.3516, 2605.2615, 2646.9883, 2661.0090, 2675.0190, 2681.3855, 2698.3696, 2705.3142, 2707.3074, 2714.3647, 2717.3394, 2719.3167, 2722.3188, 2763.3738, 2792.2446, 2826.3545, 2843.4670, 2846.4014, 2847.4082, 2872.3359, 2884.3538, 2887.3474, 2889.3481, 2892.4165, 2894.4639, 2896.4890, 2914.4551, 2987.5244, 2995.4431, 3004.5840, 3029.4812, 3054.4724, 3069.4849, 3072.4231, 3126.7327, 3166.6106, 3171.6748, 3231.5630, 3236.5791, 3243.6301, 3245.6216, 3247.5737, 3248.6313, 3254.6448, 3262.7324, 3267.7256, 3278.7546, 3321.5828, 3333.5762, 3334.6729, 3335.6787, 3338.7097, 3347.4907, 3395.6821, 3412.6426, 3428.5991, 3435.7024, 3457.6995, 3487.8022, 3490.6885, 3503.8049, 3548.6917, 3551.6768, 3552.6902, 3601.6826, 3616.7095, 3624.6824, 3639.7014, 3645.7827, 3651.7559, 3655.7354, 3667.7646, 3697.8025, 3716.7253, 3762.8396, 3929.9304, 4040.7656, 4057.9707, 4071.9524, 4092.8394, 4147.9063, 4160.9473, 4174.9575, 4193.9395, 4253.8755, 4415.9736, 4452.1865, 4465.1670, 4845.0845

4. Q3T0P6 **Mass:** 44908 **Score:** 43 **Expect:** 1.1 **Matches:** 21

Phosphoglycerate kinase 1 OS=Bos taurus GN=PGK1 PE=2 SV=3

Observed	Mr(expt)	Mr(calc)	ppm	Start	End	Miss	Peptide
734.4380	733.4308	733.4374	-9.07	92	97	0	K.DVLFLK.D
949.5414	948.5341	948.5314	2.86	265	272	1	K.IVKDLMSK.A + Oxidation (M)
974.4473	973.4400	973.4505	-10.79	124	131	0	R.FHVEEEGK.G
978.5344	977.5271	977.5294	-2.30	324	332	1	K.YAEAVARAK.Q
1161.6290	1160.6217	1160.6189	2.40	142	151	1	K.AEPTKIEAFR.A
1255.6359	1254.6286	1254.6391	-8.31	172	183	0	R.AHSSMVGVNLPK.K + Oxidation (M)
1383.7378	1382.7305	1382.7340	-2.52	172	184	1	R.AHSSMVGVNLPKK.A + Oxidation (M)
1388.7888	1387.7815	1387.7823	-0.57	140	151	2	K.VKAEPTKIEAFR.A
1634.8071	1633.7998	1633.7849	9.15	157	171	0	K.LGDVYVNDAFGTAHR.A
1647.8682	1646.8609	1646.8991	-23.20	142	156	2	K.AEPTKIEAFRASLSK.L
1769.0105	1768.0032	1767.9883	8.45	200	216	0	K.ALESPERPFLAILGGAK.V
2023.0551	2022.0478	2022.0310	8.33	280	297	1	K.ITLPVDFVTADKFDENAK.T
2032.0228	2031.0155	2030.9956	9.81	23	39	1	R.VDFNVPMKNNQITNNQR.I
2105.0796	2104.0723	2104.0531	9.16	333	350	0	K.QIVWNGPVGVFEWEAFAR.G
2246.2151	2245.2078	2245.2028	2.24	87	106	2	K.SLLGKDVLFLKDCVGPEVEK.A
2304.2087	2303.2014	2303.1851	7.08	331	350	1	R.AKQIVWNGPVGVFEWEAFAR.G
2391.2788	2390.2715	2390.2172	22.7	333	353	1	K.QIVWNGPVGVFEWEAFARGTK.A
2514.2664	2513.2591	2513.2398	7.69	383	406	1	K.WNTEDKVSHVSTGGGASLELLEGK.V
2605.2615	2604.2542	2604.2312	8.83	298	323	1	K.TGQATVASGIPAGWMGLDCGPESSKK.Y
3262.7324	3261.7251	3261.7254	-0.08	57	86	1	K.SVVLMSHLGRPDGVPMPDKYSLQPVAVELK.S
3278.7546	3277.7473	3277.7203	8.25	57	86	1	K.SVVLMSHLGRPDGVPMPDKYSLQPVAVELK.S + Oxidation (M)

No match to: 701.3809, 706.2637, 708.2758, 717.3943, 719.4086, 722.4171, 735.4089, 736.4033, 747.4274, 756.3826, 759.3994, 772.4160, 779.4522, 795.4493, 806.4573, 831.4625, 832.3028, 834.3088, 842.4966, 876.2747, 878.2808, 883.4713, 888.4968, 890.4931, 891.4778, 900.5316, 910.4130, 918.5359, 929.4340, 936.4846, 937.5071, 938.5070, 967.4175, 969.4572, 972.4759, 976.4465, 983.6115, 988.4531, 990.4647, 993.4956, 1000.5594, 1007.5680, 1019.5631, 1021.5624, 1031.5641, 1034.5791, 1036.5952, 1044.5217, 1046.5728, 1051.5135, 1056.5387, 1057.6144, 1060.5447, 1074.5956, 1077.5199, 1087.5442, 1091.5437, 1113.5406, 1122.5580, 1132.5316, 1145.6403, 1168.6013, 1171.5769, 1172.5704, 1178.6254, 1180.6395, 1181.6516, 1185.6835, 1198.7111, 1216.6992, 1234.6646, 1248.6750, 1254.6965, 1273.6595, 1278.6974, 1281.6390, 1284.6533, 1301.6256, 1304.6489, 1314.7235, 1315.6904, 1325.6581, 1336.7290, 1338.6799, 1342.6847, 1349.6997, 1351.6993, 1354.6641, 1361.6997, 1368.6772, 1370.6589, 1372.6199, 1381.7728, 1382.6112, 1386.7084, 1390.7301, 1397.7515, 1425.7671, 1439.7701, 1442.8314, 1457.7517, 1467.7510, 1473.8167, 1488.7367, 1497.8859, 1500.7335, 1513.7731, 1516.7435, 1528.7101, 1556.8929, 1559.8439, 1562.8004, 1563.8088, 1578.7965, 1580.8445, 1592.8094, 1601.9139, 1616.8481, 1623.8562, 1627.7197, 1628.7129, 1628.8722, 1638.8757, 1642.7318, 1645.7701, 1650.8612, 1673.8375, 1679.9817, 1680.8936, 1681.8372, 1696.8403, 1706.8660, 1715.8601, 1717.9037, 1730.8719, 1742.0088, 1758.9255, 1771.0138, 1774.9404, 1783.9508, 1786.9517, 1787.9071, 1790.9086, 1835.8361, 1837.8712, 1862.9679, 1928.0435, 1940.0333, 1954.0796, 1961.0397, 1970.0139, 1982.0297, 1984.0314, 2027.1292, 2061.0330, 2144.0679, 2170.0872, 2179.0752, 2211.0654, 2215.0876, 2239.1650, 2242.1501, 2251.1675, 2252.1614, 2255.1719, 2273.1008, 2289.1030, 2293.1750, 2316.2031, 2343.2134, 2354.0020, 2475.2476, 2491.2402, 2507.1975, 2542.2795, 2550.2063, 2558.3499, 2587.3516, 2646.9883, 2661.0090, 2675.0190, 2681.3855, 2698.3696, 2705.3142, 2707.3074, 2714.3647, 2717.3394, 2719.3167, 2722.3188, 2763.3738, 2792.2446, 2826.3545, 2843.4670, 2846.4014, 2847.4082, 2872.3359, 2884.3538, 2887.3474, 2889.3481, 2892.4165, 2894.4639, 2896.4890, 2914.4551, 2987.5244, 2995.4431, 3004.5840, 3029.4812, 3054.4724, 3069.4849, 3072.4231, 3101.5515, 3126.7327, 3166.6106, 3171.6748, 3183.6196, 3199.6411,

3231.5630, 3236.5791, 3243.6301, 3245.6216, 3247.5737, 3248.6313, 3254.6448, 3267.7256, 3321.5828, 3333.5762, 3334.6729, 3335.6787, 3338.7097, 3347.4907, 3395.6821, 3412.6426, 3428.5991, 3435.7024, 3457.6995, 3487.6885, 3490.6885, 3534.6365, 3548.6917, 3551.6768, 3552.6902, 3601.6826, 3616.7095, 3624.6824, 3639.7014, 3645.7827, 3651.7559, 3655.7354, 3667.7646, 3697.8025, 3716.7253, 3762.8396, 3929.9304, 4040.7656, 4057.9707, 4071.9524, 4092.8394, 4147.9063, 4160.9473, 4174.9575, 4193.9395, 4253.8755, 4415.9736, 4452.1865, 4465.1670, 4845.0845

5. F1MRD0 **Mass:** 42167 **Score:** 35 **Expect:** 8.4 **Matches:** 17

Actin, cytoplasmic 1 OS=Bos taurus GN=ACTB PE=3 SV=2

Observed	Mr(expt)	Mr(calc)	ppm	Start	End	Miss	Peptide
976.4465	975.4392	975.4410	-1.86	19 -	28	0	K.AGFAGDDAPR.A
1132.5316	1131.5243	1131.5197	4.13	197 -	206	0	R.GYSFTTTAER.E
1171.5769	1170.5696	1170.5638	4.98	40 -	50	0	R.HQGVMVGMGQK.D
1198.7111	1197.7038	1197.6982	4.67	29 -	39	0	R.AVFPSIVGRPR.H
1623.8562	1622.8489	1622.8338	9.34	178 -	191	1	R.LDLAGRDLTDYLMK.I
1790.9086	1789.9013	1789.8846	9.33	239 -	254	0	K.SYELPDGQVITIGNER.F
1862.9679	1861.9606	1861.9641	-1.87	313 -	328	2	R.MQKEINDLAPSTMKIK.I + Oxidation (M)
1954.0796	1953.0723	1953.0571	7.80	96 -	113	0	R.VAPEEHPVLLTEAPLNPK.A
2242.1501	2241.1428	2241.1753	-14.50	192 -	210	2	K.ILTERGYSFTTTAEREIVR.D
2252.1614	2251.1541	2251.1882	-15.13	178 -	196	2	R.LDLAGRDLTDYLMKILTER.G + Oxidation (M)
2273.1008	2272.0935	2272.0682	11.2	292 -	312	0	K.DLYANTVLSGGTTMYPGIEDR.M
2289.1030	2288.0957	2288.0631	14.3	292 -	312	0	K.DLYANTVLSGGTTMYPGIEDR.M + Oxidation (M)
2507.1975	2506.1902	2506.1693	8.35	40 -	62	2	R.HQGVMVGMGQKDSYVGDEAQSKR.G
2550.2063	2549.1990	2549.1665	12.8	216 -	238	0	K.LCYVALDFEQEMATAASSSSLEK.S
3183.6196	3182.6123	3182.6071	1.66	148 -	177	0	R.TTGIVMDSGDGVTHTVPIYEGYALPHAILR.L
3199.6411	3198.6338	3198.6020	9.96	148 -	177	0	R.TTGIVMDSGDGVTHTVPIYEGYALPHAILR.L + Oxidation (M)
3534.6365	3533.6292	3533.6240	1.47	255 -	284	1	R.FRCPEALFQPSFLGMESCGIHETTFNSIMK.C

No match to: 701.3809, 706.2637, 708.2758, 717.3943, 719.4086, 722.4171, 734.4380, 735.4089, 736.4033, 747.4274, 756.3826, 759.3994, 772.4160, 779.4522, 795.4493, 806.4573, 831.4625, 832.3028, 834.3088, 842.4966, 876.2747, 878.2808, 883.4713, 888.4968, 890.4931, 891.4778, 900.5316, 910.4130, 918.5359, 929.4340, 936.4846, 937.5071, 938.5070, 949.5414, 967.4175, 969.4572, 972.4759, 974.4473, 978.5344, 983.6115, 988.4531, 990.4647, 993.4956, 1000.5594, 1007.5680, 1019.5631, 1021.5624, 1031.5641, 1034.5791, 1036.5952, 1044.5217, 1046.5728, 1051.5135, 1056.5387, 1057.6144, 1060.5447, 1074.5956, 1077.5199, 1087.5442, 1091.5437, 1113.5406, 1122.5580, 1123.5852, 1145.6403, 1161.6290, 1168.6013, 1172.5704, 1178.6254, 1180.6395, 1181.6516, 1185.6835, 1216.6992, 1234.6646, 1248.6750, 1254.6965, 1255.6359, 1273.6595, 1278.6974, 1281.6390, 1284.6533, 1301.6256, 1304.6489, 1314.7235, 1315.6904, 1325.6581, 1336.7290, 1338.6799, 1342.6847, 1349.6997, 1351.6993, 1354.6641, 1361.6997, 1368.6772, 1370.6589, 1372.6199, 1381.7728, 1382.6112, 1383.7378, 1386.7084, 1388.7888, 1390.7301, 1397.7515, 1425.7671, 1439.7701, 1442.8314, 1457.7517, 1467.7510, 1473.8167, 1488.7367, 1497.8859, 1500.7335, 1513.7731, 1516.7435, 1528.7101, 1556.8929, 1559.8439, 1562.8004, 1563.8088, 1578.7965, 1580.8445, 1592.8094, 1601.9139, 1616.8481, 1627.7197, 1628.7129, 1628.8722, 1634.8071, 1638.8757, 1642.7318, 1645.7701, 1647.8682, 1650.8612, 1673.8375, 1679.9817, 1680.8936, 1681.8372, 1696.8403, 1706.8660, 1715.8601, 1717.9037, 1730.8719, 1742.0088, 1758.9255, 1769.0105, 1771.0138, 1774.9404, 1783.9508, 1786.9517, 1787.9071, 1835.8361, 1837.8712, 1928.0435, 1940.0333, 1961.0397, 1970.0139, 1982.0297, 1984.0314, 2023.0551, 2027.1292, 2032.0228, 2061.0330, 2105.0796, 2144.0679, 2170.0872, 2179.0752, 2211.0654, 2215.0876, 2246.2151, 2251.1675, 2255.1719, 2293.1750, 2304.2087, 2316.2031, 2343.2134, 2354.0020, 2391.2788, 2475.2476, 2491.2402, 2514.2664, 2542.2795, 2558.3499, 2587.3516, 2605.2615, 2646.9883, 2661.0090, 2675.0190, 2681.3855, 2698.3696, 2705.3142, 2707.3074, 2714.3647, 2717.3394, 2719.3167, 2722.3188, 2763.3738, 2792.2446, 2826.3545, 2843.4670, 2846.4014, 2847.4082, 2872.3359, 2884.3538, 2887.3474, 2889.3481, 2892.4165, 2894.4639, 2896.4890, 2914.4551, 2987.5244, 2995.4431, 3004.5840, 3029.4812, 3054.4724, 3069.4849, 3072.4231, 3101.5515, 3126.7327, 3166.6106, 3171.6748, 3231.5630, 3236.5791, 3243.6301, 3245.6216, 3247.5737, 3248.6313, 3254.6448, 3262.7324, 3267.7256, 3278.7546, 3321.5828, 3333.5762, 3334.6729, 3335.6787, 3338.7097, 3347.4907, 3395.6821, 3412.6426, 3428.5991, 3435.7024, 3457.6995, 3487.8022, 3490.6885, 3503.8049, 3548.6917, 3551.6768, 3552.6902, 3601.6826, 3616.7095, 3624.6824, 3639.7014, 3645.7827, 3651.7559, 3655.7354, 3667.7646, 3697.8025, 3716.7253, 3762.8396, 3929.9304, 4040.7656, 4057.9707, 4071.9524, 4092.8394, 4147.9063, 4160.9473, 4174.9575, 4193.9395, 4253.8755, 4415.9736, 4452.1865, 4465.1670, 4845.0845

6. F1MVK7 **Mass:** 40714 **Score:** 27 **Expect:** 51 **Matches:** 10

Uncharacterized protein OS=Bos taurus PE=4 SV=2

Observed	Mr(expt)	Mr(calc)	ppm	Start	End	Miss	Peptide
978.5344	977.5271	977.5216	5.71	247 -	255	0	R.LSQVGSMLK.T + Oxidation (M)
1273.6595	1272.6522	1272.6826	-23.88	279 -	288	1	R.QLLSDWKVER.L
1336.7290	1335.7217	1335.7220	-0.24	331 -	341	1	R.FSPLEMVIRTK.W + Oxidation (M)
1370.6589	1369.6516	1369.6489	1.99	140 -	149	0	K.FIYDHLQCFK.G
1650.8612	1649.8539	1649.8512	1.66	126 -	139	1	R.LQLFELSDKEATEK.F
2061.0330	2060.0257	2060.0367	-5.33	286 -	302	1	K.VERLFDLYFYSGQPAQK.K
2872.3359	2871.3286	2871.3709	-14.72	230 -	255	2	R.SDIGYLQWGKGTSDDDRLSQVGSMLK.T + Oxidation (M)
3236.5791	3235.5718	3235.5408	9.58	303 -	329	0	K.KPAHLTIDTHSHHWESVQHEDEHRPGR.R
4452.1865	4451.1792	4451.1366	9.57	100 -	139	2	K.ATICLITEDTYVAPTPGYSGDGFSERLQLFELSDKEATEK.F
4465.1670	4464.1597	4464.2159	-12.58	303 -	339	2	K.KPAHLTIDTHSHHWESVQHEDEHRPGRRFSPLEMVIR.T

No match to: 701.3809, 706.2637, 708.2758, 717.3943, 719.4086, 722.4171, 734.4380, 735.4089, 736.4033, 747.4274, 756.3826, 759.3994, 772.4160, 779.4522, 795.4493, 806.4573, 831.4625, 832.3028, 834.3088, 842.4966, 876.2747, 878.2808, 883.4713, 888.4968, 890.4931, 891.4778, 900.5316, 910.4130, 918.5359, 929.4340, 936.4846, 937.5071, 938.5070, 949.5414, 967.4175, 969.4572, 972.4759, 974.4473, 976.4465, 983.6115, 988.4531, 990.4647, 993.4956, 1000.5594, 1007.5680, 1019.5631, 1021.5624, 1031.5641, 1034.5791, 1036.5952, 1044.5217, 1046.5728, 1051.5135, 1056.5387, 1057.6144, 1060.5447, 1074.5956, 1077.5199, 1087.5442, 1091.5437, 1113.5406, 1122.5580, 1123.5852, 1132.5316, 1145.6403, 1161.6290, 1168.6013, 1171.5769, 1172.5704, 1178.6254, 1180.6395, 1181.6516, 1185.6835, 1198.7111, 1216.6992, 1234.6646, 1248.6750, 1254.6965, 1255.6359, 1278.6974, 1281.6390, 1284.6533, 1301.6256, 1304.6489, 1314.7235, 1315.6904, 1325.6581, 1338.6799, 1342.6847, 1349.6997, 1351.6993, 1354.6641, 1361.6997, 1368.6772, 1372.6199, 1381.7728, 1382.6112, 1383.7378, 1386.7084, 1388.7888, 1390.7301, 1397.7515, 1425.7671, 1439.7701, 1442.8314, 1457.7517, 1467.7510, 1473.8167, 1488.7367, 1497.8859, 1500.7335, 1513.7731, 1516.7435, 1528.7101, 1556.8929, 1559.8439, 1562.8004, 1563.8088, 1578.7965, 1580.8445, 1592.8094, 1601.9139, 1616.8481, 1623.8562, 1627.7197, 1628.7129, 1628.8722, 1634.8071, 1638.8757, 1642.7318, 1645.7701, 1647.8682, 1673.8375, 1679.9817, 1680.8936, 1681.8372, 1696.8403, 1706.8660, 1715.8601, 1717.9037, 1730.8719, 1742.0088, 1758.9255, 1769.0105, 1771.0138, 1774.9404, 1783.9508, 1786.9517, 1787.9071, 1790.9086, 1835.8361, 1837.8712, 1862.9679, 1928.0435, 1940.0333, 1954.0796, 1961.0397, 1970.0139, 1982.0297, 1984.0314, 2023.0551, 2027.1292, 2032.0228, 2105.0796, 2144.0679, 2170.0872, 2179.0752, 2211.0654, 2215.0876, 2239.1650, 2242.1501, 2246.2151, 2251.1675, 2252.1614, 2255.1719, 2273.1008, 2289.1030, 2293.1750, 2304.2087, 2316.2031, 2343.2134, 2354.0020, 2391.2788, 2475.2476, 2491.2402, 2507.1975, 2514.2664, 2542.2795, 2550.2063, 2558.3499, 2587.3516, 2605.2615, 2646.9883, 2661.0090, 2675.0190, 2681.3855, 2698.3696, 2705.3142, 2707.3074, 2714.3647, 2717.3394, 2719.3167, 2722.3188, 2763.3738, 2792.2446, 2826.3545, 2843.4670, 2846.4014, 2847.4082, 2884.3538, 2887.3474, 2889.3481, 2892.4165, 2894.4639, 2896.4890, 2914.4551, 2987.5244, 2995.4431, 3004.5840, 3029.4812, 3054.4724, 3069.4849, 3072.4231, 3101.5515, 3126.7327, 3166.6106, 3171.6748, 3183.6196, 3199.6411, 3231.5630, 3243.6301, 3245.6216, 3247.5737, 3248.6313, 3254.6448, 3262.7324, 3267.7256, 3278.7546, 3321.5828, 3333.5762, 3334.6729, 3335.6787, 3338.7097, 3347.4907, 3395.6821, 3412.6426, 3428.5991, 3435.7024, 3457.6995, 3487.8022, 3490.6885, 3503.8049, 3534.6365, 3548.6917, 3551.6768, 3552.6902, 3601.6826, 3616.7095, 3624.6824, 3639.7014, 3645.7827, 3651.7559, 3655.7354, 3667.7646, 3697.8025, 3716.7253, 3762.8396, 3929.9304, 4040.7656, 4057.9707, 4071.9524, 4092.8394, 4147.9063, 4160.9473, 4174.9575, 4193.9395, 4253.8755, 4415.9736, 4845.0845

7. P19879 **Mass:** 34530 **Score:** 26 **Expect:** 54 **Matches:** 13

Mimecan OS=Bos taurus GN=OGN PE=1 SV=2

Observed	Mr(expt)	Mr(calc)	ppm	Start	End	Miss	Peptide
806.4573	805.4500	805.4698	-24.54	190 -	196	0	K.LTLFNAK.Y
918.5359	917.5287	917.5334	-5.19	292 -	299	1	K.RLPIGSYI.-

972.4759	971.4686	971.4712	-2.67	121 - 128	0	K.ESAYLYAR.F
1060.5447	1059.5374	1059.5349	2.39	139 - 147	0	K.DFADIPNLR.R
1123.5852	1122.5779	1122.5921	-12.60	77 - 86	0	K.DENITPLPPK.K
1168.6013	1167.5940	1167.6135	-16.70	51 - 60	2	K.YLDGKSTKEK.E
1361.6997	1360.6924	1360.6775	11.0	121 - 131	1	K.ESAYLYARFNK.I
1473.8167	1472.8094	1472.7987	7.28	135 - 147	1	K.LTAKDFADIPNLR.R
1601.9139	1600.9066	1600.8937	8.10	134 - 147	2	K.KLTAKDFADIPNLR.R
1679.9817	1678.9744	1678.9617	7.56	268 - 282	1	R.IEEIRLEGNPVILGK.H
2170.0872	2169.0799	2169.0590	9.66	148 - 166	1	R.RLDFTGNLIEDIEDGTFSK.L
3004.5840	3003.5767	3003.5818	-1.68	213 - 238	0	K.LHNLSFLYLDHNALESVPLNLPESLR.V
3457.6995	3456.6922	3456.6732	5.50	239 - 267	2	R.VIHLQFNNITSITDDTFCKANDTSYIRDR.I

No match to: 701.3809, 706.2637, 708.2758, 717.3943, 719.4086, 722.4171, 734.4380, 735.4089, 736.4033, 747.4274, 756.3826, 759.3994, 772.4160, 779.4522, 795.4493, 831.4625, 832.3028, 834.3088, 842.4966, 876.2747, 878.2808, 883.4713, 888.4968, 890.4931, 891.4778, 900.5316, 910.4130, 929.4340, 936.4846, 937.5071, 938.5070, 949.5414, 967.4175, 969.4572, 974.4473, 976.4465, 978.5344, 983.6115, 988.4531, 990.4647, 993.4956, 1000.5594, 1007.5680, 1019.5631, 1021.5624, 1031.5641, 1034.5791, 1036.5952, 1044.5217, 1046.5728, 1051.5135, 1056.5387, 1057.6144, 1074.5956, 1077.5199, 1087.5442, 1091.5437, 1113.5406, 1122.5580, 1132.5316, 1145.6403, 1161.6290, 1171.5769, 1172.5704, 1178.6254, 1180.6395, 1181.6516, 1185.6835, 1198.7111, 1216.6992, 1234.6646, 1248.6750, 1254.6965, 1255.6359, 1273.6595, 1278.6974, 1281.6390, 1284.6533, 1301.6256, 1304.6489, 1314.7235, 1315.6904, 1325.6581, 1336.7290, 1338.6799, 1342.6847, 1349.6997, 1351.6993, 1354.6641, 1368.6772, 1370.6589, 1372.6199, 1381.7728, 1382.6112, 1383.7378, 1386.7084, 1388.7888, 1390.7301, 1397.7515, 1425.7671, 1439.7701, 1442.8314, 1457.7517, 1467.7510, 1488.7367, 1497.8859, 1500.7335, 1513.7731, 1516.7435, 1528.7101, 1556.8929, 1559.8439, 1562.8004, 1563.8088, 1578.7965, 1580.8445, 1592.8094, 1616.8481, 1623.8562, 1627.7197, 1628.7129, 1628.8722, 1634.8071, 1638.8757, 1642.7318, 1645.7701, 1647.8682, 1650.8612, 1673.8375, 1680.8936, 1681.8372, 1696.8403, 1706.8660, 1715.8601, 1717.9037, 1730.8719, 1742.0088, 1758.9255, 1769.0105, 1771.0138, 1774.9404, 1783.9508, 1786.9517, 1787.9071, 1790.9086, 1835.8361, 1837.8712, 1862.9679, 1928.0435, 1940.0333, 1954.0796, 1961.0397, 1970.0139, 1982.0297, 1984.0314, 2023.0551, 2027.1292, 2032.0228, 2061.0330, 2105.0796, 2144.0679, 2179.0752, 2211.0654, 2215.0876, 2239.1650, 2242.1501, 2246.2151, 2251.1675, 2252.1614, 2255.1719, 2273.1008, 2289.1030, 2293.1750, 2304.2087, 2316.2031, 2343.2134, 2354.0020, 2391.2788, 2475.2476, 2491.2402, 2507.1975, 2514.2664, 2542.2795, 2550.2063, 2558.3499, 2587.3516, 2605.2615, 2646.9883, 2661.0090, 2675.0190, 2681.3855, 2698.3696, 2705.3142, 2707.3074, 2714.3647, 2717.3394, 2719.3167, 2722.3188, 2763.3738, 2792.2446, 2826.3545, 2843.4670, 2846.4014, 2847.4082, 2872.3359, 2884.3538, 2887.3474, 2889.3481, 2892.4165, 2894.4639, 2896.4890, 2914.4551, 2987.5244, 2995.4431, 3029.4812, 3054.4724, 3069.4849, 3072.4231, 3101.5515, 3126.7327, 3166.6106, 3171.6748, 3183.6196, 3199.6411, 3231.5630, 3236.5791, 3243.6301, 3245.6216, 3247.5737, 3248.6313, 3254.6448, 3262.7324, 3267.7256, 3278.7546, 3321.5828, 3333.5762, 3334.6729, 3335.6787, 3338.7097, 3347.4907, 3395.6821, 3412.6426, 3428.5991, 3435.7024, 3487.8022, 3490.6885, 3503.8049, 3534.6365, 3548.6917, 3551.6768, 3552.6902, 3601.6826, 3616.7095, 3624.6824, 3639.7014, 3645.7827, 3651.7559, 3655.7354, 3667.7646, 3697.8025, 3716.7253, 3762.8396, 3929.9304, 4040.7656, 4057.9707, 4071.9524, 4092.8394, 4147.9063, 4160.9473, 4174.9575, 4193.9395, 4253.8755, 4415.9736, 4452.1865, 4465.1670, 4845.0845

8. F1N367 **Mass:** 244177 **Score:** 25 **Expect:** 77 **Matches:** 39

Aggrecan core protein OS=Bos taurus GN=ACAN PE=4 SV=2

Observed	Mr(expt)	Mr(calc)	ppm	Start - End	Miss	Peptide
722.4171	721.4098	721.4235	-19.04	2282 - 2287	0	R.HVPTIR.C
831.4625	830.4552	830.4650	-11.78	545 - 551	0	R.YPIVSPR.T
883.4713	882.4640	882.4712	-8.16	211 - 217	0	R.YPIHTPR.E
891.4778	890.4706	890.4763	-6.41	152 - 158	0	K.GIVFHYR.A
929.4340	928.4267	928.4290	-2.55	164 - 170	0	R.YTLDFDR.A
949.5414	948.5341	948.5392	-5.42	306 - 313	1	R.SVRYPISK.A
967.4175	966.4103	966.4130	-2.86	2125 - 2131	0	K.FQGHCYR.H
974.4473	973.4400	973.4341	6.06	678 - 684	0	R.HHAFCFR.G
1087.5442	1086.5369	1086.5233	12.5	498 - 506	0	R.YSLTFEEAK.Q
1168.6013	1167.5940	1167.5924	1.37	585 - 594	0	R.LEGEVFFATR.L
1185.6835	1184.6762	1184.6765	-0.22	83 - 93	0	K.EVVLLVATEGR.V
1248.6750	1247.6677	1247.6622	4.43	2262 - 2271	1	K.DRYEINALVR.Y
1273.6595	1272.6522	1272.6826	-23.89	987 - 997	1	R.ITWTSAPKVDR.L
1284.6533	1283.6460	1283.6258	15.7	164 - 173	1	R.YTLDFDRAQR.A
1301.6256	1300.6183	1300.5870	24.1	2272 - 2281	0	R.YQCTEGFIQR.H
1325.6581	1324.6508	1324.6445	4.75	2299 - 2309	1	R.ITCTDPATYKR.R
1372.6199	1371.6126	1371.5877	18.1	264 - 274	0	K.FTFQEAANECR.R
1382.6112	1381.6039	1381.5833	14.9	2288 - 2298	0	R.CQPSGHWEEPR.I
1383.7378	1382.7305	1382.7201	7.54	314 - 326	0	K.ARPNCGGNLLGVR.T
1442.8314	1441.8241	1441.8140	7.00	81 - 93	1	K.EKEVVLLVATEGR.V
1457.7517	1456.7444	1456.7310	9.19	159 - 170	1	R.AISTRYTLDFDR.A
1528.7101	1527.7028	1527.6888	9.15	264 - 275	1	K.FTFQEAANECRR.L
1578.7965	1577.7892	1577.7620	17.2	2241 - 2255	0	K.GTVACGEPPVVEHAR.I
1628.7129	1627.7056	1627.6937	7.34	218 - 231	1	R.EGCYGDKDEFPGVR.T
1628.8722	1627.8649	1627.8583	4.06	483 - 497	0	R.LPGGVVFHYRPGSSR.Y
1706.8660	1705.8587	1705.8570	1.01	2240 - 2255	1	K.KGTVACGEPPVVEHAR.I
1715.8601	1714.8528	1714.8348	10.5	498 - 511	1	R.YSLTFEEAKQACLR.T
1771.0138	1770.0065	1770.0251	-10.48	78 - 93	2	R.ISKEKEVVLLVATEGR.V
1940.0333	1939.0260	1939.0098	8.36	643 - 660	0	R.YPIVTPRPACGGDKPGVR.T
2144.0679	2143.0606	2143.0289	14.8	133 - 151	0	R.CEVMHGIEDSQATLEVVVK.G
2343.2134	2342.2061	2342.1940	5.18	104 - 124	0	K.VTLPNYPAIPSDATLEIQNMR.S
2872.3359	2871.3286	2871.3142	5.01	280 - 305	0	R.LATTGQLYLAWQGGMDMCSAGWLADR.S
3236.5791	3235.5718	3235.5291	13.2	2282 - 2308	2	R.HVPTIRCQPSGHWEEPRITCTDPATYK.R
3248.6313	3247.6240	3247.6183	1.77	96 - 124	1	R.VNSAYQDKVTLPNYPAIPSDATLEIQNMR.S
3457.6995	3456.6922	3456.6489	12.5	275 - 305	2	R.RLGARLATTGQLYLAWQGGMDMCSAGWLADR.S + 2 Oxidation (M)
3503.8049	3502.7976	3502.7878	2.80	94 - 124	2	R.VRVNSAYQDKVTLPNYPAIPSDATLEIQNMR.S
3639.7014	3638.6941	3638.6947	-0.16	512 - 544	0	R.TGAIIASPEQLQAAYEAGYEQCDAGWLQDQTVR.Y
4147.9063	4146.8990	4146.9164	-4.18	174 - 210	0	R.ACLQNSAIIATPEQLQAAYEDGFHQCDAGWLADQTVR.Y
4452.1865	4451.1792	4451.1492	6.75	512 - 551	1	R.TGAIIASPEQLQAAYEAGYEQCDAGWLQDQTVRYPIVSPR.T

No match to: 701.3809, 706.2637, 708.2758, 717.3943, 719.4086, 734.4380, 735.4089, 736.4033, 747.4274, 756.3826, 759.3994, 772.4160, 779.4522, 795.4493, 806.4573, 832.3028, 834.3088, 842.4966, 876.2747, 878.2808, 888.4968, 890.4931, 900.5316, 910.4130, 918.5359, 936.4846, 937.5071, 938.5070, 969.4572, 972.4759, 976.4465, 978.5344, 983.6115, 988.4531, 990.4647, 993.4956, 1000.5594, 1007.5680, 1019.5631, 1021.5624, 1031.5641, 1034.5791, 1036.5952, 1044.5217, 1046.5728, 1051.5135, 1056.5387, 1057.6144, 1060.5447, 1074.5956, 1077.5199, 1091.5437, 1113.5406, 1122.5580, 1123.5852, 1132.5316, 1145.6403, 1161.6290, 1171.5769, 1172.5704, 1178.6254, 1180.6395, 1181.6516, 1198.7111, 1216.6992, 1234.6646, 1254.6965, 1255.6359, 1278.6974, 1281.6390, 1304.6489, 1314.7235, 1315.6904, 1336.7290, 1338.6799, 1342.6847, 1349.6997, 1351.6993, 1354.6641, 1361.6997, 1368.6772, 1370.6589, 1381.7728, 1386.7084, 1388.7888, 1390.7301, 1397.7515, 1425.7671, 1439.7701, 1467.7510, 1473.8167, 1488.7367, 1497.8859, 1500.7335, 1513.7731, 1516.7435, 1556.8929, 1559.8439, 1562.8004, 1563.8088, 1580.8445, 1592.8094, 1601.9139, 1616.8481, 1623.8562, 1627.7197, 1634.8071, 1638.8757, 1642.7318, 1645.7701, 1647.8682, 1650.8612, 1673.8375, 1679.9817, 1680.8936, 1681.8372, 1696.8403, 1717.9037, 1730.8719, 1742.0088, 1758.9255, 1769.0105, 1774.9404, 1783.9508, 1786.9517, 1787.9071, 1790.9086, 1835.8361, 1837.8712, 1862.9679, 1928.0435, 1954.0796, 1961.0397, 1970.0139, 1982.0297, 1984.0314, 2023.0551, 2027.1292, 2032.0228, 2061.0330, 2105.0796, 2170.0872, 2179.0752, 2211.0654, 2215.0876, 2239.1650, 2242.1501, 2246.2151, 2251.1675, 2252.1614, 2255.1719, 2273.1008, 2289.1030, 2293.1750, 2304.2087, 2316.2031, 2354.0020, 2391.2788, 2475.2476, 2491.2402, 2507.1975, 2514.2664, 2542.2795, 2550.2063, 2558.3499, 2587.3516, 2605.2615, 2646.9883, 2661.0090,

2675.0190, 2681.3855, 2698.3696, 2705.3142, 2707.3074, 2714.3647, 2717.3394, 2719.3167, 2722.3188, 2763.3738, 2792.2446, 2826.3545, 2843.4670, 2846.4014, 2847.4082, 2884.3538, 2887.3474, 2889.3481, 2892.4165, 2894.4639, 2896.4890, 2914.4551, 2987.5244, 2995.4431, 3004.5840, 3029.4812, 3054.4724, 3069.4849, 3072.4231, 3101.5515, 3126.7327, 3166.6106, 3171.6748, 3183.6196, 3199.6411, 3231.5630, 3243.6301, 3245.6216, 3247.5737, 3254.6448, 3262.7324, 3267.7256, 3278.7546, 3321.5828, 3333.5762, 3334.6729, 3335.6787, 3338.7097, 3347.4907, 3395.6821, 3412.6426, 3428.5991, 3435.7024, 3487.8022, 3490.6885, 3534.6365, 3548.6917, 3551.6768, 3552.6902, 3601.6826, 3616.7095, 3624.6824, 3645.7827, 3651.7559, 3655.7354, 3667.7646, 3697.8025, 3716.7253, 3762.8396, 3929.9304, 4040.7656, 4057.9707, 4071.9524, 4092.8394, 4160.9473, 4174.9575, 4193.9395, 4253.8755, 4415.9736, 4465.1670, 4845.0845

9. F1N368 **Mass:** 248513 **Score:** 24 **Expect:** 96 **Matches:** 39

Aggrecan core protein OS=Bos taurus GN=ACAN PE=4 SV=2

Observed	Mr(expt)	Mr(calc)	ppm	Start		End	Miss	Peptide
722.4171	721.4098	721.4235	-19.04	2320	-	2325	0	R.HVPTIR.C
831.4625	830.4552	830.4650	-11.78	545	-	551	0	R.YPIVSPR.T
883.4713	882.4640	882.4712	-8.16	211	-	217	0	R.YPIHTPR.E
891.4778	890.4706	890.4763	-6.41	152	-	158	0	K.GIVFHYR.A
929.4340	928.4267	928.4290	-2.55	164	-	170	0	R.YTLDFDR.A
949.5414	948.5341	948.5392	-5.42	306	-	313	1	R.SVRYPISK.A
967.4175	966.4103	966.4130	-2.86	2163	-	2169	0	K.FQGHCYR.H
974.4473	973.4400	973.4341	6.06	678	-	684	0	R.HHAFCFR.G
1087.5442	1086.5369	1086.5233	12.5	498	-	506	0	R.YSLTFEEAK.Q
1168.6013	1167.5940	1167.5924	1.37	585	-	594	0	R.LEGEVFFATR.L
1185.6835	1184.6762	1184.6765	-0.22	83	-	93	0	K.EVVLLVATEGR.V
1248.6750	1247.6677	1247.6622	4.43	2300	-	2309	1	K.DRYEINALVR.Y
1273.6595	1272.6522	1272.6826	-23.89	987	-	997	1	R.ITWTSAPKVDR.L
1284.6533	1283.6460	1283.6258	15.7	164	-	173	1	R.YTLDFDRAQR.A
1301.6256	1300.6183	1300.5870	24.1	2310	-	2319	0	R.YQCTEGFIQR.H
1325.6581	1324.6508	1324.6445	4.75	2337	-	2347	1	R.ITCTDPATYKR.R
1372.6199	1371.6126	1371.5877	18.1	264	-	274	0	K.FTFQEAANECR.R
1382.6112	1381.6039	1381.5833	14.9	2326	-	2336	0	R.CQPSGHWEEPR.I
1383.7378	1382.7305	1382.7201	7.54	314	-	326	0	K.ARPNCGGNLLGVR.T
1442.8314	1441.8241	1441.8140	7.00	81	-	93	1	K.EKEVVLLVATEGR.V
1457.7517	1456.7444	1456.7310	9.19	159	-	170	1	R.AISTRYTLDFDR.A
1528.7101	1527.7028	1527.6888	9.15	264	-	275	1	K.FTFQEAANECRR.L
1578.7965	1577.7892	1577.7620	17.2	2279	-	2293	0	K.GTVACGEPPVVEHAR.I
1628.7129	1627.7056	1627.6937	7.34	218	-	231	1	R.EGCYGDKDEFPGVR.T
1628.8722	1627.8649	1627.8583	4.06	483	-	497	0	R.LPGGVVFHYRPGSSR.Y
1706.8660	1705.8587	1705.8570	1.01	2278	-	2293	1	K.KGTVACGEPPVVEHAR.I
1715.8601	1714.8528	1714.8348	10.5	498	-	511	1	R.YSLTFEEAKQACLR.T
1771.0138	1770.0065	1770.0251	-10.48	78	-	93	2	R.ISKEKEVVLLVATEGR.V
1940.0333	1939.0260	1939.0098	8.36	643	-	660	0	R.YPIVTPRPACGGDKPGVR.T
2144.0679	2143.0606	2143.0289	14.8	133	-	151	0	R.CEVMHGIEDSQATLEVVVK.G
2343.2134	2342.2061	2342.1940	5.18	104	-	124	0	K.VTLPNYPAIPSDATLEIQNMR.S
2872.3359	2871.3286	2871.3142	5.01	280	-	305	0	R.LATTGQLYLAWQGGMDMCSAGWLADR.S
3236.5791	3235.5718	3235.5291	13.2	2320	-	2346	2	R.HVPTIRCQPSGHWEEPRITCTDPATYK.R
3248.6313	3247.6240	3247.6183	1.77	96	-	124	1	R.VNSAYQDKVTLPNYPAIPSDATLEIQNMR.S
3457.6995	3456.6922	3456.6489	12.5	275	-	305	2	R.RLGARLATTGQLYLAWQGGMDMCSAGWLADR.S + 2 Oxidation (M)
3503.8049	3502.7976	3502.7878	2.80	94	-	124	2	R.VRVNSAYQDKVTLPNYPAIPSDATLEIQNMR.S
3639.7014	3638.6941	3638.6947	-0.16	512	-	544	0	R.TGAIIASPEQLQAAYEAGYEQCDAGWLQDQTVR.Y
4147.9063	4146.8990	4146.9164	-4.18	174	-	210	0	R.ACLQNSAIIATPEQLQAAYEDGFHQCDAGWLADQTVR.Y
4452.1865	4451.1792	4451.1492	6.75	512	-	551	1	R.TGAIIASPEQLQAAYEAGYEQCDAGWLQDQTVRYPIVSPR.T

No match to: 701.3809, 706.2637, 708.2758, 717.3943, 719.4086, 734.4380, 735.4089, 736.4033, 747.4274, 756.3826, 759.3994, 772.4160, 779.4522, 795.4493, 806.4573, 832.3028, 834.3088, 842.4966, 876.2747, 878.2808, 888.4968, 890.4931, 900.5316, 910.4130, 918.5359, 936.4846, 937.5071, 938.5070, 969.4572, 972.4759, 976.4465, 978.5344, 983.6115, 988.4531, 990.4647, 993.4956, 1000.5594, 1007.5680, 1019.5631, 1021.5624, 1031.5641, 1034.5791, 1036.5952, 1044.5217, 1046.5728, 1051.5135, 1056.5387, 1057.6144, 1060.5447, 1074.5956, 1077.5199, 1091.5437, 1113.5406, 1122.5580, 1123.5852, 1132.5316, 1145.6403, 1161.6290, 1171.5769, 1172.5704, 1178.6254, 1180.6395, 1181.6516, 1198.7111, 1216.6992, 1234.6646, 1254.6965, 1255.6359, 1278.6974, 1281.6390, 1304.6489, 1314.7235, 1315.6904, 1336.7290, 1338.6799, 1342.6847, 1349.6997, 1351.6993, 1354.6641, 1361.6997, 1368.6772, 1370.6589, 1381.7728, 1386.7084, 1388.7888, 1390.7301, 1397.7515, 1425.7671, 1439.7701, 1467.7510, 1473.8167, 1488.7367, 1497.8859, 1500.7335, 1513.7731, 1516.7435, 1556.8929, 1559.8439, 1562.8004, 1563.8088, 1580.8445, 1592.8094, 1601.9139, 1616.8481, 1623.8562, 1627.7197, 1634.8071, 1638.8757, 1642.7318, 1645.7701, 1647.8682, 1650.8612, 1673.8375, 1679.9817, 1680.8936, 1681.8372, 1696.8403, 1717.9037, 1730.8719, 1742.0088, 1758.9255, 1769.0105, 1774.9404, 1783.9508, 1786.9517, 1787.9071, 1790.9086, 1835.8361, 1837.8712, 1862.9679, 1928.0435, 1954.0796, 1961.0397, 1970.0139, 1982.0297, 1984.0314, 2023.0551, 2027.1292, 2032.0228, 2061.0330, 2105.0796, 2170.0872, 2179.0752, 2211.0654, 2215.0876, 2239.1650, 2242.1501, 2246.2151, 2251.1675, 2252.1614, 2255.1719, 2273.1008, 2289.1030, 2293.1750, 2304.2087, 2316.2031, 2354.0020, 2391.2788, 2475.2476, 2491.2402, 2507.1975, 2514.2664, 2542.2795, 2550.2063, 2558.3499, 2587.3516, 2605.2615, 2646.9883, 2661.0090, 2675.0190, 2681.3855, 2698.3696, 2705.3142, 2707.3074, 2714.3647, 2717.3394, 2719.3167, 2722.3188, 2763.3738, 2792.2446, 2826.3545, 2843.4670, 2846.4014, 2847.4082, 2884.3538, 2887.3474, 2889.3481, 2892.4165, 2894.4639, 2896.4890, 2914.4551, 2987.5244, 2995.4431, 3004.5840, 3029.4812, 3054.4724, 3069.4849, 3072.4231, 3101.5515, 3126.7327, 3166.6106, 3171.6748, 3183.6196, 3199.6411, 3231.5630, 3243.6301, 3245.6216, 3247.5737, 3254.6448, 3262.7324, 3267.7256, 3278.7546, 3321.5828, 3333.5762, 3334.6729, 3335.6787, 3338.7097, 3347.4907, 3395.6821, 3412.6426, 3428.5991, 3435.7024, 3487.8022, 3490.6885, 3534.6365, 3548.6917, 3551.6768, 3552.6902, 3601.6826, 3616.7095, 3624.6824, 3645.7827, 3651.7559, 3655.7354, 3667.7646, 3697.8025, 3716.7253, 3762.8396, 3929.9304, 4040.7656, 4057.9707, 4071.9524, 4092.8394, 4160.9473, 4174.9575, 4193.9395, 4253.8755, 4415.9736, 4465.1670, 4845.0845

10. F1MFI7 **Mass:** 57809 **Score:** 24 **Expect:** 1.1e+002 **Matches:** 10

Uncharacterized protein OS=Bos taurus GN=SYBU PE=4 SV=2

Observed	Mr(expt)	Mr(calc)	ppm	Start		End	Miss	Peptide
1248.6750	1247.6677	1247.6366	25.0	68	-	77	1	R.EKDLLAMLCR.N
1381.7728	1380.7655	1380.7401	18.4	225	-	235	1	K.YFVDINIQNKK.L
1862.9679	1861.9606	1861.9931	-17.45	203	-	218	2	K.QLKQVIETMRSSLADK.D + Oxidation (M)
2211.0654	2210.0581	2210.0577	0.21	46	-	67	2	R.GPHGRSNGASSYKSANSPPSPR.E
2542.2795	2541.2722	2541.2104	24.3	176	-	195	2	R.MREDWIEEECHRVEAQLALK.E
2705.3142	2704.3069	2704.2729	12.6	78	-	103	0	R.NQLSPVNIHPSYAPSSPSSSNSGSYK.G
3231.5630	3230.5557	3230.5237	9.91	114	-	141	1	R.SGRYMSCGENHGVKPPNPEQYLTPLQQK.E + Oxidation (M)
3278.7546	3277.7473	3277.7652	-5.46	472	-	500	1	R.YWSRSFLVDLLAVGAPVVPTVLWAFSTQR.G
3762.8396	3761.8323	3761.7772	14.6	235	-	266	2	K.KLESLLQSMEMAHSGLLRDELCLDFPCESPEK.G
4160.9473	4159.9400	4159.9644	-5.86	3	-	43	2	K.VYGAYLLSSEADFSSSSSTGSISAPEVPMSAAGSKRSSFSR.N

No match to: 701.3809, 706.2637, 708.2758, 717.3943, 719.4086, 722.4171, 734.4380, 735.4089, 736.4033, 747.4274, 756.3826, 759.3994, 772.4160, 779.4522, 795.4493, 806.4573, 831.4625, 832.3028, 834.3088, 842.4966, 876.2747, 878.2808, 883.4713, 888.4968, 890.4931, 891.4778, 900.5316, 910.4130, 918.5359, 929.4340, 936.4846, 937.5071, 938.5070, 949.5414, 967.4175, 969.4572, 972.4759, 974.4473, 976.4465, 978.5344, 983.6115, 988.4531, 990.4647, 993.4956, 1000.5594, 1007.5680, 1019.5631, 1021.5624, 1031.5641, 1034.5791, 1036.5952, 1044.5217, 1046.5728, 1051.5135, 1056.5387, 1057.6144, 1060.5447, 1074.5956, 1077.5199, 1087.5442, 1091.5437,

1113.5406, 1122.5580, 1123.5852, 1132.5316, 1145.6403, 1161.6290, 1168.6013, 1171.5769, 1172.5704, 1178.6254, 1180.6395, 1181.6516, 1185.6835, 1198.7111, 1216.6992, 1234.6646, 1254.6965, 1255.6359, 1273.6595, 1278.6974, 1281.6390, 1284.6533, 1301.6256, 1304.6489, 1314.7235, 1315.6904, 1325.6581, 1336.7290, 1338.6799, 1342.6847, 1349.6997, 1351.6993, 1354.6641, 1361.6997, 1368.6772, 1370.6589, 1372.6199, 1382.6112, 1383.7378, 1386.7084, 1388.7888, 1390.7301, 1397.7515, 1425.7671, 1439.7701, 1442.8314, 1457.7517, 1467.7510, 1473.8167, 1488.7367, 1497.8859, 1500.7335, 1513.7731, 1516.7435, 1528.7101, 1556.8929, 1559.8439, 1562.8004, 1563.8088, 1578.7965, 1580.8445, 1592.8094, 1601.9139, 1616.8481, 1623.8562, 1627.7197, 1628.7129, 1628.8722, 1634.8071, 1638.8757, 1642.7318, 1645.7701, 1647.8682, 1650.8612, 1673.8375, 1679.9817, 1680.8936, 1681.8372, 1696.8403, 1706.8660, 1715.8601, 1717.9037, 1730.8719, 1742.0088, 1758.9255, 1769.0105, 1771.0138, 1774.9404, 1783.9508, 1786.9517, 1787.9071, 1790.9086, 1835.8361, 1837.8712, 1928.0435, 1940.0333, 1954.0796, 1961.0397, 1970.0139, 1982.0297, 1984.0314, 2023.0551, 2027.1292, 2032.0228, 2061.0330, 2105.0796, 2144.0679, 2170.0872, 2179.0752, 2215.0876, 2239.1650, 2242.1501, 2246.2151, 2251.1675, 2252.1614, 2255.1719, 2273.1008, 2289.1030, 2293.1750, 2304.2087, 2316.2031, 2343.2134, 2354.0020, 2391.2788, 2475.2476, 2491.2402, 2507.1975, 2514.2664, 2550.2063, 2558.3499, 2587.3516, 2605.2615, 2646.9883, 2661.0090, 2675.0190, 2681.3855, 2698.3696, 2707.3074, 2714.3647, 2717.3394, 2719.3167, 2722.3188, 2763.3738, 2792.2446, 2826.3545, 2843.4670, 2846.4014, 2847.4082, 2872.3359, 2884.3538, 2887.3474, 2889.3481, 2892.4165, 2894.4639, 2896.4890, 2914.4551, 2987.5244, 2995.4431, 3004.5840, 3029.4812, 3054.4724, 3069.4849, 3072.4231, 3101.5515, 3126.7327, 3166.6106, 3171.6748, 3183.6196, 3199.6411, 3236.5791, 3243.6301, 3245.6216, 3247.5737, 3248.6313, 3254.6448, 3262.7324, 3267.7256, 3321.5828, 3333.5762, 3334.6729, 3335.6787, 3338.7097, 3347.4907, 3395.6821, 3412.6426, 3428.5991, 3435.7024, 3457.6995, 3487.8022, 3490.6885, 3503.8049, 3534.6365, 3548.6917, 3551.6768, 3552.6902, 3601.6826, 3616.7095, 3624.6824, 3639.7014, 3645.7827, 3651.7559, 3655.7354, 3667.7646, 3697.8025, 3716.7253, 3929.9304, 4040.7656, 4057.9707, 4071.9524, 4092.8394, 4147.9063, 4174.9575, 4193.9395, 4253.8755, 4415.9736, 4452.1865, 4465.1670, 4845.0845

11. F1MSC3 **Mass:** 264516 **Score:** 23 **Expect:** 1.3e+002 **Matches:** 34

Uncharacterized protein OS=Bos taurus GN=ACACB PE=4 SV=2

Observed	Mr(expt)	Mr(calc)	ppm	Start		End	Miss	Peptide
717.3943	716.3870	716.3969	-13.86	1338	-	1342	1	K.DRWLK.K
918.5359	917.5287	917.5408	-13.25	286	-	293	0	K.IGFPLMIK.A
1021.5624	1020.5551	1020.5352	19.5	1699	-	1708	0	R.VYLAANSGAR.I
1031.5641	1030.5568	1030.5382	18.1	1394	-	1402	0	K.MHLYLGAAR.V
1057.6144	1056.6071	1056.6080	-0.83	862	-	869	1	K.DWVQKLLR.A
1198.7111	1197.7038	1197.7193	-12.95	2121	-	2130	2	K.TIRRLDPISK.K
1368.6772	1367.6699	1367.6537	11.9	1481	-	1491	1	K.IEESVRSMVMR.Y + 2 Oxidation (M)
1388.7888	1387.7815	1387.8088	-19.67	1921	-	1932	0	R.WLLAGRPHPTLK.G
1513.7731	1512.7658	1512.7693	-2.32	1487	-	1498	2	R.SMVMRYGSRLWK.L
1563.8088	1562.8015	1562.8086	-4.55	769	-	782	1	K.MTTTLNVQESGRVK.Y
1623.8562	1622.8489	1622.8780	-17.92	1365	-	1378	0	R.HLEPALAFQLELSR.L
1647.8682	1646.8609	1646.8338	16.5	318	-	332	0	R.QVQSETPGSPVFLMK.L
1706.8660	1705.8587	1705.8900	-18.32	1138	-	1151	1	R.RGYIAYELTSLQHR.Q
1730.8719	1729.8646	1729.9039	-22.70	1732	-	1745	1	K.GIKYLYLTPQDYTR.I
1783.9508	1782.9435	1782.9087	19.5	1558	-	1573	0	K.QGPQHGMLINTPYVTK.D
1835.8361	1834.8288	1834.8461	-9.42	1717	-	1731	0	K.HMFQVAWVDPEDPHK.G
1862.9679	1861.9606	1862.0050	-23.84	302	-	317	2	K.GIRKAETAEDFPILFR.Q
1982.0297	1981.0224	1981.0415	-9.62	769	-	785	2	K.MTTTLNVQESGRVKYVR.R
2246.2151	2245.2078	2245.1565	22.8	941	-	958	1	R.EVFFMNTQSIVQLVQKYR.S + Oxidation (M)
2343.2134	2342.2061	2342.2529	-19.96	318	-	338	2	R.QVQSETPGSPVFLMKLAQRAR.H
2705.3142	2704.3069	2704.3319	-9.24	1709	-	1731	1	R.IGLAEEIKHMFQVAWVDPEDPHK.G + Oxidation (M)
2843.4670	2842.4597	2842.5262	-23.40	873	-	898	1	R.HPSLPLLELQEIMTSVSGRVPAPVEK.A + Oxidation (M)
2847.4082	2846.4009	2846.4562	-19.43	1418	-	1441	2	R.VIIRHSDLITKEASFEYLQNEGER.L
2887.3474	2886.3401	2886.4051	-22.51	1581	-	1604	1	R.FQAQSLGTTYVYDFPEMFRQALFK.M
2987.5244	2986.5171	2986.5375	-6.81	1709	-	1734	2	R.IGLAEEIKHMFQVAWVDPEDPHKGIK.Y
3054.4724	3053.4651	3053.4288	11.9	691	-	717	1	R.LNDGGLLLSCSGNSYTTYLKEEVDSYR.I
3101.5515	3100.5442	3100.5553	-3.57	1546	-	1573	1	R.SGNILFHSFGNKQGPQHGMLINTPYVTK.D + Oxidation (M)
3245.6216	3244.6143	3244.6492	-10.75	1174	-	1201	1	R.YRVVPTWGGVAYVSFLPDHCVTNPELLR.H
3490.6885	3489.6812	3489.7649	-23.98	2155	-	2184	2	K.AREDLLLPMYHQVALHFADLHDTAGRMLEK.G
3651.7559	3650.7486	3650.8212	-19.87	261	-	293	2	R.ISIPESVYNNGCVKDVDEGLEAAEKIGFPLMIK.A + Oxidation (M)
3697.8025	3696.7952	3696.7772	4.87	1461	-	1491	2	R.TDCNHIFLNFVPTVIMDPSKIEESVRSMVMR.Y + 2 Oxidation (M)
3716.7253	3715.7180	3715.7675	-13.32	1429	-	1460	1	K.EASFEYLQNEGERLLLEAMDELEVAFNNTSVR.T
3762.8396	3761.8323	3761.9008	-18.21	1615	-	1648	1	K.DILTYTELVLDPQGQLVEMNRLPGGNEVGMVAFK.M + Oxidation (M)
4147.9063	4146.8990	4146.9833	-20.32	1139	-	1173	1	R.GYIAYELTSLQHRQFPDGTCVVEFQFMLPSSHPHR.Y

No match to: 701.3809, 706.2637, 708.2758, 719.4086, 722.4171, 734.4380, 735.4089, 736.4033, 747.4274, 756.3826, 759.3994, 772.4160, 779.4522, 795.4493, 806.4573, 831.4625, 832.3028, 834.3088, 842.4966, 876.2747, 878.2808, 883.4713, 888.4968, 890.4931, 891.4778, 900.5316, 910.4130, 929.4340, 936.4846, 937.5071, 938.5070, 949.5414, 967.4175, 969.4572, 972.4759, 974.4473, 976.4465, 978.5344, 983.6115, 988.4531, 990.4647, 993.4956, 1000.5594, 1007.5680, 1019.5631, 1034.5791, 1036.5952, 1044.5217, 1046.5728, 1051.5135, 1056.5387, 1060.5447, 1074.5956, 1077.5199, 1087.5442, 1091.5437, 1113.5406, 1122.5580, 1123.5852, 1132.5316, 1145.6403, 1161.6290, 1168.6013, 1171.5769, 1172.5704, 1178.6254, 1180.6395, 1181.6516, 1185.6835, 1216.6992, 1234.6646, 1248.6750, 1254.6965, 1255.6359, 1273.6595, 1278.6974, 1281.6390, 1284.6533, 1301.6256, 1304.6489, 1314.7235, 1315.6904, 1325.6581, 1336.7290, 1338.6799, 1342.6847, 1349.6997, 1351.6993, 1354.6641, 1361.6997, 1370.6589, 1372.6199, 1381.7728, 1382.6112, 1383.7378, 1386.7084, 1390.7301, 1397.7515, 1425.7671, 1439.7701, 1442.8314, 1457.7517, 1467.7510, 1473.8167, 1488.7367, 1497.8859, 1500.7335, 1516.7435, 1528.7101, 1556.8929, 1559.8439, 1562.8004, 1578.7965, 1580.8445, 1592.8094, 1601.9139, 1616.8481, 1627.7197, 1628.7129, 1628.8722, 1634.8071, 1638.8757, 1642.7318, 1645.7701, 1650.8612, 1673.8375, 1679.9817, 1680.8936, 1681.8372, 1696.8403, 1715.8601, 1717.9037, 1742.0088, 1758.9255, 1769.0105, 1771.0138, 1774.9404, 1786.9517, 1787.9071, 1790.9086, 1837.8712, 1928.0435, 1940.0333, 1954.0796, 1961.0397, 1970.0139, 1984.0314, 2023.0551, 2027.1292, 2032.0228, 2061.0330, 2105.0796, 2144.0679, 2170.0872, 2179.0752, 2211.0654, 2215.0876, 2239.1650, 2242.1501, 2251.1675, 2252.1614, 2255.1719, 2273.1008, 2289.1030, 2293.1750, 2304.2087, 2316.2031, 2354.0020, 2391.2788, 2475.2476, 2491.2402, 2507.1975, 2514.2664, 2542.2795, 2550.2063, 2558.3499, 2587.3516, 2605.2615, 2646.9883, 2661.0090, 2675.0190, 2681.3855, 2698.3696, 2707.3074, 2714.3647, 2717.3394, 2719.3167, 2722.3188, 2763.3738, 2792.2446, 2826.3545, 2846.4014, 2872.3359, 2884.3538, 2889.3481, 2892.4165, 2894.4639, 2896.4890, 2914.4551, 2995.4431, 3004.5840, 3029.4812, 3069.4849, 3072.4231, 3126.7327, 3166.6106, 3171.6748, 3183.6196, 3199.6411, 3231.5630, 3236.5791, 3243.6301, 3247.5737, 3248.6313, 3254.6448, 3262.7324, 3267.7256, 3278.7546, 3321.5828, 3333.5762, 3334.6729, 3335.6787, 3338.7097, 3347.4907, 3395.6821, 3412.6426, 3428.5991, 3435.7024, 3457.6995, 3487.8022, 3503.8049, 3534.6365, 3548.6917, 3551.6768, 3552.6902, 3601.6826, 3616.7095, 3624.6824, 3639.7014, 3645.7827, 3655.7354, 3667.7646, 3929.9304, 4040.7656, 4057.9707, 4071.9524, 4092.8394, 4160.9473, 4174.9575, 4193.9395, 4253.8755, 4415.9736, 4452.1865, 4465.1670, 4845.0845

12. A5D9E8 **Mass:** 34517 **Score:** 22 **Expect:** 1.4e+002 **Matches:** 12

Mimecan OS=Bos taurus GN=OGN PE=1 SV=1

Observed	Mr(expt)	Mr(calc)	ppm	Start		End	Miss	Peptide
806.4573	805.4500	805.4698	-24.54	190	-	196	0	K.LTLFNAK.Y
918.5359	917.5287	917.5334	-5.19	292	-	299	1	K.RLPIGSYI.-
972.4759	971.4686	971.4712	-2.67	121	-	128	0	K.ESAYLYAR.F
1060.5447	1059.5374	1059.5349	2.39	139	-	147	0	K.DFADIPNLR.R
1168.6013	1167.5940	1167.6135	-16.70	51	-	60	2	K.YLDGKSTKEK.E
1361.6997	1360.6924	1360.6775	11.0	121	-	131	1	K.ESAYLYARFNK.I
1473.8167	1472.8094	1472.7987	7.28	135	-	147	1	K.LTAKDFADIPNLR.R
1601.9139	1600.9066	1600.8937	8.10	134	-	147	2	K.KLTAKDFADIPNLR.R

1679.9817	1678.9744	1678.9617	7.56	268 - 282	1	R.IEEIRLEGNPVILGK.H
2170.0872	2169.0799	2169.0590	9.66	148 - 166	1	R.RLDFTGNLIEDIEDGTFSK.L
3004.5840	3003.5767	3003.5818	-1.68	213 - 238	0	K.LHNLSFLYLDHNALESVPLNLPESLR.V
3457.6995	3456.6922	3456.6732	5.50	239 - 267	2	R.VIHLQFNNITSITDDTFCKANDTSYIRDR.I

No match to: 701.3809, 706.2637, 708.2758, 717.3943, 719.4086, 722.4171, 734.4380, 735.4089, 736.4033, 747.4274, 756.3826, 759.3994, 772.4160, 779.4522, 795.4493, 831.4625, 832.3028, 834.3088, 842.4966, 876.2747, 878.2808, 883.4713, 888.4968, 890.4931, 891.4778, 900.5316, 910.4130, 929.4340, 936.4846, 937.5071, 938.5070, 949.5414, 967.4175, 969.4572, 974.4473, 976.4465, 978.5344, 983.6115, 988.4531, 990.4647, 993.4956, 1000.5594, 1007.5680, 1019.5631, 1021.5624, 1031.5641, 1034.5791, 1036.5952, 1044.5217, 1046.5728, 1051.5135, 1056.5387, 1057.6144, 1074.5956, 1077.5199, 1087.5442, 1091.5437, 1113.5406, 1122.5580, 1123.5852, 1132.5316, 1145.6403, 1161.6290, 1171.5769, 1172.5704, 1178.6254, 1180.6395, 1181.6516, 1185.6835, 1198.7111, 1216.6992, 1234.6646, 1248.6750, 1254.6965, 1255.6359, 1273.6595, 1278.6974, 1281.6390, 1284.6533, 1301.6256, 1304.6489, 1314.7235, 1315.6904, 1325.6581, 1336.7290, 1338.6799, 1342.6847, 1349.6997, 1351.6993, 1354.6641, 1368.6772, 1370.6589, 1372.6199, 1381.7728, 1382.6112, 1383.7378, 1386.7084, 1388.7888, 1390.7301, 1397.7515, 1425.7671, 1439.7701, 1442.8314, 1457.7517, 1467.7510, 1488.7367, 1497.8859, 1500.7335, 1513.7731, 1516.7435, 1528.7101, 1556.8929, 1559.8439, 1562.8004, 1563.8088, 1578.7965, 1580.8445, 1592.8094, 1616.8481, 1623.8562, 1627.7197, 1628.7129, 1628.8722, 1634.8071, 1638.8757, 1642.7318, 1645.7701, 1647.8682, 1650.8612, 1673.8375, 1680.8936, 1681.8372, 1696.8403, 1706.8660, 1715.8601, 1717.9037, 1730.8719, 1742.0088, 1758.9255, 1769.0105, 1771.0138, 1774.9404, 1783.9508, 1786.9517, 1787.9071, 1790.9086, 1835.8361, 1837.8712, 1862.9679, 1928.0435, 1940.0333, 1954.0796, 1961.0397, 1970.0139, 1982.0297, 1984.0314, 2023.0551, 2027.1292, 2032.0228, 2061.0330, 2105.0796, 2144.0679, 2179.0752, 2211.0654, 2215.0876, 2239.1650, 2242.1501, 2246.2151, 2251.1675, 2252.1614, 2255.1719, 2273.1008, 2289.1030, 2293.1750, 2304.2087, 2316.2031, 2343.2134, 2354.0020, 2391.2788, 2475.2476, 2491.2402, 2507.1975, 2514.2664, 2542.2795, 2550.2063, 2558.3499, 2587.3516, 2605.2615, 2646.9883, 2661.0090, 2675.0190, 2681.3855, 2698.3696, 2705.3142, 2707.3074, 2714.3647, 2717.3394, 2719.3167, 2722.3188, 2763.3738, 2792.2446, 2826.3545, 2843.4670, 2846.4014, 2847.4082, 2872.3359, 2884.3538, 2887.3474, 2889.3481, 2892.4165, 2894.4639, 2896.4890, 2914.4551, 2987.5244, 2995.4431, 3029.4812, 3054.4724, 3069.4849, 3072.4231, 3101.5515, 3126.7327, 3166.6106, 3171.6748, 3183.6196, 3199.6411, 3231.5630, 3236.5791, 3243.6301, 3245.6216, 3247.5737, 3248.6313, 3254.6448, 3262.7324, 3267.7256, 3278.7546, 3321.5828, 3333.5762, 3334.6729, 3335.6787, 3338.7097, 3347.4907, 3395.6821, 3412.6426, 3428.5991, 3435.7024, 3487.8022, 3490.6885, 3503.8049, 3534.6365, 3548.6917, 3551.6768, 3552.6902, 3601.6826, 3616.7095, 3624.6824, 3639.7014, 3645.7827, 3651.7559, 3655.7354, 3667.7646, 3697.8025, 3716.7253, 3762.8396, 3929.9304, 4040.7656, 4057.9707, 4071.9524, 4092.8394, 4147.9063, 4160.9473, 4174.9575, 4193.9395, 4253.8755, 4415.9736, 4452.1865, 4465.1670, 4845.0845

13. F1N6I7 **Mass:** 76622 **Score:** 22 **Expect:** 1.4e+002 **Matches:** 18

Versican core protein OS=Bos taurus GN=VCAN PE=1 SV=2

Observed	Mr(expt)	Mr(calc)	ppm	Start - End	Miss	Peptide
747.4274	746.4202	746.4360	-21.26	1 - 6	0	-.MLINIK.S + Oxidation (M)
779.4522	778.4449	778.4450	-0.06	307 - 313	0	R.HPVTVAR.A
900.5316	899.5243	899.5341	-10.85	209 - 215	1	R.YPIRVPR.E
936.4846	935.4773	935.4825	-5.54	94 - 101	1	K.IGQDYKGR.V
993.4956	992.4883	992.4689	19.6	643 - 649	1	K.HDHRWSR.R
1007.5680	1006.5607	1006.5447	15.9	575 - 582	0	R.YEINSLIR.Y
1087.5442	1086.5369	1086.5604	-21.63	314 - 324	0	R.AQCGGGLLGVR.T
1314.7235	1313.7162	1313.7092	5.37	278 - 289	0	R.LATVGELQAAWR.N
1730.8719	1729.8646	1729.8457	10.9	157 - 171	1	R.AATSRYTLNFEMAQK.A
1982.0297	1981.0224	1980.9827	20.1	102 - 120	0	R.VSVPTHPEDVGDASLTMVK.L
2023.0551	2022.0478	2022.0404	3.68	593 - 609	2	R.HLPTIRCLGNGRWAMPK.I + Oxidation (M)
2211.0654	2210.0581	2210.1002	-19.02	100 - 120	1	K.GRVSVPTHPEDVGDASLTMVK.L + Oxidation (M)
2215.0876	2214.0803	2214.1004	-9.08	295 - 313	1	R.CDYGWLLDASVRHPVTVAR.A
2304.2087	2303.2014	2303.2233	-9.52	81 - 101	2	K.ETTVLVAQNGNIKIGQDYKGR.V
2475.2476	2474.2403	2474.2415	-0.46	273 - 294	2	K.TQDARLATVGELQAAWRNGFDR.C
2507.1975	2506.1902	2506.1488	16.5	479 - 498	1	R.VGHDYQWIGLNDKMFEHDFR.W
3166.6106	3165.6033	3165.5627	12.8	40 - 66	1	K.VNLPCHFSTMPTLPPSYNTTSEFLRIK.W + Oxidation (M)
4092.8394	4091.8321	4091.8630	-7.54	172 - 208	0	K.ACVDIGAVIATPEQLHAAYEDGFEQCDAGWLSDQTVR.Y

No match to: 701.3809, 706.2637, 708.2758, 717.3943, 719.4086, 722.4171, 734.4380, 735.4089, 736.4033, 756.3826, 759.3994, 772.4160, 795.4493, 806.4573, 831.4625, 832.3028, 834.3088, 842.4966, 876.2747, 878.2808, 883.4713, 888.4968, 890.4931, 891.4778, 910.4130, 918.5359, 929.4340, 937.5071, 938.5070, 949.5414, 967.4175, 969.4572, 972.4759, 974.4473, 976.4465, 978.5344, 983.6115, 988.4531, 990.4647, 1000.5594, 1019.5631, 1021.5624, 1031.5641, 1034.5791, 1036.5952, 1044.5217, 1046.5728, 1051.5135, 1056.5387, 1057.6144, 1060.5447, 1074.5956, 1077.5199, 1091.5437, 1113.5406, 1122.5580, 1123.5852, 1132.5316, 1145.6403, 1161.6290, 1168.6013, 1171.5769, 1172.5704, 1178.6254, 1180.6395, 1181.6516, 1185.6835, 1198.7111, 1216.6992, 1234.6646, 1248.6750, 1254.6965, 1255.6359, 1273.6595, 1278.6974, 1281.6390, 1284.6533, 1301.6256, 1304.6489, 1315.6904, 1325.6581, 1336.7290, 1338.6799, 1342.6847, 1349.6997, 1351.6993, 1354.6641, 1361.6997, 1368.6772, 1370.6589, 1372.6199, 1381.7728, 1382.6112, 1383.7378, 1386.7084, 1388.7888, 1390.7301, 1397.7515, 1425.7671, 1439.7701, 1442.8314, 1457.7517, 1467.7510, 1473.8167, 1488.7367, 1497.8859, 1500.7335, 1513.7731, 1516.7435, 1528.7101, 1556.8929, 1559.8439, 1562.8004, 1563.8088, 1578.7965, 1580.8445, 1592.8094, 1601.9139, 1616.8481, 1623.8562, 1627.7197, 1628.7129, 1628.8722, 1634.8071, 1638.8757, 1642.7318, 1645.7701, 1647.8682, 1650.8612, 1673.8375, 1679.9817, 1680.8936, 1681.8372, 1696.8403, 1706.8660, 1715.8601, 1717.9037, 1742.0088, 1758.9255, 1769.0105, 1771.0138, 1774.9404, 1783.9508, 1786.9517, 1787.9071, 1790.9086, 1835.8361, 1837.8712, 1862.9679, 1928.0435, 1940.0333, 1954.0796, 1961.0397, 1970.0139, 1984.0314, 2027.1292, 2032.0228, 2061.0330, 2105.0796, 2144.0679, 2170.0872, 2179.0752, 2239.1650, 2242.1501, 2246.2151, 2251.1675, 2252.1614, 2255.1719, 2273.1008, 2289.1030, 2293.1750, 2316.2031, 2343.2134, 2354.0020, 2391.2788, 2491.2402, 2514.2664, 2542.2795, 2550.2063, 2558.3499, 2587.3516, 2605.2615, 2646.9883, 2661.0090, 2675.0190, 2681.3855, 2698.3696, 2705.3142, 2707.3074, 2714.3647, 2717.3394, 2719.3167, 2722.3188, 2763.3738, 2792.2446, 2826.3545, 2843.4670, 2846.4014, 2847.4082, 2872.3359, 2884.3538, 2887.3474, 2889.3481, 2892.4165, 2894.4639, 2896.4890, 2914.4551, 2987.5244, 2995.4431, 3004.5840, 3029.4812, 3054.4724, 3069.4849, 3072.4231, 3101.5515, 3126.7327, 3171.6748, 3183.6196, 3199.6411, 3231.5630, 3236.5791, 3243.6301, 3245.6216, 3247.5737, 3248.6313, 3254.6448, 3262.7324, 3267.7256, 3278.7546, 3321.5828, 3333.5762, 3334.6729, 3335.6787, 3338.7097, 3347.4907, 3395.6821, 3412.6426, 3428.5991, 3435.7024, 3457.6995, 3487.8022, 3490.6885, 3503.8049, 3534.6365, 3548.6917, 3551.6768, 3552.6902, 3601.6826, 3616.7095, 3624.6824, 3639.7014, 3645.7827, 3651.7559, 3655.7354, 3667.7646, 3697.8025, 3716.7253, 3762.8396, 3929.9304, 4040.7656, 4057.9707, 4071.9524, 4147.9063, 4160.9473, 4174.9575, 4193.9395, 4253.8755, 4415.9736, 4452.1865, 4465.1670, 4845.0845

14. Q0VC60 **Mass:** 56014 **Score:** 22 **Expect:** 1.5e+002 **Matches:** 16

Dihydrouridine synthase 2-like, SMM1 homolog (S. cerevisiae) OS=Bos taurus GN=DUS2L PE=2 SV=1

Observed	Mr(expt)	Mr(calc)	ppm	Start - End	Miss	Peptide
759.3994	758.3921	758.4035	-15.03	326 - 331	1	R.ELDARR.A
1036.5952	1035.5879	1035.5647	22.4	355 - 362	2	K.MAIKFDRR.A
1074.5956	1073.5883	1073.5764	11.1	147 - 155	1	K.GTRRPVTCK.I
1255.6359	1254.6286	1254.6244	3.34	410 - 419	1	K.YQSTLWDKSK.K
1314.7235	1313.7162	1313.7125	2.82	421 - 432	0	K.LAEQAAAIVCLR.S
1368.6772	1367.6699	1367.6429	19.7	453 - 464	1	K.REALDQDPGDPR.T
1383.7378	1382.7305	1382.7194	8.05	410 - 420	2	K.YQSTLWDKSKK.L
1442.8314	1441.8241	1441.8075	11.5	420 - 432	1	K.KLAEQAAAIVCLR.S
2255.1719	2254.1646	2254.1328	14.1	334 - 354	1	K.LLARSPEEAEEPAEDTSGIIK.M
2698.3696	2697.3623	2697.3894	-10.05	334 - 358	2	K.LLARSPEEAEEPAEDTSGIIKMAIK.F
2714.3647	2713.3574	2713.3843	-9.92	334 - 358	2	K.LLARSPEEAEEPAEDTSGIIKMAIK.F + Oxidation (M)
2894.4639	2893.4566	2893.4393	6.00	55 - 79	2	R.VVNEALSTVDFVAPDDRVVFRTCER.E
3126.7327	3125.7254	3125.6664	18.9	2 - 28	2	M.TVNNLSLCYHNKLILAPMVRVGTLPMR.L + Oxidation (M)
3651.7559	3650.7486	3650.7266	6.03	102 - 136	2	R.LVENDVAGIDVNMGCPKEYSTKGGMGAALLSDPDK.I
3667.7646	3666.7573	3666.7215	9.76	102 - 136	2	R.LVENDVAGIDVNMGCPKEYSTKGGMGAALLSDPDK.I + Oxidation (M)
4452.1865	4451.1792	4451.2655	-19.39	202 - 243	2	K.AIAETLSIPVIANGGSHDHIQGYLDIEDFRRATAASSVMVAR.A

No match to: 701.3809, 706.2637, 708.2758, 717.3943, 719.4086, 722.4171, 734.4380, 735.4089, 736.4033, 747.4274, 756.3826, 772.4160, 779.4522, 795.4493, 806.4573, 831.4625, 832.3028, 834.3088, 842.4966, 876.2747, 878.2808, 883.4713, 888.4968, 890.4931, 891.4778, 900.5316, 910.4130, 918.5359, 929.4340, 936.4846, 937.5071, 938.5070, 949.5414, 967.4175, 969.4572, 972.4759, 974.4473, 976.4465, 978.5344, 983.6115, 988.4531, 990.4647, 993.4956, 1000.5594, 1007.5680, 1019.5631, 1021.5624, 1031.5641, 1034.5791, 1044.5217, 1046.5728, 1051.5135, 1056.5387, 1057.6144, 1060.5447, 1077.5199, 1087.5442, 1091.5437, 1113.5406, 1122.5580, 1123.5852, 1132.5316, 1145.6403, 1161.6290, 1168.6013, 1171.5769, 1172.5704, 1178.6254, 1180.6395, 1181.6516, 1185.6835, 1198.7111, 1216.6992, 1234.6646, 1248.6750, 1254.6965, 1273.6595, 1278.6974, 1281.6390, 1284.6533, 1301.6256, 1304.6489, 1315.6904, 1325.6581, 1336.7290, 1338.6799, 1342.6847, 1349.6997, 1351.6993, 1354.6641, 1361.6997, 1370.6589, 1372.6199, 1381.7728, 1382.6112, 1386.7084, 1388.7888, 1390.7301, 1397.7515, 1425.7671, 1439.7701, 1457.7517, 1467.7510, 1473.8167, 1488.7367, 1497.8859, 1500.7335, 1513.7731, 1516.7435, 1528.7101, 1556.8929, 1559.8439, 1562.8004, 1563.8088, 1578.7965, 1580.8445, 1592.8094, 1601.9139, 1616.8481, 1623.8562, 1627.7197, 1628.7129, 1628.8722, 1634.8071, 1638.8757, 1642.7318, 1645.7701, 1647.8682, 1650.8612, 1673.8375, 1679.9817, 1680.8936, 1681.8372, 1696.8403, 1706.8660, 1715.8601, 1717.9037, 1730.8719, 1742.0088, 1758.9255, 1769.0105, 1771.0138, 1774.9404, 1783.9508, 1786.9517, 1787.9071, 1790.9086, 1835.8361, 1837.8712, 1862.9679, 1928.0435, 1940.0333, 1954.0796, 1961.0397, 1970.0139, 1982.0297, 1984.0314, 2023.0551, 2027.1292, 2032.0228, 2061.0330, 2105.0796, 2144.0679, 2170.0872, 2179.0752, 2211.0654, 2215.0876, 2239.1650, 2242.1501, 2246.2151, 2251.1675, 2252.1614, 2273.1008, 2289.1030, 2293.1750, 2304.2087, 2316.2031, 2343.2134, 2354.0020, 2391.2788, 2475.2476, 2491.2402, 2507.1975, 2514.2664, 2542.2795, 2550.2063, 2558.3499, 2587.3516, 2605.2615, 2646.9883, 2661.0090, 2675.0190, 2681.3855, 2705.3142, 2707.3074, 2717.3394, 2719.3167, 2722.3188, 2763.3738, 2792.2446, 2826.3545, 2843.4670, 2846.4014, 2847.4082, 2872.3359, 2884.3538, 2887.3474, 2889.3481, 2892.4165, 2896.4890, 2914.4551, 2987.5244, 2995.4431, 3004.5840, 3029.4812, 3054.4724, 3069.4849, 3072.4231, 3101.5515, 3166.6106, 3171.6748, 3183.6196, 3199.6411, 3231.5630, 3236.5791, 3243.6301, 3245.6216, 3247.5737, 3248.6313, 3254.6448, 3262.7324, 3267.7256, 3278.7546, 3321.5828, 3333.5762, 3334.6729, 3335.6787, 3338.7097, 3347.4907, 3395.6821, 3412.6426, 3428.5991, 3435.7024, 3457.6995, 3487.8022, 3490.6885, 3503.8049, 3534.6365, 3548.6917, 3551.6768, 3552.6902, 3601.6826, 3616.7095, 3624.6824, 3639.7014, 3645.7827, 3655.7354, 3697.8025, 3716.7253, 3762.8396, 3929.9304, 4040.7656, 4057.9707, 4071.9524, 4092.8394, 4147.9063, 4160.9473, 4174.9575, 4193.9395, 4253.8755, 4415.9736, 4465.1670, 4845.0845

15. Q32LF3 **Mass:** 24094 **Score:** 22 **Expect:** 1.5e+002 **Matches:** 6

Similar to type 1 protein phosphatase inhibitor OS=Bos taurus GN=LOC520057 PE=2 SV=1

Observed	Mr(expt)	Mr(calc)	ppm	Start	End	Miss	Peptide
1336.7290	1335.7217	1335.6929	21.6	1 -	12	1	-.MSASTSSRRPIK.G + Oxidation (M)
1578.7965	1577.7892	1577.7896	-0.26	85 -	99	1	R.SAESEATALDSLTKR.L
2211.0654	2210.0581	2210.0676	-4.29	19 -	41	0	K.GSTASSVAGSAQQSGGPLQEVHR.K
3054.4724	3053.4651	3053.4901	-8.18	85 -	113	2	R.SAESEATALDSLTKRLAATHTSDSSYSVR.D
3321.5828	3320.5755	3320.5466	8.70	68 -	98	1	K.MNEPSTPQFGPQNLGERSAESEATALDSLTK.R + Oxidation (M)
3548.6917	3547.6844	3547.6927	-2.34	152 -	181	2	K.LARQLISRDLLHDYEDDNNEESSHVISHDK.T

No match to: 701.3809, 706.2637, 708.2758, 717.3943, 719.4086, 722.4171, 734.4380, 735.4089, 736.4033, 747.4274, 756.3826, 759.3994, 772.4160, 779.4522, 795.4493, 806.4573, 831.4625, 832.3028, 834.3088, 842.4966, 876.2747, 878.2808, 883.4713, 888.4968, 890.4931, 891.4778, 900.5316, 910.4130, 918.5359, 929.4340, 936.4846, 937.5071, 938.5070, 949.5414, 967.4175, 969.4572, 972.4759, 974.4473, 976.4465, 978.5344, 983.6115, 988.4531, 990.4647, 993.4956, 1000.5594, 1007.5680, 1019.5631, 1021.5624, 1031.5641, 1034.5791, 1036.5952, 1044.5217, 1046.5728, 1051.5135, 1056.5387, 1057.6144, 1060.5447, 1074.5956, 1077.5199, 1087.5442, 1091.5437, 1113.5406, 1122.5580, 1123.5852, 1132.5316, 1145.6403, 1161.6290, 1168.6013, 1171.5769, 1172.5704, 1180.6395, 1181.6516, 1185.6835, 1198.7111, 1216.6992, 1234.6646, 1248.6750, 1254.6965, 1255.6359, 1273.6595, 1278.6974, 1281.6390, 1284.6533, 1301.6256, 1304.6489, 1314.7235, 1315.6904, 1325.6581, 1338.6799, 1342.6847, 1349.6997, 1351.6993, 1354.6641, 1361.6997, 1368.6772, 1370.6589, 1372.6199, 1381.7728, 1382.6112, 1383.7378, 1386.7084, 1388.7888, 1390.7301, 1397.7515, 1425.7671, 1439.7701, 1442.8314, 1457.7517, 1467.7510, 1473.8167, 1488.7367, 1497.8859, 1500.7335, 1513.7731, 1516.7435, 1528.7101, 1556.8929, 1559.8439, 1562.8004, 1563.8088, 1580.8445, 1592.8094, 1601.9139, 1616.8481, 1623.8562, 1627.7197, 1628.7129, 1628.8722, 1634.8071, 1638.8757, 1642.7318, 1645.7701, 1647.8682, 1650.8612, 1673.8375, 1679.9817, 1680.8936, 1681.8372, 1696.8403, 1706.8660, 1715.8601, 1717.9037, 1730.8719, 1742.0088, 1758.9255, 1769.0105, 1771.0138, 1774.9404, 1783.9508, 1786.9517, 1787.9071, 1790.9086, 1835.8361, 1837.8712, 1862.9679, 1928.0435, 1940.0333, 1954.0796, 1961.0397, 1970.0139, 1982.0297, 1984.0314, 2023.0551, 2027.1292, 2032.0228, 2061.0330, 2105.0796, 2144.0679, 2170.0872, 2179.0752, 2215.0876, 2239.1650, 2242.1501, 2246.2151, 2251.1675, 2252.1614, 2255.1719, 2273.1008, 2289.1030, 2293.1750, 2304.2087, 2316.2031, 2343.2134, 2354.0020, 2391.2788, 2475.2476, 2491.2402, 2507.1975, 2514.2664, 2542.2795, 2550.2063, 2558.3499, 2587.3516, 2605.2615, 2646.9883, 2661.0090, 2675.0190, 2681.3855, 2698.3696, 2705.3142, 2707.3074, 2714.3647, 2717.3394, 2719.3167, 2722.3188, 2763.3738, 2792.2446, 2826.3545, 2843.4670, 2846.4014, 2847.4082, 2872.3359, 2884.3538, 2887.3474, 2889.3481, 2892.4165, 2894.4639, 2896.4890, 2914.4551, 2987.5244, 2995.4431, 3004.5840, 3029.4812, 3069.4849, 3072.4231, 3101.5515, 3126.7327, 3166.6106, 3171.6748, 3183.6196, 3199.6411, 3231.5630, 3236.5791, 3243.6301, 3245.6216, 3247.5737, 3248.6313, 3254.6448, 3262.7324, 3267.7256, 3278.7546, 3333.5762, 3334.6729, 3335.6787, 3338.7097, 3347.4907, 3395.6821, 3412.6426, 3428.5991, 3435.7024, 3457.6995, 3487.8022, 3490.6885, 3503.8049, 3534.6365, 3551.6768, 3552.6902, 3601.6826, 3616.7095, 3624.6824, 3639.7014, 3645.7827, 3651.7559, 3655.7354, 3667.7646, 3697.8025, 3716.7253, 3762.8396, 3929.9304, 4040.7656, 4057.9707, 4071.9524, 4092.8394, 4147.9063, 4160.9473, 4174.9575, 4193.9395, 4253.8755, 4415.9736, 4452.1865, 4465.1670, 4845.0845

16. F1MHQ4 **Mass:** 14751 **Score:** 21 **Expect:** 1.9e+002 **Matches:** 6

Fatty acid-binding protein, adipocyte OS=Bos taurus GN=FABP4 PE=1 SV=2

Observed	Mr(expt)	Mr(calc)	ppm	Start	End	Miss	Peptide
2144.0679	2143.0606	2143.0224	17.8	114 -	131	1	K.MVLECVMNGVTATRVYER.A + Oxidation (M)
2215.0876	2214.0803	2214.0595	9.39	114 -	132	2	K.MVLECVMNGVTATRVYERA.- + Oxidation (M)
2698.3696	2697.3623	2697.3288	12.4	109 -	131	2	K.LVDDKMVLECVMNGVTATRVYER.A
2714.3647	2713.3574	2713.3237	12.4	109 -	131	2	K.LVDDKMVLECVMNGVTATRVYER.A + Oxidation (M)
3126.7327	3125.7254	3125.7999	-23.82	23 -	53	2	K.EVGVGFATRKVAGMAKPTLIISLNGGVVTIK.S
3428.5991	3427.5918	3427.5853	1.89	2 -	31	2	M.CDAFVGTWKLVSSENFDDYMKEVGVGFATR.K

No match to: 701.3809, 706.2637, 708.2758, 717.3943, 719.4086, 722.4171, 734.4380, 735.4089, 736.4033, 747.4274, 756.3826, 759.3994, 772.4160, 779.4522, 795.4493, 806.4573, 831.4625, 832.3028, 834.3088, 842.4966, 876.2747, 878.2808, 883.4713, 888.4968, 890.4931, 891.4778, 900.5316, 910.4130, 918.5359, 929.4340, 936.4846, 937.5071, 938.5070, 949.5414, 967.4175, 969.4572, 972.4759, 974.4473, 976.4465, 978.5344, 983.6115, 988.4531, 990.4647, 993.4956, 1000.5594, 1007.5680, 1019.5631, 1021.5624, 1031.5641, 1034.5791, 1036.5952, 1044.5217, 1046.5728, 1051.5135, 1056.5387, 1057.6144, 1060.5447, 1074.5956, 1077.5199, 1087.5442, 1091.5437, 1113.5406, 1122.5580, 1123.5852, 1132.5316, 1145.6403, 1161.6290, 1168.6013, 1171.5769, 1172.5704, 1178.6254, 1180.6395, 1181.6516, 1185.6835, 1198.7111, 1216.6992, 1234.6646, 1248.6750, 1254.6965, 1255.6359, 1273.6595, 1278.6974, 1281.6390, 1284.6533, 1301.6256, 1304.6489, 1314.7235, 1315.6904, 1325.6581, 1336.7290, 1338.6799, 1342.6847, 1349.6997, 1351.6993, 1354.6641, 1361.6997, 1368.6772, 1370.6589, 1372.6199, 1381.7728, 1382.6112, 1383.7378, 1386.7084, 1388.7888, 1390.7301, 1397.7515, 1425.7671, 1439.7701, 1442.8314, 1457.7517, 1467.7510, 1473.8167, 1488.7367, 1497.8859, 1500.7335, 1513.7731, 1516.7435, 1528.7101, 1556.8929, 1559.8439, 1562.8004, 1563.8088, 1578.7965, 1580.8445, 1592.8094, 1601.9139, 1616.8481, 1623.8562, 1627.7197, 1628.7129, 1628.8722, 1634.8071, 1638.8757, 1642.7318, 1645.7701, 1647.8682, 1650.8612, 1673.8375, 1679.9817, 1680.8936, 1681.8372, 1696.8403, 1706.8660, 1715.8601, 1717.9037, 1730.8719, 1742.0088, 1758.9255, 1769.0105, 1771.0138, 1774.9404, 1783.9508, 1786.9517, 1787.9071, 1790.9086, 1835.8361, 1837.8712, 1862.9679, 1928.0435, 1940.0333, 1954.0796, 1961.0397, 1970.0139, 1982.0297, 1984.0314, 2023.0551, 2027.1292, 2032.0228, 2061.0330, 2105.0796, 2170.0872, 2179.0752, 2211.0654, 2239.1650, 2242.1501, 2246.2151, 2251.1675, 2252.1614, 2255.1719, 2273.1008, 2289.1030, 2293.1750, 2304.2087, 2316.2031, 2343.2134, 2354.0020, 2391.2788, 2475.2476, 2491.2402, 2507.1975, 2514.2664, 2542.2795, 2550.2063, 2558.3499, 2587.3516, 2605.2615, 2646.9883, 2661.0090, 2675.0190, 2681.3855, 2705.3142, 2707.3074, 2717.3394, 2719.3167, 2722.3188, 2763.3738, 2792.2446, 2826.3545, 2843.4670, 2846.4014, 2847.4082, 2872.3359, 2884.3538, 2887.3474, 2889.3481, 2892.4165, 2894.4639, 2896.4890, 2914.4551, 2987.5244, 2995.4431, 3004.5840, 3029.4812, 3054.4724, 3069.4849, 3072.4231, 3101.5515, 3166.6106, 3171.6748, 3183.6196, 3199.6411, 3231.5630, 3236.5791, 3243.6301, 3245.6216, 3247.5737, 3248.6313, 3254.6448, 3262.7324, 3267.7256, 3278.7546, 3321.5828, 3333.5762, 3334.6729, 3335.6787, 3338.7097, 3347.4907, 3395.6821, 3412.6426, 3435.7024, 3457.6995, 3487.8022, 3490.6885, 3503.8049, 3534.6365, 3548.6917, 3551.6768, 3552.6902, 3601.6826, 3616.7095, 3624.6824, 3639.7014, 3645.7827, 3651.7559, 3655.7354, 3667.7646, 3697.8025, 3716.7253, 3762.8396, 3929.9304, 4040.7656, 4057.9707, 4071.9524, 4092.8394, 4147.9063, 4160.9473, 4174.9575, 4193.9395, 4253.8755, 4415.9736, 4452.1865, 4465.1670, 4845.0845

17. E1BP29 **Mass:** 68878 **Score:** 21 **Expect:** 2e+002 **Matches:** 14

Protein-serine/threonine kinase OS=Bos taurus GN=GRK6 PE=3 SV=2

Observed	Mr(expt)	Mr(calc)	ppm	Start	End	Miss	Peptide
806.4573	805.4500	805.4559	-7.27	557 -	562	1	R.LFSRQR.T

1122.5580	1121.5507	1121.5573	-5.86	420 - 428	0	R.SLCTQLLCK.D
1132.5316	1131.5243	1131.5343	-8.79	434 - 445	0	R.LGCGGGGAQEVK.E
1349.6997	1348.6924	1348.7061	-10.13	348 - 360	0	R.VGTVGYMAPEVVK.N
1381.7728	1380.7655	1380.7626	2.11	184 - 194	2	K.NTFRQYRVLGK.G
1390.7301	1389.7228	1389.7180	3.47	247 - 258	0	R.FVVSLAYAYETK.D
1562.8004	1561.7931	1561.8287	-22.75	346 - 360	1	K.GRVGTVGYMAPEVVK.N
1578.7965	1577.7892	1577.8236	-21.77	346 - 360	1	K.GRVGTVGYMAPEVVK.N + Oxidation (M)
1717.9037	1716.8964	1716.9080	-6.72	228 - 242	2	R.KGEAMALNEKQILEK.V + Oxidation (M)
2514.2664	2513.2591	2513.2519	2.89	32 - 51	1	R.QMLQFPHISQCEELRLSLER.D
3248.6313	3247.6240	3247.6005	7.23	402 - 428	2	R.LVKEVPEEYSEHFSPQARSLCTQLLCK.D
3490.6885	3489.6812	3489.6996	-5.26	259 - 289	1	K.DALCLVLTLMNGGDLKFHIYHMGQAGFPEAR.A + Oxidation (M)
3762.8396	3761.8323	3761.9161	-22.26	457 - 489	2	K.RLGAGMLEPPFKPDPQAIYCKDVLDIEQFSTVK.G
4452.1865	4451.1792	4451.0767	23.0	504 - 542	0	K.FATGSVPIPWQNEMVETECFQELNVFGLDGSVPPDLDWK.G

No match to: 701.3809, 706.2637, 708.2758, 717.3943, 719.4086, 722.4171, 734.4380, 735.4089, 736.4033, 747.4274, 756.3826, 759.3994, 772.4160, 779.4522, 795.4493, 831.4625, 832.3028, 834.3088, 842.4966, 876.2747, 878.2808, 883.4713, 888.4968, 890.4931, 891.4778, 900.5316, 910.4130, 918.5359, 929.4340, 936.4846, 937.5071, 938.5070, 949.5414, 967.4175, 969.4572, 972.4759, 974.4473, 976.4465, 978.5344, 983.6115, 988.4531, 990.4647, 993.4956, 1000.5594, 1007.5680, 1019.5631, 1021.5624, 1031.5641, 1034.5791, 1036.5952, 1044.5217, 1046.5728, 1051.5135, 1056.5387, 1057.6144, 1060.5447, 1074.5956, 1077.5199, 1087.5442, 1091.5437, 1113.5406, 1123.5852, 1145.6403, 1161.6290, 1168.6013, 1171.5769, 1172.5704, 1178.6254, 1180.6395, 1181.6516, 1185.6835, 1198.7111, 1216.6992, 1234.6646, 1248.6750, 1254.6965, 1255.6359, 1273.6595, 1278.6974, 1281.6390, 1284.6533, 1301.6256, 1304.6489, 1314.7235, 1315.6904, 1325.6581, 1336.7290, 1338.6799, 1342.6847, 1351.6993, 1354.6641, 1361.6997, 1368.6772, 1370.6589, 1372.6199, 1382.6112, 1383.7378, 1386.7084, 1388.7888, 1397.7515, 1425.7671, 1439.7701, 1442.8314, 1457.7517, 1467.7510, 1473.8167, 1488.7367, 1497.8859, 1500.7335, 1513.7731, 1516.7435, 1528.7101, 1556.8929, 1559.8439, 1563.8088, 1580.8445, 1592.8094, 1601.9139, 1616.8481, 1623.8562, 1627.7197, 1628.7129, 1628.8722, 1634.8071, 1638.8757, 1642.7318, 1645.7701, 1647.8682, 1650.8612, 1673.8375, 1679.9817, 1680.8936, 1681.8372, 1696.8403, 1706.8660, 1715.8601, 1730.8719, 1742.0088, 1758.9255, 1769.0105, 1771.0138, 1774.9404, 1783.9508, 1786.9517, 1787.9071, 1790.9086, 1835.8361, 1837.8712, 1862.9679, 1928.0435, 1940.0333, 1954.0796, 1961.0397, 1970.0139, 1982.0297, 1984.0314, 2023.0551, 2027.1292, 2032.0228, 2061.0330, 2105.0796, 2144.0679, 2170.0872, 2179.0752, 2211.0654, 2215.0876, 2239.1650, 2242.1501, 2246.2151, 2251.1675, 2252.1614, 2255.1719, 2273.1008, 2289.1030, 2293.1750, 2304.2087, 2316.2031, 2343.2134, 2354.0020, 2391.2788, 2475.2476, 2491.2402, 2507.1975, 2542.2795, 2550.2063, 2558.3499, 2587.3516, 2605.2615, 2646.9883, 2661.0090, 2675.0190, 2681.3855, 2698.3696, 2705.3142, 2707.3074, 2714.3647, 2717.3394, 2719.3167, 2722.3188, 2763.3738, 2792.2446, 2826.3545, 2843.4670, 2846.4014, 2847.4082, 2872.3359, 2884.3538, 2887.3474, 2889.3481, 2892.4165, 2894.4639, 2896.4890, 2914.4551, 2987.5244, 2995.4431, 3004.5840, 3029.4812, 3054.4724, 3069.4849, 3072.4231, 3101.5515, 3126.7327, 3166.6106, 3171.6748, 3183.6196, 3199.6411, 3231.5630, 3236.5791, 3243.6301, 3245.6216, 3247.5737, 3254.6448, 3262.7324, 3267.7256, 3278.7546, 3321.5828, 3333.5762, 3334.6729, 3335.6787, 3338.7097, 3347.4907, 3395.6821, 3412.6426, 3428.5991, 3435.7024, 3457.6995, 3487.8022, 3503.8049, 3534.6365, 3548.6917, 3551.6768, 3552.6902, 3601.6826, 3616.7095, 3624.6824, 3639.7014, 3645.7827, 3651.7559, 3655.7354, 3667.7646, 3697.8025, 3716.7253, 3929.9304, 4040.7656, 4057.9707, 4071.9524, 4092.8394, 4147.9063, 4160.9473, 4174.9575, 4193.9395, 4253.8755, 4415.9736, 4465.1670, 4845.0845

18. Q32L62 **Mass:** 51390 **Score:** 21 **Expect:** 2e+002 **Matches:** 8

Hemogen OS=Bos taurus GN=HEMGN PE=2 SV=1

Observed	Mr(expt)	Mr(calc)	ppm	Start - End	Miss	Peptide
701.3809	700.3736	700.3755	-2.72	374 - 379	1	K.DKDVPK.E
1706.8660	1705.8587	1705.8266	18.8	85 - 98	1	K.VELPSQLEKEMMEK.A + Oxidation (M)
2550.2063	2549.1990	2549.2175	-7.24	78 - 98	2	R.QQNTEMKVELPSQLEKEMMEK.A
3248.6313	3247.6240	3247.5448	24.4	420 - 447	0	K.AEEPEIIEIPNVPQESNPENDIYSYVLF.-
3490.6885	3489.6812	3489.7025	-6.11	6 - 35	1	K.DQSLSKLHQTPDHHQEESHVPEVIGTWSLR.N
3651.7559	3650.7486	3650.6604	24.2	217 - 248	1	K.TSEDTTDLEECSLEAHPKPDMPKASTLETYQK.R
3716.7253	3715.7180	3715.7780	-16.15	416 - 447	1	K.ENPKAEEPEIIEIPNVPQESNPENDIYSYVLF.-
4160.9473	4159.9400	4159.9235	3.97	203 - 239	2	K.TCQEDIAAKVLSSKTSEDTTDLEECSLEAHPKPDMPK.A

No match to: 706.2637, 708.2758, 717.3943, 719.4086, 722.4171, 734.4380, 735.4089, 736.4033, 747.4274, 756.3826, 759.3994, 772.4160, 779.4522, 795.4493, 806.4573, 831.4625, 832.3028, 834.3088, 842.4966, 876.2747, 878.2808, 883.4713, 888.4968, 890.4931, 891.4778, 900.5316, 910.4130, 918.5359, 929.4340, 936.4846, 937.5071, 938.5070, 949.5414, 967.4175, 969.4572, 972.4759, 974.4473, 976.4465, 978.5344, 983.6115, 988.4531, 990.4647, 993.4956, 1000.5594, 1007.5680, 1019.5631, 1021.5624, 1031.5641, 1034.5791, 1036.5952, 1044.5217, 1046.5728, 1051.5135, 1056.5387, 1057.6144, 1060.5447, 1074.5956, 1077.5199, 1087.5442, 1091.5437, 1113.5406, 1122.5580, 1123.5852, 1132.5316, 1145.6403, 1161.6290, 1168.6013, 1171.5769, 1172.5704, 1178.6254, 1180.6395, 1181.6516, 1185.6835, 1198.7111, 1216.6992, 1234.6646, 1248.6750, 1254.6965, 1255.6359, 1273.6595, 1278.6974, 1281.6390, 1284.6533, 1301.6256, 1304.6489, 1314.7235, 1315.6904, 1325.6581, 1336.7290, 1338.6799, 1342.6847, 1349.6997, 1351.6993, 1354.6641, 1361.6997, 1368.6772, 1370.6589, 1372.6199, 1381.7728, 1382.6112, 1383.7378, 1386.7084, 1388.7888, 1390.7301, 1397.7515, 1425.7671, 1439.7701, 1442.8314, 1457.7517, 1467.7510, 1473.8167, 1488.7367, 1497.8859, 1500.7335, 1513.7731, 1516.7435, 1528.7101, 1556.8929, 1559.8439, 1562.8004, 1563.8088, 1578.7965, 1580.8445, 1592.8094, 1601.9139, 1616.8481, 1623.8562, 1627.7197, 1628.7129, 1628.8722, 1634.8071, 1638.8757, 1642.7318, 1645.7701, 1647.8682, 1650.8612, 1673.8375, 1679.9817, 1680.8936, 1681.8372, 1696.8403, 1715.8601, 1717.9037, 1730.8719, 1742.0088, 1758.9255, 1769.0105, 1771.0138, 1774.9404, 1783.9508, 1786.9517, 1787.9071, 1790.9086, 1835.8361, 1837.8712, 1862.9679, 1928.0435, 1940.0333, 1954.0796, 1961.0397, 1970.0139, 1982.0297, 1984.0314, 2023.0551, 2027.1292, 2032.0228, 2061.0330, 2105.0796, 2144.0679, 2170.0872, 2179.0752, 2211.0654, 2215.0876, 2239.1650, 2242.1501, 2246.2151, 2251.1675, 2252.1614, 2255.1719, 2273.1008, 2289.1030, 2293.1750, 2304.2087, 2316.2031, 2343.2134, 2354.0020, 2391.2788, 2475.2476, 2491.2402, 2507.1975, 2514.2664, 2542.2795, 2558.3499, 2587.3516, 2605.2615, 2646.9883, 2661.0090, 2675.0190, 2681.3855, 2698.3696, 2705.3142, 2707.3074, 2714.3647, 2717.3394, 2719.3167, 2722.3188, 2763.3738, 2792.2446, 2826.3545, 2843.4670, 2846.4014, 2847.4082, 2872.3359, 2884.3538, 2887.3474, 2889.3481, 2892.4165, 2894.4639, 2896.4890, 2914.4551, 2987.5244, 2995.4431, 3004.5840, 3029.4812, 3054.4724, 3069.4849, 3072.4231, 3101.5515, 3126.7327, 3166.6106, 3171.6748, 3183.6196, 3199.6411, 3231.5630, 3236.5791, 3243.6301, 3245.6216, 3247.5737, 3254.6448, 3262.7324, 3267.7256, 3278.7546, 3321.5828, 3333.5762, 3334.6729, 3335.6787, 3338.7097, 3347.4907, 3395.6821, 3412.6426, 3428.5991, 3435.7024, 3457.6995, 3487.8022, 3503.8049, 3534.6365, 3548.6917, 3551.6768, 3552.6902, 3601.6826, 3616.7095, 3624.6824, 3639.7014, 3645.7827, 3655.7354, 3667.7646, 3697.8025, 3762.8396, 3929.9304, 4040.7656, 4057.9707, 4071.9524, 4092.8394, 4147.9063, 4174.9575, 4193.9395, 4253.8755, 4415.9736, 4452.1865, 4465.1670, 4845.0845

19. G3N088 **Mass:** 40915 **Score:** 21 **Expect:** 2e+002 **Matches:** 13

Mimecan OS=Bos taurus GN=OGN PE=1 SV=1

Observed	Mr(expt)	Mr(calc)	ppm	Start - End	Miss	Peptide
735.4089	734.4016	734.3963	7.23	5 - 10	0	K.ISTTWK.L
806.4573	805.4500	805.4698	-24.54	247 - 253	0	K.LTLFNAK.Y
918.5359	917.5287	917.5334	-5.19	349 - 356	1	K.RLPIGSYI.-
972.4759	971.4686	971.4712	-2.67	178 - 185	0	K.ESAYLYAR.F
1060.5447	1059.5374	1059.5349	2.39	196 - 204	0	K.DFADIPNLR.R
1168.6013	1167.5940	1167.6135	-16.70	108 - 117	2	K.YLDGKSTKEK.E
1361.6997	1360.6924	1360.6775	11.0	178 - 188	1	K.ESAYLYARFNK.I
1473.8167	1472.8094	1472.7987	7.28	192 - 204	1	K.LTAKDFADIPNLR.R
1601.9139	1600.9066	1600.8937	8.10	191 - 204	2	K.KLTAKDFADIPNLR.R
1679.9817	1678.9744	1678.9617	7.56	325 - 339	1	R.IEEIRLEGNPVILGK.H
2170.0872	2169.0799	2169.0590	9.66	205 - 223	1	R.RLDFTGNLIEDIEDGTFSK.L
3004.5840	3003.5767	3003.5818	-1.68	270 - 295	0	K.LHNLSFLYLDHNALESVPLNLPESLR.V
3457.6995	3456.6922	3456.6732	5.50	296 - 324	2	R.VIHLQFNNITSITDDTFCKANDTSYIRDR.I

No match to: 701.3809, 706.2637, 708.2758, 717.3943, 719.4086, 722.4171, 734.4380, 736.4033, 747.4274, 756.3826, 759.3994, 772.4160, 779.4522, 795.4493, 831.4625, 832.3028, 834.3088, 842.4966, 876.2747, 878.2808, 883.4713, 888.4968, 890.4931, 891.4778, 900.5316, 910.4130, 929.4340, 936.4846, 937.5071, 938.5070, 949.5414, 967.4175, 969.4572, 974.4473, 976.4465, 978.5344, 983.6115, 988.4531, 990.4647, 993.4956, 1000.5594, 1007.5680, 1019.5631, 1021.5624, 1031.5641, 1034.5791, 1036.5952, 1044.5217, 1046.5728,

1051.5135, 1056.5387, 1057.6144, 1074.5956, 1077.5199, 1087.5442, 1091.5437, 1113.5406, 1122.5580, 1123.5852, 1132.5316, 1145.6403, 1161.6290, 1171.5769, 1172.5704, 1178.6254, 1180.6395, 1181.6516, 1185.6835, 1198.7111, 1216.6992, 1234.6646, 1248.6750, 1254.6965, 1255.6359, 1273.6595, 1278.6974, 1281.6390, 1284.6533, 1301.6256, 1304.6489, 1314.7235, 1315.6904, 1325.6581, 1336.7290, 1338.6799, 1342.6847, 1349.6997, 1351.6993, 1354.6641, 1368.6772, 1370.6589, 1372.6199, 1381.7728, 1382.6112, 1383.7378, 1386.7084, 1388.7888, 1390.7301, 1397.7515, 1425.7671, 1439.7701, 1442.8314, 1457.7517, 1467.7510, 1488.7367, 1497.8859, 1500.7335, 1513.7731, 1516.7435, 1528.7101, 1556.8929, 1559.8439, 1562.8004, 1563.8088, 1578.7965, 1580.8445, 1592.8094, 1616.8481, 1623.8562, 1627.7197, 1628.7129, 1628.8722, 1634.8071, 1638.8757, 1642.7318, 1645.7701, 1647.8682, 1650.8612, 1673.8375, 1680.8936, 1681.8372, 1696.8403, 1706.8660, 1715.8601, 1717.9037, 1730.8719, 1742.0088, 1758.9255, 1769.0105, 1771.0138, 1774.9404, 1783.9508, 1786.9517, 1787.9071, 1790.9086, 1835.8361, 1837.8712, 1862.9679, 1928.0435, 1940.0333, 1954.0796, 1961.0397, 1970.0139, 1982.0297, 1984.0314, 2023.0551, 2027.1292, 2032.0228, 2061.0330, 2105.0796, 2144.0679, 2179.0752, 2211.0654, 2215.0876, 2239.1650, 2242.1501, 2246.2151, 2251.1675, 2252.1614, 2255.1719, 2273.1008, 2289.1030, 2293.1750, 2304.2087, 2316.2031, 2343.2134, 2354.0020, 2391.2788, 2475.2476, 2491.2402, 2507.1975, 2514.2664, 2542.2795, 2550.2063, 2558.3499, 2587.3516, 2605.2615, 2646.9883, 2661.0090, 2675.0190, 2681.3855, 2698.3696, 2705.3142, 2707.3074, 2714.3647, 2717.3394, 2719.3167, 2722.3188, 2763.3738, 2792.2446, 2826.3545, 2843.4670, 2846.4014, 2847.4082, 2872.3359, 2884.3538, 2887.3474, 2889.3481, 2892.4165, 2894.4639, 2896.4890, 2914.4551, 2987.5244, 2995.4431, 3029.4812, 3054.4724, 3069.4849, 3072.4231, 3101.5515, 3126.7327, 3166.6106, 3171.6748, 3183.6196, 3199.6411, 3231.5630, 3236.5791, 3243.6301, 3245.6216, 3247.5737, 3248.6313, 3254.6448, 3262.7324, 3267.7256, 3278.7546, 3321.5828, 3333.5762, 3334.6729, 3335.6787, 3338.7097, 3347.4907, 3395.6821, 3412.6426, 3428.5991, 3435.7024, 3487.8022, 3490.6885, 3503.8049, 3534.6365, 3548.6917, 3551.6768, 3552.6902, 3601.6826, 3616.7095, 3624.6824, 3639.7014, 3645.7827, 3651.7559, 3655.7354, 3667.7646, 3697.8025, 3716.7253, 3762.8396, 3929.9304, 4040.7656, 4057.9707, 4071.9524, 4092.8394, 4147.9063, 4160.9473, 4174.9575, 4193.9395, 4253.8755, 4415.9736, 4452.1865, 4465.1670, 4845.0845

20. A8E4P9 **Mass:** 20587 **Score:** 20 **Expect:** 2.2e+002 **Matches:** 8

HAPLN1 protein OS=Bos taurus GN=HAPLN1 PE=2 SV=1

Observed	Mr(expt)	Mr(calc)	ppm	Start	End	Miss	Peptide
910.4130	909.4057	909.4093	-3.99	104	111	0	K.TYGGYHGR.V
1000.5594	999.5521	999.5600	-7.91	40	48	0	R.LLVEAEQAK.V
1254.6965	1253.6892	1253.6656	18.9	81	90	1	K.WTKLTSDYLK.E
1386.7084	1385.7011	1385.7052	-2.92	66	78	1	R.DPTAFGSGTHKIR.I
1397.7515	1396.7442	1396.7252	13.6	104	115	1	K.TYGGYHGRVFLK.G
2211.0654	2210.0581	2210.0339	11.0	116	136	0	K.GGSDNDASLVITDLTLEDYGR.Y
2698.3696	2697.3623	2697.3497	4.66	112	136	1	R.VFLKGGSDNDASLVITDLTLEDYGR.Y
3254.6448	3253.6375	3253.5979	12.2	2	28	1	M.KSLLLLVLISFCWADHHSDNYTVDHDR.V

No match to: 701.3809, 706.2637, 708.2758, 717.3943, 719.4086, 722.4171, 734.4380, 735.4089, 736.4033, 747.4274, 756.3826, 759.3994, 772.4160, 779.4522, 795.4493, 806.4573, 831.4625, 832.3028, 834.3088, 842.4966, 876.2747, 878.2808, 883.4713, 888.4968, 890.4931, 891.4778, 900.5316, 918.5359, 929.4340, 936.4846, 937.5071, 938.5070, 949.5414, 967.4175, 969.4572, 972.4759, 974.4473, 976.4465, 978.5344, 983.6115, 988.4531, 990.4647, 993.4956, 1007.5680, 1019.5631, 1021.5624, 1031.5641, 1034.5791, 1036.5952, 1044.5217, 1046.5728, 1051.5135, 1056.5387, 1057.6144, 1060.5447, 1074.5956, 1077.5199, 1087.5442, 1091.5437, 1113.5406, 1122.5580, 1123.5852, 1132.5316, 1145.6403, 1161.6290, 1168.6013, 1171.5769, 1172.5704, 1178.6254, 1180.6395, 1181.6516, 1185.6835, 1198.7111, 1216.6992, 1234.6646, 1248.6750, 1255.6359, 1273.6595, 1278.6974, 1281.6390, 1284.6533, 1301.6256, 1304.6489, 1314.7235, 1315.6904, 1325.6581, 1336.7290, 1338.6799, 1342.6847, 1349.6997, 1351.6993, 1354.6641, 1361.6997, 1368.6772, 1370.6589, 1372.6199, 1381.7728, 1382.6112, 1383.7378, 1388.7888, 1390.7301, 1425.7671, 1439.7701, 1442.8314, 1457.7517, 1467.7510, 1473.8167, 1488.7367, 1497.8859, 1500.7335, 1513.7731, 1516.7435, 1528.7101, 1556.8929, 1559.8439, 1562.8004, 1563.8088, 1578.7965, 1580.8445, 1592.8094, 1601.9139, 1616.8481, 1623.8562, 1627.7197, 1628.7129, 1628.8722, 1634.8071, 1638.8757, 1642.7318, 1645.7701, 1647.8682, 1650.8612, 1673.8375, 1679.9817, 1680.8936, 1681.8372, 1696.8403, 1706.8660, 1715.8601, 1717.9037, 1730.8719, 1742.0088, 1758.9255, 1769.0105, 1771.0138, 1774.9404, 1783.9508, 1786.9517, 1787.9071, 1790.9086, 1835.8361, 1837.8712, 1862.9679, 1928.0435, 1940.0333, 1954.0796, 1961.0397, 1970.0139, 1982.0297, 1984.0314, 2023.0551, 2027.1292, 2032.0228, 2061.0330, 2105.0796, 2144.0679, 2170.0872, 2179.0752, 2215.0876, 2239.1650, 2242.1501, 2246.2151, 2251.1675, 2252.1614, 2255.1719, 2273.1008, 2289.1030, 2293.1750, 2304.2087, 2316.2031, 2343.2134, 2354.0020, 2391.2788, 2475.2476, 2491.2402, 2507.1975, 2514.2664, 2542.2795, 2550.2063, 2558.3499, 2587.3516, 2605.2615, 2646.9883, 2661.0090, 2675.0190, 2681.3855, 2705.3142, 2707.3074, 2714.3647, 2717.3394, 2719.3167, 2722.3188, 2763.3738, 2792.2446, 2826.3545, 2843.4670, 2846.4014, 2847.4082, 2872.3359, 2884.3538, 2887.3474, 2889.3481, 2892.4165, 2894.4639, 2896.4890, 2914.4551, 2987.5244, 2995.4431, 3004.5840, 3029.4812, 3054.4724, 3069.4849, 3072.4231, 3101.5515, 3126.7327, 3166.6106, 3171.6748, 3183.6196, 3199.6411, 3231.5630, 3236.5791, 3243.6301, 3245.6216, 3247.5737, 3248.6313, 3262.7324, 3267.7256, 3278.7546, 3321.5828, 3333.5762, 3334.6729, 3335.6787, 3338.7097, 3347.4907, 3395.6821, 3412.6426, 3428.5991, 3435.7024, 3457.6995, 3487.8022, 3490.6885, 3503.8049, 3534.6365, 3548.6917, 3551.6768, 3552.6902, 3601.6826, 3616.7095, 3624.6824, 3639.7014, 3645.7827, 3651.7559, 3655.7354, 3667.7646, 3697.8025, 3716.7253, 3762.8396, 3929.9304, 4040.7656, 4057.9707, 4071.9524, 4092.8394, 4147.9063, 4160.9473, 4174.9575, 4193.9395, 4253.8755, 4415.9736, 4452.1865, 4465.1670, 4845.0845

21. G3N1F5 **Mass:** 34506 **Score:** 20 **Expect:** 2.4e+002 **Matches:** 8

Uncharacterized protein OS=Bos taurus PE=3 SV=1

Observed	Mr(expt)	Mr(calc)	ppm	Start	End	Miss	Peptide
974.4473	973.4400	973.4617	-22.33	189	196	0	K.EFSAEVHR.K
1338.6799	1337.6726	1337.6939	-15.93	165	178	0	K.GAVDGGLSIPHSTK.W
1386.7084	1385.7011	1385.6687	23.4	23	33	2	R.RQEGKTDYYAR.K
1954.0796	1953.0723	1953.0684	2.02	159	178	1	K.VFGALKGAVDGGLSIPHSTK.W
2550.2063	2549.1990	2549.1903	3.41	189	209	2	K.EFSAEVHRKHIMGQNVADYMR.Y + 2 Oxidation (M)
3101.5515	3100.5442	3100.4820	20.1	59	85	0	R.DIICQIAYAHIEGDMIVCAAYAHELPK.Y
3490.6885	3489.6812	3489.6590	6.38	165	196	2	K.GAVDGGLSIPHSTKWFPGYDSESKEFSAEVHR.K
3548.6917	3547.6844	3547.7302	-12.89	59	89	1	R.DIICQIAYAHIEGDMIVCAAYAHELPKYGVK.A

No match to: 701.3809, 706.2637, 708.2758, 717.3943, 719.4086, 722.4171, 734.4380, 735.4089, 736.4033, 747.4274, 756.3826, 759.3994, 772.4160, 779.4522, 795.4493, 806.4573, 831.4625, 832.3028, 834.3088, 842.4966, 876.2747, 878.2808, 883.4713, 888.4968, 890.4931, 891.4778, 900.5316, 910.4130, 918.5359, 929.4340, 936.4846, 937.5071, 938.5070, 949.5414, 967.4175, 969.4572, 972.4759, 976.4465, 978.5344, 983.6115, 988.4531, 990.4647, 993.4956, 1000.5594, 1007.5680, 1019.5631, 1021.5624, 1031.5641, 1034.5791, 1036.5952, 1044.5217, 1046.5728, 1051.5135, 1056.5387, 1057.6144, 1060.5447, 1074.5956, 1077.5199, 1087.5442, 1091.5437, 1113.5406, 1122.5580, 1123.5852, 1132.5316, 1145.6403, 1161.6290, 1168.6013, 1171.5769, 1172.5704, 1178.6254, 1180.6395, 1181.6516, 1185.6835, 1198.7111, 1216.6992, 1234.6646, 1248.6750, 1254.6965, 1255.6359, 1273.6595, 1278.6974, 1281.6390, 1284.6533, 1301.6256, 1304.6489, 1314.7235, 1315.6904, 1325.6581, 1336.7290, 1342.6847, 1349.6997, 1351.6993, 1354.6641, 1361.6997, 1368.6772, 1370.6589, 1372.6199, 1381.7728, 1382.6112, 1383.7378, 1388.7888, 1390.7301, 1397.7515, 1425.7671, 1439.7701, 1442.8314, 1457.7517, 1467.7510, 1473.8167, 1488.7367, 1497.8859, 1500.7335, 1513.7731, 1516.7435, 1528.7101, 1556.8929, 1559.8439, 1562.8004, 1563.8088, 1578.7965, 1580.8445, 1592.8094, 1601.9139, 1616.8481, 1623.8562, 1627.7197, 1628.7129, 1628.8722, 1634.8071, 1638.8757, 1642.7318, 1645.7701, 1647.8682, 1650.8612, 1673.8375, 1679.9817, 1680.8936, 1681.8372, 1696.8403, 1706.8660, 1715.8601, 1717.9037, 1730.8719, 1742.0088, 1758.9255, 1769.0105, 1771.0138, 1774.9404, 1783.9508, 1786.9517, 1787.9071, 1790.9086, 1835.8361, 1837.8712, 1862.9679, 1928.0435, 1940.0333, 1961.0397, 1970.0139, 1982.0297, 1984.0314, 2023.0551, 2027.1292, 2032.0228, 2061.0330, 2105.0796, 2144.0679, 2170.0872, 2179.0752, 2211.0654, 2215.0876, 2239.1650, 2242.1501, 2246.2151, 2251.1675, 2252.1614, 2255.1719, 2273.1008, 2289.1030, 2293.1750, 2304.2087, 2316.2031, 2343.2134, 2354.0020, 2391.2788, 2475.2476, 2491.2402, 2507.1975, 2514.2664, 2542.2795, 2558.3499, 2587.3516, 2605.2615, 2646.9883, 2661.0090, 2675.0190, 2681.3855, 2698.3696, 2705.3142, 2707.3074, 2714.3647, 2717.3394, 2719.3167, 2722.3188, 2763.3738, 2792.2446, 2826.3545, 2843.4670, 2846.4014, 2847.4082, 2872.3359, 2884.3538, 2887.3474, 2889.3481, 2892.4165, 2894.4639, 2896.4890, 2914.4551, 2987.5244, 2995.4431, 3004.5840, 3029.4812, 3054.4724, 3069.4849, 3072.4231, 3126.7327, 3166.6106, 3171.6748, 3183.6196, 3199.6411, 3231.5630, 3236.5791, 3243.6301, 3245.6216, 3247.5737, 3248.6313, 3254.6448, 3262.7324, 3267.7256, 3278.7546, 3321.5828, 3333.5762, 3334.6729, 3335.6787, 3338.7097, 3347.4907, 3395.6821, 3412.6426, 3428.5991, 3435.7024, 3457.6995, 3487.8022, 3503.8049, 3534.6365, 3551.6768, 3552.6902, 3601.6826, 3616.7095, 3624.6824, 3639.7014, 3645.7827, 3651.7559, 3655.7354, 3667.7646, 3697.8025, 3716.7253, 3762.8396, 3929.9304, 4040.7656, 4057.9707, 4071.9524, 4092.8394, 4147.9063, 4160.9473, 4174.9575, 4193.9395, 4253.8755, 4415.9736, 4452.1865, 4465.1670, 4845.0845

22. P13608 **Mass:** 248150 **Score:** 20 **Expect:** 2.7e+002 **Matches:** 36

Aggrecan core protein OS=Bos taurus GN=ACAN PE=1 SV=3

Observed	Mr(expt)	Mr(calc)	ppm	Start	End	Miss	Peptide

831.4625	830.4552	830.4650	-11.78	545 -	551	0	R.YPIVSPR.T
883.4713	882.4640	882.4712	-8.16	211 -	217	0	R.YPIHTPR.E
891.4778	890.4706	890.4763	-6.41	152 -	158	0	K.GIVFHYR.A
929.4340	928.4267	928.4290	-2.55	164 -	170	0	R.YTLDFDR.A
949.5414	948.5341	948.5392	-5.42	306 -	313	1	R.SVRYPISK.A
967.4175	966.4103	966.4130	-2.86	2161 -	2167	0	K.FQGHCYR.H
974.4473	973.4400	973.4341	6.06	678 -	684	0	R.HHAFCFR.G
1087.5442	1086.5369	1086.5233	12.5	498 -	506	0	R.YSLTFEEAK.Q
1168.6013	1167.5940	1167.5924	1.37	585 -	594	0	R.LEGEVFFATR.L
1185.6835	1184.6762	1184.6765	-0.22	83 -	93	0	K.EVVLLVATEGR.V
1248.6750	1247.6677	1247.6622	4.43	2298 -	2307	1	K.DRYEINALVR.Y
1273.6595	1272.6522	1272.6826	-23.89	987 -	997	1	R.ITWTSAPKVDR.L
1284.6533	1283.6460	1283.6258	15.7	164 -	173	1	R.YTLDFDRAQR.A
1325.6581	1324.6508	1324.6445	4.75	2335 -	2345	1	R.ITCTDPATYKR.R
1372.6199	1371.6126	1371.5877	18.1	264 -	274	0	K.FTFQEAANECR.R
1382.6112	1381.6039	1381.5833	14.9	2324 -	2334	0	R.CQPSGHWEEPR.I
1383.7378	1382.7305	1382.7201	7.54	314 -	326	0	K.ARPNCGGNLLGVR.T
1442.8314	1441.8241	1441.8140	7.00	81 -	93	1	K.EKEVVLLVATEGR.V
1457.7517	1456.7444	1456.7310	9.19	159 -	170	1	R.AISTRYTLDFDR.A
1528.7101	1527.7028	1527.6888	9.15	264 -	275	1	K.FTFQEAANECRR.L
1578.7965	1577.7892	1577.7620	17.2	2277 -	2291	0	K.GTVACGEPPVVEHAR.I
1628.7129	1627.7056	1627.6937	7.34	218 -	231	1	R.EGCYGDKDEFPGVR.T
1628.8722	1627.8649	1627.8583	4.06	483 -	497	0	R.LPGGVVFHYRPGSSR.Y
1706.8660	1705.8587	1705.8570	1.01	2276 -	2291	1	K.KGTVACGEPPVVEHAR.I
1715.8601	1714.8528	1714.8348	10.5	498 -	511	1	R.YSLTFEEAKQACLR.T
1771.0138	1770.0065	1770.0251	-10.48	78 -	93	2	R.ISKEKEVVLLVATEGR.V
1940.0333	1939.0260	1939.0098	8.36	643 -	660	0	R.YPIVTPRPACGGDKPGVR.T
2144.0679	2143.0606	2143.0289	14.8	133 -	151	0	R.CEVMHGIEDSQATLEVVVK.G
2343.2134	2342.2061	2342.1940	5.18	104 -	124	0	K.VTLPNYPAIPSDATLEIQNMR.S
2872.3359	2871.3286	2871.3142	5.01	280 -	305	0	R.LATTGQLYLAWQGGMDMCSAGWLADR.S
3248.6313	3247.6240	3247.6183	1.77	96 -	124	1	R.VNSAYQDKVTLPNYPAIPSDATLEIQNMR.S
3457.6995	3456.6922	3456.6489	12.5	275 -	305	2	R.RLGARLATTGQLYLAWQGGMDMCSAGWLADR.S + 2 Oxidation (M)
3503.8049	3502.7976	3502.7878	2.80	94 -	124	2	R.VRVNSAYQDKVTLPNYPAIPSDATLEIQNMR.S
3639.7014	3638.6941	3638.6947	-0.16	512 -	544	0	R.TGAIIASPEQLQAAYEAGYEQCDAGWLQDQTVR.Y
4147.9063	4146.8990	4146.9164	-4.18	174 -	210	0	R.ACLQNSAIIATPEQLQAAYEDGFHQCDAGWLADQTVR.Y
4452.1865	4451.1792	4451.1492	6.75	512 -	551	1	R.TGAIIASPEQLQAAYEAGYEQCDAGWLQDQTVRYPIVSPR.T

No match to: 701.3809, 706.2637, 708.2758, 717.3943, 719.4086, 722.4171, 734.4380, 735.4089, 736.4033, 747.4274, 756.3826, 759.3994, 772.4160, 779.4522, 795.4493, 806.4573, 832.3028, 834.3088, 842.4966, 876.2747, 878.2808, 888.4968, 890.4931, 900.5316, 910.4130, 918.5359, 936.4846, 937.5071, 938.5070, 969.4572, 972.4759, 976.4465, 978.5344, 983.6115, 988.4531, 990.4647, 993.4956, 1000.5594, 1007.5680, 1019.5631, 1021.5624, 1031.5641, 1034.5791, 1036.5952, 1044.5217, 1046.5728, 1051.5135, 1056.5387, 1057.6144, 1060.5447, 1074.5956, 1077.5199, 1091.5437, 1113.5406, 1122.5580, 1123.5852, 1132.5316, 1145.6403, 1161.6290, 1171.5769, 1172.5704, 1178.6254, 1180.6395, 1181.6516, 1198.7111, 1216.6992, 1234.6646, 1254.6965, 1255.6359, 1278.6974, 1281.6390, 1301.6256, 1304.6489, 1314.7235, 1315.6904, 1336.7290, 1338.6799, 1342.6847, 1349.6997, 1351.6993, 1354.6641, 1361.6997, 1368.6772, 1370.6589, 1381.7728, 1386.7084, 1388.7888, 1390.7301, 1397.7515, 1425.7671, 1439.7701, 1467.7510, 1473.8167, 1488.7367, 1497.8859, 1500.7335, 1513.7731, 1516.7435, 1556.8929, 1559.8439, 1562.8004, 1563.8088, 1580.8445, 1592.8094, 1601.9139, 1616.8481, 1623.8562, 1627.7197, 1634.8071, 1638.8757, 1642.7318, 1645.7701, 1647.8682, 1650.8612, 1673.8375, 1679.9817, 1680.8936, 1681.8372, 1696.8403, 1717.9037, 1730.8719, 1742.0088, 1758.9255, 1769.0105, 1774.9404, 1783.9508, 1786.9517, 1787.9071, 1790.9086, 1835.8361, 1837.8712, 1862.9679, 1928.0435, 1954.0796, 1961.0397, 1970.0139, 1982.0297, 1984.0314, 2023.0551, 2027.1292, 2032.0228, 2061.0330, 2105.0796, 2170.0872, 2179.0752, 2211.0654, 2215.0876, 2239.1650, 2242.1501, 2246.2151, 2251.1675, 2252.1614, 2255.1719, 2273.1008, 2289.1030, 2293.1750, 2304.2087, 2316.2031, 2354.0020, 2391.2788, 2475.2476, 2491.2402, 2507.1975, 2514.2664, 2542.2795, 2550.2063, 2558.3499, 2587.3516, 2605.2615, 2646.9883, 2661.0090, 2675.0190, 2681.3855, 2698.3696, 2705.3142, 2707.3074, 2714.3647, 2717.3394, 2719.3167, 2722.3188, 2763.3738, 2792.2446, 2826.3545, 2843.4670, 2846.4014, 2847.4082, 2884.3538, 2887.3474, 2889.3481, 2892.4165, 2894.4639, 2896.4890, 2914.4551, 2987.5244, 2995.4431, 3004.5840, 3029.4812, 3054.4724, 3069.4849, 3072.4231, 3101.5515, 3126.7327, 3166.6106, 3171.6748, 3183.6196, 3199.6411, 3231.5630, 3236.5791, 3243.6301, 3245.6216, 3247.5737, 3254.6448, 3262.7324, 3267.7256, 3278.7546, 3321.5828, 3333.5762, 3334.6729, 3335.6787, 3338.7097, 3347.4907, 3395.6821, 3412.6426, 3428.5991, 3435.7024, 3487.8022, 3490.6885, 3534.6365, 3548.6917, 3551.6768, 3552.6902, 3601.6826, 3616.7095, 3624.6824, 3645.7827, 3651.7559, 3655.7354, 3667.7646, 3697.8025, 3716.7253, 3762.8396, 3929.9304, 4040.7656, 4057.9707, 4071.9524, 4092.8394, 4160.9473, 4174.9575, 4193.9395, 4253.8755, 4415.9736, 4465.1670, 4845.0845

23. Q3T0X8 **Mass:** 57425 **Score:** 19 **Expect:** 2.8e+002 **Matches:** 18

Na(+)/H(+) exchange regulatory cofactor NHE-RF3 OS=Bos taurus GN=PDZK1 PE=2 SV=1

Observed	Mr(expt)	Mr(calc)	ppm	Start	End	Miss	Peptide
795.4493	794.4420	794.4360	7.51	134 -	139	0	R.LCYLVK.E
883.4713	882.4640	882.4745	-11.98	235 -	241	0	K.LLPCQPR.V
891.4778	890.4706	890.4862	-17.53	58 -	65	0	R.INGVFVDK.E
900.5316	899.5243	899.5076	18.6	274 -	282	1	K.SPAEKAGLK.N
1044.5217	1043.5144	1043.4924	21.1	140 -	149	0	K.EGGSYGFSLK.T
1057.6144	1056.6071	1056.5815	24.3	37 -	46	1	R.VIEKGSPAEK.A
1354.6641	1353.6568	1353.6711	-10.53	66 -	76	0	K.EEHMQVVDLVR.K
1370.6589	1369.6516	1369.6660	-10.50	66 -	76	0	K.EEHMQVVDLVR.K + Oxidation (M)
1381.7728	1380.7655	1380.7361	21.3	29 -	40	1	K.DTDGHLVRVIEK.G
1386.7084	1385.7011	1385.6787	16.2	104 -	116	0	K.ALGQSQEPSLNDK.K
1638.8757	1637.8684	1637.8988	-18.55	264 -	278	2	K.GQIIKDIDPKSPAEK.A
1715.8601	1714.8528	1714.8196	19.4	294 -	308	0	K.SVESLDHDSVVEMIR.K
1774.9404	1773.9331	1773.8911	23.7	380 -	395	1	R.LARGEDGYGFHLNAVR.G
1970.0139	1969.0066	1969.0116	-2.53	99 -	116	1	K.QVDLKALGQSQEPSLNDK.K
2105.0796	2104.0723	2104.1027	-14.43	448 -	465	2	K.TVTLLVCGKKAYDYFQAK.K
2255.1719	2254.1646	2254.1515	5.83	78 -	98	2	K.SGNSVTLLVLDGDSYEKAMKK.Q
2719.3167	2718.3094	2718.3514	-15.45	383 -	407	2	R.GEDGYGFHLNAVRGQPGSFVKEVQK.G
4160.9473	4159.9400	4160.0107	-16.99	404 -	441	2	K.EVQKGGPADLAGLEDEDVIIEVNGVNMLDESYEKVVDR.I + Oxidation (M)

No match to: 701.3809, 706.2637, 708.2758, 717.3943, 719.4086, 722.4171, 734.4380, 735.4089, 736.4033, 747.4274, 756.3826, 759.3994, 772.4160, 779.4522, 806.4573, 831.4625, 832.3028, 834.3088, 842.4966, 876.2747, 878.2808, 888.4968, 890.4931, 910.4130, 918.5359, 929.4340, 936.4846, 937.5071, 938.5070, 949.5414, 967.4175, 969.4572, 972.4759, 974.4473, 976.4465, 978.5344, 983.6115, 988.4531, 990.4647, 993.4956, 1000.5594, 1007.5680, 1019.5631, 1021.5624, 1031.5641, 1034.5791, 1036.5952, 1046.5728, 1051.5135, 1056.5387, 1060.5447, 1074.5956, 1077.5199, 1087.5442, 1091.5437, 1113.5406, 1122.5580, 1123.5852, 1132.5316, 1145.6403, 1161.6290, 1168.6013, 1171.5769, 1172.5704, 1178.6254, 1180.6395, 1181.6516, 1185.6835, 1198.7111, 1216.6992, 1234.6646, 1248.6750, 1254.6965, 1255.6359, 1273.6595, 1278.6974, 1281.6390, 1284.6533, 1301.6256, 1304.6489, 1314.7235, 1315.6904, 1325.6581, 1336.7290, 1338.6799, 1342.6847, 1349.6997, 1351.6993, 1361.6997, 1368.6772, 1372.6199, 1382.6112, 1383.7378, 1388.7888, 1390.7301, 1397.7515, 1425.7671, 1439.7701, 1442.8314, 1457.7517, 1467.7510, 1473.8167, 1488.7367, 1497.8859, 1500.7335, 1513.7731, 1516.7435, 1528.7101, 1556.8929, 1559.8439, 1562.8004, 1563.8088, 1578.7965, 1580.8445, 1592.8094, 1601.9139, 1616.8481, 1623.8562, 1627.7197, 1628.7129, 1628.8722, 1634.8071, 1642.7318, 1645.7701, 1647.8682, 1650.8612, 1673.8375, 1679.9817, 1680.8936, 1681.8372, 1696.8403, 1706.8660, 1717.9037,

1730.8719, 1742.0088, 1758.9255, 1769.0105, 1771.0138, 1783.9508, 1786.9517, 1787.9071, 1790.9086, 1835.8361, 1837.8712, 1862.9679, 1928.0435, 1940.0333, 1954.0796, 1961.0397, 1982.0297, 1984.0314, 2023.0551, 2027.1292, 2032.0228, 2061.0330, 2144.0679, 2170.0872, 2179.0752, 2211.0654, 2215.0876, 2239.1650, 2242.1501, 2246.2151, 2251.1675, 2252.1614, 2273.1008, 2289.1030, 2293.1750, 2304.2087, 2316.2031, 2343.2134, 2354.0020, 2391.2788, 2475.2476, 2491.2402, 2507.1975, 2514.2664, 2542.2795, 2550.2063, 2558.3499, 2587.3516, 2605.2615, 2646.9883, 2661.0090, 2675.0190, 2681.3855, 2698.3696, 2705.3142, 2707.3074, 2714.3647, 2717.3394, 2722.3188, 2763.3738, 2792.2446, 2826.3545, 2843.4670, 2846.4014, 2847.4082, 2872.3359, 2884.3538, 2887.3474, 2889.3481, 2892.4165, 2894.4639, 2896.4890, 2914.4551, 2987.5244, 2995.4431, 3004.5840, 3029.4812, 3054.4724, 3069.4849, 3072.4231, 3101.5515, 3126.7327, 3166.6106, 3171.6748, 3183.6196, 3199.6411, 3231.5630, 3236.5791, 3243.6301, 3245.6216, 3247.5737, 3248.6313, 3254.6448, 3262.7324, 3267.7256, 3278.7546, 3321.5828, 3333.5762, 3334.6729, 3335.6787, 3338.7097, 3347.4907, 3395.6821, 3412.6426, 3428.5991, 3435.7024, 3457.6995, 3487.8022, 3490.6885, 3503.8049, 3534.6365, 3548.6917, 3551.6768, 3552.6902, 3601.6826, 3616.7095, 3624.6824, 3639.7014, 3645.7827, 3651.7559, 3655.7354, 3667.7646, 3697.8025, 3716.7253, 3762.8396, 3929.9304, 4040.7656, 4057.9707, 4071.9524, 4092.8394, 4147.9063, 4174.9575, 4193.9395, 4253.8755, 4415.9736, 4452.1865, 4465.1670, 4845.0845

24. A5PK10 **Mass:** 43166 **Score:** 19 **Expect:** 2.8e+002 **Matches:** 7

TOM1L2 protein OS=Bos taurus GN=TOM1L2 PE=2 SV=1

Observed	Mr(expt)	Mr(calc)	ppm	Start	End	Miss	Peptide
1473.8167	1472.8094	1472.8140	-3.08	117 -	129	0	K.VLALIQAWADAFR.S
1862.9679	1861.9606	1861.9615	-0.45	256 -	270	2	R.TCRAMQQRVVELISR.V + Oxidation (M)
3166.6106	3165.6033	3165.6362	-10.38	66 -	92	2	R.EVMLALTVLETCVKNCGHRFHVLVANR.D
3199.6411	3198.6338	3198.6713	-11.72	117 -	145	1	K.VLALIQAWADAFRSSPDLTGVVHAYEELK.R
3548.6917	3547.6844	3547.6480	10.3	225 -	255	1	R.GNTKVMSEMLTEMVPGQEDSSDLELLQELHR.T + 2 Oxidation (M)
3651.7559	3650.7486	3650.7423	1.73	271 -	299	2	R.VCNEEVTEELLHVNDDLNNVFLRYERFER.Y
4147.9063	4146.8990	4146.9265	-6.61	229 -	263	2	K.VMSEMLTEMVPGQEDSSDLELLQELHRTCRAMQQR.V

No match to: 701.3809, 706.2637, 708.2758, 717.3943, 719.4086, 722.4171, 734.4380, 735.4089, 736.4033, 747.4274, 756.3826, 759.3994, 772.4160, 779.4522, 795.4493, 806.4573, 831.4625, 832.3028, 834.3088, 842.4966, 876.2747, 878.2808, 883.4713, 888.4968, 890.4931, 891.4778, 900.5316, 910.4130, 918.5359, 929.4340, 936.4846, 937.5071, 938.5070, 949.5414, 967.4175, 969.4572, 972.4759, 974.4473, 976.4465, 978.5344, 983.6115, 988.4531, 990.4647, 993.4956, 1000.5594, 1007.5680, 1019.5631, 1021.5624, 1031.5641, 1034.5791, 1036.5952, 1044.5217, 1046.5728, 1051.5135, 1056.5387, 1057.6144, 1060.5447, 1074.5956, 1077.5199, 1087.5442, 1091.5437, 1113.5406, 1122.5580, 1123.5852, 1132.5316, 1145.6403, 1161.6290, 1168.6013, 1171.5769, 1172.5704, 1178.6254, 1180.6395, 1181.6516, 1185.6835, 1198.7111, 1216.6992, 1234.6646, 1248.6750, 1254.6965, 1255.6359, 1273.6595, 1278.6974, 1281.6390, 1284.6533, 1301.6256, 1304.6489, 1314.7235, 1315.6904, 1325.6581, 1336.7290, 1338.6799, 1342.6847, 1349.6997, 1351.6993, 1354.6641, 1361.6997, 1368.6772, 1370.6589, 1372.6199, 1381.7728, 1382.6112, 1383.7378, 1386.7084, 1388.7888, 1390.7301, 1397.7515, 1425.7671, 1439.7701, 1442.8314, 1457.7517, 1467.7510, 1488.7367, 1497.8859, 1500.7335, 1513.7731, 1516.7435, 1528.7101, 1556.8929, 1559.8439, 1562.8004, 1563.8088, 1578.7965, 1580.8445, 1592.8094, 1601.9139, 1616.8481, 1623.8562, 1627.7197, 1628.7129, 1628.8722, 1634.8071, 1638.8757, 1642.7318, 1645.7701, 1647.8682, 1650.8612, 1673.8375, 1679.9817, 1680.8936, 1681.8372, 1696.8403, 1706.8660, 1715.8601, 1717.9037, 1730.8719, 1742.0088, 1758.9255, 1769.0105, 1771.0138, 1774.9404, 1783.9508, 1786.9517, 1787.9071, 1790.9086, 1835.8361, 1837.8712, 1928.0435, 1940.0333, 1954.0796, 1961.0397, 1970.0139, 1982.0297, 1984.0314, 2023.0551, 2027.1292, 2032.0228, 2061.0330, 2105.0796, 2144.0679, 2170.0872, 2179.0752, 2211.0654, 2215.0876, 2239.1650, 2242.1501, 2246.2151, 2251.1675, 2252.1614, 2255.1719, 2273.1008, 2289.1030, 2293.1750, 2304.2087, 2316.2031, 2343.2134, 2354.0020, 2391.2788, 2475.2476, 2491.2402, 2507.1975, 2514.2664, 2542.2795, 2550.2063, 2558.3499, 2587.3516, 2605.2615, 2646.9883, 2661.0090, 2675.0190, 2681.3855, 2698.3696, 2705.3142, 2707.3074, 2714.3647, 2717.3394, 2719.3167, 2722.3188, 2763.3738, 2792.2446, 2826.3545, 2843.4670, 2846.4014, 2847.4082, 2872.3359, 2884.3538, 2887.3474, 2889.3481, 2892.4165, 2894.4639, 2896.4890, 2914.4551, 2987.5244, 2995.4431, 3004.5840, 3029.4812, 3054.4724, 3069.4849, 3072.4231, 3101.5515, 3126.7327, 3171.6748, 3183.6196, 3231.5630, 3236.5791, 3243.6301, 3245.6216, 3247.5737, 3248.6313, 3254.6448, 3262.7324, 3267.7256, 3278.7546, 3321.5828, 3333.5762, 3334.6729, 3335.6787, 3338.7097, 3347.4907, 3395.6821, 3412.6426, 3428.5991, 3435.7024, 3457.6995, 3487.8022, 3490.6885, 3503.8049, 3534.6365, 3551.6768, 3552.6902, 3601.6826, 3616.7095, 3624.6824, 3639.7014, 3645.7827, 3655.7354, 3667.7646, 3697.8025, 3716.7253, 3762.8396, 3929.9304, 4040.7656, 4057.9707, 4071.9524, 4092.8394, 4160.9473, 4174.9575, 4193.9395, 4253.8755, 4415.9736, 4452.1865, 4465.1670, 4845.0845

25. Q3SYU8 **Mass:** 19979 **Score:** 19 **Expect:** 2.8e+002 **Matches:** 6

Tumor protein D52-like 2 OS=Bos taurus GN=TPD52L2 PE=2 SV=1

Observed	Mr(expt)	Mr(calc)	ppm	Start	End	Miss	Peptide
1255.6359	1254.6286	1254.6027	20.7	135 -	145	1	K.LGDMRNSATFK.S + Oxidation (M)
1301.6256	1300.6183	1300.6048	10.4	140 -	150	1	R.NSATFKSFEDR.V
1368.6772	1367.6699	1367.6715	-1.13	120 -	133	0	K.TSAALSTMGSAISR.K + Oxidation (M)
1383.7378	1382.7305	1382.6976	23.8	134 -	145	2	R.KLGDMRNSATFK.S + Oxidation (M)
1638.8757	1637.8684	1637.8671	0.79	65 -	78	2	R.QVLAAKERHCGELK.R
3601.6826	3600.6753	3600.7353	-16.65	16 -	50	0	K.GLLSDSMTDVPVDTAVAAQAPAVEGLTETEAEELR.A + Oxidation (M)

No match to: 701.3809, 706.2637, 708.2758, 717.3943, 719.4086, 722.4171, 734.4380, 735.4089, 736.4033, 747.4274, 756.3826, 759.3994, 772.4160, 779.4522, 795.4493, 806.4573, 831.4625, 832.3028, 834.3088, 842.4966, 876.2747, 878.2808, 883.4713, 888.4968, 890.4931, 891.4778, 900.5316, 910.4130, 918.5359, 929.4340, 936.4846, 937.5071, 938.5070, 949.5414, 967.4175, 969.4572, 972.4759, 974.4473, 976.4465, 978.5344, 983.6115, 988.4531, 990.4647, 993.4956, 1000.5594, 1007.5680, 1019.5631, 1021.5624, 1031.5641, 1034.5791, 1036.5952, 1044.5217, 1046.5728, 1051.5135, 1056.5387, 1057.6144, 1060.5447, 1074.5956, 1077.5199, 1087.5442, 1091.5437, 1113.5406, 1122.5580, 1123.5852, 1132.5316, 1145.6403, 1161.6290, 1168.6013, 1171.5769, 1172.5704, 1178.6254, 1180.6395, 1181.6516, 1185.6835, 1198.7111, 1216.6992, 1234.6646, 1248.6750, 1254.6965, 1273.6595, 1278.6974, 1281.6390, 1284.6533, 1304.6489, 1314.7235, 1315.6904, 1325.6581, 1336.7290, 1338.6799, 1342.6847, 1349.6997, 1351.6993, 1354.6641, 1361.6997, 1370.6589, 1372.6199, 1381.7728, 1382.6112, 1386.7084, 1388.7888, 1390.7301, 1397.7515, 1425.7671, 1439.7701, 1442.8314, 1457.7517, 1467.7510, 1473.8167, 1488.7367, 1497.8859, 1500.7335, 1513.7731, 1516.7435, 1528.7101, 1556.8929, 1559.8439, 1562.8004, 1563.8088, 1578.7965, 1580.8445, 1592.8094, 1601.9139, 1616.8481, 1623.8562, 1627.7197, 1628.7129, 1628.8722, 1634.8071, 1642.7318, 1645.7701, 1647.8682, 1650.8612, 1673.8375, 1679.9817, 1680.8936, 1681.8372, 1696.8403, 1706.8660, 1715.8601, 1717.9037, 1730.8719, 1742.0088, 1758.9255, 1769.0105, 1771.0138, 1774.9404, 1783.9508, 1786.9517, 1787.9071, 1790.9086, 1835.8361, 1837.8712, 1862.9679, 1928.0435, 1940.0333, 1954.0796, 1961.0397, 1970.0139, 1982.0297, 1984.0314, 2023.0551, 2027.1292, 2032.0228, 2061.0330, 2105.0796, 2144.0679, 2170.0872, 2179.0752, 2211.0654, 2215.0876, 2239.1650, 2242.1501, 2246.2151, 2251.1675, 2252.1614, 2255.1719, 2273.1008, 2289.1030, 2293.1750, 2304.2087, 2316.2031, 2343.2134, 2354.0020, 2391.2788, 2475.2476, 2491.2402, 2507.1975, 2514.2664, 2542.2795, 2550.2063, 2558.3499, 2587.3516, 2605.2615, 2646.9883, 2661.0090, 2675.0190, 2681.3855, 2698.3696, 2705.3142, 2707.3074, 2714.3647, 2717.3394, 2719.3167, 2722.3188, 2763.3738, 2792.2446, 2826.3545, 2843.4670, 2846.4014, 2847.4082, 2872.3359, 2884.3538, 2887.3474, 2889.3481, 2892.4165, 2894.4639, 2896.4890, 2914.4551, 2987.5244, 2995.4431, 3004.5840, 3029.4812, 3054.4724, 3069.4849, 3072.4231, 3101.5515, 3126.7327, 3166.6106, 3171.6748, 3183.6196, 3199.6411, 3231.5630, 3236.5791, 3243.6301, 3245.6216, 3247.5737, 3248.6313, 3254.6448, 3262.7324, 3267.7256, 3278.7546, 3321.5828, 3333.5762, 3334.6729, 3335.6787, 3338.7097, 3347.4907, 3395.6821, 3412.6426, 3428.5991, 3435.7024, 3457.6995, 3487.8022, 3490.6885, 3503.8049, 3534.6365, 3548.6917, 3551.6768, 3552.6902, 3616.7095, 3624.6824, 3639.7014, 3645.7827, 3651.7559, 3655.7354, 3667.7646, 3697.8025, 3716.7253, 3762.8396, 3929.9304, 4040.7656, 4057.9707, 4071.9524, 4092.8394, 4147.9063, 4160.9473, 4174.9575, 4193.9395, 4253.8755, 4415.9736, 4452.1865, 4465.1670, 4845.0845

26. E1BKZ5 **Mass:** 378904 **Score:** 19 **Expect:** 2.9e+002 **Matches:** 50

Uncharacterized protein OS=Bos taurus GN=GOLGB1 PE=4 SV=2

Observed	Mr(expt)	Mr(calc)	ppm	Start	End	Miss	Peptide
831.4625	830.4552	830.4497	6.63	1502 -	1508	1	K.EALKENK.G
888.4968	887.4896	887.4824	8.02	1201 -	1207	2	K.KAQEKER.H
993.4956	992.4883	992.4848	3.53	1894 -	1901	0	K.MLQEEVTK.I + Oxidation (M)
1046.5728	1045.5655	1045.5880	-21.46	1658 -	1666	2	K.LRSTEANKK.E
1056.5387	1055.5314	1055.5069	23.2	1078 -	1085	0	K.EMELEHIR.K
1122.5580	1121.5507	1121.5353	13.8	2929 -	2937	0	K.AQLQEYQDK.T
1132.5316	1131.5243	1131.5342	-8.75	1104 -	1112	0	K.QMNQNLQEK.T
1216.6992	1215.6919	1215.6757	13.3	2516 -	2524	2	K.LKEEIRCLR.S
1273.6595	1272.6522	1272.6826	-23.87	2212 -	2221	2	R.VIDEAKKWER.K

1284.6533	1283.6460	1283.6357	8.02	1758	1768	0	K.DSLSEEVQHLK.H
1315.6904	1314.6831	1314.6891	-4.57	3169	3178	2	R.KLLEEERDQR.A
1349.6997	1348.6924	1348.6908	1.23	1592	1602	1	K.ENLMKEIESLK.C + Oxidation (M)
1351.6993	1350.6920	1350.6779	10.4	2929	2939	1	K.AQLQEYQDKTK.T
1361.6997	1360.6924	1360.7132	-15.30	2147	2157	2	K.KNMQEKLDALR.R + Oxidation (M)
1473.8167	1472.8094	1472.7834	17.6	988	999	2	R.ELDLVKKESEQR.K
1559.8439	1558.8366	1558.8202	10.5	2597	2610	1	R.SSLALQEEKQDLAK.E
1562.8004	1561.7931	1561.7835	6.17	2310	2323	1	R.SSQDDLAKLESELK.I
1580.8445	1579.8372	1579.8167	13.0	725	737	0	K.EISMLTQLIEEFK.K
1592.8094	1591.8021	1591.7949	4.52	323	335	1	K.VLMEKMELEVAER.K + Oxidation (M)
1616.8481	1615.8408	1615.8165	15.1	2584	2596	2	K.LKESEEAREELQR.S
1642.7318	1641.7245	1641.7556	-18.93	908	921	0	K.DQQVTELSFSMTEK.M
1681.8372	1680.8299	1680.8318	-1.14	1029	1042	1	K.DEPSKEILLHESER.R
1715.8601	1714.8528	1714.8486	2.47	1133	1150	1	K.LTTGNKDAGDGNSAAPVK.E
1742.0088	1741.0015	1740.9621	22.6	2017	2030	2	K.ELQELLKEKQQEVK.Q
1769.0105	1768.0032	1767.9982	2.86	1397	1411	1	R.DLKPELDELQKLISK.K
1787.9071	1786.8998	1786.8809	10.6	1527	1542	1	K.SLADVENQVSAQNREK.D
1928.0435	1927.0362	1927.0374	-0.60	1502	1518	2	K.EALKENKGLQEELSLAR.D
1982.0297	1981.0224	1981.0116	5.46	2646	2662	2	K.EEEVQKLSTVFSSSQKR.V
1984.0314	1983.0241	1983.0207	1.72	2376	2391	2	R.QLTSRLHEEINMKEQK.I
2023.0551	2022.0478	2022.0316	8.02	374	390	2	K.HNAEMEEKTSHILSLRK.S
2179.0752	2178.0679	2178.0222	21.0	390	408	2	R.KSEQELQSACDALKDQNSK.L
2251.1675	2250.1602	2250.1426	7.82	2031	2048	2	K.QLQTDCIRYQEKISALER.T
2252.1614	2251.1541	2251.1803	-11.63	1059	1077	2	R.VTSKCQEIEMSLKLIISEK.E + Oxidation (M)
2514.2664	2513.2591	2513.2907	-12.58	382	403	2	K.TSHILSLRKSEQELQSACDALK.D
2717.3394	2716.3321	2716.2982	12.5	2238	2259	2	K.EENCSVLKDQLRQMSIHMEELK.I
2872.3359	2871.3286	2871.3855	-19.80	1	26	1	-.MLSRLSGLANVVLHELSGDDTDENMR.A
2884.3538	2883.3465	2883.4032	-19.65	1357	1381	2	K.QDLEIQSLKAESCEAEAHAETLRQK.L
2892.4165	2891.4092	2891.4301	-7.21	790	814	0	R.LDYESQTAHHDLLTEQIHSLTIEAK.S
2894.4639	2893.4566	2893.5185	-21.39	2788	2813	2	R.ERDVLVSKAAFPVISTEDNSLPHLEK.L
3247.5737	3246.5664	3246.6156	-15.16	1622	1648	1	K.EYEILLQSYENVSNEAERIQHVVETVR.Q
3248.6313	3247.6240	3247.5581	20.3	2950	2974	2	K.QENLSWQHELHQLRIEKNSWEMQER.R
3321.5828	3320.5755	3320.5136	18.7	1709	1738	2	R.AEIHPAGDTSKDSMEALLSSNSNMKEELER.V + 2 Oxidation (M)
3333.5762	3332.5689	3332.6385	-20.88	3013	3041	1	K.VQYQRQASPETSVSLNGSQNLVYETEHLR.T
3412.6426	3411.6353	3411.7191	-24.56	103	133	2	K.LTSLNKHIEEMKAQGGTVLSTEPQSEEQLSK.H
3435.7024	3434.6951	3434.7404	-13.20	2940	2966	2	K.TFQIMQEELKQENLSWQHELHQLRIEK.N
3487.8022	3486.7949	3486.7262	19.7	908	937	2	K.DQQVTELSFSMTEKMVQLNEEKFSLGVEIK.T
3503.8049	3502.7976	3502.7211	21.8	908	937	2	K.DQQVTELSFSMTEKMVQLNEEKFSLGVEIK.T + Oxidation (M)
3651.7559	3650.7486	3650.8018	-14.58	1559	1591	2	K.LIAEVDRSLMENQSLSGSCESLKLALEGLTEDK.E + Oxidation (M)
4071.9524	4070.9451	4070.9551	-2.46	896	929	2	K.DVEILQQTIEEKDQQVTELSFSMTEKMVQLNEEK.F + 2 Oxidation (M)
4452.1865	4451.1792	4451.2040	-5.56	336	373	2	R.KLSFHNLQEEMHHLLEQLEQAGQAQAELQSRHSALEQK.H + Oxidation (M)

No match to: 701.3809, 706.2637, 708.2758, 717.3943, 719.4086, 722.4171, 734.4380, 735.4089, 736.4033, 747.4274, 756.3826, 759.3994, 772.4160, 779.4522, 795.4493, 806.4573, 832.3028, 834.3088, 842.4966, 876.2747, 878.2808, 883.4713, 890.4931, 891.4778, 900.5316, 910.4130, 918.5359, 929.4340, 936.4846, 937.5071, 938.5070, 949.5414, 967.4175, 969.4572, 972.4759, 974.4473, 976.4465, 978.5344, 983.6115, 988.4531, 990.4647, 1000.5594, 1007.5680, 1019.5631, 1021.5624, 1031.5641, 1034.5791, 1036.5952, 1044.5217, 1051.5135, 1057.6144, 1060.5447, 1074.5956, 1077.5199, 1087.5442, 1091.5437, 1113.5406, 1123.5852, 1145.6403, 1161.6290, 1168.6013, 1171.5769, 1172.5704, 1178.6254, 1180.6395, 1181.6516, 1185.6835, 1198.7111, 1234.6646, 1248.6750, 1254.6965, 1255.6359, 1278.6974, 1281.6390, 1301.6256, 1304.6489, 1314.7235, 1325.6581, 1336.7290, 1338.6799, 1342.6847, 1354.6641, 1368.6772, 1370.6589, 1372.6199, 1381.7728, 1382.6112, 1383.7378, 1386.7084, 1388.7888, 1390.7301, 1397.7515, 1425.7671, 1439.7701, 1442.8314, 1457.7517, 1467.7510, 1488.7367, 1497.8859, 1500.7335, 1513.7731, 1516.7435, 1528.7101, 1556.8929, 1563.8088, 1578.7965, 1601.9139, 1623.8562, 1627.7197, 1628.7129, 1628.8722, 1634.8071, 1638.8757, 1645.7701, 1647.8682, 1650.8612, 1673.8375, 1679.9817, 1680.8936, 1696.8403, 1706.8660, 1717.9037, 1730.8719, 1758.9255, 1771.0138, 1774.9404, 1783.9508, 1786.9517, 1790.9086, 1835.8361, 1837.8712, 1862.9679, 1940.0333, 1954.0796, 1961.0397, 1970.0139, 2027.1292, 2032.0228, 2061.0330, 2105.0796, 2144.0679, 2170.0872, 2211.0654, 2215.0876, 2239.1650, 2242.1501, 2246.2151, 2255.1719, 2273.1008, 2289.1030, 2293.1750, 2304.2087, 2316.2031, 2343.2134, 2354.0020, 2391.2788, 2475.2476, 2491.2402, 2507.1975, 2542.2795, 2550.2063, 2558.3499, 2587.3516, 2605.2615, 2646.9883, 2661.0090, 2675.0190, 2681.3855, 2698.3696, 2705.3142, 2707.3074, 2714.3647, 2719.3167, 2722.3188, 2763.3738, 2792.2446, 2826.3545, 2843.4670, 2846.4014, 2847.4082, 2887.3474, 2889.3481, 2896.4890, 2914.4551, 2987.5244, 2995.4431, 3004.5840, 3029.4812, 3054.4724, 3069.4849, 3072.4231, 3101.5515, 3126.7327, 3166.6106, 3171.6748, 3183.6196, 3199.6411, 3231.5630, 3236.5791, 3243.6301, 3245.6216, 3254.6448, 3262.7324, 3267.7256, 3278.7546, 3334.6729, 3335.6787, 3338.7097, 3347.4907, 3395.6821, 3428.5991, 3457.6995, 3490.6885, 3534.6365, 3548.6917, 3551.6768, 3552.6902, 3601.6826, 3616.7095, 3624.6824, 3639.7014, 3645.7827, 3655.7354, 3667.7646, 3697.8025, 3716.7253, 3762.8396, 3929.9304, 4040.7656, 4057.9707, 4092.8394, 4147.9063, 4160.9473, 4174.9575, 4193.9395, 4253.8755, 4415.9736, 4465.1670, 4845.0845

27. E1BA62 **Mass:** 59054 **Score:** 19 **Expect:** 3e+002 **Matches:** 13

Uncharacterized protein OS=Bos taurus GN=DNM3 PE=3 SV=2

Observed	Mr(expt)	Mr(calc)	ppm	Start	End	Miss	Peptide
701.3809	700.3736	700.3656	11.4	360	364	0	R.IFHER.F
717.3943	716.3870	716.3969	-13.86	441	446	0	K.LANFPR.L
1019.5631	1018.5558	1018.5672	-11.18	224	232	1	R.RGYVGVVNR.S
1031.5641	1030.5568	1030.5659	-8.77	236	244	2	K.DIDGKKDIK.A
1168.6013	1167.5940	1167.5780	13.7	153	161	0	R.EMIMQFITR.E
1234.6646	1233.6573	1233.6652	-6.38	437	446	2	K.CTKKLANFPR.L
1351.6993	1350.6920	1350.6714	15.3	372	381	2	K.MEFNEKELRR.E
1634.8071	1633.7998	1633.7882	7.11	441	453	1	K.LANFPRLCEETER.I
1673.8375	1672.8302	1672.8171	7.87	365	377	1	R.FPFEIVKMEFNEK.E + Oxidation (M)
1783.9508	1782.9435	1782.9226	11.7	395	410	1	R.TGLFTPDMAFEAIVKK.Q + Oxidation (M)
2343.2134	2342.2061	2342.1787	11.7	162	183	0	R.ENCLILAVTPANTDLANSDALK.L
2542.2795	2541.2722	2541.3108	-15.19	202	223	2	K.LDLMDEGTDARDVLENKLLPLR.R + Oxidation (M)
3183.6196	3182.6123	3182.6360	-7.43	293	319	2	R.NKLQGQLLSIEHEVEAYKNFKPEDPTR.K

No match to: 706.2637, 708.2758, 719.4086, 722.4171, 734.4380, 735.4089, 736.4033, 747.4274, 756.3826, 759.3994, 772.4160, 779.4522, 795.4493, 806.4573, 831.4625, 832.3028, 834.3088, 842.4966, 876.2747, 878.2808, 883.4713, 888.4968, 890.4931, 891.4778, 900.5316, 910.4130, 918.5359, 929.4340, 936.4846, 937.5071, 938.5070, 949.5414, 967.4175, 969.4572, 972.4759, 974.4473, 976.4465, 978.5344, 983.6115, 988.4531, 990.4647, 993.4956, 1000.5594, 1007.5680, 1021.5624, 1034.5791, 1036.5952, 1044.5217, 1046.5728, 1051.5135, 1056.5387, 1057.6144, 1060.5447, 1074.5956, 1077.5199, 1087.5442, 1091.5437, 1113.5406, 1122.5580, 1123.5852, 1132.5316, 1145.6403, 1161.6290, 1171.5769, 1172.5704, 1178.6254, 1180.6395, 1181.6516, 1185.6835, 1198.7111, 1216.6992, 1248.6750, 1254.6965, 1255.6359, 1273.6595, 1278.6974, 1281.6390, 1284.6533, 1301.6256, 1304.6489, 1314.7235, 1315.6904, 1325.6581, 1336.7290, 1338.6799, 1342.6847, 1349.6997, 1354.6641, 1361.6997, 1368.6772, 1370.6589, 1372.6199, 1381.7728, 1382.6112, 1383.7378, 1386.7084, 1388.7888, 1390.7301, 1397.7515, 1425.7671, 1439.7701, 1442.8314, 1457.7517, 1467.7510, 1473.8167, 1488.7367, 1497.8859, 1500.7335, 1513.7731, 1516.7435, 1528.7101, 1556.8929, 1559.8439, 1562.8004, 1563.8088, 1578.7965, 1580.8445, 1592.8094, 1601.9139, 1616.8481, 1623.8562, 1627.7197, 1628.7129, 1628.8722, 1638.8757, 1642.7318, 1645.7701, 1647.8682, 1650.8612, 1679.9817, 1680.8936, 1681.8372, 1696.8403, 1706.8660, 1715.8601, 1717.9037, 1730.8719, 1742.0088, 1758.9255, 1769.0105, 1771.0138, 1774.9404, 1786.9517, 1787.9071, 1790.9086,

1835.8361, 1837.8712, 1862.9679, 1928.0435, 1940.0333, 1954.0796, 1961.0397, 1970.0139, 1982.0297, 1984.0314, 2023.0551, 2027.1292, 2032.0228, 2061.0330, 2105.0796, 2144.0679, 2170.0872, 2179.0752, 2211.0654, 2215.0876, 2239.1650, 2242.1501, 2246.2151, 2251.1675, 2252.1614, 2255.1719, 2273.1008, 2289.1030, 2293.1750, 2304.2087, 2316.2031, 2354.0020, 2391.2788, 2475.2476, 2491.2402, 2507.1975, 2514.2664, 2550.2063, 2558.3499, 2587.3516, 2605.2615, 2646.9883, 2661.0090, 2675.0190, 2681.3855, 2698.3696, 2705.3142, 2707.3074, 2714.3647, 2717.3394, 2719.3167, 2722.3188, 2763.3738, 2792.2446, 2826.3545, 2843.4670, 2846.4014, 2847.4082, 2872.3359, 2884.3538, 2887.3474, 2889.3481, 2892.4165, 2894.4639, 2896.4890, 2914.4551, 2987.5244, 2995.4431, 3004.5840, 3029.4812, 3054.4724, 3069.4849, 3072.4231, 3101.5515, 3126.7327, 3166.6106, 3171.6748, 3199.6411, 3231.5630, 3236.5791, 3243.6301, 3245.6216, 3247.5737, 3248.6313, 3254.6448, 3262.7324, 3267.7256, 3278.7546, 3321.5828, 3333.5762, 3334.6729, 3335.6787, 3338.7097, 3347.4907, 3395.6821, 3412.6426, 3428.5991, 3435.7024, 3457.6995, 3487.8022, 3490.6885, 3503.8049, 3534.6365, 3548.6917, 3551.6768, 3552.6902, 3601.6826, 3616.7095, 3624.6824, 3639.7014, 3645.7827, 3651.7559, 3655.7354, 3667.7646, 3697.8025, 3716.7253, 3762.8396, 3929.9304, 4040.7656, 4057.9707, 4071.9524, 4092.8394, 4147.9063, 4160.9473, 4174.9575, 4193.9395, 4253.8755, 4415.9736, 4452.1865, 4465.1670, 4845.0845

28. P67827 **Mass:** 37771 **Score:** 19 **Expect:** 3e+002 **Matches:** 10

Casein kinase I isoform alpha OS=Bos taurus GN=CSNK1A1 PE=1 SV=1

Observed	Mr(expt)	Mr(calc)	ppm	Start	End	Miss	Peptide
831.4625	830.4552	830.4610	-6.91	47	53	1	K.LESQKAR.H
1715.8601	1714.8528	1714.8890	-21.08	2	18	2	M.ASSSGSKAEFIVGGKYK.L
1862.9679	1861.9606	1861.9244	19.5	1	18	2	-.MASSSGSKAEFIVGGKYK.L + Oxidation (M)
2304.2087	2303.2014	2303.1521	21.4	265	282	2	R.GLRFEEAPDYMYLRQLFR.I
2605.2615	2604.2542	2604.2835	-11.24	283	302	1	R.ILFRTLNHQYDYTFDWTMLK.Q
2698.3696	2697.3623	2697.3380	9.01	131	152	2	K.NFIHRDIKPDNFLMGIGRHCNK.L + Oxidation (M)
2717.3394	2716.3321	2716.2877	16.4	84	106	0	K.DYNVLVMDLLGPSLEDLFNFCSR.R
2889.3481	2888.3408	2888.3837	-14.84	84	107	1	K.DYNVLVMDLLGPSLEDLFNFCSRR.F + Oxidation (M)
3412.6426	3411.6353	3411.6302	1.51	84	111	2	K.DYNVLVMDLLGPSLEDLFNFCSRRFTMK.T + 2 Oxidation (M)
3548.6917	3547.6844	3547.6976	-3.71	187	216	2	R.YASINAHLGIEQSRRDDMESLGYVLMYFNR.T

No match to: 701.3809, 706.2637, 708.2758, 717.3943, 719.4086, 722.4171, 734.4380, 735.4089, 736.4033, 747.4274, 756.3826, 759.3994, 772.4160, 779.4522, 795.4493, 806.4573, 832.3028, 834.3088, 842.4966, 876.2747, 878.2808, 883.4713, 888.4968, 890.4931, 891.4778, 900.5316, 910.4130, 918.5359, 929.4340, 936.4846, 937.5071, 938.5070, 949.5414, 967.4175, 969.4572, 972.4759, 974.4473, 976.4465, 978.5344, 983.6115, 988.4531, 990.4647, 993.4956, 1000.5594, 1007.5680, 1019.5631, 1021.5624, 1031.5641, 1034.5791, 1036.5952, 1044.5217, 1046.5728, 1051.5135, 1056.5387, 1057.6144, 1060.5447, 1074.5956, 1077.5199, 1087.5442, 1091.5437, 1113.5406, 1122.5580, 1123.5852, 1132.5316, 1145.6403, 1161.6290, 1168.6013, 1171.5769, 1172.5704, 1178.6254, 1180.6395, 1181.6516, 1185.6835, 1198.7111, 1216.6992, 1234.6646, 1248.6750, 1254.6965, 1255.6359, 1273.6595, 1278.6974, 1281.6390, 1284.6533, 1301.6256, 1304.6489, 1314.7235, 1315.6904, 1325.6581, 1336.7290, 1338.6799, 1342.6847, 1349.6997, 1351.6993, 1354.6641, 1361.6997, 1368.6772, 1370.6589, 1372.6199, 1381.7728, 1382.6112, 1383.7378, 1386.7084, 1388.7888, 1390.7301, 1397.7515, 1425.7671, 1439.7701, 1442.8314, 1457.7517, 1467.7510, 1473.8167, 1488.7367, 1497.8859, 1500.7335, 1513.7731, 1516.7435, 1528.7101, 1556.8929, 1559.8439, 1562.8004, 1563.8088, 1578.7965, 1580.8445, 1592.8094, 1601.9139, 1616.8481, 1623.8562, 1627.7197, 1628.7129, 1628.8722, 1634.8071, 1638.8757, 1642.7318, 1645.7701, 1647.8682, 1650.8612, 1673.8375, 1679.9817, 1680.8936, 1681.8372, 1696.8403, 1706.8660, 1717.9037, 1730.8719, 1742.0088, 1758.9255, 1769.0105, 1771.0138, 1774.9404, 1783.9508, 1786.9517, 1787.9071, 1790.9086, 1835.8361, 1837.8712, 1928.0435, 1940.0333, 1954.0796, 1961.0397, 1970.0139, 1982.0297, 1984.0314, 2023.0551, 2027.1292, 2032.0228, 2061.0330, 2105.0796, 2144.0679, 2170.0872, 2179.0752, 2211.0654, 2215.0876, 2239.1650, 2242.1501, 2246.2151, 2251.1675, 2252.1614, 2255.1719, 2273.1008, 2289.1030, 2293.1750, 2316.2031, 2343.2134, 2354.0020, 2391.2788, 2475.2476, 2491.2402, 2507.1975, 2514.2664, 2542.2795, 2550.2063, 2558.3499, 2587.3516, 2646.9883, 2661.0090, 2675.0190, 2681.3855, 2705.3142, 2707.3074, 2714.3647, 2719.3167, 2722.3188, 2763.3738, 2792.2446, 2826.3545, 2843.4670, 2846.4014, 2847.4082, 2872.3359, 2884.3538, 2887.3474, 2892.4165, 2894.4639, 2896.4890, 2914.4551, 2987.5244, 2995.4431, 3004.5840, 3029.4812, 3054.4724, 3069.4849, 3072.4231, 3101.5515, 3126.7327, 3166.6106, 3171.6748, 3183.6196, 3199.6411, 3231.5630, 3236.5791, 3243.6301, 3245.6216, 3247.5737, 3248.6313, 3254.6448, 3262.7324, 3267.7256, 3278.7546, 3321.5828, 3333.5762, 3334.6729, 3335.6787, 3338.7097, 3347.4907, 3395.6821, 3428.5991, 3435.7024, 3457.6995, 3487.8022, 3490.6885, 3503.8049, 3534.6365, 3551.6768, 3552.6902, 3601.6826, 3616.7095, 3624.6824, 3639.7014, 3645.7827, 3651.7559, 3655.7354, 3667.7646, 3697.8025, 3716.7253, 3762.8396, 3929.9304, 4040.7656, 4057.9707, 4071.9524, 4092.8394, 4147.9063, 4160.9473, 4174.9575, 4193.9395, 4253.8755, 4415.9736, 4452.1865, 4465.1670, 4845.0845

29. Q32LI4 **Mass:** 38763 **Score:** 19 **Expect:** 3e+002 **Matches:** 10

CSNK1A1 protein OS=Bos taurus GN=CSNK1A1 PE=2 SV=1

Observed	Mr(expt)	Mr(calc)	ppm	Start	End	Miss	Peptide
831.4625	830.4552	830.4610	-6.91	47	53	1	K.LESQKAR.H
1715.8601	1714.8528	1714.8890	-21.08	2	18	2	M.ASSSGSKAEFIVGGKYK.L
1862.9679	1861.9606	1861.9244	19.5	1	18	2	-.MASSSGSKAEFIVGGKYK.L + Oxidation (M)
2304.2087	2303.2014	2303.1521	21.4	265	282	2	R.GLRFEEAPDYMYLRQLFR.I
2605.2615	2604.2542	2604.2835	-11.24	283	302	1	R.ILFRTLNHQYDYTFDWTMLK.Q
2698.3696	2697.3623	2697.3380	9.01	131	152	2	K.NFIHRDIKPDNFLMGIGRHCNK.L + Oxidation (M)
2717.3394	2716.3321	2716.2877	16.4	84	106	0	K.DYNVLVMDLLGPSLEDLFNFCSR.R
2889.3481	2888.3408	2888.3837	-14.84	84	107	1	K.DYNVLVMDLLGPSLEDLFNFCSRR.F + Oxidation (M)
3412.6426	3411.6353	3411.6302	1.51	84	111	2	K.DYNVLVMDLLGPSLEDLFNFCSRRFTMK.T + 2 Oxidation (M)
3548.6917	3547.6844	3547.6976	-3.71	187	216	2	R.YASINAHLGIEQSRRDDMESLGYVLMYFNR.T

No match to: 701.3809, 706.2637, 708.2758, 717.3943, 719.4086, 722.4171, 734.4380, 735.4089, 736.4033, 747.4274, 756.3826, 759.3994, 772.4160, 779.4522, 795.4493, 806.4573, 832.3028, 834.3088, 842.4966, 876.2747, 878.2808, 883.4713, 888.4968, 890.4931, 891.4778, 900.5316, 910.4130, 918.5359, 929.4340, 936.4846, 937.5071, 938.5070, 949.5414, 967.4175, 969.4572, 972.4759, 974.4473, 976.4465, 978.5344, 983.6115, 988.4531, 990.4647, 993.4956, 1000.5594, 1007.5680, 1019.5631, 1021.5624, 1031.5641, 1034.5791, 1036.5952, 1044.5217, 1046.5728, 1051.5135, 1056.5387, 1057.6144, 1060.5447, 1074.5956, 1077.5199, 1087.5442, 1091.5437, 1113.5406, 1122.5580, 1123.5852, 1132.5316, 1145.6403, 1161.6290, 1168.6013, 1171.5769, 1172.5704, 1178.6254, 1180.6395, 1181.6516, 1185.6835, 1198.7111, 1216.6992, 1234.6646, 1248.6750, 1254.6965, 1255.6359, 1273.6595, 1278.6974, 1281.6390, 1284.6533, 1301.6256, 1304.6489, 1314.7235, 1315.6904, 1325.6581, 1336.7290, 1338.6799, 1342.6847, 1349.6997, 1351.6993, 1354.6641, 1361.6997, 1368.6772, 1370.6589, 1372.6199, 1381.7728, 1382.6112, 1383.7378, 1386.7084, 1388.7888, 1390.7301, 1397.7515, 1425.7671, 1439.7701, 1442.8314, 1457.7517, 1467.7510, 1473.8167, 1488.7367, 1497.8859, 1500.7335, 1513.7731, 1516.7435, 1528.7101, 1556.8929, 1559.8439, 1562.8004, 1563.8088, 1578.7965, 1580.8445, 1592.8094, 1601.9139, 1616.8481, 1623.8562, 1627.7197, 1628.7129, 1628.8722, 1634.8071, 1638.8757, 1642.7318, 1645.7701, 1647.8682, 1650.8612, 1673.8375, 1679.9817, 1680.8936, 1681.8372, 1696.8403, 1706.8660, 1717.9037, 1730.8719, 1742.0088, 1758.9255, 1769.0105, 1771.0138, 1774.9404, 1783.9508, 1786.9517, 1787.9071, 1790.9086, 1835.8361, 1837.8712, 1928.0435, 1940.0333, 1954.0796, 1961.0397, 1970.0139, 1982.0297, 1984.0314, 2023.0551, 2027.1292, 2032.0228, 2061.0330, 2105.0796, 2144.0679, 2170.0872, 2179.0752, 2211.0654, 2215.0876, 2239.1650, 2242.1501, 2246.2151, 2251.1675, 2252.1614, 2255.1719, 2273.1008, 2289.1030, 2293.1750, 2316.2031, 2343.2134, 2354.0020, 2391.2788, 2475.2476, 2491.2402, 2507.1975, 2514.2664, 2542.2795, 2550.2063, 2558.3499, 2587.3516, 2646.9883, 2661.0090, 2675.0190, 2681.3855, 2705.3142, 2707.3074, 2714.3647, 2719.3167, 2722.3188, 2763.3738, 2792.2446, 2826.3545, 2843.4670, 2846.4014, 2847.4082, 2872.3359, 2884.3538, 2887.3474, 2892.4165, 2894.4639, 2896.4890, 2914.4551, 2987.5244, 2995.4431, 3004.5840, 3029.4812, 3054.4724, 3069.4849, 3072.4231, 3101.5515, 3126.7327, 3166.6106, 3171.6748, 3183.6196, 3199.6411, 3231.5630, 3236.5791, 3243.6301, 3245.6216, 3247.5737, 3248.6313, 3254.6448, 3262.7324, 3267.7256, 3278.7546, 3321.5828, 3333.5762, 3334.6729, 3335.6787, 3338.7097, 3347.4907, 3395.6821, 3428.5991, 3435.7024, 3457.6995, 3487.8022, 3490.6885, 3503.8049, 3534.6365, 3551.6768, 3552.6902, 3601.6826, 3616.7095, 3624.6824, 3639.7014, 3645.7827, 3651.7559, 3655.7354, 3667.7646, 3697.8025, 3716.7253, 3762.8396, 3929.9304, 4040.7656, 4057.9707, 4071.9524, 4092.8394, 4147.9063, 4160.9473, 4174.9575, 4193.9395, 4253.8755, 4415.9736, 4452.1865, 4465.1670, 4845.0845

30. A6QP66 **Mass:** 23698 **Score:** 19 **Expect:** 3e+002 **Matches:** 7

Ras-related and estrogen-regulated growth inhibitor-like protein OS=Bos taurus GN=RERGL PE=2 SV=1

Observed	Mr(expt)	Mr(calc)	ppm	Start	End	Miss	Peptide
701.3809	700.3736	700.3868	-18.73	140	145	0	K.LALDNR.C
974.4473	973.4400	973.4539	-14.24	183	191	1	R.YSGSKSMAK.L + Oxidation (M)
1031.5641	1030.5568	1030.5659	-8.79	6	16	0	K.LTVLGGEGTGK.S

1216.6992	1215.6919	1215.7200	-23.09	192 - 201	2	K.LINNVFGKRR.K
1255.6359	1254.6286	1254.6139	11.7	101 - 110	1	R.IREPQTSHCK.R
1634.8071	1633.7998	1633.8345	-21.24	1 - 16	1	-.MNDVKLTVLGGEGTGK.S + Oxidation (M)
2032.0228	2031.0155	2031.0095	2.97	50 - 66	1	K.QLNLEIYDPCSQPQKAK.F

No match to: 706.2637, 708.2758, 717.3943, 719.4086, 722.4171, 734.4380, 735.4089, 736.4033, 747.4274, 756.3826, 759.3994, 772.4160, 779.4522, 795.4493, 806.4573, 831.4625, 832.3028, 834.3088, 842.4966, 876.2747, 878.2808, 883.4713, 888.4968, 890.4931, 891.4778, 900.5316, 910.4130, 918.5359, 929.4340, 936.4846, 937.5071, 938.5070, 949.5414, 967.4175, 969.4572, 972.4759, 976.4465, 978.5344, 983.6115, 988.4531, 990.4647, 993.4956, 1000.5594, 1007.5680, 1019.5631, 1021.5624, 1034.5791, 1036.5952, 1044.5217, 1046.5728, 1051.5135, 1056.5387, 1057.6144, 1060.5447, 1074.5956, 1077.5199, 1087.5442, 1091.5437, 1113.5406, 1122.5580, 1123.5852, 1132.5316, 1145.6403, 1161.6290, 1168.6013, 1171.5769, 1172.5704, 1178.6254, 1180.6395, 1181.6516, 1185.6835, 1198.7111, 1234.6646, 1248.6750, 1254.6965, 1273.6595, 1278.6974, 1281.6390, 1284.6533, 1301.6256, 1304.6489, 1314.7235, 1315.6904, 1325.6581, 1336.7290, 1338.6799, 1342.6847, 1349.6997, 1351.6993, 1354.6641, 1361.6997, 1368.6772, 1370.6589, 1372.6199, 1381.7728, 1382.6112, 1383.7378, 1386.7084, 1388.7888, 1390.7301, 1397.7515, 1425.7671, 1439.7701, 1442.8314, 1457.7517, 1467.7510, 1473.8167, 1488.7367, 1497.8859, 1500.7335, 1513.7731, 1516.7435, 1528.7101, 1556.8929, 1559.8439, 1562.8004, 1563.8088, 1578.7965, 1580.8445, 1592.8094, 1601.9139, 1616.8481, 1623.8562, 1627.7197, 1628.7129, 1628.8722, 1638.8757, 1642.7318, 1645.7701, 1647.8682, 1650.8612, 1673.8375, 1679.9817, 1680.8936, 1681.8372, 1696.8403, 1706.8660, 1715.8601, 1717.9037, 1730.8719, 1742.0088, 1758.9255, 1769.0105, 1771.0138, 1774.9404, 1783.9508, 1786.9517, 1787.9071, 1790.9086, 1835.8361, 1837.8712, 1862.9679, 1928.0435, 1940.0333, 1954.0796, 1961.0397, 1970.0139, 1982.0297, 1984.0314, 2023.0551, 2027.1292, 2061.0330, 2105.0796, 2144.0679, 2170.0872, 2179.0752, 2211.0654, 2215.0876, 2239.1650, 2242.1501, 2246.2151, 2251.1675, 2252.1614, 2255.1719, 2273.1008, 2289.1030, 2293.1750, 2304.2087, 2316.2031, 2343.2134, 2354.0020, 2391.2788, 2475.2476, 2491.2402, 2507.1975, 2514.2664, 2542.2795, 2550.2063, 2558.3499, 2587.3516, 2605.2615, 2646.9883, 2661.0090, 2675.0190, 2681.3855, 2698.3696, 2705.3142, 2707.3074, 2714.3647, 2717.3394, 2719.3167, 2722.3188, 2763.3738, 2792.2446, 2826.3545, 2843.4670, 2846.4014, 2847.4082, 2872.3359, 2884.3538, 2887.3474, 2889.3481, 2892.4165, 2894.4639, 2896.4890, 2914.4551, 2987.5244, 2995.4431, 3004.5840, 3029.4812, 3054.4724, 3069.4849, 3072.4231, 3101.5515, 3126.7327, 3166.6106, 3171.6748, 3183.6196, 3199.6411, 3231.5630, 3236.5791, 3243.6301, 3245.6216, 3247.5737, 3248.6313, 3254.6448, 3262.7324, 3267.7256, 3278.7546, 3321.5828, 3333.5762, 3334.6729, 3335.6787, 3338.7097, 3347.4907, 3395.6821, 3412.6426, 3428.5991, 3435.7024, 3457.6995, 3487.8022, 3490.6885, 3503.8049, 3534.6365, 3548.6917, 3551.6768, 3552.6902, 3601.6826, 3616.7095, 3624.6824, 3639.7014, 3645.7827, 3651.7559, 3655.7354, 3667.7646, 3697.8025, 3716.7253, 3762.8396, 3929.9304, 4040.7656, 4057.9707, 4071.9524, 4092.8394, 4147.9063, 4160.9473, 4174.9575, 4193.9395, 4253.8755, 4415.9736, 4452.1865, 4465.1670, 4845.0845

31. E1BEI2 **Mass:** 25560 **Score:** 19 **Expect:** 3.1e+002 **Matches:** 7
Uncharacterized protein OS=Bos taurus GN=SPCS2 PE=1 SV=1

Observed	Mr(expt)	Mr(calc)	ppm	Start - End	Miss	Peptide
1123.5852	1122.5779	1122.5921	-12.59	190 - 199	1	R.EAEFTKSIAK.F
1559.8439	1558.8366	1558.8566	-12.81	58 - 71	2	K.NSLDDSAKKVLLEK.Y
1742.0088	1741.0015	1740.9774	13.9	33 - 47	2	R.SGLLDKWKIDDKPVK.I
2289.1030	2288.0957	2288.1107	-6.54	150 - 169	2	R.KDPTGMDPDDIWQLSSSLKR.F
3236.5791	3235.5718	3235.5244	14.7	200 - 227	1	K.FFDHSGTLVMDAYEPEISRLHDSLATER.K
3334.6729	3333.6656	3333.5863	23.8	190 - 218	2	R.EAEFTKSIAKFFDHSGTLVMDAYEPEISR.L + Oxidation (M)
3651.7559	3650.7486	3650.7675	-5.17	196 - 227	2	K.SIAKFFDHSGTLVMDAYEPEISRLHDSLATER.K + Oxidation (M)

No match to: 701.3809, 706.2637, 708.2758, 717.3943, 719.4086, 722.4171, 734.4380, 735.4089, 736.4033, 747.4274, 756.3826, 759.3994, 772.4160, 779.4522, 795.4493, 806.4573, 831.4625, 832.3028, 834.3088, 842.4966, 876.2747, 878.2808, 883.4713, 888.4968, 890.4931, 891.4778, 900.5316, 910.4130, 918.5359, 929.4340, 936.4846, 937.5071, 938.5070, 949.5414, 967.4175, 969.4572, 972.4759, 974.4473, 976.4465, 978.5344, 983.6115, 988.4531, 990.4647, 993.4956, 1000.5594, 1007.5680, 1019.5631, 1021.5624, 1031.5641, 1034.5791, 1036.5952, 1044.5217, 1046.5728, 1051.5135, 1056.5387, 1057.6144, 1060.5447, 1074.5956, 1077.5199, 1087.5442, 1091.5437, 1113.5406, 1122.5580, 1132.5316, 1145.6403, 1161.6290, 1168.6013, 1171.5769, 1172.5704, 1178.6254, 1180.6395, 1181.6516, 1185.6835, 1198.7111, 1216.6992, 1234.6646, 1248.6750, 1254.6965, 1255.6359, 1273.6595, 1278.6974, 1281.6390, 1284.6533, 1301.6256, 1304.6489, 1314.7235, 1315.6904, 1325.6581, 1336.7290, 1338.6799, 1342.6847, 1349.6997, 1351.6993, 1354.6641, 1361.6997, 1368.6772, 1370.6589, 1372.6199, 1381.7728, 1382.6112, 1383.7378, 1386.7084, 1388.7888, 1390.7301, 1397.7515, 1425.7671, 1439.7701, 1442.8314, 1457.7517, 1467.7510, 1473.8167, 1488.7367, 1497.8859, 1500.7335, 1513.7731, 1516.7435, 1528.7101, 1556.8929, 1562.8004, 1563.8088, 1578.7965, 1580.8445, 1592.8094, 1601.9139, 1616.8481, 1623.8562, 1627.7197, 1628.7129, 1628.8722, 1634.8071, 1638.8757, 1642.7318, 1645.7701, 1647.8682, 1650.8612, 1673.8375, 1679.9817, 1680.8936, 1681.8372, 1696.8403, 1706.8660, 1715.8601, 1717.9037, 1730.8719, 1758.9255, 1769.0105, 1771.0138, 1774.9404, 1783.9508, 1786.9517, 1787.9071, 1790.9086, 1835.8361, 1837.8712, 1862.9679, 1928.0435, 1940.0333, 1954.0796, 1961.0397, 1970.0139, 1982.0297, 1984.0314, 2023.0551, 2027.1292, 2032.0228, 2061.0330, 2105.0796, 2144.0679, 2170.0872, 2179.0752, 2211.0654, 2215.0876, 2239.1650, 2242.1501, 2246.2151, 2251.1675, 2252.1614, 2255.1719, 2273.1008, 2293.1750, 2304.2087, 2316.2031, 2343.2134, 2354.0020, 2391.2788, 2475.2476, 2491.2402, 2507.1975, 2514.2664, 2542.2795, 2550.2063, 2558.3499, 2587.3516, 2605.2615, 2646.9883, 2661.0090, 2675.0190, 2681.3855, 2698.3696, 2705.3142, 2707.3074, 2714.3647, 2717.3394, 2719.3167, 2722.3188, 2763.3738, 2792.2446, 2826.3545, 2843.4670, 2846.4014, 2847.4082, 2872.3359, 2884.3538, 2887.3474, 2889.3481, 2892.4165, 2894.4639, 2896.4890, 2914.4551, 2987.5244, 2995.4431, 3004.5840, 3029.4812, 3054.4724, 3069.4849, 3072.4231, 3101.5515, 3126.7327, 3166.6106, 3171.6748, 3183.6196, 3199.6411, 3231.5630, 3243.6301, 3245.6216, 3247.5737, 3248.6313, 3254.6448, 3262.7324, 3267.7256, 3278.7546, 3321.5828, 3333.5762, 3335.6787, 3338.7097, 3347.4907, 3395.6821, 3412.6426, 3428.5991, 3435.7024, 3457.6995, 3487.8022, 3490.6885, 3503.8049, 3534.6365, 3548.6917, 3551.6768, 3552.6902, 3601.6826, 3616.7095, 3624.6824, 3639.7014, 3645.7827, 3655.7354, 3667.7646, 3697.8025, 3716.7253, 3762.8396, 3929.9304, 4040.7656, 4057.9707, 4071.9524, 4092.8394, 4147.9063, 4160.9473, 4174.9575, 4193.9395, 4253.8755, 4415.9736, 4452.1865, 4465.1670, 4845.0845

32. F1MWE0 **Mass:** 51365 **Score:** 19 **Expect:** 3.1e+002 **Matches:** 11
Uncharacterized protein OS=Bos taurus GN=PSMC3 PE=3 SV=2

Observed	Mr(expt)	Mr(calc)	ppm	Start - End	Miss	Peptide
735.4089	734.4016	734.3963	7.25	287 - 293	0	R.DAFALAK.E
772.4160	771.4087	771.4239	-19.63	195 - 200	0	K.QIQEVR.E
831.4625	830.4552	830.4722	-20.45	362 - 368	2	R.SGRLDRK.I
1056.5387	1055.5314	1055.5433	-11.30	63 - 71	0	R.VTHELQAMK.D
1074.5956	1073.5883	1073.6080	-18.37	46 - 54	1	R.TRLLDSEIK.I
1122.5580	1121.5507	1121.5387	10.8	37 - 45	0	K.MSTEEIIQR.T + Oxidation (M)
1315.6904	1314.6831	1314.6602	17.5	63 - 73	1	R.VTHELQAMKDK.I + Oxidation (M)
2316.2031	2315.1958	2315.1984	-1.11	229 - 250	1	K.FENLGIQPPKGVLMYGPPGTGK.T + Oxidation (M)
2717.3394	2716.3321	2716.3854	-19.62	322 - 344	1	R.EVQRTMLELLNQLDGFQPNTQVK.V + Oxidation (M)
3262.7324	3261.7251	3261.7496	-7.51	146 - 174	2	K.LKPGDLVGVNKDSYLILETLPTEYDSRVK.A
3624.6824	3623.6751	3623.6971	-6.06	15 - 45	2	R.QEKMATVWDEAEQDGIGEEVLKMSTEEIIQR.T + 2 Oxidation (M)

No match to: 701.3809, 706.2637, 708.2758, 717.3943, 719.4086, 722.4171, 734.4380, 736.4033, 747.4274, 756.3826, 759.3994, 779.4522, 795.4493, 806.4573, 832.3028, 834.3088, 842.4966, 876.2747, 878.2808, 883.4713, 888.4968, 890.4931, 891.4778, 900.5316, 910.4130, 918.5359, 929.4340, 936.4846, 937.5071, 938.5070, 949.5414, 967.4175, 969.4572, 972.4759, 974.4473, 976.4465, 978.5344, 983.6115, 988.4531, 990.4647, 993.4956, 1000.5594, 1007.5680, 1019.5631, 1021.5624, 1031.5641, 1034.5791, 1036.5952, 1044.5217, 1046.5728, 1051.5135, 1057.6144, 1060.5447, 1077.5199, 1087.5442, 1091.5437, 1113.5406, 1123.5852, 1132.5316, 1145.6403, 1161.6290, 1168.6013, 1171.5769, 1172.5704, 1178.6254, 1180.6395, 1181.6516, 1185.6835, 1198.7111, 1216.6992, 1234.6646, 1248.6750, 1254.6965, 1255.6359, 1273.6595, 1278.6974, 1281.6390, 1284.6533, 1301.6256, 1304.6489, 1314.7235, 1325.6581, 1336.7290, 1338.6799, 1342.6847, 1349.6997, 1351.6993, 1354.6641, 1361.6997, 1368.6772, 1370.6589, 1372.6199, 1381.7728, 1382.6112, 1383.7378, 1386.7084, 1388.7888, 1390.7301, 1397.7515, 1425.7671, 1439.7701, 1442.8314, 1457.7517, 1467.7510, 1473.8167, 1488.7367, 1497.8859, 1500.7335, 1513.7731, 1516.7435, 1528.7101, 1556.8929, 1559.8439, 1562.8004, 1563.8088, 1578.7965, 1580.8445, 1592.8094, 1601.9139, 1616.8481, 1623.8562, 1627.7197, 1628.7129, 1628.8722, 1634.8071, 1638.8757, 1642.7318, 1645.7701, 1647.8682, 1650.8612, 1673.8375, 1679.9817, 1680.8936, 1681.8372, 1696.8403, 1706.8660, 1715.8601, 1717.9037, 1730.8719, 1742.0088, 1758.9255, 1769.0105, 1771.0138, 1774.9404, 1783.9508, 1786.9517, 1787.9071, 1790.9086, 1835.8361, 1837.8712, 1862.9679, 1928.0435, 1940.0333, 1954.0796, 1961.0397, 1970.0139, 1982.0297, 1984.0314, 2023.0551, 2027.1292, 2032.0228, 2061.0330, 2105.0796, 2144.0679, 2170.0872, 2179.0752, 2211.0654, 2215.0876, 2239.1650, 2242.1501, 2246.2151, 2251.1675, 2252.1614, 2255.1719, 2273.1008, 2289.1030, 2293.1750, 2304.2087, 2343.2134, 2354.0020, 2391.2788, 2475.2476, 2491.2402, 2507.1975, 2514.2664, 2542.2795, 2550.2063, 2558.3499, 2587.3516, 2605.2615, 2646.9883, 2661.0090, 2675.0190, 2681.3855, 2698.3696, 2705.3142, 2707.3074, 2714.3647, 2719.3167, 2722.3188, 2763.3738, 2792.2446, 2826.3545, 2843.4670, 2846.4014,

2847.4082, 2872.3359, 2884.3538, 2887.3474, 2889.3481, 2892.4165, 2894.4639, 2896.4890, 2914.4551, 2987.5244, 2995.4431, 3004.5840, 3029.4812, 3054.4724, 3069.4849, 3072.4231, 3101.5515, 3126.7327, 3166.6106, 3171.6748, 3183.6196, 3199.6411, 3231.5630, 3236.5791, 3243.6301, 3245.6216, 3247.5737, 3248.6313, 3254.6448, 3267.7256, 3278.7546, 3321.5828, 3333.5762, 3334.6729, 3335.6787, 3338.7097, 3347.4907, 3395.6821, 3412.6426, 3428.5991, 3435.7024, 3457.6995, 3487.8022, 3490.6885, 3503.8049, 3534.6365, 3548.6917, 3551.6768, 3552.6902, 3601.6826, 3616.7095, 3639.7014, 3645.7827, 3651.7559, 3655.7354, 3667.7646, 3697.8025, 3716.7253, 3762.8396, 3929.9304, 4040.7656, 4057.9707, 4071.9524, 4092.8394, 4147.9063, 4160.9473, 4174.9575, 4193.9395, 4253.8755, 4415.9736, 4452.1865, 4465.1670, 4845.0845

33. A3KMV6 **Mass:** 71197 **Score:** 19 **Expect:** 3.2e+002 **Matches:** 13

HNRNPR protein OS=Bos taurus GN=HNRNPR PE=1 SV=1

Observed	Mr(expt)	Mr(calc)	ppm	Start	End	Miss	Peptide
772.4160	771.4087	771.4212	-16.17	448	455	2	R.GRGRGGGR.G
918.5359	917.5287	917.5480	-21.10	308	315	2	R.RLMSGKVK.V
1091.5437	1090.5364	1090.5618	-23.31	111	120	1	K.QGSKVQESTK.G
1386.7084	1385.7011	1385.7085	-5.33	1	13	0	-.MANQVNGNAVQLK.E
1439.7701	1438.7628	1438.7416	14.7	134	146	1	R.TGYTLDVTTGQRK.Y
2170.0872	2169.0799	2169.0823	-1.09	188	206	1	K.AGPIWDLRLMMDPLSGQNR.G
2507.1975	2506.1902	2506.2381	-19.08	147	171	0	K.YGGPPPDSVYSGVQPGIGTEVFVGK.I
2892.4165	2891.4092	2891.3813	9.66	424	447	2	R.QASRSTAYEDYYYHPPPRMPPPIR.G
3166.6106	3165.6033	3165.6057	-0.74	314	341	2	K.VKVWGNVVTVEWADPVEEPDPEVMAKVK.V + Oxidation (M)
3243.6301	3242.6228	3242.6050	5.50	385	414	2	R.GAAVKAMDEMNGKEIEGEEIEIVLAKPPDK.K + 2 Oxidation (M)
3248.6313	3247.6240	3247.5464	23.9	196	224	2	R.LMMDPLSGQNRGYAFITFCGKEAAQEAVK.L + Oxidation (M)
3667.7646	3666.7573	3666.7471	2.78	14	45	2	K.EEEPMDTSSVNHTEHYKTLIEAGLPQKVAER.L
4465.1670	4464.1597	4464.1503	2.11	2	41	2	M.ANQVNGNAVQLKEEEEPMDTSSVNHTEHYKTLIEAGLPQK.V + Oxidation (M)

No match to: 701.3809, 706.2637, 708.2758, 717.3943, 719.4086, 722.4171, 734.4380, 735.4089, 736.4033, 747.4274, 756.3826, 759.3994, 779.4522, 795.4493, 806.4573, 831.4625, 832.3028, 834.3088, 842.4966, 876.2747, 878.2808, 883.4713, 888.4968, 890.4931, 891.4778, 900.5316, 910.4130, 929.4340, 936.4846, 937.5071, 938.5070, 949.5414, 967.4175, 969.4572, 972.4759, 974.4473, 976.4465, 978.5344, 983.6115, 988.4531, 990.4647, 993.4956, 1000.5594, 1007.5680, 1019.5631, 1021.5624, 1031.5641, 1034.5791, 1036.5952, 1044.5217, 1046.5728, 1051.5135, 1056.5387, 1057.6144, 1060.5447, 1074.5956, 1077.5199, 1087.5442, 1113.5406, 1122.5580, 1123.5852, 1132.5316, 1145.6403, 1161.6290, 1168.6013, 1171.5769, 1172.5704, 1178.6254, 1180.6395, 1181.6516, 1185.6835, 1198.7111, 1216.6992, 1234.6646, 1248.6750, 1254.6965, 1255.6359, 1273.6595, 1278.6974, 1281.6390, 1284.6533, 1301.6256, 1304.6489, 1314.7235, 1315.6904, 1325.6581, 1336.7290, 1338.6799, 1342.6847, 1349.6997, 1351.6993, 1354.6641, 1361.6997, 1368.6772, 1370.6589, 1372.6199, 1381.7728, 1382.6112, 1383.7378, 1388.7888, 1390.7301, 1397.7515, 1425.7671, 1442.8314, 1457.7517, 1467.7510, 1473.8167, 1488.7367, 1497.8859, 1500.7335, 1513.7731, 1516.7435, 1528.7101, 1556.8929, 1559.8439, 1562.8004, 1563.8088, 1578.7965, 1580.8445, 1592.8094, 1601.9139, 1616.8481, 1623.8562, 1627.7197, 1628.7129, 1628.8722, 1634.8071, 1638.8757, 1642.7318, 1645.7701, 1647.8682, 1650.8612, 1673.8375, 1679.9817, 1680.8936, 1681.8372, 1696.8403, 1706.8660, 1715.8601, 1717.9037, 1730.8719, 1742.0088, 1758.9255, 1769.0105, 1771.0138, 1774.9404, 1783.9508, 1786.9517, 1787.9071, 1790.9086, 1835.8361, 1837.8712, 1862.9679, 1928.0435, 1940.0333, 1954.0796, 1961.0397, 1970.0139, 1982.0297, 1984.0314, 2023.0551, 2027.1292, 2032.0228, 2061.0330, 2105.0796, 2144.0679, 2179.0752, 2211.0654, 2215.0876, 2239.1650, 2242.1501, 2246.2151, 2251.1675, 2252.1614, 2255.1719, 2273.1008, 2289.1030, 2293.1750, 2304.2087, 2316.2031, 2343.2134, 2354.0020, 2391.2788, 2475.2476, 2491.2402, 2514.2664, 2542.2795, 2550.2063, 2558.3499, 2587.3516, 2605.2615, 2646.9883, 2661.0090, 2675.0190, 2681.3855, 2698.3696, 2705.3142, 2707.3074, 2714.3647, 2717.3394, 2719.3167, 2722.3188, 2763.3738, 2792.2446, 2826.3545, 2843.4670, 2846.4014, 2847.4082, 2872.3359, 2884.3538, 2887.3474, 2889.3481, 2894.4639, 2896.4890, 2914.4551, 2987.5244, 2995.4431, 3004.5840, 3029.4812, 3054.4724, 3069.4849, 3072.4231, 3101.5515, 3126.7327, 3171.6748, 3183.6196, 3199.6411, 3231.5630, 3236.5791, 3245.6216, 3247.5737, 3254.6448, 3262.7324, 3267.7256, 3278.7546, 3321.5828, 3333.5762, 3334.6729, 3335.6787, 3338.7097, 3347.4907, 3395.6821, 3412.6426, 3428.5991, 3435.7024, 3457.6995, 3487.8022, 3490.6885, 3503.8049, 3534.6365, 3548.6917, 3551.6768, 3552.6902, 3601.6826, 3616.7095, 3624.6824, 3639.7014, 3645.7827, 3651.7559, 3655.7354, 3697.8025, 3716.7253, 3762.8396, 3929.9304, 4040.7656, 4057.9707, 4071.9524, 4092.8394, 4147.9063, 4160.9473, 4174.9575, 4193.9395, 4253.8755, 4415.9736, 4452.1865, 4845.0845

34. F1N5N0 **Mass:** 72401 **Score:** 19 **Expect:** 3.2e+002 **Matches:** 12

Uncharacterized protein OS=Bos taurus GN=TRAK1 PE=4 SV=2

Observed	Mr(expt)	Mr(calc)	ppm	Start	End	Miss	Peptide
736.4033	735.3960	735.3803	21.4	568	573	0	R.SYLPEK.L
910.4130	909.4057	909.4192	-14.83	507	513	0	R.ENYLSER.R
1113.5406	1112.5333	1112.5237	8.64	236	244	0	K.TETITYEEK.E
1715.8601	1714.8528	1714.8535	-0.38	94	107	1	K.YFLLCAERVGQMTK.T
2032.0228	2031.0155	2030.9942	10.5	219	235	1	K.DLEEENVVLRSEACQLK.T
2211.0654	2210.0581	2210.0603	-1.01	198	216	1	R.NESSSSVQNYFHLDSLQKK.L
2273.1008	2272.0935	2272.1369	-19.09	531	552	0	K.GELLSGSLTPTESIMSLGTHSR.F
2289.1030	2288.0957	2288.1318	-15.78	531	552	0	K.GELLSGSLTPTESIMSLGTHSR.F + Oxidation (M)
2843.4670	2842.4597	2842.4382	7.57	526	552	1	R.ELAEKGELLSGSLTPTESIMSLGTHSR.F
3199.6411	3198.6338	3198.6190	4.63	245	272	2	K.EQQLVNDCVKELRDANVQIASISEELAK.K
3645.7827	3644.7754	3644.7702	1.44	27	57	1	R.TSSCDVCNSTDLPEVEIISLLEEQLPHYKLR.A
3716.7253	3715.7180	3715.7700	-14.00	447	480	1	R.SSFYGSDVSNVVLDNKTNSIILETESAELGNEER.S

No match to: 701.3809, 706.2637, 708.2758, 717.3943, 719.4086, 722.4171, 734.4380, 735.4089, 747.4274, 756.3826, 759.3994, 772.4160, 779.4522, 795.4493, 806.4573, 831.4625, 832.3028, 834.3088, 842.4966, 876.2747, 878.2808, 883.4713, 888.4968, 890.4931, 891.4778, 900.5316, 918.5359, 929.4340, 936.4846, 937.5071, 938.5070, 949.5414, 967.4175, 969.4572, 972.4759, 974.4473, 976.4465, 978.5344, 983.6115, 988.4531, 990.4647, 993.4956, 1000.5594, 1007.5680, 1019.5631, 1021.5624, 1031.5641, 1034.5791, 1036.5952, 1044.5217, 1046.5728, 1051.5135, 1056.5387, 1057.6144, 1060.5447, 1074.5956, 1077.5199, 1087.5442, 1091.5437, 1122.5580, 1123.5852, 1132.5316, 1145.6403, 1161.6290, 1168.6013, 1171.5769, 1172.5704, 1178.6254, 1180.6395, 1181.6516, 1185.6835, 1198.7111, 1216.6992, 1234.6646, 1248.6750, 1254.6965, 1255.6359, 1273.6595, 1278.6974, 1281.6390, 1284.6533, 1301.6256, 1304.6489, 1314.7235, 1315.6904, 1325.6581, 1336.7290, 1338.6799, 1342.6847, 1349.6997, 1351.6993, 1354.6641, 1361.6997, 1368.6772, 1370.6589, 1372.6199, 1381.7728, 1382.6112, 1383.7378, 1386.7084, 1388.7888, 1390.7301, 1397.7515, 1425.7671, 1439.7701, 1442.8314, 1457.7517, 1467.7510, 1473.8167, 1488.7367, 1497.8859, 1500.7335, 1513.7731, 1516.7435, 1528.7101, 1556.8929, 1559.8439, 1562.8004, 1563.8088, 1578.7965, 1580.8445, 1592.8094, 1601.9139, 1616.8481, 1623.8562, 1627.7197, 1628.7129, 1628.8722, 1634.8071, 1638.8757, 1642.7318, 1645.7701, 1647.8682, 1650.8612, 1673.8375, 1679.9817, 1680.8936, 1681.8372, 1696.8403, 1706.8660, 1717.9037, 1730.8719, 1742.0088, 1758.9255, 1769.0105, 1771.0138, 1774.9404, 1783.9508, 1786.9517, 1787.9071, 1790.9086, 1835.8361, 1837.8712, 1862.9679, 1928.0435, 1940.0333, 1954.0796, 1961.0397, 1970.0139, 1982.0297, 1984.0314, 2023.0551, 2027.1292, 2061.0330, 2105.0796, 2144.0679, 2170.0872, 2179.0752, 2215.0876, 2239.1650, 2242.1501, 2246.2151, 2251.1675, 2252.1614, 2255.1719, 2293.1750, 2304.2087, 2316.2031, 2343.2134, 2354.0020, 2391.2788, 2475.2476, 2491.2402, 2507.1975, 2514.2664, 2542.2795, 2550.2063, 2558.3499, 2587.3516, 2605.2615, 2646.9883, 2661.0090, 2675.0190, 2681.3855, 2698.3696, 2705.3142, 2707.3074, 2714.3647, 2717.3394, 2719.3167, 2722.3188, 2763.3738, 2792.2446, 2826.3545, 2846.4014, 2847.4082, 2872.3359, 2884.3538, 2887.3474, 2889.3481, 2892.4165, 2894.4639, 2896.4890, 2914.4551, 2987.5244, 2995.4431, 3004.5840, 3029.4812, 3054.4724, 3069.4849, 3072.4231, 3101.5515, 3126.7327, 3166.6106, 3171.6748, 3183.6196, 3231.5630, 3236.5791, 3243.6301, 3245.6216, 3247.5737, 3248.6313, 3254.6448, 3262.7324, 3267.7256, 3278.7546, 3321.5828, 3333.5762, 3334.6729, 3335.6787, 3338.7097, 3347.4907, 3395.6821, 3412.6426, 3428.5991, 3435.7024, 3457.6995, 3487.8022, 3490.6885, 3503.8049, 3534.6365, 3548.6917, 3551.6768, 3552.6902, 3601.6826, 3616.7095, 3624.6824, 3639.7014, 3651.7559, 3655.7354, 3667.7646, 3697.8025, 3762.8396, 3929.9304, 4040.7656, 4057.9707, 4071.9524, 4092.8394, 4147.9063, 4160.9473, 4174.9575, 4193.9395, 4253.8755, 4415.9736, 4452.1865, 4465.1670, 4845.0845

35. Q3SX14 **Mass:** 80966 **Score:** 19 **Expect:** 3.2e+002 **Matches:** 12

Gelsolin OS=Bos taurus GN=GSN PE=2 SV=1

Observed	Mr(expt)	Mr(calc)	ppm	Start	End	Miss	Peptide
1060.5447	1059.5374	1059.5169	19.3	198	206	2	R.DNERSGRAR.V
1254.6965	1253.6892	1253.6880	0.95	11	21	1	K.AGKEPGLQIWR.V
1717.9037	1716.8964	1716.8907	3.32	503	518	1	R.EGGQTAPASTRLFQVR.A

1787.9071	1786.8998	1786.8607	21.9	610 - 624	2	K.KMDAHPPRLFACSNK.I + Oxidation (M)
1970.0139	1969.0066	1968.9774	14.8	611 - 627	2	K.MDAHPPRLFACSNKIGR.F
2491.2402	2490.2329	2490.2173	6.28	252 - 276	0	K.VSNGAGTMSVSLVADENPFAQGALR.S
2507.1975	2506.1902	2506.2122	-8.77	252 - 276	0	K.VSNGAGTMSVSLVADENPFAQGALR.S + Oxidation (M)
2587.3516	2586.3443	2586.3814	-14.35	475 - 497	1	R.VVQGKEPAHLMSLFGGKPMIIYR.G + Oxidation (M)
2763.3738	2762.3665	2762.3300	13.2	576 - 601	1	R.AQPVQVAEGSEPDSFWEALGGKAAYR.T
4057.9707	4056.9634	4057.0029	-9.72	435 - 472	0	R.QGQIIYNWQGAQSTQDEVAASAILTAQLDEELGGTPVR.S
4092.8394	4091.8321	4091.9317	-24.34	252 - 290	2	K.VSNGAGTMSVSLVADENPFAQGALRSEDCFILDHGKDGK.I
4465.1670	4464.1597	4464.2058	-10.32	431 - 472	1	R.HGGRQGQIIYNWQGAQSTQDEVAASAILTAQLDEELGGTPVR.S

No match to: 701.3809, 706.2637, 708.2758, 717.3943, 719.4086, 722.4171, 734.4380, 735.4089, 736.4033, 747.4274, 756.3826, 759.3994, 772.4160, 779.4522, 795.4493, 806.4573, 831.4625, 832.3028, 834.3088, 842.4966, 876.2747, 878.2808, 883.4713, 888.4968, 890.4931, 891.4778, 900.5316, 910.4130, 918.5359, 929.4340, 936.4846, 937.5071, 938.5070, 949.5414, 967.4175, 969.4572, 972.4759, 974.4473, 976.4465, 978.5344, 983.6115, 988.4531, 990.4647, 993.4956, 1000.5594, 1007.5680, 1019.5631, 1021.5624, 1031.5641, 1034.5791, 1036.5952, 1044.5217, 1046.5728, 1051.5135, 1056.5387, 1057.6144, 1074.5956, 1077.5199, 1087.5442, 1091.5437, 1113.5406, 1122.5580, 1123.5852, 1132.5316, 1145.6403, 1161.6290, 1168.6013, 1171.5769, 1172.5704, 1178.6254, 1180.6395, 1181.6516, 1185.6835, 1198.7111, 1216.6992, 1234.6646, 1248.6750, 1255.6359, 1273.6595, 1278.6974, 1281.6390, 1284.6533, 1301.6256, 1304.6489, 1314.7235, 1315.6904, 1325.6581, 1336.7290, 1338.6799, 1342.6847, 1349.6997, 1351.6993, 1354.6641, 1361.6997, 1368.6772, 1370.6589, 1372.6199, 1381.7728, 1382.6112, 1383.7378, 1386.7084, 1388.7888, 1390.7301, 1397.7515, 1425.7671, 1439.7701, 1442.8314, 1457.7517, 1467.7510, 1473.8167, 1488.7367, 1497.8859, 1500.7335, 1513.7731, 1516.7435, 1528.7101, 1556.8929, 1559.8439, 1562.8004, 1563.8088, 1578.7965, 1580.8445, 1592.8094, 1601.9139, 1616.8481, 1623.8562, 1627.7197, 1628.7129, 1628.8722, 1634.8071, 1638.8757, 1642.7318, 1645.7701, 1647.8682, 1650.8612, 1673.8375, 1679.9817, 1680.8936, 1681.8372, 1696.8403, 1706.8660, 1715.8601, 1730.8719, 1742.0088, 1758.9255, 1769.0105, 1771.0138, 1774.9404, 1783.9508, 1786.9517, 1790.9086, 1835.8361, 1837.8712, 1862.9679, 1928.0435, 1940.0333, 1954.0796, 1961.0397, 1982.0297, 1984.0314, 2023.0551, 2027.1292, 2032.0228, 2061.0330, 2105.0796, 2144.0679, 2170.0872, 2179.0752, 2211.0654, 2215.0876, 2239.1650, 2242.1501, 2246.2151, 2251.1675, 2252.1614, 2255.1719, 2273.1008, 2289.1030, 2293.1750, 2304.2087, 2316.2031, 2343.2134, 2354.0020, 2391.2788, 2475.2476, 2514.2664, 2542.2795, 2550.2063, 2558.3499, 2605.2615, 2646.9883, 2661.0090, 2675.0190, 2681.3855, 2698.3696, 2705.3142, 2707.3074, 2714.3647, 2717.3394, 2719.3167, 2722.3188, 2792.2446, 2826.3545, 2843.4670, 2846.4014, 2847.4082, 2872.3359, 2884.3538, 2887.3474, 2889.3481, 2892.4165, 2894.4639, 2896.4890, 2914.4551, 2987.5244, 2995.4431, 3004.5840, 3029.4812, 3054.4724, 3069.4849, 3072.4231, 3101.5515, 3126.7327, 3166.6106, 3171.6748, 3183.6196, 3199.6411, 3231.5630, 3236.5791, 3243.6301, 3245.6216, 3247.5737, 3248.6313, 3254.6448, 3262.7324, 3267.7256, 3278.7546, 3321.5828, 3333.5762, 3334.6729, 3335.6787, 3338.7097, 3347.4907, 3395.6821, 3412.6426, 3428.5991, 3435.7024, 3457.6995, 3487.8022, 3490.6885, 3503.8049, 3534.6365, 3548.6917, 3551.6768, 3552.6902, 3601.6826, 3616.7095, 3624.6824, 3639.7014, 3645.7827, 3651.7559, 3655.7354, 3667.7646, 3697.8025, 3716.7253, 3762.8396, 3929.9304, 4040.7656, 4071.9524, 4147.9063, 4160.9473, 4174.9575, 4193.9395, 4253.8755, 4415.9736, 4452.1865, 4845.0845

36. E1BEE0 **Mass:** 55035 **Score:** 19 **Expect:** 3.3e+002 **Matches:** 9

Uncharacterized protein OS=Bos taurus GN=TOM1L2 PE=4 SV=2

Observed	Mr(expt)	Mr(calc)	ppm	Start - End	Miss	Peptide
1473.8167	1472.8094	1472.8140	-3.08	118 - 130	0	K.VLALIQAWADAFR.S
1862.9679	1861.9606	1861.9615	-0.45	257 - 271	2	R.TCRAMQQRVVELISR.V + Oxidation (M)
2542.2795	2541.2722	2541.2785	-2.47	415 - 436	0	K.QSSEVIPVVQPSVMDDIEVWLR.T + Oxidation (M)
2714.3647	2713.3574	2713.3857	-10.42	461 - 488	2	R.AKAAEMVPSLPSPPADAPAPASNPSGRK.K
3166.6106	3165.6033	3165.6362	-10.38	67 - 93	2	R.EVMLALTVLETCVKNCGHRFHVLVANR.D
3199.6411	3198.6338	3198.6713	-11.72	118 - 146	1	K.VLALIQAWADAFRSSPDLTGVVHAYEELK.R
3548.6917	3547.6844	3547.6480	10.3	226 - 256	1	R.GNTKVMSEMLTEMVPGQEDSSDLELLQELHR.T + 2 Oxidation (M)
3651.7559	3650.7486	3650.7423	1.73	272 - 300	2	R.VCNEEVTEELLHVNDDLNNVFLRYERFER.Y
4147.9063	4146.8990	4146.9265	-6.61	230 - 264	2	K.VMSEMLTEMVPGQEDSSDLELLQELHRTCRAMQQR.V

No match to: 701.3809, 706.2637, 708.2758, 717.3943, 719.4086, 722.4171, 734.4380, 735.4089, 736.4033, 747.4274, 756.3826, 759.3994, 772.4160, 779.4522, 795.4493, 806.4573, 831.4625, 832.3028, 834.3088, 842.4966, 876.2747, 878.2808, 883.4713, 888.4968, 890.4931, 891.4778, 900.5316, 910.4130, 918.5359, 929.4340, 936.4846, 937.5071, 938.5070, 949.5414, 967.4175, 969.4572, 972.4759, 974.4473, 976.4465, 978.5344, 983.6115, 988.4531, 990.4647, 993.4956, 1000.5594, 1007.5680, 1019.5631, 1021.5624, 1031.5641, 1034.5791, 1036.5952, 1044.5217, 1046.5728, 1051.5135, 1056.5387, 1057.6144, 1060.5447, 1074.5956, 1077.5199, 1087.5442, 1091.5437, 1113.5406, 1122.5580, 1123.5852, 1132.5316, 1145.6403, 1161.6290, 1168.6013, 1171.5769, 1172.5704, 1178.6254, 1180.6395, 1181.6516, 1185.6835, 1198.7111, 1216.6992, 1234.6646, 1248.6750, 1254.6965, 1255.6359, 1273.6595, 1278.6974, 1281.6390, 1284.6533, 1301.6256, 1304.6489, 1314.7235, 1315.6904, 1325.6581, 1336.7290, 1338.6799, 1342.6847, 1349.6997, 1351.6993, 1354.6641, 1361.6997, 1368.6772, 1370.6589, 1372.6199, 1381.7728, 1382.6112, 1383.7378, 1386.7084, 1388.7888, 1390.7301, 1397.7515, 1425.7671, 1439.7701, 1442.8314, 1457.7517, 1467.7510, 1488.7367, 1497.8859, 1500.7335, 1513.7731, 1516.7435, 1528.7101, 1556.8929, 1559.8439, 1562.8004, 1563.8088, 1578.7965, 1580.8445, 1592.8094, 1601.9139, 1616.8481, 1623.8562, 1627.7197, 1628.7129, 1628.8722, 1634.8071, 1638.8757, 1642.7318, 1645.7701, 1647.8682, 1650.8612, 1673.8375, 1679.9817, 1680.8936, 1681.8372, 1696.8403, 1706.8660, 1715.8601, 1717.9037, 1730.8719, 1742.0088, 1758.9255, 1769.0105, 1771.0138, 1774.9404, 1783.9508, 1786.9517, 1787.9071, 1790.9086, 1835.8361, 1837.8712, 1928.0435, 1940.0333, 1954.0796, 1961.0397, 1970.0139, 1982.0297, 1984.0314, 2023.0551, 2027.1292, 2032.0228, 2061.0330, 2105.0796, 2144.0679, 2170.0872, 2179.0752, 2211.0654, 2215.0876, 2239.1650, 2242.1501, 2246.2151, 2251.1675, 2252.1614, 2255.1719, 2273.1008, 2289.1030, 2293.1750, 2304.2087, 2316.2031, 2343.2134, 2354.0020, 2391.2788, 2475.2476, 2491.2402, 2507.1975, 2514.2664, 2550.2063, 2558.3499, 2587.3516, 2605.2615, 2646.9883, 2661.0090, 2675.0190, 2681.3855, 2698.3696, 2705.3142, 2707.3074, 2717.3394, 2719.3167, 2722.3188, 2763.3738, 2792.2446, 2826.3545, 2843.4670, 2846.4014, 2847.4082, 2872.3359, 2884.3538, 2887.3474, 2889.3481, 2892.4165, 2894.4639, 2896.4890, 2914.4551, 2987.5244, 2995.4431, 3004.5840, 3029.4812, 3054.4724, 3069.4849, 3072.4231, 3101.5515, 3126.7327, 3171.6748, 3183.6196, 3231.5630, 3236.5791, 3243.6301, 3245.6216, 3247.5737, 3248.6313, 3254.6448, 3262.7324, 3267.7256, 3278.7546, 3321.5828, 3333.5762, 3334.6729, 3335.6787, 3338.7097, 3347.4907, 3395.6821, 3412.6426, 3428.5991, 3435.7024, 3457.6995, 3487.8022, 3490.6885, 3503.8049, 3534.6365, 3551.6768, 3552.6902, 3601.6826, 3616.7095, 3624.6824, 3639.7014, 3645.7827, 3655.7354, 3667.7646, 3697.8025, 3716.7253, 3762.8396, 3929.9304, 4040.7656, 4057.9707, 4071.9524, 4092.8394, 4160.9473, 4174.9575, 4193.9395, 4253.8755, 4415.9736, 4452.1865, 4465.1670, 4845.0845

37. Q2TBL4 **Mass:** 60789 **Score:** 19 **Expect:** 3.3e+002 **Matches:** 9

DNA-directed RNA polymerase III subunit RPC3 OS=Bos taurus GN=POLR3C PE=2 SV=1

Observed	Mr(expt)	Mr(calc)	ppm	Start - End	Miss	Peptide
1057.6144	1056.6071	1056.6040	3.00	442 - 450	1	K.SVANLIERR.Q
1467.7510	1466.7437	1466.7585	-10.08	116 - 128	2	K.MTMSAVVKKVADR.L + 2 Oxidation (M)
1592.8094	1591.8021	1591.7916	6.63	379 - 392	1	R.QVEDFAMIPAKEAK.D + Oxidation (M)
1715.8601	1714.8528	1714.8486	2.49	204 - 219	2	R.SSDEDATGEPKAKRPK.Q
2847.4082	2846.4009	2846.3605	14.2	509 - 532	0	K.LDACEIQVDETIFLLESYIESTMK.R
3029.4812	3028.4739	3028.4634	3.47	269 - 295	1	R.TMLRMSEVTTPSGAPFTQPLSSNEIFR.S + 2 Oxidation (M)
3262.7324	3261.7251	3261.7172	2.42	88 - 115	2	R.YPRYIYTAKTLYSDTGELIVEELLLNGK.M
3645.7827	3644.7754	3644.7661	2.55	464 - 496	1	K.SQRVEAIIASMQATGAEEAQLQEIEEMITAPER.Q + Oxidation (M)
4071.9524	4070.9451	4071.0445	-24.42	269 - 305	2	R.TMLRMSEVTTPSGAPFTQPLSSNEIFRSLPVGYNISK.Q + Oxidation (M)

No match to: 701.3809, 706.2637, 708.2758, 717.3943, 719.4086, 722.4171, 734.4380, 735.4089, 736.4033, 747.4274, 756.3826, 759.3994, 772.4160, 779.4522, 795.4493, 806.4573, 831.4625, 832.3028, 834.3088, 842.4966, 876.2747, 878.2808, 883.4713, 888.4968, 890.4931, 891.4778, 900.5316, 910.4130, 918.5359, 929.4340, 936.4846, 937.5071, 938.5070, 949.5414, 967.4175, 969.4572, 972.4759, 974.4473, 976.4465, 978.5344, 983.6115, 988.4531, 990.4647, 993.4956, 1000.5594, 1007.5680, 1019.5631, 1021.5624, 1031.5641, 1034.5791, 1036.5952, 1044.5217, 1046.5728, 1051.5135, 1056.5387, 1060.5447, 1074.5956, 1077.5199, 1087.5442, 1091.5437, 1113.5406, 1122.5580, 1123.5852, 1132.5316, 1145.6403, 1161.6290, 1168.6013, 1171.5769, 1172.5704, 1178.6254, 1180.6395, 1181.6516, 1185.6835, 1198.7111, 1216.6992, 1234.6646, 1248.6750, 1254.6965, 1255.6359, 1273.6595, 1278.6974, 1281.6390, 1284.6533, 1301.6256, 1304.6489, 1314.7235, 1315.6904, 1325.6581, 1336.7290, 1338.6799, 1342.6847, 1349.6997, 1351.6993, 1354.6641, 1361.6997, 1368.6772, 1370.6589, 1372.6199, 1381.7728, 1382.6112, 1383.7378, 1386.7084, 1388.7888, 1390.7301, 1397.7515, 1425.7671, 1439.7701, 1442.8314, 1457.7517, 1473.8167, 1488.7367, 1497.8859, 1500.7335, 1513.7731, 1516.7435, 1528.7101, 1556.8929, 1559.8439, 1562.8004, 1563.8088, 1578.7965, 1580.8445, 1601.9139, 1616.8481, 1623.8562, 1627.7197, 1628.7129, 1628.8722, 1634.8071, 1638.8757, 1642.7318, 1645.7701, 1647.8682, 1650.8612, 1673.8375, 1679.9817, 1680.8936, 1681.8372, 1696.8403, 1706.8660, 1717.9037, 1730.8719, 1742.0088, 1758.9255, 1769.0105,

1771.0138, 1774.9404, 1783.9508, 1786.9517, 1787.9071, 1790.9086, 1835.8361, 1837.8712, 1862.9679, 1928.0435, 1940.0333, 1954.0796, 1961.0397, 1970.0139, 1982.0297, 1984.0314, 2023.0551, 2027.1292, 2032.0228, 2061.0330, 2105.0796, 2144.0679, 2170.0872, 2179.0752, 2211.0654, 2215.0876, 2239.1650, 2242.1501, 2246.2151, 2251.1675, 2252.1614, 2255.1719, 2273.1008, 2289.1030, 2293.1750, 2304.2087, 2316.2031, 2343.2134, 2354.0020, 2391.2788, 2475.2476, 2491.2402, 2507.1975, 2514.2664, 2542.2795, 2550.2063, 2558.3499, 2587.3516, 2605.2615, 2646.9883, 2661.0090, 2675.0190, 2681.3855, 2698.3696, 2705.3142, 2707.3074, 2714.3647, 2717.3394, 2719.3167, 2722.3188, 2763.3738, 2792.2446, 2826.3545, 2843.4670, 2846.4014, 2872.3359, 2884.3538, 2887.3474, 2889.3481, 2892.4165, 2894.4639, 2896.4890, 2914.4551, 2987.5244, 2995.4431, 3004.5840, 3054.4724, 3069.4849, 3072.4231, 3101.5515, 3126.7327, 3166.6106, 3171.6748, 3183.6196, 3199.6411, 3231.5630, 3236.5791, 3243.6301, 3245.6216, 3247.5737, 3248.6313, 3254.6448, 3267.7256, 3278.7546, 3321.5828, 3333.5762, 3334.6729, 3335.6787, 3338.7097, 3347.4907, 3395.6821, 3412.6426, 3428.5991, 3435.7024, 3457.6995, 3487.8022, 3490.6885, 3503.8049, 3534.6365, 3548.6917, 3551.6768, 3552.6902, 3601.6826, 3616.7095, 3624.6824, 3639.7014, 3651.7559, 3655.7354, 3667.7646, 3697.8025, 3716.7253, 3762.8396, 3929.9304, 4040.7656, 4057.9707, 4092.8394, 4147.9063, 4160.9473, 4174.9575, 4193.9395, 4253.8755, 4415.9736, 4452.1865, 4465.1670, 4845.0845

38. F1MUY3 **Mass:** 60801 **Score:** 19 **Expect:** 3.3e+002 **Matches:** 9

DNA-directed RNA polymerase III subunit RPC3 OS=Bos taurus GN=POLR3C PE=4 SV=1

Observed	Mr(expt)	Mr(calc)	ppm	Start	End	Miss	Peptide
1057.6144	1056.6071	1056.6040	3.00	442 -	450	1	K.SVANLIERR.Q
1467.7510	1466.7437	1466.7585	-10.08	116 -	128	2	K.MTMSAVVKKVADR.L + 2 Oxidation (M)
1592.8094	1591.8021	1591.7916	6.63	379 -	392	1	K.QVEDFAMIPAKEAK.D + Oxidation (M)
1715.8601	1714.8528	1714.8486	2.49	204 -	219	2	R.SSDEDATGEPKAKRPK.Q
2847.4082	2846.4009	2846.3605	14.2	509 -	532	0	K.LDACEIQVDETIFLLESYIESTMK.R
3029.4812	3028.4739	3028.4634	3.47	269 -	295	1	R.TMLRMSEVTTPSGAPFTQPLSSNEIFR.S + 2 Oxidation (M)
3262.7324	3261.7251	3261.7172	2.42	88 -	115	2	R.YPRYIYTAKTLYSDTGELIVEELLLNGK.M
3645.7827	3644.7754	3644.7661	2.55	464 -	496	1	K.SQRVEAIIASMQATGAEEAQLQEIEEMITAPER.Q + Oxidation (M)
4071.9524	4070.9451	4071.0445	-24.42	269 -	305	2	R.TMLRMSEVTTPSGAPFTQPLSSNEIFRSLPVGYNISK.Q + Oxidation (M)

No match to: 701.3809, 706.2637, 708.2758, 717.3943, 719.4086, 722.4171, 734.4380, 735.4089, 736.4033, 747.4274, 756.3826, 759.3994, 772.4160, 779.4522, 795.4493, 806.4573, 831.4625, 832.3028, 834.3088, 842.4966, 876.2747, 878.2808, 883.4713, 888.4968, 890.4931, 891.4778, 900.5316, 910.4130, 918.5359, 929.4340, 936.4846, 937.5071, 938.5070, 949.5414, 967.4175, 969.4572, 972.4759, 974.4473, 976.4465, 978.5344, 983.6115, 988.4531, 990.4647, 993.4956, 1000.5594, 1007.5680, 1019.5631, 1021.5624, 1031.5641, 1034.5791, 1036.5952, 1044.5217, 1046.5728, 1051.5135, 1056.5387, 1060.5447, 1074.5956, 1077.5199, 1087.5442, 1091.5437, 1113.5406, 1122.5580, 1123.5852, 1132.5316, 1145.6403, 1161.6290, 1168.6013, 1171.5769, 1172.5704, 1178.6254, 1180.6395, 1181.6516, 1185.6835, 1198.7111, 1216.6992, 1234.6646, 1248.6750, 1254.6965, 1255.6359, 1273.6595, 1278.6974, 1281.6390, 1284.6533, 1301.6256, 1304.6489, 1314.7235, 1315.6904, 1325.6581, 1336.7290, 1338.6799, 1342.6847, 1349.6997, 1351.6993, 1354.6641, 1361.6997, 1368.6772, 1370.6589, 1372.6199, 1381.7728, 1382.6112, 1383.7378, 1386.7084, 1388.7888, 1390.7301, 1397.7515, 1425.7671, 1439.7701, 1442.8314, 1457.7517, 1473.8167, 1488.7367, 1497.8859, 1500.7335, 1513.7731, 1516.7435, 1528.7101, 1556.8929, 1559.8439, 1562.8004, 1563.8088, 1578.7965, 1580.8445, 1601.9139, 1616.8481, 1623.8562, 1627.7197, 1628.7129, 1628.8722, 1634.8071, 1638.8757, 1642.7318, 1645.7701, 1647.8682, 1650.8612, 1673.8375, 1679.9817, 1680.8936, 1681.8372, 1696.8403, 1706.8660, 1717.9037, 1730.8719, 1742.0088, 1758.9255, 1769.0105, 1771.0138, 1774.9404, 1783.9508, 1786.9517, 1787.9071, 1790.9086, 1835.8361, 1837.8712, 1862.9679, 1928.0435, 1940.0333, 1954.0796, 1961.0397, 1970.0139, 1982.0297, 1984.0314, 2023.0551, 2027.1292, 2032.0228, 2061.0330, 2105.0796, 2144.0679, 2170.0872, 2179.0752, 2211.0654, 2215.0876, 2239.1650, 2242.1501, 2246.2151, 2251.1675, 2252.1614, 2255.1719, 2273.1008, 2289.1030, 2293.1750, 2304.2087, 2316.2031, 2343.2134, 2354.0020, 2391.2788, 2475.2476, 2491.2402, 2507.1975, 2514.2664, 2542.2795, 2550.2063, 2558.3499, 2587.3516, 2605.2615, 2646.9883, 2661.0090, 2675.0190, 2681.3855, 2698.3696, 2705.3142, 2707.3074, 2714.3647, 2717.3394, 2719.3167, 2722.3188, 2763.3738, 2792.2446, 2826.3545, 2843.4670, 2846.4014, 2872.3359, 2884.3538, 2887.3474, 2889.3481, 2892.4165, 2894.4639, 2896.4890, 2914.4551, 2987.5244, 2995.4431, 3004.5840, 3054.4724, 3069.4849, 3072.4231, 3101.5515, 3126.7327, 3166.6106, 3171.6748, 3183.6196, 3199.6411, 3231.5630, 3236.5791, 3243.6301, 3245.6216, 3247.5737, 3248.6313, 3254.6448, 3267.7256, 3278.7546, 3321.5828, 3333.5762, 3334.6729, 3335.6787, 3338.7097, 3347.4907, 3395.6821, 3412.6426, 3428.5991, 3435.7024, 3457.6995, 3487.8022, 3490.6885, 3503.8049, 3534.6365, 3548.6917, 3551.6768, 3552.6902, 3601.6826, 3616.7095, 3624.6824, 3639.7014, 3651.7559, 3655.7354, 3667.7646, 3697.8025, 3716.7253, 3762.8396, 3929.9304, 4040.7656, 4057.9707, 4092.8394, 4147.9063, 4160.9473, 4174.9575, 4193.9395, 4253.8755, 4415.9736, 4452.1865, 4465.1670, 4845.0845

39. F1MDV8 **Mass:** 53058 **Score:** 18 **Expect:** 3.4e+002 **Matches:** 12

Septin-10 OS=Bos taurus GN=SEPT10 PE=3 SV=2

Observed	Mr(expt)	Mr(calc)	ppm	Start	End	Miss	Peptide
1077.5199	1076.5126	1076.5284	-14.65	358 -	365	2	R.QRKEEEMK.Q
1315.6904	1314.6831	1314.7143	-23.69	374 -	384	2	K.EKEAILKEAER.E
1325.6581	1324.6508	1324.6584	-5.73	410 -	420	0	R.LLEEEIMAFSK.K + Oxidation (M)
1349.6997	1348.6924	1348.6636	21.4	351 -	360	2	R.HEFYGERQRK.E
1580.8445	1579.8372	1579.8028	21.8	360 -	371	2	R.KEEEMKQLFVQR.V + Oxidation (M)
2698.3696	2697.3623	2697.3207	15.4	223 -	246	0	K.LMSELVSNGVQIYQFPTDDETIAK.I
2714.3647	2713.3574	2713.3157	15.4	223 -	246	0	K.LMSELVSNGVQIYQFPTDDETIAK.I + Oxidation (M)
2887.3474	2886.3401	2886.2939	16.0	325 -	349	0	K.LEEMGFMDVGPENQPLSLQETYEAK.R + 2 Oxidation (M)
2914.4551	2913.4478	2913.4403	2.58	32 -	57	2	K.QKDSPRSLTMSGHVGFESLPDQLVNR.S + Oxidation (M)
3247.5737	3246.5664	3246.5734	-2.16	78 -	104	1	K.STLIDTLFNTNFEDHESSHFYPHVRLK.A
3278.7546	3277.7473	3277.7306	5.09	105 -	133	2	K.AQTYELQESNVRLKLTIVNTVGFGDQINK.E
3601.6826	3600.6753	3600.6726	0.76	1 -	31	1	-.VSCVDYHMLFQSHITTKTAHTSSQVSDHEQK.Q

No match to: 701.3809, 706.2637, 708.2758, 717.3943, 719.4086, 722.4171, 734.4380, 735.4089, 736.4033, 747.4274, 756.3826, 759.3994, 772.4160, 779.4522, 795.4493, 806.4573, 831.4625, 832.3028, 834.3088, 842.4966, 876.2747, 878.2808, 883.4713, 888.4968, 890.4931, 891.4778, 900.5316, 910.4130, 918.5359, 929.4340, 936.4846, 937.5071, 938.5070, 949.5414, 967.4175, 969.4572, 972.4759, 974.4473, 976.4465, 978.5344, 983.6115, 988.4531, 990.4647, 993.4956, 1000.5594, 1007.5680, 1019.5631, 1021.5624, 1031.5641, 1034.5791, 1036.5952, 1044.5217, 1046.5728, 1051.5135, 1056.5387, 1057.6144, 1060.5447, 1074.5956, 1087.5442, 1091.5437, 1113.5406, 1122.5580, 1123.5852, 1132.5316, 1145.6403, 1161.6290, 1168.6013, 1171.5769, 1172.5704, 1178.6254, 1180.6395, 1181.6516, 1185.6835, 1198.7111, 1216.6992, 1234.6646, 1248.6750, 1254.6965, 1255.6359, 1273.6595, 1278.6974, 1281.6390, 1284.6533, 1301.6256, 1304.6489, 1314.7235, 1336.7290, 1338.6799, 1342.6847, 1351.6993, 1354.6641, 1361.6997, 1368.6772, 1370.6589, 1372.6199, 1381.7728, 1382.6112, 1383.7378, 1386.7084, 1388.7888, 1390.7301, 1397.7515, 1425.7671, 1439.7701, 1442.8314, 1457.7517, 1467.7510, 1473.8167, 1488.7367, 1497.8859, 1500.7335, 1513.7731, 1516.7435, 1528.7101, 1556.8929, 1559.8439, 1562.8004, 1563.8088, 1578.7965, 1592.8094, 1601.9139, 1616.8481, 1623.8562, 1627.7197, 1628.7129, 1628.8722, 1634.8071, 1638.8757, 1642.7318, 1645.7701, 1647.8682, 1650.8612, 1673.8375, 1679.9817, 1680.8936, 1681.8372, 1696.8403, 1706.8660, 1715.8601, 1717.9037, 1730.8719, 1742.0088, 1758.9255, 1769.0105, 1771.0138, 1774.9404, 1783.9508, 1786.9517, 1787.9071, 1790.9086, 1835.8361, 1837.8712, 1862.9679, 1928.0435, 1940.0333, 1954.0796, 1961.0397, 1970.0139, 1982.0297, 1984.0314, 2023.0551, 2027.1292, 2032.0228, 2061.0330, 2105.0796, 2144.0679, 2170.0872, 2179.0752, 2211.0654, 2215.0876, 2239.1650, 2242.1501, 2246.2151, 2251.1675, 2252.1614, 2255.1719, 2273.1008, 2289.1030, 2293.1750, 2304.2087, 2316.2031, 2343.2134, 2354.0020, 2391.2788, 2475.2476, 2491.2402, 2507.1975, 2514.2664, 2542.2795, 2550.2063, 2558.3499, 2587.3516, 2605.2615, 2646.9883, 2661.0090, 2675.0190, 2681.3855, 2705.3142, 2707.3074, 2717.3394, 2719.3167, 2722.3188, 2763.3738, 2792.2446, 2826.3545, 2843.4670, 2846.4014, 2847.4082, 2872.3359, 2884.3538, 2889.3481, 2892.4165, 2894.4639, 2896.4890, 2987.5244, 2995.4431, 3004.5840, 3029.4812, 3054.4724, 3069.4849, 3072.4231, 3101.5515, 3126.7327, 3166.6106, 3171.6748, 3183.6196, 3199.6411, 3231.5630, 3236.5791, 3243.6301, 3245.6216, 3248.6313, 3254.6448, 3262.7324, 3267.7256, 3321.5828, 3333.5762, 3334.6729, 3335.6787, 3338.7097, 3347.4907, 3395.6821, 3412.6426, 3428.5991, 3435.7024, 3457.6995, 3487.8022, 3490.6885, 3503.8049, 3534.6365, 3548.6917, 3551.6768, 3552.6902, 3616.7095, 3624.6824, 3639.7014, 3645.7827, 3651.7559, 3655.7354, 3667.7646, 3697.8025, 3716.7253, 3762.8396, 3929.9304, 4040.7656, 4057.9707, 4071.9524, 4092.8394, 4147.9063, 4160.9473, 4174.9575, 4193.9395, 4253.8755, 4415.9736, 4452.1865, 4465.1670, 4845.0845

40. E1BL71 **Mass:** 84208 **Score:** 18 **Expect:** 3.4e+002 **Matches:** 20

Uncharacterized protein OS=Bos taurus GN=TYW1 PE=4 SV=2

Observed	Mr(expt)	Mr(calc)	ppm	Start	End	Miss	Peptide
719.4086	718.4013	718.3908	14.6	385 -	390	1	K.SMLRGR.G
722.4171	721.4098	721.4010	12.1	555 -	560	0	R.FLDSLK.A

735.4089	734.4016	734.3857	21.6	385	390	1	K.SMLRGR.G + Oxidation (M)
832.3028	831.2955	831.3116	-19.34	250	256	0	K.HCGGNCK.K
888.4968	887.4896	887.4899	-0.34	353	360	0	R.AMITPALR.E + Oxidation (M)
918.5359	917.5287	917.5069	23.7	122	129	0	R.LIEEVTSK.N
937.5071	936.4998	936.4851	15.7	382	388	1	R.WTKSMLR.G + Oxidation (M)
1172.5704	1171.5631	1171.5873	-20.67	81	91	0	K.IFYGSQTGTAK.G
1284.6533	1283.6460	1283.6227	18.2	385	395	2	K.SMLRGRGGCYK.H
1354.6641	1353.6568	1353.6234	24.7	467	477	1	K.EERFEEGMTVK.H
1361.6997	1360.6924	1360.6769	11.4	207	219	1	R.VMTRGEGDGNVVK.S
1370.6589	1369.6516	1369.6184	24.3	467	477	1	K.EERFEEGMTVK.H + Oxidation (M)
1592.8094	1591.8021	1591.7988	2.08	207	221	2	R.VMTRGEGDGNVVKSK.H + Oxidation (M)
1647.8682	1646.8609	1646.8475	8.15	331	345	1	K.EQLEGETSAIRSTVK.S
2251.1675	2250.1602	2250.1790	-8.35	53	72	2	K.TTPKASQNLMTNGYISRPKK.E + Oxidation (M)
2475.2476	2474.2403	2474.2701	-12.05	478	498	1	K.HCALSLVGEPIMYPEINKFLK.L + Oxidation (M)
2847.4082	2846.4009	2846.3765	8.60	438	460	2	K.MDQPEMILKEAIENHQNMIKQFK.G + 2 Oxidation (M)
2872.3359	2871.3286	2871.3273	0.48	672	696	2	R.FQELIQEYEDSGGSKTFSAKDYMAK.T
3262.7324	3261.7251	3261.6591	20.2	92	121	1	K.GFATILAEALSSLNLPVAMINLKDYDPDDR.L
3412.6426	3411.6353	3411.6917	-16.51	470	498	2	R.FEEGMTVKHCALSLVGEPIMYPEINKFLK.L + 2 Oxidation (M)

No match to: 701.3809, 706.2637, 708.2758, 717.3943, 734.4380, 736.4033, 747.4274, 756.3826, 759.3994, 772.4160, 779.4522, 795.4493, 806.4573, 831.4625, 834.3088, 842.4966, 876.2747, 878.2808, 883.4713, 890.4931, 891.4778, 900.5316, 910.4130, 929.4340, 936.4846, 938.5070, 949.5414, 967.4175, 969.4572, 972.4759, 974.4473, 976.4465, 978.5344, 983.6115, 988.4531, 990.4647, 993.4956, 1000.5594, 1007.5680, 1019.5631, 1021.5624, 1031.5641, 1034.5791, 1036.5952, 1044.5217, 1046.5728, 1051.5135, 1056.5387, 1057.6144, 1060.5447, 1074.5956, 1077.5199, 1087.5442, 1091.5437, 1113.5406, 1122.5580, 1123.5852, 1132.5316, 1145.6403, 1161.6290, 1168.6013, 1171.5769, 1178.6254, 1180.6395, 1181.6516, 1185.6835, 1198.7111, 1216.6992, 1234.6646, 1248.6750, 1254.6965, 1255.6359, 1273.6595, 1278.6974, 1281.6390, 1301.6256, 1304.6489, 1314.7235, 1315.6904, 1325.6581, 1336.7290, 1338.6799, 1342.6847, 1349.6997, 1351.6993, 1368.6772, 1372.6199, 1381.7728, 1382.6112, 1383.7378, 1386.7084, 1388.7888, 1390.7301, 1397.7515, 1425.7671, 1439.7701, 1442.8314, 1457.7517, 1467.7510, 1473.8167, 1488.7367, 1497.8859, 1500.7335, 1513.7731, 1516.7435, 1528.7101, 1556.8929, 1559.8439, 1562.8004, 1563.8088, 1578.7965, 1580.8445, 1601.9139, 1616.8481, 1623.8562, 1627.7197, 1628.7129, 1628.8722, 1634.8071, 1638.8757, 1642.7318, 1645.7701, 1650.8612, 1673.8375, 1679.9817, 1680.8936, 1681.8372, 1696.8403, 1706.8660, 1715.8601, 1717.9037, 1730.8719, 1742.0088, 1758.9255, 1769.0105, 1771.0138, 1774.9404, 1783.9508, 1786.9517, 1787.9071, 1790.9086, 1835.8361, 1837.8712, 1862.9679, 1928.0435, 1940.0333, 1954.0796, 1961.0397, 1970.0139, 1982.0297, 1984.0314, 2023.0551, 2027.1292, 2032.0228, 2061.0330, 2105.0796, 2144.0679, 2170.0872, 2179.0752, 2211.0654, 2215.0876, 2239.1650, 2242.1501, 2246.2151, 2252.1614, 2255.1719, 2273.1008, 2289.1030, 2293.1750, 2304.2087, 2316.2031, 2343.2134, 2354.0020, 2391.2788, 2491.2402, 2507.1975, 2514.2664, 2542.2795, 2550.2063, 2558.3499, 2587.3516, 2605.2615, 2646.9883, 2661.0090, 2675.0190, 2681.3855, 2698.3696, 2705.3142, 2707.3074, 2714.3647, 2717.3394, 2719.3167, 2722.3188, 2763.3738, 2792.2446, 2826.3545, 2843.4670, 2846.4014, 2884.3538, 2887.3474, 2889.3481, 2892.4165, 2894.4639, 2896.4890, 2914.4551, 2987.5244, 2995.4431, 3004.5840, 3029.4812, 3054.4724, 3069.4849, 3072.4231, 3101.5515, 3126.7327, 3166.6106, 3171.6748, 3183.6196, 3199.6411, 3231.5630, 3236.5791, 3243.6301, 3245.6216, 3247.5737, 3248.6313, 3254.6448, 3267.7256, 3278.7546, 3321.5828, 3333.5762, 3334.6729, 3335.6787, 3338.7097, 3347.4907, 3395.6821, 3428.5991, 3435.7024, 3457.6995, 3487.8022, 3490.6885, 3503.8049, 3534.6365, 3548.6917, 3551.6768, 3552.6902, 3601.6826, 3616.7095, 3624.6824, 3639.7014, 3645.7827, 3651.7559, 3655.7354, 3667.7646, 3697.8025, 3716.7253, 3762.8396, 3929.9304, 4040.7656, 4057.9707, 4071.9524, 4092.8394, 4147.9063, 4160.9473, 4174.9575, 4193.9395, 4253.8755, 4415.9736, 4452.1865, 4465.1670, 4845.0845

41. Q28088 **Mass:** 21431 **Score:** 18 **Expect:** 3.4e+002 **Matches:** 6
Gamma-crystallin C OS=Bos taurus GN=CRYGC PE=1 SV=3

Observed	Mr(expt)	Mr(calc)	ppm	Start	End	Miss	Peptide
1248.6750	1247.6677	1247.6986	-24.75	164	174	2	K.AGSLRRVVDLY.-
1349.6997	1348.6924	1348.7099	-12.93	90	99	2	R.LRLYEREDQK.G
1488.7367	1487.7294	1487.7157	9.21	4	15	1	K.ITFYEDRGFQGR.C
1673.8375	1672.8302	1672.8322	-1.15	2	15	2	M.GKITFYEDRGFQGR.C
2179.0752	2178.0679	2178.0198	22.1	100	117	1	K.GLIAELSEDCPCIQDRFR.L
2763.3738	2762.3665	2762.3480	6.72	100	122	2	K.GLIAELSEDCPCIQDRFRLSEVR.S

No match to: 701.3809, 706.2637, 708.2758, 717.3943, 719.4086, 722.4171, 734.4380, 735.4089, 736.4033, 747.4274, 756.3826, 759.3994, 772.4160, 779.4522, 795.4493, 806.4573, 831.4625, 832.3028, 834.3088, 842.4966, 876.2747, 878.2808, 883.4713, 888.4968, 890.4931, 891.4778, 900.5316, 910.4130, 918.5359, 929.4340, 936.4846, 937.5071, 938.5070, 949.5414, 967.4175, 969.4572, 972.4759, 974.4473, 976.4465, 978.5344, 983.6115, 988.4531, 990.4647, 993.4956, 1000.5594, 1007.5680, 1019.5631, 1021.5624, 1031.5641, 1034.5791, 1036.5952, 1044.5217, 1046.5728, 1051.5135, 1056.5387, 1057.6144, 1060.5447, 1074.5956, 1077.5199, 1087.5442, 1091.5437, 1113.5406, 1122.5580, 1123.5852, 1132.5316, 1145.6403, 1161.6290, 1168.6013, 1171.5769, 1172.5704, 1178.6254, 1180.6395, 1181.6516, 1185.6835, 1198.7111, 1216.6992, 1234.6646, 1254.6965, 1255.6359, 1273.6595, 1278.6974, 1281.6390, 1284.6533, 1301.6256, 1304.6489, 1314.7235, 1315.6904, 1325.6581, 1336.7290, 1338.6799, 1342.6847, 1351.6993, 1354.6641, 1361.6997, 1368.6772, 1370.6589, 1372.6199, 1381.7728, 1382.6112, 1383.7378, 1386.7084, 1388.7888, 1390.7301, 1397.7515, 1425.7671, 1439.7701, 1442.8314, 1457.7517, 1467.7510, 1473.8167, 1497.8859, 1500.7335, 1513.7731, 1516.7435, 1528.7101, 1556.8929, 1559.8439, 1562.8004, 1563.8088, 1578.7965, 1580.8445, 1592.8094, 1601.9139, 1616.8481, 1623.8562, 1627.7197, 1628.7129, 1628.8722, 1634.8071, 1638.8757, 1642.7318, 1645.7701, 1647.8682, 1650.8612, 1679.9817, 1680.8936, 1681.8372, 1696.8403, 1706.8660, 1715.8601, 1717.9037, 1730.8719, 1742.0088, 1758.9255, 1769.0105, 1771.0138, 1774.9404, 1783.9508, 1786.9517, 1787.9071, 1790.9086, 1835.8361, 1837.8712, 1862.9679, 1928.0435, 1940.0333, 1954.0796, 1961.0397, 1970.0139, 1982.0297, 1984.0314, 2023.0551, 2027.1292, 2032.0228, 2061.0330, 2105.0796, 2144.0679, 2170.0872, 2211.0654, 2215.0876, 2239.1650, 2242.1501, 2246.2151, 2251.1675, 2252.1614, 2255.1719, 2273.1008, 2289.1030, 2293.1750, 2304.2087, 2316.2031, 2343.2134, 2354.0020, 2391.2788, 2475.2476, 2491.2402, 2507.1975, 2514.2664, 2542.2795, 2550.2063, 2558.3499, 2587.3516, 2605.2615, 2646.9883, 2661.0090, 2675.0190, 2681.3855, 2698.3696, 2705.3142, 2707.3074, 2714.3647, 2717.3394, 2719.3167, 2722.3188, 2792.2446, 2826.3545, 2843.4670, 2846.4014, 2847.4082, 2872.3359, 2884.3538, 2887.3474, 2889.3481, 2892.4165, 2894.4639, 2896.4890, 2914.4551, 2987.5244, 2995.4431, 3004.5840, 3029.4812, 3054.4724, 3069.4849, 3072.4231, 3101.5515, 3126.7327, 3166.6106, 3171.6748, 3183.6196, 3199.6411, 3231.5630, 3236.5791, 3243.6301, 3245.6216, 3247.5737, 3248.6313, 3254.6448, 3262.7324, 3267.7256, 3278.7546, 3321.5828, 3333.5762, 3334.6729, 3335.6787, 3338.7097, 3347.4907, 3395.6821, 3412.6426, 3428.5991, 3435.7024, 3457.6995, 3487.8022, 3490.6885, 3503.8049, 3534.6365, 3548.6917, 3551.6768, 3552.6902, 3601.6826, 3616.7095, 3624.6824, 3639.7014, 3645.7827, 3651.7559, 3655.7354, 3667.7646, 3697.8025, 3716.7253, 3762.8396, 3929.9304, 4040.7656, 4057.9707, 4071.9524, 4092.8394, 4147.9063, 4160.9473, 4174.9575, 4193.9395, 4253.8755, 4415.9736, 4452.1865, 4465.1670, 4845.0845

42. Q58DL0 **Mass:** 26401 **Score:** 18 **Expect:** 3.5e+002 **Matches:** 10
Chromosome X open reading frame 23 OS=Bos taurus GN=CXorf23 PE=2 SV=1

Observed	Mr(expt)	Mr(calc)	ppm	Start	End	Miss	Peptide
1234.6646	1233.6573	1233.6275	24.2	89	99	0	R.IDMSLAELQSK.Q
1390.7301	1389.7228	1389.7286	-4.13	88	99	1	R.RIDMSLAELQSK.Q
1425.7671	1424.7598	1424.7736	-9.64	2	14	2	M.SHDLVAVGRKSEK.F
1497.8859	1496.8786	1496.8562	15.0	108	120	1	K.QTLVKIIDPSDLR.H
1563.8088	1562.8015	1562.8093	-4.95	213	225	1	R.RPFKEYTNIDPAI.-
1578.7965	1577.7892	1577.8161	-17.06	113	125	1	K.IIDPSDLRHDIER.R
1673.8375	1672.8302	1672.8209	5.58	49	62	0	K.ANYFPPSEITLHER.F
1982.0297	1981.0224	1980.9864	18.2	70	87	2	K.KAADANETKLSSDPEIHR.R
2343.2134	2342.2061	2342.1610	19.3	88	107	2	R.RIDMSLAELQSKQTMVYDSK.Q
3054.4724	3053.4651	3053.4115	17.6	148	173	2	R.SDQHSSFSRSKDTHVDEFQKPTSFEK.S

No match to: 701.3809, 706.2637, 708.2758, 717.3943, 719.4086, 722.4171, 734.4380, 735.4089, 736.4033, 747.4274, 756.3826, 759.3994, 772.4160, 779.4522, 795.4493, 806.4573, 831.4625, 832.3028, 834.3088, 842.4966, 876.2747, 878.2808, 883.4713, 888.4968, 890.4931, 891.4778, 900.5316, 910.4130, 918.5359, 929.4340, 936.4846, 937.5071, 938.5070, 949.5414, 967.4175, 969.4572, 972.4759, 974.4473, 976.4465, 978.5344, 983.6115, 988.4531, 990.4647, 993.4956, 1000.5594, 1007.5680, 1019.5631, 1021.5624, 1031.5641,

1034.5791, 1036.5952, 1044.5217, 1046.5728, 1051.5135, 1056.5387, 1057.6144, 1060.5447, 1074.5956, 1077.5199, 1087.5442, 1091.5437, 1113.5406, 1122.5580, 1123.5852, 1132.5316, 1145.6403, 1161.6290, 1168.6013, 1171.5769, 1172.5704, 1178.6254, 1180.6395, 1181.6516, 1185.6835, 1198.7111, 1216.6992, 1248.6750, 1254.6965, 1255.6359, 1273.6595, 1278.6974, 1281.6390, 1284.6533, 1301.6256, 1304.6489, 1314.7235, 1315.6904, 1325.6581, 1336.7290, 1338.6799, 1342.6847, 1349.6997, 1351.6993, 1354.6641, 1361.6997, 1368.6772, 1370.6589, 1372.6199, 1381.7728, 1382.6112, 1383.7378, 1386.7084, 1388.7888, 1397.7515, 1439.7701, 1442.8314, 1457.7517, 1467.7510, 1473.8167, 1488.7367, 1500.7335, 1513.7731, 1516.7435, 1528.7101, 1556.8929, 1559.8439, 1562.8004, 1580.8445, 1592.8094, 1601.9139, 1616.8481, 1623.8562, 1627.7197, 1628.7129, 1628.8722, 1634.8071, 1638.8757, 1642.7318, 1645.7701, 1647.8682, 1650.8612, 1679.9817, 1680.8936, 1681.8372, 1696.8403, 1706.8660, 1715.8601, 1717.9037, 1730.8719, 1742.0088, 1758.9255, 1769.0105, 1771.0138, 1774.9404, 1783.9508, 1786.9517, 1787.9071, 1790.9086, 1835.8361, 1837.8712, 1862.9679, 1928.0435, 1940.0333, 1954.0796, 1961.0397, 1970.0139, 1984.0314, 2023.0551, 2027.1292, 2032.0228, 2061.0330, 2105.0796, 2144.0679, 2170.0872, 2179.0752, 2211.0654, 2215.0876, 2239.1650, 2242.1501, 2246.2151, 2251.1675, 2252.1614, 2255.1719, 2273.1008, 2289.1030, 2293.1750, 2304.2087, 2316.2031, 2354.0020, 2391.2788, 2475.2476, 2491.2402, 2507.1975, 2514.2664, 2542.2795, 2550.2063, 2558.3499, 2587.3516, 2605.2615, 2646.9883, 2661.0090, 2675.0190, 2681.3855, 2698.3696, 2705.3142, 2707.3074, 2714.3647, 2717.3394, 2719.3167, 2722.3188, 2763.3738, 2792.2446, 2826.3545, 2843.4670, 2846.4014, 2847.4082, 2872.3359, 2884.3538, 2887.3474, 2889.3481, 2892.4165, 2894.4639, 2896.4890, 2914.4551, 2987.5244, 2995.4431, 3004.5840, 3029.4812, 3069.4849, 3072.4231, 3101.5515, 3126.7327, 3166.6106, 3171.6748, 3183.6196, 3199.6411, 3231.5630, 3236.5791, 3243.6301, 3245.6216, 3247.5737, 3248.6313, 3254.6448, 3262.7324, 3267.7256, 3278.7546, 3321.5828, 3333.5762, 3334.6729, 3335.6787, 3338.7097, 3347.4907, 3395.6821, 3412.6426, 3428.5991, 3435.7024, 3457.6995, 3487.8022, 3490.6885, 3503.8049, 3534.6365, 3548.6917, 3551.6768, 3552.6902, 3601.6826, 3616.7095, 3624.6824, 3639.7014, 3645.7827, 3651.7559, 3655.7354, 3667.7646, 3697.8025, 3716.7253, 3762.8396, 3929.9304, 4040.7656, 4057.9707, 4071.9524, 4092.8394, 4147.9063, 4160.9473, 4174.9575, 4193.9395, 4253.8755, 4415.9736, 4452.1865, 4465.1670, 4845.0845

43. F1MU80 **Mass:** 46943 **Score:** 18 **Expect:** 3.6e+002 **Matches:** 8

Uncharacterized protein OS=Bos taurus GN=LOC520336 PE=4 SV=2

Observed	Mr(expt)	Mr(calc)	ppm	Start	End	Miss	Peptide
1198.7111	1197.7038	1197.6870	14.1	200 -	209	1	R.EIVQHLKGFK.G
1592.8094	1591.8021	1591.8246	-14.10	49 -	61	1	K.NALDEKSIFLWEK.T
2211.0654	2210.0581	2210.0938	-16.13	55 -	73	1	K.SIFLWEKTLMSPMGSVPCK.D
2763.3738	2762.3665	2762.3673	-0.27	114 -	136	2	R.AVYMPQNVYCVHVDEKATVHFKK.S
3072.4231	3071.4158	3071.4845	-22.35	305 -	332	2	R.IPGVPGSMPNASWAGDLRAVKWLDMEDK.H + 2 Oxidation (M)
3101.5515	3100.5442	3100.5480	-1.23	89 -	113	2	K.EEAEFPLAYVMVIHKDFNTFERLFR.A
3166.6106	3165.6033	3165.5497	16.9	6 -	31	0	K.YWFFTFMGVSVVIFMIFYSGQLSQPR.S + Oxidation (M)
3624.6824	3623.6751	3623.6497	7.02	323 -	353	2	R.AVKWLDMEDKHGGCHGHYVHDICIYGNGDLK.W

No match to: 701.3809, 706.2637, 708.2758, 717.3943, 719.4086, 722.4171, 734.4380, 735.4089, 736.4033, 747.4274, 756.3826, 759.3994, 772.4160, 779.4522, 795.4493, 806.4573, 831.4625, 832.3028, 834.3088, 842.4966, 876.2747, 878.2808, 883.4713, 888.4968, 890.4931, 891.4778, 900.5316, 910.4130, 918.5359, 929.4340, 936.4846, 937.5071, 938.5070, 949.5414, 967.4175, 969.4572, 972.4759, 974.4473, 976.4465, 978.5344, 983.6115, 988.4531, 990.4647, 993.4956, 1000.5594, 1007.5680, 1019.5631, 1021.5624, 1031.5641, 1034.5791, 1036.5952, 1044.5217, 1046.5728, 1051.5135, 1056.5387, 1057.6144, 1060.5447, 1074.5956, 1077.5199, 1087.5442, 1091.5437, 1113.5406, 1122.5580, 1123.5852, 1132.5316, 1145.6403, 1161.6290, 1168.6013, 1171.5769, 1172.5704, 1178.6254, 1180.6395, 1181.6516, 1185.6835, 1216.6992, 1234.6646, 1248.6750, 1254.6965, 1255.6359, 1273.6595, 1278.6974, 1281.6390, 1284.6533, 1301.6256, 1304.6489, 1314.7235, 1315.6904, 1325.6581, 1336.7290, 1338.6799, 1342.6847, 1349.6997, 1351.6993, 1354.6641, 1361.6997, 1368.6772, 1370.6589, 1372.6199, 1381.7728, 1382.6112, 1383.7378, 1386.7084, 1388.7888, 1390.7301, 1397.7515, 1425.7671, 1439.7701, 1442.8314, 1457.7517, 1467.7510, 1473.8167, 1488.7367, 1497.8859, 1500.7335, 1513.7731, 1516.7435, 1528.7101, 1556.8929, 1559.8439, 1562.8004, 1563.8088, 1578.7965, 1580.8445, 1601.9139, 1616.8481, 1623.8562, 1627.7197, 1628.7129, 1628.8722, 1634.8071, 1638.8757, 1642.7318, 1645.7701, 1647.8682, 1650.8612, 1673.8375, 1679.9817, 1680.8936, 1681.8372, 1696.8403, 1706.8660, 1715.8601, 1717.9037, 1730.8719, 1742.0088, 1758.9255, 1769.0105, 1771.0138, 1774.9404, 1783.9508, 1786.9517, 1787.9071, 1790.9086, 1835.8361, 1837.8712, 1862.9679, 1928.0435, 1940.0333, 1954.0796, 1961.0397, 1970.0139, 1982.0297, 1984.0314, 2023.0551, 2027.1292, 2032.0228, 2061.0330, 2105.0796, 2144.0679, 2170.0872, 2179.0752, 2215.0876, 2239.1650, 2242.1501, 2246.2151, 2251.1675, 2252.1614, 2255.1719, 2273.1008, 2289.1030, 2293.1750, 2304.2087, 2316.2031, 2343.2134, 2354.0020, 2391.2788, 2475.2476, 2491.2402, 2507.1975, 2514.2664, 2542.2795, 2550.2063, 2558.3499, 2587.3516, 2605.2615, 2646.9883, 2661.0090, 2675.0190, 2681.3855, 2698.3696, 2705.3142, 2707.3074, 2714.3647, 2717.3394, 2719.3167, 2722.3188, 2792.2446, 2826.3545, 2843.4670, 2846.4014, 2847.4082, 2872.3359, 2884.3538, 2887.3474, 2889.3481, 2892.4165, 2894.4639, 2896.4890, 2914.4551, 2987.5244, 2995.4431, 3004.5840, 3029.4812, 3054.4724, 3069.4849, 3126.7327, 3171.6748, 3183.6196, 3199.6411, 3231.5630, 3236.5791, 3243.6301, 3245.6216, 3247.5737, 3248.6313, 3254.6448, 3262.7324, 3267.7256, 3278.7546, 3321.5828, 3333.5762, 3334.6729, 3335.6787, 3338.7097, 3347.4907, 3395.6821, 3412.6426, 3428.5991, 3435.7024, 3457.6995, 3487.8022, 3490.6885, 3503.8049, 3534.6365, 3548.6917, 3551.6768, 3552.6902, 3601.6826, 3616.7095, 3639.7014, 3645.7827, 3651.7559, 3655.7354, 3667.7646, 3697.8025, 3716.7253, 3762.8396, 3929.9304, 4040.7656, 4057.9707, 4071.9524, 4092.8394, 4147.9063, 4160.9473, 4174.9575, 4193.9395, 4253.8755, 4415.9736, 4452.1865, 4465.1670, 4845.0845

44. F1N3G4 **Mass:** 157905 **Score:** 18 **Expect:** 3.6e+002 **Matches:** 23

Condensin complex subunit 1 OS=Bos taurus GN=LOC509171 PE=3 SV=2

Observed	Mr(expt)	Mr(calc)	ppm	Start	End	Miss	Peptide
719.4086	718.4013	718.4047	-4.80	1120 -	1125	1	K.DMVKVK.G
735.4089	734.4016	734.3997	2.64	1120 -	1125	1	K.DMVKVK.G + Oxidation (M)
842.4966	841.4894	841.4770	14.7	1269 -	1276	1	R.RGAKPEGK.A
1031.5641	1030.5568	1030.5342	22.0	457 -	464	1	K.LQEMRAQR.R
1034.5791	1033.5718	1033.5893	-16.92	1315 -	1323	2	R.KQPAAARHR.H
1386.7084	1385.7011	1385.6973	2.77	758 -	769	1	R.ATEKVPCSPLER.C
1390.7301	1389.7228	1389.7286	-4.14	1070 -	1082	0	R.SNLMIATGDLAIR.F + Oxidation (M)
1680.8936	1679.8863	1679.8564	17.8	1341 -	1354	2	R.TTRQHPNTQQRASR.K
2027.1292	2026.1219	2026.1683	-22.87	1108 -	1125	2	K.TAALVMTHLILKDMVKVK.G + Oxidation (M)
2215.0876	2214.0803	2214.1228	-19.21	825 -	842	2	R.KPSMGERHPPFRLPQEHR.L + Oxidation (M)
2242.1501	2241.1428	2241.1614	-8.31	1324 -	1343	2	R.HVASTASDDFVTPKPRRTTR.Q
2587.3516	2586.3443	2586.3145	11.5	335 -	359	0	R.NAVLAAMAEMVLQVLSGDQLEAASR.D
2887.3474	2886.3401	2886.3094	10.6	311 -	334	0	R.IPAILMSSMCILLDHLDGENYMMR.N + 4 Oxidation (M)
2914.4551	2913.4478	2913.5151	-23.08	1083 -	1106	2	R.FPNLVDPWTPHLYARLRDPSQHVR.K
3069.4849	3068.4776	3068.4511	8.64	1240 -	1266	1	K.MLDNFDCFGDKLSDESIFSAFLSILGK.L
3072.4231	3071.4158	3071.4128	0.98	1357 -	1383	2	K.LRIVFSSDESSDEELSAEMTEDETPKK.T
3262.7324	3261.7251	3261.7146	3.23	156 -	182	1	K.ATHGFDWEEERQPILQLLTQLLQLDIR.H
3338.7097	3337.7024	3337.6363	19.8	1240 -	1268	2	K.MLDNFDCFGDKLSDESIFSAFLSILGKLR.R
3395.6821	3394.6748	3394.6095	19.3	119 -	148	1	K.MNCYALIRLLESFETTSSQTGLMDVELGGK.G + 2 Oxidation (M)
3457.6995	3456.6922	3456.6184	21.4	543 -	571	1	R.FQESEPFCHVDPDESEETRFLSLLGTIFK.G
3490.6885	3489.6812	3489.6127	19.6	488 -	519	0	K.ATLQQLLMLPQEGEGMSEGVAETETPEEVEGR.I + 2 Oxidation (M)
4092.8394	4091.8321	4091.9213	-21.80	1018 -	1054	1	K.VCNNPGLYSNPELSTAASLALGKFCMISATFCDSQLR.L
4160.9473	4159.9400	4159.9913	-12.32	299 -	334	1	K.GFAAFLTELAERIPAILMSSMCILLDHLDGENYMMR.N + 2 Oxidation (M)

No match to: 701.3809, 706.2637, 708.2758, 717.3943, 722.4171, 734.4380, 736.4033, 747.4274, 756.3826, 759.3994, 772.4160, 779.4522, 795.4493, 806.4573, 831.4625, 832.3028, 834.3088, 876.2747, 878.2808, 883.4713, 888.4968, 890.4931, 891.4778, 900.5316, 910.4130, 918.5359, 929.4340, 936.4846, 937.5071, 938.5070, 949.5414, 967.4175, 969.4572, 972.4759, 974.4473, 976.4465, 978.5344, 983.6115, 988.4531, 990.4647, 993.4956, 1000.5594, 1007.5680, 1019.5631, 1021.5624, 1036.5952, 1044.5217, 1046.5728, 1051.5135, 1056.5387, 1057.6144, 1060.5447, 1074.5956, 1077.5199, 1087.5442, 1091.5437, 1113.5406, 1122.5580, 1123.5852, 1132.5316, 1145.6403, 1161.6290, 1168.6013, 1171.5769, 1172.5704, 1178.6254, 1180.6395, 1181.6516, 1185.6835, 1198.7111, 1216.6992, 1234.6646, 1248.6750, 1254.6965, 1255.6359, 1273.6595, 1278.6974, 1281.6390, 1284.6533, 1301.6256, 1304.6489, 1314.7235, 1315.6904, 1325.6581, 1336.7290, 1338.6799, 1342.6847, 1349.6997, 1351.6993, 1354.6641, 1361.6997, 1368.6772, 1370.6589, 1372.6199, 1381.7728, 1382.6112, 1383.7378, 1388.7888, 1397.7515, 1425.7671, 1439.7701, 1442.8314, 1457.7517, 1467.7510, 1473.8167, 1488.7367, 1497.8859, 1500.7335, 1513.7731, 1516.7435, 1528.7101, 1556.8929, 1559.8439, 1562.8004, 1563.8088, 1578.7965, 1580.8445, 1592.8094, 1601.9139, 1616.8481, 1623.8562,

1627.7197, 1628.7129, 1628.8722, 1634.8071, 1638.8757, 1642.7318, 1645.7701, 1647.8682, 1650.8612, 1673.8375, 1679.9817, 1681.8372, 1696.8403, 1706.8660, 1715.8601, 1717.9037, 1730.8719, 1742.0088, 1758.9255, 1769.0105, 1771.0138, 1774.9404, 1783.9508, 1786.9517, 1787.9071, 1790.9086, 1835.8361, 1837.8712, 1862.9679, 1928.0435, 1940.0333, 1954.0796, 1961.0397, 1970.0139, 1982.0297, 1984.0314, 2023.0551, 2032.0228, 2061.0330, 2105.0796, 2144.0679, 2170.0872, 2179.0752, 2211.0654, 2239.1650, 2246.2151, 2251.1675, 2252.1614, 2255.1719, 2273.1008, 2289.1030, 2293.1750, 2304.2087, 2316.2031, 2343.2134, 2354.0020, 2391.2788, 2475.2476, 2491.2402, 2507.1975, 2514.2664, 2542.2795, 2550.2063, 2558.3499, 2605.2615, 2646.9883, 2661.0090, 2675.0190, 2681.3855, 2698.3696, 2705.3142, 2707.3074, 2714.3647, 2717.3394, 2719.3167, 2722.3188, 2763.3738, 2792.2446, 2826.3545, 2843.4670, 2846.4014, 2847.4082, 2872.3359, 2884.3538, 2889.3481, 2892.4165, 2894.4639, 2896.4890, 2987.5244, 2995.4431, 3004.5840, 3029.4812, 3054.4724, 3101.5515, 3126.7327, 3166.6106, 3171.6748, 3183.6196, 3199.6411, 3231.5630, 3236.5791, 3243.6301, 3245.6216, 3247.5737, 3248.6313, 3254.6448, 3267.7256, 3278.7546, 3321.5828, 3333.5762, 3334.6729, 3335.6787, 3347.4907, 3412.6426, 3428.5991, 3435.7024, 3487.8022, 3503.8049, 3534.6365, 3548.6917, 3551.6768, 3552.6902, 3601.6826, 3616.7095, 3624.6824, 3639.7014, 3645.7827, 3651.7559, 3655.7354, 3667.7646, 3697.8025, 3716.7253, 3762.8396, 3929.9304, 4040.7656, 4057.9707, 4071.9524, 4147.9063, 4174.9575, 4193.9395, 4253.8755, 4415.9736, 4452.1865, 4465.1670, 4845.0845

45. Q9TTS3 **Mass:** 266788 **Score:** 18 **Expect:** 3.6e+002 **Matches:** 31

Acetyl-CoA carboxylase 1 OS=Bos taurus GN=ACACA PE=2 SV=1

Observed	Mr(expt)	Mr(calc)	ppm	Start	End	Miss	Peptide
900.5316	899.5243	899.5076	18.6	1724	1731	0	R.IGLAEEIR.H
1021.5624	1020.5551	1020.5352	19.5	1714	1723	0	R.IYVAANSGAR.I
1031.5641	1030.5568	1030.5335	22.6	2269	2276	0	K.DLVEWLEK.Q
1034.5791	1033.5718	1033.5702	1.54	2132	2139	2	K.DLVKTMRR.V + Oxidation (M)
1051.5135	1050.5062	1050.4903	15.1	2337	2346	0	R.ILSTMDSPST.-
1087.5442	1086.5369	1086.5305	5.90	1789	1798	0	K.EEGLGAENLR.G
1255.6359	1254.6286	1254.6357	-5.61	2026	2035	1	K.TYQAIKDFNR.E
1349.6997	1348.6924	1348.6987	-4.62	597	607	0	K.LLETESFQLNR.I
1368.6772	1367.6699	1367.6537	11.9	1488	1498	1	K.IEESVRSMVMR.Y + 2 Oxidation (M)
1370.6589	1369.6516	1369.6626	-8.01	1362	1371	2	R.DKFEEDRIYR.H
1500.7335	1499.7262	1499.7191	4.76	1388	1400	0	R.NFDLTAIPCANHK.M
1513.7731	1512.7658	1512.7693	-2.32	1494	1505	2	R.SMVMRYGSRLWK.L
1563.8088	1562.8015	1562.7841	11.1	1157	1169	0	R.AYIAYELNSVQHR.Q
1592.8094	1591.8021	1591.8318	-18.63	729	741	2	K.EEVDRYRITIGNK.T
1650.8612	1649.8539	1649.8889	-21.20	1372	1385	0	R.HLEPALAFQLELNR.M
1673.8375	1672.8302	1672.8607	-18.21	336	350	0	R.QVQAEVPGSPIFVMR.L + Oxidation (M)
1696.8403	1695.8330	1695.8329	0.09	1335	1347	2	K.DFRKQVNYEVDQR.F
1787.9071	1786.8998	1786.8607	21.9	1386	1400	1	R.MRNFDLTAIPCANHK.M
1954.0796	1953.0723	1953.0254	24.0	1372	1387	1	R.HLEPALAFQLELNRMR.N + Oxidation (M)
2252.1614	2251.1541	2251.1307	10.4	1401	1420	1	K.MHLYLGAAKVEVGTEVTDYR.F
2293.1750	2292.1677	2292.1386	12.7	1668	1687	1	K.SPEYPDGRDIIVIGNDITYR.I
2542.2795	2541.2722	2541.2612	4.33	94	116	2	K.IDSQRDFTVASPAEFVTRFGGNK.V
2763.3738	2762.3665	2762.3003	24.0	2104	2127	2	R.HMEMYADRESRGSVLEPEGTVEIK.F
3054.4724	3053.4651	3053.4916	-8.68	171	198	0	K.MADHYVPVPGGPNNNNYANVELILDIAK.R + Oxidation (M)
3101.5515	3100.5442	3100.4845	19.3	1283	1309	1	R.DEPIHILNVAIKTDCDIEDDSLAAMFR.E
3412.6426	3411.6353	3411.6479	-3.69	756	786	1	R.SPSAGKLIQYIVEDGGHVFAGQCYAEIEVMK.M + Oxidation (M)
3435.7024	3434.6951	3434.7339	-11.30	1372	1400	2	R.HLEPALAFQLELNRMRNFDLTAIPCANHK.M + Oxidation (M)
3490.6885	3489.6812	3489.6268	15.6	473	502	2	R.IKDIRMMYGVSPWGDAPIDFENSAHVPCPR.G + 2 Oxidation (M)
3697.8025	3696.7952	3696.7772	4.87	1468	1498	2	R.TDCNHIFLNFVPTVIMDPSKIEESVRSMVMR.Y + 2 Oxidation (M)
4452.1865	4451.1792	4451.2563	-17.32	1782	1823	2	K.ITDIIGKEEGLGAENLRGSGMIAGESSLAYDEIITISLVTCR.A
4465.1670	4464.1597	4464.2576	-21.93	2172	2209	2	R.EEFLLPIYHQVAVQFADLHDTPGRMQEKGVINDILDWK.T

No match to: 701.3809, 706.2637, 708.2758, 717.3943, 719.4086, 722.4171, 734.4380, 735.4089, 736.4033, 747.4274, 756.3826, 759.3994, 772.4160, 779.4522, 795.4493, 806.4573, 831.4625, 832.3028, 834.3088, 842.4966, 876.2747, 878.2808, 883.4713, 888.4968, 890.4931, 891.4778, 910.4130, 918.5359, 929.4340, 936.4846, 937.5071, 938.5070, 949.5414, 967.4175, 969.4572, 972.4759, 974.4473, 976.4465, 978.5344, 983.6115, 988.4531, 990.4647, 993.4956, 1000.5594, 1007.5680, 1019.5631, 1036.5952, 1044.5217, 1046.5728, 1056.5387, 1057.6144, 1060.5447, 1074.5956, 1077.5199, 1091.5437, 1113.5406, 1122.5580, 1123.5852, 1132.5316, 1145.6403, 1161.6290, 1168.6013, 1171.5769, 1172.5704, 1178.6254, 1180.6395, 1181.6516, 1185.6835, 1198.7111, 1216.6992, 1234.6646, 1248.6750, 1254.6965, 1273.6595, 1278.6974, 1281.6390, 1284.6533, 1301.6256, 1304.6489, 1314.7235, 1315.6904, 1325.6581, 1336.7290, 1338.6799, 1342.6847, 1351.6993, 1354.6641, 1361.6997, 1372.6199, 1381.7728, 1382.6112, 1383.7378, 1386.7084, 1388.7888, 1390.7301, 1397.7515, 1425.7671, 1439.7701, 1442.8314, 1457.7517, 1467.7510, 1473.8167, 1488.7367, 1497.8859, 1516.7435, 1528.7101, 1556.8929, 1559.8439, 1562.8004, 1578.7965, 1580.8445, 1601.9139, 1616.8481, 1623.8562, 1627.7197, 1628.7129, 1628.8722, 1634.8071, 1638.8757, 1642.7318, 1645.7701, 1647.8682, 1679.9817, 1680.8936, 1681.8372, 1706.8660, 1715.8601, 1717.9037, 1730.8719, 1742.0088, 1758.9255, 1769.0105, 1771.0138, 1774.9404, 1783.9508, 1786.9517, 1790.9086, 1835.8361, 1837.8712, 1862.9679, 1928.0435, 1940.0333, 1961.0397, 1970.0139, 1982.0297, 1984.0314, 2023.0551, 2027.1292, 2032.0228, 2061.0330, 2105.0796, 2144.0679, 2170.0872, 2179.0752, 2211.0654, 2215.0876, 2239.1650, 2242.1501, 2246.2151, 2251.1675, 2255.1719, 2273.1008, 2289.1030, 2304.2087, 2316.2031, 2343.2134, 2354.0020, 2391.2788, 2475.2476, 2491.2402, 2507.1975, 2514.2664, 2550.2063, 2558.3499, 2587.3516, 2605.2615, 2646.9883, 2661.0090, 2675.0190, 2681.3855, 2698.3696, 2705.3142, 2707.3074, 2714.3647, 2717.3394, 2719.3167, 2722.3188, 2792.2446, 2826.3545, 2843.4670, 2846.4014, 2847.4082, 2872.3359, 2884.3538, 2887.3474, 2889.3481, 2892.4165, 2894.4639, 2896.4890, 2914.4551, 2987.5244, 2995.4431, 3004.5840, 3029.4812, 3069.4849, 3072.4231, 3126.7327, 3166.6106, 3171.6748, 3183.6196, 3199.6411, 3231.5630, 3236.5791, 3243.6301, 3245.6216, 3247.5737, 3248.6313, 3254.6448, 3262.7324, 3267.7256, 3278.7546, 3321.5828, 3333.5762, 3334.6729, 3335.6787, 3338.7097, 3347.4907, 3395.6821, 3428.5991, 3457.6995, 3487.8022, 3503.8049, 3534.6365, 3548.6917, 3551.6768, 3552.6902, 3601.6826, 3616.7095, 3624.6824, 3639.7014, 3645.7827, 3651.7559, 3655.7354, 3667.7646, 3716.7253, 3762.8396, 3929.9304, 4040.7656, 4057.9707, 4071.9524, 4092.8394, 4147.9063, 4160.9473, 4174.9575, 4193.9395, 4253.8755, 4415.9736, 4845.0845

46. F1MUT6 **Mass:** 54881 **Score:** 18 **Expect:** 3.6e+002 **Matches:** 10

Uncharacterized protein OS=Bos taurus GN=DDX19B PE=4 SV=1

Observed	Mr(expt)	Mr(calc)	ppm	Start	End	Miss	Peptide
747.4274	746.4202	746.4327	-16.76	235	240	1	K.FIDPKK.I
888.4968	887.4896	887.4759	15.3	1	9	1	-.MAGAAGRVR.G
1077.5199	1076.5126	1076.5237	-10.30	305	313	0	R.EEETLDTIK.Q
1769.0105	1768.0032	1767.9671	20.4	193	207	2	K.FYPELKLAYAVRGNK.L
2255.1719	2254.1646	2254.2184	-23.83	186	204	2	K.VIEQMGKFYPELKLAYAVR.G
2550.2063	2549.1990	2549.2083	-3.64	272	292	1	R.NCQMLLFSATFEDSVWKFAQK.V
2605.2615	2604.2542	2604.2175	14.1	268	288	1	R.MLPRNCQMLLFSATFEDSVWK.F + 2 Oxidation (M)
2843.4670	2842.4597	2842.4081	18.2	326	349	1	K.FQALCNIYGAITIAQAMIFCHTRK.T + Oxidation (M)
3183.6196	3182.6123	3182.6394	-8.49	349	377	2	R.KTASWLAAELSKEGHQVALLSGEMVVEQR.A + Oxidation (M)
3199.6411	3198.6338	3198.5776	17.6	323	349	2	R.DEKFQALCNIYGAITIAQAMIFCHTRK.T

No match to: 701.3809, 706.2637, 708.2758, 717.3943, 719.4086, 722.4171, 734.4380, 735.4089, 736.4033, 756.3826, 759.3994, 772.4160, 779.4522, 795.4493, 806.4573, 831.4625, 832.3028, 834.3088, 842.4966, 876.2747, 878.2808, 883.4713, 890.4931, 891.4778, 900.5316, 910.4130, 918.5359, 929.4340, 936.4846, 937.5071, 938.5070, 949.5414, 967.4175, 969.4572, 972.4759, 974.4473, 976.4465, 978.5344, 983.6115, 988.4531, 990.4647, 993.4956, 1000.5594, 1007.5680, 1019.5631, 1021.5624, 1031.5641, 1034.5791, 1036.5952, 1044.5217, 1046.5728, 1051.5135, 1056.5387, 1057.6144, 1060.5447, 1074.5956, 1087.5442, 1091.5437, 1113.5406, 1122.5580, 1123.5852, 1132.5316, 1145.6403, 1161.6290, 1168.6013, 1171.5769, 1172.5704, 1178.6254, 1180.6395, 1181.6516, 1185.6835, 1198.7111, 1216.6992, 1234.6646, 1248.6750, 1254.6965, 1255.6359, 1273.6595, 1278.6974, 1281.6390, 1284.6533, 1301.6256, 1304.6489, 1314.7235, 1315.6904, 1325.6581, 1336.7290, 1338.6799, 1342.6847, 1349.6997, 1351.6993, 1354.6641, 1361.6997, 1368.6772, 1370.6589, 1372.6199, 1381.7728,

1382.6112, 1383.7378, 1386.7084, 1388.7888, 1390.7301, 1397.7515, 1425.7671, 1439.7701, 1442.8314, 1457.7517, 1467.7510, 1473.8167, 1488.7367, 1497.8859, 1500.7335, 1513.7731, 1516.7435, 1528.7101, 1556.8929, 1559.8439, 1562.8004, 1563.8088, 1578.7965, 1580.8445, 1592.8094, 1601.9139, 1616.8481, 1623.8562, 1627.7197, 1628.7129, 1628.8722, 1634.8071, 1638.8757, 1642.7318, 1645.7701, 1647.8682, 1650.8612, 1673.8375, 1679.9817, 1680.8936, 1681.8372, 1696.8403, 1706.8660, 1715.8601, 1717.9037, 1730.8719, 1742.0088, 1758.9255, 1771.0138, 1774.9404, 1783.9508, 1786.9517, 1787.9071, 1790.9086, 1835.8361, 1837.8712, 1862.9679, 1928.0435, 1940.0333, 1954.0796, 1961.0397, 1970.0139, 1982.0297, 1984.0314, 2023.0551, 2027.1292, 2032.0228, 2061.0330, 2105.0796, 2144.0679, 2170.0872, 2179.0752, 2211.0654, 2215.0876, 2239.1650, 2242.1501, 2246.2151, 2251.1675, 2252.1614, 2273.1008, 2289.1030, 2293.1750, 2304.2087, 2316.2031, 2343.2134, 2354.0020, 2391.2788, 2475.2476, 2491.2402, 2507.1975, 2514.2664, 2542.2795, 2558.3499, 2587.3516, 2646.9883, 2661.0090, 2675.0190, 2681.3855, 2698.3696, 2705.3142, 2707.3074, 2714.3647, 2717.3394, 2719.3167, 2722.3188, 2763.3738, 2792.2446, 2826.3545, 2846.4014, 2847.4082, 2872.3359, 2884.3538, 2887.3474, 2889.3481, 2892.4165, 2894.4639, 2896.4890, 2914.4551, 2987.5244, 2995.4431, 3004.5840, 3029.4812, 3054.4724, 3069.4849, 3072.4231, 3101.5515, 3126.7327, 3166.6106, 3171.6748, 3231.5630, 3236.5791, 3243.6301, 3245.6216, 3247.5737, 3248.6313, 3254.6448, 3262.7324, 3267.7256, 3278.7546, 3321.5828, 3333.5762, 3334.6729, 3335.6787, 3338.7097, 3347.4907, 3395.6821, 3412.6426, 3428.5991, 3435.7024, 3457.6995, 3487.8022, 3490.6885, 3503.8049, 3534.6365, 3548.6917, 3551.6768, 3552.6902, 3601.6826, 3616.7095, 3624.6824, 3639.7014, 3645.7827, 3651.7559, 3655.7354, 3667.7646, 3697.8025, 3716.7253, 3762.8396, 3929.9304, 4040.7656, 4057.9707, 4071.9524, 4092.8394, 4147.9063, 4160.9473, 4174.9575, 4193.9395, 4253.8755, 4415.9736, 4452.1865, 4465.1670, 4845.0845

47. F1MV51 **Mass:** 314440 **Score:** 18 **Expect:** 3.7e+002 **Matches:** 32

Uncharacterized protein OS=Bos taurus GN=APC PE=4 SV=2

Observed	Mr(expt)	Mr(calc)	ppm	Start	End	Miss	Peptide
806.4573	805.4500	805.4545	-5.60	2181	2187	1	K.STLETKK.I
888.4968	887.4896	887.4712	20.6	2287	2294	0	K.SELSPVTR.Q
900.5316	899.5243	899.5413	-18.86	214	220	2	R.AQRRITR.I
1000.5594	999.5521	999.5461	6.03	1461	1469	2	K.NKAPSAEKR.E
1044.5217	1043.5144	1043.5360	-20.64	1693	1701	1	K.RDTIPTEGR.S
1056.5387	1055.5314	1055.5182	12.5	91	99	1	K.MSLRSYGSR.E
1060.5447	1059.5374	1059.5421	-4.40	849	856	2	K.DRSLERER.G
1077.5199	1076.5126	1076.5284	-14.68	2379	2387	0	R.QMSQQNLTK.Q
1091.5437	1090.5364	1090.5627	-24.08	717	727	0	K.MIAMGSAAALR.N
1123.5852	1122.5779	1122.5525	22.6	717	727	0	K.MIAMGSAAALR.N + 2 Oxidation (M)
1145.6403	1144.6330	1144.6451	-10.59	2187	2196	2	K.KIESENKGIK.G
1314.7235	1313.7162	1313.7052	8.43	2388	2400	1	K.QTGLSKNGSGIPR.S
1425.7671	1424.7598	1424.7623	-1.74	1456	1468	2	K.QEVPKNKAPSAEK.R
1500.7335	1499.7262	1499.7620	-23.86	2729	2741	0	R.LNSFIQVDPPDQK.G
1559.8439	1558.8366	1558.8103	16.9	1351	1365	2	R.HKAVEFSSGAKSPSK.S
1634.8071	1633.7998	1633.8033	-2.12	2532	2545	2	K.RHDIARSHSESPSR.L
1706.8660	1705.8587	1705.8482	6.16	2591	2606	2	K.AKSEDEKQVNSISGSK.Q
2215.0876	2214.0803	2214.0665	6.24	855	875	1	R.ERGISLGNYHPATENPGTSSK.R
2289.1030	2288.0957	2288.0880	3.37	1895	1915	1	K.ESEAKVTNHTELTSNQQSASK.T
2293.1750	2292.1677	2292.1393	12.4	2313	2333	0	R.DSTPSRPAQQPLSRPMQSPGR.N
2507.1975	2506.1902	2506.1374	21.1	791	810	2	R.HKQNLYGDYVFDTNRHDDNR.S
2558.3499	2557.3426	2557.3460	-1.31	2280	2304	2	R.GTKPSVKSELSPVTRQASQTAGSNK.G
2826.3545	2825.3472	2825.2946	18.6	2031	2056	0	R.NSSLSSLSIDSEDDLLQECISSAMPK.K
2847.4082	2846.4009	2846.3683	11.5	2825	2851	2	R.DSKPDSTEPSGTQSPKRHSGSYLVTSV.-
3004.5840	3003.5767	3003.5057	23.7	2313	2340	1	R.DSTPSRPAQQPLSRPMQSPGRNSISPGR.N
3029.4812	3028.4739	3028.5135	-13.07	877	904	1	R.GLQISTTAAQIAKVMEEVSAIHTSQEDR.S + Oxidation (M)
3054.4724	3053.4651	3053.5305	-21.41	756	782	2	R.KQKALEAELDAQHLSETFDNIDNLSPK.A
3166.6106	3165.6033	3165.6378	-10.89	624	653	1	R.SQTNTLAIIESGGGILRNVSSLIATNEDHR.Q
3457.6995	3456.6922	3456.6214	20.5	1208	1239	2	K.TEHISSSSENTSTTSSNAKRQNQLHPSSAQSR.S
4071.9524	4070.9451	4070.9167	6.97	2074	2109	1	R.NMGGILAEDLTLDLKDIQRPDSEHGLSPDSENFDWK.A + Oxidation (M)
4147.9063	4146.8990	4146.9925	-22.54	464	499	1	R.HAMNELGGLQAIAELLQVDCEMYGLTNDHYSITLRR.Y + Oxidation (M)
4452.1865	4451.1792	4451.0935	19.3	1957	1995	2	K.LQNFAIENTPVCFSRNSSLSSLSDIDQENNNNKENEPVK.E

No match to: 701.3809, 706.2637, 708.2758, 717.3943, 719.4086, 722.4171, 734.4380, 735.4089, 736.4033, 747.4274, 756.3826, 759.3994, 772.4160, 779.4522, 795.4493, 831.4625, 832.3028, 834.3088, 842.4966, 876.2747, 878.2808, 883.4713, 890.4931, 891.4778, 910.4130, 918.5359, 929.4340, 936.4846, 937.5071, 938.5070, 949.5414, 967.4175, 969.4572, 972.4759, 974.4473, 976.4465, 978.5344, 983.6115, 988.4531, 990.4647, 993.4956, 1007.5680, 1019.5631, 1021.5624, 1031.5641, 1034.5791, 1036.5952, 1046.5728, 1051.5135, 1057.6144, 1074.5956, 1087.5442, 1113.5406, 1122.5580, 1132.5316, 1161.6290, 1168.6013, 1171.5769, 1172.5704, 1178.6254, 1180.6395, 1181.6516, 1185.6835, 1198.7111, 1216.6992, 1234.6646, 1248.6750, 1254.6965, 1255.6359, 1273.6595, 1278.6974, 1281.6390, 1284.6533, 1301.6256, 1304.6489, 1315.6904, 1325.6581, 1336.7290, 1338.6799, 1342.6847, 1349.6997, 1351.6993, 1354.6641, 1361.6997, 1368.6772, 1370.6589, 1372.6199, 1381.7728, 1382.6112, 1383.7378, 1386.7084, 1388.7888, 1390.7301, 1397.7515, 1439.7701, 1442.8314, 1457.7517, 1467.7510, 1473.8167, 1488.7367, 1497.8859, 1513.7731, 1516.7435, 1528.7101, 1556.8929, 1562.8004, 1563.8088, 1578.7965, 1580.8445, 1592.8094, 1601.9139, 1616.8481, 1623.8562, 1627.7197, 1628.7129, 1628.8722, 1638.8757, 1642.7318, 1645.7701, 1647.8682, 1650.8612, 1673.8375, 1679.9817, 1680.8936, 1681.8372, 1696.8403, 1715.8601, 1717.9037, 1730.8719, 1742.0088, 1758.9255, 1769.0105, 1771.0138, 1774.9404, 1783.9508, 1786.9517, 1787.9071, 1790.9086, 1835.8361, 1837.8712, 1862.9679, 1928.0435, 1940.0333, 1954.0796, 1961.0397, 1970.0139, 1982.0297, 1984.0314, 2023.0551, 2027.1292, 2032.0228, 2061.0330, 2105.0796, 2144.0679, 2170.0872, 2179.0752, 2211.0654, 2239.1650, 2242.1501, 2246.2151, 2251.1675, 2252.1614, 2255.1719, 2273.1008, 2304.2087, 2316.2031, 2343.2134, 2354.0020, 2391.2788, 2475.2476, 2491.2402, 2514.2664, 2542.2795, 2550.2063, 2587.3516, 2605.2615, 2646.9883, 2661.0090, 2675.0190, 2681.3855, 2698.3696, 2705.3142, 2707.3074, 2714.3647, 2717.3394, 2719.3167, 2722.3188, 2763.3738, 2792.2446, 2843.4670, 2846.4014, 2872.3359, 2884.3538, 2887.3474, 2889.3481, 2892.4165, 2894.4639, 2896.4890, 2914.4551, 2987.5244, 2995.4431, 3069.4849, 3072.4231, 3101.5515, 3126.7327, 3171.6748, 3183.6196, 3199.6411, 3231.5630, 3236.5791, 3243.6301, 3245.6216, 3247.5737, 3248.6313, 3254.6448, 3262.7324, 3267.7256, 3278.7546, 3321.5828, 3333.5762, 3334.6729, 3335.6787, 3338.7097, 3347.4907, 3395.6821, 3412.6426, 3428.5991, 3435.7024, 3487.8022, 3490.6885, 3503.8049, 3534.6365, 3548.6917, 3551.6768, 3552.6902, 3601.6826, 3616.7095, 3624.6824, 3639.7014, 3645.7827, 3651.7559, 3655.7354, 3667.7646, 3697.8025, 3716.7253, 3762.8396, 3929.9304, 4040.7656, 4057.9707, 4092.8394, 4160.9473, 4174.9575, 4193.9395, 4253.8755, 4415.9736, 4465.1670, 4845.0845

48. G3N0V2 **Mass:** 63341 **Score:** 18 **Expect:** 3.7e+002 **Matches:** 13

Uncharacterized protein OS=Bos taurus GN=KRT1 PE=1 SV=1

Observed	Mr(expt)	Mr(calc)	ppm	Start	End	Miss	Peptide
888.4968	887.4896	887.5076	-20.35	66	74	0	R.SLINLGGSK.S
910.4130	909.4057	909.4079	-2.49	266	272	0	K.YEDEINK.R
1046.5728	1045.5655	1045.5768	-10.74	404	412	1	R.LRSEIDSVK.K
1123.5852	1122.5779	1122.5557	19.8	363	372	0	K.AEAEALYQTK.Y
1338.6799	1337.6726	1337.6826	-7.49	361	372	1	R.SKAEAEALYQTK.Y
1638.8757	1637.8684	1637.8525	9.70	182	195	1	K.SLNNQFASFIDKVR.F
1673.8375	1672.8302	1672.8380	-4.65	414	428	0	K.QISSLQQAISDAEQR.G
1928.0435	1927.0362	1927.0010	18.3	435	451	2	K.DAQNKLNELEDALQKAK.E
2211.0654	2210.0581	2210.0815	-10.58	582	603	2	R.ASSTKTSGGSSSVKFVSTSYSR.G
2215.0876	2214.0803	2214.0374	19.4	561	586	2	R.RGGSGGGGGSSGGSFISSGGRASSTK.T
2707.3074	2706.3001	2706.2529	17.5	13	40	2	R.CGGGGGGFSSGSAGVVSYQRRSTSSSVR.R
2884.3538	2883.3465	2883.3795	-11.44	42	74	2	R.SGGGGGRFSGGVCGGGAGGGFGSRSLINLGGSK.S
3236.5791	3235.5718	3235.5996	-8.60	363	391	2	K.AEAEALYQTKYEELQITAGQHGDSLKNTK.V

No match to: 701.3809, 706.2637, 708.2758, 717.3943, 719.4086, 722.4171, 734.4380, 735.4089, 736.4033, 747.4274, 756.3826, 759.3994, 772.4160, 779.4522, 795.4493, 806.4573, 831.4625, 832.3028, 834.3088, 842.4966, 876.2747, 878.2808, 883.4713, 890.4931, 891.4778, 900.5316, 918.5359, 929.4340, 936.4846, 937.5071, 938.5070, 949.5414, 967.4175, 969.4572, 972.4759, 974.4473, 976.4465, 978.5344, 983.6115, 988.4531, 990.4647, 993.4956, 1000.5594, 1007.5680, 1019.5631, 1021.5624, 1031.5641, 1034.5791, 1036.5952, 1044.5217, 1051.5135, 1056.5387, 1057.6144, 1060.5447, 1074.5956, 1077.5199, 1087.5442, 1091.5437, 1113.5406, 1122.5580, 1132.5316, 1145.6403, 1161.6290, 1168.6013, 1171.5769, 1172.5704, 1178.6254, 1180.6395, 1181.6516, 1185.6835, 1198.7111, 1216.6992, 1234.6646, 1248.6750, 1254.6965, 1255.6359, 1273.6595, 1278.6974, 1281.6390, 1284.6533, 1301.6256, 1304.6489, 1314.7235, 1315.6904, 1325.6581, 1336.7290, 1342.6847, 1349.6997, 1351.6993, 1354.6641, 1361.6997, 1368.6772, 1370.6589, 1372.6199, 1381.7728, 1382.6112, 1383.7378, 1386.7084, 1388.7888, 1390.7301, 1397.7515, 1425.7671, 1439.7701, 1442.8314, 1457.7517, 1467.7510, 1473.8167, 1488.7367, 1497.8859, 1500.7335, 1513.7731, 1516.7435, 1528.7101, 1556.8929, 1559.8439, 1562.8004, 1563.8088, 1578.7965, 1580.8445, 1592.8094, 1601.9139, 1616.8481, 1623.8562, 1627.7197, 1628.7129, 1628.8722, 1634.8071, 1642.7318, 1645.7701, 1647.8682, 1650.8612, 1679.9817, 1680.8936, 1681.8372, 1696.8403, 1706.8660, 1715.8601, 1717.9037, 1730.8719, 1742.0088, 1758.9255, 1769.0105, 1771.0138, 1774.9404, 1783.9508, 1786.9517, 1787.9071, 1790.9086, 1835.8361, 1837.8712, 1862.9679, 1940.0333, 1954.0796, 1961.0397, 1970.0139, 1982.0297, 1984.0314, 2023.0551, 2027.1292, 2032.0228, 2061.0330, 2105.0796, 2144.0679, 2170.0872, 2179.0752, 2239.1650, 2242.1501, 2246.2151, 2251.1675, 2252.1614, 2255.1719, 2273.1008, 2289.1030, 2293.1750, 2304.2087, 2316.2031, 2343.2134, 2354.0020, 2391.2788, 2475.2476, 2491.2402, 2507.1975, 2514.2664, 2542.2795, 2550.2063, 2558.3499, 2587.3516, 2605.2615, 2646.9883, 2661.0090, 2675.0190, 2681.3855, 2698.3696, 2705.3142, 2714.3647, 2717.3394, 2719.3167, 2722.3188, 2763.3738, 2792.2446, 2826.3545, 2843.4670, 2846.4014, 2847.4082, 2872.3359, 2887.3474, 2889.3481, 2892.4165, 2894.4639, 2896.4890, 2914.4551, 2987.5244, 2995.4431, 3004.5840, 3029.4812, 3054.4724, 3069.4849, 3072.4231, 3101.5515, 3126.7327, 3166.6106, 3171.6748, 3183.6196, 3199.6411, 3231.5630, 3243.6301, 3245.6216, 3247.5737, 3248.6313, 3254.6448, 3262.7324, 3267.7256, 3278.7546, 3321.5828, 3333.5762, 3334.6729, 3335.6787, 3338.7097, 3347.4907, 3395.6821, 3412.6426, 3428.5991, 3435.7024, 3457.6995, 3487.8022, 3490.6885, 3503.8049, 3534.6365, 3548.6917, 3551.6768, 3552.6902, 3601.6826, 3616.7095, 3624.6824, 3639.7014, 3645.7827, 3651.7559, 3655.7354, 3667.7646, 3697.8025, 3716.7253, 3762.8396, 3929.9304, 4040.7656, 4057.9707, 4071.9524, 4092.8394, 4147.9063, 4160.9473, 4174.9575, 4193.9395, 4253.8755, 4415.9736, 4452.1865, 4465.1670, 4845.0845

49. F1MHQ3 **Mass:** 40861 **Score:** 18 **Expect:** 3.8e+002 **Matches:** 9

Hexosyltransferase OS=Bos taurus PE=3 SV=1

Observed	Mr(expt)	Mr(calc)	ppm	Start	End	Miss	Peptide
1074.5956	1073.5883	1073.5618	24.7	141	149	1	R.QAIRESWGK.E
1255.6359	1254.6286	1254.6543	-20.46	100	108	1	K.DFLLYLRCR.N
1673.8375	1672.8302	1672.8632	-19.69	17	32	1	K.SSSQETNGKGEVIIPK.I
1928.0435	1927.0362	1927.0562	-10.39	1	16	0	-.LLMVNVFIYLIMEVSK.S + Oxidation (M)
2032.0228	2031.0155	2031.0650	-24.36	251	268	2	K.DLFIGDVIHNAGPHRDKK.L
2542.2795	2541.2722	2541.3049	-12.88	100	119	2	K.DFLLYLRCRNYSLLIDQPDK.C
2763.3738	2762.3665	2762.3183	17.5	291	313	0	R.LYNGTDVLLYPIDDVYTEMCLQK.L
2846.4014	2845.3941	2845.4605	-23.34	1	25	1	-.LLMVNVFIYLIMEVSKSSSQETNGK.G + Oxidation (M)
4174.9575	4173.9502	4173.9736	-5.59	145	181	2	R.ESWGKETNVGNQTVVRVFLMGQTPAEDNHPDLSDMLK.F + 2 Oxidation (M)

No match to: 701.3809, 706.2637, 708.2758, 717.3943, 719.4086, 722.4171, 734.4380, 735.4089, 736.4033, 747.4274, 756.3826, 759.3994, 772.4160, 779.4522, 795.4493, 806.4573, 831.4625, 832.3028, 834.3088, 842.4966, 876.2747, 878.2808, 883.4713, 888.4968, 890.4931, 891.4778, 900.5316, 910.4130, 918.5359, 929.4340, 936.4846, 937.5071, 938.5070, 949.5414, 967.4175, 969.4572, 972.4759, 974.4473, 976.4465, 978.5344, 983.6115, 988.4531, 990.4647, 993.4956, 1000.5594, 1007.5680, 1019.5631, 1021.5624, 1031.5641, 1034.5791, 1036.5952, 1044.5217, 1046.5728, 1051.5135, 1056.5387, 1057.6144, 1060.5447, 1077.5199, 1087.5442, 1091.5437, 1113.5406, 1122.5580, 1123.5852, 1132.5316, 1145.6403, 1161.6290, 1168.6013, 1171.5769, 1172.5704, 1178.6254, 1180.6395, 1181.6516, 1185.6835, 1198.7111, 1216.6992, 1234.6646, 1248.6750, 1254.6965, 1273.6595, 1278.6974, 1281.6390, 1284.6533, 1301.6256, 1304.6489, 1314.7235, 1315.6904, 1325.6581, 1336.7290, 1338.6799, 1342.6847, 1349.6997, 1351.6993, 1354.6641, 1361.6997, 1368.6772, 1370.6589, 1372.6199, 1381.7728, 1382.6112, 1383.7378, 1386.7084, 1388.7888, 1390.7301, 1397.7515, 1425.7671, 1439.7701, 1442.8314, 1457.7517, 1467.7510, 1473.8167, 1488.7367, 1497.8859, 1500.7335, 1513.7731, 1516.7435, 1528.7101, 1556.8929, 1559.8439, 1562.8004, 1563.8088, 1578.7965, 1580.8445, 1592.8094, 1601.9139, 1616.8481, 1623.8562, 1627.7197, 1628.7129, 1628.8722, 1634.8071, 1638.8757, 1642.7318, 1645.7701, 1647.8682, 1650.8612, 1679.9817, 1680.8936, 1681.8372, 1696.8403, 1706.8660, 1715.8601, 1717.9037, 1730.8719, 1742.0088, 1758.9255, 1769.0105, 1771.0138, 1774.9404, 1783.9508, 1786.9517, 1787.9071, 1790.9086, 1835.8361, 1837.8712, 1862.9679, 1940.0333, 1954.0796, 1961.0397, 1970.0139, 1982.0297, 1984.0314, 2023.0551, 2027.1292, 2061.0330, 2105.0796, 2144.0679, 2170.0872, 2179.0752, 2211.0654, 2215.0876, 2239.1650, 2242.1501, 2246.2151, 2251.1675, 2252.1614, 2255.1719, 2273.1008, 2289.1030, 2293.1750, 2304.2087, 2316.2031, 2343.2134, 2354.0020, 2391.2788, 2475.2476, 2491.2402, 2507.1975, 2514.2664, 2550.2063, 2558.3499, 2587.3516, 2605.2615, 2646.9883, 2661.0090, 2675.0190, 2681.3855, 2698.3696, 2705.3142, 2707.3074, 2714.3647, 2717.3394, 2719.3167, 2722.3188, 2792.2446, 2826.3545, 2843.4670, 2847.4082, 2872.3359, 2884.3538, 2887.3474, 2889.3481, 2892.4165, 2894.4639, 2896.4890, 2914.4551, 2987.5244, 2995.4431, 3004.5840, 3029.4812, 3054.4724, 3069.4849, 3072.4231, 3101.5515, 3126.7327, 3166.6106, 3171.6748, 3183.6196, 3199.6411, 3231.5630, 3236.5791, 3243.6301, 3245.6216, 3247.5737, 3248.6313, 3254.6448, 3262.7324, 3267.7256, 3278.7546, 3321.5828, 3333.5762, 3334.6729, 3335.6787, 3338.7097, 3347.4907, 3395.6821, 3412.6426, 3428.5991, 3435.7024, 3457.6995, 3487.8022, 3490.6885, 3503.8049, 3534.6365, 3548.6917, 3551.6768, 3552.6902, 3601.6826, 3616.7095, 3624.6824, 3639.7014, 3645.7827, 3651.7559, 3655.7354, 3667.7646, 3697.8025, 3716.7253, 3762.8396, 3929.9304, 4040.7656, 4057.9707, 4071.9524, 4092.8394, 4147.9063, 4160.9473, 4193.9395, 4253.8755, 4415.9736, 4452.1865, 4465.1670, 4845.0845

50. A5D7M3 **Mass:** 56128 **Score:** 18 **Expect:** 3.8e+002 **Matches:** 15

Growth arrest-specific protein 8 OS=Bos taurus GN=GAS8 PE=2 SV=1

Observed	Mr(expt)	Mr(calc)	ppm	Start	End	Miss	Peptide
806.4573	805.4500	805.4666	-20.60	192	197	2	K.KMKMLR.D
967.4175	966.4103	966.4341	-24.69	174	180	1	K.MRNDFER.Q
969.4572	968.4499	968.4716	-22.33	50	56	0	R.NYFQLER.D
1123.5852	1122.5779	1122.5920	-12.58	184	192	2	R.EIEAKYDKK.M
1161.6290	1160.6217	1160.5972	21.1	216	225	0	K.NGQITTLMQR.H
1397.7515	1396.7442	1396.7310	9.47	205	215	2	R.KTEIHEVEERK.N
1425.7671	1424.7598	1424.7372	15.9	204	214	2	R.RKTEIHEVEER.K
1715.8601	1714.8528	1714.8348	10.5	104	117	0	K.HLLYEHQSSLTEMK.A
1730.8719	1729.8646	1729.8557	5.19	12	28	1	K.GKGTPIVDGLAPEDMSK.E + Oxidation (M)
1758.9255	1757.9182	1757.9233	-2.88	147	161	1	K.VELKEQELANEVMVK.N
1774.9404	1773.9331	1773.9182	8.41	147	161	1	K.VELKEQELANEVMVK.N + Oxidation (M)
1970.0139	1969.0066	1969.0533	-23.73	323	337	2	K.SLRWEHEVLEQRFIK.V
2892.4165	2891.4092	2891.4447	-12.27	14	39	2	K.GTPIVDGLAPEDMSKEQVEEHIGRIR.E + Oxidation (M)
2987.5244	2986.5171	2986.5256	-2.83	104	129	2	K.HLLYEHQSSLTEMKAEGTVVMKLAQK.E + Oxidation (M)
3166.6106	3165.6033	3165.5982	1.63	226	252	1	R.HEEAFTDIKNYYNDITLNNLALINSLK.E

No match to: 701.3809, 706.2637, 708.2758, 717.3943, 719.4086, 722.4171, 734.4380, 735.4089, 736.4033, 747.4274, 756.3826, 759.3994, 772.4160, 779.4522, 795.4493, 831.4625, 832.3028, 834.3088, 842.4966, 876.2747, 878.2808, 883.4713, 888.4968, 890.4931, 891.4778, 900.5316, 910.4130, 918.5359, 929.4340, 936.4846, 937.5071, 938.5070, 949.5414, 972.4759, 974.4473, 976.4465, 978.5344, 983.6115, 988.4531, 990.4647, 993.4956, 1000.5594, 1007.5680, 1019.5631, 1021.5624, 1031.5641, 1034.5791, 1036.5952, 1044.5217, 1046.5728, 1051.5135, 1056.5387, 1057.6144, 1060.5447, 1074.5956, 1077.5199, 1087.5442, 1091.5437, 1113.5406, 1122.5580, 1132.5316, 1145.6403, 1168.6013, 1171.5769, 1172.5704, 1178.6254, 1180.6395, 1181.6516, 1185.6835, 1198.7111, 1216.6992, 1234.6646, 1248.6750, 1254.6965, 1255.6359, 1273.6595, 1278.6974, 1281.6390, 1284.6533, 1301.6256, 1304.6489, 1314.7235, 1315.6904, 1325.6581, 1336.7290, 1338.6799, 1342.6847, 1349.6997, 1351.6993, 1354.6641, 1361.6997, 1368.6772, 1370.6589, 1372.6199, 1381.7728, 1382.6112, 1383.7378, 1386.7084, 1388.7888, 1390.7301, 1439.7701, 1442.8314, 1457.7517, 1467.7510, 1473.8167, 1488.7367, 1497.8859, 1500.7335, 1513.7731, 1516.7435, 1528.7101, 1556.8929, 1559.8439, 1562.8004, 1563.8088, 1578.7965, 1580.8445, 1592.8094, 1601.9139, 1616.8481, 1623.8562, 1627.7197, 1628.7129, 1628.8722, 1634.8071, 1638.8757, 1642.7318, 1645.7701, 1647.8682, 1650.8612, 1673.8375, 1679.9817, 1680.8936, 1681.8372, 1696.8403, 1706.8660, 1717.9037, 1742.0088, 1769.0105, 1771.0138, 1783.9508, 1786.9517, 1787.9071, 1790.9086, 1835.8361, 1837.8712, 1862.9679, 1928.0435, 1940.0333, 1954.0796, 1961.0397, 1982.0297, 1984.0314, 2023.0551, 2027.1292, 2032.0228, 2061.0330, 2105.0796, 2144.0679, 2170.0872, 2179.0752, 2211.0654, 2215.0876, 2239.1650, 2242.1501, 2246.2151, 2251.1675, 2252.1614, 2255.1719, 2273.1008, 2289.1030, 2293.1750, 2304.2087, 2316.2031, 2343.2134, 2354.0020, 2391.2788, 2475.2476, 2491.2402, 2507.1975, 2514.2664,

2542.2795, 2550.2063, 2558.3499, 2587.3516, 2605.2615, 2646.9883, 2661.0090, 2675.0190, 2681.3855, 2698.3696, 2705.3142, 2707.3074, 2714.3647, 2717.3394, 2719.3167, 2722.3188, 2763.3738, 2792.2446, 2826.3545, 2843.4670, 2846.4014, 2847.4082, 2872.3359, 2884.3538, 2887.3474, 2889.3481, 2894.4639, 2896.4890, 2914.4551, 2995.4431, 3004.5840, 3029.4812, 3054.4724, 3069.4849, 3072.4231, 3101.5515, 3126.7327, 3171.6748, 3183.6196, 3199.6411, 3231.5630, 3236.5791, 3243.6301, 3245.6216, 3247.5737, 3248.6313, 3254.6448, 3262.7324, 3267.7256, 3278.7546, 3321.5828, 3333.5762, 3334.6729, 3335.6787, 3338.7097, 3347.4907, 3395.6821, 3412.6426, 3428.5991, 3435.7024, 3457.6995, 3487.8022, 3490.6885, 3503.8049, 3534.6365, 3548.6917, 3551.6768, 3552.6902, 3601.6826, 3616.7095, 3624.6824, 3639.7014, 3645.7827, 3651.7559, 3655.7354, 3667.7646, 3697.8025, 3716.7253, 3762.8396, 3929.9304, 4040.7656, 4057.9707, 4071.9524, 4092.8394, 4147.9063, 4160.9473, 4174.9575, 4193.9395, 4253.8755, 4415.9736, 4452.1865, 4465.1670, 4845.0845

Search Parameters

```
Type of search         : Peptide Mass Fingerprint
Enzyme                 : Trypsin
Fixed modifications    : Carbamidomethyl (C)
Variable modifications : Oxidation (M)
Mass values            : Monoisotopic
Protein Mass           : Unrestricted
Peptide Mass Tolerance : ± 25 ppm
Peptide Charge State   : 1+
Max Missed Cleavages   : 2
Number of queries      : 289
Selected for scoring   : 203
```

Mascot: http://www.matrixscience.com/

MATRIX SCIENCE Mascot Search Results

User	: 7F_(L7@52381_05Apr2017)
Email	: proteomics@i3s.up.pt
Search title	: Project: Proteomics, Spot Set: Proteomics\05Apr2017, Label: L7, Spot Id: 227017, Peak List Id: 447673, MS Job Run Id: 316
MS data file	: C:\Documents and Settings\Administrator\Desktop\xu\29JUNHO\Proteomics\05Apr2017\pmf_L7_149875820516.txt
Database	: OrganismSpecie Bos_taurus_Reference_Proteome_2016_06 (24214 sequences; 12839866 residues)
Timestamp	: 29 Jun 2017 at 18:09:31 GMT
Top Score	: 34 for F1MSR8, Collagen alpha-1(II) chain OS=Bos taurus GN=COL2A1 PE=4 SV=2

Mascot Score Histogram

Protein score is -10*Log(P), where P is the probability that the observed match is a random event.
Protein scores greater than 56 are significant (p<0.05).

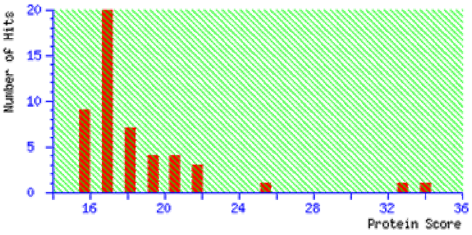

Protein Summary Report

Format As Protein Summary Help

Significance threshold p< 0.05 Max. number of hits 50

Preferred taxonomy All entries

Re-Search All Search Unmatched

Index

	Accession	Mass	Score	Description
1.	F1MSR8	134858	34	Collagen alpha-1(II) chain OS=Bos taurus GN=COL2A1 PE=4 SV=2
2.	P02459	142825	32	Collagen alpha-1(II) chain OS=Bos taurus GN=COL2A1 PE=1 SV=4
3.	F1MJH3	63488	25	Uncharacterized protein OS=Bos taurus GN=ZNF677 PE=4 SV=1
4.	P27214	54326	22	Annexin A11 OS=Bos taurus GN=ANXA11 PE=1 SV=1
5.	F1MUN7	54178	22	Annexin OS=Bos taurus GN=ANXA11 PE=3 SV=2
6.	Q0IIC4	32322	22	Zinc finger matrin-type protein 3 OS=Bos taurus GN=ZMAT3 PE=2 SV=1
7.	F1MD66	54375	21	Annexin OS=Bos taurus GN=ANXA11 PE=3 SV=2
8.	P23805	38370	20	Conglutinin OS=Bos taurus GN=CGN1 PE=1 SV=2
9.	F1MWX8	41228	20	Uncharacterized protein OS=Bos taurus GN=JSP.1 PE=3 SV=1
10.	Q5E9S8	32807	20	Uncharacterized protein C15orf41 homolog OS=Bos taurus PE=2 SV=1
11.	G5E634	53717	20	Uncharacterized protein OS=Bos taurus PE=4 SV=1
12.	G3MWR6	10152	19	Uncharacterized protein OS=Bos taurus GN=HRK PE=4 SV=1
13.	F1MCH3	61453	19	Uncharacterized protein OS=Bos taurus GN=RTP4 PE=4 SV=2
14.	Q58DF3	42440	19	TraB domain-containing protein OS=Bos taurus GN=TRABD PE=2 SV=1
15.	G3X7M1	33791	18	Uncharacterized protein OS=Bos taurus GN=SHOX PE=3 SV=1
16.	Q2KIC7	35806	18	Serine/threonine-protein phosphatase OS=Bos taurus GN=PPP6C PE=2 SV=2
17.	Q0VD05	35820	18	Voltage-dependent calcium channel gamma-3 subunit OS=Bos taurus GN=CACNG3 PE=2 SV=1
18.	F1MJI7	69096	18	Uncharacterized protein OS=Bos taurus GN=LMNB2 PE=3 SV=1
19.	F1MZ00	14021	18	Uncharacterized protein OS=Bos taurus GN=SNRPD3 PE=4 SV=2
20.	F1MYX1	71264	18	Uncharacterized protein OS=Bos taurus GN=ZNF782 PE=4 SV=2
21.	Q95LI3	92224	18	Zinc finger Y-chromosomal protein OS=Bos taurus GN=ZFY PE=2 SV=1
22.	P51867	37733	18	Tumor necrosis factor receptor superfamily member 6 OS=Bos taurus GN=FAS PE=2 SV=1
23.	E1BH62	12539	18	Uncharacterized protein OS=Bos taurus GN=XCL2 PE=4 SV=1
24.	G1K230	16435	17	Uncharacterized protein OS=Bos taurus PE=3 SV=2
25.	Q17QX9	54201	17	Peptidyl-prolyl cis-trans isomerase CWC27 homolog OS=Bos taurus GN=CWC27 PE=2 SV=1
26.	A6QPJ3	38546	17	TRABD protein OS=Bos taurus GN=TRABD PE=2 SV=1
27.	A0JNH6	63280	17	Coiled-coil domain-containing protein 102A OS=Bos taurus GN=CCDC102A PE=2 SV=1
28.	Q32LA2	40503	17	Three prime histone mRNA exonuclease 1 OS=Bos taurus GN=THEX1 PE=2 SV=1
29.	E1B821	38278	17	Uncharacterized protein OS=Bos taurus PE=3 SV=2
30.	F1N4S8	14788	17	Uncharacterized protein OS=Bos taurus GN=CCL21 PE=4 SV=1
31.	Q32LE2	41970	17	G kinase-anchoring protein 1 OS=Bos taurus GN=GKAP1 PE=2 SV=2
32.	F1MYW4	74414	17	Nucleolar GTP-binding protein 1 OS=Bos taurus GN=GTPBP4 PE=3 SV=2
33.	A2VDT9	22302	17	DNA damage-inducible transcript 4-like protein OS=Bos taurus GN=DDIT4L PE=2 SV=1
34.	Q2YDM5	18048	17	Testis, prostate and placenta-expressed protein OS=Bos taurus GN=TEPP PE=2 SV=1
35.	Q2TBQ7	47436	17	Regulator of microtubule dynamics protein 2 OS=Bos taurus GN=RMDN2 PE=2 SV=2
36.	E1BAV3	43148	17	Uncharacterized protein OS=Bos taurus GN=STOML1 PE=4 SV=2
37.	Q9NZ50	13245	17	Gamma-synuclein OS=Bos taurus GN=SNCG PE=1 SV=1
38.	F1MG09	15257	16	Uncharacterized protein OS=Bos taurus GN=RPL36AL PE=3 SV=2
39.	F1N091	43136	16	Uncharacterized protein OS=Bos taurus GN=CCDC6 PE=4 SV=1
40.	Q2YDJ9	40439	16	Mannose-1-phosphate guanyltransferase beta OS=Bos taurus GN=GMPPB PE=2 SV=1
41.	F1N7H5	40398	16	Mannose-1-phosphate guanyltransferase beta OS=Bos taurus GN=GMPPB PE=4 SV=2
42.	E1B999	83144	16	Kinesin-like protein OS=Bos taurus GN=KIF3A PE=3 SV=2
43.	F1MFY7	101515	16	Anoctamin OS=Bos taurus GN=ANO6 PE=3 SV=1
44.	E1BPA1	148570	16	Uncharacterized protein OS=Bos taurus GN=TBC1D4 PE=4 SV=1
45.	F1N6W3	25812	16	Uncharacterized protein OS=Bos taurus GN=MGC134105 PE=4 SV=1
46.	G3N3M2	148813	16	Uncharacterized protein OS=Bos taurus GN=TBC1D4 PE=4 SV=1
47.	Q9XSC3	101801	16	WD repeat-containing protein 44 OS=Bos taurus GN=WDR44 PE=1 SV=1
48.	G3X7E3	101783	16	WD repeat-containing protein 44 OS=Bos taurus GN=WDR44 PE=4 SV=1
49.	F1N0K7	15524	16	Uncharacterized protein OS=Bos taurus PE=4 SV=1
50.	Q00194	79951	16	cGMP-gated cation channel alpha-1 OS=Bos taurus GN=CNGA1 PE=1 SV=1

Results List

1. F1MSR8 **Mass:** 134858 **Score:** 34 **Expect:** 9.6 **Matches:** 29

Collagen alpha-1(II) chain OS=Bos taurus GN=COL2A1 PE=4 SV=2

Observed	Mr(expt)	Mr(calc)	ppm	Start	End	Miss	Peptide
828.4171	827.4098	827.4250	-18.28	266	275	0	R.TGPAGAAGAR.G
841.4608	840.4535	840.4566	-3.62	258	265	1	R.GLPGERGR.T
871.5104	870.5031	870.4851	20.7	1360	1366	0	R.FTYTVLK.D
935.4841	934.4768	934.4661	11.5	1265	1271	1	K.KNWWSSK.S
945.4980	944.4907	944.4676	24.5	980	989	1	R.DGAAGVKGDR.G
949.5173	948.5100	948.5029	7.48	1095	1105	0	R.GPPGPVGPSGK.D
969.4643	968.4570	968.4538	3.31	1219	1225	0	K.LCHPEWK.S
1168.5806	1167.5733	1167.5996	-22.53	69	80	2	R.GDKGEKGAPGPR.G
1173.6044	1172.5971	1172.6149	-15.17	1192	1201	0	K.SLNNQIESLR.S
1226.5743	1225.5670	1225.5939	-21.91	1181	1191	0	R.QHDAEVDATLK.S
1325.6377	1324.6304	1324.6598	-22.16	1216	1225	1	R.DLKLCHPEWK.S
1349.6682	1348.6609	1348.6445	12.2	1161	1171	1	R.EKGPDPLQYMR.A + Oxidation (M)
1427.7550	1426.7477	1426.7780	-21.20	1341	1353	0	K.ALLIQGSNDVEIR.A
1432.6827	1431.6754	1431.7068	-21.93	1360	1371	1	R.FTYTVLKDGCTK.H
1447.6959	1446.6886	1446.7103	-14.99	872	887	0	R.AGDPGLQGPAGPPGEK.G
1478.7163	1477.7090	1477.7412	-21.79	1326	1339	0	K.NSIAYLDEAAGNLK.K
1606.8096	1605.8023	1605.8086	-3.89	1372	1384	2	K.HTGKWGKTMIEYR.S
1638.8353	1637.8280	1637.8162	7.23	921	937	1	R.GERGFPGLPGPSGEPGK.Q
1650.8495	1649.8422	1649.8710	-17.43	258	275	2	R.GLPGERGRTGPAGAAGAR.G
1752.8656	1751.8583	1751.8816	-13.27	816	835	1	K.GARGAQGPPGATGFPGAAGR.V
1786.8839	1785.8766	1785.8969	-11.35	1192	1207	1	K.SLNNQIESLRSPEGSR.K
1914.9941	1913.9868	1913.9919	-2.63	1192	1208	2	K.SLNNQIESLRSPEGSRK.N
2012.9734	2011.9661	2011.9824	-8.10	506	527	0	K.VGPSGAPGEDGRPGPPGPQGAR.G
2042.0795	2041.0722	2041.0552	8.35	1341	1359	1	K.ALLIQGSNDVEIRAEGNSR.F
2070.9099	2069.9026	2069.8823	9.83	1226	1243	0	K.SGDYWIDPNQGCTLDAMK.V
2082.9165	2081.9092	2081.9549	-21.92	1038	1058	2	R.GMPGPQGPRGDKGETGEAGER.G
2086.9263	2085.9190	2085.8772	20.1	1226	1243	0	K.SGDYWIDPNQGCTLDAMK.V + Oxidation (M)
2098.9319	2097.9246	2097.9498	-11.99	1038	1058	2	R.GMPGPQGPRGDKGETGEAGER.G + Oxidation (M)
2170.1101	2169.1028	2169.1501	-21.82	1340	1359	2	K.KALLIQGSNDVEIRAEGNSR.F

No match to: 712.3012, 714.3622, 717.4536, 722.4728, 735.4618, 756.5057, 772.4633, 779.5103, 792.4439, 795.4440, 804.3116, 806.4917, 807.4467, 809.4788, 812.4258, 814.4372, 815.4436, 819.4216, 831.4965, 832.3378, 834.3423, 842.4964, 845.4365, 883.4921, 888.4818, 891.4986, 893.9725, 894.4754, 900.5499, 913.5269, 918.5532, 929.4499, 947.4821, 959.4860, 963.4728, 967.4305, 972.4830, 974.4501, 975.4561, 976.4594, 977.4788, 978.5386, 981.4812, 983.4774, 988.4600, 989.6110, 995.4939, 996.4700, 997.4576, 999.4598, 1007.5580, 1011.4736, 1018.5132, 1019.5350, 1021.5362, 1027.4982, 1032.5875, 1033.5684, 1034.5756, 1036.5582, 1044.5192, 1047.5377, 1055.5199, 1056.5244, 1057.5576, 1060.5359, 1062.5424, 1069.5072, 1074.5400, 1077.5109, 1085.5363, 1087.5255, 1091.5013, 1103.5313, 1106.5183, 1107.5238, 1111.5382, 1122.5424, 1123.5519, 1178.6016, 1179.5861, 1185.6279, 1187.6195, 1199.6720, 1207.6029, 1209.5723, 1215.6453, 1216.6425, 1234.5945, 1244.5957, 1248.6305, 1255.5890, 1295.7034, 1301.5924, 1314.6890, 1322.5939, 1328.6476, 1336.6866, 1337.6793, 1338.6221, 1342.6494, 1344.6713, 1345.6792, 1352.6564, 1353.6359, 1355.6598, 1361.6550, 1368.6198, 1370.6490, 1372.5659, 1381.7262, 1383.6938, 1386.6838, 1394.6993, 1397.7117, 1433.6854, 1442.7830, 1446.7039, 1457.7043, 1473.7709, 1497.8370, 1528.6653, 1537.7870, 1542.8158, 1550.9301, 1556.8483, 1578.7511, 1601.8662, 1615.8414, 1616.8064, 1617.7891, 1627.7168, 1628.6741, 1628.8262, 1642.6895, 1647.8445, 1679.9406, 1680.7981, 1696.8021, 1706.8348, 1715.8130, 1717.8778, 1718.8860, 1725.8102, 1727.8582, 1730.8500, 1763.7885, 1768.9357, 1769.9312, 1771.0000, 1775.8153, 1777.8390, 1788.4598, 1791.8882, 1799.8864, 1800.8976, 1809.0026, 1928.0222, 1953.8669, 1975.9434, 1982.0046, 2027.1388, 2053.0103, 2060.0637, 2084.9246, 2095.0337, 2096.9631, 2100.9241, 2136.9219, 2155.1025, 2209.0732, 2211.1094, 2239.2014, 2251.1997, 2289.1399, 2293.2251, 2324.1719, 2343.2795, 2381.2776, 2384.2988, 2398.2314, 2400.1594, 2414.1807, 2428.1990, 2431.1982, 2444.2200, 2465.3230, 2528.3064, 2542.3503, 2546.3127, 2558.3843, 2647.1560, 2661.1709, 2675.1926, 2698.5471, 2706.4133, 2708.4885, 2718.5300, 2762.5994, 2765.5881, 2767.5010, 2771.5437, 2777.7188, 2811.6846, 2814.6851, 2846.6375, 2869.6453, 2872.5757, 2884.6123, 2888.6077, 2892.6868, 2894.7185, 2902.6804, 2918.6406, 2926.6814, 2946.7546, 2994.8018, 2998.7808, 3003.8159, 3005.8979, 3054.8286, 3082.9351, 3102.9441, 3105.8228, 3127.0818, 3171.0554, 3194.9565, 3202.8757, 3207.9922, 3249.0452, 3263.1082, 3264.1118, 3266.0825, 3300.1990, 3317.0972, 3323.0837, 3331.1343, 3335.1436, 3336.1438, 3348.9949, 3354.0869, 3358.1614, 3371.1931, 3387.2393, 3454.9927, 3459.2039, 3490.2356, 3504.3723, 3518.1758, 3640.3601, 3652.4072, 3664.4243, 3822.6233, 3870.6184, 3871.6218, 3887.6787, 4089.8884, 4148.9229, 4160.9209, 4164.9634, 4175.9526, 4306.0547, 4453.3716, 4466.3892, 4680.6270, 4846.6348

2. P02459 **Mass:** 142825 **Score:** 32 **Expect:** 14 **Matches:** 30

Collagen alpha-1(II) chain OS=Bos taurus GN=COL2A1 PE=1 SV=4

Observed	Mr(expt)	Mr(calc)	ppm	Start	End	Miss	Peptide
828.4171	827.4098	827.4250	-18.28	335	344	0	R.TGPAGAAGAR.G
841.4608	840.4535	840.4566	-3.62	327	334	1	R.GLPGERGR.T
871.5104	870.5031	870.4851	20.7	1429	1435	0	R.FTYTVLK.D
935.4841	934.4768	934.4661	11.5	1334	1340	1	K.KNWWSSK.S
945.4980	944.4907	944.4676	24.5	1049	1058	1	R.DGAAGVKGDR.G
949.5173	948.5100	948.5029	7.48	1164	1174	0	R.GPPGPVGPSGK.D
969.4643	968.4570	968.4538	3.31	1288	1294	0	K.LCHPEWK.S
1168.5806	1167.5733	1167.5996	-22.53	138	149	2	R.GDKGEKGAPGPR.G
1173.6044	1172.5971	1172.6149	-15.17	1261	1270	0	K.SLNNQIESLR.S
1226.5743	1225.5670	1225.5939	-21.91	1250	1260	0	R.QHDAEVDATLK.S
1325.6377	1324.6304	1324.6598	-22.16	1285	1294	1	R.DLKLCHPEWK.S
1349.6682	1348.6609	1348.6445	12.2	1230	1240	1	R.EKGPDPLQYMR.A + Oxidation (M)
1427.7550	1426.7477	1426.7780	-21.20	1410	1422	0	K.ALLIQGSNDVEIR.A
1432.6827	1431.6754	1431.7068	-21.93	1429	1440	1	R.FTYTVLKDGCTK.H
1447.6959	1446.6886	1446.7103	-14.99	941	956	0	R.AGDPGLQGPAGPPGEK.G
1478.7163	1477.7090	1477.7412	-21.79	1395	1408	0	K.NSIAYLDEAAGNLK.K
1606.8096	1605.8023	1605.8086	-3.89	1441	1453	2	K.HTGKWGKTMIEYR.S
1638.8353	1637.8280	1637.8162	7.23	990	1006	1	R.GERGFPGLPGPSGEPGK.Q
1650.8495	1649.8422	1649.8710	-17.43	327	344	2	R.GLPGERGRTGPAGAAGAR.G
1706.8348	1705.8275	1705.7882	23.0	41	53	1	R.YNDKDVWKPEPCR.I
1752.8656	1751.8583	1751.8816	-13.27	885	904	1	K.GARGAQGPPGATGFPGAAGR.V
1786.8839	1785.8766	1785.8969	-11.35	1261	1276	1	K.SLNNQIESLRSPEGSR.K
1914.9941	1913.9868	1913.9919	-2.63	1261	1277	2	K.SLNNQIESLRSPEGSRK.N
2012.9734	2011.9661	2011.9824	-8.10	575	596	0	K.VGPSGAPGEDGRPGPPGPQGAR.G
2042.0795	2041.0722	2041.0552	8.35	1410	1428	1	K.ALLIQGSNDVEIRAEGNSR.F
2070.9099	2069.9026	2069.8823	9.83	1295	1312	0	K.SGDYWIDPNQGCTLDAMK.V
2082.9165	2081.9092	2081.9549	-21.92	1107	1127	2	R.GMPGPQGPRGDKGETGEAGER.G
2086.9263	2085.9190	2085.8772	20.1	1295	1312	0	K.SGDYWIDPNQGCTLDAMK.V + Oxidation (M)

2098.9319	2097.9246	2097.9498	-11.99	1107 - 1127	2	R.GMPGPQGPRGDKGETGEAGER.G + Oxidation (M)
2170.1101	2169.1028	2169.1501	-21.82	1409 - 1428	2	K.KALLIQGSNDVEIRAEGNSR.F

No match to: 712.3012, 714.3622, 717.4536, 722.4728, 735.4618, 756.5057, 772.4633, 779.5103, 792.4439, 795.4440, 804.3116, 806.4917, 807.4467, 809.4788, 812.4258, 814.4372, 815.4436, 819.4216, 831.4965, 832.3378, 834.3423, 842.4964, 845.4365, 883.4921, 888.4818, 891.4986, 893.9725, 894.4754, 900.5499, 913.5269, 918.5532, 929.4499, 947.4821, 959.4860, 963.4728, 967.4305, 972.4830, 974.4501, 975.4561, 976.4594, 977.4788, 978.5386, 981.4812, 983.4774, 988.4600, 989.6110, 995.4939, 996.4700, 997.4576, 999.4598, 1007.5580, 1011.4736, 1018.5132, 1019.5350, 1021.5362, 1027.4982, 1032.5875, 1033.5684, 1034.5756, 1036.5582, 1044.5192, 1047.5377, 1055.5199, 1056.5244, 1057.5576, 1060.5359, 1062.5424, 1069.5072, 1074.5400, 1077.5109, 1085.5363, 1087.5255, 1091.5013, 1103.5313, 1106.5183, 1107.5238, 1111.5382, 1122.5424, 1123.5519, 1178.6016, 1179.5861, 1185.6279, 1187.6195, 1199.6720, 1207.6029, 1209.5723, 1215.6453, 1216.6425, 1234.5945, 1244.5957, 1248.6305, 1255.5890, 1295.7034, 1301.5924, 1314.6890, 1322.5939, 1328.6476, 1336.6866, 1337.6793, 1338.6221, 1342.6494, 1344.6713, 1345.6792, 1352.6564, 1353.6359, 1355.6598, 1361.6550, 1368.6198, 1370.6490, 1372.5659, 1381.7262, 1383.6938, 1386.6838, 1394.6993, 1397.7117, 1433.6854, 1442.7830, 1446.7039, 1457.7043, 1473.7709, 1497.8370, 1528.6653, 1537.7870, 1542.8158, 1550.9301, 1556.8483, 1578.7511, 1601.8662, 1615.8414, 1616.8064, 1617.7891, 1627.7168, 1628.6741, 1628.8262, 1642.6895, 1647.8445, 1679.9406, 1680.7981, 1696.8021, 1715.8130, 1717.8778, 1718.8860, 1725.8102, 1727.8582, 1730.8500, 1763.7885, 1768.9357, 1769.9312, 1771.0000, 1775.8153, 1777.8390, 1788.4598, 1791.8882, 1799.8864, 1800.8976, 1809.0026, 1928.0222, 1953.8669, 1975.9434, 1982.0046, 2027.1388, 2053.0103, 2060.0637, 2084.9246, 2095.0337, 2096.9631, 2100.9241, 2136.9219, 2155.1025, 2209.0732, 2211.1094, 2239.2014, 2251.1997, 2289.1399, 2293.2251, 2324.1719, 2343.2795, 2381.2776, 2384.2988, 2398.2314, 2400.1594, 2414.1807, 2428.1990, 2431.1982, 2444.2200, 2465.3230, 2528.3064, 2542.3503, 2546.3127, 2558.3843, 2647.1560, 2661.1709, 2675.1926, 2698.5471, 2706.4133, 2708.4885, 2718.5300, 2762.5994, 2765.5881, 2767.5010, 2771.5437, 2777.7188, 2811.6846, 2814.6851, 2846.6375, 2869.6453, 2872.5757, 2884.6123, 2888.6077, 2892.6868, 2894.7185, 2902.6804, 2918.6406, 2926.6814, 2946.7546, 2994.8018, 2998.7808, 3003.8159, 3005.8979, 3054.8286, 3082.9351, 3102.9441, 3105.8228, 3127.0818, 3171.0554, 3194.9565, 3202.8757, 3207.9922, 3249.0452, 3263.1082, 3264.1118, 3266.0825, 3300.1990, 3317.0972, 3323.0837, 3331.1343, 3335.1436, 3336.1438, 3348.9949, 3354.0869, 3358.1614, 3371.1931, 3387.2393, 3454.9927, 3459.2039, 3490.2356, 3504.3723, 3518.1758, 3640.3601, 3652.4072, 3664.4243, 3822.6233, 3870.6184, 3871.6218, 3887.6787, 4089.8884, 4148.9229, 4160.9209, 4164.9634, 4175.9526, 4306.0547, 4453.3716, 4466.3892, 4680.6270, 4846.6348

3. F1MJH3 **Mass:** 63488 **Score:** 25 **Expect:** 73 **Matches:** 11

Uncharacterized protein OS=Bos taurus GN=ZNF677 PE=4 SV=1

Observed	Mr(expt)	Mr(calc)	ppm	Start - End	Miss	Peptide
972.4830	971.4757	971.4607	15.5	371 - 378	0	R.SNLMAHQR.I + Oxidation (M)
1074.5400	1073.5327	1073.5505	-16.58	295 - 303	1	R.VHAEEKAYK.C
1106.5183	1105.5110	1105.5226	-10.48	323 - 331	0	R.MHTGEKPYK.C + Oxidation (M)
1173.6044	1172.5971	1172.6190	-18.62	434 - 443	0	K.TIHTGEKPYK.C
1427.7550	1426.7477	1426.7252	15.8	348 - 359	1	R.HQKMHAGEKPHK.C
1606.8096	1605.8023	1605.8409	-24.03	520 - 533	1	K.MLSIINHQGNAKHK.V + Oxidation (M)
1628.8262	1627.8189	1627.8219	-1.83	394 - 406	1	K.AFIERSQLWGHER.T
1777.8390	1776.8317	1776.8240	4.34	195 - 209	1	R.FTTEEKMYGGTQLEK.S + Oxidation (M)
2546.3127	2545.3054	2545.2859	7.66	520 - 541	2	K.MLSIINHQGNAKHKVNEIPHYS.- + Oxidation (M)
4148.9229	4147.9156	4148.0023	-20.90	1 - 34	2	-.MELSQGLFTFKDVAVEFTQEEWECLDPIQRALYR.D
4164.9634	4163.9561	4163.9972	-9.87	1 - 34	2	-.MELSQGLFTFKDVAVEFTQEEWECLDPIQRALYR.D + Oxidation (M)

No match to: 712.3012, 714.3622, 717.4536, 722.4728, 735.4618, 756.5057, 772.4633, 779.5103, 792.4439, 795.4440, 804.3116, 806.4917, 807.4467, 809.4788, 812.4258, 814.4372, 815.4436, 819.4216, 828.4171, 831.4965, 832.3378, 834.3423, 841.4608, 842.4964, 845.4365, 871.5104, 883.4921, 888.4818, 891.4986, 893.9725, 894.4754, 900.5499, 913.5269, 918.5532, 929.4499, 935.4841, 945.4980, 947.4821, 949.5173, 959.4860, 963.4728, 967.4305, 969.4643, 974.4501, 975.4561, 976.4594, 977.4788, 978.5386, 981.4812, 983.4774, 988.4600, 989.6110, 995.4939, 996.4700, 997.4576, 999.4598, 1007.5580, 1011.4736, 1018.5132, 1019.5350, 1021.5362, 1027.4982, 1032.5875, 1033.5684, 1034.5756, 1036.5582, 1044.5192, 1047.5377, 1055.5199, 1056.5244, 1057.5576, 1060.5359, 1062.5424, 1069.5072, 1077.5109, 1085.5363, 1087.5255, 1091.5013, 1103.5313, 1107.5238, 1111.5382, 1122.5424, 1123.5519, 1168.5806, 1178.6016, 1179.5861, 1185.6279, 1187.6195, 1199.6720, 1207.6029, 1209.5723, 1215.6453, 1216.6425, 1226.5743, 1234.5945, 1244.5957, 1248.6305, 1255.5890, 1295.7034, 1301.5924, 1314.6890, 1322.5939, 1325.6377, 1328.6476, 1336.6866, 1337.6793, 1338.6221, 1342.6494, 1344.6713, 1345.6792, 1349.6682, 1352.6564, 1353.6359, 1355.6598, 1361.6550, 1368.6198, 1370.6490, 1372.5659, 1381.7262, 1383.6938, 1386.6838, 1394.6993, 1397.7117, 1432.6827, 1433.6854, 1442.7830, 1446.7039, 1447.6959, 1457.7043, 1473.7709, 1478.7163, 1497.8370, 1528.6653, 1537.7870, 1542.8158, 1550.9301, 1556.8483, 1578.7511, 1601.8662, 1615.8414, 1616.8064, 1617.7891, 1627.7168, 1628.6741, 1638.8353, 1642.6895, 1647.8445, 1650.8495, 1679.9406, 1680.7981, 1696.8021, 1706.8348, 1715.8130, 1717.8778, 1718.8860, 1725.8102, 1727.8582, 1730.8500, 1752.8656, 1763.7885, 1768.9357, 1769.9312, 1771.0000, 1775.8153, 1786.8839, 1788.4598, 1791.8882, 1799.8864, 1800.8976, 1809.0026, 1914.9941, 1928.0222, 1953.8669, 1975.9434, 1982.0046, 2012.9734, 2027.1388, 2042.0795, 2053.0103, 2060.0637, 2070.9099, 2082.9165, 2084.9246, 2086.9263, 2095.0337, 2096.9631, 2098.9319, 2100.9241, 2136.9219, 2155.1025, 2170.1101, 2209.0732, 2211.1094, 2239.2014, 2251.1997, 2289.1399, 2293.2251, 2324.1719, 2343.2795, 2381.2776, 2384.2988, 2398.2314, 2400.1594, 2414.1807, 2428.1990, 2431.1982, 2444.2200, 2465.3230, 2528.3064, 2542.3503, 2558.3843, 2647.1560, 2661.1709, 2675.1926, 2698.5471, 2706.4133, 2708.4885, 2718.5300, 2762.5994, 2765.5881, 2767.5010, 2771.5437, 2777.7188, 2811.6846, 2814.6851, 2846.6375, 2869.6453, 2872.5757, 2884.6123, 2888.6077, 2892.6868, 2894.7185, 2902.6804, 2918.6406, 2926.6814, 2946.7546, 2994.8018, 2998.7808, 3003.8159, 3005.8979, 3054.8286, 3082.9351, 3102.9441, 3105.8228, 3127.0818, 3171.0554, 3194.9565, 3202.8757, 3207.9922, 3249.0452, 3263.1082, 3264.1118, 3266.0825, 3300.1990, 3317.0972, 3323.0837, 3331.1343, 3335.1436, 3336.1438, 3348.9949, 3354.0869, 3358.1614, 3371.1931, 3387.2393, 3454.9927, 3459.2039, 3490.2356, 3504.3723, 3518.1758, 3640.3601, 3652.4072, 3664.4243, 3822.6233, 3870.6184, 3871.6218, 3887.6787, 4089.8884, 4160.9209, 4175.9526, 4306.0547, 4453.3716, 4466.3892, 4680.6270, 4846.6348

4. P27214 **Mass:** 54326 **Score:** 22 **Expect:** 1.7e+002 **Matches:** 8

Annexin A11 OS=Bos taurus GN=ANXA11 PE=1 SV=1

Observed	Mr(expt)	Mr(calc)	ppm	Start - End	Miss	Peptide
1062.5424	1061.5351	1061.5400	-4.59	441 - 450	2	K.AMRGAGTKDR.T
1295.7034	1294.6961	1294.6809	11.8	270 - 280	0	K.TPVLFDAYEIK.E
1349.6682	1348.6609	1348.6623	-1.03	190 - 202	0	R.GTITDASGFDPLR.D
1606.8096	1605.8023	1605.7742	17.5	411 - 425	0	R.EMSGDLEQGMLAVVK.C
2084.9246	2083.9173	2083.9667	-23.68	210 - 228	1	K.AMKGFGTDEQAIIDCLGSR.S + Oxidation (M)
2095.0337	2094.0264	2093.9865	19.1	358 - 376	1	R.DVQELYAAGENRLGTDESK.F
2289.1399	2288.1326	2288.1073	11.1	474 - 493	2	R.LYGKSLYHDITGDTSGDYRK.I
2414.1807	2413.1734	2413.1366	15.3	210 - 231	2	K.AMKGFGTDEQAIIDCLGSRSNK.Q + Oxidation (M)

No match to: 712.3012, 714.3622, 717.4536, 722.4728, 735.4618, 756.5057, 772.4633, 779.5103, 792.4439, 795.4440, 804.3116, 806.4917, 807.4467, 809.4788, 812.4258, 814.4372, 815.4436, 819.4216, 828.4171, 831.4965, 832.3378, 834.3423, 841.4608, 842.4964, 845.4365, 871.5104, 883.4921, 888.4818, 891.4986, 893.9725, 894.4754, 900.5499, 913.5269, 918.5532, 929.4499, 935.4841, 945.4980, 947.4821, 949.5173, 959.4860, 963.4728, 967.4305, 969.4643, 972.4830, 974.4501, 975.4561, 976.4594, 977.4788, 978.5386, 981.4812, 983.4774, 988.4600, 989.6110, 995.4939, 996.4700, 997.4576, 999.4598, 1007.5580, 1011.4736, 1018.5132, 1019.5350, 1021.5362, 1027.4982, 1032.5875, 1033.5684, 1034.5756, 1036.5582, 1044.5192, 1047.5377, 1055.5199, 1056.5244, 1057.5576, 1060.5359, 1069.5072, 1074.5400, 1077.5109, 1085.5363, 1087.5255, 1091.5013, 1103.5313, 1106.5183, 1107.5238, 1111.5382, 1122.5424, 1123.5519, 1168.5806, 1173.6044, 1178.6016, 1179.5861, 1185.6279, 1187.6195, 1199.6720, 1207.6029, 1209.5723, 1215.6453, 1216.6425, 1226.5743, 1234.5945, 1244.5957, 1248.6305, 1255.5890, 1301.5924, 1314.6890, 1322.5939, 1325.6377, 1328.6476, 1336.6866, 1337.6793, 1338.6221, 1342.6494, 1344.6713, 1345.6792, 1352.6564, 1353.6359, 1355.6598, 1361.6550, 1368.6198, 1370.6490, 1372.5659, 1381.7262, 1383.6938, 1386.6838, 1394.6993, 1397.7117, 1427.7550, 1432.6827, 1433.6854, 1442.7830, 1446.7039, 1447.6959, 1457.7043, 1473.7709, 1478.7163, 1497.8370, 1528.6653, 1537.7870, 1542.8158, 1550.9301, 1556.8483, 1578.7511, 1601.8662, 1615.8414, 1616.8064, 1617.7891, 1627.7168, 1628.6741, 1628.8262, 1638.8353, 1642.6895, 1647.8445, 1650.8495, 1679.9406, 1680.7981, 1696.8021, 1706.8348, 1715.8130, 1717.8778, 1718.8860, 1725.8102, 1727.8582, 1730.8500, 1752.8656, 1763.7885, 1768.9357, 1769.9312, 1771.0000, 1775.8153, 1777.8390, 1786.8839, 1788.4598, 1791.8882, 1799.8864, 1800.8976, 1809.0026, 1914.9941, 1928.0222, 1953.8669, 1975.9434, 1982.0046, 2012.9734, 2027.1388, 2042.0795, 2053.0103, 2060.0637, 2070.9099, 2082.9165, 2086.9263, 2096.9631, 2098.9319, 2100.9241, 2136.9219, 2155.1025, 2170.1101, 2209.0732, 2211.1094, 2239.2014, 2251.1997, 2293.2251, 2324.1719, 2343.2795, 2381.2776, 2384.2988, 2398.2314, 2400.1594, 2428.1990, 2431.1982, 2444.2200, 2465.3230, 2528.3064, 2542.3503, 2546.3127, 2558.3843, 2647.1560, 2661.1709, 2675.1926, 2698.5471, 2706.4133, 2708.4885, 2718.5300, 2762.5994, 2765.5881, 2767.5010, 2771.5437, 2777.7188, 2811.6846, 2814.6851, 2846.6375, 2869.6453, 2872.5757, 2884.6123, 2888.6077, 2892.6868, 2894.7185, 2902.6804, 2918.6406, 2926.6814, 2946.7546, 2994.8018, 2998.7808, 3003.8159, 3005.8979, 3054.8286,

3082.9351, 3102.9441, 3105.8228, 3127.0818, 3171.0554, 3194.9565, 3202.8757, 3207.9922, 3249.0452, 3263.1082, 3264.1118, 3266.0825, 3300.1990, 3317.0972, 3323.0837, 3331.1343, 3335.1436, 3336.1438, 3348.9949, 3354.0869, 3358.1614, 3371.1931, 3387.2393, 3454.9927, 3459.2039, 3490.2356, 3504.3723, 3518.1758, 3640.3601, 3652.4072, 3664.4243, 3822.6233, 3870.6184, 3871.6218, 3887.6787, 4089.8884, 4148.9229, 4160.9209, 4164.9634, 4175.9526, 4306.0547, 4453.3716, 4466.3892, 4680.6270, 4846.6348

5. F1MUN7 **Mass:** 54178 **Score:** 22 **Expect:** 1.7e+002 **Matches:** 7

Annexin OS=Bos taurus GN=ANXA11 PE=3 SV=2

Observed	Mr(expt)	Mr(calc)	ppm	Start	End	Miss	Peptide
1062.5424	1061.5351	1061.5400	-4.59	441	450	2	K.AMRGAGTKDR.T
1295.7034	1294.6961	1294.6809	11.8	270	280	0	K.TPVLFDAYEIK.E
1349.6682	1348.6609	1348.6623	-1.03	190	202	0	R.GTITDASGFDPLR.D
1606.8096	1605.8023	1605.7742	17.5	411	425	0	R.EMSGDLEQGMLAVVK.C
2084.9246	2083.9173	2083.9667	-23.68	210	228	1	K.AMKGFGTDEQAIIDCLGSR.S + Oxidation (M)
2289.1399	2288.1326	2288.1073	11.1	474	493	2	R.LYGKSLYHDITGDTSGDYRK.I
2414.1807	2413.1734	2413.1366	15.3	210	231	2	K.AMKGFGTDEQAIIDCLGSRSNK.Q + Oxidation (M)

No match to: 712.3012, 714.3622, 717.4536, 722.4728, 735.4618, 756.5057, 772.4633, 779.5103, 792.4439, 795.4440, 804.3116, 806.4917, 807.4467, 809.4788, 812.4258, 814.4372, 815.4436, 819.4216, 828.4171, 831.4965, 832.3378, 834.3423, 841.4608, 842.4964, 845.4365, 871.5104, 883.4921, 888.4818, 891.4986, 893.9725, 894.4754, 900.5499, 913.5269, 918.5532, 929.4499, 935.4841, 945.4980, 947.4821, 949.5173, 959.4860, 963.4728, 967.4305, 969.4643, 972.4830, 974.4501, 975.4561, 976.4594, 977.4788, 978.5386, 981.4812, 983.4774, 988.4600, 989.6110, 995.4939, 996.4700, 997.4576, 999.4598, 1007.5580, 1011.4736, 1018.5132, 1019.5350, 1021.5362, 1027.4982, 1032.5875, 1033.5684, 1034.5756, 1036.5582, 1044.5192, 1047.5377, 1055.5199, 1056.5244, 1057.5576, 1060.5359, 1069.5072, 1074.5400, 1077.5109, 1085.5363, 1087.5255, 1091.5013, 1103.5313, 1106.5183, 1107.5238, 1111.5382, 1122.5424, 1123.5519, 1168.5806, 1173.6044, 1178.6016, 1179.5861, 1185.6279, 1187.6195, 1199.6720, 1207.6029, 1209.5723, 1215.6453, 1216.6425, 1226.5743, 1234.5945, 1244.5957, 1248.6305, 1255.5890, 1301.5924, 1314.6890, 1322.5939, 1325.6377, 1328.6476, 1336.6866, 1337.6793, 1338.6221, 1342.6494, 1344.6713, 1345.6792, 1352.6564, 1353.6359, 1355.6598, 1361.6550, 1368.6198, 1370.6490, 1372.5659, 1381.7262, 1383.6938, 1386.6838, 1394.6993, 1397.7117, 1427.7550, 1432.6827, 1433.6854, 1442.7830, 1446.7039, 1447.6959, 1457.7043, 1473.7709, 1478.7163, 1497.8370, 1528.6653, 1537.7870, 1542.8158, 1550.9301, 1556.8483, 1578.7511, 1601.8662, 1615.8414, 1616.8064, 1617.7891, 1627.7168, 1628.6741, 1628.8262, 1638.8353, 1642.6895, 1647.8445, 1650.8495, 1679.9406, 1680.7981, 1696.8021, 1706.8348, 1715.8130, 1717.8778, 1718.8860, 1725.8102, 1727.8582, 1730.8500, 1752.8656, 1763.7885, 1768.9357, 1769.9312, 1771.0000, 1775.8153, 1777.8390, 1786.8839, 1788.4598, 1791.8882, 1799.8864, 1800.8976, 1809.0026, 1914.9941, 1928.0222, 1953.8669, 1975.9434, 1982.0046, 2012.9734, 2027.1388, 2042.0795, 2053.0103, 2060.0637, 2070.9099, 2082.9165, 2086.9263, 2095.0337, 2096.9631, 2098.9319, 2100.9241, 2136.9219, 2155.1025, 2170.1101, 2209.0732, 2211.1094, 2239.2014, 2251.1997, 2293.2251, 2324.1719, 2343.2795, 2381.2776, 2384.2988, 2398.2314, 2400.1594, 2428.1990, 2431.1982, 2444.2200, 2465.3230, 2528.3064, 2542.3503, 2546.3127, 2558.3843, 2647.1560, 2661.1709, 2675.1926, 2698.5471, 2706.4133, 2708.4885, 2718.5300, 2762.5994, 2765.5881, 2767.5010, 2771.5437, 2777.7188, 2811.6846, 2814.6851, 2846.6375, 2869.6453, 2872.5757, 2884.6123, 2888.6077, 2892.6868, 2894.7185, 2902.6804, 2918.6406, 2926.6814, 2946.7546, 2994.8018, 2998.7808, 3003.8159, 3005.8979, 3054.8286, 3082.9351, 3102.9441, 3105.8228, 3127.0818, 3171.0554, 3194.9565, 3202.8757, 3207.9922, 3249.0452, 3263.1082, 3264.1118, 3266.0825, 3300.1990, 3317.0972, 3323.0837, 3331.1343, 3335.1436, 3336.1438, 3348.9949, 3354.0869, 3358.1614, 3371.1931, 3387.2393, 3454.9927, 3459.2039, 3490.2356, 3504.3723, 3518.1758, 3640.3601, 3652.4072, 3664.4243, 3822.6233, 3870.6184, 3871.6218, 3887.6787, 4089.8884, 4148.9229, 4160.9209, 4164.9634, 4175.9526, 4306.0547, 4453.3716, 4466.3892, 4680.6270, 4846.6348

6. Q0IIC4 **Mass:** 32322 **Score:** 22 **Expect:** 1.7e+002 **Matches:** 4

Zinc finger matrin-type protein 3 OS=Bos taurus GN=ZMAT3 PE=2 SV=1

Observed	Mr(expt)	Mr(calc)	ppm	Start	End	Miss	Peptide
1325.6377	1324.6304	1324.6333	-2.16	140	150	0	R.VILATENDYCK.L
1368.6198	1367.6125	1367.5962	12.0	199	209	1	K.EGNEYKMMPNR.R
1528.6653	1527.6580	1527.6810	-15.01	198	209	2	R.KEGNEYKMMPNR.R + 2 Oxidation (M)
4148.9229	4147.9156	4147.9666	-12.29	100	139	1	R.NYYAANSCPPPARMSNAVEAVAAPAVSVPPQMGSFKPGGR.V + 2 Oxidation (M)

No match to: 712.3012, 714.3622, 717.4536, 722.4728, 735.4618, 756.5057, 772.4633, 779.5103, 792.4439, 795.4440, 804.3116, 806.4917, 807.4467, 809.4788, 812.4258, 814.4372, 815.4436, 819.4216, 828.4171, 831.4965, 832.3378, 834.3423, 841.4608, 842.4964, 845.4365, 871.5104, 883.4921, 888.4818, 891.4986, 893.9725, 894.4754, 900.5499, 913.5269, 918.5532, 929.4499, 935.4841, 945.4980, 947.4821, 949.5173, 959.4860, 963.4728, 967.4305, 969.4643, 972.4830, 974.4501, 975.4561, 976.4594, 977.4788, 978.5386, 981.4812, 983.4774, 988.4600, 989.6110, 995.4939, 996.4700, 997.4576, 999.4598, 1007.5580, 1011.4736, 1018.5132, 1019.5350, 1021.5362, 1027.4982, 1032.5875, 1033.5684, 1034.5756, 1036.5582, 1044.5192, 1047.5377, 1055.5199, 1056.5244, 1057.5576, 1060.5359, 1062.5424, 1069.5072, 1074.5400, 1077.5109, 1085.5363, 1087.5255, 1091.5013, 1103.5313, 1106.5183, 1107.5238, 1111.5382, 1122.5424, 1123.5519, 1168.5806, 1173.6044, 1178.6016, 1179.5861, 1185.6279, 1187.6195, 1199.6720, 1207.6029, 1209.5723, 1215.6453, 1216.6425, 1226.5743, 1234.5945, 1244.5957, 1248.6305, 1255.5890, 1295.7034, 1301.5924, 1314.6890, 1322.5939, 1328.6476, 1336.6866, 1337.6793, 1338.6221, 1342.6494, 1344.6713, 1345.6792, 1349.6682, 1352.6564, 1353.6359, 1355.6598, 1361.6550, 1370.6490, 1372.5659, 1381.7262, 1383.6938, 1386.6838, 1394.6993, 1397.7117, 1427.7550, 1432.6827, 1433.6854, 1442.7830, 1446.7039, 1447.6959, 1457.7043, 1473.7709, 1478.7163, 1497.8370, 1537.7870, 1542.8158, 1550.9301, 1556.8483, 1578.7511, 1601.8662, 1606.8096, 1615.8414, 1616.8064, 1617.7891, 1627.7168, 1628.6741, 1628.8262, 1638.8353, 1642.6895, 1647.8445, 1650.8495, 1679.9406, 1680.7981, 1696.8021, 1706.8348, 1715.8130, 1717.8778, 1718.8860, 1725.8102, 1727.8582, 1730.8500, 1752.8656, 1763.7885, 1768.9357, 1769.9312, 1771.0000, 1775.8153, 1777.8390, 1786.8839, 1788.4598, 1791.8882, 1799.8864, 1800.8976, 1809.0026, 1914.9941, 1928.0222, 1953.8669, 1975.9434, 1982.0046, 2012.9734, 2027.1388, 2042.0795, 2053.0103, 2060.0637, 2070.9099, 2082.9165, 2084.9246, 2086.9263, 2095.0337, 2096.9631, 2098.9319, 2100.9241, 2136.9219, 2155.1025, 2170.1101, 2209.0732, 2211.1094, 2239.2014, 2251.1997, 2289.1399, 2293.2251, 2324.1719, 2343.2795, 2381.2776, 2384.2988, 2398.2314, 2400.1594, 2414.1807, 2428.1990, 2431.1982, 2444.2200, 2465.3230, 2528.3064, 2542.3503, 2546.3127, 2558.3843, 2647.1560, 2661.1709, 2675.1926, 2698.5471, 2706.4133, 2708.4885, 2718.5300, 2762.5994, 2765.5881, 2767.5010, 2771.5437, 2777.7188, 2811.6846, 2814.6851, 2846.6375, 2869.6453, 2872.5757, 2884.6123, 2888.6077, 2892.6868, 2894.7185, 2902.6804, 2918.6406, 2926.6814, 2946.7546, 2994.8018, 2998.7808, 3003.8159, 3005.8979, 3054.8286, 3082.9351, 3102.9441, 3105.8228, 3127.0818, 3171.0554, 3194.9565, 3202.8757, 3207.9922, 3249.0452, 3263.1082, 3264.1118, 3266.0825, 3300.1990, 3317.0972, 3323.0837, 3331.1343, 3335.1436, 3336.1438, 3348.9949, 3354.0869, 3358.1614, 3371.1931, 3387.2393, 3454.9927, 3459.2039, 3490.2356, 3504.3723, 3518.1758, 3640.3601, 3652.4072, 3664.4243, 3822.6233, 3870.6184, 3871.6218, 3887.6787, 4089.8884, 4160.9209, 4164.9634, 4175.9526, 4306.0547, 4453.3716, 4466.3892, 4680.6270, 4846.6348

7. F1MD66 **Mass:** 54375 **Score:** 21 **Expect:** 2.1e+002 **Matches:** 7

Annexin OS=Bos taurus GN=ANXA11 PE=3 SV=2

Observed	Mr(expt)	Mr(calc)	ppm	Start	End	Miss	Peptide
1062.5424	1061.5351	1061.5400	-4.59	443	452	2	K.AMRGAGTKDR.T
1295.7034	1294.6961	1294.6809	11.8	272	282	0	K.TPVLFDAYEIK.E
1349.6682	1348.6609	1348.6623	-1.03	192	204	0	R.GTITDASGFDPLR.D
1606.8096	1605.8023	1605.7742	17.5	413	427	0	R.EMSGDLEQGMLAVVK.C
2084.9246	2083.9173	2083.9667	-23.68	212	230	1	K.AMKGFGTDEQAIIDCLGSR.S + Oxidation (M)
2289.1399	2288.1326	2288.1073	11.1	476	495	2	R.LYGKSLYHDITGDTSGDYRK.I
2414.1807	2413.1734	2413.1366	15.3	212	233	2	K.AMKGFGTDEQAIIDCLGSRSNK.Q + Oxidation (M)

No match to: 712.3012, 714.3622, 717.4536, 722.4728, 735.4618, 756.5057, 772.4633, 779.5103, 792.4439, 795.4440, 804.3116, 806.4917, 807.4467, 809.4788, 812.4258, 814.4372, 815.4436, 819.4216, 828.4171, 831.4965, 832.3378, 834.3423, 841.4608, 842.4964, 845.4365, 871.5104, 883.4921, 888.4818, 891.4986, 893.9725, 894.4754, 900.5499, 913.5269, 918.5532, 929.4499, 935.4841, 945.4980, 947.4821, 949.5173, 959.4860, 963.4728, 967.4305, 969.4643, 972.4830, 974.4501, 975.4561, 976.4594, 977.4788, 978.5386, 981.4812, 983.4774, 988.4600, 989.6110, 995.4939, 996.4700, 997.4576, 999.4598, 1007.5580, 1011.4736, 1018.5132, 1019.5350, 1021.5362, 1027.4982, 1032.5875, 1033.5684, 1034.5756, 1036.5582, 1044.5192, 1047.5377, 1055.5199, 1056.5244, 1057.5576, 1060.5359, 1069.5072, 1074.5400, 1077.5109, 1085.5363, 1087.5255, 1091.5013, 1103.5313, 1106.5183, 1107.5238, 1111.5382, 1122.5424, 1123.5519, 1168.5806, 1173.6044, 1178.6016, 1179.5861, 1185.6279, 1187.6195, 1199.6720, 1207.6029, 1209.5723, 1215.6453, 1216.6425, 1226.5743, 1234.5945, 1244.5957, 1248.6305, 1255.5890, 1301.5924, 1314.6890, 1322.5939, 1325.6377, 1328.6476, 1336.6866, 1337.6793, 1338.6221, 1342.6494, 1344.6713, 1345.6792, 1352.6564, 1353.6359, 1355.6598, 1361.6550, 1368.6198, 1370.6490, 1372.5659, 1381.7262, 1383.6938, 1386.6838, 1394.6993, 1397.7117, 1427.7550, 1432.6827, 1433.6854, 1442.7830, 1446.7039, 1447.6959, 1457.7043, 1473.7709, 1478.7163, 1497.8370, 1528.6653, 1537.7870, 1542.8158, 1550.9301, 1556.8483, 1578.7511, 1601.8662, 1615.8414, 1616.8064, 1617.7891, 1627.7168, 1628.6741,

1628.8262, 1638.8353, 1642.6895, 1647.8445, 1650.8495, 1679.9406, 1680.7981, 1696.8021, 1706.8348, 1715.8130, 1717.8778, 1718.8860, 1725.8102, 1727.8582, 1730.8500, 1752.8656, 1763.7885, 1768.9357, 1769.9312, 1771.0000, 1775.8153, 1777.8390, 1786.8839, 1788.4598, 1791.8882, 1799.8864, 1800.8976, 1809.0026, 1914.9941, 1928.0222, 1953.8669, 1975.9434, 1982.0046, 2012.9734, 2027.1388, 2042.0795, 2053.0103, 2060.0637, 2070.9099, 2082.9165, 2086.9263, 2095.0337, 2096.9631, 2098.9319, 2100.9241, 2136.9219, 2155.1025, 2170.1101, 2209.0732, 2211.1094, 2239.2014, 2251.1997, 2293.2251, 2324.1719, 2343.2795, 2381.2776, 2384.2988, 2398.2314, 2400.1594, 2428.1990, 2431.1982, 2444.2200, 2465.3230, 2528.3064, 2542.3503, 2546.3127, 2558.3843, 2647.1560, 2661.1709, 2675.1926, 2698.5471, 2706.4133, 2708.4885, 2718.5300, 2762.5994, 2765.5881, 2767.5010, 2771.5437, 2777.7188, 2811.6846, 2814.6851, 2846.6375, 2869.6453, 2872.5757, 2884.6123, 2888.6077, 2892.6868, 2894.7185, 2902.6804, 2918.6406, 2926.6814, 2946.7546, 2994.8018, 2998.7808, 3003.8159, 3005.8979, 3054.8286, 3082.9351, 3102.9441, 3105.8228, 3127.0818, 3171.0554, 3194.9565, 3202.8757, 3207.9922, 3249.0452, 3263.1082, 3264.1118, 3266.0825, 3300.1990, 3317.0972, 3323.0837, 3331.1343, 3335.1436, 3336.1438, 3348.9949, 3354.0869, 3358.1614, 3371.1931, 3387.2393, 3454.9927, 3459.2039, 3490.2356, 3504.3723, 3518.1758, 3640.3601, 3652.4072, 3664.4243, 3822.6233, 3870.6184, 3871.6218, 3887.6787, 4089.8884, 4148.9229, 4160.9209, 4164.9634, 4175.9526, 4306.0547, 4453.3716, 4466.3892, 4680.6270, 4846.6348

8. P23805 **Mass:** 38370 **Score:** 20 **Expect:** 2.2e+002 **Matches:** 7

Conglutinin OS=Bos taurus GN=CGN1 PE=1 SV=2

Observed	Mr(expt)	Mr(calc)	ppm	Start	End	Miss	Peptide
969.4643	968.4570	968.4676	-10.89	163 -	173	0	K.GAPGETGAPGR.A
1021.5362	1020.5289	1020.5314	-2.43	364 -	371	0	K.QLLVICEF.-
1187.6195	1186.6122	1186.6193	-6.00	211 -	222	0	K.GESGLAEVNALK.Q
1226.5743	1225.5670	1225.5761	-7.44	123 -	135	0	K.QGSMGPPGTPGPK.G + Oxidation (M)
2293.2251	2292.2178	2292.2127	2.23	225 -	243	2	R.VTILDGHLRRFQNAFSQYK.K
4148.9229	4147.9156	4147.9435	-6.73	18 -	56	1	R.SLGAEMTTFSQKILANACTLVMCSPLESGLPGHDGQDGR.E
4164.9634	4163.9561	4163.9384	4.25	18 -	56	1	R.SLGAEMTTFSQKILANACTLVMCSPLESGLPGHDGQDGR.E + Oxidation (M)

No match to: 712.3012, 714.3622, 717.4536, 722.4728, 735.4618, 756.5057, 772.4633, 779.5103, 792.4439, 795.4440, 804.3116, 806.4917, 807.4467, 809.4788, 812.4258, 814.4372, 815.4436, 819.4216, 828.4171, 831.4965, 832.3378, 834.3423, 841.4608, 842.4964, 845.4365, 871.5104, 883.4921, 888.4818, 891.4986, 893.9725, 894.4754, 900.5499, 913.5269, 918.5532, 929.4499, 935.4841, 945.4980, 947.4821, 949.5173, 959.4860, 963.4728, 967.4305, 972.4830, 974.4501, 975.4561, 976.4594, 977.4788, 978.5386, 981.4812, 983.4774, 988.4600, 989.6110, 995.4939, 996.4700, 997.4576, 999.4598, 1007.5580, 1011.4736, 1018.5132, 1019.5350, 1027.4982, 1032.5875, 1033.5684, 1034.5756, 1036.5582, 1044.5192, 1047.5377, 1055.5199, 1056.5244, 1057.5576, 1060.5359, 1062.5424, 1069.5072, 1074.5400, 1077.5109, 1085.5363, 1087.5255, 1091.5013, 1103.5313, 1106.5183, 1107.5238, 1111.5382, 1122.5424, 1123.5519, 1168.5806, 1173.6044, 1178.6016, 1179.5861, 1185.6279, 1199.6720, 1207.6029, 1209.5723, 1215.6453, 1216.6425, 1234.5945, 1244.5957, 1248.6305, 1255.5890, 1295.7034, 1301.5924, 1314.6890, 1322.5939, 1325.6377, 1328.6476, 1336.6866, 1337.6793, 1338.6221, 1342.6494, 1344.6713, 1345.6792, 1349.6682, 1352.6564, 1353.6359, 1355.6598, 1361.6550, 1368.6198, 1370.6490, 1372.5659, 1381.7262, 1383.6938, 1386.6838, 1394.6993, 1397.7117, 1427.7550, 1432.6827, 1433.6854, 1442.7830, 1446.7039, 1447.6959, 1457.7043, 1473.7709, 1478.7163, 1497.8370, 1528.6653, 1537.7870, 1542.8158, 1550.9301, 1556.8483, 1578.7511, 1601.8662, 1606.8096, 1615.8414, 1616.8064, 1617.7891, 1627.7168, 1628.6741, 1628.8262, 1638.8353, 1642.6895, 1647.8445, 1650.8495, 1679.9406, 1680.7981, 1696.8021, 1706.8348, 1715.8130, 1717.8778, 1718.8860, 1725.8102, 1727.8582, 1730.8500, 1752.8656, 1763.7885, 1768.9357, 1769.9312, 1771.0000, 1775.8153, 1777.8390, 1786.8839, 1788.4598, 1791.8882, 1799.8864, 1800.8976, 1809.0026, 1914.9941, 1928.0222, 1953.8669, 1975.9434, 1982.0046, 2012.9734, 2027.1388, 2042.0795, 2053.0103, 2060.0637, 2070.9099, 2082.9165, 2084.9246, 2086.9263, 2095.0337, 2096.9631, 2098.9319, 2100.9241, 2136.9219, 2155.1025, 2170.1101, 2209.0732, 2211.1094, 2239.2014, 2251.1997, 2289.1399, 2324.1719, 2343.2795, 2381.2776, 2384.2988, 2398.2314, 2400.1594, 2414.1807, 2428.1990, 2431.1982, 2444.2200, 2465.3230, 2528.3064, 2542.3503, 2546.3127, 2558.3843, 2647.1560, 2661.1709, 2675.1926, 2698.5471, 2706.4133, 2708.4885, 2718.5300, 2762.5994, 2765.5881, 2767.5010, 2771.5437, 2777.7188, 2811.6846, 2814.6851, 2846.6375, 2869.6453, 2872.5757, 2884.6123, 2888.6077, 2892.6868, 2894.7185, 2902.6804, 2918.6406, 2926.6814, 2946.7546, 2994.8018, 2998.7808, 3003.8159, 3005.8979, 3054.8286, 3082.9351, 3102.9441, 3105.8228, 3127.0818, 3171.0554, 3194.9565, 3202.8757, 3207.9922, 3249.0452, 3263.1082, 3264.1118, 3266.0825, 3300.1990, 3317.0972, 3323.0837, 3331.1343, 3335.1436, 3336.1438, 3348.9949, 3354.0869, 3358.1614, 3371.1931, 3387.2393, 3454.9927, 3459.2039, 3490.2356, 3504.3723, 3518.1758, 3640.3601, 3652.4072, 3664.4243, 3822.6233, 3870.6184, 3871.6218, 3887.6787, 4089.8884, 4160.9209, 4175.9526, 4306.0547, 4453.3716, 4466.3892, 4680.6270, 4846.6348

9. F1MWX8 **Mass:** 41228 **Score:** 20 **Expect:** 2.3e+002 **Matches:** 3

Uncharacterized protein OS=Bos taurus GN=JSP.1 PE=3 SV=1

Observed	Mr(expt)	Mr(calc)	ppm	Start	End	Miss	Peptide
1349.6682	1348.6609	1348.6623	-1.00	156 -	168	0	R.SWTAADTAAQISK.R
1628.8262	1627.8189	1627.8259	-4.31	133 -	145	1	R.LLRGFWQFGYEGR.D
4148.9229	4147.9156	4147.9393	-5.71	244 -	280	1	R.NGEDQTQDMELVETRPSGDGTFQKWVALVVPSGEEQR.Y + Oxidation (M)

No match to: 712.3012, 714.3622, 717.4536, 722.4728, 735.4618, 756.5057, 772.4633, 779.5103, 792.4439, 795.4440, 804.3116, 806.4917, 807.4467, 809.4788, 812.4258, 814.4372, 815.4436, 819.4216, 828.4171, 831.4965, 832.3378, 834.3423, 841.4608, 842.4964, 845.4365, 871.5104, 883.4921, 888.4818, 891.4986, 893.9725, 894.4754, 900.5499, 913.5269, 918.5532, 929.4499, 935.4841, 945.4980, 947.4821, 949.5173, 959.4860, 963.4728, 967.4305, 969.4643, 972.4830, 974.4501, 975.4561, 976.4594, 977.4788, 978.5386, 981.4812, 983.4774, 988.4600, 989.6110, 995.4939, 996.4700, 997.4576, 999.4598, 1007.5580, 1011.4736, 1018.5132, 1019.5350, 1021.5362, 1027.4982, 1032.5875, 1033.5684, 1034.5756, 1036.5582, 1044.5192, 1047.5377, 1055.5199, 1056.5244, 1057.5576, 1060.5359, 1062.5424, 1069.5072, 1074.5400, 1077.5109, 1085.5363, 1087.5255, 1091.5013, 1103.5313, 1106.5183, 1107.5238, 1111.5382, 1122.5424, 1123.5519, 1168.5806, 1173.6044, 1178.6016, 1179.5861, 1185.6279, 1187.6195, 1199.6720, 1207.6029, 1209.5723, 1215.6453, 1216.6425, 1226.5743, 1234.5945, 1244.5957, 1248.6305, 1255.5890, 1295.7034, 1301.5924, 1314.6890, 1322.5939, 1325.6377, 1328.6476, 1336.6866, 1337.6793, 1338.6221, 1342.6494, 1344.6713, 1345.6792, 1352.6564, 1353.6359, 1355.6598, 1361.6550, 1368.6198, 1370.6490, 1372.5659, 1381.7262, 1383.6938, 1386.6838, 1394.6993, 1397.7117, 1427.7550, 1432.6827, 1433.6854, 1442.7830, 1446.7039, 1447.6959, 1457.7043, 1473.7709, 1478.7163, 1497.8370, 1528.6653, 1537.7870, 1542.8158, 1550.9301, 1556.8483, 1578.7511, 1601.8662, 1606.8096, 1615.8414, 1616.8064, 1617.7891, 1627.7168, 1628.6741, 1638.8353, 1642.6895, 1647.8445, 1650.8495, 1679.9406, 1680.7981, 1696.8021, 1706.8348, 1715.8130, 1717.8778, 1718.8860, 1725.8102, 1727.8582, 1730.8500, 1752.8656, 1763.7885, 1768.9357, 1769.9312, 1771.0000, 1775.8153, 1777.8390, 1786.8839, 1788.4598, 1791.8882, 1799.8864, 1800.8976, 1809.0026, 1914.9941, 1928.0222, 1953.8669, 1975.9434, 1982.0046, 2012.9734, 2027.1388, 2042.0795, 2053.0103, 2060.0637, 2070.9099, 2082.9165, 2084.9246, 2086.9263, 2095.0337, 2096.9631, 2098.9319, 2100.9241, 2136.9219, 2155.1025, 2170.1101, 2209.0732, 2211.1094, 2239.2014, 2251.1997, 2289.1399, 2293.2251, 2324.1719, 2343.2795, 2381.2776, 2384.2988, 2398.2314, 2400.1594, 2414.1807, 2428.1990, 2431.1982, 2444.2200, 2465.3230, 2528.3064, 2542.3503, 2546.3127, 2558.3843, 2647.1560, 2661.1709, 2675.1926, 2698.5471, 2706.4133, 2708.4885, 2718.5300, 2762.5994, 2765.5881, 2767.5010, 2771.5437, 2777.7188, 2811.6846, 2814.6851, 2846.6375, 2869.6453, 2872.5757, 2884.6123, 2888.6077, 2892.6868, 2894.7185, 2902.6804, 2918.6406, 2926.6814, 2946.7546, 2994.8018, 2998.7808, 3003.8159, 3005.8979, 3054.8286, 3082.9351, 3102.9441, 3105.8228, 3127.0818, 3171.0554, 3194.9565, 3202.8757, 3207.9922, 3249.0452, 3263.1082, 3264.1118, 3266.0825, 3300.1990, 3317.0972, 3323.0837, 3331.1343, 3335.1436, 3336.1438, 3348.9949, 3354.0869, 3358.1614, 3371.1931, 3387.2393, 3454.9927, 3459.2039, 3490.2356, 3504.3723, 3518.1758, 3640.3601, 3652.4072, 3664.4243, 3822.6233, 3870.6184, 3871.6218, 3887.6787, 4089.8884, 4160.9209, 4164.9634, 4175.9526, 4306.0547, 4453.3716, 4466.3892, 4680.6270, 4846.6348

10. Q5E9S8 **Mass:** 32807 **Score:** 20 **Expect:** 2.4e+002 **Matches:** 6

Uncharacterized protein C15orf41 homolog OS=Bos taurus PE=2 SV=1

Observed	Mr(expt)	Mr(calc)	ppm	Start	End	Miss	Peptide
935.4841	934.4768	934.4906	-14.76	119 -	126	0	K.SVINSMLR.D + Oxidation (M)
1349.6682	1348.6609	1348.6623	-1.00	177 -	187	0	K.NLSFLDEDQLR.A
2414.1807	2413.1734	2413.2311	-23.90	79 -	101	0	K.NGAAPVLLELANEVDYAPSLMAR.I
2431.1982	2430.1909	2430.2325	-17.12	6 -	26	1	K.AQYDEIAQCLVSVPPTRQSLR.K
2542.3503	2541.3430	2541.3261	6.68	78 -	101	1	R.KNGAAPVLLELANEVDYAPSLMAR.I
2558.3843	2557.3770	2557.3210	21.9	78 -	101	1	R.KNGAAPVLLELANEVDYAPSLMAR.I + Oxidation (M)

No match to: 712.3012, 714.3622, 717.4536, 722.4728, 735.4618, 756.5057, 772.4633, 779.5103, 792.4439, 795.4440, 804.3116, 806.4917, 807.4467, 809.4788, 812.4258, 814.4372, 815.4436, 819.4216, 828.4171, 831.4965, 832.3378, 834.3423, 841.4608, 842.4964, 845.4365, 871.5104, 883.4921, 888.4818, 891.4986, 893.9725, 894.4754, 900.5499, 913.5269, 918.5532, 929.4499, 945.4980, 947.4821, 949.5173, 959.4860, 963.4728, 967.4305, 969.4643, 972.4830, 974.4501, 975.4561, 976.4594, 977.4788, 978.5386, 981.4812, 983.4774, 988.4600, 989.6110, 995.4939, 996.4700, 997.4576, 999.4598, 1007.5580, 1011.4736, 1018.5132, 1019.5350, 1021.5362, 1027.4982, 1032.5875, 1033.5684, 1034.5756, 1036.5582, 1044.5192, 1047.5377, 1055.5199, 1056.5244, 1057.5576, 1060.5359, 1062.5424, 1069.5072,

1074.5400, 1077.5109, 1085.5363, 1087.5255, 1091.5013, 1103.5313, 1106.5183, 1107.5238, 1111.5382, 1122.5424, 1123.5519, 1168.5806, 1173.6044, 1178.6016, 1179.5861, 1185.6279, 1187.6195, 1199.6720, 1207.6029, 1209.5723, 1215.6453, 1216.6425, 1226.5743, 1234.5945, 1244.5957, 1248.6305, 1255.5890, 1295.7034, 1301.5924, 1314.6890, 1322.5939, 1325.6377, 1328.6476, 1336.6866, 1337.6793, 1338.6221, 1342.6494, 1344.6713, 1345.6792, 1352.6564, 1353.6359, 1355.6598, 1361.6550, 1368.6198, 1370.6490, 1372.5659, 1381.7262, 1383.6938, 1386.6838, 1394.6993, 1397.7117, 1427.7550, 1432.6827, 1433.6854, 1442.7830, 1446.7039, 1447.6959, 1457.7043, 1473.7709, 1478.7163, 1497.8370, 1528.6653, 1537.7870, 1542.8158, 1550.9301, 1556.8483, 1578.7511, 1601.8662, 1606.8096, 1615.8414, 1616.8064, 1617.7891, 1627.7168, 1628.6741, 1628.8262, 1638.8353, 1642.6895, 1647.8445, 1650.8495, 1679.9406, 1680.7981, 1696.8021, 1706.8348, 1715.8130, 1717.8778, 1718.8860, 1725.8102, 1727.8582, 1730.8500, 1752.8656, 1763.7885, 1768.9357, 1769.9312, 1771.0000, 1775.8153, 1777.8390, 1786.8839, 1788.4598, 1791.8882, 1799.8864, 1800.8976, 1809.0026, 1914.9941, 1928.0222, 1953.8669, 1975.9434, 1982.0046, 2012.9734, 2027.1388, 2042.0795, 2053.0103, 2060.0637, 2070.9099, 2082.9165, 2084.9246, 2086.9263, 2095.0337, 2096.9631, 2098.9319, 2100.9241, 2136.9219, 2155.1025, 2170.1101, 2209.0732, 2211.1094, 2239.2014, 2251.1997, 2289.1399, 2293.2251, 2324.1719, 2343.2795, 2381.2776, 2384.2988, 2398.2314, 2400.1594, 2428.1990, 2444.2200, 2465.3230, 2528.3064, 2546.3127, 2647.1560, 2661.1709, 2675.1926, 2698.5471, 2706.4133, 2708.4885, 2718.5300, 2762.5994, 2765.5881, 2767.5010, 2771.5437, 2777.7188, 2811.6846, 2814.6851, 2846.6375, 2869.6453, 2872.5757, 2884.6123, 2888.6077, 2892.6868, 2894.7185, 2902.6804, 2918.6406, 2926.6814, 2946.7546, 2994.8018, 2998.7808, 3003.8159, 3005.8979, 3054.8286, 3082.9351, 3102.9441, 3105.8228, 3127.0818, 3171.0554, 3194.9565, 3202.8757, 3207.9922, 3249.0452, 3263.1082, 3264.1118, 3266.0825, 3300.1990, 3317.0972, 3323.0837, 3331.1343, 3335.1436, 3336.1438, 3348.9949, 3354.0869, 3358.1614, 3371.1931, 3387.2393, 3454.9927, 3459.2039, 3490.2356, 3504.3723, 3518.1758, 3640.3601, 3652.4072, 3664.4243, 3822.6233, 3870.6184, 3871.6218, 3887.6787, 4089.8884, 4148.9229, 4160.9209, 4164.9634, 4175.9526, 4306.0547, 4453.3716, 4466.3892, 4680.6270, 4846.6348

11. G5E634 **Mass:** 53717 **Score:** 20 **Expect:** 2.7e+002 **Matches:** 7

Uncharacterized protein OS=Bos taurus PE=4 SV=1

Observed	Mr(expt)	Mr(calc)	ppm	Start		End	Miss	Peptide
891.4986	890.4913	890.4974	-6.80	445	-	451	1	R.ERFAQIK.A
988.4600	987.4527	987.4630	-10.38	194	-	201	0	K.ASIHLCCK.K
1044.5192	1043.5119	1043.5334	-20.63	1	-	8	0	-.LHMLNNFR.V
1060.5359	1059.5286	1059.5284	0.24	1	-	8	0	-.LHMLNNFR.V + Oxidation (M)
1768.9357	1767.9284	1767.9229	3.11	162	-	175	1	R.RFTPEVYIELCLTK.K
2053.0103	2052.0030	2052.0417	-18.85	205	-	221	2	K.IISMSKENMEEVLRMVK.L + Oxidation (M)
2872.5757	2871.5684	2871.6004	-11.14	236	-	261	1	K.LSTLAQFAPLLGQMSNVQSLILSRIR.G + Oxidation (M)

No match to: 712.3012, 714.3622, 717.4536, 722.4728, 735.4618, 756.5057, 772.4633, 779.5103, 792.4439, 795.4440, 804.3116, 806.4917, 807.4467, 809.4788, 812.4258, 814.4372, 815.4436, 819.4216, 828.4171, 831.4965, 832.3378, 834.3423, 841.4608, 842.4964, 845.4365, 871.5104, 883.4921, 888.4818, 893.9725, 894.4754, 900.5499, 913.5269, 918.5532, 929.4499, 935.4841, 945.4980, 947.4821, 949.5173, 959.4860, 963.4728, 967.4305, 969.4643, 972.4830, 974.4501, 975.4561, 976.4594, 977.4788, 978.5386, 981.4812, 983.4774, 989.6110, 995.4939, 996.4700, 997.4576, 999.4598, 1007.5580, 1011.4736, 1018.5132, 1019.5350, 1021.5362, 1027.4982, 1032.5875, 1033.5684, 1034.5756, 1036.5582, 1047.5377, 1055.5199, 1056.5244, 1057.5576, 1062.5424, 1069.5072, 1074.5400, 1077.5109, 1085.5363, 1087.5255, 1091.5013, 1103.5313, 1106.5183, 1107.5238, 1111.5382, 1122.5424, 1123.5519, 1168.5806, 1173.6044, 1178.6016, 1179.5861, 1185.6279, 1187.6195, 1199.6720, 1207.6029, 1209.5723, 1215.6453, 1216.6425, 1226.5743, 1234.5945, 1244.5957, 1248.6305, 1255.5890, 1295.7034, 1301.5924, 1314.6890, 1322.5939, 1325.6377, 1328.6476, 1336.6866, 1337.6793, 1338.6221, 1342.6494, 1344.6713, 1345.6792, 1349.6682, 1352.6564, 1353.6359, 1355.6598, 1361.6550, 1368.6198, 1370.6490, 1372.5659, 1381.7262, 1383.6938, 1386.6838, 1394.6993, 1397.7117, 1427.7550, 1432.6827, 1433.6854, 1442.7830, 1446.7039, 1447.6959, 1457.7043, 1473.7709, 1478.7163, 1497.8370, 1528.6653, 1537.7870, 1542.8158, 1550.9301, 1556.8483, 1578.7511, 1601.8662, 1606.8096, 1615.8414, 1616.8064, 1617.7891, 1627.7168, 1628.6741, 1628.8262, 1638.8353, 1642.6895, 1647.8445, 1650.8495, 1679.9406, 1680.7981, 1696.8021, 1706.8348, 1715.8130, 1717.8778, 1718.8860, 1725.8102, 1727.8582, 1730.8500, 1752.8656, 1763.7885, 1769.9312, 1771.0000, 1775.8153, 1777.8390, 1786.8839, 1788.4598, 1791.8882, 1799.8864, 1800.8976, 1809.0026, 1914.9941, 1928.0222, 1953.8669, 1975.9434, 1982.0046, 2012.9734, 2027.1388, 2042.0795, 2060.0637, 2070.9099, 2082.9165, 2084.9246, 2086.9263, 2095.0337, 2096.9631, 2098.9319, 2100.9241, 2136.9219, 2155.1025, 2170.1101, 2209.0732, 2211.1094, 2239.2014, 2251.1997, 2289.1399, 2293.2251, 2324.1719, 2343.2795, 2381.2776, 2384.2988, 2398.2314, 2400.1594, 2414.1807, 2428.1990, 2431.1982, 2444.2200, 2465.3230, 2528.3064, 2542.3503, 2546.3127, 2558.3843, 2647.1560, 2661.1709, 2675.1926, 2698.5471, 2706.4133, 2708.4885, 2718.5300, 2762.5994, 2765.5881, 2767.5010, 2771.5437, 2777.7188, 2811.6846, 2814.6851, 2846.6375, 2869.6453, 2884.6123, 2888.6077, 2892.6868, 2894.7185, 2902.6804, 2918.6406, 2926.6814, 2946.7546, 2994.8018, 2998.7808, 3003.8159, 3005.8979, 3054.8286, 3082.9351, 3102.9441, 3105.8228, 3127.0818, 3171.0554, 3194.9565, 3202.8757, 3207.9922, 3249.0452, 3263.1082, 3264.1118, 3266.0825, 3300.1990, 3317.0972, 3323.0837, 3331.1343, 3335.1436, 3336.1438, 3348.9949, 3354.0869, 3358.1614, 3371.1931, 3387.2393, 3454.9927, 3459.2039, 3490.2356, 3504.3723, 3518.1758, 3640.3601, 3652.4072, 3664.4243, 3822.6233, 3870.6184, 3871.6218, 3887.6787, 4089.8884, 4148.9229, 4160.9209, 4164.9634, 4175.9526, 4306.0547, 4453.3716, 4466.3892, 4680.6270, 4846.6348

12. G3MWR6 **Mass:** 10152 **Score:** 19 **Expect:** 2.8e+002 **Matches:** 2

Uncharacterized protein OS=Bos taurus GN=HRK PE=4 SV=1

Observed	Mr(expt)	Mr(calc)	ppm	Start		End	Miss	Peptide
1628.8262	1627.8189	1627.7889	18.4	39	-	51	1	K.ALGDELHQRTMWR.R + Oxidation (M)
1768.9357	1767.9284	1767.8951	18.9	39	-	52	2	K.ALGDELHQRTMWRR.R

No match to: 712.3012, 714.3622, 717.4536, 722.4728, 735.4618, 756.5057, 772.4633, 779.5103, 792.4439, 795.4440, 804.3116, 806.4917, 807.4467, 809.4788, 812.4258, 814.4372, 815.4436, 819.4216, 828.4171, 831.4965, 832.3378, 834.3423, 841.4608, 842.4964, 845.4365, 871.5104, 883.4921, 888.4818, 891.4986, 893.9725, 894.4754, 900.5499, 913.5269, 918.5532, 929.4499, 935.4841, 945.4980, 947.4821, 949.5173, 959.4860, 963.4728, 967.4305, 969.4643, 972.4830, 974.4501, 975.4561, 976.4594, 977.4788, 978.5386, 981.4812, 983.4774, 988.4600, 989.6110, 995.4939, 996.4700, 997.4576, 999.4598, 1007.5580, 1011.4736, 1018.5132, 1019.5350, 1021.5362, 1027.4982, 1032.5875, 1033.5684, 1034.5756, 1036.5582, 1044.5192, 1047.5377, 1055.5199, 1056.5244, 1057.5576, 1060.5359, 1062.5424, 1069.5072, 1074.5400, 1077.5109, 1085.5363, 1087.5255, 1091.5013, 1103.5313, 1106.5183, 1107.5238, 1111.5382, 1122.5424, 1123.5519, 1168.5806, 1173.6044, 1178.6016, 1179.5861, 1185.6279, 1187.6195, 1199.6720, 1207.6029, 1209.5723, 1215.6453, 1216.6425, 1226.5743, 1234.5945, 1244.5957, 1248.6305, 1255.5890, 1295.7034, 1301.5924, 1314.6890, 1322.5939, 1325.6377, 1328.6476, 1336.6866, 1337.6793, 1338.6221, 1342.6494, 1344.6713, 1345.6792, 1349.6682, 1352.6564, 1353.6359, 1355.6598, 1361.6550, 1368.6198, 1370.6490, 1372.5659, 1381.7262, 1383.6938, 1386.6838, 1394.6993, 1397.7117, 1427.7550, 1432.6827, 1433.6854, 1442.7830, 1446.7039, 1447.6959, 1457.7043, 1473.7709, 1478.7163, 1497.8370, 1528.6653, 1537.7870, 1542.8158, 1550.9301, 1556.8483, 1578.7511, 1601.8662, 1606.8096, 1615.8414, 1616.8064, 1617.7891, 1627.7168, 1628.6741, 1638.8353, 1642.6895, 1647.8445, 1650.8495, 1679.9406, 1680.7981, 1696.8021, 1706.8348, 1715.8130, 1717.8778, 1718.8860, 1725.8102, 1727.8582, 1730.8500, 1752.8656, 1763.7885, 1769.9312, 1771.0000, 1775.8153, 1777.8390, 1786.8839, 1788.4598, 1791.8882, 1799.8864, 1800.8976, 1809.0026, 1914.9941, 1928.0222, 1953.8669, 1975.9434, 1982.0046, 2012.9734, 2027.1388, 2042.0795, 2053.0103, 2060.0637, 2070.9099, 2082.9165, 2084.9246, 2086.9263, 2095.0337, 2096.9631, 2098.9319, 2100.9241, 2136.9219, 2155.1025, 2170.1101, 2209.0732, 2211.1094, 2239.2014, 2251.1997, 2289.1399, 2293.2251, 2324.1719, 2343.2795, 2381.2776, 2384.2988, 2398.2314, 2400.1594, 2414.1807, 2428.1990, 2431.1982, 2444.2200, 2465.3230, 2528.3064, 2542.3503, 2546.3127, 2558.3843, 2647.1560, 2661.1709, 2675.1926, 2698.5471, 2706.4133, 2708.4885, 2718.5300, 2762.5994, 2765.5881, 2767.5010, 2771.5437, 2777.7188, 2811.6846, 2814.6851, 2846.6375, 2869.6453, 2872.5757, 2884.6123, 2888.6077, 2892.6868, 2894.7185, 2902.6804, 2918.6406, 2926.6814, 2946.7546, 2994.8018, 2998.7808, 3003.8159, 3005.8979, 3054.8286, 3082.9351, 3102.9441, 3105.8228, 3127.0818, 3171.0554, 3194.9565, 3202.8757, 3207.9922, 3249.0452, 3263.1082, 3264.1118, 3266.0825, 3300.1990, 3317.0972, 3323.0837, 3331.1343, 3335.1436, 3336.1438, 3348.9949, 3354.0869, 3358.1614, 3371.1931, 3387.2393, 3454.9927, 3459.2039, 3490.2356, 3504.3723, 3518.1758, 3640.3601, 3652.4072, 3664.4243, 3822.6233, 3870.6184, 3871.6218, 3887.6787, 4089.8884, 4148.9229, 4160.9209, 4164.9634, 4175.9526, 4306.0547, 4453.3716, 4466.3892, 4680.6270, 4846.6348

13. F1MCH3 **Mass:** 61453 **Score:** 19 **Expect:** 2.8e+002 **Matches:** 10

Uncharacterized protein OS=Bos taurus GN=RTP4 PE=4 SV=2

Observed	Mr(expt)	Mr(calc)	ppm	Start		End	Miss	Peptide
969.4643	968.4570	968.4749	-18.50	310	-	317	0	R.MTYSQAIR.M
1036.5582	1035.5509	1035.5648	-13.36	97	-	104	2	R.IFGQRCKK.C
1060.5359	1059.5286	1059.5495	-19.69	1	-	9	1	-.MDAKPQSKR.I
1062.5424	1061.5351	1061.5465	-10.72	353	-	362	1	R.AISRSDSQAK.G
1107.5238	1106.5165	1106.5204	-3.49	477	-	487	0	K.ATVGSDTEATR.R
1111.5382	1110.5309	1110.5570	-23.51	53	-	61	1	R.QYQQKGFGR.F
1325.6377	1324.6304	1324.6081	16.8	246	-	257	0	R.EMAQLFTAGADR.Q + Oxidation (M)

1370.6490	1369.6417	1369.6660	-17.72	340 - 352	0	K.MAEPQPTGGTIPR.A + Oxidation (M)
2398.2314	2397.2241	2397.2070	7.13	340 - 362	2	K.MAEPQPTGGTIPRAISRSDSQAK.G
2414.1807	2413.1734	2413.2020	-11.82	340 - 362	2	K.MAEPQPTGGTIPRAISRSDSQAK.G + Oxidation (M)

No match to: 712.3012, 714.3622, 717.4536, 722.4728, 735.4618, 756.5057, 772.4633, 779.5103, 792.4439, 795.4440, 804.3116, 806.4917, 807.4467, 809.4788, 812.4258, 814.4372, 815.4436, 819.4216, 828.4171, 831.4965, 832.3378, 834.3423, 841.4608, 842.4964, 845.4365, 871.5104, 883.4921, 888.4818, 891.4986, 893.9725, 894.4754, 900.5499, 913.5269, 918.5532, 929.4499, 935.4841, 945.4980, 947.4821, 949.5173, 959.4860, 963.4728, 967.4305, 972.4830, 974.4501, 975.4561, 976.4594, 977.4788, 978.5386, 981.4812, 983.4774, 988.4600, 989.6110, 995.4939, 996.4700, 997.4576, 999.4598, 1007.5580, 1011.4736, 1018.5132, 1019.5350, 1021.5362, 1027.4982, 1032.5875, 1033.5684, 1034.5756, 1044.5192, 1047.5377, 1055.5199, 1056.5244, 1057.5576, 1069.5072, 1074.5400, 1077.5109, 1085.5363, 1087.5255, 1091.5013, 1103.5313, 1106.5183, 1122.5424, 1123.5519, 1168.5806, 1173.6044, 1178.6016, 1179.5861, 1185.6279, 1187.6195, 1199.6720, 1207.6029, 1209.5723, 1215.6453, 1216.6425, 1226.5743, 1234.5945, 1244.5957, 1248.6305, 1255.5890, 1295.7034, 1301.5924, 1314.6890, 1322.5939, 1328.6476, 1336.6866, 1337.6793, 1338.6221, 1342.6494, 1344.6713, 1345.6792, 1349.6682, 1352.6564, 1353.6359, 1355.6598, 1361.6550, 1368.6198, 1372.5659, 1381.7262, 1383.6938, 1386.6838, 1394.6993, 1397.7117, 1427.7550, 1432.6827, 1433.6854, 1442.7830, 1446.7039, 1447.6959, 1457.7043, 1473.7709, 1478.7163, 1497.8370, 1528.6653, 1537.7870, 1542.8158, 1550.9301, 1556.8483, 1578.7511, 1601.8662, 1606.8096, 1615.8414, 1616.8064, 1617.7891, 1627.7168, 1628.6741, 1628.8262, 1638.8353, 1642.6895, 1647.8445, 1650.8495, 1679.9406, 1680.7981, 1696.8021, 1706.8348, 1715.8130, 1717.8778, 1718.8860, 1725.8102, 1727.8582, 1730.8500, 1752.8656, 1763.7885, 1768.9357, 1769.9312, 1771.0000, 1775.8153, 1777.8390, 1786.8839, 1788.4598, 1791.8882, 1799.8864, 1800.8976, 1809.0026, 1914.9941, 1928.0222, 1953.8669, 1975.9434, 1982.0046, 2012.9734, 2027.1388, 2042.0795, 2053.0103, 2060.0637, 2070.9099, 2082.9165, 2084.9246, 2086.9263, 2095.0337, 2096.9631, 2098.9319, 2100.9241, 2136.9219, 2155.1025, 2170.1101, 2209.0732, 2211.1094, 2239.2014, 2251.1997, 2289.1399, 2293.2251, 2324.1719, 2343.2795, 2381.2776, 2384.2988, 2400.1594, 2428.1990, 2431.1982, 2444.2200, 2465.3230, 2528.3064, 2542.3503, 2546.3127, 2558.3843, 2647.1560, 2661.1709, 2675.1926, 2698.5471, 2706.4133, 2708.4885, 2718.5300, 2762.5994, 2765.5881, 2767.5010, 2771.5437, 2777.7188, 2811.6846, 2814.6851, 2846.6375, 2869.6453, 2872.5757, 2884.6123, 2888.6077, 2892.6868, 2894.7185, 2902.6804, 2918.6406, 2926.6814, 2946.7546, 2994.8018, 2998.7808, 3003.8159, 3005.8979, 3054.8286, 3082.9351, 3102.9441, 3105.8228, 3127.0818, 3171.0554, 3194.9565, 3202.8757, 3207.9922, 3249.0452, 3263.1082, 3264.1118, 3266.0825, 3300.1990, 3317.0972, 3323.0837, 3331.1343, 3335.1436, 3336.1438, 3348.9949, 3354.0869, 3358.1614, 3371.1931, 3387.2393, 3454.9927, 3459.2039, 3490.2356, 3504.3723, 3518.1758, 3640.3601, 3652.4072, 3664.4243, 3822.6233, 3870.6184, 3871.6218, 3887.6787, 4089.8884, 4148.9229, 4160.9209, 4164.9634, 4175.9526, 4306.0547, 4453.3716, 4466.3892, 4680.6270, 4846.6348

14. Q58DF3 **Mass:** 42440 **Score:** 19 **Expect:** 3e+002 **Matches:** 10

TraB domain-containing protein OS=Bos taurus GN=TRABD PE=2 SV=1

Observed	Mr(expt)	Mr(calc)	ppm	Start	End	Miss	Peptide
1033.5684	1032.5611	1032.5386	21.8	117	- 124	1	K.MDERTLLR.E
1173.6044	1172.5971	1172.5900	6.10	260	- 268	0	R.DVYLTYMLR.Q
1606.8096	1605.8023	1605.8225	-12.56	212	- 225	0	K.LAWGLCFLSDPISK.D
1615.8414	1614.8341	1614.8188	9.50	260	- 272	1	R.DVYLTYMLRQAAR.R + Oxidation (M)
1638.8353	1637.8280	1637.8096	11.2	365	- 377	1	R.HCLQRLSDAWPQK.-
1680.7981	1679.7908	1679.8268	-21.39	75	- 89	1	R.VYVVGTAHFSDDSKR.D
2428.1990	2427.1917	2427.2355	-18.06	29	- 50	0	R.VLPGDPQNLSDVDAFNLLLEMK.L
2444.2200	2443.2127	2443.2305	-7.26	29	- 50	0	R.VLPGDPQNLSDVDAFNLLLEMK.L + Oxidation (M)
4148.9229	4147.9156	4147.9727	-13.75	235	- 268	2	K.DLLEQMMAEMVGEFPDLHRTIVSERDVYLTYMLR.Q + 3 Oxidation (M)
4164.9634	4163.9561	4163.9676	-2.75	235	- 268	2	K.DLLEQMMAEMVGEFPDLHRTIVSERDVYLTYMLR.Q + 4 Oxidation (M)

No match to: 712.3012, 714.3622, 717.4536, 722.4728, 735.4618, 756.5057, 772.4633, 779.5103, 792.4439, 795.4440, 804.3116, 806.4917, 807.4467, 809.4788, 812.4258, 814.4372, 815.4436, 819.4216, 828.4171, 831.4965, 832.3378, 834.3423, 841.4608, 842.4964, 845.4365, 871.5104, 883.4921, 888.4818, 891.4986, 893.9725, 894.4754, 900.5499, 913.5269, 918.5532, 929.4499, 935.4841, 945.4980, 947.4821, 949.5173, 959.4860, 963.4728, 967.4305, 969.4643, 972.4830, 974.4501, 975.4561, 976.4594, 977.4788, 978.5386, 981.4812, 983.4774, 988.4600, 989.6110, 995.4939, 996.4700, 997.4576, 999.4598, 1007.5580, 1011.4736, 1018.5132, 1019.5350, 1021.5362, 1027.4982, 1032.5875, 1034.5756, 1036.5582, 1044.5192, 1047.5377, 1055.5199, 1056.5244, 1057.5576, 1060.5359, 1062.5424, 1069.5072, 1074.5400, 1077.5109, 1085.5363, 1087.5255, 1091.5013, 1103.5313, 1106.5183, 1107.5238, 1111.5382, 1122.5424, 1123.5519, 1168.5806, 1178.6016, 1179.5861, 1185.6279, 1187.6195, 1199.6720, 1207.6029, 1209.5723, 1215.6453, 1216.6425, 1226.5743, 1234.5945, 1244.5957, 1248.6305, 1255.5890, 1295.7034, 1301.5924, 1314.6890, 1322.5939, 1325.6377, 1328.6476, 1336.6866, 1337.6793, 1338.6221, 1342.6494, 1344.6713, 1345.6792, 1349.6682, 1352.6564, 1353.6359, 1355.6598, 1361.6550, 1368.6198, 1370.6490, 1372.5659, 1381.7262, 1383.6938, 1386.6838, 1394.6993, 1397.7117, 1427.7550, 1432.6827, 1433.6854, 1442.7830, 1446.7039, 1447.6959, 1457.7043, 1473.7709, 1478.7163, 1497.8370, 1528.6653, 1537.7870, 1542.8158, 1550.9301, 1556.8483, 1578.7511, 1601.8662, 1616.8064, 1617.7891, 1627.7168, 1628.6741, 1628.8262, 1642.6895, 1647.8445, 1650.8495, 1679.9406, 1696.8021, 1706.8348, 1715.8130, 1717.8778, 1718.8860, 1725.8102, 1727.8582, 1730.8500, 1752.8656, 1763.7885, 1768.9357, 1769.9312, 1771.0000, 1775.8153, 1777.8390, 1786.8839, 1788.4598, 1791.8882, 1799.8864, 1800.8976, 1809.0026, 1914.9941, 1928.0222, 1953.8669, 1975.9434, 1982.0046, 2012.9734, 2027.1388, 2042.0795, 2053.0103, 2060.0637, 2070.9099, 2082.9165, 2084.9246, 2086.9263, 2095.0337, 2096.9631, 2098.9319, 2100.9241, 2136.9219, 2155.1025, 2170.1101, 2209.0732, 2211.1094, 2239.2014, 2251.1997, 2289.1399, 2293.2251, 2324.1719, 2343.2795, 2381.2776, 2384.2988, 2398.2314, 2400.1594, 2414.1807, 2431.1982, 2465.3230, 2528.3064, 2542.3503, 2546.3127, 2558.3843, 2647.1560, 2661.1709, 2675.1926, 2698.5471, 2706.4133, 2708.4885, 2718.5300, 2762.5994, 2765.5881, 2767.5010, 2771.5437, 2777.7188, 2811.6846, 2814.6851, 2846.6375, 2869.6453, 2872.5757, 2884.6123, 2888.6077, 2892.6868, 2894.7185, 2902.6804, 2918.6406, 2926.6814, 2946.7546, 2994.8018, 2998.7808, 3003.8159, 3005.8979, 3054.8286, 3082.9351, 3102.9441, 3105.8228, 3127.0818, 3171.0554, 3194.9565, 3202.8757, 3207.9922, 3249.0452, 3263.1082, 3264.1118, 3266.0825, 3300.1990, 3317.0972, 3323.0837, 3331.1343, 3335.1436, 3336.1438, 3348.9949, 3354.0869, 3358.1614, 3371.1931, 3387.2393, 3454.9927, 3459.2039, 3490.2356, 3504.3723, 3518.1758, 3640.3601, 3652.4072, 3664.4243, 3822.6233, 3870.6184, 3871.6218, 3887.6787, 4089.8884, 4160.9209, 4175.9526, 4306.0547, 4453.3716, 4466.3892, 4680.6270, 4846.6348

15. G3X7M1 **Mass:** 33791 **Score:** 18 **Expect:** 3.4e+002 **Matches:** 6

Uncharacterized protein OS=Bos taurus GN=SHOX PE=3 SV=1

Observed	Mr(expt)	Mr(calc)	ppm	Start	End	Miss	Peptide
1216.6425	1215.6352	1215.6571	-18.01	39	- 49	1	R.EVLESGLARSR.E
1349.6682	1348.6609	1348.6331	20.6	16	- 32	2	K.SKDGSGGGSGGGGGGKK.E
1606.8096	1605.8023	1605.7998	1.56	124	- 136	0	R.TNFTLEQLNELER.L
1628.8262	1627.8189	1627.8093	5.89	2	- 15	1	M.EELTAFVSKSFDQK.S
1706.8348	1705.8275	1705.8006	15.8	104	- 118	2	R.DDVKSEDEDGQTKLK.Q
1775.8153	1774.8080	1774.8447	-20.69	1	- 15	1	-.MEELTAFVSKSFDQK.S + Oxidation (M)

No match to: 712.3012, 714.3622, 717.4536, 722.4728, 735.4618, 756.5057, 772.4633, 779.5103, 792.4439, 795.4440, 804.3116, 806.4917, 807.4467, 809.4788, 812.4258, 814.4372, 815.4436, 819.4216, 828.4171, 831.4965, 832.3378, 834.3423, 841.4608, 842.4964, 845.4365, 871.5104, 883.4921, 888.4818, 891.4986, 893.9725, 894.4754, 900.5499, 913.5269, 918.5532, 929.4499, 935.4841, 945.4980, 947.4821, 949.5173, 959.4860, 963.4728, 967.4305, 969.4643, 972.4830, 974.4501, 975.4561, 976.4594, 977.4788, 978.5386, 981.4812, 983.4774, 988.4600, 989.6110, 995.4939, 996.4700, 997.4576, 999.4598, 1007.5580, 1011.4736, 1018.5132, 1019.5350, 1021.5362, 1027.4982, 1032.5875, 1033.5684, 1034.5756, 1036.5582, 1044.5192, 1047.5377, 1055.5199, 1056.5244, 1057.5576, 1060.5359, 1062.5424, 1069.5072, 1074.5400, 1077.5109, 1085.5363, 1087.5255, 1091.5013, 1103.5313, 1106.5183, 1107.5238, 1111.5382, 1122.5424, 1123.5519, 1168.5806, 1173.6044, 1178.6016, 1179.5861, 1185.6279, 1187.6195, 1199.6720, 1207.6029, 1209.5723, 1215.6453, 1226.5743, 1234.5945, 1244.5957, 1248.6305, 1255.5890, 1295.7034, 1301.5924, 1314.6890, 1322.5939, 1325.6377, 1328.6476, 1336.6866, 1337.6793, 1338.6221, 1342.6494, 1344.6713, 1345.6792, 1352.6564, 1353.6359, 1355.6598, 1361.6550, 1368.6198, 1370.6490, 1372.5659, 1381.7262, 1383.6938, 1386.6838, 1394.6993, 1397.7117, 1427.7550, 1432.6827, 1433.6854, 1442.7830, 1446.7039, 1447.6959, 1457.7043, 1473.7709, 1478.7163, 1497.8370, 1528.6653, 1537.7870, 1542.8158, 1550.9301, 1556.8483, 1578.7511, 1601.8662, 1615.8414, 1616.8064, 1617.7891, 1627.7168, 1628.6741, 1638.8353, 1642.6895, 1647.8445, 1650.8495, 1679.9406, 1680.7981, 1696.8021, 1715.8130, 1717.8778, 1718.8860, 1725.8102, 1727.8582, 1730.8500, 1752.8656, 1763.7885, 1768.9357, 1769.9312, 1771.0000, 1777.8390, 1786.8839, 1788.4598, 1791.8882, 1799.8864, 1800.8976, 1809.0026, 1914.9941, 1928.0222, 1953.8669, 1975.9434, 1982.0046, 2012.9734, 2027.1388, 2042.0795, 2053.0103, 2060.0637, 2070.9099, 2082.9165, 2084.9246, 2086.9263, 2095.0337, 2096.9631, 2098.9319, 2100.9241, 2136.9219, 2155.1025, 2170.1101, 2209.0732, 2211.1094, 2239.2014, 2251.1997, 2289.1399, 2293.2251, 2324.1719, 2343.2795, 2381.2776, 2384.2988, 2398.2314, 2400.1594, 2414.1807, 2428.1990, 2431.1982, 2444.2200, 2465.3230, 2528.3064, 2542.3503, 2546.3127, 2558.3843, 2647.1560, 2661.1709, 2675.1926, 2698.5471, 2706.4133, 2708.4885, 2718.5300, 2762.5994, 2765.5881, 2767.5010, 2771.5437, 2777.7188, 2811.6846, 2814.6851, 2846.6375, 2869.6453, 2872.5757, 2884.6123, 2888.6077, 2892.6868, 2894.7185, 2902.6804, 2918.6406, 2926.6814, 2946.7546, 2994.8018, 2998.7808, 3003.8159,

3005.8979, 3054.8286, 3082.9351, 3102.9441, 3105.8228, 3127.0818, 3171.0554, 3194.9565, 3202.8757, 3207.9922, 3249.0452, 3263.1082, 3264.1118, 3266.0825, 3300.1990, 3317.0972, 3323.0837, 3331.1343, 3335.1436, 3336.1438, 3348.9949, 3354.0869, 3358.1614, 3371.1931, 3387.2393, 3454.9927, 3459.2039, 3490.2356, 3504.3723, 3518.1758, 3640.3601, 3652.4072, 3664.4243, 3822.6233, 3870.6184, 3871.6218, 3887.6787, 4089.8884, 4148.9229, 4160.9209, 4164.9634, 4175.9526, 4306.0547, 4453.3716, 4466.3892, 4680.6270, 4846.6348

16. Q2KIC7 **Mass:** 35806 **Score:** 18 **Expect:** 3.8e+002 **Matches:** 5

Serine/threonine-protein phosphatase OS=Bos taurus GN=PPP6C PE=2 SV=2

Observed	Mr(expt)	Mr(calc)	ppm	Start	End	Miss	Peptide
832.3378	831.3306	831.3473	-20.13	246	251	0	K.FMFDEK.L + Oxidation (M)
1255.5890	1254.5817	1254.6101	-22.59	265	275	0	R.CGNIASIMVFK.D + Oxidation (M)
1295.7034	1294.6961	1294.7106	-11.15	107	117	1	R.ITLLRGNHESR.Q
1427.7550	1426.7477	1426.7569	-6.40	220	231	0	K.VTNEFVHINNLK.L
1628.8262	1627.8189	1627.7817	22.9	252	264	0	K.LVTVWSAPNYCYR.C

No match to: 712.3012, 714.3622, 717.4536, 722.4728, 735.4618, 756.5057, 772.4633, 779.5103, 792.4439, 795.4440, 804.3116, 806.4917, 807.4467, 809.4788, 812.4258, 814.4372, 815.4436, 819.4216, 828.4171, 831.4965, 834.3423, 841.4608, 842.4964, 845.4365, 871.5104, 883.4921, 888.4818, 891.4986, 893.9725, 894.4754, 900.5499, 913.5269, 918.5532, 929.4499, 935.4841, 945.4980, 947.4821, 949.5173, 959.4860, 963.4728, 967.4305, 969.4643, 972.4830, 974.4501, 975.4561, 976.4594, 977.4788, 978.5386, 981.4812, 983.4774, 988.4600, 989.6110, 995.4939, 996.4700, 997.4576, 999.4598, 1007.5580, 1011.4736, 1018.5132, 1019.5350, 1021.5362, 1027.4982, 1032.5875, 1033.5684, 1034.5756, 1036.5582, 1044.5192, 1047.5377, 1055.5199, 1056.5244, 1057.5576, 1060.5359, 1062.5424, 1069.5072, 1074.5400, 1077.5109, 1085.5363, 1087.5255, 1091.5013, 1103.5313, 1106.5183, 1107.5238, 1111.5382, 1122.5424, 1123.5519, 1168.5806, 1173.6044, 1178.6016, 1179.5861, 1185.6279, 1187.6195, 1199.6720, 1207.6029, 1209.5723, 1215.6453, 1216.6425, 1226.5743, 1234.5945, 1244.5957, 1248.6305, 1301.5924, 1314.6890, 1322.5939, 1325.6377, 1328.6476, 1336.6866, 1337.6793, 1338.6221, 1342.6494, 1344.6713, 1345.6792, 1349.6682, 1352.6564, 1353.6359, 1355.6598, 1361.6550, 1368.6198, 1370.6490, 1372.5659, 1381.7262, 1383.6938, 1386.6838, 1394.6993, 1397.7117, 1432.6827, 1433.6854, 1442.7830, 1446.7039, 1447.6959, 1457.7043, 1473.7709, 1478.7163, 1497.8370, 1528.6653, 1537.7870, 1542.8158, 1550.9301, 1556.8483, 1578.7511, 1601.8662, 1606.8096, 1615.8414, 1616.8064, 1617.7891, 1627.7168, 1628.6741, 1638.8353, 1642.6895, 1647.8445, 1650.8495, 1679.9406, 1680.7981, 1696.8021, 1706.8348, 1715.8130, 1717.8778, 1718.8860, 1725.8102, 1727.8582, 1730.8500, 1752.8656, 1763.7885, 1768.9357, 1769.9312, 1771.0000, 1775.8153, 1777.8390, 1786.8839, 1791.8882, 1799.8864, 1800.8976, 1809.0026, 1914.9941, 1928.0222, 1953.8669, 1975.9434, 1982.0046, 2012.9734, 2027.1388, 2042.0795, 2053.0103, 2060.0637, 2070.9099, 2082.9165, 2084.9246, 2086.9263, 2095.0337, 2096.9631, 2098.9319, 2100.9241, 2136.9219, 2155.1025, 2170.1101, 2209.0732, 2211.1094, 2239.2014, 2251.1997, 2289.1399, 2293.2251, 2324.1719, 2343.2795, 2381.2776, 2384.2988, 2398.2314, 2400.1594, 2414.1807, 2428.1990, 2431.1982, 2444.2200, 2465.3230, 2528.3064, 2542.3503, 2546.3127, 2558.3843, 2647.1560, 2661.1709, 2675.1926, 2698.5471, 2706.4133, 2708.4885, 2718.5300, 2762.5994, 2765.5881, 2767.5010, 2771.5437, 2777.7188, 2811.6846, 2814.6851, 2846.6375, 2869.6453, 2872.5757, 2884.6123, 2888.6077, 2892.6868, 2894.7185, 2902.6804, 2918.6406, 2926.6814, 2946.7546, 2994.8018, 2998.7808, 3003.8159, 3005.8979, 3054.8286, 3082.9351, 3102.9441, 3105.8228, 3127.0818, 3171.0554, 3194.9565, 3202.8757, 3207.9922, 3249.0452, 3263.1082, 3264.1118, 3266.0825, 3300.1990, 3317.0972, 3323.0837, 3331.1343, 3335.1436, 3336.1438, 3348.9949, 3354.0869, 3358.1614, 3371.1931, 3387.2393, 3454.9927, 3459.2039, 3490.2356, 3504.3723, 3518.1758, 3640.3601, 3652.4072, 3664.4243, 3822.6233, 3870.6184, 3871.6218, 3887.6787, 4089.8884, 4148.9229, 4160.9209, 4164.9634, 4175.9526, 4306.0547, 4453.3716, 4466.3892, 4680.6270, 4846.6348

17. Q0VD05 **Mass:** 35820 **Score:** 18 **Expect:** 3.8e+002 **Matches:** 4

Voltage-dependent calcium channel gamma-3 subunit OS=Bos taurus GN=CACNG3 PE=2 SV=1

Observed	Mr(expt)	Mr(calc)	ppm	Start	End	Miss	Peptide
1353.6359	1352.6286	1352.6532	-18.15	42	53	2	R.TKSTSDNETSRK.N
1647.8445	1646.8372	1646.8298	4.53	270	284	1	R.DPSKITMGTLLNSDR.D
4148.9229	4147.9156	4147.9992	-20.15	2	37	2	M.RMCDRGVQMLITTVGAFAAFSLMTIAVGTDYWLYSR.G + 3 Oxidation (M)
4453.3716	4452.3643	4452.3099	12.2	171	208	2	K.KSYSYGWSFYFGAFSFIIAEIVGVVAVHIYIEKHQQLR.A

No match to: 712.3012, 714.3622, 717.4536, 722.4728, 735.4618, 756.5057, 772.4633, 779.5103, 792.4439, 795.4440, 804.3116, 806.4917, 807.4467, 809.4788, 812.4258, 814.4372, 815.4436, 819.4216, 828.4171, 831.4965, 832.3378, 834.3423, 841.4608, 842.4964, 845.4365, 871.5104, 883.4921, 888.4818, 891.4986, 893.9725, 894.4754, 900.5499, 913.5269, 918.5532, 929.4499, 935.4841, 945.4980, 947.4821, 949.5173, 959.4860, 963.4728, 967.4305, 969.4643, 972.4830, 974.4501, 975.4561, 976.4594, 977.4788, 978.5386, 981.4812, 983.4774, 988.4600, 989.6110, 995.4939, 996.4700, 997.4576, 999.4598, 1007.5580, 1011.4736, 1018.5132, 1019.5350, 1021.5362, 1027.4982, 1032.5875, 1033.5684, 1034.5756, 1036.5582, 1044.5192, 1047.5377, 1055.5199, 1056.5244, 1057.5576, 1060.5359, 1062.5424, 1069.5072, 1074.5400, 1077.5109, 1085.5363, 1087.5255, 1091.5013, 1103.5313, 1106.5183, 1107.5238, 1111.5382, 1122.5424, 1123.5519, 1168.5806, 1173.6044, 1178.6016, 1179.5861, 1185.6279, 1187.6195, 1199.6720, 1207.6029, 1209.5723, 1215.6453, 1216.6425, 1226.5743, 1234.5945, 1244.5957, 1248.6305, 1255.5890, 1295.7034, 1301.5924, 1314.6890, 1322.5939, 1325.6377, 1328.6476, 1336.6866, 1337.6793, 1338.6221, 1342.6494, 1344.6713, 1345.6792, 1349.6682, 1352.6564, 1355.6598, 1361.6550, 1368.6198, 1370.6490, 1372.5659, 1381.7262, 1383.6938, 1386.6838, 1394.6993, 1397.7117, 1427.7550, 1432.6827, 1433.6854, 1442.7830, 1446.7039, 1447.6959, 1457.7043, 1473.7709, 1478.7163, 1497.8370, 1528.6653, 1537.7870, 1542.8158, 1550.9301, 1556.8483, 1578.7511, 1601.8662, 1606.8096, 1615.8414, 1616.8064, 1617.7891, 1627.7168, 1628.6741, 1628.8262, 1638.8353, 1642.6895, 1650.8495, 1679.9406, 1680.7981, 1696.8021, 1706.8348, 1715.8130, 1717.8778, 1718.8860, 1725.8102, 1727.8582, 1730.8500, 1752.8656, 1763.7885, 1768.9357, 1769.9312, 1771.0000, 1775.8153, 1777.8390, 1786.8839, 1788.4598, 1791.8882, 1799.8864, 1800.8976, 1809.0026, 1914.9941, 1928.0222, 1953.8669, 1975.9434, 1982.0046, 2012.9734, 2027.1388, 2042.0795, 2053.0103, 2060.0637, 2070.9099, 2082.9165, 2084.9246, 2086.9263, 2095.0337, 2096.9631, 2098.9319, 2100.9241, 2136.9219, 2155.1025, 2170.1101, 2209.0732, 2211.1094, 2239.2014, 2251.1997, 2289.1399, 2293.2251, 2324.1719, 2343.2795, 2381.2776, 2384.2988, 2398.2314, 2400.1594, 2414.1807, 2428.1990, 2431.1982, 2444.2200, 2465.3230, 2528.3064, 2542.3503, 2546.3127, 2558.3843, 2647.1560, 2661.1709, 2675.1926, 2698.5471, 2706.4133, 2708.4885, 2718.5300, 2762.5994, 2765.5881, 2767.5010, 2771.5437, 2777.7188, 2811.6846, 2814.6851, 2846.6375, 2869.6453, 2872.5757, 2884.6123, 2888.6077, 2892.6868, 2894.7185, 2902.6804, 2918.6406, 2926.6814, 2946.7546, 2994.8018, 2998.7808, 3003.8159, 3005.8979, 3054.8286, 3082.9351, 3102.9441, 3105.8228, 3127.0818, 3171.0554, 3194.9565, 3202.8757, 3207.9922, 3249.0452, 3263.1082, 3264.1118, 3266.0825, 3300.1990, 3317.0972, 3323.0837, 3331.1343, 3335.1436, 3336.1438, 3348.9949, 3354.0869, 3358.1614, 3371.1931, 3387.2393, 3454.9927, 3459.2039, 3490.2356, 3504.3723, 3518.1758, 3640.3601, 3652.4072, 3664.4243, 3822.6233, 3870.6184, 3871.6218, 3887.6787, 4089.8884, 4160.9209, 4164.9634, 4175.9526, 4306.0547, 4466.3892, 4680.6270, 4846.6348

18. F1MJI7 **Mass:** 69096 **Score:** 18 **Expect:** 3.8e+002 **Matches:** 14

Uncharacterized protein OS=Bos taurus GN=LMNB2 PE=3 SV=1

Observed	Mr(expt)	Mr(calc)	ppm	Start	End	Miss	Peptide
959.4860	958.4787	958.4719	7.10	56	63	0	R.ALELENDR.L
975.4561	974.4488	974.4570	-8.43	488	495	0	K.DQSLGNWR.I
1060.5359	1059.5286	1059.5382	-9.07	246	254	0	K.MAQALEELR.A
1074.5400	1073.5327	1073.5465	-12.85	96	104	1	R.VLDETARDR.A
1173.6044	1172.5971	1172.5785	15.9	38	46	1	K.EELRELNDR.L
1215.6453	1214.6380	1214.6367	1.06	128	138	1	K.REGELTVAQGR.V
1216.6425	1215.6352	1215.6360	-0.64	488	497	1	K.DQSLGNWRIK.R
1314.6890	1313.6817	1313.7052	-17.84	129	140	1	R.EGELTVAQGRVR.D
1433.6854	1432.6781	1432.6906	-8.71	281	294	1	K.LSSDQNDKAASAAR.E
1447.6959	1446.6886	1446.6950	-4.42	276	288	1	K.LDNAKLSSDQNDK.A
1768.9357	1767.9284	1767.9189	5.40	302	316	1	R.MRVESLSYQLSSLQK.Q
1769.9312	1768.9239	1768.9332	-5.26	42	55	2	R.ELNDRLAHYIDRVR.A
2042.0795	2041.0722	2041.0340	18.7	38	53	2	K.EELRELNDRLAHYIDR.V
4148.9229	4147.9156	4147.8479	16.3	569	605	2	R.TVKQSSVVRETENGEEGEDEAAEFGEEDLFHQQGDPR.T

No match to: 712.3012, 714.3622, 717.4536, 722.4728, 735.4618, 756.5057, 772.4633, 779.5103, 792.4439, 795.4440, 804.3116, 806.4917, 807.4467, 809.4788, 812.4258, 814.4372, 815.4436, 819.4216, 828.4171, 831.4965, 832.3378, 834.3423, 841.4608, 842.4964, 845.4365, 871.5104, 883.4921, 888.4818, 891.4986, 893.9725, 894.4754, 900.5499, 913.5269, 918.5532, 929.4499, 935.4841, 945.4980, 947.4821, 949.5173, 963.4728, 967.4305, 969.4643, 972.4830, 974.4501, 976.4594, 977.4788, 978.5386, 981.4812, 983.4774, 988.4600, 989.6110, 995.4939, 996.4700, 997.4576, 999.4598, 1007.5580, 1011.4736, 1018.5132, 1019.5350, 1021.5362, 1027.4982, 1032.5875, 1033.5684, 1034.5756, 1036.5582, 1044.5192, 1047.5377, 1055.5199, 1056.5244, 1057.5576, 1062.5424, 1069.5072, 1077.5109, 1085.5363,

1087.5255, 1091.5013, 1103.5313, 1106.5183, 1107.5238, 1111.5382, 1122.5424, 1123.5519, 1168.5806, 1178.6016, 1179.5861, 1185.6279, 1187.6195, 1199.6720, 1207.6029, 1209.5723, 1226.5743, 1234.5945, 1244.5957, 1248.6305, 1255.5890, 1295.7034, 1301.5924, 1322.5939, 1325.6377, 1328.6476, 1336.6866, 1337.6793, 1338.6221, 1342.6494, 1344.6713, 1345.6792, 1349.6682, 1352.6564, 1353.6359, 1355.6598, 1361.6550, 1368.6198, 1370.6490, 1372.5659, 1381.7262, 1383.6938, 1386.6838, 1394.6993, 1397.7117, 1427.7550, 1432.6827, 1442.7830, 1446.7039, 1457.7043, 1473.7709, 1478.7163, 1497.8370, 1528.6653, 1537.7870, 1542.8158, 1550.9301, 1556.8483, 1578.7511, 1601.8662, 1606.8096, 1615.8414, 1616.8064, 1617.7891, 1627.7168, 1628.6741, 1628.8262, 1638.8353, 1642.6895, 1647.8445, 1650.8495, 1679.9406, 1680.7981, 1696.8021, 1706.8348, 1715.8130, 1717.8778, 1718.8860, 1725.8102, 1727.8582, 1730.8500, 1752.8656, 1763.7885, 1771.0000, 1775.8153, 1777.8390, 1786.8839, 1788.4598, 1791.8882, 1799.8864, 1800.8976, 1809.0026, 1914.9941, 1928.0222, 1953.8669, 1975.9434, 1982.0046, 2012.9734, 2027.1388, 2053.0103, 2060.0637, 2070.9099, 2082.9165, 2084.9246, 2086.9263, 2095.0337, 2096.9631, 2098.9319, 2100.9241, 2136.9219, 2155.1025, 2170.1101, 2209.0732, 2211.1094, 2239.2014, 2251.1997, 2289.1399, 2293.2251, 2324.1719, 2343.2795, 2381.2776, 2384.2988, 2398.2314, 2400.1594, 2414.1807, 2428.1990, 2431.1982, 2444.2200, 2465.3230, 2528.3064, 2542.3503, 2546.3127, 2558.3843, 2647.1560, 2661.1709, 2675.1926, 2698.5471, 2706.4133, 2708.4885, 2718.5300, 2762.5994, 2765.5881, 2767.5010, 2771.5437, 2777.7188, 2811.6846, 2814.6851, 2846.6375, 2869.6453, 2872.5757, 2884.6123, 2888.6077, 2892.6868, 2894.7185, 2902.6804, 2918.6406, 2926.6814, 2946.7546, 2994.8018, 2998.7808, 3003.8159, 3005.8979, 3054.8286, 3082.9351, 3102.9441, 3105.8228, 3127.0818, 3171.0554, 3194.9565, 3202.8757, 3207.9922, 3249.0452, 3263.1082, 3264.1118, 3266.0825, 3300.1990, 3317.0972, 3323.0837, 3331.1343, 3335.1436, 3336.1438, 3348.9949, 3354.0869, 3358.1614, 3371.1931, 3387.2393, 3454.9927, 3459.2039, 3490.2356, 3504.3723, 3518.1758, 3640.3601, 3652.4072, 3664.4243, 3822.6233, 3870.6184, 3871.6218, 3887.6787, 4089.8884, 4160.9209, 4164.9634, 4175.9526, 4306.0547, 4453.3716, 4466.3892, 4680.6270, 4846.6348

19. F1MZ00 **Mass:** 14021 **Score:** 18 **Expect:** 4.1e+002 **Matches:** 5

Uncharacterized protein OS=Bos taurus GN=SNRPD3 PE=4 SV=2

Observed	Mr(expt)	Mr(calc)	ppm	Start	End	Miss	Peptide
1019.5350	1018.5277	1018.5303	-2.55	79 -	87	1	K.NAPMLKSMK.N
1123.5519	1122.5446	1122.5604	-14.06	115 -	124	1	R.GMGRGNIFQK.R + Oxidation (M)
1173.6044	1172.5971	1172.6010	-3.32	88 -	99	2	K.NKNQGSGAGRGK.A
1336.6866	1335.6793	1335.6830	-2.74	113 -	124	2	R.GRGMGRGNIFQK.R + Oxidation (M)
2414.1807	2413.1734	2413.1332	16.7	9 -	29	0	K.VLHEAEGHIVTCETNTGEVYR.G

No match to: 712.3012, 714.3622, 717.4536, 722.4728, 735.4618, 756.5057, 772.4633, 779.5103, 792.4439, 795.4440, 804.3116, 806.4917, 807.4467, 809.4788, 812.4258, 814.4372, 815.4436, 819.4216, 828.4171, 831.4965, 832.3378, 834.3423, 841.4608, 842.4964, 845.4365, 871.5104, 883.4921, 888.4818, 891.4986, 893.9725, 894.4754, 900.5499, 913.5269, 918.5532, 929.4499, 935.4841, 945.4980, 947.4821, 949.5173, 959.4860, 963.4728, 967.4305, 969.4643, 972.4830, 974.4501, 975.4561, 976.4594, 977.4788, 978.5386, 981.4812, 983.4774, 988.4600, 989.6110, 995.4939, 996.4700, 997.4576, 999.4598, 1007.5580, 1011.4736, 1018.5132, 1021.5362, 1027.4982, 1032.5875, 1033.5684, 1034.5756, 1036.5582, 1044.5192, 1047.5377, 1055.5199, 1056.5244, 1057.5576, 1060.5359, 1062.5424, 1069.5072, 1074.5400, 1077.5109, 1085.5363, 1087.5255, 1091.5013, 1103.5313, 1106.5183, 1107.5238, 1111.5382, 1122.5424, 1168.5806, 1178.6016, 1179.5861, 1185.6279, 1187.6195, 1199.6720, 1207.6029, 1209.5723, 1215.6453, 1216.6425, 1226.5743, 1234.5945, 1244.5957, 1248.6305, 1255.5890, 1295.7034, 1301.5924, 1314.6890, 1322.5939, 1325.6377, 1328.6476, 1337.6793, 1338.6221, 1342.6494, 1344.6713, 1345.6792, 1349.6682, 1352.6564, 1353.6359, 1355.6598, 1361.6550, 1368.6198, 1370.6490, 1372.5659, 1381.7262, 1383.6938, 1386.6838, 1394.6993, 1397.7117, 1427.7550, 1432.6827, 1433.6854, 1442.7830, 1446.7039, 1447.6959, 1457.7043, 1473.7709, 1478.7163, 1497.8370, 1528.6653, 1537.7870, 1542.8158, 1550.9301, 1556.8483, 1578.7511, 1601.8662, 1606.8096, 1615.8414, 1616.8064, 1617.7891, 1627.7168, 1628.6741, 1628.8262, 1638.8353, 1642.6895, 1647.8445, 1650.8495, 1679.9406, 1680.7981, 1696.8021, 1706.8348, 1715.8130, 1717.8778, 1718.8860, 1725.8102, 1727.8582, 1730.8500, 1752.8656, 1763.7885, 1768.9357, 1769.9312, 1771.0000, 1775.8153, 1777.8390, 1786.8839, 1788.4598, 1791.8882, 1799.8864, 1800.8976, 1809.0026, 1914.9941, 1928.0222, 1953.8669, 1975.9434, 1982.0046, 2012.9734, 2042.0795, 2053.0103, 2060.0637, 2070.9099, 2082.9165, 2084.9246, 2086.9263, 2095.0337, 2096.9631, 2098.9319, 2100.9241, 2136.9219, 2155.1025, 2170.1101, 2209.0732, 2211.1094, 2239.2014, 2251.1997, 2289.1399, 2293.2251, 2324.1719, 2343.2795, 2381.2776, 2384.2988, 2398.2314, 2400.1594, 2428.1990, 2431.1982, 2444.2200, 2465.3230, 2528.3064, 2542.3503, 2546.3127, 2558.3843, 2647.1560, 2661.1709, 2675.1926, 2698.5471, 2706.4133, 2708.4885, 2718.5300, 2762.5994, 2765.5881, 2767.5010, 2771.5437, 2777.7188, 2811.6846, 2814.6851, 2846.6375, 2869.6453, 2872.5757, 2884.6123, 2888.6077, 2892.6868, 2894.7185, 2902.6804, 2918.6406, 2926.6814, 2946.7546, 2994.8018, 2998.7808, 3003.8159, 3005.8979, 3054.8286, 3082.9351, 3102.9441, 3105.8228, 3127.0818, 3171.0554, 3194.9565, 3202.8757, 3207.9922, 3249.0452, 3263.1082, 3264.1118, 3266.0825, 3300.1990, 3317.0972, 3323.0837, 3331.1343, 3335.1436, 3336.1438, 3348.9949, 3354.0869, 3358.1614, 3371.1931, 3387.2393, 3454.9927, 3459.2039, 3490.2356, 3504.3723, 3518.1758, 3640.3601, 3652.4072, 3664.4243, 3822.6233, 3870.6184, 3871.6218, 3887.6787, 4089.8884, 4148.9229, 4160.9209, 4164.9634, 4175.9526, 4306.0547, 4453.3716, 4466.3892, 4680.6270, 4846.6348

20. F1MYX1 **Mass:** 71264 **Score:** 18 **Expect:** 4.1e+002 **Matches:** 13

Uncharacterized protein OS=Bos taurus GN=ZNF782 PE=4 SV=2

Observed	Mr(expt)	Mr(calc)	ppm	Start	End	Miss	Peptide
988.4600	987.4527	987.4410	11.9	209 -	216	0	K.FGENQHEK.S
1034.5756	1033.5683	1033.5669	1.41	438 -	446	1	K.AFGQKSQLR.G
1056.5244	1055.5171	1055.5110	5.83	201 -	208	0	K.MKPYEFNK.F
1060.5359	1059.5286	1059.5349	-5.92	563 -	571	0	R.THTGEKPYK.C
1074.5400	1073.5327	1073.5539	-19.75	274 -	282	0	K.TSCLIQPQK.S
1355.6598	1354.6525	1354.6373	11.2	404 -	414	2	K.CDECGKAFKLK.S
1381.7262	1380.7189	1380.7323	-9.67	184 -	195	1	K.DFLEKTALVMSK.S
1397.7117	1396.7044	1396.7272	-16.30	184 -	195	1	K.DFLEKTALVMSK.S + Oxidation (M)
1606.8096	1605.8023	1605.7973	3.12	196 -	208	1	K.STHPKMKPYEFNK.F
1953.8669	1952.8596	1952.8940	-17.62	254 -	269	1	K.KTQIMLKPCACNECGK.S + Oxidation (M)
2012.9734	2011.9661	2011.9382	13.9	138 -	155	2	K.DGRTNTGENSFVCSKNVK.T
2060.0637	2059.0564	2059.0824	-12.60	471 -	487	2	K.SNLRVHHRTHTGEKPYK.C
2084.9246	2083.9173	2083.9316	-6.86	238 -	253	2	K.YECTKNRNNFTCNVHK.K

No match to: 712.3012, 714.3622, 717.4536, 722.4728, 735.4618, 756.5057, 772.4633, 779.5103, 792.4439, 795.4440, 804.3116, 806.4917, 807.4467, 809.4788, 812.4258, 814.4372, 815.4436, 819.4216, 828.4171, 831.4965, 832.3378, 834.3423, 841.4608, 842.4964, 845.4365, 871.5104, 883.4921, 888.4818, 891.4986, 893.9725, 894.4754, 900.5499, 913.5269, 918.5532, 929.4499, 935.4841, 945.4980, 947.4821, 949.5173, 959.4860, 963.4728, 967.4305, 969.4643, 972.4830, 974.4501, 975.4561, 976.4594, 977.4788, 978.5386, 981.4812, 983.4774, 989.6110, 995.4939, 996.4700, 997.4576, 999.4598, 1007.5580, 1011.4736, 1018.5132, 1019.5350, 1021.5362, 1027.4982, 1032.5875, 1033.5684, 1036.5582, 1044.5192, 1047.5377, 1055.5199, 1057.5576, 1062.5424, 1069.5072, 1077.5109, 1085.5363, 1087.5255, 1091.5013, 1103.5313, 1106.5183, 1107.5238, 1111.5382, 1122.5424, 1123.5519, 1168.5806, 1173.6044, 1178.6016, 1179.5861, 1185.6279, 1187.6195, 1199.6720, 1207.6029, 1209.5723, 1215.6453, 1216.6425, 1226.5743, 1234.5945, 1244.5957, 1248.6305, 1255.5890, 1295.7034, 1301.5924, 1314.6890, 1322.5939, 1325.6377, 1328.6476, 1336.6866, 1337.6793, 1338.6221, 1342.6494, 1344.6713, 1345.6792, 1349.6682, 1352.6564, 1353.6359, 1361.6550, 1368.6198, 1370.6490, 1372.5659, 1383.6938, 1386.6838, 1394.6993, 1427.7550, 1432.6827, 1433.6854, 1442.7830, 1446.7039, 1447.6959, 1457.7043, 1473.7709, 1478.7163, 1497.8370, 1528.6653, 1537.7870, 1542.8158, 1550.9301, 1556.8483, 1578.7511, 1601.8662, 1615.8414, 1616.8064, 1617.7891, 1627.7168, 1628.6741, 1628.8262, 1638.8353, 1642.6895, 1647.8445, 1650.8495, 1679.9406, 1680.7981, 1696.8021, 1706.8348, 1715.8130, 1717.8778, 1718.8860, 1725.8102, 1727.8582, 1730.8500, 1752.8656, 1763.7885, 1768.9357, 1769.9312, 1771.0000, 1775.8153, 1777.8390, 1786.8839, 1788.4598, 1791.8882, 1799.8864, 1800.8976, 1809.0026, 1914.9941, 1928.0222, 1975.9434, 1982.0046, 2027.1388, 2042.0795, 2053.0103, 2070.9099, 2082.9165, 2086.9263, 2095.0337, 2096.9631, 2098.9319, 2100.9241, 2136.9219, 2155.1025, 2170.1101, 2209.0732, 2211.1094, 2239.2014, 2251.1997, 2289.1399, 2293.2251, 2324.1719, 2343.2795, 2381.2776, 2384.2988, 2398.2314, 2400.1594, 2414.1807, 2428.1990, 2431.1982, 2444.2200, 2465.3230, 2528.3064, 2542.3503, 2546.3127, 2558.3843, 2647.1560, 2661.1709, 2675.1926, 2698.5471, 2706.4133, 2708.4885, 2718.5300, 2762.5994, 2765.5881, 2767.5010, 2771.5437, 2777.7188, 2811.6846, 2814.6851, 2846.6375, 2869.6453, 2872.5757, 2884.6123, 2888.6077, 2892.6868, 2894.7185, 2902.6804, 2918.6406, 2926.6814, 2946.7546, 2994.8018, 2998.7808, 3003.8159, 3005.8979, 3054.8286, 3082.9351, 3102.9441, 3105.8228, 3127.0818, 3171.0554, 3194.9565, 3202.8757, 3207.9922, 3249.0452, 3263.1082, 3264.1118, 3266.0825, 3300.1990, 3317.0972, 3323.0837, 3331.1343, 3335.1436, 3336.1438, 3348.9949, 3354.0869, 3358.1614, 3371.1931, 3387.2393, 3454.9927, 3459.2039, 3490.2356, 3504.3723, 3518.1758, 3640.3601, 3652.4072, 3664.4243, 3822.6233, 3870.6184, 3871.6218, 3887.6787, 4089.8884, 4148.9229, 4160.9209, 4164.9634, 4175.9526, 4306.0547, 4453.3716, 4466.3892, 4680.6270, 4846.6348

21. Q95LI3 **Mass:** 92224 **Score:** 18 **Expect:** 4.2e+002 **Matches:** 12

Zinc finger Y-chromosomal protein OS=Bos taurus GN=ZFY PE=2 SV=1

Observed	Mr(expt)	Mr(calc)	ppm	Start	End	Miss	Peptide
792.4439	791.4366	791.4389	-2.81	476	482	1	K.LTSKSEK.A
795.4440	794.4367	794.4221	18.3	720	725	2	R.CKKGFR.Q
935.4841	934.4768	934.4695	7.84	712	718	1	K.DLPFRCK.R
969.4643	968.4570	968.4610	-4.15	733	740	1	K.HMKTHSGR.K + Oxidation (M)
1032.5875	1031.5802	1031.5698	10.1	434	441	2	R.GFLKRHMK.N + Oxidation (M)
1044.5192	1043.5119	1043.5117	0.24	682	689	2	K.GKKMHQCR.H
1060.5359	1059.5286	1059.5066	20.8	682	689	2	K.GKKMHQCR.H + Oxidation (M)
1062.5424	1061.5351	1061.5110	22.7	510	518	2	K.GASKMHKCK.F + Oxidation (M)
1314.6890	1313.6817	1313.6840	-1.74	465	475	0	K.ISLHNHLESHK.L
1352.6564	1351.6491	1351.6786	-21.78	492	503	0	K.HFSHAGALFTHK.M
1442.7830	1441.7757	1441.7790	-2.25	464	475	1	K.KISLHNHLESHK.L
4148.9229	4147.9156	4147.8876	6.75	253	290	1	K.ADPGEDDLGGTVDIVESEPENDHGVELLDQNNSIRMPR.E + Oxidation (M)

No match to: 712.3012, 714.3622, 717.4536, 722.4728, 735.4618, 756.5057, 772.4633, 779.5103, 804.3116, 806.4917, 807.4467, 809.4788, 812.4258, 814.4372, 815.4436, 819.4216, 828.4171, 831.4965, 832.3378, 834.3423, 841.4608, 842.4964, 845.4365, 871.5104, 883.4921, 888.4818, 891.4986, 893.9725, 894.4754, 900.5499, 913.5269, 918.5532, 929.4499, 945.4980, 947.4821, 949.5173, 959.4860, 963.4728, 967.4305, 972.4830, 974.4501, 975.4561, 976.4594, 977.4788, 978.5386, 981.4812, 983.4774, 988.4600, 989.6110, 995.4939, 996.4700, 997.4576, 999.4598, 1007.5580, 1011.4736, 1018.5132, 1019.5350, 1021.5362, 1027.4982, 1033.5684, 1034.5756, 1036.5582, 1047.5377, 1055.5199, 1056.5244, 1057.5576, 1069.5072, 1074.5400, 1077.5109, 1085.5363, 1087.5255, 1091.5013, 1103.5313, 1106.5183, 1107.5238, 1111.5382, 1122.5424, 1123.5519, 1168.5806, 1173.6044, 1178.6016, 1179.5861, 1185.6279, 1187.6195, 1199.6720, 1207.6029, 1209.5723, 1215.6453, 1216.6425, 1226.5743, 1234.5945, 1244.5957, 1248.6305, 1255.5890, 1295.7034, 1301.5924, 1322.5939, 1325.6377, 1328.6476, 1336.6866, 1337.6793, 1338.6221, 1342.6494, 1344.6713, 1345.6792, 1349.6682, 1353.6359, 1355.6598, 1361.6550, 1368.6198, 1370.6490, 1372.5659, 1381.7262, 1383.6938, 1386.6838, 1394.6993, 1397.7117, 1427.7550, 1432.6827, 1433.6854, 1446.7039, 1447.6959, 1457.7043, 1473.7709, 1478.7163, 1497.8370, 1528.6653, 1537.7870, 1542.8158, 1550.9301, 1556.8483, 1578.7511, 1601.8662, 1606.8096, 1615.8414, 1616.8064, 1617.7891, 1627.7168, 1628.6741, 1628.8262, 1638.8353, 1642.6895, 1647.8445, 1650.8495, 1679.9406, 1680.7981, 1696.8021, 1706.8348, 1715.8130, 1717.8778, 1718.8860, 1725.8102, 1727.8582, 1730.8500, 1752.8656, 1763.7885, 1768.9357, 1769.9312, 1771.0000, 1775.8153, 1777.8390, 1786.8839, 1788.4598, 1791.8882, 1799.8864, 1800.8976, 1809.0026, 1914.9941, 1928.0222, 1953.8669, 1975.9434, 1982.0046, 2012.9734, 2027.1388, 2042.0795, 2053.0103, 2060.0637, 2070.9099, 2082.9165, 2084.9246, 2086.9263, 2095.0337, 2096.9631, 2098.9319, 2100.9241, 2136.9219, 2155.1025, 2170.1101, 2209.0732, 2211.1094, 2239.2014, 2251.1997, 2289.1399, 2293.2251, 2324.1719, 2343.2795, 2381.2776, 2384.2988, 2398.2314, 2400.1594, 2414.1807, 2428.1990, 2431.1982, 2444.2200, 2465.3230, 2528.3064, 2542.3503, 2546.3127, 2558.3843, 2647.1560, 2661.1709, 2675.1926, 2698.5471, 2706.4133, 2708.4885, 2718.5300, 2762.5994, 2765.5881, 2767.5010, 2771.5437, 2777.7188, 2811.6846, 2814.6851, 2846.6375, 2869.6453, 2872.5757, 2884.6123, 2888.6077, 2892.6868, 2894.7185, 2902.6804, 2918.6406, 2926.6814, 2946.7546, 2994.8018, 2998.7808, 3003.8159, 3005.8979, 3054.8286, 3082.9351, 3102.9441, 3105.8228, 3127.0818, 3171.0554, 3194.9565, 3202.8757, 3207.9922, 3249.0452, 3263.1082, 3264.1118, 3266.0825, 3300.1990, 3317.0972, 3323.0837, 3331.1343, 3335.1436, 3336.1438, 3348.9949, 3354.0869, 3358.1614, 3371.1931, 3387.2393, 3454.9927, 3459.2039, 3490.2356, 3504.3723, 3518.1758, 3640.3601, 3652.4072, 3664.4243, 3822.6233, 3870.6184, 3871.6218, 3887.6787, 4089.8884, 4160.9209, 4164.9634, 4175.9526, 4306.0547, 4453.3716, 4466.3892, 4680.6270, 4846.6348

22. P51867 **Mass:** 37733 **Score:** 18 **Expect:** 4.3e+002 **Matches:** 5

Tumor necrosis factor receptor superfamily member 6 OS=Bos taurus GN=FAS PE=2 SV=1

Observed	Mr(expt)	Mr(calc)	ppm	Start	End	Miss	Peptide
935.4841	934.4768	934.4767	0.13	119	125	2	R.TRNTKCR.C
1207.6029	1206.5956	1206.6067	-9.15	224	233	0	K.YIPSIAEQMR.I
1215.6453	1214.6380	1214.6507	-10.41	213	223	0	R.QLNLTDVDLGK.Y
1606.8096	1605.8023	1605.7854	10.5	298	310	1	K.ICDIVMKDITNER.E
1628.8262	1627.8189	1627.8583	-24.18	267	279	1	K.VQLLRNWYQSHGK.K

No match to: 712.3012, 714.3622, 717.4536, 722.4728, 735.4618, 756.5057, 772.4633, 779.5103, 792.4439, 795.4440, 804.3116, 806.4917, 807.4467, 809.4788, 812.4258, 814.4372, 815.4436, 819.4216, 828.4171, 831.4965, 832.3378, 834.3423, 841.4608, 842.4964, 845.4365, 871.5104, 883.4921, 888.4818, 891.4986, 893.9725, 894.4754, 900.5499, 913.5269, 918.5532, 929.4499, 945.4980, 947.4821, 949.5173, 959.4860, 963.4728, 967.4305, 969.4643, 972.4830, 974.4501, 975.4561, 976.4594, 977.4788, 978.5386, 981.4812, 983.4774, 988.4600, 989.6110, 995.4939, 996.4700, 997.4576, 999.4598, 1007.5580, 1011.4736, 1018.5132, 1019.5350, 1021.5362, 1027.4982, 1032.5875, 1033.5684, 1034.5756, 1036.5582, 1044.5192, 1047.5377, 1055.5199, 1056.5244, 1057.5576, 1060.5359, 1062.5424, 1069.5072, 1074.5400, 1077.5109, 1085.5363, 1087.5255, 1091.5013, 1103.5313, 1106.5183, 1107.5238, 1111.5382, 1122.5424, 1123.5519, 1168.5806, 1173.6044, 1178.6016, 1179.5861, 1185.6279, 1187.6195, 1199.6720, 1209.5723, 1216.6425, 1226.5743, 1234.5945, 1244.5957, 1248.6305, 1255.5890, 1295.7034, 1301.5924, 1314.6890, 1322.5939, 1325.6377, 1328.6476, 1336.6866, 1337.6793, 1338.6221, 1342.6494, 1344.6713, 1345.6792, 1349.6682, 1352.6564, 1353.6359, 1355.6598, 1361.6550, 1368.6198, 1370.6490, 1372.5659, 1381.7262, 1383.6938, 1386.6838, 1394.6993, 1397.7117, 1427.7550, 1432.6827, 1433.6854, 1442.7830, 1446.7039, 1447.6959, 1457.7043, 1473.7709, 1478.7163, 1497.8370, 1528.6653, 1537.7870, 1542.8158, 1550.9301, 1556.8483, 1578.7511, 1601.8662, 1615.8414, 1616.8064, 1617.7891, 1627.7168, 1628.6741, 1638.8353, 1642.6895, 1647.8445, 1650.8495, 1679.9406, 1680.7981, 1696.8021, 1706.8348, 1715.8130, 1717.8778, 1718.8860, 1725.8102, 1727.8582, 1730.8500, 1752.8656, 1763.7885, 1768.9357, 1769.9312, 1771.0000, 1775.8153, 1777.8390, 1786.8839, 1788.4598, 1791.8882, 1799.8864, 1800.8976, 1809.0026, 1914.9941, 1928.0222, 1953.8669, 1975.9434, 1982.0046, 2012.9734, 2027.1388, 2042.0795, 2053.0103, 2060.0637, 2070.9099, 2082.9165, 2084.9246, 2086.9263, 2095.0337, 2096.9631, 2098.9319, 2100.9241, 2136.9219, 2155.1025, 2170.1101, 2209.0732, 2211.1094, 2239.2014, 2251.1997, 2289.1399, 2293.2251, 2324.1719, 2343.2795, 2381.2776, 2384.2988, 2398.2314, 2400.1594, 2414.1807, 2428.1990, 2431.1982, 2444.2200, 2465.3230, 2528.3064, 2542.3503, 2546.3127, 2558.3843, 2647.1560, 2661.1709, 2675.1926, 2698.5471, 2706.4133, 2708.4885, 2718.5300, 2762.5994, 2765.5881, 2767.5010, 2771.5437, 2777.7188, 2811.6846, 2814.6851, 2846.6375, 2869.6453, 2872.5757, 2884.6123, 2888.6077, 2892.6868, 2894.7185, 2902.6804, 2918.6406, 2926.6814, 2946.7546, 2994.8018, 2998.7808, 3003.8159, 3005.8979, 3054.8286, 3082.9351, 3102.9441, 3105.8228, 3127.0818, 3171.0554, 3194.9565, 3202.8757, 3207.9922, 3249.0452, 3263.1082, 3264.1118, 3266.0825, 3300.1990, 3317.0972, 3323.0837, 3331.1343, 3335.1436, 3336.1438, 3348.9949, 3354.0869, 3358.1614, 3371.1931, 3387.2393, 3454.9927, 3459.2039, 3490.2356, 3504.3723, 3518.1758, 3640.3601, 3652.4072, 3664.4243, 3822.6233, 3870.6184, 3871.6218, 3887.6787, 4089.8884, 4148.9229, 4160.9209, 4164.9634, 4175.9526, 4306.0547, 4453.3716, 4466.3892, 4680.6270, 4846.6348

23. E1BH62 **Mass:** 12539 **Score:** 18 **Expect:** 4.3e+002 **Matches:** 2

Uncharacterized protein OS=Bos taurus GN=XCL2 PE=4 SV=1

Observed	Mr(expt)	Mr(calc)	ppm	Start	End	Miss	Peptide
1173.6044	1172.5971	1172.5747	19.1	47	56	1	K.TYTIKEGSMK.A + Oxidation (M)
1325.6377	1324.6304	1324.6234	5.28	68	78	0	K.VCADPHVDWVK.K

No match to: 712.3012, 714.3622, 717.4536, 722.4728, 735.4618, 756.5057, 772.4633, 779.5103, 792.4439, 795.4440, 804.3116, 806.4917, 807.4467, 809.4788, 812.4258, 814.4372, 815.4436, 819.4216, 828.4171, 831.4965, 832.3378, 834.3423, 841.4608, 842.4964, 845.4365, 871.5104, 883.4921, 888.4818, 891.4986, 893.9725, 894.4754, 900.5499, 913.5269, 918.5532, 929.4499, 935.4841, 945.4980, 947.4821, 949.5173, 959.4860, 963.4728, 967.4305, 969.4643, 972.4830, 974.4501, 975.4561, 976.4594, 977.4788, 978.5386, 981.4812, 983.4774, 988.4600, 989.6110, 995.4939, 996.4700, 997.4576, 999.4598, 1007.5580, 1011.4736, 1018.5132, 1019.5350, 1021.5362, 1027.4982, 1032.5875, 1033.5684, 1034.5756, 1036.5582, 1044.5192, 1047.5377, 1055.5199, 1056.5244, 1057.5576, 1060.5359, 1062.5424, 1069.5072, 1074.5400, 1077.5109, 1085.5363, 1087.5255, 1091.5013, 1103.5313, 1106.5183, 1107.5238, 1111.5382, 1122.5424, 1123.5519, 1168.5806, 1178.6016, 1179.5861, 1185.6279, 1187.6195, 1199.6720, 1207.6029, 1209.5723, 1215.6453, 1216.6425, 1226.5743, 1234.5945, 1244.5957, 1248.6305, 1255.5890, 1295.7034, 1301.5924, 1314.6890, 1322.5939, 1328.6476, 1336.6866, 1337.6793, 1338.6221, 1342.6494, 1344.6713, 1345.6792, 1349.6682, 1352.6564, 1353.6359, 1355.6598, 1361.6550, 1368.6198, 1370.6490, 1372.5659, 1381.7262, 1383.6938, 1386.6838, 1394.6993, 1397.7117, 1427.7550, 1432.6827, 1433.6854, 1442.7830, 1446.7039, 1447.6959, 1457.7043, 1473.7709, 1478.7163, 1497.8370, 1528.6653, 1537.7870, 1542.8158, 1550.9301, 1556.8483, 1578.7511, 1601.8662, 1606.8096, 1615.8414, 1616.8064, 1617.7891, 1627.7168, 1628.6741, 1628.8262, 1638.8353, 1642.6895, 1647.8445, 1650.8495, 1679.9406, 1680.7981, 1696.8021, 1706.8348, 1715.8130, 1717.8778, 1718.8860, 1725.8102, 1727.8582, 1730.8500, 1752.8656, 1763.7885, 1768.9357, 1769.9312, 1771.0000, 1775.8153, 1777.8390, 1786.8839, 1788.4598, 1791.8882, 1799.8864, 1800.8976, 1809.0026, 1914.9941, 1928.0222, 1953.8669, 1975.9434, 1982.0046, 2012.9734, 2027.1388, 2042.0795, 2053.0103, 2060.0637, 2070.9099, 2082.9165, 2084.9246, 2086.9263, 2095.0337, 2096.9631, 2098.9319, 2100.9241, 2136.9219, 2155.1025, 2170.1101, 2209.0732, 2211.1094, 2239.2014, 2251.1997, 2289.1399, 2293.2251, 2324.1719, 2343.2795, 2381.2776, 2384.2988, 2398.2314, 2400.1594, 2414.1807, 2428.1990, 2431.1982, 2444.2200, 2465.3230, 2528.3064, 2542.3503, 2546.3127, 2558.3843, 2647.1560, 2661.1709, 2675.1926, 2698.5471, 2706.4133, 2708.4885, 2718.5300, 2762.5994, 2765.5881, 2767.5010, 2771.5437, 2777.7188,

2811.6846, 2814.6851, 2846.6375, 2869.6453, 2872.5757, 2884.6123, 2888.6077, 2892.6868, 2894.7185, 2902.6804, 2918.6406, 2926.6814, 2946.7546, 2994.8018, 2998.7808, 3003.8159, 3005.8979, 3054.8286, 3082.9351, 3102.9441, 3105.8228, 3127.0818, 3171.0554, 3194.9565, 3202.8757, 3207.9922, 3249.0452, 3263.1082, 3264.1118, 3266.0825, 3300.1990, 3317.0972, 3323.0837, 3331.1343, 3335.1436, 3336.1438, 3348.9949, 3354.0869, 3358.1614, 3371.1931, 3387.2393, 3454.9927, 3459.2039, 3490.2356, 3504.3723, 3518.1758, 3640.3601, 3652.4072, 3664.4243, 3822.6233, 3870.6184, 3871.6218, 3887.6787, 4089.8884, 4148.9229, 4160.9209, 4164.9634, 4175.9526, 4306.0547, 4453.3716, 4466.3892, 4680.6270, 4846.6348

24. G1K230 **Mass:** 16435 **Score:** 17 **Expect:** 4.4e+002 **Matches:** 2
Uncharacterized protein OS=Bos taurus PE=3 SV=2

Observed	Mr(expt)	Mr(calc)	ppm	Start	End	Miss	Peptide
1349.6682	1348.6609	1348.6631	-1.65	40 -	51	0	K.GVSLANWMCLAK.G
2872.5757	2871.5684	2871.5317	12.8	105 -	130	2	K.DDITQAVACAKKIVSQLGLTAWVAWK.N

No match to: 712.3012, 714.3622, 717.4536, 722.4728, 735.4618, 756.5057, 772.4633, 779.5103, 792.4439, 795.4440, 804.3116, 806.4917, 807.4467, 809.4788, 812.4258, 814.4372, 815.4436, 819.4216, 828.4171, 831.4965, 832.3378, 834.3423, 841.4608, 842.4964, 845.4365, 871.5104, 883.4921, 888.4818, 891.4986, 893.9725, 894.4754, 900.5499, 913.5269, 918.5532, 929.4499, 935.4841, 945.4980, 947.4821, 949.5173, 959.4860, 963.4728, 967.4305, 969.4643, 972.4830, 974.4501, 975.4561, 976.4594, 977.4788, 978.5386, 981.4812, 983.4774, 988.4600, 989.6110, 995.4939, 996.4700, 997.4576, 999.4598, 1007.5580, 1011.4736, 1018.5132, 1019.5350, 1021.5362, 1027.4982, 1032.5875, 1033.5684, 1034.5756, 1036.5582, 1044.5192, 1047.5377, 1055.5199, 1056.5244, 1057.5576, 1060.5359, 1062.5424, 1069.5072, 1074.5400, 1077.5109, 1085.5363, 1087.5255, 1091.5013, 1103.5313, 1106.5183, 1107.5238, 1111.5382, 1122.5424, 1123.5519, 1168.5806, 1173.6044, 1178.6016, 1179.5861, 1185.6279, 1187.6195, 1199.6720, 1207.6029, 1209.5723, 1215.6453, 1216.6425, 1226.5743, 1234.5945, 1244.5957, 1248.6305, 1255.5890, 1295.7034, 1301.5924, 1314.6890, 1322.5939, 1325.6377, 1328.6476, 1336.6866, 1337.6793, 1338.6221, 1342.6494, 1344.6713, 1345.6792, 1352.6564, 1353.6359, 1355.6598, 1361.6550, 1368.6198, 1370.6490, 1372.5659, 1381.7262, 1383.6938, 1386.6838, 1394.6993, 1397.7117, 1427.7550, 1432.6827, 1433.6854, 1442.7830, 1446.7039, 1447.6959, 1457.7043, 1473.7709, 1478.7163, 1497.8370, 1528.6653, 1537.7870, 1542.8158, 1550.9301, 1556.8483, 1578.7511, 1601.8662, 1606.8096, 1615.8414, 1616.8064, 1617.7891, 1627.7168, 1628.6741, 1628.8262, 1638.8353, 1642.6895, 1647.8445, 1650.8495, 1679.9406, 1680.7981, 1696.8021, 1706.8348, 1715.8130, 1717.8778, 1718.8860, 1725.8102, 1727.8582, 1730.8500, 1752.8656, 1763.7885, 1768.9357, 1769.9312, 1771.0000, 1775.8153, 1777.8390, 1786.8839, 1788.4598, 1791.8882, 1799.8864, 1800.8976, 1809.0026, 1914.9941, 1928.0222, 1953.8669, 1975.9434, 1982.0046, 2012.9734, 2027.1388, 2042.0795, 2053.0103, 2060.0637, 2070.9099, 2082.9165, 2084.9246, 2086.9263, 2095.0337, 2096.9631, 2098.9319, 2100.9241, 2136.9219, 2155.1025, 2170.1101, 2209.0732, 2211.1094, 2239.2014, 2251.1997, 2289.1399, 2293.2251, 2324.1719, 2343.2795, 2381.2776, 2384.2988, 2398.2314, 2400.1594, 2414.1807, 2428.1990, 2431.1982, 2444.2200, 2465.3230, 2528.3064, 2542.3503, 2546.3127, 2558.3843, 2647.1560, 2661.1709, 2675.1926, 2698.5471, 2706.4133, 2708.4885, 2718.5300, 2762.5994, 2765.5881, 2767.5010, 2771.5437, 2777.7188, 2811.6846, 2814.6851, 2846.6375, 2869.6453, 2884.6123, 2888.6077, 2892.6868, 2894.7185, 2902.6804, 2918.6406, 2926.6814, 2946.7546, 2994.8018, 2998.7808, 3003.8159, 3005.8979, 3054.8286, 3082.9351, 3102.9441, 3105.8228, 3127.0818, 3171.0554, 3194.9565, 3202.8757, 3207.9922, 3249.0452, 3263.1082, 3264.1118, 3266.0825, 3300.1990, 3317.0972, 3323.0837, 3331.1343, 3335.1436, 3336.1438, 3348.9949, 3354.0869, 3358.1614, 3371.1931, 3387.2393, 3454.9927, 3459.2039, 3490.2356, 3504.3723, 3518.1758, 3640.3601, 3652.4072, 3664.4243, 3822.6233, 3870.6184, 3871.6218, 3887.6787, 4089.8884, 4148.9229, 4160.9209, 4164.9634, 4175.9526, 4306.0547, 4453.3716, 4466.3892, 4680.6270, 4846.6348

25. Q17QX9 **Mass:** 54201 **Score:** 17 **Expect:** 4.4e+002 **Matches:** 8
Peptidyl-prolyl cis-trans isomerase CWC27 homolog OS=Bos taurus GN=CWC27 PE=2 SV=1

Observed	Mr(expt)	Mr(calc)	ppm	Start	End	Miss	Peptide
900.5499	899.5426	899.5440	-1.54	327 -	334	1	R.ELLAAKQK.K
1036.5582	1035.5509	1035.5382	12.2	460 -	467	2	R.EESKKLMR.E + Oxidation (M)
1060.5359	1059.5286	1059.5196	8.51	336 -	345	0	K.AENSAIQAEK.R
1199.6720	1198.6647	1198.6670	-1.87	227 -	237	2	K.GKSKSSHDLLK.D
1216.6425	1215.6352	1215.6207	11.9	336 -	346	1	K.AENSAIQAEKR.S
2053.0103	2052.0030	2051.9872	7.72	144 -	160	1	R.LTEVDIDDEERPRNSHK.I
4148.9229	4147.9156	4147.8804	8.49	397 -	432	1	K.LTQAIAETPENDISETEVEDDEGWMSHVLQFEDKSR.K
4164.9634	4163.9561	4163.8753	19.4	397 -	432	1	K.LTQAIAETPENDISETEVEDDEGWMSHVLQFEDKSR.K + Oxidation (M)

No match to: 712.3012, 714.3622, 717.4536, 722.4728, 735.4618, 756.5057, 772.4633, 779.5103, 792.4439, 795.4440, 804.3116, 806.4917, 807.4467, 809.4788, 812.4258, 814.4372, 815.4436, 819.4216, 828.4171, 831.4965, 832.3378, 834.3423, 841.4608, 842.4964, 845.4365, 871.5104, 883.4921, 888.4818, 891.4986, 893.9725, 894.4754, 913.5269, 918.5532, 929.4499, 935.4841, 945.4980, 947.4821, 949.5173, 959.4860, 963.4728, 967.4305, 969.4643, 972.4830, 974.4501, 975.4561, 976.4594, 977.4788, 978.5386, 981.4812, 983.4774, 988.4600, 989.6110, 995.4939, 996.4700, 997.4576, 999.4598, 1007.5580, 1011.4736, 1018.5132, 1019.5350, 1021.5362, 1027.4982, 1032.5875, 1033.5684, 1034.5756, 1044.5192, 1047.5377, 1055.5199, 1056.5244, 1057.5576, 1062.5424, 1069.5072, 1074.5400, 1077.5109, 1085.5363, 1087.5255, 1091.5013, 1103.5313, 1106.5183, 1107.5238, 1111.5382, 1122.5424, 1123.5519, 1168.5806, 1173.6044, 1178.6016, 1179.5861, 1185.6279, 1187.6195, 1207.6029, 1209.5723, 1215.6453, 1226.5743, 1234.5945, 1244.5957, 1248.6305, 1255.5890, 1295.7034, 1301.5924, 1314.6890, 1322.5939, 1325.6377, 1328.6476, 1336.6866, 1337.6793, 1338.6221, 1342.6494, 1344.6713, 1345.6792, 1349.6682, 1352.6564, 1353.6359, 1355.6598, 1361.6550, 1368.6198, 1370.6490, 1372.5659, 1381.7262, 1383.6938, 1386.6838, 1394.6993, 1397.7117, 1427.7550, 1432.6827, 1433.6854, 1442.7830, 1446.7039, 1447.6959, 1457.7043, 1473.7709, 1478.7163, 1497.8370, 1528.6653, 1537.7870, 1542.8158, 1550.9301, 1556.8483, 1578.7511, 1601.8662, 1606.8096, 1615.8414, 1616.8064, 1617.7891, 1627.7168, 1628.6741, 1628.8262, 1638.8353, 1642.6895, 1647.8445, 1650.8495, 1679.9406, 1680.7981, 1696.8021, 1706.8348, 1715.8130, 1717.8778, 1718.8860, 1725.8102, 1727.8582, 1730.8500, 1752.8656, 1763.7885, 1768.9357, 1769.9312, 1771.0000, 1775.8153, 1777.8390, 1786.8839, 1788.4598, 1791.8882, 1799.8864, 1800.8976, 1809.0026, 1914.9941, 1928.0222, 1953.8669, 1975.9434, 1982.0046, 2012.9734, 2027.1388, 2042.0795, 2060.0637, 2070.9099, 2082.9165, 2084.9246, 2086.9263, 2095.0337, 2096.9631, 2098.9319, 2100.9241, 2136.9219, 2155.1025, 2170.1101, 2209.0732, 2211.1094, 2239.2014, 2251.1997, 2289.1399, 2293.2251, 2324.1719, 2343.2795, 2381.2776, 2384.2988, 2398.2314, 2400.1594, 2414.1807, 2428.1990, 2431.1982, 2444.2200, 2465.3230, 2528.3064, 2542.3503, 2546.3127, 2558.3843, 2647.1560, 2661.1709, 2675.1926, 2698.5471, 2706.4133, 2708.4885, 2718.5300, 2762.5994, 2765.5881, 2767.5010, 2771.5437, 2777.7188, 2811.6846, 2814.6851, 2846.6375, 2869.6453, 2872.5757, 2884.6123, 2888.6077, 2892.6868, 2894.7185, 2902.6804, 2918.6406, 2926.6814, 2946.7546, 2994.8018, 2998.7808, 3003.8159, 3005.8979, 3054.8286, 3082.9351, 3102.9441, 3105.8228, 3127.0818, 3171.0554, 3194.9565, 3202.8757, 3207.9922, 3249.0452, 3263.1082, 3264.1118, 3266.0825, 3300.1990, 3317.0972, 3323.0837, 3331.1343, 3335.1436, 3336.1438, 3348.9949, 3354.0869, 3358.1614, 3371.1931, 3387.2393, 3454.9927, 3459.2039, 3490.2356, 3504.3723, 3518.1758, 3640.3601, 3652.4072, 3664.4243, 3822.6233, 3870.6184, 3871.6218, 3887.6787, 4089.8884, 4160.9209, 4175.9526, 4306.0547, 4453.3716, 4466.3892, 4680.6270, 4846.6348

26. A6QPJ3 **Mass:** 38546 **Score:** 17 **Expect:** 4.4e+002 **Matches:** 9
TRABD protein OS=Bos taurus GN=TRABD PE=2 SV=1

Observed	Mr(expt)	Mr(calc)	ppm	Start	End	Miss	Peptide
1033.5684	1032.5611	1032.5386	21.8	117 -	124	1	K.MDERTLLR.E
1173.6044	1172.5971	1172.5900	6.10	260 -	268	0	R.DVYLTYMLR.Q
1606.8096	1605.8023	1605.8225	-12.56	212 -	225	0	K.LAWGLCFLSDPISK.D
1615.8414	1614.8341	1614.8188	9.50	260 -	272	1	R.DVYLTYMLRQAAR.R + Oxidation (M)
1680.7981	1679.7908	1679.8268	-21.39	75 -	89	1	R.VYVVGTAHFSDDSKR.D
2428.1990	2427.1917	2427.2355	-18.06	29 -	50	0	R.VLPGDPQNLSDVDAFNLLLEMK.L
2444.2200	2443.2127	2443.2305	-7.26	29 -	50	0	R.VLPGDPQNLSDVDAFNLLLEMK.L + Oxidation (M)
4148.9229	4147.9156	4147.9727	-13.75	235 -	268	2	K.DLLEQMMAEMVGEFPDLHRTIVSERDVYLTYMLR.Q + 3 Oxidation (M)
4164.9634	4163.9561	4163.9676	-2.75	235 -	268	2	K.DLLEQMMAEMVGEFPDLHRTIVSERDVYLTYMLR.Q + 4 Oxidation (M)

No match to: 712.3012, 714.3622, 717.4536, 722.4728, 735.4618, 756.5057, 772.4633, 779.5103, 792.4439, 795.4440, 804.3116, 806.4917, 807.4467, 809.4788, 812.4258, 814.4372, 815.4436, 819.4216, 828.4171, 831.4965, 832.3378, 834.3423, 841.4608, 842.4964, 845.4365, 871.5104, 883.4921, 888.4818, 891.4986, 893.9725, 894.4754, 900.5499, 913.5269, 918.5532, 929.4499, 935.4841, 945.4980, 947.4821, 949.5173, 959.4860, 963.4728, 967.4305, 969.4643, 972.4830, 974.4501, 975.4561, 976.4594, 977.4788, 978.5386, 981.4812, 983.4774, 988.4600, 989.6110, 995.4939, 996.4700, 997.4576, 999.4598, 1007.5580, 1011.4736, 1018.5132, 1019.5350, 1021.5362, 1027.4982, 1032.5875, 1034.5756, 1036.5582, 1044.5192, 1047.5377, 1055.5199, 1056.5244, 1057.5576, 1060.5359, 1062.5424, 1069.5072, 1074.5400, 1077.5109, 1085.5363, 1087.5255, 1091.5013, 1103.5313, 1106.5183, 1107.5238, 1111.5382, 1122.5424, 1123.5519, 1168.5806, 1178.6016, 1179.5861, 1185.6279, 1187.6195, 1199.6720, 1207.6029, 1209.5723, 1215.6453, 1216.6425, 1226.5743, 1234.5945, 1244.5957, 1248.6305, 1255.5890, 1295.7034, 1301.5924, 1314.6890, 1322.5939, 1325.6377, 1328.6476, 1336.6866, 1337.6793, 1338.6221, 1342.6494,

1344.6713, 1345.6792, 1349.6682, 1352.6564, 1353.6359, 1355.6598, 1361.6550, 1368.6198, 1370.6490, 1372.5659, 1381.7262, 1383.6938, 1386.6838, 1394.6993, 1397.7117, 1427.7550, 1432.6827, 1433.6854, 1442.7830, 1446.7039, 1447.6959, 1457.7043, 1473.7709, 1478.7163, 1497.8370, 1528.6653, 1537.7870, 1542.8158, 1550.9301, 1556.8483, 1578.7511, 1601.8662, 1616.8064, 1617.7891, 1627.7168, 1628.6741, 1628.8262, 1638.8353, 1642.6895, 1647.8445, 1650.8495, 1679.9406, 1696.8021, 1706.8348, 1715.8130, 1717.8778, 1718.8860, 1725.8102, 1727.8582, 1730.8500, 1752.8656, 1763.7885, 1768.9357, 1769.9312, 1771.0000, 1775.8153, 1777.8390, 1786.8839, 1788.4598, 1791.8882, 1799.8864, 1800.8976, 1809.0026, 1914.9941, 1928.0222, 1953.8669, 1975.9434, 1982.0046, 2012.9734, 2027.1388, 2042.0795, 2053.0103, 2060.0637, 2070.9099, 2082.9165, 2084.9246, 2086.9263, 2095.0337, 2096.9631, 2098.9319, 2100.9241, 2136.9219, 2155.1025, 2170.1101, 2209.0732, 2211.1094, 2239.2014, 2251.1997, 2289.1399, 2293.2251, 2324.1719, 2343.2795, 2381.2776, 2384.2988, 2398.2314, 2400.1594, 2414.1807, 2431.1982, 2465.3230, 2528.3064, 2542.3503, 2546.3127, 2558.3843, 2647.1560, 2661.1709, 2675.1926, 2698.5471, 2706.4133, 2708.4885, 2718.5300, 2762.5994, 2765.5881, 2767.5010, 2771.5437, 2777.7188, 2811.6846, 2814.6851, 2846.6375, 2869.6453, 2872.5757, 2884.6123, 2888.6077, 2892.6868, 2894.7185, 2902.6804, 2918.6406, 2926.6814, 2946.7546, 2994.8018, 2998.7808, 3003.8159, 3005.8979, 3054.8286, 3082.9351, 3102.9441, 3105.8228, 3127.0818, 3171.0554, 3194.9565, 3202.8757, 3207.9922, 3249.0452, 3263.1082, 3264.1118, 3266.0825, 3300.1990, 3317.0972, 3323.0837, 3331.1343, 3335.1436, 3336.1438, 3348.9949, 3354.0869, 3358.1614, 3371.1931, 3387.2393, 3454.9927, 3459.2039, 3490.2356, 3504.3723, 3518.1758, 3640.3601, 3652.4072, 3664.4243, 3822.6233, 3870.6184, 3871.6218, 3887.6787, 4089.8884, 4160.9209, 4175.9526, 4306.0547, 4453.3716, 4466.3892, 4680.6270, 4846.6348

27. A0JNH6 **Mass:** 63280 **Score:** 17 **Expect:** 4.5e+002 **Matches:** 12

Coiled-coil domain-containing protein 102A OS=Bos taurus GN=CCDC102A PE=2 SV=1

Observed	Mr(expt)	Mr(calc)	ppm	Start	End	Miss	Peptide
807.4467	806.4394	806.4286	13.4	435 -	440	1	K.KQFQEK.V
1234.5945	1233.5872	1233.6135	-21.32	274 -	283	2	K.EREDKMALSR.N
1244.5957	1243.5884	1243.5945	-4.90	355 -	365	0	R.LQAENAAEWGR.R
1349.6682	1348.6609	1348.6591	1.36	344 -	354	2	R.KMAELRGEMER.L
1355.6598	1354.6525	1354.6333	14.2	339 -	349	2	R.SSMDRKMAELR.G + 2 Oxidation (M)
1427.7550	1426.7477	1426.7528	-3.56	385 -	397	1	R.AQVGDLEEALARR.R
1542.8158	1541.8085	1541.8161	-4.91	73 -	84	2	R.EELRLRELEEAR.A
1647.8445	1646.8372	1646.8046	19.8	79 -	92	2	R.ELEEARARAAQMEK.T + Oxidation (M)
1768.9357	1767.9284	1767.9380	-5.40	435 -	449	2	K.KQFQEKVAELAHANR.R
1786.8839	1785.8766	1785.8717	2.74	144 -	159	2	R.QEAQGESEARGRELAR.L
2084.9246	2083.9173	2083.9150	1.12	327 -	344	2	R.MSEDLEDELGARSSMDRK.M + Oxidation (M)
2100.9241	2099.9168	2099.9099	3.29	327 -	344	2	R.MSEDLEDELGARSSMDRK.M + 2 Oxidation (M)

No match to: 712.3012, 714.3622, 717.4536, 722.4728, 735.4618, 756.5057, 772.4633, 779.5103, 792.4439, 795.4440, 804.3116, 806.4917, 809.4788, 812.4258, 814.4372, 815.4436, 819.4216, 828.4171, 831.4965, 832.3378, 834.3423, 841.4608, 842.4964, 845.4365, 871.5104, 883.4921, 888.4818, 891.4986, 893.9725, 894.4754, 900.5499, 913.5269, 918.5532, 929.4499, 935.4841, 945.4980, 947.4821, 949.5173, 959.4860, 963.4728, 967.4305, 969.4643, 972.4830, 974.4501, 975.4561, 976.4594, 977.4788, 978.5386, 981.4812, 983.4774, 988.4600, 989.6110, 995.4939, 996.4700, 997.4576, 999.4598, 1007.5580, 1011.4736, 1018.5132, 1019.5350, 1021.5362, 1027.4982, 1032.5875, 1033.5684, 1034.5756, 1036.5582, 1044.5192, 1047.5377, 1055.5199, 1056.5244, 1057.5576, 1060.5359, 1062.5424, 1069.5072, 1074.5400, 1077.5109, 1085.5363, 1087.5255, 1091.5013, 1103.5313, 1106.5183, 1107.5238, 1111.5382, 1122.5424, 1123.5519, 1168.5806, 1173.6044, 1178.6016, 1179.5861, 1185.6279, 1187.6195, 1199.6720, 1207.6029, 1209.5723, 1215.6453, 1216.6425, 1226.5743, 1248.6305, 1255.5890, 1295.7034, 1301.5924, 1314.6890, 1322.5939, 1325.6377, 1328.6476, 1336.6866, 1337.6793, 1338.6221, 1342.6494, 1344.6713, 1345.6792, 1352.6564, 1353.6359, 1361.6550, 1368.6198, 1370.6490, 1372.5659, 1381.7262, 1383.6938, 1386.6838, 1394.6993, 1397.7117, 1432.6827, 1433.6854, 1442.7830, 1446.7039, 1447.6959, 1457.7043, 1473.7709, 1478.7163, 1497.8370, 1528.6653, 1537.7870, 1550.9301, 1556.8483, 1578.7511, 1601.8662, 1606.8096, 1615.8414, 1616.8064, 1617.7891, 1627.7168, 1628.6741, 1628.8262, 1638.8353, 1642.6895, 1650.8495, 1679.9406, 1680.7981, 1696.8021, 1706.8348, 1715.8130, 1717.8778, 1718.8860, 1725.8102, 1727.8582, 1730.8500, 1752.8656, 1763.7885, 1769.9312, 1771.0000, 1775.8153, 1777.8390, 1788.4598, 1791.8882, 1799.8864, 1800.8976, 1809.0026, 1914.9941, 1928.0222, 1953.8669, 1975.9434, 1982.0046, 2012.9734, 2027.1388, 2042.0795, 2053.0103, 2060.0637, 2070.9099, 2082.9165, 2086.9263, 2095.0337, 2096.9631, 2098.9319, 2136.9219, 2155.1025, 2170.1101, 2209.0732, 2211.1094, 2239.2014, 2251.1997, 2289.1399, 2293.2251, 2324.1719, 2343.2795, 2381.2776, 2384.2988, 2398.2314, 2400.1594, 2414.1807, 2428.1990, 2431.1982, 2444.2200, 2465.3230, 2528.3064, 2542.3503, 2546.3127, 2558.3843, 2647.1560, 2661.1709, 2675.1926, 2698.5471, 2706.4133, 2708.4885, 2718.5300, 2762.5994, 2765.5881, 2767.5010, 2771.5437, 2777.7188, 2811.6846, 2814.6851, 2846.6375, 2869.6453, 2872.5757, 2884.6123, 2888.6077, 2892.6868, 2894.7185, 2902.6804, 2918.6406, 2926.6814, 2946.7546, 2994.8018, 2998.7808, 3003.8159, 3005.8979, 3054.8286, 3082.9351, 3102.9441, 3105.8228, 3127.0818, 3171.0554, 3194.9565, 3202.8757, 3207.9922, 3249.0452, 3263.1082, 3264.1118, 3266.0825, 3300.1990, 3317.0972, 3323.0837, 3331.1343, 3335.1436, 3336.1438, 3348.9949, 3354.0869, 3358.1614, 3371.1931, 3387.2393, 3454.9927, 3459.2039, 3490.2356, 3504.3723, 3518.1758, 3640.3601, 3652.4072, 3664.4243, 3822.6233, 3870.6184, 3871.6218, 3887.6787, 4089.8884, 4148.9229, 4160.9209, 4164.9634, 4175.9526, 4306.0547, 4453.3716, 4466.3892, 4680.6270, 4846.6348

28. Q32LA2 **Mass:** 40503 **Score:** 17 **Expect:** 4.5e+002 **Matches:** 6

Three prime histone mRNA exonuclease 1 OS=Bos taurus GN=THEX1 PE=2 SV=1

Observed	Mr(expt)	Mr(calc)	ppm	Start	End	Miss	Peptide
935.4841	934.4768	934.4946	-19.07	209 -	215	1	K.KVIDWMK.L + Oxidation (M)
1032.5875	1031.5802	1031.5838	-3.42	210 -	217	1	K.VIDWMKLK.E
1106.5183	1105.5110	1105.5186	-6.85	41 -	49	1	R.CRLDGQETK.T
1606.8096	1605.8023	1605.7967	3.53	68 -	81	1	K.EIAITNGCINRMSK.E
1982.0046	1980.9973	1980.9501	23.9	2 -	19	1	M.EDEQTLQPGDEAVAPARR.A
2084.9246	2083.9173	2083.9341	-8.05	19 -	37	1	R.RASPGSECGDEAQRPNPEK.K

No match to: 712.3012, 714.3622, 717.4536, 722.4728, 735.4618, 756.5057, 772.4633, 779.5103, 792.4439, 795.4440, 804.3116, 806.4917, 807.4467, 809.4788, 812.4258, 814.4372, 815.4436, 819.4216, 828.4171, 831.4965, 832.3378, 834.3423, 841.4608, 842.4964, 845.4365, 871.5104, 883.4921, 888.4818, 891.4986, 893.9725, 894.4754, 900.5499, 913.5269, 918.5532, 929.4499, 945.4980, 947.4821, 949.5173, 959.4860, 963.4728, 967.4305, 969.4643, 972.4830, 974.4501, 975.4561, 976.4594, 977.4788, 978.5386, 981.4812, 983.4774, 988.4600, 989.6110, 995.4939, 996.4700, 997.4576, 999.4598, 1007.5580, 1011.4736, 1018.5132, 1019.5350, 1021.5362, 1027.4982, 1033.5684, 1034.5756, 1036.5582, 1044.5192, 1047.5377, 1055.5199, 1056.5244, 1057.5576, 1060.5359, 1062.5424, 1069.5072, 1074.5400, 1077.5109, 1085.5363, 1087.5255, 1091.5013, 1103.5313, 1107.5238, 1111.5382, 1122.5424, 1123.5519, 1168.5806, 1173.6044, 1178.6016, 1179.5861, 1185.6279, 1187.6195, 1199.6720, 1207.6029, 1209.5723, 1215.6453, 1216.6425, 1226.5743, 1234.5945, 1244.5957, 1248.6305, 1255.5890, 1295.7034, 1301.5924, 1314.6890, 1322.5939, 1325.6377, 1328.6476, 1336.6866, 1337.6793, 1338.6221, 1342.6494, 1344.6713, 1345.6792, 1349.6682, 1352.6564, 1353.6359, 1355.6598, 1361.6550, 1368.6198, 1370.6490, 1372.5659, 1381.7262, 1383.6938, 1386.6838, 1394.6993, 1397.7117, 1427.7550, 1432.6827, 1433.6854, 1442.7830, 1446.7039, 1447.6959, 1457.7043, 1473.7709, 1478.7163, 1497.8370, 1528.6653, 1537.7870, 1542.8158, 1550.9301, 1556.8483, 1578.7511, 1601.8662, 1615.8414, 1616.8064, 1617.7891, 1627.7168, 1628.6741, 1628.8262, 1638.8353, 1642.6895, 1647.8445, 1650.8495, 1679.9406, 1680.7981, 1696.8021, 1706.8348, 1715.8130, 1717.8778, 1718.8860, 1725.8102, 1727.8582, 1730.8500, 1752.8656, 1763.7885, 1768.9357, 1769.9312, 1771.0000, 1775.8153, 1777.8390, 1786.8839, 1788.4598, 1791.8882, 1799.8864, 1800.8976, 1809.0026, 1914.9941, 1928.0222, 1953.8669, 1975.9434, 2012.9734, 2027.1388, 2042.0795, 2053.0103, 2060.0637, 2070.9099, 2082.9165, 2086.9263, 2095.0337, 2096.9631, 2098.9319, 2100.9241, 2136.9219, 2155.1025, 2170.1101, 2209.0732, 2211.1094, 2239.2014, 2251.1997, 2289.1399, 2293.2251, 2324.1719, 2343.2795, 2381.2776, 2384.2988, 2398.2314, 2400.1594, 2414.1807, 2428.1990, 2431.1982, 2444.2200, 2465.3230, 2528.3064, 2542.3503, 2546.3127, 2558.3843, 2647.1560, 2661.1709, 2675.1926, 2698.5471, 2706.4133, 2708.4885, 2718.5300, 2762.5994, 2765.5881, 2767.5010, 2771.5437, 2777.7188, 2811.6846, 2814.6851, 2846.6375, 2869.6453, 2872.5757, 2884.6123, 2888.6077, 2892.6868, 2894.7185, 2902.6804, 2918.6406, 2926.6814, 2946.7546, 2994.8018, 2998.7808, 3003.8159, 3005.8979, 3054.8286, 3082.9351, 3102.9441, 3105.8228, 3127.0818, 3171.0554, 3194.9565, 3202.8757, 3207.9922, 3249.0452, 3263.1082, 3264.1118, 3266.0825, 3300.1990, 3317.0972, 3323.0837, 3331.1343, 3335.1436, 3336.1438, 3348.9949, 3354.0869, 3358.1614, 3371.1931, 3387.2393, 3454.9927, 3459.2039, 3490.2356, 3504.3723, 3518.1758, 3640.3601, 3652.4072, 3664.4243, 3822.6233, 3870.6184, 3871.6218, 3887.6787, 4089.8884, 4148.9229, 4160.9209, 4164.9634, 4175.9526, 4306.0547, 4453.3716, 4466.3892, 4680.6270, 4846.6348

29. E1B821 **Mass:** 38278 **Score:** 17 **Expect:** 4.5e+002 **Matches:** 4

Uncharacterized protein OS=Bos taurus PE=3 SV=2

Observed	Mr(expt)	Mr(calc)	ppm	Start	End	Miss	Peptide
935.4841	934.4768	934.4872	-11.15	187 -	194	0	R.YLELGGQR.F
983.4774	982.4701	982.4760	-5.99	73 -	79	1	K.YWEKETK.K

1349.6682	1348.6609	1348.6809	-14.81	174 - 185	0	K.AYLEGPCLASLR.R
1768.9357	1767.9284	1767.9631	-19.62	2 - 16	2	M.AELANLKLENKSWPR.G

No match to: 712.3012, 714.3622, 717.4536, 722.4728, 735.4618, 756.5057, 772.4633, 779.5103, 792.4439, 795.4440, 804.3116, 806.4917, 807.4467, 809.4788, 812.4258, 814.4372, 815.4436, 819.4216, 828.4171, 831.4965, 832.3378, 834.3423, 841.4608, 842.4964, 845.4365, 871.5104, 883.4921, 888.4818, 891.4986, 893.9725, 894.4754, 900.5499, 913.5269, 918.5532, 929.4499, 945.4980, 947.4821, 949.5173, 959.4860, 963.4728, 967.4305, 969.4643, 972.4830, 974.4501, 975.4561, 976.4594, 977.4788, 978.5386, 981.4812, 988.4600, 989.6110, 995.4939, 996.4700, 997.4576, 999.4598, 1007.5580, 1011.4736, 1018.5132, 1019.5350, 1021.5362, 1027.4982, 1032.5875, 1033.5684, 1034.5756, 1036.5582, 1044.5192, 1047.5377, 1055.5199, 1056.5244, 1057.5576, 1060.5359, 1062.5424, 1069.5072, 1074.5400, 1077.5109, 1085.5363, 1087.5255, 1091.5013, 1103.5313, 1106.5183, 1107.5238, 1111.5382, 1122.5424, 1123.5519, 1168.5806, 1173.6044, 1178.6016, 1179.5861, 1185.6279, 1187.6195, 1199.6720, 1207.6029, 1209.5723, 1215.6453, 1216.6425, 1226.5743, 1234.5945, 1244.5957, 1248.6305, 1255.5890, 1295.7034, 1301.5924, 1314.6890, 1322.5939, 1325.6377, 1328.6476, 1336.6866, 1337.6793, 1338.6221, 1342.6494, 1344.6713, 1345.6792, 1352.6564, 1353.6359, 1355.6598, 1361.6550, 1368.6198, 1370.6490, 1372.5659, 1381.7262, 1383.6938, 1386.6838, 1394.6993, 1397.7117, 1427.7550, 1432.6827, 1433.6854, 1442.7830, 1446.7039, 1447.6959, 1457.7043, 1473.7709, 1478.7163, 1497.8370, 1528.6653, 1537.7870, 1542.8158, 1550.9301, 1556.8483, 1578.7511, 1601.8662, 1606.8096, 1615.8414, 1616.8064, 1617.7891, 1627.7168, 1628.6741, 1628.8262, 1638.8353, 1642.6895, 1647.8445, 1650.8495, 1679.9406, 1680.7981, 1696.8021, 1706.8348, 1715.8130, 1717.8778, 1718.8860, 1725.8102, 1727.8582, 1730.8500, 1752.8656, 1763.7885, 1769.9312, 1771.0000, 1775.8153, 1777.8390, 1786.8839, 1788.4598, 1791.8882, 1799.8864, 1800.8976, 1809.0026, 1914.9941, 1928.0222, 1953.8669, 1975.9434, 1982.0046, 2012.9734, 2027.1388, 2042.0795, 2053.0103, 2060.0637, 2070.9099, 2082.9165, 2084.9246, 2086.9263, 2095.0337, 2096.9631, 2098.9319, 2100.9241, 2136.9219, 2155.1025, 2170.1101, 2209.0732, 2211.1094, 2239.2014, 2251.1997, 2289.1399, 2293.2251, 2324.1719, 2343.2795, 2381.2776, 2384.2988, 2398.2314, 2400.1594, 2414.1807, 2428.1990, 2431.1982, 2444.2200, 2465.3230, 2528.3064, 2542.3503, 2546.3127, 2558.3843, 2647.1560, 2661.1709, 2675.1926, 2698.5471, 2706.4133, 2708.4885, 2718.5300, 2762.5994, 2765.5881, 2767.5010, 2771.5437, 2777.7188, 2811.6846, 2814.6851, 2846.6375, 2869.6453, 2872.5757, 2884.6123, 2888.6077, 2892.6868, 2894.7185, 2902.6804, 2918.6406, 2926.6814, 2946.7546, 2994.8018, 2998.7808, 3003.8159, 3005.8979, 3054.8286, 3082.9351, 3102.9441, 3105.8228, 3127.0818, 3171.0554, 3194.9565, 3202.8757, 3207.9922, 3249.0452, 3263.1082, 3264.1118, 3266.0825, 3300.1990, 3317.0972, 3323.0837, 3331.1343, 3335.1436, 3336.1438, 3348.9949, 3354.0869, 3358.1614, 3371.1931, 3387.2393, 3454.9927, 3459.2039, 3490.2356, 3504.3723, 3518.1758, 3640.3601, 3652.4072, 3664.4243, 3822.6233, 3870.6184, 3871.6218, 3887.6787, 4089.8884, 4148.9229, 4160.9209, 4164.9634, 4175.9526, 4306.0547, 4453.3716, 4466.3892, 4680.6270, 4846.6348

30. F1N4S8 **Mass:** 14788 **Score:** 17 **Expect:** 4.5e+002 **Matches:** 4

Uncharacterized protein OS=Bos taurus GN=CCL21 PE=4 SV=1

Observed	Mr(expt)	Mr(calc)	ppm	Start - End	Miss	Peptide
772.4633	771.4560	771.4603	-5.55	108 - 115	2	K.GAPKSGKK.G
883.4921	882.4849	882.4923	-8.43	90 - 97	0	R.LDKPSAPR.K
1427.7550	1426.7477	1426.7714	-16.62	86 - 97	1	K.LMQRLDKPSAPR.K + Oxidation (M)
2414.1807	2413.1734	2413.1882	-6.12	70 - 89	2	R.SQPELCADPKEAWVQKLMQR.L

No match to: 712.3012, 714.3622, 717.4536, 722.4728, 735.4618, 756.5057, 779.5103, 792.4439, 795.4440, 804.3116, 806.4917, 807.4467, 809.4788, 812.4258, 814.4372, 815.4436, 819.4216, 828.4171, 831.4965, 832.3378, 834.3423, 841.4608, 842.4964, 845.4365, 871.5104, 888.4818, 891.4986, 893.9725, 894.4754, 900.5499, 913.5269, 918.5532, 929.4499, 935.4841, 945.4980, 947.4821, 949.5173, 959.4860, 963.4728, 967.4305, 969.4643, 972.4830, 974.4501, 975.4561, 976.4594, 977.4788, 978.5386, 981.4812, 983.4774, 988.4600, 989.6110, 995.4939, 996.4700, 997.4576, 999.4598, 1007.5580, 1011.4736, 1018.5132, 1019.5350, 1021.5362, 1027.4982, 1032.5875, 1033.5684, 1034.5756, 1036.5582, 1044.5192, 1047.5377, 1055.5199, 1056.5244, 1057.5576, 1060.5359, 1062.5424, 1069.5072, 1074.5400, 1077.5109, 1085.5363, 1087.5255, 1091.5013, 1103.5313, 1106.5183, 1107.5238, 1111.5382, 1122.5424, 1123.5519, 1168.5806, 1173.6044, 1178.6016, 1179.5861, 1185.6279, 1187.6195, 1199.6720, 1207.6029, 1209.5723, 1215.6453, 1216.6425, 1226.5743, 1234.5945, 1244.5957, 1248.6305, 1255.5890, 1295.7034, 1301.5924, 1314.6890, 1322.5939, 1325.6377, 1328.6476, 1336.6866, 1337.6793, 1338.6221, 1342.6494, 1344.6713, 1345.6792, 1349.6682, 1352.6564, 1353.6359, 1355.6598, 1361.6550, 1368.6198, 1370.6490, 1372.5659, 1381.7262, 1383.6938, 1386.6838, 1394.6993, 1397.7117, 1432.6827, 1433.6854, 1442.7830, 1446.7039, 1447.6959, 1457.7043, 1473.7709, 1478.7163, 1497.8370, 1528.6653, 1537.7870, 1542.8158, 1550.9301, 1556.8483, 1578.7511, 1601.8662, 1606.8096, 1615.8414, 1616.8064, 1617.7891, 1627.7168, 1628.6741, 1628.8262, 1638.8353, 1642.6895, 1647.8445, 1650.8495, 1679.9406, 1680.7981, 1696.8021, 1706.8348, 1715.8130, 1717.8778, 1718.8860, 1725.8102, 1727.8582, 1730.8500, 1752.8656, 1763.7885, 1768.9357, 1769.9312, 1771.0000, 1775.8153, 1777.8390, 1786.8839, 1788.4598, 1791.8882, 1799.8864, 1800.8976, 1809.0026, 1914.9941, 1928.0222, 1953.8669, 1975.9434, 1982.0046, 2012.9734, 2027.1388, 2042.0795, 2053.0103, 2060.0637, 2070.9099, 2082.9165, 2084.9246, 2086.9263, 2095.0337, 2096.9631, 2098.9319, 2100.9241, 2136.9219, 2155.1025, 2170.1101, 2209.0732, 2211.1094, 2239.2014, 2251.1997, 2289.1399, 2293.2251, 2324.1719, 2343.2795, 2381.2776, 2384.2988, 2398.2314, 2400.1594, 2428.1990, 2431.1982, 2444.2200, 2465.3230, 2528.3064, 2542.3503, 2546.3127, 2558.3843, 2647.1560, 2661.1709, 2675.1926, 2698.5471, 2706.4133, 2708.4885, 2718.5300, 2762.5994, 2765.5881, 2767.5010, 2771.5437, 2777.7188, 2811.6846, 2814.6851, 2846.6375, 2869.6453, 2872.5757, 2884.6123, 2888.6077, 2892.6868, 2894.7185, 2902.6804, 2918.6406, 2926.6814, 2946.7546, 2994.8018, 2998.7808, 3003.8159, 3005.8979, 3054.8286, 3082.9351, 3102.9441, 3105.8228, 3127.0818, 3171.0554, 3194.9565, 3202.8757, 3207.9922, 3249.0452, 3263.1082, 3264.1118, 3266.0825, 3300.1990, 3317.0972, 3323.0837, 3331.1343, 3335.1436, 3336.1438, 3348.9949, 3354.0869, 3358.1614, 3371.1931, 3387.2393, 3454.9927, 3459.2039, 3490.2356, 3504.3723, 3518.1758, 3640.3601, 3652.4072, 3664.4243, 3822.6233, 3870.6184, 3871.6218, 3887.6787, 4089.8884, 4148.9229, 4160.9209, 4164.9634, 4175.9526, 4306.0547, 4453.3716, 4466.3892, 4680.6270, 4846.6348

31. Q32LE2 **Mass:** 41970 **Score:** 17 **Expect:** 4.6e+002 **Matches:** 7

G kinase-anchoring protein 1 OS=Bos taurus GN=GKAP1 PE=2 SV=2

Observed	Mr(expt)	Mr(calc)	ppm	Start - End	Miss	Peptide
871.5104	870.5031	870.5174	-16.47	339 - 346	0	K.VLQAELAK.Y
900.5499	899.5426	899.5440	-1.54	255 - 261	1	R.LKLELER.K
977.4788	976.4715	976.4938	-22.75	38 - 46	1	R.NTGKSQTNK.S
997.4576	996.4504	996.4256	24.9	293 - 300	0	K.MLQEGEMK.D + 2 Oxidation (M)
2084.9246	2083.9173	2083.9368	-9.34	114 - 130	1	R.QRDEQLTSEMFEADLEK.A + Oxidation (M)
2086.9263	2085.9190	2085.9636	-21.39	145 - 162	2	K.EYENAENASTQSKVMNKK.D + Oxidation (M)
2343.2795	2342.2722	2342.2481	10.3	172 - 191	2	K.DKPLTISLKEFQSEDHISKK.T

No match to: 712.3012, 714.3622, 717.4536, 722.4728, 735.4618, 756.5057, 772.4633, 779.5103, 792.4439, 795.4440, 804.3116, 806.4917, 807.4467, 809.4788, 812.4258, 814.4372, 815.4436, 819.4216, 828.4171, 831.4965, 832.3378, 834.3423, 841.4608, 842.4964, 845.4365, 883.4921, 888.4818, 891.4986, 893.9725, 894.4754, 913.5269, 918.5532, 929.4499, 935.4841, 945.4980, 947.4821, 949.5173, 959.4860, 963.4728, 967.4305, 969.4643, 972.4830, 974.4501, 975.4561, 976.4594, 978.5386, 981.4812, 983.4774, 988.4600, 989.6110, 995.4939, 996.4700, 999.4598, 1007.5580, 1011.4736, 1018.5132, 1019.5350, 1021.5362, 1027.4982, 1032.5875, 1033.5684, 1034.5756, 1036.5582, 1044.5192, 1047.5377, 1055.5199, 1056.5244, 1057.5576, 1060.5359, 1062.5424, 1069.5072, 1074.5400, 1077.5109, 1085.5363, 1087.5255, 1091.5013, 1103.5313, 1106.5183, 1107.5238, 1111.5382, 1122.5424, 1123.5519, 1168.5806, 1173.6044, 1178.6016, 1179.5861, 1185.6279, 1187.6195, 1199.6720, 1207.6029, 1209.5723, 1215.6453, 1216.6425, 1226.5743, 1234.5945, 1244.5957, 1248.6305, 1255.5890, 1295.7034, 1301.5924, 1314.6890, 1322.5939, 1325.6377, 1328.6476, 1336.6866, 1337.6793, 1338.6221, 1342.6494, 1344.6713, 1345.6792, 1349.6682, 1352.6564, 1353.6359, 1355.6598, 1361.6550, 1368.6198, 1370.6490, 1372.5659, 1381.7262, 1383.6938, 1386.6838, 1394.6993, 1397.7117, 1427.7550, 1432.6827, 1433.6854, 1442.7830, 1446.7039, 1447.6959, 1457.7043, 1473.7709, 1478.7163, 1497.8370, 1528.6653, 1537.7870, 1542.8158, 1550.9301, 1556.8483, 1578.7511, 1601.8662, 1606.8096, 1615.8414, 1616.8064, 1617.7891, 1627.7168, 1628.6741, 1628.8262, 1638.8353, 1642.6895, 1647.8445, 1650.8495, 1679.9406, 1680.7981, 1696.8021, 1706.8348, 1715.8130, 1717.8778, 1718.8860, 1725.8102, 1727.8582, 1730.8500, 1752.8656, 1763.7885, 1768.9357, 1769.9312, 1771.0000, 1775.8153, 1777.8390, 1786.8839, 1788.4598, 1791.8882, 1799.8864, 1800.8976, 1809.0026, 1914.9941, 1928.0222, 1953.8669, 1975.9434, 1982.0046, 2012.9734, 2027.1388, 2042.0795, 2053.0103, 2060.0637, 2070.9099, 2082.9165, 2095.0337, 2096.9631, 2098.9319, 2100.9241, 2136.9219, 2155.1025, 2170.1101, 2209.0732, 2211.1094, 2239.2014, 2251.1997, 2289.1399, 2293.2251, 2324.1719, 2381.2776, 2384.2988, 2398.2314, 2400.1594, 2414.1807, 2428.1990, 2431.1982, 2444.2200, 2465.3230, 2528.3064, 2542.3503, 2546.3127, 2558.3843, 2647.1560, 2661.1709, 2675.1926, 2698.5471, 2706.4133, 2708.4885, 2718.5300, 2762.5994, 2765.5881, 2767.5010, 2771.5437, 2777.7188, 2811.6846, 2814.6851, 2846.6375, 2869.6453, 2872.5757, 2884.6123, 2888.6077, 2892.6868, 2894.7185, 2902.6804, 2918.6406, 2926.6814, 2946.7546, 2994.8018, 2998.7808, 3003.8159, 3005.8979, 3054.8286, 3082.9351, 3102.9441, 3105.8228, 3127.0818, 3171.0554, 3194.9565, 3202.8757, 3207.9922, 3249.0452, 3263.1082, 3264.1118, 3266.0825, 3300.1990, 3317.0972, 3323.0837, 3331.1343, 3335.1436, 3336.1438, 3348.9949, 3354.0869, 3358.1614, 3371.1931, 3387.2393, 3454.9927, 3459.2039, 3490.2356, 3504.3723, 3518.1758, 3640.3601, 3652.4072, 3664.4243, 3822.6233, 3870.6184, 3871.6218, 3887.6787, 4089.8884, 4148.9229, 4160.9209, 4164.9634, 4175.9526, 4306.0547, 4453.3716, 4466.3892, 4680.6270, 4846.6348

32. F1MYW4 **Mass:** 74414 **Score:** 17 **Expect:** 4.7e+002 **Matches:** 11
Nucleolar GTP-binding protein 1 OS=Bos taurus GN=GTPBP4 PE=3 SV=2

Observed	Mr(expt)	Mr(calc)	ppm	Start	End	Miss	Peptide
831.4965	830.4892	830.4796	11.6	508 -	514	2	R.MPRTAKK.V
959.4860	958.4787	958.4906	-12.34	388 -	395	1	R.MELGEPKR.R
983.4774	982.4701	982.4576	12.7	591 -	598	1	K.TMMKNAQK.K + 2 Oxidation (M)
1019.5350	1018.5277	1018.5117	15.7	518 -	525	1	R.KVLEDEMR.S
1034.5756	1033.5683	1033.5590	9.03	344 -	352	2	R.VETKMKGNK.V
1111.5382	1110.5309	1110.5525	-19.45	591 -	599	2	K.TMMKNAQKK.M + 2 Oxidation (M)
1173.6044	1172.5971	1172.6037	-5.62	556 -	566	0	R.EDSVLPASVTR.S
1314.6890	1313.6817	1313.6510	23.4	500 -	510	2	R.EKDTQGPRMPR.T
1628.8262	1627.8189	1627.8181	0.53	204 -	216	1	K.SLFVGHMDYKYLR.W
1809.0026	1807.9953	1807.9879	4.09	612 -	626	2	R.HVFDMKPKHLLSGKR.K + Oxidation (M)
2053.0103	2052.0030	2052.0283	-12.32	567 -	584	2	R.SHSCSRPPRDVSGLRDVK.M

No match to: 712.3012, 714.3622, 717.4536, 722.4728, 735.4618, 756.5057, 772.4633, 779.5103, 792.4439, 795.4440, 804.3116, 806.4917, 807.4467, 809.4788, 812.4258, 814.4372, 815.4436, 819.4216, 828.4171, 832.3378, 834.3423, 841.4608, 842.4964, 845.4365, 871.5104, 883.4921, 888.4818, 891.4986, 893.9725, 894.4754, 900.5499, 913.5269, 918.5532, 929.4499, 935.4841, 945.4980, 947.4821, 949.5173, 963.4728, 967.4305, 969.4643, 972.4830, 974.4501, 975.4561, 976.4594, 977.4788, 978.5386, 981.4812, 988.4600, 989.6110, 995.4939, 996.4700, 997.4576, 999.4598, 1007.5580, 1011.4736, 1018.5132, 1021.5362, 1027.4982, 1032.5875, 1033.5684, 1036.5582, 1044.5192, 1047.5377, 1055.5199, 1056.5244, 1057.5576, 1060.5359, 1062.5424, 1069.5072, 1074.5400, 1077.5109, 1085.5363, 1087.5255, 1091.5013, 1103.5313, 1106.5183, 1107.5238, 1122.5424, 1123.5519, 1168.5806, 1178.6016, 1179.5861, 1185.6279, 1187.6195, 1199.6720, 1207.6029, 1209.5723, 1215.6453, 1216.6425, 1226.5743, 1234.5945, 1244.5957, 1248.6305, 1255.5890, 1295.7034, 1301.5924, 1322.5939, 1325.6377, 1328.6476, 1336.6866, 1337.6793, 1338.6221, 1342.6494, 1344.6713, 1345.6792, 1349.6682, 1352.6564, 1353.6359, 1355.6598, 1361.6550, 1368.6198, 1370.6490, 1372.5659, 1381.7262, 1383.6938, 1386.6838, 1394.6993, 1397.7117, 1427.7550, 1432.6827, 1433.6854, 1442.7830, 1446.7039, 1447.6959, 1457.7043, 1473.7709, 1478.7163, 1497.8370, 1528.6653, 1537.7870, 1542.8158, 1550.9301, 1556.8483, 1578.7511, 1601.8662, 1606.8096, 1615.8414, 1616.8064, 1617.7891, 1627.7168, 1628.6741, 1638.8353, 1642.6895, 1647.8445, 1650.8495, 1679.9406, 1680.7981, 1696.8021, 1706.8348, 1715.8130, 1717.8778, 1718.8860, 1725.8102, 1727.8582, 1730.8500, 1752.8656, 1763.7885, 1768.9357, 1769.9312, 1771.0000, 1775.8153, 1777.8390, 1786.8839, 1788.4598, 1791.8882, 1799.8864, 1800.8976, 1914.9941, 1928.0222, 1953.8669, 1975.9434, 1982.0046, 2012.9734, 2027.1388, 2042.0795, 2060.0637, 2070.9099, 2082.9165, 2084.9246, 2086.9263, 2095.0337, 2096.9631, 2098.9319, 2100.9241, 2136.9219, 2155.1025, 2170.1101, 2209.0732, 2211.1094, 2239.2014, 2251.1997, 2289.1399, 2293.2251, 2324.1719, 2343.2795, 2381.2776, 2384.2988, 2398.2314, 2400.1594, 2414.1807, 2428.1990, 2431.1982, 2444.2200, 2465.3230, 2528.3064, 2542.3503, 2546.3127, 2558.3843, 2647.1560, 2661.1709, 2675.1926, 2698.5471, 2706.4133, 2708.4885, 2718.5300, 2762.5994, 2765.5881, 2767.5010, 2771.5437, 2777.7188, 2811.6846, 2814.6851, 2846.6375, 2869.6453, 2872.5757, 2884.6123, 2888.6077, 2892.6868, 2894.7185, 2902.6804, 2918.6406, 2926.6814, 2946.7546, 2994.8018, 2998.7808, 3003.8159, 3005.8979, 3054.8286, 3082.9351, 3102.9441, 3105.8228, 3127.0818, 3171.0554, 3194.9565, 3202.8757, 3207.9922, 3249.0452, 3263.1082, 3264.1118, 3266.0825, 3300.1990, 3317.0972, 3323.0837, 3331.1343, 3335.1436, 3336.1438, 3348.9949, 3354.0869, 3358.1614, 3371.1931, 3387.2393, 3454.9927, 3459.2039, 3490.2356, 3504.3723, 3518.1758, 3640.3601, 3652.4072, 3664.4243, 3822.6233, 3870.6184, 3871.6218, 3887.6787, 4089.8884, 4148.9229, 4160.9209, 4164.9634, 4175.9526, 4306.0547, 4453.3716, 4466.3892, 4680.6270, 4846.6348

33. A2VDT9 **Mass:** 22302 **Score:** 17 **Expect:** 4.9e+002 **Matches:** 4
DNA damage-inducible transcript 4-like protein OS=Bos taurus GN=DDIT4L PE=2 SV=1

Observed	Mr(expt)	Mr(calc)	ppm	Start	End	Miss	Peptide
1173.6044	1172.5971	1172.6084	-9.63	68 -	77	2	K.SKHTKLGCSR.V
1209.5723	1208.5650	1208.5893	-20.08	60 -	69	1	K.MLENCLSKSK.H
1914.9941	1913.9868	1913.9993	-6.51	88 -	104	1	R.IAQDVLRLSSTEPCGLR.G
4148.9229	4147.9156	4147.9813	-15.82	124 -	157	2	R.IVCDSSVVPTFELTLVFKQENCSWTSFRDFFFSR.G

No match to: 712.3012, 714.3622, 717.4536, 722.4728, 735.4618, 756.5057, 772.4633, 779.5103, 792.4439, 795.4440, 804.3116, 806.4917, 807.4467, 809.4788, 812.4258, 814.4372, 815.4436, 819.4216, 828.4171, 831.4965, 832.3378, 834.3423, 841.4608, 842.4964, 845.4365, 871.5104, 883.4921, 888.4818, 891.4986, 893.9725, 894.4754, 900.5499, 913.5269, 918.5532, 929.4499, 935.4841, 945.4980, 947.4821, 949.5173, 959.4860, 963.4728, 967.4305, 969.4643, 972.4830, 974.4501, 975.4561, 976.4594, 977.4788, 978.5386, 981.4812, 983.4774, 988.4600, 989.6110, 995.4939, 996.4700, 997.4576, 999.4598, 1007.5580, 1011.4736, 1018.5132, 1019.5350, 1021.5362, 1027.4982, 1032.5875, 1033.5684, 1034.5756, 1036.5582, 1044.5192, 1047.5377, 1055.5199, 1056.5244, 1057.5576, 1060.5359, 1062.5424, 1069.5072, 1074.5400, 1077.5109, 1085.5363, 1087.5255, 1091.5013, 1103.5313, 1106.5183, 1107.5238, 1111.5382, 1122.5424, 1123.5519, 1168.5806, 1178.6016, 1179.5861, 1185.6279, 1187.6195, 1199.6720, 1207.6029, 1215.6453, 1216.6425, 1226.5743, 1234.5945, 1244.5957, 1248.6305, 1255.5890, 1295.7034, 1301.5924, 1314.6890, 1322.5939, 1325.6377, 1328.6476, 1336.6866, 1337.6793, 1338.6221, 1342.6494, 1344.6713, 1345.6792, 1349.6682, 1352.6564, 1353.6359, 1355.6598, 1361.6550, 1368.6198, 1370.6490, 1372.5659, 1381.7262, 1383.6938, 1386.6838, 1394.6993, 1397.7117, 1427.7550, 1432.6827, 1433.6854, 1442.7830, 1446.7039, 1447.6959, 1457.7043, 1473.7709, 1478.7163, 1497.8370, 1528.6653, 1537.7870, 1542.8158, 1550.9301, 1556.8483, 1578.7511, 1601.8662, 1606.8096, 1615.8414, 1616.8064, 1617.7891, 1627.7168, 1628.6741, 1628.8262, 1638.8353, 1642.6895, 1647.8445, 1650.8495, 1679.9406, 1680.7981, 1696.8021, 1706.8348, 1715.8130, 1717.8778, 1718.8860, 1725.8102, 1727.8582, 1730.8500, 1752.8656, 1763.7885, 1768.9357, 1769.9312, 1771.0000, 1775.8153, 1777.8390, 1786.8839, 1788.4598, 1791.8882, 1799.8864, 1800.8976, 1809.0026, 1928.0222, 1953.8669, 1975.9434, 1982.0046, 2012.9734, 2027.1388, 2042.0795, 2053.0103, 2060.0637, 2070.9099, 2082.9165, 2084.9246, 2086.9263, 2095.0337, 2096.9631, 2098.9319, 2100.9241, 2136.9219, 2155.1025, 2170.1101, 2209.0732, 2211.1094, 2239.2014, 2251.1997, 2289.1399, 2293.2251, 2324.1719, 2343.2795, 2381.2776, 2384.2988, 2398.2314, 2400.1594, 2414.1807, 2428.1990, 2431.1982, 2444.2200, 2465.3230, 2528.3064, 2542.3503, 2546.3127, 2558.3843, 2647.1560, 2661.1709, 2675.1926, 2698.5471, 2706.4133, 2708.4885, 2718.5300, 2762.5994, 2765.5881, 2767.5010, 2771.5437, 2777.7188, 2811.6846, 2814.6851, 2846.6375, 2869.6453, 2872.5757, 2884.6123, 2888.6077, 2892.6868, 2894.7185, 2902.6804, 2918.6406, 2926.6814, 2946.7546, 2994.8018, 2998.7808, 3003.8159, 3005.8979, 3054.8286, 3082.9351, 3102.9441, 3105.8228, 3127.0818, 3171.0554, 3194.9565, 3202.8757, 3207.9922, 3249.0452, 3263.1082, 3264.1118, 3266.0825, 3300.1990, 3317.0972, 3323.0837, 3331.1343, 3335.1436, 3336.1438, 3348.9949, 3354.0869, 3358.1614, 3371.1931, 3387.2393, 3454.9927, 3459.2039, 3490.2356, 3504.3723, 3518.1758, 3640.3601, 3652.4072, 3664.4243, 3822.6233, 3870.6184, 3871.6218, 3887.6787, 4089.8884, 4160.9209, 4164.9634, 4175.9526, 4306.0547, 4453.3716, 4466.3892, 4680.6270, 4846.6348

34. Q2YDM5 **Mass:** 18048 **Score:** 17 **Expect:** 5.1e+002 **Matches:** 2
Testis, prostate and placenta-expressed protein OS=Bos taurus GN=TEPP PE=2 SV=1

Observed	Mr(expt)	Mr(calc)	ppm	Start	End	Miss	Peptide
997.4576	996.4504	996.4368	13.6	51 -	58	1	R.RMDMDSVK.A + Oxidation (M)
2872.5757	2871.5684	2871.5535	5.21	4 -	29	0	R.IIDLVPWEDGSTHVYASPAILLPIPR.R

No match to: 712.3012, 714.3622, 717.4536, 722.4728, 735.4618, 756.5057, 772.4633, 779.5103, 792.4439, 795.4440, 804.3116, 806.4917, 807.4467, 809.4788, 812.4258, 814.4372, 815.4436, 819.4216, 828.4171, 831.4965, 832.3378, 834.3423, 841.4608, 842.4964, 845.4365, 871.5104, 883.4921, 888.4818, 891.4986, 893.9725, 894.4754, 900.5499, 913.5269, 918.5532, 929.4499, 935.4841, 945.4980, 947.4821, 949.5173, 959.4860, 963.4728, 967.4305, 969.4643, 972.4830, 974.4501, 975.4561, 976.4594, 977.4788, 978.5386, 981.4812, 983.4774, 988.4600, 989.6110, 995.4939, 996.4700, 999.4598, 1007.5580, 1011.4736, 1018.5132, 1019.5350, 1021.5362, 1027.4982, 1032.5875, 1033.5684, 1034.5756, 1036.5582, 1044.5192, 1047.5377, 1055.5199, 1056.5244, 1057.5576, 1060.5359, 1062.5424, 1069.5072, 1074.5400, 1077.5109, 1085.5363, 1087.5255, 1091.5013, 1103.5313, 1106.5183, 1107.5238, 1111.5382, 1122.5424, 1123.5519, 1168.5806, 1173.6044, 1178.6016, 1179.5861, 1185.6279, 1187.6195, 1199.6720, 1207.6029, 1209.5723, 1215.6453, 1216.6425, 1226.5743, 1234.5945, 1244.5957, 1248.6305, 1255.5890, 1295.7034, 1301.5924, 1314.6890, 1322.5939, 1325.6377, 1328.6476, 1336.6866, 1337.6793, 1338.6221, 1342.6494, 1344.6713, 1345.6792, 1349.6682, 1352.6564, 1353.6359, 1355.6598, 1361.6550, 1368.6198, 1370.6490, 1372.5659, 1381.7262, 1383.6938, 1386.6838, 1394.6993, 1397.7117, 1427.7550, 1432.6827, 1433.6854, 1442.7830, 1446.7039, 1447.6959, 1457.7043, 1473.7709, 1478.7163, 1497.8370, 1528.6653, 1537.7870, 1542.8158, 1550.9301, 1556.8483, 1578.7511, 1601.8662, 1606.8096, 1615.8414, 1616.8064, 1617.7891, 1627.7168, 1628.6741, 1628.8262, 1638.8353, 1642.6895, 1647.8445, 1650.8495, 1679.9406, 1680.7981, 1696.8021, 1706.8348, 1715.8130, 1717.8778, 1718.8860, 1725.8102, 1727.8582, 1730.8500, 1752.8656, 1763.7885, 1768.9357, 1769.9312, 1771.0000, 1775.8153, 1777.8390, 1786.8839, 1788.4598, 1791.8882, 1799.8864, 1800.8976, 1809.0026, 1914.9941, 1928.0222, 1953.8669, 1975.9434, 1982.0046, 2012.9734, 2027.1388, 2042.0795, 2053.0103, 2060.0637, 2070.9099, 2082.9165, 2084.9246, 2086.9263, 2095.0337, 2096.9631, 2098.9319, 2100.9241, 2136.9219, 2155.1025, 2170.1101, 2209.0732, 2211.1094, 2239.2014, 2251.1997, 2289.1399, 2293.2251, 2324.1719, 2343.2795, 2381.2776, 2384.2988, 2398.2314, 2400.1594, 2414.1807, 2428.1990, 2431.1982, 2444.2200, 2465.3230, 2528.3064, 2542.3503, 2546.3127,

2558.3843, 2647.1560, 2661.1709, 2675.1926, 2698.5471, 2706.4133, 2708.4885, 2718.5300, 2762.5994, 2765.5881, 2767.5010, 2771.5437, 2777.7188, 2811.6846, 2814.6851, 2846.6375, 2869.6453, 2884.6123, 2888.6077, 2892.6868, 2894.7185, 2902.6804, 2918.6406, 2926.6814, 2946.7546, 2994.8018, 2998.7808, 3003.8159, 3005.8979, 3054.8286, 3082.9351, 3102.9441, 3105.8228, 3127.0818, 3171.0554, 3194.9565, 3202.8757, 3207.9922, 3249.0452, 3263.1082, 3264.1118, 3266.0825, 3300.1990, 3317.0972, 3323.0837, 3331.1343, 3335.1436, 3336.1438, 3348.9949, 3354.0869, 3358.1614, 3371.1931, 3387.2393, 3454.9927, 3459.2039, 3490.2356, 3504.3723, 3518.1758, 3640.3601, 3652.4072, 3664.4243, 3822.6233, 3870.6184, 3871.6218, 3887.6787, 4089.8884, 4148.9229, 4160.9209, 4164.9634, 4175.9526, 4306.0547, 4453.3716, 4466.3892, 4680.6270, 4846.6348

35. Q2TBQ7 **Mass:** 47436 **Score:** 17 **Expect:** 5.2e+002 **Matches:** 8

Regulator of microtubule dynamics protein 2 OS=Bos taurus GN=RMDN2 PE=2 SV=2

Observed	Mr(expt)	Mr(calc)	ppm	Start	End	Miss	Peptide
871.5104	870.5031	870.5174	-16.47	72 -	78	0	R.QLQILEK.L
1034.5756	1033.5683	1033.5590	9.03	246 -	254	1	K.TLGEKAIMR.A + Oxidation (M)
1060.5359	1059.5286	1059.5501	-20.31	282 -	289	1	K.INYGYRFK.E
1103.5313	1102.5240	1102.5229	0.98	310 -	318	1	K.GRYCYAVSK.L
1107.5238	1106.5165	1106.5396	-20.89	212 -	219	0	R.DEIEFIWR.F
1173.6044	1172.5971	1172.5898	6.27	186 -	195	1	R.ADNLRVNESR.K
1528.6653	1527.6580	1527.6697	-7.67	367 -	379	0	K.CYADLNQIDSAMK.F
1777.8390	1776.8317	1776.7876	24.8	223 -	237	0	R.AYGDMYELSTNIQEK.K + Oxidation (M)

No match to: 712.3012, 714.3622, 717.4536, 722.4728, 735.4618, 756.5057, 772.4633, 779.5103, 792.4439, 795.4440, 804.3116, 806.4917, 807.4467, 809.4788, 812.4258, 814.4372, 815.4436, 819.4216, 828.4171, 831.4965, 832.3378, 834.3423, 841.4608, 842.4964, 845.4365, 883.4921, 888.4818, 891.4986, 893.9725, 894.4754, 900.5499, 913.5269, 918.5532, 929.4499, 935.4841, 945.4980, 947.4821, 949.5173, 959.4860, 963.4728, 967.4305, 969.4643, 972.4830, 974.4501, 975.4561, 976.4594, 977.4788, 978.5386, 981.4812, 983.4774, 988.4600, 989.6110, 995.4939, 996.4700, 997.4576, 999.4598, 1007.5580, 1011.4736, 1018.5132, 1019.5350, 1021.5362, 1027.4982, 1032.5875, 1033.5684, 1036.5582, 1044.5192, 1047.5377, 1055.5199, 1056.5244, 1057.5576, 1062.5424, 1069.5072, 1074.5400, 1077.5109, 1085.5363, 1087.5255, 1091.5013, 1106.5183, 1111.5382, 1122.5424, 1123.5519, 1168.5806, 1178.6016, 1179.5861, 1185.6279, 1187.6195, 1199.6720, 1207.6029, 1209.5723, 1215.6453, 1216.6425, 1226.5743, 1234.5945, 1244.5957, 1248.6305, 1255.5890, 1295.7034, 1301.5924, 1314.6890, 1322.5939, 1325.6377, 1328.6476, 1336.6866, 1337.6793, 1338.6221, 1342.6494, 1344.6713, 1345.6792, 1349.6682, 1352.6564, 1353.6359, 1355.6598, 1361.6550, 1368.6198, 1370.6490, 1372.5659, 1381.7262, 1383.6938, 1386.6838, 1394.6993, 1397.7117, 1427.7550, 1432.6827, 1433.6854, 1442.7830, 1446.7039, 1447.6959, 1457.7043, 1473.7709, 1478.7163, 1497.8370, 1537.7870, 1542.8158, 1550.9301, 1556.8483, 1578.7511, 1601.8662, 1606.8096, 1615.8414, 1616.8064, 1617.7891, 1627.7168, 1628.6741, 1628.8262, 1638.8353, 1642.6895, 1647.8445, 1650.8495, 1679.9406, 1680.7981, 1696.8021, 1706.8348, 1715.8130, 1717.8778, 1718.8860, 1725.8102, 1727.8582, 1730.8500, 1752.8656, 1763.7885, 1768.9357, 1769.9312, 1771.0000, 1775.8153, 1786.8839, 1788.4598, 1791.8882, 1799.8864, 1800.8976, 1809.0026, 1914.9941, 1928.0222, 1953.8669, 1975.9434, 1982.0046, 2012.9734, 2027.1388, 2042.0795, 2053.0103, 2060.0637, 2070.9099, 2082.9165, 2084.9246, 2086.9263, 2095.0337, 2096.9631, 2098.9319, 2100.9241, 2136.9219, 2155.1025, 2170.1101, 2209.0732, 2211.1094, 2239.2014, 2251.1997, 2289.1399, 2293.2251, 2324.1719, 2343.2795, 2381.2776, 2384.2988, 2398.2314, 2400.1594, 2414.1807, 2428.1990, 2431.1982, 2444.2200, 2465.3230, 2528.3064, 2542.3503, 2546.3127, 2558.3843, 2647.1560, 2661.1709, 2675.1926, 2698.5471, 2706.4133, 2708.4885, 2718.5300, 2762.5994, 2765.5881, 2767.5010, 2771.5437, 2777.7188, 2811.6846, 2814.6851, 2846.6375, 2869.6453, 2872.5757, 2884.6123, 2888.6077, 2892.6868, 2894.7185, 2902.6804, 2918.6406, 2926.6814, 2946.7546, 2994.8018, 2998.7808, 3003.8159, 3005.8979, 3054.8286, 3082.9351, 3102.9441, 3105.8228, 3127.0818, 3171.0554, 3194.9565, 3202.8757, 3207.9922, 3249.0452, 3263.1082, 3264.1118, 3266.0825, 3300.1990, 3317.0972, 3323.0837, 3331.1343, 3335.1436, 3336.1438, 3348.9949, 3354.0869, 3358.1614, 3371.1931, 3387.2393, 3454.9927, 3459.2039, 3490.2356, 3504.3723, 3518.1758, 3640.3601, 3652.4072, 3664.4243, 3822.6233, 3870.6184, 3871.6218, 3887.6787, 4089.8884, 4148.9229, 4160.9209, 4164.9634, 4175.9526, 4306.0547, 4453.3716, 4466.3892, 4680.6270, 4846.6348

36. E1BAV3 **Mass:** 43148 **Score:** 17 **Expect:** 5.3e+002 **Matches:** 7

Uncharacterized protein OS=Bos taurus GN=STOML1 PE=4 SV=2

Observed	Mr(expt)	Mr(calc)	ppm	Start	End	Miss	Peptide
945.4980	944.4907	944.4716	20.3	207 -	214	0	R.AWGLEVDR.V
1007.5580	1006.5507	1006.5746	-23.72	88 -	95	1	R.MIVFRLGR.I + Oxidation (M)
1011.4736	1010.4663	1010.4525	13.7	168 -	176	0	R.MTAQNAMTK.A + Oxidation (M)
1034.5756	1033.5683	1033.5477	19.9	185 -	192	1	R.EIQMEKLK.I + Oxidation (M)
1349.6682	1348.6609	1348.6921	-23.13	366 -	377	0	R.ELRPLGAYMSGR.L
2872.5757	2871.5684	2871.5429	8.88	98 -	122	2	R.TPQGPGMVLLLPFIDSFQRVDLRTR.A + Oxidation (M)
2884.6123	2883.6050	2883.5793	8.92	96 -	120	2	R.IRTPQGPGMVLLLPFIDSFQRVDLR.T + Oxidation (M)

No match to: 712.3012, 714.3622, 717.4536, 722.4728, 735.4618, 756.5057, 772.4633, 779.5103, 792.4439, 795.4440, 804.3116, 806.4917, 807.4467, 809.4788, 812.4258, 814.4372, 815.4436, 819.4216, 828.4171, 831.4965, 832.3378, 834.3423, 841.4608, 842.4964, 845.4365, 871.5104, 883.4921, 888.4818, 891.4986, 893.9725, 894.4754, 900.5499, 913.5269, 918.5532, 929.4499, 935.4841, 947.4821, 949.5173, 959.4860, 963.4728, 967.4305, 969.4643, 972.4830, 974.4501, 975.4561, 976.4594, 977.4788, 978.5386, 981.4812, 983.4774, 988.4600, 989.6110, 995.4939, 996.4700, 997.4576, 999.4598, 1018.5132, 1019.5350, 1021.5362, 1027.4982, 1032.5875, 1033.5684, 1036.5582, 1044.5192, 1047.5377, 1055.5199, 1056.5244, 1057.5576, 1060.5359, 1062.5424, 1069.5072, 1074.5400, 1077.5109, 1085.5363, 1087.5255, 1091.5013, 1103.5313, 1106.5183, 1107.5238, 1111.5382, 1122.5424, 1123.5519, 1168.5806, 1173.6044, 1178.6016, 1179.5861, 1185.6279, 1187.6195, 1199.6720, 1207.6029, 1209.5723, 1215.6453, 1216.6425, 1226.5743, 1234.5945, 1244.5957, 1248.6305, 1255.5890, 1295.7034, 1301.5924, 1314.6890, 1322.5939, 1325.6377, 1328.6476, 1336.6866, 1337.6793, 1338.6221, 1342.6494, 1344.6713, 1345.6792, 1352.6564, 1353.6359, 1355.6598, 1361.6550, 1368.6198, 1370.6490, 1372.5659, 1381.7262, 1383.6938, 1386.6838, 1394.6993, 1397.7117, 1427.7550, 1432.6827, 1433.6854, 1442.7830, 1446.7039, 1447.6959, 1457.7043, 1473.7709, 1478.7163, 1497.8370, 1528.6653, 1537.7870, 1542.8158, 1550.9301, 1556.8483, 1578.7511, 1601.8662, 1606.8096, 1615.8414, 1616.8064, 1617.7891, 1627.7168, 1628.6741, 1628.8262, 1638.8353, 1642.6895, 1647.8445, 1650.8495, 1679.9406, 1680.7981, 1696.8021, 1706.8348, 1715.8130, 1717.8778, 1718.8860, 1725.8102, 1727.8582, 1730.8500, 1752.8656, 1763.7885, 1768.9357, 1769.9312, 1771.0000, 1775.8153, 1777.8390, 1786.8839, 1788.4598, 1791.8882, 1799.8864, 1800.8976, 1809.0026, 1914.9941, 1928.0222, 1953.8669, 1975.9434, 1982.0046, 2012.9734, 2027.1388, 2042.0795, 2053.0103, 2060.0637, 2070.9099, 2082.9165, 2084.9246, 2086.9263, 2095.0337, 2096.9631, 2098.9319, 2100.9241, 2136.9219, 2155.1025, 2170.1101, 2209.0732, 2211.1094, 2239.2014, 2251.1997, 2289.1399, 2293.2251, 2324.1719, 2343.2795, 2381.2776, 2384.2988, 2398.2314, 2400.1594, 2414.1807, 2428.1990, 2431.1982, 2444.2200, 2465.3230, 2528.3064, 2542.3503, 2546.3127, 2558.3843, 2647.1560, 2661.1709, 2675.1926, 2698.5471, 2706.4133, 2708.4885, 2718.5300, 2762.5994, 2765.5881, 2767.5010, 2771.5437, 2777.7188, 2811.6846, 2814.6851, 2846.6375, 2869.6453, 2888.6077, 2892.6868, 2894.7185, 2902.6804, 2918.6406, 2926.6814, 2946.7546, 2994.8018, 2998.7808, 3003.8159, 3005.8979, 3054.8286, 3082.9351, 3102.9441, 3105.8228, 3127.0818, 3171.0554, 3194.9565, 3202.8757, 3207.9922, 3249.0452, 3263.1082, 3264.1118, 3266.0825, 3300.1990, 3317.0972, 3323.0837, 3331.1343, 3335.1436, 3336.1438, 3348.9949, 3354.0869, 3358.1614, 3371.1931, 3387.2393, 3454.9927, 3459.2039, 3490.2356, 3504.3723, 3518.1758, 3640.3601, 3652.4072, 3664.4243, 3822.6233, 3870.6184, 3871.6218, 3887.6787, 4089.8884, 4148.9229, 4160.9209, 4164.9634, 4175.9526, 4306.0547, 4453.3716, 4466.3892, 4680.6270, 4846.6348

37. Q9NZ50 **Mass:** 13245 **Score:** 17 **Expect:** 5.3e+002 **Matches:** 2

Gamma-synuclein OS=Bos taurus GN=SNCG PE=1 SV=1

Observed	Mr(expt)	Mr(calc)	ppm	Start	End	Miss	Peptide
969.4643	968.4570	968.4637	-6.92	35 -	43	0	K.EGVMYVGAK.T + Oxidation (M)
1427.7550	1426.7477	1426.7490	-0.89	33 -	45	2	K.TKEGVMYVGAKTK.E + Oxidation (M)

No match to: 712.3012, 714.3622, 717.4536, 722.4728, 735.4618, 756.5057, 772.4633, 779.5103, 792.4439, 795.4440, 804.3116, 806.4917, 807.4467, 809.4788, 812.4258, 814.4372, 815.4436, 819.4216, 828.4171, 831.4965, 832.3378, 834.3423, 841.4608, 842.4964, 845.4365, 871.5104, 883.4921, 888.4818, 891.4986, 893.9725, 894.4754, 900.5499, 913.5269, 918.5532, 929.4499, 935.4841, 945.4980, 947.4821, 949.5173, 959.4860, 963.4728, 967.4305, 972.4830, 974.4501, 975.4561, 976.4594, 977.4788, 978.5386, 981.4812, 983.4774, 988.4600, 989.6110, 995.4939, 996.4700, 997.4576, 999.4598, 1007.5580, 1011.4736, 1018.5132, 1019.5350, 1021.5362, 1027.4982, 1032.5875, 1033.5684, 1034.5756, 1036.5582, 1044.5192, 1047.5377, 1055.5199, 1056.5244, 1057.5576, 1060.5359, 1062.5424, 1069.5072, 1074.5400, 1077.5109, 1085.5363, 1087.5255, 1091.5013, 1103.5313, 1106.5183, 1107.5238, 1111.5382, 1122.5424, 1123.5519, 1168.5806, 1173.6044, 1178.6016, 1179.5861, 1185.6279, 1187.6195, 1199.6720, 1207.6029, 1209.5723, 1215.6453, 1216.6425, 1226.5743, 1234.5945, 1244.5957, 1248.6305, 1255.5890, 1295.7034, 1301.5924, 1314.6890, 1322.5939, 1325.6377, 1328.6476, 1336.6866, 1337.6793, 1338.6221, 1342.6494, 1344.6713, 1345.6792, 1349.6682, 1352.6564, 1353.6359, 1355.6598, 1361.6550, 1368.6198, 1370.6490, 1372.5659, 1381.7262, 1383.6938, 1386.6838, 1394.6993, 1397.7117, 1432.6827, 1433.6854, 1442.7830, 1446.7039, 1447.6959, 1457.7043, 1473.7709, 1478.7163,

1497.8370, 1528.6653, 1537.7870, 1542.8158, 1550.9301, 1556.8483, 1578.7511, 1601.8662, 1606.8096, 1615.8414, 1616.8064, 1617.7891, 1627.7168, 1628.6741, 1628.8262, 1638.8353, 1642.6895, 1647.8445, 1650.8495, 1679.9406, 1680.7981, 1696.8021, 1706.8348, 1715.8130, 1717.8778, 1718.8860, 1725.8102, 1727.8582, 1730.8500, 1752.8656, 1763.7885, 1768.9357, 1769.9312, 1771.0000, 1775.8153, 1777.8390, 1786.8839, 1788.4598, 1791.8882, 1799.8864, 1800.8976, 1809.0026, 1914.9941, 1928.0222, 1953.8669, 1975.9434, 1982.0046, 2012.9734, 2027.1388, 2042.0795, 2053.0103, 2060.0637, 2070.9099, 2082.9165, 2084.9246, 2086.9263, 2095.0337, 2096.9631, 2098.9319, 2100.9241, 2136.9219, 2155.1025, 2170.1101, 2209.0732, 2211.1094, 2239.2014, 2251.1997, 2289.1399, 2293.2251, 2324.1719, 2343.2795, 2381.2776, 2384.2988, 2398.2314, 2400.1594, 2414.1807, 2428.1990, 2431.1982, 2444.2200, 2465.3230, 2528.3064, 2542.3503, 2546.3127, 2558.3843, 2647.1560, 2661.1709, 2675.1926, 2698.5471, 2706.4133, 2708.4885, 2718.5300, 2762.5994, 2765.5881, 2767.5010, 2771.5437, 2777.7188, 2811.6846, 2814.6851, 2846.6375, 2869.6453, 2872.5757, 2884.6123, 2888.6077, 2892.6868, 2894.7185, 2902.6804, 2918.6406, 2926.6814, 2946.7546, 2994.8018, 2998.7808, 3003.8159, 3005.8979, 3054.8286, 3082.9351, 3102.9441, 3105.8228, 3127.0818, 3171.0554, 3194.9565, 3202.8757, 3207.9922, 3249.0452, 3263.1082, 3264.1118, 3266.0825, 3300.1990, 3317.0972, 3323.0837, 3331.1343, 3335.1436, 3336.1438, 3348.9949, 3354.0869, 3358.1614, 3371.1931, 3387.2393, 3454.9927, 3459.2039, 3490.2356, 3504.3723, 3518.1758, 3640.3601, 3652.4072, 3664.4243, 3822.6233, 3870.6184, 3871.6218, 3887.6787, 4089.8884, 4148.9229, 4160.9209, 4164.9634, 4175.9526, 4306.0547, 4453.3716, 4466.3892, 4680.6270, 4846.6348

38. F1MG09 **Mass:** 15257 **Score:** 16 **Expect:** 5.4e+002 **Matches:** 3
Uncharacterized protein OS=Bos taurus GN=RPL36AL PE=3 SV=2

Observed	Mr(expt)	Mr(calc)	ppm	Start	End	Miss	Peptide
1349.6682	1348.6609	1348.6888	-20.64	109 -	119	2	K.RYKHFELGGDK.K
1427.7550	1426.7477	1426.7681	-14.27	41 -	52	2	R.QPHNVTQYKKGK.D
1615.8414	1614.8341	1614.8049	18.1	37 -	49	2	K.CGKRQPHNVTQYK.K

No match to: 712.3012, 714.3622, 717.4536, 722.4728, 735.4618, 756.5057, 772.4633, 779.5103, 792.4439, 795.4440, 804.3116, 806.4917, 807.4467, 809.4788, 812.4258, 814.4372, 815.4436, 819.4216, 828.4171, 831.4965, 832.3378, 834.3423, 841.4608, 842.4964, 845.4365, 871.5104, 883.4921, 888.4818, 891.4986, 893.9725, 894.4754, 900.5499, 913.5269, 918.5532, 929.4499, 935.4841, 945.4980, 947.4821, 949.5173, 959.4860, 963.4728, 967.4305, 969.4643, 972.4830, 974.4501, 975.4561, 976.4594, 977.4788, 978.5386, 981.4812, 983.4774, 988.4600, 989.6110, 995.4939, 996.4700, 997.4576, 999.4598, 1007.5580, 1011.4736, 1018.5132, 1019.5350, 1021.5362, 1027.4982, 1032.5875, 1033.5684, 1034.5756, 1036.5582, 1044.5192, 1047.5377, 1055.5199, 1056.5244, 1057.5576, 1060.5359, 1062.5424, 1069.5072, 1074.5400, 1077.5109, 1085.5363, 1087.5255, 1091.5013, 1103.5313, 1106.5183, 1107.5238, 1111.5382, 1122.5424, 1123.5519, 1168.5806, 1173.6044, 1178.6016, 1179.5861, 1185.6279, 1187.6195, 1199.6720, 1207.6029, 1209.5723, 1215.6453, 1216.6425, 1226.5743, 1234.5945, 1244.5957, 1248.6305, 1255.5890, 1295.7034, 1301.5924, 1314.6890, 1322.5939, 1325.6377, 1328.6476, 1336.6866, 1337.6793, 1338.6221, 1342.6494, 1344.6713, 1345.6792, 1352.6564, 1353.6359, 1355.6598, 1361.6550, 1368.6198, 1370.6490, 1372.5659, 1381.7262, 1383.6938, 1386.6838, 1394.6993, 1397.7117, 1432.6827, 1433.6854, 1442.7830, 1446.7039, 1447.6959, 1457.7043, 1473.7709, 1478.7163, 1497.8370, 1528.6653, 1537.7870, 1542.8158, 1550.9301, 1556.8483, 1578.7511, 1601.8662, 1606.8096, 1616.8064, 1617.7891, 1627.7168, 1628.6741, 1628.8262, 1638.8353, 1642.6895, 1647.8445, 1650.8495, 1679.9406, 1680.7981, 1696.8021, 1706.8348, 1715.8130, 1717.8778, 1718.8860, 1725.8102, 1727.8582, 1730.8500, 1752.8656, 1763.7885, 1768.9357, 1769.9312, 1771.0000, 1775.8153, 1777.8390, 1786.8839, 1788.4598, 1791.8882, 1799.8864, 1800.8976, 1809.0026, 1914.9941, 1928.0222, 1953.8669, 1975.9434, 1982.0046, 2012.9734, 2027.1388, 2042.0795, 2053.0103, 2060.0637, 2070.9099, 2082.9165, 2084.9246, 2086.9263, 2095.0337, 2096.9631, 2098.9319, 2100.9241, 2136.9219, 2155.1025, 2170.1101, 2209.0732, 2211.1094, 2239.2014, 2251.1997, 2289.1399, 2293.2251, 2324.1719, 2343.2795, 2381.2776, 2384.2988, 2398.2314, 2400.1594, 2414.1807, 2428.1990, 2431.1982, 2444.2200, 2465.3230, 2528.3064, 2542.3503, 2546.3127, 2558.3843, 2647.1560, 2661.1709, 2675.1926, 2698.5471, 2706.4133, 2708.4885, 2718.5300, 2762.5994, 2765.5881, 2767.5010, 2771.5437, 2777.7188, 2811.6846, 2814.6851, 2846.6375, 2869.6453, 2872.5757, 2884.6123, 2888.6077, 2892.6868, 2894.7185, 2902.6804, 2918.6406, 2926.6814, 2946.7546, 2994.8018, 2998.7808, 3003.8159, 3005.8979, 3054.8286, 3082.9351, 3102.9441, 3105.8228, 3127.0818, 3171.0554, 3194.9565, 3202.8757, 3207.9922, 3249.0452, 3263.1082, 3264.1118, 3266.0825, 3300.1990, 3317.0972, 3323.0837, 3331.1343, 3335.1436, 3336.1438, 3348.9949, 3354.0869, 3358.1614, 3371.1931, 3387.2393, 3454.9927, 3459.2039, 3490.2356, 3504.3723, 3518.1758, 3640.3601, 3652.4072, 3664.4243, 3822.6233, 3870.6184, 3871.6218, 3887.6787, 4089.8884, 4148.9229, 4160.9209, 4164.9634, 4175.9526, 4306.0547, 4453.3716, 4466.3892, 4680.6270, 4846.6348

39. F1N091 **Mass:** 43136 **Score:** 16 **Expect:** 5.4e+002 **Matches:** 6
Uncharacterized protein OS=Bos taurus GN=CCDC6 PE=4 SV=1

Observed	Mr(expt)	Mr(calc)	ppm	Start	End	Miss	Peptide
963.4728	962.4656	962.4742	-9.02	120 -	127	1	R.MDKLEAEK.R
977.4788	976.4715	976.4760	-4.55	207 -	213	1	K.LQREMER.R + Oxidation (M)
1034.5756	1033.5683	1033.5556	12.3	163 -	170	1	R.FLKNEVER.L
1057.5576	1056.5503	1056.5676	-16.32	198 -	205	1	R.EENLRLQR.K
1349.6682	1348.6609	1348.6339	20.0	210 -	219	2	R.EMERREALCR.Q
2414.1807	2413.1734	2413.1648	3.56	27 -	46	1	K.ETLAVNYEKEEEFLTNELSR.K

No match to: 712.3012, 714.3622, 717.4536, 722.4728, 735.4618, 756.5057, 772.4633, 779.5103, 792.4439, 795.4440, 804.3116, 806.4917, 807.4467, 809.4788, 812.4258, 814.4372, 815.4436, 819.4216, 828.4171, 831.4965, 832.3378, 834.3423, 841.4608, 842.4964, 845.4365, 871.5104, 883.4921, 888.4818, 891.4986, 893.9725, 894.4754, 900.5499, 913.5269, 918.5532, 929.4499, 935.4841, 945.4980, 947.4821, 949.5173, 959.4860, 967.4305, 969.4643, 972.4830, 974.4501, 975.4561, 976.4594, 978.5386, 981.4812, 983.4774, 988.4600, 989.6110, 995.4939, 996.4700, 997.4576, 999.4598, 1007.5580, 1011.4736, 1018.5132, 1019.5350, 1021.5362, 1027.4982, 1032.5875, 1033.5684, 1036.5582, 1044.5192, 1047.5377, 1055.5199, 1056.5244, 1060.5359, 1062.5424, 1069.5072, 1074.5400, 1077.5109, 1085.5363, 1087.5255, 1091.5013, 1103.5313, 1106.5183, 1107.5238, 1111.5382, 1122.5424, 1123.5519, 1168.5806, 1173.6044, 1178.6016, 1179.5861, 1185.6279, 1187.6195, 1199.6720, 1207.6029, 1209.5723, 1215.6453, 1216.6425, 1226.5743, 1234.5945, 1244.5957, 1248.6305, 1255.5890, 1295.7034, 1301.5924, 1314.6890, 1322.5939, 1325.6377, 1328.6476, 1336.6866, 1337.6793, 1338.6221, 1342.6494, 1344.6713, 1345.6792, 1352.6564, 1353.6359, 1355.6598, 1361.6550, 1368.6198, 1370.6490, 1372.5659, 1381.7262, 1383.6938, 1386.6838, 1394.6993, 1397.7117, 1427.7550, 1432.6827, 1433.6854, 1442.7830, 1446.7039, 1447.6959, 1457.7043, 1473.7709, 1478.7163, 1497.8370, 1528.6653, 1537.7870, 1542.8158, 1550.9301, 1556.8483, 1578.7511, 1601.8662, 1606.8096, 1615.8414, 1616.8064, 1617.7891, 1627.7168, 1628.6741, 1628.8262, 1638.8353, 1642.6895, 1647.8445, 1650.8495, 1679.9406, 1680.7981, 1696.8021, 1706.8348, 1715.8130, 1717.8778, 1718.8860, 1725.8102, 1727.8582, 1730.8500, 1752.8656, 1763.7885, 1768.9357, 1769.9312, 1771.0000, 1775.8153, 1777.8390, 1786.8839, 1788.4598, 1791.8882, 1799.8864, 1800.8976, 1809.0026, 1914.9941, 1928.0222, 1953.8669, 1975.9434, 1982.0046, 2012.9734, 2027.1388, 2042.0795, 2053.0103, 2060.0637, 2070.9099, 2082.9165, 2084.9246, 2086.9263, 2095.0337, 2096.9631, 2098.9319, 2100.9241, 2136.9219, 2155.1025, 2170.1101, 2209.0732, 2211.1094, 2239.2014, 2251.1997, 2289.1399, 2293.2251, 2324.1719, 2343.2795, 2381.2776, 2384.2988, 2398.2314, 2400.1594, 2428.1990, 2431.1982, 2444.2200, 2465.3230, 2528.3064, 2542.3503, 2546.3127, 2558.3843, 2647.1560, 2661.1709, 2675.1926, 2698.5471, 2706.4133, 2708.4885, 2718.5300, 2762.5994, 2765.5881, 2767.5010, 2771.5437, 2777.7188, 2811.6846, 2814.6851, 2846.6375, 2869.6453, 2872.5757, 2884.6123, 2888.6077, 2892.6868, 2894.7185, 2902.6804, 2918.6406, 2926.6814, 2946.7546, 2994.8018, 2998.7808, 3003.8159, 3005.8979, 3054.8286, 3082.9351, 3102.9441, 3105.8228, 3127.0818, 3171.0554, 3194.9565, 3202.8757, 3207.9922, 3249.0452, 3263.1082, 3264.1118, 3266.0825, 3300.1990, 3317.0972, 3323.0837, 3331.1343, 3335.1436, 3336.1438, 3348.9949, 3354.0869, 3358.1614, 3371.1931, 3387.2393, 3454.9927, 3459.2039, 3490.2356, 3504.3723, 3518.1758, 3640.3601, 3652.4072, 3664.4243, 3822.6233, 3870.6184, 3871.6218, 3887.6787, 4089.8884, 4148.9229, 4160.9209, 4164.9634, 4175.9526, 4306.0547, 4453.3716, 4466.3892, 4680.6270, 4846.6348

40. Q2YDJ9 **Mass:** 40439 **Score:** 16 **Expect:** 5.5e+002 **Matches:** 7
Mannose-1-phosphate guanyltransferase beta OS=Bos taurus GN=GMPPB PE=2 SV=1

Observed	Mr(expt)	Mr(calc)	ppm	Start	End	Miss	Peptide
1060.5359	1059.5286	1059.5396	-10.38	312 -	319	1	R.CRVGQWVR.M
1325.6377	1324.6304	1324.6082	16.8	144 -	155	0	K.YGVVVCEADTGR.V
1344.6713	1343.6640	1343.6618	1.67	224 -	234	0	K.DFLTGMCLFLK.S
1427.7550	1426.7477	1426.7490	-0.88	348 -	360	1	K.SIGESVPEPRIIM.-
1717.8778	1716.8705	1716.8366	19.8	144 -	158	1	K.YGVVVCEADTGRVHR.F
2095.0337	2094.0264	2094.0415	-7.21	75 -	94	0	R.ISMSHEEEPLGTAGPLALAR.D + Oxidation (M)
2209.0732	2208.0659	2208.0647	0.56	294 -	311	1	R.DAHIRSHSWLESCIVGWR.C

No match to: 712.3012, 714.3622, 717.4536, 722.4728, 735.4618, 756.5057, 772.4633, 779.5103, 792.4439, 795.4440, 804.3116, 806.4917, 807.4467, 809.4788, 812.4258, 814.4372, 815.4436, 819.4216, 828.4171, 831.4965, 832.3378, 834.3423, 841.4608, 842.4964, 845.4365, 871.5104, 883.4921, 888.4818, 891.4986, 893.9725, 894.4754, 900.5499, 913.5269, 918.5532, 929.4499, 935.4841, 945.4980, 947.4821, 949.5173, 959.4860, 963.4728, 967.4305, 969.4643, 972.4830, 974.4501, 975.4561, 976.4594, 977.4788, 978.5386, 981.4812,

983.4774, 988.4600, 989.6110, 995.4939, 996.4700, 997.4576, 999.4598, 1007.5580, 1011.4736, 1018.5132, 1019.5350, 1021.5362, 1027.4982, 1032.5875, 1033.5684, 1034.5756, 1036.5582, 1044.5192, 1047.5377, 1055.5199, 1056.5244, 1057.5576, 1062.5424, 1069.5072, 1074.5400, 1077.5109, 1085.5363, 1087.5255, 1091.5013, 1103.5313, 1106.5183, 1107.5238, 1111.5382, 1122.5424, 1123.5519, 1168.5806, 1173.6044, 1178.6016, 1179.5861, 1185.6279, 1187.6195, 1199.6720, 1207.6029, 1209.5723, 1215.6453, 1216.6425, 1226.5743, 1234.5945, 1244.5957, 1248.6305, 1255.5890, 1295.7034, 1301.5924, 1314.6890, 1322.5939, 1328.6476, 1336.6866, 1337.6793, 1338.6221, 1342.6494, 1345.6792, 1349.6682, 1352.6564, 1353.6359, 1355.6598, 1361.6550, 1368.6198, 1370.6490, 1372.5659, 1381.7262, 1383.6938, 1386.6838, 1394.6993, 1397.7117, 1432.6827, 1433.6854, 1442.7830, 1446.7039, 1447.6959, 1457.7043, 1473.7709, 1478.7163, 1497.8370, 1528.6653, 1537.7870, 1542.8158, 1550.9301, 1556.8483, 1578.7511, 1601.8662, 1606.8096, 1615.8414, 1616.8064, 1617.7891, 1627.7168, 1628.6741, 1628.8262, 1638.8353, 1642.6895, 1647.8445, 1650.8495, 1679.9406, 1680.7981, 1696.8021, 1706.8348, 1715.8130, 1718.8860, 1725.8102, 1727.8582, 1730.8500, 1752.8656, 1763.7885, 1768.9357, 1769.9312, 1771.0000, 1775.8153, 1777.8390, 1786.8839, 1788.4598, 1791.8882, 1799.8864, 1800.8976, 1809.0026, 1914.9941, 1928.0222, 1953.8669, 1975.9434, 1982.0046, 2012.9734, 2027.1388, 2042.0795, 2053.0103, 2060.0637, 2070.9099, 2082.9165, 2084.9246, 2086.9263, 2096.9631, 2098.9319, 2100.9241, 2136.9219, 2155.1025, 2170.1101, 2211.1094, 2239.2014, 2251.1997, 2289.1399, 2293.2251, 2324.1719, 2343.2795, 2381.2776, 2384.2988, 2398.2314, 2400.1594, 2414.1807, 2428.1990, 2431.1982, 2444.2200, 2465.3230, 2528.3064, 2542.3503, 2546.3127, 2558.3843, 2647.1560, 2661.1709, 2675.1926, 2698.5471, 2706.4133, 2708.4885, 2718.5300, 2762.5994, 2765.5881, 2767.5010, 2771.5437, 2777.7188, 2811.6846, 2814.6851, 2846.6375, 2869.6453, 2872.5757, 2884.6123, 2888.6077, 2892.6868, 2894.7185, 2902.6804, 2918.6406, 2926.6814, 2946.7546, 2994.8018, 2998.7808, 3003.8159, 3005.8979, 3054.8286, 3082.9351, 3102.9441, 3105.8228, 3127.0818, 3171.0554, 3194.9565, 3202.8757, 3207.9922, 3249.0452, 3263.1082, 3264.1118, 3266.0825, 3300.1990, 3317.0972, 3323.0837, 3331.1343, 3335.1436, 3336.1438, 3348.9949, 3354.0869, 3358.1614, 3371.1931, 3387.2393, 3454.9927, 3459.2039, 3490.2356, 3504.3723, 3518.1758, 3640.3601, 3652.4072, 3664.4243, 3822.6233, 3870.6184, 3871.6218, 3887.6787, 4089.8884, 4148.9229, 4160.9209, 4164.9634, 4175.9526, 4306.0547, 4453.3716, 4466.3892, 4680.6270, 4846.6348

41. F1N7H5 **Mass:** 40398 **Score:** 16 **Expect:** 5.5e+002 **Matches:** 6

Mannose-1-phosphate guanyltransferase beta OS=Bos taurus GN=GMPPB PE=4 SV=2

Observed	Mr(expt)	Mr(calc)	ppm	Start	End	Miss	Peptide
1060.5359	1059.5286	1059.5396	-10.38	312	- 319	1	R.CRVGQWVR.M
1325.6377	1324.6304	1324.6082	16.8	144	- 155	0	K.YGVVVCEADTGR.V
1427.7550	1426.7477	1426.7490	-0.88	348	- 360	1	K.SIGESVPEPRIIM.-
1717.8778	1716.8705	1716.8366	19.8	144	- 158	1	K.YGVVVCEADTGRVHR.F
2095.0337	2094.0264	2094.0415	-7.21	75	- 94	0	R.ISMSHEEEPLGTAGPLALAR.D + Oxidation (M)
2209.0732	2208.0659	2208.0647	0.56	294	- 311	1	R.DAHIRSHSWLESCIVGWR.C

No match to: 712.3012, 714.3622, 717.4536, 722.4728, 735.4618, 756.5057, 772.4633, 779.5103, 792.4439, 795.4440, 804.3116, 806.4917, 807.4467, 809.4788, 812.4258, 814.4372, 815.4436, 819.4216, 828.4171, 831.4965, 832.3378, 834.3423, 841.4608, 842.4964, 845.4365, 871.5104, 883.4921, 888.4818, 891.4986, 893.9725, 894.4754, 900.5499, 913.5269, 918.5532, 929.4499, 935.4841, 945.4980, 947.4821, 949.5173, 959.4860, 963.4728, 967.4305, 969.4643, 972.4830, 974.4501, 975.4561, 976.4594, 977.4788, 978.5386, 981.4812, 983.4774, 988.4600, 989.6110, 995.4939, 996.4700, 997.4576, 999.4598, 1007.5580, 1011.4736, 1018.5132, 1019.5350, 1021.5362, 1027.4982, 1032.5875, 1033.5684, 1034.5756, 1036.5582, 1044.5192, 1047.5377, 1055.5199, 1056.5244, 1057.5576, 1062.5424, 1069.5072, 1074.5400, 1077.5109, 1085.5363, 1087.5255, 1091.5013, 1103.5313, 1106.5183, 1107.5238, 1111.5382, 1122.5424, 1123.5519, 1168.5806, 1173.6044, 1178.6016, 1179.5861, 1185.6279, 1187.6195, 1199.6720, 1207.6029, 1209.5723, 1215.6453, 1216.6425, 1226.5743, 1234.5945, 1244.5957, 1248.6305, 1255.5890, 1295.7034, 1301.5924, 1314.6890, 1322.5939, 1328.6476, 1336.6866, 1337.6793, 1338.6221, 1342.6494, 1344.6713, 1345.6792, 1349.6682, 1352.6564, 1353.6359, 1355.6598, 1361.6550, 1368.6198, 1370.6490, 1372.5659, 1381.7262, 1383.6938, 1386.6838, 1394.6993, 1397.7117, 1432.6827, 1433.6854, 1442.7830, 1446.7039, 1447.6959, 1457.7043, 1473.7709, 1478.7163, 1497.8370, 1528.6653, 1537.7870, 1542.8158, 1550.9301, 1556.8483, 1578.7511, 1601.8662, 1606.8096, 1615.8414, 1616.8064, 1617.7891, 1627.7168, 1628.6741, 1628.8262, 1638.8353, 1642.6895, 1647.8445, 1650.8495, 1679.9406, 1680.7981, 1696.8021, 1706.8348, 1715.8130, 1718.8860, 1725.8102, 1727.8582, 1730.8500, 1752.8656, 1763.7885, 1768.9357, 1769.9312, 1771.0000, 1775.8153, 1777.8390, 1786.8839, 1788.4598, 1791.8882, 1799.8864, 1800.8976, 1809.0026, 1914.9941, 1928.0222, 1953.8669, 1975.9434, 1982.0046, 2012.9734, 2027.1388, 2042.0795, 2053.0103, 2060.0637, 2070.9099, 2082.9165, 2084.9246, 2086.9263, 2096.9631, 2098.9319, 2100.9241, 2136.9219, 2155.1025, 2170.1101, 2211.1094, 2239.2014, 2251.1997, 2289.1399, 2293.2251, 2324.1719, 2343.2795, 2381.2776, 2384.2988, 2398.2314, 2400.1594, 2414.1807, 2428.1990, 2431.1982, 2444.2200, 2465.3230, 2528.3064, 2542.3503, 2546.3127, 2558.3843, 2647.1560, 2661.1709, 2675.1926, 2698.5471, 2706.4133, 2708.4885, 2718.5300, 2762.5994, 2765.5881, 2767.5010, 2771.5437, 2777.7188, 2811.6846, 2814.6851, 2846.6375, 2869.6453, 2872.5757, 2884.6123, 2888.6077, 2892.6868, 2894.7185, 2902.6804, 2918.6406, 2926.6814, 2946.7546, 2994.8018, 2998.7808, 3003.8159, 3005.8979, 3054.8286, 3082.9351, 3102.9441, 3105.8228, 3127.0818, 3171.0554, 3194.9565, 3202.8757, 3207.9922, 3249.0452, 3263.1082, 3264.1118, 3266.0825, 3300.1990, 3317.0972, 3323.0837, 3331.1343, 3335.1436, 3336.1438, 3348.9949, 3354.0869, 3358.1614, 3371.1931, 3387.2393, 3454.9927, 3459.2039, 3490.2356, 3504.3723, 3518.1758, 3640.3601, 3652.4072, 3664.4243, 3822.6233, 3870.6184, 3871.6218, 3887.6787, 4089.8884, 4148.9229, 4160.9209, 4164.9634, 4175.9526, 4306.0547, 4453.3716, 4466.3892, 4680.6270, 4846.6348

42. E1B999 **Mass:** 83144 **Score:** 16 **Expect:** 5.8e+002 **Matches:** 13

Kinesin-like protein OS=Bos taurus GN=KIF3A PE=3 SV=2

Observed	Mr(expt)	Mr(calc)	ppm	Start	End	Miss	Peptide
815.4436	814.4363	814.4371	-0.96	198	- 204	0	R.IMTLGHK.N + Oxidation (M)
888.4818	887.4745	887.4964	-24.58	366	- 372	1	K.EIEELKK.K
974.4501	973.4428	973.4352	7.76	51	- 59	0	K.TDSSNEPPK.T
1173.6044	1172.5971	1172.6149	-15.20	159	- 168	1	R.DLLGKDQTQR.L
1185.6279	1184.6206	1184.6401	-16.41	597	- 606	0	R.EIEGLLENIR.Q
1345.6792	1344.6719	1344.6633	6.41	543	- 552	2	R.RELEEKEQER.L
1394.6993	1393.6920	1393.6581	24.3	441	- 452	1	K.VSPDKMVEMQAK.I + 2 Oxidation (M)
1478.7163	1477.7090	1477.6905	12.6	446	- 457	1	K.MVEMQAKIDEER.K
1556.8483	1555.8410	1555.8569	-10.24	713	- 726	1	K.RSAKPETVIDSLLQ.-
1606.8096	1605.8023	1605.7854	10.5	446	- 458	2	K.MVEMQAKIDEERK.A
1786.8839	1785.8766	1785.9043	-15.52	35	- 50	1	K.QAVSVDEMRGTITVHK.T + Oxidation (M)
2095.0337	2094.0264	2093.9986	13.3	648	- 665	2	K.CVAYTGNNMRKQTPVPDK.K + Oxidation (M)
2343.2795	2342.2722	2342.3057	-14.28	269	- 291	1	K.EATKINLSLSTLGNVISALVDGK.S

No match to: 712.3012, 714.3622, 717.4536, 722.4728, 735.4618, 756.5057, 772.4633, 779.5103, 792.4439, 795.4440, 804.3116, 806.4917, 807.4467, 809.4788, 812.4258, 814.4372, 819.4216, 828.4171, 831.4965, 832.3378, 834.3423, 841.4608, 842.4964, 845.4365, 871.5104, 883.4921, 891.4986, 893.9725, 894.4754, 900.5499, 913.5269, 918.5532, 929.4499, 935.4841, 945.4980, 947.4821, 949.5173, 959.4860, 963.4728, 967.4305, 969.4643, 972.4830, 975.4561, 976.4594, 977.4788, 978.5386, 981.4812, 983.4774, 988.4600, 989.6110, 995.4939, 996.4700, 997.4576, 999.4598, 1007.5580, 1011.4736, 1018.5132, 1019.5350, 1021.5362, 1027.4982, 1032.5875, 1033.5684, 1034.5756, 1036.5582, 1044.5192, 1047.5377, 1055.5199, 1056.5244, 1057.5576, 1060.5359, 1062.5424, 1069.5072, 1074.5400, 1077.5109, 1085.5363, 1087.5255, 1091.5013, 1103.5313, 1106.5183, 1107.5238, 1111.5382, 1122.5424, 1123.5519, 1168.5806, 1178.6016, 1179.5861, 1187.6195, 1199.6720, 1207.6029, 1209.5723, 1215.6453, 1216.6425, 1226.5743, 1234.5945, 1244.5957, 1248.6305, 1255.5890, 1295.7034, 1301.5924, 1314.6890, 1322.5939, 1325.6377, 1328.6476, 1336.6866, 1337.6793, 1338.6221, 1342.6494, 1344.6713, 1349.6682, 1352.6564, 1353.6359, 1355.6598, 1361.6550, 1368.6198, 1370.6490, 1372.5659, 1381.7262, 1383.6938, 1386.6838, 1397.7117, 1427.7550, 1432.6827, 1433.6854, 1442.7830, 1446.7039, 1447.6959, 1457.7043, 1473.7709, 1497.8370, 1528.6653, 1537.7870, 1542.8158, 1550.9301, 1578.7511, 1601.8662, 1615.8414, 1616.8064, 1617.7891, 1627.7168, 1628.6741, 1628.8262, 1638.8353, 1642.6895, 1647.8445, 1650.8495, 1679.9406, 1680.7981, 1696.8021, 1706.8348, 1715.8130, 1717.8778, 1718.8860, 1725.8102, 1727.8582, 1730.8500, 1752.8656, 1763.7885, 1768.9357, 1769.9312, 1771.0000, 1775.8153, 1777.8390, 1788.4598, 1791.8882, 1799.8864, 1800.8976, 1809.0026, 1914.9941, 1928.0222, 1953.8669, 1975.9434, 1982.0046, 2012.9734, 2027.1388, 2042.0795, 2053.0103, 2060.0637, 2070.9099, 2082.9165, 2084.9246, 2086.9263, 2096.9631, 2098.9319, 2100.9241, 2136.9219, 2155.1025, 2170.1101, 2209.0732, 2211.1094, 2239.2014, 2251.1997, 2289.1399, 2293.2251, 2324.1719, 2381.2776, 2384.2988, 2398.2314, 2400.1594, 2414.1807, 2428.1990, 2431.1982, 2444.2200, 2465.3230, 2528.3064, 2542.3503, 2546.3127, 2558.3843, 2647.1560, 2661.1709, 2675.1926, 2698.5471, 2706.4133, 2708.4885, 2718.5300, 2762.5994, 2765.5881, 2767.5010, 2771.5437, 2777.7188, 2811.6846, 2814.6851, 2846.6375, 2869.6453, 2872.5757, 2884.6123, 2888.6077, 2892.6868, 2894.7185, 2902.6804, 2918.6406, 2926.6814, 2946.7546, 2994.8018, 2998.7808, 3003.8159, 3005.8979, 3054.8286, 3082.9351, 3102.9441, 3105.8228, 3127.0818, 3171.0554, 3194.9565, 3202.8757, 3207.9922, 3249.0452, 3263.1082, 3264.1118, 3266.0825, 3300.1990, 3317.0972, 3323.0837, 3331.1343, 3335.1436, 3336.1438, 3348.9949, 3354.0869, 3358.1614, 3371.1931, 3387.2393, 3454.9927, 3459.2039, 3490.2356, 3504.3723, 3518.1758, 3640.3601, 3652.4072, 3664.4243, 3822.6233, 3870.6184, 3871.6218, 3887.6787, 4089.8884, 4148.9229, 4160.9209, 4164.9634, 4175.9526, 4306.0547, 4453.3716, 4466.3892, 4680.6270, 4846.6348

43. F1MFY7 **Mass:** 101515 **Score:** 16 **Expect:** 5.9e+002 **Matches:** 6

Anoctamin OS=Bos taurus GN=ANO6 PE=3 SV=1

Observed	Mr(expt)	Mr(calc)	ppm	Start	End	Miss	Peptide
935.4841	934.4768	934.4913	-15.49	391	- 398	0	R.VPFTTWGK.C
1442.7830	1441.7757	1441.7711	3.19	846	- 858	0	K.MIGAVVDNNLRPK.S + Oxidation (M)
1537.7870	1536.7797	1536.8082	-18.55	656	- 667	2	K.LTTQYRRMVPEK.A + Oxidation (M)
1628.8262	1627.8189	1627.8352	-9.98	846	- 860	1	K.MIGAVVDNNLRPKSD.-
1768.9357	1767.9284	1767.9155	7.30	18	- 31	2	R.RIDFVLVYEDESRK.E
4148.9229	4147.9156	4147.9057	2.39	193	- 226	2	K.AAFPLHDCNFSTPSEDLSCPNERYLLYREWAHPR.S

No match to: 712.3012, 714.3622, 717.4536, 722.4728, 735.4618, 756.5057, 772.4633, 779.5103, 792.4439, 795.4440, 804.3116, 806.4917, 807.4467, 809.4788, 812.4258, 814.4372, 815.4436, 819.4216, 828.4171, 831.4965, 832.3378, 834.3423, 841.4608, 842.4964, 845.4365, 871.5104, 883.4921, 888.4818, 891.4986, 893.9725, 894.4754, 900.5499, 913.5269, 918.5532, 929.4499, 945.4980, 947.4821, 949.5173, 959.4860, 963.4728, 967.4305, 969.4643, 972.4830, 974.4501, 975.4561, 976.4594, 977.4788, 978.5386, 981.4812, 983.4774, 988.4600, 989.6110, 995.4939, 996.4700, 997.4576, 999.4598, 1007.5580, 1011.4736, 1018.5132, 1019.5350, 1021.5362, 1027.4982, 1032.5875, 1033.5684, 1034.5756, 1036.5582, 1044.5192, 1047.5377, 1055.5199, 1056.5244, 1057.5576, 1060.5359, 1062.5424, 1069.5072, 1074.5400, 1077.5109, 1085.5363, 1087.5255, 1091.5013, 1103.5313, 1106.5183, 1107.5238, 1111.5382, 1122.5424, 1123.5519, 1168.5806, 1173.6044, 1178.6016, 1179.5861, 1185.6279, 1187.6195, 1199.6720, 1207.6029, 1209.5723, 1215.6453, 1216.6425, 1226.5743, 1234.5945, 1244.5957, 1248.6305, 1255.5890, 1295.7034, 1301.5924, 1314.6890, 1322.5939, 1325.6377, 1328.6476, 1336.6866, 1337.6793, 1338.6221, 1342.6494, 1344.6713, 1345.6792, 1349.6682, 1352.6564, 1353.6359, 1355.6598, 1361.6550, 1368.6198, 1370.6490, 1372.5659, 1381.7262, 1383.6938, 1386.6838, 1394.6993, 1397.7117, 1427.7550, 1432.6827, 1433.6854, 1446.7039, 1447.6959, 1457.7043, 1473.7709, 1478.7163, 1497.8370, 1528.6653, 1542.8158, 1550.9301, 1556.8483, 1578.7511, 1601.8662, 1606.8096, 1615.8414, 1616.8064, 1617.7891, 1627.7168, 1628.6741, 1638.8353, 1642.6895, 1647.8445, 1650.8495, 1679.9406, 1680.7981, 1696.8021, 1706.8348, 1715.8130, 1717.8778, 1718.8860, 1725.8102, 1727.8582, 1730.8500, 1752.8656, 1763.7885, 1769.9312, 1771.0000, 1775.8153, 1777.8390, 1786.8839, 1788.4598, 1791.8882, 1799.8864, 1800.8976, 1809.0026, 1914.9941, 1928.0222, 1953.8669, 1975.9434, 1982.0046, 2012.9734, 2027.1388, 2042.0795, 2053.0103, 2060.0637, 2070.9099, 2082.9165, 2084.9246, 2086.9263, 2095.0337, 2096.9631, 2098.9319, 2100.9241, 2136.9219, 2155.1025, 2170.1101, 2209.0732, 2211.1094, 2239.2014, 2251.1997, 2289.1399, 2293.2251, 2324.1719, 2343.2795, 2381.2776, 2384.2988, 2398.2314, 2400.1594, 2414.1807, 2428.1990, 2431.1982, 2444.2200, 2465.3230, 2528.3064, 2542.3503, 2546.3127, 2558.3843, 2647.1560, 2661.1709, 2675.1926, 2698.5471, 2706.4133, 2708.4885, 2718.5300, 2762.5994, 2765.5881, 2767.5010, 2771.5437, 2777.7188, 2811.6846, 2814.6851, 2846.6375, 2869.6453, 2872.5757, 2884.6123, 2888.6077, 2892.6868, 2894.7185, 2902.6804, 2918.6406, 2926.6814, 2946.7546, 2994.8018, 2998.7808, 3003.8159, 3005.8979, 3054.8286, 3082.9351, 3102.9441, 3105.8228, 3127.0818, 3171.0554, 3194.9565, 3202.8757, 3207.9922, 3249.0452, 3263.1082, 3264.1118, 3266.0825, 3300.1990, 3317.0972, 3323.0837, 3331.1343, 3335.1436, 3336.1438, 3348.9949, 3354.0869, 3358.1614, 3371.1931, 3387.2393, 3454.9927, 3459.2039, 3490.2356, 3504.3723, 3518.1758, 3640.3601, 3652.4072, 3664.4243, 3822.6233, 3870.6184, 3871.6218, 3887.6787, 4089.8884, 4160.9209, 4164.9634, 4175.9526, 4306.0547, 4453.3716, 4466.3892, 4680.6270, 4846.6348

44. E1BPA1 **Mass:** 148570 **Score:** 16 **Expect:** 6.1e+002 **Matches:** 14

Uncharacterized protein OS=Bos taurus GN=TBC1D4 PE=4 SV=1

Observed	Mr(expt)	Mr(calc)	ppm	Start	End	Miss	Peptide
1044.5192	1043.5119	1043.5148	-2.79	229	- 236	0	K.FSLHEQQR.L
1074.5400	1073.5327	1073.5465	-12.83	863	- 871	1	K.ENQKLEASR.D
1248.6305	1247.6232	1247.6081	12.2	840	- 848	1	K.SEELRCLWR.K
1325.6377	1324.6304	1324.6234	5.29	38	- 48	0	R.LWYVGGSCLDR.R
1349.6682	1348.6609	1348.6881	-20.18	315	- 326	1	R.SRCSSVTGVLQR.K
1478.7163	1477.7090	1477.7194	-7.05	860	- 871	2	R.MEKENQKLEASR.D + Oxidation (M)
1617.7891	1616.7818	1616.7650	10.4	908	- 920	1	K.IRCDMEDIHTSLK.E
1628.8262	1627.8189	1627.7929	16.0	36	- 48	1	R.FRLWYVGGSCLDR.R
1647.8445	1646.8372	1646.8661	-17.54	176	- 190	2	R.QLSKAAMKEDAKPSK.D + Oxidation (M)
1679.9406	1678.9333	1678.8943	23.2	928	- 940	1	R.RGEIWQFLALQYR.L
1715.8130	1714.8057	1714.8420	-21.18	673	- 687	1	R.QSSSEQCSILPPARR.M
1768.9357	1767.9284	1767.8911	21.1	64	- 80	2	R.RRSQKPEAGGCGAPAAR.E
4148.9229	4147.9156	4147.9163	-0.16	804	- 839	2	R.SELLPLSPLAPTMEEEPLMIFMSDEDDPEKGEERNK.S + 2 Oxidation (M)
4164.9634	4163.9561	4163.9112	10.8	804	- 839	2	R.SELLPLSPLAPTMEEEPLMIFMSDEDDPEKGEERNK.S + 3 Oxidation (M)

No match to: 712.3012, 714.3622, 717.4536, 722.4728, 735.4618, 756.5057, 772.4633, 779.5103, 792.4439, 795.4440, 804.3116, 806.4917, 807.4467, 809.4788, 812.4258, 814.4372, 815.4436, 819.4216, 828.4171, 831.4965, 832.3378, 834.3423, 841.4608, 842.4964, 845.4365, 871.5104, 883.4921, 888.4818, 891.4986, 893.9725, 894.4754, 900.5499, 913.5269, 918.5532, 929.4499, 935.4841, 945.4980, 947.4821, 949.5173, 959.4860, 963.4728, 967.4305, 969.4643, 972.4830, 974.4501, 975.4561, 976.4594, 977.4788, 978.5386, 981.4812, 983.4774, 988.4600, 989.6110, 995.4939, 996.4700, 997.4576, 999.4598, 1007.5580, 1011.4736, 1018.5132, 1019.5350, 1021.5362, 1027.4982, 1032.5875, 1033.5684, 1034.5756, 1036.5582, 1047.5377, 1055.5199, 1056.5244, 1057.5576, 1060.5359, 1062.5424, 1069.5072, 1077.5109, 1085.5363, 1087.5255, 1091.5013, 1103.5313, 1106.5183, 1107.5238, 1111.5382, 1122.5424, 1123.5519, 1168.5806, 1173.6044, 1178.6016, 1179.5861, 1185.6279, 1187.6195, 1199.6720, 1207.6029, 1209.5723, 1215.6453, 1216.6425, 1226.5743, 1234.5945, 1244.5957, 1255.5890, 1295.7034, 1301.5924, 1314.6890, 1322.5939, 1328.6476, 1336.6866, 1337.6793, 1338.6221, 1342.6494, 1344.6713, 1345.6792, 1352.6564, 1353.6359, 1355.6598, 1361.6550, 1368.6198, 1370.6490, 1372.5659, 1381.7262, 1383.6938, 1386.6838, 1394.6993, 1397.7117, 1427.7550, 1432.6827, 1433.6854, 1442.7830, 1446.7039, 1447.6959, 1457.7043, 1473.7709, 1497.8370, 1528.6653, 1537.7870, 1542.8158, 1550.9301, 1556.8483, 1578.7511, 1601.8662, 1606.8096, 1615.8414, 1616.8064, 1627.7168, 1628.6741, 1638.8353, 1642.6895, 1650.8495, 1680.7981, 1696.8021, 1706.8348, 1717.8778, 1718.8860, 1725.8102, 1727.8582, 1730.8500, 1752.8656, 1763.7885, 1769.9312, 1771.0000, 1775.8153, 1777.8390, 1786.8839, 1788.4598, 1791.8882, 1799.8864, 1800.8976, 1809.0026, 1914.9941, 1928.0222, 1953.8669, 1975.9434, 1982.0046, 2012.9734, 2027.1388, 2042.0795, 2053.0103, 2060.0637, 2070.9099, 2082.9165, 2084.9246, 2086.9263, 2095.0337, 2096.9631, 2098.9319, 2100.9241, 2136.9219, 2155.1025, 2170.1101, 2209.0732, 2211.1094, 2239.2014, 2251.1997, 2289.1399, 2293.2251, 2324.1719, 2343.2795, 2381.2776, 2384.2988, 2398.2314, 2400.1594, 2414.1807, 2428.1990, 2431.1982, 2444.2200, 2465.3230, 2528.3064, 2542.3503, 2546.3127, 2558.3843, 2647.1560, 2661.1709, 2675.1926, 2698.5471, 2706.4133, 2708.4885, 2718.5300, 2762.5994, 2765.5881, 2767.5010, 2771.5437, 2777.7188, 2811.6846, 2814.6851, 2846.6375, 2869.6453, 2872.5757, 2884.6123, 2888.6077, 2892.6868, 2894.7185, 2902.6804, 2918.6406, 2926.6814, 2946.7546, 2994.8018, 2998.7808, 3003.8159, 3005.8979, 3054.8286, 3082.9351, 3102.9441, 3105.8228, 3127.0818, 3171.0554, 3194.9565, 3202.8757, 3207.9922, 3249.0452, 3263.1082, 3264.1118, 3266.0825, 3300.1990, 3317.0972, 3323.0837, 3331.1343, 3335.1436, 3336.1438, 3348.9949, 3354.0869, 3358.1614, 3371.1931, 3387.2393, 3454.9927, 3459.2039, 3490.2356, 3504.3723, 3518.1758, 3640.3601, 3652.4072, 3664.4243, 3822.6233, 3870.6184, 3871.6218, 3887.6787, 4089.8884, 4160.9209, 4175.9526, 4306.0547, 4453.3716, 4466.3892, 4680.6270, 4846.6348

45. F1N6W3 **Mass:** 25812 **Score:** 16 **Expect:** 6.1e+002 **Matches:** 5

Uncharacterized protein OS=Bos taurus GN=MGC134105 PE=4 SV=1

Observed	Mr(expt)	Mr(calc)	ppm	Start	End	Miss	Peptide
900.5499	899.5426	899.5301	13.9	150	- 157	2	K.AEKRLAGR.K
1027.4982	1026.4909	1026.4982	-7.04	33	- 41	0	K.EALSEEPPR.A
1055.5199	1054.5126	1054.5018	10.2	1	- 8	1	-.MSRFSNWK.A
1628.8262	1627.8189	1627.8219	-1.83	2	- 14	2	M.SRFSNWKAYQVSR.S
4148.9229	4147.9156	4147.9898	-17.87	115	- 149	2	R.SWELTPPTANRFRIHDVHAAHLPQQGPFQNFMDMK.A + 2 Oxidation (M)

No match to: 712.3012, 714.3622, 717.4536, 722.4728, 735.4618, 756.5057, 772.4633, 779.5103, 792.4439, 795.4440, 804.3116, 806.4917, 807.4467, 809.4788, 812.4258, 814.4372, 815.4436, 819.4216, 828.4171, 831.4965, 832.3378, 834.3423, 841.4608, 842.4964, 845.4365, 871.5104, 883.4921, 888.4818, 891.4986, 893.9725, 894.4754, 913.5269, 918.5532, 929.4499, 935.4841, 945.4980, 947.4821, 949.5173, 959.4860, 963.4728, 967.4305, 969.4643, 972.4830, 974.4501, 975.4561, 976.4594, 977.4788, 978.5386, 981.4812, 983.4774, 988.4600, 989.6110, 995.4939, 996.4700, 997.4576, 999.4598, 1007.5580, 1011.4736, 1018.5132, 1019.5350, 1021.5362, 1032.5875, 1033.5684, 1034.5756, 1036.5582, 1044.5192, 1047.5377, 1056.5244, 1057.5576, 1060.5359, 1062.5424, 1069.5072, 1074.5400, 1077.5109, 1085.5363, 1087.5255, 1091.5013, 1103.5313, 1106.5183, 1107.5238, 1111.5382, 1122.5424, 1123.5519, 1168.5806, 1173.6044, 1178.6016, 1179.5861, 1185.6279, 1187.6195, 1199.6720, 1207.6029, 1209.5723, 1215.6453, 1216.6425, 1226.5743, 1234.5945, 1244.5957, 1248.6305, 1255.5890, 1295.7034, 1301.5924, 1314.6890, 1322.5939, 1325.6377, 1328.6476, 1336.6866, 1337.6793, 1338.6221, 1342.6494, 1344.6713,

1345.6792, 1349.6682, 1352.6564, 1353.6359, 1355.6598, 1361.6550, 1368.6198, 1370.6490, 1372.5659, 1381.7262, 1383.6938, 1386.6838,
1394.6993, 1397.7117, 1427.7550, 1432.6827, 1433.6854, 1442.7830, 1446.7039, 1447.6959, 1457.7043, 1473.7709, 1478.7163, 1497.8370,
1528.6653, 1537.7870, 1542.8158, 1550.9301, 1556.8483, 1578.7511, 1601.8662, 1606.8096, 1615.8414, 1616.8064, 1617.7891, 1627.7168,
1628.6741, 1638.8353, 1642.6895, 1647.8445, 1650.8495, 1679.9406, 1680.7981, 1696.8021, 1706.8348, 1715.8130, 1717.8778, 1718.8860,
1725.8102, 1727.8582, 1730.8500, 1752.8656, 1763.7885, 1768.9357, 1769.9312, 1771.0000, 1775.8153, 1777.8390, 1786.8839, 1788.4598,
1791.8882, 1799.8864, 1800.8976, 1809.0026, 1914.9941, 1928.0222, 1953.8669, 1975.9434, 1982.0046, 2012.9734, 2027.1388, 2042.0795,
2053.0103, 2060.0637, 2070.9099, 2082.9165, 2084.9246, 2086.9263, 2095.0337, 2096.9631, 2098.9319, 2100.9241, 2136.9219, 2155.1025,
2170.1101, 2209.0732, 2211.1094, 2239.2014, 2251.1997, 2289.1399, 2293.2251, 2324.1719, 2343.2795, 2381.2776, 2384.2988, 2398.2314,
2400.1594, 2414.1807, 2428.1990, 2431.1982, 2444.2200, 2465.3230, 2528.3064, 2542.3503, 2546.3127, 2558.3843, 2647.1560, 2661.1709,
2675.1926, 2698.5471, 2706.4133, 2708.4885, 2718.5300, 2762.5994, 2765.5881, 2767.5010, 2771.5437, 2777.7188, 2811.6846, 2814.6851,
2846.6375, 2869.6453, 2872.5757, 2884.6123, 2888.6077, 2892.6868, 2894.7185, 2902.6804, 2918.6406, 2926.6814, 2946.7546, 2994.8018,
2998.7808, 3003.8159, 3005.8979, 3054.8286, 3082.9351, 3102.9441, 3105.8228, 3127.0818, 3171.0554, 3194.9565, 3202.8757, 3207.9922,
3249.0452, 3263.1082, 3264.1118, 3266.0825, 3300.1990, 3317.0972, 3323.0837, 3331.1343, 3335.1436, 3336.1438, 3348.9949, 3354.0869,
3358.1614, 3371.1931, 3387.2393, 3454.9927, 3459.2039, 3490.2356, 3504.3723, 3518.1758, 3640.3601, 3652.4072, 3664.4243, 3822.6233,
3870.6184, 3871.6218, 3887.6787, 4089.8884, 4160.9209, 4164.9634, 4175.9526, 4306.0547, 4453.3716, 4466.3892, 4680.6270, 4846.6348

46. G3N3M2 **Mass:** 148813 **Score:** 16 **Expect:** 6.1e+002 **Matches:** 12

Uncharacterized protein OS=Bos taurus GN=TBC1D4 PE=4 SV=1

Observed	Mr(expt)	Mr(calc)	ppm	Start		End	Miss	Peptide
1044.5192	1043.5119	1043.5148	-2.79	229	-	236	0	K.FSLHEQQR.L
1248.6305	1247.6232	1247.6081	12.2	841	-	849	1	K.SEELRCLWR.K
1325.6377	1324.6304	1324.6234	5.29	38	-	48	0	R.LWYVGGSCLDR.R
1349.6682	1348.6609	1348.6881	-20.18	315	-	326	1	R.SRCSSVTGVLQR.K
1617.7891	1616.7818	1616.7650	10.4	910	-	922	1	K.IRCDMEDIHTSLK.E
1628.8262	1627.8189	1627.7929	16.0	36	-	48	1	R.FRLWYVGGSCLDR.R
1647.8445	1646.8372	1646.8661	-17.54	176	-	190	2	R.QLSKAAMKEDAKPSK.D + Oxidation (M)
1679.9406	1678.9333	1678.8943	23.2	930	-	942	1	R.RGEIWQFLALQYR.L
1715.8130	1714.8057	1714.8420	-21.18	673	-	687	1	R.QSSSEQCSILPPARR.M
1768.9357	1767.9284	1767.8911	21.1	64	-	80	2	R.RRSQKPEAGGCGAPAAR.E
4148.9229	4147.9156	4147.9163	-0.16	805	-	840	2	R.SELLPLSPLAPTMEEEPLMIFMSDEDDPEKGEERNK.S + 2 Oxidation (M)
4164.9634	4163.9561	4163.9112	10.8	805	-	840	2	R.SELLPLSPLAPTMEEEPLMIFMSDEDDPEKGEERNK.S + 3 Oxidation (M)

No match to: 712.3012, 714.3622, 717.4536, 722.4728, 735.4618, 756.5057, 772.4633, 779.5103, 792.4439, 795.4440, 804.3116,
806.4917, 807.4467, 809.4788, 812.4258, 814.4372, 815.4436, 819.4216, 828.4171, 831.4965, 832.3378, 834.3423, 841.4608, 842.4964,
845.4365, 871.5104, 883.4921, 888.4818, 891.4986, 893.9725, 894.4754, 900.5499, 913.5269, 918.5532, 929.4499, 935.4841, 945.4980,
947.4821, 949.5173, 959.4860, 963.4728, 967.4305, 969.4643, 972.4830, 974.4501, 975.4561, 976.4594, 977.4788, 978.5386, 981.4812,
983.4774, 988.4600, 989.6110, 995.4939, 996.4700, 997.4576, 999.4598, 1007.5580, 1011.4736, 1018.5132, 1019.5350, 1021.5362,
1027.4982, 1032.5875, 1033.5684, 1034.5756, 1036.5582, 1047.5377, 1055.5199, 1056.5244, 1057.5576, 1060.5359, 1062.5424, 1069.5072,
1074.5400, 1077.5109, 1085.5363, 1087.5255, 1091.5013, 1103.5313, 1106.5183, 1107.5238, 1111.5382, 1122.5424, 1123.5519, 1168.5806,
1173.6044, 1178.6016, 1179.5861, 1185.6279, 1187.6195, 1199.6720, 1207.6029, 1209.5723, 1215.6453, 1216.6425, 1226.5743, 1234.5945,
1244.5957, 1255.5890, 1295.7034, 1301.5924, 1314.6890, 1322.5939, 1328.6476, 1336.6866, 1337.6793, 1338.6221, 1342.6494, 1344.6713,
1345.6792, 1352.6564, 1353.6359, 1355.6598, 1361.6550, 1368.6198, 1370.6490, 1372.5659, 1381.7262, 1383.6938, 1386.6838, 1394.6993,
1397.7117, 1427.7550, 1432.6827, 1433.6854, 1442.7830, 1446.7039, 1447.6959, 1457.7043, 1473.7709, 1478.7163, 1497.8370, 1528.6653,
1537.7870, 1542.8158, 1550.9301, 1556.8483, 1578.7511, 1601.8662, 1606.8096, 1615.8414, 1616.8064, 1627.7168, 1628.6741, 1638.8353,
1642.6895, 1650.8495, 1680.7981, 1696.8021, 1706.8348, 1717.8778, 1718.8860, 1725.8102, 1727.8582, 1730.8500, 1752.8656, 1763.7885,
1769.9312, 1771.0000, 1775.8153, 1777.8390, 1786.8839, 1788.4598, 1791.8882, 1799.8864, 1800.8976, 1809.0026, 1914.9941, 1928.0222,
1953.8669, 1975.9434, 1982.0046, 2012.9734, 2027.1388, 2042.0795, 2053.0103, 2060.0637, 2070.9099, 2082.9165, 2084.9246, 2086.9263,
2095.0337, 2096.9631, 2098.9319, 2100.9241, 2136.9219, 2155.1025, 2170.1101, 2209.0732, 2211.1094, 2239.2014, 2251.1997, 2289.1399,
2293.2251, 2324.1719, 2343.2795, 2381.2776, 2384.2988, 2398.2314, 2400.1594, 2414.1807, 2428.1990, 2431.1982, 2444.2200, 2465.3230,
2528.3064, 2542.3503, 2546.3127, 2558.3843, 2647.1560, 2661.1709, 2675.1926, 2698.5471, 2706.4133, 2708.4885, 2718.5300, 2762.5994,
2765.5881, 2767.5010, 2771.5437, 2777.7188, 2811.6846, 2814.6851, 2846.6375, 2869.6453, 2872.5757, 2884.6123, 2888.6077, 2892.6868,
2894.7185, 2902.6804, 2918.6406, 2926.6814, 2946.7546, 2994.8018, 2998.7808, 3003.8159, 3005.8979, 3054.8286, 3082.9351, 3102.9441,
3105.8228, 3127.0818, 3171.0554, 3194.9565, 3202.8757, 3207.9922, 3249.0452, 3263.1082, 3264.1118, 3266.0825, 3300.1990, 3317.0972,
3323.0837, 3331.1343, 3335.1436, 3336.1438, 3348.9949, 3354.0869, 3358.1614, 3371.1931, 3387.2393, 3454.9927, 3459.2039, 3490.2356,
3504.3723, 3518.1758, 3640.3601, 3652.4072, 3664.4243, 3822.6233, 3870.6184, 3871.6218, 3887.6787, 4089.8884, 4160.9209, 4175.9526,
4306.0547, 4453.3716, 4466.3892, 4680.6270, 4846.6348

47. Q9XSC3 **Mass:** 101801 **Score:** 16 **Expect:** 6.2e+002 **Matches:** 12

WD repeat-containing protein 44 OS=Bos taurus GN=WDR44 PE=1 SV=1

Observed	Mr(expt)	Mr(calc)	ppm	Start		End	Miss	Peptide
1036.5582	1035.5509	1035.5574	-6.23	821	-	828	2	K.FTSVRRDR.N
1103.5313	1102.5240	1102.5043	17.9	440	-	448	0	K.HLAEEYGER.A
1325.6377	1324.6304	1324.6122	13.8	715	-	724	0	R.CIFYDTEHLK.Y
1336.6866	1335.6793	1335.6605	14.1	693	-	704	0	K.LITAANFCQNGK.Y
1361.6550	1360.6477	1360.6722	-17.97	74	-	85	0	K.VIQLEDDSLDSK.G
1397.7117	1396.7044	1396.7324	-20.01	725	-	735	1	K.YHTQIHVRSTR.G
1446.7039	1445.6966	1445.6998	-2.19	559	-	572	0	R.VSPSPSQESLNSSK.S
1606.8096	1605.8023	1605.8171	-9.21	346	-	359	0	R.ELTDEEILASVMIK.N + Oxidation (M)
1706.8348	1705.8275	1705.7903	21.8	870	-	885	0	K.GEDAEVLETMPSGIMK.T
2084.9246	2083.9173	2083.9294	-5.79	390	-	407	2	R.RTKEYVSNDAAQSDDEEK.L
2381.2776	2380.2703	2380.2962	-10.85	743	-	764	2	R.KITGIEPLPGENKILVTSNDSR.I
4148.9229	4147.9156	4147.9592	-10.51	791	-	825	2	K.ASFSHDFNYLVSGSEDKYVYIWSTYHDLSKFTSVR.R

No match to: 712.3012, 714.3622, 717.4536, 722.4728, 735.4618, 756.5057, 772.4633, 779.5103, 792.4439, 795.4440, 804.3116,
806.4917, 807.4467, 809.4788, 812.4258, 814.4372, 815.4436, 819.4216, 828.4171, 831.4965, 832.3378, 834.3423, 841.4608, 842.4964,
845.4365, 871.5104, 883.4921, 888.4818, 891.4986, 893.9725, 894.4754, 900.5499, 913.5269, 918.5532, 929.4499, 935.4841, 945.4980,
947.4821, 949.5173, 959.4860, 963.4728, 967.4305, 969.4643, 972.4830, 974.4501, 975.4561, 976.4594, 977.4788, 978.5386, 981.4812,
983.4774, 988.4600, 989.6110, 995.4939, 996.4700, 997.4576, 999.4598, 1007.5580, 1011.4736, 1018.5132, 1019.5350, 1021.5362,
1027.4982, 1032.5875, 1033.5684, 1034.5756, 1044.5192, 1047.5377, 1055.5199, 1056.5244, 1057.5576, 1060.5359, 1062.5424, 1069.5072,
1074.5400, 1077.5109, 1085.5363, 1087.5255, 1091.5013, 1106.5183, 1107.5238, 1111.5382, 1122.5424, 1123.5519, 1168.5806, 1173.6044,
1178.6016, 1179.5861, 1185.6279, 1187.6195, 1199.6720, 1207.6029, 1209.5723, 1215.6453, 1216.6425, 1226.5743, 1234.5945, 1244.5957,
1248.6305, 1255.5890, 1295.7034, 1301.5924, 1314.6890, 1322.5939, 1328.6476, 1337.6793, 1338.6221, 1342.6494, 1344.6713, 1345.6792,
1349.6682, 1352.6564, 1353.6359, 1355.6598, 1368.6198, 1370.6490, 1372.5659, 1381.7262, 1383.6938, 1386.6838, 1394.6993, 1427.7550,
1432.6827, 1433.6854, 1442.7830, 1447.6959, 1457.7043, 1473.7709, 1478.7163, 1497.8370, 1528.6653, 1537.7870, 1542.8158, 1550.9301,
1556.8483, 1578.7511, 1601.8662, 1615.8414, 1616.8064, 1617.7891, 1627.7168, 1628.6741, 1628.8262, 1638.8353, 1642.6895, 1647.8445,
1650.8495, 1679.9406, 1680.7981, 1696.8021, 1715.8130, 1717.8778, 1718.8860, 1725.8102, 1727.8582, 1730.8500, 1752.8656, 1763.7885,
1768.9357, 1769.9312, 1771.0000, 1775.8153, 1777.8390, 1786.8839, 1788.4598, 1791.8882, 1799.8864, 1800.8976, 1809.0026, 1914.9941,
1928.0222, 1953.8669, 1975.9434, 1982.0046, 2012.9734, 2027.1388, 2042.0795, 2053.0103, 2060.0637, 2070.9099, 2082.9165, 2086.9263,
2095.0337, 2096.9631, 2098.9319, 2100.9241, 2136.9219, 2155.1025, 2170.1101, 2209.0732, 2211.1094, 2239.2014, 2251.1997, 2289.1399,
2293.2251, 2324.1719, 2343.2795, 2384.2988, 2398.2314, 2400.1594, 2414.1807, 2428.1990, 2431.1982, 2444.2200, 2465.3230, 2528.3064,
2542.3503, 2546.3127, 2558.3843, 2647.1560, 2661.1709, 2675.1926, 2698.5471, 2706.4133, 2708.4885, 2718.5300, 2762.5994, 2765.5881,
2767.5010, 2771.5437, 2777.7188, 2811.6846, 2814.6851, 2846.6375, 2869.6453, 2872.5757, 2884.6123, 2888.6077, 2892.6868, 2894.7185,
2902.6804, 2918.6406, 2926.6814, 2946.7546, 2994.8018, 2998.7808, 3003.8159, 3005.8979, 3054.8286, 3082.9351, 3102.9441, 3105.8228,
3127.0818, 3171.0554, 3194.9565, 3202.8757, 3207.9922, 3249.0452, 3263.1082, 3264.1118, 3266.0825, 3300.1990, 3317.0972, 3323.0837,
3331.1343, 3335.1436, 3336.1438, 3348.9949, 3354.0869, 3358.1614, 3371.1931, 3387.2393, 3454.9927, 3459.2039, 3490.2356, 3504.3723,

3518.1758, 3640.3601, 3652.4072, 3664.4243, 3822.6233, 3870.6184, 3871.6218, 3887.6787, 4089.8884, 4160.9209, 4164.9634, 4175.9526, 4306.0547, 4453.3716, 4466.3892, 4680.6270, 4846.6348

48. G3X7E3 **Mass:** 101783 **Score:** 16 **Expect:** 6.2e+002 **Matches:** 11

WD repeat-containing protein 44 OS=Bos taurus GN=WDR44 PE=4 SV=1

Observed	Mr(expt)	Mr(calc)	ppm	Start	End	Miss	Peptide
1036.5582	1035.5509	1035.5574	-6.23	821	828	2	K.FTSVRRDR.N
1103.5313	1102.5240	1102.5043	17.9	440	448	0	K.HLAEEYGER.A
1325.6377	1324.6304	1324.6122	13.8	715	724	0	R.CIFYDTEHLK.Y
1336.6866	1335.6793	1335.6605	14.1	693	704	0	K.LITAANFCQNGK.Y
1361.6550	1360.6477	1360.6722	-17.97	74	85	0	K.VIQLEDDSLDSK.G
1397.7117	1396.7044	1396.7324	-20.01	725	735	1	K.YHTQIHVRSTR.G
1446.7039	1445.6966	1445.6998	-2.19	559	572	0	R.VSPSPSQESLNSSK.S
1606.8096	1605.8023	1605.8171	-9.21	346	359	0	R.ELTDEEILASVMIK.N + Oxidation (M)
2084.9246	2083.9173	2083.9294	-5.79	390	407	2	R.RTKEYVSNDAAQSDDEEK.L
2381.2776	2380.2703	2380.2962	-10.85	743	764	2	R.KITGIEPLPGENKILVTSNDSR.I
4148.9229	4147.9156	4147.9592	-10.51	791	825	2	K.ASFSHDFNYLVSGSEDKYVYIWSTYHDLSKFTSVR.R

No match to: 712.3012, 714.3622, 717.4536, 722.4728, 735.4618, 756.5057, 772.4633, 779.5103, 792.4439, 795.4440, 804.3116, 806.4917, 807.4467, 809.4788, 812.4258, 814.4372, 815.4436, 819.4216, 828.4171, 831.4965, 832.3378, 834.3423, 841.4608, 842.4964, 845.4365, 871.5104, 883.4921, 888.4818, 891.4986, 893.9725, 894.4754, 900.5499, 913.5269, 918.5532, 929.4499, 935.4841, 945.4980, 947.4821, 949.5173, 959.4860, 963.4728, 967.4305, 969.4643, 972.4830, 974.4501, 975.4561, 976.4594, 977.4788, 978.5386, 981.4812, 983.4774, 988.4600, 989.6110, 995.4939, 996.4700, 997.4576, 999.4598, 1007.5580, 1011.4736, 1018.5132, 1019.5350, 1021.5362, 1027.4982, 1032.5875, 1033.5684, 1034.5756, 1044.5192, 1047.5377, 1055.5199, 1056.5244, 1057.5576, 1060.5359, 1062.5424, 1069.5072, 1074.5400, 1077.5109, 1085.5363, 1087.5255, 1091.5013, 1106.5183, 1107.5238, 1111.5382, 1122.5424, 1123.5519, 1168.5806, 1173.6044, 1178.6016, 1179.5861, 1185.6279, 1187.6195, 1199.6720, 1207.6029, 1209.5723, 1215.6453, 1216.6425, 1226.5743, 1234.5945, 1244.5957, 1248.6305, 1255.5890, 1295.7034, 1301.5924, 1314.6890, 1322.5939, 1328.6476, 1337.6793, 1338.6221, 1342.6494, 1344.6713, 1345.6792, 1349.6682, 1352.6564, 1353.6359, 1355.6598, 1368.6198, 1370.6490, 1372.5659, 1381.7262, 1383.6938, 1386.6838, 1394.6993, 1427.7550, 1432.6827, 1433.6854, 1442.7830, 1447.6959, 1457.7043, 1473.7709, 1478.7163, 1497.8370, 1528.6653, 1537.7870, 1542.8158, 1550.9301, 1556.8483, 1578.7511, 1601.8662, 1615.8414, 1616.8064, 1617.7891, 1627.7168, 1628.6741, 1628.8262, 1638.8353, 1642.6895, 1647.8445, 1650.8495, 1679.9406, 1680.7981, 1696.8021, 1706.8348, 1715.8130, 1717.8778, 1718.8860, 1725.8102, 1727.8582, 1730.8500, 1752.8656, 1763.7885, 1768.9357, 1769.9312, 1771.0000, 1775.8153, 1777.8390, 1786.8839, 1788.4598, 1791.8882, 1799.8864, 1800.8976, 1809.0026, 1914.9941, 1928.0222, 1953.8669, 1975.9434, 1982.0046, 2012.9734, 2027.1388, 2042.0795, 2053.0103, 2060.0637, 2070.9099, 2082.9165, 2086.9263, 2095.0337, 2096.9631, 2098.9319, 2100.9241, 2136.9219, 2155.1025, 2170.1101, 2209.0732, 2211.1094, 2239.2014, 2251.1997, 2289.1399, 2293.2251, 2324.1719, 2343.2795, 2384.2988, 2398.2314, 2400.1594, 2414.1807, 2428.1990, 2431.1982, 2444.2200, 2465.3230, 2528.3064, 2542.3503, 2546.3127, 2558.3843, 2647.1560, 2661.1709, 2675.1926, 2698.5471, 2706.4133, 2708.4885, 2718.5300, 2762.5994, 2765.5881, 2767.5010, 2771.5437, 2777.7188, 2811.6846, 2814.6851, 2846.6375, 2869.6453, 2872.5757, 2884.6123, 2888.6077, 2892.6868, 2894.7185, 2902.6804, 2918.6406, 2926.6814, 2946.7546, 2994.8018, 2998.7808, 3003.8159, 3005.8979, 3054.8286, 3082.9351, 3102.9441, 3105.8228, 3127.0818, 3171.0554, 3194.9565, 3202.8757, 3207.9922, 3249.0452, 3263.1082, 3264.1118, 3266.0825, 3300.1990, 3317.0972, 3323.0837, 3331.1343, 3335.1436, 3336.1438, 3348.9949, 3354.0869, 3358.1614, 3371.1931, 3387.2393, 3454.9927, 3459.2039, 3490.2356, 3504.3723, 3518.1758, 3640.3601, 3652.4072, 3664.4243, 3822.6233, 3870.6184, 3871.6218, 3887.6787, 4089.8884, 4160.9209, 4164.9634, 4175.9526, 4306.0547, 4453.3716, 4466.3892, 4680.6270, 4846.6348

49. F1N0K7 **Mass:** 15524 **Score:** 16 **Expect:** 6.4e+002 **Matches:** 5

Uncharacterized protein OS=Bos taurus PE=4 SV=1

Observed	Mr(expt)	Mr(calc)	ppm	Start	End	Miss	Peptide
819.4216	818.4143	818.4208	-7.90	132	138	0	K.ELGLEMK.F
935.4841	934.4768	934.4794	-2.74	11	18	0	R.LLCSVSEK.R
1216.6425	1215.6352	1215.6322	2.52	132	141	1	K.ELGLEMKFIH.-
1248.6305	1247.6232	1247.6544	-24.95	1	10	2	-.IDDKVKDIMR.L + Oxidation (M)
1606.8096	1605.8023	1605.8331	-19.14	7	19	2	K.DIMRLLCSVSEKR.K

No match to: 712.3012, 714.3622, 717.4536, 722.4728, 735.4618, 756.5057, 772.4633, 779.5103, 792.4439, 795.4440, 804.3116, 806.4917, 807.4467, 809.4788, 812.4258, 814.4372, 815.4436, 828.4171, 831.4965, 832.3378, 834.3423, 841.4608, 842.4964, 845.4365, 871.5104, 883.4921, 888.4818, 891.4986, 893.9725, 894.4754, 900.5499, 913.5269, 918.5532, 929.4499, 945.4980, 947.4821, 949.5173, 959.4860, 963.4728, 967.4305, 969.4643, 972.4830, 974.4501, 975.4561, 976.4594, 977.4788, 978.5386, 981.4812, 983.4774, 988.4600, 989.6110, 995.4939, 996.4700, 997.4576, 999.4598, 1007.5580, 1011.4736, 1018.5132, 1019.5350, 1021.5362, 1027.4982, 1032.5875, 1033.5684, 1034.5756, 1036.5582, 1044.5192, 1047.5377, 1055.5199, 1056.5244, 1057.5576, 1060.5359, 1062.5424, 1069.5072, 1074.5400, 1077.5109, 1085.5363, 1087.5255, 1091.5013, 1103.5313, 1106.5183, 1107.5238, 1111.5382, 1122.5424, 1123.5519, 1168.5806, 1173.6044, 1178.6016, 1179.5861, 1185.6279, 1187.6195, 1199.6720, 1207.6029, 1209.5723, 1215.6453, 1226.5743, 1234.5945, 1244.5957, 1255.5890, 1295.7034, 1301.5924, 1314.6890, 1322.5939, 1325.6377, 1328.6476, 1336.6866, 1337.6793, 1338.6221, 1342.6494, 1344.6713, 1345.6792, 1349.6682, 1352.6564, 1353.6359, 1355.6598, 1361.6550, 1368.6198, 1370.6490, 1372.5659, 1381.7262, 1383.6938, 1386.6838, 1394.6993, 1397.7117, 1427.7550, 1432.6827, 1433.6854, 1442.7830, 1446.7039, 1447.6959, 1457.7043, 1473.7709, 1478.7163, 1497.8370, 1528.6653, 1537.7870, 1542.8158, 1550.9301, 1556.8483, 1578.7511, 1601.8662, 1615.8414, 1616.8064, 1617.7891, 1627.7168, 1628.6741, 1628.8262, 1638.8353, 1642.6895, 1647.8445, 1650.8495, 1679.9406, 1680.7981, 1696.8021, 1706.8348, 1715.8130, 1717.8778, 1718.8860, 1725.8102, 1727.8582, 1730.8500, 1752.8656, 1763.7885, 1768.9357, 1769.9312, 1771.0000, 1775.8153, 1777.8390, 1786.8839, 1788.4598, 1791.8882, 1799.8864, 1800.8976, 1809.0026, 1914.9941, 1928.0222, 1953.8669, 1975.9434, 1982.0046, 2012.9734, 2027.1388, 2042.0795, 2053.0103, 2060.0637, 2070.9099, 2082.9165, 2084.9246, 2086.9263, 2095.0337, 2096.9631, 2098.9319, 2100.9241, 2136.9219, 2155.1025, 2170.1101, 2209.0732, 2211.1094, 2239.2014, 2251.1997, 2289.1399, 2293.2251, 2324.1719, 2343.2795, 2381.2776, 2384.2988, 2398.2314, 2400.1594, 2414.1807, 2428.1990, 2431.1982, 2444.2200, 2465.3230, 2528.3064, 2542.3503, 2546.3127, 2558.3843, 2647.1560, 2661.1709, 2675.1926, 2698.5471, 2706.4133, 2708.4885, 2718.5300, 2762.5994, 2765.5881, 2767.5010, 2771.5437, 2777.7188, 2811.6846, 2814.6851, 2846.6375, 2869.6453, 2872.5757, 2884.6123, 2888.6077, 2892.6868, 2894.7185, 2902.6804, 2918.6406, 2926.6814, 2946.7546, 2994.8018, 2998.7808, 3003.8159, 3005.8979, 3054.8286, 3082.9351, 3102.9441, 3105.8228, 3127.0818, 3171.0554, 3194.9565, 3202.8757, 3207.9922, 3249.0452, 3263.1082, 3264.1118, 3266.0825, 3300.1990, 3317.0972, 3323.0837, 3331.1343, 3335.1436, 3336.1438, 3348.9949, 3354.0869, 3358.1614, 3371.1931, 3387.2393, 3454.9927, 3459.2039, 3490.2356, 3504.3723, 3518.1758, 3640.3601, 3652.4072, 3664.4243, 3822.6233, 3870.6184, 3871.6218, 3887.6787, 4089.8884, 4148.9229, 4160.9209, 4164.9634, 4175.9526, 4306.0547, 4453.3716, 4466.3892, 4680.6270, 4846.6348

50. Q00194 **Mass:** 79951 **Score:** 16 **Expect:** 6.5e+002 **Matches:** 9

cGMP-gated cation channel alpha-1 OS=Bos taurus GN=CNGA1 PE=1 SV=1

Observed	Mr(expt)	Mr(calc)	ppm	Start	End	Miss	Peptide
717.4536	716.4463	716.4432	4.34	657	662	1	R.LTKVEK.F
812.4258	811.4186	811.4149	4.46	513	518	0	R.EMYIIK.E + Oxidation (M)
1034.5756	1033.5683	1033.5590	9.03	427	434	2	K.DMEKRVIK.W + Oxidation (M)
1122.5424	1121.5351	1121.5499	-13.16	423	431	2	R.NVSKDMEKR.V + Oxidation (M)
1255.5890	1254.5817	1254.5701	9.24	65	76	0	R.SNTHGSGQPSQR.E
1325.6377	1324.6304	1324.6346	-3.17	417	426	1	K.QYMHFRNVSK.D + Oxidation (M)
1355.6598	1354.6525	1354.6438	6.42	642	652	0	R.ILAEYESMQQK.L + Oxidation (M)
1650.8495	1649.8422	1649.8407	0.94	625	638	1	K.VTRMESSVDLLQTR.F + Oxidation (M)
1768.9357	1767.9284	1767.9460	-9.97	431	443	2	K.RVIKWFDYLWTNK.K

No match to: 712.3012, 714.3622, 722.4728, 735.4618, 756.5057, 772.4633, 779.5103, 792.4439, 795.4440, 804.3116, 806.4917, 807.4467, 809.4788, 814.4372, 815.4436, 819.4216, 828.4171, 831.4965, 832.3378, 834.3423, 841.4608, 842.4964, 845.4365, 871.5104, 883.4921, 888.4818, 891.4986, 893.9725, 894.4754, 900.5499, 913.5269, 918.5532, 929.4499, 935.4841, 945.4980, 947.4821, 949.5173, 959.4860, 963.4728, 967.4305, 969.4643, 972.4830, 974.4501, 975.4561, 976.4594, 977.4788, 978.5386, 981.4812, 983.4774, 988.4600, 989.6110, 995.4939, 996.4700, 997.4576, 999.4598, 1007.5580, 1011.4736, 1018.5132, 1019.5350, 1021.5362, 1027.4982, 1032.5875, 1033.5684, 1036.5582, 1044.5192, 1047.5377, 1055.5199, 1056.5244, 1057.5576, 1060.5359, 1062.5424, 1069.5072, 1074.5400, 1077.5109, 1085.5363, 1087.5255, 1091.5013, 1103.5313, 1106.5183, 1107.5238, 1111.5382, 1123.5519, 1168.5806, 1173.6044, 1178.6016, 1179.5861,

```
1185.6279, 1187.6195, 1199.6720, 1207.6029, 1209.5723, 1215.6453, 1216.6425, 1226.5743, 1234.5945, 1244.5957, 1248.6305, 1295.7034,
1301.5924, 1314.6890, 1322.5939, 1328.6476, 1336.6866, 1337.6793, 1338.6221, 1342.6494, 1344.6713, 1345.6792, 1349.6682, 1352.6564,
1353.6359, 1361.6550, 1368.6198, 1370.6490, 1372.5659, 1381.7262, 1383.6938, 1386.6838, 1394.6993, 1397.7117, 1427.7550, 1432.6827,
1433.6854, 1442.7830, 1446.7039, 1447.6959, 1457.7043, 1473.7709, 1478.7163, 1497.8370, 1528.6653, 1537.7870, 1542.8158, 1550.9301,
1556.8483, 1578.7511, 1601.8662, 1606.8096, 1615.8414, 1616.8064, 1617.7891, 1627.7168, 1628.6741, 1628.8262, 1638.8353, 1642.6895,
1647.8445, 1679.9406, 1680.7981, 1696.8021, 1706.8348, 1715.8130, 1717.8778, 1718.8860, 1725.8102, 1727.8582, 1730.8500, 1752.8656,
1763.7885, 1769.9312, 1771.0000, 1775.8153, 1777.8390, 1786.8839, 1788.4598, 1791.8882, 1799.8864, 1800.8976, 1809.0026, 1914.9941,
1928.0222, 1953.8669, 1975.9434, 1982.0046, 2012.9734, 2027.1388, 2042.0795, 2053.0103, 2060.0637, 2070.9099, 2082.9165, 2084.9246,
2086.9263, 2095.0337, 2096.9631, 2098.9319, 2100.9241, 2136.9219, 2155.1025, 2170.1101, 2209.0732, 2211.1094, 2239.2014, 2251.1997,
2289.1399, 2293.2251, 2324.1719, 2343.2795, 2381.2776, 2384.2988, 2398.2314, 2400.1594, 2414.1807, 2428.1990, 2431.1982, 2444.2200,
2465.3230, 2528.3064, 2542.3503, 2546.3127, 2558.3843, 2647.1560, 2661.1709, 2675.1926, 2698.5471, 2706.4133, 2708.4885, 2718.5300,
2762.5994, 2765.5881, 2767.5010, 2771.5437, 2777.7188, 2811.6846, 2814.6851, 2846.6375, 2869.6453, 2872.5757, 2884.6123, 2888.6077,
2892.6868, 2894.7185, 2902.6804, 2918.6406, 2926.6814, 2946.7546, 2994.8018, 2998.7808, 3003.8159, 3005.8979, 3054.8286, 3082.9351,
3102.9441, 3105.8228, 3127.0818, 3171.0554, 3194.9565, 3202.8757, 3207.9922, 3249.0452, 3263.1082, 3264.1118, 3266.0825, 3300.1990,
3317.0972, 3323.0837, 3331.1343, 3335.1436, 3336.1438, 3348.9949, 3354.0869, 3358.1614, 3371.1931, 3387.2393, 3454.9927, 3459.2039,
3490.2356, 3504.3723, 3518.1758, 3640.3601, 3652.4072, 3664.4243, 3822.6233, 3870.6184, 3871.6218, 3887.6787, 4089.8884, 4148.9229,
4160.9209, 4164.9634, 4175.9526, 4306.0547, 4453.3716, 4466.3892, 4680.6270, 4846.6348
```

Search Parameters

```
Type of search         : Peptide Mass Fingerprint
Enzyme                 : Trypsin
Fixed modifications    : Carbamidomethyl (C)
Variable modifications : Oxidation (M)
Mass values            : Monoisotopic
Protein Mass           : Unrestricted
Peptide Mass Tolerance : ± 25 ppm
Peptide Charge State   : 1+
Max Missed Cleavages   : 2
Number of queries      : 295
Selected for scoring   : 30
```

**Mascot:** http://www.matrixscience.com/

MATRIX SCIENCE Mascot Search Results

```
User          : 8F_(L8@52381_05Apr2017)
Email         : proteomics@i3s.up.pt
Search title  : Project: Proteomics, Spot Set: Proteomics\05Apr2017, Label: L8, Spot Id: 227018, Peak List Id: 447674, MS Job Run Id: 316
MS data file  : C:\Documents and Settings\Administrator\Desktop\xu\29JUNHO\Proteomics\05Apr2017\pmf_L8_149875820517.txt
Database      : OrganismSpecie Bos_taurus_Reference_Proteome_2016_06 (24214 sequences; 12839866 residues)
Timestamp     : 29 Jun 2017 at 18:11:31 GMT
Top Score     : 73 for F1MYE4, Chondroadherin OS=Bos taurus GN=CHAD PE=4 SV=2
```

Mascot Score Histogram

Protein score is -10*Log(P), where P is the probability that the observed match is a random event.
Protein scores greater than 56 are significant (p<0.05).

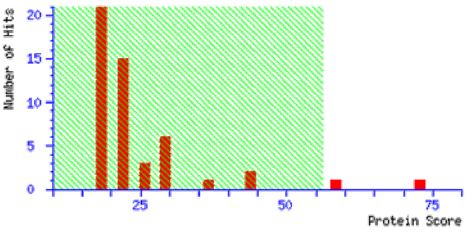

Protein Summary Report

Format As | Protein Summary | Help

Significance threshold p< 0.05 | Max. number of hits 50

Preferred taxonomy | All entries

Re-Search All | Search Unmatched

Index

	Accession	Mass	Score	Description
1.	F1MYE4	41319	73	Chondroadherin OS=Bos taurus GN=CHAD PE=4 SV=2
2.	Q27972	41373	58	Chondroadherin OS=Bos taurus GN=CHAD PE=1 SV=1
3.	E1BI02	43291	42	Fibromodulin OS=Bos taurus GN=FMOD PE=4 SV=1
4.	P13605	43410	42	Fibromodulin OS=Bos taurus GN=FMOD PE=1 SV=2
5.	P55252	40889	35	Hyaluronan and proteoglycan link protein 1 OS=Bos taurus GN=HAPLN1 PE=2 SV=1
6.	F6QVC9	36109	31	Annexin OS=Bos taurus GN=ANXA5 PE=1 SV=1
7.	P81287	36124	31	Annexin A5 OS=Bos taurus GN=ANXA5 PE=1 SV=3
8.	E1BMY3	35539	30	Olfactory receptor OS=Bos taurus GN=LOC614090 PE=3 SV=2
9.	G3N3X7	5156	30	Uncharacterized protein OS=Bos taurus PE=4 SV=1
10.	F1MSR8	134858	30	Collagen alpha-1(II) chain OS=Bos taurus GN=COL2A1 PE=4 SV=2
11.	F1MJA3	19688	29	Uncharacterized protein OS=Bos taurus GN=STK32B PE=4 SV=1
12.	P02459	142825	27	Collagen alpha-1(II) chain OS=Bos taurus GN=COL2A1 PE=1 SV=4
13.	G8JL06	14967	25	Histone H2B OS=Bos taurus GN=HIST1H2BB PE=3 SV=1
14.	Q3ZCI4	53613	25	6-phosphogluconate dehydrogenase, decarboxylating OS=Bos taurus GN=PGD PE=1 SV=2
15.	Q0VC60	56014	24	Dihydrouridine synthase 2-like, SMM1 homolog (S. cerevisiae) OS=Bos taurus GN=DUS2L PE=2 SV=1
16.	F1MEZ5	27894	23	Uncharacterized protein OS=Bos taurus GN=GZMB PE=3 SV=1
17.	Q0VCQ3	32810	23	MAX interactor 1 OS=Bos taurus GN=MXI1 PE=2 SV=1
18.	A6QQR6	12993	23	LIN52 protein OS=Bos taurus GN=LIN52 PE=2 SV=1
19.	G3N246	44905	23	Uncharacterized protein OS=Bos taurus GN=LOC101904998 PE=4 SV=1
20.	G3N2I6	40881	22	Uncharacterized protein OS=Bos taurus GN=MTERF1 PE=4 SV=1
21.	G3MZI9	42157	22	Uncharacterized protein OS=Bos taurus GN=MTERF1 PE=4 SV=1
22.	F6S1Q0	47935	22	Uncharacterized protein OS=Bos taurus GN=KRT18 PE=1 SV=1
23.	F1N024	7899	22	Uncharacterized protein OS=Bos taurus GN=FAM19A4 PE=4 SV=1
24.	G3MYS0	38145	22	Uncharacterized protein OS=Bos taurus GN=LOC508073 PE=4 SV=1
25.	E1BBR5	32112	21	Uncharacterized protein OS=Bos taurus GN=PITX3 PE=3 SV=1
26.	Q2HJB3	61006	21	Cytochrome P450, family 4, subfamily F, polypeptide 3 OS=Bos taurus GN=CYP4F3 PE=2 SV=1
27.	E1BN01	33525	20	Uncharacterized protein OS=Bos taurus GN=DNALI1 PE=4 SV=2
28.	E1BN75	19595	20	Uncharacterized protein OS=Bos taurus GN=TPRKB PE=3 SV=1
29.	Q1LZ99	53218	20	DNA methyltransferase 1 associated protein 1 OS=Bos taurus GN=DMAP1 PE=2 SV=1
30.	Q9XSK7	26702	20	Hepatoma-derived growth factor OS=Bos taurus GN=HDGF PE=2 SV=1
31.	G3X6L4	26771	20	Hepatoma-derived growth factor OS=Bos taurus GN=HDGF PE=1 SV=1
32.	G3N2D3	15087	20	Uncharacterized protein OS=Bos taurus PE=4 SV=1
33.	A0JNH6	63280	20	Coiled-coil domain-containing protein 102A OS=Bos taurus GN=CCDC102A PE=2 SV=1
34.	E1BAB2	22914	20	Uncharacterized protein OS=Bos taurus PE=4 SV=2
35.	F1MUF1	134385	20	Uncharacterized protein OS=Bos taurus GN=XPO5 PE=3 SV=1
36.	O02675	62638	19	Dihydropyrimidinase-related protein 2 OS=Bos taurus GN=DPYSL2 PE=1 SV=1
37.	E1B860	166355	19	Uncharacterized protein OS=Bos taurus GN=IFT140 PE=4 SV=1
38.	Q2KHX7	69190	19	RNA guanylyltransferase and 5'-phosphatase OS=Bos taurus GN=RNGTT PE=2 SV=1
39.	Q0P5C5	45559	19	SET and MYND domain containing 3 OS=Bos taurus GN=SMYD3 PE=2 SV=1
40.	Q0VC24	48192	19	Ribosome biogenesis protein WDR12 OS=Bos taurus GN=WDR12 PE=2 SV=1
41.	A5D7J9	59644	19	CCDC9 protein OS=Bos taurus GN=CCDC9 PE=2 SV=1
42.	F1N4W7	30110	19	Uncharacterized protein OS=Bos taurus PE=4 SV=1
43.	Q0IIE0	17671	19	Complexin-3 OS=Bos taurus GN=CPLX3 PE=2 SV=1
44.	F1MWQ0	17662	19	Complexin-3 OS=Bos taurus GN=CPLX3 PE=4 SV=1
45.	Q0VCV6	13614	19	ATPase, H+ transporting, lysosomal 13kDa, V1 subunit G2 OS=Bos taurus GN=ATP6V1G2 PE=2 SV=1
46.	Q3T0I4	26937	19	THO complex subunit 4 OS=Bos taurus GN=ALYREF PE=2 SV=1
47.	F1MSM9	64070	19	Uncharacterized protein OS=Bos taurus GN=FNBP1L PE=4 SV=2
48.	G5E5C5	52138	18	Probable tubulin polyglutamylase TTLL9 OS=Bos taurus GN=TTLL9 PE=4 SV=1
49.	A6QQQ9	48257	18	Keratin, type I cytoskeletal 20 OS=Bos taurus GN=KRT20 PE=2 SV=2
50.	P80219	27880	18	Duodenase-1 OS=Bos taurus GN=BDMD1 PE=1 SV=2

Results List

1. F1MYE4 **Mass:** 41319 **Score:** 73 **Expect:** 0.0013 **Matches:** 21

Chondroadherin OS=Bos taurus GN=CHAD PE=4 SV=2

Observed	Mr(expt)	Mr(calc)	ppm	Start		End	Miss	Peptide
713.3626	712.3553	712.3544	1.29	202	-	206	0	K.FYLDR.N
756.4689	755.4617	755.4653	-4.89	57	-	62	0	K.LLNLQR.N
837.3761	836.3688	836.3633	6.61	306	-	311	0	K.CTCQLR.G
913.5131	912.5058	912.5042	1.79	334	-	340	1	K.FRGQHIR.D
985.6076	984.6003	984.5968	3.62	220	-	227	1	K.LRVVEELK.L
1018.5203	1017.5130	1017.5104	2.57	274	-	281	0	K.HVHLENNR.L
1315.6759	1314.6686	1314.6429	19.6	336	-	346	1	R.GQHIRDTDAFR.G
1338.6461	1337.6388	1337.6364	1.80	235	-	246	0	K.SIPDNAFQSFGR.Y
1349.6952	1348.6879	1348.6888	-0.62	63	-	74	0	R.NNFPVLAANSFR.A
1355.7097	1354.7024	1354.7133	-8.02	261	-	273	0	K.FSDGAFLGVTTLK.H
1618.8162	1617.8089	1617.8124	-2.16	334	-	346	2	K.FRGQHIRDTDAFR.G
1647.8694	1646.8621	1646.8780	-9.64	102	-	114	0	K.QLIYLYLSHNDIR.V
1751.8870	1750.8797	1750.8777	1.14	247	-	260	0	R.YLETLWLDNTNLEK.F
1775.0276	1774.0203	1774.0465	-14.73	220	-	234	2	K.LRVVEELKLSHNPLK.S
2060.0461	2059.0388	2059.0374	0.68	202	-	219	1	K.FYLDRNQLSSYPSAALSK.L
2762.3970	2761.3897	2761.3810	3.16	177	-	201	0	R.WLYLSENSLSSLQPGALDDVENLAK.F
2812.4265	2811.4192	2811.4232	-1.40	282	-	305	0	R.LHQLPSNFPFDSLETLTLTNNPWK.C
2894.4741	2893.4668	2893.4498	5.89	118	-	142	1	R.AGAFDDLTELTYLYLDHNKVTELPR.G
3088.5859	3087.5786	3087.5805	-0.59	247	-	273	1	R.YLETLWLDNTNLEKFSDGAFLGVTTLK.H
3263.6973	3262.6900	3262.6604	9.08	63	-	90	1	R.NNFPVLAANSFRAMPNLVSLHLQHCQIR.E + Oxidation (M)
3456.7622	3455.7549	3455.7249	8.70	177	-	206	1	R.WLYLSENSLSSLQPGALDDVENLAKFYLDR.N

No match to: 706.2814, 707.2933, 717.4103, 719.4172, 722.4265, 735.4174, 744.3938, 768.3713, 772.4224, 779.4717, 792.4133, 804.3155, 806.4307, 807.4160, 809.4363, 812.3947, 814.4097, 815.4186, 819.3901, 828.3928, 831.4666, 832.3149, 834.3199, 841.4355, 842.4860, 845.4138, 851.3882, 871.4900, 876.2846, 878.2907, 883.4759, 891.4818, 893.9534, 894.4574, 898.4351, 910.4221, 918.5399, 929.4545, 935.4750, 937.4921, 947.4755, 959.4836, 961.4837, 963.4760, 967.4271, 969.4589, 972.4794, 974.4445, 976.4506, 977.4767, 978.5352, 981.4952, 983.4747, 988.4585, 989.6082, 997.4586, 1007.5676, 1011.4840, 1014.5074, 1019.5441, 1021.5421, 1034.5779, 1042.5320, 1044.5242, 1060.5463, 1071.5376, 1077.5208, 1087.5399, 1091.5421, 1106.5393, 1122.5577, 1156.5372, 1173.6240, 1187.6439, 1199.6992, 1215.6615, 1244.6189, 1248.6575, 1255.6039, 1276.6218, 1284.6401, 1295.7195, 1301.6177, 1313.7069, 1314.7141, 1325.6675, 1336.7151, 1337.7180, 1342.6758, 1344.7024, 1353.6804, 1356.7201, 1361.6877, 1365.6632, 1370.6783, 1372.6058, 1381.7588, 1383.7244, 1386.7131, 1394.7318, 1397.7419, 1414.6467, 1427.7865, 1432.7140, 1446.7247, 1457.7411, 1461.7784, 1473.8055, 1478.7513, 1497.8757, 1528.6993, 1542.8459, 1556.8795, 1578.7826, 1601.9006, 1606.8433, 1616.8177, 1628.7026, 1628.8568, 1642.7186, 1650.8473, 1658.8400, 1677.8625, 1680.8728, 1696.8231, 1703.8551, 1704.8862, 1710.9269, 1715.8459, 1717.8810, 1718.9111, 1725.8386, 1727.9301, 1730.8835, 1742.9099, 1768.9546, 1769.9617, 1771.0243, 1777.8676, 1786.9032, 1791.9047, 1799.9005, 1800.9124, 1915.0015, 1928.0238, 1953.9285, 1982.0009, 2012.9681, 2025.0820, 2027.1273, 2042.0642, 2052.9939, 2067.0017, 2070.8899, 2074.9629, 2082.9421, 2084.9102, 2086.9065, 2096.9797, 2100.9070, 2109.0410, 2116.0894, 2136.9121, 2155.0730, 2170.0781, 2195.0801, 2211.0686, 2238.1072, 2251.1428, 2293.1614, 2336.2495, 2343.1758, 2382.1987, 2385.2234, 2400.0720, 2414.0930, 2418.0901, 2428.1140, 2431.0991, 2465.2188, 2468.2249, 2528.1794, 2542.2344, 2560.2729, 2564.2610, 2646.9797, 2660.9968, 2675.0183, 2698.3711, 2707.3184, 2711.3606, 2712.3755, 2719.3159, 2729.3870, 2744.3770, 2767.3120, 2846.3943, 2871.4121, 2872.3569, 2873.3445, 2884.3745, 2888.3184, 2892.4063, 2893.4375, 2917.3960, 2926.4563, 2992.4536, 2995.4939, 3004.4705, 3005.5735, 3027.5032, 3054.4763, 3082.5879, 3104.5430, 3126.7307, 3170.6697, 3200.5081, 3201.4670, 3206.5745, 3207.6025, 3242.6213, 3248.6428, 3255.6025, 3300.7517, 3315.7021, 3328.7302, 3331.6746, 3334.7031, 3335.7090, 3347.5835, 3353.6013, 3356.7166, 3370.7415, 3386.7366, 3471.7375, 3487.7676, 3491.6787, 3503.8286, 3601.7178, 3639.7463, 3646.7891, 3651.8442, 3703.9653, 3716.8708, 3720.7805, 3764.8616, 3820.9673, 3869.8423, 3885.7913, 3887.9063, 3927.8738, 4148.0005, 4162.0400, 4253.0234, 4452.2729, 4464.2598, 4468.1572, 4482.1689, 4680.3301, 4845.1597

2. Q27972 **Mass:** 41373 **Score:** 58 **Expect:** 0.041 **Matches:** 19

Chondroadherin OS=Bos taurus GN=CHAD PE=1 SV=1

Observed	Mr(expt)	Mr(calc)	ppm	Start		End	Miss	Peptide
713.3626	712.3553	712.3544	1.29	202	-	206	0	K.FYLDR.N
756.4689	755.4617	755.4653	-4.89	57	-	62	0	K.LLNLQR.N
837.3761	836.3688	836.3633	6.61	306	-	311	0	K.CTCQLR.G
913.5131	912.5058	912.5042	1.79	334	-	340	1	K.FRGQHIR.D
985.6076	984.6003	984.5968	3.62	220	-	227	1	K.LRVVEELK.L
1018.5203	1017.5130	1017.5104	2.57	274	-	281	0	K.HVHLENNR.L
1315.6759	1314.6686	1314.6429	19.6	336	-	346	1	R.GQHIRDTDAFR.G
1338.6461	1337.6388	1337.6364	1.80	235	-	246	0	K.SIPDNAFQSFGR.Y
1355.7097	1354.7024	1354.7133	-8.02	261	-	273	0	K.FSDGAFLGVTTLK.H
1618.8162	1617.8089	1617.8124	-2.16	334	-	346	2	K.FRGQHIRDTDAFR.G
1647.8694	1646.8621	1646.8780	-9.64	102	-	114	0	K.QLIYLYLSHNDIR.V
1751.8870	1750.8797	1750.8777	1.14	247	-	260	0	R.YLETLWLDNTNLEK.F
1775.0276	1774.0203	1774.0465	-14.73	220	-	234	2	K.LRVVEELKLSHNPLK.S
2060.0461	2059.0388	2059.0374	0.68	202	-	219	1	K.FYLDRNQLSSYPSAALSK.L
2762.3970	2761.3897	2761.3810	3.16	177	-	201	0	R.WLYLSENSLSSLQPGALDDVENLAK.F
2812.4265	2811.4192	2811.4232	-1.40	282	-	305	0	R.LHQLPSNFPFDSLETLTLTNNPWK.C
2894.4741	2893.4668	2893.4498	5.89	118	-	142	1	R.AGAFDDLTELTYLYLDHNKVTELPR.G
3088.5859	3087.5786	3087.5805	-0.59	247	-	273	1	R.YLETLWLDNTNLEKFSDGAFLGVTTLK.H
3456.7622	3455.7549	3455.7249	8.70	177	-	206	1	R.WLYLSENSLSSLQPGALDDVENLAKFYLDR.N

No match to: 706.2814, 707.2933, 717.4103, 719.4172, 722.4265, 735.4174, 744.3938, 768.3713, 772.4224, 779.4717, 792.4133, 804.3155, 806.4307, 807.4160, 809.4363, 812.3947, 814.4097, 815.4186, 819.3901, 828.3928, 831.4666, 832.3149, 834.3199, 841.4355, 842.4860, 845.4138, 851.3882, 871.4900, 876.2846, 878.2907, 883.4759, 891.4818, 893.9534, 894.4574, 898.4351, 910.4221, 918.5399, 929.4545, 935.4750, 937.4921, 947.4755, 959.4836, 961.4837, 963.4760, 967.4271, 969.4589, 972.4794, 974.4445, 976.4506, 977.4767, 978.5352, 981.4952, 983.4747, 988.4585, 989.6082, 997.4586, 1007.5676, 1011.4840, 1014.5074, 1019.5441, 1021.5421, 1034.5779, 1042.5320, 1044.5242, 1060.5463, 1071.5376, 1077.5208, 1087.5399, 1091.5421, 1106.5393, 1122.5577, 1156.5372, 1173.6240, 1187.6439, 1199.6992, 1215.6615, 1244.6189, 1248.6575, 1255.6039, 1276.6218, 1284.6401, 1295.7195, 1301.6177, 1313.7069, 1314.7141, 1325.6675, 1336.7151, 1337.7180, 1342.6758, 1344.7024, 1349.6952, 1353.6804, 1356.7201, 1361.6877, 1365.6632, 1370.6783, 1372.6058, 1381.7588, 1383.7244, 1386.7131, 1394.7318, 1397.7419, 1414.6467, 1427.7865, 1432.7140, 1446.7247, 1457.7411, 1461.7784, 1473.8055, 1478.7513, 1497.8757, 1528.6993, 1542.8459, 1556.8795, 1578.7826, 1601.9006, 1606.8433, 1616.8177, 1628.7026, 1628.8568, 1642.7186, 1650.8473, 1658.8400, 1677.8625, 1680.8728, 1696.8231, 1703.8551, 1704.8862, 1710.9269, 1715.8459, 1717.8810, 1718.9111, 1725.8386, 1727.9301, 1730.8835, 1742.9099, 1768.9546, 1769.9617, 1771.0243, 1777.8676, 1786.9032, 1791.9047, 1799.9005, 1800.9124, 1915.0015, 1928.0238, 1953.9285, 1982.0009, 2012.9681, 2025.0820, 2027.1273, 2042.0642, 2052.9939, 2067.0017, 2070.8899, 2074.9629, 2082.9421, 2084.9102, 2086.9065, 2096.9797, 2100.9070, 2109.0410, 2116.0894, 2136.9121, 2155.0730, 2170.0781, 2195.0801, 2211.0686, 2238.1072, 2251.1428, 2293.1614, 2336.2495, 2343.1758, 2382.1987, 2385.2234, 2400.0720, 2414.0930, 2418.0901, 2428.1140, 2431.0991, 2465.2188, 2468.2249, 2528.1794, 2542.2344, 2560.2729, 2564.2610, 2646.9797, 2660.9968, 2675.0183, 2698.3711, 2707.3184, 2711.3606, 2712.3755, 2719.3159, 2729.3870, 2744.3770, 2767.3120, 2846.3943, 2871.4121, 2872.3569, 2873.3445, 2884.3745, 2888.3184, 2892.4063, 2893.4375, 2917.3960, 2926.4563, 2992.4536, 2995.4939, 3004.4705, 3005.5735, 3027.5032, 3054.4763, 3082.5879, 3104.5430, 3126.7307, 3170.6697, 3200.5081, 3201.4670, 3206.5745, 3207.6025, 3242.6213, 3248.6428, 3255.6025, 3263.6973, 3300.7517, 3315.7021, 3328.7302, 3331.6746, 3334.7031, 3335.7090, 3347.5835, 3353.6013, 3356.7166, 3370.7415, 3386.7366, 3471.7375, 3487.7676, 3491.6787, 3503.8286, 3601.7178, 3639.7463,

3646.7891, 3651.8442, 3703.9653, 3716.8708, 3720.7805, 3764.8616, 3820.9673, 3869.8423, 3885.7913, 3887.9063, 3927.8738, 4148.0005, 4162.0400, 4253.0234, 4452.2729, 4464.2598, 4468.1572, 4482.1689, 4680.3301, 4845.1597

3. E1BI02 **Mass:** 43291 **Score:** 42 **Expect:** 1.4 **Matches:** 14

Fibromodulin OS=Bos taurus GN=FMOD PE=4 SV=1

Observed	Mr(expt)	Mr(calc)	ppm	Start	End	Miss	Peptide
779.4717	778.4645	778.4701	-7.25	169	175	0	R.IPSPLPR.S
978.5352	977.5279	977.5334	-5.69	98	105	0	K.YLPFVPSR.M
1301.6177	1300.6104	1300.6268	-12.56	357	368	0	R.SAMPADAPLCLR.L
1361.6877	1360.6804	1360.6847	-3.17	179	189	0	R.ELHLDHNQISR.V
1457.7411	1456.7338	1456.7279	4.09	356	368	1	K.RSAMPADAPLCLR.L
1556.8795	1555.8722	1555.8722	0.02	223	235	0	R.SLILLDLSYNHLR.K
1717.8810	1716.8737	1716.9019	-16.43	176	189	1	R.SLRELHLDHNQISR.V
1928.0238	1927.0165	1927.0163	0.12	307	323	0	K.IPPVSTNLENLYLQGNR.I
2646.9797	2645.9724	2645.9702	0.83	74	94	0	R.DCPQECDCPPNFPTAMYCDNR.N
3206.5745	3205.5672	3205.5720	-1.50	237	264	0	K.VPDGLPSALEQLYLEHNNVFSVPDSYFR.G
3334.7031	3333.6958	3333.6670	8.65	236	264	1	R.KVPDGLPSALEQLYLEHNNVFSVPDSYFR.G
3646.7891	3645.7818	3645.8573	-20.69	190	222	1	R.VPNNALEGLENLTALYLHHNEIQEVGSSMKGLR.S
3703.9653	3702.9580	3702.8682	24.3	236	268	2	R.KVPDGLPSALEQLYLEHNNVFSVPDSYFRGSPK.L
4468.1572	4467.1499	4467.2499	-22.38	108	147	1	K.YVYFQNNQISSIQEGVFDNATGLLWIALHGNQITSDKVGK.K

No match to: 706.2814, 707.2933, 713.3626, 717.4103, 719.4172, 722.4265, 735.4174, 744.3938, 756.4689, 768.3713, 772.4224, 792.4133, 804.3155, 806.4307, 807.4160, 809.4363, 812.3947, 814.4097, 815.4186, 819.3901, 828.3928, 831.4666, 832.3149, 834.3199, 837.3761, 841.4355, 842.4860, 845.4138, 851.3882, 871.4900, 876.2846, 878.2907, 883.4759, 891.4818, 893.9534, 894.4574, 898.4351, 910.4221, 913.5131, 918.5399, 929.4545, 935.4750, 937.4921, 947.4755, 959.4836, 961.4837, 963.4760, 967.4271, 969.4589, 972.4794, 974.4445, 976.4506, 977.4767, 981.4952, 983.4747, 985.6076, 988.4585, 989.6082, 997.4586, 1007.5676, 1011.4840, 1014.5074, 1018.5203, 1019.5441, 1021.5421, 1034.5779, 1042.5320, 1044.5242, 1060.5463, 1071.5376, 1077.5208, 1087.5399, 1091.5421, 1106.5393, 1122.5577, 1156.5372, 1173.6240, 1187.6439, 1199.6992, 1215.6615, 1244.6189, 1248.6575, 1255.6039, 1276.6218, 1284.6401, 1295.7195, 1313.7069, 1314.7141, 1315.6759, 1325.6675, 1336.7151, 1337.7180, 1338.6461, 1342.6758, 1344.7024, 1349.6952, 1353.6804, 1355.7097, 1356.7201, 1365.6632, 1370.6783, 1372.6058, 1381.7588, 1383.7244, 1386.7131, 1394.7318, 1397.7419, 1414.6467, 1427.7865, 1432.7140, 1446.7247, 1461.7784, 1473.8055, 1478.7513, 1497.8757, 1528.6993, 1542.8459, 1578.7826, 1601.9006, 1606.8433, 1616.8177, 1618.8162, 1628.7026, 1628.8568, 1642.7186, 1647.8694, 1650.8473, 1658.8400, 1677.8625, 1680.8728, 1696.8231, 1703.8551, 1704.8862, 1710.9269, 1715.8459, 1718.9111, 1725.8386, 1727.9301, 1730.8835, 1742.9099, 1751.8870, 1768.9546, 1769.9617, 1771.0243, 1775.0276, 1777.8676, 1786.9032, 1791.9047, 1799.9005, 1800.9124, 1915.0015, 1953.9285, 1982.0009, 2012.9681, 2025.0820, 2027.1273, 2042.0642, 2052.9939, 2060.0461, 2067.0017, 2070.8899, 2074.9629, 2082.9421, 2084.9102, 2086.9065, 2096.9797, 2100.9070, 2109.0410, 2116.0894, 2136.9121, 2155.0730, 2170.0781, 2195.0801, 2211.0686, 2238.1072, 2251.1428, 2293.1614, 2336.2495, 2343.1758, 2382.1987, 2385.2234, 2400.0720, 2414.0930, 2418.0901, 2428.1140, 2431.0991, 2465.2188, 2468.2249, 2528.1794, 2542.2344, 2560.2729, 2564.2610, 2660.9968, 2675.0183, 2698.3711, 2707.3184, 2711.3606, 2712.3755, 2719.3159, 2729.3870, 2744.3770, 2762.3970, 2767.3120, 2812.4265, 2846.3943, 2871.4121, 2872.3569, 2873.3445, 2884.3745, 2888.3184, 2892.4063, 2893.4375, 2894.4741, 2917.3960, 2926.4563, 2992.4536, 2995.4939, 3004.4705, 3005.5735, 3027.5032, 3054.4763, 3082.5879, 3088.5859, 3104.5430, 3126.7307, 3170.6697, 3200.5081, 3201.4670, 3207.6025, 3242.6213, 3248.6428, 3255.6025, 3263.6973, 3300.7517, 3315.7021, 3328.7302, 3331.6746, 3335.7090, 3347.5835, 3353.6013, 3356.7166, 3370.7415, 3386.7366, 3456.7622, 3471.7375, 3487.7676, 3491.6787, 3503.8286, 3601.7178, 3639.7463, 3651.8442, 3716.8708, 3720.7805, 3764.8616, 3820.9673, 3869.8423, 3885.7913, 3887.9063, 3927.8738, 4148.0005, 4162.0400, 4253.0234, 4452.2729, 4464.2598, 4482.1689, 4680.3301, 4845.1597

4. P13605 **Mass:** 43410 **Score:** 42 **Expect:** 1.4 **Matches:** 14

Fibromodulin OS=Bos taurus GN=FMOD PE=1 SV=2

Observed	Mr(expt)	Mr(calc)	ppm	Start	End	Miss	Peptide
779.4717	778.4645	778.4701	-7.25	170	176	0	R.IPSPLPR.S
978.5352	977.5279	977.5334	-5.69	99	106	0	K.YLPFVPSR.M
1301.6177	1300.6104	1300.6268	-12.56	358	369	0	R.SAMPADAPLCLR.L
1361.6877	1360.6804	1360.6847	-3.17	180	190	0	R.ELHLDHNQISR.V
1457.7411	1456.7338	1456.7279	4.09	357	369	1	K.RSAMPADAPLCLR.L
1556.8795	1555.8722	1555.8722	0.02	224	236	0	R.SLILLDLSYNHLR.K
1717.8810	1716.8737	1716.9019	-16.43	177	190	1	R.SLRELHLDHNQISR.V
1928.0238	1927.0165	1927.0163	0.12	308	324	0	K.IPPVSTNLENLYLQGNR.I
2646.9797	2645.9724	2645.9702	0.83	75	95	0	R.DCPQECDCPPNFPTAMYCDNR.N
3206.5745	3205.5672	3205.5720	-1.50	238	265	0	K.VPDGLPSALEQLYLEHNNVFSVPDSYFR.G
3334.7031	3333.6958	3333.6670	8.65	237	265	1	R.KVPDGLPSALEQLYLEHNNVFSVPDSYFR.G
3646.7891	3645.7818	3645.8573	-20.69	191	223	1	R.VPNNALEGLENLTALYLHHNEIQEVGSSMKGLR.S
3703.9653	3702.9580	3702.8682	24.3	237	269	2	R.KVPDGLPSALEQLYLEHNNVFSVPDSYFRGSPK.L
4468.1572	4467.1499	4467.2499	-22.38	109	148	1	K.YVYFQNNQISSIQEGVFDNATGLLWIALHGNQITSDKVGK.K

No match to: 706.2814, 707.2933, 713.3626, 717.4103, 719.4172, 722.4265, 735.4174, 744.3938, 756.4689, 768.3713, 772.4224, 792.4133, 804.3155, 806.4307, 807.4160, 809.4363, 812.3947, 814.4097, 815.4186, 819.3901, 828.3928, 831.4666, 832.3149, 834.3199, 837.3761, 841.4355, 842.4860, 845.4138, 851.3882, 871.4900, 876.2846, 878.2907, 883.4759, 891.4818, 893.9534, 894.4574, 898.4351, 910.4221, 913.5131, 918.5399, 929.4545, 935.4750, 937.4921, 947.4755, 959.4836, 961.4837, 963.4760, 967.4271, 969.4589, 972.4794, 974.4445, 976.4506, 977.4767, 981.4952, 983.4747, 985.6076, 988.4585, 989.6082, 997.4586, 1007.5676, 1011.4840, 1014.5074, 1018.5203, 1019.5441, 1021.5421, 1034.5779, 1042.5320, 1044.5242, 1060.5463, 1071.5376, 1077.5208, 1087.5399, 1091.5421, 1106.5393, 1122.5577, 1156.5372, 1173.6240, 1187.6439, 1199.6992, 1215.6615, 1244.6189, 1248.6575, 1255.6039, 1276.6218, 1284.6401, 1295.7195, 1313.7069, 1314.7141, 1315.6759, 1325.6675, 1336.7151, 1337.7180, 1338.6461, 1342.6758, 1344.7024, 1349.6952, 1353.6804, 1355.7097, 1356.7201, 1365.6632, 1370.6783, 1372.6058, 1381.7588, 1383.7244, 1386.7131, 1394.7318, 1397.7419, 1414.6467, 1427.7865, 1432.7140, 1446.7247, 1461.7784, 1473.8055, 1478.7513, 1497.8757, 1528.6993, 1542.8459, 1578.7826, 1601.9006, 1606.8433, 1616.8177, 1618.8162, 1628.7026, 1628.8568, 1642.7186, 1647.8694, 1650.8473, 1658.8400, 1677.8625, 1680.8728, 1696.8231, 1703.8551, 1704.8862, 1710.9269, 1715.8459, 1718.9111, 1725.8386, 1727.9301, 1730.8835, 1742.9099, 1751.8870, 1768.9546, 1769.9617, 1771.0243, 1775.0276, 1777.8676, 1786.9032, 1791.9047, 1799.9005, 1800.9124, 1915.0015, 1953.9285, 1982.0009, 2012.9681, 2025.0820, 2027.1273, 2042.0642, 2052.9939, 2060.0461, 2067.0017, 2070.8899, 2074.9629, 2082.9421, 2084.9102, 2086.9065, 2096.9797, 2100.9070, 2109.0410, 2116.0894, 2136.9121, 2155.0730, 2170.0781, 2195.0801, 2211.0686, 2238.1072, 2251.1428, 2293.1614, 2336.2495, 2343.1758, 2382.1987, 2385.2234, 2400.0720, 2414.0930, 2418.0901, 2428.1140, 2431.0991, 2465.2188, 2468.2249, 2528.1794, 2542.2344, 2560.2729, 2564.2610, 2660.9968, 2675.0183, 2698.3711, 2707.3184, 2711.3606, 2712.3755, 2719.3159, 2729.3870, 2744.3770, 2762.3970, 2767.3120, 2812.4265, 2846.3943, 2871.4121, 2872.3569, 2873.3445, 2884.3745, 2888.3184, 2892.4063, 2893.4375, 2894.4741, 2917.3960, 2926.4563, 2992.4536, 2995.4939, 3004.4705, 3005.5735, 3027.5032, 3054.4763, 3082.5879, 3088.5859, 3104.5430, 3126.7307, 3170.6697, 3200.5081, 3201.4670, 3207.6025, 3242.6213, 3248.6428, 3255.6025, 3263.6973, 3300.7517, 3315.7021, 3328.7302, 3331.6746, 3335.7090, 3347.5835, 3353.6013, 3356.7166, 3370.7415, 3386.7366, 3456.7622, 3471.7375, 3487.7676, 3491.6787, 3503.8286, 3601.7178, 3639.7463, 3651.8442, 3716.8708, 3720.7805, 3764.8616, 3820.9673, 3869.8423, 3885.7913, 3887.9063, 3927.8738, 4148.0005, 4162.0400, 4253.0234, 4452.2729, 4464.2598, 4482.1689, 4680.3301, 4845.1597

5. P55252 **Mass:** 40889 **Score:** 35 **Expect:** 7.8 **Matches:** 17

Hyaluronan and proteoglycan link protein 1 OS=Bos taurus GN=HAPLN1 PE=2 SV=1

Observed	Mr(expt)	Mr(calc)	ppm	Start	End	Miss	Peptide
809.4363	808.4291	808.4120	21.2	334	340	0	R.FVGFPDK.K
910.4221	909.4148	909.4093	6.08	104	111	0	K.TYGGYHGR.V
937.4921	936.4848	936.5069	-23.62	334	341	1	R.FVGFPDKK.H
976.4506	975.4433	975.4444	-1.05	325	333	0	R.CSPSEAAVR.F

1019.5441	1018.5368	1018.5600	-22.75	289	297	0	K.VGQIFAAWK.L
1077.5208	1076.5135	1076.5113	2.03	344	351	0	K.LYGVYCFR.A
1342.6758	1341.6685	1341.6652	2.47	342	351	1	K.HKLYGVYCFR.A
1370.6783	1369.6710	1369.6409	22.0	224	236	0	R.EPCGGQNTVPGVR.N
1386.7131	1385.7058	1385.7052	0.47	66	78	1	R.DPTAFGSGTHKIR.I
1397.7419	1396.7346	1396.7252	6.77	104	115	1	K.TYGGYHGRVFLK.G
2211.0686	2210.0613	2210.0339	12.4	116	136	0	K.GGSDNDASLVITDLTLEDYGR.Y
2698.3711	2697.3638	2697.3497	5.22	112	136	1	R.VFLKGGSDNDASLVITDLTLEDYGR.Y
2707.3184	2706.3111	2706.2860	9.27	200	223	0	R.SGLDWCNAGWLSDGSVQYPITKPR.E
2893.4375	2892.4302	2892.4453	-5.22	298	322	2	K.LLGYDRCDAGWLADGSVRYPISRPR.R
3242.6213	3241.6140	3241.6118	0.70	261	288	1	R.FYYLIHPTKLTYDEAVQACLNDGAQIAK.V
3601.7178	3600.7105	3600.6368	20.5	170	199	0	R.YNLNFHEAQQACLDQDAVIASFDQLYDAWR.S
3927.8738	3926.8665	3926.8434	5.88	167	199	1	R.LGRYNLNFHEAQQACLDQDAVIASFDQLYDAWR.S

No match to: 706.2814, 707.2933, 713.3626, 717.4103, 719.4172, 722.4265, 735.4174, 744.3938, 756.4689, 768.3713, 772.4224, 779.4717, 792.4133, 804.3155, 806.4307, 807.4160, 812.3947, 814.4097, 815.4186, 819.3901, 828.3928, 831.4666, 832.3149, 834.3199, 837.3761, 841.4355, 842.4860, 845.4138, 851.3882, 871.4900, 876.2846, 878.2907, 883.4759, 891.4818, 893.9534, 894.4574, 898.4351, 913.5131, 918.5399, 929.4545, 935.4750, 947.4755, 959.4836, 961.4837, 963.4760, 967.4271, 969.4589, 972.4794, 974.4445, 977.4767, 978.5352, 981.4952, 983.4747, 985.6076, 988.4585, 989.6082, 997.4586, 1007.5676, 1011.4840, 1014.5074, 1018.5203, 1021.5421, 1034.5779, 1042.5320, 1044.5242, 1060.5463, 1071.5376, 1087.5399, 1091.5421, 1106.5393, 1122.5577, 1156.5372, 1173.6240, 1187.6439, 1199.6992, 1215.6615, 1244.6189, 1248.6575, 1255.6039, 1276.6218, 1284.6401, 1295.7195, 1301.6177, 1313.7069, 1314.7141, 1315.6759, 1325.6675, 1336.7151, 1337.7180, 1338.6461, 1344.7024, 1349.6952, 1353.6804, 1355.7097, 1356.7201, 1361.6877, 1365.6632, 1372.6058, 1381.7588, 1383.7244, 1394.7318, 1414.6467, 1427.7865, 1432.7140, 1446.7247, 1457.7411, 1461.7784, 1473.8055, 1478.7513, 1497.8757, 1528.6993, 1542.8459, 1556.8795, 1578.7826, 1601.9006, 1606.8433, 1616.8177, 1618.8162, 1628.7026, 1628.8568, 1642.7186, 1647.8694, 1650.8473, 1658.8400, 1677.8625, 1680.8728, 1696.8231, 1703.8551, 1704.8862, 1710.9269, 1715.8459, 1717.8810, 1718.9111, 1725.8386, 1727.9301, 1730.8835, 1742.9099, 1751.8870, 1768.9546, 1769.9617, 1771.0243, 1775.0276, 1777.8676, 1786.9032, 1791.9047, 1799.9005, 1800.9124, 1915.0015, 1928.0238, 1953.9285, 1982.0009, 2012.9681, 2025.0820, 2027.1273, 2042.0642, 2052.9939, 2060.0461, 2067.0017, 2070.8899, 2074.9629, 2082.9421, 2084.9102, 2086.9065, 2096.9797, 2100.9070, 2109.0410, 2116.0894, 2136.9121, 2155.0730, 2170.0781, 2195.0801, 2238.1072, 2251.1428, 2293.1614, 2336.2495, 2343.1758, 2382.1987, 2385.2234, 2400.0720, 2414.0930, 2418.0901, 2428.1140, 2431.0991, 2465.2188, 2468.2249, 2528.1794, 2542.2344, 2560.2729, 2564.2610, 2646.9797, 2660.9968, 2675.0183, 2711.3606, 2712.3755, 2719.3159, 2729.3870, 2744.3770, 2762.3970, 2767.3120, 2812.4265, 2846.3943, 2871.4121, 2872.3569, 2873.3445, 2884.3745, 2888.3184, 2892.4063, 2894.4741, 2917.3960, 2926.4563, 2992.4536, 2995.4939, 3004.4705, 3005.5735, 3027.5032, 3054.4763, 3082.5879, 3088.5859, 3104.5430, 3126.7307, 3170.6697, 3200.5081, 3201.4670, 3206.5745, 3207.6025, 3248.6428, 3255.6025, 3263.6973, 3300.7517, 3315.7021, 3328.7302, 3331.6746, 3334.7031, 3335.7090, 3347.5835, 3353.6013, 3356.7166, 3370.7415, 3386.7366, 3456.7622, 3471.7375, 3487.7676, 3491.6787, 3503.8286, 3639.7463, 3646.7891, 3651.8442, 3703.9653, 3716.8708, 3720.7805, 3764.8616, 3820.9673, 3869.8423, 3885.7913, 3887.9063, 4148.0005, 4162.0400, 4253.0234, 4452.2729, 4464.2598, 4468.1572, 4482.1689, 4680.3301, 4845.1597

6. F6QVC9 **Mass:** 36109 **Score:** 31 **Expect:** 21 **Matches:** 14

Annexin OS=Bos taurus GN=ANXA5 PE=1 SV=1

Observed	Mr(expt)	Mr(calc)	ppm	Start	End	Miss	Peptide
717.4103	716.4030	716.4003	3.75	1	6	0	-.MAQVLR.G
744.3938	743.3865	743.3813	6.96	118	123	0	R.TPEELR.A
1014.5074	1013.5001	1013.5069	-6.71	90	97	0	R.LYDAYELK.H
1122.5577	1121.5504	1121.5717	-18.93	277	285	0	R.SEIDLYNIR.K
1276.6218	1275.6145	1275.6169	-1.87	291	301	0	K.NFGTSLYSMIK.G + Oxidation (M)
1461.7784	1460.7711	1460.7735	-1.66	46	58	1	R.SNAQRQEIAVAFK.T
1704.8862	1703.8789	1703.8941	-8.93	30	45	0	K.GLGTDEESILTLLTSR.S
1710.9269	1709.9196	1709.9247	-2.94	152	166	1	R.MLVVLLQANRDPDAR.I
1799.9005	1798.8932	1798.8890	2.34	187	201	1	K.WGTDEEKFITIFGTR.S
2067.0017	2065.9944	2065.9705	11.6	7	25	1	R.GTVADFPGFDERADAETLR.K
2195.0801	2194.0728	2194.0655	3.36	7	26	2	R.GTVADFPGFDERADAETLRK.A
2385.2234	2384.2161	2384.2547	-16.17	102	123	2	K.GAGTDEKVLTEIIASRTPEELR.A
3201.4670	3200.4597	3200.3946	20.4	124	151	0	R.AIEQVYEEEYGSSLEDDVVGDTSGYYQR.M
3242.6213	3241.6140	3241.6441	-9.28	243	271	2	K.SIRSIPAYLAETLYYAMKGAGTDDHTLIR.V + Oxidation (M)

No match to: 706.2814, 707.2933, 713.3626, 719.4172, 722.4265, 735.4174, 756.4689, 768.3713, 772.4224, 779.4717, 792.4133, 804.3155, 806.4307, 807.4160, 809.4363, 812.3947, 814.4097, 815.4186, 819.3901, 828.3928, 831.4666, 832.3149, 834.3199, 837.3761, 841.4355, 842.4860, 845.4138, 851.3882, 871.4900, 876.2846, 878.2907, 883.4759, 891.4818, 893.9534, 894.4574, 898.4351, 910.4221, 913.5131, 918.5399, 929.4545, 935.4750, 937.4921, 947.4755, 959.4836, 961.4837, 963.4760, 967.4271, 969.4589, 972.4794, 974.4445, 976.4506, 977.4767, 978.5352, 981.4952, 983.4747, 985.6076, 988.4585, 989.6082, 997.4586, 1007.5676, 1011.4840, 1018.5203, 1019.5441, 1021.5421, 1034.5779, 1042.5320, 1044.5242, 1060.5463, 1071.5376, 1077.5208, 1087.5399, 1091.5421, 1106.5393, 1156.5372, 1173.6240, 1187.6439, 1199.6992, 1215.6615, 1244.6189, 1248.6575, 1255.6039, 1284.6401, 1295.7195, 1301.6177, 1313.7069, 1314.7141, 1315.6759, 1325.6675, 1336.7151, 1337.7180, 1338.6461, 1342.6758, 1344.7024, 1349.6952, 1353.6804, 1355.7097, 1356.7201, 1361.6877, 1365.6632, 1370.6783, 1372.6058, 1381.7588, 1383.7244, 1386.7131, 1394.7318, 1397.7419, 1414.6467, 1427.7865, 1432.7140, 1446.7247, 1457.7411, 1473.8055, 1478.7513, 1497.8757, 1528.6993, 1542.8459, 1556.8795, 1578.7826, 1601.9006, 1606.8433, 1616.8177, 1618.8162, 1628.7026, 1628.8568, 1642.7186, 1647.8694, 1650.8473, 1658.8400, 1677.8625, 1680.8728, 1696.8231, 1703.8551, 1715.8459, 1717.8810, 1718.9111, 1725.8386, 1727.9301, 1730.8835, 1742.9099, 1751.8870, 1768.9546, 1769.9617, 1771.0243, 1775.0276, 1777.8676, 1786.9032, 1791.9047, 1800.9124, 1915.0015, 1928.0238, 1953.9285, 1982.0009, 2012.9681, 2025.0820, 2027.1273, 2042.0642, 2052.9939, 2060.0461, 2070.8899, 2074.9629, 2082.9421, 2084.9102, 2086.9065, 2096.9797, 2100.9070, 2109.0410, 2116.0894, 2136.9121, 2155.0730, 2170.0781, 2211.0686, 2238.1072, 2251.1428, 2293.1614, 2336.2495, 2343.1758, 2382.1987, 2400.0720, 2414.0930, 2418.0901, 2428.1140, 2431.0991, 2465.2188, 2468.2249, 2528.1794, 2542.2344, 2560.2729, 2564.2610, 2646.9797, 2660.9968, 2675.0183, 2698.3711, 2707.3184, 2711.3606, 2712.3755, 2719.3159, 2729.3870, 2744.3770, 2762.3970, 2767.3120, 2812.4265, 2846.3943, 2871.4121, 2872.3569, 2873.3445, 2884.3745, 2888.3184, 2892.4063, 2893.4375, 2894.4741, 2917.3960, 2926.4563, 2992.4536, 2995.4939, 3004.4705, 3005.5735, 3027.5032, 3054.4763, 3082.5879, 3088.5859, 3104.5430, 3126.7307, 3170.6697, 3200.5081, 3206.5745, 3207.6025, 3248.6428, 3255.6025, 3263.6973, 3300.7517, 3315.7021, 3328.7302, 3331.6746, 3334.7031, 3335.7090, 3347.5835, 3353.6013, 3356.7166, 3370.7415, 3386.7366, 3456.7622, 3471.7375, 3487.7676, 3491.6787, 3503.8286, 3601.7178, 3639.7463, 3646.7891, 3651.8442, 3703.9653, 3716.8708, 3720.7805, 3764.8616, 3820.9673, 3869.8423, 3885.7913, 3887.9063, 3927.8738, 4148.0005, 4162.0400, 4253.0234, 4452.2729, 4464.2598, 4468.1572, 4482.1689, 4680.3301, 4845.1597

7. P81287 **Mass:** 36124 **Score:** 31 **Expect:** 21 **Matches:** 14

Annexin A5 OS=Bos taurus GN=ANXA5 PE=1 SV=3

Observed	Mr(expt)	Mr(calc)	ppm	Start	End	Miss	Peptide
717.4103	716.4030	716.4003	3.75	1	6	0	-.MAQVLR.G
744.3938	743.3865	743.3813	6.96	118	123	0	R.TPEELR.A
1014.5074	1013.5001	1013.5069	-6.71	90	97	0	R.LYDAYELK.H
1122.5577	1121.5504	1121.5717	-18.93	277	285	0	R.SEIDLYNIR.K
1276.6218	1275.6145	1275.6169	-1.87	291	301	0	K.NFGTSLYSMIK.G + Oxidation (M)
1461.7784	1460.7711	1460.7735	-1.66	46	58	1	R.SNAQRQEIAVAFK.T
1710.9269	1709.9196	1709.9247	-2.94	152	166	1	R.MLVVLLQANRDPDAR.I
1718.9111	1717.9038	1717.9098	-3.47	30	45	0	K.GLGTDEETILTLLTSR.S
1799.9005	1798.8932	1798.8890	2.34	187	201	1	K.WGTDEEKFITIFGTR.S
2067.0017	2065.9944	2065.9705	11.6	7	25	1	R.GTVADFPGFDERADAETLR.K
2195.0801	2194.0728	2194.0655	3.36	7	26	2	R.GTVADFPGFDERADAETLRK.A
2385.2234	2384.2161	2384.2547	-16.17	102	123	2	K.GAGTDEKVLTEIIASRTPEELR.A

3201.4670	3200.4597	3200.3946	20.4	124 - 151	0	R.AIEQVYEEEYGSSLEDDVVGDTSGYYQR.M
3242.6213	3241.6140	3241.6441	-9.28	243 - 271	2	K.SIRSIPAYLAETLYYAMKGAGTDDHTLIR.V + Oxidation (M)

No match to: 706.2814, 707.2933, 713.3626, 719.4172, 722.4265, 735.4174, 756.4689, 768.3713, 772.4224, 779.4717, 792.4133, 804.3155, 806.4307, 807.4160, 809.4363, 812.3947, 814.4097, 815.4186, 819.3901, 828.3928, 831.4666, 832.3149, 834.3199, 837.3761, 841.4355, 842.4860, 845.4138, 851.3882, 871.4900, 876.2846, 878.2907, 883.4759, 891.4818, 893.9534, 894.4574, 898.4351, 910.4221, 913.5131, 918.5399, 929.4545, 935.4750, 937.4921, 947.4755, 959.4836, 961.4837, 963.4760, 967.4271, 969.4589, 972.4794, 974.4445, 976.4506, 977.4767, 978.5352, 981.4952, 983.4747, 985.6076, 988.4585, 989.6082, 997.4586, 1007.5676, 1011.4840, 1018.5203, 1019.5441, 1021.5421, 1034.5779, 1042.5320, 1044.5242, 1060.5463, 1071.5376, 1077.5208, 1087.5399, 1091.5421, 1106.5393, 1156.5372, 1173.6240, 1187.6439, 1199.6992, 1215.6615, 1244.6189, 1248.6575, 1255.6039, 1284.6401, 1295.7195, 1301.6177, 1313.7069, 1314.7141, 1315.6759, 1325.6675, 1336.7151, 1337.7180, 1338.6461, 1342.6758, 1344.7024, 1349.6952, 1353.6804, 1355.7097, 1356.7201, 1361.6877, 1365.6632, 1370.6783, 1372.6058, 1381.7588, 1383.7244, 1386.7131, 1394.7318, 1397.7419, 1414.6467, 1427.7865, 1432.7140, 1446.7247, 1457.7411, 1473.8055, 1478.7513, 1497.8757, 1528.6993, 1542.8459, 1556.8795, 1578.7826, 1601.9006, 1606.8433, 1616.8177, 1618.8162, 1628.7026, 1628.8568, 1642.7186, 1647.8694, 1650.8473, 1658.8400, 1677.8625, 1680.8728, 1696.8231, 1703.8551, 1704.8862, 1715.8459, 1717.8810, 1725.8386, 1727.9301, 1730.8835, 1742.9099, 1751.8870, 1768.9546, 1769.9617, 1771.0243, 1775.0276, 1777.8676, 1786.9032, 1791.9047, 1800.9124, 1915.0015, 1928.0238, 1953.9285, 1982.0009, 2012.9681, 2025.0820, 2027.1273, 2042.0642, 2052.9939, 2060.0461, 2070.8899, 2074.9629, 2082.9421, 2084.9102, 2086.9065, 2096.9797, 2100.9070, 2109.0410, 2116.0894, 2136.9121, 2155.0730, 2170.0781, 2211.0686, 2238.1072, 2251.1428, 2293.1614, 2336.2495, 2343.1758, 2382.1987, 2400.0720, 2414.0930, 2418.0901, 2428.1140, 2431.0991, 2465.2188, 2468.2249, 2528.1794, 2542.2344, 2560.2729, 2564.2610, 2646.9797, 2660.9968, 2675.0183, 2698.3711, 2707.3184, 2711.3606, 2712.3755, 2719.3159, 2729.3870, 2744.3770, 2762.3970, 2767.3120, 2812.4265, 2846.3943, 2871.4121, 2872.3569, 2873.3445, 2884.3745, 2888.3184, 2892.4063, 2893.4375, 2894.4741, 2917.3960, 2926.4563, 2992.4536, 2995.4939, 3004.4705, 3005.5735, 3027.5032, 3054.4763, 3082.5879, 3088.5859, 3104.5430, 3126.7307, 3170.6697, 3200.5081, 3206.5745, 3207.6025, 3248.6428, 3255.6025, 3263.6973, 3300.7517, 3315.7021, 3328.7302, 3331.6746, 3334.7031, 3335.7090, 3347.5835, 3353.6013, 3356.7166, 3370.7415, 3386.7366, 3456.7622, 3471.7375, 3487.7676, 3491.6787, 3503.8286, 3601.7178, 3639.7463, 3646.7891, 3651.8442, 3703.9653, 3716.8708, 3720.7805, 3764.8616, 3820.9673, 3869.8423, 3885.7913, 3887.9063, 3927.8738, 4148.0005, 4162.0400, 4253.0234, 4452.2729, 4464.2598, 4468.1572, 4482.1689, 4680.3301, 4845.1597

8. E1BMY3 **Mass:** 35539 **Score:** 30 **Expect:** 22 **Matches:** 10

Olfactory receptor OS=Bos taurus GN=LOC614090 PE=3 SV=2

Observed	Mr(expt)	Mr(calc)	ppm	Start - End	Miss	Peptide
851.3882	850.3809	850.3603	24.2	228 - 234	0	R.MQSNEAR.K + Oxidation (M)
963.4760	962.4687	962.4603	8.67	228 - 235	1	R.MQSNEARK.K
1091.5421	1090.5348	1090.5553	-18.76	228 - 236	2	R.MQSNEARKK.A
1248.6575	1247.6502	1247.6193	24.8	81 - 90	1	K.MAANYLRHTR.S + Oxidation (M)
2116.0894	2115.0821	2115.1121	-14.18	123 - 139	1	R.YVAICHPLRYPILMNQK.L
3054.4763	3053.4690	3053.4966	-9.02	237 - 263	0	K.AFATCSSHLAVVGLFYGTSMFIYMRPK.S
3082.5879	3081.5806	3081.6027	-7.15	55 - 80	0	R.LHTPMYFLLSQLSLMDMMLVLTIVPK.M + 3 Oxidation (M)
3370.7415	3369.7342	3369.6771	16.9	91 - 122	0	R.SISPTGCGAQIVLLLTLGGGECFLLAAMAYDR.Y + Oxidation (M)
3764.8616	3763.8543	3763.8848	-8.10	88 - 122	1	R.HTRSISPTGCGAQIVLLLTLGGGECFLLAAMAYDR.Y + Oxidation (M)
4464.2598	4463.2525	4463.2626	-2.26	91 - 131	1	R.SISPTGCGAQIVLLLTLGGGECFLLAAMAYDRYVAICHPLR.Y

No match to: 706.2814, 707.2933, 713.3626, 717.4103, 719.4172, 722.4265, 735.4174, 744.3938, 756.4689, 768.3713, 772.4224, 779.4717, 792.4133, 804.3155, 806.4307, 807.4160, 809.4363, 812.3947, 814.4097, 815.4186, 819.3901, 828.3928, 831.4666, 832.3149, 834.3199, 837.3761, 841.4355, 842.4860, 845.4138, 871.4900, 876.2846, 878.2907, 883.4759, 891.4818, 893.9534, 894.4574, 898.4351, 910.4221, 913.5131, 918.5399, 929.4545, 935.4750, 937.4921, 947.4755, 959.4836, 961.4837, 967.4271, 969.4589, 972.4794, 974.4445, 976.4506, 977.4767, 978.5352, 981.4952, 983.4747, 985.6076, 988.4585, 989.6082, 997.4586, 1007.5676, 1011.4840, 1014.5074, 1018.5203, 1019.5441, 1021.5421, 1034.5779, 1042.5320, 1044.5242, 1060.5463, 1071.5376, 1077.5208, 1087.5399, 1106.5393, 1122.5577, 1156.5372, 1173.6240, 1187.6439, 1199.6992, 1215.6615, 1244.6189, 1255.6039, 1276.6218, 1284.6401, 1295.7195, 1301.6177, 1313.7069, 1314.7141, 1315.6759, 1325.6675, 1336.7151, 1337.7180, 1338.6461, 1342.6758, 1344.7024, 1349.6952, 1353.6804, 1355.7097, 1356.7201, 1361.6877, 1365.6632, 1370.6783, 1372.6058, 1381.7588, 1383.7244, 1386.7131, 1394.7318, 1397.7419, 1414.6467, 1427.7865, 1432.7140, 1446.7247, 1457.7411, 1461.7784, 1473.8055, 1478.7513, 1497.8757, 1528.6993, 1542.8459, 1556.8795, 1578.7826, 1601.9006, 1606.8433, 1616.8177, 1618.8162, 1628.7026, 1628.8568, 1642.7186, 1647.8694, 1650.8473, 1658.8400, 1677.8625, 1680.8728, 1696.8231, 1703.8551, 1704.8862, 1710.9269, 1715.8459, 1717.8810, 1718.9111, 1725.8386, 1727.9301, 1730.8835, 1742.9099, 1751.8870, 1768.9546, 1769.9617, 1771.0243, 1775.0276, 1777.8676, 1786.9032, 1791.9047, 1799.9005, 1800.9124, 1915.0015, 1928.0238, 1953.9285, 1982.0009, 2012.9681, 2025.0820, 2027.1273, 2042.0642, 2052.9939, 2060.0461, 2067.0017, 2070.8899, 2074.9629, 2082.9421, 2084.9102, 2086.9065, 2096.9797, 2100.9070, 2109.0410, 2136.9121, 2155.0730, 2170.0781, 2195.0801, 2211.0686, 2238.1072, 2251.1428, 2293.1614, 2336.2495, 2343.1758, 2382.1987, 2385.2234, 2400.0720, 2414.0930, 2418.0901, 2428.1140, 2431.0991, 2465.2188, 2468.2249, 2528.1794, 2542.2344, 2560.2729, 2564.2610, 2646.9797, 2660.9968, 2675.0183, 2698.3711, 2707.3184, 2711.3606, 2712.3755, 2719.3159, 2729.3870, 2744.3770, 2762.3970, 2767.3120, 2812.4265, 2846.3943, 2871.4121, 2872.3569, 2873.3445, 2884.3745, 2888.3184, 2892.4063, 2893.4375, 2894.4741, 2917.3960, 2926.4563, 2992.4536, 2995.4939, 3004.4705, 3005.5735, 3027.5032, 3088.5859, 3104.5430, 3126.7307, 3170.6697, 3200.5081, 3201.4670, 3206.5745, 3207.6025, 3242.6213, 3248.6428, 3255.6025, 3263.6973, 3300.7517, 3315.7021, 3328.7302, 3331.6746, 3334.7031, 3335.7090, 3347.5835, 3353.6013, 3356.7166, 3386.7366, 3456.7622, 3471.7375, 3487.7676, 3491.6787, 3503.8286, 3601.7178, 3639.7463, 3646.7891, 3651.8442, 3703.9653, 3716.8708, 3720.7805, 3820.9673, 3869.8423, 3885.7913, 3887.9063, 3927.8738, 4148.0005, 4162.0400, 4253.0234, 4452.2729, 4468.1572, 4482.1689, 4680.3301, 4845.1597

9. G3N3X7 **Mass:** 5156 **Score:** 30 **Expect:** 24 **Matches:** 4

Uncharacterized protein OS=Bos taurus PE=4 SV=1

Observed	Mr(expt)	Mr(calc)	ppm	Start - End	Miss	Peptide
1658.8400	1657.8327	1657.8511	-11.08	20 - 32	2	R.GHQPWLAKQMRYK.A + Oxidation (M)
1953.9285	1952.9212	1952.9176	1.84	2 - 19	0	R.GAIHHQPFPGSMHASSHR.G
2871.4121	2870.4048	2870.4048	0.01	2 - 27	1	R.GAIHHQPFPGSMHASSHRGHQPWLAK.Q
3027.5032	3026.4959	3026.5059	-3.30	1 - 27	2	-.RGAIHHQPFPGSMHASSHRGHQPWLAK.Q

No match to: 706.2814, 707.2933, 713.3626, 717.4103, 719.4172, 722.4265, 735.4174, 744.3938, 756.4689, 768.3713, 772.4224, 779.4717, 792.4133, 804.3155, 806.4307, 807.4160, 809.4363, 812.3947, 814.4097, 815.4186, 819.3901, 828.3928, 831.4666, 832.3149, 834.3199, 837.3761, 841.4355, 842.4860, 845.4138, 851.3882, 871.4900, 876.2846, 878.2907, 883.4759, 891.4818, 893.9534, 894.4574, 898.4351, 910.4221, 913.5131, 918.5399, 929.4545, 935.4750, 937.4921, 947.4755, 959.4836, 961.4837, 963.4760, 967.4271, 969.4589, 972.4794, 974.4445, 976.4506, 977.4767, 978.5352, 981.4952, 983.4747, 985.6076, 988.4585, 989.6082, 997.4586, 1007.5676, 1011.4840, 1014.5074, 1018.5203, 1019.5441, 1021.5421, 1034.5779, 1042.5320, 1044.5242, 1060.5463, 1071.5376, 1077.5208, 1087.5399, 1091.5421, 1106.5393, 1122.5577, 1156.5372, 1173.6240, 1187.6439, 1199.6992, 1215.6615, 1244.6189, 1248.6575, 1255.6039, 1276.6218, 1284.6401, 1295.7195, 1301.6177, 1313.7069, 1314.7141, 1315.6759, 1325.6675, 1336.7151, 1337.7180, 1338.6461, 1342.6758, 1344.7024, 1349.6952, 1353.6804, 1355.7097, 1356.7201, 1361.6877, 1365.6632, 1370.6783, 1372.6058, 1381.7588, 1383.7244, 1386.7131, 1394.7318, 1397.7419, 1414.6467, 1427.7865, 1432.7140, 1446.7247, 1457.7411, 1461.7784, 1473.8055, 1478.7513, 1497.8757, 1528.6993, 1542.8459, 1556.8795, 1578.7826, 1601.9006, 1606.8433, 1616.8177, 1618.8162, 1628.7026, 1628.8568, 1642.7186, 1647.8694, 1650.8473, 1677.8625, 1680.8728, 1696.8231, 1703.8551, 1704.8862, 1710.9269, 1715.8459, 1717.8810, 1718.9111, 1725.8386, 1727.9301, 1730.8835, 1742.9099, 1751.8870, 1768.9546, 1769.9617, 1771.0243, 1775.0276, 1777.8676, 1786.9032, 1791.9047, 1799.9005, 1800.9124, 1915.0015, 1928.0238, 1982.0009, 2012.9681, 2025.0820, 2027.1273, 2042.0642, 2052.9939, 2060.0461, 2067.0017, 2070.8899, 2074.9629, 2082.9421, 2084.9102, 2086.9065, 2096.9797, 2100.9070, 2109.0410, 2116.0894, 2136.9121, 2155.0730, 2170.0781, 2195.0801, 2211.0686, 2238.1072, 2251.1428, 2293.1614, 2336.2495, 2343.1758, 2382.1987, 2385.2234, 2400.0720, 2414.0930, 2418.0901, 2428.1140, 2431.0991, 2465.2188, 2468.2249, 2528.1794, 2542.2344, 2560.2729, 2564.2610, 2646.9797, 2660.9968, 2675.0183, 2698.3711, 2707.3184, 2711.3606, 2712.3755, 2719.3159, 2729.3870, 2744.3770, 2762.3970, 2767.3120, 2812.4265, 2846.3943, 2872.3569, 2873.3445, 2884.3745, 2888.3184, 2892.4063, 2893.4375, 2894.4741, 2917.3960, 2926.4563, 2992.4536, 2995.4939, 3004.4705, 3005.5735, 3054.4763, 3082.5879, 3088.5859, 3104.5430, 3126.7307, 3170.6697, 3200.5081, 3201.4670, 3206.5745, 3207.6025, 3242.6213, 3248.6428, 3255.6025, 3263.6973, 3300.7517, 3315.7021, 3328.7302, 3331.6746, 3334.7031, 3335.7090, 3347.5835, 3353.6013, 3356.7166, 3370.7415, 3386.7366, 3456.7622, 3471.7375, 3487.7676, 3491.6787, 3503.8286, 3601.7178, 3639.7463, 3646.7891, 3651.8442, 3703.9653, 3716.8708, 3720.7805, 3764.8616, 3820.9673, 3869.8423, 3885.7913, 3887.9063, 3927.8738, 4148.0005, 4162.0400, 4253.0234, 4452.2729, 4464.2598, 4468.1572, 4482.1689, 4680.3301, 4845.1597

10. F1MSR8 **Mass:** 134858 **Score:** 30 **Expect:** 27 **Matches:** 32

Collagen alpha-1(II) chain OS=Bos taurus GN=COL2A1 PE=4 SV=2

Observed	Mr(expt)	Mr(calc)	ppm	Start	End	Miss	Peptide
792.4133	791.4061	791.3960	12.7	1213	1218	1	R.TCRDLK.L
812.3947	811.3874	811.3898	-2.92	1379	1384	0	K.TMIEYR.S
815.4186	814.4114	814.3933	22.1	306	314	0	K.GEAGPTGAR.G
828.3928	827.3855	827.3847	1.00	1379	1384	0	K.TMIEYR.S + Oxidation (M)
871.4900	870.4827	870.4851	-2.75	1360	1366	0	R.FTYTVLK.D
935.4750	934.4677	934.4661	1.74	1265	1271	1	K.KNWWSSK.S
961.4837	960.4764	960.4625	14.5	66	74	2	R.GDRGDKGEK.G
969.4589	968.4516	968.4538	-2.24	1219	1225	0	K.LCHPEWK.S
1173.6240	1172.6167	1172.6149	1.54	1192	1201	0	K.SLNNQIESLR.S
1325.6675	1324.6602	1324.6598	0.34	1216	1225	1	R.DLKLCHPEWK.S
1427.7865	1426.7792	1426.7780	0.88	1341	1353	0	K.ALLIQGSNDVEIR.A
1432.7140	1431.7067	1431.7068	-0.07	1360	1371	1	R.FTYTVLKDGCTK.H
1478.7513	1477.7440	1477.7412	1.89	1326	1339	0	K.NSIAYLDEAAGNLK.K
1606.8433	1605.8360	1605.8362	-0.10	1326	1340	1	K.NSIAYLDEAAGNLKK.A
1650.8473	1649.8400	1649.8710	-18.77	258	275	2	R.GLPGERGRTGPAGAAGAR.G
1786.9032	1785.8959	1785.8969	-0.54	1192	1207	1	K.SLNNQIESLRSPEGSR.K
1915.0015	1913.9942	1913.9919	1.24	1192	1208	2	K.SLNNQIESLRSPEGSRK.N
2012.9681	2011.9608	2011.9824	-10.73	506	527	0	K.VGPSGAPGEDGRPGPPGPQGAR.G
2042.0642	2041.0569	2041.0552	0.85	1341	1359	1	K.ALLIQGSNDVEIRAEGNSR.F
2070.8899	2069.8826	2069.8823	0.17	1226	1243	0	K.SGDYWIDPNQGCTLDAMK.V
2082.9421	2081.9348	2081.9549	-9.63	1038	1058	2	R.GMPGPQGPRGDKGETGEAGER.G
2086.9065	2085.8992	2085.8772	10.6	1226	1243	0	K.SGDYWIDPNQGCTLDAMK.V + Oxidation (M)
2116.0894	2115.0821	2115.0345	22.5	561	584	1	R.GLPGKDGETGAAGPPGPAGPAGER.G
2400.0720	2399.0647	2399.0596	2.14	1244	1264	0	K.VFCNMETGETCVYPNPASVPK.K
2528.1794	2527.1721	2527.1546	6.95	1244	1265	1	K.VFCNMETGETCVYPNPASVPKK.N
2707.3184	2706.3111	2706.3726	-22.70	38	65	2	K.GEPGDIKDIVGPKGPPGPQGPAGEQGPR.G
2894.4741	2893.4668	2893.5297	-21.74	1341	1366	2	K.ALLIQGSNDVEIRAEGNSRFTYTVLK.D
2917.3960	2916.3887	2916.3468	14.4	1016	1046	2	K.QGDRGEAGAQGPMGPAGPAGARGMPGPQGPR.G + 2 Oxidation (M)
3331.6746	3330.6673	3330.6131	16.3	276	314	1	R.GNDGQPGPAGPPGPVGPAGGPGFPGAPGAKGEAGPTGAR.G
3356.7166	3355.7093	3355.6833	7.77	1388	1418	1	K.TSRLPIIDIAPMDIGGPEQEFGVDIGPVCFL.-
3869.8423	3868.8350	3868.8056	7.59	1277	1310	0	K.HIWFGETINGGFHFSYGDDNLAPNTANVQMTFLR.L
3885.7913	3884.7840	3884.8006	-4.26	1277	1310	0	K.HIWFGETINGGFHFSYGDDNLAPNTANVQMTFLR.L + Oxidation (M)

No match to: 706.2814, 707.2933, 713.3626, 717.4103, 719.4172, 722.4265, 735.4174, 744.3938, 756.4689, 768.3713, 772.4224, 779.4717, 804.3155, 806.4307, 807.4160, 809.4363, 814.4097, 819.3901, 831.4666, 832.3149, 834.3199, 837.3761, 841.4355, 842.4860, 845.4138, 851.3882, 876.2846, 878.2907, 883.4759, 891.4818, 893.9534, 894.4574, 898.4351, 910.4221, 913.5131, 918.5399, 929.4545, 937.4921, 947.4755, 959.4836, 963.4760, 967.4271, 972.4794, 974.4445, 976.4506, 977.4767, 978.5352, 981.4952, 983.4747, 985.6076, 988.4585, 989.6082, 997.4586, 1007.5676, 1011.4840, 1014.5074, 1018.5203, 1019.5441, 1021.5421, 1034.5779, 1042.5320, 1044.5242, 1060.5463, 1071.5376, 1077.5208, 1087.5399, 1091.5421, 1106.5393, 1122.5577, 1156.5372, 1187.6439, 1199.6992, 1215.6615, 1244.6189, 1248.6575, 1255.6039, 1276.6218, 1284.6401, 1295.7195, 1301.6177, 1313.7069, 1314.7141, 1315.6759, 1336.7151, 1337.7180, 1338.6461, 1342.6758, 1344.7024, 1349.6952, 1353.6804, 1355.7097, 1356.7201, 1361.6877, 1365.6632, 1370.6783, 1372.6058, 1381.7588, 1383.7244, 1386.7131, 1394.7318, 1397.7419, 1414.6467, 1446.7247, 1457.7411, 1461.7784, 1473.8055, 1497.8757, 1528.6993, 1542.8459, 1556.8795, 1578.7826, 1601.9006, 1616.8177, 1618.8162, 1628.7026, 1628.8568, 1642.7186, 1647.8694, 1658.8400, 1677.8625, 1680.8728, 1696.8231, 1703.8551, 1704.8862, 1710.9269, 1715.8459, 1717.8810, 1718.9111, 1725.8386, 1727.9301, 1730.8835, 1742.9099, 1751.8870, 1768.9546, 1769.9617, 1771.0243, 1775.0276, 1777.8676, 1791.9047, 1799.9005, 1800.9124, 1928.0238, 1953.9285, 1982.0009, 2025.0820, 2027.1273, 2052.9939, 2060.0461, 2067.0017, 2074.9629, 2084.9102, 2096.9797, 2100.9070, 2109.0410, 2136.9121, 2155.0730, 2170.0781, 2195.0801, 2211.0686, 2238.1072, 2251.1428, 2293.1614, 2336.2495, 2343.1758, 2382.1987, 2385.2234, 2414.0930, 2418.0901, 2428.1140, 2431.0991, 2465.2188, 2468.2249, 2542.2344, 2560.2729, 2564.2610, 2646.9797, 2660.9968, 2675.0183, 2698.3711, 2711.3606, 2712.3755, 2719.3159, 2729.3870, 2744.3770, 2762.3970, 2767.3120, 2812.4265, 2846.3943, 2871.4121, 2872.3569, 2873.3445, 2884.3745, 2888.3184, 2892.4063, 2893.4375, 2926.4563, 2992.4536, 2995.4939, 3004.4705, 3005.5735, 3027.5032, 3054.4763, 3082.5879, 3088.5859, 3104.5430, 3126.7307, 3170.6697, 3200.5081, 3201.4670, 3206.5745, 3207.6025, 3242.6213, 3248.6428, 3255.6025, 3263.6973, 3300.7517, 3315.7021, 3328.7302, 3334.7031, 3335.7090, 3347.5835, 3353.6013, 3370.7415, 3386.7366, 3456.7622, 3471.7375, 3487.7676, 3491.6787, 3503.8286, 3601.7178, 3639.7463, 3646.7891, 3651.8442, 3703.9653, 3716.8708, 3720.7805, 3764.8616, 3820.9673, 3887.9063, 3927.8738, 4148.0005, 4162.0400, 4253.0234, 4452.2729, 4464.2598, 4468.1572, 4482.1689, 4680.3301, 4845.1597

11. F1MJA3 **Mass:** 19688 **Score:** 29 **Expect:** 29 **Matches:** 7

Uncharacterized protein OS=Bos taurus GN=STK32B PE=4 SV=1

Observed	Mr(expt)	Mr(calc)	ppm	Start	End	Miss	Peptide
891.4818	890.4745	890.4570	19.7	135	142	2	K.NRSKDGSK.D
2155.0730	2154.0657	2154.0310	16.1	143	160	1	K.DSCPLNGHLQQCLETVRK.E
2414.0930	2413.0857	2413.1114	-10.66	139	159	1	K.DGSKDSCPLNGHLQQCLETVR.K
2542.2344	2541.2271	2541.2064	8.15	139	160	2	K.DGSKDSCPLNGHLQQCLETVRK.E
2917.3960	2916.3887	2916.4592	-24.17	21	44	1	R.GWRPYEIHSATPIDEILNMFKVER.V + Oxidation (M)
3206.5745	3205.5672	3205.5237	13.6	66	94	1	K.DPESRLSSLGDVQSAPYLADMNWDAVLEK.A
3646.7891	3645.7818	3645.8389	-15.65	71	104	1	R.LSSLGDVQSAPYLADMNWDAVLEKALTPGFVPNK.G

No match to: 706.2814, 707.2933, 713.3626, 717.4103, 719.4172, 722.4265, 735.4174, 744.3938, 756.4689, 768.3713, 772.4224, 779.4717, 792.4133, 804.3155, 806.4307, 807.4160, 809.4363, 812.3947, 814.4097, 815.4186, 819.3901, 828.3928, 831.4666, 832.3149, 834.3199, 837.3761, 841.4355, 842.4860, 845.4138, 851.3882, 871.4900, 876.2846, 878.2907, 883.4759, 893.9534, 894.4574, 898.4351, 910.4221, 913.5131, 918.5399, 929.4545, 935.4750, 937.4921, 947.4755, 959.4836, 961.4837, 963.4760, 967.4271, 972.4794, 974.4445, 976.4506, 977.4767, 978.5352, 981.4952, 983.4747, 985.6076, 988.4585, 989.6082, 997.4586, 1007.5676, 1011.4840, 1014.5074, 1018.5203, 1019.5441, 1021.5421, 1034.5779, 1042.5320, 1044.5242, 1060.5463, 1071.5376, 1077.5208, 1087.5399, 1091.5421, 1106.5393, 1122.5577, 1156.5372, 1173.6240, 1187.6439, 1199.6992, 1215.6615, 1244.6189, 1248.6575, 1255.6039, 1276.6218, 1284.6401, 1295.7195, 1301.6177, 1313.7069, 1314.7141, 1315.6759, 1325.6675, 1336.7151, 1337.7180, 1338.6461, 1342.6758, 1344.7024, 1349.6952, 1353.6804, 1355.7097, 1356.7201, 1361.6877, 1365.6632, 1370.6783, 1372.6058, 1381.7588, 1383.7244, 1386.7131, 1394.7318, 1397.7419, 1414.6467, 1427.7865, 1432.7140, 1446.7247, 1457.7411, 1461.7784, 1473.8055, 1478.7513, 1497.8757, 1528.6993, 1542.8459, 1556.8795, 1578.7826, 1601.9006, 1606.8433, 1616.8177, 1618.8162, 1628.7026, 1628.8568, 1642.7186, 1647.8694, 1650.8473, 1658.8400, 1677.8625, 1680.8728, 1696.8231, 1703.8551, 1704.8862, 1710.9269, 1715.8459, 1717.8810, 1718.9111, 1725.8386, 1727.9301, 1730.8835, 1742.9099, 1751.8870, 1768.9546, 1769.9617, 1771.0243, 1775.0276, 1777.8676, 1786.9032, 1791.9047, 1799.9005, 1800.9124, 1915.0015, 1928.0238, 1953.9285, 1982.0009, 2012.9681, 2025.0820, 2027.1273, 2042.0642, 2052.9939, 2060.0461, 2067.0017, 2070.8899, 2074.9629, 2082.9421, 2084.9102, 2086.9065, 2096.9797, 2100.9070, 2109.0410, 2116.0894, 2136.9121, 2170.0781, 2195.0801, 2211.0686, 2238.1072, 2251.1428, 2293.1614, 2336.2495, 2343.1758, 2382.1987, 2385.2234, 2400.0720, 2418.0901, 2428.1140, 2431.0991, 2465.2188, 2468.2249, 2528.1794, 2560.2729, 2564.2610, 2646.9797, 2660.9968, 2675.0183, 2698.3711, 2707.3184, 2711.3606, 2712.3755, 2719.3159, 2729.3870, 2744.3770, 2762.3970, 2767.3120, 2812.4265, 2846.3943, 2871.4121, 2872.3569, 2873.3445, 2884.3745, 2888.3184, 2892.4063, 2893.4375, 2894.4741, 2926.4563, 2992.4536, 2995.4939, 3004.4705, 3005.5735, 3027.5032, 3054.4763, 3082.5879, 3088.5859, 3104.5430, 3126.7307, 3170.6697, 3200.5081, 3201.4670, 3207.6025, 3242.6213, 3248.6428, 3255.6025, 3263.6973, 3300.7517, 3315.7021, 3328.7302, 3331.6746, 3334.7031, 3335.7090, 3347.5835, 3353.6013, 3356.7166, 3370.7415, 3386.7366, 3456.7622, 3471.7375, 3487.7676, 3491.6787, 3503.8286, 3601.7178, 3639.7463, 3651.8442, 3703.9653, 3716.8708, 3720.7805, 3764.8616, 3820.9673, 3869.8423, 3885.7913, 3887.9063, 3927.8738, 4148.0005, 4162.0400, 4253.0234, 4452.2729, 4464.2598, 4468.1572, 4482.1689, 4680.3301, 4845.1597

12. P02459 **Mass:** 142825 **Score:** 27 **Expect:** 44 **Matches:** 32

Collagen alpha-1(II) chain OS=Bos taurus GN=COL2A1 PE=1 SV=4

Observed	Mr(expt)	Mr(calc)	ppm	Start	End	Miss	Peptide
792.4133	791.4061	791.3960	12.7	1282	1287	1	R.TCRDLK.L
812.3947	811.3874	811.3898	-2.92	1448	1453	0	K.TMIEYR.S
815.4186	814.4114	814.3933	22.1	375	383	0	K.GEAGPTGAR.G
828.3928	827.3855	827.3847	1.00	1448	1453	0	K.TMIEYR.S + Oxidation (M)
871.4900	870.4827	870.4851	-2.75	1429	1435	0	R.FTYTVLK.D
935.4750	934.4677	934.4661	1.74	1334	1340	1	K.KNWWSSK.S
961.4837	960.4764	960.4625	14.5	135	143	2	R.GDRGDKGEK.G
969.4589	968.4516	968.4538	-2.24	1288	1294	0	K.LCHPEWK.S
1173.6240	1172.6167	1172.6149	1.54	1261	1270	0	K.SLNNQIESLR.S
1325.6675	1324.6602	1324.6598	0.34	1285	1294	1	R.DLKLCHPEWK.S
1427.7865	1426.7792	1426.7780	0.88	1410	1422	0	K.ALLIQGSNDVEIR.A
1432.7140	1431.7067	1431.7068	-0.07	1429	1440	1	R.FTYTVLKDGCTK.H
1478.7513	1477.7440	1477.7412	1.89	1395	1408	0	K.NSIAYLDEAAGNLK.K
1606.8433	1605.8360	1605.8362	-0.10	1395	1409	1	K.NSIAYLDEAAGNLKK.A
1650.8473	1649.8400	1649.8710	-18.77	327	344	2	R.GLPGERGRTGPAGAAGAR.G
1786.9032	1785.8959	1785.8969	-0.54	1261	1276	1	K.SLNNQIESLRSPEGSR.K
1915.0015	1913.9942	1913.9919	1.24	1261	1277	2	K.SLNNQIESLRSPEGSRK.N
2012.9681	2011.9608	2011.9824	-10.73	575	596	0	K.VGPSGAPGEDGRPGPPGPQGAR.G
2042.0642	2041.0569	2041.0552	0.85	1410	1428	1	K.ALLIQGSNDVEIRAEGNSR.F
2070.8899	2069.8826	2069.8823	0.17	1295	1312	0	K.SGDYWIDPNQGCTLDAMK.V
2082.9421	2081.9348	2081.9549	-9.63	1107	1127	2	R.GMPGPQGPRGDKGETGEAGER.G
2086.9065	2085.8992	2085.8772	10.6	1295	1312	0	K.SGDYWIDPNQGCTLDAMK.V + Oxidation (M)
2116.0894	2115.0821	2115.0345	22.5	630	653	1	R.GLPGKDGETGAAGPPGPAGPAGER.G
2400.0720	2399.0647	2399.0596	2.14	1313	1333	0	K.VFCNMETGETCVYPNPASVPK.K
2528.1794	2527.1721	2527.1546	6.95	1313	1334	1	K.VFCNMETGETCVYPNPASVPKK.N
2707.3184	2706.3111	2706.3726	-22.70	107	134	2	K.GEPGDIKDIVGPKGPPGPQGPAGEQGPR.G
2894.4741	2893.4668	2893.5297	-21.74	1410	1435	2	K.ALLIQGSNDVEIRAEGNSRFTYTVLK.D
2917.3960	2916.3887	2916.3468	14.4	1085	1115	2	K.QGDRGEAGAQGPMGPAGPAGARGMPGPQGPR.G + 2 Oxidation (M)
3331.6746	3330.6673	3330.6131	16.3	345	383	1	R.GNDGQPGPAGPPGPVGPAGGPGFPGAPGAKGEAGPTGAR.G
3356.7166	3355.7093	3355.6833	7.77	1457	1487	1	K.TSRLPIIDIAPMDIGGPEQEFGVDIGPVCFL.-
3869.8423	3868.8350	3868.8056	7.59	1346	1379	0	K.HIWFGETINGGFHFSYGDDNLAPNTANVQMTFLR.L
3885.7913	3884.7840	3884.8006	-4.26	1346	1379	0	K.HIWFGETINGGFHFSYGDDNLAPNTANVQMTFLR.L + Oxidation (M)

No match to: 706.2814, 707.2933, 713.3626, 717.4103, 719.4172, 722.4265, 735.4174, 744.3938, 756.4689, 768.3713, 772.4224, 779.4717, 804.3155, 806.4307, 807.4160, 809.4363, 814.4097, 819.3901, 831.4666, 832.3149, 834.3199, 837.3761, 841.4355, 842.4860, 845.4138, 851.3882, 876.2846, 878.2907, 883.4759, 891.4818, 893.9534, 894.4574, 898.4351, 910.4221, 913.5131, 918.5399, 929.4545, 937.4921, 947.4755, 959.4836, 963.4760, 967.4271, 972.4794, 974.4445, 976.4506, 977.4767, 978.5352, 981.4952, 983.4747, 985.6076, 988.4585, 989.6082, 997.4586, 1007.5676, 1011.4840, 1014.5074, 1018.5203, 1019.5441, 1021.5421, 1034.5779, 1042.5320, 1044.5242, 1060.5463, 1071.5376, 1077.5208, 1087.5399, 1091.5421, 1106.5393, 1122.5577, 1156.5372, 1187.6439, 1199.6992, 1215.6615, 1244.6189, 1248.6575, 1255.6039, 1276.6218, 1284.6401, 1295.7195, 1301.6177, 1313.7069, 1314.7141, 1315.6759, 1336.7151, 1337.7180, 1338.6461, 1342.6758, 1344.7024, 1349.6952, 1353.6804, 1355.7097, 1356.7201, 1361.6877, 1365.6632, 1370.6783, 1372.6058, 1381.7588, 1383.7244, 1386.7131, 1394.7318, 1397.7419, 1414.6467, 1446.7247, 1457.7411, 1461.7784, 1473.8055, 1497.8757, 1528.6993, 1542.8459, 1556.8795, 1578.7826, 1601.9006, 1616.8177, 1618.8162, 1628.7026, 1628.8568, 1642.7186, 1647.8694, 1658.8400, 1677.8625, 1680.8728, 1696.8231, 1703.8551, 1704.8862, 1710.9269, 1715.8459, 1717.8810, 1718.9111, 1725.8386, 1727.9301, 1730.8835, 1742.9099, 1751.8870, 1768.9546, 1769.9617, 1771.0243, 1775.0276, 1777.8676, 1791.9047, 1799.9005, 1800.9124, 1928.0238, 1953.9285, 1982.0009, 2025.0820, 2027.1273, 2052.9939, 2060.0461, 2067.0017, 2074.9629, 2084.9102, 2096.9797, 2100.9070, 2109.0410, 2136.9121, 2155.0730, 2170.0781, 2195.0801, 2211.0686, 2238.1072, 2251.1428, 2293.1614, 2336.2495, 2343.1758, 2382.1987, 2385.2234, 2414.0930, 2418.0901, 2428.1140, 2431.0991, 2465.2188, 2468.2249, 2542.2344, 2560.2729, 2564.2610, 2646.9797, 2660.9968, 2675.0183, 2698.3711, 2711.3606, 2712.3755, 2719.3159, 2729.3870, 2744.3770, 2762.3970, 2767.3120, 2812.4265, 2846.3943, 2871.4121, 2872.3569, 2873.3445, 2884.3745, 2888.3184, 2892.4063, 2893.4375, 2926.4563, 2992.4536, 2995.4939, 3004.4705, 3005.5735, 3027.5032, 3054.4763, 3082.5879, 3088.5859, 3104.5430, 3126.7307, 3170.6697, 3200.5081, 3201.4670, 3206.5745, 3207.6025, 3242.6213, 3248.6428, 3255.6025, 3263.6973, 3300.7517, 3315.7021, 3328.7302, 3334.7031, 3335.7090, 3347.5835, 3353.6013, 3370.7415, 3386.7366, 3456.7622, 3471.7375, 3487.7676, 3491.6787, 3503.8286, 3601.7178, 3639.7463, 3646.7891, 3651.8442, 3703.9653, 3716.8708, 3720.7805, 3764.8616, 3820.9673, 3887.9063, 3927.8738, 4148.0005, 4162.0400, 4253.0234, 4452.2729, 4464.2598, 4468.1572, 4482.1689, 4680.3301, 4845.1597

13. G8JL06 **Mass:** 14967 **Score:** 25 **Expect:** 73 **Matches:** 7

Histone H2B OS=Bos taurus GN=HIST1H2BB PE=3 SV=1

Observed	Mr(expt)	Mr(calc)	ppm	Start	End	Miss	Peptide
1014.5074	1013.5001	1013.5182	-17.84	127	135	0	K.LQGSPDIWV.-
1255.6039	1254.5966	1254.6278	-24.84	1	12	1	-.MPEPSKSAPAPK.K + Oxidation (M)
1383.7244	1382.7171	1382.7227	-4.07	1	13	2	-.MPEPSKSAPAPKK.G + Oxidation (M)
1461.7784	1460.7711	1460.7947	-16.13	88	100	1	R.STITSREIQTAVR.L
2893.4375	2892.4302	2892.3898	14.0	48	73	1	K.QVHPDTGISSKAMGIMNSFVNDIFER.I
3200.5081	3199.5008	3199.5542	-16.69	59	86	2	K.AMGIMNSFVNDIFERIASEASRLAHYNK.R + Oxidation (M)
3639.7463	3638.7390	3638.7457	-1.84	48	80	2	K.QVHPDTGISSKAMGIMNSFVNDIFERIASEASR.L + 2 Oxidation (M)

No match to: 706.2814, 707.2933, 713.3626, 717.4103, 719.4172, 722.4265, 735.4174, 744.3938, 756.4689, 768.3713, 772.4224, 779.4717, 792.4133, 804.3155, 806.4307, 807.4160, 809.4363, 812.3947, 814.4097, 815.4186, 819.3901, 828.3928, 831.4666, 832.3149, 834.3199, 837.3761, 841.4355, 842.4860, 845.4138, 851.3882, 871.4900, 876.2846, 878.2907, 883.4759, 891.4818, 893.9534, 894.4574, 898.4351, 910.4221, 913.5131, 918.5399, 929.4545, 935.4750, 937.4921, 947.4755, 959.4836, 961.4837, 963.4760, 967.4271, 969.4589, 972.4794, 974.4445, 976.4506, 977.4767, 978.5352, 981.4952, 983.4747, 985.6076, 988.4585, 989.6082, 997.4586, 1007.5676, 1011.4840, 1018.5203, 1019.5441, 1021.5421, 1034.5779, 1042.5320, 1044.5242, 1060.5463, 1071.5376, 1077.5208, 1087.5399, 1091.5421, 1106.5393, 1122.5577, 1156.5372, 1173.6240, 1187.6439, 1199.6992, 1215.6615, 1244.6189, 1248.6575, 1276.6218, 1284.6401, 1295.7195, 1301.6177, 1313.7069, 1314.7141, 1315.6759, 1325.6675, 1336.7151, 1337.7180, 1338.6461, 1342.6758, 1344.7024, 1349.6952, 1353.6804, 1355.7097, 1356.7201, 1361.6877, 1365.6632, 1370.6783, 1372.6058, 1381.7588, 1386.7131, 1394.7318, 1397.7419, 1414.6467, 1427.7865, 1432.7140, 1446.7247, 1457.7411, 1473.8055, 1478.7513, 1497.8757, 1528.6993, 1542.8459, 1556.8795, 1578.7826, 1601.9006, 1606.8433, 1616.8177, 1618.8162, 1628.7026, 1628.8568, 1642.7186, 1647.8694, 1650.8473, 1658.8400, 1677.8625, 1680.8728, 1696.8231, 1703.8551, 1704.8862, 1710.9269, 1715.8459, 1717.8810, 1718.9111, 1725.8386, 1727.9301, 1730.8835, 1742.9099, 1751.8870, 1768.9546, 1769.9617, 1771.0243, 1775.0276, 1777.8676, 1786.9032, 1791.9047, 1799.9005, 1800.9124, 1915.0015, 1928.0238, 1953.9285, 1982.0009, 2012.9681, 2025.0820, 2027.1273, 2042.0642, 2052.9939, 2060.0461, 2067.0017, 2070.8899, 2074.9629, 2082.9421, 2084.9102, 2086.9065, 2096.9797, 2100.9070, 2109.0410, 2116.0894, 2136.9121, 2155.0730, 2170.0781, 2195.0801, 2211.0686, 2238.1072, 2251.1428, 2293.1614, 2336.2495, 2343.1758, 2382.1987, 2385.2234, 2400.0720, 2414.0930, 2418.0901, 2428.1140, 2431.0991, 2465.2188, 2468.2249, 2528.1794, 2542.2344, 2560.2729, 2564.2610, 2646.9797, 2660.9968, 2675.0183, 2698.3711, 2707.3184, 2711.3606, 2712.3755, 2719.3159, 2729.3870, 2744.3770, 2762.3970, 2767.3120, 2812.4265, 2846.3943, 2871.4121, 2872.3569, 2873.3445, 2884.3745, 2888.3184, 2892.4063, 2894.4741, 2917.3960, 2926.4563, 2992.4536, 2995.4939, 3004.4705, 3005.5735, 3027.5032, 3054.4763, 3082.5879, 3088.5859, 3104.5430, 3126.7307, 3170.6697, 3201.4670, 3206.5745, 3207.6025, 3242.6213, 3248.6428, 3255.6025, 3263.6973, 3300.7517, 3315.7021, 3328.7302, 3331.6746, 3334.7031, 3335.7090, 3347.5835, 3353.6013, 3356.7166, 3370.7415, 3386.7366, 3456.7622, 3471.7375, 3487.7676, 3491.6787, 3503.8286, 3601.7178, 3646.7891, 3651.8442, 3703.9653, 3716.8708, 3720.7805, 3764.8616, 3820.9673, 3869.8423, 3885.7913, 3887.9063, 3927.8738, 4148.0005, 4162.0400, 4253.0234, 4452.2729, 4464.2598, 4468.1572, 4482.1689, 4680.3301, 4845.1597

14. Q3ZCI4 **Mass:** 53613 **Score:** 25 **Expect:** 82 **Matches:** 14

6-phosphogluconate dehydrogenase, decarboxylating OS=Bos taurus GN=PGD PE=1 SV=2

Observed	Mr(expt)	Mr(calc)	ppm	Start	End	Miss	Peptide

Observed	Mr(expt)	Mr(calc)	ppm	Start	End	Miss	Peptide
918.5399	917.5326	917.5334	-0.86	155	163	0	K.AIFQGIAAK.V
1007.5676	1006.5603	1006.5447	15.5	318	325	1	K.SFLEDIRK.A
1315.6759	1314.6686	1314.6568	8.98	306	317	1	K.GPQNVPFEGDKK.S
1427.7865	1426.7792	1426.7642	10.5	332	343	0	K.IISYAQGFMLLR.Q + Oxidation (M)
1556.8795	1555.8722	1555.8358	23.4	304	317	2	K.LKGPQNVPFEGDKK.S
1628.8568	1627.8495	1627.8603	-6.62	52	66	1	K.VLGAHSLEEMASKLK.K + Oxidation (M)
1915.0015	1913.9942	1914.0244	-15.77	49	66	2	K.GTKVLGAHSLEEMASKLK.K + Oxidation (M)
1982.0009	1980.9936	1980.9880	2.85	137	154	1	R.YGPSLMPGGNKEAWPHIK.A
2238.1072	2237.0999	2237.1117	-5.25	378	396	1	K.DAFDRNPGLQNLLLDDFFK.S
2995.4939	2994.4866	2994.4368	16.6	408	434	1	R.RAISTGVQAGIPMPCFTTALSFYDGYR.H + Oxidation (M)
3601.7178	3600.7105	3600.8003	-24.94	2	34	0	M.AQADIALIGLAVMGQNLILNMNDHGFVVCAFNR.T + Oxidation (M)
3716.8708	3715.8635	3715.8459	4.74	1	34	0	-.MAQADIALIGLAVMGQNLILNMNDHGFVVCAFNR.T
3764.8616	3763.8543	3763.8307	6.29	1	34	0	-.MAQADIALIGLAVMGQNLILNMNDHGFVVCAFNR.T + 3 Oxidation (M)
4148.0005	4146.9932	4147.0839	-21.87	1	38	1	-.MAQADIALIGLAVMGQNLILNMNDHGFVVCAFNRTVSK.V + Oxidation (M)

No match to: 706.2814, 707.2933, 713.3626, 717.4103, 719.4172, 722.4265, 735.4174, 744.3938, 756.4689, 768.3713, 772.4224, 779.4717, 792.4133, 804.3155, 806.4307, 807.4160, 809.4363, 812.3947, 814.4097, 815.4186, 819.3901, 828.3928, 831.4666, 832.3149, 834.3199, 837.3761, 841.4355, 842.4860, 845.4138, 851.3882, 871.4900, 876.2846, 878.2907, 883.4759, 891.4818, 893.9534, 894.4574, 898.4351, 910.4221, 913.5131, 929.4545, 935.4750, 937.4921, 947.4755, 959.4836, 961.4837, 963.4760, 967.4271, 969.4589, 972.4794, 974.4445, 976.4506, 977.4767, 978.5352, 981.4952, 983.4747, 985.6076, 988.4585, 989.6082, 997.4586, 1011.4840, 1014.5074, 1018.5203, 1019.5441, 1021.5421, 1034.5779, 1042.5320, 1044.5242, 1060.5463, 1071.5376, 1077.5208, 1087.5399, 1091.5421, 1106.5393, 1122.5577, 1156.5372, 1173.6240, 1187.6439, 1199.6992, 1215.6615, 1244.6189, 1248.6575, 1255.6039, 1276.6218, 1284.6401, 1295.7195, 1301.6177, 1313.7069, 1314.7141, 1325.6675, 1336.7151, 1337.7180, 1338.6461, 1342.6758, 1344.7024, 1349.6952, 1353.6804, 1355.7097, 1356.7201, 1361.6877, 1365.6632, 1370.6783, 1372.6058, 1381.7588, 1383.7244, 1386.7131, 1394.7318, 1397.7419, 1414.6467, 1432.7140, 1446.7247, 1457.7411, 1461.7784, 1473.8055, 1478.7513, 1497.8757, 1528.6993, 1542.8459, 1578.7826, 1601.9006, 1606.8433, 1616.8177, 1618.8162, 1628.7026, 1642.7186, 1647.8694, 1650.8473, 1658.8400, 1677.8625, 1680.8728, 1696.8231, 1703.8551, 1704.8862, 1710.9269, 1715.8459, 1717.8810, 1718.9111, 1725.8386, 1727.9301, 1730.8835, 1742.9099, 1751.8870, 1768.9546, 1769.9617, 1771.0243, 1775.0276, 1777.8676, 1786.9032, 1791.9047, 1799.9005, 1800.9124, 1928.0238, 1953.9285, 2012.9681, 2025.0820, 2027.1273, 2042.0642, 2052.9939, 2060.0461, 2067.0017, 2070.8899, 2074.9629, 2082.9421, 2084.9102, 2086.9065, 2096.9797, 2100.9070, 2109.0410, 2116.0894, 2136.9121, 2155.0730, 2170.0781, 2195.0801, 2211.0686, 2251.1428, 2293.1614, 2336.2495, 2343.1758, 2382.1987, 2385.2234, 2400.0720, 2414.0930, 2418.0901, 2428.1140, 2431.0991, 2465.2188, 2468.2249, 2528.1794, 2542.2344, 2560.2729, 2564.2610, 2646.9797, 2660.9968, 2675.0183, 2698.3711, 2707.3184, 2711.3606, 2712.3755, 2719.3159, 2729.3870, 2744.3770, 2762.3970, 2767.3120, 2812.4265, 2846.3943, 2871.4121, 2872.3569, 2873.3445, 2884.3745, 2888.3184, 2892.4063, 2893.4375, 2894.4741, 2917.3960, 2926.4563, 2992.4536, 3004.4705, 3005.5735, 3027.5032, 3054.4763, 3082.5879, 3088.5859, 3104.5430, 3126.7307, 3170.6697, 3200.5081, 3201.4670, 3206.5745, 3207.6025, 3242.6213, 3248.6428, 3255.6025, 3263.6973, 3300.7517, 3315.7021, 3328.7302, 3331.6746, 3334.7031, 3335.7090, 3347.5835, 3353.6013, 3356.7166, 3370.7415, 3386.7366, 3456.7622, 3471.7375, 3487.7676, 3491.6787, 3503.8286, 3639.7463, 3646.7891, 3651.8442, 3703.9653, 3720.7805, 3820.9673, 3869.8423, 3885.7913, 3887.9063, 3927.8738, 4162.0400, 4253.0234, 4452.2729, 4464.2598, 4468.1572, 4482.1689, 4680.3301, 4845.1597

15. Q0VC60 **Mass:** 56014 **Score:** 24 **Expect:** 1.1e+002 **Matches:** 11

Dihydrouridine synthase 2-like, SMM1 homolog (S. cerevisiae) OS=Bos taurus GN=DUS2L PE=2 SV=1

Observed	Mr(expt)	Mr(calc)	ppm	Start	End	Miss	Peptide
1255.6039	1254.5966	1254.6244	-22.17	410	419	1	K.YQSTLWDKSK.K
1314.7141	1313.7068	1313.7125	-4.34	421	432	0	K.LAEQAAAIVCLR.S
1383.7244	1382.7171	1382.7194	-1.64	410	420	2	K.YQSTLWDKSKK.L
2042.0642	2041.0569	2041.0956	-18.95	381	397	1	R.EKLAQPVYETVQRPLDR.L
2698.3711	2697.3638	2697.3894	-9.49	334	358	2	K.LLARSPEEAEEPAEDTSGIIKMAIK.F
2894.4741	2893.4668	2893.4393	9.52	55	79	2	R.VVNEALSTVDFVAPDDRVVFRTCER.E
3082.5879	3081.5806	3081.5766	1.30	22	48	1	R.VGTLPMRLLALDYGADIVYCEELVDLK.M + Oxidation (M)
3126.7307	3125.7234	3125.6664	18.2	2	28	2	M.TVNNLSLCYHNKLILAPMVRVGTLPMR.L + Oxidation (M)
3764.8616	3763.8543	3763.8332	5.62	84	118	2	K.VVFQMGTSDAERALAVARLVENDVAGIDVNMGCPK.E + 2 Oxidation (M)
4452.2729	4451.2656	4451.2655	0.02	202	243	2	K.AIAETLSIPVIANGGSHDHIQGYLDIEDFRRATAASSVMVAR.A
4468.1572	4467.1499	4467.2605	-24.75	202	243	2	K.AIAETLSIPVIANGGSHDHIQGYLDIEDFRRATAASSVMVAR.A + Oxidation (M)

No match to: 706.2814, 707.2933, 713.3626, 717.4103, 719.4172, 722.4265, 735.4174, 744.3938, 756.4689, 768.3713, 772.4224, 779.4717, 792.4133, 804.3155, 806.4307, 807.4160, 809.4363, 812.3947, 814.4097, 815.4186, 819.3901, 828.3928, 831.4666, 832.3149, 834.3199, 837.3761, 841.4355, 842.4860, 845.4138, 851.3882, 871.4900, 876.2846, 878.2907, 883.4759, 891.4818, 893.9534, 894.4574, 898.4351, 910.4221, 913.5131, 918.5399, 929.4545, 935.4750, 937.4921, 947.4755, 959.4836, 961.4837, 963.4760, 967.4271, 969.4589, 972.4794, 974.4445, 976.4506, 977.4767, 978.5352, 981.4952, 983.4747, 985.6076, 988.4585, 989.6082, 997.4586, 1007.5676, 1011.4840, 1014.5074, 1018.5203, 1019.5441, 1021.5421, 1034.5779, 1042.5320, 1044.5242, 1060.5463, 1071.5376, 1077.5208, 1087.5399, 1091.5421, 1106.5393, 1122.5577, 1156.5372, 1173.6240, 1187.6439, 1199.6992, 1215.6615, 1244.6189, 1248.6575, 1276.6218, 1284.6401, 1295.7195, 1301.6177, 1313.7069, 1315.6759, 1325.6675, 1336.7151, 1337.7180, 1338.6461, 1342.6758, 1344.7024, 1349.6952, 1353.6804, 1355.7097, 1356.7201, 1361.6877, 1365.6632, 1370.6783, 1372.6058, 1381.7588, 1386.7131, 1394.7318, 1397.7419, 1414.6467, 1427.7865, 1432.7140, 1446.7247, 1457.7411, 1461.7784, 1473.8055, 1478.7513, 1497.8757, 1528.6993, 1542.8459, 1556.8795, 1578.7826, 1601.9006, 1606.8433, 1616.8177, 1618.8162, 1628.7026, 1628.8568, 1642.7186, 1647.8694, 1650.8473, 1658.8400, 1677.8625, 1680.8728, 1696.8231, 1703.8551, 1704.8862, 1710.9269, 1715.8459, 1717.8810, 1718.9111, 1725.8386, 1727.9301, 1730.8835, 1742.9099, 1751.8870, 1768.9546, 1769.9617, 1771.0243, 1775.0276, 1777.8676, 1786.9032, 1791.9047, 1799.9005, 1800.9124, 1915.0015, 1928.0238, 1953.9285, 1982.0009, 2012.9681, 2025.0820, 2027.1273, 2052.9939, 2060.0461, 2067.0017, 2070.8899, 2074.9629, 2082.9421, 2084.9102, 2086.9065, 2096.9797, 2100.9070, 2109.0410, 2116.0894, 2136.9121, 2155.0730, 2170.0781, 2195.0801, 2211.0686, 2238.1072, 2251.1428, 2293.1614, 2336.2495, 2343.1758, 2382.1987, 2385.2234, 2400.0720, 2414.0930, 2418.0901, 2428.1140, 2431.0991, 2465.2188, 2468.2249, 2528.1794, 2542.2344, 2560.2729, 2564.2610, 2646.9797, 2660.9968, 2675.0183, 2707.3184, 2711.3606, 2712.3755, 2719.3159, 2729.3870, 2744.3770, 2762.3970, 2767.3120, 2812.4265, 2846.3943, 2871.4121, 2872.3569, 2873.3445, 2884.3745, 2888.3184, 2892.4063, 2893.4375, 2917.3960, 2926.4563, 2992.4536, 2995.4939, 3004.4705, 3005.5735, 3027.5032, 3054.4763, 3088.5859, 3104.5430, 3170.6697, 3200.5081, 3201.4670, 3206.5745, 3207.6025, 3242.6213, 3248.6428, 3255.6025, 3263.6973, 3300.7517, 3315.7021, 3328.7302, 3331.6746, 3334.7031, 3335.7090, 3347.5835, 3353.6013, 3356.7166, 3370.7415, 3386.7366, 3456.7622, 3471.7375, 3487.7676, 3491.6787, 3503.8286, 3601.7178, 3639.7463, 3646.7891, 3651.8442, 3703.9653, 3716.8708, 3720.7805, 3820.9673, 3869.8423, 3885.7913, 3887.9063, 3927.8738, 4148.0005, 4162.0400, 4253.0234, 4464.2598, 4482.1689, 4680.3301, 4845.1597

16. F1MEZ5 **Mass:** 27894 **Score:** 23 **Expect:** 1.1e+002 **Matches:** 9

Uncharacterized protein OS=Bos taurus GN=GZMB PE=3 SV=1

Observed	Mr(expt)	Mr(calc)	ppm	Start	End	Miss	Peptide
1077.5208	1076.5135	1076.5172	-3.43	149	158	0	R.LGVNMPSTDK.L + Oxidation (M)
2465.2188	2464.2115	2464.2420	-12.37	92	112	0	R.RPIPHPDYNDETLANDIMLLK.L
2812.4265	2811.4192	2811.4346	-5.47	123	148	1	K.VSPINLPRSLAEVKPGMMCSVAGWGR.L
3088.5859	3087.5786	3087.5216	18.5	149	175	2	R.LGVNMPSTDKLQEVDLEVQSEEKCIAR.F
3104.5430	3103.5357	3103.5165	6.18	149	175	2	R.LGVNMPSTDKLQEVDLEVQSEEKCIAR.F + Oxidation (M)
3386.7366	3385.7293	3385.7816	-15.44	84	112	1	R.TQQVIPVRRPIPHPDYNDETLANDIMLLK.L
3471.7375	3470.7302	3470.7472	-4.90	117	148	2	K.ADITDKVSPINLPRSLAEVKPGMMCSVAGWGR.L + Oxidation (M)
3487.7676	3486.7603	3486.7422	5.21	117	148	2	K.ADITDKVSPINLPRSLAEVKPGMMCSVAGWGR.L + 2 Oxidation (M)
4162.0400	4161.0327	4161.1212	-21.25	55	91	2	R.EDFVLTAAHCLGSSINVTLGAHNIMERERTQQVIPVR.R

No match to: 706.2814, 707.2933, 713.3626, 717.4103, 719.4172, 722.4265, 735.4174, 744.3938, 756.4689, 768.3713, 772.4224, 779.4717, 792.4133, 804.3155, 806.4307, 807.4160, 809.4363, 812.3947, 814.4097, 815.4186, 819.3901, 828.3928, 831.4666, 832.3149, 834.3199, 837.3761, 841.4355, 842.4860, 845.4138, 851.3882, 871.4900, 876.2846, 878.2907, 883.4759, 891.4818, 893.9534, 894.4574, 898.4351, 910.4221, 913.5131, 918.5399, 929.4545, 935.4750, 937.4921, 947.4755, 959.4836, 961.4837, 963.4760, 967.4271, 969.4589, 972.4794, 974.4445, 976.4506, 977.4767, 978.5352, 981.4952, 983.4747, 985.6076, 988.4585, 989.6082, 997.4586, 1007.5676, 1011.4840,

1014.5074, 1018.5203, 1019.5441, 1021.5421, 1034.5779, 1042.5320, 1044.5242, 1060.5463, 1071.5376, 1087.5399, 1091.5421, 1106.5393, 1122.5577, 1156.5372, 1173.6240, 1187.6439, 1199.6992, 1215.6615, 1244.6189, 1248.6575, 1255.6039, 1276.6218, 1284.6401, 1295.7195, 1301.6177, 1313.7069, 1314.7141, 1315.6759, 1325.6675, 1336.7151, 1337.7180, 1338.6461, 1342.6758, 1344.7024, 1349.6952, 1353.6804, 1355.7097, 1356.7201, 1361.6877, 1365.6632, 1370.6783, 1372.6058, 1381.7588, 1383.7244, 1386.7131, 1394.7318, 1397.7419, 1414.6467, 1427.7865, 1432.7140, 1446.7247, 1457.7411, 1461.7784, 1473.8055, 1478.7513, 1497.8757, 1528.6993, 1542.8459, 1556.8795, 1578.7826, 1601.9006, 1606.8433, 1616.8177, 1618.8162, 1628.7026, 1628.8568, 1642.7186, 1647.8694, 1650.8473, 1658.8400, 1677.8625, 1680.8728, 1696.8231, 1703.8551, 1704.8862, 1710.9269, 1715.8459, 1717.8810, 1718.9111, 1725.8386, 1727.9301, 1730.8835, 1742.9099, 1751.8870, 1768.9546, 1769.9617, 1771.0243, 1775.0276, 1777.8676, 1786.9032, 1791.9047, 1799.9005, 1800.9124, 1915.0015, 1928.0238, 1953.9285, 1982.0009, 2012.9681, 2025.0820, 2027.1273, 2042.0642, 2052.9939, 2060.0461, 2067.0017, 2070.8899, 2074.9629, 2082.9421, 2084.9102, 2086.9065, 2096.9797, 2100.9070, 2109.0410, 2116.0894, 2136.9121, 2155.0730, 2170.0781, 2195.0801, 2211.0686, 2238.1072, 2251.1428, 2293.1614, 2336.2495, 2343.1758, 2382.1987, 2385.2234, 2400.0720, 2414.0930, 2418.0901, 2428.1140, 2431.0991, 2468.2249, 2528.1794, 2542.2344, 2560.2729, 2564.2610, 2646.9797, 2660.9968, 2675.0183, 2698.3711, 2707.3184, 2711.3606, 2712.3755, 2719.3159, 2729.3870, 2744.3770, 2762.3970, 2767.3120, 2846.3943, 2871.4121, 2872.3569, 2873.3445, 2884.3745, 2888.3184, 2892.4063, 2893.4375, 2894.4741, 2917.3960, 2926.4563, 2992.4536, 2995.4939, 3004.4705, 3005.5735, 3027.5032, 3054.4763, 3082.5879, 3126.7307, 3170.6697, 3200.5081, 3201.4670, 3206.5745, 3207.6025, 3242.6213, 3248.6428, 3255.6025, 3263.6973, 3300.7517, 3315.7021, 3328.7302, 3331.6746, 3334.7031, 3335.7090, 3347.5835, 3353.6013, 3356.7166, 3370.7415, 3456.7622, 3491.6787, 3503.8286, 3601.7178, 3639.7463, 3646.7891, 3651.8442, 3703.9653, 3716.8708, 3720.7805, 3764.8616, 3820.9673, 3869.8423, 3885.7913, 3887.9063, 3927.8738, 4148.0005, 4253.0234, 4452.2729, 4464.2598, 4468.1572, 4482.1689, 4680.3301, 4845.1597

17. Q0VCQ3 **Mass:** 32810 **Score:** 23 **Expect:** 1.2e+002 **Matches:** 8

MAX interactor 1 OS=Bos taurus GN=MXI1 PE=2 SV=1

Observed	Mr(expt)	Mr(calc)	ppm	Start		End	Miss	Peptide
1383.7244	1382.7171	1382.7014	11.3	133	-	143	2	R.STHNELEKNRR.A
1457.7411	1456.7338	1456.6980	24.6	207	-	218	0	R.LEQLQGPQEMER.I
1742.9099	1741.9026	1741.8781	14.1	207	-	220	1	R.LEQLQGPQEMERIR.M + Oxidation (M)
2109.0410	2108.0337	2108.0610	-12.93	182	-	198	2	K.LEEAERKSQHQLENLER.E
2767.3120	2766.3047	2766.3119	-2.58	89	-	112	1	K.CEHGYASTFPSMPSPRLQHSKPPR.R
2992.4536	2991.4463	2991.4390	2.47	207	-	232	2	R.LEQLQGPQEMERIRMDSIGSTISSDR.S + Oxidation (M)
3206.5745	3205.5672	3205.5826	-4.79	10	-	42	1	K.EARCEGAGLAPAAPPAVTAPQPPAQPEEPAGAK.L
3334.7031	3333.6958	3333.6775	5.49	9	-	42	2	R.KEARCEGAGLAPAAPPAVTAPQPPAQPEEPAGAK.L

No match to: 706.2814, 707.2933, 713.3626, 717.4103, 719.4172, 722.4265, 735.4174, 744.3938, 756.4689, 768.3713, 772.4224, 779.4717, 792.4133, 804.3155, 806.4307, 807.4160, 809.4363, 812.3947, 814.4097, 815.4186, 819.3901, 828.3928, 831.4666, 832.3149, 834.3199, 837.3761, 841.4355, 842.4860, 845.4138, 851.3882, 871.4900, 876.2846, 878.2907, 883.4759, 891.4818, 893.9534, 894.4574, 898.4351, 910.4221, 913.5131, 918.5399, 929.4545, 935.4750, 937.4921, 947.4755, 959.4836, 961.4837, 963.4760, 967.4271, 969.4589, 972.4794, 974.4445, 976.4506, 977.4767, 978.5352, 981.4952, 983.4747, 985.6076, 988.4585, 989.6082, 997.4586, 1007.5676, 1011.4840, 1014.5074, 1018.5203, 1019.5441, 1021.5421, 1034.5779, 1042.5320, 1044.5242, 1060.5463, 1071.5376, 1077.5208, 1087.5399, 1091.5421, 1106.5393, 1122.5577, 1156.5372, 1173.6240, 1187.6439, 1199.6992, 1215.6615, 1244.6189, 1248.6575, 1255.6039, 1276.6218, 1284.6401, 1295.7195, 1301.6177, 1313.7069, 1314.7141, 1315.6759, 1325.6675, 1336.7151, 1337.7180, 1338.6461, 1342.6758, 1344.7024, 1349.6952, 1353.6804, 1355.7097, 1356.7201, 1361.6877, 1365.6632, 1370.6783, 1372.6058, 1381.7588, 1386.7131, 1394.7318, 1397.7419, 1414.6467, 1427.7865, 1432.7140, 1446.7247, 1461.7784, 1473.8055, 1478.7513, 1497.8757, 1528.6993, 1542.8459, 1556.8795, 1578.7826, 1601.9006, 1606.8433, 1616.8177, 1618.8162, 1628.7026, 1628.8568, 1642.7186, 1647.8694, 1650.8473, 1658.8400, 1677.8625, 1680.8728, 1696.8231, 1703.8551, 1704.8862, 1710.9269, 1715.8459, 1717.8810, 1718.9111, 1725.8386, 1727.9301, 1730.8835, 1751.8870, 1768.9546, 1769.9617, 1771.0243, 1775.0276, 1777.8676, 1786.9032, 1791.9047, 1799.9005, 1800.9124, 1915.0015, 1928.0238, 1953.9285, 1982.0009, 2012.9681, 2025.0820, 2027.1273, 2042.0642, 2052.9939, 2060.0461, 2067.0017, 2070.8899, 2074.9629, 2082.9421, 2084.9102, 2086.9065, 2096.9797, 2100.9070, 2116.0894, 2136.9121, 2155.0730, 2170.0781, 2195.0801, 2211.0686, 2238.1072, 2251.1428, 2293.1614, 2336.2495, 2343.1758, 2382.1987, 2385.2234, 2400.0720, 2414.0930, 2418.0901, 2428.1140, 2431.0991, 2465.2188, 2468.2249, 2528.1794, 2542.2344, 2560.2729, 2564.2610, 2646.9797, 2660.9968, 2675.0183, 2698.3711, 2707.3184, 2711.3606, 2712.3755, 2719.3159, 2729.3870, 2744.3770, 2762.3970, 2812.4265, 2846.3943, 2871.4121, 2872.3569, 2873.3445, 2884.3745, 2888.3184, 2892.4063, 2893.4375, 2894.4741, 2917.3960, 2926.4563, 2995.4939, 3004.4705, 3005.5735, 3027.5032, 3054.4763, 3082.5879, 3088.5859, 3104.5430, 3126.7307, 3170.6697, 3200.5081, 3201.4670, 3207.6025, 3242.6213, 3248.6428, 3255.6025, 3263.6973, 3300.7517, 3315.7021, 3328.7302, 3331.6746, 3335.7090, 3347.5835, 3353.6013, 3356.7166, 3370.7415, 3386.7366, 3456.7622, 3471.7375, 3487.7676, 3491.6787, 3503.8286, 3601.7178, 3639.7463, 3646.7891, 3651.8442, 3703.9653, 3716.8708, 3720.7805, 3764.8616, 3820.9673, 3869.8423, 3885.7913, 3887.9063, 3927.8738, 4148.0005, 4162.0400, 4253.0234, 4452.2729, 4464.2598, 4468.1572, 4482.1689, 4680.3301, 4845.1597

18. A6QQR6 **Mass:** 12993 **Score:** 23 **Expect:** 1.3e+002 **Matches:** 6

LIN52 protein OS=Bos taurus GN=LIN52 PE=2 SV=1

Observed	Mr(expt)	Mr(calc)	ppm	Start		End	Miss	Peptide
913.5131	912.5058	912.4916	15.6	48	-	56	0	K.SPITSSPPK.W
1677.8625	1676.8552	1676.8767	-12.80	71	-	85	1	K.ELGSLTTANLMEKVR.G + Oxidation (M)
2465.2188	2464.2115	2464.2492	-15.30	84	-	104	2	K.VRGLQNLAYQLGLDESREMTR.G + Oxidation (M)
2767.3120	2766.3047	2766.3534	-17.60	2	-	26	2	M.GWKMASPTDGTDLEASLLSFEKLDR.A
3200.5081	3199.5008	3199.5087	-2.45	57	-	83	2	K.WMAEIERDDIDMLKELGSLTTANLMEK.V + 3 Oxidation (M)
3335.7090	3334.7017	3334.7191	-5.20	71	-	100	2	K.ELGSLTTANLMEKVRGLQNLAYQLGLDESR.E + Oxidation (M)

No match to: 706.2814, 707.2933, 713.3626, 717.4103, 719.4172, 722.4265, 735.4174, 744.3938, 756.4689, 768.3713, 772.4224, 779.4717, 792.4133, 804.3155, 806.4307, 807.4160, 809.4363, 812.3947, 814.4097, 815.4186, 819.3901, 828.3928, 831.4666, 832.3149, 834.3199, 837.3761, 841.4355, 842.4860, 845.4138, 851.3882, 871.4900, 876.2846, 878.2907, 883.4759, 891.4818, 893.9534, 894.4574, 898.4351, 910.4221, 918.5399, 929.4545, 935.4750, 937.4921, 947.4755, 959.4836, 961.4837, 963.4760, 967.4271, 969.4589, 972.4794, 974.4445, 976.4506, 977.4767, 978.5352, 981.4952, 983.4747, 985.6076, 988.4585, 989.6082, 997.4586, 1007.5676, 1011.4840, 1014.5074, 1018.5203, 1019.5441, 1021.5421, 1034.5779, 1042.5320, 1044.5242, 1060.5463, 1071.5376, 1077.5208, 1087.5399, 1091.5421, 1106.5393, 1122.5577, 1156.5372, 1173.6240, 1187.6439, 1199.6992, 1215.6615, 1244.6189, 1248.6575, 1255.6039, 1276.6218, 1284.6401, 1295.7195, 1301.6177, 1313.7069, 1314.7141, 1315.6759, 1325.6675, 1336.7151, 1337.7180, 1338.6461, 1342.6758, 1344.7024, 1349.6952, 1353.6804, 1355.7097, 1356.7201, 1361.6877, 1365.6632, 1370.6783, 1372.6058, 1381.7588, 1383.7244, 1386.7131, 1394.7318, 1397.7419, 1414.6467, 1427.7865, 1432.7140, 1446.7247, 1457.7411, 1461.7784, 1473.8055, 1478.7513, 1497.8757, 1528.6993, 1542.8459, 1556.8795, 1578.7826, 1601.9006, 1606.8433, 1616.8177, 1618.8162, 1628.7026, 1628.8568, 1642.7186, 1647.8694, 1650.8473, 1658.8400, 1680.8728, 1696.8231, 1703.8551, 1704.8862, 1710.9269, 1715.8459, 1717.8810, 1718.9111, 1725.8386, 1727.9301, 1730.8835, 1742.9099, 1751.8870, 1768.9546, 1769.9617, 1771.0243, 1775.0276, 1777.8676, 1786.9032, 1791.9047, 1799.9005, 1800.9124, 1915.0015, 1928.0238, 1953.9285, 1982.0009, 2012.9681, 2025.0820, 2027.1273, 2042.0642, 2052.9939, 2060.0461, 2067.0017, 2070.8899, 2074.9629, 2082.9421, 2084.9102, 2086.9065, 2096.9797, 2100.9070, 2109.0410, 2116.0894, 2136.9121, 2155.0730, 2170.0781, 2195.0801, 2211.0686, 2238.1072, 2251.1428, 2293.1614, 2336.2495, 2343.1758, 2382.1987, 2385.2234, 2400.0720, 2414.0930, 2418.0901, 2428.1140, 2431.0991, 2468.2249, 2528.1794, 2542.2344, 2560.2729, 2564.2610, 2646.9797, 2660.9968, 2675.0183, 2698.3711, 2707.3184, 2711.3606, 2712.3755, 2719.3159, 2729.3870, 2744.3770, 2762.3970, 2812.4265, 2846.3943, 2871.4121, 2872.3569, 2873.3445, 2884.3745, 2888.3184, 2892.4063, 2893.4375, 2894.4741, 2917.3960, 2926.4563, 2992.4536, 2995.4939, 3004.4705, 3005.5735, 3027.5032, 3054.4763, 3082.5879, 3088.5859, 3104.5430, 3126.7307, 3170.6697, 3201.4670, 3206.5745, 3207.6025, 3242.6213, 3248.6428, 3255.6025, 3263.6973, 3300.7517, 3315.7021, 3328.7302, 3331.6746, 3334.7031, 3347.5835, 3353.6013, 3356.7166, 3370.7415, 3386.7366, 3456.7622, 3471.7375, 3487.7676, 3491.6787, 3503.8286, 3601.7178, 3639.7463, 3646.7891, 3651.8442, 3703.9653, 3716.8708, 3720.7805, 3764.8616, 3820.9673, 3869.8423, 3885.7913, 3887.9063, 3927.8738, 4148.0005, 4162.0400, 4253.0234, 4452.2729, 4464.2598, 4468.1572, 4482.1689, 4680.3301, 4845.1597

19. G3N246 **Mass:** 44905 **Score:** 23 **Expect:** 1.3e+002 **Matches:** 9

Uncharacterized protein OS=Bos taurus GN=LOC101904998 PE=4 SV=1

Observed	Mr(expt)	Mr(calc)	ppm	Start		End	Miss	Peptide
744.3938	743.3865	743.3926	-8.16	230	-	236	0	R.EGVNLGR.H
1647.8694	1646.8621	1646.8601	1.23	230	-	245	1	R.EGVNLGRHLAGGQSPR.R
2293.1614	2292.1541	2292.0984	24.3	174	-	194	0	R.SPYEWSAPSDPVDIMITGLSK.K
2382.1987	2381.1914	2381.2202	-12.07	387	-	406	1	R.RFTPTPLRPMHPSTEPIIYE.-
2465.2188	2464.2115	2464.1944	6.94	173	-	194	1	R.RSPYEWSAPSDPVDIMITGLSK.K + Oxidation (M)

2871.4121	2870.4048	2870.3367	23.7	139 - 166	0	R.HGEMLTGGYAPADFSIGPMTLASAGTYR.C
3201.4670	3200.4597	3200.5271	-21.04	167 - 194	2	R.CYGSLRRSPYEWSAPSDPVDIMITGLSK.K + Oxidation (M)
3639.7463	3638.7390	3638.6592	21.9	139 - 172	1	R.HGEMLTGGYAPADFSIGPMTLASAGTYRCYGSLR.R + 2 Oxidation (M)
3716.8708	3715.8635	3715.8702	-1.79	174 - 207	2	R.SPYEWSAPSDPVDIMITGLSKKPSLSAQRGPMVR.S + Oxidation (M)

No match to: 706.2814, 707.2933, 713.3626, 717.4103, 719.4172, 722.4265, 735.4174, 756.4689, 768.3713, 772.4224, 779.4717, 792.4133, 804.3155, 806.4307, 807.4160, 809.4363, 812.3947, 814.4097, 815.4186, 819.3901, 828.3928, 831.4666, 832.3149, 834.3199, 837.3761, 841.4355, 842.4860, 845.4138, 851.3882, 871.4900, 876.2846, 878.2907, 883.4759, 891.4818, 893.9534, 894.4574, 898.4351, 910.4221, 913.5131, 918.5399, 929.4545, 935.4750, 937.4921, 947.4755, 959.4836, 961.4837, 963.4760, 967.4271, 969.4589, 972.4794, 974.4445, 976.4506, 977.4767, 978.5352, 981.4952, 983.4747, 985.6076, 988.4585, 989.6082, 997.4586, 1007.5676, 1011.4840, 1014.5074, 1018.5203, 1019.5441, 1021.5421, 1034.5779, 1042.5320, 1044.5242, 1060.5463, 1071.5376, 1077.5208, 1087.5399, 1091.5421, 1106.5393, 1122.5577, 1156.5372, 1173.6240, 1187.6439, 1199.6992, 1215.6615, 1244.6189, 1248.6575, 1255.6039, 1276.6218, 1284.6401, 1295.7195, 1301.6177, 1313.7069, 1314.7141, 1315.6759, 1325.6675, 1336.7151, 1337.7180, 1338.6461, 1342.6758, 1344.7024, 1349.6952, 1353.6804, 1355.7097, 1356.7201, 1361.6877, 1365.6632, 1370.6783, 1372.6058, 1381.7588, 1383.7244, 1386.7131, 1394.7318, 1397.7419, 1414.6467, 1427.7865, 1432.7140, 1446.7247, 1457.7411, 1461.7784, 1473.8055, 1478.7513, 1497.8757, 1528.6993, 1542.8459, 1556.8795, 1578.7826, 1601.9006, 1606.8433, 1616.8177, 1618.8162, 1628.7026, 1628.8568, 1642.7186, 1650.8473, 1658.8400, 1677.8625, 1680.8728, 1696.8231, 1703.8551, 1704.8862, 1710.9269, 1715.8459, 1717.8810, 1718.9111, 1725.8386, 1727.9301, 1730.8835, 1742.9099, 1751.8870, 1768.9546, 1769.9617, 1771.0243, 1775.0276, 1777.8676, 1786.9032, 1791.9047, 1799.9005, 1800.9124, 1915.0015, 1928.0238, 1953.9285, 1982.0009, 2012.9681, 2025.0820, 2027.1273, 2042.0642, 2052.9939, 2060.0461, 2067.0017, 2070.8899, 2074.9629, 2082.9421, 2084.9102, 2086.9065, 2096.9797, 2100.9070, 2109.0410, 2116.0894, 2136.9121, 2155.0730, 2170.0781, 2195.0801, 2211.0686, 2238.1072, 2251.1428, 2336.2495, 2343.1758, 2385.2234, 2400.0720, 2414.0930, 2418.0901, 2428.1140, 2431.0991, 2468.2249, 2528.1794, 2542.2344, 2560.2729, 2564.2610, 2646.9797, 2660.9968, 2675.0183, 2698.3711, 2707.3184, 2711.3606, 2712.3755, 2719.3159, 2729.3870, 2744.3770, 2762.3970, 2767.3120, 2812.4265, 2846.3943, 2872.3569, 2873.3445, 2884.3745, 2888.3184, 2892.4063, 2893.4375, 2894.4741, 2917.3960, 2926.4563, 2992.4536, 2995.4939, 3004.4705, 3005.5735, 3027.5032, 3054.4763, 3082.5879, 3088.5859, 3104.5430, 3126.7307, 3170.6697, 3200.5081, 3206.5745, 3207.6025, 3242.6213, 3248.6428, 3255.6025, 3263.6973, 3300.7517, 3315.7021, 3328.7302, 3331.6746, 3334.7031, 3335.7090, 3347.5835, 3353.6013, 3356.7166, 3370.7415, 3386.7366, 3456.7622, 3471.7375, 3487.7676, 3491.6787, 3503.8286, 3601.7178, 3646.7891, 3651.8442, 3703.9653, 3720.7805, 3764.8616, 3820.9673, 3869.8423, 3885.7913, 3887.9063, 3927.8738, 4148.0005, 4162.0400, 4253.0234, 4452.2729, 4464.2598, 4468.1572, 4482.1689, 4680.3301, 4845.1597

20. G3N2I6 **Mass:** 40881 **Score:** 22 **Expect:** 1.4e+002 **Matches:** 11

Uncharacterized protein OS=Bos taurus GN=MTERF1 PE=4 SV=1

Observed	Mr(expt)	Mr(calc)	ppm	Start - End	Miss	Peptide
894.4574	893.4501	893.4541	-4.49	65 - 71	1	R.YPRAMTR.T
1365.6632	1364.6559	1364.6540	1.38	218 - 228	1	K.ILDLSSDCMRR.S
1658.8400	1657.8327	1657.8426	-5.93	250 - 263	0	K.FVLSYPDMIFLGEK.K
1786.9032	1785.8959	1785.9375	-23.29	250 - 264	1	K.FVLSYPDMIFLGEKK.F
1915.0015	1913.9942	1914.0325	-19.98	249 - 264	2	R.KFVLSYPDMIFLGEKK.F
2211.0686	2210.0613	2210.0603	0.45	100 - 118	1	R.SPESFFRSSDNLNLENNIK.F
2468.2249	2467.2176	2467.2780	-24.49	332 - 351	1	K.LYIEQNIVLENFSSCLKIER.-
2707.3184	2706.3111	2706.3725	-22.69	29 - 51	2	K.RQPGVFNRTDTNEQDLQTFLLSK.G
2894.4741	2893.4668	2893.4570	3.39	30 - 55	2	R.QPGVFNRTDTNEQDLQTFLLSKGASK.E
3335.7090	3334.7017	3334.7206	-5.67	150 - 177	2	K.QMVEFLQEICLSLGHNGPTDFVRKIIFK.N + Oxidation (M)
3356.7166	3355.7093	3355.6837	7.64	100 - 128	2	R.SPESFFRSSDNLNLENNIKFLYSVGLTHK.D

No match to: 706.2814, 707.2933, 713.3626, 717.4103, 719.4172, 722.4265, 735.4174, 744.3938, 756.4689, 768.3713, 772.4224, 779.4717, 792.4133, 804.3155, 806.4307, 807.4160, 809.4363, 812.3947, 814.4097, 815.4186, 819.3901, 828.3928, 831.4666, 832.3149, 834.3199, 837.3761, 841.4355, 842.4860, 845.4138, 851.3882, 871.4900, 876.2846, 878.2907, 883.4759, 891.4818, 893.9534, 898.4351, 910.4221, 913.5131, 918.5399, 929.4545, 935.4750, 937.4921, 947.4755, 959.4836, 961.4837, 963.4760, 967.4271, 969.4589, 972.4794, 974.4445, 976.4506, 977.4767, 978.5352, 981.4952, 983.4747, 985.6076, 988.4585, 989.6082, 997.4586, 1007.5676, 1011.4840, 1014.5074, 1018.5203, 1019.5441, 1021.5421, 1034.5779, 1042.5320, 1044.5242, 1060.5463, 1071.5376, 1077.5208, 1087.5399, 1091.5421, 1106.5393, 1122.5577, 1156.5372, 1173.6240, 1187.6439, 1199.6992, 1215.6615, 1244.6189, 1248.6575, 1255.6039, 1276.6218, 1284.6401, 1295.7195, 1301.6177, 1313.7069, 1314.7141, 1315.6759, 1325.6675, 1336.7151, 1337.7180, 1338.6461, 1342.6758, 1344.7024, 1349.6952, 1353.6804, 1355.7097, 1356.7201, 1361.6877, 1370.6783, 1372.6058, 1381.7588, 1383.7244, 1386.7131, 1394.7318, 1397.7419, 1414.6467, 1427.7865, 1432.7140, 1446.7247, 1457.7411, 1461.7784, 1473.8055, 1478.7513, 1497.8757, 1528.6993, 1542.8459, 1556.8795, 1578.7826, 1601.9006, 1606.8433, 1616.8177, 1618.8162, 1628.7026, 1628.8568, 1642.7186, 1647.8694, 1650.8473, 1677.8625, 1680.8728, 1696.8231, 1703.8551, 1704.8862, 1710.9269, 1715.8459, 1717.8810, 1718.9111, 1725.8386, 1727.9301, 1730.8835, 1742.9099, 1751.8870, 1768.9546, 1769.9617, 1771.0243, 1775.0276, 1777.8676, 1791.9047, 1799.9005, 1800.9124, 1928.0238, 1953.9285, 1982.0009, 2012.9681, 2025.0820, 2027.1273, 2042.0642, 2052.9939, 2060.0461, 2067.0017, 2070.8899, 2074.9629, 2082.9421, 2084.9102, 2086.9065, 2096.9797, 2100.9070, 2109.0410, 2116.0894, 2136.9121, 2155.0730, 2170.0781, 2195.0801, 2238.1072, 2251.1428, 2293.1614, 2336.2495, 2343.1758, 2382.1987, 2385.2234, 2400.0720, 2414.0930, 2418.0901, 2428.1140, 2431.0991, 2465.2188, 2528.1794, 2542.2344, 2560.2729, 2564.2610, 2646.9797, 2660.9968, 2675.0183, 2698.3711, 2711.3606, 2712.3755, 2719.3159, 2729.3870, 2744.3770, 2762.3970, 2767.3120, 2812.4265, 2846.3943, 2871.4121, 2872.3569, 2873.3445, 2884.3745, 2888.3184, 2892.4063, 2893.4375, 2917.3960, 2926.4563, 2992.4536, 2995.4939, 3004.4705, 3005.5735, 3027.5032, 3054.4763, 3082.5879, 3088.5859, 3104.5430, 3126.7307, 3170.6697, 3200.5081, 3201.4670, 3206.5745, 3207.6025, 3242.6213, 3248.6428, 3255.6025, 3263.6973, 3300.7517, 3315.7021, 3328.7302, 3331.6746, 3334.7031, 3347.5835, 3353.6013, 3370.7415, 3386.7366, 3456.7622, 3471.7375, 3487.7676, 3491.6787, 3503.8286, 3601.7178, 3639.7463, 3646.7891, 3651.8442, 3703.9653, 3716.8708, 3720.7805, 3764.8616, 3820.9673, 3869.8423, 3885.7913, 3887.9063, 3927.8738, 4148.0005, 4162.0400, 4253.0234, 4452.2729, 4464.2598, 4468.1572, 4482.1689, 4680.3301, 4845.1597

21. G3MZI9 **Mass:** 42157 **Score:** 22 **Expect:** 1.5e+002 **Matches:** 10

Uncharacterized protein OS=Bos taurus GN=MTERF1 PE=4 SV=1

Observed	Mr(expt)	Mr(calc)	ppm	Start - End	Miss	Peptide
894.4574	893.4501	893.4541	-4.49	91 - 97	1	R.YPRAMTR.T
1365.6632	1364.6559	1364.6540	1.38	244 - 254	1	K.ILDLSSDCMRR.S
1658.8400	1657.8327	1657.8426	-5.93	276 - 289	0	K.FVLSYPDMIFLGEK.K
1786.9032	1785.8959	1785.9375	-23.29	276 - 290	1	K.FVLSYPDMIFLGEKK.F
1915.0015	1913.9942	1914.0325	-19.98	275 - 290	2	R.KFVLSYPDMIFLGEKK.F
2211.0686	2210.0613	2210.0603	0.45	126 - 144	1	R.SPESFFRSSDNLNLENNIK.F
2707.3184	2706.3111	2706.3725	-22.69	55 - 77	2	K.RQPGVFNRTDTNEQDLQTFLLSK.G
2894.4741	2893.4668	2893.4570	3.39	56 - 81	2	R.QPGVFNRTDTNEQDLQTFLLSKGASK.E
3335.7090	3334.7017	3334.7206	-5.67	176 - 203	2	K.QMVEFLQEICLSLGHNGPTDFVRKIIFK.N + Oxidation (M)
3356.7166	3355.7093	3355.6837	7.64	126 - 154	2	R.SPESFFRSSDNLNLENNIKFLYSVGLTHK.D

No match to: 706.2814, 707.2933, 713.3626, 717.4103, 719.4172, 722.4265, 735.4174, 744.3938, 756.4689, 768.3713, 772.4224, 779.4717, 792.4133, 804.3155, 806.4307, 807.4160, 809.4363, 812.3947, 814.4097, 815.4186, 819.3901, 828.3928, 831.4666, 832.3149, 834.3199, 837.3761, 841.4355, 842.4860, 845.4138, 851.3882, 871.4900, 876.2846, 878.2907, 883.4759, 891.4818, 893.9534, 898.4351, 910.4221, 913.5131, 918.5399, 929.4545, 935.4750, 937.4921, 947.4755, 959.4836, 961.4837, 963.4760, 967.4271, 969.4589, 972.4794, 974.4445, 976.4506, 977.4767, 978.5352, 981.4952, 983.4747, 985.6076, 988.4585, 989.6082, 997.4586, 1007.5676, 1011.4840, 1014.5074, 1018.5203, 1019.5441, 1021.5421, 1034.5779, 1042.5320, 1044.5242, 1060.5463, 1071.5376, 1077.5208, 1087.5399, 1091.5421, 1106.5393, 1122.5577, 1156.5372, 1173.6240, 1187.6439, 1199.6992, 1215.6615, 1244.6189, 1248.6575, 1255.6039, 1276.6218, 1284.6401, 1295.7195, 1301.6177, 1313.7069, 1314.7141, 1315.6759, 1325.6675, 1336.7151, 1337.7180, 1338.6461, 1342.6758, 1344.7024, 1349.6952, 1353.6804, 1355.7097, 1356.7201, 1361.6877, 1370.6783, 1372.6058, 1381.7588, 1383.7244, 1386.7131, 1394.7318, 1397.7419, 1414.6467, 1427.7865, 1432.7140, 1446.7247, 1457.7411, 1461.7784, 1473.8055, 1478.7513, 1497.8757, 1528.6993, 1542.8459, 1556.8795, 1578.7826, 1601.9006, 1606.8433, 1616.8177, 1618.8162, 1628.7026, 1628.8568, 1642.7186, 1647.8694, 1650.8473, 1677.8625, 1680.8728, 1696.8231, 1703.8551, 1704.8862, 1710.9269, 1715.8459, 1717.8810, 1718.9111, 1725.8386, 1727.9301, 1730.8835, 1742.9099, 1751.8870, 1768.9546, 1769.9617, 1771.0243, 1775.0276, 1777.8676, 1791.9047, 1799.9005, 1800.9124, 1928.0238, 1953.9285, 1982.0009, 2012.9681, 2025.0820, 2027.1273, 2042.0642, 2052.9939, 2060.0461, 2067.0017, 2070.8899, 2074.9629, 2082.9421, 2084.9102, 2086.9065, 2096.9797, 2100.9070, 2109.0410, 2116.0894, 2136.9121, 2155.0730, 2170.0781, 2195.0801, 2238.1072, 2251.1428, 2293.1614, 2336.2495, 2343.1758, 2382.1987,

2385.2234, 2400.0720, 2414.0930, 2418.0901, 2428.1140, 2431.0991, 2465.2188, 2468.2249, 2528.1794, 2542.2344, 2560.2729, 2564.2610, 2646.9797, 2660.9968, 2675.0183, 2698.3711, 2711.3606, 2712.3755, 2719.3159, 2729.3870, 2744.3770, 2762.3970, 2767.3120, 2812.4265, 2846.3943, 2871.4121, 2872.3569, 2873.3445, 2884.3745, 2888.3184, 2892.4063, 2893.4375, 2917.3960, 2926.4563, 2992.4536, 2995.4939, 3004.4705, 3005.5735, 3027.5032, 3054.4763, 3082.5879, 3088.5859, 3104.5430, 3126.7307, 3170.6697, 3200.5081, 3201.4670, 3206.5745, 3207.6025, 3242.6213, 3248.6428, 3255.6025, 3263.6973, 3300.7517, 3315.7021, 3328.7302, 3331.6746, 3334.7031, 3347.5835, 3353.6013, 3370.7415, 3386.7366, 3456.7622, 3471.7375, 3487.7676, 3491.6787, 3503.8286, 3601.7178, 3639.7463, 3646.7891, 3651.8442, 3703.9653, 3716.8708, 3720.7805, 3764.8616, 3820.9673, 3869.8423, 3885.7913, 3887.9063, 3927.8738, 4148.0005, 4162.0400, 4253.0234, 4452.2729, 4464.2598, 4468.1572, 4482.1689, 4680.3301, 4845.1597

22. F6S1Q0 **Mass:** 47935 **Score:** 22 **Expect:** 1.6e+002 **Matches:** 10

Uncharacterized protein OS=Bos taurus GN=KRT18 PE=1 SV=1

Observed	Mr(expt)	Mr(calc)	ppm	Start	End	Miss	Peptide
807.4160	806.4087	806.3923	20.4	158 -	164	0	R.LAADDFR.V
1034.5779	1033.5706	1033.5556	14.5	158 -	166	1	R.LAADDFRVK.Y
1215.6615	1214.6542	1214.6479	5.17	97 -	106	2	K.VRSLEADNRR.L
1255.6039	1254.5966	1254.6278	-24.84	165 -	174	1	R.VKYETELAMR.Q + Oxidation (M)
1381.7588	1380.7515	1380.7725	-15.16	107 -	117	2	R.LESKIREHLEK.K
2382.1987	2381.1914	2381.2299	-16.16	175 -	195	2	R.QSVESDIHGLRKVIDDTNVTR.L
2846.3943	2845.3870	2845.4141	-9.53	50 -	80	1	R.TTSVRGGWGSGNLGAGMAGGLVGVGGIQGEK.E + Oxidation (M)
2894.4741	2893.4668	2893.4053	21.3	381 -	406	2	R.RLLEDGEDFSLGDALDSSNSRQTIQK.T
3335.7090	3334.7017	3334.7554	-16.11	222 -	252	1	K.GLQNQIANSGLTVELDAPKPQDLSKIMADIR.A
4468.1572	4467.1499	4467.1374	2.80	1 -	44	2	-.MSFSTQSTFSNYRSLGSVQSSGHRVRPVSSAASVYAGAGGSGSR.I

No match to: 706.2814, 707.2933, 713.3626, 717.4103, 719.4172, 722.4265, 735.4174, 744.3938, 756.4689, 768.3713, 772.4224, 779.4717, 792.4133, 804.3155, 806.4307, 809.4363, 812.3947, 814.4097, 815.4186, 819.3901, 828.3928, 831.4666, 832.3149, 834.3199, 837.3761, 841.4355, 842.4860, 845.4138, 851.3882, 871.4900, 876.2846, 878.2907, 883.4759, 891.4818, 893.9534, 894.4574, 898.4351, 910.4221, 913.5131, 918.5399, 929.4545, 935.4750, 937.4921, 947.4755, 959.4836, 961.4837, 963.4760, 967.4271, 969.4589, 972.4794, 974.4445, 976.4506, 977.4767, 978.5352, 981.4952, 983.4747, 985.6076, 988.4585, 989.6082, 997.4586, 1007.5676, 1011.4840, 1014.5074, 1018.5203, 1019.5441, 1021.5421, 1042.5320, 1044.5242, 1060.5463, 1071.5376, 1077.5208, 1087.5399, 1091.5421, 1106.5393, 1122.5577, 1156.5372, 1173.6240, 1187.6439, 1199.6992, 1244.6189, 1248.6575, 1276.6218, 1284.6401, 1295.7195, 1301.6177, 1313.7069, 1314.7141, 1315.6759, 1325.6675, 1336.7151, 1337.7180, 1338.6461, 1342.6758, 1344.7024, 1349.6952, 1353.6804, 1355.7097, 1356.7201, 1361.6877, 1365.6632, 1370.6783, 1372.6058, 1383.7244, 1386.7131, 1394.7318, 1397.7419, 1414.6467, 1427.7865, 1432.7140, 1446.7247, 1457.7411, 1461.7784, 1473.8055, 1478.7513, 1497.8757, 1528.6993, 1542.8459, 1556.8795, 1578.7826, 1601.9006, 1606.8433, 1616.8177, 1618.8162, 1628.7026, 1628.8568, 1642.7186, 1647.8694, 1650.8473, 1658.8400, 1677.8625, 1680.8728, 1696.8231, 1703.8551, 1704.8862, 1710.9269, 1715.8459, 1717.8810, 1718.9111, 1725.8386, 1727.9301, 1730.8835, 1742.9099, 1751.8870, 1768.9546, 1769.9617, 1771.0243, 1775.0276, 1777.8676, 1786.9032, 1791.9047, 1799.9005, 1800.9124, 1915.0015, 1928.0238, 1953.9285, 1982.0009, 2012.9681, 2025.0820, 2027.1273, 2042.0642, 2052.9939, 2060.0461, 2067.0017, 2070.8899, 2074.9629, 2082.9421, 2084.9102, 2086.9065, 2096.9797, 2100.9070, 2109.0410, 2116.0894, 2136.9121, 2155.0730, 2170.0781, 2195.0801, 2211.0686, 2238.1072, 2251.1428, 2293.1614, 2336.2495, 2343.1758, 2385.2234, 2400.0720, 2414.0930, 2418.0901, 2428.1140, 2431.0991, 2465.2188, 2468.2249, 2528.1794, 2542.2344, 2560.2729, 2564.2610, 2646.9797, 2660.9968, 2675.0183, 2698.3711, 2707.3184, 2711.3606, 2712.3755, 2719.3159, 2729.3870, 2744.3770, 2762.3970, 2767.3120, 2812.4265, 2871.4121, 2872.3569, 2873.3445, 2884.3745, 2888.3184, 2892.4063, 2893.4375, 2917.3960, 2926.4563, 2992.4536, 2995.4939, 3004.4705, 3005.5735, 3027.5032, 3054.4763, 3082.5879, 3088.5859, 3104.5430, 3126.7307, 3170.6697, 3200.5081, 3201.4670, 3206.5745, 3207.6025, 3242.6213, 3248.6428, 3255.6025, 3263.6973, 3300.7517, 3315.7021, 3328.7302, 3331.6746, 3334.7031, 3347.5835, 3353.6013, 3356.7166, 3370.7415, 3386.7366, 3456.7622, 3471.7375, 3487.7676, 3491.6787, 3503.8286, 3601.7178, 3639.7463, 3646.7891, 3651.8442, 3703.9653, 3716.8708, 3720.7805, 3764.8616, 3820.9673, 3869.8423, 3885.7913, 3887.9063, 3927.8738, 4148.0005, 4162.0400, 4253.0234, 4452.2729, 4464.2598, 4482.1689, 4680.3301, 4845.1597

23. F1N024 **Mass:** 7899 **Score:** 22 **Expect:** 1.7e+002 **Matches:** 4

Uncharacterized protein OS=Bos taurus GN=FAM19A4 PE=4 SV=1

Observed	Mr(expt)	Mr(calc)	ppm	Start	End	Miss	Peptide
1014.5074	1013.5001	1013.5155	-15.19	10 -	18	0	R.GHTGHHQIK.Q
1255.6039	1254.5966	1254.6139	-13.79	19 -	29	0	K.QGTCEVVAVHR.C
2251.1428	2250.1355	2250.1189	7.40	10 -	29	1	R.GHTGHHQIKQGTCEVVAVHR.C
3248.6428	3247.6355	3247.5952	12.4	1 -	29	2	-.MSASSQHLRGHTGHHQIKQGTCEVVAVHR.C

No match to: 706.2814, 707.2933, 713.3626, 717.4103, 719.4172, 722.4265, 735.4174, 744.3938, 756.4689, 768.3713, 772.4224, 779.4717, 792.4133, 804.3155, 806.4307, 807.4160, 809.4363, 812.3947, 814.4097, 815.4186, 819.3901, 828.3928, 831.4666, 832.3149, 834.3199, 837.3761, 841.4355, 842.4860, 845.4138, 851.3882, 871.4900, 876.2846, 878.2907, 883.4759, 891.4818, 893.9534, 894.4574, 898.4351, 910.4221, 913.5131, 918.5399, 929.4545, 935.4750, 937.4921, 947.4755, 959.4836, 961.4837, 963.4760, 967.4271, 969.4589, 972.4794, 974.4445, 976.4506, 977.4767, 978.5352, 981.4952, 983.4747, 985.6076, 988.4585, 989.6082, 997.4586, 1007.5676, 1011.4840, 1018.5203, 1019.5441, 1021.5421, 1034.5779, 1042.5320, 1044.5242, 1060.5463, 1071.5376, 1077.5208, 1087.5399, 1091.5421, 1106.5393, 1122.5577, 1156.5372, 1173.6240, 1187.6439, 1199.6992, 1215.6615, 1244.6189, 1248.6575, 1276.6218, 1284.6401, 1295.7195, 1301.6177, 1313.7069, 1314.7141, 1315.6759, 1325.6675, 1336.7151, 1337.7180, 1338.6461, 1342.6758, 1344.7024, 1349.6952, 1353.6804, 1355.7097, 1356.7201, 1361.6877, 1365.6632, 1370.6783, 1372.6058, 1381.7588, 1383.7244, 1386.7131, 1394.7318, 1397.7419, 1414.6467, 1427.7865, 1432.7140, 1446.7247, 1457.7411, 1461.7784, 1473.8055, 1478.7513, 1497.8757, 1528.6993, 1542.8459, 1556.8795, 1578.7826, 1601.9006, 1606.8433, 1616.8177, 1618.8162, 1628.7026, 1628.8568, 1642.7186, 1647.8694, 1650.8473, 1658.8400, 1677.8625, 1680.8728, 1696.8231, 1703.8551, 1704.8862, 1710.9269, 1715.8459, 1717.8810, 1718.9111, 1725.8386, 1727.9301, 1730.8835, 1742.9099, 1751.8870, 1768.9546, 1769.9617, 1771.0243, 1775.0276, 1777.8676, 1786.9032, 1791.9047, 1799.9005, 1800.9124, 1915.0015, 1928.0238, 1953.9285, 1982.0009, 2012.9681, 2025.0820, 2027.1273, 2042.0642, 2052.9939, 2060.0461, 2067.0017, 2070.8899, 2074.9629, 2082.9421, 2084.9102, 2086.9065, 2096.9797, 2100.9070, 2109.0410, 2116.0894, 2136.9121, 2155.0730, 2170.0781, 2195.0801, 2211.0686, 2238.1072, 2293.1614, 2336.2495, 2343.1758, 2382.1987, 2385.2234, 2400.0720, 2414.0930, 2418.0901, 2428.1140, 2431.0991, 2465.2188, 2468.2249, 2528.1794, 2542.2344, 2560.2729, 2564.2610, 2646.9797, 2660.9968, 2675.0183, 2698.3711, 2707.3184, 2711.3606, 2712.3755, 2719.3159, 2729.3870, 2744.3770, 2762.3970, 2767.3120, 2812.4265, 2846.3943, 2871.4121, 2872.3569, 2873.3445, 2884.3745, 2888.3184, 2892.4063, 2893.4375, 2894.4741, 2917.3960, 2926.4563, 2992.4536, 2995.4939, 3004.4705, 3005.5735, 3027.5032, 3054.4763, 3082.5879, 3088.5859, 3104.5430, 3126.7307, 3170.6697, 3200.5081, 3201.4670, 3206.5745, 3207.6025, 3242.6213, 3255.6025, 3263.6973, 3300.7517, 3315.7021, 3328.7302, 3331.6746, 3334.7031, 3335.7090, 3347.5835, 3353.6013, 3356.7166, 3370.7415, 3386.7366, 3456.7622, 3471.7375, 3487.7676, 3491.6787, 3503.8286, 3601.7178, 3639.7463, 3646.7891, 3651.8442, 3703.9653, 3716.8708, 3720.7805, 3764.8616, 3820.9673, 3869.8423, 3885.7913, 3887.9063, 3927.8738, 4148.0005, 4162.0400, 4253.0234, 4452.2729, 4464.2598, 4468.1572, 4482.1689, 4680.3301, 4845.1597

24. G3MYS0 **Mass:** 38145 **Score:** 22 **Expect:** 1.7e+002 **Matches:** 10

Uncharacterized protein OS=Bos taurus GN=LOC508073 PE=4 SV=1

Observed	Mr(expt)	Mr(calc)	ppm	Start	End	Miss	Peptide
845.4138	844.4065	844.4151	-10.20	322 -	328	1	R.DERAQAR.T
1042.5320	1041.5247	1041.5342	-9.11	12 -	20	0	K.LEEDLQAPK.E
1044.5242	1043.5169	1043.4957	20.3	288 -	296	1	R.AYAETSKMK.V + Oxidation (M)
1187.6439	1186.6366	1186.6380	-1.12	139 -	148	1	K.EPITKAEMLR.S
1337.7180	1336.7107	1336.6883	16.8	124 -	134	0	K.VAELMQFLCVK.Y
1353.6804	1352.6731	1352.6832	-7.47	124 -	134	0	K.VAELMQFLCVK.Y + Oxidation (M)
2926.4563	2925.4490	2925.4842	-12.04	210 -	234	1	K.TGFLILILGMIFMEGNCAPEEKIWK.A + Oxidation (M)
3200.5081	3199.5008	3199.5028	-0.62	153 -	178	2	R.EHKENFPEIFSKVCECMEVVFGIEVK.E + Oxidation (M)
3347.5835	3346.5762	3346.5301	13.8	7 -	35	2	K.SECCKLEEDLQAPKEAQGLECVQAPMAQK.E
3601.7178	3600.7105	3600.6745	9.99	12 -	43	2	K.LEEDLQAPKEAQGLECVQAPMAQKEEATSCPK.S + Oxidation (M)

No match to: 706.2814, 707.2933, 713.3626, 717.4103, 719.4172, 722.4265, 735.4174, 744.3938, 756.4689, 768.3713, 772.4224, 779.4717, 792.4133, 804.3155, 806.4307, 807.4160, 809.4363, 812.3947, 814.4097, 815.4186, 819.3901, 828.3928, 831.4666, 832.3149, 834.3199, 837.3761, 841.4355, 842.4860, 851.3882, 871.4900, 876.2846, 878.2907, 883.4759, 891.4818, 893.9534, 894.4574, 898.4351, 910.4221, 913.5131, 918.5399, 929.4545, 935.4750, 937.4921, 947.4755, 959.4836, 961.4837, 963.4760, 967.4271, 969.4589, 972.4794,

974.4445, 976.4506, 977.4767, 978.5352, 981.4952, 983.4747, 985.6076, 988.4585, 989.6082, 997.4586, 1007.5676, 1011.4840, 1014.5074, 1018.5203, 1019.5441, 1021.5421, 1034.5779, 1060.5463, 1071.5376, 1077.5208, 1087.5399, 1091.5421, 1106.5393, 1122.5577, 1156.5372, 1173.6240, 1199.6992, 1215.6615, 1244.6189, 1248.6575, 1255.6039, 1276.6218, 1284.6401, 1295.7195, 1301.6177, 1313.7069, 1314.7141, 1315.6759, 1325.6675, 1336.7151, 1338.6461, 1342.6758, 1344.7024, 1349.6952, 1355.7097, 1356.7201, 1361.6877, 1365.6632, 1370.6783, 1372.6058, 1381.7588, 1383.7244, 1386.7131, 1394.7318, 1397.7419, 1414.6467, 1427.7865, 1432.7140, 1446.7247, 1457.7411, 1461.7784, 1473.8055, 1478.7513, 1497.8757, 1528.6993, 1542.8459, 1556.8795, 1578.7826, 1601.9006, 1606.8433, 1616.8177, 1618.8162, 1628.7026, 1628.8568, 1642.7186, 1647.8694, 1650.8473, 1658.8400, 1677.8625, 1680.8728, 1696.8231, 1703.8551, 1704.8862, 1710.9269, 1715.8459, 1717.8810, 1718.9111, 1725.8386, 1727.9301, 1730.8835, 1742.9099, 1751.8870, 1768.9546, 1769.9617, 1771.0243, 1775.0276, 1777.8676, 1786.9032, 1791.9047, 1799.9005, 1800.9124, 1915.0015, 1928.0238, 1953.9285, 1982.0009, 2012.9681, 2025.0820, 2027.1273, 2042.0642, 2052.9939, 2060.0461, 2067.0017, 2070.8899, 2074.9629, 2082.9421, 2084.9102, 2086.9065, 2096.9797, 2100.9070, 2109.0410, 2116.0894, 2136.9121, 2155.0730, 2170.0781, 2195.0801, 2211.0686, 2238.1072, 2251.1428, 2293.1614, 2336.2495, 2343.1758, 2382.1987, 2385.2234, 2400.0720, 2414.0930, 2418.0901, 2428.1140, 2431.0991, 2465.2188, 2468.2249, 2528.1794, 2542.2344, 2560.2729, 2564.2610, 2646.9797, 2660.9968, 2675.0183, 2698.3711, 2707.3184, 2711.3606, 2712.3755, 2719.3159, 2729.3870, 2744.3770, 2762.3970, 2767.3120, 2812.4265, 2846.3943, 2871.4121, 2872.3569, 2873.3445, 2884.3745, 2888.3184, 2892.4063, 2893.4375, 2894.4741, 2917.3960, 2992.4536, 2995.4939, 3004.4705, 3005.5735, 3027.5032, 3054.4763, 3082.5879, 3088.5859, 3104.5430, 3126.7307, 3170.6697, 3201.4670, 3206.5745, 3207.6025, 3242.6213, 3248.6428, 3255.6025, 3263.6973, 3300.7517, 3315.7021, 3328.7302, 3331.6746, 3334.7031, 3335.7090, 3353.6013, 3356.7166, 3370.7415, 3386.7366, 3456.7622, 3471.7375, 3487.7676, 3491.6787, 3503.8286, 3639.7463, 3646.7891, 3651.8442, 3703.9653, 3716.8708, 3720.7805, 3764.8616, 3820.9673, 3869.8423, 3885.7913, 3887.9063, 3927.8738, 4148.0005, 4162.0400, 4253.0234, 4452.2729, 4464.2598, 4468.1572, 4482.1689, 4680.3301, 4845.1597

25. E1BBR5 **Mass:** 32112 **Score:** 21 **Expect:** 2e+002 **Matches:** 7

Uncharacterized protein OS=Bos taurus GN=PITX3 PE=3 SV=1

Observed	Mr(expt)	Mr(calc)	ppm	Start		End	Miss	Peptide
963.4760	962.4687	962.4491	20.3	123	-	130	0	R.SQQAELCK.G
1628.8568	1627.8495	1627.8417	4.80	44	-	60	1	K.ASVSLPGGSPEDGSLKK.K
1786.9032	1785.8959	1785.9373	-23.18	93	-	107	1	R.EEIAVWTNLTEARVR.V
2382.1987	2381.1914	2381.1321	24.9	86	-	105	1	R.YPDMSTREEIAVWTNLTEAR.V
3200.5081	3199.5008	3199.5105	-3.02	67	-	92	2	R.THFTSQQLQELEATFQRNRYPDMSTR.E + Oxidation (M)
3601.7178	3600.7105	3600.7341	-6.54	1	-	34	1	-.MEFGLLSEAEARSPALSLSDAGTPHPPLPEHGCK.G
4468.1572	4467.1499	4467.1037	10.4	2	-	43	2	M.EFGLLSEAEARSPALSLSDAGTPHPPLPEHGCKGQEHSDSEK.A

No match to: 706.2814, 707.2933, 713.3626, 717.4103, 719.4172, 722.4265, 735.4174, 744.3938, 756.4689, 768.3713, 772.4224, 779.4717, 792.4133, 804.3155, 806.4307, 807.4160, 809.4363, 812.3947, 814.4097, 815.4186, 819.3901, 828.3928, 831.4666, 832.3149, 834.3199, 837.3761, 841.4355, 842.4860, 845.4138, 851.3882, 871.4900, 876.2846, 878.2907, 883.4759, 891.4818, 893.9534, 894.4574, 898.4351, 910.4221, 913.5131, 918.5399, 929.4545, 935.4750, 937.4921, 947.4755, 959.4836, 961.4837, 967.4271, 969.4589, 972.4794, 974.4445, 976.4506, 977.4767, 978.5352, 981.4952, 983.4747, 985.6076, 988.4585, 989.6082, 997.4586, 1007.5676, 1011.4840, 1014.5074, 1018.5203, 1019.5441, 1021.5421, 1034.5779, 1042.5320, 1044.5242, 1060.5463, 1071.5376, 1077.5208, 1087.5399, 1091.5421, 1106.5393, 1122.5577, 1156.5372, 1173.6240, 1187.6439, 1199.6992, 1215.6615, 1244.6189, 1248.6575, 1255.6039, 1276.6218, 1284.6401, 1295.7195, 1301.6177, 1313.7069, 1314.7141, 1315.6759, 1325.6675, 1336.7151, 1337.7180, 1338.6461, 1342.6758, 1344.7024, 1349.6952, 1353.6804, 1355.7097, 1356.7201, 1361.6877, 1365.6632, 1370.6783, 1372.6058, 1381.7588, 1383.7244, 1386.7131, 1394.7318, 1397.7419, 1414.6467, 1427.7865, 1432.7140, 1446.7247, 1457.7411, 1461.7784, 1473.8055, 1478.7513, 1497.8757, 1528.6993, 1542.8459, 1556.8795, 1578.7826, 1601.9006, 1606.8433, 1616.8177, 1618.8162, 1628.7026, 1642.7186, 1647.8694, 1650.8473, 1658.8400, 1677.8625, 1680.8728, 1696.8231, 1703.8551, 1704.8862, 1710.9269, 1715.8459, 1717.8810, 1718.9111, 1725.8386, 1727.9301, 1730.8835, 1742.9099, 1751.8870, 1768.9546, 1769.9617, 1771.0243, 1775.0276, 1777.8676, 1791.9047, 1799.9005, 1800.9124, 1915.0015, 1928.0238, 1953.9285, 1982.0009, 2012.9681, 2025.0820, 2027.1273, 2042.0642, 2052.9939, 2060.0461, 2067.0017, 2070.8899, 2074.9629, 2082.9421, 2084.9102, 2086.9065, 2096.9797, 2100.9070, 2109.0410, 2116.0894, 2136.9121, 2155.0730, 2170.0781, 2195.0801, 2211.0686, 2238.1072, 2251.1428, 2293.1614, 2336.2495, 2343.1758, 2385.2234, 2400.0720, 2414.0930, 2418.0901, 2428.1140, 2431.0991, 2465.2188, 2468.2249, 2528.1794, 2542.2344, 2560.2729, 2564.2610, 2646.9797, 2660.9968, 2675.0183, 2698.3711, 2707.3184, 2711.3606, 2712.3755, 2719.3159, 2729.3870, 2744.3770, 2762.3970, 2767.3120, 2812.4265, 2846.3943, 2871.4121, 2872.3569, 2873.3445, 2884.3745, 2888.3184, 2892.4063, 2893.4375, 2894.4741, 2917.3960, 2926.4563, 2992.4536, 2995.4939, 3004.4705, 3005.5735, 3027.5032, 3054.4763, 3082.5879, 3088.5859, 3104.5430, 3126.7307, 3170.6697, 3201.4670, 3206.5745, 3207.6025, 3242.6213, 3248.6428, 3255.6025, 3263.6973, 3300.7517, 3315.7021, 3328.7302, 3331.6746, 3334.7031, 3335.7090, 3347.5835, 3353.6013, 3356.7166, 3370.7415, 3386.7366, 3456.7622, 3471.7375, 3487.7676, 3491.6787, 3503.8286, 3639.7463, 3646.7891, 3651.8442, 3703.9653, 3716.8708, 3720.7805, 3764.8616, 3820.9673, 3869.8423, 3885.7913, 3887.9063, 3927.8738, 4148.0005, 4162.0400, 4253.0234, 4452.2729, 4464.2598, 4482.1689, 4680.3301, 4845.1597

26. Q2HJB3 **Mass:** 61006 **Score:** 21 **Expect:** 2.1e+002 **Matches:** 12

Cytochrome P450, family 4, subfamily F, polypeptide 3 OS=Bos taurus GN=CYP4F3 PE=2 SV=1

Observed	Mr(expt)	Mr(calc)	ppm	Start		End	Miss	Peptide
959.4836	958.4763	958.4872	-11.38	445	-	452	1	R.FDPEAPKR.S
1342.6758	1341.6685	1341.6612	5.49	170	-	180	1	K.SADIMHAKWQR.L
1786.9032	1785.8959	1785.9196	-13.25	259	-	273	1	R.ACRLVHDFTDAIIQK.R
2052.9939	2051.9866	2051.9908	-2.01	189	-	205	0	R.LDMFEHISLMTLDSLQK.C + 2 Oxidation (M)
2170.0781	2169.0708	2169.1113	-18.64	170	-	188	2	K.SADIMHAKWQRLALEGSTR.L
2293.1614	2292.1541	2292.1804	-11.45	238	-	255	1	K.HIFLHVDFLYYLTRDGQR.F
2884.3745	2883.3672	2883.3870	-6.86	453	-	478	1	R.SPLAFIPFSAGPRNCIGQTFAMNEMK.V
3004.4705	3003.4632	3003.3960	22.4	320	-	346	0	R.AEADTFMFEGHDTTASGLSWILYNLAK.H + Oxidation (M)
3248.6428	3247.6355	3247.6026	10.1	417	-	444	0	K.GNICVISIFGIHHNPSVWPDPEVFNPFR.F
3334.7031	3333.6958	3333.6849	3.27	178	-	205	2	K.WQRLALEGSTRLDMFEHISLMTLDSLQK.C + Oxidation (M)
3335.7090	3334.7017	3334.7610	-17.77	150	-	177	2	R.LLTPAFHFEILKPYMKIFNKSADIMHAK.W + 2 Oxidation (M)
3927.8738	3926.8665	3926.8210	11.6	320	-	353	1	R.AEADTFMFEGHDTTASGLSWILYNLAKHPEYQER.C

No match to: 706.2814, 707.2933, 713.3626, 717.4103, 719.4172, 722.4265, 735.4174, 744.3938, 756.4689, 768.3713, 772.4224, 779.4717, 792.4133, 804.3155, 806.4307, 807.4160, 809.4363, 812.3947, 814.4097, 815.4186, 819.3901, 828.3928, 831.4666, 832.3149, 834.3199, 837.3761, 841.4355, 842.4860, 845.4138, 851.3882, 871.4900, 876.2846, 878.2907, 883.4759, 891.4818, 893.9534, 894.4574, 898.4351, 910.4221, 913.5131, 918.5399, 929.4545, 935.4750, 937.4921, 947.4755, 961.4837, 963.4760, 967.4271, 969.4589, 972.4794, 974.4445, 976.4506, 977.4767, 978.5352, 981.4952, 983.4747, 985.6076, 988.4585, 989.6082, 997.4586, 1007.5676, 1011.4840, 1014.5074, 1018.5203, 1019.5441, 1021.5421, 1034.5779, 1042.5320, 1044.5242, 1060.5463, 1071.5376, 1077.5208, 1087.5399, 1091.5421, 1106.5393, 1122.5577, 1156.5372, 1173.6240, 1187.6439, 1199.6992, 1215.6615, 1244.6189, 1248.6575, 1255.6039, 1276.6218, 1284.6401, 1295.7195, 1301.6177, 1313.7069, 1314.7141, 1315.6759, 1325.6675, 1336.7151, 1337.7180, 1338.6461, 1344.7024, 1349.6952, 1353.6804, 1355.7097, 1356.7201, 1361.6877, 1365.6632, 1370.6783, 1372.6058, 1381.7588, 1383.7244, 1386.7131, 1394.7318, 1397.7419, 1414.6467, 1427.7865, 1432.7140, 1446.7247, 1457.7411, 1461.7784, 1473.8055, 1478.7513, 1497.8757, 1528.6993, 1542.8459, 1556.8795, 1578.7826, 1601.9006, 1606.8433, 1616.8177, 1618.8162, 1628.7026, 1628.8568, 1642.7186, 1647.8694, 1650.8473, 1658.8400, 1677.8625, 1680.8728, 1696.8231, 1703.8551, 1704.8862, 1710.9269, 1715.8459, 1717.8810, 1718.9111, 1725.8386, 1727.9301, 1730.8835, 1742.9099, 1751.8870, 1768.9546, 1769.9617, 1771.0243, 1775.0276, 1777.8676, 1791.9047, 1799.9005, 1800.9124, 1915.0015, 1928.0238, 1953.9285, 1982.0009, 2012.9681, 2025.0820, 2027.1273, 2042.0642, 2060.0461, 2067.0017, 2070.8899, 2074.9629, 2082.9421, 2084.9102, 2086.9065, 2096.9797, 2100.9070, 2109.0410, 2116.0894, 2136.9121, 2155.0730, 2195.0801, 2211.0686, 2238.1072, 2251.1428, 2336.2495, 2343.1758, 2382.1987, 2385.2234, 2400.0720, 2414.0930, 2418.0901, 2428.1140, 2431.0991, 2465.2188, 2468.2249, 2528.1794, 2542.2344, 2560.2729, 2564.2610, 2646.9797, 2660.9968, 2675.0183, 2698.3711, 2707.3184, 2711.3606, 2712.3755, 2719.3159, 2729.3870, 2744.3770, 2762.3970, 2767.3120, 2812.4265, 2846.3943, 2871.4121, 2872.3569, 2873.3445, 2888.3184, 2892.4063, 2893.4375, 2894.4741, 2917.3960, 2926.4563, 2992.4536, 2995.4939, 3005.5735, 3027.5032, 3054.4763, 3082.5879, 3088.5859, 3104.5430, 3126.7307, 3170.6697, 3200.5081, 3201.4670, 3206.5745, 3207.6025, 3242.6213, 3255.6025, 3263.6973, 3300.7517, 3315.7021, 3328.7302, 3331.6746, 3347.5835, 3353.6013, 3356.7166, 3370.7415, 3386.7366, 3456.7622, 3471.7375, 3487.7676, 3491.6787, 3503.8286, 3601.7178, 3639.7463, 3646.7891, 3651.8442, 3703.9653, 3716.8708, 3720.7805, 3764.8616, 3820.9673, 3869.8423, 3885.7913, 3887.9063, 4148.0005, 4162.0400, 4253.0234, 4452.2729, 4464.2598, 4468.1572, 4482.1689, 4680.3301, 4845.1597

27. E1BN01 **Mass:** 33525 **Score:** 20 **Expect:** 2.2e+002 **Matches:** 9

Uncharacterized protein OS=Bos taurus GN=DNALI1 PE=4 SV=2

Observed	Mr(expt)	Mr(calc)	ppm	Start	End	Miss	Peptide
1091.5421	1090.5348	1090.5077	24.9	173	181	0	R.EVTINCAER.G
1301.6177	1300.6104	1300.6333	-17.60	87	98	0	K.LPSTSCVPDPTK.Q
1313.7069	1312.6996	1312.6888	8.28	267	276	1	K.HNEEIQFLKR.T
1457.7411	1456.7338	1456.7634	-20.28	237	248	2	R.DLERQVNEQKAK.C
1677.8625	1676.8552	1676.8846	-17.50	46	59	2	K.YDTPVLVSRNTEKR.S
2155.0730	2154.0657	2154.0125	24.7	194	212	0	R.MTIAAYQTLYESSVAFGMR.K + Oxidation (M)
2812.4265	2811.4192	2811.3571	22.1	190	213	2	R.DEIRMTIAAYQTLYESSVAFGMRK.A + 2 Oxidation (M)
2917.3960	2916.3887	2916.3456	14.8	18	45	0	R.SPCSGDNGVCWVASPAATMIPPADSLLK.Y + Oxidation (M)
4464.2598	4463.2525	4463.2543	-0.41	112	148	2	R.EWVEDTQLWIQQVSSTPSTRMDVVHLQEQLDLKLQQR.Q

No match to: 706.2814, 707.2933, 713.3626, 717.4103, 719.4172, 722.4265, 735.4174, 744.3938, 756.4689, 768.3713, 772.4224, 779.4717, 792.4133, 804.3155, 806.4307, 807.4160, 809.4363, 812.3947, 814.4097, 815.4186, 819.3901, 828.3928, 831.4666, 832.3149, 834.3199, 837.3761, 841.4355, 842.4860, 845.4138, 851.3882, 871.4900, 876.2846, 878.2907, 883.4759, 891.4818, 893.9534, 894.4574, 898.4351, 910.4221, 913.5131, 918.5399, 929.4545, 935.4750, 937.4921, 947.4755, 959.4836, 961.4837, 963.4760, 967.4271, 969.4589, 972.4794, 974.4445, 976.4506, 977.4767, 978.5352, 981.4952, 983.4747, 985.6076, 988.4585, 989.6082, 997.4586, 1007.5676, 1011.4840, 1014.5074, 1018.5203, 1019.5441, 1021.5421, 1034.5779, 1042.5320, 1044.5242, 1060.5463, 1071.5376, 1077.5208, 1087.5399, 1106.5393, 1122.5577, 1156.5372, 1173.6240, 1187.6439, 1199.6992, 1215.6615, 1244.6189, 1248.6575, 1255.6039, 1276.6218, 1284.6401, 1295.7195, 1314.7141, 1315.6759, 1325.6675, 1336.7151, 1337.7180, 1338.6461, 1342.6758, 1344.7024, 1349.6952, 1353.6804, 1355.7097, 1356.7201, 1361.6877, 1365.6632, 1370.6783, 1372.6058, 1381.7588, 1383.7244, 1386.7131, 1394.7318, 1397.7419, 1414.6467, 1427.7865, 1432.7140, 1446.7247, 1461.7784, 1473.8055, 1478.7513, 1497.8757, 1528.6993, 1542.8459, 1556.8795, 1578.7826, 1601.9006, 1606.8433, 1616.8177, 1618.8162, 1628.7026, 1628.8568, 1642.7186, 1647.8694, 1650.8473, 1658.8400, 1680.8728, 1696.8231, 1703.8551, 1704.8862, 1710.9269, 1715.8459, 1717.8810, 1718.9111, 1725.8386, 1727.9301, 1730.8835, 1742.9099, 1751.8870, 1768.9546, 1769.9617, 1771.0243, 1775.0276, 1777.8676, 1786.9032, 1791.9047, 1799.9005, 1800.9124, 1915.0015, 1928.0238, 1953.9285, 1982.0009, 2012.9681, 2025.0820, 2027.1273, 2042.0642, 2052.9939, 2060.0461, 2067.0017, 2070.8899, 2074.9629, 2082.9421, 2084.9102, 2086.9065, 2096.9797, 2100.9070, 2109.0410, 2116.0894, 2136.9121, 2170.0781, 2195.0801, 2211.0686, 2238.1072, 2251.1428, 2293.1614, 2336.2495, 2343.1758, 2382.1987, 2385.2234, 2400.0720, 2414.0930, 2418.0901, 2428.1140, 2431.0991, 2465.2188, 2468.2249, 2528.1794, 2542.2344, 2560.2729, 2564.2610, 2646.9797, 2660.9968, 2675.0183, 2698.3711, 2707.3184, 2711.3606, 2712.3755, 2719.3159, 2729.3870, 2744.3770, 2762.3970, 2767.3120, 2846.3943, 2871.4121, 2872.3569, 2873.3445, 2884.3745, 2888.3184, 2892.4063, 2893.4375, 2894.4741, 2926.4563, 2992.4536, 2995.4939, 3004.4705, 3005.5735, 3027.5032, 3054.4763, 3082.5879, 3088.5859, 3104.5430, 3126.7307, 3170.6697, 3200.5081, 3201.4670, 3206.5745, 3207.6025, 3242.6213, 3248.6428, 3255.6025, 3263.6973, 3300.7517, 3315.7021, 3328.7302, 3331.6746, 3334.7031, 3335.7090, 3347.5835, 3353.6013, 3356.7166, 3370.7415, 3386.7366, 3456.7622, 3471.7375, 3487.7676, 3491.6787, 3503.8286, 3601.7178, 3639.7463, 3646.7891, 3651.8442, 3703.9653, 3716.8708, 3720.7805, 3764.8616, 3820.9673, 3869.8423, 3885.7913, 3887.9063, 3927.8738, 4148.0005, 4162.0400, 4253.0234, 4452.2729, 4468.1572, 4482.1689, 4680.3301, 4845.1597

28. E1BN75 **Mass:** 19595 **Score:** 20 **Expect:** 2.2e+002 **Matches:** 6

Uncharacterized protein OS=Bos taurus GN=TPRKB PE=3 SV=1

Observed	Mr(expt)	Mr(calc)	ppm	Start	End	Miss	Peptide
2170.0781	2169.0708	2169.0841	-6.12	96	115	0	K.FGISANDTSILIAYIEEGEK.Q
2992.4536	2991.4463	2991.4755	-9.76	33	60	0	K.AMEGAIDGSLINPTMIVDPFQIIMAANK.A + 2 Oxidation (M)
3088.5859	3087.5786	3087.5806	-0.65	32	60	1	K.KAMEGAIDGSLINPTMIVDPFQIIMAANK.A
3104.5430	3103.5357	3103.5756	-12.84	32	60	1	K.KAMEGAIDGSLINPTMIVDPFQIIMAANK.A + Oxidation (M)
3248.6428	3247.6355	3247.6654	-9.21	31	60	2	R.KKAMEGAIDGSLINPTMIVDPFQIIMAANK.A + 2 Oxidation (M)
3703.9653	3702.9580	3702.8823	20.4	33	66	1	K.AMEGAIDGSLINPTMIVDPFQIIMAANKAVHLYK.L + 2 Oxidation (M)

No match to: 706.2814, 707.2933, 713.3626, 717.4103, 719.4172, 722.4265, 735.4174, 744.3938, 756.4689, 768.3713, 772.4224, 779.4717, 792.4133, 804.3155, 806.4307, 807.4160, 809.4363, 812.3947, 814.4097, 815.4186, 819.3901, 828.3928, 831.4666, 832.3149, 834.3199, 837.3761, 841.4355, 842.4860, 845.4138, 851.3882, 871.4900, 876.2846, 878.2907, 883.4759, 891.4818, 893.9534, 894.4574, 898.4351, 910.4221, 913.5131, 918.5399, 929.4545, 935.4750, 937.4921, 947.4755, 959.4836, 961.4837, 963.4760, 967.4271, 969.4589, 972.4794, 974.4445, 976.4506, 977.4767, 978.5352, 981.4952, 983.4747, 985.6076, 988.4585, 989.6082, 997.4586, 1007.5676, 1011.4840, 1014.5074, 1018.5203, 1019.5441, 1021.5421, 1034.5779, 1042.5320, 1044.5242, 1060.5463, 1071.5376, 1077.5208, 1087.5399, 1091.5421, 1106.5393, 1122.5577, 1156.5372, 1173.6240, 1187.6439, 1199.6992, 1215.6615, 1244.6189, 1248.6575, 1255.6039, 1276.6218, 1284.6401, 1295.7195, 1301.6177, 1313.7069, 1314.7141, 1315.6759, 1325.6675, 1336.7151, 1337.7180, 1338.6461, 1342.6758, 1344.7024, 1349.6952, 1353.6804, 1355.7097, 1356.7201, 1361.6877, 1365.6632, 1370.6783, 1372.6058, 1381.7588, 1383.7244, 1386.7131, 1394.7318, 1397.7419, 1414.6467, 1427.7865, 1432.7140, 1446.7247, 1457.7411, 1461.7784, 1473.8055, 1478.7513, 1497.8757, 1528.6993, 1542.8459, 1556.8795, 1578.7826, 1601.9006, 1606.8433, 1616.8177, 1618.8162, 1628.7026, 1628.8568, 1642.7186, 1647.8694, 1650.8473, 1658.8400, 1677.8625, 1680.8728, 1696.8231, 1703.8551, 1704.8862, 1710.9269, 1715.8459, 1717.8810, 1718.9111, 1725.8386, 1727.9301, 1730.8835, 1742.9099, 1751.8870, 1768.9546, 1769.9617, 1771.0243, 1775.0276, 1777.8676, 1786.9032, 1791.9047, 1799.9005, 1800.9124, 1915.0015, 1928.0238, 1953.9285, 1982.0009, 2012.9681, 2025.0820, 2027.1273, 2042.0642, 2052.9939, 2060.0461, 2067.0017, 2070.8899, 2074.9629, 2082.9421, 2084.9102, 2086.9065, 2096.9797, 2100.9070, 2109.0410, 2116.0894, 2136.9121, 2155.0730, 2195.0801, 2211.0686, 2238.1072, 2251.1428, 2293.1614, 2336.2495, 2343.1758, 2382.1987, 2385.2234, 2400.0720, 2414.0930, 2418.0901, 2428.1140, 2431.0991, 2465.2188, 2468.2249, 2528.1794, 2542.2344, 2560.2729, 2564.2610, 2646.9797, 2660.9968, 2675.0183, 2698.3711, 2707.3184, 2711.3606, 2712.3755, 2719.3159, 2729.3870, 2744.3770, 2762.3970, 2767.3120, 2812.4265, 2846.3943, 2871.4121, 2872.3569, 2873.3445, 2884.3745, 2888.3184, 2892.4063, 2893.4375, 2894.4741, 2917.3960, 2926.4563, 2995.4939, 3004.4705, 3005.5735, 3027.5032, 3054.4763, 3082.5879, 3126.7307, 3170.6697, 3200.5081, 3201.4670, 3206.5745, 3207.6025, 3242.6213, 3255.6025, 3263.6973, 3300.7517, 3315.7021, 3328.7302, 3331.6746, 3334.7031, 3335.7090, 3347.5835, 3353.6013, 3356.7166, 3370.7415, 3386.7366, 3456.7622, 3471.7375, 3487.7676, 3491.6787, 3503.8286, 3601.7178, 3639.7463, 3646.7891, 3651.8442, 3716.8708, 3720.7805, 3764.8616, 3820.9673, 3869.8423, 3885.7913, 3887.9063, 3927.8738, 4148.0005, 4162.0400, 4253.0234, 4452.2729, 4464.2598, 4468.1572, 4482.1689, 4680.3301, 4845.1597

29. Q1LZ99 **Mass:** 53218 **Score:** 20 **Expect:** 2.3e+002 **Matches:** 11

DNA methyltransferase 1 associated protein 1 OS=Bos taurus GN=DMAP1 PE=2 SV=1

Observed	Mr(expt)	Mr(calc)	ppm	Start	End	Miss	Peptide
717.4103	716.4030	716.3929	14.1	260	264	2	K.EREKR.S
768.3713	767.3640	767.3602	4.92	123	128	0	K.DYPFAR.F
898.4351	897.4278	897.4239	4.39	49	55	0	K.RPEGMHR.E + Oxidation (M)
1337.7180	1336.7107	1336.7139	-2.41	312	323	1	K.FPDFKSAGVTLR.S
1338.6461	1337.6388	1337.6088	22.5	107	116	1	K.DGAMFFHWRR.A + Oxidation (M)
1461.7784	1460.7711	1460.7769	-3.98	324	336	2	R.SQRMKLPSSVGQK.K + Oxidation (M)
1791.9047	1790.8974	1790.8734	13.4	41	55	1	K.SSETLTFKRPEGMHR.E + Oxidation (M)
1915.0015	1913.9942	1913.9847	4.98	67	84	1	K.DAPPLLPSDTGQGYRTVK.A
3370.7415	3369.7342	3369.6836	15.0	340	368	0	K.ALEQMLLELGVELSPTPTEELVHMFNELR.S + 2 Oxidation (M)
3716.8708	3715.8635	3715.8806	-4.58	395	434	1	R.HEALARAGVLGGPTTPASGPAPAPAEPAVSEPGLGSDPSK.D
4253.0234	4252.0161	4252.0501	-8.00	132	166	2	K.TVQVPVYSEQEYQLYLHDDAWTKAETDHLFDLSRR.F

No match to: 706.2814, 707.2933, 713.3626, 719.4172, 722.4265, 735.4174, 744.3938, 756.4689, 772.4224, 779.4717, 792.4133, 804.3155, 806.4307, 807.4160, 809.4363, 812.3947, 814.4097, 815.4186, 819.3901, 828.3928, 831.4666, 832.3149, 834.3199, 837.3761, 841.4355, 842.4860, 845.4138, 851.3882, 871.4900, 876.2846, 878.2907, 883.4759, 891.4818, 893.9534, 894.4574, 910.4221, 913.5131, 918.5399, 929.4545, 935.4750, 937.4921, 947.4755, 959.4836, 961.4837, 963.4760, 967.4271, 969.4589, 972.4794, 974.4445, 976.4506, 977.4767, 978.5352, 981.4952, 983.4747, 985.6076, 988.4585, 989.6082, 997.4586, 1007.5676, 1011.4840, 1014.5074, 1018.5203, 1019.5441, 1021.5421, 1034.5779, 1042.5320, 1044.5242, 1060.5463, 1071.5376, 1077.5208, 1087.5399, 1091.5421, 1106.5393, 1122.5577, 1156.5372, 1173.6240, 1187.6439, 1199.6992, 1215.6615, 1244.6189, 1248.6575, 1255.6039, 1276.6218, 1284.6401, 1295.7195, 1301.6177, 1313.7069, 1314.7141, 1315.6759, 1325.6675, 1336.7151, 1342.6758, 1344.7024, 1349.6952, 1353.6804, 1355.7097, 1356.7201, 1361.6877, 1365.6632, 1370.6783, 1372.6058, 1381.7588, 1383.7244, 1386.7131, 1394.7318, 1397.7419, 1414.6467, 1427.7865, 1432.7140, 1446.7247, 1457.7411, 1473.8055, 1478.7513, 1497.8757, 1528.6993, 1542.8459, 1556.8795, 1578.7826, 1601.9006, 1606.8433, 1616.8177, 1618.8162, 1628.7026, 1628.8568, 1642.7186, 1647.8694, 1650.8473, 1658.8400, 1677.8625, 1680.8728, 1696.8231, 1703.8551, 1704.8862, 1710.9269, 1715.8459, 1717.8810, 1718.9111, 1725.8386, 1727.9301, 1730.8835, 1742.9099, 1751.8870, 1768.9546, 1769.9617, 1771.0243, 1775.0276, 1777.8676, 1786.9032, 1799.9005, 1800.9124, 1928.0238, 1953.9285, 1982.0009, 2012.9681, 2025.0820, 2027.1273, 2042.0642, 2052.9939,

2060.0461, 2067.0017, 2070.8899, 2074.9629, 2082.9421, 2084.9102, 2086.9065, 2096.9797, 2100.9070, 2109.0410, 2116.0894, 2136.9121, 2155.0730, 2170.0781, 2195.0801, 2211.0686, 2238.1072, 2251.1428, 2293.1614, 2336.2495, 2343.1758, 2382.1987, 2385.2234, 2400.0720, 2414.0930, 2418.0901, 2428.1140, 2431.0991, 2465.2188, 2468.2249, 2528.1794, 2542.2344, 2560.2729, 2564.2610, 2646.9797, 2660.9968, 2675.0183, 2698.3711, 2707.3184, 2711.3606, 2712.3755, 2719.3159, 2729.3870, 2744.3770, 2762.3970, 2767.3120, 2812.4265, 2846.3943, 2871.4121, 2872.3569, 2873.3445, 2884.3745, 2888.3184, 2892.4063, 2893.4375, 2894.4741, 2917.3960, 2926.4563, 2992.4536, 2995.4939, 3004.4705, 3005.5735, 3027.5032, 3054.4763, 3082.5879, 3088.5859, 3104.5430, 3126.7307, 3170.6697, 3200.5081, 3201.4670, 3206.5745, 3207.6025, 3242.6213, 3248.6428, 3255.6025, 3263.6973, 3300.7517, 3315.7021, 3328.7302, 3331.6746, 3334.7031, 3335.7090, 3347.5835, 3353.6013, 3356.7166, 3386.7366, 3456.7622, 3471.7375, 3487.7676, 3491.6787, 3503.8286, 3601.7178, 3639.7463, 3646.7891, 3651.8442, 3703.9653, 3720.7805, 3764.8616, 3820.9673, 3869.8423, 3885.7913, 3887.9063, 3927.8738, 4148.0005, 4162.0400, 4452.2729, 4464.2598, 4468.1572, 4482.1689, 4680.3301, 4845.1597

30. Q9XSK7 **Mass:** 26702 **Score:** 20 **Expect:** 2.4e+002 **Matches:** 9
Hepatoma-derived growth factor OS=Bos taurus GN=HDGF PE=2 SV=1

Observed	Mr(expt)	Mr(calc)	ppm	Start	End	Miss	Peptide
983.4747	982.4674	982.4773	-10.11	22 -	29	0	K.GYPHWPAR.I
1044.5242	1043.5169	1043.5135	3.29	158 -	167	0	R.AGDLLEDSPK.R
1356.7201	1355.7128	1355.7157	-2.12	156 -	167	2	K.RRAGDLLEDSPK.R
1542.8459	1541.8386	1541.8300	5.56	139 -	151	2	K.LVIDEPTKEKNEK.G
2067.0017	2065.9944	2066.0043	-4.80	22 -	39	1	K.GYPHWPARIDEMPEAAVK.S
2074.9629	2073.9556	2073.9702	-7.03	127 -	146	1	K.GNAEGSSDEEGKLVIDEPTK.E
2884.3745	2883.3672	2883.4039	-12.71	81 -	106	2	K.GFSEGLWEIENNPTVKASGYQSSQKK.S
2894.4741	2893.4668	2893.4053	21.3	181 -	208	1	K.EGATLEGERPLPVEAEKNSTPSEPGSGR.G
3335.7090	3334.7017	3334.6528	14.7	168 -	197	2	K.RPKEAEDLEGEEKEGATLEGERPLPVEAEK.N

No match to: 706.2814, 707.2933, 713.3626, 717.4103, 719.4172, 722.4265, 735.4174, 744.3938, 756.4689, 768.3713, 772.4224, 779.4717, 792.4133, 804.3155, 806.4307, 807.4160, 809.4363, 812.3947, 814.4097, 815.4186, 819.3901, 828.3928, 831.4666, 832.3149, 834.3199, 837.3761, 841.4355, 842.4860, 845.4138, 851.3882, 871.4900, 876.2846, 878.2907, 883.4759, 891.4818, 893.9534, 894.4574, 898.4351, 910.4221, 913.5131, 918.5399, 929.4545, 935.4750, 937.4921, 947.4755, 959.4836, 961.4837, 963.4760, 967.4271, 969.4589, 972.4794, 974.4445, 976.4506, 977.4767, 978.5352, 981.4952, 985.6076, 988.4585, 989.6082, 997.4586, 1007.5676, 1011.4840, 1014.5074, 1018.5203, 1019.5441, 1021.5421, 1034.5779, 1042.5320, 1060.5463, 1071.5376, 1077.5208, 1087.5399, 1091.5421, 1106.5393, 1122.5577, 1156.5372, 1173.6240, 1187.6439, 1199.6992, 1215.6615, 1244.6189, 1248.6575, 1255.6039, 1276.6218, 1284.6401, 1295.7195, 1301.6177, 1313.7069, 1314.7141, 1315.6759, 1325.6675, 1336.7151, 1337.7180, 1338.6461, 1342.6758, 1344.7024, 1349.6952, 1353.6804, 1355.7097, 1361.6877, 1365.6632, 1370.6783, 1372.6058, 1381.7588, 1383.7244, 1386.7131, 1394.7318, 1397.7419, 1414.6467, 1427.7865, 1432.7140, 1446.7247, 1457.7411, 1461.7784, 1473.8055, 1478.7513, 1497.8757, 1528.6993, 1556.8795, 1578.7826, 1601.9006, 1606.8433, 1616.8177, 1618.8162, 1628.7026, 1628.8568, 1642.7186, 1647.8694, 1650.8473, 1658.8400, 1677.8625, 1680.8728, 1696.8231, 1703.8551, 1704.8862, 1710.9269, 1715.8459, 1717.8810, 1718.9111, 1725.8386, 1727.9301, 1730.8835, 1742.9099, 1751.8870, 1768.9546, 1769.9617, 1771.0243, 1775.0276, 1777.8676, 1786.9032, 1791.9047, 1799.9005, 1800.9124, 1915.0015, 1928.0238, 1953.9285, 1982.0009, 2012.9681, 2025.0820, 2027.1273, 2042.0642, 2052.9939, 2060.0461, 2070.8899, 2082.9421, 2084.9102, 2086.9065, 2096.9797, 2100.9070, 2109.0410, 2116.0894, 2136.9121, 2155.0730, 2170.0781, 2195.0801, 2211.0686, 2238.1072, 2251.1428, 2293.1614, 2336.2495, 2343.1758, 2382.1987, 2385.2234, 2400.0720, 2414.0930, 2418.0901, 2428.1140, 2431.0991, 2465.2188, 2468.2249, 2528.1794, 2542.2344, 2560.2729, 2564.2610, 2646.9797, 2660.9968, 2675.0183, 2698.3711, 2707.3184, 2711.3606, 2712.3755, 2719.3159, 2729.3870, 2744.3770, 2762.3970, 2767.3120, 2812.4265, 2846.3943, 2871.4121, 2872.3569, 2873.3445, 2888.3184, 2892.4063, 2893.4375, 2917.3960, 2926.4563, 2992.4536, 2995.4939, 3004.4705, 3005.5735, 3027.5032, 3054.4763, 3082.5879, 3088.5859, 3104.5430, 3126.7307, 3170.6697, 3200.5081, 3201.4670, 3206.5745, 3207.6025, 3242.6213, 3248.6428, 3255.6025, 3263.6973, 3300.7517, 3315.7021, 3328.7302, 3331.6746, 3334.7031, 3347.5835, 3353.6013, 3356.7166, 3370.7415, 3386.7366, 3456.7622, 3471.7375, 3487.7676, 3491.6787, 3503.8286, 3601.7178, 3639.7463, 3646.7891, 3651.8442, 3703.9653, 3716.8708, 3720.7805, 3764.8616, 3820.9673, 3869.8423, 3885.7913, 3887.9063, 3927.8738, 4148.0005, 4162.0400, 4253.0234, 4452.2729, 4464.2598, 4468.1572, 4482.1689, 4680.3301, 4845.1597

31. G3X6L4 **Mass:** 26771 **Score:** 20 **Expect:** 2.4e+002 **Matches:** 9
Hepatoma-derived growth factor OS=Bos taurus GN=HDGF PE=1 SV=1

Observed	Mr(expt)	Mr(calc)	ppm	Start	End	Miss	Peptide
983.4747	982.4674	982.4773	-10.11	22 -	29	0	K.GYPHWPAR.I
1044.5242	1043.5169	1043.5135	3.29	158 -	167	0	R.AGDLLEDSPK.R
1356.7201	1355.7128	1355.7157	-2.12	156 -	167	2	K.RRAGDLLEDSPK.R
1542.8459	1541.8386	1541.8300	5.56	139 -	151	2	K.LVIDEPTKEKNEK.G
2067.0017	2065.9944	2066.0043	-4.80	22 -	39	1	K.GYPHWPARIDEMPEAAVK.S
2074.9629	2073.9556	2073.9702	-7.03	127 -	146	1	K.GNAEGSSDEEGKLVIDEPTK.E
2884.3745	2883.3672	2883.4039	-12.71	81 -	106	2	K.GFSEGLWEIENNPTVKASGYQSSQKK.S
2894.4741	2893.4668	2893.4053	21.3	181 -	208	1	K.EGATLEGERPLPVEAEKNSTPSEPGSGR.G
3335.7090	3334.7017	3334.6528	14.7	168 -	197	2	K.RPKEAEDLEGEEKEGATLEGERPLPVEAEK.N

No match to: 706.2814, 707.2933, 713.3626, 717.4103, 719.4172, 722.4265, 735.4174, 744.3938, 756.4689, 768.3713, 772.4224, 779.4717, 792.4133, 804.3155, 806.4307, 807.4160, 809.4363, 812.3947, 814.4097, 815.4186, 819.3901, 828.3928, 831.4666, 832.3149, 834.3199, 837.3761, 841.4355, 842.4860, 845.4138, 851.3882, 871.4900, 876.2846, 878.2907, 883.4759, 891.4818, 893.9534, 894.4574, 898.4351, 910.4221, 913.5131, 918.5399, 929.4545, 935.4750, 937.4921, 947.4755, 959.4836, 961.4837, 963.4760, 967.4271, 969.4589, 972.4794, 974.4445, 976.4506, 977.4767, 978.5352, 981.4952, 985.6076, 988.4585, 989.6082, 997.4586, 1007.5676, 1011.4840, 1014.5074, 1018.5203, 1019.5441, 1021.5421, 1034.5779, 1042.5320, 1060.5463, 1071.5376, 1077.5208, 1087.5399, 1091.5421, 1106.5393, 1122.5577, 1156.5372, 1173.6240, 1187.6439, 1199.6992, 1215.6615, 1244.6189, 1248.6575, 1255.6039, 1276.6218, 1284.6401, 1295.7195, 1301.6177, 1313.7069, 1314.7141, 1315.6759, 1325.6675, 1336.7151, 1337.7180, 1338.6461, 1342.6758, 1344.7024, 1349.6952, 1353.6804, 1355.7097, 1361.6877, 1365.6632, 1370.6783, 1372.6058, 1381.7588, 1383.7244, 1386.7131, 1394.7318, 1397.7419, 1414.6467, 1427.7865, 1432.7140, 1446.7247, 1457.7411, 1461.7784, 1473.8055, 1478.7513, 1497.8757, 1528.6993, 1556.8795, 1578.7826, 1601.9006, 1606.8433, 1616.8177, 1618.8162, 1628.7026, 1628.8568, 1642.7186, 1647.8694, 1650.8473, 1658.8400, 1677.8625, 1680.8728, 1696.8231, 1703.8551, 1704.8862, 1710.9269, 1715.8459, 1717.8810, 1718.9111, 1725.8386, 1727.9301, 1730.8835, 1742.9099, 1751.8870, 1768.9546, 1769.9617, 1771.0243, 1775.0276, 1777.8676, 1786.9032, 1791.9047, 1799.9005, 1800.9124, 1915.0015, 1928.0238, 1953.9285, 1982.0009, 2012.9681, 2025.0820, 2027.1273, 2042.0642, 2052.9939, 2060.0461, 2070.8899, 2082.9421, 2084.9102, 2086.9065, 2096.9797, 2100.9070, 2109.0410, 2116.0894, 2136.9121, 2155.0730, 2170.0781, 2195.0801, 2211.0686, 2238.1072, 2251.1428, 2293.1614, 2336.2495, 2343.1758, 2382.1987, 2385.2234, 2400.0720, 2414.0930, 2418.0901, 2428.1140, 2431.0991, 2465.2188, 2468.2249, 2528.1794, 2542.2344, 2560.2729, 2564.2610, 2646.9797, 2660.9968, 2675.0183, 2698.3711, 2707.3184, 2711.3606, 2712.3755, 2719.3159, 2729.3870, 2744.3770, 2762.3970, 2767.3120, 2812.4265, 2846.3943, 2871.4121, 2872.3569, 2873.3445, 2888.3184, 2892.4063, 2893.4375, 2917.3960, 2926.4563, 2992.4536, 2995.4939, 3004.4705, 3005.5735, 3027.5032, 3054.4763, 3082.5879, 3088.5859, 3104.5430, 3126.7307, 3170.6697, 3200.5081, 3201.4670, 3206.5745, 3207.6025, 3242.6213, 3248.6428, 3255.6025, 3263.6973, 3300.7517, 3315.7021, 3328.7302, 3331.6746, 3334.7031, 3347.5835, 3353.6013, 3356.7166, 3370.7415, 3386.7366, 3456.7622, 3471.7375, 3487.7676, 3491.6787, 3503.8286, 3601.7178, 3639.7463, 3646.7891, 3651.8442, 3703.9653, 3716.8708, 3720.7805, 3764.8616, 3820.9673, 3869.8423, 3885.7913, 3887.9063, 3927.8738, 4148.0005, 4162.0400, 4253.0234, 4452.2729, 4464.2598, 4468.1572, 4482.1689, 4680.3301, 4845.1597

32. G3N2D3 **Mass:** 15087 **Score:** 20 **Expect:** 2.6e+002 **Matches:** 6
Uncharacterized protein OS=Bos taurus PE=4 SV=1

Observed	Mr(expt)	Mr(calc)	ppm	Start	End	Miss	Peptide
1461.7784	1460.7711	1460.7908	-13.49	40 -	52	0	K.ILLNLSENSVMTK.E
1928.0238	1927.0165	1926.9873	15.2	53 -	69	1	K.ELLNAKAVSEFMGLFSR.K + Oxidation (M)
2343.1758	2342.1685	2342.1787	-4.36	71 -	91	0	K.ETNDNIQVVLAMLENIGNDIK.K
3370.7415	3369.7342	3369.7676	-9.90	40 -	69	2	K.ILLNLSENSVMTKELLNAKAVSEFMGLFSR.K + Oxidation (M)
3386.7366	3385.7293	3385.7625	-9.80	40 -	69	2	K.ILLNLSENSVMTKELLNAKAVSEFMGLFSR.K + 2 Oxidation (M)
3503.8286	3502.8213	3502.8685	-13.46	4 -	34	2	R.HLTRATDYHTLVARYLSGFLLLLATGNTETR.F

No match to: 706.2814, 707.2933, 713.3626, 717.4103, 719.4172, 722.4265, 735.4174, 744.3938, 756.4689, 768.3713, 772.4224, 779.4717, 792.4133, 804.3155, 806.4307, 807.4160, 809.4363, 812.3947, 814.4097, 815.4186, 819.3901, 828.3928, 831.4666, 832.3149,

834.3199, 837.3761, 841.4355, 842.4860, 845.4138, 851.3882, 871.4900, 876.2846, 878.2907, 883.4759, 891.4818, 893.9534, 894.4574, 898.4351, 910.4221, 913.5131, 918.5399, 929.4545, 935.4750, 937.4921, 947.4755, 959.4836, 961.4837, 963.4760, 967.4271, 969.4589, 972.4794, 974.4445, 976.4506, 977.4767, 978.5352, 981.4952, 983.4747, 985.6076, 988.4585, 989.6082, 997.4586, 1007.5676, 1011.4840, 1014.5074, 1018.5203, 1019.5441, 1021.5421, 1034.5779, 1042.5320, 1044.5242, 1060.5463, 1071.5376, 1077.5208, 1087.5399, 1091.5421, 1106.5393, 1122.5577, 1156.5372, 1173.6240, 1187.6439, 1199.6992, 1215.6615, 1244.6189, 1248.6575, 1255.6039, 1276.6218, 1284.6401, 1295.7195, 1301.6177, 1313.7069, 1314.7141, 1315.6759, 1325.6675, 1336.7151, 1337.7180, 1338.6461, 1342.6758, 1344.7024, 1349.6952, 1353.6804, 1355.7097, 1356.7201, 1361.6877, 1365.6632, 1370.6783, 1372.6058, 1381.7588, 1383.7244, 1386.7131, 1394.7318, 1397.7419, 1414.6467, 1427.7865, 1432.7140, 1446.7247, 1457.7411, 1473.8055, 1478.7513, 1497.8757, 1528.6993, 1542.8459, 1556.8795, 1578.7826, 1601.9006, 1606.8433, 1616.8177, 1618.8162, 1628.7026, 1628.8568, 1642.7186, 1647.8694, 1650.8473, 1658.8400, 1677.8625, 1680.8728, 1696.8231, 1703.8551, 1704.8862, 1710.9269, 1715.8459, 1717.8810, 1718.9111, 1725.8386, 1727.9301, 1730.8835, 1742.9099, 1751.8870, 1768.9546, 1769.9617, 1771.0243, 1775.0276, 1777.8676, 1786.9032, 1791.9047, 1799.9005, 1800.9124, 1915.0015, 1953.9285, 1982.0009, 2012.9681, 2025.0820, 2027.1273, 2042.0642, 2052.9939, 2060.0461, 2067.0017, 2070.8899, 2074.9629, 2082.9421, 2084.9102, 2086.9065, 2096.9797, 2100.9070, 2109.0410, 2116.0894, 2136.9121, 2155.0730, 2170.0781, 2195.0801, 2211.0686, 2238.1072, 2251.1428, 2293.1614, 2336.2495, 2382.1987, 2385.2234, 2400.0720, 2414.0930, 2418.0901, 2428.1140, 2431.0991, 2465.2188, 2468.2249, 2528.1794, 2542.2344, 2560.2729, 2564.2610, 2646.9797, 2660.9968, 2675.0183, 2698.3711, 2707.3184, 2711.3606, 2712.3755, 2719.3159, 2729.3870, 2744.3770, 2762.3970, 2767.3120, 2812.4265, 2846.3943, 2871.4121, 2872.3569, 2873.3445, 2884.3745, 2888.3184, 2892.4063, 2893.4375, 2894.4741, 2917.3960, 2926.4563, 2992.4536, 2995.4939, 3004.4705, 3005.5735, 3027.5032, 3054.4763, 3082.5879, 3088.5859, 3104.5430, 3126.7307, 3170.6697, 3200.5081, 3201.4670, 3206.5745, 3207.6025, 3242.6213, 3248.6428, 3255.6025, 3263.6973, 3300.7517, 3315.7021, 3328.7302, 3331.6746, 3334.7031, 3335.7090, 3347.5835, 3353.6013, 3356.7166, 3456.7622, 3471.7375, 3487.7676, 3491.6787, 3601.7178, 3639.7463, 3646.7891, 3651.8442, 3703.9653, 3716.8708, 3720.7805, 3764.8616, 3820.9673, 3869.8423, 3885.7913, 3887.9063, 3927.8738, 4148.0005, 4162.0400, 4253.0234, 4452.2729, 4464.2598, 4468.1572, 4482.1689, 4680.3301, 4845.1597

33. A0JNH6 **Mass:** 63280 **Score:** 20 **Expect:** 2.7e+002 **Matches:** 19

Coiled-coil domain-containing protein 102A OS=Bos taurus GN=CCDC102A PE=2 SV=1

Observed	Mr(expt)	Mr(calc)	ppm	Start	End	Miss	Peptide
768.3713	767.3640	767.3701	-7.95	299 -	304	0	K.YEELSK.T
807.4160	806.4087	806.4286	-24.67	435 -	440	1	K.KQFQEK.V
1187.6439	1186.6366	1186.6557	-16.09	369 -	378	1	R.LETEKLGLER.E
1244.6189	1243.6116	1243.5945	13.8	355 -	365	0	R.LQAENAAEWGR.R
1349.6952	1348.6879	1348.6591	21.4	344 -	354	2	R.KMAELRGEMER.L
1365.6632	1364.6559	1364.6540	1.40	344 -	354	2	R.KMAELRGEMER.L + Oxidation (M)
1427.7865	1426.7792	1426.7528	18.5	385 -	397	1	R.AQVGDLEEALARR.R
1542.8459	1541.8386	1541.8161	14.6	73 -	84	2	R.EELRLRELEEAR.A
1768.9546	1767.9473	1767.9380	5.29	435 -	449	2	K.KQFQEKVAELAHANR.R
1786.9032	1785.8959	1785.8717	13.5	144 -	159	2	R.QEAQGESEARGRELAR.L
2067.0017	2065.9944	2065.9664	13.6	471 -	488	1	K.ELAQAEDELDEAHNQARK.L
2084.9102	2083.9029	2083.9150	-5.79	327 -	344	2	R.MSEDLEDELGARSSMDRK.M + Oxidation (M)
2100.9070	2099.8997	2099.9099	-4.85	327 -	344	2	R.MSEDLEDELGARSSMDRK.M + 2 Oxidation (M)
2195.0801	2194.0728	2194.0688	1.83	400 -	419	1	R.QTASALDCDLRASQAALFEK.N
2382.1987	2381.1914	2381.2372	-19.24	307 -	326	2	K.QEMLKQLSILKEAHQDELGR.M + Oxidation (M)
2400.0720	2399.0647	2399.0659	-0.48	318 -	338	1	K.EAHQDELGRMSEDLEDELGAR.S
2428.1140	2427.1067	2427.0670	16.3	87 -	105	2	R.AAQMEKTMRWWSDCTANWR.E
3082.5879	3081.5806	3081.5036	25.0	312 -	338	2	K.QLSILKEAHQDELGRMSEDLEDELGAR.S
3263.6973	3262.6900	3262.6654	7.55	489 -	515	2	K.LQRSLDEQTEQSENLQVQLEHVQSRLR.R

No match to: 706.2814, 707.2933, 713.3626, 717.4103, 719.4172, 722.4265, 735.4174, 744.3938, 756.4689, 772.4224, 779.4717, 792.4133, 804.3155, 806.4307, 809.4363, 812.3947, 814.4097, 815.4186, 819.3901, 828.3928, 831.4666, 832.3149, 834.3199, 837.3761, 841.4355, 842.4860, 845.4138, 851.3882, 871.4900, 876.2846, 878.2907, 883.4759, 891.4818, 893.9534, 894.4574, 898.4351, 910.4221, 913.5131, 918.5399, 929.4545, 935.4750, 937.4921, 947.4755, 959.4836, 961.4837, 963.4760, 967.4271, 969.4589, 972.4794, 974.4445, 976.4506, 977.4767, 978.5352, 981.4952, 983.4747, 985.6076, 988.4585, 989.6082, 997.4586, 1007.5676, 1011.4840, 1014.5074, 1018.5203, 1019.5441, 1021.5421, 1034.5779, 1042.5320, 1044.5242, 1060.5463, 1071.5376, 1077.5208, 1087.5399, 1091.5421, 1106.5393, 1122.5577, 1156.5372, 1173.6240, 1199.6992, 1215.6615, 1248.6575, 1255.6039, 1276.6218, 1284.6401, 1295.7195, 1301.6177, 1313.7069, 1314.7141, 1315.6759, 1325.6675, 1336.7151, 1337.7180, 1338.6461, 1342.6758, 1344.7024, 1353.6804, 1355.7097, 1356.7201, 1361.6877, 1370.6783, 1372.6058, 1381.7588, 1383.7244, 1386.7131, 1394.7318, 1397.7419, 1414.6467, 1432.7140, 1446.7247, 1457.7411, 1461.7784, 1473.8055, 1478.7513, 1497.8757, 1528.6993, 1556.8795, 1578.7826, 1601.9006, 1606.8433, 1616.8177, 1618.8162, 1628.7026, 1628.8568, 1642.7186, 1647.8694, 1650.8473, 1658.8400, 1677.8625, 1680.8728, 1696.8231, 1703.8551, 1704.8862, 1710.9269, 1715.8459, 1717.8810, 1718.9111, 1725.8386, 1727.9301, 1730.8835, 1742.9099, 1751.8870, 1769.9617, 1771.0243, 1775.0276, 1777.8676, 1791.9047, 1799.9005, 1800.9124, 1915.0015, 1928.0238, 1953.9285, 1982.0009, 2012.9681, 2025.0820, 2027.1273, 2042.0642, 2052.9939, 2060.0461, 2070.8899, 2074.9629, 2082.9421, 2086.9065, 2096.9797, 2109.0410, 2116.0894, 2136.9121, 2155.0730, 2170.0781, 2211.0686, 2238.1072, 2251.1428, 2293.1614, 2336.2495, 2343.1758, 2385.2234, 2414.0930, 2418.0901, 2431.0991, 2465.2188, 2468.2249, 2528.1794, 2542.2344, 2560.2729, 2564.2610, 2646.9797, 2660.9968, 2675.0183, 2698.3711, 2707.3184, 2711.3606, 2712.3755, 2719.3159, 2729.3870, 2744.3770, 2762.3970, 2767.3120, 2812.4265, 2846.3943, 2871.4121, 2872.3569, 2873.3445, 2884.3745, 2888.3184, 2892.4063, 2893.4375, 2894.4741, 2917.3960, 2926.4563, 2992.4536, 2995.4939, 3004.4705, 3005.5735, 3027.5032, 3054.4763, 3088.5859, 3104.5430, 3126.7307, 3170.6697, 3200.5081, 3201.4670, 3206.5745, 3207.6025, 3242.6213, 3248.6428, 3255.6025, 3300.7517, 3315.7021, 3328.7302, 3331.6746, 3334.7031, 3335.7090, 3347.5835, 3353.6013, 3356.7166, 3370.7415, 3386.7366, 3456.7622, 3471.7375, 3487.7676, 3491.6787, 3503.8286, 3601.7178, 3639.7463, 3646.7891, 3651.8442, 3703.9653, 3716.8708, 3720.7805, 3764.8616, 3820.9673, 3869.8423, 3885.7913, 3887.9063, 3927.8738, 4148.0005, 4162.0400, 4253.0234, 4452.2729, 4464.2598, 4468.1572, 4482.1689, 4680.3301, 4845.1597

34. E1BAB2 **Mass:** 22914 **Score:** 20 **Expect:** 2.7e+002 **Matches:** 7

Uncharacterized protein OS=Bos taurus PE=4 SV=2

Observed	Mr(expt)	Mr(calc)	ppm	Start	End	Miss	Peptide
809.4363	808.4291	808.4153	17.0	1 -	7	0	-.MSFSPLK.V
1187.6439	1186.6366	1186.6128	20.1	19 -	28	1	R.SMEAHSILRK.K + Oxidation (M)
1355.7097	1354.7024	1354.7106	-6.03	29 -	41	2	K.KGFSVRSFGAGSR.V
1628.8568	1627.8495	1627.8794	-18.36	74 -	87	1	R.ERYTSNGILHILGR.N
1742.9099	1741.9026	1741.9223	-11.31	76 -	90	1	R.YTSNGILHILGRNER.I
2343.1758	2342.1685	2342.2151	-19.89	162 -	182	1	R.LQQAEDMEGSLAELLLAVERK.T
2465.2188	2464.2115	2464.1807	12.5	8 -	28	2	K.VAVVCMSNMNRSMEAHSILRK.K + 2 Oxidation (M)

No match to: 706.2814, 707.2933, 713.3626, 717.4103, 719.4172, 722.4265, 735.4174, 744.3938, 756.4689, 768.3713, 772.4224, 779.4717, 792.4133, 804.3155, 806.4307, 807.4160, 812.3947, 814.4097, 815.4186, 819.3901, 828.3928, 831.4666, 832.3149, 834.3199, 837.3761, 841.4355, 842.4860, 845.4138, 851.3882, 871.4900, 876.2846, 878.2907, 883.4759, 891.4818, 893.9534, 894.4574, 898.4351, 910.4221, 913.5131, 918.5399, 929.4545, 935.4750, 937.4921, 947.4755, 959.4836, 961.4837, 963.4760, 967.4271, 969.4589, 972.4794, 974.4445, 976.4506, 977.4767, 978.5352, 981.4952, 983.4747, 985.6076, 988.4585, 989.6082, 997.4586, 1007.5676, 1011.4840, 1014.5074, 1018.5203, 1019.5441, 1021.5421, 1034.5779, 1042.5320, 1044.5242, 1060.5463, 1071.5376, 1077.5208, 1087.5399, 1091.5421, 1106.5393, 1122.5577, 1156.5372, 1173.6240, 1199.6992, 1215.6615, 1244.6189, 1248.6575, 1255.6039, 1276.6218, 1284.6401, 1295.7195, 1301.6177, 1313.7069, 1314.7141, 1315.6759, 1325.6675, 1336.7151, 1337.7180, 1338.6461, 1342.6758, 1344.7024, 1349.6952, 1353.6804, 1356.7201, 1361.6877, 1365.6632, 1370.6783, 1372.6058, 1381.7588, 1383.7244, 1386.7131, 1394.7318, 1397.7419, 1414.6467, 1427.7865, 1432.7140, 1446.7247, 1457.7411, 1461.7784, 1473.8055, 1478.7513, 1497.8757, 1528.6993, 1542.8459, 1556.8795, 1578.7826, 1601.9006, 1606.8433, 1616.8177, 1618.8162, 1628.7026, 1642.7186, 1647.8694, 1650.8473, 1658.8400, 1677.8625, 1680.8728, 1696.8231, 1703.8551, 1704.8862, 1710.9269, 1715.8459, 1717.8810, 1718.9111, 1725.8386, 1727.9301, 1730.8835, 1751.8870, 1768.9546, 1769.9617, 1771.0243, 1775.0276, 1777.8676, 1786.9032, 1791.9047, 1799.9005, 1800.9124, 1915.0015, 1928.0238, 1953.9285, 1982.0009, 2012.9681, 2025.0820, 2027.1273, 2042.0642, 2052.9939, 2060.0461, 2067.0017, 2070.8899, 2074.9629, 2082.9421, 2084.9102, 2086.9065, 2096.9797, 2100.9070, 2109.0410, 2116.0894, 2136.9121, 2155.0730, 2170.0781, 2195.0801, 2211.0686, 2238.1072, 2251.1428, 2293.1614, 2336.2495, 2382.1987, 2385.2234, 2400.0720, 2414.0930, 2418.0901, 2428.1140, 2431.0991, 2468.2249, 2528.1794, 2542.2344, 2560.2729, 2564.2610, 2646.9797, 2660.9968, 2675.0183, 2698.3711, 2707.3184, 2711.3606, 2712.3755, 2719.3159, 2729.3870, 2744.3770, 2762.3970, 2767.3120, 2812.4265,

2846.3943, 2871.4121, 2872.3569, 2873.3445, 2884.3745, 2888.3184, 2892.4063, 2893.4375, 2894.4741, 2917.3960, 2926.4563, 2992.4536, 2995.4939, 3004.4705, 3005.5735, 3027.5032, 3054.4763, 3082.5879, 3088.5859, 3104.5430, 3126.7307, 3170.6697, 3200.5081, 3201.4670, 3206.5745, 3207.6025, 3242.6213, 3248.6428, 3255.6025, 3263.6973, 3300.7517, 3315.7021, 3328.7302, 3331.6746, 3334.7031, 3335.7090, 3347.5835, 3353.6013, 3356.7166, 3370.7415, 3386.7366, 3456.7622, 3471.7375, 3487.7676, 3491.6787, 3503.8286, 3601.7178, 3639.7463, 3646.7891, 3651.8442, 3703.9653, 3716.8708, 3720.7805, 3764.8616, 3820.9673, 3869.8423, 3885.7913, 3887.9063, 3927.8738, 4148.0005, 4162.0400, 4253.0234, 4452.2729, 4464.2598, 4468.1572, 4482.1689, 4680.3301, 4845.1597

35. F1MUF1 **Mass:** 134385 **Score:** 20 **Expect:** 2.7e+002 **Matches:** 15

Uncharacterized protein OS=Bos taurus GN=XPO5 PE=3 SV=1

Observed	Mr(expt)	Mr(calc)	ppm	Start	End	Miss	Peptide
851.3882	850.3809	850.4007	-23.29	758 -	764	0	K.MAEPFTR.A
1295.7195	1294.7122	1294.7067	4.26	364 -	374	1	K.YLRASMTNLVK.M
1658.8400	1657.8327	1657.8102	13.6	10 -	23	1	K.CPVCVPCGLRLAEK.T
1680.8728	1679.8655	1679.8817	-9.63	45 -	57	2	R.WNNMSRLEKVYLK.N
1777.8676	1776.8603	1776.8725	-6.85	682 -	696	1	R.ARMSFCVYSILGVMK.R + Oxidation (M)
2195.0801	2194.0728	2194.0769	-1.85	577 -	594	0	R.DYPQLVLPSFDMLYNHVK.Q + Oxidation (M)
2251.1428	2250.1355	2250.1289	2.94	416 -	434	2	R.LACRLDPKTSFQMAGEWLK.Y
2744.3770	2743.3697	2743.3825	-4.67	628 -	650	1	R.QKVFLEELMAPVASIWLSENMHR.V + Oxidation (M)
3263.6973	3262.6900	3262.6267	19.4	624 -	650	2	K.SYERQKVFLEELMAPVASIWLSENMHR.V
3300.7517	3299.7444	3299.6635	24.5	1137 -	1166	1	K.NLPSLFKKPKPMPETDALDEEGGLATIFEP.- + Oxidation (M)
3315.7021	3314.6948	3314.6783	5.00	155 -	182	2	R.IFSFLLNTLQENVNKYQQVKTDNSQESK.A
3370.7415	3369.7342	3369.7853	-15.16	51 -	79	2	R.LEKVYLKNSVMELIANGTLNILEEENHIK.D + Oxidation (M)
3487.7676	3486.7603	3486.8367	-21.89	735 -	764	2	K.LLDNLLALIRTHNTLYAPEMLAKMAEPFTR.A + 2 Oxidation (M)
4452.2729	4451.2656	4451.2294	8.15	628 -	666	2	R.QKVFLEELMAPVASIWLSENMHRVLSDVDAFIAYVGADR.K + 2 Oxidation (M)
4464.2598	4463.2525	4463.3021	-11.12	247 -	286	2	K.LEDRKPLMVLFGDVAMHYILSAAQTADGGGLVEKHYVFLK.R + 2 Oxidation (M)

No match to: 706.2814, 707.2933, 713.3626, 717.4103, 719.4172, 722.4265, 735.4174, 744.3938, 756.4689, 768.3713, 772.4224, 779.4717, 792.4133, 804.3155, 806.4307, 807.4160, 809.4363, 812.3947, 814.4097, 815.4186, 819.3901, 828.3928, 831.4666, 832.3149, 834.3199, 837.3761, 841.4355, 842.4860, 845.4138, 871.4900, 876.2846, 878.2907, 883.4759, 891.4818, 893.9534, 894.4574, 898.4351, 910.4221, 913.5131, 918.5399, 929.4545, 935.4750, 937.4921, 947.4755, 959.4836, 961.4837, 963.4760, 967.4271, 969.4589, 972.4794, 974.4445, 976.4506, 977.4767, 978.5352, 981.4952, 983.4747, 985.6076, 988.4585, 989.6082, 997.4586, 1007.5676, 1011.4840, 1014.5074, 1018.5203, 1019.5441, 1021.5421, 1034.5779, 1042.5320, 1044.5242, 1060.5463, 1071.5376, 1077.5208, 1087.5399, 1091.5421, 1106.5393, 1122.5577, 1156.5372, 1173.6240, 1187.6439, 1199.6992, 1215.6615, 1244.6189, 1248.6575, 1255.6039, 1276.6218, 1284.6401, 1301.6177, 1313.7069, 1314.7141, 1315.6759, 1325.6675, 1336.7151, 1337.7180, 1338.6461, 1342.6758, 1344.7024, 1349.6952, 1353.6804, 1355.7097, 1356.7201, 1361.6877, 1365.6632, 1370.6783, 1372.6058, 1381.7588, 1383.7244, 1386.7131, 1394.7318, 1397.7419, 1414.6467, 1427.7865, 1432.7140, 1446.7247, 1457.7411, 1461.7784, 1473.8055, 1478.7513, 1497.8757, 1528.6993, 1542.8459, 1556.8795, 1578.7826, 1601.9006, 1606.8433, 1616.8177, 1618.8162, 1628.7026, 1628.8568, 1642.7186, 1647.8694, 1650.8473, 1677.8625, 1696.8231, 1703.8551, 1704.8862, 1710.9269, 1715.8459, 1717.8810, 1718.9111, 1725.8386, 1727.9301, 1730.8835, 1742.9099, 1751.8870, 1768.9546, 1769.9617, 1771.0243, 1775.0276, 1786.9032, 1791.9047, 1799.9005, 1800.9124, 1915.0015, 1928.0238, 1953.9285, 1982.0009, 2012.9681, 2025.0820, 2027.1273, 2042.0642, 2052.9939, 2060.0461, 2067.0017, 2070.8899, 2074.9629, 2082.9421, 2084.9102, 2086.9065, 2096.9797, 2100.9070, 2109.0410, 2116.0894, 2136.9121, 2155.0730, 2170.0781, 2211.0686, 2238.1072, 2293.1614, 2336.2495, 2343.1758, 2382.1987, 2385.2234, 2400.0720, 2414.0930, 2418.0901, 2428.1140, 2431.0991, 2465.2188, 2468.2249, 2528.1794, 2542.2344, 2560.2729, 2564.2610, 2646.9797, 2660.9968, 2675.0183, 2698.3711, 2707.3184, 2711.3606, 2712.3755, 2719.3159, 2729.3870, 2762.3970, 2767.3120, 2812.4265, 2846.3943, 2871.4121, 2872.3569, 2873.3445, 2884.3745, 2888.3184, 2892.4063, 2893.4375, 2894.4741, 2917.3960, 2926.4563, 2992.4536, 2995.4939, 3004.4705, 3005.5735, 3027.5032, 3054.4763, 3082.5879, 3088.5859, 3104.5430, 3126.7307, 3170.6697, 3200.5081, 3201.4670, 3206.5745, 3207.6025, 3242.6213, 3248.6428, 3255.6025, 3328.7302, 3331.6746, 3334.7031, 3335.7090, 3347.5835, 3353.6013, 3356.7166, 3386.7366, 3456.7622, 3471.7375, 3491.6787, 3503.8286, 3601.7178, 3639.7463, 3646.7891, 3651.8442, 3703.9653, 3716.8708, 3720.7805, 3764.8616, 3820.9673, 3869.8423, 3885.7913, 3887.9063, 3927.8738, 4148.0005, 4162.0400, 4253.0234, 4468.1572, 4482.1689, 4680.3301, 4845.1597

36. O02675 **Mass:** 62638 **Score:** 19 **Expect:** 2.8e+002 **Matches:** 13

Dihydropyrimidinase-related protein 2 OS=Bos taurus GN=DPYSL2 PE=1 SV=1

Observed	Mr(expt)	Mr(calc)	ppm	Start	End	Miss	Peptide
841.4355	840.4282	840.4164	14.1	1 -	7	1	-.MSYQGKK.N
894.4574	893.4501	893.4317	20.6	362 -	368	0	R.MSVIWDK.A + Oxidation (M)
1199.6992	1198.6919	1198.6782	11.5	7 -	16	2	K.KNIPRITSDR.L
1337.7180	1336.7107	1336.6921	13.9	1 -	11	2	-.MSYQGKKNIPR.I + Oxidation (M)
1725.8386	1724.8313	1724.8039	15.9	375 -	390	0	K.MDENQFVAVTSTNAAK.V
2086.9065	2085.8992	2085.8884	5.19	424 -	440	0	K.THNSSLEYNIFEGMECR.G
2873.3445	2872.3372	2872.3775	-14.04	147 -	171	1	K.GVQEEMEALVKDHGVNSFLVYMAFK.D + 2 Oxidation (M)
3170.6697	3169.6624	3169.6230	12.4	369 -	397	2	K.AVVTGKMDENQFVAVTSTNAAKVFNLYPR.K
3207.6025	3206.5952	3206.5918	1.07	346 -	374	2	K.DNFTLIPEGTNGTEERMSVIWDKAVVTGK.M
3869.8423	3868.8350	3868.9094	-19.23	95 -	130	1	K.AALAGGTTMIIDHVVPEPGTSLLAAFDQWREWADSK.S + Oxidation (M)
4253.0234	4252.0161	4251.9776	9.05	57 -	94	2	K.TIEAHSRMVIPGGIDVHTRFQMPDQGMTSADDFFQGTK.A + 2 Oxidation (M)
4464.2598	4463.2525	4463.2319	4.63	24 -	63	2	K.IVNDDQSFYADIYMEDGLIKQIGENLIVPGGVKTIEAHSR.M + Oxidation (M)
4468.1572	4467.1499	4467.1362	3.06	401 -	440	2	R.IAVGSDADLVIWDPDSVKTISAKTHNSSLEYNIFEGMECR.G

No match to: 706.2814, 707.2933, 713.3626, 717.4103, 719.4172, 722.4265, 735.4174, 744.3938, 756.4689, 768.3713, 772.4224, 779.4717, 792.4133, 804.3155, 806.4307, 807.4160, 809.4363, 812.3947, 814.4097, 815.4186, 819.3901, 828.3928, 831.4666, 832.3149, 834.3199, 837.3761, 842.4860, 845.4138, 851.3882, 871.4900, 876.2846, 878.2907, 883.4759, 891.4818, 893.9534, 898.4351, 910.4221, 913.5131, 918.5399, 929.4545, 935.4750, 937.4921, 947.4755, 959.4836, 961.4837, 963.4760, 967.4271, 969.4589, 972.4794, 974.4445, 976.4506, 977.4767, 978.5352, 981.4952, 983.4747, 985.6076, 988.4585, 989.6082, 997.4586, 1007.5676, 1011.4840, 1014.5074, 1018.5203, 1019.5441, 1021.5421, 1034.5779, 1042.5320, 1044.5242, 1060.5463, 1071.5376, 1077.5208, 1087.5399, 1091.5421, 1106.5393, 1122.5577, 1156.5372, 1173.6240, 1187.6439, 1215.6615, 1244.6189, 1248.6575, 1255.6039, 1276.6218, 1284.6401, 1295.7195, 1301.6177, 1313.7069, 1314.7141, 1315.6759, 1325.6675, 1336.7151, 1338.6461, 1342.6758, 1344.7024, 1349.6952, 1353.6804, 1355.7097, 1356.7201, 1361.6877, 1365.6632, 1370.6783, 1372.6058, 1381.7588, 1383.7244, 1386.7131, 1394.7318, 1397.7419, 1414.6467, 1427.7865, 1432.7140, 1446.7247, 1457.7411, 1461.7784, 1473.8055, 1478.7513, 1497.8757, 1528.6993, 1542.8459, 1556.8795, 1578.7826, 1601.9006, 1606.8433, 1616.8177, 1618.8162, 1628.7026, 1628.8568, 1642.7186, 1647.8694, 1650.8473, 1658.8400, 1677.8625, 1680.8728, 1696.8231, 1703.8551, 1704.8862, 1710.9269, 1715.8459, 1717.8810, 1718.9111, 1727.9301, 1730.8835, 1742.9099, 1751.8870, 1768.9546, 1769.9617, 1771.0243, 1775.0276, 1777.8676, 1786.9032, 1791.9047, 1799.9005, 1800.9124, 1915.0015, 1928.0238, 1953.9285, 1982.0009, 2012.9681, 2025.0820, 2027.1273, 2042.0642, 2052.9939, 2060.0461, 2067.0017, 2070.8899, 2074.9629, 2082.9421, 2084.9102, 2096.9797, 2100.9070, 2109.0410, 2116.0894, 2136.9121, 2155.0730, 2170.0781, 2195.0801, 2211.0686, 2238.1072, 2251.1428, 2293.1614, 2336.2495, 2343.1758, 2382.1987, 2385.2234, 2400.0720, 2414.0930, 2418.0901, 2428.1140, 2431.0991, 2465.2188, 2468.2249, 2528.1794, 2542.2344, 2560.2729, 2564.2610, 2646.9797, 2660.9968, 2675.0183, 2698.3711, 2707.3184, 2711.3606, 2712.3755, 2719.3159, 2729.3870, 2744.3770, 2762.3970, 2767.3120, 2812.4265, 2846.3943, 2871.4121, 2872.3569, 2884.3745, 2888.3184, 2892.4063, 2893.4375, 2894.4741, 2917.3960, 2926.4563, 2992.4536, 2995.4939, 3004.4705, 3005.5735, 3027.5032, 3054.4763, 3082.5879, 3088.5859, 3104.5430, 3126.7307, 3200.5081, 3201.4670, 3206.5745, 3242.6213, 3248.6428, 3255.6025, 3263.6973, 3300.7517, 3315.7021, 3328.7302, 3331.6746, 3334.7031, 3335.7090, 3347.5835, 3353.6013, 3356.7166, 3370.7415, 3386.7366, 3456.7622, 3471.7375, 3487.7676, 3491.6787, 3503.8286, 3601.7178, 3639.7463, 3646.7891, 3651.8442, 3703.9653, 3716.8708, 3720.7805, 3764.8616, 3820.9673, 3885.7913, 3887.9063, 3927.8738, 4148.0005, 4162.0400, 4452.2729, 4482.1689, 4680.3301, 4845.1597

37. E1B860 **Mass:** 166355 **Score:** 19 **Expect:** 2.8e+002 **Matches:** 26

Uncharacterized protein OS=Bos taurus GN=IFT140 PE=4 SV=1

Observed	Mr(expt)	Mr(calc)	ppm	Start	End	Miss	Peptide
744.3938	743.3865	743.4038	-23.27	132 -	137	1	R.LDQRGR.V
806.4307	805.4234	805.4269	-4.32	348 -	353	1	R.VAMWRK.A + Oxidation (M)

891.4818	890.4745	890.4545	22.5	346	352	1	K.GRVAMWR.K + Oxidation (M)
913.5131	912.5058	912.5215	-17.16	138	145	0	R.VQGMPLLR.H
959.4836	958.4763	958.4872	-11.40	455	463	0	K.DAVAVWNGK.Q
1019.5441	1018.5368	1018.5494	-12.38	346	353	2	K.GRVAMWRK.A + Oxidation (M)
1021.5421	1020.5348	1020.5141	20.3	1033	1040	0	R.QAVHFYTR.A
1060.5463	1059.5390	1059.5349	3.91	1308	1317	0	K.AHGALTEAYK.C
1244.6189	1243.6116	1243.5914	16.3	552	561	2	R.REAKVHCGCK.S
1717.8810	1716.8737	1716.9093	-20.75	337	352	2	K.GLLAAGTDKGRVAMWR.K + Oxidation (M)
2012.9681	2011.9608	2011.9435	8.62	900	914	2	R.HDRMHLRTTYYNYAR.H + Oxidation (M)
2042.0642	2041.0569	2041.0341	11.2	669	688	1	R.EVPGAPPQPADRNALAEGPR.Q
2116.0894	2115.0821	2115.1034	-10.06	369	386	1	K.WTLQAPTELEGNIMQIKK.W + Oxidation (M)
2155.0730	2154.0657	2154.0242	19.3	1024	1040	1	R.QYESQNEVRQAVHFYTR.A
2293.1614	2292.1541	2292.1645	-4.51	1361	1380	1	K.GSVRQCQLLLEEPHLDSGVR.V
2465.2188	2464.2115	2464.2025	3.67	1195	1215	1	R.VLLEQIASCCMRQGSYHLATK.K
2711.3606	2710.3533	2710.3353	6.66	854	876	2	R.VAMLAVQLGMLEDAEQLYRRCGR.Y + 2 Oxidation (M)
2719.3159	2718.3086	2718.2966	4.44	302	323	2	R.FWDLERGENYVLSPEEKFGFEK.G
2729.3870	2728.3797	2728.4408	-22.40	877	899	2	R.YDLLNRLLQASGQWQRAIEVAER.H
3054.4763	3053.4690	3053.5242	-18.07	779	804	2	R.DAMINFSFFLTIGDMDEAFRSIKLIK.S + 2 Oxidation (M)
3082.5879	3081.5806	3081.5156	21.1	635	660	2	K.KSQALEDERLTDLVPVSHFWDQSEPR.L
3200.5081	3199.5008	3199.5469	-14.40	94	123	0	K.EQHAVPATHTADIAILAWSPNGNCLVSGDR.L
3201.4670	3200.4597	3200.5151	-17.31	1050	1077	1	R.LCKEHGLDDQLMNLALLSSPEDMLEAAR.Y + 2 Oxidation (M)
3242.6213	3241.6140	3241.5556	18.0	1157	1183	1	K.YQEALQLCLEQNLTITEEMAEKMTVPK.D + 2 Oxidation (M)
3370.7415	3369.7342	3369.6506	24.8	1156	1183	2	K.KYQEALQLCLEQNLTITEEMAEKMTVPK.D + 2 Oxidation (M)
3646.7891	3645.7818	3645.8614	-21.82	443	476	2	R.TDMHVSGVFATKDAVAVWNGKQVVIFEPSGATLR.S + Oxidation (M)

No match to: 706.2814, 707.2933, 713.3626, 717.4103, 719.4172, 722.4265, 735.4174, 756.4689, 768.3713, 772.4224, 779.4717, 792.4133, 804.3155, 807.4160, 809.4363, 812.3947, 814.4097, 815.4186, 819.3901, 828.3928, 831.4666, 832.3149, 834.3199, 837.3761, 841.4355, 842.4860, 845.4138, 851.3882, 871.4900, 876.2846, 878.2907, 883.4759, 893.9534, 894.4574, 898.4351, 910.4221, 918.5399, 929.4545, 935.4750, 937.4921, 947.4755, 961.4837, 963.4760, 967.4271, 969.4589, 972.4794, 974.4445, 976.4506, 977.4767, 978.5352, 981.4952, 983.4747, 985.6076, 988.4585, 989.6082, 997.4586, 1007.5676, 1011.4840, 1014.5074, 1018.5203, 1034.5779, 1042.5320, 1044.5242, 1071.5376, 1077.5208, 1087.5399, 1091.5421, 1106.5393, 1122.5577, 1156.5372, 1173.6240, 1187.6439, 1199.6992, 1215.6615, 1248.6575, 1255.6039, 1276.6218, 1284.6401, 1295.7195, 1301.6177, 1313.7069, 1314.7141, 1315.6759, 1325.6675, 1336.7151, 1337.7180, 1338.6461, 1342.6758, 1344.7024, 1349.6952, 1353.6804, 1355.7097, 1356.7201, 1361.6877, 1365.6632, 1370.6783, 1372.6058, 1381.7588, 1383.7244, 1386.7131, 1394.7318, 1397.7419, 1414.6467, 1427.7865, 1432.7140, 1446.7247, 1457.7411, 1461.7784, 1473.8055, 1478.7513, 1497.8757, 1528.6993, 1542.8459, 1556.8795, 1578.7826, 1601.9006, 1606.8433, 1616.8177, 1618.8162, 1628.7026, 1628.8568, 1642.7186, 1647.8694, 1650.8473, 1658.8400, 1677.8625, 1680.8728, 1696.8231, 1703.8551, 1704.8862, 1710.9269, 1715.8459, 1718.9111, 1725.8386, 1727.9301, 1730.8835, 1742.9099, 1751.8870, 1768.9546, 1769.9617, 1771.0243, 1775.0276, 1777.8676, 1786.9032, 1791.9047, 1799.9005, 1800.9124, 1915.0015, 1928.0238, 1953.9285, 1982.0009, 2025.0820, 2027.1273, 2052.9939, 2060.0461, 2067.0017, 2070.8899, 2074.9629, 2082.9421, 2084.9102, 2086.9065, 2096.9797, 2100.9070, 2109.0410, 2136.9121, 2170.0781, 2195.0801, 2211.0686, 2238.1072, 2251.1428, 2336.2495, 2343.1758, 2382.1987, 2385.2234, 2400.0720, 2414.0930, 2418.0901, 2428.1140, 2431.0991, 2468.2249, 2528.1794, 2542.2344, 2560.2729, 2564.2610, 2646.9797, 2660.9968, 2675.0183, 2698.3711, 2707.3184, 2712.3755, 2744.3770, 2762.3970, 2767.3120, 2812.4265, 2846.3943, 2871.4121, 2872.3569, 2873.3445, 2884.3745, 2888.3184, 2892.4063, 2893.4375, 2894.4741, 2917.3960, 2926.4563, 2992.4536, 2995.4939, 3004.4705, 3005.5735, 3027.5032, 3088.5859, 3104.5430, 3126.7307, 3170.6697, 3206.5745, 3207.6025, 3248.6428, 3255.6025, 3263.6973, 3300.7517, 3315.7021, 3328.7302, 3331.6746, 3334.7031, 3335.7090, 3347.5835, 3353.6013, 3356.7166, 3386.7366, 3456.7622, 3471.7375, 3487.7676, 3491.6787, 3503.8286, 3601.7178, 3639.7463, 3651.8442, 3703.9653, 3716.8708, 3720.7805, 3764.8616, 3820.9673, 3869.8423, 3885.7913, 3887.9063, 3927.8738, 4148.0005, 4162.0400, 4253.0234, 4452.2729, 4464.2598, 4468.1572, 4482.1689, 4680.3301, 4845.1597

38. Q2KHX7 **Mass:** 69190 **Score:** 19 **Expect:** 2.8e+002 **Matches:** 15

RNA guanylyltransferase and 5'-phosphatase OS=Bos taurus GN=RNGTT PE=2 SV=1

Observed	Mr(expt)	Mr(calc)	ppm	Start	End	Miss	Peptide
845.4138	844.4065	844.4014	6.04	10	15	0	R.WLNCPR.R
891.4818	890.4745	890.4861	-13.11	424	431	0	K.LLEGNFAK.E
963.4760	962.4687	962.4821	-14.00	404	411	0	K.TQEPFSVR.H
1019.5441	1018.5368	1018.5196	16.9	291	299	1	K.VSWKADGTR.Y
1315.6759	1314.6686	1314.6965	-21.24	393	403	2	R.HEKMKTGLIDK.T + Oxidation (M)
1338.6461	1337.6388	1337.6211	13.2	209	221	1	K.ESEPGSSASFGKR.R
1394.7318	1393.7245	1393.7500	-18.25	387	397	2	R.EIINPRHEKMK.T
1397.7419	1396.7346	1396.7425	-5.61	133	144	0	R.TGFLICAFLVEK.M
1680.8728	1679.8655	1679.8705	-2.95	45	58	1	R.FHPSMLSNYLKSLK.V + Oxidation (M)
2528.1794	2527.1721	2527.1801	-3.17	35	55	1	R.YDSQVAEENRFHPSMLSNYLK.S
2711.3606	2710.3533	2710.3531	0.10	335	358	1	R.MHLSNTLLDGEMIIDRVNGQAVPR.Y + 2 Oxidation (M)
2872.3569	2871.3496	2871.3861	-12.70	35	58	2	R.YDSQVAEENRFHPSMLSNYLKSLK.V + Oxidation (M)
3716.8708	3715.8635	3715.9429	-21.37	335	366	2	R.MHLSNTLLDGEMIIDRVNGQAVPRYLIYDIIK.F + Oxidation (M)
4253.0234	4252.0161	4251.9816	8.11	295	330	2	K.ADGTRYMMLIDGTNEVFMIDRDNSVFHVSNLEFPFR.K + Oxidation (M)
4468.1572	4467.1499	4467.0756	16.6	534	573	2	K.SFPNAYNTAMAVCNSISNPVTKEMLFEFIDRCAAASQGQK.R

No match to: 706.2814, 707.2933, 713.3626, 717.4103, 719.4172, 722.4265, 735.4174, 744.3938, 756.4689, 768.3713, 772.4224, 779.4717, 792.4133, 804.3155, 806.4307, 807.4160, 809.4363, 812.3947, 814.4097, 815.4186, 819.3901, 828.3928, 831.4666, 832.3149, 834.3199, 837.3761, 841.4355, 842.4860, 851.3882, 871.4900, 876.2846, 878.2907, 883.4759, 893.9534, 894.4574, 898.4351, 910.4221, 913.5131, 918.5399, 929.4545, 935.4750, 937.4921, 947.4755, 959.4836, 961.4837, 967.4271, 969.4589, 972.4794, 974.4445, 976.4506, 977.4767, 978.5352, 981.4952, 983.4747, 985.6076, 988.4585, 989.6082, 997.4586, 1007.5676, 1011.4840, 1014.5074, 1018.5203, 1021.5421, 1034.5779, 1042.5320, 1044.5242, 1060.5463, 1071.5376, 1077.5208, 1087.5399, 1091.5421, 1106.5393, 1122.5577, 1156.5372, 1173.6240, 1187.6439, 1199.6992, 1215.6615, 1244.6189, 1248.6575, 1255.6039, 1276.6218, 1284.6401, 1295.7195, 1301.6177, 1313.7069, 1314.7141, 1325.6675, 1336.7151, 1337.7180, 1342.6758, 1344.7024, 1349.6952, 1353.6804, 1355.7097, 1356.7201, 1361.6877, 1365.6632, 1370.6783, 1372.6058, 1381.7588, 1383.7244, 1386.7131, 1414.6467, 1427.7865, 1432.7140, 1446.7247, 1457.7411, 1461.7784, 1473.8055, 1478.7513, 1497.8757, 1528.6993, 1542.8459, 1556.8795, 1578.7826, 1601.9006, 1606.8433, 1616.8177, 1618.8162, 1628.7026, 1628.8568, 1642.7186, 1647.8694, 1650.8473, 1658.8400, 1677.8625, 1696.8231, 1703.8551, 1704.8862, 1710.9269, 1715.8459, 1717.8810, 1718.9111, 1725.8386, 1727.9301, 1730.8835, 1742.9099, 1751.8870, 1768.9546, 1769.9617, 1771.0243, 1775.0276, 1777.8676, 1786.9032, 1791.9047, 1799.9005, 1800.9124, 1915.0015, 1928.0238, 1953.9285, 1982.0009, 2012.9681, 2025.0820, 2027.1273, 2042.0642, 2052.9939, 2060.0461, 2067.0017, 2070.8899, 2074.9629, 2082.9421, 2084.9102, 2086.9065, 2096.9797, 2100.9070, 2109.0410, 2116.0894, 2136.9121, 2155.0730, 2170.0781, 2195.0801, 2211.0686, 2238.1072, 2251.1428, 2293.1614, 2336.2495, 2343.1758, 2382.1987, 2385.2234, 2400.0720, 2414.0930, 2418.0901, 2428.1140, 2431.0991, 2465.2188, 2468.2249, 2542.2344, 2560.2729, 2564.2610, 2646.9797, 2660.9968, 2675.0183, 2698.3711, 2707.3184, 2712.3755, 2719.3159, 2729.3870, 2744.3770, 2762.3970, 2767.3120, 2812.4265, 2846.3943, 2871.4121, 2873.3445, 2884.3745, 2888.3184, 2892.4063, 2893.4375, 2894.4741, 2917.3960, 2926.4563, 2992.4536, 2995.4939, 3004.4705, 3005.5735, 3027.5032, 3054.4763, 3082.5879, 3088.5859, 3104.5430, 3126.7307, 3170.6697, 3200.5081, 3201.4670, 3206.5745, 3207.6025, 3242.6213, 3248.6428, 3255.6025, 3263.6973, 3300.7517, 3315.7021, 3328.7302, 3331.6746, 3334.7031, 3335.7090, 3347.5835, 3353.6013, 3356.7166, 3370.7415, 3386.7366, 3456.7622, 3471.7375, 3487.7676, 3491.6787, 3503.8286, 3601.7178, 3639.7463, 3646.7891, 3651.8442, 3703.9653, 3720.7805, 3764.8616, 3820.9673, 3869.8423, 3885.7913, 3887.9063, 3927.8738, 4148.0005, 4162.0400, 4452.2729, 4464.2598, 4482.1689, 4680.3301, 4845.1597

39. Q0P5C5 **Mass:** 45559 **Score:** 19 **Expect:** 2.9e+002 **Matches:** 11

SET and MYND domain containing 3 OS=Bos taurus GN=SMYD3 PE=2 SV=1

Observed	Mr(expt)	Mr(calc)	ppm	Start	End	Miss	Peptide
837.3761	836.3688	836.3884	-23.44	86	91	1	R.ECKCLK.S

947.4755	946.4682	946.4793	-11.79	1 -	8	1	-.MEPSKVEK.F
963.4760	962.4687	962.4743	-5.80	1 -	8	1	-.MEPSKVEK.F + Oxidation (M)
1248.6575	1247.6502	1247.6808	-24.53	355 -	364	1	K.NLRLAFDIMR.V
1276.6218	1275.6145	1275.6428	-22.15	46 -	56	1	R.GVVCDRCLLGK.E
1751.8870	1750.8797	1750.8811	-0.80	108 -	122	1	R.VVFKLMQETPSESEK.L
1928.0238	1927.0165	1926.9920	12.7	342 -	357	1	K.LQLHQGMFPQAMKNLR.L + Oxidation (M)
2067.0017	2065.9944	2066.0215	-13.11	33 -	51	2	R.SDPLAYTVSKGSRGVVCDR.C
2382.1987	2381.1914	2381.1427	20.5	157 -	177	0	R.EEIQDASQLPPSFDIFEAFAK.V
3242.6213	3241.6140	3241.6012	3.96	256 -	282	2	K.IEDLKAHWKWEQVLAMCQSIISSNAER.L
3335.7090	3334.7017	3334.6689	9.83	265 -	292	1	K.WEQVLAMCQSIISSNAERLPDINIYQLK.V + Oxidation (M)

No match to: 706.2814, 707.2933, 713.3626, 717.4103, 719.4172, 722.4265, 735.4174, 744.3938, 756.4689, 768.3713, 772.4224, 779.4717, 792.4133, 804.3155, 806.4307, 807.4160, 809.4363, 812.3947, 814.4097, 815.4186, 819.3901, 828.3928, 831.4666, 832.3149, 834.3199, 841.4355, 842.4860, 845.4138, 851.3882, 871.4900, 876.2846, 878.2907, 883.4759, 891.4818, 893.9534, 894.4574, 898.4351, 910.4221, 913.5131, 918.5399, 929.4545, 935.4750, 937.4921, 959.4836, 961.4837, 967.4271, 969.4589, 972.4794, 974.4445, 976.4506, 977.4767, 978.5352, 981.4952, 983.4747, 985.6076, 988.4585, 989.6082, 997.4586, 1007.5676, 1011.4840, 1014.5074, 1018.5203, 1019.5441, 1021.5421, 1034.5779, 1042.5320, 1044.5242, 1060.5463, 1071.5376, 1077.5208, 1087.5399, 1091.5421, 1106.5393, 1122.5577, 1156.5372, 1173.6240, 1187.6439, 1199.6992, 1215.6615, 1244.6189, 1255.6039, 1284.6401, 1295.7195, 1301.6177, 1313.7069, 1314.7141, 1315.6759, 1325.6675, 1336.7151, 1337.7180, 1338.6461, 1342.6758, 1344.7024, 1349.6952, 1353.6804, 1355.7097, 1356.7201, 1361.6877, 1365.6632, 1370.6783, 1372.6058, 1381.7588, 1383.7244, 1386.7131, 1394.7318, 1397.7419, 1414.6467, 1427.7865, 1432.7140, 1446.7247, 1457.7411, 1461.7784, 1473.8055, 1478.7513, 1497.8757, 1528.6993, 1542.8459, 1556.8795, 1578.7826, 1601.9006, 1606.8433, 1616.8177, 1618.8162, 1628.7026, 1628.8568, 1642.7186, 1647.8694, 1650.8473, 1658.8400, 1677.8625, 1680.8728, 1696.8231, 1703.8551, 1704.8862, 1710.9269, 1715.8459, 1717.8810, 1718.9111, 1725.8386, 1727.9301, 1730.8835, 1742.9099, 1768.9546, 1769.9617, 1771.0243, 1775.0276, 1777.8676, 1786.9032, 1791.9047, 1799.9005, 1800.9124, 1915.0015, 1953.9285, 1982.0009, 2012.9681, 2025.0820, 2027.1273, 2042.0642, 2052.9939, 2060.0461, 2070.8899, 2074.9629, 2082.9421, 2084.9102, 2086.9065, 2096.9797, 2100.9070, 2109.0410, 2116.0894, 2136.9121, 2155.0730, 2170.0781, 2195.0801, 2211.0686, 2238.1072, 2251.1428, 2293.1614, 2336.2495, 2343.1758, 2385.2234, 2400.0720, 2414.0930, 2418.0901, 2428.1140, 2431.0991, 2465.2188, 2468.2249, 2528.1794, 2542.2344, 2560.2729, 2564.2610, 2646.9797, 2660.9968, 2675.0183, 2698.3711, 2707.3184, 2711.3606, 2712.3755, 2719.3159, 2729.3870, 2744.3770, 2762.3970, 2767.3120, 2812.4265, 2846.3943, 2871.4121, 2872.3569, 2873.3445, 2884.3745, 2888.3184, 2892.4063, 2893.4375, 2894.4741, 2917.3960, 2926.4563, 2992.4536, 2995.4939, 3004.4705, 3005.5735, 3027.5032, 3054.4763, 3082.5879, 3088.5859, 3104.5430, 3126.7307, 3170.6697, 3200.5081, 3201.4670, 3206.5745, 3207.6025, 3248.6428, 3255.6025, 3263.6973, 3300.7517, 3315.7021, 3328.7302, 3331.6746, 3334.7031, 3347.5835, 3353.6013, 3356.7166, 3370.7415, 3386.7366, 3456.7622, 3471.7375, 3487.7676, 3491.6787, 3503.8286, 3601.7178, 3639.7463, 3646.7891, 3651.8442, 3703.9653, 3716.8708, 3720.7805, 3764.8616, 3820.9673, 3869.8423, 3885.7913, 3887.9063, 3927.8738, 4148.0005, 4162.0400, 4253.0234, 4452.2729, 4464.2598, 4468.1572, 4482.1689, 4680.3301, 4845.1597

40. Q0VC24 **Mass:** 48192 **Score:** 19 **Expect:** 3e+002 **Matches:** 9

Ribosome biogenesis protein WDR12 OS=Bos taurus GN=WDR12 PE=2 SV=1

Observed	Mr(expt)	Mr(calc)	ppm	Start	End	Miss	Peptide
717.4103	716.4030	716.3857	24.1	145 -	150	0	K.DVAWVK.K
1342.6758	1341.6685	1341.6564	9.00	377 -	388	0	K.APLYDLAAHEDK.V
1727.9301	1726.9228	1726.8825	23.4	1 -	14	2	-.MAQLQTRFFTDNKK.Y
2170.0781	2169.0708	2169.0524	8.47	350 -	368	0	K.WSPTHEQQLISGSLDNMVK.L
2729.3870	2728.3797	2728.3708	3.27	389 -	413	1	K.VLSVDWTDSGLLLSGGADNKLYSYR.Y
2995.4939	2994.4866	2994.4467	13.3	152 -	176	0	K.DSLSCLLLSASMDQTILLWEWNVER.N + Oxidation (M)
3054.4763	3053.4690	3053.4613	2.53	64 -	88	1	R.MPLFKHMELENISSEEVVELEYVEK.Y + 2 Oxidation (M)
3370.7415	3369.7342	3369.6729	18.2	377 -	408	1	K.APLYDLAAHEDKVLSVDWTDSGLLLSGGADNK.L
3639.7463	3638.7390	3638.8000	-16.76	59 -	88	2	K.GQFLRMPLFKHMELENISSEEVVELEYVEK.Y + Oxidation (M)

No match to: 706.2814, 707.2933, 713.3626, 719.4172, 722.4265, 735.4174, 744.3938, 756.4689, 768.3713, 772.4224, 779.4717, 792.4133, 804.3155, 806.4307, 807.4160, 809.4363, 812.3947, 814.4097, 815.4186, 819.3901, 828.3928, 831.4666, 832.3149, 834.3199, 837.3761, 841.4355, 842.4860, 845.4138, 851.3882, 871.4900, 876.2846, 878.2907, 883.4759, 891.4818, 893.9534, 894.4574, 898.4351, 910.4221, 913.5131, 918.5399, 929.4545, 935.4750, 937.4921, 947.4755, 959.4836, 961.4837, 963.4760, 967.4271, 969.4589, 972.4794, 974.4445, 976.4506, 977.4767, 978.5352, 981.4952, 983.4747, 985.6076, 988.4585, 989.6082, 997.4586, 1007.5676, 1011.4840, 1014.5074, 1018.5203, 1019.5441, 1021.5421, 1034.5779, 1042.5320, 1044.5242, 1060.5463, 1071.5376, 1077.5208, 1087.5399, 1091.5421, 1106.5393, 1122.5577, 1156.5372, 1173.6240, 1187.6439, 1199.6992, 1215.6615, 1244.6189, 1248.6575, 1255.6039, 1276.6218, 1284.6401, 1295.7195, 1301.6177, 1313.7069, 1314.7141, 1315.6759, 1325.6675, 1336.7151, 1337.7180, 1338.6461, 1344.7024, 1349.6952, 1353.6804, 1355.7097, 1356.7201, 1361.6877, 1365.6632, 1370.6783, 1372.6058, 1381.7588, 1383.7244, 1386.7131, 1394.7318, 1397.7419, 1414.6467, 1427.7865, 1432.7140, 1446.7247, 1457.7411, 1461.7784, 1473.8055, 1478.7513, 1497.8757, 1528.6993, 1542.8459, 1556.8795, 1578.7826, 1601.9006, 1606.8433, 1616.8177, 1618.8162, 1628.7026, 1628.8568, 1642.7186, 1647.8694, 1650.8473, 1658.8400, 1677.8625, 1680.8728, 1696.8231, 1703.8551, 1704.8862, 1710.9269, 1715.8459, 1717.8810, 1718.9111, 1725.8386, 1730.8835, 1742.9099, 1751.8870, 1768.9546, 1769.9617, 1771.0243, 1775.0276, 1777.8676, 1786.9032, 1791.9047, 1799.9005, 1800.9124, 1915.0015, 1928.0238, 1953.9285, 1982.0009, 2012.9681, 2025.0820, 2027.1273, 2042.0642, 2052.9939, 2060.0461, 2067.0017, 2070.8899, 2074.9629, 2082.9421, 2084.9102, 2086.9065, 2096.9797, 2100.9070, 2109.0410, 2116.0894, 2136.9121, 2155.0730, 2195.0801, 2211.0686, 2238.1072, 2251.1428, 2293.1614, 2336.2495, 2343.1758, 2382.1987, 2385.2234, 2400.0720, 2414.0930, 2418.0901, 2428.1140, 2431.0991, 2465.2188, 2468.2249, 2528.1794, 2542.2344, 2560.2729, 2564.2610, 2646.9797, 2660.9968, 2675.0183, 2698.3711, 2707.3184, 2711.3606, 2712.3755, 2719.3159, 2744.3770, 2762.3970, 2767.3120, 2812.4265, 2846.3943, 2871.4121, 2872.3569, 2873.3445, 2884.3745, 2888.3184, 2892.4063, 2893.4375, 2894.4741, 2917.3960, 2926.4563, 2992.4536, 3004.4705, 3005.5735, 3027.5032, 3082.5879, 3088.5859, 3104.5430, 3126.7307, 3170.6697, 3200.5081, 3201.4670, 3206.5745, 3207.6025, 3242.6213, 3248.6428, 3255.6025, 3263.6973, 3300.7517, 3315.7021, 3328.7302, 3331.6746, 3334.7031, 3335.7090, 3347.5835, 3353.6013, 3356.7166, 3386.7366, 3456.7622, 3471.7375, 3487.7676, 3491.6787, 3503.8286, 3601.7178, 3646.7891, 3651.8442, 3703.9653, 3716.8708, 3720.7805, 3764.8616, 3820.9673, 3869.8423, 3885.7913, 3887.9063, 3927.8738, 4148.0005, 4162.0400, 4253.0234, 4452.2729, 4464.2598, 4468.1572, 4482.1689, 4680.3301, 4845.1597

41. A5D7J9 **Mass:** 59644 **Score:** 19 **Expect:** 3e+002 **Matches:** 20

CCDC9 protein OS=Bos taurus GN=CCDC9 PE=2 SV=1

Observed	Mr(expt)	Mr(calc)	ppm	Start	End	Miss	Peptide
717.4103	716.4030	716.4068	-5.34	2 -	8	0	M.SAALDLK.S
871.4900	870.4827	870.5035	-23.89	28 -	34	1	K.NEALIRR.Y
963.4760	962.4687	962.4457	23.8	150 -	156	1	K.SKEWEER.R
981.4952	980.4880	980.5079	-20.37	259 -	265	1	R.SEYLRWK.Q
1091.5421	1090.5348	1090.5407	-5.37	149 -	156	2	R.KSKEWEER.R
1248.6575	1247.6502	1247.6258	19.5	301 -	312	0	K.DGPAVALEPSHR.Y
1284.6401	1283.6328	1283.6106	17.3	199 -	209	1	R.SGPLEEPERDR.R
1314.7141	1313.7068	1313.7051	1.29	269 -	278	2	R.EKIDQERLQR.H
1325.6675	1324.6602	1324.6847	-18.49	197 -	207	2	R.RRSGPLEEPER.D
1349.6952	1348.6879	1348.6782	7.18	93 -	105	1	R.IPPQHGGRAGMGR.T + Oxidation (M)
1365.6632	1364.6559	1364.6684	-9.14	34 -	43	2	R.RYQEIEEDRK.K
1394.7318	1393.7245	1393.7102	10.3	259 -	268	2	R.SEYLRWKQER.E
1616.8177	1615.8104	1615.8430	-20.18	329 -	341	2	R.EFLSQHKTEVSRR.K
1628.7026	1627.6953	1627.7300	-21.33	288 -	300	2	R.REWDAEKTDGMFK.D + Oxidation (M)
1677.8625	1676.8552	1676.8641	-5.31	93 -	108	2	R.IPPQHGGRAGMGRTSR.S
1786.9032	1785.8959	1785.8910	2.74	281 -	294	2	R.KPTGQWRREWDAEK.T
2238.1072	2237.0999	2237.0860	6.22	364 -	383	1	R.WEMKEAMSTAPEPPQPALPK.E
2428.1140	2427.1067	2427.1635	-23.37	241 -	263	2	R.AGLGGAGDMTLSMTGRERSEYLR.W
3386.7366	3385.7293	3385.6956	9.96	313 -	340	2	R.YDDQAWARPPKPPTFREFLSQHKTEVSR.R

3869.8423 3868.8350 3868.8744 -10.17 295 - 328 2 K.TDGMFKDGPAVALEPSHRYDDQAWARPPKPPTFR.E + Oxidation (M)

No match to: 706.2814, 707.2933, 713.3626, 719.4172, 722.4265, 735.4174, 744.3938, 756.4689, 768.3713, 772.4224, 779.4717, 792.4133, 804.3155, 806.4307, 807.4160, 809.4363, 812.3947, 814.4097, 815.4186, 819.3901, 828.3928, 831.4666, 832.3149, 834.3199, 837.3761, 841.4355, 842.4860, 845.4138, 851.3882, 876.2846, 878.2907, 883.4759, 891.4818, 893.9534, 894.4574, 898.4351, 910.4221, 913.5131, 918.5399, 929.4545, 935.4750, 937.4921, 947.4755, 959.4836, 961.4837, 967.4271, 969.4589, 972.4794, 974.4445, 976.4506, 977.4767, 978.5352, 983.4747, 985.6076, 988.4585, 989.6082, 997.4586, 1007.5676, 1011.4840, 1014.5074, 1018.5203, 1019.5441, 1021.5421, 1034.5779, 1042.5320, 1044.5242, 1060.5463, 1071.5376, 1077.5208, 1087.5399, 1106.5393, 1122.5577, 1156.5372, 1173.6240, 1187.6439, 1199.6992, 1215.6615, 1244.6189, 1255.6039, 1276.6218, 1295.7195, 1301.6177, 1313.7069, 1315.6759, 1336.7151, 1337.7180, 1338.6461, 1342.6758, 1344.7024, 1353.6804, 1355.7097, 1356.7201, 1361.6877, 1370.6783, 1372.6058, 1381.7588, 1383.7244, 1386.7131, 1397.7419, 1414.6467, 1427.7865, 1432.7140, 1446.7247, 1457.7411, 1461.7784, 1473.8055, 1478.7513, 1497.8757, 1528.6993, 1542.8459, 1556.8795, 1578.7826, 1601.9006, 1606.8433, 1618.8162, 1628.8568, 1642.7186, 1647.8694, 1650.8473, 1658.8400, 1680.8728, 1696.8231, 1703.8551, 1704.8862, 1710.9269, 1715.8459, 1717.8810, 1718.9111, 1725.8386, 1727.9301, 1730.8835, 1742.9099, 1751.8870, 1768.9546, 1769.9617, 1771.0243, 1775.0276, 1777.8676, 1791.9047, 1799.9005, 1800.9124, 1915.0015, 1928.0238, 1953.9285, 1982.0009, 2012.9681, 2025.0820, 2027.1273, 2042.0642, 2052.9939, 2060.0461, 2067.0017, 2070.8899, 2074.9629, 2082.9421, 2084.9102, 2086.9065, 2096.9797, 2100.9070, 2109.0410, 2116.0894, 2136.9121, 2155.0730, 2170.0781, 2195.0801, 2211.0686, 2251.1428, 2293.1614, 2336.2495, 2343.1758, 2382.1987, 2385.2234, 2400.0720, 2414.0930, 2418.0901, 2431.0991, 2465.2188, 2468.2249, 2528.1794, 2542.2344, 2560.2729, 2564.2610, 2646.9797, 2660.9968, 2675.0183, 2698.3711, 2707.3184, 2711.3606, 2712.3755, 2719.3159, 2729.3870, 2744.3770, 2762.3970, 2767.3120, 2812.4265, 2846.3943, 2871.4121, 2872.3569, 2873.3445, 2884.3745, 2888.3184, 2892.4063, 2893.4375, 2894.4741, 2917.3960, 2926.4563, 2992.4536, 2995.4939, 3004.4705, 3005.5735, 3027.5032, 3054.4763, 3082.5879, 3088.5859, 3104.5430, 3126.7307, 3170.6697, 3200.5081, 3201.4670, 3206.5745, 3207.6025, 3242.6213, 3248.6428, 3255.6025, 3263.6973, 3300.7517, 3315.7021, 3328.7302, 3331.6746, 3334.7031, 3335.7090, 3347.5835, 3353.6013, 3356.7166, 3370.7415, 3456.7622, 3471.7375, 3487.7676, 3491.6787, 3503.8286, 3601.7178, 3639.7463, 3646.7891, 3651.8442, 3703.9653, 3716.8708, 3720.7805, 3764.8616, 3820.9673, 3885.7913, 3887.9063, 3927.8738, 4148.0005, 4162.0400, 4253.0234, 4452.2729, 4464.2598, 4468.1572, 4482.1689, 4680.3301, 4845.1597

42. F1N4W7 **Mass:** 30110 **Score:** 19 **Expect:** 3e+002 **Matches:** 10

Uncharacterized protein OS=Bos taurus PE=4 SV=1

Observed	Mr(expt)	Mr(calc)	ppm	Start		End	Miss	Peptide
792.4133	791.4061	791.4211	-19.01	1	-	7	1	-.AMLEGKK.Y + Oxidation (M)
977.4767	976.4694	976.4648	4.75	66	-	73	1	K.EKEVCQGK.V
1427.7865	1426.7792	1426.7820	-1.95	22	-	34	0	K.AQVLWDLGAEVVK.G
2025.0820	2024.0747	2024.0618	6.36	212	-	229	1	K.GLYITPEAYQKLEFPGAK.E
2719.3159	2718.3086	2718.2538	20.2	223	-	244	2	K.LEFPGAKEMANMCRFYQMKPDR.D
2767.3120	2766.3047	2766.2386	23.9	223	-	244	2	K.LEFPGAKEMANMCRFYQMKPDR.D + 3 Oxidation (M)
2872.3569	2871.3496	2871.3037	16.0	230	-	251	2	K.EMANMCRFYQMKPDRDIQLTHR.L + 2 Oxidation (M)
2888.3184	2887.3111	2887.2986	4.34	230	-	251	2	K.EMANMCRFYQMKPDRDIQLTHR.L + 3 Oxidation (M)
2917.3960	2916.3887	2916.3972	-2.90	212	-	236	2	K.GLYITPEAYQKLEFPGAKEMANMCR.F
2926.4563	2925.4490	2925.4661	-5.83	41	-	67	2	K.ASVEAALKGAYGAFLVTNSWDHFSKEK.E

No match to: 706.2814, 707.2933, 713.3626, 717.4103, 719.4172, 722.4265, 735.4174, 744.3938, 756.4689, 768.3713, 772.4224, 779.4717, 804.3155, 806.4307, 807.4160, 809.4363, 812.3947, 814.4097, 815.4186, 819.3901, 828.3928, 831.4666, 832.3149, 834.3199, 837.3761, 841.4355, 842.4860, 845.4138, 851.3882, 871.4900, 876.2846, 878.2907, 883.4759, 891.4818, 893.9534, 894.4574, 898.4351, 910.4221, 913.5131, 918.5399, 929.4545, 935.4750, 937.4921, 947.4755, 959.4836, 961.4837, 963.4760, 967.4271, 969.4589, 972.4794, 974.4445, 976.4506, 978.5352, 981.4952, 983.4747, 985.6076, 988.4585, 989.6082, 997.4586, 1007.5676, 1011.4840, 1014.5074, 1018.5203, 1019.5441, 1021.5421, 1034.5779, 1042.5320, 1044.5242, 1060.5463, 1071.5376, 1077.5208, 1087.5399, 1091.5421, 1106.5393, 1122.5577, 1156.5372, 1173.6240, 1187.6439, 1199.6992, 1215.6615, 1244.6189, 1248.6575, 1255.6039, 1276.6218, 1284.6401, 1295.7195, 1301.6177, 1313.7069, 1314.7141, 1315.6759, 1325.6675, 1336.7151, 1337.7180, 1338.6461, 1342.6758, 1344.7024, 1349.6952, 1353.6804, 1355.7097, 1356.7201, 1361.6877, 1365.6632, 1370.6783, 1372.6058, 1381.7588, 1383.7244, 1386.7131, 1394.7318, 1397.7419, 1414.6467, 1432.7140, 1446.7247, 1457.7411, 1461.7784, 1473.8055, 1478.7513, 1497.8757, 1528.6993, 1542.8459, 1556.8795, 1578.7826, 1601.9006, 1606.8433, 1616.8177, 1618.8162, 1628.7026, 1628.8568, 1642.7186, 1647.8694, 1650.8473, 1658.8400, 1677.8625, 1680.8728, 1696.8231, 1703.8551, 1704.8862, 1710.9269, 1715.8459, 1717.8810, 1718.9111, 1725.8386, 1727.9301, 1730.8835, 1742.9099, 1751.8870, 1768.9546, 1769.9617, 1771.0243, 1775.0276, 1777.8676, 1786.9032, 1791.9047, 1799.9005, 1800.9124, 1915.0015, 1928.0238, 1953.9285, 1982.0009, 2012.9681, 2027.1273, 2042.0642, 2052.9939, 2060.0461, 2067.0017, 2070.8899, 2074.9629, 2082.9421, 2084.9102, 2086.9065, 2096.9797, 2100.9070, 2109.0410, 2116.0894, 2136.9121, 2155.0730, 2170.0781, 2195.0801, 2211.0686, 2238.1072, 2251.1428, 2293.1614, 2336.2495, 2343.1758, 2382.1987, 2385.2234, 2400.0720, 2414.0930, 2418.0901, 2428.1140, 2431.0991, 2465.2188, 2468.2249, 2528.1794, 2542.2344, 2560.2729, 2564.2610, 2646.9797, 2660.9968, 2675.0183, 2698.3711, 2707.3184, 2711.3606, 2712.3755, 2729.3870, 2744.3770, 2762.3970, 2812.4265, 2846.3943, 2871.4121, 2873.3445, 2884.3745, 2892.4063, 2893.4375, 2894.4741, 2992.4536, 2995.4939, 3004.4705, 3005.5735, 3027.5032, 3054.4763, 3082.5879, 3088.5859, 3104.5430, 3126.7307, 3170.6697, 3200.5081, 3201.4670, 3206.5745, 3207.6025, 3242.6213, 3248.6428, 3255.6025, 3263.6973, 3300.7517, 3315.7021, 3328.7302, 3331.6746, 3334.7031, 3335.7090, 3347.5835, 3353.6013, 3356.7166, 3370.7415, 3386.7366, 3456.7622, 3471.7375, 3487.7676, 3491.6787, 3503.8286, 3601.7178, 3639.7463, 3646.7891, 3651.8442, 3703.9653, 3716.8708, 3720.7805, 3764.8616, 3820.9673, 3869.8423, 3885.7913, 3887.9063, 3927.8738, 4148.0005, 4162.0400, 4253.0234, 4452.2729, 4464.2598, 4468.1572, 4482.1689, 4680.3301, 4845.1597

43. Q0IIE0 **Mass:** 17671 **Score:** 19 **Expect:** 3.1e+002 **Matches:** 5

Complexin-3 OS=Bos taurus GN=CPLX3 PE=2 SV=1

Observed	Mr(expt)	Mr(calc)	ppm	Start		End	Miss	Peptide
1349.6952	1348.6879	1348.6847	2.36	59	-	69	2	R.DAQFTQRKAER.A
1727.9301	1726.9228	1726.8849	21.9	139	-	154	2	K.DKAHSTLGDLKQSAEK.C
2762.3970	2761.3897	2761.3341	20.2	80	-	103	2	K.YRLPKNETDESQIQMAGGDVELPR.E + Oxidation (M)
2884.3745	2883.3672	2883.4284	-21.20	82	-	107	2	R.LPKNETDESQIQMAGGDVELPRELAK.M + Oxidation (M)
3601.7178	3600.7105	3600.7716	-16.96	108	-	140	2	K.MIEEDTEEEEERASVLGQLASLPGLDLGSLKDK.A

No match to: 706.2814, 707.2933, 713.3626, 717.4103, 719.4172, 722.4265, 735.4174, 744.3938, 756.4689, 768.3713, 772.4224, 779.4717, 792.4133, 804.3155, 806.4307, 807.4160, 809.4363, 812.3947, 814.4097, 815.4186, 819.3901, 828.3928, 831.4666, 832.3149, 834.3199, 837.3761, 841.4355, 842.4860, 845.4138, 851.3882, 871.4900, 876.2846, 878.2907, 883.4759, 891.4818, 893.9534, 894.4574, 898.4351, 910.4221, 913.5131, 918.5399, 929.4545, 935.4750, 937.4921, 947.4755, 959.4836, 961.4837, 963.4760, 967.4271, 969.4589, 972.4794, 974.4445, 976.4506, 977.4767, 978.5352, 981.4952, 983.4747, 985.6076, 988.4585, 989.6082, 997.4586, 1007.5676, 1011.4840, 1014.5074, 1018.5203, 1019.5441, 1021.5421, 1034.5779, 1042.5320, 1044.5242, 1060.5463, 1071.5376, 1077.5208, 1087.5399, 1091.5421, 1106.5393, 1122.5577, 1156.5372, 1173.6240, 1187.6439, 1199.6992, 1215.6615, 1244.6189, 1248.6575, 1255.6039, 1276.6218, 1284.6401, 1295.7195, 1301.6177, 1313.7069, 1314.7141, 1315.6759, 1325.6675, 1336.7151, 1337.7180, 1338.6461, 1342.6758, 1344.7024, 1353.6804, 1355.7097, 1356.7201, 1361.6877, 1365.6632, 1370.6783, 1372.6058, 1381.7588, 1383.7244, 1386.7131, 1394.7318, 1397.7419, 1414.6467, 1427.7865, 1432.7140, 1446.7247, 1457.7411, 1461.7784, 1473.8055, 1478.7513, 1497.8757, 1528.6993, 1542.8459, 1556.8795, 1578.7826, 1601.9006, 1606.8433, 1616.8177, 1618.8162, 1628.7026, 1628.8568, 1642.7186, 1647.8694, 1650.8473, 1658.8400, 1677.8625, 1680.8728, 1696.8231, 1703.8551, 1704.8862, 1710.9269, 1715.8459, 1717.8810, 1718.9111, 1725.8386, 1730.8835, 1742.9099, 1751.8870, 1768.9546, 1769.9617, 1771.0243, 1775.0276, 1777.8676, 1786.9032, 1791.9047, 1799.9005, 1800.9124, 1915.0015, 1928.0238, 1953.9285, 1982.0009, 2012.9681, 2025.0820, 2027.1273, 2042.0642, 2052.9939, 2060.0461, 2067.0017, 2070.8899, 2074.9629, 2082.9421, 2084.9102, 2086.9065, 2096.9797, 2100.9070, 2109.0410, 2116.0894, 2136.9121, 2155.0730, 2170.0781, 2195.0801, 2211.0686, 2238.1072, 2251.1428, 2293.1614, 2336.2495, 2343.1758, 2382.1987, 2385.2234, 2400.0720, 2414.0930, 2418.0901, 2428.1140, 2431.0991, 2465.2188, 2468.2249, 2528.1794, 2542.2344, 2560.2729, 2564.2610, 2646.9797, 2660.9968, 2675.0183, 2698.3711, 2707.3184, 2711.3606, 2712.3755, 2719.3159, 2729.3870, 2744.3770, 2767.3120, 2812.4265, 2846.3943, 2871.4121, 2872.3569, 2873.3445, 2888.3184, 2892.4063, 2893.4375, 2894.4741, 2917.3960, 2926.4563, 2992.4536, 2995.4939, 3004.4705, 3005.5735, 3027.5032, 3054.4763, 3082.5879, 3088.5859, 3104.5430, 3126.7307, 3170.6697, 3200.5081, 3201.4670, 3206.5745, 3207.6025, 3242.6213, 3248.6428, 3255.6025, 3263.6973, 3300.7517, 3315.7021, 3328.7302, 3331.6746, 3334.7031, 3335.7090, 3347.5835, 3353.6013, 3356.7166, 3370.7415, 3386.7366, 3456.7622, 3471.7375, 3487.7676, 3491.6787, 3503.8286, 3639.7463, 3646.7891, 3651.8442, 3703.9653, 3716.8708, 3720.7805, 3764.8616, 3820.9673, 3869.8423, 3885.7913, 3887.9063, 3927.8738, 4148.0005, 4162.0400, 4253.0234, 4452.2729, 4464.2598, 4468.1572, 4482.1689, 4680.3301, 4845.1597

44. F1MWQ0 **Mass:** 17662 **Score:** 19 **Expect:** 3.1e+002 **Matches:** 5

Complexin-3 OS=Bos taurus GN=CPLX3 PE=4 SV=1

Observed	Mr(expt)	Mr(calc)	ppm	Start	End	Miss	Peptide
1349.6952	1348.6879	1348.6847	2.36	59 -	69	2	R.DAQFTQRKAER.A
1718.9111	1717.9038	1717.8846	11.2	139 -	154	2	K.DKAQSTLGDLKQSAEK.C
2762.3970	2761.3897	2761.3341	20.2	80 -	103	2	K.YRLPKNETDESQIQMAGGDVELPR.E + Oxidation (M)
2884.3745	2883.3672	2883.4284	-21.20	82 -	107	2	R.LPKNETDESQIQMAGGDVELPRELAK.M + Oxidation (M)
3601.7178	3600.7105	3600.7716	-16.96	108 -	140	2	K.MIEEDTEEEEERASVLGQLASLPGLDLGSLKDK.A

No match to: 706.2814, 707.2933, 713.3626, 717.4103, 719.4172, 722.4265, 735.4174, 744.3938, 756.4689, 768.3713, 772.4224, 779.4717, 792.4133, 804.3155, 806.4307, 807.4160, 809.4363, 812.3947, 814.4097, 815.4186, 819.3901, 828.3928, 831.4666, 832.3149, 834.3199, 837.3761, 841.4355, 842.4860, 845.4138, 851.3882, 871.4900, 876.2846, 878.2907, 883.4759, 891.4818, 893.9534, 894.4574, 898.4351, 910.4221, 913.5131, 918.5399, 929.4545, 935.4750, 937.4921, 947.4755, 959.4836, 961.4837, 963.4760, 967.4271, 969.4589, 972.4794, 974.4445, 976.4506, 977.4767, 978.5352, 981.4952, 983.4747, 985.6076, 988.4585, 989.6082, 997.4586, 1007.5676, 1011.4840, 1014.5074, 1018.5203, 1019.5441, 1021.5421, 1034.5779, 1042.5320, 1044.5242, 1060.5463, 1071.5376, 1077.5208, 1087.5399, 1091.5421, 1106.5393, 1122.5577, 1156.5372, 1173.6240, 1187.6439, 1199.6992, 1215.6615, 1244.6189, 1248.6575, 1255.6039, 1276.6218, 1284.6401, 1295.7195, 1301.6177, 1313.7069, 1314.7141, 1315.6759, 1325.6675, 1336.7151, 1337.7180, 1338.6461, 1342.6758, 1344.7024, 1353.6804, 1355.7097, 1356.7201, 1361.6877, 1365.6632, 1370.6783, 1372.6058, 1381.7588, 1383.7244, 1386.7131, 1394.7318, 1397.7419, 1414.6467, 1427.7865, 1432.7140, 1446.7247, 1457.7411, 1461.7784, 1473.8055, 1478.7513, 1497.8757, 1528.6993, 1542.8459, 1556.8795, 1578.7826, 1601.9006, 1606.8433, 1616.8177, 1618.8162, 1628.7026, 1628.8568, 1642.7186, 1647.8694, 1650.8473, 1658.8400, 1677.8625, 1680.8728, 1696.8231, 1703.8551, 1704.8862, 1710.9269, 1715.8459, 1717.8810, 1725.8386, 1727.9301, 1730.8835, 1742.9099, 1751.8870, 1768.9546, 1769.9617, 1771.0243, 1775.0276, 1777.8676, 1786.9032, 1791.9047, 1799.9005, 1800.9124, 1915.0015, 1928.0238, 1953.9285, 1982.0009, 2012.9681, 2025.0820, 2027.1273, 2042.0642, 2052.9939, 2060.0461, 2067.0017, 2070.8899, 2074.9629, 2082.9421, 2084.9102, 2086.9065, 2096.9797, 2100.9070, 2109.0410, 2116.0894, 2136.9121, 2155.0730, 2170.0781, 2195.0801, 2211.0686, 2238.1072, 2251.1428, 2293.1614, 2336.2495, 2343.1758, 2382.1987, 2385.2234, 2400.0720, 2414.0930, 2418.0901, 2428.1140, 2431.0991, 2465.2188, 2468.2249, 2528.1794, 2542.2344, 2560.2729, 2564.2610, 2646.9797, 2660.9968, 2675.0183, 2698.3711, 2707.3184, 2711.3606, 2712.3755, 2719.3159, 2729.3870, 2744.3770, 2767.3120, 2812.4265, 2846.3943, 2871.4121, 2872.3569, 2873.3445, 2888.3184, 2892.4063, 2893.4375, 2894.4741, 2917.3960, 2926.4563, 2992.4536, 2995.4939, 3004.4705, 3005.5735, 3027.5032, 3054.4763, 3082.5879, 3088.5859, 3104.5430, 3126.7307, 3170.6697, 3200.5081, 3201.4670, 3206.5745, 3207.6025, 3242.6213, 3248.6428, 3255.6025, 3263.6973, 3300.7517, 3315.7021, 3328.7302, 3331.6746, 3334.7031, 3335.7090, 3347.5835, 3353.6013, 3356.7166, 3370.7415, 3386.7366, 3456.7622, 3471.7375, 3487.7676, 3491.6787, 3503.8286, 3639.7463, 3646.7891, 3651.8442, 3703.9653, 3716.8708, 3720.7805, 3764.8616, 3820.9673, 3869.8423, 3885.7913, 3887.9063, 3927.8738, 4148.0005, 4162.0400, 4253.0234, 4452.2729, 4464.2598, 4468.1572, 4482.1689, 4680.3301, 4845.1597

45. Q0VCV6 **Mass:** 13614 **Score:** 19 **Expect:** 3.3e+002 **Matches:** 5

ATPase, H+ transporting, lysosomal 13kDa, V1 subunit G2 OS=Bos taurus GN=ATP6V1G2 PE=2 SV=1

Observed	Mr(expt)	Mr(calc)	ppm	Start	End	Miss	Peptide
1276.6218	1275.6145	1275.5990	12.2	80 -	90	0	R.QVQGMQSSQQR.N
1432.7140	1431.7067	1431.7001	4.63	79 -	90	1	R.RQVQGMQSSQQR.N
1628.8568	1627.8495	1627.8529	-2.09	2 -	16	0	M.ASQSQGIQQLLQAEK.R
1982.0009	1980.9936	1980.9574	18.3	33 -	48	2	R.LKQAKEEAQMEVDQYR.R + Oxidation (M)
3005.5735	3004.5662	3004.5851	-6.29	93 -	118	2	R.ERVLAQLLGMVCDVRPQVHPNYRIAA.-

No match to: 706.2814, 707.2933, 713.3626, 717.4103, 719.4172, 722.4265, 735.4174, 744.3938, 756.4689, 768.3713, 772.4224, 779.4717, 792.4133, 804.3155, 806.4307, 807.4160, 809.4363, 812.3947, 814.4097, 815.4186, 819.3901, 828.3928, 831.4666, 832.3149, 834.3199, 837.3761, 841.4355, 842.4860, 845.4138, 851.3882, 871.4900, 876.2846, 878.2907, 883.4759, 891.4818, 893.9534, 894.4574, 898.4351, 910.4221, 913.5131, 918.5399, 929.4545, 935.4750, 937.4921, 947.4755, 959.4836, 961.4837, 963.4760, 967.4271, 969.4589, 972.4794, 974.4445, 976.4506, 977.4767, 978.5352, 981.4952, 983.4747, 985.6076, 988.4585, 989.6082, 997.4586, 1007.5676, 1011.4840, 1014.5074, 1018.5203, 1019.5441, 1021.5421, 1034.5779, 1042.5320, 1044.5242, 1060.5463, 1071.5376, 1077.5208, 1087.5399, 1091.5421, 1106.5393, 1122.5577, 1156.5372, 1173.6240, 1187.6439, 1199.6992, 1215.6615, 1244.6189, 1248.6575, 1255.6039, 1284.6401, 1295.7195, 1301.6177, 1313.7069, 1314.7141, 1315.6759, 1325.6675, 1336.7151, 1337.7180, 1338.6461, 1342.6758, 1344.7024, 1349.6952, 1353.6804, 1355.7097, 1356.7201, 1361.6877, 1365.6632, 1370.6783, 1372.6058, 1381.7588, 1383.7244, 1386.7131, 1394.7318, 1397.7419, 1414.6467, 1427.7865, 1446.7247, 1457.7411, 1461.7784, 1473.8055, 1478.7513, 1497.8757, 1528.6993, 1542.8459, 1556.8795, 1578.7826, 1601.9006, 1606.8433, 1616.8177, 1618.8162, 1628.7026, 1642.7186, 1647.8694, 1650.8473, 1658.8400, 1677.8625, 1680.8728, 1696.8231, 1703.8551, 1704.8862, 1710.9269, 1715.8459, 1717.8810, 1718.9111, 1725.8386, 1727.9301, 1730.8835, 1742.9099, 1751.8870, 1768.9546, 1769.9617, 1771.0243, 1775.0276, 1777.8676, 1786.9032, 1791.9047, 1799.9005, 1800.9124, 1915.0015, 1928.0238, 1953.9285, 2012.9681, 2025.0820, 2027.1273, 2042.0642, 2052.9939, 2060.0461, 2067.0017, 2070.8899, 2074.9629, 2082.9421, 2084.9102, 2086.9065, 2096.9797, 2100.9070, 2109.0410, 2116.0894, 2136.9121, 2155.0730, 2170.0781, 2195.0801, 2211.0686, 2238.1072, 2251.1428, 2293.1614, 2336.2495, 2343.1758, 2382.1987, 2385.2234, 2400.0720, 2414.0930, 2418.0901, 2428.1140, 2431.0991, 2465.2188, 2468.2249, 2528.1794, 2542.2344, 2560.2729, 2564.2610, 2646.9797, 2660.9968, 2675.0183, 2698.3711, 2707.3184, 2711.3606, 2712.3755, 2719.3159, 2729.3870, 2744.3770, 2762.3970, 2767.3120, 2812.4265, 2846.3943, 2871.4121, 2872.3569, 2873.3445, 2884.3745, 2888.3184, 2892.4063, 2893.4375, 2894.4741, 2917.3960, 2926.4563, 2992.4536, 2995.4939, 3004.4705, 3027.5032, 3054.4763, 3082.5879, 3088.5859, 3104.5430, 3126.7307, 3170.6697, 3200.5081, 3201.4670, 3206.5745, 3207.6025, 3242.6213, 3248.6428, 3255.6025, 3263.6973, 3300.7517, 3315.7021, 3328.7302, 3331.6746, 3334.7031, 3335.7090, 3347.5835, 3353.6013, 3356.7166, 3370.7415, 3386.7366, 3456.7622, 3471.7375, 3487.7676, 3491.6787, 3503.8286, 3601.7178, 3639.7463, 3646.7891, 3651.8442, 3703.9653, 3716.8708, 3720.7805, 3764.8616, 3820.9673, 3869.8423, 3885.7913, 3887.9063, 3927.8738, 4148.0005, 4162.0400, 4253.0234, 4452.2729, 4464.2598, 4468.1572, 4482.1689, 4680.3301, 4845.1597

46. Q3T0I4 **Mass:** 26937 **Score:** 19 **Expect:** 3.3e+002 **Matches:** 8

THO complex subunit 4 OS=Bos taurus GN=ALYREF PE=2 SV=1

Observed	Mr(expt)	Mr(calc)	ppm	Start	End	Miss	Peptide
772.4224	771.4151	771.4212	-7.95	24 -	31	2	R.GGGRGRGR.A
845.4138	844.4065	844.4264	-23.54	30 -	38	1	R.GRAGSQGGR.G
959.4836	958.4763	958.4984	-23.10	134 -	141	1	K.KAAVHYDR.S
1122.5577	1121.5504	1121.5327	15.8	205 -	217	1	R.GSGGFGGGGTRR.G
1715.8459	1714.8386	1714.8757	-21.65	190 -	204	2	R.RPAQSVNRGGMTRNR.G + Oxidation (M)
1768.9546	1767.9473	1767.9241	13.2	64 -	81	1	R.GAAGGGGRNRPAPYSRPK.Q
3703.9653	3702.9580	3702.8821	20.5	157 -	189	2	K.ADALKAMKQYNGVPLDGRPMNIQLVTSQIDTQR.R + 2 Oxidation (M)
3869.8423	3868.8350	3868.9034	-17.67	72 -	107	2	R.NRPAPYSRPKQLPDKWQHDLFDSGFGGGAGVETGGK.L

No match to: 706.2814, 707.2933, 713.3626, 717.4103, 719.4172, 722.4265, 735.4174, 744.3938, 756.4689, 768.3713, 779.4717, 792.4133, 804.3155, 806.4307, 807.4160, 809.4363, 812.3947, 814.4097, 815.4186, 819.3901, 828.3928, 831.4666, 832.3149, 834.3199, 837.3761, 841.4355, 842.4860, 851.3882, 871.4900, 876.2846, 878.2907, 883.4759, 891.4818, 893.9534, 894.4574, 898.4351, 910.4221, 913.5131, 918.5399, 929.4545, 935.4750, 937.4921, 947.4755, 961.4837, 963.4760, 967.4271, 969.4589, 972.4794, 974.4445, 976.4506, 977.4767, 978.5352, 981.4952, 983.4747, 985.6076, 988.4585, 989.6082, 997.4586, 1007.5676, 1011.4840, 1014.5074, 1018.5203, 1019.5441, 1021.5421, 1034.5779, 1042.5320, 1044.5242, 1060.5463, 1071.5376, 1077.5208, 1087.5399, 1091.5421, 1106.5393, 1156.5372, 1173.6240, 1187.6439, 1199.6992, 1215.6615, 1244.6189, 1248.6575, 1255.6039, 1276.6218, 1284.6401, 1295.7195, 1301.6177, 1313.7069, 1314.7141, 1315.6759, 1325.6675, 1336.7151, 1337.7180, 1338.6461, 1342.6758, 1344.7024, 1349.6952, 1353.6804, 1355.7097, 1356.7201, 1361.6877, 1365.6632, 1370.6783, 1372.6058, 1381.7588, 1383.7244, 1386.7131, 1394.7318, 1397.7419, 1414.6467, 1427.7865, 1432.7140, 1446.7247, 1457.7411, 1461.7784, 1473.8055, 1478.7513, 1497.8757, 1528.6993, 1542.8459, 1556.8795, 1578.7826, 1601.9006, 1606.8433, 1616.8177, 1618.8162, 1628.7026, 1628.8568, 1642.7186, 1647.8694, 1650.8473, 1658.8400, 1677.8625, 1680.8728, 1696.8231, 1703.8551, 1704.8862, 1710.9269, 1717.8810, 1718.9111, 1725.8386, 1727.9301, 1730.8835, 1742.9099, 1751.8870, 1769.9617, 1771.0243, 1775.0276, 1777.8676, 1786.9032, 1791.9047, 1799.9005, 1800.9124, 1915.0015, 1928.0238, 1953.9285, 1982.0009, 2012.9681, 2025.0820, 2027.1273, 2042.0642, 2052.9939, 2060.0461, 2067.0017, 2070.8899, 2074.9629, 2082.9421, 2084.9102, 2086.9065, 2096.9797, 2100.9070, 2109.0410, 2116.0894, 2136.9121, 2155.0730, 2170.0781, 2195.0801, 2211.0686, 2238.1072, 2251.1428, 2293.1614, 2336.2495, 2343.1758, 2382.1987, 2385.2234, 2400.0720, 2414.0930, 2418.0901, 2428.1140, 2431.0991, 2465.2188, 2468.2249, 2528.1794, 2542.2344, 2560.2729, 2564.2610, 2646.9797, 2660.9968, 2675.0183, 2698.3711, 2707.3184, 2711.3606, 2712.3755, 2719.3159, 2729.3870, 2744.3770, 2762.3970, 2767.3120, 2812.4265, 2846.3943, 2871.4121, 2872.3569, 2873.3445, 2884.3745, 2888.3184, 2892.4063, 2893.4375, 2894.4741, 2917.3960, 2926.4563, 2992.4536, 2995.4939, 3004.4705, 3005.5735, 3027.5032, 3054.4763, 3082.5879, 3088.5859, 3104.5430, 3126.7307, 3170.6697, 3200.5081, 3201.4670, 3206.5745, 3207.6025, 3242.6213, 3248.6428, 3255.6025, 3263.6973, 3300.7517, 3315.7021, 3328.7302, 3331.6746, 3334.7031, 3335.7090, 3347.5835, 3353.6013, 3356.7166, 3370.7415, 3386.7366, 3456.7622, 3471.7375, 3487.7676, 3491.6787, 3503.8286, 3601.7178,

3639.7463, 3646.7891, 3651.8442, 3716.8708, 3720.7805, 3764.8616, 3820.9673, 3885.7913, 3887.9063, 3927.8738, 4148.0005, 4162.0400, 4253.0234, 4452.2729, 4464.2598, 4468.1572, 4482.1689, 4680.3301, 4845.1597

47. F1MSM9 **Mass:** 64070 **Score:** 19 **Expect:** 3.3e+002 **Matches:** 12
Uncharacterized protein OS=Bos taurus GN=FNBP1L PE=4 SV=2

Observed	Mr(expt)	Mr(calc)	ppm	Start		End	Miss	Peptide
918.5399	917.5326	917.5182	15.7	309	-	317	1	K.VDAKTTVGK.A
961.4837	960.4764	960.4876	-11.65	304	-	312	1	K.QESGKVDAK.T
1014.5074	1013.5001	1013.5076	-7.40	114	-	121	1	K.MHLQEGRK.A + Oxidation (M)
1325.6675	1324.6602	1324.6921	-24.08	222	-	231	2	R.RTIKLSECYR.G
1338.6461	1337.6388	1337.6211	13.2	146	-	156	1	R.EAEKAQQSYER.L
1461.7784	1460.7711	1460.7405	20.9	393	-	404	1	K.LAETMNNIDRLR.M + Oxidation (M)
2109.0410	2108.0337	2108.0062	13.1	503	-	520	1	K.EGEVLYIIEEDKGDGWTR.A
2116.0894	2115.0821	2115.0564	12.1	393	-	409	2	K.LAETMNNIDRLRMEIHK.N + 2 Oxidation (M)
2542.2344	2541.2271	2541.1951	12.6	380	-	402	1	K.NPQMGDPGSLQPKLAETMNNIDR.L + Oxidation (M)
2762.3970	2761.3897	2761.3316	21.1	87	-	109	2	R.EVVAEEMAHRVYGELMRYAHDLK.T + Oxidation (M)
3353.6013	3352.5940	3352.6305	-10.89	2	-	27	2	R.NWLKYLWDQFDSLDKHTQWGIDFLER.Y
3869.8423	3868.8350	3868.8254	2.49	487	-	520	2	K.AIYPFDGHNEGTLAMKEGEVLYIIEEDKGDGWTR.A + Oxidation (M)

No match to: 706.2814, 707.2933, 713.3626, 717.4103, 719.4172, 722.4265, 735.4174, 744.3938, 756.4689, 768.3713, 772.4224, 779.4717, 792.4133, 804.3155, 806.4307, 807.4160, 809.4363, 812.3947, 814.4097, 815.4186, 819.3901, 828.3928, 831.4666, 832.3149, 834.3199, 837.3761, 841.4355, 842.4860, 845.4138, 851.3882, 871.4900, 876.2846, 878.2907, 883.4759, 891.4818, 893.9534, 894.4574, 898.4351, 910.4221, 913.5131, 929.4545, 935.4750, 937.4921, 947.4755, 959.4836, 963.4760, 967.4271, 969.4589, 972.4794, 974.4445, 976.4506, 977.4767, 978.5352, 981.4952, 983.4747, 985.6076, 988.4585, 989.6082, 997.4586, 1007.5676, 1011.4840, 1018.5203, 1019.5441, 1021.5421, 1034.5779, 1042.5320, 1044.5242, 1060.5463, 1071.5376, 1077.5208, 1087.5399, 1091.5421, 1106.5393, 1122.5577, 1156.5372, 1173.6240, 1187.6439, 1199.6992, 1215.6615, 1244.6189, 1248.6575, 1255.6039, 1276.6218, 1284.6401, 1295.7195, 1301.6177, 1313.7069, 1314.7141, 1315.6759, 1336.7151, 1337.7180, 1342.6758, 1344.7024, 1349.6952, 1353.6804, 1355.7097, 1356.7201, 1361.6877, 1365.6632, 1370.6783, 1372.6058, 1381.7588, 1383.7244, 1386.7131, 1394.7318, 1397.7419, 1414.6467, 1427.7865, 1432.7140, 1446.7247, 1457.7411, 1473.8055, 1478.7513, 1497.8757, 1528.6993, 1542.8459, 1556.8795, 1578.7826, 1601.9006, 1606.8433, 1616.8177, 1618.8162, 1628.7026, 1628.8568, 1642.7186, 1647.8694, 1650.8473, 1658.8400, 1677.8625, 1680.8728, 1696.8231, 1703.8551, 1704.8862, 1710.9269, 1715.8459, 1717.8810, 1718.9111, 1725.8386, 1727.9301, 1730.8835, 1742.9099, 1751.8870, 1768.9546, 1769.9617, 1771.0243, 1775.0276, 1777.8676, 1786.9032, 1791.9047, 1799.9005, 1800.9124, 1915.0015, 1928.0238, 1953.9285, 1982.0009, 2012.9681, 2025.0820, 2027.1273, 2042.0642, 2052.9939, 2060.0461, 2067.0017, 2070.8899, 2074.9629, 2082.9421, 2084.9102, 2086.9065, 2096.9797, 2100.9070, 2136.9121, 2155.0730, 2170.0781, 2195.0801, 2211.0686, 2238.1072, 2251.1428, 2293.1614, 2336.2495, 2343.1758, 2382.1987, 2385.2234, 2400.0720, 2414.0930, 2418.0901, 2428.1140, 2431.0991, 2465.2188, 2468.2249, 2528.1794, 2560.2729, 2564.2610, 2646.9797, 2660.9968, 2675.0183, 2698.3711, 2707.3184, 2711.3606, 2712.3755, 2719.3159, 2729.3870, 2744.3770, 2767.3120, 2812.4265, 2846.3943, 2871.4121, 2872.3569, 2873.3445, 2884.3745, 2888.3184, 2892.4063, 2893.4375, 2894.4741, 2917.3960, 2926.4563, 2992.4536, 2995.4939, 3004.4705, 3005.5735, 3027.5032, 3054.4763, 3082.5879, 3088.5859, 3104.5430, 3126.7307, 3170.6697, 3200.5081, 3201.4670, 3206.5745, 3207.6025, 3242.6213, 3248.6428, 3255.6025, 3263.6973, 3300.7517, 3315.7021, 3328.7302, 3331.6746, 3334.7031, 3335.7090, 3347.5835, 3356.7166, 3370.7415, 3386.7366, 3456.7622, 3471.7375, 3487.7676, 3491.6787, 3503.8286, 3601.7178, 3639.7463, 3646.7891, 3651.8442, 3703.9653, 3716.8708, 3720.7805, 3764.8616, 3820.9673, 3885.7913, 3887.9063, 3927.8738, 4148.0005, 4162.0400, 4253.0234, 4452.2729, 4464.2598, 4468.1572, 4482.1689, 4680.3301, 4845.1597

48. G5E5C5 **Mass:** 52138 **Score:** 18 **Expect:** 3.4e+002 **Matches:** 12
Probable tubulin polyglutamylase TTLL9 OS=Bos taurus GN=TTLL9 PE=4 SV=1

Observed	Mr(expt)	Mr(calc)	ppm	Start		End	Miss	Peptide
935.4750	934.4677	934.4582	10.1	147	-	153	1	K.DIMDWKK.G
1060.5463	1059.5390	1059.5461	-6.69	65	-	72	1	R.NHYELTRK.N
1325.6675	1324.6602	1324.6485	8.82	93	-	103	1	K.LEAAKCDFFPK.T
1349.6952	1348.6879	1348.6809	5.20	147	-	158	2	K.DIMDWKKGTAGK.K
1365.6632	1364.6559	1364.6758	-14.58	147	-	158	2	K.DIMDWKKGTAGK.K + Oxidation (M)
1432.7140	1431.7067	1431.6994	5.12	188	-	199	0	K.DEIPVENYVAQR.Y
1742.9099	1741.9026	1741.9086	-3.43	275	-	287	2	K.WMLQRFRQYLASK.H + Oxidation (M)
2027.1273	2026.1200	2026.1146	2.69	120	-	137	2	R.KNPGITWIMKPVARSQGK.G + Oxidation (M)
2343.1758	2342.1685	2342.1722	-1.59	360	-	379	1	K.TCLLEDTLHIVDMEARLTGR.E
3200.5081	3199.5008	3199.5761	-23.51	280	-	306	2	R.FRQYLASKHGPEAVETLFSDMDNIFIR.S + Oxidation (M)
3456.7622	3455.7549	3455.7222	9.46	233	-	262	2	R.DGFARFSNTRFTLNSIDDQYVHLTNVAVQK.T
3651.8442	3650.8369	3650.8403	-0.91	282	-	313	2	R.QYLASKHGPEAVETLFSDMDNIFIRSLQSVQK.V

No match to: 706.2814, 707.2933, 713.3626, 717.4103, 719.4172, 722.4265, 735.4174, 744.3938, 756.4689, 768.3713, 772.4224, 779.4717, 792.4133, 804.3155, 806.4307, 807.4160, 809.4363, 812.3947, 814.4097, 815.4186, 819.3901, 828.3928, 831.4666, 832.3149, 834.3199, 837.3761, 841.4355, 842.4860, 845.4138, 851.3882, 871.4900, 876.2846, 878.2907, 883.4759, 891.4818, 893.9534, 894.4574, 898.4351, 910.4221, 913.5131, 918.5399, 929.4545, 937.4921, 947.4755, 959.4836, 961.4837, 963.4760, 967.4271, 969.4589, 972.4794, 974.4445, 976.4506, 977.4767, 978.5352, 981.4952, 983.4747, 985.6076, 988.4585, 989.6082, 997.4586, 1007.5676, 1011.4840, 1014.5074, 1018.5203, 1019.5441, 1021.5421, 1034.5779, 1042.5320, 1044.5242, 1071.5376, 1077.5208, 1087.5399, 1091.5421, 1106.5393, 1122.5577, 1156.5372, 1173.6240, 1187.6439, 1199.6992, 1215.6615, 1244.6189, 1248.6575, 1255.6039, 1276.6218, 1284.6401, 1295.7195, 1301.6177, 1313.7069, 1314.7141, 1315.6759, 1336.7151, 1337.7180, 1338.6461, 1342.6758, 1344.7024, 1353.6804, 1355.7097, 1356.7201, 1361.6877, 1370.6783, 1372.6058, 1381.7588, 1383.7244, 1386.7131, 1394.7318, 1397.7419, 1414.6467, 1427.7865, 1446.7247, 1457.7411, 1461.7784, 1473.8055, 1478.7513, 1497.8757, 1528.6993, 1542.8459, 1556.8795, 1578.7826, 1601.9006, 1606.8433, 1616.8177, 1618.8162, 1628.7026, 1628.8568, 1642.7186, 1647.8694, 1650.8473, 1658.8400, 1677.8625, 1680.8728, 1696.8231, 1703.8551, 1704.8862, 1710.9269, 1715.8459, 1717.8810, 1718.9111, 1725.8386, 1727.9301, 1730.8835, 1751.8870, 1768.9546, 1769.9617, 1771.0243, 1775.0276, 1777.8676, 1786.9032, 1791.9047, 1799.9005, 1800.9124, 1915.0015, 1928.0238, 1953.9285, 1982.0009, 2012.9681, 2025.0820, 2042.0642, 2052.9939, 2060.0461, 2067.0017, 2070.8899, 2074.9629, 2082.9421, 2084.9102, 2086.9065, 2096.9797, 2100.9070, 2109.0410, 2116.0894, 2136.9121, 2155.0730, 2170.0781, 2195.0801, 2211.0686, 2238.1072, 2251.1428, 2293.1614, 2336.2495, 2382.1987, 2385.2234, 2400.0720, 2414.0930, 2418.0901, 2428.1140, 2431.0991, 2465.2188, 2468.2249, 2528.1794, 2542.2344, 2560.2729, 2564.2610, 2646.9797, 2660.9968, 2675.0183, 2698.3711, 2707.3184, 2711.3606, 2712.3755, 2719.3159, 2729.3870, 2744.3770, 2762.3970, 2767.3120, 2812.4265, 2846.3943, 2871.4121, 2872.3569, 2873.3445, 2884.3745, 2888.3184, 2892.4063, 2893.4375, 2894.4741, 2917.3960, 2926.4563, 2992.4536, 2995.4939, 3004.4705, 3005.5735, 3027.5032, 3054.4763, 3082.5879, 3088.5859, 3104.5430, 3126.7307, 3170.6697, 3201.4670, 3206.5745, 3207.6025, 3242.6213, 3248.6428, 3255.6025, 3263.6973, 3300.7517, 3315.7021, 3328.7302, 3331.6746, 3334.7031, 3335.7090, 3347.5835, 3353.6013, 3356.7166, 3370.7415, 3386.7366, 3471.7375, 3487.7676, 3491.6787, 3503.8286, 3601.7178, 3639.7463, 3646.7891, 3703.9653, 3716.8708, 3720.7805, 3764.8616, 3820.9673, 3869.8423, 3885.7913, 3887.9063, 3927.8738, 4148.0005, 4162.0400, 4253.0234, 4452.2729, 4464.2598, 4468.1572, 4482.1689, 4680.3301, 4845.1597

49. A6QQQ9 **Mass:** 48257 **Score:** 18 **Expect:** 3.5e+002 **Matches:** 10
Keratin, type I cytoskeletal 20 OS=Bos taurus GN=KRT20 PE=2 SV=2

Observed	Mr(expt)	Mr(calc)	ppm	Start		End	Miss	Peptide
1060.5463	1059.5390	1059.5383	0.71	141	-	149	0	R.CVLQIDNAK.L
1122.5577	1121.5504	1121.5717	-18.93	363	-	371	0	R.LEQEIATYR.R
1786.9032	1785.8959	1785.8719	13.4	150	-	163	2	K.LAMEDFRLKYEAER.G + Oxidation (M)
2109.0410	2108.0337	2108.0525	-8.89	373	-	390	1	R.LLEGEDVKEYQLSTLDEK.D
2465.2188	2464.2115	2464.2584	-19.03	373	-	393	2	R.LLEGEDVKEYQLSTLDEKDIK.K
2560.2729	2559.2656	2559.2830	-6.78	47	-	71	1	R.ISTSRHLANYGSVPAGGNLFAGNEK.M
3104.5430	3103.5357	3103.6037	-21.90	257	-	283	2	K.AKEQFEIQINDLQQQVTVSTEELKGTK.D
3263.6973	3262.6900	3262.6186	21.9	214	-	243	1	R.SLRAHLGNNVNVEVDAPPSLNLGAIMNEMR.Q + 2 Oxidation (M)

3487.7676	3486.7603	3486.7823	-6.31	214 - 245	2	R.SLRAHLGNNVNVEVDAPPSLNLGAIMNEMRQK.Y
3503.8286	3502.8213	3502.7773	12.6	214 - 245	2	R.SLRAHLGNNVNVEVDAPPSLNLGAIMNEMRQK.Y + Oxidation (M)

No match to: 706.2814, 707.2933, 713.3626, 717.4103, 719.4172, 722.4265, 735.4174, 744.3938, 756.4689, 768.3713, 772.4224, 779.4717, 792.4133, 804.3155, 806.4307, 807.4160, 809.4363, 812.3947, 814.4097, 815.4186, 819.3901, 828.3928, 831.4666, 832.3149, 834.3199, 837.3761, 841.4355, 842.4860, 845.4138, 851.3882, 871.4900, 876.2846, 878.2907, 883.4759, 891.4818, 893.9534, 894.4574, 898.4351, 910.4221, 913.5131, 918.5399, 929.4545, 935.4750, 937.4921, 947.4755, 959.4836, 961.4837, 963.4760, 967.4271, 969.4589, 972.4794, 974.4445, 976.4506, 977.4767, 978.5352, 981.4952, 983.4747, 985.6076, 988.4585, 989.6082, 997.4586, 1007.5676, 1011.4840, 1014.5074, 1018.5203, 1019.5441, 1021.5421, 1034.5779, 1042.5320, 1044.5242, 1071.5376, 1077.5208, 1087.5399, 1091.5421, 1106.5393, 1156.5372, 1173.6240, 1187.6439, 1199.6992, 1215.6615, 1244.6189, 1248.6575, 1255.6039, 1276.6218, 1284.6401, 1295.7195, 1301.6177, 1313.7069, 1314.7141, 1315.6759, 1325.6675, 1336.7151, 1337.7180, 1338.6461, 1342.6758, 1344.7024, 1349.6952, 1353.6804, 1355.7097, 1356.7201, 1361.6877, 1365.6632, 1370.6783, 1372.6058, 1381.7588, 1383.7244, 1386.7131, 1394.7318, 1397.7419, 1414.6467, 1427.7865, 1432.7140, 1446.7247, 1457.7411, 1461.7784, 1473.8055, 1478.7513, 1497.8757, 1528.6993, 1542.8459, 1556.8795, 1578.7826, 1601.9006, 1606.8433, 1616.8177, 1618.8162, 1628.7026, 1628.8568, 1642.7186, 1647.8694, 1650.8473, 1658.8400, 1677.8625, 1680.8728, 1696.8231, 1703.8551, 1704.8862, 1710.9269, 1715.8459, 1717.8810, 1718.9111, 1725.8386, 1727.9301, 1730.8835, 1742.9099, 1751.8870, 1768.9546, 1769.9617, 1771.0243, 1775.0276, 1777.8676, 1791.9047, 1799.9005, 1800.9124, 1915.0015, 1928.0238, 1953.9285, 1982.0009, 2012.9681, 2025.0820, 2027.1273, 2042.0642, 2052.9939, 2060.0461, 2067.0017, 2070.8899, 2074.9629, 2082.9421, 2084.9102, 2086.9065, 2096.9797, 2100.9070, 2116.0894, 2136.9121, 2155.0730, 2170.0781, 2195.0801, 2211.0686, 2238.1072, 2251.1428, 2293.1614, 2336.2495, 2343.1758, 2382.1987, 2385.2234, 2400.0720, 2414.0930, 2418.0901, 2428.1140, 2431.0991, 2468.2249, 2528.1794, 2542.2344, 2564.2610, 2646.9797, 2660.9968, 2675.0183, 2698.3711, 2707.3184, 2711.3606, 2712.3755, 2719.3159, 2729.3870, 2744.3770, 2762.3970, 2767.3120, 2812.4265, 2846.3943, 2871.4121, 2872.3569, 2873.3445, 2884.3745, 2888.3184, 2892.4063, 2893.4375, 2894.4741, 2917.3960, 2926.4563, 2992.4536, 2995.4939, 3004.4705, 3005.5735, 3027.5032, 3054.4763, 3082.5879, 3088.5859, 3126.7307, 3170.6697, 3200.5081, 3201.4670, 3206.5745, 3207.6025, 3242.6213, 3248.6428, 3255.6025, 3300.7517, 3315.7021, 3328.7302, 3331.6746, 3334.7031, 3335.7090, 3347.5835, 3353.6013, 3356.7166, 3370.7415, 3386.7366, 3456.7622, 3471.7375, 3491.6787, 3601.7178, 3639.7463, 3646.7891, 3651.8442, 3703.9653, 3716.8708, 3720.7805, 3764.8616, 3820.9673, 3869.8423, 3885.7913, 3887.9063, 3927.8738, 4148.0005, 4162.0400, 4253.0234, 4452.2729, 4464.2598, 4468.1572, 4482.1689, 4680.3301, 4845.1597

50. P80219 **Mass:** 27880 **Score:** 18 **Expect:** 3.5e+002 **Matches:** 6

Duodenase-1 OS=Bos taurus GN=BDMD1 PE=1 SV=2

Observed	Mr(expt)	Mr(calc)	ppm	Start - End	Miss	Peptide
2465.2188	2464.2115	2464.2420	-12.37	92 - 112	0	R.RPIPHPDYNDETLANDIMLLK.L
2812.4265	2811.4192	2811.4346	-5.47	123 - 148	1	K.VSPINLPRSLAEVKPGMMCSVAGWGR.L
3386.7366	3385.7293	3385.7816	-15.44	84 - 112	1	R.TQQVIPVRRPIPHPDYNDETLANDIMLLK.L
3471.7375	3470.7302	3470.7472	-4.90	117 - 148	2	K.ADITDKVSPINLPRSLAEVKPGMMCSVAGWGR.L + Oxidation (M)
3487.7676	3486.7603	3486.7422	5.21	117 - 148	2	K.ADITDKVSPINLPRSLAEVKPGMMCSVAGWGR.L + 2 Oxidation (M)
4162.0400	4161.0327	4161.1212	-21.25	55 - 91	2	R.EDFVLTAAHCLGSSINVTLGAHNIMERERTQQVIPVR.R

No match to: 706.2814, 707.2933, 713.3626, 717.4103, 719.4172, 722.4265, 735.4174, 744.3938, 756.4689, 768.3713, 772.4224, 779.4717, 792.4133, 804.3155, 806.4307, 807.4160, 809.4363, 812.3947, 814.4097, 815.4186, 819.3901, 828.3928, 831.4666, 832.3149, 834.3199, 837.3761, 841.4355, 842.4860, 845.4138, 851.3882, 871.4900, 876.2846, 878.2907, 883.4759, 891.4818, 893.9534, 894.4574, 898.4351, 910.4221, 913.5131, 918.5399, 929.4545, 935.4750, 937.4921, 947.4755, 959.4836, 961.4837, 963.4760, 967.4271, 969.4589, 972.4794, 974.4445, 976.4506, 977.4767, 978.5352, 981.4952, 983.4747, 985.6076, 988.4585, 989.6082, 997.4586, 1007.5676, 1011.4840, 1014.5074, 1018.5203, 1019.5441, 1021.5421, 1034.5779, 1042.5320, 1044.5242, 1060.5463, 1071.5376, 1077.5208, 1087.5399, 1091.5421, 1106.5393, 1122.5577, 1156.5372, 1173.6240, 1187.6439, 1199.6992, 1215.6615, 1244.6189, 1248.6575, 1255.6039, 1276.6218, 1284.6401, 1295.7195, 1301.6177, 1313.7069, 1314.7141, 1315.6759, 1325.6675, 1336.7151, 1337.7180, 1338.6461, 1342.6758, 1344.7024, 1349.6952, 1353.6804, 1355.7097, 1356.7201, 1361.6877, 1365.6632, 1370.6783, 1372.6058, 1381.7588, 1383.7244, 1386.7131, 1394.7318, 1397.7419, 1414.6467, 1427.7865, 1432.7140, 1446.7247, 1457.7411, 1461.7784, 1473.8055, 1478.7513, 1497.8757, 1528.6993, 1542.8459, 1556.8795, 1578.7826, 1601.9006, 1606.8433, 1616.8177, 1618.8162, 1628.7026, 1628.8568, 1642.7186, 1647.8694, 1650.8473, 1658.8400, 1677.8625, 1680.8728, 1696.8231, 1703.8551, 1704.8862, 1710.9269, 1715.8459, 1717.8810, 1718.9111, 1725.8386, 1727.9301, 1730.8835, 1742.9099, 1751.8870, 1768.9546, 1769.9617, 1771.0243, 1775.0276, 1777.8676, 1786.9032, 1791.9047, 1799.9005, 1800.9124, 1915.0015, 1928.0238, 1953.9285, 1982.0009, 2012.9681, 2025.0820, 2027.1273, 2042.0642, 2052.9939, 2060.0461, 2067.0017, 2070.8899, 2074.9629, 2082.9421, 2084.9102, 2086.9065, 2096.9797, 2100.9070, 2109.0410, 2116.0894, 2136.9121, 2155.0730, 2170.0781, 2195.0801, 2211.0686, 2238.1072, 2251.1428, 2293.1614, 2336.2495, 2343.1758, 2382.1987, 2385.2234, 2400.0720, 2414.0930, 2418.0901, 2428.1140, 2431.0991, 2468.2249, 2528.1794, 2542.2344, 2560.2729, 2564.2610, 2646.9797, 2660.9968, 2675.0183, 2698.3711, 2707.3184, 2711.3606, 2712.3755, 2719.3159, 2729.3870, 2744.3770, 2762.3970, 2767.3120, 2846.3943, 2871.4121, 2872.3569, 2873.3445, 2884.3745, 2888.3184, 2892.4063, 2893.4375, 2894.4741, 2917.3960, 2926.4563, 2992.4536, 2995.4939, 3004.4705, 3005.5735, 3027.5032, 3054.4763, 3082.5879, 3088.5859, 3104.5430, 3126.7307, 3170.6697, 3200.5081, 3201.4670, 3206.5745, 3207.6025, 3242.6213, 3248.6428, 3255.6025, 3263.6973, 3300.7517, 3315.7021, 3328.7302, 3331.6746, 3334.7031, 3335.7090, 3347.5835, 3353.6013, 3356.7166, 3370.7415, 3456.7622, 3491.6787, 3503.8286, 3601.7178, 3639.7463, 3646.7891, 3651.8442, 3703.9653, 3716.8708, 3720.7805, 3764.8616, 3820.9673, 3869.8423, 3885.7913, 3887.9063, 3927.8738, 4148.0005, 4253.0234, 4452.2729, 4464.2598, 4468.1572, 4482.1689, 4680.3301, 4845.1597

Search Parameters

Type of search : **Peptide Mass Fingerprint**
Enzyme : **Trypsin**
Fixed modifications : **Carbamidomethyl (C)**
Variable modifications : **Oxidation (M)**
Mass values : **Monoisotopic**
Protein Mass : **Unrestricted**
Peptide Mass Tolerance : **± 25 ppm**
Peptide Charge State : **1+**
Max Missed Cleavages : **2**
Number of queries : **281**
Selected for scoring : **225**

Mascot: http://www.matrixscience.com/

MATRIX SCIENCE Mascot Search Results

```
User          : 1Y_(L12@52381_05Apr2017)
Email         : proteomics@i3s.up.pt
Search title  : Project: Proteomics, Spot Set: Proteomics\05Apr2017, Label: L12, Spot Id: 226999, Peak List Id: 447678, MS Job Run Id: 31
MS data file  : C:\Documents and Settings\Administrator\Desktop\xu\29JUNHO\Proteomics\05Apr2017\pmf_L12_149875820403.txt
Database      : OrganismSpecie Bos_taurus_Reference_Proteome_2016_06 (24214 sequences; 12839866 residues)
Timestamp     : 29 Jun 2017 at 18:16:14 GMT
Top Score     : 188 for Mixture 1, F1MRZ5 + P21809
```

Mascot Score Histogram

Protein score is -10*Log(P), where P is the probability that the observed match is a random event.
Protein scores greater than 56 are significant (p<0.05).

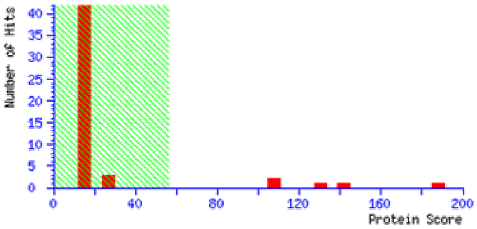

Protein Summary Report

Format As | Protein Summary | Help

Significance threshold p< 0.05 | Max. number of hits 50

Preferred taxonomy All entries

Re-Search All | Search Unmatched

Index

	Accession	Mass	Score	Description
1.	Mixture 1		188	F1MRZ5 + P21809
2.	Mixture 2		147	F1MRZ6 + P21809
3.	F1MRZ5	196677	126	Uncharacterized protein OS=Bos taurus GN=TNC PE=4 SV=1
4.	F1MRZ6	250106	108	Uncharacterized protein OS=Bos taurus GN=TNC PE=4 SV=2
5.	P21809	41963	103	Biglycan OS=Bos taurus GN=BGN PE=1 SV=3
6.	F1MVK7	40714	30	Uncharacterized protein OS=Bos taurus PE=4 SV=2
7.	G3MZZ4	7599	24	Uncharacterized protein OS=Bos taurus PE=4 SV=1
8.	F1MS89	47695	22	Uncharacterized protein OS=Bos taurus GN=FUT1 PE=4 SV=1
9.	F1MPP1	23962	20	Ras-related protein Rab-21 OS=Bos taurus GN=RAB21 PE=4 SV=2
10.	F1N451	67798	20	Uncharacterized protein OS=Bos taurus GN=GRHL3 PE=4 SV=1
11.	Q2YDN4	58176	20	Coiled-coil domain-containing protein 105 OS=Bos taurus GN=CCDC105 PE=2 SV=1
12.	F1N367	244177	20	Aggrecan core protein OS=Bos taurus GN=ACAN PE=4 SV=2
13.	Q17R06	24530	20	Ras-related protein Rab-21 OS=Bos taurus GN=RAB21 PE=2 SV=1
14.	F1N368	248513	19	Aggrecan core protein OS=Bos taurus GN=ACAN PE=4 SV=2
15.	F1MDD0	13795	19	Uncharacterized protein OS=Bos taurus GN=PSMG4 PE=4 SV=2
16.	G3N2C2	12918	19	Uncharacterized protein OS=Bos taurus GN=LOC100139826 PE=4 SV=1
17.	Q32PA3	14710	19	Magnesium-dependent phosphatase 1 OS=Bos taurus GN=MDP-1 PE=2 SV=1
18.	F1MU12	53494	19	Keratin, type II cytoskeletal 8 OS=Bos taurus GN=KRT8 PE=1 SV=1
19.	F1MDA4	51404	19	Hemogen OS=Bos taurus GN=HEMGN PE=4 SV=2
20.	Q32LF3	24094	19	Similar to type 1 protein phosphatase inhibitor OS=Bos taurus GN=LOC520057 PE=2 SV=1
21.	P13608	248150	19	Aggrecan core protein OS=Bos taurus GN=ACAN PE=1 SV=3
22.	A2VDY9	11285	18	P antigen family, member 4 (Prostate associated) OS=Bos taurus GN=PAGE4 PE=2 SV=1
23.	Q5KR49	32732	18	Tropomyosin alpha-1 chain OS=Bos taurus GN=TPM1 PE=2 SV=1
24.	P28782	10567	18	Protein S100-A8 OS=Bos taurus GN=S100A8 PE=1 SV=2
25.	E1B8L7	12965	18	Uncharacterized protein OS=Bos taurus PE=3 SV=1
26.	G3N1F5	34506	17	Uncharacterized protein OS=Bos taurus PE=3 SV=1
27.	Q0P5K1	53559	17	Vasculin OS=Bos taurus GN=GPBP1 PE=2 SV=1
28.	Q3B7N2	103486	17	Alpha-actinin-1 OS=Bos taurus GN=ACTN1 PE=2 SV=1
29.	G3N1D4	4565	17	Uncharacterized protein OS=Bos taurus PE=4 SV=1
30.	E1B8M0	54699	16	Uncharacterized protein OS=Bos taurus GN=CEP55 PE=4 SV=1
31.	G3MXA5	71155	16	Uncharacterized protein OS=Bos taurus GN=TIAM1 PE=4 SV=1
32.	Q5EA43	36620	16	Sideroflexin-2 OS=Bos taurus GN=SFXN2 PE=2 SV=2
33.	A6QR46	23561	16	Ras-related protein Rab-6B OS=Bos taurus GN=RAB6B PE=2 SV=1
34.	G8JL06	14967	16	Histone H2B OS=Bos taurus GN=HIST1H2BB PE=3 SV=1
35.	Q32L62	51390	16	Hemogen OS=Bos taurus GN=HEMGN PE=2 SV=1
36.	Q02379	12888	16	NADH dehydrogenase [ubiquinone] iron-sulfur protein 5 OS=Bos taurus GN=NDUFS5 PE=1 SV=2
37.	G3N0K0	16157	16	Uncharacterized protein OS=Bos taurus PE=4 SV=1
38.	A0A140T855	36964	16	Sideroflexin OS=Bos taurus GN=SFXN2 PE=3 SV=1
39.	E1BLR1	58429	16	Uncharacterized protein OS=Bos taurus GN=SPATA2 PE=4 SV=1
40.	G3MYV3	5981	16	Uncharacterized protein OS=Bos taurus PE=4 SV=1
41.	G3X6Z3	13501	16	Small nuclear ribonucleoprotein Sm D2 OS=Bos taurus GN=SNRPD2 PE=4 SV=1
42.	P05786	53594	16	Keratin, type II cytoskeletal 8 OS=Bos taurus GN=KRT8 PE=2 SV=3
43.	Q3T0C2	29235	16	15-hydroxyprostaglandin dehydrogenase [NAD(+)] OS=Bos taurus GN=HPGD PE=2 SV=1
44.	Q3SZF8	13632	16	Small nuclear ribonucleoprotein Sm D2 OS=Bos taurus GN=SNRPD2 PE=3 SV=1
45.	F1MYC4	31994	16	Uncharacterized protein OS=Bos taurus PE=4 SV=2
46.	G3X6N4	29159	16	Uncharacterized protein OS=Bos taurus PE=4 SV=1
47.	F6QUW4	15733	16	Uncharacterized protein OS=Bos taurus GN=BAALC PE=4 SV=1
48.	Q5E9J7	21569	15	tRNA-specific adenosine deaminase 2 OS=Bos taurus GN=DEADC1 PE=2 SV=1
49.	G3MXN2	75852	15	Uncharacterized protein OS=Bos taurus GN=ZNF282 PE=4 SV=1
50.	E1BGD1	35077	15	Uncharacterized protein OS=Bos taurus GN=TOMM34 PE=4 SV=1

Results List

1. **Mixture 1** **Total score:** 188 **Expect:** 3.8e-015 **Matches:** 96

Components: 1. F1MRZ5 Uncharacterized protein OS=Bos taurus GN=TNC PE=4 SV=1
2. P21809 Biglycan OS=Bos taurus GN=BGN PE=1 SV=3

Observed	Mr(expt)	Mr(calc)	ppm	Start	End	Miss	Comp	Peptide
735.4123	734.4050	734.4075	-3.43	178 -	184	0	2	K.GVFSGLR.N
745.4143	744.4070	744.4130	-8.09	1028 -	1033	0	1	K.EVQLTR.D
753.3587	752.3514	752.3453	8.15	918 -	924	0	1	K.AAADSYR.I
756.3719	755.3647	755.3715	-8.98	1012 -	1016	1	1	K.FDRYR.L
771.3859	770.3787	770.3752	4.54	1687 -	1692	0	1	K.GAFWYK.N
818.4813	817.4740	817.4810	-8.51	134 -	140	1	2	K.AFSPLRK.L
823.4747	822.4675	822.4824	-18.16	1060 -	1066	1	1	R.HKSKPAR.V
829.4503	828.4430	828.4494	-7.62	253 -	258	1	2	R.YSKLYR.L
889.4957	888.4884	888.4930	-5.15	1731 -	1737	0	1	K.LRPSNFR.N
909.5331	908.5259	908.5331	-7.98	293 -	301	0	2	R.VPAGLPDLK.L
922.4722	921.4650	921.4668	-2.02	763 -	769	0	1	R.RPETTYR.Q
923.5116	922.5043	922.5097	-5.80	167 -	173	1	2	R.IHDNRIR.K
938.4929	937.4856	937.4869	-1.35	1034 -	1041	0	1	R.DTTSFVLR.G
985.6010	984.5937	984.5716	22.4	1500 -	1508	0	1	R.IQALNGTLR.S
1007.5710	1006.5637	1006.5672	-3.45	259 -	267	0	2	R.LGLGHNQIR.M
1017.5679	1016.5606	1016.5655	-4.76	1412 -	1420	1	1	R.IFAEKGPQK.S
1051.5060	1050.4987	1050.4982	0.50	1428 -	1436	0	1	K.FTTDLDSPR.D
1056.4977	1055.4904	1055.4784	11.4	1576 -	1583	1	1	K.NGREDFYR.N
1118.5876	1117.5803	1117.5802	0.15	74 -	83	0	2	R.VVQCSDLGLK.A
1143.4429	1142.4356	1142.4346	0.91	563 -	572	0	1	R.CPGDCHGQGR.C
1178.6199	1177.6126	1177.6091	2.98	1609 -	1618	0	1	K.ITAQGQYELR.V
1187.7002	1186.6929	1186.7186	-21.63	134 -	143	2	2	K.AFSPLRKLQK.L
1194.5691	1193.5618	1193.5612	0.56	155 -	164	0	1	R.VDTRPFCSGR.G
1207.6023	1206.5950	1206.5881	5.76	887 -	897	0	1	K.ETFTTGLDAPR.N
1216.4987	1215.4914	1215.4873	3.38	376 -	384	1	1	R.RCPSDCHER.G
1257.6666	1256.6593	1256.6765	-13.67	1514 -	1524	0	1	K.TIFTTSGVLYR.F
1278.6932	1277.6859	1277.6840	1.50	760 -	769	1	1	K.SLRRPETTYR.Q
1299.5466	1298.5393	1298.5357	2.80	562 -	572	1	1	R.RCPGDCHGQGR.C
1312.7513	1311.7440	1311.7398	3.24	242 -	252	0	2	K.IQAIELEDLLR.Y
1338.6886	1337.6813	1337.7051	-17.79	282 -	292	1	2	R.ELHLDNNKLSR.V
1351.6915	1350.6842	1350.6819	1.69	681 -	691	0	1	R.ELEPGVEYFIR.V
1413.6514	1412.6441	1412.6507	-4.63	1719 -	1730	0	1	K.GHEHSIQFAEMK.L
1494.7429	1493.7356	1493.7337	1.27	316 -	329	0	2	K.VGVNDFCPVGFGVK.R
1562.7856	1561.7783	1561.7736	3.01	902 -	914	0	1	R.ISQTDNSITLEWR.N
1571.9341	1570.9268	1570.9334	-4.19	1314 -	1328	0	1	R.LLPGADYLVSVIALK.G
1590.7900	1589.7827	1589.8162	-21.03	887 -	900	1	1	K.ETFTTGLDAPRNLR.R
1650.8488	1649.8415	1649.8348	4.05	316 -	330	1	2	K.VGVNDFCPVGFGVKR.A
1657.9104	1656.9031	1656.9046	-0.92	798 -	812	1	1	R.VTTTRLDAPSQIEVK.D
1664.8605	1663.8532	1663.8893	-21.71	1028 -	1041	1	1	K.EVQLTRDTTSFVLR.G
1696.8295	1695.8222	1695.8216	0.34	927 -	942	0	1	K.YAPISGGDHAEVEVPR.S
1718.8820	1717.8747	1717.8747	-0.00	901 -	914	1	1	R.RISQTDNSITLEWR.N
1747.9136	1746.9063	1746.9039	1.36	1042 -	1057	0	1	R.GLEPGQEYSILLTAEK.G
1787.8756	1786.8683	1786.8849	-9.30	227 -	241	0	2	K.DLPETLNELHLDHNK.I
1870.7738	1869.7665	1869.7404	14.0	138 -	154	0	1	R.EQCTSGAGCCLQSAEGR.V
1883.7272	1882.7199	1882.7145	2.87	593 -	608	0	1	R.SCPNDCSNWGQCVSGR.C
1903.0176	1902.0103	1902.0099	0.24	1287 -	1306	0	1	R.VTYVPIAGGAPSAVTVDGTK.T
1938.0094	1937.0021	1937.0007	0.76	925 -	942	1	1	R.IKYAPISGGDHAEVEVPR.S
1941.0232	1940.0159	1939.9904	13.1	1012 -	1027	2	1	K.FDRYRLNYSLPSGQPK.E
1961.0339	1960.0266	1960.0265	0.05	1042 -	1059	1	1	R.GLEPGQEYSILLTAEKGR.H
2008.9807	2007.9734	2007.9672	3.10	1687 -	1702	2	1	K.GAFWYKNCHRVNLMGR.Y
2027.1300	2026.1227	2026.1211	0.80	149 -	166	0	2	K.NHLVEIPPNLPSSLVELR.I
2086.1130	2085.1057	2085.0994	3.03	993 -	1011	0	1	R.ISDPTETSLTLVWQTPVAK.F
2108.9956	2107.9883	2107.9844	1.85	878 -	897	1	1	R.ADMSSNPAKETFTTGLDAPR.N
2112.9822	2111.9749	2111.9694	2.60	1646 -	1665	1	1	R.LKVEGYSGTAGDSMAYHNGR.S
2245.8369	2244.8296	2244.8293	0.14	392 -	410	0	1	R.CECNDGFTGADCGELQCPR.D
2265.0969	2264.0896	2264.0855	1.81	877 -	897	2	1	R.RADMSSNPAKETFTTGLDAPR.N
2273.0667	2272.0594	2272.0509	3.76	85 -	103	0	1	R.ESFQEHTVDGENQIVFTHR.I
2286.1521	2285.1448	2285.1387	2.70	88 -	107	0	2	K.EISPDTTLLDLQNNDISELR.K
2318.1763	2317.1690	2317.1703	-0.53	1437 -	1456	0	1	R.DFTATEVQSETALLTWRPPR.A
2328.9463	2327.9390	2327.9392	-0.08	542 -	561	0	1	R.CVDGQCVCLEGFTGPDCAQR.R
2347.8403	2346.8330	2346.8246	3.58	356 -	375	0	1	R.CDEGQCVCDEGFAGPDCSER.R
2352.9194	2351.9121	2351.9141	-0.82	573 -	592	0	1	R.CVDGQCVCHEGFTGPDCAQR.S
2363.8716	2362.8643	2362.8672	-1.20	418 -	437	0	1	R.CVNGQCVCDEGYTGEDCGQR.R
2371.0796	2370.0723	2370.0686	1.59	1666 -	1686	1	1	R.SFSTFDKDTDSAITNCALSYK.G
2389.2690	2388.2617	2388.2649	-1.34	1287 -	1310	1	1	R.VTYVPIAGGAPSAVTVDGTKTQTR.L
2414.2378	2413.2305	2413.2336	-1.28	88 -	108	1	2	K.EISPDTTLLDLQNNDISELRK.D
2477.2441	2476.2368	2476.2598	-9.28	670 -	691	1	1	R.VPGDQTSATIRELEPGVEYFIR.V
2485.0479	2484.0406	2484.0403	0.12	542 -	562	1	1	R.CVDGQCVCLEGFTGPDCAQRR.C
2519.9768	2518.9695	2518.9683	0.50	418 -	438	1	1	R.CVNGQCVCDEGYTGEDCGQRR.C
2541.2239	2540.2166	2540.2257	-3.58	649 -	669	0	1	R.VTEYLIVYTPTHEDGLEMQFR.V
2542.2581	2541.2508	2541.2598	-3.55	1389 -	1411	0	1	R.TVSGNTVEYALTNLEPATEYTLR.I
2580.0098	2579.0025	2578.9968	2.23	480 -	500	1	1	R.CVNGMCVCDDAYTGEDCRELR.C
2631.4714	2630.4641	2630.4795	-5.85	144 -	166	1	2	K.LYISKNHLVEIPPNLPSSLVELR.I
2681.3691	2680.3618	2680.3919	-11.22	84 -	107	1	2	K.AVPKEISPDTTLLDLQNNDISELR.K
2695.0193	2694.0120	2694.0138	-0.67	516 -	539	0	1	R.CVCEDGFAGPDCADLACPGNCHGR.G
2798.2344	2797.2271	2797.2146	4.49	185 -	210	0	2	R.NMNCIEMGGNPLENSGFEPGAFDGLK.L
2799.2815	2798.2742	2798.3392	-23.23	58 -	84	1	1	K.LPVGSQCSVDLESASGEKDLAAPSEPR.E
2809.4841	2808.4768	2808.4869	-3.57	84 -	108	2	2	K.AVPKEISPDTTLLDLQNNDISELRK.D
2833.0999	2832.0926	2832.0779	5.20	387 -	410	1	1	R.CVDGRCECNDGFTGADCGELQCPR.D

2846.3840	2845.3767	2845.3864	-3.39	331	354	0	2	R.AYYNGISLFNNPVPYWEVQPATFR.C
2919.4407	2918.4334	2918.4509	-5.98	88	112	2	2	K.EISPDTTLLDLQNNDISELRKDDFK.G
2961.2092	2960.2019	2960.1993	0.88	268	291	1	1	R.CVCQEGFAGEDCREPLCLHNCHGR.G
3002.4895	3001.4822	3001.4875	-1.75	330	354	1	2	K.RAYYNGISLFNNPVPYWEVQPATFR.C
3029.4480	3028.4407	3028.4488	-2.66	1260	1286	0	1	K.EIIFSDITENAASVSWMAPTTQVESFR.V
3081.6030	3080.5957	3080.6142	-5.99	227	252	1	2	K.DLPETLNELHLDHNKIQAIELEDLLR.Y
3161.0999	3160.0926	3160.1158	-7.32	454	479	0	1	R.CECQPGFQGDDCGEMSCPHDCHQHGR.C
3435.6848	3434.6775	3434.6841	-1.92	847	876	0	1	R.TSIDLTHEENQYSIGNLKPDTEYEVALISR.R
3457.5947	3456.5874	3456.5900	-0.76	185	215	1	2	R.NMNCIEMGGNPLENSGFEPGAFDGLKLNYLR.I
3489.6526	3488.6453	3488.5799	18.8	185	215	1	2	R.NMNCIEMGGNPLENSGFEPGAFDGLKLNYLR.I + 2 Oxidation (M)
3528.3171	3527.3098	3527.2775	9.15	294	322	0	1	R.CVENECVCDEGFTGDDCGELVCPNDCFDR.G
3690.9905	3689.9832	3689.9992	-4.32	221	252	2	2	K.LTGIPKDLPETLNELHLDHNKIQAIELEDLLR.Y
3722.4707	3721.4634	3721.4307	8.80	231	262	0	1	K.CVDGACVCFEGYSGLDCSQETCPVPCSEEHGR.C
3741.4561	3740.4488	3740.4001	13.0	294	324	1	1	R.CVENECVCDEGFTGDDCGELVCPNDCFDRGR.C
3748.4346	3747.4273	3747.3644	16.8	449	479	1	1	R.CVDGRCECQPGFQGDDCGEMSCPHDCHQHGR.C
4075.0212	4074.0139	4073.9818	7.89	841	876	1	1	K.DVPGDRTSIDLTHEENQYSIGNLKPDTEYEVALISR.R
4690.3101	4689.3028	4689.3113	-1.81	1457	1499	1	1	R.ASVTGYLLVYESVDGTIKEVVLDPDTTSYSLTDLSPSTYYTAR.I

No match to: 701.3929, 710.3735, 712.2889, 713.2998, 717.3996, 719.4157, 720.4154, 722.4257, 729.4009, 734.4510, 738.4139, 747.4459, 779.4640, 783.3826, 805.4482, 806.4485, 831.4642, 832.3057, 834.3104, 835.3138, 840.4758, 842.5006, 874.5018, 883.4738, 891.4817, 900.5336, 925.4815, 929.4376, 936.4885, 949.5431, 958.4930, 967.4177, 969.4647, 974.4397, 978.5347, 983.6221, 988.4032, 993.4985, 1034.5784, 1036.5477, 1044.5231, 1046.5923, 1057.5691, 1058.6145, 1074.6044, 1087.5525, 1113.5417, 1123.5989, 1145.6317, 1169.4524, 1172.6801, 1185.6628, 1216.7014, 1248.6699, 1254.6930, 1255.6340, 1265.7057, 1269.6703, 1273.6517, 1281.6456, 1284.6411, 1286.7430, 1301.6044, 1304.6500, 1314.7311, 1317.7122, 1349.6870, 1354.7209, 1358.7173, 1365.5874, 1368.6487, 1371.7622, 1372.5999, 1374.6569, 1375.7383, 1382.5975, 1383.7290, 1390.6992, 1393.7732, 1397.7450, 1412.6091, 1420.7966, 1440.7816, 1442.8168, 1450.8618, 1456.7800, 1457.7509, 1467.7542, 1470.7775, 1500.7238, 1513.7605, 1528.6982, 1539.7341, 1556.7584, 1563.7985, 1570.7755, 1578.7968, 1580.8304, 1592.7916, 1616.8013, 1628.7054, 1628.8561, 1635.7781, 1642.7219, 1647.8557, 1649.8578, 1663.8551, 1706.8674, 1707.9520, 1715.8798, 1721.9437, 1730.8646, 1741.9973, 1755.8970, 1758.9091, 1771.0317, 1774.9242, 1786.9141, 1862.9530, 1895.7275, 1897.7380, 1911.7487, 1913.7377, 1928.0281, 1959.9926, 1970.0011, 1974.9122, 1979.0769, 1981.0564, 1982.0408, 2007.9467, 2055.0208, 2060.9963, 2067.1592, 2144.0425, 2181.1780, 2223.1548, 2239.1460, 2241.1360, 2251.1431, 2255.1433, 2259.8535, 2335.8999, 2343.1943, 2361.8635, 2364.8738, 2366.9121, 2467.2051, 2474.2170, 2503.0457, 2504.9548, 2505.9473, 2595.0286, 2646.9739, 2678.0037, 2684.3965, 2701.2148, 2705.2874, 2709.0286, 2717.2839, 2770.4202, 2792.2090, 2813.3928, 2816.2957, 2842.4780, 2872.3215, 2876.3640, 2885.3044, 2970.4829, 2976.2786, 2990.3098, 3071.4028, 3087.4146, 3146.5461, 3157.4587, 3184.4336, 3236.5503, 3248.6294, 3254.5583, 3265.5901, 3315.6875, 3330.5125, 3334.5588, 3335.6306, 3346.5308, 3347.4827, 3352.6125, 3410.6125, 3411.7185, 3428.5632, 3462.6028, 3471.6272, 3474.6221, 3503.7864, 3542.3335, 3552.6479, 3574.6042, 3624.6587, 3639.7046, 3645.7671, 3651.7319, 3667.7424, 3707.5422, 3716.6360, 3721.5728, 3736.5007, 3753.4888, 3764.5239, 4147.9077, 4160.8794, 4238.9067, 4253.8887, 4274.9287, 4301.2393, 4397.9902, 4415.0044, 4452.1357, 4458.2759, 4465.1338, 4631.9888, 4845.1016

2. **Mixture 2** **Total score:** **147** **Expect:** 4.8e-011 **Matches:** 98

Components: 1. F1MRZ6 Uncharacterized protein OS=Bos taurus GN=TNC PE=4 SV=2

2. P21809 Biglycan OS=Bos taurus GN=BGN PE=1 SV=3

Observed	Mr(expt)	Mr(calc)	ppm	Start	End	Miss	Comp	Peptide
735.4123	734.4050	734.4075	-3.43	178	184	0	2	K.GVFSGLR.N
745.4143	744.4070	744.4130	-8.09	1058	1063	0	1	K.EVQLTR.D
753.3587	752.3514	752.3453	8.15	948	954	0	1	K.AAADSYR.I
756.3719	755.3647	755.3715	-8.98	1042	1046	1	1	K.FDRYR.L
771.3859	770.3787	770.3752	4.54	2172	2177	0	1	K.GAFWYK.N
818.4813	817.4740	817.4810	-8.51	134	140	1	2	K.AFSPLRK.L
823.4747	822.4675	822.4824	-18.16	1090	1096	1	1	R.HKSKPAR.V
829.4503	828.4430	828.4494	-7.62	253	258	1	2	R.YSKLYR.L
889.4957	888.4884	888.4930	-5.15	2216	2222	0	1	K.LRPSNFR.N
909.5331	908.5259	908.5331	-7.98	293	301	0	2	R.VPAGLPDLK.L
922.4722	921.4650	921.4668	-2.02	793	799	0	1	R.RPETTYR.Q
923.5116	922.5043	922.5097	-5.80	167	173	1	2	R.IHDNRIR.K
938.4929	937.4856	937.4869	-1.35	1064	1071	0	1	R.DTTSFVLR.G
985.6010	984.5937	984.5716	22.4	1985	1993	0	1	R.IQALNGTLR.S
1007.5710	1006.5637	1006.5672	-3.45	259	267	0	2	R.LGLGHNQIR.M
1017.5679	1016.5606	1016.5655	-4.76	1897	1905	1	1	R.IFAEKGPQK.S
1051.5060	1050.4987	1050.4982	0.50	1913	1921	0	1	K.FTTDLDSPR.D
1056.4977	1055.4904	1055.4784	11.4	2061	2068	1	1	K.NGREDFYR.N
1118.5876	1117.5803	1117.5802	0.15	74	83	0	2	R.VVQCSDLGLK.A
1143.4429	1142.4356	1142.4346	0.91	593	602	0	1	R.CPGDCHGQGR.C
1178.6199	1177.6126	1177.6091	2.98	2094	2103	0	1	K.ITAQGQYELR.V
1187.7002	1186.6929	1186.7186	-21.63	134	143	2	2	K.AFSPLRKLQK.L
1194.5691	1193.5618	1193.5612	0.56	185	194	0	1	R.VDTRPFCSGR.G
1207.6023	1206.5950	1206.5881	5.76	917	927	0	1	K.ETFTTGLDAPR.N
1216.4987	1215.4914	1215.4873	3.38	406	414	1	1	R.RCPSDCHER.G
1257.6666	1256.6593	1256.6765	-13.67	1999	2009	0	1	K.TIFTTSGVLYR.F
1278.6932	1277.6859	1277.6840	1.50	790	799	1	1	K.SLRRPETTYR.Q
1299.5466	1298.5393	1298.5357	2.80	592	602	1	1	R.RCPGDCHGQGR.C
1312.7513	1311.7440	1311.7398	3.24	242	252	0	2	K.IQAIELEDLLR.Y
1338.6886	1337.6813	1337.7051	-17.79	282	292	1	2	R.ELHLDNNKLSR.V
1351.6915	1350.6842	1350.6819	1.69	711	721	0	1	R.ELEPGVEYFIR.V
1413.6514	1412.6441	1412.6507	-4.63	2204	2215	0	1	K.GHEHSIQFAEMK.L
1494.7429	1493.7356	1493.7337	1.27	316	329	0	2	K.VGVNDFCPVGFGVK.R
1562.7856	1561.7783	1561.7736	3.01	932	944	0	1	R.ISQTDNSITLEWR.N
1571.9341	1570.9268	1570.9334	-4.19	1799	1813	0	1	R.LLPGADYLVSVIALK.G
1578.7968	1577.7895	1577.8161	-16.87	1453	1467	1	1	R.GYRTPVLSAEASTAR.E
1590.7900	1589.7827	1589.8162	-21.03	917	930	1	1	K.ETFTTGLDAPRNLR.R
1650.8488	1649.8415	1649.8348	4.05	316	330	1	2	K.VGVNDFCPVGFGVKR.A
1657.9104	1656.9031	1656.9046	-0.92	828	842	1	1	R.VTTTRLDAPSQIEVK.D
1664.8605	1663.8532	1663.8893	-21.71	1058	1071	1	1	K.EVQLTRDTTSFVLR.G
1696.8295	1695.8222	1695.8216	0.34	957	972	0	1	K.YAPISGGDHAEVEVPR.S
1715.8798	1714.8725	1714.9036	-18.12	1728	1744	1	1	R.RSQPVTAIATAMGSPK.E
1718.8820	1717.8747	1717.8747	-0.00	931	944	1	1	R.RISQTDNSITLEWR.N
1747.9136	1746.9063	1746.9039	1.36	1072	1087	0	1	R.GLEPGQEYSILLTAEK.G
1787.8756	1786.8683	1786.8849	-9.30	227	241	0	2	K.DLPETLNELHLDHNK.I
1870.7738	1869.7665	1869.7404	14.0	168	184	0	1	R.EQCTSGAGCCLQSAEGR.V
1883.7272	1882.7199	1882.7145	2.87	623	638	0	1	R.SCPNDCSNWGQCVSGR.C

1903.0176	1902.0103	1902.0099	0.24	1772 - 1791	0	1	R.VTYVPIAGGAPSAVTVDGTK.T
1938.0094	1937.0021	1937.0007	0.76	955 - 972	1	1	R.IKYAPISGGDHAEVEVPR.S
1941.0232	1940.0159	1939.9904	13.1	1042 - 1057	2	1	K.FDRYRLNYSLPSGQPK.E
1961.0339	1960.0266	1960.0265	0.05	1072 - 1089	1	1	R.GLEPGQEYSILLTAEKGR.H
2008.9807	2007.9734	2007.9672	3.10	2172 - 2187	2	1	K.GAFWYKNCHRVNLMGR.Y
2027.1300	2026.1227	2026.1211	0.80	149 - 166	0	2	K.NHLVEIPPNLPSSLVELR.I
2086.1130	2085.1057	2085.0994	3.03	1023 - 1041	0	1	R.ISDPTETSLTLVWQTPVAK.F
2108.9956	2107.9883	2107.9844	1.85	908 - 927	1	1	R.ADMSSNPAKETFTTGLDAPR.N
2112.9822	2111.9749	2111.9694	2.60	2131 - 2150	1	1	R.LKVEGYSGTAGDSMAYHNGR.S
2245.8369	2244.8296	2244.8293	0.14	422 - 440	0	1	R.CECNDGFTGADCGELQCPR.D
2265.0969	2264.0896	2264.0855	1.81	907 - 927	2	1	R.RADMSSNPAKETFTTGLDAPR.N
2273.0667	2272.0594	2272.0509	3.76	115 - 133	0	1	R.ESFQEHTVDGENQIVFTHR.I
2286.1521	2285.1448	2285.1387	2.70	88 - 107	0	2	K.EISPDTTLLDLQNNDISELR.K
2318.1763	2317.1690	2317.1703	-0.53	1922 - 1941	0	1	R.DFTATEVQSETALLTWRPPR.A
2328.9463	2327.9390	2327.9392	-0.08	572 - 591	0	1	R.CVDGQCVCLEGFTGPDCAQR.R
2347.8403	2346.8330	2346.8246	3.58	386 - 405	0	1	R.CDEGQCVCDEGFAGPDCSER.R
2352.9194	2351.9121	2351.9141	-0.82	603 - 622	0	1	R.CVDGQCVCHEGFTGPDCAQR.S
2363.8716	2362.8643	2362.8672	-1.20	448 - 467	0	1	R.CVNGQCVCDEGYTGEDCGQR.R
2371.0796	2370.0723	2370.0686	1.59	2151 - 2171	1	1	R.SFSTFDKDTDSAITNCALSYK.G
2389.2690	2388.2617	2388.2649	-1.34	1772 - 1795	1	1	R.VTYVPIAGGAPSAVTVDGTKTQTR.L
2414.2378	2413.2305	2413.2336	-1.28	88 - 108	1	2	K.EISPDTTLLDLQNNDISELRK.D
2477.2441	2476.2368	2476.2598	-9.28	700 - 721	1	1	R.VPGDQTSATIRELEPGVEYFIR.V
2485.0479	2484.0406	2484.0403	0.12	572 - 592	1	1	R.CVDGQCVCLEGFTGPDCAQRR.C
2519.9768	2518.9695	2518.9683	0.50	448 - 468	1	1	R.CVNGQCVCDEGYTGEDCGQRR.C
2541.2239	2540.2166	2540.2257	-3.58	679 - 699	0	1	R.VTEYLIVYTPTHEDGLEMQFR.V
2542.2581	2541.2508	2541.2598	-3.55	1874 - 1896	0	1	R.TVSGNTVEYALTNLEPATEYTLR.I
2580.0098	2579.0025	2578.9968	2.23	510 - 530	1	1	R.CVNGMCVCDDAYTGEDCRELR.C
2631.4714	2630.4641	2630.4795	-5.85	144 - 166	1	2	K.LYISKNHLVEIPPNLPSSLVELR.I
2681.3691	2680.3618	2680.3919	-11.22	84 - 107	1	2	K.AVPKEISPDTTLLDLQNNDISELR.K
2695.0193	2694.0120	2694.0138	-0.67	546 - 569	0	1	R.CVCEDGFAGPDCADLACPGNCHGR.G
2798.2344	2797.2271	2797.2146	4.49	185 - 210	0	2	R.NMNCIEMGGNPLENSGFEPGAFDGLK.L
2799.2815	2798.2742	2798.3392	-23.23	88 - 114	1	1	K.LPVGSQCSVDLESASGEKDLAAPSEPR.E
2809.4841	2808.4768	2808.4869	-3.57	84 - 108	2	2	K.AVPKEISPDTTLLDLQNNDISELRK.D
2833.0999	2832.0926	2832.0779	5.20	417 - 440	1	1	R.CVDGRCECNDGFTGADCGELQCPR.D
2846.3840	2845.3767	2845.3864	-3.39	331 - 354	0	2	R.AYYNGISLFNNPVPYWEVQPATFR.C
2919.4407	2918.4334	2918.4960	-21.45	1522 - 1548	0	1	R.TAHISGLRPSNDFIVYLSGLAPSMQTK.T + Oxidation (M)
2961.2092	2960.2019	2960.1993	0.88	298 - 321	1	1	R.CVCQEGFAGEDCREPLCLHNCHGR.G
3002.4895	3001.4822	3001.4875	-1.75	330 - 354	1	2	K.RAYYNGISLFNNPVPYWEVQPATFR.C
3029.4480	3028.4407	3028.4488	-2.66	1745 - 1771	0	1	K.EIIFSDITENAASVSWMAPTTQVESFR.V
3081.6030	3080.5957	3080.5891	2.16	1320 - 1347	1	1	K.IRETDQPQEVHSLTVPGSQHSVEIPGLK.A
3161.0999	3160.0926	3160.1158	-7.32	484 - 509	0	1	R.CECQPGFQGDDCGEMSCPHDCHQHGR.C
3435.6848	3434.6775	3434.6841	-1.92	877 - 906	0	1	R.TSIDLTHEENQYSIGNLKPDTEYEVALISR.R
3457.5947	3456.5874	3456.5900	-0.76	185 - 215	1	2	R.NMNCIEMGGNPLENSGFEPGAFDGLKLNYLR.I
3489.6526	3488.6453	3488.5799	18.8	185 - 215	1	2	R.NMNCIEMGGNPLENSGFEPGAFDGLKLNYLR.I + 2 Oxidation (M)
3528.3171	3527.3098	3527.2775	9.15	324 - 352	0	1	R.CVENECVCDEGFTGDDCGELVCPNDCFDR.G
3690.9905	3689.9832	3689.9992	-4.32	221 - 252	2	2	K.LTGIPKDLPETLNELHLDHNKIQAIELEDLLR.Y
3722.4707	3721.4634	3721.4307	8.80	261 - 292	0	1	K.CVDGACVCFEGYSGLDCSQETCPVPCSEEHGR.C
3741.4561	3740.4488	3740.4001	13.0	324 - 354	1	1	R.CVENECVCDEGFTGDDCGELVCPNDCFDRGR.C
3748.4346	3747.4273	3747.3644	16.8	479 - 509	1	1	R.CVDGRCECQPGFQGDDCGEMSCPHDCHQHGR.C
4075.0212	4074.0139	4073.9818	7.89	871 - 906	1	1	K.DVPGDRTSIDLTHEENQYSIGNLKPDTEYEVALISR.R
4690.3101	4689.3028	4689.3113	-1.81	1942 - 1984	1	1	R.ASVTGYLLVYESVDGTIKEVVLDPDTTSYSLTDLSPSTYYTAR.I

No match to: 701.3929, 710.3735, 712.2889, 713.2998, 717.3996, 719.4157, 720.4154, 722.4257, 729.4009, 734.4510, 738.4139, 747.4459, 779.4640, 783.3826, 805.4482, 806.4485, 831.4642, 832.3057, 834.3104, 835.3138, 840.4758, 842.5006, 874.5018, 883.4738, 891.4817, 900.5336, 925.4815, 929.4376, 936.4885, 949.5431, 958.4930, 967.4177, 969.4647, 974.4397, 978.5347, 983.6221, 988.4032, 993.4985, 1034.5784, 1036.5477, 1044.5231, 1046.5923, 1057.5691, 1058.6145, 1074.6044, 1087.5525, 1113.5417, 1123.5989, 1145.6317, 1169.4524, 1172.6801, 1185.6628, 1216.7014, 1248.6699, 1254.6930, 1255.6340, 1265.7057, 1269.6703, 1273.6517, 1281.6456, 1284.6411, 1286.7430, 1301.6044, 1304.6500, 1314.7311, 1317.7122, 1349.6870, 1354.7209, 1358.7173, 1365.5874, 1368.6487, 1371.7622, 1372.5999, 1374.6569, 1375.7383, 1382.5975, 1383.7290, 1390.6992, 1393.7732, 1397.7450, 1412.6091, 1420.7966, 1440.7816, 1442.8168, 1450.8618, 1456.7800, 1457.7509, 1467.7542, 1470.7775, 1500.7238, 1513.7605, 1528.6982, 1539.7341, 1556.7584, 1563.7985, 1570.7755, 1580.8304, 1592.7916, 1616.8013, 1628.7054, 1628.8561, 1635.7781, 1642.7219, 1647.8557, 1649.8578, 1663.8551, 1706.8674, 1707.9520, 1721.9437, 1730.8646, 1741.9973, 1755.8970, 1758.9091, 1771.0317, 1774.9242, 1786.9141, 1862.9530, 1895.7275, 1897.7380, 1911.7487, 1913.7377, 1928.0281, 1959.9926, 1970.0011, 1974.9122, 1979.0769, 1981.0564, 1982.0408, 2007.9467, 2055.0208, 2060.9963, 2067.1592, 2144.0425, 2181.1780, 2223.1548, 2239.1460, 2241.1360, 2251.1431, 2255.1433, 2259.8535, 2335.8999, 2343.1943, 2361.8635, 2364.8738, 2366.9121, 2467.2051, 2474.2170, 2503.0457, 2504.9548, 2505.9473, 2595.0286, 2646.9739, 2678.0037, 2684.3965, 2701.2148, 2705.2874, 2709.0286, 2717.2839, 2770.4202, 2792.2090, 2813.3928, 2816.2957, 2842.4780, 2872.3215, 2876.3640, 2885.3044, 2970.4829, 2976.2786, 2990.3098, 3071.4028, 3087.4146, 3146.5461, 3157.4587, 3184.4336, 3236.5503, 3248.6294, 3254.5583, 3265.5901, 3315.6875, 3330.5125, 3334.5588, 3335.6306, 3346.5308, 3347.4827, 3352.6125, 3410.6125, 3411.7185, 3428.5632, 3462.6028, 3471.6272, 3474.6221, 3503.7864, 3542.3335, 3552.6479, 3574.6042, 3624.6587, 3639.7046, 3645.7671, 3651.7319, 3667.7424, 3707.5422, 3716.6360, 3721.5728, 3736.5007, 3753.4888, 3764.5239, 4147.9077, 4160.8794, 4238.9067, 4253.8887, 4274.9287, 4301.2393, 4397.9902, 4415.0044, 4452.1357, 4458.2759, 4465.1338, 4631.9888, 4845.1016

3. F1MRZ5 **Mass:** 196677 **Score:** **126** **Expect:** 6.1e-009 **Matches:** 69

Uncharacterized protein OS=Bos taurus GN=TNC PE=4 SV=1

Observed	Mr(expt)	Mr(calc)	ppm	Start	End	Miss	Peptide
745.4143	744.4070	744.4130	-8.09	1028 -	1033	0	K.EVQLTR.D
753.3587	752.3514	752.3453	8.15	918 -	924	0	K.AAADSYR.I
756.3719	755.3647	755.3715	-8.98	1012 -	1016	1	K.FDRYR.L
771.3859	770.3787	770.3752	4.54	1687 -	1692	0	K.GAFWYK.N
823.4747	822.4675	822.4824	-18.16	1060 -	1066	1	R.HKSKPAR.V
889.4957	888.4884	888.4930	-5.15	1731 -	1737	0	K.LRPSNFR.N
922.4722	921.4650	921.4668	-2.02	763 -	769	0	R.RPETTYR.Q
938.4929	937.4856	937.4869	-1.35	1034 -	1041	0	R.DTTSFVLR.G
985.6010	984.5937	984.5716	22.4	1500 -	1508	0	R.IQALNGTLR.S
1017.5679	1016.5606	1016.5655	-4.76	1412 -	1420	1	R.IFAEKGPQK.S
1051.5060	1050.4987	1050.4982	0.50	1428 -	1436	0	K.FTTDLDSPR.D
1056.4977	1055.4904	1055.4784	11.4	1576 -	1583	1	K.NGREDFYR.N
1143.4429	1142.4356	1142.4346	0.91	563 -	572	0	R.CPGDCHGQGR.C
1178.6199	1177.6126	1177.6091	2.98	1609 -	1618	0	K.ITAQGQYELR.V

1194.5691	1193.5618	1193.5612	0.56	155 -	164	0	R.VDTRPFCSGR.G
1207.6023	1206.5950	1206.5881	5.76	887 -	897	0	K.ETFTTGLDAPR.N
1216.4987	1215.4914	1215.4873	3.38	376 -	384	1	R.RCPSDCHER.G
1257.6666	1256.6593	1256.6765	-13.67	1514 -	1524	0	K.TIFTTSGVLYR.F
1278.6932	1277.6859	1277.6840	1.50	760 -	769	1	K.SLRRPETTYR.Q
1299.5466	1298.5393	1298.5357	2.80	562 -	572	1	R.RCPGDCHGQGR.C
1351.6915	1350.6842	1350.6819	1.69	681 -	691	0	R.ELEPGVEYFIR.V
1413.6514	1412.6441	1412.6507	-4.63	1719 -	1730	0	K.GHEHSIQFAEMK.L
1562.7856	1561.7783	1561.7736	3.01	902 -	914	0	R.ISQTDNSITLEWR.N
1571.9341	1570.9268	1570.9334	-4.19	1314 -	1328	0	R.LLPGADYLVSVIALK.G
1590.7900	1589.7827	1589.8162	-21.03	887 -	900	1	K.ETFTTGLDAPRNLR.R
1657.9104	1656.9031	1656.9046	-0.92	798 -	812	1	R.VTTTRLDAPSQIEVK.D
1664.8605	1663.8532	1663.8893	-21.71	1028 -	1041	1	K.EVQLTRDTTSFVLR.G
1696.8295	1695.8222	1695.8216	0.34	927 -	942	0	K.YAPISGGDHAEVEVPR.S
1718.8820	1717.8747	1717.8747	-0.00	901 -	914	1	R.RISQTDNSITLEWR.N
1747.9136	1746.9063	1746.9039	1.36	1042 -	1057	0	R.GLEPGQEYSILLTAEK.G
1870.7738	1869.7665	1869.7404	14.0	138 -	154	0	R.EQCTSGAGCCLQSAEGR.V
1883.7272	1882.7199	1882.7145	2.87	593 -	608	0	R.SCPNDCSNWGQCVSGR.C
1903.0176	1902.0103	1902.0099	0.24	1287 -	1306	0	R.VTYVPIAGGAPSAVTVDGTK.T
1938.0094	1937.0021	1937.0007	0.76	925 -	942	1	R.IKYAPISGGDHAEVEVPR.S
1941.0232	1940.0159	1939.9904	13.1	1012 -	1027	2	K.FDRYRLNYSLPSGQPK.E
1961.0339	1960.0266	1960.0265	0.05	1042 -	1059	1	R.GLEPGQEYSILLTAEKGR.H
2008.9807	2007.9734	2007.9672	3.10	1687 -	1702	2	K.GAFWYKNCHRVNLMGR.Y
2086.1130	2085.1057	2085.0994	3.03	993 -	1011	0	R.ISDPTETSLTLVWQTPVAK.F
2108.9956	2107.9883	2107.9844	1.85	878 -	897	1	R.ADMSSNPAKETFTTGLDAPR.N
2112.9822	2111.9749	2111.9694	2.60	1646 -	1665	1	R.LKVEGYSGTAGDSMAYHNGR.S
2245.8369	2244.8296	2244.8293	0.14	392 -	410	0	R.CECNDGFTGADCGELQCPR.D
2265.0969	2264.0896	2264.0855	1.81	877 -	897	2	R.RADMSSNPAKETFTTGLDAPR.N
2273.0667	2272.0594	2272.0509	3.76	85 -	103	0	R.ESFQEHTVDGENQIVFTHR.I
2318.1763	2317.1690	2317.1703	-0.53	1437 -	1456	0	R.DFTATEVQSETALLTWRPPR.A
2328.9463	2327.9390	2327.9392	-0.08	542 -	561	0	R.CVDGQCVCLEGFTGPDCAQR.R
2347.8403	2346.8330	2346.8246	3.58	356 -	375	0	R.CDEGQCVCDEGFAGPDCSER.R
2352.9194	2351.9121	2351.9141	-0.82	573 -	592	0	R.CVDGQCVCHEGFTGPDCAQR.S
2363.8716	2362.8643	2362.8672	-1.20	418 -	437	0	R.CVNGQCVCDEGYTGEDCGQR.R
2371.0796	2370.0723	2370.0686	1.59	1666 -	1686	1	R.SFSTFDKDTDSAITNCALSYK.G
2389.2690	2388.2617	2388.2649	-1.34	1287 -	1310	1	R.VTYVPIAGGAPSAVTVDGTKTQTR.L
2477.2441	2476.2368	2476.2598	-9.28	670 -	691	1	R.VPGDQTSATIRELEPGVEYFIR.V
2485.0479	2484.0406	2484.0403	0.12	542 -	562	1	R.CVDGQCVCLEGFTGPDCAQRR.C
2519.9768	2518.9695	2518.9683	0.50	418 -	438	1	R.CVNGQCVCDEGYTGEDCGQRR.C
2541.2239	2540.2166	2540.2257	-3.58	649 -	669	0	R.VTEYLIVYTPTHEDGLEMQFR.V
2542.2581	2541.2508	2541.2598	-3.55	1389 -	1411	0	R.TVSGNTVEYALTNLEPATEYTLR.I
2580.0098	2579.0025	2578.9968	2.23	480 -	500	1	R.CVNGMCVCDDAYTGEDCRELR.C
2695.0193	2694.0120	2694.0138	-0.67	516 -	539	0	R.CVCEDGFAGPDCADLACPGNCHGR.G
2799.2815	2798.2742	2798.3392	-23.23	58 -	84	1	K.LPVGSQCSVDLESASGEKDLAAPSEPR.E
2833.0999	2832.0926	2832.0779	5.20	387 -	410	1	R.CVDGRCECNDGFTGADCGELQCPR.D
2961.2092	2960.2019	2960.1993	0.88	268 -	291	1	R.CVCQEGFAGEDCREPLCLHNCHGR.G
3029.4480	3028.4407	3028.4488	-2.66	1260 -	1286	0	K.EIIFSDITENAASVSWMAPTTQVESFR.V
3161.0999	3160.0926	3160.1158	-7.32	454 -	479	0	R.CECQPGFQGDDCGEMSCPHDCHQHGR.C
3435.6848	3434.6775	3434.6841	-1.92	847 -	876	0	R.TSIDLTHEENQYSIGNLKPDTEYEVALISR.R
3528.3171	3527.3098	3527.2775	9.15	294 -	322	0	R.CVENECVCDEGFTGDDCGELVCPNDCFDR.G
3722.4707	3721.4634	3721.4307	8.80	231 -	262	0	K.CVDGACVCFEGYSGLDCSQETCPVPCSEEHGR.C
3741.4561	3740.4488	3740.4001	13.0	294 -	324	1	R.CVENECVCDEGFTGDDCGELVCPNDCFDRGR.C
3748.4346	3747.4273	3747.3644	16.8	449 -	479	1	R.CVDGRCECQPGFQGDDCGEMSCPHDCHQHGR.C
4075.0212	4074.0139	4073.9818	7.89	841 -	876	1	K.DVPGDRTSIDLTHEENQYSIGNLKPDTEYEVALISR.R
4690.3101	4689.3028	4689.3113	-1.81	1457 -	1499	1	R.ASVTGYLLVYESVDGTIKEVVLDPDTTSYSLTDLSPSTYYTAR.I

No match to: 701.3929, 710.3735, 712.2889, 713.2998, 717.3996, 719.4157, 720.4154, 722.4257, 729.4009, 734.4510, 735.4123, 738.4139, 747.4459, 779.4640, 783.3826, 805.4482, 806.4485, 818.4813, 829.4503, 831.4642, 832.3057, 834.3104, 835.3138, 840.4758, 842.5006, 874.5018, 883.4738, 891.4817, 900.5336, 909.5331, 923.5116, 925.4815, 929.4376, 936.4885, 949.5431, 958.4930, 967.4177, 969.4647, 974.4397, 978.5347, 983.6221, 988.4032, 993.4985, 1007.5710, 1034.5784, 1036.5477, 1044.5231, 1046.5923, 1057.5691, 1058.6145, 1074.6044, 1087.5525, 1113.5417, 1118.5876, 1123.5989, 1145.6317, 1169.4524, 1172.6801, 1185.6628, 1187.7002, 1216.7014, 1248.6699, 1254.6930, 1255.6340, 1265.7057, 1269.6703, 1273.6517, 1281.6456, 1284.6411, 1286.7430, 1301.6044, 1304.6500, 1312.7513, 1314.7311, 1317.7122, 1338.6886, 1349.6870, 1354.7209, 1358.7173, 1365.5874, 1368.6487, 1371.7622, 1372.5999, 1374.6569, 1375.7383, 1382.5975, 1383.7290, 1390.6992, 1393.7732, 1397.7450, 1412.6091, 1420.7966, 1440.7816, 1442.8168, 1450.8618, 1456.7800, 1457.7509, 1467.7542, 1470.7775, 1494.7429, 1500.7238, 1513.7605, 1528.6982, 1539.7341, 1556.7584, 1563.7985, 1570.7755, 1578.7968, 1580.8304, 1592.7916, 1616.8013, 1628.7054, 1628.8561, 1635.7781, 1642.7219, 1647.8557, 1649.8578, 1650.8488, 1663.8551, 1706.8674, 1707.9520, 1715.8798, 1721.9437, 1730.8646, 1741.9973, 1755.8970, 1758.9091, 1771.0317, 1774.9242, 1786.9141, 1787.8756, 1862.9530, 1895.7275, 1897.7380, 1911.7487, 1913.7377, 1928.0281, 1959.9926, 1970.0011, 1974.9122, 1979.0769, 1981.0564, 1982.0408, 2007.9467, 2027.1300, 2055.0208, 2060.9963, 2067.1592, 2144.0425, 2181.1780, 2223.1548, 2239.1460, 2241.1360, 2251.1431, 2255.1433, 2259.8535, 2286.1521, 2335.8999, 2343.1943, 2361.8635, 2364.8738, 2366.9121, 2414.2378, 2467.2051, 2474.2170, 2503.0457, 2504.9548, 2505.9473, 2595.0286, 2631.4714, 2646.9739, 2678.0037, 2681.3691, 2684.3965, 2701.2148, 2705.2874, 2709.0286, 2717.2839, 2770.4202, 2792.2090, 2798.2344, 2809.4841, 2813.3928, 2816.2957, 2842.4780, 2846.3840, 2872.3215, 2876.3640, 2885.3044, 2919.4407, 2970.4829, 2976.2786, 2990.3098, 3002.4895, 3071.4028, 3081.6030, 3087.4146, 3146.5461, 3157.4587, 3184.4336, 3236.5503, 3248.6294, 3254.5583, 3265.5901, 3315.6875, 3330.5125, 3334.5588, 3335.6306, 3346.5308, 3347.4827, 3352.6125, 3410.6125, 3411.7185, 3428.5632, 3457.5947, 3462.6028, 3471.6272, 3474.6221, 3489.6526, 3503.7864, 3542.3335, 3552.6479, 3574.6042, 3624.6587, 3639.7046, 3645.7671, 3651.7319, 3667.7424, 3690.9905, 3707.5422, 3716.6360, 3721.5728, 3736.5007, 3753.4888, 3764.5239, 4147.9077, 4160.8794, 4238.9067, 4253.8887, 4274.9287, 4301.2393, 4397.9902, 4415.0044, 4452.1357, 4458.2759, 4465.1338, 4631.9888, 4845.1016

4. F1MRZ6 **Mass:** 250106 **Score:** **108** **Expect:** 3.8e-007 **Matches:** 73

Uncharacterized protein OS=Bos taurus GN=TNC PE=4 SV=2

Observed	Mr(expt)	Mr(calc)	ppm	Start	End	Miss	Peptide
745.4143	744.4070	744.4130	-8.09	1058 -	1063	0	K.EVQLTR.D
753.3587	752.3514	752.3453	8.15	948 -	954	0	K.AAADSYR.I
756.3719	755.3647	755.3715	-8.98	1042 -	1046	1	K.FDRYR.L
771.3859	770.3787	770.3752	4.54	2172 -	2177	0	K.GAFWYK.N
823.4747	822.4675	822.4824	-18.16	1090 -	1096	1	R.HKSKPAR.V
889.4957	888.4884	888.4930	-5.15	2216 -	2222	0	K.LRPSNFR.N
922.4722	921.4650	921.4668	-2.02	793 -	799	0	R.RPETTYR.Q
938.4929	937.4856	937.4869	-1.35	1064 -	1071	0	R.DTTSFVLR.G

985.6010	984.5937	984.5716	22.4	1985 - 1993	0	R.IQALNGTLR.S
1017.5679	1016.5606	1016.5655	-4.76	1897 - 1905	1	R.IFAEKGPQK.S
1051.5060	1050.4987	1050.4982	0.50	1913 - 1921	0	K.FTTDLDSPR.D
1056.4977	1055.4904	1055.4784	11.4	2061 - 2068	1	K.NGREDFYR.N
1143.4429	1142.4356	1142.4346	0.91	593 - 602	0	R.CPGDCHGQGR.C
1178.6199	1177.6126	1177.6091	2.98	2094 - 2103	0	K.ITAQGQYELR.V
1194.5691	1193.5618	1193.5612	0.56	185 - 194	0	R.VDTRPFCSGR.G
1207.6023	1206.5950	1206.5881	5.76	917 - 927	0	K.ETFTTGLDAPR.N
1216.4987	1215.4914	1215.4873	3.38	406 - 414	1	R.RCPSDCHER.G
1257.6666	1256.6593	1256.6765	-13.67	1999 - 2009	0	K.TIFTTSGVLYR.F
1278.6932	1277.6859	1277.6840	1.50	790 - 799	1	K.SLRRPETTYR.Q
1299.5466	1298.5393	1298.5357	2.80	592 - 602	1	R.RCPGDCHGQGR.C
1351.6915	1350.6842	1350.6819	1.69	711 - 721	0	R.ELEPGVEYFIR.V
1413.6514	1412.6441	1412.6507	-4.63	2204 - 2215	0	K.GHEHSIQFAEMK.L
1562.7856	1561.7783	1561.7736	3.01	932 - 944	0	R.ISQTDNSITLEWR.N
1571.9341	1570.9268	1570.9334	-4.19	1799 - 1813	0	R.LLPGADYLVSVIALK.G
1578.7968	1577.7895	1577.8161	-16.87	1453 - 1467	1	R.GYRTPVLSAEASTAR.E
1590.7900	1589.7827	1589.8162	-21.03	917 - 930	1	K.ETFTTGLDAPRNLR.R
1657.9104	1656.9031	1656.9046	-0.92	828 - 842	1	R.VTTTRLDAPSQIEVK.D
1664.8605	1663.8532	1663.8893	-21.71	1058 - 1071	1	K.EVQLTRDTTSFVLR.G
1696.8295	1695.8222	1695.8216	0.34	957 - 972	0	K.YAPISGGDHAEVEVPR.S
1715.8798	1714.8725	1714.9036	-18.12	1728 - 1744	1	R.RSQPVTAIATTAMGSPK.E
1718.8820	1717.8747	1717.8747	-0.00	931 - 944	1	R.RISQTDNSITLEWR.N
1747.9136	1746.9063	1746.9039	1.36	1072 - 1087	0	R.GLEPGQEYSILLTAEK.G
1870.7738	1869.7665	1869.7404	14.0	168 - 184	0	R.EQCTSGAGCCLQSAEGR.V
1883.7272	1882.7199	1882.7145	2.87	623 - 638	0	R.SCPNDCSNWGQCVSGR.C
1903.0176	1902.0103	1902.0099	0.24	1772 - 1791	0	R.VTYVPIAGGAPSAVTVDGTK.T
1938.0094	1937.0021	1937.0007	0.76	955 - 972	1	R.IKYAPISGGDHAEVEVPR.S
1941.0232	1940.0159	1939.9904	13.1	1042 - 1057	2	K.FDRYRLNYSLPSGQPK.E
1961.0339	1960.0266	1960.0265	0.05	1072 - 1089	1	R.GLEPGQEYSILLTAEKGR.H
2008.9807	2007.9734	2007.9672	3.10	2172 - 2187	2	K.GAFWYKNCHRVNLMGR.Y
2086.1130	2085.1057	2085.0994	3.03	1023 - 1041	0	R.ISDPTETSLTLVWQTPVAK.F
2108.9956	2107.9883	2107.9844	1.85	908 - 927	1	R.ADMSSNPAKETFTTGLDAPR.N
2112.9822	2111.9749	2111.9694	2.60	2131 - 2150	1	R.LKVEGYSGTAGDSMAYHNGR.S
2245.8369	2244.8296	2244.8293	0.14	422 - 440	0	R.CECNDGFTGADCGELQCPR.D
2265.0969	2264.0896	2264.0855	1.81	907 - 927	2	R.RADMSSNPAKETFTTGLDAPR.N
2273.0667	2272.0594	2272.0509	3.76	115 - 133	0	R.ESFQEHTVDGENQIVFTHR.I
2318.1763	2317.1690	2317.1703	-0.53	1922 - 1941	0	R.DFTATEVQSETALLTWRPPR.A
2328.9463	2327.9390	2327.9392	-0.08	572 - 591	0	R.CVDGQCVCLEGFTGPDCAQR.R
2347.8403	2346.8330	2346.8246	3.58	386 - 405	0	R.CDEGQCVCDEGFAGPDCSER.R
2352.9194	2351.9121	2351.9141	-0.82	603 - 622	0	R.CVDGQCVCHEGFTGPDCAQR.S
2363.8716	2362.8643	2362.8672	-1.20	448 - 467	0	R.CVNGQCVCDEGYTGEDCGQR.R
2371.0796	2370.0723	2370.0686	1.59	2151 - 2171	1	R.SFSTFDKDTDSAITNCALSYK.G
2389.2690	2388.2617	2388.2649	-1.34	1772 - 1795	1	R.VTYVPIAGGAPSAVTVDGTKTQTR.L
2477.2441	2476.2368	2476.2598	-9.28	700 - 721	1	R.VPGDQTSATIRELEPGVEYFIR.V
2485.0479	2484.0406	2484.0403	0.12	572 - 592	1	R.CVDGQCVCLEGFTGPDCAQRR.C
2519.9768	2518.9695	2518.9683	0.50	448 - 468	1	R.CVNGQCVCDEGYTGEDCGQRR.C
2541.2239	2540.2166	2540.2257	-3.58	679 - 699	0	R.VTEYLIVYTPTHEDGLEMQFR.V
2542.2581	2541.2508	2541.2598	-3.55	1874 - 1896	0	R.TVSGNTVEYALTNLEPATEYTLR.I
2580.0098	2579.0025	2578.9968	2.23	510 - 530	1	R.CVNGMCVCDDAYTGEDCRELR.C
2695.0193	2694.0120	2694.0138	-0.67	546 - 569	0	R.CVCEDGFAGPDCADLACPGNCHGR.G
2799.2815	2798.2742	2798.3392	-23.23	88 - 114	1	K.LPVGSQCSVDLESASGEKDLAAPSEPR.E
2833.0999	2832.0926	2832.0779	5.20	417 - 440	1	R.CVDGRCECNDGFTGADCGELQCPR.D
2919.4407	2918.4334	2918.4960	-21.45	1522 - 1548	0	R.TAHISGLRPSNDFIVYLSGLAPSMQTK.T + Oxidation (M)
2961.2092	2960.2019	2960.1993	0.88	298 - 321	1	R.CVCQEGFAGEDCREPLCLHNCHGR.G
3029.4480	3028.4407	3028.4488	-2.66	1745 - 1771	0	K.EIIFSDITENAASVSVSWMAPTTQVESFR.V
3081.6030	3080.5957	3080.5891	2.16	1320 - 1347	1	K.IRETDQPQEVHSLTVPGSQHSVEIPGLK.A
3161.0999	3160.0926	3160.1158	-7.32	484 - 509	0	R.CECQPGFQGDDCGEMSCPHDCHQHGR.C
3435.6848	3434.6775	3434.6841	-1.92	877 - 906	0	R.TSIDLTHEENQYSIGNLKPDTEYEVALISR.R
3528.3171	3527.3098	3527.2775	9.15	324 - 352	0	R.CVENECVCDEGFTGDDCGELVCPNDCFDR.G
3722.4707	3721.4634	3721.4307	8.80	261 - 292	0	K.CVDGACVCFEGYSGLDCSQETCPVPCSEEHGR.C
3741.4561	3740.4488	3740.4001	13.0	324 - 354	1	R.CVENECVCDEGFTGDDCGELVCPNDCFDRGR.C
3748.4346	3747.4273	3747.3644	16.8	479 - 509	1	R.CVDGRCECQPGFQGDDCGEMSCPHDCHQHGR.C
4075.0212	4074.0139	4073.9818	7.89	871 - 906	1	K.DVPGDRTSIDLTHEENQYSIGNLKPDTEYEVALISR.R
4690.3101	4689.3028	4689.3113	-1.81	1942 - 1984	1	R.ASVTGYLLVYESVDGTIKEVVLDPDTTSYSLTDLSPSTYYTAR.I

No match to: 701.3929, 710.3735, 712.2889, 713.2998, 717.3996, 719.4157, 720.4154, 722.4257, 729.4009, 734.4510, 735.4123, 738.4139, 747.4459, 779.4640, 783.3826, 805.4482, 806.4485, 818.4813, 829.4503, 831.4642, 832.3057, 834.3104, 835.3138, 840.4758, 842.5006, 874.5018, 883.4738, 891.4817, 900.5336, 909.5331, 923.5116, 925.4815, 929.4376, 936.4885, 949.5431, 958.4930, 967.4177, 969.4647, 974.4397, 978.5347, 983.6221, 988.4032, 993.4985, 1007.5710, 1034.5784, 1036.5477, 1044.5231, 1046.5923, 1057.5691, 1058.6145, 1074.6044, 1087.5525, 1113.5417, 1118.5876, 1123.5989, 1145.6317, 1169.4524, 1172.6801, 1185.6628, 1187.7002, 1216.7014, 1248.6699, 1254.6930, 1255.6340, 1265.7057, 1269.6703, 1273.6517, 1281.6456, 1284.6411, 1286.7430, 1301.6044, 1304.6500, 1312.7513, 1314.7311, 1317.7122, 1338.6886, 1349.6870, 1354.7209, 1358.7173, 1365.5874, 1368.6487, 1371.7622, 1372.5999, 1374.6569, 1375.7383, 1382.5975, 1383.7290, 1390.6992, 1393.7732, 1397.7450, 1412.6091, 1420.7966, 1440.7816, 1442.8168, 1450.8618, 1456.7800, 1457.7509, 1467.7542, 1470.7775, 1494.7429, 1500.7238, 1513.7605, 1528.6982, 1539.7341, 1556.7584, 1563.7985, 1570.7755, 1580.8304, 1592.7916, 1616.8013, 1628.7054, 1628.8561, 1635.7781, 1642.7219, 1647.8557, 1649.8578, 1650.8488, 1663.8551, 1706.8674, 1707.9520, 1721.9437, 1730.8646, 1741.9973, 1755.8970, 1758.9091, 1771.0317, 1774.9242, 1786.9141, 1787.8756, 1862.9530, 1895.7275, 1897.7380, 1911.7487, 1913.7377, 1928.0281, 1959.9926, 1970.0011, 1974.9122, 1979.0769, 1981.0564, 1982.0408, 2007.9467, 2027.1300, 2055.0208, 2060.9963, 2067.1592, 2144.0425, 2181.1780, 2223.1548, 2239.1460, 2241.1360, 2251.1431, 2255.1433, 2259.8535, 2286.1521, 2335.8999, 2343.1943, 2361.8635, 2364.8738, 2366.9121, 2414.2378, 2467.2051, 2474.2170, 2503.0457, 2504.9548, 2505.9473, 2595.0286, 2631.4714, 2646.9739, 2678.0037, 2681.3691, 2684.3965, 2701.2148, 2705.2874, 2709.0286, 2717.2839, 2770.4202, 2792.2090, 2798.2344, 2809.4841, 2813.3928, 2816.2957, 2842.4780, 2846.3840, 2872.3215, 2876.3640, 2885.3044, 2970.4829, 2976.2786, 2990.3098, 3002.4895, 3071.4028, 3087.4146, 3146.5461, 3157.4587, 3184.4336, 3236.5503, 3248.6294, 3254.5583, 3265.5901, 3315.6875, 3330.5125, 3334.5588, 3335.6306, 3346.5308, 3347.4827, 3352.6125, 3410.6125, 3411.7185, 3428.5632, 3457.5947, 3462.6028, 3471.6272, 3474.6221, 3489.6526, 3503.7864, 3542.3335, 3552.6479, 3574.6042, 3624.6587, 3639.7046, 3645.7671, 3651.7319, 3667.7424, 3690.9905, 3707.5422, 3716.6360, 3721.5728, 3736.5007, 3753.4888, 3764.5239, 4147.9077, 4160.8794, 4238.9067, 4253.8887, 4274.9287, 4301.2393, 4397.9902, 4415.0044, 4452.1357, 4458.2759, 4465.1338, 4631.9888, 4845.1016

5. P21809 **Mass:** 41963 **Score:** **103** **Expect:** 1.2e-006 **Matches:** 27

Biglycan OS=Bos taurus GN=BGN PE=1 SV=3

Observed	Mr(expt)	Mr(calc)	ppm	Start	End	Miss	Peptide
735.4123	734.4050	734.4075	-3.43	178	184	0	K.GVFSGLR.N
818.4813	817.4740	817.4810	-8.51	134	140	1	K.AFSPLRK.L
829.4503	828.4430	828.4494	-7.62	253	258	1	R.YSKLYR.L
909.5331	908.5259	908.5331	-7.98	293	301	0	R.VPAGLPDLK.L
923.5116	922.5043	922.5097	-5.80	167	173	1	R.IHDNRIR.K
1007.5710	1006.5637	1006.5672	-3.45	259	267	0	R.LGLGHNQIR.M
1118.5876	1117.5803	1117.5802	0.15	74	83	0	R.VVQCSDLGLK.A
1187.7002	1186.6929	1186.7186	-21.63	134	143	2	K.AFSPLRKLQK.L
1312.7513	1311.7440	1311.7398	3.24	242	252	0	K.IQAIELEDLLR.Y
1338.6886	1337.6813	1337.7051	-17.79	282	292	1	R.ELHLDNNKLSR.V
1494.7429	1493.7356	1493.7337	1.27	316	329	0	K.VGVNDFCPVGFGVK.R
1650.8488	1649.8415	1649.8348	4.05	316	330	1	K.VGVNDFCPVGFGVKR.A
1787.8756	1786.8683	1786.8849	-9.30	227	241	0	K.DLPETLNELHLDHNK.I
2027.1300	2026.1227	2026.1211	0.80	149	166	0	K.NHLVEIPPNLPSSLVELR.I
2286.1521	2285.1448	2285.1387	2.70	88	107	0	K.EISPDTTLLDLQNNDISELR.K
2414.2378	2413.2305	2413.2336	-1.28	88	108	1	K.EISPDTTLLDLQNNDISELRK.D
2631.4714	2630.4641	2630.4795	-5.85	144	166	1	K.LYISKNHLVEIPPNLPSSLVELR.I
2681.3691	2680.3618	2680.3919	-11.22	84	107	1	K.AVPKEISPDTTLLDLQNNDISELR.K
2798.2344	2797.2271	2797.2146	4.49	185	210	0	R.NMNCIEMGGNPLENSGFEPGAFDGLK.L
2809.4841	2808.4768	2808.4869	-3.57	84	108	2	K.AVPKEISPDTTLLDLQNNDISELRK.D
2846.3840	2845.3767	2845.3864	-3.39	331	354	0	R.AYYNGISLFNNPVPYWEVQPATFR.C
2919.4407	2918.4334	2918.4509	-5.98	88	112	2	K.EISPDTTLLDLQNNDISELRKDDFK.G
3002.4895	3001.4822	3001.4875	-1.75	330	354	1	K.RAYYNGISLFNNPVPYWEVQPATFR.C
3081.6030	3080.5957	3080.6142	-5.99	227	252	1	K.DLPETLNELHLDHNKIQAIELEDLLR.Y
3457.5947	3456.5874	3456.5900	-0.76	185	215	1	R.NMNCIEMGGNPLENSGFEPGAFDGLKLNYLR.I
3489.6526	3488.6453	3488.5799	18.8	185	215	1	R.NMNCIEMGGNPLENSGFEPGAFDGLKLNYLR.I + 2 Oxidation (M)
3690.9905	3689.9832	3689.9992	-4.32	221	252	2	K.LTGIPKDLPETLNELHLDHNKIQAIELEDLLR.Y

No match to: 701.3929, 710.3735, 712.2889, 713.2998, 717.3996, 719.4157, 720.4154, 722.4257, 729.4009, 734.4510, 738.4139, 745.4143, 747.4459, 753.3587, 756.3719, 771.3859, 779.4640, 783.3826, 805.4482, 806.4485, 823.4747, 831.4642, 832.3057, 834.3104, 835.3138, 840.4758, 842.5006, 874.5018, 883.4738, 889.4957, 891.4817, 900.5336, 922.4722, 925.4815, 929.4376, 936.4885, 938.4929, 949.5431, 958.4930, 967.4177, 969.4647, 974.4397, 978.5347, 983.6221, 985.6010, 988.4032, 993.4985, 1017.5679, 1034.5784, 1036.5477, 1044.5231, 1046.5923, 1051.5060, 1056.4977, 1057.5691, 1058.6145, 1074.6044, 1087.5525, 1113.5417, 1123.5989, 1143.4429, 1145.6317, 1169.4524, 1172.6801, 1178.6199, 1185.6628, 1194.5691, 1207.6023, 1216.4987, 1216.7014, 1248.6699, 1254.6930, 1255.6340, 1257.6666, 1265.7057, 1269.6703, 1273.6517, 1278.6932, 1281.6456, 1284.6411, 1286.7430, 1299.5466, 1301.6044, 1304.6500, 1314.7311, 1317.7122, 1349.6870, 1351.6915, 1354.7209, 1358.7173, 1365.5874, 1368.6487, 1371.7622, 1372.5999, 1374.6569, 1375.7383, 1382.5975, 1383.7290, 1390.6992, 1393.7732, 1397.7450, 1412.6091, 1413.6514, 1420.7966, 1440.7816, 1442.8168, 1450.8618, 1456.7800, 1457.7509, 1467.7542, 1470.7775, 1500.7238, 1513.7605, 1528.6982, 1539.7341, 1556.7584, 1562.7856, 1563.7985, 1570.7755, 1571.9341, 1578.7968, 1580.8304, 1590.7900, 1592.7916, 1616.8013, 1628.7054, 1628.8561, 1635.7781, 1642.7219, 1647.8557, 1649.8578, 1657.9104, 1663.8551, 1664.8605, 1696.8295, 1706.8674, 1707.9520, 1715.8798, 1718.8820, 1721.9437, 1730.8646, 1741.9973, 1747.9136, 1755.8970, 1758.9091, 1771.0317, 1774.9242, 1786.9141, 1862.9530, 1870.7738, 1883.7272, 1895.7275, 1897.7380, 1903.0176, 1911.7487, 1913.7377, 1928.0281, 1938.0094, 1941.0232, 1959.9926, 1961.0339, 1970.0011, 1974.9122, 1979.0769, 1981.0564, 1982.0408, 2007.9467, 2008.9807, 2055.0208, 2060.9963, 2067.1592, 2086.1130, 2108.9956, 2112.9822, 2144.0425, 2181.1780, 2223.1548, 2239.1460, 2241.1360, 2245.8369, 2251.1431, 2255.1433, 2259.8535, 2265.0969, 2273.0667, 2318.1763, 2328.9463, 2335.8999, 2343.1943, 2347.8403, 2352.9194, 2361.8635, 2363.8716, 2364.8738, 2366.9121, 2371.0796, 2389.2690, 2467.2051, 2474.2170, 2477.2441, 2485.0479, 2503.0457, 2504.9548, 2505.9473, 2519.9768, 2541.2239, 2542.2581, 2580.0098, 2595.0286, 2646.9739, 2678.0037, 2684.3965, 2695.0193, 2701.2148, 2705.2874, 2709.0286, 2717.2839, 2770.4202, 2792.2090, 2799.2815, 2813.3928, 2816.2957, 2833.0999, 2842.4780, 2872.3215, 2876.3640, 2885.3044, 2961.2092, 2970.4829, 2976.2786, 2990.3098, 3029.4480, 3071.4028, 3087.4146, 3146.5461, 3157.4587, 3161.0999, 3184.4336, 3236.5503, 3248.6294, 3254.5583, 3265.5901, 3315.6875, 3330.5125, 3334.5588, 3335.6306, 3346.5308, 3347.4827, 3352.6125, 3410.6125, 3411.7185, 3428.5632, 3435.6848, 3462.6028, 3471.6272, 3474.6221, 3503.7864, 3528.3171, 3542.3335, 3552.6479, 3574.6042, 3624.6587, 3639.7046, 3645.7671, 3651.7319, 3667.7424, 3707.5422, 3716.6360, 3721.5728, 3722.4707, 3736.5007, 3741.4561, 3748.4346, 3753.4888, 3764.5239, 4075.0212, 4147.9077, 4160.8794, 4238.9067, 4253.8887, 4274.9287, 4301.2393, 4397.9902, 4415.0044, 4452.1357, 4458.2759, 4465.1338, 4631.9888, 4690.3101, 4845.1016

6. F1MVK7 **Mass:** 40714 **Score:** 30 **Expect:** 27 **Matches:** 10

Uncharacterized protein OS=Bos taurus PE=4 SV=2

Observed	Mr(expt)	Mr(calc)	ppm	Start	End	Miss	Peptide
889.4957	888.4884	888.4705	20.1	279	285	0	R.QLLSDWK.V
978.5347	977.5274	977.5216	5.97	247	255	0	R.LSQVGSMLK.T + Oxidation (M)
1650.8488	1649.8415	1649.8512	-5.85	126	139	1	R.LQLFELSDKEATEK.F
2060.9963	2059.9890	2060.0367	-23.15	286	302	1	K.VERLFDLYFYSGQPAQK.K
2241.1360	2240.1287	2240.1590	-13.51	1	19	1	-.LRQTLFGNTIQVFSSDWTK.A
2872.3215	2871.3142	2871.3709	-19.74	230	255	2	R.SDIGYLQWGKGTSDDDRLSQVGSMLK.T + Oxidation (M)
3002.4895	3001.4822	3001.4895	-2.43	126	149	2	R.LQLFELSDKEATEKFIYDHLQCFK.G
3236.5503	3235.5430	3235.5408	0.68	303	329	0	K.KPAHLTIDTHSHHWESVQHEDEHRPGR.R
4452.1357	4451.1284	4451.1366	-1.84	100	139	2	K.ATICLITEDTYVAPTPGYSGDGFSERLQLFELSDKEATEK.F
4465.1338	4464.1265	4464.2159	-20.01	303	339	2	K.KPAHLTIDTHSHHWESVQHEDEHRPGRRFSPLEMVIR.T

No match to: 701.3929, 710.3735, 712.2889, 713.2998, 717.3996, 719.4157, 720.4154, 722.4257, 729.4009, 734.4510, 735.4123, 738.4139, 745.4143, 747.4459, 753.3587, 756.3719, 771.3859, 779.4640, 783.3826, 805.4482, 806.4485, 818.4813, 823.4747, 829.4503, 831.4642, 832.3057, 834.3104, 835.3138, 840.4758, 842.5006, 874.5018, 883.4738, 891.4817, 900.5336, 909.5331, 922.4722, 923.5116, 925.4815, 929.4376, 936.4885, 938.4929, 949.5431, 958.4930, 967.4177, 969.4647, 974.4397, 983.6221, 985.6010, 988.4032, 993.4985, 1007.5710, 1017.5679, 1034.5784, 1036.5477, 1044.5231, 1046.5923, 1051.5060, 1056.4977, 1057.5691, 1058.6145, 1074.6044, 1087.5525, 1113.5417, 1118.5876, 1123.5989, 1143.4429, 1145.6317, 1169.4524, 1172.6801, 1178.6199, 1185.6628, 1187.7002, 1194.5691, 1207.6023, 1216.4987, 1216.7014, 1248.6699, 1254.6930, 1255.6340, 1257.6666, 1265.7057, 1269.6703, 1273.6517, 1278.6932, 1281.6456, 1284.6411, 1286.7430, 1299.5466, 1301.6044, 1304.6500, 1312.7513, 1314.7311, 1317.7122, 1338.6886, 1349.6870, 1351.6915, 1354.7209, 1358.7173, 1365.5874, 1368.6487, 1371.7622, 1372.5999, 1374.6569, 1375.7383, 1382.5975, 1383.7290, 1390.6992, 1393.7732, 1397.7450, 1412.6091, 1413.6514, 1420.7966, 1440.7816, 1442.8168, 1450.8618, 1456.7800, 1457.7509, 1467.7542, 1470.7775, 1494.7429, 1500.7238, 1513.7605, 1528.6982, 1539.7341, 1556.7584, 1562.7856, 1563.7985, 1570.7755, 1571.9341, 1578.7968, 1580.8304, 1590.7900, 1592.7916, 1616.8013, 1628.7054, 1628.8561, 1635.7781, 1642.7219, 1647.8557, 1649.8578, 1657.9104, 1663.8551, 1664.8605, 1696.8295, 1706.8674, 1707.9520, 1715.8798, 1718.8820, 1721.9437, 1730.8646, 1741.9973, 1747.9136, 1755.8970, 1758.9091, 1771.0317, 1774.9242, 1786.9141, 1787.8756, 1862.9530, 1870.7738, 1883.7272, 1895.7275, 1897.7380, 1903.0176, 1911.7487, 1913.7377, 1928.0281, 1938.0094, 1941.0232, 1959.9926, 1961.0339, 1970.0011, 1974.9122, 1979.0769, 1981.0564, 1982.0408, 2007.9467, 2008.9807, 2027.1300, 2055.0208, 2067.1592, 2086.1130, 2108.9956, 2112.9822, 2144.0425, 2181.1780, 2223.1548, 2239.1460, 2245.8369, 2251.1431, 2255.1433, 2259.8535, 2265.0969, 2273.0667, 2286.1521, 2318.1763, 2328.9463, 2335.8999, 2343.1943, 2347.8403, 2352.9194, 2361.8635, 2363.8716, 2364.8738, 2366.9121, 2371.0796, 2389.2690, 2414.2378, 2467.2051, 2474.2170, 2477.2441, 2485.0479, 2503.0457, 2504.9548, 2505.9473, 2519.9768, 2541.2239, 2542.2581, 2580.0098, 2595.0286, 2631.4714, 2646.9739, 2678.0037, 2681.3691, 2684.3965, 2695.0193, 2701.2148, 2705.2874, 2709.0286, 2717.2839, 2770.4202, 2792.2090, 2798.2344, 2799.2815, 2809.4841, 2813.3928, 2816.2957, 2833.0999, 2842.4780, 2846.3840, 2876.3640, 2885.3044, 2919.4407, 2961.2092, 2970.4829, 2976.2786, 2990.3098, 3029.4480, 3071.4028, 3081.6030, 3087.4146, 3146.5461, 3157.4587, 3161.0999, 3184.4336, 3248.6294, 3254.5583, 3265.5901, 3315.6875, 3330.5125, 3334.5588, 3335.6306, 3346.5308, 3347.4827, 3352.6125, 3410.6125, 3411.7185, 3428.5632, 3435.6848, 3457.5947, 3462.6028, 3471.6272, 3474.6221, 3489.6526, 3503.7864, 3528.3171, 3542.3335, 3552.6479, 3574.6042, 3624.6587, 3639.7046, 3645.7671, 3651.7319, 3667.7424, 3690.9905, 3707.5422, 3716.6360, 3721.5728, 3722.4707, 3736.5007,

3741.4561, 3748.4346, 3753.4888, 3764.5239, 4075.0212, 4147.9077, 4160.8794, 4238.9067, 4253.8887, 4274.9287, 4301.2393, 4397.9902, 4415.0044, 4458.2759, 4631.9888, 4690.3101, 4845.1016

7. G3MZZ4 **Mass:** 7599 **Score:** 24 **Expect:** 94 **Matches:** 7

Uncharacterized protein OS=Bos taurus PE=4 SV=1

Observed	Mr(expt)	Mr(calc)	ppm	Start	End	Miss	Peptide
805.4482	804.4409	804.4341	8.46	55 -	61	1	R.ESKNISK.E
1301.6044	1300.5971	1300.5717	19.5	2 -	12	0	K.MEEGHLENAQK.S + Oxidation (M)
1413.6514	1412.6441	1412.6718	-19.57	1 -	12	1	-.KMEEGHLENAQK.S
2008.9807	2007.9734	2007.9612	6.10	13 -	30	1	K.SFETTVGYFGMKPKSGEK.E + Oxidation (M)
2876.3640	2875.3567	2875.2873	24.1	27 -	49	1	K.SGEKEITPNYVFMVWYEFCSDFK.T
3002.4895	3001.4822	3001.4314	16.9	1 -	26	2	-.KMEEGHLENAQKSFETTVGYFGMKPK.S + Oxidation (M)
4465.1338	4464.1265	4464.0469	17.8	13 -	49	2	K.SFETTVGYFGMKPKSGEKEITPNYVFMVWYEFCSDFK.T + Oxidation (M)

No match to: 701.3929, 710.3735, 712.2889, 713.2998, 717.3996, 719.4157, 720.4154, 722.4257, 729.4009, 734.4510, 735.4123, 738.4139, 745.4143, 747.4459, 753.3587, 756.3719, 771.3859, 779.4640, 783.3826, 806.4485, 818.4813, 823.4747, 829.4503, 831.4642, 832.3057, 834.3104, 835.3138, 840.4758, 842.5006, 874.5018, 883.4738, 889.4957, 891.4817, 900.5336, 909.5331, 922.4722, 923.5116, 925.4815, 929.4376, 936.4885, 938.4929, 949.5431, 958.4930, 967.4177, 969.4647, 974.4397, 978.5347, 983.6221, 985.6010, 988.4032, 993.4985, 1007.5710, 1017.5679, 1034.5784, 1036.5477, 1044.5231, 1046.5923, 1051.5060, 1056.4977, 1057.5691, 1058.6145, 1074.6044, 1087.5525, 1113.5417, 1118.5876, 1123.5989, 1143.4429, 1145.6317, 1169.4524, 1172.6801, 1178.6199, 1185.6628, 1187.7002, 1194.5691, 1207.6023, 1216.4987, 1216.7014, 1248.6699, 1254.6930, 1255.6340, 1257.6666, 1265.7057, 1269.6703, 1273.6517, 1278.6932, 1281.6456, 1284.6411, 1286.7430, 1299.5466, 1304.6500, 1312.7513, 1314.7311, 1317.7122, 1338.6886, 1349.6870, 1351.6915, 1354.7209, 1358.7173, 1365.5874, 1368.6487, 1371.7622, 1372.5999, 1374.6569, 1375.7383, 1382.5975, 1383.7290, 1390.6992, 1393.7732, 1397.7450, 1412.6091, 1420.7966, 1440.7816, 1442.8168, 1450.8618, 1456.7800, 1457.7509, 1467.7542, 1470.7775, 1494.7429, 1500.7238, 1513.7605, 1528.6982, 1539.7341, 1556.7584, 1562.7856, 1563.7985, 1570.7755, 1571.9341, 1578.7968, 1580.8304, 1590.7900, 1592.7916, 1616.8013, 1628.7054, 1628.8561, 1635.7781, 1642.7219, 1647.8557, 1649.8578, 1650.8488, 1657.9104, 1663.8551, 1664.8605, 1696.8295, 1706.8674, 1707.9520, 1715.8798, 1718.8820, 1721.9437, 1730.8646, 1741.9973, 1747.9136, 1755.8970, 1758.9091, 1771.0317, 1774.9242, 1786.9141, 1787.8756, 1862.9530, 1870.7738, 1883.7272, 1895.7275, 1897.7380, 1903.0176, 1911.7487, 1913.7377, 1928.0281, 1938.0094, 1941.0232, 1959.9926, 1961.0339, 1970.0011, 1974.9122, 1979.0769, 1981.0564, 1982.0408, 2007.9467, 2027.1300, 2055.0208, 2060.9963, 2067.1592, 2086.1130, 2108.9956, 2112.9822, 2144.0425, 2181.1780, 2223.1548, 2239.1460, 2241.1360, 2245.8369, 2251.1431, 2255.1433, 2259.8535, 2265.0969, 2273.0667, 2286.1521, 2318.1763, 2328.9463, 2335.8999, 2343.1943, 2347.8403, 2352.9194, 2361.8635, 2363.8716, 2364.8738, 2366.9121, 2371.0796, 2389.2690, 2414.2378, 2467.2051, 2474.2170, 2477.2441, 2485.0479, 2503.0457, 2504.9548, 2505.9473, 2519.9768, 2541.2239, 2542.2581, 2580.0098, 2595.0286, 2631.4714, 2646.9739, 2678.0037, 2681.3691, 2684.3965, 2695.0193, 2701.2148, 2705.2874, 2709.0286, 2717.2839, 2770.4202, 2792.2090, 2798.2344, 2799.2815, 2809.4841, 2813.3928, 2816.2957, 2833.0999, 2842.4780, 2846.3840, 2872.3215, 2885.3044, 2919.4407, 2961.2092, 2970.4829, 2976.2786, 2990.3098, 3029.4480, 3071.4028, 3081.6030, 3087.4146, 3146.5461, 3157.4587, 3161.0999, 3184.4336, 3236.5503, 3248.6294, 3254.5583, 3265.5901, 3315.6875, 3330.5125, 3334.5588, 3335.6306, 3346.5308, 3347.4827, 3352.6125, 3410.6125, 3411.7185, 3428.5632, 3435.6848, 3457.5947, 3462.6028, 3471.6272, 3474.6221, 3489.6526, 3503.7864, 3528.3171, 3542.3335, 3552.6479, 3574.6042, 3624.6587, 3639.7046, 3645.7671, 3651.7319, 3667.7424, 3690.9905, 3707.5422, 3716.6360, 3721.5728, 3722.4707, 3736.5007, 3741.4561, 3748.4346, 3753.4888, 3764.5239, 4075.0212, 4147.9077, 4160.8794, 4238.9067, 4253.8887, 4274.9287, 4301.2393, 4397.9902, 4415.0044, 4452.1357, 4458.2759, 4631.9888, 4690.3101, 4845.1016

8. F1MS89 **Mass:** 47695 **Score:** 22 **Expect:** 1.7e+002 **Matches:** 10

Uncharacterized protein OS=Bos taurus GN=FUT1 PE=4 SV=1

Observed	Mr(expt)	Mr(calc)	ppm	Start	End	Miss	Peptide
891.4817	890.4744	890.4570	19.6	24 -	30	2	R.REKSTDR.S
1178.6199	1177.6126	1177.5992	11.4	16 -	24	1	K.HPQTPEWKR.E
1706.8674	1705.8601	1705.8212	22.8	10 -	23	1	R.AWPDSKHPQTPEWK.R
1862.9530	1861.9457	1861.9223	12.6	10 -	24	2	R.AWPDSKHPQTPEWKR.E
1928.0281	1927.0208	1927.0138	3.63	165 -	181	0	R.QAFIQPSMHAVLAPVFR.I + Oxidation (M)
2239.1460	2238.1387	2238.1116	12.1	290 -	308	2	K.GVVGDRAYLQQAMDWFRAR.H
2255.1433	2254.1360	2254.1066	13.1	290 -	308	2	K.GVVGDRAYLQQAMDWFRAR.H + Oxidation (M)
3002.4895	3001.4822	3001.5048	-7.53	102 -	128	2	R.SRVRSPVAILCLSGTPMNPNATFTCPR.H
3667.7424	3666.7351	3666.8055	-19.20	220 -	248	2	K.LTGFPCSWTFFHHLRDQIRSEFTLHEHLR.Q
4075.0212	4074.0139	4074.1084	-23.19	145 -	181	1	R.FGNQMGQYATLLALAQLNGRQAFIQPSMHAVLAPVFR.I + Oxidation (M)

No match to: 701.3929, 710.3735, 712.2889, 713.2998, 717.3996, 719.4157, 720.4154, 722.4257, 729.4009, 734.4510, 735.4123, 738.4139, 745.4143, 747.4459, 753.3587, 756.3719, 771.3859, 779.4640, 783.3826, 805.4482, 806.4485, 818.4813, 823.4747, 829.4503, 831.4642, 832.3057, 834.3104, 835.3138, 840.4758, 842.5006, 874.5018, 883.4738, 889.4957, 900.5336, 909.5331, 922.4722, 923.5116, 925.4815, 929.4376, 936.4885, 938.4929, 949.5431, 958.4930, 967.4177, 969.4647, 974.4397, 978.5347, 983.6221, 985.6010, 988.4032, 993.4985, 1007.5710, 1017.5679, 1034.5784, 1036.5477, 1044.5231, 1046.5923, 1051.5060, 1056.4977, 1057.5691, 1058.6145, 1074.6044, 1087.5525, 1113.5417, 1118.5876, 1123.5989, 1143.4429, 1145.6317, 1169.4524, 1172.6801, 1185.6628, 1187.7002, 1194.5691, 1207.6023, 1216.4987, 1216.7014, 1248.6699, 1254.6930, 1255.6340, 1257.6666, 1265.7057, 1269.6703, 1273.6517, 1278.6932, 1281.6456, 1284.6411, 1286.7430, 1299.5466, 1301.6044, 1304.6500, 1312.7513, 1314.7311, 1317.7122, 1338.6886, 1349.6870, 1351.6915, 1354.7209, 1358.7173, 1365.5874, 1368.6487, 1371.7622, 1372.5999, 1374.6569, 1375.7383, 1382.5975, 1383.7290, 1390.6992, 1393.7732, 1397.7450, 1412.6091, 1413.6514, 1420.7966, 1440.7816, 1442.8168, 1450.8618, 1456.7800, 1457.7509, 1467.7542, 1470.7775, 1494.7429, 1500.7238, 1513.7605, 1528.6982, 1539.7341, 1556.7584, 1562.7856, 1563.7985, 1570.7755, 1571.9341, 1578.7968, 1580.8304, 1590.7900, 1592.7916, 1616.8013, 1628.7054, 1628.8561, 1635.7781, 1642.7219, 1647.8557, 1649.8578, 1650.8488, 1657.9104, 1663.8551, 1664.8605, 1696.8295, 1707.9520, 1715.8798, 1718.8820, 1721.9437, 1730.8646, 1741.9973, 1747.9136, 1755.8970, 1758.9091, 1771.0317, 1774.9242, 1786.9141, 1787.8756, 1870.7738, 1883.7272, 1895.7275, 1897.7380, 1903.0176, 1911.7487, 1913.7377, 1938.0094, 1941.0232, 1959.9926, 1961.0339, 1970.0011, 1974.9122, 1979.0769, 1981.0564, 1982.0408, 2007.9467, 2008.9807, 2027.1300, 2055.0208, 2060.9963, 2067.1592, 2086.1130, 2108.9956, 2112.9822, 2144.0425, 2181.1780, 2223.1548, 2241.1360, 2245.8369, 2251.1431, 2259.8535, 2265.0969, 2273.0667, 2286.1521, 2318.1763, 2328.9463, 2335.8999, 2343.1943, 2347.8403, 2352.9194, 2361.8635, 2363.8716, 2364.8738, 2366.9121, 2371.0796, 2389.2690, 2414.2378, 2467.2051, 2474.2170, 2477.2441, 2485.0479, 2503.0457, 2504.9548, 2505.9473, 2519.9768, 2541.2239, 2542.2581, 2580.0098, 2595.0286, 2631.4714, 2646.9739, 2678.0037, 2681.3691, 2684.3965, 2695.0193, 2701.2148, 2705.2874, 2709.0286, 2717.2839, 2770.4202, 2792.2090, 2798.2344, 2799.2815, 2809.4841, 2813.3928, 2816.2957, 2833.0999, 2842.4780, 2846.3840, 2872.3215, 2876.3640, 2885.3044, 2919.4407, 2961.2092, 2970.4829, 2976.2786, 2990.3098, 3029.4480, 3071.4028, 3081.6030, 3087.4146, 3146.5461, 3157.4587, 3161.0999, 3184.4336, 3236.5503, 3248.6294, 3254.5583, 3265.5901, 3315.6875, 3330.5125, 3334.5588, 3335.6306, 3346.5308, 3347.4827, 3352.6125, 3410.6125, 3411.7185, 3428.5632, 3435.6848, 3457.5947, 3462.6028, 3471.6272, 3474.6221, 3489.6526, 3503.7864, 3528.3171, 3542.3335, 3552.6479, 3574.6042, 3624.6587, 3639.7046, 3645.7671, 3651.7319, 3690.9905, 3707.5422, 3716.6360, 3721.5728, 3722.4707, 3736.5007, 3741.4561, 3748.4346, 3753.4888, 3764.5239, 4147.9077, 4160.8794, 4238.9067, 4253.8887, 4274.9287, 4301.2393, 4397.9902, 4415.0044, 4452.1357, 4458.2759, 4465.1338, 4631.9888, 4690.3101, 4845.1016

9. F1MPP1 **Mass:** 23962 **Score:** 20 **Expect:** 2.2e+002 **Matches:** 7

Ras-related protein Rab-21 OS=Bos taurus GN=RAB21 PE=4 SV=2

Observed	Mr(expt)	Mr(calc)	ppm	Start	End	Miss	Peptide
1390.6992	1389.6919	1389.6922	-0.19	169 -	180	1	R.MIETAQVDERAK.G
1562.7856	1561.7783	1561.7882	-6.33	168 -	180	2	K.RMIETAQVDERAK.G + Oxidation (M)
1628.8561	1627.8488	1627.8430	3.56	56 -	69	1	K.RVNLAIWDTAGQER.F
1706.8674	1705.8601	1705.8709	-6.31	154 -	167	1	K.QNKGIEELFLDLCK.R
1862.9530	1861.9457	1861.9720	-14.10	154 -	168	2	K.QNKGIEELFLDLCKR.M
2681.3691	2680.3618	2680.3200	15.6	157 -	178	2	K.GIEELFLDLCKRMIETAQVDER.A + Oxidation (M)
3002.4895	3001.4822	3001.4711	3.69	11 -	36	2	K.VVLLGEGCVGKTSLVLCYCENKFNDK.H

No match to: 701.3929, 710.3735, 712.2889, 713.2998, 717.3996, 719.4157, 720.4154, 722.4257, 729.4009, 734.4510, 735.4123, 738.4139, 745.4143, 747.4459, 753.3587, 756.3719, 771.3859, 779.4640, 783.3826, 805.4482, 806.4485, 818.4813, 823.4747, 829.4503, 831.4642, 832.3057, 834.3104, 835.3138, 840.4758, 842.5006, 874.5018, 883.4738, 889.4957, 891.4817, 900.5336, 909.5331, 922.4722, 923.5116, 925.4815, 929.4376, 936.4885, 938.4929, 949.5431, 958.4930, 967.4177, 969.4647, 974.4397, 978.5347, 983.6221, 985.6010,

988.4032, 993.4985, 1007.5710, 1017.5679, 1034.5784, 1036.5477, 1044.5231, 1046.5923, 1051.5060, 1056.4977, 1057.5691, 1058.6145, 1074.6044, 1087.5525, 1113.5417, 1118.5876, 1123.5989, 1143.4429, 1145.6317, 1169.4524, 1172.6801, 1178.6199, 1185.6628, 1187.7002, 1194.5691, 1207.6023, 1216.4987, 1216.7014, 1248.6699, 1254.6930, 1255.6340, 1257.6666, 1265.7057, 1269.6703, 1273.6517, 1278.6932, 1281.6456, 1284.6411, 1286.7430, 1299.5466, 1301.6044, 1304.6500, 1312.7513, 1314.7311, 1317.7122, 1338.6886, 1349.6870, 1351.6915, 1354.7209, 1358.7173, 1365.5874, 1368.6487, 1371.7622, 1372.5999, 1374.6569, 1375.7383, 1382.5975, 1383.7290, 1393.7732, 1397.7450, 1412.6091, 1413.6514, 1420.7966, 1440.7816, 1442.8168, 1450.8618, 1456.7800, 1457.7509, 1467.7542, 1470.7775, 1494.7429, 1500.7238, 1513.7605, 1528.6982, 1539.7341, 1556.7584, 1563.7985, 1570.7755, 1571.9341, 1578.7968, 1580.8304, 1590.7900, 1592.7916, 1616.8013, 1628.7054, 1635.7781, 1642.7219, 1647.8557, 1649.8578, 1650.8488, 1657.9104, 1663.8551, 1664.8605, 1696.8295, 1707.9520, 1715.8798, 1718.8820, 1721.9437, 1730.8646, 1741.9973, 1747.9136, 1755.8970, 1758.9091, 1771.0317, 1774.9242, 1786.9141, 1787.8756, 1870.7738, 1883.7272, 1895.7275, 1897.7380, 1903.0176, 1911.7487, 1913.7377, 1928.0281, 1938.0094, 1941.0232, 1959.9926, 1961.0339, 1970.0011, 1974.9122, 1979.0769, 1981.0564, 1982.0408, 2007.9467, 2008.9807, 2027.1300, 2055.0208, 2060.9963, 2067.1592, 2086.1130, 2108.9956, 2112.9822, 2144.0425, 2181.1780, 2223.1548, 2239.1460, 2241.1360, 2245.8369, 2251.1431, 2255.1433, 2259.8535, 2265.0969, 2273.0667, 2286.1521, 2318.1763, 2328.9463, 2335.8999, 2343.1943, 2347.8403, 2352.9194, 2361.8635, 2363.8716, 2364.8738, 2366.9121, 2371.0796, 2389.2690, 2414.2378, 2467.2051, 2474.2170, 2477.2441, 2485.0479, 2503.0457, 2504.9548, 2505.9473, 2519.9768, 2541.2239, 2542.2581, 2580.0098, 2595.0286, 2631.4714, 2646.9739, 2678.0037, 2684.3965, 2695.0193, 2701.2148, 2705.2874, 2709.0286, 2717.2839, 2770.4202, 2792.2090, 2798.2344, 2799.2815, 2809.4841, 2813.3928, 2816.2957, 2833.0999, 2842.4780, 2846.3840, 2872.3215, 2876.3640, 2885.3044, 2919.4407, 2961.2092, 2970.4829, 2976.2786, 2990.3098, 3029.4480, 3071.4028, 3081.6030, 3087.4146, 3146.5461, 3157.4587, 3161.0999, 3184.4336, 3236.5503, 3248.6294, 3254.5583, 3265.5901, 3315.6875, 3330.5125, 3334.5588, 3335.6306, 3346.5308, 3347.4827, 3352.6125, 3410.6125, 3411.7185, 3428.5632, 3435.6848, 3457.5947, 3462.6028, 3471.6272, 3474.6221, 3489.6526, 3503.7864, 3528.3171, 3542.3335, 3552.6479, 3574.6042, 3624.6587, 3639.7046, 3645.7671, 3651.7319, 3667.7424, 3690.9905, 3707.5422, 3716.6360, 3721.5728, 3722.4707, 3736.5007, 3741.4561, 3748.4346, 3753.4888, 3764.5239, 4075.0212, 4147.9077, 4160.8794, 4238.9067, 4253.8887, 4274.9287, 4301.2393, 4397.9902, 4415.0044, 4452.1357, 4458.2759, 4465.1338, 4631.9888, 4690.3101, 4845.1016

10. F1N451 **Mass:** 67798 **Score:** 20 **Expect:** 2.3e+002 **Matches:** 14

Uncharacterized protein OS=Bos taurus GN=GRHL3 PE=4 SV=1

Observed	Mr(expt)	Mr(calc)	ppm	Start	End	Miss	Peptide
734.4510	733.4437	733.4347	12.3	412	416	2	K.QFRRK.V
1371.7622	1370.7549	1370.7340	15.3	561	572	0	R.GILVNMDNNIIR.H
1580.8304	1579.8231	1579.7916	20.0	517	529	1	R.RETEEVFDALMLK.T
1649.8578	1648.8505	1648.8137	22.3	544	556	1	K.YGFPEENIYKVYK.K
1657.9104	1656.9031	1656.9311	-16.90	6	19	2	R.SVRLLKNDPVNFQK.F
1758.9091	1757.9018	1757.9134	-6.58	237	252	1	K.AIHIKSGESPMAYLNK.G
1774.9242	1773.9169	1773.9083	4.86	237	252	1	K.AIHIKSGESPMAYLNK.G + Oxidation (M)
1787.8756	1786.8683	1786.8560	6.89	347	362	0	K.VFVGVNCLSTDFSSQK.G
2265.0969	2264.0896	2264.1364	-20.68	538	556	2	R.SAISEKYGFPEENIYKVYK.K
2701.2148	2700.2075	2700.1910	6.13	87	108	0	R.YYHGMEYEMDLTPLESPTHLMK.F + Oxidation (M)
2770.4202	2769.4129	2769.4523	-14.23	573	596	1	R.HYSNHVAFLLDMGELDGKIQIILK.E + Oxidation (M)
3411.7185	3410.7112	3410.6670	13.0	211	241	2	K.TSPEPPGPEDYPNPKSDFEYTLGSPKAIHIK.S
3503.7864	3502.7791	3502.7654	3.93	109	141	2	K.FLTENVSGTPEYPDVLKKNNLMSVEGAAPTPGK.A
4147.9077	4146.9004	4146.9991	-23.79	347	384	2	K.VFVGVNCLSTDFSSQKGKVKGVPLNLQIDTYDCGSGTER.L

No match to: 701.3929, 710.3735, 712.2889, 713.2998, 717.3996, 719.4157, 720.4154, 722.4257, 729.4009, 735.4123, 738.4139, 745.4143, 747.4459, 753.3587, 756.3719, 771.3859, 779.4640, 783.3826, 805.4482, 806.4485, 818.4813, 823.4747, 829.4503, 831.4642, 832.3057, 834.3104, 835.3138, 840.4758, 842.5006, 874.5018, 883.4738, 889.4957, 891.4817, 900.5336, 909.5331, 922.4722, 923.5116, 925.4815, 929.4376, 936.4885, 938.4929, 949.5431, 958.4930, 967.4177, 969.4647, 974.4397, 978.5347, 983.6221, 985.6010, 988.4032, 993.4985, 1007.5710, 1017.5679, 1034.5784, 1036.5477, 1044.5231, 1046.5923, 1051.5060, 1056.4977, 1057.5691, 1058.6145, 1074.6044, 1087.5525, 1113.5417, 1118.5876, 1123.5989, 1143.4429, 1145.6317, 1169.4524, 1172.6801, 1178.6199, 1185.6628, 1187.7002, 1194.5691, 1207.6023, 1216.4987, 1216.7014, 1248.6699, 1254.6930, 1255.6340, 1257.6666, 1265.7057, 1269.6703, 1273.6517, 1278.6932, 1281.6456, 1284.6411, 1286.7430, 1299.5466, 1301.6044, 1304.6500, 1312.7513, 1314.7311, 1317.7122, 1338.6886, 1349.6870, 1351.6915, 1354.7209, 1358.7173, 1365.5874, 1368.6487, 1372.5999, 1374.6569, 1375.7383, 1382.5975, 1383.7290, 1390.6992, 1393.7732, 1397.7450, 1412.6091, 1413.6514, 1420.7966, 1440.7816, 1442.8168, 1450.8618, 1456.7800, 1457.7509, 1467.7542, 1470.7775, 1494.7429, 1500.7238, 1513.7605, 1528.6982, 1539.7341, 1556.7584, 1562.7856, 1563.7985, 1570.7755, 1571.9341, 1578.7968, 1590.7900, 1592.7916, 1616.8013, 1628.7054, 1628.8561, 1635.7781, 1642.7219, 1647.8557, 1650.8488, 1663.8551, 1664.8605, 1696.8295, 1706.8674, 1707.9520, 1715.8798, 1718.8820, 1721.9437, 1730.8646, 1741.9973, 1747.9136, 1755.8970, 1771.0317, 1786.9141, 1862.9530, 1870.7738, 1883.7272, 1895.7275, 1897.7380, 1903.0176, 1911.7487, 1913.7377, 1928.0281, 1938.0094, 1941.0232, 1959.9926, 1961.0339, 1970.0011, 1974.9122, 1979.0769, 1981.0564, 1982.0408, 2007.9467, 2008.9807, 2027.1300, 2055.0208, 2060.9963, 2067.1592, 2086.1130, 2108.9956, 2112.9822, 2144.0425, 2181.1780, 2223.1548, 2239.1460, 2241.1360, 2245.8369, 2251.1431, 2255.1433, 2259.8535, 2273.0667, 2286.1521, 2318.1763, 2328.9463, 2335.8999, 2343.1943, 2347.8403, 2352.9194, 2361.8635, 2363.8716, 2364.8738, 2366.9121, 2371.0796, 2389.2690, 2414.2378, 2467.2051, 2474.2170, 2477.2441, 2485.0479, 2503.0457, 2504.9548, 2505.9473, 2519.9768, 2541.2239, 2542.2581, 2580.0098, 2595.0286, 2631.4714, 2646.9739, 2678.0037, 2681.3691, 2684.3965, 2695.0193, 2705.2874, 2709.0286, 2717.2839, 2792.2090, 2798.2344, 2799.2815, 2809.4841, 2813.3928, 2816.2957, 2833.0999, 2842.4780, 2846.3840, 2872.3215, 2876.3640, 2885.3044, 2919.4407, 2961.2092, 2970.4829, 2976.2786, 2990.3098, 3002.4895, 3029.4480, 3071.4028, 3081.6030, 3087.4146, 3146.5461, 3157.4587, 3161.0999, 3184.4336, 3236.5503, 3248.6294, 3254.5583, 3265.5901, 3315.6875, 3330.5125, 3334.5588, 3335.6306, 3346.5308, 3347.4827, 3352.6125, 3410.6125, 3428.5632, 3435.6848, 3457.5947, 3462.6028, 3471.6272, 3474.6221, 3489.6526, 3528.3171, 3542.3335, 3552.6479, 3574.6042, 3624.6587, 3639.7046, 3645.7671, 3651.7319, 3667.7424, 3690.9905, 3707.5422, 3716.6360, 3721.5728, 3722.4707, 3736.5007, 3741.4561, 3748.4346, 3753.4888, 3764.5239, 4075.0212, 4160.8794, 4238.9067, 4253.8887, 4274.9287, 4301.2393, 4397.9902, 4415.0044, 4452.1357, 4458.2759, 4465.1338, 4631.9888, 4690.3101, 4845.1016

11. Q2YDN4 **Mass:** 58176 **Score:** 20 **Expect:** 2.6e+002 **Matches:** 14

Coiled-coil domain-containing protein 105 OS=Bos taurus GN=CCDC105 PE=2 SV=1

Observed	Mr(expt)	Mr(calc)	ppm	Start	End	Miss	Peptide
922.4722	921.4650	921.4477	18.7	309	316	0	K.DTLLDMAK.N + Oxidation (M)
938.4929	937.4856	937.5055	-21.19	73	79	1	R.FRVEMLK.G + Oxidation (M)
1036.5477	1035.5404	1035.5382	2.11	204	211	2	K.EELKSMKR.K + Oxidation (M)
1178.6199	1177.6126	1177.6204	-6.57	404	414	0	R.HVGTQLPEAAR.L
1351.6915	1350.6842	1350.6748	7.00	348	358	1	K.DRLNMTLGLMR.G + 2 Oxidation (M)
1393.7732	1392.7659	1392.7473	13.3	119	131	0	R.ALQTAEVVQAHAR.G
1494.7429	1493.7356	1493.7105	16.8	213	225	1	K.MEADMEKSEALLK.T
1628.8561	1627.8488	1627.8392	5.92	367	379	1	K.FNQEMYITRGLIK.G + Oxidation (M)
1649.8578	1648.8505	1648.8896	-23.72	181	194	2	K.LRGYRPKSETISDK.A
1718.8820	1717.8747	1717.8821	-4.32	1	15	0	-.MPVLIPPVEHSQDTR.V
1938.0094	1937.0021	1937.0392	-19.16	100	115	1	K.MKPPAWHARLPLPMHR.D
1970.0011	1968.9938	1969.0291	-17.90	100	115	1	K.MKPPAWHARLPLPMHR.D + 2 Oxidation (M)
2086.1130	2085.1057	2085.0565	23.6	80	99	1	K.GGSTMEKPPPGEGVTLWKSK.M
4465.1338	4464.1265	4464.0658	13.6	27	64	2	R.ALAEEAHQLTDRCGQEAVTMWQPNESVRDPHVAHHLCR.A + Oxidation (M)

No match to: 701.3929, 710.3735, 712.2889, 713.2998, 717.3996, 719.4157, 720.4154, 722.4257, 729.4009, 734.4510, 735.4123, 738.4139, 745.4143, 747.4459, 753.3587, 756.3719, 771.3859, 779.4640, 783.3826, 805.4482, 806.4485, 818.4813, 823.4747, 829.4503, 831.4642, 832.3057, 834.3104, 835.3138, 840.4758, 842.5006, 874.5018, 883.4738, 889.4957, 891.4817, 900.5336, 909.5331, 923.5116, 925.4815, 929.4376, 936.4885, 949.5431, 958.4930, 967.4177, 969.4647, 974.4397, 978.5347, 983.6221, 985.6010, 988.4032, 993.4985, 1007.5710, 1017.5679, 1034.5784, 1044.5231, 1046.5923, 1051.5060, 1056.4977, 1057.5691, 1058.6145, 1074.6044, 1087.5525, 1113.5417, 1118.5876, 1123.5989, 1143.4429, 1145.6317, 1169.4524, 1172.6801, 1185.6628, 1187.7002, 1194.5691, 1207.6023, 1216.4987, 1216.7014, 1248.6699, 1254.6930, 1255.6340, 1257.6666, 1265.7057, 1269.6703, 1273.6517, 1278.6932, 1281.6456, 1284.6411, 1286.7430, 1299.5466, 1301.6044, 1304.6500, 1312.7513, 1314.7311, 1317.7122, 1338.6886, 1349.6870, 1354.7209, 1358.7173, 1365.5874, 1368.6487, 1371.7622, 1372.5999, 1374.6569, 1375.7383, 1382.5975, 1383.7290, 1390.6992, 1397.7450, 1412.6091, 1413.6514, 1420.7966, 1440.7816, 1442.8168, 1450.8618, 1456.7800, 1457.7509, 1467.7542, 1470.7775, 1500.7238, 1513.7605, 1528.6982, 1539.7341, 1556.7584, 1562.7856, 1563.7985,

1570.7755, 1571.9341, 1578.7968, 1580.8304, 1590.7900, 1592.7916, 1616.8013, 1628.7054, 1635.7781, 1642.7219, 1647.8557, 1650.8488, 1657.9104, 1663.8551, 1664.8605, 1696.8295, 1706.8674, 1707.9520, 1715.8798, 1721.9437, 1730.8646, 1741.9973, 1747.9136, 1755.8970, 1758.9091, 1771.0317, 1774.9242, 1786.9141, 1787.8756, 1862.9530, 1870.7738, 1883.7272, 1895.7275, 1897.7380, 1903.0176, 1911.7487, 1913.7377, 1928.0281, 1941.0232, 1959.9926, 1961.0339, 1974.9122, 1979.0769, 1981.0564, 1982.0408, 2007.9467, 2008.9807, 2027.1300, 2055.0208, 2060.9963, 2067.1592, 2108.9956, 2112.9822, 2144.0425, 2181.1780, 2223.1548, 2239.1460, 2241.1360, 2245.8369, 2251.1431, 2255.1433, 2259.8535, 2265.0969, 2273.0667, 2286.1521, 2318.1763, 2328.9463, 2335.8999, 2343.1943, 2347.8403, 2352.9194, 2361.8635, 2363.8716, 2364.8738, 2366.9121, 2371.0796, 2389.2690, 2414.2378, 2467.2051, 2474.2170, 2477.2441, 2485.0479, 2503.0457, 2504.9548, 2505.9473, 2519.9768, 2541.2239, 2542.2581, 2580.0098, 2595.0286, 2631.4714, 2646.9739, 2678.0037, 2681.3691, 2684.3965, 2695.0193, 2701.2148, 2705.2874, 2709.0286, 2717.2839, 2770.4202, 2792.2090, 2798.2344, 2799.2815, 2809.4841, 2813.3928, 2816.2957, 2833.0999, 2842.4780, 2846.3840, 2872.3215, 2876.3640, 2885.3044, 2919.4407, 2961.2092, 2970.4829, 2976.2786, 2990.3098, 3002.4895, 3029.4480, 3071.4028, 3081.6030, 3087.4146, 3146.5461, 3157.4587, 3161.0999, 3184.4336, 3236.5503, 3248.6294, 3254.5583, 3265.5901, 3315.6875, 3330.5125, 3334.5588, 3335.6306, 3346.5308, 3347.4827, 3352.6125, 3410.6125, 3411.7185, 3428.5632, 3435.6848, 3457.5947, 3462.6028, 3471.6272, 3474.6221, 3489.6526, 3503.7864, 3528.3171, 3542.3335, 3552.6479, 3574.6042, 3624.6587, 3639.7046, 3645.7671, 3651.7319, 3667.7424, 3690.9905, 3707.5422, 3716.6360, 3721.5728, 3722.4707, 3736.5007, 3741.4561, 3748.4346, 3753.4888, 3764.5239, 4075.0212, 4147.9077, 4160.8794, 4238.9067, 4253.8887, 4274.9287, 4301.2393, 4397.9902, 4415.0044, 4452.1357, 4458.2759, 4631.9888, 4690.3101, 4845.1016

12. F1N367 **Mass:** 244177 **Score:** 20 **Expect:** 2.6e+002 **Matches:** 38

Aggrecan core protein OS=Bos taurus GN=ACAN PE=4 SV=2

Observed	Mr(expt)	Mr(calc)	ppm	Start	End	Miss	Peptide
722.4257	721.4184	721.4235	-7.02	2282 -	2287	0	R.HVPTIR.C
831.4642	830.4570	830.4650	-9.72	545 -	551	0	R.YPIVSPR.T
883.4738	882.4665	882.4712	-5.33	211 -	217	0	R.YPIHTPR.E
891.4817	890.4744	890.4763	-2.09	152 -	158	0	K.GIVFHYR.A
929.4376	928.4303	928.4290	1.33	164 -	170	0	R.YTLDFDR.A
949.5431	948.5358	948.5392	-3.62	306 -	313	1	R.SVRYPISK.A
967.4177	966.4104	966.4130	-2.73	2125 -	2131	0	K.FQGHCYR.H
974.4397	973.4324	973.4341	-1.72	678 -	684	0	R.HHAFCFR.G
1087.5525	1086.5452	1086.5233	20.2	498 -	506	0	R.YSLTFEEAK.Q
1185.6628	1184.6555	1184.6765	-17.69	83 -	93	0	K.EVVLLVATEGR.V
1248.6699	1247.6626	1247.6622	0.34	2262 -	2271	1	K.DRYEINALVR.Y
1284.6411	1283.6338	1283.6258	6.22	164 -	173	1	R.YTLDFDRAQR.A
1301.6044	1300.5971	1300.5870	7.77	2272 -	2281	0	R.YQCTEGFIQR.H
1368.6487	1367.6414	1367.6292	8.93	631 -	642	0	K.CYAGWLADGSLR.Y
1372.5999	1371.5926	1371.5877	3.56	264 -	274	0	K.FTFQEAANECR.R
1382.5975	1381.5902	1381.5833	4.99	2288 -	2298	0	R.CQPSGHWEEPR.I
1383.7290	1382.7217	1382.7201	1.18	314 -	326	0	K.ARPNCGGNLLGVR.T
1442.8168	1441.8095	1441.8140	-3.12	81 -	93	1	K.EKEVVLLVATEGR.V
1457.7509	1456.7436	1456.7310	8.64	159 -	170	1	R.AISTRYTLDFDR.A
1528.6982	1527.6909	1527.6888	1.36	264 -	275	1	K.FTFQEAANECRR.L
1578.7968	1577.7895	1577.7620	17.4	2241 -	2255	0	K.GTVACGEPPVVEHAR.I
1628.7054	1627.6981	1627.6937	2.73	218 -	231	1	R.EGCYGDKDEFPGVR.T
1628.8561	1627.8488	1627.8583	-5.83	483 -	497	0	R.LPGGVVFHYRPGSSR.Y
1706.8674	1705.8601	1705.8570	1.83	2240 -	2255	1	K.KGTVACGEPPVVEHAR.I
1715.8798	1714.8725	1714.8348	22.0	498 -	511	1	R.YSLTFEEAKQACLR.T
1771.0317	1770.0244	1770.0251	-0.36	78 -	93	2	R.ISKEKEVVLLVATEGR.V
1974.9122	1973.9049	1973.8802	12.5	2132 -	2147	1	R.HFPDRATWVDAESQCR.K
2144.0425	2143.0352	2143.0289	2.93	133 -	151	0	R.CEVMHGIEDSQATLEVVVK.G
2343.1943	2342.1870	2342.1940	-2.98	104 -	124	0	K.VTLPNYPAIPSDATLEIQNMR.S
2872.3215	2871.3142	2871.3142	-0.01	280 -	305	0	R.LATTGQLYLAWQGGMDMCSAGWLADR.S
3236.5503	3235.5430	3235.5291	4.29	2282 -	2308	2	R.HVPTIRCQPSGHWEEPRITCTDPATYK.R
3248.6294	3247.6221	3247.6183	1.18	96 -	124	1	R.VNSAYQDKVTLPNYPAIPSDATLEIQNMR.S
3457.5947	3456.5874	3456.6489	-17.79	275 -	305	2	R.RLGARLATTGQLYLAWQGGMDMCSAGWLADR.S + 2 Oxidation (M)
3489.6526	3488.6453	3488.6347	3.04	566 -	594	1	R.TYGVRPPSETYDVYCYVDRLEGEVFFATR.L
3503.7864	3502.7791	3502.7878	-2.48	94 -	124	2	R.VRVNSAYQDKVTLPNYPAIPSDATLEIQNMR.S
3639.7046	3638.6973	3638.6947	0.72	512 -	544	0	R.TGAIIASPEQLQAAYEAGYEQCDAGWLQDQTVR.Y
4147.9077	4146.9004	4146.9164	-3.84	174 -	210	0	R.ACLQNSAIIATPEQLQAAYEDGFHQCDAGWLADQTVR.Y
4452.1357	4451.1284	4451.1492	-4.66	512 -	551	1	R.TGAIIASPEQLQAAYEAGYEQCDAGWLQDQTVRYPIVSPR.T

No match to: 701.3929, 710.3735, 712.2889, 713.2998, 717.3996, 719.4157, 720.4154, 729.4009, 734.4510, 735.4123, 738.4139, 745.4143, 747.4459, 753.3587, 756.3719, 771.3859, 779.4640, 783.3826, 805.4482, 806.4485, 818.4813, 823.4747, 829.4503, 832.3057, 834.3104, 835.3138, 840.4758, 842.5006, 874.5018, 889.4957, 900.5336, 909.5331, 922.4722, 923.5116, 925.4815, 936.4885, 938.4929, 958.4930, 969.4647, 978.5347, 983.6221, 985.6010, 988.4032, 993.4985, 1007.5710, 1017.5679, 1034.5784, 1036.5477, 1044.5231, 1046.5923, 1051.5060, 1056.4977, 1057.5691, 1058.6145, 1074.6044, 1113.5417, 1118.5876, 1123.5989, 1143.4429, 1145.6317, 1169.4524, 1172.6801, 1178.6199, 1187.7002, 1194.5691, 1207.6023, 1216.4987, 1216.7014, 1254.6930, 1255.6340, 1257.6666, 1265.7057, 1269.6703, 1273.6517, 1278.6932, 1281.6456, 1286.7430, 1299.5466, 1304.6500, 1312.7513, 1314.7311, 1317.7122, 1338.6886, 1349.6870, 1351.6915, 1354.7209, 1358.7173, 1365.5874, 1371.7622, 1374.6569, 1375.7383, 1390.6992, 1393.7732, 1397.7450, 1412.6091, 1413.6514, 1420.7966, 1440.7816, 1450.8618, 1456.7800, 1467.7542, 1470.7775, 1494.7429, 1500.7238, 1513.7605, 1539.7341, 1556.7584, 1562.7856, 1563.7985, 1570.7755, 1571.9341, 1580.8304, 1590.7900, 1592.7916, 1616.8013, 1635.7781, 1642.7219, 1647.8557, 1649.8578, 1650.8488, 1657.9104, 1663.8551, 1664.8605, 1696.8295, 1707.9520, 1718.8820, 1721.9437, 1730.8646, 1741.9973, 1747.9136, 1755.8970, 1758.9091, 1774.9242, 1786.9141, 1787.8756, 1862.9530, 1870.7738, 1883.7272, 1895.7275, 1897.7380, 1903.0176, 1911.7487, 1913.7377, 1928.0281, 1938.0094, 1941.0232, 1959.9926, 1961.0339, 1970.0011, 1979.0769, 1981.0564, 1982.0408, 2007.9467, 2008.9807, 2027.1300, 2055.0208, 2060.9963, 2067.1592, 2086.1130, 2108.9956, 2112.9822, 2181.1780, 2223.1548, 2239.1460, 2241.1360, 2245.8369, 2251.1431, 2255.1433, 2259.8535, 2265.0969, 2273.0667, 2286.1521, 2318.1763, 2328.9463, 2335.8999, 2347.8403, 2352.9194, 2361.8635, 2363.8716, 2364.8738, 2366.9121, 2371.0796, 2389.2690, 2414.2378, 2467.2051, 2474.2170, 2477.2441, 2485.0479, 2503.0457, 2504.9548, 2505.9473, 2519.9768, 2541.2239, 2542.2581, 2580.0098, 2595.0286, 2631.4714, 2646.9739, 2678.0037, 2681.3691, 2684.3965, 2695.0193, 2701.2148, 2705.2874, 2709.0286, 2717.2839, 2770.4202, 2792.2090, 2798.2344, 2799.2815, 2809.4841, 2813.3928, 2816.2957, 2833.0999, 2842.4780, 2846.3840, 2876.3640, 2885.3044, 2919.4407, 2961.2092, 2970.4829, 2976.2786, 2990.3098, 3002.4895, 3029.4480, 3071.4028, 3081.6030, 3087.4146, 3146.5461, 3157.4587, 3161.0999, 3184.4336, 3254.5583, 3265.5901, 3315.6875, 3330.5125, 3334.5588, 3335.6306, 3346.5308, 3347.4827, 3352.6125, 3410.6125, 3411.7185, 3428.5632, 3435.6848, 3462.6028, 3471.6272, 3474.6221, 3528.3171, 3542.3335, 3552.6479, 3574.6042, 3624.6587, 3645.7671, 3651.7319, 3667.7424, 3690.9905, 3707.5422, 3716.6360, 3721.5728, 3722.4707, 3736.5007, 3741.4561, 3748.4346, 3753.4888, 3764.5239, 4075.0212, 4160.8794, 4238.9067, 4253.8887, 4274.9287, 4301.2393, 4397.9902, 4415.0044, 4458.2759, 4465.1338, 4631.9888, 4690.3101, 4845.1016

13. Q17R06 **Mass:** 24530 **Score:** 20 **Expect:** 2.7e+002 **Matches:** 7

Ras-related protein Rab-21 OS=Bos taurus GN=RAB21 PE=2 SV=1

Observed	Mr(expt)	Mr(calc)	ppm	Start	End	Miss	Peptide
713.2998	712.2925	712.2850	10.6	36 -	40	0	R.YCENK.F
1390.6992	1389.6919	1389.6922	-0.19	177 -	188	1	R.MIETAQVDERAK.G
1562.7856	1561.7783	1561.7882	-6.33	176 -	188	2	K.RMIETAQVDERAK.G + Oxidation (M)
1628.8561	1627.8488	1627.8430	3.56	64 -	77	1	K.RVNLAIWDTAGQER.F
1706.8674	1705.8601	1705.8709	-6.31	162 -	175	1	K.QNKGIEELFLDLCK.R

1862.9530	1861.9457	1861.9720	-14.10	162 - 176	2	K.QNKGIEELFLDLCKR.M
2681.3691	2680.3618	2680.3200	15.6	165 - 186	2	K.GIEELFLDLCKRMIETAQVDER.A + Oxidation (M)

No match to: 701.3929, 710.3735, 712.2889, 717.3996, 719.4157, 720.4154, 722.4257, 729.4009, 734.4510, 735.4123, 738.4139, 745.4143, 747.4459, 753.3587, 756.3719, 771.3859, 779.4640, 783.3826, 805.4482, 806.4485, 818.4813, 823.4747, 829.4503, 831.4642, 832.3057, 834.3104, 835.3138, 840.4758, 842.5006, 874.5018, 883.4738, 889.4957, 891.4817, 900.5336, 909.5331, 922.4722, 923.5116, 925.4815, 929.4376, 936.4885, 938.4929, 949.5431, 958.4930, 967.4177, 969.4647, 974.4397, 978.5347, 983.6221, 985.6010, 988.4032, 993.4985, 1007.5710, 1017.5679, 1034.5784, 1036.5477, 1044.5231, 1046.5923, 1051.5060, 1056.4977, 1057.5691, 1058.6145, 1074.6044, 1087.5525, 1113.5417, 1118.5876, 1123.5989, 1143.4429, 1145.6317, 1169.4524, 1172.6801, 1178.6199, 1185.6628, 1187.7002, 1194.5691, 1207.6023, 1216.4987, 1216.7014, 1248.6699, 1254.6930, 1255.6340, 1257.6666, 1265.7057, 1269.6703, 1273.6517, 1278.6932, 1281.6456, 1284.6411, 1286.7430, 1299.5466, 1301.6044, 1304.6500, 1312.7513, 1314.7311, 1317.7122, 1338.6886, 1349.6870, 1351.6915, 1354.7209, 1358.7173, 1365.5874, 1368.6487, 1371.7622, 1372.5999, 1374.6569, 1375.7383, 1382.5975, 1383.7290, 1393.7732, 1397.7450, 1412.6091, 1413.6514, 1420.7966, 1440.7816, 1442.8168, 1450.8618, 1456.7800, 1457.7509, 1467.7542, 1470.7775, 1494.7429, 1500.7238, 1513.7605, 1528.6982, 1539.7341, 1556.7584, 1563.7985, 1570.7755, 1571.9341, 1578.7968, 1580.8304, 1590.7900, 1592.7916, 1616.8013, 1628.7054, 1635.7781, 1642.7219, 1647.8557, 1649.8578, 1650.8488, 1657.9104, 1663.8551, 1664.8605, 1696.8295, 1707.9520, 1715.8798, 1718.8820, 1721.9437, 1730.8646, 1741.9973, 1747.9136, 1755.8970, 1758.9091, 1771.0317, 1774.9242, 1786.9141, 1787.8756, 1870.7738, 1883.7272, 1895.7275, 1897.7380, 1903.0176, 1911.7487, 1913.7377, 1928.0281, 1938.0094, 1941.0232, 1959.9926, 1961.0339, 1970.0011, 1974.9122, 1979.0769, 1981.0564, 1982.0408, 2007.9467, 2008.9807, 2027.1300, 2055.0208, 2060.9963, 2067.1592, 2086.1130, 2108.9956, 2112.9822, 2144.0425, 2181.1780, 2223.1548, 2239.1460, 2241.1360, 2245.8369, 2251.1431, 2255.1433, 2259.8535, 2265.0969, 2273.0667, 2286.1521, 2318.1763, 2328.9463, 2335.8999, 2343.1943, 2347.8403, 2352.9194, 2361.8635, 2363.8716, 2364.8738, 2366.9121, 2371.0796, 2389.2690, 2414.2378, 2467.2051, 2474.2170, 2477.2441, 2485.0479, 2503.0457, 2504.9548, 2505.9473, 2519.9768, 2541.2239, 2542.2581, 2580.0098, 2595.0286, 2631.4714, 2646.9739, 2678.0037, 2684.3965, 2695.0193, 2701.2148, 2705.2874, 2709.0286, 2717.2839, 2770.4202, 2792.2090, 2798.2344, 2799.2815, 2809.4841, 2813.3928, 2816.2957, 2833.0999, 2842.4780, 2846.3840, 2872.3215, 2876.3640, 2885.3044, 2919.4407, 2961.2092, 2970.4829, 2976.2786, 2990.3098, 3002.4895, 3029.4480, 3071.4028, 3081.6030, 3087.4146, 3146.5461, 3157.4587, 3161.0999, 3184.4336, 3236.5503, 3248.6294, 3254.5583, 3265.5901, 3315.6875, 3330.5125, 3334.5588, 3335.6306, 3346.5308, 3347.4827, 3352.6125, 3410.6125, 3411.7185, 3428.5632, 3435.6848, 3457.5947, 3462.6028, 3471.6272, 3474.6221, 3489.6526, 3503.7864, 3528.3171, 3542.3335, 3552.6479, 3574.6042, 3624.6587, 3639.7046, 3645.7671, 3651.7319, 3667.7424, 3690.9905, 3707.5422, 3716.6360, 3721.5728, 3722.4707, 3736.5007, 3741.4561, 3748.4346, 3753.4888, 3764.5239, 4075.0212, 4147.9077, 4160.8794, 4238.9067, 4253.8887, 4274.9287, 4301.2393, 4397.9902, 4415.0044, 4452.1357, 4458.2759, 4465.1338, 4631.9888, 4690.3101, 4845.1016

14. F1N368 **Mass:** 248513 **Score:** 19 **Expect:** 2.9e+002 **Matches:** 38

Aggrecan core protein OS=Bos taurus GN=ACAN PE=4 SV=2

Observed	Mr(expt)	Mr(calc)	ppm	Start	End	Miss	Peptide
722.4257	721.4184	721.4235	-7.02	2320	2325	0	R.HVPTIR.C
831.4642	830.4570	830.4650	-9.72	545	551	0	R.YPIVSPR.T
883.4738	882.4665	882.4712	-5.33	211	217	0	R.YPIHTPR.E
891.4817	890.4744	890.4763	-2.09	152	158	0	K.GIVFHYR.A
929.4376	928.4303	928.4290	1.33	164	170	0	R.YTLDFDR.A
949.5431	948.5358	948.5392	-3.62	306	313	1	R.SVRYPISK.A
967.4177	966.4104	966.4130	-2.73	2163	2169	0	K.FQGHCYR.H
974.4397	973.4324	973.4341	-1.72	678	684	0	R.HHAFCFR.G
1087.5525	1086.5452	1086.5233	20.2	498	506	0	R.YSLTFEEAK.Q
1185.6628	1184.6555	1184.6765	-17.69	83	93	0	K.EVVLLVATEGR.V
1248.6699	1247.6626	1247.6622	0.34	2300	2309	1	K.DRYEINALVR.Y
1284.6411	1283.6338	1283.6258	6.22	164	173	1	R.YTLDFDRAQR.A
1301.6044	1300.5971	1300.5870	7.77	2310	2319	0	R.YQCTEGFIQR.H
1368.6487	1367.6414	1367.6292	8.93	631	642	0	K.CYAGWLADGSLR.Y
1372.5999	1371.5926	1371.5877	3.56	264	274	0	K.FTFQEAANECR.R
1382.5975	1381.5902	1381.5833	4.99	2326	2336	0	R.CQPSGHWEEPR.I
1383.7290	1382.7217	1382.7201	1.18	314	326	0	K.ARPNCGGNLLGVR.T
1442.8168	1441.8095	1441.8140	-3.12	81	93	1	K.EKEVVLLVATEGR.V
1457.7509	1456.7436	1456.7310	8.64	159	170	1	R.AISTRYTLDFDR.A
1528.6982	1527.6909	1527.6888	1.36	264	275	1	K.FTFQEAANECRR.L
1578.7968	1577.7895	1577.7620	17.4	2279	2293	0	K.GTVACGEPPVVEHAR.I
1628.7054	1627.6981	1627.6943	2.73	218	231	1	R.EGCYGDKDEFPGVR.T
1628.8561	1627.8488	1627.8583	-5.83	483	497	0	R.LPGGVVFHYRPGSSR.Y
1706.8674	1705.8601	1705.8570	1.83	2278	2293	1	K.KGTVACGEPPVVEHAR.I
1715.8798	1714.8725	1714.8348	22.0	498	511	1	R.YSLTFEEAKQACLR.T
1771.0317	1770.0244	1770.0251	-0.36	78	93	2	R.ISKEKEVVLLVATEGR.V
1974.9122	1973.9049	1973.8802	12.5	2170	2185	1	R.HFPDRATWVDAESQCR.K
2144.0425	2143.0352	2143.0289	2.93	133	151	0	R.CEVMHGIEDSQATLEVVVK.G
2343.1943	2342.1870	2342.1940	-2.98	104	124	0	K.VTLPNYPAIPSDATLEIQNMR.S
2872.3215	2871.3142	2871.3142	-0.01	280	305	0	R.LATTGQLYLAWQGGMDMCSAGWLADR.S
3236.5503	3235.5430	3235.5291	4.29	2320	2346	2	R.HVPTIRCQPSGHWEEPRITCTDPATYK.R
3248.6294	3247.6221	3247.6183	1.18	96	124	1	R.VNSAYQDKVTLPNYPAIPSDATLEIQNMR.S
3457.5947	3456.5874	3456.6489	-17.79	275	305	2	R.RLGARLATTGQLYLAWQGGMDMCSAGWLADR.S + 2 Oxidation (M)
3489.6526	3488.6453	3488.6347	3.04	566	594	1	R.TYGVRPPSETYDVYCYVDRLEGEVFFATR.L
3503.7864	3502.7791	3502.7878	-2.48	94	124	2	R.VRVNSAYQDKVTLPNYPAIPSDATLEIQNMR.S
3639.7046	3638.6973	3638.6947	0.72	512	544	0	R.TGAIIASPEQLQAAYEAGYEQCDAGWLQDQTVR.Y
4147.9077	4146.9004	4146.9164	-3.84	174	210	0	R.ACLQNSAIIATPEQLQAAYEDGFHQCDAGWLADQTVR.Y
4452.1357	4451.1284	4451.1492	-4.66	512	551	1	R.TGAIIASPEQLQAAYEAGYEQCDAGWLQDQTVRYPIVSPR.T

No match to: 701.3929, 710.3735, 712.2889, 713.2998, 717.3996, 719.4157, 720.4154, 729.4009, 734.4510, 735.4123, 738.4139, 745.4143, 747.4459, 753.3587, 756.3719, 771.3859, 779.4640, 783.3826, 805.4482, 806.4485, 818.4813, 823.4747, 829.4503, 832.3057, 834.3104, 835.3138, 840.4758, 842.5006, 874.5018, 889.4957, 900.5336, 909.5331, 922.4722, 923.5116, 925.4815, 936.4885, 938.4929, 958.4930, 969.4647, 978.5347, 983.6221, 985.6010, 988.4032, 993.4985, 1007.5710, 1017.5679, 1034.5784, 1036.5477, 1044.5231, 1046.5923, 1051.5060, 1056.4977, 1057.5691, 1058.6145, 1074.6044, 1113.5417, 1118.5876, 1123.5989, 1143.4429, 1145.6317, 1169.4524, 1172.6801, 1178.6199, 1187.7002, 1194.5691, 1207.6023, 1216.4987, 1216.7014, 1254.6930, 1255.6340, 1257.6666, 1265.7057, 1269.6703, 1273.6517, 1278.6932, 1281.6456, 1286.7430, 1299.5466, 1304.6500, 1312.7513, 1314.7311, 1317.7122, 1338.6886, 1349.6870, 1351.6915, 1354.7209, 1358.7173, 1365.5874, 1371.7622, 1374.6569, 1375.7383, 1390.6992, 1393.7732, 1397.7450, 1412.6091, 1413.6514, 1420.7966, 1440.7816, 1450.8618, 1456.7800, 1467.7542, 1470.7775, 1494.7429, 1500.7238, 1513.7605, 1539.7341, 1556.7584, 1562.7856, 1563.7985, 1570.7755, 1571.9341, 1580.8304, 1590.7900, 1592.7916, 1616.8013, 1635.7781, 1642.7219, 1647.8557, 1649.8578, 1650.8488, 1657.9104, 1663.8551, 1664.8605, 1696.8295, 1707.9520, 1718.8820, 1721.9437, 1730.8646, 1741.9973, 1747.9136, 1755.8970, 1758.9091, 1774.9242, 1786.9141, 1787.8756, 1862.9530, 1870.7738, 1883.7272, 1895.7275, 1897.7380, 1903.0176, 1911.7487, 1913.7377, 1928.0281, 1938.0094, 1941.0232, 1959.9926, 1961.0339, 1970.0011, 1979.0769, 1981.0564, 1982.0408, 2007.9467, 2008.9807, 2027.1300, 2055.0208, 2060.9963, 2067.1592, 2086.1130, 2108.9956, 2112.9822, 2181.1780, 2223.1548, 2239.1460, 2241.1360, 2245.8369, 2251.1431, 2255.1433, 2259.8535, 2265.0969, 2273.0667, 2286.1521, 2318.1763, 2328.9463, 2335.8999, 2347.8403, 2352.9194, 2361.8635, 2363.8716, 2364.8738, 2366.9121, 2371.0796, 2389.2690, 2414.2378, 2467.2051, 2474.2170, 2477.2441, 2485.0479, 2503.0457, 2504.9548, 2505.9473, 2519.9768, 2541.2239, 2542.2581, 2580.0098, 2595.0286, 2631.4714, 2646.9739, 2678.0037, 2681.3691, 2684.3965, 2695.0193, 2701.2148, 2705.2874, 2709.0286, 2717.2839, 2770.4202, 2792.2090, 2798.2344, 2799.2815, 2809.4841, 2813.3928, 2816.2957, 2833.0999, 2842.4780, 2846.3840, 2876.3640, 2885.3044, 2919.4407, 2961.2092, 2970.4829, 2976.2786, 2990.3098, 3002.4895, 3029.4480, 3071.4028, 3081.6030, 3087.4146, 3146.5461, 3157.4587, 3161.0999, 3184.4336, 3254.5583, 3265.5901, 3315.6875, 3330.5125, 3334.5588, 3335.6306, 3346.5308, 3347.4827, 3352.6125, 3410.6125, 3411.7185, 3428.5632, 3435.6848, 3462.6028, 3471.6272, 3474.6221, 3528.3171, 3542.3335, 3552.6479, 3574.6042, 3624.6587, 3645.7671, 3651.7319, 3667.7424, 3690.9905, 3707.5422, 3716.6360, 3721.5728, 3722.4707, 3736.5007, 3741.4561, 3748.4346, 3753.4888,

3764.5239, 4075.0212, 4160.8794, 4238.9067, 4253.8887, 4274.9287, 4301.2393, 4397.9902, 4415.0044, 4458.2759, 4465.1338, 4631.9888, 4690.3101, 4845.1016

15. F1MDD0 **Mass:** 13795 **Score:** 19 **Expect:** 3e+002 **Matches:** 6
Uncharacterized protein OS=Bos taurus GN=PSMG4 PE=4 SV=2

Observed	Mr(expt)	Mr(calc)	ppm	Start	End	Miss	Peptide
1051.5060	1050.4987	1050.4950	3.52	49 -	57	0	R.NLAVAMCTR.Y + Oxidation (M)
1349.6870	1348.6797	1348.6696	7.47	112 -	122	1	R.IKEEMQAFPEK.F
2842.4780	2841.4707	2841.4782	-2.63	33 -	57	1	R.LTDSLFLWVGATPHLRNLAVAMCTR.Y
3081.6030	3080.5957	3080.5812	4.72	58 -	86	2	R.YDSIPLSTALLGDTSDTMSTGLAQRLARK.T
3503.7864	3502.7791	3502.7429	10.3	2 -	32	1	M.ERPAAAGGGDVSLHNFSARLWEQLVHFHVMR.L + Oxidation (M)
3645.7671	3644.7598	3644.7484	3.13	49 -	82	1	R.NLAVAMCTRYDSIPLSTALLGDTSDTMSTGLAQR.L + Oxidation (M)

No match to: 701.3929, 710.3735, 712.2889, 713.2998, 717.3996, 719.4157, 720.4154, 722.4257, 729.4009, 734.4510, 735.4123, 738.4139, 745.4143, 747.4459, 753.3587, 756.3719, 771.3859, 779.4640, 783.3826, 805.4482, 806.4485, 818.4813, 823.4747, 829.4503, 831.4642, 832.3057, 834.3104, 835.3138, 840.4758, 842.5006, 874.5018, 883.4738, 889.4957, 891.4817, 900.5336, 909.5331, 922.4722, 923.5116, 925.4815, 929.4376, 936.4885, 938.4929, 949.5431, 958.4930, 967.4177, 969.4647, 974.4397, 978.5347, 983.6221, 985.6010, 988.4032, 993.4985, 1007.5710, 1017.5679, 1034.5784, 1036.5477, 1044.5231, 1046.5923, 1056.4977, 1057.5691, 1058.6145, 1074.6044, 1087.5525, 1113.5417, 1118.5876, 1123.5989, 1143.4429, 1145.6317, 1169.4524, 1172.6801, 1178.6199, 1185.6628, 1187.7002, 1194.5691, 1207.6023, 1216.4987, 1216.7014, 1248.6699, 1254.6930, 1255.6340, 1257.6666, 1265.7057, 1269.6703, 1273.6517, 1278.6932, 1281.6456, 1284.6411, 1286.7430, 1299.5466, 1301.6044, 1304.6500, 1312.7513, 1314.7311, 1317.7122, 1338.6886, 1351.6915, 1354.7209, 1358.7173, 1365.5874, 1368.6487, 1371.7622, 1372.5999, 1374.6569, 1375.7383, 1382.5975, 1383.7290, 1390.6992, 1393.7732, 1397.7450, 1412.6091, 1413.6514, 1420.7966, 1440.7816, 1442.8168, 1450.8618, 1456.7800, 1457.7509, 1467.7542, 1470.7775, 1494.7429, 1500.7238, 1513.7605, 1528.6982, 1539.7341, 1556.7584, 1562.7856, 1563.7985, 1570.7755, 1571.9341, 1578.7968, 1580.8304, 1590.7900, 1592.7916, 1616.8013, 1628.7054, 1628.8561, 1635.7781, 1642.7219, 1647.8557, 1649.8578, 1650.8488, 1657.9104, 1663.8551, 1664.8605, 1696.8295, 1706.8674, 1707.9520, 1715.8798, 1718.8820, 1721.9437, 1730.8646, 1741.9973, 1747.9136, 1755.8970, 1758.9091, 1771.0317, 1774.9242, 1786.9141, 1787.8756, 1862.9530, 1870.7738, 1883.7272, 1895.7275, 1897.7380, 1903.0176, 1911.7487, 1913.7377, 1928.0281, 1938.0094, 1941.0232, 1959.9926, 1961.0339, 1970.0011, 1974.9122, 1979.0769, 1981.0564, 1982.0408, 2007.9467, 2008.9807, 2027.1300, 2055.0208, 2060.9963, 2067.1592, 2086.1130, 2108.9956, 2112.9822, 2144.0425, 2181.1780, 2223.1548, 2239.1460, 2241.1360, 2245.8369, 2251.1431, 2255.1433, 2259.8535, 2265.0969, 2273.0667, 2286.1521, 2318.1763, 2328.9463, 2335.8999, 2343.1943, 2347.8403, 2352.9194, 2361.8635, 2363.8716, 2364.8738, 2366.9121, 2371.0796, 2389.2690, 2414.2378, 2467.2051, 2474.2170, 2477.2441, 2485.0479, 2503.0457, 2504.9548, 2505.9473, 2519.9768, 2541.2239, 2542.2581, 2580.0098, 2595.0286, 2631.4714, 2646.9739, 2678.0037, 2681.3691, 2684.3965, 2695.0193, 2701.2148, 2705.2874, 2709.0286, 2717.2839, 2770.4202, 2792.2090, 2798.2344, 2799.2815, 2809.4841, 2813.3928, 2816.2957, 2833.0999, 2846.3840, 2872.3215, 2876.3640, 2885.3044, 2919.4407, 2961.2092, 2970.4829, 2976.2786, 2990.3098, 3002.4895, 3029.4480, 3071.4028, 3087.4146, 3146.5461, 3157.4587, 3161.0999, 3184.4336, 3236.5503, 3248.6294, 3254.5583, 3265.5901, 3315.6875, 3330.5125, 3334.5588, 3335.6306, 3346.5308, 3347.4827, 3352.6125, 3410.6125, 3411.7185, 3428.5632, 3435.6848, 3457.5947, 3462.6028, 3471.6272, 3474.6221, 3489.6526, 3528.3171, 3542.3335, 3552.6479, 3574.6042, 3624.6587, 3639.7046, 3651.7319, 3667.7424, 3690.9905, 3707.5422, 3716.6360, 3721.5728, 3722.4707, 3736.5007, 3741.4561, 3748.4346, 3753.4888, 3764.5239, 4075.0212, 4147.9077, 4160.8794, 4238.9067, 4253.8887, 4274.9287, 4301.2393, 4397.9902, 4415.0044, 4452.1357, 4458.2759, 4465.1338, 4631.9888, 4690.3101, 4845.1016

16. G3N2C2 **Mass:** 12918 **Score:** 19 **Expect:** 3e+002 **Matches:** 4
Uncharacterized protein OS=Bos taurus GN=LOC100139826 PE=4 SV=1

Observed	Mr(expt)	Mr(calc)	ppm	Start	End	Miss	Peptide
938.4929	937.4856	937.4869	-1.30	105 -	112	0	K.NSLSNYLK.G
1903.0176	1902.0103	1902.0172	-3.63	1 -	17	0	-.ESTLILPFTQLPSTMPK.A
4465.1338	4464.1265	4464.1444	-4.01	66 -	104	2	R.RGLDLGRFSDTFSMNENPRPLSFQSELIASAFADYEQQK.N
4690.3101	4689.3028	4689.1969	22.6	73 -	113	2	R.FSDTFSMNENPRPLSFQSELIASAFADYEQQKNSLSNYLKG.- + Oxidation (M)

No match to: 701.3929, 710.3735, 712.2889, 713.2998, 717.3996, 719.4157, 720.4154, 722.4257, 729.4009, 734.4510, 735.4123, 738.4139, 745.4143, 747.4459, 753.3587, 756.3719, 771.3859, 779.4640, 783.3826, 805.4482, 806.4485, 818.4813, 823.4747, 829.4503, 831.4642, 832.3057, 834.3104, 835.3138, 840.4758, 842.5006, 874.5018, 883.4738, 889.4957, 891.4817, 900.5336, 909.5331, 922.4722, 923.5116, 925.4815, 929.4376, 936.4885, 949.5431, 958.4930, 967.4177, 969.4647, 974.4397, 978.5347, 983.6221, 985.6010, 988.4032, 993.4985, 1007.5710, 1017.5679, 1034.5784, 1036.5477, 1044.5231, 1046.5923, 1051.5060, 1056.4977, 1057.5691, 1058.6145, 1074.6044, 1087.5525, 1113.5417, 1118.5876, 1123.5989, 1143.4429, 1145.6317, 1169.4524, 1172.6801, 1178.6199, 1185.6628, 1187.7002, 1194.5691, 1207.6023, 1216.4987, 1216.7014, 1248.6699, 1254.6930, 1255.6340, 1257.6666, 1265.7057, 1269.6703, 1273.6517, 1278.6932, 1281.6456, 1284.6411, 1286.7430, 1299.5466, 1301.6044, 1304.6500, 1312.7513, 1314.7311, 1317.7122, 1338.6886, 1349.6870, 1351.6915, 1354.7209, 1358.7173, 1365.5874, 1368.6487, 1371.7622, 1372.5999, 1374.6569, 1375.7383, 1382.5975, 1383.7290, 1390.6992, 1393.7732, 1397.7450, 1412.6091, 1413.6514, 1420.7966, 1440.7816, 1442.8168, 1450.8618, 1456.7800, 1457.7509, 1467.7542, 1470.7775, 1494.7429, 1500.7238, 1513.7605, 1528.6982, 1539.7341, 1556.7584, 1562.7856, 1563.7985, 1570.7755, 1571.9341, 1578.7968, 1580.8304, 1590.7900, 1592.7916, 1616.8013, 1628.7054, 1628.8561, 1635.7781, 1642.7219, 1647.8557, 1649.8578, 1650.8488, 1657.9104, 1663.8551, 1664.8605, 1696.8295, 1706.8674, 1707.9520, 1715.8798, 1718.8820, 1721.9437, 1730.8646, 1741.9973, 1747.9136, 1755.8970, 1758.9091, 1771.0317, 1774.9242, 1786.9141, 1787.8756, 1862.9530, 1870.7738, 1883.7272, 1895.7275, 1897.7380, 1911.7487, 1913.7377, 1928.0281, 1938.0094, 1941.0232, 1959.9926, 1961.0339, 1970.0011, 1974.9122, 1979.0769, 1981.0564, 1982.0408, 2007.9467, 2008.9807, 2027.1300, 2055.0208, 2060.9963, 2067.1592, 2086.1130, 2108.9956, 2112.9822, 2144.0425, 2181.1780, 2223.1548, 2239.1460, 2241.1360, 2245.8369, 2251.1431, 2255.1433, 2259.8535, 2265.0969, 2273.0667, 2286.1521, 2318.1763, 2328.9463, 2335.8999, 2343.1943, 2347.8403, 2352.9194, 2361.8635, 2363.8716, 2364.8738, 2366.9121, 2371.0796, 2389.2690, 2414.2378, 2467.2051, 2474.2170, 2477.2441, 2485.0479, 2503.0457, 2504.9548, 2505.9473, 2519.9768, 2541.2239, 2542.2581, 2580.0098, 2595.0286, 2631.4714, 2646.9739, 2678.0037, 2681.3691, 2684.3965, 2695.0193, 2701.2148, 2705.2874, 2709.0286, 2717.2839, 2770.4202, 2792.2090, 2798.2344, 2799.2815, 2809.4841, 2813.3928, 2816.2957, 2833.0999, 2842.4780, 2846.3840, 2872.3215, 2876.3640, 2885.3044, 2919.4407, 2961.2092, 2970.4829, 2976.2786, 2990.3098, 3002.4895, 3029.4480, 3071.4028, 3081.6030, 3087.4146, 3146.5461, 3157.4587, 3161.0999, 3184.4336, 3236.5503, 3248.6294, 3254.5583, 3265.5901, 3315.6875, 3330.5125, 3334.5588, 3335.6306, 3346.5308, 3347.4827, 3352.6125, 3410.6125, 3411.7185, 3428.5632, 3435.6848, 3457.5947, 3462.6028, 3471.6272, 3474.6221, 3489.6526, 3503.7864, 3528.3171, 3542.3335, 3552.6479, 3574.6042, 3624.6587, 3639.7046, 3645.7671, 3651.7319, 3667.7424, 3690.9905, 3707.5422, 3716.6360, 3721.5728, 3722.4707, 3736.5007, 3741.4561, 3748.4346, 3753.4888, 3764.5239, 4075.0212, 4147.9077, 4160.8794, 4238.9067, 4253.8887, 4274.9287, 4301.2393, 4397.9902, 4415.0044, 4452.1357, 4458.2759, 4631.9888, 4845.1016

17. Q32PA3 **Mass:** 14710 **Score:** 19 **Expect:** 3e+002 **Matches:** 5
Magnesium-dependent phosphatase 1 OS=Bos taurus GN=MDP-1 PE=2 SV=1

Observed	Mr(expt)	Mr(calc)	ppm	Start	End	Miss	Peptide
1185.6628	1184.6555	1184.6414	11.9	64 -	72	1	K.VTHFERLQR.K
1562.7856	1561.7783	1561.7889	-6.76	57 -	69	1	R.EIYPGSKVTHFER.L
1959.9926	1958.9853	1959.0326	-24.14	57 -	72	2	R.EIYPGSKVTHFERLQR.K
2060.9963	2059.9890	2060.0037	-7.14	73 -	89	2	R.KTGVPFSQMIFFDDEKR.N + Oxidation (M)
2286.1521	2285.1448	2285.1514	-2.90	70 -	88	2	R.LQRKTGVPFSQMIFFDDEK.R

No match to: 701.3929, 710.3735, 712.2889, 713.2998, 717.3996, 719.4157, 720.4154, 722.4257, 729.4009, 734.4510, 735.4123, 738.4139, 745.4143, 747.4459, 753.3587, 756.3719, 771.3859, 779.4640, 783.3826, 805.4482, 806.4485, 818.4813, 823.4747, 829.4503, 831.4642, 832.3057, 834.3104, 835.3138, 840.4758, 842.5006, 874.5018, 883.4738, 889.4957, 891.4817, 900.5336, 909.5331, 922.4722, 923.5116, 925.4815, 929.4376, 936.4885, 938.4929, 949.5431, 958.4930, 967.4177, 969.4647, 974.4397, 978.5347, 983.6221, 985.6010, 988.4032, 993.4985, 1007.5710, 1017.5679, 1034.5784, 1036.5477, 1044.5231, 1046.5923, 1051.5060, 1056.4977, 1057.5691, 1058.6145, 1074.6044, 1087.5525, 1113.5417, 1118.5876, 1123.5989, 1143.4429, 1145.6317, 1169.4524, 1172.6801, 1178.6199, 1187.7002, 1194.5691, 1207.6023, 1216.4987, 1216.7014, 1248.6699, 1254.6930, 1255.6340, 1257.6666, 1265.7057, 1269.6703, 1273.6517, 1278.6932, 1281.6456, 1284.6411, 1286.7430, 1299.5466, 1301.6044, 1304.6500, 1312.7513, 1314.7311, 1317.7122, 1338.6886, 1349.6870, 1351.6915, 1354.7209, 1358.7173, 1365.5874, 1368.6487, 1371.7622, 1372.5999, 1374.6569, 1375.7383, 1382.5975, 1383.7290, 1390.6992, 1393.7732, 1397.7450, 1412.6091, 1413.6514, 1420.7966, 1440.7816, 1442.8168, 1450.8618, 1456.7800, 1457.7509, 1467.7542, 1470.7775, 1494.7429, 1500.7238, 1513.7605, 1528.6982, 1539.7341, 1556.7584, 1563.7985, 1570.7755, 1571.9341, 1578.7968, 1580.8304, 1590.7900, 1592.7916, 1616.8013, 1628.7054, 1628.8561, 1635.7781, 1642.7219, 1647.8557, 1649.8578, 1650.8488, 1657.9104, 1663.8551, 1664.8605, 1696.8295, 1706.8674, 1707.9520, 1715.8798, 1718.8820, 1721.9437, 1730.8646, 1741.9973, 1747.9136, 1755.8970, 1758.9091, 1771.0317, 1774.9242, 1786.9141, 1787.8756, 1862.9530, 1870.7738, 1883.7272, 1895.7275, 1897.7380, 1903.0176, 1911.7487, 1913.7377, 1928.0281, 1938.0094, 1941.0232,

1961.0339, 1970.0011, 1974.9122, 1979.0769, 1981.0564, 1982.0408, 2007.9467, 2008.9807, 2027.1300, 2055.0208, 2067.1592, 2086.1130, 2108.9956, 2112.9822, 2144.0425, 2181.1780, 2223.1548, 2239.1460, 2241.1360, 2245.8369, 2251.1431, 2255.1433, 2259.8535, 2265.0969, 2273.0667, 2318.1763, 2328.9463, 2335.8999, 2343.1943, 2347.8403, 2352.9194, 2361.8635, 2363.8716, 2364.8738, 2366.9121, 2371.0796, 2389.2690, 2414.2378, 2467.2051, 2474.2170, 2477.2441, 2485.0479, 2503.0457, 2504.9548, 2505.9473, 2519.9768, 2541.2239, 2542.2581, 2580.0098, 2595.0286, 2631.4714, 2646.9739, 2678.0037, 2681.3691, 2684.3965, 2695.0193, 2701.2148, 2705.2874, 2709.0286, 2717.2839, 2770.4202, 2792.2090, 2798.2344, 2799.2815, 2809.4841, 2813.3928, 2816.2957, 2833.0999, 2842.4780, 2846.3840, 2872.3215, 2876.3640, 2885.3044, 2919.4407, 2961.2092, 2970.4829, 2976.2786, 2990.3098, 3002.4895, 3029.4480, 3071.4028, 3081.6030, 3087.4146, 3146.5461, 3157.4587, 3161.0999, 3184.4336, 3236.5503, 3248.6294, 3254.5583, 3265.5901, 3315.6875, 3330.5125, 3334.5588, 3335.6306, 3346.5308, 3347.4827, 3352.6125, 3410.6125, 3411.7185, 3428.5632, 3435.6848, 3457.5947, 3462.6028, 3471.6272, 3474.6221, 3489.6526, 3503.7864, 3528.3171, 3542.3335, 3552.6479, 3574.6042, 3624.6587, 3639.7046, 3645.7671, 3651.7319, 3667.7424, 3690.9905, 3707.5422, 3716.6360, 3721.5728, 3722.4707, 3736.5007, 3741.4561, 3748.4346, 3753.4888, 3764.5239, 4075.0212, 4147.9077, 4160.8794, 4238.9067, 4253.8887, 4274.9287, 4301.2393, 4397.9902, 4415.0044, 4452.1357, 4458.2759, 4465.1338, 4631.9888, 4690.3101, 4845.1016

18. F1MU12 **Mass:** 53494 **Score:** 19 **Expect:** 3e+002 **Matches:** 14

Keratin, type II cytoskeletal 8 OS=Bos taurus GN=KRT8 PE=1 SV=1

Observed	Mr(expt)	Mr(calc)	ppm	Start		End	Miss	Peptide
717.3996	716.3923	716.3817	14.8	12	-	18	0	K.VSTSAPR.S
978.5347	977.5274	977.5328	-5.52	1	-	8	1	-.MSIRVTQK.S + Oxidation (M)
1057.5691	1056.5618	1056.5716	-9.26	130	-	137	0	K.WNLLQQQK.T
1281.6456	1280.6383	1280.6473	-7.02	12	-	23	1	K.VSTSAPRSFSSR.S
1338.6886	1337.6813	1337.6537	20.7	194	-	204	0	R.TDMENEFVIIK.K
1494.7429	1493.7356	1493.7548	-12.83	193	-	204	1	K.RTDMENEFVIIK.K
1571.9341	1570.9268	1570.9406	-8.79	451	-	464	2	R.TSSKPVVVKKIETR.D
1721.9437	1720.9364	1720.9584	-12.76	116	-	129	2	K.VRHLEQQNKVLETK.W
1959.9926	1958.9853	1958.9731	6.25	336	-	354	1	R.ASLEAAIADAEQRGEMAVK.D
1981.0564	1980.0491	1980.0462	1.46	101	-	117	2	K.IMKSVNQNLATFTDKVR.H + Oxidation (M)
2286.1521	2285.1448	2285.1249	8.73	283	-	302	1	R.AEAEAMYQIKYEELQTLAGK.H
2414.2378	2413.2305	2413.2199	4.42	380	-	399	1	R.EYQELMNVKLALDVEIATYR.K + Oxidation (M)
2474.2170	2473.2097	2473.2594	-20.09	312	-	332	2	K.TEISEMNRNINRLQAEIEGLK.G + Oxidation (M)
3335.6306	3334.6233	3334.6901	-20.04	49	-	84	0	R.GGLGTGMSMAGSYGGAPGLGGITAVTVNQSLLSPLK.L + Oxidation (M)

No match to: 701.3929, 710.3735, 712.2889, 713.2998, 719.4157, 720.4154, 722.4257, 729.4009, 734.4510, 735.4123, 738.4139, 745.4143, 747.4459, 753.3587, 756.3719, 771.3859, 779.4640, 783.3826, 805.4482, 806.4485, 818.4813, 823.4747, 829.4503, 831.4642, 832.3057, 834.3104, 835.3138, 840.4758, 842.5006, 874.5018, 883.4738, 889.4957, 891.4817, 900.5336, 909.5331, 922.4722, 923.5116, 925.4815, 929.4376, 936.4885, 938.4929, 949.5431, 958.4930, 967.4177, 969.4647, 974.4397, 983.6221, 985.6010, 988.4032, 993.4985, 1007.5710, 1017.5679, 1034.5784, 1036.5477, 1044.5231, 1046.5923, 1051.5060, 1056.4977, 1058.6145, 1074.6044, 1087.5525, 1113.5417, 1118.5876, 1123.5989, 1143.4429, 1145.6317, 1169.4524, 1172.6801, 1178.6199, 1185.6628, 1187.7002, 1194.5691, 1207.6023, 1216.4987, 1216.7014, 1248.6699, 1254.6930, 1255.6340, 1257.6666, 1265.7057, 1269.6703, 1273.6517, 1278.6932, 1284.6411, 1286.7430, 1299.5466, 1301.6044, 1304.6500, 1312.7513, 1314.7311, 1317.7122, 1349.6870, 1351.6915, 1354.7209, 1358.7173, 1365.5874, 1368.6487, 1371.7622, 1372.5999, 1374.6569, 1375.7383, 1382.5975, 1383.7290, 1390.6992, 1393.7732, 1397.7450, 1412.6091, 1413.6514, 1420.7966, 1440.7816, 1442.8168, 1450.8618, 1456.7800, 1457.7509, 1467.7542, 1470.7775, 1500.7238, 1513.7605, 1528.6982, 1539.7341, 1556.7584, 1562.7856, 1563.7985, 1570.7755, 1578.7968, 1580.8304, 1590.7900, 1592.7916, 1616.8013, 1628.7054, 1628.8561, 1635.7781, 1642.7219, 1647.8557, 1649.8578, 1650.8488, 1657.9104, 1663.8551, 1664.8605, 1696.8295, 1706.8674, 1707.9520, 1715.8798, 1718.8820, 1730.8646, 1741.9973, 1747.9136, 1755.8970, 1758.9091, 1771.0317, 1774.9242, 1786.9141, 1787.8756, 1862.9530, 1870.7738, 1883.7272, 1895.7275, 1897.7380, 1903.0176, 1911.7487, 1913.7377, 1928.0281, 1938.0094, 1941.0232, 1961.0339, 1970.0011, 1974.9122, 1979.0769, 1982.0408, 2007.9467, 2008.9807, 2027.1300, 2055.0208, 2060.9963, 2067.1592, 2086.1130, 2108.9956, 2112.9822, 2144.0425, 2181.1780, 2223.1548, 2239.1460, 2241.1360, 2245.8369, 2251.1431, 2255.1433, 2259.8535, 2265.0969, 2273.0667, 2318.1763, 2328.9463, 2335.8999, 2343.1943, 2347.8403, 2352.9194, 2361.8635, 2363.8716, 2364.8738, 2366.9121, 2371.0796, 2389.2690, 2467.2051, 2477.2441, 2485.0479, 2503.0457, 2504.9548, 2505.9473, 2519.9768, 2541.2239, 2542.2581, 2580.0098, 2595.0286, 2631.4714, 2646.9739, 2678.0037, 2681.3691, 2684.3965, 2695.0193, 2701.2148, 2705.2874, 2709.0286, 2717.2839, 2770.4202, 2792.2090, 2798.2344, 2799.2815, 2809.4841, 2813.3928, 2816.2957, 2833.0999, 2842.4780, 2846.3840, 2872.3215, 2876.3640, 2885.3044, 2919.4407, 2961.2092, 2970.4829, 2976.2786, 2990.3098, 3002.4895, 3029.4480, 3071.4028, 3081.6030, 3087.4146, 3146.5461, 3157.4587, 3161.0999, 3184.4336, 3236.5503, 3248.6294, 3254.5583, 3265.5901, 3315.6875, 3330.5125, 3334.5588, 3346.5308, 3347.4827, 3352.6125, 3410.6125, 3411.7185, 3428.5632, 3435.6848, 3457.5947, 3462.6028, 3471.6272, 3474.6221, 3489.6526, 3503.7864, 3528.3171, 3542.3335, 3552.6479, 3574.6042, 3624.6587, 3639.7046, 3645.7671, 3651.7319, 3667.7424, 3690.9905, 3707.5422, 3716.6360, 3721.5728, 3722.4707, 3736.5007, 3741.4561, 3748.4346, 3753.4888, 3764.5239, 4075.0212, 4147.9077, 4160.8794, 4238.9067, 4253.8887, 4274.9287, 4301.2393, 4397.9902, 4415.0044, 4452.1357, 4458.2759, 4465.1338, 4631.9888, 4690.3101, 4845.1016

19. F1MDA4 **Mass:** 51404 **Score:** 19 **Expect:** 3.1e+002 **Matches:** 10

Hemogen OS=Bos taurus GN=HEMGN PE=4 SV=2

Observed	Mr(expt)	Mr(calc)	ppm	Start		End	Miss	Peptide
701.3929	700.3856	700.3755	14.4	374	-	379	1	K.DKDVPK.E
1178.6199	1177.6126	1177.5873	21.5	76	-	84	2	R.KRQQNTEMK.V + Oxidation (M)
1706.8674	1705.8601	1705.8266	19.6	85	-	98	1	K.VELPSQLEKEMMEK.A + Oxidation (M)
2846.3840	2845.3767	2845.3896	-4.53	12	-	35	0	K.LHQTPDHHQEETHVPEVIGTWSLR.N
3248.6294	3247.6221	3247.5448	23.8	420	-	447	0	K.AEEPEIIEIPNVPQESNPENDIYSYVLF.-
3352.6125	3351.6052	3351.6557	-15.06	282	-	309	2	K.ELATEIHQETVEPEHFSHKIYREIAMSK.D
3645.7671	3644.7598	3644.6788	22.2	249	-	281	0	K.RPEPEEPDSETGQGIVETESFVSNTQQEMAVPK.E
3651.7319	3650.7246	3650.6604	17.6	217	-	248	1	K.TSEDTTDLEECSLEAHPKPDMPKASTLETYQK.R
3667.7424	3666.7351	3666.6553	21.8	217	-	248	1	K.TSEDTTDLEECSLEAHPKPDMPKASTLETYQK.R + Oxidation (M)
4160.8794	4159.8721	4159.9235	-12.36	203	-	239	2	K.TCQEDIAAKVLSSKTSEDTTDLEECSLEAHPKPDMPK.A

No match to: 710.3735, 712.2889, 713.2998, 717.3996, 719.4157, 720.4154, 722.4257, 729.4009, 734.4510, 735.4123, 738.4139, 745.4143, 747.4459, 753.3587, 756.3719, 771.3859, 779.4640, 783.3826, 805.4482, 806.4485, 818.4813, 823.4747, 829.4503, 831.4642, 832.3057, 834.3104, 835.3138, 840.4758, 842.5006, 874.5018, 883.4738, 889.4957, 891.4817, 900.5336, 909.5331, 922.4722, 923.5116, 925.4815, 929.4376, 936.4885, 938.4929, 949.5431, 958.4930, 967.4177, 969.4647, 974.4397, 978.5347, 983.6221, 985.6010, 988.4032, 993.4985, 1007.5710, 1017.5679, 1034.5784, 1036.5477, 1044.5231, 1046.5923, 1051.5060, 1056.4977, 1057.5691, 1058.6145, 1074.6044, 1087.5525, 1113.5417, 1118.5876, 1123.5989, 1143.4429, 1145.6317, 1169.4524, 1172.6801, 1185.6628, 1187.7002, 1194.5691, 1207.6023, 1216.4987, 1216.7014, 1248.6699, 1254.6930, 1255.6340, 1257.6666, 1265.7057, 1269.6703, 1273.6517, 1278.6932, 1281.6456, 1284.6411, 1286.7430, 1299.5466, 1301.6044, 1304.6500, 1312.7513, 1314.7311, 1317.7122, 1338.6886, 1349.6870, 1351.6915, 1354.7209, 1358.7173, 1365.5874, 1368.6487, 1371.7622, 1372.5999, 1374.6569, 1375.7383, 1382.5975, 1383.7290, 1390.6992, 1393.7732, 1397.7450, 1412.6091, 1413.6514, 1420.7966, 1440.7816, 1442.8168, 1450.8618, 1456.7800, 1457.7509, 1467.7542, 1470.7775, 1494.7429, 1500.7238, 1513.7605, 1528.6982, 1539.7341, 1556.7584, 1562.7856, 1563.7985, 1570.7755, 1571.9341, 1578.7968, 1580.8304, 1590.7900, 1592.7916, 1616.8013, 1628.7054, 1628.8561, 1635.7781, 1642.7219, 1647.8557, 1649.8578, 1650.8488, 1657.9104, 1663.8551, 1664.8605, 1696.8295, 1707.9520, 1715.8798, 1718.8820, 1721.9437, 1730.8646, 1741.9973, 1747.9136, 1755.8970, 1758.9091, 1771.0317, 1774.9242, 1786.9141, 1787.8756, 1862.9530, 1870.7738, 1883.7272, 1895.7275, 1897.7380, 1903.0176, 1911.7487, 1913.7377, 1928.0281, 1938.0094, 1941.0232, 1959.9926, 1961.0339, 1970.0011, 1974.9122, 1979.0769, 1981.0564, 1982.0408, 2007.9467, 2008.9807, 2027.1300, 2055.0208, 2060.9963, 2067.1592, 2086.1130, 2108.9956, 2112.9822, 2144.0425, 2181.1780, 2223.1548, 2239.1460, 2241.1360, 2245.8369, 2251.1431, 2255.1433, 2259.8535, 2265.0969, 2273.0667, 2286.1521, 2318.1763, 2328.9463, 2335.8999, 2343.1943, 2347.8403, 2352.9194, 2361.8635, 2363.8716, 2364.8738, 2366.9121, 2371.0796, 2389.2690, 2414.2378, 2467.2051, 2474.2170, 2477.2441, 2485.0479, 2503.0457, 2504.9548, 2505.9473, 2519.9768, 2541.2239, 2542.2581, 2580.0098, 2595.0286, 2631.4714, 2646.9739, 2678.0037, 2681.3691, 2684.3965, 2695.0193, 2701.2148, 2705.2874, 2709.0286, 2717.2839, 2770.4202, 2792.2090, 2798.2344, 2799.2815, 2809.4841, 2813.3928, 2816.2957, 2833.0999, 2842.4780, 2872.3215, 2876.3640, 2885.3044, 2919.4407, 2961.2092, 2970.4829, 2976.2786, 2990.3098, 3002.4895, 3029.4480, 3071.4028, 3081.6030, 3087.4146, 3146.5461, 3157.4587, 3161.0999, 3184.4336, 3236.5503, 3254.5583, 3265.5901, 3315.6875, 3330.5125, 3334.5588, 3335.6306, 3346.5308, 3347.4827, 3410.6125, 3411.7185, 3428.5632, 3435.6848, 3457.5947, 3462.6028, 3471.6272, 3474.6221, 3489.6526, 3503.7864, 3528.3171, 3542.3335, 3552.6479, 3574.6042, 3624.6587, 3639.7046, 3690.9905, 3707.5422, 3716.6360, 3721.5728, 3722.4707, 3736.5007, 3741.4561,

3748.4346, 3753.4888, 3764.5239, 4075.0212, 4147.9077, 4238.9067, 4253.8887, 4274.9287, 4301.2393, 4397.9902, 4415.0044, 4452.1357, 4458.2759, 4465.1338, 4631.9888, 4690.3101, 4845.1016

20. Q32LF3 **Mass:** 24094 **Score:** 19 **Expect:** 3.3e+002 **Matches:** 6

Similar to type 1 protein phosphatase inhibitor OS=Bos taurus GN=LOC520057 PE=2 SV=1

Observed	Mr(expt)	Mr(calc)	ppm	Start	End	Miss	Peptide
1494.7429	1493.7356	1493.7110	16.5	100 -	113	0	R.LAATHTSDSSYSVR.D
1578.7968	1577.7895	1577.7896	-0.07	85 -	99	1	R.SAESEATALDSLTKR.L
1650.8488	1649.8415	1649.8121	17.8	99 -	113	1	K.RLAATHTSDSSYSVR.D
2467.2051	2466.1978	2466.2575	-24.20	19 -	43	2	K.GSTASSVAGSAQQSGGPLQEVHRKK.S
2701.2148	2700.2075	2700.1948	4.70	62 -	84	1	R.DYDFMKMNEPSTPQFGPQNLGER.S
3071.4028	3070.3955	3070.4130	-5.70	44 -	67	2	K.SQKWDESNILATYRPEYRDYDFMK.M + Oxidation (M)

No match to: 701.3929, 710.3735, 712.2889, 713.2998, 717.3996, 719.4157, 720.4154, 722.4257, 729.4009, 734.4510, 735.4123, 738.4139, 745.4143, 747.4459, 753.3587, 756.3719, 771.3859, 779.4640, 783.3826, 805.4482, 806.4485, 818.4813, 823.4747, 829.4503, 831.4642, 832.3057, 834.3104, 835.3138, 840.4758, 842.5006, 874.5018, 883.4738, 889.4957, 891.4817, 900.5336, 909.5331, 922.4722, 923.5116, 925.4815, 929.4376, 936.4885, 938.4929, 949.5431, 958.4930, 967.4177, 969.4647, 974.4397, 978.5347, 983.6221, 985.6010, 988.4032, 993.4985, 1007.5710, 1017.5679, 1034.5784, 1036.5477, 1044.5231, 1046.5923, 1051.5060, 1056.4977, 1057.5691, 1058.6145, 1074.6044, 1087.5525, 1113.5417, 1118.5876, 1123.5989, 1143.4429, 1145.6317, 1169.4524, 1172.6801, 1178.6199, 1185.6628, 1187.7002, 1194.5691, 1207.6023, 1216.4987, 1216.7014, 1248.6699, 1254.6930, 1255.6340, 1257.6666, 1265.7057, 1269.6703, 1273.6517, 1278.6932, 1281.6456, 1284.6411, 1286.7430, 1299.5466, 1301.6044, 1304.6500, 1312.7513, 1314.7311, 1317.7122, 1338.6886, 1349.6870, 1351.6915, 1354.7209, 1358.7173, 1365.5874, 1368.6487, 1371.7622, 1372.5999, 1374.6569, 1375.7383, 1382.5975, 1383.7290, 1390.6992, 1393.7732, 1397.7450, 1412.6091, 1413.6514, 1420.7966, 1440.7816, 1442.8168, 1450.8618, 1456.7800, 1457.7509, 1467.7542, 1470.7775, 1500.7238, 1513.7605, 1528.6982, 1539.7341, 1556.7584, 1562.7856, 1563.7985, 1570.7755, 1571.9341, 1580.8304, 1590.7900, 1592.7916, 1616.8013, 1628.7054, 1628.8561, 1635.7781, 1642.7219, 1647.8557, 1649.8578, 1657.9104, 1663.8551, 1664.8605, 1696.8295, 1706.8674, 1707.9520, 1715.8798, 1718.8820, 1721.9437, 1730.8646, 1741.9973, 1747.9136, 1755.8970, 1758.9091, 1771.0317, 1774.9242, 1786.9141, 1787.8756, 1862.9530, 1870.7738, 1883.7272, 1895.7275, 1897.7380, 1903.0176, 1911.7487, 1913.7377, 1928.0281, 1938.0094, 1941.0232, 1959.9926, 1961.0339, 1970.0011, 1974.9122, 1979.0769, 1981.0564, 1982.0408, 2007.9467, 2008.9807, 2027.1300, 2055.0208, 2060.9963, 2067.1592, 2086.1130, 2108.9956, 2112.9822, 2144.0425, 2181.1780, 2223.1548, 2239.1460, 2241.1360, 2245.8369, 2251.1431, 2255.1433, 2259.8535, 2265.0969, 2273.0667, 2286.1521, 2318.1763, 2328.9463, 2335.8999, 2343.1943, 2347.8403, 2352.9194, 2361.8635, 2363.8716, 2364.8738, 2366.9121, 2371.0796, 2389.2690, 2414.2378, 2474.2170, 2477.2441, 2485.0479, 2503.0457, 2504.9548, 2505.9473, 2519.9768, 2541.2239, 2542.2581, 2580.0098, 2595.0286, 2631.4714, 2646.9739, 2678.0037, 2681.3691, 2684.3965, 2695.0193, 2705.2874, 2709.0286, 2717.2839, 2770.4202, 2792.2090, 2798.2344, 2799.2815, 2809.4841, 2813.3928, 2816.2957, 2833.0999, 2842.4780, 2846.3840, 2872.3215, 2876.3640, 2885.3044, 2919.4407, 2961.2092, 2970.4829, 2976.2786, 2990.3098, 3002.4895, 3029.4480, 3081.6030, 3087.4146, 3146.5461, 3157.4587, 3161.0999, 3184.4336, 3236.5503, 3248.6294, 3254.5583, 3265.5901, 3315.6875, 3330.5125, 3334.5588, 3335.6306, 3346.5308, 3347.4827, 3352.6125, 3410.6125, 3411.7185, 3428.5632, 3435.6848, 3457.5947, 3462.6028, 3471.6272, 3474.6221, 3489.6526, 3503.7864, 3528.3171, 3542.3335, 3552.6479, 3574.6042, 3624.6587, 3639.7046, 3645.7671, 3651.7319, 3667.7424, 3690.9905, 3707.5422, 3716.6360, 3721.5728, 3722.4707, 3736.5007, 3741.4561, 3748.4346, 3753.4888, 3764.5239, 4075.0212, 4147.9077, 4160.8794, 4238.9067, 4253.8887, 4274.9287, 4301.2393, 4397.9902, 4415.0044, 4452.1357, 4458.2759, 4465.1338, 4631.9888, 4690.3101, 4845.1016

21. P13608 **Mass:** 248150 **Score:** 19 **Expect:** 3.3e+002 **Matches:** 36

Aggrecan core protein OS=Bos taurus GN=ACAN PE=1 SV=3

Observed	Mr(expt)	Mr(calc)	ppm	Start	End	Miss	Peptide
831.4642	830.4570	830.4650	-9.72	545 -	551	0	R.YPIVSPR.T
883.4738	882.4665	882.4712	-5.33	211 -	217	0	R.YPIHTPR.E
891.4817	890.4744	890.4763	-2.09	152 -	158	0	K.GIVFHYR.A
929.4376	928.4303	928.4290	1.33	164 -	170	0	R.YTLDFDR.A
949.5431	948.5358	948.5392	-3.62	306 -	313	1	R.SVRYPISK.A
967.4177	966.4104	966.4130	-2.73	2161 -	2167	0	K.FQGHCYR.H
974.4397	973.4324	973.4341	-1.72	678 -	684	0	R.HHAFCFR.G
1087.5525	1086.5452	1086.5233	20.2	498 -	506	0	R.YSLTFEEAK.Q
1185.6628	1184.6555	1184.6765	-17.69	83 -	93	0	K.EVVLLVATEGR.V
1248.6699	1247.6626	1247.6622	0.34	2298 -	2307	1	K.DRYEINALVR.Y
1284.6411	1283.6338	1283.6258	6.22	164 -	173	1	R.YTLDFDRAQR.A
1368.6487	1367.6414	1367.6292	8.93	631 -	642	0	K.CYAGWLADGSLR.Y
1372.5999	1371.5926	1371.5877	3.56	264 -	274	0	K.FTFQEAANECR.R
1382.5975	1381.5902	1381.5833	4.99	2324 -	2334	0	R.CQPSGHWEEPR.I
1383.7290	1382.7217	1382.7201	1.18	314 -	326	0	K.ARPNCGGNLLGVR.T
1442.8168	1441.8095	1441.8140	-3.12	81 -	93	1	K.EKEVVLLVATEGR.V
1457.7509	1456.7436	1456.7310	8.64	159 -	170	1	R.AISTRYTLDFDR.A
1528.6982	1527.6909	1527.6888	1.36	264 -	275	1	K.FTFQEAANECRR.L
1578.7968	1577.7895	1577.7620	17.4	2277 -	2291	0	K.GTVACGEPPVVEHAR.I
1628.7054	1627.6981	1627.6937	2.73	218 -	231	1	R.EGCYGDKDEFPGVR.T
1628.8561	1627.8488	1627.8583	-5.83	483 -	497	0	R.LPGGVVFHYRPGSSR.Y
1706.8674	1705.8601	1705.8570	1.83	2276 -	2291	1	K.KGTVACGEPPVVEHAR.I
1715.8798	1714.8725	1714.8348	22.0	498 -	511	1	R.YSLTFEEAKQACLR.T
1771.0317	1770.0244	1770.0251	-0.36	78 -	93	2	R.ISKEKEVVLLVATEGR.V
1974.9122	1973.9049	1973.8802	12.5	2168 -	2183	1	R.HFPDRATWVDAESQCR.K
2144.0425	2143.0352	2143.0289	2.93	133 -	151	0	R.CEVMHGIEDSQATLEVVVK.G
2343.1943	2342.1870	2342.1940	-2.98	104 -	124	0	K.VTLPNYPAIPSDATLEIQNMR.S
2872.3215	2871.3142	2871.3142	-0.01	280 -	305	0	R.LATTGQLYLAWQGGMDMCSAGWLADR.S
3002.4895	3001.4822	3001.4485	11.2	125 -	151	1	R.SNDSGILRCEVMHGIEDSQATLEVVVK.G + Oxidation (M)
3248.6294	3247.6221	3247.6183	1.18	96 -	124	1	R.VNSAYQDKVTLPNYPAIPSDATLEIQNMR.S
3457.5947	3456.5874	3456.6489	-17.79	275 -	305	2	R.RLGARLATTGQLYLAWQGGMDMCSAGWLADR.S + 2 Oxidation (M)
3489.6526	3488.6453	3488.6347	3.04	566 -	594	1	R.TYGVRPPSETYDVYCYVDRLEGEVFFATR.L
3503.7864	3502.7791	3502.7878	-2.48	94 -	124	2	R.VRVNSAYQDKVTLPNYPAIPSDATLEIQNMR.S
3639.7046	3638.6973	3638.6947	0.72	512 -	544	0	R.TGAIIASPEQLQAAYEAGYEQCDAGWLQDQTVR.Y
4147.9077	4146.9004	4146.9164	-3.84	174 -	210	0	R.ACLQNSAIIATPEQLQAAYEDGFHQCDAGWLADQTVR.Y
4452.1357	4451.1284	4451.1492	-4.66	512 -	551	1	R.TGAIIASPEQLQAAYEAGYEQCDAGWLQDQTVRYPIVSPR.T

No match to: 701.3929, 710.3735, 712.2889, 713.2998, 717.3996, 719.4157, 720.4154, 722.4257, 729.4009, 734.4510, 735.4123, 738.4139, 745.4143, 747.4459, 753.3587, 756.3719, 771.3859, 779.4640, 783.3826, 805.4482, 806.4485, 818.4813, 823.4747, 829.4503, 832.3057, 834.3104, 835.3138, 840.4758, 842.5006, 874.5018, 889.4957, 900.5336, 909.5331, 922.4722, 923.5116, 925.4815, 936.4885, 938.4929, 958.4930, 969.4647, 978.5347, 983.6221, 985.6010, 988.4032, 993.4985, 1007.5710, 1017.5679, 1034.5784, 1036.5477, 1044.5231, 1046.5923, 1051.5060, 1056.4977, 1057.5691, 1058.6145, 1074.6044, 1113.5417, 1118.5876, 1123.5989, 1143.4429, 1145.6317, 1169.4524, 1172.6801, 1178.6199, 1187.7002, 1194.5691, 1207.6023, 1216.4987, 1216.7014, 1254.6930, 1255.6340, 1257.6666, 1265.7057, 1269.6703, 1273.6517, 1278.6932, 1281.6456, 1286.7430, 1299.5466, 1301.6044, 1304.6500, 1312.7513, 1314.7311, 1317.7122, 1338.6886, 1349.6870, 1351.6915, 1354.7209, 1358.7173, 1365.5874, 1371.7622, 1374.6569, 1375.7383, 1390.6992, 1393.7732, 1397.7450, 1412.6091, 1413.6514, 1420.7966, 1440.7816, 1450.8618, 1456.7800, 1467.7542, 1470.7775, 1494.7429, 1500.7238, 1513.7605, 1539.7341, 1556.7584, 1562.7856, 1563.7985, 1570.7755, 1571.9341, 1580.8304, 1590.7900, 1592.7916, 1616.8013, 1635.7781, 1642.7219, 1647.8557, 1649.8578, 1650.8488, 1657.9104, 1663.8551, 1664.8605, 1696.8295, 1707.9520, 1718.8820, 1721.9437, 1730.8646, 1741.9973, 1747.9136, 1755.8970, 1758.9091, 1774.9242, 1786.9141, 1787.8756, 1862.9530, 1870.7738, 1883.7272, 1895.7275, 1897.7380, 1903.0176, 1911.7487, 1913.7377, 1928.0281, 1938.0094, 1941.0232, 1959.9926, 1961.0339, 1970.0011, 1979.0769, 1981.0564, 1982.0408, 2007.9467, 2008.9807, 2027.1300,

2055.0208, 2060.9963, 2067.1592, 2086.1130, 2108.9956, 2112.9822, 2181.1780, 2223.1548, 2239.1460, 2241.1360, 2245.8369, 2251.1431, 2255.1433, 2259.8535, 2265.0969, 2273.0667, 2286.1521, 2318.1763, 2328.9463, 2335.8999, 2347.8403, 2352.9194, 2361.8635, 2363.8716, 2364.8738, 2366.9121, 2371.0796, 2389.2690, 2414.2378, 2467.2051, 2474.2170, 2477.2441, 2485.0479, 2503.0457, 2504.9548, 2505.9473, 2519.9768, 2541.2239, 2542.2581, 2580.0098, 2595.0286, 2631.4714, 2646.9739, 2678.0037, 2681.3691, 2684.3965, 2695.0193, 2701.2148, 2705.2874, 2709.0286, 2717.2839, 2770.4202, 2792.2090, 2798.2344, 2799.2815, 2809.4841, 2813.3928, 2816.2957, 2833.0999, 2842.4780, 2846.3840, 2876.3640, 2885.3044, 2919.4407, 2961.2092, 2970.4829, 2976.2786, 2990.3098, 3029.4480, 3071.4028, 3081.6030, 3087.4146, 3146.5461, 3157.4587, 3161.0999, 3184.4336, 3236.5503, 3254.5583, 3265.5901, 3315.6875, 3330.5125, 3334.5588, 3335.6306, 3346.5308, 3347.4827, 3352.6125, 3410.6125, 3411.7185, 3428.5632, 3435.6848, 3462.6028, 3471.6272, 3474.6221, 3528.3171, 3542.3335, 3552.6479, 3574.6042, 3624.6587, 3645.7671, 3651.7319, 3667.7424, 3690.9905, 3707.5422, 3716.6360, 3721.5728, 3722.4707, 3736.5007, 3741.4561, 3748.4346, 3753.4888, 3764.5239, 4075.0212, 4160.8794, 4238.9067, 4253.8887, 4274.9287, 4301.2393, 4397.9902, 4415.0044, 4458.2759, 4465.1338, 4631.9888, 4690.3101, 4845.1016

22. A2VDY9 **Mass:** 11285 **Score:** 18 **Expect:** 3.7e+002 **Matches:** 4
P antigen family, member 4 (Prostate associated) OS=Bos taurus GN=PAGE4 PE=2 SV=1

Observed	Mr(expt)	Mr(calc)	ppm	Start	End	Miss	Peptide
719.4157	718.4084	718.3908	24.5	1 -	6	1	-.MSARVR.S
978.5347	977.5274	977.5189	8.73	1 -	8	2	-.MSARVRSR.S + Oxidation (M)
2108.9956	2107.9883	2107.9804	3.76	13 -	33	2	R.GDGQESSDTAETMAARKLGGK.K
3335.6306	3334.6233	3334.5623	18.3	39 -	69	2	K.EPPAKNVDMEPGQEKEGGPSVVQEPELEGSR.Q + Oxidation (M)

No match to: 701.3929, 710.3735, 712.2889, 713.2998, 717.3996, 720.4154, 722.4257, 729.4009, 734.4510, 735.4123, 738.4139, 745.4143, 747.4459, 753.3587, 756.3719, 771.3859, 779.4640, 783.3826, 805.4482, 806.4485, 818.4813, 823.4747, 829.4503, 831.4642, 832.3057, 834.3104, 835.3138, 840.4758, 842.5006, 874.5018, 883.4738, 889.4957, 891.4817, 900.5336, 909.5331, 922.4722, 923.5116, 925.4815, 929.4376, 936.4885, 938.4929, 949.5431, 958.4930, 967.4177, 969.4647, 974.4397, 983.6221, 985.6010, 988.4032, 993.4985, 1007.5710, 1017.5679, 1034.5784, 1036.5477, 1044.5231, 1046.5923, 1051.5060, 1056.4977, 1057.5691, 1058.6145, 1074.6044, 1087.5525, 1113.5417, 1118.5876, 1123.5989, 1143.4429, 1145.6317, 1169.4524, 1172.6801, 1178.6199, 1185.6628, 1187.7002, 1194.5691, 1207.6023, 1216.4987, 1216.7014, 1248.6699, 1254.6930, 1255.6340, 1257.6666, 1265.7057, 1269.6703, 1273.6517, 1278.6932, 1281.6456, 1284.6411, 1286.7430, 1299.5466, 1301.6044, 1304.6500, 1312.7513, 1314.7311, 1317.7122, 1338.6886, 1349.6870, 1351.6915, 1354.7209, 1358.7173, 1365.5874, 1368.6487, 1371.7622, 1372.5999, 1374.6569, 1375.7383, 1382.5975, 1383.7290, 1390.6992, 1393.7732, 1397.7450, 1412.6091, 1413.6514, 1420.7966, 1440.7816, 1442.8168, 1450.8618, 1456.7800, 1457.7509, 1467.7542, 1470.7775, 1494.7429, 1500.7238, 1513.7605, 1528.6982, 1539.7341, 1556.7584, 1562.7856, 1563.7985, 1570.7755, 1571.9341, 1578.7968, 1580.8304, 1590.7900, 1592.7916, 1616.8013, 1628.7054, 1628.8561, 1635.7781, 1642.7219, 1647.8557, 1649.8578, 1650.8488, 1657.9104, 1663.8551, 1664.8605, 1696.8295, 1706.8674, 1707.9520, 1715.8798, 1718.8820, 1721.9437, 1730.8646, 1741.9973, 1747.9136, 1755.8970, 1758.9091, 1771.0317, 1774.9242, 1786.9141, 1787.8756, 1862.9530, 1870.7738, 1883.7272, 1895.7275, 1897.7380, 1903.0176, 1911.7487, 1913.7377, 1928.0281, 1938.0094, 1941.0232, 1959.9926, 1961.0339, 1970.0011, 1974.9122, 1979.0769, 1981.0564, 1982.0408, 2007.9467, 2008.9807, 2027.1300, 2055.0208, 2060.9963, 2067.1592, 2086.1130, 2112.9822, 2144.0425, 2181.1780, 2223.1548, 2239.1460, 2241.1360, 2245.8369, 2251.1431, 2255.1433, 2259.8535, 2265.0969, 2273.0667, 2286.1521, 2318.1763, 2328.9463, 2335.8999, 2343.1943, 2347.8403, 2352.9194, 2361.8635, 2363.8716, 2364.8738, 2366.9121, 2371.0796, 2389.2690, 2414.2378, 2467.2051, 2474.2170, 2477.2441, 2485.0479, 2503.0457, 2504.9548, 2505.9473, 2519.9768, 2541.2239, 2542.2581, 2580.0098, 2595.0286, 2631.4714, 2646.9739, 2678.0037, 2681.3691, 2684.3965, 2695.0193, 2701.2148, 2705.2874, 2709.0286, 2717.2839, 2770.4202, 2792.2090, 2798.2344, 2799.2815, 2809.4841, 2813.3928, 2816.2957, 2833.0999, 2842.4780, 2846.3840, 2872.3215, 2876.3640, 2885.3044, 2919.4407, 2961.2092, 2970.4829, 2976.2786, 2990.3098, 3002.4895, 3029.4480, 3071.4028, 3081.6030, 3087.4146, 3146.5461, 3157.4587, 3161.0999, 3184.4336, 3236.5503, 3248.6294, 3254.5583, 3265.5901, 3315.6875, 3330.5125, 3334.5588, 3346.5308, 3347.4827, 3352.6125, 3410.6125, 3411.7185, 3428.5632, 3435.6848, 3457.5947, 3462.6028, 3471.6272, 3474.6221, 3489.6526, 3503.7864, 3528.3171, 3542.3335, 3552.6479, 3574.6042, 3624.6587, 3639.7046, 3645.7671, 3651.7319, 3667.7424, 3690.9905, 3707.5422, 3716.6360, 3721.5728, 3722.4707, 3736.5007, 3741.4561, 3748.4346, 3753.4888, 3764.5239, 4075.0212, 4147.9077, 4160.8794, 4238.9067, 4253.8887, 4274.9287, 4301.2393, 4397.9902, 4415.0044, 4452.1357, 4458.2759, 4465.1338, 4631.9888, 4690.3101, 4845.1016

23. Q5KR49 **Mass:** 32732 **Score:** 18 **Expect:** 3.7e+002 **Matches:** 9
Tropomyosin alpha-1 chain OS=Bos taurus GN=TPM1 PE=2 SV=1

Observed	Mr(expt)	Mr(calc)	ppm	Start	End	Miss	Peptide
710.3735	709.3662	709.3646	2.21	60 -	65	0	K.YSEALK.D
938.4929	937.4856	937.5089	-24.77	6 -	12	2	K.KKMQMLK.L + 2 Oxidation (M)
1273.6517	1272.6444	1272.6561	-9.17	66 -	76	1	K.DAQEKLELAEK.K
1281.6456	1280.6383	1280.6248	10.6	60 -	70	1	K.YSEALKDAQEK.L
1314.7311	1313.7238	1313.7554	-24.06	168 -	178	1	R.KLVIIESDLER.A
1616.8013	1615.7940	1615.8278	-20.88	77 -	91	2	K.KATDAEADVASLNRR.I
1928.0281	1927.0208	1927.0374	-8.59	102 -	118	2	R.AQERLATALQKLEEAEK.A
1959.9926	1958.9853	1958.9982	-6.59	134 -	149	2	R.AQKDEEKMEIQEIQLK.E
1961.0339	1960.0266	1959.9822	22.6	137 -	152	2	K.DEEKMEIQEIQLKEAK.H

No match to: 701.3929, 712.2889, 713.2998, 717.3996, 719.4157, 720.4154, 722.4257, 729.4009, 734.4510, 735.4123, 738.4139, 745.4143, 747.4459, 753.3587, 756.3719, 771.3859, 779.4640, 783.3826, 805.4482, 806.4485, 818.4813, 823.4747, 829.4503, 831.4642, 832.3057, 834.3104, 835.3138, 840.4758, 842.5006, 874.5018, 883.4738, 889.4957, 891.4817, 900.5336, 909.5331, 922.4722, 923.5116, 925.4815, 929.4376, 936.4885, 949.5431, 958.4930, 967.4177, 969.4647, 974.4397, 978.5347, 983.6221, 985.6010, 988.4032, 993.4985, 1007.5710, 1017.5679, 1034.5784, 1036.5477, 1044.5231, 1046.5923, 1051.5060, 1056.4977, 1057.5691, 1058.6145, 1074.6044, 1087.5525, 1113.5417, 1118.5876, 1123.5989, 1143.4429, 1145.6317, 1169.4524, 1172.6801, 1178.6199, 1185.6628, 1187.7002, 1194.5691, 1207.6023, 1216.4987, 1216.7014, 1248.6699, 1254.6930, 1255.6340, 1257.6666, 1265.7057, 1269.6703, 1278.6932, 1284.6411, 1286.7430, 1299.5466, 1301.6044, 1304.6500, 1312.7513, 1317.7122, 1338.6886, 1349.6870, 1351.6915, 1354.7209, 1358.7173, 1365.5874, 1368.6487, 1371.7622, 1372.5999, 1374.6569, 1375.7383, 1382.5975, 1383.7290, 1390.6992, 1393.7732, 1397.7450, 1412.6091, 1413.6514, 1420.7966, 1440.7816, 1442.8168, 1450.8618, 1456.7800, 1457.7509, 1467.7542, 1470.7775, 1494.7429, 1500.7238, 1513.7605, 1528.6982, 1539.7341, 1556.7584, 1562.7856, 1563.7985, 1570.7755, 1571.9341, 1578.7968, 1580.8304, 1590.7900, 1592.7916, 1628.7054, 1628.8561, 1635.7781, 1642.7219, 1647.8557, 1649.8578, 1650.8488, 1657.9104, 1663.8551, 1664.8605, 1696.8295, 1706.8674, 1707.9520, 1715.8798, 1718.8820, 1721.9437, 1730.8646, 1741.9973, 1747.9136, 1755.8970, 1758.9091, 1771.0317, 1774.9242, 1786.9141, 1787.8756, 1862.9530, 1870.7738, 1883.7272, 1895.7275, 1897.7380, 1903.0176, 1911.7487, 1913.7377, 1938.0094, 1941.0232, 1970.0011, 1974.9122, 1979.0769, 1981.0564, 1982.0408, 2007.9467, 2008.9807, 2027.1300, 2055.0208, 2060.9963, 2067.1592, 2086.1130, 2108.9956, 2112.9822, 2144.0425, 2181.1780, 2223.1548, 2239.1460, 2241.1360, 2245.8369, 2251.1431, 2255.1433, 2259.8535, 2265.0969, 2273.0667, 2286.1521, 2318.1763, 2328.9463, 2335.8999, 2343.1943, 2347.8403, 2352.9194, 2361.8635, 2363.8716, 2364.8738, 2366.9121, 2371.0796, 2389.2690, 2414.2378, 2467.2051, 2474.2170, 2477.2441, 2485.0479, 2503.0457, 2504.9548, 2505.9473, 2519.9768, 2541.2239, 2542.2581, 2580.0098, 2595.0286, 2631.4714, 2646.9739, 2678.0037, 2681.3691, 2684.3965, 2695.0193, 2701.2148, 2705.2874, 2709.0286, 2717.2839, 2770.4202, 2792.2090, 2798.2344, 2799.2815, 2809.4841, 2813.3928, 2816.2957, 2833.0999, 2842.4780, 2846.3840, 2872.3215, 2876.3640, 2885.3044, 2919.4407, 2961.2092, 2970.4829, 2976.2786, 2990.3098, 3002.4895, 3029.4480, 3071.4028, 3081.6030, 3087.4146, 3146.5461, 3157.4587, 3161.0999, 3184.4336, 3236.5503, 3248.6294, 3254.5583, 3265.5901, 3315.6875, 3330.5125, 3334.5588, 3335.6306, 3346.5308, 3347.4827, 3352.6125, 3410.6125, 3411.7185, 3428.5632, 3435.6848, 3457.5947, 3462.6028, 3471.6272, 3474.6221, 3489.6526, 3503.7864, 3528.3171, 3542.3335, 3552.6479, 3574.6042, 3624.6587, 3639.7046, 3645.7671, 3651.7319, 3667.7424, 3690.9905, 3707.5422, 3716.6360, 3721.5728, 3722.4707, 3736.5007, 3741.4561, 3748.4346, 3753.4888, 3764.5239, 4075.0212, 4147.9077, 4160.8794, 4238.9067, 4253.8887, 4274.9287, 4301.2393, 4397.9902, 4415.0044, 4452.1357, 4458.2759, 4465.1338, 4631.9888, 4690.3101, 4845.1016

24. P28782 **Mass:** 10567 **Score:** 18 **Expect:** 3.7e+002 **Matches:** 4
Protein S100-A8 OS=Bos taurus GN=S100A8 PE=1 SV=2

Observed	Mr(expt)	Mr(calc)	ppm	Start	End	Miss	Peptide
1390.6992	1389.6919	1389.6888	2.24	78 -	89	1	K.VGLEAHEEIHKE.-
1539.7341	1538.7268	1538.7473	-13.29	36 -	47	1	K.QLLETECPKFMK.K + Oxidation (M)
1578.7968	1577.7895	1577.7950	-3.48	23 -	35	2	K.KGNYHAVYRDDLK.Q
2770.4202	2769.4129	2769.4081	1.75	1 -	23	2	-.MLTDLECAINSLIDVYHKYSLKK.G + Oxidation (M)

No match to: 701.3929, 710.3735, 712.2889, 713.2998, 717.3996, 719.4157, 720.4154, 722.4257, 729.4009, 734.4510, 735.4123, 738.4139, 745.4143, 747.4459, 753.3587, 756.3719, 771.3859, 779.4640, 783.3826, 805.4482, 806.4485, 818.4813, 823.4747, 829.4503, 831.4642, 832.3057, 834.3104, 835.3138, 840.4758, 842.5006, 874.5018, 883.4738, 889.4957, 891.4817, 900.5336, 909.5331, 922.4722,

923.5116, 925.4815, 929.4376, 936.4885, 938.4929, 949.5431, 958.4930, 967.4177, 969.4647, 974.4397, 978.5347, 983.6221, 985.6010, 988.4032, 993.4985, 1007.5710, 1017.5679, 1034.5784, 1036.5477, 1044.5231, 1046.5923, 1051.5060, 1056.4977, 1057.5691, 1058.6145, 1074.6044, 1087.5525, 1113.5417, 1118.5876, 1123.5989, 1143.4429, 1145.6317, 1169.4524, 1172.6801, 1178.6199, 1185.6628, 1187.7002, 1194.5691, 1207.6023, 1216.4987, 1216.7014, 1248.6699, 1254.6930, 1255.6340, 1257.6666, 1265.7057, 1269.6703, 1273.6517, 1278.6932, 1281.6456, 1284.6411, 1286.7430, 1299.5466, 1301.6044, 1304.6500, 1312.7513, 1314.7311, 1317.7122, 1338.6886, 1349.6870, 1351.6915, 1354.7209, 1358.7173, 1365.5874, 1368.6487, 1371.7622, 1372.5999, 1374.6569, 1375.7383, 1382.5975, 1383.7290, 1393.7732, 1397.7450, 1412.6091, 1413.6514, 1420.7966, 1440.7816, 1442.8168, 1450.8618, 1456.7800, 1457.7509, 1467.7542, 1470.7775, 1494.7429, 1500.7238, 1513.7605, 1528.6982, 1556.7584, 1562.7856, 1563.7985, 1570.7755, 1571.9341, 1580.8304, 1590.7900, 1592.7916, 1616.8013, 1628.7054, 1628.8561, 1635.7781, 1642.7219, 1647.8557, 1649.8578, 1650.8488, 1657.9104, 1663.8551, 1664.8605, 1696.8295, 1706.8674, 1707.9520, 1715.8798, 1718.8820, 1721.9437, 1730.8646, 1741.9973, 1747.9136, 1755.8970, 1758.9091, 1771.0317, 1774.9242, 1786.9141, 1787.8756, 1862.9530, 1870.7738, 1883.7272, 1895.7275, 1897.7380, 1903.0176, 1911.7487, 1913.7377, 1928.0281, 1938.0094, 1941.0232, 1959.9926, 1961.0339, 1970.0011, 1974.9122, 1979.0769, 1981.0564, 1982.0408, 2007.9467, 2008.9807, 2027.1300, 2055.0208, 2060.9963, 2067.1592, 2086.1130, 2108.9956, 2112.9822, 2144.0425, 2181.1780, 2223.1548, 2239.1460, 2241.1360, 2245.8369, 2251.1431, 2255.1433, 2259.8535, 2265.0969, 2273.0667, 2286.1521, 2318.1763, 2328.9463, 2335.8999, 2343.1943, 2347.8403, 2352.9194, 2361.8635, 2363.8716, 2364.8738, 2366.9121, 2371.0796, 2389.2690, 2414.2378, 2467.2051, 2474.2170, 2477.2441, 2485.0479, 2503.0457, 2504.9548, 2505.9473, 2519.9768, 2541.2239, 2542.2581, 2580.0098, 2595.0286, 2631.4714, 2646.9739, 2678.0037, 2681.3691, 2684.3965, 2695.0193, 2701.2148, 2705.2874, 2709.0286, 2717.2839, 2792.2090, 2798.2344, 2799.2815, 2809.4841, 2813.3928, 2816.2957, 2833.0999, 2842.4780, 2846.3840, 2872.3215, 2876.3640, 2885.3044, 2919.4407, 2961.2092, 2970.4829, 2976.2786, 2990.3098, 3002.4895, 3029.4480, 3071.4028, 3081.6030, 3087.4146, 3146.5461, 3157.4587, 3161.0999, 3184.4336, 3236.5503, 3248.6294, 3254.5583, 3265.5901, 3315.6875, 3330.5125, 3334.5588, 3335.6306, 3346.5308, 3347.4827, 3352.6125, 3410.6125, 3411.7185, 3428.5632, 3435.6848, 3457.5947, 3462.6028, 3471.6272, 3474.6221, 3489.6526, 3503.7864, 3528.3171, 3542.3335, 3552.6479, 3574.6042, 3624.6587, 3639.7046, 3645.7671, 3651.7319, 3667.7424, 3690.9905, 3707.5422, 3716.6360, 3721.5728, 3722.4707, 3736.5007, 3741.4561, 3748.4346, 3753.4888, 3764.5239, 4075.0212, 4147.9077, 4160.8794, 4238.9067, 4253.8887, 4274.9287, 4301.2393, 4397.9902, 4415.0044, 4452.1357, 4458.2759, 4465.1338, 4631.9888, 4690.3101, 4845.1016

25. E1B8L7 **Mass:** 12965 **Score:** 18 **Expect:** 4e+002 **Matches:** 4

Uncharacterized protein OS=Bos taurus PE=3 SV=1

Observed	Mr(expt)	Mr(calc)	ppm	Start	End	Miss	Peptide
747.4459	746.4386	746.4360	3.41	18 -	23	0	R.LQLVMK.S + Oxidation (M)
1774.9242	1773.9169	1773.9521	-19.83	18 -	32	2	R.LQLVMKSGKYMLGYK.Q + Oxidation (M)
2467.2051	2466.1978	2466.1499	19.4	69 -	90	1	K.TGVHHYGGNNIELGTACGKYYR.V
3411.7185	3410.7112	3410.6387	21.3	57 -	87	2	R.KSEIEYYAMLAKTGVHHYGGNNIELGTACGK.Y

No match to: 701.3929, 710.3735, 712.2889, 713.2998, 717.3996, 719.4157, 720.4154, 722.4257, 729.4009, 734.4510, 735.4123, 738.4139, 745.4143, 753.3587, 756.3719, 771.3859, 779.4640, 783.3826, 805.4482, 806.4485, 818.4813, 823.4747, 829.4503, 831.4642, 832.3057, 834.3104, 835.3138, 840.4758, 842.5006, 874.5018, 883.4738, 889.4957, 891.4817, 900.5336, 909.5331, 922.4722, 923.5116, 925.4815, 929.4376, 936.4885, 938.4929, 949.5431, 958.4930, 967.4177, 969.4647, 974.4397, 978.5347, 983.6221, 985.6010, 988.4032, 993.4985, 1007.5710, 1017.5679, 1034.5784, 1036.5477, 1044.5231, 1046.5923, 1051.5060, 1056.4977, 1057.5691, 1058.6145, 1074.6044, 1087.5525, 1113.5417, 1118.5876, 1123.5989, 1143.4429, 1145.6317, 1169.4524, 1172.6801, 1178.6199, 1185.6628, 1187.7002, 1194.5691, 1207.6023, 1216.4987, 1216.7014, 1248.6699, 1254.6930, 1255.6340, 1257.6666, 1265.7057, 1269.6703, 1273.6517, 1278.6932, 1281.6456, 1284.6411, 1286.7430, 1299.5466, 1301.6044, 1304.6500, 1312.7513, 1314.7311, 1317.7122, 1338.6886, 1349.6870, 1351.6915, 1354.7209, 1358.7173, 1365.5874, 1368.6487, 1371.7622, 1372.5999, 1374.6569, 1375.7383, 1382.5975, 1383.7290, 1390.6992, 1393.7732, 1397.7450, 1412.6091, 1413.6514, 1420.7966, 1440.7816, 1442.8168, 1450.8618, 1456.7800, 1457.7509, 1467.7542, 1470.7775, 1494.7429, 1500.7238, 1513.7605, 1528.6982, 1539.7341, 1556.7584, 1562.7856, 1563.7985, 1570.7755, 1571.9341, 1578.7968, 1580.8304, 1590.7900, 1592.7916, 1616.8013, 1628.7054, 1628.8561, 1635.7781, 1642.7219, 1647.8557, 1649.8578, 1650.8488, 1657.9104, 1663.8551, 1664.8605, 1696.8295, 1706.8674, 1707.9520, 1715.8798, 1718.8820, 1721.9437, 1730.8646, 1741.9973, 1747.9136, 1755.8970, 1758.9091, 1771.0317, 1774.9242, 1786.9141, 1787.8756, 1862.9530, 1870.7738, 1883.7272, 1895.7275, 1897.7380, 1903.0176, 1911.7487, 1913.7377, 1928.0281, 1938.0094, 1941.0232, 1959.9926, 1961.0339, 1970.0011, 1974.9122, 1979.0769, 1981.0564, 1982.0408, 2007.9467, 2008.9807, 2027.1300, 2055.0208, 2060.9963, 2067.1592, 2086.1130, 2108.9956, 2112.9822, 2144.0425, 2181.1780, 2223.1548, 2239.1460, 2241.1360, 2245.8369, 2251.1431, 2255.1433, 2259.8535, 2265.0969, 2273.0667, 2286.1521, 2318.1763, 2328.9463, 2335.8999, 2343.1943, 2347.8403, 2352.9194, 2361.8635, 2363.8716, 2364.8738, 2366.9121, 2371.0796, 2389.2690, 2414.2378, 2474.2170, 2477.2441, 2485.0479, 2503.0457, 2504.9548, 2505.9473, 2519.9768, 2541.2239, 2542.2581, 2580.0098, 2595.0286, 2631.4714, 2646.9739, 2678.0037, 2681.3691, 2684.3965, 2695.0193, 2701.2148, 2705.2874, 2709.0286, 2717.2839, 2770.4202, 2792.2090, 2798.2344, 2799.2815, 2809.4841, 2813.3928, 2816.2957, 2833.0999, 2842.4780, 2846.3840, 2872.3215, 2876.3640, 2885.3044, 2919.4407, 2961.2092, 2970.4829, 2976.2786, 2990.3098, 3002.4895, 3029.4480, 3071.4028, 3081.6030, 3087.4146, 3146.5461, 3157.4587, 3161.0999, 3184.4336, 3236.5503, 3248.6294, 3254.5583, 3265.5901, 3315.6875, 3330.5125, 3334.5588, 3335.6306, 3346.5308, 3347.4827, 3352.6125, 3410.6125, 3428.5632, 3435.6848, 3457.5947, 3462.6028, 3471.6272, 3474.6221, 3489.6526, 3503.7864, 3528.3171, 3542.3335, 3552.6479, 3574.6042, 3624.6587, 3639.7046, 3645.7671, 3651.7319, 3667.7424, 3690.9905, 3707.5422, 3716.6360, 3721.5728, 3722.4707, 3736.5007, 3741.4561, 3748.4346, 3753.4888, 3764.5239, 4075.0212, 4147.9077, 4160.8794, 4238.9067, 4253.8887, 4274.9287, 4301.2393, 4397.9902, 4415.0044, 4452.1357, 4458.2759, 4465.1338, 4631.9888, 4690.3101, 4845.1016

26. G3N1F5 **Mass:** 34506 **Score:** 17 **Expect:** 4.7e+002 **Matches:** 9

Uncharacterized protein OS=Bos taurus PE=3 SV=1

Observed	Mr(expt)	Mr(calc)	ppm	Start	End	Miss	Peptide
805.4482	804.4409	804.4527	-14.70	271 -	277	1	K.MSLAQKK.D
840.4758	839.4686	839.4654	3.80	16 -	21	1	R.YQVKFR.R
1265.7057	1264.6984	1264.6710	21.7	108 -	117	2	R.RLLNRFGMDK.I + Oxidation (M)
1338.6886	1337.6813	1337.6939	-9.43	165 -	178	0	K.GAVDGGLSIPHSTK.W
1358.7173	1357.7100	1357.7288	-13.86	266 -	276	1	R.WNRPKMSLAQK.K
1440.7816	1439.7743	1439.7707	2.51	44 -	54	2	K.YNTPKYRMIVR.V
1456.7800	1455.7727	1455.7656	4.88	44 -	54	2	K.YNTPKYRMIVR.V + Oxidation (M)
1562.7856	1561.7783	1561.7493	18.6	197 -	209	1	R.KHIMGQNVADYMR.Y
4415.0044	4413.9971	4414.0046	-1.69	113 -	152	1	R.FGMDKIYEGQVEVTGDEYNVESIDGQPGAFTCYLDAGLAR.T

No match to: 701.3929, 710.3735, 712.2889, 713.2998, 717.3996, 719.4157, 720.4154, 722.4257, 729.4009, 734.4510, 735.4123, 738.4139, 745.4143, 747.4459, 753.3587, 756.3719, 771.3859, 779.4640, 783.3826, 806.4485, 818.4813, 823.4747, 829.4503, 831.4642, 832.3057, 834.3104, 835.3138, 842.5006, 874.5018, 883.4738, 889.4957, 891.4817, 900.5336, 909.5331, 922.4722, 923.5116, 925.4815, 929.4376, 936.4885, 938.4929, 949.5431, 958.4930, 967.4177, 969.4647, 974.4397, 978.5347, 983.6221, 985.6010, 988.4032, 993.4985, 1007.5710, 1017.5679, 1034.5784, 1036.5477, 1044.5231, 1046.5923, 1051.5060, 1056.4977, 1057.5691, 1058.6145, 1074.6044, 1087.5525, 1113.5417, 1118.5876, 1123.5989, 1143.4429, 1145.6317, 1169.4524, 1172.6801, 1178.6199, 1185.6628, 1187.7002, 1194.5691, 1207.6023, 1216.4987, 1216.7014, 1248.6699, 1254.6930, 1255.6340, 1257.6666, 1269.6703, 1273.6517, 1278.6932, 1281.6456, 1284.6411, 1286.7430, 1299.5466, 1301.6044, 1304.6500, 1312.7513, 1314.7311, 1317.7122, 1349.6870, 1351.6915, 1354.7209, 1365.5874, 1368.6487, 1371.7622, 1372.5999, 1374.6569, 1375.7383, 1382.5975, 1383.7290, 1390.6992, 1393.7732, 1397.7450, 1412.6091, 1413.6514, 1420.7966, 1442.8168, 1450.8618, 1457.7509, 1467.7542, 1470.7775, 1494.7429, 1500.7238, 1513.7605, 1528.6982, 1539.7341, 1556.7584, 1563.7985, 1570.7755, 1571.9341, 1578.7968, 1580.8304, 1590.7900, 1592.7916, 1616.8013, 1628.7054, 1628.8561, 1635.7781, 1642.7219, 1647.8557, 1649.8578, 1650.8488, 1657.9104, 1663.8551, 1664.8605, 1696.8295, 1706.8674, 1707.9520, 1715.8798, 1718.8820, 1721.9437, 1730.8646, 1741.9973, 1747.9136, 1755.8970, 1758.9091, 1771.0317, 1774.9242, 1786.9141, 1787.8756, 1862.9530, 1870.7738, 1883.7272, 1895.7275, 1897.7380, 1903.0176, 1911.7487, 1913.7377, 1928.0281, 1938.0094, 1941.0232, 1959.9926, 1961.0339, 1970.0011, 1974.9122, 1979.0769, 1981.0564, 1982.0408, 2007.9467, 2008.9807, 2027.1300, 2055.0208, 2060.9963, 2067.1592, 2086.1130, 2108.9956, 2112.9822, 2144.0425, 2181.1780, 2223.1548, 2239.1460, 2241.1360, 2245.8369, 2251.1431, 2255.1433, 2259.8535, 2265.0969, 2273.0667, 2286.1521, 2318.1763, 2328.9463, 2335.8999, 2343.1943, 2347.8403, 2352.9194, 2361.8635, 2363.8716, 2364.8738, 2366.9121, 2371.0796, 2389.2690, 2414.2378, 2467.2051, 2474.2170, 2477.2441, 2485.0479, 2503.0457, 2504.9548, 2505.9473, 2519.9768, 2541.2239, 2542.2581, 2580.0098, 2595.0286, 2631.4714, 2646.9739, 2678.0037, 2681.3691, 2684.3965, 2695.0193, 2701.2148, 2705.2874, 2709.0286, 2717.2839, 2770.4202, 2792.2090, 2798.2344, 2799.2815, 2809.4841, 2813.3928, 2816.2957, 2833.0999, 2842.4780, 2846.3840, 2872.3215, 2876.3640, 2885.3044, 2919.4407, 2961.2092, 2970.4829, 2976.2786, 2990.3098, 3002.4895, 3029.4480, 3071.4028, 3081.6030, 3087.4146, 3146.5461, 3157.4587, 3161.0999, 3184.4336, 3236.5503, 3248.6294, 3254.5583, 3265.5901, 3315.6875, 3330.5125, 3334.5588, 3335.6306, 3346.5308, 3347.4827, 3352.6125, 3410.6125, 3411.7185, 3428.5632, 3435.6848, 3457.5947, 3462.6028, 3471.6272, 3474.6221, 3489.6526, 3503.7864, 3528.3171, 3542.3335, 3552.6479, 3574.6042, 3624.6587, 3639.7046, 3645.7671, 3651.7319, 3667.7424, 3690.9905, 3707.5422, 3716.6360, 3721.5728, 3722.4707, 3736.5007, 3741.4561, 3748.4346, 3753.4888, 3764.5239, 4075.0212, 4147.9077, 4160.8794, 4238.9067, 4253.8887, 4274.9287, 4301.2393, 4397.9902, 4452.1357, 4458.2759, 4465.1338, 4631.9888, 4690.3101, 4845.1016

27. Q0P5K1 **Mass:** 53559 **Score:** 17 **Expect:** 4.9e+002 **Matches:** 8
Vasculin OS=Bos taurus GN=GPBP1 PE=2 SV=1

Observed	Mr(expt)	Mr(calc)	ppm	Start		End	Miss	Peptide
922.4722	921.4650	921.4556	10.2	201	-	209	0	K.NGTGPSVYK.G
978.5347	977.5274	977.5155	12.2	40	-	46	2	R.YDVNRRR.H
1036.5477	1035.5404	1035.5495	-8.76	290	-	297	2	K.LTRMRTDK.K + Oxidation (M)
2060.9963	2059.9890	2059.9810	3.87	369	-	387	0	R.SSAFPQTDVLSSSLEAEHR.L
2239.1460	2238.1387	2238.1069	14.2	147	-	166	1	R.EPNQNKSLAAGVWEYPPNPK.S
2631.4714	2630.4641	2630.4544	3.70	210	-	233	2	K.GLVPKPAAPPTKPTQWKSQTKENK.V
3736.5007	3735.4934	3735.5541	-16.25	310	-	341	1	R.VEEEHEDESHVGSEKDDDSFNLHNSNSTHQER.D
4397.9902	4396.9829	4396.9078	17.1	435	-	473	2	K.NGLICDFKFGPWKNSTFKPTIENDDTETSSSDTSDDDDV.-

No match to: 701.3929, 710.3735, 712.2889, 713.2998, 717.3996, 719.4157, 720.4154, 722.4257, 729.4009, 734.4510, 735.4123, 738.4139, 745.4143, 747.4459, 753.3587, 756.3719, 771.3859, 779.4640, 783.3826, 805.4482, 806.4485, 818.4813, 823.4747, 829.4503, 831.4642, 832.3057, 834.3104, 835.3138, 840.4758, 842.5006, 874.5018, 883.4738, 889.4957, 891.4817, 900.5336, 909.5331, 923.5116, 925.4815, 929.4376, 936.4885, 938.4929, 949.5431, 958.4930, 967.4177, 969.4647, 974.4397, 983.6221, 985.6010, 988.4032, 993.4985, 1007.5710, 1017.5679, 1034.5784, 1044.5231, 1046.5923, 1051.5060, 1056.4977, 1057.5691, 1058.6145, 1074.6044, 1087.5525, 1113.5417, 1118.5876, 1123.5989, 1143.4429, 1145.6317, 1169.4524, 1172.6801, 1178.6199, 1185.6628, 1187.7002, 1194.5691, 1207.6023, 1216.4987, 1216.7014, 1248.6699, 1254.6930, 1255.6340, 1257.6666, 1265.7057, 1269.6703, 1273.6517, 1278.6932, 1281.6456, 1284.6411, 1286.7430, 1299.5466, 1301.6044, 1304.6500, 1312.7513, 1314.7311, 1317.7122, 1338.6886, 1349.6870, 1351.6915, 1354.7209, 1358.7173, 1365.5874, 1368.6487, 1371.7622, 1372.5999, 1374.6569, 1375.7383, 1382.5975, 1383.7290, 1390.6992, 1393.7732, 1397.7450, 1412.6091, 1413.6514, 1420.7966, 1440.7816, 1442.8168, 1450.8618, 1456.7800, 1457.7509, 1467.7542, 1470.7775, 1494.7429, 1500.7238, 1513.7605, 1528.6982, 1539.7341, 1556.7584, 1562.7856, 1563.7985, 1570.7755, 1571.9341, 1578.7968, 1580.8304, 1590.7900, 1592.7916, 1616.8013, 1628.7054, 1628.8561, 1635.7781, 1642.7219, 1647.8557, 1649.8578, 1650.8488, 1657.9104, 1663.8551, 1664.8605, 1696.8295, 1706.8674, 1707.9520, 1715.8798, 1718.8820, 1721.9437, 1730.8646, 1741.9973, 1747.9136, 1755.8970, 1758.9091, 1771.0317, 1774.9242, 1786.9141, 1787.8756, 1862.9530, 1870.7738, 1883.7272, 1895.7275, 1897.7380, 1903.0176, 1911.7487, 1913.7377, 1928.0281, 1938.0094, 1941.0232, 1959.9926, 1961.0339, 1970.0011, 1974.9122, 1979.0769, 1981.0564, 1982.0408, 2007.9467, 2008.9807, 2027.1300, 2055.0208, 2067.1592, 2086.1130, 2108.9956, 2112.9822, 2144.0425, 2181.1780, 2223.1548, 2241.1360, 2245.8369, 2251.1431, 2255.1433, 2259.8535, 2265.0969, 2273.0667, 2286.1521, 2318.1763, 2328.9463, 2335.8999, 2343.1943, 2347.8403, 2352.9194, 2361.8635, 2363.8716, 2364.8738, 2366.9121, 2371.0796, 2389.2690, 2414.2378, 2467.2051, 2474.2170, 2477.2441, 2485.0479, 2503.0457, 2504.9548, 2505.9473, 2519.9768, 2541.2239, 2542.2581, 2580.0098, 2595.0286, 2646.9739, 2678.0037, 2681.3691, 2684.3965, 2695.0193, 2701.2148, 2705.2874, 2709.0286, 2717.2839, 2770.4202, 2792.2090, 2798.2344, 2799.2815, 2809.4841, 2813.3928, 2816.2957, 2833.0999, 2842.4780, 2846.3840, 2872.3215, 2876.3640, 2885.3044, 2919.4407, 2961.2092, 2970.4829, 2976.2786, 2990.3098, 3002.4895, 3029.4480, 3071.4028, 3081.6030, 3087.4146, 3146.5461, 3157.4587, 3161.0999, 3184.4336, 3236.5503, 3248.6294, 3254.5583, 3265.5901, 3315.6875, 3330.5125, 3334.5588, 3335.6306, 3346.5308, 3347.4827, 3352.6125, 3410.6125, 3411.7185, 3428.5632, 3435.6848, 3457.5947, 3462.6028, 3471.6272, 3474.6221, 3489.6526, 3503.7864, 3528.3171, 3542.3335, 3552.6479, 3574.6042, 3624.6587, 3639.7046, 3645.7671, 3651.7319, 3667.7424, 3690.9905, 3707.5422, 3716.6360, 3721.5728, 3722.4707, 3741.4561, 3748.4346, 3753.4888, 3764.5239, 4075.0212, 4147.9077, 4160.8794, 4238.9067, 4253.8887, 4274.9287, 4301.2393, 4415.0044, 4452.1357, 4458.2759, 4465.1338, 4631.9888, 4690.3101, 4845.1016

28. Q3B7N2 **Mass:** 103486 **Score:** 17 **Expect:** 5.1e+002 **Matches:** 16
Alpha-actinin-1 OS=Bos taurus GN=ACTN1 PE=2 SV=1

Observed	Mr(expt)	Mr(calc)	ppm	Start		End	Miss	Peptide
891.4817	890.4744	890.4644	11.3	414	-	420	1	K.EAMLRQK.D + Oxidation (M)
1113.5417	1112.5344	1112.5251	8.42	313	-	320	1	K.LEDFRDYR.R
1185.6628	1184.6555	1184.6812	-21.63	77	-	86	2	R.LAKPERGKMR.V
1278.6932	1277.6859	1277.6728	10.3	841	-	850	1	K.NYITVDELRR.E
1301.6044	1300.5971	1300.5982	-0.85	705	-	714	0	K.HTNYTMEHIR.V
1371.7622	1370.7549	1370.7670	-8.81	282	-	292	1	K.LASDLLEWIRR.T
1730.8646	1729.8573	1729.8862	-16.68	804	-	818	0	R.LGVVTFQAFIDFMSR.E
1862.9530	1861.9457	1861.9872	-22.30	148	-	162	2	K.EGLLLWCQRKTAPYK.N
2007.9467	2006.9394	2006.9560	-8.27	237	-	254	0	K.AIMTYVSSFYHAFSGAQK.A
2067.1592	2066.1519	2066.1921	-19.46	65	-	82	1	K.LMLLLEVISGERLAKPER.G
2467.2051	2466.1978	2466.2590	-24.81	175	-	195	1	K.DGLGFCALIHRHRPELIDYGK.L
3071.4028	3070.3955	3070.3464	16.0	864	-	892	0	R.MAPYTGPDAVPGALDYMSFSTALYGESDL.- + 2 Oxidation (M)
3087.4146	3086.4073	3086.4702	-20.37	293	-	317	2	R.TIPWLENRAPENTMHAMQQKLEDFR.D + 2 Oxidation (M)
3248.6294	3247.6221	3247.6070	4.65	265	-	291	1	K.VLAVNQENEQLMEDYEKLASDLLEWIR.R
3457.5947	3456.5874	3456.6218	-9.94	804	-	834	1	R.LGVVTFQAFIDFMSRETADTDTADQVMASFK.I + Oxidation (M)
4415.0044	4413.9971	4414.0737	-17.35	437	-	475	1	K.HEAFESDLAAHQDRVEQIAAIAQELNELDYYDSPSVNAR.C

No match to: 701.3929, 710.3735, 712.2889, 713.2998, 717.3996, 719.4157, 720.4154, 722.4257, 729.4009, 734.4510, 735.4123, 738.4139, 745.4143, 747.4459, 753.3587, 756.3719, 771.3859, 779.4640, 783.3826, 805.4482, 806.4485, 818.4813, 823.4747, 829.4503, 831.4642, 832.3057, 834.3104, 835.3138, 840.4758, 842.5006, 874.5018, 883.4738, 889.4957, 900.5336, 909.5331, 922.4722, 923.5116, 925.4815, 929.4376, 936.4885, 938.4929, 949.5431, 958.4930, 967.4177, 969.4647, 974.4397, 978.5347, 983.6221, 985.6010, 988.4032, 993.4985, 1007.5710, 1017.5679, 1034.5784, 1036.5477, 1044.5231, 1046.5923, 1051.5060, 1056.4977, 1057.5691, 1058.6145, 1074.6044, 1087.5525, 1118.5876, 1123.5989, 1143.4429, 1145.6317, 1169.4524, 1172.6801, 1178.6199, 1187.7002, 1194.5691, 1207.6023, 1216.4987, 1216.7014, 1248.6699, 1254.6930, 1255.6340, 1257.6666, 1265.7057, 1269.6703, 1273.6517, 1281.6456, 1284.6411, 1286.7430, 1299.5466, 1304.6500, 1312.7513, 1314.7311, 1317.7122, 1338.6886, 1349.6870, 1351.6915, 1354.7209, 1358.7173, 1365.5874, 1368.6487, 1372.5999, 1374.6569, 1375.7383, 1382.5975, 1383.7290, 1390.6992, 1393.7732, 1397.7450, 1412.6091, 1413.6514, 1420.7966, 1440.7816, 1442.8168, 1450.8618, 1456.7800, 1457.7509, 1467.7542, 1470.7775, 1494.7429, 1500.7238, 1513.7605, 1528.6982, 1539.7341, 1556.7584, 1562.7856, 1563.7985, 1570.7755, 1571.9341, 1578.7968, 1580.8304, 1590.7900, 1592.7916, 1616.8013, 1628.7054, 1628.8561, 1635.7781, 1642.7219, 1647.8557, 1649.8578, 1650.8488, 1657.9104, 1663.8551, 1664.8605, 1696.8295, 1706.8674, 1707.9520, 1715.8798, 1718.8820, 1721.9437, 1741.9973, 1747.9136, 1755.8970, 1758.9091, 1771.0317, 1774.9242, 1786.9141, 1787.8756, 1870.7738, 1883.7272, 1895.7275, 1897.7380, 1903.0176, 1911.7487, 1913.7377, 1928.0281, 1938.0094, 1941.0232, 1959.9926, 1961.0339, 1970.0011, 1974.9122, 1979.0769, 1981.0564, 1982.0408, 2008.9807, 2027.1300, 2055.0208, 2060.9963, 2086.1130, 2108.9956, 2112.9822, 2144.0425, 2181.1780, 2223.1548, 2239.1460, 2241.1360, 2245.8369, 2251.1431, 2255.1433, 2259.8535, 2265.0969, 2273.0667, 2286.1521, 2318.1763, 2328.9463, 2335.8999, 2343.1943, 2347.8403, 2352.9194, 2361.8635, 2363.8716, 2364.8738, 2366.9121, 2371.0796, 2389.2690, 2414.2378, 2474.2170, 2477.2441, 2485.0479, 2503.0457, 2504.9548, 2505.9473, 2519.9768, 2541.2239, 2542.2581, 2580.0098, 2595.0286, 2631.4714, 2646.9739, 2678.0037, 2681.3691, 2684.3965, 2695.0193, 2701.2148, 2705.2874, 2709.0286, 2717.2839, 2770.4202, 2792.2090, 2798.2344, 2799.2815, 2809.4841, 2813.3928, 2816.2957, 2833.0999, 2842.4780, 2846.3840, 2872.3215, 2876.3640, 2885.3044, 2919.4407, 2961.2092, 2970.4829, 2976.2786, 2990.3098, 3002.4895, 3029.4480, 3081.6030, 3146.5461, 3157.4587, 3161.0999, 3184.4336, 3236.5503, 3254.5583, 3265.5901, 3315.6875, 3330.5125, 3334.5588, 3335.6306, 3346.5308, 3347.4827, 3352.6125, 3410.6125, 3411.7185, 3428.5632, 3435.6848, 3462.6028, 3471.6272, 3474.6221, 3489.6526, 3503.7864, 3528.3171, 3542.3335, 3552.6479, 3574.6042, 3624.6587, 3639.7046, 3645.7671, 3651.7319, 3667.7424, 3690.9905, 3707.5422, 3716.6360, 3721.5728, 3722.4707, 3736.5007, 3741.4561, 3748.4346, 3753.4888, 3764.5239, 4075.0212, 4147.9077, 4160.8794, 4238.9067, 4253.8887, 4274.9287, 4301.2393, 4397.9902, 4452.1357, 4458.2759, 4465.1338, 4631.9888, 4690.3101, 4845.1016

29. G3N1D4 **Mass:** 4565 **Score:** 17 **Expect:** 5.2e+002 **Matches:** 2
Uncharacterized protein OS=Bos taurus PE=4 SV=1

Observed	Mr(expt)	Mr(calc)	ppm	Start		End	Miss	Peptide
2816.2957	2815.2884	2815.2663	7.87	7	-	30	0	K.DGVICISEACEGHMVQVFACHKPR.V + Oxidation (M)
3071.4028	3070.3955	3070.4358	-13.11	7	-	32	1	K.DGVICISEACEGHMVQVFACHKPRVR.C + Oxidation (M)

No match to: 701.3929, 710.3735, 712.2889, 713.2998, 717.3996, 719.4157, 720.4154, 722.4257, 729.4009, 734.4510, 735.4123, 738.4139, 745.4143, 747.4459, 753.3587, 756.3719, 771.3859, 779.4640, 783.3826, 805.4482, 806.4485, 818.4813, 823.4747, 829.4503, 831.4642, 832.3057, 834.3104, 835.3138, 840.4758, 842.5006, 874.5018, 883.4738, 889.4957, 891.4817, 900.5336, 909.5331, 922.4722, 923.5116, 925.4815, 929.4376, 936.4885, 938.4929, 949.5431, 958.4930, 967.4177, 969.4647, 974.4397, 978.5347, 983.6221, 985.6010,

988.4032, 993.4985, 1007.5710, 1017.5679, 1034.5784, 1036.5477, 1044.5231, 1046.5923, 1051.5060, 1056.4977, 1057.5691, 1058.6145, 1074.6044, 1087.5525, 1113.5417, 1118.5876, 1123.5989, 1143.4429, 1145.6317, 1169.4524, 1172.6801, 1178.6199, 1185.6628, 1187.7002, 1194.5691, 1207.6023, 1216.4987, 1216.7014, 1248.6699, 1254.6930, 1255.6340, 1257.6666, 1265.7057, 1269.6703, 1273.6517, 1278.6932, 1281.6456, 1284.6411, 1286.7430, 1299.5466, 1301.6044, 1304.6500, 1312.7513, 1314.7311, 1317.7122, 1338.6886, 1349.6870, 1351.6915, 1354.7209, 1358.7173, 1365.5874, 1368.6487, 1371.7622, 1372.5999, 1374.6569, 1375.7383, 1382.5975, 1383.7290, 1390.6992, 1393.7732, 1397.7450, 1412.6091, 1413.6514, 1420.7966, 1440.7816, 1442.8168, 1450.8618, 1456.7800, 1457.7509, 1467.7542, 1470.7775, 1494.7429, 1500.7238, 1513.7605, 1528.6982, 1539.7341, 1556.7584, 1562.7856, 1563.7985, 1570.7755, 1571.9341, 1578.7968, 1580.8304, 1590.7900, 1592.7916, 1616.8013, 1628.7054, 1628.8561, 1635.7781, 1642.7219, 1647.8557, 1649.8578, 1650.8488, 1657.9104, 1663.8551, 1664.8605, 1696.8295, 1706.8674, 1707.9520, 1715.8798, 1718.8820, 1721.9437, 1730.8646, 1741.9973, 1747.9136, 1755.8970, 1758.9091, 1771.0317, 1774.9242, 1786.9141, 1787.8756, 1862.9530, 1870.7738, 1883.7272, 1895.7275, 1897.7380, 1903.0176, 1911.7487, 1913.7377, 1928.0281, 1938.0094, 1941.0232, 1959.9926, 1961.0339, 1970.0011, 1974.9122, 1979.0769, 1981.0564, 1982.0408, 2007.9467, 2008.9807, 2027.1300, 2055.0208, 2060.9963, 2067.1592, 2086.1130, 2108.9956, 2112.9822, 2144.0425, 2181.1780, 2223.1548, 2239.1460, 2241.1360, 2245.8369, 2251.1431, 2255.1433, 2259.8535, 2265.0969, 2273.0667, 2286.1521, 2318.1763, 2328.9463, 2335.8999, 2343.1943, 2347.8403, 2352.9194, 2361.8635, 2363.8716, 2364.8738, 2366.9121, 2371.0796, 2389.2690, 2414.2378, 2467.2051, 2474.2170, 2477.2441, 2485.0479, 2503.0457, 2504.9548, 2505.9473, 2519.9768, 2541.2239, 2542.2581, 2580.0098, 2595.0286, 2631.4714, 2646.9739, 2678.0037, 2681.3691, 2684.3965, 2695.0193, 2701.2148, 2705.2874, 2709.0286, 2717.2839, 2770.4202, 2792.2090, 2798.2344, 2799.2815, 2809.4841, 2813.3928, 2833.0999, 2842.4780, 2846.3840, 2872.3215, 2876.3640, 2885.3044, 2919.4407, 2961.2092, 2970.4829, 2976.2786, 2990.3098, 3002.4895, 3029.4480, 3081.6030, 3087.4146, 3146.5461, 3157.4587, 3161.0999, 3184.4336, 3236.5503, 3248.6294, 3254.5583, 3265.5901, 3315.6875, 3330.5125, 3334.5588, 3335.6306, 3346.5308, 3347.4827, 3352.6125, 3410.6125, 3411.7185, 3428.5632, 3435.6848, 3457.5947, 3462.6028, 3471.6272, 3474.6221, 3489.6526, 3503.7864, 3528.3171, 3542.3335, 3552.6479, 3574.6042, 3624.6587, 3639.7046, 3645.7671, 3651.7319, 3667.7424, 3690.9905, 3707.5422, 3716.6360, 3721.5728, 3722.4707, 3736.5007, 3741.4561, 3748.4346, 3753.4888, 3764.5239, 4075.0212, 4147.9077, 4160.8794, 4238.9067, 4253.8887, 4274.9287, 4301.2393, 4397.9902, 4415.0044, 4452.1357, 4458.2759, 4465.1338, 4631.9888, 4690.3101, 4845.1016

30. E1B8M0 **Mass:** 54699 **Score:** 16 **Expect:** 5.5e+002 **Matches:** 13

Uncharacterized protein OS=Bos taurus GN=CEP55 PE=4 SV=1

Observed	Mr(expt)	Mr(calc)	ppm	Start	End	Miss	Peptide
925.4815	924.4742	924.4552	20.5	268	274	1	R.KYEETQK.E
985.6010	984.5937	984.5716	22.4	63	70	1	K.IRVLEAER.E
1034.5784	1033.5711	1033.5920	-20.21	30	38	1	K.FRGEITALK.T
1087.5525	1086.5452	1086.5669	-19.94	307	315	1	R.LKEENDLAR.E
1248.6699	1247.6626	1247.6609	1.40	120	130	1	K.SLSEETDVLKK.Q
1265.7057	1264.6984	1264.6775	16.5	73	82	2	K.NVYRLTEKDK.E
1590.7900	1589.7827	1589.7910	-5.21	288	300	2	R.KADVQHLEDDRHK.T
1696.8295	1695.8222	1695.8315	-5.47	222	235	2	K.KTESEGYLQEEKQK.Y
1730.8646	1729.8573	1729.8781	-12.01	275	288	1	K.EVQDLNQLLCSQRK.A
1758.9091	1757.9018	1757.8948	4.01	14	29	1	K.WGPKPSNSKSETALEK.F
1903.0176	1902.0103	1902.0258	-8.11	371	385	1	R.QNMQHQLHVILKELR.K + Oxidation (M)
2467.2051	2466.1978	2466.2390	-16.71	247	266	1	K.DLEVERQTITQLNFELSEFR.R
4690.3101	4689.3028	4689.2724	6.49	139	180	2	R.LAELEGKANTLHLSQTVATSCFNSSMSNIHEMEVQLKDALEK.N + Oxidation (M)

No match to: 701.3929, 710.3735, 712.2889, 713.2998, 717.3996, 719.4157, 720.4154, 722.4257, 729.4009, 734.4510, 735.4123, 738.4139, 745.4143, 747.4459, 753.3587, 756.3719, 771.3859, 779.4640, 783.3826, 805.4482, 806.4485, 818.4813, 823.4747, 829.4503, 831.4642, 832.3057, 834.3104, 835.3138, 840.4758, 842.5006, 874.5018, 883.4738, 889.4957, 891.4817, 900.5336, 909.5331, 922.4722, 923.5116, 929.4376, 936.4885, 938.4929, 949.5431, 958.4930, 967.4177, 969.4647, 974.4397, 978.5347, 983.6221, 988.4032, 993.4985, 1007.5710, 1017.5679, 1036.5477, 1044.5231, 1046.5923, 1051.5060, 1056.4977, 1057.5691, 1058.6145, 1074.6044, 1113.5417, 1118.5876, 1123.5989, 1143.4429, 1145.6317, 1169.4524, 1172.6801, 1178.6199, 1185.6628, 1187.7002, 1194.5691, 1207.6023, 1216.4987, 1216.7014, 1254.6930, 1255.6340, 1257.6666, 1269.6703, 1273.6517, 1278.6932, 1281.6456, 1284.6411, 1286.7430, 1299.5466, 1301.6044, 1304.6500, 1312.7513, 1314.7311, 1317.7122, 1338.6886, 1349.6870, 1351.6915, 1354.7209, 1358.7173, 1365.5874, 1368.6487, 1371.7622, 1372.5999, 1374.6569, 1375.7383, 1382.5975, 1383.7290, 1390.6992, 1393.7732, 1397.7450, 1412.6091, 1413.6514, 1420.7966, 1440.7816, 1442.8168, 1450.8618, 1456.7800, 1457.7509, 1467.7542, 1470.7775, 1494.7429, 1500.7238, 1513.7605, 1528.6982, 1539.7341, 1556.7584, 1562.7856, 1563.7985, 1570.7755, 1571.9341, 1578.7968, 1580.8304, 1592.7916, 1616.8013, 1628.7054, 1628.8561, 1635.7781, 1642.7219, 1647.8557, 1649.8578, 1650.8488, 1657.9104, 1663.8551, 1664.8605, 1706.8674, 1707.9520, 1715.8798, 1718.8820, 1721.9437, 1741.9973, 1747.9136, 1755.8970, 1771.0317, 1774.9242, 1786.9141, 1787.8756, 1862.9530, 1870.7738, 1883.7272, 1895.7275, 1897.7380, 1911.7487, 1913.7377, 1928.0281, 1938.0094, 1941.0232, 1959.9926, 1961.0339, 1970.0011, 1974.9122, 1979.0769, 1981.0564, 1982.0408, 2007.9467, 2008.9807, 2027.1300, 2055.0208, 2060.9963, 2067.1592, 2086.1130, 2108.9956, 2112.9822, 2144.0425, 2181.1780, 2223.1548, 2239.1460, 2241.1360, 2245.8369, 2251.1431, 2255.1433, 2259.8535, 2265.0969, 2273.0667, 2286.1521, 2318.1763, 2328.9463, 2335.8999, 2343.1943, 2347.8403, 2352.9194, 2361.8635, 2363.8716, 2364.8738, 2366.9121, 2371.0796, 2389.2690, 2414.2378, 2474.2170, 2477.2441, 2485.0479, 2503.0457, 2504.9548, 2505.9473, 2519.9768, 2541.2239, 2542.2581, 2580.0098, 2595.0286, 2631.4714, 2646.9739, 2678.0037, 2681.3691, 2684.3965, 2695.0193, 2701.2148, 2705.2874, 2709.0286, 2717.2839, 2770.4202, 2792.2090, 2798.2344, 2799.2815, 2809.4841, 2813.3928, 2816.2957, 2833.0999, 2842.4780, 2846.3840, 2872.3215, 2876.3640, 2885.3044, 2919.4407, 2961.2092, 2970.4829, 2976.2786, 2990.3098, 3002.4895, 3029.4480, 3071.4028, 3081.6030, 3087.4146, 3146.5461, 3157.4587, 3161.0999, 3184.4336, 3236.5503, 3248.6294, 3254.5583, 3265.5901, 3315.6875, 3330.5125, 3334.5588, 3335.6306, 3346.5308, 3347.4827, 3352.6125, 3410.6125, 3411.7185, 3428.5632, 3435.6848, 3457.5947, 3462.6028, 3471.6272, 3474.6221, 3489.6526, 3503.7864, 3528.3171, 3542.3335, 3552.6479, 3574.6042, 3624.6587, 3639.7046, 3645.7671, 3651.7319, 3667.7424, 3690.9905, 3707.5422, 3716.6360, 3721.5728, 3722.4707, 3736.5007, 3741.4561, 3748.4346, 3753.4888, 3764.5239, 4075.0212, 4147.9077, 4160.8794, 4238.9067, 4253.8887, 4274.9287, 4301.2393, 4397.9902, 4415.0044, 4452.1357, 4458.2759, 4465.1338, 4631.9888, 4845.1016

31. G3MXA5 **Mass:** 71155 **Score:** 16 **Expect:** 5.7e+002 **Matches:** 11

Uncharacterized protein OS=Bos taurus GN=TIAM1 PE=4 SV=1

Observed	Mr(expt)	Mr(calc)	ppm	Start	End	Miss	Peptide
1118.5876	1117.5803	1117.5992	-16.90	169	177	2	K.RSKSADIWR.E
1273.6517	1272.6444	1272.6357	6.87	218	228	1	R.ASPRQLSACQR.A
1412.6091	1411.6018	1411.6323	-21.60	583	595	0	K.MGEMQLSSVTDSK.K
1500.7238	1499.7165	1499.6892	18.2	134	147	0	R.LYGDDATYLAEGGR.R
1539.7341	1538.7268	1538.7320	-3.36	570	581	2	K.LEQKIDMDEKMK.K + 2 Oxidation (M)
1556.7584	1555.7511	1555.7222	18.6	583	596	1	K.MGEMQLSSVTDSKK.K + Oxidation (M)
2286.1521	2285.1448	2285.1375	3.21	148	167	2	R.RQRPYASNGPAFMETASFKK.K
2371.0796	2370.0723	2370.0441	11.9	294	315	0	K.SHCLSEGATNPQISHSSSMQGR.R
3236.5503	3235.5430	3235.4892	16.7	178	206	0	R.EDSLEFSLSDLSQEHLTSNEEILGSAEEK.D
3639.7046	3638.6973	3638.7212	-6.56	134	166	2	R.LYGDDATYLAEGGRRQRPYASNGPAFMETASFK.K
4690.3101	4689.3028	4689.2632	8.45	511	552	1	K.KDFVFCLSNSLGDAFLFQTTSQTELENWITAIHSACAAAVAR.H

No match to: 701.3929, 710.3735, 712.2889, 713.2998, 717.3996, 719.4157, 720.4154, 722.4257, 729.4009, 734.4510, 735.4123, 738.4139, 745.4143, 747.4459, 753.3587, 756.3719, 771.3859, 779.4640, 783.3826, 805.4482, 806.4485, 818.4813, 823.4747, 829.4503, 831.4642, 832.3057, 834.3104, 835.3138, 840.4758, 842.5006, 874.5018, 883.4738, 889.4957, 891.4817, 900.5336, 909.5331, 922.4722, 923.5116, 925.4815, 929.4376, 936.4885, 938.4929, 949.5431, 958.4930, 967.4177, 969.4647, 974.4397, 978.5347, 983.6221, 985.6010, 988.4032, 993.4985, 1007.5710, 1017.5679, 1034.5784, 1036.5477, 1044.5231, 1046.5923, 1051.5060, 1056.4977, 1057.5691, 1058.6145, 1074.6044, 1087.5525, 1113.5417, 1123.5989, 1143.4429, 1145.6317, 1169.4524, 1172.6801, 1178.6199, 1185.6628, 1187.7002, 1194.5691, 1207.6023, 1216.4987, 1216.7014, 1248.6699, 1254.6930, 1255.6340, 1257.6666, 1265.7057, 1269.6703, 1278.6932, 1281.6456, 1284.6411, 1286.7430, 1299.5466, 1301.6044, 1304.6500, 1312.7513, 1314.7311, 1317.7122, 1338.6886, 1349.6870, 1351.6915, 1354.7209, 1358.7173, 1365.5874, 1368.6487, 1371.7622, 1372.5999, 1374.6569, 1375.7383, 1382.5975, 1383.7290, 1390.6992, 1393.7732, 1397.7450, 1413.6514, 1420.7966, 1440.7816, 1442.8168, 1450.8618, 1456.7800, 1457.7509, 1467.7542, 1470.7775, 1494.7429, 1513.7605, 1528.6982, 1562.7856, 1563.7985, 1570.7755, 1571.9341, 1578.7968, 1580.8304, 1590.7900, 1592.7916, 1616.8013, 1628.7054, 1628.8561, 1635.7781, 1642.7219, 1647.8557, 1649.8578, 1650.8488, 1657.9104, 1663.8551, 1664.8605, 1696.8295, 1706.8674, 1707.9520, 1715.8798, 1718.8820, 1721.9437, 1730.8646, 1741.9973, 1747.9136, 1755.8970, 1758.9091, 1771.0317, 1774.9242, 1786.9141, 1787.8756, 1862.9530, 1870.7738, 1883.7272, 1895.7275, 1897.7380, 1903.0176, 1911.7487, 1913.7377, 1928.0281, 1938.0094, 1941.0232, 1959.9926, 1961.0339, 1970.0011, 1974.9122,

1979.0769, 1981.0564, 1982.0408, 2007.9467, 2008.9807, 2027.1300, 2055.0208, 2060.9963, 2067.1592, 2086.1130, 2108.9956, 2112.9822, 2144.0425, 2181.1780, 2223.1548, 2239.1460, 2241.1360, 2245.8369, 2251.1431, 2255.1433, 2259.8535, 2265.0969, 2273.0667, 2318.1763, 2328.9463, 2335.8999, 2343.1943, 2347.8403, 2352.9194, 2361.8635, 2363.8716, 2364.8738, 2366.9121, 2389.2690, 2414.2378, 2467.2051, 2474.2170, 2477.2441, 2485.0479, 2503.0457, 2504.9548, 2505.9473, 2519.9768, 2541.2239, 2542.2581, 2580.0098, 2595.0286, 2631.4714, 2646.9739, 2678.0037, 2681.3691, 2684.3965, 2695.0193, 2701.2148, 2705.2874, 2709.0286, 2717.2839, 2770.4202, 2792.2090, 2798.2344, 2799.2815, 2809.4841, 2813.3928, 2816.2957, 2833.0999, 2842.4780, 2846.3840, 2872.3215, 2876.3640, 2885.3044, 2919.4407, 2961.2092, 2970.4829, 2976.2786, 2990.3098, 3002.4895, 3029.4480, 3071.4028, 3081.6030, 3087.4146, 3146.5461, 3157.4587, 3161.0999, 3184.4336, 3248.6294, 3254.5583, 3265.5901, 3315.6875, 3330.5125, 3334.5588, 3335.6306, 3346.5308, 3347.4827, 3352.6125, 3410.6125, 3411.7185, 3428.5632, 3435.6848, 3457.5947, 3462.6028, 3471.6272, 3474.6221, 3489.6526, 3503.7864, 3528.3171, 3542.3335, 3552.6479, 3574.6042, 3624.6587, 3645.7671, 3651.7319, 3667.7424, 3690.9905, 3707.5422, 3716.6360, 3721.5728, 3722.4707, 3736.5007, 3741.4561, 3748.4346, 3753.4888, 3764.5239, 4075.0212, 4147.9077, 4160.8794, 4238.9067, 4253.8887, 4274.9287, 4301.2393, 4397.9902, 4415.0044, 4452.1357, 4458.2759, 4465.1338, 4631.9888, 4845.1016

32. Q5EA43 **Mass:** 36620 **Score:** 16 **Expect:** 5.8e+002 **Matches:** 9
Sideroflexin-2 OS=Bos taurus GN=SFXN2 PE=2 SV=2

Observed	Mr(expt)	Mr(calc)	ppm	Start		End	Miss	Peptide
818.4813	817.4740	817.4922	-22.27	253	-	258	1	K.LRFVQR.V
1590.7900	1589.7827	1589.7872	-2.79	42	-	54	1	R.ELDWAKVMVEQSR.M
1718.8820	1717.8747	1717.8940	-11.23	309	-	322	2	K.YREPVPHVYFNKGL.-
1747.9136	1746.9063	1746.9205	-8.14	307	-	320	2	K.AKYREPVPHVYFNK.G
1941.0232	1940.0159	1940.0268	-5.63	26	-	41	1	K.HFFNITDPRTVLVPER.E
2286.1521	2285.1448	2285.1375	3.20	72	-	91	2	K.KLYDSAFHPDTGHKMNVIGR.M
2371.0796	2370.0723	2370.1211	-20.58	92	-	111	0	R.MSFQVPGGMIITGFMLQFYR.T + 3 Oxidation (M)
3489.6526	3488.6453	3488.7313	-24.65	136	-	169	1	R.NAASPTSVRQMAVSYITATTAVATAVGMNMLTK.R + 2 Oxidation (M)
3645.7671	3644.7598	3644.8324	-19.92	136	-	170	2	R.NAASPTSVRQMAVSYITATTAVATAVGMNMLTKR.A + 2 Oxidation (M)

No match to: 701.3929, 710.3735, 712.2889, 713.2998, 717.3996, 719.4157, 720.4154, 722.4257, 729.4009, 734.4510, 735.4123, 738.4139, 745.4143, 747.4459, 753.3587, 756.3719, 771.3859, 779.4640, 783.3826, 805.4482, 806.4485, 823.4747, 829.4503, 831.4642, 832.3057, 834.3104, 835.3138, 840.4758, 842.5006, 874.5018, 883.4738, 889.4957, 891.4817, 900.5336, 909.5331, 922.4722, 923.5116, 925.4815, 929.4376, 936.4885, 938.4929, 949.5431, 958.4930, 967.4177, 969.4647, 974.4397, 978.5347, 983.6221, 985.6010, 988.4032, 993.4985, 1007.5710, 1017.5679, 1034.5784, 1036.5477, 1044.5231, 1046.5923, 1051.5060, 1056.4977, 1057.5691, 1058.6145, 1074.6044, 1087.5525, 1113.5417, 1118.5876, 1123.5989, 1143.4429, 1145.6317, 1169.4524, 1172.6801, 1178.6199, 1185.6628, 1187.7002, 1194.5691, 1207.6023, 1216.4987, 1216.7014, 1248.6699, 1254.6930, 1255.6340, 1257.6666, 1265.7057, 1269.6703, 1273.6517, 1278.6932, 1281.6456, 1284.6411, 1286.7430, 1299.5466, 1301.6044, 1304.6500, 1312.7513, 1314.7311, 1317.7122, 1338.6886, 1349.6870, 1351.6915, 1354.7209, 1358.7173, 1365.5874, 1368.6487, 1371.7622, 1372.5999, 1374.6569, 1375.7383, 1382.5975, 1383.7290, 1390.6992, 1393.7732, 1397.7450, 1412.6091, 1413.6514, 1420.7966, 1440.7816, 1442.8168, 1450.8618, 1456.7800, 1457.7509, 1467.7542, 1470.7775, 1494.7429, 1500.7238, 1513.7605, 1528.6982, 1539.7341, 1556.7584, 1562.7856, 1563.7985, 1570.7755, 1571.9341, 1578.7968, 1580.8304, 1592.7916, 1616.8013, 1628.7054, 1628.8561, 1635.7781, 1642.7219, 1647.8557, 1649.8578, 1650.8488, 1657.9104, 1663.8551, 1664.8605, 1696.8295, 1706.8674, 1707.9520, 1715.8798, 1721.9437, 1730.8646, 1741.9973, 1755.8970, 1758.9091, 1771.0317, 1774.9242, 1786.9141, 1787.8756, 1862.9530, 1870.7738, 1883.7272, 1895.7275, 1897.7380, 1903.0176, 1911.7487, 1913.7377, 1928.0281, 1938.0094, 1959.9926, 1961.0339, 1970.0011, 1974.9122, 1979.0769, 1981.0564, 1982.0408, 2007.9467, 2008.9807, 2027.1300, 2055.0208, 2060.9963, 2067.1592, 2086.1130, 2108.9956, 2112.9822, 2144.0425, 2181.1780, 2223.1548, 2239.1460, 2241.1360, 2245.8369, 2251.1431, 2255.1433, 2259.8535, 2265.0969, 2273.0667, 2318.1763, 2328.9463, 2335.8999, 2343.1943, 2347.8403, 2352.9194, 2361.8635, 2363.8716, 2364.8738, 2366.9121, 2389.2690, 2414.2378, 2467.2051, 2474.2170, 2477.2441, 2485.0479, 2503.0457, 2504.9548, 2505.9473, 2519.9768, 2541.2239, 2542.2581, 2580.0098, 2595.0286, 2631.4714, 2646.9739, 2678.0037, 2681.3691, 2684.3965, 2695.0193, 2701.2148, 2705.2874, 2709.0286, 2717.2839, 2770.4202, 2792.2090, 2798.2344, 2799.2815, 2809.4841, 2813.3928, 2816.2957, 2833.0999, 2842.4780, 2846.3840, 2872.3215, 2876.3640, 2885.3044, 2919.4407, 2961.2092, 2970.4829, 2976.2786, 2990.3098, 3002.4895, 3029.4480, 3071.4028, 3081.6030, 3087.4146, 3146.5461, 3157.4587, 3161.0999, 3184.4336, 3236.5503, 3248.6294, 3254.5583, 3265.5901, 3315.6875, 3330.5125, 3334.5588, 3335.6306, 3346.5308, 3347.4827, 3352.6125, 3410.6125, 3411.7185, 3428.5632, 3435.6848, 3457.5947, 3462.6028, 3471.6272, 3474.6221, 3503.7864, 3528.3171, 3542.3335, 3552.6479, 3574.6042, 3624.6587, 3639.7046, 3651.7319, 3667.7424, 3690.9905, 3707.5422, 3716.6360, 3721.5728, 3722.4707, 3736.5007, 3741.4561, 3748.4346, 3753.4888, 3764.5239, 4075.0212, 4147.9077, 4160.8794, 4238.9067, 4253.8887, 4274.9287, 4301.2393, 4397.9902, 4415.0044, 4452.1357, 4458.2759, 4465.1338, 4631.9888, 4690.3101, 4845.1016

33. A6QR46 **Mass:** 23561 **Score:** 16 **Expect:** 5.8e+002 **Matches:** 8
Ras-related protein Rab-6B OS=Bos taurus GN=RAB6B PE=2 SV=1

Observed	Mr(expt)	Mr(calc)	ppm	Start		End	Miss	Peptide
719.4157	718.4084	718.4238	-21.43	165	-	169	1	K.QLFRR.V
1036.5477	1035.5404	1035.5270	12.9	184	-	192	1	K.SKEGMIDIK.L + Oxidation (M)
1349.6870	1348.6797	1348.6558	17.8	1	-	13	1	-.MSAGGDFGNPLRK.F
1354.7209	1353.7136	1353.6850	21.2	147	-	158	0	K.ELSVMFIETSAK.T
1358.7173	1357.7100	1357.6950	11.1	134	-	144	1	K.RQITIEEGEQR.A
1628.8561	1627.8488	1627.8352	8.39	169	-	183	1	R.RVASALPGMENVQEK.S
1647.8557	1646.8484	1646.8662	-10.76	113	-	127	1	R.TERGSDVIIMLVGNK.T + Oxidation (M)
2474.2170	2473.2097	2473.2556	-18.55	170	-	192	2	R.VASALPGMENVQEKSKEGMIDIK.L
[truncated: 4,696,154 more chars]
